# Deciphering differences in DNA methylation and transcriptome profiles of oocytes from pigs with high and low developmental competence

**DOI:** 10.1093/eep/dvaf018

**Published:** 2025-06-03

**Authors:** Laura Abril-Parreño, Jordana S Lopes, Jon Romero-Aguirregomezcorta, Antonio Galvao, Gavin Kelsey, Pilar Coy

**Affiliations:** Physiology of Reproduction Group, Department of Physiology, Faculty of Veterinary Medicine, International Excellence Campus for Higher Education and Research (Campus Mare Nostrum), University of Murcia, 30100 Murcia, Spain; Institute for Biomedical Research of Murcia, IMIB-Pascual Parrilla, 30100 Murcia, Spain; MED – Mediterranean Institute for Agriculture, Environment and Development & CHANGE – Global Change and Sustainability Institute, Institute for Advanced Studies and Research, Universidade de Évora, 7006–554 Évora, Portugal; Physiology of Reproduction Group, Department of Physiology, Faculty of Veterinary Medicine, International Excellence Campus for Higher Education and Research (Campus Mare Nostrum), University of Murcia, 30100 Murcia, Spain; Institute for Biomedical Research of Murcia, IMIB-Pascual Parrilla, 30100 Murcia, Spain; Department of Comparative Biomedical Sciences, Royal Veterinary College, London NW1 0TU, United Kingdom; Epigenetics Programme, The Babraham Institute, Cambridge CB22 3AT, United Kingdom; Epigenetics Programme, The Babraham Institute, Cambridge CB22 3AT, United Kingdom; Centre for Trophoblast Research, University of Cambridge , Cambridge CB2 3EG, United Kingdom; Metabolic Research Laboratories, Wellcome-MRC Institute of Metabolic Science, Cambridge CB2 0QQ, United Kingdom; Physiology of Reproduction Group, Department of Physiology, Faculty of Veterinary Medicine, International Excellence Campus for Higher Education and Research (Campus Mare Nostrum), University of Murcia, 30100 Murcia, Spain; Institute for Biomedical Research of Murcia, IMIB-Pascual Parrilla, 30100 Murcia, Spain

**Keywords:** oocyte maturation, porcine, epigenomic, multi-omic analysis, cellular division

## Abstract

*In vitro* maturation (IVM) is a critical step in animal *in vitro* embryo production, yet oocytes matured *in vitro* often exhibit lower developmental competence than their *in vivo* counterparts. However, the molecular mechanisms behind this observation remain unclear. This study investigated the gene expression and DNA methylation profiles in porcine oocytes with different developmental competencies. To study these differences, we used as a model oocytes from prepubertal gilts (IVM) and sows (*in vivo* matured) and assessed their developmental competence up to the blastocyst stage. We also examined their gene expression and DNA methylation profiles at single-cell resolution using RNA sequencing and bisulfite sequencing. Oocytes were obtained by aspiration of either ovarian follicles between 3 and 6 mm diameter, and the subsequent IVM, or ovarian follicles from 8 to 10 mm diameter, with no need for maturation (*in vivo* matured oocytes). Cleavage rates (58.2 ± 3.0 and 45.7 ± 4.4) and blastocyst rates (31.4 ± 3.7 and 47.5 ± 6.6) for IVM and *in vivo* groups differed significantly. Using the *in vivo* group as a reference, IVM oocytes had 1297 downregulated and 476 upregulated differentially expressed genes (DEGs), with upregulated DEGs associated with organelle organization and cell cycle processes, and downregulated genes involved in protein synthesis and metabolomic processes. While global DNA methylation levels were similar between groups, a few differentially methylated regions were found in CpG islands, promoters, and coding regions. Our integrative analysis identified key methylated regions and genes that distinguish each group, suggesting that both donor age and maturation conditions significantly influence gene expression regulation in oocytes with different developmental competencies.

## Introduction

It is estimated that the world population will increase from 7.6 to 9.8 billion by 2050, thus also increasing by 20% the *per capita* consumption of meat and milk [1]. Nevertheless, it is not a viable solution to increase livestock numbers and the use of intensive facilities to meet this demand. Instead, new technologies are required to achieve higher yields and reproductive efficiency, while simultaneously modernizing facilities to ensure animal welfare and reduce the carbon footprint and associated waste. In this context, the utilization of assisted reproductive technologies (ART) in livestock is essential to enhance meat production while concurrently contributing to a reduction in global warming through the generation of more meat from fewer animals in a shorter time. This is particularly pertinent in the context of the pig industry, which accounts for 40% of global meat consumption [2]. In light of this, the development of *in vitro* embryo production (IVP) will prove pivotal for the pig industry, as they will facilitate the global transport of genetically superior stock in a manner that ensures higher animal welfare, reduced transportation costs, and minimal risk of disease transmission.

The porcine IVP was not achieved to an acceptable level until 1989, when Mattioli et al. demonstrated that oocytes sourced from an abattoir could be matured and fertilized *in vitro*, resulting in the development of blastocysts, which led to the birth of live piglets [3]. Despite the numerous improvements that have been made to IVP systems, the success rate of IVP remains relatively low [4, 5]. Several intrinsic and extrinsic factors can affect the developmental potential of porcine oocytes and embryos, which can compromise the efficiency of IVP systems [6]. One such factor is the oocyte source; oocytes can be collected from young gilts that have not yet experienced regular estrous cycles, or from sexually mature sows. The use of ovaries from prepubertal gilts is a highly attractive proposition for research, as these have been used in >500 published studies. Gilt oocytes are more accessible from the slaughterhouse than sow oocytes, and the transfer of embryos after IVP with prepubertal gilt oocytes leads to a higher genetic gain in a breeding programme, thereby facilitating the introduction of new genetics in a more expedient manner [7]. However, some studies showed that oocytes from sows are more likely to develop to the blastocyst stage than oocytes from prepubertal gilts [8–10], which have been associated with the follicular environment from which the oocytes are recovered, where oocyte quality increases with increasing antral follicle size [11]. Indeed, numerous studies have focused on the improvement of culture conditions for prepubertal porcine oocytes to enhance their nuclear and cytoplasmic maturation more efficiently [6, 12]. Historically, the use of *in vitro* maturation (IVM) medium supplemented with follicular fluid from large follicles promoted oocyte maturation to a greater degree than IVM medium supplemented with follicular fluid from small follicles [13]. Similarly, follicular fluid from sows was superior to follicular fluid from prepubertal gilts in supporting the maturation of oocytes *in vitro* [14]. Furthermore, variation in the concentrations of any of the components of sow follicular fluid has been shown to affect IVM outcomes and oocyte developmental competence [15, 16], which are controlled by different molecular mechanisms.

The potential impact of IVM on normal deoxyribonucleic acid (DNA) methylation and transcriptomic profiles in oocytes from different species has also been discussed. For example, Saenz-De-Juano et al. (2019) reported a limited number of discrete DNA methylation differences in mouse oocytes collected from immature compared with mature females [17]. However, studies in various species, including mice, cows, pigs, and humans, have shown that IVM also affects the dynamics of epigenetic markers during early embryo development [18–21]. In mouse oocytes, it has been identified that IVM resulted in metabolic and gene expression modifications due to environmental changes compared with *in vivo* matured oocytes [22]. In cows, IVM was found to influence gene expression as compared to *in vivo* matured oocytes [23]. Recently, Takeuchi et al. (2022), using a single-cell transcriptomic analysis, identified hundreds of transcripts that were dynamically altered in their expression during the different stages of IVM of human oocytes [24].

Therefore, the objective of this study was to identify molecular markers of oocyte quality in pigs, focusing on a model that compares *in vitro* matured oocytes from prepubertal gilts, known to have reduced developmental competence, with *in vivo* matured oocytes from sows. By analysing both epigenetic and transcriptomic profiles, this research aims to uncover novel markers that can provide insights into the underlying mechanisms of reduced oocyte quality during IVM. Such markers could guide the development of improved IVM protocols, ultimately achieving oocyte quality comparable to that of *in vivo* matured counterparts. Our results indicated a discrete effect of IVM on the DNA methylation landscape in mature porcine oocytes, but these changes seem to have a greater impact on the regulation of gene expression. These molecular differences reflected the different developmental competence of the two groups of oocytes, which was higher in the *in vivo* matured oocytes.

## Methods

### Oocyte collection and *in vitro* maturation

Ovaries from Landrace–Large White prepubertal gilts and sows were collected at the slaughterhouse and transported to the laboratory (within 60 min) in a saline solution containing 100 µg/ml kanamycin sulphate at 38.5°C, washed once in 0.04% cetrimide solution (w/v), and then twice in saline. In experiment 1 (assessment of the developmental competence of *in vitro* and *in vivo* matured oocytes), all the culture media were purchased from EmbryoCloud, Murcia, Spain. Selected cumulus-oocyte complexes (COCs) from prepubertal gilts (follicles of 3–6 mm diameter) were washed twice in PIG-WASH medium and twice in NaturARTs-PIG-IVM1 medium before being placed in groups of 50–55 in four-well plates containing 500 µl NaturARTs-PIG-IVM1 medium. Oocytes were incubated for 20–22 h at 38.5°C in an atmosphere of 5% CO_2_, 7% O_2_, and saturated humidity. After incubation, COCs were washed twice in NaturARTs-PIG-IVM2 medium and placed in 4-well plates for an additional incubation of 20–22 h in NaturARTs-PIG-IVM2. In contrast, COCs from sows, aspirated from follicles between 8 and 10 mm were washed twice in NaturARTs-PIG-IVM2 medium, but the incubation in the maturation medium lasted only 4 h before *in vitro* fertilization (IVF). This 4-h waiting period has been introduced before IVF based on our previous data (nonpublished) and results from human fertility clinics that implement a waiting period of 2−6 h, ∼4 h, before performing IVF or ICSI [25].

In experiment 2 (collection of oocytes for scRNA-seq and scBS-seq), IVM protocols were the same as previously described. As for the *in vivo* matured oocytes, the contents of follicles of 8–10 mm diameter were aspirated and washed twice in Dulbecco’s PBS supplemented with 1 mg ml^−1^ PVA. All IVM and *in vivo*, matured COCs were identified under a stereomicroscope and mechanically detached from the cumulus cells using an automatic pipette set to a volume of 200 µl. To do this, the COCs were gently aspirated and repeatedly ejected into the Petri dish until the zona pellucida was visualized free of cumulus cells at 50× magnification. Only oocytes with a visible polar body under the stereomicroscope were selected and stored in 5 µl RLT buffer and snap frozen in LN_2_ and stored at −80°C until use (*n* = 20 IVM and 20 *in vivo* matured oocytes).

### *In vitro* fertilization and *in vitro* culture of putative zygotes

To assess the potential competence of both the IVM and *in vivo* matured oocytes, we used a total of *n* = 268 and *n* = 129 oocytes from IVM and *in vivo* groups, respectively. For IVF of porcine oocytes, matured COCs were washed twice in the PIG-IVF medium (EmbryoCloud) before being added to pre-equilibrated 4-well dishes containing 250 µl of PIG-IVF medium. Porcine spermatozoa were prepared using the swim-up method. A 0.5 ml straw of Landrace–Large White boar semen was thawed in a water bath at 38°C for 30 s. The thawed semen was then transferred to a sterile 5-ml tube, 2 ml of PIG-SUM medium (EmbryoCloud) was carefully added, and the mixture was gently combined. Next, 1 ml of the semen solution was carefully added to the bottom of a conical tube containing 1 ml PIG-SUM medium, ensuring no turbulence or bubbles were created. The tubes were incubated at 38°C for 20 min at a 45° angle. After incubation, 500 µl from the top of each tube were collected and transferred to a clean tube, and the sperm concentration was adjusted for IVF. Spermatozoa were then added in a volume of 250 µl, resulting in a final concentration of 1 × 10^4^ spermatozoa/ml, and co-incubation was carried out for 22 h.

For *in vitro* embryo culture (IVC) of porcine presumptive zygotes, COCs from IVF were gently stripped of cumulus cells by pipetting in IVF media. After decumulation, the presumptive zygotes were then washed twice in PIG-IVC1 medium (EmbryoCloud) and transferred in groups of 50–55 to mineral oil-covered 4-well plates containing 500 µl PIG-IVC1 medium. Zygotes were cultured for 22–24 h before checking for the first cell division. Only those divided into two cells or more were selected and transferred to a dish containing PIG-IVC2 medium (EmbryoCloud) for an additional six days of culture. These were washed twice and placed in mineral oil-covered 4-well plates containing 500 µl PIG-IVC2 medium. Blastocyst rates are calculated as the number of blastocysts relative to the number of 2-cell embryos cultured in PIG-IVC2 medium.

### Statistical analysis

All statistical analyses were conducted using IBM SPSS Statistics for Windows (IBM, Armonk, NY, USA). The data are presented as the mean ± standard error of the mean (SEM) and were tested for normality using the Kolmogorov–Smirnov test, and the homogeneity of variance was determined using the Levene test. The comparison between IVM and *in vivo* matured oocytes was performed using the Student *t*-test. Values of *P* < .05 were considered significantly different.

### RNA and DNA isolation

As described by Giaccari et al. (2024), matured oocytes were individually lysed and flash-frozen in 5 μl of RLT Plus buffer (Qiagen) at −80°C until further use [26]. The zona pellucida was removed with Tyrode’s solution (Sigma-Aldrich), and the polar body was discarded as previously described by Giaccari et al. 2024 [26]. Both DNA and RNA from lysate were separated using the G&T-seq and Smartseq2 protocols [27]. Magnetic beads (MyOne C1, Life Technologies) were washed and then annealed to oligo dTs. These were employed to capture polyadenylated mRNA from cell lysates. Subsequently, the supernatant containing the DNA was transferred to a new tube. The beads were washed a total of three times in a solution comprising 1× FSS buffer (Superscript II, Invitrogen), 10 mM dithiothreitol (DTT), 0.005% Tween-20 (Sigma) and 0.4 U/μl of RNAsin (Promega). The purpose of this was to remove all residual DNA. Each washing solution was added to the DNA tube to enhance the recovery process.

### Single-cell RNA sequencing and raw data processing

The mRNA on the beads was processed further for cDNA conversion as previously described in Giaccari et al. (2024). Briefly, this involved the resuspension in 10 μl of reverse transcriptase master mix (100 U SuperScript II (Invitrogen), 10 U RNAsin (Promega), 1× Superscript II First-Strand Buffer, 5 mM DTT (Invitrogen), 1 M betaine (Sigma), 9 mM MgCl_2_ (Invitrogen), 1 μM Template-Switching Oligo (TSO, Eurogentec), and 1 mM dNTP mix (Roche). The mRNA mixture was reverse transcribed by incubation for 60 min at 42°C followed by 30 min at 50°C and 10 min at 60°C. The cDNA obtained was subjected to PCR amplification by adding 11 μl of 2× KAPA HiFi HotStart ReadyMix and 1 μl of ISPCR primer (2 μM). The amplification was carried out in a thermocycler at 98°C for 3 min, followed by 15 cycles of 98°C for 15 s, 67°C for 20 s, 72°C for 6 min, and the final extension step at 72°C for 5 min. The amplified product was purified using Ampure XP beads with a 1:1 ratio and eluted into 20 μl of water.

Single-cell RNA-seq libraries were prepared from 100 to 400 pg of cDNA using the Nextera XT Kit (Illumina), per the manufacturer’s instructions, but with one-fifth volumes. All 96 single-cell RNA-seq libraries were pooled together and sequenced on the Illumina NextSeq platform to an average depth of 4.2 million reads, using paired-end 75 bp read-length settings. Raw sequence reads were quality trimmed (Phred score <20) using Trim Galore version 0.4.4 (http://www.bioinformatics.babraham.ac.uk/projects/trim_galore/) to remove adapter contamination and reads with poor quality defined by low PHRED score. Reads with a minimum length of 20 bp after trimming were retained for downstream processing. The trimmed sequences were aligned to the reference genome *Sus scrofa* (pig) version 11.1 (https://www.ensembl.org/Sus_scrofa/Info/Index) using the STARsolo aligner with default parameters. After a filtering step, three samples that presented <25 000 reads and <5000 genes identified after alignment with the reference genome were discarded. Then, the Seurat package from the R program [28] was also used to eliminate those cells whose values for the number of unique genes detected (nFeature_RNA < 7500), the total number of molecules detected (nCount_RNA > 2000 000), and the percentage of the mitochondrial genome (% mt > 5%) were outside the normal values. In summary, all downstream analyses were performed with a total of 32 cells, 16 of them obtained from *in vitro* culture and 16 from *in vivo*.

### Single-cell BS sequencing and raw data processing

Single-cell BS-seq libraries were prepared based on a previous method described by Clark et al. (2017). The lysate containing the DNA was purified using a 0.9:1 ratio of Ampure XP Beads (Beckman Coulter) and eluted into 10 μl of water to use for scBS-seq library preparation [29]. DNA purified from single cells was treated using the EZ Methylation Direct Kit (Zymo) for bisulfite conversion following the manufacturer’s instructions. First-strand synthesis was conducted in five iterative cycles. Initially, the bisulfite-treated DNA was mixed with 40 μl of first-strand synthesis master (1 × Blue Buffer (Enzymatics), 0.4 mM dNTP mix (Roche), and 0.4 μM 6NF oligo (IDT) and heated to 65°C for 2 min and cooled on ice. These initial cycles of amplification of the forward strand represent a preamplification step. Subsequently, 50U of exo-Klenow fragment DNA polymerase (Enzymatics) was added, and the mixture was incubated at 37°C for 30 min. This was followed by a gradual increase in temperature ramping from 4°C. This process was repeated four further times, with each additional reaction mixture being added. Then, the final round was then incubated at 37°C for 90 min. The subsequent step involved exonuclease digestion. To that end, 20 U of exonuclease I (NEB) was added to the reaction mixture, which was then diluted with water to a total volume of 100 μl. The resulting solution was incubated at 37°C for 1 h. The resulting samples were purified using AMPure XP beads with a 0.8:1 ratio. The beads were mixed with 50 μl second-strand master mix (1 × Blue Buffer (Enzymatics), 0.4 mM dNTP mix (Roche), 0.4 μM 6NF oligo (IDT), and then the mixture was heated to 98°C for 1 min and cooled on ice. Subsequently, 50 U of Klenow exo-(Enzymatics) were added, and the mixture was incubated on a thermocycler at 37°C for 90 min after slowly ramping from 4°C. The resulting samples were purified using a 0.8:1 ratio of AMPure XP beads. Adapter ligation occurred prior to library amplification, using indexed adapters compatible with Illumina sequencing. Then, libraries were amplified using a 50 μl PCR mastermix (1 × KAPA HiFi Readymix, 0.2 μM PE1.0 primer, 0.2 μM iTAG index primer). The amplification process involved a 2 min step at 95°C, followed by 14 cycles of 80 s at 94°C, 30 s at 65°C, and 30 s at 72°C, with a final extension for 3 min at 72°C. Finally, the scBS-seq libraries underwent a purification step using an AMPure XP beads solution at a 0.7:1 ratio. Following purification, the resulting libraries were eluted in 15 µl of water, combined into a single pool, and subjected to high-throughput sequencing.

Pools of 48 libraries were generated and sequenced on two lanes on the Illumina NextSeq 500 platform. Single-cell libraries were sequenced to an average of 13 million paired-end reads with a 75 bp read length. After sequencing, random primers, adapters, and bases called with poor quality (Phred score < 20) were removed using Trim Galore version 0.4.4 (https://www.bioinformatics.babraham.ac.uk/projects/trim_galore) in single-end mode. The obtained data were processed using Bismark (version 0.22.3, Babraham Institute) for read alignment, deduplication, and methylation extraction [30]. Library sequence reads were aligned to the pig (genomic assembly *Sus scrofa* 11.1), followed by deduplication (deduplicate_bismark) and methylation calling (bismark_methylation_extractor). scBS-seq libraries having <500 000 CpGs covered, highly altered methylation patterns in TSS areas, or methylation percentages in the CpG context <30% and >70% were discarded (15% and 10% of the initially established *in vitro* and *in vivo* libraries, respectively). The sequencing coverage was between 0.5 and 3X in the DNA experiment with a mapping efficiency of around 30%–40%.

For unbiased analysis, scBS software (version 0.6.0, recently renamed as MethSCAn) was used for VMRs identification [31]. These VMRs are sequence windows of differing lengths which have a high methylation variability between individual cells. Moreover, a complete division of the genome in windows of 1000 bp was used in the unbiased analysis. For the targeted analysis, different genomic features such as CGIs (as annotated in the porcine genome), promoter regions (defined as ±2 kb from the TSS), genes (coding region), transposable elements (as annotated in the porcine genome), and imprinted genes were analysed. The methylation profile of CGIs was identified for genes officially described as related to the imprinting process in pigs, using the GeneImprint database (https://www.geneimprint.com/), as well as those ‘predicted’ by previous studies from the research group [32].

### Differential gene expression analysis

Differential expression was tested using the R package DESeq2 [33]. Thus, to consider the difference in gene expression between the study groups (cluster 0 *in vitro* vs. cluster 1 physiological pattern or *in vivo*) statistically significant, DEGs with a corrected FDR *P*-value < .05 and an FC (expressed as log_2_FC) ≥ 2 or ≤−2 were considered.

### Differential methylation analysis

Once the samples that would be used for the differential methylation analysis were selected, a second filtering process was carried out that allowed us to select the methylation profile of each of the areas of interest. The hypermethylated zone was considered to have a methylation percentage >75%, while those in which the methylation percentage was <25% were called hypomethylated zones. Additional filtering steps were applied to select only the regions whose methylation levels were uniformly affected in the majority of cells. To achieve this, a script was developed in the R software that applies the following filtering process: (1) Only those cells that have a coverage of at least five reads per cytosine were selected; (2) methylation regions were selected if, at least, half of the cells in each of the analysis groups have reads in that region; (3) methylation regions were selected if, at least, the average reads of cytosines for those specific regions are >10 in each analysis group. The comparison of the percentage of methylation was performed using the ‘t.test’ function with default parameters [34] of the Student *t*-test. DMRs were those with differences that had a *P*-value < .05 and an FC > 0.1 (10%).

### GO pathway analysis

The functional enrichment or GO analysis was conducted using the ClueGO tool [35], an application developed for Cytoscape (version 3.9.1) [36]. The parameters used for ClueGO analysis were two-tailed tests (enrichment/depletion) based on the hypergeometric distribution for enrichment analysis. The *P-*value < .05 was corrected using the Bonferroni step-down correction method. Only the GO databases (Molecular Function, MF; Biological Process, BP; and Cellular Component, CC) and pathways affected were used in this analysis. Terms that exceeded the *P*-value threshold (*P* < .05) were considered significantly enriched.

### Integrative analysis of scRNA-seq and scBSseq data sets

Possible correlations between the methylation and gene expression values in the corresponding promoters (±2000 bp from the TSS), VMR, and CGIs were investigated. For the integration analysis of both omics studies, the scAI (single-cell aggregation and integration) software [37] was employed. Cells that passed the filters, determined for both scBS-seq and RNA-seq analyses, were used. Thus, 27 cells were included, with 14 cells from the IVM and 13 cells from the *in vivo* group. We used a *P*-value < .0.5 and FC > 0.25 as the thresholds to select significant correlations.

Additionally, we analysed the regulatory relationships between methylation (promoters, VMR, GGIs) and gene expression values using the inferRegulations() function of the scAI software. A threshold of an absolute value of correlation ≥0.5, a difference of correlation >0.1 and a correlation threshold >0.2 was used. We also filtered the loci with a distance of <500 000 bp between its position and the start of the transcription site.

### Regulatory transcription factors analysis

TFs are essential proteins that act as primary regulators of gene expression [38] and can control the amount of protein produced by a gene in a cell. To study the TFs that could be involved in the gene expression profiles, we used the tool ChEA3, which is based on human and mouse data sets [39]. The results from the TF target libraries of ChEA3 are ranked based on the *P*-value from the exact Fisher’s test. This provides a ranking of TFs whose presumed transcriptional targets are more similar to the query set. Lower scores indicate higher relevance of the TF.

### Data visualization

In this study, data visualization was mainly conducted by R (version 4.2.2), including the R/Bioconductor. The heatmap was generated using the online tool *Heatmapper* (www.heatmapper.ca/) [40]. Principal component analysis and UMAP were generated using the Seurat package from the R program. The ggplot2 R package (version 3.4.2) was used to generate violin plots. Volcano plots were generated using VolcaNoseR [41]. The tool iRegulon was used to map gene networks based on the FC enrichment of motifs in the overall network of significantly expressed genes. The iRegulon results were generated using the Cytoscape plugin v0.97 on Cytoscape 2.8.1. The latest version of iRegulon (v1.2) includes support for the ‘10K’ motif collection as well as track discovery features. The iRegulon plugin's source code can also be accessed through the iRegulon website (http://iregulon.aertslab.org).

## Results

This study investigates differences in gene expression and DNA methylation profiles in the *in vitro* matured porcine oocytes from prepubertal gilts compared to the *in vivo* matured oocytes from adult sows, and their relationship with embryo developmental competence. In Experiment 1, IVM and *in vivo* matured oocytes (*n* = 397) were compared for developmental potential until the blastocyst stage. Experiment 2 analysed gene expression by single-cell ribonucleic acid sequencing (scRNA-seq) and DNA methylation at the single-cell level (*n* = 40) using RNA and bisulfite sequencing (scBS-seq).

### Developmental ability of *in vitro* and *in vivo* matured oocytes

Evaluation on day 2 revealed significant differences in cleavage rates between the IVM and *in vivo* groups (58.2 ± 3.0 vs. 45.7 ± 4.4, *P* = .010), respectively. Regarding embryo development, >31% of cleaved embryos reached the blastocyst stage in the IVM and 47% in the *in vivo* group (Table 1). However, there were no significant differences in the mean number of cells between the IVM and *in vivo* matured groups (Table 1) at day 7.

**Table 1.** Comparative results of cleavage rate (at day 2), blastocyst rate (at day 7), and number of cells per blastocyst in both the IVM oocytes from prepubertal gilts and *in vivo* matured porcine oocytes from adult sows

Group	*N*	Cleavage rate (%)	Blastocyst rate (%)	Cells/blastocyst
*In vitro*	268	58.2 ± 3.0^a^ *N* = 156	31.4 ± 3.7^a^ *N* = 49	48.1 ± 1.9^a^ *N* = 49
*In vivo*	129	45.7 ± 4.4^b^ *N* = 59	47.5 ± 6.6^b^ *N* = 28	48.2 ± 2.7^a^ *N* = 28

^a, b^Different letters in the same column indicate values statistically different (*P* < .05). Data expressed as number and (mean ± SEM). Cleavage rate: percentage of cleaved embryos from *N*. Blastocyst rate: percentage of blastocysts calculated from cleaved embryos. Cell/blastocyst: mean number of cells per blastocyst.

### Differential gene expression and gene ontology analyses

**Figure 1.**
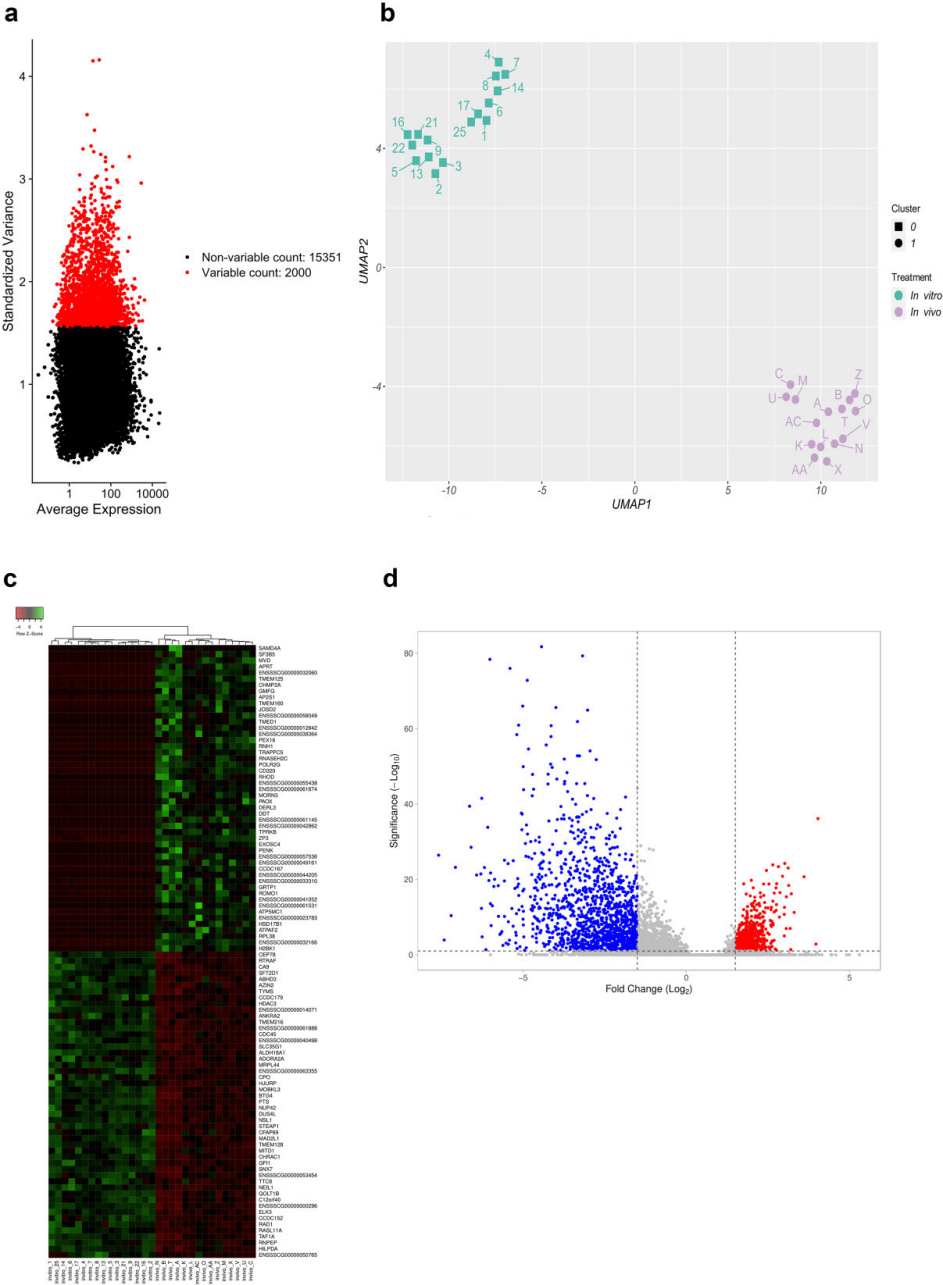
(a) Visualization of highly variable genes from the porcine oocyte pool obtained from single-cell RNA sequencing data using Seurat. Each point represents a single gene, with those in red representing the 2000 genes with the highest variability. (b) UMAP plot shows distribution of RNA-sequencing samples, where colours indicate the *in vitro* and *in vivo* treatments. (c) Heatmap showing the gene expression profile of porcine oocytes. The red colour indicates lower expression, while the green colour indicates a higher level of expression. (d) Volcano plot of DEGs in the samples obtained by *in vitro* and *in vivo* culture. Each point represents a single gene; in blue are shown those genes whose expression is decreased and, in red, those whose expression is increased when comparing *in vitro* vs. *in vivo* matured oocytes. Genes with an adjusted *P*-value < .05 and an FC > or <±1.5 were considered statistically significant.

Using the 2000 most variable genes between samples (Fig. 1a), individual oocytes from IVM (reduced developmental competence) and *in vivo* groups were clustered by Uniform Manifold Approximation and Projection (UMAP) analysis to assess the distribution of samples within treatments and between them. Figure 1b shows an evident separation in gene expression profiles between both the IVM and *in vivo* groups. Using stringent statistical filtering criteria (adjusted *P* < .05 and |Fold Change (FC)| > 2), RNA sequencing revealed a total of 1773 differentially expressed genes (DEGs) (Fig. 1c and d). It is important to note that these ‘DEGs’ represent differences in the abundance of polyadenylated transcripts between the two oocyte groups, rather than true differences in transcriptional activity. These variations may reflect differences in transcript stability, cytoplasmic polyadenylation, or degradation, and in some cases, may also involve differences in poly(A) tail length. Using the *in vivo* group as a reference level, 476 genes were upregulated and 1297 genes downregulated in the IVM group. The top five DEGs with the highest expression in the IVM group compared to the *in vivo* matured oocytes included genes such as Carboxypeptidase O (*CPO*), RAS Like Family 11 Member A (*RASL11A*), and 3 novel transcripts. The top five downregulated DEGs included H2B.K Variant Histone 1 (*H2BK1*), Proenkephalin (*PENK*), Transmembrane Protein 125 (*TMEM125*), Coiled-Coil Domain Containing 167 (*CCDC167*), and 1 novel transcript. The mapping information and the full lists of the DEGs can be found in Supplementary Tables S1–S3.

**Figure 2.**
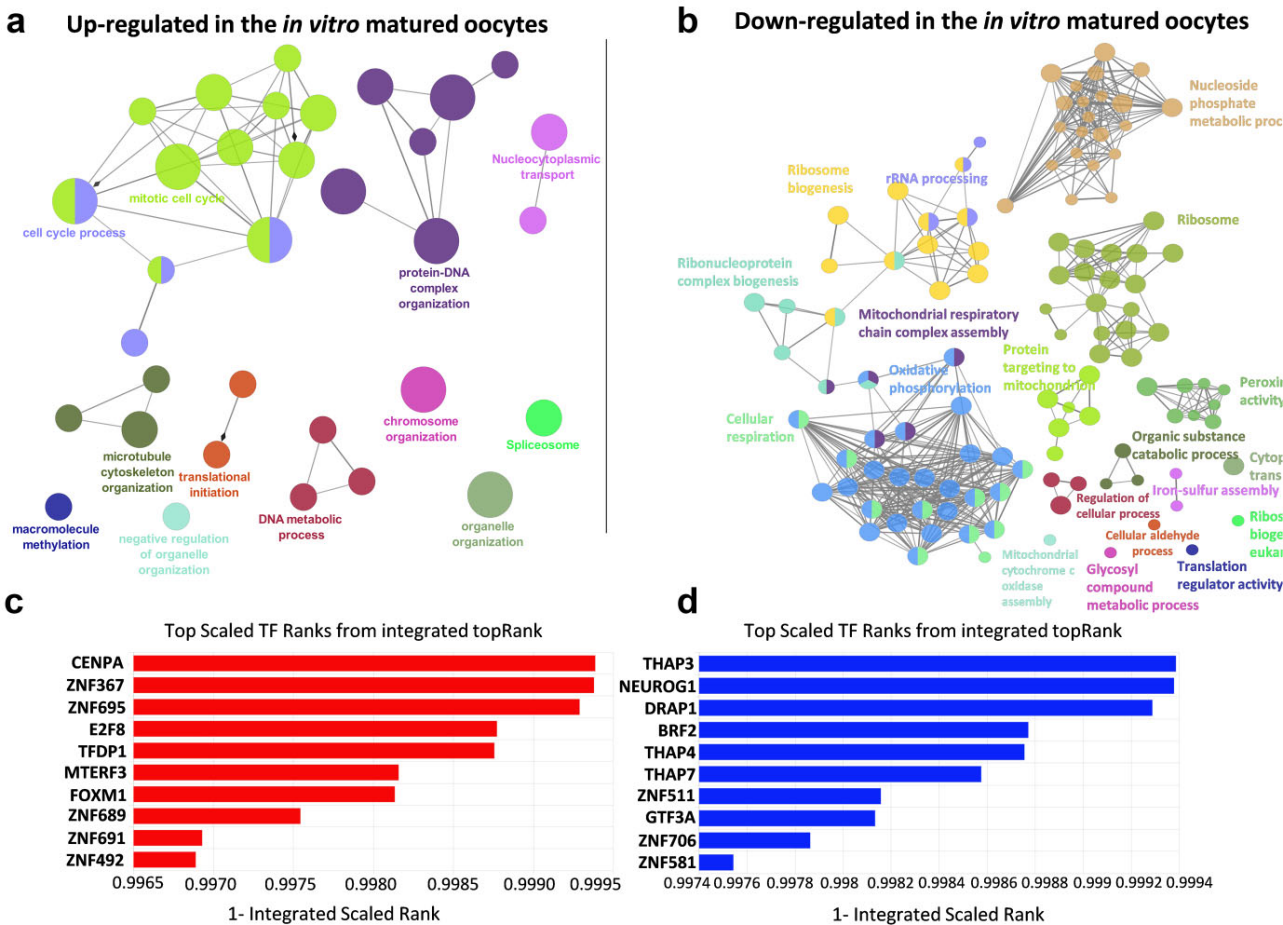
Functional enrichment analysis of (a) genes with higher expression when comparing *in vitro* vs. *in vivo* groups and (b) genes with lower expression when comparing *in vitro* vs. *in vivo* group was performed using ClueGO software within the Cytoscape environment. The analysis was performed with the ClueGO 2.1.3 plugin of the Cytoscape 3.9.1. The size of the nodules represents their significance; different pathways are represented with different colours. (c) The top 10 of upregulated and (d) downregulated TFs after comparing the gene expression profile of porcine oocytes cultured *in vitro* vs. *in vivo*.

Gene ontology (GO) analysis revealed enriched pathways in the IVM compared with the *in vivo* group (Fig. 2a and b); the majority of these were pathways involved in cell division and cell cycle processes (GO:0022402—cell cycle process; GO:1903047—mitotic cell cycle process; GO:0071824—protein–DNA complex organization), organelle and cytoskeleton regulation (GO:0006996—organelle organization; GO:0000226—microtubule cytoskeleton organization), and processes related to DNA methylation (GO:0043414—macromolecule methylation). On the other hand, identification of enriched pathways using GO and KEGG analyses revealed a repression in pathways involved in ribosomal RNA processing and synthesis and ribosome activity (GO:0006364—rRNA processing; GO:0022613—ribonucleoprotein complex biogenesis and KEGG:03010—ribosome), protein synthesis activity (GO:0002181—cytoplasmic translation; GO:0045182—translation regulator activity), the activity of cellular machinery, as indicated by processes related to oxidative phosphorylation and cellular respiration (KEGG:00190—oxidative phosphorylation and GO:0045333—cellular respiration), and activation of metabolism, through processes such as GO:1901657—glycosyl compound metabolic process and GO:0006081—cellular aldehyde metabolic process. All the enriched pathways involved in biological processes are listed in Supplementary Tables S4 and S5.

**Figure 3.**
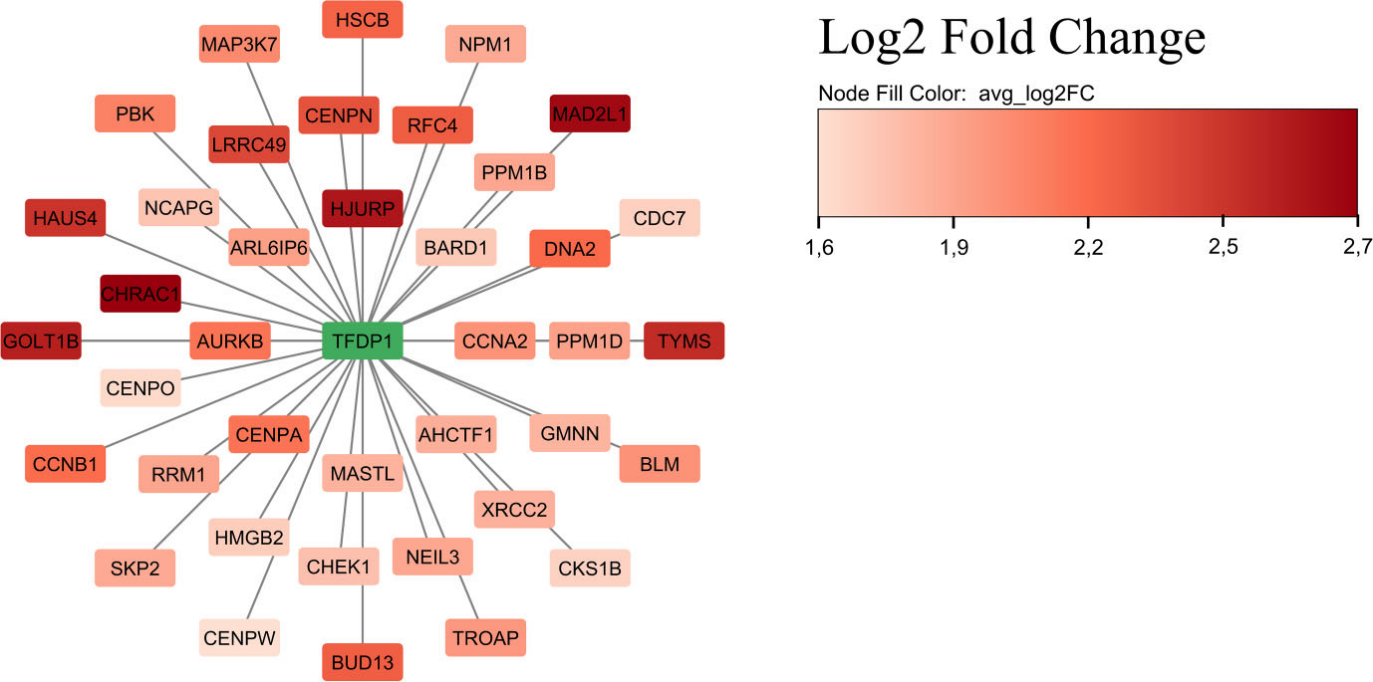
Direct targets of *TDFP1* up-expressed in the IVM porcine oocytes compared to the *in vivo* group. All genes are significantly upregulated by *TDFP1*, and are predicted as *TDFP1* targets by motif discovery in iRegulon.

In a complementary approach, we ran our list of genes through ChEA3 (ChIP-X Enrichment Analysis Version 3), which predicts regulatory transcription factors (TFs). Thus, among the TFs represented in those genes that showed higher expression when comparing the IVM vs. *in vivo* group (Fig. 2c), we identified genes such as the centromere-specific variant of histone H3 or centromere Protein A (*CENP-A*), Zinc Finger Protein 367 (*ZNF367*), Zinc Finger Protein 695 (*ZNF695*), E2F Transcription Factor 8 (*E2F8*) and Transcription Factor Dp-1 (*TFDP1*), among others. We identified 39 predicted targets of *TFDP1*, as shown in Fig. 3. These genes are predicted as *TFDP1* targets by iRegulon and showed a significant upregulation of these in the IVM compared to *in vivo* matured oocytes. Regarding the genes that decreased their expression after the comparison between IVM vs. *in vivo* matured oocytes (low vs. high developmental competence, respectively), the top five downregulated TFs included THAP Domain Containing 3 (*THAP3*), Neurogenin 1 (*NEUROG1*), DR1 Associated Protein 1 (*DRAP1*), BRF2 RNA Polymerase III Transcription Initiation Factor Subunit (*BRF2*) and THAP Domain Containing 4 (*THAP4*) (Fig. 2d). A full list of TFs can be found in Supplementary Tables S6 and S7.

### Differentially methylated regions and functional analyses

As studied in different species such as in mouse and cow oocytes, a correlation has been found between gene expression and DNA methylation [42, 43]. To identify potential correlations between gene expression and DNA methylation, scBSseq was performed on the same oocytes used for scRNA-seq libraries. The global percentage of methylation was similar between the IVM and *in vivo* groups, which is represented by no clear separation observed using Principal Components (PCs), t-distributed stochastic neighbour embedding (t-SNE), or UMAP analyses (Supplementary Fig. S1). The methylation levels were 46.59% in the *in vitro* group and 47.19% in the *in vivo* group within the CpG context.

**Figure 4.**
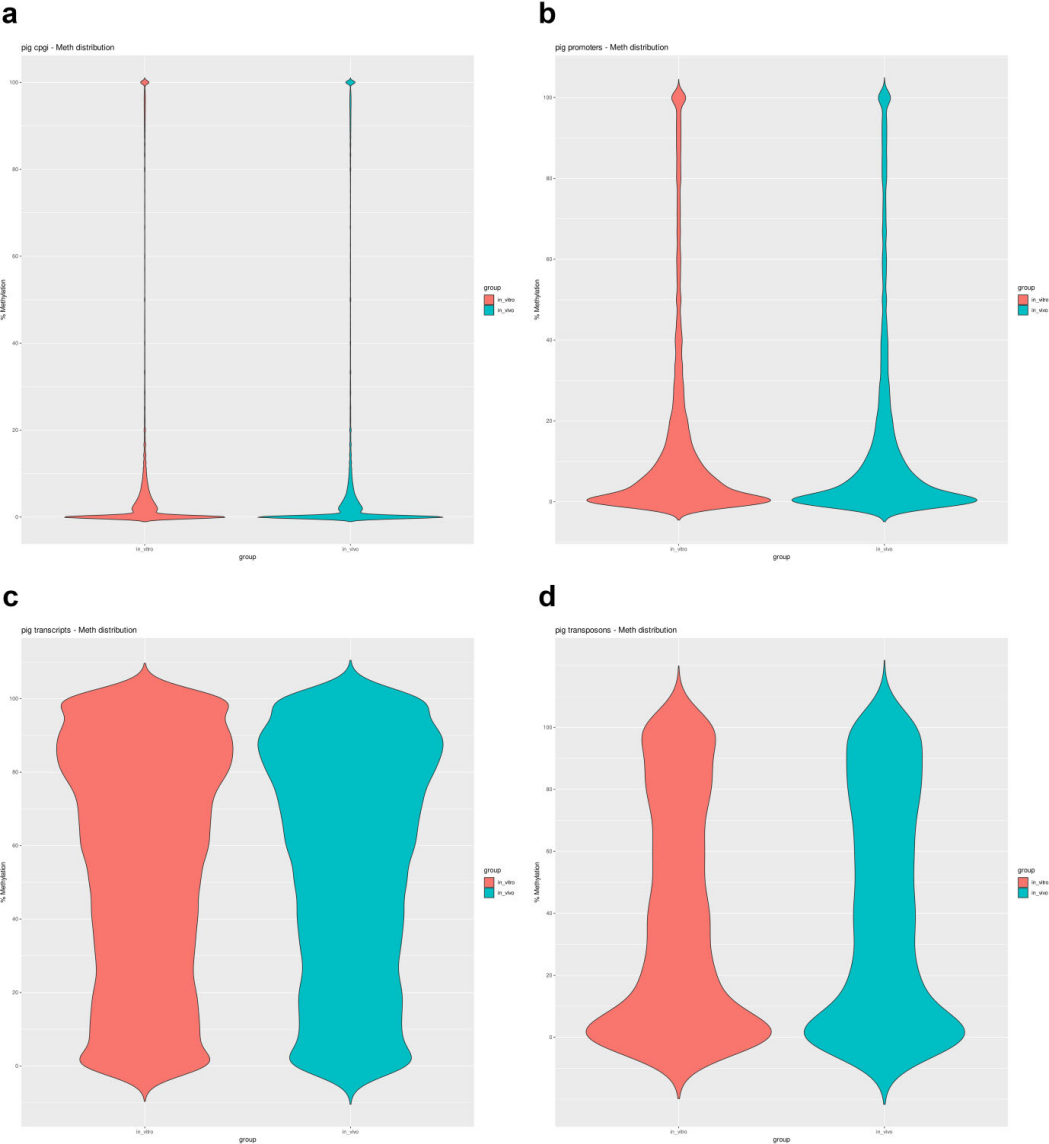
Violin plots of the distribution of average methylation values at both IVM and *in vivo* groups for the analysis performed on (a) CGIs, (b) promoters, (c) coding regions, and (d) transposons.

Differential methylation regions were annotated to various elements, including cytosine-phosphate-guanine islands (CGIs), promoters [located ±2 kb from the transcription start site (TSS)], coding regions (genes), and transposons (Fig. 4) between the IVM and *in vivo* matured oocytes. A total of 321 differentially methylated regions (DMRs) were detected (*P* < .05) in CGIs with distinct methylation patterns observed between the groups. Specifically, 42 CGIs were hypomethylated and 10 were hypermethylated in the IVM group, while in *in vivo* matured oocytes, 13 CGIs were hypomethylated and 2 were hypermethylated. When considering only the DMRs associated with CGIs, both groups exhibited a predominance of hypomethylation: 42 out of 52 CGI-related DMRs (81%) were hypomethylated in the IVM group (low developmental competence), and 13 out of 15 (87%) in the *in vivo* group. These findings indicate that hypomethylation is the dominant modification pattern in CGIs in both IVM and *in vivo* matured oocytes. Studying only the CGIs associated with promoters or coding regions where the gene was clearly described, only one DMR (from those 10 hypermethylated regions) was associated with a promoter region on the Solute Carrier Family 7 Member 14 (*SLC7A14*) gene. Interestingly, this gene was associated with hypomethylation in the *in vivo* group. On the other hand, we found a higher number of DMRs in hypomethylated CGIs (42) including a region corresponding to the 5′ UTR of the Purinergic Receptor P2Y2 (*P2RY2*) gene, an exonic region of the Protein Phosphatase 1 Regulatory Inhibitor Subunit 11 (*PPP1R11*) gene, and the promoter regions of the Claudin 14 (*CLDN14*), ssc-mir-9825, and Sperm Equatorial Segment Protein 1 (*SPESP1*) genes.

A total of 344 DMRs were detected (*P* < .05) in promoters showing distinct methylation patterns between the groups. Specifically, 64 promoters were hypomethylated and 15 were hypermethylated in the IVM group, whereas 31 promoters were hypomethylated and 10 were hypermethylated in the *in vivo* matured oocytes. GO analysis showed that hypomethylation in promoters only found in the *in vitro* group was associated with processes related to protein degradation regulation (GO:0005765; GO:0098852), while hypermethylation was associated with the transferase activity (GO:0016740) pathway in the IVM group (low developmental competence).

The analysis of coding regions (genes) refers to differences in methylation that affect areas encoding the entire gene; here, we demonstrated that the IVM group exhibits a greater number of both hypomethylated and hypermethylated DMRs in coding regions compared to *in vivo* matured oocytes. From the 843 DMRs detected (*P* < .05) in coding regions, 101 and 146 were hypomethylated and hypermethylated (respectively) in the IVM group, while 50 and 61 were hypomethylated and hypermethylated (respectively) in the *in vivo* group. GO analysis of hypomethylated coding regions in the IVM group revealed enriched pathways involved in antioxidant activity (i.e. inhibition of reactions triggered by dioxygen or peroxides). However, hypomethylation in the *in vivo* group was associated with the regulation of metabolic processes, especially of nitrogen compounds, and processes related to the regulation of transcription through RNA polymerase II. On the other hand, hypermethylation in the IVM group was related to processes that take place in the cytoplasm and nucleoplasm (cellular component function), while in the *in vivo* matured oocytes, these are related to processes that involve DNA modification.

The oocyte has functionally significant DNA methylation sites, including germline DMRs (gDMRs) that regulate imprinting [44]. We found that the gDMRs of previously described imprinted genes (https://www.geneimprint.com/) were appropriately methylated irrespective of IVM, although there were differences in methylation levels in ‘predicted’ imprinted genes [32]. For example, there was a hypomethylation of the U6 spliceosomal RNA (*U6*) gene in the IVM group, which did not occur in the *in vivo* group. Additionally, a general hypomethylation on the coding region was associated with Collagen Type XVIII Alpha 1 Chain (*COL18A1*), Ciliary Rootlet Coiled-Coil, Rootletin Family Member 2 (*CROCC2*), and Two Pore Segment Channel 2 (*TPCN2*) genes, and hypermethylation of the gene region of the Zinc Finger Protein 623 (*ZNF623*), Tribbles Pseudokinase 3 (*TRIB3*), and Cell Division Cycle Associated 7 (*CDCA7*) genes in the IVM compared to the *in vivo* group. Differential methylation events were also identified in CGIs. One of these islands, located in the gene region of the FERM, ARH/RhoGEF, and Pleckstrin Domain Protein 2 (*FARP2*) gene, was observed to be hypermethylated *in vitro*, while other islands located in the gene regions of the PPARG Coactivator 1 Beta (*PPARGC1B*) and Poly(RC) Binding Protein 3 (*PCBP3*) genes were hypomethylated also in the IVM group. Full lists of the DMRs and their pathways can be found in Supplementary Tables S8–S14.

### Integrative analysis: gene expression related to the methylation process

We performed a combined analysis of DNA methylation and transcriptomics, as DNA methylation can prevent the binding of TFs and enhancer-blocking elements, thus inhibiting gene expression [38]. In oocytes, DNA methylation patterns differ from those in somatic cells, as promoter methylation is generally low and associated with active transcription, whereas gene body methylation is more prevalent and often linked to transcriptional activity too [45]. This unique methylation landscape reflects the specialized regulatory mechanisms governing oocyte development and early embryonic competence.

**Figure 5.**
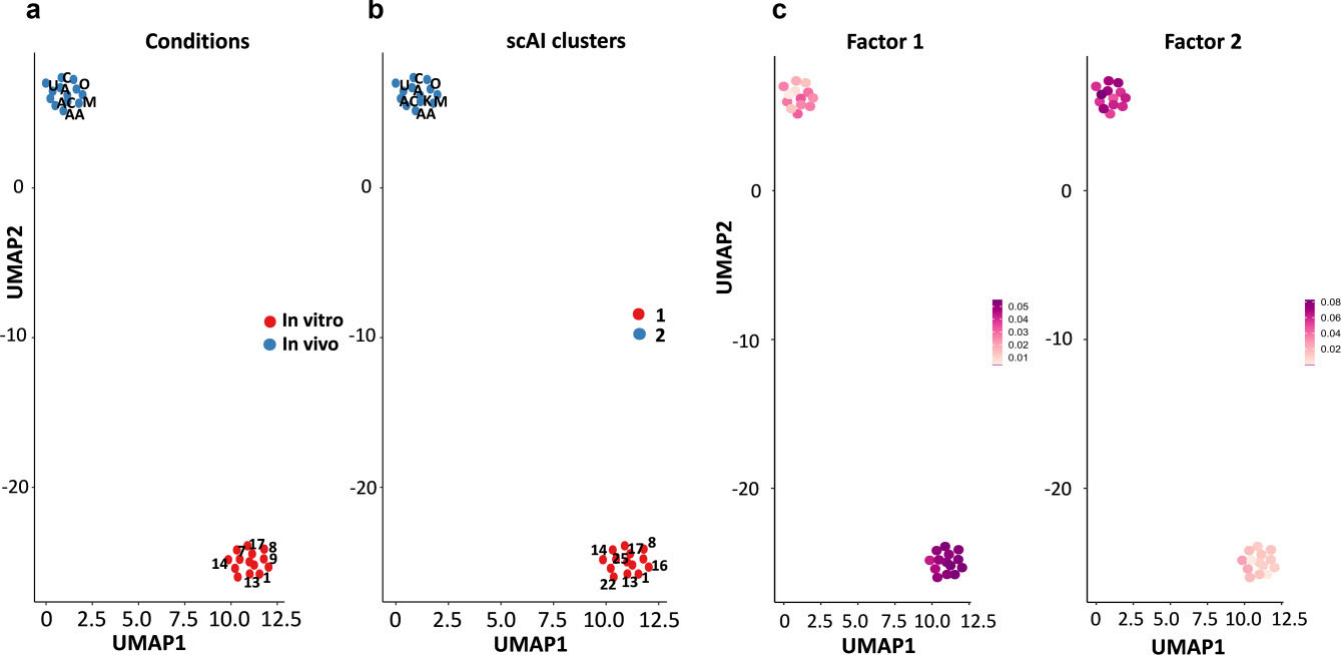
UMAP plots to visualize the intrinsic structure and relationships between samples in the dataset. The cells are coloured based on the factor they belong to (a, b) and the score of each factor in each cell (c). Each point in the plot represents a sample, and its position is determined by nonlinear dimensionality reduction.

First, we clustered the 27 oocytes used (14 oocytes from IVM and 13 from *in vivo*) by UMAP. As shown in Fig. 5a, the samples are grouped into two clearly differentiated clusters (IVM and *in vivo*, or low vs. high developmental competence, respectively). Through the algorithm designed by scAI that clusters of oocytes based on both gene expression and methylation levels, there was also a clear segregation of both groups (Fig. 5b). To complete the clustering process, we performed a new classification using the ‘factors’ generated by the algorithm to decompose the transcriptomic and methylation [variable methylated regions (VMRs), promoters, and CGIs] data. Thus, those genes and loci that are most relevant and that will allow the samples to be separated were identified. As represented in Fig. 5c, the UMAP algorithm clearly showed that the samples are grouped into two distinct clusters. It is noteworthy that the groups of samples defined by their origin (IVM or *in vivo*) coincide perfectly with the groups defined by the factors.

**Figure 6.**
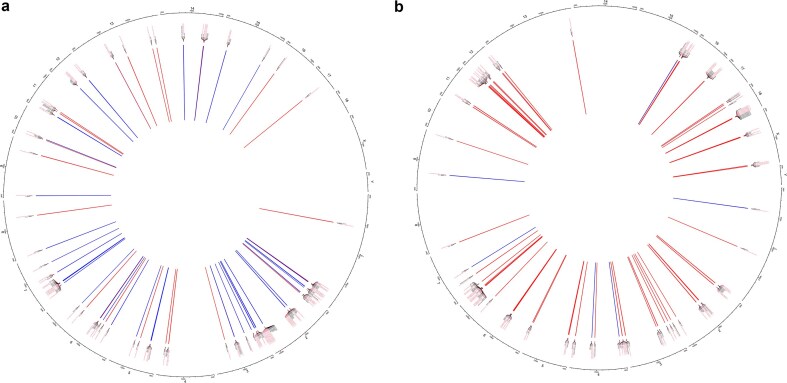
Top 100 of the regulatory relationships between gene expression (RNA-seq) and methylation (promoter regions, VMR and CGIs) in the IVM (a) and in the in *vivo* (b) matured oocytes. Methylation is represented by red labels and genes by black labels. Positive ratios are shown in red and negative ratios in blue. The thickness of the line indicates the strength of the correlation.

This integrative analysis of DNA methylation and transcriptomic data allows the identification of the main methylated regions and genes that define each group. From the 984 identified genes, 296 and 688 defined the IVM group (factor 1) and the *in vivo* group (factor 2), respectively (Supplementary Table S15). On the other hand, from the 1092 identified differential methylated loci, a total of 236 defined the IVM group, while 856 loci were related to the *in vivo* group (Supplementary Table S16). The top 20 of each is shown in Table 2A and B. Interestingly, promoter regions were not found to be represented in factor 1 or factor 2, with the CGIs and VMRs regions being the most important regions defining both factors. However, we identified CGI located in promoter regions of genes. In addition, we analysed the correlations between gene expression and DNA methylation, where a total of 1106 and 1408 were identified in the IVM and *in vivo* groups, respectively. Interestingly, in the IVM group, we found a higher number of negative correlations (803), while the *in vivo* group showed 1266 positive correlations (Fig. 6a and b). A full list of these correlations for each factor is supplied in Supplementary Tables S17 and S18.

**Table 2.** Top 20 of (A) identified genes (RNA markers) and (B) loci (DNA markers) for factor 1 (cluster *in vitro*) and factor 2 (cluster *in vivo*)

(A) Gene name	Factor	*P*-value	Fold change	
ENSSSCG00000063504	1	.030	2.047	
*ADGRL2*	1	.002	1.742	
*F2R*	1	.001	1.712	
*ALG6*	1	.000	1.691	
ENSSSCG00000047016	1	.012	1.647	
ENSSSCG00000062132	1	.000	1.630	
*CCDC179*	1	.026	1.564	
*PLGRKT*	1	.002	1.560	
*SLCO6A1*	1	.013	1.520	
*UBE2D1*	1	.006	1.516	
*RPS14*	2	.000	4.771	
ENSSSCG00000012088	2	.000	4.543	
*NPAS1*	2	.000	4.471	
*DYNC2I2*	2	.026	4.379	
*AP2S1*	2	.015	4.298	
*SYCN*	2	.000	4.288	
*TNNT1*	2	.000	4.175	
*SRM*	2	.002	4.135	
*RPS5*	2	.000	4.082	
ENSSSCG00000025928	2	.000	3.978	
**(B)**				
Loci	Genomic regions (gene + region)	Factor	*P* **-**value	Fold change
chr12.1990952.1991729_.C.	*SLC26A11*_Distal Intergenic	1	.046	1.719
chr11.5042384.5044586_.C.	*ssc-mir-9803*_Distal Intergenic	1	.026	1.657
chr1.207173457.207174369_.C.	*PSIP1*_Promoter (≤1 kb)	1	.026	1.623
chr11.9912809.9913316_.C.	*RFC3*_Distal Intergenic	1	.004	1.613
chr1.75169857.75170800_.C.	*PPIL6*_Distal Intergenic	1	.034	1.549
chr11.4879361.4880102_.C.	*ssc-mir-9803*_Distal Intergenic	1	.000	1.543
chr1.8243705.8244469_.C.	*TAGAP*_Distal Intergenic	1	.003	1.524
chr12.56329633.56330718_.C.	*ssc-mir-744*_Distal Intergenic	1	.001	1.467
chr1.130389264.130390128_.C.	*DLL4_*Distal Intergenic	1	.008	1.460
chr1.220952750.220953643_.C.	*DMRT1*_Promoter (≤1 kb)	1	.004	1.446
chr2.149531920.149535260_.V.	ENSSSCG00000029257_Distal Intergenic	2	.037	1.371
chr1.1727106.1729876_.V.	*ssc-mir-9824*_Distal Intergenic	2	.026	1.207
chr10.66844399.66847999_.V.	*KLF6*_Distal Intergenic	2	.030	1.094
chr9.136753582.136756492_.V.	*GRB10*_Distal Intergenic	2	.046	1.075
chr16.55458699.55460509_.V.	*RARS1*_Distal Intergenic	2	.042	1.060
chrX.121752258.121755268_.V.	*FATE1*_Distal Intergenic	2	.042	0.970
chr1.970705.972819_.C.	ssc-mir-9815_Distal Intergenic	2	.021	0.961
chr10.52445273.52447376_.C.	*SPAG6*_Distal Intergenic	2	.003	0.958
chr12.3166999.3170069_.V.	ENSSSCG00000017164_Distal Intergenic	2	.030	0.953
chr4.32680749.32684029_.V.	*LRP12*_Distal Intergenic	2	.016	0.941

**Figure 7.**
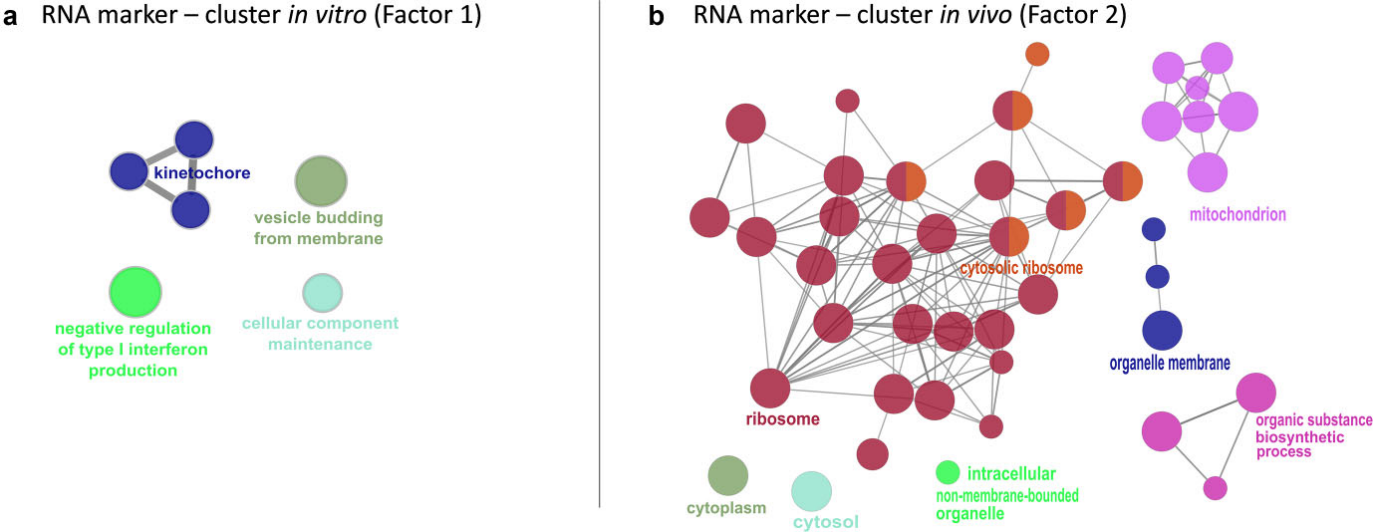
Functional enrichment analysis of (a) genes identified in the *in vitro* cluster (factor 1) and (b) genes identified in the *in vivo* cluster (factor 2) was performed using ClueGO software within the Cytoscape environment. The analysis was performed with the ClueGO 2.1.3 plugin of the Cytoscape 3.9.1. The size of the nodules represents their significance; different pathways are represented with different colours.

The analysis of the selected genes defining factor 1 revealed an enrichment in processes related to preparatory events for cell division, such as kinetochore formation and cellular component maintenance, as shown in Fig. 7a. Additionally, the analysis showed negative regulation of type I interferon production. Factor 2 enrichment analysis revealed functions and pathways related to the cytosolic ribosome, mitochondrion, and organic substance biosynthetic process (Fig. 7b).

For those loci identified in factors 1 and 2, only those loci referring to methylation processes affecting regions located in the gene region, i.e. promoters, 3′ and 5′ UTR regions, exons, and introns, were selected. The induced enriched pathways in the IVM cluster (factor 1) were related to meiotic division and regulation of the macromolecule biosynthetic process, among others. On the other hand, enriched pathways related to the *in vivo* cluster (factor 2) were involved in signalling receptor binding, cell adhesion, protein binding, regulation of miRNA transcription, and G protein receptor activity. All the enriched pathways involved are listed in Supplementary Tables S19 and S20.

## Discussion

Here, we provide a parallel and integrative, single-cell analysis of the gene expression and DNA methylation profiles in the *in vitro* matured porcine oocytes from prepubertal gilts compared to the *in vivo* matured oocytes from adult sows. The aim was to identify novel molecular markers for porcine oocyte quality, employing the model of oocytes from young animals being of reduced quality compared to those from adult sows. Previous studies have shown that the source and the process of IVM in pigs are determinant factors for the developmental rate up to the blastocyst stage [46]. Here, our results showed >45% progression of the cleaved embryos to blastocysts in the *in vivo* group, which indicates an improvement over the previous results [47]. This could be due to the novel IVP system used, with commercial media (from Embryocloud company) of already tested and standardized quality, which would give data not comparable to those obtained with homemade media. As already referred to by other authors [4, 5], the ability to develop until the blastocyst stage was higher in the *in vivo* matured oocytes than in the *in vitro* ones. The fact that cleavage rates were higher at day 2 in the IVM oocytes, but the blastocyst rate was lower, indicates that some of those cleaved embryos could not progress in the culture beyond this stage, but the molecular mechanisms involved in this failure have not been elucidated until now, and that was the aim of our second experiment.

The oocyte growth phase is followed by transcriptional silencing and the resumption of meiotic activity. During this stage, changes in gene expression primarily depend on the translation and degradation of transcripts, as major epigenetic marks are already established [48]. In the current study, results indicate overall similar DNA methylation patterns between IVM and *in vivo* matured porcine oocytes (low vs. high developmental competence). However, we identified extensive differences in the transcriptomic profile between both groups, suggesting that postgrowth transcriptional regulation, rather than methylation changes at the global level, plays a key role in mediating these differences. Although it is important to emphasize that these differences do not necessarily reflect active transcription, but rather differences in the abundance of polyadenylated transcripts, as our RNA isolation method captures only transcripts with poly(A) tails.

Notably, studies in mice have also reported gene-level differences linked to *in vitro* follicle culture and sexual maturity [17]. Similarly, previous studies have demonstrated that IVM impacts gene expression profiles in porcine, bovine, and human oocytes when compared to those matured *in vivo* [49, 23]. These findings suggest that differences between the physiological maturation environment within the follicle and the environment in the *in vitro* culture system can alter the gene expression of porcine oocytes through mechanisms that reflect developmental competence, transcript utilization, or cellular stress responses. In our model, the differences between IVM oocytes from prepubertal gilts and *in vivo* matured oocytes from adult sows could reflect the combined effects of the young gilts vs. adult status and the *in vitro* vs. *in vivo* maturation systems. Although global DNA methylation patterns appeared similar between groups, the few DMRs we identified may reflect pre-existing epigenetic differences established during the oocyte growth phase. It is possible that the methylation patterns were already distinct during the oocyte growth phase due to age-related differences, as prepubertal gilts are known to have reduced oocyte quality. These inherent differences may influence how oocytes respond to the IVM environment, leading to extensive gene expression changes while maintaining relatively stable DNA methylation patterns.

Interestingly, there were more negative correlations between DEGs and DMRs in the IVM oocytes and more positive correlations in the *in vivo* matured oocytes. However, it is known that oocytes accumulate mRNA over a long developmental period [50–52], and thus, mRNA levels in oocytes do not always correlate with gene methylation. Therefore, the integration of transcriptomic and methylome data in the oocyte must also be interpreted with caution. While such integrative approaches can be valuable for identifying potential biomarkers that distinguish oocytes of different origins or developmental competence, they do not necessarily reveal direct regulatory relationships between DNA methylation and gene expression.

The methylation patterns observed in oocytes have been demonstrated to reflect the potential for developmental processes or to regulate gene expression in subsequent stages, including fertilization and the early stages of embryonic development. For example, the hypermethylation of promoters associated with transferase activity (GO:0016740) in the IVM group (low developmental competence) suggests that genes involved in enzymatic modifications, such as phosphorylation or glycosylation, may be downregulated. These processes are crucial for cell signalling, metabolism, and structural modifications, meaning their reduced activity in IVM oocytes could impact cellular function and developmental outcomes. Consequently, the methylation marks observed in oocytes may also be better conceptualized as developmental ‘set points’ rather than as direct indicators of current expression levels. In addition, we hypothesized that those differences could be attributed to the complexity of *in vivo* environments, where additional regulatory factors such as tissue-specific signals, and hormonal influences likely contribute to a more precise coupling between DNA methylation and gene expression. In contrast, *in vitro* conditions may lack some of these key regulatory elements, leading to a more decoupled relationship between DNA methylation and gene expression.

Using the *in vivo* group (high developmental competence) as a reference, 1297 and 476 DEGs were downregulated and upregulated (respectively) in the IVM group. The genes that increased in expression in the IVM oocytes were primarily associated with mechanisms that enable the oocyte to complete maturation and fertilization, particularly genes involved in chromosome organization, meiotic nuclear division, centromere complex assembly, and cytoskeleton organization. Therefore, while our results suggest that IVM oocytes activate maturation-associated pathways, they may lack other epigenetic marks (i.e. histone modifications), synchrony between nuclear and cytoplasmic maturation or metabolic and molecular refinements that *in vivo* conditions provide, leading to reduced blastocyst formation. As discussed by Sirard (2011), even though the correct genes are expressed, post-transcriptional and translational regulation in IVM oocytes might differ from *in vivo* conditions [53]. Thus, the data presented here also helps to highlight the importance of the transcriptional phase that occurs when the oocyte reaches its fully grown stage, prior to the shutdown of transcription during oocyte maturation. This phase is essential for the accumulation of transcripts required for the resumption of meiosis and the completion of maturation. For example, the transcription factor Dp-1 (*TFDP1*) was upregulated in the IVM group and involved in cell cycle division, which is in agreement with previous studies where *TFDP1* was highly expressed in human matured oocytes and also involved in cell cycle regulation [54]. In addition, the upregulation of *TFDP1* has also been related to promoting the proliferation of goat granulosa cells, which is crucial for oocyte development and folliculogenesis [55]. Other genes involved in the meiotic resumption pathway were also upregulated in the IVM group. For instance, the gene *F2R* (Coagulation Factor II Thrombin Receptor) has also been higher expressed in matured human [56] and mouse oocytes [57] compared to germinal vesicles. The gene *APPL1* (Adaptor Protein, Phosphotyrosine Interacting with PH Domain and Leucine Zipper 1), which was also upregulated in the IVM group, was previously described by Nader et al. (2020), who investigated how membrane progesterone receptors (mPRs) facilitate frog oocyte maturation [58]. This process relies on the interaction of mPRs with APPL1, which is crucial for the proper localization and function of these receptors within signalling endosomes, promoting the progression of meiosis and oocyte development.

In addition, the cell division cycle 20 (*CDC20*) gene that was upregulated in the oocytes matured *in vitro* (low developmental competence) plays an essential role in the activation of anaphase-promoting complex/cyclosome (APC/C), which is a key step in homolog disjunction and in the transition from meiosis I to meiosis II [37, 59]. CDC20 is also involved in the degradation of cyclins, including cyclin B1, which contributes to the inactivation of the mitosis-promoting factor complex and allows progression to anaphase [60]. Previous studies in swine [61] and mice [62] have reported that CDC20 levels increase during oocyte maturation and are highest by the second meiotic metaphase. In agreement with our results, Iyyappan et al. (2023) also identified increased expression of an essential cell division factor *CENP-A* that in combination with CDC20 is essential for centromere function [63].

Interestingly, *CENP-A* is considered the primary epigenetic mark responsible for determining centromere identity during cell division [64]. It replaces the canonical histone H3 at centromeres and serves as the foundation necessary for kinetochore assembly [64, 65]. Thus, proper regulation of *CENP-A* is crucial, as defects in its expression or incorporation can lead to chromosome mis-segregation and impaired cellular growth [63]. Recent studies have demonstrated that CENP-A protein is stable and gradually deposited in starfish and mice oocytes [66–68]. However, it remains to be studied whether the mechanisms that promote the retention of CENP-A chromatin over short periods in cycling cells also contribute to extreme stability during oogenesis.

Studying more in detail other genes related with *CENP-A*, such as *CENP-N* and *CENP-V*, there was also an upregulation of both genes in the IVM group compared to the *in vivo* matured oocytes. Importantly, the absence of *CENP-V* in mouse oocytes of young adults failed to properly align chromosomes, and in the exclusion of the polar body [69]. In addition, the same authors reported an age-related reduction in CENP-V that contributed to weakened centromere cohesion and spindle assembly checkpoint function, potentially leading to either subfertility or the development of aneuploid embryos. Therefore, the upregulation of GO pathways associated with cell division and chromosome organization in the IVM group may suggest an activation of these mechanisms in an *in vitro* environment, although this is not reflected in their ability to develop to the blastocyst stage. However, it should also be noted that this upregulation may instead reflect a delay in the translation or degradation of the corresponding transcripts in the IVM group, rather than true activation of these pathways.

The primary source of the energy necessary for chromosome segregation and cytoplasmic maturation is the mitochondria, which is highly active in matured oocytes [70]. However, the imbalance between the production and elimination of the by-products of this mitochondrial activation, which are called reactive oxygen species (ROS), can negatively affect the oocyte quality [71]. As shown by Braga et al. (2019), oocytes from prepubertal gilts exhibit altered gene expression patterns related to impaired lipid metabolism, and deficient epigenetic reprogramming, all of which may contribute to reduced competence and heightened vulnerability to oxidative stress during IVM [72]. In the present study, the downregulated DEGs were mainly involved in the cytosolic ribosome and mitochondrion organization, oxidative phosphorylation, and metabolic processes (i.e. ATP, glycosyl, amide, and aldehyde metabolism). However, previous studies have revealed that the production of ROS *via* mitochondrial metabolism was higher in IVM oocytes than *in vivo* matured oocytes [73–75], arguing the negative influence of physical factors, such as exposure to cool white or warm white fluorescent lights [76]. Thus, our findings could also be interpreted as a compensatory response to oxidative stress during IVM, where mitochondrial dynamics shift to meet higher energy demands, potentially generating more ROS and affecting further embryo development. Therefore, future studies combining transcriptomic data with proteomic and functional assays are necessary to clarify whether the observed gene expression patterns correlate with mitochondrial activity or ROS levels.

In conclusion, our results demonstrate that while global DNA methylation remains stable in porcine oocytes matured *in vitro* compared to those matured *in vivo*, localized methylation differences or age-dependent epigenetic programming established during oocyte growth may contribute to the observed transcriptional changes. It is important to note that comparing oocytes from prepubertal gilts and adult sows represents a limitation in attributing observed effects exclusively to IVM conditions. Notably, we identified significant differences in the expression of genes involved in biological processes such as chromosome organization, cellular division, meiotic resumption, and mitochondrial organization. These pathways are critical for oocyte competence, and understanding them will aid in optimizing IVM systems to improve the quality of *in vitro* matured porcine oocytes. For instance, the supplementation of IVM media with CPEB-binding enhancers, mRNA stabilizers, or specific growth factors may support proper transcript storage and utilization during oocyte maturation. In addition, key genes (i.e. *F2R, TFDP1, CDC20, CENPA*) in the IVM group (low developmental competence) could be used as potential molecular targets for customising IVM conditions based on donor type (i.e. gilts vs. sows) and thus accelerating oocyte maturation *in vitro*. These findings may also have applications in human reproductive research, particularly in the development of culture systems capable of supporting the growth of immature oocytes from primordial stages to the metaphase-II stage.

## Supplementary Material

dvaf018_Supplemental_Files

## Data Availability

Single-cell BS-Seq and single-cell RNA-Seq data have been deposited under accession codes GSE274012 and GSE274092 (respectively) in the Gene Expression Omnibus database.
