# Supplementary material for: Deciphering differences in DNA methylation and transcriptome profiles of oocytes from pigs with high and low developmental competence
Source: Environ Epigenet. 2025 Jun 3;11(1):dvaf018. doi: 10.1093/eep/dvaf018 (PMC12418950; doi:10.1093/eep/dvaf018)
Supplement: dvaf018_Supplemental_Files [file dvaf018_supplemental_files.zip › Additional 1_transcrip.pdf]

Sample	Number of input reads	Uniquely mapped reads number	Uniquely mapped reads %
1	4170977	3956325	94.85
2	2834288	2598942	91.7
3	1473633	1345770	91.32
4	2624469	2401746	91.51
5	1714651	1594152	92.97
6	962416	876713	91.1
7	2455507	2226534	90.68
8	3621428	3282472	90.64
9	1808801	1662090	91.89
13	379947	345034	90.81
14	732040	661990	90.43
16	1361374	1249824	91.81
17	2206962	2059300	93.31
19	177534	145986	82.23
20	31398	23888	76.08
21	978096	891720	91.17
22	1550686	1400560	90.32
24	31	28	90.32
25	1778106	1638105	92.13
26	119330	109978	92.16
A	980739	870897	88.8
AA	1028482	946517	92.03
AB	178332	165970	93.07
AC	1027061	921536	89.73
B	382309	347373	90.86
C	1735515	1625157	93.64
J	8834	8217	93.02
K	451007	421660	93.49
L	704589	657227	93.28
M	3049665	2820073	92.47
N	1132946	1050494	92.72
O	831205	769094	92.53
Q	1502778	1335570	88.87
R	84957	75526	88.9
S	83929	78181	93.15
T	1660865	1512792	91.08
U	960394	892432	92.92
V	1246261	1169696	93.86
X	2752868	2587876	94.01
Z	1101808	1031578	93.63

Gene name	P-value	avg_log2FC	pct.1	pct.2	p_value_adj
GSTM3	1.04E-86	-4.433006734	1	1	1.80E-82
TFDP2	2.75E-84	-3.177096336	1	1	4.78E-80
ZP3	2.38E-83	-6.015396062	1	1	4.13E-79
ATP5MC1	5.83E-81	-5.399416519	0.938	1	1.01E-76
SPC24	8.57E-78	-4.870920051	0.938	1	1.49E-73
ENSSSCG00000044567	5.89E-71	-5.010647244	0.812	1	1.02E-66
STX8	1.41E-70	-3.993366908	1	1	2.44E-66
VTI1B	6.98E-70	-3.022510367	1	1	1.21E-65
PSMA6	7.79E-67	-3.33267553	1	1	1.35E-62
ATP5IF1	6.91E-66	-5.134680245	0.875	1	1.20E-61
NUDT5	9.53E-66	-4.13922368	1	1	1.65E-61
POLR2G	2.20E-63	-5.191775723	0.938	1	3.82E-59
ENSSSCG00000013386	7.35E-63	-4.143414486	0.938	1	1.28E-58
ZAR1	1.21E-60	-4.289123635	0.938	1	2.10E-56
STAT5A	1.42E-59	-4.833612988	0.812	1	2.46E-55
PSMD13	4.61E-59	-2.947201482	0.938	1	8.00E-55
SKIC8	9.03E-58	-3.334381207	0.938	1	1.57E-53
CNIH4	9.73E-58	-3.272066477	1	1	1.69E-53
RPL7L1	6.73E-57	-3.737951715	1	1	1.17E-52
ENSSSCG00000054115	9.59E-57	-2.75729124	1	1	1.66E-52
ENSSSCG00000002036	1.51E-55	-4.154829138	1	1	2.62E-51
LSM2	7.07E-55	-4.993866456	1	1	1.23E-50
C6orf52	1.10E-54	-4.108141464	1	1	1.91E-50
PIGP	2.71E-53	-3.652805147	1	1	4.70E-49
BCAP31	6.92E-53	-4.23299078	0.875	1	1.20E-48
ENSSSCG00000048076	7.84E-53	-4.707819249	0.875	1	1.36E-48
RPL6	1.38E-51	-3.963033674	1	1	2.39E-47
EMC6	5.65E-51	-3.724084303	1	1	9.81E-47
HSBP1	2.19E-50	-3.109274224	1	1	3.80E-46
MRPL37	2.25E-50	-3.359639762	1	1	3.90E-46
TLE2	2.66E-50	-3.733296946	1	1	4.62E-46
PARK7	2.84E-50	-4.237632299	1	1	4.92E-46
CRNKL1	4.54E-49	-1.49424903	1	1	7.87E-45
TUBA1B	6.94E-49	-4.676940823	1	1	1.20E-44
RPL23A	9.12E-49	-4.981852653	0.875	1	1.58E-44
ENSSSCG00000024070	2.86E-48	-3.633975668	0.938	1	4.95E-44
COPZ1	3.32E-47	-3.028014376	1	1	5.76E-43
NDUFB9	3.76E-47	-4.719499041	1	1	6.52E-43
ENSSSCG00000015217	8.26E-47	-1.861187839	1	1	1.43E-42
ENSSSCG00000032166	1.75E-46	-6.263568638	0.812	1	3.04E-42
NSDHL	2.01E-46	-2.836175198	1	1	3.48E-42
GSDMB	2.93E-46	-3.485642171	1	1	5.09E-42
ZP4	6.97E-45	-3.388611949	1	1	1.21E-40
CCDC167	2.22E-44	-6.637898252	0.375	1	3.85E-40
CDC26	2.70E-44	-3.839590794	1	1	4.68E-40
ARMC2	1.70E-43	-2.013514806	1	1	2.96E-39
ENSSSCG00000023691	1.75E-43	-2.916550707	0.938	1	3.04E-39
STYXL1	6.49E-43	-3.058661912	0.938	1	1.13E-38
NDUFA11	1.46E-42	-5.048339534	0.875	1	2.53E-38
PRDX1	1.81E-42	-2.421547541	1	1	3.15E-38
SERF1A	2.81E-42	-5.027783157	0.75	1	4.87E-38
C11orf98	3.41E-42	-3.182719114	1	1	5.92E-38
ENSSSCG00000005528	4.73E-42	-3.751728947	1	1	8.21E-38
ENSSSCG00000024588	1.37E-41	-3.330148602	1	1	2.38E-37
WDR5	1.74E-41	-3.285333073	0.875	1	3.03E-37
TIMM22	1.85E-41	-3.358127286	0.938	1	3.20E-37
CHCHD4	3.26E-41	-2.13162464	1	1	5.66E-37
RPL7	3.38E-41	-3.57740515	1	1	5.87E-37
ENSSSCG00000061888	4.35E-41	4.03180478	1	1	7.54E-37
MRPL40	4.88E-41	-3.820266461	1	1	8.46E-37
NR2C2AP	5.52E-41	-3.742923049	0.938	1	9.58E-37
NDUFB8	7.29E-41	-3.946675592	1	1	1.26E-36
ENSSSCG00000028414	1.19E-40	-3.493621187	1	1	2.06E-36
COX5B	1.81E-40	-4.20701957	1	1	3.15E-36
ENSSSCG00000035909	2.97E-40	-3.31177874	0.938	1	5.15E-36
ENSSSCG00000039544	1.91E-39	-2.718025096	1	1	3.31E-35
COX7A2	2.14E-39	-4.894811864	1	1	3.72E-35
FTL	4.04E-39	-3.506178543	1	1	7.02E-35
REC114	6.77E-39	-2.635806178	1	1	1.17E-34

ENSSSCG00000032060	8.76E-39	-6.079688889	0.688	1	1.52E-34
IDI1	9.56E-39	-2.926917122	1	1	1.66E-34
MAP1LC3B	1.76E-38	-2.022026306	1	1	3.05E-34
GINS2	1.93E-38	-2.758285966	1	1	3.36E-34
NDUFA7	3.74E-38	-5.034557222	0.875	1	6.49E-34
PSMB7	4.23E-38	-3.308349922	1	1	7.35E-34
CDIPT	4.47E-38	-3.147741853	1	1	7.75E-34
RHOC	7.04E-38	-3.987706724	0.875	1	1.22E-33
CNPY2	1.18E-37	-3.541854344	1	1	2.05E-33
MRPS25	1.38E-37	-3.21897236	1	1	2.40E-33
GDF9	1.63E-37	-2.261016009	1	1	2.83E-33
DPPA5	2.51E-37	-3.463134425	1	1	4.35E-33
ENSSSCG00000039523	3.75E-37	-3.090659313	1	1	6.51E-33
ENSSSCG00000028167	5.41E-37	-4.862881144	0.812	1	9.38E-33
HAUS1	1.20E-36	-2.349847571	1	1	2.08E-32
ENSSSCG00000007493	1.61E-36	-2.177421518	1	1	2.79E-32
NEDD8	1.95E-36	-3.312857104	1	1	3.38E-32
TFB1M	3.24E-36	-2.182425236	1	1	5.62E-32
MYL6	3.60E-36	-4.504923003	0.812	1	6.25E-32
MRPL51	3.98E-36	-2.904937803	1	1	6.91E-32
ENSSSCG00000009610	4.17E-36	-3.116655702	1	1	7.23E-32
CBR3	4.82E-36	-2.833538854	1	1	8.35E-32
GEMIN6	7.36E-36	-2.451362189	1	1	1.28E-31
ENSSSCG00000032129	8.37E-36	-3.418551983	1	1	1.45E-31
PFDN1	9.79E-36	-2.36656808	1	1	1.70E-31
POP4	1.93E-35	-3.189970944	0.938	1	3.35E-31
ENSSSCG00000009303	2.84E-35	-4.620378184	0.938	1	4.92E-31
TNNT1	5.42E-35	-4.763357093	0.625	1	9.41E-31
LSM10	6.93E-35	-3.055742552	1	1	1.20E-30
TK1	7.53E-35	-4.49966875	0.688	1	1.31E-30
NDUFA9	9.69E-35	-3.241753486	1	1	1.68E-30
POLR3H	1.13E-34	-3.287064475	1	1	1.96E-30
PPIB	1.49E-34	-3.708211765	0.938	1	2.58E-30
POLR2F	1.73E-34	-4.927685795	0.625	1	3.00E-30
ENSSSCG00000057874	1.77E-34	-3.306103128	1	1	3.08E-30
PDCD5	2.53E-34	-2.444916142	1	1	4.40E-30
SYCN	2.98E-34	-4.093109404	0.812	1	5.16E-30
ATP6V1D	7.02E-34	-1.394783349	1	1	1.22E-29
ARHGAP22	1.41E-33	-2.109076153	1	1	2.45E-29
CD320	1.76E-33	-6.590779827	0.375	1	3.05E-29
ENSSSCG00000032916	1.84E-33	-3.085020584	1	1	3.19E-29
COX5A	1.98E-33	-2.844683085	1	1	3.43E-29
BUD31	2.27E-33	-2.264183048	1	1	3.95E-29
MTRES1	2.35E-33	-2.19291522	0.938	1	4.08E-29
GGH	2.93E-33	-2.920516485	1	1	5.09E-29
GPN1	4.72E-33	-1.127382596	1	1	8.19E-29
ENSSSCG00000027041	5.02E-33	-2.339220179	1	1	8.72E-29
MRPL30	9.01E-33	-4.191843358	0.688	1	1.56E-28
EIF3M	9.09E-33	-1.01368084	1	1	1.58E-28
EEF1B2	1.22E-32	-2.816700776	1	1	2.11E-28
MRPS15	2.47E-32	-3.836501268	0.938	1	4.28E-28
ENSSSCG00000002963	3.16E-32	-3.149940627	0.938	1	5.48E-28
ENSSSCG00000015337	3.76E-32	-3.937715722	0.875	1	6.52E-28
CYTH2	4.56E-32	-3.523840711	1	1	7.91E-28
TMEM219	4.76E-32	-4.427010141	0.625	1	8.27E-28
TPI1	5.88E-32	-3.851403508	0.875	1	1.02E-27
RABAC1	7.54E-32	-4.64200265	0.75	1	1.31E-27
CUL7	8.33E-32	-3.56125824	0.938	1	1.45E-27
POLR2D	9.85E-32	-1.857845832	1	1	1.71E-27
ENSSSCG00000005217	1.01E-31	-2.325255617	0.938	1	1.75E-27
FIGLA	1.24E-31	-4.372532771	0.75	1	2.16E-27
ENSSSCG00000001696	1.25E-31	-4.375425643	0.688	1	2.16E-27
RAB7A	1.81E-31	-1.667076512	1	1	3.15E-27
ENSSSCG00000000905	1.84E-31	-3.9492467	1	1	3.19E-27
NDUFV2	1.92E-31	-2.049388027	1	1	3.33E-27
H2BK1	2.14E-31	-7.584962501	0.125	1	3.72E-27
MRPL28	4.09E-31	-4.910732662	0.625	1	7.09E-27
UQCRQ	6.43E-31	-3.437593624	1	1	1.12E-26
SHD	6.93E-31	-4.824428435	0.5	1	1.20E-26
ENSSSCG00000006081	8.10E-31	-2.750199047	1	1	1.41E-26

POLR2H	1.14E-30	-2.49986834	1	1	1.99E-26
FSD2	1.60E-30	-2.62995331	1	1	2.77E-26
POLE4	1.84E-30	-2.800647975	1	1	3.19E-26
SMIM26	1.90E-30	-4.730160416	0.375	1	3.30E-26
DAD1	1.93E-30	-3.080472579	0.938	1	3.34E-26
PRDX3	2.76E-30	-1.867487004	1	1	4.80E-26
ENSSSCG00000024520	3.07E-30	-1.706520335	1	1	5.33E-26
COX4I1	3.08E-30	-3.69743723	1	1	5.34E-26
VKORC1	3.53E-30	-3.464926375	0.75	1	6.12E-26
HAX1	4.20E-30	-1.716123211	1	1	7.28E-26
MYL12A	4.74E-30	-2.429016083	1	1	8.23E-26
CHMP2A	4.78E-30	-5.568842835	0.438	1	8.29E-26
VDAC3	5.40E-30	-2.721494185	0.938	1	9.38E-26
MRPS10	6.05E-30	-1.385167992	1	1	1.05E-25
MARS1	6.10E-30	-2.440010028	1	1	1.06E-25
EIF6	6.33E-30	-3.256408929	0.875	1	1.10E-25
AP2S1	6.45E-30	-5.299309792	0.562	1	1.12E-25
SLC25A26	8.03E-30	-1.282001028	1	1	1.39E-25
GPX4	8.51E-30	-4.381507876	0.688	1	1.48E-25
ITGB5	1.14E-29	-3.323575948	0.938	1	1.97E-25
MVP	1.19E-29	-2.662130361	1	1	2.06E-25
ENSSSCG00000017509	1.20E-29	-4.387588775	0.938	1	2.08E-25
POLR2K	1.33E-29	-2.581325519	1	1	2.31E-25
AMZ2	1.51E-29	-2.380368483	0.938	1	2.62E-25
MGMT	2.30E-29	-4.186037006	0.75	1	3.99E-25
PAIP2	2.53E-29	-3.047305715	1	1	4.40E-25
RPS5	2.64E-29	-4.450661409	0.938	1	4.59E-25
HADHA	2.75E-29	-2.194422891	1	1	4.77E-25
TMEM128	3.34E-29	3.015103393	1	1	5.80E-25
DEGS2	3.75E-29	-3.737548384	0.875	1	6.51E-25
ROMO1	3.84E-29	-5.458181991	0.438	1	6.66E-25
TIMM17B	5.42E-29	-3.693022247	0.812	1	9.41E-25
TOMM20	5.88E-29	-2.134301092	1	1	1.02E-24
COA4	7.59E-29	-2.543142325	1	1	1.32E-24
TAF1A	8.06E-29	2.647720015	1	1	1.40E-24
ENSSSCG00000040854	9.01E-29	-4.499101764	0.688	1	1.56E-24
NUDC	9.59E-29	-2.976106794	0.938	1	1.66E-24
ENSSSCG00000010058	1.41E-28	-2.457319548	1	1	2.44E-24
ENSSSCG00000013064	1.65E-28	-3.364342109	0.938	1	2.86E-24
VPS29	1.75E-28	-1.61027192	1	1	3.03E-24
PPAN	1.91E-28	-2.36143811	1	1	3.31E-24
SELENOS	1.93E-28	-2.663099398	0.938	1	3.34E-24
ENSSSCG00000014071	2.43E-28	2.820801619	1	1	4.22E-24
LALBA	2.56E-28	-3.833141302	0.812	1	4.44E-24
TMEM125	3.74E-28	-7.070389328	0.062	1	6.49E-24
RNH1	4.12E-28	-6.180572246	0.062	1	7.15E-24
CLDN10	4.78E-28	-2.1498785	1	1	8.29E-24
IMP4	5.22E-28	-3.545369368	0.812	1	9.06E-24
SFT2D1	5.58E-28	3.120036725	1	1	9.69E-24
UXT	6.01E-28	-4.46634959	0.812	1	1.04E-23
BUD23	6.03E-28	-2.697880386	0.938	1	1.05E-23
POMP	8.30E-28	-2.70286109	1	1	1.44E-23
EMG1	8.56E-28	-4.410522018	0.688	1	1.48E-23
DYDC1	8.75E-28	-2.802060622	0.938	1	1.52E-23
COMMD9	9.11E-28	-3.814644958	0.812	1	1.58E-23
ENSSSCG00000056716	9.23E-28	-3.159198595	0.875	1	1.60E-23
ENSSSCG00000038035	1.13E-27	-3.163058282	0.938	1	1.97E-23
NAXE	1.16E-27	-3.332180567	0.75	1	2.01E-23
MRPL55	1.20E-27	-4.193090285	0.875	1	2.08E-23
TLCD1	1.51E-27	-2.229753584	0.875	1	2.62E-23
PHB1	1.66E-27	-2.307003265	1	1	2.88E-23
EMC7	1.90E-27	-2.695681305	0.812	1	3.30E-23
UBL7	2.15E-27	-2.729380999	1	1	3.74E-23
HAUS4	2.44E-27	2.453180215	1	1	4.24E-23
ENSSSCG00000007826	2.94E-27	-2.505740406	1	1	5.10E-23
ARG2	3.06E-27	-3.033608393	0.875	1	5.31E-23
DPCD	3.32E-27	-3.404832717	1	1	5.76E-23
C1orf105	3.62E-27	-3.343846451	0.875	1	6.27E-23
SAT2	4.96E-27	-3.600904045	0.875	1	8.60E-23
AJUBA	5.41E-27	-2.760065277	0.938	1	9.39E-23

FLOT1	6.04E-27	-2.252063288	0.938	1	1.05E-22
GUCA1A	6.23E-27	-2.830074999	1	1	1.08E-22
GTF2F1	7.88E-27	-2.641313355	1	1	1.37E-22
SNX22	8.80E-27	-3.823502345	0.75	1	1.53E-22
TALDO1	9.07E-27	-4.133855747	0.688	1	1.57E-22
NOP10	1.05E-26	-2.281381064	1	1	1.82E-22
RPS18	1.10E-26	-3.563041882	1	1	1.90E-22
ENSSSCG00000042862	1.17E-26	-5.993976212	0.75	0.933	2.02E-22
ENSSSCG00000010056	1.26E-26	-3.254997087	1	1	2.18E-22
ADA	1.54E-26	-2.3111759	1	1	2.67E-22
ENSSSCG00000014284	1.66E-26	-2.572968905	1	1	2.88E-22
ENSSSCG00000012842	2.09E-26	-6.290817562	0.125	1	3.62E-22
NDUFA13	2.68E-26	-4.503805356	0.625	1	4.64E-22
MIEN1	3.05E-26	-4.732837709	0.562	1	5.30E-22
PTPMT1	3.09E-26	-3.107815903	0.812	1	5.37E-22
COX6A1	3.40E-26	-3.538668848	0.875	1	5.90E-22
RPL38	4.10E-26	-6.42103624	0.125	1	7.11E-22
ASB11	4.27E-26	-2.270431399	1	1	7.40E-22
CZIB	4.76E-26	-4.302042786	0.812	1	8.26E-22
IMP3	5.48E-26	-1.525461489	1	1	9.51E-22
TSPAN31	6.10E-26	-3.098110085	0.812	1	1.06E-21
SSBP1	6.24E-26	-1.543400369	1	1	1.08E-21
DNAJC15	7.29E-26	-1.999860197	1	1	1.26E-21
PDZD11	7.66E-26	-3.053363226	0.938	1	1.33E-21
ENSSSCG00000009851	8.03E-26	-2.701658725	1	1	1.39E-21
PTS	8.50E-26	2.946358034	1	1	1.48E-21
SLC25A38	9.15E-26	-2.978279803	0.875	1	1.59E-21
ENSSSCG00000000296	1.15E-25	3.605214326	1	1	2.00E-21
COPS9	1.20E-25	-4.421580345	0.625	1	2.07E-21
PTPRT	1.25E-25	-1.823371458	1	1	2.18E-21
ATP5MC2	1.27E-25	-2.416655778	1	1	2.20E-21
ENSSSCG00000045485	1.46E-25	-4.54092194	0.562	1	2.53E-21
SELENOF	1.48E-25	-1.351053043	1	1	2.56E-21
SNU13	1.51E-25	-2.076890894	1	1	2.61E-21
TMEM126A	2.18E-25	-3.275471509	0.938	1	3.78E-21
ACAD9	2.27E-25	-2.965234582	0.812	1	3.94E-21
BCKDHA	2.40E-25	-3.452259963	0.938	1	4.17E-21
TIMM13	2.95E-25	-2.714597781	0.875	1	5.11E-21
CDK5RAP3	2.97E-25	-3.986672468	0.625	1	5.14E-21
DLGAP4	3.26E-25	-1.542240102	1	1	5.65E-21
WRAP53	3.59E-25	-5.162782932	0.312	0.933	6.22E-21
ENSSSCG00000017913	4.57E-25	-2.531814801	1	1	7.94E-21
ATP5PO	4.63E-25	-2.068333059	1	1	8.04E-21
ENSSSCG00000052263	5.25E-25	-2.573374526	1	1	9.10E-21
NDUFB11	5.33E-25	-4.478540442	0.562	1	9.25E-21
NDUFA10	5.47E-25	-3.57797607	0.688	1	9.50E-21
ENSSSCG00000061874	5.56E-25	-5.870993208	0.688	1	9.65E-21
ENSSSCG00000038628	6.86E-25	-1.321073299	1	1	1.19E-20
RPS14	8.58E-25	-4.619282574	0.688	1	1.49E-20
FAM162A	8.76E-25	-2.020008913	1	1	1.52E-20
ENSSSCG00000021644	9.67E-25	1.990075288	1	1	1.68E-20
ENSSSCG00000041579	1.11E-24	-3.245833665	0.875	1	1.93E-20
ENSSSCG00000055438	1.14E-24	-6.045850652	0.625	1	1.98E-20
ENSSSCG00000028397	1.21E-24	-1.930727165	1	1	2.11E-20
MRPS33	1.31E-24	-2	1	1	2.28E-20
COPS3	1.36E-24	-2.806991385	0.938	1	2.36E-20
GLRX3	1.44E-24	-2.717600269	0.938	1	2.51E-20
TDRD10	1.45E-24	-3.101440679	0.938	1	2.52E-20
DDT	1.52E-24	-5.571156701	0.25	1	2.64E-20
HSD17B7	1.67E-24	-1.58770869	1	1	2.90E-20
ENSSSCG00000018046	2.00E-24	-3.623946691	0.875	1	3.47E-20
EBNA1BP2	2.41E-24	-2.130103612	1	1	4.18E-20
ALDOC	2.71E-24	-2.715262464	0.875	1	4.70E-20
ACAT1	3.20E-24	-1.997719059	1	1	5.55E-20
TRAPPC4	3.20E-24	-2.068810808	1	1	5.56E-20
C19orf38	3.64E-24	-4.584962501	0.375	1	6.31E-20
MANBA	4.71E-24	-1.741369417	1	1	8.17E-20
MIX23	5.17E-24	-2.010940863	1	1	8.98E-20
SUCLG1	6.13E-24	-2.486181572	0.938	1	1.06E-19
ATP5F1A	7.27E-24	-1.598644656	1	1	1.26E-19

MAPK13	7.63E-24	-3.710204526	0.688	1	1.32E-19
BTG4	8.42E-24	2.767651617	1	1	1.46E-19
PDCD6	8.68E-24	-1.213925	1	1	1.51E-19
CUTA	8.91E-24	-4.365132593	0.562	1	1.55E-19
CNBP	8.99E-24	1.758101516	1	1	1.56E-19
MRPS28	9.31E-24	-2.276470545	0.938	1	1.62E-19
LAMTOR2	9.49E-24	-3.888045067	0.75	1	1.65E-19
SLC35G1	9.66E-24	2.529820947	1	1	1.68E-19
NSMCE4A	9.70E-24	2.367134588	1	1	1.68E-19
MICOS13	9.88E-24	-5.137503524	0.312	1	1.71E-19
NDUFS3	1.12E-23	-2.803482798	0.812	1	1.95E-19
UQCRB	1.18E-23	-2.375613674	1	1	2.05E-19
SEC11C	1.46E-23	-2.531682418	1	1	2.53E-19
TMEM147	1.49E-23	-4.119909464	0.562	1	2.58E-19
WDR74	1.52E-23	-2.777013755	0.812	1	2.64E-19
GFER	1.60E-23	-4.389042291	0.375	1	2.78E-19
RPS10	1.76E-23	-3.92372842	0.938	1	3.06E-19
RPS9	1.89E-23	-4.202678914	0.938	1	3.27E-19
MRPL44	2.36E-23	3.135383216	1	0.867	4.09E-19
EEF1D	2.42E-23	-3.703351314	0.875	1	4.21E-19
DERL3	2.70E-23	-5.758445322	0.188	1	4.69E-19
MRPL54	2.88E-23	-2.796286685	0.875	1	4.99E-19
RTF2	3.19E-23	-1.445300338	1	1	5.54E-19
UQCR10	3.67E-23	-3.520970944	0.875	1	6.37E-19
EFHC1	3.76E-23	-1.765323099	1	1	6.52E-19
NR0B1	3.98E-23	-2.360289747	1	1	6.91E-19
OOEP	4.23E-23	-2.496253737	1	1	7.35E-19
EXOSC4	4.24E-23	-5.895302621	0.188	0.933	7.36E-19
ENSSSCG00000027538	4.31E-23	-3.969626351	0.625	1	7.48E-19
CDK5RAP2	4.54E-23	-2.672487787	1	1	7.88E-19
RNF26	4.85E-23	-2.253756592	1	1	8.42E-19
FIBP	4.95E-23	-4.315501826	0.312	1	8.58E-19
POLR1D	5.37E-23	-2.054281661	1	1	9.32E-19
ENSSSCG00000033310	5.53E-23	-5.173856818	0.5	1	9.59E-19
ENSSSCG00000036499	5.58E-23	-1.361992689	1	1	9.68E-19
STMP1	5.59E-23	-1.666769256	1	1	9.70E-19
GMPS	5.89E-23	-1.501278506	1	1	1.02E-18
LUC7L2	5.96E-23	-1.636956051	1	1	1.03E-18
ENSSSCG00000025673	6.86E-23	-3.097667686	0.875	1	1.19E-18
CCDC12	9.34E-23	-1.969102764	1	1	1.62E-18
ENSSSCG00000002020	1.00E-22	2.049370351	1	1	1.74E-18
GALK1	1.00E-22	-4.415037499	0.625	0.933	1.74E-18
ENSSSCG00000024974	1.04E-22	-4.60968493	0.312	1	1.80E-18
CHRAC1	1.05E-22	2.747817929	1	1	1.82E-18
COMMD4	1.11E-22	-4.653824359	0.562	0.933	1.93E-18
ENSSSCG00000011272	1.32E-22	-2.396632919	1	1	2.29E-18
ENSSSCG00000037510	1.35E-22	-3.152890633	0.938	1	2.34E-18
C11orf80	1.38E-22	-1.502180062	1	1	2.39E-18
PHB2	1.41E-22	-1.998170329	1	1	2.45E-18
TPRKB	1.43E-22	-5.24961389	0.625	1	2.48E-18
AP1M2	1.44E-22	-2.966503088	0.812	1	2.50E-18
EIF3H	1.48E-22	-1.706572228	1	1	2.56E-18
NLRP5	1.51E-22	-1.92919812	1	1	2.61E-18
FZR1	1.55E-22	-2.333531241	0.938	1	2.68E-18
AP1S1	1.69E-22	-2.55468949	0.938	1	2.93E-18
TEX35	1.86E-22	-2.928050158	0.812	1	3.24E-18
MRPL20	1.97E-22	-2.004800986	1	1	3.42E-18
FAM98A	1.98E-22	-1.385160458	1	1	3.44E-18
MRPL45	2.04E-22	-2.141318497	0.938	1	3.54E-18
FAM32A	2.21E-22	-2.258772379	1	1	3.83E-18
MEA1	2.30E-22	-2.845181891	0.938	1	3.99E-18
MALSU1	2.42E-22	-4.973527789	0.438	1	4.19E-18
GABARAP	2.51E-22	-2.632747915	0.938	1	4.36E-18
FKBP4	2.57E-22	-2.092006005	1	1	4.46E-18
TPT1	2.62E-22	-3.057143907	0.875	1	4.55E-18
SYT2	2.62E-22	-3.671377253	0.625	1	4.55E-18
ENSSSCG00000052310	3.13E-22	-3.395408693	0.812	1	5.44E-18
ERP29	3.49E-22	-3.653824359	0.812	1	6.06E-18
HSPA8	4.33E-22	-1.646974879	1	1	7.52E-18
FDPS	4.57E-22	-3.160032957	0.875	1	7.92E-18

ATOX1	4.61E-22	-4.552541023	0.562	1	8.00E-18
MRPL36	5.17E-22	-3.268648012	0.75	1	8.96E-18
DCTN3	6.64E-22	-3.398917834	0.938	1	1.15E-17
ENSSSCG00000022401	6.71E-22	-2.454930226	0.875	1	1.16E-17
ECRG4	6.96E-22	-5.006853671	0.312	1	1.21E-17
GDPD5	7.63E-22	-2.949373927	0.75	1	1.32E-17
METTL23	8.60E-22	-2.712718048	0.875	1	1.49E-17
SCNM1	9.75E-22	-3.102173487	1	1	1.69E-17
PRPF4	1.00E-21	-1.93844619	1	1	1.74E-17
WDR91	1.01E-21	-1.308978702	1	1	1.76E-17
PUF60	1.08E-21	-2.51524796	1	1	1.88E-17
RPS25	1.13E-21	-3.271284293	1	1	1.97E-17
RTN4IP1	1.14E-21	-1.682666479	0.938	1	1.97E-17
TMEM248	1.16E-21	-1.884414833	1	1	2.01E-17
ARPC3	1.22E-21	-2.343222583	1	1	2.12E-17
ENSSSCG00000008012	1.25E-21	-2.203219161	1	1	2.18E-17
MRPL17	1.28E-21	-2.91707904	0.688	1	2.22E-17
ENSSSCG00000017955	1.33E-21	-3.064780681	0.688	1	2.31E-17
ENSSSCG00000034739	1.51E-21	-1.781165398	0.938	1	2.62E-17
RPP40	1.52E-21	-3.299363179	0.812	1	2.64E-17
CCDC32	1.63E-21	-3.082248412	0.75	0.933	2.83E-17
MRPL13	1.66E-21	-1.053300551	1	1	2.88E-17
TCP1	1.70E-21	-1.320024384	1	1	2.94E-17
ILF2	1.73E-21	-1.238172684	1	1	3.00E-17
ZMAT5	1.87E-21	-2.232085818	0.938	1	3.24E-17
UST	1.89E-21	-2.360331607	0.875	1	3.28E-17
SNX7	1.90E-21	2.868352343	1	1	3.30E-17
CLPP	2.11E-21	-3.338865819	0.688	1	3.66E-17
AMFR	2.20E-21	2.2644426	1	1	3.81E-17
CEP78	2.52E-21	2.692330741	1	1	4.37E-17
MDH1	3.00E-21	-1.813769426	1	1	5.20E-17
ENSSSCG00000040498	3.81E-21	3.214415625	1	0.933	6.61E-17
HNRNPC	4.48E-21	1.833240347	1	1	7.77E-17
HSPA4	5.41E-21	-1.356605547	1	1	9.38E-17
MACROD1	6.13E-21	-3.202733896	0.75	1	1.06E-16
ENSSSCG00000058091	6.23E-21	-3.494359952	0.812	1	1.08E-16
ENSSSCG00000017971	7.07E-21	-4.284250892	0.5	1	1.23E-16
ENSSSCG00000004703	7.99E-21	-3.589785373	1	1	1.39E-16
OXA1L	8.05E-21	-1.341373156	1	1	1.40E-16
BPNT1	8.10E-21	-1.101314327	1	1	1.41E-16
CD40	8.33E-21	-2.051325934	0.938	1	1.45E-16
RPL36	8.79E-21	-3.4639471	0.75	1	1.53E-16
TAGLN	9.42E-21	-4.017921908	0.562	0.933	1.63E-16
RNF121	9.49E-21	-2.499734664	0.938	1	1.65E-16
SNX31	1.02E-20	2.372787211	1	1	1.78E-16
FIS1	1.06E-20	-5.130198723	0.188	1	1.85E-16
TMBIM6	1.09E-20	-1.862974638	1	1	1.89E-16
RPL32	1.30E-20	-2.59946207	1	1	2.26E-16
POP7	1.36E-20	-2.12015628	1	1	2.36E-16
DTYMK	1.37E-20	-3.703973769	0.625	1	2.38E-16
MELK	1.38E-20	1.956840472	1	1	2.39E-16
LHFPL1	1.43E-20	-1.620728464	1	1	2.48E-16
ENSSSCG00000046592	1.54E-20	-2.753669524	0.938	1	2.67E-16
CTNS	1.56E-20	-2.435262527	1	1	2.71E-16
DMAP1	1.58E-20	-4.627087976	0.25	1	2.74E-16
COX6C	1.59E-20	-2.84820373	1	1	2.76E-16
KCNIP4	1.74E-20	-1.505626031	1	1	3.01E-16
ZNHIT6	1.77E-20	-1.050108251	1	1	3.07E-16
MED8	1.77E-20	-2.326599535	1	1	3.08E-16
IMMP2L	2.02E-20	-2.847996907	0.938	1	3.50E-16
PET100	2.36E-20	-4.773229138	0.375	1	4.09E-16
DUS4L	2.42E-20	3.065426492	1	1	4.20E-16
TMED3	2.50E-20	-3.020647151	0.625	1	4.34E-16
ENSSSCG00000053939	2.51E-20	-3.103678646	0.875	1	4.35E-16
NGLY1	2.68E-20	2.278596252	1	1	4.65E-16
NRBF2	3.10E-20	2.095657031	1	1	5.38E-16
YIPF2	3.33E-20	-2.73231495	0.875	1	5.78E-16
ARF5	3.40E-20	-2.291379032	0.875	1	5.89E-16
SWSAP1	3.59E-20	-2.852569636	0.938	0.933	6.24E-16
MRPS9	3.89E-20	-1.597948935	1	1	6.75E-16

ARPP19	4.16E-20	-1.666789194	1	1	7.22E-16
GRHPR	4.46E-20	-2.634891081	1	1	7.75E-16
ZPR1	5.55E-20	-2.25762091	0.938	1	9.64E-16
ENSSSCG00000016100	5.59E-20	-2.576431643	0.875	1	9.70E-16
ISCA2	6.28E-20	-2.882816461	0.75	1	1.09E-15
ENSSSCG00000038475	6.36E-20	-3.756074417	0.375	1	1.10E-15
ENSSSCG00000060915	6.42E-20	-3.678071905	0.812	1	1.11E-15
ENSSSCG00000003286	6.88E-20	-2.458385042	1	1	1.19E-15
COMMD7	7.60E-20	-2.411285364	0.812	1	1.32E-15
SDF2	8.30E-20	-1.116585889	1	1	1.44E-15
PRSS2	8.43E-20	-3.304854582	0.812	1	1.46E-15
ENSSSCG00000036716	8.69E-20	-4.038732394	0.75	1	1.51E-15
LMF1	8.90E-20	-4.099108145	0.562	1	1.54E-15
PCID2	9.28E-20	-3.093109404	0.812	1	1.61E-15
ELOB	1.00E-19	-4.450661409	0.375	1	1.74E-15
ZNF821	1.09E-19	-3.080170349	0.812	1	1.89E-15
SLC35B1	1.21E-19	-2.251371488	1	1	2.10E-15
TBRG4	1.21E-19	-2.58812978	0.938	1	2.10E-15
ENSSSCG00000035997	1.24E-19	-4.636043713	0.875	1	2.16E-15
ADCK5	1.47E-19	-4.650486073	0.375	0.933	2.55E-15
RPS15A	1.58E-19	-3.761619696	0.875	1	2.74E-15
EPHX1	1.72E-19	-3.222392421	0.688	0.933	2.99E-15
DERL1	1.82E-19	-1.498522485	1	1	3.16E-15
GTF2E2	2.02E-19	-1.534452666	1	1	3.51E-15
RMDN1	2.03E-19	-1.193590797	1	1	3.52E-15
PRDX2	2.13E-19	-1.719882611	1	1	3.69E-15
RALY	2.20E-19	-1.698731376	0.938	1	3.81E-15
RPS7	2.20E-19	-2.784423886	0.875	1	3.82E-15
DUT	2.42E-19	-1.527512229	1	1	4.21E-15
RPL35	2.46E-19	-3.910732662	0.625	1	4.27E-15
FETUB	2.57E-19	-3.333021925	0.688	1	4.45E-15
APOO	2.81E-19	-1.865698908	1	1	4.87E-15
ADH5	3.25E-19	2.095906726	1	1	5.64E-15
MOBKL3	3.45E-19	2.651588877	1	1	5.99E-15
ATP5MK	3.51E-19	-2.856099887	1	1	6.09E-15
CASQ2	3.59E-19	-3.796716402	0.625	1	6.22E-15
FSTL3	3.59E-19	-2.591015171	0.875	1	6.23E-15
ENSSSCG00000006694	3.74E-19	-0.636148296	1	1	6.49E-15
PFDN5	4.69E-19	-2.375887746	1	1	8.13E-15
EXOSC8	4.86E-19	-1.736965594	1	1	8.43E-15
CDH6	4.86E-19	-3.655351829	0.562	0.933	8.44E-15
IFT57	6.40E-19	-1.71086184	1	1	1.11E-14
GLIPR1	6.86E-19	-0.984560378	1	1	1.19E-14
CD247	7.28E-19	-2.292418213	0.938	1	1.26E-14
WDR53	7.37E-19	-1.298156199	0.938	1	1.28E-14
JAM3	7.40E-19	-2.02233633	0.938	1	1.28E-14
TUBB4B	7.57E-19	-3.375663261	0.625	1	1.31E-14
TAF9	7.91E-19	2.398175692	1	1	1.37E-14
LYPLAL1	8.99E-19	-1.795723494	0.938	1	1.56E-14
C19orf53	1.02E-18	-3.597072183	0.562	1	1.78E-14
FAT2	1.03E-18	-2.374570908	0.875	0.933	1.79E-14
TMEM42	1.09E-18	-2.785600369	0.938	1	1.89E-14
ENSSSCG00000014133	1.12E-18	-4.232527869	0.812	1	1.95E-14
CDC40	1.21E-18	2.043233601	1	1	2.10E-14
AARSD1	1.28E-18	-1.67363284	1	1	2.22E-14
RFC4	1.29E-18	2.227744324	1	1	2.24E-14
PIH1D1	1.30E-18	-2.334770159	0.812	1	2.25E-14
BNIP3	1.31E-18	-2.415037499	0.938	0.933	2.28E-14
EPHB6	1.45E-18	-4.568842835	0.25	1	2.52E-14
ENSSSCG00000046487	1.49E-18	-3.034765418	0.75	1	2.59E-14
GTF2H5	1.56E-18	-2.406848101	0.938	1	2.70E-14
APRT	1.58E-18	-6	0.062	1	2.74E-14
POC1A	1.60E-18	-2.133186844	0.875	1	2.78E-14
ZNF830	1.81E-18	-0.939968987	1	1	3.14E-14
NOP2	1.84E-18	-1.805648956	0.938	1	3.19E-14
PDE8A	1.92E-18	1.993544978	1	1	3.33E-14
IDH3G	2.03E-18	-3.552541023	0.75	1	3.52E-14
SSMEM1	2.22E-18	-2.793549123	0.938	1	3.85E-14
JPT1	2.26E-18	-2.623624121	0.938	1	3.92E-14
CCT3	2.36E-18	-1.443101418	1	1	4.09E-14

RPL10A	2.38E-18	-3.538586852	0.938	1	4.13E-14
ENSSSCG00000032573	2.47E-18	-1.917113455	1	1	4.28E-14
BNIP1	2.48E-18	-2.195624315	1	1	4.30E-14
ENSSSCG00000006559	2.51E-18	-2.065364414	0.938	1	4.36E-14
CDC6	2.61E-18	-1.110381486	1	1	4.53E-14
NDUFC1	2.70E-18	-2.113008962	0.875	1	4.69E-14
NSL1	2.79E-18	2.760265682	1	1	4.85E-14
PARL	2.80E-18	2.158125	1	1	4.86E-14
VPS26C	3.05E-18	-1.414353595	1	1	5.29E-14
GADD45GIP1	3.27E-18	-2.920928429	0.688	1	5.67E-14
UMPS	3.60E-18	-1.01790306	1	1	6.25E-14
ENSSSCG00000031249	3.91E-18	-3.018204595	0.812	1	6.78E-14
SLC25A6	3.91E-18	-2.045944296	0.938	1	6.79E-14
ENSSSCG00000055313	4.01E-18	-1.760320317	0.938	1	6.95E-14
CFAP298	4.60E-18	-1.688224621	1	1	7.98E-14
ENSSSCG00000030849	4.67E-18	-3.612794188	1	1	8.10E-14
YKT6	4.80E-18	-2.434402824	0.812	1	8.33E-14
EIF4E2	4.88E-18	-1.021556143	1	1	8.47E-14
HCRT	4.93E-18	-3.95491211	0.438	1	8.56E-14
ATP6V1F	5.53E-18	-4.712019237	0.312	0.933	9.59E-14
PSMC3IP	5.85E-18	-2.117275716	0.938	1	1.01E-13
IP6K2	5.88E-18	-2.471621028	1	1	1.02E-13
ENSSSCG00000057536	5.93E-18	-5.938599455	0.062	0.933	1.03E-13
TRIM77	6.12E-18	2.249337225	1	1	1.06E-13
MKS1	6.15E-18	-3.474303449	0.625	1	1.07E-13
FTSJ3	6.22E-18	-1.657978335	1	1	1.08E-13
SPTLC1	6.24E-18	2.016150344	1	1	1.08E-13
ENSA	6.35E-18	-1.908040197	0.938	1	1.10E-13
RPS6	6.65E-18	-2.094454575	1	1	1.15E-13
ENSSSCG00000035904	6.96E-18	-1.790597077	1	1	1.21E-13
NCF1	6.96E-18	-2.35033181	0.875	1	1.21E-13
STX18	7.08E-18	-1.39509009	1	1	1.23E-13
SNX11	7.20E-18	-1.82058355	0.938	1	1.25E-13
GNL3	7.45E-18	-1.53331335	1	1	1.29E-13
SRA1	8.53E-18	-5.028569152	0.25	0.867	1.48E-13
PSME3IP1	8.71E-18	-2.032446622	1	1	1.51E-13
PATL2	9.01E-18	-1.139485211	1	1	1.56E-13
ENSSSCG00000028677	9.05E-18	-1.064485396	1	1	1.57E-13
MAP3K13	9.10E-18	1.874358342	1	1	1.58E-13
DPEP1	9.44E-18	-3.638245925	0.812	1	1.64E-13
POLE3	9.70E-18	-1.971499957	0.938	1	1.68E-13
LSM4	1.03E-17	-2.192645078	0.938	1	1.78E-13
STARD3NL	1.04E-17	2.076598078	1	1	1.81E-13
ENSSSCG00000008170	1.10E-17	-3.742034964	0.938	1	1.91E-13
IDH3B	1.16E-17	-2.108706259	0.875	1	2.01E-13
ENSSSCG00000058502	1.19E-17	-4.502500341	0.25	1	2.06E-13
AIMP2	1.20E-17	-3.779609932	0.5	1	2.07E-13
UQCC2	1.28E-17	-3.526068812	0.5	1	2.22E-13
RPLP1	1.31E-17	-3.788134272	1	1	2.27E-13
ATP5PF	1.34E-17	-4.656045599	0.875	1	2.33E-13
MRPL58	1.40E-17	-2.088909416	1	1	2.42E-13
HILPDA	1.44E-17	2.60711463	1	1	2.49E-13
NEIL1	1.51E-17	3.005693006	1	1	2.62E-13
ATP6V1G1	1.53E-17	-1.292031628	1	1	2.65E-13
CRHR1	1.60E-17	-3.137870855	0.938	1	2.78E-13
FAU	1.76E-17	-3.943965965	0.5	1	3.05E-13
PAAF1	1.81E-17	-2.18001747	1	1	3.14E-13
THOC6	1.98E-17	2.430452552	1	0.933	3.44E-13
TYMS	2.00E-17	2.501766903	1	1	3.48E-13
C4orf33	2.10E-17	-1.319365406	1	1	3.64E-13
IDH2	2.32E-17	-2.386373741	0.938	1	4.03E-13
NRG4	2.34E-17	-0.891307387	1	1	4.05E-13
ENSSSCG00000014569	2.39E-17	-3.73392514	0.875	1	4.15E-13
VPS25	2.40E-17	-2.22606081	1	1	4.17E-13
TCIRG1	2.45E-17	-2.761487913	0.75	1	4.25E-13
B4GALT4	2.45E-17	-1.875263772	0.938	1	4.25E-13
WDR54	2.50E-17	-3.832957507	0.438	1	4.33E-13
IAH1	2.55E-17	-2.655634516	0.812	1	4.43E-13
RPL11	2.59E-17	-2.846162942	1	1	4.50E-13
COX7A1	2.60E-17	-4.473931188	0.25	1	4.50E-13

LSM6	2.62E-17	-1.475579041	1	1	4.54E-13
ETFB	2.71E-17	-4.154220919	0.375	1	4.71E-13
ENSSSCG00000012088	2.78E-17	-4.310340121	0.562	0.867	4.83E-13
SCRG1	2.85E-17	-3.273018494	0.688	0.933	4.94E-13
CCT6B	2.86E-17	-1.694489665	1	1	4.96E-13
PEX16	2.86E-17	-6.273018494	0.062	0.867	4.96E-13
AIP	2.95E-17	-4.512648296	0.25	1	5.12E-13
ENSSSCG00000023783	3.06E-17	-5.341036918	0.062	0.933	5.31E-13
TCL1B	3.19E-17	-2.928167255	1	1	5.53E-13
TMEM225B	3.22E-17	-3.296049463	0.75	0.933	5.58E-13
DNAJB13	3.54E-17	-1.849440323	1	1	6.14E-13
NELFE	3.56E-17	-2.927685795	0.688	1	6.17E-13
NPC2	3.62E-17	-2.273176416	1	1	6.28E-13
MIB2	3.65E-17	-4.24838763	0.25	1	6.33E-13
ENSSSCG00000015083	3.91E-17	-2.295245716	1	1	6.79E-13
DUS1L	4.09E-17	-4.736965594	0.25	0.933	7.09E-13
NDUFV1	4.10E-17	-4.714347292	0.188	1	7.11E-13
ETHE1	4.51E-17	-2.696735749	0.875	0.933	7.82E-13
ENSSSCG00000021624	4.85E-17	-3.560467939	0.625	1	8.41E-13
BAG2	4.86E-17	-2.508146904	0.875	1	8.43E-13
NUP35	5.30E-17	2.084742217	1	1	9.19E-13
RPS15	5.49E-17	-4.754174884	0.625	1	9.52E-13
WTAP	5.88E-17	1.757829777	1	1	1.02E-12
ELK3	6.17E-17	2.593203241	1	1	1.07E-12
ENSSSCG00000034019	6.27E-17	-2.968973104	1	1	1.09E-12
EMC3	6.36E-17	-2.957295549	0.812	1	1.10E-12
TSPAN1	6.45E-17	-2.543599989	0.938	1	1.12E-12
C15orf48	6.51E-17	-4.062735755	0.375	1	1.13E-12
PNLDC1	6.76E-17	-1.684330896	0.938	1	1.17E-12
SPACA3	7.40E-17	-5.126532406	0.125	0.933	1.28E-12
RPS19	7.58E-17	-3.917776875	0.875	1	1.31E-12
LAT2	7.70E-17	-1.132782712	1	1	1.34E-12
MTMR14	7.71E-17	-2.494039957	0.938	1	1.34E-12
ENSSSCG00000058371	8.03E-17	-1.668662061	0.938	1	1.39E-12
SLC25A17	9.35E-17	-1.087601426	1	1	1.62E-12
CA9	9.64E-17	2.749230138	1	1	1.67E-12
PLA2G12A	9.78E-17	-2.116760672	0.938	1	1.70E-12
CRB3	9.87E-17	-2.010235222	0.938	1	1.71E-12
ISOC1	1.01E-16	2.154508152	1	1	1.75E-12
NDUFC2	1.02E-16	-3.492640317	0.625	1	1.78E-12
CARMIL1	1.07E-16	1.99051427	1	1	1.86E-12
ENSSSCG00000007646	1.13E-16	-4.712019237	0.25	1	1.96E-12
YTHDF1	1.35E-16	-2.817706441	0.688	1	2.35E-12
TOMM34	1.40E-16	-1.774667485	1	1	2.43E-12
ENSSSCG00000013232	1.52E-16	-0.691034212	1	1	2.63E-12
PQBP1	1.52E-16	-3.21649182	0.75	1	2.63E-12
MAST2	1.54E-16	-2.915111102	0.812	1	2.68E-12
ENSSSCG00000011875	1.56E-16	-1.200731638	1	1	2.71E-12
ANKRD40CL	1.63E-16	-1.765534746	0.938	1	2.83E-12
POLR2I	1.65E-16	-4.987927168	0.188	1	2.86E-12
ENSSSCG00000005440	1.73E-16	1.853706272	1	1	3.00E-12
OXSM	1.74E-16	-2.003842066	0.875	1	3.02E-12
GPS1	1.82E-16	-2.887525271	0.75	1	3.15E-12
EIPR1	1.92E-16	-3.450661409	0.562	1	3.34E-12
METTL9	1.94E-16	2.35331022	1	1	3.37E-12
MAP7	1.95E-16	-1.481446976	1	1	3.38E-12
BCAS2	2.11E-16	-1.99342083	1	1	3.66E-12
PRR13	2.12E-16	-2.113287286	0.938	1	3.67E-12
RNF31	2.21E-16	-3.449802917	0.688	1	3.84E-12
MRPL22	2.27E-16	-1.754741673	1	1	3.94E-12
ALKBH7	2.33E-16	-2.647045009	0.75	1	4.05E-12
NUP42	2.42E-16	2.699171005	1	1	4.21E-12
TMA7	2.44E-16	-2.407982742	1	1	4.24E-12
BORCS5	2.46E-16	-1.652671688	0.938	1	4.26E-12
AMN1	2.47E-16	-1.458962642	1	1	4.28E-12
DDX39B	2.50E-16	-1.359725826	1	1	4.34E-12
MAD2L1	2.58E-16	2.690511219	1	1	4.47E-12
SQLE	2.58E-16	-1.545038281	1	1	4.48E-12
DYNLL1	2.84E-16	-2.064648741	1	1	4.93E-12
COA8	3.18E-16	-2.718429643	0.75	1	5.51E-12

LRRC47	3.27E-16	-3.007777351	0.625	1	5.67E-12
OTUB1	3.36E-16	-2.148001535	1	1	5.83E-12
RASL11A	3.38E-16	3.302713962	1	0.867	5.86E-12
SWI5	3.39E-16	-2.528566445	1	1	5.88E-12
SRPX	3.66E-16	2.397800932	1	1	6.35E-12
WDR20	4.23E-16	1.861195383	1	1	7.34E-12
DDX11	4.56E-16	-1.861645078	0.938	1	7.91E-12
TSPAN14	4.66E-16	-1.477232261	1	1	8.08E-12
ATP6V0B	5.16E-16	-1.637587842	1	1	8.95E-12
AIDA	5.19E-16	-1.193952163	1	1	9.01E-12
GSTK1	5.21E-16	-3.977632187	0.5	0.933	9.04E-12
TIMM17A	5.27E-16	-2.499101764	0.688	1	9.15E-12
SDF4	5.43E-16	-4.074962058	0.438	1	9.42E-12
MED31	5.49E-16	-2.09797516	1	1	9.52E-12
ATG7	5.66E-16	-0.878058958	1	1	9.82E-12
RPL18A	5.73E-16	-4.178449074	0.562	1	9.94E-12
MCRS1	5.99E-16	-2.463567393	0.938	1	1.04E-11
GLRX5	6.01E-16	-1.056467561	1	1	1.04E-11
SLC17A5	7.19E-16	2.258422404	1	1	1.25E-11
JOSD2	7.34E-16	-5.201633861	0.125	0.933	1.27E-11
NUDT2	7.53E-16	-2.915839552	0.75	0.933	1.31E-11
AVPI1	8.33E-16	-4.180572246	0.438	0.867	1.45E-11
DNAJC14	8.40E-16	-0.933766302	1	1	1.46E-11
CCT4	8.40E-16	-1.673513623	1	1	1.46E-11
TUBGCP2	8.77E-16	-3.195724471	0.688	0.933	1.52E-11
RPL34	8.78E-16	-3.9510904	0.312	1	1.52E-11
PPP2R2B	9.03E-16	-1.301552267	1	1	1.57E-11
THYN1	9.25E-16	-1.089464261	1	1	1.61E-11
MEPCE	9.54E-16	-2.019777666	0.938	1	1.65E-11
SNPH	9.57E-16	-1.649743182	0.875	1	1.66E-11
ENSSSCG00000053447	9.85E-16	-3.481806511	0.438	1	1.71E-11
FHIT	1.00E-15	-1.865241853	0.938	1	1.74E-11
RFC3	1.01E-15	-1.74080766	1	1	1.76E-11
GFI1	1.11E-15	2.99906044	1	0.867	1.92E-11
H2AJ	1.14E-15	-4.20461772	0.312	0.933	1.98E-11
ENSSSCG00000052759	1.22E-15	-4.351843673	0.188	1	2.12E-11
ENSSSCG00000040571	1.25E-15	-1.640434248	1	1	2.16E-11
ENSSSCG00000002995	1.28E-15	-3.865698908	0.625	1	2.23E-11
SBDS	1.30E-15	1.867298417	1	1	2.26E-11
GPATCH11	1.33E-15	-1.565817451	1	1	2.30E-11
RBX1	1.34E-15	-0.741466986	1	1	2.33E-11
TEX264	1.43E-15	-2.735787403	0.875	1	2.48E-11
LMNA	1.46E-15	-1.562344198	0.938	1	2.53E-11
AVEN	1.47E-15	-1.551344593	1	1	2.56E-11
ENSSSCG00000037514	1.51E-15	-1.286678991	0.938	1	2.63E-11
CDK10	1.53E-15	-3.973527789	0.375	0.933	2.66E-11
FXR2	1.56E-15	-1.988412026	0.938	1	2.71E-11
ENSSSCG00000049464	1.58E-15	-2.124587636	1	1	2.74E-11
BTF3L4	1.62E-15	-1.468400725	1	1	2.81E-11
GCA	1.63E-15	2.219736947	1	1	2.84E-11
NDUFS4	1.66E-15	-1.886810959	1	1	2.87E-11
PAQR7	1.68E-15	-2.349449158	0.938	0.933	2.91E-11
RPS4X	1.74E-15	-3.062348377	1	1	3.02E-11
ENSSSCG00000031117	1.80E-15	-2.726981506	0.938	1	3.12E-11
ZMAT2	1.81E-15	-1.19088462	1	1	3.14E-11
ENSSSCG00000002379	1.84E-15	-2.188266637	1	1	3.19E-11
ARMC12	1.91E-15	-2.685807554	0.688	1	3.32E-11
TMEM179B	1.97E-15	-3.298223834	0.438	1	3.41E-11
NDUFS5	2.00E-15	-2.674904626	1	1	3.47E-11
TULP3	2.01E-15	-1.672596775	1	1	3.49E-11
IER2	2.07E-15	-2.263034406	0.875	0.933	3.59E-11
ENSSSCG00000031730	2.07E-15	-2.27897595	1	1	3.60E-11
CRYBB3	2.15E-15	-4.121015401	0.5	0.933	3.73E-11
DTD1	2.20E-15	-1.443541016	1	1	3.82E-11
NLRP8	2.26E-15	-1.143542393	1	1	3.93E-11
ENSSSCG00000061145	2.40E-15	-7.201633861	0	0.733	4.17E-11
ENSSSCG00000025928	2.44E-15	-4.180572246	0.562	1	4.23E-11
RHOT1	2.53E-15	1.728831319	1	1	4.39E-11
RCN2	2.55E-15	2.125407063	1	1	4.43E-11
RPS13	2.62E-15	-4.119909464	0.938	1	4.55E-11

ESYT1	2.69E-15	-1.310138641	1	1	4.66E-11
ENSSSCG00000033926	2.81E-15	-3.532221039	0.438	1	4.88E-11
LENG1	2.84E-15	-3.431911318	0.5	1	4.93E-11
CALR	2.95E-15	-1.254413923	1	1	5.12E-11
CARS2	3.24E-15	-1.946719917	0.875	1	5.62E-11
PSENEN	3.53E-15	-3.135995217	0.875	1	6.13E-11
RCBTB2	3.69E-15	1.998683967	1	1	6.41E-11
RNASEH2C	3.72E-15	-5.751320887	0.062	0.933	6.45E-11
CCNB1	3.78E-15	2.140053101	1	1	6.56E-11
GET1	3.90E-15	-0.943336134	1	1	6.76E-11
ENSSSCG00000038364	3.92E-15	-5.568842835	0.062	0.933	6.80E-11
B3GNTL1	4.18E-15	-3.90902634	0.375	0.933	7.25E-11
FBL	4.19E-15	-3.793549123	0.375	1	7.27E-11
COPS6	4.19E-15	-1.959092056	1	1	7.27E-11
SYTL3	4.31E-15	-1.186424189	1	1	7.48E-11
ENSSSCG00000056015	4.35E-15	-3.204140717	0.75	1	7.56E-11
ENSSSCG00000053570	4.36E-15	-1.292801985	1	1	7.57E-11
SLC25A31	4.46E-15	2.339218765	1	1	7.73E-11
RBPMS2	4.46E-15	-1.834323334	1	1	7.74E-11
ZNHIT1	4.49E-15	-4.653824359	0.188	0.867	7.79E-11
TMEM186	4.75E-15	-1.528365183	0.938	1	8.24E-11
SMPD2	4.85E-15	-3.254101281	0.75	0.933	8.41E-11
CHST10	4.93E-15	-1.27551518	1	1	8.56E-11
RPL4	5.15E-15	-1.626541604	1	1	8.94E-11
ENSSSCG00000031756	5.20E-15	-2.312467766	0.875	1	9.02E-11
RARS2	5.21E-15	2.350834658	1	1	9.05E-11
COX14	5.33E-15	-3.124587636	0.688	1	9.25E-11
SIL1	5.45E-15	-1.880000224	1	1	9.45E-11
RPS11	5.46E-15	-3.850539101	0.938	1	9.47E-11
RTRAF	5.75E-15	2.84955432	1	1	9.97E-11
LONP1	5.86E-15	-2.147901877	0.938	1	1.02E-10
MKKS	6.19E-15	-1.580224582	1	1	1.07E-10
KLHL7	6.63E-15	2.105913653	1	1	1.15E-10
FAM168B	7.21E-15	1.836792626	1	1	1.25E-10
VPS28	7.67E-15	-2.443606651	0.875	1	1.33E-10
ENSSSCG00000035007	8.18E-15	-3.748106152	1	1	1.42E-10
ACYP2	8.21E-15	2.185044731	1	1	1.42E-10
GSR	8.34E-15	1.886583089	1	1	1.45E-10
LRPAP1	8.61E-15	-3.040641984	0.5	0.933	1.49E-10
PDIA3	8.77E-15	-1.09427569	1	1	1.52E-10
C11orf58	9.04E-15	-0.843417126	1	1	1.57E-10
ENSSSCG00000038027	9.19E-15	-2.603724564	0.562	1	1.59E-10
CMC4	9.23E-15	-2.563429339	0.812	1	1.60E-10
FTSJ1	9.45E-15	-1.256608137	1	1	1.64E-10
MRPS11	9.65E-15	-4.21649182	0.25	1	1.68E-10
ENSSSCG00000012100	9.90E-15	-3.075995384	0.688	1	1.72E-10
POP5	1.00E-14	-1.812888488	1	1	1.74E-10
SNRPC	1.03E-14	2.25801286	1	1	1.79E-10
RPS12	1.06E-14	-3.920321745	0.875	1	1.84E-10
GPD1	1.08E-14	-2.863351153	0.812	1	1.87E-10
LDHC	1.10E-14	-1.241591694	1	1	1.91E-10
SPATA16	1.12E-14	-4.606178987	0.25	1	1.95E-10
ENSSSCG00000047189	1.15E-14	-2.878984599	0.688	1	2.00E-10
TMEM165	1.16E-14	-0.878370556	0.938	1	2.01E-10
PRIM2	1.23E-14	-0.860505178	1	1	2.13E-10
TMCC1	1.23E-14	1.811684666	1	1	2.14E-10
VPS4A	1.28E-14	-1.604720163	1	1	2.21E-10
MRPL46	1.45E-14	-1.621604953	1	1	2.52E-10
RNF170	1.45E-14	-1.429987841	1	1	2.52E-10
ZBTB48	1.48E-14	-3.699133521	0.375	0.933	2.56E-10
DRG1	1.59E-14	-2.094757257	1	1	2.76E-10
COQ3	1.61E-14	-1.980256065	0.875	1	2.79E-10
COX17	1.63E-14	-2.935568127	0.688	1	2.83E-10
ENSSSCG00000027573	1.70E-14	-2.993113614	0.875	1	2.95E-10
ESD	1.79E-14	-1.565990978	1	1	3.10E-10
MT3	1.82E-14	-4.362570079	0.125	0.933	3.15E-10
NDUFB2	1.83E-14	-2.607348581	1	1	3.18E-10
TIMM10	1.85E-14	-4.561878888	0.188	0.933	3.22E-10
CTDNEP1	1.97E-14	-2.315501826	0.875	1	3.42E-10
ENSSSCG00000060974	2.01E-14	-1.526354522	1	1	3.49E-10

MAMDC4	2.07E-14	-5	0.062	1	3.59E-10
SRM	2.14E-14	-3.341036918	0.625	0.933	3.72E-10
DNAJC11	2.15E-14	-1.827819025	0.938	1	3.74E-10
NLRP9	2.19E-14	-1.389012091	1	1	3.80E-10
ELP2	2.28E-14	-0.872110218	1	1	3.96E-10
ENSSSCG00000016990	2.34E-14	-1.64548461	1	1	4.07E-10
ENSSSCG00000052208	2.45E-14	-2.441631709	0.562	1	4.24E-10
C8orf76	2.88E-14	-1.710986528	1	1	5.00E-10
ENSSSCG00000032082	3.07E-14	-1.781165398	0.875	1	5.33E-10
CHMP4A	3.23E-14	-1.978938384	0.812	1	5.61E-10
IQCG	3.37E-14	-1.484578433	1	1	5.85E-10
ENSSSCG00000059201	3.37E-14	-1.801454321	1	1	5.85E-10
LRSAM1	3.48E-14	-2.581395886	0.812	1	6.04E-10
PSMB4	3.49E-14	-1.913559984	0.938	1	6.05E-10
NUDT16L1	3.53E-14	-4.113008962	0.375	0.867	6.12E-10
HSD17B10	3.53E-14	-2.988772745	0.812	0.933	6.13E-10
USE1	3.82E-14	-4.394278939	0.375	0.933	6.64E-10
WDFY2	3.86E-14	-1.424998609	1	1	6.69E-10
LGALS8	3.92E-14	-1.276937169	0.938	1	6.80E-10
TNNC2	4.13E-14	-2.935869663	0.625	0.933	7.16E-10
EZR	4.31E-14	-0.497619192	1	1	7.48E-10
ENSSSCG00000059584	4.39E-14	-4.546567122	0.375	0.933	7.62E-10
NOTO	4.49E-14	-1.614346308	0.938	1	7.79E-10
GOLT1B	4.50E-14	2.570474352	1	1	7.80E-10
UPB1	4.50E-14	-2.087118967	0.938	1	7.81E-10
GON7	4.89E-14	-1.900464326	1	1	8.48E-10
PARD6B	4.99E-14	1.85297505	1	1	8.66E-10
MRTO4	5.24E-14	-2.294743266	0.812	1	9.10E-10
APIP	5.29E-14	2.391364415	1	1	9.17E-10
PEX19	5.35E-14	-2.717018185	0.875	0.933	9.29E-10
PSMC4	5.38E-14	-1.646923207	1	1	9.34E-10
C1orf54	5.53E-14	-2.584962501	0.812	1	9.60E-10
ATRAID	5.55E-14	-2.14286244	0.875	1	9.63E-10
STX7	5.74E-14	1.989783604	1	1	9.97E-10
TOMM22	5.90E-14	-1.365635259	1	1	1.02E-09
ENSSSCG00000005006	6.08E-14	-2.117183539	1	1	1.06E-09
DHDDS	6.14E-14	-1.362156187	0.938	1	1.07E-09
PNP	6.61E-14	2.379167825	1	1	1.15E-09
UBE2J2	6.64E-14	-1.917259569	0.875	1	1.15E-09
TCP11	6.92E-14	-1.526412235	1	1	1.20E-09
CNBD2	6.97E-14	-1.957846984	1	1	1.21E-09
PIP4P1	7.40E-14	-2.144421495	0.938	1	1.28E-09
ALAD	7.55E-14	-2.805827452	0.688	1	1.31E-09
AZIN2	7.60E-14	2.618820598	1	1	1.32E-09
PMF1	7.90E-14	-2.264589872	0.938	1	1.37E-09
FOXJ3	8.06E-14	-0.829294953	1	1	1.40E-09
DUSP16	8.09E-14	-0.601232924	1	1	1.40E-09
TRIP4	8.22E-14	-1.103173088	1	1	1.43E-09
MMAA	8.46E-14	-0.828089183	1	1	1.47E-09
RPL26	8.55E-14	-4.500073603	0.312	1	1.48E-09
ECHS1	8.66E-14	-2.760964913	0.75	0.933	1.50E-09
RAB4B	8.69E-14	-3.058005603	0.75	1	1.51E-09
NOP14	8.87E-14	-2.080170349	0.812	1	1.54E-09
ISCA1	9.52E-14	-1.523955925	1	1	1.65E-09
ENSSSCG00000061531	1.02E-13	-5.450661409	0.062	1	1.77E-09
P2RX5	1.04E-13	-1.870407093	0.938	1	1.80E-09
EIF1B	1.04E-13	-1.350302932	1	1	1.81E-09
ATPSCKMT	1.06E-13	-1.688294765	1	1	1.84E-09
ENSSSCG00000047605	1.10E-13	-0.871722696	1	1	1.90E-09
SLC47A1	1.11E-13	-2.925999419	0.562	0.933	1.92E-09
UBXN6	1.16E-13	-1.810641025	0.875	0.933	2.01E-09
ENSSSCG00000021161	1.16E-13	-1.588162104	1	1	2.02E-09
NDUFAF1	1.23E-13	-1.306218585	1	1	2.14E-09
AIMP1	1.25E-13	-0.859603526	1	1	2.16E-09
CSTB	1.25E-13	-3.137503524	0.75	0.933	2.17E-09
ABHD17A	1.28E-13	-2.704544116	0.688	0.933	2.22E-09
ENSSSCG00000033814	1.34E-13	2.358864381	1	1	2.33E-09
CDA	1.37E-13	-2.119104613	0.75	1	2.38E-09
MRPL2	1.38E-13	-2.875517969	0.688	0.867	2.40E-09
FHIP1A	1.42E-13	-0.742019857	1	1	2.46E-09

CNN3	1.50E-13	2.168456354	1	1	2.60E-09
ME2	1.50E-13	1.873282203	1	1	2.60E-09
CALM3	1.54E-13	-1.32443495	1	1	2.67E-09
CCDC172	1.55E-13	-2.449594721	0.75	1	2.69E-09
DONSON	1.58E-13	1.848430865	1	1	2.73E-09
TMEM138	1.58E-13	-0.915111102	1	1	2.75E-09
SMIM12	1.61E-13	-2.78181983	0.812	0.933	2.80E-09
SERPINB1	1.67E-13	-2.193303692	0.938	1	2.91E-09
COPA	1.70E-13	1.701163208	1	1	2.95E-09
ENSSSCG00000011294	1.72E-13	-2.51731855	0.75	1	2.98E-09
TRAK2	1.73E-13	-1.96437609	0.938	1	3.00E-09
ENSSSCG00000025940	1.76E-13	1.932878128	1	1	3.05E-09
BCS1L	1.77E-13	-3.635636639	0.562	1	3.07E-09
SCPEP1	1.81E-13	-2.252449655	0.875	1	3.14E-09
EPB41L5	1.83E-13	2.057190776	1	1	3.18E-09
TXNL4A	1.84E-13	-1.207106175	1	1	3.19E-09
GPC3	1.86E-13	-1.87175303	0.75	1	3.23E-09
SNRNP25	1.92E-13	-3.315501826	0.438	0.933	3.33E-09
CEP44	1.98E-13	1.830519326	1	1	3.43E-09
RIMBP2	2.01E-13	2.3474175	1	1	3.49E-09
SDHAF1	2.14E-13	-3.228268988	0.375	1	3.71E-09
SNRPD2	2.19E-13	-2.793549123	0.812	1	3.80E-09
COA3	2.24E-13	-2.493647334	0.625	1	3.89E-09
SAR1A	2.52E-13	-0.785820052	1	1	4.38E-09
STIMATE	2.63E-13	-0.831947413	1	1	4.56E-09
LYRM4	2.63E-13	-2.840343334	0.688	0.933	4.57E-09
LYRM1	2.70E-13	-3.943965965	0.312	0.867	4.68E-09
C12orf40	2.93E-13	2.6555913	1	1	5.09E-09
GSK3B	3.02E-13	-1.035667512	1	1	5.24E-09
KHDRBS1	3.08E-13	2.138454663	1	1	5.34E-09
COG3	3.15E-13	1.938844457	1	1	5.47E-09
EDRF1	3.19E-13	1.765957912	1	1	5.53E-09
MCM5	3.22E-13	-1.503608696	0.938	1	5.59E-09
ENSSSCG00000029003	3.25E-13	-2.769219793	0.812	1	5.64E-09
TBC1D7	3.29E-13	-1.541397278	0.938	1	5.71E-09
FAM110A	3.31E-13	-1.68953102	0.938	1	5.74E-09
IK	3.42E-13	-0.779834621	1	1	5.93E-09
UQCR11	3.54E-13	-4.701918647	0.125	0.8	6.14E-09
PDE6D	3.66E-13	-1.28769646	1	1	6.34E-09
NSMAF	3.70E-13	-1.553301871	1	1	6.42E-09
NECAP1	3.98E-13	-0.986301941	1	1	6.90E-09
PSMD14	4.16E-13	-0.747475954	1	1	7.22E-09
SIRT5	4.23E-13	-3.621488377	0.438	0.933	7.33E-09
ENSSSCG00000004151	4.34E-13	2.261529099	1	1	7.53E-09
CCNA2	4.53E-13	1.946110343	1	1	7.86E-09
DDX49	4.59E-13	-1.860089979	1	1	7.97E-09
JPT2	4.63E-13	-2.613773412	0.75	0.933	8.03E-09
HACE1	4.63E-13	2.273501405	1	1	8.03E-09
CTSF	4.80E-13	-3.335184192	0.375	0.933	8.33E-09
C5orf34	4.84E-13	2.024233184	1	1	8.40E-09
SGSM2	5.05E-13	-1.707100954	0.938	1	8.76E-09
DYNLL2	5.21E-13	-1.215750078	1	1	9.05E-09
ACTR1B	5.30E-13	-3.067114196	0.562	1	9.19E-09
DMWD	5.31E-13	-2.583075391	0.75	1	9.21E-09
GFUS	5.56E-13	-2.352976531	0.812	1	9.64E-09
KANK1	5.60E-13	1.714174324	1	1	9.72E-09
ENSSSCG00000039815	5.75E-13	-2.239950793	0.688	1	9.97E-09
FANK1	5.86E-13	-1.436272141	1	1	1.02E-08
EIF2AK4	5.93E-13	1.685963583	1	1	1.03E-08
FAM104A	5.96E-13	-1.116087968	1	1	1.03E-08
PPIF	6.06E-13	-1.094876331	1	1	1.05E-08
SLC8B1	6.32E-13	-1.538169387	1	1	1.10E-08
ALDH18A1	6.44E-13	2.657370996	1	1	1.12E-08
SECISBP2	6.79E-13	1.947391555	1	1	1.18E-08
CLDN1	6.82E-13	2.013764314	1	1	1.18E-08
RAD1	7.00E-13	2.588906089	1	1	1.21E-08
TMEM131L	7.02E-13	1.628625223	1	1	1.22E-08
PBDC1	7.03E-13	-1.191673239	1	1	1.22E-08
RPS26	7.27E-13	-4.990810829	0.375	1	1.26E-08
ENSSSCG00000039972	7.28E-13	-1.341929951	0.938	1	1.26E-08

SLC25A14	7.34E-13	-2.697971463	0.75	0.933	1.27E-08
ATP6AP1	7.37E-13	-2.016873819	1	0.933	1.28E-08
TK2	7.48E-13	-2.156351838	1	1	1.30E-08
VPS37A	7.53E-13	2.011686786	1	1	1.31E-08
EXOSC5	7.59E-13	-1.427375834	1	1	1.32E-08
PSMC3	7.80E-13	-1.071840788	1	1	1.35E-08
NUDCD3	7.80E-13	-2.333669948	0.875	1	1.35E-08
HIGD2A	7.92E-13	-2.398038496	1	1	1.37E-08
MESD	7.99E-13	-2.409684265	0.75	1	1.39E-08
TANGO2	8.01E-13	-2.269432177	0.938	1	1.39E-08
MED10	8.09E-13	-2.337034987	0.75	1	1.40E-08
SCGB3A2	8.09E-13	-4.263034406	0.125	0.933	1.40E-08
ENSSSCG00000008996	8.14E-13	2.081859803	1	1	1.41E-08
FCGR1A	8.37E-13	-0.903610119	1	1	1.45E-08
C17orf80	8.39E-13	-0.775515625	1	1	1.46E-08
MIF	8.42E-13	-3.300704824	0.625	1	1.46E-08
EIF3G	8.46E-13	-3.557481764	0.688	1	1.47E-08
CBX8	8.54E-13	-1.779517169	0.938	1	1.48E-08
KIF4A	8.66E-13	1.840404308	1	1	1.50E-08
CERS4	9.10E-13	-2.911414626	0.938	1	1.58E-08
COPB1	9.11E-13	2.214169759	1	1	1.58E-08
TIMM50	9.32E-13	-2.434146322	0.812	1	1.62E-08
ENSSSCG00000034853	9.52E-13	-3.242856524	0.625	1	1.65E-08
ENSSSCG00000052760	9.88E-13	-1.060102299	1	1	1.71E-08
WDR48	9.90E-13	1.382770612	1	1	1.72E-08
LMO2	1.01E-12	-3.310340121	0.375	1	1.75E-08
TMEM160	1.03E-12	-5.975752454	0	0.933	1.79E-08
C9orf40	1.11E-12	2.077886243	1	1	1.92E-08
TMEM251	1.13E-12	-1.568688446	0.938	1	1.97E-08
YWHAQ	1.15E-12	-0.751953103	1	1	2.00E-08
ENSSSCG00000060365	1.20E-12	-1.334313562	1	1	2.08E-08
NRBP1	1.22E-12	-0.623062958	1	1	2.12E-08
ENSSSCG00000052671	1.23E-12	-3.261271797	0.938	1	2.14E-08
NARF	1.24E-12	-1.584458854	0.938	1	2.15E-08
ZNF212	1.26E-12	-1.852496293	0.75	1	2.19E-08
NKIRAS1	1.27E-12	-0.886596597	1	1	2.20E-08
ELAC2	1.38E-12	-2.918275603	0.625	1	2.40E-08
RDH13	1.42E-12	-2.435215381	0.812	0.933	2.46E-08
NSFL1C	1.43E-12	-2.447337991	0.75	1	2.49E-08
RNFT1	1.45E-12	2.348841623	1	1	2.52E-08
ERCC1	1.47E-12	-3.169925001	0.438	1	2.55E-08
ATPAF2	1.52E-12	-5.30256277	0.062	0.867	2.65E-08
DRC7	1.53E-12	-3.137503524	0.438	1	2.65E-08
HMGB2	1.53E-12	1.647952264	1	1	2.66E-08
COX7C	1.57E-12	-1.795937409	1	1	2.72E-08
SVOPL	1.59E-12	-0.970810921	1	1	2.75E-08
PPM1G	1.62E-12	-1.310667052	0.938	1	2.82E-08
MTUS2	1.64E-12	-1.453211228	0.938	1	2.85E-08
ENSSSCG00000036197	1.65E-12	-1.608466438	1	1	2.86E-08
GPR108	1.68E-12	-2.451301043	0.562	1	2.91E-08
RNF181	1.69E-12	-2.13492958	0.875	1	2.94E-08
SUGT1	1.72E-12	-0.833994037	1	1	2.98E-08
CDC45	1.74E-12	2.605578832	1	1	3.01E-08
SGMS1	1.75E-12	1.862972371	1	1	3.03E-08
DNAJB6	1.76E-12	-0.709277773	1	1	3.06E-08
ENSSSCG00000057138	1.78E-12	-2.292090439	0.75	0.933	3.08E-08
MYCBP	1.80E-12	-1.269030734	1	1	3.13E-08
RPS27A	1.84E-12	-2.369440633	1	1	3.19E-08
ATG4A	1.87E-12	-0.549425205	1	1	3.24E-08
MOCS2	1.90E-12	2.104562594	1	1	3.29E-08
SLC25A19	1.92E-12	-4.263034406	0.312	0.8	3.32E-08
TUBB2A	1.93E-12	-1.636452816	1	1	3.35E-08
FUNDC2	1.96E-12	-2.485426827	0.812	1	3.41E-08
ENSSSCG00000021991	2.03E-12	-4.013674937	0.312	0.867	3.52E-08
CMTM8	2.04E-12	2.305542121	1	1	3.54E-08
GALNT6	2.05E-12	-2.297799824	0.812	1	3.56E-08
TAB2	2.06E-12	1.831676759	1	1	3.57E-08
AGA	2.08E-12	-2.831751576	0.562	1	3.61E-08
CRYZ	2.14E-12	2.094147155	1	1	3.72E-08
ANKRD39	2.18E-12	-1.929610672	0.938	1	3.78E-08

ENSSSCG00000034927	2.21E-12	-1.033109415	1	1	3.84E-08
DCAF11	2.24E-12	-3.194647431	0.75	0.933	3.88E-08
PGLS	2.24E-12	-4.565597176	0.25	0.867	3.89E-08
POC1B	2.28E-12	2.351408963	1	1	3.95E-08
HAUS7	2.38E-12	-2.678071905	0.688	0.933	4.13E-08
ENSSSCG00000003930	2.38E-12	-2.964510915	0.938	1	4.13E-08
LGI1	2.64E-12	-3.987927168	0.25	0.867	4.59E-08
ATG3	2.72E-12	-0.761903496	0.938	1	4.73E-08
CHST7	2.84E-12	-1.636099635	0.688	1	4.92E-08
ENSSSCG00000032216	2.92E-12	-1.047562811	1	1	5.07E-08
ENSSSCG00000059649	2.95E-12	-0.626933056	1	1	5.12E-08
PPCDC	2.97E-12	-1.227212826	1	1	5.15E-08
ENSSSCG00000001075	3.01E-12	2.009949005	1	1	5.22E-08
DUSP5	3.04E-12	-1.17533253	1	1	5.28E-08
ARRDC4	3.11E-12	-2.41130478	1	1	5.40E-08
ENSSSCG00000006996	3.29E-12	-1.08435693	1	1	5.70E-08
ENSSSCG00000005596	3.33E-12	-1.281150321	0.938	1	5.78E-08
ARFGAP2	3.56E-12	-1.205430429	1	1	6.18E-08
PRDX5	3.61E-12	-2.501691882	0.812	1	6.26E-08
SPPL2A	3.61E-12	1.941824386	1	1	6.27E-08
CCDC137	3.66E-12	-3.693022247	0.25	0.933	6.35E-08
SCAND1	3.74E-12	-1.900464326	0.875	0.933	6.50E-08
MOSPD1	3.78E-12	-1.281099122	1	1	6.56E-08
FBXO43	4.20E-12	2.108918286	1	1	7.29E-08
ENSSSCG00000010719	4.26E-12	-1.016488123	1	1	7.39E-08
MPP7	4.53E-12	1.98179201	1	1	7.87E-08
PIM1	4.59E-12	2.316732069	1	1	7.97E-08
MAP3K7	4.76E-12	1.989567618	1	1	8.26E-08
MOK	4.81E-12	-2.093109404	0.75	0.933	8.35E-08
GRPEL2	4.84E-12	1.613787476	1	1	8.41E-08
BPGM	4.91E-12	-2.044453598	0.938	1	8.51E-08
RNF8	5.01E-12	-0.556955188	1	1	8.69E-08
SGCD	5.08E-12	-1.067114196	1	1	8.82E-08
GHITM	5.14E-12	-2.154565608	1	1	8.91E-08
ENSSSCG00000037143	5.17E-12	-0.482950751	1	1	8.97E-08
PSMD7	5.17E-12	-0.682340237	1	1	8.98E-08
ZNF235	5.18E-12	-1.52554995	0.938	1	8.99E-08
MRPS7	5.21E-12	-2.236945177	0.625	1	9.05E-08
SSNA1	5.49E-12	-1.729734025	0.875	1	9.52E-08
TBC1D1	5.58E-12	-1.547466069	1	1	9.68E-08
CRLS1	5.59E-12	2.064678618	1	1	9.70E-08
CAPZB	5.67E-12	-1.650415562	1	1	9.83E-08
SLC25A20	5.68E-12	1.82527683	1	1	9.86E-08
NUDT21	5.69E-12	1.464466188	1	1	9.87E-08
PRDX4	5.76E-12	-2.251741236	1	1	9.99E-08
C19orf67	5.84E-12	-1.564180939	1	1	1.01E-07
GALNT11	5.89E-12	2.112237651	1	1	1.02E-07
TMEM213	5.98E-12	-2.138005942	0.875	1	1.04E-07
ENSSSCG00000063355	6.18E-12	3.181226319	1	0.867	1.07E-07
C19orf81	6.24E-12	-4.230612928	0.188	0.8	1.08E-07
ENSSSCG00000052241	6.25E-12	-1.25918208	0.938	1	1.08E-07
MRPL19	6.28E-12	-1.271647536	0.938	1	1.09E-07
ARR3	6.35E-12	-3.033730857	0.625	0.933	1.10E-07
EIF4G2	6.39E-12	1.935715115	1	1	1.11E-07
PRR5L	6.39E-12	-1.6405972	1	1	1.11E-07
CCDC152	6.47E-12	3.060365076	1	0.867	1.12E-07
OSBPL2	6.47E-12	-1.265062087	1	1	1.12E-07
NRAP	6.58E-12	-2.267883805	0.625	1	1.14E-07
TXNDC12	6.68E-12	-2.313157885	0.688	1	1.16E-07
NUDT14	6.94E-12	-4.552541023	0.188	0.933	1.20E-07
MRPL50	7.19E-12	-0.983069604	1	1	1.25E-07
ENSSSCG00000052096	7.38E-12	-3.915111102	0.312	0.933	1.28E-07
CACYBP	7.73E-12	-1.033166864	1	1	1.34E-07
CCDC124	7.78E-12	-3.074000581	0.625	0.933	1.35E-07
HMGCR	7.90E-12	-1.494816573	1	1	1.37E-07
ENSSSCG00000021591	8.01E-12	-1.43641115	1	1	1.39E-07
OAZ1	8.02E-12	2.063053848	1	1	1.39E-07
TNK1	8.24E-12	-3.793549123	0.188	1	1.43E-07
PLIN5	8.63E-12	-2.247437551	0.875	1	1.50E-07
TPCN1	8.66E-12	-2.557390423	0.625	1	1.50E-07

PFKM	8.73E-12	-2.009586049	0.938	1	1.52E-07
RPS17	8.76E-12	-3.150011796	1	1	1.52E-07
ETFA	8.85E-12	1.951779687	1	1	1.53E-07
IER3IP1	8.87E-12	2.431573399	1	1	1.54E-07
ENSSSCG00000011147	9.26E-12	-2.074493726	0.812	1	1.61E-07
NAPRT	9.33E-12	-4.693022247	0.062	0.867	1.62E-07
NR2C1	9.34E-12	2.051872623	1	1	1.62E-07
KBTBD13	9.60E-12	-0.094786469	1	1	1.67E-07
DR1	9.91E-12	1.810473113	1	1	1.72E-07
MAPKAPK2	1.04E-11	-2.545621609	0.625	1	1.81E-07
NAXD	1.06E-11	-1.635258822	0.875	1	1.84E-07
PDLIM2	1.11E-11	-3.385891154	0.312	0.933	1.92E-07
SLC25A39	1.13E-11	-2.802060622	0.688	0.933	1.97E-07
SSR1	1.14E-11	-0.519348645	1	1	1.99E-07
ENSSSCG00000044108	1.18E-11	-2.026346871	1	1	2.04E-07
LEPROT	1.21E-11	2.055395592	1	1	2.10E-07
HECTD2	1.22E-11	2.316742956	1	1	2.12E-07
ENSSSCG00000056174	1.23E-11	2.124121312	1	1	2.13E-07
TMEM11	1.24E-11	-3.802060622	0.312	0.933	2.15E-07
ERGIC3	1.26E-11	-1.738223471	1	1	2.19E-07
CDH22	1.34E-11	-3.192645078	0.5	0.933	2.32E-07
ENSSSCG00000056323	1.34E-11	-2.156219448	0.812	1	2.33E-07
ADIPOR2	1.36E-11	-0.566082469	1	1	2.35E-07
LSG1	1.40E-11	-1.391549146	1	1	2.43E-07
SRP14	1.41E-11	-0.698551297	1	1	2.44E-07
ATP5ME	1.44E-11	-3.013674937	0.938	1	2.49E-07
PRKCI	1.47E-11	1.805465923	1	1	2.55E-07
CHID1	1.50E-11	-3.989273593	0.375	0.867	2.60E-07
PSMA2	1.51E-11	1.984092766	1	1	2.62E-07
NUCB1	1.51E-11	-2.322355497	0.812	1	2.63E-07
VEZF1	1.57E-11	1.870521458	1	1	2.73E-07
SDHA	1.59E-11	-0.927627927	1	1	2.75E-07
RFC2	1.65E-11	-1.369838549	1	1	2.87E-07
STK16	1.67E-11	-1.743363365	0.875	0.933	2.89E-07
PPFIA1	1.67E-11	1.752353315	1	1	2.90E-07
DMGDH	1.68E-11	-2.818694771	0.875	1	2.91E-07
MLH1	1.70E-11	-1.342920115	1	1	2.95E-07
ENSSSCG00000034524	1.72E-11	-1.224706287	0.938	1	2.98E-07
NAT10	1.74E-11	-0.990372648	1	1	3.02E-07
PRKAR1A	1.78E-11	1.594419608	1	1	3.09E-07
SF3B6	1.81E-11	2.330102026	1	1	3.15E-07
BCAP29	1.91E-11	-0.919510082	1	1	3.32E-07
EIF4A1	1.91E-11	-0.905834422	1	1	3.32E-07
RAF1	1.94E-11	1.845181503	1	1	3.37E-07
C2orf76	2.04E-11	-1.64345815	1	1	3.54E-07
SORD	2.08E-11	-4.450661409	0.188	0.8	3.61E-07
CLN8	2.09E-11	-1.620151929	0.938	1	3.62E-07
ENSSSCG00000056896	2.13E-11	-1.571156701	0.875	1	3.69E-07
NOL4L	2.19E-11	-2.093109404	0.625	0.933	3.80E-07
TMUB1	2.21E-11	-1.926537838	0.938	1	3.83E-07
TRAFD1	2.24E-11	1.86773925	1	1	3.89E-07
ENSSSCG00000056114	2.25E-11	-2.601450624	0.75	0.933	3.90E-07
ENSSSCG00000052306	2.30E-11	-0.894780688	1	1	3.99E-07
XRCC6	2.31E-11	-0.634027572	1	1	4.01E-07
ENSSSCG00000030908	2.39E-11	-1.324735106	1	1	4.15E-07
GREB1L	2.40E-11	-1.004036441	1	1	4.16E-07
SLC38A8	2.46E-11	-2.168599604	0.875	0.933	4.28E-07
DUS3L	2.47E-11	-2.50589093	0.625	0.933	4.29E-07
PRXL2A	2.51E-11	2.245797026	1	1	4.36E-07
ZNF280D	2.57E-11	2.159583057	1	0.933	4.46E-07
PLEKHB1	2.63E-11	-2.039069565	0.875	0.933	4.56E-07
NECAP2	2.67E-11	-0.765136267	1	1	4.64E-07
ENSSSCG00000029160	2.69E-11	-1.438389371	1	1	4.66E-07
ENSSSCG00000045681	2.84E-11	-2.17625064	0.875	1	4.92E-07
TBCE	2.89E-11	-0.879645855	1	1	5.01E-07
GRIN3B	2.90E-11	-2.970426885	0.562	0.933	5.03E-07
SRP19	2.91E-11	-0.663309844	1	1	5.05E-07
SIVA1	2.93E-11	-3.169925001	0.438	1	5.08E-07
MVK	3.02E-11	-2.61667136	0.625	0.933	5.25E-07
NEIL3	3.06E-11	1.847480932	1	1	5.31E-07

GSTZ1	3.10E-11	-3.152003093	0.375	1	5.39E-07
NPAS1	3.20E-11	-3.275312736	0.5	0.933	5.56E-07
KAT5	3.23E-11	-3.166358386	0.5	0.867	5.61E-07
PLIN3	3.27E-11	-2.541569905	0.875	1	5.68E-07
CCER2	3.27E-11	-2.628072519	1	1	5.68E-07
CYC1	3.32E-11	-1.560888366	1	1	5.75E-07
LAPTM4A	3.37E-11	1.806840594	1	1	5.84E-07
MRPL16	3.38E-11	2.258610984	1	1	5.87E-07
FYB2	3.41E-11	1.827074642	1	1	5.93E-07
NDUFAB1	3.42E-11	-3.428093652	0.625	0.933	5.93E-07
ENSSSCG00000009015	3.42E-11	-1.38502493	0.875	0.933	5.94E-07
C5orf49	3.47E-11	-2.405387329	0.938	1	6.02E-07
BLMH	3.47E-11	-0.4012317	1	1	6.02E-07
FOXN2	3.54E-11	1.964627887	1	1	6.14E-07
MTO1	3.63E-11	-1.76087221	0.875	1	6.31E-07
ENSSSCG00000026248	3.72E-11	-3.30256277	0.562	0.8	6.46E-07
TUFM	3.82E-11	-1.767516686	0.938	1	6.63E-07
SPOUT1	3.82E-11	-3.707819249	0.312	0.933	6.63E-07
CTH	3.88E-11	-1.952065523	0.812	0.933	6.73E-07
IGFBP6	3.90E-11	-3.450661409	0.375	0.933	6.77E-07
UROD	3.96E-11	-2.774933444	0.688	1	6.87E-07
LAMTOR1	3.98E-11	-4.263034406	0.125	1	6.91E-07
NXF1	4.07E-11	-0.968382924	1	1	7.06E-07
ENSSSCG00000041052	4.14E-11	-5.647698256	0	0.933	7.19E-07
SLC25A11	4.15E-11	-1.833141302	0.875	1	7.21E-07
CCDC63	4.18E-11	-2.437937901	0.438	1	7.25E-07
ATXN10	4.41E-11	-1.797866418	1	1	7.66E-07
ATP5F1D	4.43E-11	-3.462343214	0.375	0.867	7.69E-07
NDUFA2	4.44E-11	-4.072931522	0.438	1	7.70E-07
B3GNT3	4.47E-11	-1.475966498	0.875	1	7.75E-07
SH3GL2	4.60E-11	-0.648841194	1	1	7.98E-07
SEH1L	4.62E-11	1.726191123	1	1	8.01E-07
SMAD1	4.63E-11	2.335374792	1	1	8.03E-07
TMEM200C	4.64E-11	-2.669458775	0.5	0.867	8.05E-07
ENSSSCG00000030478	4.68E-11	-1.974102224	1	1	8.12E-07
ARMC8	4.72E-11	1.716357809	1	1	8.19E-07
FGFR1OP2	4.84E-11	2.131716682	1	1	8.40E-07
CTBS	5.13E-11	1.870909175	1	1	8.90E-07
RAD51D	5.36E-11	-1.751418055	0.938	1	9.30E-07
PSMC5	5.37E-11	-1.466397921	0.938	1	9.32E-07
AAAS	5.44E-11	-1.57589151	1	1	9.43E-07
CASP6	5.45E-11	1.956892733	1	1	9.45E-07
PBK	5.45E-11	2.029968463	1	1	9.46E-07
OOSP3	5.47E-11	2.24828312	1	1	9.50E-07
AIFM1	5.51E-11	-1.443606651	0.938	1	9.56E-07
LUC7L	5.63E-11	-3.468148836	0.312	0.933	9.76E-07
STAT4	5.83E-11	-0.879899096	1	1	1.01E-06
ERAP2	5.85E-11	-1.788094692	0.938	1	1.02E-06
CEP131	5.88E-11	-4.201633861	0.188	0.933	1.02E-06
MAP2K1	5.89E-11	2.030832606	1	1	1.02E-06
RRM1	5.90E-11	1.850669584	1	1	1.02E-06
LIN37	6.05E-11	-2.584249883	0.75	1	1.05E-06
ENSSSCG00000022738	6.09E-11	-2.173674886	0.812	0.933	1.06E-06
EHD1	6.19E-11	-2.315501826	0.625	1	1.07E-06
TPST1	6.31E-11	2.195676406	1	1	1.10E-06
WNT7A	6.35E-11	-1.715449515	0.875	1	1.10E-06
ENSSSCG00000047619	6.38E-11	-1.271446646	1	1	1.11E-06
FBXO31	6.39E-11	-2.229375094	0.812	0.933	1.11E-06
PSMD6	6.42E-11	-0.666940898	1	1	1.11E-06
PPP1R11	6.46E-11	-1.690044547	1	1	1.12E-06
ENSSSCG00000049161	6.46E-11	-5.222392421	0.062	0.733	1.12E-06
HERPUD2	6.47E-11	1.786094079	1	1	1.12E-06
UNC13B	6.53E-11	1.93477371	1	1	1.13E-06
ENSSSCG00000008016	6.55E-11	-3.081036572	0.438	1	1.14E-06
MLYCD	6.65E-11	-2.72537762	0.438	1	1.15E-06
ENSSSCG00000059061	6.76E-11	-2.689476668	0.562	0.933	1.17E-06
TMEM50A	6.85E-11	-0.669411567	1	1	1.19E-06
DNPH1	6.90E-11	-3.660793914	0.25	0.933	1.20E-06
NID2	6.95E-11	1.627675754	1	1	1.21E-06
ENY2	6.96E-11	1.859062071	1	1	1.21E-06

CIB2	7.21E-11	-3.081036572	0.375	0.933	1.25E-06
KMT2E	7.24E-11	1.646338602	1	1	1.26E-06
RNF2	7.30E-11	1.480553423	1	1	1.27E-06
ZDHHC4	7.52E-11	-1.159488244	1	1	1.31E-06
LSM3	7.53E-11	-1.200750128	1	1	1.31E-06
BIRC5	7.79E-11	-0.996147675	1	1	1.35E-06
SHANK2	8.01E-11	-2.651104858	0.688	0.933	1.39E-06
FAM3A	8.03E-11	-4.485426827	0.125	0.867	1.39E-06
MRPL9	8.20E-11	-1.272507158	1	1	1.42E-06
ENSSSCG00000037399	8.20E-11	-4.468148836	0.188	0.867	1.42E-06
HDAC3	8.35E-11	2.492707015	1	1	1.45E-06
ENSSSCG00000028786	8.48E-11	-1.236067358	1	1	1.47E-06
YBX2	8.59E-11	-2.202231127	0.875	1	1.49E-06
EEF1AKMT1	8.60E-11	-1.888289613	0.812	0.933	1.49E-06
DSN1	8.61E-11	-0.722499371	1	1	1.49E-06
DHPS	8.79E-11	-2.198462405	0.688	0.933	1.52E-06
CIRBP	9.02E-11	-1.533093261	0.938	1	1.56E-06
TBL3	9.08E-11	-3.623624121	0.188	0.933	1.58E-06
ADCK2	9.27E-11	-1.66780357	0.875	0.933	1.61E-06
ARL2	9.27E-11	-3.401862111	0.25	0.933	1.61E-06
NKIRAS2	9.39E-11	-1.607682577	1	1	1.63E-06
PPM1B	9.48E-11	1.844984691	1	1	1.65E-06
CDC42	9.51E-11	-0.672497035	1	1	1.65E-06
CMAS	9.56E-11	1.797178592	1	1	1.66E-06
CNOT8	9.69E-11	1.983875399	1	1	1.68E-06
GPN2	9.73E-11	-2.380391356	0.812	0.933	1.69E-06
SLC25A1	9.77E-11	-4.108059746	0.25	0.867	1.70E-06
ENSSSCG00000053454	9.89E-11	2.494450208	1	1	1.72E-06
ASPA	1.01E-10	-1.835613182	0.812	1	1.76E-06
SLC25A12	1.01E-10	-1.399536406	0.875	1	1.76E-06
PRMT6	1.03E-10	-0.643876985	1	1	1.78E-06
HDGFL2	1.04E-10	-1.786682403	0.938	1	1.81E-06
CLTA	1.10E-10	-0.620554795	1	1	1.91E-06
MORN3	1.14E-10	-5.678071905	0	0.933	1.98E-06
CDKN2AIPNL	1.14E-10	-1.346865997	1	1	1.98E-06
NUP43	1.15E-10	2.099140144	1	1	2.00E-06
DNASE2	1.16E-10	-2.873328197	0.438	0.933	2.01E-06
PGS1	1.21E-10	-1.862728216	1	1	2.10E-06
AKR1A1	1.21E-10	-2.316912775	0.812	0.933	2.10E-06
TXNDC17	1.23E-10	-1.740473974	1	1	2.13E-06
MRPS6	1.25E-10	-1.879402711	0.875	0.933	2.17E-06
KDM8	1.27E-10	-2.58185659	0.688	0.933	2.21E-06
MBD3	1.29E-10	-3.728697978	0.25	0.867	2.24E-06
CCNB2	1.30E-10	-0.152153171	1	1	2.25E-06
NEGR1	1.30E-10	-0.814941695	1	1	2.26E-06
POLB	1.30E-10	2.278539969	1	1	2.26E-06
CENPS	1.31E-10	-1.014383918	1	1	2.28E-06
TM2D3	1.32E-10	-0.7314716	1	1	2.29E-06
ENSSSCG00000001064	1.33E-10	-1.114923766	1	1	2.30E-06
ENSSSCG00000052822	1.37E-10	-1.61667136	1	1	2.38E-06
STX6	1.37E-10	1.558227658	1	1	2.39E-06
PCOLCE2	1.39E-10	1.637581225	1	1	2.41E-06
SPRING1	1.45E-10	-0.763188519	1	1	2.52E-06
MAPK7	1.46E-10	-2.40780593	0.812	1	2.53E-06
UTS2	1.49E-10	-3.804604311	0.188	0.933	2.58E-06
SKA2	1.53E-10	-1.219987467	0.938	1	2.65E-06
UBTD2	1.55E-10	-1.167266339	0.938	1	2.69E-06
ENSSSCG00000033744	1.56E-10	-3.27897595	0.312	0.933	2.70E-06
FAM219B	1.59E-10	-1.847244131	0.938	1	2.75E-06
NDUFB7	1.63E-10	-3.900464326	0.25	0.867	2.82E-06
PIGH	1.63E-10	-1.969726989	0.812	0.933	2.83E-06
ENSSSCG00000061183	1.64E-10	-4.595609745	0.125	0.8	2.85E-06
H1-8	1.67E-10	2.061878384	1	1	2.89E-06
CDK5RAP1	1.71E-10	-1.19005496	0.938	1	2.97E-06
ENSSSCG00000052576	1.74E-10	-3.308122295	0.375	0.867	3.02E-06
ENSSSCG00000032953	1.79E-10	-2.051289229	0.812	1	3.10E-06
HEXIM2	1.79E-10	-1.832518174	0.75	0.933	3.10E-06
ENSSSCG00000039612	1.80E-10	-3.068306013	0.562	1	3.13E-06
TMED9	1.82E-10	-1.949745229	0.812	1	3.15E-06
DNAJB11	1.82E-10	-0.838816224	1	1	3.16E-06

HSF4	1.84E-10	-3.325770161	0.438	0.8	3.20E-06
SPOCK1	1.85E-10	-1.280581387	0.938	1	3.20E-06
MFF	1.85E-10	1.8252513	1	1	3.21E-06
MRPL15	1.87E-10	1.780585735	1	1	3.25E-06
PCGF6	1.90E-10	2.258577835	1	1	3.30E-06
LRCH4	1.93E-10	-2.054635257	0.75	0.933	3.35E-06
TOMM40L	1.94E-10	-2.330148602	0.5	1	3.36E-06
VPS41	1.96E-10	-0.928255392	1	1	3.40E-06
USP33	1.96E-10	1.897881896	1	1	3.40E-06
ENSSSCG00000052254	1.96E-10	-2.197446064	0.5	0.933	3.40E-06
VPS26B	1.96E-10	-1.541498852	1	1	3.41E-06
FCF1	1.97E-10	-1.825629094	1	1	3.42E-06
TXN2	2.06E-10	-1.933168492	0.938	0.933	3.58E-06
WNT2B	2.08E-10	-1.013674937	0.938	1	3.61E-06
PELO	2.08E-10	-0.58236383	1	1	3.61E-06
RAB43	2.12E-10	-1.345027778	0.875	1	3.67E-06
ENSSSCG00000002707	2.13E-10	-0.517339328	1	1	3.69E-06
RIDA	2.14E-10	1.872844123	1	1	3.71E-06
HYI	2.14E-10	-3.595609745	0.25	0.933	3.71E-06
DARS1	2.17E-10	1.98681924	1	1	3.76E-06
TMEM216	2.17E-10	2.540692234	1	0.933	3.76E-06
NACC2	2.22E-10	-3.941106311	0.188	0.8	3.86E-06
TRAPPC5	2.23E-10	-5.707819249	0	0.867	3.87E-06
RPS29	2.25E-10	-2.984960601	0.75	1	3.90E-06
AURKAIP1	2.28E-10	-2.111968432	0.75	1	3.95E-06
SAT1	2.31E-10	2.065794191	1	1	4.00E-06
NCOA3	2.35E-10	1.885400313	1	1	4.08E-06
UXS1	2.38E-10	1.553617912	1	1	4.12E-06
NENF	2.38E-10	-3.180572246	0.188	0.933	4.13E-06
ENSSSCG00000008147	2.39E-10	-2.185758692	0.812	0.933	4.15E-06
ENSSSCG00000029830	2.42E-10	-2.521203056	0.938	1	4.19E-06
BCAR3	2.42E-10	-0.961319532	0.938	1	4.19E-06
SNX25	2.48E-10	1.606274683	1	1	4.30E-06
CENPA	2.48E-10	2.102857731	1	1	4.30E-06
SIAE	2.50E-10	-1.256083616	1	1	4.34E-06
SMS	2.52E-10	-0.544063405	1	1	4.38E-06
ENSSSCG00000043434	2.52E-10	-2.728697978	0.438	1	4.38E-06
MAP4K1	2.53E-10	-2.768674454	0.625	0.933	4.40E-06
NME2	2.55E-10	-2.082248412	1	1	4.42E-06
EMC4	2.55E-10	-1.415037499	1	1	4.43E-06
ENSSSCG00000051057	2.58E-10	-1.368743847	0.938	1	4.47E-06
SCCPDH	2.63E-10	1.722895174	1	1	4.56E-06
POLM	2.69E-10	-2.435501602	0.75	0.867	4.67E-06
DPYS	2.73E-10	1.71334129	1	1	4.74E-06
UQCRC1	2.79E-10	-2.004001931	0.875	1	4.84E-06
ENSSSCG00000053185	2.81E-10	-1.509273569	0.938	1	4.88E-06
NDC1	2.85E-10	1.65327006	1	1	4.94E-06
ENSSSCG00000010531	2.94E-10	-1.639852583	0.875	1	5.10E-06
KIAA1143	2.95E-10	-1.640302862	1	1	5.12E-06
DYNC2I2	2.97E-10	-2.830074999	0.5	0.867	5.16E-06
ENSSSCG00000003612	2.99E-10	-1.142455458	1	1	5.19E-06
NDUFB10	3.01E-10	-2.790546634	0.75	0.933	5.22E-06
SNURF	3.01E-10	-0.971171787	1	1	5.22E-06
NAA10	3.03E-10	-2.415037499	0.75	1	5.25E-06
TIMM23B	3.10E-10	-1.24691474	1	1	5.37E-06
ABCF1	3.10E-10	-0.934593004	1	1	5.37E-06
MAF	3.11E-10	-2.212408333	0.562	1	5.40E-06
SNRPN	3.18E-10	-0.776925292	1	1	5.52E-06
IRAK2	3.24E-10	2.135768962	1	1	5.62E-06
RNF141	3.24E-10	-2.74080766	0.5	0.933	5.63E-06
ENSSSCG00000057224	3.28E-10	-2.32443495	0.75	1	5.69E-06
STK35	3.30E-10	1.655013158	1	1	5.73E-06
TRAF4	3.34E-10	2.377825621	1	1	5.79E-06
RAB5C	3.35E-10	-1.195359463	0.938	1	5.81E-06
NUP133	3.35E-10	1.747885009	1	1	5.82E-06
HSPE1	3.36E-10	-1.085000647	1	1	5.82E-06
DPP7	3.39E-10	-3.446746359	0.312	0.867	5.89E-06
POLE2	3.55E-10	2.208871282	1	1	6.17E-06
IQCC	3.56E-10	-1.942732799	0.938	1	6.18E-06
LRRC49	3.58E-10	2.341827652	1	0.933	6.21E-06

EIF3F	3.66E-10	-3.036525876	0.312	0.933	6.35E-06
ENSSSCG00000056638	3.67E-10	-0.983136562	0.875	1	6.37E-06
MFSD12	3.70E-10	-3.047305715	0.625	0.867	6.42E-06
POLA2	3.78E-10	-1.662805148	0.938	1	6.56E-06
ACTL7B	3.83E-10	-3.121306296	0.375	0.933	6.65E-06
DYNC1I2	3.84E-10	1.357296662	1	1	6.67E-06
GDF3	3.85E-10	-3.793549123	0.5	0.8	6.67E-06
ENSSSCG00000047706	3.87E-10	-2.117771459	0.75	0.933	6.72E-06
SAMD4A	4.16E-10	-5.208586622	0.125	0.933	7.22E-06
SLC35A1	4.17E-10	1.971502958	1	1	7.23E-06
PSMA5	4.23E-10	2.050838491	1	1	7.35E-06
CCDC59	4.27E-10	-1.285458818	1	1	7.41E-06
DERL2	4.30E-10	-0.590104436	1	1	7.46E-06
THOC3	4.30E-10	1.840075642	1	1	7.47E-06
ARHGEF28	4.31E-10	-2.300704824	0.562	1	7.47E-06
SAMM50	4.32E-10	-2.119909464	0.812	1	7.49E-06
ENSSSCG00000038404	4.46E-10	-2.815575429	0.875	1	7.74E-06
NDUFA4	4.53E-10	-2.391682103	1	1	7.86E-06
ENSSSCG00000004623	4.55E-10	2.1452581	1	1	7.89E-06
AMDHD2	4.61E-10	-1.982926487	0.812	1	7.99E-06
GNL3L	4.62E-10	-1.949518551	0.75	1	8.02E-06
ASGR2	4.65E-10	-3.294743266	0.312	0.867	8.07E-06
ENSSSCG00000034188	4.70E-10	-2.736965594	0.438	1	8.15E-06
CFAP44	4.75E-10	-0.327618112	1	1	8.24E-06
RPA3	4.82E-10	2.155821258	1	1	8.37E-06
MORC2	4.99E-10	-0.684243872	1	1	8.66E-06
ZNF584	5.10E-10	-1.524375365	0.875	1	8.85E-06
EIF2A	5.14E-10	1.683206594	1	1	8.91E-06
DNAJA3	5.15E-10	-0.968208933	1	1	8.93E-06
NDUFS2	5.21E-10	-1.334825089	1	1	9.04E-06
ENSSSCG00000026229	5.28E-10	-1.240815562	1	1	9.17E-06
THAP7	5.29E-10	-2.296981738	0.688	1	9.17E-06
ARHGAP27	5.29E-10	-1.143067563	0.938	1	9.19E-06
HSPA5	5.33E-10	-1.064144386	1	1	9.25E-06
ENSSSCG00000055905	5.57E-10	-2.154509949	0.625	1	9.66E-06
UCHL3	5.60E-10	-0.605922119	1	1	9.71E-06
PIGO	5.62E-10	-1.522725369	0.875	0.933	9.75E-06
STEAP1	5.75E-10	3.000194315	1	0.8	9.98E-06
CARD19	5.80E-10	-2.36159824	0.812	1	1.01E-05
ENSSSCG00000061274	5.91E-10	-2.719892081	0.5	0.933	1.02E-05
SLC25A32	6.05E-10	1.924195482	1	1	1.05E-05
PMM1	6.12E-10	-2.900464326	0.312	0.933	1.06E-05
PSTPIP1	6.20E-10	-2.449802917	0.688	0.933	1.08E-05
LIMS2	6.29E-10	-1.565659362	0.938	1	1.09E-05
ENSSSCG00000054196	6.33E-10	-1.31817596	1	1	1.10E-05
ASTL	6.42E-10	-1.568770273	1	1	1.11E-05
GNL2	6.51E-10	-1.132339179	1	1	1.13E-05
GDPGP1	6.54E-10	-2.354069482	0.688	1	1.14E-05
EIF2S2	6.64E-10	-0.466033103	1	1	1.15E-05
CCDC127	6.66E-10	1.975345898	1	1	1.16E-05
SNX3	6.73E-10	-1.052314178	1	1	1.17E-05
PEX3	6.74E-10	2.089246271	1	1	1.17E-05
ENSSSCG00000021236	6.79E-10	-1.427528443	0.938	1	1.18E-05
C15orf40	7.17E-10	-3.074962058	0.375	0.867	1.24E-05
GABARAPL1	7.28E-10	-2.783424905	0.438	0.933	1.26E-05
NUB1	7.32E-10	-0.237291677	1	1	1.27E-05
ALKBH3	7.46E-10	-0.911030203	1	1	1.30E-05
ACAA1	7.54E-10	-2.03284888	0.75	0.933	1.31E-05
MRGBP	7.61E-10	-2.975752454	0.438	0.8	1.32E-05
RNF4	7.76E-10	-1.214258891	0.938	1	1.35E-05
EDF1	7.78E-10	-2.923184403	0.438	1	1.35E-05
RAB6B	7.93E-10	-1.217367507	1	1	1.38E-05
NUBP2	7.95E-10	-3.504042505	0.375	0.867	1.38E-05
LETM2	7.95E-10	-0.952931746	1	1	1.38E-05
CHN2	8.02E-10	-0.336995358	1	1	1.39E-05
SNF8	8.05E-10	-2.322843801	0.812	1	1.40E-05
SPG7	8.35E-10	-4.707819249	0.062	0.867	1.45E-05
ZNF207	8.44E-10	1.261019582	1	1	1.46E-05
KLHDC2	8.47E-10	1.706456971	1	1	1.47E-05
PSMC1	8.49E-10	-0.980799188	1	1	1.47E-05

PLPP4	8.60E-10	-2.423258006	0.688	1	1.49E-05
LMBR1L	8.63E-10	-2.168397532	0.812	0.933	1.50E-05
SLC16A5	8.65E-10	-2.145197916	0.625	1	1.50E-05
MGST2	8.77E-10	-1.570099394	1	1	1.52E-05
PPOX	8.81E-10	-2.285754482	0.5	0.933	1.53E-05
BRCC3	8.97E-10	-1.093109404	0.938	1	1.56E-05
ENSSSCG00000035728	9.07E-10	-2.175571565	0.812	1	1.57E-05
MASTL	9.12E-10	1.783366188	1	1	1.58E-05
PPEF2	9.30E-10	-1.678071905	0.938	0.933	1.61E-05
SUGP1	9.41E-10	-3	0.5	0.933	1.63E-05
SLC12A8	9.46E-10	1.938439431	1	1	1.64E-05
CD164	9.63E-10	1.693993495	1	1	1.67E-05
GATD1	9.67E-10	-2.542416806	0.625	0.933	1.68E-05
TOP1MT	1.00E-09	-1.983880335	0.812	0.933	1.74E-05
PSME2	1.00E-09	-2.938599455	0.438	0.933	1.74E-05
TKT	1.01E-09	-1.683332422	1	1	1.75E-05
ATG4B	1.01E-09	-1.418375785	1	1	1.75E-05
ENSSSCG00000044205	1.02E-09	-6.251538767	0.125	0.8	1.77E-05
PAFAH1B3	1.03E-09	-4.975752454	0.062	0.733	1.79E-05
ENSSSCG00000001769	1.04E-09	1.634028348	1	1	1.80E-05
RNF220	1.04E-09	-0.895401246	1	1	1.80E-05
ENSSSCG00000027506	1.05E-09	-0.585589642	1	1	1.81E-05
NAA20	1.05E-09	-1.199202794	1	1	1.82E-05
PDHX	1.05E-09	1.840777924	1	1	1.82E-05
ENSSSCG00000033019	1.06E-09	-3.061400545	0.812	1	1.84E-05
ZNF621	1.06E-09	-2.670538232	0.562	0.867	1.84E-05
MCMBP	1.07E-09	1.667316889	1	1	1.86E-05
ENSSSCG00000013768	1.08E-09	-2.678071905	0.562	0.933	1.87E-05
KCTD20	1.08E-09	-1.126024027	1	1	1.88E-05
ZCCHC17	1.09E-09	-0.981765817	1	1	1.89E-05
GEMIN2	1.09E-09	-2.111394757	0.812	0.933	1.90E-05
AUP1	1.10E-09	-1.801220136	0.938	1	1.90E-05
ZNF606	1.11E-09	1.927610269	1	1	1.93E-05
ENSSSCG00000003903	1.14E-09	-1.828958361	1	1	1.97E-05
PHYHIPL	1.15E-09	2.058262372	1	1	2.00E-05
MYBPC3	1.19E-09	-3.310340121	0.375	0.867	2.06E-05
TTC5	1.19E-09	1.896446704	1	1	2.07E-05
SAL1	1.21E-09	-2.982926487	0.562	1	2.10E-05
NUDT9	1.22E-09	-0.657124967	1	1	2.12E-05
EMC2	1.23E-09	2.026801923	1	1	2.14E-05
ALG8	1.27E-09	-1.195093437	1	1	2.20E-05
RPGRIP1	1.27E-09	-0.743969791	1	1	2.21E-05
UBA52	1.28E-09	-2.72416832	0.875	1	2.21E-05
TBCA	1.28E-09	-1.640750279	1	1	2.22E-05
MYL6B	1.31E-09	-1.331681753	0.938	1	2.27E-05
ATP5PB	1.32E-09	-0.475626662	1	1	2.28E-05
MAD2L1BP	1.32E-09	-1.250961574	0.938	1	2.30E-05
EIF3B	1.33E-09	-1.90902634	0.938	0.933	2.30E-05
CIDEA	1.34E-09	-2.345113482	0.938	1	2.33E-05
ENSSSCG00000012452	1.37E-09	2.175952849	1	1	2.39E-05
OOSP2	1.39E-09	1.988350296	1	1	2.40E-05
ENSSSCG00000050347	1.39E-09	-2.717600269	0.375	0.933	2.40E-05
PSTK	1.40E-09	-1.234328822	1	1	2.43E-05
RPS16	1.41E-09	-2.962322797	0.938	1	2.45E-05
LNX1	1.45E-09	1.930084168	1	1	2.52E-05
GLT8D1	1.46E-09	-1.879705766	0.875	0.933	2.53E-05
TUBA4A	1.46E-09	-1.273468407	0.938	1	2.54E-05
DDRGK1	1.53E-09	-2.810966176	0.438	0.933	2.66E-05
FNTB	1.54E-09	-0.854471653	1	1	2.67E-05
ENSSSCG00000058768	1.61E-09	-4.093109404	0.188	0.733	2.79E-05
BUD13	1.63E-09	2.217581719	1	1	2.82E-05
ANKLE2	1.65E-09	1.600442284	1	1	2.87E-05
MPV17	1.65E-09	-2.20461772	0.938	0.933	2.87E-05
RTCB	1.66E-09	-1.233444519	1	1	2.89E-05
UPP1	1.69E-09	-4.180572246	0.188	0.667	2.93E-05
ICA1	1.74E-09	1.782374089	1	1	3.02E-05
TDP2	1.75E-09	1.688618558	1	1	3.03E-05
ENSSSCG00000038630	1.75E-09	1.715600797	1	1	3.04E-05
XRCC2	1.76E-09	1.800268176	1	1	3.05E-05
GLP2R	1.79E-09	-0.711334359	1	1	3.11E-05

ENSSSCG00000061349	1.85E-09	-2.291048782	0.688	0.867	3.22E-05
CFDP1	1.86E-09	-1.167877173	1	1	3.22E-05
MTM1	1.91E-09	-0.685293132	1	1	3.32E-05
COQ10B	1.93E-09	1.786147538	1	1	3.35E-05
ENSSSCG00000039222	1.99E-09	-0.792683211	1	1	3.45E-05
KCNN2	2.01E-09	-1.546700144	0.938	1	3.50E-05
LZIC	2.08E-09	-0.198772777	1	1	3.61E-05
EAF1	2.08E-09	-0.993573731	0.938	1	3.62E-05
OXLD1	2.09E-09	-3.847996907	0.125	0.867	3.63E-05
GSE1	2.11E-09	-1.774933444	0.875	1	3.66E-05
CD274	2.16E-09	-1.567949091	1	1	3.74E-05
ENSSSCG00000062187	2.20E-09	-4.595609745	0.125	0.8	3.83E-05
SMYD3	2.27E-09	-2.515066318	0.812	1	3.95E-05
TMEM205	2.31E-09	-2.783424905	0.688	0.867	4.00E-05
ENSSSCG00000007203	2.32E-09	1.54863596	1	1	4.03E-05
KLHDC4	2.33E-09	-2.336563441	0.875	0.867	4.05E-05
NLRP4	2.34E-09	1.649275169	1	1	4.05E-05
MCAT	2.40E-09	-2.815575429	0.625	0.867	4.17E-05
SAP30BP	2.43E-09	-1.524955191	0.938	1	4.22E-05
NDUFB4	2.43E-09	-2.441837559	0.812	1	4.22E-05
TNIP1	2.47E-09	-3.334117504	0.25	0.867	4.28E-05
BRF2	2.48E-09	-1.399471242	0.938	1	4.31E-05
TUT1	2.54E-09	-3.793549123	0.188	0.867	4.42E-05
MIER1	2.57E-09	2.027383612	1	1	4.45E-05
CCDC28B	2.65E-09	-3.629162305	0.25	0.867	4.60E-05
SEPTIN2	2.69E-09	2.029509882	1	1	4.66E-05
SLC24A3	2.72E-09	-1.697808123	0.875	1	4.72E-05
PSMG3	2.83E-09	-1.220390068	1	1	4.91E-05
GRIN3A	2.86E-09	-1.103717504	0.938	1	4.96E-05
GSPT1	2.87E-09	-0.303046827	1	1	4.98E-05
RNASEH2A	2.89E-09	-1.078210528	0.938	1	5.02E-05
SMIM11	2.92E-09	2.316614295	1	0.933	5.06E-05
NAALAD2	2.92E-09	-0.530542495	1	1	5.06E-05
TCTN2	2.92E-09	1.790823198	1	1	5.06E-05
RFK	2.93E-09	2.394409312	1	1	5.08E-05
HSCB	2.93E-09	2.185191758	1	1	5.09E-05
NMD3	2.95E-09	1.935699219	1	1	5.11E-05
RPP25L	3.03E-09	1.970249993	1	1	5.26E-05
SLC16A13	3.09E-09	-1.236528652	1	1	5.36E-05
NOP9	3.11E-09	-1.110964769	1	1	5.40E-05
CHST3	3.16E-09	-1.051993551	0.938	1	5.49E-05
MLST8	3.18E-09	-2.285754482	0.75	0.933	5.52E-05
ENSSSCG00000060912	3.23E-09	-2.604071324	0.438	0.933	5.61E-05
GTSF1	3.24E-09	-0.613139669	1	1	5.62E-05
MTMR2	3.27E-09	-0.521088936	1	1	5.68E-05
MTA3	3.30E-09	1.706193245	1	1	5.72E-05
LIG1	3.33E-09	-1.163498732	0.938	1	5.78E-05
CARHSP1	3.38E-09	-1.925381959	0.75	0.933	5.86E-05
FBXO25	3.38E-09	1.451249671	1	1	5.87E-05
KAT2A	3.45E-09	2.307467282	1	1	5.98E-05
NPM1	3.50E-09	1.827574231	1	1	6.06E-05
CDC20	3.53E-09	2.325557038	1	1	6.12E-05
GPR160	3.61E-09	1.929964619	1	1	6.27E-05
METTL3	3.72E-09	2.364976417	1	1	6.45E-05
NUS1	3.78E-09	1.951599199	1	1	6.56E-05
SHFL	3.86E-09	-3.415037499	0.25	0.867	6.70E-05
ENSSSCG00000027374	3.88E-09	-0.675180732	1	1	6.74E-05
ENSSSCG00000062406	3.92E-09	-0.510404482	1	1	6.81E-05
ARL6IP4	3.99E-09	-2.078609835	0.688	0.933	6.93E-05
UBR2	4.04E-09	1.601089374	1	1	7.01E-05
ARV1	4.04E-09	-0.835721562	1	1	7.01E-05
ARHGAP9	4.13E-09	-1.452190498	0.875	0.933	7.16E-05
RND2	4.14E-09	-3.180572246	0.25	0.933	7.18E-05
CDH7	4.27E-09	1.586386027	1	1	7.42E-05
PADI3	4.28E-09	-2.055495113	0.75	1	7.43E-05
SPAG8	4.38E-09	-2.511610759	0.625	0.933	7.59E-05
TEX10	4.46E-09	1.587813114	1	1	7.73E-05
CHCHD3	4.60E-09	-0.507036711	1	1	7.98E-05
ENSSSCG00000039658	4.61E-09	2.01283646	1	1	8.00E-05
CHCHD5	4.67E-09	-1.608530972	0.938	1	8.10E-05

PYCR2	4.71E-09	2.209207973	1	1	8.18E-05
MMAB	4.73E-09	-2.519374159	0.5	0.933	8.20E-05
PAPLN	4.84E-09	-2.393504338	0.562	1	8.39E-05
FBXO7	4.86E-09	-0.98579638	1	1	8.44E-05
CCNI	4.98E-09	-0.404282534	1	1	8.63E-05
PDIA2	5.09E-09	-3.499101764	0.25	0.867	8.84E-05
ENSSSCG00000031636	5.20E-09	-0.248545069	1	1	9.02E-05
PSMA7	5.29E-09	-1.231888803	1	1	9.18E-05
ENSSSCG00000053801	5.31E-09	-3.606178987	0.312	0.8	9.22E-05
FAM50A	5.35E-09	-1.99492901	0.812	1	9.28E-05
PADI6	5.51E-09	-1.104206143	1	1	9.55E-05
MRPL3	5.55E-09	-0.316356756	1	1	9.63E-05
MAF1	5.56E-09	-1.547675268	0.812	1	9.64E-05
CDKN3	5.57E-09	-1.27104622	1	1	9.66E-05
SUPT3H	5.58E-09	-0.718579629	1	1	9.68E-05
GPANK1	5.63E-09	-1.276555545	1	1	9.76E-05
PALLD	5.70E-09	-1.798584712	1	1	9.88E-05
ENSSSCG00000060926	5.81E-09	-1.033576081	0.875	1	0.000100881
CCNL2	5.82E-09	-1.747334568	0.875	1	0.000100907
TBCC	5.82E-09	-1.181532655	1	1	0.000100917
SLC23A2	5.82E-09	1.516889647	1	1	0.000100922
ENSSSCG00000029257	5.98E-09	-1.35614381	0.875	1	0.000103762
NDFIP2	6.08E-09	2.000421286	1	1	0.000105492
TUBB	6.16E-09	-1.160028996	1	1	0.000106846
COL4A6	6.25E-09	-1.327019669	1	1	0.000108376
STOML2	6.26E-09	2.118982423	1	1	0.000108682
MCEE	6.27E-09	-1.614346308	0.938	1	0.000108723
MRM3	6.39E-09	-1.276081476	1	1	0.00011083
THBS3	6.39E-09	-2.212848649	0.625	1	0.000110875
ENSSSCG00000013613	6.40E-09	-2.188266637	0.688	1	0.000111102
PRPF6	6.41E-09	-1.555900035	0.875	1	0.00011122
ABI1	6.42E-09	1.29785963	1	1	0.000111428
PIGC	6.51E-09	-0.846536245	1	1	0.000112946
RPL23	6.56E-09	-3.379266368	0.812	1	0.000113782
SPIRE1	6.69E-09	1.840365135	1	1	0.00011607
PADI4	6.70E-09	-1.511822562	0.938	1	0.000116325
ZP2	6.75E-09	-0.806185294	1	1	0.000117114
RBP2	6.76E-09	-2.639597757	0.562	0.867	0.000117304
CFAP276	6.76E-09	-3.143735477	0.25	0.933	0.000117337
UBC	6.82E-09	2.248487915	1	1	0.000118316
SIAH3	6.84E-09	-2.516320835	0.688	0.933	0.000118658
EIF4EBP1	6.88E-09	-2.443606651	0.562	1	0.000119369
CHMP1A	7.01E-09	-3.629162305	0.125	0.867	0.000121637
RND1	7.11E-09	-1.047828525	1	1	0.000123304
ZFAND3	7.12E-09	-0.816485934	1	1	0.00012346
ENSSSCG00000031299	7.12E-09	-1.705552642	0.875	1	0.000123493
PENK	7.19E-09	-7.415037499	0	0.8	0.000124726
ENSSSCG00000061194	7.30E-09	-1.200024608	0.938	1	0.000126578
MST1	7.39E-09	-2.847996907	0.812	0.867	0.000128151
U2AF1L4	7.50E-09	-3.113008962	0.375	0.933	0.000130079
DTX2	7.69E-09	-0.756455166	1	1	0.000133488
KLK15	7.81E-09	-3.475579041	0.125	0.867	0.000135582
MVD	7.93E-09	-5.415037499	0	0.8	0.00013762
BBS5	7.96E-09	2.191781311	1	1	0.000138141
RNF115	8.03E-09	1.307835866	1	1	0.000139284
TTC7A	8.26E-09	-1.664461474	0.812	1	0.000143268
MCOLN1	8.27E-09	2.305169577	1	0.933	0.000143576
CAAP1	8.28E-09	1.905272317	1	1	0.000143675
UPK1A	8.31E-09	-2.344070978	0.625	0.867	0.000144219
GGA1	8.32E-09	-1.881605299	0.688	0.933	0.000144286
TMEM156	8.33E-09	-3.761487913	0.25	0.733	0.000144606
PCCB	8.40E-09	-0.586723668	1	1	0.000145774
SMYD2	8.45E-09	-0.953810367	0.938	1	0.000146609
ENSSSCG00000012235	8.55E-09	2.069324598	1	1	0.00014832
FLAD1	8.56E-09	-2.709780765	0.438	0.933	0.000148536
NHEJ1	8.78E-09	-1.487793842	0.875	1	0.000152356
CCDC179	8.85E-09	2.688785533	1	0.867	0.000153589
PSMG1	8.86E-09	1.422742753	1	1	0.000153662
SKA1	8.98E-09	-1.11137418	1	1	0.000155895
CHEK1	9.02E-09	1.717060111	1	1	0.000156589

ERH	9.16E-09	-0.923184403	1	1	0.000158857
ATRIP	9.18E-09	-1.62203787	0.875	1	0.000159234
MLLT11	9.36E-09	1.841624824	1	1	0.00016234
PCYOX1	9.55E-09	-0.992832176	1	1	0.000165667
REX1BD	9.58E-09	-2.719892081	0.438	1	0.000166203
PITRM1	9.67E-09	2.466180149	1	1	0.000167698
NPEPL1	9.80E-09	-3.821029859	0.125	0.8	0.000170093
GLMP	9.88E-09	-1.753259402	0.938	0.933	0.000171479
GAPDH	9.91E-09	-2.591295462	0.938	1	0.000172017
TRAP1	1.01E-08	-0.82930944	0.938	1	0.000174444
ITPRID2	1.02E-08	1.555273235	1	1	0.000176242
AHCTF1	1.02E-08	1.805775963	1	1	0.000176586
LANCL2	1.03E-08	-1.328557979	1	1	0.000177994
PRMT2	1.04E-08	2.121224571	1	1	0.000180156
CORO6	1.04E-08	-2.678071905	0.438	1	0.000180574
PPHLN1	1.04E-08	1.664068197	1	1	0.000180661
MEST	1.04E-08	1.874618149	1	1	0.000181144
ADRM1	1.05E-08	-1.744035167	0.812	0.933	0.000181866
MROH7	1.05E-08	-1.276114233	0.875	1	0.000182888
SFN	1.08E-08	-1.981464049	0.75	1	0.000186784
TIMD4	1.10E-08	-1.351177557	0.938	1	0.00019051
STMN1	1.12E-08	-1.81232633	1	1	0.000194453
CEP57L1	1.13E-08	2.106650827	1	1	0.000196073
REV1	1.13E-08	1.482413868	1	1	0.00019628
RPL15	1.13E-08	-0.465519923	1	1	0.000196294
ENSSSCG00000033189	1.15E-08	-1.891475543	0.875	1	0.000199362
KNG1	1.17E-08	-0.581762098	1	1	0.000202447
PRPF19	1.18E-08	-1.200024608	0.938	0.933	0.000204877
KCNAB3	1.19E-08	-1.467304809	1	1	0.000205751
ANTKMT	1.19E-08	-3.180572246	0.188	0.933	0.000205969
UBXN1	1.19E-08	-3.093109404	0.312	0.733	0.000205971
UNC50	1.20E-08	-0.594022028	1	1	0.00020741
EFCAB7	1.20E-08	-0.841346111	1	1	0.000207425
MITD1	1.20E-08	2.631992368	1	0.933	0.000207638
MFSD13A	1.21E-08	-2.432959407	0.625	0.933	0.000210064
ENSSSCG00000053975	1.22E-08	-3.208586622	0.25	0.933	0.000211856
ENSSSCG00000059210	1.23E-08	-2.341036918	0.5	0.933	0.000213461
ENSSSCG00000052563	1.23E-08	-3.159198595	0.25	0.867	0.000213917
OGFOD1	1.25E-08	-0.512664405	1	1	0.000217009
RDX	1.26E-08	1.554478431	1	1	0.000219143
USP27X	1.28E-08	-0.917382117	1	1	0.000221728
ZNHIT3	1.28E-08	-0.639471852	1	1	0.000222035
EPB41L2	1.29E-08	1.40721507	1	1	0.000223191
PPP1CC	1.29E-08	1.806780231	1	1	0.000223787
MBOAT1	1.29E-08	-0.368743847	1	1	0.00022394
USP28	1.30E-08	1.623563571	1	1	0.000225147
ZNF567	1.30E-08	2.117845904	1	1	0.00022568
ARL6IP6	1.30E-08	1.881799615	1	1	0.000225803
SUPV3L1	1.30E-08	2.075816056	1	1	0.000226395
NOLC1	1.31E-08	-0.494649119	1	1	0.000227016
MMD2	1.31E-08	-2.03866162	0.562	0.933	0.00022718
SMARCE1	1.34E-08	-0.544858682	1	1	0.000232963
SAP30	1.38E-08	1.879861364	1	1	0.000238832
ENSSSCG00000016475	1.38E-08	-1.335470242	1	0.933	0.000239913
WDR89	1.41E-08	1.980186105	1	1	0.000244806
PCBP1	1.42E-08	1.818636586	1	1	0.000245923
FKBP9	1.43E-08	-0.634882948	1	1	0.000247606
ENSSSCG00000042361	1.43E-08	-1.947258538	0.75	0.933	0.000247769
ZNF410	1.43E-08	-1.439434154	0.875	1	0.00024809
TARBP2	1.43E-08	-2.823502345	0.5	0.8	0.000248448
MFSD2A	1.44E-08	-1.924354947	0.812	1	0.000249106
ADPGK	1.45E-08	1.785155183	1	1	0.000252168
CLPTM1L	1.49E-08	2.196498097	1	1	0.0002587
ENSSSCG00000062012	1.50E-08	-2.719892081	0.562	0.8	0.000259453
PPP1R7	1.51E-08	-1.495741883	0.812	1	0.000261312
OMA1	1.52E-08	1.859597987	1	1	0.000263857
GLIPR2	1.52E-08	-1.189111224	1	1	0.000264251
TADA1	1.54E-08	1.708423358	1	1	0.000267767
WBP2	1.56E-08	-1.462343214	1	1	0.000270333
NCLN	1.57E-08	-2.012832815	0.875	0.933	0.000271961

BLM	1.58E-08	1.952296575	1	1	0.000274322
POLG2	1.60E-08	2.043271108	1	1	0.000276763
ECSIT	1.62E-08	-1.411380476	0.938	1	0.00028069
ENSSSCG00000036988	1.62E-08	-1.252307999	0.875	1	0.000280732
CTSD	1.63E-08	-2.910732662	0.375	0.933	0.000282431
DDX23	1.65E-08	-1.222877279	0.938	1	0.0002869
ENSSSCG00000043578	1.66E-08	-4.208586622	0.25	0.8	0.000287211
MPHOSPH8	1.66E-08	-0.403505662	1	1	0.000288422
JOSD1	1.67E-08	-0.864400137	0.938	1	0.00028996
PPIE	1.67E-08	-0.593538395	1	1	0.000290113
TMEM41A	1.68E-08	-0.283197445	1	1	0.000291124
RAB2B	1.69E-08	-0.368091664	1	1	0.00029342
ENSSSCG00000047379	1.70E-08	-2.171111916	0.625	1	0.000294962
CRYM	1.72E-08	-1.002200908	1	1	0.000298507
AKTIP	1.73E-08	2.009272088	1	1	0.000299952
ENSSSCG00000051352	1.78E-08	-2.118644496	0.75	0.867	0.000308745
CYFIP1	1.78E-08	1.938169762	1	1	0.000309135
CAPNS1	1.79E-08	-1.734974296	0.688	0.933	0.000311229
EIF1	1.84E-08	2.397698817	1	1	0.000319391
NME7	1.85E-08	1.914351909	1	1	0.00032113
CBFB	1.85E-08	1.910510911	1	1	0.000321405
ENSSSCG00000060529	1.86E-08	1.882935863	1	1	0.000322913
CHMP6	1.89E-08	-4.180572246	0.125	0.667	0.00032737
EIF2B2	1.89E-08	-2.561878888	0.625	0.933	0.000328771
CAPN8	1.91E-08	-1.915111102	0.625	1	0.000331314
HJURP	1.92E-08	2.624119972	1	0.933	0.000333931
ATL1	1.96E-08	1.876654589	1	1	0.000340165
LRRC61	1.96E-08	-2.877380713	0.312	0.933	0.000340228
PSEN1	1.96E-08	-0.417905003	1	1	0.000340793
ATP6V0A1	1.99E-08	-0.364227248	1	1	0.000345582
KCNMB4	2.00E-08	-0.836991861	1	1	0.000347219
ENSSSCG00000008335	2.02E-08	1.8217351	1	1	0.000349837
ENSSSCG00000033421	2.05E-08	-1.522974321	0.875	0.933	0.000355958
ENSSSCG00000059232	2.06E-08	-2.001961516	0.812	1	0.000357801
MRPS18C	2.07E-08	-2.013256097	0.875	1	0.000359545
MRPL35	2.07E-08	-0.786341117	0.938	1	0.00035971
CENPN	2.10E-08	2.279793831	1	1	0.000364593
COPS4	2.13E-08	-0.408894306	1	1	0.000368808
BRD9	2.15E-08	-0.993810064	1	1	0.000372688
PLD6	2.15E-08	-3.396890153	0.25	0.867	0.00037365
THEM5	2.18E-08	-2.093109404	0.562	1	0.000377851
GNB4	2.19E-08	1.716366119	1	1	0.000379406
SLC4A4	2.20E-08	1.822708174	1	1	0.000381831
MKRN1	2.22E-08	-0.754669598	1	1	0.000385886
EIF2S3B	2.25E-08	-0.477925205	1	1	0.000390186
TCEANC	2.27E-08	-3.351843673	0.25	0.733	0.000393546
TTC1	2.31E-08	-0.822171113	1	1	0.000400683
ENSSSCG00000051162	2.31E-08	-1.04800915	0.938	1	0.000401598
ENSSSCG00000015390	2.32E-08	1.209560482	1	1	0.000401959
UBAC2	2.32E-08	1.563842443	1	1	0.000403121
TXNRD3	2.35E-08	-2.183307213	0.688	0.933	0.000408179
CCDC3	2.37E-08	-1.632930774	1	1	0.000411688
PTP4A2	2.38E-08	-0.458319747	1	1	0.00041285
ENSSSCG00000043116	2.38E-08	-0.675166174	1	1	0.000413171
DOC2B	2.39E-08	-0.915785126	1	1	0.00041399
SSX2IP	2.40E-08	1.686716974	1	1	0.000416763
THOP1	2.45E-08	-3.508146904	0.312	0.867	0.000425137
UBE2D1	2.46E-08	2.371476543	1	0.933	0.000427081
ANXA5	2.47E-08	-0.587953107	1	1	0.000428998
CTDSP2	2.52E-08	-1.686789122	0.812	1	0.00043688
HNF1B	2.53E-08	-0.665304609	1	1	0.000438138
OARD1	2.57E-08	-0.935568127	0.875	1	0.000445342
SMG1	2.57E-08	-0.171291378	1	1	0.000446598
HARS1	2.60E-08	-1.375748368	0.812	1	0.000451205
ENSSSCG00000049122	2.65E-08	-4.584962501	0.062	0.8	0.000460665
DERA	2.67E-08	2.11585031	1	0.933	0.000462818
YJU2B	2.69E-08	-1.697489359	0.875	1	0.00046631
RXYLT1	2.73E-08	2.173398388	1	1	0.00047284
RAB5IF	2.74E-08	-0.964254339	0.938	1	0.000475238
ENSSSCG00000048122	2.75E-08	-3.415037499	0.188	0.867	0.00047779

ENSSSCG00000041692	2.83E-08	-2.185031894	0.688	0.867	0.000491629
DPM1	2.85E-08	-0.677186001	1	1	0.000495332
CFL1	2.88E-08	-1.015328283	1	1	0.000500276
APH1A	2.91E-08	1.849088499	1	1	0.000504213
ACOT13	2.98E-08	-3.010647244	0.375	0.867	0.000517156
TAF5	2.99E-08	1.810392008	1	1	0.00051917
MFSD11	2.99E-08	-0.979627156	1	1	0.000519259
KANK3	3.00E-08	-2.805827452	0.438	0.867	0.000519988
CDT1	3.01E-08	-3.306103128	0.25	0.867	0.000522439
SNRNP40	3.01E-08	1.932165403	1	1	0.000522748
FBXL20	3.03E-08	1.420819564	1	1	0.000525735
CALML4	3.07E-08	-2.12963528	0.75	1	0.000533479
OSER1	3.08E-08	-1.024677679	1	1	0.000535115
ENSSSCG00000063358	3.10E-08	-3.35614381	0.125	0.867	0.000537218
DAPK3	3.17E-08	-1.610508621	0.75	0.933	0.000550771
ACBD7	3.19E-08	-0.850084959	0.938	1	0.000552688
QPRT	3.21E-08	-3.756074417	0.062	0.867	0.000557806
ENSSSCG00000031991	3.26E-08	-0.968446831	1	1	0.000565058
C9orf72	3.27E-08	1.766723691	1	1	0.000568012
ENSSSCG00000032073	3.28E-08	-3.874469118	0.125	0.8	0.000568608
PPIL2	3.30E-08	-0.985040403	1	1	0.000573051
FRA10AC1	3.40E-08	-0.763869778	1	1	0.000589952
NPLOC4	3.46E-08	-1.332779817	0.875	1	0.000600957
SELENON	3.47E-08	-1.343246816	1	1	0.000601253
FAM174C	3.47E-08	-2.900464326	0.375	0.867	0.000602641
COPE	3.55E-08	-1.085949613	0.938	1	0.000616151
MRPS18A	3.59E-08	-3.552541023	0.188	0.8	0.000622699
PLEKHO1	3.61E-08	-1.208586622	0.938	1	0.000625613
DNPEP	3.61E-08	-2.164975391	1	1	0.000626059
ENSSSCG00000005649	3.66E-08	-1.709780765	0.875	1	0.000634529
SYCP3	3.67E-08	-1.07189795	1	1	0.00063663
FBXO17	3.70E-08	-1.390495315	0.812	1	0.000641464
MAPK6	3.71E-08	1.776318141	1	1	0.000643971
TMED5	3.73E-08	1.737527032	1	1	0.000647763
KDELR2	3.75E-08	1.62028149	1	1	0.000651357
FUNDC1	3.78E-08	1.710902779	1	1	0.000655535
RPF2	3.78E-08	-1.287506773	1	1	0.000656317
ENSSSCG00000010489	3.78E-08	-1.809316438	0.625	1	0.000656601
ENSSSCG00000027349	3.89E-08	1.870043711	1	1	0.000674888
GAB1	3.90E-08	1.629387342	1	1	0.000677459
ENSSSCG00000062560	3.94E-08	-0.802251316	1	1	0.000684344
COPS7B	3.98E-08	-1.283905584	1	0.933	0.000690438
VWA7	3.99E-08	2.382848703	1	1	0.000692398
IFT88	4.06E-08	-0.746855887	1	1	0.000704714
CBR4	4.07E-08	1.851964385	1	1	0.000705637
ENSSSCG00000057916	4.10E-08	-1.265659875	1	1	0.000711876
SNRPE	4.13E-08	1.809716512	1	1	0.000717241
HSD17B1	4.19E-08	-5.222392421	0	0.8	0.000727491
SPATA24	4.34E-08	-3.394278939	0.188	0.733	0.000753104
ZNF75D	4.37E-08	-0.510918653	1	1	0.000757648
TPGS2	4.40E-08	-0.877849776	0.938	1	0.000763194
VIRMA	4.42E-08	1.533280993	1	1	0.00076639
SLC28A3	4.47E-08	-1.447291698	0.812	1	0.000775123
SOS2	4.48E-08	1.777892239	1	1	0.000777224
DRAP1	4.48E-08	-1.475883054	0.938	1	0.000777337
RAP2C	4.49E-08	1.888796802	1	1	0.000779408
PPP6C	4.50E-08	1.683483653	1	1	0.000781549
ENSSSCG00000054294	4.50E-08	-1.485426827	0.625	1	0.000781598
ZC3H13	4.60E-08	-0.297718674	1	1	0.000797904
ENSSSCG00000062784	4.67E-08	-2.277533976	0.562	1	0.000810684
DDX28	4.69E-08	-1.155231116	0.938	1	0.000813766
CAPN7	4.72E-08	1.511395335	1	1	0.000819713
SLC37A4	4.80E-08	-1.000850897	1	0.933	0.000832331
MRPS14	4.80E-08	-0.574340501	1	1	0.00083264
SF3B5	4.81E-08	-5.519374159	0	0.667	0.000834372
MFAP1	4.91E-08	-0.266278777	1	1	0.00085178
ENSSSCG00000041717	4.91E-08	-1.633677786	0.938	1	0.000852766
SSR3	4.93E-08	-0.89426806	1	1	0.00085567
PRRT3	4.95E-08	-2.765534746	0.375	0.867	0.000858689
ENSSSCG00000011447	4.95E-08	-0.780545526	1	1	0.000858761

ASPDH	4.99E-08	-3.236067358	0.312	0.8	0.000866098
SSR2	5.09E-08	-1.479658347	1	0.933	0.000883038
NFATC2	5.17E-08	-0.563875051	1	1	0.000897696
DNMT3B	5.21E-08	1.861357454	1	1	0.000903329
MYOM1	5.21E-08	-1.77663574	0.812	0.933	0.000903873
CIAPIN1	5.21E-08	-1.574978412	0.938	1	0.000904518
HAT1	5.23E-08	-0.306923251	1	1	0.000907967
RCHY1	5.24E-08	1.778530344	1	1	0.000909226
PRSS8	5.26E-08	-1.199560346	0.938	1	0.000912608
ISYNA1	5.28E-08	-2.22059914	0.688	0.867	0.000915432
PIP4K2B	5.39E-08	-0.838925916	1	0.933	0.000935456
RABGAP1	5.44E-08	1.336867759	1	1	0.000944688
ENSSSCG00000044054	5.55E-08	-2.118644496	0.688	1	0.000962625
ANKRA2	5.57E-08	2.588196965	1	0.933	0.00096612
ALG14	5.57E-08	-0.783488205	1	0.933	0.000966633
ACSL5	5.60E-08	-0.444460047	1	1	0.000971096
UBE2R2	5.60E-08	-0.659212878	1	1	0.000972239
PATE1	5.67E-08	-4.378511623	0.062	0.8	0.000983854
MTHFS	5.67E-08	-1.513012659	0.75	1	0.000984381
ENSSSCG00000017907	5.73E-08	-1.2264715	1	1	0.00099503
AP3S2	5.74E-08	-1.910732662	0.562	1	0.000995144
SLC38A5	5.83E-08	-2.489999557	0.625	0.8	0.001012363
TTC32	5.87E-08	-1.072850565	1	1	0.001018402
TM9SF1	5.94E-08	1.939411738	1	1	0.001030794
ENSSSCG00000026746	5.97E-08	2.142680165	1	1	0.001035336
TTLL3	6.01E-08	-0.737743118	1	1	0.001042393
PTPN13	6.02E-08	1.270783108	1	1	0.001045285
LDHA	6.08E-08	-1.084296335	0.938	1	0.001054667
TMEM178A	6.19E-08	-2.703162886	0.375	0.933	0.001074113
GFRA4	6.20E-08	-1.965648495	0.625	0.933	0.001075958
UBXN8	6.21E-08	-1.079116132	1	0.933	0.001077879
SSR4	6.27E-08	-1.613263609	0.938	1	0.001087508
DNAJC3	6.33E-08	-0.682758059	1	1	0.001098572
ENSSSCG00000050296	6.34E-08	-3.629162305	0.125	0.867	0.001099562
ENSSSCG00000009422	6.39E-08	-0.945175975	1	1	0.001108813
PRPSAP1	6.45E-08	-1.370748223	1	0.933	0.00111983
ATP6V0E2	6.47E-08	-1.712019237	0.875	1	0.001122959
TRMT11	6.63E-08	2.138802484	1	0.933	0.001149704
TRPT1	6.65E-08	-3.040641984	0.188	0.867	0.001154201
LACTB2	6.74E-08	2.026037861	1	0.867	0.001169959
ENSSSCG00000037808	6.77E-08	-2.202338474	0.938	1	0.001175196
ENSSSCG00000028962	6.78E-08	-2.239950793	0.438	0.933	0.001175709
ARSL	6.84E-08	-3.385891154	0.25	0.8	0.001186894
MKLN1	6.86E-08	1.373074128	1	1	0.001189545
DPH7	6.91E-08	-1.949373927	0.688	0.933	0.001198132
CENPF	6.99E-08	-0.527420916	1	1	0.001213647
PXMP2	7.15E-08	-1.423417165	1	0.933	0.001241326
PRPF31	7.30E-08	-1.878984599	0.562	1	0.001267487
SMARCAL1	7.34E-08	-1.279730695	0.875	1	0.001273135
FAAP100	7.40E-08	-3.208586622	0.25	0.733	0.001284215
WDR47	7.41E-08	1.628470444	1	1	0.00128495
NUP107	7.44E-08	1.658083293	1	1	0.001290833
ACTL8	7.46E-08	2.184703057	1	1	0.001294641
ORMDL1	7.47E-08	1.813531352	1	1	0.001296922
PRKAG1	7.52E-08	-1.910564916	0.812	0.933	0.00130444
NELFCD	7.66E-08	-2.469485283	0.562	0.867	0.00132971
TAOK3	7.71E-08	1.78495202	1	1	0.001337646
PPP1CB	7.77E-08	1.543635638	1	1	0.001347377
NDRG3	7.80E-08	1.677781191	1	1	0.001353322
TRAIP	7.82E-08	-1.305262641	0.938	1	0.001356035
PPM1J	8.05E-08	-2.552541023	0.438	0.867	0.001396809
LDLRAP1	8.11E-08	-0.594507188	0.938	1	0.001407053
CCT5	8.13E-08	-0.254544523	1	1	0.00141008
SLAIN2	8.18E-08	1.523380394	1	1	0.001419624
SKP2	8.22E-08	1.834397152	1	1	0.00142677
RBBP7	8.23E-08	1.462745729	1	1	0.001428216
INSL6	8.26E-08	-3.152003093	0.375	0.8	0.00143348
POLR3C	8.28E-08	-0.748259753	1	1	0.001437235
PPP4R4	8.28E-08	1.405882705	1	1	0.001437339
CIAO2A	8.32E-08	1.707494476	1	1	0.001444283

ADI1	8.36E-08	-3.070389328	0.312	0.867	0.001450794
MON1A	8.37E-08	-1.238251305	0.875	1	0.001451877
PDCD1LG2	8.43E-08	-1.190650339	0.938	1	0.001462317
CPO	8.62E-08	3.969900393	1	0.333	0.001495808
RPS19BP1	8.70E-08	-4	0.125	0.8	0.001510242
PALB2	8.70E-08	1.494613246	1	1	0.001510305
COPS7A	8.76E-08	-2.105933445	0.562	0.933	0.001520427
SPATA7	8.82E-08	1.643024426	1	1	0.001529632
KNSTRN	8.82E-08	1.650188891	1	1	0.001529992
GNAI1	8.83E-08	1.492281641	1	1	0.001532561
ENSSSCG00000000576	8.88E-08	1.995947086	1	1	0.001540106
MAP2K2	8.96E-08	-2.428712436	0.5	0.867	0.001554729
DVL1	9.03E-08	-4.222392421	0.062	0.8	0.001567021
INPP1	9.04E-08	-0.130005882	1	1	0.001569273
RPL3	9.12E-08	-0.988772745	1	1	0.001582244
TM4SF5	9.21E-08	-3.756074417	0.125	0.867	0.00159808
TENT4A	9.33E-08	1.764018514	1	1	0.001619618
SLC41A3	9.38E-08	-0.817851743	1	1	0.001627598
TMEM233	9.42E-08	-0.994194378	0.938	1	0.001634981
COMMD1	9.46E-08	-2.366127899	0.5	0.867	0.001642246
ENSSSCG00000058049	9.54E-08	-5.450661409	0	0.733	0.001655271
ATG101	9.63E-08	-1.342723294	0.938	1	0.001671144
PCDHGA4	9.65E-08	-0.772700736	1	1	0.001673657
RBM17	9.77E-08	1.885682937	1	1	0.001694563
TFR2	9.77E-08	-1.790546634	0.812	1	0.001695803
PWP1	9.79E-08	-0.130594414	1	1	0.00169889
INTS14	9.97E-08	1.526377673	1	1	0.001729272
UBE2B	1.00E-07	-1.005646563	0.938	1	0.001736186
NOL7	1.01E-07	-0.954441948	0.938	1	0.00174761
ENSSSCG00000042939	1.01E-07	-1.858644151	0.562	0.933	0.001757709
BAZ1A	1.01E-07	1.66310998	1	1	0.001760857
ENSSSCG00000045404	1.02E-07	-1.477773255	0.938	0.933	0.001770807
BMAL1	1.03E-07	2.088060354	1	1	0.001783321
DCXR	1.03E-07	-3.462343214	0.25	0.667	0.001784623
TRIM4	1.03E-07	-2.24961389	0.562	0.8	0.001788072
NOC4L	1.03E-07	-2.13492958	0.625	0.933	0.001793292
NAA35	1.05E-07	1.675133385	1	1	0.001825756
CLINT1	1.05E-07	1.304413648	1	1	0.001829781
WAPL	1.06E-07	1.456452254	1	1	0.001840963
ENSSSCG00000049957	1.07E-07	-3.847996907	0.062	0.733	0.001851898
SART3	1.07E-07	-0.45429693	1	1	0.001852775
YIF1A	1.07E-07	-3.010647244	0.5	0.867	0.001863935
FAAP24	1.11E-07	-0.744992503	1	1	0.001923506
ENSSSCG00000063521	1.11E-07	2.33229481	1	0.933	0.001934452
KANSL2	1.13E-07	-0.20932751	1	1	0.001954415
DHX30	1.14E-07	2.133710104	1	1	0.001976659
SPDYC	1.14E-07	-0.770822028	1	1	0.001984676
ENDOU	1.15E-07	-1.790546634	0.562	0.933	0.001992765
ATP5F1C	1.15E-07	1.669914851	1	1	0.002002283
POLD1	1.17E-07	-1.816591769	0.812	0.933	0.002029088
LRRFIP2	1.18E-07	-0.009335781	1	1	0.002043169
PSMA3	1.18E-07	-0.384846706	1	1	0.002054864
FFAR4	1.19E-07	2.364059163	1	0.933	0.002058847
ARL4A	1.20E-07	-0.111073154	1	1	0.002083142
FBXO16	1.20E-07	-1.248626982	0.875	1	0.002086164
SFT2D2	1.22E-07	-1.003354752	1	1	0.002113404
GARS1	1.22E-07	-1.072645302	0.938	1	0.002119738
PLPP1	1.23E-07	-0.889178519	0.938	1	0.002137081
CTNND1	1.24E-07	1.418153376	1	1	0.002158661
TMEM120A	1.24E-07	-4.30256277	0.062	0.733	0.002159744
PPM1D	1.24E-07	1.872215328	1	1	0.002159884
CNOT7	1.25E-07	1.763788602	1	1	0.002167964
TXNDC5	1.25E-07	-1.825413621	1	0.933	0.002174115
TMEM223	1.25E-07	-2.208586622	0.625	0.867	0.002176893
XKRX	1.26E-07	-2.552541023	0.562	0.933	0.002184646
TSEN54	1.26E-07	-2.485426827	0.5	0.8	0.002192351
NCBP3	1.28E-07	-0.480953941	1	1	0.002215081
SURF2	1.29E-07	-3.366127899	0.188	0.733	0.002230672
DDB2	1.32E-07	-1.05403984	0.875	1	0.002295406
SLC30A4	1.34E-07	1.640024328	1	1	0.002320256

CTNNB1	1.34E-07	1.277044708	1	1	0.002328767
NQO2	1.34E-07	-1.809316438	0.625	1	0.002329011
GMPPA	1.36E-07	-2.437937901	0.5	0.867	0.002354595
BUB3	1.37E-07	-0.531026191	1	1	0.002375438
BLOC1S1	1.37E-07	-2.398917834	0.375	0.933	0.002378694
RAP1A	1.37E-07	1.759994393	1	1	0.002380976
ENSSSCG00000039365	1.40E-07	-3.471621028	0.25	0.667	0.002424684
TJP3	1.42E-07	-1.961864871	0.625	0.933	0.002472323
ENSSSCG00000032413	1.43E-07	-2.093109404	0.688	0.933	0.002483484
PPP2R1B	1.43E-07	1.371955583	1	1	0.002488651
C2orf88	1.44E-07	-0.445647895	1	1	0.002499494
PSMD8	1.45E-07	1.758339245	1	1	0.002524324
HMOX2	1.46E-07	-1.396387442	1	1	0.002538015
ENSSSCG00000048488	1.47E-07	-2.929610672	0.25	0.8	0.002553335
ZKSCAN4	1.48E-07	-1.868755467	0.875	1	0.002562623
NMT1	1.49E-07	-0.882665826	1	1	0.002576639
STX5	1.49E-07	-1.607682577	0.812	0.933	0.002587367
HEMK1	1.49E-07	-2.458758877	0.688	0.867	0.002588197
EAPP	1.49E-07	-0.909863024	1	1	0.002588854
DCAF6	1.50E-07	1.597061978	1	1	0.002596012
HAUS2	1.50E-07	-0.922268697	0.875	1	0.002601125
PIWIL1	1.50E-07	1.390492381	1	1	0.002602272
C10orf90	1.51E-07	1.653462185	1	1	0.002612155
ASRGL1	1.51E-07	-1.334117504	0.812	1	0.002616998
CDCA7L	1.52E-07	-0.385795866	0.938	1	0.002635562
NOX4	1.52E-07	-0.526511671	1	1	0.002636304
ENSSSCG00000056486	1.52E-07	-0.974014347	0.875	1	0.00264599
EIF2AK2	1.53E-07	-0.631629957	1	1	0.002658911
ZNRD2	1.55E-07	-2.485426827	0.5	0.867	0.002693137
NDUFS8	1.59E-07	-2.803602787	0.25	0.8	0.002762909
SRRD	1.60E-07	-1.71871389	0.688	0.933	0.002776821
WARS1	1.61E-07	-0.921222887	1	1	0.002786989
B4GALT7	1.62E-07	-2.2255597	0.625	0.867	0.002812829
SLC9A3R1	1.64E-07	-1.723159795	0.75	0.933	0.002849015
PROM2	1.67E-07	-2.27897595	0.5	0.8	0.002892421
PCDH12	1.67E-07	-1.860663318	0.75	1	0.002901763
CENPW	1.70E-07	1.557784573	1	1	0.002948231
CLN6	1.72E-07	-1.756074417	0.875	0.933	0.002982613
NDUFS1	1.73E-07	-1.165865747	0.938	1	0.00300361
ZNF79	1.75E-07	-0.075746696	1	1	0.003041681
HENMT1	1.76E-07	1.722047403	1	1	0.00304964
ENSSSCG00000044533	1.79E-07	2.391276142	1	1	0.003108455
GLCE	1.79E-07	2.224849788	1	0.933	0.00310916
PIN1	1.81E-07	-3.152003093	0.25	0.867	0.003140481
KLF1	1.83E-07	-2.30461351	0.5	0.933	0.003179516
STOM	1.85E-07	2.095073819	1	1	0.003217219
TMED1	1.87E-07	-5.61667136	0	0.733	0.003244314
ADAD1	1.88E-07	-0.289374798	1	1	0.003253645
ERI3	1.92E-07	-1.821029859	0.625	1	0.003338236
GM2A	1.93E-07	-1.25624524	0.875	1	0.003349115
CRY2	1.99E-07	-1.335859539	0.938	0.933	0.003453865
TMEM69	2.01E-07	-1.093109404	0.938	1	0.003480418
PDE6C	2.01E-07	-0.650169631	1	1	0.003493691
NTPCR	2.02E-07	-0.883846255	1	1	0.003503376
ENSSSCG00000056719	2.06E-07	-1.48436952	0.938	0.933	0.003578
SDHAF2	2.07E-07	-1.299560282	0.875	1	0.003583779
VWA3A	2.07E-07	-1.358699515	0.938	1	0.003598868
SEZ6L2	2.14E-07	-0.824378268	1	1	0.00370762
RAE1	2.16E-07	1.769979241	1	1	0.003740694
HSD17B12	2.16E-07	1.427998763	1	1	0.003746467
SNAPC2	2.16E-07	-0.451802311	1	1	0.003754215
ENSSSCG00000057665	2.17E-07	-1.088448027	0.938	1	0.003767757
SLC6A12	2.19E-07	-2.989273593	0.25	0.8	0.003792175
YEATS4	2.22E-07	-0.53470773	1	1	0.003845501
ACVR1B	2.25E-07	1.958460944	1	1	0.003900831
ENSSSCG00000043568	2.26E-07	-0.680697375	1	1	0.003925795
CFI	2.27E-07	-1.493040011	0.812	1	0.00394466
PEX1	2.28E-07	1.518716759	1	1	0.003951587
PDXP	2.29E-07	-1.367402928	0.938	0.933	0.003965373
SWT1	2.29E-07	1.611131884	1	1	0.003969631

ECD	2.31E-07	-0.304877643	1	1	0.004010403
CABLES1	2.34E-07	-1.735952821	0.875	1	0.00406362
ENSSSCG00000058372	2.35E-07	-2.805827452	0.438	0.867	0.00408124
CTHRC1	2.35E-07	2.178695211	1	0.8	0.004084409
LONP2	2.36E-07	1.72055616	1	1	0.004087095
ENSSSCG00000060526	2.36E-07	-2.36159824	0.312	0.933	0.004099612
GFM2	2.38E-07	-0.774040964	1	1	0.00413364
PLEK2	2.39E-07	-0.340989311	1	1	0.004148814
LRRC42	2.39E-07	2.031924912	1	1	0.00415109
SMYD5	2.40E-07	-1.925999419	0.625	0.8	0.004155848
ENSSSCG00000051290	2.40E-07	1.730291251	1	1	0.004166719
ENSSSCG00000050177	2.46E-07	-3.933212909	0.562	0.867	0.004276993
POLL	2.48E-07	-2.728697978	0.438	0.867	0.004300999
XPO4	2.48E-07	-1.579807889	0.812	1	0.004310781
CHEK2	2.49E-07	-1.375622818	0.875	1	0.004323089
ENSSSCG00000003234	2.56E-07	-2.388565288	0.438	0.933	0.004444173
DDX25	2.56E-07	-1.634128558	0.875	0.933	0.004449233
PFDN6	2.58E-07	-0.779609932	1	1	0.004484278
ENSSSCG00000059558	2.60E-07	-1.527512229	0.75	0.933	0.00450815
ENSSSCG00000013395	2.60E-07	1.597238307	1	1	0.004512775
ENSSSCG00000052707	2.60E-07	-0.465420878	1	1	0.004513583
SLC35B4	2.60E-07	-1.089702803	0.938	1	0.004515083
ENSSSCG00000051526	2.64E-07	-0.987022146	1	1	0.004576071
SLC39A12	2.70E-07	1.751436077	1	1	0.004680292
FERMT2	2.71E-07	1.330585528	1	1	0.004698905
CDC7	2.71E-07	1.638829373	1	1	0.004707555
CIAO2B	2.71E-07	-1.602123052	1	1	0.004708391
AURKB	2.75E-07	2.103927443	1	1	0.004769222
NT5DC2	2.75E-07	-2.146976276	0.75	0.933	0.004778405
ARL8B	2.76E-07	1.482679972	1	1	0.004783589
ENSSSCG00000057727	2.81E-07	-0.562322044	1	1	0.004872946
GLS2	2.83E-07	1.613110664	1	1	0.004916252
DNA2	2.84E-07	2.159083794	1	1	0.004921645
HABP4	2.86E-07	-0.973439174	1	1	0.004955696
CFAP36	2.92E-07	-1.387556763	0.938	1	0.005060204
ENSSSCG00000040118	2.95E-07	-2.809316438	0.312	0.867	0.005123008
IFRD2	2.96E-07	1.906890596	1	1	0.005128258
TMEM134	2.99E-07	-2.9861942	0.188	0.867	0.005190446
INTS11	2.99E-07	-2.385891154	0.562	0.933	0.005193427
GTF2IRD1	3.03E-07	-2.388565288	0.5	0.933	0.005252371
BTBD3	3.04E-07	1.410824552	1	1	0.005273494
ZNF16	3.14E-07	2.156169942	1	1	0.005445552
KLF16	3.15E-07	-3.263034406	0.25	0.733	0.005461918
SARS1	3.15E-07	-1.683718468	0.875	1	0.005466171
ID3	3.15E-07	1.827864414	1	1	0.005471755
ARRDC1	3.17E-07	-1.836333989	0.5	1	0.005507499
PITPNA	3.22E-07	-1.081968847	1	1	0.005585122
GNB1L	3.23E-07	-2.777607579	0.312	0.8	0.005607337
CLPS	3.23E-07	-2.635163314	0.688	1	0.005611858
ENSSSCG00000012125	3.24E-07	-0.459840707	1	1	0.005624378
CCS	3.28E-07	-2.862496476	0.25	0.867	0.005691302
LIG3	3.30E-07	-0.506088674	1	1	0.005724372
ADAD2	3.32E-07	-2.934411658	0.25	0.867	0.005752004
ENSSSCG00000051497	3.32E-07	2.241175897	1	0.867	0.005765392
TRMT2B	3.36E-07	2.240314329	1	0.8	0.005827112
CYB561D2	3.42E-07	-2.582494245	0.438	0.8	0.005927883
RMC1	3.42E-07	2.306702555	1	1	0.005937686
FHIP2B	3.46E-07	-2.77118131	0.375	0.733	0.006008928
NDUFA6	3.47E-07	-1.485299835	1	1	0.00602515
SPATA19	3.48E-07	-2.9861942	0.375	0.733	0.006034942
ABHD3	3.49E-07	2.628589433	1	0.8	0.00605455
WEE2	3.53E-07	1.900622379	1	1	0.006120335
MATCAP1	3.59E-07	-2.600904045	0.562	0.667	0.00622084
ENSSSCG00000044091	3.60E-07	-3.485426827	0.125	0.8	0.006239949
ETF1	3.61E-07	-0.760534065	1	1	0.006256611
NCF2	3.64E-07	-1.027046765	0.938	1	0.006315
BAG5	3.65E-07	-1.938134744	0.688	0.933	0.006328532
LY6G6D	3.66E-07	-4.341036918	0.062	0.733	0.006347928
DNAJA4	3.71E-07	-1.389158867	0.938	1	0.006428797
TNN	3.75E-07	-0.649270382	1	1	0.00650922

SAP18	3.77E-07	-1.089337651	1	1	0.006533722
ENSSSCG00000058562	3.77E-07	-1.281554494	0.875	0.933	0.00654411
ENSSSCG00000058326	3.79E-07	-0.86097087	1	1	0.006583778
FN3KRP	3.79E-07	-2.647698256	0.312	1	0.0065843
ASCC1	3.81E-07	-0.924555135	1	0.933	0.006610226
KCNN3	3.87E-07	-0.783781346	0.938	1	0.006706767
TMEM266	3.94E-07	-1.703835001	0.812	0.867	0.006839773
NSMCE1	3.97E-07	-1.779801792	0.812	0.933	0.006896021
MYOF	4.02E-07	-0.421320541	1	1	0.006975427
ARL13B	4.06E-07	2.108876806	1	0.8	0.007045686
MORF4L2	4.10E-07	1.680893083	1	1	0.007108665
TMEM45A	4.12E-07	-0.610815467	1	1	0.007145467
RIC1	4.15E-07	1.595086011	1	1	0.007209207
FKBP2	4.19E-07	-3.471621028	0.188	0.667	0.007265236
CD164L2	4.21E-07	-5.093109404	0	0.733	0.007300304
ENSSSCG00000056768	4.22E-07	-1.15787966	1	1	0.007329621
ENSSSCG00000003303	4.25E-07	1.411726362	1	1	0.007366974
FAM98C	4.25E-07	-3.793549123	0.125	0.8	0.007376677
RPS20	4.31E-07	-1.12145403	1	1	0.007475784
RGS11	4.35E-07	-3.552541023	0.188	0.733	0.007553073
HMMR	4.37E-07	1.617624648	1	1	0.007576955
NDUFA5	4.37E-07	-1.835951565	0.875	0.933	0.007590397
PMVK	4.39E-07	-3.385891154	0.125	0.8	0.007621551
ENSSSCG00000016678	4.40E-07	-1.019688335	0.938	1	0.007642081
MAPKBP1	4.41E-07	1.983921081	1	1	0.0076442
ENSSSCG00000038506	4.41E-07	-1.229130199	1	1	0.007648256
TP63	4.52E-07	0.034871345	1	1	0.007847919
AQP6	4.56E-07	-2.255380833	0.625	0.867	0.007907673
DTWD1	4.64E-07	1.916617142	1	1	0.008054912
ENSSSCG00000054357	4.65E-07	-3.294743266	0.25	0.8	0.008070855
LETMD1	4.70E-07	-0.433733593	1	1	0.008147322
LYSMD2	4.70E-07	-1.347923303	0.75	0.933	0.008150097
FBXW8	4.73E-07	-0.282722741	1	1	0.008215459
ALG3	4.74E-07	-2.678071905	0.438	0.867	0.008221613
FAM151B	4.79E-07	1.5710144	1	1	0.008302828
USP25	4.80E-07	1.607903722	1	1	0.008321593
TSR3	4.82E-07	-3.230612928	0.25	0.8	0.00836956
GPN3	4.84E-07	-0.655055252	1	1	0.008402802
USP36	4.86E-07	-1.03441541	0.938	1	0.008425773
ENSSSCG00000022773	4.87E-07	-1.377562794	0.938	1	0.008454279
GLYR1	4.91E-07	-0.812026362	1	1	0.008523416
CRY1	4.93E-07	1.930658762	1	1	0.008546989
ENSSSCG00000031705	5.03E-07	1.649493895	1	1	0.008734038
MTSS1	5.05E-07	1.695794892	1	1	0.008755967
ENSSSCG00000003282	5.05E-07	-1.94458688	0.312	0.867	0.008766849
TMSB10	5.15E-07	-3.440877504	0.812	1	0.008934754
RNPEP	5.17E-07	2.536391492	1	1	0.008962419
FAN1	5.19E-07	-0.661448075	1	1	0.00900548
ENSSSCG00000056115	5.21E-07	-3.192645078	0.25	0.667	0.009036211
NCOA1	5.25E-07	1.389367417	1	1	0.009105661
SPR	5.26E-07	-1.130198723	1	1	0.009124608
ENSSSCG00000033451	5.26E-07	-3.471621028	0.188	0.733	0.009125773
SRSF7	5.30E-07	2.201166457	1	1	0.009187886
ASTN1	5.34E-07	-0.662662466	1	1	0.009257207
GTF2B	5.37E-07	2.005660936	1	1	0.00932055
ELP6	5.41E-07	-1.334117504	0.875	1	0.009393972
CHIC2	5.43E-07	-0.455254599	1	1	0.009421176
PIN4	5.45E-07	-0.780226449	0.938	1	0.009449064
ITM2B	5.51E-07	-0.464949214	1	1	0.009561531
SCAMP4	5.52E-07	-2.282143229	0.562	0.867	0.009579811
PHLPP2	5.58E-07	1.284378753	1	1	0.009679752
ENSSSCG00000004983	5.59E-07	1.871744033	1	1	0.009706062
ETFDH	5.60E-07	2.449998253	1	0.933	0.009710765
TMEM50B	5.60E-07	-0.417682221	1	1	0.009718761
DBF4B	5.61E-07	-1.589940995	0.812	0.933	0.009736986
ASIP	5.61E-07	-3.128733314	0.25	0.733	0.009738643
GLA	5.71E-07	1.585964555	1	1	0.009911142
CFAP69	5.87E-07	2.572665142	1	0.667	0.010193507
XAB2	5.98E-07	-1.222151349	0.938	0.933	0.010375571
MAT2A	6.04E-07	-0.059391289	1	1	0.010480238

ENSSSCG00000028479	6.12E-07	-0.524721128	1	1	0.010612105
IFT81	6.13E-07	1.629034865	1	1	0.010641767
LIMA1	6.18E-07	1.904321236	1	0.933	0.010714595
HAS3	6.22E-07	1.668601566	1	1	0.010797688
PSMD3	6.29E-07	2.102635153	1	1	0.010907434
CENPO	6.40E-07	1.590260181	1	1	0.011097784
SLIT3	6.49E-07	-0.783504588	1	1	0.011269044
GNPTG	6.55E-07	-1.360589715	0.938	1	0.011361162
ARL3	6.55E-07	-1.668611576	0.938	1	0.011368771
BARD1	6.58E-07	1.674551523	1	1	0.01141825
TOE1	6.63E-07	-1.403835152	0.875	1	0.011503437
ENSSSCG00000001934	6.69E-07	1.54760354	1	1	0.011604171
IQCD	6.71E-07	-2.656045599	0.25	0.867	0.011636202
ITGB7	6.72E-07	-4.047305715	0.062	0.8	0.011655077
CNOT11	6.72E-07	-0.291005617	1	1	0.011658104
TAB3	6.91E-07	1.574600749	1	1	0.011988563
TP53INP2	6.92E-07	-1.423417165	0.938	1	0.012001392
SIKE1	6.94E-07	2.120486069	1	1	0.012045213
SMU1	6.98E-07	1.547856577	1	1	0.012110939
BMPR2	7.16E-07	1.410657013	1	1	0.012429407
CSPP1	7.19E-07	1.664133138	1	1	0.012480951
INO80B	7.27E-07	-2.9510904	0.188	0.867	0.012614057
WASHC3	7.35E-07	-2.5981036	0.562	0.933	0.012745113
MSL1	7.39E-07	-0.765961138	0.938	1	0.012823961
RPS21	7.39E-07	-3.296049463	0.625	0.867	0.012830063
FKBP1B	7.44E-07	-2.415037499	0.438	0.867	0.012901357
RBM22	7.44E-07	-0.693738502	1	1	0.012913496
SDS	7.48E-07	-3	0.188	0.8	0.01298216
TANK	7.69E-07	1.9021989	1	1	0.013349352
CDCA7	7.72E-07	1.892909841	1	1	0.013395458
DIP2B	7.72E-07	1.420817834	1	1	0.013398722
ZFYVE1	7.86E-07	-0.613876145	1	1	0.01363043
STRAP	7.93E-07	1.728606527	1	1	0.013753482
ENSSSCG00000010791	8.00E-07	-4.900464326	0	0.8	0.013888609
TMEM245	8.08E-07	-0.355736901	1	1	0.014024073
CLK3	8.11E-07	2.164121796	1	1	0.014078249
WWC3	8.14E-07	-2.188266637	0.438	0.867	0.014117306
C1orf174	8.17E-07	-1.895428737	0.875	0.933	0.014173403
DUSP12	8.21E-07	2.365391743	1	0.933	0.014243074
MND1	8.22E-07	2.09025786	1	0.933	0.014268021
MFGE8	8.26E-07	-3.180572246	0.188	0.733	0.014334346
LRIG3	8.26E-07	1.689172706	1	1	0.014334579
ENSSSCG00000060068	8.27E-07	-2.958179824	0.125	0.933	0.014348589
RPL28	8.31E-07	-2.160016063	0.875	1	0.014415636
THOC7	8.37E-07	1.637647225	1	1	0.014525265
NOCT	8.37E-07	1.27941248	1	1	0.014526955
TROAP	8.43E-07	1.920986939	1	1	0.014626141
PKN1	8.46E-07	-2.24838763	0.5	0.933	0.014683238
ENSSSCG00000022295	8.48E-07	-3.678071905	0.062	0.8	0.014719965
ENSSSCG00000006581	8.50E-07	-2.9510904	0.188	0.867	0.014742939
ENSSSCG00000006344	8.51E-07	1.56233476	1	1	0.01476067
SLC17A2	8.57E-07	2.063111982	1	1	0.014876895
HESX1	8.70E-07	2.14112463	1	0.8	0.015101778
STRN	8.72E-07	1.366006146	1	1	0.015128942
AKAIN1	8.73E-07	2.045505167	1	1	0.015150217
NCAPG	8.73E-07	1.697768694	1	1	0.015154501
UHRF1	8.74E-07	1.641121222	1	1	0.015165766
DCLK1	8.86E-07	-0.271165307	1	1	0.015367144
RSL24D1	9.00E-07	-1.462866024	1	1	0.015620425
PRKAG2	9.03E-07	-1.093109404	0.938	1	0.015663092
RIOK3	9.06E-07	1.705759832	1	1	0.01571312
CREG1	9.10E-07	1.776561601	1	1	0.015786707
SYCP2L	9.11E-07	-1.027020214	0.938	1	0.015800017
UTRN	9.13E-07	1.524803518	1	1	0.015846826
TVP23A	9.18E-07	-2.315501826	0.438	0.8	0.015924332
EIF4B	9.19E-07	-0.151258284	1	1	0.01594539
CAMTA1	9.19E-07	-0.753903318	1	1	0.015951316
SLC19A2	9.21E-07	1.687488217	1	1	0.015977283
RHBG	9.29E-07	-1.9861942	0.812	0.867	0.016123459
NANS	9.31E-07	-3.093109404	0.5	0.733	0.016158842

PELP1	9.42E-07	-1.574236094	0.688	1	0.016336806
ENSSSCG00000050758	9.54E-07	-1.218640286	0.875	1	0.016548929
ENSSSCG00000042524	9.58E-07	-1.084794136	0.938	1	0.016616612
HOATZ	9.82E-07	-2.61667136	0.312	0.8	0.01704113
SRRT	9.85E-07	-1.086930829	1	1	0.017086339
RFXANK	9.87E-07	-3.298223834	0.125	0.867	0.017128917
ENSSSCG00000009178	1.01E-06	1.989057724	1	1	0.017542931
ENSSSCG00000001776	1.04E-06	-2.323407024	0.438	0.867	0.017974564
ENSSSCG00000046622	1.05E-06	-1.609359155	0.625	0.867	0.018222684
ACTL6A	1.07E-06	1.483581834	1	1	0.018487178
NUDT18	1.07E-06	-3.013674937	0.312	0.733	0.018549614
SLC17A1	1.07E-06	-0.741441355	1	1	0.018603451
MPV17L2	1.08E-06	-2.865698908	0.25	0.867	0.018653594
ANO4	1.09E-06	-0.572030268	1	1	0.018861652
PYGB	1.09E-06	-0.855529184	1	1	0.018861771
TSPAN13	1.09E-06	-0.994194378	0.938	1	0.018923548
BORA	1.09E-06	2.040817399	1	1	0.018928374
FIGNL1	1.11E-06	-0.649057923	1	1	0.019251517
CYP2E1	1.12E-06	-1.39176772	0.688	1	0.019403534
RESF1	1.13E-06	1.646133374	1	1	0.019551068
GMNN	1.13E-06	1.776971761	1	1	0.01956387
WSB2	1.13E-06	-1.472455197	0.875	0.933	0.019619436
YIPF5	1.13E-06	-0.296158366	1	1	0.019642152
ENSSSCG00000050595	1.15E-06	-2.830074999	0.312	0.733	0.019875044
PEDS1	1.16E-06	-0.69366876	1	1	0.020041297
UFC1	1.16E-06	-0.96848275	1	1	0.02018581
PHYKPL	1.16E-06	-1.756074417	0.562	0.867	0.020198252
ASH2L	1.17E-06	-0.218408789	1	1	0.020296565
SERHL2	1.18E-06	-1.050040682	0.938	1	0.020409495
TMED4	1.18E-06	-0.491032883	1	1	0.020439474
PDLIM7	1.18E-06	-2.862496476	0.375	0.8	0.020443019
TM2D2	1.19E-06	-1.186891551	0.938	0.933	0.020570959
ARL14EPL	1.19E-06	1.544011951	1	1	0.020597373
IPO9	1.19E-06	-1.701155518	0.938	1	0.020616435
ABLIM3	1.20E-06	-1.697971463	0.875	0.933	0.020760594
TMEM86A	1.20E-06	-3.126124462	0.312	0.933	0.020824999
DNAJC18	1.21E-06	-0.604025554	1	1	0.020936913
ENSSSCG00000061760	1.21E-06	-1.71871389	0.812	0.933	0.02094427
YIF1B	1.21E-06	-2.208586622	0.438	0.933	0.020981163
PRMT1	1.21E-06	2.066235664	1	1	0.021007243
ENSSSCG00000049777	1.21E-06	-1.745186101	0.75	0.933	0.021069645
ARF4	1.22E-06	1.496439937	1	1	0.021167942
TTC8	1.22E-06	2.557655155	1	0.667	0.0211717
ENTREP1	1.23E-06	-1.062735755	0.938	1	0.021405049
TFPT	1.24E-06	-2.712019237	0.25	0.867	0.021533377
GRTP1	1.25E-06	-5.180572246	0	0.667	0.021630765
MRM1	1.25E-06	-1.903070822	0.812	0.933	0.021698092
MIOS	1.25E-06	-0.363279087	1	1	0.021720935
WAC	1.28E-06	1.359306738	1	1	0.022181346
GMFG	1.30E-06	-5.180572246	0	0.733	0.022583802
DCLRE1A	1.31E-06	1.892656985	1	1	0.022664081
CDCP1	1.31E-06	1.923077642	1	1	0.022767816
KRR1	1.31E-06	1.476972397	1	1	0.022775628
DCAF13	1.32E-06	-0.551130074	1	1	0.022961175
SMURF2	1.32E-06	1.302431308	1	1	0.022966123
ENSSSCG00000027270	1.33E-06	-0.762960803	1	1	0.023066082
AHSA1	1.34E-06	1.726522106	1	1	0.023237526
MRPS12	1.34E-06	-2.803602787	0.312	0.867	0.023333024
SEPTIN10	1.35E-06	1.471618613	1	1	0.023341009
DCTD	1.37E-06	1.711179215	1	1	0.02370505
RMND5A	1.37E-06	1.322632364	1	1	0.023798091
ENSSSCG00000048663	1.37E-06	-3.137503524	0.188	0.8	0.023812538
ACTR2	1.38E-06	1.360790027	1	1	0.023965323
USP11	1.39E-06	-1.371316122	0.938	1	0.024160544
ENSSSCG00000026520	1.40E-06	-1.019860422	1	1	0.024257867
MAP4K4	1.41E-06	-0.364234398	1	1	0.024391068
LAMA3	1.41E-06	-0.788015748	0.938	1	0.024515866
ENSSSCG00000021041	1.42E-06	-2.783424905	0.312	0.733	0.024657452
MRPS23	1.42E-06	-0.455504091	1	1	0.02466459
ENSSSCG00000040146	1.42E-06	-3.044199804	0.438	0.933	0.02470248

RPSA	1.42E-06	-0.638310836	1	1	0.02471224
DIMT1	1.43E-06	1.802513979	1	1	0.024792884
PAFAH2	1.44E-06	-0.607188419	1	1	0.02502292
ENSSSCG00000009327	1.44E-06	-0.025250546	1	1	0.025030087
TOGARAM2	1.45E-06	2.266786541	1	0.867	0.025094473
CPSF3	1.45E-06	-0.444581775	1	1	0.025183898
PCNX4	1.45E-06	1.437220647	1	1	0.025211619
NARS1	1.47E-06	-0.542980385	1	1	0.025467526
NIT1	1.47E-06	-1.72914609	0.688	0.933	0.025556536
LGR4	1.48E-06	1.622604417	1	1	0.025713213
CDKN2AIP	1.49E-06	1.522488781	1	1	0.025850132
PM20D2	1.49E-06	1.638073837	1	1	0.025853483
ENSSSCG00000052480	1.49E-06	-3.03170886	0.25	0.8	0.0259386
PCIF1	1.50E-06	-2.054635257	0.5	0.867	0.02595232
WIPF2	1.53E-06	-0.338761732	1	1	0.026620242
MAPK3	1.54E-06	-2.093109404	0.5	0.867	0.02671252
C14orf93	1.55E-06	-2.315501826	0.375	0.933	0.026980752
ATG5	1.56E-06	1.398267139	1	1	0.027063631
SLC2A8	1.56E-06	-0.847439041	1	1	0.027151158
OCRL	1.58E-06	1.532661013	1	1	0.027397493
ENSSSCG00000017061	1.58E-06	-1.328325866	0.75	1	0.027448638
CKS1B	1.59E-06	1.62728767	1	1	0.027525321
LDAF1	1.60E-06	1.95473037	1	1	0.027709869
TOLLIP	1.60E-06	-1.135753742	0.812	1	0.027762462
VBP1	1.64E-06	-0.516320835	1	1	0.028502808
ENSSSCG00000059030	1.65E-06	-4	0.062	0.733	0.028547392
ENSSSCG00000063102	1.65E-06	-1.8372705	0.5	0.8	0.028550015
ADORA2A	1.66E-06	2.725052273	1	0.6	0.028768688
PKIG	1.67E-06	-0.588211293	1	1	0.028931735
SLC25A10	1.67E-06	-2.35614381	0.188	0.933	0.028966505
ENSSSCG00000025913	1.69E-06	-0.926459535	1	0.933	0.02929923
NSG1	1.69E-06	-0.803227036	1	1	0.029355248
ENSSSCG00000027124	1.70E-06	-0.978825977	0.938	1	0.029497892
ABR	1.71E-06	-0.851011574	0.938	1	0.029704378
SYPL1	1.72E-06	1.724134845	1	1	0.029860201
HYAL3	1.75E-06	-3.341036918	0.188	0.733	0.030282698
RHPN2	1.75E-06	1.715332294	1	1	0.030306698
TPM1	1.76E-06	-0.523097245	1	1	0.030591207
ZNRF2	1.78E-06	1.94035188	1	1	0.030954364
FAM114A2	1.79E-06	-0.748350601	1	1	0.030991266
TMEM181	1.80E-06	-0.040734909	1	1	0.031269265
UBR1	1.80E-06	1.476235981	1	1	0.031287808
F3	1.81E-06	1.600852475	1	1	0.031403994
ACAD8	1.81E-06	-0.491088162	1	1	0.031409223
GUSB	1.82E-06	-3.987927168	0.188	0.733	0.031602718
ATF4	1.88E-06	-0.472370674	1	1	0.032533271
LRRC36	1.88E-06	-2.338221902	0.5	0.933	0.032575999
TENM4	1.88E-06	-1.478763097	0.812	0.933	0.03260626
YARS1	1.89E-06	-0.829001964	0.938	1	0.032843893
TAX1BP3	1.94E-06	-1.222989687	1	1	0.03366211
HSD17B2	1.95E-06	-3.415037499	0.125	0.8	0.033765532
ENSSSCG00000053208	1.97E-06	-3	0.312	0.733	0.03417565
ENSSSCG00000050083	1.98E-06	-0.394032934	1	1	0.034271292
RACK1	1.98E-06	-2.902380256	0.938	1	0.034348173
ENSSSCG00000033181	1.99E-06	2.400136927	0.938	0.8	0.034503582
SAR1B	1.99E-06	-0.920175653	1	1	0.034578546
BBS4	2.00E-06	2.326666014	1	0.733	0.034729117
CCDC9	2.00E-06	-2.571156701	0.375	0.867	0.034738138
ANAPC1	2.01E-06	1.160299843	1	1	0.03489341
NPDC1	2.01E-06	-3.552541023	0.125	0.733	0.034958267
LRRC59	2.02E-06	-0.793549123	0.938	1	0.035010006
NEU1	2.02E-06	-2.742202243	0.375	0.8	0.03501214
ENSSSCG00000051717	2.03E-06	-1.034215715	0.938	1	0.035167065
C6orf120	2.05E-06	-0.151673297	1	1	0.035645692
SDHAF4	2.07E-06	2.288139781	1	0.8	0.035939686
TRMT5	2.07E-06	1.784555053	1	1	0.035940575
FBXO34	2.07E-06	-1.259467791	0.938	1	0.035977983
GBA2	2.11E-06	-2.247437551	0.625	0.933	0.036532621
TSTD1	2.11E-06	-1.498821111	1	1	0.03657423
GZMA	2.12E-06	1.627940593	1	1	0.036828202

PRXL2B	2.16E-06	-2.874469118	0.375	0.733	0.037449795
ADD3	2.16E-06	1.617232363	1	1	0.037454415
DHCR24	2.17E-06	-1.217845632	0.938	0.933	0.037678632
MTERF3	2.18E-06	1.643119933	1	1	0.037747134
TCP11L1	2.20E-06	-0.243563045	1	1	0.038227094
B4GAT1	2.21E-06	-1.35938947	0.875	0.867	0.038422858
ENSSSCG00000033697	2.22E-06	-1.110140485	1	1	0.038483298
MTAP	2.23E-06	1.390063936	1	1	0.038701872
ENSSSCG00000027723	2.23E-06	-1.840343334	0.75	1	0.038729951
PTPN2	2.23E-06	1.207960086	1	1	0.038778272
TRMT10A	2.27E-06	-0.280861804	1	1	0.03938029
ZNF526	2.28E-06	-1.216167801	0.75	1	0.039571689
SF3B3	2.31E-06	-0.653409851	1	1	0.040006968
ENSSSCG00000057721	2.32E-06	-2.777607579	0.25	0.733	0.040188179
RIMS3	2.32E-06	-1.666294737	0.75	0.867	0.040224855
SMARCD3	2.36E-06	-2.180572246	0.375	0.867	0.040942038
VAPA	2.36E-06	1.564283981	1	1	0.041020352
HBEGF	2.38E-06	1.568922489	1	1	0.04127448
SPAG16	2.41E-06	1.622184163	1	1	0.041876173
ENSSSCG00000050765	2.43E-06	3.19865672	1	0.667	0.04212399
SRI	2.43E-06	-0.935403952	1	1	0.042147976
UTP23	2.43E-06	-0.663848491	1	1	0.04214827
SVOP	2.44E-06	-2.228624375	0.688	0.867	0.042398324
GFPT2	2.45E-06	2.148524367	1	1	0.042474278
KATNB1	2.45E-06	-1.311944006	0.812	1	0.042491403
TRNT1	2.46E-06	-0.354954449	1	1	0.04275075
RHOD	2.50E-06	-6.137503524	0	0.667	0.043342858
ENSSSCG00000016385	2.50E-06	-1.259467791	0.812	0.933	0.043432992
ENKD1	2.51E-06	-2.234465254	0.5	0.867	0.043503533
ENSSSCG00000030671	2.51E-06	2.077654617	1	0.933	0.043528982
RALA	2.51E-06	-0.107867594	1	1	0.04356708
EDC3	2.52E-06	-0.729536548	1	1	0.043669749
TIMP2	2.56E-06	-2.323407024	0.5	0.8	0.04440117
REN	2.56E-06	-3.047305715	0.188	0.8	0.044487341
SEPTIN8	2.57E-06	-0.701127141	1	1	0.044575922
LRMDA	2.58E-06	-0.693258309	0.938	1	0.04483446
ENSSSCG00000015011	2.60E-06	-0.340729471	1	1	0.045064088
SKA3	2.60E-06	-0.944146765	0.938	1	0.045089369
CYTH3	2.60E-06	-0.766299088	1	0.933	0.045174604
SLC4A1AP	2.63E-06	-0.226102741	1	1	0.045708593
TCEA3	2.64E-06	-0.660720571	1	1	0.045859029
DNAJB2	2.66E-06	-1.187746279	0.688	1	0.046236516
RNF166	2.68E-06	-2.840343334	0.312	0.733	0.046450154
DBT	2.68E-06	1.485016912	1	1	0.046461175
CTNNBL1	2.68E-06	1.989957207	1	1	0.046509475
SNRPA1	2.68E-06	1.685968961	1	1	0.046522886
TMEM163	2.68E-06	-1.009693396	1	1	0.046536988
DNAI1	2.70E-06	-2.325770161	0.438	0.867	0.046933095
ENSSSCG00000006084	2.71E-06	1.410299263	1	1	0.046964512
DBNL	2.83E-06	-1.782769284	0.562	1	0.049186401
CCL22	2.84E-06	-0.686292412	0.938	1	0.049357611
GORASP1	2.85E-06	-1.687290435	0.812	0.933	0.049447247
ENSSSCG00000053039	2.85E-06	1.810366527	1	1	0.04952177
TMCO6	2.87E-06	1.771904551	1	1	0.049723937
ODC1	2.90E-06	1.624044755	1	1	0.050316624
ENSSSCG00000063054	2.94E-06	-1.941106311	0.562	0.8	0.05101326
ENSSSCG00000057488	2.98E-06	-3.35614381	0.188	0.6	0.051635479
UBE4A	2.98E-06	1.61609715	1	1	0.051705333
MTARC2	2.98E-06	2.553053253	1	0.867	0.051718237
MAP1LC3A	2.99E-06	-1.726570423	0.688	0.933	0.051954671
ZNF789	3.00E-06	-1.529973266	0.688	0.933	0.051982289
FAM227B	3.01E-06	-0.309984821	1	1	0.052221614
BNIP3L	3.02E-06	-0.286651402	1	1	0.052466515
USP5	3.03E-06	-0.598259323	1	1	0.052507171
PPP1R36	3.06E-06	-0.431431938	1	1	0.05307027
AMN	3.08E-06	-2.858644151	0.188	0.867	0.053447624
OPA1	3.10E-06	1.340475394	1	1	0.053834068
PHC1	3.11E-06	-0.966352262	0.938	1	0.054019741
PABPC1	3.12E-06	-1.906340893	0.75	0.867	0.054179985
RPL24	3.13E-06	-2.019108823	0.875	1	0.054222088

ELP4	3.14E-06	1.893428336	1	1	0.054503179
MRPL52	3.19E-06	-1.25180715	0.688	1	0.055274611
ENSSSCG00000055613	3.19E-06	-3.197446064	0.188	0.733	0.055355867
CHIC1	3.21E-06	-0.882027078	0.938	1	0.055718106
RRP1	3.22E-06	-3.137503524	0.188	0.8	0.055840625
DPY30	3.26E-06	1.47066258	1	1	0.056481462
ENSSSCG00000061799	3.26E-06	-1.839922785	0.812	0.933	0.056492193
GSTO2	3.27E-06	-2.066142357	0.625	0.733	0.056674187
SCAF4	3.28E-06	1.586035535	1	1	0.056923755
PLAGL1	3.30E-06	1.540433277	1	1	0.057251611
INO80E	3.30E-06	-1.300434378	0.938	0.933	0.057314188
LTF	3.32E-06	-1.282933963	0.75	0.933	0.057667967
LATS1	3.34E-06	1.65593296	1	1	0.057874737
SHMT1	3.34E-06	1.950579413	1	1	0.057903108
SPAG5	3.36E-06	-1.025266759	1	1	0.058216688
CXorf38	3.36E-06	-1.843131151	0.688	0.867	0.058244754
ENSSSCG00000025759	3.38E-06	-0.489919808	1	1	0.05861693
C1QTNF2	3.40E-06	-2.315501826	0.438	0.8	0.058917784
ENSSSCG00000009496	3.41E-06	2.760341932	1	0.4	0.059195868
MRPS24	3.42E-06	-0.733980646	1	1	0.059376638
WDR37	3.47E-06	-1.072548082	1	0.933	0.060278624
SESTD1	3.48E-06	1.400197946	1	1	0.060424219
CHP1	3.50E-06	-0.22732119	1	1	0.060721184
ENSSSCG00000039286	3.50E-06	-0.441159924	1	1	0.060732361
RPL27	3.50E-06	-1.45602944	1	1	0.060767789
MEX3C	3.51E-06	1.421272417	1	1	0.060880459
MSH2	3.52E-06	1.416000874	1	1	0.061014938
RAD9A	3.53E-06	-1.363963315	0.812	0.933	0.061187754
CRYBB1	3.55E-06	-2.093109404	0.562	0.867	0.061550612
VAMP8	3.57E-06	-2.595609745	0.312	0.867	0.061883655
CCDC180	3.59E-06	-0.714597781	0.938	1	0.062309603
B3GNT2	3.61E-06	1.519333833	1	1	0.062560737
IPO8	3.63E-06	1.218662285	1	1	0.062969515
WFDC1	3.64E-06	-3.847996907	0.062	0.733	0.063171705
PDHB	3.69E-06	-0.612219358	1	0.933	0.064015493
ENSSSCG00000014969	3.72E-06	1.636489703	1	1	0.064506468
PSMD9	3.76E-06	1.881866572	1	1	0.065275542
ENSSSCG00000039753	3.78E-06	1.600037821	1	1	0.065574189
DOCK9	3.79E-06	1.649977811	1	1	0.065706539
MRPL47	3.79E-06	-0.802123252	1	1	0.065820034
TRPC4AP	3.81E-06	1.546486712	1	1	0.066066187
TSPAN17	3.81E-06	-0.599359929	0.938	1	0.066082557
MAGEB3	3.81E-06	-0.099629504	1	1	0.066180283
GBX1	3.83E-06	-4.9510904	0	0.667	0.066480121
C1orf74	3.87E-06	-2.491658781	0.312	0.8	0.067101582
CCDC60	3.88E-06	-0.848424308	0.938	1	0.067341218
ENSSSCG00000016719	3.90E-06	-0.543336332	1	1	0.06770951
HMGA1	3.93E-06	-1.781165398	0.625	0.867	0.06818686
RIPOR1	3.94E-06	2.119632847	1	0.933	0.068437936
ENSSSCG00000024144	3.98E-06	-3.093109404	0.188	0.733	0.068999159
LAMC1	4.00E-06	1.3163563	1	1	0.069320646
GTF3A	4.02E-06	-2.044199804	0.562	0.8	0.069765629
TMEM59	4.03E-06	-0.297349958	1	1	0.069892645
USPL1	4.06E-06	1.662715217	1	0.933	0.070467694
PARVA	4.08E-06	-1.05994254	1	0.933	0.070712151
PUS3	4.08E-06	-0.888524713	0.938	1	0.070750191
SUV39H1	4.08E-06	-2.074000581	0.5	0.867	0.070822558
BRMS1	4.08E-06	-1.803602787	0.75	1	0.070840656
ORC4	4.12E-06	-0.756628554	1	1	0.071507495
ENSSSCG00000016543	4.13E-06	-0.123502444	1	1	0.07164778
ENSSSCG00000023467	4.15E-06	-4.180572246	0.062	0.667	0.072055746
EIF3L	4.20E-06	-0.30705589	1	1	0.072900798
NUP37	4.22E-06	1.739054833	1	1	0.073166715
PSKH1	4.22E-06	-0.729986674	1	1	0.07320503
SSH2	4.23E-06	1.4086114	1	1	0.073318661
G3BP2	4.24E-06	-0.385772905	1	1	0.073565777
PPP1R12A	4.24E-06	1.567635022	1	1	0.073579464
PRMT7	4.27E-06	-1.698391889	0.75	1	0.074003933
XRCC5	4.27E-06	-0.672718671	1	1	0.074020008
CDK17	4.30E-06	1.521139843	1	1	0.074540894

IQGAP2	4.31E-06	1.329618456	1	1	0.074860495
TMEM63A	4.32E-06	-0.897653698	0.938	1	0.074913545
NIPA1	4.32E-06	1.555319515	1	1	0.074946764
TAF12	4.34E-06	1.707909561	1	1	0.075373317
ENSSSCG00000058767	4.39E-06	-4.9510904	0	0.667	0.076158333
HIGD1C	4.39E-06	-1.047305715	1	1	0.076255797
PI4K2B	4.46E-06	1.501284322	1	1	0.077315752
YBEY	4.48E-06	-1.799378201	0.625	0.8	0.077660018
FUS	4.49E-06	-1.106284793	1	1	0.077846101
EOGT	4.50E-06	1.591845066	1	1	0.078110842
LMOD3	4.51E-06	-1.042836198	0.938	1	0.078277989
TSHZ1	4.52E-06	1.630645316	1	1	0.078440395
ENSSSCG00000034715	4.55E-06	-1.813001485	0.75	0.8	0.0789516
SHLD2	4.57E-06	1.50701664	1	1	0.079282147
SIRT4	4.59E-06	2.125425541	1	0.933	0.079687753
SMARCB1	4.60E-06	-0.635705874	1	1	0.079771429
ENSSSCG00000017446	4.62E-06	-2.036525876	0.375	0.8	0.080236047
TCEANC2	4.63E-06	-1.934411658	0.562	0.867	0.080362685
MCM6	4.64E-06	1.327981215	1	1	0.080458304
GTPBP8	4.65E-06	-1.789103218	0.438	0.933	0.080727494
RBFOX2	4.69E-06	-0.417978775	1	1	0.081297554
METTL1	4.72E-06	-1.123195131	1	1	0.08188664
MYO5B	4.74E-06	-0.245063342	1	1	0.082188924
NICN1	4.77E-06	-1.523097245	0.75	0.867	0.082682479
PIM3	4.79E-06	2.171773426	1	1	0.083140178
KCTD9	4.83E-06	1.585869588	1	1	0.083852803
C19orf47	4.94E-06	-1.660149997	0.812	0.867	0.085728539
MYBPC2	4.95E-06	-3.197446064	0.125	0.667	0.085958801
DDB1	5.01E-06	1.925299062	1	1	0.086995594
ATP23	5.02E-06	-0.345088622	1	1	0.087070302
PRELID1	5.02E-06	-0.179584228	1	1	0.087109387
SKI	5.03E-06	-0.984037705	1	1	0.087293648
RANBP17	5.04E-06	1.651299173	1	1	0.087500185
ANAPC13	5.11E-06	-1.50157425	0.938	1	0.088611212
PTPRK	5.11E-06	2.424166289	1	0.933	0.088745602
ENSSSCG00000054608	5.18E-06	-0.468930507	1	1	0.089951688
TEX261	5.23E-06	-2.023846742	0.5	0.867	0.090721101
ASF1A	5.26E-06	-0.344542861	1	1	0.091221257
MARK3	5.29E-06	-0.623279432	0.938	1	0.09185179
RABEP2	5.33E-06	-2.044199804	0.438	0.8	0.092476015
LAMB1	5.37E-06	1.587081366	1	1	0.093176526
MB21D2	5.38E-06	-0.563247159	1	1	0.093316361
TRIM32	5.40E-06	-0.567243775	0.938	1	0.093621556
RRP36	5.44E-06	-1.373217324	0.75	1	0.094459297
SLC9B1	5.48E-06	1.94862116	1	0.933	0.09505672
RPN2	5.49E-06	-0.690744122	1	1	0.09525848
TRMT10B	5.58E-06	-0.347210685	1	1	0.096765827
ENSSSCG00000050918	5.58E-06	-0.647624289	1	1	0.096844174
ADGRA2	5.58E-06	-1.931358334	0.688	0.933	0.096879079
COL23A1	5.60E-06	-1.152003093	0.812	1	0.09714056
ENSSSCG00000004415	5.60E-06	-0.181973391	1	1	0.097140785
ABCF3	5.60E-06	-1.383786565	0.75	0.933	0.097233154
RASSF9	5.62E-06	-1.130103612	0.812	0.933	0.097435029
ENSSSCG00000008892	5.63E-06	1.600097018	1	0.933	0.09764421
DOK4	5.63E-06	-0.280322375	1	1	0.097724259
RHNO1	5.69E-06	1.482701398	1	1	0.09878976
BRIX1	5.70E-06	-0.533159438	1	1	0.098943654
ATP11C	5.73E-06	1.437953047	1	1	0.099382803
ENSSSCG00000004760	5.75E-06	-3.152003093	0.188	0.733	0.099779512
UTP25	5.77E-06	-1.30461351	0.812	0.933	0.100169888
TFDP1	5.81E-06	2.512317199	1	0.8	0.10082446
RBM28	5.83E-06	-0.23405592	1	1	0.101133341
ENSSSCG00000059371	5.87E-06	-1.468618539	0.75	0.933	0.101843389
ENSSSCG00000033298	5.88E-06	1.885516945	1	0.867	0.101983606
SAMD12	5.89E-06	-0.220659454	1	1	0.102225934
MRPL12	5.91E-06	-3.093109404	0.188	0.733	0.102531251
RABEP1	5.92E-06	-0.157165578	1	1	0.102802981
MAN1A2	5.95E-06	1.415231815	1	1	0.103250363
PFDN2	5.96E-06	-1.0131411	1	1	0.103470404
ZCWPW1	6.00E-06	-0.693433958	1	1	0.104027733

ZC3H7A	6.02E-06	1.959185103	1	0.933	0.104436735
ENSSSCG00000040187	6.02E-06	1.683939748	1	1	0.104469182
CYB5R3	6.05E-06	-2.078002512	0.625	0.933	0.104932374
ENSSSCG00000009926	6.11E-06	-1.878984599	0.625	0.8	0.105932118
UBB	6.13E-06	-0.680652651	1	1	0.106339925
NAGA	6.26E-06	-3.308122295	0.25	0.6	0.108603732
MEAF6	6.26E-06	1.559577182	1	1	0.10865816
UBE2M	6.27E-06	-3.415037499	0.188	0.6	0.108767834
LTV1	6.28E-06	1.540651385	1	1	0.10904125
STX17	6.29E-06	-1.375405712	1	1	0.109138232
POLR3E	6.30E-06	-0.675525223	1	1	0.109375687
A1CF	6.35E-06	1.669905979	1	1	0.110244179
BOLA1	6.38E-06	-2.756074417	0.188	0.733	0.110654941
C5orf24	6.42E-06	1.995619514	1	0.8	0.111362488
CCDC88C	6.46E-06	-1.115305151	0.875	1	0.112164404
POLD2	6.47E-06	-1.36994961	0.75	0.933	0.112178855
ENSSSCG00000058574	6.47E-06	-0.982685415	0.938	1	0.112238653
C3orf49	6.48E-06	1.383671445	1	1	0.112460654
ENSSSCG00000020808	6.58E-06	-0.429672846	1	1	0.11412395
ING1	6.61E-06	-0.636141225	1	1	0.114629241
TTLL12	6.61E-06	-1.89219671	0.625	0.867	0.114684448
KIF18A	6.62E-06	1.796320221	1	1	0.114839599
NUP155	6.62E-06	0.047371819	1	1	0.114870676
ENSSSCG00000061886	6.62E-06	-1.870716983	0.625	0.933	0.114903783
MARCHF2	6.63E-06	-2.61667136	0.375	0.667	0.115008207
TBC1D32	6.63E-06	1.432401636	1	1	0.115086521
ENSSSCG00000060138	6.64E-06	-0.893171834	0.875	1	0.115253447
MCC	6.66E-06	1.641434544	1	1	0.115608831
POMGNT1	6.69E-06	-1.635003183	0.625	1	0.116102622
ENSSSCG00000042487	6.69E-06	-1.183874868	0.812	1	0.116164099
ENSSSCG00000047692	6.72E-06	-1.173032949	0.938	0.933	0.116680191
SMC3	6.74E-06	1.495782688	1	1	0.1169753
CDKN2B	6.82E-06	-0.77325266	0.938	1	0.118394095
SERP1	6.84E-06	1.339885816	1	1	0.118630724
SNAP23	6.87E-06	-0.113249727	1	1	0.119202655
USP13	6.88E-06	1.628615446	1	1	0.119302802
SCN5A	6.91E-06	2.040289721	1	0.867	0.119923276
ALG6	6.92E-06	2.510389048	1	0.933	0.120079177
ZDHHC5	6.93E-06	1.016229444	1	1	0.12015659
HMGXB4	6.97E-06	1.42477075	1	1	0.120973287
TFG	7.00E-06	1.500313216	1	1	0.121448024
C5orf15	7.00E-06	2.372705831	1	0.867	0.121461535
CTSB	7.03E-06	-0.502837101	1	1	0.122032622
WRAP73	7.06E-06	-0.722740954	1	1	0.122424534
ENSSSCG00000060885	7.08E-06	-1.741637034	0.75	0.933	0.12290054
RUVBL2	7.09E-06	-2.800062429	0.312	0.8	0.122984666
CHORDC1	7.10E-06	1.527411478	1	1	0.123154277
ENSSSCG00000039915	7.14E-06	-3	0.125	0.867	0.123859431
UQCRFS1	7.24E-06	-0.28825699	1	1	0.125646163
EPS8L3	7.36E-06	-1.691010961	0.688	0.867	0.127732893
PAPSS1	7.39E-06	1.620859443	1	1	0.128274129
EXOSC6	7.42E-06	-2.093109404	0.438	0.867	0.128701956
AKIRIN2	7.43E-06	1.18639133	1	1	0.128868583
STAMBP	7.48E-06	1.891569796	1	1	0.129706761
ELMOD3	7.51E-06	-1.642447995	0.75	0.867	0.130362085
ENSSSCG00000016075	7.51E-06	1.204339007	1	1	0.130371259
TAFA4	7.54E-06	1.393952205	1	1	0.130829822
CD47	7.58E-06	1.480723282	1	1	0.131484959
ADSL	7.63E-06	-0.825217435	1	1	0.132433935
TOB2	7.72E-06	-0.913288367	0.938	1	0.133928702
SNAPC3	7.81E-06	1.351654834	1	1	0.135580815
HMCES	7.82E-06	1.757656172	1	1	0.135638171
DUSP22	7.82E-06	-2.678071905	0.25	0.8	0.135668465
FOSL2	7.84E-06	-1.919080005	0.562	0.867	0.136005484
PDHA1	7.86E-06	-0.171700411	1	1	0.136330553
RAB36	7.99E-06	-2.552541023	0.25	0.733	0.138671353
WIPI2	8.03E-06	1.968577991	1	1	0.139327235
FAM210B	8.05E-06	-0.761076432	1	1	0.139717935
NUP50	8.07E-06	-0.024226929	1	1	0.139972574
ENSSSCG00000004811	8.15E-06	-3.047305715	0.188	0.733	0.141481272

MRPL38	8.16E-06	-1.567040593	0.812	0.867	0.141530143
FBXO33	8.17E-06	1.421076416	1	1	0.141744759
CEBPG	8.20E-06	-0.267560189	1	1	0.142243276
ENSSSCG00000011307	8.27E-06	-0.618428959	0.875	1	0.143530623
MYO3B	8.33E-06	-0.311110057	1	1	0.144605225
ENSSSCG00000000298	8.40E-06	-3.678071905	0.062	0.733	0.145728037
ENSSSCG00000041524	8.41E-06	-0.910245347	0.875	1	0.145872557
ENSSSCG00000032959	8.42E-06	-2.11417102	0.562	0.867	0.146081944
CGNL1	8.43E-06	-0.161434677	1	1	0.146329231
YPEL5	8.44E-06	1.258112997	1	1	0.14650352
FGGY	8.45E-06	-1.157239742	0.812	0.933	0.146633848
ENSSSCG00000007733	8.49E-06	1.430046314	1	1	0.147362298
TMEM126B	8.55E-06	-0.275634443	1	1	0.148377317
ENSSSCG00000031854	8.57E-06	2.088384986	1	0.933	0.148677091
ILRUN	8.58E-06	-0.470368869	1	1	0.148868144
CCP110	8.58E-06	1.516170892	1	1	0.14895208
ZHX1	8.63E-06	2.220430905	1	0.933	0.149753533
LSR	8.65E-06	1.678957933	1	1	0.150001115
MXD1	8.71E-06	-0.191417775	1	1	0.151195181
ABCB8	8.79E-06	-3.197446064	0.125	0.733	0.152497441
FNDC3B	8.81E-06	1.354180133	1	1	0.152848068
DIO1	8.83E-06	-1.505235308	0.75	0.867	0.153225704
NUDT15	8.85E-06	1.521664771	1	1	0.153495479
SHROOM1	8.87E-06	-1.955605881	0.625	0.867	0.153962842
UCHL1	8.89E-06	-0.460931198	1	1	0.154184087
MKNK1	8.90E-06	1.624241808	1	1	0.154478092
KDM4A	9.02E-06	-0.785785722	0.875	1	0.156488642
CDIP1	9.11E-06	-2.376043368	0.375	0.867	0.157996959
SESN2	9.12E-06	-1.062735755	0.938	1	0.158224336
TOMM40	9.18E-06	-2.637429921	0.25	0.8	0.159279996
CCDC42	9.24E-06	-4.047305715	0.062	0.667	0.160404928
IFT43	9.25E-06	2.23457796	0.938	0.867	0.160482249
ENSSSCG00000061796	9.32E-06	-1.697180728	0.562	1	0.161646616
ENSSSCG00000056538	9.34E-06	1.607260273	1	1	0.162005649
ENSSSCG00000053692	9.42E-06	-0.868403117	1	1	0.163502347
GSTO1	9.42E-06	-0.978339217	0.938	1	0.163532763
C9orf78	9.53E-06	-0.669361267	0.938	1	0.165404741
GTF3C6	9.59E-06	-0.51077716	1	1	0.166357984
HROB	9.64E-06	-0.938266359	1	0.933	0.167177469
FMNL3	9.74E-06	-0.948266103	1	1	0.169069999
LRATD2	9.75E-06	1.39143531	1	1	0.1691432
ENSSSCG00000001970	9.78E-06	1.541386482	1	1	0.169684094
CHST12	9.81E-06	-1.678071905	0.625	0.933	0.170127645
USP45	9.82E-06	1.695136695	1	1	0.170452705
AHSG	9.88E-06	-1.166571566	0.938	0.867	0.171348957
ENSSSCG00000033293	9.92E-06	1.557114516	1	1	0.172070709
SEMA4B	9.97E-06	2.056637715	1	1	0.172932521
TAF7	9.99E-06	-0.225439415	1	1	0.173338345
ARPIN	1.00E-05	2.388583826	1	1	0.173453804
AKIRIN1	1.00E-05	1.533783777	1	1	0.173717986
NMB	1.00E-05	-3.450661409	0.125	0.667	0.174281517
TCL1A	1.01E-05	-0.876900855	1	1	0.174462939
DGAT1	1.01E-05	-1.535113951	0.625	0.933	0.175796819
VCPKMT	1.02E-05	2.868822555	0.938	0.6	0.177724516
PCYT1A	1.04E-05	1.45876231	1	1	0.179613237
CELA2A	1.05E-05	-2.277533976	0.312	0.8	0.18266326
MARCHF9	1.05E-05	-3.697971463	0.125	0.667	0.182720291
PLCG2	1.06E-05	-0.969374036	0.938	1	0.184039029
WDR18	1.07E-05	-3.462343214	0.062	0.733	0.185697734
PABPC1L	1.07E-05	-0.56056782	1	1	0.185909271
ZNF3	1.08E-05	-2.062735755	0.75	0.867	0.186764075
RGP1	1.09E-05	-1.15787966	0.938	0.933	0.189218064
ENSSSCG00000050302	1.09E-05	-2.595609745	0.375	0.667	0.189398155
DLST	1.10E-05	-0.283384719	1	1	0.190193076
COX8A	1.11E-05	-1.215966152	1	1	0.19193035
OSBPL6	1.11E-05	-0.298091142	1	1	0.191957823
BAIAP2L1	1.11E-05	-0.604811205	0.938	1	0.19307945
TSN	1.12E-05	0.025162037	1	1	0.193810016
SLC26A3	1.12E-05	-0.390328512	1	1	0.194081438
TRAPPC2L	1.13E-05	-4.159198595	0.062	0.667	0.195245832

CENPI	1.14E-05	1.698391566	1	1	0.197143822
ENO1	1.15E-05	-2.146220741	1	1	0.198932444
ENSSSCG00000058414	1.15E-05	-2.062735755	0.562	0.8	0.199677779
ENSSSCG00000061173	1.15E-05	-1.576431643	0.5	1	0.200264183
ZFYVE21	1.16E-05	-0.311454993	1	1	0.200405593
ITPR3	1.16E-05	-0.923897021	0.938	1	0.200877868
CDK8	1.16E-05	-0.463520924	1	1	0.20187421
ENSSSCG00000030013	1.18E-05	-0.756975933	1	1	0.204092632
OSGEPL1	1.18E-05	1.836501268	1	0.933	0.205458389
PIGT	1.18E-05	-1.810966176	0.688	0.8	0.20555731
ENSSSCG00000041032	1.19E-05	-3.793549123	0.062	0.667	0.206930247
ADCY9	1.19E-05	-0.511213207	1	1	0.206960238
ST7L	1.20E-05	-1.172543872	0.938	0.933	0.207818482
ENSSSCG00000013150	1.21E-05	-2.858644151	0.125	0.733	0.209369617
UFM1	1.21E-05	-0.296788533	1	1	0.210156641
ENSSSCG00000055548	1.21E-05	-2.571156701	0.375	0.733	0.21050367
DACT1	1.22E-05	1.837285128	1	0.8	0.211674209
SIRT7	1.22E-05	-1.183307213	0.875	0.867	0.211744675
ASB3	1.22E-05	1.314421112	1	1	0.211768201
MAGT1	1.22E-05	1.567340582	1	1	0.211919388
FABP3	1.24E-05	-0.915430177	1	1	0.215506789
PSD2	1.24E-05	-3.137503524	0.125	0.733	0.215676068
DNAH1	1.24E-05	-2.32443495	0.562	0.8	0.215981067
TBC1D8	1.26E-05	-0.486024093	1	1	0.218174944
ENSSSCG00000055956	1.26E-05	-2.132637769	0.5	0.933	0.219182824
ENSSSCG00000045719	1.26E-05	-2.341036918	0.312	0.8	0.219421208
ATP2C2	1.27E-05	-1.100488935	1	1	0.220717493
RUVBL1	1.27E-05	-0.763884719	1	1	0.221140499
TMEM109	1.28E-05	1.838729518	1	1	0.22149184
SHE	1.28E-05	1.546806787	1	1	0.221500709
LRRC17	1.29E-05	-0.375675067	1	1	0.223575058
TEK	1.29E-05	-0.595609745	1	1	0.223709651
KPNA1	1.29E-05	-0.567206888	1	1	0.224058403
GPR107	1.29E-05	-0.940421108	0.938	0.933	0.224112018
GNAI3	1.31E-05	-0.077661405	1	1	0.226932088
USP48	1.31E-05	1.422590434	1	1	0.227022507
MYEF2	1.32E-05	1.489892913	1	1	0.22837116
QSOX1	1.32E-05	-0.658014253	1	1	0.228413602
FASTKD2	1.32E-05	1.780110086	1	1	0.228664061
NLRP13	1.32E-05	-0.506108911	1	1	0.229019579
ANKRD24	1.32E-05	-2.858644151	0.188	0.667	0.229372822
TWF1	1.32E-05	1.470362568	1	1	0.229780442
ANKRD27	1.33E-05	-0.557414449	0.938	1	0.230688807
RNF146	1.33E-05	1.308131097	1	1	0.230923506
SP3	1.33E-05	1.674752061	1	1	0.231133265
ENSSSCG00000060152	1.34E-05	-1.117771459	0.875	1	0.231708892
GATM	1.34E-05	3.661778098	0.938	0.2	0.231997314
HSPBP1	1.35E-05	-1.423754716	0.688	0.867	0.233514616
CCM2	1.35E-05	-3.462343214	0.125	0.667	0.234459311
ATP6V0D1	1.36E-05	-0.63076619	1	1	0.236515802
NUTM1	1.36E-05	-2.167109986	0.438	0.867	0.2367438
RPLP0	1.36E-05	-0.253807453	1	1	0.236773708
KIF20A	1.37E-05	1.7062503	1	1	0.237143844
ACP2	1.37E-05	-2.019108823	0.5	0.867	0.237265453
SDAD1	1.37E-05	-0.336502124	1	1	0.237817793
ENSSSCG00000002907	1.38E-05	-1.580491545	1	1	0.239584594
NOM1	1.39E-05	1.637069481	1	1	0.241602856
ENTPD6	1.40E-05	-1.432739501	0.875	1	0.242238128
UEVLD	1.40E-05	2.127848213	1	1	0.24258544
TRIP10	1.41E-05	-1.122856748	0.875	1	0.244557192
ENSSSCG00000002850	1.41E-05	-4.047305715	0.062	0.6	0.245142351
PLS3	1.42E-05	1.366619047	1	1	0.245556762
NME1	1.43E-05	-3.180572246	0.312	0.8	0.247614796
ENSSSCG00000053231	1.43E-05	2.677408749	1	0.533	0.247934937
HDLBP	1.43E-05	-1.023475217	1	0.933	0.248239889
ENSSSCG00000012202	1.45E-05	-0.733007581	0.938	1	0.251882491
F8	1.46E-05	-1.335470242	0.812	0.867	0.25332453
CD72	1.47E-05	-1.386555123	0.875	0.933	0.255131508
ROPN1L	1.47E-05	-1.625604485	0.5	0.867	0.255669235
CTPS1	1.47E-05	-0.460078464	1	1	0.255848997

ZDHHC1	1.48E-05	2.136624992	1	0.733	0.256441191
ZC3H8	1.48E-05	1.356165997	1	1	0.256719681
SEMA4D	1.48E-05	2.059773113	1	0.933	0.256862392
ENSSSCG00000013992	1.48E-05	-0.175075004	1	1	0.257447926
ARHGDIA	1.49E-05	-1.854949667	0.625	0.933	0.258414323
TSC2	1.49E-05	-0.9861942	0.938	1	0.258704692
PRKCH	1.50E-05	1.495874549	1	1	0.25999483
ENSSSCG00000059900	1.50E-05	-2.900464326	0.125	0.867	0.260529518
SEC13	1.51E-05	1.729238232	1	1	0.261650911
APMAP	1.52E-05	-0.249862897	1	1	0.263452331
AQP11	1.53E-05	-1.69214709	0.562	0.933	0.265570194
NFIL3	1.53E-05	-0.408959329	1	1	0.265576897
ENSSSCG00000006621	1.53E-05	-0.828896807	0.938	0.933	0.265602649
HMGN3	1.54E-05	2.003147611	1	1	0.266884815
MAD2L2	1.55E-05	-1.3910776	0.938	1	0.268145831
YIPF3	1.55E-05	-1.709780765	0.75	0.8	0.269019104
PSMB1	1.56E-05	-0.285075669	1	1	0.271540006
ASB6	1.57E-05	-1.26895924	0.938	1	0.272638145
DIABLO	1.58E-05	-1.002200908	1	0.933	0.27403966
KCNMA1	1.59E-05	-0.897189194	0.875	1	0.276036496
IZUMO4	1.59E-05	-2.941106311	0.125	0.667	0.276052857
GALE	1.59E-05	-2.446746359	0.375	0.667	0.276196214
RNASEH2B	1.60E-05	-1.053849974	0.875	1	0.278295075
SMUG1	1.61E-05	-1.678071905	0.5	0.933	0.278737568
MCOLN2	1.61E-05	3.994353437	0.938	0.4	0.278897384
NXN	1.61E-05	-1.184257292	0.75	1	0.278926894
YWHAH	1.61E-05	1.585927837	1	1	0.280195518
LOXL2	1.62E-05	-1.292418213	0.688	0.933	0.281479455
ENSSSCG00000009670	1.63E-05	1.506701959	1	1	0.282043995
BANP	1.63E-05	1.853600893	1	1	0.282255094
ROS1	1.66E-05	3.157117225	0.938	0.4	0.288345814
HELQ	1.68E-05	1.70555979	1	1	0.291285595
E2F2	1.69E-05	-1.77118131	0.688	0.867	0.292621772
STT3A	1.69E-05	-0.101800365	1	1	0.293786936
ALDOB	1.70E-05	-1.085804604	0.875	0.933	0.294502154
ENSSSCG00000016226	1.70E-05	-0.435094511	1	1	0.295382318
PTCD2	1.70E-05	-0.942775131	0.875	0.933	0.295460605
ENSSSCG00000055543	1.73E-05	2.501837185	1	0.933	0.299675214
ATP6V0A4	1.75E-05	1.877890724	1	1	0.303124562
FRMD5	1.75E-05	-0.345208333	1	1	0.304463075
PFKP	1.76E-05	-0.659264637	1	1	0.304917465
DESI2	1.76E-05	-0.51481712	1	1	0.305027174
SRSF5	1.77E-05	-0.895170027	1	1	0.306539471
BAP1	1.77E-05	-1.087716146	0.938	1	0.307752445
CSNK1D	1.77E-05	-1.021216486	0.75	1	0.30779613
ABCG2	1.78E-05	-2.349449158	0.438	0.8	0.308496819
POLR1H	1.80E-05	-1.140415119	0.812	1	0.313016643
ENSSSCG00000003253	1.82E-05	-1.189970944	0.688	0.933	0.315652473
SS18L2	1.83E-05	-0.195868979	1	1	0.318251194
PHF24	1.84E-05	-0.659073803	0.938	1	0.318484576
PI4KB	1.84E-05	1.885321298	1	1	0.318720697
STK38	1.84E-05	1.482529159	1	1	0.318876216
EMC10	1.84E-05	-2.678071905	0.25	0.8	0.320109266
CELA1	1.85E-05	-4.678071905	0	0.667	0.321216314
MVB12A	1.86E-05	-3.128733314	0.125	0.667	0.322480245
MTMR1	1.87E-05	-0.354576515	1	1	0.323684676
NOL12	1.87E-05	-1.77118131	0.625	0.933	0.324045517
GRID2	1.87E-05	1.757225744	1	0.867	0.324568596
ODF2	1.88E-05	-0.628659711	0.938	1	0.326233217
PNPT1	1.88E-05	1.560534387	1	1	0.327025232
STAG2	1.89E-05	1.874900481	1	1	0.327529593
ARL5B	1.89E-05	1.5129088	1	1	0.3280715
APLF	1.89E-05	-0.070918831	1	1	0.328488559
NDST2	1.89E-05	-0.815201722	0.875	1	0.328661877
AGAP3	1.92E-05	-1.482151695	0.438	0.933	0.333211916
NLK	1.93E-05	-0.419073013	1	1	0.335001044
ARHGEF7	1.93E-05	-0.251371488	1	1	0.33561975
GRAMD1A	1.95E-05	-1.941106311	0.5	0.933	0.337806456
DNAAF10	1.95E-05	1.536172482	1	1	0.337902933
AKR7A2	1.97E-05	-1.974464908	0.438	0.933	0.341663967

NFXL1	1.97E-05	1.423801928	1	1	0.342618772
CC2D1A	1.98E-05	-1.852101305	0.5	0.867	0.343912106
KLHDC3	2.02E-05	-0.806991385	1	1	0.350129039
ZNF146	2.02E-05	1.914190116	1	1	0.351110907
CHTOP	2.03E-05	-0.430931146	0.938	1	0.351545738
TRMU	2.04E-05	-2.315501826	0.25	0.8	0.354032759
LIN7B	2.04E-05	-3.366127899	0.125	0.6	0.354072342
LEPROTL1	2.05E-05	-0.744238829	1	1	0.354829537
ZNRF1	2.06E-05	-1.040641984	1	0.933	0.357439182
BRK1	2.06E-05	-0.500363097	1	1	0.357761039
EIF2B3	2.08E-05	-0.932644732	1	0.933	0.360080037
IQCB1	2.08E-05	1.545006115	1	1	0.360397673
CPSF4	2.08E-05	1.82572945	1	1	0.360783421
SYT14	2.08E-05	1.340932701	1	1	0.361414202
ENSSSCG00000062804	2.09E-05	-1.182746617	0.625	1	0.362921467
RBM7	2.09E-05	-2.135753742	0.562	1	0.362972688
TYRO3	2.09E-05	-1.888968688	0.688	0.8	0.363241435
TNKS2	2.10E-05	1.43272344	1	1	0.364501063
ENSSSCG00000041142	2.10E-05	-0.631074426	0.938	1	0.364556308
ENSSSCG00000033805	2.11E-05	2.886712714	0.875	0.467	0.365276351
GBX2	2.11E-05	-2.495207848	0.375	0.733	0.365708323
ELP5	2.11E-05	-1.528046461	0.688	0.867	0.366185552
HHAT	2.11E-05	-0.413590463	1	1	0.366254438
NUP54	2.11E-05	1.51971371	1	1	0.366815484
HHEX	2.12E-05	1.397667455	1	1	0.367839196
ENSSSCG00000000908	2.15E-05	-0.256739446	1	1	0.372378212
NFYC	2.17E-05	-0.649695421	1	1	0.377102771
NPRL3	2.20E-05	-3.047305715	0.188	0.667	0.380911257
ENSSSCG00000058051	2.20E-05	-0.555289887	1	1	0.381807355
MAP7D1	2.20E-05	-0.975053643	0.875	0.933	0.382283439
RNF111	2.22E-05	1.335716203	1	1	0.38491094
NUDCD1	2.22E-05	1.379378367	1	1	0.385022286
ENSSSCG00000057586	2.23E-05	-1.131583552	0.875	1	0.386382757
KHK	2.23E-05	-2.865698908	0.25	0.667	0.387456083
GNAZ	2.23E-05	-3	0.125	0.733	0.387503965
ENSSSCG00000062136	2.25E-05	-2.036525876	0.438	0.867	0.389739592
HMG20A	2.25E-05	1.407266468	1	1	0.390304178
NUP88	2.26E-05	1.546921748	1	1	0.391286648
ENSSSCG00000012703	2.28E-05	-0.459152982	1	1	0.394839141
ENSSSCG00000055095	2.28E-05	-1.750221691	0.562	0.867	0.395473368
CKM	2.29E-05	-1.5360529	0.812	0.933	0.397585142
SS18	2.30E-05	1.430233978	1	1	0.399010406
RNASEH1	2.31E-05	-0.46844678	1	1	0.400625093
TSACC	2.31E-05	-3.208586622	0.062	0.733	0.401279085
DLG5	2.32E-05	1.597457891	1	1	0.401990183
QPCTL	2.32E-05	-1.9510904	0.562	0.867	0.402477828
ADGRL2	2.32E-05	2.233784943	1	0.667	0.402946353
DNAJC22	2.33E-05	-0.769045565	0.938	0.933	0.403684658
RANGRF	2.33E-05	-1.9510904	0.75	0.867	0.404051025
EDARADD	2.33E-05	-1.175571565	0.875	0.933	0.404443265
HOXD9	2.34E-05	-0.957992473	0.938	1	0.405357919
GNPNAT1	2.35E-05	1.568729619	1	1	0.407838233
SLC1A2	2.37E-05	-0.731313926	1	1	0.410758969
ENSSSCG00000016902	2.38E-05	2.13941054	1	1	0.412115224
NR2F2	2.38E-05	-1.967578522	0.625	0.8	0.412164867
ENSSSCG00000027491	2.39E-05	-0.636849242	1	1	0.414435956
MBOAT7	2.40E-05	-1.793549123	0.562	0.867	0.417246429
NAT8L	2.42E-05	-3.263034406	0.125	0.667	0.419938708
FNDC5	2.44E-05	-0.941106311	0.812	1	0.422868605
HDAC1	2.45E-05	1.898556112	1	1	0.425092722
FHAD1	2.45E-05	-1.167109986	0.688	0.933	0.425250116
ENSSSCG00000004404	2.45E-05	1.740880644	1	0.867	0.425919757
ZFP36L2	2.46E-05	-1.050465067	1	1	0.426120398
TLK1	2.46E-05	-0.657607803	1	1	0.426576905
ENSSSCG00000060282	2.48E-05	-1.888491685	0.75	1	0.4299434
GGA2	2.48E-05	-0.657238402	0.938	1	0.429999473
PAICS	2.49E-05	-0.171647455	1	1	0.431583562
TXNL1	2.49E-05	1.416099775	1	1	0.43181051
MED14	2.49E-05	1.22661442	1	1	0.432158728
SCG3	2.50E-05	-0.503197688	1	1	0.434083234

H6PD	2.51E-05	-3.9510904	0.062	0.6	0.434871351
STAG1	2.51E-05	1.419873145	1	1	0.435894025
KXD1	2.52E-05	-1.360589715	0.75	0.933	0.436499317
JAZF1	2.55E-05	-0.415307895	1	1	0.442972565
DNAJC4	2.57E-05	-3.128733314	0.125	0.667	0.445062713
ENSSSCG00000054785	2.57E-05	-0.966104128	0.938	0.933	0.446785315
OGFOD2	2.58E-05	-1.298223834	0.75	0.933	0.448094226
GABRR3	2.58E-05	3.728892294	0.75	0.2	0.448187857
ENSSSCG00000040885	2.60E-05	-1.582494245	0.562	0.733	0.451181727
CPT2	2.61E-05	-0.41439473	1	1	0.452383815
SMCO4	2.61E-05	2.531201764	1	0.933	0.453096133
RRP8	2.61E-05	-2.815575429	0.188	0.667	0.453098364
DOCK6	2.62E-05	-1.062735755	0.938	1	0.454288753
CAMK1	2.64E-05	1.850028259	1	1	0.457348269
CCT8	2.67E-05	1.528097269	1	1	0.463202576
HOMER3	2.68E-05	-3.152003093	0.062	0.8	0.465041135
MTERF1	2.68E-05	2.28771238	1	0.6	0.465759783
KNTC1	2.69E-05	-0.34316109	1	1	0.467386294
YPEL1	2.71E-05	-0.642843417	1	1	0.470515814
ENSSSCG00000046461	2.72E-05	-2.678071905	0.125	0.8	0.472298726
RIMKLA	2.73E-05	-0.673570513	1	1	0.473577194
IPO5	2.74E-05	-0.855825993	0.938	0.933	0.474911912
ENSSSCG00000042921	2.74E-05	-3.9510904	0.062	0.6	0.475747195
ENSSSCG00000051835	2.74E-05	2.5980525	0.938	0.333	0.475978512
EIF3E	2.75E-05	1.528509488	1	1	0.476507584
GBF1	2.75E-05	1.357023763	1	1	0.476852874
CDC123	2.75E-05	1.42475451	1	1	0.477073016
STX3	2.75E-05	1.683558614	1	1	0.477750001
OST4	2.77E-05	-1.423754716	0.812	0.867	0.479847451
TMEM39A	2.78E-05	1.386033416	1	1	0.481818525
OGA	2.78E-05	-0.854007741	0.875	1	0.482609398
RIOK1	2.79E-05	-0.383099904	1	1	0.483842942
DLAT	2.79E-05	1.668358093	1	1	0.484199125
SH2B2	2.79E-05	-1.865698908	0.438	0.733	0.484632499
RPUSD2	2.80E-05	-1.526068812	0.75	0.933	0.485901193
SMARCD2	2.83E-05	-0.779951519	0.938	0.933	0.490244378
FAM3B	2.83E-05	-4.793549123	0	0.667	0.490330769
ACOT6	2.84E-05	-0.610026518	1	1	0.493077843
SCYL2	2.86E-05	1.594134911	1	1	0.49577656
ENSSSCG00000011569	2.86E-05	-1.1602236	0.688	1	0.496228548
ENSSSCG00000052460	2.87E-05	-1.972815171	0.75	0.867	0.498221891
TMEM104	2.88E-05	-0.953706348	0.938	1	0.498874227
FGF16	2.88E-05	1.666576266	1	1	0.499243784
FBXO3	2.89E-05	-0.772831047	1	1	0.500775768
ENSSSCG00000062912	2.89E-05	0.096487628	1	1	0.501318599
PYGO1	2.89E-05	1.397667455	1	1	0.502262564
ENSSSCG00000059772	2.90E-05	-0.723652939	0.938	1	0.502385916
NEDD1	2.94E-05	1.498315347	1	1	0.509792009
CCDC97	2.95E-05	-1.477773255	0.688	0.933	0.512567136
SCAMP5	2.96E-05	-0.784423886	0.938	1	0.51343221
UBALD2	2.96E-05	-2.678071905	0.25	0.733	0.514095737
TMEM259	2.97E-05	-1.714597781	0.688	0.8	0.515039147
EIF5A2	2.99E-05	1.952214586	1	0.933	0.518649928
RBM34	3.02E-05	-0.85025288	0.938	0.933	0.524758051
ENSSSCG00000044694	3.04E-05	-0.199657183	1	1	0.527783734
CLIP1	3.05E-05	1.353839336	1	1	0.529362766
MRPL48	3.07E-05	-1.601256308	0.75	0.933	0.532837335
SURF1	3.08E-05	-2.145576824	0.438	0.8	0.534950396
ENSSSCG00000038518	3.08E-05	-0.518716145	0.938	1	0.534974254
TPP2	3.10E-05	1.290219235	1	1	0.537289584
CAPN2	3.10E-05	-0.23336729	1	1	0.53783869
BCLAF3	3.11E-05	1.580878757	1	1	0.538910035
FAM184A	3.11E-05	-0.268476491	1	1	0.539328386
R3HDM4	3.12E-05	1.943274746	1	1	0.541184323
TMCO4	3.14E-05	-2.036525876	0.438	0.867	0.545404808
ANKIB1	3.17E-05	1.29351763	1	1	0.549237721
EXOC1	3.17E-05	1.287555495	1	1	0.549781107
N4BP2	3.17E-05	1.47255949	1	1	0.549872307
GEM	3.17E-05	3.637283536	0.75	0.4	0.550712375
GOLM1	3.18E-05	-0.341188469	1	1	0.551929529

CGGBP1	3.23E-05	1.423132121	1	1	0.560283559
PIGS	3.24E-05	-0.612483563	0.938	0.933	0.561341605
METTL26	3.24E-05	-2.815575429	0.188	0.667	0.561471445
FAM118B	3.24E-05	-0.338498004	1	1	0.562665232
MRPL11	3.28E-05	-0.442063982	1	1	0.569879232
MIS12	3.29E-05	-0.194670132	1	1	0.571073583
C2CD2	3.29E-05	-1.449253215	0.562	0.933	0.571262195
DDX51	3.31E-05	-2.351843673	0.438	0.6	0.573630491
SPCS3	3.31E-05	-1.368344264	0.875	1	0.574120442
ARPP21	3.32E-05	-0.481223619	1	1	0.576870398
LMBRD1	3.34E-05	1.729181788	1	1	0.579471589
SIGMAR1	3.35E-05	-0.451839463	1	1	0.581257657
SMARCD1	3.38E-05	1.673070554	1	1	0.585965376
ECPAS	3.38E-05	-0.104266116	1	1	0.586398491
PHF20L1	3.38E-05	1.381648306	1	1	0.586970451
TBC1D15	3.40E-05	1.574329721	1	1	0.589434339
RAB1B	3.40E-05	-1.320178313	0.688	0.933	0.590424547
POLN	3.42E-05	1.568786832	1	1	0.592681923
KANSL3	3.42E-05	-0.663043696	0.938	1	0.593524241
ARMC10	3.44E-05	1.308989039	1	1	0.596577232
NOP53	3.44E-05	-2.330148602	0.312	0.733	0.597164486
ZNF280C	3.45E-05	2.264047179	1	0.733	0.599261524
BIRC6	3.46E-05	1.187556432	1	1	0.600272388
CPN1	3.46E-05	-1.967578522	0.625	0.8	0.601099192
SPNS3	3.47E-05	-2.477773255	0.375	0.667	0.602273866
PARP14	3.48E-05	1.653886555	1	1	0.603041858
MCRIP2	3.48E-05	-3.678071905	0.062	0.733	0.603738221
ZNF106	3.48E-05	1.214756045	1	1	0.604495232
RECQL5	3.49E-05	-0.880888788	0.875	1	0.605745272
ATP6V0A2	3.50E-05	1.265874556	1	1	0.60712944
F12	3.50E-05	-2.840343334	0.188	0.667	0.607138967
ENSSSCG00000054645	3.51E-05	2.4247389	1	0.6	0.608464903
HEXA	3.51E-05	-2.310340121	0.312	0.8	0.608560428
RHOT2	3.53E-05	-2.571156701	0.312	0.667	0.612868985
CINP	3.55E-05	-2.485426827	0.188	0.8	0.616052547
CTTN	3.55E-05	2.028585249	1	1	0.616457097
ENSSSCG00000058443	3.56E-05	-3.793549123	0.312	0.667	0.617246091
ENSSSCG00000001057	3.56E-05	2.522067709	0.875	0.933	0.618212818
LINGO2	3.57E-05	1.885086225	1	0.867	0.619168484
RNF180	3.58E-05	1.665882496	1	1	0.621581357
SGK2	3.59E-05	-2.455679484	0.188	0.8	0.622512068
RAB3B	3.60E-05	-0.299850826	1	1	0.624757547
SPRY2	3.61E-05	-1.587218475	0.812	0.8	0.626432117
LNX2	3.61E-05	-0.315129084	1	1	0.62683197
ENSSSCG00000062132	3.66E-05	2.362955363	1	0.8	0.635855237
ENSSSCG00000016869	3.67E-05	2.155918143	1	0.867	0.636693384
HIF1AN	3.69E-05	-0.299846332	1	1	0.639766784
ENSSSCG00000015034	3.70E-05	-0.566614327	1	1	0.642190943
PER1	3.73E-05	-0.749456538	0.938	1	0.646747966
FZD6	3.73E-05	1.443943111	1	1	0.646812644
GPD2	3.73E-05	2.649956389	0.938	0.6	0.647209828
ENSSSCG00000042028	3.76E-05	-1.651104858	0.625	0.867	0.651835491
DNLZ	3.76E-05	-5.137503524	0	0.667	0.652444844
HNF4G	3.76E-05	2.184529414	1	0.933	0.652526394
ENSSSCG00000005959	3.78E-05	1.513431348	1	1	0.656265328
CCNDBP1	3.78E-05	-0.914772163	0.938	1	0.656537805
HSP90AB1	3.79E-05	1.535407009	1	1	0.657914966
COQ4	3.87E-05	-1.4639471	0.688	0.8	0.671485657
TRMT10C	3.87E-05	-0.894448911	0.938	1	0.671874203
CHODL	3.88E-05	-0.031490286	1	1	0.672798881
PDPN	3.88E-05	-3.124587636	0.375	0.8	0.673276335
RRP1B	3.90E-05	-1.05704015	0.812	1	0.676601635
ENSSSCG00000031170	3.90E-05	1.574058891	1	1	0.676713122
ENSSSCG00000031398	3.92E-05	-1.916231642	0.5	0.933	0.679426822
CFAP58	3.94E-05	-0.386678178	1	1	0.683010677
FKBPL	3.94E-05	-2.481379994	0.312	0.733	0.683105802
TRPC7	3.94E-05	-1.706086281	0.625	0.8	0.68347366
DHCR7	3.97E-05	-2.707819249	0.188	0.667	0.688364429
L3MBTL3	3.98E-05	-0.010030365	1	1	0.689716632
SLC6A20	3.98E-05	-0.296069882	1	1	0.690520821

SLC7A7	3.99E-05	-0.598504648	1	1	0.692144201
KDF1	4.00E-05	-2.443606651	0.312	0.733	0.69353746
SYAP1	4.02E-05	-0.205584134	1	1	0.697072022
TIMM8B	4.02E-05	-0.652780986	1	1	0.697563475
PPP1CA	4.02E-05	-0.718260995	0.938	1	0.697634069
RANGAP1	4.03E-05	-1.171111916	0.688	0.933	0.698710361
TRADD	4.03E-05	-3.047305715	0.125	0.667	0.698731881
ENSSSCG00000016215	4.03E-05	-1.830074999	0.375	0.867	0.699995639
FAM98B	4.05E-05	-0.581687369	1	1	0.702002551
ENSSSCG00000053662	4.07E-05	1.622732898	1	0.933	0.705901918
AZIN1	4.09E-05	1.5203258	1	1	0.709035039
ENSSSCG00000038304	4.10E-05	1.869864739	1	1	0.711053595
TECPR1	4.10E-05	-3.678071905	0.062	0.667	0.711779288
RRP12	4.13E-05	-1.467084247	1	0.867	0.716262642
MAG	4.14E-05	-2.900464326	0.188	0.6	0.719178295
UBE2E1	4.16E-05	1.40844769	1	1	0.72180288
CACNA2D3	4.17E-05	-1.756074417	0.625	0.867	0.723601988
DOK6	4.17E-05	-1.399770743	0.688	0.933	0.724133605
CHST14	4.18E-05	2.796707678	0.875	0.4	0.72523318
KCTD2	4.19E-05	-0.605444105	1	0.933	0.726618369
SLC36A3	4.20E-05	-0.796716402	0.938	0.933	0.727963141
ENSSSCG00000006556	4.21E-05	-2.309427311	0.312	0.867	0.730071529
FANCD2	4.24E-05	-0.330497874	1	1	0.735900804
PTCD3	4.24E-05	-0.418232838	0.938	0.933	0.736086627
DPH1	4.24E-05	-0.418566311	1	1	0.736109371
NPHP3	4.29E-05	1.408309456	1	1	0.744056099
MAP2K3	4.29E-05	-0.42365063	0.875	1	0.744388843
TRMT2A	4.32E-05	-1.093109404	0.688	1	0.749589405
ARRDC2	4.33E-05	-0.515174171	0.938	1	0.751439456
C19orf12	4.33E-05	-1.560235415	0.438	0.933	0.751589107
ZNF664	4.33E-05	1.275523783	1	1	0.75167213
C18orf21	4.36E-05	-2.745186101	0.312	0.6	0.756216771
ATL2	4.36E-05	1.373788448	1	1	0.756478072
ORAI1	4.40E-05	-0.960659108	0.812	0.933	0.762711892
ANKRD13C	4.40E-05	1.82336724	1	1	0.763140072
ESRRG	4.41E-05	1.510450904	1	1	0.765950669
PALS2	4.42E-05	-0.16871209	1	1	0.76756659
FBXW9	4.43E-05	-1.508146904	0.875	0.867	0.768754708
NEDD4L	4.49E-05	1.198456389	1	1	0.77846021
ENSSSCG00000057751	4.51E-05	-0.926212096	0.875	1	0.781779164
BRPF1	4.51E-05	-0.860549957	0.938	1	0.783146132
ATAD1	4.52E-05	1.602394804	1	1	0.783457849
GRK6	4.54E-05	-1.799378201	0.375	0.8	0.788492163
ENSSSCG00000005508	4.59E-05	1.587737531	1	1	0.796051599
SLC10A3	4.59E-05	-1.30256277	0.5	0.867	0.796562751
ZNF529	4.64E-05	-1.179009478	0.938	1	0.805865151
RHBDD2	4.66E-05	-2.093109404	0.312	0.8	0.808416751
YIPF1	4.72E-05	-0.868593811	1	1	0.818244495
RBIS	4.73E-05	-1.581047451	0.938	0.933	0.821555299
FBXW11	4.74E-05	1.49878359	1	1	0.822421045
TINF2	4.75E-05	-0.716931684	0.938	0.933	0.824831125
HEATR5B	4.76E-05	1.21802541	1	1	0.826324195
CHMP4B	4.78E-05	-0.519202092	1	0.933	0.829996563
SLC4A7	4.80E-05	1.504478579	1	1	0.833382314
LPIN2	4.83E-05	-0.036049216	1	1	0.83750145
HIP1R	4.83E-05	-0.72416832	1	1	0.838194973
ENSSSCG00000003261	4.83E-05	-2.415037499	0.188	0.733	0.838228413
CRLF3	4.83E-05	1.310377472	1	1	0.83831394
REEP2	4.83E-05	-2.230612928	0.438	0.733	0.838417625
AP1G2	4.83E-05	-1.536716056	0.75	0.867	0.838453046
CIART	4.84E-05	1.797883564	1	0.933	0.840398834
ENSSSCG00000011549	4.85E-05	2.27462238	1	0.8	0.840982701
NGRN	4.86E-05	-0.451254035	0.938	1	0.843309104
REEP4	4.86E-05	-0.207726082	1	1	0.843861489
SERBP1	4.87E-05	1.118702702	1	1	0.845581917
FNBP4	4.88E-05	1.667048415	1	0.933	0.846684536
RRP7A	4.90E-05	-2.382616022	0.375	0.733	0.85018905
TRIM41	4.92E-05	-0.589262638	1	1	0.853890734
ENSSSCG00000031818	4.96E-05	-1.678071905	0.625	0.933	0.860755978
RFC5	4.98E-05	-0.509487076	0.938	1	0.864883749

GNL1	5.00E-05	-0.956292715	0.812	1	0.867341974
PPP4R2	5.05E-05	1.375877194	1	1	0.87619451
ENSSSCG00000028501	5.05E-05	-3.30256277	0.188	0.533	0.876330863
CSE1L	5.07E-05	-0.4045705	1	1	0.880168077
OS9	5.08E-05	-0.440242839	1	1	0.882004682
GPR137B	5.09E-05	0.002328806	1	1	0.882648147
VWC2L	5.09E-05	-0.830074999	1	1	0.882732502
SINHCAF	5.09E-05	1.754566225	1	1	0.883260165
ENSSSCG00000003792	5.10E-05	1.982981097	1	1	0.885225281
CCBE1	5.12E-05	-0.487388344	1	1	0.887702756
ENSSSCG00000012879	5.12E-05	1.519867472	1	1	0.888195147
RASAL2	5.18E-05	1.752283694	1	1	0.897961776
CDV3	5.20E-05	-1.301508554	1	1	0.903067764
ENSSSCG00000048769	5.23E-05	-0.445094733	1	1	0.907569397
CMPK1	5.27E-05	1.400177116	1	1	0.914028765
MAPKAPK5	5.28E-05	1.829486092	1	1	0.915323501
ENSSSCG00000009578	5.28E-05	-1.117356951	0.812	0.933	0.915386192
NFKBIB	5.28E-05	-2.495207848	0.312	0.733	0.915543583
NCAPG2	5.29E-05	1.615789908	1	1	0.918704649
ENSSSCG00000000703	5.30E-05	-1.840343334	0.438	0.867	0.919040688
TTLL4	5.32E-05	-0.784837754	0.938	1	0.922828823
ARFIP2	5.35E-05	-2.934411658	0.125	0.6	0.927555152
HMG20B	5.36E-05	-1.04580369	0.875	1	0.930808579
RUFY1	5.39E-05	-0.580096746	0.938	1	0.93445473
DUS2	5.46E-05	-1.458758877	0.375	0.933	0.947929314
GFOD2	5.48E-05	-1.877380713	0.438	0.8	0.950866611
CKAP2	5.50E-05	1.487014153	1	1	0.955059829
SUCLG2	5.51E-05	1.497862546	1	1	0.955954601
SLCO6A1	5.52E-05	2.457087678	1	0.8	0.958052417
PPP1R8	5.54E-05	-0.198505182	1	1	0.961870178
PAOX	5.55E-05	-6	0	0.6	0.962383406
ENSSSCG00000060900	5.58E-05	-2.980634675	0.062	0.667	0.968106166
NOP16	5.58E-05	-1.125052297	0.938	0.933	0.968970544
ARHGAP10	5.65E-05	-0.178627476	1	1	0.980656936
TMEM70	5.66E-05	1.555071874	1	1	0.98197382
ARID5A	5.66E-05	-2.77118131	0.188	0.667	0.982632268
ARAP1	5.67E-05	-1.571156701	0.562	0.867	0.983825953
PCBD1	5.71E-05	-0.339561335	1	1	0.990596896
ENSSSCG00000009963	5.76E-05	-1.977632187	0.312	0.733	0.999038947
CLASP2	5.76E-05	1.226568581	1	1	1
NDUFA1	5.80E-05	-0.837372871	1	1	1
HSD17B4	5.82E-05	-0.276958222	1	1	1
HSDL1	5.85E-05	-0.283474743	1	1	1
RPS6KA6	5.85E-05	2.444547382	0.938	0.4	1
PIGV	5.87E-05	-0.52770917	1	1	1
ABHD17B	5.88E-05	1.723157762	1	1	1
CMTM4	5.88E-05	1.401029504	1	1	1
LIN9	5.89E-05	2.269987879	1	0.667	1
KCMF1	5.90E-05	-0.068127048	1	1	1
KLF8	5.97E-05	3.272540068	0.75	0.6	1
FAM13C	5.99E-05	1.440706477	1	1	1
ARF3	6.00E-05	-0.643306487	1	1	1
MFSD4A	6.02E-05	-0.191222442	1	1	1
TMPRSS7	6.07E-05	4.994353437	0.688	0.067	1
ZNF692	6.08E-05	-2.315501826	0.438	0.733	1
PDLIM5	6.13E-05	-0.153526646	1	1	1
MAP3K12	6.16E-05	-1.254860474	0.688	0.933	1
C8orf58	6.26E-05	-1.631529319	0.562	0.867	1
MVB12B	6.28E-05	-0.464707642	0.938	1	1
ALDH3A2	6.29E-05	-2.140415119	0.562	0.667	1
ENSSSCG00000007520	6.32E-05	-1.121964267	1	0.933	1
TIMMDC1	6.33E-05	-1.114642566	1	1	1
SEC61G	6.34E-05	-1.206671963	0.938	1	1
PPFIBP2	6.38E-05	-0.990175882	0.812	1	1
MGARP	6.42E-05	1.583000984	1	0.867	1
RANBP1	6.45E-05	-0.765223854	0.938	1	1
PANK2	6.47E-05	-0.197195037	0.938	1	1
DNAJC30	6.49E-05	-0.680801698	0.938	0.933	1
CYB5A	6.54E-05	1.413631237	1	1	1
ENSSSCG00000040577	6.55E-05	-1.693501946	0.75	0.867	1

USP54	6.56E-05	1.62324116	1	1	1
NUP93	6.56E-05	1.562409027	1	1	1
ENSSSCG00000036122	6.57E-05	-0.77685006	1	0.933	1
STARD3	6.57E-05	-2.900464326	0.125	0.667	1
SLC2A6	6.57E-05	-3.152003093	0.062	0.667	1
TRAPPC6B	6.58E-05	1.443184971	1	1	1
ENSSSCG00000032110	6.61E-05	-1.701918647	0.75	0.867	1
ENSSSCG00000048711	6.67E-05	-1.793549123	0.375	1	1
PINK1	6.68E-05	-0.83719441	1	1	1
CARS1	6.69E-05	-3.30256277	0.125	0.533	1
ENSSSCG00000063468	6.69E-05	-2.093109404	0.438	0.733	1
IRF6	6.72E-05	-0.604609743	1	1	1
MED19	6.75E-05	-1.034215715	0.875	0.933	1
MCM2	6.75E-05	1.68896422	1	1	1
CPA1	6.77E-05	-2.900464326	0.188	0.6	1
EZH2	6.77E-05	-0.047987937	1	1	1
ENSSSCG00000003348	6.79E-05	-0.73792176	0.938	1	1
RAB3A	6.81E-05	-1.279987539	0.875	0.867	1
ENSSSCG00000054958	6.83E-05	-1.536716056	0.5	0.867	1
PPT2	6.84E-05	1.834684375	1	1	1
PEPD	6.86E-05	1.806037994	1	1	1
TSPAN7	6.86E-05	-0.978938384	0.938	1	1
ORC1	6.90E-05	-0.943161996	1	1	1
PIBF1	6.94E-05	1.872264252	1	1	1
FAM83F	6.97E-05	-2.637429921	0.188	0.667	1
MAP4K3	6.97E-05	1.559619653	1	1	1
ATP13A3	6.99E-05	1.353508165	1	1	1
FREM2	7.01E-05	1.524441023	1	1	1
EEFSEC	7.04E-05	-1.427528443	0.562	0.867	1
ANXA7	7.05E-05	1.664830319	1	1	1
RIIAD1	7.05E-05	-0.411732355	1	1	1
COL14A1	7.06E-05	0.044947925	1	1	1
HNRNPA2B1	7.06E-05	0.064431873	1	1	1
ZNF652	7.10E-05	-1.454107064	0.875	1	1
TANC1	7.11E-05	1.235626162	1	1	1
ENSSSCG00000029285	7.13E-05	-0.569547448	0.938	1	1
EHD4	7.13E-05	-0.565460356	1	1	1
MRPL24	7.19E-05	-1.241501244	0.812	0.8	1
NFYB	7.19E-05	1.436083558	1	1	1
LHX6	7.24E-05	-1.145104042	0.875	0.933	1
DMAC2	7.25E-05	-1.159604816	0.875	0.867	1
CCR5	7.27E-05	-1.25214609	0.938	1	1
SMIM4	7.27E-05	-2.1740294	0.438	0.733	1
CDKL1	7.29E-05	-1.001686377	0.938	1	1
MARCHF5	7.31E-05	1.490295951	1	1	1
STPG4	7.32E-05	-2.446746359	0.188	0.8	1
TNFSF12	7.33E-05	1.846853013	1	0.933	1
ago-02	7.36E-05	1.290906069	1	1	1
RAVER1	7.38E-05	1.896203917	1	1	1
HLTF	7.39E-05	1.740260674	1	1	1
KRAS	7.41E-05	1.5212479	1	1	1
SEMA3A	7.43E-05	1.300598032	1	1	1
HMBOX1	7.44E-05	-0.363561282	1	1	1
SDCCAG8	7.45E-05	1.367562176	1	1	1
ALKBH2	7.50E-05	-0.392669686	0.938	1	1
VAPB	7.53E-05	-0.306590772	1	1	1
SMIM8	7.55E-05	-0.67189333	1	1	1
G6PD	7.56E-05	-1.169460291	0.875	0.867	1
UNG	7.58E-05	1.310224834	1	1	1
BOP1	7.59E-05	-1.750221691	0.438	0.8	1
TWSG1	7.60E-05	-1.99122979	0.562	0.8	1
ENSSSCG00000036877	7.61E-05	4.065319958	0.625	0.267	1
SEC23B	7.61E-05	-0.121203618	1	1	1
FBXO30	7.72E-05	1.273850163	1	1	1
ALPK2	7.82E-05	-0.740228381	0.938	1	1
CDK3	7.83E-05	-1.620356407	0.5	0.867	1
DKK3	7.85E-05	-1.078002512	1	1	1
MTG1	7.92E-05	-3.847996907	0.062	0.667	1
BRD2	7.98E-05	1.844785094	1	0.933	1
SNCA	7.98E-05	-0.284535475	1	1	1

TNRC6A	7.99E-05	1.357153097	1	1	1
ARAF	7.99E-05	-1.367731785	0.688	0.8	1
ENSSSCG00000059019	8.01E-05	-0.458564875	1	1	1
EXOSC9	8.10E-05	-0.273939182	1	1	1
TMEM39B	8.15E-05	-1.082617031	0.75	0.933	1
USP39	8.20E-05	-0.79406842	0.938	1	1
MACROD2	8.24E-05	-0.106915204	1	1	1
PLCD3	8.26E-05	-1.703162886	0.375	0.8	1
TMEM167A	8.27E-05	-1.221539345	0.938	1	1
CCDC136	8.30E-05	-0.53337288	0.938	1	1
ENSSSCG00000043562	8.36E-05	-0.386840607	1	1	1
POLR2E	8.38E-05	1.53843989	1	1	1
PPIL1	8.41E-05	-0.129384791	1	1	1
SPICE1	8.46E-05	1.302360391	1	1	1
SYTL5	8.46E-05	1.06536036	1	1	1
INVS	8.47E-05	-0.439667166	1	1	1
ENSSSCG00000035762	8.48E-05	-0.65846362	1	1	1
ENSSSCG00000040582	8.51E-05	-2.900464326	0.188	0.6	1
AP2M1	8.51E-05	-0.086871463	1	1	1
ENSSSCG00000005453	8.54E-05	1.425627752	1	1	1
PSMB10	8.54E-05	-2.607682577	0.25	0.6	1
CCDC107	8.55E-05	-2.72537762	0.25	0.667	1
ZNF638	8.58E-05	1.506769506	1	1	1
ENSSSCG00000042603	8.62E-05	-0.575556994	1	1	1
UBE3D	8.63E-05	-0.109621116	1	1	1
MBD6	8.66E-05	-3.462343214	0.125	0.6	1
NUBP1	8.69E-05	-0.346001385	1	1	1
TMEM38B	8.70E-05	2.323381692	0.938	0.667	1
ENSSSCG00000054639	8.73E-05	-1.495207848	0.562	0.867	1
ZNF329	8.74E-05	-0.786596362	1	1	1
EYA1	8.75E-05	1.271463028	1	1	1
DOHH	8.76E-05	-0.811927652	0.875	0.867	1
BMS1	8.78E-05	-0.094617711	1	1	1
CNDP2	8.79E-05	-0.891150575	0.938	1	1
ENSSSCG00000063338	8.83E-05	1.889042594	1	0.867	1
ENSSSCG00000057940	8.85E-05	-1.116193018	0.75	0.867	1
PPEF1	8.90E-05	-0.699109895	0.938	1	1
CABP1	8.92E-05	-1.916231642	0.375	0.8	1
NDUFS6	8.92E-05	-1.656045599	0.562	0.867	1
ENSSSCG00000046083	8.92E-05	-1.109788146	0.875	1	1
SLC35A4	8.98E-05	-1.604071324	0.5	0.733	1
PPCS	8.98E-05	-0.275973462	0.938	1	1
PSRC1	9.02E-05	-0.613941568	1	1	1
NUP160	9.05E-05	1.2242826	1	1	1
GPR39	9.14E-05	-0.490180357	1	0.933	1
NXT2	9.15E-05	1.155449679	1	1	1
RCN3	9.15E-05	-2.552541023	0.312	0.533	1
HDAC11	9.16E-05	-2.285754482	0.188	0.8	1
STUB1	9.16E-05	-1.538909157	0.562	0.8	1
OTUD5	9.21E-05	-0.301759513	1	1	1
ENSSSCG00000063033	9.22E-05	1.539434161	1	1	1
KIN	9.24E-05	-0.219781208	0.938	1	1
GLS	9.29E-05	1.32042607	1	1	1
ENSSSCG00000002790	9.31E-05	-2.1655179	0.562	0.933	1
SNX17	9.37E-05	-0.654688978	1	1	1
UPF1	9.37E-05	1.645416704	1	1	1
MCM4	9.41E-05	1.573073346	1	1	1
ENSSSCG00000004806	9.43E-05	1.25409107	1	1	1
PPARG	9.49E-05	-0.384029934	1	1	1
ERMP1	9.49E-05	1.370145426	1	1	1
LARS1	9.55E-05	-0.342993704	1	1	1
ENSSSCG00000057441	9.56E-05	4.579315938	0.75	0.067	1
ENSSSCG00000049247	9.60E-05	-1.093109404	0.875	0.933	1
GEMIN7	9.61E-05	-3.462343214	0.062	0.533	1
INTS12	9.66E-05	-0.250980726	1	1	1
SCYL1	9.69E-05	-0.738014446	0.875	0.933	1
CTIF	9.74E-05	-2.192645078	0.25	0.667	1
FAM76B	9.82E-05	1.603142517	1	1	1
DMXL1	9.84E-05	1.226802658	1	1	1
CCN6	9.86E-05	-1.701918647	0.5	0.8	1

MOV10	9.87E-05	-0.99034983	0.875	1	1
HNRNPA1	9.89E-05	1.312182931	1	1	1
PIGU	9.93E-05	-0.16923585	1	1	1
PNISR	9.98E-05	1.467346923	1	1	1
CNEP1R1	0.000100492	1.577304997	1	0.933	1
CTU2	0.000100947	-1.607682577	0.5	0.867	1
LRRC8C	0.000102045	1.321928095	1	1	1
MSANTD3	0.00010237	-1.048255169	0.875	1	1
ENSSSCG00000056164	0.000102457	-0.363910693	0.938	1	1
JMJD6	0.000102699	1.909969989	1	0.867	1
ENSSSCG00000041572	0.000102748	2.845490051	0.938	0.4	1
SNX27	0.000103047	1.212604914	1	1	1
ELOVL4	0.000103461	1.459021704	1	1	1
SIRT6	0.000103626	-1.334117504	0.562	0.933	1
ENSSSCG00000034966	0.00010405	-1.734655433	0.562	0.733	1
ROR1	0.000104176	3.154818109	0.875	0.267	1
C2orf74	0.00010456	-0.747218066	1	1	1
NOS2	0.000105138	-1.499101764	0.688	0.8	1
ATP6V1B2	0.000105199	0.003425934	1	1	1
VIPAS39	0.000105593	-0.425185455	1	1	1
LHFPL6	0.000105897	-0.119052672	1	1	1
PARD6G	0.000106085	1.742079726	1	0.933	1
SEC61B	0.000106327	1.597206096	1	1	1
NCCRP1	0.000106348	-0.589785373	0.938	1	1
ENSSSCG00000053304	0.000106665	-1.267138804	0.562	1	1
ENSSSCG00000059681	0.000106674	-0.508146904	1	1	1
DPY19L3	0.000106757	1.899937835	1	0.8	1
CLCN3	0.00010726	1.234796214	1	1	1
CEP76	0.000107371	1.844705764	1	1	1
IPPK	0.000107676	-0.242165214	1	1	1
SELENOW	0.000107677	-0.717600269	1	1	1
KDM1A	0.000107784	1.40284809	1	1	1
ASIC1	0.000107808	-1.394278939	0.562	0.8	1
NFIB	0.000108014	-0.379482452	1	1	1
TBC1D25	0.000108556	-1.762960803	0.562	0.867	1
ENSSSCG00000033395	0.000108665	2.115477217	1	0.867	1
RBM45	0.000108721	2.141355849	1	0.867	1
ENSSSCG00000057847	0.000109467	-1.175571565	0.625	1	1
FLT3	0.000109679	1.607330314	1	1	1
CD28	0.000109771	-0.87435556	1	0.933	1
POLR2B	0.000110199	-1.264068225	1	1	1
ENSSSCG00000061752	0.000110284	1.450226001	1	1	1
ZNF577	0.000111227	1.521680663	1	1	1
U2AF1	0.000111502	1.600519782	1	1	1
TRAF1	0.00011157	-2.208586622	0.25	0.733	1
ZFAND2B	0.000112298	-1.777607579	0.438	0.8	1
CDC25A	0.000112309	-0.313046174	1	1	1
GNA14	0.00011272	-0.705038953	0.812	1	1
RXRG	0.000112739	1.666981692	1	1	1
CYCS	0.000112817	0.022983534	1	1	1
SNAP29	0.00011318	-1.058343986	0.688	1	1
ABHD12	0.000113338	-1.200024608	0.75	0.933	1
ENSSSCG00000062032	0.000113954	-3.093109404	0.125	0.533	1
AP1AR	0.000115037	1.454168621	1	0.933	1
MED23	0.000115513	1.501754954	1	1	1
NDUFB6	0.000115926	-0.954403134	0.938	1	1
RSPO2	0.000116395	-0.156946143	1	1	1
CNIH1	0.000116433	1.453490852	1	1	1
FSD1	0.000116586	-0.941106311	0.812	0.867	1
TRIR	0.00011704	-2.05246742	0.438	0.733	1
SOCS5	0.000117047	-0.307428525	1	1	1
ENSSSCG00000047468	0.000117267	-1.093109404	0.75	1	1
KRT25	0.000117414	-3.595609745	0.062	0.6	1
NCKAP1L	0.000118419	-0.262109305	1	1	1
RPS3	0.000118778	-0.571308924	1	1	1
SEC23IP	0.000118836	1.526893444	1	1	1
GPT2	0.000118937	1.316994502	1	1	1
SIGIRR	0.00011913	-3.485426827	0.062	0.733	1
CSMD3	0.000119507	1.366371427	1	1	1
CEP295	0.000119764	1.321115538	1	1	1

CD302	0.00012093	-1.508146904	0.688	0.867	1
CDH3	0.000121388	1.636089307	1	1	1
SLC25A5	0.00012171	-0.188820604	1	1	1
ENSSSCG00000052597	0.000122773	3.22881869	0.812	0.2	1
METTL13	0.000122903	-1.326599535	0.688	0.933	1
CCDC61	0.000123081	-1.47498004	0.625	0.933	1
TIPARP	0.00012319	1.961429872	1	0.867	1
RPL22	0.000123545	-1.422173681	1	1	1
CHAC1	0.000123787	-0.626915392	1	1	1
MTX1	0.000123886	-2.315501826	0.312	0.733	1
ENSSSCG00000027428	0.000124	-4.678071905	0	0.533	1
ENSSSCG00000046886	0.000124037	-0.714100214	0.938	1	1
ITGA11	0.00012452	-0.659740082	1	1	1
ENSSSCG00000062439	0.00012455	-0.846194664	1	0.933	1
F2R	0.00012488	3.233183883	1	0.6	1
ENSSSCG00000008899	0.000125124	-1.155393683	0.75	0.8	1
HEPACAM2	0.000125369	2.861086906	0.812	0.533	1
AFF1	0.000125478	-0.389648718	1	1	1
ENSSSCG00000061331	0.000126219	-0.756826017	0.875	1	1
PARP12	0.000126862	1.492625045	1	1	1
MPPED1	0.000127003	-0.6405972	0.875	1	1
MRPL41	0.000127106	-1.235128409	0.562	1	1
AVL9	0.000127489	1.131583966	1	1	1
GSTCD	0.000127808	1.456630623	1	1	1
DPYSL2	0.000128498	-0.729673102	0.938	1	1
MYO1B	0.000129246	1.178727157	1	1	1
FAM210A	0.000129932	-0.267899297	1	1	1
ACO2	0.000130072	-0.72603723	1	1	1
NDUFB5	0.000130124	-0.582549294	1	1	1
APPL2	0.000130513	1.336100342	1	1	1
ASNS	0.000130694	-0.369871017	1	1	1
ENSSSCG00000058013	0.000131035	-4.61667136	0	0.6	1
REPS1	0.000131486	1.363073115	1	1	1
ACCS	0.000132065	-2.171111916	0.312	0.667	1
STOML3	0.000132217	-1.462343214	0.625	0.933	1
XDH	0.000132477	-1.330148602	0.688	0.933	1
COL19A1	0.000133089	2.691722501	1	0.733	1
ENSSSCG00000060497	0.000133118	2.60249716	0.875	0.333	1
DNAJB12	0.000133464	-1.188266637	0.812	0.867	1
ZNF140	0.00013348	1.352393376	1	1	1
ENSSSCG00000040389	0.000133702	1.584438931	1	1	1
ENSSSCG00000040707	0.00013373	-2.125530882	0.312	0.8	1
TP53RK	0.000135136	-0.347050662	1	1	1
CORIN	0.000135278	-0.42241703	1	1	1
LEF1	0.000136039	1.447265262	1	1	1
IGSF3	0.000136182	-0.921997488	0.75	0.933	1
PLIN2	0.00013622	1.472895197	1	1	1
COBLL1	0.000136475	1.384074944	1	1	1
ABCB7	0.000136505	0.06211615	1	1	1
POLR3K	0.000136928	1.416362897	1	1	1
TCTN3	0.000137277	-0.511899039	1	0.933	1
SLC46A2	0.00013728	-0.118077485	1	1	1
ING3	0.000137526	1.544320516	1	1	1
ENSSSCG00000062067	0.000137771	-2.578536232	0.25	0.6	1
GGT6	0.000139195	-0.688407922	1	1	1
GLIS3	0.000139764	1.440542744	1	1	1
ALKBH5	0.000140193	-0.66195224	1	1	1
LRRC34	0.000140642	2.607330314	1	0.667	1
MLX	0.000142625	1.484933682	1	1	1
CLDN4	0.000142671	-1.40053793	0.625	0.8	1
UIMC1	0.00014317	-0.519000729	0.938	1	1
PRPSAP2	0.000143323	1.452364079	1	1	1
MAP4K2	0.000143502	-1.502500341	0.75	0.8	1
NAT9	0.000143869	-2.77118131	0.25	0.6	1
MSMO1	0.000144187	1.564790165	1	1	1
LEO1	0.000144569	-0.093109404	1	1	1
RHCG	0.000144943	-1.813001485	0.438	0.867	1
PAFAH1B2	0.000145015	1.277214871	1	1	1
ENSSSCG00000032016	0.000145813	-0.405917121	1	1	1
BDH1	0.000146158	-1.161280907	0.688	0.867	1

IQGAP1	0.000146715	1.407788832	1	1	1
KIAA2012	0.000147109	-1.360589715	0.812	0.867	1
ENSSSCG00000042533	0.000147266	-2.019108823	0.312	0.8	1
CASTOR1	0.000148126	1.92270572	1	1	1
H2AC1	0.000149959	-1.032988412	0.812	1	1
XPNPEP3	0.00015088	2.009857175	1	0.933	1
ENSSSCG00000057950	0.00015096	-0.699766976	1	1	1
ALOX15	0.000151932	-2.651104858	0.312	0.533	1
YAP1	0.000152191	1.360699139	1	1	1
TERF1	0.000152254	1.381748181	1	1	1
ENSSSCG00000006679	0.000152538	-0.789055208	0.938	1	1
DCP1A	0.00015316	1.416525846	1	1	1
ADGB	0.000153897	5.299208018	0.625	0.067	1
CMTM7	0.000154415	2.147699083	1	0.933	1
LCA5	0.000154858	1.586690225	1	1	1
RASL10B	0.000154993	-2.629162305	0.188	0.667	1
AKAP8	0.00015508	-0.197639988	1	1	1
IL6ST	0.000155158	-0.906373504	1	1	1
CALU	0.00015537	-0.084108604	1	1	1
AP2A2	0.000155719	-1.732519689	0.625	0.867	1
JCHAIN	0.000156012	-0.490701769	1	1	1
FH	0.00015623	-0.126740182	1	1	1
DGKZ	0.000157049	-1.040641984	0.688	0.867	1
ENSSSCG00000010161	0.000157272	-1.462343214	0.812	1	1
KLHL11	0.000157458	1.391627963	1	1	1
SLC38A1	0.000157716	-2.171111916	0.625	0.867	1
ENSSSCG00000060134	0.000158563	2.000402482	1	0.867	1
CYBC1	0.000158823	-1.138913094	0.75	0.867	1
GPR137	0.000159104	-1.607682577	0.562	0.8	1
MGME1	0.000159454	-0.561364873	1	1	1
RNF7	0.000159665	1.724747642	1	1	1
INPP5B	0.000160237	-0.309872656	1	1	1
KIF5C	0.000160511	-0.303768197	0.938	1	1
DEDD	0.00016067	-0.076586496	1	1	1
XPOT	0.00016145	1.571798324	1	1	1
BZW1	0.000162194	1.275076137	1	1	1
ENSSSCG00000046452	0.00016284	-1.923184403	0.375	0.8	1
ENSSSCG00000048017	0.000163843	-0.537894247	1	1	1
TMEM106B	0.000164559	1.50463037	1	1	1
NDUFAF5	0.000165106	-1.073209847	0.812	1	1
CTSK	0.000165205	1.878667436	1	1	1
SCAF11	0.000165268	1.597423016	1	1	1
ENSSSCG00000053738	0.000165533	-1.476438044	0.625	0.867	1
KIF3C	0.000165732	-0.417460162	1	0.933	1
ENSSSCG00000027988	0.000166973	-2.125530882	0.375	0.733	1
C1D	0.000168664	1.865311492	1	1	1
RBMS3	0.000168773	1.45571931	1	1	1
AMPD3	0.000170467	1.26388081	1	1	1
CAMKK2	0.00017196	-1.058062457	0.875	1	1
PTDSS2	0.000172685	-1.629162305	0.375	0.8	1
UNC5B	0.000173062	2.427722759	1	0.533	1
SLC35C2	0.000173481	-1.020959619	0.75	0.867	1
ENSSSCG00000054543	0.000173554	-1.113358088	0.938	0.933	1
CLTRN	0.0001737	1.782008845	1	0.933	1
DDX19A	0.000174178	-0.208034394	1	1	1
ENSSSCG00000037376	0.000174347	-1.03170886	0.5	0.933	1
LAS1L	0.000174397	-0.418648753	1	1	1
TMEM115	0.000174398	-3.047305715	0.188	0.533	1
RPIA	0.000175297	1.381162862	1	1	1
MOGAT1	0.000175342	-0.496377127	1	1	1
S100A11	0.000176223	-0.932356049	1	1	1
TREH	0.000176362	-2.315501826	0.312	0.6	1
ABT1	0.00017685	1.431989969	1	1	1
MPZL2	0.000176914	-0.594462472	1	1	1
ENSSSCG00000039407	0.00017727	-4.61667136	0	0.6	1
LTO1	0.000177887	-1.886658527	0.5	0.733	1
POGLUT2	0.000178744	2.208306093	1	0.867	1
NR1H3	0.000179224	-0.695213038	0.938	0.933	1
VTCN1	0.00017931	1.688889943	1	1	1
ENSSSCG00000043136	0.000179678	-1.299560282	0.625	0.867	1

ARHGAP18	0.000179919	1.390112493	1	1	1
PSMD5	0.000180227	1.285948798	1	1	1
PIK3R3	0.000181703	0.103754312	1	1	1
ENSSSCG00000014043	0.000182126	-3.263034406	0.125	0.6	1
MSH3	0.000183093	1.305354197	1	1	1
ENSSSCG00000043245	0.000184512	-1.595609745	0.438	0.733	1
EPN2	0.000185551	-0.402793904	1	1	1
SPINDOC	0.000187013	2.054232312	1	0.933	1
CROCC	0.00018776	-2.074962058	0.562	0.867	1
ZNF827	0.0001878	2	1	0.667	1
MCU	0.000188399	1.352629134	1	1	1
PLEKHA8	0.000188808	1.335943827	1	1	1
CEND1	0.00018881	-2.61667136	0.125	0.667	1
PSME4	0.00019068	1.254241287	1	1	1
HEATR6	0.000191499	1.379289342	1	1	1
GTPBP3	0.000191706	-1.297467903	0.5	0.867	1
GNG10	0.000192024	1.513403774	1	1	1
ENSSSCG00000024791	0.000192895	-0.545331824	1	1	1
KIF15	0.00019403	1.396089526	1	1	1
PIGF	0.000194115	1.868168598	1	1	1
NT5E	0.000194386	1.938368827	1	0.933	1
SCO2	0.000194416	-4.61667136	0	0.533	1
MKRN2	0.000195079	-0.769954006	0.938	1	1
ENSSSCG00000033766	0.000195283	-1.375509135	0.75	0.8	1
MBD5	0.000195706	1.671221089	1	1	1
PDAP1	0.000196443	-0.678071905	1	0.933	1
ENSSSCG00000059543	0.000196893	-0.939758627	0.812	1	1
BACE1	0.000196953	-2.651104858	0.25	0.533	1
ENSSSCG00000059729	0.000197345	-1.704544116	0.562	0.667	1
ENSSSCG00000057663	0.000197539	-0.647845118	1	1	1
ENSSSCG00000010888	0.000197663	-0.844081857	1	0.933	1
PTDSS1	0.000198039	-0.585757155	0.938	1	1
HMGN1	0.000198428	1.167950059	1	1	1
TBCEL	0.000198552	-0.143685712	1	1	1
SCN1A	0.000198672	1.712604537	1	1	1
ENSSSCG00000059603	0.000198791	1.526846702	1	1	1
PDS5A	0.000199009	1.17658648	1	1	1
NDC80	0.000199749	1.335046187	1	1	1
HEY1	0.000201355	1.450032921	1	1	1
ZWINT	0.000201381	1.669521073	1	1	1
DHRS13	0.000201534	-3.03170886	0.125	0.533	1
TPD52	0.000202108	-1.325770161	0.75	0.867	1
RMI2	0.000202332	-1.479167837	0.812	0.867	1
PCLAF	0.000202605	-2.584962501	0.188	0.733	1
SCFD2	0.000202813	-0.267652301	1	1	1
CCDC34	0.000203161	1.275592872	1	1	1
L2HGDH	0.00020326	-2.132637769	0.375	0.733	1
ZNF436	0.000204118	-0.119391869	1	1	1
DNAJC24	0.000206071	1.705977902	1	0.867	1
HNRNPR	0.000206285	1.251665759	1	1	1
IKZF4	0.000206872	2.581490309	0.938	0.467	1
ZDHHC6	0.000207053	1.655897749	1	0.933	1
GPBP1	0.000208	-0.168581373	1	1	1
LCA5L	0.000208484	-2.285754482	0.25	0.667	1
LPAR2	0.000208754	-1.805827452	0.562	0.8	1
RNF216	0.00020914	1.506543739	1	1	1
CCNC	0.000209177	2.197109831	1	0.733	1
RWDD3	0.000209228	-1.389350856	0.812	1	1
SETD6	0.000209347	-0.674156855	0.938	1	1
C5orf63	0.000210984	3.116343961	0.812	0.333	1
ENSSSCG00000050380	0.000211134	1.445932764	1	1	1
PMS1	0.000211815	1.599593156	1	1	1
RPP21	0.000211841	-0.872609042	1	1	1
ENSSSCG00000038327	0.000212721	-0.525189388	0.938	1	1
TRNAU1AP	0.000212824	1.63610669	1	1	1
ZC3H14	0.000213595	1.190448172	1	1	1
SYCP2	0.000214069	1.444596971	1	1	1
MAPK14	0.000214924	1.396084726	1	1	1
F13B	0.000215791	-0.600995682	0.875	1	1
RYK	0.000216761	1.50902824	1	1	1

RNPC3	0.00021795	1.181421931	1	1	1
DAAM2	0.000218448	-1.330148602	0.75	0.867	1
SLC2A4	0.00021874	-2.678071905	0.188	0.667	1
BEX3	0.000219957	-1.163498732	0.75	1	1
TXNDC11	0.000220694	2.362342363	1	1	1
POGK	0.000220761	1.243925583	1	1	1
HAS1	0.000221501	2.321928095	1	0.867	1
LLGL1	0.000221691	2.266360653	1	1	1
NEO1	0.000221892	1.171405448	1	1	1
UBR5	0.000222538	1.176444195	1	1	1
PEF1	0.000222615	1.53466618	1	0.933	1
CDIN1	0.00022345	1.287487318	1	1	1
ENSSSCG00000061064	0.000223816	-1.15787966	0.812	0.933	1
NSRP1	0.000224956	0.053905625	1	1	1
ASTN2	0.000225324	2.032146548	1	1	1
IDE	0.000225538	1.323269335	1	1	1
ERN1	0.000225718	1.268445765	1	1	1
ZNF398	0.000226557	-0.011639916	1	1	1
ANKRD46	0.000227368	1.763449516	1	1	1
SH3GLB2	0.000227622	-1.180572246	0.688	0.933	1
GAB2	0.000227958	-0.941106311	0.875	1	1
MRPL10	0.000228643	-1.361361519	0.625	0.933	1
PGD	0.000229152	1.498021105	1	1	1
DGUOK	0.000229399	1.80743568	1	0.933	1
CDK9	0.000229845	1.904531323	1	1	1
C4orf3	0.000229979	1.692078629	1	1	1
USP31	0.000230126	-0.645650427	0.875	1	1
GPLD1	0.000230794	-0.531656588	1	1	1
FBXW5	0.000231456	-4.30256277	0.062	0.533	1
MYL3	0.000231608	-1.818934441	0.5	0.733	1
MBTD1	0.000232555	1.393277939	1	1	1
ASAH1	0.000232649	-0.192514393	1	1	1
DLEC1	0.000233631	-0.122809639	1	1	1
ACP7	0.000234735	-1.73947245	0.625	0.867	1
XIAP	0.000235501	1.191168157	1	1	1
FTO	0.000236453	2.906890596	0.875	0.6	1
TM7SF2	0.000236911	-2.9510904	0.062	0.6	1
ENSSSCG00000039635	0.000237551	-2.366127899	0.125	0.733	1
KHDC4	0.000238037	-0.381752908	1	1	1
ENSSSCG00000063116	0.000238186	-0.07221418	0.938	1	1
CLPX	0.000239289	1.154140542	1	1	1
CD2BP2	0.000241659	-0.848305736	1	1	1
ENSSSCG00000057760	0.000241823	1.544120406	1	1	1
SIK2	0.000243004	-0.197217808	1	1	1
TDRD7	0.000243336	1.120529181	1	1	1
YJU2	0.00024335	-0.853921741	0.938	0.933	1
WBP1L	0.000243351	-1.479167837	0.812	0.867	1
C19orf54	0.00024336	-0.592098682	0.938	1	1
IREB2	0.000244702	1.513375895	1	1	1
CAP1	0.000244995	-0.393011193	1	1	1
NR3C1	0.000245576	-0.107629822	1	1	1
RAB11FIP4	0.000246384	-1.843131151	0.25	0.8	1
DBX2	0.000246825	-0.09683249	1	1	1
AP3S1	0.000247361	1.631286094	1	1	1
SBK1	0.000248073	-2.61667136	0.188	0.6	1
ARHGEF1	0.000248911	-0.845799351	0.875	0.8	1
TCAIM	0.00025006	-0.160814264	1	1	1
ARID2	0.000250568	1.198586195	1	1	1
SCML2	0.000251565	1.390863713	1	1	1
ENSSSCG00000049125	0.000252136	-0.075298128	1	1	1
TXNL4B	0.000252509	-0.585223127	0.875	1	1
ENSSSCG00000016107	0.000252847	-0.752684966	0.938	1	1
WDR25	0.000253733	-1.334117504	0.625	0.933	1
FRZB	0.000254755	-0.399480218	1	1	1
KRTCAP2	0.000255551	-1.409442036	0.875	1	1
CFAP61	0.000255601	-1.006952761	0.938	0.867	1
ENSSSCG00000058505	0.000257219	-1.016147423	0.875	0.933	1
KDM4C	0.000257924	1.725147547	1	0.867	1
DXO	0.000258246	-4.415037499	0	0.6	1
EXT1	0.000259196	-0.133207473	1	1	1

ENSSSCG00000055752	0.000260001	-1.150442579	0.688	0.933	1
ARHGEF18	0.000260194	-1.263034406	0.625	0.867	1
ENSSSCG00000056225	0.000261168	-1.595609745	0.5	1	1
GIPC2	0.000261602	-0.248659384	1	1	1
NFKB1	0.000261642	1.534202449	1	1	1
AKT1S1	0.00026174	-1.730539325	0.312	0.867	1
ENSSSCG00000052841	0.000262114	-0.764160882	1	1	1
FEN1	0.000262486	-0.268476491	1	1	1
TMC4	0.000264726	-1.131583552	0.938	0.867	1
ZNF74	0.000266246	-1.346865997	0.688	0.867	1
LYPD6B	0.000266795	-2.135278859	0.625	0.8	1
OR8S1	0.000268853	-1.133186844	0.688	0.933	1
CCSAP	0.000269807	1.563614322	1	1	1
CMTR2	0.000270367	1.529671646	1	0.867	1
GPR32	0.000270381	-1.308377391	0.938	0.933	1
ESF1	0.000270519	1.360889987	1	1	1
ENSSSCG00000028717	0.000271781	-1.169058258	0.688	0.933	1
KIAA0586	0.00027196	1.252072263	1	1	1
PRRT1B	0.000272442	-0.196000819	1	1	1
ENSSSCG00000063078	0.000274314	-2.366127899	0.062	0.667	1
NOBOX	0.000274455	-0.58435747	1	1	1
CPLANE1	0.000274909	1.171804289	1	1	1
CISD3	0.000274926	-3.137503524	0.125	0.6	1
ENSSSCG00000059138	0.000275304	-0.967578522	0.75	0.933	1
NDUFAF3	0.000275482	-1.517607233	0.625	0.8	1
DAP3	0.000275903	-0.444908029	1	0.933	1
NCOA4	0.000276086	0.023297249	1	1	1
ANP32B	0.000277572	1.424558259	1	1	1
LMAN2L	0.000277862	-1.911271081	0.375	0.667	1
ACAA2	0.000279905	-1.497499659	0.5	0.8	1
VPS35	0.000280546	1.364321168	1	1	1
ENSSSCG00000042029	0.00028063	-1.169058258	0.75	1	1
ACADS	0.000282663	-1.626541604	0.375	0.667	1
GEMIN4	0.000283082	-0.044813847	1	1	1
ENSSSCG00000045343	0.00028365	-1.558772977	0.688	0.933	1
MED27	0.000284188	1.472774004	1	1	1
ENSSSCG00000008769	0.000284721	-4.341036918	0	0.6	1
PIK3C2A	0.000284763	1.445955047	1	1	1
RBM23	0.000286378	-0.705293374	0.938	1	1
RBBP9	0.000287207	-1.72782494	0.625	0.733	1
HORMAD1	0.000287396	1.459237591	1	1	1
HNRNPH1	0.000287536	1.641366638	1	1	1
RP9	0.000288198	-0.798302653	0.938	1	1
ADIPOR1	0.000288959	-0.213224688	1	1	1
FANCG	0.000289129	1.637530552	1	0.933	1
SNRNP35	0.000289455	-0.545046486	0.812	1	1
SLC30A6	0.000290525	1.872889924	1	1	1
ENSSSCG00000030015	0.000290733	1.558721664	1	0.933	1
ST6GALNAC4	0.000291467	-2.629162305	0.188	0.6	1
RNF122	0.00029179	-0.539388814	1	1	1
ENSSSCG00000062841	0.000292238	-1.298223834	0.812	0.933	1
FBXO21	0.000294864	1.074460176	1	1	1
BLOC1S4	0.000294955	-1.311014435	0.875	0.867	1
ELL	0.000296536	-0.163213643	1	1	1
ACOT8	0.000297106	-1.730539325	0.375	0.867	1
ENSSSCG00000053955	0.00029721	1.590917224	1	1	1
ANO5	0.000300394	2.346759948	1	0.733	1
CETN2	0.000300735	1.268115048	1	1	1
XXYLT1	0.000302717	-1.27368165	0.562	0.733	1
ENSSSCG00000028159	0.000305069	-0.600646933	0.875	1	1
STN1	0.000305186	-1.076989739	0.688	0.8	1
WDFY4	0.000306248	2.89112328	0.938	0.533	1
CD3E	0.000306569	-1.607682577	0.438	0.733	1
BCR	0.00030726	-0.809861778	0.938	0.867	1
EFCAB12	0.000308799	-1.601256308	0.75	0.933	1
ENSSSCG00000048408	0.000309219	-1.678071905	0.375	0.8	1
RBL2	0.000309822	1.254303662	1	1	1
DKK4	0.000310125	-1.644124573	0.625	0.733	1
GPAA1	0.000310702	-1.967578522	0.438	0.8	1
SLC44A1	0.000310999	2.440322796	0.875	0.467	1

RAB35	0.000313255	-0.199394198	1	1	1
LLPH	0.000313721	-0.294865533	1	1	1
HAUS8	0.000314327	-0.568237354	1	1	1
MIER3	0.000314501	1.294271752	1	1	1
CAMLG	0.00031501	-0.073209847	1	1	1
PPIP5K2	0.000316745	1.430521133	1	1	1
SELENOO	0.000318099	-4.485426827	0	0.533	1
ENSSSCG00000055918	0.000318587	-1.30256277	0.625	0.933	1
ENSSSCG00000047248	0.000321623	2.237807474	0.875	0.667	1
ENSSSCG00000063044	0.000321717	-3.152003093	0.062	0.667	1
PTPN5	0.000321826	1.679604432	1	1	1
ENSSSCG00000057278	0.000322263	-4.485426827	0	0.533	1
ENSSSCG00000031083	0.000322687	-0.983484913	0.688	0.867	1
ENSSSCG00000061676	0.000323174	-2.093109404	0.25	0.667	1
SMC4	0.000323841	1.540549201	1	1	1
C18orf54	0.000323955	-1.649159213	0.688	1	1
ENSSSCG00000054866	0.000324164	-1.438884241	0.562	0.867	1
ENSSSCG00000050152	0.000325005	-0.856409146	0.938	1	1
ZDHHC13	0.000325052	1.589025149	1	1	1
ENSSSCG00000053718	0.000325064	2.087956155	0.938	0.4	1
HRH1	0.000325435	-1.773229138	0.688	0.933	1
DGKK	0.00032663	-0.317750766	1	1	1
MYH11	0.000327135	-0.285196056	1	1	1
CHRNA3	0.000327787	0.033667713	1	1	1
AMPD2	0.000328004	2.162953041	1	0.867	1
RBM25	0.00032861	-0.074936658	1	1	1
TNPO1	0.000328992	1.288719693	1	1	1
JKAMP	0.000329965	-0.372041437	1	1	1
VPS51	0.000330785	-1.42281485	0.562	0.867	1
SIDT2	0.000332603	-0.720450507	1	0.867	1
CFAP97	0.000333337	1.410404895	1	1	1
CHI3L1	0.000334352	-1.629162305	0.438	0.8	1
ENSSSCG00000016810	0.000334888	1.130190658	1	1	1
DCLK3	0.000337034	2.888743249	0.875	0.4	1
RIOX2	0.000337126	-0.218229204	1	1	1
OXTR	0.000338464	-0.608874395	0.875	1	1
SMIM14	0.000338836	1.403136421	1	1	1
RLF	0.000340558	1.237349154	1	1	1
TMEM242	0.000341061	-0.149596596	1	1	1
ATL3	0.000341409	1.160464672	1	1	1
CDC42SE1	0.000342563	1.31585286	1	1	1
GCN1	0.000343661	1.714245518	1	0.933	1
KIF23	0.000343829	1.08448661	1	1	1
DDIT4	0.000345571	-1.830074999	0.5	0.667	1
GABRA3	0.00034611	-0.430260322	1	1	1
FYB1	0.000347067	3.491853096	0.75	0.2	1
VPS11	0.000348657	-0.617445546	0.938	1	1
ENSSSCG00000049664	0.000349292	-1.220864952	0.688	0.867	1
TARS1	0.000349307	1.12654562	1	1	1
PCM1	0.000349857	1.30168359	1	1	1
ITSN2	0.00035234	1.398066964	1	1	1
PLAUR	0.00035235	2.349408831	1	0.8	1
CNOT6	0.000353657	1.316751061	1	1	1
CPEB4	0.000354003	1.296194126	1	1	1
PRKX	0.00035485	-0.967578522	0.75	0.867	1
MACROH2A1	0.00035534	-0.352113933	1	1	1
ENSSSCG00000062072	0.000357302	-1.093109404	0.688	0.867	1
CWC25	0.00035741	-0.20744208	1	1	1
MED30	0.000357645	1.371215033	1	1	1
CAMK2N2	0.00035907	-4.341036918	0	0.667	1
OCEL1	0.000360794	-1.526068812	0.438	0.667	1
CHMP2B	0.000361744	1.397004038	1	1	1
ATG2B	0.000361881	1.217067205	1	1	1
SLC12A5	0.000362223	1.693275744	1	1	1
ISY1	0.000363018	1.781833116	1	1	1
ENSSSCG00000057519	0.000363805	-0.814573905	0.875	0.933	1
SRPRA	0.000366089	-0.577952263	1	1	1
ENSSSCG00000061564	0.000366537	-1.109169255	0.812	1	1
MFSD14A	0.000367865	1.49739128	1	1	1
PIGW	0.000370598	-1.119581616	0.938	0.933	1

GPKOW	0.000370642	-1.517607233	0.562	0.8	1
ENSSSCG00000043636	0.000371579	-0.803602787	0.5	1	1
PCMTD1	0.00037235	1.373912443	1	1	1
OTUB2	0.000372551	-1.489999557	0.562	0.8	1
NT5C3A	0.000372641	-0.724546335	0.938	1	1
UBIAD1	0.000373253	1.547268264	1	1	1
FAM13B	0.000373401	1.314575114	1	1	1
DNAL1	0.000373509	1.496702496	1	1	1
GET3	0.000373922	-2.736965594	0.188	0.533	1
EOMES	0.000373932	1.920965781	1	0.667	1
E2F6	0.000374073	-3.180572246	0.125	0.533	1
STEAP2	0.00037491	1.862496476	0.938	1	1
FGF9	0.000375278	-0.443711808	1	1	1
NFE2L2	0.000375971	1.471247465	1	1	1
ZNF45	0.000376517	-0.670997379	0.938	1	1
TBX18	0.000376647	1.682901389	1	1	1
GNG12	0.000376898	-0.028665696	1	1	1
ENSSSCG00000050581	0.000377726	-3.9510904	0.062	0.6	1
RALGDS	0.000377967	-1.053849974	0.875	1	1
ZFAND5	0.000379471	1.139399817	1	1	1
SYTL4	0.000382978	1.47972265	1	1	1
ENSSSCG00000039538	0.000383518	-1.074962058	0.5	0.867	1
HOMER2	0.000385387	-0.220148593	1	1	1
ANGPTL6	0.000385928	-4.341036918	0	0.6	1
SORBS1	0.000386136	-0.217500267	1	1	1
YBX1	0.000386727	1.698665506	1	1	1
ENSSSCG00000060173	0.000386945	-0.757060201	0.812	1	1
NES	0.000387117	-3.197446064	0.188	0.533	1
ACRV1	0.000387442	-2.526068812	0.188	0.6	1
STX4	0.000394128	-1.388565288	0.562	0.733	1
AGFG2	0.000394485	-1.241208043	0.688	0.8	1
BBS2	0.000397272	-0.088196007	1	1	1
DNAJC8	0.000398465	0.067717539	1	1	1
DEF6	0.000398568	-1.205002284	0.75	0.933	1
BCL2L14	0.000398604	0.006086772	1	1	1
ZNF268	0.000398705	-2.495207848	0.188	0.533	1
ENSSSCG00000006738	0.000398946	-1.341036918	0.75	0.8	1
DICER1	0.00039931	1.451875795	1	1	1
ALDH4A1	0.000399511	-0.919777802	0.688	1	1
CETN3	0.000399677	1.627112926	1	1	1
HOMEZ	0.000400312	-0.093109404	1	1	1
RNASEK	0.000400498	-2.05246742	0.25	0.733	1
RBM42	0.000401769	-0.503058027	1	1	1
PRKCQ	0.00040219	2.139551352	1	0.733	1
STRN3	0.000402251	1.52823603	1	1	1
SHARPIN	0.000404701	-1.277533976	0.625	0.8	1
TLE7	0.000404813	1.997719574	1	1	1
VPS52	0.000406673	-0.701392979	0.938	1	1
IL17D	0.000407036	-3.208586622	0.125	0.6	1
FRMD7	0.000409274	1.354564243	1	0.933	1
KIF18B	0.000409524	2.703357202	0.938	0.6	1
BCL7B	0.000413736	-0.238070339	1	1	1
BZW2	0.000415523	1.362586521	1	1	1
RPUSD3	0.000417486	1.915254069	1	0.8	1
GPX3	0.000417566	-1.002294241	1	1	1
PIWIL3	0.000418695	-0.19548154	1	1	1
ENSSSCG00000009374	0.000420465	-0.493349902	0.938	1	1
ENSSSCG00000022164	0.000420809	1.710908519	1	1	1
ENSSSCG00000027916	0.000421401	-1.399770743	0.688	0.733	1
MFN1	0.000421961	1.277285169	1	1	1
ENSSSCG00000063088	0.000422443	-1.396890153	0.5	0.867	1
HMGCS1	0.000422704	-0.146503708	1	1	1
ENSSSCG00000062491	0.000423786	-1.74080766	0.5	0.8	1
TMED7	0.000423865	1.539255966	1	1	1
KAZN	0.000424643	-0.453469811	0.938	1	1
TOMM7	0.000425189	1.41737488	1	1	1
CFL2	0.000427877	-0.634935402	1	1	1
TSPAN5	0.000427904	-0.238960271	1	0.933	1
MEI1	0.000428439	-1.13965199	0.625	0.867	1
DPPA2	0.000431484	-1.269186633	0.875	0.867	1

ENSSSCG00000036736	0.000432388	-1.249228606	0.562	0.933	1
STAT6	0.000434835	-3.03170886	0.062	0.6	1
ENSSSCG00000008097	0.000435644	-1.394278939	0.562	0.867	1
ENSSSCG00000054830	0.000436805	-1.415037499	0.562	0.933	1
GALT	0.000437214	-2.8372705	0.312	0.6	1
SRSF3	0.0004378	1.309480261	1	1	1
CDKN2D	0.000437819	-0.617677307	0.938	1	1
APBA2	0.000438272	-1.438884241	0.312	0.867	1
ENSSSCG00000058154	0.000438564	-2.830074999	0.25	0.533	1
ENSSSCG00000015691	0.000443444	-0.517862059	0.938	1	1
CCNJL	0.000444221	-1.768269714	0.75	0.867	1
UBE2J1	0.000444741	-1.523743759	0.688	0.8	1
MFSD5	0.000444924	-2.495207848	0.188	0.6	1
ENSSSCG00000050221	0.000445555	-1.929610672	0.375	0.667	1
SNX2	0.000447045	1.347057803	1	1	1
MPC1L	0.000450171	-3.485426827	0.062	0.533	1
MECP2	0.000450377	-1.870716983	0.25	0.733	1
NUFIP1	0.000451153	1.182174898	1	1	1
ENSSSCG00000043676	0.000452591	-0.59171162	1	1	1
NELL1	0.000453226	-0.145013783	1	1	1
PTGR2	0.000454166	1.623642969	1	1	1
HK1	0.000458667	-2.552541023	0.188	0.533	1
TBCD	0.000459934	-0.785927895	0.812	1	1
ZYG11B	0.000460226	1.27059603	1	1	1
PUSL1	0.000460826	-2.230612928	0.25	0.733	1
SNRPA	0.00046402	-1.247437551	0.75	0.867	1
SLC7A6OS	0.000464628	1.267804459	1	1	1
ST6GALNAC1	0.000465678	3.661778098	0.75	0.133	1
PCGF3	0.00046724	-0.849838253	0.688	1	1
UPF3B	0.000468171	0.141686099	1	1	1
LY75	0.000468479	1.410498401	1	1	1
AK6	0.000468876	1.34351632	1	1	1
NF2	0.000469113	1.317271469	1	1	1
ZMAT3	0.000469148	1.344864906	1	1	1
CHAF1A	0.000469222	0.106170317	1	1	1
CCDC158	0.000469303	3.025285296	0.812	0.333	1
ENSSSCG00000045223	0.000470539	-1.227758931	0.688	0.867	1
AKAP12	0.000471689	-1.407217995	0.625	0.867	1
GART	0.000472104	1.875914946	1	0.867	1
ENSSSCG00000005101	0.000472359	0.090962171	1	1	1
FANCA	0.000474326	-1.196202897	0.562	0.8	1
SMARCA5	0.000474576	1.375405822	1	1	1
SVIL	0.000475786	1.280348991	1	1	1
ENSSSCG00000032891	0.000476116	-2.900464326	0.062	0.667	1
VRK1	0.000476833	-0.393475256	1	1	1
TUBB2B	0.000477982	-1.065095028	0.75	0.8	1
GPAM	0.000479065	-0.813331735	0.938	1	1
CDPF1	0.000481198	-1.373217324	0.5	0.867	1
TAF6L	0.000481406	-1.20987647	0.875	1	1
RCCD1	0.000481905	-0.752072487	0.875	1	1
CABLES2	0.000483517	-0.315501826	1	1	1
GJA5	0.000484741	-0.936707847	0.75	0.867	1
MCF2	0.000486204	1.869949017	1	0.8	1
TIAL1	0.000486384	1.415786438	1	1	1
DEF8	0.000486992	-1.157239742	0.688	0.933	1
ENSSSCG00000059847	0.000488266	-1.728697978	0.688	0.733	1
MAPKAPK3	0.000489374	1.610497593	1	1	1
PRELID2	0.000489554	-0.758690365	1	1	1
ZNF770	0.000490056	1.90172889	1	0.933	1
PGAM5	0.000491219	1.804042188	0.938	0.933	1
RNF34	0.00049181	1.171105654	1	1	1
SLC25A4	0.000492923	-0.480995297	1	1	1
RHOV	0.000493004	-2.678071905	0.188	0.533	1
ENSSSCG00000013387	0.000493894	-1.415037499	0.625	0.8	1
TAB1	0.000498203	-0.645013617	0.938	0.933	1
SLK	0.000499098	-1.736965594	0.75	0.867	1
NAB1	0.000501197	1.464886049	1	1	1
SRARP	0.000504586	-1.272815427	0.812	0.8	1
TEAD4	0.000506683	-2.415037499	0.125	0.667	1
KCNH3	0.000506825	-0.496114348	1	0.933	1

ENSSSCG00000032501	0.000506883	-4.415037499	0	0.533	1
CNRIP1	0.000507484	2.318316841	0.938	0.6	1
ENSSSCG00000051302	0.000508731	-0.454538111	1	1	1
CCDC138	0.000510607	-1.99492901	0.5	0.8	1
MDP1	0.000511815	-1.373217324	0.5	0.933	1
ENSSSCG00000008942	0.000512223	-0.482958572	1	1	1
VTI1A	0.000514539	-1.378511623	0.5	0.8	1
GTF3C3	0.000515429	1.594721467	1	0.933	1
ENSSSCG00000061608	0.000515542	-2.141472426	0.375	0.867	1
ENSSSCG00000013556	0.000517069	-2.019108823	0.188	0.6	1
VCL	0.000517339	1.163530416	1	1	1
PITPNC1	0.000523012	-1.777607579	0.438	0.733	1
MYH15	0.000523701	-0.053436097	1	1	1
HSD17B8	0.000527059	-1.929610672	0.25	0.667	1
TSNAXIP1	0.000527978	-2.533681996	0.25	0.533	1
MAL2	0.00052804	3.6794801	0.812	0.133	1
LAMTOR3	0.000530842	1.220099308	1	1	1
SERPINA11	0.000531678	-2.294743266	0.25	0.6	1
ENSSSCG00000041453	0.000533875	-2.093109404	0.312	0.667	1
HDHD3	0.000533902	-2.023846742	0.375	0.733	1
PLEKHG3	0.000534176	-1.793549123	0.75	0.733	1
ST6GAL1	0.000534901	1.611647609	1	0.933	1
UCHL5	0.000535917	0.179212003	1	1	1
ENSSSCG00000011324	0.000536765	-2.010647244	0.312	0.6	1
EIF4E3	0.000537382	1.401956476	1	1	1
ENSSSCG00000027746	0.00053835	-0.01939047	1	1	1
ADPRM	0.000538948	1.454776925	1	1	1
CRACD	0.000539483	2.157627643	1	0.733	1
SLF1	0.000542239	1.358987152	1	1	1
SYNE3	0.000542283	-1.945552216	0.5	0.733	1
SLC25A40	0.000542514	1.252128533	1	1	1
STRIT1	0.000544452	-2.180572246	0.188	0.667	1
CRAT	0.000545229	-1.076621282	0.75	0.933	1
ENSSSCG00000053787	0.000548205	-2.315501826	0.188	0.6	1
ENSSSCG00000055413	0.000551048	-2.30256277	0.25	0.667	1
PDCL2	0.000552205	-1.427528443	0.438	0.867	1
SOD1	0.000552328	-0.156655221	1	1	1
TSPAN15	0.000552379	1.612218049	1	0.933	1
RAB9A	0.000554094	0.040500203	1	1	1
TBC1D23	0.000558067	1.489352758	1	1	1
DMXL2	0.000558259	-0.728046547	0.938	1	1
COPB2	0.000560905	-0.059444993	1	1	1
WDR26	0.000561165	0.973806823	1	1	1
PSIP1	0.000563245	1.440964386	1	1	1
RNF38	0.000564654	1.228011102	1	1	1
EXOSC2	0.000564693	-1.338865819	0.625	0.867	1
PHAX	0.000566721	1.173826859	1	1	1
SCYL3	0.000567572	1.363530857	1	1	1
ENSSSCG00000029043	0.000569805	-2.35614381	0.25	0.6	1
ADIRF	0.00057061	-3.263034406	0.062	0.6	1
CIT	0.000570644	-0.016561391	1	1	1
ENSSSCG00000056619	0.000570911	-1.263034406	0.688	0.867	1
ABI2	0.000571004	-0.953946657	1	0.933	1
ENSSSCG00000057571	0.000571379	-1.068018423	0.688	0.933	1
WDR55	0.000571482	-1.379990552	0.625	0.8	1
DPF2	0.000576062	1.592524566	1	1	1
RAB37	0.000578401	-3.552541023	0.062	0.6	1
TMEM150A	0.000581442	1.911979472	1	1	1
ENSSSCG00000006153	0.000581501	-1.117771459	0.625	0.933	1
CENPM	0.000584473	-0.696219252	0.938	0.933	1
ENSSSCG00000056500	0.000584645	-0.340082349	1	1	1
NFAT5	0.000585339	-0.900464326	0.812	0.8	1
ENSSSCG00000012165	0.000587751	1.126517643	1	1	1
TMEM218	0.000588584	-0.379080622	0.938	1	1
AKAP8L	0.000592001	-0.742202243	0.938	0.933	1
CHRNE	0.000594655	-0.188855031	0.938	1	1
ACTR1A	0.000594994	-0.166781316	1	1	1
ILVBL	0.000596073	-1.285754482	0.562	0.867	1
CDH8	0.000596465	-0.036340308	0.875	1	1
PXK	0.000597508	1.414286844	1	1	1

TEX2	0.00059795	0.094362579	1	1	1
FBXO42	0.000601359	1.244285492	1	1	1
TFPI2	0.000602127	2.609563012	1	1	1
TAF8	0.000604426	1.783048064	1	1	1
ENSSSCG00000050213	0.000604514	-0.04257667	1	1	1
C6orf136	0.000605255	-0.36518895	1	0.933	1
KICS2	0.000605311	-0.403177344	0.938	1	1
ELOVL1	0.000606538	-1.039867383	0.875	0.933	1
GLO1	0.000607928	-0.143735477	1	1	1
ARMH1	0.000611156	-2.143735477	0.25	0.667	1
ENSSSCG00000045152	0.000611983	-1.929610672	0.312	0.667	1
ZNF226	0.000612123	-0.909312228	0.938	1	1
DLL3	0.000612323	-4.415037499	0	0.533	1
C20orf204	0.000612661	-4.093109404	0	0.6	1
ZNF496	0.000614879	-0.285078905	1	1	1
ENSSSCG00000018027	0.000616294	-0.138150369	1	1	1
DCC	0.000617313	1.38051191	1	1	1
MFSD1	0.000620956	1.352233727	1	1	1
ENSSSCG00000058521	0.000622587	-1.157681406	0.938	1	1
SGO2	0.000623039	1.512149901	1	1	1
CDKL2	0.000623256	-0.753259402	0.75	0.867	1
PLGRKT	0.000625321	1.721858702	0.938	0.733	1
HSP90AA1	0.000625918	-0.142229271	1	1	1
METTL25B	0.00062593	-2.05246742	0.25	0.667	1
ENSSSCG00000046397	0.00062604	-2.03170886	0.188	0.667	1
ENSSSCG00000012219	0.000627472	1.983511877	0.938	0.8	1
ENSSSCG00000007481	0.000630042	-0.491018105	1	0.933	1
ENSSSCG00000061765	0.000631218	-3.595609745	0.125	0.533	1
ENSSSCG00000002349	0.00063223	-1.637429921	0.5	0.867	1
FOXRED1	0.000635787	-1.709780765	0.375	0.8	1
RAVER2	0.000636343	-0.533093261	0.938	1	1
TJAP1	0.000637848	-0.748461233	0.875	1	1
XPO1	0.000637928	-0.444396419	1	1	1
SNUPN	0.000639955	-1.275312736	0.812	0.867	1
SALL2	0.000642335	1.460062029	1	1	1
BRAP	0.000642807	-0.212925558	1	1	1
ENSSSCG00000007947	0.0006431	-1.56125824	0.75	0.867	1
ZNF688	0.000643242	-4.485426827	0	0.533	1
ENSSSCG00000045539	0.000643468	-4.263034406	0	0.6	1
UBE2V1	0.000644706	0.201118095	1	1	1
ENSSSCG00000007719	0.000646271	-0.805307093	0.75	0.867	1
SOX30	0.00064707	1.466318004	1	1	1
ENSSSCG00000050661	0.000648463	-1.415037499	0.5	0.8	1
FBXL4	0.000648596	1.447978287	1	1	1
IL10RB	0.000649494	2.129283017	1	0.8	1
SPECC1L	0.000657149	1.513729977	1	1	1
ULK2	0.00065723	-0.854949667	1	0.867	1
CDKL5	0.000659206	1.10567046	1	1	1
APOD	0.000660482	-1.784987109	0.562	0.667	1
TMEM192	0.000661475	-0.638105498	0.938	1	1
BIN2	0.000662012	2.14671761	1	0.933	1
SPAST	0.000663816	1.309740839	1	1	1
PLA2G15	0.000663842	-0.490344081	0.875	1	1
ENSSSCG00000041170	0.000666533	-0.888750906	0.75	0.867	1
ENSSSCG00000053691	0.000668068	-2.208586622	0.25	0.533	1
IFI35	0.000668231	-3.263034406	0.062	0.533	1
MAGI3	0.000668859	1.3084434	1	1	1
AFG3L2	0.000670213	-0.210292944	1	1	1
STK17A	0.000670708	-0.242753025	1	1	1
NEMP2	0.000670965	1.601627954	1	0.933	1
ATP10D	0.000671201	0.071014458	1	1	1
ENSSSCG00000007963	0.000671928	-2.147557188	0.312	0.6	1
BCL2L1	0.000672	0.039043341	1	1	1
FAM221A	0.000673083	-0.563251503	0.938	1	1
DPY19L4	0.000675752	1.609740832	1	0.867	1
BTRC	0.000677092	-0.152720419	1	1	1
CDS2	0.000678022	-0.453951944	1	1	1
ENSSSCG00000059437	0.000681487	-2.263034406	0.188	0.6	1
IRAK3	0.000681568	-0.0806018	1	1	1
CSNK1G3	0.000683759	1.337674759	1	1	1

PFDN4	0.000684789	1.243717567	1	1	1
TNFRSF11A	0.000685386	-1.562594688	0.25	0.667	1
EFR3A	0.000685913	1.273579676	1	1	1
STIL	0.000685928	1.406350482	1	1	1
ENSSSCG00000041827	0.000690233	-0.744444927	0.938	1	1
PROP1	0.000693949	-0.735952821	0.812	1	1
ZNF740	0.000694531	1.519246863	1	1	1
ENSSSCG00000061304	0.000694837	-2.571156701	0.188	0.6	1
NRDC	0.000695472	1.313807633	1	1	1
ENSSSCG00000041998	0.000696586	-2.239950793	0.25	0.667	1
TMTC1	0.000696607	1.888274917	0.938	0.933	1
JAKMIP2	0.000700114	-0.214813897	1	1	1
AKAP1	0.000704486	-0.349449158	0.938	1	1
DTL	0.00070453	1.382643347	1	1	1
CYP39A1	0.000705062	-2.143735477	0.25	0.533	1
TMEM9B	0.000708447	-0.175145173	0.938	1	1
VAT1	0.000711482	-0.411185174	1	0.933	1
ENSSSCG00000002270	0.000712078	-3.093109404	0.125	0.6	1
AEN	0.000713612	-1.164900087	0.625	0.8	1
SHCBP1	0.000715425	1.092087416	1	1	1
MINDY2	0.000715447	1.591248764	0.938	0.933	1
SIDT1	0.000716013	-1.638543541	0.5	0.8	1
PHF5A	0.00071891	1.293383219	1	1	1
NAPA	0.000720076	-0.774747925	0.812	0.933	1
TECPR2	0.000721799	-1.255380833	0.75	0.8	1
ENSSSCG00000015632	0.000723596	-0.716039755	0.812	0.933	1
TOP2B	0.000723644	1.371270096	1	1	1
ENSSSCG00000050259	0.000726572	-0.847996907	0.875	0.933	1
STK25	0.000728071	-1.415037499	0.938	0.8	1
ENSSSCG00000053510	0.000728241	-2.263034406	0.25	0.6	1
DDX56	0.000731212	-1.06185847	0.875	1	1
NRAS	0.000733025	1.235347341	1	1	1
P3H1	0.000737995	-1.415037499	0.625	0.8	1
ARPC1B	0.000738599	-0.513842509	0.938	1	1
ENSSSCG00000061352	0.000739964	-0.819733054	0.938	0.867	1
FBXO48	0.000743156	-1.196732035	0.562	0.933	1
SCRN1	0.000744113	-0.581210366	0.875	0.933	1
ENSSSCG00000059315	0.000744431	-1.607682577	0.812	0.667	1
ENSSSCG00000010084	0.000746283	-2.736965594	0.125	0.533	1
HASPIN	0.000752432	-0.482034063	0.938	1	1
ZNF599	0.000753604	1.27964295	1	1	1
CYB5R4	0.000755481	-0.617712942	0.938	1	1
MYG1	0.000755484	-2.578536232	0.062	0.6	1
ENSSSCG00000041889	0.000755928	-2.056583528	0.375	0.6	1
CLP1	0.00075641	1.93004156	1	0.933	1
ENSSSCG00000036102	0.000756927	-2.27368165	0.188	0.8	1
FEM1C	0.000757181	1.064054142	1	1	1
DNAJB4	0.000757435	1.310354129	1	1	1
NIPAL3	0.000758901	-0.821029859	0.75	0.933	1
ENSSSCG00000051117	0.000759684	-1.736965594	0.375	0.733	1
ACADM	0.000762174	1.231338054	1	1	1
RPF1	0.000762492	-0.272725322	1	1	1
ENOX2	0.000763435	-0.723271325	0.938	1	1
PRR7	0.000764214	1.87036472	1	1	1
ENSSSCG00000032422	0.000764857	-1.397963986	0.375	0.667	1
ENSSSCG00000009435	0.000765006	1.325843145	1	1	1
TAF1C	0.000766409	-1.471621028	0.5	0.667	1
KDM3B	0.00076681	1.742852437	1	0.933	1
RDH11	0.000769184	1.808470186	1	0.933	1
SLC10A7	0.00077137	-0.110024155	1	1	1
ACADVL	0.000773399	-0.261863862	1	1	1
GALNTL6	0.000773429	-0.176913161	1	1	1
TBPL1	0.000775009	1.423636628	1	1	1
LRPPRC	0.000776369	1.366484057	1	1	1
LIG4	0.00077647	1.40107198	1	1	1
FOXP1	0.000777659	-0.053893008	1	1	1
RPTOR	0.000779638	-0.919777802	0.875	0.933	1
ANKRD12	0.000779718	1.378233501	1	1	1
GCNT1	0.000781596	1.296613242	1	1	1
SRP9	0.000782156	1.277354528	1	1	1

RPAP3	0.000787091	1.334692046	1	1	1
TCFL5	0.000788593	-0.783424905	0.812	0.933	1
ZWILCH	0.000789522	1.76858325	1	1	1
SYCE1	0.000791982	-3.047305715	0.25	0.533	1
RHOF	0.000792614	1.697070374	1	1	1
ENSSSCG00000031622	0.000793271	-0.886658527	0.438	0.933	1
ZNF593	0.000794205	-0.210679001	1	1	1
RFWD3	0.000795153	0.103240202	1	1	1
ZC3H4	0.000796745	-0.31188151	0.938	1	1
LYPLA2	0.000800299	-0.45369912	1	0.867	1
JAG2	0.000800785	-2.315501826	0.25	0.6	1
KIF22	0.000808309	-0.519958102	0.938	1	1
CD2	0.000809844	-1.015704901	0.875	0.933	1
AFG1L	0.000810602	2.113341473	0.875	0.6	1
PEX2	0.000811276	-0.148250959	1	1	1
NUP62	0.00082122	-0.870716983	0.812	0.867	1
CPB2	0.000821569	-1.590609064	0.5	0.733	1
ENSSSCG00000028423	0.000822164	1.360323168	1	1	1
LYZL4	0.000822907	-2.157239742	0.312	0.667	1
RHOBTB1	0.000823608	2.123546647	1	0.867	1
RBM39	0.000829394	-0.125385807	0.938	1	1
ENSSSCG00000008383	0.000829713	-0.071765869	1	1	1
TTLL13	0.000834385	-1.204140717	0.625	0.867	1
PRR19	0.000835321	-0.923184403	0.812	0.933	1
ABRACL	0.000836095	-1.075407403	0.812	1	1
ENSSSCG00000025041	0.000838314	-1.717600269	0.5	0.733	1
TMEM167B	0.000845595	-0.996894089	0.625	0.933	1
HCN2	0.000849037	-0.998717034	0.938	1	1
RSU1	0.000849626	1.302140923	1	1	1
HADH	0.000851424	0.072871467	1	1	1
AOAH	0.000853442	-1.645650427	0.375	0.667	1
STT3B	0.000853846	1.252179633	1	1	1
ISCU	0.000854578	-0.516790998	1	1	1
ENSSSCG00000062306	0.000856644	-2.415037499	0.125	0.6	1
CCSER2	0.000856716	1.214709795	1	1	1
MORC3	0.000859241	1.162220942	1	1	1
ENSSSCG00000007284	0.000860106	1.383073102	1	1	1
DDX4	0.000863773	-1.370447348	0.938	0.933	1
TMEM176B	0.000864313	-1.867549704	0.5	0.8	1
PRADC1	0.00086473	-0.393941664	1	1	1
SPIDR	0.000864896	-0.174618347	1	1	1
SF3A3	0.000864965	1.337838245	1	1	1
HSPBAP1	0.000866219	1.343482238	1	1	1
CEP89	0.000867009	0.138121678	1	1	1
CEACAM20	0.000868305	-1.407217995	0.562	0.933	1
LDOC1	0.000869578	-0.493647334	1	1	1
MAN2B1	0.00087253	-1.187436787	0.625	0.933	1
ATP5MC3	0.000878126	-0.251417075	1	1	1
SLC10A4	0.000879286	-2.250650681	0.312	0.667	1
TLR10	0.000881658	3.285402219	0.625	0.467	1
IGFBP7	0.000881807	-0.650850436	1	1	1
PDGFA	0.000882517	-0.162151049	0.938	1	1
SETD1B	0.000882539	-0.269987166	1	1	1
KLHL13	0.000886791	2.306821202	0.938	0.533	1
ZNF239	0.000888499	-0.457550927	0.938	1	1
WDR13	0.000890113	-1.900464326	0.375	0.733	1
ZNF200	0.000890231	-2.351843673	0.375	0.6	1
ENSSSCG00000014015	0.000890676	-0.638758981	1	1	1
ENSSSCG00000053482	0.000892019	-0.480132528	0.938	1	1
ZNF367	0.000892944	1.580662363	1	1	1
GNG4	0.000893945	-0.418595319	0.938	1	1
BCAS4	0.000896041	-0.363237108	1	1	1
XIRP2	0.000896813	1.284775096	1	1	1
PALM	0.000896875	-0.858644151	0.5	0.867	1
WDR5B	0.00090326	-2.036525876	0.188	0.667	1
ENSSSCG00000011317	0.000904488	1.902738952	1	1	1
COLGALT2	0.00090607	0.034532261	1	1	1
EPSTI1	0.000907967	1.590517745	1	1	1
ENSSSCG00000043931	0.000910965	-1.222392421	0.5	0.933	1
PDIA4	0.000911184	1.183957906	1	1	1

UBE2G1	0.00091324	1.385858473	1	1	1
AKR1B1	0.000914455	-0.345651436	1	1	1
ENSSSCG00000039695	0.000915867	-0.162974211	1	1	1
ENSSSCG00000028892	0.000916405	-1.093109404	0.75	1	1
ALOX15B	0.000916792	-1.548788888	0.562	0.733	1
SLC44A2	0.000918781	3.607330314	0.75	0.2	1
GLYCTK	0.000919386	-1.415037499	0.625	0.733	1
ZNF689	0.000919592	-1.620356407	0.562	0.733	1
ENSSSCG00000043990	0.000919607	-2.678071905	0.125	0.533	1
ENSSSCG00000052039	0.00092359	-2.047305715	0.25	0.6	1
APOBEC2	0.00092707	-1.325770161	0.562	0.8	1
IFFO1	0.000928854	-1.840343334	0.438	0.8	1
NPFFR2	0.000929599	-0.441371647	0.938	1	1
ENSSSCG00000044936	0.000931438	-1.344648171	0.562	0.933	1
GHRH	0.000936865	-3.485426827	0.062	0.533	1
DMAC2L	0.000938632	-1.571156701	0.5	0.667	1
VARS1	0.000938637	-2.208586622	0.125	0.667	1
TTC26	0.000941119	-0.809316438	0.75	0.933	1
CSTF1	0.00094141	-0.002528025	1	1	1
USP7	0.000943811	0.204855401	1	1	1
DENND3	0.000945346	-1.13492958	0.625	0.867	1
ITCH	0.000945523	1.269086678	1	1	1
CPEB1	0.000949263	-0.037179899	1	1	1
GAS7	0.000953265	-0.538141644	0.938	1	1
TAF13	0.00095587	1.484630578	1	1	1
ENSSSCG00000061086	0.000960396	-1.395672174	0.562	0.8	1
ARSI	0.000960803	-3.093109404	0.062	0.6	1
GPX1	0.000961328	-0.794669662	0.812	0.867	1
AKAP10	0.000961384	-0.034765418	1	1	1
B3GNT4	0.000962447	-0.096018065	1	1	1
MED9	0.000962805	-1.070025791	0.812	0.8	1
CENPJ	0.00096292	1.250059838	1	1	1
ENSSSCG00000057960	0.000964759	-1.35614381	0.5	0.8	1
BHMT	0.000966039	-1.678071905	0.188	0.733	1
ITGA8	0.000966332	-1.592680414	0.688	0.667	1
ING4	0.000967208	-0.299560282	1	1	1
ARSJ	0.000967261	1.314248597	1	1	1
KIF5B	0.000970432	1.581699714	1	1	1
WNK1	0.000973348	1.277508183	1	1	1
PHRF1	0.000978639	-1.034215715	0.812	0.933	1
ENSSSCG00000051427	0.000982939	-1.847996907	0.312	0.867	1
ENSSSCG00000053205	0.000987893	-0.929610672	0.5	0.933	1
SETD7	0.000987954	-0.782321867	0.938	1	1
ZRANB1	0.00098806	0.05489651	1	1	1
FIG4	0.00098867	-0.168904755	1	1	1
FASTKD5	0.000990118	-0.347952475	1	1	1
ENSSSCG00000017324	0.000991067	-1.415037499	0.5	0.8	1
ABHD18	0.000992322	-0.251021888	0.938	1	1
PLEKHG5	0.000992586	-1.756074417	0.375	0.867	1
GATAD1	0.000993329	-1.392669686	0.688	0.8	1
PEA15	0.000998209	-2.35614381	0.25	0.6	1
FKTN	0.000998416	1.151829281	1	1	1
GHR	0.000998964	1.218932342	1	1	1
ZNF174	0.00099919	-1.328325866	0.75	0.733	1
NIF3L1	0.00100021	-0.079941532	1	1	1
FBXO4	0.001001872	1.641918386	1	1	1
EML3	0.001008736	-1.277533976	0.5	0.8	1
COL4A4	0.001012807	-0.863627558	0.75	0.867	1
FCHO2	0.001016431	1.249964499	1	1	1
WSCD1	0.001017597	-1.781165398	0.188	0.733	1
ATAD2	0.001019111	1.225563878	1	1	1
TMEFF1	0.001020792	1.224136132	1	1	1
DHRS4	0.001024092	-1.426533138	0.562	0.8	1
MED28	0.001025746	0.087034915	1	1	1
MPHOSPH9	0.001028571	1.255924756	1	1	1
PTAR1	0.001041682	-1.557777671	0.562	0.8	1
AFF4	0.001049292	1.310622915	1	1	1
CAMSAP2	0.001052328	-1.192645078	0.5	0.867	1
ENSSSCG00000052268	0.001054024	-0.576192291	0.938	1	1
LRRC8D	0.001055866	1.091922489	1	1	1

ENSSSCG00000046125	0.001056881	-0.426403754	1	0.933	1
CD37	0.001058254	-0.541867522	0.875	0.933	1
ENSSSCG00000035992	0.001058718	-0.44503686	1	1	1
ITGA6	0.001063845	1.041036586	1	1	1
PISD	0.001064151	-0.17989749	1	1	1
ZNF524	0.001066351	-1.721140627	0.375	0.667	1
RAB3GAP2	0.001067506	1.102762544	1	1	1
IYD	0.001068566	-1.471621028	0.438	0.6	1
ENSSSCG00000053414	0.001070328	-3.415037499	0.062	0.533	1
MTFMT	0.001073564	1.664776841	0.938	1	1
FRMD4A	0.00107809	-0.299013703	0.938	1	1
DSCC1	0.001078871	1.442862799	1	1	1
ENSSSCG00000051230	0.001091966	-2.61667136	0.125	0.533	1
ENSSSCG00000057212	0.001092492	-1.519374159	0.438	0.8	1
SCN9A	0.001092805	1.319643542	1	0.933	1
CCDC117	0.001093834	1.204813227	1	1	1
PGAP3	0.001095092	-0.847996907	0.812	0.867	1
UTP18	0.001097131	0.050423831	1	1	1
KMT5B	0.001102479	0.227800198	1	1	1
QARS1	0.001103429	-0.738576644	0.875	1	1
ENSSSCG00000055443	0.001103919	-1.678071905	0.438	0.667	1
ENSSSCG00000062695	0.001117417	-2.019108823	0.375	0.6	1
DUSP11	0.001119864	1.543596671	1	1	1
ENSSSCG00000031838	0.001124191	-1.891065628	0.562	0.933	1
HARS2	0.001125861	-1.033166864	0.812	0.867	1
ZAR1L	0.00112657	0.174378046	1	1	1
HMGN4	0.001127421	2.861086906	0.688	0.2	1
EIF2B5	0.001128106	-1.4012317	0.688	0.867	1
NIPSNAP1	0.00112895	-0.468320923	1	1	1
MNAT1	0.001131737	1.126870483	1	1	1
ESRRA	0.001133258	-3.263034406	0.062	0.6	1
ZNF202	0.00113352	-0.916231642	1	0.933	1
CLCN4	0.001134044	-0.234592302	1	1	1
LIPI	0.001143647	3.321928095	0.75	0.2	1
RPS28	0.001146096	-1.14303463	1	1	1
GTF2H1	0.001149189	-0.42281485	0.875	1	1
SCAP	0.001150327	-0.556554086	1	1	1
SDHB	0.001153414	-0.089593207	1	1	1
MAPK8	0.001159485	1.129956607	1	1	1
CDC25B	0.001164362	-0.21183794	0.938	1	1
HMGCL	0.001167503	-0.152305197	1	0.933	1
ENSSSCG00000034572	0.001168727	-0.317930197	1	1	1
ACP3	0.001171109	-1.923184403	0.25	0.6	1
PTBP2	0.001172393	-0.040933939	1	1	1
HSF1	0.001182156	-0.62814668	0.875	0.933	1
UFD1	0.00118232	1.315740382	1	1	1
ENSSSCG00000042257	0.001183216	-2.040641984	0.375	0.6	1
ENSSSCG00000016982	0.001187593	1.348724522	1	1	1
XPC	0.001190451	-0.078065524	1	1	1
NYX	0.001194312	-1.929610672	0.25	0.667	1
EFCAB14	0.001194396	1.399314279	1	1	1
ENSSSCG00000011862	0.001199183	2.269460675	0.938	0.467	1
ENSSSCG00000038264	0.001200015	-3.180572246	0.062	0.533	1
COG8	0.001200803	-0.682872891	1	0.933	1
CLMP	0.001202286	-1.604071324	0.562	0.8	1
TEX12	0.001202805	1.895208791	1	0.933	1
FSIP2	0.001207015	-0.402640473	1	1	1
UCKL1	0.001209479	-1.341036918	0.375	0.667	1
HRAS	0.001210593	-4.093109404	0	0.533	1
CASK	0.001212202	1.095690045	1	1	1
ENSSSCG00000031897	0.001214961	-0.699340522	1	1	1
DEUP1	0.001216337	1.292425345	1	1	1
LSM7	0.001220509	-0.703973769	1	1	1
TMEM237	0.001223788	1.416085589	1	1	1
GIT1	0.001224559	-0.455679484	1	1	1
ENSSSCG00000059385	0.001228973	-1.075621978	0.938	0.867	1
TRA2A	0.00122946	1.238900458	1	1	1
ENSSSCG00000049223	0.001230046	-0.564861295	0.938	0.933	1
COMMD10	0.001231038	1.62058641	1	1	1
ENSSSCG00000041310	0.001236521	-0.593802988	0.875	0.933	1

MAMDC2	0.001238297	1.945571064	0.938	0.933	1
DYNC2I1	0.001239803	-1.967578522	0.25	0.6	1
ENSSSCG00000008056	0.001241669	-0.660704773	0.938	0.933	1
IER3	0.001242559	-0.52554995	0.938	1	1
ENSSSCG00000056962	0.001251866	-1.30461351	0.375	0.8	1
GDA	0.001252569	1.445579244	1	0.933	1
RSBN1L	0.001254002	1.197633985	1	1	1
ENSSSCG00000026486	0.001254163	1.744044787	1	1	1
EMILIN1	0.001255365	-1.397963986	0.562	0.867	1
ABCF2	0.00125861	-0.112474729	1	1	1
TNPO3	0.001258848	-0.278236131	1	1	1
EEF1AKMT4	0.001260248	-1.30461351	0.625	0.733	1
PLXDC2	0.001268147	-1.781165398	0.375	0.6	1
ENSSSCG00000012602	0.001271501	1.576288245	1	1	1
ENSSSCG00000048413	0.001274372	-1.027521063	0.562	0.8	1
ART3	0.001280065	1.440376879	1	1	1
ECI1	0.001282018	-1.714597781	0.438	0.733	1
SUB1	0.001282201	-0.263034406	1	1	1
ARHGDIG	0.001286582	-2.294743266	0.188	0.6	1
ERO1A	0.001286612	1.291096229	1	1	1
CEP85	0.00128735	1.226839345	1	1	1
NEK9	0.001288442	-1.803602787	0.5	0.533	1
LGALS3	0.001290641	1.275102822	1	1	1
TBC1D9	0.001291417	1.237949702	1	1	1
POC5	0.001292994	1.172588556	1	1	1
ENSSSCG00000012152	0.001293503	1.59373271	1	1	1
ACCSL	0.001293634	1.732786937	1	1	1
DHX58	0.001295128	-0.248432856	1	1	1
ATP2B1	0.001299043	1.258192314	1	1	1
ATG12	0.001299151	0.970941831	1	1	1
PPME1	0.001299629	-0.122661152	1	1	1
APBA3	0.001303534	-1.035776229	0.938	1	1
MMS22L	0.001311599	1.110478616	1	1	1
GPR19	0.001312493	-0.582494245	0.812	1	1
UBL5	0.001315608	-0.943791758	1	1	1
SLU7	0.001316041	1.154745861	1	1	1
RAB31	0.001326514	1.574127099	1	0.8	1
GAA	0.001328528	-0.403628336	1	1	1
ENSSSCG00000042996	0.001331415	3.951284715	0.688	0.067	1
DDX59	0.001331566	1.32369849	1	1	1
BRF1	0.00133451	-3.137503524	0.125	0.533	1
ZFP1	0.001339492	1.234130317	1	1	1
KCTD21	0.001340112	-0.987756404	0.688	1	1
ENSSSCG00000042842	0.001353441	-0.493647334	0.812	1	1
TCTN1	0.001353928	-0.465078182	1	1	1
BCL7A	0.001355083	-0.40861123	0.875	1	1
TM7SF3	0.001355528	1.859584881	1	0.8	1
TTC21A	0.001355603	-1.214124805	0.688	0.8	1
DDX46	0.001359858	0.011074553	1	1	1
AASS	0.001361161	1.626127687	1	1	1
SLC25A13	0.001361405	1.225043807	1	1	1
ENSSSCG00000013471	0.001361491	-0.471389048	0.938	1	1
STK10	0.001368389	-0.148250959	0.938	1	1
ENSSSCG00000044253	0.001370903	-1.36159824	0.625	0.667	1
FLVCR2	0.001372361	2.11146174	0.938	0.933	1
NABP2	0.001374907	-1.578536232	0.438	0.8	1
RIT1	0.001377128	-1.678071905	0.5	0.733	1
RTEL1	0.001388983	-2.756074417	0.125	0.533	1
EIF4H	0.001396339	1.128722856	1	1	1
PPIL4	0.00139645	1.325119758	1	1	1
DYNC1H1	0.001407447	-0.604822923	0.938	1	1
MMS19	0.001408874	1.436308974	1	1	1
GLB1	0.001410176	2.880895387	0.812	0.2	1
STRADB	0.001414933	1.505660065	1	1	1
RRAGC	0.001419506	0.00590939	1	1	1
LPAR3	0.001419778	2.259050892	1	0.867	1
ENSSSCG00000036748	0.001420254	-1.170588157	0.875	1	1
DPH2	0.001420968	-1.843131151	0.312	0.667	1
CCAR1	0.001426439	1.256297308	1	1	1
CHADL	0.001431625	-1.239950793	0.438	0.8	1

MED11	0.001431889	-1.543770813	0.562	0.733	1
SCG5	0.001432532	-1.352496033	0.812	1	1
CATSPERD	0.001433181	-2.967578522	0.062	0.533	1
GYG2	0.001435761	-2.736965594	0.125	0.533	1
ENSSSCG00000040835	0.001437667	-0.158785579	1	1	1
CEP68	0.001441325	-0.250078751	1	1	1
ENSSSCG00000060817	0.001444297	-1.323407024	0.438	0.8	1
ENSSSCG00000051966	0.001445443	-1.858644151	0.438	0.667	1
EGLN1	0.001448621	-0.732519689	0.812	0.933	1
DAPL1	0.001449243	-0.415037499	0.938	1	1
ENSSSCG00000053444	0.001451966	-1.9861942	0.312	0.667	1
SMG9	0.001456373	-1.629162305	0.25	0.733	1
AFF2	0.001466947	-0.440460096	0.938	1	1
ZMIZ2	0.001469163	-0.554243318	0.938	0.933	1
LMCD1	0.001472075	-0.233157364	1	1	1
GOLGA5	0.001474193	1.169203834	1	1	1
PRR3	0.001476568	1.975413565	1	1	1
SCAF8	0.001476784	1.309172087	1	1	1
HPS3	0.001477131	1.320229807	1	1	1
ATG16L2	0.001479128	-2.03170886	0.188	0.533	1
NEXN	0.001484305	1.407460909	1	1	1
ENSSSCG00000058485	0.001486762	-1.167109986	0.625	0.733	1
ENSSSCG00000062490	0.001488796	-1.042483331	0.688	0.867	1
TIGD7	0.001493801	1.263290908	1	1	1
KANSL1L	0.001496343	1.452378813	1	1	1
ENSSSCG00000037714	0.001496805	1.867896464	1	0.6	1
CCDC22	0.001499299	-2.678071905	0.062	0.533	1
ENSSSCG00000015814	0.001500564	1.432978637	1	1	1
FAM240B	0.001511034	-1.368116452	0.938	0.867	1
C6orf62	0.001516186	-0.907097419	1	0.933	1
ANXA9	0.001519795	-0.690044547	0.688	0.933	1
NFKBIE	0.001522973	-1.282933963	0.625	1	1
ENSSSCG00000003825	0.001524351	-0.802519276	0.688	0.867	1
CTNNA1	0.001529383	1.486344518	1	1	1
VSTM5	0.001533132	-0.395916616	1	1	1
ANAPC10	0.001535155	1.503325881	1	1	1
IRF5	0.001535512	3.638694485	0.75	0.2	1
HERPUD1	0.001537907	-1.803602787	0.812	0.533	1
ENSSSCG00000043234	0.001538685	-0.993573731	0.688	0.8	1
DIRAS2	0.001538794	1.233281404	1	1	1
ENSSSCG00000006176	0.001541211	1.246377062	1	1	1
ITGAD	0.001546073	-0.324166687	1	1	1
CEP20	0.001548215	1.217947955	1	1	1
IFRD1	0.00155116	0.00061746	1	1	1
MOCOS	0.001553908	-1.224353938	0.5	0.733	1
PPP1R10	0.001555607	-0.647210941	1	1	1
ENSSSCG00000057427	0.001559171	-0.500109585	0.938	0.933	1
ENSSSCG00000013715	0.001563839	1.328508792	1	1	1
MX1	0.001564555	1.668494844	1	1	1
NUDT17	0.001565701	-0.343846451	0.938	1	1
ZC2HC1A	0.001567378	1.22424914	1	1	1
ZHX2	0.001567488	1.695866988	1	0.933	1
ATG2A	0.001568119	-0.990015911	0.75	0.8	1
ENSSSCG00000058514	0.001569467	-2.415037499	0.25	0.6	1
MYSM1	0.001570562	1.298461413	1	1	1
MCL1	0.001575231	-0.522893489	1	0.933	1
MRPS2	0.00157801	-1.870716983	0.25	0.533	1
ORC5	0.001578219	1.10617339	1	1	1
CPNE2	0.001585845	1.747987	1	1	1
THAP2	0.001586233	1.909200756	1	0.933	1
LAT	0.001592028	-1.633677786	0.312	0.667	1
FUT2	0.001593268	-0.734655433	0.875	0.933	1
CLYBL	0.001593946	-0.650798975	0.938	1	1
CCDC113	0.001596974	-0.279877916	0.938	1	1
ENSSSCG00000063039	0.001597824	-4.415037499	0	0.533	1
EHMT2	0.001598526	1.677626926	1	0.933	1
GNPDA2	0.001598602	-0.111031312	1	1	1
ENSSSCG00000009182	0.001599231	-1.591915261	0.688	0.6	1
ZKSCAN5	0.001603033	-0.411964665	0.875	1	1
HYCC2	0.001604589	1.108303962	1	1	1

GJC1	0.001605385	-0.133373275	1	1	1
ENSSSCG00000016414	0.001614891	-2.03170886	0.312	0.6	1
SUPT16H	0.00161667	0.161030546	1	1	1
NADK	0.001619834	1.737403239	1	0.933	1
ENSSSCG00000059593	0.00162146	-2	0.25	0.6	1
PLB1	0.0016293	-0.254698547	1	1	1
RPS6KA5	0.001633482	1.275397057	1	0.933	1
DIAPH2	0.001635332	1.340287913	1	1	1
ENSSSCG00000028345	0.00163747	-1.067114196	0.5	0.867	1
TSR2	0.001651765	1.59844573	1	0.733	1
EPHA7	0.001653762	1.55488256	1	0.933	1
SLC35F6	0.001654312	-1.047305715	0.688	0.8	1
ENSSSCG00000057787	0.001668203	-1.415037499	0.562	0.733	1
EEF2	0.001669633	-0.177603882	1	1	1
FBXO38	0.001673163	-0.567551191	0.875	1	1
MED20	0.001678784	1.611434712	1	1	1
TBPL2	0.001679404	-1.197446064	0.812	1	1
TTC4	0.001680144	-0.234045194	1	1	1
ENSSSCG00000011068	0.001680231	1.121719536	1	1	1
ND4	0.001681275	1.826567838	1	1	1
BCDIN3D	0.001681591	-0.842301301	0.75	0.933	1
DYNC1LI1	0.001683799	-1.603593689	0.812	0.8	1
ATP10B	0.001687702	1.10839565	1	1	1
BET1L	0.001690318	-0.204418988	1	1	1
ENSSSCG00000017202	0.001694324	1.092137738	1	1	1
ENSSSCG00000035776	0.001695405	-1.171111916	0.625	0.8	1
CA5A	0.001695941	-0.313708544	0.812	0.933	1
RABEPK	0.001697418	1.947047722	1	0.867	1
UBE2W	0.001702935	1.204659418	1	1	1
P4HA3	0.001721933	2.248581945	0.812	0.4	1
NSUN5	0.001722774	-3.493040011	0.188	0.667	1
SOCS4	0.001723395	1.329477193	1	1	1
SEC24A	0.001723403	1.245302805	1	1	1
ENSSSCG00000039009	0.001726796	-0.138706271	1	1	1
ZBTB38	0.001731007	-0.202943956	1	1	1
ENSSSCG00000055388	0.001753278	0.038941751	1	1	1
SLC45A4	0.001760879	-0.49119377	0.938	0.933	1
ENSSSCG00000057821	0.001760892	-3.847996907	0.062	0.533	1
ENSSSCG00000059229	0.001762165	-1.900464326	0.188	0.667	1
ENSSSCG00000056422	0.001765862	-3.263034406	0.062	0.533	1
ACY1	0.001768244	-1.518415239	0.625	0.733	1
IMPA2	0.001777827	-1.830074999	0.25	0.733	1
ENSSSCG00000054032	0.001779439	-1.263034406	0.562	0.8	1
OTX2	0.001781526	-0.513154043	1	1	1
ENSSSCG00000034393	0.001785265	-0.945883451	0.812	0.933	1
HERC5	0.001791577	-1.009277814	0.875	1	1
SGK3	0.001802584	1.226237137	1	1	1
POPDC3	0.00180351	3.011227255	0.812	0.133	1
FBXL7	0.001806236	2.478432581	0.812	0.667	1
SHMT2	0.001809686	1.251217449	1	1	1
ENSSSCG00000054611	0.001811748	-1.967578522	0.312	0.6	1
RBMS2	0.001816125	0.126030661	1	1	1
SPI1	0.00181777	-2.093109404	0.25	0.6	1
TRIM14	0.001819295	-0.400164223	1	0.933	1
CLCC1	0.001819782	1.082110504	1	1	1
PLAGL2	0.001821929	0.998464983	1	1	1
CDO1	0.001829161	1.12307213	1	1	1
LYAR	0.001833956	-0.123287553	1	1	1
EEF1AKMT2	0.001834159	-0.808132446	0.875	0.867	1
PPFIA4	0.001837937	-2.446746359	0.25	0.6	1
RAD23A	0.001842833	2.382624027	1	0.933	1
ENSSSCG00000055595	0.00184384	-2.03170886	0.188	0.667	1
LYPD3	0.001845424	-0.516320835	0.938	0.933	1
CSGALNACT2	0.001850122	1.332368516	1	1	1
FITM2	0.001851643	-0.163905183	1	1	1
TMEM9	0.001852398	-4.900464326	0	0.533	1
COG1	0.001853129	-0.792449926	0.875	0.867	1
MLXIPL	0.001854344	-1.900464326	0.312	0.6	1
STAT3	0.001854529	1.378751664	1	1	1
PITPNM3	0.001865102	1.678528767	1	1	1

YDJC	0.001865437	-1.438884241	0.688	0.733	1
PLA2G4A	0.001870123	1.260314543	1	1	1
REL	0.001878266	-0.024513358	1	1	1
PDK2	0.001879081	-0.894563725	0.875	0.8	1
SLC25A48	0.001883479	-1.934411658	0.375	0.667	1
RAB40C	0.001884507	-0.343087657	1	0.867	1
ENSSSCG00000012886	0.001891292	-0.565862402	1	0.933	1
DIRAS1	0.001893362	-1.552541023	0.562	0.667	1
RETREG2	0.001907241	-0.347292893	1	0.933	1
FSTL1	0.001912845	2.274866058	1	0.867	1
SLC35D1	0.001915407	1.322994783	1	1	1
C4orf19	0.001917169	-0.93363119	0.625	0.933	1
NSUN6	0.00191912	1.272762038	1	1	1
ENSSSCG00000033787	0.001926275	-0.809316438	0.688	0.933	1
VLDLR	0.001933589	-0.309445776	1	1	1
SLC45A2	0.001939175	-0.816591769	0.75	0.8	1
USP15	0.001939519	1.090455274	1	1	1
DCUN1D2	0.001950888	-2.263034406	0.125	0.6	1
CEP126	0.001953998	1.639951735	1	0.733	1
P4HB	0.001966428	-0.389502407	0.938	1	1
ADAMTS12	0.00197353	-0.411697926	1	1	1
DYRK1A	0.001976112	-0.216211595	1	1	1
UBA1	0.00197791	1.508780424	1	1	1
ENSSSCG00000062820	0.001981727	-0.445990428	0.938	1	1
DNAH12	0.001983799	-0.207117375	1	1	1
BAG1	0.00198551	-0.205292454	1	1	1
REV3L	0.001988957	1.514814245	1	1	1
GINS1	0.002001299	-0.63584367	0.875	1	1
NRP2	0.002001995	1.788431762	1	0.8	1
RNF6	0.002006593	-0.848872594	0.688	0.867	1
ENSSSCG00000050594	0.002016825	1.397722457	1	1	1
NT5C1A	0.002026701	-4.093109404	0	0.533	1
SBF1	0.002035947	-1.140415119	0.312	0.733	1
TRIML2	0.00203605	-0.440004415	1	1	1
TUBE1	0.002036332	1.412594235	1	0.933	1
FZD7	0.002038389	-0.949745229	0.688	0.867	1
ENSSSCG00000011253	0.002045363	1.178858126	1	1	1
ENSSSCG00000049691	0.002047606	-0.730539325	0.5	0.867	1
DDHD2	0.002048831	1.179596302	1	1	1
SERPINI1	0.002050299	-3.109411217	0.312	0.6	1
ZNF34	0.002053408	1.568831912	1	1	1
TRAPPC2	0.002055838	-0.965101792	0.938	0.933	1
ARHGEF26	0.002061725	0.02710523	1	1	1
HIKESHI	0.002069165	-0.116555669	1	1	1
MTCL1	0.002084426	-1.211290831	0.625	0.867	1
ENSSSCG00000027557	0.002086787	-1.415037499	0.438	0.733	1
ARMT1	0.002090044	1.406928999	1	1	1
EIF2D	0.002090542	-0.568688446	0.875	1	1
MAD1L1	0.002095055	-1.208586622	0.562	0.8	1
ENSSSCG00000057403	0.002095847	-0.382616022	1	1	1
NBEAL1	0.002098841	1.150143437	1	1	1
CFAP92	0.00210167	-1.730539325	0.312	0.667	1
DHRS7	0.002104998	-0.275312736	1	0.933	1
SMYD4	0.00211439	-1.217098122	0.562	0.933	1
ARPC1A	0.002114903	-0.00904514	1	1	1
SLC25A16	0.002122331	1.693675311	1	0.8	1
RAB3C	0.002127106	-0.228726524	1	1	1
DDX41	0.002138854	-0.996894089	0.812	0.933	1
RBM5	0.002144802	-0.616574408	0.938	1	1
CDH15	0.002146592	-1.254572827	0.562	0.8	1
ENSSSCG00000043700	0.002149391	-0.277533976	1	1	1
HNRNPUL2	0.002157124	-0.363523341	1	1	1
NOPCHAP1	0.002158227	-0.53562764	0.875	0.933	1
HHATL	0.002158671	-2.294743266	0.062	0.6	1
SSUH2	0.002159926	-1.323407024	0.625	0.667	1
VPS4B	0.002161997	1.266121329	1	1	1
BMPR1A	0.002164878	1.426826953	1	1	1
UBE2Z	0.002166291	-0.709780765	0.688	0.867	1
USP53	0.002168241	1.455806755	1	1	1
CSKMT	0.002169932	-1.445411148	0.5	0.867	1

DVL2	0.002171952	2.246740598	0.875	0.8	1
PTPN23	0.002173558	-0.196055706	1	1	1
SARS2	0.002174878	-1.128733314	0.688	0.867	1
ENSSSCG00000012264	0.002176474	-0.556416113	0.938	1	1
NODAL	0.002180269	-1.637429921	0.375	0.667	1
NPTX2	0.002188264	-1.349449158	0.625	0.667	1
COQ8A	0.002195842	1.660411615	1	1	1
HNRNPK	0.002198658	0.927338124	1	1	1
ENSSSCG00000049460	0.002199454	-1.717600269	0.438	0.667	1
INO80D	0.002207992	-0.282399731	0.938	1	1
POLR2C	0.002211863	1.609767299	1	1	1
ENSSSCG00000060368	0.002212085	2.020101206	0.875	0.867	1
NT5DC1	0.002212154	-0.67320615	1	0.933	1
EPB41L3	0.002219417	-0.267022114	0.938	1	1
SFXN4	0.002219726	-1.159198595	0.625	0.8	1
LNPK	0.002229954	1.554588852	1	1	1
ENSSSCG00000007527	0.002230423	-1.900464326	0.188	0.667	1
CUEDC2	0.002235075	-2.315501826	0.25	0.867	1
MINDY3	0.002241749	0.141389891	1	1	1
FANCL	0.002246509	1.449038671	1	1	1
NUAK2	0.002256887	-1.730539325	0.312	0.6	1
RAB8A	0.002257746	-0.46723371	0.938	1	1
ENSSSCG00000002536	0.002267231	-2.341036918	0.188	0.533	1
SLC37A2	0.002270608	-0.124270336	1	1	1
TCF19	0.002271228	-1.793549123	0.375	0.6	1
METTL14	0.002283478	-0.224905197	1	1	1
SPECC1	0.002283501	-0.443606651	0.625	0.933	1
NOX3	0.002284131	-1.500284786	0.562	0.667	1
ENSSSCG00000006682	0.002297812	-0.171465042	0.938	1	1
ENSSSCG00000052302	0.002301072	-1.214932333	0.938	1	1
RAD51AP1	0.002304006	1.173297099	1	1	1
ABHD1	0.002306841	-1.552541023	0.438	0.6	1
DUSP28	0.002309557	-1.299560282	0.562	0.8	1
MROH1	0.002309851	-2.093109404	0.25	0.533	1
ENSSSCG00000062959	0.002310533	-0.422057927	1	0.867	1
IKBKE	0.002313142	-0.71871389	0.688	0.933	1
ENSSSCG00000060307	0.002317974	-3	0.062	0.6	1
GPATCH2L	0.002318625	-0.306939812	1	0.933	1
IVNS1ABP	0.002321743	1.024806667	1	1	1
RASSF8	0.002328162	1.201982943	1	1	1
GOLGA3	0.002330213	1.246377062	1	1	1
VPS26A	0.002333087	-0.559462018	1	1	1
FGB	0.002336931	-3.093109404	0.062	0.533	1
ENSSSCG00000047092	0.002347602	-1.730539325	0.25	0.6	1
MEX3D	0.002347777	3.076815597	0.812	0.133	1
RPL26L1	0.002351347	1.188010989	1	1	1
RRAGA	0.002356005	1.046551306	1	1	1
CYB5D1	0.002361347	-0.091690127	1	1	1
EHF	0.002364524	-0.047987937	1	1	1
ENSSSCG00000037274	0.002368693	-0.661393164	1	1	1
EIF2B4	0.002370094	-0.707819249	0.875	0.933	1
ENSSSCG00000041961	0.002379678	-1.04580369	0.688	0.933	1
GALNT7	0.002384689	1.34129342	1	1	1
PTPN9	0.002385685	-0.229394264	0.938	1	1
MRPL33	0.002389548	-0.38036269	1	1	1
ENSSSCG00000013600	0.002394591	-1.431911318	0.438	0.733	1
ENSSSCG00000042498	0.002396951	1.637500695	0.875	0.933	1
SYT5	0.002397748	-0.559890479	0.875	0.933	1
ENSSSCG00000029600	0.002414902	1.447797916	1	1	1
ENSSSCG00000014037	0.002418527	-1.706086281	0.375	0.733	1
ACP4	0.002420275	-2.341036918	0.062	0.533	1
ASB7	0.002427989	-0.086836807	1	1	1
ENSSSCG00000056648	0.002429314	-2.595609745	0.125	0.6	1
ENSSSCG00000033340	0.002432919	1.799975392	0.938	0.6	1
HCFC2	0.002435678	0.955899398	1	1	1
TRIM36	0.002439732	1.245812975	1	1	1
ENSSSCG00000062045	0.002440071	-1.415037499	0.438	0.733	1
CCDC150	0.002441316	0.113267144	1	1	1
MTMR3	0.002443444	1.132584204	1	1	1
NUP210	0.002449481	1.948443085	1	0.867	1

IRF3	0.00245263	-2.61667136	0.062	0.6	1
HEATR1	0.002459427	-0.199061487	1	1	1
ZNF250	0.002460837	-1.050674138	0.938	0.933	1
LRRC72	0.002466301	-1.321928095	0.625	0.8	1
ENSSSCG00000001396	0.0024671	-1.540568381	0.25	0.667	1
SMAD3	0.002473678	-0.540568381	0.875	0.933	1
SPDYA	0.002475669	1.491853096	1	0.867	1
CNIH3	0.002477367	-2.341036918	0.188	0.533	1
FOXN3	0.002477589	-0.105207547	1	1	1
CHRNA5	0.002478111	-0.326500825	0.938	1	1
ENSSSCG00000002532	0.002479398	-0.489038081	0.688	0.933	1
PAK4	0.00248282	-0.9861942	0.625	0.8	1
SNX12	0.002483053	-0.082865373	1	1	1
MAPK8IP3	0.002488697	-1.415037499	0.312	0.667	1
ENSSSCG00000010077	0.002491907	-1.967578522	0.25	0.6	1
DOCK7	0.002491938	1.229004953	1	1	1
ENSSSCG00000008121	0.002495553	-0.57589151	0.812	1	1
TAF15	0.002496621	-1.060687927	0.812	0.933	1
HSD11B1L	0.002499533	-2.093109404	0.312	0.533	1
YPEL4	0.002504365	-1.263034406	0.562	0.733	1
FCHO1	0.00250606	2.094517599	0.938	0.867	1
ERG28	0.002516538	0.096551815	1	1	1
PAXIP1	0.002524492	1.823474587	1	0.933	1
UBE2C	0.002530474	1.301463992	1	1	1
ENSSSCG00000055583	0.002534577	-3	0.062	0.6	1
RARS1	0.002535846	-0.086725788	1	1	1
NOC2L	0.002548354	-1.285754482	0.562	0.8	1
CAMKMT	0.002554095	-0.583744523	0.938	0.933	1
RAD51C	0.002558778	1.160647188	1	1	1
TP53BP1	0.002561505	1.083410786	1	1	1
SKIC2	0.002567599	-1.847996907	0.25	0.6	1
ENSSSCG00000017754	0.002570983	-1.830074999	0.312	0.667	1
INTS1	0.002578974	-1.714597781	0.375	0.6	1
GSS	0.002579709	-0.949518551	0.938	1	1
AHSP	0.002589442	-5.047305715	0	0.533	1
ENSSSCG00000050627	0.002590688	0.23166937	1	1	1
RNF215	0.002597316	1.67688499	1	0.933	1
ENSSSCG00000009692	0.002602077	-0.051256437	1	1	1
TGM7	0.002602379	-0.559194509	0.938	0.867	1
ENTPD4	0.002605775	-0.406837018	1	0.933	1
ZNF618	0.002607059	1.845490051	0.938	0.8	1
SURF4	0.00261036	-0.532641466	1	1	1
PIGQ	0.002616868	-1.602123052	0.312	0.667	1
SPSB3	0.002618279	-1.543770813	0.5	0.733	1
ENSSSCG00000061525	0.002619694	-2.455679484	0.312	0.533	1
ENSSSCG00000035336	0.002629758	-2.093109404	0.25	0.6	1
NEK6	0.002644661	-0.465078182	1	1	1
TPRG1L	0.002655145	-1.578536232	0.375	0.6	1
ENSSSCG00000012382	0.002657053	0.182012284	1	1	1
EIF3D	0.002659462	-0.921997488	1	1	1
RAD50	0.002660182	0.049835239	1	1	1
POLDIP2	0.002668171	-0.467712039	1	1	1
PDZK1	0.002671628	0.009263536	1	1	1
RANBP9	0.002680659	1.026593327	1	1	1
RSRC2	0.00268927	0.155977884	1	1	1
ASF1B	0.002690298	1.69698539	1	1	1
KARS1	0.002695551	1.436431505	1	1	1
ENSSSCG00000014540	0.002696532	-1.23413826	0.938	1	1
MSX1	0.002703947	1.756750246	1	0.8	1
PHKA2	0.002706709	0.011552957	1	1	1
SNX1	0.002715843	-0.548191623	0.875	1	1
ENSSSCG00000023603	0.002720472	-1.143735477	0.562	0.8	1
DENND4C	0.002725606	1.115473298	1	1	1
CHURC1	0.002727202	-0.000109832	1	1	1
DPPA4	0.002728882	2.182168597	1	0.733	1
C8orf34	0.002729071	1.741347662	1	0.867	1
PRSS23	0.002730919	-1.029544276	0.938	0.867	1
CAST	0.002734537	-0.462148479	0.875	1	1
IGF1R	0.002741866	-0.070986593	0.938	1	1
ELAVL1	0.002747541	1.294342263	1	1	1

FRRS1	0.002748165	-2.208586622	0.438	0.533	1
ENSSSCG00000043543	0.002753399	-2.485426827	0.062	0.667	1
ENSSSCG00000040775	0.002754117	-1.578536232	0.375	0.733	1
ACSS2	0.002754475	-0.291544571	1	1	1
SIRT2	0.00275651	-1.437063806	0.25	0.733	1
TTC6	0.002760885	-0.10295719	0.938	1	1
PARD6A	0.002764164	-2.047305715	0.188	0.6	1
PLPPR3	0.00277029	-1.198462405	0.5	0.8	1
PNRC1	0.002774922	-0.845181891	0.875	0.933	1
COMMD5	0.002784343	-1.620356407	0.5	0.733	1
SPATA5	0.002787572	1.282359375	1	1	1
DCTN4	0.00278762	-0.003746289	1	1	1
EFNA5	0.002791778	0.011881623	1	1	1
NAA60	0.002793938	-0.360456803	1	1	1
RPL35A	0.002796017	-0.938300255	1	1	1
RRAGB	0.002796695	-1.587874096	0.5	0.733	1
PDE3A	0.002797504	1.059660876	1	1	1
SLITRK4	0.002806168	1.428006614	1	0.933	1
RNF17	0.00280644	1.101408628	1	1	1
SLC25A33	0.002808313	0.033475694	1	1	1
ZNF32	0.00281029	-1.540568381	0.562	0.8	1
DNAAF2	0.002815552	1.534719746	1	0.933	1
KHDRBS2	0.002816552	-0.065887936	1	1	1
SRSF4	0.002821385	2.418212334	0.938	0.933	1
NOA1	0.002836838	-0.298383292	0.875	1	1
ENSSSCG00000062919	0.002840953	2.392317423	0.938	0.267	1
NUCKS1	0.002841192	-0.823502345	0.938	0.933	1
PEAK1	0.002841621	1.260364405	1	1	1
ENSSSCG00000053385	0.002845029	2.062168821	1	0.667	1
APCS	0.002867139	-1.519374159	0.375	0.667	1
YES1	0.002869704	1.254164657	1	1	1
CACNA2D2	0.002872048	-0.29127571	0.875	1	1
AGBL5	0.002873488	-0.347155027	0.938	0.933	1
BTBD18	0.002874978	1.626186441	1	1	1
GOLGA7	0.002884253	0.042624455	1	1	1
ODF2L	0.002885353	1.677545414	1	1	1
ZNF133	0.002890556	1.591054774	1	1	1
MEIKIN	0.002894882	-0.284927012	0.938	1	1
TM2D1	0.002895143	1.124197728	1	1	1
CDYL	0.002899997	1.790566478	1	0.933	1
ZNF408	0.002909701	-1.900464326	0.375	0.533	1
RPAP2	0.002916288	-0.061454066	1	1	1
THTPA	0.002920492	-0.94308396	0.812	0.933	1
CDK4	0.002922825	-0.090310771	1	1	1
TIGD2	0.002922939	-1.208586622	0.438	0.733	1
RAD51AP2	0.002927644	-1.607682577	0.25	0.6	1
TOMM70	0.002931983	-0.166884688	1	1	1
FAM135A	0.002940007	1.431126866	1	1	1
ZNF383	0.002942501	1.811733363	0.938	0.733	1
FBLN5	0.002942605	-0.399139872	1	1	1
SUN2	0.002947978	1.586972292	1	1	1
BARX2	0.002950413	1.812031409	1	0.933	1
COPRS	0.002954027	1.594304535	1	1	1
BUB1B	0.002957314	1.009320407	1	1	1
PIERCE1	0.002960432	2.064431873	1	0.867	1
DDIT3	0.00296383	-0.732391546	1	1	1
C22orf23	0.002966131	-2.263034406	0.125	0.533	1
ENSSSCG00000038150	0.002969411	-1.327574658	0.312	0.733	1
SERTAD1	0.002982407	-2.208586622	0.312	0.667	1
CHCHD6	0.002994975	-1.809316438	0.25	0.6	1
ENSSSCG00000015293	0.003001345	1.335363609	1	1	1
PNKP	0.003006059	-1.587874096	0.312	0.6	1
CARM1	0.003013252	-0.27716869	1	1	1
COX15	0.00301432	-0.255006032	0.938	1	1
MAX	0.003021309	-0.0199747	1	1	1
CTNNA3	0.003025658	0.161011946	1	1	1
HINT1	0.003027103	-0.737525755	1	1	1
ENSSSCG00000061957	0.003027791	-0.012340458	1	1	1
HYLS1	0.003034895	1.085536142	1	1	1
MPPE1	0.003038397	-1.049388027	0.75	0.867	1

IGF2R	0.003047981	1.552560356	1	1	1
TONSL	0.003054519	-1.263034406	0.312	0.667	1
ENSSSCG00000047552	0.003054988	3.813781191	0.688	0.067	1
METTL17	0.003075504	-0.473988042	0.938	1	1
MAPRE2	0.003076712	0.003427941	1	1	1
CUX1	0.003078112	-0.782769284	0.562	0.867	1
DNTTIP1	0.003080449	-0.176940995	1	0.933	1
HACD4	0.003082424	-0.314773376	1	1	1
ANKRD40	0.003083817	-0.265132872	1	1	1
CCDC77	0.003088143	1.286215998	1	1	1
OXR1	0.003091919	1.463670329	1	1	1
PLOD1	0.003100194	-0.297283968	1	1	1
CERCAM	0.003104146	-1.941106311	0.188	0.533	1
ENSSSCG00000060272	0.003104791	-0.109503843	0.938	1	1
ENSSSCG00000036445	0.003106706	1.783809111	1	1	1
APOE	0.003124447	-0.535331733	0.812	0.867	1
UBE2O	0.003134833	-0.607682577	0.938	0.933	1
LRRCC1	0.003134947	1.411759505	1	1	1
ENSSSCG00000012218	0.003165645	-1.306103128	0.25	0.733	1
IST1	0.003181505	0.010344864	1	1	1
PPP2R5C	0.003183784	0.175197429	1	1	1
PAIP2B	0.003195947	-0.161055556	1	1	1
LMNB2	0.00320227	1.389910307	1	1	1
ABHD2	0.003206231	-0.778236067	0.812	0.867	1
KCNQ1	0.003213231	-0.529208519	0.625	0.933	1
COQ9	0.003214892	-0.051202453	1	0.933	1
ZMYM2	0.003222513	1.200824448	1	1	1
VPS50	0.003239198	1.215978489	1	1	1
CLPTM1	0.003252446	-0.607682577	0.875	0.933	1
PROSER1	0.003252519	1.477187553	1	1	1
ZCCHC9	0.003261717	1.38436867	1	1	1
TBC1D10B	0.003264119	-1.029915578	0.688	0.733	1
NFU1	0.003272404	1.527433076	1	1	1
ENSSSCG00000040260	0.003275881	1.553253641	1	0.933	1
MGAT5	0.003278352	1.048801221	1	1	1
PTCHD3	0.003280556	-0.433083955	0.875	1	1
ENSSSCG00000046754	0.003284252	-2.093109404	0.188	0.6	1
SMPD1	0.003286849	-2.208586622	0.125	0.533	1
PSMA8	0.003289207	1.165229713	1	1	1
EXOC7	0.00330562	-1.881605299	0.375	0.6	1
CAMK1D	0.003308974	-0.844430292	0.75	0.933	1
HTR2A	0.003314899	-1.870716983	0.188	0.533	1
OCLN	0.003315386	1.258982702	1	1	1
RAB32	0.003316061	-0.076558228	1	1	1
AHCYL1	0.003318696	-1.602123052	0.562	0.733	1
CNOT6L	0.003321023	1.086049704	1	1	1
ZC3H15	0.003332213	1.088827148	1	1	1
CYLD	0.003333682	1.263034406	1	1	1
RAB4A	0.00333707	1.593524514	1	1	1
BPIFB3	0.00334031	-3	0.062	0.533	1
RADIL	0.003340936	1.574710029	1	1	1
TAGAP	0.003345055	-2.040641984	0.25	0.6	1
PLCD1	0.003346755	-2	0.188	0.533	1
ENSSSCG00000035645	0.003355656	-1.830074999	0.25	0.667	1
DHRS1	0.00335568	-0.576192291	0.938	0.867	1
MINDY1	0.003362145	-0.846826363	0.938	0.867	1
SFSWAP	0.003364212	0.006937772	1	1	1
E2F4	0.003368995	1.723473358	1	1	1
UFSP2	0.003379566	1.615841814	1	1	1
ENSSSCG00000025565	0.003384383	-0.367825615	1	1	1
AMBRA1	0.003385814	-0.175571565	1	1	1
ARMH3	0.003387423	-0.093109404	1	1	1
DIRAS3	0.0033939	-1.075407403	0.812	0.667	1
FBXL3	0.003394151	1.098640222	1	1	1
SNX24	0.003395123	1.228145949	1	1	1
ENSSSCG00000052085	0.003403266	-0.876298015	0.875	0.8	1
C4orf36	0.003407158	-1.331269142	0.875	0.6	1
IGHMBP2	0.003411726	-1.346865997	0.25	0.667	1
RNF157	0.003423326	-0.36994961	1	1	1
ENSSSCG00000006899	0.0034338	-0.120014362	1	1	1

ENSSSCG00000018055	0.003440821	-3.093109404	0.062	0.533	1
CCDC70	0.003451056	-1.633677786	0.438	0.667	1
KCNN4	0.003455423	-0.952931746	0.688	0.8	1
MTHFD1	0.003456934	-0.425953277	1	1	1
ENSSSCG00000056241	0.003472451	1.745954377	1	0.933	1
CDC37	0.003472899	-0.462632244	1	1	1
GRHL2	0.003476762	0.190683562	1	1	1
RECQL4	0.003481945	-0.707366212	0.812	0.933	1
ARFRP1	0.003495641	-2.093109404	0.125	0.533	1
ZFPL1	0.003499934	-0.8372705	0.75	1	1
CIB1	0.003509375	-0.40019899	1	0.933	1
PTCH1	0.003509959	2.047864223	1	0.933	1
ATXN1	0.003511234	1.312405321	1	1	1
TMEM101	0.003512418	-1.200024608	0.562	0.8	1
MEN1	0.003524306	-1.721140627	0.438	0.667	1
NR1H4	0.00352516	1.549127902	1	1	1
SUPT6H	0.003526454	-0.182208451	1	1	1
RAPGEF6	0.003529486	1.152967673	1	1	1
ENSSSCG00000055037	0.003554471	-1.678071905	0.312	0.733	1
CYSTM1	0.003556037	-0.292004644	1	1	1
DDX52	0.003571542	-0.484447068	0.938	1	1
ARFGEF2	0.003575271	0.094554913	1	1	1
TAPT1	0.003577088	1.107071417	1	1	1
CCNYL1	0.003578541	1.268773408	1	1	1
SYT16	0.003601286	1.267513981	1	0.933	1
PRR14	0.003606817	-0.534481051	0.875	1	1
PDP1	0.003608342	-0.24307191	0.938	1	1
KDM3A	0.003608406	1.295292968	1	1	1
ENSSSCG00000036117	0.003612109	-2.35614381	0.188	0.533	1
FBRS	0.003614807	-1.941106311	0.25	0.6	1
ENSSSCG00000043719	0.003620154	-0.689212463	0.75	1	1
KAT14	0.003622771	-0.941106311	0.812	0.867	1
CCDC126	0.003638713	1.213470726	1	1	1
ENSSSCG00000053468	0.003643463	2.994353437	0.688	0.133	1
ND3	0.003647303	1.903446038	1	1	1
NRDE2	0.003655863	-0.867227997	0.812	1	1
FOXM1	0.00365656	-0.250078751	0.938	1	1
ENSSSCG00000051926	0.003658365	-1.540568381	0.25	0.667	1
LYZL6	0.00366004	-2.010647244	0.25	0.6	1
SPRTN	0.003672097	0.075741982	1	1	1
ENSSSCG00000038171	0.003675617	2.379826479	0.812	0.4	1
ABHD5	0.003676693	-0.344334508	0.938	0.933	1
THPO	0.003684568	4.461479447	0.562	0.067	1
DHX37	0.00369846	-0.77118131	0.75	0.733	1
ENSSSCG00000042380	0.003718659	-0.330148602	0.812	1	1
ENSSSCG00000060794	0.003719697	-0.528324786	1	0.933	1
DBN1	0.003722907	-0.063114664	0.938	0.933	1
BOC	0.003736522	0.165202591	1	1	1
GNA13	0.003750963	0.992938723	1	1	1
SYT6	0.003754827	-0.540568381	0.625	0.933	1
FRMD4B	0.003765099	1.56139403	1	0.933	1
AMMECR1	0.003778771	-0.699445556	0.875	0.933	1
ZW10	0.0037897	-0.034264746	1	1	1
CHFR	0.003794691	-0.334117504	0.938	0.933	1
NTHL1	0.003797173	-0.520530628	0.938	0.867	1
ZACN	0.003801953	-1.508146904	0.312	0.667	1
TET3	0.003807019	1.161034496	1	1	1
ENSSSCG00000025525	0.003809767	-2.471621028	0.125	0.533	1
SMIM1	0.003814297	-3.793549123	0	0.533	1
PRMT9	0.003815807	1.603498452	1	1	1
PDE7A	0.003816812	1.587822155	1	0.867	1
SYT17	0.003830005	-1.567040593	0.25	0.533	1
ETV6	0.00383129	-0.486930418	0.938	1	1
VAC14	0.003837938	-0.971803108	0.562	0.867	1
ENSSSCG00000049047	0.003845953	-1.382616022	0.688	0.8	1
ENOX1	0.003848627	1.416400868	1	1	1
TSKU	0.003854453	-2.415037499	0.125	0.6	1
SLC16A10	0.003864344	1.321071133	1	1	1
SH3BP5	0.003874812	1.695344306	1	1	1
ENSSSCG00000055400	0.003889725	-2.163498732	0.25	0.6	1

DDR1	0.003892094	1.75227865	1	0.933	1
GRK7	0.003895052	1.443892058	1	0.933	1
CCNF	0.003898147	-0.811696469	0.812	0.933	1
TSR1	0.00390173	0.095126006	1	1	1
RTTN	0.003906411	1.106919538	1	1	1
BPNT2	0.003908411	1.100648435	1	1	1
CD99L2	0.003913237	-1.756074417	0.25	0.667	1
ENSSSCG00000060667	0.003926691	-1.005646563	0.562	0.867	1
DCAF5	0.003930267	1.009919479	1	1	1
RGS16	0.003934752	1.392214266	1	1	1
TMOD1	0.003936761	-0.166065438	1	1	1
ENSSSCG00000037703	0.003949153	1.62309763	0.938	0.733	1
CYFIP2	0.003950291	-0.082001837	1	1	1
TOR1B	0.003958137	1.760501109	1	0.867	1
HFM1	0.003960092	1.487514161	1	0.733	1
CERS2	0.003962174	1.428048282	1	1	1
ARL10	0.003963497	-0.132667878	1	1	1
RWDD2A	0.003964215	0.111941817	1	1	1
U2SURP	0.003967972	1.203810637	1	1	1
ENSSSCG00000020830	0.003969552	-0.159656666	1	1	1
COTL1	0.003972061	-0.451156925	1	0.933	1
DEPTOR	0.003980075	1.159583057	1	1	1
ENSSSCG00000055009	0.003985808	-0.379291838	1	0.933	1
PRKCA	0.003986832	-0.470179053	0.938	1	1
VWA5A	0.003996502	-0.827172191	1	0.933	1
DCAF8	0.003997394	1.214390052	1	1	1
CYTH1	0.004002644	-1.237499314	0.625	0.733	1
PPP5C	0.004004554	1.827078014	1	0.867	1
USP44	0.004066362	1.065578002	1	1	1
BST1	0.004073574	-1.877380713	0.25	0.6	1
GALK2	0.004084168	0.160761248	1	1	1
PRIM1	0.004085167	-0.423074818	0.938	1	1
ENSSSCG00000036013	0.004108756	-1.491658781	0.312	0.667	1
ENSSSCG00000004392	0.004116138	1.186605232	1	1	1
ENSSSCG00000056738	0.004118914	1.674056428	1	0.8	1
SPATS2	0.00412589	1.111801256	1	1	1
ENSSSCG00000045298	0.004134115	-1.929610672	0.312	0.6	1
ENSSSCG00000031300	0.004137477	-0.638543541	0.625	0.867	1
WDSUB1	0.004138511	1.13524704	1	1	1
EPHB1	0.004142522	3.076815597	0.812	0.2	1
TRIM33	0.004143069	1.311194433	1	1	1
TLN2	0.004144264	-0.524000855	0.938	1	1
SFMBT1	0.004160949	1.105427033	1	1	1
TOR3A	0.004165347	-0.815575429	0.75	0.933	1
PAIP1	0.00416589	1.133607905	1	1	1
NKAPD1	0.004170086	0.008292628	1	1	1
SUSD4	0.004170691	-1.093109404	0.812	0.867	1
DSCAML1	0.004179731	-0.736965594	0.812	0.933	1
ENSSSCG00000031365	0.004179786	-0.442916532	0.812	1	1
ENSSSCG00000059837	0.00420086	-0.542094236	0.875	0.933	1
EPHB2	0.004208899	-0.693501946	0.688	0.867	1
CAV2	0.004210329	2.555983434	0.812	0.2	1
ENSSSCG00000056139	0.00421262	-1.700791982	0.5	0.667	1
ENSSSCG00000002855	0.004212799	1.42096961	1	1	1
PLK1	0.004213681	0.066533751	1	1	1
COX19	0.00423249	-1.30256277	0.375	0.667	1
COX16	0.00424075	-0.76747099	1	1	1
TMEM97	0.004245652	-0.416486716	1	1	1
ENSSSCG00000049366	0.004249699	-2.093109404	0.25	0.533	1
TUBGCP5	0.004250061	1.097744618	1	1	1
PRTG	0.004253135	1.240959253	1	0.933	1
ZMYND11	0.004257926	1.203106442	1	1	1
YBX3	0.004259547	-0.443866152	1	1	1
C7orf50	0.004263598	-2.263034406	0.062	0.6	1
CC2D1B	0.004281894	-0.551539499	0.875	0.867	1
MESP2	0.004282651	-0.329307625	0.938	0.933	1
PIP5K1C	0.00429615	-0.079125175	1	0.933	1
ANO10	0.004318358	-0.172082579	0.938	1	1
SIN3B	0.00432188	1.41564253	1	0.933	1
SCAMP2	0.004329533	-0.249305511	1	1	1

WDR12	0.004345062	0.123267602	1	1	1
ENSSSCG00000050702	0.004359009	-1.607682577	0.25	0.667	1
EVI5L	0.004359258	-0.540568381	0.438	0.8	1
EPHA6	0.004363273	-0.005236648	1	1	1
ACER3	0.004363713	-0.04449946	1	1	1
CTSZ	0.004375029	-1.629162305	0.188	0.733	1
PRMT5	0.004386024	-0.115248382	1	1	1
C21orf91	0.004387499	1.58176149	1	1	1
GRK4	0.004392869	-0.870716983	0.5	0.867	1
ENSSSCG00000049490	0.004399501	3.965784285	0.562	0.133	1
RMI1	0.00441071	1.483167368	1	1	1
DAZAP1	0.00442163	-0.688267672	0.812	0.933	1
SLC46A3	0.004424853	-0.481179856	0.812	1	1
TELO2	0.004428386	-4.552541023	0	0.533	1
ENSSSCG00000005908	0.004444505	-1.678071905	0.312	0.533	1
ENSSSCG00000024263	0.004446487	-0.602304397	1	1	1
ENSSSCG00000048448	0.004455114	-1.756074417	0.25	0.667	1
ENSSSCG00000032840	0.004468855	-1.533681996	0.375	0.6	1
DCN	0.004477309	-1.415037499	0.25	0.667	1
CFAP100	0.004488589	-1.137503524	0.438	0.867	1
ENSSSCG00000059579	0.004488653	-1.809316438	0.25	0.533	1
HNRNPH3	0.004495153	0.218284396	1	1	1
BIRC3	0.004509153	1.335189994	1	1	1
MTRFR	0.004515436	-0.576533879	0.938	1	1
AUH	0.004519207	1.213061505	1	1	1
ZMYM3	0.004520551	1.276124405	1	1	1
KIFC1	0.00454935	1.63462835	1	1	1
ENSSSCG00000008850	0.004558646	1.491853096	1	1	1
SYCP1	0.004561086	1.491853096	1	1	1
ENSSSCG00000061649	0.004561682	2.025285296	0.812	0.667	1
UBE2D4	0.00456437	-1.900464326	0.125	0.533	1
TMA16	0.004568599	-0.357351592	1	1	1
DPP8	0.004570584	1.430602966	1	1	1
TBL2	0.004585269	-0.252013	0.875	1	1
ENSSSCG00000050522	0.004596683	-0.958179824	0.562	0.867	1
HINT3	0.004611473	-0.124405535	1	1	1
ENSSSCG00000052071	0.004614814	-0.9510904	0.688	0.867	1
SLC8A1	0.004616304	0.124312201	1	1	1
DTX3	0.00462001	1.807354922	1	0.733	1
STRIP2	0.00462387	1.27293544	1	1	1
ENSSSCG00000012654	0.004645889	1.317433078	1	1	1
YOD1	0.004662139	1.291838261	1	1	1
DKC1	0.004668682	1.360566774	1	1	1
HSPA14	0.004674531	1.111267567	1	1	1
TXNDC15	0.004695706	-1.643306487	0.438	0.667	1
SEL1L	0.004701149	0.15259286	1	1	1
RPS6KB1	0.00470322	-0.222946842	0.938	1	1
MAP3K15	0.004704048	0.91847857	1	1	1
GUCD1	0.004713396	2.121903487	0.938	0.933	1
ENSSSCG00000007749	0.004742423	1.048959768	1	1	1
RMND5B	0.004747565	-0.643923683	0.875	0.933	1
CENPE	0.004771629	1.456258982	1	1	1
MRAP2	0.004783019	-1.093109404	0.812	1	1
SNX30	0.004806712	-0.348106491	0.938	0.933	1
FAIM	0.004808346	0.053224939	1	1	1
CALCOCO1	0.004814925	1.767487539	1	0.867	1
B4GALT3	0.004815501	1.681123447	1	1	1
TERF2IP	0.004821764	0.116206035	1	1	1
VPS33B	0.004835043	-0.701648842	0.938	0.867	1
THAP5	0.004835657	1.315395125	1	1	1
DOCK3	0.004837583	1.289557848	1	1	1
ENSSSCG00000044306	0.004838811	-1.192645078	0.375	0.667	1
CYP2D6	0.004849954	-2.809316438	0.125	0.533	1
GPAT4	0.004880329	-0.040160524	1	0.933	1
SAV1	0.004882146	1.176322773	1	1	1
ENSSSCG00000053483	0.004886867	-2.24961389	0.688	0.467	1
ECT2	0.004887427	1.273988395	1	1	1
CS	0.004891176	1.146013232	1	1	1
LIMD2	0.004906286	-0.623624121	0.875	1	1
ENSSSCG00000053875	0.004906928	2.87036472	0.75	0.2	1

ENSSSCG00000044627	0.004913522	-0.270936273	1	1	1
SERPINE3	0.004922378	-0.798366139	0.75	0.8	1
ENSSSCG00000058938	0.004926601	-1.00904514	0.812	0.8	1
CDK1	0.004927524	-0.566246126	0.938	1	1
DCAF10	0.004943984	-0.805532814	0.75	0.933	1
ENSSSCG00000055605	0.004944753	-1.093109404	0.562	0.867	1
LIFR	0.004944861	-0.018943386	1	1	1
H2BC1	0.004947739	-0.420962333	0.938	1	1
LHX1	0.00494933	1.557942287	1	0.867	1
CLOCK	0.004957714	1.014081575	1	1	1
IFT20	0.00497319	-0.247437551	1	1	1
PIP4K2A	0.004975443	-0.600713022	0.938	0.933	1
LPP	0.004977295	1.405696453	1	0.733	1
ENSSSCG00000035622	0.004983301	1.392791136	1	1	1
SCN4A	0.00499137	-0.617268626	0.938	1	1
ENSSSCG00000007847	0.004996897	1.506352666	0.938	0.8	1
KCNK13	0.005009519	-1.366127899	0.438	0.733	1
ZNF248	0.005034404	1.310740414	1	1	1
ND4L	0.005053496	1.859288913	1	1	1
HLX	0.005058039	-1.186218809	0.438	0.8	1
ENSSSCG00000060401	0.00506609	-1.736965594	0.188	0.533	1
ENSSSCG00000048380	0.00508099	-1.35614381	0.5	0.733	1
PSME1	0.005084239	-2.359895945	0.125	0.933	1
ENSSSCG00000045735	0.005085673	-1.010647244	0.688	0.867	1
UTP3	0.00508885	-0.238208298	1	1	1
ENSSSCG00000060030	0.005091424	-0.252013	0.938	1	1
CNOT2	0.005103703	1.216283055	1	1	1
ENSSSCG00000042822	0.005104484	-1.678071905	0.25	0.533	1
KBTBD8	0.005107408	1.190062647	1	0.933	1
SELENOT	0.005109147	0.065783906	1	1	1
ENSSSCG00000059519	0.005110852	-0.913288367	0.812	1	1
FAM177A1	0.005123195	1.114998791	1	1	1
PRICKLE2	0.005123249	0.977229813	1	1	1
SLC26A9	0.005127875	1.967772838	0.938	0.667	1
LRP11	0.005128565	2.321928095	0.812	0.4	1
RBMS1	0.005142862	1.554411409	1	1	1
ADGRA3	0.005153442	0.137472707	1	1	1
GBE1	0.005157857	1.270619001	1	1	1
MFSD14B	0.005171349	1.112345022	1	1	1
SLC7A5	0.005176572	-1.545621609	0.5	0.667	1
ENSSSCG00000011121	0.005217488	-0.013765973	1	1	1
EFNA1	0.005218028	1.490233001	1	0.933	1
ENSSSCG00000042788	0.005235956	-0.334117504	0.938	0.933	1
C10orf143	0.00523728	-0.144639705	1	1	1
PRTFDC1	0.005241011	3.491853096	0.562	0.133	1
ARL6	0.005241351	-0.451902793	1	1	1
DNAJA2	0.005242834	0.062202624	1	1	1
FKBP5	0.005256536	0.241541542	1	1	1
QDPR	0.005269388	-0.301597987	1	1	1
ENSSSCG00000014242	0.005278195	-0.595609745	0.812	0.933	1
DHRS11	0.005286664	-0.941106311	0.562	0.733	1
ARID3B	0.005289192	-0.043356369	0.938	1	1
ENSSSCG00000061655	0.005291905	-0.881605299	0.812	0.933	1
SYS1	0.005292468	-0.068115025	1	0.933	1
AP5S1	0.005304202	-1.578536232	0.312	0.667	1
ELL2	0.005311439	-0.406863568	1	1	1
TUBA3C	0.005320275	1.438215133	1	0.933	1
DYNC2LI1	0.00532711	1.539729451	1	1	1
SUMF1	0.005328766	-0.093109404	1	1	1
FAM178B	0.005336157	-1.508146904	0.312	0.533	1
BLCAP	0.00534171	1.924812504	0.938	0.8	1
PKP2	0.005344945	3.74819285	0.562	0.067	1
IKBKG	0.005358922	-1.552541023	0.438	0.667	1
RBM15B	0.005359852	-0.641730058	0.812	0.933	1
RETREG3	0.005361475	-1.00843908	1	0.933	1
RTN2	0.005379844	-0.652536813	0.938	0.867	1
POFUT1	0.005407976	1.259515488	1	1	1
CDCA3	0.005421415	1.717073072	1	1	1
FAM193A	0.005423346	-0.286989138	0.938	0.933	1
TRAPPC9	0.005430356	-0.351711564	1	1	1

PBLD	0.005432964	-0.599569931	1	1	1
C12orf43	0.005442949	-0.877380713	0.812	0.933	1
TMEM254	0.005470772	-0.716039755	0.875	0.867	1
ITIH2	0.005472225	-1.127056736	0.375	0.667	1
HERC4	0.0054773	1.136535133	1	1	1
PIP5K1B	0.005481814	1.329666605	1	1	1
BLVRA	0.005489233	-0.9861942	0.5	0.733	1
HPGDS	0.005495061	1.094053307	1	1	1
KAT8	0.005509312	-0.100359143	1	1	1
SEC14L2	0.005512227	-1.236067358	0.625	0.867	1
FDXR	0.005524215	-2.211290831	0.438	0.933	1
STK39	0.005532642	0.053947225	1	1	1
RELA	0.005539256	-1.05994254	0.5	0.8	1
MTHFD1L	0.005540365	-0.443606651	0.938	0.933	1
MAPK1IP1L	0.005580373	-0.304216018	1	1	1
PRRG1	0.005597382	1.151396925	1	1	1
ENSSSCG00000044155	0.005602329	-0.706086281	0.75	0.933	1
ENSSSCG00000057365	0.005616955	-0.053226889	1	1	1
TTLL11	0.005622202	-0.858644151	0.625	0.733	1
GINM1	0.005622399	1.364265041	1	1	1
FLOT2	0.005637594	-0.644519345	0.75	0.8	1
ZNF462	0.005639236	1.134502947	1	1	1
ENSSSCG00000033707	0.005640175	-0.761903496	0.875	1	1
ENSSSCG00000063070	0.005641162	-0.967578522	0.688	0.733	1
DROSHA	0.005671023	0.042829253	1	1	1
GCAT	0.005671757	-2.595609745	0.125	0.533	1
FAM8A1	0.005672375	-0.703162886	0.812	1	1
CDK6	0.0056762	-2.571156701	0.125	0.6	1
ENSSSCG00000050357	0.005683974	-1.455679484	0.375	0.533	1
SLCO3A1	0.00568573	-0.739800895	1	0.867	1
E2F8	0.005703716	1.283680626	1	1	1
ENSSSCG00000035091	0.005704734	-0.809316438	0.688	0.8	1
TFE3	0.005715325	-0.260051781	1	1	1
MTMR8	0.005727756	-0.545621609	0.875	1	1
USP46	0.005734949	0.099011343	1	1	1
ENSSSCG00000002709	0.005736097	-0.125450124	1	1	1
ATIC	0.005745059	1.126662749	1	1	1
NUDT22	0.005752809	-1.567040593	0.188	0.533	1
PDCD10	0.005770791	1.168410588	1	1	1
PDCD7	0.005775975	1.281127564	1	1	1
ENSSSCG00000059276	0.00577862	-0.62814668	0.625	0.867	1
TMEM107	0.005783607	1.32349539	1	1	1
DCTPP1	0.005784441	1.27330723	1	1	1
PSMD11	0.005793122	1.131586109	1	1	1
ILKAP	0.005804703	-0.10718459	0.938	1	1
FEM1A	0.005844526	-0.753622938	0.562	0.8	1
TASP1	0.005847952	-0.900464326	0.875	0.733	1
APLP2	0.005864213	0.10671416	1	1	1
WDR19	0.005892945	1.118286568	1	1	1
ENSSSCG00000039249	0.005912017	1.12026654	1	1	1
TRAF6	0.005944952	1.218492838	1	1	1
ENSSSCG00000047668	0.005945823	-1.595609745	0.188	0.733	1
CASTOR2	0.005948285	-0.391632895	0.875	0.933	1
HNRNPLL	0.005971441	1.791710933	1	0.8	1
KYAT3	0.005975677	-0.530730189	0.938	0.933	1
MCCC1	0.005976278	1.147065047	1	1	1
GOLGA1	0.005980579	0.103815206	1	1	1
ENSSSCG00000050056	0.005990304	1.411565307	1	1	1
ENSSSCG00000004814	0.005996519	0.072186495	1	1	1
LUZP1	0.00599683	1.072080505	1	1	1
CD63	0.006002124	-1.569324242	0.938	1	1
TTC19	0.006009942	-1.830074999	0.312	0.533	1
MRC1	0.006037231	2.039340892	0.812	0.4	1
FAM209B	0.006040547	-1.967578522	0.188	0.6	1
ENSSSCG00000005457	0.006079988	1.555000815	1	0.867	1
C14orf119	0.006099237	1.290854135	1	1	1
UGP2	0.006102108	1.044777809	1	1	1
ILDR2	0.006106369	1.315331398	1	1	1
RABGGTA	0.006119668	-0.580461108	1	1	1
TMEM131	0.006133844	1.060022654	1	1	1

REPS2	0.006134178	-0.116140373	1	0.933	1
ENSSSCG00000036792	0.006140201	-0.249699767	1	1	1
DGKB	0.006145983	1.328454029	1	0.933	1
F2RL1	0.00616401	0.085860737	1	1	1
INTS13	0.006172333	0.005396141	1	1	1
ENSSSCG00000051336	0.006229765	-1.530514717	0.438	0.733	1
RING1	0.006237745	-3.793549123	0	0.533	1
UBE3B	0.006255331	-0.276139393	1	1	1
MAP3K20	0.006258928	1.329833293	1	0.933	1
MBLAC1	0.00627138	-0.7355574	0.75	0.8	1
PLA2G4D	0.00627531	1.269121736	1	1	1
ENSSSCG00000012541	0.006283995	-0.9861942	0.312	0.8	1
ZNF691	0.006296909	-1.220864952	0.625	0.533	1
RNPS1	0.006297178	1.239770693	1	1	1
SLC9A6	0.006321817	-0.074342375	0.938	1	1
KREMEN1	0.006330265	-0.206003461	1	1	1
NEK11	0.0063524	1.266934063	1	0.867	1
DCLRE1C	0.006364363	-1.032567862	0.938	0.867	1
C17orf107	0.006365252	-1.415037499	0.312	0.6	1
TSEN34	0.006369861	-1.736965594	0.125	0.6	1
ENSSSCG00000014031	0.006376259	1.520493144	1	1	1
SASS6	0.00638171	1.241090493	1	1	1
ENSSSCG00000017032	0.006385684	-0.407893937	1	1	1
P2RX4	0.006389982	-1.257496222	0.5	0.733	1
SUGP2	0.006406737	1.58739743	1	1	1
FAM102B	0.006422957	1.227046389	1	1	1
MYH3	0.006423398	-1.382616022	0.312	0.6	1
ACBD5	0.006444325	1.220390068	1	1	1
ENSSSCG00000060966	0.006447721	-1.973527789	0.5	0.667	1
ADAM10	0.00647117	0.960543575	1	1	1
CPNE4	0.006472851	1.761039729	1	0.667	1
HELZ	0.006482316	1.148965383	1	1	1
MGAT1	0.006489943	-0.278921468	1	1	1
MYOCD	0.006496678	1.906890596	0.875	0.667	1
ENSSSCG00000054140	0.006562858	-0.615530439	0.812	0.933	1
ATP2A2	0.006593387	1.004811316	1	1	1
SETD5	0.006595634	1.564002882	1	1	1
MAP2K4	0.006608175	1.136304089	1	1	1
TGFBR3	0.006642223	-1.093109404	0.625	0.733	1
ARHGEF39	0.006654822	1.882643049	0.938	0.667	1
SEMA3C	0.006656298	1.3534546	0.938	0.933	1
RGL1	0.006657928	1.322363205	1	1	1
GABBR2	0.006680015	-0.426846802	0.812	1	1
ZNF510	0.006696715	1.631046383	1	0.867	1
GALNTL5	0.006731749	-0.492716863	0.75	0.867	1
ENSSSCG00000055570	0.006733857	1.878876219	0.875	0.4	1
TMEM33	0.006744906	1.133594438	1	1	1
ASIC3	0.006757782	-1.900464326	0.125	0.533	1
SLC41A2	0.006760532	1.245885688	1	1	1
POGZ	0.006764842	1.120863413	1	1	1
RASGRP1	0.006772245	-0.417460162	0.938	1	1
GTF2E1	0.006778353	0.863482585	1	1	1
ENSSSCG00000012746	0.006813925	0.147533861	1	1	1
NOL6	0.006821983	-0.730539325	0.5	0.867	1
LMO7	0.00682268	0.290594888	1	1	1
WHAMM	0.006823959	1.232575012	1	1	1
ENSSSCG00000021767	0.006834887	0.026876452	1	1	1
HIRIP3	0.006836261	0.067762349	0.938	1	1
COMMD3	0.006838131	-0.044310106	1	1	1
ENSSSCG00000044382	0.006843274	-0.813001485	0.75	0.8	1
C18orf25	0.006843589	0.162005338	1	1	1
SEC22C	0.006847258	-0.121678557	1	1	1
SLC35F5	0.006856356	1.491853096	1	0.733	1
GUCY1B1	0.006870875	3.845490051	0.5	0.067	1
THBS4	0.006887517	1.349574513	1	1	1
LXN	0.006896796	-0.561298382	1	1	1
SCFD1	0.00693904	0.147663911	1	1	1
TEPSIN	0.006950135	-0.852101305	0.688	0.733	1
ITGA9	0.006962833	1.45462019	1	1	1
ENSSSCG00000020870	0.006969622	1.17077892	1	1	1

SERPINE2	0.006985018	-0.214950527	0.938	1	1
TMEM63B	0.00699644	-0.350267244	0.938	1	1
EMILIN2	0.006998782	-0.058663258	1	1	1
RANBP10	0.007002369	-0.259985774	0.938	1	1
AAR2	0.007050229	-0.268427665	0.875	0.933	1
TACR1	0.007059722	-0.761700256	0.75	1	1
RHEB	0.0070691	1.298346245	1	1	1
ENSSSCG00000058759	0.007126074	-0.701341684	0.938	0.867	1
PSMD10	0.007152424	1.066128376	1	1	1
FDX2	0.007154693	-1.781165398	0.25	0.667	1
CLK4	0.007159509	-0.161127828	1	1	1
ATP6AP2	0.00716564	0.244100955	1	1	1
DCBLD2	0.007192128	1.340776292	1	1	1
MPI	0.007194023	-1.458758877	0.438	0.667	1
ENSSSCG00000024765	0.007202274	-0.483315051	0.938	1	1
CFTR	0.007239212	1.315187949	1	0.867	1
MED22	0.007275677	-1.040641984	0.625	0.6	1
WWOX	0.007290126	0.11914457	1	1	1
TKFC	0.00729195	-1.61667136	0.438	0.533	1
ENSSSCG00000063021	0.007296875	-0.332297068	0.875	1	1
TSPAN3	0.007304546	1.089898012	1	1	1
C8orf88	0.00730874	-1.533681996	0.562	0.467	1
MRTFB	0.007313886	0.08628835	1	1	1
TIMM44	0.00733504	-0.751418055	0.875	1	1
TBC1D31	0.007340612	0.129563779	1	1	1
CHSY3	0.007351416	-0.551539499	1	1	1
ATG16L1	0.007359026	1.310389596	1	1	1
C17orf49	0.007383154	1.519867472	1	0.867	1
ALS2	0.007383888	1.172522113	1	1	1
ENSSSCG00000045482	0.007405644	1.966294883	1	0.933	1
MIER2	0.007407082	1.417421372	1	1	1
ZBTB1	0.007423155	1.614297999	1	0.733	1
EIF2AK3	0.007425416	-0.874469118	0.312	0.8	1
ENSSSCG00000055921	0.007425654	-1.607682577	0.25	0.6	1
PPIL6	0.007425687	-1.392669686	0.375	0.667	1
ENSSSCG00000056286	0.007447523	-1.629162305	0.375	0.6	1
ENSSSCG00000062599	0.007456053	1.126244484	1	1	1
UBXN2A	0.007456584	1.509351687	1	1	1
DAZAP2	0.007470889	1.190522872	1	1	1
PCSK4	0.007485478	-0.68975371	0.938	0.733	1
ADAMTS7	0.00749284	-0.337416602	0.938	1	1
ENSSSCG00000003421	0.007496963	-1.941106311	0.188	0.533	1
LIN7C	0.007504088	1.10206983	1	1	1
CLASP1	0.007504358	-0.120721636	0.875	0.933	1
BLOC1S2	0.007518544	-0.317982815	1	0.933	1
FBXO28	0.007519606	-0.13517904	1	1	1
FCSK	0.007520955	-1.678071905	0.438	0.533	1
RNF13	0.007536222	1.141044568	1	1	1
SPATA22	0.007537142	-0.457293688	0.938	0.933	1
GTF3C1	0.007554823	1.35108881	1	1	1
PBRM1	0.007561767	1.157894664	1	1	1
ASPHD2	0.007564091	-0.32287434	1	1	1
PCNP	0.007591315	1.453472005	1	1	1
FHOD1	0.00762823	1.611647609	1	0.8	1
ENSSSCG00000057584	0.007640168	-1.446746359	0.312	0.533	1
RECK	0.007643923	1.360847084	1	1	1
DZANK1	0.00764772	-0.462343214	1	0.867	1
ENSSSCG00000061166	0.00765453	-1.552541023	0.25	0.733	1
WIF1	0.007663325	1.37627507	1	1	1
ENSSSCG00000051948	0.007666266	-1.61667136	0.312	0.533	1
P2RY14	0.007713201	-0.996894089	0.625	0.667	1
IFTAP	0.007718904	-0.775919229	0.875	0.867	1
DUSP23	0.007722562	-1.392669686	0.5	0.667	1
GHRHR	0.007723854	-1.334117504	0.25	0.667	1
TMEM243	0.007726216	1.322578984	1	1	1
VPS33A	0.007731314	-1.05994254	0.688	0.8	1
KIAA0825	0.007731594	0.963834392	1	1	1
CDK2AP1	0.007744263	1.260000346	1	1	1
SMDT1	0.007780534	-1.600069393	0.375	0.867	1
SRGN	0.007804	-0.415037499	0.875	0.933	1

ZNF784	0.007818807	-2.093109404	0.125	0.533	1
FAF1	0.007846159	-0.263536824	1	0.933	1
MID1	0.007846521	-0.158134564	1	1	1
SCAMP3	0.007847933	-0.309317954	1	0.933	1
UBXN2B	0.007849614	1.206590127	1	1	1
SH2D5	0.007859236	-1.325770161	0.5	0.667	1
PRPF4B	0.007861151	0.201057619	1	1	1
ENSSSCG00000007188	0.007894975	1.396241185	1	1	1
KDM6A	0.007905764	1.204206136	1	1	1
JMJD7	0.007926179	-1.127056736	0.125	0.733	1
KLC1	0.007945081	1.259824075	1	1	1
TRMT112	0.007948199	-0.248792483	1	1	1
ENSSSCG00000032311	0.007952043	-0.093109404	0.938	1	1
CLGN	0.007970298	1.220618209	1	1	1
ZFP2	0.007989201	-0.186580886	1	1	1
PTPRM	0.008019595	-0.527512229	0.875	0.933	1
FASTKD1	0.008020315	1.329779267	1	1	1
PI4KA	0.008040544	-0.139279586	1	0.933	1
VWA2	0.008042179	-2.900464326	0.062	0.533	1
PGM5	0.008074074	1.512701653	1	0.933	1
NUDT6	0.008107561	-0.527512229	0.812	0.933	1
ENSSSCG00000057830	0.008107682	-1.736965594	0.25	0.6	1
ENSSSCG00000061141	0.008125764	1.479469372	0.938	0.867	1
MYORG	0.008128682	-0.600478784	1	1	1
ENSSSCG00000014075	0.008149589	-0.509879871	1	1	1
THEG	0.008195424	-1.441032708	0.25	0.667	1
COX18	0.008214384	-0.396890153	0.688	0.933	1
ATRN	0.008223796	1.337555209	1	1	1
CFAP410	0.008229363	-1.900464326	0.188	0.733	1
OTUD1	0.008250976	1.497434129	1	0.933	1
ENSSSCG00000042022	0.008257761	-0.136502	1	1	1
SLC29A4	0.00826434	-0.977632187	0.375	0.533	1
ZNF696	0.008274044	-0.963048864	0.688	0.8	1
PTPA	0.008324418	-2.128733314	0.188	0.667	1
NSUN7	0.00833371	1.035840998	1	1	1
RBBP5	0.008342716	0.920939673	1	1	1
PIGK	0.00834454	1.420280747	1	1	1
CAGE1	0.008359536	-0.212498134	1	1	1
SNAPIN	0.008361958	-0.315501826	1	1	1
KNOP1	0.008374206	-1.415037499	0.5	0.6	1
STX16	0.008376227	-1.587874096	0.25	0.733	1
IP6K1	0.008382438	-0.109199107	1	1	1
CUL4A	0.008390774	-0.115220106	1	1	1
GSN	0.008391142	2.540762697	0.875	0.6	1
KIAA1191	0.008405816	-0.056274188	1	1	1
PSMB2	0.008409854	-0.109597527	1	1	1
TMPRSS11E	0.008411896	2.528378972	0.688	0.333	1
RRP9	0.008421105	-0.447642165	0.938	1	1
CNTN4	0.008422065	1.571641122	1	0.867	1
ENSSSCG00000010440	0.008422372	-0.08323916	1	1	1
CCDC50	0.008425082	1.074874207	1	1	1
ENSSSCG00000002811	0.008446167	-1.830074999	0.125	0.533	1
PHEX	0.008456296	1.024221383	1	1	1
MED12	0.008463695	-0.304983954	0.938	0.933	1
RALGPS1	0.008476109	-1.337034987	0.562	0.733	1
ACSL1	0.008493716	0.04342228	1	1	1
CYB5R1	0.008494978	-1.11417102	0.688	0.933	1
JUP	0.008498157	2.5987683	0.688	0.4	1
ENSSSCG00000062217	0.008499836	-0.707819249	0.875	0.867	1
HMGXB3	0.008527872	-0.140588115	1	0.933	1
CXCL9	0.008542785	-0.444581775	0.875	0.933	1
CCNO	0.00856579	-0.912537159	1	0.867	1
ENSSSCG00000049325	0.008568402	-1.137503524	0.312	0.867	1
LARP6	0.008574291	1.63895739	1	0.8	1
EHD2	0.008589437	-1.607682577	0.25	0.667	1
ENSSSCG00000034353	0.008594299	1.821160722	1	0.667	1
CHMP3	0.008604423	0.16817297	1	1	1
ENSSSCG00000009523	0.008607321	1.359202468	1	1	1
KAT7	0.008617584	-0.118898086	1	1	1
ENSSSCG00000033770	0.008620243	2.091765939	1	0.733	1

NMRAL1	0.008634753	3.076815597	0.75	0.067	1
MST1R	0.008654325	-1.155393683	0.562	0.8	1
ENSSSCG00000037652	0.008676467	-0.578536232	0.938	1	1
ORC3	0.008679872	-0.004434723	1	1	1
NIBAN1	0.008690764	-1.019108823	0.625	0.667	1
TLCD5	0.00873065	1.349834091	1	1	1
PAK1IP1	0.008735031	0.037954351	1	1	1
ENSSSCG00000039774	0.008744576	-0.957225976	0.812	0.933	1
ENSSSCG00000061987	0.008777565	1.884864289	0.938	0.733	1
SHTN1	0.008783507	-0.186744477	1	1	1
TAF1D	0.008794031	-0.284250892	1	0.933	1
ETV3	0.008802665	-0.127056736	1	1	1
ELAPOR1	0.008832487	-0.075674811	0.938	1	1
ENSSSCG00000060630	0.008835699	-1.093109404	0.438	0.733	1
LPCAT4	0.008863533	-2.315501826	0.125	0.533	1
SLC48A1	0.008871185	-0.595609745	0.75	0.933	1
MAP10	0.008893822	-1.18932472	0.5	0.8	1
PPIL3	0.008931824	-1.256608137	0.688	1	1
VAMP7	0.008933744	1.140974982	1	1	1
YAF2	0.008944984	1.421072741	1	0.867	1
MARCHF7	0.008951602	1.101400699	1	1	1
ARL4D	0.008965862	1.29687293	1	1	1
CKAP4	0.008971613	-0.141364573	0.875	0.933	1
ENSSSCG00000057252	0.008990667	2.594946589	0.75	0.2	1
RSRC1	0.008994867	1.026001807	1	1	1
CCDC191	0.009000426	-0.378086363	0.938	1	1
ERFL	0.009002402	-0.929610672	0.5	0.733	1
KIZ	0.00900671	1.700039025	1	1	1
SRGAP1	0.009015529	-0.099350344	0.938	1	1
KLHL25	0.009024724	1.464169538	1	1	1
ENSSSCG00000011243	0.009072507	0.042338789	1	1	1
CCDC141	0.009076424	-0.900464326	0.688	0.733	1
MRPS27	0.009111366	-0.181918671	1	1	1
MOCS1	0.009120395	-0.127874823	0.875	0.933	1
ENSSSCG00000044312	0.009123229	-1.180572246	0.438	0.667	1
SLC6A6	0.00913751	1.121939343	1	1	1
C15orf39	0.009141976	-0.343336034	0.938	0.867	1
LIPE	0.009199204	1.949959318	0.938	0.733	1
SENP1	0.00921318	-0.208586622	0.938	1	1
ARHGEF37	0.009215453	-1.341036918	0.25	0.533	1
NOB1	0.009280988	0.181897643	1	1	1
MIIP	0.009301737	-0.604362219	0.938	0.933	1
IFT122	0.009314346	-0.683289614	0.875	1	1
IRAK1BP1	0.009317523	-0.638105498	0.938	0.8	1
ASNSD1	0.009339908	-1.18932472	0.688	0.733	1
ENSSSCG00000035290	0.009345975	-0.678071905	0.812	0.8	1
ENSSSCG00000052712	0.009352073	-0.227868926	1	1	1
RTN4	0.009368772	-0.745186101	0.75	0.8	1
ENSSSCG00000047723	0.009393826	3.550746785	0.688	0.067	1
CREB1	0.009414355	-0.588434693	0.812	1	1
ENSSSCG00000007639	0.009468773	3.861086906	0.562	0.067	1
ALB	0.009474861	-0.65870658	0.812	0.867	1
SDR42E1	0.009476	-1.707819249	0.438	0.6	1
AGFG1	0.00948893	-0.09230185	1	1	1
ESRRB	0.009496742	-1.415037499	0.312	0.6	1
TM9SF2	0.009511636	1.097805666	1	1	1
SNX29	0.009520239	-0.230612928	0.812	0.933	1
B3GLCT	0.009537811	-0.116760672	1	1	1
NAA50	0.009565712	1.027701716	1	1	1
RCC1L	0.009586893	1.75406098	1	1	1
ENSSSCG00000057040	0.009644749	-2.629162305	0.375	0.6	1
APOB	0.009649302	-0.358162978	0.812	0.933	1
ZBTB37	0.00965654	1.127686232	1	1	1
KYAT1	0.009674193	1.538980491	1	0.933	1
KCNMB3	0.009696457	-1.678071905	0.188	0.533	1
AP1S2	0.009697095	1.303863379	1	1	1
ZNF449	0.009714157	1.120260014	1	1	1
ENSSSCG00000048494	0.009715503	-1.607682577	0.375	0.533	1
FBXL2	0.009727541	-0.212949174	1	1	1
FAM53C	0.009755217	0.160324671	1	1	1

TRPV6	0.009769658	-1.607682577	0.188	0.6	1
TTC21B	0.009792474	1.182895784	1	1	1
STIM1	0.009825555	1.861903141	1	1	1
GGT7	0.009829074	-1.341036918	0.5	0.667	1
ABL1	0.009851161	1.096551815	1	1	1
ZNF354A	0.009863822	-0.08901665	1	1	1
CASP3	0.009905165	-0.27690658	1	1	1
CCDC92	0.009909554	-0.852101305	0.562	0.8	1
NUDT3	0.009919234	0.164090376	1	1	1
KIFBP	0.009921398	-0.289506617	1	1	1
VDAC2	0.009963083	-0.858644151	0.938	0.8	1
GCDH	0.009975813	-0.228886183	0.812	0.933	1
RPAIN	0.009995982	1.182525038	1	1	1
FKBP8	0.009996734	-0.900464326	0.625	0.8	1
ZNF655	0.010011132	1.0172456	1	1	1
ZNF713	0.010035345	1.558450845	0.938	0.933	1
ENSSSCG00000009333	0.01005876	-1.230612928	0.375	0.667	1
ENSSSCG00000060591	0.010073103	-1.497499659	0.5	0.6	1
ENSSSCG00000062462	0.010080175	-1.277533976	0.312	0.6	1
TRMT61A	0.010095336	-1.77118131	0.188	0.533	1
C11orf54	0.010098715	1.346513733	1	1	1
CARF	0.010106734	2.072228328	0.812	0.4	1
SC5D	0.010107106	-0.514936069	0.812	0.733	1
PRPS1	0.010126225	0.291008456	1	1	1
DNAJC2	0.010140525	0.160384234	1	1	1
SMC1A	0.010143871	0.245782697	1	1	1
MPC2	0.010159635	1.193533629	1	1	1
ENSSSCG00000059490	0.010162469	-1.299560282	0.375	0.533	1
ENSSSCG00000059628	0.010182865	-0.09965225	1	1	1
GPRASP2	0.010185205	1.370123784	1	1	1
TRAPPC3	0.01022478	1.137465129	1	1	1
HNRNPU	0.01023144	0.204345133	1	1	1
RAB8B	0.010242291	-0.157862197	1	1	1
ENSSSCG00000059905	0.01027855	2.316281532	0.812	0.467	1
SH3BGRL2	0.010288232	0.969024031	1	1	1
ZNF483	0.010342221	-0.180307846	1	1	1
TYSND1	0.010345805	-1.540568381	0.312	0.733	1
PWP2	0.010347576	-0.793549123	0.75	0.933	1
RO60	0.010388587	-0.316580476	1	1	1
ZNF668	0.010399104	-1.230612928	0.375	0.6	1
TMEM47	0.010470909	1.448351458	1	1	1
DDX24	0.010492246	-0.063337505	1	1	1
INTS2	0.010517815	-0.04608407	1	1	1
ZSWIM8	0.010530459	-0.330860446	1	1	1
WWP1	0.01055146	1.186697011	1	1	1
ENSSSCG00000061694	0.010565786	-0.704544116	0.688	0.867	1
BLTP1	0.010594311	0.951586053	1	1	1
ENSSSCG00000046323	0.010595497	1.446049407	1	0.867	1
ENKUR	0.010605179	-0.076929477	0.938	1	1
SLC5A2	0.010609115	-0.53562764	0.875	0.867	1
EHD3	0.010624948	-1.858644151	0.062	0.6	1
CNNM2	0.010636661	0.134864893	1	1	1
CDH18	0.010650361	2.417852515	0.75	0.333	1
KLHDC10	0.010668548	0.905725252	1	1	1
RBM11	0.01069402	-1.299560282	0.5	0.6	1
ZGLP1	0.010716376	-1.137503524	0.5	0.6	1
EXOC2	0.010731129	-0.076989739	1	1	1
STK19	0.01076807	-0.611576493	0.625	0.867	1
ENSSSCG00000004572	0.010779353	-1.277533976	0.188	0.6	1
ENSSSCG00000018034	0.010796718	1.35720357	1	1	1
RALGPS2	0.010801694	1.004049506	1	1	1
TRAF3IP1	0.010805838	-0.967578522	0.5	0.867	1
MYBBP1A	0.010807099	-0.516917113	0.938	1	1
ENSSSCG00000009802	0.010809493	-1.18932472	0.625	0.733	1
ENSSSCG00000052522	0.01084831	-0.937458534	0.562	0.733	1
CENPT	0.010850042	-0.033196503	0.938	1	1
PTPN20	0.010871208	1.452631787	1	0.933	1
EHBP1	0.010903262	1.365858698	1	1	1
KCTD3	0.0109093	0.178052882	1	1	1
MBNL3	0.010911503	1.243362748	1	1	1

FANCM	0.010940286	-0.276615973	0.938	1	1
MAP3K4	0.010946288	1.099149048	1	1	1
EAF2	0.01094979	1.106572784	1	1	1
NCSTN	0.010950731	1.16458258	1	1	1
COPS5	0.01097474	-0.441288391	0.938	1	1
ENSSSCG00000036529	0.010987272	-0.28530877	1	1	1
MTMR7	0.011027648	1.217476004	1	1	1
ENSSSCG00000058915	0.011070854	-0.323889611	0.812	0.933	1
ENSSSCG00000059428	0.011083863	-1.721140627	0.312	0.667	1
PDCD6IP	0.011094472	0.945709844	1	1	1
ENSSSCG00000001917	0.011106772	-0.931358334	0.688	0.8	1
ENSSSCG00000056992	0.0111121	-1.441032708	0.438	0.6	1
HECTD3	0.011113634	-1.075407403	0.5	0.667	1
TUT4	0.011124939	-0.972815171	0.812	0.8	1
SPATA2	0.011125741	0.340072756	1	1	1
SREBF2	0.011128994	1.493979397	1	0.933	1
ENSSSCG00000039144	0.011131566	-1.552541023	0.375	0.533	1
F11R	0.011165284	1.484399699	1	1	1
SNRPD1	0.011168055	0.052445169	1	1	1
ENSSSCG00000056070	0.011173031	-0.723159795	0.875	0.8	1
ARRB2	0.01117823	-1.870716983	0.25	0.533	1
PRPF3	0.011202839	0.975630186	1	1	1
ENSSSCG00000051889	0.011211861	0.012907484	1	1	1
RUNX1	0.011272417	-1.044199804	0.438	0.667	1
RCAN3	0.01133349	1.118629221	1	1	1
BET1	0.011387088	-0.188707502	1	1	1
PRKN	0.011400256	-0.19363628	1	0.933	1
DFFA	0.011405652	-0.19363628	0.938	1	1
PC	0.011416011	-1.050040682	0.5	0.667	1
ANKS3	0.01142995	-0.678071905	0.375	0.667	1
BCL2L13	0.011459069	1.311537649	1	1	1
ENSSSCG00000058426	0.011478566	2.584962501	0.688	0.2	1
ENSSSCG00000038362	0.011481419	-0.312890612	0.875	1	1
KRT10	0.011488455	-1.245112498	0.312	0.6	1
ENSSSCG00000039731	0.011516984	-0.060596591	1	0.933	1
SLC35B3	0.011556873	-0.853158612	0.625	0.867	1
TBC1D22A	0.011583123	-0.65870658	0.562	0.733	1
BCAS1	0.011604549	0.250502517	1	1	1
SKOR1	0.011645253	-1.062735755	0.562	0.8	1
CEP63	0.011655665	0.032421478	1	1	1
UROC1	0.011659996	-1.093109404	0.562	0.667	1
ERMARD	0.011670405	-0.529208519	0.562	0.8	1
TASOR2	0.011696611	0.912725879	1	1	1
ENSSSCG00000005371	0.011705797	-0.556241192	1	1	1
ABCC6	0.011719887	-0.543630617	1	1	1
MYH14	0.011790242	-1.595609745	0.188	0.6	1
EXTL2	0.011798274	-0.550959987	0.938	0.867	1
SNRK	0.011807638	0.887019674	1	1	1
ENSSSCG00000053759	0.011833258	-1.434146322	0.312	0.6	1
AGPS	0.011838706	1.147336394	1	1	1
ENSSSCG00000041959	0.01187174	-1.508146904	0.25	0.6	1
ENSSSCG00000057344	0.01192454	-0.384875529	0.75	0.933	1
APEX1	0.011925524	1.032421478	1	1	1
PLA2G12B	0.011933174	-1.093109404	0.562	0.733	1
ENSSSCG00000013990	0.011962502	-0.16930903	1	1	1
ARMC1	0.011976172	1.086924934	1	1	1
ZSWIM3	0.011997334	1.555677641	1	1	1
SLC1A4	0.012010982	0.019200812	0.938	1	1
ZNF165	0.012011737	-0.176674121	0.938	1	1
TGFBR1	0.012012429	-0.227671653	1	0.933	1
ZNF514	0.012038223	1.211698057	1	1	1
DISP1	0.012056176	-0.315501826	0.875	0.867	1
SCAPER	0.012057978	0.961644554	1	1	1
ENSSSCG00000047974	0.012081627	-1.678071905	0.188	0.6	1
ENSSSCG00000046630	0.012115437	2.366322214	0.688	0.4	1
BTBD6	0.012130485	2.027905997	1	0.733	1
ARID5B	0.012227974	-0.326828243	1	0.933	1
HCCS	0.012235556	1.449727621	1	1	1
ENSSSCG00000063163	0.012253825	0.029747343	1	1	1
GAP43	0.01225872	-0.272079545	0.75	1	1

ENSSSCG00000029696	0.012275246	-0.59400764	0.75	0.8	1
ADAMTS9	0.012281162	-1.13058411	0.625	0.6	1
ENSSSCG00000016119	0.012346359	-0.044942122	1	1	1
CCDC62	0.012410298	1.276124405	1	0.733	1
TREX1	0.012456663	-1.455679484	0.125	0.6	1
DCLRE1B	0.012461246	-0.421945869	0.812	1	1
BRDT	0.012461438	1.137210464	1	1	1
LIMD1	0.012462778	-0.496174262	0.812	0.933	1
ABCG5	0.012469728	-0.011809302	1	1	1
ENSSSCG00000053393	0.012470409	-1.27368165	0.5	0.533	1
ENSSSCG00000059780	0.012486272	-0.870716983	0.438	0.733	1
EXOC3L1	0.012491994	-1.678071905	0.062	0.667	1
NR4A3	0.01251167	2.534921818	0.75	0.333	1
DLG2	0.012521815	0.054622012	1	1	1
ANKRD44	0.012530839	0.997533482	1	1	1
ZNF281	0.012535079	1.189483778	1	1	1
NTMT1	0.012550984	-1.533681996	0.312	0.533	1
GALM	0.012555968	2.314975334	0.625	0.467	1
ENSSSCG00000048417	0.012602228	-0.312226694	1	0.933	1
ETV5	0.012610702	-0.348870575	0.938	1	1
ENSSSCG00000062960	0.012635362	-1.595609745	0.312	0.533	1
CAMSAP1	0.012659165	-0.274716211	0.875	0.867	1
SSBP3	0.012687898	-0.257496222	0.875	0.933	1
DCTN1	0.012692453	-0.353636955	0.875	0.933	1
TMPRSS9	0.012697286	-1.793549123	0.188	0.533	1
ENSSSCG00000054992	0.012725431	-1.188266637	0.5	0.8	1
DNAJC27	0.012778923	1.305637111	1	1	1
DPP6	0.012811877	-0.52279368	0.75	0.733	1
GLB1L3	0.012823835	3.344295908	0.5	0.267	1
SEC61A2	0.012826556	1.269396451	1	1	1
CNKSR1	0.012863242	0.107287949	1	1	1
SANBR	0.012875343	1.24710838	1	1	1
KMT2C	0.012905324	1.063528195	1	1	1
TENT2	0.012914465	1.389529605	1	1	1
ENSSSCG00000057240	0.012939502	2.254813899	0.812	0.4	1
TRIP11	0.012940883	0.095755469	1	1	1
ZNF286A	0.012964043	1.16040769	1	1	1
ENSSSCG00000055852	0.012971061	-1.093109404	0.438	0.733	1
PIAS1	0.012993877	1.178543238	1	1	1
ASPSCR1	0.013025065	-1.533681996	0.25	0.533	1
KDM2A	0.013032504	1.436926886	1	0.933	1
EGR1	0.013042966	-0.19345993	1	1	1
TIMP3	0.013073995	1.843164998	0.938	0.933	1
RPAP1	0.013116739	1.337734191	1	1	1
WASHC4	0.013125395	1.045983731	1	1	1
FOCAD	0.01312834	0.174785712	1	1	1
MOB3A	0.013160386	-1.020353062	0.75	0.867	1
ENPP4	0.013183414	-1.13492958	0.5	0.6	1
PUS10	0.013211612	1.329313022	1	1	1
LMO1	0.013231246	-0.364411426	0.812	0.933	1
TGDS	0.013249263	1.426884172	1	1	1
ENSSSCG00000042047	0.013253102	-1.678071905	0.25	0.533	1
ILF3	0.013258097	1.293618337	1	1	1
WWP2	0.013258777	-0.078801568	1	1	1
ORC2	0.013285693	1.064178292	1	1	1
UBA5	0.013296286	-0.05900155	1	1	1
SIGLEC1	0.013309049	1.276515433	1	1	1
ITPRID1	0.013320709	-0.496074071	0.812	0.933	1
ENSSSCG00000056033	0.013332756	1.790866799	0.938	0.667	1
ENSSSCG00000057312	0.013349228	-0.582147485	0.688	0.867	1
TMEM117	0.013351665	0.097555309	1	1	1
CDON	0.013358256	1.369033199	1	0.933	1
CCNE2	0.013366184	-1.495207848	0.5	0.467	1
TBRG1	0.01338026	1.205382135	1	1	1
EIF4E	0.013386209	0.005996832	1	1	1
ENSSSCG00000018064	0.013393554	1.794070765	1	0.933	1
RASSF1	0.013438765	-1.093109404	0.188	0.6	1
VSIG1	0.013451987	4.906890596	0.5	0	1
KIF12	0.01347262	-0.717600269	0.688	0.667	1
MAGEF1	0.013487277	-2.019108823	0.125	0.533	1

TNFRSF1A	0.013547623	-1.158697746	0.688	0.8	1
ENSSSCG00000048914	0.013565235	-0.11417102	1	1	1
RBMX2	0.013571103	-0.37996243	1	1	1
BOLL	0.01357797	-0.811927652	0.75	0.8	1
MYO19	0.013584993	-0.445682817	1	0.933	1
PDCL3	0.013585894	1.058326225	1	1	1
NTN1	0.013590561	-1.341036918	0.312	0.6	1
ACACA	0.013594705	0.259041632	1	1	1
HYKK	0.013603371	-0.466233262	0.875	0.933	1
THOC2	0.013613892	1.254344562	1	1	1
ENSSSCG00000045522	0.013618164	-0.182746617	0.938	1	1
SSB	0.013628834	0.054801153	1	1	1
PER2	0.013644639	1.672425342	0.938	0.467	1
TLCD2	0.01367414	-1.35614381	0.312	0.6	1
FLNB	0.013708519	-0.075161771	1	1	1
CDC42EP4	0.013723093	-0.521952703	0.625	0.867	1
SYBU	0.013734949	1.965784285	0.938	0.6	1
INTU	0.0137433	1.483316414	0.938	0.867	1
ENSSSCG00000049063	0.013745856	-1.334117504	0.25	0.667	1
PSMA1	0.013762313	1.146436363	1	1	1
DELE1	0.013781254	-0.745186101	0.5	0.667	1
RANBP2	0.013829883	0.981803636	1	1	1
HACL1	0.013830716	-1.239950793	0.25	0.667	1
ENSSSCG00000032946	0.013833412	1.36757076	0.938	0.933	1
PECR	0.01383761	1.412589818	1	1	1
HYPK	0.013844405	-0.254321974	1	1	1
ARRDC3	0.01384768	-0.272877024	0.938	1	1
VPS13B	0.013848144	0.878328789	1	1	1
PPID	0.01388368	1.289235932	1	0.933	1
RSRP1	0.013913257	-0.350907162	0.875	0.933	1
CREB3	0.01391657	-0.020353062	1	1	1
ENSSSCG00000056777	0.013944345	1.131720002	1	1	1
CIITA	0.013947547	-1.036525876	0.25	0.733	1
MEMO1	0.013958083	0.977670951	1	1	1
PARP2	0.013962328	1.749482698	1	0.733	1
ENSSSCG00000023195	0.013964652	-0.179266048	0.875	1	1
TTC9C	0.013984371	-0.056939668	0.938	1	1
SARAF	0.013997222	0.090769363	1	1	1
ADAM12	0.014008563	0.051280505	1	1	1
SSRP1	0.014015572	1.44578564	1	1	1
CILK1	0.014020198	1.381617534	1	0.867	1
SYTL2	0.014029323	0.959278622	1	1	1
MAIP1	0.014060373	-0.177010662	1	1	1
DND1	0.014066194	-0.02193064	1	1	1
KLK5	0.014069416	-0.809316438	0.625	0.8	1
ENSSSCG00000055200	0.014075347	-0.752072487	0.688	0.733	1
ENSSSCG00000055973	0.014104393	2.491853096	0.75	0.267	1
ARHGAP44	0.01418232	-0.263034406	0.812	1	1
GTF3C2	0.014194882	0.057974482	1	1	1
COMT	0.014195324	-1.589535231	0.562	0.8	1
SMG6	0.014210764	-0.095946564	1	1	1
ENSSSCG00000000148	0.014211157	-0.341036918	0.562	0.933	1
TXNRD1	0.014238034	-0.157526133	1	1	1
MRPS18B	0.01426208	-0.136698673	1	1	1
LGMN	0.014300191	1.263149412	1	1	1
NBR1	0.014325307	1.140536545	1	1	1
SLBP	0.014335463	1.290288394	1	1	1
APPL1	0.014347101	1.169521507	1	1	1
ARHGAP42	0.014363791	0.911936753	1	1	1
QKI	0.01438018	1.09703623	1	1	1
ENSSSCG00000039259	0.014382828	-0.9510904	0.5	0.733	1
PLEKHB2	0.014392212	0.278412854	1	1	1
TRMT6	0.014407743	1.181594482	1	1	1
C1orf87	0.014420524	-0.712019237	0.688	0.667	1
BLZF1	0.014430276	-0.166945285	0.938	1	1
HNRNPD	0.014442274	-0.381090167	1	1	1
SERPING1	0.014496038	-2.35614381	0.062	0.533	1
ENSSSCG00000045125	0.014522997	-0.54830403	1	0.867	1
TPD52L2	0.014523865	0.292544288	1	1	1
ZNF622	0.014551116	-1.349449158	0.25	0.533	1

ADAM3A	0.014556352	-0.37064338	0.812	0.933	1
DPAGT1	0.014557072	-0.200912694	1	1	1
ERAP1	0.014577948	1.067725256	0.938	0.933	1
LIN28A	0.014584435	1.213137068	1	1	1
CDK5R2	0.01458586	-1.341036918	0.188	0.533	1
CCDC86	0.014589784	1.582268392	1	1	1
ERCC5	0.014590048	-0.37690237	1	0.867	1
ZCCHC8	0.014621425	1.197847303	1	1	1
ENSSSCG00000052755	0.014630291	-0.334867151	1	0.933	1
ZNF777	0.014645877	-0.578536232	0.812	0.867	1
NUP214	0.014651229	1.558534875	1	1	1
VGLL4	0.014715507	-0.175051018	1	1	1
CTDSPL2	0.014729975	1.120747433	1	1	1
TIMM29	0.014739147	-0.257228784	0.938	1	1
NLRC5	0.014743129	-0.923184403	0.688	0.8	1
ENSSSCG00000001979	0.014778436	-0.93363119	0.875	0.8	1
ENSSSCG00000058487	0.014789665	-1.455679484	0.312	0.533	1
KCNG3	0.014807359	1.18730708	1	0.933	1
MTRF1	0.014812582	-0.521443726	1	0.867	1
TOM1	0.014813802	-0.941106311	0.875	0.8	1
APEX2	0.014844439	1.2306385	1	1	1
SNX9	0.014852698	0.09847697	1	1	1
CAPN12	0.014885653	0.135966723	1	1	1
ZFAND6	0.014916606	1.000446309	1	1	1
ENSSSCG00000012709	0.014931041	1.906890596	0.938	0.4	1
ENSSSCG00000045128	0.01494854	-1.027020214	0.875	0.8	1
MGST1	0.014953091	2.977279923	0.625	0.267	1
PHKG2	0.015020072	-1.489999557	0.562	0.6	1
L3MBTL2	0.015025709	-0.086054647	0.875	1	1
ATP8B2	0.015026486	-1.208586622	0.25	0.6	1
PACSIN3	0.015039994	-0.712019237	0.75	0.667	1
SLC30A5	0.015056542	1.1864724	1	1	1
ZCCHC10	0.0150575	1.353776961	1	1	1
GPR22	0.015084472	0.985364654	1	0.933	1
ENSSSCG00000058007	0.015102014	1.060325645	1	1	1
LAPTM4B	0.015106796	-0.128237628	1	1	1
NCAPH2	0.015143258	-0.756074417	0.375	0.8	1
BAALC	0.015154916	-0.319617934	1	1	1
SMARCA1	0.015157505	-0.757242119	0.5	0.8	1
JADE3	0.015158101	1.207345326	1	1	1
DAG1	0.015160165	1.202190537	1	1	1
ZKSCAN1	0.015161726	-0.717600269	1	0.933	1
ZDHHC3	0.015167713	0.051409756	1	1	1
ENSSSCG00000058720	0.015201121	-0.583435031	0.75	0.8	1
CNKSR3	0.01524067	-2.208586622	0.125	0.533	1
RABIF	0.015297166	1.991424106	0.938	0.533	1
C2CD3	0.01530118	-0.136178126	1	1	1
ENSSSCG00000007692	0.015315899	0.192138027	1	1	1
NCOR1	0.015358676	1.266416885	1	1	1
GTDC1	0.015364319	2.587010329	0.812	0.467	1
TMEM209	0.015397453	1.093073479	1	1	1
METTL18	0.015416189	-0.049630768	1	1	1
DNAJC1	0.015418173	1.038360097	1	1	1
TSNAX	0.015497949	-0.17420783	1	1	1
TADA2A	0.015513539	1.102125521	1	1	1
RRM2B	0.01551439	1.041075159	1	1	1
ENSSSCG00000055703	0.015524324	-1.285754482	0.438	0.6	1
PIK3R1	0.01555057	-0.191388922	1	1	1
PGBD1	0.015591116	-0.596875822	0.938	0.933	1
NDUFAF2	0.015592984	-0.929610672	0.312	0.867	1
ROCK2	0.015657789	1.083338327	1	1	1
ENSSSCG00000059383	0.015669557	-0.35614381	1	1	1
SEPSECS	0.015672327	1.154652672	1	1	1
ENSSSCG00000012961	0.015685987	-0.143963313	1	1	1
SH3BGR	0.015689397	-0.664266106	0.688	0.867	1
GORAB	0.015721035	2.994353437	0.688	0.2	1
NFKBIA	0.015725702	1.236930442	1	1	1
ZNF703	0.015753655	-0.535113951	0.812	0.933	1
ANGEL1	0.015805528	1.21888747	1	1	1
PPP2R1A	0.015806927	1.590126845	1	0.933	1

SPTAN1	0.015814572	-0.041163189	1	1	1
ATP9A	0.015831745	-0.107323264	0.938	1	1
CDH24	0.015842695	-0.485426827	0.688	0.8	1
ENSSSCG00000007065	0.015857923	-1.093109404	0.312	0.733	1
ENSSSCG00000017577	0.015906335	-0.463477854	0.875	0.867	1
TMEM169	0.015908945	1.524274574	0.938	0.733	1
GDAP2	0.015958714	1.039315807	1	1	1
TARDBP	0.01603271	1.083008806	1	1	1
ENSSSCG00000006771	0.016117054	1.17228408	1	1	1
TOP2A	0.016117722	1.048864334	1	1	1
BEND7	0.016144048	-0.549967079	0.875	0.933	1
KIAA1522	0.016152459	-0.098712111	1	1	1
ELP1	0.016158775	-0.867613299	0.938	0.933	1
INTS7	0.016159169	1.215158141	1	1	1
MUTYH	0.016174778	1.470319935	1	0.8	1
PERP	0.016182829	-0.154509949	0.812	0.867	1
ENSSSCG00000040466	0.016188813	2.54630088	0.812	0.4	1
ENSSSCG00000014062	0.016200796	-0.159451899	0.875	0.933	1
MATCAP2	0.016217371	-0.394393189	1	1	1
UBLCP1	0.016251866	-0.152097538	1	1	1
ANGPT1	0.016260743	1.32074118	1	1	1
MAST4	0.016274776	0.092336182	0.938	1	1
CCDC47	0.016275733	1.358174661	1	1	1
ANKDD1A	0.016276387	-1.446746359	0.375	0.533	1
ENSSSCG00000033490	0.016287399	-0.703162886	0.625	0.8	1
BIN3	0.016313553	1.402486094	1	1	1
GPC2	0.016324713	-1.157239742	0.312	0.667	1
SLC37A3	0.016351423	-0.326308581	0.938	1	1
ENSSSCG00000047398	0.016364047	-1.368743847	0.438	0.667	1
ZNF283	0.016365222	1.30959364	1	1	1
TIMP4	0.016370141	-0.879211777	0.938	0.8	1
BTBD8	0.01637505	-0.182235295	0.938	1	1
GPR158	0.016380771	0.309302612	0.938	1	1
ENSSSCG00000032999	0.01641747	1.332899213	0.938	1	1
MAML3	0.01642058	-0.249349491	0.938	1	1
ENSSSCG00000048278	0.016455348	-0.977632187	0.5	0.667	1
GNB2	0.016481903	2.140009424	0.938	0.733	1
ACTN2	0.016527983	-1.025995209	0.625	0.667	1
ENSSSCG00000052892	0.016532478	2.62935662	0.688	0.267	1
FAM199X	0.016587122	-0.045214714	0.938	1	1
ENSSSCG00000030260	0.016604761	0.034260753	1	1	1
ZNF135	0.016618851	-0.874469118	0.5	0.667	1
KLHDC1	0.016657494	-0.592680414	0.938	1	1
SH3BP5L	0.016674286	-0.156955886	0.875	0.933	1
EBP	0.016733128	-0.226375935	0.938	1	1
BAX	0.016755719	-1.093109404	0.562	0.533	1
ADCYAP1	0.016769435	-1.508146904	0.25	0.6	1
MALRD1	0.016778693	1.107317685	1	1	1
ENSSSCG00000003965	0.016791533	-1.180572246	0.312	0.6	1
ENSSSCG00000013607	0.016821837	1.669851398	0.938	0.6	1
MCAM	0.016823088	2.491853096	0.625	0.2	1
H2AZ2	0.016823742	0.230394545	1	1	1
SPINT1	0.016831402	-0.89061654	0.688	0.733	1
GPRC5A	0.016866661	-0.961864871	0.375	0.733	1
SLAIN1	0.016877534	-0.150537684	1	0.933	1
TNRC6B	0.01697714	0.185985966	1	1	1
NMRK1	0.016990301	-0.549369566	0.938	1	1
MAEA	0.017016918	0.01282404	1	1	1
SENP3	0.017060946	-0.335679708	0.875	0.933	1
CWC22	0.017065429	1.130251431	1	1	1
ATP1A1	0.017067232	1.584336881	1	1	1
FLII	0.017104058	-0.213963799	1	1	1
UNC45A	0.017149014	-1.230612928	0.25	0.6	1
RALGAPA2	0.017210266	1.220129743	1	1	1
PABPC4	0.017231932	1.075048671	1	1	1
ENSSSCG00000045932	0.017234501	-1.137503524	0.312	0.533	1
SLC23A1	0.017244561	-1.291048782	0.562	0.467	1
TAFAZZIN	0.017345766	1.200086972	1	1	1
KIAA2013	0.017385771	-0.419337637	0.938	0.933	1
ENSSSCG00000061039	0.017397118	1.837627933	0.875	0.467	1

ZIC2	0.017410692	-0.974464908	0.438	0.867	1
VASP	0.017492813	-0.477773255	0.812	0.733	1
CALD1	0.017542924	-0.420199204	0.938	0.933	1
ENSSSCG00000060891	0.017558153	-0.781165398	0.438	0.867	1
OSBPL3	0.017575431	-0.121214742	1	1	1
ZNF131	0.017628475	-1.469485283	0.812	0.467	1
ZFP28	0.017675661	-0.170391325	0.938	1	1
RAD21	0.017766166	-0.009550793	1	1	1
DHX38	0.01778314	1.309579702	1	1	1
KIF2A	0.0177863	0.942133754	1	1	1
ENSSSCG00000059539	0.0178	-0.732519689	0.938	0.8	1
NRXN1	0.017822621	0.975743916	1	1	1
ENSSSCG00000052641	0.017823781	-0.299560282	0.75	0.933	1
ENSSSCG00000014884	0.017833517	-0.003332697	1	1	1
ENSSSCG00000062144	0.017838429	-0.941106311	0.562	0.8	1
CCDC106	0.017840343	-1.093109404	0.25	0.6	1
DYNLT2B	0.017848166	-0.319880266	0.875	0.933	1
CSPG5	0.017865976	-1.552541023	0.25	0.6	1
ENSSSCG00000011162	0.017881877	0.10712295	1	1	1
ENSSSCG00000061116	0.017905635	-0.912537159	0.5	0.667	1
C12orf29	0.017975151	-0.574236094	0.875	0.867	1
FAM124A	0.017977472	-0.941106311	0.5	0.533	1
MTRF1L	0.018017353	-0.183711953	0.875	1	1
LMBRD2	0.018035993	1.340689023	1	1	1
ADCK1	0.018059391	-0.193730219	1	1	1
TLK2	0.018061726	1.047632728	1	1	1
MARVELD2	0.018116617	1.07710579	1	1	1
ANKRD54	0.018157752	-0.323407024	0.688	0.933	1
KPNA7	0.018169403	1.666609228	1	1	1
ZNF384	0.018184646	-1.341036918	0.312	0.667	1
CLIC4	0.018190635	0.987119621	1	1	1
ENSSSCG00000051957	0.018191093	1.82336724	0.875	0.667	1
TEDC2	0.018256515	-2.341036918	0.125	0.8	1
DDX54	0.018266454	0.005380459	0.938	1	1
GYG1	0.018273756	-0.860132232	0.625	0.733	1
DNTTIP2	0.018313341	1.177413775	1	1	1
FGL1	0.018337909	-0.385891154	0.438	0.733	1
ENSSSCG00000016733	0.018338051	-0.471621028	0.938	0.867	1
PSEN2	0.018351014	1.474111514	1	0.933	1
STAM2	0.018393072	1.160009533	1	1	1
TACSTD2	0.018393508	1.195877697	1	1	1
STAU1	0.018429427	1.197111828	1	1	1
DHX9	0.018443451	-0.197320399	1	1	1
AARS1	0.01844604	-0.399212532	0.938	1	1
ARMC9	0.01849303	-0.47689597	0.875	0.933	1
ARL15	0.01849749	0.950825021	1	1	1
STEAP3	0.018533634	-0.315501826	1	1	1
COMTD1	0.018579096	1.982178723	0.875	0.4	1
MIPEP	0.01858398	-0.346228341	0.812	0.867	1
OBSCN	0.018604277	-0.818934441	0.75	0.733	1
DYM	0.018624467	0.998813085	1	1	1
METAP2	0.018644615	0.246224967	1	1	1
ENSSSCG00000033366	0.018647786	1.236961864	1	1	1
ENSSSCG00000042658	0.018648807	-0.275700818	1	1	1
ANGEL2	0.018686503	-0.010647244	1	1	1
ZMYND19	0.018698795	-1.143735477	0.375	0.667	1
ENSSSCG00000047302	0.018744033	-0.016147423	1	1	1
MSH6	0.018878232	1.148524367	1	1	1
PGAP2	0.018879838	1.935841969	0.938	0.667	1
ZSWIM6	0.018898169	-0.688719149	0.812	0.733	1
EPDR1	0.018968256	-1.415037499	0.625	0.6	1
BRMS1L	0.018974934	1.321928095	1	0.933	1
PIH1D2	0.018992002	-0.384158186	0.875	0.8	1
PAN3	0.019004057	1.013652101	1	1	1
ENSSSCG00000028572	0.0190137	-0.522502197	0.812	0.8	1
B3GALNT2	0.019023627	0.206297955	1	1	1
CCDC160	0.019041329	-0.41975991	0.938	1	1
DOK5	0.019105579	-0.704544116	0.875	1	1
FREM1	0.019178358	-0.313235219	0.875	0.933	1
ENSSSCG00000062937	0.01918945	1.994353437	0.812	0.467	1

ENSSSCG00000054552	0.019230765	1.353025392	0.938	0.933	1
BYSL	0.019273462	1.526410318	1	0.867	1
NPAS3	0.019306139	-0.001235713	1	1	1
PTRH2	0.019324866	-3.415037499	0	0.533	1
SUSD3	0.019332627	1.373300197	1	1	1
WDR1	0.01936322	-0.340490546	0.938	0.933	1
ENSSSCG00000021322	0.019374633	0.312956589	1	1	1
GOLIM4	0.019423828	1.625531532	1	1	1
MME	0.019516039	2.254813899	0.75	0.333	1
IRS1	0.019527208	-0.471621028	0.875	0.933	1
RAD51B	0.019533463	-0.01160662	1	1	1
CDCA8	0.019541786	1.107553428	1	1	1
ARID1A	0.019542556	1.217428052	1	1	1
GTF2F2	0.019549983	-0.240916	1	1	1
ENSSSCG00000062092	0.019552084	-1.192645078	0.375	0.667	1
DCAF17	0.019558283	-0.486342533	0.875	0.933	1
ENSSSCG00000032202	0.019579766	0.180795737	1	1	1
NFKB2	0.019582478	-0.806805219	0.375	0.667	1
POLR3A	0.019589998	-0.232173442	1	1	1
BLOC1S6	0.019616824	1.242493627	1	0.867	1
UBXN4	0.019627226	0.945990415	1	1	1
ENSSSCG00000036812	0.019634682	-0.601256308	0.75	0.8	1
ENSSSCG00000041031	0.019658732	1.130156055	1	0.933	1
KIF26B	0.019673128	-0.214671384	0.875	1	1
HTR1B	0.019796211	-1.029915578	0.75	0.8	1
LIN28B	0.019809087	1.025210563	1	1	1
GRM5	0.019843019	1.124970024	1	1	1
ENSSSCG00000043207	0.019843921	-0.955605881	0.312	0.533	1
CALM2	0.019943439	-0.023933828	1	1	1
ENSSSCG00000016704	0.019966803	-0.815575429	0.562	0.667	1
CTC1	0.019995338	-0.009693396	1	1	1
ENSSSCG00000005656	0.0200676	0.167306646	1	1	1
PHC3	0.020073943	0.127578711	1	1	1
ENSSSCG00000007066	0.020075457	1.274866058	0.938	1	1
MAP1A	0.020077122	-0.717600269	0.562	0.8	1
GINS3	0.020079862	1.220706045	1	1	1
RDH10	0.020132936	0.894553936	1	1	1
ENSSSCG00000037473	0.020134344	-1.533681996	0.125	0.667	1
SF3B4	0.020163363	-0.030373649	0.938	1	1
SLC17A3	0.020210388	1.330790664	1	0.933	1
ZDHHC20	0.020241758	1.152593444	1	1	1
MYO5A	0.020242336	1.252755356	1	1	1
SIRT1	0.020260139	1.051735037	1	1	1
ENSSSCG00000059059	0.020277997	-1.093109404	0.562	0.6	1
GCG	0.020288164	-0.736965594	0.875	0.933	1
GGA3	0.020302755	-0.398735687	0.812	1	1
GSK3A	0.020312693	-0.208586622	1	1	1
CAMKK1	0.020313852	-0.30565404	0.75	0.867	1
ZNF37A	0.020337668	-1.063362061	0.812	0.733	1
DZIP3	0.020352045	1.093285934	1	1	1
GLTP	0.020375724	1.416143437	1	1	1
XPA	0.020393155	1.218351691	1	0.933	1
ENSSSCG00000054646	0.020393292	2.104829973	0.75	0.2	1
ENSSSCG00000055001	0.020403279	1.972182056	0.875	0.667	1
SHB	0.020424974	-1.455679484	0.25	0.533	1
ENSSSCG00000053064	0.02044332	1.781359714	0.938	0.6	1
CEP135	0.020448494	1.259515488	1	1	1
NSUN4	0.020458967	0.089137826	0.938	1	1
ATP1A3	0.020501466	-0.784987109	0.438	0.867	1
SAE1	0.020508451	1.521710708	1	1	1
ENSSSCG00000032978	0.02054667	-0.525060287	0.812	0.933	1
SLC12A4	0.02054701	-0.297265052	1	1	1
SMARCC1	0.020551205	0.100379437	1	1	1
PSAT1	0.020602663	-0.23397194	0.938	1	1
ENSSSCG00000001931	0.020645526	0.049914521	1	1	1
ENSSSCG00000060436	0.020666294	-1.140415119	0.562	0.6	1
SCAI	0.020666752	1.169925001	1	1	1
ENSSSCG00000051978	0.020689842	-0.678071905	0.625	0.933	1
ENSSSCG00000040337	0.020698397	1.218768811	1	1	1
ENSSSCG00000038960	0.020713711	-0.372085354	1	1	1

TARBP1	0.020734282	1.282589947	0.938	0.867	1
ANKS1B	0.020756831	0.923946557	1	1	1
ZBTB17	0.020796194	-0.571763003	0.812	0.667	1
BMP2	0.020820589	1.615124471	0.938	0.933	1
ONECUT2	0.020850419	1.40598098	1	1	1
ZNF793	0.020872988	0.969927257	1	1	1
LHX2	0.020879729	-0.467504919	0.625	0.8	1
IRGQ	0.020891856	-0.963826387	0.625	0.667	1
SLC6A5	0.020928641	0.236359985	1	1	1
MORN4	0.020950131	-0.239950793	0.438	0.8	1
ARMCX5	0.020950342	1.423140346	1	1	1
SLC38A6	0.020970471	1.242713132	1	1	1
C12orf4	0.020975145	0.030598742	0.938	1	1
NT5C3B	0.02104992	-0.808379927	0.688	0.733	1
TRMT1	0.021088507	-0.858644151	0.5	0.867	1
CDH1	0.021113033	0.960646246	1	1	1
TCEA1	0.021132172	1.021883118	1	1	1
GRIN2C	0.021166149	-1.578536232	0.188	0.6	1
FUBP1	0.021191015	0.983953929	1	1	1
NCL	0.021244979	1.207607492	1	1	1
EZH1	0.02124766	0.105272043	0.938	1	1
CERS6	0.02124899	1.583775586	1	0.733	1
ACADL	0.021277202	-0.489038081	0.75	0.8	1
ING2	0.021303736	-0.874469118	0.688	0.733	1
ZC3H18	0.021305701	-0.476438044	0.375	0.867	1
TMEM196	0.021315177	-1.35614381	0.25	0.667	1
DBP	0.021350868	-1.578536232	0.25	0.533	1
EFHD2	0.021355534	-1.245112498	0.438	0.533	1
CXADR	0.021373682	1.457905764	1	1	1
ENSSSCG00000003990	0.021379891	-1.127056736	0.562	0.733	1
RORB	0.021402209	0.022113566	1	1	1
ENSSSCG00000038326	0.021417175	1.441667339	1	0.867	1
CLDND1	0.021444191	-0.117513205	1	1	1
SNTA1	0.021467762	-0.623624121	0.5	0.667	1
CDK18	0.021498363	-1.056583528	0.438	0.667	1
ENSSSCG00000061538	0.02152849	-1.140415119	0.5	0.467	1
ENSSSCG00000061401	0.021545362	-1.192645078	0.312	0.533	1
ENSSSCG00000063544	0.021545444	1.106631251	1	1	1
CCNG1	0.021545852	0.202721024	1	1	1
EEF1E1	0.021586545	-0.390577307	1	1	1
PDPR	0.021587179	1.253630446	1	1	1
WDR35	0.021620811	1.753345337	1	0.733	1
UCK1	0.021803824	-1.093109404	0.312	0.667	1
PLXNB2	0.021813103	-0.9861942	0.438	0.667	1
IVD	0.021826408	-0.357293826	1	1	1
ENSSSCG00000009348	0.021833341	-0.745186101	0.812	0.867	1
CFAP206	0.02192581	0.035405336	1	1	1
TAF4B	0.021925979	1.256447447	1	1	1
LACTB	0.021929059	0.100732104	1	1	1
RWDD1	0.021941096	0.024290979	1	1	1
ENSSSCG00000032364	0.021988107	-0.034970968	1	1	1
EI24	0.022007511	-0.005646563	1	1	1
APBB1IP	0.022027624	3.22881869	0.562	0.133	1
DOLPP1	0.022037026	-0.678071905	0.5	0.8	1
NEK5	0.022061599	-0.415037499	0.938	1	1
POLI	0.022128883	0.007960977	0.938	1	1
VPS36	0.022188066	0.051031571	1	1	1
SLC24A1	0.022192514	-0.020353062	0.938	1	1
ARHGAP12	0.02226128	1.044022179	1	1	1
NUDT19	0.022268494	1.504102993	1	1	1
ENSSSCG00000008845	0.022287783	1.259450309	1	1	1
ENSSSCG00000037475	0.022291329	-0.334117504	0.75	0.733	1
LLGL2	0.022311768	1.326882977	1	1	1
SPNS1	0.022332347	-0.266174113	0.875	1	1
NPTN	0.022335336	1.052794662	1	1	1
NAGPA	0.02233941	-0.070025791	0.938	0.867	1
UGT8	0.022365941	1.177571813	1	1	1
PRKCSH	0.022377159	-0.843131151	0.75	0.8	1
OPTN	0.022380429	-0.047594745	1	1	1
ENSSSCG00000022842	0.022413607	-0.854949667	0.562	0.8	1

SORCS1	0.022426471	0.946920374	1	1	1
FAR1	0.022455702	-0.107944446	0.938	1	1
ENSSSCG00000034914	0.022468099	4.430452552	0.562	0	1
CEP192	0.022508333	0.888210641	1	1	1
CTNND2	0.022559829	-1.508146904	0.312	0.6	1
TMBIM4	0.022562451	1.599912842	0.938	0.667	1
BLOC1S5	0.022628017	0.281379365	1	1	1
ENSSSCG00000048787	0.02263035	-0.155037154	0.938	1	1
TMEM231	0.022630493	-0.498365883	0.688	0.867	1
NOP56	0.022646939	-0.191513108	1	1	1
S100PBP	0.022671903	0.314316709	1	1	1
ENSSSCG00000005971	0.022683204	-0.402156208	0.938	1	1
THOC5	0.022708039	-0.166873459	1	1	1
CSK	0.022716466	-0.097926421	1	1	1
SNX16	0.022745152	1.844063758	1	0.667	1
SGO1	0.022825873	1.141977292	1	1	1
ENSSSCG00000021891	0.022835093	1.463283944	0.938	1	1
PLLP	0.022844721	1.18893539	1	1	1
ENSSSCG00000038487	0.022916995	1.62935662	1	0.667	1
KLF2	0.022925547	-0.487388344	0.875	0.8	1
DGKG	0.022953247	0.068543859	1	0.933	1
TCERG1	0.022984364	1.085408208	1	1	1
USF2	0.022994578	1.730012834	1	0.733	1
GLUD1	0.023009587	1.170782727	1	1	1
MLF1	0.02302587	0.882643049	1	1	1
INTS9	0.023029964	1.18924186	1	1	1
RASAL1	0.023053399	-1.450661409	0.375	0.6	1
FMC1	0.023069525	1.180392	1	0.933	1
ZBTB24	0.023075143	-0.297467903	0.812	1	1
ENSSSCG00000000104	0.023211311	-0.02923958	1	1	1
RFX5	0.023211484	1.179049462	1	1	1
GNAT2	0.023220817	1.738767837	0.875	0.533	1
RARB	0.023246226	-0.359624379	1	1	1
IQCE	0.02324818	-1.455679484	0.188	0.533	1
ENSSSCG00000057725	0.023259404	-0.032263849	1	1	1
DOP1B	0.0232776	0.031435694	1	1	1
PLA2G4E	0.023277981	1.180336049	1	1	1
DNAJA1	0.023280555	1.173775486	1	1	1
TMPRSS15	0.023290561	0.978165284	1	1	1
ENSSSCG00000048343	0.023296127	-0.027307346	1	1	1
ARHGAP19	0.023331104	-0.131841799	1	1	1
GPR182	0.023340269	0.968153891	1	1	1
LNPEP	0.023377874	1.134711263	1	1	1
TNFAIP8	0.023388345	0.334873673	1	1	1
RB1	0.023399055	1.283509332	1	1	1
SLC25A3	0.02344446	-0.509909512	1	1	1
PIGA	0.023515009	0.925873699	1	1	1
ENSSSCG00000003461	0.023527611	1.148316888	1	1	1
ITSN1	0.023531316	0.000576041	1	0.933	1
CYP26A1	0.023546975	1.346261242	1	0.933	1
ENSSSCG00000037476	0.02355105	-0.941106311	0.5	0.6	1
VPS13C	0.023576279	0.233918227	1	1	1
EEF2K	0.023609579	-0.269987166	0.938	1	1
ENSSSCG00000060951	0.023627364	2.089093927	0.812	0.4	1
CAT	0.023656365	0.924072558	1	1	1
VPS9D1	0.023725653	-1.678071905	0.125	0.533	1
ENSSSCG00000059863	0.023756654	-1.415037499	0.188	0.6	1
ENSSSCG00000035160	0.023758365	-1.142019005	0.812	0.8	1
ZSCAN26	0.023792611	0.933739224	1	1	1
ALG13	0.023816827	0.344933756	1	1	1
URI1	0.023825164	-2.11417102	0.5	0.4	1
ENSSSCG00000010221	0.023857608	1.314394422	1	0.867	1
FANCC	0.023871576	-0.026558211	1	0.933	1
DCPS	0.023894349	1.458362031	0.938	1	1
PTCH2	0.023931896	-0.30256277	0.812	0.867	1
IQSEC3	0.024018386	-1.010647244	0.562	0.667	1
RB1CC1	0.02414826	1.217952454	1	1	1
ACBD3	0.02416921	0.141666741	1	1	1
KATNBL1	0.024213874	0.012243596	1	1	1
NSD2	0.02422268	0.093149231	1	1	1

TRIM39	0.024275126	0.153456691	1	1	1
CALM1	0.024276455	0.216813049	1	1	1
TBC1D2	0.024290838	-0.466924241	0.875	0.8	1
SPTLC2	0.024315865	0.079001263	1	1	1
METAP1	0.024318544	-1.072931522	0.625	0.6	1
ENSSSCG00000042348	0.024337656	-1	0.562	0.667	1
SV2C	0.024480839	-0.568447414	0.812	0.8	1
HPRT1	0.024499583	0.276044577	1	1	1
SUSD1	0.024577759	-0.347292893	0.938	0.933	1
DHRS7B	0.024579096	-0.809316438	0.688	0.867	1
MDM2	0.024590078	-0.421163602	0.938	1	1
ZBTB10	0.024619221	-0.025404545	1	1	1
NCAPH	0.024655061	-0.324859536	1	1	1
DHX32	0.024713794	1.373342642	1	1	1
MTRR	0.024746635	-0.206003461	0.812	1	1
PARP11	0.024818197	-1.157239742	0.312	0.533	1
HNMT	0.024901534	2.137188215	0.75	0.267	1
KCNT2	0.024904795	1.461202883	1	0.933	1
CCDC39	0.024908331	1.446410126	0.875	0.933	1
NUTF2	0.024910952	-0.001415606	1	1	1
TMIGD1	0.025012166	-0.941106311	0.875	0.867	1
ERGIC2	0.02501359	1.071448575	1	1	1
SUPT5H	0.025017039	1.246395827	1	1	1
ANKEF1	0.025021919	-0.224353938	0.938	0.933	1
RPGRIP1L	0.025039463	0.954110877	1	1	1
MBOAT2	0.025074633	-0.159707153	1	1	1
ZYG11A	0.025133663	0.126090891	1	1	1
SNAPC5	0.025143502	1.665882496	1	0.933	1
ZNF574	0.025181826	-0.300648698	1	0.933	1
TMC1	0.025222512	-1.415037499	0.312	0.6	1
MBL2	0.025247975	-0.415037499	0.75	0.867	1
NPSR1	0.025251976	-1.03170886	0.188	0.533	1
ABCD3	0.025286899	0.408754967	1	1	1
SORCS3	0.025304202	-0.221842719	1	1	1
RNF14	0.025357724	0.23639191	1	1	1
PRRC2B	0.025372915	-0.35614381	0.938	0.933	1
CALHM6	0.025389813	-0.845181891	0.562	0.8	1
SHOX2	0.025414357	-0.565177849	0.812	0.867	1
EMD	0.0254659	-0.190806078	0.938	1	1
PPM1A	0.025486612	1.0887579	1	1	1
TGIF1	0.025493967	3.33315535	0.5	0.133	1
SERINC3	0.025511419	0.329041198	1	1	1
LRIG2	0.02556471	-0.195207592	1	1	1
OLFML3	0.025631851	-0.508146904	0.312	0.667	1
SEMA3D	0.025662059	1.059439028	1	1	1
ZCCHC7	0.02569299	1.168905789	1	1	1
GIGYF2	0.025723928	0.150725354	1	1	1
IPMK	0.025735402	1.531632998	1	1	1
SACM1L	0.02573724	1.22063633	1	1	1
NFX1	0.02576411	1.196397213	1	0.8	1
TMEM94	0.025776928	1.298256184	1	1	1
FPGS	0.025812825	-0.607682577	0.75	0.733	1
RHBDF2	0.025862538	1.935459748	0.938	0.667	1
TMEM183A	0.025947101	0.150406309	1	1	1
ENSSSCG00000060015	0.025966547	0.050085231	0.938	1	1
SLC35B2	0.025975147	1.192068904	1	1	1
SCN11A	0.025979584	-0.814573905	0.875	0.867	1
TRAF2	0.026087105	-0.35614381	0.75	0.867	1
COG5	0.026128531	0.296934313	1	1	1
CACTIN	0.026143402	1.941378972	0.938	0.533	1
ENSSSCG00000029449	0.026223386	1.285402219	0.875	0.4	1
SPACA9	0.026233627	-0.974464908	0.438	0.8	1
MYOZ1	0.026242449	-0.281181753	0.938	0.867	1
ENSSSCG00000057683	0.026283025	-0.485426827	0.625	0.733	1
UTP11	0.026297188	1.059028514	1	1	1
ENSSSCG00000052863	0.026339242	1.717620244	1	0.867	1
AFF3	0.026460563	1.287812737	1	1	1
ENSSSCG00000052562	0.026502642	-0.886658527	0.5	0.6	1
NXPH4	0.026560198	-1.010647244	0.375	0.533	1
ENSSSCG00000055609	0.026601156	-1.040641984	0.312	0.6	1

BSDC1	0.026658116	0.086685841	1	0.933	1
FRS3	0.026667736	-0.993573731	0.5	0.8	1
ENSSSCG00000041066	0.026712917	2.396275436	0.75	0.467	1
ENSSSCG00000007435	0.026734318	-0.442443657	0.875	0.867	1
NAF1	0.026735805	0.878221253	1	1	1
ENSSSCG00000044013	0.026759255	-0.052258167	1	1	1
CLVS2	0.02677932	0.026146152	1	1	1
PLEKHJ1	0.02684552	1.290856649	1	1	1
LSAMP	0.02691409	-0.337034987	0.812	0.867	1
PITHD1	0.026949073	1.26693143	1	1	1
S100Z	0.026953129	-0.861783858	0.688	0.733	1
ABRAXAS1	0.026972869	-2.03170886	0.438	0.6	1
ENSSSCG00000061680	0.026985755	-1.245112498	0.25	0.533	1
AGR2	0.027014233	-0.305479906	1	1	1
ENSSSCG00000038924	0.027077152	2.906890596	0.562	0.133	1
TTC28	0.027081792	1.180500203	1	0.933	1
MSL3	0.027088962	0.013199498	1	1	1
SFR1	0.027124498	-0.348542087	1	1	1
MYNN	0.027171937	1.18255029	1	1	1
GPR4	0.027225391	0.035405336	1	1	1
LARP1B	0.027237832	0.935947608	1	1	1
KPNA6	0.02728806	0.272508541	1	1	1
DHX40	0.027347372	0.299913182	1	1	1
MARK4	0.027359535	-0.863627558	0.625	0.6	1
WDR82	0.027435219	0.21673554	1	1	1
USP16	0.027470937	0.171504149	1	1	1
ENSSSCG00000051232	0.027484399	-0.752072487	0.5	0.8	1
WDHD1	0.027687355	1.19106151	1	1	1
PAK3	0.027710935	1.176872475	1	1	1
CEP95	0.027720231	1.020276069	1	1	1
AREL1	0.027737042	-0.033667713	1	1	1
SMURF1	0.027748773	-0.814393376	0.625	0.667	1
FARP2	0.027757241	-0.600069393	0.688	0.8	1
SLC35F2	0.027784752	1.272625175	1	1	1
ENSSSCG00000006395	0.027809439	1.533075759	1	1	1
PAGE2B	0.027840339	1.590961241	0.938	0.667	1
RRBP1	0.027841581	-0.329176763	0.875	0.867	1
C1orf112	0.027875371	-0.088216143	0.938	1	1
ENSSSCG00000021069	0.027929977	-0.452190498	0.688	0.8	1
TRHR	0.027950312	1.109198771	1	1	1
ENSSSCG00000002034	0.027981656	1.750489038	0.938	0.933	1
ENSSSCG00000050888	0.027997117	-1.192645078	0.25	0.533	1
CUL3	0.028004169	-0.015642748	1	1	1
PRR16	0.02804283	0.922183245	1	1	1
MAP4	0.028084185	1.346097018	1	1	1
ENSSSCG00000049849	0.028108694	-0.649502753	0.438	0.8	1
ENSSSCG00000021343	0.028108837	-1.598344713	0.438	0.667	1
SUV39H2	0.02815471	0.024837625	1	1	1
PLEKHM2	0.028161394	-0.054115273	1	0.933	1
ZNF451	0.028238218	0.997498752	1	1	1
PCSK5	0.028252749	1.267535798	1	1	1
KIFAP3	0.028258033	1.051562805	1	1	1
HPSE	0.028259943	3.461479447	0.5	0.133	1
RPS6KC1	0.028280677	0.932776056	1	1	1
SPATC1L	0.028306664	-3.263034406	0	0.533	1
FAM76A	0.028393649	-0.237499314	0.938	1	1
ENSSSCG00000051511	0.028423937	-0.834191107	0.75	0.8	1
SCP2D1	0.028446633	-1.521952703	0.25	0.533	1
HCN4	0.028472864	0.00507099	1	1	1
TAF1B	0.028482248	0.136580173	1	1	1
FNDC1	0.028490603	-0.319880266	0.875	1	1
PSPH	0.028534806	1.093057032	1	1	1
MRPL34	0.028563998	1.285402219	1	0.867	1
SUMO1	0.028613037	0.976326142	1	1	1
ENSSSCG00000035459	0.028664221	-1.068861858	0.5	0.733	1
SAYSD1	0.028690558	-1.77118131	0.125	0.533	1
PDIK1L	0.028702879	1.578267848	1	0.467	1
MANEA	0.028763458	1.211147664	1	0.933	1
NCAPD3	0.028775394	1.282940806	1	0.933	1
HEG1	0.028791445	-1.076235586	0.75	0.8	1

AATF	0.028826634	0.259241611	1	1	1
NCK2	0.028873734	0.226333427	1	1	1
GFRA3	0.028924753	-0.782769284	0.812	0.733	1
ILDR1	0.028936908	0.020402288	1	1	1
XRCC1	0.028980515	-0.466924241	0.812	0.733	1
SPART	0.029002135	1.210648936	1	1	1
IFNLR1	0.029138978	2.936637939	0.562	0.2	1
ST8SIA4	0.029152727	-0.289115059	1	1	1
ZNF397	0.02916413	-1.006397771	0.812	0.533	1
ENSSSCG00000045465	0.029168157	-0.65701029	0.625	0.867	1
BBX	0.029219628	1.408316303	1	1	1
MFSD6	0.029246824	1.042242449	1	1	1
ENSSSCG00000000563	0.029258298	1.016484057	1	1	1
STX12	0.029355286	1.11782289	1	1	1
ENSSSCG00000053769	0.029422199	2.63481105	0.625	0.333	1
HIBADH	0.029442759	1.350130729	1	1	1
ZFR	0.029474621	0.986787185	1	1	1
ENSSSCG00000060175	0.029502864	-1.093109404	0.188	0.6	1
ZFTRAF1	0.029521589	-0.945552216	0.688	0.867	1
ENSSSCG00000061948	0.029529414	-0.955605881	0.375	0.6	1
ZNF558	0.029568953	1.09978612	1	1	1
ENSSSCG00000054038	0.029681221	-1.093109404	0.312	0.533	1
SDR42E2	0.029688286	-0.678071905	0.5	0.467	1
GRB2	0.029715672	-0.57068837	0.812	0.867	1
ALG11	0.029779982	-0.281871048	0.938	0.933	1
NAALADL2	0.029824237	1.1205658	1	1	1
ADAMTSL3	0.029835564	-0.967578522	0.375	0.733	1
SLC26A4	0.029881097	1.128957131	1	1	1
SUMO2	0.029908953	1.040568166	1	1	1
ENSSSCG00000009240	0.029940743	-0.555452618	0.938	0.667	1
BIVM	0.0300522	1.394991557	0.938	0.933	1
ENSSSCG00000061723	0.030062196	0.979905393	1	0.933	1
ENSSSCG00000015193	0.030071012	-1.093109404	0.5	0.667	1
RSL1D1	0.030150788	0.125735596	1	1	1
HECA	0.030188104	-0.111927433	1	1	1
DKK1	0.030223897	-0.220221322	0.938	0.933	1
TRIO	0.030336736	-0.109692289	1	1	1
EXO5	0.030367451	1.397525714	1	0.933	1
CDHR3	0.030379083	-0.297727124	0.875	0.933	1
IL34	0.030408328	-0.601256308	0.812	0.667	1
ENSSSCG00000058307	0.03048722	-0.692571475	0.938	0.733	1
IFT22	0.030491741	-0.10718459	0.812	0.933	1
ENSSSCG00000052763	0.03051887	-0.654988292	0.438	0.733	1
SHC1	0.030591396	1.190820226	1	1	1
VGLL3	0.030622003	1.09427151	1	1	1
APPBP2	0.030629913	0.979540718	1	1	1
FAM169A	0.03071155	0.8498451	1	1	1
NR1I3	0.030745366	-1.315501826	0.188	0.533	1
TAP1	0.030748496	-0.9510904	0.625	0.667	1
PPP1R14B	0.030758345	1.475733431	1	0.667	1
DCUN1D3	0.030851825	-0.448590059	0.875	0.867	1
ZNF22	0.030851886	0.048458236	1	1	1
SLC38A2	0.031005857	-0.254915673	1	1	1
ENSSSCG00000047016	0.031008553	1.491853096	0.812	0.733	1
ENSSSCG00000058777	0.03101315	-0.752072487	0.625	0.667	1
EPS8	0.031028357	-0.651104858	0.875	0.733	1
C9orf85	0.031038125	-0.849838253	0.688	0.6	1
DNM3	0.03104416	1.144140976	1	1	1
NDST1	0.031121789	-0.613941568	0.438	0.6	1
AP3M1	0.031135634	0.916686375	1	1	1
ENSSSCG00000057928	0.031135731	1.190986617	0.938	1	1
MRM2	0.031235894	1.196268876	1	1	1
ELP3	0.03130955	0.302885792	1	1	1
SRSF12	0.031337517	-0.384875529	0.938	1	1
WDTC1	0.031384548	-0.639199013	0.875	0.867	1
RIBC1	0.031529674	-1.005646563	0.438	0.667	1
SPTY2D1	0.031570508	-0.678071905	0.875	0.867	1
DCLK2	0.031633894	1.083553032	1	1	1
ENSSSCG00000058221	0.031645597	-0.852101305	0.625	0.533	1
PLXNB1	0.031697262	-2	0.188	0.533	1

FAM168A	0.03170874	-0.279987539	1	1	1
SMC1B	0.031721722	0.299602036	1	1	1
ENSSSCG00000057350	0.031766091	2.607330314	0.688	0.2	1
SPC25	0.031777555	-0.023815499	1	1	1
VPS72	0.031834591	1.334237236	1	1	1
UNK	0.031859455	-0.10433666	1	1	1
ADARB1	0.031905639	-1.208586622	0.188	0.6	1
RAB2A	0.031918056	1.065738803	1	1	1
ALG5	0.031944519	1.654703572	1	0.733	1
PLXNC1	0.031956791	-0.31890441	0.938	0.933	1
EXOC3L4	0.032011687	-1.341036918	0.125	0.533	1
SH3BGRL3	0.032068046	-1.552541023	0.188	0.6	1
FAHD1	0.032095514	-0.578536232	0.688	0.8	1
EIF4EBP2	0.032167927	0.181397786	0.938	1	1
ATXN1L	0.032177137	1.144396156	1	1	1
ENSSSCG00000062485	0.032269609	1.484199524	1	0.933	1
NBAS	0.032300779	1.080431472	1	1	1
DSTN	0.032305942	0.999537014	1	1	1
CADPS2	0.032386364	0.981278854	1	1	1
PNRC2	0.032427817	1.091661719	1	1	1
ENSSSCG00000049230	0.032475589	-0.289506617	0.938	0.867	1
KIAA1958	0.032476897	1.027283303	1	1	1
ENSSSCG00000059101	0.032496867	-0.254390311	0.875	0.867	1
LIF	0.032539833	1.770829046	1	0.733	1
ENSSSCG00000059082	0.032544448	2.845490051	0.688	0.067	1
ACSBG1	0.032585224	-0.749155003	0.688	0.733	1
FARS2	0.032699651	-1.028979067	0.75	0.667	1
CMSS1	0.032705593	-0.982078092	0.438	0.667	1
PRKDC	0.032709021	0.123919832	1	1	1
AMOT	0.032732558	-0.087736232	1	1	1
PDK1	0.032762191	1.374158054	1	1	1
TUBGCP4	0.032799038	-0.286960245	0.938	0.933	1
RCBTB1	0.032809971	1.166777944	1	1	1
APAF1	0.032828785	-0.149526956	1	1	1
GRAMD2B	0.03287133	1.090838164	1	1	1
ANAPC4	0.032877752	1.426635437	1	1	1
ENSSSCG00000042690	0.03288025	-0.093109404	0.875	0.933	1
VWA3B	0.032923056	-0.529208519	0.75	0.8	1
SLC66A2	0.033127534	1.303586459	1	1	1
TPX2	0.033147481	0.977645579	1	1	1
SEC22A	0.03314977	0.355711971	1	1	1
SPG21	0.033167806	0.301132895	1	1	1
KLHL2	0.033267461	1.209453366	1	0.8	1
SEPHS1	0.033309726	1.837627933	1	0.6	1
CCDC91	0.033417377	1.217230716	1	0.933	1
CABIN1	0.033438121	1.272112201	1	0.8	1
OTULINL	0.033456822	-0.24691474	0.812	0.867	1
COPS8	0.033542125	0.359592168	1	1	1
ZNF326	0.033586792	1.544061574	1	1	1
DDX3X	0.033595246	1.015760038	1	1	1
FBXL16	0.033651284	-0.455679484	0.75	0.867	1
ENSSSCG00000052025	0.033775213	-1.00737953	0.75	0.667	1
PPP1R27	0.033806223	2.192292814	0.812	0.333	1
RBM18	0.033808562	1.216073462	1	1	1
ENSSSCG00000056205	0.033819025	-0.847996907	0.688	0.533	1
EPB41L4B	0.033838901	0.292274026	1	1	1
ENSSSCG00000060362	0.033857131	-0.595609745	0.375	0.6	1
DNAAF4	0.0339206	-0.912537159	0.5	0.667	1
MED1	0.0339719	0.85076664	1	1	1
COQ5	0.033997108	1.313845749	1	1	1
SAMHD1	0.034053004	1.572367702	1	1	1
INPP5F	0.034053032	1.042848283	1	1	1
ENSSSCG00000032119	0.034115759	1.054535913	1	1	1
GMPPB	0.034138429	-0.385891154	0.938	1	1
ENSSSCG00000038396	0.034333818	1.906890596	0.875	0.267	1
SENP7	0.034380632	1.237144623	1	1	1
ENSSSCG00000034843	0.034393031	-0.678071905	0.812	0.867	1
JUN	0.03441827	1.805010982	1	0.733	1
ARHGAP39	0.034477462	1.730012834	1	0.667	1
CDKN1A	0.034500102	-0.064540252	0.938	1	1

MTCH1	0.03451355	0.085060351	0.938	1	1
ETNK2	0.034527295	-0.299560282	0.875	0.8	1
LIPA	0.034570818	1.342372198	1	1	1
VMAC	0.034633615	-0.73947245	0.562	0.667	1
CPSF6	0.03463908	1.0408363	1	1	1
CALCOCO2	0.034685479	0.207030002	1	1	1
NFE2L1	0.034695482	0.109489148	1	1	1
OXNAD1	0.034720754	0.997711823	1	1	1
NCOA5	0.034737791	-0.112050968	0.938	1	1
ICE1	0.034897799	1.338606836	1	1	1
PLEKHA3	0.034948459	-0.617771395	0.75	0.8	1
TCP11L2	0.03503198	-0.647698256	0.5	0.733	1
ZNF541	0.035031985	1.984893108	0.812	0.4	1
CUX2	0.035041526	1.051172652	1	1	1
USP35	0.035076008	-0.88957601	0.5	0.733	1
OPN4	0.035143155	-1.044199804	0.438	0.733	1
NBN	0.035149894	0.027604461	1	1	1
ENSSSCG00000034174	0.03524116	1.638073837	0.875	0.8	1
CRYZL1	0.035261286	-0.123062626	0.938	1	1
MEAK7	0.035267945	-1.294743266	0.438	0.533	1
DALRD3	0.035275245	-0.79193987	0.688	0.933	1
HINFP	0.035314208	-0.73617486	0.812	0.8	1
SZT2	0.03531996	-0.633677786	0.375	0.8	1
ENSSSCG00000035376	0.035333853	0.227125768	1	1	1
TAOK2	0.035370512	-0.549487699	0.75	0.867	1
DGKE	0.035372801	1.027629237	1	1	1
SDC2	0.035382687	1.085961242	1	1	1
LIAS	0.035393673	-0.158165288	1	1	1
IDH3A	0.035470547	1.227524213	1	1	1
ENSSSCG00000046071	0.035476051	-1.471621028	0.125	0.533	1
PAXBP1	0.035482489	-0.410591594	1	1	1
KPNA4	0.035488299	-1.055438986	0.875	0.733	1
CCDC90B	0.035489516	-0.111056084	1	1	1
REEP1	0.03551577	-0.587874096	0.312	0.733	1
C1orf131	0.035530602	-0.721140627	0.625	0.733	1
SLC45A3	0.035556045	1.407650862	1	0.933	1
PGAP1	0.035633443	1.231392634	1	1	1
GTF2H4	0.035710065	2.476256241	0.875	0.6	1
ENSSSCG00000058744	0.035765519	-1.093109404	0.312	0.667	1
SMAGP	0.035850343	1.421463768	1	0.8	1
BICRA	0.035880463	-0.567040593	0.5	0.667	1
C18orf32	0.035931901	0.943141122	1	1	1
KCTD16	0.036036376	0.15368436	1	1	1
AP5M1	0.036111884	-0.091030593	1	1	1
ENSSSCG00000059840	0.036235085	1.33752495	1	0.867	1
ENSSSCG00000047186	0.036240881	-1.455679484	0.25	0.533	1
UBR7	0.036358415	0.943027924	1	1	1
PTPRG	0.03647229	0.039671709	1	1	1
ENSSSCG00000053618	0.036485966	-0.736965594	0.438	0.667	1
BCAS3	0.036528062	-0.259119356	1	0.933	1
CERK	0.036537715	1.501951544	1	1	1
IPO11	0.036552581	0.973659608	1	1	1
PDZRN4	0.036557597	-0.280736408	0.875	1	1
SMAD2	0.03658482	0.200921595	1	1	1
CAMTA2	0.036653837	-0.700791982	0.625	0.733	1
ERLIN2	0.036695281	0.994154677	1	1	1
ENSSSCG00000036022	0.036728179	1.805010982	1	0.6	1
SYCE2	0.036737586	-0.045412662	1	1	1
CDKL3	0.036771924	1.566815154	0.938	0.6	1
SLC6A4	0.036782732	-0.13076753	1	1	1
ENSSSCG00000054177	0.036786998	1.232507925	1	1	1
UVRAG	0.03683489	0.279877291	1	1	1
INTS3	0.036836396	1.397609295	1	1	1
ENSSSCG00000053387	0.036849578	-0.019647242	0.938	1	1
TXLNA	0.036859702	1.457279948	1	0.933	1
ERI2	0.036859961	1.534261244	1	1	1
SPCS2	0.03689336	1.07525667	1	1	1
AP1M1	0.036900088	-0.20475476	0.938	0.933	1
VOPP1	0.036909539	-0.877380713	0.5	0.467	1
PTGER2	0.036937984	3.661778098	0.5	0.067	1

GPR173	0.036966559	-0.9861942	0.375	0.533	1
MIPOL1	0.036967201	1.023808347	1	0.933	1
SHF	0.037029225	-1.093109404	0.188	0.6	1
AAMP	0.037065652	1.651794009	1	0.933	1
MEF2D	0.037089565	-0.339749372	0.812	0.867	1
GIT2	0.037150922	0.194871359	1	1	1
ENSSSCG00000058517	0.037205685	-0.186218809	1	0.933	1
ZNF354C	0.037208435	-0.909397451	0.688	0.733	1
ZNF471	0.037285105	-0.716546053	0.438	0.733	1
UBE2G2	0.037286352	1.352537521	1	0.933	1
SUPT20H	0.037307491	0.109295183	1	1	1
CELSR1	0.037390399	-0.706086281	0.375	0.667	1
FRYL	0.037478497	1.021066452	1	1	1
H1-0	0.037541727	-1.093109404	0.312	0.6	1
MGAT4A	0.037548048	0.845444956	1	1	1
ENSSSCG00000039545	0.037594895	-0.177998302	0.812	0.867	1
KLF11	0.037753418	0.088410736	1	1	1
GINS4	0.037785304	1.363480584	1	1	1
ENSSSCG00000002877	0.037788361	1.187733721	1	1	1
MMP1	0.037841609	-0.439759929	0.812	0.933	1
QSOX2	0.037896092	-1.415037499	0.25	0.533	1
MTERF4	0.037906075	-1.171111916	0.438	0.533	1
LRRC58	0.037909018	-0.230612928	1	0.733	1
ZSCAN12	0.037924956	0.067054717	0.938	1	1
CLCN2	0.037937673	1.393449392	1	0.933	1
ENSSSCG00000053345	0.037961601	-1.678071905	0.25	0.6	1
DDIT4L	0.037984569	1.158265411	1	1	1
MTHFSD	0.038144455	-0.218640286	0.938	0.8	1
ENSSSCG00000046327	0.038164111	-0.36104261	0.938	0.867	1
PDCD4	0.038207202	0.271660912	1	1	1
SRR	0.038227511	-0.205375637	0.875	0.867	1
SLC22A23	0.038248246	-0.186976328	1	1	1
ZBBX	0.038251224	1.067355268	1	1	1
NME3	0.038284029	1.796707678	0.938	0.6	1
FKBP6	0.038299463	0.313794237	1	1	1
EID1	0.038378964	-0.331269142	0.938	0.867	1
CPNE3	0.038396244	1.102578693	1	1	1
PRSS22	0.038434016	4.154818109	0.625	0	1
USP3	0.038479426	-0.12855824	1	1	1
KBTBD6	0.038528074	-0.242856524	0.688	0.867	1
ADNP	0.038536514	1.887154378	0.938	0.933	1
ZPBP2	0.038570798	1.519074565	1	1	1
MLLT1	0.0386133	1.339850003	1	0.867	1
ENSSSCG00000063245	0.03868756	-0.283212288	1	1	1
ENSSSCG00000031855	0.038747945	2	0.812	0.533	1
CCL17	0.038750312	-1	0.375	0.667	1
RBM27	0.038868685	0.997552319	1	1	1
MRPS36	0.038912115	0.065821678	1	1	1
ATP2C1	0.038983915	0.089543902	1	1	1
BCAT2	0.03899998	-0.035393907	0.875	0.933	1
ABHD12B	0.039000419	1.236717705	1	1	1
SLC26A6	0.039024629	1.32011998	1	1	1
NKAPL	0.03904782	-0.006694653	1	1	1
ENSSSCG00000056471	0.039052872	-1.015106892	0.5	0.667	1
TRAM2	0.039055604	0.235743745	1	1	1
CDR2L	0.039100123	-1.093109404	0.375	0.667	1
HIC2	0.039216932	1.95419631	0.75	0.533	1
TDRD9	0.039225133	-0.555452618	0.688	0.867	1
ENSSSCG00000056411	0.03926358	1.017692015	1	1	1
ACTR3	0.039290903	0.30934125	1	1	1
ENSSSCG00000059570	0.039302428	-0.654988292	0.625	0.667	1
UNC13D	0.03933527	-0.689212463	0.875	0.867	1
IFIT5	0.039352693	1.014950341	1	1	1
SLC37A1	0.039377941	-0.134136673	0.938	1	1
DYRK2	0.039436336	-1.093109404	0.688	0.733	1
ENSSSCG00000033894	0.039586426	0.013199498	1	1	1
FAM102A	0.0396821	-0.176767333	0.875	1	1
TM9SF4	0.039698279	1.249577251	1	1	1
CCNQ	0.039779764	2.22881869	0.688	0.267	1
TRPC3	0.039803374	0.40272621	1	1	1

PRRG4	0.039935978	1.441927871	1	0.933	1
KIAA1549	0.03993892	-0.018341636	1	1	1
TBXA2R	0.040022109	-0.040160524	1	1	1
CEP164	0.040143412	-0.063091259	1	1	1
ENSSSCG00000023865	0.040173757	-0.863627558	0.438	0.533	1
NAPB	0.040224892	-0.471621028	0.75	0.867	1
CCDC28A	0.040296503	1.638073837	1	0.867	1
ABHD10	0.040315157	1.254241287	1	0.933	1
RSPH3	0.040441647	0.022367813	0.875	0.933	1
TUBGCP3	0.040496916	1.375794967	1	1	1
PCF11	0.040500435	0.19661818	1	1	1
ATP6V1H	0.040531335	-0.03729219	1	1	1
ENSSSCG00000037445	0.040532421	1.043156286	1	1	1
SAG	0.04054574	-1.093109404	0.25	0.667	1
ATP9B	0.0405476	1.513069582	0.812	0.667	1
DSE	0.040570717	1.536641018	1	0.933	1
UBA3	0.040580233	0.848943246	1	1	1
ADNP2	0.040603054	0.163512263	1	1	1
COX7B2	0.04065802	-0.512485839	1	1	1
PACC1	0.040787212	-0.217736059	1	0.867	1
GSKIP	0.040835877	1.152284842	1	1	1
YTHDC1	0.040874154	0.242110187	1	1	1
CSTF3	0.040902039	1.164351157	1	1	1
INPP5E	0.040972668	1.999336844	0.875	0.733	1
GK5	0.041018731	-0.024803391	0.938	0.933	1
PPP2CB	0.041022106	0.976070623	1	1	1
KPNB1	0.041079488	0.066781004	1	1	1
ENSSSCG00000055817	0.041178292	-1.208586622	0.188	0.533	1
SPG11	0.04119017	0.981022325	1	1	1
AAGAB	0.041216439	0.128234318	1	1	1
MAP2K6	0.041245839	-1.678071905	0.25	0.533	1
MTREX	0.041317858	0.029323367	1	1	1
AKAP11	0.041355159	0.953898822	1	1	1
PHF11	0.041386291	1.15254972	1	1	1
ZFP91	0.041436049	0.890211854	1	1	1
TRIM11	0.041441311	1.459431619	0.875	0.6	1
PHF19	0.041492443	0.089794851	1	1	1
CNOT10	0.041496602	1.038223472	1	1	1
GAR1	0.041530763	1.312488399	1	1	1
ENSSSCG00000000746	0.041554072	-0.326599535	0.938	0.933	1
SLX4	0.041603243	-0.449253215	0.5	0.6	1
ENSSSCG00000026454	0.041617104	-0.118644496	0.75	0.933	1
ND5	0.041688592	1.671794797	1	1	1
CRELD1	0.04175462	-0.455679484	0.875	0.8	1
BVES	0.041757761	-0.050465067	1	1	1
POT1	0.041791472	-0.274174963	1	1	1
MTMR10	0.041877432	1.457254435	1	1	1
NUP188	0.041902285	0.978275229	1	1	1
COG4	0.041928056	-0.642882477	0.938	0.8	1
MLEC	0.041937656	0.29053196	1	1	1
EGFL6	0.042010135	1.091315167	1	1	1
BRI3	0.042024693	1.05166212	1	0.867	1
YPEL2	0.042043461	1.209307867	1	1	1
ANKRD11	0.042174586	-0.540568381	0.5	0.6	1
SEC62	0.042184853	0.265082234	1	1	1
INIP	0.042196702	0.166628373	1	1	1
CDC34	0.042236836	0.22743746	1	1	1
CLPB	0.042367913	-0.200912694	0.938	1	1
TFRC	0.042402459	-0.411606594	1	1	1
UMAD1	0.042438842	0.240677774	1	1	1
ACAT2	0.042467195	-0.723875595	0.812	0.733	1
TFIP11	0.042482226	0.068058979	1	1	1
TLE4	0.042511092	-0.121201711	1	1	1
GDI1	0.042601517	0.017921908	1	0.933	1
RPRD1A	0.0426483	1.225252075	1	1	1
SYNRG	0.042699904	0.28171874	1	1	1
ENSSSCG00000040973	0.042716332	1.36833856	1	0.8	1
SLC5A6	0.042766315	-0.910906073	0.812	0.933	1
GRIK2	0.042776482	-0.151605054	0.875	0.933	1
PLCB1	0.042856141	-0.445781023	0.812	0.933	1

ISL2	0.042877151	-0.912537159	0.625	0.6	1
NDEL1	0.042939118	0.304028822	1	1	1
NSD1	0.042987848	0.898515919	1	1	1
TMLHE	0.04302367	1.164021717	1	1	1
ARHGAP31	0.043077731	1.829722735	0.875	0.467	1
EML1	0.043122317	2.330102026	0.562	0.333	1
TRAPPC13	0.043134153	0.093871809	1	1	1
C16orf46	0.043146656	-0.545621609	0.5	0.733	1
SNAP47	0.043239207	0.107156901	1	1	1
GCNT3	0.043284895	0.010171805	1	1	1
EPS15L1	0.043296683	0.159612627	1	1	1
RNF149	0.043326989	0.010144557	1	1	1
COX1	0.043376122	1.736681473	1	1	1
ENSSSCG00000057034	0.043419411	-0.578536232	0.938	0.933	1
LIMK1	0.043485024	-0.431525622	0.938	0.933	1
TGFBI	0.043522634	-0.2255597	0.812	0.933	1
RBM6	0.043599715	0.299317583	1	1	1
BICC1	0.043750503	0.960636167	1	1	1
SLC3A2	0.043798902	-0.584095757	0.688	0.733	1
DENR	0.0438554	-1.093109404	0.5	0.533	1
RHOBTB3	0.043890564	-0.267607136	0.875	0.933	1
ENSSSCG00000026719	0.043932244	1.253121938	1	1	1
GRSF1	0.043950991	1.00476625	1	1	1
PPIG	0.043971732	1.082365823	1	1	1
ENSSSCG00000054218	0.044117122	-0.242487028	0.688	0.933	1
CDC16	0.044166311	1.218658824	1	1	1
PRKCD	0.044344735	1.312032059	1	1	1
ADAMTSL5	0.044366316	-0.531230517	0.812	0.667	1
SYF2	0.044374137	0.003566615	1	1	1
BSCL2	0.04437603	0.018638953	0.938	0.933	1
BRD8	0.044377458	0.787050826	1	1	1
GPAT3	0.044387011	1.637283536	0.812	0.667	1
PLEKHM1	0.044421405	1.159533396	1	1	1
ZNF227	0.044428341	1.208804901	1	1	1
METTL5	0.044433241	-0.396387442	1	0.933	1
ZC4H2	0.044510271	1.540159293	0.938	1	1
NT5DC3	0.044579544	-0.109983223	0.938	1	1
GEN1	0.044595483	-0.303876501	0.875	0.867	1
PDE5A	0.044614949	0.005970369	1	1	1
KLHL8	0.044686951	1.096715154	1	1	1
DDX55	0.044694485	1.250222542	0.938	1	1
KCNK9	0.044815527	-0.552541023	0.875	0.667	1
GRIN2A	0.044821034	1.370291116	0.938	0.867	1
SEC24B	0.044873665	-0.170242875	1	1	1
SMG7	0.044917339	0.187316801	1	1	1
ALDH5A1	0.044953015	1.951284715	0.688	0.267	1
CDC14A	0.045069625	2.154818109	0.688	0.533	1
NUP85	0.045082517	1.193585413	1	1	1
CMKLR1	0.045083473	1.56872186	0.875	0.8	1
SH2B1	0.045121314	1.600787468	1	0.8	1
WDR36	0.045144147	1.003675658	1	1	1
SRP72	0.045157948	-0.55468949	1	0.933	1
ENSSSCG00000062130	0.045177003	-1.35614381	0.188	0.533	1
DZIP1	0.045275784	1.170655665	1	1	1
ITK	0.045322767	-1.093109404	0.5	0.533	1
ACD	0.045451999	-0.213403638	1	0.8	1
DYRK3	0.045491002	1.140282016	1	1	1
PMP22	0.045523773	-0.384010604	0.875	0.933	1
KLRG2	0.045548638	-0.977632187	0.438	0.6	1
PKNOX1	0.045644561	0.225920742	1	1	1
ENSSSCG00000059754	0.045659117	1.321928095	1	0.933	1
ENSSSCG00000041267	0.045823678	2.22881869	0.688	0.267	1
SRP68	0.04583928	0.114344897	1	1	1
KIF16B	0.045940838	0.34307587	1	1	1
ZNF330	0.046034984	1.274930615	1	0.933	1
ENSSSCG00000007688	0.046070596	0.883558092	1	1	1
SPDL1	0.046100854	-0.064091304	1	1	1
EIF5B	0.046192783	0.447486904	1	1	1
PREX1	0.046196128	1.613159393	0.938	0.733	1
ENSSSCG00000021534	0.046207414	-1.230612928	0.125	0.6	1

SNRNP27	0.046270531	1.137422362	1	1	1
H2AC4	0.046271683	-0.458393868	0.75	0.733	1
RNF10	0.046290538	1.516804943	1	1	1
SLC40A1	0.046336986	-0.158327064	0.938	0.867	1
ENSSSCG00000061793	0.04635005	1.122396631	1	0.867	1
PHLDA1	0.0463882	0.071949842	0.938	0.933	1
TMEM150C	0.046434136	0.046963479	1	1	1
PIGN	0.046435274	1.019365325	1	1	1
C14orf39	0.046466188	1.534921818	0.812	0.667	1
FAM217B	0.046516056	-0.923184403	0.25	0.533	1
HEY2	0.046543887	0.91411699	1	1	1
WASF3	0.046569349	1.088546963	1	1	1
ACTG1	0.046582719	0.317806106	1	1	1
ENSSSCG00000042700	0.046686412	2.714245518	0.5	0.133	1
LDLRAD4	0.046696072	1.578657924	0.938	0.733	1
HPS1	0.046725972	-0.625983394	0.75	0.6	1
ENSSSCG00000061446	0.046775415	-0.435997118	0.812	0.8	1
EFCAB10	0.046800942	-0.295530405	0.938	1	1
RNF227	0.046903353	-0.760534065	0.562	0.667	1
TXLNG	0.046919942	0.362085221	1	1	1
NTAN1	0.0469341	1.095335685	1	0.8	1
PDE11A	0.046936096	-0.4639471	0.688	0.867	1
APBB2	0.046944995	0.211994068	0.938	1	1
PECAM1	0.046992337	1.301750213	0.938	0.667	1
PSMD4	0.047130311	1.234695257	1	1	1
LTN1	0.047130891	0.947001803	1	1	1
POMT2	0.047178394	-0.63673111	0.625	0.867	1
CCL25	0.047195093	-0.493647334	0.812	0.8	1
ENSSSCG00000008984	0.047207785	-0.471621028	0.875	0.867	1
PIKFYVE	0.047221969	-0.150140349	0.938	0.933	1
ARPC2	0.047251296	0.399207546	1	1	1
STK4	0.047258137	1.021308488	1	1	1
ETFRF1	0.047314811	-0.098110085	0.938	1	1
ELOVL6	0.047410704	0.29192645	1	1	1
NDE1	0.047474319	0.288253561	1	1	1
RYR3	0.047489651	0.196909443	1	1	1
FRMD6	0.047540474	0.945341437	1	1	1
ENSSSCG00000014314	0.047550226	-0.178276788	0.875	1	1
PPAT	0.047593162	1.029617552	0.938	1	1
TET1	0.047716667	2.070389328	0.75	0.667	1
CD151	0.047720493	-0.721140627	0.438	0.533	1
DDOST	0.047757641	0.201633861	1	1	1
UVSSA	0.047780765	-0.166358386	1	0.933	1
COQ8B	0.047809506	-1.066142357	0.5	0.733	1
ENSSSCG00000017507	0.047905541	0.263498078	1	1	1
STK38L	0.047906177	1.199283181	1	1	1
ENSSSCG00000003102	0.047942635	-0.967578522	0.375	0.533	1
ATP1B1	0.047948708	-0.027421501	0.875	1	1
ENSSSCG00000055321	0.047980653	1.369517553	0.875	0.867	1
M6PR	0.048023192	0.191458163	1	1	1
TRAF7	0.048101892	1.38466385	1	0.933	1
PTK7	0.048154875	2.430452552	0.625	0.267	1
PAN2	0.048176091	1.082252408	1	1	1
MYO9B	0.048183459	-0.455679484	0.938	0.867	1
DCTN6	0.048199093	0.852709557	1	1	1
XPR1	0.048288385	0.927749201	1	1	1
ENSSSCG00000045808	0.048297671	1.037227795	1	1	1
USP38	0.048343521	0.937717122	1	1	1
NKAIN2	0.048363453	-0.924986646	0.812	0.733	1
PHTF2	0.048397312	1.093626496	1	1	1
PREP	0.048398878	1.044712162	1	1	1
MAP2K5	0.048606473	-0.220488711	0.875	0.8	1
PPT1	0.048667446	0.338505964	1	1	1
ENSSSCG00000049958	0.048720064	-1.277533976	0.312	0.533	1
ITGB1BP1	0.048725898	-0.10137702	0.938	1	1
DOT1L	0.048732575	0.059593857	0.938	1	1
ENSSSCG00000061001	0.048854711	1.294494865	0.938	0.6	1
FAM163A	0.048984046	-0.587874096	0.688	0.8	1
ENSSSCG00000062471	0.049024065	-0.9861942	0.375	0.667	1
SPTB	0.049060227	1.383442484	1	1	1

GSX2	0.049076626	1.350725359	1	1	1
VPS16	0.049119582	1.323661062	1	1	1
SEZ6L	0.049124515	-1.388565288	0.562	0.467	1
R3HDM2	0.049137306	0.135019386	0.938	1	1
ENSSSCG00000036144	0.049305232	-0.256608137	0.812	0.933	1
STAT5B	0.04935411	1.391090119	0.938	0.933	1
MFN2	0.049355576	-0.062412607	1	0.867	1
METTL8	0.049431224	-0.211753901	0.562	0.667	1
ENSSSCG00000049427	0.04945828	-0.852101305	0.25	0.733	1
ADAR	0.049513065	0.941872792	1	1	1
ENSSSCG00000061041	0.049624469	-0.423258006	0.875	0.8	1
AMMECR1L	0.049686609	0.114556502	0.938	1	1
ARL14EP	0.049690895	1.105770994	1	1	1
ABRAXAS2	0.049776677	0.241406512	1	1	1
BCL2L2	0.049806585	-0.77118131	0.5	0.6	1
KDM5B	0.049834997	-1.749155003	0.625	0.533	1
ACOX1	0.049836478	1.055527091	1	1	1
SESN1	0.049850534	1.253776113	1	1	1
ENSSSCG00000044685	0.049873702	-1.230612928	0.188	0.533	1
CNOT3	0.049952273	1.743391863	1	0.933	1
ESPL1	0.049992993	1.076744948	1	1	1
ENSSSCG00000061301	0.049994377	-0.809316438	0.312	0.667	1
SQSTM1	0.050024742	1.201857503	1	1	1
TMEM201	0.050056228	-0.34836646	0.625	0.733	1
PCSK1	0.05006224	2.658963082	0.75	0.6	1
GABPA	0.050143386	0.941576824	1	1	1
TAF5L	0.050149719	0.098405773	1	1	1
ENSSSCG00000042411	0.05021929	2.321928095	0.625	0.2	1
MYO10	0.050269654	0.175222682	1	1	1
MTF2	0.05029783	0.94473488	1	1	1
KIF20B	0.050301479	1.13767323	1	1	1
RAB6A	0.050309173	0.891293741	1	1	1
C5	0.050320434	-0.779951519	0.688	0.6	1
E2F7	0.050323806	-0.093109404	0.875	1	1
NEPRO	0.050353471	-0.054635257	0.938	0.933	1
ENSSSCG00000041243	0.050358258	3.076815597	0.562	0.067	1
POLR1C	0.050392918	-1.096747981	1	0.733	1
ENSSSCG00000040773	0.050581634	1.217859479	0.938	0.933	1
GUF1	0.050643294	-0.026189812	0.938	1	1
NAP1L3	0.050690037	-0.112940576	1	1	1
ENSSSCG00000004246	0.050787972	1.369417021	0.938	0.867	1
ENSSSCG00000051194	0.050794418	-1	0.312	0.6	1
ENSSSCG00000048507	0.050826526	-0.607682577	0.75	0.8	1
MARCKS	0.051032683	1.516781493	1	0.867	1
ENSSSCG00000056774	0.051053445	-0.47498004	0.438	0.6	1
GREB1	0.051074094	-0.815575429	0.438	0.733	1
ZFP62	0.05112333	1.282399731	0.938	0.867	1
WDR72	0.051147878	1.100662339	1	1	1
APP	0.051149789	-0.4639471	0.625	0.8	1
SKIDA1	0.051265435	-0.77118131	0.5	0.533	1
OR51Q1	0.051274421	1.656912343	0.812	0.267	1
ENSSSCG00000062038	0.051304872	-0.883656039	0.625	0.467	1
GNPAT	0.051382311	1.067643951	1	1	1
AMIGO1	0.051488547	-0.065453061	0.875	0.933	1
ENSSSCG00000048831	0.051507794	-0.830074999	0.25	0.667	1
ENSSSCG00000055578	0.051513756	-0.585505786	0.875	0.933	1
WDCP	0.0515304	-0.088260005	1	1	1
ZNF473	0.051638893	-0.363963315	0.875	0.8	1
ENSSSCG00000007088	0.051642522	0.371164306	1	1	1
IPCEF1	0.051841153	-0.052082136	0.875	0.933	1
TUBG2	0.051930853	1.216575095	1	1	1
TUBAL3	0.052051178	0.897904923	1	1	1
TESC	0.05228381	1.153233766	1	1	1
SLC44A5	0.052300007	0.215322848	1	1	1
COX7A2L	0.052346008	0.323881021	1	1	1
ENSSSCG00000051466	0.052414724	-0.382616022	0.562	0.733	1
NPR3	0.052460616	0.067355268	1	1	1
TRIM44	0.052488514	0.026921299	0.938	0.933	1
PRKACB	0.052503927	1.189926954	1	1	1
FOXK1	0.052529832	1.491853096	0.938	0.533	1

CCIN	0.052547222	-0.075727326	0.875	0.933	1
AP3B1	0.052547491	0.81606443	1	1	1
ENSSSCG00000043595	0.052552205	-0.804604311	0.688	0.867	1
TMEM127	0.052557273	-0.204140717	0.75	0.8	1
PABPN1L	0.05257893	1.986375379	1	0.933	1
TNIK	0.052611317	1.183788482	1	1	1
BID	0.052655863	-2.341036918	0.188	0.667	1
IMPA1	0.052727857	1.081780535	1	1	1
ZSCAN23	0.052817609	0.098189248	0.875	1	1
ZSCAN16	0.052818505	-0.111256751	1	0.933	1
RBCK1	0.05282138	0.289610375	1	0.933	1
ENSSSCG00000052558	0.052940743	-0.35614381	0.625	0.933	1
VAMP4	0.053025157	-0.361857461	0.938	0.933	1
ENSSSCG00000053698	0.05312643	-0.14286244	0.938	0.933	1
RBPJ	0.053174403	1.103369232	1	1	1
ENSSSCG00000032337	0.053181365	-0.253574077	0.875	0.933	1
CMIP	0.053182484	-0.449253215	0.625	0.733	1
TTC14	0.053275831	1.633325522	0.938	0.933	1
ENSSSCG00000061435	0.053411562	-0.800928653	0.438	0.6	1
PRMT3	0.053504303	1.392317423	0.938	0.8	1
SLC39A5	0.053516202	-0.079812582	0.875	0.933	1
CAPRIN1	0.053558484	-0.120177648	1	0.933	1
ANGPTL1	0.053558861	-0.295926287	0.812	0.933	1
ENSSSCG00000032445	0.053642861	1.751526534	1	0.933	1
NUP98	0.053677943	0.974142842	1	1	1
LPAR6	0.053723569	1.567404129	0.875	0.8	1
ENSSSCG00000032456	0.05373808	-0.745186101	0.438	0.667	1
DYNLRB2	0.053857023	-0.877380713	0.5	0.6	1
ENSSSCG00000061311	0.053945311	-0.205584134	0.875	0.867	1
ADGRG1	0.054052292	0.206292121	1	1	1
ENSSSCG00000043560	0.054067552	1.845490051	0.812	0.467	1
DDHD1	0.054079846	0.151309323	1	1	1
WDR83OS	0.054188173	-0.152216022	1	1	1
TPR	0.054200283	1.176270513	1	1	1
SUN1	0.054216592	1.299901789	1	1	1
BAG4	0.054242741	0.26709366	1	1	1
COMMD2	0.05438539	0.951114596	1	1	1
CAP2	0.054460977	1.396037309	1	0.933	1
PAQR3	0.054499354	1.054989235	1	1	1
ENSSSCG00000051907	0.054609406	-0.967578522	0.625	0.6	1
ENSSSCG00000058908	0.054610608	1.701306462	0.688	0.533	1
RNF11	0.054634939	-0.048715285	1	1	1
PIMREG	0.054637434	-0.312890612	0.688	0.733	1
ATG4C	0.054666781	1.435269568	0.938	0.467	1
TJP1	0.054682366	0.985235486	1	1	1
GALNT2	0.054724939	-0.499101764	0.812	0.8	1
ENSSSCG00000011905	0.054873241	1.704397732	0.688	0.667	1
TBC1D2B	0.054944295	0.171718283	1	1	1
ENSSSCG00000004573	0.054945587	-0.706086281	0.5	0.733	1
THG1L	0.055057323	0.078501974	1	1	1
ENSSSCG00000052350	0.055073862	0.074639735	1	1	1
CYP20A1	0.05517065	1.142662047	1	1	1
CPEB2	0.055213505	1.080707412	1	1	1
ENSSSCG00000055258	0.055259269	-1.35614381	0.125	0.533	1
RIC8B	0.055323678	-0.162372067	0.875	1	1
MXI1	0.055366328	1.018783476	1	1	1
CEP43	0.055372106	0.416000874	1	1	1
NIN	0.055494541	1.038135129	1	1	1
MICU1	0.05573889	-0.300704824	0.938	0.867	1
CDR2	0.05580099	0.014950341	1	1	1
RASIP1	0.055811559	-0.495207848	0.625	0.667	1
SUDS3	0.055833361	0.290052753	1	1	1
TACO1	0.055841609	-0.062412607	1	0.867	1
WDR46	0.055931553	-0.578536232	0.562	0.6	1
ND1	0.055989398	1.596896499	1	1	1
SLC16A7	0.056104961	2.19865672	0.812	0.667	1
DUSP6	0.056126894	-0.497499659	0.562	0.933	1
HNRNPDL	0.056238512	0.999979205	1	1	1
AEBP2	0.056289459	-1.54830403	0.688	0.467	1
SLC27A6	0.056357448	2.621136113	0.562	0.467	1

SAFB	0.056633834	0.337219747	0.938	1	1
MYL12B	0.056637916	0.114503297	1	1	1
ENSSSCG00000062276	0.056796836	-0.578536232	0.875	0.733	1
KIF17	0.056813054	-1.378511623	0.438	0.6	1
HACD1	0.05683109	1.602884409	0.875	0.467	1
SARNP	0.056832071	1.084027222	1	1	1
YIPF6	0.056834228	1.180494243	1	0.867	1
RINT1	0.056858212	0.160838429	1	1	1
EWSR1	0.057058721	-0.021556143	1	1	1
UBE3A	0.057068788	1.289063461	1	1	1
STAG3	0.05714234	-0.061676897	0.875	1	1
RNF169	0.057214201	-0.113968009	1	1	1
USP37	0.057245388	0.240815036	1	1	1
ENSSSCG00000041164	0.057281314	1.302819272	0.875	0.733	1
TRAM1L1	0.057338023	-0.101146723	1	0.933	1
KRIT1	0.057365217	1.237807474	1	0.933	1
PTPRA	0.057380602	0.261692988	1	1	1
IFT172	0.057403917	-0.849838253	0.625	0.8	1
DOP1A	0.05745075	0.139946631	1	1	1
ARID4A	0.057452687	1.065327804	1	1	1
ENSSSCG00000027232	0.057546205	2.17990909	0.625	0.2	1
NETO2	0.057654598	1.024585638	1	1	1
ago-03	0.057655801	-0.147951475	1	1	1
RNF185	0.057703584	-0.027602298	0.938	1	1
CHKB	0.057814415	-0.623624121	0.625	0.733	1
RRS1	0.05782403	0.204434532	1	1	1
RAB23	0.057859488	1.164558492	1	1	1
TPMT	0.057884299	-0.253574077	0.5	0.733	1
ENSSSCG00000003989	0.057934428	1.617860981	0.938	0.933	1
ENSSSCG00000056123	0.058004475	-0.338221902	0.938	1	1
SNX21	0.058064803	-1.010647244	0.188	0.533	1
BTBD9	0.058086798	0.226926631	0.938	1	1
BBS9	0.058105352	1.558967292	0.875	0.4	1
ESRP1	0.058126029	0.358465299	1	1	1
MACO1	0.058148008	0.052549227	0.938	1	1
ZFP30	0.058149706	0.948158614	1	1	1
SCN10A	0.058195618	-0.065394026	1	1	1
ENSSSCG00000061639	0.058206395	-0.734655433	0.75	0.733	1
ORMDL3	0.05826311	0.213221835	1	0.933	1
HMCN1	0.058443404	0.188012474	1	1	1
ENSSSCG00000056598	0.058456599	-0.279730695	0.875	1	1
SPNS2	0.058487563	-0.707819249	0.438	0.6	1
SLC46A1	0.058493827	-0.415037499	0.875	0.733	1
KIF7	0.058498024	1.271084417	1	0.933	1
COPG1	0.058571776	1.240651054	1	1	1
TIMM10B	0.058753294	0.324714405	1	1	1
MTMR6	0.058782509	0.330744242	1	1	1
SCUBE3	0.058813618	2.044394119	0.688	0.333	1
ALDH1B1	0.058846164	-0.050040682	0.875	0.933	1
ENSSSCG00000038540	0.058864271	-0.437063806	0.75	0.933	1
ENSSSCG00000045852	0.058867806	1.029509882	0.938	0.733	1
CSNK1G1	0.058878331	0.932653692	1	1	1
ENSSSCG00000058423	0.058902081	-0.721140627	0.375	0.533	1
DNAJC9	0.058929926	0.441767865	1	1	1
MSTO1	0.05908921	-0.508146904	0.75	0.933	1
ENSSSCG00000003746	0.059136109	-0.923184403	0.375	0.6	1
ZKSCAN8	0.05919456	0.881093716	1	1	1
FECH	0.059239535	1.260037421	1	0.933	1
ENSSSCG00000044152	0.059298776	-0.678071905	0.5	0.667	1
ENSSSCG00000056615	0.059300978	-0.745186101	0.375	0.533	1
FGF20	0.059475363	1.934371332	0.812	0.333	1
SETD3	0.059544756	1.089176256	1	1	1
SULT4A1	0.059569285	1.725052273	0.938	0.667	1
ENTHD1	0.059649598	-0.318669105	0.75	0.667	1
SH3PXD2A	0.059674649	1.191134897	1	1	1
SHQ1	0.059708473	1.155719624	1	1	1
RFLNB	0.059763675	-0.590609064	0.5	0.8	1
NDUFAF7	0.059820701	0.019274012	1	1	1
PTGFRN	0.059853403	-0.546827372	0.812	0.667	1
ENSSSCG00000029203	0.059900398	0.12889556	1	1	1

KLHL41	0.059941014	-0.923184403	0.562	0.6	1
KLHL15	0.060038348	1.014429788	1	1	1
ENSSSCG00000022039	0.060043215	-0.556081381	0.625	0.667	1
ENSSSCG00000045442	0.060055052	-0.693501946	0.75	0.867	1
RHEBL1	0.06011643	0.321073164	1	1	1
ZDHHC18	0.060134998	1.337318403	1	1	1
RAB21	0.060201722	0.149500051	1	1	1
KHDRBS3	0.060216416	-0.05173897	1	1	1
MET	0.060427628	-0.347923303	0.812	0.867	1
ENSSSCG00000004252	0.060432569	-0.254611489	1	1	1
ENSSSCG00000041452	0.06071077	-0.900464326	0.312	0.667	1
FBXO9	0.060895069	0.279475953	1	1	1
RIC8A	0.060944781	-0.863627558	0.312	0.533	1
TNFRSF19	0.060967955	1.581795222	0.875	0.733	1
ENSSSCG00000027907	0.060992393	0.094823546	1	0.933	1
CAVIN4	0.061052881	1.401655287	1	0.8	1
SLC25A24	0.061093437	0.087217193	1	1	1
AGGF1	0.06116062	1.096846029	1	1	1
EPS15	0.061199777	1.280948203	1	1	1
RAD18	0.061346366	0.202875423	1	1	1
ZFP69B	0.061372485	0.820733952	1	1	1
ATP8A1	0.061406636	-0.303327112	0.938	0.933	1
ENSSSCG00000022044	0.061422197	0.069443608	0.688	0.933	1
SND1	0.061447688	0.272035193	1	1	1
BMP6	0.061575518	0.119298928	1	1	1
ACP1	0.061641711	0.901846202	1	1	1
DET1	0.061643493	0.391301798	1	1	1
AASDHPPT	0.061706132	0.906426731	1	1	1
TBCK	0.061776206	1.118594009	1	1	1
RFESD	0.061813427	0.963167526	1	1	1
MRPL57	0.06181725	-0.495207848	0.75	0.8	1
SLC8A2	0.061887224	-0.803602787	0.25	0.533	1
ENSSSCG00000062875	0.061932252	-0.395672174	0.562	0.8	1
PABPC5	0.061966914	2.845490051	0.5	0.133	1
GPA33	0.062160992	1.078671154	1	1	1
NIT2	0.062167959	0.401154786	1	1	1
FANCI	0.062210822	0.903984864	1	1	1
ABCC10	0.062292706	-0.697180728	0.5	0.733	1
TTC9	0.062320922	-0.533681996	0.688	0.667	1
CAND1	0.062526553	0.402046583	1	1	1
KIF21A	0.062530583	1.27462238	1	0.933	1
AKIP1	0.062542306	1.22881869	1	1	1
ENSSSCG00000032146	0.062564407	1.329248781	1	0.933	1
ENSSSCG00000037720	0.062632797	-0.803602787	0.375	0.6	1
PRPF40B	0.062651581	-0.22521294	0.75	0.867	1
ENSSSCG00000060990	0.062652333	-0.495207848	0.625	0.667	1
OLIG1	0.062667315	2.299208018	0.5	0.2	1
PXN	0.062702838	1.189648093	1	1	1
ACSL4	0.063154754	0.138279375	1	1	1
GBP5	0.063195017	-1.044199804	0.562	0.6	1
OSBPL7	0.063196318	-0.166172867	0.875	0.867	1
MEIS2	0.063254965	-0.475579041	0.875	0.867	1
MPHOSPH10	0.063288257	0.183661615	1	1	1
PRKAA1	0.063359114	-0.449253215	0.875	0.867	1
AKR1C8	0.063398628	-0.105182237	1	1	1
RBM4	0.063471341	0.352374773	1	1	1
ENSSSCG00000015151	0.063471583	-0.870716983	0.125	0.733	1
ENSSSCG00000054797	0.06349402	-0.693501946	0.688	0.667	1
TMEM74	0.063533072	0.336355166	1	1	1
ELOVL5	0.063759217	-0.090886453	1	1	1
NAA30	0.063824863	1.041395099	1	1	1
ENSSSCG00000055086	0.063961888	1.143929793	1	1	1
UFL1	0.064001561	0.952096792	1	1	1
EFNB1	0.064076269	0.057622032	1	1	1
ENSSSCG00000055357	0.06409534	1.672425342	0.625	0.267	1
ENSSSCG00000036416	0.064124584	-0.923184403	0.375	0.533	1
DECR1	0.064428566	0.999222476	1	1	1
DENND2A	0.064433131	-0.183874868	0.938	0.8	1
ENSSSCG00000061594	0.064630368	-0.405053411	0.812	0.933	1
EN2	0.06469455	-0.858644151	0.312	0.667	1

ENSSSCG00000010391	0.064741862	1.700439718	0.875	0.533	1
GOLGB1	0.064773442	0.05735809	1	1	1
ECH1	0.064777397	-0.518415239	1	0.867	1
AXIN2	0.064921089	-0.374880373	0.75	0.8	1
NAGLU	0.064946766	1.419703311	1	0.533	1
CD2AP	0.065101735	1.046790892	1	1	1
NADK2	0.065130831	1.115379178	1	1	1
ENSSSCG00000052804	0.065140812	2.64385619	0.5	0.133	1
ZNF18	0.06515961	-0.552541023	0.375	0.6	1
TMTC3	0.065164541	1.066522143	1	1	1
ACAP2	0.065384452	1.132229549	1	1	1
AFAP1L1	0.065531232	0.132657744	0.938	1	1
PLK2	0.065557144	1.866248611	0.75	0.467	1
ENSSSCG00000044479	0.065701569	1.926790153	0.688	0.533	1
SUOX	0.065753918	-0.26021939	0.875	0.933	1
ATP6	0.065770207	1.447202939	1	1	1
PBX3	0.066084808	1.194623771	0.938	0.933	1
ENSSSCG00000053554	0.066168582	-1.230612928	0.188	0.533	1
MAN1A1	0.066253887	1.166757722	1	0.867	1
CPNE1	0.066255883	0.247414373	1	1	1
LRP2BP	0.066261401	-0.462343214	0.625	0.667	1
PDP2	0.066307345	1.158429363	1	1	1
FMNL1	0.066337065	1.369322621	1	0.933	1
ENSSSCG00000041892	0.066427086	-0.385891154	0.812	0.8	1
PPP4R3B	0.066435939	1.054653583	1	1	1
DTD2	0.066479295	1.186464678	1	0.933	1
PAX5	0.066521894	0.016301812	0.938	1	1
ENSSSCG00000036659	0.066691128	1.094663598	1	1	1
CSNK1E	0.066798707	0.977880035	1	1	1
ACP6	0.066805033	1.141174138	1	1	1
ARF6	0.067024329	0.098983234	0.938	1	1
LRIG1	0.067045148	1.383643879	1	0.933	1
GOSR2	0.067117061	1.049676256	1	1	1
GRIA1	0.067124008	-0.491658781	0.5	0.8	1
MYLK	0.067143531	1.102810806	1	1	1
AMIGO3	0.067199638	-1.010647244	0.312	0.533	1
G3BP1	0.067213429	-0.199042849	1	1	1
TIMM8A	0.067266971	-1.093109404	0.375	0.533	1
ENSSSCG00000058942	0.067280288	0.929078921	1	1	1
SHROOM2	0.067356506	1.05572641	1	1	1
ENSSSCG00000015383	0.067396764	-0.313878083	1	0.933	1
STAM	0.067431842	0.773044694	1	1	1
GOLPH3L	0.067489624	1.394005773	1	1	1
DOCK4	0.067595128	1.170604077	1	0.933	1
ENSSSCG00000063011	0.067631192	-0.590609064	0.438	0.6	1
ZYX	0.067766593	-0.529208519	0.562	0.6	1
ZSCAN30	0.067792224	1.653134003	0.938	0.8	1
LARP7	0.06779352	-0.108706259	0.938	1	1
SLC12A2	0.067842774	-0.226965151	0.875	0.8	1
EDAR	0.067853591	-0.18473988	0.812	0.867	1
PKN2	0.067861994	0.124721132	1	1	1
DGKD	0.06794729	1.109141879	1	1	1
ENSSSCG00000055280	0.067973875	-0.290146251	0.875	0.933	1
ZNF883	0.068062351	1.465686434	1	1	1
RPRD2	0.068079327	0.297893163	1	1	1
TMEM184B	0.068095417	1.336574871	0.938	0.867	1
SMNDC1	0.068158316	1.126335733	1	1	1
SERINC5	0.068289747	-0.477932291	0.938	0.867	1
SMAD4	0.06831736	-0.415037499	0.75	0.8	1
SEC11A	0.06835519	0.400142859	1	1	1
ENSSSCG00000022206	0.068492751	1.041540122	1	0.933	1
SGSM3	0.068519208	0.062419376	1	1	1
CACHD1	0.068685377	0.360544296	1	1	1
ERO1B	0.068836551	0.854397021	1	1	1
ARHGAP5	0.068886308	1.078285485	1	1	1
VPS13A	0.068994631	0.9354255	1	1	1
STK11	0.069056289	-0.0596347	0.938	0.933	1
VKORC1L1	0.0690904	1.041331884	1	1	1
SRCAP	0.069184229	1.166534412	1	1	1
PTBP3	0.069192212	0.930779741	1	1	1

SLC25A30	0.069207613	0.475338621	1	1	1
LYRM9	0.069241847	1.33315535	1	0.933	1
TYR	0.0692984	1.991424106	0.688	0.733	1
ENSSSCG00000054996	0.069375291	0.102881701	1	1	1
CERS3	0.069423038	1.138414029	0.938	1	1
DDX21	0.069423715	0.987183941	1	1	1
PLOD3	0.06948533	-0.584962501	0.5	0.733	1
PLEKHD1	0.069544478	-0.208586622	0.875	0.933	1
CCDC85C	0.069664994	-0.385891154	0.75	0.733	1
ENSSSCG00000042319	0.069678483	-0.654988292	0.562	0.533	1
ENSSSCG00000053904	0.069714671	1.824428435	0.625	0.333	1
POLR1B	0.069746166	0.954837447	1	1	1
CNDP1	0.069829396	-0.048290901	1	0.933	1
FER	0.070079374	0.938599455	1	1	1
ENSSSCG00000052117	0.070203944	0.139775391	0.938	0.933	1
TOM1L1	0.070215281	0.128793387	1	0.933	1
SPRYD7	0.070258916	1.139867768	1	1	1
NPTXR	0.070308	-0.046815752	0.875	0.933	1
ENSSSCG00000026446	0.070325959	1.308023574	1	1	1
ENSSSCG00000056195	0.070331033	1.951284715	0.688	0.333	1
SNRNP200	0.070355552	0.073009379	1	1	1
WDR24	0.070369483	-0.376043368	0.812	0.867	1
ENSSSCG00000011567	0.070428435	0.965809473	1	1	1
SLMAP	0.070495942	0.968181522	1	1	1
EXOC6B	0.070583814	0.201390862	1	1	1
ENSSSCG00000032894	0.070598579	1.481584761	1	0.733	1
CCDC81	0.070694621	2.269460675	0.688	0.2	1
ARHGEF11	0.070769996	-0.188868387	0.938	0.933	1
ZNF389	0.070805522	1.009984089	0.938	0.4	1
TTC39A	0.070811019	1.447772028	0.938	0.933	1
PLEKHF2	0.070825112	1.200141366	1	1	1
PRPS2	0.070949302	1.103691303	1	1	1
LAMC3	0.071005548	-0.607682577	0.625	0.6	1
MFAP2	0.071011778	-0.487388344	0.812	0.667	1
ENSSSCG00000004963	0.071016021	0.207338963	1	1	1
TOP3B	0.071189005	-1.05246742	0.375	0.6	1
PPP1R15B	0.071492396	-0.287756835	0.688	0.667	1
SLC24A4	0.071516188	0.072669068	1	1	1
DAPK2	0.071551634	1.569855608	0.625	0.333	1
NEK3	0.071568733	-1.044199804	0.812	0.733	1
ENSSSCG00000014132	0.071626454	0.162572335	1	1	1
GPC6	0.071735125	-0.159451899	0.875	1	1
AGPAT4	0.07185345	-0.236700258	0.875	1	1
PLEKHH1	0.071865332	0.051280505	1	1	1
MUS81	0.07189278	-0.100845053	0.938	0.867	1
ZNF786	0.07195202	-1.192645078	0.438	0.667	1
ENSSSCG00000063225	0.072017599	2.036173613	0.625	0.2	1
SUZ12	0.072039635	-0.214610409	1	0.933	1
TMCC3	0.072085185	0.082980277	1	1	1
TTC24	0.072088191	0.040157126	0.875	0.933	1
SRD5A1	0.072146323	1.303549566	1	0.933	1
TMEM268	0.072281323	-0.116696225	0.875	0.933	1
CCDC115	0.072341416	0.333094013	1	1	1
IMPDH1	0.072415343	-0.977632187	0.5	0.467	1
ENSSSCG00000063446	0.072427393	-1.157239742	0.312	0.6	1
ENSSSCG00000025060	0.072436547	-0.351642418	1	1	1
MED18	0.07244769	0.038621294	1	1	1
ZDHHC7	0.072528037	-0.248760275	0.875	0.867	1
ENSSSCG00000035307	0.072734178	-0.56125824	0.75	0.867	1
SENP2	0.072844349	0.08367679	1	1	1
COG2	0.072948775	-0.12217187	1	1	1
TRPM4	0.073095024	-0.037766371	0.938	1	1
CST3	0.073105149	-0.289506617	1	0.933	1
ENSSSCG00000020777	0.073133154	1.74819285	0.938	0.867	1
CBX5	0.073374588	0.964261775	1	1	1
ENSSSCG00000034625	0.073630953	1.300628693	1	1	1
TDRD5	0.073639212	0.148747003	1	1	1
CCPG1	0.073782179	0.425550726	1	1	1
PLK3	0.073802653	1.513831465	1	0.933	1
PFKFB4	0.074014809	-0.02406776	0.875	1	1

LIN54	0.074086022	-0.023985044	0.938	0.933	1
CCNY	0.074272939	0.39243472	1	1	1
HPSE2	0.074342665	1.965784285	0.562	0.267	1
SLC39A6	0.074396236	0.948493951	1	1	1
REXO5	0.074419818	-0.143735477	0.938	1	1
ATM	0.074711717	-0.089478459	1	1	1
KCNK2	0.074723941	0.205156013	1	1	1
SOAT1	0.074727639	0.413145356	1	1	1
LSM11	0.074820494	1.096134517	1	1	1
MARCHF8	0.074825835	0.225959036	1	1	1
PCDH10	0.074909657	-0.830074999	0.438	0.533	1
PRR11	0.074975308	-0.678071905	0.625	0.667	1
SEMA3E	0.075126393	-0.550657199	0.938	0.933	1
ENSSSCG00000057777	0.075313186	-0.678071905	0.375	0.533	1
RAB14	0.075402587	1.207844561	1	0.933	1
PMFBP1	0.075415629	-0.071495591	1	1	1
ARHGEF3	0.075546289	0.019365325	1	1	1
ENSSSCG00000029803	0.075558888	-0.282933963	0.812	0.867	1
TAF9B	0.075561702	0.968818345	1	1	1
MOGS	0.075603484	1.736965594	0.75	0.333	1
ZNF346	0.075679646	0.282399731	0.688	0.933	1
ENSSSCG00000018085	0.075766291	-1.327574658	0.438	0.6	1
SLC43A1	0.075864468	-0.119340946	0.875	0.867	1
SMC6	0.075876661	1.06799945	1	1	1
ENSSSCG00000037885	0.075896931	-0.003472192	1	0.933	1
CERS5	0.075901252	0.415468205	1	1	1
XRRA1	0.075907787	-0.0717884	0.938	1	1
CSRNP1	0.076005544	-0.309920793	0.75	0.667	1
LYRM7	0.076037587	0.985415776	1	1	1
DNAJC7	0.076057587	0.474443697	1	1	1
KLF4	0.076199512	1.059175438	0.938	1	1
ENSSSCG00000001594	0.076229289	2.64385619	0.5	0.133	1
CHD7	0.076298221	0.19958285	1	1	1
RCC2	0.076327864	0.37997989	1	1	1
PPP1R12B	0.076530664	1.271164981	1	0.933	1
CERKL	0.076579409	-0.001428714	1	1	1
BRWD3	0.076606641	0.091466623	1	1	1
DNMT3A	0.076619643	0.894280547	1	1	1
ENSSSCG00000041063	0.076833241	0.918553513	1	1	1
FGFR2	0.077102476	1.064803079	1	1	1
ZNF454	0.077141926	-0.233878998	0.875	1	1
CDK19	0.077161919	-1.058343986	0.438	0.533	1
ENSSSCG00000003826	0.07722116	1.010900068	1	1	1
JMJD1C	0.077289518	1.168654464	1	1	1
ENSSSCG00000037359	0.077315238	1.308989039	0.938	0.8	1
FMO5	0.077575361	1.555816155	0.938	0.933	1
PAF1	0.077629755	0.008299323	1	1	1
TASL	0.077761178	-0.793549123	0.562	0.6	1
ANXA2	0.07776732	1.651585463	1	1	1
PEX11A	0.077813807	2.698303974	0.625	0.2	1
CUL1	0.077927589	1.088167065	1	1	1
PRKRA	0.078079093	1.326115892	1	1	1
ENSSSCG00000050494	0.078122157	-0.77118131	0.312	0.6	1
C18orf63	0.078128137	1.014079842	1	1	1
MICAL1	0.078255111	-0.959842874	0.375	0.533	1
ZBTB39	0.078327129	1.001862158	1	1	1
ENSSSCG00000056588	0.078330506	2.906890596	0.5	0.067	1
SGTB	0.078381191	1.185932878	0.938	1	1
TRERF1	0.078390516	-0.607682577	0.375	0.667	1
ENSSSCG00000061444	0.078422145	1.371558863	1	0.733	1
ARCN1	0.078470473	0.989209265	1	1	1
DDX20	0.078555778	1.18509388	1	1	1
C5orf58	0.078586602	-0.389502407	0.938	1	1
ENSSSCG00000041434	0.078660673	-0.929610672	0.375	0.6	1
MRE11	0.078665432	-0.046780353	1	1	1
ENSSSCG00000046164	0.078752093	1.321928095	0.75	0.4	1
SON	0.07875698	0.818563126	1	1	1
ENSSSCG00000039529	0.078892971	1.271463028	0.875	0.867	1
CYP51A1	0.078995835	0.146941683	1	1	1
GLE1	0.079054723	1.119084367	1	1	1

GRWD1	0.079062397	-0.041314789	1	1	1
ENSSSCG00000035105	0.079118236	0.16754235	0.875	1	1
ENSSSCG00000035856	0.079145154	-0.27368165	0.625	0.733	1
TTC23L	0.079161373	1.048343105	1	1	1
ZNF532	0.079247233	0.928980138	1	1	1
ENSSSCG00000000854	0.079325406	-0.05994254	0.562	0.667	1
USP21	0.079452408	1.243022554	1	1	1
TMX2	0.079579236	0.169183447	1	1	1
TMEM87B	0.079678596	1.018005788	1	1	1
ENSSSCG00000062377	0.0797576	-0.113768875	0.938	1	1
ENSSSCG00000056505	0.07978251	1.714245518	0.75	0.4	1
ENSSSCG00000063426	0.079822727	1.015247774	1	1	1
SPOCK3	0.079867814	1.501634117	1	0.733	1
ANKZF1	0.079920877	-0.912537159	0.438	0.533	1
PEX11B	0.079962975	0.807217333	1	1	1
MED24	0.079986339	1.1109822	1	1	1
ENSSSCG00000048962	0.080162772	2.392317423	0.562	0.267	1
ZFYVE27	0.080194636	0.319829629	1	0.933	1
PLOD2	0.080361516	1.05197597	1	1	1
FGD1	0.080475579	0.053560245	1	1	1
ENSSSCG00000018063	0.080481015	1.220141466	1	1	1
ENSSSCG00000023569	0.080520719	0.097221808	0.938	1	1
ENSSSCG00000052847	0.080541478	1.607330314	0.75	0.533	1
LDAH	0.080545937	-0.900464326	0.625	0.533	1
ENSSSCG00000002653	0.080631081	1.767123231	1	1	1
CFAP54	0.080688546	0.324371271	1	1	1
WDR11	0.080783876	1.084871814	1	1	1
ENSSSCG00000015182	0.080814968	-0.312422433	0.688	0.867	1
ISL1	0.080847551	0.880010931	1	1	1
TMCO1	0.081029763	0.83662826	1	1	1
FOXR2	0.081157725	2.321928095	0.625	0.2	1
NEK4	0.08117301	1.277605764	1	0.933	1
EP400	0.081305765	-0.721140627	0.625	0.4	1
ENSSSCG00000047440	0.081332546	2.169925001	0.562	0.2	1
KCNAB1	0.081370497	-0.015506586	1	1	1
TCF7L2	0.081414523	-0.37941359	0.938	0.867	1
OBI1	0.081557501	1.1109822	1	1	1
HAAO	0.081768246	-0.756074417	0.5	0.4	1
BCAN	0.081934618	-0.923184403	0.25	0.6	1
CECR2	0.081962947	1.181619719	1	1	1
STEEP1	0.081965085	0.90459392	1	1	1
ENSSSCG00000054384	0.082132855	0.006426269	0.625	0.933	1
FAM172A	0.082185017	1.040309387	1	1	1
PLPPR4	0.082240352	-0.621488377	0.625	0.867	1
ENSSSCG00000042623	0.08226403	1.876516947	0.75	0.533	1
CYB561D1	0.082338392	-0.765534746	0.562	0.8	1
ENSSSCG00000057171	0.082342093	-0.830074999	0.625	0.6	1
AHCY	0.08237323	0.248447497	1	1	1
LAMP1	0.082440805	0.007584595	0.938	0.867	1
IL1R1	0.082590498	1.312843901	1	1	1
ENSSSCG00000034866	0.082591604	1.618765209	0.812	0.933	1
ENSSSCG00000053746	0.082613738	-0.645650427	0.5	0.533	1
IL6	0.082708685	2.299208018	0.562	0.2	1
TAF2	0.082723283	0.921574641	1	0.933	1
ENSSSCG00000057642	0.082800447	0.208919133	0.938	1	1
AGL	0.083127629	0.971403357	1	1	1
GPX8	0.083194783	0.074490544	0.938	0.933	1
CENPP	0.083221001	-0.150065289	0.938	0.933	1
CSRNP2	0.083248831	0.272817883	0.938	1	1
ENSSSCG00000033601	0.083277003	0.227571224	1	1	1
GXYLT2	0.083328745	-0.400290914	1	0.867	1
RANBP3	0.083521666	0.201337954	1	1	1
DPP9	0.083541277	1.296661336	1	0.933	1
CACNA1E	0.083550129	-0.029684024	0.938	0.933	1
ZXDC	0.083568695	0.134301092	0.938	0.933	1
FAM117B	0.083617151	0.140539472	1	1	1
GPR63	0.083630575	0.146021815	1	1	1
ENSSSCG00000053851	0.083669239	-0.492379588	0.875	0.8	1
IL12B	0.083891536	1.018152205	1	1	1
PPP2R5B	0.08396385	1.771961016	0.625	0.533	1

DENND5B	0.084056258	-0.325770161	1	0.933	1
PAK5	0.084123301	0.951284715	1	1	1
LIN52	0.084286513	0.034513098	1	1	1
ENSSSCG00000049258	0.08442163	2.714245518	0.5	0.133	1
GPR161	0.08448126	1.111721905	1	1	1
TEKT1	0.084678817	-0.886658527	0.5	0.333	1
SALL4	0.08468827	1.029993645	1	1	1
PRORP	0.084738795	-0.176767333	0.875	0.8	1
PODN	0.084762736	0.048246445	0.875	0.933	1
FAM228B	0.084971968	-0.326308581	0.75	0.733	1
APC	0.085141434	0.942041051	1	1	1
CTSS	0.08515204	0.113935053	1	1	1
ENSSSCG00000007023	0.085358519	0.95298701	1	1	1
MPG	0.08537407	1.282757498	1	0.867	1
PCNX1	0.085692208	0.348764793	1	1	1
TIMM21	0.085748339	0.098095139	1	1	1
TMEM129	0.08581133	1.528198924	1	1	1
ENSSSCG00000059540	0.085827607	0.176588732	1	1	1
C3orf70	0.085998886	0.346979467	1	1	1
SYNPR	0.086050557	1.584962501	0.812	0.467	1
TAMM41	0.086064775	1.04615794	1	1	1
RPRM	0.086189986	-0.678071905	0.438	0.6	1
CRKL	0.086192682	0.846190728	1	1	1
MRPS5	0.086314722	1.192562805	1	0.933	1
CENATAC	0.086457737	1.518325308	0.938	0.4	1
SMAP2	0.086464472	-0.779743323	0.938	0.8	1
MAN2A2	0.086677798	1.302375297	1	0.933	1
ARAP3	0.086751987	-0.256608137	0.5	0.6	1
PTPRU	0.086847085	-0.067460817	1	1	1
API5	0.086866817	1.30232736	1	1	1
ENSSSCG00000055073	0.086878848	-0.629162305	0.375	0.533	1
ZNF318	0.087014869	0.90971664	1	1	1
ENSSSCG00000053065	0.087156679	-0.500284786	0.75	0.867	1
CIAO1	0.087313874	0.376272456	1	1	1
ENSSSCG00000007482	0.087327958	1.107140134	1	1	1
ENSSSCG00000053776	0.087358252	1.491853096	0.812	0.733	1
SULF2	0.087374686	0.059258882	1	1	1
CATSPER2	0.08737837	0.264784188	0.875	1	1
PIGBOS1	0.087431432	-0.678071905	0.75	0.8	1
PUM3	0.087569067	0.157157705	1	1	1
ENSSSCG00000003984	0.087571589	0.301422439	1	1	1
PARP1	0.087634385	0.29759211	1	1	1
LTBP1	0.087755063	0.876592736	1	1	1
PHF20	0.087768463	0.975993619	1	1	1
METTL6	0.087848533	1.230119428	1	1	1
ENSSSCG00000051414	0.087879616	2.64385619	0.5	0.133	1
H1-6	0.087957661	1.305439972	0.75	0.467	1
ENSSSCG00000008990	0.088196389	-0.455679484	0.562	0.733	1
SLC1A5	0.08820269	1.130677262	1	1	1
ASPM	0.088231129	0.280159813	1	1	1
PDE8B	0.088370905	0.220716518	1	1	1
TMEM263	0.088396999	1.049243519	1	1	1
ZBTB18	0.088420491	-0.618200449	0.812	0.8	1
VIT	0.088597609	0.911772817	1	1	1
ERBB4	0.088677323	1.247927513	1	0.867	1
PAPOLG	0.088769236	1.027184829	1	1	1
ENSSSCG00000003076	0.088946576	-0.703162886	0.812	0.8	1
ENSSSCG00000045269	0.089111997	-0.9861942	0.438	0.533	1
FAM120A	0.089161781	1.034152781	1	1	1
AAK1	0.089244755	0.298315538	1	1	1
CCDC15	0.089280614	0.937385714	1	1	1
TRPV3	0.089340197	1.112005026	1	0.867	1
GALNT17	0.089349585	-0.784987109	0.562	0.533	1
SPATA9	0.08935253	-0.645650427	0.625	0.6	1
ENSSSCG00000059452	0.089392684	1.866248611	0.75	0.333	1
ENSSSCG00000062636	0.089558941	1.510961919	0.812	0.533	1
PPM1H	0.089618844	-0.023312437	1	1	1
CA12	0.089694035	-0.745186101	0.625	0.533	1
NUFIP2	0.08973896	0.259340815	1	1	1
TOR2A	0.089774479	-0.239950793	0.875	0.667	1

EDIL3	0.089783646	0.894043716	1	1	1
ENSSSCG00000058178	0.089788075	0.007819504	1	1	1
ENSSSCG00000044640	0.089819464	1.539158811	1	0.933	1
GCLM	0.089871118	1.278012188	0.938	0.933	1
ZMPSTE24	0.089887696	1.086283687	1	1	1
CPQ	0.089923927	-0.385891154	0.688	0.6	1
ENSSSCG00000053788	0.090031296	-0.299560282	0.5	0.8	1
ENSSSCG00000023596	0.090071282	1.356693513	0.938	0.867	1
ENSSSCG00000000887	0.090073745	1.560894741	0.812	0.8	1
PGPEP1	0.090074184	-0.03500645	0.812	0.933	1
DMTF1	0.090124457	1.019899557	1	0.933	1
VEPH1	0.090188387	1.204571144	0.75	0.467	1
ZNF251	0.090199263	0.278859373	0.438	0.867	1
ENSSSCG00000021024	0.090220912	-0.250650681	0.562	0.8	1
ILK	0.090269167	-0.449253215	0.562	0.667	1
STMND1	0.090286413	-0.607682577	0.562	0.533	1
TBC1D12	0.090385101	-0.531230517	0.812	0.733	1
PKD1L2	0.090797753	-0.632268215	0.688	0.933	1
LYSMD3	0.090913005	-0.104080522	0.938	0.8	1
NCALD	0.091005617	-0.169058258	0.812	0.933	1
ANKMY2	0.091030774	0.957777106	1	1	1
ZNF484	0.091035382	1.714245518	0.75	0.6	1
KIAA0319	0.091048992	1.428993072	1	0.8	1
TRHDE	0.091075183	1.297805301	1	0.867	1
ITGAE	0.09113626	1.152674254	1	1	1
PEX5L	0.091202323	0.24391206	1	1	1
SLC1A1	0.091238555	-0.253574077	0.688	0.667	1
IWS1	0.09135124	0.59363488	1	1	1
ATF7IP2	0.091385376	-0.082011696	1	1	1
ENSSSCG00000006510	0.09172587	0.150106714	1	1	1
SLC35F4	0.091731348	1.053975621	1	1	1
TUBD1	0.091733956	-0.229926094	0.938	1	1
KIF2C	0.091942478	1.041837477	1	1	1
SEPTIN7	0.092135908	0.132657744	1	1	1
RER1	0.092282322	0.351736904	0.938	1	1
NDNF	0.092354361	1.148328773	0.938	0.8	1
USO1	0.092357127	0.994002922	1	1	1
ATP6V1C1	0.092357853	0.208217804	1	1	1
TOR1A	0.092408701	-0.03451475	1	1	1
ENSSSCG00000013052	0.092549046	0.246655376	1	1	1
CSTPP1	0.092557214	-0.392669686	0.562	0.733	1
CEP170	0.092597512	0.928734417	1	1	1
FXR1	0.092638171	0.913349997	1	1	1
ENSSSCG00000009497	0.092782141	1.564002882	0.75	0.6	1
DYNC1LI2	0.09284445	0.277957852	1	1	1
SHOC2	0.092909667	-1.484300162	0.875	0.533	1
ENSSSCG00000061630	0.092917949	-0.552541023	0.625	0.6	1
UBASH3B	0.092961909	0.254813899	1	1	1
ABHD14A	0.093100646	-0.363198568	0.688	0.667	1
ZFAND1	0.093126305	1.09085343	1	0.933	1
ESYT2	0.093234621	0.220198208	1	1	1
TRIM50	0.093421474	1.104829973	1	0.933	1
LYSMD1	0.093530482	0.109707479	0.688	0.867	1
ENSSSCG00000040413	0.093544672	-1.054635257	0.312	0.533	1
PPP2R3A	0.093570684	1.238096504	1	1	1
ENSSSCG00000055939	0.093594279	-1.143735477	0.5	0.667	1
CDC42SE2	0.093622531	1.089748087	1	1	1
DLGAP1	0.093704764	-1.093109404	0.125	0.533	1
SIM2	0.093774926	-0.446746359	0.5	0.667	1
ENSSSCG00000026817	0.093907634	-0.350907162	0.688	0.867	1
FAM204A	0.093962551	0.346526349	1	1	1
ENSSSCG00000011106	0.094022518	0.20407607	1	1	1
SGPL1	0.094060946	-0.219321767	1	1	1
BNC2	0.094083066	0.887185186	1	1	1
TSC22D2	0.094246777	0.868318475	1	1	1
ENSSSCG00000009499	0.09443057	-0.177998302	0.75	0.8	1
CREB3L4	0.094492386	1.906890596	0.812	0.533	1
SF3B2	0.094494526	1.411803005	1	1	1
SPINK5	0.094497579	0.313058562	1	1	1
MSRB2	0.094548857	-0.881605299	0.562	0.6	1

CCDC87	0.09458273	-0.721140627	0.312	0.533	1
TAFA1	0.094745757	-0.714597781	0.375	0.6	1
SYVN1	0.094813775	1.564279199	1	0.933	1
AFAP1	0.094816951	-0.465078182	0.438	0.533	1
PHACTR3	0.094850043	0.243498586	1	1	1
NFATC2IP	0.094903514	-0.058343986	0.625	0.8	1
KCTD13	0.09490543	1.34488208	1	1	1
TENM3	0.094921579	1.600787468	0.812	0.667	1
LCLAT1	0.094939278	0.03562391	1	1	1
MYO6	0.095069177	0.872789988	1	1	1
ARHGEF33	0.09516669	0.108524457	0.812	1	1
HIRA	0.095346481	1.432959407	1	0.8	1
ENSSSCG00000023791	0.095438684	0.00877021	0.938	0.867	1
TARS2	0.095617344	-0.90669628	0.562	0.467	1
SNRPF	0.096011003	0.964725784	1	1	1
ENSSSCG00000054135	0.096108516	0.0138058	0.875	0.933	1
PES1	0.096248603	-0.043204499	0.938	1	1
ENSSSCG00000059368	0.096481511	1.548997003	0.938	0.733	1
TESK2	0.096640731	1.416751641	1	0.933	1
ENSSSCG00000042179	0.096778386	1.544320516	0.75	0.4	1
ENSSSCG00000045166	0.096835788	1.442943496	0.938	0.733	1
SRSF1	0.096836305	0.227904338	1	1	1
PRICKLE1	0.096888017	1.016780296	1	1	1
IRF8	0.09694836	0.120210935	1	1	1
GARRE1	0.09700304	1.052088511	1	1	1
TOMM20L	0.097036929	-0.449253215	0.5	0.667	1
GFPT1	0.097157278	0.83351337	1	1	1
GALNT10	0.097191285	-0.135922488	0.875	0.933	1
GALNT3	0.09732238	1.415037499	0.938	0.733	1
SLC43A2	0.097373788	0.076815597	0.938	0.933	1
CLCN5	0.097619453	0.231617324	0.938	0.933	1
MMP13	0.09767939	0.913962644	1	1	1
NKAP	0.097717417	0.144354177	1	1	1
CDC25C	0.097737762	1.017591648	1	1	1
JADE1	0.097804485	0.973774274	1	1	1
ENSSSCG00000007003	0.097841827	-0.678071905	0.562	0.533	1
ENSSSCG00000037257	0.097916474	-0.852101305	0.312	0.533	1
EIF3A	0.098093396	0.127619968	1	1	1
RNF20	0.098121817	0.258499326	1	1	1
ENSSSCG00000061205	0.098262881	-0.533681996	0.25	0.533	1
DPP3	0.09831783	-0.4040385	0.688	0.8	1
CCAR2	0.09834675	-0.001961516	0.938	0.933	1
ASCC2	0.098425859	-0.154509949	0.938	0.867	1
COG7	0.098438562	1.667702932	0.625	0.467	1
RGS18	0.098557348	2.539158811	0.562	0.133	1
DPYD	0.098629215	1.058503719	1	0.933	1
ENSSSCG00000055749	0.098752737	-0.397963986	0.5	0.733	1
TC2N	0.098868025	1.096482631	1	1	1
ENSSSCG00000058193	0.098904872	1.52160044	0.688	0.267	1
THOC1	0.098987899	-0.033520608	1	1	1
IL1RAPL1	0.099161768	0.967586527	1	0.933	1
HTATIP2	0.099283461	0.040746342	1	1	1
ENSSSCG00000034311	0.099296748	1.906890596	0.625	0.267	1
ASXL3	0.099606266	1.146302303	1	1	1
GPM6A	0.09962406	2.327222395	0.5	0.267	1
CYP7A1	0.099707346	1.813781191	0.625	0.4	1
DNAJB1	0.099718795	0.165890676	1	1	1
SEC63	0.099732762	0.379459093	1	1	1
ZBTB22	0.099753351	2.764871591	0.562	0.2	1
ENSSSCG00000062914	0.099758872	-0.693501946	0.75	0.667	1
BNIP2	0.099785018	1.048246445	1	1	1
IQUB	0.099797417	1.136372442	0.938	0.8	1
CROT	0.099809563	0.975906582	1	1	1
ZDHHC2	0.099951057	-0.263034406	1	0.867	1
RFC1	0.100007724	0.788467248	1	1	1
RRP15	0.100193912	0.269365876	1	1	1
ENSSSCG00000061277	0.100217901	-0.508146904	0.5	0.667	1
CCDC93	0.100323869	0.287849539	1	1	1
COIL	0.100385218	-0.43824489	0.75	0.867	1
ENSSSCG00000010734	0.100441194	1.454231798	1	1	1

BORCS7	0.100618654	-0.830074999	0.688	0.867	1
SLC31A2	0.100699885	0.876231112	1	1	1
ENSSSCG00000041731	0.100740432	-0.533681996	0.562	1	1
PGM2	0.100907277	1.126884521	1	1	1
SLC49A4	0.100942349	1.026899925	1	1	1
SNAP91	0.101057095	0.241240249	1	1	1
IARS1	0.101154429	1.346932101	1	1	1
DPH6	0.101209369	0.829722735	1	1	1
ADCY7	0.101226191	1.491853096	0.562	0.4	1
HIBCH	0.101332676	1.004309089	1	1	1
CDS1	0.101416046	0.160745575	0.938	1	1
LRWD1	0.101658214	1.611647609	0.938	0.8	1
ERAL1	0.10167379	0.098114999	0.938	0.933	1
ATXN2L	0.101673792	1.022184065	1	1	1
EXOSC1	0.101744966	0.3447539	1	1	1
ZNF879	0.101913549	-0.35614381	0.75	0.8	1
LAMB4	0.102038459	1.288418833	0.938	0.8	1
NXPE3	0.102064971	0.253693359	1	1	1
PRCP	0.102195319	-0.540568381	0.812	0.933	1
ZC3H12C	0.102199146	-0.076868763	1	1	1
NCKAP5L	0.102469582	-0.315501826	0.688	0.733	1
ENSSSCG00000046222	0.10250396	-0.321378392	0.75	0.733	1
UQCC1	0.102653192	-0.025995209	0.938	0.933	1
UBA6	0.102694699	0.102928437	1	1	1
THUMPD1	0.102750221	1.089065057	1	1	1
DHX35	0.102838471	0.844847036	1	1	1
ENSSSCG00000042522	0.102875958	0.899710442	1	1	1
CUL4B	0.102884339	0.861276847	1	1	1
PCMT1	0.103035061	0.906890596	1	1	1
PANK1	0.103129744	0.108097687	0.938	1	1
MANF	0.103154739	0.163736055	1	1	1
ENSSSCG00000051934	0.103180197	3.076815597	0.562	0.067	1
NASP	0.103207106	1.339476948	1	1	1
PAFAH1B1	0.103387506	0.121385032	1	1	1
ENSSSCG00000059776	0.10352622	-0.709780765	0.438	0.667	1
SLC36A4	0.103614106	0.099535674	1	0.933	1
YWHAZ	0.103714509	0.303761196	1	1	1
C1orf43	0.103865033	0.157174299	0.938	1	1
ENSSSCG00000059823	0.103958125	0.926790153	1	1	1
ENSSSCG00000062865	0.104095874	0.998346752	1	1	1
KIAA1328	0.104272725	0.023535514	1	1	1
ENSSSCG00000000547	0.10443827	0.884039656	1	0.933	1
PMS2	0.104478466	-0.32443495	0.688	0.867	1
ZNF521	0.104540028	1.183837308	0.938	1	1
DCDC1	0.104600335	-0.529208519	0.625	0.667	1
ALKBH1	0.104623108	1.102020937	0.938	0.867	1
ANKHD1	0.104862031	0.782788478	1	1	1
ENSSSCG00000055582	0.10491072	1.666939803	0.812	0.733	1
ENSSSCG00000061549	0.105108365	2.076815597	0.562	0.2	1
SNX13	0.105125498	1.07753425	1	1	1
GPR151	0.105201234	0.119634591	1	1	1
MYO1D	0.105202873	0.34298971	1	1	1
MIB1	0.105242358	0.881714867	1	1	1
PI4K2A	0.105271394	1.165117862	0.938	1	1
RAB11FIP2	0.105428771	1.079592682	0.938	1	1
TXNDC16	0.105625952	1.299874852	1	0.933	1
ENSSSCG00000054995	0.105681684	-0.540568381	0.375	0.667	1
TTC3	0.105712206	1.030149825	1	1	1
DLG1	0.105719504	0.970722207	1	1	1
LANCL1	0.105827676	0.057740916	1	1	1
LRR1	0.105893414	0.252487596	1	1	1
LAMTOR5	0.105949237	0.003105911	1	1	1
CHPT1	0.10629476	1.121177967	1	1	1
PLA2G6	0.106403268	1.362570079	1	0.667	1
CCDC196	0.106421925	-0.434146322	0.312	0.533	1
FBXO22	0.106462057	0.151712198	0.938	1	1
BANK1	0.106464595	1.023214714	1	1	1
ENSSSCG00000062612	0.106531359	-0.607682577	0.5	0.533	1
RHOH	0.106557797	2.169925001	0.5	0.267	1
FHL1	0.106671703	1.156599434	1	1	1

NUMB	0.106856627	0.328431615	1	1	1
PPP1R13B	0.106941023	1.05254496	1	1	1
ATXN7L2	0.107001693	-0.338221902	0.75	0.733	1
CDCA2	0.107017788	1.111977125	1	1	1
OSGEP	0.107135292	-0.040958122	1	1	1
ENSSSCG00000058436	0.107139155	-0.313075088	0.875	0.867	1
FANCE	0.107148711	-0.228461257	0.688	0.733	1
B4GALT6	0.107177009	0.753515094	1	1	1
C7orf31	0.107222996	0.049848549	0.812	0.933	1
CORO2A	0.107234525	2.22881869	0.625	0.333	1
CDC27	0.107251743	0.766587484	1	1	1
ATRNL1	0.107426319	1.074000581	0.938	0.667	1
ALKBH8	0.107450249	1.329123596	0.938	0.8	1
SUFU	0.107450407	1.754887502	0.75	0.4	1
TMEM62	0.107542146	-0.042483331	0.938	0.933	1
PLXNA4	0.107552956	2.053731984	0.625	0.2	1
PIAS2	0.107571971	0.966398661	1	1	1
ENSSSCG00000046945	0.107616094	1.41550221	0.938	0.8	1
LHX4	0.107704226	-0.272079545	0.812	0.733	1
AXIN1	0.107760264	1.688889943	1	0.8	1
PMPCA	0.107935232	-0.030645317	0.938	1	1
ATG13	0.108250031	1.156000355	1	1	1
ENSSSCG00000057596	0.108338494	-0.557056504	0.625	0.667	1
ALDH1A2	0.108421437	1.194692907	0.875	0.867	1
ENSSSCG00000047655	0.108431022	-0.093109404	1	1	1
RAD17	0.108450292	0.957236212	1	1	1
GGCT	0.108450412	1.009847786	1	1	1
ENSSSCG00000008582	0.108520481	-0.567040593	0.5	0.533	1
UNC80	0.108550259	0.454668176	1	1	1
NPC1	0.108611035	0.750197985	1	1	1
RAB3IP	0.108619511	0.425021588	1	1	1
CPEB3	0.10862231	1.08813091	1	0.933	1
HSF5	0.108701584	-0.392669686	0.812	0.667	1
LAX1	0.108795149	1.316636569	1	0.867	1
ZNF804B	0.108843179	0.952453173	1	1	1
TAPBPL	0.108965575	-0.497499659	0.75	0.6	1
MYZAP	0.109115349	-0.093109404	0.375	0.667	1
KTI12	0.109134488	-0.134529332	0.875	0.867	1
MED13	0.109378929	0.825516931	1	1	1
RNF182	0.109417698	1.124569863	1	1	1
BEND3	0.109622198	-0.131583552	0.875	0.867	1
SMCR8	0.109632964	1.111657346	1	1	1
ENSSSCG00000063154	0.10963922	1.510961919	0.938	0.733	1
ENSSSCG00000036554	0.109696671	0.847474897	0.938	1	1
CSRP2	0.109815931	1.155918143	1	0.8	1
UNC5A	0.109851489	0.949156323	1	0.933	1
ENSSSCG00000053400	0.109877881	0.910226308	1	0.933	1
SLC36A1	0.110107081	0.086440016	1	0.867	1
DIS3	0.110118067	0.172702086	1	1	1
KDSR	0.110445065	0.419340597	1	0.933	1
ENSSSCG00000061303	0.110492103	0.108524457	0.875	0.8	1
ATRX	0.110606834	1.098531966	1	1	1
ENSSSCG00000058286	0.110682269	1.244760234	1	0.867	1
APOOL	0.110821103	1.120195144	1	0.867	1
USP49	0.111006569	1.094962944	0.875	0.733	1
SLC17A4	0.111089197	-0.297467903	0.688	0.733	1
ALG2	0.111259027	1.24046905	1	0.8	1
ENSSSCG00000055798	0.11131108	-0.583435031	0.438	0.667	1
TAF4	0.111345981	1.119501996	1	1	1
JRKL	0.111387755	-0.050465067	0.875	1	1
JAK2	0.111464382	0.297805301	0.938	0.933	1
DENND1A	0.111502253	0.154515562	1	1	1
CGAS	0.111552723	-0.13058411	0.875	0.8	1
BFAR	0.11158924	1.100770329	1	1	1
VRK3	0.111658396	-0.584962501	0.562	0.533	1
CHD2	0.111743749	0.404849055	1	1	1
ENSSSCG00000029430	0.111811889	-0.144508557	0.75	0.933	1
CCDC125	0.111920115	0.249880306	1	1	1
NIBAN2	0.111937941	0.204571144	0.938	1	1
ENSSSCG00000055828	0.111970692	0.886072124	1	1	1

ADAM17	0.112115246	0.333536229	1	1	1
BPHL	0.112363166	1.555983434	1	1	1
PTPN1	0.112441249	0.218351691	1	1	1
ENSSSCG00000028944	0.112452658	-0.080857144	1	1	1
HDAC8	0.112607949	1.074000581	0.938	0.867	1
PDE1A	0.112907471	1.558967292	0.875	0.6	1
ENSSSCG00000055365	0.112918334	1.61214733	0.875	0.733	1
SGMS2	0.113120238	-0.042777729	0.875	1	1
PPRC1	0.113211096	0.963815631	1	1	1
VPS53	0.113211744	0.210467664	1	0.933	1
MANSC4	0.113237254	-0.649502753	0.562	0.467	1
FURIN	0.113268044	0.993047239	1	0.933	1
ATAT1	0.113429763	2.339850003	0.5	0.4	1
RNF113B	0.113448685	0.12389647	1	1	1
CASC3	0.113617677	1.057597682	1	1	1
ENSSSCG00000055233	0.113704762	-0.086932386	1	1	1
REXO2	0.113887302	1.041181883	1	1	1
RAP2A	0.113889683	0.176730179	1	1	1
DBI	0.113973304	-0.16250047	1	1	1
ENSSSCG00000010203	0.113987564	0.360214195	1	1	1
ENSSSCG00000022634	0.1142109	0.971020933	1	0.8	1
SH2B3	0.114342778	0.361033886	1	1	1
CCDC43	0.114395705	0.865398467	1	1	1
MMUT	0.11440239	0.397852864	1	1	1
TM6SF1	0.114468835	1.317522916	0.875	0.733	1
MRGPRF	0.114475498	-0.182376742	0.688	0.8	1
STOX1	0.114541989	-0.523743759	0.875	0.8	1
ENSSSCG00000007000	0.114555035	0.782071172	1	1	1
INSIG2	0.114595285	0.96274383	1	1	1
IL10RA	0.114597454	-0.427528443	0.688	0.6	1
RAB28	0.114732406	1.000070774	1	1	1
SNX10	0.114753031	1.216854683	1	1	1
RNF126	0.114909824	1.558967292	0.938	0.933	1
JMY	0.114931959	0.967011588	1	1	1
PET117	0.115102379	-0.222392421	0.75	0.8	1
TMUB2	0.115413143	-0.093109404	0.625	0.667	1
ZNF277	0.115520498	0.178043082	1	1	1
AKAP6	0.11564896	-0.1740294	0.938	0.933	1
RIPK2	0.115698752	2.22881869	0.562	0.133	1
SLC31A1	0.115901601	0.918773587	1	1	1
MCM3	0.116161462	0.886880635	1	1	1
FAM133B	0.116291438	1.005755092	1	1	1
MEIOC	0.116334034	1.137934887	0.938	0.933	1
ENSSSCG00000041238	0.116347907	-0.245112498	0.562	0.533	1
BRCA2	0.116375234	0.897348091	1	1	1
TCF12	0.116382368	0.28273632	1	1	1
PHLDB3	0.116590855	-0.288365696	0.75	0.8	1
TANC2	0.116806652	0.308193069	1	1	1
ENSSSCG00000061887	0.116850723	1.714245518	0.688	0.533	1
MTURN	0.116981657	2.006426269	0.5	0.267	1
RNF183	0.117053329	0.073540465	0.938	1	1
THSD1	0.117177954	0.25883126	1	1	1
MCF2L2	0.117220316	1.117457582	0.938	0.733	1
TRPM7	0.117302807	0.877382291	1	1	1
ABCD4	0.117361507	1.060365076	0.938	0.867	1
ENSSSCG00000053298	0.117457313	-0.253574077	0.688	0.8	1
ENSSSCG00000029261	0.117472186	0.4147487	1	1	1
CHSY1	0.117545491	0.149068448	1	1	1
ENSSSCG00000061577	0.117616279	0.901273346	1	1	1
UACA	0.117905125	0.417743882	1	1	1
GSDME	0.117980354	0.459178403	1	1	1
ENSSSCG00000050482	0.117980412	2.22881869	0.562	0.2	1
TLCD3A	0.118078916	-0.803602787	0.562	0.533	1
ENSSSCG00000011729	0.118083373	1.326604582	1	0.867	1
ENSSSCG00000061482	0.118128823	1.482202926	0.938	0.867	1
SYMPK	0.118273302	-0.7355574	0.625	0.733	1
LYPD1	0.118444561	-0.366127899	0.5	0.8	1
ITGA4	0.118610869	0.778314729	1	1	1
TMEM67	0.118713158	1.117310375	0.938	0.867	1
USP24	0.118734692	0.842079186	1	1	1

ENSSSCG00000031893	0.118971709	-1.30461351	0.25	0.533	1
ENSSSCG00000012322	0.119013355	-0.230612928	0.688	0.667	1
ABCC2	0.119172251	0.81983067	1	1	1
CFAP20	0.119186334	1.650262316	1	0.867	1
RAP1B	0.119326667	0.900549069	1	1	1
IQCM	0.119704823	-0.042483331	1	1	1
SGF29	0.119744448	0.232826798	1	1	1
ENSSSCG00000063276	0.120210872	-0.678071905	0.438	0.6	1
ENSSSCG00000054327	0.120223584	1.374496146	0.938	0.867	1
GGCX	0.120232728	0.001306198	1	0.933	1
HBS1L	0.120266837	1.26052755	1	0.933	1
PELI1	0.120453748	0.89796708	1	1	1
MAEL	0.120467805	0.901730429	1	1	1
SEPTIN11	0.120545454	0.233131472	1	1	1
SAMD8	0.120687345	0.045986205	1	1	1
ENSSSCG00000031169	0.120874806	1.202088325	1	0.933	1
ZDHHC16	0.120906414	-0.179914232	0.938	1	1
MMADHC	0.120919685	0.884522783	1	1	1
ENSSSCG00000047735	0.121035671	-0.349449158	0.812	0.933	1
ENSSSCG00000049449	0.121241516	0.301169535	0.938	0.933	1
ENSSSCG00000056134	0.121389336	1.006426269	0.875	0.6	1
RNaseP-nuc.3	0.121394418	1.885516945	0.75	0.4	1
STRADA	0.1215471	0.158173996	1	1	1
QSER1	0.121724262	1.029194693	1	1	1
PHF10	0.121748784	1.11803826	1	1	1
ZNF445	0.121859528	0.768470427	1	1	1
PRKRIP1	0.1218632	0.419798159	1	1	1
ENSSSCG00000042151	0.121977932	-0.232272152	0.812	1	1
MITF	0.121985789	-0.492716863	0.812	0.667	1
PIK3CB	0.122013227	-0.281554494	0.688	0.733	1
GLCCI1	0.122038622	0.077849417	1	0.933	1
ENSSSCG00000005241	0.12237996	0.319107576	1	1	1
KLF3	0.122424268	0.836602152	1	1	1
CBFA2T2	0.122437135	0.690411087	1	1	1
PAX6	0.122470636	0.353706295	1	1	1
MISP	0.122551555	-0.858644151	0.25	0.6	1
IRAK4	0.122607302	1.289924778	0.938	1	1
SLC25A36	0.122610452	0.928293072	1	1	1
CCNT2	0.122624296	1.026604151	1	0.933	1
HSPA12B	0.122748723	-0.415037499	0.312	0.6	1
CISD2	0.122783261	1.037964668	1	1	1
CEBPZOS	0.122930373	0.941257255	1	1	1
ENSSSCG00000049042	0.122991127	-0.312675782	0.938	0.867	1
CCNT1	0.123096986	0.935683352	1	1	1
PBXIP1	0.123159116	1.217977131	1	1	1
ETV1	0.123307781	0.75445883	1	1	1
RAB15	0.123415572	1.278694597	1	1	1
SLC17A6	0.123749532	-0.707819249	0.688	0.533	1
CENPH	0.123823092	0.990836247	1	1	1
RELN	0.124093212	-0.958179824	0.562	0.4	1
CCDC69	0.124207665	-0.216257635	0.938	0.867	1
ZNF583	0.12440798	1.192977223	0.938	1	1
ENSSSCG00000054606	0.124532309	-0.562594688	0.312	0.667	1
ENSSSCG00000002259	0.12457517	0.996037327	1	1	1
ZNF76	0.124801413	1.194692907	1	0.733	1
SETDB2	0.12481778	0.231415802	0.938	1	1
ENSSSCG00000021560	0.124922666	0.989808272	1	1	1
PUS7L	0.12505572	1.225338122	1	0.733	1
ULK3	0.125232657	-0.042483331	0.438	0.6	1
KITLG	0.125341015	0.81563823	1	1	1
ZFAT	0.12544874	-0.480132528	0.75	0.867	1
TMTC4	0.125629179	0.2644426	1	1	1
ENSSSCG00000003240	0.125658532	-0.065275196	0.875	0.933	1
ENSSSCG00000054793	0.125699288	0.980600282	1	0.933	1
AGTR1	0.125729599	-0.242856524	0.875	0.8	1
CMTR1	0.125791972	0.807779008	1	1	1
STXBP5L	0.12657988	1.054232312	1	0.8	1
TICRR	0.126624794	0.20994098	1	1	1
WDR3	0.126641634	0.393241339	1	1	1
RBM15	0.126657485	-0.077427782	0.938	1	1

MON1B	0.126662447	1.02722737	1	1	1
USP9X	0.126867961	0.851741146	1	1	1
DDX27	0.127003182	0.299533573	1	1	1
SOCS2	0.127231026	-0.093109404	0.938	0.867	1
ENSSSCG00000041766	0.127256027	-0.441032708	0.5	0.667	1
ZNF502	0.127332196	-0.110596831	0.938	0.867	1
ENSSSCG00000030362	0.127412911	0.129876841	1	1	1
COA5	0.12750547	0.936006339	1	1	1
ZUP1	0.127835072	0.914075858	1	1	1
RBL1	0.127896439	1.142957954	1	1	1
NAMPT	0.127909091	0.291623423	1	1	1
SYN2	0.127983457	2.22881869	0.5	0.067	1
PDCD11	0.128090597	0.063796651	0.938	1	1
IGSF11	0.128244255	1.13138454	1	0.933	1
CLSTN1	0.128285556	1.224400019	1	0.933	1
BCL6	0.128288498	-0.607682577	0.438	0.6	1
ENSSSCG00000039370	0.128434177	0.910439663	1	1	1
ALAS1	0.128437401	1.639194812	0.938	0.6	1
ENSSSCG00000062273	0.128438398	-0.281554494	0.562	0.867	1
ELMOD2	0.128477368	0.956268962	1	0.933	1
MAP2K7	0.128730778	1.299997455	1	1	1
DUSP18	0.128867414	0.8881038	1	1	1
THUMPD3	0.128868684	0.058893689	0.938	0.867	1
MLXIP	0.128899349	1.487221648	1	0.867	1
ZIC1	0.12902575	0.071949842	0.938	0.933	1
ENSSSCG00000055045	0.12906513	-0.149692933	0.625	0.8	1
GADD45A	0.129131864	1.327222395	0.875	0.733	1
IRF1	0.129150949	1.002206045	1	1	1
UNC119B	0.129302308	-0.249228606	0.812	1	1
MTX3	0.129356727	0.407095478	0.938	1	1
SLCO4C1	0.12957691	0.855631089	1	1	1
ETAA1	0.129880806	0.849521227	1	1	1
ERBB3	0.129999433	-0.180572246	0.312	0.6	1
ACBD6	0.130090867	0.034269902	0.875	0.867	1
WBP4	0.130326179	0.11564334	0.938	1	1
ENSSSCG00000016828	0.130448499	0.311893078	1	1	1
PYROXD1	0.130450622	-0.335810808	0.938	0.933	1
CCNH	0.13065334	0.07945341	1	1	1
ZKSCAN7	0.130707025	0.878508794	1	0.933	1
CHM	0.130742168	1.110188785	1	1	1
ITGB8	0.131214832	1.553253641	0.688	0.667	1
PRELID3A	0.131362137	0.375443604	1	1	1
ENSSSCG00000034545	0.131427111	0.038135129	0.938	0.933	1
PTPRR	0.13181801	1.12701641	0.938	1	1
GRK5	0.131843956	-0.247437551	0.812	0.867	1
ERC1	0.131946115	0.522077018	1	1	1
CWC15	0.131951257	0.925702781	1	1	1
PPP3CB	0.131994422	-0.131244533	1	1	1
TRPC6	0.132138767	0.097113011	1	1	1
CBX1	0.132403633	0.269116644	1	1	1
PCDHAC2	0.132416217	0.20968683	1	1	1
DTNBP1	0.132460971	-0.043068722	0.875	0.933	1
LMBR1	0.132639211	0.040157126	0.938	0.867	1
STARD7	0.132692765	0.984543151	1	1	1
HCFC1R1	0.132798982	-0.529208519	0.625	0.6	1
ENSSSCG00000026564	0.132924268	1.491853096	0.812	0.6	1
ENSSSCG00000035901	0.132962627	0.347252251	1	1	1
TRIT1	0.133013556	1.568610295	0.938	0.733	1
ENSSSCG00000052027	0.133206883	2.044394119	0.5	0.2	1
KLHL21	0.133218159	0.717229376	1	0.867	1
HSD17B11	0.133268796	-0.239658068	1	1	1
ATP7A	0.133312257	-0.149692933	0.938	0.933	1
ENSSSCG00000059134	0.133489196	0.903332776	1	1	1
SFI1	0.133707142	0.032421478	0.875	1	1
PGBD5	0.133932861	0.306346554	1	1	1
DPH3	0.134043107	1.124764558	1	1	1
ENSSSCG00000011059	0.134095836	0.039645804	0.938	0.8	1
ENSSSCG00000013281	0.134122177	1.197853745	1	1	1
DENND2C	0.134201481	0.674943868	1	1	1
AFAP1L2	0.134233002	-0.195207592	0.938	0.867	1

ENSSSCG00000061235	0.134410303	0.904402475	1	1	1
RHOBTB2	0.134579441	1.269136146	1	1	1
ENSSSCG00000041888	0.134621971	-0.292418213	0.438	0.733	1
ENSSSCG00000026996	0.134870149	-0.489038081	0.438	0.6	1
HIGD1A	0.134965364	0.26216246	1	1	1
ENSSSCG00000054771	0.135023227	-0.562594688	0.375	0.6	1
DPF1	0.135063082	1.46982679	0.938	0.8	1
LRP8	0.135330552	0.006426269	0.875	0.867	1
NEK1	0.135426442	0.940423977	1	1	1
PDE4C	0.135514925	-0.784987109	0.312	0.533	1
FYN	0.13552643	0.857980995	1	1	1
MTFR1L	0.135608497	0.991659063	1	1	1
ZSWIM1	0.135740098	0.212516878	0.812	0.933	1
ICE2	0.135744328	0.85680062	1	1	1
TRIM59	0.135748689	1.11038918	1	1	1
LONRF1	0.135809138	0.870885642	1	1	1
ENSSSCG00000004687	0.135896125	-0.327574658	0.312	0.533	1
SLC25A46	0.13603294	1.028196892	0.938	0.933	1
ZBTB14	0.136165567	0.020547377	0.625	0.8	1
HSPA4L	0.136414163	0.841086772	1	1	1
SERTAD2	0.136436892	0.91034781	1	1	1
PTPRN	0.136507585	-0.623624121	0.125	0.533	1
RETSAT	0.13679959	-0.063536289	1	0.933	1
ENSSSCG00000015565	0.137298199	0.928023408	1	1	1
IFNAR1	0.137553043	0.864356552	1	1	1
HSPA12A	0.137566813	0.905564792	1	1	1
ENSSSCG00000046421	0.137692354	-0.470344871	0.938	0.933	1
DHTKD1	0.137896123	-0.259119356	0.688	0.667	1
MOSPD2	0.137923798	0.231538192	1	1	1
ENSSSCG00000028092	0.137976765	0.449551154	1	1	1
PCSK7	0.138087384	-0.587874096	0.5	0.533	1
MMD	0.138147356	1.093163591	0.938	0.933	1
TNRC18	0.138216681	0.058893689	0.75	0.867	1
OLA1	0.138290887	0.179093317	1	1	1
ENSSSCG00000008981	0.138405543	-0.736965594	0.375	0.533	1
GABPB2	0.138405626	1.178850231	0.938	1	1
ADGRG7	0.138482796	0.299473244	0.938	1	1
C12orf73	0.138509644	0.341827652	0.688	0.933	1
ZNF772	0.139147426	-0.101671418	0.938	0.933	1
SBNO2	0.139312982	0.293913719	0.875	1	1
ENSSSCG00000054839	0.139373539	2.108524457	0.5	0.2	1
BRCA1	0.13939757	0.206938671	1	1	1
BTBD7	0.139441199	0.715672751	1	1	1
KLK4	0.139478386	-0.714597781	0.5	0.467	1
CPS1	0.139519781	1.794415866	0.688	0.267	1
PAK1	0.139574443	0.411471107	1	1	1
CYTB	0.139748639	1.472024351	1	1	1
PDIA5	0.139762784	1.460826201	1	1	1
CA13	0.140090332	0.000503646	0.938	0.867	1
HDGF	0.140118544	1.442943496	0.938	0.467	1
FBXO41	0.140247462	-0.335679708	0.812	0.867	1
NLRP3	0.140327107	1.14964073	0.938	1	1
ENSSSCG00000032196	0.140404997	-0.048885404	0.938	0.933	1
DVL3	0.140522399	0.335964155	1	1	1
ZMAT1	0.140632222	1.444156354	0.812	0.667	1
RILPL1	0.140674669	-0.745186101	0.312	0.533	1
ENSSSCG00000036417	0.140707851	1.153051183	0.938	0.533	1
ASAP1	0.140861536	0.972186506	1	1	1
ENSSSCG00000046239	0.141012243	2.044394119	0.562	0.133	1
MNT	0.141399609	-0.154509949	0.625	0.667	1
ENSSSCG00000050485	0.141659047	-1.019108823	0.188	0.533	1
C4orf46	0.141662888	-0.299560282	0.562	0.6	1
RTCA	0.141665467	0.938194716	1	1	1
ZCCHC4	0.141672712	-0.099395667	0.938	0.933	1
MBNL1	0.141767262	-0.264720782	1	0.733	1
ACSS3	0.141826295	0.274195484	0.938	1	1
ATG14	0.141927395	0.116646862	1	1	1
ENSSSCG00000062271	0.141984139	-0.071735754	0.812	0.8	1
WDR31	0.141989634	1.293599372	1	0.8	1
ENSSSCG00000052396	0.142179796	1.182525038	0.688	0.267	1

KIF1B	0.14219709	0.888743249	1	1	1
UBE2Q2	0.142330537	0.903372815	1	1	1
ZNF566	0.14237909	-0.10433666	0.938	0.867	1
NUDCD2	0.142656649	0.95922206	1	1	1
POLDIP3	0.142661917	0.409069688	1	1	1
DPY19L1	0.14270741	1.152829008	1	1	1
NCOA2	0.142779749	0.946600305	1	1	1
GNGT1	0.142932414	-0.327574658	0.625	0.867	1
SPO11	0.142932997	-0.010647244	0.875	0.933	1
PCOLCE	0.142991706	-0.529208519	0.625	0.533	1
MCTP1	0.143114383	0.924623682	1	1	1
BTBD1	0.14326733	0.016863438	0.938	0.933	1
ENSSSCG00000054288	0.143323427	1.799975392	0.5	0.2	1
ENSSSCG00000032549	0.143348379	0.297680549	1	1	1
WNT5A	0.143386794	-0.552541023	0.438	0.6	1
TRIM13	0.14350416	1.136896201	1	1	1
ENSSSCG00000026365	0.143628688	1.672425342	0.812	0.267	1
MFHAS1	0.143698477	0.215965165	1	1	1
SF3A2	0.143704375	0.213551934	0.875	1	1
RTKN2	0.143707577	0.793813688	1	1	1
BTBD2	0.143746074	1.251225104	1	0.933	1
ENSSSCG00000036465	0.143827023	1.764871591	0.75	0.2	1
GJD2	0.1438851	1.054561444	1	1	1
RBM26	0.144207735	0.973192966	1	1	1
PHACTR1	0.14423576	0.17990909	0.938	0.933	1
ENSSSCG00000022331	0.144271768	-0.830074999	0.25	0.6	1
NDUFAF4	0.144435127	0.806286655	1	1	1
POLQ	0.144504739	0.993602229	1	1	1
RNF130	0.144571227	0.906890596	1	1	1
FAM118A	0.144583344	1.286550927	1	1	1
SORL1	0.144959148	0.792074104	1	1	1
LGALSL	0.145303274	1.541606132	0.812	0.533	1
APOF	0.145540629	-0.294743266	0.312	0.533	1
SMTNL2	0.145632627	0.389452089	0.938	1	1
SHROOM4	0.145641265	0.866701505	0.938	0.8	1
SCN3B	0.145898366	0.172053394	1	1	1
PDE1B	0.14600354	-0.847996907	0.375	0.533	1
PANX1	0.146064842	0.139383607	1	1	1
ENSSSCG00000059731	0.146072046	-0.900464326	0.375	0.533	1
SLC38A3	0.146963976	-0.638543541	0.625	0.733	1
ENSSSCG00000055296	0.147046703	-1.093109404	0.5	0.6	1
ENSSSCG00000059103	0.147123402	1.480075929	0.875	0.667	1
COL12A1	0.147141062	0.927249683	1	0.933	1
NOVA1	0.147288771	0.171485515	0.938	1	1
ENSSSCG00000038556	0.147335713	0.37895904	1	1	1
UBP1	0.147382669	1.081141522	1	1	1
ENSSSCG00000057625	0.147456677	1.861086906	0.688	0.2	1
CACNB1	0.147494784	-0.540568381	0.375	0.6	1
ENSSSCG00000056100	0.147629126	1.093163591	1	1	1
LAMA5	0.147635111	-0.245112498	0.438	0.6	1
LRRC57	0.147646832	-0.649502753	0.5	0.533	1
UCK2	0.147716933	0.032421478	1	1	1
ATP13A2	0.147728261	-0.550316358	0.75	0.733	1
MTBP	0.147752682	1.09592442	1	1	1
ATAD5	0.14776766	0.954135948	1	1	1
RC3H1	0.148128017	0.914534031	1	1	1
GCLC	0.148235447	0.937730215	1	1	1
RGS6	0.148261684	-0.285754482	0.438	0.667	1
TJP2	0.148283807	0.95155511	1	1	1
ATOSA	0.148300068	0.28101045	1	1	1
FADS1	0.14869998	-0.009819061	0.938	0.933	1
CTDP1	0.148756257	1.619314006	1	1	1
TTC29	0.148810329	0.02768874	1	0.933	1
UROS	0.148918534	0.111249094	0.688	0.8	1
ENSSSCG00000056001	0.148960815	-0.594272678	0.938	1	1
ENSSSCG00000014165	0.149166555	0.286687089	0.938	1	1
CCDC88A	0.149304763	0.910632772	1	1	1
PHLPP1	0.149334227	0.128569694	1	1	1
ENSSSCG00000061233	0.149594729	1.832890014	0.562	0.4	1
GPS2	0.14986372	-0.033403158	0.875	0.933	1

ENSSSCG00000012362	0.149888618	1.188460953	0.812	0.6	1
ACAD11	0.149904586	0.315289453	0.938	1	1
RAC3	0.14997162	1.64385619	0.75	0.467	1
FLT1	0.150004642	0.464516012	1	1	1
ERCC3	0.150106626	1.275612902	1	0.933	1
ADGRB3	0.15017488	1.114998791	1	0.733	1
NANP	0.150174949	-0.424952968	0.625	0.667	1
ENSSSCG00000049146	0.150371176	1.05166212	0.875	0.667	1
EFNA3	0.150582756	-0.30723421	0.438	0.733	1
SHLD3	0.150616173	1.164188632	1	1	1
ENSSSCG00000060358	0.150727435	0.067355268	0.875	0.533	1
ADARB2	0.15073226	-0.250650681	0.812	0.867	1
ENSSSCG00000052364	0.151201049	0.197708158	1	1	1
ENSSSCG00000048319	0.151491602	-0.279522529	0.688	0.6	1
DAGLB	0.151620752	0.370345224	1	0.933	1
ENSSSCG00000053028	0.151664611	2.108524457	0.5	0.267	1
UPRT	0.151757325	1.06161319	1	0.8	1
NOL4	0.151861762	0.727834582	1	1	1
LYST	0.152430524	0.898425198	1	1	1
ENSSSCG00000055282	0.15243186	-0.242856524	0.938	0.867	1
DDX18	0.15293913	0.828363791	1	1	1
SIN3A	0.15300743	0.855442585	1	1	1
MICU2	0.153380643	1.118508545	1	1	1
MCPH1	0.153467174	0.747692001	1	1	1
CSDE1	0.153551409	0.346345021	1	1	1
PTPDC1	0.153559293	1.134158638	1	0.933	1
CCDC120	0.153608313	-0.175571565	0.812	0.733	1
ALMS1	0.153768217	0.596036936	1	1	1
LHX8	0.153828625	0.863314717	1	1	1
KCNN1	0.153930532	-0.197446064	0.812	0.733	1
ENSSSCG00000005255	0.153935404	0.961664818	1	1	1
SLC27A3	0.15408703	1.62309763	0.812	0.533	1
ACTC1	0.154565599	2.409390936	0.5	0.333	1
NKAIN1	0.154678277	0.032421478	0.75	0.733	1
CMKLR2	0.154678331	1.714245518	0.5	0.2	1
ENSSSCG00000004170	0.154693716	0.271654888	0.938	0.933	1
ENSSSCG00000054874	0.154751232	-0.093109404	0.75	0.533	1
ENSSSCG00000052077	0.155290246	-0.745186101	0.125	0.533	1
IDH1	0.15559963	0.339779971	1	1	1
NLN	0.15561847	0.328779441	1	1	1
ENSSSCG00000050064	0.15562824	1.528378972	0.812	0.267	1
METTL4	0.155661499	0.894489129	1	1	1
RAB3D	0.155698485	-0.526068812	0.562	0.4	1
ENSSSCG00000027046	0.155729582	1.173677136	0.812	0.667	1
ENSSSCG00000059407	0.156076752	1.558967292	0.625	0.267	1
ENSSSCG00000000611	0.156086352	0.943321052	1	1	1
UTP6	0.156145295	0.39662852	1	1	1
ANLN	0.156240248	0.676203738	1	1	1
IMPACT	0.156242311	1.245059332	1	0.933	1
HHLA2	0.156252964	0.091984476	0.938	1	1
RCOR2	0.156708408	1.394828642	1	0.8	1
NEFH	0.156768335	-0.044900311	1	0.933	1
AXDND1	0.156782702	-0.113008962	0.875	0.8	1
ADD1	0.156897374	1.066130049	1	1	1
ENSSSCG00000007848	0.157395801	0.979791143	1	1	1
TAF10	0.157405345	-0.093109404	0.938	0.933	1
ENSSSCG00000062863	0.157436532	1.69538649	0.625	0.4	1
GNPTAB	0.157524764	0.441023152	1	1	1
PCK2	0.157668342	-0.545621609	0.375	0.8	1
ATF1	0.157791472	1.082332602	1	1	1
SRPK1	0.157879238	0.692705012	1	1	1
ABHD13	0.157976638	0.372828993	1	1	1
MOB3C	0.158265447	-0.35614381	0.375	0.6	1
MALT1	0.158303972	0.01995326	0.875	0.933	1
COASY	0.158459933	-0.2255597	0.688	0.667	1
THNSL1	0.15847327	0.030879313	0.875	0.667	1
ENSSSCG00000062141	0.158677262	1.039340892	0.938	0.8	1
ZFP82	0.158682301	-0.112671671	0.938	1	1
KDM7A	0.158729477	0.928089761	1	1	1
KLK7	0.15878457	1.584962501	0.5	0.467	1

ENSSSCG00000021579	0.158859737	1.421463768	0.812	0.533	1
COG6	0.158967085	0.385870992	1	1	1
PDE4B	0.159006388	1.409390936	0.938	0.8	1
GABRB2	0.15923696	0.863519743	1	1	1
GNAQ	0.159313355	1.06801621	1	1	1
RASSF3	0.159342043	0.858934552	1	1	1
ATE1	0.159668474	0.426897654	1	1	1
ZNF687	0.159967904	-0.455679484	0.562	0.333	1
RCAN1	0.160005031	0.434710974	1	1	1
SRRM2	0.160144987	0.923964109	1	1	1
KLHL14	0.160479248	0.877265426	0.938	0.8	1
ENSSSCG00000049984	0.160487278	-0.285754482	0.5	0.533	1
ENSSSCG00000056769	0.160631114	0.969069587	1	1	1
ABCA5	0.160649556	0.268856405	1	1	1
ENO3	0.160848647	-1.947258538	0.062	0.667	1
CCND3	0.161008167	1.122143157	1	0.933	1
HAUS6	0.16120839	-0.166732268	0.875	0.933	1
CAMKV	0.161279744	0.193470429	1	1	1
BCCIP	0.161314596	1.30448296	1	0.933	1
ENSSSCG00000050974	0.161497421	-0.186218809	1	0.933	1
ANKRD28	0.161506396	0.834078605	1	1	1
STK36	0.161531619	-0.434146322	0.562	0.867	1
CHERP	0.161684634	1.197492356	1	1	1
ZNF184	0.161863618	0.779056928	1	1	1
ZSCAN25	0.161956792	1.624747367	0.875	0.667	1
RTP1	0.162338188	0.063759444	0.875	0.867	1
NATD1	0.162363944	0.169925001	0.75	0.6	1
SP2	0.162412135	1.468769483	1	0.867	1
RIOX1	0.162501256	0.285735443	1	1	1
C16orf87	0.162538165	0.276124405	1	1	1
CNOT1	0.162629092	0.155495443	1	1	1
ENSSSCG00000006685	0.162637701	-0.386840607	0.688	0.6	1
ENSSSCG00000059696	0.162676278	-0.157239742	0.688	0.8	1
NALCN	0.162799775	-0.318401716	0.812	0.8	1
ATXN2	0.16310826	0.969418279	1	1	1
ZSWIM2	0.16313828	-0.331269142	0.75	0.667	1
CEPT1	0.163183963	0.844812507	1	1	1
ENSSSCG00000051458	0.163218316	-0.77118131	0.625	0.4	1
ENSSSCG00000010426	0.163292483	0.734945484	1	1	1
TCF20	0.163473132	0.913352846	1	1	1
RAPGEF1	0.163496166	0.230893019	0.938	1	1
MED12L	0.163496706	0.370788489	1	1	1
MED25	0.163544827	1.11461763	1	1	1
ENSSSCG00000044654	0.163550578	2.076815597	0.5	0.133	1
ENSSSCG00000054102	0.163641233	-0.285754482	0.688	0.733	1
DNER	0.163813221	1.399930607	0.938	0.733	1
FMO2	0.163957623	0.951838002	1	0.933	1
ZBED9	0.164070409	1.142518844	0.938	0.6	1
TMEM170B	0.1641406	1.025614516	1	1	1
PSMA4	0.164316961	0.448494566	1	1	1
KDM5A	0.164490328	0.474621927	1	1	1
BAG3	0.164579801	0.16307133	1	1	1
CEP19	0.164978712	-0.489038081	0.5	0.4	1
SLC7A11	0.165024124	1.005476815	1	1	1
ENSSSCG00000050495	0.165032294	-0.27368165	0.562	0.6	1
IGF2BP3	0.165099071	0.379367454	1	1	1
CAMK4	0.165405683	1.186998515	0.938	0.533	1
ARHGEF25	0.165431382	1.386883537	0.875	0.533	1
PLEKHG1	0.165451448	0.925278402	1	1	1
PPA2	0.165485129	1.348996338	0.875	0.667	1
STON1	0.165537074	0.288285251	1	1	1
ITPR2	0.165557752	1.473931188	0.938	0.933	1
IFIH1	0.166106585	1.106199404	0.938	0.533	1
HSP90B1	0.166139028	0.416738738	1	1	1
MYH9	0.166235828	-0.120330873	0.938	0.933	1
ERI1	0.166252538	0.788814276	1	1	1
CLTC	0.166266406	0.846678425	1	1	1
EGFLAM	0.166338556	0.265812898	1	1	1
PURA	0.166346661	0.036173613	0.875	0.933	1
RAB29	0.166709322	0.364827658	1	1	1

MAT2B	0.166874528	0.149707354	1	1	1
MYO1E	0.167049912	0.383520475	1	0.933	1
MAPRE1	0.167214574	0.845684064	1	1	1
CELF6	0.167374108	0.018207618	0.938	1	1
SPINT2	0.167411734	1.001465409	1	1	1
ENSSSCG00000063457	0.167577792	0.970750538	1	1	1
TOPBP1	0.167893969	0.761931926	1	1	1
FMNL2	0.167906729	0.944696468	1	1	1
ENSSSCG00000063474	0.16792142	-0.415037499	0.562	0.467	1
RHOQ	0.168030299	0.979040381	0.938	0.667	1
DNAJC28	0.168045342	1.209453366	0.625	0.267	1
ENSSSCG00000062434	0.168056934	-0.315501826	0.625	0.667	1
DLX4	0.168113522	-0.480132528	0.5	0.667	1
MAZ	0.168513391	1.423466121	0.938	0.867	1
ENSSSCG00000050531	0.16859401	0.316360768	1	1	1
ENSSSCG00000028693	0.168640662	1.252665432	0.938	0.667	1
LRCH3	0.168783389	0.869817163	1	1	1
AP4S1	0.168895323	1.311280851	0.688	0.533	1
PCBP2	0.168901478	0.683136177	1	1	1
C8B	0.168964117	1.564002882	0.625	0.2	1
EYA3	0.169034667	0.344505282	1	1	1
IL6R	0.169141326	-0.344648171	0.5	0.467	1
MYBL2	0.169749473	0.152515323	1	1	1
RNF19A	0.169854032	0.227699889	1	1	1
ENSSSCG00000058608	0.170028918	-0.441032708	0.375	0.533	1
CENPQ	0.170110053	0.210631901	1	1	1
YTHDF2	0.170128155	1.20330367	1	1	1
ENSSSCG00000005869	0.17047391	1.152003093	0.875	0.533	1
ENSSSCG00000062392	0.170524797	-0.4012317	0.438	0.733	1
TRIM24	0.170573071	-0.13112273	1	1	1
TSPYL5	0.170591347	-0.441032708	0.875	0.867	1
HUNK	0.170867286	0.022367813	0.438	0.533	1
ENSSSCG00000016486	0.171160163	-0.480132528	0.25	0.533	1
ESCO1	0.171250675	1.079875397	1	0.8	1
ADSS2	0.171313561	0.255344985	1	0.933	1
MED7	0.171687874	0.437323529	1	1	1
ENSSSCG00000005982	0.171791229	0.816233073	1	1	1
SYDE2	0.171812612	2.094517599	0.625	0.2	1
SLC4A2	0.172003841	1.491853096	0.625	0.4	1
AASDH	0.172194466	0.271046926	1	1	1
ENSSSCG00000035527	0.172367751	0.232290882	0.938	0.867	1
EXD2	0.172615238	1.374016606	0.875	0.867	1
SPRED2	0.172659997	0.90366349	1	1	1
RBM12B	0.172794694	0.860849848	1	1	1
DNM1L	0.172866896	0.849760488	1	1	1
ENSSSCG00000007355	0.172917253	0.859573387	1	1	1
ENSSSCG00000005424	0.172955038	-0.280736408	0.688	0.733	1
LTA4H	0.173125181	0.020547377	0.625	0.667	1
ANKRD37	0.173441965	0.944487382	1	1	1
ALG9	0.173464078	0.276536192	0.75	0.867	1
KCTD10	0.17349381	0.485426827	1	1	1
PEG10	0.173580792	0.155030786	1	1	1
ODAD3	0.17363725	-1.062735755	0.562	0.733	1
NRG3	0.173896106	1.188965044	1	1	1
TSC1	0.174043815	0.103713768	0.938	1	1
EPB42	0.174153297	-0.388565288	0.625	0.733	1
IL1RAPL2	0.174310594	1.246096682	1	0.933	1
CSNK1G2	0.174330926	0.154818109	0.875	0.933	1
NDUFAF6	0.174341494	0.0138058	0.875	0.933	1
CPPED1	0.174347319	-0.9861942	0.312	0.533	1
ENSSSCG00000002749	0.174499776	0.366322214	0.312	0.8	1
GTPBP1	0.174611532	1.051441385	1	1	1
SOWAHA	0.174689039	1.161074084	0.938	0.867	1
C1orf109	0.174998722	-0.023846742	0.875	0.8	1
ELF1	0.175085725	0.931409959	1	1	1
ENSSSCG00000048585	0.175113382	1.360256214	1	0.933	1
ENSSSCG00000042520	0.175158452	-0.415037499	0.375	0.533	1
ENSSSCG00000061821	0.175228795	1.906890596	0.625	0.267	1
ASZ1	0.175642602	0.665335917	1	0.867	1
RNF41	0.175723672	0.751392665	1	1	1

ENSSSCG00000057006	0.17576282	-0.055634699	0.875	0.8	1
ZNF614	0.175844571	1.539158811	0.875	0.867	1
WNT16	0.175967925	-0.397963986	0.625	0.733	1
ENSSSCG00000057680	0.175975445	-0.230612928	0.5	0.6	1
ENSSSCG00000062750	0.176158661	0.952453173	0.875	0.933	1
VCPIP1	0.176220908	0.917207887	1	1	1
EXOSC7	0.176246836	0.757747156	1	0.933	1
ZBTB43	0.176317378	0.024727086	0.875	0.867	1
HDAC2	0.176318172	0.199402203	1	1	1
ST6GALNAC3	0.17637609	0.59248018	1	1	1
ENSSSCG00000047687	0.176383074	1.491853096	0.688	0.333	1
GPX6	0.176836482	-0.805827452	0.438	0.6	1
RALB	0.177083834	-0.294743266	0.688	0.733	1
ZMYND8	0.177125082	0.85828457	1	1	1
WWC2	0.177141081	0.964223771	0.875	0.733	1
TUSC3	0.17732396	1.031691358	1	1	1
TPRG1	0.177428869	2.154818109	0.5	0.133	1
ANKRD42	0.177432124	0.9919565	1	1	1
NAT14	0.177461961	1.62309763	0.812	0.467	1
HYOU1	0.177499304	1.346028003	1	1	1
RWDD4	0.177511173	-0.024396654	0.812	0.733	1
TCERG1L	0.177543953	1.321928095	1	0.867	1
SPAG1	0.177548536	0.359476882	1	1	1
ENSSSCG00000032299	0.177607126	1.714245518	0.625	0.4	1
LEKR1	0.177980154	1.32439635	1	0.933	1
LRBA	0.178395729	0.833680749	1	1	1
TCF15	0.178454812	1.661778098	0.75	0.133	1
ENSSSCG00000050757	0.17846551	-0.093109404	0.688	0.867	1
NOL9	0.178475029	0.418643249	0.938	1	1
SIRT3	0.17859048	0.161311871	1	0.867	1
TANGO6	0.178708922	-0.17663276	0.938	0.933	1
ENSSSCG00000059556	0.178880965	-0.392669686	0.5	0.6	1
ENSSSCG00000051017	0.178908357	1.22881869	0.75	0.4	1
USP40	0.178948621	0.154018611	1	1	1
SEMA7A	0.179042198	2.22881869	0.5	0.4	1
DIS3L	0.179223703	1.027771172	1	0.933	1
MTFR1	0.17942505	0.873841924	1	1	1
DDX60	0.179578098	0.939495261	1	1	1
IMMT	0.179665093	0.095318055	1	1	1
CFAP70	0.179676125	-0.291048782	0.562	0.6	1
CCDC186	0.179862424	0.984781204	1	1	1
ENSSSCG00000014098	0.179874489	1.067355268	1	0.867	1
ARID1B	0.179910374	1.040414268	1	1	1
TATDN1	0.180138502	0.327076642	1	1	1
STRIP1	0.180164787	1.147939465	1	1	1
ENSSSCG00000048231	0.180229973	1.046615359	0.875	0.6	1
TCEA2	0.180310426	-0.327574658	0.5	0.467	1
ESR2	0.180376157	1.279755656	1	1	1
ENSSSCG00000050484	0.180418356	-0.415037499	0.312	0.6	1
CACNB2	0.180540566	0.324617182	0.938	1	1
ENSSSCG00000062745	0.180791545	-0.415037499	0.5	0.467	1
UTP15	0.180865845	0.208470045	0.938	1	1
APBA1	0.181220921	0.436827194	1	1	1
ENSSSCG00000011623	0.181265122	1.154157686	0.938	0.933	1
TGS1	0.181265411	0.958151611	1	1	1
CNNM4	0.18135723	0.070389328	0.812	0.867	1
IER5L	0.181398928	-0.334117504	0.125	0.533	1
UNC119	0.181429665	0.22881869	0.938	0.933	1
PPARD	0.181554785	1.089267338	1	0.933	1
MTERF2	0.181587292	-0.390789953	0.688	0.6	1
FSTL4	0.18191422	0.268214873	1	1	1
ENSSSCG00000047937	0.182092969	-0.213403638	0.5	0.667	1
SLC29A1	0.182502341	1.162545471	1	0.8	1
MAPKAP1	0.182520935	0.437603471	1	1	1
ENSSSCG00000046110	0.182521048	2.714245518	0.5	0.067	1
MINPP1	0.182559143	0.877295813	1	0.933	1
DNAJC19	0.182794978	0.873467594	0.938	0.933	1
FDFT1	0.182874899	0.974908581	1	1	1
ARHGAP24	0.182977956	1.301750213	0.938	0.733	1
SLC9A7	0.18321079	0.885597246	1	1	1

UGGT1	0.183215545	-0.034481674	1	1	1
BORCS8	0.183376023	-0.437063806	0.625	0.533	1
PIK3C2B	0.183396448	1.109982461	1	0.733	1
ENSSSCG00000035799	0.183738914	1.977279923	0.625	0.267	1
ENSSSCG00000060443	0.183934429	0.129283017	0.938	0.733	1
RBM19	0.184044013	-0.019108823	0.75	0.733	1
PATL1	0.184051628	1.199780986	1	1	1
PCCA	0.184404614	0.155803893	0.938	0.933	1
ALDH1A1	0.184418818	-0.415037499	0.312	0.533	1
STPG2	0.184536224	0.710999858	1	1	1
IKZF5	0.184754425	0.96829114	1	0.933	1
TRIM21	0.184761124	1.240791332	0.938	0.867	1
ENSSSCG00000009495	0.184843907	0.315586568	1	1	1
ENSSSCG00000063436	0.185031299	-0.1241363	0.875	0.733	1
PWWP3A	0.185162055	-0.529208519	0.875	0.867	1
GEMIN8	0.185193138	1.238433779	1	1	1
ENSSSCG00000023850	0.18526282	0.008674334	1	1	1
UNC79	0.18559125	1.994353437	0.562	0.4	1
COL11A1	0.185746163	0.250502517	1	1	1
DENND11	0.18574864	0.358457907	1	1	1
GXYLT1	0.185808948	0.375241868	1	1	1
SEC22B	0.18585631	0.804719341	1	1	1
ENSSSCG00000061915	0.186249626	0.027184829	0.312	0.667	1
PLPPR5	0.186380334	2.058893689	0.625	0.267	1
SLC39A13	0.186387197	0.343530349	1	0.867	1
LARS2	0.18650185	1.194467186	1	1	1
USP34	0.186507441	0.758306802	1	1	1
CNOT4	0.186610806	0.755297417	1	1	1
MLF2	0.186694739	1.343432935	1	1	1
HECW1	0.186746806	0.81998253	1	1	1
ENSSSCG00000059022	0.186873176	-0.093109404	0.812	0.6	1
TNFAIP1	0.187056086	1.842760258	0.688	0.533	1
ANAPC16	0.187060762	0.469571649	1	1	1
TNS2	0.187233323	1.746425923	0.812	0.667	1
ENSSSCG00000059416	0.187389541	1.617383978	0.688	0.533	1
PDRG1	0.187568283	0.805010982	1	1	1
BRAF	0.187604926	0.805059921	1	1	1
PLXNA1	0.187649543	0.079727192	1	0.933	1
SYNE2	0.187695131	0.310698708	1	1	1
IFT46	0.187974262	0.993146329	1	1	1
MARK2	0.188080407	1.324787749	1	1	1
ZNF518B	0.188118342	0.178521025	1	1	1
ENSSSCG00000036083	0.188222525	1.198302264	1	0.933	1
NARS2	0.188236862	0.311133779	1	1	1
DCTN2	0.18857274	-0.870716983	0.5	0.733	1
ENSSSCG00000009281	0.188686894	0.300711609	1	0.867	1
DDAH1	0.188691328	0.296302287	0.812	0.933	1
POU2F1	0.18870108	1.272540068	0.938	0.933	1
ENSSSCG00000050878	0.188810219	-0.810966176	0.812	0.667	1
ENSSSCG00000016191	0.188860752	0.020348645	1	0.933	1
CEP162	0.189172247	0.859362226	1	1	1
DHODH	0.189216548	0.897730596	1	1	1
VDR	0.189558728	-0.093109404	0.75	0.933	1
RBPMS	0.18959106	1.111482729	1	1	1
ZRANB2	0.189749592	0.918525296	1	1	1
SMPD4	0.189765539	-0.150824902	0.875	0.933	1
RAD51	0.189820972	0.877046085	1	1	1
SREK1IP1	0.189944072	0.860843228	1	1	1
HK2	0.190130762	0.922974319	1	1	1
COP1	0.190181486	0.190762464	1	1	1
RALBP1	0.190207661	0.338093682	1	1	1
GALC	0.190243214	1.114215569	1	0.933	1
NOL10	0.190364792	1.096507981	1	1	1
ERCC8	0.190375199	0.071949842	1	0.933	1
ENSSSCG00000060577	0.190950324	1.854423176	0.625	0.2	1
PRDM10	0.191169299	1.069162025	1	0.733	1
ENSSSCG00000055078	0.191210642	1.134696513	0.812	0.667	1
PCBP4	0.191331229	0.095335685	0.75	0.867	1
MAGI1	0.191333696	0.399930607	1	1	1
SCD	0.19144109	0.379911279	1	1	1

CAPS2	0.191699077	0.243416067	0.875	0.933	1
SGTA	0.191831265	0.351629217	1	1	1
ADAM9	0.191831275	0.176979759	0.688	0.867	1
GCH1	0.191926015	0.216079579	1	1	1
IPO7	0.1920306	0.998633164	1	1	1
SNX14	0.192267714	1.071991678	1	1	1
FBXO10	0.192313226	0.702837218	1	1	1
ENSSSCG00000048283	0.192421606	-0.397963986	0.625	0.467	1
ZBTB5	0.192598982	-0.339749372	1	0.933	1
ENSSSCG00000061997	0.192774725	-0.228764504	1	0.8	1
CNTLN	0.192789543	1.152135688	1	1	1
MAU2	0.192797855	0.892256201	1	1	1
XYLB	0.192948377	1.525800428	0.812	0.467	1
NAGK	0.193001696	0.276311295	1	1	1
ENSSSCG00000061607	0.193125401	1.070389328	0.688	0.4	1
EFHB	0.193219562	2.22881869	0.5	0.133	1
BICD2	0.193371787	0.892996154	1	1	1
TCN2	0.193427714	-0.63076619	0.562	0.6	1
SH3BP1	0.193538764	1.607330314	0.688	0.333	1
PHF14	0.194008491	0.465889077	1	1	1
NUDT13	0.194106658	1.038257158	1	0.933	1
GRIA4	0.194354663	0.304312552	1	1	1
PICALM	0.194601187	0.809700668	1	1	1
NEU3	0.194605582	-0.043068722	0.688	0.6	1
SLC35E3	0.194748831	0.91580532	1	0.933	1
PLAC9	0.194754049	-0.437063806	0.5	0.6	1
ATP13A4	0.194802572	1.568305157	0.812	0.733	1
FBXL12	0.194857188	0.445531528	1	1	1
NREP	0.195077249	0.852326235	1	1	1
CA14	0.195123351	1.743391863	0.562	0.267	1
UBE2H	0.195401281	0.512635903	1	1	1
SERPINB5	0.195695158	1.541606132	0.688	0.533	1
NUDT16	0.195853082	0.309437151	0.938	0.8	1
MUC15	0.196107969	0.980139578	0.938	0.867	1
TTI1	0.196338726	0.047488926	0.875	1	1
PDS5B	0.196464138	0.881193058	1	1	1
ZMYM5	0.196506011	0.35132188	1	1	1
ARHGAP17	0.196595682	0.120486069	1	0.867	1
CNGA3	0.196631233	-0.253574077	0.5	0.6	1
RPA1	0.196816107	0.938769896	1	1	1
EXD1	0.196888645	0.958658657	1	1	1
ENSSSCG00000062995	0.196923727	1.089093927	1	1	1
PDE3B	0.196935206	1.054320959	1	0.933	1
PRRC2A	0.197086308	-0.042923647	0.938	0.933	1
CD81	0.19723949	0.266886731	1	0.933	1
USP1	0.197576473	0.754887502	1	1	1
ENSSSCG00000056876	0.197628862	1.343407822	0.812	0.867	1
ME3	0.197668607	0.088337467	0.812	0.867	1
BMPER	0.197907856	0.861123695	1	1	1
PUS1	0.198197612	1.534921818	0.562	0.333	1
XKR8	0.198224865	-0.366127899	0.562	0.467	1
ENSSSCG00000055999	0.198673324	1.085227837	0.625	0.267	1
RIPPLY2	0.198931464	1.321928095	0.562	0.4	1
KLHL20	0.198937264	0.845810045	1	1	1
SMCHD1	0.199079493	0.879145605	1	1	1
RNF25	0.199416943	-0.246519319	0.938	0.933	1
ENSSSCG00000058314	0.199446284	0.091630475	0.875	1	1
MMRN1	0.199488925	-0.16525919	0.812	0.733	1
USB1	0.199545872	1.137503524	1	0.933	1
ENSSSCG00000058343	0.19961959	-0.1271904	1	1	1
MIS18BP1	0.199677019	0.946968035	1	0.933	1
WIPF3	0.199715262	0.265150903	1	0.933	1
ENSSSCG00000017038	0.199788153	0.438046653	1	1	1
BDNF	0.19992184	1.10938346	0.812	0.933	1
RBM41	0.200013435	0.101086125	1	1	1
CDK5R1	0.200193228	1.531381461	0.875	0.867	1
NEURL3	0.20021715	0.190683562	0.938	0.867	1
MAGOH	0.200454989	0.732794179	1	1	1
AP5B1	0.20089276	-0.388565288	0.5	0.467	1
FYTTD1	0.201232376	0.851894027	1	1	1

ENSSSCG00000059637	0.201273691	0.165237996	1	1	1
POMGNT2	0.201333006	-0.433449536	0.875	0.933	1
CEP152	0.201598316	0.950825021	1	1	1
ENSSSCG00000044105	0.201602385	1.845490051	0.5	0.267	1
GEMIN5	0.201816472	0.252387162	1	1	1
C1QBP	0.202086102	0.948994627	1	1	1
SCOC	0.202266365	0.138216142	0.938	1	1
TMEM222	0.202575854	-0.044199804	0.688	0.733	1
ENSSSCG00000060099	0.202590506	-0.186218809	0.688	0.733	1
OXCT1	0.202595342	0.20523187	0.938	0.933	1
PHLDB1	0.20280384	0.238872355	1	0.933	1
VSIG2	0.202942105	0.868416448	0.938	0.867	1
ENSSSCG00000051245	0.203081196	1.118729239	0.938	0.933	1
PTGIS	0.203316128	1.035505028	1	1	1
ENSSSCG00000057811	0.20342235	0.781359714	1	0.867	1
ENSSSCG00000022423	0.203500093	1.017534659	0.938	0.667	1
ENSSSCG00000052887	0.203508211	-0.508146904	0.312	0.6	1
TMEM232	0.2036905	0.074257302	1	1	1
TLE1	0.203795397	0.220999186	1	0.867	1
TBCB	0.203906422	0.886280316	1	1	1
ENSSSCG00000056414	0.204003132	1.441064986	1	0.867	1
RTBDN	0.204036801	0.0489096	0.75	0.667	1
MESP1	0.204088878	1.159656666	0.938	1	1
HERC1	0.204140377	0.365456136	1	1	1
RAP1GDS1	0.204179848	1.118532848	1	1	1
RBM44	0.204181196	1.030648371	1	1	1
KLHL36	0.204271718	1.441479574	1	0.867	1
ENSSSCG00000058222	0.204279791	-0.206320015	0.625	0.867	1
PML	0.204774173	-0.250650681	0.438	0.6	1
ADAMTS6	0.204921733	0.306034448	1	1	1
OSBP	0.205004531	0.311262686	1	1	1
LEMD3	0.205135157	0.927424604	1	1	1
PDE4DIP	0.205182546	0.388367537	1	1	1
SYNGR2	0.205216598	-0.392669686	0.5	0.467	1
KCNH1	0.205520206	0.452422952	1	1	1
CXXC4	0.205698181	1.430452552	0.625	0.2	1
USF1	0.205875762	-0.043356369	0.75	0.733	1
ENSSSCG00000060568	0.206088399	0.179262866	0.938	0.867	1
EFTUD2	0.206238852	0.304237619	1	1	1
PPP2R3B	0.206296088	1.231482948	1	1	1
SNX19	0.206553302	-0.076989739	0.812	0.933	1
ENSSSCG00000059277	0.206823995	1.401655287	0.75	0.2	1
SPHK2	0.206970948	-0.338221902	0.812	0.6	1
STK3	0.207789314	0.346247774	0.938	0.933	1
USP8	0.207920789	0.760230302	1	1	1
PAPOLA	0.208042787	0.953618076	1	1	1
JARID2	0.208281945	1.023517371	1	1	1
MRPS30	0.208289002	0.483140665	1	1	1
MLH3	0.208611483	0.003808739	1	0.933	1
ENSSSCG00000054702	0.208720604	1.201221132	0.938	0.933	1
ENSSSCG00000058499	0.208818943	-0.115135711	0.75	0.867	1
PRKCE	0.208920415	0.968951564	0.938	0.867	1
TMEM64	0.208946635	1.027642755	1	0.867	1
GDAP1	0.208949024	-0.37690237	0.5	0.6	1
EFCAB5	0.20955957	0.154328146	1	1	1
ENSSSCG00000063005	0.209698396	0.815410435	1	1	1
FKBP14	0.209702479	0.529327802	0.812	0.933	1
ENSSSCG00000012192	0.209936408	0.203640127	1	1	1
ATP13A1	0.209955685	1.327877361	1	1	1
ZNF711	0.2101833	1.277780991	1	1	1
HIPK1	0.210203238	0.857963805	1	1	1
ZFAND2A	0.210271797	1.262302389	1	1	1
ZC3H10	0.21030983	0.240016897	0.875	0.933	1
LRRC70	0.210382824	1.034002514	0.812	0.667	1
CHPF	0.210529997	0.254813899	0.812	0.867	1
STXBP3	0.210561085	0.060435854	1	1	1
RIF1	0.21064945	0.810909554	1	1	1
TM9SF3	0.211049913	0.330304517	1	1	1
AGTPBP1	0.211198568	0.840893037	1	1	1
ENSSSCG00000059564	0.211204248	0.225075826	1	1	1

ACLY	0.211224955	-0.007479333	1	1	1
FAM171B	0.211252023	0.94061747	1	1	1
LRP2	0.211291695	1.282757498	0.812	0.867	1
SLC7A1	0.21130234	1.265971689	0.938	0.933	1
NUP153	0.21149216	0.758020075	1	1	1
CRYBG2	0.211529031	0.233700912	1	1	1
RAB12	0.211547348	0.993544978	1	0.933	1
ENSSSCG00000061018	0.211551284	-0.1602236	0.625	0.533	1
RCC1	0.211708	1.125070766	1	0.933	1
NMT2	0.211769206	1.047753131	0.875	0.6	1
PKDCC	0.211785534	-0.093109404	0.625	0.333	1
ZNF365	0.211819836	1.352690349	0.875	0.6	1
ADM	0.211850349	-0.034215715	0.688	0.467	1
VEZT	0.211872472	0.978374325	0.938	1	1
RABGAP1L	0.212003036	0.674506403	1	1	1
PLBD1	0.212055654	-0.366127899	0.875	0.6	1
FBXW4	0.212080592	1.218476746	1	0.867	1
ZMYM6	0.212210606	0.311607289	1	1	1
NEK7	0.212423298	0.224860677	0.875	0.867	1
ENSSSCG00000040433	0.212494276	-0.137503524	0.938	0.667	1
ENSSSCG00000059817	0.212648634	-0.167109986	0.5	0.733	1
ENSSSCG00000015027	0.21265691	-0.134332067	0.875	0.933	1
POLR3B	0.212704003	0.192292814	0.938	0.933	1
PGP	0.212710535	0.1661826	0.938	0.933	1
NOD2	0.212734443	-0.218640286	0.25	0.533	1
OGT	0.21277482	0.332775477	1	1	1
C1orf56	0.212868879	-0.077679364	1	0.867	1
HCFC1	0.213045696	1.106114322	1	1	1
SULT1C3	0.213197601	0.091315167	0.688	0.8	1
SURF6	0.213401352	0.053731984	0.812	0.533	1
C1QTNF3	0.213438117	1.807354922	0.5	0.2	1
OSR2	0.213513988	0.286082896	1	0.933	1
POLR1G	0.213566074	0.085693748	0.875	0.933	1
ENSSSCG00000048082	0.214191974	-0.467504919	0.438	0.6	1
PRPF8	0.214239257	0.928529075	1	1	1
ELK1	0.214654366	0.481584761	0.875	0.867	1
SELENOI	0.214827301	0.824001489	1	1	1
NOP58	0.214832665	1.072305076	1	1	1
TBCCD1	0.215688267	0.982178723	0.75	0.533	1
ENSSSCG00000063326	0.215714821	1.534921818	0.688	0.333	1
ENSSSCG00000060264	0.215737952	-0.043356369	0.938	0.867	1
ENSSSCG00000018068	0.215894606	0.041191687	0.875	0.667	1
FAM180A	0.215896517	0.017921908	0.562	0.4	1
SP4	0.216030834	-0.382616022	0.875	0.867	1
EXOC3	0.216535944	-0.176940995	0.75	0.933	1
DARS2	0.216556896	-0.136611043	0.75	0.667	1
YRDC	0.216668398	0.339850003	0.938	0.933	1
ENSSSCG00000046312	0.216691588	0.032421478	0.625	0.533	1
ZNF300	0.217055602	1.54059743	0.938	0.867	1
LBH	0.217380437	0.240134004	1	0.933	1
TLDC2	0.217496413	1.335733894	0.75	0.4	1
NEMP1	0.217617288	0.321128153	1	1	1
ENSSSCG00000061203	0.21768749	0.886623197	1	1	1
ENSSSCG00000059967	0.217927306	1.34298971	0.812	0.467	1
CFAP20DC	0.21803128	0.966762052	1	1	1
ENSSSCG00000055335	0.218199384	-0.062082509	0.812	0.733	1
SF3A1	0.218451241	0.430452552	1	1	1
BPTF	0.21868868	0.837958932	1	1	1
ENSSSCG00000015988	0.21870024	0.343474534	1	1	1
ITPRIP	0.218707135	1.529820947	0.75	0.4	1
FNBP1	0.21876728	1.965784285	0.625	0.133	1
AP3D1	0.218857606	0.302819272	1	1	1
ITPR1	0.218886298	0.323392314	1	1	1
UPK1B	0.219076648	1.866248611	0.562	0.333	1
MTMR9	0.219117176	0.790499528	1	1	1
ENSSSCG00000033390	0.219182322	0.877546634	1	1	1
ENSSSCG00000029920	0.219303368	1.324125651	0.875	0.8	1
IFN-OMEGA-6	0.219410362	-0.03866162	0.5	0.533	1
FOXP2	0.219599636	1.321928095	0.625	0.4	1
GRAMD4	0.219636108	0.438935212	1	1	1

SLC38A7	0.219776094	1.491853096	0.625	0.533	1
SLC16A9	0.219895855	-0.128733314	0.438	0.533	1
ENSSSCG00000000377	0.219931137	0.544899331	1	1	1
ZDHHC17	0.220055136	1.303780748	0.812	0.467	1
PLD5	0.220100404	0.282589947	1	0.933	1
HDDC2	0.220305609	0.894917954	0.938	1	1
CNP	0.220314448	0.266746929	1	1	1
PSMD1	0.220316856	0.993256226	1	1	1
KLF6	0.220342798	-0.497499659	0.5	0.6	1
FAM161B	0.220482438	0.123701985	0.875	1	1
CFB	0.220589893	-0.180572246	0.438	0.533	1
PRKACA	0.220973157	1.558967292	0.875	0.6	1
LARGE1	0.22101771	0.262827583	1	0.933	1
ZNF512	0.221025229	-0.072645302	0.938	0.867	1
SPON1	0.221138343	0.131013905	1	1	1
CFAP65	0.221662227	-0.315501826	0.5	0.4	1
ENSSSCG00000051090	0.221674928	0.838719093	0.938	0.8	1
WLS	0.221737489	0.484131075	1	1	1
SLC29A3	0.221933091	0.183408231	1	0.933	1
PTPRS	0.221957438	-0.471621028	0.562	0.8	1
PPIH	0.221978239	0.774720516	1	0.933	1
DRD3	0.222028545	1.754887502	0.5	0.133	1
SOCS7	0.222042769	0.75130248	1	1	1
ZNF395	0.222348589	0.385135698	1	1	1
CD19	0.222394678	-0.044199804	0.875	0.867	1
NEURL1	0.222455515	0.478199034	1	1	1
DDX31	0.222599146	1.326604582	0.938	1	1
ENSSSCG00000009770	0.222750445	1.011720569	1	1	1
ELOVL2	0.222791383	0.07756657	1	1	1
GCC1	0.222904695	0.908215993	1	1	1
ENSSSCG00000034690	0.222929047	0.950833943	0.938	0.867	1
ENSSSCG00000058715	0.223196147	0.036049076	1	0.933	1
ENSSSCG00000052974	0.223311942	1.076815597	1	0.933	1
LHX5	0.223408589	1.951284715	0.562	0.333	1
GGPS1	0.223486227	0.156270447	1	1	1
MORC1	0.223734258	1.339850003	0.938	0.733	1
LSM12	0.223758202	-0.019108823	0.938	0.867	1
ENSSSCG00000016426	0.224188931	1.209389756	1	1	1
PPP2R5E	0.224260706	0.856264523	1	1	1
ZNF335	0.224384993	0.159871337	1	0.933	1
ENSSSCG00000008058	0.224398694	1.140120489	1	1	1
ENSSSCG00000038679	0.224509618	-0.022720077	0.562	0.667	1
FAM20B	0.224565984	0.318086029	0.812	0.867	1
MRRF	0.224677606	0.808262563	1	1	1
DIP2A	0.224713855	1.165624864	0.812	0.733	1
DDX5	0.224722119	0.452590854	1	1	1
RNF214	0.224765651	0.285225212	1	1	1
PPP4R3A	0.224779091	0.824905123	1	1	1
ADGRG6	0.224839315	2.464886049	0.625	0.133	1
ANKRD49	0.225223595	0.075881512	1	1	1
ENSSSCG00000040203	0.225236126	1.307428525	1	1	1
TRIM37	0.225316283	0.770289532	1	1	1
ENSSSCG00000027515	0.225468106	-0.055876498	0.812	0.867	1
STRC	0.225647265	1.190683562	0.75	0.533	1
TGIF2	0.225681648	0.17139041	1	0.867	1
SDE2	0.22573086	0.955823563	1	1	1
SRBD1	0.226108749	0.894421244	1	1	1
ENSSSCG00000043051	0.22623927	0.86466236	1	1	1
PIAS3	0.226259617	1.161512762	1	0.933	1
DCAF1	0.226364972	0.865408216	1	1	1
OPA3	0.226736629	0.174823801	0.875	0.8	1
LEAP2	0.226897432	0.945109918	1	1	1
SENP8	0.226910012	0.280204683	1	1	1
CRHBP	0.227183425	0.185650831	1	1	1
ENSSSCG00000046546	0.227284115	1.3594028	0.75	0.533	1
DGCR8	0.227563662	0.027584413	1	1	1
RFT1	0.227745768	1.280621121	0.875	0.8	1
FGD6	0.227759324	1.020648886	1	1	1
PALM3	0.227885804	0.427722759	0.5	0.667	1
FOXR1	0.227916171	0.404421629	1	1	1

GPC4	0.228154532	0.399675781	1	1	1
CDCA4	0.228344453	1.691161905	0.5	0.4	1
MYCBPAP	0.228697799	0.052321035	0.812	0.8	1
ENSSSCG00000059707	0.22893925	-0.063963059	0.875	0.8	1
GYS1	0.228999813	0.016768213	1	0.867	1
NR6A1	0.229262595	0.895674978	1	1	1
DHX16	0.229439155	0.230667115	1	1	1
KLHL42	0.229835539	0.295509747	1	1	1
PEX7	0.229855066	0.993783765	1	0.933	1
ENSSSCG00000003555	0.229914941	0.788507548	1	1	1
RP2	0.230081469	0.268609276	1	1	1
ENSSSCG00000000635	0.230099641	1.083388251	0.938	1	1
UTP20	0.230279398	0.777403871	0.938	1	1
POU6F1	0.230344948	-0.039670145	1	1	1
SPEF2	0.230352706	1.096715154	0.938	0.733	1
FAM237B	0.230371479	1.854423176	0.5	0.267	1
BCKDK	0.230664292	0.383643879	1	0.933	1
CEP97	0.231015718	1.034269902	1	1	1
ZNF366	0.231210079	1.573647187	0.938	0.733	1
ENSSSCG00000055760	0.231457999	0.625119627	0.938	0.867	1
MYO1H	0.231475703	-0.175571565	0.562	0.667	1
NPL	0.23151208	1.018311878	1	0.867	1
THRA	0.231536714	0.172624151	0.938	0.933	1
SLITRK6	0.231817573	1.047753131	0.938	0.533	1
IL18R1	0.231920533	-0.2255597	0.875	0.667	1
PROSER3	0.231999954	0.343954401	0.938	0.867	1
DCDC2C	0.232032845	1.994353437	0.562	0.133	1
GBGT1	0.23214006	-0.315501826	0.5	0.533	1
RFTN1	0.232141051	1.419960178	0.812	0.867	1
PCED1A	0.232624152	0	0.625	0.8	1
RACGAP1	0.232886684	0.165500992	1	1	1
HECW2	0.232970762	0.234055339	1	1	1
CNTROB	0.233299118	0.789320999	1	0.933	1
PNPO	0.233477246	0.025535092	0.5	0.8	1
PAQR8	0.233532859	-0.520530628	0.625	0.6	1
DNAI7	0.233546576	0.65150664	1	1	1
RNF123	0.233695812	0.059720212	0.938	0.933	1
ENSSSCG00000039073	0.233917093	1.661778098	0.625	0.333	1
CRYBG3	0.233982068	0.844201052	1	1	1
TMOD3	0.233999426	0.668662606	1	1	1
THAP11	0.23424815	0.886022848	1	1	1
PLCG1	0.234261718	0.845634907	1	1	1
ANKDD1B	0.234323084	0.96916374	1	1	1
MYO5C	0.23446579	0.296876133	1	1	1
COLEC12	0.234697266	-0.455679484	0.5	0.467	1
GALNT14	0.234732776	0.278314308	0.938	1	1
ENSSSCG00000053748	0.234816045	1.558967292	0.5	0.333	1
ENSSSCG00000052642	0.234893012	-0.051289229	0.75	0.8	1
DOCK5	0.234938264	0.945544027	1	1	1
ENSSSCG00000008677	0.235093513	1.319787788	1	1	1
NSUN3	0.235132736	-0.483055923	0.625	0.6	1
KCTD19	0.235178184	1.222392421	1	0.733	1
TCF7	0.235247763	1.047753131	1	1	1
SGCE	0.235620162	1.004378441	1	1	1
SCLT1	0.235641836	0.985245414	1	1	1
ARMH4	0.235801051	0.571974398	1	1	1
ZNF829	0.235972465	0.151073222	1	1	1
HYCC1	0.236409086	0.732519689	1	1	1
ENSSSCG00000055844	0.23642498	-0.32443495	0.5	0.6	1
IRF2BP2	0.236802705	1.015666942	0.938	0.667	1
ZNF581	0.237063482	-0.192645078	0.312	0.533	1
HCN1	0.237095725	1.252026082	0.875	0.867	1
CCDC18	0.237808735	1.012367996	1	1	1
THAP6	0.237833331	1.06667104	1	0.867	1
MAPK9	0.237923344	0.320484678	1	1	1
NPM3	0.238265256	1.16753157	1	1	1
DLG3	0.238372835	-0.781165398	0.375	0.667	1
SOS1	0.238399853	0.856114234	1	1	1
LIMK2	0.2387265	0.20968683	0.875	0.933	1
ENSSSCG00000051170	0.238911409	0.098189248	1	1	1

ENSSSCG00000052437	0.238937255	-0.163498732	0.312	0.6	1
ENSSSCG00000052253	0.23894299	1.569855608	0.625	0.2	1
ENSSSCG00000016224	0.239106608	1.080538683	0.875	0.8	1
MASP1	0.239233592	-0.346865997	0.875	0.667	1
SLC25A22	0.239234907	0.252026082	0.875	0.8	1
ENSSSCG00000050796	0.239406404	1.491853096	0.625	0.333	1
TRIM28	0.23941248	0.530585491	1	0.933	1
ENSSSCG00000062625	0.239594957	1.22881869	0.562	0.4	1
COX2	0.239731717	1.186670972	1	1	1
ENSSSCG00000037812	0.240185673	1.236596041	1	0.667	1
DNAJC25	0.240344703	-0.093109404	0.25	0.533	1
ENSSSCG00000058062	0.240742975	1.824428435	0.5	0.4	1
TSTD2	0.240756104	0.92346482	1	1	1
VPS54	0.240827805	-0.182192948	1	0.867	1
CUL5	0.240837582	0.919121609	1	1	1
KLHL32	0.241001262	0.629321601	1	1	1
ENSSSCG00000060550	0.241190334	0.991206882	1	0.933	1
METTL21A	0.241472657	1.029287227	1	1	1
ZNF24	0.24156702	0.903681039	1	1	1
CENPK	0.241615314	0.978876708	1	0.867	1
ENSSSCG00000052279	0.241731237	0.0138058	0.875	0.733	1
DMRT2	0.241762029	0.136587818	0.875	1	1
TMEM14A	0.242168668	0.767834952	1	1	1
ENSSSCG00000042369	0.24224415	-0.306103128	0.625	0.733	1
MICU3	0.242261912	1.791413378	0.625	0.333	1
ENSSSCG00000010432	0.242569416	0.546715031	1	1	1
ENSSSCG00000059054	0.24270276	1.447458977	0.562	0.333	1
ENSSSCG00000044869	0.242806451	1.315355441	0.875	0.8	1
KIAA1549L	0.243004585	0.16754235	0.938	0.933	1
ENSSSCG00000021676	0.243592056	0.239465935	1	0.867	1
RAB33B	0.243691566	0.979040381	0.938	0.8	1
TRMT61B	0.243698723	0.352301744	0.938	1	1
RNF103	0.244400915	0.19479297	1	1	1
ENSSSCG00000057932	0.244804756	-0.562594688	0.5	0.6	1
ENSSSCG00000063049	0.245022836	-0.263034406	0.438	0.533	1
KIT	0.245597534	0.755578779	1	1	1
CDKAL1	0.245604845	0.367599953	1	1	1
PNPLA2	0.24597869	0.078656944	1	0.8	1
RASGEF1A	0.246120479	0.205709577	0.938	1	1
FAM114A1	0.246166615	1.058562758	1	1	1
NEB	0.246265262	0.326917966	1	1	1
SLC9A8	0.24639597	0.112359714	0.938	0.933	1
EIF4E1B	0.246984902	1.022367813	1	1	1
MIGA2	0.247280559	0.217230716	0.438	0.667	1
DAAM1	0.247312694	0.788065299	1	1	1
ASXL1	0.247342423	0.010601945	0.938	0.867	1
NCK1	0.247427255	0.485870938	1	1	1
GCC2	0.247660119	0.898941842	1	1	1
LARP4	0.247777204	0.691328274	1	1	1
CFAP418	0.247857205	1.384507687	1	0.867	1
RNF114	0.248018912	1.220656602	1	1	1
ENSSSCG00000051861	0.248234062	0.894612266	0.938	1	1
MYDGF	0.248704834	1.011511558	1	1	1
MUL1	0.248754635	-0.102154544	0.75	0.733	1
ZDHHC12	0.249017123	0.200621799	0.938	0.933	1
CFAP95	0.249126785	0.906890596	0.875	0.733	1
KAT6B	0.249318762	0.853719937	1	1	1
PLCE1	0.249642144	0.947798539	1	0.933	1
TRANK1	0.249956554	1.158808969	1	0.933	1
PDSS2	0.250227194	0.226169112	0.938	0.933	1
KIF27	0.250596744	0.461479447	1	1	1
PNMA1	0.250752164	0.958504703	1	1	1
TENT5C	0.250860439	0.870030666	1	1	1
CCT7	0.251242129	0.888248189	1	1	1
ENSSSCG00000035836	0.251301216	1.531381461	0.562	0.333	1
ATXN7	0.251360326	0.26524996	1	1	1
MLLT10	0.251591345	0.879409859	1	0.8	1
ENSSSCG00000014779	0.251615399	0.09330372	0.875	0.8	1
GPD1L	0.252005406	0.854646728	1	1	1
ABCA1	0.252014251	0.394747877	1	1	1

ENSSSCG00000042120	0.252043789	-0.415037499	0.375	0.533	1
ENSSSCG00000035561	0.252157745	1.007893914	1	0.933	1
GSC	0.2522768	1.321928095	0.75	0.533	1
TMX3	0.252280255	0.342475472	1	1	1
STX11	0.252390718	0.872457099	0.938	0.867	1
OSBPL9	0.252538532	0.264634391	1	1	1
SEC16A	0.252538802	0.509743255	1	1	1
KIAA0513	0.25259866	0.087462841	1	0.733	1
SEC24C	0.25282545	0.386883537	1	0.933	1
ENSSSCG00000005475	0.252875931	1.564002882	0.5	0.333	1
GAS2	0.253010493	0.317678622	0.938	0.867	1
MARCKSL1	0.25307656	1.987810591	0.688	0.4	1
METTL16	0.253149082	0.659413211	1	1	1
FRAT2	0.253189613	1.299208018	0.812	0.533	1
GOT1	0.253463754	1.249122787	1	0.867	1
HACD2	0.25352632	0.716959902	1	1	1
ENSSSCG00000032896	0.253561488	1.271463028	0.875	0.733	1
EIF4G1	0.25358693	0.340400755	1	0.933	1
DDX19B	0.253724065	0.283453947	0.688	0.933	1
ADAMTS2	0.253753677	1.035720576	1	0.933	1
KMT5C	0.253845006	1.154818109	0.938	0.867	1
ERCC6L2	0.254031956	0.472319209	1	1	1
TTYH3	0.254042972	0.186998515	0.75	0.6	1
C5AR1	0.254119861	0.431157165	0.938	1	1
SIPA1L1	0.254432653	0.242904447	1	1	1
ENSSSCG00000045643	0.254502947	1.362955363	0.875	0.8	1
ENSSSCG00000041350	0.254657374	2.813781191	0.5	0.067	1
ZNF169	0.254806717	0.480625841	1	0.867	1
CLNS1A	0.25480999	0.955389113	1	1	1
SLC16A11	0.254895635	-0.285754482	0.562	0.6	1
GALNT13	0.254904032	0.885453425	1	1	1
ENSSSCG00000056346	0.25491165	-0.093109404	0.75	0.733	1
CHRNA1	0.254929255	-0.16663844	1	1	1
LIX1	0.254930653	0.906890596	0.812	0.4	1
KIF11	0.254945951	0.406611758	1	1	1
THEMIS	0.255051954	1.491853096	0.625	0.4	1
TRIM23	0.255082306	0.159755369	0.938	0.867	1
MIA3	0.255307934	0.249458457	1	1	1
PDE4A	0.255452462	1.409390936	0.562	0.333	1
LRP6	0.255540037	0.810859769	1	1	1
MCM8	0.255841833	0.131013905	1	1	1
ARFGEF1	0.255935723	0.780077508	1	1	1
GPC5	0.256018594	0.280348991	0.812	0.733	1
RIGI	0.256088616	0.632048459	1	1	1
ENSSSCG00000062580	0.256612387	1.417852515	0.688	0.533	1
ENSSSCG00000060507	0.256629895	1.398743692	0.875	0.6	1
POLR2A	0.256729038	1.309393741	1	1	1
AARD	0.25709506	-0.093109404	0.688	0.733	1
C4orf17	0.257643267	0.376375879	0.938	0.933	1
DYNC2H1	0.257753741	1.199071347	1	1	1
NOS1	0.2580049	0.138216142	0.438	0.533	1
THAP4	0.258152376	-0.093109404	0.625	0.6	1
PKD1	0.258374627	-0.049607766	0.812	0.867	1
ENSSSCG00000051679	0.258739246	-0.200024608	0.438	0.533	1
LMTK2	0.258978437	-0.222392421	0.625	0.667	1
PRR14L	0.259259163	0.852255339	1	1	1
C11orf87	0.259304751	1.252665432	0.75	0.667	1
SCAMP1	0.259311411	0.520151708	1	1	1
ENTREP3	0.259359996	-0.034215715	0.438	0.533	1
ZNF461	0.259461475	-0.545621609	0.625	0.6	1
ENSSSCG00000033232	0.259538649	-0.093109404	0.625	0.733	1
SYT11	0.25984394	0.0138058	0.75	0.867	1
PGRMC1	0.259846376	1.024542184	1	1	1
IGDCC4	0.260234941	1.607330314	0.5	0.4	1
TRAF3	0.260680924	0.423681594	1	1	1
ZNF513	0.260913502	1.083213368	1	0.533	1
THSD7A	0.260985786	0.499524576	1	1	1
ENSSSCG00000060492	0.261006015	0.906890596	1	1	1
ZBTB2	0.261055575	0.809866141	1	0.933	1
CNKSR2	0.261186942	1.047068254	0.938	0.667	1

LPCAT3	0.261531754	-0.451563375	0.625	0.667	1
SERTAD3	0.261581802	1.125900378	1	1	1
DIPK2A	0.261643532	1.053600883	1	1	1
ENSSSCG00000062726	0.261691006	0.136896201	1	0.933	1
ENSSSCG00000041277	0.261902402	-0.130723696	1	1	1
SETD2	0.262005631	-0.718069973	0.75	0.533	1
MSANTD7	0.262685962	0.803472836	1	1	1
ZIC3	0.262749964	0.082570272	1	1	1
FRMPD1	0.262767385	1.111249094	0.938	0.8	1
ZDHHC8	0.262806432	0.254813899	0.812	0.733	1
CIC	0.263097737	1.33315535	0.812	0.467	1
ENSSSCG00000018079	0.263133769	0.099535674	1	0.933	1
USP42	0.263174424	0.043603818	0.938	1	1
ANTXR2	0.263215404	0.445058885	1	0.933	1
TSPYL2	0.263315304	0.378196315	0.75	0.867	1
ENSSSCG00000061416	0.263424666	0.769387072	0.938	0.867	1
PPP3CC	0.263498426	0.405800652	1	0.933	1
DDX42	0.263516464	0.871073044	1	1	1
ENSSSCG00000055991	0.263776996	1.305439972	0.625	0.4	1
ATF2	0.264040348	1.079499682	1	1	1
ZSWIM5	0.264241005	-0.174409506	0.812	0.8	1
ADCY3	0.264347299	0.820682454	1	1	1
ENSSSCG00000062269	0.26443004	1.218092284	0.875	0.667	1
NPRL2	0.264636743	0.922093002	1	1	1
CYBRD1	0.264655073	1.723178642	0.688	0.467	1
LSM14A	0.264665496	0.79470045	1	1	1
ENSSSCG00000025711	0.264813444	0.34227674	1	1	1
RPUSD4	0.26486518	0.226827393	1	1	1
C15orf61	0.265283257	-0.128733314	0.812	0.867	1
VPS37B	0.265374188	-0.093109404	1	0.933	1
GNE	0.265436508	0.856280241	1	1	1
ENSSSCG00000063549	0.26560288	1.26052755	0.875	0.533	1
ACACB	0.265618655	1.076815597	1	0.667	1
ENSSSCG00000022957	0.265668019	0.219274918	0.875	0.933	1
RBAK	0.265818123	-0.480132528	0.5	0.4	1
PRPF39	0.266156638	0.472794761	0.875	0.933	1
ENSSSCG00000057934	0.266181237	-0.253574077	0.562	0.467	1
TIAM2	0.26646926	0.388423507	1	1	1
PTGDR	0.266644651	1.17990909	0.938	0.867	1
NINJ1	0.266900174	0.824428435	0.938	0.533	1
ENSSSCG00000057414	0.267029528	-0.163498732	0.75	0.667	1
BTG1	0.267260351	1.001749782	0.938	0.867	1
GPATCH4	0.267426923	0.062541466	0.812	0.933	1
RUBCNL	0.267440596	-0.017821277	0.75	0.8	1
DHX29	0.26766562	0.13289427	1	1	1
EPB41	0.267674594	0.956875345	1	1	1
ENSSSCG00000009341	0.267780601	0.293242352	1	1	1
COL1A2	0.268054818	1.030273011	1	0.933	1
INO80	0.268056091	0.890173201	1	1	1
ENSSSCG00000037609	0.268113333	0.33752495	0.5	0.6	1
PHOSPHO2	0.268138885	0.805305409	1	1	1
LARP1	0.268708042	0.147657771	1	1	1
CPSF2	0.268815675	0.750000112	1	1	1
NPHP1	0.268885262	1.380821784	0.625	0.267	1
NMI	0.268921508	-0.855417847	0.75	0.533	1
ENSSSCG00000006324	0.26894876	0.961052175	1	1	1
ZNF143	0.268981617	0.747442311	1	1	1
CCDC146	0.269060996	-0.035393907	0.875	1	1
ENSSSCG00000018077	0.269300584	-0.386840607	0.75	0.533	1
COLGALT1	0.269308928	0.419740118	1	0.933	1
TMEM87A	0.269934871	0.916481955	1	1	1
AGPAT5	0.269955513	0.429103831	1	1	1
CDK2AP2	0.269963971	0.473970391	1	1	1
TLR7	0.26996786	0.408717252	0.938	1	1
ARHGAP35	0.270091465	0.753846221	1	1	1
POLA1	0.270138414	0.719846724	1	1	1
ADAMTS3	0.270269252	1.17990909	0.625	0.267	1
SECISBP2L	0.270318836	0.744787649	1	1	1
XK	0.270328548	0.445866891	1	1	1
DHX8	0.270556809	0.268056934	1	1	1

ENSSSCG00000042928	0.270652161	-0.093109404	0.5	0.6	1
VXN	0.270684695	0.884170519	1	1	1
PIM2	0.270686322	0.415795018	1	1	1
GTPBP2	0.270786395	0.82799505	1	1	1
DDX1	0.271012196	0.287759733	1	1	1
ENSSSCG00000047889	0.271202977	0.174112798	1	1	1
CDC73	0.271339261	0.97549441	1	0.933	1
HM13	0.271478253	0.829928614	0.938	0.867	1
ZNF217	0.271511324	0.922386932	1	1	1
MGRN1	0.271531582	1.483651283	1	0.933	1
NIP7	0.271818024	0.700807143	1	1	1
MDFIC	0.271915434	0.273948066	1	0.867	1
UNC5C	0.271927317	0.076075264	1	1	1
ATF6	0.272482124	0.800517741	1	1	1
PPM1K	0.272671744	-0.35614381	0.5	0.6	1
C1orf35	0.27301546	0.89077093	1	1	1
LSM5	0.273150324	0.693445879	1	1	1
LMAN2	0.273253839	0.600009052	1	1	1
TEFM	0.273301943	1.097712938	0.875	0.8	1
BAZ1B	0.273602313	0.889179724	1	1	1
ENSSSCG00000056010	0.273642128	0.009984089	0.438	0.533	1
ENSSSCG00000062352	0.273747484	-0.042483331	0.375	0.6	1
AADAC	0.273813099	1.147898695	0.875	0.6	1
EMC1	0.273982263	0.885426086	1	1	1
CLUAP1	0.274055002	1.067674943	1	1	1
DNAI3	0.274211628	-0.025995209	0.5	0.733	1
TDRD6	0.274436666	0.810525498	1	1	1
LBR	0.27463775	0.751169214	1	1	1
HIPK3	0.27475068	0.390099598	1	1	1
TECTA	0.274917686	0.418940725	1	1	1
ENSSSCG00000045922	0.27502312	0.121015401	0.5	0.667	1
EMSY	0.275131109	0.818582177	1	1	1
ENSSSCG00000051999	0.275187527	-0.253574077	0.438	0.6	1
PLA2G10	0.275292261	1.409390936	0.5	0.2	1
SIK1	0.275428062	0.129283017	0.562	0.6	1
AKAP7	0.275626804	-0.093109404	0.562	0.533	1
PLP2	0.275723861	-0.455679484	0.25	0.533	1
MAP3K14	0.275774549	-0.327574658	0.438	0.533	1
RAI14	0.275783019	0.430551641	1	1	1
TDRD1	0.275801329	0.762880293	1	1	1
DISP2	0.275845586	0.15264701	0.938	0.933	1
PHKA1	0.275847084	0.91906531	1	0.667	1
YWHAE	0.276697255	0.297259747	1	1	1
RNF144B	0.276704824	0.426897654	1	1	1
ENSSSCG00000045636	0.276745756	-0.66247505	0.5	0.533	1
PJA2	0.276997838	0.293583469	1	1	1
ENSSSCG00000041432	0.277124506	1.087462841	0.75	0.333	1
IGF2BP2	0.27718135	0.27409957	0.938	0.8	1
ENSSSCG00000063045	0.27721485	-0.108791027	1	0.933	1
ENSSSCG00000015401	0.277228091	0.297266041	1	1	1
MLLT3	0.277262417	0.315793978	1	1	1
TMEM35B	0.277368439	1.076815597	0.812	0.8	1
TOP3A	0.277717289	-0.526068812	0.438	0.533	1
ENSSSCG00000007919	0.277872668	-0.042483331	0.562	0.6	1
IL1R2	0.278299645	0.624608305	0.938	0.933	1
AP3B2	0.278410894	0.428843299	1	0.933	1
ASAP2	0.278470324	1.171718283	1	0.933	1
MCUB	0.278516857	1.0878928	1	1	1
ENSSSCG00000053166	0.278779587	-0.35614381	0.5	0.6	1
MDH2	0.278842136	1.156527853	1	1	1
KCNQ5	0.278871311	1.331388424	0.75	0.333	1
MTR	0.278928534	0.201565081	1	1	1
KLHL28	0.278971172	1.256873421	1	1	1
RLIM	0.279172133	0.189577059	1	1	1
ITGA2	0.279190532	0.805403528	1	1	1
CASP7	0.279319208	0.270669975	1	1	1
ENSSSCG00000060901	0.279327681	1.299208018	0.625	0.333	1
FAM120B	0.279536985	0.860616087	1	1	1
DAXX	0.280165571	1.181253007	1	0.8	1
FDXACB1	0.280368428	-0.275312736	0.438	0.533	1

PLPP2	0.280447641	-0.204140717	0.5	0.667	1
LRP4	0.280453206	0.130946833	0.938	1	1
CHMP7	0.280745417	0.742287465	1	1	1
CHD6	0.280821552	0.428699368	1	1	1
ITPA	0.280830047	0.859193854	0.938	1	1
ENSSSCG00000039473	0.28095802	0.729366381	1	1	1
RC3H2	0.281076532	-0.104514168	0.938	0.933	1
STIP1	0.281100753	0.777625634	1	1	1
PCSK6	0.281164991	1.196397213	0.75	0.467	1
CHD1L	0.281202159	0.412189569	1	1	1
ENSSSCG00000052814	0.281323033	0.33149598	1	1	1
TMEM19	0.281323772	0.052321035	0.875	0.733	1
DOCK8	0.281472572	0.29281969	1	1	1
ME1	0.281545222	0.398205568	1	1	1
CDC42BPA	0.281937911	0.400655306	1	1	1
ENSSSCG00000048959	0.282201369	-0.489038081	0.5	0.667	1
ENSSSCG00000042915	0.282392073	-0.315501826	0.375	0.6	1
ENSSSCG00000018061	0.282392881	0.928451584	1	1	1
DCP2	0.282584302	0.45888605	1	1	1
HHIP	0.28263564	0.791898073	1	1	1
STAT1	0.282652483	0.370616458	1	1	1
MAP3K9	0.282742821	0.396695863	0.75	0.667	1
ST13	0.282859138	0.889995977	1	1	1
PTPN11	0.283278947	0.575896692	1	1	1
DTNA	0.28360545	0.782711184	1	0.933	1
LYRM2	0.283649096	-0.062521085	0.875	0.933	1
SS18L1	0.283770377	1.147898695	0.625	0.333	1
FNIP2	0.284416233	0.28128611	0.812	1	1
HOMER1	0.284557306	-0.018762063	0.938	0.933	1
MAP9	0.284642576	1.043786797	1	1	1
UHRF2	0.284679909	0.542275785	1	1	1
KDELR1	0.284819239	0.651188836	0.938	1	1
MSR1	0.285210057	1.049848549	0.938	0.933	1
CTCF	0.285355723	0.519499344	1	1	1
GNAI2	0.285379681	0.651893509	1	1	1
TTLL1	0.285509951	0.339586761	1	0.933	1
MT1X	0.285734279	0.196397213	1	1	1
ENSSSCG00000062174	0.285760516	0.748785122	1	1	1
ENSSSCG00000063055	0.285832527	0.781359714	0.938	0.467	1
GJB2	0.285991449	0.058893689	0.5	0.533	1
NHSL1	0.286024445	0.755966556	1	1	1
PHF3	0.286300431	0.707766705	1	1	1
ENSSSCG00000061382	0.28644733	-0.093109404	0.5	0.667	1
PHACTR4	0.286643641	0.185191758	1	0.933	1
COPG2	0.287082146	0.950239503	1	1	1
C1orf185	0.287094032	1.491853096	0.5	0.133	1
ARHGAP26	0.287183973	0.366009163	1	1	1
ENSSSCG00000051054	0.287188161	1.282181916	0.875	0.667	1
AKAP17A	0.287241533	0.334687326	1	1	1
ENSSSCG00000008459	0.287441269	0.913744267	0.938	0.867	1
PATZ1	0.287520541	0.269460675	0.562	0.733	1
CRTC3	0.287763655	0.016012318	0.938	0.867	1
LRRC41	0.288159957	1.576741994	0.562	0.333	1
CFAP300	0.288216252	0.857980995	0.938	1	1
ENSSSCG00000053107	0.288339993	0.845490051	0.688	0.267	1
ZNF619	0.288831396	-0.706086281	0.5	0.6	1
INPPL1	0.288838024	1.458300537	0.812	0.733	1
ENSSSCG00000037699	0.288868392	-0.245112498	0.5	0.4	1
SLC39A14	0.289196561	0.45538154	1	1	1
C2orf68	0.289361579	-0.093109404	0.5	0.4	1
TNPO2	0.28941721	0.921277701	0.938	1	1
AVPR1A	0.289459742	-0.817474962	0.562	0.267	1
ENSSSCG00000027301	0.289501684	0.924145626	1	1	1
HDGFL3	0.289607509	0.84395934	1	1	1
GRAMD1B	0.289886546	-0.150824902	0.625	0.8	1
RNF187	0.289950187	0.0138058	0.438	0.533	1
AGBL1	0.290022722	0.309523074	0.938	0.933	1
REXO1	0.290027181	1.176979759	1	0.867	1
GTPBP4	0.290031343	0.40092413	0.938	1	1
ENSSSCG00000058386	0.290062368	1.64385619	0.562	0.533	1

TSSK4	0.290078201	0.212993723	0.875	0.933	1
ENSSSCG00000052824	0.29007964	-0.093109404	0.688	0.667	1
CREBL2	0.290294367	1.198056437	0.938	0.933	1
MRPL4	0.290327844	0.451855028	1	1	1
MSL2	0.290442996	0.365661099	1	1	1
CD83	0.29044769	-0.590609064	0.188	0.533	1
SPATA17	0.291070525	-0.093109404	0.812	0.667	1
ENSSSCG00000045074	0.291084631	-0.12963528	0.562	0.6	1
SPPL3	0.291112838	0.631163776	1	1	1
ENSSSCG00000002937	0.29143356	-0.081925666	0.938	0.867	1
ZNF630	0.29159468	0.787949523	0.875	0.8	1
ENSSSCG00000044342	0.292155352	0.099535674	0.562	0.6	1
SUCO	0.292733165	0.915286517	1	1	1
SMG5	0.292887821	-0.215099929	0.875	1	1
FARP1	0.292909602	0.525349366	1	1	1
ENSSSCG00000042401	0.293098446	-0.623624121	0.625	0.667	1
STIM2	0.293196061	0.22881869	0.875	0.867	1
GTF2A1	0.293419143	0.32214024	1	1	1
ENSSSCG00000003741	0.293453897	1.021710998	0.938	0.867	1
ENSSSCG00000052854	0.29397635	-0.441032708	0.438	0.533	1
BECN1	0.29425058	0.868416448	1	1	1
ARSB	0.294445649	0.338606836	0.875	0.8	1
ATP6V1A	0.294723542	0.389518789	1	1	1
FGL2	0.294745809	0.867719998	0.938	0.933	1
CCNL1	0.294845234	0.367405725	1	1	1
SNRPG	0.294866862	0.737089959	1	1	1
SMOX	0.294922384	-0.363198568	0.5	0.533	1
ENSSSCG00000009399	0.294970021	-0.134332067	0.75	0.733	1
ENSSSCG00000055398	0.295112929	0.027184829	0.625	0.533	1
ENSSSCG00000005643	0.295125139	0.386331128	1	1	1
CDK2	0.295187455	0.254458512	1	1	1
MYD88	0.29522353	1.196397213	0.75	0.6	1
ADCY6	0.295466727	1.186250617	1	1	1
SAAL1	0.29578646	0.348596045	0.875	0.933	1
PITPNM2	0.295895121	-0.167109986	0.438	0.6	1
TADA2B	0.29609508	1.036173613	0.875	0.667	1
BRD4	0.296222053	0.956478605	1	1	1
SMAD5	0.296347389	0.827496454	1	1	1
SLITRK1	0.296575942	1.94641896	0.5	0.267	1
FZD5	0.297052865	0.372265744	1	1	1
FBXO45	0.297112314	0.022367813	1	0.867	1
MYLK3	0.297119427	0.022367813	0.812	0.6	1
PEX14	0.297404756	0.150816178	0.875	0.867	1
TTLL5	0.297479619	1.108297992	1	1	1
CASP2	0.297506333	0.513439959	1	0.933	1
ENSSSCG00000014219	0.297614427	1.442943496	0.562	0.333	1
ENSSSCG00000056190	0.297732599	1.366322214	0.5	0.4	1
PKP4	0.297961268	0.269460675	1	1	1
SCML4	0.297972239	0.780133453	1	1	1
ACADSB	0.298456891	-0.322591251	0.812	0.667	1
FEZ1	0.298495424	0.901199484	1	1	1
ENSSSCG00000046349	0.298701139	0.285402219	0.438	0.533	1
ENSSSCG00000059120	0.298719716	0.345774837	0.938	0.933	1
EPCAM	0.298764215	0.324623882	1	1	1
INPP5D	0.299159151	0.123208503	0.938	0.8	1
QTRT2	0.299431664	1.133166451	1	0.733	1
LRRIQ4	0.299573789	-0.405053411	0.688	0.6	1
PLK4	0.299709641	0.725994476	1	1	1
ENSSSCG00000053762	0.299710802	0.516944077	0.438	0.533	1
KATNAL1	0.29976718	0.31187443	1	0.933	1
ABHD17C	0.299839241	0.462105753	1	1	1
BLTP3B	0.300000167	0.898458689	1	1	1
MACC1	0.300077361	0.878719923	0.938	0.933	1
UBE2Q1	0.300276478	0.309969631	1	1	1
RECQL	0.300369388	0.18758637	0.938	1	1
MOSMO	0.300379373	-0.552541023	0.5	0.4	1
ENSSSCG00000056369	0.300414899	1.33752495	0.5	0.467	1
CMBL	0.300604163	0.890649955	1	1	1
PHETA2	0.300721466	0.062168821	0.75	0.733	1
MOS	0.300873027	0.539087595	1	1	1

ENSSSCG00000009672	0.301119367	0.41612577	1	0.933	1
DOLK	0.30113252	0.931373281	1	1	1
RRN3	0.301146327	0.636213495	1	1	1
ZNF112	0.301370957	0.076815597	0.625	0.6	1
BMP2K	0.301626678	0.852882493	1	1	1
TRIP13	0.301767293	1.074618041	1	1	1
RLBP1	0.302239216	-0.490444902	0.688	0.6	1
GFM1	0.30242208	0.923569337	1	1	1
KBTBD3	0.302777059	-0.165621904	0.938	0.867	1
SYNCRIP	0.30299733	0.90194399	1	1	1
PRPF40A	0.303139849	0.906407793	1	1	1
OTUD7B	0.303301153	0.0138058	0.938	0.867	1
ZNF667	0.30364198	0.459919098	1	1	1
ZNF648	0.303746032	-0.200024608	0.5	0.4	1
KCNH2	0.303872984	1.299208018	0.688	0.533	1
GNB5	0.304167617	0.921733932	1	1	1
ENSSSCG00000058262	0.304307346	1.072008212	1	0.867	1
TMEM214	0.304320122	0.163766209	1	0.933	1
GABRR1	0.304597062	0.967482001	1	1	1
SLITRK3	0.304905469	-0.341036918	0.75	0.867	1
LSM8	0.305022669	0.340075442	1	1	1
SLC33A1	0.305099749	0.993086247	0.938	0.867	1
COPS2	0.305310773	0.148057679	1	1	1
NPNT	0.305441491	-0.256608137	0.562	0.467	1
IARS2	0.305695484	0.954023615	1	1	1
IL13RA1	0.30576602	0.465275845	1	1	1
SAP30L	0.306077193	0.764033837	1	1	1
ENSSSCG00000063185	0.306895864	1.138216142	0.625	0.333	1
SPAG6	0.307054419	0.087462841	0.812	0.8	1
WDR59	0.307112658	0.11042399	1	1	1
BAG6	0.307507088	0.889417289	1	1	1
FHIP1B	0.307762577	0.279802619	1	1	1
ENSSSCG00000063467	0.307819845	0.696714124	1	1	1
CDH13	0.308427551	0.429763092	1	1	1
GDNF	0.308481647	-0.093109404	0.688	0.733	1
DENND1B	0.308858377	0.101063143	1	1	1
HSPB8	0.308928144	1.327466278	0.938	0.867	1
PDCL	0.309043576	0.420468463	1	1	1
ABHD11	0.309212199	0.142778859	1	0.933	1
ENSSSCG00000040688	0.309261843	0.775061943	1	1	1
THAP1	0.309305897	0.406637094	1	1	1
SLC30A1	0.309701621	0.79058171	1	1	1
PPP2R5D	0.309769083	0.436143664	1	1	1
TOR1AIP1	0.309985423	0.918712304	1	1	1
ENSSSCG00000053805	0.31010221	-0.186218809	0.5	0.533	1
AZI2	0.310459266	0.328717261	0.938	0.933	1
PDPK1	0.310495448	0.413953337	1	1	1
ENSSSCG00000044256	0.310566186	0.141355849	0.688	0.733	1
RTF1	0.310944288	0.25402403	1	1	1
MTF1	0.310951697	0.247304261	1	1	1
CREB3L2	0.311111953	0.878656915	1	0.933	1
EIF4ENIF1	0.31145534	0.873345396	1	1	1
DSP	0.31158528	1.150619479	0.938	0.933	1
GPATCH2	0.311683389	0.449683642	0.938	1	1
ENSSSCG00000055825	0.311895074	-0.397963986	0.625	0.533	1
TMF1	0.312066984	0.873925832	1	1	1
DLGAP5	0.31241498	0.528867939	1	1	1
PLEKHM3	0.312621215	0.407544877	1	1	1
WDR45	0.312673214	0.777677802	1	1	1
ENSSSCG00000050930	0.312749597	1.154818109	0.688	0.467	1
AGPAT1	0.312985764	1.0138058	1	0.733	1
MTPAP	0.313058339	0.750040936	1	1	1
HEBP1	0.313106186	1.22881869	0.5	0.267	1
FHOD3	0.313396889	1.113935053	0.938	0.733	1
WDFY1	0.313487425	0.243864985	1	1	1
MDN1	0.313508243	0.372924949	1	1	1
PHAF1	0.313563208	0.997887708	0.938	1	1
CRACDL	0.313602824	-0.493647334	0.5	0.533	1
HAPSTR1	0.314066285	0.284064532	1	1	1
SMC2	0.314141109	0.78696344	1	1	1

ENSSSCG00000052455	0.314225279	1.254813899	0.562	0.4	1
CLK2	0.314493315	0.978443857	1	1	1
SPPL2B	0.314503656	0.308730793	1	0.933	1
ENSSSCG00000058381	0.314752006	-0.032567862	0.625	0.867	1
ENSSSCG00000054974	0.315381845	-0.35614381	0.5	0.4	1
DHX33	0.315396035	-0.021674266	1	0.867	1
TMTC2	0.315705291	0.948274516	1	1	1
SH3KBP1	0.315776017	0.914204655	1	1	1
ZBED5	0.315879541	0.093511886	0.938	1	1
RANBP6	0.315921107	1.181065559	0.875	0.667	1
ABTB3	0.316032426	0.261650083	1	1	1
ENSSSCG00000042640	0.316201241	1.108524457	0.938	0.8	1
ENSSSCG00000035362	0.316260626	0.34298971	0.625	0.867	1
LDHB	0.316456117	1.26052755	1	0.667	1
ZNF791	0.316758601	-0.332297068	0.812	0.6	1
URB2	0.316893552	0.91675634	1	1	1
CAPN13	0.316981374	0.47217073	1	1	1
PIAS4	0.31707595	1.103963386	1	1	1
CDK13	0.317137458	0.347039873	1	1	1
FASTKD3	0.317191327	0.051280505	0.75	0.8	1
CAB39L	0.31792647	0.847389584	1	1	1
COL15A1	0.317946852	0.913616459	0.812	0.867	1
DSTYK	0.318016115	-0.32443495	0.562	0.533	1
SIX5	0.318366477	1.569855608	0.562	0.333	1
PSMF1	0.318481852	0.168430755	0.938	0.867	1
PDGFRA	0.318543624	-0.037614292	0.875	0.6	1
YARS2	0.319093258	1.041090867	1	0.8	1
THRAP3	0.319109678	0.923764414	1	1	1
ENSSSCG00000060599	0.320257036	-0.346865997	0.5	0.6	1
AP3M2	0.3203323	0.234944793	0.938	1	1
TIMELESS	0.320739774	0.803990688	0.938	1	1
BMT2	0.32074761	1.048551745	0.938	0.8	1
SLTM	0.320792569	0.455863239	1	1	1
POLR1F	0.321226553	0.25100131	1	1	1
PARD3	0.321253476	0.974209705	1	1	1
COL24A1	0.321838011	0.764150423	1	1	1
CADM2	0.321875025	1.401655287	0.688	0.333	1
BMF	0.321915653	0.334752135	1	1	1
CENPC	0.321970223	0.746865361	1	1	1
SLC9A9	0.322526223	0.418536419	0.938	1	1
VPS13D	0.323027347	0.881712033	1	1	1
INTS5	0.323096791	1.129283017	0.562	0.333	1
PIK3R4	0.323298143	0.748157717	1	1	1
ENSSSCG00000031883	0.323318485	1.183730801	0.812	0.6	1
AKT2	0.323592772	0.47436567	1	1	1
ENSSSCG00000061502	0.32366712	1.053731984	0.688	0.267	1
OTULIN	0.323697489	0.410028873	1	1	1
TOPORS	0.323786451	0.833796372	1	1	1
MTIF2	0.323872231	-0.180572246	0.75	0.6	1
FAM117A	0.323888783	0.284257677	0.938	1	1
SCMH1	0.324312871	1.100901216	0.938	0.933	1
ENSSSCG00000030335	0.324464559	0.767673657	1	1	1
ENSSSCG00000059464	0.324487124	0.070389328	0.688	0.667	1
ZFP64	0.324616906	0.038135129	0.875	0.667	1
KBTBD2	0.324704761	0.830718915	0.938	1	1
ENSSSCG00000010278	0.324814929	0.312147074	0.938	1	1
TP53BP2	0.324916037	1.043688817	1	0.933	1
SRSF2	0.325284457	1.189824559	0.938	0.733	1
ENSSSCG00000061969	0.325296095	1.347463187	0.562	0.467	1
TUFT1	0.32557816	0.395006093	1	1	1
EPC1	0.325754346	0.881279952	1	1	1
GZF1	0.325818197	-0.019108823	0.5	0.667	1
FAM120C	0.325838977	-0.15298086	0.875	0.933	1
IFFO2	0.325896042	0.121015401	0.812	0.867	1
LHFPL3	0.325944435	1.006426269	0.75	0.8	1
NAV2	0.326128306	0.884067825	1	1	1
LMLN	0.326295846	0.123701985	0.875	0.867	1
METTL22	0.326545421	0.168665556	1	0.933	1
RSF1	0.326727494	1.001012773	0.938	0.933	1
UQCRC2	0.326802273	-0.054635257	0.812	0.8	1

B4GALNT1	0.326917635	-0.064540252	0.688	0.733	1
STON2	0.327062453	0.443376023	1	1	1
FGFR1	0.327104801	0.906890596	1	0.733	1
ENSSSCG00000053970	0.327144052	0.413075983	0.938	1	1
PITPNB	0.327204092	0.438002699	1	1	1
ZBTB33	0.327205552	0.759167529	1	1	1
COQ7	0.327497209	0.215143754	1	1	1
STAU2	0.327511828	0.837181624	1	0.933	1
PDIA6	0.327638933	1.154464897	1	1	1
HRG	0.327803932	-0.072645302	0.75	0.867	1
KIF14	0.327817256	0.939123103	1	1	1
ENSSSCG00000039071	0.327868257	1.435819061	0.875	0.733	1
LETM1	0.327885866	0.344099476	1	0.933	1
CAPN14	0.328116515	-0.483055923	0.75	0.6	1
ENSSSCG00000024411	0.328145075	0.893215659	1	0.933	1
UBE3C	0.328241873	1.202346479	0.5	0.4	1
YTHDC2	0.328408733	0.984166309	1	1	1
SAMD4B	0.328765974	0.505242474	1	1	1
PATJ	0.329093237	0.759761164	1	1	1
ENSSSCG00000055520	0.329535245	0.030273011	0.75	0.733	1
DYNLT1	0.329571747	0.232405999	1	1	1
BMP4	0.329703022	0.5585134	1	1	1
C20orf27	0.329998607	0.412125904	1	0.933	1
TLCD4	0.330148505	0.343697996	1	1	1
N4BP2L1	0.330229164	0.193771743	1	1	1
DDIAS	0.330408787	0.522628475	1	1	1
ENSSSCG00000055646	0.33046609	1.321928095	0.625	0.533	1
ENSSSCG00000028810	0.330731924	0.091315167	0.688	0.533	1
PLPPR2	0.330822385	0.491853096	0.875	0.933	1
CWC27	0.331057682	0.704654371	1	1	1
ENSSSCG00000003791	0.331153215	0.79733067	1	1	1
ZNF662	0.331323024	0.342029301	1	1	1
ENSSSCG00000051810	0.331361582	0.850307067	0.938	0.8	1
KCTD6	0.331752179	-0.093109404	0.875	0.733	1
ANAPC5	0.331813056	1.271586976	1	0.933	1
ENSSSCG00000014186	0.332030086	0.032421478	0.625	0.667	1
ENSSSCG00000011489	0.332531776	1.046053343	0.938	0.733	1
ZKSCAN2	0.332664562	-0.321378392	0.75	0.733	1
ENSSSCG00000059700	0.332715776	1.186998515	0.812	0.8	1
ENSSSCG00000053210	0.332752084	0.141355849	0.562	0.733	1
USP43	0.332813137	1.115477217	0.938	0.867	1
NLE1	0.333261305	0.153530563	0.875	0.867	1
ENSSSCG00000032483	0.333464509	1.124695747	0.875	1	1
USP10	0.333713104	0.922312782	1	1	1
FUCA2	0.333852274	0.300035489	1	0.933	1
ENSSSCG00000055963	0.333934013	0.846769603	0.938	0.933	1
ENSSSCG00000049656	0.334567707	0.447023315	1	1	1
TSPAN2	0.334733431	0.932686596	1	1	1
ASTE1	0.334891979	0.360608563	0.812	0.933	1
ZNF177	0.335118984	-0.163498732	0.625	0.733	1
ENSSSCG00000035223	0.335225678	-0.180572246	0.5	0.4	1
CRELD2	0.335245953	1.146124006	1	1	1
EPRS1	0.335354828	0.419564236	1	1	1
ENSSSCG00000031706	0.335487769	0.289772857	1	1	1
ITGBL1	0.33556332	0.512611656	0.812	0.467	1
MBD1	0.335855659	1.099535674	1	0.933	1
LRP1B	0.335926932	0.976003767	1	0.933	1
RIMS2	0.335933447	0.455390646	1	1	1
FBXL5	0.336114056	0.617863222	1	1	1
ADAM29	0.336136374	-0.446746359	0.5	0.4	1
ITGB1	0.336277203	0.262071696	1	1	1
P4HA1	0.336828777	0.52968517	1	1	1
DUSP3	0.336895927	0.696967526	1	1	1
MTUS1	0.336904725	0.71052704	1	1	1
ACSL3	0.337127139	0.700798448	1	1	1
NME6	0.337490061	0.162147651	0.875	0.933	1
RMND1	0.337638824	1.134996668	1	1	1
ENSSSCG00000056149	0.337714418	1.106199404	0.875	0.867	1
ENSSSCG00000047137	0.337900003	-0.042483331	0.625	0.467	1
GPR180	0.338002856	0.922157352	1	0.867	1

UBE2T	0.338003099	0.4606772	1	1	1
TRPM3	0.338159665	0.675697456	1	1	1
ENC1	0.338229336	0.171075017	1	0.933	1
CRMP1	0.338255983	1.076815597	0.875	0.667	1
ENSSSCG00000043804	0.338341742	0.087462841	0.5	0.667	1
ENSSSCG00000056589	0.338674541	-0.526068812	0.438	0.6	1
GLYATL3	0.338785592	0.780537196	1	1	1
SLC15A4	0.338865451	1.023614202	1	0.933	1
TPGS1	0.33910096	-0.180572246	0.562	0.533	1
ENSSSCG00000026045	0.339320997	0.759628105	0.938	1	1
ENSSSCG00000062614	0.339336875	0.823232667	0.938	0.933	1
HEXB	0.33934963	0.445088629	1	0.933	1
ITGB6	0.339718569	0.906890596	0.562	0.2	1
FAM234B	0.340158884	-0.093109404	0.5	0.333	1
C2	0.34051464	0.172624151	1	0.933	1
ENSSSCG00000056226	0.340530528	0.25354112	0.938	0.933	1
FUBP3	0.340794248	0.984893108	0.875	0.6	1
PKMYT1	0.341048917	1.335733894	0.688	0.467	1
CHRDL1	0.341442252	0.238096504	0.75	0.733	1
PALM2AKAP2	0.341537091	0.285923141	0.938	1	1
RHOU	0.341728698	0.083768358	0.938	1	1
ENSSSCG00000045748	0.342194678	1.714245518	0.5	0.133	1
CEP104	0.342251914	0.983616386	1	0.8	1
ENSSSCG00000042530	0.342279823	0.34298971	0.625	0.667	1
ENSSSCG00000056152	0.342568488	1.398743692	0.688	0.533	1
ZBED6	0.342910057	-0.069650432	0.812	0.733	1
ENSSSCG00000007004	0.343156592	0.878350182	1	1	1
ZSWIM4	0.343324158	0.963474124	0.938	0.6	1
MTA1	0.343688196	-0.093109404	0.375	0.533	1
NONO	0.343762532	0.182806293	1	1	1
INTS6	0.344150424	0.909843913	1	0.933	1
SPTSSA	0.344476877	0.454953514	1	0.8	1
TDRD3	0.344733029	0.185426095	0.875	0.933	1
NFRKB	0.345082243	0.971407937	1	1	1
ATF7IP	0.345115733	0.842660061	1	1	1
KMO	0.345123978	1.206450877	0.688	0.6	1
GSDMA	0.345723854	-0.415037499	0.312	0.533	1
ZNF550	0.345898296	-0.192645078	0.562	0.533	1
ARHGEF10L	0.345990079	0.145050333	0.812	0.6	1
ZMYM4	0.346013792	0.187490874	0.938	1	1
ANKRD16	0.346349952	1.196397213	0.875	0.733	1
TRIM71	0.346422792	0.31046898	1	0.933	1
NEDD9	0.346544848	0.084982918	0.938	0.867	1
VDAC1	0.346740253	1.110685798	1	1	1
ENSSSCG00000038757	0.347136233	0.735812821	1	0.933	1
MINAR1	0.34716615	1.861086906	0.562	0.267	1
ENSSSCG00000048697	0.347405963	-0.140415119	0.562	0.733	1
DCT	0.347569653	0.900662485	1	1	1
ENSSSCG00000032353	0.347795473	1.273672926	0.812	0.533	1
KIF3B	0.348029949	-0.03196911	1	1	1
ENSSSCG00000036234	0.348163954	0.64385619	1	0.933	1
ENSSSCG00000062901	0.348550069	-0.373217324	0.5	0.333	1
CDC20B	0.348633298	0.445663463	0.812	0.867	1
PTH2R	0.348652708	-0.590609064	0.625	0.4	1
NTNG1	0.348857174	1.065588342	0.938	0.733	1
AP2B1	0.349542821	0.872412395	1	1	1
SLC35A3	0.349686922	0.758188865	1	0.933	1
TNFRSF1B	0.350008557	1.491853096	0.562	0.2	1
CPD	0.350049255	0.788702672	1	1	1
VASH2	0.350093336	0.569855608	0.938	0.8	1
LMF2	0.350490629	-0.196202897	0.5	0.733	1
TNNI3K	0.350702195	0.427722759	0.438	0.667	1
SLC34A1	0.350943917	0.051280505	0.5	0.533	1
RAD23B	0.351143921	1.100062878	1	1	1
TRMT1L	0.351153481	0.853064743	1	1	1
SUCLA2	0.351159824	0.526033943	1	1	1
UBAP1	0.351311458	0.445511081	1	1	1
HIF1A	0.351752601	0.846902594	1	1	1
LRRIQ1	0.35199449	1.106199404	0.938	0.533	1
ENSSSCG00000048161	0.352199555	0.409390936	0.688	0.8	1

CHST11	0.352306345	0.123701985	0.938	1	1
ENSSSCG00000059156	0.352344403	0.269460675	0.562	0.4	1
B3GALNT1	0.352461083	0.390017085	1	1	1
FCHSD2	0.35257296	0.190423312	1	0.933	1
TIPRL	0.352635576	0.357142787	1	1	1
ABHD4	0.352822414	0.466370049	1	1	1
DGKI	0.352838313	0.914100481	1	1	1
DYDC2	0.353131616	0.126456973	0.875	0.8	1
PELI2	0.353311561	0.193771743	0.875	0.933	1
ENSSSCG00000059909	0.353386353	1.033117054	1	1	1
OIP5	0.35357007	0.784998291	1	1	1
NET1	0.353650849	0.276399755	1	1	1
ACKR2	0.354167155	-0.08152143	0.938	0.933	1
ENSSSCG00000062401	0.354250831	0.83336156	1	0.933	1
BRI3BP	0.354285091	0.726248904	1	1	1
ENSSSCG00000061431	0.354590249	-0.167109986	0.5	0.4	1
ENSSSCG00000002935	0.354675776	0.705256734	1	1	1
EPG5	0.354916012	0.240846787	0.938	0.933	1
F2	0.355118254	1.046723532	1	1	1
FASLG	0.355132618	0.214072105	0.938	0.667	1
YEATS2	0.355965326	0.783407542	1	1	1
THAP12	0.356560843	0.150816178	0.75	0.733	1
FUT10	0.356706216	1.321928095	0.5	0.333	1
DCBLD1	0.356813837	1.018083583	1	1	1
POLH	0.356871543	0.424922088	0.938	0.933	1
XPO5	0.356893177	0.304515563	1	1	1
LRRK2	0.35711136	0.832890014	1	0.933	1
HSPA9	0.357214113	0.898614467	1	1	1
ENSSSCG00000041471	0.357767025	1.062168821	0.812	0.867	1
ENSSSCG00000054707	0.358064403	1.0138058	0.812	0.6	1
UBE2A	0.358090886	0.809775396	1	1	1
ABCE1	0.35813538	0.856791984	1	1	1
SYNJ2BP	0.358437264	1.039176795	1	1	1
HSPB11	0.358526541	1.321928095	0.75	0.533	1
ENSSSCG00000051601	0.35872325	0.810814624	1	1	1
MMGT1	0.358791298	0.935255487	1	1	1
TRPM6	0.358851735	0.655828831	0.938	1	1
PPP1R13L	0.359106978	1.202346479	0.75	0.4	1
CDK16	0.359256376	0.283631575	1	0.933	1
ENSSSCG00000015823	0.359503139	0.768156103	1	1	1
FBLN2	0.359528044	0.842760258	0.875	0.8	1
ANKRD52	0.359537451	0.800360675	1	1	1
HECTD4	0.359600397	0.443625984	1	1	1
IGFBP3	0.359602251	1.594946589	0.562	0.267	1
ALDH1L2	0.359651674	0.655351829	1	0.933	1
PPWD1	0.359736312	0.848278435	1	1	1
HAVCR2	0.359828559	-0.285754482	0.875	0.733	1
MYO18B	0.35992481	1.246740598	0.812	0.8	1
ENSSSCG00000047145	0.360078173	0.578468739	1	1	1
MAP3K11	0.360111837	0.022367813	0.5	0.6	1
CENPU	0.360167595	0.393413279	1	1	1
ENSSSCG00000057494	0.360397109	-0.208586622	0.688	0.667	1
CTBP2	0.360562027	1.057777052	1	1	1
FMN1	0.360601191	0.233239618	1	1	1
PHF8	0.360665934	0.799541259	1	1	1
PGGT1B	0.361018492	0.383643879	1	1	1
ACTB	0.361439063	0.421463768	1	0.933	1
COQ2	0.362008037	0.533212126	1	1	1
ZCRB1	0.362086317	0.865785392	0.875	0.8	1
PPA1	0.362117966	0.918374299	1	1	1
MRPS22	0.362190394	0.3819102	1	1	1
CCT2	0.3622233	0.416933361	1	1	1
FLT4	0.362323681	0.26052755	0.938	1	1
BBS12	0.3625601	-0.925381959	0.5	0.467	1
ENSSSCG00000030800	0.362928472	0.717066037	1	0.733	1
FUCA1	0.363029075	0.987858661	1	1	1
KIF24	0.363179993	0.439001214	1	1	1
ENSSSCG00000055922	0.363197611	0.129283017	0.688	0.8	1
CPSF7	0.363382236	-0.560235415	0.438	0.8	1
RPRD1B	0.363445552	0.114695481	0.688	0.867	1

DSCAM	0.363575292	0.762500686	0.688	0.4	1
MZT1	0.363650857	0.781849011	1	1	1
EHMT1	0.364063226	0.870862286	1	0.933	1
CYB561A3	0.364210479	0.941346669	1	1	1
OSBPL10	0.364697464	0.657883894	1	1	1
CCSER1	0.364763823	0.738480235	1	1	1
ATXN7L1	0.365099161	0.60225139	1	1	1
PTOV1	0.365122478	1.117945473	1	0.933	1
ENSSSCG00000042273	0.365170722	0.054989235	0.75	0.733	1
ENSSSCG00000036685	0.365342663	-0.022720077	0.562	0.6	1
TYK2	0.365677938	-0.042483331	0.75	0.867	1
POLE	0.365810833	-0.043121269	0.875	1	1
CHD8	0.366140852	0.754322957	0.938	0.933	1
BRINP2	0.366198067	0.468006354	1	1	1
TMEM108	0.366295702	0.774135387	1	1	1
RBM10	0.36647435	0.813781191	0.875	0.733	1
TRIM8	0.36647601	0.842760258	0.938	1	1
ENSSSCG00000049674	0.366580017	1.02888112	0.688	0.733	1
CHMP5	0.366677275	0.938344745	1	1	1
KLF7	0.367117436	0.332766075	1	1	1
TRPS1	0.367167367	0.758791957	0.875	0.6	1
NPHS1	0.367207266	-0.093109404	0.5	0.4	1
ENSSSCG00000062186	0.367284219	0.361456459	0.562	0.6	1
CDC5L	0.36729102	0.402047138	1	1	1
DLGAP2	0.367589343	-0.093109404	0.5	0.6	1
CELSR2	0.367645001	0.089093927	0.688	0.8	1
OSTC	0.367931845	0.152153617	1	1	1
CKAP2L	0.367972344	0.268603204	1	1	1
FAM219A	0.368032602	0.52160044	0.75	0.867	1
ENSSSCG00000044910	0.368134262	0.413236796	1	1	1
DDX50	0.368137114	0.442943496	1	1	1
DERPC	0.368368194	0.176979759	0.812	0.6	1
ENSSSCG00000059270	0.36839058	-0.9861942	0.5	0.467	1
TTBK2	0.368652768	1.162323279	1	0.933	1
ZCCHC3	0.368748908	-0.206766186	0.75	0.867	1
ENSSSCG00000037996	0.368877761	1.017921908	0.812	0.467	1
STRA6	0.368897015	0.208546295	0.812	0.8	1
H1-3	0.369044003	0.129283017	0.75	0.867	1
EDC4	0.369267006	0.81721855	1	1	1
ZNF10	0.369376634	0.240791332	0.938	0.933	1
MSH5	0.370078084	1.206876138	0.938	0.867	1
GRIPAP1	0.371027723	0.316003261	0.875	0.8	1
EPAS1	0.371183937	0.805961687	1	1	1
PPP1R35	0.371237926	0.289834465	1	1	1
RUNDC3B	0.371669227	0.824165673	1	1	1
ARSK	0.372295119	0.883806982	0.938	0.8	1
XRN1	0.37239821	0.96144133	1	1	1
ABCG8	0.372401267	-0.093109404	0.688	0.533	1
ENSSSCG00000055840	0.372576823	0.530922661	0.812	0.667	1
CISH	0.37296398	-0.285754482	0.562	0.533	1
ENSSSCG00000009125	0.373000434	0.461819211	1	1	1
ATP8B4	0.373150574	0.795607262	1	1	1
NR4A2	0.373171878	1.525800428	0.562	0.4	1
ENSSSCG00000045408	0.373389226	0.176077228	0.875	0.8	1
GFI1B	0.373432798	0.994353437	0.688	0.533	1
ENSSSCG00000059736	0.374375153	-0.397963986	0.562	0.533	1
FAM83C	0.37455346	0.87036472	0.75	0.333	1
ENSSSCG00000011676	0.374566922	0.901951063	1	1	1
COL2A1	0.374610453	0.301169535	1	0.867	1
FAM167A	0.374844874	0.360608563	0.75	0.867	1
GANC	0.374931585	0.882368276	0.938	0.867	1
TUBG1	0.374993039	1.031948075	1	0.933	1
YWHAB	0.375713006	0.885603745	1	1	1
DNAH11	0.375825039	0.383486207	1	1	1
EGLN3	0.376426349	0.355390854	1	1	1
ENSSSCG00000061675	0.377343316	1.069162025	0.875	0.667	1
ENSSSCG00000055262	0.377372051	-0.540568381	0.5	0.467	1
STAT2	0.377434797	0.218834602	1	1	1
INSIG1	0.377510078	0.977973694	1	1	1
ENSSSCG00000063364	0.377722528	1.118394701	0.938	0.867	1

TTI2	0.377879841	0.276671814	1	1	1
CCNJ	0.377892945	0.777782993	1	1	1
MRPS31	0.378006029	0.785430778	1	1	1
ENSSSCG00000052954	0.378207546	0.041191687	0.938	0.867	1
BIRC2	0.378331554	0.90456179	1	1	1
RPL37	0.378535286	-0.185969013	1	1	1
MNS1	0.378843756	0.929988423	1	1	1
URB1	0.37885876	1.672425342	0.625	0.467	1
FUT11	0.379191828	0.945364743	0.938	0.8	1
CENPL	0.379615922	0.484486127	1	1	1
IL12RB2	0.379725876	1.331388424	0.562	0.4	1
HGS	0.379752955	0.274261661	0.938	0.933	1
CHCHD2	0.38009765	0.440480995	1	1	1
TICAM1	0.380317396	1.392317423	0.938	0.6	1
TCAF1	0.380574392	0.673218249	1	0.933	1
HDDC3	0.380838949	1.491853096	0.625	0.467	1
PPP1R15A	0.381122342	1.179262866	1	1	1
PCMTD2	0.381214251	0.478410513	1	1	1
GAK	0.381391362	0.175142711	1	1	1
PAM	0.381476662	1.49833713	1	0.733	1
SOCS1	0.381506547	0.820475844	1	0.8	1
BLTP2	0.381649376	0.279510654	0.938	0.933	1
UBE2S	0.382077919	1.111822047	0.938	0.933	1
ATP8B1	0.382253754	0.948331398	0.938	1	1
ENSSSCG00000004287	0.382596826	0.779384183	1	1	1
PAPPA2	0.382708198	0.295730016	1	1	1
FLVCR1	0.382834845	1.19865672	0.812	0.6	1
ENSSSCG00000038187	0.382889236	-0.071083098	0.875	0.6	1
PAX9	0.382895092	1.202346479	0.875	0.867	1
ENSSSCG00000059653	0.383102427	0.301422439	0.938	1	1
ATXN3	0.383118301	0.819844497	1	1	1
OSBPL1A	0.383626191	0.791738859	1	1	1
DNAJB9	0.384001562	0.354545046	1	1	1
ENSSSCG00000037332	0.384031403	0.083768358	1	1	1
FAM133A	0.384279712	1.063009798	0.812	0.6	1
DYNC1I1	0.384805174	0.414752128	1	1	1
RIBC2	0.385130377	0.489133595	0.875	1	1
ENSSSCG00000041773	0.385159195	0.200621799	0.688	0.733	1
ZNF605	0.385182328	0.973110282	1	1	1
STXBP1	0.385202179	0.112671076	1	0.933	1
UBFD1	0.385536208	0.962017021	1	1	1
ENSSSCG00000048686	0.386076626	0.038994132	0.938	0.8	1
DIAPH3	0.38649005	0.823635092	1	1	1
NFYA	0.387303536	0.871719817	1	1	1
DNAJC17	0.387330783	0.928029363	1	1	1
ENSSSCG00000047853	0.38810005	1.139551352	0.75	0.667	1
UBN2	0.388902872	0.474880018	1	1	1
MRI1	0.38898151	0.955800196	0.938	0.6	1
ENSSSCG00000041180	0.389218423	0.696535264	1	1	1
BACH2	0.389604118	0.021061616	1	0.867	1
TRPC4	0.389631832	0.609599884	1	1	1
PUM1	0.390010284	1.012759231	1	1	1
KIAA0319L	0.390094577	0.829147612	1	1	1
ENSSSCG00000053589	0.390770845	-0.136178126	0.625	0.6	1
ALYREF	0.390882251	0.067355268	0.75	0.867	1
TMOD2	0.391253752	0.733775907	1	1	1
ENSSSCG00000044577	0.39143649	0.251106758	1	1	1
RELB	0.391493615	1.617383978	0.75	0.4	1
ENSSSCG00000038188	0.391527581	0.097412502	0.938	0.8	1
ACTN4	0.391636735	1.188303531	1	1	1
PTGES3L	0.391645917	0.125070766	0.75	0.867	1
CTBP1	0.391923639	1.354349573	0.625	0.267	1
TSEN15	0.392016236	0.756119187	1	1	1
FAM131B	0.392093864	-0.093109404	0.5	0.333	1
UAP1	0.392190533	0.652573407	0.938	0.8	1
HTATSF1	0.392574545	0.376375879	1	0.933	1
PSMB3	0.39261118	0.09330372	0.875	1	1
ENSSSCG00000059580	0.393085212	0.247927513	1	1	1
SMPDL3A	0.393502146	1.28771238	0.812	0.667	1
DST	0.393647914	0.475197933	1	1	1

HSD17B6	0.393703402	1.531381461	0.688	0.467	1
ENSSSCG00000055079	0.393821769	1.006426269	0.562	0.4	1
PRKAB1	0.394539837	1.036242656	1	1	1
ENSSSCG00000053714	0.395102749	0.868416448	0.875	0.733	1
ENSSSCG00000026529	0.395201443	-0.093109404	0.688	0.667	1
ENSSSCG00000061829	0.396122703	0.740351409	0.938	0.933	1
PRDX6	0.39614732	0.319934045	1	0.933	1
HVCN1	0.396317414	0.362401845	1	1	1
MYO1C	0.396752762	0.267888255	1	1	1
NIM1K	0.396870806	0.7508994	1	1	1
MDGA2	0.397061786	0.391469028	1	1	1
ENSSSCG00000061357	0.39727748	1.06608919	0.812	0.933	1
NT5C2	0.397443793	0.850118114	1	1	1
MATN2	0.397539214	0.009984089	0.5	0.467	1
ENSSSCG00000061672	0.39776516	0.152003093	0.5	0.4	1
TUB	0.397990183	0.299208018	0.625	0.467	1
GLRA2	0.398080861	-0.373217324	0.5	0.467	1
ENSSSCG00000061319	0.398189205	0.22881869	0.625	0.667	1
FAM91A1	0.398229585	0.380821784	1	0.667	1
IZUMO1R	0.398371384	1.154818109	0.938	0.867	1
OR10A4	0.398539777	1.169925001	0.75	0.2	1
RNASE4	0.398991248	1.335733894	0.688	0.333	1
EXOSC10	0.399049718	0.049848549	0.75	0.733	1
GATAD2A	0.399131617	0.502500341	1	1	1
FZD3	0.399182548	0.574468242	1	1	1
POP1	0.399319569	0.785584299	0.938	0.733	1
FANCF	0.399360523	0.888178335	1	1	1
ENSSSCG00000058981	0.399411667	0.080657663	0.938	0.733	1
ENSSSCG00000053175	0.399510011	-0.082138287	0.938	0.8	1
FAF2	0.399571567	0.857980995	1	1	1
RPN1	0.399598844	0.858372659	1	1	1
CSNK1A1	0.399878124	0.553998038	1	1	1
TBC1D30	0.399909223	0.108818914	0.938	1	1
ENSSSCG00000050566	0.400075413	0.083768358	0.562	0.6	1
ENSSSCG00000046897	0.400119015	1.003105911	0.688	0.467	1
PPP1R3D	0.400167653	1.144958086	0.938	0.933	1
BCL2L11	0.400637705	-0.138913094	0.625	0.4	1
PTK2	0.401455893	0.904484098	1	1	1
CCDC112	0.40155891	0.980891177	0.688	0.6	1
INTS15	0.401674215	1.233700912	1	1	1
ODAD2	0.401877958	0.302510641	1	0.933	1
ENSSSCG00000052080	0.401952566	-0.294743266	0.562	0.6	1
ENSSSCG00000012832	0.402107712	1.714245518	0.5	0.4	1
FST	0.40245818	1.049413649	1	1	1
ENSSSCG00000047367	0.402771042	0.584962501	0.938	0.667	1
SEC31A	0.402882167	0.90265609	1	1	1
ENSSSCG00000052019	0.402998235	1.22881869	0.562	0.333	1
FMR1	0.403198764	0.756731341	1	1	1
FARSA	0.404042295	0.87389232	1	0.933	1
ALOX12	0.404270727	-0.175571565	0.562	0.333	1
USP19	0.404289198	0.894447533	1	1	1
SNX4	0.404539492	0.945304479	1	1	1
CLN3	0.404544497	0.539158811	0.938	1	1
ENSSSCG00000060014	0.405008917	1.186998515	0.5	0.267	1
IKZF2	0.405233028	0.189824559	0.875	0.8	1
UPF3A	0.405469968	0.547532869	1	1	1
STYX	0.405561488	0.946857003	1	0.933	1
PDXDC1	0.405830723	1.273672926	0.812	0.8	1
RAB27A	0.405873061	0.520299784	1	1	1
ENSSSCG00000040690	0.405874717	0.64385619	0.375	0.6	1
ENSSSCG00000040742	0.406447178	1.036173613	0.688	0.467	1
MTX2	0.406466091	0.952199757	1	1	1
ARIH2	0.406870528	0.362763009	1	1	1
MYO18A	0.407043467	0.358269942	1	0.933	1
ENSSSCG00000043175	0.407722373	-0.1602236	0.562	0.533	1
GID4	0.408010678	1.252026082	0.938	0.8	1
ENSSSCG00000011531	0.408055929	-0.093109404	0.5	0.533	1
ENSSSCG00000041123	0.408091934	0.017921908	0.5	0.467	1
SH3RF1	0.408136379	0.906890596	0.875	1	1
HNRNPH2	0.408268488	0.289971328	1	1	1

DIAPH1	0.408468908	0.924612366	1	1	1
MIDEAS	0.408581272	0.854423176	0.938	0.933	1
BMAL2	0.408894631	0.39702955	0.938	1	1
RELCH	0.409077812	0.429412522	0.938	0.933	1
CEP85L	0.409275654	0.454739923	1	1	1
ENSSSCG00000032301	0.409405549	1.104829973	0.938	0.667	1
TRIM35	0.409426011	1.305439972	0.562	0.267	1
GREM1	0.409731113	0.308363367	0.938	0.933	1
ENSSSCG00000057851	0.409839619	0.518325308	0.625	0.8	1
ENAH	0.410219054	0.369990336	1	1	1
ENSSSCG00000007645	0.410250569	0.022367813	0.625	0.467	1
TRPV5	0.410510923	1.26052755	0.562	0.2	1
SNW1	0.410681271	0.455063066	1	1	1
TAT	0.410920682	-0.061948473	0.938	1	1
DHX15	0.411107439	1.071587082	1	0.933	1
ZFPM2	0.411237429	0.26689873	1	1	1
PTPN14	0.411628706	0.576655937	1	1	1
STK24	0.411719352	0.669076769	1	1	1
ST3GAL6	0.411748312	0.787151351	1	1	1
LFNG	0.411959799	0.22881869	0.812	0.6	1
H2BC18	0.412218432	-0.334117504	0.5	0.6	1
ENSSSCG00000062858	0.412441893	0.553300037	1	1	1
ZC3HC1	0.41254911	0.750378573	1	1	1
TMEM116	0.412598773	1.129283017	0.688	0.467	1
FNDC9	0.413000875	1.194467186	0.875	0.533	1
KCNH7	0.413124384	0.521471863	1	1	1
MED15	0.41339073	0.944770479	1	1	1
ENSSSCG00000038275	0.413393805	0.09330372	0.812	0.667	1
ENSSSCG00000024459	0.413581485	0.376375879	0.812	0.667	1
DNAAF11	0.413654443	-0.005646563	0.5	0.4	1
HIVEP1	0.413828455	0.434519921	0.938	0.8	1
MLKL	0.413881148	0.612386762	0.938	1	1
LYSMD4	0.413884092	0.346422657	1	1	1
IFT74	0.414465841	0.646898247	1	1	1
DEPDC7	0.414551663	0.321235969	1	1	1
ENSSSCG00000060818	0.414646918	0.950592302	1	1	1
LIX1L	0.41478612	0.776944423	1	1	1
CUBN	0.415153258	0.389042291	0.812	0.867	1
GAREM1	0.41531151	0.566815154	0.75	0.733	1
CCNP	0.415316661	0.914795794	1	0.867	1
THAP9	0.416402475	-0.329809662	0.875	0.733	1
MBTPS1	0.416525084	1.163230349	0.875	0.8	1
ENSSSCG00000034963	0.416555385	0.058893689	0.5	0.533	1
SEC61A1	0.417140302	0.761383617	1	1	1
NMNAT1	0.417159382	0.883633321	1	1	1
WASL	0.417527893	0.750976794	1	1	1
RBM12	0.417555497	0.630605166	1	0.8	1
BCL10	0.417559437	0.766429124	1	1	1
SSTR1	0.417875453	1.044394119	0.812	0.733	1
DENND6A	0.41793856	0.380701804	0.938	1	1
TRIQK	0.41816939	1.62935662	0.625	0.467	1
RNF145	0.418335026	0.730745807	1	1	1
SDHC	0.418385505	0.660196369	1	1	1
EBPL	0.418634378	0.566334927	1	1	1
KHSRP	0.419169419	0.328892043	1	1	1
ENSSSCG00000051049	0.419338465	0.260942894	0.938	1	1
CANT1	0.419493751	1.26237125	1	0.867	1
PARPBP	0.419697638	0.875181736	1	0.933	1
ARL5A	0.419860317	0.821921035	1	1	1
PACS1	0.419978209	1.083270619	1	0.933	1
ENSSSCG00000052587	0.420026934	0.215012891	0.5	0.667	1
PARD3B	0.420125526	0.590719771	1	1	1
CELF1	0.420170189	0.438758425	1	1	1
JAG1	0.420344622	0.816761002	1	1	1
ENSSSCG00000032067	0.420767733	0.497462774	1	1	1
CYP2C49	0.420901006	0.186998515	0.5	0.467	1
TIFA	0.421109944	1.347463187	0.5	0.4	1
PIWIL2	0.421332095	0.654596689	1	1	1
LYVE1	0.421448112	0.249778309	0.875	0.933	1
ENSSSCG00000057459	0.421749676	0.697172004	1	0.933	1

ENSSSCG00000063375	0.421757741	0	0.688	0.533	1
PIK3C3	0.422340442	0.680180879	0.938	1	1
VSTM1	0.422429735	0.121015401	0.75	0.6	1
ENSSSCG00000059862	0.422699101	2.022367813	0.5	0.133	1
PTPN4	0.422854924	-0.075621978	0.875	0.6	1
SBF2	0.422948789	0.934088737	0.938	1	1
ANXA6	0.422984696	0.094517599	0.688	0.733	1
CCNB1IP1	0.423145229	0.729431921	1	0.667	1
PSMD2	0.423578529	0.795972254	1	1	1
GRK2	0.423653601	0.186998515	0.562	0.467	1
OSMR	0.42371644	0.705163534	1	0.933	1
ENSSSCG00000062788	0.423816132	0.072476661	0.812	0.667	1
WDR75	0.423873624	0.721273808	1	1	1
RBM14	0.424071695	0.265344567	0.938	0.933	1
RAPGEF2	0.424256147	0.179724141	1	1	1
PEX26	0.424450095	1.00023685	1	1	1
ENSSSCG00000060636	0.424505793	0.735124247	0.938	0.8	1
GOT2	0.424676521	0.520162855	1	1	1
IFNGR1	0.424753345	0.080919995	0.875	0.867	1
CFAP46	0.42521589	-0.04580369	0.75	0.6	1
LZTFL1	0.425489919	1.129283017	1	1	1
PPP1R3C	0.425738346	0.664917805	1	1	1
WIZ	0.425761747	0.31676639	0.938	0.867	1
ENSSSCG00000027115	0.425981344	0.265885687	0.938	1	1
ENSSSCG00000001393	0.426373963	0.99305565	1	1	1
DENND5A	0.426560812	0.74130453	1	1	1
OSTM1	0.427391346	-0.516917113	0.562	0.667	1
HDHD5	0.427552481	1.392317423	0.5	0.267	1
RNF24	0.427589492	0.452196896	1	0.933	1
ENSSSCG00000040632	0.427593526	-0.218640286	0.5	0.4	1
AGK	0.427917567	0.783924704	1	1	1
SLC44A3	0.428169919	0.057307438	0.75	0.867	1
FGD5	0.428284478	0.055141554	0.938	0.933	1
WDR70	0.428292132	0.36496011	1	1	1
MBD4	0.428298998	0.827383149	1	1	1
CLSPN	0.428340213	0.867845863	1	1	1
FIGN	0.428610051	1.044394119	0.938	0.8	1
ENSSSCG00000059998	0.428617594	0.610497593	0.812	0.8	1
ENSSSCG00000044589	0.428663727	0.661778098	0.812	0.733	1
ENSSSCG00000059284	0.428829459	-0.093109404	0.5	0.533	1
ENPP1	0.429313469	0.356803136	0.938	1	1
PDGFRL	0.429380118	0.079357792	0.938	0.933	1
ENSSSCG00000059777	0.429398331	0.164048435	0.875	0.933	1
VMP1	0.429752931	0.941378972	1	1	1
SLC25A27	0.429912236	0.718033778	1	1	1
ENSSSCG00000058479	0.430018461	0.754887502	0.688	0.467	1
ZNF341	0.430135765	0.26052755	0.688	0.733	1
ENSSSCG00000002251	0.430230812	0.957516669	0.625	0.4	1
ABCC1	0.430231087	0.875366507	1	1	1
STK33	0.430719975	0.147898695	0.5	0.533	1
ENTPD7	0.431180766	0.647914406	1	1	1
SMARCA4	0.431263884	0.335733894	1	0.933	1
PARG	0.431319185	0.56175511	1	1	1
SDC3	0.431501573	0.96133838	0.812	0.6	1
ENSSSCG00000045095	0.431672504	-0.049388027	0.938	0.867	1
DCAF12	0.432752886	0.871615121	1	0.933	1
ENSSSCG00000057903	0.432902726	0.211745177	0.562	0.467	1
ZBTB6	0.432921971	-0.093109404	0.875	0.733	1
TRBV27	0.433276093	0.217678133	0.938	0.933	1
PRKD3	0.434225912	0.632836376	1	1	1
MTCP1	0.434339182	0.378642486	1	0.933	1
VPS8	0.43454555	0.365440401	1	1	1
PLAA	0.43509955	0.59017693	1	1	1
ITPRIPL1	0.435300347	0.880706664	1	1	1
ENSSSCG00000063016	0.435313568	1.516944077	0.625	0.6	1
CTDSPL	0.435352637	0.801280408	1	0.933	1
TASOR	0.435539807	0.633255885	1	1	1
SLC15A2	0.435567244	1.376375879	0.562	0.333	1
HNRNPM	0.435825884	0.973495994	1	1	1
ZSCAN20	0.436638897	0.198494154	1	0.8	1

ENSSSCG00000056610	0.436747981	0.376551764	1	1	1
TRAPPC14	0.436987213	-0.093109404	0.5	0.533	1
ENSSSCG00000044591	0.437045332	0.856617389	0.938	0.733	1
EXTL3	0.437125439	0.621509896	1	1	1
WDR43	0.437775918	0.921104455	1	1	1
NSD3	0.437878379	0.41043592	1	1	1
ENSSSCG00000060593	0.438106177	1.196397213	0.562	0.4	1
NUF2	0.438234108	0.871809944	1	1	1
ENSSSCG00000047048	0.43830258	1.730860232	0.75	0.533	1
WDR73	0.43880272	0.113341473	0.875	0.6	1
TXLNB	0.438922153	0.311280851	1	0.867	1
ENSSSCG00000037602	0.438946018	0.601758653	1	1	1
DUSP1	0.439117293	0.50907951	1	1	1
SEC24D	0.439319624	0.509274531	1	1	1
CHD9	0.439336504	-0.13965199	0.875	0.533	1
ENSSSCG00000048249	0.439584974	0.50084188	1	1	1
CYP2R1	0.440006478	0.196397213	1	0.933	1
ENSSSCG00000032352	0.440150357	0.235839118	0.688	0.667	1
ENSSSCG00000035650	0.440612295	0.194871359	1	0.8	1
PPP3CA	0.440750276	-0.03170886	0.562	0.533	1
ENSSSCG00000053249	0.441321911	1.032421478	0.625	0.467	1
HEXIM1	0.441616331	0.407319586	0.875	0.733	1
ENSSSCG00000062400	0.441722151	0.448476034	0.938	1	1
ACVR2B	0.442071006	0.603498452	1	1	1
ZNF546	0.44213655	0.885516945	0.938	0.733	1
TLR5	0.442213641	-0.040641984	0.5	0.667	1
HTD2	0.442277516	0.69619233	1	0.933	1
ENSSSCG00000056294	0.442282087	1.607330314	0.5	0.2	1
ARHGEF5	0.442289489	0.237901795	0.938	1	1
SSH1	0.442422491	1.019100099	1	0.933	1
SLC35E1	0.442837758	0.399594588	0.938	0.933	1
NCKAP5	0.442947154	0.698949331	1	1	1
TUBB3	0.442980033	0.070389328	0.812	0.4	1
CCDC38	0.443072154	0.984893108	0.562	0.267	1
PFKFB2	0.443082605	0.987509056	0.875	0.933	1
MED4	0.443123445	0.415732463	1	1	1
ENSSSCG00000008849	0.443407036	0.338606836	0.812	0.8	1
SLC18A2	0.443521263	0.349123022	1	1	1
MAN2A1	0.443594174	0.837181624	1	1	1
UBAP2	0.443706953	0.78508774	1	1	1
COX3	0.443870451	1.169709762	1	1	1
NFE2L3	0.444002925	0.356234217	1	1	1
TAFA2	0.444205602	0.47436567	1	0.933	1
MPDZ	0.444976817	0.528551396	1	1	1
CSNK2A2	0.445024989	0.383047889	1	1	1
CREB3L3	0.445144763	0.540644535	1	1	1
KDM1B	0.445514907	0.784935168	1	1	1
ENSSSCG00000014198	0.445792845	0.622813774	1	1	1
ZBTB44	0.445832367	0.639870842	1	1	1
ANKRD17	0.445879002	0.467523767	1	1	1
TTC17	0.446043136	0.809965545	1	1	1
LAMP2	0.446305005	0.774112576	1	1	1
BMP15	0.446413656	0.377368237	1	1	1
ENSSSCG00000003847	0.446454459	1.594946589	0.562	0.333	1
KIF13B	0.446579256	0.35638509	1	1	1
TBC1D24	0.446732749	0.542734265	1	1	1
NMNAT2	0.446805474	0.334061851	0.875	0.933	1
ENSSSCG00000009211	0.447216371	0.512582076	1	1	1
STRBP	0.447400412	0.901119808	1	1	1
ZFP36	0.447445805	0.497499659	0.938	0.933	1
RRAGD	0.447491411	0.432734881	0.938	1	1
ENSSSCG00000047947	0.447546308	0.129283017	0.875	0.867	1
MFAP3	0.447568882	0.734224688	1	1	1
ENSSSCG00000052153	0.447611347	1.076815597	0.5	0.267	1
RUSC2	0.447671214	0.189620803	0.938	1	1
LIPH	0.447757649	0.307428525	0.875	0.6	1
ENSSSCG00000009105	0.448077347	1.392317423	0.562	0.133	1
METTL15	0.448079533	1.00877021	0.875	0.6	1
CSTF2	0.448243019	0.743992861	1	0.8	1
ADAL	0.448738733	0.031070007	0.938	0.933	1

APTX	0.448772241	0.586695468	1	1	1
AUTS2	0.448908019	0.978443857	0.938	0.933	1
MRS2	0.44919284	0.188845863	1	1	1
ENSSSCG00000045959	0.449240557	1.569855608	0.5	0.2	1
MORC4	0.449538932	-0.003472192	0.812	0.867	1
POLR3D	0.449578459	0.917317249	1	1	1
C2CD5	0.449602545	0.692879983	1	1	1
PKLR	0.449618769	0.0138058	0.5	0.333	1
RITA1	0.449694789	0.951037699	1	1	1
CAMK2D	0.449916816	0.816125132	1	1	1
CYB561	0.450158655	0.427147407	0.938	1	1
ENSSSCG00000059573	0.45019858	0.239087026	0.938	1	1
ENSSSCG00000012517	0.450291771	0.837524651	1	0.933	1
SLC26A2	0.450332774	1.347463187	0.5	0.2	1
KLHL5	0.450540788	0.525327801	1	1	1
BBOX1	0.450752986	0.649941915	1	1	1
ENSSSCG00000032596	0.450898225	0.994353437	0.688	0.533	1
BRD1	0.451113391	0.909641207	1	1	1
CAVIN2	0.451215059	0.866248611	0.938	0.8	1
CHL1	0.451499004	0.402480665	1	1	1
AKAP9	0.451622169	0.816550218	1	1	1
BSPRY	0.451671142	-0.200024608	0.5	0.333	1
ENSSSCG00000060888	0.452000136	0.01268526	1	1	1
ND6	0.452121073	1.129150854	1	1	1
EYA2	0.452137713	0.054989235	0.688	0.867	1
SLC26A8	0.452189659	1.22881869	0.75	0.533	1
ENSSSCG00000054171	0.452254898	-0.093109404	0.5	0.467	1
KATNA1	0.45232848	0.904047846	1	1	1
USP4	0.452466982	0.188122109	1	0.933	1
PRKAR2A	0.452755042	0.491853096	0.938	0.8	1
RAB39A	0.452850596	0.856264523	0.938	1	1
ENSSSCG00000055276	0.45290027	0.838801606	1	0.933	1
FRMD8	0.453087142	1.096493082	1	1	1
PALS1	0.453189321	0.479833514	1	1	1
ENSSSCG00000055511	0.453492399	0.769387072	0.688	0.4	1
TAX1BP1	0.453549763	0.733714577	1	1	1
MYF6	0.453652205	0.491853096	0.875	0.733	1
SENP6	0.453680788	0.450153805	1	1	1
FAM186B	0.454205647	1.023404604	0.938	1	1
LYPD6	0.454617115	1.507283137	0.688	0.733	1
ARFGAP3	0.455359308	0.530979428	1	1	1
DEGS1	0.455436494	1.343661617	1	0.933	1
ENSSSCG00000002938	0.455592875	0.791988501	0.938	1	1
PMPCB	0.455688775	0.399717984	1	1	1
KIF9	0.455738964	0.269460675	0.812	0.733	1
RAI2	0.455779612	0.106199404	0.625	0.6	1
HDHD2	0.455863563	0.704778654	1	1	1
STRN4	0.455919143	1.109830654	1	1	1
BCL9	0.456467654	0.424166289	1	1	1
ENSSSCG00000052282	0.456512324	0.160647188	0.875	0.8	1
PABPN1	0.456645506	0.696703569	1	1	1
ENSSSCG00000059954	0.456882089	0.64385619	0.938	0.8	1
ENSSSCG00000045434	0.457280412	1.242493627	0.812	0.733	1
GNA12	0.457321598	0.500676947	0.938	0.933	1
ENSSSCG00000001038	0.457687289	1.001217978	1	1	1
PRDM5	0.457689868	0.529430554	1	1	1
PLPPR1	0.458619444	0.278859373	0.5	0.467	1
CFAP299	0.458643772	1.002047829	1	0.867	1
LMAN1	0.45929146	1.041615698	1	0.933	1
ATP11A	0.459460665	0.26052755	0.875	0.733	1
TBC1D8B	0.459563002	0.816203853	0.938	1	1
LSM14B	0.459696484	0.214072105	0.938	0.733	1
GIMAP8	0.460093782	0.380821784	0.5	0.6	1
USF3	0.460334398	0.203176807	1	1	1
ENSSSCG00000049992	0.460377553	1.594946589	0.562	0.133	1
DDX47	0.460386142	0.337034987	0.875	1	1
RASGRF2	0.46056112	1.243925583	0.875	0.733	1
ENSSSCG00000057066	0.461059079	-0.093109404	0.625	0.667	1
EPHA2	0.461092222	1.103691303	0.938	0.6	1
ENSSSCG00000061060	0.461288346	-0.127874823	0.812	0.467	1

DUOX2	0.461404226	1.392317423	0.562	0.333	1
ENSSSCG00000055040	0.461724284	0.415904243	0.812	0.8	1
FBXO15	0.463126485	0.844105321	1	1	1
AHI1	0.4634218	0.770132458	1	1	1
FBH1	0.463495666	0.934635586	1	0.933	1
TLR1	0.464031559	1.137188215	0.625	0.667	1
ENSSSCG00000046579	0.464066403	1.076815597	0.688	0.467	1
EBF2	0.464134388	0.027905997	0.875	0.8	1
IMMP1L	0.46429822	0.726101322	1	1	1
ODR4	0.464409023	0.485752897	0.75	0.933	1
ERCC2	0.464637923	0.252914366	1	0.933	1
MRPL18	0.465192601	0.881210049	1	1	1
CCDC134	0.465350734	0.401655287	0.938	0.8	1
CACNA2D1	0.465611744	0.955191928	1	1	1
RCOR3	0.465710226	0.243072674	1	1	1
ENSSSCG00000021380	0.465835015	1.136024595	0.812	0.6	1
AGBL3	0.465840114	0.423373358	0.938	0.933	1
ENSSSCG00000059042	0.465945686	1.05691604	1	0.933	1
GPR156	0.466033291	0.291808107	0.938	0.933	1
ASCC3	0.46616508	0.526110479	1	1	1
CEP128	0.467118404	0.549034757	1	1	1
MSN	0.467161334	1.321928095	0.875	0.667	1
ENOSF1	0.467667255	1.160647188	0.625	0.533	1
ZBTB34	0.468004459	0.807998839	1	1	1
SETD4	0.468267513	0.858635427	0.938	0.867	1
TBK1	0.46833407	0.828035175	0.938	0.733	1
ENSSSCG00000061302	0.468389814	-0.093109404	0.688	0.733	1
TBC1D20	0.468831583	0.785295758	1	1	1
ENSSSCG00000056401	0.468852772	0.206450877	0.625	0.6	1
ENSSSCG00000056542	0.468883604	0.495715736	1	1	1
GPATCH1	0.469018814	0.715591943	1	1	1
TNKS	0.469094083	0.641290746	1	1	1
TENT4B	0.469168011	0.8470812	1	0.933	1
ENSSSCG00000027421	0.469182065	1.354349573	0.5	0.4	1
SPATS2L	0.469335467	1.186998515	0.812	0.533	1
CRYBG1	0.469747024	0.321928095	1	1	1
DNM2	0.470241591	0.390099598	0.875	0.867	1
RSBN1	0.470436373	0.22336426	0.812	0.667	1
SNIP1	0.47071785	0.22881869	0.812	0.733	1
ENSSSCG00000013880	0.4709294	1.160647188	0.562	0.333	1
TCEAL9	0.471030095	0.716919652	1	0.867	1
WDR45B	0.471042215	0.88472327	1	1	1
YAE1	0.471055385	0.367731785	0.938	1	1
STX2	0.471072017	0.615427781	1	0.867	1
PTGES3	0.471433498	0.921587107	1	0.933	1
POR	0.471523308	0.906890596	0.938	1	1
B4GALT5	0.471631206	1.064122204	1	1	1
ARGLU1	0.471967289	0.89566334	1	0.933	1
ALDH16A1	0.472181313	1.00877021	0.938	0.8	1
ETS2	0.472258191	0.099535674	0.938	0.6	1
TBX3	0.472282616	0.906890596	0.688	0.6	1
GORASP2	0.472365978	0.631763897	1	1	1
N4BP1	0.472410655	0.189290326	1	0.867	1
ENSSSCG00000043817	0.472564835	0.491853096	0.938	0.8	1
ENSSSCG00000038066	0.472857781	0.526738614	1	1	1
TENM2	0.473197443	0.381027135	1	1	1
EPB41L4A	0.473767645	0.614337103	0.938	1	1
ARHGAP29	0.473897522	0.680585574	1	1	1
CHAF1B	0.475001291	0.297072906	1	1	1
DUSP19	0.4753741	0.902375114	1	1	1
CIP2A	0.475665192	0.472184936	0.938	1	1
C17orf58	0.475718187	0.468769483	0.688	0.8	1
NF1	0.475856975	0.571990204	1	1	1
SGPP2	0.476139595	0.174270472	1	1	1
BABAM2	0.476397952	0.079727192	0.812	0.733	1
ENSSSCG00000004583	0.4764599	0.62548294	1	1	1
SUPT4H1	0.476482753	0.137188215	0.688	0.667	1
ARPC5	0.47653955	0.494684687	1	1	1
SUSD6	0.476573633	0.682613909	1	1	1
NANOS1	0.477514598	0.293913719	0.562	0.6	1

RNFT2	0.477947505	0.353224225	1	1	1
TEC	0.477973669	0.328276061	1	1	1
B3GNT5	0.478051765	-0.194989018	0.562	0.667	1
ENSSSCG00000007525	0.478269659	0.906890596	0.562	0.267	1
CUL2	0.478339694	0.102441405	0.875	0.867	1
PTPRQ	0.478739259	0.814444347	0.938	1	1
PARP6	0.478746128	0.150816178	0.938	0.867	1
PSAP	0.478798331	0.033916034	1	1	1
ENSSSCG00000054758	0.478962265	0.103927443	0.75	0.533	1
ACAD10	0.47996241	0.951978485	0.875	0.733	1
ENSSSCG00000045708	0.480206429	1.154818109	0.562	0.4	1
UBE2V2	0.480632015	0.848156771	1	1	1
ENSSSCG00000062427	0.480644296	0.664771769	1	1	1
ENSSSCG00000035439	0.481123683	0.076815597	0.5	0.4	1
MYOG	0.481509712	0.129283017	0.625	0.667	1
XRN2	0.481677421	0.393532836	1	1	1
RAG1	0.481841805	1.044394119	0.625	0.267	1
BABAM1	0.482130766	0.812285395	1	1	1
TBC1D5	0.482477892	0.771163946	1	0.867	1
HS2ST1	0.482589818	0.385290156	1	1	1
HTR4	0.482669475	0.496425826	0.812	0.867	1
SCO1	0.482681476	0.361456459	1	0.933	1
C9orf64	0.482724364	0.925175949	1	0.933	1
KCTD12	0.482843238	0.583691963	1	0.933	1
ANKFY1	0.482914106	0.560170039	1	1	1
SLC35E2B	0.483029408	0.470791481	0.875	0.867	1
PTBP1	0.483379654	0.593543032	1	1	1
MRPS26	0.48359105	0.587915818	1	1	1
RMDN3	0.483723921	0.229700803	0.938	1	1
CBL	0.483921014	0.316145742	0.938	1	1
TMPPE	0.484373137	2.518325308	0.5	0.133	1
HERC2	0.484442603	0.757225744	1	1	1
SNRNP70	0.484574259	0.022367813	0.375	0.533	1
ADK	0.48457505	0.750771394	0.875	0.667	1
ENSSSCG00000043661	0.484669994	0.822409873	1	1	1
ENSSSCG00000058363	0.484889373	0.252026082	0.688	0.533	1
CEP120	0.484920868	0.65250806	1	1	1
RALGAPB	0.484931886	0.769431217	1	1	1
ZDHHC23	0.485024929	0.019899557	0.938	0.867	1
ZNF518A	0.485081966	0.234008247	1	0.933	1
ATG9A	0.485327772	0.142268659	0.812	0.933	1
CCNB3	0.486866039	0.269120055	1	1	1
SCEL	0.486902991	1.409390936	0.5	0.267	1
HPS4	0.487077551	1.038135129	0.875	0.8	1
BDP1	0.487129853	0.50846781	1	1	1
SMIM7	0.487242365	0.681330895	1	1	1
DOCK11	0.487307359	0.872125177	0.875	0.6	1
PHF6	0.487505899	0.35979628	1	1	1
ENSSSCG00000052578	0.487524116	1.0138058	0.562	0.333	1
ESYT3	0.487969464	0.423965793	1	1	1
SENP5	0.488045358	0.756995165	1	1	1
CBLL1	0.488250277	0.660422982	1	1	1
ENSSSCG00000060231	0.488547153	0.903817762	0.938	0.933	1
CEMIP2	0.488997682	0.40050415	1	1	1
RYBP	0.489180668	0.267621675	1	1	1
IFT52	0.489327828	0.331068749	1	1	1
UBQLN1	0.489331435	0.586153066	1	1	1
ENSSSCG00000048597	0.489336731	1.055753982	0.75	0.467	1
ENSSSCG00000039095	0.489357938	0.437405312	0.812	0.667	1
SLC1A7	0.489398569	0.154818109	0.562	0.467	1
RASGEF1B	0.489438718	0.375443604	0.875	0.933	1
SPTBN1	0.489519999	0.575890595	1	1	1
RNF40	0.49024897	0.79524524	1	1	1
NR3C2	0.490555332	0.835765011	1	1	1
ENSSSCG00000048640	0.490889179	0.418390934	0.75	0.867	1
SPAG7	0.490923835	0.392317423	0.938	0.8	1
SLC30A9	0.491658971	0.262567482	1	1	1
ENSSSCG00000012523	0.491933016	0.389973482	1	0.933	1
ZNF609	0.492006983	1.025285296	1	0.933	1
PPP6R2	0.492028239	0.380821784	0.938	0.8	1

TMEM168	0.492078759	0.90875334	1	1	1
KLHL18	0.492086552	0.762320572	1	1	1
CREBRF	0.492801457	-0.100971522	0.938	0.867	1
NELL2	0.492942506	0.732212064	0.812	0.667	1
EEF1AKMT3	0.493030189	0.182525038	0.625	0.333	1
WDR77	0.49376678	0.908926863	1	1	1
NUP58	0.494092162	0.781051083	1	1	1
DNAH9	0.494216336	0.540762697	1	0.8	1
ZNF644	0.494229803	0.36225255	1	1	1
PSMD12	0.494414823	0.367931667	1	1	1
XKR9	0.495015945	0.5980525	1	1	1
ZNF569	0.495165909	0.683914442	0.938	1	1
TAOK1	0.495588333	0.573280696	1	1	1
ENSSSCG00000045014	0.495741511	0.971020933	0.562	0.467	1
CNPPD1	0.49600904	1.066290417	1	0.933	1
ENSSSCG00000027890	0.496180412	1.491853096	0.5	0.2	1
ENSSSCG00000063156	0.496485066	0.147898695	0.938	0.8	1
SOCS6	0.496967876	0.67071565	1	1	1
PNPLA1	0.497089158	0.05626822	0.688	0.733	1
ENSSSCG00000031239	0.497097596	0.006426269	0.5	0.467	1
ZCCHC14	0.497272595	0.830269314	1	0.867	1
PTPRJ	0.497668369	0.841795567	1	1	1
DNAJC13	0.497698904	0.437949774	1	1	1
ENSSSCG00000053722	0.498383551	1.209453366	0.75	0.733	1
SLA2	0.49861774	0.036658471	0.812	0.8	1
RGS2	0.499110823	0.632802534	1	1	1
PPP2R5A	0.499376522	0.446723721	1	1	1
ENSSSCG00000016384	0.499410327	0.285402219	0.625	0.867	1
TMEM267	0.499501503	0.941067711	0.938	0.933	1
POLG	0.50010242	0.186998515	0.438	0.6	1
MED6	0.50020598	0.798329001	1	1	1
ARHGEF38	0.500401649	0.723726571	0.875	0.8	1
ENSSSCG00000057369	0.500698372	0.817623258	0.75	0.533	1
PIF1	0.500751359	0.003105911	0.562	0.6	1
TRMT13	0.500815746	0.906890596	0.938	0.8	1
TMSB4X	0.500849614	0.377279328	1	1	1
SNX18	0.501198077	0.616762529	1	1	1
NAA25	0.501280472	0.824428435	1	0.733	1
LAP3	0.501726699	0.678071905	1	1	1
ZC3H6	0.50181996	0.401087632	0.938	0.867	1
UBE2E2	0.502307324	0.830269314	0.875	0.667	1
CRK	0.502433745	0.733316212	1	1	1
SRSF6	0.502747977	0.323268267	1	1	1
AP4B1	0.502883797	0.22881869	0.938	0.933	1
UBN1	0.503118635	1.116790208	1	1	1
CASP8AP2	0.503471408	0.78722566	1	1	1
ZFYVE9	0.503528619	0.506972655	1	1	1
MORN2	0.503897152	0.692490965	1	1	1
ENSSSCG00000059507	0.504690198	0.754887502	0.812	0.333	1
ENSSSCG00000053580	0.504829036	0.276124405	0.938	0.933	1
EXOC8	0.505211265	0.107587946	0.875	0.667	1
LONRF2	0.505408984	0.577001215	1	1	1
PGM3	0.505935009	0.6794801	0.812	0.533	1
PTPRB	0.506412333	0.92347348	1	0.933	1
SNRPB	0.506977442	0.027184829	0.812	0.8	1
EIF1AX	0.507246587	0.700690208	1	1	1
ERLEC1	0.507379424	0.653499449	1	1	1
ENSSSCG00000060137	0.507931127	1.254813899	0.688	0.667	1
WDR33	0.508274472	0.671821198	1	1	1
MON2	0.50869214	0.717794585	1	1	1
SWAP70	0.508721058	0.855201119	1	1	1
ENSSSCG00000044572	0.508830565	0.254813899	0.625	0.667	1
ENSSSCG00000026233	0.508887725	0.482802282	1	1	1
CASR	0.509162946	0.652317769	0.938	0.867	1
ATP11B	0.509643804	0.775492706	1	1	1
B4GALT1	0.509805298	0.568634331	0.938	0.933	1
EGFL8	0.510195795	0.118394701	0.562	0.533	1
ENSSSCG00000056580	0.510716354	0.827323338	0.938	1	1
SCARB2	0.511221178	0.354211377	1	0.933	1
DNAH17	0.511305988	0.0489096	0.625	0.6	1

ENSSSCG00000056336	0.511487444	0.922157352	0.938	0.867	1
ANKRD50	0.51152759	0.425776064	1	1	1
PYCR3	0.511807481	1.169925001	0.625	0.467	1
TERF2	0.511967703	0.412046893	1	1	1
ENSSSCG00000027741	0.512734221	0.319740536	1	1	1
ENSSSCG00000036356	0.513030671	0.129283017	0.438	0.533	1
ATG4D	0.513456666	0.169925001	0.938	0.8	1
ENSSSCG00000024021	0.513741484	1.299208018	0.812	0.667	1
IRAG1	0.513844522	0.046615359	0.75	0.6	1
ENSSSCG00000044174	0.513870006	0.977279923	0.625	0.4	1
ZNF84	0.514734415	1.010513227	0.938	0.733	1
PTPRD	0.515313229	0.532824877	1	1	1
ZBTB21	0.515667116	0.468769483	0.625	0.667	1
PABIR2	0.515966161	0.595304445	1	1	1
ZC2HC1C	0.516227012	0.8758637	0.875	0.8	1
PLS1	0.516639243	0.925269125	0.688	0.667	1
HUS1	0.51720295	0.38283637	1	1	1
NEK10	0.517298618	0.563111779	0.938	0.933	1
SLC2A1	0.517317636	0.379643593	0.875	0.867	1
CPM	0.517506361	1.004933569	1	1	1
ATXN7L3B	0.51757809	0.356491482	1	1	1
RIPK4	0.517950569	1.130134416	1	0.933	1
SOGA1	0.518555408	0.918295359	1	0.867	1
ENSSSCG00000045070	0.518760014	0.607330314	0.562	0.467	1
CPNE8	0.518944536	0.775646062	0.625	0.667	1
ENSSSCG00000061161	0.519253122	1.154818109	0.562	0.267	1
RUBCN	0.519260703	0.776448407	1	1	1
ENSSSCG00000062358	0.519536807	0.951284715	0.688	0.6	1
CNTN5	0.519823319	0.467523767	1	1	1
SLC24A2	0.519934571	0.356197997	1	1	1
LCP1	0.519938305	0.202346479	0.438	0.533	1
TTC39B	0.520199983	0.649502753	1	0.867	1
U2AF2	0.520210602	0.928159212	1	0.867	1
DSG1	0.520262518	0.824428435	0.688	0.467	1
KSR2	0.520765564	0.437752887	1	1	1
ZNF12	0.520810135	-0.224353938	0.688	0.6	1
NAPEPLD	0.520923491	0.770255904	1	1	1
ENSSSCG00000055882	0.521149341	0.837627933	1	1	1
LINS1	0.521235549	0.372055978	1	1	1
S100A14	0.521322927	1.285402219	0.562	0.267	1
ZSCAN31	0.522126487	0.220048481	0.875	0.667	1
DPY19L2	0.522311865	0.752168001	0.875	0.667	1
TINAGL1	0.522370372	1.160647188	0.688	0.267	1
SLC66A1	0.52276555	0.215012891	0.625	0.533	1
BAIAP2	0.523163549	0.560326231	1	1	1
ENSSSCG00000052619	0.523164755	0.852028661	0.938	0.733	1
ATF6B	0.523176005	0.155711142	1	0.867	1
CLDN20	0.52317747	0.94295985	0.938	0.8	1
CCDC40	0.523362219	1.089862667	1	1	1
AP2A1	0.523367075	0.380821784	0.625	0.533	1
RPS6KL1	0.523415657	0.206450877	0.562	0.533	1
PALD1	0.524007217	1.217230716	0.688	0.667	1
MAN1C1	0.524126015	0.929258409	0.938	0.533	1
ZNF592	0.524139321	0.459838872	1	1	1
PEX12	0.524812015	0.61363636	1	1	1
PON2	0.524868071	0.233700912	0.812	0.867	1
RFX7	0.524918973	0.87734632	1	1	1
ZNF215	0.524995511	0.758765717	1	1	1
PORCN	0.525517203	1.305439972	0.625	0.333	1
ENSSSCG00000018060	0.525646902	0.769387072	1	0.6	1
AKT3	0.525707835	0.977312268	1	1	1
FNBP1L	0.526014971	0.733919888	1	1	1
MCFD2	0.526308981	0.633826835	1	1	1
RPH3AL	0.526673627	0.2644426	0.875	0.933	1
PLXND1	0.526774305	0.847777502	1	0.933	1
ENSSSCG00000059811	0.527525497	0.62309763	0.562	0.333	1
GID8	0.528310414	1.053208984	1	0.867	1
TEAD3	0.52833829	0.301169535	0.75	0.733	1
SLC4A8	0.528347722	0.702351839	0.875	0.733	1
SLC35A5	0.528480006	0.850307067	1	1	1

CAMK2G	0.528523254	0.778205092	1	1	1
MKNK2	0.528644666	0.906890596	1	0.933	1
TRRAP	0.528845491	0.864035117	1	1	1
PGK1	0.528964414	0.536438263	1	1	1
ENSSSCG00000062615	0.529124964	0.458132478	0.812	0.867	1
KDM6B	0.529139053	0.468769483	0.938	0.8	1
C8orf48	0.530079797	0.040157126	0.875	0.6	1
PUS7	0.530295089	0.534921818	1	1	1
PRC1	0.530361138	0.696427021	1	1	1
RIMOC1	0.530540027	0.744240258	1	1	1
CTTNBP2	0.530662899	0.618850763	1	1	1
PGAP4	0.530960239	1.206450877	0.5	0.133	1
ST3GAL5	0.531171481	0.513745702	1	0.933	1
GMDS	0.531580352	0.750771394	0.875	0.733	1
MGAT5B	0.531614291	0.973192966	1	1	1
ENSSSCG00000055759	0.532133543	0.006426269	0.375	0.533	1
SAP130	0.532567575	0.817474182	1	1	1
RAB5B	0.533099737	0.86476512	0.938	1	1
RASL12	0.533170639	0.705293917	1	1	1
MSX2	0.533338533	0.09330372	0.875	0.733	1
ANXA11	0.53407429	0.513795649	0.938	1	1
TMEM135	0.534212135	0.407674062	1	1	1
CD109	0.534298563	0.73470962	1	1	1
ENSSSCG00000010212	0.534312763	0.681598284	0.938	0.933	1
KMT2B	0.534652402	0.892293219	1	1	1
QRSL1	0.534688211	0.839427628	1	1	1
TTK	0.534746354	0.66264644	1	1	1
GATAD2B	0.534897788	0.420207612	1	1	1
SEC14L1	0.535082939	1.257387843	0.875	0.8	1
ZFAND4	0.535494543	1.26052755	0.562	0.333	1
PDZRN3	0.535834741	0.192292814	0.812	0.667	1
PACSIN2	0.536183301	0.841524316	1	1	1
ENSSSCG00000062318	0.536237572	-0.520530628	0.562	0.4	1
SCRN3	0.536322562	0.862768042	0.938	0.8	1
SNX15	0.536414137	0.3594028	0.625	0.467	1
ANO6	0.536509715	0.455750818	1	1	1
ZNF236	0.536667552	0.924099886	1	1	1
PON1	0.53730346	1.108524457	0.75	0.6	1
CDC42EP3	0.537370033	0.314065977	1	0.933	1
CLHC1	0.537379852	0.665882496	0.812	0.533	1
ANKRD10	0.537425864	0.887562335	1	1	1
UBR4	0.537443019	0.371154597	1	1	1
ENSSSCG00000035663	0.537891786	0.7426612	1	1	1
ENSSSCG00000040103	0.538058629	0.6036671	1	1	1
PAH	0.538270993	0.397069793	1	1	1
SLC7A8	0.538328105	0.403043829	1	1	1
ENSSSCG00000029199	0.538479614	0.818970747	1	1	1
ENSSSCG00000061287	0.538675136	0.083768358	0.875	0.667	1
ZNRF3	0.538770737	0.512611656	0.75	1	1
MCM7	0.53894917	1.03092031	1	1	1
ENSSSCG00000053596	0.538961482	0.713286598	1	1	1
ENSSSCG00000041876	0.539517931	0.169925001	0.938	0.667	1
ALDH7A1	0.539579627	0.640810922	1	1	1
SCN8A	0.540505495	0.377271129	1	1	1
PDE12	0.540658101	0.695850827	1	0.867	1
ENSSSCG00000034367	0.54106977	-0.006521719	0.938	0.933	1
PHF1	0.54179138	1.430452552	0.938	0.8	1
PJVK	0.54180117	0.236198221	0.812	0.733	1
NR2E3	0.541928671	0.211745177	0.562	0.533	1
NDUFA4L2	0.542123111	0.147898695	0.5	0.4	1
GFOD1	0.542313233	0.841795567	1	1	1
ENSSSCG00000056431	0.54232635	1.366322214	0.75	0.267	1
ENSSSCG00000012126	0.542581519	0.552459861	1	1	1
MAGI2	0.542746923	0.496885173	1	1	1
IL17RD	0.542813657	0.849405101	1	0.933	1
KPNA2	0.542949066	0.715784997	1	1	1
RBM8A	0.542986461	0.167481826	1	1	1
NOL11	0.544197302	0.690144737	1	1	1
ANKS4B	0.544254392	0.649633199	1	1	1
RAB10	0.544272804	0.629766785	1	1	1

SFPQ	0.544807901	0.631792371	1	1	1
LUC7L3	0.545029337	0.875752084	1	1	1
ENSSSCG00000055060	0.545248521	0.434628228	0.938	0.667	1
ARIH1	0.54528304	0.689188583	1	1	1
PTPN12	0.545697844	0.453172628	0.938	0.933	1
CLUL1	0.546519479	0.863821874	1	0.933	1
TFCP2	0.546777626	0.965784285	0.75	0.733	1
BCL7C	0.547762932	1.044394119	0.625	0.4	1
ZNFX1	0.548096303	0.905546678	1	1	1
ABHD16A	0.548147788	0.704073713	1	1	1
ENTPD5	0.548331886	0.824805771	1	1	1
ENSSSCG00000034696	0.548754796	0.261985554	0.75	0.8	1
ENSSSCG00000048793	0.548764435	0.88582898	1	1	1
AMER1	0.548948054	0.819427754	0.625	0.4	1
ENSSSCG00000056041	0.549537876	0.169925001	0.5	0.333	1
GIMAP4	0.549740286	0.022367813	0.375	0.533	1
PLCH1	0.549802547	0.665200425	1	1	1
NAV3	0.55029098	0.404601903	1	1	1
SLC12A9	0.550417296	1.206450877	0.75	0.733	1
ENSSSCG00000037928	0.550548433	0.906890596	0.812	0.667	1
ENSSSCG00000042741	0.550591229	0.104829973	0.688	0.6	1
ANKRD31	0.550721962	0.34639709	1	1	1
PPP6R3	0.550873128	0.552026651	1	1	1
ACTR6	0.550971081	0.677933239	1	1	1
SMAP1	0.550999582	0.797266104	1	0.933	1
CEP350	0.551320803	0.435831713	1	1	1
ITGAV	0.551528685	1.067836985	1	1	1
RICTOR	0.551549789	0.651496252	1	1	1
ENSSSCG00000053830	0.551781015	1.006426269	0.5	0.533	1
EIF3J	0.552347459	0.422439331	1	1	1
ENSSSCG00000056613	0.55262235	0.321928095	1	0.933	1
VPS39	0.552846961	0.400599871	1	1	1
HBP1	0.552851714	0.874415055	1	1	1
ENSSSCG00000033497	0.552858561	0.662505446	1	1	1
TAF6	0.55301546	0.72597481	1	1	1
ITFG1	0.553135518	0.733956844	1	1	1
SLC20A1	0.553400514	0.700085602	1	1	1
C9orf131	0.553833162	0.196397213	0.688	0.733	1
ENSSSCG00000023531	0.554268952	0.428427716	1	0.933	1
GRHL1	0.554345022	0.951284715	0.75	0.8	1
CLIP4	0.554391994	0.533478642	1	1	1
DDX39A	0.554568415	0.770241749	1	1	1
ARID4B	0.55463036	0.872443103	1	1	1
ENSSSCG00000033786	0.555235577	0.397216222	1	1	1
RAB11FIP5	0.555332958	0.321928095	0.75	0.6	1
KBTBD4	0.555375274	0.64542861	1	1	1
RBM38	0.55545798	1.073248982	1	0.867	1
ENSSSCG00000007705	0.555506944	0.739002746	1	1	1
AFDN	0.555582266	0.912515145	1	1	1
NAA11	0.555629448	0.984893108	0.75	0.667	1
LPGAT1	0.556465898	0.712791576	1	1	1
SPOP	0.556686804	0.606093965	1	1	1
ING5	0.556805815	0.903252019	1	1	1
FRY	0.556909798	0.539636868	1	1	1
HSPH1	0.557106199	0.494902449	1	1	1
TMEM174	0.557265009	0.738136138	0.875	0.867	1
ZMYM1	0.557653715	0.694995039	1	1	1
ENSSSCG00000054251	0.557751368	-0.015106892	0.438	0.533	1
ENSSSCG00000035428	0.557807648	0.875729664	0.938	1	1
DNAH14	0.558050907	0.6987824	1	1	1
CEP41	0.558396944	0.94753258	0.688	0.533	1
SLC39A8	0.558800267	0.35359601	0.938	1	1
ENSSSCG00000055775	0.559335408	-0.373217324	0.375	0.533	1
DNAJC16	0.559635061	0.755966556	1	1	1
ENSSSCG00000052335	0.559998399	0.67507492	0.938	0.733	1
PCP4L1	0.560041138	0.058893689	0.75	0.467	1
SP1	0.560182247	0.270638866	1	0.867	1
DCUN1D4	0.560378982	0.586651741	1	1	1
ENSSSCG00000053584	0.560650302	0.690398776	1	0.8	1
ZNF654	0.560774611	0.727003405	1	1	1

ENSSSCG00000045310	0.560897853	1.022367813	0.5	0.2	1
HSPD1	0.561000398	0.616634721	1	1	1
ZNF570	0.561029458	0.819040298	0.938	1	1
ENSSSCG00000037372	0.561077957	0.248996572	1	1	1
ENSSSCG00000015826	0.561407825	0.337130502	0.875	0.933	1
GNS	0.561525285	0.59101847	1	1	1
ROBO1	0.56153483	0.860383735	1	1	1
SIK3	0.561571943	0.733493402	1	1	1
CSNK2B	0.561769361	1.399758806	1	0.933	1
KATNAL2	0.561909445	0.536591411	1	1	1
MBD2	0.562089981	0.353989304	1	1	1
RUFY3	0.562206407	0.309971121	1	1	1
ERBIN	0.562242006	0.419929077	1	1	1
ENSSSCG00000058114	0.562585922	0.154818109	0.5	0.533	1
PRKG1	0.562886565	0.664320292	1	1	1
ENSSSCG00000055627	0.563201282	0.765714533	1	0.733	1
FAM222B	0.563265873	0.724026538	0.875	0.733	1
HPF1	0.563403905	0.681127885	1	1	1
ENSSSCG00000040442	0.563727494	0.661778098	0.688	0.467	1
SLC6A15	0.563775548	0.857397322	1	1	1
ENSSSCG00000008488	0.563857227	0.507534719	1	0.867	1
RPA2	0.564017406	0.573199051	1	1	1
ENSSSCG00000053892	0.564032345	0.906890596	0.562	0.4	1
IBTK	0.564081433	0.542262311	1	1	1
MCM3AP	0.564122619	0.093976148	0.938	1	1
ENSSSCG00000057567	0.56468102	0.22881869	0.75	0.8	1
MICAL3	0.564718789	0.809972452	1	1	1
ST3GAL3	0.565074956	0.994353437	0.562	0.333	1
RDH14	0.565123342	0.979040381	1	0.933	1
UBAP2L	0.565154069	0.622478677	1	1	1
NCOA7	0.565369125	0.215335461	1	1	1
DCAF7	0.566371734	0.657921911	1	1	1
ENSSSCG00000063104	0.566483093	0.827456128	0.625	0.267	1
BUB1	0.567381465	0.727471468	1	1	1
DMD	0.56779201	0.770829046	1	1	1
ALDH6A1	0.567811435	0.49842575	0.812	0.867	1
EDEM2	0.567848124	0.29620553	1	1	1
RTL6	0.567881807	0.437405312	0.625	0.533	1
TMEM184C	0.568320759	0.862240838	1	1	1
VPS18	0.568358332	0.332545202	1	1	1
NEIL2	0.568406287	-0.015106892	0.75	0.467	1
SRRM1	0.568589597	0.617383978	1	1	1
VPS35L	0.56869713	0.469904123	1	0.933	1
ENSSSCG00000054089	0.568944432	0.390212834	0.938	1	1
PRKAR2B	0.568964842	1.015294188	1	0.933	1
SBNO1	0.569125612	0.691331867	1	1	1
KANK2	0.569797064	0.512424706	1	1	1
ENSSSCG00000055266	0.569852879	0.276124405	0.938	0.8	1
ELMO1	0.569857184	0.743391863	0.75	0.4	1
CRTC1	0.569870104	0.788246099	0.75	0.533	1
ENSSSCG00000053847	0.570116124	0.57758297	0.812	0.867	1
WASF2	0.570357932	0.497897073	1	1	1
NAPG	0.57052315	0.534921818	1	1	1
ANKRD6	0.571011205	0.657001566	1	1	1
JPH1	0.571509492	0.590197038	1	1	1
ENSSSCG00000059296	0.571914085	1.067355268	0.75	0.6	1
DUSP7	0.572108436	0.757693523	1	1	1
WSB1	0.572265565	0.748888762	1	1	1
ENSSSCG00000059326	0.572433736	-0.27368165	0.688	0.467	1
ZNF280B	0.572705436	0.437613508	1	1	1
SLC25A15	0.572737226	0.382469637	0.875	0.8	1
PCDHB1	0.572808157	0.399671874	0.938	1	1
ENSSSCG00000057818	0.573127028	0.791413378	0.438	0.533	1
ENSSSCG00000017906	0.573275624	0.288761231	0.812	0.867	1
TMEM265	0.573723218	0.27462238	0.75	0.667	1
SLC25A35	0.573790848	0.364572432	0.812	0.867	1
FAM81A	0.574067633	0.100977648	0.938	0.933	1
PDF	0.57407776	0.283744901	0.812	0.8	1
EP300	0.574215042	0.510697633	1	1	1
MSH4	0.574237128	0.642928113	1	1	1

ENSSSCG00000007135	0.57425743	1.154515562	1	1	1
VANGL1	0.574391637	0.530585491	1	1	1
EXO1	0.574471725	0.377826955	1	1	1
SPRYD4	0.574868589	0.4559064	1	0.933	1
ELOVL7	0.575043944	0.254813899	0.562	0.8	1
AP4E1	0.575060743	0.451019379	1	0.933	1
ENSSSCG00000007589	0.575152002	0.788627714	0.938	0.867	1
ENSSSCG00000059288	0.575179447	0.540762697	0.938	0.867	1
MADD	0.575225714	0.377755129	1	1	1
ROCK1	0.57527551	0.935459748	1	1	1
CNTNAP5	0.575664207	0.719805043	0.875	0.8	1
TYW5	0.576175447	0.138972073	1	0.933	1
ADAMTS17	0.57643328	0.802193217	0.812	0.733	1
CTNNBIP1	0.576729345	0.321928095	0.375	0.667	1
ENSSSCG00000000405	0.576973201	0.706799333	1	0.933	1
LRRC8B	0.577073704	0.584962501	1	1	1
CNST	0.577640449	0.270379004	1	1	1
ENSSSCG00000062102	0.578245278	1.269460675	0.5	0.267	1
ZNF639	0.578706187	0.647478985	1	1	1
TATDN3	0.578720368	1.096715154	0.938	0.6	1
TTF2	0.579449978	0.329232896	0.938	1	1
KPNA5	0.579892792	0.848787641	1	0.8	1
KANTR	0.580163562	1.147898695	0.562	0.467	1
ENSSSCG00000053935	0.581586161	0.781359714	0.562	0.533	1
SLC39A7	0.581684667	0.966596842	0.938	0.933	1
GMEB1	0.581758079	0.402418013	0.938	0.867	1
YPEL3	0.581758619	0.452736966	1	0.933	1
ENSSSCG00000052555	0.581884138	0.785584299	0.938	0.8	1
DUSP10	0.581926853	0.186998515	0.75	0.8	1
CCDC181	0.582431488	0.387780642	0.938	0.8	1
GKAP1	0.582798639	0.723709497	1	1	1
ENSSSCG00000060864	0.583101496	0.206450877	0.5	0.533	1
TMCO3	0.583103048	0.967125703	0.938	1	1
ADCY10	0.583131639	0.002649578	0.875	0.733	1
COX10	0.58337544	0.217230716	0.812	0.667	1
ENSSSCG00000061843	0.584327156	0.628589433	1	0.933	1
MRAS	0.584374232	0.749534268	1	0.933	1
ENSSSCG00000041019	0.584522629	0.875181736	0.875	0.6	1
STK40	0.584630916	0.681123447	1	1	1
ENSSSCG00000037413	0.584695485	0.123701985	0.625	0.667	1
ARHGAP21	0.584963088	0.692482263	1	1	1
GALNT1	0.585154471	0.665882496	1	1	1
PDLIM1	0.585199165	0.819427754	0.938	0.8	1
PPP2CA	0.585483095	0.601953938	1	1	1
ENSSSCG00000008137	0.585684765	-0.491658781	0.562	0.667	1
REPIN1	0.585863333	1.312602303	1	1	1
ENSSSCG00000039046	0.586016726	0.12701641	0.875	0.867	1
THSD7B	0.586582	0.488844346	1	1	1
ENSSSCG00000035073	0.587023288	0.492938237	1	1	1
ZNF175	0.587024383	1.058893689	0.625	0.8	1
ENSSSCG00000054299	0.587427643	0.635588574	0.625	0.4	1
ENSSSCG00000062996	0.587443074	0.906890596	0.938	1	1
TMX1	0.587496863	0.614024966	1	0.933	1
GPR137C	0.58762996	0.185791407	1	1	1
SART1	0.587630324	0.771961016	0.938	0.933	1
ZRSR2	0.588742009	0.37185332	0.938	0.867	1
ST8SIA6	0.588816679	0.576741994	1	1	1
RBSN	0.588979699	0.259906368	1	1	1
CEP290	0.589189884	0.641574621	1	1	1
NCBP2	0.589321302	0.557504953	1	1	1
HIPK2	0.590087737	0.366322214	0.938	0.867	1
INSC	0.590202077	0.63907905	1	1	1
ENSSSCG00000056178	0.590597337	1.049848549	0.562	0.4	1
RIPK1	0.590671358	0.782776899	1	1	1
C10orf105	0.591093228	0.736965594	0.562	0.333	1
ENSSSCG00000054636	0.591152599	0.392317423	0.75	0.667	1
ENSSSCG00000034711	0.592055551	0.758040636	1	1	1
WDR7	0.592402153	0.082740431	0.938	0.867	1
PIEZO1	0.592451662	0.321928095	0.812	0.867	1
ANAPC7	0.59255778	0.615395425	1	1	1

MINK1	0.592572841	0.94641896	0.562	0.467	1
ENSSSCG00000048555	0.593228532	1.076815597	0.562	0.267	1
ENSSSCG00000043473	0.593298763	0.040157126	0.688	0.6	1
ENSSSCG00000054056	0.593465463	1.196397213	0.625	0.533	1
ENSSSCG00000031741	0.59356104	0.949451058	1	1	1
PREPL	0.593593932	0.679182345	1	1	1
MED21	0.593603269	0.59047402	1	1	1
MAP7D2	0.593793919	0.57766591	0.938	1	1
TEX9	0.594145195	0.314314911	0.938	0.933	1
RNF139	0.594646489	0.514706182	1	1	1
ENSSSCG00000050691	0.59477878	0.761039729	0.875	0.733	1
AIG1	0.595030345	0.26052755	0.5	0.4	1
MDM4	0.595044628	0.679810684	1	1	1
SNX8	0.595267106	0.760170723	1	1	1
PXYLP1	0.59539086	-0.147557188	0.812	0.733	1
ENSSSCG00000055510	0.595433083	1.169925001	0.562	0.4	1
UNC5D	0.595667038	0.798691855	1	0.933	1
RSPRY1	0.596006979	0.103287808	0.625	0.533	1
NAP1L2	0.596627095	0.206450877	0.75	0.733	1
PARN	0.597197241	0.669561904	1	1	1
MDH1B	0.597388041	0.169925001	0.562	0.4	1
SOX4	0.597782409	0.906890596	0.812	0.4	1
CHPF2	0.598111154	0.453633774	0.938	0.867	1
SLC17A7	0.598221368	0.984893108	0.562	0.267	1
GRM8	0.598340973	0.658277307	1	1	1
ZNF391	0.598777459	-0.042923647	0.875	0.867	1
MAP3K7CL	0.598840502	0.363128955	1	1	1
XPO7	0.598904384	0.650375193	1	1	1
ZNF596	0.599016857	0.534437489	1	1	1
ZNF580	0.59904807	0.070389328	0.75	0.867	1
SFT2D3	0.599121072	0.531381461	0.75	0.8	1
PHC2	0.599260372	0.52501996	1	0.867	1
MMACHC	0.599335282	-0.03866162	0.625	0.667	1
DEPDC1	0.600524141	0.752856967	0.938	0.933	1
RAN	0.601119387	0.557450587	1	1	1
NRIP1	0.601348359	0.710667107	1	0.8	1
MAPRE3	0.601399403	0.824428435	0.562	0.6	1
RNF19B	0.601907733	0.628558301	1	1	1
ABLIM1	0.602140514	0.744444968	0.938	1	1
ZNF613	0.60310775	-0.047305715	0.875	0.6	1
ENSSSCG00000005880	0.603153543	0.427722759	0.438	0.6	1
ENSSSCG00000045157	0.603245434	0.994353437	0.625	0.2	1
ACOT9	0.603543096	0.577042837	1	0.867	1
WNK3	0.603607565	0.459431619	1	1	1
TTLL7	0.603662141	0.403722186	0.938	0.867	1
ENSSSCG00000053756	0.603967807	0.906890596	0.625	0.333	1
MMP9	0.604124534	0.211745177	0.5	0.4	1
HOOK3	0.60505202	0.749490575	1	1	1
ZDHHC21	0.605187851	0.835099913	0.875	0.8	1
ENSSSCG00000047436	0.60561398	0.54630088	1	0.933	1
ENSSSCG00000048636	0.606081024	0.564784619	0.75	0.667	1
TLN1	0.606203636	0.78730898	0.938	0.867	1
PIGB	0.606256655	0.305986551	1	0.867	1
ADGRV1	0.606300461	0.668279664	1	1	1
TFAP2C	0.606450467	1.07534421	1	1	1
NFILZ	0.606708483	1.26052755	0.5	0.267	1
ENSSSCG00000046422	0.606755623	0.810214576	0.875	0.733	1
ENSSSCG00000057139	0.60683477	0.584962501	0.688	0.6	1
DGCR2	0.607021179	0.639194812	1	0.933	1
ST7	0.607470041	0.369183002	1	1	1
GNB1	0.607774925	0.783686016	1	1	1
LHFPL2	0.60832416	0.562349932	1	1	1
PITX2	0.608548179	0.269460675	0.562	0.6	1
PRRC2C	0.608728443	0.762450796	1	1	1
TCF3	0.608977717	0.636801432	0.938	1	1
ACTR10	0.610130682	0.457087678	1	1	1
DYNLT3	0.610392951	0.656912343	0.875	0.867	1
TMEM236	0.61046928	0.724026538	0.562	0.533	1
MSANTD4	0.61055229	0.166757722	0.875	0.733	1
UGGT2	0.610671736	0.257046981	1	1	1

DNAL4	0.61079047	0.67577137	1	1	1
TADA3	0.610863276	1.086514083	1	1	1
ENSSSCG00000020988	0.610912432	-0.4012317	0.5	0.4	1
ZFYVE16	0.610950618	0.235513343	0.625	0.4	1
ATP7B	0.611381642	0.551103697	1	1	1
C2orf69	0.611589679	0.582946155	1	0.933	1
JMJD4	0.611845474	0.802110258	1	1	1
ENSSSCG00000063532	0.612643102	0.619480615	0.938	0.933	1
ENSSSCG00000053530	0.61403575	0.708455429	1	1	1
SVIP	0.614800867	0.923569337	0.812	0.733	1
ENSSSCG00000062546	0.615078376	0.076815597	0.562	0.4	1
TAF11	0.615164197	0.502936595	1	1	1
EML6	0.615191582	0.509555098	1	1	1
ABI3BP	0.616020492	0.496537175	1	1	1
ENSSSCG00000056714	0.616487851	0.442943496	0.688	0.533	1
CNOT9	0.616510727	0.600253408	1	1	1
SERPINA6	0.616930915	0.555418225	0.875	0.667	1
TEX30	0.617111266	0.745252205	1	1	1
ELF5	0.617759433	1.193771743	0.875	0.6	1
ZSCAN29	0.618095215	0.539158811	0.5	0.533	1
ENSSSCG00000012074	0.61829248	0.993768046	1	1	1
SLC22A15	0.618303174	1.055753982	0.938	0.933	1
PLEKHA1	0.618377289	0.812613895	1	1	1
ENSSSCG00000055399	0.618568056	0.669496011	0.938	0.867	1
ENSSSCG00000028195	0.619076279	0.387516437	0.812	0.733	1
DCTN5	0.619187351	0.538957331	1	1	1
C14orf28	0.619312233	0.770569124	0.938	0.933	1
TMEM161B	0.619485374	0.730567823	0.875	0.867	1
C2orf42	0.620062271	0.411909462	1	1	1
SPRYD3	0.620104039	0.354349573	0.875	1	1
CLCN6	0.620165702	0.532495081	0.75	0.733	1
PRSS12	0.620289976	0.437405312	0.688	0.667	1
TRIM65	0.620451935	0.121015401	0.562	0.733	1
CLUH	0.62053959	0.894893063	1	1	1
MYLIP	0.620959878	0.823059005	1	1	1
PPFIA2	0.621294228	0.486624582	1	1	1
ENSSSCG00000056325	0.621372231	0.409390936	0.5	0.533	1
ASMT	0.62153154	0.701571688	0.938	0.733	1
PLEKHA2	0.621674208	0.760109258	1	1	1
FAM216A	0.621757454	0.372554168	0.688	0.533	1
FN1	0.622253576	0.505149919	0.688	0.733	1
IGSF9	0.622573503	1.285402219	0.562	0.267	1
ZNF385B	0.62319137	0.906890596	0.75	0.667	1
FADD	0.623200517	0.476256241	0.625	0.667	1
ZNF148	0.623243855	0.682184308	1	0.733	1
SYNPO2	0.623922409	0.735124247	0.812	0.933	1
BMERB1	0.623940233	0.483815777	0.938	0.867	1
NEK2	0.624049125	0.676827161	1	1	1
LTBP2	0.624129456	1.321928095	0.562	0.333	1
ITGB4	0.624226044	0.256224848	0.75	0.733	1
NUP205	0.625136272	0.489572155	1	1	1
POLK	0.625345371	0.685475279	1	0.8	1
CDK12	0.625668033	0.530087315	1	1	1
RPS6KA3	0.625974526	0.568177916	1	1	1
SKAP2	0.626247792	1.073787904	1	1	1
GABRR2	0.626273533	0.817005092	0.938	0.933	1
FBXW7	0.626350213	0.721535554	1	0.933	1
RGS22	0.626471043	0.562445064	1	1	1
GABPB1	0.626596532	0.71897567	1	1	1
COCH	0.627218264	0.568490066	1	1	1
SLC20A2	0.627935112	0.807354922	0.812	0.8	1
AGBL2	0.628378153	0.3594028	0.875	1	1
CCR1	0.62838867	0.65011518	0.938	1	1
ATP2B4	0.628610199	0.60694087	1	1	1
THSD4	0.628732013	1.006426269	0.625	0.4	1
ENSSSCG00000037963	0.628776172	0.685676365	1	1	1
NAA15	0.628789607	0.392964033	1	1	1
MRPL39	0.629263997	0.458193785	1	1	1
ENSSSCG00000035908	0.629319724	0.595935033	1	1	1
RBM48	0.629669362	0.314768303	1	1	1

MAP2	0.629772887	0.461479447	0.938	0.8	1
SLC43A3	0.630176404	0.881579507	1	1	1
QRFPR	0.630269601	0.293913719	0.5	0.333	1
ENSSSCG00000047090	0.630355821	0.965784285	0.875	0.6	1
ENSSSCG00000058385	0.630423441	0.576465324	1	1	1
SLC25A53	0.630746769	0.312882955	0.688	0.6	1
CORO1C	0.630751553	0.702598293	1	1	1
ENSSSCG00000061837	0.63125623	1.036173613	0.688	0.667	1
CDYL2	0.631723903	0.63481105	1	1	1
PRKD2	0.631762467	0.613159393	0.562	0.6	1
LMNB1	0.631948608	0.69930442	1	1	1
ICMT	0.631965543	0.954141942	1	1	1
ENSSSCG00000004489	0.632251862	0.520571407	1	1	1
EIF2S1	0.632498249	0.339385292	1	1	1
GRM7	0.632555555	0.033087388	1	1	1
STXBP5	0.63267688	0.916015832	1	1	1
ZNF292	0.632881779	0.166494096	1	1	1
FERD3L	0.632922143	0.984893108	0.562	0.2	1
MAML1	0.632957141	0.68061474	1	1	1
GLMN	0.633336235	0.392317423	1	0.867	1
PRDM6	0.633608883	0.574212891	1	1	1
GSAP	0.633652711	0.524274574	0.812	0.8	1
UBE2K	0.633825933	0.536849267	1	1	1
RAB18	0.633963601	0.57505611	1	1	1
HS6ST1	0.634342227	0.430452552	0.5	0.4	1
CACNA2D4	0.634372486	0.705274519	1	1	1
C3orf38	0.634612645	0.509887019	1	1	1
CNNM3	0.634725339	0.757302055	1	1	1
ADAMTS18	0.634904184	0.624082516	1	0.933	1
ABCC5	0.634990332	0.394684437	0.812	0.867	1
MECR	0.635169875	0.3594028	0.75	0.733	1
MRPL43	0.63523784	0.795036291	1	1	1
GRAMD1C	0.63579771	0.058893689	0.5	0.533	1
TIGD4	0.636267878	0.761285273	0.938	0.933	1
CXCR4	0.636665477	0.789054105	0.875	0.533	1
TDRKH	0.636916581	0.182455186	0.938	1	1
KASH5	0.636927439	0.440322796	0.812	0.6	1
DNAJC6	0.636928725	0.484393787	1	1	1
ENSSSCG00000009157	0.637082468	0.345846722	1	1	1
HARBI1	0.637203589	0.710140058	0.938	1	1
ENSSSCG00000055074	0.637350469	0.509555098	0.625	0.733	1
PPIP5K1	0.637716007	0.927952211	0.625	0.667	1
ENSSSCG00000010884	0.638060224	1.044394119	0.688	0.6	1
ENSSSCG00000059944	0.638360715	0.563359061	1	1	1
GTF2A1L	0.638509838	0.163104187	1	1	1
ZCWPW2	0.638607752	0.799281622	0.938	0.933	1
MFSD4B	0.638659102	0.680324357	1	1	1
ENSSSCG00000058576	0.638678839	0.769387072	0.5	0.267	1
ENSSSCG00000058300	0.638750572	0.653771659	0.875	0.8	1
TMEM35A	0.638979338	0.805010982	0.688	0.6	1
SLF2	0.639270791	0.539833125	1	1	1
ENSSSCG00000051837	0.639497406	0.411884792	1	0.933	1
HLF	0.639730796	0.825753833	0.938	1	1
E2F5	0.639800421	0.211745177	0.5	0.533	1
CNN2	0.639942778	0.357153757	1	1	1
CANX	0.640434965	0.679294694	1	1	1
PLEKHA5	0.640517502	0.807354922	0.875	0.533	1
ASXL2	0.640523415	0.897283268	1	1	1
GPSM2	0.640602959	0.540216118	1	1	1
RAD9B	0.640775692	1.055282436	0.875	0.8	1
TMEM51	0.64106893	0.762500686	0.938	1	1
DUSP14	0.64154995	0.773349745	1	1	1
PEX6	0.641555279	0.211745177	0.5	0.533	1
ENSSSCG00000055034	0.641565026	0.064431873	0.5	0.6	1
TMEM18	0.641983049	0.619314006	1	0.933	1
ENSSSCG00000062177	0.64215586	0.850307067	0.562	0.4	1
PRCC	0.642172138	0.648697453	1	1	1
RAB11A	0.642318895	0.712561703	1	1	1
ACO1	0.64262078	0.484435625	0.938	0.867	1
KTN1	0.642647571	0.623388711	1	1	1

SMARCAD1	0.6429263	0.579595991	1	1	1
ENSSSCG00000047287	0.642999974	0.321928095	1	1	1
TTF1	0.643116892	0.915352174	0.875	1	1
NFATC3	0.643699498	0.711166125	1	0.867	1
ENSSSCG00000063479	0.643875218	0.52931011	1	1	1
DNAH6	0.644509139	0.748805924	1	1	1
RNMT	0.644650049	0.688374229	1	1	1
ENSSSCG00000037401	0.644773354	0.837627933	0.75	0.6	1
CAD	0.644779652	0.466318004	0.625	0.6	1
DNAJB14	0.644924089	0.7570668	1	1	1
NID1	0.645037734	0.480075929	0.812	1	1
ORC6	0.645085941	0.573845582	1	1	1
ADAM19	0.645113587	0.674282491	1	1	1
ENSSSCG00000045838	0.645654305	0.250844997	0.625	0.467	1
ENSSSCG00000051325	0.64575708	0.526859546	0.938	1	1
POU3F2	0.645799032	0.421463768	0.688	0.467	1
ABAT	0.645846029	-0.093109404	0.562	0.733	1
ENSSSCG00000057133	0.645967641	0.867719998	0.812	0.733	1
ENSSSCG00000044626	0.646182527	0.929258409	0.875	0.6	1
ACTN1	0.64632393	0.984893108	0.875	0.733	1
BAZ2B	0.646517313	-0.005291526	0.938	0.733	1
OR10G3	0.64693414	0.965784285	0.625	0.4	1
CELF3	0.647129357	0.514573173	0.75	0.533	1
GCFC2	0.64724192	0.67507492	0.812	1	1
PGM2L1	0.647451792	0.539021882	1	1	1
ENSSSCG00000054374	0.647557933	0.347463187	0.625	0.667	1
ENSSSCG00000059141	0.647920355	0.72631835	0.438	0.533	1
CCDC174	0.648024082	0.47720632	0.938	0.867	1
PAWR	0.648398431	0.769049954	1	1	1
PCNX3	0.648555878	0.351192689	0.938	0.867	1
CYP2U1	0.64874598	0.184424571	0.688	0.6	1
COX11	0.648800238	0.209453366	0.625	0.533	1
IL17RB	0.649545187	0.508154909	0.875	0.933	1
GADL1	0.649557043	0.792431871	0.875	0.8	1
ENSSSCG00000054723	0.64990478	0.354349573	0.625	0.8	1
PIK3C2G	0.65012851	0.453633774	0.938	0.933	1
STK11IP	0.650290948	0.881445579	1	0.8	1
ENSSSCG00000055210	0.650419069	0.638862	0.938	1	1
KIAA2026	0.650552665	0.324448087	0.938	1	1
IFT80	0.650770576	0.517323784	1	1	1
RABL3	0.650923876	0.214522845	1	1	1
TWNK	0.651087684	0.636243006	0.938	1	1
NOC3L	0.651575246	0.998038484	0.875	0.933	1
TRIM66	0.651645888	0.321928095	0.688	0.6	1
FAM174A	0.653209025	1.491853096	0.5	0.467	1
ZBTB40	0.653276303	0.814133455	1	1	1
GPNMB	0.653362736	0.298023528	1	1	1
ENSSSCG00000043807	0.653663861	0.113341473	0.625	0.467	1
MCTP2	0.653817424	0.49816687	0.875	0.867	1
ENSSSCG00000054366	0.654496989	0.378196315	0.875	0.867	1
SOWAHB	0.654608026	0.293913719	0.75	0.6	1
LVRN	0.654705857	0.427722759	1	0.933	1
HERC3	0.654856902	0.757195445	1	1	1
ZNF527	0.654954516	0.468317582	0.938	1	1
GMPR2	0.655011438	0.797266104	0.875	0.667	1
MED17	0.655197918	0.8560152	1	1	1
GBA1	0.65541859	0.042242449	0.812	0.733	1
LDLR	0.655952459	0.639162314	1	1	1
ELAVL3	0.656075788	1.169925001	0.5	0.133	1
FANCB	0.656165384	0.643414255	1	1	1
COL17A1	0.656417081	0.245692509	0.75	0.8	1
ENSSSCG00000053550	0.656741728	1.09330372	0.812	0.733	1
SH3GLB1	0.656777059	0.620081839	1	1	1
SVEP1	0.65689782	0.541073171	1	1	1
RAPGEF4	0.656982393	0.519867472	0.875	0.933	1
MLIP	0.657404268	0.347463187	0.75	0.8	1
ENSSSCG00000058582	0.657971604	0.906890596	0.562	0.667	1
NABP1	0.658063325	0.880579318	1	1	1
SEMA6D	0.658255797	1.432352085	0.812	0.6	1
HADHB	0.658521456	0.649224364	1	1	1

NLGN1	0.658674512	1.169925001	0.75	0.667	1
TSPYL1	0.658992986	0.516944077	1	1	1
HCN3	0.659441398	0.727341175	0.938	0.867	1
WRNIP1	0.659497514	0.731119336	1	0.933	1
ZNF438	0.659665597	0.321928095	0.75	0.6	1
ENSSSCG00000044651	0.660324762	0.906890596	0.5	0.133	1
DEPDC5	0.660352498	0.641508435	1	0.867	1
ENSSSCG00000053141	0.660516911	0.38332864	0.75	0.867	1
ENSSSCG00000045328	0.660879908	0.285402219	0.375	0.6	1
PPM1E	0.66090447	0.498269838	1	1	1
PURG	0.661387384	0.099535674	0.812	0.533	1
UBAC1	0.661394433	0.669851398	0.875	0.6	1
TGFBRAP1	0.661579014	0.750634898	1	1	1
NRF1	0.661599416	0.727283041	1	1	1
RUNX1T1	0.661748801	0.5493026	1	1	1
SLC35A2	0.661782599	0.895575282	1	0.867	1
SCIN	0.662375918	0.6622055	0.938	1	1
SPAG9	0.662423562	0.822935073	1	1	1
ENSSSCG00000056189	0.662552415	0.64385619	0.188	0.533	1
GPR12	0.662855656	0.781359714	0.75	0.8	1
RAD54L2	0.662973103	0.732507724	1	1	1
CALB1	0.663236637	0.584962501	0.5	0.733	1
DAPP1	0.663429634	0.641500048	1	1	1
BRINP1	0.663634726	0.083768358	0.625	0.533	1
EIF4G3	0.663814198	0.475094183	1	1	1
MDM1	0.663893999	0.74624586	1	1	1
PSME3	0.6641385	0.429161318	1	1	1
SLC2A5	0.664214203	0.427722759	0.625	0.467	1
ENSSSCG00000051151	0.664300951	0.62935662	0.562	0.733	1
TRIM42	0.664447281	1.519867472	0.562	0.467	1
KLF5	0.66464179	0.167757165	1	1	1
GOSR1	0.664951403	0.529874941	1	1	1
ENSSSCG00000034192	0.664999383	0.655537995	1	1	1
ENSSSCG00000056202	0.665359338	0.421463768	0.5	0.667	1
ENSSSCG00000036124	0.665495695	0.096551815	1	1	1
ENSSSCG00000063471	0.665647326	0.510468774	0.875	0.8	1
SLC38A9	0.665848169	0.811706174	1	1	1
ENSSSCG00000063173	0.6658642	0.332816411	1	0.933	1
ZNF197	0.665969836	0.553253641	0.5	0.533	1
ITPK1	0.66670331	0.210282739	0.75	0.8	1
ENSSSCG00000032297	0.666705397	0.680254951	1	1	1
CHUK	0.666816821	0.679154853	1	1	1
TLNRD1	0.666912416	0.706865987	1	1	1
ENSSSCG00000059771	0.667170341	0.406461605	0.938	0.933	1
ENSSSCG00000052502	0.667785639	0.74819285	0.312	0.533	1
PBX2	0.667986452	1.022367813	0.5	0.333	1
COL21A1	0.668123596	0.276124405	0.562	0.6	1
TIGD3	0.668233407	0.169925001	0.5	0.333	1
ZHX3	0.668391412	0.242310927	1	1	1
ZFP90	0.668598009	0.299976268	1	1	1
SLC25A42	0.668879791	0.457905764	0.75	0.667	1
FAM3C	0.669248628	0.789342298	1	1	1
CRBN	0.669475841	0.480861081	1	1	1
SH3D19	0.669758294	0.392317423	0.75	0.533	1
HSDL2	0.669798034	0.588613355	1	1	1
RIPOR2	0.669804645	0.659975855	1	0.867	1
NOTCH2	0.669827019	0.811733363	1	0.8	1
ABL2	0.670046728	0.680832838	1	1	1
ENSSSCG00000055153	0.670212813	0.906890596	0.875	0.8	1
RALGAPA1	0.670441821	0.603498452	1	1	1
SLC10A6	0.670488906	0.743391863	0.625	0.533	1
NAP1L4	0.670561144	0.753195038	1	1	1
SLC2A13	0.670715343	0.670247689	1	1	1
DDR2	0.670934815	0.945025724	1	1	1
ZNF26	0.671274212	0.619743734	1	1	1
ENSSSCG00000045607	0.67154914	0.773624065	0.562	0.6	1
SDF2L1	0.671949294	0.595761902	1	0.933	1
FEM1B	0.671994918	0.381286158	1	1	1
ENSSSCG00000053861	0.672000305	0.1627295	1	0.933	1
DNAH10	0.672204059	0.824428435	0.812	0.733	1

BTG3	0.672381255	0.886712714	0.75	0.467	1
JAK1	0.673417755	0.646743376	1	1	1
SEC31B	0.673458879	0.646922493	0.875	1	1
RGS7	0.673634122	0.94641896	0.625	0.467	1
MARK1	0.673788887	0.609504685	1	1	1
ENSSSCG00000049364	0.674289085	0.321928095	0.625	0.533	1
CHRM2	0.674438194	0.349248701	1	0.933	1
MPZL3	0.674637185	0.957516669	0.938	0.867	1
CARNMT1	0.675430445	0.526986633	1	1	1
PLBD2	0.6759078	0.794997716	0.875	1	1
ENSSSCG00000046103	0.676128651	0.906890596	0.75	0.533	1
POGLUT3	0.676452024	1.136896201	1	0.733	1
ENSSSCG00000032439	0.676480452	0.254813899	0.562	0.333	1
NDFIP1	0.677119266	0.732743256	1	1	1
HTR7	0.677230403	0.305439972	0.625	0.467	1
FKBP15	0.677610254	0.126271126	1	0.933	1
ENSSSCG00000045184	0.678463074	0.099535674	0.688	0.8	1
ENSSSCG00000031911	0.678480754	1.064431873	0.625	0.467	1
NELFB	0.678491057	0.797266104	0.938	0.667	1
DNAAF5	0.678853469	0.899801171	0.938	0.8	1
MTTP	0.679027333	1.058893689	0.5	0.267	1
MCUR1	0.679627607	0.585961251	1	1	1
RUSF1	0.679773832	0.512611656	0.688	0.8	1
ENSSSCG00000052959	0.679828498	0.178192617	0.875	0.667	1
ENSSSCG00000021757	0.679881118	0.327176173	0.938	1	1
PRPF18	0.680580808	0.436759251	1	1	1
ENSSSCG00000058255	0.680703075	0.774618136	1	1	1
ZNF800	0.681156052	0.593474001	0.938	0.933	1
IDNK	0.681284371	0.888628389	0.938	1	1
TMEM164	0.681322715	0.632952444	1	1	1
LSM1	0.681567189	0.438337587	1	0.867	1
ZNF7	0.681818545	0.745455007	0.938	1	1
EEA1	0.681870635	0.74199799	1	1	1
MTPN	0.681893393	0.745491984	1	1	1
ENSSSCG00000002202	0.682007042	1.299208018	0.5	0.2	1
EXOG	0.682066322	0.336283388	0.875	0.8	1
ENSSSCG00000054944	0.682496378	0.380821784	0.562	0.4	1
DNAH7	0.682732169	0.602538083	1	1	1
DCAF15	0.683148082	0.588361077	1	0.867	1
ARL1	0.683738813	0.639410285	1	1	1
ENSSSCG00000060078	0.683915244	0.22881869	0.688	0.533	1
ANKS1A	0.684149826	0.847996907	1	0.933	1
RAB30	0.684523868	0.636969804	0.938	1	1
UNKL	0.68482678	0.957280876	1	1	1
TTC31	0.685134506	0.754887502	0.688	0.4	1
ABCB10	0.68530049	1.026800059	1	1	1
BNIPL	0.685336806	-0.005646563	0.5	0.533	1
CAPN15	0.685370437	1.22881869	0.5	0.2	1
PRICKLE4	0.685593792	0.756421352	1	1	1
FBLIM1	0.685887011	0.676770812	1	0.933	1
ENSSSCG00000023320	0.685975672	0.780653869	1	1	1
HYAL2	0.686092377	0.908506156	1	1	1
CBLB	0.686233972	0.369739575	1	1	1
GPATCH3	0.686523323	0.554588852	0.812	0.8	1
HOXA7	0.686713375	0.793233814	0.938	0.733	1
ENSSSCG00000038192	0.686883349	0.321928095	0.625	0.4	1
SLC5A3	0.686910793	-0.377676972	0.625	0.667	1
RNF44	0.687048817	0.432959407	0.938	0.933	1
ATR	0.687111401	0.547934648	1	1	1
NECAB1	0.687173136	0.509298231	0.938	0.867	1
RBBP6	0.68726885	0.637062457	1	1	1
THUMPD2	0.687298021	0.581795222	0.875	0.8	1
PRPF38A	0.687510673	0.528378972	1	1	1
CAPN10	0.687569621	1.030625964	0.938	0.8	1
FGF11	0.688181111	0.988684687	0.75	0.733	1
PACRGL	0.6885506	0.513795649	0.938	0.867	1
PLCL2	0.68861493	0.056699476	0.938	1	1
UBE2E3	0.688722005	0.703053993	1	1	1
RGMB	0.689049918	0.564498398	0.938	0.867	1
PTPRZ1	0.68950452	0.832350117	1	1	1

NEDD4	0.689780819	0.812344959	1	0.933	1
ENSSSCG00000002657	0.689960526	0.491853096	0.688	0.533	1
ENSSSCG00000039823	0.690652111	0.646922493	1	1	1
ENSSSCG00000004387	0.690731503	0.437977243	1	1	1
ENSSSCG00000032084	0.691138408	0.312882955	0.875	0.667	1
SLC41A1	0.691208385	0.22881869	0.75	0.533	1
ENSSSCG00000002520	0.692116989	1.058893689	0.688	0.467	1
ABTB2	0.69222528	0.904634622	1	1	1
LRP5	0.692976202	0.34298971	0.938	0.933	1
YTHDF3	0.693190475	0.827354555	1	1	1
F9	0.693461127	0.764871591	0.812	0.6	1
ENSSSCG00000016548	0.693540728	0.484330138	1	1	1
SASH1	0.693719228	0.583276124	0.938	0.867	1
ASPH	0.693790311	0.841302254	0.812	0.733	1
MTMR12	0.693880681	0.672834023	1	1	1
MDC1	0.693880908	0.044394119	0.75	0.533	1
PAQR5	0.69425312	0.451944375	1	0.933	1
ENSSSCG00000055221	0.694670521	0.575158627	1	1	1
BCKDHB	0.695463636	0.533766738	0.938	0.867	1
PPP3R1	0.69560038	0.458706828	1	1	1
OSGIN2	0.695823762	0.635468404	1	0.867	1
LSMEM2	0.696329096	0.421463768	0.5	0.4	1
FBXO6	0.696766158	0.633872101	0.688	0.8	1
TMEM80	0.697105977	0.361456459	0.75	0.6	1
ENSSSCG00000047109	0.697155743	0.417085328	0.812	0.8	1
WRN	0.697195159	0.290563924	1	1	1
GPBP1L1	0.697378523	0.714717064	1	1	1
PHIP	0.697568358	0.073669042	0.938	0.867	1
FAM83D	0.698057427	0.633740846	0.938	1	1
MEGF10	0.698059526	0.562674434	1	0.933	1
OTUD3	0.698355272	0.600705237	1	0.933	1
SOX5	0.698527848	0.64385619	0.625	0.467	1
CACUL1	0.699129036	0.313688809	1	1	1
ENSSSCG00000028674	0.699399229	-0.637429921	0.5	0.333	1
TMEM255A	0.69995396	0.767993745	1	1	1
KDM4B	0.70094424	0.979299091	1	0.8	1
FAM13A	0.701228391	0.649007478	1	1	1
SMARCC2	0.701378172	0.789054105	1	1	1
ENSSSCG00000055878	0.701394186	0.129283017	0.5	0.4	1
TXNDC9	0.701714038	0.926057569	1	1	1
ZNF624	0.702573289	0.631487632	0.938	1	1
EBF1	0.702592967	0.8518673	1	1	1
ENSSSCG00000057227	0.703426205	-0.03170886	0.688	0.533	1
TBL1XR1	0.703441707	0.577315042	1	1	1
AUNIP	0.70347692	0.354349573	1	0.8	1
KRI1	0.703805552	0.290219235	0.5	0.6	1
PYM1	0.70394556	0.497370101	1	0.8	1
EML4	0.703952226	0.668399579	1	1	1
CDC23	0.704473089	1.165624864	0.5	0.467	1
GRK3	0.704670657	0.454763778	1	1	1
RYR2	0.704712284	0.627402258	1	1	1
ZIC5	0.705016487	0.43198164	0.812	0.6	1
TRAPPC11	0.705156924	0.408798818	0.938	1	1
FBXO32	0.705583224	1.372554168	0.688	0.533	1
ENSSSCG00000026253	0.705842762	0.819427754	0.5	0.4	1
SDK1	0.705890342	0.62309763	0.812	0.667	1
MAP3K5	0.706100684	0.313515855	0.812	0.867	1
ENSSSCG00000050890	0.706159543	0.930737338	0.75	0.533	1
PHTF1	0.706451941	0.654283561	1	1	1
YIPF4	0.706704943	0.736356219	1	1	1
ENSSSCG00000052917	0.707230908	0.687880814	0.938	0.867	1
SNTB2	0.707339865	0.556393349	0.875	0.933	1
DMRT1	0.707536171	0.489133595	1	1	1
KCNV1	0.707784622	0.447458977	0.75	0.933	1
HCK	0.708251428	0.778069644	1	0.933	1
GAPVD1	0.708416712	0.430066325	1	1	1
HAUS5	0.708506916	0.784649342	1	1	1
SEPTIN3	0.708539826	0.712404171	0.938	1	1
FBXO8	0.708709472	0.518213683	1	1	1
MAST3	0.709351668	0.425855538	1	0.933	1

RPS27L	0.709760079	0.465924631	1	1	1
ENSSSCG00000061734	0.70983615	0.299208018	0.812	0.533	1
TMED10	0.709899238	0.623542425	1	1	1
PNPLA8	0.710536325	0.46001183	1	1	1
TSFM	0.71154598	0.452324732	0.938	0.733	1
LMX1A	0.711620133	0.765846714	0.875	0.8	1
ENSSSCG00000010401	0.711686397	0.55288253	1	1	1
PPTC7	0.711767969	1.219059724	0.875	0.933	1
GPI	0.711976443	0.520264709	1	1	1
ENSSSCG00000007143	0.712092123	0.402659367	1	0.933	1
CCDC6	0.712121553	0.546811741	1	1	1
EXOSC3	0.712166816	0.399930607	0.938	0.8	1
NRGN	0.712291006	0.762500686	0.938	0.667	1
ENSSSCG00000061246	0.7126964	0.794415866	0.875	0.867	1
RASSF5	0.712780083	0.269460675	0.5	0.4	1
ENSSSCG00000059615	0.712781157	0.684235651	1	1	1
ADAP1	0.713305301	0.74819285	1	1	1
ENSSSCG00000046060	0.714065065	0.209453366	0.5	0.6	1
GTSE1	0.714123813	0.693896872	0.938	0.733	1
MEGF11	0.71418488	0.509131088	1	0.8	1
MED26	0.714220585	0.108524457	0.438	0.533	1
LRRC8A	0.714272712	0.816125132	0.812	0.933	1
MOCS3	0.714702919	0.436670181	1	1	1
ANP32E	0.714875638	1.213551934	0.812	0.733	1
HES6	0.715106994	1.012542383	1	0.933	1
RASA1	0.715324211	0.494780288	1	1	1
SSBP2	0.715379506	0.711702532	1	1	1
RASGRP3	0.715706302	0.380000352	1	1	1
ECHDC1	0.715895075	0.473931188	0.812	0.733	1
BRD3	0.716228273	0.517848305	1	1	1
UBE2D2	0.716309012	0.769609008	1	1	1
SATB2	0.716977517	0.242493627	0.875	0.6	1
MATR3	0.71741442	0.59886779	1	1	1
RAB22A	0.717522287	0.99993932	1	1	1
SAXO2	0.717596484	0.645327577	1	1	1
NCBP1	0.718181729	0.629844099	1	1	1
GTF3C4	0.718256874	0.588068412	0.938	0.867	1
IQSEC2	0.718655659	0.854423176	0.688	0.4	1
CTSV	0.719419683	1.233784943	0.938	0.8	1
RXFP2	0.719493385	0.684498174	0.938	0.8	1
H3-3A	0.719609182	0.41603244	1	1	1
SERINC1	0.719727161	0.588334039	1	1	1
GABRA4	0.719846401	0.478242665	0.938	0.8	1
DCUN1D5	0.719867203	0.809711527	1	1	1
ENSSSCG00000052540	0.719913988	0.451886689	0.688	0.733	1
CMC1	0.720205176	0.311944006	1	1	1
ELAVL4	0.720456943	0.475174355	0.812	0.667	1
ENSSSCG00000032495	0.720993584	0.333705263	0.938	0.8	1
CACNG8	0.721012818	0.658963082	0.75	0.4	1
TAF3	0.721444538	0.860635005	1	1	1
ENSSSCG00000059577	0.722364492	0.872125177	0.812	0.733	1
IL27RA	0.722619028	0.906890596	0.875	0.6	1
GJA8	0.722814498	0.354349573	0.5	0.533	1
BICRAL	0.72300819	0.3594028	0.875	0.8	1
ENSSSCG00000054421	0.723456942	0.006426269	0.562	0.533	1
LINGO3	0.723504007	0.854423176	0.938	0.8	1
NIPAL2	0.723518067	0.321928095	0.5	0.4	1
PHKB	0.723720162	0.493694443	1	1	1
TMEM25	0.72410875	0.942895549	1	1	1
C1orf146	0.72425827	0.676211563	1	1	1
ENSSSCG00000052265	0.724544982	0.764871591	0.625	0.6	1
SF1	0.724648069	0.63907905	1	0.933	1
PDZD9	0.724649997	0.868416448	0.75	0.733	1
PTPN3	0.724980091	0.554472133	1	1	1
NISCH	0.725066236	0.794415866	1	1	1
PDE4D	0.725912598	0.576741994	0.812	0.6	1
FGD4	0.726698572	0.594128132	1	1	1
TCF4	0.726713636	0.824428435	0.5	0.333	1
TXNIP	0.726811145	0.138432454	0.812	0.8	1
CAPN3	0.72717625	0.289360232	0.812	0.733	1

EDNRB	0.727442576	0.123284276	0.875	1	1
KLHL3	0.727917412	0.781990124	1	1	1
ENSSSCG00000059602	0.728120675	0.491853096	0.562	0.267	1
C7orf25	0.729584944	0.716919652	0.938	1	1
KLC4	0.729760094	0.836501268	1	0.733	1
TRIM45	0.730312654	0.187292133	1	1	1
SELENOK	0.730791931	0.276753168	1	1	1
PDCD2L	0.730992751	0.500182081	1	1	1
SLC9A1	0.731916047	0.639410285	0.812	0.933	1
DENND10	0.731943985	0.981389718	1	1	1
MRTFA	0.732582213	0.569855608	0.938	1	1
OGFRL1	0.732728568	0.75802721	0.938	0.8	1
ENSSSCG00000054437	0.733211598	0.044394119	0.688	0.667	1
LDB1	0.73380596	0.539471117	1	1	1
MYO9A	0.733846605	0.358532513	0.938	1	1
ANKRD45	0.733966848	0.052968855	1	0.933	1
TERB1	0.734192235	0.743941357	1	1	1
SH2D3C	0.734285781	0.404390255	0.5	0.533	1
IPP	0.7342987	0.635260166	1	1	1
GALNT4	0.734393626	0.459431619	0.625	0.333	1
TRBV9	0.73467249	0.531381461	0.688	0.667	1
SORT1	0.734726508	0.643378595	1	1	1
ENSSSCG00000041810	0.734875681	0.452052088	0.938	0.8	1
ENSSSCG00000054816	0.735056334	0.222392421	0.938	0.733	1
ENSSSCG00000055326	0.735132631	0.584962501	0.75	0.467	1
ZNF182	0.736486937	0.467847633	1	0.933	1
FDX1	0.736574402	0.602803813	1	1	1
FOXK2	0.736805101	0.537244999	1	1	1
TXK	0.737312736	0.321928095	0.75	0.667	1
ENSSSCG00000046648	0.737961174	0.017921908	0.75	0.6	1
DHX57	0.73896282	0.710781662	1	1	1
KPNA3	0.739154724	0.697662633	1	1	1
E2F3	0.73932422	0.314065977	1	1	1
FLCN	0.739412664	1.052968855	0.938	0.8	1
OCIAD1	0.73952599	0.530528506	1	1	1
VCP	0.740203139	0.563124918	1	1	1
LIN7A	0.740537299	0.807354922	0.5	0.533	1
ENSSSCG00000010943	0.740600316	0.658606641	1	1	1
GATA4	0.740897475	0.716311923	1	1	1
CDC42BPG	0.741656558	0.321928095	0.688	0.667	1
LATS2	0.741915155	0.86561432	1	1	1
ENSSSCG00000014822	0.741949359	0.558967292	0.562	0.467	1
GRPR	0.742080328	0.0138058	0.5	0.533	1
DNAH5	0.742927213	0.141355849	0.812	0.667	1
SLC25A44	0.743029912	0.679394063	0.938	1	1
L3HYPDH	0.743373897	0.884864289	0.875	0.733	1
PPP2R2A	0.743945631	0.667982087	1	0.933	1
ASH1L	0.743977192	0.557898431	1	1	1
ENSSSCG00000042032	0.743985431	1.22881869	0.5	0.2	1
NNT	0.744106289	0.694049917	1	1	1
FGF2	0.744213317	0.758791957	0.938	0.867	1
NCAPD2	0.74424237	0.716990894	0.938	1	1
FAM161A	0.74427144	0.716538786	1	1	1
AMDHD1	0.744459007	0.684498174	0.688	0.333	1
PNN	0.744645602	0.746459125	1	1	1
NEBL	0.744888391	0.440587318	1	1	1
DDA1	0.744998332	0.473023028	1	1	1
GIGYF1	0.745041048	0.723178642	1	0.8	1
ENSSSCG00000063417	0.745305742	0.413850584	0.938	0.933	1
ZFYVE19	0.745917533	0.36289674	1	1	1
BROX	0.746205983	0.910532847	1	1	1
BAZ2A	0.746478198	0.777717895	0.938	1	1
LDLRAD3	0.747135705	0.463283944	0.938	0.533	1
MPZL1	0.74741741	0.635901805	1	1	1
ZNF287	0.747433962	0.549136506	0.938	1	1
DENND2B	0.747652888	0.189290326	0.75	0.867	1
ELK4	0.748438525	0.588730971	1	1	1
SGSM1	0.748520284	0.651051691	0.75	0.733	1
ENOPH1	0.748809045	0.632030754	0.875	1	1
ENSSSCG00000003352	0.748854607	0.767123231	1	1	1

ENSSSCG00000051454	0.748986725	1.076815597	0.562	0.4	1
HACD3	0.749226734	0.571922296	1	1	1
SLC15A1	0.749402872	0.44467116	1	1	1
TDP1	0.750015183	0.652250631	1	1	1
GAN	0.750242279	0.441729617	1	0.933	1
CSMD1	0.751032131	0.33752495	0.875	0.867	1
ENSSSCG00000041656	0.751226697	0.906890596	0.625	0.533	1
EXOC5	0.751374835	0.848303569	1	1	1
TMEM41B	0.751782358	0.609653665	1	1	1
ENSSSCG00000060025	0.752185713	0.470791481	0.75	0.6	1
FOXN1	0.752717665	0.86507042	0.812	0.867	1
ENSSSCG00000063030	0.75281997	0.514573173	1	1	1
MYO7A	0.753109756	0.488664819	0.875	1	1
COA7	0.753178856	0.452446056	1	1	1
TOPAZ1	0.753360693	0.723178642	1	1	1
CACNA1D	0.753434638	0.633325522	0.812	0.6	1
CERT1	0.753921045	0.597906604	1	1	1
TRIB1	0.754281394	1.169925001	0.5	0.267	1
ENSSSCG00000042386	0.754487425	0.550746785	0.938	0.733	1
FNIP1	0.75449222	0.638614105	1	1	1
BOD1L1	0.754636526	0.735124247	1	1	1
ENSSSCG00000059073	0.754857885	0.384937892	0.812	0.467	1
ENSSSCG00000036608	0.754989398	0.405159624	0.938	1	1
DAP	0.755143977	1.039085208	1	0.867	1
ATAD3A	0.755179372	0.540108265	0.75	0.667	1
ENSSSCG00000038151	0.755322482	0.786596362	0.75	0.733	1
BRIP1	0.755508387	0.518776381	0.938	1	1
TUBA1A	0.756560237	0.425855538	0.75	0.933	1
VPS37C	0.756947121	0.673691419	0.938	0.933	1
SIM1	0.757207333	0.247927513	0.625	0.6	1
CLK1	0.757291717	0.753878977	0.938	0.8	1
ARFGEF3	0.757830154	0.547706331	1	0.933	1
ATOSB	0.757959253	0.754887502	0.375	0.533	1
ENSSSCG00000035142	0.758161137	0.650399036	1	1	1
ENSSSCG00000050552	0.758432801	0.652135668	1	0.933	1
G2E3	0.75844302	0.446175522	1	1	1
ZNF507	0.758576013	0.54819108	1	1	1
NR1H2	0.758603649	0.906890596	1	0.933	1
NRIP3	0.7586988	0.878754806	1	1	1
ENSSSCG00000042371	0.758956328	-0.292418213	0.562	0.467	1
EFHC2	0.759045038	0.658813127	1	1	1
CCDC25	0.759763476	0.633452094	1	1	1
MEIS3	0.760876634	0.366322214	0.812	0.733	1
ENSSSCG00000038009	0.761087454	0.538578172	1	1	1
ZBTB41	0.761234695	0.777053158	0.938	0.933	1
ZRANB3	0.761388834	0.591585443	0.938	0.933	1
GNAL	0.761419032	0.906890596	0.5	0.333	1
MPV17L	0.762273514	0.500570313	0.812	0.8	1
EML2	0.762286399	1.32679385	0.875	0.8	1
HUWE1	0.762370975	0.376087956	1	1	1
TES	0.762769778	0.591828762	1	1	1
ENSSSCG00000018083	0.762950123	0.354349573	0.5	0.4	1
NUMA1	0.763017871	0.746036585	1	0.933	1
METTL24	0.763214839	0.726736462	1	1	1
NOL8	0.76355267	0.560652093	1	1	1
KCND2	0.763641504	0.491853096	0.562	0.4	1
ALCAM	0.763908902	0.710625202	0.875	0.8	1
SNTN	0.763923819	0.458631677	1	1	1
GNA11	0.764112801	0.547348209	0.875	0.667	1
PRUNE1	0.764152144	0.736072561	1	1	1
ENSSSCG00000024860	0.764315102	0.787050826	0.75	0.8	1
ENSSSCG00000008576	0.764537798	0.64385619	0.938	0.933	1
SLC39A10	0.764600851	0.687467702	1	1	1
DNMBP	0.765219196	0.705541449	1	1	1
WDPCP	0.765444147	0.547562529	1	1	1
HTT	0.765465652	0.757648642	1	1	1
EIF1AD	0.765529639	0.639530264	1	1	1
ENSSSCG00000059679	0.76559041	0.829722735	0.688	0.4	1
ADAMTSL4	0.76577751	0.509555098	0.875	0.933	1
ANGPTL2	0.766028747	1.058893689	0.75	0.667	1

CUTC	0.766222789	0.022367813	0.688	0.4	1
ZNF565	0.766766333	0.792855352	0.875	0.933	1
SLC6A11	0.766800058	0.874829386	1	1	1
ZNF773	0.76693679	0.611692866	1	1	1
GDI2	0.767004805	0.623263474	1	1	1
ENSSSCG00000052280	0.767105856	0.174948617	1	1	1
SETX	0.76713029	0.652387738	1	1	1
FOXA1	0.767131823	0.736965594	0.5	0.267	1
RAD54B	0.767179995	0.561678276	1	1	1
ENSSSCG00000063161	0.767279642	0.34129342	0.625	0.467	1
WDR62	0.768115612	0.566815154	0.812	0.733	1
MANEAL	0.768227807	0.538351558	0.938	0.933	1
PRKAB2	0.768261085	0.719805043	1	0.933	1
TIAM1	0.768345681	0.556523525	1	1	1
DENND4A	0.768361885	0.405103578	1	1	1
PRRC1	0.768380766	0.65905148	1	1	1
STK17B	0.768513071	-0.1602236	0.875	0.667	1
CFAP210	0.768745078	0.784555053	1	1	1
SIPA1L3	0.768754784	0.866248611	0.938	0.733	1
CHD4	0.768997293	0.79105381	1	1	1
UNC13C	0.769200352	0.510824853	1	1	1
CDADC1	0.76936314	0.676592976	1	1	1
ENSSSCG00000053778	0.769444562	0.206450877	0.875	0.533	1
ZNF511	0.769520589	0.362955363	1	0.733	1
ENSSSCG00000045377	0.769580201	0.410933101	0.938	0.8	1
HRH2	0.769856108	0.34298971	0.562	0.467	1
ENSSSCG00000059162	0.770124595	0.69743723	0.875	0.667	1
ENSSSCG00000048556	0.770565873	0.006426269	0.812	0.733	1
ENSSSCG00000025500	0.77083601	0.796600665	1	1	1
ENSSSCG00000062556	0.771033134	0.993651908	0.938	1	1
ANKRD33B	0.771454099	0.404009747	0.875	0.933	1
ACVR2A	0.771887733	0.411682748	1	1	1
NUSAP1	0.771916993	0.619199433	1	1	1
ENDOV	0.772642169	0.386883537	0.938	0.8	1
CCDC85A	0.772645899	0.458466928	1	1	1
RAB3GAP1	0.77275902	0.782594158	1	1	1
ZNF304	0.773037255	0.423949032	0.938	0.867	1
ENSSSCG00000061085	0.773279455	0.154818109	0.625	0.4	1
ENSSSCG00000032554	0.773580825	0.752760556	1	1	1
DNAJC10	0.773581752	0.546008866	1	1	1
ENSSSCG00000033241	0.773718347	0.637985602	1	1	1
RPUSD1	0.773838997	-0.167109986	0.438	0.6	1
CEBPZ	0.774045584	0.547735181	1	1	1
TTC39C	0.774476307	0.769808851	0.938	0.933	1
UBE4B	0.774599389	0.828562235	1	1	1
ENSSSCG00000056080	0.774760256	0.799975392	0.75	0.4	1
ENSSSCG00000013120	0.775150097	0.571706404	0.875	0.933	1
PTPN21	0.775481017	0.578457585	1	1	1
MEF2A	0.77551373	0.64385619	1	0.733	1
SEMA6A	0.775904015	0.52501996	0.812	0.733	1
ENSSSCG00000056025	0.776001812	0.356693513	0.812	0.733	1
CKAP5	0.776056534	0.673498479	1	1	1
ENSSSCG00000016093	0.776078137	0.759333407	0.812	0.8	1
RABGEF1	0.776738017	0.584340515	1	1	1
NVL	0.77700832	0.557655155	1	1	1
CCDC82	0.777321187	0.186998515	1	0.733	1
PIGL	0.778024072	0.607330314	0.5	0.4	1
IQSEC1	0.77810387	0.714245518	0.812	0.6	1
KLHL40	0.778731278	0.906890596	0.812	0.667	1
ENSSSCG00000031776	0.778793965	0.830787617	1	1	1
GAD2	0.778807084	0.531381461	0.562	0.4	1
SMIM29	0.779123769	0.392317423	1	0.733	1
ZNF81	0.779163615	0.687267263	1	1	1
ENSSSCG00000045006	0.779501776	0.449667866	1	1	1
GABRG2	0.78100983	0.675866585	1	1	1
NCKAP1	0.781230561	0.474575105	1	1	1
FBXO11	0.781526425	0.487434425	1	0.933	1
FBXO47	0.781745656	0.269460675	0.625	0.733	1
CCDC51	0.78177527	0.541373232	1	0.8	1
AMER3	0.781787269	0.781359714	0.625	0.533	1

ENSSSCG00000060228	0.782329496	1.058893689	0.5	0.333	1
ENSSSCG00000047466	0.782550004	0.761039729	1	0.867	1
ENSSSCG00000002260	0.783135215	0.531606829	1	1	1
FHIP2A	0.78326006	0.844606317	0.938	1	1
ARNT	0.783405427	0.497566748	1	0.867	1
LRRTM1	0.783579483	-0.093109404	0.5	0.533	1
ENSSSCG00000030140	0.783726151	0.451211112	0.625	0.667	1
ENSSSCG00000062147	0.784720462	0.710970386	1	1	1
NFS1	0.784791796	0.624747367	1	1	1
ENSSSCG00000042074	0.785680338	0.34298971	0.562	0.467	1
SLC27A1	0.785870091	0.472487771	0.688	0.6	1
KLHL26	0.785966012	0.292180751	0.688	0.6	1
ENSSSCG00000056845	0.787303786	0.536299824	1	1	1
ENSSSCG00000055269	0.787480356	0.714245518	0.5	0.467	1
TRIB3	0.787700286	0.597562538	0.688	0.867	1
SMIM35	0.787717637	0.743952024	0.875	0.8	1
MEOX2	0.787762512	0.312882955	0.875	0.533	1
ENSSSCG00000057514	0.788067242	0.087462841	0.375	0.533	1
ENSSSCG00000060154	0.788315524	0.689299161	0.875	0.6	1
SPATA6	0.788332913	0.664320292	0.875	0.867	1
WDR17	0.788641741	1.426264755	0.5	0.4	1
PHF13	0.788710441	0.682103659	1	1	1
MAPK1	0.788720037	0.611015361	1	1	1
GATC	0.789096928	0.609449328	1	1	1
HOXD8	0.789568853	0.739290647	1	1	1
NPAS2	0.789610597	0.87036472	0.688	0.467	1
TLR8	0.789727965	0.520937589	1	1	1
ENSSSCG00000055025	0.789964348	0.243925583	0.688	0.267	1
KLF12	0.789976723	0.632110982	1	0.933	1
ENSSSCG00000045742	0.790753782	0.440322796	0.938	0.8	1
ERRFI1	0.791048959	1.22881869	0.5	0.333	1
ZNF382	0.791175002	0.414685236	0.875	0.8	1
BARHL2	0.791343292	0.482750889	0.938	0.867	1
ENSSSCG00000022345	0.791576536	1.081977302	0.812	0.733	1
DHX34	0.793283553	0.530327244	0.688	0.6	1
SLC25A21	0.793326157	0.461264776	0.938	0.867	1
ENSSSCG00000055926	0.793352953	0.752168001	0.812	0.667	1
SOHLH2	0.794215646	0.669391282	1	0.6	1
LRRC1	0.794674634	0.867719998	1	1	1
ENSSSCG00000015489	0.794936449	0.631256153	0.625	0.467	1
ENSSSCG00000017834	0.795095846	0.129283017	0.562	0.333	1
RARRES1	0.795453768	0.870023942	1	1	1
GIN1	0.795613009	0.435269568	0.75	0.733	1
ENSSSCG00000034749	0.795617569	0.82130204	1	1	1
METTL25	0.796643834	0.288761231	0.812	0.667	1
TMEM185B	0.797075109	0.600105987	1	0.933	1
ENSSSCG00000062704	0.797513707	0.69538649	0.75	0.733	1
RBBP8	0.797647537	0.440743141	1	1	1
AKT1	0.797870837	0.660730008	0.938	0.867	1
UBQLN2	0.798102709	0.766960334	1	0.933	1
ENSSSCG00000047003	0.79858679	0.663221515	0.938	0.867	1
ENSSSCG00000038289	0.799085437	0.714725856	1	1	1
RFX1	0.799097285	0.668730858	0.75	0.533	1
ENSSSCG00000049335	0.799201347	0.375039431	0.938	0.8	1
ENSSSCG00000038337	0.799356855	0.714245518	0.812	0.533	1
CCDC122	0.799637634	0.730012834	0.625	0.467	1
SDCBP	0.799696198	0.794737317	1	1	1
ENSSSCG00000031787	0.799753369	0.666064139	1	1	1
EFCAB11	0.80002839	0.906890596	0.875	0.933	1
PRIMPOL	0.800088442	0.601335312	1	1	1
UHMK1	0.800157814	0.603022472	1	1	1
DRAM2	0.800216197	0.578836398	1	1	1
PDK3	0.800623972	0.703643224	1	1	1
PLD1	0.800861935	0.328717261	0.688	0.733	1
EPC2	0.801575286	0.484075746	1	0.933	1
P4HA2	0.802428518	0.658543633	0.938	0.933	1
HECTD1	0.80258547	0.524809129	1	1	1
INTS8	0.802602716	0.329581667	0.938	0.667	1
CCDC14	0.803220316	0.280348991	0.875	0.667	1
ERCC4	0.803427252	0.635818871	1	1	1

GOPC	0.80358512	0.613279876	1	1	1
ENSSSCG00000000327	0.803618105	0.206450877	0.875	0.667	1
ENSSSCG00000003322	0.804193373	0.399930607	0.938	0.933	1
ENSSSCG00000055908	0.804460834	0.8397764	0.75	0.533	1
GLYAT	0.804685911	1.046565218	1	1	1
C6orf89	0.805059112	0.620305524	1	1	1
ENSSSCG00000057246	0.805424297	0.534573971	0.938	0.933	1
HELB	0.805665323	0.514623602	1	1	1
FAR2	0.805675509	0.487265827	0.875	0.8	1
ZNF555	0.805737628	0.431775276	0.938	1	1
ENTR1	0.805986611	0.527301932	0.938	0.867	1
ENSSSCG00000046607	0.806032425	0.672425342	0.562	0.4	1
EXOC6	0.806166519	0.628749297	1	1	1
ZNF396	0.806474912	0.770829046	0.875	0.667	1
DHX36	0.806585603	0.55316365	1	1	1
ENSSSCG00000048605	0.806927934	0.539158811	0.688	0.467	1
ENSSSCG00000062678	0.806948762	0.755428953	1	1	1
TRAPPC10	0.807173757	0.820475844	0.938	0.933	1
DEPDC1B	0.807350788	0.439119815	1	1	1
ADGRG2	0.807570044	0.269460675	0.688	0.733	1
EME1	0.807857602	0.737264134	1	1	1
GTPBP10	0.807871236	0.439937626	1	1	1
PLCB2	0.807893247	0.491853096	0.812	0.933	1
ENSSSCG00000063050	0.808465927	0.478432581	1	1	1
ARAP2	0.80861427	0.493457874	1	1	1
ENSSSCG00000028004	0.808727587	0.269460675	1	1	1
OSTF1	0.808894928	0.631064636	1	1	1
ENSSSCG00000026271	0.809083213	0.554009572	1	0.867	1
RUNDC1	0.809237454	0.762500686	0.562	0.4	1
ENSSSCG00000040056	0.809334657	0.394019049	1	1	1
ENSSSCG00000015545	0.809692463	0.663793781	1	1	1
ENSSSCG00000060644	0.809730921	0.051625689	1	0.933	1
ZEB1	0.810238322	0.626782676	0.938	0.933	1
ENSSSCG00000038702	0.811654498	0.52501996	0.812	0.8	1
ENSSSCG00000046699	0.811718545	0.607330314	0.562	0.2	1
ENSSSCG00000053808	0.811972461	0.463283944	0.562	0.333	1
RFX3	0.812243004	0.219678037	0.875	0.8	1
FAM170A	0.812682028	0.74819285	0.438	0.6	1
MAK16	0.812684097	0.515716989	1	1	1
ENSSSCG00000057502	0.812809763	0.913082429	1	1	1
ENSSSCG00000015810	0.813138217	0.614108846	0.875	0.8	1
IL31RA	0.813362619	-0.32443495	0.625	0.4	1
ENSSSCG00000032113	0.814614059	0.852442812	0.75	0.6	1
MAPT	0.814732059	0.856797256	1	1	1
MGST3	0.81482354	0.599566914	1	1	1
FLNC	0.815245304	0.566757905	0.938	1	1
ENSSSCG00000008241	0.815392045	0.22881869	0.688	0.6	1
FNTA	0.815551322	0.526443025	1	1	1
PHF23	0.815606496	0.638872782	1	1	1
ACVR1	0.815615318	0.525853768	1	1	1
EHHADH	0.815850811	0.586961683	1	1	1
HDAC6	0.815960554	0.69276579	0.562	0.733	1
CCDC171	0.816065995	0.725216635	1	1	1
DNMT1	0.816542679	0.726406141	1	1	1
CFAP91	0.816676214	1.089093927	0.625	0.533	1
ENSSSCG00000055112	0.816806975	0.652317769	0.75	0.6	1
APH1B	0.817075112	0.861228042	1	1	1
FILIP1L	0.817129367	0.211745177	0.562	0.8	1
MIS18A	0.817265103	0.707882476	1	1	1
NFIC	0.817597801	0.602884409	0.875	0.867	1
SRSF10	0.817721554	0.565304352	1	1	1
CLIP3	0.818403916	1.120669887	0.875	0.867	1
LRRC4C	0.818681513	0.97593224	0.938	0.867	1
YLPM1	0.819653776	0.688250309	1	0.933	1
HP1BP3	0.819740657	0.527450369	1	1	1
ENSSSCG00000057417	0.819997558	0.635614812	1	1	1
TMEM260	0.820600904	0.668730858	0.812	0.733	1
S1PR2	0.820605887	0.459431619	0.812	0.733	1
RFFL	0.821178262	0.792530275	1	1	1
ENSSSCG00000033643	0.821180349	0.491853096	0.5	0.4	1

BRPF3	0.821508559	0.473237418	0.938	1	1
ENSSSCG00000023172	0.821701732	0.813781191	0.75	0.6	1
ADCY5	0.821802136	0.156868849	0.625	0.6	1
BCLAF1	0.821957267	0.617579452	1	1	1
GRIP2	0.822060114	0.765490729	1	1	1
ATPAF1	0.82250466	0.508455064	1	0.933	1
DNAAF9	0.822539006	0.801045508	1	1	1
TENT5B	0.822630236	0.96281601	1	1	1
RCOR1	0.823060505	0.678179372	1	1	1
ABCA2	0.823064066	0.5987683	0.625	0.467	1
SLN	0.823400019	0.22336426	0.875	0.8	1
NCEH1	0.82343434	0.714245518	0.688	0.667	1
ENSSSCG00000057038	0.823709982	0.287980763	0.812	0.667	1
ZCCHC2	0.823719279	0.601403519	1	1	1
ENSSSCG00000000540	0.824098092	0.399072811	1	1	1
BCORL1	0.824662219	0.73586556	0.938	1	1
CALCR	0.824703127	0.658160649	1	0.933	1
WDFY3	0.825301966	0.53162156	1	1	1
DPH5	0.825429593	0.638260727	1	1	1
LRIF1	0.826224233	0.35974956	1	1	1
NIPBL	0.826637762	0.571848583	1	1	1
POLR2M	0.826731876	0.820755755	1	1	1
ZNF260	0.826846373	0.213868349	0.938	0.8	1
FZD4	0.827004191	0.957516669	0.875	0.4	1
ENSSSCG00000052612	0.827223824	0.542479169	1	1	1
INPP4A	0.827282376	0.615427781	0.938	0.867	1
ZC3H12A	0.827309609	0.392317423	0.5	0.267	1
HSD11B2	0.827454287	0.671359705	1	1	1
RNGTT	0.82748998	0.459910204	1	1	1
MAP4K5	0.827524796	0.656673987	1	1	1
TMEM43	0.827748853	0.807303241	1	1	1
RAD52	0.828042934	0.516171727	0.938	1	1
ENSSSCG00000009498	0.828063439	0.714245518	0.75	0.667	1
ZNF658	0.828409571	-0.228461257	0.688	0.333	1
ENSSSCG00000062885	0.828564409	0.994353437	0.562	0.467	1
ERC2	0.828566684	0.651486631	1	1	1
CCDC65	0.828885087	0.684498174	0.938	1	1
ENSSSCG00000050278	0.829154889	0.451211112	0.688	0.467	1
OGDH	0.829618556	0.737390854	0.938	1	1
ENSSSCG00000000194	0.830212199	0.890666558	1	1	1
ENSSSCG00000034578	0.830326295	0.719805043	0.938	0.733	1
SRGAP3	0.830401549	0.749349319	0.562	0.8	1
CIPC	0.830456274	0.873930397	1	1	1
CPE	0.830481537	0.639786525	0.812	0.6	1
FEZ2	0.830529051	0.459041226	1	1	1
ENSSSCG00000042218	0.830636588	0.607330314	0.812	0.467	1
ENSSSCG00000041874	0.830812947	0.773624065	0.75	0.6	1
DTWD2	0.831887225	-0.183307213	0.562	0.6	1
EVI5	0.831989968	0.645710554	1	0.933	1
ENSSSCG00000055415	0.832135093	0.679975616	1	1	1
ALPK1	0.832391876	0.57758297	0.75	0.733	1
ZFC3H1	0.832696415	0.654310547	1	1	1
RAC1	0.832877464	0.601952811	1	1	1
CEP55	0.833159641	0.460193941	1	1	1
SRPK2	0.83365742	0.711034532	1	1	1
NR2F1	0.83373653	0.5360529	0.938	0.867	1
CTR9	0.833924899	0.535673374	1	1	1
ENSSSCG00000053016	0.834364923	0.537656786	0.875	0.6	1
ARHGAP32	0.834551146	0.475733431	0.938	0.933	1
ZNF71	0.834827538	0.555418225	0.938	0.733	1
ENSSSCG00000055301	0.835107014	1.016515087	0.688	0.667	1
ENSSSCG00000031890	0.835202718	0.46760555	1	0.933	1
GPCPD1	0.835521341	0.692181782	1	1	1
SLC13A2	0.835622146	0.575684687	0.625	0.667	1
FOXO1	0.836039108	0.589281107	1	1	1
C1orf52	0.836434025	0.525800428	0.75	0.667	1
SETDB1	0.836597746	0.453448557	1	1	1
CXCL10	0.836673127	0.087462841	0.625	0.467	1
PANK3	0.836864442	0.650157116	1	1	1
ENSSSCG00000039706	0.837259235	0.421463768	0.625	0.8	1

ND2	0.837406115	0.627053486	1	1	1
PAPSS2	0.837423297	0.384469561	1	1	1
C17orf75	0.837600489	0.22881869	0.812	0.467	1
ENSSSCG00000042026	0.837654344	0.584962501	0.875	0.733	1
EMP2	0.837705057	0.862947248	0.875	0.733	1
PJA1	0.837761381	0.854423176	0.562	0.467	1
ZNF2	0.838012515	0.651196199	0.938	1	1
ENSSSCG00000038455	0.83801781	0.866248611	0.75	0.333	1
NSMCE2	0.838093085	0.64385619	0.875	0.867	1
BLTP3A	0.838102065	0.476256241	0.625	0.6	1
DLD	0.838703926	0.572288671	1	0.933	1
OAZ2	0.83883902	0.587385533	1	1	1
SLC25A23	0.838919077	0.584962501	0.812	0.733	1
MEI4	0.839162922	0.22881869	0.75	0.467	1
GLT8D2	0.839263259	0.728399223	1	1	1
SETD1A	0.83936481	0.906890596	1	1	1
ARFIP1	0.839449736	0.692987026	1	1	1
UBTF	0.839827353	0.625403427	1	1	1
PLEKHG2	0.840016966	0.37895904	0.75	0.733	1
ENSSSCG00000044943	0.840061606	0.514573173	0.938	1	1
ZDHHC14	0.840622592	0.769387072	0.625	0.467	1
LACC1	0.840977004	0.663884969	1	1	1
KIF3A	0.841176593	0.540895719	1	1	1
ENSSSCG00000038144	0.841294382	0.796466606	0.938	0.867	1
POGLUT1	0.841362205	0.409390936	0.625	0.533	1
ZNF782	0.841453803	0.467191042	1	0.867	1
ENSSSCG00000059077	0.842013774	0.421463768	0.562	0.4	1
PEX5	0.842070283	0.529224526	1	1	1
MARF1	0.842113398	0.434137598	1	1	1
ZNF710	0.842405821	0.8397764	0.812	0.6	1
RBFOX1	0.842453891	0.652915529	1	1	1
GABRA1	0.842636085	0.454378391	1	0.867	1
MCCC2	0.842656457	0.637283536	1	1	1
PPL	0.842791035	0.725443724	0.938	0.933	1
HSPB1	0.843517473	0.661778098	0.562	0.533	1
ENSSSCG00000005121	0.843924053	0.459431619	0.562	0.533	1
RWDD2B	0.843949795	0.823232667	1	1	1
ENSSSCG00000061420	0.843953653	0.206450877	0.5	0.267	1
SCN2A	0.843988138	-0.125530882	0.688	0.533	1
ENSSSCG00000040367	0.844012367	0.514573173	0.75	0.467	1
STXBP6	0.844419425	0.798987718	1	1	1
MBIP	0.844550902	0.616300467	1	0.933	1
NR2C2	0.844708777	0.604058012	1	1	1
BACH1	0.844781856	0.588920514	1	1	1
XPO6	0.845236785	0.734779929	1	1	1
OSBP2	0.84552927	0.058893689	0.375	0.533	1
ENSSSCG00000043563	0.845987592	0.64385619	0.875	0.533	1
ELAPOR2	0.84643165	0.515552932	1	1	1
CCDC142	0.846689645	0.169925001	0.562	0.267	1
ENSSSCG00000047832	0.846805569	0.730012834	0.812	0.733	1
CLCN7	0.846815727	0.752242781	1	1	1
RAG2	0.847066842	0.26052755	0.5	0.533	1
ENSSSCG00000021941	0.847197556	0.613339763	1	1	1
ARHGAP20	0.847233557	0.776830055	0.688	0.467	1
ENSSSCG00000034709	0.847679711	0.566275457	1	1	1
SATB1	0.847873894	0.354349573	1	1	1
ENSSSCG00000041398	0.847963921	0.293913719	0.375	0.533	1
HTR3B	0.848343771	0.26052755	0.875	0.667	1
FOXJ2	0.848437018	0.661778098	0.75	0.533	1
ZFP36L1	0.848548446	0.475551284	1	1	1
ANKH	0.848924031	0.650325685	0.812	0.933	1
RTL4	0.848942341	0.235513343	0.938	0.8	1
ENSSSCG00000043724	0.849252129	0.380821784	0.562	0.4	1
ENSSSCG00000062411	0.849262195	0.607330314	0.688	0.4	1
PGR	0.849522042	0.680244846	1	1	1
EIF4A3	0.849776409	0.343084746	1	1	1
ENSSSCG00000052923	0.850029203	0.51793375	1	1	1
CAND2	0.850282693	0.603022472	1	1	1
KMT5A	0.850371431	0.621829883	1	1	1
SLC39A1	0.850578338	0.531851164	1	0.933	1

MS4A2	0.850678041	0.542734265	0.75	0.667	1
RHOA	0.850770231	0.679895738	1	1	1
BMPR1B	0.850934399	0.689546748	1	1	1
PDSS1	0.850988805	0.627273306	1	1	1
C5orf22	0.851223195	0.624015277	1	0.933	1
ENSSSCG00000025590	0.851499962	0.432959407	0.875	0.6	1
ENSSSCG00000054550	0.851506458	0.956359272	0.688	0.533	1
POU4F2	0.851632791	0.813781191	0.562	0.333	1
CCNK	0.852710443	0.516581898	1	1	1
PRPH	0.852839337	0.906890596	0.688	0.467	1
RAB5A	0.853148643	0.341518823	1	0.867	1
ENSSSCG00000061091	0.853220408	0.432959407	0.625	0.467	1
ENSSSCG00000054193	0.853439914	0.703357202	0.625	0.6	1
PPP1R9A	0.853567115	0.827089884	1	1	1
RAB27B	0.853651466	-0.215099929	0.75	0.4	1
CLDN7	0.854158535	0.625550035	1	1	1
CYP1A1	0.854531266	0.550746785	0.688	0.467	1
RGS14	0.855058509	0.805010982	0.75	0.667	1
TRIM52	0.855260953	0.617383978	0.938	0.8	1
ENSSSCG00000017285	0.855499511	0.804710201	1	0.933	1
TRIP12	0.855570236	0.716113181	1	1	1
TRIM38	0.855769942	0.65137304	1	0.933	1
GTF2H3	0.855987485	0.648156327	0.875	0.667	1
CASD1	0.855997187	0.485146774	1	1	1
ABTB1	0.856700307	0.425357685	0.812	0.867	1
ENSSSCG00000057242	0.857107705	0.732212064	0.938	0.933	1
ACTR5	0.857207871	0.601264313	0.875	0.8	1
TIPIN	0.857263076	0.642474959	1	1	1
ENSSSCG00000055690	0.8576402	0.574315257	0.625	0.6	1
SHISA2	0.858044041	0.547996174	1	1	1
ACTR8	0.858431415	0.525985781	1	1	1
ENSSSCG00000047157	0.858659207	1.0138058	0.875	0.8	1
MTOR	0.858870164	0.614877123	1	1	1
COQ10A	0.859640858	1.006426269	0.688	0.733	1
ENSSSCG00000057507	0.859817322	0.742259894	0.75	0.733	1
TBC1D22B	0.859861917	0.986117287	1	0.933	1
NLRP11	0.859873533	0.598113868	1	1	1
RBP1	0.860020199	0.047068254	0.375	0.733	1
C19orf44	0.860250654	0.736965594	0.5	0.267	1
KLC2	0.860418553	0.99095486	0.938	0.867	1
CLN5	0.860466453	0.702200176	1	1	1
ENSSSCG00000002427	0.860560649	0.706865987	0.938	0.933	1
PSD3	0.861440581	0.340349292	1	1	1
ENSSSCG00000052590	0.861563501	0.5987683	0.75	0.8	1
IL2RA	0.861718842	0.80786122	1	1	1
ENSSSCG00000026167	0.86197738	0.239465935	0.812	0.533	1
DIP2C	0.862013055	0.757512972	1	1	1
ENSSSCG00000021656	0.862031232	0.454378391	0.562	0.467	1
ARHGEF10	0.862041938	0.799975392	0.75	0.4	1
ELAVL2	0.862203744	0.669347851	1	1	1
SLC4A11	0.862217445	0.738013684	0.938	0.8	1
ENSSSCG00000053948	0.862429468	0.965784285	0.75	0.4	1
EDEM1	0.862439856	0.606877277	1	0.933	1
MAB21L3	0.862839522	0.762500686	0.562	0.533	1
MYCBP2	0.86285274	0.566769763	1	1	1
TMEM120B	0.863081213	0.872125177	0.938	0.867	1
HEATR3	0.863378538	0.396695863	0.688	0.533	1
SIAH2	0.863642775	0.707581787	0.812	0.8	1
DDX6	0.863684511	0.519995222	1	1	1
DNAJC12	0.863977322	0.616479751	1	1	1
TMC7	0.864101604	0.857980995	0.625	0.6	1
FRMD3	0.864116418	0.534124749	1	1	1
DESI1	0.864308999	0.794891297	1	1	1
ENSSSCG00000009419	0.864405869	0.732190487	1	1	1
ENSSSCG00000005232	0.864725862	0.462105753	0.938	0.6	1
ANKRD13A	0.865162687	0.693606679	1	1	1
BRWD1	0.865323531	0.633768535	1	1	1
PCDHA13	0.865404784	0.321928095	0.5	0.267	1
SPOPL	0.865424594	0.59373271	1	1	1
ENSSSCG00000058419	0.865611053	0.552225714	0.688	0.6	1

SYNJ1	0.865621475	0.798787988	0.938	1	1
MRPL49	0.86566591	0.710198685	1	0.933	1
UBOX5	0.865733381	0.533273024	0.938	1	1
PPFIBP1	0.865805901	0.417628511	1	0.8	1
ENSSSCG00000034769	0.865884484	0.544320516	0.625	0.333	1
ANAPC15	0.866004079	0.606231117	1	1	1
CYB5B	0.866013565	0.624219525	1	1	1
CXXC1	0.86676485	0.850981854	1	1	1
SLC26A5	0.866906881	0.760596824	1	1	1
PLEKHA7	0.867097877	0.656359722	1	1	1
CLASRP	0.867536612	0.727566896	1	1	1
NELFA	0.867828703	0.77131305	1	1	1
TEX14	0.867880669	0.906890596	0.812	0.4	1
RNF207	0.868151287	0.769387072	0.938	0.8	1
ENSSSCG00000061293	0.868241569	0.22881869	0.688	0.733	1
ENSSSCG00000049199	0.868417492	0.536940986	0.938	0.867	1
B3GAT2	0.868542216	0.671176796	1	1	1
ENSSSCG00000052142	0.868607729	0.64385619	1	1	1
ZNF385C	0.868692696	0.921537372	0.875	0.867	1
YY1	0.868693399	0.463643077	1	1	1
RASA2	0.868985314	0.5017959	1	1	1
TRDMT1	0.869030159	0.645775306	1	0.933	1
ENSSSCG00000038269	0.8694568	0.64385619	0.562	0.333	1
S1PR1	0.869563113	1.030879313	0.938	0.733	1
HDAC9	0.869797711	0.524643031	1	1	1
CAPZA1	0.869990935	0.465598456	1	1	1
ENSSSCG00000052972	0.870864524	0.5987683	0.562	0.333	1
ENSSSCG00000063274	0.871529747	0.665882496	0.5	0.4	1
LYPLA1	0.871541629	0.572195205	1	1	1
WDR44	0.871565935	0.680298186	1	1	1
ENSSSCG00000062082	0.872039277	0.468769483	0.875	0.8	1
CWF19L1	0.872555421	0.927952211	0.938	0.8	1
NSUN2	0.873399218	0.69538649	0.625	0.467	1
RHBDD1	0.873622054	0.668777565	1	1	1
ENSSSCG00000022176	0.873696393	0.746425923	0.812	0.4	1
ENSSSCG00000016737	0.873945525	0.50754662	1	1	1
ENSSSCG00000061941	0.873974522	0.722134789	1	1	1
ZZZ3	0.873976363	0.749198117	1	1	1
UPF2	0.874023975	0.655592298	1	1	1
LCOR	0.874503102	0.511613797	1	1	1
PIP5K1A	0.874599311	0.631256153	0.75	0.6	1
ENSSSCG00000013380	0.874995316	0.64385619	1	1	1
TMX4	0.875912245	0.597068129	1	1	1
ENSSSCG00000034743	0.876245248	0.458794808	1	0.933	1
NCKIPSD	0.87642152	0.766712938	1	1	1
ZGRF1	0.876519141	0.517658514	0.938	1	1
MTHFD2	0.876855992	0.541012709	1	1	1
PCGF2	0.877246155	0.611434712	0.875	0.667	1
IQGAP3	0.877396373	0.493300859	1	1	1
ZNF598	0.877497199	0.825147446	1	0.933	1
GTF2I	0.877565669	0.675489474	1	1	1
ENSSSCG00000060843	0.877930905	0.678138009	1	1	1
ATP8	0.87800678	0.659693116	1	1	1
COQ6	0.878216466	0.646706663	1	1	1
ENSSSCG00000028035	0.87826176	0.59980101	1	0.933	1
ENSSSCG00000009595	0.878320862	0.542734265	0.938	0.867	1
ENSSSCG00000047290	0.878823007	0.380821784	0.688	0.467	1
POMK	0.878982834	0.674229839	0.75	0.6	1
ATF7	0.879356534	0.677498115	1	1	1
ZZEF1	0.879486123	0.561655127	0.938	0.933	1
CRTAP	0.879699942	0.68061474	0.812	0.6	1
ENSSSCG00000045286	0.879790859	0.606082847	1	1	1
CCDC73	0.880005176	0.651923471	1	1	1
GPHN	0.880471948	0.541241123	0.875	0.733	1
ENSSSCG00000006205	0.880586198	0.637832402	0.938	0.8	1
GABRA2	0.880648794	0.672425342	1	0.933	1
CTTNBP2NL	0.880679303	0.312529397	0.938	0.8	1
ENSSSCG00000061109	0.881335684	0.417628511	0.812	0.933	1
ENSSSCG00000004410	0.881538069	0.514573173	0.625	0.6	1
ELOVL3	0.881597818	1.006426269	0.625	0.6	1

TEAD1	0.881609471	0.677975568	1	1	1
FRS2	0.88200597	0.990658953	0.875	0.8	1
CHCHD1	0.882058033	0.857980995	1	0.8	1
NMNAT3	0.882851458	0.50153565	1	0.867	1
OTUD4	0.882869425	0.7367343	1	1	1
AP1B1	0.882971342	0.733496245	1	1	1
ID1	0.882994028	0.658963082	0.75	0.467	1
EBF3	0.883088622	0.376375879	0.562	0.467	1
VPS45	0.883193531	0.701000097	1	1	1
ARHGEF12	0.883202879	0.597346225	0.938	0.867	1
ENSSSCG00000043429	0.883744171	0.202346479	0.5	0.333	1
UGDH	0.883849016	0.552549028	1	1	1
PTP4A1	0.883964606	0.731701007	1	1	1
MOB3B	0.884251663	0.761039729	0.75	0.533	1
SIAH1	0.884593278	0.636951781	1	1	1
CAMSAP3	0.884651845	0.424289813	1	0.867	1
TMEM185A	0.884717567	0.703357202	0.688	0.533	1
SEMA3F	0.88555638	0.457087678	0.625	0.467	1
ENSSSCG00000044144	0.885570974	0.689299161	0.625	0.6	1
DENND4B	0.886036125	0.677408749	0.625	0.6	1
SHISA5	0.886071642	0.731671921	1	1	1
MCM10	0.886268287	0.636376225	1	1	1
KLKB1	0.886386214	0.781359714	0.5	0.667	1
INCENP	0.886454829	0.765336451	1	1	1
ENSSSCG00000010893	0.886613778	0.432518957	0.938	0.733	1
HNRNPL	0.886700765	0.62639522	1	1	1
USP12	0.886787411	0.247230728	0.938	0.8	1
NHLRC3	0.886881617	0.818638126	1	1	1
CDK7	0.887280689	0.285874171	1	1	1
WFS1	0.887334966	0.895208791	0.75	0.867	1
HORMAD2	0.887955183	0.558967292	0.562	0.467	1
ENSSSCG00000047289	0.888040831	0.521339861	0.938	0.867	1
ADAT1	0.888165649	0.715557655	0.812	0.867	1
OTP	0.888326198	0.781359714	0.625	0.667	1
ENSSSCG00000052175	0.889533632	0.88582898	0.938	0.933	1
CAB39	0.889631945	0.386294016	1	1	1
NWD2	0.889917131	0.881799615	0.938	1	1
ENSSSCG00000061191	0.890161107	0.534921818	0.5	0.467	1
TRAM1	0.890345625	0.713780207	1	1	1
TRA2B	0.890365197	0.686634648	1	1	1
ENSSSCG00000057241	0.890425714	0.37895904	0.562	0.6	1
E2F1	0.890487137	0.662653785	1	1	1
NKX2-8	0.890817075	0.754887502	0.688	0.467	1
ENSSSCG00000048684	0.891270448	0.761039729	0.75	0.667	1
FBXL18	0.891697145	1.150161747	0.875	0.867	1
C2CD2L	0.891941726	0.409390936	0.5	0.333	1
NIPA2	0.892180115	0.589041015	1	1	1
NHLRC2	0.892191691	0.691067503	1	1	1
PKHD1	0.892676093	0.475174355	0.5	0.533	1
TNK2	0.892824456	0.613159393	0.562	0.267	1
ZNF333	0.892970256	0.489980682	1	1	1
ENSSSCG00000061077	0.893109709	0.196397213	0.562	0.267	1
SNAPC1	0.893289441	0.481496304	1	0.933	1
MAP1B	0.893577065	0.575821398	1	1	1
NAP1L1	0.893660515	0.7409819	1	1	1
CACNB4	0.895213427	0.153805336	0.938	0.8	1
LSS	0.895311358	0.647131322	0.938	0.933	1
ENSSSCG00000010952	0.89613669	0.636903429	0.938	1	1
CILP	0.896162558	0.426724285	0.938	0.933	1
C12orf76	0.89616845	0.923316144	1	1	1
SRFBP1	0.89645862	0.639870842	1	0.933	1
MGAM2	0.896688699	0.539102223	1	1	1
NDUFB1	0.896872807	0.317078695	1	0.933	1
ENSSSCG00000044127	0.897111236	0.654124525	0.875	0.867	1
SREK1	0.897218311	0.82536071	1	1	1
TRPV4	0.897443438	0.78754965	1	1	1
MELTF	0.897606147	0.464580166	1	0.933	1
ENSSSCG00000061869	0.897902111	0.546008866	1	0.933	1
ENSSSCG00000055563	0.897947153	0.512611656	0.875	0.733	1
GAS2L3	0.897998102	0.41550221	0.938	0.733	1

ENSSSCG00000045906	0.898271311	0.376375879	0.5	0.467	1
TRIM26	0.898275429	0.278859373	0.812	0.733	1
ROBO2	0.898427104	0.75987091	1	1	1
ENSSSCG00000045464	0.898612802	0.714245518	0.5	0.4	1
PRDM1	0.899613051	0.416564969	0.938	0.733	1
ENSSSCG00000054101	0.899954937	0.64385619	0.562	0.467	1
NAAA	0.900242287	0.673093411	0.938	0.867	1
ENSSSCG00000035000	0.90026724	0.569587275	1	1	1
ENSSSCG00000012006	0.900435588	0.398702217	0.75	1	1
ENSSSCG00000034540	0.901130226	0.378440116	0.938	0.933	1
NPEPPS	0.901418229	0.661210013	1	1	1
CHST4	0.901751779	0.708344916	0.688	0.667	1
PHF2	0.902152866	0.847184349	0.875	0.867	1
HIP1	0.90217591	0.547251335	1	1	1
TRIM68	0.902655669	0.452324732	0.875	0.6	1
OTUD6B	0.903369376	0.305439972	1	0.533	1
ARHGAP15	0.903940713	0.64385619	0.562	0.533	1
NRXN3	0.904033876	0.662552283	1	1	1
RNLS	0.904582811	0.476256241	0.812	0.6	1
KMT2A	0.904725163	0.631436931	1	1	1
NAA80	0.904930031	0.145050333	0.75	0.867	1
IGSF10	0.905066511	0.362707945	1	1	1
KIF13A	0.905483924	0.547476603	1	1	1
LRRIQ3	0.906848539	0.483162136	1	1	1
RIMS4	0.907380568	0.515699838	0.875	0.667	1
KEAP1	0.907617013	0.485063931	0.875	0.733	1
ENSSSCG00000061934	0.907687767	0.529626439	0.938	1	1
TPK1	0.908065374	0.655734946	1	1	1
ERCC6L	0.90819168	0.676032081	1	1	1
MYBL1	0.908570225	0.51737146	1	1	1
ESS2	0.908809943	0.691430888	1	1	1
ARL2BP	0.908818962	0.598266231	1	1	1
USP14	0.909110295	0.653288355	1	1	1
CDC37L1	0.909790246	0.524435919	1	1	1
SMIM19	0.910022284	0.556393349	1	1	1
HELLS	0.910376458	0.67507492	1	1	1
EGF	0.910452161	0.585689482	1	1	1
ADCYAP1R1	0.910604767	0.72744282	1	1	1
CIR1	0.911249372	0.709698728	1	1	1
ENSSSCG00000058555	0.911408241	0.186998515	0.938	0.733	1
PSPC1	0.911475914	0.655828831	0.938	1	1
ZNF354B	0.911898941	0.378341404	0.938	0.933	1
C11orf65	0.912136111	0.505336326	0.875	0.867	1
BLNK	0.912172751	0.781359714	0.625	0.4	1
ENSSSCG00000044841	0.9123598	0.719467004	1	1	1
AP1G1	0.913155265	0.719643261	1	0.933	1
AHR	0.913273396	0.546596061	1	1	1
ENSSSCG00000035189	0.913880899	0.72631835	0.625	0.267	1
TDRD12	0.914125169	0.673803573	1	1	1
MEGF9	0.914156207	0.531109309	1	1	1
SERAC1	0.914707313	0.483082887	0.938	0.933	1
ENSSSCG00000060381	0.915016413	0.321928095	0.812	0.6	1
PIK3CA	0.915369628	0.334553776	1	1	1
DAB2IP	0.915892995	0.557962892	1	1	1
KANSL1	0.915964435	0.452584453	1	1	1
CABCOCO1	0.916146847	0.736965594	0.812	0.667	1
ENSSSCG00000041329	0.917018406	0.491853096	0.875	0.867	1
ZFYVE26	0.917073109	0.567305325	0.938	0.867	1
MARS2	0.917274356	0.92347348	1	0.933	1
SLC12A6	0.917399292	0.546008866	1	1	1
ENSSSCG00000047646	0.917624267	0.339850003	0.5	0.467	1
ENSSSCG00000056848	0.918109612	0.38332864	0.562	0.467	1
CPOX	0.918628963	0.439385676	0.75	0.733	1
XPNPEP1	0.918644621	0.347463187	0.625	0.533	1
SLC39A9	0.918748869	0.573617527	1	1	1
HAPLN1	0.918937798	-0.005646563	0.125	0.533	1
PHF12	0.919042629	0.43650068	1	1	1
AGMO	0.919287078	0.574459468	1	1	1
TSGA10	0.919476545	0.582480292	1	1	1
TOGARAM1	0.91958071	0.542326466	1	1	1

ENSSSCG00000004520	0.91978427	0.698806786	0.938	1	1
ENSSSCG00000044331	0.919844368	0.670190338	1	0.933	1
ODAD4	0.920692944	0.632201348	0.938	1	1
AFTPH	0.920972415	0.594862268	1	1	1
ENSSSCG00000003654	0.921064984	0.493378951	1	1	1
SPRY1	0.921294806	0.60232269	1	1	1
ATAD2B	0.921522168	0.673492424	1	1	1
ENSSSCG00000060388	0.921983071	0.556648219	0.938	1	1
ZNF180	0.922240999	0.493378951	0.938	1	1
SACS	0.923171642	0.736271824	1	0.867	1
MIGA1	0.923211512	0.637667978	1	1	1
ELMO2	0.92372534	0.627153311	1	1	1
ENSSSCG00000057454	0.923967887	0.301608111	0.938	0.933	1
TMED8	0.924533372	0.47023452	1	1	1
PIP4P2	0.924571734	0.511589314	0.938	0.8	1
TMEM199	0.924649502	0.715359138	1	0.933	1
CREB5	0.924654995	0.309855263	0.938	0.867	1
BNC1	0.924658793	0.693304679	1	1	1
ENSSSCG00000056312	0.925030473	0.64385619	0.438	0.533	1
PYGL	0.925124971	0.491853096	1	0.8	1
PSMC2	0.925534184	0.496179022	1	1	1
NECTIN3	0.925669873	0.220551075	0.938	1	1
BRINP3	0.925728257	0.754887502	1	1	1
PPP2R3C	0.926420651	0.469666333	1	1	1
IGSF9B	0.926454559	0.285402219	0.75	0.467	1
ENSSSCG00000036656	0.926624742	0.479959418	0.812	1	1
ENSSSCG00000016085	0.926704482	0.504792152	0.938	0.933	1
NPAT	0.926710692	0.507158416	1	1	1
CTSH	0.92692105	0.863821874	1	1	1
ENSSSCG00000012627	0.92745233	0.633776984	1	1	1
ENSSSCG00000010669	0.927526827	0.461899874	1	1	1
ADAM22	0.927601085	-0.093109404	0.688	0.533	1
CRPPA	0.927854534	0.599256192	1	1	1
PLAG1	0.927912274	0.714245518	1	0.933	1
CDKN1C	0.928454231	0.447458977	0.5	0.467	1
MGA	0.928987194	0.627891636	1	1	1
FGF12	0.92899307	0.508531837	1	0.867	1
FBXO5	0.929315137	0.488253107	1	1	1
TMEM178B	0.929502285	0.299208018	0.625	0.467	1
WASHC5	0.930924374	0.616240978	1	1	1
IMPDH2	0.931290283	0.733028068	1	1	1
TBC1D10A	0.931364668	0.776529796	1	1	1
ZNF470	0.931582867	0.604327826	1	0.933	1
ENSSSCG00000060892	0.931693244	0.836501268	0.5	0.533	1
SUPT7L	0.931788004	0.620397217	0.938	1	1
OSBPL8	0.932188435	0.415732463	1	1	1
LENG8	0.932932399	0.441227023	0.938	0.867	1
MRO	0.933066518	0.681882226	0.938	1	1
CEP83	0.933070097	0.765622053	1	1	1
MFSD9	0.933177166	0.672425342	0.625	0.667	1
ENSSSCG00000060691	0.933395131	0.87036472	0.5	0.533	1
AFMID	0.933939884	0.616032508	0.938	1	1
CRIM1	0.934020185	0.472728441	1	1	1
CMYA5	0.93423218	0.671870566	1	0.933	1
NEMF	0.934260412	0.627623163	1	1	1
PPP4R1	0.934475234	0.584223226	1	1	1
MIF4GD	0.934692785	0.772113916	1	0.933	1
PPP1R21	0.934983138	0.5585134	1	1	1
ENSSSCG00000058394	0.934998218	0.582247201	1	1	1
KIDINS220	0.935383246	0.728355919	1	1	1
GLDC	0.935390633	0.62309763	0.938	0.733	1
JCAD	0.935889269	0.607330314	0.5	0.333	1
ZBTB26	0.936085015	-0.001478929	0.812	0.467	1
ULK4	0.936240726	0.726082307	1	1	1
EDEM3	0.936769925	0.584962501	0.938	0.667	1
CIZ1	0.936971436	0.564002882	0.75	0.8	1
PPP4C	0.937137488	0.340877198	1	1	1
ENSSSCG00000053348	0.937604177	0.196397213	0.625	0.533	1
NUBPL	0.937875152	0.593615812	1	1	1
ENSSSCG00000059926	0.938090956	0.633325522	1	0.8	1

DIPK1A	0.938561951	0.736965594	1	1	1
TECTB	0.938669285	0.304974962	0.875	0.933	1
LPIN1	0.938764231	0.330102026	0.938	0.733	1
MED16	0.938939161	0.626782676	1	0.933	1
ENSSSCG00000015876	0.939220091	0.432302293	1	0.8	1
C2orf49	0.939744957	0.438413837	1	1	1
PHF7	0.940082692	0.728553354	0.812	0.8	1
NIFK	0.940208361	0.496006578	1	1	1
ENSSSCG00000058823	0.940241775	0.673913424	0.688	0.8	1
PIGG	0.941273423	0.349408831	0.875	0.867	1
RBM20	0.941359727	0.733291273	1	0.933	1
KIAA1217	0.941430438	0.906890596	0.938	0.867	1
ENSSSCG00000040412	0.941559967	0.447458977	0.5	0.4	1
SYNJ2	0.941563858	0.609504685	1	1	1
AQR	0.941963988	0.491285218	1	1	1
ENSSSCG00000054931	0.942013839	0.64385619	0.562	0.467	1
FSIP1	0.942143669	0.940837928	1	1	1
GOLM2	0.942144108	0.747110152	0.875	0.867	1
PSMC6	0.94236755	0.432959407	1	0.867	1
TAPBP	0.942541818	0.427722759	0.75	0.733	1
MED13L	0.942620493	0.613125097	1	1	1
ZBTB11	0.943066412	0.531381461	0.938	0.933	1
PRDM2	0.943115667	0.823078608	1	1	1
ENSSSCG00000062978	0.943364782	0.591794534	1	0.933	1
KLHL9	0.943411495	0.139551352	0.5	0.6	1
POLR1A	0.943774424	0.430109747	1	0.933	1
FRMPD4	0.943895199	0.794232795	1	1	1
SLC50A1	0.945105904	0.302819272	0.562	0.4	1
CTPS2	0.945242672	0.611434712	0.688	0.6	1
DFFB	0.945245552	0.468769483	0.562	0.4	1
ENSSSCG00000059588	0.94561861	0.906890596	0.562	0.6	1
NUAK1	0.945643912	0.646299366	1	1	1
TBC1D9B	0.946012426	0.285402219	0.75	0.667	1
CRTC2	0.946772465	0.771235496	0.938	0.8	1
ORMDL2	0.946813198	0.665882496	1	0.933	1
ENSSSCG00000050165	0.947291699	0.64385619	0.688	0.467	1
Vault.2	0.947327102	0.491853096	0.5	0.4	1
ENSSSCG00000057557	0.947554151	0.475174355	0.688	0.467	1
MYBPC1	0.948008361	0.384469561	0.938	0.867	1
CAMK2N1	0.948255031	0.219274918	1	1	1
ZNF684	0.94841773	0.156868849	0.625	0.6	1
ENSSSCG00000057597	0.948870221	0.69276579	0.75	0.6	1
ENSSSCG00000053625	0.948917506	0.863821874	0.562	0.467	1
GALNT12	0.948927937	0.335127593	1	1	1
SPRED1	0.949267239	0.331925684	1	1	1
WNT3	0.949324834	0.776775776	1	1	1
CHD1	0.949330178	0.71104009	1	1	1
GRIP1	0.949355488	0.60118352	1	1	1
MEP1B	0.949593091	0.653471279	1	1	1
OGDHL	0.950530948	0.385751672	0.938	1	1
ENSSSCG00000057119	0.950701371	0.549374315	1	1	1
ENSSSCG00000039618	0.950909216	0.361005617	1	1	1
ENSSSCG00000033351	0.951276894	0.622051819	0.938	0.8	1
CLEC16A	0.951450872	0.797661526	1	1	1
MTMR4	0.951772693	0.300554444	0.875	0.867	1
GJA10	0.951809901	0.719377179	0.938	1	1
VRK2	0.951922934	0.674552987	1	1	1
CDK14	0.952052906	0.568872798	1	1	1
IDS	0.952058636	0.757460298	1	1	1
MFAP3L	0.952112595	0.701421477	0.938	0.933	1
ENSSSCG00000063513	0.952440453	0.202346479	0.625	0.467	1
ENSSSCG00000056036	0.952904661	0.366322214	0.562	0.333	1
ENSSSCG00000056261	0.95297842	0.944365301	0.812	0.867	1
GMFB	0.953076449	0.664264621	1	1	1
FBLN1	0.95309568	0.415037499	0.812	0.867	1
ENSSSCG00000055669	0.953334444	1.036173613	0.5	0.4	1
PDE1C	0.953787937	0.736490945	1	1	1
ENSSSCG00000053386	0.953845626	0.491853096	0.5	0.467	1
PREB	0.953863173	0.749534268	1	0.933	1
ENSSSCG00000011803	0.953867903	0.771815941	1	1	1

MAP3K3	0.954516751	0.870053827	1	1	1
SOD2	0.955402246	0.570173278	1	1	1
FUT8	0.955891839	0.495934273	0.938	1	1
ENSSSCG00000060561	0.956515391	0.534921818	0.562	0.333	1
TYW3	0.956536612	0.148476582	1	1	1
ENSSSCG00000053433	0.957088358	0.68195598	0.938	1	1
RAB1A	0.957370362	0.714511428	1	1	1
ENSSSCG00000060721	0.957472784	0.668730858	0.5	0.533	1
WARS2	0.957474039	0.49330523	1	1	1
ENSSSCG00000042615	0.957766382	0.531381461	1	0.867	1
POLR1E	0.95779739	0.520638226	1	1	1
LCORL	0.957860957	0.468440551	1	1	1
MTFR2	0.957898217	0.604543863	1	1	1
COMMD8	0.958199267	0.469510895	1	1	1
FBXL17	0.958211823	0.558967292	1	0.867	1
CWF19L2	0.958307693	0.654090904	1	1	1
MGAT4B	0.958313429	0.754887502	0.812	0.667	1
CDCA5	0.958321929	0.867362231	0.938	0.933	1
SPAG17	0.959104485	0.706953025	1	1	1
ENSSSCG00000057954	0.959123807	0.491853096	0.5	0.4	1
SHPRH	0.959672068	0.5987683	0.938	0.8	1
RNF5	0.959898008	0.836501268	0.75	0.8	1
ENSSSCG00000033117	0.960230012	0.455679484	1	1	1
USP2	0.960629115	0.648031866	1	1	1
HOXA9	0.960638	0.728892294	0.938	0.8	1
ENSSSCG00000053300	0.960777829	0.491853096	0.938	0.8	1
CBX3	0.96097963	0.497089745	1	1	1
ALX1	0.961133499	0.425528337	1	1	1
STK26	0.962156649	0.61381643	1	1	1
MFSD8	0.96226215	0.911203588	1	0.933	1
ABCA4	0.962302079	0.347463187	0.625	0.467	1
ZNF317	0.962399378	0.500525398	1	1	1
ZFHX2	0.962611928	0.676006713	1	1	1
RGS10	0.962860769	0.186998515	0.625	0.4	1
ENSSSCG00000055215	0.962911244	0.516944077	0.688	0.467	1
EFL1	0.962977889	0.622583672	1	1	1
KLHL12	0.963153657	0.770073906	0.875	0.933	1
HID1	0.963177918	0.81688043	1	0.867	1
RPP30	0.963399779	0.741552864	1	1	1
MEIS1	0.964051382	0.613159393	0.688	0.733	1
PIGX	0.964054576	0.420377885	1	1	1
NBEA	0.964338127	0.671735222	1	0.867	1
PRDM4	0.964369463	0.686017125	1	1	1
BICDL1	0.964477308	0.689299161	0.875	0.667	1
PUM2	0.964896941	0.619808732	1	1	1
TMEM30A	0.965043877	0.548360193	1	0.933	1
ZNF23	0.965079697	0.549734012	0.938	0.867	1
TNFSF18	0.965835464	0.581314724	1	1	1
MYPN	0.965905716	0.454378391	0.562	0.467	1
SAFB2	0.965995843	0.798514435	0.938	0.8	1
WDR76	0.966300978	0.564097981	1	1	1
BBOF1	0.966369935	0.951284715	0.5	0.467	1
ETNK1	0.966569078	0.550034167	0.938	0.933	1
HNRNPUL1	0.966855499	0.801793533	1	1	1
SLC7A3	0.967004332	0.404606211	1	1	1
UBR3	0.967176055	0.462370075	1	1	1
ENSSSCG00000041163	0.967496068	0.454378391	0.5	0.267	1
RCE1	0.967832436	0.836117521	1	0.867	1
CAPRIN2	0.967938317	0.634197046	1	1	1
ENSSSCG00000012520	0.968184736	0.578205135	1	1	1
SHISA9	0.968511072	0.673691419	0.938	0.933	1
ENSSSCG00000060472	0.969410112	0.64385619	0.625	0.4	1
TBC1D13	0.969904497	1.002649578	0.875	0.933	1
TRAF3IP2	0.97076885	0.5987683	0.5	0.333	1
GLG1	0.970947837	0.503441071	1	1	1
BBS7	0.971318749	0.461479447	1	0.8	1
FAM185A	0.972298556	0.371294172	0.875	0.8	1
LRATD1	0.972466494	0.661778098	0.562	0.467	1
PGRMC2	0.973044098	0.576319886	1	1	1
TP53INP1	0.973137093	0.600768001	1	1	1

ENSSSCG00000059586	0.973176458	0.564002882	0.625	0.533	1
SYT1	0.973658713	0.736965594	0.5	0.333	1
DPP10	0.973684385	0.523561956	0.562	0.6	1
EBAG9	0.973890907	0.692219211	1	1	1
DHRS9	0.974422692	0.656912343	0.562	0.467	1
USP6NL	0.974756401	0.321928095	1	1	1
CFAP43	0.975415668	0.491853096	0.625	0.467	1
SNX6	0.975570776	0.608558314	1	1	1
MARCHF6	0.975581117	0.727470816	1	1	1
KLHL22	0.97580569	0.588638159	1	1	1
TRIM9	0.97627211	0.69538649	0.5	0.333	1
MAFG	0.976448927	0.785328616	1	1	1
ENSSSCG00000058281	0.976783926	0.154818109	0.5	0.4	1
SLC5A8	0.977042585	0.769387072	0.75	0.533	1
SH3GL1	0.977373553	0.627273306	1	1	1
ITGAM	0.977475515	0.587272661	1	0.933	1
SAXO1	0.977583191	0.564654775	1	1	1
FAT4	0.977929577	0.267832351	1	0.933	1
MTIF3	0.977946167	0.650550842	0.875	0.667	1
DAB1	0.977956418	0.594946589	1	1	1
TOR1AIP2	0.978395547	0.369862572	0.938	0.933	1
ENSSSCG00000050726	0.979005602	0.617383978	0.625	0.4	1
ADAMTS14	0.979800484	0.743391863	0.75	0.667	1
HLCS	0.979897675	0.80580447	1	1	1
PAMR1	0.979914447	0.343954401	1	1	1
CCDC66	0.980233522	0.588059422	1	1	1
ENSSSCG00000025440	0.980383672	0.318817923	1	1	1
ENSSSCG00000009356	0.980754103	0.521434087	1	0.933	1
USP30	0.980857304	0.6036671	1	1	1
ENSSSCG00000003248	0.981013784	0.392317423	0.625	0.4	1
TFCP2L1	0.981856625	0.392317423	0.688	0.4	1
GGNBP2	0.982764364	0.792821887	1	1	1
ZNF544	0.982904557	0.700439718	1	1	1
ENSSSCG00000049625	0.983472309	0.594946589	0.75	0.4	1
TMEM106A	0.983966506	0.909129068	1	1	1
ENSSSCG00000013760	0.983978893	0.642771049	1	1	1
SLC11A2	0.984393529	0.623373929	1	1	1
MYOC	0.984527	0.491853096	0.625	0.533	1
ZBTB9	0.984556359	0.956643631	0.938	1	1
USP47	0.984869382	0.580662363	1	1	1
R3HDM1	0.985323775	0.544518675	1	1	1
EARS2	0.985389259	0.340306624	1	0.8	1
POLRMT	0.985498709	-0.028979067	0.562	0.267	1
ADAMTS19	0.98555962	0.564784619	0.875	0.667	1
CREBBP	0.985636884	0.596004672	1	1	1
ENSSSCG00000038359	0.986013408	0.661149335	1	1	1
GANAB	0.986188112	0.75276745	1	1	1
ENSSSCG00000053419	0.986445117	0.718128952	0.938	0.933	1
NSF	0.986583048	0.636283873	1	1	1
SRSF9	0.987248779	0.673815892	1	1	1
ZBTB47	0.987352322	0.769387072	0.812	0.8	1
SFXN2	0.987380232	0.933984256	1	1	1
SGCB	0.987711551	0.441227023	0.625	0.4	1
BTAF1	0.987857497	0.637077657	1	1	1
PIP4K2C	0.988373159	0.730899146	1	0.933	1
TDRP	0.989059141	0.738858647	1	1	1
OSBPL11	0.989075999	0.590389573	1	1	1
PCYT1B	0.989548294	0.633872101	1	0.933	1
ENSSSCG00000056449	0.989917424	0.64385619	0.5	0.4	1
PAK2	0.990126339	0.641977855	1	1	1
RTN1	0.990219874	0.34298971	0.875	0.867	1
UTP4	0.990981414	0.540594639	1	1	1
TERB2	0.991706536	0.596550475	0.938	0.8	1
ENSSSCG00000014896	0.992288255	0.832890014	0.5	0.4	1
ENSSSCG00000062223	0.992716475	0.722807531	0.875	0.933	1
OCA2	0.992782799	0.22881869	0.5	0.4	1
ENSSSCG00000031683	0.992799746	0.689299161	0.75	0.4	1
RAD54L	0.992860428	0.639118271	1	1	1
SLC26A11	0.992944293	0.829353995	1	1	1
SRP54	0.992947569	0.721198278	1	1	1

C4orf47	0.993002995	0.526204601	0.938	0.867	1
FBN1	0.993150706	0.496386039	1	1	1
ULK1	0.993235394	0.548997003	1	1	1
ENSSSCG00000026430	0.994277712	0.626338429	1	1	1
ADAMTSL1	0.994366355	0.402522205	1	0.933	1
GPATCH8	0.994440873	0.527765219	1	1	1
TRAPPC8	0.99477295	0.531845072	1	1	1
DIS3L2	0.995294971	0.583913649	0.938	1	1
ENSSSCG00000050748	0.995585647	0.392317423	0.688	0.467	1
ENSSSCG00000034356	0.995596654	0.544320516	0.625	0.4	1
ENSSSCG00000060962	0.995966054	0.553253641	0.625	0.4	1
ENSSSCG00000045200	0.996004633	0.437405312	0.562	0.267	1
ZNF189	0.996330671	0.549848648	1	1	1
SHKBP1	0.996444095	0.417085328	0.75	0.733	1
NAA40	0.99658086	0.806779111	1	1	1
FNDC3A	0.996595735	0.548825282	1	1	1
ABHD6	0.996873564	0.665882496	0.812	0.667	1
ARHGAP1	0.997071351	0.780793062	1	1	1
DSEL	0.997109775	0.523561956	0.5	0.267	1
ENSSSCG00000053448	0.997315713	0.62309763	0.688	0.533	1
GNPDA1	0.997779038	0.750771394	0.812	0.8	1
ICA1L	0.997812987	0.608466145	1	1	1
TTC27	0.997917571	0.743004511	1	0.867	1
NKTR	0.997996955	0.566217031	1	1	1
ENSSSCG00000033739	0.998016592	0.508341219	0.938	0.733	1
ATP8A2	0.998022064	0.545567552	1	1	1
ZFHX3	0.998208452	0.857027319	1	1	1
USP22	0.998292863	0.822847	1	1	1
EPHX2	0.998823691	0.64385619	0.75	0.6	1
VTA1	0.999118589	0.580061324	1	1	1
ENSSSCG00000059963	0.99935236	0.508341219	0.812	0.8	1
PHF21A	0.999991502	0.54630088	0.938	0.933	1
ENSSSCG00000036114	NA	-0.780321963	0.25	1	NA
ENSSSCG00000045657	NA	-0.694560028	0.25	0.733	NA
ENSSSCG00000044939	NA	-1.756074417	0.125	0.6	NA
ENSSSCG00000063504	NA	2.845490051	1	0.533	NA
RAB13	NA	-1.543770813	0.562	1	NA
SDCBP2	NA	0.565853678	0.562	0.133	NA
ENSSSCG00000018067	NA	2.714245518	0.688	0.267	NA
TMEM98	NA	0.296837114	0.25	0.667	NA
NR1D2	NA	-1.030825126	0.75	0.333	NA
ENSSSCG00000056354	NA	-0.802330008	0.75	0.333	NA
ENSSSCG00000061722	NA	-1.269432177	0.812	0.4	NA
GSTA4	NA	3.293913719	0.812	0.4	NA
INHBA	NA	-1.230612928	0.188	0.6	NA
GPRASP1	NA	3.192292814	0.875	0.467	NA
ENSSSCG00000054266	NA	3.154818109	0.5	0.133	NA
LRCH2	NA	2.086214295	0.5	0.133	NA
ENSSSCG00000013849	NA	0.249282793	0.438	0.8	NA
WWTR1	NA	-0.158697746	0.375	0.733	NA
EED	NA	-0.119104613	0.312	0.667	NA
PROS1	NA	0.651051691	0.75	0.4	NA
SBSN	NA	-2.714597781	0.25	0.6	NA
TEX47	NA	1.347463187	0.25	0.6	NA
SERPINE1	NA	-3.710270728	0.25	0.6	NA
ZBTB8OS	NA	-0.678071905	0.188	0.533	NA
BTG2	NA	-1.900464326	0.188	0.533	NA
CFAP52	NA	1.25334101	0.812	0.467	NA
TBC1D4	NA	0.025072022	0.875	0.533	NA
ZC3HAV1	NA	-0.165865747	0.875	0.533	NA
FBN2	NA	-0.595609745	0.938	0.6	NA
HSF2	NA	-1.425953277	1	0.667	NA
POF1B	NA	3.366322214	0.5	0.2	NA
PAG1	NA	1.781359714	0.562	0.267	NA
ENSSSCG00000018076	NA	1.186998515	0.75	0.467	NA
RPS6KA1	NA	-0.280194957	0.812	0.533	NA
ENSSSCG00000060412	NA	-1.286790529	0.938	0.667	NA
CAPZA2	NA	0.704530363	0.938	0.667	NA
ABCA7	NA	-1.563429339	0.562	0.8	NA
GNG11	NA	1.138972073	0.5	0.733	NA

ENSSSCG00000002623	NA	3.831483193	0.5	0.267	NA
GABRB1	NA	-0.208586622	0.562	0.333	NA
GRIA3	NA	0.054989235	0.438	0.667	NA
CCNG2	NA	0.803054785	0.625	0.4	NA
ENSSSCG00000032381	NA	-2.526068812	0.375	0.6	NA
TIMP1	NA	-0.66247505	0.375	0.6	NA
CHAC2	NA	1.506352666	0.688	0.467	NA
SKIL	NA	-0.03170886	0.812	0.6	NA
MOB1A	NA	-1.093109404	0.875	0.667	NA
RUFY2	NA	1.577322439	0.938	0.733	NA
SESN3	NA	0.266432982	0.562	0.733	NA
ALDOA	NA	-0.526458782	0.562	0.733	NA
RGS5	NA	0.760638423	0.5	0.667	NA
ENSSSCG00000057898	NA	-1.012189409	0.562	0.4	NA
VIM	NA	-0.232272152	0.375	0.533	NA
ENSSSCG00000048190	NA	-0.001961516	0.688	0.533	NA
SERPINF1	NA	1.939312073	0.688	0.533	NA
ENSSSCG00000009772	NA	-0.53006168	0.75	0.6	NA
THBS1	NA	-3.390657772	0.812	0.667	NA
ALDH2	NA	-0.191194404	0.812	0.933	NA
SRPRB	NA	-0.900464326	0.562	0.667	NA
ENSSSCG00000022925	NA	-2.370267629	0.5	0.6	NA
HSPG2	NA	-0.093109404	0.438	0.533	NA
BMP1	NA	-0.208586622	0.438	0.533	NA
ENSSSCG00000061178	NA	-2.575502171	0.562	0.467	NA
KIAA0930	NA	2.049848549	0.562	0.467	NA
PHLDB2	NA	-1.05246742	0.625	0.533	NA
ENSSSCG00000031640	NA	1.22881869	0.625	0.533	NA
ENTPD1	NA	1.457905764	0.625	0.533	NA
ZNF25	NA	0.836501268	0.688	0.6	NA
ENSSSCG00000048856	NA	-1.703351314	0.812	0.733	NA
CTSC	NA	1.705541449	0.812	0.733	NA
MAGED1	NA	0.311586151	0.875	0.8	NA
RPS3A	NA	0.893215659	1	0.933	NA
CFAP57	NA	2.851451609	0.938	1	NA
FGF1	NA	0.158429363	0.688	0.733	NA
PIK3IP1	NA	0.989352756	0.5	0.467	NA
SLC30A7	NA	-0.689212463	0.562	0.533	NA
ENSSSCG00000004081	NA	0.828888084	0.625	0.6	NA
ENSSSCG00000061243	NA	-0.392669686	0.625	0.6	NA
TIA1	NA	0.352301744	0.75	0.733	NA
RETREG1	NA	-0.747334568	0.812	0.8	NA
PKM	NA	-0.380596854	0.938	0.933	NA
FKBP3	NA	-1.285754482	1	1	NA
ERP44	NA	0.897688103	1	1	NA
RNF138	NA	2.14974712	1	1	NA
VCAN	NA	1.156385161	1	1	NA
TMEM60	NA	1.963673317	1	1	NA
SERPINH1	NA	0.842978832	1	1	NA
ENSSSCG00000016970	NA	1.686780978	1	1	NA

Gene	p_val	avg_log2FC	pct.1	pct.2	p_val_adj
ENSSSCG00000061888	4.35E-41	4.03180478	1	1	7.54E-37
CPO	8.62E-08	3.969900393	1	0.333	0.001495808
ENSSSCG00000000296	1.15E-25	3.605214326	1	1	2.00E-21
RASL11A	3.38E-16	3.302713962	1	0.867	5.86E-12
ENSSSCG00000040498	3.81E-21	3.214415625	1	0.933	6.61E-17
ENSSSCG00000050765	2.43E-06	3.19865672	1	0.667	0.04212399
ENSSSCG00000063355	6.18E-12	3.181226319	1	0.867	1.07E-07
MRPL44	2.36E-23	3.135383216	1	0.867	4.09E-19
SFT2D1	5.58E-28	3.120036725	1	1	9.69E-24
DUS4L	2.42E-20	3.065426492	1	1	4.20E-16
CCDC152	6.47E-12	3.060365076	1	0.867	1.12E-07
TMEM128	3.34E-29	3.015103393	1	1	5.80E-25
NEIL1	1.51E-17	3.005693006	1	1	2.62E-13
STEAP1	5.75E-10	3.000194315	1	0.8	9.98E-06
GFI1	1.11E-15	2.99906044	1	0.867	1.92E-11
PTS	8.50E-26	2.946358034	1	1	1.48E-21
SNX7	1.90E-21	2.868352343	1	1	3.30E-17
RTRAF	5.75E-15	2.84955432	1	1	9.97E-11
ENSSSCG00000014071	2.43E-28	2.820801619	1	1	4.22E-24
BTG4	8.42E-24	2.767651617	1	1	1.46E-19
NSL1	2.79E-18	2.760265682	1	1	4.85E-14
CA9	9.64E-17	2.749230138	1	1	1.67E-12
CHRAC1	1.05E-22	2.747817929	1	1	1.82E-18
ADORA2A	1.66E-06	2.725052273	1	0.6	0.028768688
NUP42	2.42E-16	2.699171005	1	1	4.21E-12
CEP78	2.52E-21	2.692330741	1	1	4.37E-17
MAD2L1	2.58E-16	2.690511219	1	1	4.47E-12
CCDC179	8.85E-09	2.688785533	1	0.867	0.000153589
ALDH18A1	6.44E-13	2.657370996	1	1	1.12E-08
C12orf40	2.93E-13	2.6555913	1	1	5.09E-09
MOBKL3	3.45E-19	2.651588877	1	1	5.99E-15
TAF1A	8.06E-29	2.647720015	1	1	1.40E-24
MITD1	1.20E-08	2.631992368	1	0.933	0.000207638
ABHD3	3.49E-07	2.628589433	1	0.8	0.00605455
HJURP	1.92E-08	2.624119972	1	0.933	0.000333931
AZIN2	7.60E-14	2.618820598	1	1	1.32E-09
HILPDA	1.44E-17	2.60711463	1	1	2.49E-13
CDC45	1.74E-12	2.605578832	1	1	3.01E-08
ELK3	6.17E-17	2.593203241	1	1	1.07E-12
RAD1	7.00E-13	2.588906089	1	1	1.21E-08
ANKRA2	5.57E-08	2.588196965	1	0.933	0.00096612
CFAP69	5.87E-07	2.572665142	1	0.667	0.010193507
GOLT1B	4.50E-14	2.570474352	1	1	7.80E-10
TTC8	1.22E-06	2.557655155	1	0.667	0.0211717
TMEM216	2.17E-10	2.540692234	1	0.933	3.76E-06
RNPEP	5.17E-07	2.536391492	1	1	0.008962419
SLC35G1	9.66E-24	2.529820947	1	1	1.68E-19
TYMS	2.00E-17	2.501766903	1	1	3.48E-13
ENSSSCG00000053454	9.89E-11	2.494450208	1	1	1.72E-06
HDAC3	8.35E-11	2.492707015	1	1	1.45E-06
PITRM1	9.67E-09	2.466180149	1	1	0.000167698
HAUS4	2.44E-27	2.453180215	1	1	4.24E-23
ETFDH	5.60E-07	2.449998253	1	0.933	0.009710765
IER3IP1	8.87E-12	2.431573399	1	1	1.54E-07
THOC6	1.98E-17	2.430452552	1	0.933	3.44E-13
ENSSSCG00000033181	1.99E-06	2.400136927	0.938	0.8	0.034503582
TAF9	7.91E-19	2.398175692	1	1	1.37E-14
SRPX	3.66E-16	2.397800932	1	1	6.35E-12
EIF1	1.84E-08	2.397698817	1	1	0.000319391
RFK	2.93E-09	2.394409312	1	1	5.08E-05
APIP	5.29E-14	2.391364415	1	1	9.17E-10
ENSSSCG00000044533	1.79E-07	2.391276142	1	1	0.003108455
VWA7	3.99E-08	2.382848703	1	1	0.000692398
PNP	6.61E-14	2.379167825	1	1	1.15E-09
TRAF4	3.34E-10	2.377825621	1	1	5.79E-06

SNX31	1.02E-20	2.372787211	1	1	1.78E-16
UBE2D1	2.46E-08	2.371476543	1	0.933	0.000427081
NSMCE4A	9.70E-24	2.367134588	1	1	1.68E-19
DUSP12	8.21E-07	2.365391743	1	0.933	0.014243074
METTL3	3.72E-09	2.364976417	1	1	6.45E-05
FFAR4	1.19E-07	2.364059163	1	0.933	0.002058847
ENSSSCG00000033814	1.34E-13	2.358864381	1	1	2.33E-09
METTL9	1.94E-16	2.35331022	1	1	3.37E-12
POC1B	2.28E-12	2.351408963	1	1	3.95E-08
RARS2	5.21E-15	2.350834658	1	1	9.05E-11
RNFT1	1.45E-12	2.348841623	1	1	2.52E-08
RIMBP2	2.01E-13	2.3474175	1	1	3.49E-09
LRRC49	3.58E-10	2.341827652	1	0.933	6.21E-06
SLC25A31	4.46E-15	2.339218765	1	1	7.73E-11
SMAD1	4.63E-11	2.335374792	1	1	8.03E-07
ENSSSCG00000063521	1.11E-07	2.33229481	1	0.933	0.001934452
SF3B6	1.81E-11	2.330102026	1	1	3.15E-07
BBS4	2.00E-06	2.326666014	1	0.733	0.034729117
CDC20	3.53E-09	2.325557038	1	1	6.12E-05
HECTD2	1.22E-11	2.316742956	1	1	2.12E-07
PIM1	4.59E-12	2.316732069	1	1	7.97E-08
SMIM11	2.92E-09	2.316614295	1	0.933	5.06E-05
KAT2A	3.45E-09	2.307467282	1	1	5.98E-05
RMC1	3.42E-07	2.306702555	1	1	0.005937686
CMTM8	2.04E-12	2.305542121	1	1	3.54E-08
MCOLN1	8.27E-09	2.305169577	1	0.933	0.000143576
SDHAF4	2.07E-06	2.288139781	1	0.8	0.035939686
CENPN	2.10E-08	2.279793831	1	1	0.000364593
NGLY1	2.68E-20	2.278596252	1	1	4.65E-16
POLB	1.30E-10	2.278539969	1	1	2.26E-06
HACE1	4.63E-13	2.273501405	1	1	8.03E-09
TOGARAM2	1.45E-06	2.266786541	1	0.867	0.025094473
AMFR	2.20E-21	2.2644426	1	1	3.81E-17
ENSSSCG00000004151	4.34E-13	2.261529099	1	1	7.53E-09
MRPL16	3.38E-11	2.258610984	1	1	5.87E-07
PCGF6	1.90E-10	2.258577835	1	1	3.30E-06
SLC17A5	7.19E-16	2.258422404	1	1	1.25E-11
SNRPC	1.03E-14	2.25801286	1	1	1.79E-10
TRIM77	6.12E-18	2.249337225	1	1	1.06E-13
UBC	6.82E-09	2.248487915	1	1	0.000118316
OOSP3	5.47E-11	2.24828312	1	1	9.50E-07
PRXL2A	2.51E-11	2.245797026	1	1	4.36E-07
ENSSSCG00000051497	3.32E-07	2.241175897	1	0.867	0.005765392
TRMT2B	3.36E-07	2.240314329	1	0.8	0.005827112
RFC4	1.29E-18	2.227744324	1	1	2.24E-14
GLCE	1.79E-07	2.224849788	1	0.933	0.00310916
GCA	1.63E-15	2.219736947	1	1	2.84E-11
BUD13	1.63E-09	2.217581719	1	1	2.82E-05
COPB1	9.11E-13	2.214169759	1	1	1.58E-08
PYCR2	4.71E-09	2.209207973	1	1	8.18E-05
POLE2	3.55E-10	2.208871282	1	1	6.17E-06
SRSF7	5.30E-07	2.201166457	1	1	0.009187886
CLPTM1L	1.49E-08	2.196498097	1	1	0.0002587
TPST1	6.31E-11	2.195676406	1	1	1.10E-06
BBS5	7.96E-09	2.191781311	1	1	0.000138141
HSCB	2.93E-09	2.185191758	1	1	5.09E-05
ACYP2	8.21E-15	2.185044731	1	1	1.42E-10
ACTL8	7.46E-08	2.184703057	1	1	0.001294641
CTHRC1	2.35E-07	2.178695211	1	0.8	0.004084409
ENSSSCG00000012452	1.37E-09	2.175952849	1	1	2.39E-05
RXYLT1	2.73E-08	2.173398388	1	1	0.00047284
CNN3	1.50E-13	2.168456354	1	1	2.60E-09
CLK3	8.11E-07	2.164121796	1	1	0.014078249
ZNF280D	2.57E-11	2.159583057	1	0.933	4.46E-07
DNA2	2.84E-07	2.159083794	1	1	0.004921645
PARL	2.80E-18	2.158125	1	1	4.86E-14

ZNF16	3.14E-07	2.156169942	1	1	0.005445552
RPA3	4.82E-10	2.155821258	1	1	8.37E-06
ISOC1	1.01E-16	2.154508152	1	1	1.75E-12
GFPT2	2.45E-06	2.148524367	1	1	0.042474278
ENSSSCG00000004623	4.55E-10	2.1452581	1	1	7.89E-06
ENSSSCG00000026746	5.97E-08	2.142680165	1	1	0.001035336
HESX1	8.70E-07	2.14112463	1	0.8	0.015101778
CCNB1	3.78E-15	2.140053101	1	1	6.56E-11
TRMT11	6.63E-08	2.138802484	1	0.933	0.001149704
KHDRBS1	3.08E-13	2.138454663	1	1	5.34E-09
IRAK2	3.24E-10	2.135768962	1	1	5.62E-06
DHX30	1.14E-07	2.133710104	1	1	0.001976659
FGFR1OP2	4.84E-11	2.131716682	1	1	8.40E-07
RCN2	2.55E-15	2.125407063	1	1	4.43E-11
ENSSSCG00000056174	1.23E-11	2.124121312	1	1	2.13E-07
PRMT2	1.04E-08	2.121224571	1	1	0.000180156
SIKE1	6.94E-07	2.120486069	1	1	0.012045213
STOML2	6.26E-09	2.118982423	1	1	0.000108682
ZNF567	1.30E-08	2.117845904	1	1	0.00022568
DERA	2.67E-08	2.11585031	1	0.933	0.000462818
GALNT11	5.89E-12	2.112237651	1	1	1.02E-07
FBXO43	4.20E-12	2.108918286	1	1	7.29E-08
ARL13B	4.06E-07	2.108876806	1	0.8	0.007045686
CEP57L1	1.13E-08	2.106650827	1	1	0.000196073
KLHL7	6.63E-15	2.105913653	1	1	1.15E-10
MOCS2	1.90E-12	2.104562594	1	1	3.29E-08
AURKB	2.75E-07	2.103927443	1	1	0.004769222
CENPA	2.48E-10	2.102857731	1	1	4.30E-06
PSMD3	6.29E-07	2.102635153	1	1	0.010907434
NUP43	1.15E-10	2.099140144	1	1	2.00E-06
ADH5	3.25E-19	2.095906726	1	1	5.64E-15
NRBF2	3.10E-20	2.095657031	1	1	5.38E-16
STOM	1.85E-07	2.095073819	1	1	0.003217219
CRYZ	2.14E-12	2.094147155	1	1	3.72E-08
MND1	8.22E-07	2.09025786	1	0.933	0.014268021
PEX3	6.74E-10	2.089246271	1	1	1.17E-05
BMAL1	1.03E-07	2.088060354	1	1	0.001783321
NUP35	5.30E-17	2.084742217	1	1	9.19E-13
ENSSSCG00000008996	8.14E-13	2.081859803	1	1	1.41E-08
C9orf40	1.11E-12	2.077886243	1	1	1.92E-08
ENSSSCG00000030671	2.51E-06	2.077654617	1	0.933	0.043528982
STARD3NL	1.04E-17	2.076598078	1	1	1.81E-13
SUPV3L1	1.30E-08	2.075816056	1	1	0.000226395
ENSSSCG00000012235	8.55E-09	2.069324598	1	1	0.00014832
PRMT1	1.21E-06	2.066235664	1	1	0.021007243
SAT1	2.31E-10	2.065794191	1	1	4.00E-06
CRLS1	5.59E-12	2.064678618	1	1	9.70E-08
SLC17A2	8.57E-07	2.063111982	1	1	0.014876895
OAZ1	8.02E-12	2.063053848	1	1	1.39E-07
H1-8	1.67E-10	2.061878384	1	1	2.89E-06
PHYHIPL	1.15E-09	2.058262372	1	1	2.00E-05
EPB41L5	1.83E-13	2.057190776	1	1	3.18E-09
LEPROT	1.21E-11	2.055395592	1	1	2.10E-07
NR2C1	9.34E-12	2.051872623	1	1	1.62E-07
PSMA5	4.23E-10	2.050838491	1	1	7.35E-06
ENSSSCG00000002020	1.00E-22	2.049370351	1	1	1.74E-18
AKAIN1	8.73E-07	2.045505167	1	1	0.015150217
POLG2	1.60E-08	2.043271108	1	1	0.000276763
CDC40	1.21E-18	2.043233601	1	1	2.10E-14
BORA	1.09E-06	2.040817399	1	1	0.018928374
LRRC42	2.39E-07	2.031924912	1	1	0.00415109
MAP2K1	5.89E-11	2.030832606	1	1	1.02E-06
PBK	5.45E-11	2.029968463	1	1	9.46E-07
SEPTIN2	2.69E-09	2.029509882	1	1	4.66E-05
MIER1	2.57E-09	2.027383612	1	1	4.45E-05
EMC2	1.23E-09	2.026801923	1	1	2.14E-05

LACTB2	6.74E-08	2.026037861	1	0.867	0.001169959
C5orf34	4.84E-13	2.024233184	1	1	8.40E-09
SPTLC1	6.24E-18	2.016150344	1	1	1.08E-13
CLDN1	6.82E-13	2.013764314	1	1	1.18E-08
ENSSSCG00000039658	4.61E-09	2.01283646	1	1	8.00E-05
VPS37A	7.53E-13	2.011686786	1	1	1.31E-08
ENSSSCG00000001075	3.01E-12	2.009949005	1	1	5.22E-08
AKTIP	1.73E-08	2.009272088	1	1	0.000299952
GTF2B	5.37E-07	2.005660936	1	1	0.00932055
NDFIP2	6.08E-09	2.000421286	1	1	0.000105492
RCBTB2	3.69E-15	1.998683967	1	1	6.41E-11
ENSSSCG00000000576	8.88E-08	1.995947086	1	1	0.001540106
PDE8A	1.92E-18	1.993544978	1	1	3.33E-14
CARMIL1	1.07E-16	1.99051427	1	1	1.86E-12
ENSSSCG00000021644	9.67E-25	1.990075288	1	1	1.68E-20
CTNNBL1	2.68E-06	1.989957207	1	1	0.046509475
STX7	5.74E-14	1.989783604	1	1	9.97E-10
MAP3K7	4.76E-12	1.989567618	1	1	8.26E-08
ENSSSCG00000009178	1.01E-06	1.989057724	1	1	0.017542931
OOSP2	1.39E-09	1.988350296	1	1	2.40E-05
DARS1	2.17E-10	1.98681924	1	1	3.76E-06
PSMA2	1.51E-11	1.984092766	1	1	2.62E-07
MAPKBP1	4.41E-07	1.983921081	1	1	0.0076442
CNOT8	9.69E-11	1.983875399	1	1	1.68E-06
MPP7	4.53E-12	1.98179201	1	1	7.87E-08
WDR89	1.41E-08	1.980186105	1	1	0.000244806
CCDC127	6.66E-10	1.975345898	1	1	1.16E-05
SLC35A1	4.17E-10	1.971502958	1	1	7.23E-06
RPP25L	3.03E-09	1.970249993	1	1	5.26E-05
FOXN2	3.54E-11	1.964627887	1	1	6.14E-07
ACVR1B	2.25E-07	1.958460944	1	1	0.003900831
CASP6	5.45E-11	1.956892733	1	1	9.45E-07
MELK	1.38E-20	1.956840472	1	1	2.39E-16
LDAF1	1.60E-06	1.95473037	1	1	0.027709869
BLM	1.58E-08	1.952296575	1	1	0.000274322
ETFA	8.85E-12	1.951779687	1	1	1.53E-07
NUS1	3.78E-09	1.951599199	1	1	6.56E-05
SECISBP2	6.79E-13	1.947391555	1	1	1.18E-08
CCNA2	4.53E-13	1.946110343	1	1	7.86E-09
SPPL2A	3.61E-12	1.941824386	1	1	6.27E-08
ZNRF2	1.78E-06	1.94035188	1	1	0.030954364
TM9SF1	5.94E-08	1.939411738	1	1	0.001030794
COG3	3.15E-13	1.938844457	1	1	5.47E-09
SLC12A8	9.46E-10	1.938439431	1	1	1.64E-05
CYFIP1	1.78E-08	1.938169762	1	1	0.000309135
EIF4G2	6.39E-12	1.935715115	1	1	1.11E-07
NMD3	2.95E-09	1.935699219	1	1	5.11E-05
UNC13B	6.53E-11	1.93477371	1	1	1.13E-06
ENSSSCG00000025940	1.76E-13	1.932878128	1	1	3.05E-09
SNRNP40	3.01E-08	1.932165403	1	1	0.000522748
CRY1	4.93E-07	1.930658762	1	1	0.008546989
LNX1	1.45E-09	1.930084168	1	1	2.52E-05
GPR160	3.61E-09	1.929964619	1	1	6.27E-05
ZNF606	1.11E-09	1.927610269	1	1	1.93E-05
SLC25A32	6.05E-10	1.924195482	1	1	1.05E-05
CDCP1	1.31E-06	1.923077642	1	1	0.022767816
TROAP	8.43E-07	1.920986939	1	1	0.014626141
DTWD1	4.64E-07	1.916617142	1	1	0.008054912
NME7	1.85E-08	1.914351909	1	1	0.00032113
CBFB	1.85E-08	1.910510911	1	1	0.000321405
IFRD2	2.96E-07	1.906890596	1	1	0.005128258
CAAP1	8.28E-09	1.905272317	1	1	0.000143675
LIMA1	6.18E-07	1.904321236	1	0.933	0.010714595
TANK	7.69E-07	1.9021989	1	1	0.013349352
WEE2	3.53E-07	1.900622379	1	1	0.006120335
USP33	1.96E-10	1.897881896	1	1	3.40E-06

TTC5	1.19E-09	1.896446704	1	1	2.07E-05
CDCA7	7.72E-07	1.892909841	1	1	0.013395458
DCLRE1A	1.31E-06	1.892656985	1	1	0.022664081
RAP2C	4.49E-08	1.888796802	1	1	0.000779408
GSR	8.34E-15	1.886583089	1	1	1.45E-10
RBM17	9.77E-08	1.885682937	1	1	0.001694563
NCOA3	2.35E-10	1.885400313	1	1	4.08E-06
ENSSSCG00000060529	1.86E-08	1.882935863	1	1	0.000322913
ARL6IP6	1.30E-08	1.881799615	1	1	0.000225803
SAP30	1.38E-08	1.879861364	1	1	0.000238832
ATL1	1.96E-08	1.876654589	1	1	0.000340165
MEST	1.04E-08	1.874618149	1	1	0.000181144
MAP3K13	9.10E-18	1.874358342	1	1	1.58E-13
ME2	1.50E-13	1.873282203	1	1	2.60E-09
RIDA	2.14E-10	1.872844123	1	1	3.71E-06
PPM1D	1.24E-07	1.872215328	1	1	0.002159884
ENSSSCG00000004983	5.59E-07	1.871744033	1	1	0.009706062
CTBS	5.13E-11	1.870909175	1	1	8.90E-07
VEZF1	1.57E-11	1.870521458	1	1	2.73E-07
ENSSSCG00000027349	3.89E-08	1.870043711	1	1	0.000674888
TRAFD1	2.24E-11	1.86773925	1	1	3.89E-07
SBDS	1.30E-15	1.867298417	1	1	2.26E-11
SGMS1	1.75E-12	1.862972371	1	1	3.03E-08
DNMT3B	5.21E-08	1.861357454	1	1	0.000903329
WDR20	4.23E-16	1.861195383	1	1	7.34E-12
OMA1	1.52E-08	1.859597987	1	1	0.000263857
ENY2	6.96E-11	1.859062071	1	1	1.21E-06
ENSSSCG00000005440	1.73E-16	1.853706272	1	1	3.00E-12
PARD6B	4.99E-14	1.85297505	1	1	8.66E-10
CBR4	4.07E-08	1.851964385	1	1	0.000705637
RRM1	5.90E-11	1.850669584	1	1	1.02E-06
APH1A	2.91E-08	1.849088499	1	1	0.000504213
DONSON	1.58E-13	1.848430865	1	1	2.73E-09
NEIL3	3.06E-11	1.847480932	1	1	5.31E-07
RAF1	1.94E-11	1.845181503	1	1	3.37E-07
PPM1B	9.48E-11	1.844984691	1	1	1.65E-06
MLLT11	9.36E-09	1.841624824	1	1	0.00016234
PDHX	1.05E-09	1.840777924	1	1	1.82E-05
KIF4A	8.66E-13	1.840404308	1	1	1.50E-08
SPIRE1	6.69E-09	1.840365135	1	1	0.00011607
THOC3	4.30E-10	1.840075642	1	1	7.47E-06
FAM168B	7.21E-15	1.836792626	1	1	1.25E-10
SKP2	8.22E-08	1.834397152	1	1	0.00142677
HNRNPC	4.48E-21	1.833240347	1	1	7.77E-17
TAB2	2.06E-12	1.831676759	1	1	3.57E-08
CEP44	1.98E-13	1.830519326	1	1	3.43E-09
ID3	3.15E-07	1.827864414	1	1	0.005471755
NPM1	3.50E-09	1.827574231	1	1	6.06E-05
FYB2	3.41E-11	1.827074642	1	1	5.93E-07
SLC25A20	5.68E-12	1.82527683	1	1	9.86E-08
MFF	1.85E-10	1.8252513	1	1	3.21E-06
SLC4A4	2.20E-08	1.822708174	1	1	0.000381831
ENSSSCG00000008335	2.02E-08	1.8217351	1	1	0.000349837
PCBP1	1.42E-08	1.818636586	1	1	0.000245923
ORMDL1	7.47E-08	1.813531352	1	1	0.001296922
TMCC1	1.23E-14	1.811684666	1	1	2.14E-10
DR1	9.91E-12	1.810473113	1	1	1.72E-07
TAF5	2.99E-08	1.810392008	1	1	0.00051917
ENSSSCG00000053039	2.85E-06	1.810366527	1	1	0.04952177
SNRPE	4.13E-08	1.809716512	1	1	0.000717241
LAPTM4A	3.37E-11	1.806840594	1	1	5.84E-07
PPP1CC	1.29E-08	1.806780231	1	1	0.000223787
AHCTF1	1.02E-08	1.805775963	1	1	0.000176586
PRKCI	1.47E-11	1.805465923	1	1	2.55E-07
DIMT1	1.43E-06	1.802513979	1	1	0.024792884
XRCC2	1.76E-09	1.800268176	1	1	3.05E-05

CMAS	9.56E-11	1.797178592	1	1	1.66E-06
TCTN2	2.92E-09	1.790823198	1	1	5.06E-05
COQ10B	1.93E-09	1.786147538	1	1	3.35E-05
HERPUD2	6.47E-11	1.786094079	1	1	1.12E-06
ADPGK	1.45E-08	1.785155183	1	1	0.000252168
TAOK3	7.71E-08	1.78495202	1	1	0.001337646
TRMT5	2.07E-06	1.784555053	1	1	0.035940575
MASTL	9.12E-10	1.783366188	1	1	1.58E-05
ICA1	1.74E-09	1.782374089	1	1	3.02E-05
MRPL15	1.87E-10	1.780585735	1	1	3.25E-06
RCHY1	5.24E-08	1.778530344	1	1	0.000909226
SOS2	4.48E-08	1.777892239	1	1	0.000777224
GMNN	1.13E-06	1.776971761	1	1	0.01956387
CREG1	9.10E-07	1.776561601	1	1	0.015786707
MAPK6	3.71E-08	1.776318141	1	1	0.000643971
TMCO6	2.87E-06	1.771904551	1	1	0.049723937
RAE1	2.16E-07	1.769979241	1	1	0.003740694
C9orf72	3.27E-08	1.766723691	1	1	0.000568012
EDRF1	3.19E-13	1.765957912	1	1	5.53E-09
TENT4A	9.33E-08	1.764018514	1	1	0.001619618
CNOT7	1.25E-07	1.763788602	1	1	0.002167964
RAP1A	1.37E-07	1.759994393	1	1	0.002380976
PSMD8	1.45E-07	1.758339245	1	1	0.002524324
CNBP	8.99E-24	1.758101516	1	1	1.56E-19
WTAP	5.88E-17	1.757829777	1	1	1.02E-12
PPFIA1	1.67E-11	1.752353315	1	1	2.90E-07
SLC39A12	2.70E-07	1.751436077	1	1	0.004680292
NUP133	3.35E-10	1.747885009	1	1	5.82E-06
TMED5	3.73E-08	1.737527032	1	1	0.000647763
ENSSSCG00000051290	2.40E-07	1.730291251	1	1	0.004166719
RHOT1	2.53E-15	1.728831319	1	1	4.39E-11
STRAP	7.93E-07	1.728606527	1	1	0.013753482
AHSA1	1.34E-06	1.726522106	1	1	0.023237526
SEH1L	4.62E-11	1.726191123	1	1	8.01E-07
SYPL1	1.72E-06	1.724134845	1	1	0.029860201
SCCPDH	2.63E-10	1.722895174	1	1	4.56E-06
HENMT1	1.76E-07	1.722047403	1	1	0.00304964
LONP2	2.36E-07	1.72055616	1	1	0.004087095
CHEK1	9.02E-09	1.717060111	1	1	0.000156589
GNB4	2.19E-08	1.716366119	1	1	0.000379406
ARMC8	4.72E-11	1.716357809	1	1	8.19E-07
ENSSSCG00000038630	1.75E-09	1.715600797	1	1	3.04E-05
RHPN2	1.75E-06	1.715332294	1	1	0.030306698
KANK1	5.60E-13	1.714174324	1	1	9.72E-09
DPYS	2.73E-10	1.71334129	1	1	4.74E-06
DCTD	1.37E-06	1.711179215	1	1	0.02370505
FUNDC1	3.78E-08	1.710902779	1	1	0.000655535
TADA1	1.54E-08	1.708423358	1	1	0.000267767
CIAO2A	8.32E-08	1.707494476	1	1	0.001444283
KLHDC2	8.47E-10	1.706456971	1	1	1.47E-05
MTA3	3.30E-09	1.706193245	1	1	5.72E-05
RIOK3	9.06E-07	1.705759832	1	1	0.01571312
COPA	1.70E-13	1.701163208	1	1	2.95E-09
NCAPG	8.73E-07	1.697768694	1	1	0.015154501
MTSS1	5.05E-07	1.695794892	1	1	0.008755967
CD164	9.63E-10	1.693993495	1	1	1.67E-05
LRIG3	8.26E-07	1.689172706	1	1	0.014334579
TDP2	1.75E-09	1.688618558	1	1	3.03E-05
SLC19A2	9.21E-07	1.687488217	1	1	0.015977283
SSX2IP	2.40E-08	1.686716974	1	1	0.000416763
SNRPA1	2.68E-06	1.685968961	1	1	0.046522886
EIF2AK4	5.93E-13	1.685963583	1	1	1.03E-08
PPP6C	4.50E-08	1.683483653	1	1	0.000781549
EIF2A	5.14E-10	1.683206594	1	1	8.91E-06
MORF4L2	4.10E-07	1.680893083	1	1	0.007108665
NDRG3	7.80E-08	1.677781191	1	1	0.001353322

NAA35	1.05E-07	1.675133385	1	1	0.001825756
BARD1	6.58E-07	1.674551523	1	1	0.01141825
ATP5F1C	1.15E-07	1.669914851	1	1	0.002002283
HAS3	6.22E-07	1.668601566	1	1	0.010797688
MCMBP	1.07E-09	1.667316889	1	1	1.86E-05
CSPP1	7.19E-07	1.664133138	1	1	0.012480951
PPHLN1	1.04E-08	1.664068197	1	1	0.000180661
BAZ1A	1.01E-07	1.66310998	1	1	0.001760857
NUP107	7.44E-08	1.658083293	1	1	0.001290833
STK35	3.30E-10	1.655013158	1	1	5.73E-06
C10orf90	1.51E-07	1.653462185	1	1	0.002612155
NDC1	2.85E-10	1.65327006	1	1	4.94E-06
KNSTRN	8.82E-08	1.650188891	1	1	0.001529992
ENSSSCG00000031705	5.03E-07	1.649493895	1	1	0.008734038
NLRP4	2.34E-09	1.649275169	1	1	4.05E-05
HMGB2	1.53E-12	1.647952264	1	1	2.66E-08
KMT2E	7.24E-11	1.646338602	1	1	1.26E-06
RESF1	1.13E-06	1.646133374	1	1	0.019551068
MTERF3	2.18E-06	1.643119933	1	1	0.037747134
SPATA7	8.82E-08	1.643024426	1	1	0.001529632
UHRF1	8.74E-07	1.641121222	1	1	0.015165766
SLC30A4	1.34E-07	1.640024328	1	1	0.002320256
CDC7	2.71E-07	1.638829373	1	1	0.004707555
PM20D2	1.49E-06	1.638073837	1	1	0.025853483
THOC7	8.37E-07	1.637647225	1	1	0.014525265
PCOLCE2	1.39E-10	1.637581225	1	1	2.41E-06
ENSSSCG00000001769	1.04E-09	1.634028348	1	1	1.80E-05
GAB1	3.90E-08	1.629387342	1	1	0.000677459
IFT81	6.13E-07	1.629034865	1	1	0.010641767
TMEM131L	7.02E-13	1.628625223	1	1	1.22E-08
WDR47	7.41E-08	1.628470444	1	1	0.00128495
GZMA	2.12E-06	1.627940593	1	1	0.036828202
NID2	6.95E-11	1.627675754	1	1	1.21E-06
CKS1B	1.59E-06	1.62728767	1	1	0.027525321
USP28	1.30E-08	1.623563571	1	1	0.000225147
LGR4	1.48E-06	1.622604417	1	1	0.025713213
SPAG16	2.41E-06	1.622184163	1	1	0.041876173
KDELR2	3.75E-08	1.62028149	1	1	0.000651357
HMMR	4.37E-07	1.617624648	1	1	0.007576955
ADD3	2.16E-06	1.617232363	1	1	0.037454415
GRPEL2	4.84E-12	1.613787476	1	1	8.41E-08
GLS2	2.83E-07	1.613110664	1	1	0.004916252
SWT1	2.29E-07	1.611131884	1	1	0.003969631
USP25	4.80E-07	1.607903722	1	1	0.008321593
SNX25	2.48E-10	1.606274683	1	1	4.30E-06
UBR2	4.04E-09	1.601089374	1	1	7.01E-05
F3	1.81E-06	1.600852475	1	1	0.031403994
ANKLE2	1.65E-09	1.600442284	1	1	2.87E-05
ENSSSCG00000013395	2.60E-07	1.597238307	1	1	0.004512775
DCAF6	1.50E-07	1.597061978	1	1	0.002596012
RIC1	4.15E-07	1.595086011	1	1	0.007209207
PRKAR1A	1.78E-11	1.594419608	1	1	3.09E-07
CENPO	6.40E-07	1.590260181	1	1	0.011097784
TEX10	4.46E-09	1.587813114	1	1	7.73E-05
CDH7	4.27E-09	1.586386027	1	1	7.42E-05
GLA	5.71E-07	1.585964555	1	1	0.009911142
TAB3	6.91E-07	1.574600749	1	1	0.011988563
FAM151B	4.79E-07	1.5710144	1	1	0.008302828
HBEGF	2.38E-06	1.568922489	1	1	0.04127448
VAPA	2.36E-06	1.564283981	1	1	0.041020352
UBAC2	2.32E-08	1.563842443	1	1	0.000403121
ENSSSCG00000006344	8.51E-07	1.56233476	1	1	0.01476067
STX6	1.37E-10	1.558227658	1	1	2.39E-06
CENPW	1.70E-07	1.557784573	1	1	0.002948231
ITPRID2	1.02E-08	1.555273235	1	1	0.000176242
RDX	1.26E-08	1.554478431	1	1	0.000219143

UXS1	2.38E-10	1.553617912	1	1	4.12E-06
ENSSSCG00000007203	2.32E-09	1.54863596	1	1	4.03E-05
SMU1	6.98E-07	1.547856577	1	1	0.012110939
ENSSSCG00000001934	6.69E-07	1.54760354	1	1	0.011604171
ARL14EPL	1.19E-06	1.544011951	1	1	0.020597373
PPP1CB	7.77E-08	1.543635638	1	1	0.001347377
VIRMA	4.42E-08	1.533280993	1	1	0.00076639
OCRL	1.58E-06	1.532661013	1	1	0.027397493
INTS14	9.97E-08	1.526377673	1	1	0.001729272
UTRN	9.13E-07	1.524803518	1	1	0.015846826
SLAIN2	8.18E-08	1.523380394	1	1	0.001419624
CDKN2AIP	1.49E-06	1.522488781	1	1	0.025850132
PEX1	2.28E-07	1.518716759	1	1	0.003951587
SLC23A2	5.82E-09	1.516889647	1	1	0.000100922
CAPN7	4.72E-08	1.511395335	1	1	0.000819713
ARF4	1.22E-06	1.496439937	1	1	0.021167942
PALB2	8.70E-08	1.494613246	1	1	0.001510305
GNAI1	8.83E-08	1.492281641	1	1	0.001532561
DBT	2.68E-06	1.485016912	1	1	0.046461175
ACTL6A	1.07E-06	1.483581834	1	1	0.018487178
ARL8B	2.76E-07	1.482679972	1	1	0.004783589
REV1	1.13E-08	1.482413868	1	1	0.00019628
RNF2	7.30E-11	1.480553423	1	1	1.27E-06
KRR1	1.31E-06	1.476972397	1	1	0.022775628
UBR1	1.80E-06	1.476235981	1	1	0.031287808
SEPTIN10	1.35E-06	1.471618613	1	1	0.023341009
NUDT21	5.69E-12	1.464466188	1	1	9.87E-08
RBBP7	8.23E-08	1.462745729	1	1	0.001428216
WAPL	1.06E-07	1.456452254	1	1	0.001840963
FBXO25	3.38E-09	1.451249671	1	1	5.87E-05
PCNX4	1.45E-06	1.437220647	1	1	0.025211619
HSD17B12	2.16E-07	1.427998763	1	1	0.003746467
PSMG1	8.86E-09	1.422742753	1	1	0.000153662
FBXL20	3.03E-08	1.420819564	1	1	0.000525735
DIP2B	7.72E-07	1.420817834	1	1	0.013398722
CTNND1	1.24E-07	1.418153376	1	1	0.002158661
ENSSSCG00000003303	4.25E-07	1.411726362	1	1	0.007366974
BTBD3	3.04E-07	1.410824552	1	1	0.005273494
BMPR2	7.16E-07	1.410657013	1	1	0.012429407
ENSSSCG00000006084	2.71E-06	1.410299263	1	1	0.046964512
EPB41L2	1.29E-08	1.40721507	1	1	0.000223191
PPP4R4	8.28E-08	1.405882705	1	1	0.001437339
ATG5	1.56E-06	1.398267139	1	1	0.027063631
PIWIL1	1.50E-07	1.390492381	1	1	0.002602272
MTAP	2.23E-06	1.390063936	1	1	0.038701872
NCOA1	5.25E-07	1.389367417	1	1	0.009105661
WDR48	9.90E-13	1.382770612	1	1	1.72E-08
MKLN1	6.86E-08	1.373074128	1	1	0.001189545
PPP2R1B	1.43E-07	1.371955583	1	1	0.002488651
STRN	8.72E-07	1.366006146	1	1	0.015128942
ACTR2	1.38E-06	1.360790027	1	1	0.023965323
WAC	1.28E-06	1.359306738	1	1	0.022181346
DYNC1I2	3.84E-10	1.357296662	1	1	6.67E-06
RABGAP1	5.44E-08	1.336867759	1	1	0.000944688
FERMT2	2.71E-07	1.330585528	1	1	0.004698905
RMND5A	1.37E-06	1.322632364	1	1	0.023798091
RNF115	8.03E-09	1.307835866	1	1	0.000139284
CLINT1	1.05E-07	1.304413648	1	1	0.001829781
SMURF2	1.32E-06	1.302431308	1	1	0.022966123
ABI1	6.42E-09	1.29785963	1	1	0.000111428
PHLPP2	5.58E-07	1.284378753	1	1	0.009679752
NOCT	8.37E-07	1.27941248	1	1	0.014526955
CTNNB1	1.34E-07	1.277044708	1	1	0.002328767
PTPN13	6.02E-08	1.270783108	1	1	0.001045285
ZNF207	8.44E-10	1.261019582	1	1	1.46E-05
ENSSSCG00000015390	2.32E-08	1.209560482	1	1	0.000401959

PTPN2	2.23E-06	1.207960086	1	1	0.038778272
ANAPC1	2.01E-06	1.160299843	1	1	0.03489341
TP63	4.52E-07	0.034871345	1	1	0.007847919
LRRFIP2	1.18E-07	-0.009335781	1	1	0.002043169
ENSSSCG00000009327	1.44E-06	-0.025250546	1	1	0.025030087
TMEM181	1.80E-06	-0.040734909	1	1	0.031269265
MAT2A	6.04E-07	-0.059391289	1	1	0.010480238
ZNF79	1.75E-07	-0.075746696	1	1	0.003041681
KBTBD13	9.60E-12	-0.094786469	1	1	1.67E-07
RALA	2.51E-06	-0.107867594	1	1	0.04356708
ARL4A	1.20E-07	-0.111073154	1	1	0.002083142
INPP1	9.04E-08	-0.130005882	1	1	0.001569273
PWP1	9.79E-08	-0.130594414	1	1	0.00169889
EIF4B	9.19E-07	-0.151258284	1	1	0.01594539
C6orf120	2.05E-06	-0.151673297	1	1	0.035645692
CCNB2	1.30E-10	-0.152153171	1	1	2.25E-06
SMG1	2.57E-08	-0.171291378	1	1	0.000446598
LZIC	2.08E-09	-0.198772777	1	1	3.61E-05
KANSL2	1.13E-07	-0.20932751	1	1	0.001954415
ASH2L	1.17E-06	-0.218408789	1	1	0.020296565
SLC4A1AP	2.63E-06	-0.226102741	1	1	0.045708593
NUB1	7.32E-10	-0.237291677	1	1	1.27E-05
TCP11L1	2.20E-06	-0.243563045	1	1	0.038227094
ENSSSCG00000031636	5.20E-09	-0.248545069	1	1	9.02E-05
CCT5	8.13E-08	-0.254544523	1	1	0.00141008
MFAP1	4.91E-08	-0.266278777	1	1	0.00085178
DCLK1	8.86E-07	-0.271165307	1	1	0.015367144
TRMT10A	2.27E-06	-0.280861804	1	1	0.03938029
FBXW8	4.73E-07	-0.282722741	1	1	0.008215459
TMEM41A	1.68E-08	-0.283197445	1	1	0.000291124
ADAD1	1.88E-07	-0.289374798	1	1	0.003253645
CNOT11	6.72E-07	-0.291005617	1	1	0.011658104
YIPF5	1.13E-06	-0.296158366	1	1	0.019642152
ZC3H13	4.60E-08	-0.297718674	1	1	0.000797904
GSPT1	2.87E-09	-0.303046827	1	1	4.98E-05
ECD	2.31E-07	-0.304877643	1	1	0.004010403
HAT1	5.23E-08	-0.306923251	1	1	0.000907967
MRPL3	5.55E-09	-0.316356756	1	1	9.63E-05
CFAP44	4.75E-10	-0.327618112	1	1	8.24E-06
CHN2	8.02E-10	-0.336995358	1	1	1.39E-05
WIPF2	1.53E-06	-0.338761732	1	1	0.026620242
ENSSSCG00000015011	2.60E-06	-0.340729471	1	1	0.045064088
PLEK2	2.39E-07	-0.340989311	1	1	0.004148814
TRNT1	2.46E-06	-0.354954449	1	1	0.04275075
TMEM245	8.08E-07	-0.355736901	1	1	0.014024073
MIOS	1.25E-06	-0.363279087	1	1	0.021720935
ATP6V0A1	1.99E-08	-0.364227248	1	1	0.000345582
MAP4K4	1.41E-06	-0.364234398	1	1	0.024391068
RAB2B	1.69E-08	-0.368091664	1	1	0.00029342
MBOAT1	1.29E-08	-0.368743847	1	1	0.00022394
PSMA3	1.18E-07	-0.384846706	1	1	0.002054864
CDCA7L	1.52E-07	-0.385795866	0.938	1	0.002635562
ENSSSCG00000050083	1.98E-06	-0.394032934	1	1	0.034271292
BLMH	3.47E-11	-0.4012317	1	1	6.02E-07
MPHOSPH8	1.66E-08	-0.403505662	1	1	0.000288422
CCNI	4.98E-09	-0.404282534	1	1	8.63E-05
COPS4	2.13E-08	-0.408894306	1	1	0.000368808
TMEM50B	5.60E-07	-0.417682221	1	1	0.009718761
PSEN1	1.96E-08	-0.417905003	1	1	0.000340793
MYOF	4.02E-07	-0.421320541	1	1	0.006975427
LETMD1	4.70E-07	-0.433733593	1	1	0.008147322
ACSL5	5.60E-08	-0.444460047	1	1	0.000971096
CPSF3	1.45E-06	-0.444581775	1	1	0.025183898
C2orf88	1.44E-07	-0.445647895	1	1	0.002499494
SNAPC2	2.16E-07	-0.451802311	1	1	0.003754215
SART3	1.07E-07	-0.45429693	1	1	0.001852775

CHIC2	5.43E-07	-0.455254599	1	1	0.009421176
MRPS23	1.42E-06	-0.455504091	1	1	0.02466459
PTP4A2	2.38E-08	-0.458319747	1	1	0.00041285
ENSSSCG00000012125	3.24E-07	-0.459840707	1	1	0.005624378
ITM2B	5.51E-07	-0.464949214	1	1	0.009561531
ENSSSCG00000052707	2.60E-07	-0.465420878	1	1	0.004513583
RPL15	1.13E-08	-0.465519923	1	1	0.000196294
EIF2S2	6.64E-10	-0.466033103	1	1	1.15E-05
ATF4	1.88E-06	-0.472370674	1	1	0.032533271
ATP5PB	1.32E-09	-0.475626662	1	1	2.28E-05
EIF2S3B	2.25E-08	-0.477925205	1	1	0.000390186
NCBP3	1.28E-07	-0.480953941	1	1	0.002215081
ENSSSCG00000037143	5.17E-12	-0.482950751	1	1	8.97E-08
TMED4	1.18E-06	-0.491032883	1	1	0.020439474
ACAD8	1.81E-06	-0.491088162	1	1	0.031409223
NOLC1	1.31E-08	-0.494649119	1	1	0.000227016
EZR	4.31E-14	-0.497619192	1	1	7.48E-10
LIG3	3.30E-07	-0.506088674	1	1	0.005724372
CHCHD3	4.60E-09	-0.507036711	1	1	7.98E-05
ENSSSCG00000062406	3.92E-09	-0.510404482	1	1	6.81E-05
ZNF75D	4.37E-08	-0.510918653	1	1	0.000757648
OGFOD1	1.25E-08	-0.512664405	1	1	0.000217009
VBP1	1.64E-06	-0.516320835	1	1	0.028502808
ENSSSCG00000002707	2.13E-10	-0.517339328	1	1	3.69E-06
SSR1	1.14E-11	-0.519348645	1	1	1.99E-07
MTMR2	3.27E-09	-0.521088936	1	1	5.68E-05
TPM1	1.76E-06	-0.523097245	1	1	0.030591207
ENSSSCG00000028479	6.12E-07	-0.524721128	1	1	0.010612105
NOX4	1.52E-07	-0.526511671	1	1	0.002636304
CENPF	6.99E-08	-0.527420916	1	1	0.001213647
NAALAD2	2.92E-09	-0.530542495	1	1	5.06E-05
BUB3	1.37E-07	-0.531026191	1	1	0.002375438
YEATS4	2.22E-07	-0.53470773	1	1	0.003845501
NARS1	1.47E-06	-0.542980385	1	1	0.025467526
SMS	2.52E-10	-0.544063405	1	1	4.38E-06
SMARCE1	1.34E-08	-0.544858682	1	1	0.000232963
ATG4A	1.87E-12	-0.549425205	1	1	3.24E-08
DCAF13	1.32E-06	-0.551130074	1	1	0.022961175
RNF8	5.01E-12	-0.556955188	1	1	8.69E-08
ENSSSCG00000057727	2.81E-07	-0.562322044	1	1	0.004872946
NFATC2	5.17E-08	-0.563875051	1	1	0.000897696
ADIPOR2	1.36E-11	-0.566082469	1	1	2.35E-07
ANO4	1.09E-06	-0.572030268	1	1	0.018861652
MRPS14	4.80E-08	-0.574340501	1	1	0.00083264
KNG1	1.17E-08	-0.581762098	1	1	0.000202447
PELO	2.08E-10	-0.58236383	1	1	3.61E-06
ENSSSCG00000027506	1.05E-09	-0.585589642	1	1	1.81E-05
PCCB	8.40E-09	-0.586723668	1	1	0.000145774
ANXA5	2.47E-08	-0.587953107	1	1	0.000428998
PKIG	1.67E-06	-0.588211293	1	1	0.028931735
DERL2	4.30E-10	-0.590104436	1	1	7.46E-06
PPIE	1.67E-08	-0.593538395	1	1	0.000290113
UNC50	1.20E-08	-0.594022028	1	1	0.00020741
LDLRAP1	8.11E-08	-0.594507188	0.938	1	0.001407053
DUSP16	8.09E-14	-0.601232924	1	1	1.40E-09
DNAJC18	1.21E-06	-0.604025554	1	1	0.020936913
UCHL3	5.60E-10	-0.605922119	1	1	9.71E-06
PAFAH2	1.44E-06	-0.607188419	1	1	0.02502292
TMEM45A	4.12E-07	-0.610815467	1	1	0.007145467
GTSF1	3.24E-09	-0.613139669	1	1	5.62E-05
ZFYVE1	7.86E-07	-0.613876145	1	1	0.01363043
CLTA	1.10E-10	-0.620554795	1	1	1.91E-06
NRBP1	1.22E-12	-0.623062958	1	1	2.12E-08
ENSSSCG00000059649	2.95E-12	-0.626933056	1	1	5.12E-08
EIF2AK2	1.53E-07	-0.631629957	1	1	0.002658911
XRCC6	2.31E-11	-0.634027572	1	1	4.01E-07

FKBP9	1.43E-08	-0.634882948	1	1	0.000247606
ENSSSCG00000006694	3.74E-19	-0.636148296	1	1	6.49E-15
RPSA	1.42E-06	-0.638310836	1	1	0.02471224
ZNHIT3	1.28E-08	-0.639471852	1	1	0.000222035
PRMT6	1.03E-10	-0.643876985	1	1	1.78E-06
SH3GL2	4.60E-11	-0.648841194	1	1	7.98E-07
FIGNL1	1.11E-06	-0.649057923	1	1	0.019251517
TNN	3.75E-07	-0.649270382	1	1	0.00650922
PDE6C	2.01E-07	-0.650169631	1	1	0.003493691
SF3B3	2.31E-06	-0.653409851	1	1	0.040006968
GPN3	4.84E-07	-0.655055252	1	1	0.008402802
NUDT9	1.22E-09	-0.657124967	1	1	2.12E-05
UBE2R2	5.60E-08	-0.659212878	1	1	0.000972239
TCEA3	2.64E-06	-0.660720571	1	1	0.045859029
FAN1	5.19E-07	-0.661448075	1	1	0.00900548
ASTN1	5.34E-07	-0.662662466	1	1	0.009257207
SRP19	2.91E-11	-0.663309844	1	1	5.05E-07
UTP23	2.43E-06	-0.663848491	1	1	0.04214827
HNF1B	2.53E-08	-0.665304609	1	1	0.000438138
PSMD6	6.42E-11	-0.666940898	1	1	1.11E-06
TMEM50A	6.85E-11	-0.669411567	1	1	1.19E-06
CDC42	9.51E-11	-0.672497035	1	1	1.65E-06
ENSSSCG00000043116	2.38E-08	-0.675166174	1	1	0.000413171
ENSSSCG00000027374	3.88E-09	-0.675180732	1	1	6.74E-05
DPM1	2.85E-08	-0.677186001	1	1	0.000495332
ENSSSCG00000043568	2.26E-07	-0.680697375	1	1	0.003925795
PSMD7	5.17E-12	-0.682340237	1	1	8.98E-08
DNAJC3	6.33E-08	-0.682758059	1	1	0.001098572
MORC2	4.99E-10	-0.684243872	1	1	8.66E-06
MTM1	1.91E-09	-0.685293132	1	1	3.32E-05
CCL22	2.84E-06	-0.686292412	0.938	1	0.049357611
ENSSSCG00000013232	1.52E-16	-0.691034212	1	1	2.63E-12
LRMDA	2.58E-06	-0.693258309	0.938	1	0.04483446
PEDS1	1.16E-06	-0.69366876	1	1	0.020041297
RBM22	7.44E-07	-0.693738502	1	1	0.012913496
SRP14	1.41E-11	-0.698551297	1	1	2.44E-07
SEPTIN8	2.57E-06	-0.701127141	1	1	0.044575922
DNAJB6	1.76E-12	-0.709277773	1	1	3.06E-08
GLP2R	1.79E-09	-0.711334359	1	1	3.11E-05
SUPT3H	5.58E-09	-0.718579629	1	1	9.68E-05
DSN1	8.61E-11	-0.722499371	1	1	1.49E-06
EDC3	2.52E-06	-0.729536548	1	1	0.043669749
TM2D3	1.32E-10	-0.7314716	1	1	2.29E-06
TTLL3	6.01E-08	-0.737743118	1	1	0.001042393
SLC17A1	1.07E-06	-0.741441355	1	1	0.018603451
RBX1	1.34E-15	-0.741466986	1	1	2.33E-11
FHIP1A	1.42E-13	-0.742019857	1	1	2.46E-09
RPGRIP1	1.27E-09	-0.743969791	1	1	2.21E-05
FAAP24	1.11E-07	-0.744992503	1	1	0.001923506
IFT88	4.06E-08	-0.746855887	1	1	0.000704714
PSMD14	4.16E-13	-0.747475954	1	1	7.22E-09
POLR3C	8.28E-08	-0.748259753	1	1	0.001437235
FAM114A2	1.79E-06	-0.748350601	1	1	0.030991266
YWHAQ	1.15E-12	-0.751953103	1	1	2.00E-08
CAMTA1	9.19E-07	-0.753903318	1	1	0.015951316
MKRN1	2.22E-08	-0.754669598	1	1	0.000385886
DTX2	7.69E-09	-0.756455166	1	1	0.000133488
ETF1	3.61E-07	-0.760534065	1	1	0.006256611
ATG3	2.72E-12	-0.761903496	0.938	1	4.73E-08
ENSSSCG00000027270	1.33E-06	-0.762960803	1	1	0.023066082
SPRING1	1.45E-10	-0.763188519	1	1	2.52E-06
FRA10AC1	3.40E-08	-0.763869778	1	1	0.000589952
NECAP2	2.67E-11	-0.765136267	1	1	4.64E-07
MSL1	7.39E-07	-0.765961138	0.938	1	0.012823961
CYTH3	2.60E-06	-0.766299088	1	0.933	0.045174604
SPDYC	1.14E-07	-0.770822028	1	1	0.001984676

PCDHGA4	9.65E-08	-0.772700736	1	1	0.001673657
GFM2	2.38E-07	-0.774040964	1	1	0.00413364
C17orf80	8.39E-13	-0.775515625	1	1	1.46E-08
SNRPN	3.18E-10	-0.776925292	1	1	5.52E-06
PFDN6	2.58E-07	-0.779609932	1	1	0.004484278
IK	3.42E-13	-0.779834621	1	1	5.93E-09
PIN4	5.45E-07	-0.780226449	0.938	1	0.009449064
ENSSSCG00000011447	4.95E-08	-0.780545526	1	1	0.000858761
ALG14	5.57E-08	-0.783488205	1	0.933	0.000966633
SLIT3	6.49E-07	-0.783504588	1	1	0.011269044
KCNN3	3.87E-07	-0.783781346	0.938	1	0.006706767
SAR1A	2.52E-13	-0.785820052	1	1	4.38E-09
MRPL35	2.07E-08	-0.786341117	0.938	1	0.00035971
LAMA3	1.41E-06	-0.788015748	0.938	1	0.024515866
ENSSSCG00000039222	1.99E-09	-0.792683211	1	1	3.45E-05
LRRC59	2.02E-06	-0.793549123	0.938	1	0.035010006
ENSSSCG00000062560	3.94E-08	-0.802251316	1	1	0.000684344
NSG1	1.69E-06	-0.803227036	1	1	0.029355248
ZP2	6.75E-09	-0.806185294	1	1	0.000117114
GLYR1	4.91E-07	-0.812026362	1	1	0.008523416
NEGR1	1.30E-10	-0.814941695	1	1	2.26E-06
ZFAND3	7.12E-09	-0.816485934	1	1	0.00012346
SLC41A3	9.38E-08	-0.817851743	1	1	0.001627598
TTC1	2.31E-08	-0.822171113	1	1	0.000400683
SEZ6L2	2.14E-07	-0.824378268	1	1	0.00370762
MMAA	8.46E-14	-0.828089183	1	1	1.47E-09
YARS1	1.89E-06	-0.829001964	0.938	1	0.032843893
FOXJ3	8.06E-14	-0.829294953	1	1	1.40E-09
TRAP1	1.01E-08	-0.82930944	0.938	1	0.000174444
STIMATE	2.63E-13	-0.831947413	1	1	4.56E-09
SUGT1	1.72E-12	-0.833994037	1	1	2.98E-08
ARV1	4.04E-09	-0.835721562	1	1	7.01E-05
KCNMB4	2.00E-08	-0.836991861	1	1	0.000347219
DNAJB11	1.82E-10	-0.838816224	1	1	3.16E-06
PIP4K2B	5.39E-08	-0.838925916	1	0.933	0.000935456
EFCAB7	1.20E-08	-0.841346111	1	1	0.000207425
C11orf58	9.04E-15	-0.843417126	1	1	1.57E-10
PIGC	6.51E-09	-0.846536245	1	1	0.000112946
SLC2A8	1.56E-06	-0.847439041	1	1	0.027151158
ACBD7	3.19E-08	-0.850084959	0.938	1	0.000552688
ABR	1.71E-06	-0.851011574	0.938	1	0.029704378
FNTB	1.54E-09	-0.854471653	1	1	2.67E-05
PYGB	1.09E-06	-0.855529184	1	1	0.018861771
AIMP1	1.25E-13	-0.859603526	1	1	2.16E-09
PRIM2	1.23E-14	-0.860505178	1	1	2.13E-10
ENSSSCG00000058326	3.79E-07	-0.86097087	1	1	0.006583778
JOSD1	1.67E-08	-0.864400137	0.938	1	0.00028996
ENSSSCG00000047605	1.10E-13	-0.871722696	1	1	1.90E-09
ELP2	2.28E-14	-0.872110218	1	1	3.96E-10
TPGS2	4.40E-08	-0.877849776	0.938	1	0.000763194
ATG7	5.66E-16	-0.878058958	1	1	9.82E-12
TMEM165	1.16E-14	-0.878370556	0.938	1	2.01E-10
TBCE	2.89E-11	-0.879645855	1	1	5.01E-07
STAT4	5.83E-11	-0.879899096	1	1	1.01E-06
NMT1	1.49E-07	-0.882665826	1	1	0.002576639
NTPCR	2.02E-07	-0.883846255	1	1	0.003503376
NKIRAS1	1.27E-12	-0.886596597	1	1	2.20E-08
PLPP1	1.23E-07	-0.889178519	0.938	1	0.002137081
NRG4	2.34E-17	-0.891307387	1	1	4.05E-13
SSR3	4.93E-08	-0.89426806	1	1	0.00085567
ENSSSCG00000052306	2.30E-11	-0.894780688	1	1	3.99E-07
RNF220	1.04E-09	-0.895401246	1	1	1.80E-05
FCGR1A	8.37E-13	-0.903610119	1	1	1.45E-08
EIF4A1	1.91E-11	-0.905834422	1	1	3.32E-07
EAPP	1.49E-07	-0.909863024	1	1	0.002588854
ALKBH3	7.46E-10	-0.911030203	1	1	1.30E-05

TMEM138	1.58E-13	-0.915111102	1	1	2.75E-09
DOC2B	2.39E-08	-0.915785126	1	1	0.00041399
USP27X	1.28E-08	-0.917382117	1	1	0.000221728
BCAP29	1.91E-11	-0.919510082	1	1	3.32E-07
SAR1B	1.99E-06	-0.920175653	1	1	0.034578546
WARS1	1.61E-07	-0.921222887	1	1	0.002786989
HAUS2	1.50E-07	-0.922268697	0.875	1	0.002601125
ERH	9.16E-09	-0.923184403	1	1	0.000158857
ASCC1	3.81E-07	-0.924555135	1	0.933	0.006610226
ENSSSCG00000025913	1.69E-06	-0.926459535	1	0.933	0.02929923
SDHA	1.59E-11	-0.927627927	1	1	2.75E-07
VPS41	1.96E-10	-0.928255392	1	1	3.40E-06
DNAJC14	8.40E-16	-0.933766302	1	1	1.46E-11
ABCF1	3.10E-10	-0.934593004	1	1	5.37E-06
SRI	2.43E-06	-0.935403952	1	1	0.042147976
OARD1	2.57E-08	-0.935568127	0.875	1	0.000445342
ZNF830	1.81E-18	-0.939968987	1	1	3.14E-14
GET1	3.90E-15	-0.943336134	1	1	6.76E-11
SKA3	2.60E-06	-0.944146765	0.938	1	0.045089369
ENSSSCG00000009422	6.39E-08	-0.945175975	1	1	0.001108813
LETM2	7.95E-10	-0.952931746	1	1	1.38E-05
SMYD2	8.45E-09	-0.953810367	0.938	1	0.000146609
NOL7	1.01E-07	-0.954441948	0.938	1	0.00174761
BCAR3	2.42E-10	-0.961319532	0.938	1	4.19E-06
RAB5IF	2.74E-08	-0.964254339	0.938	1	0.000475238
DNAJA3	5.15E-10	-0.968208933	1	1	8.93E-06
NXF1	4.07E-11	-0.968382924	1	1	7.06E-07
ENSSSCG00000031991	3.26E-08	-0.968446831	1	1	0.000565058
UFC1	1.16E-06	-0.96848275	1	1	0.02018581
SVOPL	1.59E-12	-0.970810921	1	1	2.75E-08
SNURF	3.01E-10	-0.971171787	1	1	5.22E-06
HABP4	2.86E-07	-0.973439174	1	1	0.004955696
ENSSSCG00000056486	1.52E-07	-0.974014347	0.875	1	0.00264599
ENSSSCG00000027124	1.70E-06	-0.978825977	0.938	1	0.029497892
MFSD11	2.99E-08	-0.979627156	1	1	0.000519259
PSMC1	8.49E-10	-0.980799188	1	1	1.47E-05
ZCCHC17	1.09E-09	-0.981765817	1	1	1.89E-05
MRPL50	7.19E-12	-0.983069604	1	1	1.25E-07
ENSSSCG00000056638	3.67E-10	-0.983136562	0.875	1	6.37E-06
GLIPR1	6.86E-19	-0.984560378	1	1	1.19E-14
PPIL2	3.30E-08	-0.985040403	1	1	0.000573051
FBXO7	4.86E-09	-0.98579638	1	1	8.44E-05
NECAP1	3.98E-13	-0.986301941	1	1	6.90E-09
ENSSSCG00000051526	2.64E-07	-0.987022146	1	1	0.004576071
RPL3	9.12E-08	-0.988772745	1	1	0.001582244
NAT10	1.74E-11	-0.990372648	1	1	3.02E-07
PCYOX1	9.55E-09	-0.992832176	1	1	0.000165667
EAF1	2.08E-09	-0.993573731	0.938	1	3.62E-05
BRD9	2.15E-08	-0.993810064	1	1	0.000372688
TMEM233	9.42E-08	-0.994194378	0.938	1	0.001634981
TSPAN13	1.09E-06	-0.994194378	0.938	1	0.018923548
BIRC5	7.79E-11	-0.996147675	1	1	1.35E-06
SLC37A4	4.80E-08	-1.000850897	1	0.933	0.000832331
CRYM	1.72E-08	-1.002200908	1	1	0.000298507
SFT2D2	1.22E-07	-1.003354752	1	1	0.002113404
GREB1L	2.40E-11	-1.004036441	1	1	4.16E-07
UBE2B	1.00E-07	-1.005646563	0.938	1	0.001736186
TMEM163	2.68E-06	-1.009693396	1	1	0.046536988
WNT2B	2.08E-10	-1.013674937	0.938	1	3.61E-06
EIF3M	9.09E-33	-1.01368084	1	1	1.58E-28
CENPS	1.31E-10	-1.014383918	1	1	2.28E-06
CFL1	2.88E-08	-1.015328283	1	1	0.000500276
ENSSSCG00000010719	4.26E-12	-1.016488123	1	1	7.39E-08
UMPS	3.60E-18	-1.01790306	1	1	6.25E-14
ENSSSCG00000016678	4.40E-07	-1.019688335	0.938	1	0.007642081
ENSSSCG00000026520	1.40E-06	-1.019860422	1	1	0.024257867

EIF4E2	4.88E-18	-1.021556143	1	1	8.47E-14
OSER1	3.08E-08	-1.024677679	1	1	0.000535115
SYCP2L	9.11E-07	-1.027020214	0.938	1	0.015800017
NCF2	3.64E-07	-1.027046765	0.938	1	0.006315
ENSSSCG00000034927	2.21E-12	-1.033109415	1	1	3.84E-08
CACYBP	7.73E-12	-1.033166864	1	1	1.34E-07
ENSSSCG00000060926	5.81E-09	-1.033576081	0.875	1	0.000100881
ENSSSCG00000051717	2.03E-06	-1.034215715	0.938	1	0.035167065
USP36	4.86E-07	-1.03441541	0.938	1	0.008425773
GSK3B	3.02E-13	-1.035667512	1	1	5.24E-09
ENSSSCG00000032216	2.92E-12	-1.047562811	1	1	5.07E-08
RND1	7.11E-09	-1.047828525	1	1	0.000123304
ENSSSCG00000051162	2.31E-08	-1.04800915	0.938	1	0.000401598
SERHL2	1.18E-06	-1.050040682	0.938	1	0.020409495
ZNHIT6	1.77E-20	-1.050108251	1	1	3.07E-16
CHST3	3.16E-09	-1.051993551	0.938	1	5.49E-05
SNX3	6.73E-10	-1.052314178	1	1	1.17E-05
MRPL13	1.66E-21	-1.053300551	1	1	2.88E-17
DDB2	1.32E-07	-1.05403984	0.875	1	0.002295406
GLRX5	6.01E-16	-1.056467561	1	1	1.04E-11
ENSSSCG00000052760	9.88E-13	-1.060102299	1	1	1.71E-08
ENTREP1	1.23E-06	-1.062735755	0.938	1	0.021405049
HSPA5	5.33E-10	-1.064144386	1	1	9.25E-06
ENSSSCG00000028677	9.05E-18	-1.064485396	1	1	1.57E-13
SGCD	5.08E-12	-1.067114196	1	1	8.82E-08
PSMC3	7.80E-13	-1.071840788	1	1	1.35E-08
SYCP3	3.67E-08	-1.07189795	1	1	0.00063663
GARS1	1.22E-07	-1.072645302	0.938	1	0.002119738
TTC32	5.87E-08	-1.072850565	1	1	0.001018402
RNASEH2A	2.89E-09	-1.078210528	0.938	1	5.02E-05
UBXN8	6.21E-08	-1.079116132	1	0.933	0.001077879
PITPNA	3.22E-07	-1.081968847	1	1	0.005585122
LDHA	6.08E-08	-1.084296335	0.938	1	0.001054667
ENSSSCG00000006996	3.29E-12	-1.08435693	1	1	5.70E-08
ENSSSCG00000042524	9.58E-07	-1.084794136	0.938	1	0.016616612
HSPE1	3.36E-10	-1.085000647	1	1	5.82E-06
COPE	3.55E-08	-1.085949613	0.938	1	0.000616151
SRRT	9.85E-07	-1.086930829	1	1	0.017086339
SLC25A17	9.35E-17	-1.087601426	1	1	1.62E-12
ENSSSCG00000057665	2.17E-07	-1.088448027	0.938	1	0.003767757
SAP18	3.77E-07	-1.089337651	1	1	0.006533722
THYN1	9.25E-16	-1.089464261	1	1	1.61E-11
SLC35B4	2.60E-07	-1.089702803	0.938	1	0.004515083
BRCC3	8.97E-10	-1.093109404	0.938	1	1.56E-05
TMEM69	2.01E-07	-1.093109404	0.938	1	0.003480418
PRKAG2	9.03E-07	-1.093109404	0.938	1	0.015663092
PDIA3	8.77E-15	-1.09427569	1	1	1.52E-10
PPIF	6.06E-13	-1.094876331	1	1	1.05E-08
BPNT1	8.10E-21	-1.101314327	1	1	1.41E-16
TRIP4	8.22E-14	-1.103173088	1	1	1.43E-09
GRIN3A	2.86E-09	-1.103717504	0.938	1	4.96E-05
PADI6	5.51E-09	-1.104206143	1	1	9.55E-05
ENSSSCG00000033697	2.22E-06	-1.110140485	1	1	0.038483298
CDC6	2.61E-18	-1.110381486	1	1	4.53E-14
NOP9	3.11E-09	-1.110964769	1	1	5.40E-05
SKA1	8.98E-09	-1.11137418	1	1	0.000155895
ENSSSCG00000001064	1.33E-10	-1.114923766	1	1	2.30E-06
FAM104A	5.96E-13	-1.116087968	1	1	1.03E-08
SDF2	8.30E-20	-1.116585889	1	1	1.44E-15
RPS20	4.31E-07	-1.12145403	1	1	0.007475784
KCTD20	1.08E-09	-1.126024027	1	1	1.88E-05
GPN1	4.72E-33	-1.127382596	1	1	8.19E-29
SPR	5.26E-07	-1.130198723	1	1	0.009124608
GNL2	6.51E-10	-1.132339179	1	1	1.13E-05
LAT2	7.70E-17	-1.132782712	1	1	1.34E-12
TOLLIP	1.60E-06	-1.135753742	0.812	1	0.027762462

PATL2	9.01E-18	-1.139485211	1	1	1.56E-13
ENSSSCG00000003612	2.99E-10	-1.142455458	1	1	5.19E-06
ARHGAP27	5.29E-10	-1.143067563	0.938	1	9.19E-06
NLRP8	2.26E-15	-1.143542393	1	1	3.93E-11
DDX28	4.69E-08	-1.155231116	0.938	1	0.000813766
ENSSSCG00000056768	4.22E-07	-1.15787966	1	1	0.007329621
ZDHHC4	7.52E-11	-1.159488244	1	1	1.31E-06
TUBB	6.16E-09	-1.160028996	1	1	0.000106846
LIG1	3.33E-09	-1.163498732	0.938	1	5.78E-05
NDUFS1	1.73E-07	-1.165865747	0.938	1	0.00300361
UBTD2	1.55E-10	-1.167266339	0.938	1	2.69E-06
CFDP1	1.86E-09	-1.167877173	1	1	3.22E-05
DUSP5	3.04E-12	-1.17533253	1	1	5.28E-08
TBCC	5.82E-09	-1.181532655	1	1	0.000100917
SYTL3	4.31E-15	-1.186424189	1	1	7.48E-11
TM2D2	1.19E-06	-1.186891551	0.938	0.933	0.020570959
DNAJB2	2.66E-06	-1.187746279	0.688	1	0.046236516
GLIPR2	1.52E-08	-1.189111224	1	1	0.000264251
CDK5RAP1	1.71E-10	-1.19005496	0.938	1	2.97E-06
PDCD1LG2	8.43E-08	-1.190650339	0.938	1	0.001462317
ZMAT2	1.81E-15	-1.19088462	1	1	3.14E-11
PBDC1	7.03E-13	-1.191673239	1	1	1.22E-08
RMDN1	2.03E-19	-1.193590797	1	1	3.52E-15
AIDA	5.19E-16	-1.193952163	1	1	9.01E-12
ALG8	1.27E-09	-1.195093437	1	1	2.20E-05
RAB5C	3.35E-10	-1.195359463	0.938	1	5.81E-06
NAA20	1.05E-09	-1.199202794	1	1	1.82E-05
PRSS8	5.26E-08	-1.199560346	0.938	1	0.000912608
ENSSSCG00000061194	7.30E-09	-1.200024608	0.938	1	0.000126578
PRPF19	1.18E-08	-1.200024608	0.938	0.933	0.000204877
ENSSSCG00000011875	1.56E-16	-1.200731638	1	1	2.71E-12
LSM3	7.53E-11	-1.200750128	1	1	1.31E-06
ARFGAP2	3.56E-12	-1.205430429	1	1	6.18E-08
TXNL4A	1.84E-13	-1.207106175	1	1	3.19E-09
PLEKHO1	3.61E-08	-1.208586622	0.938	1	0.000625613
PDCD6	8.68E-24	-1.213925	1	1	1.51E-19
RNF4	7.76E-10	-1.214258891	0.938	1	1.35E-05
DYNLL2	5.21E-13	-1.215750078	1	1	9.05E-09
ZNF526	2.28E-06	-1.216167801	0.75	1	0.039571689
RAB6B	7.93E-10	-1.217367507	1	1	1.38E-05
DHCR24	2.17E-06	-1.217845632	0.938	0.933	0.037678632
ENSSSCG00000050758	9.54E-07	-1.218640286	0.875	1	0.016548929
SKA2	1.53E-10	-1.219987467	0.938	1	2.65E-06
PSMG3	2.83E-09	-1.220390068	1	1	4.91E-05
XAB2	5.98E-07	-1.222151349	0.938	0.933	0.010375571
DDX23	1.65E-08	-1.222877279	0.938	1	0.0002869
TAX1BP3	1.94E-06	-1.222989687	1	1	0.03366211
ENSSSCG00000034524	1.72E-11	-1.224706287	0.938	1	2.98E-07
ENSSSCG00000017907	5.73E-08	-1.2264715	1	1	0.00099503
PPCDC	2.97E-12	-1.227212826	1	1	5.15E-08
ENSSSCG00000038506	4.41E-07	-1.229130199	1	1	0.007648256
PSMA7	5.29E-09	-1.231888803	1	1	9.18E-05
RTCB	1.66E-09	-1.233444519	1	1	2.89E-05
PSTK	1.40E-09	-1.234328822	1	1	2.43E-05
ENSSSCG00000028786	8.48E-11	-1.236067358	1	1	1.47E-06
SLC16A13	3.09E-09	-1.236528652	1	1	5.36E-05
ILF2	1.73E-21	-1.238172684	1	1	3.00E-17
MON1A	8.37E-08	-1.238251305	0.875	1	0.001451877
ENSSSCG00000026229	5.28E-10	-1.240815562	1	1	9.17E-06
LDHC	1.10E-14	-1.241591694	1	1	1.91E-10
TIMM23B	3.10E-10	-1.24691474	1	1	5.37E-06
FBXO16	1.20E-07	-1.248626982	0.875	1	0.002086164
MAD2L1BP	1.32E-09	-1.250961574	0.938	1	2.30E-05
ENSSSCG00000036988	1.62E-08	-1.252307999	0.875	1	0.000280732
CALR	2.95E-15	-1.254413923	1	1	5.12E-11
SIAE	2.50E-10	-1.256083616	1	1	4.34E-06

GM2A	1.93E-07	-1.25624524	0.875	1	0.003349115
FTSJ1	9.45E-15	-1.256608137	1	1	1.64E-10
ENSSSCG00000052241	6.25E-12	-1.25918208	0.938	1	1.08E-07
FBXO34	2.07E-06	-1.259467791	0.938	1	0.035977983
ENSSSCG00000016385	2.50E-06	-1.259467791	0.812	0.933	0.043432992
OSBPL2	6.47E-12	-1.265062087	1	1	1.12E-07
ENSSSCG00000057916	4.10E-08	-1.265659875	1	1	0.000711876
MYCBP	1.80E-12	-1.269030734	1	1	3.13E-08
CDKN3	5.57E-09	-1.27104622	1	1	9.66E-05
ENSSSCG00000047619	6.38E-11	-1.271446646	1	1	1.11E-06
MRPL19	6.28E-12	-1.271647536	0.938	1	1.09E-07
MRPL9	8.20E-11	-1.272507158	1	1	1.42E-06
TUBA4A	1.46E-09	-1.273468407	0.938	1	2.54E-05
CHST10	4.93E-15	-1.27551518	1	1	8.56E-11
MRM3	6.39E-09	-1.276081476	1	1	0.00011083
MROH7	1.05E-08	-1.276114233	0.875	1	0.000182888
GPANK1	5.63E-09	-1.276555545	1	1	9.76E-05
LGALS8	3.92E-14	-1.276937169	0.938	1	6.80E-10
SMARCAL1	7.34E-08	-1.279730695	0.875	1	0.001273135
SPOCK1	1.85E-10	-1.280581387	0.938	1	3.20E-06
MOSPD1	3.78E-12	-1.281099122	1	1	6.56E-08
ENSSSCG00000005596	3.33E-12	-1.281150321	0.938	1	5.78E-08
ENSSSCG00000058562	3.77E-07	-1.281554494	0.875	0.933	0.00654411
SLC25A26	8.03E-30	-1.282001028	1	1	1.39E-25
COPS7B	3.98E-08	-1.283905584	1	0.933	0.000690438
CCDC59	4.27E-10	-1.285458818	1	1	7.41E-06
ENSSSCG00000037514	1.51E-15	-1.286678991	0.938	1	2.63E-11
RPF2	3.78E-08	-1.287506773	1	1	0.000656317
PDE6D	3.66E-13	-1.28769646	1	1	6.34E-09
ATP6V1G1	1.53E-17	-1.292031628	1	1	2.65E-13
ENSSSCG00000053570	4.36E-15	-1.292801985	1	1	7.57E-11
WDR53	7.37E-19	-1.298156199	0.938	1	1.28E-14
SDHAF2	2.07E-07	-1.299560282	0.875	1	0.003583779
PPP2R2B	9.03E-16	-1.301552267	1	1	1.57E-11
TRAIP	7.82E-08	-1.305262641	0.938	1	0.001356035
NDUFAF1	1.23E-13	-1.306218585	1	1	2.14E-09
WDR91	1.01E-21	-1.308978702	1	1	1.76E-17
ESYT1	2.69E-15	-1.310138641	1	1	4.66E-11
PPM1G	1.62E-12	-1.310667052	0.938	1	2.82E-08
KATNB1	2.45E-06	-1.311944006	0.812	1	0.042491403
ENSSSCG00000054196	6.33E-10	-1.31817596	1	1	1.10E-05
C4orf33	2.10E-17	-1.319365406	1	1	3.64E-13
TCP1	1.70E-21	-1.320024384	1	1	2.94E-17
ENSSSCG00000038628	6.86E-25	-1.321073299	1	1	1.19E-20
CALM3	1.54E-13	-1.32443495	1	1	2.67E-09
ENSSSCG00000030908	2.39E-11	-1.324735106	1	1	4.15E-07
COL4A6	6.25E-09	-1.327019669	1	1	0.000108376
ENSSSCG00000017061	1.58E-06	-1.328325866	0.75	1	0.027448638
LANCL2	1.03E-08	-1.328557979	1	1	0.000177994
MYL6B	1.31E-09	-1.331681753	0.938	1	2.27E-05
NPLOC4	3.46E-08	-1.332779817	0.875	1	0.000600957
ASRGL1	1.51E-07	-1.334117504	0.812	1	0.002616998
ELP6	5.41E-07	-1.334117504	0.875	1	0.009393972
ENSSSCG00000060365	1.20E-12	-1.334313562	1	1	2.08E-08
NDUFS2	5.21E-10	-1.334825089	1	1	9.04E-06
ENSSSCG00000016475	1.38E-08	-1.335470242	1	0.933	0.000239913
CRY2	1.99E-07	-1.335859539	0.938	0.933	0.003453865
OXA1L	8.05E-21	-1.341373156	1	1	1.40E-16
ENSSSCG00000039972	7.28E-13	-1.341929951	0.938	1	1.26E-08
ATG101	9.63E-08	-1.342723294	0.938	1	0.001671144
MLH1	1.70E-11	-1.342920115	1	1	2.95E-07
SELENON	3.47E-08	-1.343246816	1	1	0.000601253
RAB43	2.12E-10	-1.345027778	0.875	1	3.67E-06
CDKN2AIPNL	1.14E-10	-1.346865997	1	1	1.98E-06
LYSMD2	4.70E-07	-1.347923303	0.75	0.933	0.008150097
EIF1B	1.04E-13	-1.350302932	1	1	1.81E-09

SELENOF	1.48E-25	-1.351053043	1	1	2.56E-21
TIMD4	1.10E-08	-1.351177557	0.938	1	0.00019051
ENSSSCG00000029257	5.98E-09	-1.35614381	0.875	1	0.000103762
HSPA4	5.41E-21	-1.356605547	1	1	9.38E-17
VWA3A	2.07E-07	-1.358699515	0.938	1	0.003598868
B4GAT1	2.21E-06	-1.35938947	0.875	0.867	0.038422858
DDX39B	2.50E-16	-1.359725826	1	1	4.34E-12
GNPTG	6.55E-07	-1.360589715	0.938	1	0.011361162
ENSSSCG00000036499	5.58E-23	-1.361992689	1	1	9.68E-19
DHDDS	6.14E-14	-1.362156187	0.938	1	1.07E-09
TOMM22	5.90E-14	-1.365635259	1	1	1.02E-09
PDXP	2.29E-07	-1.367402928	0.938	0.933	0.003965373
ENSSSCG00000051057	2.58E-10	-1.368743847	0.938	1	4.47E-06
RFC2	1.65E-11	-1.369838549	1	1	2.87E-07
PRPSAP1	6.45E-08	-1.370748223	1	0.933	0.00111983
USP11	1.39E-06	-1.371316122	0.938	1	0.024160544
CHEK2	2.49E-07	-1.375622818	0.875	1	0.004323089
HARS1	2.60E-08	-1.375748368	0.812	1	0.000451205
ENSSSCG00000022773	4.87E-07	-1.377562794	0.938	1	0.008454279
ENSSSCG00000009015	3.42E-11	-1.38502493	0.875	0.933	5.94E-07
FAM98A	1.98E-22	-1.385160458	1	1	3.44E-18
MRPS10	6.05E-30	-1.385167992	1	1	1.05E-25
CFAP36	2.92E-07	-1.387556763	0.938	1	0.005060204
NLRP9	2.19E-14	-1.389012091	1	1	3.80E-10
DNAJA4	3.71E-07	-1.389158867	0.938	1	0.006428797
FBXO17	3.70E-08	-1.390495315	0.812	1	0.000641464
LSG1	1.40E-11	-1.391549146	1	1	2.43E-07
CYP2E1	1.12E-06	-1.39176772	0.688	1	0.019403534
ATP6V1D	7.02E-34	-1.394783349	1	1	1.22E-29
STX18	7.08E-18	-1.39509009	1	1	1.23E-13
HMOX2	1.46E-07	-1.396387442	1	1	0.002538015
BRF2	2.48E-09	-1.399471242	0.938	1	4.31E-05
SLC25A12	1.01E-10	-1.399536406	0.875	1	1.76E-06
TOE1	6.63E-07	-1.403835152	0.875	1	0.011503437
ECSIT	1.62E-08	-1.411380476	0.938	1	0.00028069
VPS26C	3.05E-18	-1.414353595	1	1	5.29E-14
EMC4	2.55E-10	-1.415037499	1	1	4.43E-06
ATG4B	1.01E-09	-1.418375785	1	1	1.75E-05
PXMP2	7.15E-08	-1.423417165	1	0.933	0.001241326
TP53INP2	6.92E-07	-1.423417165	0.938	1	0.012001392
WDFY2	3.86E-14	-1.424998609	1	1	6.69E-10
EXOSC5	7.59E-13	-1.427375834	1	1	1.32E-08
ENSSSCG00000021236	6.79E-10	-1.427528443	0.938	1	1.18E-05
RNF170	1.45E-14	-1.429987841	1	1	2.52E-10
FANK1	5.86E-13	-1.436272141	1	1	1.02E-08
ENSSSCG00000021591	8.01E-12	-1.43641115	1	1	1.39E-07
ENSSSCG00000029160	2.69E-11	-1.438389371	1	1	4.66E-07
ZNF410	1.43E-08	-1.439434154	0.875	1	0.00024809
CCT3	2.36E-18	-1.443101418	1	1	4.09E-14
DTD1	2.20E-15	-1.443541016	1	1	3.82E-11
AIFM1	5.51E-11	-1.443606651	0.938	1	9.56E-07
RTF2	3.19E-23	-1.445300338	1	1	5.54E-19
SLC28A3	4.47E-08	-1.447291698	0.812	1	0.000775123
ARHGAP9	4.13E-09	-1.452190498	0.875	0.933	7.16E-05
MTUS2	1.64E-12	-1.453211228	0.938	1	2.85E-08
AMN1	2.47E-16	-1.458962642	1	1	4.28E-12
WBP2	1.56E-08	-1.462343214	1	1	0.000270333
RSL24D1	9.00E-07	-1.462866024	1	1	0.015620425
PSMC5	5.37E-11	-1.466397921	0.938	1	9.32E-07
KCNAB3	1.19E-08	-1.467304809	1	1	0.000205751
BTF3L4	1.62E-15	-1.468400725	1	1	2.81E-11
WSB2	1.13E-06	-1.472455197	0.875	0.933	0.019619436
LSM6	2.62E-17	-1.475579041	1	1	4.54E-13
DRAP1	4.48E-08	-1.475883054	0.938	1	0.000777337
B3GNT3	4.47E-11	-1.475966498	0.875	1	7.75E-07
TSPAN14	4.66E-16	-1.477232261	1	1	8.08E-12

ENSSSCG00000045404	1.02E-07	-1.477773255	0.938	0.933	0.001770807
TENM4	1.88E-06	-1.478763097	0.812	0.933	0.03260626
SSR2	5.09E-08	-1.479658347	1	0.933	0.000883038
MAP7	1.95E-16	-1.481446976	1	1	3.38E-12
ENSSSCG00000056719	2.06E-07	-1.48436952	0.938	0.933	0.003578
IQCG	3.37E-14	-1.484578433	1	1	5.85E-10
NDUFA6	3.47E-07	-1.485299835	1	1	0.00602515
ENSSSCG00000054294	4.50E-08	-1.485426827	0.625	1	0.000781598
NHEJ1	8.78E-09	-1.487793842	0.875	1	0.000152356
CFI	2.27E-07	-1.493040011	0.812	1	0.00394466
CRNKL1	4.54E-49	-1.49424903	1	1	7.87E-45
HMGCR	7.90E-12	-1.494816573	1	1	1.37E-07
PPP1R7	1.51E-08	-1.495741883	0.812	1	0.000261312
DERL1	1.82E-19	-1.498522485	1	1	3.16E-15
TSTD1	2.11E-06	-1.498821111	1	1	0.03657423
GMPS	5.89E-23	-1.501278506	1	1	1.02E-18
C11orf80	1.38E-22	-1.502180062	1	1	2.39E-18
MCM5	3.22E-13	-1.503608696	0.938	1	5.59E-09
KCNIP4	1.74E-20	-1.505626031	1	1	3.01E-16
ENSSSCG00000053185	2.81E-10	-1.509273569	0.938	1	4.88E-06
PADI4	6.70E-09	-1.511822562	0.938	1	0.000116325
MTHFS	5.67E-08	-1.513012659	0.75	1	0.000984381
PIGO	5.62E-10	-1.522725369	0.875	0.933	9.75E-06
ENSSSCG00000033421	2.05E-08	-1.522974321	0.875	0.933	0.000355958
ISCA1	9.52E-14	-1.523955925	1	1	1.65E-09
ZNF584	5.10E-10	-1.524375365	0.875	1	8.85E-06
SAP30BP	2.43E-09	-1.524955191	0.938	1	4.22E-05
IMP3	5.48E-26	-1.525461489	1	1	9.51E-22
ZNF235	5.18E-12	-1.52554995	0.938	1	8.99E-08
ENSSSCG00000060974	2.01E-14	-1.526354522	1	1	3.49E-10
TCP11	6.92E-14	-1.526412235	1	1	1.20E-09
DUT	2.42E-19	-1.527512229	1	1	4.21E-15
ENSSSCG00000059558	2.60E-07	-1.527512229	0.75	0.933	0.00450815
TMEM186	4.75E-15	-1.528365183	0.938	1	8.24E-11
CIRBP	9.02E-11	-1.533093261	0.938	1	1.56E-06
GNL3	7.45E-18	-1.53331335	1	1	1.29E-13
GTF2E2	2.02E-19	-1.534452666	1	1	3.51E-15
SLC8B1	6.32E-13	-1.538169387	1	1	1.10E-08
TBC1D7	3.29E-13	-1.541397278	0.938	1	5.71E-09
VPS26B	1.96E-10	-1.541498852	1	1	3.41E-06
DLGAP4	3.26E-25	-1.542240102	1	1	5.65E-21
SSBP1	6.24E-26	-1.543400369	1	1	1.08E-21
SQLE	2.58E-16	-1.545038281	1	1	4.48E-12
KCNN2	2.01E-09	-1.546700144	0.938	1	3.50E-05
TBC1D1	5.58E-12	-1.547466069	1	1	9.68E-08
MAF1	5.56E-09	-1.547675268	0.812	1	9.64E-05
AVEN	1.47E-15	-1.551344593	1	1	2.56E-11
NSMAF	3.70E-13	-1.553301871	1	1	6.42E-09
PRPF6	6.41E-09	-1.555900035	0.875	1	0.00011122
CYC1	3.32E-11	-1.560888366	1	1	5.75E-07
LMNA	1.46E-15	-1.562344198	0.938	1	2.53E-11
C19orf67	5.84E-12	-1.564180939	1	1	1.01E-07
LIMS2	6.29E-10	-1.565659362	0.938	1	1.09E-05
GPATCH11	1.33E-15	-1.565817451	1	1	2.30E-11
ESD	1.79E-14	-1.565990978	1	1	3.10E-10
CD274	2.16E-09	-1.567949091	1	1	3.74E-05
TMEM251	1.13E-12	-1.568688446	0.938	1	1.97E-08
ASTL	6.42E-10	-1.568770273	1	1	1.11E-05
MGST2	8.77E-10	-1.570099394	1	1	1.52E-05
ENSSSCG00000056896	2.13E-11	-1.571156701	0.875	1	3.69E-07
PELP1	9.42E-07	-1.574236094	0.688	1	0.016336806
CIAPIN1	5.21E-08	-1.574978412	0.938	1	0.000904518
AAAS	5.44E-11	-1.57589151	1	1	9.43E-07
XPO4	2.48E-07	-1.579807889	0.812	1	0.004310781
MKKS	6.19E-15	-1.580224582	1	1	1.07E-10
NARF	1.24E-12	-1.584458854	0.938	1	2.15E-08

HSD17B7	1.67E-24	-1.58770869	1	1	2.90E-20
ENSSSCG00000021161	1.16E-13	-1.588162104	1	1	2.02E-09
DBF4B	5.61E-07	-1.589940995	0.812	0.933	0.009736986
MRPS9	3.89E-20	-1.597948935	1	1	6.75E-16
ATP5F1A	7.27E-24	-1.598644656	1	1	1.26E-19
CIAO2B	2.71E-07	-1.602123052	1	1	0.004708391
VPS4A	1.28E-14	-1.604720163	1	1	2.21E-10
NKIRAS2	9.39E-11	-1.607682577	1	1	1.63E-06
STX5	1.49E-07	-1.607682577	0.812	0.933	0.002587367
ENSSSCG00000036197	1.65E-12	-1.608466438	1	1	2.86E-08
CHCHD5	4.67E-09	-1.608530972	0.938	1	8.10E-05
ENSSSCG00000046622	1.05E-06	-1.609359155	0.625	0.867	0.018222684
VPS29	1.75E-28	-1.61027192	1	1	3.03E-24
DAPK3	3.17E-08	-1.610508621	0.75	0.933	0.000550771
SSR4	6.27E-08	-1.613263609	0.938	1	0.001087508
NOTO	4.49E-14	-1.614346308	0.938	1	7.79E-10
MCEE	6.27E-09	-1.614346308	0.938	1	0.000108723
ENSSSCG00000052822	1.37E-10	-1.61667136	1	1	2.38E-06
CLN8	2.09E-11	-1.620151929	0.938	1	3.62E-07
LHFPL1	1.43E-20	-1.620728464	1	1	2.48E-16
MRPL46	1.45E-14	-1.621604953	1	1	2.52E-10
ATRIP	9.18E-09	-1.62203787	0.875	1	0.000159234
RPL4	5.15E-15	-1.626541604	1	1	8.94E-11
CCDC3	2.37E-08	-1.632930774	1	1	0.000411688
ENSSSCG00000041717	4.91E-08	-1.633677786	0.938	1	0.000852766
DDX25	2.56E-07	-1.634128558	0.875	0.933	0.004449233
NAXD	1.06E-11	-1.635258822	0.875	1	1.84E-07
CHST7	2.84E-12	-1.636099635	0.688	1	4.92E-08
TUBB2A	1.93E-12	-1.636452816	1	1	3.35E-08
LUC7L2	5.96E-23	-1.636956051	1	1	1.03E-18
ATP6V0B	5.16E-16	-1.637587842	1	1	8.95E-12
ENSSSCG00000010531	2.94E-10	-1.639852583	0.875	1	5.10E-06
KIAA1143	2.95E-10	-1.640302862	1	1	5.12E-06
ENSSSCG00000040571	1.25E-15	-1.640434248	1	1	2.16E-11
PRR5L	6.39E-12	-1.6405972	1	1	1.11E-07
TBCA	1.28E-09	-1.640750279	1	1	2.22E-05
C2orf76	2.04E-11	-1.64345815	1	1	3.54E-07
ENSSSCG00000016990	2.34E-14	-1.64548461	1	1	4.07E-10
PSMC4	5.38E-14	-1.646923207	1	1	9.34E-10
HSPA8	4.33E-22	-1.646974879	1	1	7.52E-18
SNPH	9.57E-16	-1.649743182	0.875	1	1.66E-11
CAPZB	5.67E-12	-1.650415562	1	1	9.83E-08
BORCS5	2.46E-16	-1.652671688	0.938	1	4.26E-12
FTSJ3	6.22E-18	-1.657978335	1	1	1.08E-13
POLA2	3.78E-10	-1.662805148	0.938	1	6.56E-06
TTC7A	8.26E-09	-1.664461474	0.812	1	0.000143268
RIMS3	2.32E-06	-1.666294737	0.75	0.867	0.040224855
STMP1	5.59E-23	-1.666769256	1	1	9.70E-19
ARPP19	4.16E-20	-1.666789194	1	1	7.22E-16
RAB7A	1.81E-31	-1.667076512	1	1	3.15E-27
ADCK2	9.27E-11	-1.66780357	0.875	0.933	1.61E-06
ARL3	6.55E-07	-1.668611576	0.938	1	0.011368771
ENSSSCG00000058371	8.03E-17	-1.668662061	0.938	1	1.39E-12
TULP3	2.01E-15	-1.672596775	1	1	3.49E-11
CCT4	8.40E-16	-1.673513623	1	1	1.46E-11
AARSD1	1.28E-18	-1.67363284	1	1	2.22E-14
PPEF2	9.30E-10	-1.678071905	0.938	0.933	1.61E-05
RTN4IP1	1.14E-21	-1.682666479	0.938	1	1.97E-17
TKT	1.01E-09	-1.683332422	1	1	1.75E-05
SARS1	3.15E-07	-1.683718468	0.875	1	0.005466171
PNLDC1	6.76E-17	-1.684330896	0.938	1	1.17E-12
CTDSP2	2.52E-08	-1.686789122	0.812	1	0.00043688
GORASP1	2.85E-06	-1.687290435	0.812	0.933	0.049447247
CFAP298	4.60E-18	-1.688224621	1	1	7.98E-14
ATPSCKMT	1.06E-13	-1.688294765	1	1	1.84E-09
FAM110A	3.31E-13	-1.68953102	0.938	1	5.74E-09

PPP1R11	6.46E-11	-1.690044547	1	1	1.12E-06
CCT6B	2.86E-17	-1.694489665	1	1	4.96E-13
YJU2B	2.69E-08	-1.697489359	0.875	1	0.00046631
SLC24A3	2.72E-09	-1.697808123	0.875	1	4.72E-05
ABLIM3	1.20E-06	-1.697971463	0.875	0.933	0.020760594
RALY	2.20E-19	-1.698731376	0.938	1	3.81E-15
IPO9	1.19E-06	-1.701155518	0.938	1	0.020616435
TMEM266	3.94E-07	-1.703835001	0.812	0.867	0.006839773
ENSSSCG00000031299	7.12E-09	-1.705552642	0.875	1	0.000123493
ENSSSCG00000024520	3.07E-30	-1.706520335	1	1	5.33E-26
EIF3H	1.48E-22	-1.706572228	1	1	2.56E-18
SGSM2	5.05E-13	-1.707100954	0.938	1	8.76E-09
ENSSSCG00000005649	3.66E-08	-1.709780765	0.875	1	0.000634529
IFT57	6.40E-19	-1.71086184	1	1	1.11E-14
C8orf76	2.88E-14	-1.710986528	1	1	5.00E-10
ATP6V0E2	6.47E-08	-1.712019237	0.875	1	0.001122959
WNT7A	6.35E-11	-1.715449515	0.875	1	1.10E-06
HAX1	4.20E-30	-1.716123211	1	1	7.28E-26
SRRD	1.60E-07	-1.71871389	0.688	0.933	0.002776821
ENSSSCG00000061760	1.21E-06	-1.71871389	0.812	0.933	0.02094427
PRDX2	2.13E-19	-1.719882611	1	1	3.69E-15
SLC9A3R1	1.64E-07	-1.723159795	0.75	0.933	0.002849015
NIT1	1.47E-06	-1.72914609	0.688	0.933	0.025556536
SSNA1	5.49E-12	-1.729734025	0.875	1	9.52E-08
CAPNS1	1.79E-08	-1.734974296	0.688	0.933	0.000311229
CABLES1	2.34E-07	-1.735952821	0.875	1	0.00406362
EXOSC8	4.86E-19	-1.736965594	1	1	8.43E-15
ERGIC3	1.26E-11	-1.738223471	1	1	2.19E-07
TXNDC17	1.23E-10	-1.740473974	1	1	2.13E-06
RFC3	1.01E-15	-1.74080766	1	1	1.76E-11
MANBA	4.71E-24	-1.741369417	1	1	8.17E-20
STK16	1.67E-11	-1.743363365	0.875	0.933	2.89E-07
ADRM1	1.05E-08	-1.744035167	0.812	0.933	0.000181866
ENSSSCG00000049777	1.21E-06	-1.745186101	0.75	0.933	0.021069645
CCNL2	5.82E-09	-1.747334568	0.875	1	0.000100907
RAD51D	5.36E-11	-1.751418055	0.938	1	9.30E-07
GLMP	9.88E-09	-1.753259402	0.938	0.933	0.000171479
MRPL22	2.27E-16	-1.754741673	1	1	3.94E-12
CLN6	1.72E-07	-1.756074417	0.875	0.933	0.002982613
PHYKPL	1.16E-06	-1.756074417	0.562	0.867	0.020198252
ENSSSCG00000055313	4.01E-18	-1.760320317	0.938	1	6.95E-14
MTO1	3.63E-11	-1.76087221	0.875	1	6.31E-07
EFHC1	3.76E-23	-1.765323099	1	1	6.52E-19
ANKRD40CL	1.63E-16	-1.765534746	0.938	1	2.83E-12
TUFM	3.82E-11	-1.767516686	0.938	1	6.63E-07
TOMM34	1.40E-16	-1.774667485	1	1	2.43E-12
GSE1	2.11E-09	-1.774933444	0.875	1	3.66E-05
MYOM1	5.21E-08	-1.77663574	0.812	0.933	0.000903873
CBX8	8.54E-13	-1.779517169	0.938	1	1.48E-08
NSMCE1	3.97E-07	-1.779801792	0.812	0.933	0.006896021
ENSSSCG00000034739	1.51E-21	-1.781165398	0.938	1	2.62E-17
ENSSSCG00000032082	3.07E-14	-1.781165398	0.875	1	5.33E-10
DBNL	2.83E-06	-1.782769284	0.562	1	0.049186401
HDGFL2	1.04E-10	-1.786682403	0.938	1	1.81E-06
ERAP2	5.85E-11	-1.788094692	0.938	1	1.02E-06
TFR2	9.77E-08	-1.790546634	0.812	1	0.001695803
ENDOU	1.15E-07	-1.790546634	0.562	0.933	0.001992765
ENSSSCG00000035904	6.96E-18	-1.790597077	1	1	1.21E-13
LYPLAL1	8.99E-19	-1.795723494	0.938	1	1.56E-14
COX7C	1.57E-12	-1.795937409	1	1	2.72E-08
ATXN10	4.41E-11	-1.797866418	1	1	7.66E-07
PALLD	5.70E-09	-1.798584712	1	1	9.88E-05
AUP1	1.10E-09	-1.801220136	0.938	1	1.90E-05
ENSSSCG00000059201	3.37E-14	-1.801454321	1	1	5.85E-10
NOP2	1.84E-18	-1.805648956	0.938	1	3.19E-14
ENSSSCG00000010489	3.78E-08	-1.809316438	0.625	1	0.000656601

NQO2	1.34E-07	-1.809316438	0.625	1	0.002329011
UBXN6	1.16E-13	-1.810641025	0.875	0.933	2.01E-09
STMN1	1.12E-08	-1.81232633	1	1	0.000194453
POP5	1.00E-14	-1.812888488	1	1	1.74E-10
MDH1	3.00E-21	-1.813769426	1	1	5.20E-17
POLD1	1.17E-07	-1.816591769	0.812	0.933	0.002029088
SNX11	7.20E-18	-1.82058355	0.938	1	1.25E-13
ERI3	1.92E-07	-1.821029859	0.625	1	0.003338236
PTPRT	1.25E-25	-1.823371458	1	1	2.18E-21
TXNDC5	1.25E-07	-1.825413621	1	0.933	0.002174115
FCF1	1.97E-10	-1.825629094	1	1	3.42E-06
DNAJC11	2.15E-14	-1.827819025	0.938	1	3.74E-10
ENSSSCG00000003903	1.14E-09	-1.828958361	1	1	1.97E-05
HEXIM2	1.79E-10	-1.832518174	0.75	0.933	3.10E-06
SLC25A11	4.15E-11	-1.833141302	0.875	1	7.21E-07
RBPMS2	4.46E-15	-1.834323334	1	1	7.74E-11
ASPA	1.01E-10	-1.835613182	0.812	1	1.76E-06
NDUFA5	4.37E-07	-1.835951565	0.875	0.933	0.007590397
ARRDC1	3.17E-07	-1.836333989	0.5	1	0.005507499
ENSSSCG00000063102	1.65E-06	-1.8372705	0.5	0.8	0.028550015
ENSSSCG00000027723	2.23E-06	-1.840343334	0.75	1	0.038729951
FAM219B	1.59E-10	-1.847244131	0.938	1	2.75E-06
DNAJB13	3.54E-17	-1.849440323	1	1	6.14E-13
ZNF212	1.26E-12	-1.852496293	0.75	1	2.19E-08
POLR2D	9.85E-32	-1.857845832	1	1	1.71E-27
ENSSSCG00000042939	1.01E-07	-1.858644151	0.562	0.933	0.001757709
DDX49	4.59E-13	-1.860089979	1	1	7.97E-09
PCDH12	1.67E-07	-1.860663318	0.75	1	0.002901763
ENSSSCG00000015217	8.26E-47	-1.861187839	1	1	1.43E-42
DDX11	4.56E-16	-1.861645078	0.938	1	7.91E-12
PGS1	1.21E-10	-1.862728216	1	1	2.10E-06
TMBIM6	1.09E-20	-1.862974638	1	1	1.89E-16
FHIT	1.00E-15	-1.865241853	0.938	1	1.74E-11
APOO	2.81E-19	-1.865698908	1	1	4.87E-15
PRDX3	2.76E-30	-1.867487004	1	1	4.80E-26
ZKSCAN4	1.48E-07	-1.868755467	0.875	1	0.002562623
P2RX5	1.04E-13	-1.870407093	0.938	1	1.80E-09
GPC3	1.86E-13	-1.87175303	0.75	1	3.23E-09
B4GALT4	2.45E-17	-1.875263772	0.938	1	4.25E-13
PRPF31	7.30E-08	-1.878984599	0.562	1	0.001267487
MRPS6	1.25E-10	-1.879402711	0.875	0.933	2.17E-06
GLT8D1	1.46E-09	-1.879705766	0.875	0.933	2.53E-05
SIL1	5.45E-15	-1.880000224	1	1	9.45E-11
GGA1	8.32E-09	-1.881605299	0.688	0.933	0.000144286
TMEM248	1.16E-21	-1.884414833	1	1	2.01E-17
NDUFS4	1.66E-15	-1.886810959	1	1	2.87E-11
EEF1AKMT1	8.60E-11	-1.888289613	0.812	0.933	1.49E-06
ENSSSCG00000033189	1.15E-08	-1.891475543	0.875	1	0.000199362
C1orf174	8.17E-07	-1.895428737	0.875	0.933	0.014173403
GON7	4.89E-14	-1.900464326	1	1	8.48E-10
SCAND1	3.74E-12	-1.900464326	0.875	0.933	6.50E-08
MRM1	1.25E-06	-1.903070822	0.812	0.933	0.021698092
ENSA	6.35E-18	-1.908040197	0.938	1	1.10E-13
EIF3B	1.33E-09	-1.90902634	0.938	0.933	2.30E-05
PRKAG1	7.52E-08	-1.910564916	0.812	0.933	0.00130444
AP3S2	5.74E-08	-1.910732662	0.562	1	0.000995144
PSMB4	3.49E-14	-1.913559984	0.938	1	6.05E-10
CAPN8	1.91E-08	-1.915111102	0.625	1	0.000331314
ENSSSCG00000032573	2.47E-18	-1.917113455	1	1	4.28E-14
UBE2J2	6.64E-14	-1.917259569	0.875	1	1.15E-09
MFSD2A	1.44E-08	-1.924354947	0.812	1	0.000249106
CARHSP1	3.38E-09	-1.925381959	0.75	0.933	5.86E-05
SMYD5	2.40E-07	-1.925999419	0.625	0.8	0.004155848
TMUB1	2.21E-11	-1.926537838	0.938	1	3.83E-07
NLRP5	1.51E-22	-1.92919812	1	1	2.61E-18
ANKRD39	2.18E-12	-1.929610672	0.938	1	3.78E-08

ENSSSCG00000028397	1.21E-24	-1.930727165	1	1	2.11E-20
TXN2	2.06E-10	-1.933168492	0.938	0.933	3.58E-06
BAG5	3.65E-07	-1.938134744	0.688	0.933	0.006328532
PRPF4	1.00E-21	-1.93844619	1	1	1.74E-17
IQCC	3.56E-10	-1.942732799	0.938	1	6.18E-06
ENSSSCG00000003282	5.05E-07	-1.94458688	0.312	0.867	0.008766849
CARS2	3.24E-15	-1.946719917	0.875	1	5.62E-11
ENSSSCG00000042361	1.43E-08	-1.947258538	0.75	0.933	0.000247769
DPH7	6.91E-08	-1.949373927	0.688	0.933	0.001198132
GNL3L	4.62E-10	-1.949518551	0.75	1	8.02E-06
TMED9	1.82E-10	-1.949745229	0.812	1	3.15E-06
CTH	3.88E-11	-1.952065523	0.812	0.933	6.73E-07
CNBD2	6.97E-14	-1.957846984	1	1	1.21E-09
COPS6	4.19E-15	-1.959092056	1	1	7.27E-11
TJP3	1.42E-07	-1.961864871	0.625	0.933	0.002472323
TRAK2	1.73E-13	-1.96437609	0.938	1	3.00E-09
GFRA4	6.20E-08	-1.965648495	0.625	0.933	0.001075958
CCDC12	9.34E-23	-1.969102764	1	1	1.62E-18
PIGH	1.63E-10	-1.969726989	0.812	0.933	2.83E-06
POLE3	9.70E-18	-1.971499957	0.938	1	1.68E-13
ENSSSCG00000030478	4.68E-11	-1.974102224	1	1	8.12E-07
CHMP4A	3.23E-14	-1.978938384	0.812	1	5.61E-10
COQ3	1.61E-14	-1.980256065	0.875	1	2.79E-10
SFN	1.08E-08	-1.981464049	0.75	1	0.000186784
AMDHD2	4.61E-10	-1.982926487	0.812	1	7.99E-06
TOP1MT	1.00E-09	-1.983880335	0.812	0.933	1.74E-05
RHBG	9.29E-07	-1.9861942	0.812	0.867	0.016123459
FXR2	1.56E-15	-1.988412026	0.938	1	2.71E-11
BCAS2	2.11E-16	-1.99342083	1	1	3.66E-12
FAM50A	5.35E-09	-1.99492901	0.812	1	9.28E-05
ACAT1	3.20E-24	-1.997719059	1	1	5.55E-20
PHB2	1.41E-22	-1.998170329	1	1	2.45E-18
DNAJC15	7.29E-26	-1.999860197	1	1	1.26E-21
MRPS33	1.31E-24	-2	1	1	2.28E-20
ENSSSCG00000059232	2.06E-08	-2.001961516	0.812	1	0.000357801
OXSM	1.74E-16	-2.003842066	0.875	1	3.02E-12
UQCRC1	2.79E-10	-2.004001931	0.875	1	4.84E-06
MRPL20	1.97E-22	-2.004800986	1	1	3.42E-18
PFKM	8.73E-12	-2.009586049	0.938	1	1.52E-07
CRB3	9.87E-17	-2.010235222	0.938	1	1.71E-12
MIX23	5.17E-24	-2.010940863	1	1	8.98E-20
NCLN	1.57E-08	-2.012832815	0.875	0.933	0.000271961
MRPS18C	2.07E-08	-2.013256097	0.875	1	0.000359545
ARMC2	1.70E-43	-2.013514806	1	1	2.96E-39
ATP6AP1	7.37E-13	-2.016873819	1	0.933	1.28E-08
MEPCE	9.54E-16	-2.019777666	0.938	1	1.65E-11
FAM162A	8.76E-25	-2.020008913	1	1	1.52E-20
MAP1LC3B	1.76E-38	-2.022026306	1	1	3.05E-34
JAM3	7.40E-19	-2.02233633	0.938	1	1.28E-14
ENSSSCG00000044108	1.18E-11	-2.026346871	1	1	2.04E-07
PSME3IP1	8.71E-18	-2.032446622	1	1	1.51E-13
ACAA1	7.54E-10	-2.03284888	0.75	0.933	1.31E-05
MMD2	1.31E-08	-2.03866162	0.562	0.933	0.00022718
PLEKHB1	2.63E-11	-2.039069565	0.875	0.933	4.56E-07
BPGM	4.91E-12	-2.044453598	0.938	1	8.51E-08
SLC25A6	3.91E-18	-2.045944296	0.938	1	6.79E-14
NDUFV2	1.92E-31	-2.049388027	1	1	3.33E-27
ENSSSCG00000032953	1.79E-10	-2.051289229	0.812	1	3.10E-06
CD40	8.33E-21	-2.051325934	0.938	1	1.45E-16
POLR1D	5.37E-23	-2.054281661	1	1	9.32E-19
LRCH4	1.93E-10	-2.054635257	0.75	0.933	3.35E-06
PCIF1	1.50E-06	-2.054635257	0.5	0.867	0.02595232
PADI3	4.28E-09	-2.055495113	0.75	1	7.43E-05
DYNLL1	2.84E-16	-2.064648741	1	1	4.93E-12
ENSSSCG00000006559	2.51E-18	-2.065364414	0.938	1	4.36E-14
ATP5PO	4.63E-25	-2.068333059	1	1	8.04E-21

TRAPPC4	3.20E-24	-2.068810808	1	1	5.56E-20
ENSSSCG00000011147	9.26E-12	-2.074493726	0.812	1	1.61E-07
SNU13	1.51E-25	-2.076890894	1	1	2.61E-21
ARL6IP4	3.99E-09	-2.078609835	0.688	0.933	6.93E-05
NOP14	8.87E-14	-2.080170349	0.812	1	1.54E-09
NME2	2.55E-10	-2.082248412	1	1	4.42E-06
UPB1	4.50E-14	-2.087118967	0.938	1	7.81E-10
MRPL58	1.40E-17	-2.088909416	1	1	2.42E-13
FKBP4	2.57E-22	-2.092006005	1	1	4.46E-18
MOK	4.81E-12	-2.093109404	0.75	0.933	8.35E-08
NOL4L	2.19E-11	-2.093109404	0.625	0.933	3.80E-07
THEM5	2.18E-08	-2.093109404	0.562	1	0.000377851
ENSSSCG00000032413	1.43E-07	-2.093109404	0.688	0.933	0.002483484
MAPK3	1.54E-06	-2.093109404	0.5	0.867	0.02671252
RPS6	6.65E-18	-2.094454575	1	1	1.15E-13
DRG1	1.59E-14	-2.094757257	1	1	2.76E-10
MED31	5.49E-16	-2.09797516	1	1	9.52E-12
COPS7A	8.76E-08	-2.105933445	0.562	0.933	0.001520427
IDH3B	1.16E-17	-2.108706259	0.875	1	2.01E-13
ARHGAP22	1.41E-33	-2.109076153	1	1	2.45E-29
GEMIN2	1.09E-09	-2.111394757	0.812	0.933	1.90E-05
AURKAIP1	2.28E-10	-2.111968432	0.75	1	3.95E-06
NDUFC1	2.70E-18	-2.113008962	0.875	1	4.69E-14
PRR13	2.12E-16	-2.113287286	0.938	1	3.67E-12
PLA2G12A	9.78E-17	-2.116760672	0.938	1	1.70E-12
ENSSSCG00000005006	6.08E-14	-2.117183539	1	1	1.06E-09
PSMC3IP	5.85E-18	-2.117275716	0.938	1	1.01E-13
ENSSSCG00000047706	3.87E-10	-2.117771459	0.75	0.933	6.72E-06
ENSSSCG00000051352	1.78E-08	-2.118644496	0.75	0.867	0.000308745
ENSSSCG00000044054	5.55E-08	-2.118644496	0.688	1	0.000962625
CDA	1.37E-13	-2.119104613	0.75	1	2.38E-09
SAMM50	4.32E-10	-2.119909464	0.812	1	7.49E-06
POP7	1.36E-20	-2.12015628	1	1	2.36E-16
ENSSSCG00000049464	1.58E-15	-2.124587636	1	1	2.74E-11
CALML4	3.07E-08	-2.12963528	0.75	1	0.000533479
EBNA1BP2	2.41E-24	-2.130103612	1	1	4.18E-20
CHCHD4	3.26E-41	-2.13162464	1	1	5.66E-37
POC1A	1.60E-18	-2.133186844	0.875	1	2.78E-14
TOMM20	5.88E-29	-2.134301092	1	1	1.02E-24
RNF181	1.69E-12	-2.13492958	0.875	1	2.94E-08
NOC4L	1.03E-07	-2.13492958	0.625	0.933	0.001793292
TMEM213	5.98E-12	-2.138005942	0.875	1	1.04E-07
MRPL45	2.04E-22	-2.141318497	0.938	1	3.54E-18
ATRAID	5.55E-14	-2.14286244	0.875	1	9.63E-10
PIP4P1	7.40E-14	-2.144421495	0.938	1	1.28E-09
SLC16A5	8.65E-10	-2.145197916	0.625	1	1.50E-05
NT5DC2	2.75E-07	-2.146976276	0.75	0.933	0.004778405
LONP1	5.86E-15	-2.147901877	0.938	1	1.02E-10
OTUB1	3.36E-16	-2.148001535	1	1	5.83E-12
CLDN10	4.78E-28	-2.1498785	1	1	8.29E-24
ENSSSCG00000055905	5.57E-10	-2.154509949	0.625	1	9.66E-06
GHITM	5.14E-12	-2.154565608	1	1	8.91E-08
ENSSSCG00000056323	1.34E-11	-2.156219448	0.812	1	2.33E-07
TK2	7.48E-13	-2.156351838	1	1	1.30E-08
RPL28	8.31E-07	-2.160016063	0.875	1	0.014415636
DNPEP	3.61E-08	-2.164975391	1	1	0.000626059
LMBR1L	8.63E-10	-2.168397532	0.812	0.933	1.50E-05
SLC38A8	2.46E-11	-2.168599604	0.875	0.933	4.28E-07
ENSSSCG00000047379	1.70E-08	-2.171111916	0.625	1	0.000294962
ENSSSCG00000022738	6.09E-11	-2.173674886	0.812	0.933	1.06E-06
ENSSSCG00000035728	9.07E-10	-2.175571565	0.812	1	1.57E-05
ENSSSCG00000045681	2.84E-11	-2.17625064	0.875	1	4.92E-07
ENSSSCG00000007493	1.61E-36	-2.177421518	1	1	2.79E-32
PAAF1	1.81E-17	-2.18001747	1	1	3.14E-13
SMARCD3	2.36E-06	-2.180572246	0.375	0.867	0.040942038
TFB1M	3.24E-36	-2.182425236	1	1	5.62E-32

TXNRD3	2.35E-08	-2.183307213	0.688	0.933	0.000408179
ENSSSCG00000041692	2.83E-08	-2.185031894	0.688	0.867	0.000491629
ENSSSCG00000008147	2.39E-10	-2.185758692	0.812	0.933	4.15E-06
ENSSSCG00000002379	1.84E-15	-2.188266637	1	1	3.19E-11
ENSSSCG00000013613	6.40E-09	-2.188266637	0.688	1	0.000111102
WWC3	8.14E-07	-2.188266637	0.438	0.867	0.014117306
LSM4	1.03E-17	-2.192645078	0.938	1	1.78E-13
MTRES1	2.35E-33	-2.19291522	0.938	1	4.08E-29
SERPINB1	1.67E-13	-2.193303692	0.938	1	2.91E-09
HADHA	2.75E-29	-2.194422891	1	1	4.77E-25
BNIP1	2.48E-18	-2.195624315	1	1	4.30E-14
ENSSSCG00000052254	1.96E-10	-2.197446064	0.5	0.933	3.40E-06
DHPS	8.79E-11	-2.198462405	0.688	0.933	1.52E-06
YBX2	8.59E-11	-2.202231127	0.875	1	1.49E-06
ENSSSCG00000037808	6.77E-08	-2.202338474	0.938	1	0.001175196
ENSSSCG00000008012	1.25E-21	-2.203219161	1	1	2.18E-17
MPV17	1.65E-09	-2.20461772	0.938	0.933	2.87E-05
TMEM223	1.25E-07	-2.208586622	0.625	0.867	0.002176893
YIF1B	1.21E-06	-2.208586622	0.438	0.933	0.020981163
MAF	3.11E-10	-2.212408333	0.562	1	5.40E-06
THBS3	6.39E-09	-2.212848649	0.625	1	0.000110875
ISYNA1	5.28E-08	-2.22059914	0.688	0.867	0.000915432
B4GALT7	1.62E-07	-2.2255597	0.625	0.867	0.002812829
VPS25	2.40E-17	-2.22606081	1	1	4.17E-13
SVOP	2.44E-06	-2.228624375	0.688	0.867	0.042398324
FBXO31	6.39E-11	-2.229375094	0.812	0.933	1.11E-06
TLCD1	1.51E-27	-2.229753584	0.875	1	2.62E-23
ZMAT5	1.87E-21	-2.232085818	0.938	1	3.24E-17
ENKD1	2.51E-06	-2.234465254	0.5	0.867	0.043503533
MRPS7	5.21E-12	-2.236945177	0.625	1	9.05E-08
ENSSSCG00000039815	5.75E-13	-2.239950793	0.688	1	9.97E-09
ENSSSCG00000028962	6.78E-08	-2.239950793	0.438	0.933	0.001175709
PLIN5	8.63E-12	-2.247437551	0.875	1	1.50E-07
GBA2	2.11E-06	-2.247437551	0.625	0.933	0.036532621
PKN1	8.46E-07	-2.24838763	0.5	0.933	0.014683238
TRIM4	1.03E-07	-2.24961389	0.562	0.8	0.001788072
SLC35B1	1.21E-19	-2.251371488	1	1	2.10E-15
PRDX4	5.76E-12	-2.251741236	1	1	9.99E-08
FLOT1	6.04E-27	-2.252063288	0.938	1	1.05E-22
SCPEP1	1.81E-13	-2.252449655	0.875	1	3.14E-09
RNF26	4.85E-23	-2.253756592	1	1	8.42E-19
AQP6	4.56E-07	-2.255380833	0.625	0.867	0.007907673
ZPR1	5.55E-20	-2.25762091	0.938	1	9.64E-16
FAM32A	2.21E-22	-2.258772379	1	1	3.83E-18
GDF9	1.63E-37	-2.261016009	1	1	2.83E-33
IER2	2.07E-15	-2.263034406	0.875	0.933	3.59E-11
BUD31	2.27E-33	-2.264183048	1	1	3.95E-29
PMF1	7.90E-14	-2.264589872	0.938	1	1.37E-09
NRAP	6.58E-12	-2.267883805	0.625	1	1.14E-07
TANGO2	8.01E-13	-2.269432177	0.938	1	1.39E-08
ASB11	4.27E-26	-2.270431399	1	1	7.40E-22
NPC2	3.62E-17	-2.273176416	1	1	6.28E-13
MRPS28	9.31E-24	-2.276470545	0.938	1	1.62E-19
ENSSSCG00000062784	4.67E-08	-2.277533976	0.562	1	0.000810684
ENSSSCG00000031730	2.07E-15	-2.27897595	1	1	3.60E-11
PROM2	1.67E-07	-2.27897595	0.5	0.8	0.002892421
NOP10	1.05E-26	-2.281381064	1	1	1.82E-22
SCAMP4	5.52E-07	-2.282143229	0.562	0.867	0.009579811
PPOX	8.81E-10	-2.285754482	0.5	0.933	1.53E-05
MLST8	3.18E-09	-2.285754482	0.75	0.933	5.52E-05
ENSSSCG00000061349	1.85E-09	-2.291048782	0.688	0.867	3.22E-05
ARF5	3.40E-20	-2.291379032	0.875	1	5.89E-16
ENSSSCG00000057138	1.78E-12	-2.292090439	0.75	0.933	3.08E-08
CD247	7.28E-19	-2.292418213	0.938	1	1.26E-14
MRTO4	5.24E-14	-2.294743266	0.812	1	9.10E-10
ENSSSCG00000015083	3.91E-17	-2.295245716	1	1	6.79E-13

THAP7	5.29E-10	-2.296981738	0.688	1	9.17E-06
GALNT6	2.05E-12	-2.297799824	0.812	1	3.56E-08
ARHGEF28	4.31E-10	-2.300704824	0.562	1	7.47E-06
KLF1	1.83E-07	-2.30461351	0.5	0.933	0.003179516
PHB1	1.66E-27	-2.307003265	1	1	2.88E-23
ADA	1.54E-26	-2.3111759	1	1	2.67E-22
ENSSSCG00000031756	5.20E-15	-2.312467766	0.875	1	9.02E-11
TXNDC12	6.68E-12	-2.313157885	0.688	1	1.16E-07
CTDNEP1	1.97E-14	-2.315501826	0.875	1	3.42E-10
EHD1	6.19E-11	-2.315501826	0.625	1	1.07E-06
TVP23A	9.18E-07	-2.315501826	0.438	0.8	0.015924332
C14orf93	1.55E-06	-2.315501826	0.375	0.933	0.026980752
AKR1A1	1.21E-10	-2.316912775	0.812	0.933	2.10E-06
NUCB1	1.51E-11	-2.322355497	0.812	1	2.63E-07
SNF8	8.05E-10	-2.322843801	0.812	1	1.40E-05
ENSSSCG00000001776	1.04E-06	-2.323407024	0.438	0.867	0.017974564
TIMP2	2.56E-06	-2.323407024	0.5	0.8	0.04440117
ENSSSCG00000057224	3.28E-10	-2.32443495	0.75	1	5.69E-06
ENSSSCG00000005217	1.01E-31	-2.325255617	0.938	1	1.75E-27
DNAI1	2.70E-06	-2.325770161	0.438	0.867	0.046933095
MED8	1.77E-20	-2.326599535	1	1	3.08E-16
TOMM40L	1.94E-10	-2.330148602	0.5	1	3.36E-06
FZR1	1.55E-22	-2.333531241	0.938	1	2.68E-18
NUDCD3	7.80E-13	-2.333669948	0.875	1	1.35E-08
PIH1D1	1.30E-18	-2.334770159	0.812	1	2.25E-14
KLHDC4	2.33E-09	-2.336563441	0.875	0.867	4.05E-05
MED10	8.09E-13	-2.337034987	0.75	1	1.40E-08
LRRC36	1.88E-06	-2.338221902	0.5	0.933	0.032575999
ENSSSCG00000027041	5.02E-33	-2.339220179	1	1	8.72E-29
ENSSSCG00000059210	1.23E-08	-2.341036918	0.5	0.933	0.000213461
ARPC3	1.22E-21	-2.343222583	1	1	2.12E-17
UPK1A	8.31E-09	-2.344070978	0.625	0.867	0.000144219
CIDEA	1.34E-09	-2.345113482	0.938	1	2.33E-05
PAQR7	1.68E-15	-2.349449158	0.938	0.933	2.91E-11
HAUS1	1.20E-36	-2.349847571	1	1	2.08E-32
NCF1	6.96E-18	-2.35033181	0.875	1	1.21E-13
GFUS	5.56E-13	-2.352976531	0.812	1	9.64E-09
GDPGP1	6.54E-10	-2.354069482	0.688	1	1.14E-05
SLC25A10	1.67E-06	-2.35614381	0.188	0.933	0.028966505
NR0B1	3.98E-23	-2.360289747	1	1	6.91E-19
UST	1.89E-21	-2.360331607	0.875	1	3.28E-17
PPAN	1.91E-28	-2.36143811	1	1	3.31E-24
CARD19	5.80E-10	-2.36159824	0.812	1	1.01E-05
ENSSSCG00000060526	2.36E-07	-2.36159824	0.312	0.933	0.004099612
COMMD1	9.46E-08	-2.366127899	0.5	0.867	0.001642246
PFDN1	9.79E-36	-2.36656808	1	1	1.70E-31
RPS27A	1.84E-12	-2.369440633	1	1	3.19E-08
FAT2	1.03E-18	-2.374570908	0.875	0.933	1.79E-14
UQCRB	1.18E-23	-2.375613674	1	1	2.05E-19
PFDN5	4.69E-19	-2.375887746	1	1	8.13E-15
AMZ2	1.51E-29	-2.380368483	0.938	1	2.62E-25
GPN2	9.73E-11	-2.380391356	0.812	0.933	1.69E-06
INTS11	2.99E-07	-2.385891154	0.562	0.933	0.005193427
IDH2	2.32E-17	-2.386373741	0.938	1	4.03E-13
ENSSSCG00000003234	2.56E-07	-2.388565288	0.438	0.933	0.004444173
GTF2IRD1	3.03E-07	-2.388565288	0.5	0.933	0.005252371
NDUFA4	4.53E-10	-2.391682103	1	1	7.86E-06
PAPLN	4.84E-09	-2.393504338	0.562	1	8.39E-05
ENSSSCG00000011272	1.32E-22	-2.396632919	1	1	2.29E-18
HIGD2A	7.92E-13	-2.398038496	1	1	1.37E-08
BLOC1S1	1.37E-07	-2.398917834	0.375	0.933	0.002378694
C5orf49	3.47E-11	-2.405387329	0.938	1	6.02E-07
GTF2H5	1.56E-18	-2.406848101	0.938	1	2.70E-14
MAPK7	1.46E-10	-2.40780593	0.812	1	2.53E-06
TMA7	2.44E-16	-2.407982742	1	1	4.24E-12
MESD	7.99E-13	-2.409684265	0.75	1	1.39E-08

COMMD7	7.60E-20	-2.411285364	0.812	1	1.32E-15
ARRDC4	3.11E-12	-2.41130478	1	1	5.40E-08
BNIP3	1.31E-18	-2.415037499	0.938	0.933	2.28E-14
NAA10	3.03E-10	-2.415037499	0.75	1	5.25E-06
FKBP1B	7.44E-07	-2.415037499	0.438	0.867	0.012901357
ATP5MC2	1.27E-25	-2.416655778	1	1	2.20E-21
PRDX1	1.81E-42	-2.421547541	1	1	3.15E-38
PLPP4	8.60E-10	-2.423258006	0.688	1	1.49E-05
MAP2K2	8.96E-08	-2.428712436	0.5	0.867	0.001554729
MYL12A	4.74E-30	-2.429016083	1	1	8.23E-26
MFSD13A	1.21E-08	-2.432959407	0.625	0.933	0.000210064
TIMM50	9.32E-13	-2.434146322	0.812	1	1.62E-08
YKT6	4.80E-18	-2.434402824	0.812	1	8.33E-14
RDH13	1.42E-12	-2.435215381	0.812	0.933	2.46E-08
CTNS	1.56E-20	-2.435262527	1	1	2.71E-16
POLM	2.69E-10	-2.435501602	0.75	0.867	4.67E-06
CCDC63	4.18E-11	-2.437937901	0.438	1	7.25E-07
GMPPA	1.36E-07	-2.437937901	0.5	0.867	0.002354595
MARS1	6.10E-30	-2.440010028	1	1	1.06E-25
ENSSSCG00000052208	2.45E-14	-2.441631709	0.562	1	4.24E-10
NDUFB4	2.43E-09	-2.441837559	0.812	1	4.22E-05
VPS28	7.67E-15	-2.443606651	0.875	1	1.33E-10
EIF4EBP1	6.88E-09	-2.443606651	0.562	1	0.000119369
PDCD5	2.53E-34	-2.444916142	1	1	4.40E-30
NSFL1C	1.43E-12	-2.447337991	0.75	1	2.49E-08
CCDC172	1.55E-13	-2.449594721	0.75	1	2.69E-09
PSTPIP1	6.20E-10	-2.449802917	0.688	0.933	1.08E-05
GPR108	1.68E-12	-2.451301043	0.562	1	2.91E-08
GEMIN6	7.36E-36	-2.451362189	1	1	1.28E-31
ENSSSCG00000022401	6.71E-22	-2.454930226	0.875	1	1.16E-17
ENSSSCG00000010058	1.41E-28	-2.457319548	1	1	2.44E-24
ENSSSCG00000003286	6.88E-20	-2.458385042	1	1	1.19E-15
HEMK1	1.49E-07	-2.458758877	0.688	0.867	0.002588197
MCRS1	5.99E-16	-2.463567393	0.938	1	1.04E-11
NELFCD	7.66E-08	-2.469485283	0.562	0.867	0.00132971
IP6K2	5.88E-18	-2.471621028	1	1	1.02E-13
FUNDC2	1.96E-12	-2.485426827	0.812	1	3.41E-08
TSEN54	1.26E-07	-2.485426827	0.5	0.8	0.002192351
ZNRD2	1.55E-07	-2.485426827	0.5	0.867	0.002693137
SUCLG1	6.13E-24	-2.486181572	0.938	1	1.06E-19
SLC38A5	5.83E-08	-2.489999557	0.625	0.8	0.001012363
COA3	2.24E-13	-2.493647334	0.625	1	3.89E-09
MTMR14	7.71E-17	-2.494039957	0.938	1	1.34E-12
OOEP	4.23E-23	-2.496253737	1	1	7.35E-19
TIMM17A	5.27E-16	-2.499101764	0.688	1	9.15E-12
RNF121	9.49E-21	-2.499734664	0.938	1	1.65E-16
POLR2H	1.14E-30	-2.49986834	1	1	1.99E-26
PRDX5	3.61E-12	-2.501691882	0.812	1	6.26E-08
ENSSSCG00000007826	2.94E-27	-2.505740406	1	1	5.10E-23
DUS3L	2.47E-11	-2.50589093	0.625	0.933	4.29E-07
BAG2	4.86E-17	-2.508146904	0.875	1	8.43E-13
SPAG8	4.38E-09	-2.511610759	0.625	0.933	7.59E-05
SMYD3	2.27E-09	-2.515066318	0.812	1	3.95E-05
PUF60	1.08E-21	-2.51524796	1	1	1.88E-17
SIAH3	6.84E-09	-2.516320835	0.688	0.933	0.000118658
ENSSSCG00000011294	1.72E-13	-2.51731855	0.75	1	2.98E-09
MMAB	4.73E-09	-2.519374159	0.5	0.933	8.20E-05
ENSSSCG00000029830	2.42E-10	-2.521203056	0.938	1	4.19E-06
SWI5	3.39E-16	-2.528566445	1	1	5.88E-12
SEC11C	1.46E-23	-2.531682418	1	1	2.53E-19
ENSSSCG00000017913	4.57E-25	-2.531814801	1	1	7.94E-21
PLIN3	3.27E-11	-2.541569905	0.875	1	5.68E-07
GATD1	9.67E-10	-2.542416806	0.625	0.933	1.68E-05
COA4	7.59E-29	-2.543142325	1	1	1.32E-24
TSPAN1	6.45E-17	-2.543599989	0.938	1	1.12E-12
MAPKAPK2	1.04E-11	-2.545621609	0.625	1	1.81E-07

PPM1J	8.05E-08	-2.552541023	0.438	0.867	0.001396809
XKRX	1.26E-07	-2.552541023	0.562	0.933	0.002184646
AP1S1	1.69E-22	-2.55468949	0.938	1	2.93E-18
TPCN1	8.66E-12	-2.557390423	0.625	1	1.50E-07
EIF2B2	1.89E-08	-2.561878888	0.625	0.933	0.000328771
CMC4	9.23E-15	-2.563429339	0.812	1	1.60E-10
CCDC9	2.00E-06	-2.571156701	0.375	0.867	0.034738138
ENSSSCG00000014284	1.66E-26	-2.572968905	1	1	2.88E-22
ENSSSCG00000052263	5.25E-25	-2.573374526	1	1	9.10E-21
ENSSSCG00000016100	5.59E-20	-2.576431643	0.875	1	9.70E-16
POLR2K	1.33E-29	-2.581325519	1	1	2.31E-25
LRSAM1	3.48E-14	-2.581395886	0.812	1	6.04E-10
KDM8	1.27E-10	-2.58185659	0.688	0.933	2.21E-06
CYB561D2	3.42E-07	-2.582494245	0.438	0.8	0.005927883
DMWD	5.31E-13	-2.583075391	0.75	1	9.21E-09
LIN37	6.05E-11	-2.584249883	0.75	1	1.05E-06
C1orf54	5.53E-14	-2.584962501	0.812	1	9.60E-10
TBRG4	1.21E-19	-2.58812978	0.938	1	2.10E-15
FSTL3	3.59E-19	-2.591015171	0.875	1	6.23E-15
GAPDH	9.91E-09	-2.591295462	0.938	1	0.000172017
WASHC3	7.35E-07	-2.5981036	0.562	0.933	0.012745113
RPL32	1.30E-20	-2.59946207	1	1	2.26E-16
MATCAP1	3.59E-07	-2.600904045	0.562	0.667	0.00622084
ENSSSCG00000056114	2.25E-11	-2.601450624	0.75	0.933	3.90E-07
ENSSSCG00000038027	9.19E-15	-2.603724564	0.562	1	1.59E-10
ENSSSCG00000060912	3.23E-09	-2.604071324	0.438	0.933	5.61E-05
NDUFB2	1.83E-14	-2.607348581	1	1	3.18E-10
JPT2	4.63E-13	-2.613773412	0.75	0.933	8.03E-09
MVK	3.02E-11	-2.61667136	0.625	0.933	5.25E-07
HOATZ	9.82E-07	-2.61667136	0.312	0.8	0.01704113
JPT1	2.26E-18	-2.623624121	0.938	1	3.92E-14
CCER2	3.27E-11	-2.628072519	1	1	5.68E-07
FSD2	1.60E-30	-2.62995331	1	1	2.77E-26
GABARAP	2.51E-22	-2.632747915	0.938	1	4.36E-18
GRHPR	4.46E-20	-2.634891081	1	1	7.75E-16
CLPS	3.23E-07	-2.635163314	0.688	1	0.005611858
REC114	6.77E-39	-2.635806178	1	1	1.17E-34
RBP2	6.76E-09	-2.639597757	0.562	0.867	0.000117304
GTF2F1	7.88E-27	-2.641313355	1	1	1.37E-22
ALKBH7	2.33E-16	-2.647045009	0.75	1	4.05E-12
FN3KRP	3.79E-07	-2.647698256	0.312	1	0.0065843
SHANK2	8.01E-11	-2.651104858	0.688	0.933	1.39E-06
IAH1	2.55E-17	-2.655634516	0.812	1	4.43E-13
IQCD	6.71E-07	-2.656045599	0.25	0.867	0.011636202
MVP	1.19E-29	-2.662130361	1	1	2.06E-25
SELENOS	1.93E-28	-2.663099398	0.938	1	3.34E-24
TMEM200C	4.64E-11	-2.669458775	0.5	0.867	8.05E-07
ZNF621	1.06E-09	-2.670538232	0.562	0.867	1.84E-05
CDK5RAP2	4.54E-23	-2.672487787	1	1	7.88E-19
NDUFS5	2.00E-15	-2.674904626	1	1	3.47E-11
HAUS7	2.38E-12	-2.678071905	0.688	0.933	4.13E-08
ENSSSCG00000013768	1.08E-09	-2.678071905	0.562	0.933	1.87E-05
CORO6	1.04E-08	-2.678071905	0.438	1	0.000180574
ALG3	4.74E-07	-2.678071905	0.438	0.867	0.008221613
ARMC12	1.91E-15	-2.685807554	0.688	1	3.32E-11
ENSSSCG00000059061	6.76E-11	-2.689476668	0.562	0.933	1.17E-06
EMC7	1.90E-27	-2.695681305	0.812	1	3.30E-23
ETHE1	4.51E-17	-2.696735749	0.875	0.933	7.82E-13
BUD23	6.03E-28	-2.697880386	0.938	1	1.05E-23
SLC25A14	7.34E-13	-2.697971463	0.75	0.933	1.27E-08
ENSSSCG00000009851	8.03E-26	-2.701658725	1	1	1.39E-21
POMP	8.30E-28	-2.70286109	1	1	1.44E-23
TMEM178A	6.19E-08	-2.703162886	0.375	0.933	0.001074113
ABHD17A	1.28E-13	-2.704544116	0.688	0.933	2.22E-09
FLAD1	8.56E-09	-2.709780765	0.438	0.933	0.000148536
TFPT	1.24E-06	-2.712019237	0.25	0.867	0.021533377

METTL23	8.60E-22	-2.712718048	0.875	1	1.49E-17
TIMM13	2.95E-25	-2.714597781	0.875	1	5.11E-21
ALDOC	2.71E-24	-2.715262464	0.875	1	4.70E-20
PEX19	5.35E-14	-2.717018185	0.875	0.933	9.29E-10
GLRX3	1.44E-24	-2.717600269	0.938	1	2.51E-20
ENSSSCG00000050347	1.39E-09	-2.717600269	0.375	0.933	2.40E-05
ENSSSCG00000039544	1.91E-39	-2.718025096	1	1	3.31E-35
COA8	3.18E-16	-2.718429643	0.75	1	5.51E-12
ENSSSCG00000061274	5.91E-10	-2.719892081	0.5	0.933	1.02E-05
REX1BD	9.58E-09	-2.719892081	0.438	1	0.000166203
ENSSSCG00000062012	1.50E-08	-2.719892081	0.562	0.8	0.000259453
VDAC3	5.40E-30	-2.721494185	0.938	1	9.38E-26
UBA52	1.28E-09	-2.72416832	0.875	1	2.21E-05
MLYCD	6.65E-11	-2.72537762	0.438	1	1.15E-06
ENSSSCG00000031117	1.80E-15	-2.726981506	0.938	1	3.12E-11
ENSSSCG00000043434	2.52E-10	-2.728697978	0.438	1	4.38E-06
POLL	2.48E-07	-2.728697978	0.438	0.867	0.004300999
UBL7	2.15E-27	-2.729380999	1	1	3.74E-23
YIPF2	3.33E-20	-2.73231495	0.875	1	5.78E-16
TEX264	1.43E-15	-2.735787403	0.875	1	2.48E-11
ENSSSCG00000034188	4.70E-10	-2.736965594	0.438	1	8.15E-06
RNF141	3.24E-10	-2.74080766	0.5	0.933	5.63E-06
NEU1	2.02E-06	-2.742202243	0.375	0.8	0.03501214
ENSSSCG00000006081	8.10E-31	-2.750199047	1	1	1.41E-26
ENSSSCG00000046592	1.54E-20	-2.753669524	0.938	1	2.67E-16
ENSSSCG00000054115	9.59E-57	-2.75729124	1	1	1.66E-52
GINS2	1.93E-38	-2.758285966	1	1	3.36E-34
AJUBA	5.41E-27	-2.760065277	0.938	1	9.39E-23
ECHS1	8.66E-14	-2.760964913	0.75	0.933	1.50E-09
TCIRG1	2.45E-17	-2.761487913	0.75	1	4.25E-13
PRRT3	4.95E-08	-2.765534746	0.375	0.867	0.000858689
MAP4K1	2.53E-10	-2.768674454	0.625	0.933	4.40E-06
ENSSSCG00000029003	3.25E-13	-2.769219793	0.812	1	5.64E-09
FHIP2B	3.46E-07	-2.77118131	0.375	0.733	0.006008928
UROD	3.96E-11	-2.774933444	0.688	1	6.87E-07
WDR74	1.52E-23	-2.777013755	0.812	1	2.64E-19
GNB1L	3.23E-07	-2.777607579	0.312	0.8	0.005607337
ENSSSCG00000057721	2.32E-06	-2.777607579	0.25	0.733	0.040188179
SMIM12	1.61E-13	-2.78181983	0.812	0.933	2.80E-09
GABARAPL1	7.28E-10	-2.783424905	0.438	0.933	1.26E-05
TMEM205	2.31E-09	-2.783424905	0.688	0.867	4.00E-05
ENSSSCG00000021041	1.42E-06	-2.783424905	0.312	0.733	0.024657452
RPS7	2.20E-19	-2.784423886	0.875	1	3.82E-15
TMEM42	1.09E-18	-2.785600369	0.938	1	1.89E-14
NDUFB10	3.01E-10	-2.790546634	0.75	0.933	5.22E-06
SSMEM1	2.22E-18	-2.793549123	0.938	1	3.85E-14
SNRPD2	2.19E-13	-2.793549123	0.812	1	3.80E-09
MRPL54	2.88E-23	-2.796286685	0.875	1	4.99E-19
POLE4	1.84E-30	-2.800647975	1	1	3.19E-26
DYDC1	8.75E-28	-2.802060622	0.938	1	1.52E-23
SLC25A39	1.13E-11	-2.802060622	0.688	0.933	1.97E-07
NDUFS3	1.12E-23	-2.803482798	0.812	1	1.95E-19
NDUFS8	1.59E-07	-2.803602787	0.25	0.8	0.002762909
MRPS12	1.34E-06	-2.803602787	0.312	0.867	0.023333024
ALAD	7.55E-14	-2.805827452	0.688	1	1.31E-09
KANK3	3.00E-08	-2.805827452	0.438	0.867	0.000519988
ENSSSCG00000058372	2.35E-07	-2.805827452	0.438	0.867	0.00408124
COPS3	1.36E-24	-2.806991385	0.938	1	2.36E-20
ENSSSCG00000040118	2.95E-07	-2.809316438	0.312	0.867	0.005123008
DDRGK1	1.53E-09	-2.810966176	0.438	0.933	2.66E-05
ENSSSCG00000038404	4.46E-10	-2.815575429	0.875	1	7.74E-06
MCAT	2.40E-09	-2.815575429	0.625	0.867	4.17E-05
EEF1B2	1.22E-32	-2.816700776	1	1	2.11E-28
YTHDF1	1.35E-16	-2.817706441	0.688	1	2.35E-12
DMGDH	1.68E-11	-2.818694771	0.875	1	2.91E-07
TARBP2	1.43E-08	-2.823502345	0.5	0.8	0.000248448

GUCA1A	6.23E-27	-2.830074999	1	1	1.08E-22
DYNC2I2	2.97E-10	-2.830074999	0.5	0.867	5.16E-06
ENSSSCG00000050595	1.15E-06	-2.830074999	0.312	0.733	0.019875044
AGA	2.08E-12	-2.831751576	0.562	1	3.61E-08
CBR3	4.82E-36	-2.833538854	1	1	8.35E-32
NSDHL	2.01E-46	-2.836175198	1	1	3.48E-42
LYRM4	2.63E-13	-2.840343334	0.688	0.933	4.57E-09
RNF166	2.68E-06	-2.840343334	0.312	0.733	0.046450154
COX5A	1.98E-33	-2.844683085	1	1	3.43E-29
MEA1	2.30E-22	-2.845181891	0.938	1	3.99E-18
RPL11	2.59E-17	-2.846162942	1	1	4.50E-13
IMMP2L	2.02E-20	-2.847996907	0.938	1	3.50E-16
MST1	7.39E-09	-2.847996907	0.812	0.867	0.000128151
COX6C	1.59E-20	-2.84820373	1	1	2.76E-16
SWSAP1	3.59E-20	-2.852569636	0.938	0.933	6.24E-16
ATP5MK	3.51E-19	-2.856099887	1	1	6.09E-15
CCS	3.28E-07	-2.862496476	0.25	0.867	0.005691302
PDLIM7	1.18E-06	-2.862496476	0.375	0.8	0.020443019
GPD1	1.08E-14	-2.863351153	0.812	1	1.87E-10
MPV17L2	1.08E-06	-2.865698908	0.25	0.867	0.018653594
DNASE2	1.16E-10	-2.873328197	0.438	0.933	2.01E-06
PRXL2B	2.16E-06	-2.874469118	0.375	0.733	0.037449795
MRPL2	1.38E-13	-2.875517969	0.688	0.867	2.40E-09
LRRC61	1.96E-08	-2.877380713	0.312	0.933	0.000340228
ENSSSCG00000047189	1.15E-14	-2.878984599	0.688	1	2.00E-10
ISCA2	6.28E-20	-2.882816461	0.75	1	1.09E-15
GPS1	1.82E-16	-2.887525271	0.75	1	3.15E-12
PMM1	6.12E-10	-2.900464326	0.312	0.933	1.06E-05
FAM174C	3.47E-08	-2.900464326	0.375	0.867	0.000602641
RACK1	1.98E-06	-2.902380256	0.938	1	0.034348173
MRPL51	3.98E-36	-2.904937803	1	1	6.91E-32
CTSD	1.63E-08	-2.910732662	0.375	0.933	0.000282431
CERS4	9.10E-13	-2.911414626	0.938	1	1.58E-08
MAST2	1.54E-16	-2.915111102	0.812	1	2.68E-12
NUDT2	7.53E-16	-2.915839552	0.75	0.933	1.31E-11
ENSSSCG00000023691	1.75E-43	-2.916550707	0.938	1	3.04E-39
MRPL17	1.28E-21	-2.91707904	0.688	1	2.22E-17
ELAC2	1.38E-12	-2.918275603	0.625	1	2.40E-08
GGH	2.93E-33	-2.920516485	1	1	5.09E-29
GADD45GIP1	3.27E-18	-2.920928429	0.688	1	5.67E-14
EDF1	7.78E-10	-2.923184403	0.438	1	1.35E-05
SLC47A1	1.11E-13	-2.925999419	0.562	0.933	1.92E-09
IDI1	9.56E-39	-2.926917122	1	1	1.66E-34
NELFE	3.56E-17	-2.927685795	0.688	1	6.17E-13
TEX35	1.86E-22	-2.928050158	0.812	1	3.24E-18
TCL1B	3.19E-17	-2.928167255	1	1	5.53E-13
ENSSSCG00000048488	1.47E-07	-2.929610672	0.25	0.8	0.002553335
ADAD2	3.32E-07	-2.934411658	0.25	0.867	0.005752004
COX17	1.63E-14	-2.935568127	0.688	1	2.83E-10
TNNC2	4.13E-14	-2.935869663	0.625	0.933	7.16E-10
PSME2	1.00E-09	-2.938599455	0.438	0.933	1.74E-05
PSMD13	4.61E-59	-2.947201482	0.938	1	8.00E-55
GDPD5	7.63E-22	-2.949373927	0.75	1	1.32E-17
INO80B	7.27E-07	-2.9510904	0.188	0.867	0.012614057
ENSSSCG00000006581	8.50E-07	-2.9510904	0.188	0.867	0.014742939
EMC3	6.36E-17	-2.957295549	0.812	1	1.10E-12
ENSSSCG00000060068	8.27E-07	-2.958179824	0.125	0.933	0.014348589
RPS16	1.41E-09	-2.962322797	0.938	1	2.45E-05
ENSSSCG00000003930	2.38E-12	-2.964510915	0.938	1	4.13E-08
ACAD9	2.27E-25	-2.965234582	0.812	1	3.94E-21
AP1M2	1.44E-22	-2.966503088	0.812	1	2.50E-18
ENSSSCG00000034019	6.27E-17	-2.968973104	1	1	1.09E-12
GRIN3B	2.90E-11	-2.970426885	0.562	0.933	5.03E-07
MRGBP	7.61E-10	-2.975752454	0.438	0.8	1.32E-05
NUDC	9.59E-29	-2.976106794	0.938	1	1.66E-24
SLC25A38	9.15E-26	-2.978279803	0.875	1	1.59E-21

SAL1	1.21E-09	-2.982926487	0.562	1	2.10E-05
RPS29	2.25E-10	-2.984960601	0.75	1	3.90E-06
TMEM134	2.99E-07	-2.9861942	0.188	0.867	0.005190446
SPATA19	3.48E-07	-2.9861942	0.375	0.733	0.006034942
HSD17B10	3.53E-14	-2.988772745	0.812	0.933	6.13E-10
SLC6A12	2.19E-07	-2.989273593	0.25	0.8	0.003792175
ENSSSCG00000027573	1.70E-14	-2.993113614	0.875	1	2.95E-10
SUGP1	9.41E-10	-3	0.5	0.933	1.63E-05
SDS	7.48E-07	-3	0.188	0.8	0.01298216
ENSSSCG00000053208	1.97E-06	-3	0.312	0.733	0.03417565
LRRC47	3.27E-16	-3.007777351	0.625	1	5.67E-12
ACOT13	2.98E-08	-3.010647244	0.375	0.867	0.000517156
YIF1A	1.07E-07	-3.010647244	0.5	0.867	0.001863935
ATP5ME	1.44E-11	-3.013674937	0.938	1	2.49E-07
NUDT18	1.07E-06	-3.013674937	0.312	0.733	0.018549614
ENSSSCG00000031249	3.91E-18	-3.018204595	0.812	1	6.78E-14
TMED3	2.50E-20	-3.020647151	0.625	1	4.34E-16
VTI1B	6.98E-70	-3.022510367	1	1	1.21E-65
COPZ1	3.32E-47	-3.028014376	1	1	5.76E-43
ENSSSCG00000052480	1.49E-06	-3.03170886	0.25	0.8	0.0259386
ARG2	3.06E-27	-3.033608393	0.875	1	5.31E-23
ARR3	6.35E-12	-3.033730857	0.625	0.933	1.10E-07
ENSSSCG00000046487	1.49E-18	-3.034765418	0.75	1	2.59E-14
EIF3F	3.66E-10	-3.036525876	0.312	0.933	6.35E-06
LRPAP1	8.61E-15	-3.040641984	0.5	0.933	1.49E-10
TRPT1	6.65E-08	-3.040641984	0.188	0.867	0.001154201
ENSSSCG00000040146	1.42E-06	-3.044199804	0.438	0.933	0.02470248
PAIP2	2.53E-29	-3.047305715	1	1	4.40E-25
MFSD12	3.70E-10	-3.047305715	0.625	0.867	6.42E-06
REN	2.56E-06	-3.047305715	0.188	0.8	0.044487341
PDZD11	7.66E-26	-3.053363226	0.938	1	1.33E-21
LSM10	6.93E-35	-3.055742552	1	1	1.20E-30
TPT1	2.62E-22	-3.057143907	0.875	1	4.55E-18
RAB4B	8.69E-14	-3.058005603	0.75	1	1.51E-09
STYXL1	6.49E-43	-3.058661912	0.938	1	1.13E-38
ENSSSCG00000033019	1.06E-09	-3.061400545	0.812	1	1.84E-05
RPS4X	1.74E-15	-3.062348377	1	1	3.02E-11
ENSSSCG00000017955	1.33E-21	-3.064780681	0.688	1	2.31E-17
ACTR1B	5.30E-13	-3.067114196	0.562	1	9.19E-09
ENSSSCG00000039612	1.80E-10	-3.068306013	0.562	1	3.13E-06
ADI1	8.36E-08	-3.070389328	0.312	0.867	0.001450794
CCDC124	7.78E-12	-3.074000581	0.625	0.933	1.35E-07
C15orf40	7.17E-10	-3.074962058	0.375	0.867	1.24E-05
ENSSSCG00000012100	9.90E-15	-3.075995384	0.688	1	1.72E-10
ZNF821	1.09E-19	-3.080170349	0.812	1	1.89E-15
DAD1	1.93E-30	-3.080472579	0.938	1	3.34E-26
ENSSSCG00000008016	6.55E-11	-3.081036572	0.438	1	1.14E-06
CIB2	7.21E-11	-3.081036572	0.375	0.933	1.25E-06
CCDC32	1.63E-21	-3.082248412	0.75	0.933	2.83E-17
ENSSSCG00000032916	1.84E-33	-3.085020584	1	1	3.19E-29
ENSSSCG00000039523	3.75E-37	-3.090659313	1	1	6.51E-33
PCID2	9.28E-20	-3.093109404	0.812	1	1.61E-15
UBXN1	1.19E-08	-3.093109404	0.312	0.733	0.000205971
NANS	9.31E-07	-3.093109404	0.5	0.733	0.016158842
ENSSSCG00000025673	6.86E-23	-3.097667686	0.875	1	1.19E-18
TSPAN31	6.10E-26	-3.098110085	0.812	1	1.06E-21
TDRD10	1.45E-24	-3.101440679	0.938	1	2.52E-20
SCNM1	9.75E-22	-3.102173487	1	1	1.69E-17
ENSSSCG00000053939	2.51E-20	-3.103678646	0.875	1	4.35E-16
PTPMT1	3.09E-26	-3.107815903	0.812	1	5.37E-22
HSBP1	2.19E-50	-3.109274224	1	1	3.80E-46
U2AF1L4	7.50E-09	-3.113008962	0.375	0.933	0.000130079
ENSSSCG00000009610	4.17E-36	-3.116655702	1	1	7.23E-32
ACTL7B	3.83E-10	-3.121306296	0.375	0.933	6.65E-06
COX14	5.33E-15	-3.124587636	0.688	1	9.25E-11
TMEM86A	1.20E-06	-3.126124462	0.312	0.933	0.020824999

ASIP	5.61E-07	-3.128733314	0.25	0.733	0.009738643
PSENEN	3.53E-15	-3.135995217	0.875	1	6.13E-11
CSTB	1.25E-13	-3.137503524	0.75	0.933	2.17E-09
DRC7	1.53E-12	-3.137503524	0.438	1	2.65E-08
ENSSSCG00000048663	1.37E-06	-3.137503524	0.188	0.8	0.023812538
CRHR1	1.60E-17	-3.137870855	0.938	1	2.78E-13
CFAP276	6.76E-09	-3.143735477	0.25	0.933	0.000117337
CDIPT	4.47E-38	-3.147741853	1	1	7.75E-34
ENSSSCG00000002963	3.16E-32	-3.149940627	0.938	1	5.48E-28
RPS17	8.76E-12	-3.150011796	1	1	1.52E-07
GSTZ1	3.10E-11	-3.152003093	0.375	1	5.39E-07
INSL6	8.26E-08	-3.152003093	0.375	0.8	0.00143348
PIN1	1.81E-07	-3.152003093	0.25	0.867	0.003140481
ENSSSCG00000037510	1.35E-22	-3.152890633	0.938	1	2.34E-18
ENSSSCG00000056716	9.23E-28	-3.159198595	0.875	1	1.60E-23
ENSSSCG00000052563	1.23E-08	-3.159198595	0.25	0.867	0.000213917
FDPS	4.57E-22	-3.160032957	0.875	1	7.92E-18
ENSSSCG00000038035	1.13E-27	-3.163058282	0.938	1	1.97E-23
KAT5	3.23E-11	-3.166358386	0.5	0.867	5.61E-07
ERCC1	1.47E-12	-3.169925001	0.438	1	2.55E-08
SIVA1	2.93E-11	-3.169925001	0.438	1	5.08E-07
TFDP2	2.75E-84	-3.177096336	1	1	4.78E-80
NENF	2.38E-10	-3.180572246	0.188	0.933	4.13E-06
RND2	4.14E-09	-3.180572246	0.25	0.933	7.18E-05
ANTKMT	1.19E-08	-3.180572246	0.188	0.933	0.000205969
MFGE8	8.26E-07	-3.180572246	0.188	0.733	0.014334346
C11orf98	3.41E-42	-3.182719114	1	1	5.92E-38
POP4	1.93E-35	-3.189970944	0.938	1	3.35E-31
CDH22	1.34E-11	-3.192645078	0.5	0.933	2.32E-07
ENSSSCG00000056115	5.21E-07	-3.192645078	0.25	0.667	0.009036211
DCAF11	2.24E-12	-3.194647431	0.75	0.933	3.88E-08
TUBGCP2	8.77E-16	-3.195724471	0.688	0.933	1.52E-11
MACROD1	6.13E-21	-3.202733896	0.75	1	1.06E-16
ENSSSCG00000056015	4.35E-15	-3.204140717	0.75	1	7.56E-11
ENSSSCG00000053975	1.22E-08	-3.208586622	0.25	0.933	0.000211856
FAAP100	7.40E-08	-3.208586622	0.25	0.733	0.001284215
PQBP1	1.52E-16	-3.21649182	0.75	1	2.63E-12
MRPS25	1.38E-37	-3.21897236	1	1	2.40E-33
EPHX1	1.72E-19	-3.222392421	0.688	0.933	2.99E-15
SDHAF1	2.14E-13	-3.228268988	0.375	1	3.71E-09
TSR3	4.82E-07	-3.230612928	0.25	0.8	0.00836956
ASPDH	4.99E-08	-3.236067358	0.312	0.8	0.000866098
NDUFA9	9.69E-35	-3.241753486	1	1	1.68E-30
ENSSSCG00000034853	9.52E-13	-3.242856524	0.625	1	1.65E-08
ENSSSCG00000041579	1.11E-24	-3.245833665	0.875	1	1.93E-20
SMPD2	4.85E-15	-3.254101281	0.75	0.933	8.41E-11
ENSSSCG00000010056	1.26E-26	-3.254997087	1	1	2.18E-22
EIF6	6.33E-30	-3.256408929	0.875	1	1.10E-25
ENSSSCG00000052671	1.23E-12	-3.261271797	0.938	1	2.14E-08
KLF16	3.15E-07	-3.263034406	0.25	0.733	0.005461918
MRPL36	5.17E-22	-3.268648012	0.75	1	8.96E-18
RPS25	1.13E-21	-3.271284293	1	1	1.97E-17
CNIH4	9.73E-58	-3.272066477	1	1	1.69E-53
SCRG1	2.85E-17	-3.273018494	0.688	0.933	4.94E-13
NPAS1	3.20E-11	-3.275312736	0.5	0.933	5.56E-07
TMEM126A	2.18E-25	-3.275471509	0.938	1	3.78E-21
ENSSSCG00000033744	1.56E-10	-3.27897595	0.312	0.933	2.70E-06
WDR5	1.74E-41	-3.285333073	0.875	1	3.03E-37
POLR3H	1.13E-34	-3.287064475	1	1	1.96E-30
ASGR2	4.65E-10	-3.294743266	0.312	0.867	8.07E-06
ENSSSCG00000054357	4.65E-07	-3.294743266	0.25	0.8	0.008070855
TMEM225B	3.22E-17	-3.296049463	0.75	0.933	5.58E-13
RPS21	7.39E-07	-3.296049463	0.625	0.867	0.012830063
TMEM179B	1.97E-15	-3.298223834	0.438	1	3.41E-11
RFXANK	9.87E-07	-3.298223834	0.125	0.867	0.017128917
RPP40	1.52E-21	-3.299363179	0.812	1	2.64E-17

MIF	8.42E-13	-3.300704824	0.625	1	1.46E-08
ENSSSCG00000026248	3.72E-11	-3.30256277	0.562	0.8	6.46E-07
PRSS2	8.43E-20	-3.304854582	0.812	1	1.46E-15
ENSSSCG00000057874	1.77E-34	-3.306103128	1	1	3.08E-30
CDT1	3.01E-08	-3.306103128	0.25	0.867	0.000522439
ENSSSCG00000052576	1.74E-10	-3.308122295	0.375	0.867	3.02E-06
PSMB7	4.23E-38	-3.308349922	1	1	7.35E-34
LMO2	1.01E-12	-3.310340121	0.375	1	1.75E-08
MYBPC3	1.19E-09	-3.310340121	0.375	0.867	2.06E-05
ENSSSCG00000035909	2.97E-40	-3.31177874	0.938	1	5.15E-36
NEDD8	1.95E-36	-3.312857104	1	1	3.38E-32
SNRNP25	1.92E-13	-3.315501826	0.438	0.933	3.33E-09
ITGB5	1.14E-29	-3.323575948	0.938	1	1.97E-25
HSF4	1.84E-10	-3.325770161	0.438	0.8	3.20E-06
ENSSSCG00000024588	1.37E-41	-3.330148602	1	1	2.38E-37
NAXE	1.16E-27	-3.332180567	0.75	1	2.01E-23
PSMA6	7.79E-67	-3.33267553	1	1	1.35E-62
FETUB	2.57E-19	-3.333021925	0.688	1	4.45E-15
TNIP1	2.47E-09	-3.334117504	0.25	0.867	4.28E-05
SKIC8	9.03E-58	-3.334381207	0.938	1	1.57E-53
CTSF	4.80E-13	-3.335184192	0.375	0.933	8.33E-09
CLPP	2.11E-21	-3.338865819	0.688	1	3.66E-17
SRM	2.14E-14	-3.341036918	0.625	0.933	3.72E-10
HYAL3	1.75E-06	-3.341036918	0.188	0.733	0.030282698
C1orf105	3.62E-27	-3.343846451	0.875	1	6.27E-23
TCEANC	2.27E-08	-3.351843673	0.25	0.733	0.000393546
ENSSSCG00000063358	3.10E-08	-3.35614381	0.125	0.867	0.000537218
TIMM22	1.85E-41	-3.358127286	0.938	1	3.20E-37
MRPL37	2.25E-50	-3.359639762	1	1	3.90E-46
ENSSSCG00000013064	1.65E-28	-3.364342109	0.938	1	2.86E-24
SURF2	1.29E-07	-3.366127899	0.188	0.733	0.002230672
TUBB4B	7.57E-19	-3.375663261	0.625	1	1.31E-14
RPL23	6.56E-09	-3.379266368	0.812	1	0.000113782
PDLIM2	1.11E-11	-3.385891154	0.312	0.933	1.92E-07
ARSL	6.84E-08	-3.385891154	0.25	0.8	0.001186894
PMVK	4.39E-07	-3.385891154	0.125	0.8	0.007621551
ZP4	6.97E-45	-3.388611949	1	1	1.21E-40
SPATA24	4.34E-08	-3.394278939	0.188	0.733	0.000753104
ENSSSCG00000052310	3.13E-22	-3.395408693	0.812	1	5.44E-18
PLD6	2.15E-08	-3.396890153	0.25	0.867	0.00037365
DCTN3	6.64E-22	-3.398917834	0.938	1	1.15E-17
ARL2	9.27E-11	-3.401862111	0.25	0.933	1.61E-06
DPCD	3.32E-27	-3.404832717	1	1	5.76E-23
SHFL	3.86E-09	-3.415037499	0.25	0.867	6.70E-05
ENSSSCG00000048122	2.75E-08	-3.415037499	0.188	0.867	0.00047779
HSD17B2	1.95E-06	-3.415037499	0.125	0.8	0.033765532
ENSSSCG00000032129	8.37E-36	-3.418551983	1	1	1.45E-31
NDUFAB1	3.42E-11	-3.428093652	0.625	0.933	5.93E-07
LENG1	2.84E-15	-3.431911318	0.5	1	4.93E-11
UQCRQ	6.43E-31	-3.437593624	1	1	1.12E-26
TMSB10	5.15E-07	-3.440877504	0.812	1	0.008934754
DPP7	3.39E-10	-3.446746359	0.312	0.867	5.89E-06
RNF31	2.21E-16	-3.449802917	0.688	1	3.84E-12
EIPR1	1.92E-16	-3.450661409	0.562	1	3.34E-12
IGFBP6	3.90E-11	-3.450661409	0.375	0.933	6.77E-07
BCKDHA	2.40E-25	-3.452259963	0.938	1	4.17E-21
ATP5F1D	4.43E-11	-3.462343214	0.375	0.867	7.69E-07
DCXR	1.03E-07	-3.462343214	0.25	0.667	0.001784623
DPPA5	2.51E-37	-3.463134425	1	1	4.35E-33
RPL36	8.79E-21	-3.4639471	0.75	1	1.53E-16
VKORC1	3.53E-30	-3.464926375	0.75	1	6.12E-26
LUC7L	5.63E-11	-3.468148836	0.312	0.933	9.76E-07
ENSSSCG00000039365	1.40E-07	-3.471621028	0.25	0.667	0.002424684
FKBP2	4.19E-07	-3.471621028	0.188	0.667	0.007265236
ENSSSCG00000033451	5.26E-07	-3.471621028	0.188	0.733	0.009125773
MKS1	6.15E-18	-3.474303449	0.625	1	1.07E-13

KLK15	7.81E-09	-3.475579041	0.125	0.867	0.000135582
ENSSSCG00000053447	9.85E-16	-3.481806511	0.438	1	1.71E-11
ENSSSCG00000044091	3.60E-07	-3.485426827	0.125	0.8	0.006239949
GSDMB	2.93E-46	-3.485642171	1	1	5.09E-42
NDUFC2	1.02E-16	-3.492640317	0.625	1	1.78E-12
ENSSSCG00000028414	1.19E-40	-3.493621187	1	1	2.06E-36
ENSSSCG00000058091	6.23E-21	-3.494359952	0.812	1	1.08E-16
PDIA2	5.09E-09	-3.499101764	0.25	0.867	8.84E-05
NUBP2	7.95E-10	-3.504042505	0.375	0.867	1.38E-05
FTL	4.04E-39	-3.506178543	1	1	7.02E-35
THOP1	2.45E-08	-3.508146904	0.312	0.867	0.000425137
UQCR10	3.67E-23	-3.520970944	0.875	1	6.37E-19
CYTH2	4.56E-32	-3.523840711	1	1	7.91E-28
UQCC2	1.28E-17	-3.526068812	0.5	1	2.22E-13
ENSSSCG00000033926	2.81E-15	-3.532221039	0.438	1	4.88E-11
RPL10A	2.38E-18	-3.538586852	0.938	1	4.13E-14
COX6A1	3.40E-26	-3.538668848	0.875	1	5.90E-22
CNPY2	1.18E-37	-3.541854344	1	1	2.05E-33
IMP4	5.22E-28	-3.545369368	0.812	1	9.06E-24
IDH3G	2.03E-18	-3.552541023	0.75	1	3.52E-14
MRPS18A	3.59E-08	-3.552541023	0.188	0.8	0.000622699
RGS11	4.35E-07	-3.552541023	0.188	0.733	0.007553073
NPDC1	2.01E-06	-3.552541023	0.125	0.733	0.034958267
EIF3G	8.46E-13	-3.557481764	0.688	1	1.47E-08
ENSSSCG00000021624	4.85E-17	-3.560467939	0.625	1	8.41E-13
CUL7	8.33E-32	-3.56125824	0.938	1	1.45E-27
RPS18	1.10E-26	-3.563041882	1	1	1.90E-22
RPL7	3.38E-41	-3.57740515	1	1	5.87E-37
NDUFA10	5.47E-25	-3.57797607	0.688	1	9.50E-21
ENSSSCG00000004703	7.99E-21	-3.589785373	1	1	1.39E-16
HYI	2.14E-10	-3.595609745	0.25	0.933	3.71E-06
C19orf53	1.02E-18	-3.597072183	0.562	1	1.78E-14
SAT2	4.96E-27	-3.600904045	0.875	1	8.60E-23
ENSSSCG00000053801	5.31E-09	-3.606178987	0.312	0.8	9.22E-05
ENSSSCG00000030849	4.67E-18	-3.612794188	1	1	8.10E-14
SIRT5	4.23E-13	-3.621488377	0.438	0.933	7.33E-09
TBL3	9.08E-11	-3.623624121	0.188	0.933	1.58E-06
ENSSSCG00000018046	2.00E-24	-3.623946691	0.875	1	3.47E-20
CCDC28B	2.65E-09	-3.629162305	0.25	0.867	4.60E-05
CHMP1A	7.01E-09	-3.629162305	0.125	0.867	0.000121637
ENSSSCG00000050296	6.34E-08	-3.629162305	0.125	0.867	0.001099562
ENSSSCG00000024070	2.86E-48	-3.633975668	0.938	1	4.95E-44
BCS1L	1.77E-13	-3.635636639	0.562	1	3.07E-09
DPEP1	9.44E-18	-3.638245925	0.812	1	1.64E-13
PIGP	2.71E-53	-3.652805147	1	1	4.70E-49
ERP29	3.49E-22	-3.653824359	0.812	1	6.06E-18
CDH6	4.86E-19	-3.655351829	0.562	0.933	8.44E-15
DNPH1	6.90E-11	-3.660793914	0.25	0.933	1.20E-06
SYT2	2.62E-22	-3.671377253	0.625	1	4.55E-18
ENSSSCG00000060915	6.42E-20	-3.678071905	0.812	1	1.11E-15
ENSSSCG00000022295	8.48E-07	-3.678071905	0.062	0.8	0.014719965
TIMM17B	5.42E-29	-3.693022247	0.812	1	9.41E-25
CCDC137	3.66E-12	-3.693022247	0.25	0.933	6.35E-08
COX4I1	3.08E-30	-3.69743723	1	1	5.34E-26
ZBTB48	1.48E-14	-3.699133521	0.375	0.933	2.56E-10
EEF1D	2.42E-23	-3.703351314	0.875	1	4.21E-19
DTYMK	1.37E-20	-3.703973769	0.625	1	2.38E-16
SPOUT1	3.82E-11	-3.707819249	0.312	0.933	6.63E-07
PPIB	1.49E-34	-3.708211765	0.938	1	2.58E-30
MAPK13	7.63E-24	-3.710204526	0.688	1	1.32E-19
EMC6	5.65E-51	-3.724084303	1	1	9.81E-47
MBD3	1.29E-10	-3.728697978	0.25	0.867	2.24E-06
TLE2	2.66E-50	-3.733296946	1	1	4.62E-46
ENSSSCG00000014569	2.39E-17	-3.73392514	0.875	1	4.15E-13
DEGS2	3.75E-29	-3.737548384	0.875	1	6.51E-25
RPL7L1	6.73E-57	-3.737951715	1	1	1.17E-52

ENSSSCG00000008170	1.10E-17	-3.742034964	0.938	1	1.91E-13
NR2C2AP	5.52E-41	-3.742923049	0.938	1	9.58E-37
ENSSSCG00000035007	8.18E-15	-3.748106152	1	1	1.42E-10
ENSSSCG00000005528	4.73E-42	-3.751728947	1	1	8.21E-38
ENSSSCG00000038475	6.36E-20	-3.756074417	0.375	1	1.10E-15
QPRT	3.21E-08	-3.756074417	0.062	0.867	0.000557806
TM4SF5	9.21E-08	-3.756074417	0.125	0.867	0.00159808
TMEM156	8.33E-09	-3.761487913	0.25	0.733	0.000144606
RPS15A	1.58E-19	-3.761619696	0.875	1	2.74E-15
AIMP2	1.20E-17	-3.779609932	0.5	1	2.07E-13
RPLP1	1.31E-17	-3.788134272	1	1	2.27E-13
FBL	4.19E-15	-3.793549123	0.375	1	7.27E-11
TNK1	8.24E-12	-3.793549123	0.188	1	1.43E-07
GDF3	3.85E-10	-3.793549123	0.5	0.8	6.67E-06
TUT1	2.54E-09	-3.793549123	0.188	0.867	4.42E-05
FAM98C	4.25E-07	-3.793549123	0.125	0.8	0.007376677
CASQ2	3.59E-19	-3.796716402	0.625	1	6.22E-15
TMEM11	1.24E-11	-3.802060622	0.312	0.933	2.15E-07
UTS2	1.49E-10	-3.804604311	0.188	0.933	2.58E-06
COMMD9	9.11E-28	-3.814644958	0.812	1	1.58E-23
MRPL40	4.88E-41	-3.820266461	1	1	8.46E-37
NPEPL1	9.80E-09	-3.821029859	0.125	0.8	0.000170093
SNX22	8.80E-27	-3.823502345	0.75	1	1.53E-22
WDR54	2.50E-17	-3.832957507	0.438	1	4.33E-13
LALBA	2.56E-28	-3.833141302	0.812	1	4.44E-24
MRPS15	2.47E-32	-3.836501268	0.938	1	4.28E-28
CDC26	2.70E-44	-3.839590794	1	1	4.68E-40
OXLD1	2.09E-09	-3.847996907	0.125	0.867	3.63E-05
ENSSSCG00000049957	1.07E-07	-3.847996907	0.062	0.733	0.001851898
RPS11	5.46E-15	-3.850539101	0.938	1	9.47E-11
TPI1	5.88E-32	-3.851403508	0.875	1	1.02E-27
ENSSSCG00000002995	1.28E-15	-3.865698908	0.625	1	2.23E-11
ENSSSCG00000032073	3.28E-08	-3.874469118	0.125	0.8	0.000568608
LAMTOR2	9.49E-24	-3.888045067	0.75	1	1.65E-19
NDUFB7	1.63E-10	-3.900464326	0.25	0.867	2.82E-06
B3GNTL1	4.18E-15	-3.90902634	0.375	0.933	7.25E-11
RPL35	2.46E-19	-3.910732662	0.625	1	4.27E-15
ENSSSCG00000052096	7.38E-12	-3.915111102	0.312	0.933	1.28E-07
RPS19	7.58E-17	-3.917776875	0.875	1	1.31E-12
RPS12	1.06E-14	-3.920321745	0.875	1	1.84E-10
RPS10	1.76E-23	-3.92372842	0.938	1	3.06E-19
ENSSSCG00000050177	2.46E-07	-3.933212909	0.562	0.867	0.004276993
ENSSSCG00000015337	3.76E-32	-3.937715722	0.875	1	6.52E-28
NACC2	2.22E-10	-3.941106311	0.188	0.8	3.86E-06
FAU	1.76E-17	-3.943965965	0.5	1	3.05E-13
LYRM1	2.70E-13	-3.943965965	0.312	0.867	4.68E-09
NDUFB8	7.29E-41	-3.946675592	1	1	1.26E-36
ENSSSCG00000000905	1.84E-31	-3.9492467	1	1	3.19E-27
RPL34	8.78E-16	-3.9510904	0.312	1	1.52E-11
HCRT	4.93E-18	-3.95491211	0.438	1	8.56E-14
RPL6	1.38E-51	-3.963033674	1	1	2.39E-47
ENSSSCG00000027538	4.31E-23	-3.969626351	0.625	1	7.48E-19
CDK10	1.53E-15	-3.973527789	0.375	0.933	2.66E-11
GSTK1	5.21E-16	-3.977632187	0.5	0.933	9.04E-12
CDK5RAP3	2.97E-25	-3.986672468	0.625	1	5.14E-21
RHOC	7.04E-38	-3.987706724	0.875	1	1.22E-33
LGI1	2.64E-12	-3.987927168	0.25	0.867	4.59E-08
GUSB	1.82E-06	-3.987927168	0.188	0.733	0.031602718
CHID1	1.50E-11	-3.989273593	0.375	0.867	2.60E-07
STX8	1.41E-70	-3.993366908	1	1	2.44E-66
RPS19BP1	8.70E-08	-4	0.125	0.8	0.001510242
ENSSSCG00000059030	1.65E-06	-4	0.062	0.733	0.028547392
ENSSSCG00000021991	2.03E-12	-4.013674937	0.312	0.867	3.52E-08
TAGLN	9.42E-21	-4.017921908	0.562	0.933	1.63E-16
ENSSSCG00000036716	8.69E-20	-4.038732394	0.75	1	1.51E-15
ITGB7	6.72E-07	-4.047305715	0.062	0.8	0.011655077

C15orf48	6.51E-17	-4.062735755	0.375	1	1.13E-12
NDUFA2	4.44E-11	-4.072931522	0.438	1	7.70E-07
SDF4	5.43E-16	-4.074962058	0.438	1	9.42E-12
SYCN	2.98E-34	-4.093109404	0.812	1	5.16E-30
ENSSSCG00000058768	1.61E-09	-4.093109404	0.188	0.733	2.79E-05
LMF1	8.90E-20	-4.099108145	0.562	1	1.54E-15
SLC25A1	9.77E-11	-4.108059746	0.25	0.867	1.70E-06
C6orf52	1.10E-54	-4.108141464	1	1	1.91E-50
NUDT16L1	3.53E-14	-4.113008962	0.375	0.867	6.12E-10
TMEM147	1.49E-23	-4.119909464	0.562	1	2.58E-19
RPS13	2.62E-15	-4.119909464	0.938	1	4.55E-11
CRYBB3	2.15E-15	-4.121015401	0.5	0.933	3.73E-11
TALDO1	9.07E-27	-4.133855747	0.688	1	1.57E-22
NUDT5	9.53E-66	-4.13922368	1	1	1.65E-61
ENSSSCG00000013386	7.35E-63	-4.143414486	0.938	1	1.28E-58
ETFB	2.71E-17	-4.154220919	0.375	1	4.71E-13
ENSSSCG00000002036	1.51E-55	-4.154829138	1	1	2.62E-51
RPL18A	5.73E-16	-4.178449074	0.562	1	9.94E-12
AVPI1	8.33E-16	-4.180572246	0.438	0.867	1.45E-11
ENSSSCG00000025928	2.44E-15	-4.180572246	0.562	1	4.23E-11
UPP1	1.69E-09	-4.180572246	0.188	0.667	2.93E-05
CHMP6	1.89E-08	-4.180572246	0.125	0.667	0.00032737
MGMT	2.30E-29	-4.186037006	0.75	1	3.99E-25
MRPL30	9.01E-33	-4.191843358	0.688	1	1.56E-28
MRPL55	1.20E-27	-4.193090285	0.875	1	2.08E-23
CEP131	5.88E-11	-4.201633861	0.188	0.933	1.02E-06
RPS9	1.89E-23	-4.202678914	0.938	1	3.27E-19
H2AJ	1.14E-15	-4.20461772	0.312	0.933	1.98E-11
COX5B	1.81E-40	-4.20701957	1	1	3.15E-36
ENSSSCG00000043578	1.66E-08	-4.208586622	0.25	0.8	0.000287211
MRPS11	9.65E-15	-4.21649182	0.25	1	1.68E-10
DVL1	9.03E-08	-4.222392421	0.062	0.8	0.001567021
C19orf81	6.24E-12	-4.230612928	0.188	0.8	1.08E-07
ENSSSCG00000014133	1.12E-18	-4.232527869	0.812	1	1.95E-14
BCAP31	6.92E-53	-4.23299078	0.875	1	1.20E-48
PARK7	2.84E-50	-4.237632299	1	1	4.92E-46
MIB2	3.65E-17	-4.24838763	0.25	1	6.33E-13
SCGB3A2	8.09E-13	-4.263034406	0.125	0.933	1.40E-08
SLC25A19	1.92E-12	-4.263034406	0.312	0.8	3.32E-08
LAMTOR1	3.98E-11	-4.263034406	0.125	1	6.91E-07
ENSSSCG00000017971	7.07E-21	-4.284250892	0.5	1	1.23E-16
ZAR1	1.21E-60	-4.289123635	0.938	1	2.10E-56
CZIB	4.76E-26	-4.302042786	0.812	1	8.26E-22
TMEM120A	1.24E-07	-4.30256277	0.062	0.733	0.002159744
ENSSSCG00000012088	2.78E-17	-4.310340121	0.562	0.867	4.83E-13
FIBP	4.95E-23	-4.315501826	0.312	1	8.58E-19
LY6G6D	3.66E-07	-4.341036918	0.062	0.733	0.006347928
ENSSSCG00000052759	1.22E-15	-4.351843673	0.188	1	2.12E-11
MT3	1.82E-14	-4.362570079	0.125	0.933	3.15E-10
CUTA	8.91E-24	-4.365132593	0.562	1	1.55E-19
FIGLA	1.24E-31	-4.372532771	0.75	1	2.16E-27
ENSSSCG00000001696	1.25E-31	-4.375425643	0.688	1	2.16E-27
PATE1	5.67E-08	-4.378511623	0.062	0.8	0.000983854
GPX4	8.51E-30	-4.381507876	0.688	1	1.48E-25
ENSSSCG00000017509	1.20E-29	-4.387588775	0.938	1	2.08E-25
GFER	1.60E-23	-4.389042291	0.375	1	2.78E-19
USE1	3.82E-14	-4.394278939	0.375	0.933	6.64E-10
EMG1	8.56E-28	-4.410522018	0.688	1	1.48E-23
GALK1	1.00E-22	-4.415037499	0.625	0.933	1.74E-18
COPS9	1.20E-25	-4.421580345	0.625	1	2.07E-21
TMEM219	4.76E-32	-4.427010141	0.625	1	8.27E-28
GSTM3	1.04E-86	-4.433006734	1	1	1.80E-82
RPS5	2.64E-29	-4.450661409	0.938	1	4.59E-25
ELOB	1.00E-19	-4.450661409	0.375	1	1.74E-15
SORD	2.08E-11	-4.450661409	0.188	0.8	3.61E-07
UXT	6.01E-28	-4.46634959	0.812	1	1.04E-23

ENSSSCG00000037399	8.20E-11	-4.468148836	0.188	0.867	1.42E-06
COX7A1	2.60E-17	-4.473931188	0.25	1	4.50E-13
NDUFB11	5.33E-25	-4.478540442	0.562	1	9.25E-21
FAM3A	8.03E-11	-4.485426827	0.125	0.867	1.39E-06
ENSSSCG00000040854	9.01E-29	-4.499101764	0.688	1	1.56E-24
TK1	7.53E-35	-4.49966875	0.688	1	1.31E-30
RPL26	8.55E-14	-4.500073603	0.312	1	1.48E-09
ENSSSCG00000058502	1.19E-17	-4.502500341	0.25	1	2.06E-13
NDUFA13	2.68E-26	-4.503805356	0.625	1	4.64E-22
MYL6	3.60E-36	-4.504923003	0.812	1	6.25E-32
AIP	2.95E-17	-4.512648296	0.25	1	5.12E-13
ENSSSCG00000045485	1.46E-25	-4.54092194	0.562	1	2.53E-21
ENSSSCG00000059584	4.39E-14	-4.546567122	0.375	0.933	7.62E-10
ATOX1	4.61E-22	-4.552541023	0.562	1	8.00E-18
NUDT14	6.94E-12	-4.552541023	0.188	0.933	1.20E-07
TIMM10	1.85E-14	-4.561878888	0.188	0.933	3.22E-10
PGLS	2.24E-12	-4.565597176	0.25	0.867	3.89E-08
EPHB6	1.45E-18	-4.568842835	0.25	1	2.52E-14
C19orf38	3.64E-24	-4.584962501	0.375	1	6.31E-20
ENSSSCG00000049122	2.65E-08	-4.584962501	0.062	0.8	0.000460665
ENSSSCG00000061183	1.64E-10	-4.595609745	0.125	0.8	2.85E-06
ENSSSCG00000062187	2.20E-09	-4.595609745	0.125	0.8	3.83E-05
SPATA16	1.12E-14	-4.606178987	0.25	1	1.95E-10
ENSSSCG00000024974	1.04E-22	-4.60968493	0.312	1	1.80E-18
RPS14	8.58E-25	-4.619282574	0.688	1	1.49E-20
ENSSSCG00000009303	2.84E-35	-4.620378184	0.938	1	4.92E-31
DMAP1	1.58E-20	-4.627087976	0.25	1	2.74E-16
ENSSSCG00000035997	1.24E-19	-4.636043713	0.875	1	2.16E-15
RABAC1	7.54E-32	-4.64200265	0.75	1	1.31E-27
ADCK5	1.47E-19	-4.650486073	0.375	0.933	2.55E-15
COMMD4	1.11E-22	-4.653824359	0.562	0.933	1.93E-18
ZNHIT1	4.49E-15	-4.653824359	0.188	0.867	7.79E-11
ATP5PF	1.34E-17	-4.656045599	0.875	1	2.33E-13
TUBA1B	6.94E-49	-4.676940823	1	1	1.20E-44
NAPRT	9.33E-12	-4.693022247	0.062	0.867	1.62E-07
UQCR11	3.54E-13	-4.701918647	0.125	0.8	6.14E-09
ENSSSCG00000048076	7.84E-53	-4.707819249	0.875	1	1.36E-48
SPG7	8.35E-10	-4.707819249	0.062	0.867	1.45E-05
ATP6V1F	5.53E-18	-4.712019237	0.312	0.933	9.59E-14
ENSSSCG00000007646	1.13E-16	-4.712019237	0.25	1	1.96E-12
NDUFV1	4.10E-17	-4.714347292	0.188	1	7.11E-13
NDUFB9	3.76E-47	-4.719499041	1	1	6.52E-43
SMIM26	1.90E-30	-4.730160416	0.375	1	3.30E-26
MIEN1	3.05E-26	-4.732837709	0.562	1	5.30E-22
DUS1L	4.09E-17	-4.736965594	0.25	0.933	7.09E-13
RPS15	5.49E-17	-4.754174884	0.625	1	9.52E-13
TNNT1	5.42E-35	-4.763357093	0.625	1	9.41E-31
PET100	2.36E-20	-4.773229138	0.375	1	4.09E-16
SHD	6.93E-31	-4.824428435	0.5	1	1.20E-26
STAT5A	1.42E-59	-4.833612988	0.812	1	2.46E-55
ENSSSCG00000028167	5.41E-37	-4.862881144	0.812	1	9.38E-33
SPC24	8.57E-78	-4.870920051	0.938	1	1.49E-73
COX7A2	2.14E-39	-4.894811864	1	1	3.72E-35
ENSSSCG00000010791	8.00E-07	-4.900464326	0	0.8	0.013888609
MRPL28	4.09E-31	-4.910732662	0.625	1	7.09E-27
POLR2F	1.73E-34	-4.927685795	0.625	1	3.00E-30
MALSU1	2.42E-22	-4.973527789	0.438	1	4.19E-18
PAFAH1B3	1.03E-09	-4.975752454	0.062	0.733	1.79E-05
RPL23A	9.12E-49	-4.981852653	0.875	1	1.58E-44
POLR2I	1.65E-16	-4.987927168	0.188	1	2.86E-12
RPS26	7.27E-13	-4.990810829	0.375	1	1.26E-08
LSM2	7.07E-55	-4.993866456	1	1	1.23E-50
MAMDC4	2.07E-14	-5	0.062	1	3.59E-10
ECRG4	6.96E-22	-5.006853671	0.312	1	1.21E-17
ENSSSCG00000044567	5.89E-71	-5.010647244	0.812	1	1.02E-66
SERF1A	2.81E-42	-5.027783157	0.75	1	4.87E-38

SRA1	8.53E-18	-5.028569152	0.25	0.867	1.48E-13
NDUFA7	3.74E-38	-5.034557222	0.875	1	6.49E-34
NDUFA11	1.46E-42	-5.048339534	0.875	1	2.53E-38
CD164L2	4.21E-07	-5.093109404	0	0.733	0.007300304
SPACA3	7.40E-17	-5.126532406	0.125	0.933	1.28E-12
FIS1	1.06E-20	-5.130198723	0.188	1	1.85E-16
ATP5IF1	6.91E-66	-5.134680245	0.875	1	1.20E-61
MICOS13	9.88E-24	-5.137503524	0.312	1	1.71E-19
WRAP53	3.59E-25	-5.162782932	0.312	0.933	6.22E-21
ENSSSCG00000033310	5.53E-23	-5.173856818	0.5	1	9.59E-19
GRTP1	1.25E-06	-5.180572246	0	0.667	0.021630765
GMFG	1.30E-06	-5.180572246	0	0.733	0.022583802
POLR2G	2.20E-63	-5.191775723	0.938	1	3.82E-59
JOSD2	7.34E-16	-5.201633861	0.125	0.933	1.27E-11
SAMD4A	4.16E-10	-5.208586622	0.125	0.933	7.22E-06
ENSSSCG00000049161	6.46E-11	-5.222392421	0.062	0.733	1.12E-06
HSD17B1	4.19E-08	-5.222392421	0	0.8	0.000727491
TPRKB	1.43E-22	-5.24961389	0.625	1	2.48E-18
AP2S1	6.45E-30	-5.299309792	0.562	1	1.12E-25
ATPAF2	1.52E-12	-5.30256277	0.062	0.867	2.65E-08
ENSSSCG00000023783	3.06E-17	-5.341036918	0.062	0.933	5.31E-13
ATP5MC1	5.83E-81	-5.399416519	0.938	1	1.01E-76
MVD	7.93E-09	-5.415037499	0	0.8	0.00013762
ENSSSCG00000061531	1.02E-13	-5.450661409	0.062	1	1.77E-09
ENSSSCG00000058049	9.54E-08	-5.450661409	0	0.733	0.001655271
ROMO1	3.84E-29	-5.458181991	0.438	1	6.66E-25
SF3B5	4.81E-08	-5.519374159	0	0.667	0.000834372
CHMP2A	4.78E-30	-5.568842835	0.438	1	8.29E-26
ENSSSCG00000038364	3.92E-15	-5.568842835	0.062	0.933	6.80E-11
DDT	1.52E-24	-5.571156701	0.25	1	2.64E-20
TMED1	1.87E-07	-5.61667136	0	0.733	0.003244314
ENSSSCG00000041052	4.14E-11	-5.647698256	0	0.933	7.19E-07
MORN3	1.14E-10	-5.678071905	0	0.933	1.98E-06
TRAPPC5	2.23E-10	-5.707819249	0	0.867	3.87E-06
RNASEH2C	3.72E-15	-5.751320887	0.062	0.933	6.45E-11
DERL3	2.70E-23	-5.758445322	0.188	1	4.69E-19
ENSSSCG00000061874	5.56E-25	-5.870993208	0.688	1	9.65E-21
EXOSC4	4.24E-23	-5.895302621	0.188	0.933	7.36E-19
ENSSSCG00000057536	5.93E-18	-5.938599455	0.062	0.933	1.03E-13
TMEM160	1.03E-12	-5.975752454	0	0.933	1.79E-08
ENSSSCG00000042862	1.17E-26	-5.993976212	0.75	0.933	2.02E-22
APRT	1.58E-18	-6	0.062	1	2.74E-14
ZP3	2.38E-83	-6.015396062	1	1	4.13E-79
ENSSSCG00000055438	1.14E-24	-6.045850652	0.625	1	1.98E-20
ENSSSCG00000032060	8.76E-39	-6.079688889	0.688	1	1.52E-34
RHOD	2.50E-06	-6.137503524	0	0.667	0.043342858
RNH1	4.12E-28	-6.180572246	0.062	1	7.15E-24
ENSSSCG00000044205	1.02E-09	-6.251538767	0.125	0.8	1.77E-05
ENSSSCG00000032166	1.75E-46	-6.263568638	0.812	1	3.04E-42
PEX16	2.86E-17	-6.273018494	0.062	0.867	4.96E-13
ENSSSCG00000012842	2.09E-26	-6.290817562	0.125	1	3.62E-22
RPL38	4.10E-26	-6.42103624	0.125	1	7.11E-22
CD320	1.76E-33	-6.590779827	0.375	1	3.05E-29
CCDC167	2.22E-44	-6.637898252	0.375	1	3.85E-40
TMEM125	3.74E-28	-7.070389328	0.062	1	6.49E-24
ENSSSCG00000061145	2.40E-15	-7.201633861	0	0.733	4.17E-11
PENK	7.19E-09	-7.415037499	0	0.8	0.000124726
H2BK1	2.14E-31	-7.584962501	0.125	1	3.72E-27

ID	Term	Ontology Source	Term P-Value	Term P-Value Corrected with Bonferroni step	Group P-Valu	Group P-Value Corrected with	GO Levels	GO Group	% Associated Genes	Numbe r of	Associated Genes Found
[illegible]	cytoplasmic translation	[illegible]	0.00	0.00	0.00	0.00	[5, 6, 7, 8]	Group00	29.76	25.00	[illegible]
[illegible]	mitochondrial cytochrome c oxidase assembly	[illegible]	0.00	0.03	0.00	0.00	[7, 8]	Group02	50.00	6.00	[illegible]
[illegible]	translation regulator activity	[illegible]	0.00	0.03	0.00	0.00	[6, 7, 8, 9, 10]	Group03	16.39	20.00	[illegible]
[illegible]	glycosyl compound metabolic process	[illegible]	0.00	0.03	0.00	0.00	[4]	Group04	23.53	12.00	[illegible]
[illegible]	cellular aldehyde metabolic process	[illegible]	0.00	0.03	0.00	0.00	[3]	Group05	22.41	13.00	[illegible]
[illegible]	metallo-sulfur cluster assembly	[illegible]	0.00	0.05	0.00	0.00	[5]	Group06	33.33	8.00	[illegible]
[illegible]	iron-sulfur cluster assembly	[illegible]	0.00	0.05	0.00	0.00	[6]	Group06	33.33	8.00	[illegible]
[illegible]	positive regulation of biological process	[illegible]	0.00	0.00	0.00	0.00	[1, 2, 3]	Group07	4.66	226.00	[illegible]
[illegible]	regulation of cellular process	[illegible]	0.00	0.00	0.00	0.00	[2, 3]	Group07	4.44	418.00	[illegible]
[illegible]	positive regulation of cellular process	[illegible]	0.00	0.00	0.00	0.00	[2, 3, 4]	Group07	4.58	203.00	[illegible]
[illegible]	cellular catabolic process	[illegible]	0.00	0.04	0.00	0.00	[3]	Group08	8.76	106.00	[illegible]
[illegible]	organic substance catabolic process	[illegible]	0.00	0.00	0.00	0.00	[3]	Group08	8.71	144.00	[illegible]
[illegible]	organonitrogen compound catabolic process	[illegible]	0.00	0.02	0.00	0.00	[4]	Group08	9.08	103.00	[illegible]
[illegible]	ribosomal large subunit biogenesis	[illegible]	0.00	0.00	0.00	0.00	[5, 6]	Group09	25.00	14.00	[illegible]
[illegible]	rRNA metabolic process	[illegible]	0.00	0.00	0.00	0.00	[7, 8]	Group09	20.00	39.00	[illegible]
[illegible]	rRNA processing	[illegible]	0.00	0.00	0.00	0.00	[6, 8, 9]	Group09	23.31	38.00	[illegible]
[illegible]	maturation of LSU-rRNA	[illegible]	0.00	0.01	0.00	0.00	[6, 7, 9, 10]	Group09	36.00	9.00	[illegible]
[illegible]	organelle organization	[illegible]	0.00	0.00	0.00	0.00	[4]	Group10	7.89	220.00	[illegible]
[illegible]	mitochondrion organization	[illegible]	0.00	0.00	0.00	0.00	[5]	Group10	18.87	70.00	[illegible]
[illegible]	NADH dehydrogenase complex assembly	[illegible]	0.00	0.00	0.00	0.00	[6]	Group10	44.23	23.00	[illegible]
[illegible]	mitochondrial respiratory chain complex assembly	[illegible]	0.00	0.00	0.00	0.00	[6]	Group10	42.67	32.00	[illegible]
[illegible]	mitochondrial respiratory chain complex I assembly	[illegible]	0.00	0.00	0.00	0.00	[7]	Group10	44.23	23.00	[illegible]
[illegible]	mitochondrial transport	[illegible]	0.00	0.01	0.00	0.00	[4]	Group11	17.19	22.00	[illegible]
[illegible]	mitochondrial membrane organization	[illegible]	0.00	0.00	0.00	0.00	[5, 6]	Group11	23.68	18.00	[illegible]
[illegible]	protein localization to mitochondrion	[illegible]	0.00	0.00	0.00	0.00	[6]	Group11	25.33	19.00	[illegible]
[illegible]	establishment of protein localization to mitochondrion	[illegible]	0.00	0.00	0.00	0.00	[5, 7]	Group11	27.14	19.00	[illegible]
[illegible]	protein targeting to mitochondrion	[illegible]	0.00	0.00	0.00	0.00	[5, 6, 7, 8]	Group11	31.03	18.00	[illegible]
[illegible]	inner mitochondrial membrane organization	[illegible]	0.00	0.00	0.00	0.00	[6, 7]	Group11	34.48	10.00	[illegible]
[illegible]	cellular component biogenesis	[illegible]	0.00	0.00	0.00	0.00	[3]	Group12	9.70	243.00	[illegible]
[illegible]	organelle organization	[illegible]	0.00	0.00	0.00	0.00	[4]	Group12	7.89	220.00	[illegible]
[illegible]	cellular component assembly	[illegible]	0.00	0.00	0.00	0.00	[4]	Group12	8.29	188.00	[illegible]
[illegible]	ribonucleoprotein complex biogenesis	[illegible]	0.00	0.00	0.00	0.00	[4]	Group12	21.24	79.00	[illegible]
[illegible]	protein-containing complex organization	[illegible]	0.00	0.00	0.00	0.00	[4]	Group12	8.56	150.00	[illegible]
[illegible]	mitochondrion organization	[illegible]	0.00	0.00	0.00	0.00	[5]	Group12	18.87	70.00	[illegible]
[illegible]	protein-containing complex assembly	[illegible]	0.00	0.00	0.00	0.00	[5]	Group12	10.41	121.00	[illegible]
[illegible]	detoxification	[illegible]	0.00	0.02	0.00	0.00	[1, 4]	Group13	18.37	18.00	[illegible]
[illegible]	cell redox homeostasis	[illegible]	0.00	0.03	0.00	0.00	[3]	Group13	34.78	8.00	[illegible]
[illegible]	cellular detoxification	[illegible]	0.00	0.00	0.00	0.00	[2, 5]	Group13	20.22	18.00	[illegible]
[illegible]	cellular response to toxic substance	[illegible]	0.00	0.01	0.00	0.00	[4]	Group13	19.15	18.00	[illegible]
[illegible]	cellular oxidant detoxification	[illegible]	0.00	0.02	0.00	0.00	[3, 6]	Group13	20.55	15.00	[illegible]
[illegible]	peroxiredoxin activity	[illegible]	0.00	0.00	0.00	0.00	[6, 9]	Group13	85.71	6.00	[illegible]
[illegible]	thioredoxin-dependent peroxiredoxin activity	[illegible]	0.00	0.00	0.00	0.00	[7, 10]	Group13	100.00	5.00	[illegible]
[illegible]	thioredoxin peroxidase activity	[illegible]	0.00	0.00	0.00	0.00	[8, 11]	Group13	100.00	5.00	[illegible]
[illegible]	electron transport chain	[illegible]	0.00	0.00	0.00	0.00	[4]	Group14	32.61	30.00	[illegible]
[illegible]	cellular respiration	[illegible]	0.00	0.00	0.00	0.00	[5]	Group14	25.64	40.00	[illegible]
[illegible]	respiratory electron transport chain	[illegible]	0.00	0.00	0.00	0.00	[5, 6]	Group14	33.78	25.00	[illegible]
[illegible]	aerobic respiration	[illegible]	0.00	0.00	0.00	0.00	[6]	Group14	28.93	35.00	[illegible]
[illegible]	oxidative phosphorylation	[illegible]	0.00	0.00	0.00	0.00	[7]	Group14	34.57	28.00	[illegible]
[illegible]	aerobic electron transport chain	[illegible]	0.00	0.00	0.00	0.00	[6, 7, 8]	Group14	42.22	19.00	[illegible]
[illegible]	ATP synthesis coupled electron transport	[illegible]	0.00	0.00	0.00	0.00	[6, 7, 8]	Group14	37.50	21.00	[illegible]
[illegible]	NADH dehydrogenase (ubiquinone) activity	[illegible]	0.00	0.03	0.00	0.00	[7, 9]	Group14	41.18	7.00	[illegible]
[illegible]	mitochondrial ATP synthesis coupled electron transport	[illegible]	0.00	0.00	0.00	0.00	[7, 8, 9]	Group14	38.46	20.00	[illegible]
[illegible]	mitochondrial electron transport, NADH to ubiquinone	[illegible]	0.00	0.00	0.00	0.00	[7, 8, 9, 10]	Group14	45.83	11.00	[illegible]
[illegible]	cellular component biogenesis	[illegible]	0.00	0.00	0.00	0.00	[3]	Group15	9.70	243.00	[illegible]
[illegible]	ribonucleoprotein complex biogenesis	[illegible]	0.00	0.00	0.00	0.00	[4]	Group15	21.24	79.00	[illegible]
[illegible]	ribosome biogenesis	[illegible]	0.00	0.00	0.00	0.00	[5]	Group15	23.74	61.00	[illegible]
[illegible]	protein-RNA complex organization	[illegible]	0.00	0.00	0.00	0.00	[5]	Group15	17.69	26.00	[illegible]
[illegible]	protein-RNA complex assembly	[illegible]	0.00	0.00	0.00	0.00	[5, 6]	Group15	18.71	26.00	[illegible]
[illegible]	ribosomal large subunit biogenesis	[illegible]	0.00	0.00	0.00	0.00	[5, 6]	Group15	25.00	14.00	[illegible]
[illegible]	ribosomal small subunit biogenesis	[illegible]	0.00	0.00	0.00	0.00	[5, 6]	Group15	34.09	30.00	[illegible]
[illegible]	RNA processing	[illegible]	0.00	0.00	0.00	0.00	[6, 7]	Group15	11.89	88.00	[illegible]
[illegible]	ncRNA metabolic process	[illegible]	0.00	0.00	0.00	0.00	[6, 7]	Group15	12.33	65.00	[illegible]
[illegible]	rRNA metabolic process	[illegible]	0.00	0.00	0.00	0.00	[7, 8]	Group15	20.00	39.00	[illegible]
[illegible]	ncRNA processing	[illegible]	0.00	0.00	0.00	0.00	[7, 8]	Group15	16.81	57.00	[illegible]
[illegible]	rRNA processing	[illegible]	0.00	0.00	0.00	0.00	[6, 8, 9]	Group15	23.31	38.00	[illegible]
[illegible]	cellular nitrogen compound metabolic process	[illegible]	0.00	0.00	0.00	0.00	[3]	Group16	8.28	401.00	[illegible]
[illegible]	macromolecule metabolic process	[illegible]	0.00	0.00	0.00	0.00	[3]	Group16	7.38	504.00	[illegible]
[illegible]	amide metabolic process	[illegible]	0.00	0.00	0.00	0.00	[3]	Group16	16.48	150.00	[illegible]
[illegible]	cellular biosynthetic process	[illegible]	0.00	0.00	0.00	0.00	[3]	Group16	7.73	451.00	[illegible]
[illegible]	organonitrogen compound metabolic process	[illegible]	0.00	0.00	0.00	0.00	[3]	Group16	9.46	377.00	[illegible]
[illegible]	organic substance biosynthetic process	[illegible]	0.00	0.00	0.00	0.00	[3]	Group16	7.81	461.00	[illegible]
[illegible]	protein metabolic process	[illegible]	0.00	0.00	0.00	0.00	[3, 4]	Group16	9.28	292.00	[illegible]
[illegible]	peptide metabolic process	[illegible]	0.00	0.00	0.00	0.00	[4]	Group16	18.16	126.00	[illegible]
[illegible]	macromolecule biosynthetic process	[illegible]	0.00	0.00	0.00	0.00	[4]	Group16	7.29	378.00	[illegible]
[illegible]	cellular nitrogen compound biosynthetic process	[illegible]	0.00	0.00	0.00	0.00	[4]	Group16	7.82	280.00	[illegible]
[illegible]	organonitrogen compound biosynthetic process	[illegible]	0.00	0.00	0.00	0.00	[4]	Group16	13.50	172.00	[illegible]
[illegible]	amide biosynthetic process	[illegible]	0.00	0.00	0.00	0.00	[4, 5]	Group16	18.58	120.00	[illegible]
[illegible]	gene expression	[illegible]	0.00	0.00	0.00	0.00	[5]	Group16	7.39	356.00	[illegible]
[illegible]	translation	[illegible]	0.00	0.00	0.00	0.00	[4, 5, 6, 7]	Group16	20.95	110.00	[illegible]
[illegible]	peptide biosynthetic process	[illegible]	0.00	0.00	0.00	0.00	[5, 6]	Group16	20.55	112.00	[illegible]
[illegible]	regulation of RNA biosynthetic process	[illegible]	0.00	0.01	0.00	0.00	[5, 6, 7, 8]	Group16	4.13	106.00	[illegible]
[illegible]	regulation of DNA-templated transcription	[illegible]	0.00	0.01	0.00	0.00	[6, 7, 8, 9]	Group16	4.14	106.00	[illegible]
[illegible]	proton transmembrane ATP synthase activity, rotational mechani	[illegible]	0.00	0.01	0.00	0.00	[illegible]	Group17	60.00	6.00	[illegible]
[illegible]	proton motive force-driven mitochondrial ATP synthesis	[illegible]	0.00	0.01	0.00	0.00	[illegible]	Group17	60.00	6.00	[illegible]
[illegible]	organophosphate metabolic process	[illegible]	0.00	0.00	0.00	0.00	[3, 4]	Group17	10.76	85.00	[illegible]
[illegible]	nucleobase-containing small molecule metabolic process	[illegible]	0.00	0.00	0.00	0.00	[3, 4, 5]	Group17	12.81	62.00	[illegible]
[illegible]	purine-containing compound metabolic process	[illegible]	0.00	0.01	0.00	0.00	[4]	Group17	11.88	48.00	[illegible]
[illegible]	ribose phosphate metabolic process	[illegible]	0.00	0.00	0.00	0.00	[4, 5]	Group17	12.65	43.00	[illegible]
[illegible]	organophosphate biosynthetic process	[illegible]	0.00	0.00	0.00	0.00	[4, 5]	Group17	12.25	49.00	[illegible]
[illegible]	nucleoside phosphate metabolic process	[illegible]	0.00	0.00	0.00	0.00	[4, 5, 6]	Group17	13.76	60.00	[illegible]
[illegible]	nucleotide metabolic process	[illegible]	0.00	0.00	0.00	0.00	[5, 6, 7]	Group17	13.75	59.00	[illegible]
[illegible]	nucleoside triphosphate metabolic process	[illegible]	0.00	0.00	0.00	0.00	[5, 6, 7]	Group17	15.38	28.00	[illegible]
[illegible]	nucleoside phosphate biosynthetic process	[illegible]	0.00	0.01	0.00	0.00	[5, 6, 7]	Group17	14.84	27.00	[illegible]
[illegible]	purine nucleotide metabolic process	[illegible]	0.00	0.00	0.00	0.00	[5, 6, 7, 8]	Group17	12.30	47.00	[illegible]
[illegible]	ribonucleotide metabolic process	[illegible]	0.00	0.02	0.00	0.00	[5, 6, 7, 8]	Group17	12.12	40.00	[illegible]
[illegible]	purine nucleoside triphosphate metabolic process	[illegible]	0.00	0.01	0.00	0.00	[6, 7, 8]	Group17	15.66	26.00	[illegible]
[illegible]	nucleotide biosynthetic process	[illegible]	0.00	0.01	0.00	0.00	[6, 7, 8]	Group17	14.92	27.00	[illegible]
[illegible]	ribonucleoside triphosphate metabolic process	[illegible]	0.00	0.02	0.00	0.00	[6, 7, 8]	Group17	14.88	25.00	[illegible]
[illegible]	proton transmembrane transporter activity	[illegible]	0.00	0.04	0.00	0.00	[6, 8]	Group17	17.14	18.00	[illegible]
[illegible]	purine nucleoside triphosphate biosynthetic process	[illegible]	0.00	0.04	0.00	0.00	[7, 8, 9]	Group17	25.00	11.00	[illegible]
[illegible]	purine ribonucleoside triphosphate metabolic process	[illegible]	0.00	0.01	0.00	0.00	[7, 8, 9]	Group17	15.53	25.00	[illegible]
[illegible]	ATP metabolic process	[illegible]	0.00	0.00	0.00	0.00	[7, 8, 9, 10]	Group17	17.27	24.00	[illegible]
[illegible]	purine ribonucleoside triphosphate biosynthetic process	[illegible]	0.00	0.03	0.00	0.00	[8, 9, 10]	Group17	25.58	11.00	[illegible]
[illegible]	ATP biosynthetic process	[illegible]	0.00	0.01	0.00	0.00	[8, 9, 10, 11]	Group17	30.30	10.00	[illegible]
[illegible]	generation of precursor metabolites and energy	[illegible]	0.00	0.00	0.00	0.00	[3]	Group18	17.01	58.00	[illegible]
[illegible]	energy derivation by oxidation of organic compounds	[illegible]	0.00	0.00	0.00	0.00	[4]	Group18	18.70	43.00	[illegible]
[illegible]	electron transport chain	[illegible]	0.00	0.00	0.00	0.00	[4]	Group18	32.61	30.00	[illegible]
[illegible]	mitochondrion organization	[illegible]	0.00	0.00	0.00	0.00	[5]	Group18	18.87	70.00	[illegible]
[illegible]	cellular respiration	[illegible]	0.00	0.00	0.00	0.00	[5]	Group18	25.64	40.00	[illegible]
[illegible]	respiratory electron transport chain	[illegible]	0.00	0.00	0.00	0.00	[5, 6]	Group18	33.78	25.00	[illegible]
[illegible]	aerobic respiration	[illegible]	0.00	0.00	0.00	0.00	[6]	Group18	28.93	35.00	[illegible]
[illegible]	NADH dehydrogenase complex assembly	[illegible]	0.00	0.00	0.00	0.00	[6]	Group18	44.23	23.00	[illegible]
[illegible]	mitochondrial respiratory chain complex assembly	[illegible]	0.00	0.00	0.00	0.00	[6]	Group18	42.67	32.00	[illegible]
[illegible]	oxidative phosphorylation	[illegible]	0.00	0.00	0.00	0.00	[7]	Group18	34.57	28.00	[illegible]
[illegible]	aerobic electron transport chain	[illegible]	0.00	0.00	0.00	0.00	[6, 7, 8]	Group18	42.22	19.00	[illegible]
[illegible]	mitochondrial respiratory chain complex I assembly	[illegible]	0.00	0.00	0.00	0.00	[7]	Group18	44.23	23.00	[illegible]
[illegible]	ATP synthesis coupled electron transport	[illegible]	0.00	0.00	0.00	0.00	[6, 7, 8]	Group18	37.50	21.00	[illegible]
[illegible]	mitochondrial ATP synthesis coupled electron transport	[illegible]	0.00	0.00	0.00	0.00	[7, 8, 9]	Group18	38.46	20.00	[illegible]
[illegible]	mitochondrial electron transport, NADH to ubiquinone	[illegible]	0.00	0.00	0.00	0.00	[7, 8, 9, 10]	Group18	45.83	11.00	[illegible]

NL3L, GPC3, GPD1, GPR108, GTF2F1, HAX1, HCRT, HDGFL2, HSPA8, HYAL3, IER2, IGFBP6, INO80B, IP6K2, ITGB7, JAM3, KAT5, KDM8, KLF1, LAMTOR1, LAMTOR2, LAMTOR4, LGI1, LIMS2, LMNA, LMO2, LRCH4, LRSAM1, LSM10, LYPLAL1, MAF, MAP2K2, MAP4K1, MAPK13, MAPK3, MAPK7, MAPKAPK2, MCRS1, MED10, MEPCE, MESD, METTL23, MFSD2A, MGMT, MIEN1, MIF, MKKS, MLST8, MLYCD, MMD2, MPV17L2, MRGBP, MRPS6, MST1, MT3, MVD, MYOM1, NACC2, NCF1, NDUFS4, NENF, NME2, NOP2, NPM2, NSFL1C, NSMAF, OTUB1, PAIP2
COX7A1, COX7A2, CRB3, CRHR1, CRSP3, CTDNEP1, CTDSP2, CTH, CTNS, CTSD, CUL7, CYTH2, DAD1, DAPK3, DBF4B, DBNL, DDRGK1, DDX11, DDX25, DDX49, DERL3, DHFR, DHPS, DMAP1, DNAI1, DNAJC15, DPEP1, DPPA5, DRG1, DUS3L, DVL1, DYNLL1, EDF1, EEF1B2, EEF1D, EEF1G, EFHC1, EHD1, EIF2B2, EIF3B, EIF3F, EIF3G, EIF3H, EIF3K, EIF4EBP1, EIF6, EIPR1, ELOB, ENSA, EPHB6, ERCC1, ERP29, EXOSC4, FAM107A, FAM162A, FAM50A, FBXO31, FHIT, FHL2, FIGLA, FIS1, FKBP1B, FLOT1, FSTL3, FUNDC2, FXR2, FZR1, GAPDH, G
76, INO80B, IP6K2, ITGB7, JAM3, KAT5, KDM8, KLF1, LAMTOR1, LAMTOR2, LAMTOR4, LGI1, LIMS2, LMNA, LMO2, LRCH4, LRSAM1, LSM10, MAF, MAP2K2, MAP4K1, MAPK13, MAPK3, MAPK7, MAPKAPK2, MCRS1, MED10, MEPCE, MESD, METTL23, MFSD2A, MGMT, MIEN1, MIF, MLST8, MLYCD, MMD2, MPV17L2, MRGBP, MRPS6, MST1, MT3, MVD, MYOM1, NACC2, NCF1, NDUFS4, NENF, NME2, NOP2, NPM2, NSFL1C, OTUB1, PAIP2, PARK7, PCID2, PCIF1, PDCD5, PELP1, PFKM, PHB2, PIH1D1, PIN1, PIP4P1, PKN1, PLD6, PNLDC1, POLR2G, PQ

SM4, MANBA, MAPKAPK2, MFSD2A, MGST2, MLYCD, MTHFS, NEDD8, NEU1, NQO2, NSFL1C, NUDT16L1, NUDT18, NUDT5, PARK7, PCID2, PDLIM2, PFKM, PIN1, PLA2G12A, PLD6, PNLDC1, PNPLA4, POLR2G, PPP1R11, PRR5L, PSMA6, PSMB4, PSMB6, PSMB7, PSMD13, PSME2, QPRT, RAB7A, RACK1, RNASEH2C, RNF166, RNF26, RPL11, RPS27A, SAMD4A, SDS, SELENOS, SIAH3, SLIRP, SMPD2, SNF8, SORD, STX5, TBRG4, TCIRG1, TIMP2, TNIP1, TPI1, UBE2J2, UBXN1, UBXN6, UPB1, UPP1, UROD, VPS25, VPS28, VPS4A, YTHDF1, ZPR1]

[illegible]

ABARAP, GBA2, GEMIN2, GEMIN6, GLRX3, GNL3L, GPX4, GTF2H5, HAUS1, HAUS7, HAX1, HIGD2A, HSD17B10, HSPA8, IFT57, IMMP2L, IMP3, IMP4, ISCA1, ISCA2, ITGB5, JAM3, KANK3, LOC100738684, LOC100739087, LOC102162486, LONP1, LRCH4, LRSAM1, LSM2, LUC7L, LYRM4, MALSU1, MAP1LC3B, MFSD2A, MIEN1, MIF, MKKS, MKS1, MLST8, MPV17L2, MRM1, MRPS7, MRTO4, NACC2, NCLN, NDUFA10, NDUFA11, NDUFA12, NDUFA5, NDUFA9, NDUFAB1, NDUFB10, NDUFB11, NDUFB2, NDUFB4, NDUFB7, NDUFB8, NDUFB9, NDUFC1, NDUF
[illegible]
7, HAX1, HIGD2A, HSD17B10, HSPA8, IFT57, IMMP2L, ISCA1, ISCA2, ITGB5, JAM3, KANK3, LOC100738684, LOC102162486, LONP1, LRCH4, LRSAM1, LSM2, LUC7L, LYRM4, MAP1LC3B, MFSD2A, MIEN1, MIF, MKKS, MKS1, MLST8, MPV17L2, MRPS7, NACC2, NCLN, NDUFA10, NDUFA11, NDUFA12, NDUFA5, NDUFA9, NDUFAB1, NDUFB10, NDUFB11, NDUFB2, NDUFB4, NDUFB7, NDUFB8, NDUFB9, NDUFC1, NDUFC2, NDUFS3, NDUFS4, NDUFS5, NDUFS8, NOTO, NSFL1C, NUBP2, NUDCD3, PADI4, PARK7, PDZD11, PET100, PIH1D1, PIP4P1, PL

8, MRPS7, NACC2, NCLN, NDUFA10, NDUFA11, NDUFA12, NDUFA5, NDUFA9, NDUFAB1, NDUFB10, NDUFB11, NDUFB2, NDUFB4, NDUFB7, NDUFB8, NDUFB9, NDUFC1, NDUFC2, NDUFS3, NDUFS4, NDUFS5, NDUFS8, NPM2, NUDT5, PADI4, PARK7, PCID2, PDZD11, PET100, PIH1D1, PIP4P1, PKN1, POLE3, POLR2D, POMP, PRPF31, PRPF6, PSMG2, PUF60, RACK1, RHOC, RPL38, RPS15, RPS19, RPS5, SAMM50, SDHAF1, SEM1, SMARCD3, SMIM20, SMYD3, SMYD5, SNRPD2, SNRPD3, SSBP1, SSNA1, STMN1, STMP1, TARBP2, TBCA, TFPT,

ABARAP, GBA2, GEMIN2, GEMIN6, GLRX3, GNL3L, GPX4, GTF2H5, HAUS1, HAUS7, HAX1, HIGD2A, HSD17B10, HSPA8, IFT57, IMMP2L, IMP3, IMP4, ISCA1, ISCA2, ITGB5, JAM3, KANK3, LOC100738684, LOC100739087, LOC102162486, LONP1, LRCH4, LRSAM1, LSM2, LUC7L, LYRM4, MALSU1, MAP1LC3B, MFSD2A, MIEN1, MIF, MKKS, MKS1, MLST8, MPV17L2, MRM1, MRPS7, MRTO4, NACC2, NCLN, NDUFA10, NDUFA11, NDUFA12, NDUFA5, NDUFA9, NDUFAB1, NDUFB10, NDUFB11, NDUFB2, NDUFB4, NDUFB7, NDUFB8, NDUFB9, NDUFC1, NDUF

L7, DAD1, DAPK3, DBF4B, DCAF11, DDRGK1, DDX11, DDX25, DDX49, DERL3, DMAP1, DNAJB13, DNASE2, DNPEP, DPEP1, DPH7, DPP7, DPPA5, DRG1, DUS1L, DUS3L, DVL1, EDF1, EEF1B2, EEF1D, EEF1G, EIF2B2, EIF3B, EIF3F, EIF3G, EIF3H, EIF3K, EIF4EBP1, EIF6, EIPR1, ELAC2, ELOB, ELOF1, EMG1, ENDOU, ENSA, ERAP2, ERCC1, ERI3, ERP29, EXOSC4, EXOSC8, FAAP100, FAM50A, FAU, FBL, FBXO31, FCF1, FHIT, FHL2, FIGLA, FKBP2, FKBP4, FLOT1, FSTL3, FTSJ3, FXR2, FZR1, GALNT6, GAPDH, GDF9, GEMIN2, GEMIN6, GGA1, G

, DMAP1, DNAJB13, DNPH1, DPPA5, DRG1, DTYMK, DUS1L, DUS3L, DUT, DVL1, EDF1, EEF1B2, EEF1D, EEF1G, EIF2B2, EIF3B, EIF3F, EIF3G, EIF3H, EIF3K, EIF4EBP1, EIF6, ELAC2, ELOB, ELOF1, EMG1, ERCC1, ERI3, ERP29, EXOSC4, EXOSC8, FAM50A, FAU, FBL, FCF1, FDPS, FHL2, FIGLA, FKBP2, FKBP4, FLAD1, FLOT1, FSTL3, FTSJ3, FXR2, FZR1, GALNT6, GAPDH, GATD1, GEMIN2, GEMIN6, GGT1, GLMP, GLRX3, GMPS, GNL3, GNL3L, GON7, GORASP1, GTF2E2, GTF2F1, GTF2H5, GTF2IRD1, HAX1, HEXIM2, HSBP1, HSD17B1, HSD17B
B, CTDSP2, CTH, CTNS, CTSD, CTSF, CUL7, DAD1, DAPK3, DCAF11, DDRGK1, DDX25, DEGS2, DERL3, DHFR, DHPS, DMGDH, DNAJB13, DNPEP, DNPH1, DPEP1, DPH7, DPP7, DRG1, DTYMK, DUS3L, DUT, DVL1, EEF1B2, EEF1D, EEF1G, EIF2B2, EIF3B, EIF3F, EIF3G, EIF3H, EIF3K, EIF4EBP1, EIF6, EIPR1, ENDOU, ENSA, ERAP2, ERP29, ETFB, ETHE1, FAU, FBXO31, FHIT, FIS1, FKBP2, FKBP4, FLAD1, FLOT1, FXR2, FZR1, GALK1, GALNT6, GAPDH, GATD1, GBA2, GDF9, GGA1, GGH, GGT1, GLRX3, GMPS, GNL3, GNL3L, GORASP1, GPC3, GPD
S2, DERL3, DHFR, DMAP1, DNAJB13, DNPH1, DPPA5, DRG1, DTYMK, DUS1L, DUS3L, DUT, DVL1, EDF1, EEF1B2, EEF1D, EEF1G, EIF2B2, EIF3B, EIF3F, EIF3G, EIF3H, EIF3K, EIF4EBP1, EIF6, ELAC2, ELOB, ELOF1, EMG1, ERCC1, ERI3, ERP29, EXOSC4, EXOSC8, FAM50A, FAU, FBL, FCF1, FDPS, FHL2, FIGLA, FKBP2, FKBP4, FLAD1, FLOT1, FSTL3, FTSJ3, FXR2, FZR1, GALNT6, GAPDH, GATD1, GEMIN2, GEMIN6, GGT1, GLMP, GLRX3, GMPS, GNL3, GNL3L, GON7, GORASP1, GPD1, GTF2E2, GTF2F1, GTF2H5, GTF2IRD1, HAX1, HEXIM2, H
3F, EIF3G, EIF3H, EIF3K, EIF4EBP1, EIF6, EIPR1, ENDOU, ENSA, ERAP2, ERP29, FAU, FBXO31, FHIT, FKBP2, FKBP4, FLOT1, FXR2, FZR1, GALNT6, GAPDH, GDF9, GGA1, GGT1, GLRX3, GNL3, GNL3L, GORASP1, GPC3, GPS1, GUSB, HAX1, HSPA8, IMMP2L, IP6K2, ISCA2, JOSD2, KAT5, KLK15, LMF1, LOC100517502, LOC100519130, LOC100738684, LOC100739087, LONP1, LRRC47, LRSAM1, MALSU1, MANBA, MAPK7, MESD, MIB2, MIF, MKKS, MLST8, MPV17L2, MRPL14, MRPL17, MRPL2, MRPL20, MRPL22, MRPL23, MRPL27, MRPL30, MRPL

F2F1, GTF2H5, GTF2IRD1, HAX1, HEXIM2, HSBP1, HSPA8, IER2, INO80B, ITGB7, KAT5, KDM8, KLF1, KLF16, LIN37, LMO2, LOC100519130, LOC100523672, LOC100623639, LOC100738684, LOC100739087, LRRC47, MAF, MAF1, MALSU1, MAP2K2, MAPK3, MAPK7, MBD3, MCRS1, MED10, MED31, MED8, MEPCE, METTL23, MGST2, MLYCD, MPV17L2, MRGBP, MRPL14, MRPL17, MRPL2, MRPL20, MRPL22, MRPL23, MRPL27, MRPL30, MRPL36, MRPL37, MRPL51, MRPL58, MRPS11, MRPS12, MRPS15, MRPS18A, MRPS18C, MRPS28, MRPS34, MRPS6, M

ID	Term	Ontology Source	Term PValue	Term P-Value Corrected with [illegible]	Group P-Value	Group P-Value Corrected [illegible]	GO Levels	GO Group	%	Number	Associated Genes Found
GO:0006996	organelle organization	[illegible]	0.00	[illegible]	0.00	0.00	[illegible]	[illegible]	[illegible]	[illegible]	[illegible]
GO:0010639	negative regulation of organelle organization	[illegible]	0.00	[illegible]	0.00	0.00	[illegible]	[illegible]	[illegible]	[illegible]	[illegible]
GO:0043414	macromolecule methylation	[illegible]	0.00	[illegible]	0.00	0.00	[illegible]	[illegible]	[illegible]	[illegible]	[illegible]
GO:0051276	chromosome organization	[illegible]	0.00	[illegible]	0.00	0.00	[illegible]	[illegible]	[illegible]	[illegible]	[illegible]
GO:0006413	translational initiation	[illegible]	0.00	[illegible]	0.00	0.00	[illegible]	[illegible]	[illegible]	[illegible]	[illegible]
GO:0006446	regulation of translational initiation	[illegible]	0.00	[illegible]	0.00	0.00	[illegible]	[illegible]	[illegible]	[illegible]	[illegible]
GO:0006999	nuclear pore organization	[illegible]	0.00	[illegible]	0.00	0.00	[illegible]	[illegible]	[illegible]	[illegible]	[illegible]
GO:0006259	DNA metabolic process	[illegible]	0.00	[illegible]	0.00	0.00	[illegible]	[illegible]	[illegible]	[illegible]	[illegible]
GO:0006260	DNA replication	[illegible]	0.00	[illegible]	0.00	0.00	[illegible]	[illegible]	[illegible]	[illegible]	[illegible]
GO:0006261	DNA-templated DNA replication	[illegible]	0.00	[illegible]	0.00	0.00	[illegible]	[illegible]	[illegible]	[illegible]	[illegible]
GO:0000226	microtubule cytoskeleton organization	[illegible]	0.00	[illegible]	0.00	0.00	[illegible]	[illegible]	[illegible]	[illegible]	[illegible]
GO:0070925	organelle assembly	[illegible]	0.00	[illegible]	0.00	0.00	[illegible]	[illegible]	[illegible]	[illegible]	[illegible]
GO:0140694	non-membrane-bounded organelle assembly	[illegible]	0.00	[illegible]	0.00	0.00	[illegible]	[illegible]	[illegible]	[illegible]	[illegible]
GO:0022402	cell cycle process	[illegible]	0.00	[illegible]	0.00	0.00	[illegible]	[illegible]	[illegible]	[illegible]	[illegible]
GO:1903047	mitotic cell cycle process	[illegible]	0.00	[illegible]	0.00	0.00	[illegible]	[illegible]	[illegible]	[illegible]	[illegible]
GO:0048285	organelle fission	[illegible]	[illegible]	[illegible]	[illegible]	[illegible]	[illegible]	[illegible]	[illegible]	[illegible]	[illegible]
GO:0000280	nuclear division	[illegible]	0.00	[illegible]	0.00	0.00	[illegible]	[illegible]	[illegible]	[illegible]	[illegible]
GO:0043933	protein-containing complex organization	[illegible]	0.00	[illegible]	0.00	0.00	[illegible]	[illegible]	[illegible]	[illegible]	[illegible]
GO:0065003	protein-containing complex assembly	[illegible]	[illegible]	[illegible]	[illegible]	[illegible]	[illegible]	[illegible]	[illegible]	[illegible]	[illegible]
GO:0071824	protein-DNA complex organization	[illegible]	0.00	[illegible]	0.00	0.00	[illegible]	[illegible]	[illegible]	[illegible]	[illegible]
GO:0006325	chromatin organization	[illegible]	0.00	[illegible]	0.00	0.00	[illegible]	[illegible]	[illegible]	[illegible]	[illegible]
GO:0065004	protein-DNA complex assembly	[illegible]	0.00	[illegible]	0.00	0.00	[illegible]	[illegible]	[illegible]	[illegible]	[illegible]
GO:0006338	chromatin remodeling	[illegible]	0.00	[illegible]	0.00	0.00	[illegible]	[illegible]	[illegible]	[illegible]	[illegible]
GO:0022402	cell cycle process	[illegible]	0.00	[illegible]	0.00	0.00	[illegible]	[illegible]	[illegible]	[illegible]	[illegible]
GO:0000278	mitotic cell cycle	[illegible]	0.00	[illegible]	0.00	0.00	[illegible]	[illegible]	[illegible]	[illegible]	[illegible]
GO:0044770	cell cycle phase transition	[illegible]	0.00	[illegible]	0.00	0.00	[illegible]	[illegible]	[illegible]	[illegible]	[illegible]
GO:0051726	regulation of cell cycle	[illegible]	0.00	[illegible]	0.00	0.00	[illegible]	[illegible]	[illegible]	[illegible]	[illegible]
GO:1903047	mitotic cell cycle process	[illegible]	0.00	[illegible]	0.00	0.00	[illegible]	[illegible]	[illegible]	[illegible]	[illegible]
GO:0010564	regulation of cell cycle process	[illegible]	0.00	[illegible]	0.00	0.00	[illegible]	[illegible]	[illegible]	[illegible]	[illegible]
GO:0044772	mitotic cell cycle phase transition	[illegible]	0.00	[illegible]	0.00	0.00	[illegible]	[illegible]	[illegible]	[illegible]	[illegible]
GO:0044839	cell cycle G2/M phase transition	[illegible]	0.00	[illegible]	0.00	0.00	[illegible]	[illegible]	[illegible]	[illegible]	[illegible]
GO:0000086	G2/M transition of mitotic cell cycle	[illegible]	0.00	[illegible]	0.00	0.00	[illegible]	[illegible]	[illegible]	[illegible]	[illegible]
GO:0000280	nuclear division	[illegible]	0.00	[illegible]	0.00	0.00	[illegible]	[illegible]	[illegible]	[illegible]	[illegible]
GO:1902749	regulation of cell cycle G2/M phase transition	[illegible]	0.00	[illegible]	0.00	0.00	[illegible]	[illegible]	[illegible]	[illegible]	[illegible]

RAD1, RAE1, RAF1, RCHY1, RDX, RESF1, RFC4, RHOT1, RHPN2, RRM1, SBDS, SDHAF4, SEC23A, SLAIN2, SLC25A31, SLC39A12, SNX7, SPAG16, SPATA7, SPIRE1, SRPX, SSX2IP, STOML2, STX6, STX7, SUPV3L1, TCTN2, TMCC1, TMED5, TMEM216, TOGARAM2, TTC8, UBR2, UHRF1, UNC13B, USP33, VAPA, WDR47, WEE2, X

Rank	TF	Score	Library
1	THAP3	6.14E-04	ARCHS4 Coexpression,6.143E-4
2	NEUROG1	6.22E-04	GTEx Coexpression,6.223E-4
3	DRAP1	7.12E-04	Enrichr Queries,7.123E-4
4	BRF2	0.001229	ARCHS4 Coexpression,0.001229
5	THAP4	0.001245	GTEx Coexpression,0.001245
6	THAP7	0.001425	Enrichr Queries,0.001425
7	ZNF511	0.001843	ARCHS4 Coexpression,0.001843
8	GTF3A	0.001867	GTEx Coexpression,0.001867
9	ZNF706	0.002137	Enrichr Queries,0.002137
10	ZNF581	0.002457	ARCHS4 Coexpression,0.002457
11	NME2	0.003071	ARCHS4 Coexpression,0.003071
12	ELK1	0.003367	ReMap ChIP-seq,0.003367
13	YBX1	0.003561	Enrichr Queries,0.003561
14	ESRRA	0.003734	GTEx Coexpression,0.003734
15	ZNF428	0.004274	Enrichr Queries,0.004274
16	THAP8	0.0043	ARCHS4 Coexpression,0.0043
17	NRL	0.004356	GTEx Coexpression,0.004356
18	DNTTIP1	0.004914	ARCHS4 Coexpression,0.004914
19	ZNF414	0.004978	GTEx Coexpression,0.004978
20	CREB3	0.005528	ARCHS4 Coexpression,0.005528
21	REPIN1	0.0056	GTEx Coexpression,0.0056
22	THAP11	0.005698	Enrichr Queries,0.005698
23	MYC	0.006098	Literature ChIP-seq,0.006098
24	ALX1	0.006143	ARCHS4 Coexpression,0.006143
25	HMGN3	0.006223	GTEx Coexpression,0.006223
26	MBD3	0.00641	Enrichr Queries,0.00641
27	GABPA	0.006734	ReMap ChIP-seq,0.006734
28	CENPS	0.006757	ARCHS4 Coexpression,0.006757
29	SNAPC5	0.006845	GTEx Coexpression,0.006845
30	ZNF787	0.007123	Enrichr Queries,0.007123
31	CHCHD3	0.007371	ARCHS4 Coexpression,0.007371
32	ZNF32	0.007467	GTEx Coexpression,0.007467
33	ATF4	0.007835	Enrichr Queries,0.007835
34	ZNF672	0.00809	GTEx Coexpression,0.00809
35	SIX5	0.008475	ENCODE ChIP-seq,0.008475
36	ZNF576	0.0086	ARCHS4 Coexpression,0.0086
37	TCF24	0.008712	GTEx Coexpression,0.008712
38	ZNF232	0.009214	ARCHS4 Coexpression,0.009214
39	CENPX	0.009334	GTEx Coexpression,0.009334
40	PIN1	0.009828	ARCHS4 Coexpression,0.009828
41	TCF15	0.009956	GTEx Coexpression,0.009956
42	PREB	0.009972	Enrichr Queries,0.009972
43	SP2	0.0101	ReMap ChIP-seq,0.0101
44	THYN1	0.01044	ARCHS4 Coexpression,0.01044
45	ARGFX	0.01058	GTEx Coexpression,0.01058
46	ARNT	0.01106	ARCHS4 Coexpression,0.01106
47	ZNF524	0.0114	Enrichr Queries,0.0114
48	HOXC9	0.01167	ARCHS4 Coexpression,0.01167
49	NKX25	0.01182	GTEx Coexpression,0.01182
50	E2F1	0.0122	Literature ChIP-seq,0.0122
51	ZNF408	0.01229	ARCHS4 Coexpression,0.01229
52	ZNF837	0.01245	GTEx Coexpression,0.01245

53	CENPB	0.01282	Enrichr Queries,0.01282
54	NR2C2	0.01347	ReMap ChIP-seq,0.01347
55	ZNF574	0.01353	Enrichr Queries,0.01353
56	MLX	0.01369	GTEx Coexpression,0.01369
57	ZNF395	0.01431	GTEx Coexpression,0.01431
58	FOXB1	0.01474	ARCHS4 Coexpression,0.01474
59	ZNF598	0.01496	Enrichr Queries,0.01496
60	ZNF691	0.01536	ARCHS4 Coexpression,0.01536
61	ESRRB	0.01556	GTEx Coexpression,0.01556
62	IRF3	0.01567	Enrichr Queries,0.01567
63	HESX1	0.01597	ARCHS4 Coexpression,0.01597
64	ZNF768	0.01618	GTEx Coexpression,0.01618
65	ISL2	0.01658	ARCHS4 Coexpression,0.01658
66	SOX4	0.0168	GTEx Coexpression,0.0168
67	MXI1	0.01684	ReMap ChIP-seq,0.01684
68	JUND	0.01709	Enrichr Queries,0.01709
69	SMYD3	0.0172	ARCHS4 Coexpression,0.0172
70	ZBTB45	0.01742	GTEx Coexpression,0.01742
71	POU5F1B	0.01781	ARCHS4 Coexpression,0.01781
72	KLF1	0.01829	Literature ChIP-seq,0.01829
73	ZNF174	0.01843	ARCHS4 Coexpression,0.01843
74	HMG20B	0.01852	Enrichr Queries,0.01852
75	GATA4	0.01867	GTEx Coexpression,0.01867
76	NR1H2	0.01923	Enrichr Queries,0.01923
77	SNAPC2	0.01966	ARCHS4 Coexpression,0.01966
78	ZNF668	0.01994	Enrichr Queries,0.01994
79	PRDM2	0.02054	GTEx Coexpression,0.02054
80	HOXA7	0.02088	ARCHS4 Coexpression,0.02088
81	MTERF3	0.02116	GTEx Coexpression,0.02116
82	ZNF3	0.0215	ARCHS4 Coexpression,0.0215
83	RBPJ	0.02178	GTEx Coexpression,0.02178
84	ZNF749	0.02211	ARCHS4 Coexpression,0.02211
85	SPEN	0.0224	GTEx Coexpression,0.0224
86	REXO4	0.02273	ARCHS4 Coexpression,0.02273
87	HMGA1	0.02279	Enrichr Queries,0.02279
88	CXXC1	0.0235	Enrichr Queries,0.0235
89	ELF1	0.02357	ReMap ChIP-seq,0.02357
90	GLMP	0.02396	ARCHS4 Coexpression,0.02396
91	ZNF579	0.02422	Enrichr Queries,0.02422
92	ZSCAN5C	0.02427	GTEx Coexpression,0.02427
93	ETS1	0.02439	Literature ChIP-seq,0.02439
94	FAM200B	0.02457	ARCHS4 Coexpression,0.02457
95	TP53	0.02489	GTEx Coexpression,0.02489
96	TBP	0.02542	ENCODE ChIP-seq,0.02542
97	ZNF784	0.02551	GTEx Coexpression,0.02551
98	ZNF653	0.02564	Enrichr Queries,0.02564
99	ZNF830	0.0258	ARCHS4 Coexpression,0.0258
100	ESRRG	0.02614	GTEx Coexpression,0.02614
101	ZNF358	0.02635	Enrichr Queries,0.02635
102	TIGD5	0.02676	GTEx Coexpression,0.02676
103	HES4	0.02703	ARCHS4 Coexpression,0.02703
104	TBX5	0.02738	GTEx Coexpression,0.02738
105	HES1	0.02764	ARCHS4 Coexpression,0.02764

106	SLC2A4RG	0.02778	Enrichr Queries,0.02778
107	TFAP4	0.028	GTEx Coexpression,0.028
108	ZKSCAN8	0.02826	ARCHS4 Coexpression,0.02826
109	USF2	0.02849	Enrichr Queries,0.02849
110	ZNF580	0.02887	ARCHS4 Coexpression,0.02887
111	XBP1	0.0292	Enrichr Queries,0.0292
112	ZNF747	0.02925	GTEx Coexpression,0.02925
113	NPAS1	0.02948	ARCHS4 Coexpression,0.02948
114	ZNF865	0.02987	GTEx Coexpression,0.02987
115	E4F1	0.02991	Enrichr Queries,0.02991
116	MESP1	0.0301	ARCHS4 Coexpression,0.0301
117	NRF1	0.0303	ReMap ChIP-seq,0.0303
118	CEBPB	0.03049	Literature ChIP-seq,0.03049
119	NKX26	0.03049	GTEx Coexpression,0.03049
120	ZGPAT	0.03063	Enrichr Queries,0.03063
121	HOXC5	0.03133	ARCHS4 Coexpression,0.03133
122	MAZ	0.03134	Enrichr Queries,0.03134
123	TBX20	0.03174	GTEx Coexpression,0.03174
124	ZNF207	0.03205	Enrichr Queries,0.03205
125	ZNF771	0.03256	ARCHS4 Coexpression,0.03256
126	ZNF688	0.03276	Enrichr Queries,0.03276
127	BAZ2B	0.03298	GTEx Coexpression,0.03298
128	VAX2	0.03317	ARCHS4 Coexpression,0.03317
129	ZNF444	0.03348	Enrichr Queries,0.03348
130	ELK4	0.03367	ReMap ChIP-seq,0.03367
131	ZNF721	0.03419	Enrichr Queries,0.03419
132	ATF6B	0.03423	GTEx Coexpression,0.03423
133	PRDM13	0.0344	ARCHS4 Coexpression,0.0344
134	HSFX1	0.03485	GTEx Coexpression,0.03485
135	MXD3	0.03501	ARCHS4 Coexpression,0.03501
136	E2F4	0.03561	Enrichr Queries,0.03561
137	TTF1	0.03624	ARCHS4 Coexpression,0.03624
138	VDR	0.03659	Literature ChIP-seq,0.03659
139	ZNF670	0.03686	ARCHS4 Coexpression,0.03686
140	SMAD5	0.03704	ReMap ChIP-seq,0.03704
141	NR2F6	0.03704	Enrichr Queries,0.03704
142	DPF3	0.03734	GTEx Coexpression,0.03734
143	NFAT5	0.03747	ARCHS4 Coexpression,0.03747
144	KLF16	0.03775	Enrichr Queries,0.03775
145	CREB1	0.03808	ARCHS4 Coexpression,0.03808
146	FIZ1	0.03846	Enrichr Queries,0.03846
147	HOXB7	0.0387	ARCHS4 Coexpression,0.0387
148	GLI4	0.0392	GTEx Coexpression,0.0392
149	HMX1	0.03931	ARCHS4 Coexpression,0.03931
150	ZNF584	0.03983	GTEx Coexpression,0.03983
151	RXRB	0.03989	Enrichr Queries,0.03989
152	MYNN	0.0404	ReMap ChIP-seq,0.0404
153	CEBPZ	0.0406	Enrichr Queries,0.0406
154	ZNF692	0.04131	Enrichr Queries,0.04131
155	ZNF513	0.04231	GTEx Coexpression,0.04231
156	MAX	0.04237	ENCODE ChIP-seq,0.04237
157	TEAD4	0.04238	ARCHS4 Coexpression,0.04238
158	ZNF205	0.04274	Enrichr Queries,0.04274

159	LBX2	0.043	ARCHS4 Coexpression,0.043
160	ZNF446	0.04345	Enrichr Queries,0.04345
161	E2F8	0.04356	GTEx Coexpression,0.04356
162	NKX28	0.04361	ARCHS4 Coexpression,0.04361
163	ZNF76	0.04416	Enrichr Queries,0.04416
164	ZNF480	0.04418	GTEx Coexpression,0.04418
165	THAP10	0.04423	ARCHS4 Coexpression,0.04423
166	RBCK1	0.0448	GTEx Coexpression,0.0448
167	ZBED1	0.04543	GTEx Coexpression,0.04543
168	HIVEP1	0.04545	ARCHS4 Coexpression,0.04545
169	CENPT	0.04558	Enrichr Queries,0.04558
170	DMTF1	0.04605	GTEx Coexpression,0.04605
171	DLX4	0.04607	ARCHS4 Coexpression,0.04607
172	HES6	0.0463	Enrichr Queries,0.0463
173	GMEB2	0.04667	GTEx Coexpression,0.04667
174	ZNF623	0.04668	ARCHS4 Coexpression,0.04668
175	DEAF1	0.04701	Enrichr Queries,0.04701
176	POU2F2	0.04714	ReMap ChIP-seq,0.04714
177	ZFP3	0.04729	GTEx Coexpression,0.04729
178	ZNF212	0.0473	ARCHS4 Coexpression,0.0473
179	TSC22D1	0.04772	Enrichr Queries,0.04772
180	TIGD6	0.04791	ARCHS4 Coexpression,0.04791
181	CDC5L	0.04843	Enrichr Queries,0.04843
182	FOXJ2	0.04853	ARCHS4 Coexpression,0.04853
183	ZFPM1	0.04854	GTEx Coexpression,0.04854
184	CDX4	0.04914	ARCHS4 Coexpression,0.04914
185	ZBTB22	0.04915	Enrichr Queries,0.04915
186	AKAP8L	0.04986	Enrichr Queries,0.04986
187	ZBTB47	0.0504	GTEx Coexpression,0.0504
188	THAP1	0.05051	ReMap ChIP-seq,0.05051
189	GATA1	0.05085	ENCODE ChIP-seq,0.05085
190	ZBTB11	0.05098	ARCHS4 Coexpression,0.05098
191	NFE2L1	0.05128	Enrichr Queries,0.05128
192	ZBTB9	0.0516	ARCHS4 Coexpression,0.0516
193	SMAD4	0.05221	ARCHS4 Coexpression,0.05221
194	ZBTB42	0.05227	GTEx Coexpression,0.05227
195	PRMT3	0.05271	Enrichr Queries,0.05271
196	ZNF296	0.05283	ARCHS4 Coexpression,0.05283
197	ASH1L	0.05289	GTEx Coexpression,0.05289
198	ANKZF1	0.05342	Enrichr Queries,0.05342
199	NFYA	0.05387	ReMap ChIP-seq,0.05387
200	ZNF410	0.05413	Enrichr Queries,0.05413
201	MZF1	0.05414	GTEx Coexpression,0.05414
202	TMF1	0.05467	ARCHS4 Coexpression,0.05467
203	ZNF48	0.05484	Enrichr Queries,0.05484
204	ZFP42	0.05488	Literature ChIP-seq,0.05488
205	PA2G4	0.05528	ARCHS4 Coexpression,0.05528
206	SREBF1	0.05556	Enrichr Queries,0.05556
207	DMRTA1	0.0559	ARCHS4 Coexpression,0.0559
208	ZBTB17	0.056	GTEx Coexpression,0.056
209	ZNF547	0.05651	ARCHS4 Coexpression,0.05651
210	ZNF777	0.05663	GTEx Coexpression,0.05663
211	OTX1	0.05713	ARCHS4 Coexpression,0.05713

212	ZFX	0.05724	ReMap ChIP-seq,0.05724
213	ZNF558	0.05725	GTEx Coexpression,0.05725
214	SREBF2	0.05769	Enrichr Queries,0.05769
215	OLIG3	0.05787	GTEx Coexpression,0.05787
216	BARX1	0.05835	ARCHS4 Coexpression,0.05835
217	ZBTB48	0.0584	Enrichr Queries,0.0584
218	ZNF761	0.05849	GTEx Coexpression,0.05849
219	GBX1	0.05912	GTEx Coexpression,0.05912
220	NR5A1	0.05912	Enrichr Queries,0.05912
221	YY1	0.05932	ENCODE ChIP-seq,0.05932
222	ZNF57	0.05958	ARCHS4 Coexpression,0.05958
223	ZSCAN2	0.05974	GTEx Coexpression,0.05974
224	ZNF213	0.05983	Enrichr Queries,0.05983
225	NKX32	0.0602	ARCHS4 Coexpression,0.0602
226	PPARA	0.06036	GTEx Coexpression,0.06036
227	USF1	0.06054	Enrichr Queries,0.06054
228	ZNF629	0.06098	GTEx Coexpression,0.06098
229	NFYC	0.06125	Enrichr Queries,0.06125
230	ZNF148	0.06143	ARCHS4 Coexpression,0.06143
231	ZNF121	0.06161	GTEx Coexpression,0.06161
232	TFDP1	0.06197	Enrichr Queries,0.06197
233	GATA2	0.06204	ARCHS4 Coexpression,0.06204
234	RUNX1	0.06265	ARCHS4 Coexpression,0.06265
235	ZNF628	0.06268	Enrichr Queries,0.06268
236	ZNF117	0.06327	ARCHS4 Coexpression,0.06327
237	MECP2	0.06347	GTEx Coexpression,0.06347
238	ZNF282	0.06409	GTEx Coexpression,0.06409
239	ZNF674	0.0641	Enrichr Queries,0.0641
240	RLF	0.0645	ARCHS4 Coexpression,0.0645
241	ZNF335	0.06472	GTEx Coexpression,0.06472
242	CENPBD1	0.06511	ARCHS4 Coexpression,0.06511
243	ZNF707	0.06534	GTEx Coexpression,0.06534
244	NCOA3	0.06553	Enrichr Queries,0.06553
245	ZSCAN29	0.06572	ARCHS4 Coexpression,0.06572
246	FOXK2	0.06596	GTEx Coexpression,0.06596
247	ZNF22	0.06624	Enrichr Queries,0.06624
248	ZNF324	0.06634	ARCHS4 Coexpression,0.06634
249	ZNF202	0.06658	GTEx Coexpression,0.06658
250	ZNF160	0.06695	ARCHS4 Coexpression,0.06695
251	BAZ2A	0.06721	GTEx Coexpression,0.06721
252	GATAD2A	0.06766	Enrichr Queries,0.06766
253	FLI1	0.0678	ENCODE ChIP-seq,0.0678
254	GZF1	0.06783	GTEx Coexpression,0.06783
255	MYBL2	0.06818	ARCHS4 Coexpression,0.06818
256	KCMF1	0.06838	Enrichr Queries,0.06838
257	FIGLA	0.06845	GTEx Coexpression,0.06845
258	BBX	0.0688	ARCHS4 Coexpression,0.0688
259	ZNF700	0.06907	GTEx Coexpression,0.06907
260	HSF1	0.06941	ARCHS4 Coexpression,0.06941
261	HOXD13	0.07002	ARCHS4 Coexpression,0.07002
262	ZNF764	0.07032	GTEx Coexpression,0.07032
263	CREB3L4	0.07064	ARCHS4 Coexpression,0.07064
264	ZBTB33	0.07071	ReMap ChIP-seq,0.07071

265	ZNF705E	0.07094	GTEx Coexpression,0.07094
266	SNAPC4	0.07123	Enrichr Queries,0.07123
267	ZNF791	0.07156	GTEx Coexpression,0.07156
268	ZNF775	0.07194	Enrichr Queries,0.07194
269	TCF3	0.07265	Enrichr Queries,0.07265
270	POU3F1	0.0731	ARCHS4 Coexpression,0.0731
271	SPI1	0.07317	Literature ChIP-seq,0.07317
272	ZNF512	0.07336	Enrichr Queries,0.07336
273	NANOG	0.07343	GTEx Coexpression,0.07343
274	LMX1A	0.07371	ARCHS4 Coexpression,0.07371
275	NKRF	0.07407	Enrichr Queries,0.07407
276	ATF1	0.07407	ReMap ChIP-seq,0.07407
277	ZNF696	0.07432	ARCHS4 Coexpression,0.07432
278	ZNF79	0.07467	GTEx Coexpression,0.07467
279	RELA	0.07479	Enrichr Queries,0.07479
280	CEBPG	0.07494	ARCHS4 Coexpression,0.07494
281	ETV4	0.0753	GTEx Coexpression,0.0753
282	AKAP8	0.0755	Enrichr Queries,0.0755
283	ZNF175	0.07678	ARCHS4 Coexpression,0.07678
284	ZNF549	0.07692	Enrichr Queries,0.07692
285	RARA	0.07744	ReMap ChIP-seq,0.07744
286	DBP	0.07764	Enrichr Queries,0.07764
287	RORC	0.07778	GTEx Coexpression,0.07778
288	FOXN3	0.07801	ARCHS4 Coexpression,0.07801
289	MAFG	0.07835	Enrichr Queries,0.07835
290	HOXB2	0.07862	ARCHS4 Coexpression,0.07862
291	ZNF582	0.07924	ARCHS4 Coexpression,0.07924
292	ZNF208	0.07965	GTEx Coexpression,0.07965
293	ATF5	0.07977	Enrichr Queries,0.07977
294	MTERF2	0.08027	GTEx Coexpression,0.08027
295	VEZF1	0.08047	ARCHS4 Coexpression,0.08047
296	MXD4	0.08048	Enrichr Queries,0.08048
297	MYCN	0.08081	ReMap ChIP-seq,0.08081
298	ZNF211	0.0809	GTEx Coexpression,0.0809
299	FOXK1	0.08108	ARCHS4 Coexpression,0.08108
300	ZNF875	0.08152	GTEx Coexpression,0.08152
301	ZNF680	0.0817	ARCHS4 Coexpression,0.0817
302	ZNF689	0.08191	Enrichr Queries,0.08191
303	NKX23	0.08231	ARCHS4 Coexpression,0.08231
304	ZNF600	0.08276	GTEx Coexpression,0.08276
305	HOXA9	0.08292	ARCHS4 Coexpression,0.08292
306	ZNF655	0.08339	GTEx Coexpression,0.08339
307	ZNF346	0.08401	GTEx Coexpression,0.08401
308	ETV1	0.08418	ReMap ChIP-seq,0.08418
309	ZNF75D	0.08463	GTEx Coexpression,0.08463
310	NFE2L2	0.08476	Enrichr Queries,0.08476
311	ZNF841	0.08477	ARCHS4 Coexpression,0.08477
312	ZNF317	0.08525	GTEx Coexpression,0.08525
313	CREM	0.08537	Literature ChIP-seq,0.08537
314	E2F6	0.08538	ARCHS4 Coexpression,0.08538
315	TEAD2	0.08618	Enrichr Queries,0.08618
316	CREBZF	0.0865	GTEx Coexpression,0.0865
317	SRF	0.08689	Enrichr Queries,0.08689

318	ZNF142	0.08761	Enrichr Queries,0.08761
319	ZNF189	0.08784	ARCHS4 Coexpression,0.08784
320	HSF4	0.08836	GTEx Coexpression,0.08836
321	FOXJ3	0.08845	ARCHS4 Coexpression,0.08845
322	NR1H4	0.08907	ARCHS4 Coexpression,0.08907
323	SON	0.08961	GTEx Coexpression,0.08961
324	ZNF276	0.08974	Enrichr Queries,0.08974
325	HOXC13	0.09029	ARCHS4 Coexpression,0.09029
326	PLAGL1	0.09085	GTEx Coexpression,0.09085
327	KMT2B	0.09091	ReMap ChIP-seq,0.09091
328	ZNF473	0.09117	Enrichr Queries,0.09117
329	CTCF	0.09146	Literature ChIP-seq,0.09146
330	ZNF44	0.09147	GTEx Coexpression,0.09147
331	HOXC10	0.09152	ARCHS4 Coexpression,0.09152
332	ZNF263	0.0921	GTEx Coexpression,0.0921
333	ZFP57	0.09214	ARCHS4 Coexpression,0.09214
334	STAT3	0.09259	Enrichr Queries,0.09259
335	ATF7	0.09272	GTEx Coexpression,0.09272
336	RFX7	0.09275	ARCHS4 Coexpression,0.09275
337	ATF3	0.09322	ENCODE ChIP-seq,0.09322
338	ZNF16	0.0933	Enrichr Queries,0.0933
339	ZNF783	0.09402	Enrichr Queries,0.09402
340	CTCFL	0.09428	ReMap ChIP-seq,0.09428
341	ZNF333	0.09459	GTEx Coexpression,0.09459
342	NFKB2	0.09473	Enrichr Queries,0.09473
343	SP3	0.09521	ARCHS4 Coexpression,0.09521
344	ZNF7	0.09544	Enrichr Queries,0.09544
345	MEF2A	0.09582	ARCHS4 Coexpression,0.09582
346	NR1I3	0.09615	Enrichr Queries,0.09615
347	USF3	0.09644	ARCHS4 Coexpression,0.09644
348	ZSCAN5B	0.09687	Enrichr Queries,0.09687
349	TBX3	0.09708	GTEx Coexpression,0.09708
350	TET1	0.09756	Literature ChIP-seq,0.09756
351	PBX2	0.09758	Enrichr Queries,0.09758
352	SP1	0.09764	ReMap ChIP-seq,0.09764
353	DDIT3	0.09767	ARCHS4 Coexpression,0.09767
354	STAT2	0.0977	GTEx Coexpression,0.0977
355	ZBTB44	0.09889	ARCHS4 Coexpression,0.09889
356	FOXM1	0.099	Enrichr Queries,0.099
357	ZKSCAN2	0.09956	GTEx Coexpression,0.09956
358	ZBED5	0.1001	ARCHS4 Coexpression,0.1001
359	MSANTD3	0.1002	GTEx Coexpression,0.1002
360	ZNF526	0.1004	Enrichr Queries,0.1004
361	FOXA2	0.1007	ARCHS4 Coexpression,0.1007
362	IRX1	0.1008	GTEx Coexpression,0.1008
363	CEBPD	0.101	ReMap ChIP-seq,0.101
364	ZNF514	0.1014	ARCHS4 Coexpression,0.1014
365	RXRA	0.1014	GTEx Coexpression,0.1014
366	ZNF430	0.1019	Enrichr Queries,0.1019
367	TCF4	0.1021	GTEx Coexpression,0.1021
368	ZC3H8	0.1026	Enrichr Queries,0.1026
369	TCFL5	0.1033	Enrichr Queries,0.1033
370	SAFB	0.1033	GTEx Coexpression,0.1033

371	TAL1	0.1037	Literature ChIP-seq,0.1037
372	ZNF35	0.1038	ARCHS4 Coexpression,0.1038
373	ZNF146	0.104	Enrichr Queries,0.104
374	ZHX2	0.1044	ReMap ChIP-seq,0.1044
375	ZBTB10	0.1044	ARCHS4 Coexpression,0.1044
376	ZNF704	0.1045	GTEx Coexpression,0.1045
377	MEF2B	0.1047	Enrichr Queries,0.1047
378	HOXA10	0.105	ARCHS4 Coexpression,0.105
379	KLF11	0.1052	GTEx Coexpression,0.1052
380	ZNF251	0.1054	Enrichr Queries,0.1054
381	ZNF219	0.1061	Enrichr Queries,0.1061
382	MYSM1	0.107	GTEx Coexpression,0.107
383	HOXA1	0.1075	ARCHS4 Coexpression,0.1075
384	YBX2	0.1075	Enrichr Queries,0.1075
385	ZBTB40	0.1077	GTEx Coexpression,0.1077
386	STAT6	0.1083	Enrichr Queries,0.1083
387	TCF7L2	0.1089	GTEx Coexpression,0.1089
388	NFKB1	0.109	Enrichr Queries,0.109
389	FOXD2	0.1093	ARCHS4 Coexpression,0.1093
390	THAP9	0.1095	GTEx Coexpression,0.1095
391	DACH1	0.1098	Literature ChIP-seq,0.1098
392	ZNF451	0.11	ARCHS4 Coexpression,0.11
393	ZNF280A	0.1101	GTEx Coexpression,0.1101
394	SIX3	0.1106	ARCHS4 Coexpression,0.1106
395	HSF2	0.1108	GTEx Coexpression,0.1108
396	DPRX	0.1112	ARCHS4 Coexpression,0.1112
397	ZUP1	0.1118	ARCHS4 Coexpression,0.1118
398	E2F2	0.1118	Enrichr Queries,0.1118
399	ZNF740	0.112	GTEx Coexpression,0.112
400	TFE3	0.1125	Enrichr Queries,0.1125
401	CREBL2	0.1126	GTEx Coexpression,0.1126
402	ZSCAN21	0.113	ARCHS4 Coexpression,0.113
403	RFX5	0.1132	Enrichr Queries,0.1132
404	ATF2	0.1136	ARCHS4 Coexpression,0.1136
405	ERG	0.1145	ReMap ChIP-seq,0.1145
406	ZNF326	0.1147	Enrichr Queries,0.1147
407	ZHX1	0.1151	GTEx Coexpression,0.1151
408	ZNF324B	0.1155	ARCHS4 Coexpression,0.1155
409	ZNF592	0.1157	GTEx Coexpression,0.1157
410	HOXC4	0.1161	ARCHS4 Coexpression,0.1161
411	ZNF394	0.1161	Enrichr Queries,0.1161
412	NKX11	0.1164	GTEx Coexpression,0.1164
413	GATA3	0.1167	ARCHS4 Coexpression,0.1167
414	ZNF41	0.117	GTEx Coexpression,0.117
415	HES5	0.1173	ARCHS4 Coexpression,0.1173
416	FOXR1	0.1175	Enrichr Queries,0.1175
417	MAFK	0.1176	GTEx Coexpression,0.1176
418	CENPA	0.1179	ARCHS4 Coexpression,0.1179
419	BPTF	0.1186	ARCHS4 Coexpression,0.1186
420	UBP1	0.1189	GTEx Coexpression,0.1189
421	ZNF367	0.1189	Enrichr Queries,0.1189
422	MSX1	0.1204	ARCHS4 Coexpression,0.1204
423	PLAGL2	0.1207	GTEx Coexpression,0.1207

424	CBX2	0.121	ARCHS4 Coexpression,0.121
425	NR1D1	0.1211	Enrichr Queries,0.1211
426	TGIF2	0.1212	ReMap ChIP-seq,0.1212
427	CEBPA	0.1213	GTEx Coexpression,0.1213
428	ZBTB3	0.1218	Enrichr Queries,0.1218
429	FOXP3	0.122	Literature ChIP-seq,0.122
430	MBD6	0.1226	GTEx Coexpression,0.1226
431	ZNF33A	0.1229	ARCHS4 Coexpression,0.1229
432	ZNF33B	0.1235	ARCHS4 Coexpression,0.1235
433	ZBTB12	0.1239	Enrichr Queries,0.1239
434	HOXA5	0.1241	ARCHS4 Coexpression,0.1241
435	ZNF512B	0.1245	GTEx Coexpression,0.1245
436	CREB3L3	0.1246	Enrichr Queries,0.1246
437	GPBP1	0.1247	ARCHS4 Coexpression,0.1247
438	YBX3	0.1251	GTEx Coexpression,0.1251
439	ZNF500	0.1254	Enrichr Queries,0.1254
440	ZNF843	0.1263	GTEx Coexpression,0.1263
441	HMBOX1	0.1265	ARCHS4 Coexpression,0.1265
442	FOXN4	0.1269	GTEx Coexpression,0.1269
443	ZKSCAN5	0.1276	GTEx Coexpression,0.1276
444	FOXO3	0.1278	ARCHS4 Coexpression,0.1278
445	CREB3L1	0.1279	ReMap ChIP-seq,0.1279
446	HOXB4	0.128	Literature ChIP-seq,0.128
447	ZNF710	0.1282	GTEx Coexpression,0.1282
448	ZNF12	0.1288	GTEx Coexpression,0.1288
449	WIZ	0.1289	Enrichr Queries,0.1289
450	HOXA11	0.129	ARCHS4 Coexpression,0.129
451	SOX15	0.1296	ARCHS4 Coexpression,0.1296
452	ZNF385A	0.1296	Enrichr Queries,0.1296
453	ZXDB	0.1302	ARCHS4 Coexpression,0.1302
454	PHF1	0.1307	GTEx Coexpression,0.1307
455	ELF5	0.1308	ARCHS4 Coexpression,0.1308
456	TIGD3	0.1311	Enrichr Queries,0.1311
457	ZNF662	0.1313	GTEx Coexpression,0.1313
458	EGR1	0.1313	ReMap ChIP-seq,0.1313
459	CC2D1A	0.1314	ARCHS4 Coexpression,0.1314
460	PCGF6	0.1318	Enrichr Queries,0.1318
461	ZSCAN25	0.1319	GTEx Coexpression,0.1319
462	ZBED6	0.1321	ARCHS4 Coexpression,0.1321
463	ZNF131	0.1325	Enrichr Queries,0.1325
464	ELF2	0.1325	GTEx Coexpression,0.1325
465	ZNF138	0.1327	ARCHS4 Coexpression,0.1327
466	ZNF81	0.1333	ARCHS4 Coexpression,0.1333
467	ZNF550	0.1338	GTEx Coexpression,0.1338
468	ZFP62	0.1344	GTEx Coexpression,0.1344
469	LCOR	0.1345	ARCHS4 Coexpression,0.1345
470	ZNF266	0.1346	Enrichr Queries,0.1346
471	PBX3	0.1347	ReMap ChIP-seq,0.1347
472	REST	0.1356	ENCODE ChIP-seq,0.1356
473	ZNF560	0.1363	GTEx Coexpression,0.1363
474	EEA1	0.1364	ARCHS4 Coexpression,0.1364
475	RAX2	0.1368	Enrichr Queries,0.1368
476	TAL2	0.1369	GTEx Coexpression,0.1369

477	SCX	0.137	ARCHS4 Coexpression,0.137
478	ETV2	0.1375	Enrichr Queries,0.1375
479	NCOA2	0.1375	GTEx Coexpression,0.1375
480	JAZF1	0.1376	ARCHS4 Coexpression,0.1376
481	SP4	0.138	ReMap ChIP-seq,0.138
482	NFYB	0.1382	ARCHS4 Coexpression,0.1382
483	KLF9	0.1388	GTEx Coexpression,0.1388
484	ZNF746	0.1389	Enrichr Queries,0.1389
485	ZNF772	0.1394	GTEx Coexpression,0.1394
486	TEF	0.1396	Enrichr Queries,0.1396
487	NEUROG3	0.14	ARCHS4 Coexpression,0.14
488	KLF6	0.141	Enrichr Queries,0.141
489	NR0B1	0.1413	GTEx Coexpression,0.1413
490	NCOA1	0.1413	ARCHS4 Coexpression,0.1413
491	ZNF34	0.1417	Enrichr Queries,0.1417
492	ZNF518A	0.1419	GTEx Coexpression,0.1419
493	MNX1	0.1419	ARCHS4 Coexpression,0.1419
494	ZNF23	0.1425	Enrichr Queries,0.1425
495	ZNF133	0.1425	GTEx Coexpression,0.1425
496	ZNF300	0.1431	GTEx Coexpression,0.1431
497	ZSCAN10	0.1432	Enrichr Queries,0.1432
498	HBP1	0.1437	ARCHS4 Coexpression,0.1437
499	TBX1	0.1437	GTEx Coexpression,0.1437
500	HNF4A	0.1439	Enrichr Queries,0.1439
501	PITX1	0.1443	ARCHS4 Coexpression,0.1443
502	LHX3	0.1444	GTEx Coexpression,0.1444
503	ZNF789	0.1446	Enrichr Queries,0.1446
504	DMRTA2	0.145	ARCHS4 Coexpression,0.145
505	DBX1	0.145	GTEx Coexpression,0.145
506	HOXA4	0.1456	ARCHS4 Coexpression,0.1456
507	MYB	0.146	Enrichr Queries,0.146
508	ZNF503	0.1462	ARCHS4 Coexpression,0.1462
509	RFX1	0.1462	GTEx Coexpression,0.1462
510	POU5F1	0.1467	Enrichr Queries,0.1467
511	ZNF292	0.1468	ARCHS4 Coexpression,0.1468
512	HOXD11	0.1474	ARCHS4 Coexpression,0.1474
513	RARG	0.1474	Enrichr Queries,0.1474
514	ZXDC	0.1475	GTEx Coexpression,0.1475
515	ZNF83	0.1481	GTEx Coexpression,0.1481
516	ZNF143	0.1481	ReMap ChIP-seq,0.1481
517	HIF1A	0.1481	Enrichr Queries,0.1481
518	ZSCAN9	0.1487	GTEx Coexpression,0.1487
519	SNAI3	0.1489	Enrichr Queries,0.1489
520	ZZZ3	0.1493	ARCHS4 Coexpression,0.1493
521	ASCL5	0.1493	GTEx Coexpression,0.1493
522	ERF	0.1496	Enrichr Queries,0.1496
523	ZNF594	0.1499	ARCHS4 Coexpression,0.1499
524	ZNF541	0.15	GTEx Coexpression,0.15
525	MBD4	0.1503	Enrichr Queries,0.1503
526	ZBTB43	0.1505	ARCHS4 Coexpression,0.1505
527	ELF3	0.151	Enrichr Queries,0.151
528	MGA	0.1511	ARCHS4 Coexpression,0.1511
529	KLF13	0.1515	ReMap ChIP-seq,0.1515

530	MBD1	0.1517	Enrichr Queries,0.1517
531	RXRG	0.1518	GTEx Coexpression,0.1518
532	ZBTB49	0.1529	ARCHS4 Coexpression,0.1529
533	ZNF778	0.1531	Enrichr Queries,0.1531
534	FOSL1	0.1536	ARCHS4 Coexpression,0.1536
535	ZNF215	0.1542	ARCHS4 Coexpression,0.1542
536	ZNF496	0.1546	Enrichr Queries,0.1546
537	TFDP2	0.1548	ARCHS4 Coexpression,0.1548
538	ESR2	0.1549	GTEx Coexpression,0.1549
539	ZNF416	0.1554	ARCHS4 Coexpression,0.1554
540	SOHLH1	0.156	Enrichr Queries,0.156
541	ZNF575	0.1562	GTEx Coexpression,0.1562
542	MNT	0.1567	Enrichr Queries,0.1567
543	SAFB2	0.1568	GTEx Coexpression,0.1568
544	ZNF544	0.1572	ARCHS4 Coexpression,0.1572
545	MEF2D	0.1574	Enrichr Queries,0.1574
546	HOMEZ	0.1574	GTEx Coexpression,0.1574
547	KLF12	0.1579	ARCHS4 Coexpression,0.1579
548	STAT5B	0.1581	Enrichr Queries,0.1581
549	HOXC12	0.1585	ARCHS4 Coexpression,0.1585
550	RARB	0.1585	Literature ChIP-seq,0.1585
551	SOX6	0.1587	GTEx Coexpression,0.1587
552	ZNF776	0.1591	ARCHS4 Coexpression,0.1591
553	ZNF853	0.1593	GTEx Coexpression,0.1593
554	ZXDA	0.1597	ARCHS4 Coexpression,0.1597
555	SRCAP	0.1599	GTEx Coexpression,0.1599
556	ZBTB7B	0.1603	Enrichr Queries,0.1603
557	BHLHA15	0.1609	ARCHS4 Coexpression,0.1609
558	ZKSCAN3	0.1617	Enrichr Queries,0.1617
559	ZNF485	0.1618	GTEx Coexpression,0.1618
560	ZNF236	0.1628	ARCHS4 Coexpression,0.1628
561	SETDB1	0.163	GTEx Coexpression,0.163
562	ZNF341	0.1631	Enrichr Queries,0.1631
563	FOXD4L4	0.1634	ARCHS4 Coexpression,0.1634
564	LBX1	0.1643	GTEx Coexpression,0.1643
565	FOXE3	0.1652	ARCHS4 Coexpression,0.1652
566	AIRE	0.1652	Enrichr Queries,0.1652
567	ZNF384	0.1655	GTEx Coexpression,0.1655
568	TBPL1	0.1658	ARCHS4 Coexpression,0.1658
569	ZNF497	0.166	Enrichr Queries,0.166
570	ZNF354B	0.1661	GTEx Coexpression,0.1661
571	ZNF654	0.1665	ARCHS4 Coexpression,0.1665
572	NR1D2	0.1667	Enrichr Queries,0.1667
573	ZNF587	0.1668	GTEx Coexpression,0.1668
574	ZNF714	0.1671	ARCHS4 Coexpression,0.1671
575	ZNF362	0.1674	Enrichr Queries,0.1674
576	MAFA	0.1674	GTEx Coexpression,0.1674
577	LCORL	0.1683	ARCHS4 Coexpression,0.1683
578	ARID2	0.1689	ARCHS4 Coexpression,0.1689
579	PAX1	0.1693	GTEx Coexpression,0.1693
580	ZNF260	0.1695	ARCHS4 Coexpression,0.1695
581	ZNF695	0.1701	ARCHS4 Coexpression,0.1701
582	MLXIPL	0.1702	Enrichr Queries,0.1702

583	ZNF19	0.1705	GTEx Coexpression,0.1705
584	TERF2	0.1709	Enrichr Queries,0.1709
585	ZBTB4	0.1711	GTEx Coexpression,0.1711
586	ZNF280C	0.1714	ARCHS4 Coexpression,0.1714
587	FOXP2	0.1717	ReMap ChIP-seq,0.1717
588	ZNF565	0.1717	GTEx Coexpression,0.1717
589	MTF2	0.172	ARCHS4 Coexpression,0.172
590	JUNB	0.1731	Enrichr Queries,0.1731
591	SPDEF	0.1738	Enrichr Queries,0.1738
592	ZNF197	0.1738	ARCHS4 Coexpression,0.1738
593	RFX4	0.1742	GTEx Coexpression,0.1742
594	BCL11A	0.1749	GTEx Coexpression,0.1749
595	ZNF460	0.1751	ARCHS4 Coexpression,0.1751
596	IRF2	0.1751	ReMap ChIP-seq,0.1751
597	ZBTB7A	0.1752	Enrichr Queries,0.1752
598	ZNF66	0.1755	GTEx Coexpression,0.1755
599	CLOCK	0.1757	ARCHS4 Coexpression,0.1757
600	KLF2	0.1759	Enrichr Queries,0.1759
601	PDX1	0.1761	GTEx Coexpression,0.1761
602	ZNF10	0.1763	ARCHS4 Coexpression,0.1763
603	NOBOX	0.1767	GTEx Coexpression,0.1767
604	KLF4	0.1768	Literature ChIP-seq,0.1768
605	FOXD4L1	0.1769	ARCHS4 Coexpression,0.1769
606	FOXA3	0.1773	GTEx Coexpression,0.1773
607	ZNF277	0.1774	Enrichr Queries,0.1774
608	ZNF615	0.1775	ARCHS4 Coexpression,0.1775
609	JUN	0.178	ENCODE ChIP-seq,0.178
610	MITF	0.178	GTEx Coexpression,0.178
611	ZBTB37	0.1781	ARCHS4 Coexpression,0.1781
612	CIC	0.1786	GTEx Coexpression,0.1786
613	KLF10	0.1788	Enrichr Queries,0.1788
614	MYOG	0.1792	GTEx Coexpression,0.1792
615	TFEB	0.1795	Enrichr Queries,0.1795
616	MBD2	0.1802	Enrichr Queries,0.1802
617	ZNF639	0.1809	Enrichr Queries,0.1809
618	PITX2	0.1811	GTEx Coexpression,0.1811
619	ZFP90	0.1816	Enrichr Queries,0.1816
620	DMRTB1	0.1817	GTEx Coexpression,0.1817
621	GSC	0.1818	ARCHS4 Coexpression,0.1818
622	ZMAT1	0.1824	ARCHS4 Coexpression,0.1824
623	FOXP1	0.1829	Literature ChIP-seq,0.1829
624	CGGBP1	0.1829	GTEx Coexpression,0.1829
625	ZNF74	0.1837	ARCHS4 Coexpression,0.1837
626	ZNF274	0.1838	Enrichr Queries,0.1838
627	ZBTB32	0.1845	Enrichr Queries,0.1845
628	KIN	0.1852	Enrichr Queries,0.1852
629	PITX3	0.1854	GTEx Coexpression,0.1854
630	XPA	0.1855	ARCHS4 Coexpression,0.1855
631	ZNF780B	0.1861	GTEx Coexpression,0.1861
632	NR1H3	0.1861	ARCHS4 Coexpression,0.1861
633	CAMTA2	0.1866	Enrichr Queries,0.1866
634	FOXP4	0.1867	GTEx Coexpression,0.1867
635	ZNF233	0.1873	GTEx Coexpression,0.1873

636	ZNF821	0.1873	Enrichr Queries,0.1873
637	FOXD4	0.1873	ARCHS4 Coexpression,0.1873
638	ZNF275	0.1879	GTEx Coexpression,0.1879
639	HOXC11	0.188	ARCHS4 Coexpression,0.188
640	ZNF664	0.188	Enrichr Queries,0.188
641	ZFP91	0.1886	GTEx Coexpression,0.1886
642	SOX3	0.1886	ARCHS4 Coexpression,0.1886
643	ZNF687	0.1887	Enrichr Queries,0.1887
644	FEV	0.1892	GTEx Coexpression,0.1892
645	ZNF519	0.1895	Enrichr Queries,0.1895
646	LIN28B	0.1898	GTEx Coexpression,0.1898
647	SOX2	0.1898	ARCHS4 Coexpression,0.1898
648	ZNF217	0.1902	Enrichr Queries,0.1902
649	ZNF546	0.191	GTEx Coexpression,0.191
650	ZNF530	0.1916	ARCHS4 Coexpression,0.1916
651	ZNF852	0.1923	ARCHS4 Coexpression,0.1923
652	ZNF516	0.1923	GTEx Coexpression,0.1923
653	MTERF1	0.1929	ARCHS4 Coexpression,0.1929
654	ZNF467	0.193	Enrichr Queries,0.193
655	ZNF195	0.1935	ARCHS4 Coexpression,0.1935
656	GLI3	0.1935	GTEx Coexpression,0.1935
657	TBX10	0.1937	Enrichr Queries,0.1937
658	ZNF222	0.1941	ARCHS4 Coexpression,0.1941
659	KMT2A	0.1942	GTEx Coexpression,0.1942
660	ZNF551	0.1944	Enrichr Queries,0.1944
661	ZNF140	0.1947	ARCHS4 Coexpression,0.1947
662	ZFAT	0.1948	GTEx Coexpression,0.1948
663	STAT1	0.1952	Enrichr Queries,0.1952
664	ZKSCAN1	0.1953	ReMap ChIP-seq,0.1953
665	ZSCAN16	0.1953	ARCHS4 Coexpression,0.1953
666	MYF6	0.1954	GTEx Coexpression,0.1954
667	TEAD3	0.1959	Enrichr Queries,0.1959
668	ZNF573	0.1966	ARCHS4 Coexpression,0.1966
669	TERF1	0.1966	Enrichr Queries,0.1966
670	ZNF200	0.1966	GTEx Coexpression,0.1966
671	HHEX	0.1972	ARCHS4 Coexpression,0.1972
672	ZNF316	0.1973	GTEx Coexpression,0.1973
673	ZNF589	0.1973	Enrichr Queries,0.1973
674	NFIB	0.1979	GTEx Coexpression,0.1979
675	KLF15	0.198	Enrichr Queries,0.198
676	SMAD9	0.1984	ARCHS4 Coexpression,0.1984
677	GTF2IRD2B	0.1985	GTEx Coexpression,0.1985
678	NEUROD1	0.1987	ReMap ChIP-seq,0.1987
679	NFXL1	0.199	ARCHS4 Coexpression,0.199
680	HSFY1	0.1991	GTEx Coexpression,0.1991
681	ZNF669	0.1994	Enrichr Queries,0.1994
682	MYOD1	0.1998	GTEx Coexpression,0.1998
683	ZNF223	0.2001	Enrichr Queries,0.2001
684	ZNF391	0.2002	ARCHS4 Coexpression,0.2002
685	ARID3B	0.2004	GTEx Coexpression,0.2004
686	PPARD	0.2009	Enrichr Queries,0.2009
687	E2F5	0.2009	ARCHS4 Coexpression,0.2009
688	ZNF646	0.2016	Enrichr Queries,0.2016

689	PAX7	0.2022	GTEx Coexpression,0.2022
690	RBPJL	0.2027	ARCHS4 Coexpression,0.2027
691	KDM2A	0.2029	GTEx Coexpression,0.2029
692	HNF1A	0.203	Enrichr Queries,0.203
693	IRF1	0.2034	ENCODE ChIP-seq,0.2034
694	PATZ1	0.2037	Enrichr Queries,0.2037
695	ZNF703	0.2039	ARCHS4 Coexpression,0.2039
696	RHOXF2	0.2044	Enrichr Queries,0.2044
697	ZNF417	0.2045	ARCHS4 Coexpression,0.2045
698	HES7	0.2051	Enrichr Queries,0.2051
699	ZNF626	0.2052	ARCHS4 Coexpression,0.2052
700	MAF	0.2054	ReMap ChIP-seq,0.2054
701	ZNF432	0.206	GTEx Coexpression,0.206
702	FEZF1	0.207	ARCHS4 Coexpression,0.207
703	ZNF786	0.2072	GTEx Coexpression,0.2072
704	ATMIN	0.208	Enrichr Queries,0.208
705	DNMT1	0.2082	ARCHS4 Coexpression,0.2082
706	ZIM3	0.2085	GTEx Coexpression,0.2085
707	ZNF517	0.2087	Enrichr Queries,0.2087
708	NR2F1	0.2088	ReMap ChIP-seq,0.2088
709	BORCS8MEF2B	0.2088	ARCHS4 Coexpression,0.2088
710	LYL1	0.2091	GTEx Coexpression,0.2091
711	RELB	0.2094	Enrichr Queries,0.2094
712	ZNF71	0.2095	ARCHS4 Coexpression,0.2095
713	SKOR1	0.2101	ARCHS4 Coexpression,0.2101
714	HMX2	0.2107	ARCHS4 Coexpression,0.2107
715	ARID3A	0.2108	Enrichr Queries,0.2108
716	ZNF658	0.2113	ARCHS4 Coexpression,0.2113
717	GFI1B	0.2121	ReMap ChIP-seq,0.2121
718	SP5	0.2122	GTEx Coexpression,0.2122
719	ZNF552	0.2125	ARCHS4 Coexpression,0.2125
720	ZFP14	0.2128	GTEx Coexpression,0.2128
721	ZNF554	0.213	Enrichr Queries,0.213
722	ZNF718	0.2131	ARCHS4 Coexpression,0.2131
723	ZNF77	0.2137	Enrichr Queries,0.2137
724	ZNF433	0.2138	ARCHS4 Coexpression,0.2138
725	NR2E3	0.2144	ARCHS4 Coexpression,0.2144
726	SOX12	0.2144	Enrichr Queries,0.2144
727	GPBP1L1	0.2147	GTEx Coexpression,0.2147
728	ZNF2	0.2151	Enrichr Queries,0.2151
729	ZNF226	0.2156	ARCHS4 Coexpression,0.2156
730	SIX1	0.2159	GTEx Coexpression,0.2159
731	ZNF155	0.2162	ARCHS4 Coexpression,0.2162
732	DMRTC2	0.2172	GTEx Coexpression,0.2172
733	ZNF586	0.2172	Enrichr Queries,0.2172
734	ZNF69	0.2174	ARCHS4 Coexpression,0.2174
735	DOT1L	0.2184	GTEx Coexpression,0.2184
736	NR2C1	0.2187	ARCHS4 Coexpression,0.2187
737	FBXL19	0.2193	ARCHS4 Coexpression,0.2193
738	ZSCAN18	0.2194	Enrichr Queries,0.2194
739	SOX17	0.2195	Literature ChIP-seq,0.2195
740	ZBED9	0.2197	GTEx Coexpression,0.2197
741	IKZF5	0.2199	ARCHS4 Coexpression,0.2199

742	OVOL3	0.2203	GTEx Coexpression,0.2203
743	ZNF737	0.2205	ARCHS4 Coexpression,0.2205
744	RAG1	0.2209	GTEx Coexpression,0.2209
745	ZNF319	0.2215	Enrichr Queries,0.2215
746	HIC2	0.2215	GTEx Coexpression,0.2215
747	TGIF1	0.2217	ARCHS4 Coexpression,0.2217
748	BHLHE40	0.2222	ReMap ChIP-seq,0.2222
749	ZFP41	0.2222	Enrichr Queries,0.2222
750	ZNF625	0.223	ARCHS4 Coexpression,0.223
751	FOXD1	0.2236	ARCHS4 Coexpression,0.2236
752	MSANTD4	0.224	GTEx Coexpression,0.224
753	ZNF606	0.2242	ARCHS4 Coexpression,0.2242
754	ZNF354A	0.2244	Enrichr Queries,0.2244
755	FAM170A	0.2253	GTEx Coexpression,0.2253
756	MBNL2	0.2259	GTEx Coexpression,0.2259
757	OVOL2	0.2265	Enrichr Queries,0.2265
758	OTX2	0.2267	ARCHS4 Coexpression,0.2267
759	NFIC	0.2272	Enrichr Queries,0.2272
760	ZNF599	0.2278	GTEx Coexpression,0.2278
761	THRA	0.2279	Enrichr Queries,0.2279
762	ARHGAP35	0.2284	GTEx Coexpression,0.2284
763	E2F3	0.2286	Enrichr Queries,0.2286
764	ZNF808	0.2291	ARCHS4 Coexpression,0.2291
765	ZNF577	0.2296	GTEx Coexpression,0.2296
766	ZNF846	0.2297	ARCHS4 Coexpression,0.2297
767	ZNF90	0.2303	ARCHS4 Coexpression,0.2303
768	FOXO4	0.2308	Enrichr Queries,0.2308
769	DMRT1	0.2309	GTEx Coexpression,0.2309
770	ETS2	0.2315	Enrichr Queries,0.2315
771	NFATC3	0.2315	GTEx Coexpression,0.2315
772	ZNF75A	0.2316	ARCHS4 Coexpression,0.2316
773	E2F7	0.2322	Enrichr Queries,0.2322
774	SNAI2	0.2323	ReMap ChIP-seq,0.2323
775	GFI1	0.2328	ARCHS4 Coexpression,0.2328
776	GLIS2	0.2329	Enrichr Queries,0.2329
777	SIX2	0.2334	GTEx Coexpression,0.2334
778	POGK	0.2336	Enrichr Queries,0.2336
779	POU5F2	0.234	GTEx Coexpression,0.234
780	ASCL2	0.234	ARCHS4 Coexpression,0.234
781	TIGD2	0.2343	Enrichr Queries,0.2343
782	HOXC6	0.2346	ARCHS4 Coexpression,0.2346
783	PRR12	0.2352	GTEx Coexpression,0.2352
784	GATAD2B	0.2353	ARCHS4 Coexpression,0.2353
785	JDP2	0.2358	Enrichr Queries,0.2358
786	FOXF2	0.2359	ARCHS4 Coexpression,0.2359
787	EBF4	0.2365	GTEx Coexpression,0.2365
788	ETV5	0.2365	Enrichr Queries,0.2365
789	IRX5	0.2371	ARCHS4 Coexpression,0.2371
790	HINFP	0.2377	GTEx Coexpression,0.2377
791	SRY	0.2378	Literature ChIP-seq,0.2378
792	ZNF652	0.2383	ARCHS4 Coexpression,0.2383
793	POU1F1	0.2383	GTEx Coexpression,0.2383
794	NEUROD4	0.239	GTEx Coexpression,0.239

795	ZEB1	0.2391	ReMap ChIP-seq,0.2391
796	STAT5A	0.2393	Enrichr Queries,0.2393
797	TBX15	0.2396	GTEx Coexpression,0.2396
798	ZBTB2	0.24	Enrichr Queries,0.24
799	ZNF699	0.2402	ARCHS4 Coexpression,0.2402
800	MYF5	0.2402	GTEx Coexpression,0.2402
801	ZNF239	0.2407	Enrichr Queries,0.2407
802	ZNF345	0.2408	GTEx Coexpression,0.2408
803	ZFY	0.2414	ARCHS4 Coexpression,0.2414
804	ZNF20	0.2414	GTEx Coexpression,0.2414
805	ZNF716	0.2421	GTEx Coexpression,0.2421
806	ZNF564	0.2422	Enrichr Queries,0.2422
807	TLX2	0.2426	ARCHS4 Coexpression,0.2426
808	ZNF684	0.2427	GTEx Coexpression,0.2427
809	ADNP2	0.2429	Enrichr Queries,0.2429
810	NFE2L3	0.2433	GTEx Coexpression,0.2433
811	ZNF415	0.2439	ARCHS4 Coexpression,0.2439
812	SIX4	0.2439	GTEx Coexpression,0.2439
813	FOXH1	0.245	Enrichr Queries,0.245
814	KAT7	0.2452	GTEx Coexpression,0.2452
815	SPIC	0.2457	ARCHS4 Coexpression,0.2457
816	KDM5B	0.2458	ReMap ChIP-seq,0.2458
817	FOS	0.2463	ARCHS4 Coexpression,0.2463
818	ZSCAN22	0.2464	GTEx Coexpression,0.2464
819	ZNF180	0.2469	ARCHS4 Coexpression,0.2469
820	TFCP2	0.2472	Enrichr Queries,0.2472
821	SOX18	0.2475	ARCHS4 Coexpression,0.2475
822	MESP2	0.2477	GTEx Coexpression,0.2477
823	MAFF	0.2482	ARCHS4 Coexpression,0.2482
824	THAP5	0.2483	GTEx Coexpression,0.2483
825	ZNF141	0.2488	ARCHS4 Coexpression,0.2488
826	PRDM15	0.2489	GTEx Coexpression,0.2489
827	CXXC5	0.2494	ARCHS4 Coexpression,0.2494
828	ZNF587B	0.2495	GTEx Coexpression,0.2495
829	CEBPE	0.25	Enrichr Queries,0.25
830	ZNF256	0.25	ARCHS4 Coexpression,0.25
831	RFX3	0.2502	GTEx Coexpression,0.2502
832	ZNF254	0.2506	ARCHS4 Coexpression,0.2506
833	NR3C2	0.2508	GTEx Coexpression,0.2508
834	TOPORS	0.2512	ARCHS4 Coexpression,0.2512
835	ZNF486	0.2514	Enrichr Queries,0.2514
836	ZNF597	0.2521	Enrichr Queries,0.2521
837	ZNF813	0.2525	ARCHS4 Coexpression,0.2525
838	ZNF253	0.2531	ARCHS4 Coexpression,0.2531
839	FOXL1	0.2537	ARCHS4 Coexpression,0.2537
840	OLIG1	0.2543	Enrichr Queries,0.2543
841	ZGLP1	0.2543	ARCHS4 Coexpression,0.2543
842	ZNF644	0.2549	ARCHS4 Coexpression,0.2549
843	ZSCAN12	0.2555	ARCHS4 Coexpression,0.2555
844	ETV6	0.2558	GTEx Coexpression,0.2558
845	GTF2B	0.2559	ReMap ChIP-seq,0.2559
846	ZFP82	0.2561	ARCHS4 Coexpression,0.2561
847	ZNF627	0.2568	ARCHS4 Coexpression,0.2568

848	CRX	0.257	GTEx Coexpression,0.257
849	NFIX	0.2578	Enrichr Queries,0.2578
850	ZNF98	0.2582	GTEx Coexpression,0.2582
851	HOXA6	0.2586	ARCHS4 Coexpression,0.2586
852	ONECUT3	0.2589	GTEx Coexpression,0.2589
853	ZIK1	0.2593	Enrichr Queries,0.2593
854	KLF5	0.2593	ReMap ChIP-seq,0.2593
855	PTF1A	0.2595	GTEx Coexpression,0.2595
856	SALL2	0.26	Enrichr Queries,0.26
857	ZSCAN5A	0.2614	Enrichr Queries,0.2614
858	TBX6	0.262	GTEx Coexpression,0.262
859	FOXO1	0.2621	Enrichr Queries,0.2621
860	SMAD1	0.2626	ReMap ChIP-seq,0.2626
861	THAP6	0.2635	ARCHS4 Coexpression,0.2635
862	ZNF561	0.2641	ARCHS4 Coexpression,0.2641
863	NPAS4	0.2642	Enrichr Queries,0.2642
864	ZFP2	0.2647	ARCHS4 Coexpression,0.2647
865	ZNF250	0.265	Enrichr Queries,0.265
866	SOX30	0.2651	GTEx Coexpression,0.2651
867	ZFP1	0.2657	Enrichr Queries,0.2657
868	HOXA2	0.2663	GTEx Coexpression,0.2663
869	ASCL3	0.2664	Enrichr Queries,0.2664
870	ZNF566	0.2666	ARCHS4 Coexpression,0.2666
871	DMBX1	0.267	GTEx Coexpression,0.267
872	GRHL2	0.2671	Enrichr Queries,0.2671
873	ZNF165	0.2676	GTEx Coexpression,0.2676
874	IRF6	0.2678	Enrichr Queries,0.2678
875	ZNF620	0.2678	ARCHS4 Coexpression,0.2678
876	SCMH1	0.2682	GTEx Coexpression,0.2682
877	ZNF429	0.2684	ARCHS4 Coexpression,0.2684
878	RFX2	0.2685	Enrichr Queries,0.2685
879	SOHLH2	0.2688	GTEx Coexpression,0.2688
880	DLX5	0.269	ARCHS4 Coexpression,0.269
881	PAX5	0.2694	ReMap ChIP-seq,0.2694
882	PRDM4	0.2699	Enrichr Queries,0.2699
883	FOXL2	0.2707	GTEx Coexpression,0.2707
884	GSX2	0.2726	GTEx Coexpression,0.2726
885	MEIS3	0.2735	Enrichr Queries,0.2735
886	HSF5	0.2738	GTEx Coexpression,0.2738
887	SOX9	0.2744	Literature ChIP-seq,0.2744
888	TP73	0.2746	ARCHS4 Coexpression,0.2746
889	PKNOX1	0.2749	Enrichr Queries,0.2749
890	ZNF709	0.275	GTEx Coexpression,0.275
891	ZNF136	0.2752	ARCHS4 Coexpression,0.2752
892	ZNF641	0.2757	GTEx Coexpression,0.2757
893	ZEB2	0.2758	ARCHS4 Coexpression,0.2758
894	ADNP	0.2764	Enrichr Queries,0.2764
895	FOXC1	0.2764	ARCHS4 Coexpression,0.2764
896	ZKSCAN4	0.2769	GTEx Coexpression,0.2769
897	TLX1	0.277	ARCHS4 Coexpression,0.277
898	ZNF93	0.2771	Enrichr Queries,0.2771
899	KLF7	0.2776	ARCHS4 Coexpression,0.2776
900	FOXA1	0.2789	ARCHS4 Coexpression,0.2789

901	AHCTF1	0.2799	Enrichr Queries,0.2799
902	ZNF671	0.2801	ARCHS4 Coexpression,0.2801
903	TRAFD1	0.2806	Enrichr Queries,0.2806
904	TGIF2LX	0.2807	ARCHS4 Coexpression,0.2807
905	FOXI1	0.2813	ARCHS4 Coexpression,0.2813
906	ZNF385C	0.2813	Enrichr Queries,0.2813
907	ZNF630	0.2819	ARCHS4 Coexpression,0.2819
908	DMRT3	0.2825	GTEx Coexpression,0.2825
909	DR1	0.2828	Enrichr Queries,0.2828
910	TBX19	0.2831	GTEx Coexpression,0.2831
911	TFAP2D	0.2832	ARCHS4 Coexpression,0.2832
912	ZBTB1	0.2838	GTEx Coexpression,0.2838
913	FLYWCH1	0.2844	GTEx Coexpression,0.2844
914	SIX6	0.285	GTEx Coexpression,0.285
915	POU2F1	0.285	ARCHS4 Coexpression,0.285
916	ETV3	0.2856	Enrichr Queries,0.2856
917	ZNF548	0.2856	GTEx Coexpression,0.2856
918	TCF12	0.2862	ReMap ChIP-seq,0.2862
919	ZNF337	0.2863	Enrichr Queries,0.2863
920	ZNF681	0.2869	ARCHS4 Coexpression,0.2869
921	ZNF816	0.2875	ARCHS4 Coexpression,0.2875
922	BATF2	0.2875	GTEx Coexpression,0.2875
923	IRF9	0.2877	Enrichr Queries,0.2877
924	ZNF235	0.2881	ARCHS4 Coexpression,0.2881
925	ZNF569	0.2881	GTEx Coexpression,0.2881
926	ZNF484	0.2893	ARCHS4 Coexpression,0.2893
927	ZNF648	0.2894	GTEx Coexpression,0.2894
928	MSANTD1	0.2899	ARCHS4 Coexpression,0.2899
929	NR2E1	0.2905	ARCHS4 Coexpression,0.2905
930	FOSL2	0.2906	GTEx Coexpression,0.2906
931	ZNF799	0.2912	ARCHS4 Coexpression,0.2912
932	ZNF616	0.2913	Enrichr Queries,0.2913
933	ZBED3	0.2918	ARCHS4 Coexpression,0.2918
934	ZBTB14	0.2918	GTEx Coexpression,0.2918
935	ZNF177	0.292	Enrichr Queries,0.292
936	HDX	0.2924	ARCHS4 Coexpression,0.2924
937	ZNF91	0.2934	Enrichr Queries,0.2934
938	PHF20	0.2936	ARCHS4 Coexpression,0.2936
939	ARNTL	0.2942	Enrichr Queries,0.2942
940	ZNF708	0.2948	ARCHS4 Coexpression,0.2948
941	ZNF624	0.2955	ARCHS4 Coexpression,0.2955
942	ZNF107	0.2961	ARCHS4 Coexpression,0.2961
943	SALL4	0.2963	Enrichr Queries,0.2963
944	KLF17	0.297	Enrichr Queries,0.297
945	RAX	0.2979	ARCHS4 Coexpression,0.2979
946	ZNF181	0.2981	GTEx Coexpression,0.2981
947	ZBTB5	0.2984	Enrichr Queries,0.2984
948	PPARG	0.2988	Literature ChIP-seq,0.2988
949	ZNF556	0.2993	GTEx Coexpression,0.2993
950	CXXC4	0.2997	ReMap ChIP-seq,0.2997
951	ZNF850	0.2998	ARCHS4 Coexpression,0.2998
952	TERB1	0.2999	GTEx Coexpression,0.2999
953	ZNF765	0.3004	ARCHS4 Coexpression,0.3004

954	POU4F2	0.3006	GTEx Coexpression,0.3006
955	ZNF320	0.301	ARCHS4 Coexpression,0.301
956	HOXB1	0.3012	GTEx Coexpression,0.3012
957	GMEB1	0.3018	GTEx Coexpression,0.3018
958	CARF	0.3022	ARCHS4 Coexpression,0.3022
959	CCDC17	0.3024	GTEx Coexpression,0.3024
960	ZNF442	0.3027	Enrichr Queries,0.3027
961	ZNF880	0.3028	ARCHS4 Coexpression,0.3028
962	ZNF343	0.303	GTEx Coexpression,0.303
963	SATB1	0.3037	GTEx Coexpression,0.3037
964	PRDM10	0.3047	ARCHS4 Coexpression,0.3047
965	ZNF182	0.3049	GTEx Coexpression,0.3049
966	SETDB2	0.3053	ARCHS4 Coexpression,0.3053
967	NFATC1	0.3056	Enrichr Queries,0.3056
968	ZNF273	0.3065	ARCHS4 Coexpression,0.3065
969	ZBTB8A	0.3068	GTEx Coexpression,0.3068
970	CREB3L2	0.307	Enrichr Queries,0.307
971	SMAD3	0.3077	Enrichr Queries,0.3077
972	ZNF610	0.3077	ARCHS4 Coexpression,0.3077
973	TGIF2LY	0.308	GTEx Coexpression,0.308
974	SALL3	0.3099	GTEx Coexpression,0.3099
975	HOXD9	0.3105	GTEx Coexpression,0.3105
976	TCF7L1	0.3105	Enrichr Queries,0.3105
977	MEIS1	0.311	Literature ChIP-seq,0.311
978	MYPOP	0.3111	GTEx Coexpression,0.3111
979	GTF2IRD1	0.3118	GTEx Coexpression,0.3118
980	FOXJ1	0.312	Enrichr Queries,0.312
981	ZSCAN1	0.3127	ARCHS4 Coexpression,0.3127
982	ZBTB16	0.3131	ReMap ChIP-seq,0.3131
983	ZNF607	0.3136	GTEx Coexpression,0.3136
984	RREB1	0.3145	ARCHS4 Coexpression,0.3145
985	NR6A1	0.3149	GTEx Coexpression,0.3149
986	CDX1	0.3155	Enrichr Queries,0.3155
987	SOX13	0.3161	GTEx Coexpression,0.3161
988	ZNF487	0.3162	Enrichr Queries,0.3162
989	IRX3	0.3163	ARCHS4 Coexpression,0.3163
990	ZNF559	0.317	ARCHS4 Coexpression,0.317
991	MSC	0.3176	ARCHS4 Coexpression,0.3176
992	POU4F1	0.318	GTEx Coexpression,0.318
993	TBXT	0.3182	ARCHS4 Coexpression,0.3182
994	NR4A1	0.3184	Enrichr Queries,0.3184
995	LIN28A	0.3192	GTEx Coexpression,0.3192
996	ZNF491	0.3194	ARCHS4 Coexpression,0.3194
997	TFAP2A	0.3199	ReMap ChIP-seq,0.3199
998	ZNF287	0.3205	GTEx Coexpression,0.3205
999	HOXD1	0.3206	ARCHS4 Coexpression,0.3206
1000	ZNF334	0.3211	GTEx Coexpression,0.3211
1001	ZFHX3	0.3212	Enrichr Queries,0.3212
1002	ELF4	0.3234	Enrichr Queries,0.3234
1003	DMRT2	0.3236	GTEx Coexpression,0.3236
1004	ZNF790	0.3237	ARCHS4 Coexpression,0.3237
1005	SP140L	0.3243	ARCHS4 Coexpression,0.3243
1006	ZNF555	0.3249	ARCHS4 Coexpression,0.3249

1007	ZNF543	0.3256	ARCHS4 Coexpression,0.3256
1008	ZNF449	0.3261	GTEx Coexpression,0.3261
1009	ZNF814	0.3262	ARCHS4 Coexpression,0.3262
1010	CUX1	0.3266	ReMap ChIP-seq,0.3266
1011	ZNF418	0.3268	ARCHS4 Coexpression,0.3268
1012	ANHX	0.3286	GTEx Coexpression,0.3286
1013	MLXIP	0.3292	GTEx Coexpression,0.3292
1014	ZNF682	0.3292	ARCHS4 Coexpression,0.3292
1015	OVOL1	0.3298	Enrichr Queries,0.3298
1016	ZNF227	0.3299	ARCHS4 Coexpression,0.3299
1017	ZNF214	0.3305	ARCHS4 Coexpression,0.3305
1018	ZNF43	0.3311	ARCHS4 Coexpression,0.3311
1019	PAX8	0.3312	Enrichr Queries,0.3312
1020	ZNF727	0.3317	ARCHS4 Coexpression,0.3317
1021	ZNF532	0.3323	GTEx Coexpression,0.3323
1022	ZBTB25	0.3329	ARCHS4 Coexpression,0.3329
1023	PAX6	0.3333	ReMap ChIP-seq,0.3333
1024	ZNF280B	0.3333	Enrichr Queries,0.3333
1025	ZNF570	0.3335	ARCHS4 Coexpression,0.3335
1026	CSRNP2	0.3335	GTEx Coexpression,0.3335
1027	TBPL2	0.3354	GTEx Coexpression,0.3354
1028	ZNF100	0.336	ARCHS4 Coexpression,0.336
1029	GTF2IRD2	0.3362	Enrichr Queries,0.3362
1030	ZNF678	0.3366	ARCHS4 Coexpression,0.3366
1031	ZNF462	0.3367	GTEx Coexpression,0.3367
1032	ATOH8	0.3372	ARCHS4 Coexpression,0.3372
1033	ZNF518B	0.3378	ARCHS4 Coexpression,0.3378
1034	ZNF883	0.3385	ARCHS4 Coexpression,0.3385
1035	PRDM5	0.3391	ARCHS4 Coexpression,0.3391
1036	ZNF423	0.3391	GTEx Coexpression,0.3391
1037	BNC1	0.3398	GTEx Coexpression,0.3398
1038	FOXS1	0.3403	ARCHS4 Coexpression,0.3403
1039	GLYR1	0.341	GTEx Coexpression,0.341
1040	ZNF101	0.3412	Enrichr Queries,0.3412
1041	ZNF705A	0.3419	Enrichr Queries,0.3419
1042	FOXR2	0.3426	Enrichr Queries,0.3426
1043	ZNF829	0.3428	ARCHS4 Coexpression,0.3428
1044	ONECUT1	0.3429	GTEx Coexpression,0.3429
1045	ZNF14	0.3433	Enrichr Queries,0.3433
1046	NR1I2	0.3435	GTEx Coexpression,0.3435
1047	ZNF426	0.344	ARCHS4 Coexpression,0.344
1048	PRDM14	0.344	Enrichr Queries,0.344
1049	ZBTB39	0.3441	GTEx Coexpression,0.3441
1050	LHX8	0.3452	ARCHS4 Coexpression,0.3452
1051	ZFP28	0.3454	GTEx Coexpression,0.3454
1052	ZNF350	0.3454	Enrichr Queries,0.3454
1053	ZNF675	0.3464	ARCHS4 Coexpression,0.3464
1054	ZNF608	0.3466	GTEx Coexpression,0.3466
1055	PGR	0.3477	ARCHS4 Coexpression,0.3477
1056	ETV7	0.3479	GTEx Coexpression,0.3479
1057	ZNF383	0.3483	ARCHS4 Coexpression,0.3483
1058	ARID5B	0.3489	ARCHS4 Coexpression,0.3489
1059	ZNF304	0.349	Enrichr Queries,0.349

1060	SOX5	0.3491	GTEx Coexpression,0.3491
1061	ZNF585A	0.3495	ARCHS4 Coexpression,0.3495
1062	HNF4G	0.3502	ReMap ChIP-seq,0.3502
1063	OSR2	0.3503	GTEx Coexpression,0.3503
1064	ZMAT4	0.3504	Enrichr Queries,0.3504
1065	ZNF25	0.3511	Enrichr Queries,0.3511
1066	ZNF766	0.3514	ARCHS4 Coexpression,0.3514
1067	ZNF385B	0.3516	GTEx Coexpression,0.3516
1068	SOX10	0.3519	Enrichr Queries,0.3519
1069	ESR1	0.352	ARCHS4 Coexpression,0.352
1070	ZNF169	0.3526	Enrichr Queries,0.3526
1071	HLF	0.3533	Enrichr Queries,0.3533
1072	TCF7	0.3537	Literature ChIP-seq,0.3537
1073	ZNF431	0.3544	ARCHS4 Coexpression,0.3544
1074	ZNF436	0.3547	Enrichr Queries,0.3547
1075	ZNF676	0.3553	GTEx Coexpression,0.3553
1076	ZNF891	0.3557	ARCHS4 Coexpression,0.3557
1077	TCF23	0.3561	Enrichr Queries,0.3561
1078	HOXB5	0.3563	ARCHS4 Coexpression,0.3563
1079	ZNF184	0.3569	ARCHS4 Coexpression,0.3569
1080	ZNF736	0.3572	GTEx Coexpression,0.3572
1081	GLI1	0.3575	Enrichr Queries,0.3575
1082	ZNF836	0.3578	GTEx Coexpression,0.3578
1083	NPAS3	0.3584	GTEx Coexpression,0.3584
1084	NR3C1	0.359	Enrichr Queries,0.359
1085	ZNF679	0.3591	GTEx Coexpression,0.3591
1086	ZNF683	0.3597	GTEx Coexpression,0.3597
1087	NR5A2	0.3606	ARCHS4 Coexpression,0.3606
1088	NKX24	0.3609	GTEx Coexpression,0.3609
1089	EPAS1	0.3611	Enrichr Queries,0.3611
1090	ZFP30	0.3612	ARCHS4 Coexpression,0.3612
1091	PHOX2A	0.3624	ARCHS4 Coexpression,0.3624
1092	MYBL1	0.3625	Enrichr Queries,0.3625
1093	ZNF729	0.3628	GTEx Coexpression,0.3628
1094	ZNF461	0.363	ARCHS4 Coexpression,0.363
1095	KLF8	0.3632	Enrichr Queries,0.3632
1096	HIF3A	0.3634	GTEx Coexpression,0.3634
1097	NR2F2	0.3636	ReMap ChIP-seq,0.3636
1098	ZNF474	0.3636	ARCHS4 Coexpression,0.3636
1099	TFCP2L1	0.364	Enrichr Queries,0.364
1100	ALX4	0.364	GTEx Coexpression,0.364
1101	HMX3	0.3643	ARCHS4 Coexpression,0.3643
1102	ZNF281	0.3654	Enrichr Queries,0.3654
1103	SOX11	0.3659	Literature ChIP-seq,0.3659
1104	PRDM9	0.3659	GTEx Coexpression,0.3659
1105	DPF1	0.3661	Enrichr Queries,0.3661
1106	TIGD1	0.3665	GTEx Coexpression,0.3665
1107	TLX3	0.3667	ARCHS4 Coexpression,0.3667
1108	WT1	0.3668	Enrichr Queries,0.3668
1109	ZNF525	0.3671	GTEx Coexpression,0.3671
1110	FOXD3	0.3673	ARCHS4 Coexpression,0.3673
1111	CUX2	0.3675	Enrichr Queries,0.3675
1112	HAND1	0.3679	ARCHS4 Coexpression,0.3679

1113	TPRX1	0.3682	Enrichr Queries,0.3682
1114	ZNF774	0.3703	GTEx Coexpression,0.3703
1115	ZNF763	0.3704	ARCHS4 Coexpression,0.3704
1116	TSHZ1	0.3709	GTEx Coexpression,0.3709
1117	RBSN	0.371	ARCHS4 Coexpression,0.371
1118	ELK3	0.3711	Enrichr Queries,0.3711
1119	ZNF407	0.3715	GTEx Coexpression,0.3715
1120	ZNF347	0.3721	GTEx Coexpression,0.3721
1121	ZNF17	0.3732	Enrichr Queries,0.3732
1122	IRX2	0.374	GTEx Coexpression,0.374
1123	ZFPM2	0.3741	ARCHS4 Coexpression,0.3741
1124	ARID3C	0.3746	GTEx Coexpression,0.3746
1125	BCL6	0.3746	Enrichr Queries,0.3746
1126	ZNF438	0.3761	Enrichr Queries,0.3761
1127	PAX9	0.3765	GTEx Coexpression,0.3765
1128	ZNF697	0.3768	Enrichr Queries,0.3768
1129	ZNF613	0.3771	GTEx Coexpression,0.3771
1130	DBX2	0.3775	Enrichr Queries,0.3775
1131	ZNF827	0.3778	ARCHS4 Coexpression,0.3778
1132	TP63	0.378	Literature ChIP-seq,0.378
1133	HMG20A	0.3782	Enrichr Queries,0.3782
1134	RFX6	0.3789	Enrichr Queries,0.3789
1135	SOX8	0.379	ARCHS4 Coexpression,0.379
1136	ZNF583	0.3796	GTEx Coexpression,0.3796
1137	AHR	0.3796	Enrichr Queries,0.3796
1138	NKX63	0.3802	ARCHS4 Coexpression,0.3802
1139	NFE2	0.3805	ReMap ChIP-seq,0.3805
1140	TIGD4	0.3814	ARCHS4 Coexpression,0.3814
1141	NAIF1	0.3815	GTEx Coexpression,0.3815
1142	ZNF785	0.3827	ARCHS4 Coexpression,0.3827
1143	PAX2	0.3827	GTEx Coexpression,0.3827
1144	ZNF468	0.3833	ARCHS4 Coexpression,0.3833
1145	SOX21	0.3839	GTEx Coexpression,0.3839
1146	ZBTB7C	0.3846	GTEx Coexpression,0.3846
1147	ZNF28	0.3851	ARCHS4 Coexpression,0.3851
1148	ZNF18	0.386	Enrichr Queries,0.386
1149	LIN54	0.3864	ARCHS4 Coexpression,0.3864
1150	BACH2	0.3864	GTEx Coexpression,0.3864
1151	NFIL3	0.3868	Enrichr Queries,0.3868
1152	ZFP64	0.387	ARCHS4 Coexpression,0.387
1153	TRPS1	0.3882	ARCHS4 Coexpression,0.3882
1154	MYCL	0.3883	GTEx Coexpression,0.3883
1155	ZBTB41	0.3888	ARCHS4 Coexpression,0.3888
1156	MXD1	0.3896	Enrichr Queries,0.3896
1157	ZNF425	0.3902	GTEx Coexpression,0.3902
1158	HES2	0.3908	GTEx Coexpression,0.3908
1159	POU6F1	0.391	Enrichr Queries,0.391
1160	PAX4	0.3914	GTEx Coexpression,0.3914
1161	STAT4	0.3927	GTEx Coexpression,0.3927
1162	FOXN2	0.3931	ARCHS4 Coexpression,0.3931
1163	HIVEP3	0.3939	Enrichr Queries,0.3939
1164	ZNF490	0.3953	Enrichr Queries,0.3953
1165	NPAS2	0.3964	GTEx Coexpression,0.3964

1166	NKX21	0.3976	GTEx Coexpression,0.3976
1167	ZBTB46	0.3989	Enrichr Queries,0.3989
1168	ZNF24	0.3996	Enrichr Queries,0.3996
1169	ZNF157	0.401	Enrichr Queries,0.401
1170	SOX7	0.4011	ARCHS4 Coexpression,0.4011
1171	L3MBTL4	0.4014	GTEx Coexpression,0.4014
1172	SEBOX	0.4017	Enrichr Queries,0.4017
1173	DLX6	0.4023	ARCHS4 Coexpression,0.4023
1174	TFAP2E	0.4024	Enrichr Queries,0.4024
1175	SPIB	0.4029	ARCHS4 Coexpression,0.4029
1176	GATA6	0.4032	GTEx Coexpression,0.4032
1177	ZNF529	0.4036	ARCHS4 Coexpression,0.4036
1178	KLF3	0.4038	Enrichr Queries,0.4038
1179	ZNF419	0.4042	ARCHS4 Coexpression,0.4042
1180	POU3F2	0.4045	GTEx Coexpression,0.4045
1181	PROX2	0.4046	Enrichr Queries,0.4046
1182	ZNF92	0.4054	ARCHS4 Coexpression,0.4054
1183	ZBTB20	0.4057	GTEx Coexpression,0.4057
1184	ZNF398	0.4067	Enrichr Queries,0.4067
1185	ZNF311	0.407	GTEx Coexpression,0.407
1186	TFDP3	0.4081	Enrichr Queries,0.4081
1187	GATA5	0.4082	GTEx Coexpression,0.4082
1188	HOXD4	0.4085	ARCHS4 Coexpression,0.4085
1189	ZFHX4	0.4088	GTEx Coexpression,0.4088
1190	CBLL2	0.4095	GTEx Coexpression,0.4095
1191	ZNF563	0.4101	GTEx Coexpression,0.4101
1192	ZNF557	0.4103	Enrichr Queries,0.4103
1193	SHOX	0.4103	ARCHS4 Coexpression,0.4103
1194	LHX2	0.4107	GTEx Coexpression,0.4107
1195	SPZ1	0.4119	GTEx Coexpression,0.4119
1196	ZNF397	0.4122	ARCHS4 Coexpression,0.4122
1197	ZBED4	0.4124	Enrichr Queries,0.4124
1198	AR	0.4126	GTEx Coexpression,0.4126
1199	ZBTB8B	0.4134	ARCHS4 Coexpression,0.4134
1200	BATF	0.4138	Enrichr Queries,0.4138
1201	ZNF750	0.4141	ReMap ChIP-seq,0.4141
1202	SKIL	0.4144	GTEx Coexpression,0.4144
1203	IRF8	0.4146	Literature ChIP-seq,0.4146
1204	HOXD8	0.4151	GTEx Coexpression,0.4151
1205	ZNF280D	0.4157	GTEx Coexpression,0.4157
1206	ZNF8	0.4165	ARCHS4 Coexpression,0.4165
1207	IRF5	0.4167	Enrichr Queries,0.4167
1208	ZSCAN26	0.4171	ARCHS4 Coexpression,0.4171
1209	RUNX3	0.4174	Enrichr Queries,0.4174
1210	ZSCAN32	0.4177	ARCHS4 Coexpression,0.4177
1211	NFX1	0.4188	Enrichr Queries,0.4188
1212	MTERF4	0.4194	GTEx Coexpression,0.4194
1213	ISX	0.4195	Enrichr Queries,0.4195
1214	SETBP1	0.42	GTEx Coexpression,0.42
1215	ZIC5	0.4201	ARCHS4 Coexpression,0.4201
1216	ZNF705G	0.4207	GTEx Coexpression,0.4207
1217	EOMES	0.4207	Literature ChIP-seq,0.4207
1218	ZNF568	0.4214	ARCHS4 Coexpression,0.4214

1219	ZNF331	0.4217	Enrichr Queries,0.4217
1220	MEF2C	0.4219	GTEx Coexpression,0.4219
1221	HES3	0.4226	ARCHS4 Coexpression,0.4226
1222	ZNF595	0.4238	Enrichr Queries,0.4238
1223	ARID5A	0.4238	ARCHS4 Coexpression,0.4238
1224	EBF1	0.4242	ReMap ChIP-seq,0.4242
1225	ZNF711	0.425	GTEx Coexpression,0.425
1226	PROP1	0.4256	GTEx Coexpression,0.4256
1227	ETV3L	0.4257	ARCHS4 Coexpression,0.4257
1228	TBX22	0.4269	GTEx Coexpression,0.4269
1229	ZKSCAN7	0.4269	ARCHS4 Coexpression,0.4269
1230	ZNF728	0.4275	GTEx Coexpression,0.4275
1231	FOXO6	0.4281	GTEx Coexpression,0.4281
1232	VENTX	0.4295	Enrichr Queries,0.4295
1233	CREB5	0.4302	Enrichr Queries,0.4302
1234	ASCL1	0.4306	GTEx Coexpression,0.4306
1235	ZNF618	0.4309	Enrichr Queries,0.4309
1236	HOXB6	0.4312	ARCHS4 Coexpression,0.4312
1237	ZNF567	0.4318	ARCHS4 Coexpression,0.4318
1238	LHX6	0.4323	Enrichr Queries,0.4323
1239	NFIA	0.4337	GTEx Coexpression,0.4337
1240	ATOH1	0.4343	GTEx Coexpression,0.4343
1241	NFATC4	0.4356	GTEx Coexpression,0.4356
1242	AHRR	0.4367	ARCHS4 Coexpression,0.4367
1243	ZNF502	0.4387	Enrichr Queries,0.4387
1244	GRHL1	0.4393	GTEx Coexpression,0.4393
1245	ZNF649	0.4395	Enrichr Queries,0.4395
1246	HOXB9	0.44	GTEx Coexpression,0.44
1247	ARX	0.4402	Enrichr Queries,0.4402
1248	ZBED2	0.4409	Enrichr Queries,0.4409
1249	ZNF660	0.4416	ARCHS4 Coexpression,0.4416
1250	ZNF528	0.4423	ARCHS4 Coexpression,0.4423
1251	HIC1	0.443	Enrichr Queries,0.443
1252	ZNF701	0.4441	ARCHS4 Coexpression,0.4441
1253	IRX4	0.4443	GTEx Coexpression,0.4443
1254	ZNF443	0.4444	Enrichr Queries,0.4444
1255	ZNF614	0.4453	ARCHS4 Coexpression,0.4453
1256	FOXI3	0.4456	GTEx Coexpression,0.4456
1257	ZNF420	0.4459	ARCHS4 Coexpression,0.4459
1258	FOXF1	0.4466	ARCHS4 Coexpression,0.4466
1259	MAFB	0.4468	GTEx Coexpression,0.4468
1260	EGR4	0.4473	Enrichr Queries,0.4473
1261	ZNF804B	0.4474	GTEx Coexpression,0.4474
1262	SALL1	0.448	GTEx Coexpression,0.448
1263	IRX6	0.4484	ARCHS4 Coexpression,0.4484
1264	ZNF229	0.449	ARCHS4 Coexpression,0.449
1265	ONECUT2	0.4512	GTEx Coexpression,0.4512
1266	TFAP2C	0.4512	ReMap ChIP-seq,0.4512
1267	HOXD10	0.4515	ARCHS4 Coexpression,0.4515
1268	ZNF284	0.4527	ARCHS4 Coexpression,0.4527
1269	ZNF713	0.4533	ARCHS4 Coexpression,0.4533
1270	ZNF483	0.4536	GTEx Coexpression,0.4536
1271	ZNF224	0.4537	Enrichr Queries,0.4537

1272	ZNF823	0.4539	ARCHS4 Coexpression,0.4539
1273	ZNF793	0.4552	ARCHS4 Coexpression,0.4552
1274	NANOGP8	0.4574	GTEx Coexpression,0.4574
1275	BACH1	0.4579	ReMap ChIP-seq,0.4579
1276	EMX2	0.458	Enrichr Queries,0.458
1277	ZNF471	0.458	GTEx Coexpression,0.458
1278	ZNF501	0.4582	ARCHS4 Coexpression,0.4582
1279	EN1	0.4605	GTEx Coexpression,0.4605
1280	PBX4	0.4611	GTEx Coexpression,0.4611
1281	ZNF221	0.4619	ARCHS4 Coexpression,0.4619
1282	ZNF492	0.4638	ARCHS4 Coexpression,0.4638
1283	MSGN1	0.4642	GTEx Coexpression,0.4642
1284	FOXI2	0.4661	GTEx Coexpression,0.4661
1285	SIM2	0.4667	GTEx Coexpression,0.4667
1286	ZNF135	0.4679	Enrichr Queries,0.4679
1287	ZNF878	0.468	GTEx Coexpression,0.468
1288	UNCX	0.4693	ARCHS4 Coexpression,0.4693
1289	IRF7	0.4694	Enrichr Queries,0.4694
1290	ZNF264	0.4699	ARCHS4 Coexpression,0.4699
1291	ZNF234	0.4708	Enrichr Queries,0.4708
1292	GRHL3	0.4717	GTEx Coexpression,0.4717
1293	ZNF257	0.4717	ARCHS4 Coexpression,0.4717
1294	EHF	0.4729	GTEx Coexpression,0.4729
1295	CAMTA1	0.4729	Enrichr Queries,0.4729
1296	BARX2	0.4742	GTEx Coexpression,0.4742
1297	SP100	0.4742	ARCHS4 Coexpression,0.4742
1298	ZFP92	0.4744	Enrichr Queries,0.4744
1299	ZNF479	0.4748	GTEx Coexpression,0.4748
1300	NACC2	0.4754	GTEx Coexpression,0.4754
1301	ZNF844	0.4754	ARCHS4 Coexpression,0.4754
1302	HEY1	0.476	GTEx Coexpression,0.476
1303	EMX1	0.4773	ARCHS4 Coexpression,0.4773
1304	VAX1	0.4779	GTEx Coexpression,0.4779
1305	ZNF860	0.4785	ARCHS4 Coexpression,0.4785
1306	ZNF773	0.4793	Enrichr Queries,0.4793
1307	ZNF605	0.4804	GTEx Coexpression,0.4804
1308	ZNF677	0.4808	Enrichr Queries,0.4808
1309	ZNF84	0.481	ARCHS4 Coexpression,0.481
1310	RHOXF1	0.4822	Enrichr Queries,0.4822
1311	ZNF154	0.4834	ARCHS4 Coexpression,0.4834
1312	REL	0.4836	Enrichr Queries,0.4836
1313	TSHZ3	0.4841	GTEx Coexpression,0.4841
1314	IRF4	0.4843	Enrichr Queries,0.4843
1315	ZNF619	0.4846	ARCHS4 Coexpression,0.4846
1316	TSHZ2	0.4848	GTEx Coexpression,0.4848
1317	AEBP2	0.4854	GTEx Coexpression,0.4854
1318	CPXCR1	0.4858	Enrichr Queries,0.4858
1319	ZNF665	0.4859	ARCHS4 Coexpression,0.4859
1320	LTF	0.4866	GTEx Coexpression,0.4866
1321	TBX21	0.4871	ARCHS4 Coexpression,0.4871
1322	MECOM	0.4878	Literature ChIP-seq,0.4878
1323	ZFHX2	0.4879	GTEx Coexpression,0.4879
1324	ZNF85	0.4879	Enrichr Queries,0.4879

1325	ZNF506	0.4885	GTEx Coexpression,0.4885
1326	ZNF114	0.4902	ARCHS4 Coexpression,0.4902
1327	LEUTX	0.4908	ARCHS4 Coexpression,0.4908
1328	HOXB13	0.4915	Enrichr Queries,0.4915
1329	ESX1	0.4935	GTEx Coexpression,0.4935
1330	SKOR2	0.4939	ARCHS4 Coexpression,0.4939
1331	ZBTB24	0.4941	GTEx Coexpression,0.4941
1332	HOXA3	0.4947	GTEx Coexpression,0.4947
1333	DLX3	0.4957	ARCHS4 Coexpression,0.4957
1334	GLI2	0.4957	Enrichr Queries,0.4957
1335	NANOGNB	0.496	GTEx Coexpression,0.496
1336	FOXB2	0.4963	ARCHS4 Coexpression,0.4963
1337	ZNF788P	0.4964	Enrichr Queries,0.4964
1338	MEOX2	0.4972	Enrichr Queries,0.4972
1339	LMX1B	0.4988	ARCHS4 Coexpression,0.4988
1340	ZNF800	0.4991	GTEx Coexpression,0.4991
1341	ZNF782	0.4997	GTEx Coexpression,0.4997
1342	OTP	0.5006	ARCHS4 Coexpression,0.5006
1343	MIXL1	0.5007	Enrichr Queries,0.5007
1344	HNF1B	0.5014	Enrichr Queries,0.5014
1345	TRERF1	0.5047	GTEx Coexpression,0.5047
1346	ZNF609	0.5053	GTEx Coexpression,0.5053
1347	ZFP37	0.5057	Enrichr Queries,0.5057
1348	NHLH1	0.5065	GTEx Coexpression,0.5065
1349	ZSCAN30	0.5072	GTEx Coexpression,0.5072
1350	ZNF286A	0.5078	Enrichr Queries,0.5078
1351	CDX2	0.5084	GTEx Coexpression,0.5084
1352	ZNF45	0.5085	Enrichr Queries,0.5085
1353	MSX2	0.5092	ARCHS4 Coexpression,0.5092
1354	FOXG1	0.5093	Enrichr Queries,0.5093
1355	ZNF441	0.5098	ARCHS4 Coexpression,0.5098
1356	FOXE1	0.5103	GTEx Coexpression,0.5103
1357	ZNF724	0.5104	ARCHS4 Coexpression,0.5104
1358	ZNF302	0.5115	GTEx Coexpression,0.5115
1359	ZNF225	0.5117	ARCHS4 Coexpression,0.5117
1360	ZNF562	0.514	GTEx Coexpression,0.514
1361	ZNF454	0.516	ARCHS4 Coexpression,0.516
1362	ZNF124	0.5165	GTEx Coexpression,0.5165
1363	PROX1	0.5177	GTEx Coexpression,0.5177
1364	LHX5	0.5178	ARCHS4 Coexpression,0.5178
1365	HOXB3	0.5184	GTEx Coexpression,0.5184
1366	IKZF4	0.5184	ARCHS4 Coexpression,0.5184
1367	NOTO	0.519	ARCHS4 Coexpression,0.519
1368	ZNF527	0.5199	Enrichr Queries,0.5199
1369	ZNF540	0.5203	ARCHS4 Coexpression,0.5203
1370	NKX12	0.5208	GTEx Coexpression,0.5208
1371	PRDM8	0.5221	Enrichr Queries,0.5221
1372	GBX2	0.5221	ARCHS4 Coexpression,0.5221
1373	MEOX1	0.5233	ARCHS4 Coexpression,0.5233
1374	L3MBTL3	0.5246	ARCHS4 Coexpression,0.5246
1375	SATB2	0.5246	GTEx Coexpression,0.5246
1376	TEAD1	0.5249	Enrichr Queries,0.5249
1377	ZNF726	0.5252	GTEx Coexpression,0.5252

1378	EGR3	0.5258	GTEx Coexpression,0.5258
1379	FOXN1	0.5271	Enrichr Queries,0.5271
1380	ZNF879	0.5276	ARCHS4 Coexpression,0.5276
1381	PRRX2	0.5278	Enrichr Queries,0.5278
1382	ZNF283	0.5289	ARCHS4 Coexpression,0.5289
1383	NR4A3	0.5296	GTEx Coexpression,0.5296
1384	ZIC3	0.5321	Enrichr Queries,0.5321
1385	HELT	0.5327	GTEx Coexpression,0.5327
1386	ZNF322	0.5332	ARCHS4 Coexpression,0.5332
1387	ZNF99	0.535	ARCHS4 Coexpression,0.535
1388	ZNF404	0.5352	GTEx Coexpression,0.5352
1389	ZNF70	0.5356	ARCHS4 Coexpression,0.5356
1390	ZNF440	0.5358	GTEx Coexpression,0.5358
1391	ZNF717	0.5362	ARCHS4 Coexpression,0.5362
1392	SP7	0.5381	ARCHS4 Coexpression,0.5381
1393	EVX1	0.5383	GTEx Coexpression,0.5383
1394	POU2F3	0.5385	Enrichr Queries,0.5385
1395	HLX	0.5392	Enrichr Queries,0.5392
1396	ZSCAN23	0.5393	ARCHS4 Coexpression,0.5393
1397	FOXD4L5	0.5408	GTEx Coexpression,0.5408
1398	ZNF285	0.5412	ARCHS4 Coexpression,0.5412
1399	HMGA2	0.5413	Enrichr Queries,0.5413
1400	ZNF329	0.5418	ARCHS4 Coexpression,0.5418
1401	ZNF286B	0.5442	ARCHS4 Coexpression,0.5442
1402	FEZF2	0.5445	GTEx Coexpression,0.5445
1403	AHDC1	0.5463	Enrichr Queries,0.5463
1404	HOXB8	0.547	GTEx Coexpression,0.547
1405	ZNF585B	0.547	Enrichr Queries,0.547
1406	ARNTL2	0.5477	Enrichr Queries,0.5477
1407	POU3F3	0.5482	GTEx Coexpression,0.5482
1408	DLX1	0.5485	ARCHS4 Coexpression,0.5485
1409	ZNF705B	0.5488	GTEx Coexpression,0.5488
1410	ZNF667	0.5498	ARCHS4 Coexpression,0.5498
1411	GSC2	0.5513	GTEx Coexpression,0.5513
1412	FOSB	0.552	Enrichr Queries,0.552
1413	ZBTB21	0.5522	ARCHS4 Coexpression,0.5522
1414	RHOXF2B	0.5532	GTEx Coexpression,0.5532
1415	TCF21	0.5549	Literature ChIP-seq,0.5549
1416	MYRFL	0.5551	GTEx Coexpression,0.5551
1417	TBR1	0.5553	ARCHS4 Coexpression,0.5553
1418	BATF3	0.5556	Enrichr Queries,0.5556
1419	HOXA13	0.5588	GTEx Coexpression,0.5588
1420	THAP12	0.559	ARCHS4 Coexpression,0.559
1421	NEUROD2	0.561	Literature ChIP-seq,0.561
1422	HAND2	0.5619	GTEx Coexpression,0.5619
1423	ZNF382	0.562	ARCHS4 Coexpression,0.562
1424	ZNF792	0.5625	GTEx Coexpression,0.5625
1425	INSM1	0.5632	GTEx Coexpression,0.5632
1426	THRB	0.5644	GTEx Coexpression,0.5644
1427	EGR2	0.5681	GTEx Coexpression,0.5681
1428	TCF20	0.5726	Enrichr Queries,0.5726
1429	ZBTB26	0.5734	Enrichr Queries,0.5734
1430	BCL11B	0.5737	GTEx Coexpression,0.5737

1431	RFX8	0.5756	ARCHS4 Coexpression,0.5756
1432	POU3F4	0.578	ARCHS4 Coexpression,0.578
1433	ZNF572	0.5783	Enrichr Queries,0.5783
1434	BHLHE22	0.5791	ReMap ChIP-seq,0.5791
1435	HEY2	0.5805	ARCHS4 Coexpression,0.5805
1436	CASZ1	0.5805	Enrichr Queries,0.5805
1437	PRDM1	0.5812	GTEx Coexpression,0.5812
1438	KDM2B	0.5817	ARCHS4 Coexpression,0.5817
1439	DACH2	0.5826	Enrichr Queries,0.5826
1440	MYT1	0.5833	Enrichr Queries,0.5833
1441	HEYL	0.5835	ARCHS4 Coexpression,0.5835
1442	ZNF37A	0.5843	GTEx Coexpression,0.5843
1443	ZNF596	0.5848	ARCHS4 Coexpression,0.5848
1444	DLX2	0.5849	GTEx Coexpression,0.5849
1445	ATF6	0.5855	Enrichr Queries,0.5855
1446	TIGD7	0.5862	GTEx Coexpression,0.5862
1447	SCML4	0.5869	Enrichr Queries,0.5869
1448	ZSCAN20	0.5876	Enrichr Queries,0.5876
1449	TBX4	0.5878	ARCHS4 Coexpression,0.5878
1450	POU2AF1	0.5887	GTEx Coexpression,0.5887
1451	PLSCR1	0.5897	ARCHS4 Coexpression,0.5897
1452	ZNF488	0.5905	Enrichr Queries,0.5905
1453	SHOX2	0.5933	Enrichr Queries,0.5933
1454	OSR1	0.5977	ARCHS4 Coexpression,0.5977
1455	ZNF845	0.5983	ARCHS4 Coexpression,0.5983
1456	YY2	0.5989	ARCHS4 Coexpression,0.5989
1457	PAX3	0.5993	GTEx Coexpression,0.5993
1458	ZNF439	0.6004	Enrichr Queries,0.6004
1459	PKNOX2	0.6019	Enrichr Queries,0.6019
1460	THAP2	0.6032	ARCHS4 Coexpression,0.6032
1461	ZNF469	0.605	ARCHS4 Coexpression,0.605
1462	ZNF835	0.6057	ARCHS4 Coexpression,0.6057
1463	GLIS1	0.6075	ARCHS4 Coexpression,0.6075
1464	ZIM2	0.6086	GTEx Coexpression,0.6086
1465	IKZF1	0.6093	ARCHS4 Coexpression,0.6093
1466	EVX2	0.6098	GTEx Coexpression,0.6098
1467	ARNT2	0.6104	Enrichr Queries,0.6104
1468	IKZF3	0.6111	Enrichr Queries,0.6111
1469	ZNF735	0.6118	ARCHS4 Coexpression,0.6118
1470	ZNF26	0.613	ARCHS4 Coexpression,0.613
1471	GSX1	0.6143	ARCHS4 Coexpression,0.6143
1472	ZNF132	0.6161	ARCHS4 Coexpression,0.6161
1473	HOXC8	0.6161	Enrichr Queries,0.6161
1474	ZNF611	0.6173	ARCHS4 Coexpression,0.6173
1475	SNAI1	0.6175	Enrichr Queries,0.6175
1476	PRDM16	0.6192	GTEx Coexpression,0.6192
1477	VSX2	0.6198	GTEx Coexpression,0.6198
1478	MEIS2	0.6204	GTEx Coexpression,0.6204
1479	ZNF267	0.6218	Enrichr Queries,0.6218
1480	ZNF396	0.6222	ARCHS4 Coexpression,0.6222
1481	RUNX2	0.6225	Enrichr Queries,0.6225
1482	ZNF268	0.6229	ARCHS4 Coexpression,0.6229
1483	NKX31	0.6229	GTEx Coexpression,0.6229

1484	ZSCAN4	0.6235	ARCHS4 Coexpression,0.6235
1485	HOXD3	0.6241	GTEx Coexpression,0.6241
1486	ZNF831	0.6253	ARCHS4 Coexpression,0.6253
1487	HIVEP2	0.6254	Enrichr Queries,0.6254
1488	ZSCAN31	0.6259	ARCHS4 Coexpression,0.6259
1489	GCM2	0.626	GTEx Coexpression,0.626
1490	SP6	0.6265	ARCHS4 Coexpression,0.6265
1491	DUXA	0.6271	ARCHS4 Coexpression,0.6271
1492	NEUROD6	0.6296	ARCHS4 Coexpression,0.6296
1493	LEF1	0.6297	GTEx Coexpression,0.6297
1494	ZNF805	0.6311	Enrichr Queries,0.6311
1495	AEBP1	0.6314	ARCHS4 Coexpression,0.6314
1496	FOXQ1	0.6316	GTEx Coexpression,0.6316
1497	ZHX3	0.6329	GTEx Coexpression,0.6329
1498	ZBTB38	0.6333	ARCHS4 Coexpression,0.6333
1499	NR4A2	0.6341	GTEx Coexpression,0.6341
1500	CSRNP1	0.6345	ARCHS4 Coexpression,0.6345
1501	C11ORF95	0.6353	GTEx Coexpression,0.6353
1502	PRDM6	0.636	GTEx Coexpression,0.636
1503	ZNF534	0.6388	ARCHS4 Coexpression,0.6388
1504	BHLHE23	0.6407	ARCHS4 Coexpression,0.6407
1505	DZIP1	0.6432	Enrichr Queries,0.6432
1506	MTF1	0.6441	GTEx Coexpression,0.6441
1507	SP8	0.6447	GTEx Coexpression,0.6447
1508	POU6F2	0.645	ARCHS4 Coexpression,0.645
1509	TFAP2B	0.6486	ARCHS4 Coexpression,0.6486
1510	RORB	0.651	Enrichr Queries,0.651
1511	ZNF493	0.6515	GTEx Coexpression,0.6515
1512	RORA	0.6528	GTEx Coexpression,0.6528
1513	EN2	0.6529	ARCHS4 Coexpression,0.6529
1514	ZNF318	0.6532	ReMap ChIP-seq,0.6532
1515	ZNF536	0.6546	Enrichr Queries,0.6546
1516	POU4F3	0.6548	ARCHS4 Coexpression,0.6548
1517	ZNF705D	0.6567	Enrichr Queries,0.6567
1518	ST18	0.6579	ARCHS4 Coexpression,0.6579
1519	SCRT2	0.661	Enrichr Queries,0.661
1520	ZIC2	0.6667	Enrichr Queries,0.6667
1521	ZNF365	0.6674	Enrichr Queries,0.6674
1522	FOXC2	0.6677	ARCHS4 Coexpression,0.6677
1523	OLIG2	0.6683	ARCHS4 Coexpression,0.6683
1524	ASCL4	0.6696	GTEx Coexpression,0.6696
1525	ZNF134	0.6714	ARCHS4 Coexpression,0.6714
1526	ZNF730	0.672	ARCHS4 Coexpression,0.672
1527	INSM2	0.6752	Enrichr Queries,0.6752
1528	ZNF470	0.6763	ARCHS4 Coexpression,0.6763
1529	ZBTB34	0.6766	Enrichr Queries,0.6766
1530	ZNF571	0.6769	ARCHS4 Coexpression,0.6769
1531	GLIS3	0.6781	ARCHS4 Coexpression,0.6781
1532	ZNF445	0.6787	ARCHS4 Coexpression,0.6787
1533	MKX	0.6795	GTEx Coexpression,0.6795
1534	JRKL	0.6814	GTEx Coexpression,0.6814
1535	ZNF230	0.683	Enrichr Queries,0.683
1536	ZNF30	0.6843	ARCHS4 Coexpression,0.6843

1537	KCNIP3	0.6845	GTEx Coexpression,0.6845
1538	TBX18	0.6882	GTEx Coexpression,0.6882
1539	SCRT1	0.6902	Enrichr Queries,0.6902
1540	PLAG1	0.691	ARCHS4 Coexpression,0.691
1541	ZNF621	0.6923	Enrichr Queries,0.6923
1542	LHX1	0.6947	ARCHS4 Coexpression,0.6947
1543	PURB	0.699	ARCHS4 Coexpression,0.699
1544	BNC2	0.6994	Enrichr Queries,0.6994
1545	FERD3L	0.6996	ARCHS4 Coexpression,0.6996
1546	CPEB1	0.7002	ARCHS4 Coexpression,0.7002
1547	SP110	0.7023	Enrichr Queries,0.7023
1548	ZNF510	0.7051	Enrichr Queries,0.7051
1549	GTF2I	0.7064	ARCHS4 Coexpression,0.7064
1550	PHOX2B	0.707	ARCHS4 Coexpression,0.707
1551	ZNF521	0.7076	ARCHS4 Coexpression,0.7076
1552	GCM1	0.708	Enrichr Queries,0.708
1553	TBX2	0.7106	GTEx Coexpression,0.7106
1554	ZBTB6	0.7131	GTEx Coexpression,0.7131
1555	VSX1	0.7131	ARCHS4 Coexpression,0.7131
1556	TFEC	0.7144	ARCHS4 Coexpression,0.7144
1557	SKI	0.7156	GTEx Coexpression,0.7156
1558	ZNF507	0.7165	Enrichr Queries,0.7165
1559	ALX3	0.7181	GTEx Coexpression,0.7181
1560	EBF2	0.7211	ARCHS4 Coexpression,0.7211
1561	ATOH7	0.7222	Enrichr Queries,0.7222
1562	ZIC1	0.7229	Enrichr Queries,0.7229
1563	SIM1	0.7236	Enrichr Queries,0.7236
1564	ZNF770	0.7244	Enrichr Queries,0.7244
1565	NKX61	0.725	GTEx Coexpression,0.725
1566	ZNF780A	0.7251	Enrichr Queries,0.7251
1567	PRRX1	0.7272	Enrichr Queries,0.7272
1568	BCL6B	0.7291	ARCHS4 Coexpression,0.7291
1569	ZNF732	0.7293	GTEx Coexpression,0.7293
1570	TWIST2	0.7316	ARCHS4 Coexpression,0.7316
1571	PBX1	0.735	Enrichr Queries,0.735
1572	NEUROG2	0.7362	GTEx Coexpression,0.7362
1573	ZNF112	0.7371	ARCHS4 Coexpression,0.7371
1574	NKX62	0.7383	ARCHS4 Coexpression,0.7383
1575	ZFP69B	0.7439	ARCHS4 Coexpression,0.7439
1576	NFE4	0.7449	GTEx Coexpression,0.7449
1577	HOXD12	0.7461	GTEx Coexpression,0.7461
1578	ZNF248	0.7472	Enrichr Queries,0.7472
1579	TWIST1	0.7508	ReMap ChIP-seq,0.7508
1580	EBF3	0.7543	Enrichr Queries,0.7543
1581	NFATC2	0.7549	ARCHS4 Coexpression,0.7549
1582	BHLHA9	0.7567	GTEx Coexpression,0.7567
1583	HSFY2	0.7598	ARCHS4 Coexpression,0.7598
1584	SP9	0.7654	GTEx Coexpression,0.7654
1585	ZNF366	0.7666	GTEx Coexpression,0.7666
1586	NKX22	0.7685	GTEx Coexpression,0.7685
1587	PCGF2	0.7709	ARCHS4 Coexpression,0.7709
1588	SOX1	0.7758	ARCHS4 Coexpression,0.7758
1589	ZNF354C	0.7778	Enrichr Queries,0.7778

1590	CHAMP1	0.7785	GTEx Coexpression,0.7785
1591	JRK	0.7797	GTEx Coexpression,0.7797
1592	ZNF80	0.7801	ARCHS4 Coexpression,0.7801
1593	RBAK	0.7863	Enrichr Queries,0.7863
1594	ZNF578	0.7893	ARCHS4 Coexpression,0.7893
1595	BARHL2	0.7897	GTEx Coexpression,0.7897
1596	PRDM12	0.7903	GTEx Coexpression,0.7903
1597	PURG	0.7912	ARCHS4 Coexpression,0.7912
1598	MYT1L	0.797	Enrichr Queries,0.797
1599	PHF21A	0.7978	GTEx Coexpression,0.7978
1600	ZNF385D	0.7979	ARCHS4 Coexpression,0.7979
1601	L3MBTL1	0.8034	ARCHS4 Coexpression,0.8034
1602	ZNF781	0.8053	ARCHS4 Coexpression,0.8053
1603	SP140	0.8059	ARCHS4 Coexpression,0.8059
1604	IKZF2	0.8063	Enrichr Queries,0.8063
1605	AKNA	0.8077	Enrichr Queries,0.8077
1606	ZNF888	0.8139	GTEx Coexpression,0.8139
1607	DUX4	0.8151	ARCHS4 Coexpression,0.8151
1608	SOX14	0.8182	ARCHS4 Coexpression,0.8182
1609	FOXD4L3	0.8191	Enrichr Queries,0.8191
1610	KLF14	0.8213	ARCHS4 Coexpression,0.8213
1611	ZBTB18	0.8214	GTEx Coexpression,0.8214
1612	ZIC4	0.8219	Enrichr Queries,0.8219
1613	BARHL1	0.8264	GTEx Coexpression,0.8264
1614	TET3	0.8307	GTEx Coexpression,0.8307
1615	DRGX	0.8378	ARCHS4 Coexpression,0.8378
1616	ZFP69	0.8444	GTEx Coexpression,0.8444
1617	ISL1	0.8482	GTEx Coexpression,0.8482
1618	SGSM2	0.8507	ARCHS4 Coexpression,0.8507
1619	LHX4	0.8682	Enrichr Queries,0.8682
1620	NHLH2	0.8724	GTEx Coexpression,0.8724
1621	PURA	0.8787	GTEx Coexpression,0.8787
1622	CSRNP3	0.8805	GTEx Coexpression,0.8805
1623	ZNF804A	0.8808	ARCHS4 Coexpression,0.8808
1624	BHLHE41	0.89	ARCHS4 Coexpression,0.89
1625	MYRF	0.8998	GTEx Coexpression,0.8998
1626	LHX9	0.9042	GTEx Coexpression,0.9042
1627	BSX	0.9117	Enrichr Queries,0.9117
1628	PEG3	0.916	GTEx Coexpression,0.916
1629	HSFX2	0.9429	ARCHS4 Coexpression,0.9429
1630	CCDC169SOHLH2	0.9459	ARCHS4 Coexpression,0.9459
1631	FOXD4L6	0.9793	Enrichr Queries,0.9793
1632	TET2	0.9844	GTEx Coexpression,0.9844

Rank	TF	Score	Library
1	CENPA	6.14E-04	ARCHS4 Coexpression,6.143E-4
2	ZNF367	6.22E-04	GTEx Coexpression,6.223E-4
3	ZNF695	7.12E-04	Enrichr Queries,7.123E-4
4	E2F8	0.001229	ARCHS4 Coexpression,0.001229
5	TFDP1	0.001245	GTEx Coexpression,0.001245
6	MTERF3	0.001843	ARCHS4 Coexpression,0.001843
7	FOXM1	0.001867	GTEx Coexpression,0.001867
8	ZNF689	0.002457	ARCHS4 Coexpression,0.002457
9	ZNF691	0.003071	ARCHS4 Coexpression,0.003071
10	ZNF492	0.003111	GTEx Coexpression,0.003111
11	GLYR1	0.003367	ReMap ChIP-seq,0.003367
12	E2F7	0.003561	Enrichr Queries,0.003561
13	PRMT3	0.003686	ARCHS4 Coexpression,0.003686
14	DNMT1	0.003734	GTEx Coexpression,0.003734
15	MYBL2	0.004274	Enrichr Queries,0.004274
16	ZNF227	0.0043	ARCHS4 Coexpression,0.0043
17	HMGA1	0.004356	GTEx Coexpression,0.004356
18	SOHLH2	0.004914	ARCHS4 Coexpression,0.004914
19	LCORL	0.004978	GTEx Coexpression,0.004978
20	PCGF6	0.004986	Enrichr Queries,0.004986
21	ZNF670	0.005528	ARCHS4 Coexpression,0.005528
22	PA2G4	0.0056	GTEx Coexpression,0.0056
23	ZNF519	0.005698	Enrichr Queries,0.005698
24	CREM	0.006098	Literature ChIP-seq,0.006098
25	LIN28B	0.006143	ARCHS4 Coexpression,0.006143
26	ZNF215	0.006223	GTEx Coexpression,0.006223
27	MYBL1	0.00641	Enrichr Queries,0.00641
28	KMT2A	0.006734	ReMap ChIP-seq,0.006734
29	E2F2	0.007123	Enrichr Queries,0.007123
30	ZNF146	0.007371	ARCHS4 Coexpression,0.007371
31	ZNF726	0.00809	GTEx Coexpression,0.00809
32	MAX	0.008475	ENCODE ChIP-seq,0.008475
33	MXD3	0.008547	Enrichr Queries,0.008547
34	TGIF2	0.0086	ARCHS4 Coexpression,0.0086
35	ZNF239	0.009214	ARCHS4 Coexpression,0.009214
36	CBX2	0.009259	Enrichr Queries,0.009259
37	ZNF124	0.009334	GTEx Coexpression,0.009334
38	ZNF732	0.009828	ARCHS4 Coexpression,0.009828
39	ZNF724	0.009956	GTEx Coexpression,0.009956
40	E2F1	0.009972	Enrichr Queries,0.009972
41	NCOA3	0.0101	ReMap ChIP-seq,0.0101
42	NR6A1	0.01044	ARCHS4 Coexpression,0.01044
43	ZNF93	0.01068	Enrichr Queries,0.01068
44	ZNF155	0.01106	ARCHS4 Coexpression,0.01106
45	ZNF625	0.0112	GTEx Coexpression,0.0112
46	OTX2	0.01167	ARCHS4 Coexpression,0.01167
47	ZFP69B	0.01182	GTEx Coexpression,0.01182
48	ZFX	0.0122	Literature ChIP-seq,0.0122
49	ZNF281	0.01229	ARCHS4 Coexpression,0.01229
50	AHRR	0.01245	GTEx Coexpression,0.01245
51	ZNF85	0.01282	Enrichr Queries,0.01282
52	ALX1	0.0129	ARCHS4 Coexpression,0.0129

53	ETV4	0.01307	GTEx Coexpression,0.01307
54	GATA2	0.01347	ReMap ChIP-seq,0.01347
55	ZNF878	0.01351	ARCHS4 Coexpression,0.01351
56	ZNF530	0.01353	Enrichr Queries,0.01353
57	MTERF1	0.01369	GTEx Coexpression,0.01369
58	THAP10	0.01425	Enrichr Queries,0.01425
59	ZNF850	0.01431	GTEx Coexpression,0.01431
60	ZIC2	0.01474	ARCHS4 Coexpression,0.01474
61	IKZF2	0.01493	GTEx Coexpression,0.01493
62	CENPT	0.01496	Enrichr Queries,0.01496
63	LEUTX	0.01556	GTEx Coexpression,0.01556
64	ZNF273	0.01567	Enrichr Queries,0.01567
65	ZNF888	0.01618	GTEx Coexpression,0.01618
66	ZNF788P	0.01638	Enrichr Queries,0.01638
67	ZNF286A	0.01658	ARCHS4 Coexpression,0.01658
68	ZNF92	0.0168	GTEx Coexpression,0.0168
69	HMBOX1	0.01684	ReMap ChIP-seq,0.01684
70	E2F4	0.01695	ENCODE ChIP-seq,0.01695
71	MYB	0.01709	Enrichr Queries,0.01709
72	STAT1	0.01742	GTEx Coexpression,0.01742
73	E2F3	0.01781	Enrichr Queries,0.01781
74	ZNF749	0.01781	ARCHS4 Coexpression,0.01781
75	ZNF257	0.01805	GTEx Coexpression,0.01805
76	KDM5B	0.01829	Literature ChIP-seq,0.01829
77	HOXA7	0.01843	ARCHS4 Coexpression,0.01843
78	ZNF473	0.01852	Enrichr Queries,0.01852
79	ZSCAN5C	0.01867	GTEx Coexpression,0.01867
80	ZNF560	0.01904	ARCHS4 Coexpression,0.01904
81	ZNF714	0.01923	Enrichr Queries,0.01923
82	MBD4	0.01994	Enrichr Queries,0.01994
83	CEBPG	0.0202	ReMap ChIP-seq,0.0202
84	CENPS	0.02027	ARCHS4 Coexpression,0.02027
85	ZNF100	0.02054	GTEx Coexpression,0.02054
86	ZNF511	0.02066	Enrichr Queries,0.02066
87	NKX12	0.02088	ARCHS4 Coexpression,0.02088
88	ZNF90	0.02116	GTEx Coexpression,0.02116
89	ZC3H8	0.02137	Enrichr Queries,0.02137
90	HESX1	0.0215	ARCHS4 Coexpression,0.0215
91	ZNF813	0.02178	GTEx Coexpression,0.02178
92	ZNF256	0.02211	ARCHS4 Coexpression,0.02211
93	ZNF26	0.0224	GTEx Coexpression,0.0224
94	SKIL	0.02273	ARCHS4 Coexpression,0.02273
95	DR1	0.02279	Enrichr Queries,0.02279
96	ZUP1	0.02302	GTEx Coexpression,0.02302
97	TERF1	0.0235	Enrichr Queries,0.0235
98	DUX4	0.02357	ReMap ChIP-seq,0.02357
99	FIGLA	0.02396	ARCHS4 Coexpression,0.02396
100	ZNF22	0.02422	Enrichr Queries,0.02422
101	ZNF717	0.02427	GTEx Coexpression,0.02427
102	CDX4	0.02457	ARCHS4 Coexpression,0.02457
103	HMX3	0.02489	GTEx Coexpression,0.02489
104	HOXA1	0.02518	ARCHS4 Coexpression,0.02518
105	GABPA	0.02542	ENCODE ChIP-seq,0.02542

106	IRF4	0.02551	GTEx Coexpression,0.02551
107	ZNF684	0.02564	Enrichr Queries,0.02564
108	CEBPZ	0.0258	ARCHS4 Coexpression,0.0258
109	ZBED4	0.02614	GTEx Coexpression,0.02614
110	KIN	0.02635	Enrichr Queries,0.02635
111	HMX2	0.02676	GTEx Coexpression,0.02676
112	HOXB13	0.02694	ReMap ChIP-seq,0.02694
113	CHCHD3	0.02703	ARCHS4 Coexpression,0.02703
114	ZNF232	0.02707	Enrichr Queries,0.02707
115	ZNF624	0.02738	GTEx Coexpression,0.02738
116	ZNF229	0.02764	ARCHS4 Coexpression,0.02764
117	ZIK1	0.02778	Enrichr Queries,0.02778
118	RFX8	0.028	GTEx Coexpression,0.028
119	ZNF214	0.02826	ARCHS4 Coexpression,0.02826
120	ZNF639	0.02849	Enrichr Queries,0.02849
121	ZNF277	0.02862	GTEx Coexpression,0.02862
122	ZNF280C	0.02887	ARCHS4 Coexpression,0.02887
123	ZNF77	0.02948	ARCHS4 Coexpression,0.02948
124	BHLHE23	0.02987	GTEx Coexpression,0.02987
125	THAP9	0.02991	Enrichr Queries,0.02991
126	ZNF35	0.0301	ARCHS4 Coexpression,0.0301
127	MAFG	0.0303	ReMap ChIP-seq,0.0303
128	GFI1B	0.03049	Literature ChIP-seq,0.03049
129	ZNF131	0.03049	GTEx Coexpression,0.03049
130	CREB3L4	0.03063	Enrichr Queries,0.03063
131	ZNF416	0.03071	ARCHS4 Coexpression,0.03071
132	ZNF891	0.03111	GTEx Coexpression,0.03111
133	TTF1	0.03133	ARCHS4 Coexpression,0.03133
134	ARNTL2	0.03134	Enrichr Queries,0.03134
135	PRDM13	0.03174	GTEx Coexpression,0.03174
136	HMGA2	0.03236	GTEx Coexpression,0.03236
137	E2F5	0.03276	Enrichr Queries,0.03276
138	TOPORS	0.03298	GTEx Coexpression,0.03298
139	ZNF449	0.03317	ARCHS4 Coexpression,0.03317
140	THAP1	0.03348	Enrichr Queries,0.03348
141	CREB1	0.0336	GTEx Coexpression,0.0336
142	POU2F1	0.03367	ReMap ChIP-seq,0.03367
143	ZBTB49	0.03378	ARCHS4 Coexpression,0.03378
144	MYC	0.0339	ENCODE ChIP-seq,0.0339
145	ZNF107	0.03419	Enrichr Queries,0.03419
146	ZNF567	0.03423	GTEx Coexpression,0.03423
147	ZBTB2	0.0344	ARCHS4 Coexpression,0.0344
148	MLX	0.03485	GTEx Coexpression,0.03485
149	YY2	0.0349	Enrichr Queries,0.0349
150	ZNF770	0.03501	ARCHS4 Coexpression,0.03501
151	TP53	0.03547	GTEx Coexpression,0.03547
152	ZSCAN21	0.03563	ARCHS4 Coexpression,0.03563
153	CHAMP1	0.03609	GTEx Coexpression,0.03609
154	NFYB	0.03624	ARCHS4 Coexpression,0.03624
155	MYCN	0.03659	Literature ChIP-seq,0.03659
156	ZNF543	0.03671	GTEx Coexpression,0.03671
157	ZNF597	0.03686	ARCHS4 Coexpression,0.03686
158	NFX1	0.03734	GTEx Coexpression,0.03734

159	TET1	0.03747	ARCHS4 Coexpression,0.03747
160	ZNF789	0.03775	Enrichr Queries,0.03775
161	MIXL1	0.03796	GTEx Coexpression,0.03796
162	LIN28A	0.03808	ARCHS4 Coexpression,0.03808
163	ADNP2	0.0387	ARCHS4 Coexpression,0.0387
164	ZNF410	0.03917	Enrichr Queries,0.03917
165	MSANTD4	0.03931	ARCHS4 Coexpression,0.03931
166	BACH1	0.03983	GTEx Coexpression,0.03983
167	ATF1	0.03989	Enrichr Queries,0.03989
168	ZNF304	0.03993	ARCHS4 Coexpression,0.03993
169	ONECUT1	0.0404	ReMap ChIP-seq,0.0404
170	HIF1A	0.04045	GTEx Coexpression,0.04045
171	TFAP2D	0.04054	ARCHS4 Coexpression,0.04054
172	ZNF654	0.04107	GTEx Coexpression,0.04107
173	ZFAT	0.04169	GTEx Coexpression,0.04169
174	HMG20A	0.04177	ARCHS4 Coexpression,0.04177
175	ZNF200	0.04202	Enrichr Queries,0.04202
176	ZNF69	0.04231	GTEx Coexpression,0.04231
177	HES6	0.04274	Enrichr Queries,0.04274
178	SETDB1	0.04294	GTEx Coexpression,0.04294
179	ZNF443	0.043	ARCHS4 Coexpression,0.043
180	SALL4	0.04345	Enrichr Queries,0.04345
181	ZNF468	0.04356	GTEx Coexpression,0.04356
182	TCF7L2	0.04361	ARCHS4 Coexpression,0.04361
183	FOXA1	0.04377	ReMap ChIP-seq,0.04377
184	ZBED2	0.04416	Enrichr Queries,0.04416
185	ZNF845	0.04418	GTEx Coexpression,0.04418
186	ZNF311	0.0448	GTEx Coexpression,0.0448
187	ADNP	0.04484	ARCHS4 Coexpression,0.04484
188	ZNF300	0.04487	Enrichr Queries,0.04487
189	ZNF28	0.04543	GTEx Coexpression,0.04543
190	LHX8	0.04545	ARCHS4 Coexpression,0.04545
191	TEAD4	0.04558	Enrichr Queries,0.04558
192	ZNF268	0.04605	GTEx Coexpression,0.04605
193	ZNF287	0.04607	ARCHS4 Coexpression,0.04607
194	E2F6	0.0463	Enrichr Queries,0.0463
195	ZNF572	0.04667	GTEx Coexpression,0.04667
196	ZNF57	0.04668	ARCHS4 Coexpression,0.04668
197	ZFP1	0.04701	Enrichr Queries,0.04701
198	HHEX	0.04714	ReMap ChIP-seq,0.04714
199	POU5F1B	0.04791	ARCHS4 Coexpression,0.04791
200	TIGD6	0.04792	GTEx Coexpression,0.04792
201	ZNF680	0.04843	Enrichr Queries,0.04843
202	ZNF578	0.04854	GTEx Coexpression,0.04854
203	ZFP69	0.04914	ARCHS4 Coexpression,0.04914
204	MBD2	0.04915	Enrichr Queries,0.04915
205	ZNF699	0.04916	GTEx Coexpression,0.04916
206	NANOGP8	0.04975	ARCHS4 Coexpression,0.04975
207	ZBED1	0.04978	GTEx Coexpression,0.04978
208	ZNF318	0.0504	GTEx Coexpression,0.0504
209	SOX2	0.05051	ReMap ChIP-seq,0.05051
210	GTF3A	0.05057	Enrichr Queries,0.05057
211	FLI1	0.05085	ENCODE ChIP-seq,0.05085

212	KLF1	0.05098	ARCHS4 Coexpression,0.05098
213	ZNF507	0.05103	GTEx Coexpression,0.05103
214	ZNF620	0.05128	Enrichr Queries,0.05128
215	FOXE3	0.0516	ARCHS4 Coexpression,0.0516
216	ZNF202	0.05165	GTEx Coexpression,0.05165
217	TFAP4	0.05199	Enrichr Queries,0.05199
218	FOXN4	0.05221	ARCHS4 Coexpression,0.05221
219	ARNTL	0.05227	GTEx Coexpression,0.05227
220	FOXN2	0.05271	Enrichr Queries,0.05271
221	ATF6B	0.05289	GTEx Coexpression,0.05289
222	HMGN3	0.05344	ARCHS4 Coexpression,0.05344
223	KLF3	0.05387	ReMap ChIP-seq,0.05387
224	NR2E1	0.05405	ARCHS4 Coexpression,0.05405
225	ZNF551	0.05413	Enrichr Queries,0.05413
226	ZNF649	0.05414	GTEx Coexpression,0.05414
227	AEBP2	0.05467	ARCHS4 Coexpression,0.05467
228	ZNF790	0.05476	GTEx Coexpression,0.05476
229	ZNF721	0.05484	Enrichr Queries,0.05484
230	ERG	0.05488	Literature ChIP-seq,0.05488
231	FOXO1	0.05538	GTEx Coexpression,0.05538
232	TEAD2	0.05556	Enrichr Queries,0.05556
233	ZNF823	0.0559	ARCHS4 Coexpression,0.0559
234	ZNF730	0.056	GTEx Coexpression,0.056
235	ZNF66	0.05663	GTEx Coexpression,0.05663
236	HINFP	0.05724	ReMap ChIP-seq,0.05724
237	KCMF1	0.05769	Enrichr Queries,0.05769
238	ZNF404	0.05774	ARCHS4 Coexpression,0.05774
239	ZBTB9	0.05835	ARCHS4 Coexpression,0.05835
240	TIGD3	0.0584	Enrichr Queries,0.0584
241	POU2AF1	0.05849	GTEx Coexpression,0.05849
242	ZFP42	0.05897	ARCHS4 Coexpression,0.05897
243	ZFP91	0.05912	Enrichr Queries,0.05912
244	NRF1	0.05932	ENCODE ChIP-seq,0.05932
245	ZIC5	0.05958	ARCHS4 Coexpression,0.05958
246	ZNF765	0.05974	GTEx Coexpression,0.05974
247	VEZF1	0.05983	Enrichr Queries,0.05983
248	NCOA1	0.0602	ARCHS4 Coexpression,0.0602
249	KLF10	0.06036	GTEx Coexpression,0.06036
250	PREB	0.06054	Enrichr Queries,0.06054
251	KLF11	0.06081	ARCHS4 Coexpression,0.06081
252	KLF4	0.06098	Literature ChIP-seq,0.06098
253	RAG1	0.06098	GTEx Coexpression,0.06098
254	THAP5	0.06125	Enrichr Queries,0.06125
255	JRKL	0.06161	GTEx Coexpression,0.06161
256	ZNF121	0.06197	Enrichr Queries,0.06197
257	ZFPM2	0.06204	ARCHS4 Coexpression,0.06204
258	SOX15	0.06265	ARCHS4 Coexpression,0.06265
259	SNAPC5	0.06285	GTEx Coexpression,0.06285
260	HIC2	0.06327	ARCHS4 Coexpression,0.06327
261	ZNF195	0.06339	Enrichr Queries,0.06339
262	ZNF573	0.06347	GTEx Coexpression,0.06347
263	ZBTB16	0.06409	GTEx Coexpression,0.06409
264	ZNF138	0.0641	Enrichr Queries,0.0641

265	CGGBP1	0.0645	ARCHS4 Coexpression,0.0645
266	ZKSCAN4	0.06472	GTEx Coexpression,0.06472
267	ZNF143	0.06481	Enrichr Queries,0.06481
268	TIGD7	0.06511	ARCHS4 Coexpression,0.06511
269	ZNF675	0.06534	GTEx Coexpression,0.06534
270	TCF3	0.06553	Enrichr Queries,0.06553
271	ZNF518B	0.06572	ARCHS4 Coexpression,0.06572
272	ZNF17	0.06596	GTEx Coexpression,0.06596
273	CDC5L	0.06624	Enrichr Queries,0.06624
274	ZNF544	0.06658	GTEx Coexpression,0.06658
275	SMYD3	0.06695	ARCHS4 Coexpression,0.06695
276	FOXO3	0.06707	Literature ChIP-seq,0.06707
277	ZNF354C	0.06721	GTEx Coexpression,0.06721
278	FOXP1	0.06734	ReMap ChIP-seq,0.06734
279	ZNF91	0.06757	ARCHS4 Coexpression,0.06757
280	CTCF	0.06766	Enrichr Queries,0.06766
281	ZFY	0.06783	GTEx Coexpression,0.06783
282	GSX2	0.06818	ARCHS4 Coexpression,0.06818
283	ZNF317	0.06838	Enrichr Queries,0.06838
284	GLIS3	0.06845	GTEx Coexpression,0.06845
285	PRDM14	0.0688	ARCHS4 Coexpression,0.0688
286	ZNF706	0.06909	Enrichr Queries,0.06909
287	ZNF581	0.06941	ARCHS4 Coexpression,0.06941
288	RBAK	0.0697	GTEx Coexpression,0.0697
289	ZNF45	0.07002	ARCHS4 Coexpression,0.07002
290	ZNF565	0.07032	GTEx Coexpression,0.07032
291	ZNF589	0.07064	ARCHS4 Coexpression,0.07064
292	ZNF398	0.07094	GTEx Coexpression,0.07094
293	UBP1	0.07123	Enrichr Queries,0.07123
294	ZBED5	0.07125	ARCHS4 Coexpression,0.07125
295	ZNF681	0.07156	GTEx Coexpression,0.07156
296	ZBTB39	0.07187	ARCHS4 Coexpression,0.07187
297	ZZZ3	0.07194	Enrichr Queries,0.07194
298	ARID5B	0.07218	GTEx Coexpression,0.07218
299	POU5F1	0.07248	ARCHS4 Coexpression,0.07248
300	ZNF800	0.07265	Enrichr Queries,0.07265
301	ZNF7	0.07281	GTEx Coexpression,0.07281
302	ZNF292	0.0731	ARCHS4 Coexpression,0.0731
303	SPI1	0.07317	Literature ChIP-seq,0.07317
304	ZNF705E	0.07343	GTEx Coexpression,0.07343
305	ZNF502	0.07371	ARCHS4 Coexpression,0.07371
306	BNC1	0.07405	GTEx Coexpression,0.07405
307	YY1	0.07407	Enrichr Queries,0.07407
308	MAFB	0.07407	ReMap ChIP-seq,0.07407
309	DMRTB1	0.07432	ARCHS4 Coexpression,0.07432
310	NEUROG2	0.07467	GTEx Coexpression,0.07467
311	ZNF207	0.07479	Enrichr Queries,0.07479
312	ZNF664	0.0755	Enrichr Queries,0.0755
313	ARID2	0.07617	ARCHS4 Coexpression,0.07617
314	ZNF682	0.07621	Enrichr Queries,0.07621
315	BHLHE40	0.07627	ENCODE ChIP-seq,0.07627
316	FOXJ2	0.07654	GTEx Coexpression,0.07654
317	TBP	0.07678	ARCHS4 Coexpression,0.07678

318	THAP6	0.07716	GTEx Coexpression,0.07716
319	ZNF181	0.0774	ARCHS4 Coexpression,0.0774
320	NFE2L1	0.07744	ReMap ChIP-seq,0.07744
321	TCFL5	0.07764	Enrichr Queries,0.07764
322	ZNF569	0.07801	ARCHS4 Coexpression,0.07801
323	SP3	0.07835	Enrichr Queries,0.07835
324	ZNF480	0.07841	GTEx Coexpression,0.07841
325	LIN54	0.07862	ARCHS4 Coexpression,0.07862
326	FOXN3	0.07903	GTEx Coexpression,0.07903
327	ZNF322	0.07924	ARCHS4 Coexpression,0.07924
328	FOXP3	0.07927	Literature ChIP-seq,0.07927
329	ZNF678	0.07985	ARCHS4 Coexpression,0.07985
330	KLF15	0.08027	GTEx Coexpression,0.08027
331	ZNF134	0.08047	ARCHS4 Coexpression,0.08047
332	YBX1	0.08048	Enrichr Queries,0.08048
333	FOXK1	0.08081	ReMap ChIP-seq,0.08081
334	NFYC	0.0812	Enrichr Queries,0.0812
335	ELK3	0.08152	GTEx Coexpression,0.08152
336	ZNF112	0.0817	ARCHS4 Coexpression,0.0817
337	TCF12	0.08191	Enrichr Queries,0.08191
338	PRRX1	0.08214	GTEx Coexpression,0.08214
339	DLX2	0.08231	ARCHS4 Coexpression,0.08231
340	ZSCAN12	0.08262	Enrichr Queries,0.08262
341	ZNF644	0.08276	GTEx Coexpression,0.08276
342	ISL2	0.08292	ARCHS4 Coexpression,0.08292
343	ZNF197	0.08333	Enrichr Queries,0.08333
344	ZNF830	0.08339	GTEx Coexpression,0.08339
345	TAL2	0.08354	ARCHS4 Coexpression,0.08354
346	ZNF12	0.08405	Enrichr Queries,0.08405
347	ZNF395	0.08415	ARCHS4 Coexpression,0.08415
348	ZKSCAN2	0.08463	GTEx Coexpression,0.08463
349	REST	0.08475	ENCODE ChIP-seq,0.08475
350	GTF2B	0.08477	ARCHS4 Coexpression,0.08477
351	TBX5	0.08537	Literature ChIP-seq,0.08537
352	ZNF30	0.08538	ARCHS4 Coexpression,0.08538
353	CLOCK	0.08587	GTEx Coexpression,0.08587
354	TPRX1	0.086	ARCHS4 Coexpression,0.086
355	ZBTB47	0.0865	GTEx Coexpression,0.0865
356	SOX6	0.08661	ARCHS4 Coexpression,0.08661
357	MAZ	0.08689	Enrichr Queries,0.08689
358	ZNF697	0.08712	GTEx Coexpression,0.08712
359	NFXL1	0.08722	ARCHS4 Coexpression,0.08722
360	CEBPA	0.08754	ReMap ChIP-seq,0.08754
361	FOXK2	0.08774	GTEx Coexpression,0.08774
362	PRDM9	0.08784	ARCHS4 Coexpression,0.08784
363	NR3C1	0.08836	GTEx Coexpression,0.08836
364	ZSCAN5B	0.08845	ARCHS4 Coexpression,0.08845
365	TIGD2	0.08903	Enrichr Queries,0.08903
366	MBD3	0.08974	Enrichr Queries,0.08974
367	SNAI1	0.09023	GTEx Coexpression,0.09023
368	ZNF177	0.09029	ARCHS4 Coexpression,0.09029
369	TFDP2	0.09046	Enrichr Queries,0.09046
370	ZNF485	0.09085	GTEx Coexpression,0.09085

371	GATA3	0.09091	ReMap ChIP-seq,0.09091
372	VDR	0.09146	Literature ChIP-seq,0.09146
373	ZFP64	0.09147	GTEx Coexpression,0.09147
374	TFAP2C	0.09152	ARCHS4 Coexpression,0.09152
375	ZNF512	0.09188	Enrichr Queries,0.09188
376	TFCP2	0.0921	GTEx Coexpression,0.0921
377	ARNT	0.09214	ARCHS4 Coexpression,0.09214
378	FOSL1	0.09259	Enrichr Queries,0.09259
379	ZBTB24	0.09272	GTEx Coexpression,0.09272
380	ZNF114	0.0933	Enrichr Queries,0.0933
381	ZNF117	0.09337	ARCHS4 Coexpression,0.09337
382	ZNF43	0.09396	GTEx Coexpression,0.09396
383	ZSCAN10	0.09398	ARCHS4 Coexpression,0.09398
384	PRDM4	0.09402	Enrichr Queries,0.09402
385	AHR	0.09428	ReMap ChIP-seq,0.09428
386	ZNF383	0.09459	GTEx Coexpression,0.09459
387	RFX6	0.09459	ARCHS4 Coexpression,0.09459
388	ZNF165	0.09473	Enrichr Queries,0.09473
389	ZNF837	0.09521	GTEx Coexpression,0.09521
390	ZNF574	0.09582	ARCHS4 Coexpression,0.09582
391	XPA	0.09583	GTEx Coexpression,0.09583
392	RBPJ	0.09615	Enrichr Queries,0.09615
393	TBXT	0.09644	ARCHS4 Coexpression,0.09644
394	ZNF407	0.09645	GTEx Coexpression,0.09645
395	ZNF217	0.09764	ReMap ChIP-seq,0.09764
396	ZNF71	0.09767	ARCHS4 Coexpression,0.09767
397	ZNF491	0.09828	ARCHS4 Coexpression,0.09828
398	NR2C1	0.09829	Enrichr Queries,0.09829
399	PRDM15	0.09832	GTEx Coexpression,0.09832
400	CXXC4	0.09889	ARCHS4 Coexpression,0.09889
401	ZNF669	0.09894	GTEx Coexpression,0.09894
402	RXRB	0.099	Enrichr Queries,0.099
403	NFIL3	0.09956	GTEx Coexpression,0.09956
404	SKOR1	0.1001	ARCHS4 Coexpression,0.1001
405	ZNF764	0.1002	GTEx Coexpression,0.1002
406	ZNF24	0.1004	Enrichr Queries,0.1004
407	PRDM1	0.101	ReMap ChIP-seq,0.101
408	SP4	0.1017	ENCODE ChIP-seq,0.1017
409	ZNF586	0.1026	Enrichr Queries,0.1026
410	ZNF852	0.1026	ARCHS4 Coexpression,0.1026
411	MITF	0.1027	GTEx Coexpression,0.1027
412	ZNF280B	0.1033	Enrichr Queries,0.1033
413	ZNF766	0.1033	GTEx Coexpression,0.1033
414	PRDM5	0.1037	Literature ChIP-seq,0.1037
415	NFKB1	0.1039	GTEx Coexpression,0.1039
416	ZNF562	0.104	Enrichr Queries,0.104
417	POU4F2	0.1044	ReMap ChIP-seq,0.1044
418	TIGD4	0.1044	ARCHS4 Coexpression,0.1044
419	ZNF211	0.105	ARCHS4 Coexpression,0.105
420	SMAD1	0.1052	GTEx Coexpression,0.1052
421	MYNN	0.1054	Enrichr Queries,0.1054
422	ZNF260	0.1057	ARCHS4 Coexpression,0.1057
423	SMAD4	0.1064	GTEx Coexpression,0.1064

424	ZNF222	0.1069	ARCHS4 Coexpression,0.1069
425	GZF1	0.1075	Enrichr Queries,0.1075
426	RFX7	0.1077	GTEx Coexpression,0.1077
427	ZNF384	0.1077	ReMap ChIP-seq,0.1077
428	ZFP62	0.1081	ARCHS4 Coexpression,0.1081
429	ZNF334	0.1087	ARCHS4 Coexpression,0.1087
430	ZNF883	0.1093	ARCHS4 Coexpression,0.1093
431	ZNF358	0.1095	GTEx Coexpression,0.1095
432	CREBZF	0.1097	Enrichr Queries,0.1097
433	THAP11	0.11	ARCHS4 Coexpression,0.11
434	ESR2	0.1101	GTEx Coexpression,0.1101
435	ZNF326	0.1104	Enrichr Queries,0.1104
436	ZNF136	0.1108	GTEx Coexpression,0.1108
437	ZNF83	0.1111	ReMap ChIP-seq,0.1111
438	SETDB2	0.1111	Enrichr Queries,0.1111
439	ZBTB26	0.1114	GTEx Coexpression,0.1114
440	NME2	0.112	GTEx Coexpression,0.112
441	ZNF337	0.1132	Enrichr Queries,0.1132
442	ZNF616	0.1133	GTEx Coexpression,0.1133
443	HOXD8	0.1139	GTEx Coexpression,0.1139
444	ZNF486	0.114	Enrichr Queries,0.114
445	EGR1	0.1143	ARCHS4 Coexpression,0.1143
446	LHX2	0.1145	ReMap ChIP-seq,0.1145
447	MSC	0.1145	GTEx Coexpression,0.1145
448	ZNF488	0.1147	Enrichr Queries,0.1147
449	DUXA	0.1149	ARCHS4 Coexpression,0.1149
450	HSF2	0.1155	ARCHS4 Coexpression,0.1155
451	KLF7	0.1157	GTEx Coexpression,0.1157
452	MSANTD3	0.117	GTEx Coexpression,0.117
453	ZNF160	0.1175	Enrichr Queries,0.1175
454	ZNF718	0.1176	GTEx Coexpression,0.1176
455	ELF5	0.1178	ReMap ChIP-seq,0.1178
456	PDX1	0.1179	ARCHS4 Coexpression,0.1179
457	IRF3	0.1182	GTEx Coexpression,0.1182
458	HES3	0.1186	ARCHS4 Coexpression,0.1186
459	ZNF799	0.1189	GTEx Coexpression,0.1189
460	SOX11	0.1189	Enrichr Queries,0.1189
461	ZNF534	0.1192	ARCHS4 Coexpression,0.1192
462	ZNF264	0.1195	GTEx Coexpression,0.1195
463	HBP1	0.1198	ARCHS4 Coexpression,0.1198
464	TSC22D1	0.1204	Enrichr Queries,0.1204
465	NOBOX	0.1204	ARCHS4 Coexpression,0.1204
466	ZSCAN20	0.1207	GTEx Coexpression,0.1207
467	ZBTB6	0.1211	Enrichr Queries,0.1211
468	HNF1B	0.1212	ReMap ChIP-seq,0.1212
469	ZNF84	0.1213	GTEx Coexpression,0.1213
470	SIX6	0.1216	ARCHS4 Coexpression,0.1216
471	POU3F2	0.122	Literature ChIP-seq,0.122
472	ZBTB1	0.122	GTEx Coexpression,0.122
473	ZNF223	0.1225	Enrichr Queries,0.1225
474	THYN1	0.1229	ARCHS4 Coexpression,0.1229
475	ZNF782	0.1232	GTEx Coexpression,0.1232
476	DPF1	0.1232	Enrichr Queries,0.1232

477	EBF2	0.1235	ARCHS4 Coexpression,0.1235
478	ZNF75D	0.1238	GTEx Coexpression,0.1238
479	ZBED3	0.1239	Enrichr Queries,0.1239
480	TEAD1	0.1245	GTEx Coexpression,0.1245
481	POGK	0.1246	Enrichr Queries,0.1246
482	ZNF354B	0.1251	GTEx Coexpression,0.1251
483	RHOXF2	0.1253	ARCHS4 Coexpression,0.1253
484	ZNF559	0.1254	Enrichr Queries,0.1254
485	ZBTB12	0.1261	Enrichr Queries,0.1261
486	ZNF484	0.1265	ARCHS4 Coexpression,0.1265
487	ATF4	0.1268	Enrichr Queries,0.1268
488	SP1	0.1271	ENCODE ChIP-seq,0.1271
489	ZNF594	0.1271	ARCHS4 Coexpression,0.1271
490	THAP4	0.1275	Enrichr Queries,0.1275
491	ZNF662	0.1276	GTEx Coexpression,0.1276
492	GATA1	0.1279	ReMap ChIP-seq,0.1279
493	MLXIP	0.1282	GTEx Coexpression,0.1282
494	ZNF3	0.1282	Enrichr Queries,0.1282
495	ZNF614	0.1284	ARCHS4 Coexpression,0.1284
496	ZNF584	0.1288	GTEx Coexpression,0.1288
497	ZNF420	0.129	ARCHS4 Coexpression,0.129
498	ZNF773	0.1294	GTEx Coexpression,0.1294
499	CREBL2	0.1296	ARCHS4 Coexpression,0.1296
500	ETV3L	0.1301	GTEx Coexpression,0.1301
501	ZNF711	0.1302	ARCHS4 Coexpression,0.1302
502	ZBTB11	0.1307	GTEx Coexpression,0.1307
503	ZNF254	0.1308	ARCHS4 Coexpression,0.1308
504	YBX2	0.1311	Enrichr Queries,0.1311
505	ZNF674	0.1313	GTEx Coexpression,0.1313
506	FOXA2	0.1313	ReMap ChIP-seq,0.1313
507	ZNF587B	0.1314	ARCHS4 Coexpression,0.1314
508	ZFP28	0.1318	Enrichr Queries,0.1318
509	NR2C2	0.1319	GTEx Coexpression,0.1319
510	MTF2	0.1321	ARCHS4 Coexpression,0.1321
511	IKZF4	0.1332	GTEx Coexpression,0.1332
512	ZNF490	0.1332	Enrichr Queries,0.1332
513	ELK4	0.1339	Enrichr Queries,0.1339
514	ZSCAN9	0.1339	ARCHS4 Coexpression,0.1339
515	TAL1	0.1341	Literature ChIP-seq,0.1341
516	PAX5	0.1344	GTEx Coexpression,0.1344
517	STAT2	0.1347	ReMap ChIP-seq,0.1347
518	ZNF570	0.135	GTEx Coexpression,0.135
519	ZNF708	0.1351	ARCHS4 Coexpression,0.1351
520	IRF1	0.1356	ENCODE ChIP-seq,0.1356
521	ZSCAN23	0.1357	ARCHS4 Coexpression,0.1357
522	MSGN1	0.1364	ARCHS4 Coexpression,0.1364
523	ZNF141	0.1368	Enrichr Queries,0.1368
524	ZSCAN16	0.137	ARCHS4 Coexpression,0.137
525	IKZF5	0.1375	Enrichr Queries,0.1375
526	ZNF772	0.1375	GTEx Coexpression,0.1375
527	ZNF529	0.1382	Enrichr Queries,0.1382
528	ZNF627	0.1382	ARCHS4 Coexpression,0.1382
529	SIM1	0.1394	GTEx Coexpression,0.1394

530	GLI4	0.14	GTEx Coexpression,0.14
531	PURG	0.14	ARCHS4 Coexpression,0.14
532	SRF	0.1402	Literature ChIP-seq,0.1402
533	AKAP8	0.1403	Enrichr Queries,0.1403
534	ZNF880	0.1406	GTEx Coexpression,0.1406
535	MTERF2	0.1407	ARCHS4 Coexpression,0.1407
536	SALL3	0.1413	ARCHS4 Coexpression,0.1413
537	ZNF394	0.1417	Enrichr Queries,0.1417
538	ZNF665	0.1419	GTEx Coexpression,0.1419
539	ATF3	0.1419	ARCHS4 Coexpression,0.1419
540	ZFP41	0.1425	Enrichr Queries,0.1425
541	ZNF14	0.1425	GTEx Coexpression,0.1425
542	ZNF10	0.1425	ARCHS4 Coexpression,0.1425
543	TGIF1	0.1431	ARCHS4 Coexpression,0.1431
544	ZBTB8A	0.1431	GTEx Coexpression,0.1431
545	SLC2A4RG	0.1432	Enrichr Queries,0.1432
546	ZNF442	0.1437	GTEx Coexpression,0.1437
547	ZNF606	0.1443	ARCHS4 Coexpression,0.1443
548	ZNF623	0.1444	GTEx Coexpression,0.1444
549	ZNF709	0.145	ARCHS4 Coexpression,0.145
550	MGA	0.145	GTEx Coexpression,0.145
551	ZNF585A	0.1456	ARCHS4 Coexpression,0.1456
552	DLX6	0.1462	ARCHS4 Coexpression,0.1462
553	SHOX2	0.1462	GTEx Coexpression,0.1462
554	ZNF354A	0.1467	Enrichr Queries,0.1467
555	JAZF1	0.1468	ARCHS4 Coexpression,0.1468
556	ZNF221	0.1469	GTEx Coexpression,0.1469
557	ZNF679	0.1474	ARCHS4 Coexpression,0.1474
558	NFATC3	0.1475	GTEx Coexpression,0.1475
559	KMT2B	0.1481	ReMap ChIP-seq,0.1481
560	RAX2	0.1481	Enrichr Queries,0.1481
561	MEF2A	0.1486	ARCHS4 Coexpression,0.1486
562	SNAI2	0.1489	Enrichr Queries,0.1489
563	PRDM10	0.1499	ARCHS4 Coexpression,0.1499
564	GTF2IRD2	0.15	GTEx Coexpression,0.15
565	ZNF44	0.1506	GTEx Coexpression,0.1506
566	ZFP57	0.151	Enrichr Queries,0.151
567	ZNF346	0.1511	ARCHS4 Coexpression,0.1511
568	BAZ2A	0.1512	GTEx Coexpression,0.1512
569	TCF7	0.1515	ReMap ChIP-seq,0.1515
570	ZNF280A	0.1517	Enrichr Queries,0.1517
571	ZNF184	0.1517	ARCHS4 Coexpression,0.1517
572	ZSCAN4	0.1523	ARCHS4 Coexpression,0.1523
573	RUNX1	0.1524	Literature ChIP-seq,0.1524
574	NFIC	0.1525	GTEx Coexpression,0.1525
575	CEBPB	0.1525	ENCODE ChIP-seq,0.1525
576	FOXR1	0.1529	ARCHS4 Coexpression,0.1529
577	ZNF496	0.1531	GTEx Coexpression,0.1531
578	THAP12	0.1536	ARCHS4 Coexpression,0.1536
579	DLX5	0.1542	ARCHS4 Coexpression,0.1542
580	ZNF180	0.1546	Enrichr Queries,0.1546
581	ZBTB44	0.1548	ARCHS4 Coexpression,0.1548
582	SOX10	0.1549	ReMap ChIP-seq,0.1549

583	NKX21	0.1554	ARCHS4 Coexpression,0.1554
584	ZNF829	0.156	Enrichr Queries,0.156
585	ZSCAN32	0.156	ARCHS4 Coexpression,0.156
586	ZNF816	0.1562	GTEx Coexpression,0.1562
587	GSC	0.1566	ARCHS4 Coexpression,0.1566
588	ZBTB33	0.1567	Enrichr Queries,0.1567
589	PPARA	0.1568	GTEx Coexpression,0.1568
590	SOX4	0.1574	Enrichr Queries,0.1574
591	CARF	0.1574	GTEx Coexpression,0.1574
592	GTF2IRD2B	0.1581	GTEx Coexpression,0.1581
593	ZNF576	0.1581	Enrichr Queries,0.1581
594	ZIC3	0.1585	ARCHS4 Coexpression,0.1585
595	ZHX3	0.1587	GTEx Coexpression,0.1587
596	ATMIN	0.1588	Enrichr Queries,0.1588
597	PAX6	0.1591	ARCHS4 Coexpression,0.1591
598	ZNF619	0.1595	Enrichr Queries,0.1595
599	ZNF525	0.1599	GTEx Coexpression,0.1599
600	ZNF692	0.1603	Enrichr Queries,0.1603
601	ZNF821	0.1603	ARCHS4 Coexpression,0.1603
602	XBP1	0.1609	ARCHS4 Coexpression,0.1609
603	RFX5	0.161	ENCODE ChIP-seq,0.161
604	ZNF700	0.1612	GTEx Coexpression,0.1612
605	TCF15	0.1615	ARCHS4 Coexpression,0.1615
606	NR1H3	0.1616	ReMap ChIP-seq,0.1616
607	ZXDB	0.1617	Enrichr Queries,0.1617
608	PHF20	0.1618	GTEx Coexpression,0.1618
609	TGIF2LX	0.1622	ARCHS4 Coexpression,0.1622
610	ZNF740	0.1624	Enrichr Queries,0.1624
611	DNTTIP1	0.1628	ARCHS4 Coexpression,0.1628
612	ZHX1	0.1631	Enrichr Queries,0.1631
613	ZNF582	0.1634	ARCHS4 Coexpression,0.1634
614	ZNF225	0.1637	GTEx Coexpression,0.1637
615	ZNF235	0.1638	Enrichr Queries,0.1638
616	NKRF	0.164	ARCHS4 Coexpression,0.164
617	HIVEP1	0.1643	GTEx Coexpression,0.1643
618	ZNF2	0.1645	Enrichr Queries,0.1645
619	EOMES	0.1646	Literature ChIP-seq,0.1646
620	ZSCAN25	0.1649	GTEx Coexpression,0.1649
621	ZBTB14	0.1655	GTEx Coexpression,0.1655
622	ZNF415	0.1658	ARCHS4 Coexpression,0.1658
623	GLMP	0.1661	GTEx Coexpression,0.1661
624	BAZ2B	0.1665	ARCHS4 Coexpression,0.1665
625	KLF16	0.1667	Enrichr Queries,0.1667
626	ZXDA	0.1668	GTEx Coexpression,0.1668
627	L3MBTL3	0.1671	ARCHS4 Coexpression,0.1671
628	AKAP8L	0.1674	Enrichr Queries,0.1674
629	CSRNP2	0.1674	GTEx Coexpression,0.1674
630	MSANTD1	0.1677	ARCHS4 Coexpression,0.1677
631	CEBPD	0.168	GTEx Coexpression,0.168
632	ATF2	0.1681	Enrichr Queries,0.1681
633	HOXC11	0.1684	ReMap ChIP-seq,0.1684
634	ZNF224	0.1686	GTEx Coexpression,0.1686
635	ZNF433	0.1689	ARCHS4 Coexpression,0.1689

636	ZNF226	0.1695	Enrichr Queries,0.1695
637	PPARG	0.1707	Literature ChIP-seq,0.1707
638	ZNF786	0.1709	Enrichr Queries,0.1709
639	ZNF501	0.172	ARCHS4 Coexpression,0.172
640	ZNF430	0.1731	Enrichr Queries,0.1731
641	ZNF253	0.1732	ARCHS4 Coexpression,0.1732
642	ZNF844	0.1736	GTEx Coexpression,0.1736
643	ZNF761	0.1738	Enrichr Queries,0.1738
644	ZBED6	0.1738	ARCHS4 Coexpression,0.1738
645	ZNF676	0.1744	ARCHS4 Coexpression,0.1744
646	ZBTB41	0.1751	ARCHS4 Coexpression,0.1751
647	NCOA2	0.1751	ReMap ChIP-seq,0.1751
648	ZNF879	0.1761	GTEx Coexpression,0.1761
649	GATA4	0.1768	Literature ChIP-seq,0.1768
650	JUN	0.1769	ARCHS4 Coexpression,0.1769
651	NR5A1	0.1774	Enrichr Queries,0.1774
652	ZNF737	0.1775	ARCHS4 Coexpression,0.1775
653	ESRRA	0.178	GTEx Coexpression,0.178
654	PAX8	0.1785	ReMap ChIP-seq,0.1785
655	ZNF561	0.1788	Enrichr Queries,0.1788
656	ZNF234	0.1792	GTEx Coexpression,0.1792
657	EEA1	0.1794	ARCHS4 Coexpression,0.1794
658	ZNF132	0.1805	GTEx Coexpression,0.1805
659	ZNF735	0.1806	ARCHS4 Coexpression,0.1806
660	ZFP82	0.1809	Enrichr Queries,0.1809
661	BBX	0.1816	Enrichr Queries,0.1816
662	MAFK	0.1818	ReMap ChIP-seq,0.1818
663	DLX1	0.1818	ARCHS4 Coexpression,0.1818
664	TMF1	0.1823	GTEx Coexpression,0.1823
665	HES1	0.1823	Enrichr Queries,0.1823
666	SOX17	0.1829	Literature ChIP-seq,0.1829
667	EMX1	0.183	ARCHS4 Coexpression,0.183
668	ZBTB5	0.183	Enrichr Queries,0.183
669	CREB3L2	0.1836	GTEx Coexpression,0.1836
670	TP73	0.1838	Enrichr Queries,0.1838
671	ZNF860	0.1843	ARCHS4 Coexpression,0.1843
672	ZNF33A	0.1848	GTEx Coexpression,0.1848
673	ZNF302	0.1852	Enrichr Queries,0.1852
674	STAT5B	0.1852	ReMap ChIP-seq,0.1852
675	MBNL2	0.1861	GTEx Coexpression,0.1861
676	REXO4	0.1867	GTEx Coexpression,0.1867
677	ZNF599	0.1873	GTEx Coexpression,0.1873
678	ZNF33B	0.1873	Enrichr Queries,0.1873
679	RARB	0.1879	GTEx Coexpression,0.1879
680	USF3	0.188	ARCHS4 Coexpression,0.188
681	ZNF781	0.1886	ARCHS4 Coexpression,0.1886
682	ZNF808	0.1887	Enrichr Queries,0.1887
683	DDIT3	0.1892	ARCHS4 Coexpression,0.1892
684	NFIA	0.1895	Enrichr Queries,0.1895
685	FOXD1	0.1898	GTEx Coexpression,0.1898
686	ZNF655	0.1902	Enrichr Queries,0.1902
687	ZBTB3	0.1909	Enrichr Queries,0.1909
688	HOXC6	0.191	GTEx Coexpression,0.191

689	MXI1	0.1916	ARCHS4 Coexpression,0.1916
690	DMTF1	0.1917	GTEx Coexpression,0.1917
691	ZNF23	0.1923	ARCHS4 Coexpression,0.1923
692	DRAP1	0.1923	Enrichr Queries,0.1923
693	ZNF577	0.1929	GTEx Coexpression,0.1929
694	ZFP30	0.1935	ARCHS4 Coexpression,0.1935
695	CREB3L1	0.1935	GTEx Coexpression,0.1935
696	REPIN1	0.1942	GTEx Coexpression,0.1942
697	ZNF34	0.1944	Enrichr Queries,0.1944
698	ATF6	0.1947	ARCHS4 Coexpression,0.1947
699	NR2F1	0.1948	GTEx Coexpression,0.1948
700	FOS	0.1949	ENCODE ChIP-seq,0.1949
701	NANOG	0.1951	Literature ChIP-seq,0.1951
702	ZNF174	0.1959	ARCHS4 Coexpression,0.1959
703	FOXD3	0.1966	ARCHS4 Coexpression,0.1966
704	THAP7	0.1972	ARCHS4 Coexpression,0.1972
705	ZKSCAN5	0.1973	GTEx Coexpression,0.1973
706	HEY2	0.1978	ARCHS4 Coexpression,0.1978
707	ZBTB21	0.1979	GTEx Coexpression,0.1979
708	ZNF563	0.198	Enrichr Queries,0.198
709	CREB3	0.1984	ARCHS4 Coexpression,0.1984
710	ZNF451	0.1985	GTEx Coexpression,0.1985
711	ZNF446	0.1987	Enrichr Queries,0.1987
712	ZNF747	0.1991	GTEx Coexpression,0.1991
713	ZBTB40	0.1994	Enrichr Queries,0.1994
714	ZKSCAN1	0.1996	ARCHS4 Coexpression,0.1996
715	ZNF250	0.2001	Enrichr Queries,0.2001
716	LHX6	0.2002	ARCHS4 Coexpression,0.2002
717	LCOR	0.2004	GTEx Coexpression,0.2004
718	TERF2	0.2009	Enrichr Queries,0.2009
719	ISL1	0.2009	ARCHS4 Coexpression,0.2009
720	HOXB4	0.2012	Literature ChIP-seq,0.2012
721	ZNF408	0.2015	ARCHS4 Coexpression,0.2015
722	ZNF140	0.2016	Enrichr Queries,0.2016
723	NRL	0.2016	GTEx Coexpression,0.2016
724	DEAF1	0.202	ReMap ChIP-seq,0.202
725	ZNF763	0.2021	ARCHS4 Coexpression,0.2021
726	ZBTB43	0.2023	Enrichr Queries,0.2023
727	ZNF652	0.2027	ARCHS4 Coexpression,0.2027
728	ZNF592	0.2029	GTEx Coexpression,0.2029
729	ZNF506	0.203	Enrichr Queries,0.203
730	MSX2	0.2033	ARCHS4 Coexpression,0.2033
731	ZNF585B	0.2037	Enrichr Queries,0.2037
732	ZNF441	0.2039	ARCHS4 Coexpression,0.2039
733	ZNF135	0.2041	GTEx Coexpression,0.2041
734	PITX1	0.2044	Enrichr Queries,0.2044
735	HOXB2	0.2045	ARCHS4 Coexpression,0.2045
736	ZEB2	0.2054	ReMap ChIP-seq,0.2054
737	SP9	0.2058	ARCHS4 Coexpression,0.2058
738	ZNF462	0.2058	Enrichr Queries,0.2058
739	WT1	0.206	GTEx Coexpression,0.206
740	NFE2L3	0.2073	Enrichr Queries,0.2073
741	ETS1	0.2073	Literature ChIP-seq,0.2073

742	ZNF607	0.2076	ARCHS4 Coexpression,0.2076
743	JUND	0.2078	GTEx Coexpression,0.2078
744	OTX1	0.2082	ARCHS4 Coexpression,0.2082
745	GATAD2A	0.2085	GTEx Coexpression,0.2085
746	NFYA	0.2087	Enrichr Queries,0.2087
747	ETV6	0.2088	ReMap ChIP-seq,0.2088
748	MEF2D	0.2091	GTEx Coexpression,0.2091
749	ZNF658	0.2095	ARCHS4 Coexpression,0.2095
750	RUNX2	0.2097	GTEx Coexpression,0.2097
751	RLF	0.2101	ARCHS4 Coexpression,0.2101
752	BCL6B	0.2107	ARCHS4 Coexpression,0.2107
753	IKZF3	0.2116	GTEx Coexpression,0.2116
754	HOXB7	0.2121	ReMap ChIP-seq,0.2121
755	ZNF778	0.2122	GTEx Coexpression,0.2122
756	ZNF320	0.2123	Enrichr Queries,0.2123
757	HDX	0.2125	ARCHS4 Coexpression,0.2125
758	SIX3	0.2131	ARCHS4 Coexpression,0.2131
759	HNF4A	0.2134	Literature ChIP-seq,0.2134
760	ZNF391	0.2134	GTEx Coexpression,0.2134
761	DPRX	0.2144	ARCHS4 Coexpression,0.2144
762	ZNF79	0.2144	Enrichr Queries,0.2144
763	ZNF426	0.2151	Enrichr Queries,0.2151
764	HSFX1	0.2153	GTEx Coexpression,0.2153
765	PLSCR1	0.2172	GTEx Coexpression,0.2172
766	RREB1	0.2172	Enrichr Queries,0.2172
767	POU1F1	0.2174	ARCHS4 Coexpression,0.2174
768	ZNF558	0.2178	GTEx Coexpression,0.2178
769	CUX1	0.2195	Literature ChIP-seq,0.2195
770	ZNF266	0.2197	GTEx Coexpression,0.2197
771	HOXA9	0.2199	ARCHS4 Coexpression,0.2199
772	ZNF16	0.2203	GTEx Coexpression,0.2203
773	MECP2	0.2205	ARCHS4 Coexpression,0.2205
774	ZHX2	0.2209	GTEx Coexpression,0.2209
775	ZNF596	0.2222	GTEx Coexpression,0.2222
776	RELA	0.2222	Enrichr Queries,0.2222
777	BACH2	0.2222	ReMap ChIP-seq,0.2222
778	GMEB2	0.2229	Enrichr Queries,0.2229
779	ZXDC	0.2236	Enrichr Queries,0.2236
780	TSHZ1	0.2242	ARCHS4 Coexpression,0.2242
781	ELF2	0.2251	Enrichr Queries,0.2251
782	ZNF703	0.2253	GTEx Coexpression,0.2253
783	ZNF280D	0.2254	ARCHS4 Coexpression,0.2254
784	TFEB	0.2256	Literature ChIP-seq,0.2256
785	HMG20B	0.2258	Enrichr Queries,0.2258
786	BARX1	0.226	ARCHS4 Coexpression,0.226
787	KLF9	0.2265	GTEx Coexpression,0.2265
788	STAT3	0.2271	GTEx Coexpression,0.2271
789	ASCL4	0.2297	ARCHS4 Coexpression,0.2297
790	ZKSCAN8	0.2302	GTEx Coexpression,0.2302
791	ZNF263	0.2308	Enrichr Queries,0.2308
792	TCF4	0.231	ARCHS4 Coexpression,0.231
793	PHF21A	0.2315	Enrichr Queries,0.2315
794	ZBTB37	0.2316	ARCHS4 Coexpression,0.2316

795	AR	0.2317	Literature ChIP-seq,0.2317
796	SATB1	0.2321	GTEx Coexpression,0.2321
797	AHCTF1	0.2322	Enrichr Queries,0.2322
798	MAFF	0.2323	ReMap ChIP-seq,0.2323
799	ZEB1	0.2334	GTEx Coexpression,0.2334
800	ESR1	0.2334	ARCHS4 Coexpression,0.2334
801	NR0B1	0.2352	GTEx Coexpression,0.2352
802	ZNF547	0.2353	ARCHS4 Coexpression,0.2353
803	LEF1	0.2357	ReMap ChIP-seq,0.2357
804	CXXC1	0.2358	Enrichr Queries,0.2358
805	TWIST1	0.2365	GTEx Coexpression,0.2365
806	HOXC5	0.2371	GTEx Coexpression,0.2371
807	TWIST2	0.2377	GTEx Coexpression,0.2377
808	PLAGL2	0.2379	Enrichr Queries,0.2379
809	ZSCAN29	0.2386	Enrichr Queries,0.2386
810	ZBTB38	0.239	GTEx Coexpression,0.239
811	MEIS2	0.2391	ReMap ChIP-seq,0.2391
812	ZSCAN26	0.2396	ARCHS4 Coexpression,0.2396
813	ZNF148	0.2396	GTEx Coexpression,0.2396
814	ZNF610	0.24	Enrichr Queries,0.24
815	NEUROG3	0.2402	ARCHS4 Coexpression,0.2402
816	SALL2	0.2408	ARCHS4 Coexpression,0.2408
817	ZNF248	0.2414	ARCHS4 Coexpression,0.2414
818	SCMH1	0.2414	GTEx Coexpression,0.2414
819	ANKZF1	0.2415	Enrichr Queries,0.2415
820	ANHX	0.242	ARCHS4 Coexpression,0.242
821	ZNF212	0.2422	Enrichr Queries,0.2422
822	TET3	0.2426	ARCHS4 Coexpression,0.2426
823	RFX4	0.2432	ARCHS4 Coexpression,0.2432
824	HES4	0.2436	Enrichr Queries,0.2436
825	ELK1	0.2439	Literature ChIP-seq,0.2439
826	ZNF736	0.2439	GTEx Coexpression,0.2439
827	ZNF836	0.2446	GTEx Coexpression,0.2446
828	HOXC9	0.2451	ARCHS4 Coexpression,0.2451
829	ZNF331	0.2457	Enrichr Queries,0.2457
830	HAND2	0.2463	ARCHS4 Coexpression,0.2463
831	PRDM2	0.2469	ARCHS4 Coexpression,0.2469
832	ZNF32	0.247	GTEx Coexpression,0.247
833	NFE2L2	0.2475	ARCHS4 Coexpression,0.2475
834	TSHZ2	0.2477	GTEx Coexpression,0.2477
835	ZNF641	0.2479	Enrichr Queries,0.2479
836	KLF14	0.2482	ARCHS4 Coexpression,0.2482
837	HOXA4	0.2483	GTEx Coexpression,0.2483
838	YBX3	0.2489	GTEx Coexpression,0.2489
839	MEIS1	0.2492	ReMap ChIP-seq,0.2492
840	ZNF189	0.2493	Enrichr Queries,0.2493
841	POU3F4	0.2494	ARCHS4 Coexpression,0.2494
842	THAP3	0.25	ARCHS4 Coexpression,0.25
843	ZNF514	0.25	Enrichr Queries,0.25
844	TBX3	0.25	Literature ChIP-seq,0.25
845	ZNF521	0.2507	Enrichr Queries,0.2507
846	HAND1	0.2512	ARCHS4 Coexpression,0.2512
847	ZNF696	0.2514	GTEx Coexpression,0.2514

848	ETV5	0.2518	ARCHS4 Coexpression,0.2518
849	TBX21	0.2525	ReMap ChIP-seq,0.2525
850	ZNF236	0.2526	GTEx Coexpression,0.2526
851	ZNF296	0.2528	Enrichr Queries,0.2528
852	PLAGL1	0.2531	ARCHS4 Coexpression,0.2531
853	ZNF608	0.2543	Enrichr Queries,0.2543
854	ZNF660	0.2545	GTEx Coexpression,0.2545
855	ZNF169	0.255	Enrichr Queries,0.255
856	ZNF843	0.2551	GTEx Coexpression,0.2551
857	ZNF805	0.2555	ARCHS4 Coexpression,0.2555
858	PBX1	0.2557	Enrichr Queries,0.2557
859	NR4A1	0.2559	ReMap ChIP-seq,0.2559
860	ARID3B	0.2561	ARCHS4 Coexpression,0.2561
861	ZIM2	0.2574	ARCHS4 Coexpression,0.2574
862	FOXO6	0.2576	GTEx Coexpression,0.2576
863	HOXB9	0.2578	Enrichr Queries,0.2578
864	EGR2	0.258	ARCHS4 Coexpression,0.258
865	ZNF600	0.2582	GTEx Coexpression,0.2582
866	PBX3	0.2585	Enrichr Queries,0.2585
867	TIGD5	0.2589	GTEx Coexpression,0.2589
868	ARX	0.2592	ARCHS4 Coexpression,0.2592
869	ZNF362	0.2593	Enrichr Queries,0.2593
870	ZNF791	0.26	Enrichr Queries,0.26
871	POU4F1	0.2601	GTEx Coexpression,0.2601
872	ZBTB20	0.2607	Enrichr Queries,0.2607
873	ARGFX	0.2607	GTEx Coexpression,0.2607
874	TCF7L1	0.2617	ARCHS4 Coexpression,0.2617
875	NFIB	0.2621	Enrichr Queries,0.2621
876	NKX28	0.2623	ARCHS4 Coexpression,0.2623
877	ZNF727	0.2632	GTEx Coexpression,0.2632
878	ZFP90	0.2638	GTEx Coexpression,0.2638
879	ZNF80	0.2641	ARCHS4 Coexpression,0.2641
880	NEUROD1	0.2647	ARCHS4 Coexpression,0.2647
881	HIC1	0.265	Enrichr Queries,0.265
882	ZNF283	0.2651	GTEx Coexpression,0.2651
883	ZNF571	0.2657	GTEx Coexpression,0.2657
884	FOSB	0.2663	GTEx Coexpression,0.2663
885	ZNF397	0.267	GTEx Coexpression,0.267
886	ZNF436	0.2672	ARCHS4 Coexpression,0.2672
887	FOXC2	0.2678	Enrichr Queries,0.2678
888	ZNF629	0.2682	GTEx Coexpression,0.2682
889	FEV	0.269	ARCHS4 Coexpression,0.269
890	SAFB	0.2694	GTEx Coexpression,0.2694
891	PROX2	0.2701	GTEx Coexpression,0.2701
892	ZFP37	0.2703	ARCHS4 Coexpression,0.2703
893	SIX4	0.2707	Enrichr Queries,0.2707
894	ZNF347	0.2713	GTEx Coexpression,0.2713
895	HIVEP2	0.2714	Enrichr Queries,0.2714
896	DBX2	0.2715	ARCHS4 Coexpression,0.2715
897	RAX	0.2719	GTEx Coexpression,0.2719
898	ZSCAN5A	0.2721	Enrichr Queries,0.2721
899	TCF24	0.2726	GTEx Coexpression,0.2726
900	ZBTB42	0.2738	GTEx Coexpression,0.2738

901	ZNF701	0.274	ARCHS4 Coexpression,0.274
902	FOXP2	0.2744	Literature ChIP-seq,0.2744
903	ASH1L	0.2744	GTEx Coexpression,0.2744
904	ZNF418	0.2746	ARCHS4 Coexpression,0.2746
905	TFCP2L1	0.275	GTEx Coexpression,0.275
906	TFAP2A	0.2756	Enrichr Queries,0.2756
907	GPBP1	0.2757	GTEx Coexpression,0.2757
908	ZNF438	0.2758	ARCHS4 Coexpression,0.2758
909	NFATC1	0.2763	GTEx Coexpression,0.2763
910	PATZ1	0.2785	Enrichr Queries,0.2785
911	NR2F6	0.2792	Enrichr Queries,0.2792
912	HEY1	0.2795	ARCHS4 Coexpression,0.2795
913	ARID3A	0.2797	ENCODE ChIP-seq,0.2797
914	ZBTB4	0.28	GTEx Coexpression,0.28
915	MSX1	0.2806	Enrichr Queries,0.2806
916	RBPJL	0.2806	GTEx Coexpression,0.2806
917	STAT6	0.2813	GTEx Coexpression,0.2813
918	ZNF324B	0.2819	GTEx Coexpression,0.2819
919	ZNF550	0.2825	GTEx Coexpression,0.2825
920	ZNF432	0.2828	Enrichr Queries,0.2828
921	ZNF487	0.2856	Enrichr Queries,0.2856
922	MYSM1	0.2856	GTEx Coexpression,0.2856
923	SNAPC4	0.2862	ReMap ChIP-seq,0.2862
924	ZBTB10	0.2863	Enrichr Queries,0.2863
925	PROP1	0.2869	ARCHS4 Coexpression,0.2869
926	FOXA3	0.2869	GTEx Coexpression,0.2869
927	RORA	0.2875	ARCHS4 Coexpression,0.2875
928	FOXJ3	0.2875	GTEx Coexpression,0.2875
929	FOXG1	0.2881	ARCHS4 Coexpression,0.2881
930	SOX9	0.2885	Enrichr Queries,0.2885
931	KLF17	0.2893	ARCHS4 Coexpression,0.2893
932	ZNF284	0.2894	GTEx Coexpression,0.2894
933	THAP2	0.2899	Enrichr Queries,0.2899
934	ZNF780B	0.2899	ARCHS4 Coexpression,0.2899
935	TIGD1	0.2905	ARCHS4 Coexpression,0.2905
936	ZNF566	0.2906	Enrichr Queries,0.2906
937	CTCFL	0.2912	ARCHS4 Coexpression,0.2912
938	NFAT5	0.2913	Enrichr Queries,0.2913
939	ZNF333	0.2918	GTEx Coexpression,0.2918
940	ZBTB25	0.292	Enrichr Queries,0.292
941	ZNF527	0.2925	GTEx Coexpression,0.2925
942	DACH1	0.2927	Literature ChIP-seq,0.2927
943	ZNF792	0.2927	Enrichr Queries,0.2927
944	ZNF471	0.2936	ARCHS4 Coexpression,0.2936
945	ZNF133	0.2942	Enrichr Queries,0.2942
946	ZNF615	0.2943	GTEx Coexpression,0.2943
947	USF2	0.2949	Enrichr Queries,0.2949
948	ZNF444	0.295	GTEx Coexpression,0.295
949	RORC	0.2962	GTEx Coexpression,0.2962
950	ZNF460	0.2963	Enrichr Queries,0.2963
951	PLAG1	0.2968	GTEx Coexpression,0.2968
952	MBD1	0.2977	Enrichr Queries,0.2977
953	DMRTA1	0.2979	ARCHS4 Coexpression,0.2979

954	GATA6	0.2988	Literature ChIP-seq,0.2988
955	TET2	0.2991	ARCHS4 Coexpression,0.2991
956	ZBTB7A	0.2991	Enrichr Queries,0.2991
957	MKX	0.2993	GTEx Coexpression,0.2993
958	SMAD9	0.2998	ARCHS4 Coexpression,0.2998
959	ZNF469	0.2999	GTEx Coexpression,0.2999
960	ZNF425	0.3006	Enrichr Queries,0.3006
961	ZNF728	0.301	ARCHS4 Coexpression,0.301
962	HOXC4	0.3012	GTEx Coexpression,0.3012
963	HOXD4	0.3016	ARCHS4 Coexpression,0.3016
964	PKNOX1	0.302	Enrichr Queries,0.302
965	RFX1	0.303	ReMap ChIP-seq,0.303
966	HOXA2	0.303	GTEx Coexpression,0.303
967	ZNF564	0.3041	Enrichr Queries,0.3041
968	ZNF729	0.3047	ARCHS4 Coexpression,0.3047
969	BCL11A	0.3053	ARCHS4 Coexpression,0.3053
970	SREBF1	0.3055	GTEx Coexpression,0.3055
971	ZNF335	0.3062	GTEx Coexpression,0.3062
972	ZSCAN30	0.3096	ARCHS4 Coexpression,0.3096
973	ZBTB46	0.3098	Enrichr Queries,0.3098
974	ZNF512B	0.3099	GTEx Coexpression,0.3099
975	ZNF37A	0.3102	ARCHS4 Coexpression,0.3102
976	NEUROD6	0.3108	ARCHS4 Coexpression,0.3108
977	BPTF	0.3118	GTEx Coexpression,0.3118
978	ZNF774	0.312	Enrichr Queries,0.312
979	ZNF182	0.312	ARCHS4 Coexpression,0.312
980	HLF	0.3124	GTEx Coexpression,0.3124
981	OVOL1	0.3127	Enrichr Queries,0.3127
982	MEF2B	0.3131	ReMap ChIP-seq,0.3131
983	KLF13	0.3134	Enrichr Queries,0.3134
984	USF1	0.3141	Enrichr Queries,0.3141
985	GSC2	0.3143	GTEx Coexpression,0.3143
986	ZNF251	0.3145	ARCHS4 Coexpression,0.3145
987	FOXD4L4	0.3149	GTEx Coexpression,0.3149
988	ZNF780A	0.3161	GTEx Coexpression,0.3161
989	ZNF20	0.3162	Enrichr Queries,0.3162
990	GCM2	0.317	ARCHS4 Coexpression,0.317
991	SALL1	0.3171	Literature ChIP-seq,0.3171
992	NR3C2	0.3176	ARCHS4 Coexpression,0.3176
993	ZNF552	0.3177	Enrichr Queries,0.3177
994	PHOX2A	0.3182	ARCHS4 Coexpression,0.3182
995	RXRA	0.3184	Enrichr Queries,0.3184
996	EBF1	0.3188	ARCHS4 Coexpression,0.3188
997	ZNF230	0.3198	Enrichr Queries,0.3198
998	ZNF516	0.3199	GTEx Coexpression,0.3199
999	TBX22	0.32	ARCHS4 Coexpression,0.32
1000	HIF3A	0.3205	GTEx Coexpression,0.3205
1001	TRERF1	0.3205	Enrichr Queries,0.3205
1002	SIX2	0.3211	GTEx Coexpression,0.3211
1003	ZFP3	0.3219	ARCHS4 Coexpression,0.3219
1004	RFX2	0.3225	ARCHS4 Coexpression,0.3225
1005	SMAD5	0.323	GTEx Coexpression,0.323
1006	ATOH7	0.3231	ARCHS4 Coexpression,0.3231

1007	ESX1	0.3236	GTEx Coexpression,0.3236
1008	CPEB1	0.3237	ARCHS4 Coexpression,0.3237
1009	ZNF316	0.3242	GTEx Coexpression,0.3242
1010	ZFHX4	0.3243	ARCHS4 Coexpression,0.3243
1011	ZNF503	0.3248	GTEx Coexpression,0.3248
1012	NEUROG1	0.3255	GTEx Coexpression,0.3255
1013	ZBTB32	0.3255	Enrichr Queries,0.3255
1014	NANOGNB	0.3261	GTEx Coexpression,0.3261
1015	BRF2	0.3266	ReMap ChIP-seq,0.3266
1016	FEZF1	0.3268	ARCHS4 Coexpression,0.3268
1017	TBX15	0.3279	GTEx Coexpression,0.3279
1018	NR4A2	0.328	ARCHS4 Coexpression,0.328
1019	ZNF526	0.3283	Enrichr Queries,0.3283
1020	SHOX	0.3286	GTEx Coexpression,0.3286
1021	SNAPC2	0.3286	ARCHS4 Coexpression,0.3286
1022	DOT1L	0.3291	Enrichr Queries,0.3291
1023	EGR4	0.3292	ARCHS4 Coexpression,0.3292
1024	ETV3	0.3298	Enrichr Queries,0.3298
1025	ZNF345	0.3298	GTEx Coexpression,0.3298
1026	ZNF274	0.3299	ARCHS4 Coexpression,0.3299
1027	SPIB	0.33	ReMap ChIP-seq,0.33
1028	POU2F2	0.3305	ENCODE ChIP-seq,0.3305
1029	JRK	0.3311	GTEx Coexpression,0.3311
1030	RFX3	0.3311	ARCHS4 Coexpression,0.3311
1031	ZNF341	0.3326	Enrichr Queries,0.3326
1032	ZNF555	0.3329	GTEx Coexpression,0.3329
1033	SRCAP	0.3333	Enrichr Queries,0.3333
1034	NR1H2	0.3335	GTEx Coexpression,0.3335
1035	NR1H4	0.3342	ARCHS4 Coexpression,0.3342
1036	ZNF536	0.3348	ARCHS4 Coexpression,0.3348
1037	THRB	0.3348	GTEx Coexpression,0.3348
1038	HOXC8	0.3354	ARCHS4 Coexpression,0.3354
1039	ZNF549	0.3355	Enrichr Queries,0.3355
1040	KLF6	0.336	ARCHS4 Coexpression,0.336
1041	NKX22	0.3366	ARCHS4 Coexpression,0.3366
1042	ZNF587	0.3367	GTEx Coexpression,0.3367
1043	MNX1	0.3376	Enrichr Queries,0.3376
1044	ZNF528	0.3378	ARCHS4 Coexpression,0.3378
1045	ZNF175	0.3379	GTEx Coexpression,0.3379
1046	OSR2	0.3383	Enrichr Queries,0.3383
1047	NR1D2	0.3385	ARCHS4 Coexpression,0.3385
1048	GCM1	0.339	Enrichr Queries,0.339
1049	REL	0.3404	GTEx Coexpression,0.3404
1050	ZNF814	0.3409	ARCHS4 Coexpression,0.3409
1051	FOXS1	0.3419	Enrichr Queries,0.3419
1052	MEOX2	0.3426	Enrichr Queries,0.3426
1053	ZNF439	0.3433	Enrichr Queries,0.3433
1054	ZKSCAN3	0.3441	GTEx Coexpression,0.3441
1055	NPAS2	0.3447	Enrichr Queries,0.3447
1056	ZNF350	0.3454	Enrichr Queries,0.3454
1057	TCF20	0.3466	GTEx Coexpression,0.3466
1058	SOX13	0.3468	ReMap ChIP-seq,0.3468
1059	CRX	0.3469	Enrichr Queries,0.3469

1060	SMAD3	0.3476	Literature ChIP-seq,0.3476
1061	PURA	0.3503	GTEx Coexpression,0.3503
1062	ZNF618	0.3511	Enrichr Queries,0.3511
1063	ELF1	0.3516	GTEx Coexpression,0.3516
1064	TRPS1	0.352	ARCHS4 Coexpression,0.352
1065	NR5A2	0.3522	GTEx Coexpression,0.3522
1066	ZNF382	0.3526	Enrichr Queries,0.3526
1067	ZSCAN31	0.3528	GTEx Coexpression,0.3528
1068	SP7	0.3532	ARCHS4 Coexpression,0.3532
1069	ZNF275	0.354	Enrichr Queries,0.354
1070	ZNF423	0.3541	GTEx Coexpression,0.3541
1071	ZNF81	0.355	ARCHS4 Coexpression,0.355
1072	ZNF428	0.3554	Enrichr Queries,0.3554
1073	ZNF285	0.3563	ARCHS4 Coexpression,0.3563
1074	EHF	0.3569	ReMap ChIP-seq,0.3569
1075	SKOR2	0.3572	GTEx Coexpression,0.3572
1076	PRRX2	0.3575	ARCHS4 Coexpression,0.3575
1077	GLIS1	0.3581	ARCHS4 Coexpression,0.3581
1078	ZNF583	0.3583	Enrichr Queries,0.3583
1079	ZNF98	0.3584	GTEx Coexpression,0.3584
1080	ZNF286B	0.3587	ARCHS4 Coexpression,0.3587
1081	ETV2	0.359	Enrichr Queries,0.359
1082	ZFPM1	0.3597	GTEx Coexpression,0.3597
1083	GLI1	0.3597	Enrichr Queries,0.3597
1084	MECOM	0.3598	Literature ChIP-seq,0.3598
1085	ZNF713	0.3603	GTEx Coexpression,0.3603
1086	ZNF841	0.3606	ARCHS4 Coexpression,0.3606
1087	ZFP14	0.3628	GTEx Coexpression,0.3628
1088	STAT5A	0.3634	GTEx Coexpression,0.3634
1089	PAX4	0.3636	ARCHS4 Coexpression,0.3636
1090	ZNF419	0.364	Enrichr Queries,0.364
1091	RUNX3	0.3644	ENCODE ChIP-seq,0.3644
1092	POU5F2	0.3661	ARCHS4 Coexpression,0.3661
1093	ZFP2	0.3668	Enrichr Queries,0.3668
1094	ZNF343	0.3678	GTEx Coexpression,0.3678
1095	ZNF282	0.369	GTEx Coexpression,0.369
1096	ZNF267	0.3697	Enrichr Queries,0.3697
1097	RELB	0.3703	GTEx Coexpression,0.3703
1098	BATF	0.3704	ReMap ChIP-seq,0.3704
1099	HOXA5	0.3715	GTEx Coexpression,0.3715
1100	SIM2	0.3722	ARCHS4 Coexpression,0.3722
1101	ZNF518A	0.3725	Enrichr Queries,0.3725
1102	KLF8	0.3732	Enrichr Queries,0.3732
1103	ELF3	0.3737	ReMap ChIP-seq,0.3737
1104	SOX7	0.3739	Enrichr Queries,0.3739
1105	ZNF787	0.3746	Enrichr Queries,0.3746
1106	RBSN	0.3752	GTEx Coexpression,0.3752
1107	ZNF532	0.3754	Enrichr Queries,0.3754
1108	HOXA10	0.3759	ARCHS4 Coexpression,0.3759
1109	HSF5	0.3768	Enrichr Queries,0.3768
1110	FOXP4	0.3777	GTEx Coexpression,0.3777
1111	ZNF429	0.3778	ARCHS4 Coexpression,0.3778
1112	OLIG2	0.378	Literature ChIP-seq,0.378

1113	SIX5	0.3783	GTEx Coexpression,0.3783
1114	RXRG	0.3796	Enrichr Queries,0.3796
1115	ZNF707	0.3802	GTEx Coexpression,0.3802
1116	PTF1A	0.3802	ARCHS4 Coexpression,0.3802
1117	HOXC12	0.3808	ARCHS4 Coexpression,0.3808
1118	ONECUT3	0.3833	ARCHS4 Coexpression,0.3833
1119	ZNF776	0.3839	ARCHS4 Coexpression,0.3839
1120	SETBP1	0.3839	GTEx Coexpression,0.3839
1121	CSRNP1	0.3845	ARCHS4 Coexpression,0.3845
1122	SPDEF	0.3846	GTEx Coexpression,0.3846
1123	ZSCAN22	0.3846	Enrichr Queries,0.3846
1124	PPARD	0.3853	Enrichr Queries,0.3853
1125	ZNF677	0.3864	ARCHS4 Coexpression,0.3864
1126	HIVEP3	0.387	ARCHS4 Coexpression,0.387
1127	GTF2IRD1	0.3871	GTEx Coexpression,0.3871
1128	FOXD4L5	0.3876	ARCHS4 Coexpression,0.3876
1129	ZMAT1	0.3882	ARCHS4 Coexpression,0.3882
1130	DLX4	0.3888	ARCHS4 Coexpression,0.3888
1131	IRX1	0.3889	GTEx Coexpression,0.3889
1132	TFDP3	0.3902	GTEx Coexpression,0.3902
1133	CDX2	0.3906	ReMap ChIP-seq,0.3906
1134	DMRTC2	0.3919	ARCHS4 Coexpression,0.3919
1135	ZNF579	0.392	GTEx Coexpression,0.392
1136	NR4A3	0.3925	ARCHS4 Coexpression,0.3925
1137	NKX31	0.3943	ARCHS4 Coexpression,0.3943
1138	DMRT2	0.3946	Enrichr Queries,0.3946
1139	FAM200B	0.395	ARCHS4 Coexpression,0.395
1140	HOXB1	0.3956	ARCHS4 Coexpression,0.3956
1141	TBX10	0.3958	GTEx Coexpression,0.3958
1142	POU3F1	0.3963	Literature ChIP-seq,0.3963
1143	ZNF557	0.3964	GTEx Coexpression,0.3964
1144	SP2	0.3967	Enrichr Queries,0.3967
1145	ZKSCAN7	0.3976	GTEx Coexpression,0.3976
1146	FOXH1	0.398	ARCHS4 Coexpression,0.398
1147	INSM2	0.3986	ARCHS4 Coexpression,0.3986
1148	MESP1	0.3989	GTEx Coexpression,0.3989
1149	MYF6	0.3995	GTEx Coexpression,0.3995
1150	KLF5	0.4003	Enrichr Queries,0.4003
1151	PHOX2B	0.4005	ARCHS4 Coexpression,0.4005
1152	ZNF827	0.4007	GTEx Coexpression,0.4007
1153	LMX1A	0.402	GTEx Coexpression,0.402
1154	ZNF846	0.4024	Enrichr Queries,0.4024
1155	ZNF454	0.4026	GTEx Coexpression,0.4026
1156	VAX1	0.4036	ARCHS4 Coexpression,0.4036
1157	ZSCAN2	0.4039	GTEx Coexpression,0.4039
1158	RARG	0.4046	Enrichr Queries,0.4046
1159	ZFP92	0.4051	GTEx Coexpression,0.4051
1160	ASCL3	0.406	Enrichr Queries,0.406
1161	LBX2	0.406	ARCHS4 Coexpression,0.406
1162	JUNB	0.4081	Enrichr Queries,0.4081
1163	OTP	0.4088	GTEx Coexpression,0.4088
1164	EBF3	0.4095	GTEx Coexpression,0.4095
1165	GRHL2	0.4095	Enrichr Queries,0.4095

1166	IRF6	0.4097	ARCHS4 Coexpression,0.4097
1167	BSX	0.4101	GTEx Coexpression,0.4101
1168	TFEC	0.4103	ARCHS4 Coexpression,0.4103
1169	ZNF671	0.4109	ARCHS4 Coexpression,0.4109
1170	ZNF548	0.4113	GTEx Coexpression,0.4113
1171	BHLHE41	0.4122	ARCHS4 Coexpression,0.4122
1172	TBPL1	0.4128	ARCHS4 Coexpression,0.4128
1173	HOXC13	0.4134	ARCHS4 Coexpression,0.4134
1174	TEAD3	0.4138	GTEx Coexpression,0.4138
1175	ZNF41	0.4144	GTEx Coexpression,0.4144
1176	FOXB1	0.4152	ARCHS4 Coexpression,0.4152
1177	ST18	0.4158	ARCHS4 Coexpression,0.4158
1178	SPEN	0.4169	GTEx Coexpression,0.4169
1179	TP63	0.4171	ARCHS4 Coexpression,0.4171
1180	MEF2C	0.4175	ReMap ChIP-seq,0.4175
1181	ZNF653	0.4181	Enrichr Queries,0.4181
1182	TLX2	0.4183	ARCHS4 Coexpression,0.4183
1183	NR2F2	0.4188	GTEx Coexpression,0.4188
1184	SIX1	0.4188	Enrichr Queries,0.4188
1185	PITX2	0.4189	ARCHS4 Coexpression,0.4189
1186	PEG3	0.4194	GTEx Coexpression,0.4194
1187	PRDM16	0.4195	ARCHS4 Coexpression,0.4195
1188	DPF3	0.42	GTEx Coexpression,0.42
1189	KLF12	0.4202	Enrichr Queries,0.4202
1190	NFATC4	0.4207	GTEx Coexpression,0.4207
1191	ESRRB	0.4207	Literature ChIP-seq,0.4207
1192	DBX1	0.422	ARCHS4 Coexpression,0.422
1193	ZNF609	0.4231	GTEx Coexpression,0.4231
1194	KDM2A	0.4238	GTEx Coexpression,0.4238
1195	ZNF598	0.4256	GTEx Coexpression,0.4256
1196	SPIC	0.4257	ARCHS4 Coexpression,0.4257
1197	ERF	0.4263	GTEx Coexpression,0.4263
1198	ZNF750	0.4266	Enrichr Queries,0.4266
1199	ZNF417	0.4269	GTEx Coexpression,0.4269
1200	GBX2	0.4275	ARCHS4 Coexpression,0.4275
1201	ZNF672	0.4295	Enrichr Queries,0.4295
1202	BHLHA15	0.4306	GTEx Coexpression,0.4306
1203	BCL6	0.4312	GTEx Coexpression,0.4312
1204	BATF3	0.4319	GTEx Coexpression,0.4319
1205	EPAS1	0.4331	GTEx Coexpression,0.4331
1206	FOSL2	0.4343	ReMap ChIP-seq,0.4343
1207	SREBF2	0.4359	Enrichr Queries,0.4359
1208	TBX20	0.4361	ARCHS4 Coexpression,0.4361
1209	NKX63	0.4367	ARCHS4 Coexpression,0.4367
1210	ZNF483	0.4368	GTEx Coexpression,0.4368
1211	FOXF1	0.4386	ARCHS4 Coexpression,0.4386
1212	PROX1	0.4393	GTEx Coexpression,0.4393
1213	HOXA6	0.44	GTEx Coexpression,0.44
1214	ZNF431	0.4409	Enrichr Queries,0.4409
1215	ZNF25	0.4418	GTEx Coexpression,0.4418
1216	MAF	0.4423	ARCHS4 Coexpression,0.4423
1217	HOMEZ	0.4423	Enrichr Queries,0.4423
1218	ZNF613	0.4429	ARCHS4 Coexpression,0.4429

1219	SP5	0.4449	GTEx Coexpression,0.4449
1220	TCF23	0.4453	ARCHS4 Coexpression,0.4453
1221	ZNF99	0.4459	ARCHS4 Coexpression,0.4459
1222	SATB2	0.4474	GTEx Coexpression,0.4474
1223	ZGLP1	0.4478	ARCHS4 Coexpression,0.4478
1224	L3MBTL4	0.4484	ARCHS4 Coexpression,0.4484
1225	DZIP1	0.4505	GTEx Coexpression,0.4505
1226	PAX3	0.4512	GTEx Coexpression,0.4512
1227	E4F1	0.4512	Literature ChIP-seq,0.4512
1228	TFE3	0.4523	Enrichr Queries,0.4523
1229	ZNF875	0.453	Enrichr Queries,0.453
1230	ATOH8	0.453	GTEx Coexpression,0.453
1231	ZNF621	0.4537	Enrichr Queries,0.4537
1232	GMEB1	0.4549	GTEx Coexpression,0.4549
1233	ZNF440	0.4555	GTEx Coexpression,0.4555
1234	ZNF324	0.4573	Enrichr Queries,0.4573
1235	DMRTA2	0.4574	GTEx Coexpression,0.4574
1236	FOXD4L1	0.4582	ARCHS4 Coexpression,0.4582
1237	ZNF385D	0.4588	ARCHS4 Coexpression,0.4588
1238	PRDM8	0.4592	GTEx Coexpression,0.4592
1239	ZNF445	0.4595	ARCHS4 Coexpression,0.4595
1240	EVX1	0.4599	GTEx Coexpression,0.4599
1241	NPAS1	0.4601	ARCHS4 Coexpression,0.4601
1242	CC2D1A	0.4611	GTEx Coexpression,0.4611
1243	ZBTB17	0.4615	Enrichr Queries,0.4615
1244	GATA5	0.4619	ARCHS4 Coexpression,0.4619
1245	ZBTB48	0.4623	Enrichr Queries,0.4623
1246	GATAD2B	0.463	Enrichr Queries,0.463
1247	ZNF784	0.4644	Enrichr Queries,0.4644
1248	DRGX	0.4655	GTEx Coexpression,0.4655
1249	FOXD4L6	0.4665	Enrichr Queries,0.4665
1250	THAP8	0.4667	GTEx Coexpression,0.4667
1251	ZNF667	0.4672	Enrichr Queries,0.4672
1252	ZNF611	0.4681	ARCHS4 Coexpression,0.4681
1253	MBD6	0.4686	GTEx Coexpression,0.4686
1254	FOXO4	0.4698	GTEx Coexpression,0.4698
1255	ZNF595	0.4708	Enrichr Queries,0.4708
1256	MEOX1	0.4722	Enrichr Queries,0.4722
1257	NKX25	0.4723	GTEx Coexpression,0.4723
1258	SON	0.4729	GTEx Coexpression,0.4729
1259	ZNF396	0.4748	ARCHS4 Coexpression,0.4748
1260	ESRRG	0.4754	GTEx Coexpression,0.4754
1261	ZNF233	0.476	GTEx Coexpression,0.476
1262	MYOD1	0.4772	Enrichr Queries,0.4772
1263	ZNF630	0.4773	GTEx Coexpression,0.4773
1264	FOXB2	0.4792	GTEx Coexpression,0.4792
1265	FIZ1	0.4803	ARCHS4 Coexpression,0.4803
1266	ARHGAP35	0.4804	GTEx Coexpression,0.4804
1267	AIRE	0.4822	Enrichr Queries,0.4822
1268	DMBX1	0.4828	ARCHS4 Coexpression,0.4828
1269	HOXD3	0.4829	GTEx Coexpression,0.4829
1270	ZNF497	0.4829	Enrichr Queries,0.4829
1271	ZNF768	0.4835	GTEx Coexpression,0.4835

1272	ZNF853	0.4848	GTEx Coexpression,0.4848
1273	OVOL2	0.4872	Enrichr Queries,0.4872
1274	TBX1	0.4879	GTEx Coexpression,0.4879
1275	MYF5	0.4879	Enrichr Queries,0.4879
1276	SOX5	0.4889	ARCHS4 Coexpression,0.4889
1277	NFIX	0.4893	Enrichr Queries,0.4893
1278	ZFHX3	0.491	GTEx Coexpression,0.491
1279	LHX3	0.4922	GTEx Coexpression,0.4922
1280	ZFHX2	0.4928	GTEx Coexpression,0.4928
1281	ZNF208	0.4939	ARCHS4 Coexpression,0.4939
1282	RHOXF2B	0.4941	GTEx Coexpression,0.4941
1283	MYRFL	0.4947	GTEx Coexpression,0.4947
1284	NFE2	0.4949	ReMap ChIP-seq,0.4949
1285	ZNF467	0.495	Enrichr Queries,0.495
1286	ZNF710	0.496	GTEx Coexpression,0.496
1287	GLI3	0.4966	GTEx Coexpression,0.4966
1288	ZSCAN1	0.4975	ARCHS4 Coexpression,0.4975
1289	NR1D1	0.4986	Enrichr Queries,0.4986
1290	LHX9	0.4988	ARCHS4 Coexpression,0.4988
1291	CENPBD1	0.4991	GTEx Coexpression,0.4991
1292	ZNF461	0.5	ARCHS4 Coexpression,0.5
1293	FOXQ1	0.5003	GTEx Coexpression,0.5003
1294	ZNF568	0.5018	ARCHS4 Coexpression,0.5018
1295	FOXF2	0.5021	Enrichr Queries,0.5021
1296	NEUROD4	0.5028	GTEx Coexpression,0.5028
1297	THRA	0.5028	Enrichr Queries,0.5028
1298	ZNF74	0.5031	ARCHS4 Coexpression,0.5031
1299	ZNF626	0.5037	ARCHS4 Coexpression,0.5037
1300	TBX2	0.504	GTEx Coexpression,0.504
1301	CREB5	0.5043	ARCHS4 Coexpression,0.5043
1302	HOXC10	0.5064	Enrichr Queries,0.5064
1303	GLI2	0.5074	ARCHS4 Coexpression,0.5074
1304	HNF4G	0.5078	GTEx Coexpression,0.5078
1305	ZNF746	0.5078	Enrichr Queries,0.5078
1306	ATOH1	0.5103	GTEx Coexpression,0.5103
1307	VSX2	0.5104	ARCHS4 Coexpression,0.5104
1308	ZNF75A	0.5107	Enrichr Queries,0.5107
1309	DACH2	0.5117	ARCHS4 Coexpression,0.5117
1310	MLXIPL	0.5121	Enrichr Queries,0.5121
1311	CREB3L3	0.5129	ARCHS4 Coexpression,0.5129
1312	BATF2	0.514	GTEx Coexpression,0.514
1313	SOX14	0.5152	GTEx Coexpression,0.5152
1314	ZNF804A	0.5166	ARCHS4 Coexpression,0.5166
1315	HOXD1	0.5171	GTEx Coexpression,0.5171
1316	ALX3	0.5177	GTEx Coexpression,0.5177
1317	ETS2	0.5178	Enrichr Queries,0.5178
1318	TCF21	0.5183	Literature ChIP-seq,0.5183
1319	GLIS2	0.5185	Enrichr Queries,0.5185
1320	ETV1	0.5196	GTEx Coexpression,0.5196
1321	MAFA	0.5199	Enrichr Queries,0.5199
1322	RARA	0.5207	Enrichr Queries,0.5207
1323	ISX	0.5208	GTEx Coexpression,0.5208
1324	HELT	0.5221	ARCHS4 Coexpression,0.5221

1325	GRHL1	0.5235	Enrichr Queries,0.5235
1326	ARID3C	0.5246	GTEx Coexpression,0.5246
1327	MYOG	0.5254	ENCODE ChIP-seq,0.5254
1328	OLIG3	0.5264	ARCHS4 Coexpression,0.5264
1329	SRY	0.5264	GTEx Coexpression,0.5264
1330	SP100	0.5277	GTEx Coexpression,0.5277
1331	ZNF205	0.5283	GTEx Coexpression,0.5283
1332	TBX19	0.5285	Enrichr Queries,0.5285
1333	NHLH2	0.5289	ARCHS4 Coexpression,0.5289
1334	ZNF19	0.5289	GTEx Coexpression,0.5289
1335	CDX1	0.5299	Enrichr Queries,0.5299
1336	LTF	0.5301	ARCHS4 Coexpression,0.5301
1337	FOXE1	0.5302	GTEx Coexpression,0.5302
1338	ONECUT2	0.5307	ARCHS4 Coexpression,0.5307
1339	NKX61	0.5308	GTEx Coexpression,0.5308
1340	TFAP2E	0.5314	GTEx Coexpression,0.5314
1341	ZNF804B	0.532	GTEx Coexpression,0.532
1342	NFKB2	0.5328	Enrichr Queries,0.5328
1343	ATF5	0.5335	Enrichr Queries,0.5335
1344	HOXD11	0.5338	ARCHS4 Coexpression,0.5338
1345	INSM1	0.5342	Enrichr Queries,0.5342
1346	HOXB8	0.535	ARCHS4 Coexpression,0.535
1347	SP6	0.5356	Enrichr Queries,0.5356
1348	PKNOX2	0.5362	ARCHS4 Coexpression,0.5362
1349	ZNF777	0.5383	GTEx Coexpression,0.5383
1350	ZNF385C	0.5389	GTEx Coexpression,0.5389
1351	CSRNP3	0.5405	ARCHS4 Coexpression,0.5405
1352	HES5	0.5406	Enrichr Queries,0.5406
1353	EGR3	0.5413	Enrichr Queries,0.5413
1354	NPAS4	0.542	GTEx Coexpression,0.542
1355	SOX21	0.543	ARCHS4 Coexpression,0.543
1356	GPBP1L1	0.5436	ARCHS4 Coexpression,0.5436
1357	TRAFD1	0.5445	GTEx Coexpression,0.5445
1358	AHDC1	0.5449	Enrichr Queries,0.5449
1359	PBX4	0.5461	ARCHS4 Coexpression,0.5461
1360	HOXB5	0.547	GTEx Coexpression,0.547
1361	ZNF605	0.547	Enrichr Queries,0.547
1362	ZNF540	0.5485	ARCHS4 Coexpression,0.5485
1363	ZBTB7B	0.5488	GTEx Coexpression,0.5488
1364	SNAI3	0.5499	Enrichr Queries,0.5499
1365	LHX4	0.5501	GTEx Coexpression,0.5501
1366	OLIG1	0.5516	ARCHS4 Coexpression,0.5516
1367	OSR1	0.5528	ARCHS4 Coexpression,0.5528
1368	GRHL3	0.5534	Enrichr Queries,0.5534
1369	ZNF556	0.5557	GTEx Coexpression,0.5557
1370	ZNF18	0.557	Enrichr Queries,0.557
1371	PRDM6	0.5571	ARCHS4 Coexpression,0.5571
1372	ZNF329	0.5577	Enrichr Queries,0.5577
1373	ZNF479	0.5577	ARCHS4 Coexpression,0.5577
1374	IRF9	0.5589	ReMap ChIP-seq,0.5589
1375	NR2E3	0.559	ARCHS4 Coexpression,0.559
1376	MXD1	0.5591	Enrichr Queries,0.5591
1377	ZNF785	0.5596	ARCHS4 Coexpression,0.5596

1378	ZNF793	0.562	ARCHS4 Coexpression,0.562
1379	NFE4	0.5625	GTEx Coexpression,0.5625
1380	ZNF783	0.5638	GTEx Coexpression,0.5638
1381	TBX18	0.5645	ARCHS4 Coexpression,0.5645
1382	ZNF365	0.5648	Enrichr Queries,0.5648
1383	CENPB	0.5657	ARCHS4 Coexpression,0.5657
1384	STAT4	0.5671	Literature ChIP-seq,0.5671
1385	FOXL1	0.5676	ARCHS4 Coexpression,0.5676
1386	ZNF668	0.5677	Enrichr Queries,0.5677
1387	ZNF76	0.5684	Enrichr Queries,0.5684
1388	ZNF142	0.5698	Enrichr Queries,0.5698
1389	ZNF835	0.5706	ARCHS4 Coexpression,0.5706
1390	ZNF500	0.5725	ARCHS4 Coexpression,0.5725
1391	CAMTA1	0.5726	Enrichr Queries,0.5726
1392	FBXL19	0.5731	ARCHS4 Coexpression,0.5731
1393	ZNF154	0.5734	Enrichr Queries,0.5734
1394	SOX12	0.5737	GTEx Coexpression,0.5737
1395	ZNF213	0.5741	Enrichr Queries,0.5741
1396	HOXA11	0.575	GTEx Coexpression,0.575
1397	ZNF157	0.5755	Enrichr Queries,0.5755
1398	RBCK1	0.5806	GTEx Coexpression,0.5806
1399	ZNF470	0.5811	ARCHS4 Coexpression,0.5811
1400	MNT	0.5812	Enrichr Queries,0.5812
1401	PGR	0.5817	ARCHS4 Coexpression,0.5817
1402	ATF7	0.5825	ReMap ChIP-seq,0.5825
1403	ASCL2	0.5826	Enrichr Queries,0.5826
1404	ZNF385B	0.5831	GTEx Coexpression,0.5831
1405	ZNF101	0.5842	ARCHS4 Coexpression,0.5842
1406	NOTO	0.5843	GTEx Coexpression,0.5843
1407	BNC2	0.5848	ARCHS4 Coexpression,0.5848
1408	FERD3L	0.5862	GTEx Coexpression,0.5862
1409	ZNF580	0.5881	GTEx Coexpression,0.5881
1410	MTERF4	0.5912	GTEx Coexpression,0.5912
1411	CASZ1	0.5912	Enrichr Queries,0.5912
1412	RHOXF1	0.5919	Enrichr Queries,0.5919
1413	WIZ	0.5921	ARCHS4 Coexpression,0.5921
1414	POU4F3	0.5924	GTEx Coexpression,0.5924
1415	ZSCAN18	0.5926	Enrichr Queries,0.5926
1416	ZNF493	0.5946	ARCHS4 Coexpression,0.5946
1417	DMRT1	0.5949	GTEx Coexpression,0.5949
1418	ZNF319	0.5962	Enrichr Queries,0.5962
1419	CEBPE	0.5969	Enrichr Queries,0.5969
1420	KAT7	0.5971	ARCHS4 Coexpression,0.5971
1421	NKX24	0.5974	GTEx Coexpression,0.5974
1422	GBX1	0.5993	GTEx Coexpression,0.5993
1423	ZNF546	0.5995	ARCHS4 Coexpression,0.5995
1424	SCML4	0.6004	Enrichr Queries,0.6004
1425	GFI1	0.6019	Enrichr Queries,0.6019
1426	SOX18	0.6024	GTEx Coexpression,0.6024
1427	L3MBTL1	0.6026	ARCHS4 Coexpression,0.6026
1428	IRF2	0.6027	ReMap ChIP-seq,0.6027
1429	IRF8	0.6037	Literature ChIP-seq,0.6037
1430	BCL11B	0.6038	ARCHS4 Coexpression,0.6038

1431	ZBTB8B	0.6044	ARCHS4 Coexpression,0.6044
1432	ASCL1	0.6055	GTEx Coexpression,0.6055
1433	PRR12	0.6061	GTEx Coexpression,0.6061
1434	POU3F3	0.6069	ARCHS4 Coexpression,0.6069
1435	ZNF705A	0.6075	Enrichr Queries,0.6075
1436	HOXA13	0.608	GTEx Coexpression,0.608
1437	ZNF688	0.609	Enrichr Queries,0.609
1438	HOXA3	0.6092	GTEx Coexpression,0.6092
1439	ZNF524	0.6123	GTEx Coexpression,0.6123
1440	FOXD4	0.6125	Enrichr Queries,0.6125
1441	DMRT3	0.6136	ARCHS4 Coexpression,0.6136
1442	ZNF510	0.6161	Enrichr Queries,0.6161
1443	ZNF716	0.6179	GTEx Coexpression,0.6179
1444	ZNF8	0.6179	ARCHS4 Coexpression,0.6179
1445	HSF1	0.6186	ARCHS4 Coexpression,0.6186
1446	ZNF474	0.6189	Enrichr Queries,0.6189
1447	NKX26	0.6192	GTEx Coexpression,0.6192
1448	C11ORF95	0.6204	GTEx Coexpression,0.6204
1449	FOXI3	0.621	ARCHS4 Coexpression,0.621
1450	FOXR2	0.6217	GTEx Coexpression,0.6217
1451	PAX7	0.6235	GTEx Coexpression,0.6235
1452	TSHZ3	0.6254	Enrichr Queries,0.6254
1453	ZGPAT	0.6266	GTEx Coexpression,0.6266
1454	EMX2	0.6278	ARCHS4 Coexpression,0.6278
1455	MESP2	0.6279	GTEx Coexpression,0.6279
1456	NKX23	0.6285	GTEx Coexpression,0.6285
1457	LHX1	0.6296	ARCHS4 Coexpression,0.6296
1458	LYL1	0.6296	ReMap ChIP-seq,0.6296
1459	VSX1	0.6304	GTEx Coexpression,0.6304
1460	ZNF705B	0.6321	ARCHS4 Coexpression,0.6321
1461	ZNF648	0.6322	GTEx Coexpression,0.6322
1462	IRX2	0.6325	Enrichr Queries,0.6325
1463	ETV7	0.6333	ARCHS4 Coexpression,0.6333
1464	LMX1B	0.6339	ARCHS4 Coexpression,0.6339
1465	BARHL2	0.6341	GTEx Coexpression,0.6341
1466	SP8	0.6347	GTEx Coexpression,0.6347
1467	FOXD4L3	0.6351	ARCHS4 Coexpression,0.6351
1468	UNCX	0.636	GTEx Coexpression,0.636
1469	ZIC1	0.6396	Enrichr Queries,0.6396
1470	ZNF366	0.6403	Enrichr Queries,0.6403
1471	POU2F3	0.6403	GTEx Coexpression,0.6403
1472	PCGF2	0.6407	ARCHS4 Coexpression,0.6407
1473	ZNF704	0.6409	GTEx Coexpression,0.6409
1474	ZNF70	0.6434	GTEx Coexpression,0.6434
1475	PAX9	0.6447	GTEx Coexpression,0.6447
1476	ARID5A	0.6459	GTEx Coexpression,0.6459
1477	KDM2B	0.6468	ARCHS4 Coexpression,0.6468
1478	FOXI1	0.6474	Enrichr Queries,0.6474
1479	NR1I3	0.6484	GTEx Coexpression,0.6484
1480	GTF2I	0.6493	ARCHS4 Coexpression,0.6493
1481	POU6F2	0.6499	ARCHS4 Coexpression,0.6499
1482	MYT1	0.6515	GTEx Coexpression,0.6515
1483	ARNT2	0.6524	Enrichr Queries,0.6524

1484	ZBTB45	0.6529	ARCHS4 Coexpression,0.6529
1485	IKZF1	0.6532	ReMap ChIP-seq,0.6532
1486	TLX1	0.6536	ARCHS4 Coexpression,0.6536
1487	JDP2	0.654	GTEx Coexpression,0.654
1488	HOXB6	0.6574	Enrichr Queries,0.6574
1489	ZBTB34	0.6602	GTEx Coexpression,0.6602
1490	ZNF48	0.6609	ARCHS4 Coexpression,0.6609
1491	SOX1	0.6634	ARCHS4 Coexpression,0.6634
1492	ZNF646	0.6646	GTEx Coexpression,0.6646
1493	NEUROD2	0.6658	GTEx Coexpression,0.6658
1494	HOXB3	0.6665	GTEx Coexpression,0.6665
1495	MYCL	0.6671	ARCHS4 Coexpression,0.6671
1496	DBP	0.6674	Enrichr Queries,0.6674
1497	PRDM12	0.6689	ARCHS4 Coexpression,0.6689
1498	NPAS3	0.6744	ARCHS4 Coexpression,0.6744
1499	HOXD13	0.6745	GTEx Coexpression,0.6745
1500	EN1	0.6777	GTEx Coexpression,0.6777
1501	ZNF775	0.6781	Enrichr Queries,0.6781
1502	ZBTB18	0.6801	GTEx Coexpression,0.6801
1503	ZBTB7C	0.6802	Enrichr Queries,0.6802
1504	ZNF541	0.6806	ARCHS4 Coexpression,0.6806
1505	TEF	0.6809	Enrichr Queries,0.6809
1506	NR1I2	0.6812	ARCHS4 Coexpression,0.6812
1507	CXXC5	0.6814	GTEx Coexpression,0.6814
1508	ZIC4	0.6845	GTEx Coexpression,0.6845
1509	NFATC2	0.6859	Enrichr Queries,0.6859
1510	PURB	0.6864	GTEx Coexpression,0.6864
1511	MZF1	0.688	Enrichr Queries,0.688
1512	IRX6	0.6886	ARCHS4 Coexpression,0.6886
1513	IRF5	0.6895	GTEx Coexpression,0.6895
1514	HOXD12	0.6907	GTEx Coexpression,0.6907
1515	ELF4	0.6926	GTEx Coexpression,0.6926
1516	ZNF575	0.6929	ARCHS4 Coexpression,0.6929
1517	OVOL3	0.6951	GTEx Coexpression,0.6951
1518	HLX	0.6973	Enrichr Queries,0.6973
1519	ZNF865	0.6994	GTEx Coexpression,0.6994
1520	TLX3	0.7007	GTEx Coexpression,0.7007
1521	TERB1	0.7009	ARCHS4 Coexpression,0.7009
1522	TBPL2	0.7015	ARCHS4 Coexpression,0.7015
1523	NACC2	0.7026	GTEx Coexpression,0.7026
1524	FOXC1	0.705	GTEx Coexpression,0.705
1525	RORB	0.7075	GTEx Coexpression,0.7075
1526	SOX3	0.7082	GTEx Coexpression,0.7082
1527	ALX4	0.7082	ARCHS4 Coexpression,0.7082
1528	SAFB2	0.71	GTEx Coexpression,0.71
1529	ZBTB22	0.7115	Enrichr Queries,0.7115
1530	PITX3	0.7119	GTEx Coexpression,0.7119
1531	GSX1	0.7144	ARCHS4 Coexpression,0.7144
1532	FOXD2	0.7144	Enrichr Queries,0.7144
1533	PAX1	0.7174	ARCHS4 Coexpression,0.7174
1534	FOXL2	0.7175	GTEx Coexpression,0.7175
1535	CUX2	0.7187	GTEx Coexpression,0.7187
1536	VENTX	0.7193	ARCHS4 Coexpression,0.7193

1537	ZNF414	0.7201	Enrichr Queries,0.7201
1538	HOXD9	0.7212	GTEx Coexpression,0.7212
1539	TBR1	0.7224	ARCHS4 Coexpression,0.7224
1540	ZNF705D	0.723	ARCHS4 Coexpression,0.723
1541	KLF2	0.7236	Enrichr Queries,0.7236
1542	IRX3	0.7265	Enrichr Queries,0.7265
1543	VAX2	0.7285	ARCHS4 Coexpression,0.7285
1544	HES2	0.7293	Enrichr Queries,0.7293
1545	SOX8	0.7322	Enrichr Queries,0.7322
1546	EBF4	0.7337	GTEx Coexpression,0.7337
1547	TGIF2LY	0.7353	ARCHS4 Coexpression,0.7353
1548	TBX4	0.7355	GTEx Coexpression,0.7355
1549	AEBP1	0.7371	ARCHS4 Coexpression,0.7371
1550	CBLL2	0.7372	Enrichr Queries,0.7372
1551	HSF4	0.74	Enrichr Queries,0.74
1552	LBX1	0.7405	GTEx Coexpression,0.7405
1553	PBX2	0.7429	Enrichr Queries,0.7429
1554	ZNF219	0.7445	ARCHS4 Coexpression,0.7445
1555	MXD4	0.7455	GTEx Coexpression,0.7455
1556	ZNF385A	0.7472	Enrichr Queries,0.7472
1557	SEBOX	0.7479	Enrichr Queries,0.7479
1558	MTF1	0.748	GTEx Coexpression,0.748
1559	NHLH1	0.75	ARCHS4 Coexpression,0.75
1560	PIN1	0.7537	ARCHS4 Coexpression,0.7537
1561	NKX32	0.7579	GTEx Coexpression,0.7579
1562	ZNF771	0.758	ARCHS4 Coexpression,0.758
1563	BHLHE22	0.7586	GTEx Coexpression,0.7586
1564	HNF1A	0.7604	GTEx Coexpression,0.7604
1565	SCRT2	0.761	GTEx Coexpression,0.761
1566	AKNA	0.7614	Enrichr Queries,0.7614
1567	CIC	0.7628	Enrichr Queries,0.7628
1568	ZIM3	0.7629	ARCHS4 Coexpression,0.7629
1569	CPXCR1	0.7635	ARCHS4 Coexpression,0.7635
1570	CAMTA2	0.7642	Enrichr Queries,0.7642
1571	PAX2	0.7666	GTEx Coexpression,0.7666
1572	ZNF687	0.7685	Enrichr Queries,0.7685
1573	EVX2	0.7707	Enrichr Queries,0.7707
1574	EN2	0.771	GTEx Coexpression,0.771
1575	HMX1	0.7714	Enrichr Queries,0.7714
1576	BARX2	0.7746	ARCHS4 Coexpression,0.7746
1577	IRX5	0.7758	ARCHS4 Coexpression,0.7758
1578	ZNF554	0.7772	GTEx Coexpression,0.7772
1579	CENPX	0.7778	GTEx Coexpression,0.7778
1580	ZNF831	0.7799	Enrichr Queries,0.7799
1581	IRX4	0.785	ARCHS4 Coexpression,0.785
1582	ZMAT4	0.7869	ARCHS4 Coexpression,0.7869
1583	SOX30	0.7885	Enrichr Queries,0.7885
1584	ZNF705G	0.7899	ARCHS4 Coexpression,0.7899
1585	FOXI2	0.7984	Enrichr Queries,0.7984
1586	BARHL1	0.799	GTEx Coexpression,0.799
1587	ZNF628	0.8016	ARCHS4 Coexpression,0.8016
1588	HES7	0.8052	GTEx Coexpression,0.8052
1589	ZBED9	0.8083	GTEx Coexpression,0.8083

1590	LHX5	0.8084	ARCHS4 Coexpression,0.8084
1591	NAIF1	0.8108	ARCHS4 Coexpression,0.8108
1592	IRF7	0.8114	GTEx Coexpression,0.8114
1593	TBX6	0.8152	GTEx Coexpression,0.8152
1594	MYT1L	0.8177	GTEx Coexpression,0.8177
1595	HOXD10	0.8194	ARCHS4 Coexpression,0.8194
1596	SGSM2	0.8219	ARCHS4 Coexpression,0.8219
1597	SKI	0.8237	ARCHS4 Coexpression,0.8237
1598	MEIS3	0.8239	GTEx Coexpression,0.8239
1599	ZNF513	0.8283	Enrichr Queries,0.8283
1600	BHLHA9	0.8289	GTEx Coexpression,0.8289
1601	FOXN1	0.8291	Enrichr Queries,0.8291
1602	MYPOP	0.8299	ARCHS4 Coexpression,0.8299
1603	NKX62	0.8307	GTEx Coexpression,0.8307
1604	HEYL	0.8376	GTEx Coexpression,0.8376
1605	FOXJ1	0.8376	Enrichr Queries,0.8376
1606	SP110	0.855	ARCHS4 Coexpression,0.855
1607	NKX11	0.8556	GTEx Coexpression,0.8556
1608	HSFY1	0.8563	GTEx Coexpression,0.8563
1609	DLX3	0.8568	Enrichr Queries,0.8568
1610	PHF1	0.8569	GTEx Coexpression,0.8569
1611	FLYWCH1	0.8575	GTEx Coexpression,0.8575
1612	SP140	0.86	ARCHS4 Coexpression,0.86
1613	POU6F1	0.8618	Enrichr Queries,0.8618
1614	FAM170A	0.8667	ARCHS4 Coexpression,0.8667
1615	HSFY2	0.8704	ARCHS4 Coexpression,0.8704
1616	ASCL5	0.8718	GTEx Coexpression,0.8718
1617	SPZ1	0.8821	ARCHS4 Coexpression,0.8821
1618	ZNF276	0.8889	Enrichr Queries,0.8889
1619	ZNF683	0.8939	Enrichr Queries,0.8939
1620	TFAP2B	0.8986	GTEx Coexpression,0.8986
1621	ORCS8MEF2	0.8993	ARCHS4 Coexpression,0.8993
1622	CCDC17	0.9115	ARCHS4 Coexpression,0.9115
1623	KCNIP3	0.9197	GTEx Coexpression,0.9197
1624	HSFX2	0.9201	ARCHS4 Coexpression,0.9201
1625	FEZF2	0.9203	GTEx Coexpression,0.9203
1626	MYRF	0.9247	GTEx Coexpression,0.9247
1627	DC169SOHL	0.9281	ARCHS4 Coexpression,0.9281
1628	ZNF517	0.9459	Enrichr Queries,0.9459
1629	SOHLH1	0.9544	Enrichr Queries,0.9544
1630	SP140L	0.9546	GTEx Coexpression,0.9546
1631	SCX	0.9693	ARCHS4 Coexpression,0.9693
1632	SCRT1	0.9939	ARCHS4 Coexpression,0.9939
