# Supplementary material for: Deciphering differences in DNA methylation and transcriptome profiles of oocytes from pigs with high and low developmental competence
Source: Environ Epigenet. 2025 Jun 3;11(1):dvaf018. doi: 10.1093/eep/dvaf018 (PMC12418950; doi:10.1093/eep/dvaf018)
Supplement: dvaf018_Supplemental_Files [file dvaf018_supplemental_files.zip › Additional 3 integration.pdf]

Gene	factors	features	factors	pvalues	log2FC
ENSSSCG00000063504	1	ENSSSCG00000063504	1	0.030	2.047
ADGRL2	1	ADGRL2	1	0.002	1.742
F2R	1	F2R	1	0.001	1.712
ALG6	1	ALG6	1	0.000	1.691
ENSSSCG00000047016	1	ENSSSCG00000047016	1	0.012	1.647
ENSSSCG00000062132	1	ENSSSCG00000062132	1	0.000	1.630
CCDC179	1	CCDC179	1	0.026	1.564
PLGRKT	1	PLGRKT	1	0.002	1.560
SLCO6A1	1	SLCO6A1	1	0.013	1.520
UBE2D1	1	UBE2D1	1	0.006	1.516
ENSSSCG00000011905	1	ENSSSCG00000011905	1	0.002	1.483
ANO5	1	ANO5	1	0.018	1.421
ABHD3	1	ABHD3	1	0.000	1.365
ENSSSCG00000061888	1	ENSSSCG00000061888	1	0.026	1.350
POLE2	1	POLE2	1	0.009	1.337
C5orf24	1	C5orf24	1	0.013	1.313
COL19A1	1	COL19A1	1	0.003	1.273
ENSSSCG00000055582	1	ENSSSCG00000055582	1	0.037	1.273
ZNF383	1	ZNF383	1	0.000	1.271
ENSSSCG00000055001	1	ENSSSCG00000055001	1	0.015	1.265
ZNF770	1	ZNF770	1	0.005	1.246
TMEM38B	1	TMEM38B	1	0.000300279	1.233064099
LRRC34	1	LRRC34	1	0.000710659	1.226103314
GSN	1	GSN	1	0.020719161	1.185448197
DPY19L3	1	DPY19L3	1	0.006935744	1.177066991
GLCE	1	GLCE	1	1.73E-06	1.166784317
ZSCAN30	1	ZSCAN30	1	1.56E-05	1.163086444
RANBP6	1	RANBP6	1	0.001283029	1.147517459
ENSSSCG00000031854	1	ENSSSCG00000031854	1	0.006935744	1.147499263
RFK	1	RFK	1	0.000710659	1.129886334
ENSSSCG00000003792	1	ENSSSCG00000003792	1	0.026428998	1.12135498
ENSSSCG00000011549	1	ENSSSCG00000011549	1	0.033340064	1.118788769
HMGN3	1	HMGN3	1	2.90E-05	1.11172896
SDHAF4	1	SDHAF4	1	0.00023867	1.109885068
GADD45A	1	GADD45A	1	0.029724008	1.100845535
GART	1	GART	1	0.023433173	1.060485223
DACT1	1	DACT1	1	0.000577892	1.052463634
AFG1L	1	AFG1L	1	6.90E-07	1.050985246
IER3IP1	1	IER3IP1	1	0.042987587	1.041777864
TMEM216	1	TMEM216	1	0.0160561	1.019540819
CEP78	1	CEP78	1	0.024049126	1.015628569
PHYHIPL	1	PHYHIPL	1	0.029724008	1.007955604
BMAL1	1	BMAL1	1	0.001058683	0.994226651
ELP4	1	ELP4	1	0.003702112	0.990222261
ZNF567	1	ZNF567	1	0.037294767	0.986675778
COMMD10	1	COMMD10	1	0.001843636	0.970959474
ZNF146	1	ZNF146	1	0.046305078	0.956657315
ZNF614	1	ZNF614	1	0.026428998	0.955062187
HLTF	1	HLTF	1	0.018265564	0.946501491
ZNF618	1	ZNF618	1	0.001547182	0.928846544
DTWD1	1	DTWD1	1	0.020719161	0.927490208
ENSSSCG00000034174	1	ENSSSCG00000034174	1	0.005104217	0.914106433
MINDY2	1	MINDY2	1	0.046305078	0.912043916
CRYZ	1	CRYZ	1	0.005960184	0.906554876
ENSSSCG00000061001	1	ENSSSCG00000061001	1	0.0160561	0.900741308
NMD3	1	NMD3	1	0.005960184	0.895155276
TOR1B	1	TOR1B	1	0.000467215	0.890212744
MAN1A1	1	MAN1A1	1	0.010706368	0.886369793
SLC25A16	1	SLC25A16	1	0.003821092	0.865543654
ALAS1	1	ALAS1	1	0.000869715	0.865025582
PTPRK	1	PTPRK	1	0.011624617	0.862017709
DNAJC24	1	DNAJC24	1	2.59E-06	0.861773336
ENSSSCG00000004623	1	ENSSSCG00000004623	1	0.021418193	0.860289115
APIP	1	APIP	1	0.010135106	0.859481268
TBX18	1	TBX18	1	0.004354844	0.857079652
PBX3	1	PBX3	1	0.008042288	0.856719862
MORC1	1	MORC1	1	0.044753595	0.854811375
THOC7	1	THOC7	1	0.008042288	0.845059325
CACTIN	1	CACTIN	1	0.001857873	0.832350927
RARS2	1	RARS2	1	0.000710659	0.826513574
CNTN4	1	CNTN4	1	0.009294944	0.824418571
ENSSSCG00000053039	1	ENSSSCG00000053039	1	0.002644062	0.81414714
SOS2	1	SOS2	1	0.041610781	0.813886409
A1CF	1	A1CF	1	0.001547182	0.813252869
SLC17A5	1	SLC17A5	1	0.000467215	0.807756201
KDM3B	1	KDM3B	1	0.00023867	0.799512977
GPR160	1	GPR160	1	0.000493572	0.797553281
MAP3K7	1	MAP3K7	1	0.010706368	0.795623107
CLP1	1	CLP1	1	0.026781675	0.791260392
SCN5A	1	SCN5A	1	0.023433173	0.779344403
TYMS	1	TYMS	1	0.015287539	0.775140505
CFAP95	1	CFAP95	1	0.026428998	0.752058849
AMFR	1	AMFR	1	0.001547182	0.74635386
ENSSSCG00000023596	1	ENSSSCG00000023596	1	0.041610781	0.74565271
ENSSSCG00000056241	1	ENSSSCG00000056241	1	0.014070638	0.741448572
GNPNAT1	1	GNPNAT1	1	0.0160561	0.736335434
CEP76	1	CEP76	1	2.90E-05	0.734287924
HAS1	1	HAS1	1	0.001283029	0.729883287
ENSSSCG00000014071	1	ENSSSCG00000014071	1	0.00023867	0.726622214
GOLIM4	1	GOLIM4	1	0.009294944	0.723268813
DPP8	1	DPP8	1	0.012293472	0.722788406
CPNE4	1	CPNE4	1	0.000577892	0.72181781
RFC4	1	RFC4	1	0.001283029	0.715206704
UTRN	1	UTRN	1	1.12E-05	0.714944766
ENSSSCG00000040389	1	ENSSSCG00000040389	1	0.046305078	0.710208087
PRKCI	1	PRKCI	1	0.002644062	0.708579579
GNB4	1	GNB4	1	4.03E-07	0.70711296
IRAK2	1	IRAK2	1	5.18E-05	0.705268277
KIF18A	1	KIF18A	1	0.029724008	0.702028271

Gene	factors	features	factors	pvalues	log2FC
RPS14	2	RPS14	2	0.000	4.771
ENSSSCG00000012088	2	ENSSSCG0000	2	0.000	4.543
NPAS1	2	NPAS1	2	0.000	4.471
DYNC2I2	2	DYNC2I2	2	0.026	4.379
AP2S1	2	AP2S1	2	0.015	4.298
SYCN	2	SYCN	2	0.000	4.288
TNNT1	2	TNNT1	2	0.000	4.175
SRM	2	SRM	2	0.002	4.135
RPS5	2	RPS5	2	0.000	4.082
ENSSSCG00000025928	2	ENSSSCG0000	2	0.000	3.978
ENSSSCG00000032060	2	ENSSSCG0000	2	0.000	3.944
RBP2	2	RBP2	2	0.001	3.890
CSTB	2	CSTB	2	0.001	3.878
TMEM200C	2	TMEM200C	2	0.030	3.866
RPS12	2	RPS12	2	0.003	3.737
ENSSSCG00000002995	2	ENSSSCG0000	2	0.000	3.632
RPS9	2	RPS9	2	0.000	3.615
SMPD2	2	SMPD2	2	0.002	3.593
PKN1	2	PKN1	2	0.000	3.589
COMMD9	2	COMMD9	2	0.000	3.588
SNX22	2	SNX22	2	0.008942176	3.55092969
MATCAP1	2	MATCAP1	2	0.000242075	3.481716925
TMEM147	2	TMEM147	2	5.75E-08	3.448252344
EIF3G	2	EIF3G	2	0.00023303	3.431200392
RPL35	2	RPL35	2	0.002644062	3.402098086
ENSSSCG00000027573	2	ENSSSCG0000	2	4.03E-07	3.34357831
CYTH2	2	CYTH2	2	0.001058683	3.343305743
ENSSSCG00000033019	2	ENSSSCG0000	2	0.001116287	3.295989996
GPN2	2	GPN2	2	0.008492143	3.277081
ENSSSCG00000013768	2	ENSSSCG0000	2	0.000467215	3.262406389
WDR5	2	WDR5	2	3.02E-05	3.247335448
MAST2	2	MAST2	2	2.30E-07	3.247045506
ATP5IF1	2	ATP5IF1	2	2.30E-07	3.239566125
NUDC	2	NUDC	2	3.89E-05	3.236394733
SMIM12	2	SMIM12	2	7.84E-06	3.231532559
RPS29	2	RPS29	2	4.03E-07	3.229519311
BCKDHA	2	BCKDHA	2	0.037294767	3.221482921
LRSAM1	2	LRSAM1	2	0.002220853	3.212403176
ENSSSCG00000052208	2	ENSSSCG0000	2	0.039026116	3.209331726
ACAD9	2	ACAD9	2	0.003486015	3.203303543
ALAD	2	ALAD	2	0.000300279	3.176632975
ENSSSCG00000003930	2	ENSSSCG0000	2	2.59E-06	3.139558193
ITGB5	2	ITGB5	2	0.001857873	3.137182123
ETHE1	2	ETHE1	2	4.03E-07	3.135513596
RPS16	2	RPS16	2	3.85E-06	3.131390814
ENSSSCG00000058091	2	ENSSSCG0000	2	0.033801955	3.128714119
RDH13	2	RDH13	2	2.14E-05	3.117481928
NANS	2	NANS	2	0.005960184	3.098562382
ENSSSCG00000052671	2	ENSSSCG0000	2	0.017803159	3.084589491
COX17	2	COX17	2	2.30E-07	3.081658172
TEX264	2	TEX264	2	3.89E-05	3.08091552
ENSSSCG00000031249	2	ENSSSCG0000	2	0.000710659	3.078643027
EMC3	2	EMC3	2	0.039026116	3.072591366
INTS11	2	INTS11	2	0.000869715	3.071859464
CDC26	2	CDC26	2	2.59E-05	3.056341135
ENSSSCG00000062012	2	ENSSSCG0000	2	0.008042288	3.041425186
LRRC47	2	LRRC47	2	0.0160561	3.024468735
COX7A2	2	COX7A2	2	9.68E-06	3.015625108
ENSSSCG00000046487	2	ENSSSCG0000	2	0.018756024	3.00207513
MAF	2	MAF	2	0.000586505	2.988984361
GRHPR	2	GRHPR	2	1.21E-05	2.971967578
KLHDC4	2	KLHDC4	2	1.12E-05	2.970291211
ENSSSCG00000005528	2	ENSSSCG0000	2	9.64E-05	2.960624846
DMAC2L	2	DMAC2L	2	0.006345009	2.959191008
MRPS15	2	MRPS15	2	0.006859771	2.956327729
UPK1A	2	UPK1A	2	2.90E-05	2.952009115
CBR3	2	CBR3	2	0.03165226	2.95181193
CTU2	2	CTU2	2	0.000167265	2.951478213
MRPS25	2	MRPS25	2	2.10E-05	2.946117282
CCN6	2	CCN6	2	1.09E-06	2.935287687
CZIB	2	CZIB	2	0.006935744	2.934931393
RPS13	2	RPS13	2	0.001857873	2.934359144
SPAG8	2	SPAG8	2	0.000345248	2.912644112
ZNF821	2	ZNF821	2	5.75E-08	2.911910723
ENSSSCG00000002963	2	ENSSSCG0000	2	8.00E-06	2.908519625
SNRPD2	2	SNRPD2	2	0.000114934	2.907740745
HSPBP1	2	HSPBP1	2	1.09E-06	2.895482928
SLC25A38	2	SLC25A38	2	0.000586505	2.89395266
PPIB	2	PPIB	2	0.001967605	2.890813165
RNF141	2	RNF141	2	1.15E-07	2.885727349
ENSSSCG00000014133	2	ENSSSCG0000	2	0.012386865	2.86853382
UROD	2	UROD	2	2.10E-05	2.861648132
PIH1D1	2	PIH1D1	2	0.001575813	2.850352694
NDUFS5	2	NDUFS5	2	1.15E-07	2.846399771
TMEM42	2	TMEM42	2	0.006146611	2.833404085
ENSSSCG00000054958	2	ENSSSCG0000	2	5.75E-08	2.831622392
HEMK1	2	HEMK1	2	0.003702112	2.829270072
LIN37	2	LIN37	2	3.40E-05	2.816353008
ENSSSCG00000004703	2	ENSSSCG0000	2	0.003702112	2.812243447
ENSSSCG00000013386	2	ENSSSCG0000	2	5.18E-05	2.794163336
FBXO31	2	FBXO31	2	1.09E-06	2.766831975
KLF1	2	KLF1	2	0.000464153	2.752238727
MYOM1	2	MYOM1	2	0.023997922	2.750513199
POMGNT1	2	POMGNT1	2	0.003702112	2.738004653
LENG1	2	LENG1	2	0.017930259	2.727152926
DMWD	2	DMWD	2	0.001704943	2.717436167
TIMM50	2	TIMM50	2	0.005201637	2.714183366
ENSSSCG00000035007	2	ENSSSCG0000	2	0.001857873	2.712581716
ENSSSCG00000057874	2	ENSSSCG0000	2	0.020373546	2.709233713

PPP1CC	1	PPP1CC	1	0.001547182	0.697273134	ENSSSCG00000038035	2	ENSSSCG0000	2	0.000577892	2.707482244
ZMAT3	1	ZMAT3	1	0.009294944	0.695210242	POC1A	2	POC1A	2	0.005184075	2.70222323
CD47	1	CD47	1	0.000147723	0.690115574	TXNRD3	2	TXNRD3	2	0.001143623	2.690857064
TTC14	1	TTC14	1	0.023997922	0.671265986	MBOAT7	2	MBOAT7	2	0.030129455	2.690046706
EPHA7	1	EPHA7	1	2.14E-05	0.668647457	ENSSSCG00000047379	2	ENSSSCG0000	2	0.005960184	2.685718055
DNAJB4	1	DNAJB4	1	0.014070638	0.667898044	SAL1	2	SAL1	2	1.56E-05	2.681906447
NGLY1	1	NGLY1	1	0.018902624	0.659272327	ENSSSCG00000055939	2	ENSSSCG0000	2	0.003275881	2.676854203
LATS1	1	LATS1	1	0.021370608	0.658634701	LSM10	2	LSM10	2	0.002152224	2.676639835
DNAAF2	1	DNAAF2	1	2.90E-05	0.65590683	ACAA1	2	ACAA1	2	0.003486015	2.663489379
ENSSSCG00000003989	1	ENSSSCG00000003989	1	2.14E-05	0.650956768	ENKD1	2	ENKD1	2	2.59E-06	2.661407637
SACM1L	1	SACM1L	1	6.81E-05	0.650675323	AKR1A1	2	AKR1A1	2	0.00023303	2.657794795
TM2D1	1	TM2D1	1	1.73E-06	0.648441473	COQ4	2	COQ4	2	1.21E-05	2.650922275
SLC30A4	1	SLC30A4	1	3.89E-05	0.644136299	PPAN	2	PPAN	2	0.012293472	2.646468824
ENSSSCG00000059603	1	ENSSSCG00000059603	1	0.022143274	0.641319582	PAQR7	2	PAQR7	2	0.000155629	2.637175551
RIPOR1	1	RIPOR1	1	0.046305078	0.640211665	MTMR14	2	MTMR14	2	0.003366418	2.634110789
CMPK1	1	CMPK1	1	0.02694642	0.623999145	ENSSSCG00000014284	2	ENSSSCG0000	2	1.73E-06	2.632605928
SLC4A7	1	SLC4A7	1	0.029724008	0.619447839	NDUFB4	2	NDUFB4	2	0.004230034	2.617315442
SNRPA1	1	SNRPA1	1	0.000695383	0.618636354	RPLP1	2	RPLP1	2	1.73E-06	2.611872948
FBXL4	1	FBXL4	1	8.89E-05	0.618303335	CAPNS1	2	CAPNS1	2	0.0079516	2.600607336
TAFA4	1	TAFA4	1	0.022553484	0.617850382	CCDC32	2	CCDC32	2	5.75E-08	2.597682742
FECH	1	FECH	1	0.008042288	0.615122639	SLC38A8	2	SLC38A8	2	0.048462232	2.596855973
PDHX	1	PDHX	1	0.003702112	0.610946054	ATP5PF	2	ATP5PF	2	0.000467215	2.578980579
ABHD17B	1	ABHD17B	1	0.003134574	0.609786255	PSMB7	2	PSMB7	2	0.001343923	2.574705426
PPP5C	1	PPP5C	1	0.046305078	0.609173362	CCER2	2	CCER2	2	5.58E-06	2.572756741
SCAF4	1	SCAF4	1	0.006935744	0.60598753	ENSSSCG00000011294	2	ENSSSCG0000	2	0.000188336	2.562967259
ENSSSCG00000045482	1	ENSSSCG00000045482	1	2.90E-05	0.605021644	GINS2	2	GINS2	2	0.02019671	2.562256943
ABHD10	1	ABHD10	1	2.14E-05	0.59642415	ENSSSCG00000056114	2	ENSSSCG0000	2	4.03E-07	2.558353774
RAB31	1	RAB31	1	0.002220853	0.592242194	PIGP	2	PIGP	2	0.013473887	2.556847288
TRAPPC6B	1	TRAPPC6B	1	0.000375808	0.575077023	ENSSSCG00000029003	2	ENSSSCG0000	2	0.000164126	2.549790632
CAPN7	1	CAPN7	1	0.001283029	0.57202104	PADI3	2	PADI3	2	8.89E-05	2.531689146
SLC38A6	1	SLC38A6	1	0.026598818	0.570167986	ENO1	2	ENO1	2	0.003895995	2.526883511
HPS3	1	HPS3	1	0.008042288	0.566055616	ENSSSCG00000031083	2	ENSSSCG0000	2	1.56E-05	2.510937591
TSHZ1	1	TSHZ1	1	1.09E-06	0.562144458	TXNDC12	2	TXNDC12	2	0.009294944	2.509567714
PRTG	1	PRTG	1	0.041610781	0.560964894	CC2D1A	2	CC2D1A	2	6.81E-05	2.50493293
TTC39A	1	TTC39A	1	0.001547182	0.559476548	ENSSSCG00000032082	2	ENSSSCG0000	2	1.73E-06	2.497919322
KDM4C	1	KDM4C	1	0.012293472	0.55832896	SHROOM1	2	SHROOM1	2	0.004354844	2.47432108
ENSSSCG00000022206	1	ENSSSCG00000022206	1	8.89E-05	0.557154196	UQCRQ	2	UQCRQ	2	0.000519525	2.459774572
SNRNP40	1	SNRNP40	1	0.001058683	0.554490909	NOX3	2	NOX3	2	0.003138614	2.451240524
PPIL4	1	PPIL4	1	0.026428998	0.553328493	PSENEN	2	PSENEN	2	5.11E-05	2.439589248
FNBP4	1	FNBP4	1	0.006935744	0.552838505	UST	2	UST	2	4.03E-07	2.434224186
SSX2IP	1	SSX2IP	1	0.000375808	0.549417501	CCDC97	2	CCDC97	2	0.000532133	2.420667958
ENSSSCG00000030015	1	ENSSSCG00000030015	1	0.008042288	0.548943426	BDH1	2	BDH1	2	0.002127872	2.405670594
PPFIA1	1	PPFIA1	1	0.046305078	0.548710592	ENSSSCG00000060282	2	ENSSSCG0000	2	2.30E-07	2.396740432
SNAPC3	1	SNAPC3	1	8.89E-05	0.538953241	FAM32A	2	FAM32A	2	6.81E-05	2.387624988
MAP2K1	1	MAP2K1	1	2.14E-05	0.535755601	ENSSSCG00000032110	2	ENSSSCG0000	2	1.51E-05	2.384985097
FRMD4B	1	FRMD4B	1	0.020719161	0.529087064	ACY1	2	ACY1	2	0.000188336	2.384195949
ENSSSCG00000041063	1	ENSSSCG00000041063	1	0.023433173	0.528991288	RPL32	2	RPL32	2	0.005451491	2.371142276
ITFG1	1	ITFG1	1	0.046305078	0.527071421	TYRO3	2	TYRO3	2	0.002483651	2.363371332
ESCO1	1	ESCO1	1	0.026428998	0.526920052	GSE1	2	GSE1	2	0.002218129	2.358340176
ZWINT	1	ZWINT	1	0.010706368	0.524658824	RIMS3	2	RIMS3	2	0.000656469	2.357607667
USP45	1	USP45	1	0.001058683	0.519601946	TK2	2	TK2	2	3.85E-06	2.355787949
KIAA0586	1	KIAA0586	1	0.026428998	0.516479653	ENSSSCG00000028414	2	ENSSSCG0000	2	9.45E-05	2.355652589
RAVER1	1	RAVER1	1	2.30E-07	0.514860203	MED8	2	MED8	2	0.000375808	2.352077977
ZNF300	1	ZNF300	1	0.005960184	0.514348595	PRMT7	2	PRMT7	2	1.73E-06	2.351582342
VGLL3	1	VGLL3	1	0.000147723	0.51335142	UQCRC1	2	UQCRC1	2	0.001316174	2.350245247
RYK	1	RYK	1	8.89E-05	0.508078651	SWI5	2	SWI5	2	1.21E-05	2.335789636
VPS4B	1	VPS4B	1	0.000467215	0.505442812	ENSSSCG00000013387	2	ENSSSCG0000	2	0.014070638	2.335617702
DMXL1	1	DMXL1	1	0.014813318	0.501738266	EBNA1BP2	2	EBNA1BP2	2	1.51E-05	2.332954538
CTBS	1	CTBS	1	5.18E-05	0.501722345	RPL24	2	RPL24	2	6.22E-06	2.331431853
MAPKAPK3	1	MAPKAPK3	1	0.000869715	0.500172527	AEN	2	AEN	2	3.89E-05	2.328560771
E2F4	1	E2F4	1	0.029724008	0.496883051	SIDT1	2	SIDT1	2	5.18E-05	2.324005628
TMCO6	1	TMCO6	1	0.001058683	0.495823152	ERI3	2	ERI3	2	0.000115366	2.321087514
CENPW	1	CENPW	1	0.004354844	0.492719315	ENSSSCG00000005217	2	ENSSSCG0000	2	0.001054086	2.300694782
UNC13B	1	UNC13B	1	0.000300279	0.491359655	SIL1	2	SIL1	2	0.000179675	2.299619836
TUBE1	1	TUBE1	1	0.041610781	0.489440673	CDA	2	CDA	2	2.30E-07	2.29865076
BLOC1S6	1	BLOC1S6	1	0.037294767	0.489257354	HSBP1	2	HSBP1	2	0.000188336	2.295004402
MTAP	1	MTAP	1	0.020719161	0.480753513	ENSSSCG00000022401	2	ENSSSCG0000	2	0.003151667	2.294912046
MEAF6	1	MEAF6	1	0.047914559	0.480205857	HARS1	2	HARS1	2	0.001547182	2.290261834
ANXA2	1	ANXA2	1	0.001575813	0.479090923	ENSSSCG00000027916	2	ENSSSCG0000	2	9.51E-06	2.288470773
SLC35D1	1	SLC35D1	1	8.89E-05	0.4783096	IQCC	2	IQCC	2	5.75E-08	2.275679813
RBL2	1	RBL2	1	0.000375808	0.477498841	P3H1	2	P3H1	2	0.000242075	2.274844991
ENSSSCG00000005440	1	ENSSSCG00000005440	1	0.010706368	0.477183714	MYL3	2	MYL3	2	0.000147723	2.270271281
SLC25A46	1	SLC25A46	1	0.001058683	0.474927366	CACNA2D3	2	CACNA2D3	2	0.020038076	2.270190983
GRPEL2	1	GRPEL2	1	0.000577892	0.471640846	LRRC36	2	LRRC36	2	0.000869715	2.266448653
CYLD	1	CYLD	1	0.026428998	0.470697153	VPS4A	2	VPS4A	2	0.000114934	2.26119956
ZDHHC13	1	ZDHHC13	1	0.000577892	0.466020254	SLC35A4	2	SLC35A4	2	0.00023303	2.259319404
ARMC8	1	ARMC8	1	0.007828194	0.464726683	IP6K2	2	IP6K2	2	2.30E-07	2.25590192
TBC1D32	1	TBC1D32	1	0.002220853	0.462561858	ENSSSCG00000002790	2	ENSSSCG0000	2	9.05E-05	2.253247068
YTHDC2	1	YTHDC2	1	5.18E-05	0.460635874	GRAMD1A	2	GRAMD1A	2	5.29E-06	2.246685356
C18orf63	1	C18orf63	1	8.89E-05	0.458730381	TFDP2	2	TFDP2	2	0.000114934	2.241245509
ME2	1	ME2	1	0.003702112	0.457356595	CLN6	2	CLN6	2	2.90E-05	2.240405797
VAPA	1	VAPA	1	0.023433173	0.456413689	RALGPS1	2	RALGPS1	2	0.00758365	2.238368607
NUP93	1	NUP93	1	0.029724008	0.454916562	ENSSSCG00000023691	2	ENSSSCG0000	2	4.03E-07	2.220562489
MKNK1	1	MKNK1	1	0.041610781	0.453943293	DNAJC11	2	DNAJC11	2	0.018013828	2.2195001
CDH7	1	CDH7	1	0.001058683	0.45373598	ENSSSCG00000048380	2	ENSSSCG0000	2	2.14E-05	2.218241816
GCNT1	1	GCNT1	1	0.006795087	0.452831578	ENSSSCG00000033189	2	ENSSSCG0000	2	5.58E-06	2.216434105
CSNK1G3	1	CSNK1G3	1	2.90E-05	0.452676284	POLR2H	2	POLR2H	2	0.034751397	2.21525966
CTTN	1	CTTN	1	5.23E-05	0.452504263	AURKAIP1	2	AURKAIP1	2	5.58E-06	2.212671401
ATP13A3	1	ATP13A3	1	0.041610781	0.451604457	MTRES1	2	MTRES1	2	0.000188336	2.204463101
PNISR	1	PNISR	1	0.005104217	0.451253846	RPL28	2	RPL28	2	8.89E-05	2.200079182
TRNAU1AP	1	TRNAU1AP	1	0.002220853	0.45053886	XXYLT1	2	XXYLT1	2	0.014255672	2.198964066
WTAP	1	WTAP	1	0.037294767	0.446685073	EEFSEC	2	EEFSEC	2	5.75E-08	2.196959212
PSMD5	1	PSMD5	1	3.85E-06	0.4459708	CIDEA	2	CIDEA	2	3.72E-05	2.18447743
CDCP1	1	CDCP1	1	0.011268715	0.445375357	DELE1	2	DELE1	2	3.89E-05	2.182546833
ZCCHC9	1	ZCCHC9	1	0.037294767	0.441184136	ENSSSCG00000032916	2	ENSSSCG0000	2	0.002218129	2.181534585
ENSSSCG00000005453	1	ENSSSCG00000005453	1	0.018265564	0.438602148	C1orf174	2	C1orf174	2	0.002238832	2.178044792
GPT2	1	GPT2	1	0.046305078	0.437266705	PCDH12	2	PCDH12	2	2.30E-07	2.176842707
APPL1	1	APPL1	1	0.004354844	0.433293232	TMA7	2	TMA7	2	5.75E-08	2.17626291
ZFP30	1	ZFP30	1	0.029953228	0.433283243	ENSSSCG00000005649	2	ENSSSCG0000	2	2.30E-07	2.173495111
UBIAD1	1	UBIAD1	1	0.018265564	0.431913344	TSPAN1	2	TSPAN1	2	2.90E-05	2.17110149

CDIN1	1	CDIN1	1	2.14E-05	0.430631061	TCEANC2	2	TCEANC2	2	0.020719161	2.170427484
PEF1	1	PEF1	1	0.016138618	0.430431756	C19orf67	2	C19orf67	2	0.015767149	2.168392949
YES1	1	YES1	1	4.03E-07	0.429801382	ILVBL	2	ILVBL	2	5.92E-06	2.165852055
SLF1	1	SLF1	1	0.014070638	0.429201161	ACP7	2	ACP7	2	4.03E-07	2.165436792
CNOT10	1	CNOT10	1	0.009294944	0.426728569	ENSSSCG00000052522	2	ENSSSCG0000C	2	1.15E-05	2.163858107
NDC1	1	NDC1	1	0.003134574	0.424837023	GLT8D1	2	GLT8D1	2	1.73E-06	2.162540403
PTPN5	1	PTPN5	1	0.000810525	0.422226299	DIO1	2	DIO1	2	5.58E-06	2.162447623
CLASP2	1	CLASP2	1	0.001058683	0.42035115	MAP7D1	2	MAP7D1	2	6.38E-05	2.158396912
LSR	1	LSR	1	0.006935744	0.418584266	NDUFAF3	2	NDUFAF3	2	2.80E-05	2.156563633
SNX14	1	SNX14	1	0.002220853	0.41609226	SELENOS	2	SELENOS	2	5.92E-06	2.151725045
ANKRD12	1	ANKRD12	1	0.001283029	0.415567237	ENSSSCG00000058485	2	ENSSSCG0000C	2	6.38E-05	2.151186948
NETO2	1	NETO2	1	0.026428998	0.413445358	GORASP1	2	GORASP1	2	2.59E-06	2.145980509
SEC13	1	SEC13	1	0.041610781	0.412009083	RPUSD2	2	RPUSD2	2	0.006345009	2.145053111
PCOLCE2	1	PCOLCE2	1	0.011624617	0.407206985	ENSSSCG00000031730	2	ENSSSCG0000C	2	0.000300279	2.139766686
E2F8	1	E2F8	1	0.003702112	0.407045239	RPL11	2	RPL11	2	1.56E-05	2.13881208
HSD17B12	1	HSD17B12	1	0.023895579	0.406859799	ENSSSCG00000045343	2	ENSSSCG0000C	2	8.89E-05	2.138005391
HNRNPH1	1	HNRNPH1	1	0.000300279	0.405844375	ENSSSCG00000005006	2	ENSSSCG0000C	2	1.56E-05	2.135761748
ATG5	1	ATG5	1	2.90E-05	0.404233789	ENSSSCG00000056323	2	ENSSSCG0000C	2	1.15E-07	2.134728792
ELOVL4	1	ELOVL4	1	0.026428998	0.402725928	GLYCTK	2	GLYCTK	2	0.004514803	2.128110615
PIK3C2A	1	PIK3C2A	1	3.89E-05	0.402057565	ZNRF1	2	ZNRF1	2	2.30E-07	2.125815841
UFD1	1	UFD1	1	0.0160561	0.400173971	GBA2	2	GBA2	2	7.61E-05	2.123773309
STT3B	1	STT3B	1	0.001857873	0.395507603	WNT7A	2	WNT7A	2	0.000330324	2.120611315
INTS14	1	INTS14	1	6.81E-05	0.39452233	NUCB1	2	NUCB1	2	0.000300279	2.120514434
MORC3	1	MORC3	1	0.046305078	0.394325439	FHIT	2	FHIT	2	1.29E-05	2.116338147
AFF4	1	AFF4	1	0.014042022	0.393343456	COQ3	2	COQ3	2	5.75E-08	2.115162961
PCMT1	1	PCMT1	1	0.001058683	0.39272279	CCDC61	2	CCDC61	2	0.000412155	2.107397788
ZNF793	1	ZNF793	1	0.001283029	0.389449965	CCDC12	2	CCDC12	2	2.14E-05	2.105679268
MYSM1	1	MYSM1	1	0.037294767	0.387626879	ENSSSCG00000059558	2	ENSSSCG0000C	2	0.02400302	2.097713686
FUBP1	1	FUBP1	1	0.001058683	0.386851264	ASB6	2	ASB6	2	0.022143274	2.081313944
ZFP1	1	ZFP1	1	0.000710659	0.385917216	APOD	2	APOD	2	0.000114934	2.080476228
RTTN	1	RTTN	1	0.018265564	0.384777886	CHCHD4	2	CHCHD4	2	5.92E-06	2.080367439
NUDT21	1	NUDT21	1	3.89E-05	0.383399961	CDK5RAP2	2	CDK5RAP2	2	5.92E-06	2.074420849
SESN1	1	SESN1	1	5.75E-08	0.383032568	IRGQ	2	IRGQ	2	0.003366418	2.052138015
MELK	1	MELK	1	8.89E-05	0.381120602	NOC2L	2	NOC2L	2	0.004230034	2.050722503
DCP1A	1	DCP1A	1	0.000467215	0.380462111	TAF1C	2	TAF1C	2	0.012014747	2.049945832
PTPN2	1	PTPN2	1	0.000577892	0.377337683	DMAC2	2	DMAC2	2	0.006723538	2.040193276
TRAFD1	1	TRAFD1	1	0.002220853	0.376215665	FBXO17	2	FBXO17	2	3.89E-05	2.039575273
USP48	1	USP48	1	0.000467215	0.374710385	ABCF3	2	ABCF3	2	0.000375808	2.028697014
BANP	1	BANP	1	0.041610781	0.374222229	ENSSSCG00000062784	2	ENSSSCG0000C	2	6.90E-07	2.020647373
PIGN	1	PIGN	1	0.020719161	0.372411712	CDH15	2	CDH15	2	0.020038076	2.019208789
NAALADL2	1	NAALADL2	1	0.000375808	0.370227404	DYNC1LI1	2	DYNC1LI1	2	0.000188336	2.006426906
TFG	1	TFG	1	0.000467215	0.367655619	PDCD5	2	PDCD5	2	1.15E-07	2.001307442
RNF13	1	RNF13	1	0.010706368	0.36486569	HARS2	2	HARS2	2	5.75E-08	1.997720914
CMTM4	1	CMTM4	1	0.000710659	0.363940775	UBE2J1	2	UBE2J1	2	3.85E-06	1.995782952
DPF2	1	DPF2	1	0.001857873	0.361260014	PNRC1	2	PNRC1	2	0.029724008	1.994643568
ZNF283	1	ZNF283	1	0.001857873	0.359170089	YJU2B	2	YJU2B	2	4.03E-07	1.993440399
TRIM36	1	TRIM36	1	0.005960184	0.358848145	CD274	2	CD274	2	0.000485666	1.987871372
MFSD1	1	MFSD1	1	4.03E-07	0.358162593	FBXW9	2	FBXW9	2	0.000745249	1.984630173
PSIP1	1	PSIP1	1	0.029724008	0.354744161	KATNB1	2	KATNB1	2	5.92E-06	1.976278114
SAV1	1	SAV1	1	8.00E-06	0.354696214	ABLIM3	2	ABLIM3	2	0.005979933	1.975054823
CLPX	1	CLPX	1	8.89E-05	0.354249815	PRR5L	2	PRR5L	2	0.006838179	1.956512359
STX3	1	STX3	1	0.000577892	0.353759101	TMEM39B	2	TMEM39B	2	0.000300279	1.956244794
FERMT2	1	FERMT2	1	1.73E-06	0.353183481	CEACAM20	2	CEACAM20	2	0.001547182	1.955811621
SEC24A	1	SEC24A	1	0.008042288	0.350940634	C19orf47	2	C19orf47	2	5.75E-08	1.953088177
ZYG11B	1	ZYG11B	1	0.003134574	0.347373195	NFAT5	2	NFAT5	2	0.000778876	1.951946614
ENSSSCG00000005255	1	ENSSSCG00000005255	1	0.014070638	0.345073538	TRAIP	2	TRAIP	2	0.000317541	1.95158895
PHLPP2	1	PHLPP2	1	0.026428998	0.344495029	MAD2L2	2	MAD2L2	2	2.25E-05	1.9497997
PSMD8	1	PSMD8	1	2.14E-05	0.344345313	PIGO	2	PIGO	2	0.003370113	1.947391106
WDR48	1	WDR48	1	0.011425232	0.343886235	ENSSSCG00000039972	2	ENSSSCG0000C	2	4.03E-07	1.942138508
NAA30	1	NAA30	1	2.90E-05	0.342204718	GTF2H5	2	GTF2H5	2	8.00E-06	1.940837163
ENSSSCG00000056411	1	ENSSSCG00000056411	1	6.90E-07	0.341022172	RNASEH2A	2	RNASEH2A	2	5.58E-06	1.939586805
PCYT1A	1	PCYT1A	1	0.029724008	0.338184681	EPHB2	2	EPHB2	2	3.89E-05	1.939155865
KDM1A	1	KDM1A	1	0.014826327	0.337908458	SH3GLB2	2	SH3GLB2	2	1.85E-05	1.936148836
ENSSSCG00000013395	1	ENSSSCG00000013395	1	0.037294767	0.334331964	ENSSSCG00000041170	2	ENSSSCG0000C	2	0.004354844	1.932376355
DOCK7	1	DOCK7	1	0.02694642	0.333902014	EXOSC5	2	EXOSC5	2	0.000710659	1.931591147
REPS1	1	REPS1	1	5.58E-06	0.333606727	FNDC5	2	FNDC5	2	0.000200216	1.927441986
RSRC1	1	RSRC1	1	0.000188336	0.332999086	MRPS6	2	MRPS6	2	0.000193726	1.925054781
FAM13C	1	FAM13C	1	0.030129455	0.329743314	PFDN1	2	PFDN1	2	0.030070698	1.921367532
H1-8	1	H1-8	1	0.001857873	0.328570795	DEF8	2	DEF8	2	0.00014874	1.907848376
AMPD3	1	AMPD3	1	0.016138618	0.328204736	ENSSSCG00000043636	2	ENSSSCG0000C	2	0.007852036	1.907532687
ENSSSCG00000002855	1	ENSSSCG00000002855	1	6.90E-07	0.323483852	CIAPIN1	2	CIAPIN1	2	0.000109805	1.903076981
NUP160	1	NUP160	1	0.001283029	0.320201597	E2F2	2	E2F2	2	8.00E-06	1.896952357
PDP2	1	PDP2	1	5.58E-06	0.319841525	ENSSSCG00000031299	2	ENSSSCG0000C	2	0.031964545	1.896027712
CNTLN	1	CNTLN	1	0.000300279	0.316815007	RPS6	2	RPS6	2	0.000290242	1.892190055
MMS22L	1	MMS22L	1	0.018265564	0.315864806	IFT57	2	IFT57	2	0.001283029	1.890484087
NDC80	1	NDC80	1	0.000869715	0.315851874	MRPL46	2	MRPL46	2	5.58E-06	1.888418596
MCC	1	MCC	1	0.000710659	0.315555919	CABLES1	2	CABLES1	2	0.002096524	1.887478172
POLR2E	1	POLR2E	1	0.006935744	0.312559311	ENSSSCG00000056225	2	ENSSSCG0000C	2	6.81E-05	1.883526666
ZNF599	1	ZNF599	1	0.009294944	0.310820335	OXSM	2	OXSM	2	8.00E-06	1.882538383
TTC3	1	TTC3	1	1.09E-06	0.308905063	TOE1	2	TOE1	2	0.001682964	1.882326123
DPH3	1	DPH3	1	0.006935744	0.300304171	PSMC4	2	PSMC4	2	0.020038076	1.881064175
SPINDOC	1	SPINDOC	1	0.002220853	0.297929983	TTC21A	2	TTC21A	2	7.72E-05	1.879455282
ENSSSCG00000011253	1	ENSSSCG00000011253	1	5.75E-08	0.291770548	GLIPR2	2	GLIPR2	2	2.59E-06	1.874648487
HEY2	1	HEY2	1	0.013088729	0.284932958	RAB6B	2	RAB6B	2	5.75E-08	1.871913396
IQGAP2	1	IQGAP2	1	0.008042288	0.283832249	MIX23	2	MIX23	2	6.27E-06	1.86516469
ADAM10	1	ADAM10	1	0.037294767	0.282588995	TMC4	2	TMC4	2	0.000467215	1.862378304
TP53BP1	1	TP53BP1	1	0.005104217	0.281968323	ENSSSCG00000045223	2	ENSSSCG0000C	2	0.000467215	1.859487371
PSMG1	1	PSMG1	1	0.0160561	0.281895331	PRPF4	2	PRPF4	2	0.036678068	1.842943415
HAS3	1	HAS3	1	0.00896843	0.279718799	MPPE1	2	MPPE1	2	0.001283029	1.840367269
ATG12	1	ATG12	1	0.003134574	0.279571027	CAPZB	2	CAPZB	2	0.003134574	1.838211434
NDUFAF4	1	NDUFAF4	1	0.009294944	0.278841425	AKAP12	2	AKAP12	2	5.75E-08	1.835594494
ID3	1	ID3	1	0.004354844	0.278591379	MTO1	2	MTO1	2	8.00E-06	1.829015927
TOP2B	1	TOP2B	1	0.000147723	0.270949368	ENSSSCG00000011569	2	ENSSSCG0000C	2	8.00E-06	1.827926806
DCC	1	DCC	1	0.000147723	0.263536812	C2CD2	2	C2CD2	2	0.001084316	1.827862226
ENSSSCG00000061752	1	ENSSSCG00000061752	1	0.010706368	0.25943532	MTCL1	2	MTCL1	2	0.000710659	1.821550476
ENSSSCG00000062865	1	ENSSSCG00000062865	1	0.000114934	0.254886861	MON1A	2	MON1A	2	0.000710659	1.818949886
NEDD4L	1	NEDD4L	1	3.85E-06	0.250323485	DOLPP1	2	DOLPP1	2	0.028330535	1.818219293
						PAIP2	2	PAIP2	2	1.10E-05	1.809085288
						ENSSSCG00000056205	2	ENSSSCG0000C	2	1.09E-06	1.806918768
						B4GALT4	2	B4GALT4	2	5.75E-08	1.805785191

ENSSSCG00000050758	2	ENSSSCG0000	2	3.85E-06	1.800719611
RTN4IP1	2	RTN4IP1	2	4.76E-05	1.793193803
ENSSSCG00000005596	2	ENSSSCG0000	2	1.15E-07	1.791449221
KDM4A	2	KDM4A	2	5.58E-06	1.785248444
MROH7	2	MROH7	2	0.00023867	1.785063005
SELENON	2	SELENON	2	6.81E-05	1.784116461
PSME3IP1	2	PSME3IP1	2	2.90E-05	1.781140969
MED22	2	MED22	2	0.0136361	1.779475101
QPCTL	2	QPCTL	2	0.001547182	1.777672457
ENSSSCG00000011272	2	ENSSSCG0000	2	0.003341253	1.77726592
ENSSSCG00000002907	2	ENSSSCG0000	2	6.81E-05	1.773770793
EEF1AKMT4	2	EEF1AKMT4	2	2.83E-05	1.770069738
IFTAP	2	IFTAP	2	1.09E-06	1.769379638
CRAT	2	CRAT	2	0.025884322	1.758662936
LTF	2	LTF	2	0.013911861	1.755683833
ENSSSCG00000053185	2	ENSSSCG0000	2	2.90E-05	1.753104781
ENSSSCG00000003286	2	ENSSSCG0000	2	3.89E-05	1.75133582
APOE	2	APOE	2	0.005960184	1.749556909
ENSSSCG00000035904	2	ENSSSCG0000	2	9.23E-06	1.748022035
PRDX1	2	PRDX1	2	0.002127872	1.747111668
NDUFAF1	2	NDUFAF1	2	7.40E-06	1.743921647
SEC11C	2	SEC11C	2	2.59E-06	1.739976363
TFB1M	2	TFB1M	2	6.90E-07	1.73939628
AHSG	2	AHSG	2	0.002096524	1.738982106
PADI4	2	PADI4	2	2.59E-05	1.736629282
ENSSSCG00000059847	2	ENSSSCG0000	2	0.025433593	1.734229629
HAUS1	2	HAUS1	2	5.58E-06	1.733247139
ATRIP	2	ATRIP	2	0.01228243	1.728744913
NDUFV2	2	NDUFV2	2	5.75E-08	1.728274582
PDCD1LG2	2	PDCD1LG2	2	1.20E-05	1.726257204
CFAP298	2	CFAP298	2	0.000577892	1.722513381
PTAR1	2	PTAR1	2	0.022143274	1.720416652
PAK4	2	PAK4	2	0.006146611	1.719191283
ENSSSCG00000037514	2	ENSSSCG0000	2	5.19E-05	1.716299478
SRARP	2	SRARP	2	0.014701667	1.716291315
NIPAL3	2	NIPAL3	2	8.60E-05	1.713377107
DNAAF4	2	DNAAF4	2	9.76E-06	1.710117365
POLE3	2	POLE3	2	0.001048664	1.708842345
ENSSSCG00000058562	2	ENSSSCG0000	2	0.000577367	1.704062729
ZNF235	2	ZNF235	2	4.03E-07	1.701088887
ENSSSCG00000061086	2	ENSSSCG0000	2	0.000869715	1.700975007
ARHGEF1	2	ARHGEF1	2	5.58E-06	1.700351489
RBPMS2	2	RBPMS2	2	0.004354844	1.697218042
SRPRB	2	SRPRB	2	6.90E-07	1.695257269
IQCG	2	IQCG	2	0.01955007	1.68507954
CALM3	2	CALM3	2	5.92E-06	1.681546509
GEMIN2	2	GEMIN2	2	2.59E-06	1.679653649
PARVA	2	PARVA	2	0.000493572	1.677337926
ZNF584	2	ZNF584	2	3.85E-06	1.676652493
CROCC	2	CROCC	2	6.83E-05	1.673411488
ENSSSCG00000054830	2	ENSSSCG0000	2	0.022553484	1.672991422
LIG1	2	LIG1	2	0.0009192	1.672306911
ENSSSCG00000055918	2	ENSSSCG0000	2	5.75E-08	1.670737929
CIAO2B	2	CIAO2B	2	0.014255672	1.669756828
ENSSSCG00000035776	2	ENSSSCG0000	2	0.001138311	1.665866529
CKM	2	CKM	2	2.59E-06	1.663834283
DUT	2	DUT	2	5.58E-06	1.65889096
C18orf54	2	C18orf54	2	2.14E-05	1.639787308
PKD1L2	2	PKD1L2	2	0.014901282	1.63958932
SPOCK1	2	SPOCK1	2	0.000114934	1.636965917
ELP6	2	ELP6	2	5.75E-08	1.636146486
ENSSSCG00000062612	2	ENSSSCG0000	2	0.001235481	1.635741613
ALPK2	2	ALPK2	2	5.58E-06	1.632873939
LYPD3	2	LYPD3	2	9.76E-06	1.62953718
CD72	2	CD72	2	1.85E-05	1.62470669
DHCR24	2	DHCR24	2	5.75E-08	1.62420491
GPR32	2	GPR32	2	3.92E-05	1.623101919
NAPA	2	NAPA	2	1.15E-07	1.619165786
MCEE	2	MCEE	2	0.013473887	1.618787046
ENSSSCG00000029257	2	ENSSSCG0000	2	0.00023303	1.616491017
FHAD1	2	FHAD1	2	2.14E-05	1.611248441
GNA14	2	GNA14	2	0.000300279	1.611069815
PNLDC1	2	PNLDC1	2	0.000638806	1.610115358
KCNN4	2	KCNN4	2	0.005370656	1.60170355
ENSSSCG00000026229	2	ENSSSCG0000	2	0.002496949	1.598950597
DOK6	2	DOK6	2	0.004230034	1.597120339
CDKN2AIPNL	2	CDKN2AIPNL	2	0.003996747	1.596192418
ENSSSCG00000028717	2	ENSSSCG0000	2	0.032471124	1.594759183
NLRC5	2	NLRC5	2	0.003821092	1.590672171
HECTD3	2	HECTD3	2	6.22E-06	1.588023283
NCCRP1	2	NCCRP1	2	1.09E-06	1.584377455
BTF3L4	2	BTF3L4	2	0.010135106	1.57901755
GNL3	2	GNL3	2	8.00E-06	1.577589672
CFDP1	2	CFDP1	2	0.001002284	1.57631324
RAB7A	2	RAB7A	2	0.008492143	1.574978225
VAC14	2	VAC14	2	0.004354844	1.55222936
RBM15B	2	RBM15B	2	4.03E-07	1.551564475
CA5A	2	CA5A	2	1.15E-07	1.547402665
DHDDS	2	DHDDS	2	0.00910266	1.546727063
ENSSSCG00000029696	2	ENSSSCG0000	2	0.005184075	1.544858789
ARMC2	2	ARMC2	2	0.003134574	1.543855652
ENSSSCG00000034393	2	ENSSSCG0000	2	0.014701667	1.537510555
MYL12A	2	MYL12A	2	7.77E-06	1.532052005
RNF220	2	RNF220	2	0.002220853	1.53200526
ENSSSCG00000061446	2	ENSSSCG0000	2	3.19E-05	1.531510219
SCG5	2	SCG5	2	0.003366418	1.530644883
ENSSSCG00000004573	2	ENSSSCG0000	2	5.75E-08	1.530488532
SKOR1	2	SKOR1	2	0.033340064	1.529277586
ENSSSCG00000061116	2	ENSSSCG0000	2	0.000425076	1.509336669
ATP2C2	2	ATP2C2	2	0.000288555	1.508580339

HAUS2	2	HAUS2	2	6.22E-06	1.50682925
PLCG2	2	PLCG2	2	0.027810754	1.506024915
ENSSSCG00000053570	2	ENSSSCG0000	2	0.012293472	1.496596652
SESN2	2	SESN2	2	0.000147723	1.494349058
LYSMD2	2	LYSMD2	2	0.000288555	1.490755927
MKRN2	2	MKRN2	2	9.51E-06	1.489882143
ENSSSCG00000022773	2	ENSSSCG0000	2	0.006723538	1.489594797
PAFAH2	2	PAFAH2	2	5.18E-05	1.488435857
TWSG1	2	TWSG1	2	0.005184075	1.486505953
SYT5	2	SYT5	2	0.007828194	1.484526694
ENSSSCG00000011875	2	ENSSSCG0000	2	1.15E-07	1.481565969
MRPL37	2	MRPL37	2	1.12E-05	1.479046029
DDX28	2	DDX28	2	0.020525443	1.473568218
ENTREP1	2	ENTREP1	2	0.001547182	1.472224919
ENSSSCG00000060152	2	ENSSSCG0000	2	0.005960184	1.472132872
ENSSSCG00000056638	2	ENSSSCG0000	2	5.58E-06	1.471746775
DKK3	2	DKK3	2	0.018536686	1.47031349
SNRPA	2	SNRPA	2	5.75E-08	1.463548255
TBPL2	2	TBPL2	2	0.037294767	1.462442153
ENSSSCG00000003903	2	ENSSSCG0000	2	0.000188336	1.460438287
MRPL50	2	MRPL50	2	2.14E-05	1.460228321
NUP62	2	NUP62	2	0.00727314	1.459607201
ENSSSCG00000052760	2	ENSSSCG0000	2	0.020719161	1.458482732
ENSSSCG00000057916	2	ENSSSCG0000	2	5.75E-08	1.457254155
CC2D1B	2	CC2D1B	2	0.000290242	1.453507105
ALDOB	2	ALDOB	2	0.011517462	1.453054389
COQ8B	2	COQ8B	2	0.008492143	1.450474032
SEPTIN8	2	SEPTIN8	2	0.027810754	1.449685891
ATP5PO	2	ATP5PO	2	0.008042288	1.440102775
ENSSSCG00000053298	2	ENSSSCG0000	2	4.03E-07	1.43831803
NLRP5	2	NLRP5	2	1.18E-05	1.434969626
ENSSSCG00000028892	2	ENSSSCG0000	2	0.000330324	1.431563509
ENSSSCG00000046083	2	ENSSSCG0000	2	5.75E-08	1.421055217
GSK3B	2	GSK3B	2	0.001283029	1.418412318
EIF2B3	2	EIF2B3	2	0.002902456	1.417572785
SYTL3	2	SYTL3	2	0.011425232	1.417288242
EFCAB12	2	EFCAB12	2	3.72E-05	1.415973069
FBXO34	2	FBXO34	2	0.006935744	1.413282879
ZMAT2	2	ZMAT2	2	0.044056742	1.406198907
EAF1	2	EAF1	2	0.006723538	1.405231216
RRP1B	2	RRP1B	2	6.10E-05	1.40441348
ENSSSCG00000062804	2	ENSSSCG0000	2	4.84E-06	1.403857177
CACNA2D2	2	CACNA2D2	2	0.001176694	1.400559606
EIF1B	2	EIF1B	2	0.002323459	1.39662373
LYPLA2	2	LYPLA2	2	0.02694642	1.395511635
SLC2A8	2	SLC2A8	2	0.004527302	1.390351403
CCL22	2	CCL22	2	1.73E-06	1.388771482
ANAPC13	2	ANAPC13	2	0.005366052	1.387878631
SKI	2	SKI	2	1.12E-05	1.386829892
CCR5	2	CCR5	2	0.004514803	1.385750634
PRDX2	2	PRDX2	2	0.000279674	1.376172295
ENSSSCG00000045922	2	ENSSSCG0000	2	1.15E-07	1.375733569
ALDH4A1	2	ALDH4A1	2	0.043160388	1.372998791
LSAMP	2	LSAMP	2	0.000114934	1.372374935
NEGR1	2	NEGR1	2	0.023997922	1.368678821
RIMKLA	2	RIMKLA	2	6.27E-06	1.368278206
MAP1LC3B	2	MAP1LC3B	2	8.00E-06	1.365776131
STMN1	2	STMN1	2	0.000147723	1.362902082
TGM7	2	TGM7	2	1.73E-06	1.360211401
WDR53	2	WDR53	2	0.000714435	1.354999803
QARS1	2	QARS1	2	7.84E-06	1.35468464
ENSSSCG00000058326	2	ENSSSCG0000	2	9.68E-06	1.35383227
ARPP19	2	ARPP19	2	0.010812995	1.352929507
TCP1	2	TCP1	2	0.010192667	1.349667307
ZBTB24	2	ZBTB24	2	0.044056742	1.346615709
SARS2	2	SARS2	2	0.002913699	1.34266802
LIMD1	2	LIMD1	2	8.00E-06	1.341002094
SLC25A26	2	SLC25A26	2	9.51E-06	1.338686811
ENSSSCG00000003612	2	ENSSSCG0000	2	0.026428998	1.338335502
CNDP2	2	CNDP2	2	0.003821092	1.336611347
RPL4	2	RPL4	2	0.004514803	1.336382427
LMOD3	2	LMOD3	2	0.001316174	1.33200618
ENSSSCG00000062560	2	ENSSSCG0000	2	1.15E-07	1.329981109
MSANTD3	2	MSANTD3	2	0.000197764	1.329214135
TXNL4A	2	TXNL4A	2	1.21E-05	1.324073908
CDKN3	2	CDKN3	2	0.006935744	1.323935101
ELOVL1	2	ELOVL1	2	1.86E-05	1.322874087
COTL1	2	COTL1	2	0.001283029	1.322246435
ENSSSCG00000062439	2	ENSSSCG0000	2	0.030129455	1.312984338
NLRP8	2	NLRP8	2	0.013791034	1.303981419
COG8	2	COG8	2	0.0160561	1.302128346
SIM2	2	SIM2	2	0.009435702	1.301357203
MRAP2	2	MRAP2	2	5.18E-05	1.292690096
COX7C	2	COX7C	2	5.75E-08	1.290254563
ENSSSCG00000032978	2	ENSSSCG0000	2	0.004354844	1.288974145
RBM42	2	RBM42	2	0.014070638	1.281485497
RSL24D1	2	RSL24D1	2	0.001508638	1.278613284
KPNA1	2	KPNA1	2	0.000300279	1.269567036
OOEP	2	OOEP	2	0.000881027	1.269461385
YIPF1	2	YIPF1	2	8.89E-05	1.269141854
KIAA1143	2	KIAA1143	2	3.85E-06	1.261807817
ENSSSCG00000056768	2	ENSSSCG0000	2	0.000224278	1.259832557
STIMATE	2	STIMATE	2	1.73E-06	1.258500515
CPT2	2	CPT2	2	4.81E-05	1.256148539
FBXO38	2	FBXO38	2	0.00896843	1.25500264
TIMMDC1	2	TIMMDC1	2	0.001036934	1.254871574
UBE2B	2	UBE2B	2	0.004202746	1.253977556
DYNLT2B	2	DYNLT2B	2	2.90E-05	1.25184219
ATP6V0D1	2	ATP6V0D1	2	0.014515303	1.249139332
LDHA	2	LDHA	2	0.000300279	1.243971721

MST1R	2	MST1R	2	6.90E-07	1.24219797
ENSSSCG00000054140	2	ENSSSCG0000	2	0.00023867	1.241535419
ENSSSCG00000042842	2	ENSSSCG0000	2	3.85E-06	1.239515977
ENSSSCG00000028572	2	ENSSSCG0000	2	0.00055248	1.238328615
GAP43	2	GAP43	2	6.81E-05	1.232369003
LDLRAP1	2	LDLRAP1	2	2.27E-05	1.228888178
ENSSSCG00000061564	2	ENSSSCG0000	2	4.03E-07	1.228366506
PTCH2	2	PTCH2	2	3.40E-05	1.223422765
ENSSSCG00000003348	2	ENSSSCG0000	2	5.58E-06	1.221747192
LSG1	2	LSG1	2	4.03E-07	1.2202597
SNX3	2	SNX3	2	8.89E-05	1.217832725
MLH1	2	MLH1	2	1.73E-06	1.211534448
SLC1A2	2	SLC1A2	2	0.003176925	1.211165756
PRR19	2	PRR19	2	0.00271136	1.207794096
HSPA4	2	HSPA4	2	4.03E-07	1.207407388
LMCD1	2	LMCD1	2	0.002238832	1.204184875
TMEM69	2	TMEM69	2	8.00E-06	1.200915787
TTLL3	2	TTLL3	2	1.12E-05	1.199549374
OXTR	2	OXTR	2	0.004158027	1.192454268
C19orf54	2	C19orf54	2	2.59E-06	1.191246383
NECAP2	2	NECAP2	2	5.58E-06	1.190925172
TRIP4	2	TRIP4	2	0.035483329	1.187458309
ABRACL	2	ABRACL	2	8.00E-06	1.186427853
ENSSSCG00000062072	2	ENSSSCG0000	2	0.002496949	1.181082418
EXOSC2	2	EXOSC2	2	0.006935744	1.179454176
BRPF1	2	BRPF1	2	0.000586505	1.176469561
ETF1	2	ETF1	2	3.85E-06	1.173719341
CHSY3	2	CHSY3	2	6.81E-05	1.17266738
PCDHGA4	2	PCDHGA4	2	0.00023867	1.170432214
INVS	2	INVS	2	0.022035366	1.169960541
ENSSSCG00000032216	2	ENSSSCG0000	2	5.75E-08	1.16919894
FKBP3	2	FKBP3	2	0.001575813	1.164870403
DCAF10	2	DCAF10	2	3.02E-05	1.158574877
YARS1	2	YARS1	2	5.65E-06	1.158092951
ENSSSCG00000002709	2	ENSSSCG0000	2	0.000347213	1.156136454
RUVBL1	2	RUVBL1	2	6.90E-07	1.152525376
CALHM6	2	CALHM6	2	0.046408879	1.152006234
TGFBI	2	TGFBI	2	0.012293472	1.150917084
ENSSSCG00000059138	2	ENSSSCG0000	2	2.30E-07	1.150797155
ENSSSCG00000042524	2	ENSSSCG0000	2	0.002506937	1.148769649
CAMTA1	2	CAMTA1	2	0.000188336	1.146167721
FTL	2	FTL	2	1.86E-05	1.14091622
GNL2	2	GNL2	2	0.000577892	1.140487509
TRIM32	2	TRIM32	2	1.12E-05	1.139559293
EHD4	2	EHD4	2	5.18E-05	1.137394148
SSR3	2	SSR3	2	0.000869715	1.135638224
ATP5F1A	2	ATP5F1A	2	5.75E-08	1.135158809
ENSSSCG00000057950	2	ENSSSCG0000	2	6.90E-07	1.129491255
ATG3	2	ATG3	2	0.018938621	1.128328052
PRRC2B	2	PRRC2B	2	7.84E-06	1.128166781
STX17	2	STX17	2	0.009294944	1.127818029
CTH	2	CTH	2	5.75E-08	1.127753214
HINT1	2	HINT1	2	2.30E-07	1.125474037
SCAP	2	SCAP	2	6.27E-06	1.123376106
PWP2	2	PWP2	2	0.006468794	1.120901803
PINK1	2	PINK1	2	0.003702112	1.111940579
ENSSSCG00000026454	2	ENSSSCG0000	2	0.000288555	1.111396057
LDHC	2	LDHC	2	0.000881027	1.111234422
ATP6V1G1	2	ATP6V1G1	2	3.89E-05	1.110541239
SAR1B	2	SAR1B	2	1.21E-05	1.109128742
ENSSSCG00000057571	2	ENSSSCG0000	2	0.0398414	1.104862299
CTPS1	2	CTPS1	2	1.73E-06	1.10360096
ENSSSCG00000042603	2	ENSSSCG0000	2	0.025420547	1.102082621
HRH1	2	HRH1	2	6.90E-07	1.100873167
ZNF593	2	ZNF593	2	0.000188336	1.099134089
CCDC191	2	CCDC191	2	0.002496949	1.098952766
MTHFD1L	2	MTHFD1L	2	0.00094184	1.098077785
RAB8A	2	RAB8A	2	0.001622797	1.097277987
GABBR2	2	GABBR2	2	1.73E-06	1.096262746
HMGCL	2	HMGCL	2	0.021370608	1.095324494
MAP1A	2	MAP1A	2	0.001993285	1.094786406
PHF24	2	PHF24	2	1.73E-06	1.094413314
KAZN	2	KAZN	2	8.00E-06	1.09252484
TRMT10C	2	TRMT10C	2	0.00014156	1.091593139
ENSSSCG00000028677	2	ENSSSCG0000	2	4.03E-07	1.087095894
ZNF529	2	ZNF529	2	0.001547182	1.086544761
MVB12B	2	MVB12B	2	0.000147723	1.084890692
NFYC	2	NFYC	2	3.89E-05	1.082563168
CATSPER2	2	CATSPER2	2	0.000290242	1.077709621
ENSSSCG00000044155	2	ENSSSCG0000	2	0.000147723	1.077698313
GREB1L	2	GREB1L	2	1.10E-05	1.075579533
PSKH1	2	PSKH1	2	0.000132983	1.071096316
SH3GL2	2	SH3GL2	2	0.004337661	1.067454219
RPSA	2	RPSA	2	9.23E-06	1.064996523
SNX30	2	SNX30	2	0.012293472	1.061137177
ATG7	2	ATG7	2	4.03E-07	1.055882031
PLOD1	2	PLOD1	2	0.003394756	1.054741412
PDIA3	2	PDIA3	2	0.000317541	1.053781989
PIGV	2	PIGV	2	0.000300279	1.051270288
TXNL4B	2	TXNL4B	2	0.000506537	1.050032804
CDKL1	2	CDKL1	2	2.90E-05	1.045790254
DNAJB11	2	DNAJB11	2	0.00693054	1.043680565
TMEM231	2	TMEM231	2	0.010039451	1.043536476
RSRP1	2	RSRP1	2	5.75E-08	1.038247749
KLF2	2	KLF2	2	0.013911861	1.03781185
ZNF45	2	ZNF45	2	0.002703418	1.034270622
ENSSSCG00000054543	2	ENSSSCG0000	2	0.008042288	1.033572402
IK	2	IK	2	0.003341253	1.031648998
ENSSSCG00000011307	2	ENSSSCG0000	2	6.81E-05	1.031516239
SCG3	2	SCG3	2	2.90E-05	1.025955764

HEG1	2	HEG1	2	5.18E-05	1.018255418
TPM1	2	TPM1	2	0.012803237	1.011500186
FOXJ3	2	FOXJ3	2	2.59E-06	1.00662578
CCBE1	2	CCBE1	2	2.90E-05	1.005129841
SYMPK	2	SYMPK	2	0.000347213	1.003872123
MAPK1IP1L	2	MAPK1IP1L	2	2.30E-07	1.003769054
MRPL47	2	MRPL47	2	0.003366418	1.003160148
NKIRAS1	2	NKIRAS1	2	0.00156892	1.001670083
HSPA5	2	HSPA5	2	2.54E-05	0.997030293
TLN2	2	TLN2	2	5.58E-06	0.994647163
MYCBP	2	MYCBP	2	0.000167265	0.993451203
TCEA3	2	TCEA3	2	0.002842896	0.992763761
TPGS2	2	TPGS2	2	2.59E-06	0.992707438
COQ9	2	COQ9	2	8.74E-05	0.983922381
AFG3L2	2	AFG3L2	2	4.03E-07	0.982448205
C9orf78	2	C9orf78	2	0.005104217	0.980133796
PSMD7	2	PSMD7	2	7.92E-05	0.974222213
DOK4	2	DOK4	2	0.000147723	0.969402241
ENSSSCG00000027374	2	ENSSSCG0000	2	0.000577892	0.965492506
CHAC1	2	CHAC1	2	0.001967605	0.964192997
PPIE	2	PPIE	2	2.30E-07	0.958394284
HMGXB3	2	HMGXB3	2	0.004595792	0.957718423
ENSSSCG00000027041	2	ENSSSCG0000	2	6.81E-05	0.955265372
GMPPB	2	GMPPB	2	1.73E-06	0.949568702
ENSSSCG00000048914	2	ENSSSCG0000	2	8.89E-05	0.946314411
CCIN	2	CCIN	2	0.000577892	0.944940237
RBM22	2	RBM22	2	0.046730083	0.943987956
SURF4	2	SURF4	2	0.000467215	0.942629854
CLTA	2	CLTA	2	2.28E-05	0.940613985
NUDT16	2	NUDT16	2	0.002506937	0.937351418
SSBP3	2	SSBP3	2	1.56E-05	0.933170872
ENSSSCG00000011447	2	ENSSSCG0000	2	5.75E-08	0.929179061
CCNDBP1	2	CCNDBP1	2	0.000189312	0.925200645
ENSSSCG00000063245	2	ENSSSCG0000	2	0.000167265	0.92509126
GET1	2	GET1	2	2.59E-06	0.921046694
ECH1	2	ECH1	2	0.001283029	0.917766423
PRKN	2	PRKN	2	0.025420547	0.909056305
CCDC113	2	CCDC113	2	1.15E-07	0.9088996
RASGRP1	2	RASGRP1	2	0.018265564	0.907310697
HYPK	2	HYPK	2	5.75E-08	0.903926497
MTRF1L	2	MTRF1L	2	5.18E-05	0.90000919
CENPS	2	CENPS	2	0.016840103	0.897266243
ENSSSCG00000032016	2	ENSSSCG0000	2	0.000467215	0.896161051
ENSSSCG00000027270	2	ENSSSCG0000	2	0.000188336	0.895683741
PTPRU	2	PTPRU	2	2.30E-07	0.890856896
ENSSSCG00000058051	2	ENSSSCG0000	2	0.010763018	0.88922514
PRELID2	2	PRELID2	2	0.000529683	0.884316966
DALRD3	2	DALRD3	2	0.001383405	0.875216128
ODF2	2	ODF2	2	6.90E-07	0.873739646
NAT10	2	NAT10	2	2.90E-05	0.869786868
TMEM245	2	TMEM245	2	0.009435702	0.868941115
MFAP1	2	MFAP1	2	0.02907723	0.868560917
ENSSSCG00000047735	2	ENSSSCG0000	2	0.000114538	0.861228893
RPL35A	2	RPL35A	2	4.03E-07	0.858692022
AFAP1L1	2	AFAP1L1	2	4.03E-07	0.857233634
LAMA3	2	LAMA3	2	0.000188336	0.856790075
TRIM44	2	TRIM44	2	0.00023867	0.856025973
ZCCHC17	2	ZCCHC17	2	0.000467215	0.856008324
CAPN12	2	CAPN12	2	1.73E-06	0.849145802
DND1	2	DND1	2	0.014070638	0.845735571
ENSSSCG00000043568	2	ENSSSCG0000	2	1.15E-07	0.844999974
CDH8	2	CDH8	2	0.00403509	0.843781174
PTPN23	2	PTPN23	2	0.00758365	0.837890518
GSK3A	2	GSK3A	2	6.10E-06	0.837007671
UBE3D	2	UBE3D	2	3.85E-06	0.832448178
PCCB	2	PCCB	2	7.61E-05	0.83077398
FAAP24	2	FAAP24	2	1.56E-05	0.829346597
SLC24A1	2	SLC24A1	2	0.012293472	0.822662074
ENSSSCG00000005101	2	ENSSSCG0000	2	0.000656469	0.814079336
PPP1R8	2	PPP1R8	2	0.000446586	0.813221056
IP6K1	2	IP6K1	2	0.003529038	0.811404446
ENSSSCG00000059837	2	ENSSSCG0000	2	0.022990185	0.811114911
FNDC1	2	FNDC1	2	2.81E-05	0.804517704
ENSSSCG00000050083	2	ENSSSCG0000	2	0.000135902	0.802568042
SLC37A1	2	SLC37A1	2	5.58E-06	0.790124902
WDTC1	2	WDTC1	2	0.003370113	0.783734868
SMIM8	2	SMIM8	2	0.001283029	0.778699065
KIAA2013	2	KIAA2013	2	0.001547182	0.77626083
ZNF574	2	ZNF574	2	0.03701085	0.773401114
RER1	2	RER1	2	0.031834316	0.753008259
ENSSSCG00000014242	2	ENSSSCG0000	2	1.09E-06	0.73855496
ENSSSCG00000024791	2	ENSSSCG0000	2	2.59E-06	0.726808072
TMEM268	2	TMEM268	2	2.90E-05	0.718318943
BOC	2	BOC	2	7.84E-06	0.704664306
PAX5	2	PAX5	2	5.75E-08	0.688171235
CENPT	2	CENPT	2	0.033618093	0.675176039
DFFA	2	DFFA	2	0.000710659	0.660111053
PLEKHM2	2	PLEKHM2	2	0.02694642	0.614550112
ENSSSCG00000003825	2	ENSSSCG0000	2	0.01955007	0.547781614

Loci	factors	features	pvalues	log2FC
chr12.1990952.1991729_.C.	1	chr12.1990952.1991729_.C.	0.046	1.719
chr11.5042384.5044586_.C.	1	chr11.5042384.5044586_.C.	0.026	1.657
chr1.207173457.207174369_.C.	1	chr1.207173457.207174369_.C	0.026	1.623
chr11.9912809.9913316_.C.	1	chr11.9912809.9913316_.C.	0.004	1.613
chr1.75169857.75170800_.C.	1	chr1.75169857.75170800_.C.	0.034	1.549
chr11.4879361.4880102_.C.	1	chr11.4879361.4880102_.C.	0.000	1.543
chr1.8243705.8244469_.C.	1	chr1.8243705.8244469_.C.	0.003	1.524
chr12.56329633.56330718_.C.	1	chr12.56329633.56330718_.C.	0.001	1.467
chr1.130389264.130390128_.C.	1	chr1.130389264.130390128_.C	0.008	1.460
chr1.220952750.220953643_.C.	1	chr1.220952750.220953643_.C	0.004	1.446
chr1.271103816.271104402_.C.	1	chr1.271103816.271104402_.C	0.026	1.444
chr10.14183392.14184638_.C.	1	chr10.14183392.14184638_.C.	0.002	1.438
chr12.16254662.16256264_.C.	1	chr12.16254662.16256264_.C.	0.018	1.413
chr13.22124847.22125444_.C.	1	chr13.22124847.22125444_.C.	0.026	1.388
chr1.128641100.128642036_.C.	1	chr1.128641100.128642036_.C	0.030	1.377
chr1.239492188.239493251_.C.	1	chr1.239492188.239493251_.C	0.000	1.373
chr13.27647771.27648483_.C.	1	chr13.27647771.27648483_.C.	0.030	1.365
chr13.31308410.31309009_.C.	1	chr13.31308410.31309009_.C.	0.012	1.364
chr13.22883055.22883603_.C.	1	chr13.22883055.22883603_.C.	0.009	1.349
chr1.81996855.81997907_.C.	1	chr1.81996855.81997907_.C.	0.002	1.346
chr11.64308939.64309682_.C.	1	chr11.64308939.64309682_.C.	0.046	1.341
chr1.239285331.239286111_.C.	1	chr1.239285331.239286111_.C	0.008	1.318
chr1.185397819.185398991_.C.	1	chr1.185397819.185398991_.C	0.006	1.302
chr10.38889028.38889711_.C.	1	chr10.38889028.38889711_.C.	0.00693574	1.27880782
chr1.17136658.17138462_.C.	1	chr1.17136658.17138462_.C.	0.00313457	1.27634052
chr13.31627106.31627958_.C.	1	chr13.31627106.31627958_.C.	0.00435484	1.25460317
chr1.255168342.255169051_.C.	1	chr1.255168342.255169051_.C	0.026429	1.25419353
chr12.42849222.42850200_.C.	1	chr12.42849222.42850200_.C.	0.00370211	1.25255761
chr1.267964175.267965046_.C.	1	chr1.267964175.267965046_.C	0.00804229	1.24202681
chr10.31416365.31417437_.C.	1	chr10.31416365.31417437_.C.	0.00435484	1.22367144
chr1.98568735.98569606_.C.	1	chr1.98568735.98569606_.C.	0.00510422	1.22072674
chr1.217236861.217237767_.C.	1	chr1.217236861.217237767_.C	0.00105868	1.21666831
chr11.67710536.67711157_.C.	1	chr11.67710536.67711157_.C.	0.00222085	1.2141646
chr1.57128007.57129548_.C.	1	chr1.57128007.57129548_.C.	0.04630508	1.21296807
chr10.53602092.53603913_.C.	1	chr10.53602092.53603913_.C.	0.04630508	1.20323248
chr12.24679720.24680359_.C.	1	chr12.24679720.24680359_.C.	0.00929494	1.1965288
chr1.27247922.27250919_.C.	1	chr1.27247922.27250919_.C.	0.00596018	1.1929535
chr10.27953468.27954613_.C.	1	chr10.27953468.27954613_.C.	0.00011493	1.18978539
chr11.77354566.77356438_.C.	1	chr11.77354566.77356438_.C.	0.03729477	1.17550303
chr12.37693741.37694252_.C.	1	chr12.37693741.37694252_.C.	0.04630508	1.16547883
chr12.18257274.18258147_.C.	1	chr12.18257274.18258147_.C.	0.00510422	1.16470891
chr1.181681232.181681657_.C.	1	chr1.181681232.181681657_.C	0.0160561	1.15835622
chr10.10156482.10157235_.C.	1	chr10.10156482.10157235_.C.	0.00370211	1.15724159
chr1.186025525.186026828_.C.	1	chr1.186025525.186026828_.C	8.89E-05	1.14673863
chr1.142566853.142567341_.C.	1	chr1.142566853.142567341_.C	0.00693574	1.1215105
chr11.9737631.9738333_.C.	1	chr11.9737631.9738333_.C.	0.00693574	1.11077704
chr1.31796141.31797056_.C.	1	chr1.31796141.31797056_.C.	0.02389558	1.11018134
chr11.6762590.6763775_.C.	1	chr11.6762590.6763775_.C.	0.00313457	1.10663286
chr13.27320290.27324941_.C.	1	chr13.27320290.27324941_.C.	0.00596018	1.10091313
chr1.268465007.268466088_.C.	1	chr1.268465007.268466088_.C	0.00222085	1.10060799
chr12.52426498.52428609_.C.	1	chr12.52426498.52428609_.C.	0.00086971	1.09657216
chr11.52157608.52160831_.C.	1	chr11.52157608.52160831_.C.	0.00313457	1.08798001
chr11.68838249.68842592_.C.	1	chr11.68838249.68842592_.C.	0.01229347	1.08714829
chr11.21928915.21930059_.C.	1	chr11.21928915.21930059_.C.	0.02546328	1.08354888
chr12.52627516.52629243_.C.	1	chr12.52627516.52629243_.C.	0.00086971	1.08084507
chr1.242682071.242683303_.C.	1	chr1.242682071.242683303_.C	0.00154718	1.08051533
chr11.17947617.17948835_.C.	1	chr11.17947617.17948835_.C.	2.90E-05	1.06889115
chr11.66678135.66679652_.C.	1	chr11.66678135.66679652_.C.	0.00370211	1.06386069
chr1.120398506.120399196_.C.	1	chr1.120398506.120399196_.C	0.03334006	1.05674523
chr12.39739482.39740013_.C.	1	chr12.39739482.39740013_.C.	0.02071916	1.05389523
chr1.187718846.187720384_.C.	1	chr1.187718846.187720384_.C	0.02071916	1.05219254
chr1.270179344.270180174_.C.	1	chr1.270179344.270180174_.C	0.026429	1.04966525
chr11.15734905.15735621_.C.	1	chr11.15734905.15735621_.C.	0.00128303	1.0439389
chr1.123461438.123462218_.C.	1	chr1.123461438.123462218_.C	0.00071066	1.04206221
chr1.121067661.121068085_.C.	1	chr1.121067661.121068085_.C	0.00804229	1.03660377
chr13.23279037.23279516_.C.	1	chr13.23279037.23279516_.C.	0.01407064	1.0325477
chr10.46934776.46936137_.C.	1	chr10.46934776.46936137_.C.	0.02343317	1.02675134
chr1.239918638.239919296_.C.	1	chr1.239918638.239919296_.C	0.01407064	1.02634233
chr1.252323217.252324977_.C.	1	chr1.252323217.252324977_.C	0.0160561	1.02325563
chr1.7650678.7652010_.C.	1	chr1.7650678.7652010_.C.	0.01407064	1.00404516
chr11.65229604.65230677_.C.	1	chr11.65229604.65230677_.C.	0.00105868	1.00288754
chr1.267391415.267392150_.C.	1	chr1.267391415.267392150_.C	0.01229347	0.99995591
chr1.166465252.166466453_.C.	1	chr1.166465252.166466453_.C	3.89E-05	0.99906238
chr1.15328656.15330141_.C.	1	chr1.15328656.15330141_.C.	0.00086971	0.99797791
chr12.36831969.36832389_.C.	1	chr12.36831969.36832389_.C.	0.00105868	0.99691554
chr1.144787214.144788783_.C.	1	chr1.144787214.144788783_.C	0.01826556	0.99379972
chr1.268641282.268643052_.C.	1	chr1.268641282.268643052_.C	0.04630508	0.99031579
chr10.53314006.53315553_.C.	1	chr10.53314006.53315553_.C.	0.00264406	0.9822102
chr1.240494463.240496777_.C.	1	chr1.240494463.240496777_.C	0.00071066	0.97867901
chr1.249905208.249905824_.C.	1	chr1.249905208.249905824_.C	0.02270155	0.95752616
chr12.7637681.7639269_.C.	1	chr12.7637681.7639269_.C.	0.00313457	0.94163973
chr1.215806690.215808328_.C.	1	chr1.215806690.215808328_.C	0.00018834	0.94147572
chr10.31276259.31277324_.C.	1	chr10.31276259.31277324_.C.	0.00510422	0.93983135
chr1.147781309.147782595_.C.	1	chr1.147781309.147782595_.C	0.026429	0.92786864
chr1.272961037.272962500_.C.	1	chr1.272961037.272962500_.C	0.00222085	0.92726065
chr13.9213718.9214343_.C.	1	chr13.9213718.9214343_.C.	5.58E-06	0.92241828
chr11.12864358.12865079_.C.	1	chr11.12864358.12865079_.C.	0.01070637	0.92064019
chr1.7679156.7680433_.C.	1	chr1.7679156.7680433_.C.	0.00510422	0.91957312
chr13.32329740.32330722_.C.	1	chr13.32329740.32330722_.C.	0.01407064	0.90603459
chr11.3835568.3836152_.C.	1	chr11.3835568.3836152_.C.	0.02343317	0.90118143
chr12.8243844.8245010_.C.	1	chr12.8243844.8245010_.C.	0.00929494	0.90073338
chr11.16911110.16912081_.C.	1	chr11.16911110.16912081_.C.	0.00105868	0.89791535
chr1.239109878.239110333_.C.	1	chr1.239109878.239110333_.C	0.01826556	0.89511426
chr12.27439282.27440471_.C.	1	chr12.27439282.27440471_.C.	0.01826556	0.89475714
chr1.271462633.271464147_.C.	1	chr1.271462633.271464147_.C	0.03729477	0.88739475
chr11.15408631.15411432_.C.	1	chr11.15408631.15411432_.C.	0.00154718	0.88462581
chr10.33865976.33868184_.C.	1	chr10.33865976.33868184_.C.	0.026429	0.87837593
chr11.5332263.5334043_.C.	1	chr11.5332263.5334043_.C.	0.04630508	0.87393409
chr1.237785173.237786908_.C.	1	chr1.237785173.237786908_.C	0.00370211	0.87265859
chr1.268115922.268117045_.C.	1	chr1.268115922.268117045_.C	0.01070637	0.86985046
chr1.163525770.163526535_.C.	1	chr1.163525770.163526535_.C	0.00023867	0.86805451
chr1.241569484.241573110_.C.	1	chr1.241569484.241573110_.C	0.0160561	0.86349379
chr13.6984630.6986375_.C.	1	chr13.6984630.6986375_.C.	0.00370211	0.86319622
chr1.140566769.140568093_.C.	1	chr1.140566769.140568093_.C	0.02343317	0.86228602
chr1.108514583.108515849_.C.	1	chr1.108514583.108515849_.C	0.02972401	0.86131419
chr10.14294698.14296364_.C.	1	chr10.14294698.14296364_.C.	0.04159344	0.85673432

Loci	factors	features	pvalues	log2FC
chr2.149531920.149535260_.V.	2	chr2.149531920.149535260_.V.	0.037	1.371
chr1.1727106.1729876_.V.	2	chr1.1727106.1729876_.V.	0.026	1.207
chr10.66844399.66847999_.V.	2	chr10.66844399.66847999_.V.	0.030	1.094
chr9.136753582.136756492_.V.	2	chr9.136753582.136756492_.V.	0.046	1.075
chr16.55458699.55460509_.V.	2	chr16.55458699.55460509_.V.	0.042	1.060
chrX.121752258.121755268_.V.	2	chrX.121752258.121755268_.V.	0.042	0.970
chr1.970705.972819_.C.	2	chr1.970705.972819_.C.	0.021	0.961
chr10.52445273.52447376_.C.	2	chr10.52445273.52447376_.C.	0.003	0.958
chr12.3166999.3170069_.V.	2	chr12.3166999.3170069_.V.	0.030	0.953
chr4.32680749.32684029_.V.	2	chr4.32680749.32684029_.V.	0.016	0.941
chr15.139048928.139052328_.V.	2	chr15.139048928.139052328_.V.	0.021	0.929
chr13.200128785.200129482_.C.	2	chr13.200128785.200129482_.C.	0.014	0.917
chr1.119948447.119948891_.C.	2	chr1.119948447.119948891_.C.	0.030	0.908
chr2.3694300.3695680_.V.	2	chr2.3694300.3695680_.V.	0.000	0.902
chr1.200792376.200792811_.C.	2	chr1.200792376.200792811_.C.	0.007	0.902
chr14.141098189.141104699_.V.	2	chr14.141098189.141104699_.V.	0.030	0.889
chr2.76277430.76279160_.V.	2	chr2.76277430.76279160_.V.	0.000	0.888
chr3.25891176.25896476_.V.	2	chr3.25891176.25896476_.V.	0.033	0.884
chr5.3952758.3955988_.V.	2	chr5.3952758.3955988_.V.	0.006	0.879
chr6.162378686.162382916_.V.	2	chr6.162378686.162382916_.V.	0.023	0.877
chr10.39874289.39876849_.V.	2	chr10.39874289.39876849_.V.	0.001	0.858
chr16.54267269.54269829_.V.	2	chr16.54267269.54269829_.V.	0.042	0.857
chr4.59549079.59551499_.V.	2	chr4.59549079.59551499_.V.	0.000	0.851
chr4.76065169.76068979_.V.	2	chr4.76065169.76068979_.V.	0.002	0.850
chr4.128935039.128939899_.V.	2	chr4.128935039.128939899_.V.	0.002	0.845
chr9.258992.261072_.V.	2	chr9.258992.261072_.V.	0.0003124	0.84244517
chr16.79741769.79744539_.V.	2	chr16.79741769.79744539_.V.	0.00105868	0.83256699
chr18.52624905.52628135_.V.	2	chr18.52624905.52628135_.V.	0.01407064	0.82969591
chr13.206843554.206847737_.C.	2	chr13.206843554.206847737_.C.	0.00313457	0.82654598
chr9.10731572.10734082_.V.	2	chr9.10731572.10734082_.V.	0.04886078	0.81978776
chr18.840575.842495_.V.	2	chr18.840575.842495_.V.	0.00071066	0.81834193
chr18.815975.818965_.V.	2	chr18.815975.818965_.V.	0.00222085	0.81772034
chr7.41816429.41818709_.V.	2	chr7.41816429.41818709_.V.	0.04852935	0.81634442
chr15.138868278.138870448_.V.	2	chr15.138868278.138870448_.V.	0.01826556	0.81216265
chr1.363396.374996_.V.	2	chr1.363396.374996_.V.	0.00023867	0.80881599
chr14.140303169.140305619_.V.	2	chr14.140303169.140305619_.V.	0.00313457	0.80486894
chr1.1921361.1921991_.C.	2	chr1.1921361.1921991_.C.	0.0413817	0.78926828
chr1.1776886.1789336_.V.	2	chr1.1776886.1789336_.V.	0.00222085	0.78874526
chr6.89558216.89560016_.V.	2	chr6.89558216.89560016_.V.	0.00264406	0.78726497
chr12.2141178.2144859_.C.	2	chr12.2141178.2144859_.C.	0.00030028	0.78266732
chr2.4256940.4259620_.V.	2	chr2.4256940.4259620_.V.	0.00222085	0.77042265
chr14.141392439.141394999_.V.	2	chr14.141392439.141394999_.V.	0.02343317	0.76310825
chr3.89803706.89805516_.V.	2	chr3.89803706.89805516_.V.	0.00929494	0.75836251
chr4.4245539.4248379_.V.	2	chr4.4245539.4248379_.V.	0.01070637	0.75779159
chr7.882149.884899_.V.	2	chr7.882149.884899_.V.	0.00435484	0.75749209
chr12.18868719.18875189_.V.	2	chr12.18868719.18875189_.V.	0.00804229	0.75720785
chr16.78729349.78736299_.V.	2	chr16.78729349.78736299_.V.	0.03334006	0.75645214
chr18.1701815.1705065_.V.	2	chr18.1701815.1705065_.V.	0.00510422	0.75520111
chr11.25643532.25644840_.C.	2	chr11.25643532.25644840_.C.	0.00086971	0.75501136
chr9.139054472.139056352_.V.	2	chr9.139054472.139056352_.V.	0.00929494	0.7507372
chr3.23198926.23200886_.V.	2	chr3.23198926.23200886_.V.	0.02071916	0.74819679
chr10.24458712.24459669_.C.	2	chr10.24458712.24459669_.C.	0.04152286	0.74675826
chr3.49441556.49445936_.V.	2	chr3.49441556.49445936_.V.	0.00264406	0.74449459
chr14.140087869.140090589_.V.	2	chr14.140087869.140090589_.V.	0.04161078	0.7390735
chr6.166829006.166831646_.V.	2	chr6.166829006.166831646_.V.	0.03334006	0.73841248
chr11.5241105.5241689_.C.	2	chr11.5241105.5241689_.C.	0.00804229	0.73748073
chr5.1771438.1776178_.V.	2	chr5.1771438.1776178_.V.	0.04630508	0.73711452
chr9.138947412.138952722_.V.	2	chr9.138947412.138952722_.V.	0.00435484	0.73496171
chr7.161119.166329_.V.	2	chr7.161119.166329_.V.	0.02594227	0.73290416
chr12.7581599.7584879_.V.	2	chr12.7581599.7584879_.V.	0.01229347	0.73073467
chr7.79413829.79415779_.V.	2	chr7.79413829.79415779_.V.	0.00370211	0.73064261
chr18.5873195.5876335_.V.	2	chr18.5873195.5876335_.V.	0.01826556	0.73051492
chrX.125184248.125185808_.V.	2	chrX.125184248.125185808_.V.	0.01407064	0.72665939
chr8.55570718.55572198_.V.	2	chr8.55570718.55572198_.V.	0.0269349	0.72620066
chr14.92224439.92226169_.V.	2	chr14.92224439.92226169_.V.	0.00370211	0.72573849
chr13.206865565.206869585_.V.	2	chr13.206865565.206869585_.V.	0.01826556	0.72477738
chr7.7795369.7798579_.V.	2	chr7.7795369.7798579_.V.	0.0160561	0.72460129
chr12.33861199.33867119_.V.	2	chr12.33861199.33867119_.V.	0.00693574	0.72152538
chr2.2723860.2733450_.V.	2	chr2.2723860.2733450_.V.	0.00693574	0.71713057
chr10.8917679.8921219_.V.	2	chr10.8917679.8921219_.V.	0.00057789	0.71580496
chr7.4273899.4274969_.V.	2	chr7.4273899.4274969_.V.	0.01498872	0.7155158
chr6.167656186.167665426_.V.	2	chr6.167656186.167665426_.V.	0.03334006	0.71429149
chr1.400186.404786_.V.	2	chr1.400186.404786_.V.	0.00037581	0.71198167
chr1.731516.740006_.V.	2	chr1.731516.740006_.V.	0.00510422	0.71193408
chr3.131726366.131731426_.V.	2	chr3.131726366.131731426_.V.	0.01826556	0.71173613
chr11.2602205.2603499_.C.	2	chr11.2602205.2603499_.C.	0.04630508	0.70797367
chr12.448754.450833_.C.	2	chr12.448754.450833_.C.	0.01229347	0.70764168
chr1.1717576.1722746_.V.	2	chr1.1717576.1722746_.V.	0.00037581	0.70667967
chr2.2705840.2713480_.V.	2	chr2.2705840.2713480_.V.	0.01826556	0.70361713
chr5.75448848.75450858_.V.	2	chr5.75448848.75450858_.V.	0.026429	0.70341598
chr12.6336839.6340259_.V.	2	chr12.6336839.6340259_.V.	0.04161078	0.70132991
chr9.11174532.11178572_.V.	2	chr9.11174532.11178572_.V.	0.01826556	0.69356819
chr1.379816.389776_.V.	2	chr1.379816.389776_.V.	0.00086971	0.69326083
chr18.3040845.3044465_.V.	2	chr18.3040845.3044465_.V.	0.00128303	0.6923778
chr12.1760259.1764969_.V.	2	chr12.1760259.1764969_.V.	0.026429	0.69067308
chr4.1900849.1906229_.V.	2	chr4.1900849.1906229_.V.	0.01229347	0.68859612
chr10.66708949.66710689_.V.	2	chr10.66708949.66710689_.V.	0.026429	0.68801853
chr18.472955.475115_.V.	2	chr18.472955.475115_.V.	0.02972401	0.68639619
chr4.128389539.128391119_.V.	2	chr4.128389539.128391119_.V.	0.00313457	0.68593859
chrX.88048758.88052718_.V.	2	chrX.88048758.88052718_.V.	0.02264068	0.68395965
chr15.139822668.139834538_.V.	2	chr15.139822668.139834538_.V.	0.00071066	0.68222241
chr18.3716145.3718925_.V.	2	chr18.3716145.3718925_.V.	0.00046721	0.68068933
chr7.802119.805159_.V.	2	chr7.802119.805159_.V.	0.00014772	0.68028295
chr7.867759.873679_.V.	2	chr7.867759.873679_.V.	0.01407064	0.67953931
chr11.74017450.74021200_.V.	2	chr11.74017450.74021200_.V.	0.00435484	0.67838132
chr17.61321802.61328422_.V.	2	chr17.61321802.61328422_.V.	0.00929494	0.67726224
chr1.66515495.66516311_.C.	2	chr1.66515495.66516311_.C.	0.00046721	0.67725387
chr12.1743839.1753369_.V.	2	chr12.1743839.1753369_.V.	0.04161078	0.67333283
chr18.1154575.1159705_.V.	2	chr18.1154575.1159705_.V.	0.00929494	0.67122901
chr12.6214528.6215144_.C.	2	chr12.6214528.6215144_.C.	0.02972401	0.66945925
chr12.1304812.1306458_.C.	2	chr12.1304812.1306458_.C.	0.00510422	0.66857989
chr12.43165819.43169679_.V.	2	chr12.43165819.43169679_.V.	0.026429	0.66765897
chr18.1148775.1153895_.V.	2	chr18.1148775.1153895_.V.	0.026429	0.66553301
chr12.51843603.51844550_.C.	2	chr12.51843603.51844550_.C.	0.00264406	0.66345042
chr13.206034618.206037701_.C.	2	chr13.206034618.206037701_.C.	0.04630508	0.66276978
chr14.140414459.140419889_.V.	2	chr14.140414459.140419889_.V.	0.00804229	0.66261548

chr1.272954630.272955607_.C.	1	chr1.272954630.272955607_.C	0.01229347	0.84699072	chr14.137927429.137930519_.V.	2	chr14.137927429.137930519_.V.	0.03729477	0.66171087
chr12.41725597.41727813_.C.	1	chr12.41725597.41727813_.C.	0.0028429	0.84489714	chr10.66111510.66112730_.C.	2	chr10.66111510.66112730_.C.	0.00370211	0.65965599
chr1.109010167.109010965_.C.	1	chr1.109010167.109010965_.C	0.02343317	0.84351927	chr12.50728138.50729135_.C.	2	chr12.50728138.50729135_.C.	0.00128303	0.65885268
chr1.268652871.268653576_.C.	1	chr1.268652871.268653576_.C	0.01070637	0.83903114	chr16.77381779.77384759_.V.	2	chr16.77381779.77384759_.V.	0.00130583	0.65584085
chr10.12557515.12558552_.C.	1	chr10.12557515.12558552_.C.	0.00370211	0.82911634	chr6.65005436.65012316_.V.	2	chr6.65005436.65012316_.V.	0.01229347	0.65560797
chr1.228044376.228045374_.C.	1	chr1.228044376.228045374_.C	0.00929494	0.82497827	chr1.286656.289836_.V.	2	chr1.286656.289836_.V.	0.00105868	0.65469983
chr12.47124265.47125516_.C.	1	chr12.47124265.47125516_.C.	0.00057789	0.81925448	chr9.138513222.138516292_.V.	2	chr9.138513222.138516292_.V.	0.02694642	0.65432946
chr1.32418386.32419693_.C.	1	chr1.32418386.32419693_.C.	0.00510422	0.81885789	chrX.122227078.122232108_.V.	2	chrX.122227078.122232108_.V.	0.04161078	0.65276169
chr12.53553347.53554403_.C.	1	chr12.53553347.53554403_.C.	0.00370211	0.81882767	chr11.5623444.5624326_.C.	2	chr11.5623444.5624326_.C.	0.00185787	0.65268502
chr1.6996235.6997358_.C.	1	chr1.6996235.6997358_.C.	0.00264406	0.8178679	chr4.76733289.76735109_.V.	2	chr4.76733289.76735109_.V.	0.00014772	0.65246856
chr13.11358483.11360380_.C.	1	chr13.11358483.11360380_.C.	0.03729477	0.81611549	chr4.7238559.7243049_.V.	2	chr4.7238559.7243049_.V.	0.026429	0.65234466
chr1.3118820.3122474_.C.	1	chr1.3118820.3122474_.C.	0.02343317	0.81531691	chr6.64710876.64718966_.V.	2	chr6.64710876.64718966_.V.	0.01826556	0.64979602
chr1.25480793.25481989_.C.	1	chr1.25480793.25481989_.C.	0.02972401	0.8114562	chr15.139052628.139057078_.V.	2	chr15.139052628.139057078_.V.	0.00011493	0.6479606
chr1.121413109.121414480_.C.	1	chr1.121413109.121414480_.C	0.04630508	0.80878873	chr14.140746869.140750169_.V.	2	chr14.140746869.140750169_.V.	0.02071916	0.64567714
chr1.158860711.158862808_.C.	1	chr1.158860711.158862808_.C	0.00929494	0.8049158	chr1.2003255.2004766_.C.	2	chr1.2003255.2004766_.C.	0.01090343	0.64399029
chr1.128305408.128305965_.C.	1	chr1.128305408.128305965_.C	0.026429	0.7901294	chr1.273651376.273656236_.V.	2	chr1.273651376.273656236_.V.	0.03729477	0.64375096
chr11.23237035.23238925_.C.	1	chr11.23237035.23238925_.C.	0.00105868	0.78916675	chr7.121088159.121093409_.V.	2	chr7.121088159.121093409_.V.	0.01070637	0.64364981
chr11.19106367.19107200_.C.	1	chr11.19106367.19107200_.C.	0.02343317	0.78548294	chr3.126148606.126153916_.V.	2	chr3.126148606.126153916_.V.	0.026429	0.64307555
chr1.264195399.264196413_.C.	1	chr1.264195399.264196413_.C	0.03334006	0.78487665	chr15.137692318.137698258_.V.	2	chr15.137692318.137698258_.V.	0.00030028	0.64233066
chr13.21877378.21878749_.C.	1	chr13.21877378.21878749_.C.	0.01229347	0.78431854	chr1.986216.988616_.V.	2	chr1.986216.988616_.V.	0.00057789	0.64188183
chr1.265934267.265935360_.C.	1	chr1.265934267.265935360_.C	0.00046721	0.77801248	chr12.32816299.32818319_.V.	2	chr12.32816299.32818319_.V.	0.00510422	0.64022619
chr1.26254832.26255836_.C.	1	chr1.26254832.26255836_.C.	0.01070637	0.77611307	chr6.64663816.64666816_.V.	2	chr6.64663816.64666816_.V.	0.01159305	0.64011072
chr11.17689461.17691697_.C.	1	chr11.17689461.17691697_.C.	0.00185787	0.76972999	chr8.4901688.4905898_.V.	2	chr8.4901688.4905898_.V.	0.03729477	0.63790019
chr1.118913210.118914612_.C.	1	chr1.118913210.118914612_.C	0.02972401	0.76576357	chr5.5025318.5031868_.V.	2	chr5.5025318.5031868_.V.	0.026429	0.63729535
chr13.12574830.12575751_.C.	1	chr13.12574830.12575751_.C.	0.00804229	0.75985991	chr16.78552969.78556889_.V.	2	chr16.78552969.78556889_.V.	0.01229347	0.63640626
chr13.18371847.18373032_.C.	1	chr13.18371847.18373032_.C.	0.04630508	0.75922379	chr14.140038389.140042669_.V.	2	chr14.140038389.140042669_.V.	0.00071066	0.63622251
chr11.49807826.49808981_.C.	1	chr11.49807826.49808981_.C.	0.00014772	0.75895284	chr7.1463629.1466979_.V.	2	chr7.1463629.1466979_.V.	0.00189937	0.63538756
chr10.65471676.65472178_.C.	1	chr10.65471676.65472178_.C.	0.00370211	0.75701238	chr11.77426533.77428310_.C.	2	chr11.77426533.77428310_.C.	0.02972401	0.63326349
chr1.109921476.109922526_.C.	1	chr1.109921476.109922526_.C	0.00510422	0.75356931	chr18.1416435.1421805_.V.	2	chr18.1416435.1421805_.V.	0.00596018	0.63194184
chr15.76991098.76995238_.V.	1	chr15.76991098.76995238_.V.	0.00929494	0.75113167	chr18.3673835.3675515_.V.	2	chr18.3673835.3675515_.V.	0.00804229	0.63059599
chr1.236340984.236341976_.C.	1	chr1.236340984.236341976_.C	0.01229347	0.74651503	chr11.78891930.78896300_.V.	2	chr11.78891930.78896300_.V.	0.02972401	0.62914037
chr1.251570577.251571193_.C.	1	chr1.251570577.251571193_.C	0.02071916	0.7456884	chr17.61222882.61224592_.V.	2	chr17.61222882.61224592_.V.	0.02071916	0.62730045
chr1.97734395.97735500_.C.	1	chr1.97734395.97735500_.C.	0.00264406	0.74562468	chr1.3066316.3070096_.V.	2	chr1.3066316.3070096_.V.	0.00510422	0.62512877
chr1.119758703.119759757_.C.	1	chr1.119758703.119759757_.C	0.03729477	0.73880982	chr8.131031428.131035218_.V.	2	chr8.131031428.131035218_.V.	0.02343317	0.62479483
chr1.230906557.230908239_.C.	1	chr1.230906557.230908239_.C	0.00929494	0.73801718	chr1.969496.973026_.V.	2	chr1.969496.973026_.V.	0.00023867	0.62439519
chr1.57423069.57424350_.C.	1	chr1.57423069.57424350_.C.	0.00596018	0.73743776	chr14.139651639.139655749_.V.	2	chr14.139651639.139655749_.V.	0.02972401	0.62434269
chr13.40768236.40768750_.C.	1	chr13.40768236.40768750_.C.	0.00154718	0.73354073	chr10.66654679.66661289_.V.	2	chr10.66654679.66661289_.V.	0.00264406	0.62432655
chr11.13959329.13960821_.C.	1	chr11.13959329.13960821_.C.	0.03334006	0.7300881	chr13.206321005.206328245_.V.	2	chr13.206321005.206328245_.V.	0.00037581	0.62368869
chr1.25735449.25736948_.C.	1	chr1.25735449.25736948_.C.	0.00596018	0.7271449	chr16.76625789.76633749_.V.	2	chr16.76625789.76633749_.V.	0.04161078	0.62212869
chr1.242169220.242169827_.C.	1	chr1.242169220.242169827_.C	0.00037581	0.71578232	chr18.2235895.2238315_.V.	2	chr18.2235895.2238315_.V.	0.00370211	0.62182785
chr1.250680302.250681256_.C.	1	chr1.250680302.250681256_.C	0.0160561	0.70492106	chr3.132616516.132619386_.V.	2	chr3.132616516.132619386_.V.	0.01826556	0.6195149
chr1.137855508.137856731_.C.	1	chr1.137855508.137856731_.C	0.03729477	0.69907477	chr11.76710950.76713460_.V.	2	chr11.76710950.76713460_.V.	0.02972401	0.61906318
chr1.221581373.221582232_.C.	1	chr1.221581373.221582232_.C	0.01407064	0.69280121	chr14.21009729.21013389_.V.	2	chr14.21009729.21013389_.V.	0.00804229	0.61854195
chr13.35179148.35181193_.C.	1	chr13.35179148.35181193_.C.	0.03334006	0.68988919	chr17.60916312.60920102_.V.	2	chr17.60916312.60920102_.V.	0.00693574	0.6184615
chr10.19888436.19889542_.C.	1	chr10.19888436.19889542_.C.	0.00046721	0.68583108	chr1.90466.92736_.V.	2	chr1.90466.92736_.V.	0.00071066	0.61696186
chr1.228854463.228855381_.C.	1	chr1.228854463.228855381_.C	0.00057789	0.67823531	chr18.48989585.48995365_.V.	2	chr18.48989585.48995365_.V.	0.00030028	0.61689
chr1.270363751.270364715_.C.	1	chr1.270363751.270364715_.C	0.0160561	0.67777022	chr1.188729585.188729940_.C.	2	chr1.188729585.188729940_.C.	0.00370211	0.61641898
chr11.75544552.75545320_.C.	1	chr11.75544552.75545320_.C.	0.00804229	0.67622062	chr13.206309755.206315565_.V.	2	chr13.206309755.206315565_.V.	0.00435484	0.61405647
chr1.145707133.145707900_.C.	1	chr1.145707133.145707900_.C	0.00804229	0.6706088	chr14.137175889.137179879_.V.	2	chr14.137175889.137179879_.V.	6.81E-05	0.61322703
chr11.5793860.5797121_.C.	1	chr11.5793860.5797121_.C.	0.00435484	0.66348787	chr1.2044406.2047956_.V.	2	chr1.2044406.2047956_.V.	0.02343317	0.61170037
chr11.77979282.77981624_.C.	1	chr11.77979282.77981624_.C.	0.00154718	0.66104928	chr18.50064445.50066235_.V.	2	chr18.50064445.50066235_.V.	0.00313457	0.61079055
chr1.115219826.115220413_.C.	1	chr1.115219826.115220413_.C	0.00693574	0.65539857	chr9.10448262.10452072_.V.	2	chr9.10448262.10452072_.V.	0.00046721	0.60841888
chr1.262203524.262204368_.C.	1	chr1.262203524.262204368_.C	0.00370211	0.65135442	chr9.135368712.135371122_.V.	2	chr9.135368712.135371122_.V.	0.03729477	0.60671156
chr10.68769934.68770936_.C.	1	chr10.68769934.68770936_.C.	0.00086971	0.64099712	chr11.65200901.65201681_.C.	2	chr11.65200901.65201681_.C.	0.00154718	0.60621835
chr1.96839460.96842049_.C.	1	chr1.96839460.96842049_.C.	0.02071916	0.64021417	chr18.52549975.52553555_.V.	2	chr18.52549975.52553555_.V.	0.00030028	0.60588649
chr1.130384819.130388088_.C.	1	chr1.130384819.130388088_.C	0.00264406	0.63208304	chr5.3279228.3281228_.V.	2	chr5.3279228.3281228_.V.	0.026429	0.60494376
chr1.180591892.180593286_.C.	1	chr1.180591892.180593286_.C	0.00154718	0.6310611	chr4.7447329.7451569_.V.	2	chr4.7447329.7451569_.V.	0.01826556	0.6049043
chr1.52997619.52998959_.C.	1	chr1.52997619.52998959_.C.	0.00222085	0.62385119	chrX.35573888.35577558_.V.	2	chrX.35573888.35577558_.V.	0.02071916	0.60411535
chr10.54675617.54677199_.C.	1	chr10.54675617.54677199_.C.	0.00185787	0.62053857	chr10.64054469.64079119_.V.	2	chr10.64054469.64079119_.V.	0.02343317	0.6036888
chr1.4882178.4885075_.C.	1	chr1.4882178.4885075_.C.	0.00804229	0.61682269	chr17.60939522.60943472_.V.	2	chr17.60939522.60943472_.V.	0.00370211	0.60357346
chr1.238825022.238826808_.C.	1	chr1.238825022.238826808_.C	0.00014772	0.61565831	chr5.26146488.26152818_.V.	2	chr5.26146488.26152818_.V.	0.00071066	0.6028681
chr12.52440268.52442734_.C.	1	chr12.52440268.52442734_.C.	0.00185787	0.61520707	chr18.50056015.50059125_.V.	2	chr18.50056015.50059125_.V.	0.02343317	0.60202666
chr1.95727119.95728266_.C.	1	chr1.95727119.95728266_.C.	0.00023867	0.61073149	chr17.57631282.57634562_.V.	2	chr17.57631282.57634562_.V.	0.00510422	0.60073059
chr1.188597843.188598626_.C.	1	chr1.188597843.188598626_.C	2.90E-05	0.60573349	chr13.208214115.208217535_.V.	2	chr13.208214115.208217535_.V.	0.00023867	0.6004645
chr13.22706057.22708111_.C.	1	chr13.22706057.22708111_.C.	0.02343317	0.60238158	chr3.126639236.126644176_.V.	2	chr3.126639236.126644176_.V.	0.00071066	0.60032808
chr13.5415652.5417586_.C.	1	chr13.5415652.5417586_.C.	0.01407064	0.60081657	chr13.206031271.206032506_.C.	2	chr13.206031271.206032506_.C.	0.04630508	0.59896621
chr13.1127397.1128336_.C.	1	chr13.1127397.1128336_.C.	0.00154718	0.60074304	chr14.138182749.138186599_.V.	2	chr14.138182749.138186599_.V.	0.00370211	0.59765548
chr15.48693778.48695388_.V.	1	chr15.48693778.48695388_.V.	0.01826556	0.59255948	chr12.18864249.18866079_.V.	2	chr12.18864249.18866079_.V.	0.00804229	0.5976492
chr1.72815404.72815846_.C.	1	chr1.72815404.72815846_.C.	0.01229347	0.58602689	chr18.50062425.50064345_.V.	2	chr18.50062425.50064345_.V.	0.00128303	0.59744979
chr10.68528038.68529249_.C.	1	chr10.68528038.68529249_.C.	0.00154718	0.58369463	chr5.3321948.3323428_.V.	2	chr5.3321948.3323428_.V.	0.00086971	0.59557879
chr11.46459363.46461173_.C.	1	chr11.46459363.46461173_.C.	0.02071916	0.57564938	chr3.15937626.15941906_.V.	2	chr3.15937626.15941906_.V.	0.00596018	0.59556455
chr1.83849134.83850970_.C.	1	chr1.83849134.83850970_.C.	0.00264406	0.57374206	chr11.1673848.1674791_.C.	2	chr11.1673848.1674791_.C.	0.00086971	0.59442824
chr13.30601700.30602411_.C.	1	chr13.30601700.30602411_.C.	0.00128303	0.56972592	chr10.1075259.1078819_.V.	2	chr10.1075259.1078819_.V.	0.02343317	0.59401662
chr13.31990619.31991906_.C.	1	chr13.31990619.31991906_.C.	2.90E-05	0.56653958	chr14.28802819.28808389_.V.	2	chr14.28802819.28808389_.V.	0.00693574	0.59396114
chr10.68621049.68622278_.C.	1	chr10.68621049.68622278_.C.	0.00693574	0.5629601	chr3.125782346.125785516_.V.	2	chr3.125782346.125785516_.V.	0.01229347	0.59382968
chr10.34001454.34002137_.C.	1	chr10.34001454.34002137_.C.	0.00435484	0.56013601	chr9.136453712.136457442_.V.	2	chr9.136453712.136457442_.V.	0.01407064	0.59155893
chr1.198992230.198993785_.C.	1	chr1.198992230.198993785_.C	0.00014772	0.55656681	chr3.126125186.126128636_.V.	2	chr3.126125186.126128636_.V.	0.04161078	0.59098428
chr1.14866348.14867856_.C.	1	chr1.14866348.14867856_.C.	0.00105868	0.55256361	chr12.26745423.26746618_.C.	2	chr12.26745423.26746618_.C.	0.02343317	0.5908833
chr1.164997476.164999127_.C.	1	chr1.164997476.164999127_.C	0.00313457	0.55161347	chr11.78263210.78271840_.V.	2	chr11.78263210.78271840_.V.	0.00023867	0.59062497
chr1.138340690.138342678_.C.	1	chr1.138340690.138342678_.C	0.04852935	0.54476134	chr5.837998.842798_.V.	2	chr5.837998.842798_.V.	0.00154718	0.59056886
chr1.181771496.181773290_.C.	1	chr1.181771496.181773290_.C	0.04161078	0.54008294	chr5.5176438.5181598_.V.	2	chr5.5176438.5181598_.V.	0.02972401	0.58996226
chr1.150709729.150712378_.C.	1	chr1.150709729.150712378_.C	0.01826556	0.53871568	chr7.1440269.1447389_.V.	2	chr7.1440269.1447389_.V.	0.01875602	0.58949916
chr10.51453624.51455597_.C.	1	chr10.51453624.51455597_.C.	0.00435484	0.538111	chr14.7359889.7361529_.V.	2	chr14.7359889.7361529_.V.	0.02071916	0.58598634
chr12.1317683.1322503_.C.	1	chr12.1317683.1322503_.C.	0.04161078	0.52999762	chr3.125787726.125792026_.V.	2	chr3.125787726.125792026_.V.	0.00023867	0.58507097
chr11.49029946.49031917_.C.	1	chr11.49029946.49031917_.C.	0.03729477	0.52688895	chr18.851535.853675_.V.	2	chr18.851535.853675_.V.	0.00086971	0.58456723
chr1.37911315.37912718_.C.	1	chr1.37911315.37912718_.C.	0.00804229	0.52653583	chr12.40492457.40493372_.C.	2	chr12.40492457.40493372_.C.	0.0160561	0.58367293
chr9.68507352.68510302_.V.	1	chr9.68507352.68510302_.V.	0.01826556	0.52491738	chr14.136710069.136717369_.V.	2	chr14.136710069.136717369_.V.	5.18E-05	0.58273044
chr12.45760921.45763958_.C.	1	chr12.45760921.45763958_.C.	0.01826556	0.52488549	chr10.67517009.67525249_.V.	2	chr10.67517009.67525249_.V.	0.00037581	0.58135611
chr1.31184928.31185414_.C.	1	chr1.31184928.31185414_.C.	0.03729477	0.52185475	chr16.76567329.76569229_.V.	2	chr16.76567329.76569229_.V.	0.00804229	0.58042609
chr11.12576418.12577666_.C.	1	chr11.12576418.12577666_.C.	0.00693574	0.51882478	chr2.1598720.1611190_.V.	2	chr2.1598720.1611190_.V.	0.026429	0.57920753
chr10.20732446.20733451_.C.	1	chr10.20732446.20733451_.C.	0.02071916	0.51080266	chr14.136545109.136548939_.V.	2	chr14.136545109.136548939_.V.	0.00014772	0.578981
chr7.13377029.13380019_.V.	1	chr7.13377029.13380019_.V.	0.00154718	0.50404716	chr16.77903549.77906319_.V.	2	chr16.77903549.77906319_.V.	0.00071066	0.57851221
chr13.18030794.18031998_.C.	1	chr13.18030794.18031998_.C.	0.00222085	0.47839168	chr14.141175729.141179889_.V.	2	chr14.141175729.141179889_.V.	0.01070637	0.57734921
chr11.862370.863216_.C.	1	chr11.862370.863216_.C.	0.01070637	0.47387007	chr4.129774569.129779739_.V.	2	chr4.129774569.129779739_.V.	0.01070637	0.57622243
chr1.16139115.16140322_.C.	1	chr1.16139115.16140322_.C.	0.026429	0.4712128	chr1.807820.809441_.C.	2	chr1.807820.809441_.C.	0.01470167	0.57543925
chr8.120370758.120373218_.V.	1	chr8.120370758.120373218_.V	0.00596018	0.45417238	chr15.139604568.139621818_.V.	2	chr15.139604568.139621818_.V.	0.00185787	0.57535986
chr11.60731373.60733345_.C.	1	chr11.60731373.60733345_.C.	0.03729477	0.44985925	chr13.206209785.206213475_.V.	2	chr13.206209785.206213475_.V.	0.00011493	0.57373562
chr1.181995066.181995661_.C.	1	chr1.181995066.181995661_.C	0.00313457	0.44816134	chr12.3215499.3219479_.V.	2	chr12.3215499.3219479_.V.	0.04630508	0.57364645
chr1.237777497.237779049_.C.	1	chr1.237777497.237779049_.C	0.03729477	0.44726661	chr10.5725169.5727699_.V.	2	chr10.5725169.5727699_.V.	0.026429	0.57308846
chr11.64042445.64043710_.C.	1	chr11.64042445.64043710_.C.	0.00693574	0.44549445	chr15.109247828.109253198_.V.	2	chr15.109247828.109253198_.V.	0.00057789	0.57303158
chr1.147461608.147463377_.C.	1	chr1.147461608.147463377_.C	0.02972401	0.43370471	chr16.78852469.78857009_.V.	2	chr16.78852469.78857009_.V.	0.00510422	0.57267732
chr11.51494201.51494648_.C.	1	chr11.51494201.51494648_.C.	0.026429	0.42219284	chr4.76888489.76894389_.V.	2	chr4.76888489.76894389_.V.	0.01826556	0.57086179
chr1.256544838.256545500_.C.	1	chr1.256544838.256545500_.C	0.03729477	0.40915993	chr18.1516805.1521415_.V.	2	chr18.1516805.1521415_.V.	0.00804229	0.57063189
chr2.66344600.66347110_.V.	1	chr2.66344600.66347110_.V.	0.00596018	0.40873798	chr15.132128328.132133218_.V.	2	chr15.132128328.132133218_.V.	0.00057789	0.57057448
chr16.52819079.52825559_.V.	1	chr16.52819079.52825559_.V.	0.00250694	0.38708922	chr3.125810976.125812866_.V.	2	chr3.125810976.125812866_.V.	0.026429	0.57043868
chr6.31746036.31749326_.V.	1	chr6.31746036.31749326_.V.	0.02404913	0.38530218	chrX.42955808.42959908_.V.	2	chrX.42955808.42959908_.V.	0.02071916	0.56978568
chr10.53065708.53067189_.C.	1	chr10.53065708.53067189_.C.	0.02071916	0.37893328	chr14.138976219.138981529_.V.	2	chr14.138976219.138981529_.V.	0.02343317	0.56856511

chr18.18978175.18981285_.V.	1	chr18.18978175.18981285_.V.	0.00264406	0.37720222
chrX.118824848.118830998_.V.	1	chrX.118824848.118830998_.V	0.00557874	0.37254679
chr1.261898896.261900556_.V.	1	chr1.261898896.261900556_.V	0.04161078	0.35472034
chr14.105010519.105014699_.V.	1	chr14.105010519.105014699_.'	0.02071916	0.35364905
chr2.142577020.142579530_.V.	1	chr2.142577020.142579530_.V	0.02071916	0.35103601
chr6.137522376.137525646_.V.	1	chr6.137522376.137525646_.V	0.04161078	0.33727316
chr10.11334439.11336379_.V.	1	chr10.11334439.11336379_.V.	0.00128303	0.3343449
chr4.130422279.130428829_.V.	1	chr4.130422279.130428829_.V	0.04159344	0.33288593
chrX.10217608.10219668_.V.	1	chrX.10217608.10219668_.V.	0.00105868	0.33012925
chr2.137765440.137768060_.V.	1	chr2.137765440.137768060_.V	2.14E-05	0.32883584
chr4.122826159.122828559_.V.	1	chr4.122826159.122828559_.V	0.00105868	0.31889841
chr13.65994525.65997625_.V.	1	chr13.65994525.65997625_.V.	0.04381021	0.31703106
chr9.133385662.133389422_.V.	1	chr9.133385662.133389422_.V	0.04159344	0.30479574
chr6.55198826.55201756_.V.	1	chr6.55198826.55201756_.V.	0.026429	0.30381661
chr2.142639970.142642910_.V.	1	chr2.142639970.142642910_.V	0.01826556	0.30283191
chr3.30269976.30274766_.V.	1	chr3.30269976.30274766_.V.	0.026429	0.30157507
chr7.17694559.17696599_.V.	1	chr7.17694559.17696599_.V.	0.00222085	0.28905052
chr2.8410290.8416580_.V.	1	chr2.8410290.8416580_.V.	0.00693574	0.27774353
chr18.14065305.14068395_.V.	1	chr18.14065305.14068395_.V.	0.0160561	0.2775579
chr9.107744692.107748482_.V.	1	chr9.107744692.107748482_.V	0.01070637	0.27389805
chr6.56225666.56227716_.V.	1	chr6.56225666.56227716_.V.	0.02343317	0.27207396
chr12.1120907.1129651_.C.	1	chr12.1120907.1129651_.C.	0.00222085	0.26739823
chr18.51493135.51495445_.V.	1	chr18.51493135.51495445_.V.	0.00037581	0.25838689

chr14.141119109.141125409_.V.	2	chr14.141119109.141125409_.V.	0.00435484	0.56846529
chr1.7364266.7367856_.V.	2	chr1.7364266.7367856_.V.	0.02972401	0.56719267
chr1.267741236.267743686_.V.	2	chr1.267741236.267743686_.V.	6.81E-05	0.56677237
chr4.79393579.79401379_.V.	2	chr4.79393579.79401379_.V.	0.01407064	0.56643625
chr9.10001892.10004452_.V.	2	chr9.10001892.10004452_.V.	0.0160561	0.56611268
chr1.1017936.1021376_.V.	2	chr1.1017936.1021376_.V.	0.00128303	0.5655216
chr18.52395835.52398155_.V.	2	chr18.52395835.52398155_.V.	0.00046721	0.56528697
chr13.205508615.205514475_.V.	2	chr13.205508615.205514475_.V.	0.01407064	0.5645838
chr14.139000749.139004889_.V.	2	chr14.139000749.139004889_.V.	0.00313457	0.56429381
chr5.39884708.39889348_.V.	2	chr5.39884708.39889348_.V.	0.00086971	0.56235362
chr1.228205316.228208576_.V.	2	chr1.228205316.228208576_.V.	0.026429	0.5622716
chr14.141145779.141148769_.V.	2	chr14.141145779.141148769_.V.	0.03729477	0.56006256
chr18.55603325.55605505_.V.	2	chr18.55603325.55605505_.V.	0.01229347	0.55934667
chr1.1712876.1715666_.V.	2	chr1.1712876.1715666_.V.	2.14E-05	0.55875554
chr2.4377340.4383010_.V.	2	chr2.4377340.4383010_.V.	0.01070637	0.55663694
chr15.139858888.139868418_.V.	2	chr15.139858888.139868418_.V.	0.01407064	0.55636126
chr11.5415520.5417840_.V.	2	chr11.5415520.5417840_.V.	0.00105868	0.55627677
chr14.141151169.141169699_.V.	2	chr14.141151169.141169699_.V.	0.00030028	0.55607947
chr16.78837739.78842699_.V.	2	chr16.78837739.78842699_.V.	0.04161078	0.55602778
chr17.30801572.30807492_.V.	2	chr17.30801572.30807492_.V.	0.00510422	0.55549011
chr16.77740359.77743999_.V.	2	chr16.77740359.77743999_.V.	0.02071916	0.55541143
chr9.10408852.10411382_.V.	2	chr9.10408852.10411382_.V.	0.00023867	0.55472856
chr1.975216.986006_.V.	2	chr1.975216.986006_.V.	0.01229347	0.55451669
chr10.66739959.66744679_.V.	2	chr10.66739959.66744679_.V.	0.00030028	0.55410377
chr9.136285762.136289922_.V.	2	chr9.136285762.136289922_.V.	0.017307	0.55364062
chr18.52610855.52613855_.V.	2	chr18.52610855.52613855_.V.	0.04630508	0.55287415
chr1.1805526.1808086_.V.	2	chr1.1805526.1808086_.V.	0.00435484	0.55229936
chr15.135898088.135904648_.V.	2	chr15.135898088.135904648_.V.	0.00154718	0.55126624
chr1.274078796.274082926_.V.	2	chr1.274078796.274082926_.V.	0.026429	0.54881902
chr17.60443102.60447102_.V.	2	chr17.60443102.60447102_.V.	0.00071066	0.54790939
chr14.139834989.139836949_.V.	2	chr14.139834989.139836949_.V.	0.00596018	0.54730686
chr17.62489512.62496852_.V.	2	chr17.62489512.62496852_.V.	0.0160561	0.54675472
chr12.54676329.54677869_.V.	2	chr12.54676329.54677869_.V.	0.00128303	0.54600073
chr8.135203218.135209618_.V.	2	chr8.135203218.135209618_.V.	0.04161078	0.54522232
chr17.61208112.61211942_.V.	2	chr17.61208112.61211942_.V.	0.03334006	0.54459806
chr12.31277011.31277760_.C.	2	chr12.31277011.31277760_.C.	2.14E-05	0.54436095
chr16.79746909.79748689_.V.	2	chr16.79746909.79748689_.V.	0.00337904	0.5435097
chr7.57099289.57103789_.V.	2	chr7.57099289.57103789_.V.	0.00264406	0.54332043
chrX.122392378.122394028_.V.	2	chrX.122392378.122394028_.V.	0.0029137	0.54311409
chr18.53310715.53313395_.V.	2	chr18.53310715.53313395_.V.	0.00596018	0.54260916
chr14.140288349.140293009_.V.	2	chr14.140288349.140293009_.V.	0.02343317	0.54168438
chrX.89994848.89998308_.V.	2	chrX.89994848.89998308_.V.	0.00439723	0.541277
chr3.125110276.125116666_.V.	2	chr3.125110276.125116666_.V.	0.02071916	0.54118246
chr11.77160680.77173220_.V.	2	chr11.77160680.77173220_.V.	0.00596018	0.54098945
chr6.68107426.68110016_.V.	2	chr6.68107426.68110016_.V.	0.01407064	0.54018424
chr3.132151636.132158906_.V.	2	chr3.132151636.132158906_.V.	0.02972401	0.53892377
chr6.40152416.40156126_.V.	2	chr6.40152416.40156126_.V.	0.00128303	0.5380479
chr1.100288868.100289605_.C.	2	chr1.100288868.100289605_.C.	0.01826556	0.5371381
chr4.4638639.4643039_.V.	2	chr4.4638639.4643039_.V.	0.04161078	0.5363836
chr7.121486789.121489109_.V.	2	chr7.121486789.121489109_.V.	0.00086971	0.53623097
chr15.139477888.139482538_.V.	2	chr15.139477888.139482538_.V.	5.18E-05	0.53619122
chr6.65312326.65316126_.V.	2	chr6.65312326.65316126_.V.	0.00804229	0.53538947
chr1.267569346.267574476_.V.	2	chr1.267569346.267574476_.V.	0.0160561	0.53501837
chr7.112479109.112481399_.V.	2	chr7.112479109.112481399_.V.	0.00071066	0.53320711
chr8.39675128.39679828_.V.	2	chr8.39675128.39679828_.V.	0.03946843	0.53033051
chr12.53006559.53008829_.V.	2	chr12.53006559.53008829_.V.	0.00014772	0.5301089
chr3.127539586.127542766_.V.	2	chr3.127539586.127542766_.V.	0.02343317	0.52976797
chr15.138509228.138517658_.V.	2	chr15.138509228.138517658_.V.	0.01826556	0.5294264
chr14.113597569.113600209_.V.	2	chr14.113597569.113600209_.V.	0.03729477	0.5280042
chr1.460776.465756_.V.	2	chr1.460776.465756_.V.	0.03334006	0.52678546
chr14.138425199.138429809_.V.	2	chr14.138425199.138429809_.V.	8.89E-05	0.52633954
chrX.35662488.35666898_.V.	2	chrX.35662488.35666898_.V.	0.04612132	0.52593051
chr1.1528376.1534766_.V.	2	chr1.1528376.1534766_.V.	2.90E-05	0.52588287
chr14.141184479.141191799_.V.	2	chr14.141184479.141191799_.V.	2.14E-05	0.52462518
chr18.899435.902215_.V.	2	chr18.899435.902215_.V.	0.02343317	0.52445197
chr5.10811288.10814498_.V.	2	chr5.10811288.10814498_.V.	0.00030028	0.52385828
chr13.207882785.207886425_.V.	2	chr13.207882785.207886425_.V.	0.00693574	0.52385791
chr4.129795209.129797689_.V.	2	chr4.129795209.129797689_.V.	0.00086971	0.52362324
chr3.26464566.26466636_.V.	2	chr3.26464566.26466636_.V.	0.02972401	0.52304956
chr12.7934529.7938329_.V.	2	chr12.7934529.7938329_.V.	0.02343317	0.5230405
chr1.1277966.1282036_.V.	2	chr1.1277966.1282036_.V.	0.00222085	0.52265673
chr13.206033565.206037935_.V.	2	chr13.206033565.206037935_.V.	0.00023867	0.52245594
chr12.453099.460989_.V.	2	chr12.453099.460989_.V.	0.03729477	0.52151098
chr10.64015149.64023309_.V.	2	chr10.64015149.64023309_.V.	0.00057789	0.52106697
chr6.1017706.1025116_.V.	2	chr6.1017706.1025116_.V.	0.02071916	0.52069544
chr14.109581859.109584609_.V.	2	chr14.109581859.109584609_.V.	0.01826556	0.52031992
chr12.10353259.10356689_.V.	2	chr12.10353259.10356689_.V.	0.0160561	0.51985418
chr5.590338.593108_.V.	2	chr5.590338.593108_.V.	0.04630508	0.51864915
chr12.51975989.51978009_.V.	2	chr12.51975989.51978009_.V.	0.02071916	0.51766358
chr4.76143499.76145939_.V.	2	chr4.76143499.76145939_.V.	0.04161078	0.51543573
chr5.953598.957878_.V.	2	chr5.953598.957878_.V.	0.02343317	0.51528147
chr9.136622922.136626462_.V.	2	chr9.136622922.136626462_.V.	0.03361809	0.51318616
chr15.138292798.138294848_.V.	2	chr15.138292798.138294848_.V.	0.02071916	0.51289098
chr5.991728.995108_.V.	2	chr5.991728.995108_.V.	0.00011493	0.51209412
chr14.141114989.141118819_.V.	2	chr14.141114989.141118819_.V.	0.03729477	0.51194455
chr6.170242316.170248076_.V.	2	chr6.170242316.170248076_.V.	0.00441537	0.51180658
chr17.60471462.60473942_.V.	2	chr17.60471462.60473942_.V.	0.00185787	0.51058711
chr6.71017576.71021076_.V.	2	chr6.71017576.71021076_.V.	0.02486316	0.51056893
chr18.13503165.13506645_.V.	2	chr18.13503165.13506645_.V.	0.04161078	0.51021893
chr7.987719.991469_.V.	2	chr7.987719.991469_.V.	0.00222085	0.50979124
chr11.76631880.76635410_.V.	2	chr11.76631880.76635410_.V.	0.00264406	0.50844058
chr9.137555712.137557602_.V.	2	chr9.137555712.137557602_.V.	0.01407064	0.50739288
chr12.45504906.45505816_.C.	2	chr12.45504906.45505816_.C.	0.00046721	0.50684134
chr1.2414426.2422746_.V.	2	chr1.2414426.2422746_.V.	0.00037581	0.50606984
chr12.57609809.57612209_.V.	2	chr12.57609809.57612209_.V.	0.00222085	0.50591021
chr11.77808350.77813860_.V.	2	chr11.77808350.77813860_.V.	0.04161078	0.50510827
chr2.143486850.143494100_.V.	2	chr2.143486850.143494100_.V.	0.00023867	0.5036336
chr9.133306452.133308672_.V.	2	chr9.133306452.133308672_.V.	0.02071916	0.50346762
chr1.1112056.1117736_.V.	2	chr1.1112056.1117736_.V.	3.89E-05	0.50314176
chr1.427666.430856_.V.	2	chr1.427666.430856_.V.	0.00596018	0.50289053
chr12.18036976.18040754_.C.	2	chr12.18036976.18040754_.C.	0.02404913	0.50279179
chr12.42492019.42494339_.V.	2	chr12.42492019.42494339_.V.	0.0160561	0.50234863
chr6.69507746.69511246_.V.	2	chr6.69507746.69511246_.V.	0.04161078	0.50224366
chr14.140075189.140077539_.V.	2	chr14.140075189.140077539_.V.	0.00105868	0.50160351
chr1.231142443.231143232_.C.	2	chr1.231142443.231143232_.C.	0.04630508	0.50106283
chr17.46638022.46640212_.V.	2	chr17.46638022.46640212_.V.	0.00011493	0.50091352
chr11.66330220.66335220_.V.	2	chr11.66330220.66335220_.V.	0.00264406	0.4989312

chr1.217287626.217288039_.C.	2	chr1.217287626.217288039_.C.	0.00510422	0.49817951
chr16.78255669.78257569_.V.	2	chr16.78255669.78257569_.V.	0.0160561	0.49781144
chr10.66532856.66534574_.C.	2	chr10.66532856.66534574_.C.	0.00086971	0.49668221
chr1.270259836.270264736_.V.	2	chr1.270259836.270264736_.V.	0.02972401	0.49614452
chr1.270558716.270565436_.V.	2	chr1.270558716.270565436_.V.	0.00023867	0.49534936
chr5.588968.590088_.V.	2	chr5.588968.590088_.V.	0.00264406	0.49530906
chrX.35621628.35625368_.V.	2	chrX.35621628.35625368_.V.	0.03334006	0.49502348
chr7.74957429.74959919_.V.	2	chr7.74957429.74959919_.V.	0.01070637	0.49384122
chr1.96302626.96303169_.C.	2	chr1.96302626.96303169_.C.	0.026429	0.49343833
chr17.62701912.62704932_.V.	2	chr17.62701912.62704932_.V.	0.00185787	0.49273039
chr7.771569.775979_.V.	2	chr7.771569.775979_.V.	0.00370211	0.49023971
chr18.53162845.53165055_.V.	2	chr18.53162845.53165055_.V.	0.01826556	0.48997299
chr10.66695569.66700939_.V.	2	chr10.66695569.66700939_.V.	0.00071066	0.4890556
chr1.2030956.2036246_.V.	2	chr1.2030956.2036246_.V.	0.01070637	0.48871806
chr13.206030935.206033335_.V.	2	chr13.206030935.206033335_.V.	0.0160561	0.48856377
chr5.809138.813688_.V.	2	chr5.809138.813688_.V.	0.00037581	0.48813731
chr6.1409866.1413016_.V.	2	chr6.1409866.1413016_.V.	0.00154718	0.48784507
chr16.76432009.76435519_.V.	2	chr16.76432009.76435519_.V.	0.0160561	0.48744873
chr13.206853755.206856525_.V.	2	chr13.206853755.206856525_.V.	0.00264406	0.48587494
chr13.206214145.206223085_.V.	2	chr13.206214145.206223085_.V.	0.01407064	0.48573877
chr1.1268876.1273716_.V.	2	chr1.1268876.1273716_.V.	0.00596018	0.48528231
chr3.17803416.17807526_.V.	2	chr3.17803416.17807526_.V.	0.01407064	0.4831861
chr1.249876176.249878826_.V.	2	chr1.249876176.249878826_.V.	0.00804229	0.4831269
chr1.1027006.1030186_.V.	2	chr1.1027006.1030186_.V.	0.00264406	0.48289685
chr15.136299588.136302168_.V.	2	chr15.136299588.136302168_.V.	0.02972401	0.48272882
chr16.79544639.79547779_.V.	2	chr16.79544639.79547779_.V.	0.00693574	0.48184007
chr11.76898450.76905200_.V.	2	chr11.76898450.76905200_.V.	0.00023867	0.48170763
chr11.2467950.2471110_.V.	2	chr11.2467950.2471110_.V.	0.00037581	0.48079874
chr6.1563686.1569256_.V.	2	chr6.1563686.1569256_.V.	0.00264406	0.4806649
chr5.1238268.1243888_.V.	2	chr5.1238268.1243888_.V.	0.01407064	0.47944673
chr14.140407549.140410609_.V.	2	chr14.140407549.140410609_.V.	0.00185787	0.47939509
chr8.3279248.3282578_.V.	2	chr8.3279248.3282578_.V.	0.00105868	0.47916678
chr15.139284398.139289478_.V.	2	chr15.139284398.139289478_.V.	6.81E-05	0.47901899
chr13.207836074.207837600_.C.	2	chr13.207836074.207837600_.C.	0.00804229	0.47893742
chr16.79653319.79662869_.V.	2	chr16.79653319.79662869_.V.	0.02071916	0.47806511
chr5.3395198.3407528_.V.	2	chr5.3395198.3407528_.V.	0.04630508	0.4770394
chr9.139070772.139077652_.V.	2	chr9.139070772.139077652_.V.	0.00313457	0.47658184
chr6.169777346.169781966_.V.	2	chr6.169777346.169781966_.V.	0.01407064	0.4764615
chr5.902058.909388_.V.	2	chr5.902058.909388_.V.	0.00313457	0.47632295
chr9.134787102.134794662_.V.	2	chr9.134787102.134794662_.V.	0.02037355	0.4761884
chr5.1412758.1419198_.V.	2	chr5.1412758.1419198_.V.	0.02972401	0.4757619
chr14.109576279.109579779_.V.	2	chr14.109576279.109579779_.V.	0.01070637	0.47434992
chr6.48150486.48153526_.V.	2	chr6.48150486.48153526_.V.	0.02972401	0.4740234
chr2.1534830.1536310_.V.	2	chr2.1534830.1536310_.V.	0.00264406	0.47401726
chr17.46566172.46571562_.V.	2	chr17.46566172.46571562_.V.	0.00086971	0.47288897
chr1.933856.938486_.V.	2	chr1.933856.938486_.V.	0.00435484	0.47162705
chr15.137259558.137263238_.V.	2	chr15.137259558.137263238_.V.	0.00046721	0.47148056
chr9.138855342.138860072_.V.	2	chr9.138855342.138860072_.V.	0.00037581	0.47064887
chr5.3908018.3915028_.V.	2	chr5.3908018.3915028_.V.	0.00037581	0.47025436
chr18.49181915.49186015_.V.	2	chr18.49181915.49186015_.V.	0.02972401	0.4696046
chr18.1185005.1195565_.V.	2	chr18.1185005.1195565_.V.	0.00037581	0.4694849
chr11.76880640.76886850_.V.	2	chr11.76880640.76886850_.V.	0.01407064	0.46899752
chr12.50736832.50738032_.C.	2	chr12.50736832.50738032_.C.	0.026429	0.46778608
chr16.79064329.79067429_.V.	2	chr16.79064329.79067429_.V.	0.02343317	0.46699627
chr1.266084036.266090246_.V.	2	chr1.266084036.266090246_.V.	0.04161078	0.46674509
chr16.79552959.79554459_.V.	2	chr16.79552959.79554459_.V.	0.00435484	0.46491533
chr9.135169882.135172302_.V.	2	chr9.135169882.135172302_.V.	0.00030028	0.46384569
chr6.53428926.53431616_.V.	2	chr6.53428926.53431616_.V.	0.04604676	0.46317524
chr9.7690052.7697432_.V.	2	chr9.7690052.7697432_.V.	2.90E-05	0.46265587
chr12.40489799.40494329_.V.	2	chr12.40489799.40494329_.V.	0.00596018	0.46232537
chr9.3473252.3475242_.V.	2	chr9.3473252.3475242_.V.	0.01229347	0.46125878
chr12.57766699.57770849_.V.	2	chr12.57766699.57770849_.V.	0.00596018	0.46062082
chr12.8447539.8451929_.V.	2	chr12.8447539.8451929_.V.	0.00435484	0.45989336
chr12.59454279.59457869_.V.	2	chr12.59454279.59457869_.V.	0.00014772	0.45950432
chr9.138319262.138322222_.V.	2	chr9.138319262.138322222_.V.	0.00804229	0.45896752
chr5.39771978.39802708_.V.	2	chr5.39771978.39802708_.V.	0.03729477	0.45857115
chr11.2606080.2608500_.V.	2	chr11.2606080.2608500_.V.	0.00014772	0.45803768
chr9.10752072.10753202_.V.	2	chr9.10752072.10753202_.V.	0.02343317	0.45548206
chr13.200641940.200643216_.C.	2	chr13.200641940.200643216_.C.	0.00693574	0.45434333
chr11.77568280.77572640_.V.	2	chr11.77568280.77572640_.V.	0.01407064	0.4531319
chr17.46302122.46309462_.V.	2	chr17.46302122.46309462_.V.	0.00370211	0.45264016
chr9.139254192.139257322_.V.	2	chr9.139254192.139257322_.V.	0.04161078	0.45263397
chr2.130230.133660_.V.	2	chr2.130230.133660_.V.	0.01407064	0.45243927
chrX.121948228.121951038_.V.	2	chrX.121948228.121951038_.V.	0.00264406	0.45160952
chr10.58075410.58076488_.C.	2	chr10.58075410.58076488_.C.	0.02972401	0.45096336
chr12.59899359.59903719_.V.	2	chr12.59899359.59903719_.V.	0.00929494	0.44996929
chr16.77099889.77104129_.V.	2	chr16.77099889.77104129_.V.	0.00037581	0.44974775
chr9.139341082.139344902_.V.	2	chr9.139341082.139344902_.V.	0.00929494	0.44914755
chr10.63368609.63375269_.V.	2	chr10.63368609.63375269_.V.	0.00086971	0.44476107
chr16.79537609.79543449_.V.	2	chr16.79537609.79543449_.V.	0.00011493	0.4443883
chr13.203618615.203628075_.V.	2	chr13.203618615.203628075_.V.	0.00071066	0.44415676
chr14.138986709.138990689_.V.	2	chr14.138986709.138990689_.V.	0.026429	0.44411583
chr2.151555380.151563830_.V.	2	chr2.151555380.151563830_.V.	0.04161078	0.44308183
chr11.16514780.16518890_.V.	2	chr11.16514780.16518890_.V.	0.04630508	0.4423573
chr12.57589829.57593429_.V.	2	chr12.57589829.57593429_.V.	0.00596018	0.44071134
chr2.1030190.1033250_.V.	2	chr2.1030190.1033250_.V.	0.02972401	0.44040687
chr1.2504186.2516876_.V.	2	chr1.2504186.2516876_.V.	0.00086971	0.43971353
chr11.76463940.76465870_.V.	2	chr11.76463940.76465870_.V.	0.00264406	0.43947603
chr11.845120.847250_.V.	2	chr11.845120.847250_.V.	0.03729477	0.43919321
chr13.203800275.203802845_.V.	2	chr13.203800275.203802845_.V.	0.00804229	0.43919064
chr18.2191015.2194725_.V.	2	chr18.2191015.2194725_.V.	0.00804229	0.43916119
chr13.205687055.205689635_.V.	2	chr13.205687055.205689635_.V.	0.0160561	0.43904612
chr13.206831545.206840435_.V.	2	chr13.206831545.206840435_.V.	0.03334006	0.43793242
chr13.208227875.208231895_.V.	2	chr13.208227875.208231895_.V.	0.00046721	0.43685897
chr1.1283336.1285656_.V.	2	chr1.1283336.1285656_.V.	0.00264406	0.43526845
chr5.4656128.4660808_.V.	2	chr5.4656128.4660808_.V.	0.01070637	0.43454676
chr1.1905726.1911696_.V.	2	chr1.1905726.1911696_.V.	0.00018834	0.43399778
chr1.2423266.2427186_.V.	2	chr1.2423266.2427186_.V.	0.00154718	0.43279851
chr9.139078692.139082692_.V.	2	chr9.139078692.139082692_.V.	0.00185787	0.43235319
chr15.138150828.138155938_.V.	2	chr15.138150828.138155938_.V.	0.00804229	0.43221271
chr7.896039.903859_.V.	2	chr7.896039.903859_.V.	0.00057789	0.43210588
chr7.117463509.117468069_.V.	2	chr7.117463509.117468069_.V.	0.00804229	0.43205534
chr13.206519035.206525405_.V.	2	chr13.206519035.206525405_.V.	0.026429	0.43079764
chr3.1066576.1070166_.V.	2	chr3.1066576.1070166_.V.	0.00030028	0.43071738
chr4.104218519.104225309_.V.	2	chr4.104218519.104225309_.V.	0.00804229	0.43040061
chr7.55363709.55366089_.V.	2	chr7.55363709.55366089_.V.	0.00071066	0.42975401
chr15.137398238.137401498_.V.	2	chr15.137398238.137401498_.V.	0.00105868	0.42965389

chr4.7275539.7280759_.V.	2	chr4.7275539.7280759_.V.	0.00510422	0.42933059
chr6.67759306.67762556_.V.	2	chr6.67759306.67762556_.V.	0.00023867	0.42931402
chr11.78179830.78183890_.V.	2	chr11.78179830.78183890_.V.	0.00929494	0.42928764
chr1.1444436.1454966_.V.	2	chr1.1444436.1454966_.V.	0.00154718	0.42913162
chr16.75575719.75578049_.V.	2	chr16.75575719.75578049_.V.	0.00313457	0.42841241
chr14.138275659.138279459_.V.	2	chr14.138275659.138279459_.V.	0.02071916	0.42782383
chr7.739009.745339_.V.	2	chr7.739009.745339_.V.	0.026429	0.42698932
chr3.25495726.25499196_.V.	2	chr3.25495726.25499196_.V.	0.01407064	0.42694329
chr17.55527122.55531472_.V.	2	chr17.55527122.55531472_.V.	0.00313457	0.42671292
chr8.129867778.129871438_.V.	2	chr8.129867778.129871438_.V.	0.00313457	0.42663397
chr4.12230849.12234179_.V.	2	chr4.12230849.12234179_.V.	0.00057789	0.42554375
chr18.50125275.50128495_.V.	2	chr18.50125275.50128495_.V.	0.00215222	0.42518266
chr13.196955955.196959185_.V.	2	chr13.196955955.196959185_.V.	0.00804229	0.42472019
chr14.141133839.141138489_.V.	2	chr14.141133839.141138489_.V.	0.04161078	0.42422265
chr15.138676278.138679518_.V.	2	chr15.138676278.138679518_.V.	0.04161078	0.42421465
chr12.34584909.34591689_.V.	2	chr12.34584909.34591689_.V.	0.00018834	0.42325957
chr2.6895080.6899700_.V.	2	chr2.6895080.6899700_.V.	0.00370211	0.42305711
chr12.18875419.18877149_.V.	2	chr12.18875419.18877149_.V.	0.00128303	0.42275402
chr1.9350656.9354186_.V.	2	chr1.9350656.9354186_.V.	0.03334006	0.42231599
chr16.79511609.79518859_.V.	2	chr16.79511609.79518859_.V.	0.00313457	0.4222298
chr16.74548109.74550049_.V.	2	chr16.74548109.74550049_.V.	8.89E-05	0.422052
chr16.79495219.79497289_.V.	2	chr16.79495219.79497289_.V.	0.00313457	0.42055136
chr15.137290218.137298818_.V.	2	chr15.137290218.137298818_.V.	0.00128303	0.41950195
chr7.49445909.49448319_.V.	2	chr7.49445909.49448319_.V.	0.01407064	0.41869289
chr10.9861359.9866989_.V.	2	chr10.9861359.9866989_.V.	0.00510422	0.41837935
chr11.77835410.77839800_.V.	2	chr11.77835410.77839800_.V.	0.00046721	0.41762711
chr13.202561255.202564075_.V.	2	chr13.202561255.202564075_.V.	0.00510422	0.41743237
chr9.138424712.138428072_.V.	2	chr9.138424712.138428072_.V.	0.00037581	0.41719982
chr1.1457826.1464396_.V.	2	chr1.1457826.1464396_.V.	0.00018834	0.41669193
chr16.75617129.75620139_.V.	2	chr16.75617129.75620139_.V.	0.0160561	0.41628192
chr7.3064599.3070619_.V.	2	chr7.3064599.3070619_.V.	0.03334006	0.41576571
chr13.206403635.206406165_.V.	2	chr13.206403635.206406165_.V.	0.00804229	0.41525413
chr1.1262166.1268546_.V.	2	chr1.1262166.1268546_.V.	0.01826556	0.41505732
chr10.66317839.66322719_.V.	2	chr10.66317839.66322719_.V.	0.0160561	0.41468343
chr4.782159.793879_.V.	2	chr4.782159.793879_.V.	0.00929494	0.41289312
chr1.149625936.149627336_.V.	2	chr1.149625936.149627336_.V.	0.00510422	0.41260461
chr13.206018845.206024715_.V.	2	chr13.206018845.206024715_.V.	0.02071916	0.4123329
chr5.66303788.66315038_.V.	2	chr5.66303788.66315038_.V.	5.18E-05	0.4122466
chr1.187726376.187728896_.V.	2	chr1.187726376.187728896_.V.	0.00510422	0.41189792
chr3.131724226.131726126_.V.	2	chr3.131724226.131726126_.V.	0.00510422	0.4118156
chr14.137241859.137244129_.V.	2	chr14.137241859.137244129_.V.	0.00222085	0.41142597
chr8.2189548.2191558_.V.	2	chr8.2189548.2191558_.V.	0.0045148	0.41130663
chr9.137768342.137770952_.V.	2	chr9.137768342.137770952_.V.	0.02972401	0.41060147
chr12.2560207.2562838_.C.	2	chr12.2560207.2562838_.C.	0.0160561	0.41045303
chr13.206428405.206434395_.V.	2	chr13.206428405.206434395_.V.	0.00030028	0.41019036
chr17.58667572.58669912_.V.	2	chr17.58667572.58669912_.V.	0.00435484	0.40994483
chr2.9195750.9198850_.V.	2	chr2.9195750.9198850_.V.	0.00264406	0.4093364
chr18.1691365.1694665_.V.	2	chr18.1691365.1694665_.V.	0.00018834	0.40909298
chr16.79436079.79447039_.V.	2	chr16.79436079.79447039_.V.	0.01229347	0.40802827
chr15.127185678.127188448_.V.	2	chr15.127185678.127188448_.V.	0.02972401	0.4080199
chr7.120897229.120900569_.V.	2	chr7.120897229.120900569_.V.	0.00071066	0.4077604
chr15.133473548.133476628_.V.	2	chr15.133473548.133476628_.V.	8.89E-05	0.40688045
chr1.4939266.4942916_.V.	2	chr1.4939266.4942916_.V.	0.02071916	0.40648909
chr7.2489439.2492099_.V.	2	chr7.2489439.2492099_.V.	0.02071916	0.4063178
chr2.9293700.9298410_.V.	2	chr2.9293700.9298410_.V.	0.00071066	0.40559646
chr7.2669839.2672659_.V.	2	chr7.2669839.2672659_.V.	0.00046721	0.4054317
chr6.66883866.66887526_.V.	2	chr6.66883866.66887526_.V.	5.18E-05	0.40538268
chr1.147683576.147685686_.V.	2	chr1.147683576.147685686_.V.	0.00105868	0.40537491
chr10.66891569.66904269_.V.	2	chr10.66891569.66904269_.V.	0.02343317	0.40461496
chr13.205667665.205671515_.V.	2	chr13.205667665.205671515_.V.	0.00037581	0.404403
chr13.208182365.208185485_.V.	2	chr13.208182365.208185485_.V.	0.04161078	0.40429904
chr11.6410690.6413660_.V.	2	chr11.6410690.6413660_.V.	0.00804229	0.40428444
chr13.34088194.34089168_.C.	2	chr13.34088194.34089168_.C.	0.04161078	0.40374023
chr2.143041670.143043540_.V.	2	chr2.143041670.143043540_.V.	0.026429	0.40334454
chr13.203619487.203620128_.C.	2	chr13.203619487.203620128_.C.	0.0160561	0.40259307
chr14.137108879.137114009_.V.	2	chr14.137108879.137114009_.V.	0.03334006	0.40257483
chr3.22414746.22421516_.V.	2	chr3.22414746.22421516_.V.	0.00222085	0.40177578
chr12.15360359.15364249_.V.	2	chr12.15360359.15364249_.V.	0.02972401	0.40017281
chr11.1559030.1562300_.V.	2	chr11.1559030.1562300_.V.	0.02343317	0.40009462
chr9.134571202.134575572_.V.	2	chr9.134571202.134575572_.V.	0.00804229	0.39930419
chr2.150981810.150986810_.V.	2	chr2.150981810.150986810_.V.	0.01229347	0.39873189
chr18.3880605.3883325_.V.	2	chr18.3880605.3883325_.V.	0.00023867	0.39868012
chr7.1727489.1732119_.V.	2	chr7.1727489.1732119_.V.	0.02343317	0.39848011
chr7.654719.658879_.V.	2	chr7.654719.658879_.V.	2.14E-05	0.39774609
chr14.140273699.140278569_.V.	2	chr14.140273699.140278569_.V.	0.00370211	0.39706852
chr11.78901410.78910190_.V.	2	chr11.78901410.78910190_.V.	0.00264406	0.3968574
chr9.25422002.25424452_.V.	2	chr9.25422002.25424452_.V.	0.04630508	0.39671504
chr7.3528039.3537739_.V.	2	chr7.3528039.3537739_.V.	3.89E-05	0.39636241
chr2.1016580.1019990_.V.	2	chr2.1016580.1019990_.V.	0.01229347	0.39591456
chr18.49977355.49983615_.V.	2	chr18.49977355.49983615_.V.	0.03334006	0.39557096
chr18.2415625.2424205_.V.	2	chr18.2415625.2424205_.V.	0.00313457	0.39546576
chr9.41102112.41107602_.V.	2	chr9.41102112.41107602_.V.	0.01885471	0.39473646
chr1.1474166.1483956_.V.	2	chr1.1474166.1483956_.V.	0.00435484	0.39433058
chr10.12941809.12947419_.V.	2	chr10.12941809.12947419_.V.	0.00037581	0.39420404
chr10.66760219.66770069_.V.	2	chr10.66760219.66770069_.V.	0.00435484	0.39401918
chr1.1201636.1207326_.V.	2	chr1.1201636.1207326_.V.	0.02071916	0.39347887
chr12.20957709.20961019_.V.	2	chr12.20957709.20961019_.V.	0.00046721	0.39304067
chr13.131327565.131331315_.V.	2	chr13.131327565.131331315_.V.	0.00510422	0.392567
chr15.135905038.135911398_.V.	2	chr15.135905038.135911398_.V.	0.00929494	0.39195999
chr13.206045005.206050015_.V.	2	chr13.206045005.206050015_.V.	0.03729477	0.39155619
chr13.208115935.208121455_.V.	2	chr13.208115935.208121455_.V.	0.00435484	0.39062899
chr6.33740746.33747206_.V.	2	chr6.33740746.33747206_.V.	0.02972401	0.39039019
chr1.825426.843926_.V.	2	chr1.825426.843926_.V.	0.03729477	0.39035163
chr15.138715498.138719278_.V.	2	chr15.138715498.138719278_.V.	0.01229347	0.39010451
chr10.24069239.24076199_.V.	2	chr10.24069239.24076199_.V.	0.04630508	0.38989418
chr12.3434269.3437599_.V.	2	chr12.3434269.3437599_.V.	0.00046721	0.38956664
chr13.131343175.131346375_.V.	2	chr13.131343175.131346375_.V.	0.026429	0.38953514
chr11.75991550.75995620_.V.	2	chr11.75991550.75995620_.V.	0.00313457	0.38789047
chr14.140755239.140764049_.V.	2	chr14.140755239.140764049_.V.	0.00105868	0.38770639
chr11.7214710.7217740_.V.	2	chr11.7214710.7217740_.V.	0.0160561	0.38716261
chr10.66740987.66741547_.C.	2	chr10.66740987.66741547_.C.	0.00105868	0.38705686
chr15.137538308.137541858_.V.	2	chr15.137538308.137541858_.V.	6.81E-05	0.38688664
chr16.79604119.79606079_.V.	2	chr16.79604119.79606079_.V.	0.02071916	0.38679623
chr6.1505426.1510766_.V.	2	chr6.1505426.1510766_.V.	0.04161078	0.38641533
chr13.206690975.206698715_.V.	2	chr13.206690975.206698715_.V.	0.03729477	0.38593347
chr17.59125342.59128692_.V.	2	chr17.59125342.59128692_.V.	0.00046721	0.385891
chr3.131714146.131717146_.V.	2	chr3.131714146.131717146_.V.	0.04630508	0.38585009

chr15.139705558.139712278_.V.	2	chr15.139705558.139712278_.V.	0.00014772	0.38568522
chr3.114933746.114935236_.V.	2	chr3.114933746.114935236_.V.	0.02343317	0.38507198
chr14.138102289.138110389_.V.	2	chr14.138102289.138110389_.V.	0.00313457	0.38494163
chr1.804766.814136_.V.	2	chr1.804766.814136_.V.	0.00929494	0.38489038
chr11.78912650.78918030_.V.	2	chr11.78912650.78918030_.V.	0.00510422	0.38471242
chr7.121706089.121709769_.V.	2	chr7.121706089.121709769_.V.	0.00435484	0.38379781
chr12.7561259.7563859_.V.	2	chr12.7561259.7563859_.V.	0.00435484	0.38359996
chr3.34564836.34572766_.V.	2	chr3.34564836.34572766_.V.	0.00018834	0.38251458
chr18.1936845.1942035_.V.	2	chr18.1936845.1942035_.V.	0.026429	0.38161652
chr15.136720368.136729028_.V.	2	chr15.136720368.136729028_.V.	0.00264406	0.37986907
chr14.138088909.138095629_.V.	2	chr14.138088909.138095629_.V.	0.02071916	0.37956487
chr6.66859326.66863106_.V.	2	chr6.66859326.66863106_.V.	0.00313457	0.37948596
chr11.5844260.5847130_.V.	2	chr11.5844260.5847130_.V.	0.03729477	0.37869927
chr5.1324428.1331418_.V.	2	chr5.1324428.1331418_.V.	0.00510422	0.37840865
chr12.5432569.5435759_.V.	2	chr12.5432569.5435759_.V.	0.00128303	0.37757036
chr17.60929292.60934082_.V.	2	chr17.60929292.60934082_.V.	0.01826556	0.37754806
chr7.1736929.1740429_.V.	2	chr7.1736929.1740429_.V.	0.02137061	0.37750196
chr6.41320436.41326606_.V.	2	chr6.41320436.41326606_.V.	0.03729477	0.37742284
chr15.136304968.136307978_.V.	2	chr15.136304968.136307978_.V.	0.00929494	0.37737739
chr1.1237976.1243796_.V.	2	chr1.1237976.1243796_.V.	0.00435484	0.37734
chr10.68757659.68760869_.V.	2	chr10.68757659.68760869_.V.	0.04630508	0.37654782
chr13.205782805.205785835_.V.	2	chr13.205782805.205785835_.V.	0.00011493	0.37573611
chr16.79898299.79904919_.V.	2	chr16.79898299.79904919_.V.	0.00693574	0.37550253
chr10.66461799.66469199_.V.	2	chr10.66461799.66469199_.V.	0.00370211	0.37539682
chr16.73950759.73954099_.V.	2	chr16.73950759.73954099_.V.	0.01229347	0.37494711
chrX.6047358.6051308_.V.	2	chrX.6047358.6051308_.V.	0.00071066	0.37485715
chr18.1082315.1084085_.V.	2	chr18.1082315.1084085_.V.	0.00510422	0.3746286
chr18.1138935.1142095_.V.	2	chr18.1138935.1142095_.V.	0.03334006	0.37414426
chr6.40231656.40233506_.V.	2	chr6.40231656.40233506_.V.	0.02486316	0.37345699
chr4.128568059.128570239_.V.	2	chr4.128568059.128570239_.V.	0.02071916	0.37343969
chr17.60849952.60853722_.V.	2	chr17.60849952.60853722_.V.	0.026429	0.37328918
chrX.120778.124128_.V.	2	chrX.120778.124128_.V.	0.01407064	0.37316171
chr4.1476009.1485859_.V.	2	chr4.1476009.1485859_.V.	0.01070637	0.37293576
chr10.56527899.56531299_.V.	2	chr10.56527899.56531299_.V.	0.04630508	0.37275808
chr7.1028279.1030329_.V.	2	chr7.1028279.1030329_.V.	0.00154718	0.37164451
chr1.270552826.270555986_.V.	2	chr1.270552826.270555986_.V.	0.00222085	0.3710881
chr18.55614125.55615445_.V.	2	chr18.55614125.55615445_.V.	0.00057789	0.37070809
chr12.56278059.56280549_.V.	2	chr12.56278059.56280549_.V.	6.81E-05	0.36941762
chr9.138614132.138616202_.V.	2	chr9.138614132.138616202_.V.	0.00370211	0.3691465
chr10.66882359.66885469_.V.	2	chr10.66882359.66885469_.V.	0.00370211	0.3685992
chr14.138075609.138081359_.V.	2	chr14.138075609.138081359_.V.	0.00071066	0.36724736
chr6.79866836.79874666_.V.	2	chr6.79866836.79874666_.V.	0.026429	0.36706461
chr2.2449990.2453940_.V.	2	chr2.2449990.2453940_.V.	0.00510422	0.366279
chr1.148077526.148080116_.V.	2	chr1.148077526.148080116_.V.	0.00086971	0.36530431
chr8.11108868.11113758_.V.	2	chr8.11108868.11113758_.V.	0.00046721	0.36525824
chrX.111740818.111745788_.V.	2	chrX.111740818.111745788_.V.	0.00929494	0.36462701
chr12.509229.513189_.V.	2	chr12.509229.513189_.V.	0.02071916	0.36452297
chr11.811360.813820_.V.	2	chr11.811360.813820_.V.	0.02071916	0.36319696
chr13.205896555.205903535_.V.	2	chr13.205896555.205903535_.V.	0.00596018	0.36301778
chr10.24047609.24053319_.V.	2	chr10.24047609.24053319_.V.	0.00370211	0.36299258
chr16.78603069.78605119_.V.	2	chr16.78603069.78605119_.V.	0.00071066	0.36249923
chr2.2743030.2750430_.V.	2	chr2.2743030.2750430_.V.	0.00596018	0.36245492
chr17.58657742.58667192_.V.	2	chr17.58657742.58667192_.V.	0.00105868	0.36185132
chr14.141458889.141460269_.V.	2	chr14.141458889.141460269_.V.	0.02343317	0.36184579
chr18.1237085.1239895_.V.	2	chr18.1237085.1239895_.V.	0.03334006	0.36155842
chr4.72945129.72947739_.V.	2	chr4.72945129.72947739_.V.	0.02343317	0.36149733
chrX.122073748.122076198_.V.	2	chrX.122073748.122076198_.V.	0.00313457	0.36050899
chr11.78253260.78255430_.V.	2	chr11.78253260.78255430_.V.	0.026429	0.35929452
chr9.134830922.134834712_.V.	2	chr9.134830922.134834712_.V.	0.00446757	0.35868074
chr9.137104532.137107162_.V.	2	chr9.137104532.137107162_.V.	0.04630508	0.35810353
chr6.51276966.51282366_.V.	2	chr6.51276966.51282366_.V.	0.01070637	0.35806113
chr6.170187216.170189606_.V.	2	chr6.170187216.170189606_.V.	0.01070637	0.3579996
chr6.54761096.54766246_.V.	2	chr6.54761096.54766246_.V.	0.02343317	0.35774835
chr6.1783616.1785936_.V.	2	chr6.1783616.1785936_.V.	0.03334006	0.3576321
chr8.135663648.135670928_.V.	2	chr8.135663648.135670928_.V.	0.00435484	0.35740402
chr14.139346949.139351589_.V.	2	chr14.139346949.139351589_.V.	0.00435484	0.35693638
chr15.139380268.139387828_.V.	2	chr15.139380268.139387828_.V.	6.81E-05	0.3561817
chr3.127889316.127891806_.V.	2	chr3.127889316.127891806_.V.	5.18E-05	0.35458992
chr12.2095212.2099139_.C.	2	chr12.2095212.2099139_.C.	0.00018834	0.35458535
chr16.78148089.78155199_.V.	2	chr16.78148089.78155199_.V.	0.00370211	0.35440291
chr8.76308.78318_.V.	2	chr8.76308.78318_.V.	0.00370211	0.35432587
chr5.626048.631888_.V.	2	chr5.626048.631888_.V.	0.00264406	0.3538141
chr14.140466979.140475139_.V.	2	chr14.140466979.140475139_.V.	0.00222085	0.35315553
chr14.140817589.140835029_.V.	2	chr14.140817589.140835029_.V.	0.00222085	0.35274892
chr16.79037029.79040119_.V.	2	chr16.79037029.79040119_.V.	8.00E-06	0.35270089
chr16.75648289.75652069_.V.	2	chr16.75648289.75652069_.V.	0.00086971	0.35247192
chr15.135078578.135083508_.V.	2	chr15.135078578.135083508_.V.	0.00086971	0.35221472
chr12.58732849.58735679_.V.	2	chr12.58732849.58735679_.V.	0.00435484	0.35220883
chr10.23813989.23817179_.V.	2	chr10.23813989.23817179_.V.	0.00313457	0.35202447
chr18.1071575.1077335_.V.	2	chr18.1071575.1077335_.V.	0.02343317	0.35139895
chr11.77154080.77160330_.V.	2	chr11.77154080.77160330_.V.	0.02071916	0.35103129
chr2.5070700.5072860_.V.	2	chr2.5070700.5072860_.V.	0.00023867	0.34971228
chr9.138932632.138938622_.V.	2	chr9.138932632.138938622_.V.	0.00435484	0.3496949
chr2.143317870.143325360_.V.	2	chr2.143317870.143325360_.V.	0.00435484	0.34957217
chr3.3231956.3244946_.V.	2	chr3.3231956.3244946_.V.	0.00086971	0.34943262
chr9.136707472.136712522_.V.	2	chr9.136707472.136712522_.V.	0.00030028	0.34806256
chr11.73941850.73946820_.V.	2	chr11.73941850.73946820_.V.	0.00804229	0.34779209
chr9.136471012.136473532_.V.	2	chr9.136471012.136473532_.V.	0.00071066	0.34681354
chr8.11288408.11292708_.V.	2	chr8.11288408.11292708_.V.	0.00222085	0.34656557
chrX.6057518.6067148_.V.	2	chrX.6057518.6067148_.V.	6.81E-05	0.3457608
chr13.204802515.204805145_.V.	2	chr13.204802515.204805145_.V.	0.00596018	0.34545511
chr17.60364542.60368132_.V.	2	chr17.60364542.60368132_.V.	0.00046721	0.34495957
chr18.3331635.3335665_.V.	2	chr18.3331635.3335665_.V.	0.00105868	0.34490871
chr12.15396499.15400959_.V.	2	chr12.15396499.15400959_.V.	0.00071066	0.34470431
chr5.1560988.1565728_.V.	2	chr5.1560988.1565728_.V.	0.01229347	0.34381377
chr13.207369155.207370835_.V.	2	chr13.207369155.207370835_.V.	0.00185787	0.34365186
chr10.64038209.64045349_.V.	2	chr10.64038209.64045349_.V.	0.02972401	0.34344942
chr12.56728579.56731699_.V.	2	chr12.56728579.56731699_.V.	0.00693574	0.34342354
chr7.1618809.1622759_.V.	2	chr7.1618809.1622759_.V.	0.03729477	0.3433779
chr13.204314425.204317845_.V.	2	chr13.204314425.204317845_.V.	6.81E-05	0.34336703
chr3.131304376.131310136_.V.	2	chr3.131304376.131310136_.V.	0.00023867	0.34199998
chr15.138476328.138484028_.V.	2	chr15.138476328.138484028_.V.	0.00596018	0.34156662
chr1.272559146.272562386_.V.	2	chr1.272559146.272562386_.V.	0.00046721	0.34154717
chr5.4826248.4832618_.V.	2	chr5.4826248.4832618_.V.	0.00435484	0.34149315
chr13.206885775.206888865_.V.	2	chr13.206885775.206888865_.V.	0.01070637	0.34139877
chr18.2154505.2156285_.V.	2	chr18.2154505.2156285_.V.	0.02343317	0.34098194
chr7.9189169.9194929_.V.	2	chr7.9189169.9194929_.V.	0.00507745	0.34054433

chr17.30109252.30112132_.V.	2	chr17.30109252.30112132_.V.	0.00693574	0.34036161
chr18.410315.414525_.V.	2	chr18.410315.414525_.V.	0.02071916	0.34008411
chr17.36232622.36235622_.V.	2	chr17.36232622.36235622_.V.	0.00046721	0.33995379
chr2.150286910.150293060_.V.	2	chr2.150286910.150293060_.V.	0.00086971	0.33984734
chr11.68334910.68338470_.V.	2	chr11.68334910.68338470_.V.	0.00105868	0.33935455
chr6.80503476.80507176_.V.	2	chr6.80503476.80507176_.V.	0.00057789	0.3390701
chr7.3052479.3057429_.V.	2	chr7.3052479.3057429_.V.	0.01229347	0.33901233
chr15.71705338.71707328_.V.	2	chr15.71705338.71707328_.V.	0.00435484	0.33893367
chr12.8281079.8284709_.V.	2	chr12.8281079.8284709_.V.	0.00018834	0.33885328
chr9.133422932.133427412_.V.	2	chr9.133422932.133427412_.V.	0.04630508	0.33878185
chr11.1606840.1609800_.V.	2	chr11.1606840.1609800_.V.	0.00222085	0.33796031
chr9.25375202.25380322_.V.	2	chr9.25375202.25380322_.V.	0.00086971	0.33768779
chr15.135013718.135019108_.V.	2	chr15.135013718.135019108_.V.	0.03334006	0.33707773
chr12.56539319.56542159_.V.	2	chr12.56539319.56542159_.V.	0.00264406	0.33705752
chr1.269901146.269907206_.V.	2	chr1.269901146.269907206_.V.	0.00011493	0.33703202
chr11.826930.828910_.V.	2	chr11.826930.828910_.V.	0.04630508	0.33691988
chr12.27097716.27099057_.C.	2	chr12.27097716.27099057_.C.	0.026429	0.33635999
chr17.30237192.30243162_.V.	2	chr17.30237192.30243162_.V.	0.04630508	0.33588551
chr5.814468.818758_.V.	2	chr5.814468.818758_.V.	0.04161078	0.33587453
chr14.28107019.28115119_.V.	2	chr14.28107019.28115119_.V.	0.00128303	0.33538803
chr4.12235139.12242019_.V.	2	chr4.12235139.12242019_.V.	0.0160561	0.33527049
chr6.168524156.168530576_.V.	2	chr6.168524156.168530576_.V.	0.00596018	0.33514373
chr5.10586208.10589518_.V.	2	chr5.10586208.10589518_.V.	0.02343317	0.33505767
chr8.54586638.54587668_.V.	2	chr8.54586638.54587668_.V.	0.01319203	0.33486828
chr11.1661120.1663910_.V.	2	chr11.1661120.1663910_.V.	0.0160561	0.33338465
chr5.40197598.40200348_.V.	2	chr5.40197598.40200348_.V.	0.02071916	0.33312482
chr18.48737495.48741355_.V.	2	chr18.48737495.48741355_.V.	0.01407064	0.33295487
chr15.138862548.138866848_.V.	2	chr15.138862548.138866848_.V.	0.0160561	0.33129171
chr15.138240648.138243538_.V.	2	chr15.138240648.138243538_.V.	0.04161078	0.33122277
chr2.6526990.6534360_.V.	2	chr2.6526990.6534360_.V.	0.03334006	0.33027619
chr14.136293439.136297589_.V.	2	chr14.136293439.136297589_.V.	0.01229347	0.33009934
chr14.139120929.139123949_.V.	2	chr14.139120929.139123949_.V.	0.04630508	0.32967956
chr6.88049896.88059876_.V.	2	chr6.88049896.88059876_.V.	0.00596018	0.32934807
chrX.124234838.124236418_.V.	2	chrX.124234838.124236418_.V.	0.03355544	0.32901079
chr2.151552720.151555320_.V.	2	chr2.151552720.151555320_.V.	0.026429	0.32883903
chr9.138909332.138913472_.V.	2	chr9.138909332.138913472_.V.	0.0160561	0.32823008
chr17.57662942.57667932_.V.	2	chr17.57662942.57667932_.V.	0.02071916	0.32820502
chr18.3151255.3153755_.V.	2	chr18.3151255.3153755_.V.	0.00435484	0.3277411
chr17.60866642.60878492_.V.	2	chr17.60866642.60878492_.V.	0.03334006	0.32742911
chr18.857695.860645_.V.	2	chr18.857695.860645_.V.	0.00018834	0.32673336
chr14.132330909.132335069_.V.	2	chr14.132330909.132335069_.V.	0.03334006	0.3259163
chr13.206205555.206209275_.V.	2	chr13.206205555.206209275_.V.	0.00929494	0.32583135
chr5.2580368.2583668_.V.	2	chr5.2580368.2583668_.V.	0.00693574	0.32474608
chr6.82550096.82554946_.V.	2	chr6.82550096.82554946_.V.	0.00154718	0.32443482
chr1.1530866.1535259_.C.	2	chr1.1530866.1535259_.C.	0.00071066	0.32400201
chrY.5151990.5158280_.V.	2	chrY.5151990.5158280_.V.	0.04564902	0.32316975
chr15.126444208.126453028_.V.	2	chr15.126444208.126453028_.V.	0.0160561	0.32107043
chr13.201976615.201980625_.V.	2	chr13.201976615.201980625_.V.	0.01070637	0.32088492
chr6.89908806.89912726_.V.	2	chr6.89908806.89912726_.V.	0.03334006	0.32083458
chr15.121565378.121578088_.V.	2	chr15.121565378.121578088_.V.	5.18E-05	0.32068734
chr14.140130749.140134879_.V.	2	chr14.140130749.140134879_.V.	0.00128303	0.32050256
chr3.31796726.31803986_.V.	2	chr3.31796726.31803986_.V.	0.03334006	0.31996464
chr15.138739508.138741938_.V.	2	chr15.138739508.138741938_.V.	0.00086971	0.31961896
chrX.97102018.97105678_.V.	2	chrX.97102018.97105678_.V.	0.00435484	0.31918128
chr13.201935711.201936265_.C.	2	chr13.201935711.201936265_.C.	0.00596018	0.31898324
chr5.197378.203528_.V.	2	chr5.197378.203528_.V.	0.00037581	0.3188685
chr14.51195729.51198409_.V.	2	chr14.51195729.51198409_.V.	0.00222085	0.3187173
chr9.53389332.53391822_.V.	2	chr9.53389332.53391822_.V.	5.18E-05	0.31841297
chr14.133028839.133031539_.V.	2	chr14.133028839.133031539_.V.	0.03334006	0.31815767
chr1.245176.249636_.V.	2	chr1.245176.249636_.V.	0.026429	0.31789683
chr3.33496746.33499166_.V.	2	chr3.33496746.33499166_.V.	0.00046721	0.3176639
chr4.1291569.1292569_.V.	2	chr4.1291569.1292569_.V.	0.02343317	0.31760669
chr6.163921196.163925556_.V.	2	chr6.163921196.163925556_.V.	0.02343317	0.31671469
chr5.2571708.2576618_.V.	2	chr5.2571708.2576618_.V.	0.00264406	0.31658878
chr4.125366989.125370459_.V.	2	chr4.125366989.125370459_.V.	0.00105868	0.31598191
chr14.74883799.74887899_.V.	2	chr14.74883799.74887899_.V.	0.00510422	0.31594977
chr9.135272762.135281422_.V.	2	chr9.135272762.135281422_.V.	0.0160561	0.31559024
chr1.205976.211656_.V.	2	chr1.205976.211656_.V.	0.02071916	0.31496914
chr1.2616166.2621206_.V.	2	chr1.2616166.2621206_.V.	0.03334006	0.31411353
chr18.17987535.17989915_.V.	2	chr18.17987535.17989915_.V.	0.03334006	0.31388941
chr18.50233045.50234445_.V.	2	chr18.50233045.50234445_.V.	0.00057789	0.31375094
chr11.76212570.76214054_.C.	2	chr11.76212570.76214054_.C.	0.026429	0.31363847
chr11.806250.810370_.V.	2	chr11.806250.810370_.V.	0.00023867	0.31320816
chr16.74667349.74670539_.V.	2	chr16.74667349.74670539_.V.	0.00693574	0.31311752
chr5.729828.732748_.V.	2	chr5.729828.732748_.V.	0.00105868	0.31297446
chr4.110584109.110585339_.V.	2	chr4.110584109.110585339_.V.	0.00057789	0.31292405
chr4.103092789.103096679_.V.	2	chr4.103092789.103096679_.V.	0.03729477	0.31275421
chr1.9513086.9516486_.V.	2	chr1.9513086.9516486_.V.	0.00313457	0.31248155
chr11.76748420.76752040_.V.	2	chr11.76748420.76752040_.V.	0.01826556	0.31103118
chr18.2272575.2275685_.V.	2	chr18.2272575.2275685_.V.	0.01070637	0.31020466
chr18.1095905.1098925_.V.	2	chr18.1095905.1098925_.V.	0.00030028	0.31012796
chr12.47291739.47297129_.V.	2	chr12.47291739.47297129_.V.	0.00435484	0.30992663
chr12.33896719.33901049_.V.	2	chr12.33896719.33901049_.V.	0.0300707	0.3095163
chr7.3101659.3103669_.V.	2	chr7.3101659.3103669_.V.	0.026429	0.3091028
chr10.66374829.66380089_.V.	2	chr10.66374829.66380089_.V.	0.01070637	0.3089603
chr4.801299.805749_.V.	2	chr4.801299.805749_.V.	0.0160561	0.30893018
chr3.11050706.11053846_.V.	2	chr3.11050706.11053846_.V.	0.00510422	0.30847105
chr4.108614479.108617509_.V.	2	chr4.108614479.108617509_.V.	0.00370211	0.30843168
chr7.2614139.2616239_.V.	2	chr7.2614139.2616239_.V.	0.03334006	0.30725197
chr13.207851085.207852185_.C.	2	chr13.207851085.207852185_.C.	0.00086971	0.30709286
chr8.138963068.138964698_.V.	2	chr8.138963068.138964698_.V.	0.00596018	0.30675458
chr11.4043250.4045240_.V.	2	chr11.4043250.4045240_.V.	0.00596018	0.30649318
chr4.1395609.1398549_.V.	2	chr4.1395609.1398549_.V.	0.0160561	0.30491988
chr15.134599548.134604448_.V.	2	chr15.134599548.134604448_.V.	0.00057789	0.30484147
chr13.137917235.137921315_.V.	2	chr13.137917235.137921315_.V.	0.00222085	0.30433275
chr9.7757462.7760202_.V.	2	chr9.7757462.7760202_.V.	0.00693574	0.30329662
chr12.53957499.53961049_.V.	2	chr12.53957499.53961049_.V.	0.04630508	0.30323107
chr13.122482235.122484295_.V.	2	chr13.122482235.122484295_.V.	0.00313457	0.30281478
chr12.9250039.9255929_.V.	2	chr12.9250039.9255929_.V.	0.00313457	0.30268836
chr10.66270579.66275109_.V.	2	chr10.66270579.66275109_.V.	0.03334006	0.30266926
chr11.78167005.78170890_.C.	2	chr11.78167005.78170890_.C.	0.00222085	0.30264089
chr11.78799490.78805060_.V.	2	chr11.78799490.78805060_.V.	0.02972401	0.30255054
chr9.133343502.133348402_.V.	2	chr9.133343502.133348402_.V.	0.00185787	0.30212572
chr6.64597076.64601866_.V.	2	chr6.64597076.64601866_.V.	0.00086971	0.3018733
chrX.110961228.110965038_.V.	2	chrX.110961228.110965038_.V.	0.02972401	0.30177179
chr13.207920015.207924845_.V.	2	chr13.207920015.207924845_.V.	0.00105868	0.30120672
chrX.15086758.15090018_.V.	2	chrX.15086758.15090018_.V.	0.01713848	0.30110605

chr1.779396.791206_.V.	2	chr1.779396.791206_.V.	0.03334006	0.30017978
chr13.207334585.207337535_.V.	2	chr13.207334585.207337535_.V.	0.00154718	0.30005073
chr3.9597376.9609166_.V.	2	chr3.9597376.9609166_.V.	0.01070637	0.29949604
chr12.1733187.1756485_.C.	2	chr12.1733187.1756485_.C.	0.00105868	0.29944461
chr6.164089606.164094406_.V.	2	chr6.164089606.164094406_.V.	0.03729477	0.299168
chr2.70657400.70660160_.V.	2	chr2.70657400.70660160_.V.	0.00510422	0.29879002
chr2.2420990.2422920_.V.	2	chr2.2420990.2422920_.V.	0.03334006	0.29869891
chr18.4943185.4946905_.V.	2	chr18.4943185.4946905_.V.	0.026429	0.29841688
chr8.11799898.11805648_.V.	2	chr8.11799898.11805648_.V.	0.04433484	0.29816036
chr6.12871866.12878766_.V.	2	chr6.12871866.12878766_.V.	0.03369858	0.29802484
chr15.135928008.135935298_.V.	2	chr15.135928008.135935298_.V.	0.03334006	0.29801697
chr1.1338296.1346816_.V.	2	chr1.1338296.1346816_.V.	0.02071916	0.29789612
chr7.116584369.116587509_.V.	2	chr7.116584369.116587509_.V.	0.01070637	0.29758719
chr12.17970399.17973599_.V.	2	chr12.17970399.17973599_.V.	0.01407064	0.29755765
chr2.71110600.71114230_.V.	2	chr2.71110600.71114230_.V.	0.01229347	0.29704815
chr1.4913236.4916166_.V.	2	chr1.4913236.4916166_.V.	0.01070637	0.29653821
chr6.66851756.66855146_.V.	2	chr6.66851756.66855146_.V.	0.02343317	0.29597412
chr9.131817522.131821372_.V.	2	chr9.131817522.131821372_.V.	0.00222085	0.29562139
chr17.61283952.61295862_.V.	2	chr17.61283952.61295862_.V.	0.00929494	0.29531657
chr18.48938595.48941875_.V.	2	chr18.48938595.48941875_.V.	0.03729477	0.29498063
chr11.78138900.78143630_.V.	2	chr11.78138900.78143630_.V.	0.01070637	0.29492483
chr3.113718876.113725716_.V.	2	chr3.113718876.113725716_.V.	0.02071916	0.29454586
chr4.96775389.96779099_.V.	2	chr4.96775389.96779099_.V.	0.00123548	0.29362419
chr3.113473226.113476906_.V.	2	chr3.113473226.113476906_.V.	5.18E-05	0.29291322
chr7.2794199.2797759_.V.	2	chr7.2794199.2797759_.V.	0.00046721	0.29284816
chr3.113513086.113521056_.V.	2	chr3.113513086.113521056_.V.	0.04630508	0.292605
chr9.136680792.136687932_.V.	2	chr9.136680792.136687932_.V.	0.00804229	0.29235852
chr12.58106109.58112419_.V.	2	chr12.58106109.58112419_.V.	0.02972401	0.29212916
chr17.60235822.60239962_.V.	2	chr17.60235822.60239962_.V.	0.00370211	0.29201358
chr5.880898.887848_.V.	2	chr5.880898.887848_.V.	0.00071066	0.29175154
chr2.69172580.69174080_.V.	2	chr2.69172580.69174080_.V.	0.00105868	0.29021164
chr5.4929778.4944218_.V.	2	chr5.4929778.4944218_.V.	1.56E-05	0.29021025
chr1.266090536.266096266_.V.	2	chr1.266090536.266096266_.V.	0.02071916	0.29013549
chr12.3887149.3894119_.V.	2	chr12.3887149.3894119_.V.	0.00693574	0.28978162
chr18.39828045.39831105_.V.	2	chr18.39828045.39831105_.V.	0.00046721	0.28975455
chr6.1574996.1579556_.V.	2	chr6.1574996.1579556_.V.	0.00154718	0.28950281
chr13.207825375.207830235_.V.	2	chr13.207825375.207830235_.V.	0.00037581	0.28945639
chr5.1267908.1273458_.V.	2	chr5.1267908.1273458_.V.	0.00018834	0.28922197
chr5.4775208.4785358_.V.	2	chr5.4775208.4785358_.V.	0.00105868	0.28859441
chr11.7706050.7708880_.V.	2	chr11.7706050.7708880_.V.	0.02343317	0.28852968
chr9.65018732.65022572_.V.	2	chr9.65018732.65022572_.V.	0.02141819	0.288052
chr14.139444509.139447079_.V.	2	chr14.139444509.139447079_.V.	0.00510422	0.28796551
chr6.167368936.167377426_.V.	2	chr6.167368936.167377426_.V.	0.026429	0.28785488
chr1.862256.875336_.V.	2	chr1.862256.875336_.V.	0.00929494	0.28771325
chr10.66516689.66523159_.V.	2	chr10.66516689.66523159_.V.	0.00435484	0.28674378
chr4.123218039.123221889_.V.	2	chr4.123218039.123221889_.V.	0.01407064	0.28627162
chr12.56286169.56291389_.V.	2	chr12.56286169.56291389_.V.	0.00018834	0.2857546
chr17.47653472.47658062_.V.	2	chr17.47653472.47658062_.V.	0.00804229	0.28534825
chr15.135844108.135850298_.V.	2	chr15.135844108.135850298_.V.	0.02972401	0.28508487
chr11.78896710.78901170_.V.	2	chr11.78896710.78901170_.V.	0.00313457	0.28453184
chr3.125602766.125605436_.V.	2	chr3.125602766.125605436_.V.	0.01407064	0.28333631
chr12.1708838.1725965_.C.	2	chr12.1708838.1725965_.C.	0.00128303	0.2822725
chr2.1526300.1530330_.V.	2	chr2.1526300.1530330_.V.	0.03729477	0.28149825
chr12.57315389.57318049_.V.	2	chr12.57315389.57318049_.V.	0.00105868	0.28123952
chr18.2129945.2132905_.V.	2	chr18.2129945.2132905_.V.	0.00370211	0.28004139
chr4.15793249.15797329_.V.	2	chr4.15793249.15797329_.V.	0.00105868	0.27982809
chr10.67435519.67446299_.V.	2	chr10.67435519.67446299_.V.	0.00046721	0.27971733
chr1.272535826.272540916_.V.	2	chr1.272535826.272540916_.V.	0.026429	0.27957797
chr9.136634302.136640792_.V.	2	chr9.136634302.136640792_.V.	0.04564902	0.27950006
chr2.17969090.17975170_.V.	2	chr2.17969090.17975170_.V.	0.00057789	0.27915832
chr11.93490.98460_.V.	2	chr11.93490.98460_.V.	0.00596018	0.27827421
chr14.139222899.139228039_.V.	2	chr14.139222899.139228039_.V.	0.04161078	0.27752037
chr3.9503526.9507376_.V.	2	chr3.9503526.9507376_.V.	0.02343317	0.27749442
chr5.1531118.1548328_.V.	2	chr5.1531118.1548328_.V.	0.04161078	0.27621628
chr12.1783879.1788199_.V.	2	chr12.1783879.1788199_.V.	0.02071916	0.27544612
chr11.78677137.78678566_.C.	2	chr11.78677137.78678566_.C.	0.00313457	0.27541618
chr5.69443068.69451418_.V.	2	chr5.69443068.69451418_.V.	0.01826556	0.27513134
chr4.753289.757889_.V.	2	chr4.753289.757889_.V.	0.0160561	0.27370834
chr11.3807702.3808665_.C.	2	chr11.3807702.3808665_.C.	0.03729477	0.27347736
chrX.122078398.122081848_.V.	2	chrX.122078398.122081848_.V.	0.03729477	0.27253489
chr9.8358382.8363692_.V.	2	chr9.8358382.8363692_.V.	0.0160561	0.27249669
chr9.2192002.2194782_.V.	2	chr9.2192002.2194782_.V.	0.00264406	0.27090784
chr14.137231709.137236249_.V.	2	chr14.137231709.137236249_.V.	0.00057789	0.27084087
chr14.140244459.140248619_.V.	2	chr14.140244459.140248619_.V.	0.00313457	0.27079611
chr12.1396069.1402069_.V.	2	chr12.1396069.1402069_.V.	0.01826556	0.27027625
chr17.46316722.46322122_.V.	2	chr17.46316722.46322122_.V.	0.02343317	0.26989909
chr2.71159280.71162450_.V.	2	chr2.71159280.71162450_.V.	0.0160561	0.26932365
chr17.10765752.10768522_.V.	2	chr17.10765752.10768522_.V.	0.01826556	0.26907147
chr12.48069459.48073839_.V.	2	chr12.48069459.48073839_.V.	0.00435484	0.26887756
chr5.1519708.1528168_.V.	2	chr5.1519708.1528168_.V.	0.01070637	0.26878775
chr12.54553579.54559169_.V.	2	chr12.54553579.54559169_.V.	0.00693574	0.26824007
chr9.134087302.134092522_.V.	2	chr9.134087302.134092522_.V.	0.01070637	0.26823875
chr3.25474506.25477466_.V.	2	chr3.25474506.25477466_.V.	0.00510422	0.2675033
chr4.74619249.74623839_.V.	2	chr4.74619249.74623839_.V.	0.00596018	0.26519686
chr10.64948929.64955329_.V.	2	chr10.64948929.64955329_.V.	0.01070637	0.26462176
chr7.805219.809829_.V.	2	chr7.805219.809829_.V.	0.00018834	0.26327131
chr3.41248196.41252396_.V.	2	chr3.41248196.41252396_.V.	0.00929494	0.26265179
chr2.2591680.2597420_.V.	2	chr2.2591680.2597420_.V.	0.026429	0.26243443
chr5.4259958.4262828_.V.	2	chr5.4259958.4262828_.V.	0.03729477	0.26220571
chr3.127556286.127558286_.V.	2	chr3.127556286.127558286_.V.	0.04161078	0.26171827
chr11.78534516.78538856_.C.	2	chr11.78534516.78538856_.C.	0.02071916	0.26163626
chr7.7807889.7811419_.V.	2	chr7.7807889.7811419_.V.	0.04612132	0.26112565
chr1.1252336.1261106_.V.	2	chr1.1252336.1261106_.V.	0.00086971	0.26080103
chr13.207881641.207883728_.C.	2	chr13.207881641.207883728_.C.	0.00435484	0.26025796
chr12.54155339.54159459_.V.	2	chr12.54155339.54159459_.V.	0.04630508	0.26007399
chr12.59630459.59633979_.V.	2	chr12.59630459.59633979_.V.	0.00929494	0.25934959
chr11.78946300.78950400_.V.	2	chr11.78946300.78950400_.V.	0.04161078	0.25864145
chr7.2747059.2750209_.V.	2	chr7.2747059.2750209_.V.	0.04829971	0.25702535
chr5.2506818.2512008_.V.	2	chr5.2506818.2512008_.V.	0.00046721	0.25611666
chr14.137556249.137557679_.V.	2	chr14.137556249.137557679_.V.	0.00037581	0.25598495
chr7.546619.550269_.V.	2	chr7.546619.550269_.V.	0.02905462	0.25525948
chr14.72869909.72873509_.V.	2	chr14.72869909.72873509_.V.	0.01070637	0.25508886
chr12.6025019.6028749_.V.	2	chr12.6025019.6028749_.V.	0.00185787	0.25435947
chr7.1388459.1392379_.V.	2	chr7.1388459.1392379_.V.	0.00313457	0.25404977
chr5.982098.986558_.V.	2	chr5.982098.986558_.V.	0.00086971	0.25313463
chr10.23622739.23625309_.V.	2	chr10.23622739.23625309_.V.	0.0160561	0.25312394
chr5.9839198.9845318_.V.	2	chr5.9839198.9845318_.V.	0.00264406	0.25251462

chr7.2693029.2695299_.V.	2	chr7.2693029.2695299_.V.	0.01826556	0.25147723

Gene	Gene-Chr	Gene-ini	Gene-end	Loci	Loci-Chr	Loci-ini	Loci-end	cor
RNASEH2A	chr2	66193197	66207697	344600.66347	chr2	66344600	66347110	-0.8136199
PFDN6	chr7	29653679	29654945	643130.29647	chr7	29643130	29647130	-0.7907699
RPS18	chr7	29638833	29642912	643130.29647	chr7	29643130	29647130	-0.7601996
PRPF4	chr1	253906717	253925046	076730.25408	chr1	254076730	254080730	-0.7574917
RNASEH2A	chr2	66193197	66207697	345269.66346	chr2	66345269	66346152	-0.7529248
SLC46A3	chr11	5982443	6000701	793860.5797	chr11	5793860	5797121	-0.7376826
DND1	chr2	142384532	142387199	578003.14257	chr2	142578003	142579756	-0.7359584
POLA2	chr2	6922387	6950179	079608.70836	chr2	7079608	7083608	-0.7211563
CDC26	chr1	253895885	253906632	076730.25408	chr1	254076730	254080730	-0.7210019
GPR39	chr15	19557893	19799040	520828.1952	chr15	19520828	19524828	-0.7152016
PRPF4	chr1	253906717	253925046	569456.25357	chr1	253569456	253571216	-0.7135366
DDIT3	chr5	22785445	22789829	056982.23060	chr5	23056982	23060982	-0.7116108
ATG2A	chr2	7262031	7282347	079608.70836	chr2	7079608	7083608	-0.7071768
CNPY2	chr5	21707834	21711292	557029.21561	chr5	21557029	21561029	-0.7064878
RAB7A	chr13	71777424	71852694	558012.7156	chr13	71558012	71562012	-0.7036052
CUTA	chr7	29720094	29721769	643130.29647	chr7	29643130	29647130	-0.7020374
MTUS2	chr11	6197740	6532924	793860.5797	chr11	5793860	5797121	-0.700481
MRPL20	chr6	63670755	63675839	862113.63863	chr6	63862113	63863570	-0.6988649
GPR39	chr15	19557893	19799040	521903.1952	chr15	19521903	19524191	-0.6988223
ILK	chr9	3145608	3159858	376207.33802	chr9	3376207	3380207	-0.6985952
COQ4	chr1	268756685	268768343	615087.26861	chr1	268615087	268619087	-0.6983036
POP7	chr3	8605018	8610218	904591.89085	chr3	8904591	8908591	-0.696298
SLC35A4	chr2	142323341	142325335	577020.14257	chr2	142577020	142579530	-0.6936215
RPS10	chr7	30435150	30442886	513925.30517	chr7	30513925	30517925	-0.6906976
VPS52	chr7	29624230	29639140	643130.29647	chr7	29643130	29647130	-0.6894168
WDR55	chr2	142378462	142384677	577020.14257	chr2	142577020	142579530	-0.6877988
ZDHHC4	chr3	4628385	4643691	132177.41361	chr3	4132177	4136177	-0.6876099
POP7	chr3	8605018	8610218	886506.88905	chr3	8886506	8890506	-0.6861088
ENSSSCG00000057727	chr3	68572415	68573980	570415.68574	chr3	68570415	68574415	-0.6815488
TMEM63A	chr10	13769605	13814020	939003.1394	chr10	13939003	13940930	-0.6808646
KXD1	chr2	59242310	59247713	826251.58830	chr2	58826251	58830251	-0.6793992
ENSSSCG00000033697	chr3	40001326	40003107	776754.39780	chr3	39776754	39780754	-0.676876
NELFE	chr7	24040525	24047025	836996.23840	chr7	23836996	23840996	-0.6747608
CDC26	chr1	253895885	253906632	569435.25357	chr1	253569435	253570326	-0.6745479
UQCC2	chr7	29899043	29929795	643130.29647	chr7	29643130	29647130	-0.6741012
COQ4	chr1	268756685	268768343	614034.26861	chr1	268614034	268618034	-0.6738121
CYTH3	chr3	4900570	5002194	992827.49946	chr3	4992827	4994602	-0.6735548
EIF4A1	chr12	52868434	52874249	627516.5262	chr12	52627516	52629243	-0.6716959
TCP11	chr7	31007474	31114734	513925.30517	chr7	30513925	30517925	-0.6702733
WDR55	chr2	142378462	142384677	578003.14257	chr2	142578003	142579756	-0.6700052
CRAT	chr1	269386650	269401331	332838.26933	chr1	269332838	269336838	-0.6693265
TRMT1	chr2	65940125	65949324	344600.66347	chr2	66344600	66347110	-0.6689601
ENSSSCG00000010058	chr14	49585326	49598007	792613.4979	chr14	49792613	49793506	-0.6685373
WDR55	chr2	142378462	142384677	639970.14264	chr2	142639970	142642910	-0.6682313
PPCDC	chr7	58525143	58549430	826727.58830	chr7	58826727	58830727	-0.6664395
ENSSSCG00000060885	chr15	120345579	120348027	343579.1203	chr15	120343579	120347579	-0.6662964
NOP14	chr8	1716826	1737902	386266.13902	chr8	1386266	1390266	-0.665622
RNASEH2A	chr2	66193197	66207697	950519.65954	chr2	65950519	65954519	-0.6651774
RPL27	chr12	19892217	19894504	256373.2026	chr12	20256373	20260373	-0.6649229
USP5	chr5	63843745	63858716	114150.64118	chr5	64114150	64118150	-0.6642902
TRMT1	chr2	65940125	65949324	055483.66056	chr2	66055483	66056550	-0.6641826
ENSSSCG00000035904	chr1	272959831	272965634	961551.27296	chr1	272961551	272965551	-0.6635291
FUT2	chr6	54034684	54047224	809477.53810	chr6	53809477	53810078	-0.6627397
TXNL4A	chr6	127974543	127991177	958266.12796	chr6	127958266	127962266	-0.6619466
PSMC3IP	chr12	20244304	20250159	256373.2026	chr12	20256373	20260373	-0.6610793
SLC35A4	chr2	142323341	142325335	578003.14257	chr2	142578003	142579756	-0.6591694
FAU	chr2	7070906	7072809	079608.70836	chr2	7079608	7083608	-0.6584998
DND1	chr2	142384532	142387199	577020.14257	chr2	142577020	142579530	-0.6582568
EDC3	chr7	58828727	58897919	908967.58910	chr7	58908967	58910668	-0.6579982
AP1S1	chr3	8881107	8887566	315140.93191	chr3	9315140	9319140	-0.6577859
ENSSSCG00000033310	chr5	21506554	21507767	557029.21561	chr5	21557029	21561029	-0.6551344

ATP6V1D	chr7	91064396	91084913	955179.90956	chr7	90955179	90956189	-0.6550597
WDR55	chr2	142378462	142384677	641101.14264	chr2	142641101	142642772	-0.6550202
CALR	chr2	66098229	66102132	344600.66347	chr2	66344600	66347110	-0.6538977
CALR	chr2	66098229	66102132	345269.66346	chr2	66345269	66346152	-0.6532716
CIAO2B	chr6	27610470	27612592	081955.28085	chr6	28081955	28085955	-0.6531807
PER1	chr12	53361889	53374248	109256.5311	chr12	53109256	53113256	-0.6528601
AARSD1	chr12	19918248	19926825	256373.2026	chr12	20256373	20260373	-0.6527317
TUFM	chr3	18521128	18524959	118581.18122	chr3	18118581	18122581	-0.6515957
UBE2J2	chr6	63555488	63568017	862113.63863	chr6	63862113	63863570	-0.6514578
ENSSSCG00000057727	chr3	68572415	68573980	020319.69021	chr3	69020319	69021858	-0.6500186
YPEL1	chr14	50190798	50208256	792613.4979	chr14	49792613	49793506	-0.6497723
MYOF	chr14	104770515	104941109	010519.1050	chr14	105010519	105014699	-0.6492483
LSM2	chr7	23900395	23908490	836996.23840	chr7	23836996	23840996	-0.6485954
PYGB	chr17	30940452	30995691	.194616.3119	chr17	31194616	31195316	-0.6479662
EHD1	chr2	7298208	7321576	079608.70836	chr2	7079608	7083608	-0.6479379
PCDHGA4	chr2	142993554	143156556	124047.14312	chr2	143124047	143126730	-0.6475112
OTUB1	chr2	8087829	8095609	359774.83637	chr2	8359774	8363774	-0.6469739
DYNC2I2	chr1	269002261	269030719	614034.26861	chr1	268614034	268618034	-0.6468438
DRAP1	chr2	6409655	6412395	561091.65650	chr2	6561091	6565091	-0.6455566
IK	chr2	142361155	142376333	578003.14257	chr2	142578003	142579756	-0.6452791
SRRT	chr3	8717785	8731201	904591.89085	chr3	8904591	8908591	-0.6441181
CDIPT	chr3	18084549	18091798	175737.18179	chr3	18175737	18179737	-0.6432599
GFRA4	chr17	32040235	32046105	.868517.3186	chr17	31868517	31869375	-0.6425129
DRAP1	chr2	6409655	6412395	409833.64138	chr2	6409833	6413833	-0.6419435
CTDNEP1	chr12	52599226	52606059	399810.5240	chr12	52399810	52403810	-0.6414976
BRMS1	chr2	6046098	6053667	044098.60480	chr2	6044098	6048098	-0.6414968
SCAMP4	chr2	76615000	76634264	996982.77000	chr2	76996982	77000982	-0.6409534
PSKH1	chr6	28508823	28544062	081955.28085	chr6	28081955	28085955	-0.6403351
PRPF4	chr1	253906717	253925046	569435.25357	chr1	253569435	253570326	-0.6402758
ENSSSCG00000027573	chr13	34119539	34121859	663957.3366	chr13	33663957	33666213	-0.6401848
DNAJB2	chr15	121319982	121335991	.210881.1212	chr15	121210881	121214881	-0.6392163
FAM219B	chr7	58650754	58659479	826727.58830	chr7	58826727	58830727	-0.637818
TNNT1	chr6	59347582	59365284	850751.58854	chr6	58850751	58854751	-0.6375082
PRDX2	chr2	66207828	66212009	344600.66347	chr2	66344600	66347110	-0.6373143
IDH2	chr7	55651609	55675554	237411.55238	chr7	55237411	55238015	-0.6364029
FSD2	chr7	52144506	52194133	254159.52258	chr7	52254159	52258159	-0.6348018
GRK4	chr8	1738333	1792374	386266.13902	chr8	1386266	1390266	-0.6344396
NOTO	chr3	69567449	69571100	234351.69238	chr3	69234351	69238351	-0.6342576
ENSSSCG00000044567	chr17	31217462	31238052	.194616.3119	chr17	31194616	31195316	-0.6341606
DYNLL1	chr14	40455985	40459104	217722.4021	chr14	40217722	40218386	-0.6334011
SPDYC	chr2	7030733	7058967	561091.65650	chr2	6561091	6565091	-0.632104
DDX39B	chr7	23658088	23670031	836996.23840	chr7	23836996	23840996	-0.6316889
POLE3	chr1	254033385	254035978	076730.25408	chr1	254076730	254080730	-0.6316266
PPCDC	chr7	58525143	58549430	908967.58910	chr7	58908967	58910668	-0.6315353
OTUB1	chr2	8087829	8095609	410290.84165	chr2	8410290	8416580	-0.6312373
ZKSCAN5	chr3	6442391	6466430	141962.61429	chr3	6141962	6142972	-0.6301048
TRMT1	chr2	65940125	65949324	345269.66346	chr2	66345269	66346152	-0.6299274
DRAP1	chr2	6409655	6412395	428153.64321	chr2	6428153	6432153	-0.62969
SDHA	chr16	79834044	79862524	471745.7947	chr16	79471745	79475745	-0.6294945
MYOF	chr14	104770515	104941109	011963.1050	chr14	105011963	105012625	-0.6291878
EIF4A1	chr12	52868434	52874249	399810.5240	chr12	52399810	52403810	-0.628687
POLA2	chr2	6922387	6950179	537993.65396	chr2	6537993	6539697	-0.6285497
C6orf136	chr7	23185599	23190299	062338.23066	chr7	23062338	23066338	-0.6282814
CREB3	chr1	236450573	236456375	378019.23638	chr1	236378019	236382019	-0.6273878
IFT22	chr3	8976306	8981510	315140.93191	chr3	9315140	9319140	-0.626371
ENSSSCG00000057727	chr3	68572415	68573980	496471.68497	chr3	68496471	68497933	-0.625159
RNF4	chr8	1353658	1380634	386266.13902	chr8	1386266	1390266	-0.6248329
ENSSSCG00000009374	chr11	15550286	15628604	734905.1573	chr11	15734905	15735621	-0.6241543
RPL7L1	chr7	37940834	37951324	388035.38389	chr7	38388035	38389283	-0.6230995
DRAP1	chr2	6409655	6412395	044098.60480	chr2	6044098	6048098	-0.6224658
DNAJC14	chr5	21264664	21272646	557029.21561	chr5	21557029	21561029	-0.6222813
IMP3	chr7	58023368	58024496	702066.57703	chr7	57702066	57703313	-0.621759

ZNF410	chr7	97195975	97243614	123873.97124	chr7	97123873	97124625	-0.6214527
PCDHGA4	chr2	142993554	143156556	578003.14257	chr2	142578003	142579756	-0.6209353
ENSSSCG00000010888	chr10	19585162	19586394	286367.1929	chr10	19286367	19290367	-0.6206572
MTMR14	chr13	65948544	65994820	994525.6599	chr13	65994525	65997625	-0.6205725
MYL6	chr5	21559029	21562413	557029.21561	chr5	21557029	21561029	-0.6190794
DEF8	chr6	140908	160692	.63387.64608	chr6	63387	64608	-0.6184721
SPDYC	chr2	7030733	7058967	079608.70836	chr2	7079608	7083608	-0.6176907
ENSSSCG00000034927	chr3	18468001	18489099	175737.18179	chr3	18175737	18179737	-0.6176402
DYNLL1	chr14	40455985	40459104	561159.4056	chr14	40561159	40565159	-0.6172405
COX8A	chr2	8101613	8103285	410290.84165	chr2	8410290	8416580	-0.6167249
ATG4B	chr15	140223904	140246321	131918.1401	chr15	140131918	140133932	-0.6164811
ESYT1	chr5	21514510	21535929	557029.21561	chr5	21557029	21561029	-0.6164586
ENSSSCG00000017907	chr12	51961952	51965464	399810.5240	chr12	52399810	52403810	-0.6164153
POLE3	chr1	254033385	254035978	569435.25357	chr1	253569435	253570326	-0.6163757
COPS3	chr12	60994310	61024846	.029157.6103	chr12	61029157	61033157	-0.616279
FXR2	chr12	52882870	52903450	399810.5240	chr12	52399810	52403810	-0.6160764
RNF4	chr8	1353658	1380634	038195.10396	chr8	1038195	1039688	-0.6160232
ARHGAP22	chr14	89113768	89331274	192374.8919	chr14	89192374	89193566	-0.6159656
STK16	chr15	121284920	121288473	.210881.1212	chr15	121210881	121214881	-0.6157955
MEPCE	chr3	8357289	8364661	356207.83580	chr3	8356207	8358016	-0.6157666
ANKRD39	chr3	56762386	56775004	810364.56812	chr3	56810364	56812121	-0.6145532
PCDH12	chr2	143550849	143565033	124047.14312	chr2	143124047	143126730	-0.6142234
ENSSSCG00000057727	chr3	68572415	68573980	519015.68520	chr3	68519015	68520573	-0.6137421
PRPF4	chr1	253906717	253925046	973973.25397	chr1	253973973	253977973	-0.6134243
RNF4	chr8	1353658	1380634	053894.10572	chr8	1053894	1057212	-0.6134077
CD63	chr5	21172283	21176232	272511.21276	chr5	21272511	21276511	-0.613395
ZNRD2	chr2	6683057	6684494	079608.70836	chr2	7079608	7083608	-0.6130591
ENSSSCG00000035909	chr15	76958516	76972527	991098.7699	chr15	76991098	76995238	-0.6124565
SLC35A4	chr2	142323341	142325335	882155.14188	chr2	141882155	141886155	-0.6115984
HAX1	chr4	95467632	95470290	903054.95907	chr4	95903054	95907054	-0.6106257
ENSSSCG00000013064	chr2	9163317	9174468	979963.89839	chr2	8979963	8983963	-0.610397
STX18	chr8	5890765	6004399	888765.58927	chr8	5888765	5892765	-0.6103423
CDIPT	chr3	18084549	18091798	118581.18122	chr3	18118581	18122581	-0.6095811
HIGD2A	chr2	81486890	81488045	285759.81289	chr2	81285759	81289759	-0.6094224
MVP	chr3	18057177	18081155	175737.18179	chr3	18175737	18179737	-0.6091438
TMEM219	chr3	18167923	18176563	118581.18122	chr3	18118581	18122581	-0.608817
POMP	chr11	5953743	5974060	000360.6001	chr11	6000360	6001074	-0.6085742
ZNF584	chr6	62992506	63005691	690463.62691	chr6	62690463	62691254	-0.6084842
TTLL4	chr15	120773185	120793194	343579.1203	chr15	120343579	120347579	-0.6084296
UBE2J2	chr6	63555488	63568017	654119.63658	chr6	63654119	63658119	-0.6082249
USP5	chr5	63843745	63858716	116264.64117	chr5	64116264	64117021	-0.6074977
EMG1	chr5	63745476	63751469	114150.64118	chr5	64114150	64118150	-0.6072481
CCBE1	chr1	161321273	161556537	720590.16172	chr1	161720590	161721936	-0.6069404
CD40	chr17	48286029	48298528	081366.4808	chr17	48081366	48082939	-0.6064718
PRPSAP1	chr12	5144069	5174382	521113.5525	chr12	5521113	5525113	-0.6061228
SLC25A11	chr12	51970806	51975548	399810.5240	chr12	52399810	52403810	-0.6056563
MRPL20	chr6	63670755	63675839	654119.63658	chr6	63654119	63658119	-0.6054911
CFL1	chr2	6469254	6475035	409833.64138	chr2	6409833	6413833	-0.6054236
NSG1	chr8	6000057	6024769	888765.58927	chr8	5888765	5892765	-0.6051511
SEZ6L2	chr3	18098102	18119558	118581.18122	chr3	18118581	18122581	-0.6048354
SNAPC2	chr2	71291257	71294006	519081.71523	chr2	71519081	71523081	-0.6044325
SNX30	chr1	253428508	253550375	569456.25357	chr1	253569456	253571216	-0.6040337
TTLL4	chr15	120773185	120793194	.024245.1210	chr15	121024245	121028245	-0.603985
RECQL5	chr12	5732348	5770133	938647.5942	chr12	5938647	5942647	-0.6028024
PRPF6	chr17	62815085	62858382	754250.6275	chr17	62754250	62755455	-0.6022651
HARS1	chr2	142385872	142401208	577020.14257	chr2	142577020	142579530	-0.6021938
C9orf78	chr1	269986391	269995395	190175.27019	chr1	270190175	270190671	-0.6018442
C9orf78	chr1	269986391	269995395	363751.27036	chr1	270363751	270364715	-0.6016402
FTL	chr6	54231172	54232750	705237.54709	chr6	54705237	54709237	-0.601155
DHRS1	chr7	74992115	75002148	027923.75031	chr7	75027923	75031923	-0.6011487
NR1H3	chr2	15315282	15334820	379707.15383	chr2	15379707	15383707	-0.6001341
KCNN3	chr4	94915965	95113275	495309.94499	chr4	94495309	94499039	-0.6000853

POLA2	chr2	6922387	6950179	561091.6565(	chr2	6561091	6565091	-0.5996739
THBS3	chr4	94611593	94623902	830270.94834	chr4	94830270	94834270	-0.5987967
ARHGAP9	chr5	22747339	22762049	056982.23060	chr5	23056982	23060982	-0.5987328
ZDHHC4	chr3	4628385	4643691	992827.49946	chr3	4992827	4994602	-0.5982771
ENSSSCG00000017955	chr12	52878764	52882863	627516.5262	chr12	52627516	52629243	-0.5976937
TUFM	chr3	18521128	18524959	505886.18507	chr3	18505886	18507873	-0.5974875
ACAD9	chr13	71374944	71427126	.558012.7156	chr13	71558012	71562012	-0.597401
DND1	chr2	142384532	142387199	383680.14188	chr2	141883680	141886890	-0.5971154
CIAPIN1	chr6	19356069	19373060	535476.19539	chr6	19535476	19539846	-0.5970716
PRDX2	chr2	66207828	66212009	345269.66346	chr2	66345269	66346152	-0.596858
ENSSSCG00000050152	chr15	78669325	78674715	502078.7850	chr15	78502078	78506078	-0.5964322
FAAP24	chr6	42815528	42819623	558113.42562	chr6	42558113	42562113	-0.5959355
P4HB	chr12	1121824	1131289	.120907.1129	chr12	1120907	1129651	-0.5948861
DNPEP	chr15	121391746	121412864	.253797.1212	chr15	121253797	121254795	-0.5948016
CCNL2	chr6	63659054	63668047	862113.63863	chr6	63862113	63863570	-0.5943175
RTN4IP1	chr1	72762855	72815540	697571.72701	chr1	72697571	72701571	-0.5938757
FCGR1A	chr4	99233611	99242586	052426.99056	chr4	99052426	99056426	-0.5936167
LIG1	chr6	53620483	53686562	809477.53810	chr6	53809477	53810078	-0.5932922
TUBA1B	chr5	15149099	15153620	027205.15029	chr5	15027205	15029851	-0.5928971
ENSSSCG00000011147	chr10	65576464	65595000	266094.6526	chr10	65266094	65267932	-0.5928257
IK	chr2	142361155	142376333	376462.14238	chr2	142376462	142380462	-0.5927184
UBE2O	chr12	5055065	5114123	116522.5120	chr12	5116522	5120486	-0.5924159
MVP	chr3	18057177	18081155	118581.18122	chr3	18118581	18122581	-0.5923195
HIRIP3	chr3	18198807	18202089	505886.18507	chr3	18505886	18507873	-0.5922887
SCN4A	chr12	15002175	15047582	571199.1457	chr12	14571199	14573341	-0.5922403
SWI5	chr1	268720355	268726521	614034.26861	chr1	268614034	268618034	-0.5920443
ZNF212	chr18	55528138	55541841	887653.5589	chr18	55887653	55891653	-0.5920294
BRD9	chr16	79473745	79492635	471745.7947	chr16	79471745	79475745	-0.5919773
MLH1	chr13	21791203	21877757	124847.2212	chr13	22124847	22125444	-0.5916907
RECQL5	chr12	5732348	5770133	359854.5363	chr12	5359854	5363459	-0.591557
MYOZ1	chr14	76444557	76452799	496827.7650	chr14	76496827	76500827	-0.5914014
SETD7	chr8	87478173	87527188	478129.87480	chr8	87478129	87480437	-0.5913755
ARHGDIA	chr12	1113379	1119315	.120907.1129	chr12	1120907	1129651	-0.5909331
POP7	chr3	8605018	8610218	356207.83580	chr3	8356207	8358016	-0.5906605
LRCH4	chr3	8499285	8511262	904591.89085	chr3	8904591	8908591	-0.590564
KXD1	chr2	59242310	59247713	221175.59223	chr2	59221175	59223270	-0.5903451
RAB7A	chr13	71777424	71852694	.760305.7176	chr13	71760305	71762524	-0.5900438
GAPDH	chr5	64129679	64133991	114150.64118	chr5	64114150	64118150	-0.5898319
RECQL4	chr4	287214	293913	429062.43018	chr4	429062	430184	-0.5897395
SH3BP5L	chr2	52323048	52339665	311759.52312	chr2	52311759	52312351	-0.5890496
SNRNP35	chr14	29471429	29477011	630876.2963	chr14	29630876	29632175	-0.5888387
IDH2	chr7	55651609	55675554	573336.55577	chr7	55573336	55577336	-0.5886929
FSD2	chr7	52144506	52194133	302472.52306	chr7	52302472	52306472	-0.5886347
GMPPB	chr13	32248268	32253155	329740.3233	chr13	32329740	32330722	-0.5885462
TRAF3IP1	chr15	137863609	137912477	636504.1376	chr15	137636504	137637619	-0.5879761
RAB43	chr13	71562350	71596249	.620954.7162	chr13	71620954	71624954	-0.587822
DNAJC30	chr3	10955877	10959916	419176.11420	chr3	11419176	11420477	-0.5877794
EIF4A1	chr12	52868434	52874249	923693.5292	chr12	52923693	52927693	-0.5877607
BRMS1	chr2	6046098	6053667	428153.64321	chr2	6428153	6432153	-0.5874354
SLC41A3	chr7	53672130	53748727	639893.53641	chr7	53639893	53641617	-0.5868779
NXF1	chr2	8969581	8981984	982070.89825	chr2	8982070	8982593	-0.5863793
CTDSP2	chr5	23105172	23132586	056982.23060	chr5	23056982	23060982	-0.5851124
MPHOSPH8	chr11	447052	480121	.778458.7802	chr11	778458	780289	-0.5849253
CYBC1	chr12	656347	662513	.120907.1129	chr12	1120907	1129651	-0.5846726
GTF2E2	chr15	54444470	54531213	380567.5438	chr15	54380567	54384567	-0.5846211
MYL6B	chr5	21556061	21558758	557029.21561	chr5	21557029	21561029	-0.5842679
SLC35A4	chr2	142323341	142325335	641101.14264	chr2	142641101	142642772	-0.5837174
MIF	chr14	49840305	49841068	792613.4979	chr14	49792613	49793506	-0.5834302
PFKM	chr5	78476123	78526997	717866.78718	chr5	78717866	78718572	-0.5833976
RTN4IP1	chr1	72762855	72815540	699835.72701	chr1	72699835	72701157	-0.583113
SPDYC	chr2	7030733	7058967	537993.65396	chr2	6537993	6539697	-0.5825746
MEA1	chr7	38074439	38081897	388035.38389	chr7	38388035	38389283	-0.5824303

COA3	chr12	20062826	20063980	256373.2026	chr12	20256373	20260373	-0.5821994
KANSL3	chr3	56959110	57005310	810364.5681	chr3	56810364	56812121	-0.5818803
RECQL4	chr4	287214	293913	134107.1381	chr4	134107	138107	-0.5818614
ARV1	chr14	59396962	59408958	407123.5941	chr14	59407123	59411123	-0.5812798
ENSSSCG00000011272	chr13	24691893	24699363	345660.2434	chr13	24345660	24349660	-0.5811991
MRPL28	chr3	41329928	41334451	480363.4148	chr3	41480363	41484363	-0.5811514
SSR1	chr7	4652921	4685901	057871.5060	chr7	5057871	5060641	-0.5811048
METTL13	chr9	114202484	114218368	774648.1137	chr9	113774648	113775299	-0.5809223
SLC41A3	chr7	53672130	53748727	638266.5364	chr7	53638266	53642266	-0.5803257
COPS6	chr3	7982892	8005633	356207.8358	chr3	8356207	8358016	-0.5800385
NXF1	chr2	8969581	8981984	898604.8899	chr2	8898604	8899470	-0.5799899
RAB43	chr13	71562350	71596249	558012.7156	chr13	71558012	71562012	-0.5798942
FDPS	chr4	94500141	94518408	495309.9449	chr4	94495309	94499039	-0.579573
SLC5A6	chr3	111922848	111930940	769239.1117	chr3	111769239	111773239	-0.578656
LALBA	chr5	79214505	79216791	591915.7959	chr5	79591915	79595915	-0.5786496
SNRNP35	chr14	29471429	29477011	629084.2963	chr14	29629084	29633084	-0.5785235
YIPF1	chr6	158403072	158445511	535465.1585	chr6	158535465	158539465	-0.5784176
RECQL4	chr4	287214	293913	205764.2070	chr4	205764	207099	-0.5782232
TLE2	chr2	75510004	75536804	162787.7516	chr2	75162787	75166787	-0.5781202
WDR55	chr2	142378462	142384677	882155.1418	chr2	141882155	141886155	-0.5778382
SLC43A2	chr12	47791913	47839012	737633.4774	chr12	47737633	47741633	-0.5777508
ENSSSCG00000015083	chr9	45182400	45218254	783701.4478	chr9	44783701	44787701	-0.5772667
ILF2	chr4	95980918	95987867	903054.9590	chr4	95903054	95907054	-0.5770224
MYOF	chr14	104770515	104941109	009942.1050	chr14	105009942	105013942	-0.5762827
TUBA1B	chr5	15149099	15153620	024080.1502	chr5	15024080	15028080	-0.5760855
NXF1	chr2	8969581	8981984	979963.8983	chr2	8979963	8983963	-0.5760526
LRCH4	chr3	8499285	8511262	886506.8890	chr3	8886506	8890506	-0.5755274
TIMM44	chr2	71271805	71287817	519081.7152	chr2	71519081	71523081	-0.5753799
TSPAN31	chr5	23029306	23038977	881828.2288	chr5	22881828	22885548	-0.5753181
WDR53	chr13	133561021	133574376	155321.1331	chr13	133155321	133159321	-0.5749248
BRK1	chr13	66315315	66324641	994525.6599	chr13	65994525	65997625	-0.5746727
FAM219B	chr7	58650754	58659479	908967.5891	chr7	58908967	58910668	-0.5746431
GJA5	chr4	99854392	99871286	477372.9948	chr4	99477372	99481372	-0.5739763
FXR2	chr12	52882870	52903450	109256.5311	chr12	53109256	53113256	-0.5736373
FUS	chr3	17314332	17326637	619269.1762	chr3	17619269	17623269	-0.5729049
TPT1	chr11	21929221	21932691	499492.2150	chr11	21499492	21503492	-0.5726883
UTP25	chr9	133105063	133141217	385662.1333	chr9	133385662	133389422	-0.5726585
MAPK13	chr7	31880638	31889455	622450.3162	chr7	31622450	31626450	-0.5726368
RNF8	chr7	32931786	32970585	786291.3279	chr7	32786291	32790291	-0.5723996
OTUB1	chr2	8087829	8095609	281049.8282	chr2	8281049	8282602	-0.5723933
PELP1	chr12	52177094	52232420	929802.5193	chr12	51929802	51933802	-0.5723396
MAPKAPK2	chr9	67314484	67365059	679953.6768	chr9	67679953	67683953	-0.5723043
PCDHGA4	chr2	142993554	143156556	577020.1425	chr2	142577020	142579530	-0.5721196
NAPA	chr6	53246968	53275265	078236.5308	chr6	53078236	53082236	-0.5719308
TRMT1	chr2	65940125	65949324	271286.6627	chr2	66271286	66272770	-0.5717489
CRY2	chr2	16587109	16620385	657423.1665	chr2	16657423	16658649	-0.5717216
ENSSSCG00000032082	chr6	95322453	95366673	321439.9532	chr6	95321439	95322907	-0.5716742
ENSSSCG00000007719	chr3	11419446	11505406	419176.1142	chr3	11419176	11420477	-0.5716566
POLA2	chr2	6922387	6950179	755635.6759	chr2	6755635	6759635	-0.5714933
SNPH	chr17	33986513	34043995	867527.3387	chr17	33867527	33871527	-0.5707108
MRPL17	chr9	3075995	3083580	177192.3179	chr9	3177192	3179272	-0.5705516
BRMS1	chr2	6046098	6053667	409833.6413	chr2	6409833	6413833	-0.5701469
TK2	chr6	27226425	27255574	546706.2755	chr6	27546706	27550706	-0.5698032
FSD2	chr7	52144506	52194133	256466.5225	chr7	52256466	52256966	-0.5697309
MAF1	chr4	597472	600447	134107.1381	chr4	134107	138107	-0.5697103
SWI5	chr1	268720355	268726521	615087.2686	chr1	268615087	268619087	-0.5695375
ITGB5	chr13	135467798	135587473	467337.1354	chr13	135467337	135468789	-0.5694365
PPP1R7	chr15	139917257	139941251	131918.1401	chr15	140131918	140133932	-0.5694039
RAB8A	chr2	61429523	61450413	268199.6127	chr2	61268199	61272199	-0.5692465
LMBR1L	chr5	15121554	15135252	024080.1502	chr5	15024080	15028080	-0.5692413
CYTH3	chr3	4900570	5002194	992886.4995	chr3	4992886	4995036	-0.5688288
PRDX1	chr6	165824395	165861442	670840.1656	chr6	165670840	165671536	-0.5686771

TTLL4	chr15	120773185	120793194	.210881.1212	chr15	121210881	121214881	-0.5686736
STX5	chr2	8937778	8968932	979963.89839	chr2	8979963	8983963	-0.5686483
RPL7	chr4	62601569	62605892	610903.62611	chr4	62610903	62611910	-0.5685873
RND1	chr5	14914645	14922038	027205.15029	chr5	15027205	15029851	-0.5685396
LIMS2	chr15	59672540	59709379	9639470.5964	chr15	59639470	59643470	-0.5682205
RAB43	chr13	71562350	71596249	.730277.7173	chr13	71730277	71734277	-0.5680726
CDA	chr6	78824270	78849547	786138.78790	chr6	78786138	78790138	-0.5678987
OS9	chr5	22957875	23004458	880669.22884	chr5	22880669	22884669	-0.5677578
RNASEH2B	chr11	16825128	16911969	6374841.1637	chr11	16374841	16377014	-0.567746
POLD2	chr18	51038358	51046779	0698457.5070	chr18	50698457	50700032	-0.5676414
NOP9	chr7	74985753	74992946	027923.75031	chr7	75027923	75031923	-0.5674237
ENSSSCG00000036812	chr12	61077631	61186002	.029157.6103	chr12	61029157	61033157	-0.5670786
PFDN1	chr2	142011063	142077925	376462.14238	chr2	142376462	142380462	-0.5669644
TBCC	chr7	37822767	37824953	211487.38215	chr7	38211487	38215487	-0.5663492
ACAT1	chr9	36525261	36545633	878970.36879	chr9	36878970	36879685	-0.5662269
GNL3	chr13	34814901	34828517	4837384.3484	chr13	34837384	34841384	-0.5661993
YBX2	chr12	52635385	52641651	2627516.5262	chr12	52627516	52629243	-0.5659514
SENP3	chr12	52765187	52867576	2627516.5262	chr12	52627516	52629243	-0.5654556
LSM10	chr6	92489927	92493665	413296.92414	chr6	92413296	92414105	-0.5654079
ALAD	chr1	254012850	254027508	076730.25408	chr1	254076730	254080730	-0.5650151
IK	chr2	142361155	142376333	882155.14188	chr2	141882155	141886155	-0.5650079
STAT5A	chr12	20474227	20499138	0256373.2026	chr12	20256373	20260373	-0.5645638
DNPEP	chr15	121391746	121412864	0983257.1209	chr15	120983257	120987257	-0.5643595
CCNL2	chr6	63659054	63668047	657054.63661	chr6	63657054	63661054	-0.5640838
CPB2	chr11	21234447	21298241	.499492.2150	chr11	21499492	21503492	-0.5640685
NDUFA6	chr5	6562011	6567647	560011.65640	chr5	6560011	6564011	-0.5637958
IK	chr2	142361155	142376333	577020.14257	chr2	142577020	142579530	-0.563687
ENSSSCG00000042487	chr7	58998922	59008157	826727.58830	chr7	58826727	58830727	-0.5635724
DRAP1	chr2	6409655	6412395	537993.65396	chr2	6537993	6539697	-0.563504
ENSSSCG00000044155	chr2	142321430	142324016	639970.14264	chr2	142639970	142642910	-0.5631496
ENSSSCG00000029160	chr7	23914891	23928175	836996.23840	chr7	23836996	23840996	-0.5631398
CRAT	chr1	269386650	269401331	207693.26921	chr1	269207693	269211693	-0.5629926
RDH13	chr6	59274387	59292765	850751.58854	chr6	58850751	58854751	-0.5629583
MRPL17	chr9	3075995	3083580	376207.33802	chr9	3376207	3380207	-0.5626073
PHB2	chr5	63751558	63756478	114150.64118	chr5	64114150	64118150	-0.5625091
EFHC1	chr7	46239230	46348667	185166.46186	chr7	46185166	46186082	-0.5623721
STX18	chr8	5890765	6004399	890392.58913	chr8	5890392	5891389	-0.5622893
TTLL3	chr13	66056890	66123977	5994525.6599	chr13	65994525	65997625	-0.5622186
SRSF5	chr7	93393426	93403011	927069.92931	chr7	92927069	92931069	-0.561861
ZNRD2	chr2	6683057	6684494	755635.67596	chr2	6755635	6759635	-0.5615868
MAP4K2	chr2	7365246	7378996	057462.70614	chr2	7057462	7061462	-0.5615375
POP7	chr3	8605018	8610218	618508.86225	chr3	8618508	8622508	-0.5615054
AP1S1	chr3	8881107	8887566	904591.89085	chr3	8904591	8908591	-0.5613932
ENSSSCG00000006559	chr4	95713742	95716153	903054.95907	chr4	95903054	95907054	-0.56138
RPS3	chr9	9624990	9630401	624152.96281	chr9	9624152	9628152	-0.5613396
ALAD	chr1	254012850	254027508	973973.25397	chr1	253973973	253977973	-0.5611594
HNRNPUL2	chr2	9034667	9048768	982070.89825	chr2	8982070	8982593	-0.5607342
RFC3	chr11	10146531	10165172	9912809.9913	chr11	9912809	9913316	-0.5605942
SAT2	chr12	52911725	52913363	2627516.5262	chr12	52627516	52629243	-0.5601329
TUBA1B	chr5	15149099	15153620	493157.15497	chr5	15493157	15497157	-0.5600486
ENKD1	chr6	28283117	28287306	463208.28467	chr6	28463208	28467208	-0.5599447
FTL	chr6	54231172	54232750	231050.54235	chr6	54231050	54235050	-0.5599257
ATP5F1A	chr1	95733090	95750841	727119.95728	chr1	95727119	95728266	-0.5590927
MRPL37	chr6	158090970	158143500	535465.15853	chr6	158535465	158539465	-0.5588973
SNAPC2	chr2	71291257	71294006	952896.70956	chr2	70952896	70956896	-0.5586925
NAA60	chr3	38763447	38781988	644938.38648	chr3	38644938	38648938	-0.5584047
SCAMP2	chr7	58672664	58699142	826727.58830	chr7	58826727	58830727	-0.5583892
ENSSSCG00000037510	chr17	37923475	37944393	7955507.3795	chr17	37955507	37959507	-0.5583113
FSTL3	chr2	77656663	77662613	943841.77947	chr2	77943841	77947841	-0.5567232
TAGLN	chr9	44560785	44567385	783701.44787	chr9	44783701	44787701	-0.5566371
ENSSSCG00000051162	chr8	44145897	44147246	447290.44451	chr8	44447290	44451290	-0.5561126
MRPS33	chr18	8883459	8897952	8842902.8845	chr18	8842902	8845088	-0.555909

TUBB	chr7	23247850	23252233	062338.23066	chr7	23062338	23066338	-0.5557856
C19orf67	chr2	65100755	65104628	094877.65098	chr2	65094877	65098877	-0.5553905
RNF121	chr9	6488806	6587324	064374.60656	chr9	6064374	6065627	-0.5549373
YBX2	chr12	52635385	52641651	399810.5240	chr12	52399810	52403810	-0.5549265
LONP1	chr2	73266258	73286773	060896.73064	chr2	73060896	73064896	-0.5548652
ARHGAP9	chr5	22747339	22762049	880669.22884	chr5	22880669	22884669	-0.5548549
ENSSSCG00000014540	chr2	9535148	9537974	734823.97360	chr2	9734823	9736008	-0.5543057
LGALS8	chr14	54867021	54892049	891100.5489	chr14	54891100	54892064	-0.5542413
MON1A	chr13	32426736	32437862	990619.3199	chr13	31990619	31991906	-0.5542379
NAT10	chr2	26786177	26826992	146241.27147	chr2	27146241	27147074	-0.5541599
MAF1	chr4	597472	600447	157303.16130	chr4	157303	161303	-0.5537305
ENSSSCG00000003286	chr6	59113039	59134353	850751.58854	chr6	58850751	58854751	-0.5536627
CCNDBP1	chr1	128253035	128269452	641100.12864	chr1	128641100	128642036	-0.5535038
CDH6	chr16	17531952	17660079	7938135.1794	chr16	17938135	17942135	-0.5533442
GABARAP	chr12	52596543	52598194	627516.5262	chr12	52627516	52629243	-0.5532962
ATG101	chr5	17418121	17425624	312276.17316	chr5	17312276	17316276	-0.5532318
GMPPB	chr13	32248268	32253155	251515.3225	chr13	32251515	32255515	-0.5529424
POLA2	chr2	6922387	6950179	057462.70614	chr2	7057462	7061462	-0.5528455
NLRP5	chr6	60379623	60409465	845868.60846	chr6	60845868	60846733	-0.5528294
AUP1	chr3	68519969	68523073	519015.68520	chr3	68519015	68520573	-0.5525421
ENSSSCG00000029830	chr4	136107	138652	205764.20709	chr4	205764	207099	-0.5522319
PRR14	chr3	17745892	17751099	902442.17906	chr3	17902442	17906442	-0.552103
ZFPL1	chr2	7098766	7103536	079608.70836	chr2	7079608	7083608	-0.5519783
NRAP	chr14	123926927	124018440	017164.1240	chr14	124017164	124021164	-0.5514752
CDIPT	chr3	18084549	18091798	902442.17906	chr3	17902442	17906442	-0.5514335
EIF3G	chr2	68966222	68970614	109309.69113	chr2	69109309	69113309	-0.5513657
RNF157	chr12	5225849	5304458	116522.5120	chr12	5116522	5120486	-0.550983
MRPL40	chr14	51102613	51105767	695047.5069	chr14	50695047	50699047	-0.5507743
ARHGEF18	chr2	71651100	71747409	519081.71523	chr2	71519081	71523081	-0.5503763
FAM104A	chr12	7727796	7749240	7637681.7639	chr12	7637681	7639269	-0.5502372
DGAT1	chr4	452662	466684	157303.16130	chr4	157303	161303	-0.550135
FAU	chr2	7070906	7072809	057462.70614	chr2	7057462	7061462	-0.5500568
DDX49	chr2	58922988	58930716	826251.58830	chr2	58826251	58830251	-0.5499689
RAB5C	chr12	20613996	20636073	0256958.2025	chr12	20256958	20257834	-0.5499425
AHSG	chr13	124426308	124438840	582327.1245	chr13	124582327	124586327	-0.5498232
ENSSSCG00000032082	chr6	95322453	95366673	978926.94980	chr6	94978926	94980105	-0.5496076
TIMM29	chr2	69649712	69652362	058193.70062	chr2	70058193	70062193	-0.5495765
RAB43	chr13	71562350	71596249	760305.7176	chr13	71760305	71762524	-0.5494234
IK	chr2	142361155	142376333	350030.14235	chr2	142350030	142354030	-0.5492364
BPNT1	chr10	9659487	9683912	0156482.1015	chr10	10156482	10157235	-0.549184
UPB1	chr14	49533231	49565075	792613.4979	chr14	49792613	49793506	-0.5487969
TPT1	chr11	21929221	21932691	927221.2193	chr11	21927221	21931221	-0.5485086
ENSSSCG00000006081	chr4	38701720	38720780	489285.38493	chr4	38489285	38493285	-0.5483764
VPS28	chr4	362912	370588	157303.16130	chr4	157303	161303	-0.5483047
RIMKLA	chr6	168916725	168950046	994003.16899	chr6	168994003	168995215	-0.5479569
CDC26	chr1	253895885	253906632	973973.25397	chr1	253973973	253977973	-0.5479352
STK25	chr15	140144336	140154366	131918.1401	chr15	140131918	140133932	-0.5478377
KRTCAP2	chr4	94642942	94646436	495309.94499	chr4	94495309	94499039	-0.5476644
ENSSSCG00000029830	chr4	136107	138652	429062.43018	chr4	429062	430184	-0.5476072
ENSSSCG00000010056	chr14	49619838	49655206	792613.4979	chr14	49792613	49793506	-0.5475469
ENSSSCG00000044155	chr2	142321430	142324016	578003.14257	chr2	142578003	142579756	-0.5473172
ENSSSCG00000005101	chr1	191102213	191102539	533301.19153	chr1	191533301	191537301	-0.5473093
NSMAF	chr4	74236826	74298193	234826.74238	chr4	74234826	74238826	-0.5472742
TMED4	chr18	50699470	50702705	456411.5046	chr18	50456411	50460411	-0.5469519
ARMC12	chr7	31586797	31603600	549482.31550	chr7	31549482	31550858	-0.5469262
EDC3	chr7	58828727	58897919	826727.58830	chr7	58826727	58830727	-0.5468872
GMPPB	chr13	32248268	32253155	989610.3199	chr13	31989610	31993610	-0.5468018
ENSSSCG00000001064	chr7	12254388	12299531	116919.12117	chr7	12116919	12117991	-0.5466769
OSBPL2	chr17	61643377	61685819	551971.6155	chr17	61551971	61555971	-0.5466645
NRBP1	chr3	111720379	111733304	044398.11204	chr3	112044398	112048398	-0.5465362
STX5	chr2	8937778	8968932	982070.89825	chr2	8982070	8982593	-0.5461965
ENSSSCG00000044567	chr17	31217462	31238052	941728.3094	chr17	30941728	30942375	-0.5459805

MCRS1	chr5	15517032	15526510	024080.15028	chr5	15024080	15028080	-0.5456753
PMP22	chr12	58679775	58707925	3962479.5896	chr12	58962479	58966479	-0.5454875
ENSSSCG00000035904	chr1	272959831	272965634	835449.27283	chr1	272835449	272836800	-0.5453309
ZKSCAN5	chr3	6442391	6466430	373763.63747	chr3	6373763	6374740	-0.5451982
VPS11	chr9	46285739	46297474	362936.46366	chr9	46362936	46366936	-0.5451903
NRBP1	chr3	111720379	111733304	045290.11204	chr3	112045290	112049290	-0.5450511
NHEJ1	chr15	121100628	121190062	.025670.1210	chr15	121025670	121027619	-0.5447932
ARMC12	chr7	31586797	31603600	622450.31626	chr7	31622450	31626450	-0.5447823
ZNRD2	chr2	6683057	6684494	537993.65396	chr2	6537993	6539697	-0.5447018
DDIT3	chr5	22785445	22789829	852316.22856	chr5	22852316	22856316	-0.5445063
SWI5	chr1	268720355	268726521	464700.26846	chr1	268464700	268468700	-0.5444983
EIF4EBP1	chr15	48422816	48443399	3693778.4869	chr15	48693778	48695388	-0.5443341
ZP3	chr3	9934761	9944941	132373.10132	chr3	10132373	10132908	-0.544217
RETREG2	chr15	121212881	121218812	.210881.1212	chr15	121210881	121214881	-0.5441268
FTL	chr6	54231172	54232750	601535.54602	chr6	54601535	54602403	-0.5441124
AURKAIP1	chr6	63656119	63657472	654119.63658	chr6	63654119	63658119	-0.544009
REEP4	chr14	6411513	6415590	6833996.6835	chr14	6833996	6835311	-0.544007
RAB7A	chr13	71777424	71852694	.758467.7176	chr13	71758467	71762467	-0.5439882
ENSSSCG00000059201	chr4	90261918	90263262	107057.90111	chr4	90107057	90111057	-0.5435551
ENSSSCG00000061760	chr7	31573753	31585356	622450.31626	chr7	31622450	31626450	-0.5435545
CALR	chr2	66098229	66102132	271286.66272	chr2	66271286	66272770	-0.5434511
DRG1	chr14	48126863	48157906	3049624.4805	chr14	48049624	48050222	-0.5433151
CIAO2B	chr6	27610470	27612592	546706.27550	chr6	27546706	27550706	-0.5432665
NDUFAF1	chr1	129968502	130009391	110847.13011	chr1	130110847	130114847	-0.5431694
TXNL4A	chr6	127974543	127991177	028715.12803	chr6	128028715	128032715	-0.5427004
ZMAT2	chr2	142411089	142419137	376462.14238	chr2	142376462	142380462	-0.5425257
TOMM20	chr14	56113192	56127677	5923940.5592	chr14	55923940	55925554	-0.5423766
MTCH1	chr7	32617426	32635575	786291.32790	chr7	32786291	32790291	-0.5423135
PCDHGA4	chr2	142993554	143156556	639970.14264	chr2	142639970	142642910	-0.5418157
MAP2K3	chr12	61396369	61417321	.029157.6103	chr12	61029157	61033157	-0.541763
THBS3	chr4	94611593	94623902	495309.94499	chr4	94495309	94499039	-0.5415974
SARS1	chr4	110857089	110874815	006543.11100	chr4	111006543	111007819	-0.5411731
METTL23	chr12	4804037	4808913	5053065.5057	chr12	5053065	5057065	-0.541115
ARHGAP22	chr14	89113768	89331274	3986949.8899	chr14	88986949	88990949	-0.5410575
TINF2	chr7	75056798	75063979	027923.75031	chr7	75027923	75031923	-0.5409273
ARV1	chr14	59396962	59408958	9408985.5940	chr14	59408985	59409978	-0.5408055
RAB5C	chr12	20613996	20636073	0256373.2026	chr12	20256373	20260373	-0.5406804
MON1A	chr13	32426736	32437862	2251515.3225	chr13	32251515	32255515	-0.5406138
GREB1L	chr6	106419943	106691625	314117.10631	chr6	106314117	106315302	-0.540485
TCFL5	chr17	62093907	62115650	2558809.6255	chr17	62558809	62559642	-0.5404718
COX5B	chr3	56629272	56632119	810364.56812	chr3	56810364	56812121	-0.5402128
RABAC1	chr6	49933434	49936372	931434.49935	chr6	49931434	49935434	-0.5400383
NUCB1	chr6	54180038	54209930	231050.54235	chr6	54231050	54235050	-0.5398066
TMEM219	chr3	18167923	18176563	175737.18179	chr3	18175737	18179737	-0.5396107
ALG8	chr9	12497721	12532307	850636.12851	chr9	12850636	12851763	-0.5395501
MRAP2	chr1	53256006	53318107	997619.52998	chr1	52997619	52998959	-0.53916
TTLL4	chr15	120773185	120793194	.025670.1210	chr15	121025670	121027619	-0.5391548
RRP9	chr13	34067520	34075430	3663957.3366	chr13	33663957	33666213	-0.5391285
AURKAIP1	chr6	63656119	63657472	657054.63661	chr6	63657054	63661054	-0.5390541
CALR	chr2	66098229	66102132	055483.66056	chr2	66055483	66056550	-0.5387027
YBX2	chr12	52635385	52641651	3109256.5311	chr12	53109256	53113256	-0.5386852
GINS1	chr17	31060650	31080314	0941728.3094	chr17	30941728	30942375	-0.5385876
STK10	chr16	52003887	52140367	2003268.5200	chr16	52003268	52004540	-0.5385431
TFR2	chr3	8539320	8578763	904591.89085	chr3	8904591	8908591	-0.5382372
ENSSSCG00000054785	chrX	9898365	9905146	217608.10219	chrX	10217608	10219668	-0.5380615
PIH1D1	chr6	54524172	54535249	705237.54709	chr6	54705237	54709237	-0.537957
ENSSSCG00000054115	chr5	69746689	69772024	395938.69400	chr5	69395938	69400178	-0.537842
LONP1	chr2	73266258	73286773	294501.73295	chr2	73294501	73295458	-0.537621
PSMC3	chr2	15166306	15193047	379707.15383	chr2	15379707	15383707	-0.5376192
ZFTRAF1	chr4	335301	346534	157303.16130	chr4	157303	161303	-0.5375792
MRPL38	chr12	5514415	5521664	5521113.5525	chr12	5521113	5525113	-0.5367771
RAB8A	chr2	61429523	61450413	271040.61273	chr2	61271040	61273371	-0.5367029

CDK4	chr5	23038891	23042061	)56982.2306(	chr5	23056982	23060982	-0.5366568
MED11	chr12	52150448	52152204	!627516.5262	chr12	52627516	52629243	-0.5364409
MAML3	chr8	86879974	87323117	546273.8654(	chr8	86546273	86546957	-0.5364265
ENSSSCG00000038518	chr3	6790314	6819644	373763.6374(	chr3	6373763	6374740	-0.536261
DAPK3	chr2	74759004	74775330	162787.7516(	chr2	75162787	75166787	-0.5360875
VPS52	chr7	29624230	29639140	641182.2964!	chr7	29641182	29645182	-0.5360502
CUL7	chr7	38097854	38115822	388035.3838(	chr7	38388035	38389283	-0.535896
PPAN	chr2	68958906	68962657	109309.6911:	chr2	69109309	69113309	-0.53587
BIRC5	chr12	3747279	3755215	354245.3358	chr12	3354245	3358245	-0.535291
PFDN5	chr5	18512697	18517174	485052.1848(	chr5	18485052	18489052	-0.5352802
ENSSSCG00000031756	chr14	40455954	40458063	)003385.4000	chr14	40003385	40004961	-0.5351359
TCP11	chr7	31007474	31114734	160689.3116:	chr7	31160689	31161712	-0.53507
MST1R	chr13	32409555	32421920	!329740.3233	chr13	32329740	32330722	-0.5349315
TIMM44	chr2	71271805	71287817	952896.7095(	chr2	70952896	70956896	-0.534803
ZP3	chr3	9934761	9944941	130093.1013(	chr3	10130093	10134093	-0.5346545
DND1	chr2	142384532	142387199	539970.1426(	chr2	142639970	142642910	-0.5345429
TUFM	chr3	18521128	18524959	175737.1817(	chr3	18175737	18179737	-0.5344882
TBCC	chr7	37822767	37824953	)51598.3805!	chr7	38051598	38052427	-0.5337695
C9orf78	chr1	269986391	269995395	995271.2699(	chr1	269995271	269995975	-0.5332196
NEDD8	chr7	75070161	75085899	972308.7497(	chr7	74972308	74976308	-0.533177
CTDNEP1	chr12	52599226	52606059	!627516.5262	chr12	52627516	52629243	-0.5330405
TMED4	chr18	50699470	50702705	)698457.5070	chr18	50698457	50700032	-0.5329305
ENSSSCG00000061760	chr7	31573753	31585356	160689.3116:	chr7	31160689	31161712	-0.5328909
ACTR1A	chr14	113480196	113498740	228965.1132	chr14	113228965	113232965	-0.5327616
ODF2	chr1	268856766	268893387	332838.2693:	chr1	269332838	269336838	-0.5325888
MRPL54	chr2	74925070	74928167	162787.7516(	chr2	75162787	75166787	-0.5325683
SMYD4	chr12	47944148	48009586	'859043.4786	chr12	47859043	47863043	-0.5324743
HEXIM2	chr12	18251428	18258295	257274.1825	chr12	18257274	18258147	-0.5321694
LSM4	chr2	59499564	59513246	529154.5953:	chr2	59529154	59533154	-0.5320492
DNPEP	chr15	121391746	121412864	.024245.1210	chr15	121024245	121028245	-0.5320003
DPCD	chr14	112617398	112637913	!214732.1122	chr14	112214732	112218732	-0.5317909
HIGD2A	chr2	81486890	81488045	287760.8128(	chr2	81287760	81288900	-0.5317734
MOB3A	chr2	76475530	76496060	954111.7695(	chr2	76954111	76958111	-0.5316908
HSPA8	chr9	49982284	49990508	628793.4963!	chr9	49628793	49632793	-0.5316243
ITGA11	chr1	166186613	166310800	463953.1664(	chr1	166463953	166467953	-0.5315468
MEPCE	chr3	8357289	8364661	918675.7922(	chr3	7918675	7922675	-0.5314875
HIP1R	chr14	29993655	30024216	)630876.2963	chr14	29630876	29632175	-0.53141
COG8	chr6	17645590	17650481	643590.1764(	chr6	17643590	17647590	-0.5312004
RPL10A	chr7	31327440	31333638	360867.3136!	chr7	31360867	31362660	-0.5311163
GMPPA	chr15	121507564	121515300	.024245.1210	chr15	121024245	121028245	-0.5310398
DUSP5	chr14	120906343	120919384	.062346.1210	chr14	121062346	121066346	-0.530976
COX6A1	chr14	40408631	40412870	)003385.4000	chr14	40003385	40004961	-0.5309282
ENSSSCG00000025928	chr6	53974309	53978544	229172.5423:	chr6	54229172	54233172	-0.5308004
TBCE	chr14	55816668	55901171	923940.5592	chr14	55923940	55925554	-0.5306604
SWI5	chr1	268720355	268726521	718355.2687!	chr1	268718355	268722355	-0.5305004
NTPCR	chr14	57802565	57833151	237469.5823	chr14	58237469	58238478	-0.5304377
MDP1	chr7	75087468	75089227	)27923.7503:	chr7	75027923	75031923	-0.5304143
ENSSSCG00000013064	chr2	9163317	9174468	982070.8982!	chr2	8982070	8982593	-0.5303604
TMEM97	chr12	44604021	44609999	634379.4463	chr12	44634379	44638379	-0.5303489
ADIPOR2	chr5	68755811	68807235	541988.6865(	chr5	68641988	68650108	-0.5300266
B3GNT3	chr2	59934958	59952859	394810.6039(	chr2	60394810	60396270	-0.5299897
CDKN2AIPNL	chr2	136677034	136685955	383860.1363(	chr2	136383860	136386180	-0.5297242
CSNK1D	chr12	768865	796595	.120907.1129	chr12	1120907	1129651	-0.5295604
ATP5PF	chr13	189278945	189290229	)163975.1891	chr13	189163975	189167975	-0.5294723
OS9	chr5	22957875	23004458	895808.2289(	chr5	22895808	22897067	-0.5293381
DYNC2I2	chr1	269002261	269030719	615087.2686:	chr1	268615087	268619087	-0.529171
ENSSSCG00000002790	chr6	27853452	27867226	546706.2755(	chr6	27546706	27550706	-0.5291204
NARF	chr12	632363	651817	.076166.1080	chr12	1076166	1080166	-0.5290763
C9orf78	chr1	269986391	269995395	179344.2701(	chr1	270179344	270180174	-0.5289522
GALK1	chr12	5642025	5651183	521113.5525	chr12	5521113	5525113	-0.5285715
TUBA4A	chr15	121288957	121294853	.210881.1212	chr15	121210881	121214881	-0.5285595
COL23A1	chr2	79766150	80141293	668479.7967:	chr2	79668479	79672479	-0.528528

DRAP1	chr2	6409655	6412395	755635.67596	chr2	6755635	6759635	-0.5284759
CYC1	chr4	606516	608996	157303.16130	chr4	157303	161303	-0.5280437
PRADC1	chr3	69547649	69555085	234351.69238	chr3	69234351	69238351	-0.5280404
MRPL11	chr2	5978483	5981449	409833.64138	chr2	6409833	6413833	-0.5279854
PALM	chr2	77619199	77644673	943841.77947	chr2	77943841	77947841	-0.5279766
LYPLA2	chr6	81601486	81605912	564801.81568	chr6	81564801	81568801	-0.5278629
ENSSSCG00000052071	chr7	64532731	64535636	507971.64508	chr7	64507971	64508850	-0.5277665
RAB43	chr13	71562350	71596249	.982067.7198	chr13	71982067	71986067	-0.5277008
PSMD13	chr2	55108	74829	396766.40076	chr2	396766	400766	-0.5275404
TTLL13	chr7	55811855	55827077	573336.55577	chr7	55573336	55577336	-0.5275073
PDCD5	chr6	42483449	42491591	558113.42562	chr6	42558113	42562113	-0.5274417
ENSSSCG00000036812	chr12	61077631	61186002	.247989.6125	chr12	61247989	61253489	-0.5274227
LOXL2	chr14	7435867	7540704	7388061.7392	chr14	7388061	7392061	-0.5271798
SLC35A4	chr2	142323341	142325335	639970.14264	chr2	142639970	142642910	-0.5268386
DRG1	chr14	48126863	48157906	7638106.4763	chr14	47638106	47638634	-0.5267212
RNASEH2A	chr2	66193197	66207697	055483.66056	chr2	66055483	66056550	-0.5265992
GABBR2	chr1	240107466	240496101	918638.23991	chr1	239918638	239919296	-0.526588
RRP36	chr7	38087258	38094548	211487.38215	chr7	38211487	38215487	-0.5263858
CHCHD4	chr13	70417326	70428922	0536894.7054	chr13	70536894	70540894	-0.5263126
POLR1D	chr11	5044329	5089766	4879361.4880	chr11	4879361	4880102	-0.5261941
SLC46A3	chr11	5982443	6000701	6000360.6001	chr11	6000360	6001074	-0.5259805
DND1	chr2	142384532	142387199	641101.14264	chr2	142641101	142642772	-0.5258789
SRRT	chr3	8717785	8731201	356207.83580	chr3	8356207	8358016	-0.5258444
PARK7	chr6	68629214	68645516	487103.68487	chr6	68487103	68487955	-0.5258248
RBM15B	chr13	33664472	33667153	3663957.3366	chr13	33663957	33666213	-0.5254259
POLD1	chr6	55247880	55272085	198826.55201	chr6	55198826	55201756	-0.5253934
MRM3	chr12	47080367	47087405	7123236.4712	chr12	47123236	47127236	-0.5253763
OOEP	chr1	53006761	53007877	997619.52998	chr1	52997619	52998959	-0.5251799
SLC25A11	chr12	51970806	51975548	2102504.5210	chr12	52102504	52106504	-0.5251168
SSR2	chr4	94011527	94021962	495309.94499	chr4	94495309	94499039	-0.5251098
CPSF3	chr3	126868467	126905746	510477.12651	chr3	126510477	126511819	-0.5251006
DRAP1	chr2	6409655	6412395	471205.64727	chr2	6471205	6472742	-0.5251004
PIGH	chr7	91306121	91316278	955179.90956	chr7	90955179	90956189	-0.5250002
FZR1	chr2	75121835	75146835	162787.75166	chr2	75162787	75166787	-0.5249679
ENSSSCG00000014284	chr2	135074111	135078541	700543.13470	chr2	134700543	134701416	-0.5248231
ENSSSCG00000017971	chr12	53111256	53112484	3109256.5311	chr12	53109256	53113256	-0.5247182
ENSSSCG00000052760	chr1	208706991	208710843	179467.20918	chr1	209179467	209183467	-0.524577
ATRAID	chr3	111914708	111919946	647539.11165	chr3	111647539	111651539	-0.5245292
MRPL51	chr5	64167200	64168983	114150.64118	chr5	64114150	64118150	-0.524459
BRF2	chr15	48568202	48573029	3693951.4869	chr15	48693951	48697396	-0.5244019
PRPF6	chr17	62815085	62858382	2609909.6261	chr17	62609909	62613909	-0.5242235
ENSSSCG00000053570	chr6	62255210	62260695	690463.62691	chr6	62690463	62691254	-0.5237526
MMD2	chr3	3729531	3775205	798186.38021	chr3	3798186	3802186	-0.5237251
PRR14	chr3	17745892	17751099	118581.18122	chr3	18118581	18122581	-0.5233472
ARMC2	chr1	74923868	75043060	169857.75170	chr1	75169857	75170800	-0.523326
ENSSSCG00000013064	chr2	9163317	9174468	981759.89857	chr2	8981759	8985759	-0.5231207
MRPL51	chr5	64167200	64168983	841745.63845	chr5	63841745	63845745	-0.5230699
FXR2	chr12	52882870	52903450	2627516.5262	chr12	52627516	52629243	-0.5228692
NDUFA6	chr5	6562011	6567647	081444.60854	chr5	6081444	6085444	-0.5227748
CLN8	chr15	33288301	33304631	3478739.3347	chr15	33478739	33479825	-0.5225681
TMC4	chr6	55955991	55970280	189076.56190	chr6	56189076	56190746	-0.5225057
TBC1D10B	chr3	17944629	17956069	118581.18122	chr3	18118581	18122581	-0.5224226
TRIM14	chr1	239912126	239945073	492188.23949	chr1	239492188	239493251	-0.5221891
IK	chr2	142361155	142376333	883680.14188	chr2	141883680	141886890	-0.5221795
GGH	chr4	70996755	71021838	959465.70963	chr4	70959465	70963465	-0.5221587
AIDA	chr10	11428399	11466643	.334439.1133	chr10	11334439	11336379	-0.5221542
ARID3B	chr7	58932461	58998711	908967.58910	chr7	58908967	58910668	-0.5219025
POP7	chr3	8605018	8610218	916872.89208	chr3	8916872	8920872	-0.5218946
MDP1	chr7	75087468	75089227	972308.74976	chr7	74972308	74976308	-0.5218121
DNPEP	chr15	121391746	121412864	.282920.1212	chr15	121282920	121286920	-0.5215438
AAAS	chr5	18526076	18537830	409267.18410	chr5	18409267	18410275	-0.5213368
TRAPPC4	chr9	46242171	46246343	362936.46366	chr9	46362936	46366936	-0.5211443

TTLL13	chr7	55811855	55827077	575340.55576	chr7	55575340	55576442	-0.5211433
PDCD5	chr6	42483449	42491591	587556.42591	chr6	42587556	42591556	-0.5211366
MARS1	chr5	22752374	22785501	881828.22885	chr5	22881828	22885548	-0.5209745
PCDHGA4	chr2	142993554	143156556	641101.14264	chr2	142641101	142642772	-0.5206271
SPR	chr3	69889452	69894680	655696.69659	chr3	69655696	69659696	-0.5205829
COQ4	chr1	268756685	268768343	465007.26846	chr1	268465007	268466088	-0.5204881
RAB43	chr13	71562350	71596249	.758467.7176	chr13	71758467	71762467	-0.5204536
ENSSSCG00000003253	chr6	56236311	56244185	225666.56227	chr6	56225666	56227716	-0.5198653
TUBB2A	chr7	1910269	1914761	130734.21330	chr7	2130734	2133042	-0.5198322
NFATC2IP	chr3	18609912	18622640	522820.18526	chr3	18522820	18526820	-0.5196812
CAPN8	chr10	19665855	19732139	286367.1929	chr10	19286367	19290367	-0.5194498
RIMKLA	chr6	168916725	168950046	875249.16887	chr6	168875249	168879249	-0.5193333
CAPNS1	chr6	45511295	45518610	304692.45306	chr6	45304692	45306421	-0.5191693
ENSSSCG00000037143	chr14	50264868	50315717	792613.4979	chr14	49792613	49793506	-0.5187888
ENSSSCG00000029830	chr4	136107	138652	157303.16130	chr4	157303	161303	-0.5186895
NDUFS3	chr2	15031149	15036880	379707.15383	chr2	15379707	15383707	-0.5184486
ALAD	chr1	254012850	254027508	569435.25357	chr1	253569435	253570326	-0.5181611
HIRIP3	chr3	18198807	18202089	522820.18526	chr3	18522820	18526820	-0.5181405
MCRS1	chr5	15517032	15526510	027205.15029	chr5	15027205	15029851	-0.5181042
ARMC2	chr1	74923868	75043060	169061.75173	chr1	75169061	75173061	-0.5176447
KAT5	chr2	6560513	6572466	755635.67596	chr2	6755635	6759635	-0.5176156
MTUS2	chr11	6197740	6532924	000360.6001	chr11	6000360	6001074	-0.5174102
ENSSSCG00000032060	chr6	7255169	7279427	418790.74227	chr6	7418790	7422790	-0.5173239
NXN	chr12	46904407	47075432	123236.4712	chr12	47123236	47127236	-0.5172687
ENSSSCG00000037514	chr6	95566555	95599254	321439.95322	chr6	95321439	95322907	-0.5171928
DPPA5	chr1	52986462	52987590	090036.53094	chr1	53090036	53094036	-0.5170537
ADAMTS7	chr7	47929033	47994453	447036.47451	chr7	47447036	47451036	-0.5168752
ARHGAP9	chr5	22747339	22762049	881828.22885	chr5	22881828	22885548	-0.5168721
STK16	chr15	121284920	121288473	.282920.1212	chr15	121282920	121286920	-0.5168434
ENSSSCG00000058051	chr1	52996822	52999185	997619.52998	chr1	52997619	52998959	-0.5166043
SEC61G	chr9	139128296	139135727	988480.13899	chr9	138988480	138992480	-0.5162653
POLR1D	chr11	5044329	5089766	042329.5046	chr11	5042329	5046329	-0.516258
NHEJ1	chr15	121100628	121190062	.024245.1210	chr15	121024245	121028245	-0.5161625
TPI1	chr5	63838506	63843134	841745.63845	chr5	63841745	63845745	-0.5160653
FTL	chr6	54231172	54232750	142020.54146	chr6	54142020	54146020	-0.5160283
MRPL20	chr6	63670755	63675839	657054.63661	chr6	63657054	63661054	-0.5158789
POLR1D	chr11	5044329	5089766	042384.5044	chr11	5042384	5044586	-0.5158702
AUP1	chr3	68519969	68523073	570415.68574	chr3	68570415	68574415	-0.5155819
YIF1A	chr2	6095877	6102037	304558.63059	chr2	6304558	6305906	-0.5155625
ENSSSCG00000040854	chr3	68883051	68898008	234351.69238	chr3	69234351	69238351	-0.5155294
MAN2B1	chr2	66329045	66348056	271286.66272	chr2	66271286	66272770	-0.5155154
OS9	chr5	22957875	23004458	056982.23060	chr5	23056982	23060982	-0.5152797
ATP5MC2	chr5	18871026	18879609	165157.19169	chr5	19165157	19169157	-0.5151403
NLRP8	chr6	60350622	60370529	845868.60846	chr6	60845868	60846733	-0.5149519
CLTA	chr1	236971505	237001731	917879.23692	chr1	236917879	236921879	-0.5148332
OTUB1	chr2	8087829	8095609	828920.78329	chr2	7828920	7832920	-0.5148231
PPIL2	chr14	50208374	50232830	695047.5069	chr14	50695047	50699047	-0.5148056
ENSSSCG00000061760	chr7	31573753	31585356	925961.31927	chr7	31925961	31927012	-0.5146009
PYGB	chr17	30940452	30995691	941728.3094	chr17	30941728	30942375	-0.5143061
NDUFS5	chr6	94913076	94919452	321439.95322	chr6	95321439	95322907	-0.5142001
KXD1	chr2	59242310	59247713	828025.58828	chr2	58828025	58828910	-0.5140146
SEC61G	chr9	139128296	139135727	990014.13899	chr9	138990014	138990729	-0.5138481
PIP4P1	chr7	78447369	78451846	434300.78434	chr7	78434300	78434853	-0.5138167
GPX4	chr2	77320464	77323931	776470.77777	chr2	77776470	77777125	-0.5136688
ZDHHC4	chr3	4628385	4643691	992886.49950	chr3	4992886	4995036	-0.5134837
ENSSSCG00000005528	chr1	262236482	262264097	898896.26190	chr1	261898896	261900556	-0.5133871
APMAP	chr17	30768488	30795755	.194616.3119	chr17	31194616	31195316	-0.5132692
ESYT1	chr5	21514510	21535929	493944.21497	chr5	21493944	21497944	-0.5131017
ENSSSCG00000035904	chr1	272959831	272965634	953473.27295	chr1	272953473	272957473	-0.5128877
ZNF584	chr6	62992506	63005691	965994.62969	chr6	62965994	62969994	-0.5128364
UMPS	chr13	135610058	135650603	465798.1354	chr13	135465798	135469798	-0.5128279
DPPA5	chr1	52986462	52987590	997619.52998	chr1	52997619	52998959	-0.5128055

CDC25B	chr17	31910825	31929242	.591390.3159	chr17	31591390	31592201	-0.5127743
ENSSSCG00000024588	chr2	75538090	75542189	162787.7516	chr2	75162787	75166787	-0.5126896
CFL1	chr2	6469254	6475035	428153.6432	chr2	6428153	6432153	-0.5126814
BNIP1	chr16	51126926	51140394	.075321.5107	chr16	51075321	51077044	-0.5126089
PROP1	chr2	79627603	79631270	668479.7967	chr2	79668479	79672479	-0.5122525
MVP	chr3	18057177	18081155	902442.1790	chr3	17902442	17906442	-0.5122086
TPI1	chr5	63838506	63843134	114150.6411	chr5	64114150	64118150	-0.512189
RPL10A	chr7	31327440	31333638	622450.3162	chr7	31622450	31626450	-0.5121603
UQCRB	chr4	40403109	40409057	472366.4047	chr4	40472366	40476373	-0.5121089
CDIPT	chr3	18084549	18091798	701901.1770	chr3	17701901	17702971	-0.5120239
BLVRA	chr18	51131584	51185435	.493135.5149	chr18	51493135	51495445	-0.5116482
MFSD11	chr12	4774678	4801456	807118.4811	chr12	4807118	4811118	-0.5114535
TCP11	chr7	31007474	31114734	666632.3066	chr7	30666632	30667390	-0.5113506
POLE3	chr1	254033385	254035978	569456.2535	chr1	253569456	253571216	-0.511234
FAM193A	chr8	1388266	1523928	386266.1390	chr8	1386266	1390266	-0.5110404
PSMD7	chr6	17047430	17057176	231858.1723	chr6	17231858	17235858	-0.5109959
ATP5MK	chr14	114321622	114327485	435045.1144	chr14	114435045	114439045	-0.5109323
POLR1D	chr11	5044329	5089766	332263.5334	chr11	5332263	5334043	-0.5109169
SLC25A11	chr12	51970806	51975548	440268.5244	chr12	52440268	52442734	-0.5109115
ATP5MC2	chr5	18871026	18879609	409267.1841	chr5	18409267	18410275	-0.5109039
UMPS	chr13	135610058	135650603	467337.1354	chr13	135467337	135468789	-0.5107639
GEMIN2	chr1	169676156	169701481	723649.1697	chr1	169723649	169727649	-0.5107283
ARFGAP2	chr2	15390415	15401227	379707.1538	chr2	15379707	15383707	-0.5107191
HOXD9	chr15	81924142	81926274	.881154.8188	chr15	81881154	81884936	-0.5107011
OS9	chr5	22957875	23004458	881828.2288	chr5	22881828	22885548	-0.5105651
ENSSSCG00000007947	chr3	37989180	37998365	553394.3755	chr3	37553394	37557394	-0.5104924
DPH1	chr12	48146417	48161770	859199.4786	chr12	47859199	47861179	-0.5104508
MARS1	chr5	22752374	22785501	044561.2304	chr5	23044561	23048561	-0.5104342
PCDHGA4	chr2	142993554	143156556	300062.1433	chr2	143300062	143304062	-0.5103728
HEXIM2	chr12	18251428	18258295	766111.1777	chr12	17766111	17770111	-0.5102865
TUFM	chr3	18521128	18524959	903945.1890	chr3	18903945	18905665	-0.5102605
TRMT1	chr2	65940125	65949324	950519.6595	chr2	65950519	65954519	-0.5102092
CALR	chr2	66098229	66102132	153750.6615	chr2	66153750	66154740	-0.51018
ENSSSCG00000032573	chr4	75636278	75646153	645455.7564	chr4	75645455	75646908	-0.510076
RND1	chr5	14914645	14922038	024080.1502	chr5	15024080	15028080	-0.5100229
RAB8A	chr2	61429523	61450413	138907.6113	chr2	61138907	61139628	-0.5096221
TTLL4	chr15	120773185	120793194	.253797.1212	chr15	121253797	121254795	-0.5094652
PCID2	chr11	78541318	78556733	753533.7875	chr11	78753533	78757533	-0.5093164
TMEM192	chr8	43960709	43990093	447290.4445	chr8	44447290	44451290	-0.5092215
ILK	chr9	3145608	3159858	073995.3077	chr9	3073995	3077995	-0.5092189
VPS29	chr14	31859067	31869579	311154.3231	chr14	32311154	32312445	-0.5088397
TMEM231	chr6	12140687	12164140	369369.1237	chr6	12369369	12373369	-0.508758
RNF141	chr2	48985511	49028486	082751.4908	chr2	49082751	49083432	-0.5087488
RPS19	chr6	50000948	50010341	931434.4993	chr6	49931434	49935434	-0.5086643
FSD2	chr7	52144506	52194133	730443.5173	chr7	51730443	51731835	-0.5084374
ZFTRAF1	chr4	335301	346534	429062.4301	chr4	429062	430184	-0.5084023
SMYD2	chr9	129263526	129316349	054886.1290	chr9	129054886	129056417	-0.5083802
PSKH1	chr6	28508823	28544062	463208.2846	chr6	28463208	28467208	-0.508099
DNPEP	chr15	121391746	121412864	.025670.1210	chr15	121025670	121027619	-0.5080382
DYNC2I2	chr1	269002261	269030719	207693.2692	chr1	269207693	269211693	-0.5077809
HSPA5	chr1	265930045	265934894	931340.2659	chr1	265931340	265935340	-0.5076678
ENSSSCG00000005101	chr1	191102213	191102539	533757.1915	chr1	191533757	191536473	-0.507657
PRDX2	chr2	66207828	66212009	055483.6605	chr2	66055483	66056550	-0.5076416
ENSSSCG00000042487	chr7	58998922	59008157	908967.5891	chr7	58908967	58910668	-0.5076
ENSSSCG00000037143	chr14	50264868	50315717	695047.5069	chr14	50695047	50699047	-0.5074999
ACTR1B	chr3	56610304	56620164	810364.5681	chr3	56810364	56812121	-0.5074984
RALGDS	chr1	272776846	272824184	953473.2729	chr1	272953473	272957473	-0.5074342
STYXL1	chr3	10132093	10205179	132373.1013	chr3	10132373	10132908	-0.5073261
DNPEP	chr15	121391746	121412864	.210881.1212	chr15	121210881	121214881	-0.5071022
MAD2L1BP	chr7	38613440	38623832	388035.3838	chr7	38388035	38389283	-0.5070741
PDE6D	chr15	132375743	132426683	354132.1323	chr15	132354132	132358935	-0.5068769
MRPL50	chr1	243100372	243107084	682071.2426	chr1	242682071	242683303	-0.5068698

SAT2	chr12	52911725	52913363	109256.5311	chr12	53109256	53113256	-0.5068159
HAUS1	chr1	95756267	95780758	727119.9572	chr1	95727119	95728266	-0.5064424
SCYL1	chr2	6716115	6733438	755635.6759	chr2	6755635	6759635	-0.5061493
KIF22	chr3	18023096	18041730	742406.1774	chr3	17742406	17744406	-0.5060779
TMEM223	chr2	8981963	8983263	098702.9102	chr2	9098702	9102702	-0.5058906
TRMT1	chr2	65940125	65949324	153750.6615	chr2	66153750	66154740	-0.5058697
LDLRAP1	chr6	83030702	83054377	361071.8336	chr6	83361071	83362136	-0.505591
ENSSSCG00000037143	chr14	50264868	50315717	838305.4984	chr14	49838305	49842305	-0.5055616
YPEL1	chr14	50190798	50208256	020243.5002	chr14	50020243	50020763	-0.5055393
YJU2B	chr2	65353578	65372085	094877.6509	chr2	65094877	65098877	-0.5055255
TOMM40L	chr4	89228554	89235347	258461.8926	chr4	89258461	89262461	-0.5053252
SUSD4	chr10	19421565	19551840	286367.1929	chr10	19286367	19290367	-0.5052528
ADA	chr17	47044497	47072245	385600.4738	chr17	47385600	47389600	-0.5048283
STX18	chr8	5890765	6004399	022066.6024	chr8	6022066	6024012	-0.5047072
TRAP1	chr3	38536668	38603015	644938.3864	chr3	38644938	38648938	-0.504638
FSD2	chr7	52144506	52194133	729077.5173	chr7	51729077	51733077	-0.5044355
PHB2	chr5	63751558	63756478	841745.6384	chr5	63841745	63845745	-0.504348
LHX6	chr1	262295353	262322530	203524.2622	chr1	262203524	262204368	-0.5043083
MMD2	chr3	3729531	3775205	132177.4136	chr3	4132177	4136177	-0.5042865
TTLL4	chr15	120773185	120793194	983257.1209	chr15	120983257	120987257	-0.5040979
OTUB1	chr2	8087829	8095609	280127.8284	chr2	8280127	8284127	-0.503853
RIOK1	chr7	4750752	4782448	057871.5060	chr7	5057871	5060641	-0.5038513
AKR1A1	chr6	165811247	165824896	669174.1656	chr6	165669174	165673174	-0.5037728
NOC2L	chr6	63307695	63335201	657054.6366	chr6	63657054	63661054	-0.5037713
CCDC180	chr1	239209387	239270803	490721.2394	chr1	239490721	239494721	-0.5036278
AP1S1	chr3	8881107	8887566	886506.8890	chr3	8886506	8890506	-0.5035874
TMBIM6	chr5	15674057	15692936	964706.1596	chr5	15964706	15965446	-0.5035858
DOC2B	chr12	47529367	47562373	737633.4774	chr12	47737633	47741633	-0.5034787
GADD45GIP1	chr2	66087453	66090710	950519.6595	chr2	65950519	65954519	-0.5033008
COX6A1	chr14	40408631	40412870	561159.4056	chr14	40561159	40565159	-0.5032749
DPCD	chr14	112617398	112637913	217181.1122	chr14	112217181	112217863	-0.5032387
DYNLL1	chr14	40455985	40459104	003385.4000	chr14	40003385	40004961	-0.5031805
ADGRA2	chr15	48573197	48610668	693778.4869	chr15	48693778	48695388	-0.503092
FUS	chr3	17314332	17326637	742406.1774	chr3	17742406	17744406	-0.5029966
MAP4K2	chr2	7365246	7378996	079608.7083	chr2	7079608	7083608	-0.5029829
ISCU	chr14	42216111	42222711	221964.4222	chr14	42221964	42225964	-0.5028285
CYB5R1	chr10	24925410	24931917	960329.2496	chr10	24960329	24962069	-0.5027073
CAPN8	chr10	19665855	19732139	888436.1988	chr10	19888436	19889542	-0.502577
ENSSSCG00000060152	chr1	166102570	166106753	463953.1664	chr1	166463953	166467953	-0.5025653
INO80E	chr3	18202134	18212186	505886.1850	chr3	18505886	18507873	-0.5025119
ENSSSCG00000061655	chr3	42976793	42982961	287523.4328	chr3	43287523	43288290	-0.5023955
RETREG2	chr15	121212881	121218812	024245.1210	chr15	121024245	121028245	-0.5023587
KCNH3	chr5	15495157	15517022	024080.1502	chr5	15024080	15028080	-0.5023096
ENSSSCG00000031299	chr2	64723435	64731124	094877.6509	chr2	65094877	65098877	-0.5020929
NSG1	chr8	6000057	6024769	125965.6129	chr8	6125965	6129965	-0.5020096
ENSSSCG00000056719	chr6	61903216	61907832	556435.6156	chr6	61556435	61560435	-0.5019157
ENSSSCG00000014569	chr9	707693	711219	710255.7116	chr9	710255	711662	-0.5018803
PCDH12	chr2	143550849	143565033	300062.1433	chr2	143300062	143304062	-0.5018672
PKIG	chr17	47008035	47044049	385600.4738	chr17	47385600	47389600	-0.5014392
COQ4	chr1	268756685	268768343	718355.2687	chr1	268718355	268722355	-0.5013632
TEPSIN	chr12	1519760	1529542	120907.1129	chr12	1120907	1129651	-0.5012121
USP5	chr5	63843745	63858716	841745.6384	chr5	63841745	63845745	-0.5011129
ATG4B	chr15	140223904	140246321	262766.1402	chr15	140262766	140266766	-0.5009992
TSPAN31	chr5	23029306	23038977	852316.2285	chr5	22852316	22856316	-0.5008934
GPN1	chr3	111537737	111572692	647539.1116	chr3	111647539	111651539	-0.5008267
ZMAT2	chr2	142411089	142419137	577020.1425	chr2	142577020	142579530	-0.5006975
ATP6V0D1	chr6	28090183	28131985	682030.2768	chr6	27682030	27686030	-0.5006007
PPP1R8	chr6	84966791	84990663	305189.8530	chr6	85305189	85309189	-0.5004546
SRRT	chr3	8717785	8731201	916872.8920	chr3	8916872	8920872	-0.5003733
DNPEP	chr15	121391746	121412864	241116.1212	chr15	121241116	121245116	-0.5001085
ZC3H14	chr7	110463517	110513257	461517.1104	chr7	110461517	110465517	0.50003984
XPOT	chr5	28828566	28875325	247852.2924	chr5	29247852	29248866	0.50059333

ENSSSCG00000040187	chr2	66650451	66674686	344600.66347	chr2	66344600	66347110	0.50103846
DGUOK	chr3	69054761	69091341	235860.69236	chr3	69235860	69236713	0.50136356
RPA3	chr9	77910881	77937060	509110.77510	chr9	77509110	77510115	0.50190571
SMC3	chr14	120969631	121021497	062346.1210	chr14	121062346	121066346	0.50198142
CBR4	chr14	20621981	20682240	420267.2042	chr14	20420267	20421437	0.502194
RHOT1	chr12	42711233	42783421	931445.4293	chr12	42931445	42935445	0.50281285
ZNRF2	chr18	42549846	42651807	408792.4241	chr18	42408792	42412792	0.50307483
PPP5C	chr6	52377979	52402045	660294.52661	chr6	52660294	52661512	0.50322247
ISOC1	chr2	131857168	131877631	369532.13137	chr2	131369532	131371871	0.50349924
OSGEPL1	chr15	94331064	94351564	259905.9426	chr15	94259905	94263905	0.50358203
MELK	chr1	237317191	237431589	754070.23775	chr1	237754070	237754508	0.5040461
ATL2	chr3	101957209	102029667	601707.10160	chr3	101601707	101605707	0.50450844
WASHC4	chr5	79417130	79474609	591915.79595	chr5	79591915	79595915	0.50492583
SRSF7	chr3	101603707	101613237	208841.10120	chr3	101208841	101209896	0.50540475
DUSP12	chr4	88809565	88818411	258461.89262	chr4	89258461	89262461	0.5058066
F3	chr4	122826644	122837666	824644.12282	chr4	122824644	122828644	0.50606663
RHPN2	chr6	42820568	42891346	587556.42591	chr6	42587556	42591556	0.50608514
MSH3	chr2	89250003	89453936	043362.89047	chr2	89043362	89047362	0.50686941
GHR	chr16	27126734	27421449	124734.2712	chr16	27124734	27128734	0.50689121
MAD2L1	chr8	103927353	103936664	268881.10427	chr8	104268881	104272881	0.50708667
CHRAC1	chr4	3025928	3028725	577459.25785	chr4	2577459	2578563	0.50758206
LEPROT	chr6	146974639	146987242	428927.14742	chr6	147428927	147429746	0.50768426
ADPGK	chr7	60584051	60623929	582051.60586	chr7	60582051	60586051	0.50773097
LRRC42	chr6	158316226	158338122	535465.15853	chr6	158535465	158539465	0.50844904
AKTIP	chr6	31747606	31758279	746036.31749	chr6	31746036	31749326	0.50894198
ALG6	chr6	149027980	149112665	153739.14915	chr6	149153739	149156202	0.50922109
PSMA5	chr4	110662692	110717290	694958.11069	chr4	110694958	110695918	0.50940143
FASTKD1	chr15	75895526	75929017	088477.7608	chr15	76088477	76089243	0.50944326
ZNF146	chr6	45520895	45599452	182499.45186	chr6	45182499	45186499	0.50958581
NID2	chr1	181699106	181815167	337919.18133	chr1	181337919	181339414	0.5097096
CNOT6	chr2	78132939	78202492	283203.78287	chr2	78283203	78287203	0.51020838
ENSSSCG00000016869	chr16	27803439	27805161	856157.2786	chr16	27856157	27860157	0.5105588
RPA3	chr9	77910881	77937060	217717.78218	chr9	78217717	78218825	0.51116167
ARF4	chr13	39409376	39430129	569981.3957	chr13	39569981	39573981	0.51127709
WDR47	chr4	111006968	111076918	004968.11100	chr4	111004968	111008968	0.51216197
ENSSSCG00000026746	chr13	79305680	79386940	980710.7898	chr13	78980710	78984710	0.51267181
RHEB	chr18	5779212	5822257	241524.6245	chr18	6241524	6245524	0.5140666
ANKLE2	chr14	22745641	22771727	379509.2238	chr14	22379509	22380259	0.51518843
SLC35A1	chr1	55859614	55895784	406112.55410	chr1	55406112	55410112	0.51555008
RRM1	chr9	6025073	6065143	064374.60656	chr9	6064374	6065627	0.51561817
ANP32B	chr1	239842067	239868403	492188.23949	chr1	239492188	239493251	0.5159734
GPD2	chr15	63619762	63728633	482472.6348	chr15	63482472	63482999	0.51674295
POLB	chr17	11356129	11388821	410074.1141	chr17	11410074	11411067	0.5170283
ABT1	chr7	21024635	21027794	996957.21000	chr7	20996957	21000957	0.51719872
KIAA0586	chr1	187504638	187642492	718846.18772	chr1	187718846	187720384	0.5175175
MORF4L2	chrX	84553774	84569030	173200.84177	chrX	84173200	84177200	0.5183264
GAR1	chr8	112400005	112414033	865664.11286	chr8	112865664	112869664	0.51833499
BAZ1A	chr7	64969640	65069889	238220.65242	chr7	65238220	65242220	0.51861066
ENSSSCG00000033293	chr12	4535650	4593526	798624.4802	chr12	4798624	4802624	0.51881771
ACTL6A	chr13	117598496	117628488	370272.1173	chr13	117370272	117371363	0.51882763
FFAR4	chr14	105011942	105037498	009942.1050	chr14	105009942	105013942	0.51884293
STX12	chr6	84918678	84960720	964791.84968	chr6	84964791	84968791	0.51928099
RHEB	chr18	5779212	5822257	242381.6244	chr18	6242381	6244253	0.51941386
CSGALNACT2	chr14	61372318	61415620	666240.6166	chr14	61666240	61668094	0.51960137
RHOT1	chr12	42711233	42783421	848632.4285	chr12	42848632	42852632	0.5203465
C5orf34	chr16	28058264	28094452	856157.2786	chr16	27856157	27860157	0.52063863
PARD6B	chr17	52195041	52213843	721967.5172	chr17	51721967	51724107	0.52066013
CRY1	chr5	13275545	13364593	365438.13369	chr5	13365438	13369438	0.52124197
GPR160	chr13	108748145	108793348	486808.1084	chr13	108486808	108487146	0.52144416
ENSSSCG00000016869	chr16	27803439	27805161	133077.2813	chr16	28133077	28134347	0.52168258
PARP14	chr13	137809510	137865106	453346.1374	chr13	137453346	137457346	0.52203945
PRKCI	chr13	108913173	108997931	486808.1084	chr13	108486808	108487146	0.52250181

TRMT5	chr1	189909660	189923234	471076.18947	chr1	189471076	189471705	0.52287273
STAMBP	chr3	69133978	69188192	545649.69549	chr3	69545649	69549649	0.52290808
DUSP11	chr3	69207475	69225729	020319.69021	chr3	69020319	69021858	0.52334054
FBXO4	chr16	26707470	26752024	7124734.2712	chr16	27124734	27128734	0.52398815
CREG1	chr4	83458242	83469930	067748.83071	chr4	83067748	83071748	0.52402087
LACTB2	chr4	64710771	64748936	768402.64769	chr4	64768402	64769676	0.52416787
FLT3	chr11	5370496	5455358	5793860.5797	chr11	5793860	5797121	0.52467386
TET3	chr3	68918933	69020986	570415.68574	chr3	68570415	68574415	0.52486412
SLC17A2	chr7	20644244	20663423	902846.20906	chr7	20902846	20906846	0.52509367
CENPW	chr1	36685265	36695955	022295.37026	chr1	37022295	37026295	0.52528366
RBM17	chr10	64885870	64908963	5266094.6526	chr10	65266094	65267932	0.52539222
ZNF606	chr6	62695610	62719642	965994.62969	chr6	62965994	62969994	0.52555352
ENSSSCG00000004151	chr1	26043665	26044123	254832.26255	chr1	26254832	26255836	0.52595937
TMED5	chr4	124009816	124030773	834362.12383	chr4	123834362	123836334	0.52633368
STK38	chr7	32206541	32253624	924308.31928	chr7	31924308	31928308	0.52714871
PPP6C	chr1	265870477	265898124	934267.26593	chr1	265934267	265935360	0.52791739
FBXO33	chr1	169877197	169915798	725317.16972	chr1	169725317	169726241	0.52815686
SLC25A40	chr9	92481564	92528186	270443.92271	chr9	92270443	92271593	0.52826257
RHEB	chr18	5779212	5822257	5690552.5691	chr18	5690552	5691835	0.52826568
POLE2	chr1	179495214	179529896	711299.17971	chr1	179711299	179715299	0.52851396
SHLD2	chr14	88005611	88121352	3473596.8847	chr14	88473596	88474327	0.529699
SEPTIN2	chr15	140024049	140058289	9540602.1395	chr15	139540602	139544602	0.53014808
MIER1	chr6	145609626	145680005	673985.14567	chr6	145673985	145674943	0.53030888
HHEX	chr14	104194343	104200219	3835011.1038	chr14	103835011	103835799	0.53042563
DPP8	chr1	163446899	163517413	525770.16352	chr1	163525770	163526535	0.53097142
RNF216	chr3	4158450	4329378	089899.40938	chr3	4089899	4093899	0.53102358
TIGD7	chr3	38893694	38900327	128777.39132	chr3	39128777	39132777	0.53115907
CEP20	chr3	6977215	6996022	441088.74415	chr3	7441088	7441520	0.53159846
KLHL7	chr9	91924495	91971695	270443.92271	chr9	92270443	92271593	0.53184771
MTA3	chr3	97601412	97782863	199101.97202	chr3	97199101	97202900	0.53279023
DTL	chr9	131227705	131275451	631585.13163	chr9	131631585	131635585	0.53299363
TTC8	chr7	110731731	110784853	463371.11046	chr7	110463371	110463994	0.53302695
CNOT6	chr2	78132939	78202492	943841.77947	chr2	77943841	77947841	0.53378942
F2R	chr2	85641474	85656636	086481.86087	chr2	86086481	86087566	0.53405007
TNFSF12	chr12	52843026	52853325	2399810.5240	chr12	52399810	52403810	0.53460197
TRAPPC6B	chr1	169709558	169722735	723649.16972	chr1	169723649	169727649	0.53517396
RHNO1	chr5	67239202	67246124	445777.67446	chr5	67445777	67446595	0.53530361
SNAPC3	chr1	207215738	207252396	173457.20717	chr1	207173457	207174369	0.53565477
WDR48	chr13	23735567	23786308	3235699.2323	chr13	23235699	23239699	0.53580917
CDK17	chr5	87179615	87295167	567798.87571	chr5	87567798	87571798	0.53594851
LIN9	chr10	14109768	14184586	4293023.1429	chr10	14293023	14297023	0.53607949
ENSSSCG00000008845	chr8	42024370	42044719	022370.42026	chr8	42022370	42026370	0.53620344
SGO2	chr15	104099384	104150720	4523451.1045	chr15	104523451	104527451	0.5363233
CNBP	chr13	71630566	71646563	1760305.7176	chr13	71760305	71762524	0.53700858
NRAS	chr4	105845872	105858227	056109.10606	chr4	106056109	106060109	0.5377546
DTL	chr9	131227705	131275451	784511.13078	chr9	130784511	130786914	0.53786428
SLC17A5	chr1	92286841	92342776	421305.92422	chr1	92421305	92422860	0.53804229
ENSSSCG00000040187	chr2	66650451	66674686	345269.66346	chr2	66345269	66346152	0.53818461
ENSSSCG00000008996	chr8	74614893	74633102	662598.74666	chr8	74662598	74666598	0.53922939
XPA	chr1	239532272	239568570	490721.23949	chr1	239490721	239494721	0.53999146
ST6GAL1	chr13	124791751	124837841	4582327.1245	chr13	124582327	124586327	0.54059367
ENSSSCG00000000296	chr5	19672068	19673217	483096.19487	chr5	19483096	19487096	0.54063428
RHOT1	chr12	42711233	42783421	2849222.4285	chr12	42849222	42850200	0.54127065
SLC25A20	chr13	31523821	31553286	1989610.3199	chr13	31989610	31993610	0.54164196
CD47	chr13	151429288	151488544	1427288.1514	chr13	151427288	151431288	0.54250926
ZNF140	chr14	22533916	22542728	2650568.2265	chr14	22650568	22654568	0.54273829
ENSSSCG00000028423	chr6	88987099	89006811	530010.88531	chr6	88530010	88531426	0.54317893
PBX3	chr1	266382640	266610640	931340.26593	chr1	265931340	265935340	0.5432107
MYEF2	chr1	123600908	123638236	461438.12346	chr1	123461438	123462218	0.54543884
CDK17	chr5	87179615	87295167	485168.87489	chr5	87485168	87489478	0.54581902
PARP14	chr13	137809510	137865106	7455216.1374	chr13	137455216	137457020	0.5466584
ZNF484	chr3	41893285	41918685	476270.41480	chr3	41476270	41480270	0.54666879

CD164	chr1	75441575	75458145	169857.7517(	chr1	75169857	75170800	0.54720609
PPIL4	chr1	16400983	16446854	139115.1614(	chr1	16139115	16140322	0.54760463
LONP2	chr6	36372599	36466190	340872.36344	chr6	36340872	36344872	0.54762582
PRDM10	chr9	56657270	56749482	748541.5675(	chr9	56748541	56750127	0.54836367
KAT2A	chr12	20638497	20647461	256958.2025	chr12	20256958	20257834	0.54839773
KLHL11	chr12	20842393	20852980	937554.2094	chr12	20937554	20941554	0.54853451
EIF4G2	chr2	48666520	48678950	082751.49083	chr2	49082751	49083432	0.54908594
RPA3	chr9	77910881	77937060	215213.78219	chr9	78215213	78219213	0.54924098
SCAI	chr1	265714211	265867101	931340.26593	chr1	265931340	265935340	0.54924588
ENSSSCG00000059603	chr14	16624296	16629222	680792.1668	chr14	16680792	16682843	0.54979668
YAP1	chr9	32811416	32925603	437726.32439	chr9	32437726	32439106	0.551888
DCLRE1A	chr14	124144273	124162102	399551.1244	chr14	124399551	124401921	0.55229274
INTS14	chr1	163567777	163603184	525770.16352	chr1	163525770	163526535	0.55249305
RANBP17	chr16	52836297	53160162	821657.5282	chr16	52821657	52825657	0.55253747
SNAPC5	chr1	164467599	164475011	273811.16427	chr1	164273811	164275327	0.55256524
NUP133	chr14	60353474	60408828	528065.6052	chr14	60528065	60529075	0.55261551
TMCC1	chr13	69036428	69290098	402021.6940	chr13	69402021	69406021	0.55310246
EIF5A2	chr13	109510812	109530413	229745.1092	chr13	109229745	109232545	0.55339481
PTGR2	chr7	97174219	97195595	821130.96825	chr7	96821130	96825130	0.55379687
MELK	chr1	237317191	237431589	737586.23774	chr1	237737586	237740566	0.55381341
CD164	chr1	75441575	75458145	169061.75173	chr1	75169061	75173061	0.55445465
COG3	chr11	21759180	21826913	499492.2150	chr11	21499492	21503492	0.55542923
TOR1B	chr1	269967008	269973693	190175.27019	chr1	270190175	270190671	0.5572611
CTNNBL1	chr17	40774720	40949022	092032.4109	chr17	41092032	41096032	0.55737868
CD164	chr1	75441575	75458145	522280.75523	chr1	75522280	75523303	0.55765999
WDR47	chr4	111006968	111076918	006543.1110(	chr4	111006543	111007819	0.55769063
MAPK8	chr14	88988949	89108874	192374.8919	chr14	89192374	89193566	0.55853329
SNX7	chr4	119035133	119255877	947829.11894	chr4	118947829	118949869	0.5585929
CEP135	chr8	55251005	55323350	727116.55731	chr8	55727116	55731116	0.55950743
ENSSSCG00000001769	chr7	48050045	48075795	837392.47841	chr7	47837392	47841392	0.55952271
SGO2	chr15	104099384	104150720	879439.1038	chr15	103879439	103880711	0.55971288
DTL	chr9	131227705	131275451	784592.13078	chr9	130784592	130788222	0.56034727
RNFT1	chr12	36162444	36174851	846540.3585	chr12	35846540	35850540	0.5603877
PPP1R12A	chr5	101490341	101639429	691246.10169	chr5	101691246	101695246	0.56132716
AZIN1	chr4	34234679	34257134	221012.34222	chr4	34221012	34222788	0.56162286
SEPTIN2	chr15	140024049	140058289	131918.1401	chr15	140131918	140133932	0.56180485
TMBIM4	chr5	30488328	30511533	186885.3019(	chr5	30186885	30190885	0.56236104
POLE2	chr1	179495214	179529896	475992.17947	chr1	179475992	179479992	0.56252956
OMA1	chr6	153882451	153949468	859444.15386	chr6	153859444	153860669	0.56348625
TET3	chr3	68918933	69020986	519015.6852(	chr3	68519015	68520573	0.56387796
CBFB	chr6	27684030	27750852	682030.27686	chr6	27682030	27686030	0.56482307
ENSSSCG00000004983	chr1	169777596	169870892	723649.16972	chr1	169723649	169727649	0.56583454
ACTL6A	chr13	117598496	117628488	368544.1173	chr13	117368544	117372544	0.5661877
ZNF567	chr6	45707074	45737962	741855.45745	chr6	45741855	45745855	0.56682419
ELK3	chr5	87285238	87354208	567798.87571	chr5	87567798	87571798	0.56686195
KDM1A	chr6	80903424	80971873	516429.80517	chr6	80516429	80517008	0.56885468
LIN9	chr10	14109768	14184586	939003.1394	chr10	13939003	13940930	0.56952503
NPM1	chr16	52765298	52781771	819079.5282	chr16	52819079	52825559	0.56991719
LONP2	chr6	36372599	36466190	342480.36343	chr6	36342480	36343795	0.57243952
ENSSSCG00000002877	chr6	44345463	44357772	933498.43934	chr6	43933498	43934468	0.57259566
PRKCI	chr13	108913173	108997931	229745.1092	chr13	109229745	109232545	0.57289011
NFYB	chr5	80334748	80353768	408372.80412	chr5	80408372	80412372	0.57312201
TOR1B	chr1	269967008	269973693	995271.26999	chr1	269995271	269995975	0.57315533
CNN3	chr4	122504433	122528348	824644.12282	chr4	122824644	122828644	0.57338829
MSX1	chr8	5628380	5632607	022066.6024(	chr8	6022066	6024012	0.5734783
ABT1	chr7	21024635	21027794	997828.20999	chr7	20997828	20999197	0.57376178
TET3	chr3	68918933	69020986	496471.68497	chr3	68496471	68497933	0.57398683
DNAAF2	chr1	179482208	179491142	711299.17971	chr1	179711299	179715299	0.57411462
CREG1	chr4	83458242	83469930	068725.8307(	chr4	83068725	83070535	0.57456547
NUDT19	chr6	42574071	42583926	558113.42562	chr6	42558113	42562113	0.57466513
SIKE1	chr4	105779814	105789389	057753.10605	chr4	106057753	106059218	0.57512503
AP3S1	chr2	119965539	120039947	803806.1198(	chr2	119803806	119804690	0.57531405

POLN	chr8	1073889	1197086	053894.10572	chr8	1053894	1057212	0.57661166
RCN2	chr7	57041003	57059330	040612.57041	chr7	57040612	57041309	0.57691049
BANP	chr6	1421462	1495486	494442.14961	chr6	1494442	1496104	0.57776088
GALNT11	chr18	5289917	5348058	992746.4995	chr18	4992746	4995436	0.57840763
TMEM128	chr8	6115002	6125139	890392.58913	chr8	5890392	5891389	0.57870468
PSMA5	chr4	110662692	110717290	004968.11100	chr4	111004968	111008968	0.57884564
TOR1B	chr1	269967008	269973693	965008.26996	chr1	269965008	269969008	0.57971741
AURKB	chr12	53410177	53423225	925589.5292	chr12	52925589	52926771	0.58315633
MKNK1	chr6	164887748	164933521	132690.16513	chr6	165132690	165136690	0.58325068
CIAO2A	chr1	107951646	107969298	995293.10799	chr1	107995293	107999293	0.58462101
MAP3K20	chr15	79173033	79380993	773073.7877	chr15	78773073	78773732	0.58480105
SIRT4	chr14	40290288	40303879	561159.4056	chr14	40561159	40565159	0.58792532
LEKR1	chr13	96652441	96879731	651679.9665	chr13	96651679	96652236	0.58800498
TOR1B	chr1	269967008	269973693	993520.26999	chr1	269993520	269997520	0.58875147
ENSSSCG00000032353	chr8	55787215	55830694	192440.56196	chr8	56192440	56196440	0.58891815
SLAIN2	chr8	38241598	38322031	934847.37938	chr8	37934847	37938847	0.5890656
STX12	chr6	84918678	84960720	467181.84468	chr6	84467181	84468278	0.59128119
ENSSSCG00000026746	chr13	79305680	79386940	707967.7971	chr13	79707967	79710829	0.59145859
ALG6	chr6	149027980	149112665	160941.14916	chr6	149160941	149161931	0.59177529
ENSSSCG00000014071	chr2	82660495	82668374	535019.82537	chr2	82535019	82537507	0.59202955
TTC8	chr7	110731731	110784853	461517.11046	chr7	110461517	110465517	0.59249548
DCLRE1A	chr14	124144273	124162102	017164.1240	chr14	124017164	124021164	0.59295273
TMEM128	chr8	6115002	6125139	888765.58927	chr8	5888765	5892765	0.5935677
CNOT8	chr16	68329790	68350260	675963.6867	chr16	68675963	68679963	0.59388871
PDE8A	chr7	52410779	52557645	302472.52306	chr7	52302472	52306472	0.59389945
ENSSSCG00000011253	chr13	23020584	23116955	236881.2323	chr13	23236881	23238804	0.59508785
SLC41A2	chr5	79593915	79720895	591915.79595	chr5	79591915	79595915	0.59547787
ENSSSCG00000053231	chr14	61024449	61030097	677026.6067	chr14	60677026	60678251	0.59587208
NSL1	chr9	130622331	130668936	784592.13078	chr9	130784592	130788222	0.5964835
SRSF7	chr3	101603707	101613237	456930.10146	chr3	101456930	101460930	0.59700064
STAG1	chr13	77321872	77863491	749660.7775	chr13	77749660	77751338	0.59806475
F2R	chr2	85641474	85656636	642347.85643	chr2	85642347	85643055	0.59823178
AZIN1	chr4	34234679	34257134	429189.34431	chr4	34429189	34431968	0.59887249
RHNO1	chr5	67239202	67246124	237801.67238	chr5	67237801	67238687	0.59979188
SIKE1	chr4	105779814	105789389	056109.10606	chr4	106056109	106060109	0.59987244
IMPA1	chr4	54935860	54984238	281589.55282	chr4	55281589	55282904	0.59995662
SPPL2A	chr1	121067454	121134888	411506.12141	chr1	121411506	121415506	0.60020067
GALNT11	chr18	5289917	5348058	474457.5476	chr18	5474457	5476891	0.60195752
ENSSSCG00000059603	chr14	16624296	16629222	708864.1671	chr14	16708864	16710745	0.60401696
CNOT8	chr16	68329790	68350260	678219.6868	chr16	68678219	68680897	0.60431704
DNAL1	chr7	96999602	97047090	123873.97124	chr7	97123873	97124625	0.60694396
DTX3	chr5	22867711	22873393	044561.23048	chr5	23044561	23048561	0.60781905
ENSSSCG00000016869	chr16	27803439	27805161	639997.2764	chr16	27639997	27640867	0.60851947
NSL1	chr9	130622331	130668936	784511.13078	chr9	130784511	130786914	0.60882922
USP25	chr13	180571342	180712435	569342.1805	chr13	180569342	180573342	0.60934512
C12orf40	chr5	71185592	71270728	672760.71673	chr5	71672760	71673927	0.60944687
SNX7	chr4	119035133	119255877	945440.11894	chr4	118945440	118949440	0.60951009
INTS14	chr1	163567777	163603184	435340.16343	chr1	163435340	163436160	0.60991858
GNG10	chr1	252453107	252460315	323217.25232	chr1	252323217	252324977	0.60993059
RDX	chr9	38220447	38322097	580776.38582	chr9	38580776	38582849	0.61121691
ANKRD13C	chr6	142516426	142616328	666213.14267	chr6	142666213	142670213	0.61234725
CAMK4	chr2	116000791	116229758	285018.11628	chr2	116285018	116289018	0.61249143
SLC35G1	chr14	105335160	105345274	011963.1050	chr14	105011963	105012625	0.61273086
PRKCI	chr13	108913173	108997931	230698.1092	chr13	109230698	109231563	0.61376732
ITSN2	chr3	114363529	114508444	576199.11458	chr3	114576199	114580199	0.61446313
ENSSSCG00000013715	chr2	66829154	66853255	345269.66346	chr2	66345269	66346152	0.61534714
FFAR4	chr14	105011942	105037498	010519.1050	chr14	105010519	105014699	0.61542434
ENSSSCG00000033395	chr10	55709135	55820506	129857.5613	chr10	56129857	56130854	0.6170523
POLB	chr17	11356129	11388821	166763.1116	chr17	11166763	11167738	0.61815382
MOCS2	chr16	32436205	32462521	334292.3233	chr16	32334292	32338292	0.61854928
C12orf40	chr5	71185592	71270728	028825.71030	chr5	71028825	71030050	0.61874379
NPM1	chr16	52765298	52781771	821657.5282	chr16	52821657	52825657	0.62013788

STT3B	chr13	17534171	17635035	030794.1803	chr13	18030794	18031998	0.620307
TBC1D15	chr5	35827473	35908385	670415.35671	chr5	35670415	35671285	0.62129833
RANBP17	chr16	52836297	53160162	819079.5282	chr16	52819079	52825559	0.6244887
CRYZ	chr6	138435392	138460975	433392.13843	chr6	138433392	138437392	0.62608409
XRCC2	chr18	4808470	4835802	991227.4995	chr18	4991227	4995227	0.62656765
LDAF1	chr3	24876637	24910760	285886.25286	chr3	25285886	25286690	0.62674791
ENSSSCG00000033814	chr16	39954354	39958520	103948.4010	chr16	40103948	40107227	0.62687614
GPR160	chr13	108748145	108793348	229745.1092	chr13	109229745	109232545	0.62753431
TRAPPC6B	chr1	169709558	169722735	725317.16972	chr1	169725317	169726241	0.62871238
THOC6	chr3	39161434	39164707	128777.39132	chr3	39128777	39132777	0.62961313
CMAS	chr5	51438020	51458615	808943.51812	chr5	51808943	51812943	0.63083549
ENSSSCG00000013715	chr2	66829154	66853255	344600.66347	chr2	66344600	66347110	0.63127817
POLN	chr8	1073889	1197086	386266.13902	chr8	1386266	1390266	0.63131038
ENSSSCG00000026746	chr13	79305680	79386940	706693.7971	chr13	79706693	79710693	0.63247036
PSMA5	chr4	110662692	110717290	006543.1110	chr4	111006543	111007819	0.63331214
ENSSSCG00000063355	chr15	133206674	133208289	376576.1333	chr15	133376576	133378660	0.63529405
XRCC2	chr18	4808470	4835802	992746.4995	chr18	4992746	4995436	0.63625907
HHEX	chr14	104194343	104200219	549119.1045	chr14	104549119	104554279	0.63863861
SCAF11	chr5	76809514	76848660	126152.77127	chr5	77126152	77127879	0.63881303
ENSSSCG00000026746	chr13	79305680	79386940	610176.7961	chr13	79610176	79611044	0.63917338
TMBIM4	chr5	30488328	30511533	187944.3019	chr5	30187944	30190487	0.64236645
LIMA1	chr5	16074438	16177103	964706.15965	chr5	15964706	15965446	0.64320963
PPP6C	chr1	265870477	265898124	931340.26593	chr1	265931340	265935340	0.64539516
PDE8A	chr7	52410779	52557645	254159.52258	chr7	52254159	52258159	0.64746632
ANKLE2	chr14	22745641	22771727	650568.2265	chr14	22650568	22654568	0.6481573
ALG6	chr6	149027980	149112665	152033.14915	chr6	149152033	149156033	0.64868141
LRPPRC	chr3	96474549	96595143	472549.96476	chr3	96472549	96476549	0.65192153
SNX7	chr4	119035133	119255877	947397.11894	chr4	118947397	118948903	0.65267695
HHEX	chr14	104194343	104200219	549134.1045	chr14	104549134	104553134	0.65465189
DNM3	chr9	114262791	114797674	200484.1142	chr9	114200484	114204484	0.65731675
CCT8	chr13	192406281	192423413	247246.1922	chr13	192247246	192251246	0.65787705
USP25	chr13	180571342	180712435	570640.1805	chr13	180570640	180571603	0.65865279
SS18	chr6	110710569	110790379	147751.11115	chr6	111147751	111151751	0.6649037
TAF1A	chr10	11340642	11362432	334439.1133	chr10	11334439	11336379	0.66751822
ZNF567	chr6	45707074	45737962	304692.45306	chr6	45304692	45306421	0.66999751
COG3	chr11	21759180	21826913	927221.2193	chr11	21927221	21931221	0.67239613
ENSSSCG00000051290	chr11	17731530	17762314	945850.1794	chr11	17945850	17949850	0.67292346
ZNF606	chr6	62695610	62719642	690463.62691	chr6	62690463	62691254	0.67295123
TMEM216	chr2	10115607	10121649	733322.97373	chr2	9733322	9737322	0.67330523
CNBP	chr13	71630566	71646563	558012.7156	chr13	71558012	71562012	0.67397995
SLC35G1	chr14	105335160	105345274	009942.1050	chr14	105009942	105013942	0.68072485
GPR160	chr13	108748145	108793348	230698.1092	chr13	109230698	109231563	0.68473123
SCLT1	chr8	95949572	96199110	267859.96269	chr8	96267859	96269407	0.68664357
GNG10	chr1	252453107	252460315	323757.25232	chr1	252323757	252327757	0.6887167
STAMBP	chr3	69133978	69188192	020319.69021	chr3	69020319	69021858	0.69179529
ENSSSCG00000042498	chr16	18299051	18305299	537410.1853	chr16	18537410	18538073	0.69291809
ENSSSCG00000051290	chr11	17731530	17762314	947617.1794	chr11	17947617	17948835	0.69644294
ITCH	chr17	37810235	37918209	955507.3795	chr17	37955507	37959507	0.70575892
SLC35G1	chr14	105335160	105345274	010519.1050	chr14	105010519	105014699	0.71118119
HENMT1	chr4	111364966	111382572	004968.1110	chr4	111004968	111008968	0.71473632
CEP57L1	chr1	75171061	75227022	522280.75523	chr1	75522280	75523303	0.72679978
HENMT1	chr4	111364966	111382572	006543.1110	chr4	111006543	111007819	0.73024919
GOLT1B	chr5	51939485	51953315	808943.51812	chr5	51808943	51812943	0.75170073

Gene	Gene-Chr	Gene-ini	Gene-end	Loci	Loci-Chr	Loci-ini	Loci-end	cor
EFR3A	chr4	9053086	9137265	051086.90550	chr4	9051086	9055086	-0.7608383
TEX12	chr9	39864148	39869979	158462.40161	chr9	40158462	40161372	-0.7431674
BLM	chr7	53317540	53412645	285609.53288	chr7	53285609	53288709	-0.7349497
TADA1	chr4	84174476	84187514	108767.84112	chr4	84108767	84112767	-0.7044881
TADA1	chr4	84174476	84187514	111766.84112	chr4	84111766	84112592	-0.6831932
ENSSSCG00000063355	chr15	133206674	133208289	473548.1334	chr15	133473548	133476628	-0.6716355
RARS2	chr1	55893480	55982068	376101.56380	chr1	56376101	56380101	-0.6667698
ZNF300	chr2	151842009	151856042	555380.15156	chr2	151555380	151563830	-0.6636798
VPS37A	chr17	4949644	4988074	4947644.4951	chr17	4947644	4951644	-0.6574041
TOR1B	chr1	269967008	269973693	598798.26960	chr1	269598798	269602798	-0.6548929
ZNF449	chrX	110928720	110948614	247183.11124	chrX	111247183	111248501	-0.6508967
VEZF1	chr12	34245884	34263538	3896719.3390	chr12	33896719	33901049	-0.650731
ZFP1	chr6	12480278	12531530	550006.12551	chr6	12550006	12551126	-0.6494921
GALNT11	chr18	5289917	5348058	4980080.4981	chr18	4980080	4981148	-0.6392882
NCAPG2	chr18	653224	717200	1130327.1131	chr18	1130327	1131165	-0.637561
PTS	chr9	39920273	39928634	158462.40161	chr9	40158462	40161372	-0.6351548
CFAP57	chr6	167988092	168049263	986876.16798	chr6	167986876	167989686	-0.6326948
ENSSSCG00000001769	chr7	48050045	48075795	008589.48012	chr7	48008589	48012589	-0.6304671
SYCP2	chr17	59875993	59955209	0235822.6023	chr17	60235822	60239962	-0.6292017
ROCK2	chr3	125352920	125493362	103753.12510	chr3	125103753	125104644	-0.6241188
DSCC1	chr4	19161226	19178568	317810.19321	chr4	19317810	19321810	-0.6239561
POLN	chr8	1073889	1197086	702463.70552	chr8	702463	705529	-0.6227604
CNOT7	chr17	4933453	4950346	4947644.4951	chr17	4947644	4951644	-0.6224747
MPP7	chr10	39532370	39798356	9874289.3987	chr10	39874289	39876849	-0.6180078
CEP295	chr9	26103283	26155076	546594.26550	chr9	26546594	26550594	-0.6176766
ATP13A3	chr13	131391798	131493623	1383044.1313	chr13	131383044	131387044	-0.6171484
SEPTIN2	chr15	140024049	140058289	9858888.1398	chr15	139858888	139868418	-0.6171017
ZNF664	chr14	29031605	29068864	3802819.2880	chr14	28802819	28808389	-0.614801
APPBP2	chr12	37613770	37678708	3059927.3806	chr12	38059927	38063927	-0.6141951
RNF2	chr9	126172301	126287603	172009.12617	chr9	126172009	126172651	-0.6078482
KIF4A	chrX	56319529	56447553	132853.56136	chrX	56132853	56136853	-0.6065243
DONSON	chr13	197193658	197203932	7465015.1974	chr13	197465015	197468485	-0.6055793
ATP13A3	chr13	131391798	131493623	1341940.1313	chr13	131341940	131345940	-0.6050735
NXT2	chrX	89684671	89691805	994848.89998	chrX	89994848	89998308	-0.6020805
AFF4	chr2	135200076	135283675	137272.13514	chr2	135137272	135141272	-0.5992346
LAPTM4A	chr3	118152587	118170278	444356.11844	chr3	118444356	118447936	-0.5982496
ENSSSCG00000056411	chr13	205345690	205349487	5423454.2054	chr13	205423454	205427454	-0.5982
ENSSSCG00000060529	chr11	25541019	25595113	5903366.2590	chr11	25903366	25907366	-0.5978392
ZDHHC20	chr11	1328110	1405872	1836951.8409	chr11	836951	840951	-0.5976899
RHEB	chr18	5779212	5822257	5873195.5876	chr18	5873195	5876335	-0.5973598
FUNDC1	chrX	39724998	39737532	255608.39260	chrX	39255608	39260588	-0.5972778
MKLN1	chr18	17501857	17832503	7986022.1799	chr18	17986022	17990022	-0.5972483
MPP7	chr10	39532370	39798356	9875244.3987	chr10	39875244	39875701	-0.5950923
TAF1A	chr10	11340642	11362432	0870521.1087	chr10	10870521	10871478	-0.5926971
ENSSSCG00000002020	chr7	75618988	75629447	005976.76006	chr7	76005976	76006524	-0.5916653
MAPK6	chr1	119725029	119771312	597054.11959	chr1	119597054	119597567	-0.5895446
LACTB2	chr4	64710771	64748936	953940.64956	chr4	64953940	64956027	-0.5885036
EIF5A2	chr13	109510812	109530413	9529851.1095	chr13	109529851	109530793	-0.5869992
CNN3	chr4	122504433	122528348	771109.12277	chr4	122771109	122773489	-0.5867099
TOR1B	chr1	269967008	269973693	573594.26957	chr1	269573594	269577594	-0.5861427
HAUS4	chr7	76085608	76097953	005976.76006	chr7	76005976	76006524	-0.5855849
ATP13A3	chr13	131391798	131493623	1327565.1313	chr13	131327565	131331315	-0.585302
PITRM1	chr10	66996954	67047235	5811869.6681	chr10	66811869	66815749	-0.5831604
SEPTIN2	chr15	140024049	140058289	9865812.1398	chr15	139865812	139867602	-0.5821924
KHDRBS1	chr6	88530385	88572463	808587.88809	chr6	88808587	88809709	-0.580337
PITRM1	chr10	66996954	67047235	7435519.6744	chr10	67435519	67446299	-0.5796333
PTS	chr9	39920273	39928634	790010.39790	chr9	39790010	39790626	-0.5780028
RABGAP1	chr1	263857513	264030574	731927.26373	chr1	263731927	263735927	-0.5767059
WTAP	chr1	7622547	7651961	364266.73678	chr1	7364266	7367856	-0.5756975
HDAC3	chr2	143273192	143287399	317870.14332	chr2	143317870	143325360	-0.5733276
ATL2	chr3	101957209	102029667	193741.10219	chr3	102193741	102197741	-0.5726689

TOR1B	chr1	269967008	269973693	259836.27026	chr1	270259836	270264736	-0.5715756
PPFIA1	chr2	3073241	3164235	723860.27334	chr2	2723860	2733450	-0.5713659
PSMG1	chr13	202762512	202775642	561255.2025	chr13	202561255	202564075	-0.5699792
SMC3	chr14	120969631	121021497	347059.1213	chr14	121347059	121351059	-0.5685397
XRCC2	chr18	4808470	4835802	943185.4946	chr18	4943185	4946905	-0.5674818
PRDM10	chr9	56657270	56749482	098862.57101	chr9	57098862	57101522	-0.5674284
GRPEL2	chr2	150511178	150520462	981810.15098	chr2	150981810	150986810	-0.567368
ENSSSCG00000063355	chr15	133206674	133208289	933798.1329	chr15	132933798	132935628	-0.5670037
TBC1D23	chr13	158665077	158729274	326227.1583	chr13	158326227	158326985	-0.5661287
NXT2	chrX	89684671	89691805	739190.89743	chrX	89739190	89743190	-0.5658678
ENSSSCG00000005440	chr1	249898712	249905370	876176.24987	chr1	249876176	249878826	-0.5655663
SEC23IP	chr14	129758288	129810642	577544.1295	chr14	129577544	129578376	-0.5628973
SEPTIN2	chr15	140024049	140058289	764414.1397	chr15	139764414	139768414	-0.5625779
TFG	chr13	158288579	158326885	326227.1583	chr13	158326227	158326985	-0.5609832
ENSSSCG00000033293	chr12	4535650	4593526	005799.5007	chr12	5005799	5007699	-0.560497
STAG2	chrX	101478247	101617563	477610.10147	chrX	101477610	101479275	-0.5590943
TIGD7	chr3	38893694	38900327	260264.39264	chr3	39260264	39264264	-0.5573602
UBIAD1	chr6	71419602	71433210	017576.71021	chr6	71017576	71021076	-0.5572284
UBE2G1	chr12	50266696	50348689	736832.5073	chr12	50736832	50738032	-0.55527
ZCCHC10	chr2	135305852	135328527	147046.13515	chr2	135147046	135151046	-0.5546122
FERMT2	chr1	182519684	182606869	357377.18235	chr1	182357377	182358281	-0.552894
DTL	chr9	131227705	131275451	707002.13170	chr9	131707002	131709562	-0.5519138
ENSSSCG00000033293	chr12	4535650	4593526	908039.4909	chr12	4908039	4909329	-0.5507279
ENSSSCG00000012165	chrX	18013930	18054247	839324.17842	chrX	17839324	17842394	-0.549302
ZDHHC20	chr11	1328110	1405872	559030.1562	chr11	1559030	1562300	-0.5489007
WAC	chr10	39932599	40022338	875244.3987	chr10	39875244	39875701	-0.5486428
MORC3	chr13	199997156	200041236	128785.2001	chr13	200128785	200129482	-0.5468321
UBR2	chr7	37655926	37784359	097569.38102	chr7	38097569	38102239	-0.5465325
ADH5	chr8	121266764	121282310	794058.12079	chr8	120794058	120796588	-0.5455828
KHDRBS1	chr6	88530385	88572463	049896.88059	chr6	88049896	88059876	-0.5455723
ENSSSCG00000014071	chr2	82660495	82668374	713416.82717	chr2	82713416	82717416	-0.5445592
CCDC127	chr16	79862686	79870755	877864.7987	chr16	79877864	79878678	-0.5400646
GALNT11	chr18	5289917	5348058	943185.4946	chr18	4943185	4946905	-0.5400341
MEIOC	chr12	18651511	18671987	465382.1846	chr12	18465382	18469382	-0.5383009
ENSSSCG00000012152	chrX	14210164	14222047	247778.14250	chrX	14247778	14250728	-0.5377026
FNBP4	chr2	14896175	14932183	164306.15168	chr2	15164306	15168306	-0.5376022
SWT1	chr9	126347517	126481803	172009.12617	chr9	126172009	126172651	-0.5364748
ZNF606	chr6	62695610	62719642	745816.62746	chr6	62745816	62746568	-0.5355229
WDR47	chr4	111006968	111076918	584109.11058	chr4	110584109	110585339	-0.5329223
DONSON	chr13	197193658	197203932	955955.1969	chr13	196955955	196959185	-0.5325581
PSMG1	chr13	202762512	202775642	234243.2032	chr13	203234243	203238243	-0.5313164
CLK3	chr7	58895419	58909551	068728.59072	chr7	59068728	59072728	-0.5308227
F3	chr4	122826644	122837666	218039.12322	chr4	123218039	123221889	-0.529741
ATP13A3	chr13	131391798	131493623	343175.1313	chr13	131343175	131346375	-0.5295176
TFDP1	chr11	78755533	78778309	645567.7864	chr11	78645567	78646089	-0.5294319
ENSSSCG00000002020	chr7	75618988	75629447	003612.76007	chr7	76003612	76007612	-0.5284715
RNF216	chr3	4158450	4329378	044136.40488	chr3	4044136	4048850	-0.5264299
CDC123	chr10	59718340	59765621	587169.5959	chr10	59587169	59591069	-0.5254505
GALNT11	chr18	5289917	5348058	977436.4981	chr18	4977436	4981436	-0.5250574
WTAP	chr1	7622547	7651961	517526.75263	chr1	7517526	7526346	-0.5248272
MEST	chr18	18327536	18345843	817641.1882	chr18	18817641	18821641	-0.5237521
SCML2	chrX	14717606	14823413	086758.15090	chrX	15086758	15090018	-0.520721
ENSSSCG00000026719	chr5	40416335	40423016	197598.40200	chr5	40197598	40200348	-0.5202416
MAP2K1	chr1	164381845	164471226	734118.16473	chr1	164734118	164734722	-0.5201875
PTGR2	chr7	97174219	97195595	314711.97318	chr7	97314711	97318711	-0.5186577
CDC7	chr4	125432772	125459288	366989.12537	chr4	125366989	125370459	-0.5179807
HSD17B12	chr2	18456019	18611782	969090.17975	chr2	17969090	17975170	-0.5178353
ATAD2	chr4	16012561	16082638	790997.15794	chr4	15790997	15794997	-0.5170517
PDP2	chr6	27565450	27575455	825350.27829	chr6	27825350	27829350	-0.5164717
FLT3	chr11	5370496	5455358	241105.5241	chr11	5241105	5241689	-0.5158095
ARPIN	chr7	55468568	55483536	363709.55366	chr7	55363709	55366089	-0.5157043
MRPL15	chr4	77069092	77083723	888489.76894	chr4	76888489	76894389	-0.5142664

DLAT	chr9	39738564	39796806	696254.39697	chr9	39696254	39697000	-0.5121136
SEPSECS	chr8	19097456	19136222	797198.18803	chr8	18797198	18803368	-0.509522
ZNF606	chr6	62695610	62719642	054906.63058	chr6	63054906	63058226	-0.5085161
ZHX2	chr4	16374902	16553002	527039.16532	chr4	16527039	16532019	-0.5084073
SPTLC1	chr14	3081972	3138103	2802061.2806	chr14	2802061	2806061	-0.5081514
CFAP57	chr6	167988092	168049263	986092.16799	chr6	167986092	167990092	-0.5079123
ZC3H8	chr3	44161310	44184683	973665.43974	chr3	43973665	43974341	-0.5055722
ZDHHC20	chr11	1328110	1405872	1661120.1663	chr11	1661120	1663910	-0.5052623
GALNT11	chr18	5289917	5348058	4978355.4984	chr18	4978355	4984775	-0.5044738
CFAP57	chr6	167988092	168049263	801936.16780	chr6	167801936	167808346	-0.5042291
SRPX	chrX	34075579	34198467	296220.34296	chrX	34296220	34296757	-0.5040744
STX12	chr6	84918678	84960720	918888.84919	chr6	84918888	84919217	-0.5038682
NCAPG	chr8	12759641	12807163	689067.12689	chr8	12689067	12689731	-0.5037506
XRCC2	chr18	4808470	4835802	4977436.4981	chr18	4977436	4981436	-0.5036917
UBXN2B	chr4	74405902	74437615	619249.74623	chr4	74619249	74623839	-0.5029863
ZDHHC20	chr11	1328110	1405872	1606840.1609	chr11	1606840	1609800	-0.5012085
ODF2L	chr4	129721291	129758495	795209.12979	chr4	129795209	129797689	-0.5005479
SCML2	chrX	14717606	14823413	821461.14822	chrX	14821461	14822726	-0.500383
CYLD	chr6	34059081	34121264	740746.33747	chr6	33740746	33747206	-0.5003599
DGAT1	chr4	452662	466684	801299.80574	chr4	801299	805749	0.50000674
PSMA7	chr17	61566373	61572438	1331052.6133	chr17	61331052	61338302	0.50008182
LYRM4	chr7	3070470	3192922	614139.26162	chr7	2614139	2616239	0.50009639
NAPA	chr6	53246968	53275265	504410.53508	chr6	53504410	53508410	0.50009878
TMEM147	chr6	44979190	44981049	805436.44807	chr6	44805436	44807246	0.50028825
CSTB	chr13	206706063	206710646	6843554.2068	chr13	206843554	206847737	0.50045765
CLN8	chr15	33288301	33304631	3261878.3326	chr15	33261878	33268778	0.50054852
FBXO31	chr6	1949276	1995462	574996.15795	chr6	1574996	1579556	0.50056098
FAM50A	chrX	124967510	124973483	271488.12527	chrX	125271488	125274598	0.50077667
LAT2	chr3	11352579	11372092	052516.11053	chr3	11052516	11053085	0.50082614
PIP4K2B	chr12	23317208	23346424	3729110.2373	chr12	23729110	23733110	0.50083627
TUBB2A	chr7	1910269	1914761	230059.22352	chr7	2230059	2235289	0.50085304
ENSSSCG00000018046	chr12	59977028	60019814	9576119.5957	chr12	59576119	59578709	0.50092662
STX8	chr12	54296995	54544646	4052571.5405	chr12	54052571	54056571	0.50093964
MRPL36	chr16	79001549	79004358	9037029.7904	chr16	79037029	79040119	0.50105766
DBNL	chr18	48713340	48725697	9159885.4917	chr18	49159885	49170155	0.50112392
OSBPL2	chr17	61643377	61685819	1331052.6133	chr17	61331052	61338302	0.5012067
WDR25	chr7	121238268	121384292	895092.12089	chr7	120895092	120899092	0.5012257
RANGRF	chr12	53485265	53486713	3904511.5390	chr12	53904511	53908511	0.50142196
PKN1	chr2	64800721	64824957	716906.64720	chr2	64716906	64720906	0.5014298
ENSSSCG00000003253	chr6	56236311	56244185	389302.56390	chr6	56389302	56390042	0.50143371
MTM1	chrX	122286916	122379299	707368.12270	chrX	122707368	122709996	0.50143999
TMEM39B	chr6	88585291	88606906	808587.88809	chr6	88808587	88809709	0.50153383
SDHA	chr16	79834044	79862524	9891263.7989	chr16	79891263	79895263	0.50157517
BRD9	chr16	79473745	79492635	9511609.7951	chr16	79511609	79518859	0.50175323
GABARAP	chr12	52596543	52598194	3006559.5300	chr12	53006559	53008829	0.50182234
KCNAB3	chr12	53171319	53180823	3006559.5300	chr12	53006559	53008829	0.50193236
POLR2G	chr2	9004390	9017779	193765.91977	chr2	9193765	9197765	0.50199102
PPP1CA	chr2	5119939	5123617	099970.51007	chr2	5099970	5100709	0.50199432
BNIP3	chr14	140362127	140371907	0430596.1404	chr14	140430596	140434596	0.50200926
XRCC6	chr5	6903495	6929052	576101.65771	chr5	6576101	6577139	0.50207552
TMC4	chr6	55955991	55970280	781617.55782	chr6	55781617	55782322	0.50212511
RPS9	chr6	55920934	55927280	781617.55782	chr6	55781617	55782322	0.50217799
TSPAN7	chrX	34522933	34660212	296220.34296	chrX	34296220	34296757	0.5023116
PSENEN	chr6	45171825	45174102	805436.44807	chr6	44805436	44807246	0.50231956
FIGNL1	chr9	136484216	136488250	707472.13671	chr9	136707472	136712522	0.50250495
FTSJ3	chr12	15106719	15114398	5380779.1538	chr12	15380779	15384509	0.50253526
EBNA1BP2	chr6	168049758	168061013	524156.16853	chr6	168524156	168530576	0.50298445
WARS1	chr7	121212981	121238113	705122.12170	chr7	121705122	121709122	0.50305032
RPTOR	chr12	1709128	1991574	1783700.1786	chr12	1783700	1786995	0.50320053
RIOK1	chr7	4750752	4782448	273899.42749	chr7	4273899	4274969	0.50332105
ZNF212	chr18	55528138	55541841	5614429.5561	chr18	55614429	55616784	0.50337021
CARS2	chr11	77229971	77265272	6748420.7675	chr11	76748420	76752040	0.50350034

CFL1	chr2	6469254	6475035	395080.68997	chr2	6895080	6899700	0.50361707
RPP40	chr7	2987271	3005271	064599.30706	chr7	3064599	3070619	0.50366147
ENSSSCG00000038404	chr7	1752689	1776087	748321.17494	chr7	1748321	1749415	0.50367391
FSD1	chr2	74471744	74485786	040989.74044	chr2	74040989	74044989	0.50388422
EIF4E2	chr15	133060247	133103719	118093.1331	chr15	133118093	133122093	0.50409348
ENSSSCG00000012088	chr13	207801096	207818130	214622.2082	chr13	208214622	208222830	0.50421833
ENSSSCG00000027723	chr15	137567231	137621880	444508.1374	chr15	137444508	137445469	0.50427649
NCF1	chr3	11820266	11839354	038056.12042	chr3	12038056	12042056	0.5043675
CLDN10	chr11	65121607	65145072	200901.6520	chr11	65200901	65201681	0.50453632
POLM	chr18	51079503	51088781	951075.5095	chr18	50951075	50958745	0.50453741
GGA1	chr5	10190367	10213477	587800.10588	chr5	10587800	10588562	0.50456488
ENSSSCG00000013064	chr2	9163317	9174468	082256.90862	chr2	9082256	9086256	0.50461283
EEF1D	chr4	969527	983270	785393.79847	chr4	785393	798477	0.50467174
ENSSSCG00000013064	chr2	9163317	9174468	293700.92984	chr2	9293700	9298410	0.50468397
DOK5	chr17	55390802	55583858	527122.5553	chr17	55527122	55531472	0.50468574
RNF220	chr6	166666138	166910646	808576.16681	chr6	166808576	166811236	0.50492836
SEC61G	chr9	139128296	139135727	147222.13915	chr9	139147222	139153542	0.50500076
CFDP1	chr6	12223748	12354177	054571.12058	chr6	12054571	12058571	0.50500872
MRPL36	chr16	79001549	79004358	168279.7917	chr16	79168279	79175129	0.50504884
ADAM3A	chr17	9088860	9166340	596773.8597	chr17	8596773	8597366	0.50515698
C1orf174	chr6	65323756	65333886	005436.65012	chr6	65005436	65012316	0.50520894
TCP1	chr1	7590140	7601795	527865.75286	chr1	7527865	7528661	0.505368
GTF3A	chr11	4857023	4869272	241105.5241	chr11	5241105	5241689	0.50543436
NPC2	chr7	97730516	97740331	314711.97318	chr7	97314711	97318711	0.50556104
WARS1	chr7	121212981	121238113	706792.12171	chr7	121706792	121711117	0.5055859
ILK	chr9	3145608	3159858	486681.34906	chr9	3486681	3490681	0.50570011
NIPAL3	chr6	82111626	82167383	533691.82534	chr6	82533691	82534381	0.50570957
ANKRD40CL	chr12	27038840	27043741	745423.2674	chr12	26745423	26746618	0.50576719
G6PD	chrX	125029150	125041040	129837.12513	chrX	125129837	125130618	0.50582588
FBXO31	chr6	1949276	1995462	565705.15725	chr6	1565705	1572513	0.50584116
CYP2E1	chr14	141690426	141736817	189763.1411	chr14	141189763	141193763	0.50585596
PHRF1	chr2	340528	371692	212170.21617	chr2	212170	216170	0.50590849
ENSSSCG00000031249	chr13	207493659	207499867	375502.2073	chr13	207375502	207379502	0.50610465
PSENEN	chr6	45171825	45174102	344637.45348	chr6	45344637	45348637	0.50611296
TRAF3IP1	chr15	137863609	137912477	707322.1377	chr15	137707322	137711322	0.50628195
ATP6V0B	chr6	167316947	167320212	943409.16694	chr6	166943409	166947409	0.50629958
SKA3	chr11	1302886	1320981	836951.8409	chr11	836951	840951	0.50630869
TUBGCP2	chr14	141233856	141253359	396639.1414	chr14	141396639	141400649	0.50632115
RPP40	chr7	2987271	3005271	720009.27279	chr7	2720009	2727929	0.50634095
MVP	chr3	18057177	18081155	803416.17807	chr3	17803416	17807526	0.50639999
MYL6	chr5	21559029	21562413	663252.21667	chr5	21663252	21667252	0.50641257
TRAF3IP1	chr15	137863609	137912477	032918.1380	chr15	138032918	138036878	0.50642963
TUBB2A	chr7	1910269	1914761	325359.23277	chr7	2325359	2327729	0.50662191
ZCCHC17	chr6	87879830	87942014	049896.88059	chr6	88049896	88059876	0.50678635
ACO2	chr5	7008719	7071025	576101.65771	chr5	6576101	6577139	0.50679911
SRRT	chr3	8717785	8731201	492530.84965	chr3	8492530	8496530	0.50698296
ENSSSCG00000036988	chr7	1980292	1988727	618809.16227	chr7	1618809	1622759	0.5071619
AFAP1L1	chr2	150445126	150510002	88203.15059	chr2	150588203	150592203	0.50718958
CARS2	chr11	77229971	77265272	586457.7758	chr11	77586457	77587718	0.50733521
NAXD	chr11	77210228	77230680	710558.7671	chr11	76710558	76711935	0.50738322
ENSSSCG00000062841	chr15	115446825	115450516	349501.1153	chr15	115349501	115353501	0.50776866
RNF220	chr6	166666138	166910646	943423.16694	chr6	166943423	166947423	0.50803725
PSMG3	chr3	1052191	1054688	312596.13169	chr3	1312596	1316956	0.50809838
PDZD11	chrX	56312754	56319405	132853.56136	chrX	56132853	56136853	0.50830571
ENSSSCG00000061173	chrX	110774840	110792950	959577.11096	chrX	110959577	110960315	0.50838345
AP1S1	chr3	8881107	8887566	836970.88376	chr3	8836970	8837652	0.50839745
NDUFA7	chr2	70954896	70963322	110600.71114	chr2	71110600	71114230	0.5085734
GET1	chr13	202962664	202979902	234243.2032	chr13	203234243	203238243	0.50863114
HARS2	chr2	142401328	142409619	547516.14255	chr2	142547516	142551516	0.50873003
TIMM17B	chrX	42970108	42975955	680269.42684	chrX	42680269	42684269	0.50880048
CENPS	chr6	70701694	70711717	017576.71021	chr6	71017576	71021076	0.50882847
DDX56	chr18	50705632	50715033	750846.5075	chr18	50750846	50751473	0.50888834

GLRX3	chr14	139072575	139111249	121536.1391	chr14	139121536	139122737	0.50898985
LIG1	chr6	53620483	53686562	428926.53431	chr6	53428926	53431616	0.5092411
PTPRS	chr2	73574854	73680686	042210.74044	chr2	74042210	74044130	0.50928474
GLP2R	chr12	54680057	54738102	553579.5455	chr12	54553579	54559169	0.50936787
HIRIP3	chr3	18198807	18202089	809718.1781	chr3	17809718	17813718	0.50951693
SELENOF	chr4	129258944	129287720	841529.1288	chr4	128841529	128852539	0.50959297
MYBBP1A	chr12	50491044	50506050	728138.5072	chr12	50728138	50729135	0.50970756
ENSSSCG00000008056	chr3	39532921	39538681	117229.3911	chr3	39117229	39118770	0.50974388
LYRM4	chr7	3070470	3192922	053192.3057	chr7	3053192	3057192	0.50978655
GAS7	chr12	54752507	54973515	676329.5467	chr12	54676329	54677869	0.50994078
LIG1	chr6	53620483	53686562	070265.5407	chr6	54070265	54074265	0.50998012
CMC4	chrX	125372941	125376999	903258.1249	chrX	124903258	124909988	0.5103378
NARF	chr12	632363	651817	145208.1492	chr12	145208	149208	0.51041531
ENSSSCG00000052263	chr4	98779348	98794830	803588.9880	chr4	98803588	98807588	0.51046384
PPP1R7	chr15	139917257	139941251	479553.1394	chr15	139479553	139483165	0.51050545
RAB5C	chr12	20613996	20636073	957709.2096	chr12	20957709	20961019	0.51065012
ENO1	chr6	69385879	69401151	723206.6972	chr6	69723206	69728846	0.5106923
NDUFA10	chr15	138986421	139031897	477888.1394	chr15	139477888	139482538	0.51093089
TUBA4A	chr15	121288957	121294853	560936.1215	chr15	121560936	121562466	0.51104698
PYGB	chr17	30940452	30995691	801572.3080	chr17	30801572	30807492	0.5114659
SEPTIN8	chr2	135077170	135104471	147046.1351	chr2	135147046	135151046	0.51155086
BCAP31	chrX	124457001	124484743	423872.1244	chrX	124423872	124427872	0.51158641
SYT5	chr6	59381642	59389298	950381.5895	chr6	58950381	58954381	0.51159494
ENSSSCG00000035728	chr12	1095096	1099180	162187.1166	chr12	1162187	1166187	0.51177015
ZNRD2	chr2	6683057	6684494	526990.6534	chr2	6526990	6534360	0.5118706
ENSSSCG00000057427	chr18	2568274	2595510	949925.2952	chr18	2949925	2952915	0.51198271
NDUFA10	chr15	138986421	139031897	497388.1385	chr15	138497388	138508728	0.5120235
MRPL36	chr16	79001549	79004358	436079.7944	chr16	79436079	79447039	0.51204008
EIPR1	chr3	131360883	131435579	304376.1313	chr3	131304376	131310136	0.51210564
ARL3	chr14	113665270	113707591	597569.1136	chr14	113597569	113600209	0.51211669
ENSSSCG00000032916	chr1	2541383	2552858	044406.2047	chr1	2044406	2047956	0.51225485
MRPL36	chr16	79001549	79004358	206448.7920	chr16	79206448	79209364	0.51226536
ABHD17A	chr2	76658068	76666552	276671.7627	chr2	76276671	76278423	0.51230706
BRD9	chr16	79473745	79492635	792309.7979	chr16	79792309	79794329	0.51235785
SCPEP1	chr12	33265562	33298142	816761.3281	chr12	32816761	32817407	0.51253477
RPP40	chr7	2987271	3005271	566999.2568	chr7	2566999	2568269	0.51268833
FANK1	chr14	135197450	135302516	863359.1348	chr14	134863359	134867349	0.51295349
ENSSSCG00000017913	chr12	52095888	52098231	975989.5197	chr12	51975989	51978009	0.51309724
TMEM176B	chr18	6342611	6348867	873195.5876	chr18	5873195	5876335	0.51310615
PIH1D1	chr6	54524172	54535249	346936.5435	chr6	54346936	54350546	0.51316769
RPS11	chr6	54577667	54580846	346936.5435	chr6	54346936	54350546	0.51319936
NRAP	chr14	123926927	124018440	871319.1238	chr14	123871319	123873859	0.5132491
FIGNL1	chr9	136484216	136488250	627192.1366	chr9	136627192	136633732	0.51335565
NLRP8	chr6	60350622	60370529	594958.6059	chr6	60594958	60596272	0.51335783
CTPS1	chr6	170201751	170233016	242316.1702	chr6	170242316	170248076	0.51336712
PFKP	chr10	67022655	67082449	844399.6684	chr10	66844399	66847999	0.51341766
STX5	chr2	8937778	8968932	293700.9298	chr2	9293700	9298410	0.51357003
RPS16	chr6	48085678	48088423	969275.4797	chr6	47969275	47973275	0.51367911
MED8	chr6	167861373	167869411	435586.1674	chr6	167435586	167437366	0.51370804
MPPED1	chr5	5417833	5486726	176438.5181	chr5	5176438	5181598	0.51373803
TBC1D10B	chr3	17944629	17956069	021096.1802	chr3	18021096	18025096	0.51374393
HADHA	chr3	112752865	112797733	698376.1127	chr3	112698376	112701986	0.51379208
DAD1	chr7	76432051	76457192	003612.7600	chr7	76003612	76007612	0.51382328
ZFTRAF1	chr4	335301	346534	377258.3783	chr4	377258	378347	0.51386505
ENSSSCG00000044567	chr17	31217462	31238052	516319.3151	chr17	31516319	31518091	0.51401734
TUBGCP2	chr14	141233856	141253359	391083.1413	chr14	141391083	141395083	0.51406757
MRPL28	chr3	41329928	41334451	248196.4125	chr3	41248196	41252396	0.5142642
GPR32	chr6	55519957	55525919	717729.5572	chr6	55717729	55721729	0.51435754
ENSSSCG00000035997	chrX	124926772	124929791	717923.1247	chrX	124717923	124727079	0.51436274
RPS7	chr3	131258722	131263256	623366.1316	chr3	131623366	131629856	0.51436863
TIMM44	chr2	71271805	71287817	951708.7095	chr2	70951708	70955708	0.51455845
PMF1	chr4	93810507	93837652	460308.9346	chr4	93460308	93464308	0.51458671

ENSSSCG00000047692	chr16	55617518	55623070	458699.5546	chr16	55458699	55460509	0.51468387
NEDD8	chr7	75070161	75085899	957429.74959	chr7	74957429	74959919	0.51475079
G6PD	chrX	125029150	125041040	148743.12515	chrX	125148743	125152743	0.51489119
ENSSSCG00000044567	chr17	31217462	31238052	801572.3080	chr17	30801572	30807492	0.51497821
PTPRS	chr2	73574854	73680686	485267.73486	chr2	73485267	73486251	0.51499488
ATP6AP1	chrX	124952500	124960343	123038.12512	chrX	125123038	125124988	0.51513724
ENSSSCG00000051526	chrX	34521562	34522768	296220.34296	chrX	34296220	34296757	0.51516774
ACTR1A	chr14	113480196	113498740	597569.1136	chr14	113597569	113600209	0.51523478
MRPL36	chr16	79001549	79004358	896319.7890	chr16	78896319	78901689	0.51526249
ENSSSCG00000049777	chr7	121082820	121091813	397229.12090	chr7	120897229	120900569	0.5152775
MRPS24	chr18	48825782	48830609	708347.4871	chr18	48708347	48712347	0.51540298
PSMC3	chr2	15166306	15193047	164306.15168	chr2	15164306	15168306	0.51541941
TRAF3IP1	chr15	137863609	137912477	442855.1374	chr15	137442855	137446855	0.51566455
GPR32	chr6	55519957	55525919	781617.55782	chr6	55781617	55782322	0.51589277
MAF1	chr4	597472	600447	441311.44531	chr4	441311	445311	0.51589792
TCP1	chr1	7590140	7601795	525861.75298	chr1	7525861	7529861	0.51591619
STK25	chr15	140144336	140154366	764414.1397	chr15	139764414	139768414	0.51615608
PCDH12	chr2	143550849	143565033	317870.14332	chr2	143317870	143325360	0.51616497
MEI1	chr5	6800961	6871625	576101.65771	chr5	6576101	6577139	0.51629113
STX8	chr12	54296995	54544646	904511.5390	chr12	53904511	53908511	0.51632745
BRCC3	chrX	125383414	125439082	903258.12490	chrX	124903258	124909988	0.51660714
RPS20	chr4	75762209	75769955	739459.75740	chr4	75739459	75740629	0.51686651
ERI3	chr6	166957316	167082631	329006.16683	chr6	166829006	166831646	0.51697639
BCKDHA	chr6	49381967	49398743	708933.49712	chr6	49708933	49712933	0.51713243
MRPL36	chr16	79001549	79004358	036641.7904	chr16	79036641	79043345	0.51716344
TK1	chr12	3786031	3795917	800240.3804	chr12	3800240	3804240	0.51716758
ENSSSCG00000036812	chr12	61077631	61186002	918169.6092	chr12	60918169	60920239	0.51716852
COA4	chr9	8278311	8281153	358382.83636	chr9	8358382	8363692	0.51719055
TUBA4A	chr15	121288957	121294853	428658.1214	chr15	121428658	121429211	0.51721807
NAA10	chrX	124658158	124662702	148743.12515	chrX	125148743	125152743	0.51722388
NPAS1	chr6	52845207	52875240	634601.52635	chr6	52634601	52635934	0.51723848
SDHA	chr16	79834044	79862524	552127.7955	chr16	79552127	79553615	0.51733153
SCAMP4	chr2	76615000	76634264	277430.76279	chr2	76277430	76279160	0.51739809
ENSSSCG00000035728	chr12	1095096	1099180	396069.1402	chr12	1396069	1402069	0.5174014
IDH3G	chrX	124528585	124537575	826225.12483	chrX	124826225	124830225	0.51744946
HSPBP1	chr6	59452935	59464533	233436.59233	chr6	59233436	59233978	0.5174505
WRAP73	chr6	65155209	65167079	316926.65322	chr6	65316926	65322026	0.51747686
ORAI1	chr14	31011644	31027993	522232.3052	chr14	30522232	30526232	0.51764001
CCNL2	chr6	63659054	63668047	991306.63993	chr6	63991306	63993056	0.5177015
RPS14	chr2	151430049	151433856	981810.15098	chr2	150981810	150986810	0.51771337
MTM1	chrX	122286916	122379299	392921.12239	chrX	122392921	122396921	0.51775255
WARS1	chr7	121212981	121238113	707640.12171	chr7	121707640	121711640	0.51813455
ENSSSCG00000042487	chr7	58998922	59008157	068728.59072	chr7	59068728	59072728	0.51817739
LRCH4	chr3	8499285	8511262	688988.86902	chr3	8688988	8690214	0.51837661
SPECC1	chr12	59466791	59654192	895496.5989	chr12	59895496	59899496	0.51840878
GALNTL5	chr18	5353769	5433814	977436.4981	chr18	4977436	4981436	0.5185821
ENSSSCG00000024070	chr18	6166918	6171153	312474.6314	chr18	6312474	6314041	0.51863555
PDCD6	chr16	79818958	79835746	638839.7964	chr16	79638839	79642899	0.51866894
CBX8	chr12	2562374	2565780	095212.2099	chr12	2095212	2099139	0.51879534
RDH13	chr6	59274387	59292765	713636.59717	chr6	59713636	59717636	0.51881418
ENSSSCG00000052671	chr6	54564793	54568340	761096.54766	chr6	54761096	54766246	0.51885425
UBXN6	chr2	74357517	74378592	040989.74044	chr2	74040989	74044989	0.51889691
NCF2	chr9	124776534	124812923	492652.12449	chr9	124492652	124494632	0.51895452
RPS4X	chrX	58149318	58155157	182285.58184	chrX	58182285	58184738	0.51900865
POP7	chr3	8605018	8610218	492530.84965	chr3	8492530	8496530	0.51904072
BRD9	chr16	79473745	79492635	898299.7990	chr16	79898299	79904919	0.51905163
STK10	chr16	52003887	52140367	831879.5183	chr16	51831879	51834749	0.51907348
MAF1	chr4	597472	600447	785393.79847	chr4	785393	798477	0.519091
SDHA	chr16	79834044	79862524	605069.7960	chr16	79605069	79606470	0.51910301
NOP14	chr8	1716826	1737902	223429.12274	chr8	1223429	1227429	0.5191605
IAH1	chr3	126849443	126865734	639236.12664	chr3	126639236	126644176	0.51923908
BIRC5	chr12	3747279	3755215	867743.3868	chr12	3867743	3868767	0.51924806

MEPCE	chr3	8357289	8364661	492530.84965	chr3	8492530	8496530	0.51928547
C6orf52	chr7	7430542	7437789	795369.77985	chr7	7795369	7798579	0.51942831
DBNL	chr18	48713340	48725697	737495.4874	chr18	48737495	48741355	0.51950556
RIMS3	chr6	170506426	170546933	401157.17040	chr6	170401157	170403038	0.51974957
AP1S1	chr3	8881107	8887566	832274.88362	chr3	8832274	8836274	0.51977185
GADD45GIP1	chr2	66087453	66090710	910372.65911	chr2	65910372	65911524	0.51977657
TIMP2	chr12	3254493	3300834	166999.3170	chr12	3166999	3170069	0.51981153
FANK1	chr14	135197450	135302516	822589.1348	chr14	134822589	134825479	0.51982803
ZMAT2	chr2	142411089	142419137	906041.14291	chr2	142906041	142910041	0.51989381
RPTOR	chr12	1709128	1991574	141178.2144	chr12	2141178	2144859	0.52002705
DUSP28	chr15	139537326	139539438	477888.1394	chr15	139477888	139482538	0.52002966
GMPPA	chr15	121507564	121515300	560936.1215	chr15	121560936	121562466	0.52014159
GLP2R	chr12	54680057	54738102	207189.5421	chr12	54207189	54212279	0.5203872
COPS9	chr15	139260478	139264211	705558.1397	chr15	139705558	139712278	0.52043602
ENSSSCG00000025928	chr6	53974309	53978544	346936.54350	chr6	54346936	54350546	0.52059584
ALG8	chr9	12497721	12532307	477907.12481	chr9	12477907	12481907	0.52059654
FTL	chr6	54231172	54232750	461759.54465	chr6	54461759	54465759	0.52062525
KXD1	chr2	59242310	59247713	406904.59410	chr2	59406904	59410904	0.52067443
DPCD	chr14	112617398	112637913	079981.1130	chr14	113079981	113083981	0.52071123
ENSSSCG00000038506	chr9	135025443	135078913	787102.13479	chr9	134787102	134794662	0.52072361
OXA1L	chr7	76230889	76266429	003612.76007	chr7	76003612	76007612	0.52073889
ENSSSCG00000056015	chr6	61202803	61213758	473531.61474	chr6	61473531	61474411	0.52080201
ENSSSCG00000052671	chr6	54564793	54568340	070265.54074	chr6	54070265	54074265	0.52113399
TUBGCP2	chr14	141233856	141253359	746869.1407	chr14	140746869	140750169	0.52120259
STX5	chr2	8937778	8968932	295283.92992	chr2	9295283	9299283	0.52127304
CD63	chr5	21172283	21176232	622762.21626	chr5	21622762	21626762	0.52136598
ENSSSCG00000032916	chr1	2541383	2552858	935566.29385	chr1	2935566	2938576	0.52147688
RDH13	chr6	59274387	59292765	733286.59736	chr6	59733286	59736796	0.52158185
PI4KA	chr14	50390410	50491944	810984.5081	chr14	50810984	50814984	0.52177215
TUBB2A	chr7	1910269	1914761	618809.16227	chr7	1618809	1622759	0.52197834
NHEJ1	chr15	121100628	121190062	923499.1209	chr15	120923499	120927499	0.52208267
ECHS1	chr14	141339364	141348994	278460.1412	chr14	141278460	141282460	0.52208666
PIH1D1	chr6	54524172	54535249	070265.54074	chr6	54070265	54074265	0.52208954
RECQL5	chr12	5732348	5770133	025019.6028	chr12	6025019	6028749	0.52217004
UBE2J2	chr6	63555488	63568017	487976.63500	chr6	63487976	63500036	0.52232789
C11orf98	chr2	9084256	9090037	293700.92984	chr2	9293700	9298410	0.52249004
C19orf54	chr6	48962838	48970959	219763.49223	chr6	49219763	49223763	0.52254731
PRPSAP1	chr12	5144069	5174382	914069.4923	chr12	4914069	4923019	0.52256184
MRPL36	chr16	79001549	79004358	729349.7873	chr16	78729349	78736299	0.52266332
RPL28	chr6	59556687	59562147	703779.59705	chr6	59703779	59705229	0.52275536
ENSSSCG00000013613	chr2	70193730	70199975	656247.70660	chr2	70656247	70660247	0.52287213
ZNRF1	chr6	12555664	12668804	550006.12551	chr6	12550006	12551126	0.5228789
NCLN	chr2	75369955	75390545	396091.75397	chr2	75396091	75397399	0.52304049
ENSSSCG00000015632	chr9	136893356	137056101	634302.13664	chr9	136634302	136640792	0.52317741
SAT2	chr12	52911725	52913363	006559.5300	chr12	53006559	53008829	0.5232029
WARS1	chr7	121212981	121238113	706089.12170	chr7	121706089	121709769	0.5232042
ARHGDIA	chr12	1113379	1119315	304812.1306	chr12	1304812	1306458	0.52333909
WARS1	chr7	121212981	121238113	684359.12168	chr7	121684359	121686349	0.5233768
NFYC	chr6	170413638	170485840	193186.17019	chr6	170193186	170197086	0.52342571
EBNA1BP2	chr6	168049758	168061013	922536.16792	chr6	167922536	167926936	0.52347481
ENSSSCG00000024070	chr18	6166918	6171153	310855.6315	chr18	6310855	6315595	0.52351695
NFYC	chr6	170413638	170485840	192822.17019	chr6	170192822	170196822	0.52354745
PRDX5	chr2	7798670	7802952	286187.82881	chr2	8286187	8288147	0.52365841
RPS15A	chr3	26776780	26784124	464566.26466	chr3	26464566	26466636	0.52371736
BRD9	chr16	79473745	79492635	206448.7920	chr16	79206448	79209364	0.52376007
PWP2	chr13	207015125	207031879	843554.2068	chr13	206843554	206847737	0.52380474
AURKAIP1	chr6	63656119	63657472	991306.63993	chr6	63991306	63993056	0.52389039
PRRC2B	chr1	271336818	271430916	678866.27168	chr1	271678866	271684276	0.52439351
MRPS24	chr18	48825782	48830609	989585.4899	chr18	48989585	48995365	0.52440963
LANCL2	chr18	48550944	48593455	989585.4899	chr18	48989585	48995365	0.52441312
DBNL	chr18	48713340	48725697	708347.4871	chr18	48708347	48712347	0.5245005
ATP6V0B	chr6	167316947	167320212	796056.16779	chr6	167796056	167799916	0.5248027

ENSSSCG00000035997	chrX	124926772	124929791	636523.12464	chrX	124636523	124640523	0.52501132
ZFTRAF1	chr4	335301	346534	785393.79847	chr4	785393	798477	0.52510368
EEF1AKMT1	chr11	1067061	1082591	826930.8289	chr11	826930	828910	0.52516538
BRD9	chr16	79473745	79492635	767469.7977	chr16	79767469	79770829	0.52544745
SERPINB1	chr7	1684215	1693299	727489.17321	chr7	1727489	1732119	0.52572626
ZNF212	chr18	55528138	55541841	612730.5561	chr18	55612730	55616730	0.52579488
NDUFA7	chr2	70954896	70963322	658392.70659	chr2	70658392	70659484	0.52583317
SLC16A5	chr12	6185528	6200001	336839.6340	chr12	6336839	6340259	0.52593189
ING1	chr11	77270320	77277068	898450.7690	chr11	76898450	76905200	0.52605818
UBE2J2	chr6	63555488	63568017	991306.63993	chr6	63991306	63993056	0.52640759
C11orf98	chr2	9084256	9090037	195750.91988	chr2	9195750	9198850	0.52642381
RPP40	chr7	2987271	3005271	616409.26226	chr7	2616409	2622659	0.52647051
RANGRF	chr12	53485265	53486713	006559.5300	chr12	53006559	53008829	0.52674876
LSM2	chr7	23900395	23908490	086873.24090	chr7	24086873	24090873	0.52676878
RPP40	chr7	2987271	3005271	053192.30571	chr7	3053192	3057192	0.52680743
SKI	chr6	64188236	64242560	637886.64641	chr6	64637886	64641676	0.52699707
NXF1	chr2	8969581	8981984	293700.92984	chr2	9293700	9298410	0.5270416
GUCA1A	chr7	37282700	37296717	828394.36832	chr7	36828394	36832394	0.5272499
COTL1	chr6	4099414	4147469	710866.37171	chr6	3710866	3717166	0.52741897
PTCH2	chr6	166495763	166512648	829006.16683	chr6	166829006	166831646	0.52752519
ZDHHC16	chr14	108842966	108852789	884753.1088	chr14	108884753	108888753	0.5276122
ATP5MC2	chr5	18871026	18879609	149084.19153	chr5	19149084	19153084	0.52761379
ENSSSCG00000036988	chr7	1980292	1988727	229293.22332	chr7	2229293	2233293	0.52767183
CA5A	chr6	1506042	1538777	409866.14130	chr6	1409866	1413016	0.52771303
LYRM4	chr7	3070470	3192922	102006.31031	chr7	3102006	3103105	0.52799365
THAP7	chr14	50591410	50594105	810984.5081	chr14	50810984	50814984	0.52807641
PSMD13	chr2	55108	74829	403950.40533	chr2	403950	405332	0.52808079
ENSSSCG00000061886	chr11	66802860	66809872	330220.6633	chr11	66330220	66335220	0.52826769
MRPL10	chr12	24075632	24083052	729110.2373	chr12	23729110	23733110	0.52848389
ATP6AP1	chrX	124952500	124960343	271488.12527	chrX	125271488	125274598	0.52868592
LRRC47	chr6	65260793	65271729	025442.65029	chr6	65025442	65029442	0.52870864
GLRX3	chr14	139072575	139111249	120929.1391	chr14	139120929	139123949	0.52882812
CCDC136	chr18	19793781	19824453	165695.2016	chr18	20165695	20169695	0.52884597
DGAT1	chr4	452662	466684	204188.20818	chr4	204188	208188	0.52897145
PFKP	chr10	67022655	67082449	819339.6682	chr10	66819339	66823389	0.52903392
KCTD2	chr12	6226376	6241318	214528.6215	chr12	6214528	6215144	0.52917708
NELFE	chr7	24040525	24047025	086873.24090	chr7	24086873	24090873	0.52967701
YJU2	chr2	74509739	74528300	040989.74044	chr2	74040989	74044989	0.52971301
PRR14	chr3	17745892	17751099	613617.17614	chr3	17613617	17614750	0.52983872
ZNF410	chr7	97195975	97243614	316238.97316	chr7	97316238	97316776	0.52992871
SUV39H1	chrX	42797828	42811461	680269.42684	chrX	42680269	42684269	0.52998238
ATG2A	chr2	7262031	7282347	895080.68997	chr2	6895080	6899700	0.53000416
PLIN5	chr2	74300619	74314315	042210.74044	chr2	74042210	74044130	0.53049926
ENSSSCG00000030908	chrX	110622132	110658827	832171.11083	chrX	110832171	110836171	0.53052897
SPECC1	chr12	59466791	59654192	454279.5945	chr12	59454279	59457869	0.53058304
ENSSSCG00000063021	chr12	10292427	10297310	353259.1035	chr12	10353259	10356689	0.53063389
OXA1L	chr7	76230889	76266429	005976.76006	chr7	76005976	76006524	0.53066505
BRD9	chr16	79473745	79492635	037029.7904	chr16	79037029	79040119	0.53089968
SLC38A5	chrX	42590607	42601502	955808.42959	chrX	42955808	42959908	0.53090434
MOSPD1	chrX	110693552	110720762	961228.11096	chrX	110961228	110965038	0.53097298
PDE6D	chr15	132375743	132426683	220868.1322	chr15	132220868	132223458	0.53112209
ENSSSCG00000018046	chr12	59977028	60019814	531479.5953	chr12	59531479	59536229	0.53124279
MED10	chr16	75517023	75524758	908265.7590	chr16	75908265	75908603	0.53128086
TRAF3IP1	chr15	137863609	137912477	398238.1374	chr15	137398238	137401498	0.53130852
POLM	chr18	51079503	51088781	728428.5072	chr18	50728428	50729797	0.53154847
PCDHGA4	chr2	142993554	143156556	906041.14291	chr2	142906041	142910041	0.53164704
ENSSSCG00000002995	chr6	49002854	49010040	659926.48665	chr6	48659926	48665966	0.53186809
RPS20	chr4	75762209	75769955	926549.75930	chr4	75926549	75930959	0.5318856
SLC35A4	chr2	142323341	142325335	804530.14280	chr2	142804530	142808530	0.5319672
DBNL	chr18	48713340	48725697	984125.4898	chr18	48984125	48986935	0.53205397
DAD1	chr7	76432051	76457192	012901.76016	chr7	76012901	76016901	0.53209338
DEGS2	chr7	121058105	121076328	397229.12090	chr7	120897229	120900569	0.53217571

HSPBP1	chr6	59452935	59464533	733286.59736	chr6	59733286	59736796	0.53232239
DBNL	chr18	48713340	48725697	189535.4919	chr18	49189535	49195785	0.53232668
UTP25	chr9	133105063	133141217	306452.13330	chr9	133306452	133308672	0.53236994
BRD9	chr16	79473745	79492635	436079.7944	chr16	79436079	79447039	0.5324678
HSD17B10	chrX	46218548	46220883	223748.46227	chrX	46223748	46227988	0.53258344
SMARCD2	chr12	15091729	15101899	380779.1538	chr12	15380779	15384509	0.53259797
HDLBP	chr15	139957715	140022302	858888.1398	chr15	139858888	139868418	0.53264763
HADHA	chr3	112752865	112797733	042836.11304	chr3	113042836	113046836	0.53265238
C8orf76	chr4	16137193	16158255	527039.16532	chr4	16527039	16532019	0.53287522
RAB4B	chr6	48989172	48999608	219763.49223	chr6	49219763	49223763	0.53292992
CARS2	chr11	77229971	77265272	880640.7688	chr11	76880640	76886850	0.53299832
PLEKHO1	chr4	98923996	98934189	803588.98807	chr4	98803588	98807588	0.53303962
RPS5	chr6	62967994	62974350	444151.63445	chr6	63444151	63445363	0.533063
HARS2	chr2	142401328	142409619	483896.14248	chr2	142483896	142487896	0.53309271
CYBC1	chr12	656347	662513	.448428.4524	chr12	448428	452428	0.53318831
CSNK1D	chr12	768865	796595	.453099.4609	chr12	453099	460989	0.53323847
COG1	chr12	7743274	7760504	178209.8181	chr12	8178209	8181929	0.53325537
ASB6	chr1	269828985	269834271	259836.27026	chr1	270259836	270264736	0.53332795
SDHA	chr16	79834044	79862524	538042.7953	chr16	79538042	79539074	0.53336396
SRM	chr6	71246876	71253508	997366.71001	chr6	70997366	71001106	0.53336484
HMG20B	chr2	75093075	75098536	729195.74730	chr2	74729195	74730347	0.53338253
RECQL5	chr12	5732348	5770133	214528.6215	chr12	6214528	6215144	0.53379775
CYC1	chr4	606516	608996	782159.79387	chr4	782159	793879	0.53391011
TBCD	chr12	329714	449556	.453099.4609	chr12	453099	460989	0.53399912
MRPL20	chr6	63670755	63675839	679085.63683	chr6	63679085	63683085	0.53406859
DEF8	chr6	140908	160692	179225.18322	chr6	179225	183225	0.53410557
NSDHL	chrX	123906199	123929117	025315.12402	chrX	124025315	124028273	0.5343479
VKORC1	chr3	17386245	17389905	809718.17813	chr3	17809718	17813718	0.53436439
CARS2	chr11	77229971	77265272	887510.7689	chr11	76887510	76890510	0.53450439
ENSSSCG00000036988	chr7	1980292	1988727	417517.24215	chr7	2417517	2421517	0.53452282
SDHA	chr16	79834044	79862524	495219.7949	chr16	79495219	79497289	0.53459167
SHANK2	chr2	2864331	3015314	705840.27134	chr2	2705840	2713480	0.53461656
XAB2	chr2	71521081	71530929	110600.71114	chr2	71110600	71114230	0.53500607
BRMS1	chr2	6046098	6053667	456140.64608	chr2	6456140	6460880	0.5350433
ENSSSCG00000061352	chr7	85598675	85602661	919594.85923	chr7	85919594	85923594	0.53511015
FIGNL1	chr9	136484216	136488250	649752.13665	chr9	136649752	136654312	0.53557732
MED8	chr6	167861373	167869411	796056.16779	chr6	167796056	167799916	0.53566685
ATP5ME	chr8	110605	113510	.79738.81698	chr8	79738	81698	0.535678
MRPL36	chr16	79001549	79004358	888489.7889	chr16	78888489	78894099	0.53568679
MMD2	chr3	3729531	3775205	231956.32449	chr3	3231956	3244946	0.53579904
MRPL36	chr16	79001549	79004358	539179.7854	chr16	78539179	78544609	0.535807
AURKAIP1	chr6	63656119	63657472	453866.63457	chr6	63453866	63457336	0.53589457
NSDHL	chrX	123906199	123929117	024145.12402	chrX	124024145	124028145	0.53605231
GPS1	chr12	951867	957080	.304812.1306	chr12	1304812	1306458	0.53628037
TUBA4A	chr15	121288957	121294853	.425642.1214	chr15	121425642	121429642	0.53632002
FCGR1A	chr4	99233611	99242586	803588.98807	chr4	98803588	98807588	0.53634347
JOSD1	chr5	9321640	9335041	545177.95491	chr5	9545177	9549177	0.53644404
CCNL2	chr6	63659054	63668047	453866.63457	chr6	63453866	63457336	0.53674096
GLRX3	chr14	139072575	139111249	444509.1394	chr14	139444509	139447079	0.53676653
PCCB	chr13	77217428	77316568	943395.7694	chr13	76943395	76947395	0.53676791
C1orf174	chr6	65323756	65333886	025442.65029	chr6	65025442	65029442	0.53680423
ENSSSCG00000051057	chr4	129797110	129860442	319459.12932	chr4	129319459	129323069	0.53685647
MYL6	chr5	21559029	21562413	622762.21626	chr5	21622762	21626762	0.53696559
ENSSSCG00000032573	chr4	75636278	75646153	737402.75741	chr4	75737402	75741402	0.53704743
BRD9	chr16	79473745	79492635	295297.7930	chr16	79295297	79302303	0.53705833
TUBGCP2	chr14	141233856	141253359	812715.1408	chr14	140812715	140816165	0.537164
FAM50A	chrX	124967510	124973483	148743.12515	chrX	125148743	125152743	0.53726157
NIT1	chr4	89320712	89327066	972183.88973	chr4	88972183	88973295	0.53737082
CYC1	chr4	606516	608996	441311.44531	chr4	441311	445311	0.53742293
BUD23	chr3	10960092	10973082	052516.11053	chr3	11052516	11053085	0.53745427
TUBGCP2	chr14	141233856	141253359	.151169.1411	chr14	141151169	141169699	0.53746361
DFFA	chr6	70724590	70736236	017576.71021	chr6	71017576	71021076	0.53748173

FXR2	chr12	52882870	52903450	006559.5300	chr12	53006559	53008829	0.53759659
VPS28	chr4	362912	370588	441311.44531	chr4	441311	445311	0.53771373
PSMA7	chr17	61566373	61572438	208112.6121	chr17	61208112	61211942	0.53773369
MAPK3	chr3	18291445	18299567	812944.17813	chr3	17812944	17813910	0.53774549
POP4	chr6	39734559	39760953	129356.40134	chr6	40129356	40134356	0.53780331
SLC35B1	chr12	25881256	25888293	153561.2615	chr12	26153561	26157561	0.53781795
SLC41A3	chr7	53672130	53748727	285609.53288	chr7	53285609	53288709	0.53785134
ENSSSCG00000012886	chr2	4682315	4695942	256940.42596	chr2	4256940	4259620	0.53790284
SLC7A7	chr7	76198256	76235402	003612.76007	chr7	76003612	76007612	0.53797299
FUS	chr3	17314332	17326637	803416.17807	chr3	17803416	17807526	0.53799843
PUF60	chr4	812131	824318	377258.37834	chr4	377258	378347	0.5380596
OTUD5	chrX	43000231	43029468	651789.42655	chrX	42651789	42655789	0.53818186
JPT1	chr12	6139428	6158452	337182.6338	chr12	6337182	6338139	0.53822821
STAT5A	chr12	20474227	20499138	166659.2016	chr12	20166659	20168579	0.53829482
DPEP1	chr6	361197	376616	180690.18282	chr6	180690	182821	0.53830504
NDUFA10	chr15	138986421	139031897	052628.1390	chr15	139052628	139057078	0.53836647
NDUFS6	chr16	78993913	79000006	729349.7873	chr16	78729349	78736299	0.53846001
USP11	chrX	41851602	41866355	174600.42175	chrX	42174600	42175367	0.53872469
ZP3	chr3	9934761	9944941	460014.94610	chr3	9460014	9461065	0.53872605
TCL1B	chr7	116912976	116917934	469559.11647	chr7	116469559	116474519	0.53879973
PDCD6	chr16	79818958	79835746	550266.7955	chr16	79550266	79551345	0.53884908
ATP6V0E2	chr18	55877504	55882204	614429.5561	chr18	55614429	55616784	0.5388568
MRM1	chr12	38124400	38144083	059927.3806	chr12	38059927	38063927	0.53891321
ENSSSCG00000056015	chr6	61202803	61213758	857817.60859	chr6	60857817	60859961	0.53913922
C1orf54	chr4	98805588	98814563	803588.98807	chr4	98803588	98807588	0.5392271
BRD9	chr16	79473745	79492635	741769.7974	chr16	79741769	79744539	0.53929051
ENSSSCG00000062841	chr15	115446825	115450516	351188.1153	chr15	115351188	115353598	0.53955518
ENSSSCG00000045223	chr6	166945409	166957165	435586.16743	chr6	167435586	167437366	0.53995638
PALLD	chr14	20678452	21020125	009729.2101	chr14	21009729	21013389	0.54026638
ASB11	chrX	11842855	11868793	091242.12095	chrX	12091242	12095242	0.54063687
GLRX3	chr14	139072575	139111249	976219.1389	chr14	138976219	138981529	0.54070636
ENSSSCG00000032959	chrX	125001675	125002920	180305.12518	chrX	125180305	125182027	0.54083612
FANCA	chr6	253207	300152	179225.18322	chr6	179225	183225	0.54086445
DNAJB6	chr18	1444739	1499887	095905.1098	chr18	1095905	1098925	0.54099538
SHANK2	chr2	2864331	3015314	723860.27334	chr2	2723860	2733450	0.54110353
ATP5PO	chr13	197509331	197519588	465015.1974	chr13	197465015	197468485	0.54134651
DUSP28	chr15	139537326	139539438	044907.1390	chr15	139044907	139048907	0.54154236
NUDT9	chr8	131443997	131472696	031428.13103	chr8	131031428	131035218	0.54177871
BNIP3	chr14	140362127	140371907	087869.1400	chr14	140087869	140090589	0.54193834
SYTL3	chr1	8453283	8546530	357656.83601	chr1	8357656	8360166	0.541971
ENSSSCG00000057427	chr18	2568274	2595510	272575.2275	chr18	2272575	2275685	0.54210694
DHRS7B	chr12	61320730	61350219	055733.6105	chr12	61055733	61059733	0.54247094
NAA10	chrX	124658158	124662702	407289.12441	chrX	124407289	124411289	0.54252078
SAL1	chr1	253638015	253642866	792913.25379	chr1	253792913	253796913	0.54265573
EEF1B2	chr15	109451514	109455525	247828.1092	chr15	109247828	109253198	0.54271004
SDHA	chr16	79834044	79862524	653319.7966	chr16	79653319	79662869	0.54271483
SRM	chr6	71246876	71253508	017576.71021	chr6	71017576	71021076	0.54275172
MAF1	chr4	597472	600447	377258.37834	chr4	377258	378347	0.54282148
RPS14	chr2	151430049	151433856	153756.15115	chr2	151153756	151157756	0.54284761
ARHGAP27	chr12	18037138	18082348	036976.1804	chr12	18036976	18040754	0.5429086
C19orf67	chr2	65100755	65104628	716906.64720	chr2	64716906	64720906	0.54298512
ENSSSCG00000035997	chrX	124926772	124929791	427335.12442	chrX	124427335	124429654	0.54312973
VPS41	chr18	55301624	55466628	271610.5527	chr18	55271610	55272585	0.54341333
SENP3	chr12	52765187	52867576	006559.5300	chr12	53006559	53008829	0.54350578
NARF	chr12	632363	651817	740005.7440	chr12	740005	744005	0.54363308
ENSSSCG00000038506	chr9	135025443	135078913	368712.13537	chr9	135368712	135371122	0.54368106
SDHA	chr16	79834044	79862524	898299.7990	chr16	79898299	79904919	0.54372731
ENSSSCG00000031249	chr13	207493659	207499867	225755.2072	chr13	207225755	207228460	0.54379102
AP2A2	chr2	580182	628087	212170.21617	chr2	212170	216170	0.5441471
PEX19	chr4	90195852	90205215	288394.90292	chr4	90288394	90292394	0.54431172
EDC3	chr7	58828727	58897919	068728.59072	chr7	59068728	59072728	0.54445019
IDH3G	chrX	124528585	124537575	642648.12464	chrX	124642648	124648648	0.54446954

IPO9	chr10	24110064	24150291	458712.2445	chr10	24458712	24459669	0.54449769
ENSSSCG00000038506	chr9	135025443	135078913	794722.1348(	chr9	134794722	134800842	0.54452022
RPS3	chr9	9624990	9630401	335482.9841(	chr9	9835482	9841672	0.54452419
RNF4	chr8	1353658	1380634	223429.1227	chr8	1223429	1227429	0.54459844
VAT1	chr12	19870312	19879230	166659.2016	chr12	20166659	20168579	0.54462021
AJUBA	chr7	76063058	76076585	005976.7600(	chr7	76005976	76006524	0.54468546
TUBGCP2	chr14	141233856	141253359	.189763.1411	chr14	141189763	141193763	0.54493513
PRR14	chr3	17745892	17751099	445606.1744	chr3	17445606	17447676	0.54497317
PPP1CA	chr2	5119939	5123617	070700.5072	chr2	5070700	5072860	0.54505907
GATD1	chr2	474296	480736	212170.2161	chr2	212170	216170	0.54530335
ZMIZ2	chr18	50579655	50598562	125275.5012	chr18	50125275	50128495	0.54538327
ENSSSCG00000033019	chr1	267961404	267964881	094326.2681(	chr1	268094326	268100136	0.54541037
BRD9	chr16	79473745	79492635	653319.7966	chr16	79653319	79662869	0.54555733
UBE2J2	chr6	63555488	63568017	017978.6402(	chr6	64017978	64020207	0.54558668
ENSSSCG00000052671	chr6	54564793	54568340	346936.5435(	chr6	54346936	54350546	0.54560674
BRD9	chr16	79473745	79492635	808628.7980	chr16	79808628	79809073	0.54563902
CSTB	chr13	206706063	206710646	906685.2069	chr13	206906685	206910205	0.54571052
PRPF31	chr6	55995091	56011441	701622.5570	chr6	55701622	55705622	0.5457222
TIMM44	chr2	71271805	71287817	110600.7111	chr2	71110600	71114230	0.54573221
ENSSSCG00000015632	chr9	136893356	137056101	578362.1365	chr9	136578362	136582362	0.54578086
OXTR	chr13	65134057	65153487	365608.6536	chr13	65365608	65369608	0.5457842
NSDHL	chrX	123906199	123929117	232736.1242	chrX	124232736	124236736	0.54581341
ZNRD2	chr2	6683057	6684494	456140.6460	chr2	6456140	6460880	0.5458315
MED8	chr6	167861373	167869411	684752.1676	chr6	167684752	167687008	0.5458463
FIGNL1	chr9	136484216	136488250	578362.1365	chr9	136578362	136582362	0.545961
CNPY2	chr5	21707834	21711292	622762.2162	chr5	21622762	21626762	0.54601761
MEA1	chr7	38074439	38081897	097569.3810	chr7	38097569	38102239	0.54635327
CDK5RAP3	chr12	24224263	24234467	729110.2373	chr12	23729110	23733110	0.54650476
ENSSSCG00000012088	chr13	207801096	207818130	369155.2073	chr13	207369155	207370835	0.54660728
DUSP28	chr15	139537326	139539438	822668.1398	chr15	139822668	139834538	0.54666899
COQ4	chr1	268756685	268768343	689502.2686	chr1	268689502	268693502	0.5466948
ENSSSCG00000033019	chr1	267961404	267964881	741236.2677	chr1	267741236	267743686	0.54692572
KAT5	chr2	6560513	6572466	395080.6899	chr2	6895080	6899700	0.54722324
ECHS1	chr14	141339364	141348994	.098189.1411	chr14	141098189	141104699	0.54736434
WDR55	chr2	142378462	142384677	804530.1428(	chr2	142804530	142808530	0.54750039
ENSSSCG00000038506	chr9	135025443	135078913	847862.1348	chr9	134847862	134850842	0.54751135
VPS25	chr12	20082865	20088566	166659.2016	chr12	20166659	20168579	0.54756374
PSMA7	chr17	61566373	61572438	.683622.6168	chr17	61683622	61687972	0.54759059
EIF3B	chr3	1645706	1666091	228872.1232	chr3	1228872	1232833	0.54777146
IPO9	chr10	24110064	24150291	179769.2418	chr10	24179769	24184649	0.5478604
AGFG2	chr3	8468044	8490831	836970.8837	chr3	8836970	8837652	0.54803353
RIMKLA	chr6	168916725	168950046	524156.1685	chr6	168524156	168530576	0.54815207
SLC38A8	chr6	4564533	4594520	640764.4644	chr6	4640764	4644764	0.54835215
ENSSSCG00000061173	chrX	110774840	110792950	247183.1112	chrX	111247183	111248501	0.5483628
TBRG4	chr18	50365988	50378084	977355.4998	chr18	49977355	49983615	0.54838003
DGAT1	chr4	452662	466684	441311.4453	chr4	441311	445311	0.54843792
PCDHGA4	chr2	142993554	143156556	004701.1430(	chr2	143004701	143005341	0.54884774
MRPS6	chr13	197738890	197756785	465015.1974	chr13	197465015	197468485	0.5488949
PRPF4	chr1	253906717	253925046	792913.2537	chr1	253792913	253796913	0.54913215
RPS20	chr4	75762209	75769955	065169.7606	chr4	76065169	76068979	0.54913987
CCER2	chr6	47712765	47715620	969275.4797	chr6	47969275	47973275	0.54924195
UQCRQ	chr2	135186501	135188293	137272.1351	chr2	135137272	135141272	0.54930102
ENSSSCG00000051352	chr14	73350356	73357245	180892.7318	chr14	73180892	73184892	0.54931473
TUBB2A	chr7	1910269	1914761	470509.1472	chr7	1470509	1472579	0.54939831
ATP6V0B	chr6	167316947	167320212	190926.1672(	chr6	167190926	167200676	0.54953565
ENSSSCG00000035997	chrX	124926772	124929791	271488.1252	chrX	125271488	125274598	0.54963476
RETREG2	chr15	121212881	121218812	.428658.1214	chr15	121428658	121429211	0.54965147
PGS1	chr12	3589541	3628961	166999.3170	chr12	3166999	3170069	0.54971832
ENSSSCG00000038506	chr9	135025443	135078913	571202.1345	chr9	134571202	134575572	0.54971868
COX5A	chr7	58622964	58644021	068728.5907	chr7	59068728	59072728	0.54974281
GPR137	chr2	7826456	7830969	286187.8288	chr2	8286187	8288147	0.54976823
AIFM1	chrX	106670520	106708317	659892.1066	chrX	106659892	106661951	0.54980808

NAXD	chr11	77210228	77230680	887510.7689	chr11	76887510	76890510	0.54981995
BNIP3	chr14	140362127	140371907	746869.1407	chr14	140746869	140750169	0.54997229
AFAP1L1	chr2	150445126	150510002	286910.1502	chr2	150286910	150293060	0.54999166
CYC1	chr4	606516	608996	801299.8057	chr4	801299	805749	0.55001263
FBXO17	chr6	47732303	47759606	969275.4797	chr6	47969275	47973275	0.55016519
ENSSSCG00000033707	chr12	61514310	61563818	055733.6105	chr12	61055733	61059733	0.55030448
PDXP	chr5	10162643	10169512	587800.1058	chr5	10587800	10588562	0.55039504
ENSSSCG00000032959	chrX	125001675	125002920	903258.1249	chrX	124903258	124909988	0.55050598
ENSSSCG00000029830	chr4	136107	138652	441311.4453	chr4	441311	445311	0.55053187
NCCRP1	chr6	47915118	47929937	969275.4797	chr6	47969275	47973275	0.55055763
LIG1	chr6	53620483	53686562	761062.5376	chr6	53761062	53765062	0.55058254
SSR1	chr7	4652921	4685901	504349.4508	chr7	4504349	4508659	0.55067993
LYPLAL1	chr10	8946487	9062412	917679.8921	chr10	8917679	8921219	0.55071555
POLD1	chr6	55247880	55272085	717729.5572	chr6	55717729	55721729	0.55076377
ENSSSCG00000038506	chr9	135025443	135078913	373142.1353	chr9	135373142	135380772	0.55096902
C11orf98	chr2	9084256	9090037	082256.9086	chr2	9082256	9086256	0.55098826
ENSSSCG00000025928	chr6	53974309	53978544	070265.5407	chr6	54070265	54074265	0.55110835
IDH3G	chrX	124528585	124537575	822947.1248	chrX	124822947	124824355	0.5512602
RPS14	chr2	151430049	151433856	555380.1515	chr2	151555380	151563830	0.55127291
SELENOF	chr4	129258944	129287720	319564.1293	chr4	129319564	129323564	0.55147144
YJU2	chr2	74509739	74528300	042210.7404	chr2	74042210	74044130	0.55149865
ILK	chr9	3145608	3159858	120522.3124	chr9	3120522	3124932	0.55158371
ENSSSCG00000003612	chr6	88695926	88708678	808587.8880	chr6	88808587	88809709	0.55171031
ATP5ME	chr8	110605	113510	444863.4507	chr8	444863	450757	0.55178795
TMEM147	chr6	44979190	44981049	344637.4534	chr6	45344637	45348637	0.55181077
ENSSSCG00000057427	chr18	2568274	2595510	154505.2156	chr18	2154505	2156285	0.5521237
ENSSSCG00000027723	chr15	137567231	137621880	673710.1376	chr15	137673710	137677710	0.55221869
ENSSSCG00000032959	chrX	125001675	125002920	271488.1252	chrX	125271488	125274598	0.55228562
POLA2	chr2	6922387	6950179	363246.7367	chr2	7363246	7367246	0.55228949
ENSSSCG00000035997	chrX	124926772	124929791	148743.1251	chrX	125148743	125152743	0.55245627
POLR3H	chr5	6997939	7014421	431892.7432	chr5	7431892	7432609	0.5525008
DRAP1	chr2	6409655	6412395	526990.6534	chr2	6526990	6534360	0.55255674
PRR14	chr3	17745892	17751099	803416.1780	chr3	17803416	17807526	0.55271988
NDUFA6	chr5	6562011	6567647	576101.6577	chr5	6576101	6577139	0.55294099
EEF1AKMT1	chr11	1067061	1082591	559030.1562	chr11	1559030	1562300	0.55303463
SLC35A4	chr2	142323341	142325335	483896.1424	chr2	142483896	142487896	0.55308376
FIGNL1	chr9	136484216	136488250	285762.1362	chr9	136285762	136289922	0.55331543
NARF	chr12	632363	651817	448428.4524	chr12	448428	452428	0.55334668
MRPL10	chr12	24075632	24083052	579721.2358	chr12	23579721	23583721	0.55346414
TRAF3IP1	chr15	137863609	137912477	555738.1375	chr15	137555738	137558808	0.55364174
PRR14	chr3	17745892	17751099	812944.1781	chr3	17812944	17813910	0.55369908
MRPL11	chr2	5978483	5981449	456140.6460	chr2	6456140	6460880	0.55393088
ENSSSCG00000021624	chr10	23767206	23778722	626979.2363	chr10	23626979	23630979	0.55394607
CYBC1	chr12	656347	662513	053218.1057	chr12	1053218	1057218	0.55449361
NLRP8	chr6	60350622	60370529	422807.6042	chr6	60422807	60424327	0.55476375
EHD1	chr2	7298208	7321576	895080.6899	chr2	6895080	6899700	0.55479646
YKT6	chr18	50960113	50971384	750846.5075	chr18	50750846	50751473	0.55479802
SERPINB1	chr7	1684215	1693299	748321.1749	chr7	1748321	1749415	0.55486155
MRPL36	chr16	79001549	79004358	911469.7891	chr16	78911469	78914430	0.55526387
BCAP31	chrX	124457001	124484743	822947.1248	chrX	124822947	124824355	0.55529231
MRPL45	chr12	23751926	23766459	729110.2373	chr12	23729110	23733110	0.55535801
PDGFA	chr3	301584	321712	343167.3503	chr3	343167	350359	0.55564531
AAAS	chr5	18526076	18537830	586758.1859	chr5	18586758	18590758	0.55575749
CDIPT	chr3	18084549	18091798	021096.1802	chr3	18021096	18025096	0.55579009
LRPAP1	chr8	2125405	2145647	299768.2308	chr8	2299768	2308478	0.55593366
ENSSSCG00000032166	chr12	6240277	6247343	214528.6215	chr12	6214528	6215144	0.55649769
MED8	chr6	167861373	167869411	922536.1679	chr6	167922536	167926936	0.55657829
MRPS23	chr12	34122020	34129548	861199.3386	chr12	33861199	33867119	0.55665497
APOE	chr6	51372292	51375330	402558.5140	chr6	51402558	51406558	0.55665581
FAM104A	chr12	7727796	7749240	581599.7584	chr12	7581599	7584879	0.55670805
ENSSSCG00000014540	chr2	9535148	9537974	195750.9198	chr2	9195750	9198850	0.5567856
RPL27	chr12	19892217	19894504	166659.2016	chr12	20166659	20168579	0.55691018

DYNLL2	chr12	34349294	34356472	861199.3386	chr12	33861199	33867119	0.55699282
DAD1	chr7	76432051	76457192	005976.7600	chr7	76005976	76006524	0.55707608
POLR2G	chr2	9004390	9017779	195750.9198	chr2	9195750	9198850	0.55708001
NARF	chr12	632363	651817	448754.4508	chr12	448754	450833	0.55715847
SAP18	chr11	1291202	1298983	656336.1660	chr11	1656336	1660336	0.55717074
RPS11	chr6	54577667	54580846	761096.5476	chr6	54761096	54766246	0.55719862
PRSS8	chr3	17357802	17362220	613617.1761	chr3	17613617	17614750	0.55733807
NR2C2AP	chr2	58675990	58678130	631062.5863	chr2	58631062	58632193	0.55751787
ENSSSCG00000012088	chr13	207801096	207818130	293451.2082	chr13	208293451	208297451	0.55765363
HIRIP3	chr3	18198807	18202089	812944.1781	chr3	17812944	17813910	0.55765939
ABHD17A	chr2	76658068	76666552	277430.7627	chr2	76277430	76279160	0.55768875
MRPL36	chr16	79001549	79004358	295297.7930	chr16	79295297	79302303	0.55775887
SDHA	chr16	79834044	79862524	797679.7980	chr16	79797679	79802279	0.55783532
EBNA1BP2	chr6	168049758	168061013	796056.1677	chr6	167796056	167799916	0.55792196
BNIP3	chr14	140362127	140371907	835389.1408	chr14	140835389	140842459	0.55792744
ENSSSCG00000044155	chr2	142321430	142324016	483896.1424	chr2	142483896	142487896	0.5580582
MRPL40	chr14	51102613	51105767	810984.5081	chr14	50810984	50814984	0.55808061
TUBB2A	chr7	1910269	1914761	748321.1749	chr7	1748321	1749415	0.55844079
TTLL13	chr7	55811855	55827077	363709.5536	chr7	55363709	55366089	0.55857924
RPP40	chr7	2987271	3005271	614139.2616	chr7	2614139	2616239	0.55874749
ENSSSCG00000060915	chr17	59172433	59175227	150116.5915	chr17	59150116	59154116	0.55884548
STMP1	chr18	13641541	13665565	502464.1350	chr18	13502464	13506464	0.55888532
PFDN2	chr4	89326798	89342325	972183.8897	chr4	88972183	88973295	0.55906371
OSBPL2	chr17	61643377	61685819	208112.6121	chr17	61208112	61211942	0.55914218
ZMAT2	chr2	142411089	142419137	473803.1424	chr2	142473803	142474940	0.55948621
PRR13	chr5	18660814	18665925	671981.1867	chr5	18671981	18675981	0.55948841
RECQL4	chr4	287214	293913	441311.4453	chr4	441311	445311	0.55966617
PKIG	chr17	47008035	47044049	638022.4664	chr17	46638022	46640212	0.55994523
DNAJC11	chr6	67463374	67529930	938806.6794	chr6	67938806	67942416	0.5600792
PIMREG	chr12	50898501	50903464	728138.5072	chr12	50728138	50729135	0.56014722
TBC1D10B	chr3	17944629	17956069	812944.1781	chr3	17812944	17813910	0.56022838
TCL1B	chr7	116912976	116917934	476479.1164	chr7	116476479	116479379	0.56029444
GMPPA	chr15	121507564	121515300	428658.1214	chr15	121428658	121429211	0.56033968
WRAP73	chr6	65155209	65167079	005436.6501	chr6	65005436	65012316	0.56039754
COX8A	chr2	8101613	8103285	286187.8288	chr2	8286187	8288147	0.56050985
TMED4	chr18	50699470	50702705	728428.5072	chr18	50728428	50729797	0.56073392
TUBGCP2	chr14	141233856	141253359	133839.1411	chr14	141133839	141138489	0.56081882
AARSD1	chr12	19918248	19926825	166659.2016	chr12	20166659	20168579	0.56093877
CYP2E1	chr14	141690426	141736817	450562.1414	chr14	141450562	141451766	0.56094546
ENSSSCG00000045223	chr6	166945409	166957165	907657.1669	chr6	166907657	166910521	0.56096581
ARHGEF7	chr11	77427368	77530993	854120.7785	chr11	77854120	77859120	0.56115705
DPEP1	chr6	361197	376616	251207.2552	chr6	251207	255207	0.56120157
ENSSSCG00000031249	chr13	207493659	207499867	369155.2073	chr13	207369155	207370835	0.56133248
CLN6	chr1	166107228	166126808	599696.1666	chr1	166599696	166602986	0.56139457
VPS28	chr4	362912	370588	785393.7984	chr4	785393	798477	0.56141534
NLRP8	chr6	60350622	60370529	420391.6042	chr6	60420391	60424391	0.5614315
VKORC1	chr3	17386245	17389905	445606.1744	chr3	17445606	17447676	0.56145055
DNPEP	chr15	121391746	121412864	428658.1214	chr15	121428658	121429211	0.56201278
ENSSSCG00000037652	chr3	65951	140492	341139.3451	chr3	341139	345139	0.56205899
TBC1D10B	chr3	17944629	17956069	809718.1781	chr3	17809718	17813718	0.56215758
CNPY2	chr5	21707834	21711292	663252.2166	chr5	21663252	21667252	0.56216945
ENSSSCG00000037652	chr3	65951	140492	343167.3503	chr3	343167	350359	0.56253788
TRMT2A	chr14	51489892	51494190	291092.5129	chr14	51291092	51297177	0.56256684
ZNF212	chr18	55528138	55541841	271610.5527	chr18	55271610	55272585	0.56265652
HDGFL2	chr2	74332106	74357040	042210.7404	chr2	74042210	74044130	0.56294116
NSDHL	chrX	123906199	123929117	165690.1241	chrX	124165690	124169690	0.56296957
DGAT1	chr4	452662	466684	785393.7984	chr4	785393	798477	0.5630065
GTPBP3	chr2	60352026	60357176	740996.6074	chr2	60740996	60744996	0.56308258
PSMC5	chr12	15101859	15106213	392487.1539	chr12	15392487	15396487	0.56316451
MRPL20	chr6	63670755	63675839	017978.6402	chr6	64017978	64020207	0.56338479
FTSJ3	chr12	15106719	15114398	360359.1536	chr12	15360359	15364249	0.56358682
ENSSSCG00000048787	chr15	137765933	137767998	701312.1377	chr15	137701312	137702888	0.56416953

TALDO1	chr2	464728	472835	709596.71359	chr2	709596	713596	0.56433856
RDH13	chr6	59274387	59292765	233436.59233	chr6	59233436	59233978	0.56442977
GPS1	chr12	951867	957080	453099.4609	chr12	453099	460989	0.56450912
VAT1	chr12	19870312	19879230	529603.1953	chr12	19529603	19533603	0.56453066
ENSSSCG00000038506	chr9	135025443	135078913	113078.13511	chr9	135113078	135117078	0.56459463
GINS2	chr6	3218702	3243899	710866.37171	chr6	3710866	3717166	0.56465036
OTUD5	chrX	43000231	43029468	680269.42684	chrX	42680269	42684269	0.56475683
FEM1A	chr2	74042989	74044983	042210.74044	chr2	74042210	74044130	0.56479159
TUBGCP2	chr14	141233856	141253359	098189.1411	chr14	141098189	141104699	0.56479376
C10orf143	chr14	139013253	139050019	346949.1393	chr14	139346949	139351589	0.56484406
COG1	chr12	7743274	7760504	170949.8173	chr12	8170949	8173959	0.56485153
CUL7	chr7	38097854	38115822	097569.38102	chr7	38097569	38102239	0.56499039
CA5A	chr6	1506042	1538777	020937.10249	chr6	1020937	1024937	0.56536382
SEZ6L2	chr3	18098102	18119558	812944.17813	chr3	17812944	17813910	0.56539839
SLC16A5	chr12	6185528	6200001	214528.6215	chr12	6214528	6215144	0.56541379
DGAT1	chr4	452662	466684	377258.37834	chr4	377258	378347	0.56544107
WRAP73	chr6	65155209	65167079	710876.64718	chr6	64710876	64718966	0.56548402
BRD9	chr16	79473745	79492635	438153.7943	chr16	79438153	79439277	0.56549666
MTMR1	chrX	122394921	122453905	002378.12200	chrX	122002378	122004278	0.56578428
MRPL54	chr2	74925070	74928167	729195.74730	chr2	74729195	74730347	0.56586628
PCID2	chr11	78541318	78556733	167005.7817	chr11	78167005	78170890	0.56588715
TXNL4A	chr6	127974543	127991177	712046.12771	chr6	127712046	127713246	0.56595289
SERPINB1	chr7	1684215	1693299	618809.16227	chr7	1618809	1622759	0.56614374
SELENOF	chr4	129258944	129287720	319459.12932	chr4	129319459	129323069	0.56622214
FIGNL1	chr9	136484216	136488250	240759.13624	chr9	136240759	136244759	0.56634823
TBCD	chr12	329714	449556	145208.1492	chr12	145208	149208	0.5663549
MGMT	chr14	138499309	138771540	976219.1389	chr14	138976219	138981529	0.56644054
NDUFA13	chr2	58401551	58411981	631062.58632	chr2	58631062	58632193	0.56656624
NLRP5	chr6	60379623	60409465	857817.60859	chr6	60857817	60859961	0.5666867
PRSS8	chr3	17357802	17362220	809718.17813	chr3	17809718	17813718	0.5673088
ENSSSCG00000035728	chr12	1095096	1099180	304812.1306	chr12	1304812	1306458	0.56731674
ENSSSCG00000046487	chr6	48052136	48059215	150486.48153	chr6	48150486	48153526	0.56732759
ENSSSCG00000060915	chr17	59172433	59175227	145902.5914	chr17	59145902	59148352	0.56754017
NSDHL	chrX	123906199	123929117	234696.12423	chrX	124234696	124236903	0.56754628
POLR2F	chr5	9855983	9917726	547161.95483	chr5	9547161	9548308	0.56756201
GAA	chr12	2314591	2336988	095212.2099	chr12	2095212	2099139	0.56756714
TNNT1	chr6	59347582	59365284	733286.59736	chr6	59733286	59736796	0.56756727
ENSSSCG00000016990	chr16	51225724	51260716	049039.5105	chr16	51049039	51051709	0.56766568
TEPSIN	chr12	1519760	1529542	304812.1306	chr12	1304812	1306458	0.56772734
ENKD1	chr6	28283117	28287306	851452.27855	chr6	27851452	27855452	0.56788634
HAUS7	chrX	124234736	124258098	423872.12442	chrX	124423872	124427872	0.56802394
ENSSSCG00000057427	chr18	2568274	2595510	415625.2424	chr18	2415625	2424205	0.56842325
SMARCD2	chr12	15091729	15101899	360359.1536	chr12	15360359	15364249	0.56865289
LYRM4	chr7	3070470	3192922	623009.26262	chr7	2623009	2626219	0.56875354
DGAT1	chr4	452662	466684	782159.79387	chr4	782159	793879	0.56888147
BRD9	chr16	79473745	79492635	538042.7953	chr16	79538042	79539074	0.56907898
STMP1	chr18	13641541	13665565	995535.1399	chr18	13995535	13998965	0.56914218
MRPL17	chr9	3075995	3083580	120522.31249	chr9	3120522	3124932	0.5693029
ENSSSCG00000032573	chr4	75636278	75646153	828169.75830	chr4	75828169	75830959	0.56930972
BCAP31	chrX	124457001	124484743	814014.12481	chrX	124814014	124818014	0.56937721
FTL	chr6	54231172	54232750	761062.53765	chr6	53761062	53765062	0.56960777
TRAF3IP1	chr15	137863609	137912477	673710.1376	chr15	137673710	137677710	0.56963996
RPS16	chr6	48085678	48088423	150486.48153	chr6	48150486	48153526	0.56966637
SERPINB1	chr7	1684215	1693299	470509.14725	chr7	1470509	1472579	0.56975322
TRAF3IP1	chr15	137863609	137912477	383458.1373	chr15	137383458	137390388	0.56975985
ZNHIT6	chr4	130298280	130386955	737319.13073	chr4	130737319	130739599	0.56977868
ENSSSCG00000021624	chr10	23767206	23778722	786279.2379	chr10	23786279	23790279	0.56998971
CAMTA1	chr6	67605790	68471920	759306.67762	chr6	67759306	67762556	0.57011172
C9orf78	chr1	269986391	269995395	259836.27026	chr1	270259836	270264736	0.57017726
TUBB2B	chr7	1951407	1956119	325359.23277	chr7	2325359	2327729	0.57021992
IDH3G	chrX	124528585	124537575	903258.12490	chrX	124903258	124909988	0.57042121
TMED4	chr18	50699470	50702705	750846.5075	chr18	50750846	50751473	0.57074481

FZR1	chr2	75121835	75146835	395470.75398	chr2	75395470	75398190	0.57080292
MRPS7	chr12	6052063	6055668	6337182.6338	chr12	6337182	6338139	0.57084564
SYT2	chr10	24638704	24669846	684409.2468	chr10	24684409	24686519	0.57104889
TAF1C	chr6	4448778	4457088	640764.46447	chr6	4640764	4644764	0.57147479
PLIN3	chr2	73969862	73995529	485267.73486	chr2	73485267	73486251	0.57171739
TMEM9B	chr9	495693	521013	258992.26107	chr9	258992	261072	0.57180935
ENO1	chr6	69385879	69401151	685646.69696	chr6	69685646	69696186	0.57187461
PDGFA	chr3	301584	321712	345186.35211	chr3	345186	352116	0.5719748
FTL	chr6	54231172	54232750	070265.54074	chr6	54070265	54074265	0.57246413
SDHA	chr16	79834044	79862524	544639.7954	chr16	79544639	79547779	0.57253467
ENSSSCG00000014540	chr2	9535148	9537974	082256.90862	chr2	9082256	9086256	0.57272141
CSNK1D	chr12	768865	796595	162187.1166	chr12	1162187	1166187	0.57285938
POLA2	chr2	6922387	6950179	526990.65343	chr2	6526990	6534360	0.57349107
DYNLL2	chr12	34349294	34356472	896719.3390	chr12	33896719	33901049	0.57357762
PDCD6	chr16	79818958	79835746	877864.7987	chr16	79877864	79878678	0.57361288
MRPL40	chr14	51102613	51105767	291092.5129	chr14	51291092	51297177	0.57361816
COMT	chr14	51385738	51403998	291092.5129	chr14	51291092	51297177	0.57444171
CDIPT	chr3	18084549	18091798	303416.17807	chr3	17803416	17807526	0.5745527
PHRF1	chr2	340528	371692	709596.71359	chr2	709596	713596	0.57459909
SLC3A2	chr2	8892089	8919072	293700.92984	chr2	9293700	9298410	0.57484705
HMG20B	chr2	75093075	75098536	395470.75398	chr2	75395470	75398190	0.57485258
ATP6V0B	chr6	167316947	167320212	684752.16768	chr6	167684752	167687008	0.57515746
ENSSSCG00000045735	chr18	50458411	50463097	056015.5005	chr18	50056015	50059125	0.5752046
UBE2J2	chr6	63555488	63568017	054906.63058	chr6	63054906	63058226	0.57547638
IDH3G	chrX	124528585	124537575	427335.12442	chrX	124427335	124429654	0.57549522
ZFPL1	chr2	7098766	7103536	395080.68997	chr2	6895080	6899700	0.57561321
FAM104A	chr12	7727796	7749240	170949.8173	chr12	8170949	8173959	0.57594963
EIF4E2	chr15	133060247	133103719	933798.1329	chr15	132933798	132935628	0.57641797
AKR1C8	chr10	65559508	65572895	314989.6532	chr10	65314989	65324449	0.57652357
TMED3	chr7	48431375	48442645	558753.48562	chr7	48558753	48562753	0.57656393
DTYMK	chr15	140248079	140255805	822668.1398	chr15	139822668	139834538	0.5773297
MEPCE	chr3	8357289	8364661	836970.88376	chr3	8836970	8837652	0.57749647
ENSSSCG00000038506	chr9	135025443	135078913	272762.13528	chr9	135272762	135281422	0.57764353
NAXD	chr11	77210228	77230680	710950.7671	chr11	76710950	76713460	0.57789813
ENSSSCG00000014569	chr9	707693	711219	258992.26107	chr9	258992	261072	0.57796599
ZNF75D	chrX	110890341	110904062	961228.11096	chrX	110961228	110965038	0.57829006
PCID2	chr11	78541318	78556733	891930.7889	chr11	78891930	78896300	0.57846477
LDLRAP1	chr6	83030702	83054377	550096.82554	chr6	82550096	82554946	0.57875547
SLC43A2	chr12	47791913	47839012	166900.4817	chr12	48166900	48170900	0.57910722
DUSP28	chr15	139537326	139539438	048928.1390	chr15	139048928	139052328	0.57911832
ZNF584	chr6	62992506	63005691	745816.62746	chr6	62745816	62746568	0.57941254
NAA10	chrX	124658158	124662702	424348.12442	chrX	124424348	124429148	0.57964139
HARS1	chr2	142385872	142401208	804530.14280	chr2	142804530	142808530	0.57969612
BCAP31	chrX	124457001	124484743	903258.12490	chrX	124903258	124909988	0.57981062
PRKCA	chr12	12882064	13263554	390875.1239	chr12	12390875	12394875	0.5798825
TIMM17B	chrX	42970108	42975955	357991.43361	chrX	43357991	43361991	0.57991301
ATP6AP1	chrX	124952500	124960343	903258.12490	chrX	124903258	124909988	0.58012472
MRPL36	chr16	79001549	79004358	603069.7860	chr16	78603069	78605119	0.5801346
CBR3	chr13	199830668	199840442	126477.2001	chr13	200126477	200130477	0.58040513
ARMC12	chr7	31586797	31603600	159320.31163	chr7	31159320	31163320	0.58043745
PLIN3	chr2	73969862	73995529	042210.74044	chr2	74042210	74044130	0.58053354
BNIP3	chr14	140362127	140371907	303169.1403	chr14	140303169	140305619	0.58054351
BRD9	chr16	79473745	79492635	036641.7904	chr16	79036641	79043345	0.58065802
MTM1	chrX	122286916	122379299	898508.12190	chrX	121898508	121904128	0.58099862
MRPL2	chr7	38116179	38121756	097569.38102	chr7	38097569	38102239	0.58153705
MRPS7	chr12	6052063	6055668	6336839.6340	chr12	6336839	6340259	0.58180368
CERS4	chr2	71004420	71048248	110600.71114	chr2	71110600	71114230	0.58182598
VPS28	chr4	362912	370588	204188.20818	chr4	204188	208188	0.5818983
CCER2	chr6	47712765	47715620	150486.48153	chr6	48150486	48153526	0.58191951
VKORC1	chr3	17386245	17389905	613617.17614	chr3	17613617	17614750	0.58254931
BCAP31	chrX	124457001	124484743	234696.12423	chrX	124234696	124236903	0.58282769
UXT	chrX	42176145	42184079	174600.42175	chrX	42174600	42175367	0.58283111

RPS20	chr4	75762209	75769955	328169.75830	chr4	75828169	75830959	0.58288746
ENSSSCG00000061173	chrX	110774840	110792950	890031.11089	chrX	110890031	110890938	0.58313362
PSMA7	chr17	61566373	61572438	.222882.6122	chr17	61222882	61224592	0.58336845
ECHS1	chr14	141339364	141348994	.450562.1414	chr14	141450562	141451766	0.5833742
NDUFA10	chr15	138986421	139031897	044907.1390	chr15	139044907	139048907	0.58341362
COPS9	chr15	139260478	139264211	868278.1388	chr15	138868278	138870448	0.58343469
JPT1	chr12	6139428	6158452	336839.6340	chr12	6336839	6340259	0.58415541
LSM7	chr2	76247058	76252598	276671.76278	chr2	76276671	76278423	0.58430861
SELENOF	chr4	129258944	129287720	968789.12897	chr4	128968789	128973549	0.58434362
ENSSSCG00000038506	chr9	135025443	135078913	273944.13527	chr9	135273944	135274674	0.58455484
EEF1D	chr4	969527	983270	321556.13255	chr4	1321556	1325556	0.58476967
TMEM223	chr2	8981963	8983263	082256.90862	chr2	9082256	9086256	0.58493363
BNIP3	chr14	140362127	140371907	414459.1404	chr14	140414459	140419889	0.58497522
GFUS	chr4	949867	959744	321556.13255	chr4	1321556	1325556	0.58503387
BCAP31	chrX	124457001	124484743	232736.12423	chrX	124232736	124236736	0.58514475
ENSSSCG00000017971	chr12	53111256	53112484	493453.5349	chr12	53493453	53497453	0.58520561
TNNT1	chr6	59347582	59365284	703779.59705	chr6	59703779	59705229	0.58539601
CTPS1	chr6	170201751	170233016	194906.17019	chr6	170194906	170197814	0.58561752
TUFM	chr3	18521128	18524959	021096.18025	chr3	18021096	18025096	0.58565902
ATP6V0B	chr6	167316947	167320212	435586.16743	chr6	167435586	167437366	0.58585155
MRPL20	chr6	63670755	63675839	974346.63978	chr6	63974346	63978346	0.58586126
PSMG3	chr3	1052191	1054688	066576.10701	chr3	1066576	1070166	0.58587245
COPS9	chr15	139260478	139264211	477888.1394	chr15	139477888	139482538	0.58592289
SEZ6L2	chr3	18098102	18119558	303416.17807	chr3	17803416	17807526	0.58651537
PBDC1	chrX	60907404	61019857	424975.60428	chrX	60424975	60428975	0.58655731
FAM110A	chr17	34425617	34437721	911812.3491	chr17	34911812	34917302	0.58701873
TUBB2B	chr7	1951407	1956119	424299.24311	chr7	2424299	2431169	0.58724528
NUDCD3	chr18	50759221	50830332	890905.5089	chr18	50890905	50896275	0.587393
UBL7	chr7	59070728	59084112	068728.59072	chr7	59068728	59072728	0.58750767
MRPL36	chr16	79001549	79004358	854486.7885	chr16	78854486	78856078	0.5877441
ENSSSCG00000045735	chr18	50458411	50463097	890905.5089	chr18	50890905	50896275	0.58776359
TNNT1	chr6	59347582	59365284	713636.59717	chr6	59713636	59717636	0.58815467
CBX8	chr12	2562374	2565780	2133160.2137	chr12	2133160	2137160	0.5881729
COX4I1	chr6	3131019	3137530	658189.26621	chr6	2658189	2662189	0.58843614
WARS1	chr7	121212981	121238113	665209.12166	chr7	121665209	121667959	0.58852723
NCLN	chr2	75369955	75390545	395470.75398	chr2	75395470	75398190	0.58872563
SYCN	chr6	47931042	47932708	150486.48153	chr6	48150486	48153526	0.5888219
ARID3B	chr7	58932461	58998711	068728.59072	chr7	59068728	59072728	0.58891294
MVP	chr3	18057177	18081155	613617.17614	chr3	17613617	17614750	0.5889809
ARHGEF7	chr11	77427368	77530993	7854226.7785	chr11	77854226	77857606	0.58900305
METTL23	chr12	4804037	4808913	914069.4923	chr12	4914069	4923019	0.58917951
FTL	chr6	54231172	54232750	346936.54350	chr6	54346936	54350546	0.58922646
MRPL36	chr16	79001549	79004358	495219.7949	chr16	79495219	79497289	0.58926132
ENSSSCG00000002036	chr7	76014901	76021919	003612.76007	chr7	76003612	76007612	0.58952982
FSD1	chr2	74471744	74485786	729195.74730	chr2	74729195	74730347	0.58969367
ENSSSCG00000027491	chr18	51220948	51310989	750846.5075	chr18	50750846	50751473	0.58998893
TUBB2A	chr7	1910269	1914761	259389.22659	chr7	2259389	2265929	0.58999184
DUSP28	chr15	139537326	139539438	479553.1394	chr15	139479553	139483165	0.59015734
POLR2H	chr13	122250020	122256195	482235.1224	chr13	122482235	122484295	0.59020001
ENSSSCG00000013064	chr2	9163317	9174468	195750.91988	chr2	9195750	9198850	0.59021289
ENSSSCG00000056719	chr6	61903216	61907832	473531.61474	chr6	61473531	61474411	0.5903009
VPS28	chr4	362912	370588	377258.37834	chr4	377258	378347	0.59031429
ENSSSCG00000053570	chr6	62255210	62260695	138474.62142	chr6	62138474	62142474	0.59048538
SHD	chr2	74497351	74504659	729195.74730	chr2	74729195	74730347	0.59058514
NDUFS6	chr16	78993913	79000006	837739.7884	chr16	78837739	78842699	0.59066725
ATP6V0E2	chr18	55877504	55882204	612730.5561	chr18	55612730	55616730	0.59086354
MED8	chr6	167861373	167869411	986876.16798	chr6	167986876	167989686	0.5910029
PPP1R7	chr15	139917257	139941251	865812.1398	chr15	139865812	139867602	0.59112572
SHANK2	chr2	2864331	3015314	577970.25863	chr2	2577970	2586340	0.59113815
ENSSSCG00000017907	chr12	51961952	51965464	975989.5197	chr12	51975989	51978009	0.59120892
SARS2	chr6	47716679	47728310	969275.47973	chr6	47969275	47973275	0.59126492
CYC1	chr4	606516	608996	204188.20818	chr4	204188	208188	0.59126801

BRD9	chr16	79473745	79492635	048763.7905	chr16	79048763	79050221	0.59133039
ECHS1	chr14	141339364	141348994	145779.1411	chr14	141145779	141148769	0.59139196
CDPF1	chr5	3292022	3296307	395198.34075	chr5	3395198	3407528	0.59195367
KDM4A	chr6	167597956	167642333	301936.16780	chr6	167801936	167808346	0.59208203
SEC61G	chr9	139128296	139135727	932632.13893	chr9	138932632	138938622	0.59220119
TOP1MT	chr4	1148890	1188130	476009.14858	chr4	1476009	1485859	0.59222441
ATG4B	chr15	140223904	140246321	764414.1397	chr15	139764414	139768414	0.59232392
CIAO2B	chr6	27610470	27612592	825350.27829	chr6	27825350	27829350	0.59239624
MRPL52	chr7	76187446	76191901	005976.76006	chr7	76005976	76006524	0.59308625
TMEM219	chr3	18167923	18176563	021096.18025	chr3	18021096	18025096	0.59321038
COPS9	chr15	139260478	139264211	984421.1389	chr15	138984421	138988421	0.59344437
ENSSSCG00000015632	chr9	136893356	137056101	753582.13675	chr9	136753582	136756492	0.59370223
BCAP31	chrX	124457001	124484743	214324.12421	chrX	124214324	124218324	0.59378382
SHANK2	chr2	2864331	3015314	743030.27504	chr2	2743030	2750430	0.59403428
MEPCE	chr3	8357289	8364661	832274.88362	chr3	8832274	8836274	0.59409516
VKORC1	chr3	17386245	17389905	803416.17807	chr3	17803416	17807526	0.59429022
STK25	chr15	140144336	140154366	865812.1398	chr15	139865812	139867602	0.59430337
CCNL2	chr6	63659054	63668047	679085.63683	chr6	63679085	63683085	0.59431239
TCP1	chr1	7590140	7601795	364266.73678	chr1	7364266	7367856	0.59436885
CSNK1D	chr12	768865	796595	448754.4508	chr12	448754	450833	0.59456726
ENSSSCG00000048787	chr15	137765933	137767998	240648.1382	chr15	138240648	138243538	0.59478412
LRCH4	chr3	8499285	8511262	836970.88376	chr3	8836970	8837652	0.59525021
TRAF3IP1	chr15	137863609	137912477	692318.1376	chr15	137692318	137698258	0.59542482
ENSSSCG00000045735	chr18	50458411	50463097	750846.5075	chr18	50750846	50751473	0.59563294
ATP6V0E2	chr18	55877504	55882204	493159.5549	chr18	55493159	55497159	0.5957259
TUBGCP2	chr14	141233856	141253359	119109.1411	chr14	141119109	141125409	0.59594717
MGMT	chr14	138499309	138771540	182749.1381	chr14	138182749	138186599	0.59595627
ATP5MC2	chr5	18871026	18879609	586758.18590	chr5	18586758	18590758	0.59613086
ENSSSCG00000017971	chr12	53111256	53112484	006559.5300	chr12	53006559	53008829	0.59665652
IDH3G	chrX	124528585	124537575	214324.12421	chrX	124214324	124218324	0.59674
LIG1	chr6	53620483	53686562	423936.53426	chr6	53423936	53426996	0.59716377
ATG4B	chr15	140223904	140246321	865812.1398	chr15	139865812	139867602	0.59736715
SEZ6L2	chr3	18098102	18119558	613617.17614	chr3	17613617	17614750	0.59742771
GLRX3	chr14	139072575	139111249	003294.1390	chr14	139003294	139003884	0.59759271
ENSSSCG00000012088	chr13	207801096	207818130	285845.2082	chr13	208285845	208289845	0.59789176
IDH3G	chrX	124528585	124537575	232736.12423	chrX	124232736	124236736	0.59848227
SPDYC	chr2	7030733	7058967	895080.68997	chr2	6895080	6899700	0.59852107
DNAJC30	chr3	10955877	10959916	052516.11053	chr3	11052516	11053085	0.5989485
SYT2	chr10	24638704	24669846	179769.2418	chr10	24179769	24184649	0.59901834
ENSSSCG00000027491	chr18	51220948	51310989	890905.5089	chr18	50890905	50896275	0.59913056
THEM5	chr4	97332432	97359428	041069.97043	chr4	97041069	97043669	0.59922048
NDUFS6	chr16	78993913	79000006	168279.7917	chr16	79168279	79175129	0.59930833
DND1	chr2	142384532	142387199	473803.14247	chr2	142473803	142474940	0.59947044
RPS20	chr4	75762209	75769955	737402.75741	chr4	75737402	75741402	0.5995187
SNU13	chr5	6885067	6900022	576101.65771	chr5	6576101	6577139	0.59953926
COA3	chr12	20062826	20063980	166659.2016	chr12	20166659	20168579	0.59961866
HDGFL2	chr2	74332106	74357040	729195.74730	chr2	74729195	74730347	0.59980321
UBE2J2	chr6	63555488	63568017	679085.63683	chr6	63679085	63683085	0.60058703
ZNHIT6	chr4	130298280	130386955	781369.13078	chr4	130781369	130787289	0.60070945
PSMC4	chr6	48406376	48416215	659926.48665	chr6	48659926	48665966	0.60085288
COPS3	chr12	60994310	61024846	055733.6105	chr12	61055733	61059733	0.60093978
IK	chr2	142361155	142376333	473803.14247	chr2	142473803	142474940	0.60101538
NDUFA10	chr15	138986421	139031897	479553.1394	chr15	139479553	139483165	0.60107334
DUSP28	chr15	139537326	139539438	865812.1398	chr15	139865812	139867602	0.60112904
HAUS7	chrX	124234736	124258098	232736.12423	chrX	124232736	124236736	0.60121607
ENSSSCG00000031249	chr13	207493659	207499867	882785.2078	chr13	207882785	207886425	0.60141066
NDUFB11	chrX	41770941	41774245	174600.42175	chrX	42174600	42175367	0.60176322
NCCRP1	chr6	47915118	47929937	150486.48153	chr6	48150486	48153526	0.60177491
NHEJ1	chr15	121100628	121190062	560936.1215	chr15	121560936	121562466	0.60208018
BRD9	chr16	79473745	79492635	891263.7989	chr16	79891263	79895263	0.60208524
NELFE	chr7	24040525	24047025	907960.23911	chr7	23907960	23911960	0.60221231
HAUS7	chrX	124234736	124258098	234696.12423	chrX	124234696	124236903	0.6022447

ILK	chr9	3145608	3159858	293892.32964	chr9	3293892	3296422	0.60231479
PFKM	chr5	78476123	78526997	139236.78143	chr5	78139236	78143236	0.6024654
MGMT	chr14	138499309	138771540	075609.1380	chr14	138075609	138081359	0.60248039
VPS41	chr18	55301624	55466628	493159.5549	chr18	55493159	55497159	0.60250653
POLR1D	chr11	5044329	5089766	390050.5395	chr11	5390050	5395160	0.60257696
ENSSSCG00000036988	chr7	1980292	1988727	748321.17494	chr7	1748321	1749415	0.60280072
PRPF31	chr6	55995091	56011441	020256.56024	chr6	56020256	56024256	0.60327201
BRD9	chr16	79473745	79492635	537609.7954	chr16	79537609	79543449	0.60336703
UBE2J2	chr6	63555488	63568017	444151.63445	chr6	63444151	63445363	0.60394538
BNIP1	chr16	51126926	51140394	049039.5105	chr16	51049039	51051709	0.60404173
ENSSSCG00000027723	chr15	137567231	137621880	045088.1380	chr15	138045088	138051538	0.60423485
RPS5	chr6	62967994	62974350	054906.63058	chr6	63054906	63058226	0.60433242
EBNA1BP2	chr6	168049758	168061013	684752.16768	chr6	167684752	167687008	0.60448992
BNIP3	chr14	140362127	140371907	308619.1403	chr14	140308619	140313739	0.60449267
ENSSSCG00000045735	chr18	50458411	50463097	095885.5009	chr18	50095885	50097985	0.60451262
LYRM4	chr7	3070470	3192922	573209.25814	chr7	2573209	2581459	0.60480084
ENSSSCG00000013613	chr2	70193730	70199975	658392.70659	chr2	70658392	70659484	0.60488483
TUBGCP2	chr14	141233856	141253359	114989.1411	chr14	141114989	141118819	0.60522832
MRPL20	chr6	63670755	63675839	444151.63445	chr6	63444151	63445363	0.60522996
FAU	chr2	7070906	7072809	895080.68997	chr2	6895080	6899700	0.60532661
CD63	chr5	21172283	21176232	189251.21193	chr5	21189251	21193251	0.60561382
DTYMK	chr15	140248079	140255805	858888.1398	chr15	139858888	139868418	0.60569098
NLRP5	chr6	60379623	60409465	855442.60859	chr6	60855442	60859442	0.60602808
LIN37	chr6	45175500	45181391	344637.45348	chr6	45344637	45348637	0.6061176
ENSSSCG00000002036	chr7	76014901	76021919	005976.76006	chr7	76005976	76006524	0.60658629
INO80E	chr3	18202134	18212186	809718.17813	chr3	17809718	17813718	0.60666152
FUT2	chr6	54034684	54047224	078981.54081	chr6	54078981	54081394	0.60679641
EIPR1	chr3	131360883	131435579	726366.13173	chr3	131726366	131731426	0.60685699
SEPTIN8	chr2	135077170	135104471	137272.13514	chr2	135137272	135141272	0.6069075
ENSSSCG00000035997	chrX	124926772	124929791	822947.12482	chrX	124822947	124824355	0.60692815
FZR1	chr2	75121835	75146835	729195.74730	chr2	74729195	74730347	0.60694698
TUBB2B	chr7	1951407	1956119	498649.15032	chr7	1498649	1503249	0.60699706
ELAC2	chr12	57206600	57238444	589920.5759	chr12	57589920	57593920	0.60702292
ZNF212	chr18	55528138	55541841	493159.5549	chr18	55493159	55497159	0.60704577
NUDCD3	chr18	50759221	50830332	750846.5075	chr18	50750846	50751473	0.60704752
DDX39B	chr7	23658088	23670031	907960.23911	chr7	23907960	23911960	0.60755314
TMEM219	chr3	18167923	18176563	812944.17813	chr3	17812944	17813910	0.60778423
POLR3H	chr5	6997939	7014421	576101.65771	chr5	6576101	6577139	0.60789796
ZNF574	chr6	49834650	49840391	568032.49572	chr6	49568032	49572032	0.60793427
SAP18	chr11	1291202	1298983	836951.8409	chr11	836951	840951	0.60795498
MTMR1	chrX	122394921	122453905	227078.12223	chrX	122227078	122232108	0.6088662
ENSSSCG00000012088	chr13	207801096	207818130	267585.2082	chr13	208267585	208270895	0.60900349
MRPS24	chr18	48825782	48830609	737495.4874	chr18	48737495	48741355	0.60905804
MED8	chr6	167861373	167869411	656186.16766	chr6	167656186	167665426	0.60906528
MRPS24	chr18	48825782	48830609	159885.4917	chr18	49159885	49170155	0.60965293
TBRG4	chr18	50365988	50378084	750846.5075	chr18	50750846	50751473	0.60986694
HDLBP	chr15	139957715	140022302	865812.1398	chr15	139865812	139867602	0.61007464
TBRG4	chr18	50365988	50378084	064445.5006	chr18	50064445	50066235	0.6100771
PSMG3	chr3	1052191	1054688	228872.12328	chr3	1228872	1232833	0.61008443
TMED4	chr18	50699470	50702705	479985.5048	chr18	50479985	50482895	0.61017668
BCAP31	chrX	124457001	124484743	427335.12442	chrX	124427335	124429654	0.61073077
ENOX2	chrX	107085503	107368473	659892.10666	chrX	106659892	106661951	0.61091157
RPL3	chr5	8922665	8929767	775965.87799	chr5	8775965	8779965	0.61138919
ENSSSCG00000027723	chr15	137567231	137621880	442855.1374	chr15	137442855	137446855	0.61141285
GAA	chr12	2314591	2336988	133160.2137	chr12	2133160	2137160	0.61152473
CYC1	chr4	606516	608996	785393.79847	chr4	785393	798477	0.61158116
PER1	chr12	53361889	53374248	493453.5349	chr12	53493453	53497453	0.61162567
LSM7	chr2	76247058	76252598	277430.76279	chr2	76277430	76279160	0.6120501
MRPS24	chr18	48825782	48830609	189535.4919	chr18	49189535	49195785	0.61270546
TMED4	chr18	50699470	50702705	890905.5089	chr18	50890905	50896275	0.61294932
HAUS7	chrX	124234736	124258098	424348.12442	chrX	124424348	124429148	0.6131717
HAUS7	chrX	124234736	124258098	427335.12442	chrX	124427335	124429654	0.61321331

PHRF1	chr2	340528	371692	2.90704.93254	chr2	90704	93254	0.61334754
FIGNL1	chr9	136484216	136488250	386442.13639	chr9	136386442	136391772	0.61375906
UBXN6	chr2	74357517	74378592	414316.74414	chr2	74414316	74414825	0.61376143
ZMAT2	chr2	142411089	142419137	483896.14248	chr2	142483896	142487896	0.61379893
TSTD1	chr4	89401159	89403277	972183.88973	chr4	88972183	88973295	0.61380181
USP27X	chrX	43555005	43556321	230192.43234	chrX	43230192	43234192	0.61395846
ATP6V0B	chr6	167316947	167320212	801936.16780	chr6	167801936	167808346	0.61426986
GINS2	chr6	3218702	3243899	309516.28170	chr6	2809516	2817016	0.61460198
KAT5	chr2	6560513	6572466	456140.64608	chr2	6456140	6460880	0.61464641
OSBPL2	chr17	61643377	61685819	.683622.6168	chr17	61683622	61687972	0.61470004
CIAO2B	chr6	27610470	27612592	851452.27855	chr6	27851452	27855452	0.61480605
CFL1	chr2	6469254	6475035	456140.64608	chr2	6456140	6460880	0.61481097
OSBPL2	chr17	61643377	61685819	.321802.6132	chr17	61321802	61328422	0.61485534
WRAP73	chr6	65155209	65167079	025442.65029	chr6	65025442	65029442	0.61507068
ENSSSCG00000012088	chr13	207801096	207818130	7882785.2078	chr13	207882785	207886425	0.61511142
ENSSSCG00000057427	chr18	2568274	2595510	.235895.2238	chr18	2235895	2238315	0.6152794
POLA2	chr2	6922387	6950179	395080.68997	chr2	6895080	6899700	0.61528543
ENSSSCG00000035904	chr1	272959831	272965634	535826.27254	chr1	272535826	272540916	0.61530478
NFYC	chr6	170413638	170485840	194906.17019	chr6	170194906	170197814	0.61570665
RNF220	chr6	166666138	166910646	593803.16659	chr6	166593803	166595225	0.61589578
FTSJ3	chr12	15106719	15114398	5392487.1539	chr12	15392487	15396487	0.6161268
FSD1	chr2	74471744	74485786	042210.74044	chr2	74042210	74044130	0.61619759
MRPL20	chr6	63670755	63675839	991306.63993	chr6	63991306	63993056	0.61635627
ENSSSCG00000035997	chrX	124926772	124929791	814014.12481	chrX	124814014	124818014	0.61650738
GALNTL5	chr18	5353769	5433814	4980080.4981	chr18	4980080	4981148	0.61673354
ING1	chr11	77270320	77277068	5880640.7688	chr11	76880640	76886850	0.61686474
LENG1	chr6	55970668	55976735	389302.56390	chr6	56389302	56390042	0.61708113
MRPL36	chr16	79001549	79004358	9011667.7901	chr16	79011667	79012408	0.61739899
TUBGCP2	chr14	141233856	141253359	.450562.1414	chr14	141450562	141451766	0.6174596
TLE2	chr2	75510004	75536804	395470.75398	chr2	75395470	75398190	0.61747485
TNNT1	chr6	59347582	59365284	233436.59233	chr6	59233436	59233978	0.61753749
BRD9	chr16	79473745	79492635	9552127.7955	chr16	79552127	79553615	0.61757612
NAPA	chr6	53246968	53275265	431154.53431	chr6	53431154	53431639	0.61792426
MED8	chr6	167861373	167869411	658726.16766	chr6	167658726	167662326	0.61804788
TUBGCP2	chr14	141233856	141253359	.115481.1411	chr14	141115481	141118289	0.61805999
MED8	chr6	167861373	167869411	801936.16780	chr6	167801936	167808346	0.61829373
CKM	chr6	51700507	51714814	601726.51606	chr6	51601726	51606356	0.61889501
DNPEP	chr15	121391746	121412864	.560936.1215	chr15	121560936	121562466	0.61901668
ZNRF1	chr6	12555664	12668804	054571.12058	chr6	12054571	12058571	0.61908404
CYC1	chr4	606516	608996	377258.37834	chr4	377258	378347	0.61911379
GLRX3	chr14	139072575	139111249	9222899.1392	chr14	139222899	139228039	0.61923061
ATG4B	chr15	140223904	140246321	9858888.1398	chr15	139858888	139868418	0.61971078
MRPL54	chr2	74925070	74928167	395470.75398	chr2	75395470	75398190	0.62015638
NFYC	chr6	170413638	170485840	242316.17024	chr6	170242316	170248076	0.62037007
ENSSSCG00000027723	chr15	137567231	137621880	7692318.1376	chr15	137692318	137698258	0.62052314
PRKAG2	chr18	5475077	5744657	5873195.5876	chr18	5873195	5876335	0.62065909
IDH3G	chrX	124528585	124537575	234696.12423	chrX	124234696	124236903	0.62111631
BRD9	chr16	79473745	79492635	9168279.7917	chr16	79168279	79175129	0.6211819
ENSSSCG00000021624	chr10	23767206	23778722	4179769.2418	chr10	24179769	24184649	0.62143718
ENSSSCG00000029830	chr4	136107	138652	204188.20818	chr4	204188	208188	0.62150646
CDIPT	chr3	18084549	18091798	613617.17614	chr3	17613617	17614750	0.62203075
TALDO1	chr2	464728	472835	130230.13366	chr2	130230	133660	0.62203579
NDUFA10	chr15	138986421	139031897	8868278.1388	chr15	138868278	138870448	0.62276313
ENSSSCG00000032573	chr4	75636278	75646153	065169.76068	chr4	76065169	76068979	0.62289896
IDH3G	chrX	124528585	124537575	814014.12481	chrX	124814014	124818014	0.62299352
TCP11	chr7	31007474	31114734	159320.31163	chr7	31159320	31163320	0.62317185
BNIP3	chr14	140362127	140371907	0038389.1400	chr14	140038389	140042669	0.6231784
BRD9	chr16	79473745	79492635	9544639.7954	chr16	79544639	79547779	0.62324754
SNAPC2	chr2	71291257	71294006	110600.71114	chr2	71110600	71114230	0.62420458
VKORC1	chr3	17386245	17389905	812944.17813	chr3	17812944	17813910	0.62546863
MRPL58	chr12	6255342	6266163	6214528.6215	chr12	6214528	6215144	0.62555166
CCNF	chr3	39585607	39603079	260264.39264	chr3	39260264	39264264	0.62595017

ENSSSCG00000057427	chr18	2568274	2595510	040845.3044	chr18	3040845	3044465	0.62669555
EBNA1BP2	chr6	168049758	168061013	301936.1678	chr6	167801936	167808346	0.62670863
COX17	chr13	140584363	140587621	0599515.1406	chr13	140599515	140603515	0.62675423
WRAP73	chr6	65155209	65167079	563816.6466	chr6	64663816	64666816	0.62684515
NKIRAS2	chr12	20717547	20723704	0957709.2096	chr12	20957709	20961019	0.62751063
CDIPT	chr3	18084549	18091798	809718.1781	chr3	17809718	17813718	0.628387
PRPSAP1	chr12	5144069	5174382	908039.4909	chr12	4908039	4909329	0.62873277
ALG8	chr9	12497721	12532307	479472.1248	chr9	12479472	12481122	0.62899978
RPL10A	chr7	31327440	31333638	159320.3116	chr7	31159320	31163320	0.62912911
BRD9	chr16	79473745	79492635	797679.7980	chr16	79797679	79802279	0.62946501
NAA10	chrX	124658158	124662702	427335.1244	chrX	124427335	124429654	0.62947083
STX8	chr12	54296995	54544646	676329.5467	chr12	54676329	54677869	0.62955289
FIGNL1	chr9	136484216	136488250	457852.1364	chr9	136457852	136463142	0.62966831
TXN2	chr5	11264645	11283395	811288.1081	chr5	10811288	10814498	0.6296892
ENKD1	chr6	28283117	28287306	825350.2782	chr6	27825350	27829350	0.62990907
GLRX3	chr14	139072575	139111249	000749.1390	chr14	139000749	139004889	0.63016751
RNF181	chr3	59171082	59174229	183735.5918	chr3	59183735	59187735	0.6304201
ZFTRAF1	chr4	335301	346534	441311.4453	chr4	441311	445311	0.63049005
NAA10	chrX	124658158	124662702	214324.1242	chrX	124214324	124218324	0.63089559
FIGNL1	chr9	136484216	136488250	753582.1367	chr9	136753582	136756492	0.63094928
ATP6AP1	chrX	124952500	124960343	148743.1251	chrX	125148743	125152743	0.63110078
PSMD13	chr2	55108	74829	.90704.9325	chr2	90704	93254	0.63164546
RNASEH1	chr3	131268926	131281930	726366.1317	chr3	131726366	131731426	0.63180074
HAUS7	chrX	124234736	124258098	407289.1244	chrX	124407289	124411289	0.63232911
IDH3G	chrX	124528585	124537575	423872.1244	chrX	124423872	124427872	0.63238889
POLR2F	chr5	9855983	9917726	545177.9549	chr5	9545177	9549177	0.63250637
FIGNL1	chr9	136484216	136488250	721192.1367	chr9	136721192	136724212	0.63308962
SAP18	chr11	1291202	1298983	559030.1562	chr11	1559030	1562300	0.63364155
LSM2	chr7	23900395	23908490	907960.2391	chr7	23907960	23911960	0.63383771
MGMT	chr14	138499309	138771540	239999.1382	chr14	138239999	138242709	0.63414926
TMEM213	chr18	10862206	10868435	0593024.1059	chr18	10593024	10594111	0.63498042
TMEM219	chr3	18167923	18176563	809718.1781	chr3	17809718	17813718	0.63565135
YKT6	chr18	50960113	50971384	0890905.5089	chr18	50890905	50896275	0.6359835
SHD	chr2	74497351	74504659	414316.7441	chr2	74414316	74414825	0.63609098
ENSSSCG00000030849	chrX	98178155	98181598	565158.9856	chrX	98565158	98569158	0.63628892
PFDN1	chr2	142011063	142077925	483896.1424	chr2	142483896	142487896	0.63632779
WDR55	chr2	142378462	142384677	483896.1424	chr2	142483896	142487896	0.63650116
SLIT3	chr16	54553426	55220296	267269.5426	chr16	54267269	54269829	0.63752746
IPO9	chr10	24110064	24150291	786279.2379	chr10	23786279	23790279	0.6379153
POLA2	chr2	6922387	6950179	456140.6460	chr2	6456140	6460880	0.63814434
ENSSSCG00000035997	chrX	124926772	124929791	903258.1249	chrX	124903258	124909988	0.63865776
PRSS8	chr3	17357802	17362220	812944.1781	chr3	17812944	17813910	0.63873939
ENSSSCG00000024588	chr2	75538090	75542189	395470.7539	chr2	75395470	75398190	0.63888577
BRD9	chr16	79473745	79492635	011667.7901	chr16	79011667	79012408	0.63903109
SURF4	chr1	272975160	272988688	535826.2725	chr1	272535826	272540916	0.63913154
RALGDS	chr1	272776846	272824184	535826.2725	chr1	272535826	272540916	0.63916941
C9orf78	chr1	269986391	269995395	329336.2703	chr1	270329336	270333876	0.63923939
AP1S1	chr3	8881107	8887566	688988.8690	chr3	8688988	8690214	0.63925804
FBXO17	chr6	47732303	47759606	150486.4815	chr6	48150486	48153526	0.63972231
NDUFA10	chr15	138986421	139031897	984421.1389	chr15	138984421	138988421	0.6402958
ARV1	chr14	59396962	59408958	543459.5954	chr14	59543459	59546189	0.64068788
ENSSSCG00000015632	chr9	136893356	137056101	457852.1364	chr9	136457852	136463142	0.64132685
IDH3G	chrX	124528585	124537575	407289.1244	chrX	124407289	124411289	0.6413458
ENSSSCG00000045223	chr6	166945409	166957165	285396.1672	chr6	167285396	167289306	0.64135058
GAA	chr12	2314591	2336988	134182.2135	chr12	2134182	2135153	0.64165141
TOLLIP	chr2	851512	864483	709596.7135	chr2	709596	713596	0.64185865
RPS20	chr4	75762209	75769955	143499.7614	chr4	76143499	76145939	0.64219784
LRCH4	chr3	8499285	8511262	832274.8836	chr3	8832274	8836274	0.64236013
USP36	chr12	3309237	3348525	166999.3170	chr12	3166999	3170069	0.64243882
HSF1	chr4	463393	486473	295751.2997	chr4	295751	299751	0.64245398
ADRM1	chr17	61692945	61698144	222882.6122	chr17	61222882	61224592	0.64258802
TALDO1	chr2	464728	472835	212170.2161	chr2	212170	216170	0.64367032

ENSSSCG00000056719	chr6	61903216	61907832	977286.61979	chr6	61977286	61979696	0.64378879
MRPL36	chr16	79001549	79004358	8971273.7897	chr16	78971273	78974922	0.64380668
MVP	chr3	18057177	18081155	809718.17813	chr3	17809718	17813718	0.64395202
SDHA	chr16	79834044	79862524	9638839.7964	chr16	79638839	79642899	0.64411369
CBX8	chr12	2562374	2565780	2134182.2135	chr12	2134182	2135153	0.64435511
BRD9	chr16	79473745	79492635	9495219.7949	chr16	79495219	79497289	0.64452001
ENSSSCG00000012088	chr13	207801096	207818130	7959056.2079	chr13	207959056	207961576	0.64454797
PFDN1	chr2	142011063	142077925	473803.14247	chr2	142473803	142474940	0.64494302
CYTH2	chr6	53868409	53878856	431154.53431	chr6	53431154	53431639	0.64508286
ENSSSCG00000047605	chr4	128894115	128943092	935039.12893	chr4	128935039	128939899	0.64536438
TMUB1	chr18	6142023	6144894	5873195.5876	chr18	5873195	5876335	0.64599879
POLR1D	chr11	5044329	5089766	5415520.5417	chr11	5415520	5417840	0.64635036
BCAP31	chrX	124457001	124484743	424348.12442	chrX	124424348	124429148	0.64641803
TIMM13	chr2	76146867	76155729	277430.76279	chr2	76277430	76279160	0.64672607
PSMD13	chr2	55108	74829	212170.21617	chr2	212170	216170	0.64704802
HAUS7	chrX	124234736	124258098	214324.12421	chrX	124214324	124218324	0.6470684
UBE2J2	chr6	63555488	63568017	974346.63978	chr6	63974346	63978346	0.64759216
KAZN	chr6	74236437	74378956	378056.74379	chr6	74378056	74379376	0.64792319
MVP	chr3	18057177	18081155	812944.17813	chr3	17812944	17813910	0.648515
NAA10	chrX	124658158	124662702	903258.12490	chrX	124903258	124909988	0.64856113
IDH3G	chrX	124528585	124537575	424348.12442	chrX	124424348	124429148	0.64877218
PDGFA	chr3	301584	321712	341139.34513	chr3	341139	345139	0.64948696
BNIP3	chr14	140362127	140371907	0407549.1404	chr14	140407549	140410609	0.6494879
NAXD	chr11	77210228	77230680	6880640.7688	chr11	76880640	76886850	0.64952754
RPTOR	chr12	1709128	1991574	1304812.1306	chr12	1304812	1306458	0.64968944
ENSSSCG00000003286	chr6	59113039	59134353	233436.59233	chr6	59233436	59233978	0.65012544
OSBPL2	chr17	61643377	61685819	1222882.6122	chr17	61222882	61224592	0.65049635
RIMKLA	chr6	168916725	168950046	469856.16847	chr6	168469856	168474916	0.65055991
ENSSSCG00000059201	chr4	90261918	90263262	288394.90292	chr4	90288394	90292394	0.6508391
RIMS3	chr6	170506426	170546933	187216.17018	chr6	170187216	170189606	0.65089203
ENSSSCG00000021624	chr10	23767206	23778722	3966485.2396	chr10	23966485	23968979	0.65118455
ENSSSCG00000029830	chr4	136107	138652	295751.29975	chr4	295751	299751	0.65162601
METTL23	chr12	4804037	4808913	4908039.4909	chr12	4908039	4909329	0.65164323
EBNA1BP2	chr6	168049758	168061013	469856.16847	chr6	168469856	168474916	0.65166356
GALNTL5	chr18	5353769	5433814	4978355.4984	chr18	4978355	4984775	0.65212508
HNRNPUL2	chr2	9034667	9048768	082256.90862	chr2	9082256	9086256	0.65255724
EBNA1BP2	chr6	168049758	168061013	656186.16766	chr6	167656186	167665426	0.65259418
COPS9	chr15	139260478	139264211	9479553.1394	chr15	139479553	139483165	0.65271633
IK	chr2	142361155	142376333	483896.14248	chr2	142483896	142487896	0.65291751
TUBB2B	chr7	1951407	1956119	470509.14725	chr7	1470509	1472579	0.6530349
SEZ6L2	chr3	18098102	18119558	021096.18025	chr3	18021096	18025096	0.6533819
WDR5	chr1	273560283	273578063	651376.27365	chr1	273651376	273656236	0.65345299
POLR1D	chr11	5044329	5089766	5241105.5241	chr11	5241105	5241689	0.65368869
CBX8	chr12	2562374	2565780	2141178.2144	chr12	2141178	2144859	0.6537205
SSR4	chrX	124537540	124541637	423872.12442	chrX	124423872	124427872	0.65411663
MRPS24	chr18	48825782	48830609	9267135.4926	chr18	49267135	49269745	0.65421461
MRPL36	chr16	79001549	79004358	8970059.7897	chr16	78970059	78976349	0.65431604
RPL7L1	chr7	37940834	37951324	097569.38102	chr7	38097569	38102239	0.65449679
BRD9	chr16	79473745	79492635	9638839.7964	chr16	79638839	79642899	0.65453587
SSR4	chrX	124537540	124541637	407289.12441	chrX	124407289	124411289	0.65487882
ATG4B	chr15	140223904	140246321	9822668.1398	chr15	139822668	139834538	0.65488313
STK25	chr15	140144336	140154366	9858888.1398	chr15	139858888	139868418	0.65503532
ENSSSCG00000017955	chr12	52878764	52882863	3006559.5300	chr12	53006559	53008829	0.6555723
MRPL52	chr7	76187446	76191901	003612.76007	chr7	76003612	76007612	0.6563334
LIG1	chr6	53620483	53686562	431154.53431	chr6	53431154	53431639	0.65650402
ENSSSCG00000045223	chr6	166945409	166957165	829006.16683	chr6	166829006	166831646	0.65676563
NDUFA10	chr15	138986421	139031897	8787006.1387	chr15	138787006	138788821	0.65684593
ENSSSCG00000035997	chrX	124926772	124929791	180305.12518	chrX	125180305	125182027	0.65741725
MRPS23	chr12	34122020	34129548	3896719.3390	chr12	33896719	33901049	0.65744412
ENSSSCG00000031249	chr13	207493659	207499867	7959056.2079	chr13	207959056	207961576	0.65778265
RPP40	chr7	2987271	3005271	573209.25814	chr7	2573209	2581459	0.65899626
MAF1	chr4	597472	600447	295751.29975	chr4	295751	299751	0.65900329

BRD9	chr16	79473745	79492635	605069.7960	chr16	79605069	79606470	0.65948601
POP7	chr3	8605018	8610218	836970.88376	chr3	8836970	8837652	0.65959159
COX4I1	chr6	3131019	3137530	309516.28170	chr6	2809516	2817016	0.65982335
CBR3	chr13	199830668	199840442	126305.2001	chr13	200126305	200129795	0.66021308
DUSP28	chr15	139537326	139539438	858888.1398	chr15	139858888	139868418	0.66043559
PSMC3IP	chr12	20244304	20250159	166659.2016	chr12	20166659	20168579	0.66069029
MGMT	chr14	138499309	138771540	416319.1384	chr14	138416319	138425189	0.66172784
RPS7	chr3	131258722	131263256	728816.13173	chr3	131728816	131731070	0.66192542
ENSSSCG00000036812	chr12	61077631	61186002	.055733.6105	chr12	61055733	61059733	0.66205794
TUBB2B	chr7	1951407	1956119	259389.22659	chr7	2259389	2265929	0.66243374
TBCC	chr7	37822767	37824953	097569.38102	chr7	38097569	38102239	0.66350949
VPS26C	chr13	200869167	200926108	.197137.2012	chr13	201197137	201201137	0.66441309
P4HB	chr12	1121824	1131289	.304812.1306	chr12	1304812	1306458	0.6647682
CTPS1	chr6	170201751	170233016	907106.16991	chr6	169907106	169912386	0.66560373
SSR1	chr7	4652921	4685901	273899.42749	chr7	4273899	4274969	0.66567638
ENSSSCG00000057427	chr18	2568274	2595510	2176050.2180	chr18	2176050	2180050	0.66596772
RPL4	chr1	164475357	164481534	734118.16473	chr1	164734118	164734722	0.66598741
ENSSSCG00000047605	chr4	128894115	128943092	095199.12909	chr4	129095199	129098159	0.66602911
HEXIM2	chr12	18251428	18258295	8036976.1804	chr12	18036976	18040754	0.66631135
ENSSSCG00000045735	chr18	50458411	50463097	246693.5025	chr18	50246693	50250693	0.66640116
JPT1	chr12	6139428	6158452	6214528.6215	chr12	6214528	6215144	0.66693713
COPS9	chr15	139260478	139264211	048928.1390	chr15	139048928	139052328	0.66762411
RPS5	chr6	62967994	62974350	745816.62746	chr6	62745816	62746568	0.66786857
ENSSSCG00000045735	chr18	50458411	50463097	233045.5023	chr18	50233045	50234445	0.66838024
ENSSSCG00000008097	chr3	43977111	44022535	973665.43974	chr3	43973665	43974341	0.66870514
NFYC	chr6	170413638	170485840	187216.17018	chr6	170187216	170189606	0.66877654
MRPL36	chr16	79001549	79004358	715419.7872	chr16	78715419	78721319	0.66934382
FAM104A	chr12	7727796	7749240	7934529.7938	chr12	7934529	7938329	0.6695177
ENSSSCG00000045735	chr18	50458411	50463097	064445.5006	chr18	50064445	50066235	0.6696349
RNASEH2A	chr2	66193197	66207697	910372.65911	chr2	65910372	65911524	0.67036194
VPS28	chr4	362912	370588	295751.29975	chr4	295751	299751	0.67290356
POP7	chr3	8605018	8610218	832274.88362	chr3	8832274	8836274	0.6729648
USP36	chr12	3309237	3348525	215499.3219	chr12	3215499	3219479	0.67319271
GATD1	chr2	474296	480736	130230.13366	chr2	130230	133660	0.67397093
SELENOF	chr4	129258944	129287720	935039.12893	chr4	128935039	128939899	0.67404559
CTDNEP1	chr12	52599226	52606059	006559.5300	chr12	53006559	53008829	0.67469679
MRPL36	chr16	79001549	79004358	8837739.7884	chr16	78837739	78842699	0.67522116
DND1	chr2	142384532	142387199	483896.14248	chr2	142483896	142487896	0.67586187
ENSSSCG00000037652	chr3	65951	140492	345186.35211	chr3	345186	352116	0.67608237
CYC1	chr4	606516	608996	295751.29975	chr4	295751	299751	0.67660555
CYBC1	chr12	656347	662513	453099.4609	chr12	453099	460989	0.67677096
CDIPT	chr3	18084549	18091798	812944.17813	chr3	17812944	17813910	0.67697382
GAA	chr12	2314591	2336988	2141178.2144	chr12	2141178	2144859	0.67830672
CHCHD5	chr3	43908122	43915709	973665.43974	chr3	43973665	43974341	0.67855284
CCNL2	chr6	63659054	63668047	444151.63445	chr6	63444151	63445363	0.67888401
ENSSSCG00000045735	chr18	50458411	50463097	977355.4998	chr18	49977355	49983615	0.6800394
USP11	chrX	41851602	41866355	488466.41489	chrX	41488466	41489630	0.68083023
FIGNL1	chr9	136484216	136488250	460695.13646	chr9	136460695	136461442	0.68088733
MED8	chr6	167861373	167869411	986092.16799	chr6	167986092	167990092	0.68159164
CTPS1	chr6	170201751	170233016	187216.17018	chr6	170187216	170189606	0.68172316
PCGF3	chr8	140446	182261	444863.45075	chr8	444863	450757	0.68188859
NARF	chr12	632363	651817	.143994.1479	chr12	143994	147994	0.68219344
MRPL36	chr16	79001549	79004358	534549.7853	chr16	78534549	78538079	0.68234122
MAP2K3	chr12	61396369	61417321	.055733.6105	chr12	61055733	61059733	0.68236055
NDUFA10	chr15	138986421	139031897	509228.1385	chr15	138509228	138517658	0.68250111
MRPL36	chr16	79001549	79004358	203589.7921	chr16	79203589	79211199	0.68271127
ENSSSCG00000024070	chr18	6166918	6171153	873195.5876	chr18	5873195	5876335	0.68388541
WDR91	chr18	14092322	14126475	995535.1399	chr18	13995535	13998965	0.68464662
ENSSSCG00000031299	chr2	64723435	64731124	716906.64720	chr2	64716906	64720906	0.68562524
ZNF250	chr4	66932	84170	295751.29975	chr4	295751	299751	0.68648929
DTYMK	chr15	140248079	140255805	865812.1398	chr15	139865812	139867602	0.6866551
ARAF	chrX	42078206	42090250	174600.42175	chrX	42174600	42175367	0.68752819

BRD9	chr16	79473745	79492635	203589.7921	chr16	79203589	79211199	0.68890032
ENSSSCG00000029830	chr4	136107	138652	377258.37834	chr4	377258	378347	0.68991788
TXNL4A	chr6	127974543	127991177	713256.12771	chr6	127713256	127715196	0.69087878
MRPL36	chr16	79001549	79004358	048763.7905	chr16	79048763	79050221	0.69121762
DGAT1	chr4	452662	466684	295751.29975	chr4	295751	299751	0.69189783
SSR4	chrX	124537540	124541637	424348.12442	chrX	124424348	124429148	0.69224072
MRPS24	chr18	48825782	48830609	225095.4923	chr18	49225095	49233665	0.69253218
ENSSSCG00000003930	chr6	166549949	166553230	829006.16683	chr6	166829006	166831646	0.6935188
ATP6V0B	chr6	167316947	167320212	658726.16766	chr6	167658726	167662326	0.69605677
DPEP1	chr6	361197	376616	179225.18322	chr6	179225	183225	0.69622281
BCAP31	chrX	124457001	124484743	407289.12441	chrX	124407289	124411289	0.69664106
EBNA1BP2	chr6	168049758	168061013	986876.16798	chr6	167986876	167989686	0.69691195
MTMR1	chrX	122394921	122453905	707368.12270	chrX	122707368	122709996	0.69733178
TXN2	chr5	11264645	11283395	980748.10985	chr5	10980748	10985038	0.69801193
UBXN6	chr2	74357517	74378592	729195.74730	chr2	74729195	74730347	0.70026247
TBCD	chr12	329714	449556	143994.1479	chr12	143994	147994	0.70064283
EBNA1BP2	chr6	168049758	168061013	658726.16766	chr6	167658726	167662326	0.70324623
ATP6V0B	chr6	167316947	167320212	656186.16766	chr6	167656186	167665426	0.7032465
DRAP1	chr2	6409655	6412395	895080.68997	chr2	6895080	6899700	0.7051387
SHD	chr2	74497351	74504659	042210.74044	chr2	74042210	74044130	0.70644813
COX4I1	chr6	3131019	3137530	669978.26733	chr6	2669978	2673391	0.70704147
NAA60	chr3	38763447	38781988	260264.39264	chr3	39260264	39264264	0.70853161
CPB2	chr11	21234447	21298241	743220.2074	chr11	20743220	20746240	0.70914911
ENSSSCG00000045735	chr18	50458411	50463097	125275.5012	chr18	50125275	50128495	0.70992137
ENSSSCG00000061760	chr7	31573753	31585356	159320.31163	chr7	31159320	31163320	0.71034255
NARF	chr12	632363	651817	453099.4609	chr12	453099	460989	0.71049724
NDUFA10	chr15	138986421	139031897	048928.1390	chr15	139048928	139052328	0.71732611
GATD1	chr2	474296	480736	.90704.93254	chr2	90704	93254	0.72243002
ENSSSCG00000032573	chr4	75636278	75646153	739459.75740	chr4	75739459	75740629	0.72259276
WRAP73	chr6	65155209	65167079	662641.64666	chr6	64662641	64666641	0.72476121
ATP6V0B	chr6	167316947	167320212	829006.16683	chr6	166829006	166831646	0.72832481
UBXN6	chr2	74357517	74378592	042210.74044	chr2	74042210	74044130	0.72942238
WDFY2	chr11	16092698	16195134	514780.1651	chr11	16514780	16518890	0.73000259
EBNA1BP2	chr6	168049758	168061013	986092.16799	chr6	167986092	167990092	0.73386015
OTUD5	chrX	43000231	43029468	357991.43361	chrX	43357991	43361991	0.74125253
DRAP1	chr2	6409655	6412395	456140.64608	chr2	6456140	6460880	0.74426475
GLP2R	chr12	54680057	54738102	676329.5467	chr12	54676329	54677869	0.74778092
TALDO1	chr2	464728	472835	.90704.93254	chr2	90704	93254	0.75405653
RNF220	chr6	166666138	166910646	829006.16683	chr6	166829006	166831646	0.7546161
STAT5A	chr12	20474227	20499138	957709.2096	chr12	20957709	20961019	0.75871512
RPS7	chr3	131258722	131263256	726366.13173	chr3	131726366	131731426	0.76106648
CBR3	chr13	199830668	199840442	128785.2001	chr13	200128785	200129482	0.76292937
TOMM22	chr5	9336130	9350015	545177.95491	chr5	9545177	9549177	0.77525101
EIF4E2	chr15	133060247	133103719	473548.1334	chr15	133473548	133476628	0.79026245
WRAP73	chr6	65155209	65167079	664948.64665	chr6	64664948	64665830	0.7915506

ID	Term	Ontology Source	Term PValue	Term P-Value Corrected with Bonferroni step down	Group PValue	Group P-Value Corrected with Bonferroni step down	GO Levels	GO Groups	% Associated Genes	Number of Genes	Associated Genes Found
GO:0006900	vesicle budding from membrane	GO_BiologicalProcess-EBI-UniProt-GOA-ACAP-ARAP_23.01.2024_00h00	0.00	0.00	0.00	0.00	[3, 5, 6]	Group0	12.24	6.00	[ANXA2, PEF1, SEC13, SEC24A, TFG, VAPA]
GO:0032480	negative regulation of type I interferon production	GO_BiologicalProcess-EBI-UniProt-GOA-ACAP-ARAP_23.01.2024_00h00	0.00	0.00	0.00	0.00	[4, 5, 6, 7, 8, 9, 10]	Group1	16.67	5.00	[ATG12, ATG5, CACTIN, MORC3, UFD1]
GO:0043954	cellular component maintenance	GO_BiologicalProcess-EBI-UniProt-GOA-ACAP-ARAP_23.01.2024_00h00	0.00	0.03	0.00	0.00	[4]	Group2	10.87	5.00	[ABHD17B, APPL1, CTTN, F2R, FERMT2]
GO:0098687	chromosomal region	GO_CellularComponent-EBI-UniProt-GOA-ACAP-ARAP_23.01.2024_00h00	0.00	0.01	0.00	0.00	[2, 6]	Group3	4.83	13.00	[AURKC, CENPW, CLASP2, KDM1A, MEAF6, NDC80, PPP1CC, SEC13, SMC5, THOC7, TP53BP1, ZNF618, ZWINT]
GO:0000775	chromosome, centromeric region	GO_CellularComponent-EBI-UniProt-GOA-ACAP-ARAP_23.01.2024_00h00	0.00	0.02	0.00	0.00	[3, 7]	Group3	5.56	10.00	[AURKC, CENPW, CLASP2, MEAF6, NDC80, PPP1CC, SEC13, TP53BP1, ZNF618, ZWINT]
GO:0000776	kinetochore	GO_CellularComponent-EBI-UniProt-GOA-ACAP-ARAP_23.01.2024_00h00	0.00	0.01	0.00	0.00	[3, 5, 8, 9]	Group3	6.92	9.00	[AURKC, CENPW, CLASP2, MEAF6, NDC80, PPP1CC, SEC13, TP53BP1, ZWINT]

ID	Term	Ontology Source	Term PValue	Term PValue Corrected with Bonferroni step down	Group PValue	Group PValue Corrected with Bonferroni step down	GO Levels	GO Groups	% Associated Genes	Nr. of Genes	Associated Genes Found
[illegible]	cytoplasm	[illegible]	0.00	[illegible]	0.00	[illegible]	[illegible]	[illegible]	4.15	368.00	[ABLIM3, ACAA1, ACAD9, ACY1, AFAP1L1, AFG3L2, AHSG, AKAP12, AKR1A1, ALAD, ALDH4A1, ALDOB, AP2S1, APOD, APOE, ARHGEF1, ARPC5L, ARPP19, ATG3, ATG7, ATP2C2, ATP5F1A, ATP5IF1, ATP5PF, ATP5PO, [illegible]
[illegible]	intracellular non-membrane-bounded organelle	[illegible]	0.00	[illegible]	0.00	[illegible]	[illegible]	[illegible]	4.18	153.00	[ABLIM3, AEN, AFAP1L1, ALDOB, APOD, ARPC5L, ATP6V0D1, BRPF1, CALM3, CAMTA1, CAPZB, CC2D1A, CCDC113, CCDC61, CD274, CDK5RAP2, CENPT, CFDP1, CIAO2B, CIAPIN1, CIDEA, CLN6, CPT2, CROCC, CSTB, [illegible]
[illegible]	cytosol	[illegible]	0.00	[illegible]	0.00	[illegible]	[illegible]	[illegible]	4.71	145.00	[AFAP1L1, AKAP12, AKR1A1, ALAD, ALDH4A1, ALDOB, APOD, ARHGEF1, ATG3, BRPF1, CABLES1, CAMTA1, CAPNS1, CBR3, CC2D1A, CC2D1B, CDA, CDK5RAP2, CDKN3, CHAC1, CIAO2B, CNDP2, COMMD9, COTL1, [illegible]
[illegible]	nuclear outer membrane-endoplasmic reticulum membrane network	[illegible]	0.00	[illegible]	0.00	[illegible]	[illegible]	[illegible]	5.94	42.00	[DIO1, DOLPP1, ELOVL1, EMC3, ENO1, GBA2, GRAMD1A, HSPA5, ILVBL, PDIA3, PIGO, PIGP, PIGV, PLOD1, RASGRP1, RPL11, RPL14, RPL29, RPL32, RPL35, RPS23, RPS29, RPSA, SAR1B, SCAP, SEC11C, SELENON, [illegible]
[illegible]	organelle membrane	[illegible]	0.00	[illegible]	0.00	[illegible]	[illegible]	[illegible]	5.26	122.00	[ACAD9, AEN, AFG3L2, AP2S1, ATP2C2, ATP5F1A, ATP5PF, ATP5PO, ATP6V0D1, ATP6V1G1, B4GALT4, BDH1, CC2D1A, CC2D1B, CD274, CHSY3, CKMT1A, CLTA, COG8, COQ3, COQ4, COQ9, COX6B, COX7A2, COX7C, [illegible]
[illegible]	[illegible]	[illegible]	[illegible]	[illegible]	[illegible]	[illegible]	[illegible]	[illegible]	5.29	68.00	[APOD, APOE, ATP2C2, CLN6, DHCR24, DIO1, DNAJB11, DOLPP1, EHD4, ELOVL1, EMC3, GBA2, GRAMD1A, HSPA5, HSPBP1, ILVBL, LAMA3, LSG1, MBOAT7, MRAP2, NKIRAS1, P3H1, PDIA3, PIGO, PIGP, PIGV, PINK1, [illegible]
[illegible]	cellular nitrogen compound metabolic process	[illegible]	0.00	[illegible]	0.00	[illegible]	[illegible]	[illegible]	4.00	194.00	[ABLIM3, AEN, ALAD, ALDOB, APOE, ATMIN, ATP5F1A, ATP5IF1, ATP5PF, ATP5PO, B4GALT4, BRPF1, CAMTA1, CC2D1A, CC2D1B, CD274, CDA, CDK5RAP2, CIDEA, CPT2, CRAT, CTH, CTPS1, CTU2, DALRD3, DFFA, DKK3, [illegible]
[illegible]	cellular biosynthetic process	[illegible]	0.00	[illegible]	0.00	[illegible]	[illegible]	[illegible]	4.08	238.00	[ABLIM3, AFG3L2, AKR1A1, ALAD, ALPK2, APOD, APOE, ARPP19, ATMIN, ATP5F1A, ATP5IF1, ATP5PF, ATP5PO, B4GALT4, BRPF1, CAMTA1, CC2D1A, CC2D1B, CCBE1, CD274, CDA, CDK5RAP2, CHAC1, CHCHD4, CHSY3, [illegible]
[illegible]	organic substance biosynthetic process	[illegible]	0.00	[illegible]	0.00	[illegible]	[illegible]	[illegible]	4.08	241.00	[ABLIM3, AFG3L2, AKR1A1, ALAD, ALPK2, APOD, APOE, ARPP19, ATMIN, ATP5F1A, ATP5IF1, ATP5PF, ATP5PO, B4GALT4, BRPF1, CAMTA1, CC2D1A, CC2D1B, CCBE1, CD274, CDA, CDK5RAP2, CHAC1, CHCHD4, CHSY3, [illegible]
[illegible]	cytosolic ribosome	[illegible]	0.00	[illegible]	0.00	[illegible]	[illegible]	[illegible]	25.71	27.00	[APOD, ETF1, LOC100739087, RPL11, RPL12, RPL14, RPL17, RPL24, RPL28, RPL29, RPL32, RPL35, RPL4, RPL7A, RPLP1, RPS12, RPS13, RPS14, RPS16, RPS23, RPS29, RPS5, RPS6, RPS8, RPS9, RPSA, ZCCHC17]
[illegible]	peptide biosynthetic process	[illegible]	0.00	[illegible]	0.00	[illegible]	[illegible]	[illegible]	9.36	51.00	[DALRD3, EEFSEC, EIF1B, EIF2B3, EIF3G, EIF3I, EIF3K, ETF1, GMPPB, LIMD1, LOC100521256, LOC100739087, LRRC47, MRPL37, MRPL47, MRPS15, MRPS6, MTRF1L, NAT10, PAIP2, PINK1, RPL11, RPL12, RPL14, [illegible]
[illegible]	cytoplasmic translation	[illegible]	0.00	[illegible]	0.00	[illegible]	[illegible]	[illegible]	16.67	14.00	[EIF2B3, EIF3G, EIF3I, EIF3K, ETF1, RPL17, RPL24, RPL29, RPL32, RPLP1, RPS14, RPS23, RPS29, RPSA]
[illegible]	rough endoplasmic reticulum membrane	[illegible]	0.00	[illegible]	0.00	[illegible]	[illegible]	[illegible]	27.03	10.00	[PLOD1, RPL11, RPL14, RPL29, RPL32, RPL35, RPS23, RPS29, RPSA, SRPRB]
[illegible]	cytoplasmic side of rough endoplasmic reticulum membrane	[illegible]	0.00	[illegible]	0.00	[illegible]	[illegible]	[illegible]	36.36	8.00	[RPL11, RPL14, RPL29, RPL32, RPL35, RPS23, RPS29, RPSA]
[illegible]	translation regulator activity	[illegible]	0.00	[illegible]	0.00	[illegible]	[illegible]	[illegible]	10.66	13.00	[EEFSEC, EIF1B, EIF2B3, EIF3G, EIF3I, EIF3K, ETF1, GMPPB, MTRF1L, PAIP2, RPS14, RPS9, TCEANC2]
[illegible]	Oxidative phosphorylation	[illegible]	0.00	[illegible]	0.00	[illegible]	[illegible]	[illegible]	10.22	14.00	[ATP5F1A, ATP5PF, ATP5PO, ATP6V0D1, ATP6V1G1, COX17, COX6B, COX7A2, COX7C, LOC100524873, NDUFA8, NDUFB4, NDUFS5, NDUFV2]
[illegible]	mitochondrion	[illegible]	0.00	[illegible]	0.00	[illegible]	[illegible]	[illegible]	6.31	82.00	[ACAD9, AFG3L2, ALDH4A1, ATP5F1A, ATP5IF1, ATP5PF, ATP5PO, AURKAIP1, BCKDHA, BDH1, CASA, CCNG, CHCHD4, CIAPIN1, CIDEA, CKMT1A, CMC2, COQ3, COQ4, COQ8B, COQ9, COX17, COX6B, COX7A2, COX7C, [illegible]
[illegible]	mitochondrial protein-containing complex	[illegible]	0.00	[illegible]	0.00	[illegible]	[illegible]	[illegible]	9.79	23.00	[AFG3L2, ATP5F1A, ATP5PF, ATP5PO, BCKDHA, COX6B, COX7A2, COX7C, DMAC2, DNAJC11, LOC100524873, MRPL37, MRPL46, MRPL47, MRPL50, MRPS15, MRPS6, NDUFA8, NDUFB4, NDUFS5, NDUFV2, TIMM50, TRMT10C]
[illegible]	mitochondrial envelope	[illegible]	0.00	[illegible]	0.00	[illegible]	[illegible]	[illegible]	7.63	38.00	[ACAD9, AFG3L2, ATP5F1A, ATP5PF, ATP5PO, BDH1, CHCHD4, CIAPIN1, CIDEA, CKMT1A, COQ3, COQ4, COQ9, COX17, COX6B, COX7A2, COX7C, CPT2, DELE1, DMAC2, DNAJC11, LOC100524873, MPC1, NDUFA8, NDUFAF3, NDUFB4, NDUFS5, NDUFV2, PINK1, PRELID2, RAB7A, RDH13, RTN4IP1, SLC25A26, SLC25A38, TIMM50, TIMM9, TIMMDC1]
[illegible]	mitochondrial membrane	[illegible]	0.00	[illegible]	0.00	[illegible]	[illegible]	[illegible]	7.24	33.00	[ACAD9, AFG3L2, ATP5F1A, ATP5PF, ATP5PO, BDH1, CKMT1A, COQ3, COQ4, COQ9, COX6B, COX7A2, COX7C, CPT2, DELE1, DMAC2, DNAJC11, LOC100524873, MPC1, NDUFA8, NDUFAF3, NDUFB4, NDUFS5, NDUFV2, PINK1, RAB7A, RDH13, RTN4IP1, SLC25A26, SLC25A38, TIMM50, TIMM9, TIMMDC1]
[illegible]	inner mitochondrial membrane protein complex	[illegible]	0.00	[illegible]	0.00	[illegible]	[illegible]	[illegible]	12.50	15.00	[AFG3L2, ATP5F1A, ATP5PF, ATP5PO, COX6B, COX7A2, COX7C, DMAC2, DNAJC11, LOC100524873, NDUFA8, NDUFB4, NDUFS5, NDUFV2, TIMM50]
[illegible]	mitochondrial inner membrane	[illegible]	0.00	[illegible]	0.00	[illegible]	[illegible]	[illegible]	10.11	28.00	[AFG3L2, ATP5F1A, ATP5PF, ATP5PO, BDH1, CKMT1A, COQ3, COQ4, COQ9, COX6B, COX7A2, COX7C, CPT2, DMAC2, DNAJC11, LOC100524873, MPC1, NDUFA8, NDUFAF3, NDUFB4, NDUFS5, NDUFV2, PINK1, RDH13, SLC25A26, SLC25A38, TIMM50, TIMM9]
[illegible]	preribosome	[illegible]	0.00	[illegible]	0.00	[illegible]	[illegible]	[illegible]	12.36	11.00	[NAT10, NOC2L, PPAN, PWP2, RPS12, RPS13, RPS14, RPS23, RPS5, RPS9, RRP1B]
[illegible]	amide metabolic process	[illegible]	0.00	[illegible]	0.00	[illegible]	[illegible]	[illegible]	7.36	67.00	[ALDOB, APOE, B4GALT4, CHAC1, CLN6, DALRD3, EEFSEC, EIF1B, EIF2B3, EIF3G, EIF3I, EIF3K, ELOVL1, ETF1, ETHE1, GBA2, GMPPB, GSK3A, LIMD1, LOC100521256, LOC100739087, LRRC47, MCEE, MRPL37, MRPL47, [illegible]
[illegible]	cellular component biogenesis	[illegible]	0.00	[illegible]	0.00	[illegible]	[illegible]	[illegible]	4.71	118.00	[ABLIM3, ACAD9, ALAD, ALDOB, APOD, APOE, ARMC2, ARPC5L, ATG3, ATG7, CAPZB, CCDC113, CCDC61, CDH15, CDK5RAP2, CENPT, CFAP298, CIAO2B, CIAPIN1, CLTA, COX17, COX7A2, CROCC, CTH, DDX28, DEF8, [illegible]
[illegible]	organonitrogen compound metabolic process	[illegible]	0.00	[illegible]	0.00	[illegible]	[illegible]	[illegible]	4.64	185.00	[AFG3L2, ALAD, ALDH4A1, ALDOB, ANAPC13, APOD, APOE, ARPP19, ASB6, ATG3, ATG7, ATP5F1A, ATP5IF1, ATP5PF, ATP5PO, AURKAIP1, B4GALT4, BCKDHA, CALM3, CAMTA1, CAPN12, CCBE1, CDA, CDC26, CHAC1, [illegible]
[illegible]	protein metabolic process	[illegible]	0.00	[illegible]	0.00	[illegible]	[illegible]	[illegible]	4.93	155.00	[AFG3L2, ALAD, ANAPC13, APOD, APOE, ARPP19, ASB6, ATG3, ATG7, ATP5IF1, AURKAIP1, B4GALT4, CALM3, CAMTA1, CAPN12, CCBE1, CDC26, CHAC1, CHCHD4, CHSY3, CIAO2B, CLN6, CNDP2, CSTB, CTH, CTU2, [illegible]
[illegible]	RNA binding	[illegible]	0.00	[illegible]	0.00	[illegible]	[illegible]	[illegible]	5.47	51.00	[CTU2, DALRD3, DDX28, DND1, EEFSEC, EIF1B, EIF3G, EIF3I, EIF3K, ETF1, EXOSC2, GNL3, LOC100521256, LOC100739087, LRRC47, LSM10, MRPS6, NAT10, NUDT16, OOEP, PAIP2, PNLDC1, PPAN, PPIE, PPP1R8, [illegible]
[illegible]	peptide metabolic process	[illegible]	0.00	[illegible]	0.00	[illegible]	[illegible]	[illegible]	8.50	59.00	[APOE, CHAC1, DALRD3, EEFSEC, EIF1B, EIF2B3, EIF3G, EIF3I, EIF3K, ETF1, ETHE1, GMPPB, GSK3A, LIMD1, LOC100521256, LOC100739087, LRRC47, MRPL37, MRPL47, MRPS15, MRPS6, MTRF1L, NAT10, PAIP2, [illegible]
[illegible]	ribonucleoprotein complex biogenesis	[illegible]	0.00	[illegible]	0.00	[illegible]	[illegible]	[illegible]	9.95	37.00	[DDX28, EBNA1BP2, EIF3G, EIF3I, EIF3K, ERI3, EXOSC2, GEMIN2, LOC100739087, LSG1, NAT10, NOC2L, PIH1D1, PPAN, PRMT7, PWP2, RCL1, RPL14, RPL35, RPL7A, RPS12, RPS13, RPS14, RPS16, RPS23, RPS5, RPS6, [illegible]
[illegible]	[illegible]	[illegible]	[illegible]	[illegible]	[illegible]	[illegible]	[illegible]	[illegible]	6.83	87.00	[ALAD, APOE, ATP5F1A, ATP5IF1, ATP5PF, ATP5PO, B4GALT4, CDA, CHSY3, CKM, CKMT1A, CTH, CTPS1, DALRD3, DHDDS, DOLPP1, DUT, EEFSEC, EIF1B, EIF2B3, EIF3G, EIF3I, EIF3K, ELOVL1, ENO1, ETF1, GMPPB, [illegible]
[illegible]	cytosolic ribosome	[illegible]	0.00	[illegible]	0.00	[illegible]	[illegible]	[illegible]	25.71	27.00	[APOD, ETF1, LOC100739087, RPL11, RPL12, RPL14, RPL17, RPL24, RPL28, RPL29, RPL32, RPL35, RPL4, RPL7A, RPLP1, RPS12, RPS13, RPS14, RPS16, RPS23, RPS29, RPS5, RPS6, RPS8, RPS9, RPSA, ZCCHC17]
[illegible]	amide biosynthetic process	[illegible]	0.00	[illegible]	0.00	[illegible]	[illegible]	[illegible]	8.36	54.00	[B4GALT4, DALRD3, EEFSEC, EIF1B, EIF2B3, EIF3G, EIF3I, EIF3K, ELOVL1, ETF1, GMPPB, LIMD1, LOC100521256, LOC100739087, LRRC47, MRPL37, MRPL47, MRPS15, MRPS6, MTRF1L, NAT10, PAIP2, PINK1, RPL11, [illegible]
[illegible]	[illegible]	[illegible]	[illegible]	[illegible]	[illegible]	[illegible]	[illegible]	[illegible]	19.25	31.00	[LOC100739087, MRPL37, MRPL46, MRPL47, MRPL50, MRPS15, MRPS6, RPL11, RPL12, RPL14, RPL17, RPL24, RPL28, RPL29, RPL32, RPL35, RPL4, RPL7A, RPLP1, RPS12, RPS13, RPS14, RPS16, RPS23, RPS29, [illegible]
[illegible]	rough endoplasmic reticulum	[illegible]	0.00	[illegible]	0.00	[illegible]	[illegible]	[illegible]	20.34	12.00	[PLOD1, RPL11, RPL14, RPL18, RPL29, RPL32, RPL35, RPL4, RPS23, RPS29, RPSA, SRPRB]
[illegible]	ribosome	[illegible]	0.00	[illegible]	0.00	[illegible]	[illegible]	[illegible]	16.74	36.00	[APOD, ETF1, LOC100739087, MRPL37, MRPL46, MRPL47, MRPL50, MRPS15, MRPS25, MRPS6, RPL11, RPL12, RPL14, RPL17, RPL18, RPL24, RPL28, RPL29, RPL32, RPL35, RPL4, RPL7A, RPLP1, RPS12, RPS13, [illegible]
[illegible]	ribosome biogenesis	[illegible]	0.00	[illegible]	0.00	[illegible]	[illegible]	[illegible]	11.28	29.00	[DDX28, EBNA1BP2, ERI3, EXOSC2, LOC100739087, LSG1, NAT10, NOC2L, PIH1D1, PPAN, PWP2, RCL1, RPL14, RPL35, RPL7A, RPS12, RPS13, RPS14, RPS16, RPS23, RPS5, RPS6, RPS8, RPS9, RPSA, RRP1B, RSL24D1, [illegible]
[illegible]	large ribosomal subunit	[illegible]	0.00	[illegible]	0.00	[illegible]	[illegible]	[illegible]	18.00	18.00	[LOC100739087, MRPL37, MRPL46, MRPL47, MRPL50, RPL11, RPL12, RPL14, RPL17, RPL24, RPL28, RPL29, RPL32, RPL35, RPL4, RPL7A, RPLP1, ZCCHC17]
[illegible]	small ribosomal subunit	[illegible]	0.00	[illegible]	0.00	[illegible]	[illegible]	[illegible]	20.97	13.00	[MRPS15, MRPS6, RPS12, RPS13, RPS14, RPS16, RPS23, RPS29, RPS5, RPS6, RPS8, RPS9, RPSA]
[illegible]	ribosomal small subunit biogenesis	[illegible]	0.00	[illegible]	0.00	[illegible]	[illegible]	[illegible]	13.64	12.00	[NAT10, PWP2, RPS12, RPS13, RPS14, RPS16, RPS23, RPS5, RPS6, RPS8, RPS9, RPSA]
[illegible]	peptide biosynthetic process	[illegible]	0.00	[illegible]	0.00	[illegible]	[illegible]	[illegible]	9.36	51.00	[DALRD3, EEFSEC, EIF1B, EIF2B3, EIF3G, EIF3I, EIF3K, ETF1, GMPPB, LIMD1, LOC100521256, LOC100739087, LRRC47, MRPL37, MRPL47, MRPS15, MRPS6, MTRF1L, NAT10, PAIP2, PINK1, RPL11, RPL12, RPL14, [illegible]
[illegible]	cytosolic large ribosomal subunit	[illegible]	0.00	[illegible]	0.00	[illegible]	[illegible]	[illegible]	27.45	14.00	[LOC100739087, RPL11, RPL12, RPL14, RPL17, RPL24, RPL28, RPL29, RPL32, RPL35, RPL4, RPL7A, RPLP1, ZCCHC17]
[illegible]	cytosolic small ribosomal subunit	[illegible]	0.00	[illegible]	0.00	[illegible]	[illegible]	[illegible]	28.95	11.00	[RPS12, RPS13, RPS14, RPS16, RPS23, RPS29, RPS5, RPS6, RPS8, RPS9, RPSA]
[illegible]	cytoplasmic translation	[illegible]	0.00	[illegible]	0.00	[illegible]	[illegible]	[illegible]	16.67	14.00	[EIF2B3, EIF3G, EIF3I, EIF3K, ETF1, RPL17, RPL24, RPL29, RPL32, RPLP1, RPS14, RPS23, RPS29, RPSA]
[illegible]	rough endoplasmic reticulum membrane	[illegible]	0.00	[illegible]	0.00	[illegible]	[illegible]	[illegible]	27.03	10.00	[PLOD1, RPL11, RPL14, RPL29, RPL32, RPL35, RPS23, RPS29, RPSA, SRPRB]
[illegible]	cytoplasmic side of rough endoplasmic reticulum membrane	[illegible]	0.00	[illegible]	0.00	[illegible]	[illegible]	[illegible]	36.36	8.00	[RPL11, RPL14, RPL29, RPL32, RPL35, RPS23, RPS29, RPSA]
