# Supplementary material for: Deciphering differences in DNA methylation and transcriptome profiles of oocytes from pigs with high and low developmental competence
Source: Environ Epigenet. 2025 Jun 3;11(1):dvaf018. doi: 10.1093/eep/dvaf018 (PMC12418950; doi:10.1093/eep/dvaf018)
Supplement: dvaf018_Supplemental_Files [file dvaf018_supplemental_files.zip › Sup table 1.pdf]

Sample	Number of input reads	Uniquely mapped reads number	Uniquely mapped reads %
1	4170977	3956325	94.85
2	2834288	2598942	91.7
3	1473633	1345770	91.32
4	2624469	2401746	91.51
5	1714651	1594152	92.97
6	962416	876713	91.1
7	2455507	2226534	90.68
8	3621428	3282472	90.64
9	1808801	1662090	91.89
13	379947	345034	90.81
14	732040	661990	90.43
16	1361374	1249824	91.81
17	2206962	2059300	93.31
19	177534	145986	82.23
20	31398	23888	76.08
21	978096	891720	91.17
22	1550686	1400560	90.32
24	31	28	90.32
25	1778106	1638105	92.13
26	119330	109978	92.16
A	980739	870897	88.8
AA	1028482	946517	92.03
AB	178332	165970	93.07
AC	1027061	921536	89.73
B	382309	347373	90.86
C	1735515	1625157	93.64
J	8834	8217	93.02
K	451007	421660	93.49
L	704589	657227	93.28
M	3049665	2820073	92.47
N	1132946	1050494	92.72
O	831205	769094	92.53
Q	1502778	1335570	88.87
R	84957	75526	88.9
S	83929	78181	93.15
T	1660865	1512792	91.08
U	960394	892432	92.92
V	1246261	1169696	93.86
X	2752868	2587876	94.01
Z	1101808	1031578	93.63
