# Supplementary material for: Deciphering differences in DNA methylation and transcriptome profiles of oocytes from pigs with high and low developmental competence
Source: Environ Epigenet. 2025 Jun 3;11(1):dvaf018. doi: 10.1093/eep/dvaf018 (PMC12418950; doi:10.1093/eep/dvaf018)
Supplement: dvaf018_Supplemental_Files [file dvaf018_supplemental_files.zip › Sup table 10.pdf]

chr	start	end	group1	group2	n1	n2	estimate1	estimate2	estimate	statistic	p-value	adj.p.value	name
chr6	64160768	64165101	in_vitro	in_vivo	9	10	70.1177778	23.572	46.5457778	c(t = 3.58234	0.0038	0.90887448	FAAP20
chr7	17695258	17696497	in_vitro	in_vivo	9	10	68.4455556	21.307	47.1385556	c(t = 3.29109	0.00597	0.90887448	ENSSSCG00000001083
chr13	133545417	133560499	in_vitro	in_vivo	9	9	64.4077778	20.8888889	43.5188889	c(t = 3.85756	0.00285	0.90887448	FBXO45
chr1	209620907	209668913	in_vitro	in_vivo	15	14	54.5413333	23.7292857	30.8120476	c(t = 3.53351	0.00195	0.90887448	LURAP1L
chr4	136107	138652	in_vitro	in_vivo	9	12	50.7233333	20.2833333	30.44	c(t = 3.00839	0.00854	0.90887448	ENSSSCG00000029830
chr9	53358534	53365653	in_vitro	in_vivo	10	13	49.486	17.8392308	31.6467692	c(t = 3.15921	0.00953	0.90887448	SRPRA
chrX	111144233	111158335	in_vitro	in_vivo	11	10	49.4136364	23.28	26.1336364	c(t = 2.69841	0.0196	0.90887448	MMGT1
chr6	101758014	101764531	in_vitro	in_vivo	11	12	48.1081818	24.29	23.8181818	c(t = 2.75536	0.0132	0.90887448	ZBTB14
chr1	248604820	248609035	in_vitro	in_vivo	16	14	47.47375	19.645	27.82875	c(t = 3.31065	0.00314	0.90887448	KLF4
chr15	78608432	78652962	in_vitro	in_vivo	11	13	47.2545455	17.0853846	30.1691608	c(t = 2.65403	0.021	0.90887448	PDK1
chr6	18901297	18913424	in_vitro	in_vivo	12	11	46.9158333	19.2545455	27.6612879	c(t = 2.53747	0.0195	0.90887448	HERPUD1
chr2	66084668	66096329	in_vitro	in_vivo	13	12	46.3523077	22.8608333	23.4914744	c(t = 2.35813	0.0301	0.90887448	RAD23A
chr13	110983298	111038324	in_vitro	in_vivo	15	14	45.9093333	23.9492857	21.9600476	c(t = 2.55005	0.0199	0.90887448	GHSR
chr2	88022936	88089403	in_vitro	in_vivo	16	16	45.22	23.465	21.755	c(t = 2.06467	0.0482	0.90887448	JMY
chr15	94359358	94377303	in_vitro	in_vivo	10	12	45.045	15.405	29.64	c(t = 2.86930	0.0141	0.90887448	ORMDL1
chr4	83069748	83096990	in_vitro	in_vivo	14	12	44.8392857	23.5975	21.2417857	c(t = 2.59554	0.0159	0.90887448	MPC2
chr2	80527446	80544828	in_vitro	in_vivo	15	12	44.494	19.1658333	25.3281667	c(t = 2.72051	0.0132	0.90887448	F12
chr4	51478050	51512718	in_vitro	in_vivo	13	15	44.2992308	21.622	22.6772308	c(t = 2.06171	0.0498	0.90887448	E2F5
chr3	17904442	17915347	in_vitro	in_vivo	13	17	43.6992308	17.3558824	26.3433484	c(t = 2.51317	0.0245	0.90887448	ZNF771
chr9	72142835	72185661	in_vitro	in_vivo	11	13	43.1154545	21.3023077	21.8131469	c(t = 2.59151	0.0216	0.90887448	KRIT1
chr10	25202389	25209210	in_vitro	in_vivo	10	11	42.83	16.1390909	26.6909091	c(t = 2.63348	0.0209	0.90887448	CTSV
chr13	90818395	90842363	in_vitro	in_vivo	12	12	42.2566667	19.0791667	23.1775	c(t = 2.27948	0.0368	0.90887448	SELENOT
chr2	59771113	59778354	in_vitro	in_vivo	13	16	40.5353846	18.535	22.0003846	c(t = 2.21086	0.0426	0.90887448	ARRDC2
chr2	64758957	64765485	in_vitro	in_vivo	13	13	40.3284615	8.86153846	31.4669231	c(t = 3.34153	0.00499	0.90887448	DNAJB1
chr16	27985167	28010858	in_vitro	in_vivo	12	10	40.2658333	20.873	19.3928333	c(t = 2.58158	0.0189	0.90887448	CCL28
chr13	60393650	60432802	in_vitro	in_vivo	12	15	40.2058333	16.3566667	23.8491667	c(t = 2.41385	0.03	0.90887448	LRRN1
chr15	46193746	46200987	in_vitro	in_vivo	11	10	40.2036364	15.913	24.2906364	c(t = 2.59872	0.0205	0.90887448	SLC25A4
chr2	62142769	62153799	in_vitro	in_vivo	12	14	39.8283333	20.575	19.2533333	c(t = 3.11853	0.0068	0.90887448	RASAL3
chr14	89884129	89891328	in_vitro	in_vivo	9	10	39.2055556	20.841	18.3645556	c(t = 2.22789	0.043	0.90887448	ENSSSCG00000062957
chr6	60685345	60707151	in_vitro	in_vivo	13	12	37.9576923	15.7133333	22.244359	c(t = 2.27213	0.0374	0.90887448	ZNF583
chr11	75547747	75570263	in_vitro	in_vivo	13	16	37.5276923	21.251875	16.2758173	c(t = 2.46502	0.024	0.90887448	ABHD13
chr2	76680020	76690841	in_vitro	in_vivo	13	14	35.1615385	14.0457143	21.1158242	c(t = 2.36804	0.0323	0.90887448	KLF16
chr5	3621844	3625453	in_vitro	in_vivo	12	11	34.9066667	12.3063636	22.600303	c(t = 2.22274	0.0391	0.90887448	ENSSSCG00000058612
chr13	25937645	25945793	in_vitro	in_vivo	9	11	34.5433333	11.1136364	23.429697	c(t = 2.30115	0.0441	0.90887448	CCK
chr1	251263175	251277988	in_vitro	in_vivo	12	14	34.3783333	7.11142857	27.2669048	c(t = 2.69160	0.0192	0.90887448	ENSSSCG00000005453
chr14	57424906	57481823	in_vitro	in_vivo	16	17	33.6975	18.6688235	15.0286765	c(t = 2.47900	0.0215	0.90887448	MAP3K21
chr1	267942101	267966192	in_vitro	in_vivo	14	12	33.625	21.3858333	12.2391667	c(t = 2.37448	0.0272	0.90887448	ZNF79
chr17	40313537	40322842	in_vitro	in_vivo	12	11	31.8533333	7.48545455	24.3678788	c(t = 2.37877	0.0342	0.90887448	ENSSSCG00000035214
chr6	45847013	45880709	in_vitro	in_vivo	13	12	31.3053846	20.9	10.4053846	c(t = 2.08842	0.0488	0.90887448	ZNF260
chr13	132459983	132488943	in_vitro	in_vivo	13	14	30.8261538	14.06	16.7661538	c(t = 2.93578	0.00808	0.90887448	ENSSSCG00000039249
chr11	23141133	23159649	in_vitro	in_vivo	13	15	30.5992308	11.0766667	19.5225641	c(t = 2.63958	0.0177	0.90887448	LACC1
chr2	119306665	119352465	in_vitro	in_vivo	16	15	30.41375	18.0026667	12.4110833	c(t = 2.38457	0.0267	0.90887448	TRIM36
chr6	42483449	42491591	in_vitro	in_vivo	15	11	30.2546667	3.29909091	26.9555758	c(t = 2.75521	0.0151	0.90887448	PDCD5
chr2	66144777	66148517	in_vitro	in_vivo	14	11	28.9792857	5.87272727	23.1065584	c(t = 2.88026	0.0118	0.90887448	KLF1
chr1	241570010	241611727	in_vitro	in_vivo	14	14	28.72	13.0757143	15.6442857	c(t = 2.56306	0.0207	0.90887448	NR4A3
chr4	65271315	65295178	in_vitro	in_vivo	15	16	27.8026667	9.395	18.4076667	c(t = 3.23571	0.00482	0.90887448	PRDM14
chr5	12505654	12526232	in_vitro	in_vivo	13	14	27.7276923	14.8542857	12.8734066	c(t = 2.48340	0.021	0.90887448	FBXO7
chr4	94710647	94719248	in_vitro	in_vivo	13	11	27.4361538	11.4445455	15.9916084	c(t = 2.16629	0.0463	0.90887448	EFNA3
chr4	51193756	51211259	in_vitro	in_vivo	9	9	26.2133333	6.09111111	20.1222222	c(t = 2.37707	0.0423	0.90887448	CA2
chr14	24311577	24317789	in_vitro	in_vivo	9	13	26.1777778	8.77769231	17.4000855	c(t = 2.76111	0.0192	0.90887448	RAN
chr3	25286018	25295347	in_vitro	in_vivo	12	12	24.3166667	7.54916667	16.7675	c(t = 2.93815	0.01	0.90887448	THUMPD1
chr4	56136033	56175296	in_vitro	in_vivo	16	15	23.299375	9.48733333	13.8120417	c(t = 2.44088	0.0256	0.90887448	ZBTB10
chr2	142947343	142952505	in_vitro	in_vivo	10	12	23.279	9.65666667	13.6223333	c(t = 2.26499	0.0396	0.90887448	TAF7
chr9	65056398	65066775	in_vitro	in_vivo	11	12	22.8954545	7.62083333	15.2746212	c(t = 2.22035	0.0485	0.90887448	PPP1R15B
chr13	31650967	31656306	in_vitro	in_vivo	13	14	19.2923077	7.43785714	11.8544505	c(t = 2.22761	0.0424	0.90887448	DALRD3
chr1	181955339	181966178	in_vitro	in_vivo	15	14	16.692	6.65642857	10.0355714	c(t = 2.10183	0.0491	0.90887448	PTGDR
chr18	32927748	32957960	in_vitro	in_vivo	13	13	16.6646154	7.92692308	8.73769231	c(t = 2.37394	0.0262	0.90887448	TMEM168
chrX	41770941	41774245	in_vitro	in_vivo	11	10	15.2336364	4.756	10.4776364	c(t = 2.36000	0.0357	0.90887448	NDUFB11
chr7	22888906	22891657	in_vitro	in_vivo	11	11	14.2227273	1.93727273	12.2854545	c(t = 2.73148	0.0187	0.90887448	ENSSSCG00000062469
chr16	37299119	37305154	in_vitro	in_vivo	13	13	14.16	3.88846154	10.2715385	c(t = 2.39864	0.0309	0.90887448	PLK2
chr4	88833651	88839308	in_vitro	in_vivo	9	13	13.8	4.32461538	9.47538462	c(t = 2.59057	0.0261	0.90887448	FCRLB
chr14	112291135	112297898	in_vitro	in_vivo	10	12	12.552	23.495	-10.943	c(t = -2.16583	0.0448	0.90887448	ENSSSCG00000062688
chr3	8888506	8891601	in_vitro	in_vivo	12	15	11.1783333	3.10666667	8.07166667	c(t = 2.74933	0.0149	0.90887448	VGF
chr5	19151084	19158646	in_vitro	in_vivo	13	14	10.9776923	5.07	5.90769231	c(t = 2.22442	0.0357	0.90887448	HOXC13
chr14	16470323	16475595	in_vitro	in_vivo	11	14	10.4109091	3.07214286	7.33876623	c(t = 2.50628	0.0258	0.90887448	ENSSSCG00000058445
chr14	113081981	113084534	in_vitro	in_vivo	14	12	10.3764286	21.9416667	-11.565238	c(t = -2.31952	0.0339	0.90887448	ENSSSCG00000010573
chr2	57310541	57323153	in_vitro	in_vivo	14	15	10.285	22.468	-12.183	c(t = -2.16983	0.0437	0.90887448	TRIM7
chr6	28465208	28467074	in_vitro	in_vivo	11	13	9.81181818	2.08384615	7.72797203	c(t = 2.63867	0.021	0.90887448	THAP11
chr4	119790185	119800629	in_vitro	in_vivo	11	14	9.67818182	2.30285714	7.37532468	c(t = 2.31758	0.0379	0.90887448	ENSSSCG00000045465
chrX	107506188	107537747	in_vitro	in_vivo	16	17	9.615	18.0111765	-8.3961765	c(t = -2.24027	0.0335	0.90887448	ENSSSCG00000012669
chr18	2743509	2750814	in_vitro	in_vivo	11	10	8.85363636	3.134	5.71963636	c(t = 2.19864	0.0448	0.90887448	ENSSSCG00000043069
chr1	268408448	268417944	in_vitro	in_vivo	13	9	8.71923077	20.4133333	-11.694103	c(t = -2.22485	0.0402	0.90887448	PIP5KL1
chr13	207148401	207158532	in_vitro	in_vivo	13	14	8.53923077	23.2771429	-14.737912	c(t = -2.39742	0.0275	0.90887448	AIRE
chr1	92421230	92425044	in_vitro	in_vivo	11	15	8.27454545	3.31	4.96454545	c(t = 2.27055	0.0362	0.90887448	ENSSSCG00000004489
chr18	45393885	45403257	in_vitro	in_vivo	14	14	7.98	2.68357143	5.29642857	c(t = 2.24736	0.041	0.90887448	HOXA10
chr12	53163866	53166440	in_vitro	in_vivo	10	11	7.914	3.66727273	4.24672727	c(t = 2.40488	0.0352	0.90887448	RNF227
chr3	78896675	78903122	in_vitro	in_vivo	15	15	7.78933333	2.81133333	4.978	c(t = 2.84290	0.0116	0.90887448	OTX1
chr6	85307189	85309215	in_vitro	in_vivo	13	9	7.76846154	2.01444444	5.75401709	c(t = 2.48245	0.0237	0.90887448	ATP5IF1
chr16	34376959	34384453	in_vitro	in_vivo	14	16	7.69357143	3.68375	4.00982143	c(t = 2.44942	0.0217	0.90887448	MCIDAS
chrX	90559021	90565035	in_vitro	in_vivo	12	12	7.68583333	24.5208333	-16.835	c(t = -2.26949	0.0382	0.90887448	RTL9
chr6	42560113	42565154	in_vitro	in_vivo	12	10	6.12416667	2.832	3.29216667	c(t = 2.39175	0.0271	0.90887448	RGS9BP
chr1	221578077	221579911	in_vitro	in_vivo	13	14	6.12384615	1.40785714	4.71598901	c(t = 2.21991	0.0404	0.90887448	ENSSSCG00000041279
chr8	40881552	40883995	in_vitro	in_vivo	13	14	6.12230769	1.09571429	5.02659341	c(t = 3.24807	0.00538	0.90887448	GSX2
chr17	58425081	58434669	in_vitro	in_vivo	15	15	5.914	17.5933333	-11.679333	c(t = -2.29021	0.0339	0.90887448	ANKRD60
chr6	101214902	101216782	in_vitro	in_vivo	10	11	5.739	0.95181818	4.78718182	c(t = 2.39686	0.0362	0.90887448	TMEM200C
chr7	48010589	48012003	in_vitro	in_vivo	14	14	5.60285714	19.1364286	-13.533571	c(t = -2.16306	0.0461	0.90887448	ENSSSCG00000041217
chr8	137605600	137612651	in_vitro	in_vivo	14	15	5.43571429	1.10266667	4.33304762	c(t = 2.26812	0.0402	0.90887448	PRDM8
chr14	32916798	32926224	in_vitro	in_vivo	9	11	5.24777778	2.03818182	3.20959596	c(t = 2.56481	0.0204	0.90887448	ENSSSCG00000052272
chr5	19167157	19169795	in_vitro	in_vivo	12	12	5.195	1.05833333	4.13666667	c(t = 2.64799	0.0195	0.90887448	HOXC12
chr4	42980616	42985459	in_vitro	in_vivo	10	11	5.131	0.61454545	4.51645455	c(t = 2.32223	0.0419	0.90887448	ENSSSCG00000037963
chr1	27246752	27250254	in_vitro	in_vivo	14	14	4.63214286	1.56357143	3.06857143	c(t = 2.15035	0.0448	0.90887448	SLC35D3
chr14	124400330	124401739	in_vitro	in_vivo	15	13	4.34666667	1.75692308	2.58974359	c(t = 2.73630	0.0114	0.90887448	ADRB1
chrX	124409289	124414212	in_vitro	in_vivo	9	13	4.32888889	23.9246154	-19.595726	c(t = -2.44795	0.0296	0.90887448	DUSP9

chr15	133034255	133039367	in_vitro	in_vivo	10	13	3.998	12.1146154	-8.1166154	c(t = -2.5166	0.0222	0.90887448	PRSS56
chr3	41490328	41492120	in_vitro	in_vivo	11	11	3.81	13.8827273	-10.072727	c(t = -2.2086	0.0438	0.90887448	HBZ
chr5	59667585	59673649	in_vitro	in_vivo	14	11	3.48785714	11.2236364	-7.7357792	c(t = -2.3360	0.0352	0.90887448	APOLD1
chr7	664373	665563	in_vitro	in_vivo	11	12	3.32181818	0.75666667	2.56515152	c(t = 2.37198	0.0361	0.90887448	FOXQ1
chr3	59776236	59777252	in_vitro	in_vivo	11	11	2.48545455	0.46090909	2.02454545	c(t = 2.41072	0.0312	0.90887448	TMSB10
chr14	51289321	51296725	in_vitro	in_vivo	16	14	2.28	7.02071429	-4.7407143	c(t = -2.1425	0.0495	0.90887448	TBX1
chr1	65835995	65837323	in_vitro	in_vivo	16	13	2.189375	0.33846154	1.85091346	c(t = 2.21980	0.0408	0.90887448	POU3F2
chr3	68531962	68534543	in_vitro	in_vivo	11	12	2.13909091	6.58583333	-4.4467424	c(t = -2.8680	0.00949	0.90887448	TLX2
chr11	77986200	77987351	in_vitro	in_vivo	11	11	2.13272727	0.58636364	1.54636364	c(t = 2.66312	0.0184	0.90887448	SOX1
chr17	51722426	51724305	in_vitro	in_vivo	12	15	1.9875	0.686	1.3015	c(t = 2.54238	0.0177	0.90887448	CEBPB
chr1	141494692	141495449	in_vitro	in_vivo	12	13	0.86333333	3.23384615	-2.3705128	c(t = -2.1687	0.0473	0.90887448	ENSSSCG00000043519
chr2	68706197	68708011	in_vitro	in_vivo	11	9	0	8.17555556	-8.1755556	c(t = -2.4052	0.0428	0.90887448	UBL5
chr8	76482844	76699348	in_vitro	in_vivo	16	17	74.7575	84.5182353	-9.7607353	c(t = -2.7020	0.0115	0.90887448	FBXW7
chr4	10179758	10451929	in_vitro	in_vivo	17	17	73.8911765	80.6235294	-6.7323529	c(t = -2.2912	0.0299	0.90887448	ASAP1
chr7	97382342	97491672	in_vitro	in_vivo	15	16	73.1	84.485	-11.385	c(t = -2.6696	0.0124	0.90887448	LIN52
chr10	5754156	5815086	in_vitro	in_vivo	17	17	72.7964706	86.4811765	-13.684706	c(t = -2.5054	0.0207	0.90887448	KCTD3
chr3	650704	662486	in_vitro	in_vivo	12	13	72.11	89.41	-17.3	c(t = -2.3324	0.0363	0.90887448	C7orf50
chr4	117269074	117306188	in_vitro	in_vivo	10	11	72.01	87.9627273	-15.952727	c(t = -2.5030	0.0223	0.90887448	DPH5
chr5	96297661	96341750	in_vitro	in_vivo	13	9	71.4953846	83.0844444	-11.58906	c(t = -2.2038	0.045	0.90887448	RASSF9
chr9	131354773	131362090	in_vitro	in_vivo	12	12	71.3266667	92.2466667	-20.92	c(t = -3.0013	0.0101	0.90887448	ENSSSCG00000061543
chr13	117283631	117309863	in_vitro	in_vivo	11	14	71.2936364	90.3785714	-19.084935	c(t = -2.1710	0.0483	0.90887448	KCNMB3
chr3	4741747	4779557	in_vitro	in_vivo	15	17	70.426	84.1076471	-13.681647	c(t = -2.4488	0.0237	0.90887448	DAGLB
chr6	29842658	29873792	in_vitro	in_vivo	14	10	70.2378571	83.536	-13.298143	c(t = -2.2951	0.0318	0.90887448	CES5A
chr4	127742349	127760675	in_vitro	in_vivo	13	15	69.5161538	82.4733333	-12.957179	c(t = -2.1214	0.0461	0.90887448	ENSSSCG00000049063
chr18	48713340	48725697	in_vitro	in_vivo	14	15	69.015	82.776	-13.761	c(t = -2.1083	0.0491	0.90887448	DBNL
chr4	34645809	34793940	in_vitro	in_vivo	15	15	68.9926667	82.8766667	-13.884	c(t = -2.7320	0.0119	0.90887448	UBR5
chr12	40153397	40183622	in_vitro	in_vivo	12	15	68.6266667	82.5973333	-13.970667	c(t = -2.3044	0.036	0.90887448	LIG3
chr4	45448889	45637389	in_vitro	in_vivo	12	15	68.27	80.4253333	-12.155333	c(t = -2.6952	0.0133	0.90887448	SLC26A7
chr9	118318195	118361953	in_vitro	in_vivo	11	12	68.23	80.6625	-12.4325	c(t = -2.2651	0.0342	0.90887448	ENSSSCG00000049556
chr15	60053877	60384655	in_vitro	in_vivo	17	17	68.0535294	78.8023529	-10.748824	c(t = -2.5014	0.0202	0.90887448	FMNL2
chr4	30327589	30455456	in_vitro	in_vivo	13	12	67.91	80.5158333	-12.605833	c(t = -2.1368	0.048	0.90887448	ENSSSCG00000051734
chr9	44597007	44642109	in_vitro	in_vivo	13	13	67.7553846	86.1707692	-18.415385	c(t = -2.8221	0.0103	0.90887448	RNF214
chr15	24503108	24541283	in_vitro	in_vivo	15	13	67.7186667	81.1861538	-13.467487	c(t = -2.4561	0.022	0.90887448	MARCO
chr15	31064355	31122621	in_vitro	in_vivo	15	16	66.4106667	79.741875	-13.331208	c(t = -2.1839	0.0376	0.90887448	RALB
chr6	57144107	57180662	in_vitro	in_vivo	15	14	66.332	81.9307143	-15.598714	c(t = -2.4422	0.0246	0.90887448	ENSSSCG00000045482
chr14	76473776	76499125	in_vitro	in_vivo	13	11	66.2792308	84.5472727	-18.268042	c(t = -2.2855	0.0344	0.90887448	SEC24C
chr7	76432051	76457192	in_vitro	in_vivo	12	12	65.8225	81.03	-15.2075	c(t = -2.3383	0.0337	0.90887448	DAD1
chr13	39789739	39938746	in_vitro	in_vivo	17	17	65.57	82.7335294	-17.163529	c(t = -2.6290	0.015	0.90887448	FLNB
chr4	99257227	99277788	in_vitro	in_vivo	11	11	64.9718182	84.0327273	-19.060909	c(t = -2.3293	0.0304	0.90887448	ENSSSCG00000006679
chrX	42722730	42730199	in_vitro	in_vivo	15	13	64.7206667	82.6092308	-17.888564	c(t = -2.0969	0.0459	0.90887448	WDR13
chr13	83065957	83174477	in_vitro	in_vivo	15	15	64.2066667	83.188	-18.981333	c(t = -4.2278	0.000353	0.90887448	XRN1
chr2	62051993	62070952	in_vitro	in_vivo	9	10	64.12	85.658	-21.538	c(t = -2.2193	0.0444	0.90887448	ENSSSCG00000062952
chr2	120238614	120445021	in_vitro	in_vivo	15	17	64.0293333	75.4111765	-11.381843	c(t = -2.0696	0.0479	0.90887448	COMMD10
chr5	87522542	87547455	in_vitro	in_vivo	11	16	64.0145455	76.56375	-12.549205	c(t = -2.3614	0.0291	0.90887448	HAL
chr17	38885117	38899138	in_vitro	in_vivo	10	13	63.704	84.8376923	-21.133692	c(t = -2.4103	0.0339	0.90887448	ERGIC3
chr1	53945719	53985482	in_vitro	in_vivo	11	9	63.6490909	78.1411111	-14.49202	c(t = -2.3942	0.0278	0.90887448	ENSSSCG00000041858
chr14	88338810	88379687	in_vitro	in_vivo	11	13	63.6372727	75.0269231	-11.38965	c(t = -2.1986	0.0418	0.90887448	ENSSSCG00000043778
chr1	121273043	121307075	in_vitro	in_vivo	13	9	63.5138462	77.6144444	-14.100598	c(t = -2.2575	0.0404	0.90887448	USP50
chr5	35692309	35719346	in_vitro	in_vivo	12	13	62.3433333	85.9784615	-23.635128	c(t = -3.9817	0.000611	0.90887448	TMEM19
chr10	32436656	32443968	in_vitro	in_vivo	11	10	62.2309091	81.663	-19.432091	c(t = -2.1548	0.0482	0.90887448	MYORG
chr15	13134225	13207731	in_vitro	in_vivo	13	12	61.9207692	81.315	-19.394231	c(t = -2.5531	0.0189	0.90887448	SPOPL
chr13	133193252	133269447	in_vitro	in_vivo	15	14	61.3013333	78.25	-16.948667	c(t = -2.7502	0.0105	0.90887448	SENP5
chr4	19180320	19267071	in_vitro	in_vivo	13	15	60.9130769	81.122	-20.208923	c(t = -3.0771	0.00566	0.90887448	TAF2
chr8	56084512	56132296	in_vitro	in_vivo	14	15	60.0185714	76.816	-16.797429	c(t = -2.4245	0.0226	0.90887448	ENSSSCG00000036736
chr9	114202484	114218368	in_vitro	in_vivo	14	14	59.9578571	80.4828571	-20.525	c(t = -2.2080	0.0405	0.90887448	METTL13
chr6	17778556	17818132	in_vitro	in_vivo	14	14	59.8928571	77.8471429	-17.954286	c(t = -2.5246	0.018	0.90887448	UTP4
chr3	17731764	17742921	in_vitro	in_vivo	11	17	59.6818182	82.29	-22.608182	c(t = -2.2972	0.0337	0.90887448	FBRS
chr9	3272798	3282366	in_vitro	in_vivo	13	14	59.4207692	79.0085714	-19.587802	c(t = -2.4339	0.0227	0.90887448	TRIM3
chr14	98908630	98987524	in_vitro	in_vivo	13	11	59.3984615	80.3672727	-20.968811	c(t = -3.3423	0.00335	0.90887448	A1CF
chrX	110622132	110658827	in_vitro	in_vivo	15	13	58.1226667	80.4269231	-22.304256	c(t = -2.5358	0.019	0.90887448	ENSSSCG00000030908
chr15	110021469	110043116	in_vitro	in_vivo	11	11	57.9554545	90.4854545	-32.53	c(t = -3.1112	0.00782	0.90887448	FASTKD2
chr14	87492458	87588543	in_vitro	in_vivo	17	15	57.5476471	76.3286667	-18.78102	c(t = -2.6538	0.0127	0.90887448	WAPL
chr14	60353474	60408828	in_vitro	in_vivo	14	16	56.3535714	79.23375	-22.880179	c(t = -2.9181	0.0078	0.90887448	NUP133
chr15	39581856	39632308	in_vitro	in_vivo	11	13	56.0318182	80.7515385	-24.71972	c(t = -2.1606	0.0486	0.90887448	NEIL3
chr7	43597246	43624671	in_vitro	in_vivo	13	12	53.8469231	77.8175	-23.970577	c(t = -2.9385	0.00811	0.90887448	MMUT
chr14	14945135	14966291	in_vitro	in_vivo	13	14	52.76	78.4328571	-25.672857	c(t = -2.6421	0.0166	0.90887448	NEIL2
chr6	67410574	67418293	in_vitro	in_vivo	11	13	52.3681818	80.0669231	-27.698741	c(t = -2.7261	0.0132	0.90887448	ZBTB48
chr1	189923323	189988552	in_vitro	in_vivo	13	11	52.2084615	75.3936364	-23.185175	c(t = -2.4370	0.0234	0.90887448	SLC38A6
chr16	47353324	47378745	in_vitro	in_vivo	12	13	51.2091667	81.6869231	-30.477756	c(t = -3.0235	0.00656	0.90887448	CENPH
chr7	42218290	42332518	in_vitro	in_vivo	16	15	51.001875	75.7146667	-24.712792	c(t = -3.0337	0.00509	0.90887448	CD2AP
chr13	90761465	90804274	in_vitro	in_vivo	13	10	50.2153846	79.392	-29.176615	c(t = -2.8677	0.00945	0.90887448	EIF2A
chr18	18046463	18054979	in_vitro	in_vivo	9	13	46.9866667	75.4792308	-28.492564	c(t = -2.8337	0.0104	0.90887448	ENSSSCG00000058154
chr8	52326545	52329300	in_vitro	in_vivo	9	12	41.6577778	86.1325	-44.474722	c(t = -2.9906	0.0137	0.90887448	TKTL2
chr14	48819370	48820324	in_vitro	in_vivo	11	10	38.1627273	77.663	-39.500273	c(t = -3.9548	0.000978	0.90887448	ENSSSCG00000032042
chr2	15036903	15043824	in_vitro	in_vivo	13	9	37.6361538	84.3655556	-46.729402	c(t = -5.5207	2.65E-05	0.4443785	KBTBD4
chr14	29645603	29658899	in_vitro	in_vivo	12	10	74.9216667	50.542	24.3796667	c(t = 2.54041	0.0195	0.90887448	MTRFR
chr13	109115006	109231433	in_vitro	in_vivo	15	15	74.8693333	59.1653333	15.704	c(t = 2.07142	0.0485	0.90887448	SLC7A14
chr7	38507073	38537943	in_vitro	in_vivo	16	14	74.835625	53.0471429	21.7884821	c(t = 2.96870	0.00611	0.90887448	POLR1C
chrX	58455939	58583216	in_vitro	in_vivo	15	15	74.69	54.8486667	19.8413333	c(t = 2.50843	0.0184	0.90887448	PHKA1
chr8	55251005	55323350	in_vitro	in_vivo	15	17	74.6626667	56.0923529	18.5703137	c(t = 2.60711	0.0141	0.90887448	CEP135
chr13	26125072	26174511	in_vitro	in_vivo	15	15	74.4493333	46.1213333	28.328	c(t = 3.20555	0.00347	0.90887448	NKTR
chr2	11268904	11291153	in_vitro	in_vivo	13	11	74.4169231	38.4009091	36.016014	c(t = 3.76017	0.00175	0.90887448	ENSSSCG00000022114
chr15	82061385	82139072	in_vitro	in_vivo	14	16	74.4142857	54.205	20.2092857	c(t = 2.22738	0.0343	0.90887448	MTX2
chr9	104275423	104827405	in_vitro	in_vivo	16	16	74.24625	65.859375	8.386875	c(t = 2.15673	0.0401	0.90887448	LHFPL3
chr9	26443815	26491002	in_vitro	in_vivo	16	17	74.1425	65.6182353	8.52426471	c(t = 2.12999	0.0418	0.90887448	PANX1
chr15	57046309	57087226	in_vitro	in_vivo	12	12	74.1358333	55.3016667	18.8341667	c(t = 2.72209	0.013	0.90887448	ENSSSCG00000049871
chr14	105219896	105256648	in_vitro	in_vivo	11	9	74.1318182	60.0822222	14.049596	c(t = 2.30782	0.0331	0.90887448	LGI1
chr12	14764508	14869217	in_vitro	in_vivo	15	16	73.9606667	61.825	12.1356667	c(t = 2.96849	0.0066	0.90887448	TEX2
chr1	175401390	175448276	in_vitro	in_vivo	12	12	73.8791667	50.0925	23.7866667	c(t = 2.45039	0.0229	0.90887448	MIS18BP1
chr15	48624837	48636589	in_vitro	in_vivo	11	11	73.7781818	48.6090909	25.1690909	c(t = 2.10361	0.049	0.90887448	ENSSSCG00000015826
chr13	140364075	140406112	in_vitro	in_vivo	14	15	73.77	62.1113333	11.6586667	c(t = 2.35959	0.0265	0.90887448	NR1I2
chr3	72240600	72277296	in_vitro	in_vivo	13	16	73.72	41.311875	32.408125	c(t = 3.31405	0.00263	0.90887448	TIA1
chr8	27998500	28072309	in_vitro	in_vivo	11	12	73.6736364	57.6641667	16.0094697	c(t = 2.28972	0.033	0.90887448	DTHD1
chr2	112534949	112815827	in_vitro	in_vivo	17	17	73.59	59.6752941	13.9147059	c(t = 2.77482	0.00916	0.90887448	EFNA5

chr4	95542473	95572938	in_vitro	in_vivo	13	16	73.5830769	55.10875	18.4743269	c(t = 2.38571	0.0244	0.90887448	ENSSSCG00000006556
chr9	8689481	8761709	in_vitro	in_vivo	13	14	73.4638462	53.9957143	19.4681319	c(t = 2.48592	0.0214	0.90887448	PGM2L1
chrX	67236473	67415010	in_vitro	in_vivo	17	16	73.4070588	53.156875	20.2501838	c(t = 2.71696	0.0112	0.90887448	RPS6KA6
chr3	51511436	51528048	in_vitro	in_vivo	14	15	73.3871429	53.2314286	20.1557143	c(t = 2.57305	0.0163	0.90887448	MFSD9
chr5	21936260	21983032	in_vitro	in_vivo	14	14	73.3842857	53.1271429	20.2571429	c(t = 2.59839	0.0152	0.90887448	BAZ2A
chr5	36235989	36274087	in_vitro	in_vivo	11	11	73.2881818	46.5881818	26.7	c(t = 2.29946	0.0332	0.90887448	ENSSSCG00000054950
chr2	80452706	80472658	in_vitro	in_vivo	13	12	73.2323077	53.1733333	20.0589744	c(t = 2.31366	0.0302	0.90887448	PDLIM7
chr7	52304472	52383732	in_vitro	in_vivo	16	16	73.209375	55.408125	17.80125	c(t = 2.74824	0.0101	0.90887448	CPEB1
chr5	46824150	46860553	in_vitro	in_vivo	12	12	73.18	43.8075	29.3725	c(t = 2.68891	0.0135	0.90887448	INTS13
chr17	40194950	40279156	in_vitro	in_vivo	13	16	73.1792308	56.139375	17.0398558	c(t = 2.10389	0.045	0.90887448	RBL1
chr13	82680498	82759966	in_vitro	in_vivo	16	15	73.169375	48.8186667	24.3507083	c(t = 2.77582	0.0103	0.90887448	ENSSSCG00000011676
chrX	37167577	37536160	in_vitro	in_vivo	16	16	73.1475	59.661875	13.485625	c(t = 2.77376	0.00955	0.90887448	CASK
chr6	34059081	34121264	in_vitro	in_vivo	14	15	73.095	54.3473333	18.7476667	c(t = 2.38321	0.0245	0.90887448	CYLD
chr16	13944245	14076030	in_vitro	in_vivo	15	13	73.0266667	58.5930769	14.4335897	c(t = 2.21813	0.0395	0.90887448	CDH9
chr5	9273240	9293254	in_vitro	in_vivo	12	14	72.9225	56.5807143	16.3417857	c(t = 2.18202	0.0394	0.90887448	SUN2
chr13	195528739	195663902	in_vitro	in_vivo	15	17	72.8873333	59.1611765	13.7261569	c(t = 2.32576	0.0277	0.90887448	HUNK
chrX	100827461	101109207	in_vitro	in_vivo	17	16	72.8294118	60.814375	12.0150368	c(t = 2.42303	0.0215	0.90887448	GRIA3
chr16	250217	321929	in_vitro	in_vivo	16	16	72.680625	60.48625	12.194375	c(t = 2.43607	0.0212	0.90887448	DAP
chr8	1583193	1619037	in_vitro	in_vivo	14	17	72.6071429	59.3482353	13.2589076	c(t = 2.22333	0.0342	0.90887448	SH3BP2
chrX	8542382	9438821	in_vitro	in_vivo	17	17	72.5152941	62.9441176	9.57117647	c(t = 2.87067	0.00767	0.90887448	FRMPD4
chr15	121254355	121265569	in_vitro	in_vivo	16	13	72.446875	50.7223077	21.7245673	c(t = 2.67482	0.0137	0.90887448	ATG9A
chr14	82249452	82271214	in_vitro	in_vivo	15	15	72.342	48.966	23.376	c(t = 2.62425	0.0139	0.90887448	PRXL2A
chr3	24107877	24176166	in_vitro	in_vivo	15	16	72.3106667	46.445	25.8656667	c(t = 3.53334	0.00154	0.90887448	MOSMO
chr1	64306073	64459904	in_vitro	in_vivo	15	14	72.2706667	58.9935714	13.2770952	c(t = 2.52303	0.0178	0.90887448	MMS22L
chrX	87217424	87291037	in_vitro	in_vivo	14	15	72.2492857	55.1406667	17.108619	c(t = 2.13635	0.0422	0.90887448	TBC1D8B
chr5	46290001	46482461	in_vitro	in_vivo	15	17	72.1326667	54.4323529	17.7003137	c(t = 3.15357	0.00383	0.90887448	BMAL2
chr2	5576622	5580009	in_vitro	in_vivo	12	14	72.0358333	46.825	25.2108333	c(t = 2.23486	0.035	0.90887448	LRFN4
chr2	76658068	76666552	in_vitro	in_vivo	11	10	71.9518182	37.041	34.9108182	c(t = 2.88974	0.00969	0.90887448	ABHD17A
chr3	92696441	92792616	in_vitro	in_vivo	12	16	71.7258333	56.179375	15.5464583	c(t = 2.06561	0.049	0.90887448	FBXO11
chr17	3312741	3507729	in_vitro	in_vivo	14	16	71.43	57.153125	14.276875	c(t = 2.14351	0.0409	0.90887448	TUSC3
chr12	50454063	50491164	in_vitro	in_vivo	13	13	71.0369231	54.4776923	16.5592308	c(t = 2.73464	0.0118	0.90887448	SPNS2
chr1	20514260	21048484	in_vitro	in_vivo	17	16	70.9923529	62.123125	8.86922794	c(t = 2.28262	0.0323	0.90887448	UTRN
chr4	82759245	82794714	in_vitro	in_vivo	12	15	70.98	43.2546667	27.7253333	c(t = 2.75220	0.0114	0.90887448	TIPRL
chr2	61743537	61760247	in_vitro	in_vivo	11	10	70.9390909	40.025	30.9140909	c(t = 2.77866	0.0136	0.90887448	CYP4F3
chr3	6977215	6996022	in_vitro	in_vivo	9	15	70.9333333	40.064	30.8693333	c(t = 2.87038	0.00907	0.90887448	CEP20
chr10	17220207	17259182	in_vitro	in_vivo	13	15	70.9292308	49.3973333	21.5318974	c(t = 2.20814	0.0367	0.90887448	ADSS2
chr14	130511592	130674774	in_vitro	in_vivo	16	17	70.925	61.1523529	9.77264706	c(t = 2.13368	0.0421	0.90887448	WDR11
chr9	130772262	130785506	in_vitro	in_vivo	12	12	70.7725	36.7208333	34.0516667	c(t = 3.58000	0.00169	0.90887448	ATF3
chr2	117897260	118092175	in_vitro	in_vivo	17	17	70.7523529	56.2035294	14.5488235	c(t = 2.68295	0.0119	0.90887448	YTHDC2
chr17	61553971	61564431	in_vitro	in_vivo	13	12	70.7053846	47.3908333	23.3145513	c(t = 2.46418	0.0222	0.90887448	LSM14B
chr6	17233858	17245451	in_vitro	in_vivo	13	13	70.6	50.3146154	20.2853846	c(t = 2.15319	0.0416	0.90887448	NOB1
chr7	13014741	13039651	in_vitro	in_vivo	13	13	70.5976923	49.9292308	20.6684615	c(t = 2.25728	0.0334	0.90887448	STMND1
chr1	240107466	240496101	in_vitro	in_vivo	17	15	70.5829412	60.618	9.96494118	c(t = 3.06158	0.00466	0.90887448	GABBR2
chr9	16097069	16178947	in_vitro	in_vivo	14	12	70.5214286	56.51	14.0114286	c(t = 2.10196	0.0465	0.90887448	ENSSSCG00000058844
chr3	10120083	10132752	in_vitro	in_vivo	15	17	70.5033333	51.2982353	19.205098	c(t = 2.23505	0.033	0.90887448	MDH2
chr7	19870966	19955759	in_vitro	in_vivo	16	16	70.42625	51.495625	18.930625	c(t = 3.65197	0.000985	0.90887448	ENSSSCG00000001099
chr13	162569479	163443646	in_vitro	in_vivo	16	17	70.264375	58.0405882	12.2237868	c(t = 2.07372	0.0472	0.90887448	EPHA6
chr17	59625982	59862928	in_vitro	in_vivo	15	17	70.1833333	61.5429412	8.64039216	c(t = 2.54296	0.0175	0.90887448	PHACTR3
chr14	34609075	34632269	in_vitro	in_vivo	13	14	70.1730769	39.6435714	30.5295055	c(t = 3.42286	0.00216	0.90887448	WSB2
chr14	71552121	71610740	in_vitro	in_vivo	16	15	70.078125	55.364	14.714125	c(t = 2.05950	0.0488	0.90887448	DNA2
chr2	70813745	70862586	in_vitro	in_vivo	15	15	69.862	47.4173333	22.4446667	c(t = 3.02316	0.00533	0.90887448	HNRNPM
chr7	15532015	15643692	in_vitro	in_vivo	16	16	69.754375	58.24625	11.508125	c(t = 2.34192	0.0261	0.90887448	MBOAT1
chr4	123394740	123414511	in_vitro	in_vivo	14	13	69.69	46.8584615	22.8315385	c(t = 3.47122	0.00217	0.90887448	DNTTIP2
chr1	221256009	221491953	in_vitro	in_vivo	17	16	69.4858824	57.6225	11.8633824	c(t = 2.19844	0.0364	0.90887448	DOCK8
chr3	126087242	126096051	in_vitro	in_vivo	11	15	69.47	45.132	24.338	c(t = 2.37563	0.026	0.90887448	ODC1
chr17	16831932	16881350	in_vitro	in_vivo	15	15	69.344	40.146	29.198	c(t = 3.54761	0.00144	0.90887448	TMX4
chr7	5579152	5606531	in_vitro	in_vivo	15	14	69.1773333	44.79	24.3873333	c(t = 2.91829	0.00771	0.90887448	SLC35B3
chr5	22882669	22889542	in_vitro	in_vivo	13	13	69.0192308	39.9961538	29.0230769	c(t = 2.84262	0.00914	0.90887448	ENSSSCG00000036029
chr1	25155664	25160279	in_vitro	in_vivo	11	9	68.9490909	29.4977778	39.4513131	c(t = 2.18865	0.0431	0.90887448	CITED2
chr5	79593915	79720895	in_vitro	in_vivo	15	14	68.934	51.5128571	17.4211429	c(t = 2.13584	0.0419	0.90887448	SLC41A2
chr4	100646935	100863250	in_vitro	in_vivo	17	16	68.8317647	59.76625	9.06551471	c(t = 2.06980	0.0474	0.90887448	PDE4DIP
chr14	104326827	104549636	in_vitro	in_vivo	16	16	68.28625	52.769375	15.516875	c(t = 2.08994	0.0454	0.90887448	EXOC6
chr11	14184090	14209137	in_vitro	in_vivo	10	13	68.265	47.1323077	21.1326923	c(t = 2.57840	0.0177	0.90887448	NHLRC3
chr12	11067500	11152688	in_vitro	in_vivo	16	15	67.896875	42.5493333	25.3475417	c(t = 2.87913	0.00794	0.90887448	ABCA5
chr3	65951	140492	in_vitro	in_vivo	16	16	67.881875	48.490625	19.39125	c(t = 2.76489	0.01	0.90887448	ENSSSCG00000037652
chr8	86879974	87323117	in_vitro	in_vivo	17	17	67.7482353	62.5435294	5.20470588	c(t = 2.07833	0.0458	0.90887448	MAML3
chr14	46624727	46641438	in_vitro	in_vivo	9	12	67.7188889	39.7375	27.9813889	c(t = 3.24530	0.00427	0.90887448	NIPSNAP1
chr16	28157318	28250961	in_vitro	in_vivo	15	17	67.6833333	51.8388235	15.8445098	c(t = 2.21066	0.0361	0.90887448	NNT
chr16	26512401	26659808	in_vitro	in_vivo	16	15	67.39	50.4473333	16.9426667	c(t = 2.70945	0.0112	0.90887448	OXCT1
chr16	17940135	18250794	in_vitro	in_vivo	15	17	67.386	52.9347059	14.4512941	c(t = 3.38835	0.00201	0.90887448	ENSSSCG00000058255
chr9	60918345	60940093	in_vitro	in_vivo	14	17	67.295	44.2847059	23.0102941	c(t = 2.94365	0.00634	0.90887448	GLB1L3
chr1	74139330	74181084	in_vitro	in_vivo	13	13	67.2230769	41.3776923	25.8453846	c(t = 2.77441	0.0106	0.90887448	OSTM1
chr14	51403923	51440058	in_vitro	in_vivo	16	14	67.134375	48.6592857	18.4750893	c(t = 2.81986	0.00876	0.90887448	ARVCF
chr2	4898409	4909856	in_vitro	in_vivo	9	15	66.9833333	39.5286667	27.4546667	c(t = 2.37131	0.0311	0.90887448	UNC93B1
chr14	111399071	111460995	in_vitro	in_vivo	15	14	66.9653333	51.7707143	15.194619	c(t = 2.86831	0.00846	0.90887448	PKD2L1
chr6	146166688	146719682	in_vitro	in_vivo	17	17	66.9147059	59.5876471	7.32705882	c(t = 2.03904	0.0498	0.90887448	PDE4B
chr11	69710364	70022727	in_vitro	in_vivo	17	17	66.8876471	55.4164706	11.4711765	c(t = 2.20900	0.0346	0.90887448	NALCN
chr2	72707158	72713475	in_vitro	in_vivo	10	10	66.862	36.375	30.487	c(t = 2.17026	0.0456	0.90887448	CLPP
chr1	216115820	216226154	in_vitro	in_vivo	16	14	66.814375	46.6757143	20.1386607	c(t = 2.23365	0.0337	0.90887448	KIAA2026
chr2	136302191	136396301	in_vitro	in_vivo	17	17	66.4017647	51.7129412	14.6888235	c(t = 3.03332	0.00526	0.90887448	VDAC1
chr13	140839268	140948303	in_vitro	in_vivo	15	16	66.372	52.2525	14.1195	c(t = 2.50654	0.0189	0.90887448	ARHGAP31
chr9	105325763	105388928	in_vitro	in_vivo	14	16	66.3478571	46.395625	19.9522321	c(t = 2.45345	0.0209	0.90887448	PUS7
chr13	54324998	54378276	in_vitro	in_vivo	12	16	66.2883333	34.28625	32.0020833	c(t = 3.84517	0.000719	0.90887448	PPP4R2
chr1	179907590	180015220	in_vitro	in_vivo	16	15	66.25125	46.1506667	20.1005833	c(t = 2.10102	0.0445	0.90887448	SOS2
chr13	71892002	71908932	in_vitro	in_vivo	13	13	66.2469231	30.2823077	35.9646154	c(t = 4.18902	0.000357	0.90887448	RPN1
chr3	37684207	37693652	in_vitro	in_vivo	13	14	66.2192308	45.0128571	21.2063736	c(t = 2.21118	0.0368	0.90887448	NUDT16L1
chr17	37923475	37944393	in_vitro	in_vivo	11	12	65.7645455	37.8266667	27.9378788	c(t = 2.44308	0.0235	0.90887448	ENSSSCG00000037510
chr13	13752467	13930483	in_vitro	in_vivo	15	16	65.6793333	54.21	11.4693333	c(t = 2.59688	0.0147	0.90887448	NEK10
chr5	18642823	18648848	in_vitro	in_vivo	14	9	65.545	33.01	32.535	c(t = 3.32425	0.00334	0.90887448	AMHR2
chr6	60889358	60903636	in_vitro	in_vivo	10	12	65.502	32.4691667	33.0328333	c(t = 2.73407	0.0156	0.90887448	ZNF470
chr2	41028693	41056420	in_vitro	in_vivo	13	12	65.4846154	40.2391667	25.2454487	c(t = 2.15601	0.0418	0.90887448	SAAL1
chr1	163396234	163436063	in_vitro	in_vivo	17	16	65.1682353	47.503125	17.6651103	c(t = 2.65745	0.0124	0.90887448	IGDCC4
chr16	44041490	44080492	in_vitro	in_vivo	12	12	65.0416667	39.7816667	25.26	c(t = 2.15224	0.0426	0.90887448	CENPK
chr3	39130777	39142303	in_vitro	in_vivo	15	14	64.9133333	44.5064286	20.4069048	c(t = 2.42922	0.0221	0.90887448	MMP25

chr13	32002660	32007217	in_vitro	in_vivo	10	10	64.622	40.833	23.789	c(t = 2.14647	0.0464	0.90887448	NICN1
chr14	88710824	88717130	in_vitro	in_vivo	9	10	64.6	32.109	32.491	c(t = 2.67377	0.016	0.90887448	ENSSSCG00000052288
chr2	55821915	55860382	in_vitro	in_vivo	15	13	64.3153333	43.1369231	21.1784103	c(t = 2.06564	0.0493	0.90887448	TRIM58
chr1	232355959	232504763	in_vitro	in_vivo	15	15	64.2333333	45.8653333	18.368	c(t = 2.16298	0.0397	0.90887448	TLE4
chr9	46300050	46308868	in_vitro	in_vivo	10	14	64.058	45.0657143	18.9922857	c(t = 2.12307	0.0476	0.90887448	HMBS
chr1	198964132	199135811	in_vitro	in_vivo	16	16	64.051875	42.87875	21.173125	c(t = 3.55358	0.00131	0.90887448	ELAVL2
chr7	112027921	112041489	in_vitro	in_vivo	12	13	63.9116667	41.0538462	22.8578205	c(t = 2.67124	0.0155	0.90887448	PSMC1
chr18	25569536	25612458	in_vitro	in_vivo	14	14	63.285	41.09	22.195	c(t = 2.24514	0.0341	0.90887448	FAM3C
chr9	124498109	124696194	in_vitro	in_vivo	15	17	63.2613333	72.8247059	-9.5633725	c(t = -2.55812	0.0158	0.90887448	NMNAT2
chr1	168350557	168453344	in_vitro	in_vivo	15	14	62.7593333	33.4885714	29.2707619	c(t = 3.84122	0.000703	0.90887448	UACA
chr12	55046888	55103400	in_vitro	in_vivo	14	16	62.4628571	44.98625	17.4766071	c(t = 2.08260	0.0466	0.90887448	MYH13
chrX	5166827	5363066	in_vitro	in_vivo	16	13	62.4025	35.3084615	27.0940385	c(t = 2.92427	0.00698	0.90887448	ENSSSCG00000012101
chr13	53083645	53124240	in_vitro	in_vivo	14	16	62.3071429	39.7875	22.5196429	c(t = 2.17434	0.0387	0.90887448	EIF4E3
chr7	76143726	76150147	in_vitro	in_vivo	9	13	62.15	36.1730769	25.9769231	c(t = 2.44181	0.0249	0.90887448	REM2
chr1	205081231	205285364	in_vitro	in_vivo	17	15	62.0894118	39.172	22.9174118	c(t = 2.71452	0.011	0.90887448	SH3GL2
chr12	26978215	26995714	in_vitro	in_vivo	11	12	62.0854545	35.5008333	26.5846212	c(t = 2.36760	0.028	0.90887448	ANKRD40
chr8	87885420	87909241	in_vitro	in_vivo	13	15	61.9476923	41.5966667	20.3510256	c(t = 2.34953	0.0279	0.90887448	NOCT
chr6	119945052	120018097	in_vitro	in_vivo	14	17	61.9	39.7994118	22.1005882	c(t = 3.33947	0.00251	0.90887448	MOCOS
chr13	35025769	35180042	in_vitro	in_vivo	15	17	61.804	43.2488235	18.5551765	c(t = 2.20345	0.0358	0.90887448	SFMBT1
chr4	129104606	129258377	in_vitro	in_vivo	16	17	61.675	74.3794118	-12.704412	c(t = -2.28623	0.0308	0.90887448	HS2ST1
chr13	90870496	90911109	in_vitro	in_vivo	16	12	61.618125	40.6825	20.935625	c(t = 2.44355	0.0224	0.90887448	ERICH6
chr13	60370351	60916325	in_vitro	in_vivo	15	16	61.6166667	49.53875	12.0779167	c(t = 2.19793	0.0363	0.90887448	ENSSSCG00000041465
chr7	93084336	93143168	in_vitro	in_vivo	15	16	61.318	74.789375	-13.471375	c(t = -2.20396	0.036	0.90887448	SLC39A9
chr13	151722098	152005045	in_vitro	in_vivo	15	17	60.9666667	45.9464706	15.0201961	c(t = 2.79413	0.00905	0.90887448	BBX
chr7	8093890	8127414	in_vitro	in_vivo	13	14	60.96	27.195	33.765	c(t = 3.19720	0.0038	0.90887448	TMEM170B
chr5	5742185	5751456	in_vitro	in_vivo	14	16	60.8028571	40.510625	20.2922321	c(t = 2.15107	0.0407	0.90887448	MCAT
chr18	11533183	11666113	in_vitro	in_vivo	17	17	60.7476471	71.2147059	-10.467059	c(t = -2.31980	0.0269	0.90887448	CREB3L2
chr3	53298811	53490574	in_vitro	in_vivo	17	17	60.7270588	72.0994118	-11.372353	c(t = -2.19238	0.0388	0.90887448	NPAS2
chr6	129395698	129440039	in_vitro	in_vivo	12	12	60.4841667	42.7366667	17.7475	c(t = 2.43101	0.0257	0.90887448	SAMD13
chr13	117436673	117513392	in_vitro	in_vivo	16	17	60.05125	42.8935294	17.1577206	c(t = 2.15956	0.04	0.90887448	GNB4
chr3	45906673	46000115	in_vitro	in_vivo	16	17	59.270625	45.3594118	13.9112132	c(t = 2.35865	0.0251	0.90887448	KCNIP3
chr17	51672757	51697981	in_vitro	in_vivo	14	17	59.0757143	74.0782353	-15.002521	c(t = -2.14270	0.0407	0.90887448	PEDS1
chr8	55862197	55901633	in_vitro	in_vivo	14	17	58.9285714	39.4329412	19.4956303	c(t = 2.81455	0.00894	0.90887448	ENSSSCG00000008898
chr1	52435235	52939823	in_vitro	in_vivo	17	16	58.8588235	47.01125	11.8475735	c(t = 2.22371	0.0342	0.90887448	KCNQ5
chr12	13288544	13328017	in_vitro	in_vivo	13	16	58.7661538	42.4375	16.3286538	c(t = 2.15092	0.0429	0.90887448	CACNG5
chr6	135606192	135741487	in_vitro	in_vivo	16	16	58.458125	42.605	15.853125	c(t = 2.57873	0.0154	0.90887448	ZZZ3
chr15	76135494	76360823	in_vitro	in_vivo	16	16	58.43	73.00125	-14.57125	c(t = -3.03713	0.00502	0.90887448	UBR3
chr13	120915730	121106395	in_vitro	in_vivo	15	17	58.4286667	73.3623529	-14.933686	c(t = -2.89055	0.00727	0.90887448	MCCC1
chr1	13715174	13932123	in_vitro	in_vivo	17	17	58.2605882	46.3652941	11.8952941	c(t = 2.22790	0.0335	0.90887448	ENSSSCG00000004081
chr8	96209079	96270889	in_vitro	in_vivo	15	16	58.2093333	39.54125	18.6680833	c(t = 2.45983	0.0203	0.90887448	JADE1
chr15	76991928	77034812	in_vitro	in_vivo	13	16	58.1969231	34.563125	23.6337981	c(t = 2.78712	0.0103	0.90887448	GAD1
chr18	7414007	7596236	in_vitro	in_vivo	17	15	58.1041176	66.386	-8.2818824	c(t = -2.32894	0.0277	0.90887448	ENSSSCG00000044150
chr14	6321870	6346865	in_vitro	in_vivo	12	17	58.0483333	41.8994118	16.1489216	c(t = 2.21728	0.0354	0.90887448	DMTN
chr15	65431111	65680887	in_vitro	in_vivo	16	16	57.8675	71.696875	-13.829375	c(t = -2.36384	0.0249	0.90887448	PKP4
chr14	10427957	10545704	in_vitro	in_vivo	16	17	57.505625	42.28	15.225625	c(t = 2.48443	0.0187	0.90887448	DPYSL2
chr5	17268130	17286276	in_vitro	in_vivo	12	14	57.2833333	39.64	17.6433333	c(t = 2.18097	0.0412	0.90887448	ACVRL1
chr4	61577112	61596049	in_vitro	in_vivo	13	14	57.2415385	31.8521429	25.3893956	c(t = 2.93718	0.00702	0.90887448	GDAP1
chr12	49778800	49796110	in_vitro	in_vivo	16	14	57.02	74.95	-17.93	c(t = -2.46378	0.0202	0.90887448	P2RX5
chr5	22854316	22867028	in_vitro	in_vivo	12	11	56.8441667	25.71	31.1341667	c(t = 3.39665	0.00305	0.90887448	PIP4K2C
chr1	141570493	141667338	in_vitro	in_vivo	17	16	56.8323529	70.798125	-13.965772	c(t = -2.24279	0.0324	0.90887448	ENSSSCG00000004830
chr3	113816772	113857113	in_vitro	in_vivo	14	14	56.7471429	72.9314286	-16.184286	c(t = -2.08480	0.0486	0.90887448	DNAJC27
chr14	6711442	6794667	in_vitro	in_vivo	15	16	56.402	40.665625	15.736375	c(t = 2.09326	0.0471	0.90887448	PPP3CC
chr18	42549846	42651807	in_vitro	in_vivo	16	15	56.301875	39.726	16.575875	c(t = 2.07381	0.0476	0.90887448	ZNRF2
chr14	14458042	14504449	in_vitro	in_vivo	14	14	56.2814286	35.2642857	21.0171429	c(t = 2.61598	0.0147	0.90887448	MTMR9
chrX	92819976	93084780	in_vitro	in_vivo	17	15	55.8129412	66.6153333	-10.802392	c(t = -2.19935	0.0376	0.90887448	ENSSSCG00000047539
chr5	67550289	67572588	in_vitro	in_vivo	14	15	55.7478571	72.6926667	-16.94481	c(t = -2.35669	0.0276	0.90887448	SLC6A12
chr6	94785910	94802988	in_vitro	in_vivo	11	14	55.6754545	28.78	26.8954545	c(t = 2.52269	0.0242	0.90887448	RRAGC
chr1	252781491	252956179	in_vitro	in_vivo	17	16	55.5905882	66.09375	-10.503162	c(t = -2.24718	0.0323	0.90887448	SUSD1
chr1	72311880	72335466	in_vitro	in_vivo	14	12	55.5821429	25.4791667	30.1029762	c(t = 3.79913	0.00128	0.90887448	PRDM1
chr4	118177377	118269101	in_vitro	in_vivo	12	15	55.5241667	73.4166667	-17.8925	c(t = -2.52265	0.0185	0.90887448	AGL
chr3	41410818	41437203	in_vitro	in_vivo	13	15	55.4823077	71.678	-16.195692	c(t = -2.55233	0.0196	0.90887448	FAM234A
chr2	73401028	73411574	in_vitro	in_vivo	11	14	55.4463636	28.0135714	27.4327922	c(t = 2.69122	0.0181	0.90887448	TINCR
chr8	30289407	30342600	in_vitro	in_vivo	13	15	55.3569231	70.704	-15.347077	c(t = -2.12092	0.0441	0.90887448	TMEM156
chr1	248448265	248493647	in_vitro	in_vivo	15	16	55.244	34.014375	21.229625	c(t = 2.35880	0.0253	0.90887448	RAD23B
chr9	7136644	7227964	in_vitro	in_vivo	16	17	55.2175	45.3558824	9.86161765	c(t = 2.14229	0.0431	0.90887448	PDE2A
chr14	29800281	29885475	in_vitro	in_vivo	14	16	55.2092857	65.399375	-10.190089	c(t = -2.07391	0.0474	0.90887448	PITPNM2
chr7	24188030	24200704	in_vitro	in_vivo	12	14	55.1566667	33.9314286	21.2252381	c(t = 2.51169	0.021	0.90887448	PPT2
chrX	42917964	42941887	in_vitro	in_vivo	16	16	54.826875	72.94125	-18.114375	c(t = -2.60279	0.0144	0.90887448	HDAC6
chr2	138287710	138344762	in_vitro	in_vivo	16	14	54.76125	31.4371429	23.3241071	c(t = 2.42539	0.0221	0.90887448	SMAD5
chr13	31331574	31357469	in_vitro	in_vivo	16	16	54.71625	69.190625	-14.474375	c(t = -2.06034	0.0488	0.90887448	CELSR3
chr14	22890332	22910848	in_vitro	in_vivo	13	15	54.7130769	34.1653333	20.5477436	c(t = 2.31519	0.0318	0.90887448	ENSSSCG00000055956
chr4	127327527	127345678	in_vitro	in_vivo	12	15	54.61	71.9926667	-17.382667	c(t = -2.84765	0.00899	0.90887448	ENSSSCG00000063342
chr11	65516097	66187242	in_vitro	in_vivo	17	17	54.5458824	45.5276471	9.01823529	c(t = 2.21058	0.0344	0.90887448	HS6ST3
chr14	62525903	62627586	in_vitro	in_vivo	14	14	54.5264286	33.5421429	20.9842857	c(t = 2.21300	0.036	0.90887448	PHYHIPL
chr1	159023135	159096960	in_vitro	in_vivo	17	13	54.5241176	68.8807692	-14.356652	c(t = -2.68286	0.0128	0.90887448	ENSSSCG00000039179
chr6	54250515	54266893	in_vitro	in_vivo	12	15	54.425	73.2686667	-18.843667	c(t = -2.51993	0.0203	0.90887448	RUVBL2
chr12	43032915	43089205	in_vitro	in_vivo	15	16	54.328	38.948125	15.379875	c(t = 2.24907	0.0334	0.90887448	UTP6
chr14	29477281	29523166	in_vitro	in_vivo	16	15	54.136875	38.4613333	15.6755417	c(t = 2.36057	0.0253	0.90887448	RILPL2
chr18	28946326	28993961	in_vitro	in_vivo	14	17	53.9242857	42.7317647	11.192521	c(t = 2.14245	0.0407	0.90887448	WNT2
chr1	184745799	184792502	in_vitro	in_vivo	12	12	53.8683333	74.5883333	-20.72	c(t = -2.22712	0.0372	0.90887448	TBPL2
chr2	6560513	6572466	in_vitro	in_vivo	14	15	53.6957143	35.422	18.2737143	c(t = 2.70116	0.013	0.90887448	KAT5
chr6	167896287	167908625	in_vitro	in_vivo	15	13	53.6766667	71.2369231	-17.560256	c(t = -2.10464	0.0451	0.90887448	MPL
chr14	16485081	16583552	in_vitro	in_vivo	17	17	53.5588235	69.6535294	-16.094706	c(t = -2.39489	0.025	0.90887448	ENSSSCG00000043271
chr1	184199679	184257315	in_vitro	in_vivo	16	16	53.551875	35.145	18.406875	c(t = 2.12283	0.0425	0.90887448	GCH1
chr6	82861650	82878951	in_vitro	in_vivo	10	11	53.514	32.6045455	20.9094545	c(t = 2.13870	0.0499	0.90887448	TMEM50A
chr2	150927770	150985318	in_vitro	in_vivo	15	17	53.3646667	70.1270588	-16.762392	c(t = -2.16972	0.0413	0.90887448	PDE6A
chr6	86094423	86131682	in_vitro	in_vivo	13	15	53.2853846	70.4133333	-17.127949	c(t = -2.48486	0.0213	0.90887448	MECR
chr18	10268960	10333156	in_vitro	in_vivo	14	16	53.27	70.53875	-17.26875	c(t = -2.18251	0.0388	0.90887448	LUC7L2
chr2	60187747	60197648	in_vitro	in_vivo	12	14	53.2158333	28.5414286	24.6744048	c(t = 2.36810	0.0296	0.90887448	PGLS
chr10	49553495	49623656	in_vitro	in_vivo	14	16	53.0864286	69.679375	-16.592946	c(t = -2.11291	0.0438	0.90887448	ENSSSCG00000011059
chr14	54867021	54892049	in_vitro	in_vivo	15	14	52.56	33.9366667	18.6233333	c(t = 2.23218	0.0341	0.90887448	LGALS8
chr17	9789826	10196187	in_vitro	in_vivo	16	17	52.52	39.7558824	12.7641176	c(t = 3.12972	0.0038	0.90887448	ZMAT4
chr12	4535650	4593526	in_vitro	in_vivo	15	16	52.4806667	68.046875	-15.566208	c(t = -2.50805	0.0204	0.90887448	ENSSSCG00000033293
chr2	7030733	7058967	in_vitro	in_vivo	14	12	52.425	70.4641667	-18.039167	c(t = -2.41968	0.0252	0.90887448	SPDYC

chr14	38515847	38543561	in_vitro	in_vivo	12	16	52.4091667	72.654375	-20.245208	c(t = -2.8889	0.00942	0.90887448	PLBD2
chr14	113154819	113166906	in_vitro	in_vivo	11	13	52.0881818	72.6976923	-20.60951	c(t = -2.5902	0.018	0.90887448	NOLC1
chr9	36776897	36849710	in_vitro	in_vivo	14	15	51.6721429	70.914	-19.241857	c(t = -2.0963	0.046	0.90887448	C11orf65
chr5	45973693	46030453	in_vitro	in_vivo	13	14	51.5038462	29.5378571	21.965989	c(t = 2.19395	0.0411	0.90887448	ENSSSCG00000000547
chr9	7116324	7132404	in_vitro	in_vivo	13	14	51.1215385	68.425	-17.303462	c(t = -2.1712	0.0401	0.90887448	ENSSSCG00000045388
chr3	44027262	44062808	in_vitro	in_vivo	10	17	51.056	31.9694118	19.0865882	c(t = 2.30608	0.0341	0.90887448	ENSSSCG00000048125
chr14	141360608	141369641	in_vitro	in_vivo	13	15	50.8961538	73.116	-22.219846	c(t = -2.1194	0.0467	0.90887448	PAOX
chr4	54935860	54984238	in_vitro	in_vivo	10	13	50.836	29.4146154	21.4213846	c(t = 2.45924	0.0266	0.90887448	IMPA1
chr6	52405290	52417695	in_vitro	in_vivo	12	12	50.7858333	28.7675	22.0183333	c(t = 2.42023	0.0242	0.90887448	ENSSSCG00000057046
chr1	43003390	43113631	in_vitro	in_vivo	16	16	50.23125	26.04125	24.19	c(t = 3.01093	0.00724	0.90887448	FAM184A
chr6	62833585	62859304	in_vitro	in_vivo	14	16	50.1335714	67.5575	-17.423929	c(t = -2.0568	0.0498	0.90887448	ENSSSCG00000003998
chr3	56528434	56541740	in_vitro	in_vivo	16	16	49.981875	27.1475	22.834375	c(t = 2.80084	0.00963	0.90887448	ENSSSCG00000008193
chr12	60507667	60529733	in_vitro	in_vivo	15	14	49.8466667	73.085	-23.238333	c(t = -2.1016	0.0495	0.90887448	ALKBH5
chr6	37443699	37505736	in_vitro	in_vivo	15	12	49.7466667	32	17.7466667	c(t = 2.17536	0.0412	0.90887448	NETO2
chr18	19678687	19691243	in_vitro	in_vivo	15	11	49.6433333	27.8863636	21.7569697	c(t = 2.65637	0.0139	0.90887448	IRF5
chr14	113434465	113464028	in_vitro	in_vivo	14	15	49.2292857	31.07	18.1592857	c(t = 2.54678	0.0178	0.90887448	CUEDC2
chr6	110799283	110837255	in_vitro	in_vivo	14	14	49.1078571	72.2835714	-23.175714	c(t = -2.7141	0.0125	0.90887448	PSMA8
chr1	239912126	239945073	in_vitro	in_vivo	15	14	49.03	32.42	16.61	c(t = 2.23375	0.034	0.90887448	TRIM14
chr9	66448953	66458724	in_vitro	in_vivo	9	12	48.8733333	70.0308333	-21.1575	c(t = -2.1592	0.0439	0.90887448	RAB29
chr9	45565513	45578185	in_vitro	in_vivo	11	11	48.8054545	27.0609091	21.7445455	c(t = 2.23522	0.0372	0.90887448	MPZL2
chr17	35914246	35961522	in_vitro	in_vivo	17	16	48.7176471	69.50125	-20.783603	c(t = -2.4454	0.0205	0.90887448	KIF3B
chr2	30296444	30311521	in_vitro	in_vivo	12	9	48.6658333	30.3277778	18.3380556	c(t = 2.39019	0.0279	0.90887448	ARL14EP
chr6	145533537	145596066	in_vitro	in_vivo	17	16	48.4829412	62.671875	-14.188934	c(t = -2.1572	0.039	0.90887448	SLC35D1
chr1	18231494	18290021	in_vitro	in_vivo	15	14	48.278	29.1128571	19.1651429	c(t = 2.22102	0.0356	0.90887448	SAMD5
chr3	10974127	10991873	in_vitro	in_vivo	14	14	47.9621429	68.0128571	-20.050714	c(t = -2.3399	0.0293	0.90887448	STX1A
chr4	48556967	48862985	in_vitro	in_vivo	14	16	47.7042857	62.890625	-15.186339	c(t = -2.2608	0.032	0.90887448	MMP16
chr13	138018407	138103838	in_vitro	in_vivo	14	16	47.5585714	68.4925	-20.933929	c(t = -2.1182	0.0442	0.90887448	KPNA1
chr6	31748739	31802022	in_vitro	in_vivo	15	13	47.4286667	66.2646154	-18.835949	c(t = -2.1221	0.0459	0.90887448	RBL2
chr4	51590298	51606440	in_vitro	in_vivo	9	9	47.3477778	74.8266667	-27.478889	c(t = -2.1883	0.0472	0.90887448	ENSSSCG00000006147
chr9	126172301	126287603	in_vitro	in_vivo	15	16	47.3386667	33.285625	14.0530417	c(t = 2.09303	0.0452	0.90887448	RNF2
chr16	34858368	34983699	in_vitro	in_vivo	15	17	47.29	65.9764706	-18.686471	c(t = -2.2987	0.0301	0.90887448	DDX4
chr9	3075995	3083580	in_vitro	in_vivo	12	13	47.2608333	25.57	21.6908333	c(t = 2.40270	0.0272	0.90887448	MRPL17
chr14	32140517	32203935	in_vitro	in_vivo	14	16	46.6835714	59.908125	-13.224554	c(t = -2.1513	0.0404	0.90887448	CCDC63
chr2	33561008	33613549	in_vitro	in_vivo	12	12	46.4691667	65.3675	-18.898333	c(t = -2.1411	0.0441	0.90887448	SLC5A12
chr2	142460178	142688089	in_vitro	in_vivo	17	16	46.4629412	38.019375	8.44356618	c(t = 2.25198	0.0317	0.90887448	PCDHAC2
chr3	58436324	58558934	in_vitro	in_vivo	15	17	46.034	58.0494118	-12.015412	c(t = -2.5439	0.0165	0.90887448	REEP1
chr13	104951884	105007553	in_vitro	in_vivo	10	10	45.983	74.837	-28.854	c(t = -2.3006	0.0336	0.90887448	ENSSSCG00000033350
chr12	50645094	50659642	in_vitro	in_vivo	13	13	45.4546154	68.2153846	-22.760769	c(t = -2.7724	0.0109	0.90887448	XAF1
chr6	88139498	88154848	in_vitro	in_vivo	16	13	45.139375	65.6730769	-20.533702	c(t = -2.9177	0.00706	0.90887448	HCRTR1
chr1	86358483	86481776	in_vitro	in_vivo	16	16	44.328125	60.801875	-16.47375	c(t = -2.6830	0.0118	0.90887448	SH3BGRL2
chr3	9317140	9669998	in_vitro	in_vivo	17	17	43.9952941	56.3429412	-12.347647	c(t = -2.4925	0.0182	0.90887448	CUX1
chrX	103844933	103931646	in_vitro	in_vivo	12	14	43.6958333	55.975	-12.279167	c(t = -2.1149	0.0451	0.90887448	ENSSSCG00000054975
chr2	62601758	62623371	in_vitro	in_vivo	16	15	43.67875	27.5713333	16.1074167	c(t = 2.06428	0.0481	0.90887448	SLC1A6
chr10	27724648	27954350	in_vitro	in_vivo	15	17	43.3566667	57.3823529	-14.015686	c(t = -2.4070	0.0228	0.90887448	DAPK1
chr13	26202677	26211522	in_vitro	in_vivo	9	14	42.9077778	63.4064286	-20.498651	c(t = -2.0958	0.0498	0.90887448	KLHL40
chr7	75140207	75158905	in_vitro	in_vivo	16	16	42.704375	61.89875	-19.194375	c(t = -2.4764	0.0191	0.90887448	RNF31
chr7	37790178	37805142	in_vitro	in_vivo	14	14	42.6471429	62.6521429	-20.005	c(t = -2.6167	0.0168	0.90887448	PRPH2
chr13	106308394	106343518	in_vitro	in_vivo	11	10	42.5636364	72.641	-30.077364	c(t = -2.9875	0.00954	0.90887448	SERPINI2
chr6	72514079	72521001	in_vitro	in_vivo	10	12	42.536	68.8808333	-26.344833	c(t = -2.1939	0.0429	0.90887448	ENSSSCG00000059184
chr6	14836752	14911311	in_vitro	in_vivo	17	15	42.3370588	55.2553333	-12.918275	c(t = -2.4779	0.0209	0.90887448	IST1
chr7	31361873	31379204	in_vitro	in_vivo	14	12	42.1607143	26.5566667	15.6040476	c(t = 2.08909	0.0485	0.90887448	TULP1
chr9	7302814	7332770	in_vitro	in_vivo	12	16	42.0191667	58.150625	-16.131458	c(t = -2.6011	0.0153	0.90887448	STARD10
chr6	18542386	18575514	in_vitro	in_vivo	12	14	41.5991667	66.055	-24.455833	c(t = -2.6820	0.014	0.90887448	BBS2
chr13	29884719	29996359	in_vitro	in_vivo	14	15	41.5835714	63.1526667	-21.569095	c(t = -2.6123	0.0145	0.90887448	SETD2
chrX	88105155	88109120	in_vitro	in_vivo	10	10	41.539	64.953	-23.414	c(t = -2.2120	0.0406	0.90887448	ENSSSCG00000057809
chr5	62676225	62715067	in_vitro	in_vivo	11	13	41.42	67.9807692	-26.560769	c(t = -2.5343	0.019	0.90887448	RIMKLB
chr9	36288558	36341284	in_vitro	in_vivo	14	17	41.2307143	63.3005882	-22.069874	c(t = -2.7799	0.00956	0.90887448	SLC35F2
chr12	3747279	3755215	in_vitro	in_vivo	13	12	41.2261538	65.7258333	-24.499679	c(t = -2.3176	0.0302	0.90887448	BIRC5
chr8	18563807	18620909	in_vitro	in_vivo	14	15	40.9835714	67.4106667	-26.427095	c(t = -2.4388	0.0217	0.90887448	DHX15
chrX	125014714	125025104	in_vitro	in_vivo	13	11	40.8730769	70.1709091	-29.297832	c(t = -3.6721	0.00138	0.90887448	FAM3A
chr15	68224395	68656309	in_vitro	in_vivo	17	17	40.87	53.6370588	-12.767059	c(t = -2.0457	0.0493	0.90887448	SLC4A10
chrX	70334774	70435109	in_vitro	in_vivo	15	13	40.4873333	56.5169231	-16.02959	c(t = -2.1263	0.0432	0.90887448	KLHL4
chr2	2119592	2163343	in_vitro	in_vivo	16	15	40.28875	57.482	-17.19325	c(t = -3.1094	0.00423	0.90887448	CARS1
chr1	235878355	235887993	in_vitro	in_vivo	15	15	40.0153333	62.0113333	-21.996	c(t = -2.5261	0.0175	0.90887448	PIGO
chrX	68503179	68579893	in_vitro	in_vivo	15	12	39.914	58.1133333	-18.199333	c(t = -2.6171	0.0168	0.90887448	POF1B
chr6	115351925	115383743	in_vitro	in_vivo	14	12	39.6978571	60.2266667	-20.52881	c(t = -2.1539	0.0418	0.90887448	DSG3
chr6	53620483	53686562	in_vitro	in_vivo	16	16	39.6525	58.81125	-19.15875	c(t = -2.9763	0.00589	0.90887448	LIG1
chr2	151202777	151216988	in_vitro	in_vivo	14	12	39.3707143	61.9166667	-22.545952	c(t = -2.2732	0.0324	0.90887448	CDX1
chr16	51733135	51858772	in_vitro	in_vivo	17	16	39.3552941	57.925625	-18.570331	c(t = -2.3681	0.0245	0.90887448	SH3PXD2B
chr4	15154331	15197511	in_vitro	in_vivo	16	17	39.325625	25.1664706	14.1591544	c(t = 2.22167	0.0341	0.90887448	RNF139
chr16	34294272	34349242	in_vitro	in_vivo	13	13	39.2538462	58.7384615	-19.484615	c(t = -2.3458	0.0276	0.90887448	CDC20B
chr4	89355407	89374358	in_vitro	in_vivo	11	15	39.25	60.1306667	-20.880667	c(t = -2.2793	0.038	0.90887448	NECTIN4
chr1	76896756	76909749	in_vitro	in_vivo	13	10	39.1930769	68.539	-29.345923	c(t = -2.5100	0.0204	0.90887448	GTF3C6
chr6	169450777	169464322	in_vitro	in_vivo	15	14	39.0666667	58.255	-19.188333	c(t = -2.1931	0.0371	0.90887448	ENSSSCG00000050157
chr6	61800671	61810486	in_vitro	in_vivo	10	10	38.942	61.505	-22.563	c(t = -2.3991	0.0275	0.90887448	ENSSSCG00000033232
chr18	3148483	4054118	in_vitro	in_vivo	17	17	38.8870588	49.8935294	-11.006471	c(t = -2.2524	0.0313	0.90887448	DPP6
chr13	3330023	3371091	in_vitro	in_vivo	14	14	38.6885714	57.2114286	-18.522857	c(t = -2.6762	0.013	0.90887448	GALNT15
chr1	116449079	116492907	in_vitro	in_vivo	11	10	38.4645455	67.254	-28.789455	c(t = -2.3964	0.0271	0.90887448	CCPG1
chr12	44174948	44218146	in_vitro	in_vivo	15	15	38.3093333	58.0933333	-19.784	c(t = -2.9041	0.00716	0.90887448	NOS2
chr6	64374	76394	in_vitro	in_vivo	14	13	37.7414286	60.8853846	-23.143956	c(t = -2.5698	0.0166	0.90887448	PRDM7
chr7	36959468	36969478	in_vitro	in_vivo	13	13	37.5330769	60.2507692	-22.717692	c(t = -2.1638	0.0416	0.90887448	FRS3
chr15	112236119	112462929	in_vitro	in_vivo	15	13	37.522	53.5892308	-16.067231	c(t = -2.0825	0.0496	0.90887448	MAP2
chr3	43869767	43876257	in_vitro	in_vivo	9	9	37.4588889	66.3133333	-28.854444	c(t = -2.9958	0.00949	0.90887448	ENSSSCG00000045765
chr2	151314507	151320814	in_vitro	in_vivo	9	11	37.2355556	59.0009091	-21.765354	c(t = -2.3073	0.0332	0.90887448	ARSI
chr7	22540381	22578619	in_vitro	in_vivo	14	16	37.025	54.90125	-17.87625	c(t = -3.2754	0.00296	0.90887448	GABBR1
chr14	14967074	15016227	in_vitro	in_vivo	15	15	36.732	54.1766667	-17.444667	c(t = -2.4598	0.0219	0.90887448	FDFT1
chr15	64241738	64282664	in_vitro	in_vivo	11	15	36.4827273	59.42	-22.937273	c(t = -2.6039	0.0162	0.90887448	GALNT5
chr18	42764386	42769920	in_vitro	in_vivo	10	9	36.201	62.8222222	-26.621222	c(t = -2.1215	0.0489	0.90887448	ENSSSCG00000061806
chr1	188598501	188624892	in_vitro	in_vivo	13	10	36.01	60.118	-24.108	c(t = -2.7778	0.0116	0.90887448	JKAMP
chr14	99685578	99808381	in_vitro	in_vivo	15	15	35.5906667	48.4073333	-12.816667	c(t = -2.0701	0.0483	0.90887448	PAPSS2
chr4	19319810	19433488	in_vitro	in_vivo	13	17	35.5007692	50.3817647	-14.880995	c(t = -2.2379	0.0334	0.90887448	ENPP2
chr2	10989969	11007621	in_vitro	in_vivo	13	13	35.2284615	49.7115385	-14.483077	c(t = -2.1012	0.0474	0.90887448	MS4A8
chr18	6429887	6452966	in_vitro	in_vivo	16	16	35.14625	56.04625	-20.9	c(t = -3.0455	0.00481	0.90887448	GIMAP4
chr12	60652189	60729259	in_vitro	in_vivo	16	15	35.051875	51.1253333	-16.073458	c(t = -2.2437	0.033	0.90887448	TOM1L2

chr2	62435155	62451371	in_vitro	in_vivo	14	15	34.6092857	50.1373333	-15.528048	c(t = -2.52759	0.0177	0.90887448	ILVBL
chr14	114899147	114955070	in_vitro	in_vivo	13	13	34.6023077	62.7338462	-28.131538	c(t = -2.74172	0.0117	0.90887448	SLK
chr1	1556460	1560657	in_vitro	in_vivo	9	10	33.8811111	62.376	-28.494889	c(t = -2.13066	0.0485	0.90887448	ENSSSCG00000054333
chr1	248233437	248239225	in_vitro	in_vivo	10	9	33.321	62.0411111	-28.720111	c(t = -2.78062	0.0143	0.90887448	ENSSSCG00000057298
chr2	75408724	75433814	in_vitro	in_vivo	14	14	33.13	47.3142857	-14.184286	c(t = -2.06154	0.0499	0.90887448	GNA15
chr15	92911054	93252180	in_vitro	in_vivo	17	15	32.8117647	46.492	-13.680235	c(t = -2.29922	0.0297	0.90887448	GULP1
chr5	82290385	82325562	in_vitro	in_vivo	14	15	32.5757143	46.81	-14.234286	c(t = -2.12018	0.0441	0.90887448	DRAM1
chr15	131704814	131724448	in_vitro	in_vivo	13	13	32.2230769	57.4869231	-25.263846	c(t = -2.88621	0.00813	0.90887448	ENSSSCG00000050110
chr6	79849698	79958334	in_vitro	in_vivo	17	17	32.1817647	46.9305882	-14.748824	c(t = -2.35558	0.0267	0.90887448	HSPG2
chr6	46761846	46786246	in_vitro	in_vivo	14	14	31.8214286	58.7771429	-26.955714	c(t = -2.95761	0.0066	0.90887448	ZFP30
chr15	118208684	118273680	in_vitro	in_vivo	13	16	31.5107692	45.3075	-13.796731	c(t = -2.16035	0.0405	0.90887448	MREG
chr1	270274562	270334907	in_vitro	in_vivo	17	15	31.3688235	62.184	-30.815176	c(t = -4.41202	0.000125	0.90887448	NCS1
chr1	269647591	269653388	in_vitro	in_vivo	13	10	31.3646154	57.797	-26.432385	c(t = -2.24110	0.036	0.90887448	ENSSSCG00000060776
chr9	74173931	74210397	in_vitro	in_vivo	11	10	31.2127273	61.02	-29.807273	c(t = -2.40164	0.0281	0.90887448	COL1A2
chr10	32211777	32251503	in_vitro	in_vivo	13	15	31.0530769	46.0433333	-14.990256	c(t = -2.49585	0.0197	0.90887448	CNTFR
chr5	78141236	78150583	in_vitro	in_vivo	10	13	30.908	68.8915385	-37.983538	c(t = -3.70641	0.0021	0.90887448	SLC48A1
chr12	1239834	1245181	in_vitro	in_vivo	9	11	30.65	59.1836364	-28.533636	c(t = -3.12149	0.00683	0.90887448	TSPAN10
chr13	204752196	204796777	in_vitro	in_vivo	15	17	29.7366667	46.7988235	-17.062157	c(t = -2.11400	0.0436	0.90887448	FAM3B
chr13	136954016	136971224	in_vitro	in_vivo	14	13	29.7085714	45.6469231	-15.938352	c(t = -2.20529	0.0371	0.90887448	ENSSSCG00000043024
chr15	131655663	131673693	in_vitro	in_vivo	13	12	29.6276923	55.7833333	-26.155641	c(t = -2.38769	0.0263	0.90887448	GPR55
chr13	89480260	89513351	in_vitro	in_vivo	13	15	29.3269231	53.674	-24.347077	c(t = -2.51319	0.0185	0.90887448	CPHL1
chr7	37282700	37296717	in_vitro	in_vivo	15	15	29.1873333	46.0653333	-16.878	c(t = -2.36074	0.0277	0.90887448	GUCA1A
chr6	168765539	168870281	in_vitro	in_vivo	17	17	28.7323529	43.5317647	-14.799412	c(t = -2.22106	0.0347	0.90887448	ENSSSCG00000003965
chr15	79935994	80025850	in_vitro	in_vivo	16	17	28.5875	43.8264706	-15.238971	c(t = -2.26640	0.0314	0.90887448	SP3
chr14	13229780	13240405	in_vitro	in_vivo	9	9	28.4322222	55.0833333	-26.651111	c(t = -2.60215	0.0199	0.90887448	ENSSSCG00000031914
chr12	48386881	48408039	in_vitro	in_vivo	11	15	28.3736364	50.9166667	-22.54303	c(t = -2.73753	0.0123	0.90887448	SRR
chr5	11570812	11619664	in_vitro	in_vivo	13	14	27.9623077	41.7928571	-13.830549	c(t = -2.15344	0.0414	0.90887448	ENSSSCG00000048676
chr16	79134465	79173772	in_vitro	in_vivo	13	13	27.9292308	44.7223077	-16.793077	c(t = -2.10527	0.046	0.90887448	LPCAT1
chr3	56610304	56620164	in_vitro	in_vivo	12	13	27.6941667	65.5246154	-37.830449	c(t = -4.12399	0.000533	0.90887448	ACTR1B
chr12	51954613	51961496	in_vitro	in_vivo	9	12	27.3755556	56.6283333	-29.252778	c(t = -3.09909	0.00592	0.90887448	ENO3
chr9	65279485	65352945	in_vitro	in_vivo	16	15	26.9925	43.0813333	-16.088833	c(t = -2.38864	0.0248	0.90887448	LRRN2
chr17	34558048	34571168	in_vitro	in_vivo	13	14	26.7776923	46.9257143	-20.148022	c(t = -2.45794	0.0234	0.90887448	SCRT2
chr4	101444693	101478144	in_vitro	in_vivo	14	15	26.6128571	42.1713333	-15.558476	c(t = -2.34768	0.0266	0.90887448	PHGDH
chr13	205425454	205491629	in_vitro	in_vivo	15	15	26.33	39.5066667	-13.176667	c(t = -2.26061	0.0327	0.90887448	UMODL1
chr13	208094877	208259369	in_vitro	in_vivo	15	17	25.5986667	38.9623529	-13.363686	c(t = -2.10186	0.0448	0.90887448	PCBP3
chr9	64731969	64780942	in_vitro	in_vivo	15	15	24.8753333	38.7886667	-13.913333	c(t = -2.28156	0.0317	0.90887448	SOX13
chr15	133468293	133477110	in_vitro	in_vivo	12	11	24.7408333	58.1918182	-33.450985	c(t = -3.36494	0.00337	0.90887448	NEU2
chr12	24280665	24292892	in_vitro	in_vivo	9	13	24.6722222	51.68	-27.007778	c(t = -2.73651	0.0127	0.90887448	COPZ2
chr2	323647	329660	in_vitro	in_vivo	9	13	24.6444444	50.9846154	-26.340171	c(t = -2.83225	0.0106	0.90887448	LMNTD2
chr3	5414074	5442943	in_vitro	in_vivo	15	16	24.5453333	41.99	-17.444667	c(t = -2.77783	0.0112	0.90887448	TECPR1
chr12	58964479	58982312	in_vitro	in_vivo	10	13	24.146	46.2376923	-22.091692	c(t = -2.28823	0.0362	0.90887448	ZNF286A
chr6	95442661	95459575	in_vitro	in_vivo	14	15	23.6907143	36.9226667	-13.231952	c(t = -2.34434	0.0267	0.90887448	HEYL
chr4	7195093	7374012	in_vitro	in_vivo	15	16	23.6066667	40.2825	-16.675833	c(t = -2.34003	0.0289	0.90887448	ENSSSCG00000055353
chr16	28098581	28133788	in_vitro	in_vivo	12	11	23.5775	48.0572727	-24.479773	c(t = -2.27909	0.0349	0.90887448	PAIP1
chr3	5624889	5628768	in_vitro	in_vivo	10	11	23.315	50.5	-27.185	c(t = -2.56328	0.0206	0.90887448	ENSSSCG00000062108
chr1	30961583	30970544	in_vitro	in_vivo	10	9	23.264	54.1277778	-30.863778	c(t = -2.47253	0.0282	0.90887448	ENSSSCG00000004179
chr6	168060631	168072904	in_vitro	in_vivo	14	14	23.0685714	42.775	-19.706429	c(t = -3.03264	0.00564	0.90887448	FAM183A
chr14	134002228	134082462	in_vitro	in_vivo	14	14	22.8671429	46.7228571	-23.855714	c(t = -3.41544	0.00289	0.90887448	FAM53B
chr12	57380486	57385554	in_vitro	in_vivo	9	14	22.7466667	49.7242857	-26.977619	c(t = -2.47550	0.0219	0.90887448	ENSSSCG00000054081
chr8	86568711	86614124	in_vitro	in_vivo	13	13	22.6869231	37.5661538	-14.879231	c(t = -2.48658	0.0222	0.90887448	MGAT4D
chr6	53583965	53603366	in_vitro	in_vivo	12	14	22.6691667	45.7192857	-23.050119	c(t = -2.63334	0.0162	0.90887448	ELSPBP1
chr8	131208282	131241266	in_vitro	in_vivo	13	12	22.4538462	46.41	-23.956154	c(t = -2.25045	0.037	0.90887448	IBSP
chr12	37685325	37694239	in_vitro	in_vivo	13	12	21.85	41.4341667	-19.584167	c(t = -2.30396	0.0336	0.90887448	ENSSSCG00000017679
chr17	34459359	34481026	in_vitro	in_vivo	14	16	21.7264286	36.82625	-15.099821	c(t = -2.14843	0.0422	0.90887448	SLC52A3
chr6	59715636	59719183	in_vitro	in_vivo	10	9	21.706	53.9688889	-32.262889	c(t = -2.52873	0.0233	0.90887448	FIZ1
chr5	17555728	17570581	in_vitro	in_vivo	13	14	21.6692308	49.7364286	-28.067198	c(t = -2.72949	0.0116	0.90887448	KRT7
chr1	66459987	66467850	in_vitro	in_vivo	9	10	21.4377778	48.827	-27.389222	c(t = -2.64921	0.0174	0.90887448	ENSSSCG00000038757
chrX	87321152	87355721	in_vitro	in_vivo	14	16	21.4071429	43.404375	-21.997232	c(t = -2.95625	0.00664	0.90887448	CLDN2
chr3	37961432	37972114	in_vitro	in_vivo	11	13	21.2472727	42.9369231	-21.68965	c(t = -2.57303	0.0177	0.90887448	VASN
chr17	33818816	33834134	in_vitro	in_vivo	10	13	21.201	51.2930769	-30.092077	c(t = -3.33214	0.00441	0.90887448	ENSSSCG00000063284
chr1	272995842	273011443	in_vitro	in_vivo	14	14	20.9164286	40.1357143	-19.219286	c(t = -2.17360	0.0409	0.90887448	STKLD1
chr1	270258549	270264566	in_vitro	in_vivo	12	11	20.3991667	53.4645455	-33.065379	c(t = -2.47572	0.0239	0.90887448	ENSSSCG00000051973
chr2	4069280	4105042	in_vitro	in_vivo	15	16	20.3486667	39.4575	-19.108833	c(t = -2.12457	0.0439	0.90887448	TPCN2
chr1	269575594	269610029	in_vitro	in_vivo	17	15	19.7594118	37.5513333	-17.791922	c(t = -2.21900	0.0375	0.90887448	ENSSSCG00000046947
chr12	145994	151821	in_vitro	in_vivo	12	14	19.71	33.9478571	-14.237857	c(t = -2.14437	0.0428	0.90887448	ENSSSCG00000048083
chr6	169069	178410	in_vitro	in_vivo	12	14	19.42	40.4985714	-21.078571	c(t = -2.14171	0.0457	0.90887448	TUBB3
chr6	58365160	58388252	in_vitro	in_vivo	14	12	19.4157143	33.0125	-13.596786	c(t = -2.10242	0.0488	0.90887448	ENSSSCG00000003236
chr1	98514267	98542382	in_vitro	in_vivo	16	13	19.20875	33.1030769	-13.894327	c(t = -2.20935	0.0389	0.90887448	SMAD7
chr16	51536573	51581126	in_vitro	in_vivo	17	17	19.1882353	36.5652941	-17.377059	c(t = -2.65232	0.013	0.90887448	NEURL1B
chr1	166818037	166841693	in_vitro	in_vivo	13	11	19.1592308	42.2981818	-23.138951	c(t = -2.91843	0.0115	0.90887448	SPESP1
chr1	268653070	268702222	in_vitro	in_vivo	15	16	18.9206667	30.43375	-11.513083	c(t = -2.26136	0.0325	0.90887448	DNM1
chr13	195950949	195973243	in_vitro	in_vivo	12	15	18.6333333	38.524	-19.890667	c(t = -2.41302	0.0251	0.90887448	ENSSSCG00000040843
chr9	453200	459215	in_vitro	in_vivo	9	12	18.6255556	41.9191667	-23.293611	c(t = -2.16782	0.0448	0.90887448	ENSSSCG00000059027
chr16	78981476	78987766	in_vitro	in_vivo	12	13	18.2083333	41.1753846	-22.967051	c(t = -2.57656	0.0176	0.90887448	ENSSSCG00000048696
chr4	125905770	125979865	in_vitro	in_vivo	15	16	17.4393333	28.593125	-11.153792	c(t = -2.31193	0.0302	0.90887448	ENSSSCG00000061951
chr2	6334197	6340112	in_vitro	in_vivo	10	13	17.333	31.1938462	-13.860846	c(t = -2.11019	0.0477	0.90887448	EIF1AD
chr2	8479315	8500019	in_vitro	in_vivo	15	13	17.1333333	38.2630769	-21.129744	c(t = -2.23552	0.0402	0.90887448	PLAAT5
chr3	125239594	125249231	in_vitro	in_vivo	9	15	16.9255556	37.0833333	-20.157778	c(t = -3.08996	0.00549	0.90887448	ENSSSCG00000041285
chr5	403765	416798	in_vitro	in_vivo	11	15	16.8436364	46.428	-29.584364	c(t = -2.65224	0.0154	0.90887448	PLXNB2
chr3	59438924	59458963	in_vitro	in_vivo	11	13	16.7872727	46.7938462	-30.006573	c(t = -3.43521	0.00322	0.90887448	ENSSSCG00000008241
chr12	60261555	60281883	in_vitro	in_vivo	11	15	16.4609091	37.5866667	-21.125758	c(t = -2.42143	0.0238	0.90887448	ENSSSCG00000018050
chr2	2201385	2261927	in_vitro	in_vivo	13	14	16.4138462	36.1142857	-19.70044	c(t = -2.37812	0.0271	0.90887448	OSBPL5
chr15	129220190	129271241	in_vitro	in_vivo	14	14	16.3207143	36.8642857	-20.543571	c(t = -2.59662	0.0183	0.90887448	DAW1
chr5	202137	203951	in_vitro	in_vivo	12	12	16.1108333	43.8775	-27.766667	c(t = -2.37120	0.0296	0.90887448	ENSSSCG00000000960
chr3	113515116	113522952	in_vitro	in_vivo	11	13	15.6672727	43.4315385	-27.764266	c(t = -2.99775	0.00888	0.90887448	ENSSSCG00000044418
chr2	143475935	143501487	in_vitro	in_vivo	14	14	15.6607143	34.7307143	-19.07	c(t = -2.20855	0.041	0.90887448	PCDH1
chr8	18796637	18802152	in_vitro	in_vivo	11	15	15.34	39.55	-24.21	c(t = -2.13571	0.0436	0.90887448	SOD3
chr7	114345305	114358075	in_vitro	in_vivo	12	14	15.3066667	32.2785714	-16.971905	c(t = -2.28085	0.0344	0.90887448	CHGA
chr3	125099935	125171036	in_vitro	in_vivo	15	17	15.1486667	32.6952941	-17.546627	c(t = -2.10322	0.0466	0.90887448	GREB1
chrX	39208119	39235758	in_vitro	in_vivo	13	13	14.9823077	33.7346154	-18.752308	c(t = -2.77285	0.0134	0.90887448	NDP
chr15	139766414	139811999	in_vitro	in_vivo	14	16	14.8028571	32.96375	-18.160893	c(t = -2.10617	0.0477	0.90887448	CROCC2
chr2	2028396	2055750	in_vitro	in_vivo	14	14	14.6885714	36.395	-21.706429	c(t = -2.14097	0.0467	0.90887448	SLC22A18
chr12	15436803	15447100	in_vitro	in_vivo	12	15	13.9775	29.658	-15.6805	c(t = -2.09678	0.0463	0.90887448	CYB561
chr8	35167622	35190584	in_vitro	in_vivo	12	9	13.845	47.3255556	-33.480556	c(t = -2.37918	0.0414	0.90887448	GUF1

chr6	58321856	58331573	in_vitro	in_vivo	13	14	13.6338462	28.83	-15.196154	c(t = -2.29185	0.0316	0.90887448	SPACA6
chr2	1584842	1602888	in_vitro	in_vivo	11	14	13.3345455	33.6885714	-20.354026	c(t = -2.14579	0.0467	0.90887448	ENSSSCG00000033880
chr14	132259062	132266058	in_vitro	in_vivo	12	10	13.2258333	27.461	-14.235167	c(t = -2.86108	0.0104	0.90887448	ENSSSCG00000021947
chr13	207666921	207676660	in_vitro	in_vivo	12	14	12.9841667	26.4671429	-13.482976	c(t = -2.26046	0.0377	0.90887448	ENSSSCG00000048463
chr1	7340126	7365520	in_vitro	in_vivo	16	15	12.894375	32.8133333	-19.918958	c(t = -2.66567	0.0146	0.90887448	SLC22A1
chrX	125546467	125570523	in_vitro	in_vivo	14	14	12.7578571	29.1171429	-16.359286	c(t = -2.41367	0.0251	0.90887448	CLIC2
chr12	60117461	60126173	in_vitro	in_vivo	13	14	12.5992308	33.3564286	-20.757198	c(t = -2.46872	0.0257	0.90887448	RNF112
chr10	67766648	67772587	in_vitro	in_vivo	10	9	12.515	32.5144444	-19.999444	c(t = -2.43060	0.0299	0.90887448	ENSSSCG00000059046
chr13	204798629	204831957	in_vitro	in_vivo	14	16	12.4128571	31.713125	-19.300268	c(t = -2.62658	0.0152	0.90887448	MX2
chr12	59897496	59905897	in_vitro	in_vivo	11	13	12.3854545	42.11	-29.724545	c(t = -2.72661	0.0127	0.90887448	ALDH3A1
chr12	48168900	48172636	in_vitro	in_vivo	13	13	12.3684615	34.9730769	-22.604615	c(t = -2.43317	0.0294	0.90887448	HIC1
chr6	54463759	54468361	in_vitro	in_vivo	12	11	12.2541667	25.8554545	-13.601288	c(t = -2.42308	0.0265	0.90887448	DKKL1
chr8	517290	523252	in_vitro	in_vivo	10	13	11.825	34.4084615	-22.583462	c(t = -2.43411	0.0268	0.90887448	ENSSSCG00000057151
chr15	136370530	136382553	in_vitro	in_vivo	12	15	11.6966667	29.7013333	-18.004667	c(t = -2.15469	0.0453	0.90887448	ACKR3
chr5	3275391	3282162	in_vitro	in_vivo	13	16	11.6761538	28.93125	-17.255096	c(t = -2.48340	0.0227	0.90887448	ENSSSCG00000035583
chr4	76724604	76734610	in_vitro	in_vivo	13	11	11.6515385	40.7163636	-29.064825	c(t = -2.32478	0.0377	0.90887448	RP1
chr10	39096782	39172198	in_vitro	in_vivo	15	16	11.1893333	25.434375	-14.245042	c(t = -2.48200	0.021	0.90887448	MKX
chr2	4233099	4293911	in_vitro	in_vivo	17	17	11.1229412	29.1576471	-18.034706	c(t = -2.84063	0.00971	0.90887448	CPT1A
chr11	18095974	18099819	in_vitro	in_vivo	9	12	10.7866667	40.8583333	-30.071667	c(t = -2.72767	0.0162	0.90887448	ARL11
chr1	224948963	224951669	in_vitro	in_vivo	11	10	10.2845455	45.024	-34.739455	c(t = -2.32989	0.0429	0.90887448	ENSSSCG00000049381
chr1	273230375	273294621	in_vitro	in_vivo	15	16	10.04	29.511875	-19.471875	c(t = -2.64000	0.0178	0.90887448	SARDH
chr13	205938348	206034976	in_vitro	in_vivo	15	17	10.0386667	27.3294118	-17.290745	c(t = -2.30006	0.0321	0.90887448	PDE9A
chr12	2447307	2455837	in_vitro	in_vivo	10	14	9.957	28.085	-18.128	c(t = -2.14578	0.0459	0.90887448	ENSSSCG00000017159
chr13	207940948	207982938	in_vitro	in_vivo	13	16	9.78230769	29.87375	-20.091442	c(t = -2.82004	0.0111	0.90887448	COL18A1
chr2	1603133	1610326	in_vitro	in_vivo	11	14	9.69636364	36.8092857	-27.112922	c(t = -2.47628	0.0243	0.90887448	ENSSSCG00000052617
chr6	94805640	94814337	in_vitro	in_vivo	11	13	9.52454545	30.7769231	-21.252378	c(t = -2.78615	0.0129	0.90887448	MYCBP
chr13	205669014	205672324	in_vitro	in_vivo	12	11	9.50583333	31.4827273	-21.976894	c(t = -2.09999	0.0496	0.90887448	TFF2
chr3	132384583	132409208	in_vitro	in_vivo	12	15	9.08916667	26.3833333	-17.294167	c(t = -2.23808	0.0396	0.90887448	TPO
chr2	1262079	1295887	in_vitro	in_vivo	13	14	8.95461538	25.6992857	-16.74467	c(t = -2.36977	0.0312	0.90887448	LSP1
chr3	132306447	132362841	in_vitro	in_vivo	13	15	8.57615385	25.6326667	-17.056513	c(t = -2.26065	0.0373	0.90887448	PXDN
chr5	17608637	17614227	in_vitro	in_vivo	11	13	8.47909091	30.9538462	-22.474755	c(t = -2.89459	0.0101	0.90887448	ENSSSCG00000052051
chr4	372660	378516	in_vitro	in_vivo	9	14	8.42555556	25.9157143	-17.490159	c(t = -2.56990	0.0208	0.90887448	SLC39A4
chr13	207119051	207132538	in_vitro	in_vivo	13	13	8.40769231	26.7423077	-18.334615	c(t = -2.53690	0.0245	0.90887448	DNMT3L
chr18	48972543	48981438	in_vitro	in_vivo	12	13	7.07833333	28.3584615	-21.280128	c(t = -2.50999	0.026	0.90887448	ENSSSCG00000059146
chr12	27093679	27100409	in_vitro	in_vivo	11	15	6.86090909	33.2213333	-26.360424	c(t = -3.10895	0.00619	0.90887448	WFIKKN2
chr2	1602943	1607931	in_vitro	in_vivo	11	14	6.72272727	34.8242857	-28.101558	c(t = -2.38714	0.0309	0.90887448	ENSSSCG00000057653
chr3	113478726	113483698	in_vitro	in_vivo	11	14	6.36272727	26.5207143	-20.157987	c(t = -3.02785	0.00725	0.90887448	ENSSSCG00000051648
chr14	141424468	141426136	in_vitro	in_vivo	12	11	5.99166667	26.0345455	-20.042879	c(t = -2.23126	0.0466	0.90887448	ENSSSCG00000059266
chr14	141016347	141022910	in_vitro	in_vivo	10	14	5.497	31.3185714	-25.821571	c(t = -3.26593	0.0054	0.90887448	ENSSSCG00000061990
chr2	33328253	33331593	in_vitro	in_vivo	12	10	5.14833333	29.721	-24.572667	c(t = -2.34438	0.0422	0.90887448	FIBIN
chr12	61188448	61193550	in_vitro	in_vivo	9	13	5.04222222	31.1830769	-26.140855	c(t = -2.26442	0.0396	0.90887448	ENSSSCG00000054406
chr6	2656336	2659383	in_vitro	in_vivo	9	11	2.97444444	31.0081818	-28.033737	c(t = -2.47250	0.0313	0.90887448	ENSSSCG00000062702
chr3	77554268	77560273	in_vitro	in_vivo	10	9	98.503	86.4322222	12.0707778	c(t = 3.12891	0.0118	0.90887448	ENSSSCG00000049149
chr6	169273019	169278454	in_vitro	in_vivo	12	11	97.1008333	91.8118182	5.28901515	c(t = 2.10797	0.0489	0.90887448	ENSSSCG00000041384
chr5	103776528	103782870	in_vitro	in_vivo	10	11	96.614	83.1054545	13.5085455	c(t = 2.88067	0.0132	0.90887448	ENSSSCG00000053082
chr2	986735	991848	in_vitro	in_vivo	9	10	96.1788889	81.835	14.3438889	c(t = 2.43192	0.0365	0.90887448	ENSSSCG00000057599
chr11	2332086	2380577	in_vitro	in_vivo	17	17	95.8376471	90.72	5.11764706	c(t = 2.45184	0.0209	0.90887448	ENSSSCG00000009281
chr11	77532266	77545913	in_vitro	in_vivo	14	16	95.5685714	84.896875	10.6716964	c(t = 2.17310	0.0433	0.90887448	TEX29
chr14	82051451	82067976	in_vitro	in_vivo	14	12	95.4478571	86.1283333	9.31952381	c(t = 2.49194	0.0258	0.90887448	SFTPD
chr14	49640586	49663003	in_vitro	in_vivo	12	16	94.9875	87.096875	7.890625	c(t = 2.54160	0.0175	0.90887448	GGT5
chr1	166664260	166675079	in_vitro	in_vivo	12	11	94.9108333	80.7063636	14.2044697	c(t = 2.87815	0.0126	0.90887448	ENSSSCG00000004963
chr14	43209311	43220837	in_vitro	in_vivo	11	11	94.8236364	84.9645455	9.85909091	c(t = 2.27711	0.0384	0.90887448	CRYBB2
chr14	19768776	19779256	in_vitro	in_vivo	14	14	94.6528571	82.7342857	11.9185714	c(t = 2.86257	0.0111	0.90887448	AADAT
chr2	18271070	18281431	in_vitro	in_vivo	11	11	94.3418182	82.0690909	12.2727273	c(t = 3.11003	0.00809	0.90887448	ACCSL
chr4	72573453	72694146	in_vitro	in_vivo	16	16	94.011875	89.128125	4.88375	c(t = 2.14191	0.0424	0.90887448	CHD7
chr7	95022924	95167107	in_vitro	in_vivo	17	17	93.6164706	90.1794118	3.43705882	c(t = 2.55696	0.0155	0.90887448	SIPA1L1
chr6	2561212	2586626	in_vitro	in_vivo	14	14	93.3528571	78.0185714	15.3342857	c(t = 2.74188	0.0147	0.90887448	MTHFSD
chr2	142011063	142077925	in_vitro	in_vivo	16	15	93.27375	85.8146667	7.45908333	c(t = 2.73765	0.0106	0.90887448	PFDN1
chrX	10217924	10283810	in_vitro	in_vivo	15	15	93.2726667	79.492	13.7806667	c(t = 2.73002	0.0123	0.90887448	EGFL6
chr1	160629223	160749506	in_vitro	in_vivo	12	13	93.2466667	81.9830769	11.2635897	c(t = 3.11476	0.005	0.90887448	ENSSSCG00000036234
chrX	14952225	15078855	in_vitro	in_vivo	16	15	93.18875	84.6686667	8.52008333	c(t = 2.53714	0.0206	0.90887448	CDKL5
chr17	11780129	11816049	in_vitro	in_vivo	13	15	93.1869231	84.9693333	8.21758974	c(t = 2.91271	0.0083	0.90887448	RNF170
chrX	56104509	56114744	in_vitro	in_vivo	12	13	93.1791667	76.5684615	16.6107051	c(t = 2.87802	0.0103	0.90887448	AWAT2
chr7	59175758	59188478	in_vitro	in_vivo	10	10	92.986	97.895	-4.909	c(t = -2.26976	0.0419	0.90887448	CYP11A1
chr2	137986828	138035578	in_vitro	in_vivo	15	15	92.9026667	88.946	3.95666667	c(t = 2.17424	0.0388	0.90887448	SLC25A48
chr6	455851	612868	in_vitro	in_vivo	16	15	92.718125	86.388	6.330125	c(t = 2.97525	0.00609	0.90887448	ANKRD11
chr7	74705409	74708790	in_vitro	in_vivo	9	14	92.5244444	83.0521429	9.47230159	c(t = 2.30893	0.0312	0.90887448	GZMB
chr13	133157321	133180262	in_vitro	in_vivo	14	15	92.49	79.2893333	13.2006667	c(t = 2.49131	0.0193	0.90887448	ENSSSCG00000029291
chr10	41282412	41462764	in_vitro	in_vivo	16	15	92.138125	86.8533333	5.28479167	c(t = 2.17709	0.0386	0.90887448	SVIL
chr10	2301520	2487093	in_vitro	in_vivo	15	15	91.8806667	78.5493333	13.3313333	c(t = 2.54097	0.0188	0.90887448	ENSSSCG00000050890
chr12	53271583	53281123	in_vitro	in_vivo	11	13	91.7309091	79.8469231	11.883986	c(t = 2.17039	0.0418	0.90887448	ALOX15B
chr2	150445126	150510002	in_vitro	in_vivo	15	17	91.304	85.8135294	5.49047059	c(t = 2.10549	0.0437	0.90887448	AFAP1L1
chr14	21083350	21163561	in_vitro	in_vivo	16	15	91.054375	82.12	8.934375	c(t = 2.38287	0.0256	0.90887448	DDX60
chr5	48649077	48766390	in_vitro	in_vivo	15	15	91.0026667	81.924	9.07866667	c(t = 3.07632	0.00515	0.90887448	ENSSSCG00000000563
chr3	40257685	40308089	in_vitro	in_vivo	14	16	90.9757143	79.17375	11.8019643	c(t = 2.60895	0.0149	0.90887448	CRAMP1
chr18	19711112	19741637	in_vitro	in_vivo	16	15	90.939375	81.528	9.411375	c(t = 2.48048	0.0204	0.90887448	KCP
chr3	5146479	5199273	in_vitro	in_vivo	15	16	90.9113333	80.013125	10.8982083	c(t = 2.71707	0.0122	0.90887448	ENSSSCG00000007589
chr5	5853377	5985169	in_vitro	in_vivo	16	17	90.836875	83.4905882	7.34628676	c(t = 2.45822	0.02	0.90887448	PACSIN2
chr1	189407490	189470541	in_vitro	in_vivo	9	12	90.8333333	78.7141667	12.1191667	c(t = 2.77387	0.0129	0.90887448	C14orf39
chr17	41047411	41078506	in_vitro	in_vivo	12	14	90.58	81.0264286	9.55357143	c(t = 2.57075	0.0176	0.90887448	TTI1
chr3	38763447	38781988	in_vitro	in_vivo	13	16	90.5792308	78.320625	12.2586058	c(t = 2.35257	0.0265	0.90887448	NAA60
chr6	27108597	27142177	in_vitro	in_vivo	16	14	90.161875	82.2314286	7.93044643	c(t = 2.67684	0.0127	0.90887448	CDH5
chr2	59166126	59193948	in_vitro	in_vivo	15	16	90.1466667	79.40625	10.7404167	c(t = 2.58249	0.0153	0.90887448	KLHL26
chr14	20572871	20617556	in_vitro	in_vivo	13	15	89.8492308	83.7686667	6.0805641	c(t = 2.13543	0.0425	0.90887448	ENSSSCG00000056141
chr15	121604166	121619063	in_vitro	in_vivo	12	14	89.4308333	78.7535714	10.6772619	c(t = 2.80869	0.0106	0.90887448	STK11IP
chr1	269209693	269273521	in_vitro	in_vivo	15	13	89.2073333	78.6953846	10.5119487	c(t = 2.62480	0.0148	0.90887448	LRRC8A
chr18	1542163	1653982	in_vitro	in_vivo	17	17	88.9870588	84.0088235	4.97823529	c(t = 2.14881	0.0395	0.90887448	UBE3C
chr18	46664376	47031582	in_vitro	in_vivo	17	17	88.94	84.7117647	4.22823529	c(t = 2.63445	0.0131	0.90887448	ENSSSCG00000061302
chr16	23509491	23606924	in_vitro	in_vivo	16	17	88.640625	80.0470588	8.59356618	c(t = 2.12245	0.0441	0.90887448	EGFLAM
chr13	29099738	29142879	in_vitro	in_vivo	13	16	88.5576923	78.726875	9.83081731	c(t = 3.24867	0.00311	0.90887448	ENSSSCG00000011317
chr16	71742090	71754884	in_vitro	in_vivo	12	13	88.4666667	77.4276923	11.0389744	c(t = 2.38896	0.0261	0.90887448	GM2A
chr6	147328418	147336903	in_vitro	in_vivo	9	9	88.14	95.68	-7.54	c(t = -2.45428	0.0293	0.90887448	ENSSSCG00000041959
chr16	69332587	69655380	in_vitro	in_vivo	16	16	88.12125	78.2525	9.86875	c(t = 2.33519	0.0311	0.90887448	GRIA1
chr4	119035133	119255877	in_vitro	in_vivo	16	16	87.69875	76.65625	11.0425	c(t = 2.85153	0.00798	0.90887448	SNX7

chr2	83518878	83618490	in_vitro	in_vivo	16	16	87.5975	77.7525	9.845	c(t = 2.18306	0.0393	0.90887448	ENSSSCG00000061538
chr2	138373189	138510728	in_vitro	in_vivo	16	16	87.581875	79.99625	7.585625	c(t = 2.24991	0.032	0.90887448	TRPC7
chr3	11252648	11279284	in_vitro	in_vivo	15	16	86.782	76.388125	10.393875	c(t = 2.11444	0.0451	0.90887448	LIMK1
chr8	114252416	114373427	in_vitro	in_vivo	15	17	86.7313333	75.4394118	11.2919216	c(t = 2.76463	0.00973	0.90887448	PAPSS1
chr4	98622494	98635271	in_vitro	in_vivo	9	14	86.6622222	75.1614286	11.5007937	c(t = 2.20676	0.0388	0.90887448	TARS2
chr2	76339470	76401965	in_vitro	in_vivo	17	17	86.3870588	76.4429412	9.94411765	c(t = 2.19039	0.0362	0.90887448	DOT1L
chr2	48745371	48930883	in_vitro	in_vivo	17	16	85.9135294	78.45125	7.46227941	c(t = 2.33736	0.0261	0.90887448	IRAG1
chr15	69059870	69555049	in_vitro	in_vivo	15	17	85.7093333	78.1588235	7.5505098	c(t = 2.46514	0.0212	0.90887448	KCNH7
chr14	40627730	40638493	in_vitro	in_vivo	14	11	84.7971429	96.0936364	-11.296494	c(t = -2.90201	0.0101	0.90887448	UNC119B
chr6	19753036	19825631	in_vitro	in_vivo	17	15	84.7311765	77.9186667	6.8125098	c(t = 2.18666	0.0382	0.90887448	ENSSSCG00000002811
chr5	86792450	87102623	in_vitro	in_vivo	16	17	84.586875	75.5441176	9.04275735	c(t = 2.78438	0.00933	0.90887448	CFAP54
chr11	52175808	52356741	in_vitro	in_vivo	16	16	84.4725	79.423125	5.049375	c(t = 2.34017	0.0264	0.90887448	ENSSSCG00000050522
chr6	155908424	156023632	in_vitro	in_vivo	17	15	84.4141176	75.472	8.94211765	c(t = 2.89138	0.0089	0.90887448	ENSSSCG00000041720
chrX	6939387	7019738	in_vitro	in_vivo	17	16	84.0505882	77.06125	6.98933824	c(t = 2.16491	0.0388	0.90887448	CLCN4
chr15	14088999	15046042	in_vitro	in_vivo	16	17	83.994375	77.1111765	6.88319853	c(t = 2.55246	0.0161	0.90887448	THSD7B
chr6	155908144	156311025	in_vitro	in_vivo	17	17	83.8888235	77.3358824	6.55294118	c(t = 3.02418	0.005	0.90887448	ENSSSCG00000043918
chrX	12710603	12818681	in_vitro	in_vivo	17	15	83.7882353	77.0366667	6.75156863	c(t = 2.11362	0.043	0.90887448	ENSSSCG00000051458
chr6	7661402	8013338	in_vitro	in_vivo	17	16	83.1523529	77.483125	5.66922794	c(t = 2.66113	0.0122	0.90887448	ENSSSCG00000042603
chr7	100777144	100902948	in_vitro	in_vivo	17	17	82.3829412	75.7047059	6.67823529	c(t = 2.09001	0.045	0.90887448	ADCK1
chr5	79788572	80067795	in_vitro	in_vivo	16	16	82.348125	75.755625	6.5925	c(t = 2.17438	0.0385	0.90887448	CHST11
chrX	56544560	56925779	in_vitro	in_vivo	17	17	82.3170588	75.9423529	6.37470588	c(t = 2.26235	0.0306	0.90887448	TEX11
chr2	149674448	149722865	in_vitro	in_vivo	16	15	81.744375	92.4393333	-10.694958	c(t = -2.15669	0.0439	0.90887448	FBXO38
chr8	1809785	1934024	in_vitro	in_vivo	14	17	79.795	87.0947059	-7.2997059	c(t = -2.21206	0.0369	0.90887448	HTT
chr5	7008719	7071025	in_vitro	in_vivo	17	15	79.7841176	86.8986667	-7.114549	c(t = -2.33322	0.0273	0.90887448	ACO2
chr4	29933135	30233960	in_vitro	in_vivo	15	15	79.642	88.1493333	-8.5073333	c(t = -2.69884	0.0129	0.90887448	ANGPT1
chr6	6654411	6881813	in_vitro	in_vivo	17	15	78.5511765	85.692	-7.1408235	c(t = -2.45843	0.0208	0.90887448	CMIP
chr8	66191841	66236143	in_vitro	in_vivo	11	13	77.5845455	90.6984615	-13.113916	c(t = -2.45756	0.0268	0.90887448	ENSSSCG00000034735
chr14	39695516	39806773	in_vitro	in_vivo	14	15	76.4692857	87.2306667	-10.761381	c(t = -2.14691	0.045	0.90887448	TMEM116
chr8	1388266	1523928	in_vitro	in_vivo	17	17	75.8882353	90.1452941	-14.257059	c(t = -2.24336	0.0386	0.90887448	FAM193A
chr3	40835413	40934253	in_vitro	in_vivo	15	16	75.4633333	88.86375	-13.400417	c(t = -3.15549	0.00604	0.90887448	LMF1
chr1	253656010	253657160	in_vitro	in_vivo	9	14	94.2633333	72.2435714	22.0197619	c(t = 3.20174	0.00671	0.90887448	ENSSSCG00000043950
chr15	32408511	32419636	in_vitro	in_vivo	12	11	93.8133333	68.4454545	25.3678788	c(t = 2.23416	0.0474	0.90887448	TDRP
chr7	36410749	36449010	in_vitro	in_vivo	14	13	90.5535714	74.2823077	16.2712637	c(t = 2.72863	0.0147	0.90887448	ENSSSCG00000001612
chr3	31461709	31469293	in_vitro	in_vivo	11	11	90.5381818	72.4909091	18.0472727	c(t = 3.09354	0.00585	0.90887448	ENSSSCG00000041169
chr2	103414746	103448260	in_vitro	in_vivo	11	10	89.8881818	62.802	27.0861818	c(t = 2.35365	0.0393	0.90887448	ENSSSCG00000046723
chr3	132667045	132684674	in_vitro	in_vivo	12	15	89.255	63.76	25.495	c(t = 3.41695	0.00227	0.90887448	ACP1
chr9	80678207	80866146	in_vitro	in_vivo	14	14	89.2442857	74.4992857	14.745	c(t = 2.23879	0.0402	0.90887448	PHF14
chr6	1817744	1823542	in_vitro	in_vivo	9	9	88.9355556	59.7255556	29.21	c(t = 3.07299	0.00905	0.90887448	ENSSSCG00000053834
chr6	1817744	1823542	in_vitro	in_vivo	9	9	88.9355556	59.7255556	29.21	c(t = 3.07299	0.00905	0.90887448	ENSSSCG00000053834
chrX	124952500	124960343	in_vitro	in_vivo	14	10	88.2414286	67.545	20.6964286	c(t = 2.46950	0.0265	0.90887448	ATP6AP1
chr9	22067266	22106722	in_vitro	in_vivo	9	10	88.1133333	68.69	19.4233333	c(t = 2.86546	0.0127	0.90887448	ENSSSCG00000052702
chr8	130889684	130945393	in_vitro	in_vivo	14	16	88.0271429	72.65	15.3771429	c(t = 2.64777	0.0139	0.90887448	ABCG2
chr1	184501182	184549993	in_vitro	in_vivo	14	11	87.6292857	66.6254545	21.0038312	c(t = 2.44033	0.0295	0.90887448	DLGAP5
chr6	45482371	45492627	in_vitro	in_vivo	9	14	87.4522222	59.1978571	28.2543651	c(t = 2.63947	0.0161	0.90887448	TBCB
chr6	61929587	61941939	in_vitro	in_vivo	13	15	87.3576923	65.756	21.6016923	c(t = 2.58845	0.0175	0.90887448	ZNF773
chr2	103255750	103378623	in_vitro	in_vivo	16	17	86.99875	73.1188235	13.8799265	c(t = 2.41441	0.0257	0.90887448	CAST
chr2	144092030	144097060	in_vitro	in_vivo	10	9	86.901	59.5166667	27.3843333	c(t = 2.45895	0.0353	0.90887448	ENSSSCG00000062276
chr4	93899019	93926320	in_vitro	in_vivo	14	16	86.5235714	71.25	15.2735714	c(t = 2.34641	0.0287	0.90887448	LMNA
chr18	31027056	31125465	in_vitro	in_vivo	14	15	86.4514286	74.1093333	12.3420952	c(t = 2.40356	0.026	0.90887448	MDFIC
chr3	31606214	31782894	in_vitro	in_vivo	16	17	86.4325	63.3182353	23.1142647	c(t = 4.25133	0.000221	0.90887448	ENSSSCG00000007899
chr15	79432667	79447568	in_vitro	in_vivo	10	14	86.413	68.4164286	17.9965714	c(t = 2.27311	0.0383	0.90887448	CDCA7
chr15	125669842	125720158	in_vitro	in_vivo	15	13	86.1853333	71.3423077	14.8430256	c(t = 2.26403	0.038	0.90887448	WDFY1
chrX	92223747	92335430	in_vitro	in_vivo	16	15	86.183125	74.0613333	12.1217917	c(t = 2.30274	0.0312	0.90887448	RTL4
chr12	38461830	38564306	in_vitro	in_vivo	15	17	85.9766667	73.9094118	12.0672549	c(t = 2.29141	0.0308	0.90887448	AATF
chr14	51029126	51102409	in_vitro	in_vivo	16	15	85.97	69.138	16.832	c(t = 2.79319	0.00915	0.90887448	HIRA
chr1	57660224	57826350	in_vitro	in_vivo	16	17	85.97	67.9188235	18.0511765	c(t = 2.67815	0.0133	0.90887448	MDN1
chr11	6538045	6604658	in_vitro	in_vivo	17	17	85.9282353	74.4376471	11.4905882	c(t = 2.60573	0.0142	0.90887448	SLC7A1
chr6	83823229	83857092	in_vitro	in_vivo	15	12	85.8586667	72.1708333	13.6878333	c(t = 2.32173	0.032	0.90887448	DHDDS
chr1	106933269	106963960	in_vitro	in_vivo	12	11	85.615	74.5545455	11.0604545	c(t = 2.45666	0.0249	0.90887448	ENSSSCG00000057874
chr7	100350460	100392332	in_vitro	in_vivo	15	17	85.5773333	64.55	21.0273333	c(t = 3.29727	0.00277	0.90887448	POMT2
chr9	26281103	26293538	in_vitro	in_vivo	11	11	85.5727273	63.5454545	22.0272727	c(t = 2.84010	0.0103	0.90887448	ENSSSCG00000041237
chr8	543291	568452	in_vitro	in_vivo	15	16	85.5326667	73.944375	11.5882917	c(t = 2.16286	0.0391	0.90887448	MAEA
chr7	23147478	23165689	in_vitro	in_vivo	13	12	85.3707692	69.385	15.9857692	c(t = 2.22086	0.0418	0.90887448	PPP1R10
chr3	31376510	31445632	in_vitro	in_vivo	15	17	85.2826667	68.5858824	16.6967843	c(t = 3.24305	0.00327	0.90887448	TXNDC11
chr5	17248070	17250958	in_vitro	in_vivo	12	11	85.2691667	65.9254545	19.3437121	c(t = 2.16194	0.0469	0.90887448	ENSSSCG00000000231
chr1	129968502	130009391	in_vitro	in_vivo	15	13	85.2553333	69.7761538	15.4791795	c(t = 2.16671	0.0416	0.90887448	NDUFAF1
chr17	7311007	7326119	in_vitro	in_vivo	10	11	85.069	62.4745455	22.5944545	c(t = 2.35570	0.0317	0.90887448	TRIML2
chrX	92582047	92647857	in_vitro	in_vivo	17	15	85.0205882	71.9513333	13.0692549	c(t = 2.07982	0.0463	0.90887448	AMOT
chr13	71374944	71427126	in_vitro	in_vivo	16	13	84.8275	68.7892308	16.0382692	c(t = 2.40185	0.0279	0.90887448	ACAD9
chr2	140263301	140283040	in_vitro	in_vivo	11	14	84.7572727	63.9378571	20.8194156	c(t = 2.12774	0.0454	0.90887448	GFRA3
chr10	60241016	60391642	in_vitro	in_vivo	15	17	84.6093333	72.0982353	12.511098	c(t = 2.43134	0.0239	0.90887448	USP6NL
chr1	11566245	11653871	in_vitro	in_vivo	16	16	84.593125	73.22375	11.369375	c(t = 2.79195	0.00903	0.90887448	TFB1M
chr1	18870884	19029417	in_vitro	in_vivo	16	15	84.535625	72.9986667	11.5369583	c(t = 2.77678	0.00966	0.90887448	ADGB
chr6	75251467	75279314	in_vitro	in_vivo	14	16	84.3792857	60.75875	23.6205357	c(t = 2.96472	0.00645	0.90887448	EPHA2
chr9	92037509	92071499	in_vitro	in_vivo	11	11	84.3236364	73.0945455	11.2290909	c(t = 2.26740	0.0347	0.90887448	GPNMB
chr4	18532041	18608602	in_vitro	in_vivo	13	16	84.1661538	69.80875	14.3574038	c(t = 2.83558	0.00866	0.90887448	MTBP
chr2	77945841	77957313	in_vitro	in_vivo	13	14	84.1423077	65.6328571	18.5094505	c(t = 2.20837	0.0385	0.90887448	PLPP2
chr4	124690317	124773887	in_vitro	in_vivo	15	15	84.074	70.4013333	13.6726667	c(t = 2.81844	0.00933	0.90887448	RPAP2
chr13	151156800	151298330	in_vitro	in_vivo	13	15	84.0153846	73.0926667	10.9227179	c(t = 2.06884	0.0494	0.90887448	HHLA2
chr3	1905656	1918227	in_vitro	in_vivo	15	16	84.0093333	68.378125	15.6312083	c(t = 2.78888	0.00924	0.90887448	BRAT1
chr1	238232867	238308625	in_vitro	in_vivo	17	15	83.9141176	63.42	20.4941176	c(t = 2.59227	0.0182	0.90887448	FBXO10
chr14	95530787	96386699	in_vitro	in_vivo	17	17	83.8376471	72.1976471	11.64	c(t = 2.26256	0.0345	0.90887448	ENSSSCG00000010426
chr2	37809008	38754525	in_vitro	in_vivo	17	17	83.8029412	74.8558824	8.94705882	c(t = 2.37791	0.0239	0.90887448	NELL1
chr12	34745236	34771167	in_vitro	in_vivo	14	16	83.8007143	71.483125	12.3175893	c(t = 2.15397	0.04	0.90887448	MTMR4
chr14	29095490	29249833	in_vitro	in_vivo	16	16	83.7875	72.445625	11.341875	c(t = 2.13881	0.0439	0.90887448	DNAH10
chr5	64065992	64105173	in_vitro	in_vivo	15	16	83.504	70.05875	13.44525	c(t = 2.38602	0.024	0.90887448	CHD4
chrX	43100032	43116227	in_vitro	in_vivo	15	15	83.4326667	65.1606667	18.272	c(t = 2.75971	0.0106	0.90887448	TFE3
chr3	41190830	41208653	in_vitro	in_vivo	12	13	83.3358333	67.2584615	16.0773718	c(t = 2.42940	0.0236	0.90887448	CAPN15
chr14	65408598	65598485	in_vitro	in_vivo	17	17	83.2105882	73.7958824	9.41470588	c(t = 2.37486	0.0237	0.90887448	ARID5B
chr5	376551	389330	in_vitro	in_vivo	10	15	83.145	65.274	17.871	c(t = 2.12015	0.0465	0.90887448	DENND6B
chr13	35910698	35948683	in_vitro	in_vivo	14	14	83.0478571	53.29	29.7578571	c(t = 3.53838	0.00174	0.90887448	CHDH
chr8	96405386	96608368	in_vitro	in_vivo	14	17	83.0314286	73.1805882	9.85084034	c(t = 2.15685	0.0396	0.90887448	ENSSSCG00000058289
chr16	27858157	27930361	in_vitro	in_vivo	14	15	83.0285714	65.8593333	17.1692381	c(t = 2.60029	0.0168	0.90887448	NIM1K
chr12	632363	651817	in_vitro	in_vivo	12	15	83.0216667	70.6286667	12.393	c(t = 2.35651	0.0266	0.90887448	NARF

chr14	91833844	92178913	in_vitro	in_vivo	16	16	82.99625	67.164375	15.831875	c(t = 3.10447	0.00496	0.90887448	ENSSSCG00000057641
chr1	87347418	87495090	in_vitro	in_vivo	15	13	82.9913333	68.4530769	14.5382564	c(t = 2.36172	0.0311	0.90887448	ENSSSCG00000053341
chr3	55950591	56171718	in_vitro	in_vivo	16	17	82.889375	74.6288235	8.26055147	c(t = 2.29518	0.0287	0.90887448	VWA3B
chr15	4096647	4171398	in_vitro	in_vivo	15	12	82.862	62.505	20.357	c(t = 2.56857	0.0179	0.90887448	ORC4
chr11	15997827	16034395	in_vitro	in_vivo	16	16	82.75625	74.510625	8.245625	c(t = 2.43298	0.0221	0.90887448	TMEM272
chr5	98697035	99229550	in_vitro	in_vivo	17	17	82.5941176	72.3664706	10.2276471	c(t = 2.45812	0.0212	0.90887448	TMTC2
chr13	204600735	204695316	in_vitro	in_vivo	15	17	82.4513333	58.9729412	23.4783922	c(t = 3.38967	0.00221	0.90887448	ENSSSCG00000012074
chr14	90207442	90236369	in_vitro	in_vivo	17	17	82.3817647	54.8247059	27.5570588	c(t = 3.45109	0.00214	0.90887448	OGDHL
chr17	32023091	32037880	in_vitro	in_vivo	14	14	82.3721429	58.045	24.3271429	c(t = 2.96096	0.00845	0.90887448	ADAM33
chrX	60186814	60313064	in_vitro	in_vivo	16	14	82.3425	66.1435714	16.1989286	c(t = 2.60437	0.0146	0.90887448	ABCB7
chr12	47845555	47856401	in_vitro	in_vivo	11	15	82.2718182	57.2753333	24.9964848	c(t = 2.77202	0.0119	0.90887448	SCARF1
chr6	83318411	83329639	in_vitro	in_vivo	12	13	82.23	55.4276923	26.8023077	c(t = 2.99165	0.00686	0.90887448	PAQR7
chr5	77009862	77058427	in_vitro	in_vivo	15	14	82.2073333	73.2292857	8.97804762	c(t = 2.11626	0.0439	0.90887448	SLC38A1
chr3	103566228	103615769	in_vitro	in_vivo	13	14	82.18	68.5507143	13.6292857	c(t = 2.17242	0.0426	0.90887448	FEZ2
chr14	77636149	77737654	in_vitro	in_vivo	16	14	82.09125	71.5742857	10.5169643	c(t = 2.47776	0.0203	0.90887448	SAMD8
chr6	87879830	87942014	in_vitro	in_vivo	15	14	82.042	66.7342857	15.3077143	c(t = 2.25205	0.0355	0.90887448	ZCCHC17
chr13	68758734	68787192	in_vitro	in_vivo	15	14	81.9013333	63.7607143	18.140619	c(t = 2.88887	0.00864	0.90887448	CAND2
chr7	52144506	52194133	in_vitro	in_vivo	16	16	81.779375	69.408125	12.37125	c(t = 2.61357	0.0147	0.90887448	FSD2
chr1	253559546	253570113	in_vitro	in_vivo	12	12	81.71	68.6441667	13.0658333	c(t = 2.17725	0.0436	0.90887448	SLC46A2
chr7	97174219	97195595	in_vitro	in_vivo	11	11	81.6009091	61.1609091	20.44	c(t = 2.70909	0.0163	0.90887448	PTGR2
chr7	68318417	68422394	in_vitro	in_vivo	14	14	81.2457143	56.1928571	25.0528571	c(t = 3.50283	0.00187	0.90887448	HECTD1
chr17	50631092	50732243	in_vitro	in_vivo	14	17	81.2207143	69.6129412	11.6077731	c(t = 2.43730	0.0214	0.90887448	ARFGEF2
chr6	155387145	155453438	in_vitro	in_vivo	15	15	81.0133333	71.318	9.69533333	c(t = 2.27311	0.0312	0.90887448	C8B
chr18	51387836	51802945	in_vitro	in_vivo	17	17	80.8458824	73.2411765	7.60470588	c(t = 2.37058	0.0245	0.90887448	HECW1
chr3	103112058	103230551	in_vitro	in_vivo	15	16	80.8233333	66.515625	14.3077083	c(t = 2.38175	0.024	0.90887448	HEATR5B
chr9	36620658	36759552	in_vitro	in_vivo	13	17	80.8146154	65.2558824	15.558733	c(t = 2.45274	0.0207	0.90887448	ATM
chr11	70943141	71009627	in_vitro	in_vivo	17	17	80.7058824	63.7529412	16.9529412	c(t = 2.24914	0.0325	0.90887448	TPP2
chr14	74120050	74205263	in_vitro	in_vivo	16	15	80.613125	64.1826667	16.4304583	c(t = 2.35037	0.0273	0.90887448	UNC5B
chr16	22599936	22895796	in_vitro	in_vivo	16	17	80.60625	69.1705882	11.4356618	c(t = 2.25497	0.0331	0.90887448	WDR70
chr3	98005812	98155846	in_vitro	in_vivo	16	13	80.541875	65.6007692	14.9411058	c(t = 2.28310	0.0317	0.90887448	EML4
chr2	66329045	66348056	in_vitro	in_vivo	15	16	80.4466667	63.15875	17.2879167	c(t = 2.25418	0.0328	0.90887448	MAN2B1
chr6	4099414	4147469	in_vitro	in_vivo	14	15	80.4328571	69.8393333	10.5935238	c(t = 2.69052	0.0136	0.90887448	COTL1
chrX	25441980	25544855	in_vitro	in_vivo	12	13	80.405	62.1838462	18.2211538	c(t = 2.80301	0.0104	0.90887448	ENSSSCG00000054121
chrX	27028231	28383840	in_vitro	in_vivo	17	17	80.3105882	71.1188235	9.19176471	c(t = 2.29122	0.0287	0.90887448	DMD
chr9	1936953	1951889	in_vitro	in_vivo	13	14	80.2130769	49.5707143	30.6423626	c(t = 3.81609	0.000808	0.90887448	OVCH2
chr11	55640568	55696261	in_vitro	in_vivo	14	12	80.1328571	64.16	15.9728571	c(t = 2.41165	0.0245	0.90887448	ENSSSCG00000042996
chr12	18915422	18919757	in_vitro	in_vivo	9	13	79.9155556	45.5761538	34.3394017	c(t = 2.88838	0.00921	0.90887448	SLC25A39
chr17	31548636	31591975	in_vitro	in_vivo	14	16	79.7264286	64.219375	15.5070536	c(t = 2.09683	0.0467	0.90887448	SMOX
chr3	32450604	32492721	in_vitro	in_vivo	14	16	79.6542857	58.635	21.0192857	c(t = 3.57639	0.00136	0.90887448	EMP2
chr4	100460304	100478501	in_vitro	in_vivo	15	14	79.6433333	68.0807143	11.562619	c(t = 2.51944	0.018	0.90887448	ENSSSCG00000060248
chr17	34807582	34816451	in_vitro	in_vivo	11	11	79.51	57.5418182	21.9681818	c(t = 2.30041	0.034	0.90887448	TRIB3
chrX	24409327	25806680	in_vitro	in_vivo	17	17	79.44	69.2394118	10.2005882	c(t = 2.23194	0.0348	0.90887448	IL1RAPL1
chr1	165458258	165740297	in_vitro	in_vivo	17	17	79.4005882	63.1011765	16.2994118	c(t = 2.78921	0.0114	0.90887448	MAP2K5
chr2	79766150	80141293	in_vitro	in_vivo	17	17	79.3817647	68.6341176	10.7476471	c(t = 2.07545	0.0497	0.90887448	COL23A1
chr6	30516948	30572591	in_vitro	in_vivo	12	13	79.1975	54.1830769	25.0144231	c(t = 2.51235	0.0205	0.90887448	ENSSSCG00000047270
chr9	56934112	56940461	in_vitro	in_vivo	9	10	79.1822222	65.233	13.9492222	c(t = 2.31814	0.0332	0.90887448	ENSSSCG00000058826
chr1	190735881	191031684	in_vitro	in_vivo	16	17	79.140625	67.9252941	11.2153309	c(t = 2.94173	0.00613	0.90887448	SYT16
chr12	61366741	61376873	in_vitro	in_vivo	12	13	79.0541667	49.2738462	29.7803205	c(t = 3.06179	0.00565	0.90887448	NATD1
chrX	47256505	47284520	in_vitro	in_vivo	15	11	78.774	64.45	14.324	c(t = 2.22171	0.0375	0.90887448	GNL3L
chr4	928798	940679	in_vitro	in_vivo	13	14	78.69	60.7257143	17.9642857	c(t = 2.30633	0.03	0.90887448	ZNF623
chr9	133164440	133185628	in_vitro	in_vivo	12	13	78.6566667	59.5161538	19.1405128	c(t = 2.16796	0.0411	0.90887448	IRF6
chr3	55560118	55666193	in_vitro	in_vivo	15	16	78.636	51.40125	27.23475	c(t = 3.27093	0.00278	0.90887448	MGAT4A
chr11	18040011	18072633	in_vitro	in_vivo	13	16	78.6046154	62.3125	16.2921154	c(t = 2.42625	0.0222	0.90887448	EBPL
chr1	236919879	236943823	in_vitro	in_vivo	15	16	78.3593333	58.770625	19.5887083	c(t = 2.52469	0.0177	0.90887448	GLIPR2
chr10	15317581	15359839	in_vitro	in_vivo	12	13	78.2075	51.2761538	26.9313462	c(t = 2.79848	0.0108	0.90887448	EXO1
chr12	37962943	37972509	in_vitro	in_vivo	13	14	78.19	56.2392857	21.9507143	c(t = 2.88540	0.00808	0.90887448	CA4
chr9	65833054	65891999	in_vitro	in_vivo	12	15	78.0541667	64.396	13.6581667	c(t = 2.08406	0.0475	0.90887448	DSTYK
chr14	129381208	129423702	in_vitro	in_vivo	15	15	78.022	66.8766667	11.1453333	c(t = 2.27339	0.0309	0.90887448	RGS10
chr1	63638596	63700476	in_vitro	in_vivo	13	13	77.8607692	56.3692308	21.4915385	c(t = 2.36518	0.0283	0.90887448	UFL1
chr1	166925031	167002317	in_vitro	in_vivo	15	15	77.7246667	66.8606667	10.864	c(t = 2.28863	0.03	0.90887448	ENSSSCG00000040442
chr6	42820568	42891346	in_vitro	in_vivo	16	14	77.628125	65.9921429	11.6359821	c(t = 2.07777	0.0479	0.90887448	RHPN2
chr11	7110533	7144050	in_vitro	in_vivo	14	16	77.6071429	63.201875	14.4052679	c(t = 2.23141	0.034	0.90887448	ENSSSCG00000043013
chr6	67463374	67529930	in_vitro	in_vivo	16	16	77.6025	65.061875	12.540625	c(t = 2.32048	0.0289	0.90887448	DNAJC11
chr4	41821768	41878690	in_vitro	in_vivo	12	14	77.5108333	64.305	13.2058333	c(t = 2.12013	0.0445	0.90887448	INTS8
chr2	59637371	59652073	in_vitro	in_vivo	15	12	77.2986667	56.6458333	20.6528333	c(t = 2.11081	0.0471	0.90887448	PIK3R2
chr10	64885870	64908963	in_vitro	in_vivo	13	16	77.2830769	57.205	20.0780769	c(t = 2.37812	0.0262	0.90887448	RBM17
chr5	8922665	8929767	in_vitro	in_vivo	12	13	77.1616667	56.4407692	20.7208974	c(t = 2.26944	0.0332	0.90887448	RPL3
chr14	73090944	73108156	in_vitro	in_vivo	11	12	77.1090909	50.09	27.0190909	c(t = 2.71103	0.0139	0.90887448	SAR1A
chr2	29305417	29365184	in_vitro	in_vivo	13	10	77.0669231	53.294	23.7729231	c(t = 2.43263	0.0291	0.90887448	DNAJC24
chr14	106285173	106612455	in_vitro	in_vivo	16	16	77.014375	65.553125	11.46125	c(t = 2.22004	0.0342	0.90887448	CYP2C49
chr13	82752458	82937668	in_vitro	in_vivo	15	17	77.008	63.0735294	13.9344706	c(t = 3.00921	0.00533	0.90887448	TFDP2
chr14	9817002	10025164	in_vitro	in_vivo	16	17	76.88875	64.0405882	12.8481618	c(t = 2.72485	0.0105	0.90887448	EBF2
chr10	19758757	19820246	in_vitro	in_vivo	15	17	76.8266667	60.8405882	15.9860784	c(t = 2.29572	0.029	0.90887448	CAPN2
chr1	265577704	265604502	in_vitro	in_vivo	15	15	76.7573333	57.1813333	19.576	c(t = 2.16922	0.0391	0.90887448	OLFML2A
chr1	237111012	237235485	in_vitro	in_vivo	16	15	76.560625	59.708	16.852625	c(t = 2.54124	0.0178	0.90887448	RNF38
chr7	26561359	26793724	in_vitro	in_vivo	16	16	76.55	66.40625	10.14375	c(t = 2.22133	0.0381	0.90887448	MLIP
chr6	128776430	128836951	in_vitro	in_vivo	15	15	76.4433333	55.7786667	20.6646667	c(t = 2.99598	0.00579	0.90887448	MCOLN2
chr2	11177412	11193661	in_vitro	in_vivo	14	13	76.3792857	55.8184615	20.5608242	c(t = 2.56253	0.0178	0.90887448	MS4A12
chr17	36300087	36324918	in_vitro	in_vivo	15	15	76.254	52.0933333	24.1606667	c(t = 2.65439	0.0136	0.90887448	COMMD7
chr3	96872157	96915327	in_vitro	in_vivo	13	12	76.0953846	54.6116667	21.4837179	c(t = 2.67509	0.0139	0.90887448	ENSSSCG00000008459
chr4	118947440	119065184	in_vitro	in_vivo	16	17	76.04375	59.6658824	16.3778676	c(t = 2.77400	0.00938	0.90887448	PLPPR5
chr9	1291661	1297972	in_vitro	in_vivo	11	16	75.9854545	48.845	27.1404545	c(t = 2.91301	0.00743	0.90887448	EIF3F
chrY	2945460	3310365	in_vitro	in_vivo	15	14	75.7166667	61.7192857	13.997381	c(t = 2.21329	0.0355	0.90887448	ENSSSCG00000054294
chr12	5940647	6010835	in_vitro	in_vivo	16	16	75.708125	60.355	15.353125	c(t = 2.21303	0.0348	0.90887448	GRB2
chr15	66254948	66571077	in_vitro	in_vivo	15	17	75.5973333	59.6647059	15.9326275	c(t = 2.55526	0.0168	0.90887448	BAZ2B
chr6	69385879	69401151	in_vitro	in_vivo	14	14	75.5785714	55.63	19.9485714	c(t = 2.50563	0.019	0.90887448	ENO1
chr14	51298845	51302954	in_vitro	in_vivo	10	9	75.563	44.3433333	31.2196667	c(t = 2.56457	0.0205	0.90887448	ENSSSCG00000061046
chr9	85422317	85710750	in_vitro	in_vivo	16	16	75.47	60.615625	14.854375	c(t = 2.40897	0.0224	0.90887448	CRPPA
chr6	2003704	2190874	in_vitro	in_vivo	16	15	75.280625	60.68	14.600625	c(t = 3.56819	0.00129	0.90887448	ENSSSCG00000002657
chr12	59431513	59449672	in_vitro	in_vivo	13	16	75.2246154	57.31625	17.9083654	c(t = 2.38043	0.0251	0.90887448	ADORA2B
chr3	96709059	96833410	in_vitro	in_vivo	16	14	75.053125	59.6078571	15.4452679	c(t = 2.23416	0.0337	0.90887448	PLEKHH2
chr1	163894312	164275442	in_vitro	in_vivo	17	16	75.0370588	68.73625	6.30080882	c(t = 3.14857	0.00369	0.90887448	MEGF11
