# Supplementary material for: Deciphering differences in DNA methylation and transcriptome profiles of oocytes from pigs with high and low developmental competence
Source: Environ Epigenet. 2025 Jun 3;11(1):dvaf018. doi: 10.1093/eep/dvaf018 (PMC12418950; doi:10.1093/eep/dvaf018)
Supplement: dvaf018_Supplemental_Files [file dvaf018_supplemental_files.zip › Sup table 11.pdf]

	chr	start	end	group1	group2	n1	n2	estimate1	estimate2	estimate	statistic	p-value	adj.p.value	name
Promoter	chr6	17231858	17235858	in_vitro	in_vivo	10	11	8.936	2.10454546	6.83145455	c(t = 2.45606	0.0321	0.89488417	NOB1
Promoter	chr2	1673360	1677360	in_vitro	in_vivo	9	10	7.40555556	24.856	-17.450444	c(t = -2.2545[illegible]	0.0411	0.89488417	KCNQ1
Transcript	chr9	8689481	8761709	in_vitro	in_vivo	13	14	73.4638462	53.9957143	19.4681319	c(t = 2.48592[illegible]	0.0214	0.90887448	PGM2L1
Transcript	chr6	17233858	17245451	in_vitro	in_vivo	13	13	70.6	50.3146154	20.2853846	c(t = 2.15319[illegible]	0.0416	0.90887448	NOB1
