# Supplementary material for: Deciphering differences in DNA methylation and transcriptome profiles of oocytes from pigs with high and low developmental competence
Source: Environ Epigenet. 2025 Jun 3;11(1):dvaf018. doi: 10.1093/eep/dvaf018 (PMC12418950; doi:10.1093/eep/dvaf018)
Supplement: dvaf018_Supplemental_Files [file dvaf018_supplemental_files.zip › Sup table 12.pdf]

	chr	start	end	group1	group2	n1	n2	estimate1	estimate2	estimate	statistic	p	adj.p.value	name
Promoters	chr7	9733660	9737660	in_vitro	in_vivo	9	9	89.6544444	98.46555556	-8.811111111	c(t = -2.43423584200277)	0.0367	0.89488417	GFOD1
Promoters	chr14	140430596	140434596	in_vitro	in_vivo	9	9	16.8188889	50.58444444	-33.76555556	c(t = -2.5586157678357)	0.0226	0.89488417	U6
Promoters	chr2	1673360	1677360	in_vitro	in_vivo	9	10	7.40555556	24.856	-17.45044444	c(t = -2.25452231583328)	0.0411	0.89488417	KCNQ1
Promoters	chr10	63875586	63879586	in_vitro	in_vivo	11	11	0.31909091	5.490909091	-5.171818182	c(t = -2.69390117824639)	0.0216	0.89488417	SFMBT2
Transcripts	chr13	207940948	207982938	in_vitro	in_vivo	13	16	9.78230769	29.87375	-20.09144231	c(t = -2.82004604981061)	0.0111	0.90887448	COL18A1
Transcripts	chr15	139766414	139811999	in_vitro	in_vivo	14	16	14.8028571	32.96375	-18.16089286	c(t = -2.10617013968425)	0.0477	0.90887448	CROCC2
Transcripts	chr2	4069280	4105042	in_vitro	in_vivo	15	16	20.3486667	39.4575	-19.10883333	c(t = -2.12457037342831)	0.0439	0.90887448	TPCN2
Transcripts	chr13	208094877	208259369	in_vitro	in_vivo	15	17	25.5986667	38.96235294	-13.36368627	c(t = -2.10186538519328)	0.0448	0.90887448	PCBP3
Transcripts	chr15	131655663	131673693	in_vitro	in_vivo	13	12	29.6276923	55.78333333	-26.15564103	c(t = -2.38769215966156)	0.0263	0.90887448	GPR55
Transcripts	chr12	1239834	1245181	in_vitro	in_vivo	9	11	30.65	59.18363636	-28.53363636	c(t = -3.1214991585093)	0.00683	0.90887448	TSPAN10
Transcripts	chr18	3148483	4054118	in_vitro	in_vivo	17	17	38.8870588	49.89352941	-11.00647059	c(t = -2.25246427752213)	0.0313	0.90887448	DPP6
Transcripts	chr2	142460178	142688089	in_vitro	in_vivo	17	16	46.4629412	38.019375	8.443566176	c(t = 2.25198234489141)	0.0317	0.90887448	PCDHAC2
Transcripts	chr4	928798	940679	in_vitro	in_vivo	13	14	78.69	60.72571429	17.96428571	c(t = 2.3063313515242)	0.03	0.90887448	ZNF623
Transcripts	chr17	34807582	34816451	in_vitro	in_vivo	11	11	79.51	57.54181818	21.96818182	c(t = 2.3004137338063)	0.034	0.90887448	TRIB3
Transcripts	chr15	79432667	79447568	in_vitro	in_vivo	10	14	86.413	68.41642857	17.99657143	c(t = 2.27311834195357)	0.0383	0.90887448	CDCA7
Transcripts	chr18	1542163	1653982	in_vitro	in_vivo	17	17	88.9870588	84.00882353	4.978235294	c(t = 2.14881315937008)	0.0395	0.90887448	UBE3C
