# Supplementary material for: Deciphering differences in DNA methylation and transcriptome profiles of oocytes from pigs with high and low developmental competence
Source: Environ Epigenet. 2025 Jun 3;11(1):dvaf018. doi: 10.1093/eep/dvaf018 (PMC12418950; doi:10.1093/eep/dvaf018)
Supplement: dvaf018_Supplemental_Files [file dvaf018_supplemental_files.zip › Sup table 13.pdf]

	source	term_name	term_id	adjusted_p_value	negative_log10_of_adjusted_p_value	term_size	query_size	intersection_size	effective_domain_size	intersections
Hyper in vitro	GO:MF	transferase activity	GO:0016740	0.001528058	2.815860125	2171	9	7	18002	1.00624E+62
Hypo in vitro	GO:MF	amino acid binding	GO:0016597	0.005334885	2.272874934	37	25	3	18002	3.97181E+20
Hypo in vitro	GO:CC	lysosomal membrane	GO:0005765	0.021889263	1.659768858	170	30	4	20315	1.00621E+35
Hypo in vitro	GO:CC	lytic vacuole membrane	GO:0098852	0.021889263	1.659768858	170	30	4	20315	1.00621E+35
