# Supplementary material for: Deciphering differences in DNA methylation and transcriptome profiles of oocytes from pigs with high and low developmental competence
Source: Environ Epigenet. 2025 Jun 3;11(1):dvaf018. doi: 10.1093/eep/dvaf018 (PMC12418950; doi:10.1093/eep/dvaf018)
Supplement: dvaf018_Supplemental_Files [file dvaf018_supplemental_files.zip › Sup table 14.pdf]

	source	term_name	term_id	adjusted_p_value	negative_log10_of_adjusted_p_value	term_size	query_size	intersection_size	effective_domain_size	intersections
Hypo in vitro	GO:MF	antioxidant activity	GO:0016209	0.034506784	1.462095518	68	59	4	18002	1.00516E+29
Hyper in vitro	GO:CC	cytoplasm	GO:0005737	0.001960768	2.70757375	9163	105	70	20315	1.00514E+33
Hyper in vitro	GO:CC	nucleoplasm	GO:0005654	0.047283674	1.325288786	3109	105	31	20315	1.01E+27
