# Supplementary material for: Deciphering differences in DNA methylation and transcriptome profiles of oocytes from pigs with high and low developmental competence
Source: Environ Epigenet. 2025 Jun 3;11(1):dvaf018. doi: 10.1093/eep/dvaf018 (PMC12418950; doi:10.1093/eep/dvaf018)
Supplement: dvaf018_Supplemental_Files [file dvaf018_supplemental_files.zip › Sup table 15.pdf]

Gene	factors	features	factors	pvalues	log2FC
ENSSSCG00000063504	1	ENSSSCG00000063504	1	0.030	2.047
ADGRL2	1	ADGRL2	1	0.002	1.742
F2R	1	F2R	1	0.001	1.712
ALG6	1	ALG6	1	0.000	1.691
ENSSSCG00000047016	1	ENSSSCG00000047016	1	0.012	1.647
ENSSSCG00000062132	1	ENSSSCG00000062132	1	0.000	1.630
CCDC179	1	CCDC179	1	0.026	1.564
PLGRKT	1	PLGRKT	1	0.002	1.560
SLCO6A1	1	SLCO6A1	1	0.013	1.520
UBE2D1	1	UBE2D1	1	0.006	1.516
ENSSSCG00000011905	1	ENSSSCG00000011905	1	0.002	1.483
ANO5	1	ANO5	1	0.018	1.421
ABHD3	1	ABHD3	1	0.000	1.365
ENSSSCG00000061888	1	ENSSSCG00000061888	1	0.026	1.350
POLE2	1	POLE2	1	0.009	1.337
C5orf24	1	C5orf24	1	0.013	1.313
COL19A1	1	COL19A1	1	0.003	1.273
ENSSSCG00000055582	1	ENSSSCG00000055582	1	0.037	1.273
ZNF383	1	ZNF383	1	0.000	1.271
ENSSSCG00000055001	1	ENSSSCG00000055001	1	0.015	1.265
ZNF770	1	ZNF770	1	0.005	1.246
TMEM38B	1	TMEM38B	1	0.00030028	1.2330641
LRRC34	1	LRRC34	1	0.00071066	1.22610331
GSN	1	GSN	1	0.02071916	1.1854482
DPY19L3	1	DPY19L3	1	0.00693574	1.17706699
GLCE	1	GLCE	1	1.73E-06	1.16678432
ZSCAN30	1	ZSCAN30	1	1.56E-05	1.16308644
RANBP6	1	RANBP6	1	0.00128303	1.14751746
ENSSSCG00000031854	1	ENSSSCG00000031854	1	0.00693574	1.14749926
RFK	1	RFK	1	0.00071066	1.12988633
ENSSSCG00000003792	1	ENSSSCG00000003792	1	0.026429	1.12135498
ENSSSCG00000011549	1	ENSSSCG00000011549	1	0.03334006	1.11878877
HMGN3	1	HMGN3	1	2.90E-05	1.11172896
SDHAF4	1	SDHAF4	1	0.00023867	1.10988507
GADD45A	1	GADD45A	1	0.02972401	1.10084554
GART	1	GART	1	0.02343317	1.06048522
DACT1	1	DACT1	1	0.00057789	1.05246363
AFG1L	1	AFG1L	1	6.90E-07	1.05098525
IER3IP1	1	IER3IP1	1	0.04298759	1.04177786
TMEM216	1	TMEM216	1	0.0160561	1.01954082
CEP78	1	CEP78	1	0.02404913	1.01562857
PHYHIPL	1	PHYHIPL	1	0.02972401	1.0079556
BMAL1	1	BMAL1	1	0.00105868	0.99422665
ELP4	1	ELP4	1	0.00370211	0.99022226
ZNF567	1	ZNF567	1	0.03729477	0.98667578
COMMD10	1	COMMD10	1	0.00184364	0.97095947
ZNF146	1	ZNF146	1	0.04630508	0.95665731
ZNF614	1	ZNF614	1	0.026429	0.95506219
HLTF	1	HLTF	1	0.01826556	0.94650149
ZNF618	1	ZNF618	1	0.00154718	0.92884654
DTWD1	1	DTWD1	1	0.02071916	0.92749021
ENSSSCG00000034174	1	ENSSSCG00000034174	1	0.00510422	0.91410643
MINDY2	1	MINDY2	1	0.04630508	0.91204392
CRYZ	1	CRYZ	1	0.00596018	0.90655488
ENSSSCG00000061001	1	ENSSSCG00000061001	1	0.0160561	0.90074131
NMD3	1	NMD3	1	0.00596018	0.89515528
TOR1B	1	TOR1B	1	0.00046721	0.89021274
MAN1A1	1	MAN1A1	1	0.01070637	0.88636979
SLC25A16	1	SLC25A16	1	0.00382109	0.86554365
ALAS1	1	ALAS1	1	0.00086971	0.86502558
PTPRK	1	PTPRK	1	0.01162462	0.86201771
DNAJC24	1	DNAJC24	1	2.59E-06	0.86177334
ENSSSCG00000004623	1	ENSSSCG00000004623	1	0.02141819	0.86028912
APIP	1	APIP	1	0.01013511	0.85948127
TBX18	1	TBX18	1	0.00435484	0.85707965
PBX3	1	PBX3	1	0.00804229	0.85671986
MORC1	1	MORC1	1	0.0447536	0.85481138
THOC7	1	THOC7	1	0.00804229	0.84505932
CACTIN	1	CACTIN	1	0.00185787	0.83235093
RARS2	1	RARS2	1	0.00071066	0.82651357
CNTN4	1	CNTN4	1	0.00929494	0.82441857
ENSSSCG00000053039	1	ENSSSCG00000053039	1	0.00264406	0.81414714
SOS2	1	SOS2	1	0.04161078	0.81388641
A1CF	1	A1CF	1	0.00154718	0.81325287
SLC17A5	1	SLC17A5	1	0.00046721	0.8077562
KDM3B	1	KDM3B	1	0.00023867	0.79951298
GPR160	1	GPR160	1	0.00049357	0.79755328
MAP3K7	1	MAP3K7	1	0.01070637	0.79562311
CLP1	1	CLP1	1	0.02678167	0.79126039
SCN5A	1	SCN5A	1	0.02343317	0.7793444
TYMS	1	TYMS	1	0.01528754	0.77514051
CFAP95	1	CFAP95	1	0.026429	0.75205885
AMFR	1	AMFR	1	0.00154718	0.74635386
ENSSSCG00000023596	1	ENSSSCG00000023596	1	0.04161078	0.74565271
ENSSSCG00000056241	1	ENSSSCG00000056241	1	0.01407064	0.74144857
GNPNAT1	1	GNPNAT1	1	0.0160561	0.73633543
CEP76	1	CEP76	1	2.90E-05	0.73428792
HAS1	1	HAS1	1	0.00128303	0.72988329
ENSSSCG00000014071	1	ENSSSCG00000014071	1	0.00023867	0.72662221
GOLIM4	1	GOLIM4	1	0.00929494	0.72326881
DPP8	1	DPP8	1	0.01229347	0.72278841
CPNE4	1	CPNE4	1	0.00057789	0.72181781
RFC4	1	RFC4	1	0.00128303	0.7152067
UTRN	1	UTRN	1	1.12E-05	0.71494477
ENSSSCG00000040389	1	ENSSSCG00000040389	1	0.04630508	0.71020809
PRKCI	1	PRKCI	1	0.00264406	0.70857958
GNB4	1	GNB4	1	4.03E-07	0.70711296
IRAK2	1	IRAK2	1	5.18E-05	0.70526828
KIF18A	1	KIF18A	1	0.02972401	0.70202827
PPP1CC	1	PPP1CC	1	0.00154718	0.69727313

Gene	factors	features	factors	pvalues	log2FC
RPS14	2	RPS14	2	0.000	4.771
ENSSSCG00000012088	2	ENSSSCG0000	2	0.000	4.543
NPAS1	2	NPAS1	2	0.000	4.471
DYNC2I2	2	DYNC2I2	2	0.026	4.379
AP2S1	2	AP2S1	2	0.015	4.298
SYCN	2	SYCN	2	0.000	4.288
TNNT1	2	TNNT1	2	0.000	4.175
SRM	2	SRM	2	0.002	4.135
RPS5	2	RPS5	2	0.000	4.082
ENSSSCG00000025928	2	ENSSSCG0000	2	0.000	3.978
ENSSSCG00000032060	2	ENSSSCG0000	2	0.000	3.944
RBP2	2	RBP2	2	0.001	3.890
CSTB	2	CSTB	2	0.001	3.878
TMEM200C	2	TMEM200C	2	0.030	3.866
RPS12	2	RPS12	2	0.003	3.737
ENSSSCG00000002995	2	ENSSSCG0000	2	0.000	3.632
RPS9	2	RPS9	2	0.000	3.615
SMPD2	2	SMPD2	2	0.002	3.593
PKN1	2	PKN1	2	0.000	3.589
COMMD9	2	COMMD9	2	0.000	3.588
SNX22	2	SNX22	2	0.00894218	3.55092969
MATCAP1	2	MATCAP1	2	0.00024208	3.48171692
TMEM147	2	TMEM147	2	5.75E-08	3.44825234
EIF3G	2	EIF3G	2	0.00023303	3.43120039
RPL35	2	RPL35	2	0.00264406	3.40209809
ENSSSCG00000027573	2	ENSSSCG0000	2	4.03E-07	3.34357831
CYTH2	2	CYTH2	2	0.00105868	3.34330574
ENSSSCG00000033019	2	ENSSSCG0000	2	0.00111629	3.29599
GPN2	2	GPN2	2	0.00849214	3.277081
ENSSSCG00000013768	2	ENSSSCG0000	2	0.00046721	3.26240639
WDR5	2	WDR5	2	3.02E-05	3.24733545
MAST2	2	MAST2	2	2.30E-07	3.24704551
ATP5IF1	2	ATP5IF1	2	2.30E-07	3.23956612
NUDC	2	NUDC	2	3.89E-05	3.23639473
SMIM12	2	SMIM12	2	7.84E-06	3.23153256
RPS29	2	RPS29	2	4.03E-07	3.22951931
BCKDHA	2	BCKDHA	2	0.03729477	3.22148292
LRSAM1	2	LRSAM1	2	0.00222085	3.21240318
ENSSSCG00000052208	2	ENSSSCG0000	2	0.03902612	3.20933173
ACAD9	2	ACAD9	2	0.00348602	3.20330354
ALAD	2	ALAD	2	0.00030028	3.17663297
ENSSSCG00000003930	2	ENSSSCG0000	2	2.59E-06	3.13955819
ITGB5	2	ITGB5	2	0.00185787	3.13718212
ETHE1	2	ETHE1	2	4.03E-07	3.1355136
RPS16	2	RPS16	2	3.85E-06	3.13139081
ENSSSCG00000058091	2	ENSSSCG0000	2	0.03380196	3.12871412
RDH13	2	RDH13	2	2.14E-05	3.11748193
NANS	2	NANS	2	0.00596018	3.09856238
ENSSSCG00000052671	2	ENSSSCG0000	2	0.01780316	3.08458949
COX17	2	COX17	2	2.30E-07	3.08165817
TEX264	2	TEX264	2	3.89E-05	3.08091552
ENSSSCG00000031249	2	ENSSSCG0000	2	0.00071066	3.07864303
EMC3	2	EMC3	2	0.03902612	3.07259137
INTS11	2	INTS11	2	0.00086971	3.07185946
CDC26	2	CDC26	2	2.59E-05	3.05634114
ENSSSCG00000062012	2	ENSSSCG0000	2	0.00804229	3.04142519
LRRC47	2	LRRC47	2	0.0160561	3.02446874
COX7A2	2	COX7A2	2	9.68E-06	3.01562511
ENSSSCG00000046487	2	ENSSSCG0000	2	0.01875602	3.00207513
MAF	2	MAF	2	0.0005865	2.98898436
GRHPR	2	GRHPR	2	1.21E-05	2.97196758
KLHDC4	2	KLHDC4	2	1.12E-05	2.97029121
ENSSSCG00000005528	2	ENSSSCG0000	2	9.64E-05	2.96062485
DMAC2L	2	DMAC2L	2	0.00634501	2.95919101
MRPS15	2	MRPS15	2	0.00685977	2.95632773
UPK1A	2	UPK1A	2	2.90E-05	2.95200912
CBR3	2	CBR3	2	0.03165226	2.95181193
CTU2	2	CTU2	2	0.00016726	2.95147821
MRPS25	2	MRPS25	2	2.10E-05	2.94611728
CCN6	2	CCN6	2	1.09E-06	2.93528769
CZIB	2	CZIB	2	0.00693574	2.93493139
RPS13	2	RPS13	2	0.00185787	2.93435914
SPAG8	2	SPAG8	2	0.00034525	2.91264411
ZNF821	2	ZNF821	2	5.75E-08	2.91191072
ENSSSCG00000002963	2	ENSSSCG0000	2	8.00E-06	2.90851962
SNRPD2	2	SNRPD2	2	0.00011493	2.90774075
HSPBP1	2	HSPBP1	2	1.09E-06	2.89548293
SLC25A38	2	SLC25A38	2	0.0005865	2.89395266
PPIB	2	PPIB	2	0.0019676	2.89081317
RNF141	2	RNF141	2	1.15E-07	2.88572735
ENSSSCG00000014133	2	ENSSSCG0000	2	0.01238686	2.86853382
UROD	2	UROD	2	2.10E-05	2.86164813
PIH1D1	2	PIH1D1	2	0.00157581	2.85035269
NDUFS5	2	NDUFS5	2	1.15E-07	2.84639977
TMEM42	2	TMEM42	2	0.00614661	2.83340408
ENSSSCG00000054958	2	ENSSSCG0000	2	5.75E-08	2.83162239
HEMK1	2	HEMK1	2	0.00370211	2.82927007
LIN37	2	LIN37	2	3.40E-05	2.81635301
ENSSSCG00000004703	2	ENSSSCG0000	2	0.00370211	2.81224345
ENSSSCG00000013386	2	ENSSSCG0000	2	5.18E-05	2.79416334
FBXO31	2	FBXO31	2	1.09E-06	2.76683198
KLF1	2	KLF1	2	0.00046415	2.75223873
MYOM1	2	MYOM1	2	0.02399792	2.7505132
POMGNT1	2	POMGNT1	2	0.00370211	2.73800465
LENG1	2	LENG1	2	0.01793026	2.72715293
DMWD	2	DMWD	2	0.00170494	2.71743617
TIMM50	2	TIMM50	2	0.00520164	2.71418337
ENSSSCG00000035007	2	ENSSSCG0000	2	0.00185787	2.71258172
ENSSSCG00000057874	2	ENSSSCG0000	2	0.02037355	2.70923371
ENSSSCG00000038035	2	ENSSSCG0000	2	0.00057789	2.70748224

ZMAT3	1	ZMAT3	1	0.00929494	0.69521024
CD47	1	CD47	1	0.00014772	0.69011557
TTC14	1	TTC14	1	0.02399792	0.67126599
EPHA7	1	EPHA7	1	2.14E-05	0.66864746
DNAJB4	1	DNAJB4	1	0.01407064	0.66789804
NGLY1	1	NGLY1	1	0.01890262	0.65927233
LATS1	1	LATS1	1	0.02137061	0.6586347
DNAAF2	1	DNAAF2	1	2.90E-05	0.65590683
ENSSSCG00000003989	1	ENSSSCG00000003989	1	2.14E-05	0.65095677
SACM1L	1	SACM1L	1	6.81E-05	0.65067532
TM2D1	1	TM2D1	1	1.73E-06	0.64844147
SLC30A4	1	SLC30A4	1	3.89E-05	0.6441363
ENSSSCG00000059603	1	ENSSSCG00000059603	1	0.02214327	0.64131958
RIPOR1	1	RIPOR1	1	0.04630508	0.64021166
CMPK1	1	CMPK1	1	0.02694642	0.62399915
SLC4A7	1	SLC4A7	1	0.02972401	0.61944784
SNRPA1	1	SNRPA1	1	0.00069538	0.61863635
FBXL4	1	FBXL4	1	8.89E-05	0.61830334
TAFA4	1	TAFA4	1	0.02255348	0.61785038
FECH	1	FECH	1	0.00804229	0.61512264
PDHX	1	PDHX	1	0.00370211	0.61094605
ABHD17B	1	ABHD17B	1	0.00313457	0.60978626
PPP5C	1	PPP5C	1	0.04630508	0.60917336
SCAF4	1	SCAF4	1	0.00693574	0.60598753
ENSSSCG00000045482	1	ENSSSCG00000045482	1	2.90E-05	0.60502164
ABHD10	1	ABHD10	1	2.14E-05	0.59642415
RAB31	1	RAB31	1	0.00222085	0.59224219
TRAPPC6B	1	TRAPPC6B	1	0.00037581	0.57507702
CAPN7	1	CAPN7	1	0.00128303	0.57202104
SLC38A6	1	SLC38A6	1	0.02659882	0.57016799
HPS3	1	HPS3	1	0.00804229	0.56605562
TSHZ1	1	TSHZ1	1	1.09E-06	0.56214446
PRTG	1	PRTG	1	0.04161078	0.56096489
TTC39A	1	TTC39A	1	0.00154718	0.55947655
KDM4C	1	KDM4C	1	0.01229347	0.55832896
ENSSSCG00000022206	1	ENSSSCG00000022206	1	8.89E-05	0.5571542
SNRNP40	1	SNRNP40	1	0.00105868	0.55449091
PPIL4	1	PPIL4	1	0.026429	0.55332849
FNBP4	1	FNBP4	1	0.00693574	0.5528385
SSX2IP	1	SSX2IP	1	0.00037581	0.5494175
ENSSSCG00000030015	1	ENSSSCG00000030015	1	0.00804229	0.54894343
PPFIA1	1	PPFIA1	1	0.04630508	0.54871059
SNAPC3	1	SNAPC3	1	8.89E-05	0.53895324
MAP2K1	1	MAP2K1	1	2.14E-05	0.5357556
FRMD4B	1	FRMD4B	1	0.02071916	0.52908706
ENSSSCG00000041063	1	ENSSSCG00000041063	1	0.02343317	0.52899129
ITFG1	1	ITFG1	1	0.04630508	0.52707142
ESCO1	1	ESCO1	1	0.026429	0.52692005
ZWINT	1	ZWINT	1	0.01070637	0.52465882
USP45	1	USP45	1	0.00105868	0.51960195
KIAA0586	1	KIAA0586	1	0.026429	0.51647965
RAVER1	1	RAVER1	1	2.30E-07	0.5148602
ZNF300	1	ZNF300	1	0.00596018	0.51434859
VGLL3	1	VGLL3	1	0.00014772	0.51335142
RYK	1	RYK	1	8.89E-05	0.50807865
VPS4B	1	VPS4B	1	0.00046721	0.50544281
DMXL1	1	DMXL1	1	0.01481332	0.50173827
CTBS	1	CTBS	1	5.18E-05	0.50172234
MAPKAPK3	1	MAPKAPK3	1	0.00086971	0.50017253
E2F4	1	E2F4	1	0.02972401	0.49688305
TMCO6	1	TMCO6	1	0.00105868	0.49582315
CENPW	1	CENPW	1	0.00435484	0.49271932
UNC13B	1	UNC13B	1	0.00030028	0.49135965
TUBE1	1	TUBE1	1	0.04161078	0.48944067
BLOC1S6	1	BLOC1S6	1	0.03729477	0.48925735
MTAP	1	MTAP	1	0.02071916	0.48075351
MEAF6	1	MEAF6	1	0.04791456	0.48020586
ANXA2	1	ANXA2	1	0.00157581	0.47909092
SLC35D1	1	SLC35D1	1	8.89E-05	0.4783096
RBL2	1	RBL2	1	0.00037581	0.47749884
ENSSSCG00000005440	1	ENSSSCG00000005440	1	0.01070637	0.47718371
SLC25A46	1	SLC25A46	1	0.00105868	0.47492737
GRPEL2	1	GRPEL2	1	0.00057789	0.47164085
CYLD	1	CYLD	1	0.026429	0.47069715
ZDHHC13	1	ZDHHC13	1	0.00057789	0.46602025
ARMC8	1	ARMC8	1	0.00782819	0.46472668
TBC1D32	1	TBC1D32	1	0.00222085	0.46256186
YTHDC2	1	YTHDC2	1	5.18E-05	0.46063587
C18orf63	1	C18orf63	1	8.89E-05	0.45873038
ME2	1	ME2	1	0.00370211	0.4573566
VAPA	1	VAPA	1	0.02343317	0.45641369
NUP93	1	NUP93	1	0.02972401	0.45491656
MKNK1	1	MKNK1	1	0.04161078	0.45394329
CDH7	1	CDH7	1	0.00105868	0.45373598
GCNT1	1	GCNT1	1	0.00679509	0.45283158
CSNK1G3	1	CSNK1G3	1	2.90E-05	0.45267628
CTTN	1	CTTN	1	5.23E-05	0.45250426
ATP13A3	1	ATP13A3	1	0.04161078	0.45160446
PNISR	1	PNISR	1	0.00510422	0.45125385
TRNAU1AP	1	TRNAU1AP	1	0.00222085	0.45053886
WTAP	1	WTAP	1	0.03729477	0.44668507
PSMD5	1	PSMD5	1	3.85E-06	0.4459708
CDCP1	1	CDCP1	1	0.01126872	0.44537536
ZCCHC9	1	ZCCHC9	1	0.03729477	0.44118414
ENSSSCG00000005453	1	ENSSSCG00000005453	1	0.01826556	0.43860215
GPT2	1	GPT2	1	0.04630508	0.43726671
APPL1	1	APPL1	1	0.00435484	0.43329323
ZFP30	1	ZFP30	1	0.02995323	0.43328324
UBIAD1	1	UBIAD1	1	0.01826556	0.43191334
CDIN1	1	CDIN1	1	2.14E-05	0.43063106
PEF1	1	PEF1	1	0.01613862	0.43043176

POC1A	2	POC1A	2	0.00518408	2.70222323
TXNRD3	2	TXNRD3	2	0.00114362	2.69085706
MBOAT7	2	MBOAT7	2	0.03012945	2.69004671
ENSSSCG00000047379	2	ENSSSCG0000	2	0.00596018	2.68571805
SAL1	2	SAL1	2	1.56E-05	2.68190645
ENSSSCG00000055939	2	ENSSSCG0000	2	0.00327588	2.6768542
LSM10	2	LSM10	2	0.00215222	2.67663984
ACAA1	2	ACAA1	2	0.00348602	2.66348938
ENKD1	2	ENKD1	2	2.59E-06	2.66140764
AKR1A1	2	AKR1A1	2	0.00023303	2.65779479
COQ4	2	COQ4	2	1.21E-05	2.65092228
PPAN	2	PPAN	2	0.01229347	2.64646882
PAQR7	2	PAQR7	2	0.00015563	2.63717555
MTMR14	2	MTMR14	2	0.00336642	2.63411079
ENSSSCG00000014284	2	ENSSSCG0000	2	1.73E-06	2.63260593
NDUFB4	2	NDUFB4	2	0.00423003	2.61731544
RPLP1	2	RPLP1	2	1.73E-06	2.61187295
CAPNS1	2	CAPNS1	2	0.0079516	2.60060734
CCDC32	2	CCDC32	2	5.75E-08	2.59768274
SLC38A8	2	SLC38A8	2	0.04846223	2.59685597
ATP5PF	2	ATP5PF	2	0.00046721	2.57898058
PSMB7	2	PSMB7	2	0.00134392	2.57470543
CCER2	2	CCER2	2	5.58E-06	2.57275674
ENSSSCG00000011294	2	ENSSSCG0000	2	0.00018834	2.56296726
GINS2	2	GINS2	2	0.02019671	2.56225694
ENSSSCG00000056114	2	ENSSSCG0000	2	4.03E-07	2.55835377
PIGP	2	PIGP	2	0.01347389	2.55684729
ENSSSCG00000029003	2	ENSSSCG0000	2	0.00016413	2.54979063
PADI3	2	PADI3	2	8.89E-05	2.53168915
ENO1	2	ENO1	2	0.003896	2.52688351
ENSSSCG00000031083	2	ENSSSCG0000	2	1.56E-05	2.51093759
TXNDC12	2	TXNDC12	2	0.00929494	2.50956771
CC2D1A	2	CC2D1A	2	6.81E-05	2.50493293
ENSSSCG00000032082	2	ENSSSCG0000	2	1.73E-06	2.49791932
SHROOM1	2	SHROOM1	2	0.00435484	2.47432108
UQCRQ	2	UQCRQ	2	0.00051953	2.45977457
NOX3	2	NOX3	2	0.00313861	2.45124052
PSENEN	2	PSENEN	2	5.11E-05	2.43958925
UST	2	UST	2	4.03E-07	2.43422419
CCDC97	2	CCDC97	2	0.00053213	2.42066796
BDH1	2	BDH1	2	0.00212787	2.40567059
ENSSSCG00000060282	2	ENSSSCG0000	2	2.30E-07	2.39674043
FAM32A	2	FAM32A	2	6.81E-05	2.38762499
ENSSSCG00000032110	2	ENSSSCG0000	2	1.51E-05	2.3849851
ACY1	2	ACY1	2	0.00018834	2.38419595
RPL32	2	RPL32	2	0.00545149	2.37114228
TYRO3	2	TYRO3	2	0.00248365	2.36337133
GSE1	2	GSE1	2	0.00221813	2.35834018
RIMS3	2	RIMS3	2	0.00065647	2.35760767
TK2	2	TK2	2	3.85E-06	2.35578795
ENSSSCG00000028414	2	ENSSSCG0000	2	9.45E-05	2.35565259
MED8	2	MED8	2	0.00037581	2.35207798
PRMT7	2	PRMT7	2	1.73E-06	2.35158234
UQCRC1	2	UQCRC1	2	0.00131617	2.35024525
SWI5	2	SWI5	2	1.21E-05	2.33578964
ENSSSCG00000013387	2	ENSSSCG0000	2	0.01407064	2.3356177
EBNA1BP2	2	EBNA1BP2	2	1.51E-05	2.33295454
RPL24	2	RPL24	2	6.22E-06	2.33143185
AEN	2	AEN	2	3.89E-05	2.32856077
SIDT1	2	SIDT1	2	5.18E-05	2.32400563
ERI3	2	ERI3	2	0.00011537	2.32108751
ENSSSCG00000005217	2	ENSSSCG0000	2	0.00105409	2.30069478
SIL1	2	SIL1	2	0.00017967	2.29961984
CDA	2	CDA	2	2.30E-07	2.29865076
HSBP1	2	HSBP1	2	0.00018834	2.2950044
ENSSSCG00000022401	2	ENSSSCG0000	2	0.00315167	2.29491205
HARS1	2	HARS1	2	0.00154718	2.29026183
ENSSSCG00000027916	2	ENSSSCG0000	2	9.51E-06	2.28847077
IQCC	2	IQCC	2	5.75E-08	2.27567981
P3H1	2	P3H1	2	0.00024208	2.27484499
MYL3	2	MYL3	2	0.00014772	2.27027128
CACNA2D3	2	CACNA2D3	2	0.02003808	2.27019098
LRRC36	2	LRRC36	2	0.00086971	2.26644865
VPS4A	2	VPS4A	2	0.00011493	2.26119956
SLC35A4	2	SLC35A4	2	0.00023303	2.2593194
IP6K2	2	IP6K2	2	2.30E-07	2.25590192
ENSSSCG00000002790	2	ENSSSCG0000	2	9.05E-05	2.25324707
GRAMD1A	2	GRAMD1A	2	5.29E-06	2.24668536
TFDP2	2	TFDP2	2	0.00011493	2.24124551
CLN6	2	CLN6	2	2.90E-05	2.2404058
RALGPS1	2	RALGPS1	2	0.00758365	2.23836861
ENSSSCG00000023691	2	ENSSSCG0000	2	4.03E-07	2.22056249
DNAJC11	2	DNAJC11	2	0.01801383	2.2195001
ENSSSCG00000048380	2	ENSSSCG0000	2	2.14E-05	2.21824182
ENSSSCG00000033189	2	ENSSSCG0000	2	5.58E-06	2.21643411
POLR2H	2	POLR2H	2	0.0347514	2.21525966
AURKAIP1	2	AURKAIP1	2	5.58E-06	2.2126714
MTRES1	2	MTRES1	2	0.00018834	2.2044631
RPL28	2	RPL28	2	8.89E-05	2.20007918
XXYLT1	2	XXYLT1	2	0.01425567	2.19896407
EEFSEC	2	EEFSEC	2	5.75E-08	2.19695921
CIDEA	2	CIDEA	2	3.72E-05	2.18447743
DELE1	2	DELE1	2	3.89E-05	2.18254683
ENSSSCG00000032916	2	ENSSSCG0000	2	0.00221813	2.18153459
C1orf174	2	C1orf174	2	0.00223883	2.17804479
PCDH12	2	PCDH12	2	2.30E-07	2.17684271
TMA7	2	TMA7	2	5.75E-08	2.17626291
ENSSSCG00000005649	2	ENSSSCG0000	2	2.30E-07	2.17349511
TSPAN1	2	TSPAN1	2	2.90E-05	2.17110149
TCEANC2	2	TCEANC2	2	0.02071916	2.17042748
C19orf67	2	C19orf67	2	0.01576715	2.16839295

YES1	1	YES1	1	4.03E-07	0.42980138
SLF1	1	SLF1	1	0.01407064	0.42920116
CNOT10	1	CNOT10	1	0.00929494	0.42672857
NDC1	1	NDC1	1	0.00313457	0.42483702
PTPN5	1	PTPN5	1	0.00081053	0.4222263
CLASP2	1	CLASP2	1	0.00105868	0.42035115
LSR	1	LSR	1	0.00693574	0.41858427
SNX14	1	SNX14	1	0.00222085	0.41609226
ANKRD12	1	ANKRD12	1	0.00128303	0.41556724
NETO2	1	NETO2	1	0.026429	0.41344536
SEC13	1	SEC13	1	0.04161078	0.41200908
PCOLCE2	1	PCOLCE2	1	0.01162462	0.40720699
E2F8	1	E2F8	1	0.00370211	0.40704524
HSD17B12	1	HSD17B12	1	0.02389558	0.4068598
HNRNPH1	1	HNRNPH1	1	0.00030028	0.40584438
ATG5	1	ATG5	1	2.90E-05	0.40423379
ELOVL4	1	ELOVL4	1	0.026429	0.40272593
PIK3C2A	1	PIK3C2A	1	3.89E-05	0.40205756
UFD1	1	UFD1	1	0.0160561	0.40017397
STT3B	1	STT3B	1	0.00185787	0.3955076
INTS14	1	INTS14	1	6.81E-05	0.39452233
MORC3	1	MORC3	1	0.04630508	0.39432544
AFF4	1	AFF4	1	0.01404202	0.39334346
PCMT1	1	PCMT1	1	0.00105868	0.39272279
ZNF793	1	ZNF793	1	0.00128303	0.38944996
MYSM1	1	MYSM1	1	0.03729477	0.38762688
FUBP1	1	FUBP1	1	0.00105868	0.38685126
ZFP1	1	ZFP1	1	0.00071066	0.38591722
RTTN	1	RTTN	1	0.01826556	0.38477789
NUDT21	1	NUDT21	1	3.89E-05	0.38339996
SESN1	1	SESN1	1	5.75E-08	0.38303257
MELK	1	MELK	1	8.89E-05	0.3811206
DCP1A	1	DCP1A	1	0.00046721	0.38046211
PTPN2	1	PTPN2	1	0.00057789	0.37733768
TRAFD1	1	TRAFD1	1	0.00222085	0.37621566
USP48	1	USP48	1	0.00046721	0.37471038
BANP	1	BANP	1	0.04161078	0.37422223
PIGN	1	PIGN	1	0.02071916	0.37241171
NAALADL2	1	NAALADL2	1	0.00037581	0.3702274
TFG	1	TFG	1	0.00046721	0.36765562
RNF13	1	RNF13	1	0.01070637	0.36486569
CMTM4	1	CMTM4	1	0.00071066	0.36394077
DPF2	1	DPF2	1	0.00185787	0.36126001
ZNF283	1	ZNF283	1	0.00185787	0.35917009
TRIM36	1	TRIM36	1	0.00596018	0.35884814
MFSD1	1	MFSD1	1	4.03E-07	0.35816259
PSIP1	1	PSIP1	1	0.02972401	0.35474416
SAV1	1	SAV1	1	8.00E-06	0.35469621
CLPX	1	CLPX	1	8.89E-05	0.35424981
STX3	1	STX3	1	0.00057789	0.3537591
FERMT2	1	FERMT2	1	1.73E-06	0.35318348
SEC24A	1	SEC24A	1	0.00804229	0.35094063
ZYG11B	1	ZYG11B	1	0.00313457	0.3473732
ENSSSCG00000005255	1	ENSSSCG00000005255	1	0.01407064	0.34507354
PHLPP2	1	PHLPP2	1	0.026429	0.34449503
PSMD8	1	PSMD8	1	2.14E-05	0.34434531
WDR48	1	WDR48	1	0.01142523	0.34388624
NAA30	1	NAA30	1	2.90E-05	0.34220472
ENSSSCG00000056411	1	ENSSSCG00000056411	1	6.90E-07	0.34102217
PCYT1A	1	PCYT1A	1	0.02972401	0.33818468
KDM1A	1	KDM1A	1	0.01482633	0.33790846
ENSSSCG00000013395	1	ENSSSCG00000013395	1	0.03729477	0.33433196
DOCK7	1	DOCK7	1	0.02694642	0.33390201
REPS1	1	REPS1	1	5.58E-06	0.33360673
RSRC1	1	RSRC1	1	0.00018834	0.33299909
FAM13C	1	FAM13C	1	0.03012945	0.32974331
H1-8	1	H1-8	1	0.00185787	0.32857079
AMPD3	1	AMPD3	1	0.01613862	0.32820474
ENSSSCG00000002855	1	ENSSSCG00000002855	1	6.90E-07	0.32348385
NUP160	1	NUP160	1	0.00128303	0.3202016
PDP2	1	PDP2	1	5.58E-06	0.31984153
CNTLN	1	CNTLN	1	0.00030028	0.31681501
MMS22L	1	MMS22L	1	0.01826556	0.31586481
NDC80	1	NDC80	1	0.00086971	0.31585187
MCC	1	MCC	1	0.00071066	0.31555592
POLR2E	1	POLR2E	1	0.00693574	0.31255931
ZNF599	1	ZNF599	1	0.00929494	0.31082034
TTC3	1	TTC3	1	1.09E-06	0.30890506
DPH3	1	DPH3	1	0.00693574	0.30030417
SPINDOC	1	SPINDOC	1	0.00222085	0.29792998
ENSSSCG00000011253	1	ENSSSCG00000011253	1	5.75E-08	0.29177055
HEY2	1	HEY2	1	0.01308873	0.28493296
IQGAP2	1	IQGAP2	1	0.00804229	0.28383225
ADAM10	1	ADAM10	1	0.03729477	0.28258899
TP53BP1	1	TP53BP1	1	0.00510422	0.28196832
PSMG1	1	PSMG1	1	0.0160561	0.28189533
HAS3	1	HAS3	1	0.00896843	0.2797188
ATG12	1	ATG12	1	0.00313457	0.27957103
NDUFAF4	1	NDUFAF4	1	0.00929494	0.27884142
ID3	1	ID3	1	0.00435484	0.27859138
TOP2B	1	TOP2B	1	0.00014772	0.27094937
DCC	1	DCC	1	0.00014772	0.26353681
ENSSSCG00000061752	1	ENSSSCG00000061752	1	0.01070637	0.25943532
ENSSSCG00000062865	1	ENSSSCG00000062865	1	0.00011493	0.25488686
NEDD4L	1	NEDD4L	1	3.85E-06	0.25032349
ILVBL	2	ILVBL	2	5.92E-06	2.16585205
ACP7	2	ACP7	2	4.03E-07	2.16543679
ENSSSCG00000052522	2	ENSSSCG0000	2	1.15E-05	2.16385811
GLT8D1	2	GLT8D1	2	1.73E-06	2.1625404
DIO1	2	DIO1	2	5.58E-06	2.16244762
MAP7D1	2	MAP7D1	2	6.38E-05	2.15839691
NDUFAF3	2	NDUFAF3	2	2.80E-05	2.15656363
SELENOS	2	SELENOS	2	5.92E-06	2.15172504
ENSSSCG00000058485	2	ENSSSCG0000	2	6.38E-05	2.15118695
GORASP1	2	GORASP1	2	2.59E-06	2.14598051
RPUSD2	2	RPUSD2	2	0.00634501	2.14505311
ENSSSCG00000031730	2	ENSSSCG0000	2	0.00030028	2.13976669
RPL11	2	RPL11	2	1.56E-05	2.13881208
ENSSSCG00000045343	2	ENSSSCG0000	2	8.89E-05	2.13800539
ENSSSCG00000005006	2	ENSSSCG0000	2	1.56E-05	2.13576175
ENSSSCG00000056323	2	ENSSSCG0000	2	1.15E-07	2.13472879
GLYCTK	2	GLYCTK	2	0.0045148	2.12811062
ZNRF1	2	ZNRF1	2	2.30E-07	2.12581584
GBA2	2	GBA2	2	7.61E-05	2.12377331
WNT7A	2	WNT7A	2	0.00033032	2.12061131
NUCB1	2	NUCB1	2	0.00030028	2.12051443
FHIT	2	FHIT	2	1.29E-05	2.11633815
COQ3	2	COQ3	2	5.75E-08	2.11516296
CCDC61	2	CCDC61	2	0.00041215	2.10739779
CCDC12	2	CCDC12	2	2.14E-05	2.10567927
ENSSSCG00000059558	2	ENSSSCG0000	2	0.02400302	2.09771369
ASB6	2	ASB6	2	0.02214327	2.08131394
APOD	2	APOD	2	0.00011493	2.08047623
CHCHD4	2	CHCHD4	2	5.92E-06	2.08036744
CDK5RAP2	2	CDK5RAP2	2	5.92E-06	2.07442085
IRGQ	2	IRGQ	2	0.00336642	2.05213801
NOC2L	2	NOC2L	2	0.00423003	2.0507225
TAF1C	2	TAF1C	2	0.01201475	2.04994583
DMAC2	2	DMAC2	2	0.00672354	2.04019328
FBXO17	2	FBXO17	2	3.89E-05	2.03957527
ABCF3	2	ABCF3	2	0.00037581	2.02869701
ENSSSCG00000062784	2	ENSSSCG0000	2	6.90E-07	2.02064737
CDH15	2	CDH15	2	0.02003808	2.01920879
DYNC1LI1	2	DYNC1LI1	2	0.00018834	2.00642691
PDCD5	2	PDCD5	2	1.15E-07	2.00130744
HARS2	2	HARS2	2	5.75E-08	1.99772091
UBE2J1	2	UBE2J1	2	3.85E-06	1.99578295
PNRC1	2	PNRC1	2	0.02972401	1.99464357
YJU2B	2	YJU2B	2	4.03E-07	1.9934404
CD274	2	CD274	2	0.00048567	1.98787137
FBXW9	2	FBXW9	2	0.00074525	1.98463017
KATNB1	2	KATNB1	2	5.92E-06	1.97627811
ABLIM3	2	ABLIM3	2	0.00597993	1.97505482
PRR5L	2	PRR5L	2	0.00683818	1.95651236
TMEM39B	2	TMEM39B	2	0.00030028	1.95624479
CEACAM20	2	CEACAM20	2	0.00154718	1.95581162
C19orf47	2	C19orf47	2	5.75E-08	1.95308818
NFAT5	2	NFAT5	2	0.00077888	1.95194661
TRAIP	2	TRAIP	2	0.00031754	1.95158895
MAD2L2	2	MAD2L2	2	2.25E-05	1.9497997
PIGO	2	PIGO	2	0.00337011	1.94739111
ENSSSCG00000039972	2	ENSSSCG0000	2	4.03E-07	1.94213851
GTF2H5	2	GTF2H5	2	8.00E-06	1.94083716
RNASEH2A	2	RNASEH2A	2	5.58E-06	1.93958681
EPHB2	2	EPHB2	2	3.89E-05	1.93915587
SH3GLB2	2	SH3GLB2	2	1.85E-05	1.93614884
ENSSSCG00000041170	2	ENSSSCG0000	2	0.00435484	1.93237635
EXOSC5	2	EXOSC5	2	0.00071066	1.93159115
FNDC5	2	FNDC5	2	0.00020022	1.92744199
MRPS6	2	MRPS6	2	0.00019373	1.92505478
PFDN1	2	PFDN1	2	0.0300707	1.92136753
DEF8	2	DEF8	2	0.00014874	1.90784838
ENSSSCG00000043636	2	ENSSSCG0000	2	0.00785204	1.90753269
CIAPIN1	2	CIAPIN1	2	0.00010981	1.90307698
E2F2	2	E2F2	2	8.00E-06	1.89695236
ENSSSCG00000031299	2	ENSSSCG0000	2	0.03196455	1.89602771
RPS6	2	RPS6	2	0.00029024	1.89219006
IFT57	2	IFT57	2	0.00128303	1.89048409
MRPL46	2	MRPL46	2	5.58E-06	1.8884186
CABLES1	2	CABLES1	2	0.00209652	1.88747817
ENSSSCG00000056225	2	ENSSSCG0000	2	6.81E-05	1.88352667
OXSM	2	OXSM	2	8.00E-06	1.88253838
TOE1	2	TOE1	2	0.00168296	1.88232612
PSMC4	2	PSMC4	2	0.02003808	1.88106418
TTC21A	2	TTC21A	2	7.72E-05	1.87945528
GLIPR2	2	GLIPR2	2	2.59E-06	1.87464849
RAB6B	2	RAB6B	2	5.75E-08	1.8719134
MIX23	2	MIX23	2	6.27E-06	1.86516469
TMC4	2	TMC4	2	0.00046721	1.8623783
ENSSSCG00000045223	2	ENSSSCG0000	2	0.00046721	1.85948737
PRPF4	2	PRPF4	2	0.03667807	1.84294341
MPPE1	2	MPPE1	2	0.00128303	1.84036727
CAPZB	2	CAPZB	2	0.00313457	1.83821143
AKAP12	2	AKAP12	2	5.75E-08	1.83559449
MTO1	2	MTO1	2	8.00E-06	1.82901593
ENSSSCG00000011569	2	ENSSSCG0000	2	8.00E-06	1.82792681
C2CD2	2	C2CD2	2	0.00108432	1.82786223
MTCL1	2	MTCL1	2	0.00071066	1.82155048
MON1A	2	MON1A	2	0.00071066	1.81894989
DOLPP1	2	DOLPP1	2	0.02833053	1.81821929
PAIP2	2	PAIP2	2	1.10E-05	1.80908529
ENSSSCG00000056205	2	ENSSSCG0000	2	1.09E-06	1.80691877
B4GALT4	2	B4GALT4	2	5.75E-08	1.80578519
ENSSSCG00000050758	2	ENSSSCG0000	2	3.85E-06	1.80071961
RTN4IP1	2	RTN4IP1	2	4.76E-05	1.7931938
ENSSSCG00000005596	2	ENSSSCG0000	2	1.15E-07	1.79144922

KDM4A	2	KDM4A	2	5.58E-06	1.78524844
MROH7	2	MROH7	2	0.00023867	1.785063
SELENON	2	SELENON	2	6.81E-05	1.78411646
PSME3IP1	2	PSME3IP1	2	2.90E-05	1.78114097
MED22	2	MED22	2	0.0136361	1.7794751
QPCTL	2	QPCTL	2	0.00154718	1.77767246
ENSSSCG00000011272	2	ENSSSCG0000(	2	0.00334125	1.77726592
ENSSSCG00000002907	2	ENSSSCG0000(	2	6.81E-05	1.77377079
EEF1AKMT4	2	EEF1AKMT4	2	2.83E-05	1.77006974
IFTAP	2	IFTAP	2	1.09E-06	1.76937964
CRAT	2	CRAT	2	0.02588432	1.75866294
LTF	2	LTF	2	0.01391186	1.75568383
ENSSSCG00000053185	2	ENSSSCG0000(	2	2.90E-05	1.75310478
ENSSSCG00000003286	2	ENSSSCG0000(	2	3.89E-05	1.75133582
APOE	2	APOE	2	0.00596018	1.74955691
ENSSSCG00000035904	2	ENSSSCG0000(	2	9.23E-06	1.74802204
PRDX1	2	PRDX1	2	0.00212787	1.74711167
NDUFAF1	2	NDUFAF1	2	7.40E-06	1.74392165
SEC11C	2	SEC11C	2	2.59E-06	1.73997636
TFB1M	2	TFB1M	2	6.90E-07	1.73939628
AHSG	2	AHSG	2	0.00209652	1.73898211
PADI4	2	PADI4	2	2.59E-05	1.73662928
ENSSSCG00000059847	2	ENSSSCG0000(	2	0.02543359	1.73422963
HAUS1	2	HAUS1	2	5.58E-06	1.73324714
ATRIP	2	ATRIP	2	0.01228243	1.72874491
NDUFV2	2	NDUFV2	2	5.75E-08	1.72827458
PDCD1LG2	2	PDCD1LG2	2	1.20E-05	1.7262572
CFAP298	2	CFAP298	2	0.00057789	1.72251338
PTAR1	2	PTAR1	2	0.02214327	1.72041665
PAK4	2	PAK4	2	0.00614661	1.71919128
ENSSSCG00000037514	2	ENSSSCG0000(	2	5.19E-05	1.71629948
SRARP	2	SRARP	2	0.01470167	1.71629131
NIPAL3	2	NIPAL3	2	8.60E-05	1.71337711
DNAAF4	2	DNAAF4	2	9.76E-06	1.71011737
POLE3	2	POLE3	2	0.00104866	1.70884235
ENSSSCG00000058562	2	ENSSSCG0000(	2	0.00057737	1.70406273
ZNF235	2	ZNF235	2	4.03E-07	1.70108889
ENSSSCG00000061086	2	ENSSSCG0000(	2	0.00086971	1.70097501
ARHGEF1	2	ARHGEF1	2	5.58E-06	1.70035149
RBPMS2	2	RBPMS2	2	0.00435484	1.69721804
SRPRB	2	SRPRB	2	6.90E-07	1.69525727
IQCG	2	IQCG	2	0.01955007	1.68507954
CALM3	2	CALM3	2	5.92E-06	1.68154651
GEMIN2	2	GEMIN2	2	2.59E-06	1.67965365
PARVA	2	PARVA	2	0.00049357	1.67733793
ZNF584	2	ZNF584	2	3.85E-06	1.67665249
CROCC	2	CROCC	2	6.83E-05	1.67341149
ENSSSCG00000054830	2	ENSSSCG0000(	2	0.02255348	1.67299142
LIG1	2	LIG1	2	0.0009192	1.67230691
ENSSSCG00000055918	2	ENSSSCG0000(	2	5.75E-08	1.67073793
CIAO2B	2	CIAO2B	2	0.01425567	1.66975683
ENSSSCG00000035776	2	ENSSSCG0000(	2	0.00113831	1.66586653
CKM	2	CKM	2	2.59E-06	1.66383428
DUT	2	DUT	2	5.58E-06	1.65889096
C18orf54	2	C18orf54	2	2.14E-05	1.63978731
PKD1L2	2	PKD1L2	2	0.01490128	1.63958932
SPOCK1	2	SPOCK1	2	0.00011493	1.63696592
ELP6	2	ELP6	2	5.75E-08	1.63614649
ENSSSCG00000062612	2	ENSSSCG0000(	2	0.00123548	1.63574161
ALPK2	2	ALPK2	2	5.58E-06	1.63287394
LYPD3	2	LYPD3	2	9.76E-06	1.62953718
CD72	2	CD72	2	1.85E-05	1.62470669
DHCR24	2	DHCR24	2	5.75E-08	1.62420491
GPR32	2	GPR32	2	3.92E-05	1.62310192
NAPA	2	NAPA	2	1.15E-07	1.61916579
MCEE	2	MCEE	2	0.01347389	1.61878705
ENSSSCG00000029257	2	ENSSSCG0000(	2	0.00023303	1.61649102
FHAD1	2	FHAD1	2	2.14E-05	1.61124844
GNA14	2	GNA14	2	0.00030028	1.61106982
PNLDC1	2	PNLDC1	2	0.00063881	1.61011536
KCNN4	2	KCNN4	2	0.00537066	1.60170355
ENSSSCG00000026229	2	ENSSSCG0000(	2	0.00249695	1.5989506
DOK6	2	DOK6	2	0.00423003	1.59712034
CDKN2AIPNL	2	CDKN2AIPNL	2	0.00399675	1.59619242
ENSSSCG00000028717	2	ENSSSCG0000(	2	0.03247112	1.59475918
NLRC5	2	NLRC5	2	0.00382109	1.59067217
HECTD3	2	HECTD3	2	6.22E-06	1.58802328
NCCRP1	2	NCCRP1	2	1.09E-06	1.58437746
BTF3L4	2	BTF3L4	2	0.01013511	1.57901755
GNL3	2	GNL3	2	8.00E-06	1.57758967
CFDP1	2	CFDP1	2	0.00100228	1.57631324
RAB7A	2	RAB7A	2	0.00849214	1.57497822
VAC14	2	VAC14	2	0.00435484	1.55222936
RBM15B	2	RBM15B	2	4.03E-07	1.55156447
CA5A	2	CA5A	2	1.15E-07	1.54740266
DHDDS	2	DHDDS	2	0.00910266	1.54672706
ENSSSCG00000029696	2	ENSSSCG0000(	2	0.00518408	1.54485879
ARMC2	2	ARMC2	2	0.00313457	1.54385565
ENSSSCG00000034393	2	ENSSSCG0000(	2	0.01470167	1.53751055
MYL12A	2	MYL12A	2	7.77E-06	1.532052
RNF220	2	RNF220	2	0.00222085	1.53200526
ENSSSCG00000061446	2	ENSSSCG0000(	2	3.19E-05	1.53151022
SCG5	2	SCG5	2	0.00336642	1.53064488
ENSSSCG00000004573	2	ENSSSCG0000(	2	5.75E-08	1.53048853
SKOR1	2	SKOR1	2	0.03334006	1.52927759
ENSSSCG00000061116	2	ENSSSCG0000(	2	0.00042508	1.50933667
ATP2C2	2	ATP2C2	2	0.00028856	1.50858034
HAUS2	2	HAUS2	2	6.22E-06	1.50682925
PLCG2	2	PLCG2	2	0.02781075	1.50602491
ENSSSCG00000053570	2	ENSSSCG0000(	2	0.01229347	1.49659665
SESN2	2	SESN2	2	0.00014772	1.49434906

LYSMD2	2	LYSMD2	2	0.00028856	1.49075593
MKRN2	2	MKRN2	2	9.51E-06	1.48988214
ENSSSCG00000022773	2	ENSSSCG0000	2	0.00672354	1.4895948
PAFAH2	2	PAFAH2	2	5.18E-05	1.48843586
TWSG1	2	TWSG1	2	0.00518408	1.48650595
SYT5	2	SYT5	2	0.00782819	1.48452669
ENSSSCG00000011875	2	ENSSSCG0000	2	1.15E-07	1.48156597
MRPL37	2	MRPL37	2	1.12E-05	1.47904603
DDX28	2	DDX28	2	0.02052544	1.47356822
ENTREP1	2	ENTREP1	2	0.00154718	1.47222492
ENSSSCG00000060152	2	ENSSSCG0000	2	0.00596018	1.47213287
ENSSSCG00000056638	2	ENSSSCG0000	2	5.58E-06	1.47174677
DKK3	2	DKK3	2	0.01853669	1.47031349
SNRPA	2	SNRPA	2	5.75E-08	1.46354826
TBPL2	2	TBPL2	2	0.03729477	1.46244215
ENSSSCG00000003903	2	ENSSSCG0000	2	0.00018834	1.46043829
MRPL50	2	MRPL50	2	2.14E-05	1.46022832
NUP62	2	NUP62	2	0.00727314	1.4596072
ENSSSCG00000052760	2	ENSSSCG0000	2	0.02071916	1.45848273
ENSSSCG00000057916	2	ENSSSCG0000	2	5.75E-08	1.45725416
CC2D1B	2	CC2D1B	2	0.00029024	1.4535071
ALDOB	2	ALDOB	2	0.01151746	1.45305439
COQ8B	2	COQ8B	2	0.00849214	1.45047403
SEPTIN8	2	SEPTIN8	2	0.02781075	1.44968589
ATP5PO	2	ATP5PO	2	0.00804229	1.44010278
ENSSSCG00000053298	2	ENSSSCG0000	2	4.03E-07	1.43831803
NLRP5	2	NLRP5	2	1.18E-05	1.43496963
ENSSSCG00000028892	2	ENSSSCG0000	2	0.00033032	1.43156351
ENSSSCG00000046083	2	ENSSSCG0000	2	5.75E-08	1.42105522
GSK3B	2	GSK3B	2	0.00128303	1.41841232
EIF2B3	2	EIF2B3	2	0.00290246	1.41757279
SYTL3	2	SYTL3	2	0.01142523	1.41728824
EFCAB12	2	EFCAB12	2	3.72E-05	1.41597307
FBXO34	2	FBXO34	2	0.00693574	1.41328288
ZMAT2	2	ZMAT2	2	0.04405674	1.40619891
EAF1	2	EAF1	2	0.00672354	1.40523122
RRP1B	2	RRP1B	2	6.10E-05	1.40441348
ENSSSCG00000062804	2	ENSSSCG0000	2	4.84E-06	1.40385718
CACNA2D2	2	CACNA2D2	2	0.00117669	1.40055961
EIF1B	2	EIF1B	2	0.00232346	1.39662373
LYPLA2	2	LYPLA2	2	0.02694642	1.39551164
SLC2A8	2	SLC2A8	2	0.0045273	1.3903514
CCL22	2	CCL22	2	1.73E-06	1.38877148
ANAPC13	2	ANAPC13	2	0.00536605	1.38787863
SKI	2	SKI	2	1.12E-05	1.38682989
CCR5	2	CCR5	2	0.0045148	1.38575063
PRDX2	2	PRDX2	2	0.00027967	1.3761723
ENSSSCG00000045922	2	ENSSSCG0000	2	1.15E-07	1.37573357
ALDH4A1	2	ALDH4A1	2	0.04316039	1.37299879
LSAMP	2	LSAMP	2	0.00011493	1.37237493
NEGR1	2	NEGR1	2	0.02399792	1.36867882
RIMKLA	2	RIMKLA	2	6.27E-06	1.36827821
MAP1LC3B	2	MAP1LC3B	2	8.00E-06	1.36577613
STMN1	2	STMN1	2	0.00014772	1.36290208
TGM7	2	TGM7	2	1.73E-06	1.3602114
WDR53	2	WDR53	2	0.00071443	1.3549998
QARS1	2	QARS1	2	7.84E-06	1.35468464
ENSSSCG00000058326	2	ENSSSCG0000	2	9.68E-06	1.35383227
ARPP19	2	ARPP19	2	0.01081299	1.35292951
TCP1	2	TCP1	2	0.01019267	1.34966731
ZBTB24	2	ZBTB24	2	0.04405674	1.34661571
SARS2	2	SARS2	2	0.0029137	1.34266802
LIMD1	2	LIMD1	2	8.00E-06	1.34100209
SLC25A26	2	SLC25A26	2	9.51E-06	1.33868681
ENSSSCG00000003612	2	ENSSSCG0000	2	0.026429	1.3383355
CNDP2	2	CNDP2	2	0.00382109	1.33661135
RPL4	2	RPL4	2	0.0045148	1.33638243
LMOD3	2	LMOD3	2	0.00131617	1.33200618
ENSSSCG00000062560	2	ENSSSCG0000	2	1.15E-07	1.32998111
MSANTD3	2	MSANTD3	2	0.00019776	1.32921414
TXNL4A	2	TXNL4A	2	1.21E-05	1.32407391
CDKN3	2	CDKN3	2	0.00693574	1.3239351
ELOVL1	2	ELOVL1	2	1.86E-05	1.32287409
COTL1	2	COTL1	2	0.00128303	1.32224643
ENSSSCG00000062439	2	ENSSSCG0000	2	0.03012945	1.31298434
NLRP8	2	NLRP8	2	0.01379103	1.30398142
COG8	2	COG8	2	0.0160561	1.30212835
SIM2	2	SIM2	2	0.0094357	1.3013572
MRAP2	2	MRAP2	2	5.18E-05	1.2926901
COX7C	2	COX7C	2	5.75E-08	1.29025456
ENSSSCG00000032978	2	ENSSSCG0000	2	0.00435484	1.28897415
RBM42	2	RBM42	2	0.01407064	1.2814855
RSL24D1	2	RSL24D1	2	0.00150864	1.27861328
KPNA1	2	KPNA1	2	0.00030028	1.26956704
OOEP	2	OOEP	2	0.00088103	1.26946139
YIPF1	2	YIPF1	2	8.89E-05	1.26914185
KIAA1143	2	KIAA1143	2	3.85E-06	1.26180782
ENSSSCG00000056768	2	ENSSSCG0000	2	0.00022428	1.25983256
STIMATE	2	STIMATE	2	1.73E-06	1.25850051
CPT2	2	CPT2	2	4.81E-05	1.25614854
FBXO38	2	FBXO38	2	0.00896843	1.25500264
TIMMDC1	2	TIMMDC1	2	0.00103693	1.25487157
UBE2B	2	UBE2B	2	0.00420275	1.25397756
DYNLT2B	2	DYNLT2B	2	2.90E-05	1.25184219
ATP6V0D1	2	ATP6V0D1	2	0.0145153	1.24913933
LDHA	2	LDHA	2	0.00030028	1.24397172
MST1R	2	MST1R	2	6.90E-07	1.24219797
ENSSSCG00000054140	2	ENSSSCG0000	2	0.00023867	1.24153542
ENSSSCG00000042842	2	ENSSSCG0000	2	3.85E-06	1.23951598
ENSSSCG00000028572	2	ENSSSCG0000	2	0.00055248	1.23832862
GAP43	2	GAP43	2	6.81E-05	1.232369

LDLRAP1	2	LDLRAP1	2	2.27E-05	1.22888818
ENSSSCG00000061564	2	ENSSSCG0000(	2	4.03E-07	1.22836651
PTCH2	2	PTCH2	2	3.40E-05	1.22342277
ENSSSCG00000003348	2	ENSSSCG0000(	2	5.58E-06	1.22174719
LSG1	2	LSG1	2	4.03E-07	1.2202597
SNX3	2	SNX3	2	8.89E-05	1.21783272
MLH1	2	MLH1	2	1.73E-06	1.21153445
SLC1A2	2	SLC1A2	2	0.00317692	1.21116576
PRR19	2	PRR19	2	0.00271136	1.2077941
HSPA4	2	HSPA4	2	4.03E-07	1.20740739
LMCD1	2	LMCD1	2	0.00223883	1.20418487
TMEM69	2	TMEM69	2	8.00E-06	1.20091579
TTLL3	2	TTLL3	2	1.12E-05	1.19954937
OXTR	2	OXTR	2	0.00415803	1.19245427
C19orf54	2	C19orf54	2	2.59E-06	1.19124638
NECAP2	2	NECAP2	2	5.58E-06	1.19092517
TRIP4	2	TRIP4	2	0.03548333	1.18745831
ABRACL	2	ABRACL	2	8.00E-06	1.18642785
ENSSSCG00000062072	2	ENSSSCG0000(	2	0.00249695	1.18108242
EXOSC2	2	EXOSC2	2	0.00693574	1.17945418
BRPF1	2	BRPF1	2	0.0005865	1.17646956
ETF1	2	ETF1	2	3.85E-06	1.17371934
CHSY3	2	CHSY3	2	6.81E-05	1.17266738
PCDHGA4	2	PCDHGA4	2	0.00023867	1.17043221
INVS	2	INVS	2	0.02203537	1.16996054
ENSSSCG00000032216	2	ENSSSCG0000(	2	5.75E-08	1.16919894
FKBP3	2	FKBP3	2	0.00157581	1.1648704
DCAF10	2	DCAF10	2	3.02E-05	1.15857488
YARS1	2	YARS1	2	5.65E-06	1.15809295
ENSSSCG00000002709	2	ENSSSCG0000(	2	0.00034721	1.15613645
RUVBL1	2	RUVBL1	2	6.90E-07	1.15252538
CALHM6	2	CALHM6	2	0.04640888	1.15200623
TGFBI	2	TGFBI	2	0.01229347	1.15091708
ENSSSCG00000059138	2	ENSSSCG0000(	2	2.30E-07	1.15079715
ENSSSCG00000042524	2	ENSSSCG0000(	2	0.00250694	1.14876965
CAMTA1	2	CAMTA1	2	0.00018834	1.14616772
FTL	2	FTL	2	1.86E-05	1.14091622
GNL2	2	GNL2	2	0.00057789	1.14048751
TRIM32	2	TRIM32	2	1.12E-05	1.13955929
EHD4	2	EHD4	2	5.18E-05	1.13739415
SSR3	2	SSR3	2	0.00086971	1.13563822
ATP5F1A	2	ATP5F1A	2	5.75E-08	1.13515881
ENSSSCG00000057950	2	ENSSSCG0000(	2	6.90E-07	1.12949125
ATG3	2	ATG3	2	0.01893862	1.12832805
PRRC2B	2	PRRC2B	2	7.84E-06	1.12816678
STX17	2	STX17	2	0.00929494	1.12781803
CTH	2	CTH	2	5.75E-08	1.12775321
HINT1	2	HINT1	2	2.30E-07	1.12547404
SCAP	2	SCAP	2	6.27E-06	1.12337611
PWP2	2	PWP2	2	0.00646879	1.1209018
PINK1	2	PINK1	2	0.00370211	1.11194058
ENSSSCG00000026454	2	ENSSSCG0000(	2	0.00028856	1.11139606
LDHC	2	LDHC	2	0.00088103	1.11123442
ATP6V1G1	2	ATP6V1G1	2	3.89E-05	1.11054124
SAR1B	2	SAR1B	2	1.21E-05	1.10912874
ENSSSCG00000057571	2	ENSSSCG0000(	2	0.0398414	1.1048623
CTPS1	2	CTPS1	2	1.73E-06	1.10360096
ENSSSCG00000042603	2	ENSSSCG0000(	2	0.02542055	1.10208262
HRH1	2	HRH1	2	6.90E-07	1.10087317
ZNF593	2	ZNF593	2	0.00018834	1.09913409
CCDC191	2	CCDC191	2	0.00249695	1.09895277
MTHFD1L	2	MTHFD1L	2	0.00094184	1.09807779
RAB8A	2	RAB8A	2	0.0016228	1.09727799
GABBR2	2	GABBR2	2	1.73E-06	1.09626275
HMGCL	2	HMGCL	2	0.02137061	1.09532449
MAP1A	2	MAP1A	2	0.00199328	1.09478641
PHF24	2	PHF24	2	1.73E-06	1.09441331
KAZN	2	KAZN	2	8.00E-06	1.09252484
TRMT10C	2	TRMT10C	2	0.00014156	1.09159314
ENSSSCG00000028677	2	ENSSSCG0000(	2	4.03E-07	1.08709589
ZNF529	2	ZNF529	2	0.00154718	1.08654476
MVB12B	2	MVB12B	2	0.00014772	1.08489069
NFYC	2	NFYC	2	3.89E-05	1.08256317
CATSPER2	2	CATSPER2	2	0.00029024	1.07770962
ENSSSCG00000044155	2	ENSSSCG0000(	2	0.00014772	1.07769831
GREB1L	2	GREB1L	2	1.10E-05	1.07557953
PSKH1	2	PSKH1	2	0.00013298	1.07109632
SH3GL2	2	SH3GL2	2	0.00433766	1.06745422
RPSA	2	RPSA	2	9.23E-06	1.06499652
SNX30	2	SNX30	2	0.01229347	1.06113718
ATG7	2	ATG7	2	4.03E-07	1.05588203
PLOD1	2	PLOD1	2	0.00339476	1.05474141
PDIA3	2	PDIA3	2	0.00031754	1.05378199
PIGV	2	PIGV	2	0.00030028	1.05127029
TXNL4B	2	TXNL4B	2	0.00050654	1.0500328
CDKL1	2	CDKL1	2	2.90E-05	1.04579025
DNAJB11	2	DNAJB11	2	0.00693054	1.04368056
TMEM231	2	TMEM231	2	0.01003945	1.04353648
RSRP1	2	RSRP1	2	5.75E-08	1.03824775
KLF2	2	KLF2	2	0.01391186	1.03781185
ZNF45	2	ZNF45	2	0.00270342	1.03427062
ENSSSCG00000054543	2	ENSSSCG0000(	2	0.00804229	1.0335724
IK	2	IK	2	0.00334125	1.031649
ENSSSCG00000011307	2	ENSSSCG0000(	2	6.81E-05	1.03151624
SCG3	2	SCG3	2	2.90E-05	1.02595576
HEG1	2	HEG1	2	5.18E-05	1.01825542
TPM1	2	TPM1	2	0.01280324	1.01150019
FOXJ3	2	FOXJ3	2	2.59E-06	1.00662578
CCBE1	2	CCBE1	2	2.90E-05	1.00512984
SYMPK	2	SYMPK	2	0.00034721	1.00387212
MAPK1IP1L	2	MAPK1IP1L	2	2.30E-07	1.00376905

MRPL47	2	MRPL47	2	0.00336642	1.00316015
NKIRAS1	2	NKIRAS1	2	0.00156892	1.00167008
HSPA5	2	HSPA5	2	2.54E-05	0.99703029
TLN2	2	TLN2	2	5.58E-06	0.99464716
MYCBP	2	MYCBP	2	0.00016726	0.9934512
TCEA3	2	TCEA3	2	0.0028429	0.99276376
TPGS2	2	TPGS2	2	2.59E-06	0.99270744
COQ9	2	COQ9	2	8.74E-05	0.98392238
AFG3L2	2	AFG3L2	2	4.03E-07	0.9824482
C9orf78	2	C9orf78	2	0.00510422	0.9801338
PSMD7	2	PSMD7	2	7.92E-05	0.97422221
DOK4	2	DOK4	2	0.00014772	0.96940224
ENSSSCG00000027374	2	ENSSSCG0000	2	0.00057789	0.96549251
CHAC1	2	CHAC1	2	0.0019676	0.964193
PPIE	2	PPIE	2	2.30E-07	0.95839428
HMGXB3	2	HMGXB3	2	0.00459579	0.95771842
ENSSSCG00000027041	2	ENSSSCG0000	2	6.81E-05	0.95526537
GMPPB	2	GMPPB	2	1.73E-06	0.9495687
ENSSSCG00000048914	2	ENSSSCG0000	2	8.89E-05	0.94631441
CCIN	2	CCIN	2	0.00057789	0.94494024
RBM22	2	RBM22	2	0.04673008	0.94398796
SURF4	2	SURF4	2	0.00046721	0.94262985
CLTA	2	CLTA	2	2.28E-05	0.94061399
NUDT16	2	NUDT16	2	0.00250694	0.93735142
SSBP3	2	SSBP3	2	1.56E-05	0.93317087
ENSSSCG00000011447	2	ENSSSCG0000	2	5.75E-08	0.92917906
CCNDBP1	2	CCNDBP1	2	0.00018931	0.92520065
ENSSSCG00000063245	2	ENSSSCG0000	2	0.00016726	0.92509126
GET1	2	GET1	2	2.59E-06	0.92104669
ECH1	2	ECH1	2	0.00128303	0.91776642
PRKN	2	PRKN	2	0.02542055	0.90905631
CCDC113	2	CCDC113	2	1.15E-07	0.9088996
RASGRP1	2	RASGRP1	2	0.01826556	0.9073107
HYPK	2	HYPK	2	5.75E-08	0.9039265
MTRF1L	2	MTRF1L	2	5.18E-05	0.90000919
CENPS	2	CENPS	2	0.0168401	0.89726624
ENSSSCG00000032016	2	ENSSSCG0000	2	0.00046721	0.89616105
ENSSSCG00000027270	2	ENSSSCG0000	2	0.00018834	0.89568374
PTPRU	2	PTPRU	2	2.30E-07	0.8908569
ENSSSCG00000058051	2	ENSSSCG0000	2	0.01076302	0.88922514
PRELID2	2	PRELID2	2	0.00052968	0.88431697
DALRD3	2	DALRD3	2	0.00138341	0.87521613
ODF2	2	ODF2	2	6.90E-07	0.87373965
NAT10	2	NAT10	2	2.90E-05	0.86978687
TMEM245	2	TMEM245	2	0.0094357	0.86894111
MFAP1	2	MFAP1	2	0.02907723	0.86856092
ENSSSCG00000047735	2	ENSSSCG0000	2	0.00011454	0.86122889
RPL35A	2	RPL35A	2	4.03E-07	0.85869202
AFAP1L1	2	AFAP1L1	2	4.03E-07	0.85723363
LAMA3	2	LAMA3	2	0.00018834	0.85679007
TRIM44	2	TRIM44	2	0.00023867	0.85602597
ZCCHC17	2	ZCCHC17	2	0.00046721	0.85600832
CAPN12	2	CAPN12	2	1.73E-06	0.8491458
DND1	2	DND1	2	0.01407064	0.84573557
ENSSSCG00000043568	2	ENSSSCG0000	2	1.15E-07	0.84499997
CDH8	2	CDH8	2	0.00403509	0.84378117
PTPN23	2	PTPN23	2	0.00758365	0.83789052
GSK3A	2	GSK3A	2	6.10E-06	0.83700767
UBE3D	2	UBE3D	2	3.85E-06	0.83244818
PCCB	2	PCCB	2	7.61E-05	0.83077398
FAAP24	2	FAAP24	2	1.56E-05	0.8293466
SLC24A1	2	SLC24A1	2	0.01229347	0.82266207
ENSSSCG00000005101	2	ENSSSCG0000	2	0.00065647	0.81407934
PPP1R8	2	PPP1R8	2	0.00044659	0.81322106
IP6K1	2	IP6K1	2	0.00352904	0.81140445
ENSSSCG00000059837	2	ENSSSCG0000	2	0.02299018	0.81111491
FNDC1	2	FNDC1	2	2.81E-05	0.8045177
ENSSSCG00000050083	2	ENSSSCG0000	2	0.0001359	0.80256804
SLC37A1	2	SLC37A1	2	5.58E-06	0.7901249
WDTC1	2	WDTC1	2	0.00337011	0.78373487
SMIM8	2	SMIM8	2	0.00128303	0.77869906
KIAA2013	2	KIAA2013	2	0.00154718	0.77626083
ZNF574	2	ZNF574	2	0.03701085	0.77340111
RER1	2	RER1	2	0.03183432	0.75300826
ENSSSCG00000014242	2	ENSSSCG0000	2	1.09E-06	0.73855496
ENSSSCG00000024791	2	ENSSSCG0000	2	2.59E-06	0.72680807
TMEM268	2	TMEM268	2	2.90E-05	0.71831894
BOC	2	BOC	2	7.84E-06	0.70466431
PAX5	2	PAX5	2	5.75E-08	0.68817123
CENPT	2	CENPT	2	0.03361809	0.67517604
DFFA	2	DFFA	2	0.00071066	0.66011105
PLEKHM2	2	PLEKHM2	2	0.02694642	0.61455011
ENSSSCG00000003825	2	ENSSSCG0000	2	0.01955007	0.54778161
