# Supplementary material for: Deciphering differences in DNA methylation and transcriptome profiles of oocytes from pigs with high and low developmental competence
Source: Environ Epigenet. 2025 Jun 3;11(1):dvaf018. doi: 10.1093/eep/dvaf018 (PMC12418950; doi:10.1093/eep/dvaf018)
Supplement: dvaf018_Supplemental_Files [file dvaf018_supplemental_files.zip › Sup table 16.pdf]

Loci	factors	features	pvalues	log2FC
chr12.1990952.1991729_.C.	1	chr12.1990952.1991729_.C.	0.046	1.719
chr11.5042384.5044586_.C.	1	chr11.5042384.5044586_.C.	0.026	1.657
chr1.207173457.207174369_.C.	1	chr1.207173457.207174369_.C	0.026	1.623
chr11.9912809.9913316_.C.	1	chr11.9912809.9913316_.C.	0.004	1.613
chr1.75169857.75170800_.C.	1	chr1.75169857.75170800_.C.	0.034	1.549
chr11.4879361.4880102_.C.	1	chr11.4879361.4880102_.C.	0.000	1.543
chr1.8243705.8244469_.C.	1	chr1.8243705.8244469_.C.	0.003	1.524
chr12.56329633.56330718_.C.	1	chr12.56329633.56330718_.C.	0.001	1.467
chr1.130389264.130390128_.C.	1	chr1.130389264.130390128_.C	0.008	1.460
chr1.220952750.220953643_.C.	1	chr1.220952750.220953643_.C	0.004	1.446
chr1.271103816.271104402_.C.	1	chr1.271103816.271104402_.C	0.026	1.444
chr10.14183392.14184638_.C.	1	chr10.14183392.14184638_.C.	0.002	1.438
chr12.16254662.16256264_.C.	1	chr12.16254662.16256264_.C.	0.018	1.413
chr13.22124847.22125444_.C.	1	chr13.22124847.22125444_.C.	0.026	1.388
chr1.128641100.128642036_.C.	1	chr1.128641100.128642036_.C	0.030	1.377
chr1.239492188.239493251_.C.	1	chr1.239492188.239493251_.C	0.000	1.373
chr13.27647771.27648483_.C.	1	chr13.27647771.27648483_.C.	0.030	1.365
chr13.31308410.31309009_.C.	1	chr13.31308410.31309009_.C.	0.012	1.364
chr13.22883055.22883603_.C.	1	chr13.22883055.22883603_.C.	0.009	1.349
chr1.81996855.81997907_.C.	1	chr1.81996855.81997907_.C.	0.002	1.346
chr11.64308939.64309682_.C.	1	chr11.64308939.64309682_.C.	0.046	1.341
chr1.239285331.239286111_.C.	1	chr1.239285331.239286111_.C	0.008	1.318
chr1.185397819.185398991_.C.	1	chr1.185397819.185398991_.C	0.006	1.302
chr10.38889028.38889711_.C.	1	chr10.38889028.38889711_.C.	0.00693574	1.27880782
chr1.17136658.17138462_.C.	1	chr1.17136658.17138462_.C.	0.00313457	1.27634052
chr13.31627106.31627958_.C.	1	chr13.31627106.31627958_.C.	0.00435484	1.25460317
chr1.255168342.255169051_.C.	1	chr1.255168342.255169051_.C	0.026429	1.25419353
chr12.42849222.42850200_.C.	1	chr12.42849222.42850200_.C.	0.00370211	1.25255761
chr1.267964175.267965046_.C.	1	chr1.267964175.267965046_.C	0.00804229	1.24202681
chr10.31416365.31417437_.C.	1	chr10.31416365.31417437_.C.	0.00435484	1.22367144
chr1.98568735.98569606_.C.	1	chr1.98568735.98569606_.C.	0.00510422	1.22072674
chr1.217236861.217237767_.C.	1	chr1.217236861.217237767_.C	0.00105868	1.21666831
chr11.67710536.67711157_.C.	1	chr11.67710536.67711157_.C.	0.00222085	1.2141646
chr1.57128007.57129548_.C.	1	chr1.57128007.57129548_.C.	0.04630508	1.21296807
chr10.53602092.53603913_.C.	1	chr10.53602092.53603913_.C.	0.04630508	1.20323248
chr12.24679720.24680359_.C.	1	chr12.24679720.24680359_.C.	0.00929494	1.1965288
chr1.27247922.27250919_.C.	1	chr1.27247922.27250919_.C.	0.00596018	1.1929535
chr10.27953468.27954613_.C.	1	chr10.27953468.27954613_.C.	0.00011493	1.18978539
chr11.77354566.77356438_.C.	1	chr11.77354566.77356438_.C.	0.03729477	1.17550303
chr12.37693741.37694252_.C.	1	chr12.37693741.37694252_.C.	0.04630508	1.16547883
chr12.18257274.18258147_.C.	1	chr12.18257274.18258147_.C.	0.00510422	1.16470891
chr1.181681232.181681657_.C.	1	chr1.181681232.181681657_.C	0.0160561	1.15835622
chr10.10156482.10157235_.C.	1	chr10.10156482.10157235_.C.	0.00370211	1.15724159
chr1.186025525.186026828_.C.	1	chr1.186025525.186026828_.C	8.89E-05	1.14673863
chr1.142566853.142567341_.C.	1	chr1.142566853.142567341_.C	0.00693574	1.1215105
chr11.9737631.9738333_.C.	1	chr11.9737631.9738333_.C.	0.00693574	1.11077704
chr1.31796141.31797056_.C.	1	chr1.31796141.31797056_.C.	0.02389558	1.11018134
chr11.6762590.6763775_.C.	1	chr11.6762590.6763775_.C.	0.00313457	1.10663286
chr13.27320290.27324941_.C.	1	chr13.27320290.27324941_.C.	0.00596018	1.10091313
chr1.268465007.268466088_.C.	1	chr1.268465007.268466088_.C	0.00222085	1.10060799
chr12.52426498.52428609_.C.	1	chr12.52426498.52428609_.C.	0.00086971	1.09657216
chr11.52157608.52160831_.C.	1	chr11.52157608.52160831_.C.	0.00313457	1.08798001
chr11.68838249.68842592_.C.	1	chr11.68838249.68842592_.C.	0.01229347	1.08714829
chr11.21928915.21930059_.C.	1	chr11.21928915.21930059_.C.	0.02546328	1.08354888
chr12.52627516.52629243_.C.	1	chr12.52627516.52629243_.C.	0.00086971	1.08084507
chr1.242682071.242683303_.C.	1	chr1.242682071.242683303_.C	0.00154718	1.08051533
chr11.17947617.17948835_.C.	1	chr11.17947617.17948835_.C.	2.90E-05	1.06889115
chr11.66678135.66679652_.C.	1	chr11.66678135.66679652_.C.	0.00370211	1.06386069
chr1.120398506.120399196_.C.	1	chr1.120398506.120399196_.C	0.03334006	1.05674523
chr12.39739482.39740013_.C.	1	chr12.39739482.39740013_.C.	0.02071916	1.05389523
chr1.187718846.187720384_.C.	1	chr1.187718846.187720384_.C	0.02071916	1.05219254
chr1.270179344.270180174_.C.	1	chr1.270179344.270180174_.C	0.026429	1.04966525
chr11.15734905.15735621_.C.	1	chr11.15734905.15735621_.C.	0.00128303	1.0439389
chr1.123461438.123462218_.C.	1	chr1.123461438.123462218_.C	0.00071066	1.04206221
chr1.121067661.121068085_.C.	1	chr1.121067661.121068085_.C	0.00804229	1.03660377
chr13.23279037.23279516_.C.	1	chr13.23279037.23279516_.C.	0.01407064	1.0325477
chr10.46934776.46936137_.C.	1	chr10.46934776.46936137_.C.	0.02343317	1.02675134
chr1.239918638.239919296_.C.	1	chr1.239918638.239919296_.C	0.01407064	1.02634233
chr1.252323217.252324977_.C.	1	chr1.252323217.252324977_.C	0.0160561	1.02325563
chr1.7650678.7652010_.C.	1	chr1.7650678.7652010_.C.	0.01407064	1.00404516
chr11.65229604.65230677_.C.	1	chr11.65229604.65230677_.C.	0.00105868	1.00288754
chr1.267391415.267392150_.C.	1	chr1.267391415.267392150_.C	0.01229347	0.99995591
chr1.166465252.166466453_.C.	1	chr1.166465252.166466453_.C	3.89E-05	0.99906238
chr1.15328656.15330141_.C.	1	chr1.15328656.15330141_.C.	0.00086971	0.99797791
chr12.36831969.36832389_.C.	1	chr12.36831969.36832389_.C.	0.00105868	0.99691554
chr1.144787214.144788783_.C.	1	chr1.144787214.144788783_.C	0.01826556	0.99379972
chr1.268641282.268643052_.C.	1	chr1.268641282.268643052_.C	0.04630508	0.99031579
chr10.53314006.53315553_.C.	1	chr10.53314006.53315553_.C.	0.00264406	0.9822102
chr1.240494463.240496777_.C.	1	chr1.240494463.240496777_.C	0.00071066	0.97867901
chr1.249905208.249905824_.C.	1	chr1.249905208.249905824_.C	0.02270155	0.95752616
chr12.7637681.7639269_.C.	1	chr12.7637681.7639269_.C.	0.00313457	0.94163973
chr1.215806690.215808328_.C.	1	chr1.215806690.215808328_.C	0.00018834	0.94147572
chr10.31276259.31277324_.C.	1	chr10.31276259.31277324_.C.	0.00510422	0.93983135
chr1.147781309.147782595_.C.	1	chr1.147781309.147782595_.C	0.026429	0.92786864
chr1.272961037.272962500_.C.	1	chr1.272961037.272962500_.C	0.00222085	0.92726065
chr13.9213718.9214343_.C.	1	chr13.9213718.9214343_.C.	5.58E-06	0.92241828
chr11.12864358.12865079_.C.	1	chr11.12864358.12865079_.C.	0.01070637	0.92064019
chr1.7679156.7680433_.C.	1	chr1.7679156.7680433_.C.	0.00510422	0.91957312
chr13.32329740.32330722_.C.	1	chr13.32329740.32330722_.C.	0.01407064	0.90603459
chr11.3835568.3836152_.C.	1	chr11.3835568.3836152_.C.	0.02343317	0.90118143
chr12.8243844.8245010_.C.	1	chr12.8243844.8245010_.C.	0.00929494	0.90073338
chr11.16911110.16912081_.C.	1	chr11.16911110.16912081_.C.	0.00105868	0.89791535
chr1.239109878.239110333_.C.	1	chr1.239109878.239110333_.C	0.01826556	0.89511426
chr12.27439282.27440471_.C.	1	chr12.27439282.27440471_.C.	0.01826556	0.89475714
chr1.271462633.271464147_.C.	1	chr1.271462633.271464147_.C	0.03729477	0.88739475
chr11.15408631.15411432_.C.	1	chr11.15408631.15411432_.C.	0.00154718	0.88462581
chr10.33865976.33868184_.C.	1	chr10.33865976.33868184_.C.	0.026429	0.87837593
chr11.5332263.5334043_.C.	1	chr11.5332263.5334043_.C.	0.04630508	0.87393409
chr1.237785173.237786908_.C.	1	chr1.237785173.237786908_.C	0.00370211	0.87265859
chr1.268115922.268117045_.C.	1	chr1.268115922.268117045_.C	0.01070637	0.86985046
chr1.163525770.163526535_.C.	1	chr1.163525770.163526535_.C	0.00023867	0.86805451
chr1.241569484.241573110_.C.	1	chr1.241569484.241573110_.C	0.0160561	0.86349379
chr13.6984630.6986375_.C.	1	chr13.6984630.6986375_.C.	0.00370211	0.86319622
chr1.140566769.140568093_.C.	1	chr1.140566769.140568093_.C	0.02343317	0.86228602

Loci	factors	features	pvalues	log2FC
chr2.149531920.149535260_.V.	2	chr2.149531920.149535260_.V.	0.037	1.371
chr1.1727106.1729876_.V.	2	chr1.1727106.1729876_.V.	0.026	1.207
chr10.66844399.66847999_.V.	2	chr10.66844399.66847999_.V.	0.030	1.094
chr9.136753582.136756492_.V.	2	chr9.136753582.136756492_.V.	0.046	1.075
chr16.55458699.55460509_.V.	2	chr16.55458699.55460509_.V.	0.042	1.060
chrX.121752258.121755268_.V.	2	chrX.121752258.121755268_.V.	0.042	0.970
chr1.970705.972819_.C.	2	chr1.970705.972819_.C.	0.021	0.961
chr10.52445273.52447376_.C.	2	chr10.52445273.52447376_.C.	0.003	0.958
chr12.3166999.3170069_.V.	2	chr12.3166999.3170069_.V.	0.030	0.953
chr4.32680749.32684029_.V.	2	chr4.32680749.32684029_.V.	0.016	0.941
chr15.139048928.139052328_.V.	2	chr15.139048928.139052328_.V.	0.021	0.929
chr13.200128785.200129482_.C.	2	chr13.200128785.200129482_.C.	0.014	0.917
chr1.119948447.119948891_.C.	2	chr1.119948447.119948891_.C.	0.030	0.908
chr2.3694300.3695680_.V.	2	chr2.3694300.3695680_.V.	0.000	0.902
chr1.200792376.200792811_.C.	2	chr1.200792376.200792811_.C.	0.007	0.902
chr14.141098189.141104699_.V.	2	chr14.141098189.141104699_.V.	0.030	0.889
chr2.76277430.76279160_.V.	2	chr2.76277430.76279160_.V.	0.000	0.888
chr3.25891176.25896476_.V.	2	chr3.25891176.25896476_.V.	0.033	0.884
chr5.3952758.3955988_.V.	2	chr5.3952758.3955988_.V.	0.006	0.879
chr6.162378686.162382916_.V.	2	chr6.162378686.162382916_.V.	0.023	0.877
chr10.39874289.39876849_.V.	2	chr10.39874289.39876849_.V.	0.001	0.858
chr16.54267269.54269829_.V.	2	chr16.54267269.54269829_.V.	0.042	0.857
chr4.59549079.59551499_.V.	2	chr4.59549079.59551499_.V.	0.000	0.851
chr4.76065169.76068979_.V.	2	chr4.76065169.76068979_.V.	0.002	0.850
chr4.128935039.128939899_.V.	2	chr4.128935039.128939899_.V.	0.002	0.845
chr9.258992.261072_.V.	2	chr9.258992.261072_.V.	0.0003124	0.84244517
chr16.79741769.79744539_.V.	2	chr16.79741769.79744539_.V.	0.00105868	0.83256699
chr18.52624905.52628135_.V.	2	chr18.52624905.52628135_.V.	0.01407064	0.82969591
chr13.206843554.206847737_.C.	2	chr13.206843554.206847737_.C.	0.00313457	0.82654598
chr9.10731572.10734082_.V.	2	chr9.10731572.10734082_.V.	0.04886078	0.81978776
chr18.840575.842495_.V.	2	chr18.840575.842495_.V.	0.00071066	0.81834193
chr18.815975.818965_.V.	2	chr18.815975.818965_.V.	0.00222085	0.81772034
chr7.41816429.41818709_.V.	2	chr7.41816429.41818709_.V.	0.04852935	0.81634442
chr15.138868278.138870448_.V.	2	chr15.138868278.138870448_.V.	0.01826556	0.81216265
chr1.363396.374996_.V.	2	chr1.363396.374996_.V.	0.00023867	0.80881599
chr14.140303169.140305619_.V.	2	chr14.140303169.140305619_.V.	0.00313457	0.80486894
chr1.1921361.1921991_.C.	2	chr1.1921361.1921991_.C.	0.0413817	0.78926828
chr1.1776886.1789336_.V.	2	chr1.1776886.1789336_.V.	0.00222085	0.78874526
chr6.89558216.89560016_.V.	2	chr6.89558216.89560016_.V.	0.00264406	0.78726497
chr12.2141178.2144859_.C.	2	chr12.2141178.2144859_.C.	0.00030028	0.78266732
chr2.4256940.4259620_.V.	2	chr2.4256940.4259620_.V.	0.00222085	0.77042265
chr14.141392439.141394999_.V.	2	chr14.141392439.141394999_.V.	0.02343317	0.76310825
chr3.89803706.89805516_.V.	2	chr3.89803706.89805516_.V.	0.00929494	0.75836251
chr4.4245539.4248379_.V.	2	chr4.4245539.4248379_.V.	0.01070637	0.75779159
chr7.882149.884899_.V.	2	chr7.882149.884899_.V.	0.00435484	0.75749209
chr12.18868719.18875189_.V.	2	chr12.18868719.18875189_.V.	0.00804229	0.75720785
chr16.78729349.78736299_.V.	2	chr16.78729349.78736299_.V.	0.03334006	0.75645214
chr18.1701815.1705065_.V.	2	chr18.1701815.1705065_.V.	0.00510422	0.75520111
chr11.25643532.25644840_.C.	2	chr11.25643532.25644840_.C.	0.00086971	0.75501136
chr9.139054472.139056352_.V.	2	chr9.139054472.139056352_.V.	0.00929494	0.7507372
chr3.23198926.23200886_.V.	2	chr3.23198926.23200886_.V.	0.02071916	0.74819679
chr10.24458712.24459669_.C.	2	chr10.24458712.24459669_.C.	0.04152286	0.74675826
chr3.49441556.49445936_.V.	2	chr3.49441556.49445936_.V.	0.00264406	0.74449459
chr14.140087869.140090589_.V.	2	chr14.140087869.140090589_.V.	0.04161078	0.7390735
chr6.166829006.166831646_.V.	2	chr6.166829006.166831646_.V.	0.03334006	0.73841248
chr11.5241105.5241689_.C.	2	chr11.5241105.5241689_.C.	0.00804229	0.73748073
chr5.1771438.1776178_.V.	2	chr5.1771438.1776178_.V.	0.04630508	0.73711452
chr9.138947412.138952722_.V.	2	chr9.138947412.138952722_.V.	0.00435484	0.73496171
chr7.161119.166329_.V.	2	chr7.161119.166329_.V.	0.02594227	0.73290416
chr12.7581599.7584879_.V.	2	chr12.7581599.7584879_.V.	0.01229347	0.73073467
chr7.79413829.79415779_.V.	2	chr7.79413829.79415779_.V.	0.00370211	0.73064261
chr18.5873195.5876335_.V.	2	chr18.5873195.5876335_.V.	0.01826556	0.73051492
chrX.125184248.125185808_.V.	2	chrX.125184248.125185808_.V.	0.01407064	0.72665939
chr8.55570718.55572198_.V.	2	chr8.55570718.55572198_.V.	0.0269349	0.72620066
chr14.92224439.92226169_.V.	2	chr14.92224439.92226169_.V.	0.00370211	0.72573849
chr13.206865565.206869585_.V.	2	chr13.206865565.206869585_.V.	0.01826556	0.72477738
chr7.7795369.7798579_.V.	2	chr7.7795369.7798579_.V.	0.0160561	0.72460129
chr12.33861199.33867119_.V.	2	chr12.33861199.33867119_.V.	0.00693574	0.72152538
chr2.2723860.2733450_.V.	2	chr2.2723860.2733450_.V.	0.00693574	0.71713057
chr10.8917679.8921219_.V.	2	chr10.8917679.8921219_.V.	0.00057789	0.71580496
chr7.4273899.4274969_.V.	2	chr7.4273899.4274969_.V.	0.01498872	0.7155158
chr6.167656186.167665426_.V.	2	chr6.167656186.167665426_.V.	0.03334006	0.71429149
chr1.400186.404786_.V.	2	chr1.400186.404786_.V.	0.00037581	0.71198167
chr1.731516.740006_.V.	2	chr1.731516.740006_.V.	0.00510422	0.71193408
chr3.131726366.131731426_.V.	2	chr3.131726366.131731426_.V.	0.01826556	0.71173613
chr11.2602205.2603499_.C.	2	chr11.2602205.2603499_.C.	0.04630508	0.70797367
chr12.448754.450833_.C.	2	chr12.448754.450833_.C.	0.01229347	0.70764168
chr1.1717576.1722746_.V.	2	chr1.1717576.1722746_.V.	0.00037581	0.70667967
chr2.2705840.2713480_.V.	2	chr2.2705840.2713480_.V.	0.01826556	0.70361713
chr5.75448848.75450858_.V.	2	chr5.75448848.75450858_.V.	0.026429	0.70341598
chr12.6336839.6340259_.V.	2	chr12.6336839.6340259_.V.	0.04161078	0.70132991
chr9.11174532.11178572_.V.	2	chr9.11174532.11178572_.V.	0.01826556	0.69356819
chr1.379816.389776_.V.	2	chr1.379816.389776_.V.	0.00086971	0.69326083
chr18.3040845.3044465_.V.	2	chr18.3040845.3044465_.V.	0.00128303	0.6923778
chr12.1760259.1764969_.V.	2	chr12.1760259.1764969_.V.	0.026429	0.6907308
chr4.1900849.1906229_.V.	2	chr4.1900849.1906229_.V.	0.01229347	0.68859612
chr10.66708949.66710689_.V.	2	chr10.66708949.66710689_.V.	0.026429	0.68801853
chr18.472955.475115_.V.	2	chr18.472955.475115_.V.	0.02972401	0.68639619
chr4.128389539.128391119_.V.	2	chr4.128389539.128391119_.V.	0.00313457	0.68593859
chrX.88048758.88052718_.V.	2	chrX.88048758.88052718_.V.	0.02264068	0.68395965
chr15.139822668.139834538_.V.	2	chr15.139822668.139834538_.V.	0.00071066	0.68222241
chr18.3716145.3718925_.V.	2	chr18.3716145.3718925_.V.	0.00046721	0.68068933
chr7.802119.805159_.V.	2	chr7.802119.805159_.V.	0.00014772	0.68028295
chr7.867759.873679_.V.	2	chr7.867759.873679_.V.	0.01407064	0.67953931
chr11.74017450.74021200_.V.	2	chr11.74017450.74021200_.V.	0.00435484	0.67838132
chr17.61321802.61328422_.V.	2	chr17.61321802.61328422_.V.	0.00929494	0.67726224
chr1.66515495.66516311_.C.	2	chr1.66515495.66516311_.C.	0.00046721	0.67725387
chr12.1743839.1753369_.V.	2	chr12.1743839.1753369_.V.	0.04161078	0.67333283
chr18.1154575.1159705_.V.	2	chr18.1154575.1159705_.V.	0.00929494	0.67122901
chr12.6214528.6215144_.C.	2	chr12.6214528.6215144_.C.	0.02972401	0.66945925
chr12.1304812.1306458_.C.	2	chr12.1304812.1306458_.C.	0.00510422	0.66857989
chr12.43165819.43169679_.V.	2	chr12.43165819.43169679_.V.	0.026429	0.66765897
chr18.1148775.1153895_.V.	2	chr18.1148775.1153895_.V.	0.026429	0.66553301
chr12.51843603.51844550_.C.	2	chr12.51843603.51844550_.C.	0.00264406	0.66345042

chr1.108514583.108515849_.C.	1	chr1.108514583.108515849_.C	0.02972401	0.86131419
chr10.14294698.14296364_.C.	1	chr10.14294698.14296364_.C.	0.04159344	0.85673432
chr1.272954630.272955607_.C.	1	chr1.272954630.272955607_.C	0.01229347	0.84699072
chr12.41725597.41727813_.C.	1	chr12.41725597.41727813_.C.	0.0028429	0.84489714
chr1.109010167.109010965_.C.	1	chr1.109010167.109010965_.C	0.02343317	0.84351927
chr1.268652871.268653576_.C.	1	chr1.268652871.268653576_.C	0.01070637	0.83903114
chr10.12557515.12558552_.C.	1	chr10.12557515.12558552_.C.	0.00370211	0.82911634
chr1.228044376.228045374_.C.	1	chr1.228044376.228045374_.C	0.00929494	0.82497827
chr12.47124265.47125516_.C.	1	chr12.47124265.47125516_.C.	0.00057789	0.81925448
chr1.32418386.32419693_.C.	1	chr1.32418386.32419693_.C.	0.00510422	0.81885789
chr12.53553347.53554403_.C.	1	chr12.53553347.53554403_.C.	0.00370211	0.81882767
chr1.6996235.6997358_.C.	1	chr1.6996235.6997358_.C.	0.00264406	0.8178679
chr13.11358483.11360380_.C.	1	chr13.11358483.11360380_.C.	0.03729477	0.81611549
chr1.3118820.3122474_.C.	1	chr1.3118820.3122474_.C.	0.02343317	0.81531691
chr1.25480793.25481989_.C.	1	chr1.25480793.25481989_.C.	0.02972401	0.8114562
chr1.121413109.121414480_.C.	1	chr1.121413109.121414480_.C	0.04630508	0.80878873
chr1.158860711.158862808_.C.	1	chr1.158860711.158862808_.C	0.00929494	0.8049158
chr1.128305408.128305965_.C.	1	chr1.128305408.128305965_.C	0.026429	0.7901294
chr11.23237035.23238925_.C.	1	chr11.23237035.23238925_.C.	0.00105868	0.78916675
chr11.19106367.19107200_.C.	1	chr11.19106367.19107200_.C.	0.02343317	0.78548294
chr1.264195399.264196413_.C.	1	chr1.264195399.264196413_.C	0.03334006	0.78487665
chr13.21877378.21878749_.C.	1	chr13.21877378.21878749_.C.	0.01229347	0.78431854
chr1.265934267.265935360_.C.	1	chr1.265934267.265935360_.C	0.00046721	0.77801248
chr1.26254832.26255836_.C.	1	chr1.26254832.26255836_.C.	0.01070637	0.77611307
chr11.17689461.17691697_.C.	1	chr11.17689461.17691697_.C.	0.00185787	0.76972999
chr1.118913210.118914612_.C.	1	chr1.118913210.118914612_.C	0.02972401	0.76576357
chr13.12574830.12575751_.C.	1	chr13.12574830.12575751_.C.	0.00804229	0.75985991
chr13.18371847.18373032_.C.	1	chr13.18371847.18373032_.C.	0.04630508	0.75922379
chr11.49807826.49808981_.C.	1	chr11.49807826.49808981_.C.	0.00014772	0.75895284
chr10.65471676.65472178_.C.	1	chr10.65471676.65472178_.C.	0.00370211	0.75701238
chr1.109921476.109922526_.C.	1	chr1.109921476.109922526_.C	0.00510422	0.75356931
chr15.76991098.76995238_.V.	1	chr15.76991098.76995238_.V.	0.00929494	0.75113167
chr1.236340984.236341976_.C.	1	chr1.236340984.236341976_.C	0.01229347	0.74651503
chr1.251570577.251571193_.C.	1	chr1.251570577.251571193_.C	0.02071916	0.7456884
chr1.97734395.97735500_.C.	1	chr1.97734395.97735500_.C.	0.00264406	0.74562468
chr1.119758703.119759757_.C.	1	chr1.119758703.119759757_.C	0.03729477	0.73880982
chr1.230906557.230908239_.C.	1	chr1.230906557.230908239_.C	0.00929494	0.73801718
chr1.57423069.57424350_.C.	1	chr1.57423069.57424350_.C.	0.00596018	0.73743776
chr13.40768236.40768750_.C.	1	chr13.40768236.40768750_.C.	0.00154718	0.73354073
chr11.13959329.13960821_.C.	1	chr11.13959329.13960821_.C.	0.03334006	0.7300881
chr1.25735449.25736948_.C.	1	chr1.25735449.25736948_.C.	0.00596018	0.7271449
chr1.242169220.242169827_.C.	1	chr1.242169220.242169827_.C	0.00037581	0.71578232
chr1.250680302.250681256_.C.	1	chr1.250680302.250681256_.C	0.0160561	0.70492106
chr1.137855508.137856731_.C.	1	chr1.137855508.137856731_.C	0.03729477	0.69907477
chr1.221581373.221582232_.C.	1	chr1.221581373.221582232_.C	0.01407064	0.69280121
chr13.35179148.35181193_.C.	1	chr13.35179148.35181193_.C.	0.03334006	0.68988919
chr10.19888436.19889542_.C.	1	chr10.19888436.19889542_.C.	0.00046721	0.68583108
chr1.228854463.228855381_.C.	1	chr1.228854463.228855381_.C	0.00057789	0.67823531
chr1.270363751.270364715_.C.	1	chr1.270363751.270364715_.C	0.0160561	0.67777022
chr11.75544552.75545320_.C.	1	chr11.75544552.75545320_.C.	0.00804229	0.67622062
chr1.145707133.145707900_.C.	1	chr1.145707133.145707900_.C	0.00804229	0.6706088
chr11.5793860.5797121_.C.	1	chr11.5793860.5797121_.C.	0.00435484	0.66348787
chr11.77979282.77981624_.C.	1	chr11.77979282.77981624_.C.	0.00154718	0.66104928
chr1.115219826.115220413_.C.	1	chr1.115219826.115220413_.C	0.00693574	0.65539857
chr1.262203524.262204368_.C.	1	chr1.262203524.262204368_.C	0.00370211	0.65135442
chr10.68769934.68770936_.C.	1	chr10.68769934.68770936_.C.	0.00086971	0.64099712
chr1.96839460.96842049_.C.	1	chr1.96839460.96842049_.C.	0.02071916	0.64021417
chr1.130384819.130388088_.C.	1	chr1.130384819.130388088_.C	0.00264406	0.63208304
chr1.180591892.180593286_.C.	1	chr1.180591892.180593286_.C	0.00154718	0.6310611
chr1.52997619.52998959_.C.	1	chr1.52997619.52998959_.C.	0.00222085	0.62385119
chr10.54675617.54677199_.C.	1	chr10.54675617.54677199_.C.	0.00185787	0.62053857
chr1.4882178.4885075_.C.	1	chr1.4882178.4885075_.C.	0.00804229	0.61682269
chr1.238825022.238826808_.C.	1	chr1.238825022.238826808_.C	0.00014772	0.61565831
chr12.52440268.52442734_.C.	1	chr12.52440268.52442734_.C.	0.00185787	0.61520707
chr1.95727119.95728266_.C.	1	chr1.95727119.95728266_.C.	0.00023867	0.61073149
chr1.188597843.188598626_.C.	1	chr1.188597843.188598626_.C	2.90E-05	0.60573349
chr13.22706057.22708111_.C.	1	chr13.22706057.22708111_.C.	0.02343317	0.60238158
chr13.5415652.5417586_.C.	1	chr13.5415652.5417586_.C.	0.01407064	0.60081657
chr13.1127397.1128336_.C.	1	chr13.1127397.1128336_.C.	0.00154718	0.60074304
chr15.48693778.48695388_.V.	1	chr15.48693778.48695388_.V.	0.01826556	0.59255948
chr1.72815404.72815846_.C.	1	chr1.72815404.72815846_.C.	0.01229347	0.58602689
chr10.68528038.68529249_.C.	1	chr10.68528038.68529249_.C.	0.00154718	0.58369463
chr11.46459363.46461173_.C.	1	chr11.46459363.46461173_.C.	0.02071916	0.57564938
chr1.83849134.83850970_.C.	1	chr1.83849134.83850970_.C.	0.00264406	0.57374206
chr13.30601700.30602411_.C.	1	chr13.30601700.30602411_.C.	0.00128303	0.56972592
chr13.31990619.31991906_.C.	1	chr13.31990619.31991906_.C.	2.90E-05	0.56653958
chr10.68621049.68622278_.C.	1	chr10.68621049.68622278_.C.	0.00693574	0.5629601
chr10.34001454.34002137_.C.	1	chr10.34001454.34002137_.C.	0.00435484	0.56013601
chr1.198992230.198993785_.C.	1	chr1.198992230.198993785_.C	0.00014772	0.55656681
chr1.14866348.14867856_.C.	1	chr1.14866348.14867856_.C.	0.00105868	0.55256361
chr1.164997476.164999127_.C.	1	chr1.164997476.164999127_.C	0.00313457	0.55161347
chr1.138340690.138342678_.C.	1	chr1.138340690.138342678_.C	0.04852935	0.54476134
chr1.181771496.181773290_.C.	1	chr1.181771496.181773290_.C	0.04161078	0.54008294
chr1.150709729.150712378_.C.	1	chr1.150709729.150712378_.C	0.01826556	0.53871568
chr10.51453624.51455597_.C.	1	chr10.51453624.51455597_.C.	0.00435484	0.538111
chr12.1317683.1322503_.C.	1	chr12.1317683.1322503_.C.	0.04161078	0.52999762
chr11.49029946.49031917_.C.	1	chr11.49029946.49031917_.C.	0.03729477	0.52688895
chr1.37911315.37912718_.C.	1	chr1.37911315.37912718_.C.	0.00804229	0.52653583
chr9.68507352.68510302_.V.	1	chr9.68507352.68510302_.V.	0.01826556	0.52491738
chr12.45760921.45763958_.C.	1	chr12.45760921.45763958_.C.	0.01826556	0.52488549
chr1.31184928.31185414_.C.	1	chr1.31184928.31185414_.C.	0.03729477	0.52185475
chr11.12576418.12577666_.C.	1	chr11.12576418.12577666_.C.	0.00693574	0.51882478
chr10.20732446.20733451_.C.	1	chr10.20732446.20733451_.C.	0.02071916	0.51080266
chr7.13377029.13380019_.V.	1	chr7.13377029.13380019_.V.	0.00154718	0.50404716
chr13.18030794.18031998_.C.	1	chr13.18030794.18031998_.C.	0.00222085	0.47839168
chr11.862370.863216_.C.	1	chr11.862370.863216_.C.	0.01070637	0.47387007
chr1.16139115.16140322_.C.	1	chr1.16139115.16140322_.C.	0.026429	0.4712128
chr8.120370758.120373218_.V.	1	chr8.120370758.120373218_.V	0.00596018	0.45417238
chr11.60731373.60733345_.C.	1	chr11.60731373.60733345_.C.	0.03729477	0.44985925
chr1.181995066.181995661_.C.	1	chr1.181995066.181995661_.C	0.00313457	0.44816134
chr1.237777497.237779049_.C.	1	chr1.237777497.237779049_.C	0.03729477	0.44726661
chr11.64042445.64043710_.C.	1	chr11.64042445.64043710_.C.	0.00693574	0.44549445
chr1.147461608.147463377_.C.	1	chr1.147461608.147463377_.C	0.02972401	0.43370471
chr11.51494201.51494648_.C.	1	chr11.51494201.51494648_.C.	0.026429	0.42219284
chr1.256544838.256545500_.C.	1	chr1.256544838.256545500_.C	0.03729477	0.40915993

chr13.206034618.206037701_.C.	2	chr13.206034618.206037701_.C.	0.04630508	0.66276978
chr14.140414459.140419889_.V.	2	chr14.140414459.140419889_.V.	0.00804229	0.66261548
chr14.137927429.137930519_.V.	2	chr14.137927429.137930519_.V.	0.03729477	0.66171087
chr10.66111510.66112730_.C.	2	chr10.66111510.66112730_.C.	0.00370211	0.65965599
chr12.50728138.50729135_.C.	2	chr12.50728138.50729135_.C.	0.00128303	0.65885268
chr16.77381779.77384759_.V.	2	chr16.77381779.77384759_.V.	0.00130583	0.65584085
chr6.65005436.65012316_.V.	2	chr6.65005436.65012316_.V.	0.01229347	0.65560797
chr1.286656.289836_.V.	2	chr1.286656.289836_.V.	0.00105868	0.65469983
chr9.138513222.138516292_.V.	2	chr9.138513222.138516292_.V.	0.02694642	0.65432946
chrX.122227078.122232108_.V.	2	chrX.122227078.122232108_.V.	0.04161078	0.65276169
chr11.5623444.5624326_.C.	2	chr11.5623444.5624326_.C.	0.00185787	0.65268502
chr4.76733289.76735109_.V.	2	chr4.76733289.76735109_.V.	0.00014772	0.65246856
chr4.7238559.7243049_.V.	2	chr4.7238559.7243049_.V.	0.026429	0.65234466
chr6.64710876.64718966_.V.	2	chr6.64710876.64718966_.V.	0.01826556	0.64979602
chr15.139052628.139057078_.V.	2	chr15.139052628.139057078_.V.	0.00011493	0.6479606
chr14.140746869.140750169_.V.	2	chr14.140746869.140750169_.V.	0.02071916	0.64567714
chr1.2003255.2004766_.C.	2	chr1.2003255.2004766_.C.	0.01090343	0.64399029
chr1.273651376.273656236_.V.	2	chr1.273651376.273656236_.V.	0.03729477	0.64375096
chr7.121088159.121093409_.V.	2	chr7.121088159.121093409_.V.	0.01070637	0.64364981
chr3.126148606.126153916_.V.	2	chr3.126148606.126153916_.V.	0.026429	0.64307555
chr15.137692318.137698258_.V.	2	chr15.137692318.137698258_.V.	0.00030028	0.64233066
chr1.986216.988616_.V.	2	chr1.986216.988616_.V.	0.00057789	0.64188183
chr12.32816299.32818319_.V.	2	chr12.32816299.32818319_.V.	0.00510422	0.64022619
chr6.64663816.64666816_.V.	2	chr6.64663816.64666816_.V.	0.01159305	0.64011072
chr8.4901688.4905898_.V.	2	chr8.4901688.4905898_.V.	0.03729477	0.63790019
chr5.5025318.5031868_.V.	2	chr5.5025318.5031868_.V.	0.026429	0.63729535
chr16.78552969.78556889_.V.	2	chr16.78552969.78556889_.V.	0.01229347	0.63640626
chr14.140038389.140042669_.V.	2	chr14.140038389.140042669_.V.	0.00071066	0.63622251
chr7.1463629.1466979_.V.	2	chr7.1463629.1466979_.V.	0.00189937	0.63538756
chr11.77426533.77428310_.C.	2	chr11.77426533.77428310_.C.	0.02972401	0.63326349
chr18.1416435.1421805_.V.	2	chr18.1416435.1421805_.V.	0.00596018	0.63194184
chr18.3673835.3675515_.V.	2	chr18.3673835.3675515_.V.	0.00804229	0.63059599
chr11.78891930.78896300_.V.	2	chr11.78891930.78896300_.V.	0.02972401	0.62914037
chr17.61222882.61224592_.V.	2	chr17.61222882.61224592_.V.	0.02071916	0.62730045
chr1.3066316.3070096_.V.	2	chr1.3066316.3070096_.V.	0.00510422	0.62512877
chr8.131031428.131035218_.V.	2	chr8.131031428.131035218_.V.	0.02343317	0.62479483
chr1.969496.973026_.V.	2	chr1.969496.973026_.V.	0.00023867	0.62439519
chr14.139651639.139655749_.V.	2	chr14.139651639.139655749_.V.	0.02972401	0.62434269
chr10.66654679.66661289_.V.	2	chr10.66654679.66661289_.V.	0.00264406	0.62432655
chr13.206321005.206328245_.V.	2	chr13.206321005.206328245_.V.	0.00037581	0.62368869
chr16.76625789.76633749_.V.	2	chr16.76625789.76633749_.V.	0.04161078	0.62212869
chr18.2235895.2238315_.V.	2	chr18.2235895.2238315_.V.	0.00370211	0.62182785
chr3.132616516.132619386_.V.	2	chr3.132616516.132619386_.V.	0.01826556	0.6195149
chr11.76710950.76713460_.V.	2	chr11.76710950.76713460_.V.	0.02972401	0.61906318
chr14.21009729.21013389_.V.	2	chr14.21009729.21013389_.V.	0.00804229	0.61854195
chr17.60916312.60920102_.V.	2	chr17.60916312.60920102_.V.	0.00693574	0.6184615
chr1.90466.92736_.V.	2	chr1.90466.92736_.V.	0.00071066	0.61696186
chr18.48989585.48995365_.V.	2	chr18.48989585.48995365_.V.	0.00030028	0.61689
chr1.188729585.188729940_.C.	2	chr1.188729585.188729940_.C.	0.00370211	0.61641898
chr13.206309755.206315565_.V.	2	chr13.206309755.206315565_.V.	0.00435484	0.61405647
chr14.137175889.137179879_.V.	2	chr14.137175889.137179879_.V.	6.81E-05	0.61322703
chr1.2044406.2047956_.V.	2	chr1.2044406.2047956_.V.	0.02343317	0.61170037
chr18.50064445.50066235_.V.	2	chr18.50064445.50066235_.V.	0.00313457	0.61079055
chr9.10448262.10452072_.V.	2	chr9.10448262.10452072_.V.	0.00046721	0.60841888
chr9.135368712.135371122_.V.	2	chr9.135368712.135371122_.V.	0.03729477	0.60671156
chr11.65200901.65201681_.C.	2	chr11.65200901.65201681_.C.	0.00154718	0.60621835
chr18.52549975.52553555_.V.	2	chr18.52549975.52553555_.V.	0.00030028	0.60588649
chr5.3279228.3281228_.V.	2	chr5.3279228.3281228_.V.	0.026429	0.60494376
chr4.7447329.7451569_.V.	2	chr4.7447329.7451569_.V.	0.01826556	0.6049043
chrX.35573888.35577558_.V.	2	chrX.35573888.35577558_.V.	0.02071916	0.60411535
chr10.64054469.64079119_.V.	2	chr10.64054469.64079119_.V.	0.02343317	0.6036888
chr17.60939522.60943472_.V.	2	chr17.60939522.60943472_.V.	0.00370211	0.60357346
chr5.26146488.26152818_.V.	2	chr5.26146488.26152818_.V.	0.00071066	0.6028681
chr18.50056015.50059125_.V.	2	chr18.50056015.50059125_.V.	0.02343317	0.60202666
chr17.57631282.57634562_.V.	2	chr17.57631282.57634562_.V.	0.00510422	0.60073059
chr13.208214115.208217535_.V.	2	chr13.208214115.208217535_.V.	0.00023867	0.6004645
chr3.126639236.126644176_.V.	2	chr3.126639236.126644176_.V.	0.00071066	0.60032808
chr13.206031271.206032506_.C.	2	chr13.206031271.206032506_.C.	0.04630508	0.59896621
chr14.138182749.138186599_.V.	2	chr14.138182749.138186599_.V.	0.00370211	0.59765548
chr12.18864249.18866079_.V.	2	chr12.18864249.18866079_.V.	0.00804229	0.5976492
chr18.50062425.50064345_.V.	2	chr18.50062425.50064345_.V.	0.00128303	0.59744979
chr5.3321948.3323428_.V.	2	chr5.3321948.3323428_.V.	0.00086971	0.59557879
chr3.15937626.15941906_.V.	2	chr3.15937626.15941906_.V.	0.00596018	0.59556455
chr11.1673848.1674791_.C.	2	chr11.1673848.1674791_.C.	0.00086971	0.59442824
chr10.1075259.1078819_.V.	2	chr10.1075259.1078819_.V.	0.02343317	0.59401662
chr14.28802819.28808389_.V.	2	chr14.28802819.28808389_.V.	0.00693574	0.59396114
chr3.125782346.125785516_.V.	2	chr3.125782346.125785516_.V.	0.01229347	0.59382968
chr9.136453712.136457442_.V.	2	chr9.136453712.136457442_.V.	0.01407064	0.59155893
chr3.126125186.126128636_.V.	2	chr3.126125186.126128636_.V.	0.04161078	0.59098428
chr12.26745423.26746618_.C.	2	chr12.26745423.26746618_.C.	0.02343317	0.5908833
chr11.78263210.78271840_.V.	2	chr11.78263210.78271840_.V.	0.00023867	0.59062497
chr5.837998.842798_.V.	2	chr5.837998.842798_.V.	0.00154718	0.59056886
chr5.5176438.5181598_.V.	2	chr5.5176438.5181598_.V.	0.02972401	0.58996226
chr7.1440269.1447389_.V.	2	chr7.1440269.1447389_.V.	0.01875602	0.58949916
chr14.7359889.7361529_.V.	2	chr14.7359889.7361529_.V.	0.02071916	0.58598634
chr3.125787726.125792026_.V.	2	chr3.125787726.125792026_.V.	0.00023867	0.58507097
chr18.851535.853675_.V.	2	chr18.851535.853675_.V.	0.00086971	0.58456723
chr12.40492457.40493372_.C.	2	chr12.40492457.40493372_.C.	0.0160561	0.58367293
chr14.136710069.136717369_.V.	2	chr14.136710069.136717369_.V.	5.18E-05	0.58273044
chr10.67517009.67525249_.V.	2	chr10.67517009.67525249_.V.	0.00037581	0.58135611
chr16.76567329.76569229_.V.	2	chr16.76567329.76569229_.V.	0.00804229	0.58042609
chr2.1598720.1611190_.V.	2	chr2.1598720.1611190_.V.	0.026429	0.57920753
chr14.136545109.136548939_.V.	2	chr14.136545109.136548939_.V.	0.00014772	0.578981
chr16.77903549.77906319_.V.	2	chr16.77903549.77906319_.V.	0.00071066	0.57851221
chr14.141175729.141179889_.V.	2	chr14.141175729.141179889_.V.	0.01070637	0.57734921
chr4.129774569.129779739_.V.	2	chr4.129774569.129779739_.V.	0.01070637	0.57622243
chr1.807820.809441_.C.	2	chr1.807820.809441_.C.	0.01470167	0.57543925
chr15.139604568.139621818_.V.	2	chr15.139604568.139621818_.V.	0.00185787	0.57535986
chr13.206209785.206213475_.V.	2	chr13.206209785.206213475_.V.	0.00011493	0.57373562
chr12.3215499.3219479_.V.	2	chr12.3215499.3219479_.V.	0.04630508	0.57364645
chr10.5725169.5727699_.V.	2	chr10.5725169.5727699_.V.	0.026429	0.57308846
chr15.109247828.109253198_.V.	2	chr15.109247828.109253198_.V.	0.00057789	0.57303158
chr16.78852469.78857009_.V.	2	chr16.78852469.78857009_.V.	0.00510422	0.57267732
chr4.76888489.76894389_.V.	2	chr4.76888489.76894389_.V.	0.01826556	0.57086179
chr18.1516805.1521415_.V.	2	chr18.1516805.1521415_.V.	0.00804229	0.57063189

chr2.66344600.66347110_.V.	1	chr2.66344600.66347110_.V.	0.00596018	0.40873798
chr16.52819079.52825559_.V.	1	chr16.52819079.52825559_.V.	0.00250694	0.38708922
chr6.31746036.31749326_.V.	1	chr6.31746036.31749326_.V.	0.02404913	0.38530218
chr10.53065708.53067189_.C.	1	chr10.53065708.53067189_.C.	0.02071916	0.37893328
chr18.18978175.18981285_.V.	1	chr18.18978175.18981285_.V.	0.00264406	0.37720222
chrX.118824848.118830998_.V.	1	chrX.118824848.118830998_.V	0.00557874	0.37254679
chr1.261898896.261900556_.V.	1	chr1.261898896.261900556_.V	0.04161078	0.35472034
chr14.105010519.105014699_.V.	1	chr14.105010519.105014699_.	0.02071916	0.35364905
chr2.142577020.142579530_.V.	1	chr2.142577020.142579530_.V	0.02071916	0.35103601
chr6.137522376.137525646_.V.	1	chr6.137522376.137525646_.V	0.04161078	0.33727316
chr10.11334439.11336379_.V.	1	chr10.11334439.11336379_.V.	0.00128303	0.3343449
chr4.130422279.130428829_.V.	1	chr4.130422279.130428829_.V	0.04159344	0.33288593
chrX.10217608.10219668_.V.	1	chrX.10217608.10219668_.V.	0.00105868	0.33012925
chr2.137765440.137768060_.V.	1	chr2.137765440.137768060_.V	2.14E-05	0.32883584
chr4.122826159.122828559_.V.	1	chr4.122826159.122828559_.V	0.00105868	0.31889841
chr13.65994525.65997625_.V.	1	chr13.65994525.65997625_.V.	0.04381021	0.31703106
chr9.133385662.133389422_.V.	1	chr9.133385662.133389422_.V	0.04159344	0.30479574
chr6.55198826.55201756_.V.	1	chr6.55198826.55201756_.V.	0.026429	0.30381661
chr2.142639970.142642910_.V.	1	chr2.142639970.142642910_.V	0.01826556	0.30283191
chr3.30269976.30274766_.V.	1	chr3.30269976.30274766_.V.	0.026429	0.30157507
chr7.17694559.17696599_.V.	1	chr7.17694559.17696599_.V.	0.00222085	0.28905052
chr2.8410290.8416580_.V.	1	chr2.8410290.8416580_.V.	0.00693574	0.27774353
chr18.14065305.14068395_.V.	1	chr18.14065305.14068395_.V.	0.0160561	0.2775579
chr9.107744692.107748482_.V.	1	chr9.107744692.107748482_.V	0.01070637	0.27389805
chr6.56225666.56227716_.V.	1	chr6.56225666.56227716_.V.	0.02343317	0.27207396
chr12.1120907.1129651_.C.	1	chr12.1120907.1129651_.C.	0.00222085	0.26739823
chr18.51493135.51495445_.V.	1	chr18.51493135.51495445_.V.	0.00037581	0.25838689

chr15.132128328.132133218_.V.	2	chr15.132128328.132133218_.V.	0.00057789	0.57057448
chr3.125810976.125812866_.V.	2	chr3.125810976.125812866_.V.	0.026429	0.57043868
chrX.42955808.42959908_.V.	2	chrX.42955808.42959908_.V.	0.02071916	0.56978568
chr14.138976219.138981529_.V.	2	chr14.138976219.138981529_.V.	0.02343317	0.56856511
chr14.141119109.141125409_.V.	2	chr14.141119109.141125409_.V.	0.00435484	0.56846529
chr1.7364266.7367856_.V.	2	chr1.7364266.7367856_.V.	0.02972401	0.56719267
chr1.267741236.267743686_.V.	2	chr1.267741236.267743686_.V.	6.81E-05	0.56677237
chr4.79393579.79401379_.V.	2	chr4.79393579.79401379_.V.	0.01407064	0.56643625
chr9.10001892.10004452_.V.	2	chr9.10001892.10004452_.V.	0.0160561	0.56611268
chr1.1017936.1021376_.V.	2	chr1.1017936.1021376_.V.	0.00128303	0.5655216
chr18.52395835.52398155_.V.	2	chr18.52395835.52398155_.V.	0.00046721	0.56528697
chr13.205508615.205514475_.V.	2	chr13.205508615.205514475_.V.	0.01407064	0.5645838
chr14.139000749.139004889_.V.	2	chr14.139000749.139004889_.V.	0.00313457	0.56429381
chr5.39884708.39889348_.V.	2	chr5.39884708.39889348_.V.	0.00086971	0.56235362
chr1.228205316.228208576_.V.	2	chr1.228205316.228208576_.V.	0.026429	0.5622716
chr14.141145779.141148769_.V.	2	chr14.141145779.141148769_.V.	0.03729477	0.56006256
chr18.55603325.55605505_.V.	2	chr18.55603325.55605505_.V.	0.01229347	0.55934667
chr1.1712876.1715666_.V.	2	chr1.1712876.1715666_.V.	2.14E-05	0.55875554
chr2.4377340.4383010_.V.	2	chr2.4377340.4383010_.V.	0.01070637	0.55663694
chr15.139858888.139868418_.V.	2	chr15.139858888.139868418_.V.	0.01407064	0.55636126
chr11.5415520.5417840_.V.	2	chr11.5415520.5417840_.V.	0.00105868	0.55627677
chr14.141151169.141169699_.V.	2	chr14.141151169.141169699_.V.	0.00030028	0.55607947
chr16.78837739.78842699_.V.	2	chr16.78837739.78842699_.V.	0.04161078	0.55602778
chr17.30801572.30807492_.V.	2	chr17.30801572.30807492_.V.	0.00510422	0.55549011
chr16.77740359.77743999_.V.	2	chr16.77740359.77743999_.V.	0.02071916	0.55541143
chr9.10408852.10411382_.V.	2	chr9.10408852.10411382_.V.	0.00023867	0.55472856
chr1.975216.986006_.V.	2	chr1.975216.986006_.V.	0.01229347	0.55451669
chr10.66739959.66744679_.V.	2	chr10.66739959.66744679_.V.	0.00030028	0.55410377
chr9.136285762.136289922_.V.	2	chr9.136285762.136289922_.V.	0.017307	0.55364062
chr18.52610855.52613855_.V.	2	chr18.52610855.52613855_.V.	0.04630508	0.55287415
chr1.1805526.1808086_.V.	2	chr1.1805526.1808086_.V.	0.00435484	0.55229936
chr15.135898088.135904648_.V.	2	chr15.135898088.135904648_.V.	0.00154718	0.55126624
chr1.274078796.274082926_.V.	2	chr1.274078796.274082926_.V.	0.026429	0.54881902
chr17.60443102.60447102_.V.	2	chr17.60443102.60447102_.V.	0.00071066	0.54790939
chr14.139834989.139836949_.V.	2	chr14.139834989.139836949_.V.	0.00596018	0.54730686
chr17.62489512.62496852_.V.	2	chr17.62489512.62496852_.V.	0.0160561	0.54675472
chr12.54676329.54677869_.V.	2	chr12.54676329.54677869_.V.	0.00128303	0.54600073
chr8.135203218.135209618_.V.	2	chr8.135203218.135209618_.V.	0.04161078	0.54522232
chr17.61208112.61211942_.V.	2	chr17.61208112.61211942_.V.	0.03334006	0.54459806
chr12.31277011.31277760_.C.	2	chr12.31277011.31277760_.C.	2.14E-05	0.54436095
chr16.79746909.79748689_.V.	2	chr16.79746909.79748689_.V.	0.00337904	0.5435097
chr7.57099289.57103789_.V.	2	chr7.57099289.57103789_.V.	0.00264406	0.54332043
chrX.122392378.122394028_.V.	2	chrX.122392378.122394028_.V.	0.0029137	0.54311409
chr18.53310715.53313395_.V.	2	chr18.53310715.53313395_.V.	0.00596018	0.54260916
chr14.140288349.140293009_.V.	2	chr14.140288349.140293009_.V.	0.02343317	0.54168438
chrX.89994848.89998308_.V.	2	chrX.89994848.89998308_.V.	0.00439723	0.541277
chr3.125110276.125116666_.V.	2	chr3.125110276.125116666_.V.	0.02071916	0.54118246
chr11.77160680.77173220_.V.	2	chr11.77160680.77173220_.V.	0.00596018	0.54098945
chr6.68107426.68110016_.V.	2	chr6.68107426.68110016_.V.	0.01407064	0.54018424
chr3.132151636.132158906_.V.	2	chr3.132151636.132158906_.V.	0.02972401	0.53892377
chr6.40152416.40156126_.V.	2	chr6.40152416.40156126_.V.	0.00128303	0.5380479
chr1.100288868.100289605_.C.	2	chr1.100288868.100289605_.C.	0.01826556	0.5371381
chr4.4638639.4643039_.V.	2	chr4.4638639.4643039_.V.	0.04161078	0.5363836
chr7.121486789.121489109_.V.	2	chr7.121486789.121489109_.V.	0.00086971	0.53623097
chr15.139477888.139482538_.V.	2	chr15.139477888.139482538_.V.	5.18E-05	0.53619122
chr6.65312326.65316126_.V.	2	chr6.65312326.65316126_.V.	0.00804229	0.53538947
chr1.267569346.267574476_.V.	2	chr1.267569346.267574476_.V.	0.0160561	0.53501837
chr7.112479109.112481399_.V.	2	chr7.112479109.112481399_.V.	0.00071066	0.53320711
chr8.39675128.39679828_.V.	2	chr8.39675128.39679828_.V.	0.03946843	0.53033051
chr12.53006559.53008829_.V.	2	chr12.53006559.53008829_.V.	0.00014772	0.5301089
chr3.127539586.127542766_.V.	2	chr3.127539586.127542766_.V.	0.02343317	0.52976797
chr15.138509228.138517658_.V.	2	chr15.138509228.138517658_.V.	0.01826556	0.5294264
chr14.113597569.113600209_.V.	2	chr14.113597569.113600209_.V.	0.03729477	0.5280042
chr1.460776.465756_.V.	2	chr1.460776.465756_.V.	0.03334006	0.52678546
chr14.138425199.138429809_.V.	2	chr14.138425199.138429809_.V.	8.89E-05	0.52633954
chrX.35662488.35666898_.V.	2	chrX.35662488.35666898_.V.	0.04612132	0.52593051
chr1.1528376.1534766_.V.	2	chr1.1528376.1534766_.V.	2.90E-05	0.52588287
chr14.141184479.141191799_.V.	2	chr14.141184479.141191799_.V.	2.14E-05	0.52462518
chr18.899435.902215_.V.	2	chr18.899435.902215_.V.	0.02343317	0.52445197
chr5.10811288.10814498_.V.	2	chr5.10811288.10814498_.V.	0.00030028	0.52385828
chr13.207882785.207886425_.V.	2	chr13.207882785.207886425_.V.	0.00693574	0.52385791
chr4.129795209.129797689_.V.	2	chr4.129795209.129797689_.V.	0.00086971	0.52362324
chr3.26464566.26466636_.V.	2	chr3.26464566.26466636_.V.	0.02972401	0.52304956
chr12.7934529.7938329_.V.	2	chr12.7934529.7938329_.V.	0.02343317	0.5230405
chr1.1277966.1282036_.V.	2	chr1.1277966.1282036_.V.	0.00222085	0.52265673
chr13.206033565.206037935_.V.	2	chr13.206033565.206037935_.V.	0.00023867	0.52245594
chr12.453099.460989_.V.	2	chr12.453099.460989_.V.	0.03729477	0.52151098
chr10.64015149.64023309_.V.	2	chr10.64015149.64023309_.V.	0.00057789	0.52106697
chr6.1017706.1025116_.V.	2	chr6.1017706.1025116_.V.	0.02071916	0.52069544
chr14.109581859.109584609_.V.	2	chr14.109581859.109584609_.V.	0.01826556	0.52031992
chr12.10353259.10356689_.V.	2	chr12.10353259.10356689_.V.	0.0160561	0.51985418
chr5.590338.593108_.V.	2	chr5.590338.593108_.V.	0.04630508	0.51864915
chr12.51975989.51978009_.V.	2	chr12.51975989.51978009_.V.	0.02071916	0.51766358
chr4.76143499.76145939_.V.	2	chr4.76143499.76145939_.V.	0.04161078	0.51543573
chr5.953598.957878_.V.	2	chr5.953598.957878_.V.	0.02343317	0.51528147
chr9.136622922.136626462_.V.	2	chr9.136622922.136626462_.V.	0.03361809	0.51318616
chr15.138292798.138294848_.V.	2	chr15.138292798.138294848_.V.	0.02071916	0.51289098
chr5.991728.995108_.V.	2	chr5.991728.995108_.V.	0.00011493	0.51209412
chr14.141114989.141118819_.V.	2	chr14.141114989.141118819_.V.	0.03729477	0.51194455
chr6.170242316.170248076_.V.	2	chr6.170242316.170248076_.V.	0.00441537	0.51180658
chr17.60471462.60473942_.V.	2	chr17.60471462.60473942_.V.	0.00185787	0.51058711
chr6.71017576.71021076_.V.	2	chr6.71017576.71021076_.V.	0.02486316	0.51056893
chr18.13503165.13506645_.V.	2	chr18.13503165.13506645_.V.	0.04161078	0.51021893
chr7.987719.991469_.V.	2	chr7.987719.991469_.V.	0.00222085	0.50979124
chr11.76631880.76635410_.V.	2	chr11.76631880.76635410_.V.	0.00264406	0.50844058
chr9.137555712.137557602_.V.	2	chr9.137555712.137557602_.V.	0.01407064	0.50739288
chr12.45504906.45505816_.C.	2	chr12.45504906.45505816_.C.	0.00046721	0.50684134
chr1.2414426.2422746_.V.	2	chr1.2414426.2422746_.V.	0.00037581	0.50606984
chr12.57609809.57612209_.V.	2	chr12.57609809.57612209_.V.	0.00222085	0.50591021
chr11.77808350.77813860_.V.	2	chr11.77808350.77813860_.V.	0.04161078	0.50510827
chr2.143486850.143494100_.V.	2	chr2.143486850.143494100_.V.	0.00023867	0.5036336
chr9.133306452.133308672_.V.	2	chr9.133306452.133308672_.V.	0.02071916	0.50346762
chr1.1112056.1117736_.V.	2	chr1.1112056.1117736_.V.	3.89E-05	0.50314176
chr1.427666.430856_.V.	2	chr1.427666.430856_.V.	0.00596018	0.50289053
chr12.18036976.18040754_.C.	2	chr12.18036976.18040754_.C.	0.02404913	0.50279179

chr12.42492019.42494339_.V.	2	chr12.42492019.42494339_.V.	0.0160561	0.50234863
chr6.69507746.69511246_.V.	2	chr6.69507746.69511246_.V.	0.04161078	0.50224366
chr14.140075189.140077539_.V.	2	chr14.140075189.140077539_.V.	0.00105868	0.50160351
chr1.231142443.231143232_.C.	2	chr1.231142443.231143232_.C.	0.04630508	0.50106283
chr17.46638022.46640212_.V.	2	chr17.46638022.46640212_.V.	0.00011493	0.50091352
chr11.66330220.66335220_.V.	2	chr11.66330220.66335220_.V.	0.00264406	0.4989312
chr1.217287626.217288039_.C.	2	chr1.217287626.217288039_.C.	0.00510422	0.49817951
chr16.78255669.78257569_.V.	2	chr16.78255669.78257569_.V.	0.0160561	0.49781144
chr10.66532856.66534574_.C.	2	chr10.66532856.66534574_.C.	0.00086971	0.49668221
chr1.270259836.270264736_.V.	2	chr1.270259836.270264736_.V.	0.02972401	0.49614452
chr1.270558716.270565436_.V.	2	chr1.270558716.270565436_.V.	0.00023867	0.49534936
chr5.588968.590088_.V.	2	chr5.588968.590088_.V.	0.00264406	0.49530906
chrX.35621628.35625368_.V.	2	chrX.35621628.35625368_.V.	0.03334006	0.49502348
chr7.74957429.74959919_.V.	2	chr7.74957429.74959919_.V.	0.01070637	0.49384122
chr1.96302626.96303169_.C.	2	chr1.96302626.96303169_.C.	0.026429	0.49343833
chr17.62701912.62704932_.V.	2	chr17.62701912.62704932_.V.	0.00185787	0.49273039
chr7.771569.775979_.V.	2	chr7.771569.775979_.V.	0.00370211	0.49023971
chr18.53162845.53165055_.V.	2	chr18.53162845.53165055_.V.	0.01826556	0.48997299
chr10.66695569.66700939_.V.	2	chr10.66695569.66700939_.V.	0.00071066	0.4890556
chr1.2030956.2036246_.V.	2	chr1.2030956.2036246_.V.	0.01070637	0.48871806
chr13.206030935.206033335_.V.	2	chr13.206030935.206033335_.V.	0.0160561	0.48856377
chr5.809138.813688_.V.	2	chr5.809138.813688_.V.	0.00037581	0.48813731
chr6.1409866.1413016_.V.	2	chr6.1409866.1413016_.V.	0.00154718	0.48784507
chr16.76432009.76435519_.V.	2	chr16.76432009.76435519_.V.	0.0160561	0.48744873
chr13.206853755.206856525_.V.	2	chr13.206853755.206856525_.V.	0.00264406	0.48587494
chr13.206214145.206223085_.V.	2	chr13.206214145.206223085_.V.	0.01407064	0.48573877
chr1.1268876.1273716_.V.	2	chr1.1268876.1273716_.V.	0.00596018	0.48528231
chr3.17803416.17807526_.V.	2	chr3.17803416.17807526_.V.	0.01407064	0.4831861
chr1.249876176.249878826_.V.	2	chr1.249876176.249878826_.V.	0.00804229	0.4831269
chr1.1027006.1030186_.V.	2	chr1.1027006.1030186_.V.	0.00264406	0.48289685
chr15.136299588.136302168_.V.	2	chr15.136299588.136302168_.V.	0.02972401	0.48272882
chr16.79544639.79547779_.V.	2	chr16.79544639.79547779_.V.	0.00693574	0.48184007
chr11.76898450.76905200_.V.	2	chr11.76898450.76905200_.V.	0.00023867	0.48170763
chr11.2467950.2471110_.V.	2	chr11.2467950.2471110_.V.	0.00037581	0.48079874
chr6.1563686.1569256_.V.	2	chr6.1563686.1569256_.V.	0.00264406	0.4806649
chr5.1238268.1243888_.V.	2	chr5.1238268.1243888_.V.	0.01407064	0.47944673
chr14.140407549.140410609_.V.	2	chr14.140407549.140410609_.V.	0.00185787	0.47939509
chr8.3279248.3282578_.V.	2	chr8.3279248.3282578_.V.	0.00105868	0.47916678
chr15.139284398.139289478_.V.	2	chr15.139284398.139289478_.V.	6.81E-05	0.47901899
chr13.207836074.207837600_.C.	2	chr13.207836074.207837600_.C.	0.00804229	0.47893742
chr16.79653319.79662869_.V.	2	chr16.79653319.79662869_.V.	0.02071916	0.47806511
chr5.3395198.3407528_.V.	2	chr5.3395198.3407528_.V.	0.04630508	0.4770394
chr9.139070772.139077652_.V.	2	chr9.139070772.139077652_.V.	0.00313457	0.47658184
chr6.169777346.169781966_.V.	2	chr6.169777346.169781966_.V.	0.01407064	0.4764615
chr5.902058.909388_.V.	2	chr5.902058.909388_.V.	0.00313457	0.47632295
chr9.134787102.134794662_.V.	2	chr9.134787102.134794662_.V.	0.02037355	0.4761884
chr5.1412758.1419198_.V.	2	chr5.1412758.1419198_.V.	0.02972401	0.4757619
chr14.109576279.109579779_.V.	2	chr14.109576279.109579779_.V.	0.01070637	0.47434992
chr6.48150486.48153526_.V.	2	chr6.48150486.48153526_.V.	0.02972401	0.4740234
chr2.1534830.1536310_.V.	2	chr2.1534830.1536310_.V.	0.00264406	0.47401726
chr17.46566172.46571562_.V.	2	chr17.46566172.46571562_.V.	0.00086971	0.47288897
chr1.933856.938486_.V.	2	chr1.933856.938486_.V.	0.00435484	0.47162705
chr15.137259558.137263238_.V.	2	chr15.137259558.137263238_.V.	0.00046721	0.47148056
chr9.138855342.138860072_.V.	2	chr9.138855342.138860072_.V.	0.00037581	0.47064887
chr5.3908018.3915028_.V.	2	chr5.3908018.3915028_.V.	0.00037581	0.47025436
chr18.49181915.49186015_.V.	2	chr18.49181915.49186015_.V.	0.02972401	0.4696046
chr18.1185005.1195565_.V.	2	chr18.1185005.1195565_.V.	0.00037581	0.4694849
chr11.76880640.76886850_.V.	2	chr11.76880640.76886850_.V.	0.01407064	0.46899752
chr12.50736832.50738032_.C.	2	chr12.50736832.50738032_.C.	0.026429	0.46778608
chr16.79064329.79067429_.V.	2	chr16.79064329.79067429_.V.	0.02343317	0.46699627
chr1.266084036.266090246_.V.	2	chr1.266084036.266090246_.V.	0.04161078	0.46674509
chr16.79552959.79554459_.V.	2	chr16.79552959.79554459_.V.	0.00435484	0.46491533
chr9.135169882.135172302_.V.	2	chr9.135169882.135172302_.V.	0.00030028	0.46384569
chr6.53428926.53431616_.V.	2	chr6.53428926.53431616_.V.	0.04604676	0.46317524
chr9.7690052.7697432_.V.	2	chr9.7690052.7697432_.V.	2.90E-05	0.46265587
chr12.40489799.40494329_.V.	2	chr12.40489799.40494329_.V.	0.00596018	0.46232537
chr9.3473252.3475242_.V.	2	chr9.3473252.3475242_.V.	0.01229347	0.46125878
chr12.57766699.57770849_.V.	2	chr12.57766699.57770849_.V.	0.00596018	0.46062082
chr12.8447539.8451929_.V.	2	chr12.8447539.8451929_.V.	0.00435484	0.45989336
chr12.59454279.59457869_.V.	2	chr12.59454279.59457869_.V.	0.00014772	0.45950432
chr9.138319262.138322222_.V.	2	chr9.138319262.138322222_.V.	0.00804229	0.45896752
chr5.39771978.39802708_.V.	2	chr5.39771978.39802708_.V.	0.03729477	0.45857115
chr11.2606080.2608500_.V.	2	chr11.2606080.2608500_.V.	0.00014772	0.45803768
chr9.10752072.10753202_.V.	2	chr9.10752072.10753202_.V.	0.02343317	0.45548206
chr13.200641940.200643216_.C.	2	chr13.200641940.200643216_.C.	0.00693574	0.45434333
chr11.77568280.77572640_.V.	2	chr11.77568280.77572640_.V.	0.01407064	0.4531319
chr17.46302122.46309462_.V.	2	chr17.46302122.46309462_.V.	0.00370211	0.45264016
chr9.139254192.139257322_.V.	2	chr9.139254192.139257322_.V.	0.04161078	0.45263397
chr2.130230.133660_.V.	2	chr2.130230.133660_.V.	0.01407064	0.45243927
chrX.121948228.121951038_.V.	2	chrX.121948228.121951038_.V.	0.00264406	0.45160952
chr10.58075410.58076488_.C.	2	chr10.58075410.58076488_.C.	0.02972401	0.45096336
chr12.59899359.59903719_.V.	2	chr12.59899359.59903719_.V.	0.00929494	0.44996929
chr16.77099889.77104129_.V.	2	chr16.77099889.77104129_.V.	0.00037581	0.44974775
chr9.139341082.139344902_.V.	2	chr9.139341082.139344902_.V.	0.00929494	0.44914755
chr10.63368609.63375269_.V.	2	chr10.63368609.63375269_.V.	0.00086971	0.44476107
chr16.79537609.79543449_.V.	2	chr16.79537609.79543449_.V.	0.00011493	0.4443883
chr13.203618615.203628075_.V.	2	chr13.203618615.203628075_.V.	0.00071066	0.44415676
chr14.138986709.138990689_.V.	2	chr14.138986709.138990689_.V.	0.026429	0.44411583
chr2.151555380.151563830_.V.	2	chr2.151555380.151563830_.V.	0.04161078	0.44308183
chr11.16514780.16518890_.V.	2	chr11.16514780.16518890_.V.	0.04630508	0.4423573
chr12.57589829.57593429_.V.	2	chr12.57589829.57593429_.V.	0.00596018	0.44071134
chr2.1030190.1033250_.V.	2	chr2.1030190.1033250_.V.	0.02972401	0.44040687
chr1.2504186.2516876_.V.	2	chr1.2504186.2516876_.V.	0.00086971	0.43971353
chr11.76463940.76465870_.V.	2	chr11.76463940.76465870_.V.	0.00264406	0.43947603
chr11.845120.847250_.V.	2	chr11.845120.847250_.V.	0.03729477	0.43919321
chr13.203800275.203802845_.V.	2	chr13.203800275.203802845_.V.	0.00804229	0.43919064
chr18.2191015.2194725_.V.	2	chr18.2191015.2194725_.V.	0.00804229	0.43916119
chr13.205687055.205689635_.V.	2	chr13.205687055.205689635_.V.	0.0160561	0.43904612
chr13.206831545.206840435_.V.	2	chr13.206831545.206840435_.V.	0.03334006	0.43793242
chr13.208227875.208231895_.V.	2	chr13.208227875.208231895_.V.	0.00046721	0.43685897
chr1.1283336.1285656_.V.	2	chr1.1283336.1285656_.V.	0.00264406	0.43526845
chr5.4656128.4660808_.V.	2	chr5.4656128.4660808_.V.	0.01070637	0.43454676
chr1.1905726.1911696_.V.	2	chr1.1905726.1911696_.V.	0.00018834	0.43399778
chr1.2423266.2427186_.V.	2	chr1.2423266.2427186_.V.	0.00154718	0.43279851
chr9.139078692.139082692_.V.	2	chr9.139078692.139082692_.V.	0.00185787	0.43235319

chr15.138150828.138155938_.V.	2	chr15.138150828.138155938_.V.	0.00804229	0.43221271
chr7.896039.903859_.V.	2	chr7.896039.903859_.V.	0.00057789	0.43210588
chr7.117463509.117468069_.V.	2	chr7.117463509.117468069_.V.	0.00804229	0.43205534
chr13.206519035.206525405_.V.	2	chr13.206519035.206525405_.V.	0.026429	0.43079764
chr3.1066576.1070166_.V.	2	chr3.1066576.1070166_.V.	0.00030028	0.43071738
chr4.104218519.104225309_.V.	2	chr4.104218519.104225309_.V.	0.00804229	0.43040061
chr7.55363709.55366089_.V.	2	chr7.55363709.55366089_.V.	0.00071066	0.42975401
chr15.137398238.137401498_.V.	2	chr15.137398238.137401498_.V.	0.00105868	0.42965389
chr4.7275539.7280759_.V.	2	chr4.7275539.7280759_.V.	0.00510422	0.42933059
chr6.67759306.67762556_.V.	2	chr6.67759306.67762556_.V.	0.00023867	0.42931402
chr11.78179830.78183890_.V.	2	chr11.78179830.78183890_.V.	0.00929494	0.42928764
chr1.1444436.1454966_.V.	2	chr1.1444436.1454966_.V.	0.00154718	0.42913162
chr16.75575719.75578049_.V.	2	chr16.75575719.75578049_.V.	0.00313457	0.42841241
chr14.138275659.138279459_.V.	2	chr14.138275659.138279459_.V.	0.02071916	0.42782383
chr7.739009.745339_.V.	2	chr7.739009.745339_.V.	0.026429	0.42698932
chr3.25495726.25499196_.V.	2	chr3.25495726.25499196_.V.	0.01407064	0.42694329
chr17.55527122.55531472_.V.	2	chr17.55527122.55531472_.V.	0.00313457	0.42671292
chr8.129867778.129871438_.V.	2	chr8.129867778.129871438_.V.	0.00313457	0.42663397
chr4.12230849.12234179_.V.	2	chr4.12230849.12234179_.V.	0.00057789	0.42554375
chr18.50125275.50128495_.V.	2	chr18.50125275.50128495_.V.	0.00215222	0.42518266
chr13.196955955.196959185_.V.	2	chr13.196955955.196959185_.V.	0.00804229	0.42472019
chr14.141133839.141138489_.V.	2	chr14.141133839.141138489_.V.	0.04161078	0.42422265
chr15.138676278.138679518_.V.	2	chr15.138676278.138679518_.V.	0.04161078	0.42421465
chr12.34584909.34591689_.V.	2	chr12.34584909.34591689_.V.	0.00018834	0.42325957
chr2.6895080.6899700_.V.	2	chr2.6895080.6899700_.V.	0.00370211	0.42305711
chr12.18875419.18877149_.V.	2	chr12.18875419.18877149_.V.	0.00128303	0.42275402
chr1.9350656.9354186_.V.	2	chr1.9350656.9354186_.V.	0.03334006	0.42231599
chr16.79511609.79518859_.V.	2	chr16.79511609.79518859_.V.	0.00313457	0.4222298
chr16.74548109.74550049_.V.	2	chr16.74548109.74550049_.V.	8.89E-05	0.422052
chr16.79495219.79497289_.V.	2	chr16.79495219.79497289_.V.	0.00313457	0.42055136
chr15.137290218.137298818_.V.	2	chr15.137290218.137298818_.V.	0.00128303	0.41950195
chr7.49445909.49448319_.V.	2	chr7.49445909.49448319_.V.	0.01407064	0.41869289
chr10.9861359.9866989_.V.	2	chr10.9861359.9866989_.V.	0.00510422	0.41837935
chr11.77835410.77839800_.V.	2	chr11.77835410.77839800_.V.	0.00046721	0.41762711
chr13.202561255.202564075_.V.	2	chr13.202561255.202564075_.V.	0.00510422	0.41743237
chr9.138424712.138428072_.V.	2	chr9.138424712.138428072_.V.	0.00037581	0.41719982
chr1.1457826.1464396_.V.	2	chr1.1457826.1464396_.V.	0.00018834	0.41669193
chr16.75617129.75620139_.V.	2	chr16.75617129.75620139_.V.	0.0160561	0.41628192
chr7.3064599.3070619_.V.	2	chr7.3064599.3070619_.V.	0.03334006	0.41576571
chr13.206403635.206406165_.V.	2	chr13.206403635.206406165_.V.	0.00804229	0.41525413
chr1.1262166.1268546_.V.	2	chr1.1262166.1268546_.V.	0.01826556	0.41505732
chr10.66317839.66322719_.V.	2	chr10.66317839.66322719_.V.	0.0160561	0.41468343
chr4.782159.793879_.V.	2	chr4.782159.793879_.V.	0.00929494	0.41289312
chr1.149625936.149627336_.V.	2	chr1.149625936.149627336_.V.	0.00510422	0.41260461
chr13.206018845.206024715_.V.	2	chr13.206018845.206024715_.V.	0.02071916	0.4123329
chr5.66303788.66315038_.V.	2	chr5.66303788.66315038_.V.	5.18E-05	0.4122466
chr1.187726376.187728896_.V.	2	chr1.187726376.187728896_.V.	0.00510422	0.41189792
chr3.131724226.131726126_.V.	2	chr3.131724226.131726126_.V.	0.00510422	0.4118156
chr14.137241859.137244129_.V.	2	chr14.137241859.137244129_.V.	0.00222085	0.41142597
chr8.2189548.2191558_.V.	2	chr8.2189548.2191558_.V.	0.0045148	0.41130663
chr9.137768342.137770952_.V.	2	chr9.137768342.137770952_.V.	0.02972401	0.41060147
chr12.2560207.2562838_.C.	2	chr12.2560207.2562838_.C.	0.0160561	0.41045303
chr13.206428405.206434395_.V.	2	chr13.206428405.206434395_.V.	0.00030028	0.41019036
chr17.58667572.58669912_.V.	2	chr17.58667572.58669912_.V.	0.00435484	0.40994483
chr2.9195750.9198850_.V.	2	chr2.9195750.9198850_.V.	0.00264406	0.4093364
chr18.1691365.1694665_.V.	2	chr18.1691365.1694665_.V.	0.00018834	0.40909298
chr16.79436079.79447039_.V.	2	chr16.79436079.79447039_.V.	0.01229347	0.40802827
chr15.127185678.127188448_.V.	2	chr15.127185678.127188448_.V.	0.02972401	0.4080199
chr7.120897229.120900569_.V.	2	chr7.120897229.120900569_.V.	0.00071066	0.4077604
chr15.133473548.133476628_.V.	2	chr15.133473548.133476628_.V.	8.89E-05	0.40688045
chr1.4939266.4942916_.V.	2	chr1.4939266.4942916_.V.	0.02071916	0.40648909
chr7.2489439.2492099_.V.	2	chr7.2489439.2492099_.V.	0.02071916	0.4063178
chr2.9293700.9298410_.V.	2	chr2.9293700.9298410_.V.	0.00071066	0.40559646
chr7.2669839.2672659_.V.	2	chr7.2669839.2672659_.V.	0.00046721	0.4054317
chr6.66883866.66887526_.V.	2	chr6.66883866.66887526_.V.	5.18E-05	0.40538268
chr1.147683576.147685686_.V.	2	chr1.147683576.147685686_.V.	0.00105868	0.40537491
chr10.66891569.66904269_.V.	2	chr10.66891569.66904269_.V.	0.02343317	0.40461496
chr13.205667665.205671515_.V.	2	chr13.205667665.205671515_.V.	0.00037581	0.404403
chr13.208182365.208185485_.V.	2	chr13.208182365.208185485_.V.	0.04161078	0.40429904
chr11.6410690.6413660_.V.	2	chr11.6410690.6413660_.V.	0.00804229	0.40428444
chr13.34088194.34089168_.C.	2	chr13.34088194.34089168_.C.	0.04161078	0.40374023
chr2.143041670.143043540_.V.	2	chr2.143041670.143043540_.V.	0.026429	0.40334454
chr13.203619487.203620128_.C.	2	chr13.203619487.203620128_.C.	0.0160561	0.40259307
chr14.137108879.137114009_.V.	2	chr14.137108879.137114009_.V.	0.03334006	0.40257483
chr3.22414746.22421516_.V.	2	chr3.22414746.22421516_.V.	0.00222085	0.40177578
chr12.15360359.15364249_.V.	2	chr12.15360359.15364249_.V.	0.02972401	0.40017281
chr11.1559030.1562300_.V.	2	chr11.1559030.1562300_.V.	0.02343317	0.40009462
chr9.134571202.134575572_.V.	2	chr9.134571202.134575572_.V.	0.00804229	0.39930419
chr2.150981810.150986810_.V.	2	chr2.150981810.150986810_.V.	0.01229347	0.39873189
chr18.3880605.3883325_.V.	2	chr18.3880605.3883325_.V.	0.00023867	0.39868012
chr7.1727489.1732119_.V.	2	chr7.1727489.1732119_.V.	0.02343317	0.39848011
chr7.654719.658879_.V.	2	chr7.654719.658879_.V.	2.14E-05	0.39774609
chr14.140273699.140278569_.V.	2	chr14.140273699.140278569_.V.	0.00370211	0.39706852
chr11.78901410.78910190_.V.	2	chr11.78901410.78910190_.V.	0.00264406	0.3968574
chr9.25422002.25424452_.V.	2	chr9.25422002.25424452_.V.	0.04630508	0.39671504
chr7.3528039.3537739_.V.	2	chr7.3528039.3537739_.V.	3.89E-05	0.39636241
chr2.1016580.1019990_.V.	2	chr2.1016580.1019990_.V.	0.01229347	0.39591456
chr18.49977355.49983615_.V.	2	chr18.49977355.49983615_.V.	0.03334006	0.39557096
chr18.2415625.2424205_.V.	2	chr18.2415625.2424205_.V.	0.00313457	0.39546576
chr9.41102112.41107602_.V.	2	chr9.41102112.41107602_.V.	0.01885471	0.39473646
chr1.1474166.1483956_.V.	2	chr1.1474166.1483956_.V.	0.00435484	0.39433058
chr10.12941809.12947419_.V.	2	chr10.12941809.12947419_.V.	0.00037581	0.39420404
chr10.66760219.66770069_.V.	2	chr10.66760219.66770069_.V.	0.00435484	0.39401918
chr1.1201636.1207326_.V.	2	chr1.1201636.1207326_.V.	0.02071916	0.39347887
chr12.20957709.20961019_.V.	2	chr12.20957709.20961019_.V.	0.00046721	0.39304067
chr13.131327565.131331315_.V.	2	chr13.131327565.131331315_.V.	0.00510422	0.392567
chr15.135905038.135911398_.V.	2	chr15.135905038.135911398_.V.	0.00929494	0.39195999
chr13.206045005.206050015_.V.	2	chr13.206045005.206050015_.V.	0.03729477	0.39155619
chr13.208115935.208121455_.V.	2	chr13.208115935.208121455_.V.	0.00435484	0.39062899
chr6.33740746.33747206_.V.	2	chr6.33740746.33747206_.V.	0.02972401	0.39039019
chr1.825426.843926_.V.	2	chr1.825426.843926_.V.	0.03729477	0.39035163
chr15.138715498.138719278_.V.	2	chr15.138715498.138719278_.V.	0.01229347	0.39010451
chr10.24069239.24076199_.V.	2	chr10.24069239.24076199_.V.	0.04630508	0.38989418
chr12.3434269.3437599_.V.	2	chr12.3434269.3437599_.V.	0.00046721	0.38956664
chr13.131343175.131346375_.V.	2	chr13.131343175.131346375_.V.	0.026429	0.38953514

chr11.75991550.75995620_.V.	2	chr11.75991550.75995620_.V.	0.00313457	0.38789047
chr14.140755239.140764049_.V.	2	chr14.140755239.140764049_.V.	0.00105868	0.38770639
chr11.7214710.7217740_.V.	2	chr11.7214710.7217740_.V.	0.0160561	0.38716261
chr10.66740987.66741547_.C.	2	chr10.66740987.66741547_.C.	0.00105868	0.38705686
chr15.137538308.137541858_.V.	2	chr15.137538308.137541858_.V.	6.81E-05	0.38688664
chr16.79604119.79606079_.V.	2	chr16.79604119.79606079_.V.	0.02071916	0.38679623
chr6.1505426.1510766_.V.	2	chr6.1505426.1510766_.V.	0.04161078	0.38641533
chr13.206690975.206698715_.V.	2	chr13.206690975.206698715_.V.	0.03729477	0.38593347
chr17.59125342.59128692_.V.	2	chr17.59125342.59128692_.V.	0.00046721	0.385891
chr3.131714146.131717146_.V.	2	chr3.131714146.131717146_.V.	0.04630508	0.38585009
chr15.139705558.139712278_.V.	2	chr15.139705558.139712278_.V.	0.00014772	0.38568522
chr3.114933746.114935236_.V.	2	chr3.114933746.114935236_.V.	0.02343317	0.38507198
chr14.138102289.138110389_.V.	2	chr14.138102289.138110389_.V.	0.00313457	0.38494163
chr1.804766.814136_.V.	2	chr1.804766.814136_.V.	0.00929494	0.38489038
chr11.78912650.78918030_.V.	2	chr11.78912650.78918030_.V.	0.00510422	0.38471242
chr7.121706089.121709769_.V.	2	chr7.121706089.121709769_.V.	0.00435484	0.38379781
chr12.7561259.7563859_.V.	2	chr12.7561259.7563859_.V.	0.00435484	0.38359996
chr3.34564836.34572766_.V.	2	chr3.34564836.34572766_.V.	0.00018834	0.38251458
chr18.1936845.1942035_.V.	2	chr18.1936845.1942035_.V.	0.026429	0.38161652
chr15.136720368.136729028_.V.	2	chr15.136720368.136729028_.V.	0.00264406	0.37986907
chr14.138088909.138095629_.V.	2	chr14.138088909.138095629_.V.	0.02071916	0.37956487
chr6.66859326.66863106_.V.	2	chr6.66859326.66863106_.V.	0.00313457	0.37948596
chr11.5844260.5847130_.V.	2	chr11.5844260.5847130_.V.	0.03729477	0.37869927
chr5.1324428.1331418_.V.	2	chr5.1324428.1331418_.V.	0.00510422	0.37840865
chr12.5432569.5435759_.V.	2	chr12.5432569.5435759_.V.	0.00128303	0.37757036
chr17.60929292.60934082_.V.	2	chr17.60929292.60934082_.V.	0.01826556	0.37754806
chr7.1736929.1740429_.V.	2	chr7.1736929.1740429_.V.	0.02137061	0.37750196
chr6.41320436.41326606_.V.	2	chr6.41320436.41326606_.V.	0.03729477	0.37742284
chr15.136304968.136307978_.V.	2	chr15.136304968.136307978_.V.	0.00929494	0.37737739
chr1.1237976.1243796_.V.	2	chr1.1237976.1243796_.V.	0.00435484	0.37734
chr10.68757659.68760869_.V.	2	chr10.68757659.68760869_.V.	0.04630508	0.37654782
chr13.205782805.205785835_.V.	2	chr13.205782805.205785835_.V.	0.00011493	0.37573611
chr16.79898299.79904919_.V.	2	chr16.79898299.79904919_.V.	0.00693574	0.37550253
chr10.66461799.66469199_.V.	2	chr10.66461799.66469199_.V.	0.00370211	0.37539682
chr16.73950759.73954099_.V.	2	chr16.73950759.73954099_.V.	0.01229347	0.37494711
chrX.6047358.6051308_.V.	2	chrX.6047358.6051308_.V.	0.00071066	0.37485715
chr18.1082315.1084085_.V.	2	chr18.1082315.1084085_.V.	0.00510422	0.3746286
chr18.1138935.1142095_.V.	2	chr18.1138935.1142095_.V.	0.03334006	0.37414426
chr6.40231656.40233506_.V.	2	chr6.40231656.40233506_.V.	0.02486316	0.37345699
chr4.128568059.128570239_.V.	2	chr4.128568059.128570239_.V.	0.02071916	0.37343969
chr17.60849952.60853722_.V.	2	chr17.60849952.60853722_.V.	0.026429	0.37328918
chrX.120778.124128_.V.	2	chrX.120778.124128_.V.	0.01407064	0.37316171
chr4.1476009.1485859_.V.	2	chr4.1476009.1485859_.V.	0.01070637	0.37293576
chr10.56527899.56531299_.V.	2	chr10.56527899.56531299_.V.	0.04630508	0.37275808
chr7.1028279.1030329_.V.	2	chr7.1028279.1030329_.V.	0.00154718	0.37164451
chr1.270552826.270555986_.V.	2	chr1.270552826.270555986_.V.	0.00222085	0.3710881
chr18.55614125.55615445_.V.	2	chr18.55614125.55615445_.V.	0.00057789	0.37070809
chr12.56278059.56280549_.V.	2	chr12.56278059.56280549_.V.	6.81E-05	0.36941762
chr9.138614132.138616202_.V.	2	chr9.138614132.138616202_.V.	0.00370211	0.3691465
chr10.66882359.66885469_.V.	2	chr10.66882359.66885469_.V.	0.00370211	0.3685992
chr14.138075609.138081359_.V.	2	chr14.138075609.138081359_.V.	0.00071066	0.36724736
chr6.79866836.79874666_.V.	2	chr6.79866836.79874666_.V.	0.026429	0.36706461
chr2.2449990.2453940_.V.	2	chr2.2449990.2453940_.V.	0.00510422	0.366279
chr1.148077526.148080116_.V.	2	chr1.148077526.148080116_.V.	0.00086971	0.36530431
chr8.11108868.11113758_.V.	2	chr8.11108868.11113758_.V.	0.00046721	0.36525824
chrX.111740818.111745788_.V.	2	chrX.111740818.111745788_.V.	0.00929494	0.36462701
chr12.509229.513189_.V.	2	chr12.509229.513189_.V.	0.02071916	0.36452297
chr11.811360.813820_.V.	2	chr11.811360.813820_.V.	0.02071916	0.36319696
chr13.205896555.205903535_.V.	2	chr13.205896555.205903535_.V.	0.00596018	0.36301778
chr10.24047609.24053319_.V.	2	chr10.24047609.24053319_.V.	0.00370211	0.36299258
chr16.78603069.78605119_.V.	2	chr16.78603069.78605119_.V.	0.00071066	0.36249923
chr2.2743030.2750430_.V.	2	chr2.2743030.2750430_.V.	0.00596018	0.36245492
chr17.58657742.58667192_.V.	2	chr17.58657742.58667192_.V.	0.00105868	0.36185132
chr14.141458889.141460269_.V.	2	chr14.141458889.141460269_.V.	0.02343317	0.36184579
chr18.1237085.1239895_.V.	2	chr18.1237085.1239895_.V.	0.03334006	0.36155842
chr4.72945129.72947739_.V.	2	chr4.72945129.72947739_.V.	0.02343317	0.36149733
chrX.122073748.122076198_.V.	2	chrX.122073748.122076198_.V.	0.00313457	0.36050899
chr11.78253260.78255430_.V.	2	chr11.78253260.78255430_.V.	0.026429	0.35929452
chr9.134830922.134834712_.V.	2	chr9.134830922.134834712_.V.	0.00446757	0.35868074
chr9.137104532.137107162_.V.	2	chr9.137104532.137107162_.V.	0.04630508	0.35810353
chr6.51276966.51282366_.V.	2	chr6.51276966.51282366_.V.	0.01070637	0.35806113
chr6.170187216.170189606_.V.	2	chr6.170187216.170189606_.V.	0.01070637	0.3579996
chr6.54761096.54766246_.V.	2	chr6.54761096.54766246_.V.	0.02343317	0.35774835
chr6.1783616.1785936_.V.	2	chr6.1783616.1785936_.V.	0.03334006	0.3576321
chr8.135663648.135670928_.V.	2	chr8.135663648.135670928_.V.	0.00435484	0.35740402
chr14.139346949.139351589_.V.	2	chr14.139346949.139351589_.V.	0.00435484	0.35693638
chr15.139380268.139387828_.V.	2	chr15.139380268.139387828_.V.	6.81E-05	0.3561817
chr3.127889316.127891806_.V.	2	chr3.127889316.127891806_.V.	5.18E-05	0.35458992
chr12.2095212.2099139_.C.	2	chr12.2095212.2099139_.C.	0.00018834	0.35458535
chr16.78148089.78155199_.V.	2	chr16.78148089.78155199_.V.	0.00370211	0.35440291
chr8.76308.78318_.V.	2	chr8.76308.78318_.V.	0.00370211	0.35432587
chr5.626048.631888_.V.	2	chr5.626048.631888_.V.	0.00264406	0.3538141
chr14.140466979.140475139_.V.	2	chr14.140466979.140475139_.V.	0.00222085	0.35315553
chr14.140817589.140835029_.V.	2	chr14.140817589.140835029_.V.	0.00222085	0.35274892
chr16.79037029.79040119_.V.	2	chr16.79037029.79040119_.V.	8.00E-06	0.35270089
chr16.75648289.75652069_.V.	2	chr16.75648289.75652069_.V.	0.00086971	0.35247192
chr15.135078578.135083508_.V.	2	chr15.135078578.135083508_.V.	0.00086971	0.35221472
chr12.58732849.58735679_.V.	2	chr12.58732849.58735679_.V.	0.00435484	0.35220883
chr10.23813989.23817179_.V.	2	chr10.23813989.23817179_.V.	0.00313457	0.35202447
chr18.1071575.1077335_.V.	2	chr18.1071575.1077335_.V.	0.02343317	0.35139895
chr11.77154080.77160330_.V.	2	chr11.77154080.77160330_.V.	0.02071916	0.35103129
chr2.5070700.5072860_.V.	2	chr2.5070700.5072860_.V.	0.00023867	0.34971228
chr9.138932632.138938622_.V.	2	chr9.138932632.138938622_.V.	0.00435484	0.3496949
chr2.143317870.143325360_.V.	2	chr2.143317870.143325360_.V.	0.00435484	0.34957217
chr3.3231956.3244946_.V.	2	chr3.3231956.3244946_.V.	0.00086971	0.34943262
chr9.136707472.136712522_.V.	2	chr9.136707472.136712522_.V.	0.00030028	0.34806256
chr11.73941850.73946820_.V.	2	chr11.73941850.73946820_.V.	0.00804229	0.34779209
chr9.136471012.136473532_.V.	2	chr9.136471012.136473532_.V.	0.00071066	0.34681354
chr8.11288408.11292708_.V.	2	chr8.11288408.11292708_.V.	0.00222085	0.34656557
chrX.6057518.6067148_.V.	2	chrX.6057518.6067148_.V.	6.81E-05	0.3457608
chr13.204802515.204805145_.V.	2	chr13.204802515.204805145_.V.	0.00596018	0.34545511
chr17.60364542.60368132_.V.	2	chr17.60364542.60368132_.V.	0.00046721	0.34495957
chr18.3331635.3335665_.V.	2	chr18.3331635.3335665_.V.	0.00105868	0.34490871
chr12.15396499.15400959_.V.	2	chr12.15396499.15400959_.V.	0.00071066	0.34470431
chr5.1560988.1565728_.V.	2	chr5.1560988.1565728_.V.	0.01229347	0.34381377

chr13.207369155.207370835_.V.	2	chr13.207369155.207370835_.V.	0.00185787	0.34365186
chr10.64038209.64045349_.V.	2	chr10.64038209.64045349_.V.	0.02972401	0.34344942
chr12.56728579.56731699_.V.	2	chr12.56728579.56731699_.V.	0.00693574	0.34342354
chr7.1618809.1622759_.V.	2	chr7.1618809.1622759_.V.	0.03729477	0.3433779
chr13.204314425.204317845_.V.	2	chr13.204314425.204317845_.V.	6.81E-05	0.34336703
chr3.131304376.131310136_.V.	2	chr3.131304376.131310136_.V.	0.00023867	0.34199998
chr15.138476328.138484028_.V.	2	chr15.138476328.138484028_.V.	0.00596018	0.34156662
chr1.272559146.272562386_.V.	2	chr1.272559146.272562386_.V.	0.00046721	0.34154717
chr5.4826248.4832618_.V.	2	chr5.4826248.4832618_.V.	0.00435484	0.34149315
chr13.206885775.206888865_.V.	2	chr13.206885775.206888865_.V.	0.01070637	0.34139877
chr18.2154505.2156285_.V.	2	chr18.2154505.2156285_.V.	0.02343317	0.34098194
chr7.9189169.9194929_.V.	2	chr7.9189169.9194929_.V.	0.00507745	0.34054433
chr17.30109252.30112132_.V.	2	chr17.30109252.30112132_.V.	0.00693574	0.34036161
chr18.410315.414525_.V.	2	chr18.410315.414525_.V.	0.02071916	0.34008411
chr17.36232622.36235622_.V.	2	chr17.36232622.36235622_.V.	0.00046721	0.33995379
chr2.150286910.150293060_.V.	2	chr2.150286910.150293060_.V.	0.00086971	0.33984734
chr11.68334910.68338470_.V.	2	chr11.68334910.68338470_.V.	0.00105868	0.33935455
chr6.80503476.80507176_.V.	2	chr6.80503476.80507176_.V.	0.00057789	0.3390701
chr7.3052479.3057429_.V.	2	chr7.3052479.3057429_.V.	0.01229347	0.33901233
chr15.71705338.71707328_.V.	2	chr15.71705338.71707328_.V.	0.00435484	0.33893367
chr12.8281079.8284709_.V.	2	chr12.8281079.8284709_.V.	0.00018834	0.33885328
chr9.133422932.133427412_.V.	2	chr9.133422932.133427412_.V.	0.04630508	0.33878185
chr11.1606840.1609800_.V.	2	chr11.1606840.1609800_.V.	0.00222085	0.33796031
chr9.25375202.25380322_.V.	2	chr9.25375202.25380322_.V.	0.00086971	0.33768779
chr15.135013718.135019108_.V.	2	chr15.135013718.135019108_.V.	0.03334006	0.33707773
chr12.56539319.56542159_.V.	2	chr12.56539319.56542159_.V.	0.00264406	0.33705752
chr1.269901146.269907206_.V.	2	chr1.269901146.269907206_.V.	0.00011493	0.33703202
chr11.826930.828910_.V.	2	chr11.826930.828910_.V.	0.04630508	0.33691988
chr12.27097716.27099057_.C.	2	chr12.27097716.27099057_.C.	0.026429	0.33635999
chr17.30237192.30243162_.V.	2	chr17.30237192.30243162_.V.	0.04630508	0.33588551
chr5.814468.818758_.V.	2	chr5.814468.818758_.V.	0.04161078	0.33587453
chr14.28107019.28115119_.V.	2	chr14.28107019.28115119_.V.	0.00128303	0.33538803
chr4.12235139.12242019_.V.	2	chr4.12235139.12242019_.V.	0.0160561	0.33527049
chr6.168524156.168530576_.V.	2	chr6.168524156.168530576_.V.	0.00596018	0.33514373
chr5.10586208.10589518_.V.	2	chr5.10586208.10589518_.V.	0.02343317	0.33505767
chr8.54586638.54587668_.V.	2	chr8.54586638.54587668_.V.	0.01319203	0.33486828
chr11.1661120.1663910_.V.	2	chr11.1661120.1663910_.V.	0.0160561	0.33338465
chr5.40197598.40200348_.V.	2	chr5.40197598.40200348_.V.	0.02071916	0.33312482
chr18.48737495.48741355_.V.	2	chr18.48737495.48741355_.V.	0.01407064	0.33295487
chr15.138862548.138866848_.V.	2	chr15.138862548.138866848_.V.	0.0160561	0.33129171
chr15.138240648.138243538_.V.	2	chr15.138240648.138243538_.V.	0.04161078	0.33122277
chr2.6526990.6534360_.V.	2	chr2.6526990.6534360_.V.	0.03334006	0.33027619
chr14.136293439.136297589_.V.	2	chr14.136293439.136297589_.V.	0.01229347	0.33009934
chr14.139120929.139123949_.V.	2	chr14.139120929.139123949_.V.	0.04630508	0.32967956
chr6.88049896.88059876_.V.	2	chr6.88049896.88059876_.V.	0.00596018	0.32934807
chrX.124234838.124236418_.V.	2	chrX.124234838.124236418_.V.	0.03355544	0.32901079
chr2.151552720.151555320_.V.	2	chr2.151552720.151555320_.V.	0.026429	0.32883903
chr9.138909332.138913472_.V.	2	chr9.138909332.138913472_.V.	0.0160561	0.32823008
chr17.57662942.57667932_.V.	2	chr17.57662942.57667932_.V.	0.02071916	0.32820502
chr18.3151255.3153755_.V.	2	chr18.3151255.3153755_.V.	0.00435484	0.3277411
chr17.60866642.60878492_.V.	2	chr17.60866642.60878492_.V.	0.03334006	0.32742911
chr18.857695.860645_.V.	2	chr18.857695.860645_.V.	0.00018834	0.32673336
chr14.132330909.132335069_.V.	2	chr14.132330909.132335069_.V.	0.03334006	0.3259163
chr13.206205555.206209275_.V.	2	chr13.206205555.206209275_.V.	0.00929494	0.32583135
chr5.2580368.2583668_.V.	2	chr5.2580368.2583668_.V.	0.00693574	0.32474608
chr6.82550096.82554946_.V.	2	chr6.82550096.82554946_.V.	0.00154718	0.32443482
chr1.1530866.1535259_.C.	2	chr1.1530866.1535259_.C.	0.00071066	0.32400201
chrY.5151990.5158280_.V.	2	chrY.5151990.5158280_.V.	0.04564902	0.32316975
chr15.126444208.126453028_.V.	2	chr15.126444208.126453028_.V.	0.0160561	0.32107043
chr13.201976615.201980625_.V.	2	chr13.201976615.201980625_.V.	0.01070637	0.32088492
chr6.89908806.89912726_.V.	2	chr6.89908806.89912726_.V.	0.03334006	0.32083458
chr15.121565378.121578088_.V.	2	chr15.121565378.121578088_.V.	5.18E-05	0.32068734
chr14.140130749.140134879_.V.	2	chr14.140130749.140134879_.V.	0.00128303	0.32050256
chr3.31796726.31803986_.V.	2	chr3.31796726.31803986_.V.	0.03334006	0.31996464
chr15.138739508.138741938_.V.	2	chr15.138739508.138741938_.V.	0.00086971	0.31961896
chrX.97102018.97105678_.V.	2	chrX.97102018.97105678_.V.	0.00435484	0.31918128
chr13.201935711.201936265_.C.	2	chr13.201935711.201936265_.C.	0.00596018	0.31898324
chr5.197378.203528_.V.	2	chr5.197378.203528_.V.	0.00037581	0.3188685
chr14.51195729.51198409_.V.	2	chr14.51195729.51198409_.V.	0.00222085	0.3187173
chr9.53389332.53391822_.V.	2	chr9.53389332.53391822_.V.	5.18E-05	0.31841297
chr14.133028839.133031539_.V.	2	chr14.133028839.133031539_.V.	0.03334006	0.31815767
chr1.245176.249636_.V.	2	chr1.245176.249636_.V.	0.026429	0.31789683
chr3.33496746.33499166_.V.	2	chr3.33496746.33499166_.V.	0.00046721	0.3176639
chr4.1291569.1292569_.V.	2	chr4.1291569.1292569_.V.	0.02343317	0.31760669
chr6.163921196.163925556_.V.	2	chr6.163921196.163925556_.V.	0.02343317	0.31671469
chr5.2571708.2576618_.V.	2	chr5.2571708.2576618_.V.	0.00264406	0.31658878
chr4.125366989.125370459_.V.	2	chr4.125366989.125370459_.V.	0.00105868	0.31598191
chr14.74883799.74887899_.V.	2	chr14.74883799.74887899_.V.	0.00510422	0.31594977
chr9.135272762.135281422_.V.	2	chr9.135272762.135281422_.V.	0.0160561	0.31559024
chr1.205976.211656_.V.	2	chr1.205976.211656_.V.	0.02071916	0.31496914
chr1.2616166.2621206_.V.	2	chr1.2616166.2621206_.V.	0.03334006	0.31411353
chr18.17987535.17989915_.V.	2	chr18.17987535.17989915_.V.	0.03334006	0.31388941
chr18.50233045.50234445_.V.	2	chr18.50233045.50234445_.V.	0.00057789	0.31375094
chr11.76212570.76214054_.C.	2	chr11.76212570.76214054_.C.	0.026429	0.31363847
chr11.806250.810370_.V.	2	chr11.806250.810370_.V.	0.00023867	0.31320816
chr16.74667349.74670539_.V.	2	chr16.74667349.74670539_.V.	0.00693574	0.31311752
chr5.729828.732748_.V.	2	chr5.729828.732748_.V.	0.00105868	0.31297446
chr4.110584109.110585339_.V.	2	chr4.110584109.110585339_.V.	0.00057789	0.31292405
chr4.103092789.103096679_.V.	2	chr4.103092789.103096679_.V.	0.03729477	0.31275421
chr1.9513086.9516486_.V.	2	chr1.9513086.9516486_.V.	0.00313457	0.31248155
chr11.76748420.76752040_.V.	2	chr11.76748420.76752040_.V.	0.01826556	0.31103118
chr18.2272575.2275685_.V.	2	chr18.2272575.2275685_.V.	0.01070637	0.31020466
chr18.1095905.1098925_.V.	2	chr18.1095905.1098925_.V.	0.00030028	0.31012796
chr12.47291739.47297129_.V.	2	chr12.47291739.47297129_.V.	0.00435484	0.30992663
chr12.33896719.33901049_.V.	2	chr12.33896719.33901049_.V.	0.0300707	0.3095163
chr7.3101659.3103669_.V.	2	chr7.3101659.3103669_.V.	0.026429	0.3091028
chr10.66374829.66380089_.V.	2	chr10.66374829.66380089_.V.	0.01070637	0.3089603
chr4.801299.805749_.V.	2	chr4.801299.805749_.V.	0.0160561	0.30893018
chr3.11050706.11053846_.V.	2	chr3.11050706.11053846_.V.	0.00510422	0.30847105
chr4.108614479.108617509_.V.	2	chr4.108614479.108617509_.V.	0.00370211	0.30843168
chr7.2614139.2616239_.V.	2	chr7.2614139.2616239_.V.	0.03334006	0.30725197
chr13.207851085.207852185_.C.	2	chr13.207851085.207852185_.C.	0.00086971	0.30709286
chr8.138963068.138964698_.V.	2	chr8.138963068.138964698_.V.	0.00596018	0.30675458
chr11.4043250.4045240_.V.	2	chr11.4043250.4045240_.V.	0.00596018	0.30649318
chr4.1395609.1398549_.V.	2	chr4.1395609.1398549_.V.	0.0160561	0.30491988

chr15.134599548.134604448_.V.	2	chr15.134599548.134604448_.V.	0.00057789	0.30484147
chr13.137917235.137921315_.V.	2	chr13.137917235.137921315_.V.	0.00222085	0.30433275
chr9.7757462.7760202_.V.	2	chr9.7757462.7760202_.V.	0.00693574	0.30329662
chr12.53957499.53961049_.V.	2	chr12.53957499.53961049_.V.	0.04630508	0.30323107
chr13.122482235.122484295_.V.	2	chr13.122482235.122484295_.V.	0.00313457	0.30281478
chr12.9250039.9255929_.V.	2	chr12.9250039.9255929_.V.	0.00313457	0.30268836
chr10.66270579.66275109_.V.	2	chr10.66270579.66275109_.V.	0.03334006	0.30266926
chr11.78167005.78170890_.C.	2	chr11.78167005.78170890_.C.	0.00222085	0.30264089
chr11.78799490.78805060_.V.	2	chr11.78799490.78805060_.V.	0.02972401	0.30255054
chr9.133343502.133348402_.V.	2	chr9.133343502.133348402_.V.	0.00185787	0.30212572
chr6.64597076.64601866_.V.	2	chr6.64597076.64601866_.V.	0.00086971	0.3018733
chrX.110961228.110965038_.V.	2	chrX.110961228.110965038_.V.	0.02972401	0.30177179
chr13.207920015.207924845_.V.	2	chr13.207920015.207924845_.V.	0.00105868	0.30120672
chrX.15086758.15090018_.V.	2	chrX.15086758.15090018_.V.	0.01713848	0.30110605
chr1.779396.791206_.V.	2	chr1.779396.791206_.V.	0.03334006	0.30017978
chr13.207334585.207337535_.V.	2	chr13.207334585.207337535_.V.	0.00154718	0.30005073
chr3.9597376.9609166_.V.	2	chr3.9597376.9609166_.V.	0.01070637	0.29949604
chr12.1733187.1756485_.C.	2	chr12.1733187.1756485_.C.	0.00105868	0.29944461
chr6.164089606.164094406_.V.	2	chr6.164089606.164094406_.V.	0.03729477	0.299168
chr2.70657400.70660160_.V.	2	chr2.70657400.70660160_.V.	0.00510422	0.29879002
chr2.2420990.2422920_.V.	2	chr2.2420990.2422920_.V.	0.03334006	0.29869891
chr18.4943185.4946905_.V.	2	chr18.4943185.4946905_.V.	0.026429	0.29841688
chr8.11799898.11805648_.V.	2	chr8.11799898.11805648_.V.	0.04433484	0.29816036
chr6.12871866.12878766_.V.	2	chr6.12871866.12878766_.V.	0.03369858	0.29802484
chr15.135928008.135935298_.V.	2	chr15.135928008.135935298_.V.	0.03334006	0.29801697
chr1.1338296.1346816_.V.	2	chr1.1338296.1346816_.V.	0.02071916	0.29789612
chr7.116584369.116587509_.V.	2	chr7.116584369.116587509_.V.	0.01070637	0.29758719
chr12.17970399.17973599_.V.	2	chr12.17970399.17973599_.V.	0.01407064	0.29755765
chr2.71110600.71114230_.V.	2	chr2.71110600.71114230_.V.	0.01229347	0.29704815
chr1.4913236.4916166_.V.	2	chr1.4913236.4916166_.V.	0.01070637	0.29653821
chr6.66851756.66855146_.V.	2	chr6.66851756.66855146_.V.	0.02343317	0.29597412
chr9.131817522.131821372_.V.	2	chr9.131817522.131821372_.V.	0.00222085	0.29562139
chr17.61283952.61295862_.V.	2	chr17.61283952.61295862_.V.	0.00929494	0.29531657
chr18.48938595.48941875_.V.	2	chr18.48938595.48941875_.V.	0.03729477	0.29498063
chr11.78138900.78143630_.V.	2	chr11.78138900.78143630_.V.	0.01070637	0.29492483
chr3.113718876.113725716_.V.	2	chr3.113718876.113725716_.V.	0.02071916	0.29454586
chr4.96775389.96779099_.V.	2	chr4.96775389.96779099_.V.	0.00123548	0.29362419
chr3.113473226.113476906_.V.	2	chr3.113473226.113476906_.V.	5.18E-05	0.29291322
chr7.2794199.2797759_.V.	2	chr7.2794199.2797759_.V.	0.00046721	0.29284816
chr3.113513086.113521056_.V.	2	chr3.113513086.113521056_.V.	0.04630508	0.292605
chr9.136680792.136687932_.V.	2	chr9.136680792.136687932_.V.	0.00804229	0.29235852
chr12.58106109.58112419_.V.	2	chr12.58106109.58112419_.V.	0.02972401	0.29212916
chr17.60235822.60239962_.V.	2	chr17.60235822.60239962_.V.	0.00370211	0.29201358
chr5.880898.887848_.V.	2	chr5.880898.887848_.V.	0.00071066	0.29175154
chr2.69172580.69174080_.V.	2	chr2.69172580.69174080_.V.	0.00105868	0.29021164
chr5.4929778.4944218_.V.	2	chr5.4929778.4944218_.V.	1.56E-05	0.29021025
chr1.266090536.266096266_.V.	2	chr1.266090536.266096266_.V.	0.02071916	0.29013549
chr12.3887149.3894119_.V.	2	chr12.3887149.3894119_.V.	0.00693574	0.28978162
chr18.39828045.39831105_.V.	2	chr18.39828045.39831105_.V.	0.00046721	0.28975455
chr6.1574996.1579556_.V.	2	chr6.1574996.1579556_.V.	0.00154718	0.28950281
chr13.207825375.207830235_.V.	2	chr13.207825375.207830235_.V.	0.00037581	0.28945639
chr5.1267908.1273458_.V.	2	chr5.1267908.1273458_.V.	0.00018834	0.28922197
chr5.4775208.4785358_.V.	2	chr5.4775208.4785358_.V.	0.00105868	0.28859441
chr11.7706050.7708880_.V.	2	chr11.7706050.7708880_.V.	0.02343317	0.28852968
chr9.65018732.65022572_.V.	2	chr9.65018732.65022572_.V.	0.02141819	0.288052
chr14.139444509.139447079_.V.	2	chr14.139444509.139447079_.V.	0.00510422	0.28796551
chr6.167368936.167377426_.V.	2	chr6.167368936.167377426_.V.	0.026429	0.28785488
chr1.862256.875336_.V.	2	chr1.862256.875336_.V.	0.00929494	0.28771325
chr10.66516689.66523159_.V.	2	chr10.66516689.66523159_.V.	0.00435484	0.28674378
chr4.123218039.123221889_.V.	2	chr4.123218039.123221889_.V.	0.01407064	0.28627162
chr12.56286169.56291389_.V.	2	chr12.56286169.56291389_.V.	0.00018834	0.2857546
chr17.47653472.47658062_.V.	2	chr17.47653472.47658062_.V.	0.00804229	0.28534825
chr15.135844108.135850298_.V.	2	chr15.135844108.135850298_.V.	0.02972401	0.28508487
chr11.78896710.78901170_.V.	2	chr11.78896710.78901170_.V.	0.00313457	0.28453184
chr3.125602766.125605436_.V.	2	chr3.125602766.125605436_.V.	0.01407064	0.28333631
chr12.1708838.1725965_.C.	2	chr12.1708838.1725965_.C.	0.00128303	0.2822725
chr2.1526300.1530330_.V.	2	chr2.1526300.1530330_.V.	0.03729477	0.28149825
chr12.57315389.57318049_.V.	2	chr12.57315389.57318049_.V.	0.00105868	0.28123952
chr18.2129945.2132905_.V.	2	chr18.2129945.2132905_.V.	0.00370211	0.28004139
chr4.15793249.15797329_.V.	2	chr4.15793249.15797329_.V.	0.00105868	0.27982809
chr10.67435519.67446299_.V.	2	chr10.67435519.67446299_.V.	0.00046721	0.27971733
chr1.272535826.272540916_.V.	2	chr1.272535826.272540916_.V.	0.026429	0.27957797
chr9.136634302.136640792_.V.	2	chr9.136634302.136640792_.V.	0.04564902	0.27950006
chr2.17969090.17975170_.V.	2	chr2.17969090.17975170_.V.	0.00057789	0.27915832
chr11.93490.98460_.V.	2	chr11.93490.98460_.V.	0.00596018	0.27827421
chr14.139222899.139228039_.V.	2	chr14.139222899.139228039_.V.	0.04161078	0.27752037
chr3.9503526.9507376_.V.	2	chr3.9503526.9507376_.V.	0.02343317	0.27749442
chr5.1531118.1548328_.V.	2	chr5.1531118.1548328_.V.	0.04161078	0.27621628
chr12.1783879.1788199_.V.	2	chr12.1783879.1788199_.V.	0.02071916	0.27544612
chr11.78677137.78678566_.C.	2	chr11.78677137.78678566_.C.	0.00313457	0.27541618
chr5.69443068.69451418_.V.	2	chr5.69443068.69451418_.V.	0.01826556	0.27513134
chr4.753289.757889_.V.	2	chr4.753289.757889_.V.	0.0160561	0.27370834
chr11.3807702.3808665_.C.	2	chr11.3807702.3808665_.C.	0.03729477	0.27347736
chrX.122078398.122081848_.V.	2	chrX.122078398.122081848_.V.	0.03729477	0.27253489
chr9.8358382.8363692_.V.	2	chr9.8358382.8363692_.V.	0.0160561	0.27249669
chr9.2192002.2194782_.V.	2	chr9.2192002.2194782_.V.	0.00264406	0.27090784
chr14.137231709.137236249_.V.	2	chr14.137231709.137236249_.V.	0.00057789	0.27084087
chr14.140244459.140248619_.V.	2	chr14.140244459.140248619_.V.	0.00313457	0.27079611
chr12.1396069.1402069_.V.	2	chr12.1396069.1402069_.V.	0.01826556	0.27027625
chr17.46316722.46322122_.V.	2	chr17.46316722.46322122_.V.	0.02343317	0.26989909
chr2.71159280.71162450_.V.	2	chr2.71159280.71162450_.V.	0.0160561	0.26932365
chr17.10765752.10768522_.V.	2	chr17.10765752.10768522_.V.	0.01826556	0.26907147
chr12.48069459.48073839_.V.	2	chr12.48069459.48073839_.V.	0.00435484	0.26887756
chr5.1519708.1528168_.V.	2	chr5.1519708.1528168_.V.	0.01070637	0.26878775
chr12.54553579.54559169_.V.	2	chr12.54553579.54559169_.V.	0.00693574	0.26824007
chr9.134087302.134092522_.V.	2	chr9.134087302.134092522_.V.	0.01070637	0.26823875
chr3.25474506.25477466_.V.	2	chr3.25474506.25477466_.V.	0.00510422	0.2675033
chr4.74619249.74623839_.V.	2	chr4.74619249.74623839_.V.	0.00596018	0.26519686
chr10.64948929.64955329_.V.	2	chr10.64948929.64955329_.V.	0.01070637	0.26462176
chr7.805219.809829_.V.	2	chr7.805219.809829_.V.	0.00018834	0.26327131
chr3.41248196.41252396_.V.	2	chr3.41248196.41252396_.V.	0.00929494	0.26265179
chr2.2591680.2597420_.V.	2	chr2.2591680.2597420_.V.	0.026429	0.26243443
chr5.4259958.4262828_.V.	2	chr5.4259958.4262828_.V.	0.03729477	0.26220571
chr3.127556286.127558286_.V.	2	chr3.127556286.127558286_.V.	0.04161078	0.26171827
chr11.78534516.78538856_.C.	2	chr11.78534516.78538856_.C.	0.02071916	0.26163626

chr7.7807889.7811419_.V.	2	chr7.7807889.7811419_.V.	0.04612132	0.26112565
chr1.1252336.1261106_.V.	2	chr1.1252336.1261106_.V.	0.00086971	0.26080103
chr13.207881641.207883728_.C.	2	chr13.207881641.207883728_.C.	0.00435484	0.26025796
chr12.54155339.54159459_.V.	2	chr12.54155339.54159459_.V.	0.04630508	0.26007399
chr12.59630459.59633979_.V.	2	chr12.59630459.59633979_.V.	0.00929494	0.25934959
chr11.78946300.78950400_.V.	2	chr11.78946300.78950400_.V.	0.04161078	0.25864145
chr7.2747059.2750209_.V.	2	chr7.2747059.2750209_.V.	0.04829971	0.25702535
chr5.2506818.2512008_.V.	2	chr5.2506818.2512008_.V.	0.00046721	0.25611666
chr14.137556249.137557679_.V.	2	chr14.137556249.137557679_.V.	0.00037581	0.25598495
chr7.546619.550269_.V.	2	chr7.546619.550269_.V.	0.02905462	0.25525948
chr14.72869909.72873509_.V.	2	chr14.72869909.72873509_.V.	0.01070637	0.25508886
chr12.6025019.6028749_.V.	2	chr12.6025019.6028749_.V.	0.00185787	0.25435947
chr7.1388459.1392379_.V.	2	chr7.1388459.1392379_.V.	0.00313457	0.25404977
chr5.982098.986558_.V.	2	chr5.982098.986558_.V.	0.00086971	0.25313463
chr10.23622739.23625309_.V.	2	chr10.23622739.23625309_.V.	0.0160561	0.25312394
chr5.9839198.9845318_.V.	2	chr5.9839198.9845318_.V.	0.00264406	0.25251462
chr7.2693029.2695299_.V.	2	chr7.2693029.2695299_.V.	0.01826556	0.25147723
