# Supplementary material for: Deciphering differences in DNA methylation and transcriptome profiles of oocytes from pigs with high and low developmental competence
Source: Environ Epigenet. 2025 Jun 3;11(1):dvaf018. doi: 10.1093/eep/dvaf018 (PMC12418950; doi:10.1093/eep/dvaf018)
Supplement: dvaf018_Supplemental_Files [file dvaf018_supplemental_files.zip › Sup table 17.pdf]

Gene	Gene-Chr	Gene-ini	Gene-end	Loci	Loci-Chr	Loci-ini	Loci-end	cor
RNASEH2A	chr2	66193197	66207697	344600.66347	chr2	66344600	66347110	-0.8136199
PFDN6	chr7	29653679	29654945	643130.29647	chr7	29643130	29647130	-0.7907699
RPS18	chr7	29638833	29642912	643130.29647	chr7	29643130	29647130	-0.7601996
PRPF4	chr1	253906717	253925046	076730.25408	chr1	254076730	254080730	-0.7574917
RNASEH2A	chr2	66193197	66207697	345269.66346	chr2	66345269	66346152	-0.7529248
SLC46A3	chr11	5982443	6000701	793860.5797	chr11	5793860	5797121	-0.7376826
DND1	chr2	142384532	142387199	578003.14257	chr2	142578003	142579756	-0.7359584
POLA2	chr2	6922387	6950179	079608.70836	chr2	7079608	7083608	-0.7211563
CDC26	chr1	253895885	253906632	076730.25408	chr1	254076730	254080730	-0.7210019
GPR39	chr15	19557893	19799040	520828.1952	chr15	19520828	19524828	-0.7152016
PRPF4	chr1	253906717	253925046	569456.25357	chr1	253569456	253571216	-0.7135366
DDIT3	chr5	22785445	22789829	056982.23060	chr5	23056982	23060982	-0.7116108
ATG2A	chr2	7262031	7282347	079608.70836	chr2	7079608	7083608	-0.7071768
CNPY2	chr5	21707834	21711292	557029.21561	chr5	21557029	21561029	-0.7064878
RAB7A	chr13	71777424	71852694	558012.7156	chr13	71558012	71562012	-0.7036052
CUTA	chr7	29720094	29721769	643130.29647	chr7	29643130	29647130	-0.7020374
MTUS2	chr11	6197740	6532924	793860.5797	chr11	5793860	5797121	-0.700481
MRPL20	chr6	63670755	63675839	862113.63863	chr6	63862113	63863570	-0.6988649
GPR39	chr15	19557893	19799040	521903.1952	chr15	19521903	19524191	-0.6988223
ILK	chr9	3145608	3159858	376207.33802	chr9	3376207	3380207	-0.6985952
COQ4	chr1	268756685	268768343	615087.26861	chr1	268615087	268619087	-0.6983036
POP7	chr3	8605018	8610218	904591.89085	chr3	8904591	8908591	-0.696298
SLC35A4	chr2	142323341	142325335	577020.14257	chr2	142577020	142579530	-0.6936215
RPS10	chr7	30435150	30442886	513925.30517	chr7	30513925	30517925	-0.6906976
VPS52	chr7	29624230	29639140	643130.29647	chr7	29643130	29647130	-0.6894168
WDR55	chr2	142378462	142384677	577020.14257	chr2	142577020	142579530	-0.6877988
ZDHHC4	chr3	4628385	4643691	132177.41361	chr3	4132177	4136177	-0.6876099
POP7	chr3	8605018	8610218	886506.88905	chr3	8886506	8890506	-0.6861088
ENSSSCG00000057727	chr3	68572415	68573980	570415.68574	chr3	68570415	68574415	-0.6815488
TMEM63A	chr10	13769605	13814020	939003.1394	chr10	13939003	13940930	-0.6808646
KXD1	chr2	59242310	59247713	826251.58830	chr2	58826251	58830251	-0.6793992
ENSSSCG00000033697	chr3	40001326	40003107	776754.39780	chr3	39776754	39780754	-0.676876
NELFE	chr7	24040525	24047025	836996.23840	chr7	23836996	23840996	-0.6747608
CDC26	chr1	253895885	253906632	569435.25357	chr1	253569435	253570326	-0.6745479
UQCC2	chr7	29899043	29929795	643130.29647	chr7	29643130	29647130	-0.6741012
COQ4	chr1	268756685	268768343	614034.26861	chr1	268614034	268618034	-0.6738121
CYTH3	chr3	4900570	5002194	992827.49946	chr3	4992827	4994602	-0.6735548
EIF4A1	chr12	52868434	52874249	627516.5262	chr12	52627516	52629243	-0.6716959
TCP11	chr7	31007474	31114734	513925.30517	chr7	30513925	30517925	-0.6702733
WDR55	chr2	142378462	142384677	578003.14257	chr2	142578003	142579756	-0.6700052
CRAT	chr1	269386650	269401331	332838.26933	chr1	269332838	269336838	-0.6693265
TRMT1	chr2	65940125	65949324	344600.66347	chr2	66344600	66347110	-0.6689601
ENSSSCG00000010058	chr14	49585326	49598007	792613.4979	chr14	49792613	49793506	-0.6685373
WDR55	chr2	142378462	142384677	639970.14264	chr2	142639970	142642910	-0.6682313
PPCDC	chr7	58525143	58549430	826727.58830	chr7	58826727	58830727	-0.6664395
ENSSSCG00000060885	chr15	120345579	120348027	343579.1203	chr15	120343579	120347579	-0.6662964
NOP14	chr8	1716826	1737902	386266.13902	chr8	1386266	1390266	-0.665622
RNASEH2A	chr2	66193197	66207697	950519.65954	chr2	65950519	65954519	-0.6651774
RPL27	chr12	19892217	19894504	256373.2026	chr12	20256373	20260373	-0.6649229
USP5	chr5	63843745	63858716	114150.64118	chr5	64114150	64118150	-0.6642902
TRMT1	chr2	65940125	65949324	055483.66056	chr2	66055483	66056550	-0.6641826
ENSSSCG00000035904	chr1	272959831	272965634	961551.27296	chr1	272961551	272965551	-0.6635291
FUT2	chr6	54034684	54047224	809477.53810	chr6	53809477	53810078	-0.6627397
TXNL4A	chr6	127974543	127991177	958266.12796	chr6	127958266	127962266	-0.6619466
PSMC3IP	chr12	20244304	20250159	256373.2026	chr12	20256373	20260373	-0.6610793
SLC35A4	chr2	142323341	142325335	578003.14257	chr2	142578003	142579756	-0.6591694
FAU	chr2	7070906	7072809	079608.70836	chr2	7079608	7083608	-0.6584998
DND1	chr2	142384532	142387199	577020.14257	chr2	142577020	142579530	-0.6582568
EDC3	chr7	58828727	58897919	908967.58910	chr7	58908967	58910668	-0.6579982
AP1S1	chr3	8881107	8887566	315140.93191	chr3	9315140	9319140	-0.6577859
ENSSSCG00000033310	chr5	21506554	21507767	557029.21561	chr5	21557029	21561029	-0.6551344
ATP6V1D	chr7	91064396	91084913	955179.90956	chr7	90955179	90956189	-0.6550597

WDR55	chr2	142378462	142384677	541101.14264	chr2	142641101	142642772	-0.6550202
CALR	chr2	66098229	66102132	344600.66347	chr2	66344600	66347110	-0.6538977
CALR	chr2	66098229	66102132	345269.66346	chr2	66345269	66346152	-0.6532716
CIAO2B	chr6	27610470	27612592	081955.28085	chr6	28081955	28085955	-0.6531807
PER1	chr12	53361889	53374248	109256.5311	chr12	53109256	53113256	-0.6528601
AARSD1	chr12	19918248	19926825	0256373.2026	chr12	20256373	20260373	-0.6527317
TUFM	chr3	18521128	18524959	118581.18122	chr3	18118581	18122581	-0.6515957
UBE2J2	chr6	63555488	63568017	362113.63863	chr6	63862113	63863570	-0.6514578
ENSSSCG00000057727	chr3	68572415	68573980	020319.69021	chr3	69020319	69021858	-0.6500186
YPEL1	chr14	50190798	50208256	792613.4979	chr14	49792613	49793506	-0.6497723
MYOF	chr14	104770515	104941109	010519.1050	chr14	105010519	105014699	-0.6492483
LSM2	chr7	23900395	23908490	836996.23840	chr7	23836996	23840996	-0.6485954
PYGB	chr17	30940452	30995691	194616.3119	chr17	31194616	31195316	-0.6479662
EHD1	chr2	7298208	7321576	079608.70836	chr2	7079608	7083608	-0.6479379
PCDHGA4	chr2	142993554	143156556	124047.14312	chr2	143124047	143126730	-0.6475112
OTUB1	chr2	8087829	8095609	359774.83637	chr2	8359774	8363774	-0.6469739
DYNC2I2	chr1	269002261	269030719	614034.26861	chr1	268614034	268618034	-0.6468438
DRAP1	chr2	6409655	6412395	561091.65650	chr2	6561091	6565091	-0.6455566
IK	chr2	142361155	142376333	578003.14257	chr2	142578003	142579756	-0.6452791
SRRT	chr3	8717785	8731201	904591.89085	chr3	8904591	8908591	-0.6441181
CDIPT	chr3	18084549	18091798	175737.18179	chr3	18175737	18179737	-0.6432599
GFRA4	chr17	32040235	32046105	868517.3186	chr17	31868517	31869375	-0.6425129
DRAP1	chr2	6409655	6412395	409833.64138	chr2	6409833	6413833	-0.6419435
CTDNEP1	chr12	52599226	52606059	399810.5240	chr12	52399810	52403810	-0.6414976
BRMS1	chr2	6046098	6053667	044098.60480	chr2	6044098	6048098	-0.6414968
SCAMP4	chr2	76615000	76634264	996982.77000	chr2	76996982	77000982	-0.6409534
PSKH1	chr6	28508823	28544062	081955.28085	chr6	28081955	28085955	-0.6403351
PRPF4	chr1	253906717	253925046	569435.25357	chr1	253569435	253570326	-0.6402758
ENSSSCG00000027573	chr13	34119539	34121859	663957.3366	chr13	33663957	33666213	-0.6401848
DNAJB2	chr15	121319982	121335991	210881.1212	chr15	121210881	121214881	-0.6392163
FAM219B	chr7	58650754	58659479	826727.58830	chr7	58826727	58830727	-0.637818
TNNT1	chr6	59347582	59365284	850751.58854	chr6	58850751	58854751	-0.6375082
PRDX2	chr2	66207828	66212009	344600.66347	chr2	66344600	66347110	-0.6373143
IDH2	chr7	55651609	55675554	237411.55238	chr7	55237411	55238015	-0.6364029
FSD2	chr7	52144506	52194133	254159.52258	chr7	52254159	52258159	-0.6348018
GRK4	chr8	1738333	1792374	386266.13902	chr8	1386266	1390266	-0.6344396
NOTO	chr3	69567449	69571100	234351.69238	chr3	69234351	69238351	-0.6342576
ENSSSCG00000044567	chr17	31217462	31238052	194616.3119	chr17	31194616	31195316	-0.6341606
DYNLL1	chr14	40455985	40459104	0217722.4021	chr14	40217722	40218386	-0.6334011
SPDYC	chr2	7030733	7058967	561091.65650	chr2	6561091	6565091	-0.632104
DDX39B	chr7	23658088	23670031	836996.23840	chr7	23836996	23840996	-0.6316889
POLE3	chr1	254033385	254035978	076730.25408	chr1	254076730	254080730	-0.6316266
PPCDC	chr7	58525143	58549430	908967.58910	chr7	58908967	58910668	-0.6315353
OTUB1	chr2	8087829	8095609	410290.84165	chr2	8410290	8416580	-0.6312373
ZKSCAN5	chr3	6442391	6466430	141962.61429	chr3	6141962	6142972	-0.6301048
TRMT1	chr2	65940125	65949324	345269.66346	chr2	66345269	66346152	-0.6299274
DRAP1	chr2	6409655	6412395	428153.64321	chr2	6428153	6432153	-0.62969
SDHA	chr16	79834044	79862524	471745.7947	chr16	79471745	79475745	-0.6294945
MYOF	chr14	104770515	104941109	011963.1050	chr14	105011963	105012625	-0.6291878
EIF4A1	chr12	52868434	52874249	399810.5240	chr12	52399810	52403810	-0.628687
POLA2	chr2	6922387	6950179	537993.65396	chr2	6537993	6539697	-0.6285497
C6orf136	chr7	23185599	23190299	062338.23066	chr7	23062338	23066338	-0.6282814
CREB3	chr1	236450573	236456375	378019.23638	chr1	236378019	236382019	-0.6273878
IFT22	chr3	8976306	8981510	315140.93191	chr3	9315140	9319140	-0.626371
ENSSSCG00000057727	chr3	68572415	68573980	496471.68497	chr3	68496471	68497933	-0.625159
RNF4	chr8	1353658	1380634	386266.13902	chr8	1386266	1390266	-0.6248329
ENSSSCG00000009374	chr11	15550286	15628604	734905.1573	chr11	15734905	15735621	-0.6241543
RPL7L1	chr7	37940834	37951324	388035.38389	chr7	38388035	38389283	-0.6230995
DRAP1	chr2	6409655	6412395	044098.60480	chr2	6044098	6048098	-0.6224658
DNAJC14	chr5	21264664	21272646	557029.21561	chr5	21557029	21561029	-0.6222813
IMP3	chr7	58023368	58024496	702066.57703	chr7	57702066	57703313	-0.621759
ZNF410	chr7	97195975	97243614	123873.97124	chr7	97123873	97124625	-0.6214527
PCDHGA4	chr2	142993554	143156556	578003.14257	chr2	142578003	142579756	-0.6209353

ENSSSCG00000010888	chr10	19585162	19586394	286367.1929	chr10	19286367	19290367	-0.6206572
MTMR14	chr13	65948544	65994820	994525.6599	chr13	65994525	65997625	-0.6205725
MYL6	chr5	21559029	21562413	557029.21561	chr5	21557029	21561029	-0.6190794
DEF8	chr6	140908	160692	63387.64608	chr6	63387	64608	-0.6184721
SPDYC	chr2	7030733	7058967	079608.70836	chr2	7079608	7083608	-0.6176907
ENSSSCG00000034927	chr3	18468001	18489099	175737.18179	chr3	18175737	18179737	-0.6176402
DYNLL1	chr14	40455985	40459104	561159.4056	chr14	40561159	40565159	-0.6172405
COX8A	chr2	8101613	8103285	410290.84165	chr2	8410290	8416580	-0.6167249
ATG4B	chr15	140223904	140246321	131918.1401	chr15	140131918	140133932	-0.6164811
ESYT1	chr5	21514510	21535929	557029.21561	chr5	21557029	21561029	-0.6164586
ENSSSCG00000017907	chr12	51961952	51965464	399810.5240	chr12	52399810	52403810	-0.6164153
POLE3	chr1	254033385	254035978	569435.25357	chr1	253569435	253570326	-0.6163757
COPS3	chr12	60994310	61024846	029157.6103	chr12	61029157	61033157	-0.616279
FXR2	chr12	52882870	52903450	399810.5240	chr12	52399810	52403810	-0.6160764
RNF4	chr8	1353658	1380634	038195.10396	chr8	1038195	1039688	-0.6160232
ARHGAP22	chr14	89113768	89331274	192374.8919	chr14	89192374	89193566	-0.6159656
STK16	chr15	121284920	121288473	210881.1212	chr15	121210881	121214881	-0.6157955
MEPCE	chr3	8357289	8364661	356207.83580	chr3	8356207	8358016	-0.6157666
ANKRD39	chr3	56762386	56775004	810364.56812	chr3	56810364	56812121	-0.6145532
PCDH12	chr2	143550849	143565033	124047.14312	chr2	143124047	143126730	-0.6142234
ENSSSCG00000057727	chr3	68572415	68573980	519015.68520	chr3	68519015	68520573	-0.6137421
PRPF4	chr1	253906717	253925046	973973.25397	chr1	253973973	253977973	-0.6134243
RNF4	chr8	1353658	1380634	053894.10572	chr8	1053894	1057212	-0.6134077
CD63	chr5	21172283	21176232	272511.21276	chr5	21272511	21276511	-0.613395
ZNRD2	chr2	6683057	6684494	079608.70836	chr2	7079608	7083608	-0.6130591
ENSSSCG00000035909	chr15	76958516	76972527	991098.7699	chr15	76991098	76995238	-0.6124565
SLC35A4	chr2	142323341	142325335	882155.14188	chr2	141882155	141886155	-0.6115984
HAX1	chr4	95467632	95470290	903054.95907	chr4	95903054	95907054	-0.6106257
ENSSSCG00000013064	chr2	9163317	9174468	979963.89839	chr2	8979963	8983963	-0.610397
STX18	chr8	5890765	6004399	888765.58927	chr8	5888765	5892765	-0.6103423
CDIPT	chr3	18084549	18091798	118581.18122	chr3	18118581	18122581	-0.6095811
HIGD2A	chr2	81486890	81488045	285759.81289	chr2	81285759	81289759	-0.6094224
MVP	chr3	18057177	18081155	175737.18179	chr3	18175737	18179737	-0.6091438
TMEM219	chr3	18167923	18176563	118581.18122	chr3	18118581	18122581	-0.608817
POMP	chr11	5953743	5974060	000360.6001	chr11	6000360	6001074	-0.6085742
ZNF584	chr6	62992506	63005691	590463.62691	chr6	62690463	62691254	-0.6084842
TTLL4	chr15	120773185	120793194	343579.1203	chr15	120343579	120347579	-0.6084296
UBE2J2	chr6	63555488	63568017	654119.63658	chr6	63654119	63658119	-0.6082249
USP5	chr5	63843745	63858716	116264.64117	chr5	64116264	64117021	-0.6074977
EMG1	chr5	63745476	63751469	114150.64118	chr5	64114150	64118150	-0.6072481
CCBE1	chr1	161321273	161556537	720590.16172	chr1	161720590	161721936	-0.6069404
CD40	chr17	48286029	48298528	081366.4808	chr17	48081366	48082939	-0.6064718
PRPSAP1	chr12	5144069	5174382	521113.5525	chr12	5521113	5525113	-0.6061228
SLC25A11	chr12	51970806	51975548	399810.5240	chr12	52399810	52403810	-0.6056563
MRPL20	chr6	63670755	63675839	654119.63658	chr6	63654119	63658119	-0.6054911
CFL1	chr2	6469254	6475035	409833.64138	chr2	6409833	6413833	-0.6054236
NSG1	chr8	6000057	6024769	888765.58927	chr8	5888765	5892765	-0.6051511
SEZ6L2	chr3	18098102	18119558	118581.18122	chr3	18118581	18122581	-0.6048354
SNAPC2	chr2	71291257	71294006	519081.71523	chr2	71519081	71523081	-0.6044325
SNX30	chr1	253428508	253550375	569456.25357	chr1	253569456	253571216	-0.6040337
TTLL4	chr15	120773185	120793194	024245.1210	chr15	121024245	121028245	-0.603985
RECQL5	chr12	5732348	5770133	938647.5942	chr12	5938647	5942647	-0.6028024
PRPF6	chr17	62815085	62858382	754250.6275	chr17	62754250	62755455	-0.6022651
HARS1	chr2	142385872	142401208	577020.14257	chr2	142577020	142579530	-0.6021938
C9orf78	chr1	269986391	269995395	190175.27019	chr1	270190175	270190671	-0.6018442
C9orf78	chr1	269986391	269995395	363751.27036	chr1	270363751	270364715	-0.6016402
FTL	chr6	54231172	54232750	705237.54709	chr6	54705237	54709237	-0.601155
DHRS1	chr7	74992115	75002148	027923.75031	chr7	75027923	75031923	-0.6011487
NR1H3	chr2	15315282	15334820	379707.15383	chr2	15379707	15383707	-0.6001341
KCNN3	chr4	94915965	95113275	495309.94499	chr4	94495309	94499039	-0.6000853
POLA2	chr2	6922387	6950179	561091.65650	chr2	6561091	6565091	-0.5996739
THBS3	chr4	94611593	94623902	830270.94834	chr4	94830270	94834270	-0.5987967
ARHGAP9	chr5	22747339	22762049	056982.23060	chr5	23056982	23060982	-0.5987328

ZDHHC4	chr3	4628385	4643691	992827.49946	chr3	4992827	4994602	-0.5982771
ENSSSCG00000017955	chr12	52878764	52882863	627516.5262	chr12	52627516	52629243	-0.5976937
TUFM	chr3	18521128	18524959	505886.18507	chr3	18505886	18507873	-0.5974875
ACAD9	chr13	71374944	71427126	558012.7156	chr13	71558012	71562012	-0.597401
DND1	chr2	142384532	142387199	383680.14188	chr2	141883680	141886890	-0.5971154
CIAPIN1	chr6	19356069	19373060	535476.19539	chr6	19535476	19539846	-0.5970716
PRDX2	chr2	66207828	66212009	345269.66346	chr2	66345269	66346152	-0.596858
ENSSSCG00000050152	chr15	78669325	78674715	502078.7850	chr15	78502078	78506078	-0.5964322
FAAP24	chr6	42815528	42819623	558113.42562	chr6	42558113	42562113	-0.5959355
P4HB	chr12	1121824	1131289	120907.1129	chr12	1120907	1129651	-0.5948861
DNPEP	chr15	121391746	121412864	253797.1212	chr15	121253797	121254795	-0.5948016
CCNL2	chr6	63659054	63668047	862113.63863	chr6	63862113	63863570	-0.5943175
RTN4IP1	chr1	72762855	72815540	697571.72701	chr1	72697571	72701571	-0.5938757
FCGR1A	chr4	99233611	99242586	052426.99056	chr4	99052426	99056426	-0.5936167
LIG1	chr6	53620483	53686562	809477.53810	chr6	53809477	53810078	-0.5932922
TUBA1B	chr5	15149099	15153620	027205.15029	chr5	15027205	15029851	-0.5928971
ENSSSCG00000011147	chr10	65576464	65595000	266094.6526	chr10	65266094	65267932	-0.5928257
IK	chr2	142361155	142376333	376462.14238	chr2	142376462	142380462	-0.5927184
UBE2O	chr12	5055065	5114123	116522.5120	chr12	5116522	5120486	-0.5924159
MVP	chr3	18057177	18081155	118581.18122	chr3	18118581	18122581	-0.5923195
HIRIP3	chr3	18198807	18202089	505886.18507	chr3	18505886	18507873	-0.5922887
SCN4A	chr12	15002175	15047582	571199.1457	chr12	14571199	14573341	-0.5922403
SWI5	chr1	268720355	268726521	614034.26861	chr1	268614034	268618034	-0.5920443
ZNF212	chr18	55528138	55541841	887653.5589	chr18	55887653	55891653	-0.5920294
BRD9	chr16	79473745	79492635	471745.7947	chr16	79471745	79475745	-0.5919773
MLH1	chr13	21791203	21877757	124847.2212	chr13	22124847	22125444	-0.5916907
RECQL5	chr12	5732348	5770133	359854.5363	chr12	5359854	5363459	-0.591557
MYOZ1	chr14	76444557	76452799	496827.7650	chr14	76496827	76500827	-0.5914014
SETD7	chr8	87478173	87527188	478129.87480	chr8	87478129	87480437	-0.5913755
ARHGDIA	chr12	1113379	1119315	120907.1129	chr12	1120907	1129651	-0.5909331
POP7	chr3	8605018	8610218	356207.83580	chr3	8356207	8358016	-0.5906605
LRCH4	chr3	8499285	8511262	904591.89085	chr3	8904591	8908591	-0.590564
KXD1	chr2	59242310	59247713	221175.59223	chr2	59221175	59223270	-0.5903451
RAB7A	chr13	71777424	71852694	760305.7176	chr13	71760305	71762524	-0.5900438
GAPDH	chr5	64129679	64133991	114150.64118	chr5	64114150	64118150	-0.5898319
RECQL4	chr4	287214	293913	429062.43018	chr4	429062	430184	-0.5897395
SH3BP5L	chr2	52323048	52339665	311759.52312	chr2	52311759	52312351	-0.5890496
SNRNP35	chr14	29471429	29477011	630876.2963	chr14	29630876	29632175	-0.5888387
IDH2	chr7	55651609	55675554	573336.55577	chr7	55573336	55577336	-0.5886929
FSD2	chr7	52144506	52194133	302472.52306	chr7	52302472	52306472	-0.5886347
GMPPB	chr13	32248268	32253155	329740.3233	chr13	32329740	32330722	-0.5885462
TRAF3IP1	chr15	137863609	137912477	636504.1376	chr15	137636504	137637619	-0.5879761
RAB43	chr13	71562350	71596249	620954.7162	chr13	71620954	71624954	-0.587822
DNAJC30	chr3	10955877	10959916	419176.11420	chr3	11419176	11420477	-0.5877794
EIF4A1	chr12	52868434	52874249	923693.5292	chr12	52923693	52927693	-0.5877607
BRMS1	chr2	6046098	6053667	428153.64321	chr2	6428153	6432153	-0.5874354
SLC41A3	chr7	53672130	53748727	639893.53641	chr7	53639893	53641617	-0.5868779
NXF1	chr2	8969581	8981984	982070.89825	chr2	8982070	8982593	-0.5863793
CTDSP2	chr5	23105172	23132586	056982.23060	chr5	23056982	23060982	-0.5851124
MPHOSPH8	chr11	447052	480121	778458.7802	chr11	778458	780289	-0.5849253
CYBC1	chr12	656347	662513	120907.1129	chr12	1120907	1129651	-0.5846726
GTF2E2	chr15	54444470	54531213	380567.5438	chr15	54380567	54384567	-0.5846211
MYL6B	chr5	21556061	21558758	557029.21561	chr5	21557029	21561029	-0.5842679
SLC35A4	chr2	142323341	142325335	641101.14264	chr2	142641101	142642772	-0.5837174
MIF	chr14	49840305	49841068	792613.4979	chr14	49792613	49793506	-0.5834302
PFKM	chr5	78476123	78526997	717866.78718	chr5	78717866	78718572	-0.5833976
RTN4IP1	chr1	72762855	72815540	699835.72701	chr1	72699835	72701157	-0.583113
SPDYC	chr2	7030733	7058967	537993.65396	chr2	6537993	6539697	-0.5825746
MEA1	chr7	38074439	38081897	388035.38389	chr7	38388035	38389283	-0.5824303
COA3	chr12	20062826	20063980	256373.2026	chr12	20256373	20260373	-0.5821994
KANSL3	chr3	56959110	57005310	810364.56812	chr3	56810364	56812121	-0.5818803
RECQL4	chr4	287214	293913	134107.13810	chr4	134107	138107	-0.5818614
ARV1	chr14	59396962	59408958	407123.5941	chr14	59407123	59411123	-0.5812798

ENSSSCG00000011272	chr13	24691893	24699363	345660.2434	chr13	24345660	24349660	-0.5811991
MRPL28	chr3	41329928	41334451	480363.41484	chr3	41480363	41484363	-0.5811514
SSR1	chr7	4652921	4685901	057871.50606	chr7	5057871	5060641	-0.5811048
METTL13	chr9	114202484	114218368	774648.11377	chr9	113774648	113775299	-0.5809223
SLC41A3	chr7	53672130	53748727	638266.53642	chr7	53638266	53642266	-0.5803257
COPS6	chr3	7982892	8005633	356207.83580	chr3	8356207	8358016	-0.5800385
NXF1	chr2	8969581	8981984	898604.88994	chr2	8898604	8899470	-0.5799899
RAB43	chr13	71562350	71596249	558012.7156	chr13	71558012	71562012	-0.5798942
FDPS	chr4	94500141	94518408	495309.94499	chr4	94495309	94499039	-0.579573
SLC5A6	chr3	111922848	111930940	769239.11177	chr3	111769239	111773239	-0.578656
LALBA	chr5	79214505	79216791	591915.79595	chr5	79591915	79595915	-0.5786496
SNRNP35	chr14	29471429	29477011	629084.2963	chr14	29629084	29633084	-0.5785235
YIPF1	chr6	158403072	158445511	535465.15853	chr6	158535465	158539465	-0.5784176
RECQL4	chr4	287214	293913	205764.20709	chr4	205764	207099	-0.5782232
TLE2	chr2	75510004	75536804	162787.75166	chr2	75162787	75166787	-0.5781202
WDR55	chr2	142378462	142384677	882155.14188	chr2	141882155	141886155	-0.5778382
SLC43A2	chr12	47791913	47839012	737633.4774	chr12	47737633	47741633	-0.5777508
ENSSSCG00000015083	chr9	45182400	45218254	783701.44787	chr9	44783701	44787701	-0.5772667
ILF2	chr4	95980918	95987867	903054.95907	chr4	95903054	95907054	-0.5770224
MYOF	chr14	104770515	104941109	009942.1050	chr14	105009942	105013942	-0.5762827
TUBA1B	chr5	15149099	15153620	024080.15028	chr5	15024080	15028080	-0.5760855
NXF1	chr2	8969581	8981984	979963.89839	chr2	8979963	8983963	-0.5760526
LRCH4	chr3	8499285	8511262	886506.88905	chr3	8886506	8890506	-0.5755274
TIMM44	chr2	71271805	71287817	519081.71523	chr2	71519081	71523081	-0.5753799
TSPAN31	chr5	23029306	23038977	881828.22885	chr5	22881828	22885548	-0.5753181
WDR53	chr13	133561021	133574376	155321.1331	chr13	133155321	133159321	-0.5749248
BRK1	chr13	66315315	66324641	994525.6599	chr13	65994525	65997625	-0.5746727
FAM219B	chr7	58650754	58659479	908967.58910	chr7	58908967	58910668	-0.5746431
GJA5	chr4	99854392	99871286	477372.99481	chr4	99477372	99481372	-0.5739763
FXR2	chr12	52882870	52903450	109256.5311	chr12	53109256	53113256	-0.5736373
FUS	chr3	17314332	17326637	619269.17623	chr3	17619269	17623269	-0.5729049
TPT1	chr11	21929221	21932691	499492.2150	chr11	21499492	21503492	-0.5726883
UTP25	chr9	133105063	133141217	385662.13338	chr9	133385662	133389422	-0.5726585
MAPK13	chr7	31880638	31889455	622450.31626	chr7	31622450	31626450	-0.5726368
RNF8	chr7	32931786	32970585	786291.32790	chr7	32786291	32790291	-0.5723996
OTUB1	chr2	8087829	8095609	281049.82826	chr2	8281049	8282602	-0.5723933
PELP1	chr12	52177094	52232420	929802.5193	chr12	51929802	51933802	-0.5723396
MAPKAPK2	chr9	67314484	67365059	679953.67683	chr9	67679953	67683953	-0.5723043
PCDHGA4	chr2	142993554	143156556	577020.14257	chr2	142577020	142579530	-0.5721196
NAPA	chr6	53246968	53275265	078236.53082	chr6	53078236	53082236	-0.5719308
TRMT1	chr2	65940125	65949324	271286.66272	chr2	66271286	66272770	-0.5717489
CRY2	chr2	16587109	16620385	657423.16658	chr2	16657423	16658649	-0.5717216
ENSSSCG00000032082	chr6	95322453	95366673	321439.95322	chr6	95321439	95322907	-0.5716742
ENSSSCG00000007719	chr3	11419446	11505406	419176.11420	chr3	11419176	11420477	-0.5716566
POLA2	chr2	6922387	6950179	755635.67596	chr2	6755635	6759635	-0.5714933
SNPH	chr17	33986513	34043995	867527.3387	chr17	33867527	33871527	-0.5707108
MRPL17	chr9	3075995	3083580	177192.31792	chr9	3177192	3179272	-0.5705516
BRMS1	chr2	6046098	6053667	409833.64138	chr2	6409833	6413833	-0.5701469
TK2	chr6	27226425	27255574	546706.27550	chr6	27546706	27550706	-0.5698032
FSD2	chr7	52144506	52194133	256466.52256	chr7	52256466	52256966	-0.5697309
MAF1	chr4	597472	600447	134107.13810	chr4	134107	138107	-0.5697103
SWI5	chr1	268720355	268726521	615087.26861	chr1	268615087	268619087	-0.5695375
ITGB5	chr13	135467798	135587473	467337.1354	chr13	135467337	135468789	-0.5694365
PPP1R7	chr15	139917257	139941251	131918.1401	chr15	140131918	140133932	-0.5694039
RAB8A	chr2	61429523	61450413	268199.61272	chr2	61268199	61272199	-0.5692465
LMBR1L	chr5	15121554	15135252	024080.15028	chr5	15024080	15028080	-0.5692413
CYTH3	chr3	4900570	5002194	992886.49950	chr3	4992886	4995036	-0.5688288
PRDX1	chr6	165824395	165861442	570840.16567	chr6	165670840	165671536	-0.5686771
TTLL4	chr15	120773185	120793194	210881.1212	chr15	121210881	121214881	-0.5686736
STX5	chr2	8937778	8968932	979963.89839	chr2	8979963	8983963	-0.5686483
RPL7	chr4	62601569	62605892	610903.62611	chr4	62610903	62611910	-0.5685873
RND1	chr5	14914645	14922038	027205.15029	chr5	15027205	15029851	-0.5685396
LIMS2	chr15	59672540	59709379	639470.5964	chr15	59639470	59643470	-0.5682205

RAB43	chr13	71562350	71596249	730277.7173	chr13	71730277	71734277	-0.5680726
CDA	chr6	78824270	78849547	786138.7879	chr6	78786138	78790138	-0.5678987
OS9	chr5	22957875	23004458	880669.2288	chr5	22880669	22884669	-0.5677578
RNASEH2B	chr11	16825128	16911969	374841.1637	chr11	16374841	16377014	-0.567746
POLD2	chr18	51038358	51046779	698457.5070	chr18	50698457	50700032	-0.5676414
NOP9	chr7	74985753	74992946	027923.7503	chr7	75027923	75031923	-0.5674237
ENSSSCG00000036812	chr12	61077631	61186002	029157.6103	chr12	61029157	61033157	-0.5670786
PFDN1	chr2	142011063	142077925	376462.1423	chr2	142376462	142380462	-0.5669644
TBCC	chr7	37822767	37824953	211487.3821	chr7	38211487	38215487	-0.5663492
ACAT1	chr9	36525261	36545633	878970.3687	chr9	36878970	36879685	-0.5662269
GNL3	chr13	34814901	34828517	837384.3484	chr13	34837384	34841384	-0.5661993
YBX2	chr12	52635385	52641651	627516.5262	chr12	52627516	52629243	-0.5659514
SENP3	chr12	52765187	52867576	627516.5262	chr12	52627516	52629243	-0.5654556
LSM10	chr6	92489927	92493665	413296.9241	chr6	92413296	92414105	-0.5654079
ALAD	chr1	254012850	254027508	076730.2540	chr1	254076730	254080730	-0.5650151
IK	chr2	142361155	142376333	882155.1418	chr2	141882155	141886155	-0.5650079
STAT5A	chr12	20474227	20499138	256373.2026	chr12	20256373	20260373	-0.5645638
DNPEP	chr15	121391746	121412864	983257.1209	chr15	120983257	120987257	-0.5643595
CCNL2	chr6	63659054	63668047	657054.6366	chr6	63657054	63661054	-0.5640838
CPB2	chr11	21234447	21298241	499492.2150	chr11	21499492	21503492	-0.5640685
NDUFA6	chr5	6562011	6567647	560011.6564	chr5	6560011	6564011	-0.5637958
IK	chr2	142361155	142376333	577020.1425	chr2	142577020	142579530	-0.563687
ENSSSCG00000042487	chr7	58998922	59008157	826727.5883	chr7	58826727	58830727	-0.5635724
DRAP1	chr2	6409655	6412395	537993.6539	chr2	6537993	6539697	-0.563504
ENSSSCG00000044155	chr2	142321430	142324016	639970.1426	chr2	142639970	142642910	-0.5631496
ENSSSCG00000029160	chr7	23914891	23928175	836996.2384	chr7	23836996	23840996	-0.5631398
CRAT	chr1	269386650	269401331	207693.2692	chr1	269207693	269211693	-0.5629926
RDH13	chr6	59274387	59292765	850751.5885	chr6	58850751	58854751	-0.5629583
MRPL17	chr9	3075995	3083580	376207.3380	chr9	3376207	3380207	-0.5626073
PHB2	chr5	63751558	63756478	114150.6411	chr5	64114150	64118150	-0.5625091
EFHC1	chr7	46239230	46348667	185166.4618	chr7	46185166	46186082	-0.5623721
STX18	chr8	5890765	6004399	890392.5891	chr8	5890392	5891389	-0.5622893
TTLL3	chr13	66056890	66123977	994525.6599	chr13	65994525	65997625	-0.5622186
SRSF5	chr7	93393426	93403011	927069.9293	chr7	92927069	92931069	-0.561861
ZNRD2	chr2	6683057	6684494	755635.6759	chr2	6755635	6759635	-0.5615868
MAP4K2	chr2	7365246	7378996	057462.7061	chr2	7057462	7061462	-0.5615375
POP7	chr3	8605018	8610218	618508.8622	chr3	8618508	8622508	-0.5615054
AP1S1	chr3	8881107	8887566	904591.8908	chr3	8904591	8908591	-0.5613932
ENSSSCG00000006559	chr4	95713742	95716153	903054.9590	chr4	95903054	95907054	-0.56138
RPS3	chr9	9624990	9630401	624152.9628	chr9	9624152	9628152	-0.5613396
ALAD	chr1	254012850	254027508	973973.2539	chr1	253973973	253977973	-0.5611594
HNRNPUL2	chr2	9034667	9048768	982070.8982	chr2	8982070	8982593	-0.5607342
RFC3	chr11	10146531	10165172	912809.9913	chr11	9912809	9913316	-0.5605942
SAT2	chr12	52911725	52913363	627516.5262	chr12	52627516	52629243	-0.5601329
TUBA1B	chr5	15149099	15153620	493157.1549	chr5	15493157	15497157	-0.5600486
ENKD1	chr6	28283117	28287306	463208.2846	chr6	28463208	28467208	-0.5599447
FTL	chr6	54231172	54232750	231050.5423	chr6	54231050	54235050	-0.5599257
ATP5F1A	chr1	95733090	95750841	727119.9572	chr1	95727119	95728266	-0.5590927
MRPL37	chr6	158090970	158143500	535465.1585	chr6	158535465	158539465	-0.5588973
SNAPC2	chr2	71291257	71294006	952896.7095	chr2	70952896	70956896	-0.5586925
NAA60	chr3	38763447	38781988	644938.3864	chr3	38644938	38648938	-0.5584047
SCAMP2	chr7	58672664	58699142	826727.5883	chr7	58826727	58830727	-0.5583892
ENSSSCG00000037510	chr17	37923475	37944393	955507.3795	chr17	37955507	37959507	-0.5583113
FSTL3	chr2	77656663	77662613	943841.7794	chr2	77943841	77947841	-0.5567232
TAGLN	chr9	44560785	44567385	783701.4478	chr9	44783701	44787701	-0.5566371
ENSSSCG00000051162	chr8	44145897	44147246	447290.4445	chr8	44447290	44451290	-0.5561126
MRPS33	chr18	8883459	8897952	842902.8845	chr18	8842902	8845088	-0.555909
TUBB	chr7	23247850	23252233	062338.2306	chr7	23062338	23066338	-0.5557856
C19orf67	chr2	65100755	65104628	094877.6509	chr2	65094877	65098877	-0.5553905
RNF121	chr9	6488806	6587324	064374.6065	chr9	6064374	6065627	-0.5549373
YBX2	chr12	52635385	52641651	399810.5240	chr12	52399810	52403810	-0.5549265
LONP1	chr2	73266258	73286773	060896.7306	chr2	73060896	73064896	-0.5548652
ARHGAP9	chr5	22747339	22762049	880669.2288	chr5	22880669	22884669	-0.5548549

ENSSSCG00000014540	chr2	9535148	9537974	734823.97360	chr2	9734823	9736008	-0.5543057
LGALS8	chr14	54867021	54892049	891100.5489	chr14	54891100	54892064	-0.5542413
MON1A	chr13	32426736	32437862	990619.3199	chr13	31990619	31991906	-0.5542379
NAT10	chr2	26786177	26826992	146241.27147	chr2	27146241	27147074	-0.5541599
MAF1	chr4	597472	600447	157303.16130	chr4	157303	161303	-0.5537305
ENSSSCG00000003286	chr6	59113039	59134353	850751.58854	chr6	58850751	58854751	-0.5536627
CCNDBP1	chr1	128253035	128269452	641100.12864	chr1	128641100	128642036	-0.5535038
CDH6	chr16	17531952	17660079	7938135.1794	chr16	17938135	17942135	-0.5533442
GABARAP	chr12	52596543	52598194	627516.5262	chr12	52627516	52629243	-0.5532962
ATG101	chr5	17418121	17425624	312276.17316	chr5	17312276	17316276	-0.5532318
GMPPB	chr13	32248268	32253155	251515.3225	chr13	32251515	32255515	-0.5529424
POLA2	chr2	6922387	6950179	057462.70614	chr2	7057462	7061462	-0.5528455
NLRP5	chr6	60379623	60409465	845868.60846	chr6	60845868	60846733	-0.5528294
AUP1	chr3	68519969	68523073	519015.68520	chr3	68519015	68520573	-0.5525421
ENSSSCG00000029830	chr4	136107	138652	205764.20709	chr4	205764	207099	-0.5522319
PRR14	chr3	17745892	17751099	902442.17906	chr3	17902442	17906442	-0.552103
ZFPL1	chr2	7098766	7103536	079608.70836	chr2	7079608	7083608	-0.5519783
NRAP	chr14	123926927	124018440	017164.1240	chr14	124017164	124021164	-0.5514752
CDIPT	chr3	18084549	18091798	902442.17906	chr3	17902442	17906442	-0.5514335
EIF3G	chr2	68966222	68970614	109309.69113	chr2	69109309	69113309	-0.5513657
RNF157	chr12	5225849	5304458	116522.5120	chr12	5116522	5120486	-0.550983
MRPL40	chr14	51102613	51105767	0695047.5069	chr14	50695047	50699047	-0.5507743
ARHGEF18	chr2	71651100	71747409	519081.71523	chr2	71519081	71523081	-0.5503763
FAM104A	chr12	7727796	7749240	637681.7639	chr12	7637681	7639269	-0.5502372
DGAT1	chr4	452662	466684	157303.16130	chr4	157303	161303	-0.550135
FAU	chr2	7070906	7072809	057462.70614	chr2	7057462	7061462	-0.5500568
DDX49	chr2	58922988	58930716	826251.58830	chr2	58826251	58830251	-0.5499689
RAB5C	chr12	20613996	20636073	0256958.2025	chr12	20256958	20257834	-0.5499425
AHSG	chr13	124426308	124438840	582327.1245	chr13	124582327	124586327	-0.5498232
ENSSSCG00000032082	chr6	95322453	95366673	978926.94980	chr6	94978926	94980105	-0.5496076
TIMM29	chr2	69649712	69652362	058193.70062	chr2	70058193	70062193	-0.5495765
RAB43	chr13	71562350	71596249	760305.7176	chr13	71760305	71762524	-0.5494234
IK	chr2	142361155	142376333	350030.14235	chr2	142350030	142354030	-0.5492364
BPNT1	chr10	9659487	9683912	0156482.1015	chr10	10156482	10157235	-0.549184
UPB1	chr14	49533231	49565075	9792613.4979	chr14	49792613	49793506	-0.5487969
TPT1	chr11	21929221	21932691	927221.2193	chr11	21927221	21931221	-0.5485086
ENSSSCG00000006081	chr4	38701720	38720780	489285.38493	chr4	38489285	38493285	-0.5483764
VPS28	chr4	362912	370588	157303.16130	chr4	157303	161303	-0.5483047
RIMKLA	chr6	168916725	168950046	994003.16899	chr6	168994003	168995215	-0.5479569
CDC26	chr1	253895885	253906632	973973.25397	chr1	253973973	253977973	-0.5479352
STK25	chr15	140144336	140154366	0131918.1401	chr15	140131918	140133932	-0.5478377
KRTCAP2	chr4	94642942	94646436	495309.94499	chr4	94495309	94499039	-0.5476644
ENSSSCG00000029830	chr4	136107	138652	429062.43018	chr4	429062	430184	-0.5476072
ENSSSCG00000010056	chr14	49619838	49655206	9792613.4979	chr14	49792613	49793506	-0.5475469
ENSSSCG00000044155	chr2	142321430	142324016	578003.14257	chr2	142578003	142579756	-0.5473172
ENSSSCG00000005101	chr1	191102213	191102539	533301.19153	chr1	191533301	191537301	-0.5473093
NSMAF	chr4	74236826	74298193	234826.74238	chr4	74234826	74238826	-0.5472742
TMED4	chr18	50699470	50702705	0456411.5046	chr18	50456411	50460411	-0.5469519
ARMC12	chr7	31586797	31603600	549482.31550	chr7	31549482	31550858	-0.5469262
EDC3	chr7	58828727	58897919	826727.58830	chr7	58826727	58830727	-0.5468872
GMPPB	chr13	32248268	32253155	989610.3199	chr13	31989610	31993610	-0.5468018
ENSSSCG00000001064	chr7	12254388	12299531	116919.12117	chr7	12116919	12117991	-0.5466769
OSBPL2	chr17	61643377	61685819	551971.6155	chr17	61551971	61555971	-0.5466645
NRBP1	chr3	111720379	111733304	044398.11204	chr3	112044398	112048398	-0.5465362
STX5	chr2	8937778	8968932	982070.89825	chr2	8982070	8982593	-0.5461965
ENSSSCG00000044567	chr17	31217462	31238052	0941728.3094	chr17	30941728	30942375	-0.5459805
MCRS1	chr5	15517032	15526510	024080.15028	chr5	15024080	15028080	-0.5456753
PMP22	chr12	58679775	58707925	962479.5896	chr12	58962479	58966479	-0.5454875
ENSSSCG00000035904	chr1	272959831	272965634	835449.27283	chr1	272835449	272836800	-0.5453309
ZKSCAN5	chr3	6442391	6466430	373763.63747	chr3	6373763	6374740	-0.5451982
VPS11	chr9	46285739	46297474	362936.46366	chr9	46362936	46366936	-0.5451903
NRBP1	chr3	111720379	111733304	045290.11204	chr3	112045290	112049290	-0.5450511
NHEJ1	chr15	121100628	121190062	025670.1210	chr15	121025670	121027619	-0.5447932

ARMC12	chr7	31586797	31603600	522450.3162	chr7	31622450	31626450	-0.5447823
ZNRD2	chr2	6683057	6684494	537993.6539	chr2	6537993	6539697	-0.5447018
DDIT3	chr5	22785445	22789829	852316.2285	chr5	22852316	22856316	-0.5445063
SWI5	chr1	268720355	268726521	464700.2684	chr1	268464700	268468700	-0.5444983
EIF4EBP1	chr15	48422816	48443399	693778.4869	chr15	48693778	48695388	-0.5443341
ZP3	chr3	9934761	9944941	132373.1013	chr3	10132373	10132908	-0.544217
RETREG2	chr15	121212881	121218812	210881.1212	chr15	121210881	121214881	-0.5441268
FTL	chr6	54231172	54232750	601535.5460	chr6	54601535	54602403	-0.5441124
AURKAIP1	chr6	63656119	63657472	654119.6365	chr6	63654119	63658119	-0.544009
REEP4	chr14	6411513	6415590	833996.6835	chr14	6833996	6835311	-0.544007
RAB7A	chr13	71777424	71852694	758467.7176	chr13	71758467	71762467	-0.5439882
ENSSSCG00000059201	chr4	90261918	90263262	107057.9011	chr4	90107057	90111057	-0.5435551
ENSSSCG00000061760	chr7	31573753	31585356	522450.3162	chr7	31622450	31626450	-0.5435545
CALR	chr2	66098229	66102132	271286.6627	chr2	66271286	66272770	-0.5434511
DRG1	chr14	48126863	48157906	049624.4805	chr14	48049624	48050222	-0.5433151
CIAO2B	chr6	27610470	27612592	546706.2755	chr6	27546706	27550706	-0.5432665
NDUFAF1	chr1	129968502	130009391	110847.1301	chr1	130110847	130114847	-0.5431694
TXNL4A	chr6	127974543	127991177	028715.1280	chr6	128028715	128032715	-0.5427004
ZMAT2	chr2	142411089	142419137	376462.1423	chr2	142376462	142380462	-0.5425257
TOMM20	chr14	56113192	56127677	923940.5592	chr14	55923940	55925554	-0.5423766
MTCH1	chr7	32617426	32635575	786291.3279	chr7	32786291	32790291	-0.5423135
PCDHGA4	chr2	142993554	143156556	639970.1426	chr2	142639970	142642910	-0.5418157
MAP2K3	chr12	61396369	61417321	029157.6103	chr12	61029157	61033157	-0.541763
THBS3	chr4	94611593	94623902	495309.9449	chr4	94495309	94499039	-0.5415974
SARS1	chr4	110857089	110874815	006543.1110	chr4	111006543	111007819	-0.5411731
METTL23	chr12	4804037	4808913	053065.5057	chr12	5053065	5057065	-0.541115
ARHGAP22	chr14	89113768	89331274	986949.8899	chr14	88986949	88990949	-0.5410575
TINF2	chr7	75056798	75063979	027923.7503	chr7	75027923	75031923	-0.5409273
ARV1	chr14	59396962	59408958	408985.5940	chr14	59408985	59409978	-0.5408055
RAB5C	chr12	20613996	20636073	256373.2026	chr12	20256373	20260373	-0.5406804
MON1A	chr13	32426736	32437862	251515.3225	chr13	32251515	32255515	-0.5406138
GREB1L	chr6	106419943	106691625	314117.1063	chr6	106314117	106315302	-0.540485
TCFL5	chr17	62093907	62115650	558809.6255	chr17	62558809	62559642	-0.5404718
COX5B	chr3	56629272	56632119	810364.5681	chr3	56810364	56812121	-0.5402128
RABAC1	chr6	49933434	49936372	931434.4993	chr6	49931434	49935434	-0.5400383
NUCB1	chr6	54180038	54209930	231050.5423	chr6	54231050	54235050	-0.5398066
TMEM219	chr3	18167923	18176563	175737.1817	chr3	18175737	18179737	-0.5396107
ALG8	chr9	12497721	12532307	850636.1285	chr9	12850636	12851763	-0.5395501
MRAP2	chr1	53256006	53318107	997619.5299	chr1	52997619	52998959	-0.53916
TTLL4	chr15	120773185	120793194	025670.1210	chr15	121025670	121027619	-0.5391548
RRP9	chr13	34067520	34075430	663957.3366	chr13	33663957	33666213	-0.5391285
AURKAIP1	chr6	63656119	63657472	657054.6366	chr6	63657054	63661054	-0.5390541
CALR	chr2	66098229	66102132	055483.6605	chr2	66055483	66056550	-0.5387027
YBX2	chr12	52635385	52641651	109256.5311	chr12	53109256	53113256	-0.5386852
GINS1	chr17	31060650	31080314	941728.3094	chr17	30941728	30942375	-0.5385876
STK10	chr16	52003887	52140367	003268.5200	chr16	52003268	52004540	-0.5385431
TFR2	chr3	8539320	8578763	904591.8908	chr3	8904591	8908591	-0.5382372
ENSSSCG00000054785	chrX	9898365	9905146	217608.1021	chrX	10217608	10219668	-0.5380615
PIH1D1	chr6	54524172	54535249	705237.5470	chr6	54705237	54709237	-0.537957
ENSSSCG00000054115	chr5	69746689	69772024	395938.6940	chr5	69395938	69400178	-0.537842
LONP1	chr2	73266258	73286773	294501.7329	chr2	73294501	73295458	-0.537621
PSMC3	chr2	15166306	15193047	379707.1538	chr2	15379707	15383707	-0.5376192
ZFTRAF1	chr4	335301	346534	157303.1613	chr4	157303	161303	-0.5375792
MRPL38	chr12	5514415	5521664	521113.5525	chr12	5521113	5525113	-0.5367771
RAB8A	chr2	61429523	61450413	271040.6127	chr2	61271040	61273371	-0.5367029
CDK4	chr5	23038891	23042061	056982.2306	chr5	23056982	23060982	-0.5366568
MED11	chr12	52150448	52152204	627516.5262	chr12	52627516	52629243	-0.5364409
MAML3	chr8	86879974	87323117	546273.8654	chr8	86546273	86546957	-0.5364265
ENSSSCG00000038518	chr3	6790314	6819644	373763.6374	chr3	6373763	6374740	-0.536261
DAPK3	chr2	74759004	74775330	162787.7516	chr2	75162787	75166787	-0.5360875
VPS52	chr7	29624230	29639140	641182.2964	chr7	29641182	29645182	-0.5360502
CUL7	chr7	38097854	38115822	388035.3838	chr7	38388035	38389283	-0.535896
PPAN	chr2	68958906	68962657	109309.6911	chr2	69109309	69113309	-0.53587

BIRC5	chr12	3747279	3755215	354245.3358	chr12	3354245	3358245	-0.535291
PFDN5	chr5	18512697	18517174	485052.18489	chr5	18485052	18489052	-0.5352802
ENSSSCG00000031756	chr14	40455954	40458063	003385.4000	chr14	40003385	40004961	-0.5351359
TCP11	chr7	31007474	31114734	160689.31161	chr7	31160689	31161712	-0.53507
MST1R	chr13	32409555	32421920	329740.3233	chr13	32329740	32330722	-0.5349315
TIMM44	chr2	71271805	71287817	952896.70956	chr2	70952896	70956896	-0.534803
ZP3	chr3	9934761	9944941	130093.10134	chr3	10130093	10134093	-0.5346545
DND1	chr2	142384532	142387199	639970.14264	chr2	142639970	142642910	-0.5345429
TUFM	chr3	18521128	18524959	175737.18179	chr3	18175737	18179737	-0.5344882
TBCC	chr7	37822767	37824953	051598.38052	chr7	38051598	38052427	-0.5337695
C9orf78	chr1	269986391	269995395	995271.26999	chr1	269995271	269995975	-0.5332196
NEDD8	chr7	75070161	75085899	972308.74976	chr7	74972308	74976308	-0.533177
CTDNEP1	chr12	52599226	52606059	627516.5262	chr12	52627516	52629243	-0.5330405
TMED4	chr18	50699470	50702705	698457.5070	chr18	50698457	50700032	-0.5329305
ENSSSCG00000061760	chr7	31573753	31585356	160689.31161	chr7	31160689	31161712	-0.5328909
ACTR1A	chr14	113480196	113498740	228965.1132	chr14	113228965	113232965	-0.5327616
ODF2	chr1	268856766	268893387	332838.26933	chr1	269332838	269336838	-0.5325888
MRPL54	chr2	74925070	74928167	162787.75166	chr2	75162787	75166787	-0.5325683
SMYD4	chr12	47944148	48009586	859043.4786	chr12	47859043	47863043	-0.5324743
HEXIM2	chr12	18251428	18258295	257274.1825	chr12	18257274	18258147	-0.5321694
LSM4	chr2	59499564	59513246	529154.59533	chr2	59529154	59533154	-0.5320492
DNPEP	chr15	121391746	121412864	.024245.1210	chr15	121024245	121028245	-0.5320003
DPCD	chr14	112617398	112637913	214732.1122	chr14	112214732	112218732	-0.5317909
HIGD2A	chr2	81486890	81488045	287760.81288	chr2	81287760	81288900	-0.5317734
MOB3A	chr2	76475530	76496060	954111.76958	chr2	76954111	76958111	-0.5316908
HSPA8	chr9	49982284	49990508	628793.49632	chr9	49628793	49632793	-0.5316243
ITGA11	chr1	166186613	166310800	463953.16646	chr1	166463953	166467953	-0.5315468
MEPCE	chr3	8357289	8364661	918675.79226	chr3	7918675	7922675	-0.5314875
HIP1R	chr14	29993655	30024216	630876.2963	chr14	29630876	29632175	-0.53141
COG8	chr6	17645590	17650481	643590.17647	chr6	17643590	17647590	-0.5312004
RPL10A	chr7	31327440	31333638	360867.31362	chr7	31360867	31362660	-0.5311163
GMPPA	chr15	121507564	121515300	.024245.1210	chr15	121024245	121028245	-0.5310398
DUSP5	chr14	120906343	120919384	.062346.1210	chr14	121062346	121066346	-0.530976
COX6A1	chr14	40408631	40412870	003385.4000	chr14	40003385	40004961	-0.5309282
ENSSSCG00000025928	chr6	53974309	53978544	229172.54233	chr6	54229172	54233172	-0.5308004
TBCE	chr14	55816668	55901171	923940.5592	chr14	55923940	55925554	-0.5306604
SWI5	chr1	268720355	268726521	718355.26872	chr1	268718355	268722355	-0.5305004
NTPCR	chr14	57802565	57833151	237469.5823	chr14	58237469	58238478	-0.5304377
MDP1	chr7	75087468	75089227	027923.75031	chr7	75027923	75031923	-0.5304143
ENSSSCG00000013064	chr2	9163317	9174468	982070.89825	chr2	8982070	8982593	-0.5303604
TMEM97	chr12	44604021	44609999	634379.4463	chr12	44634379	44638379	-0.5303489
ADIPOR2	chr5	68755811	68807235	641988.68650	chr5	68641988	68650108	-0.5300266
B3GNT3	chr2	59934958	59952859	394810.60396	chr2	60394810	60396270	-0.5299897
CDKN2AIPNL	chr2	136677034	136685955	383860.13638	chr2	136383860	136386180	-0.5297242
CSNK1D	chr12	768865	796595	.120907.1129	chr12	1120907	1129651	-0.5295604
ATP5PF	chr13	189278945	189290229	163975.1891	chr13	189163975	189167975	-0.5294723
OS9	chr5	22957875	23004458	895808.22897	chr5	22895808	22897067	-0.5293381
DYNC2I2	chr1	269002261	269030719	615087.26861	chr1	268615087	268619087	-0.529171
ENSSSCG00000002790	chr6	27853452	27867226	546706.27550	chr6	27546706	27550706	-0.5291204
NARF	chr12	632363	651817	.076166.1080	chr12	1076166	1080166	-0.5290763
C9orf78	chr1	269986391	269995395	179344.27018	chr1	270179344	270180174	-0.5289522
GALK1	chr12	5642025	5651183	521113.5525	chr12	5521113	5525113	-0.5285715
TUBA4A	chr15	121288957	121294853	.210881.1212	chr15	121210881	121214881	-0.5285595
COL23A1	chr2	79766150	80141293	668479.79672	chr2	79668479	79672479	-0.528528
DRAP1	chr2	6409655	6412395	755635.67596	chr2	6755635	6759635	-0.5284759
CYC1	chr4	606516	608996	157303.16130	chr4	157303	161303	-0.5280437
PRADC1	chr3	69547649	69555085	234351.69238	chr3	69234351	69238351	-0.5280404
MRPL11	chr2	5978483	5981449	409833.64138	chr2	6409833	6413833	-0.5279854
PALM	chr2	77619199	77644673	943841.77947	chr2	77943841	77947841	-0.5279766
LYPLA2	chr6	81601486	81605912	564801.81568	chr6	81564801	81568801	-0.5278629
ENSSSCG00000052071	chr7	64532731	64535636	507971.64508	chr7	64507971	64508850	-0.5277665
RAB43	chr13	71562350	71596249	.982067.7198	chr13	71982067	71986067	-0.5277008
PSMD13	chr2	55108	74829	396766.40076	chr2	396766	400766	-0.5275404

TTLL13	chr7	55811855	55827077	573336.55577	chr7	55573336	55577336	-0.5275073
PDCD5	chr6	42483449	42491591	558113.42562	chr6	42558113	42562113	-0.5274417
ENSSSCG00000036812	chr12	61077631	61186002	247989.6125	chr12	61247989	61253489	-0.5274227
LOXL2	chr14	7435867	7540704	388061.7392	chr14	7388061	7392061	-0.5271798
SLC35A4	chr2	142323341	142325335	639970.14264	chr2	142639970	142642910	-0.5268386
DRG1	chr14	48126863	48157906	638106.4763	chr14	47638106	47638634	-0.5267212
RNASEH2A	chr2	66193197	66207697	055483.66056	chr2	66055483	66056550	-0.5265992
GABBR2	chr1	240107466	240496101	918638.23991	chr1	239918638	239919296	-0.526588
RRP36	chr7	38087258	38094548	211487.38215	chr7	38211487	38215487	-0.5263858
CHCHD4	chr13	70417326	70428922	536894.7054	chr13	70536894	70540894	-0.5263126
POLR1D	chr11	5044329	5089766	879361.4880	chr11	4879361	4880102	-0.5261941
SLC46A3	chr11	5982443	6000701	000360.6001	chr11	6000360	6001074	-0.5259805
DND1	chr2	142384532	142387199	641101.14264	chr2	142641101	142642772	-0.5258789
SRRT	chr3	8717785	8731201	356207.83580	chr3	8356207	8358016	-0.5258444
PARK7	chr6	68629214	68645516	487103.68487	chr6	68487103	68487955	-0.5258248
RBM15B	chr13	33664472	33667153	663957.3366	chr13	33663957	33666213	-0.5254259
POLD1	chr6	55247880	55272085	198826.55201	chr6	55198826	55201756	-0.5253934
MRM3	chr12	47080367	47087405	123236.4712	chr12	47123236	47127236	-0.5253763
OOEP	chr1	53006761	53007877	997619.52998	chr1	52997619	52998959	-0.5251799
SLC25A11	chr12	51970806	51975548	102504.5210	chr12	52102504	52106504	-0.5251168
SSR2	chr4	94011527	94021962	495309.94499	chr4	94495309	94499039	-0.5251098
CPSF3	chr3	126868467	126905746	510477.12651	chr3	126510477	126511819	-0.5251006
DRAP1	chr2	6409655	6412395	471205.64727	chr2	6471205	6472742	-0.5251004
PIGH	chr7	91306121	91316278	955179.90956	chr7	90955179	90956189	-0.5250002
FZR1	chr2	75121835	75146835	162787.75166	chr2	75162787	75166787	-0.5249679
ENSSSCG00000014284	chr2	135074111	135078541	700543.13470	chr2	134700543	134701416	-0.5248231
ENSSSCG00000017971	chr12	53111256	53112484	109256.5311	chr12	53109256	53113256	-0.5247182
ENSSSCG00000052760	chr1	208706991	208710843	179467.20918	chr1	209179467	209183467	-0.524577
ATRAID	chr3	111914708	111919946	647539.11165	chr3	111647539	111651539	-0.5245292
MRPL51	chr5	64167200	64168983	114150.64118	chr5	64114150	64118150	-0.524459
BRF2	chr15	48568202	48573029	693951.4869	chr15	48693951	48697396	-0.5244019
PRPF6	chr17	62815085	62858382	609909.6261	chr17	62609909	62613909	-0.5242235
ENSSSCG00000053570	chr6	62255210	62260695	690463.62691	chr6	62690463	62691254	-0.5237526
MMD2	chr3	3729531	3775205	798186.38021	chr3	3798186	3802186	-0.5237251
PRR14	chr3	17745892	17751099	118581.18122	chr3	18118581	18122581	-0.5233472
ARMC2	chr1	74923868	75043060	169857.75170	chr1	75169857	75170800	-0.523326
ENSSSCG00000013064	chr2	9163317	9174468	981759.89857	chr2	8981759	8985759	-0.5231207
MRPL51	chr5	64167200	64168983	841745.63845	chr5	63841745	63845745	-0.5230699
FXR2	chr12	52882870	52903450	627516.5262	chr12	52627516	52629243	-0.5228692
NDUFA6	chr5	6562011	6567647	081444.60854	chr5	6081444	6085444	-0.5227748
CLN8	chr15	33288301	33304631	478739.3347	chr15	33478739	33479825	-0.5225681
TMC4	chr6	55955991	55970280	189076.56190	chr6	56189076	56190746	-0.5225057
TBC1D10B	chr3	17944629	17956069	118581.18122	chr3	18118581	18122581	-0.5224226
TRIM14	chr1	239912126	239945073	492188.23949	chr1	239492188	239493251	-0.5221891
IK	chr2	142361155	142376333	883680.14188	chr2	141883680	141886890	-0.5221795
GGH	chr4	70996755	71021838	959465.70963	chr4	70959465	70963465	-0.5221587
AIDA	chr10	11428399	11466643	334439.1133	chr10	11334439	11336379	-0.5221542
ARID3B	chr7	58932461	58998711	908967.58910	chr7	58908967	58910668	-0.5219025
POP7	chr3	8605018	8610218	916872.89208	chr3	8916872	8920872	-0.5218946
MDP1	chr7	75087468	75089227	972308.74976	chr7	74972308	74976308	-0.5218121
DNPEP	chr15	121391746	121412864	282920.1212	chr15	121282920	121286920	-0.5215438
AAAS	chr5	18526076	18537830	409267.18410	chr5	18409267	18410275	-0.5213368
TRAPPC4	chr9	46242171	46246343	362936.46366	chr9	46362936	46366936	-0.5211443
TTLL13	chr7	55811855	55827077	575340.55576	chr7	55575340	55576442	-0.5211433
PDCD5	chr6	42483449	42491591	587556.42591	chr6	42587556	42591556	-0.5211366
MARS1	chr5	22752374	22785501	881828.22885	chr5	22881828	22885548	-0.5209745
PCDHGA4	chr2	142993554	143156556	641101.14264	chr2	142641101	142642772	-0.5206271
SPR	chr3	69889452	69894680	655696.69659	chr3	69655696	69659696	-0.5205829
COQ4	chr1	268756685	268768343	465007.26846	chr1	268465007	268466088	-0.5204881
RAB43	chr13	71562350	71596249	758467.7176	chr13	71758467	71762467	-0.5204536
ENSSSCG00000003253	chr6	56236311	56244185	225666.56227	chr6	56225666	56227716	-0.5198653
TUBB2A	chr7	1910269	1914761	130734.21330	chr7	2130734	2133042	-0.5198322
NFATC2IP	chr3	18609912	18622640	522820.18526	chr3	18522820	18526820	-0.5196812

CAPN8	chr10	19665855	19732139	286367.1929	chr10	19286367	19290367	-0.5194498
RIMKLA	chr6	168916725	168950046	875249.16887	chr6	168875249	168879249	-0.5193333
CAPNS1	chr6	45511295	45518610	304692.45306	chr6	45304692	45306421	-0.5191693
ENSSSCG00000037143	chr14	50264868	50315717	792613.4979	chr14	49792613	49793506	-0.5187888
ENSSSCG00000029830	chr4	136107	138652	157303.16130	chr4	157303	161303	-0.5186895
NDUFS3	chr2	15031149	15036880	379707.15383	chr2	15379707	15383707	-0.5184486
ALAD	chr1	254012850	254027508	569435.25357	chr1	253569435	253570326	-0.5181611
HIRIP3	chr3	18198807	18202089	522820.18526	chr3	18522820	18526820	-0.5181405
MCRS1	chr5	15517032	15526510	027205.15029	chr5	15027205	15029851	-0.5181042
ARMC2	chr1	74923868	75043060	169061.75173	chr1	75169061	75173061	-0.5176447
KAT5	chr2	6560513	6572466	755635.67596	chr2	6755635	6759635	-0.5176156
MTUS2	chr11	6197740	6532924	000360.6001	chr11	6000360	6001074	-0.5174102
ENSSSCG00000032060	chr6	7255169	7279427	418790.74227	chr6	7418790	7422790	-0.5173239
NXN	chr12	46904407	47075432	123236.4712	chr12	47123236	47127236	-0.5172687
ENSSSCG00000037514	chr6	95566555	95599254	321439.95322	chr6	95321439	95322907	-0.5171928
DPPA5	chr1	52986462	52987590	090036.53094	chr1	53090036	53094036	-0.5170537
ADAMTS7	chr7	47929033	47994453	447036.47451	chr7	47447036	47451036	-0.5168752
ARHGAP9	chr5	22747339	22762049	881828.22885	chr5	22881828	22885548	-0.5168721
STK16	chr15	121284920	121288473	282920.1212	chr15	121282920	121286920	-0.5168434
ENSSSCG00000058051	chr1	52996822	52999185	997619.52998	chr1	52997619	52998959	-0.5166043
SEC61G	chr9	139128296	139135727	988480.13899	chr9	138988480	138992480	-0.5162653
POLR1D	chr11	5044329	5089766	042329.5046	chr11	5042329	5046329	-0.516258
NHEJ1	chr15	121100628	121190062	024245.1210	chr15	121024245	121028245	-0.5161625
TPI1	chr5	63838506	63843134	841745.63845	chr5	63841745	63845745	-0.5160653
FTL	chr6	54231172	54232750	142020.54146	chr6	54142020	54146020	-0.5160283
MRPL20	chr6	63670755	63675839	657054.63661	chr6	63657054	63661054	-0.5158789
POLR1D	chr11	5044329	5089766	042384.5044	chr11	5042384	5044586	-0.5158702
AUP1	chr3	68519969	68523073	570415.68574	chr3	68570415	68574415	-0.5155819
YIF1A	chr2	6095877	6102037	304558.63059	chr2	6304558	6305906	-0.5155625
ENSSSCG00000040854	chr3	68883051	68898008	234351.69238	chr3	69234351	69238351	-0.5155294
MAN2B1	chr2	66329045	66348056	271286.66272	chr2	66271286	66272770	-0.5155154
OS9	chr5	22957875	23004458	056982.23060	chr5	23056982	23060982	-0.5152797
ATP5MC2	chr5	18871026	18879609	165157.19169	chr5	19165157	19169157	-0.5151403
NLRP8	chr6	60350622	60370529	845868.60846	chr6	60845868	60846733	-0.5149519
CLTA	chr1	236971505	237001731	917879.23692	chr1	236917879	236921879	-0.5148332
OTUB1	chr2	8087829	8095609	828920.78329	chr2	7828920	7832920	-0.5148231
PPIL2	chr14	50208374	50232830	695047.5069	chr14	50695047	50699047	-0.5148056
ENSSSCG00000061760	chr7	31573753	31585356	925961.31927	chr7	31925961	31927012	-0.5146009
PYGB	chr17	30940452	30995691	941728.3094	chr17	30941728	30942375	-0.5143061
NDUFS5	chr6	94913076	94919452	321439.95322	chr6	95321439	95322907	-0.5142001
KXD1	chr2	59242310	59247713	828025.58828	chr2	58828025	58828910	-0.5140146
SEC61G	chr9	139128296	139135727	990014.13899	chr9	138990014	138990729	-0.5138481
PIP4P1	chr7	78447369	78451846	434300.78434	chr7	78434300	78434853	-0.5138167
GPX4	chr2	77320464	77323931	776470.77777	chr2	77776470	77777125	-0.5136688
ZDHHC4	chr3	4628385	4643691	992886.49950	chr3	4992886	4995036	-0.5134837
ENSSSCG00000005528	chr1	262236482	262264097	898896.26190	chr1	261898896	261900556	-0.5133871
APMAP	chr17	30768488	30795755	194616.3119	chr17	31194616	31195316	-0.5132692
ESYT1	chr5	21514510	21535929	493944.21497	chr5	21493944	21497944	-0.5131017
ENSSSCG00000035904	chr1	272959831	272965634	953473.27295	chr1	272953473	272957473	-0.5128877
ZNF584	chr6	62992506	63005691	965994.62969	chr6	62965994	62969994	-0.5128364
UMPS	chr13	135610058	135650603	465798.1354	chr13	135465798	135469798	-0.5128279
DPPA5	chr1	52986462	52987590	997619.52998	chr1	52997619	52998959	-0.5128055
CDC25B	chr17	31910825	31929242	591390.3159	chr17	31591390	31592201	-0.5127743
ENSSSCG00000024588	chr2	75538090	75542189	162787.75166	chr2	75162787	75166787	-0.5126896
CFL1	chr2	6469254	6475035	428153.64321	chr2	6428153	6432153	-0.5126814
BNIP1	chr16	51126926	51140394	075321.5107	chr16	51075321	51077044	-0.5126089
PROP1	chr2	79627603	79631270	668479.79672	chr2	79668479	79672479	-0.5122525
MVP	chr3	18057177	18081155	902442.17906	chr3	17902442	17906442	-0.5122086
TPI1	chr5	63838506	63843134	114150.64118	chr5	64114150	64118150	-0.512189
RPL10A	chr7	31327440	31333638	622450.31626	chr7	31622450	31626450	-0.5121603
UQCRB	chr4	40403109	40409057	472366.40476	chr4	40472366	40476373	-0.5121089
CDIPT	chr3	18084549	18091798	701901.17702	chr3	17701901	17702971	-0.5120239
BLVRA	chr18	51131584	51185435	493135.5149	chr18	51493135	51495445	-0.5116482

MFSD11	chr12	4774678	4801456	807118.4811	chr12	4807118	4811118	-0.5114535
TCP11	chr7	31007474	31114734	566632.3066	chr7	30666632	30667390	-0.5113506
POLE3	chr1	254033385	254035978	569456.2535	chr1	253569456	253571216	-0.511234
FAM193A	chr8	1388266	1523928	386266.1390	chr8	1386266	1390266	-0.5110404
PSMD7	chr6	17047430	17057176	231858.1723	chr6	17231858	17235858	-0.5109959
ATP5MK	chr14	114321622	114327485	435045.1144	chr14	114435045	114439045	-0.5109323
POLR1D	chr11	5044329	5089766	332263.5334	chr11	5332263	5334043	-0.5109169
SLC25A11	chr12	51970806	51975548	440268.5244	chr12	52440268	52442734	-0.5109115
ATP5MC2	chr5	18871026	18879609	409267.1841	chr5	18409267	18410275	-0.5109039
UMPS	chr13	135610058	135650603	467337.1354	chr13	135467337	135468789	-0.5107639
GEMIN2	chr1	169676156	169701481	723649.1697	chr1	169723649	169727649	-0.5107283
ARFGAP2	chr2	15390415	15401227	379707.1538	chr2	15379707	15383707	-0.5107191
HOXD9	chr15	81924142	81926274	881154.8188	chr15	81881154	81884936	-0.5107011
OS9	chr5	22957875	23004458	881828.2288	chr5	22881828	22885548	-0.5105651
ENSSSCG00000007947	chr3	37989180	37998365	553394.3755	chr3	37553394	37557394	-0.5104924
DPH1	chr12	48146417	48161770	859199.4786	chr12	47859199	47861179	-0.5104508
MARS1	chr5	22752374	22785501	044561.2304	chr5	23044561	23048561	-0.5104342
PCDHGA4	chr2	142993554	143156556	300062.1433	chr2	143300062	143304062	-0.5103728
HEXIM2	chr12	18251428	18258295	766111.1777	chr12	17766111	17770111	-0.5102865
TUFM	chr3	18521128	18524959	903945.1890	chr3	18903945	18905665	-0.5102605
TRMT1	chr2	65940125	65949324	950519.6595	chr2	65950519	65954519	-0.5102092
CALR	chr2	66098229	66102132	153750.6615	chr2	66153750	66154740	-0.51018
ENSSSCG00000032573	chr4	75636278	75646153	645455.7564	chr4	75645455	75646908	-0.510076
RND1	chr5	14914645	14922038	024080.1502	chr5	15024080	15028080	-0.5100229
RAB8A	chr2	61429523	61450413	138907.6113	chr2	61138907	61139628	-0.5096221
TTLL4	chr15	120773185	120793194	253797.1212	chr15	121253797	121254795	-0.5094652
PCID2	chr11	78541318	78556733	753533.7875	chr11	78753533	78757533	-0.5093164
TMEM192	chr8	43960709	43990093	447290.4445	chr8	44447290	44451290	-0.5092215
ILK	chr9	3145608	3159858	073995.3077	chr9	3073995	3077995	-0.5092189
VPS29	chr14	31859067	31869579	311154.3231	chr14	32311154	32312445	-0.5088397
TMEM231	chr6	12140687	12164140	369369.1237	chr6	12369369	12373369	-0.508758
RNF141	chr2	48985511	49028486	082751.4908	chr2	49082751	49083432	-0.5087488
RPS19	chr6	50000948	50010341	931434.4993	chr6	49931434	49935434	-0.5086643
FSD2	chr7	52144506	52194133	730443.5173	chr7	51730443	51731835	-0.5084374
ZFTRAF1	chr4	335301	346534	429062.4301	chr4	429062	430184	-0.5084023
SMYD2	chr9	129263526	129316349	054886.1290	chr9	129054886	129056417	-0.5083802
PSKH1	chr6	28508823	28544062	463208.2846	chr6	28463208	28467208	-0.508099
DNPEP	chr15	121391746	121412864	025670.1210	chr15	121025670	121027619	-0.5080382
DYNC2I2	chr1	269002261	269030719	207693.2692	chr1	269207693	269211693	-0.5077809
HSPA5	chr1	265930045	265934894	931340.2659	chr1	265931340	265935340	-0.5076678
ENSSSCG00000005101	chr1	191102213	191102539	533757.1915	chr1	191533757	191536473	-0.507657
PRDX2	chr2	66207828	66212009	055483.6605	chr2	66055483	66056550	-0.5076416
ENSSSCG00000042487	chr7	58998922	59008157	908967.5891	chr7	58908967	58910668	-0.5076
ENSSSCG00000037143	chr14	50264868	50315717	695047.5069	chr14	50695047	50699047	-0.5074999
ACTR1B	chr3	56610304	56620164	810364.5681	chr3	56810364	56812121	-0.5074984
RALGDS	chr1	272776846	272824184	953473.2729	chr1	272953473	272957473	-0.5074342
STYXL1	chr3	10132093	10205179	132373.1013	chr3	10132373	10132908	-0.5073261
DNPEP	chr15	121391746	121412864	210881.1212	chr15	121210881	121214881	-0.5071022
MAD2L1BP	chr7	38613440	38623832	388035.3838	chr7	38388035	38389283	-0.5070741
PDE6D	chr15	132375743	132426683	354132.1323	chr15	132354132	132358935	-0.5068769
MRPL50	chr1	243100372	243107084	682071.2426	chr1	242682071	242683303	-0.5068698
SAT2	chr12	52911725	52913363	109256.5311	chr12	53109256	53113256	-0.5068159
HAUS1	chr1	95756267	95780758	727119.9572	chr1	95727119	95728266	-0.5064424
SCYL1	chr2	6716115	6733438	755635.6759	chr2	6755635	6759635	-0.5061493
KIF22	chr3	18023096	18041730	742406.1774	chr3	17742406	17744406	-0.5060779
TMEM223	chr2	8981963	8983263	098702.9102	chr2	9098702	9102702	-0.5058906
TRMT1	chr2	65940125	65949324	153750.6615	chr2	66153750	66154740	-0.5058697
LDLRAP1	chr6	83030702	83054377	361071.8336	chr6	83361071	83362136	-0.505591
ENSSSCG00000037143	chr14	50264868	50315717	838305.4984	chr14	49838305	49842305	-0.5055616
YPEL1	chr14	50190798	50208256	020243.5002	chr14	50020243	50020763	-0.5055393
YJU2B	chr2	65353578	65372085	094877.6509	chr2	65094877	65098877	-0.5055255
TOMM40L	chr4	89228554	89235347	258461.8926	chr4	89258461	89262461	-0.5053252
SUSD4	chr10	19421565	19551840	286367.1929	chr10	19286367	19290367	-0.5052528

ADA	chr17	47044497	47072245	385600.4738	chr17	47385600	47389600	-0.5048283
STX18	chr8	5890765	6004399	022066.6024	chr8	6022066	6024012	-0.5047072
TRAP1	chr3	38536668	38603015	644938.3864	chr3	38644938	38648938	-0.504638
FSD2	chr7	52144506	52194133	729077.5173	chr7	51729077	51733077	-0.5044355
PHB2	chr5	63751558	63756478	841745.6384	chr5	63841745	63845745	-0.504348
LHX6	chr1	262295353	262322530	203524.2622	chr1	262203524	262204368	-0.5043083
MMD2	chr3	3729531	3775205	132177.4136	chr3	4132177	4136177	-0.5042865
TTLL4	chr15	120773185	120793194	983257.1209	chr15	120983257	120987257	-0.5040979
OTUB1	chr2	8087829	8095609	280127.8284	chr2	8280127	8284127	-0.503853
RIOK1	chr7	4750752	4782448	057871.5060	chr7	5057871	5060641	-0.5038513
AKR1A1	chr6	165811247	165824896	669174.1656	chr6	165669174	165673174	-0.5037728
NOC2L	chr6	63307695	63335201	657054.6366	chr6	63657054	63661054	-0.5037713
CCDC180	chr1	239209387	239270803	490721.2394	chr1	239490721	239494721	-0.5036278
AP1S1	chr3	8881107	8887566	886506.8890	chr3	8886506	8890506	-0.5035874
TMBIM6	chr5	15674057	15692936	964706.1596	chr5	15964706	15965446	-0.5035858
DOC2B	chr12	47529367	47562373	737633.4774	chr12	47737633	47741633	-0.5034787
GADD45GIP1	chr2	66087453	66090710	950519.6595	chr2	65950519	65954519	-0.5033008
COX6A1	chr14	40408631	40412870	561159.4056	chr14	40561159	40565159	-0.5032749
DPCD	chr14	112617398	112637913	217181.1122	chr14	112217181	112217863	-0.5032387
DYNLL1	chr14	40455985	40459104	003385.4000	chr14	40003385	40004961	-0.5031805
ADGRA2	chr15	48573197	48610668	693778.4869	chr15	48693778	48695388	-0.503092
FUS	chr3	17314332	17326637	742406.1774	chr3	17742406	17744406	-0.5029966
MAP4K2	chr2	7365246	7378996	079608.7083	chr2	7079608	7083608	-0.5029829
ISCU	chr14	42216111	42222711	221964.4222	chr14	42221964	42225964	-0.5028285
CYB5R1	chr10	24925410	24931917	960329.2496	chr10	24960329	24962069	-0.5027073
CAPN8	chr10	19665855	19732139	888436.1988	chr10	19888436	19889542	-0.502577
ENSSSCG00000060152	chr1	166102570	166106753	463953.1664	chr1	166463953	166467953	-0.5025653
INO80E	chr3	18202134	18212186	505886.1850	chr3	18505886	18507873	-0.5025119
ENSSSCG00000061655	chr3	42976793	42982961	287523.4328	chr3	43287523	43288290	-0.5023955
RETREG2	chr15	121212881	121218812	024245.1210	chr15	121024245	121028245	-0.5023587
KCNH3	chr5	15495157	15517022	024080.1502	chr5	15024080	15028080	-0.5023096
ENSSSCG00000031299	chr2	64723435	64731124	094877.6509	chr2	65094877	65098877	-0.5020929
NSG1	chr8	6000057	6024769	125965.6129	chr8	6125965	6129965	-0.5020096
ENSSSCG00000056719	chr6	61903216	61907832	556435.6156	chr6	61556435	61560435	-0.5019157
ENSSSCG00000014569	chr9	707693	711219	710255.7116	chr9	710255	711662	-0.5018803
PCDH12	chr2	143550849	143565033	300062.1433	chr2	143300062	143304062	-0.5018672
PKIG	chr17	47008035	47044049	385600.4738	chr17	47385600	47389600	-0.5014392
COQ4	chr1	268756685	268768343	718355.2687	chr1	268718355	268722355	-0.5013632
TEPSIN	chr12	1519760	1529542	120907.1129	chr12	1120907	1129651	-0.5012121
USP5	chr5	63843745	63858716	841745.6384	chr5	63841745	63845745	-0.5011129
ATG4B	chr15	140223904	140246321	262766.1402	chr15	140262766	140266766	-0.5009992
TSPAN31	chr5	23029306	23038977	852316.2285	chr5	22852316	22856316	-0.5008934
GPN1	chr3	111537737	111572692	647539.1116	chr3	111647539	111651539	-0.5008267
ZMAT2	chr2	142411089	142419137	577020.1425	chr2	142577020	142579530	-0.5006975
ATP6V0D1	chr6	28090183	28131985	682030.2768	chr6	27682030	27686030	-0.5006007
PPP1R8	chr6	84966791	84990663	305189.8530	chr6	85305189	85309189	-0.5004546
SRRT	chr3	8717785	8731201	916872.8920	chr3	8916872	8920872	-0.5003733
DNPEP	chr15	121391746	121412864	241116.1212	chr15	121241116	121245116	-0.5001085
ZC3H14	chr7	110463517	110513257	461517.1104	chr7	110461517	110465517	0.50003984
XPOT	chr5	28828566	28875325	247852.2924	chr5	29247852	29248866	0.50059333
ENSSSCG00000040187	chr2	66650451	66674686	344600.6634	chr2	66344600	66347110	0.50103846
DGUOK	chr3	69054761	69091341	235860.6923	chr3	69235860	69236713	0.50136356
RPA3	chr9	77910881	77937060	509110.7751	chr9	77509110	77510115	0.50190571
SMC3	chr14	120969631	121021497	062346.1210	chr14	121062346	121066346	0.50198142
CBR4	chr14	20621981	20682240	420267.2042	chr14	20420267	20421437	0.502194
RHOT1	chr12	42711233	42783421	931445.4293	chr12	42931445	42935445	0.50281285
ZNRF2	chr18	42549846	42651807	408792.4241	chr18	42408792	42412792	0.50307483
PPP5C	chr6	52377979	52402045	660294.5266	chr6	52660294	52661512	0.50322247
ISOC1	chr2	131857168	131877631	369532.1313	chr2	131369532	131371871	0.50349924
OSGEPL1	chr15	94331064	94351564	259905.9426	chr15	94259905	94263905	0.50358203
MELK	chr1	237317191	237431589	754070.2377	chr1	237754070	237754508	0.5040461
ATL2	chr3	101957209	102029667	601707.1016	chr3	101601707	101605707	0.50450844
WASHC4	chr5	79417130	79474609	591915.7959	chr5	79591915	79595915	0.50492583

SRSF7	chr3	101603707	101613237	208841.10120	chr3	101208841	101209896	0.50540475
DUSP12	chr4	88809565	88818411	258461.89262	chr4	89258461	89262461	0.5058066
F3	chr4	122826644	122837666	824644.12282	chr4	122824644	122828644	0.50606663
RHPN2	chr6	42820568	42891346	587556.42591	chr6	42587556	42591556	0.50608514
MSH3	chr2	89250003	89453936	043362.89047	chr2	89043362	89047362	0.50686941
GHR	chr16	27126734	27421449	124734.2712	chr16	27124734	27128734	0.50689121
MAD2L1	chr8	103927353	103936664	268881.10427	chr8	104268881	104272881	0.50708667
CHRAC1	chr4	3025928	3028725	577459.25785	chr4	2577459	2578563	0.50758206
LEPROT	chr6	146974639	146987242	428927.14742	chr6	147428927	147429746	0.50768426
ADPGK	chr7	60584051	60623929	582051.60586	chr7	60582051	60586051	0.50773097
LRRC42	chr6	158316226	158338122	535465.15853	chr6	158535465	158539465	0.50844904
AKTIP	chr6	31747606	31758279	746036.31749	chr6	31746036	31749326	0.50894198
ALG6	chr6	149027980	149112665	153739.14915	chr6	149153739	149156202	0.50922109
PSMA5	chr4	110662692	110717290	594958.11069	chr4	110694958	110695918	0.50940143
FASTKD1	chr15	75895526	75929017	088477.7608	chr15	76088477	76089243	0.50944326
ZNF146	chr6	45520895	45599452	182499.45186	chr6	45182499	45186499	0.50958581
NID2	chr1	181699106	181815167	337919.18133	chr1	181337919	181339414	0.5097096
CNOT6	chr2	78132939	78202492	283203.78287	chr2	78283203	78287203	0.51020838
ENSSSCG00000016869	chr16	27803439	27805161	856157.2786	chr16	27856157	27860157	0.5105588
RPA3	chr9	77910881	77937060	217717.78218	chr9	78217717	78218825	0.51116167
ARF4	chr13	39409376	39430129	569981.3957	chr13	39569981	39573981	0.51127709
WDR47	chr4	111006968	111076918	004968.11100	chr4	111004968	111008968	0.51216197
ENSSSCG00000026746	chr13	79305680	79386940	980710.7898	chr13	78980710	78984710	0.51267181
RHEB	chr18	5779212	5822257	241524.6245	chr18	6241524	6245524	0.5140666
ANKLE2	chr14	22745641	22771727	379509.2238	chr14	22379509	22380259	0.51518843
SLC35A1	chr1	55859614	55895784	406112.55410	chr1	55406112	55410112	0.51555008
RRM1	chr9	6025073	6065143	064374.60656	chr9	6064374	6065627	0.51561817
ANP32B	chr1	239842067	239868403	492188.23949	chr1	239492188	239493251	0.5159734
GPD2	chr15	63619762	63728633	482472.6348	chr15	63482472	63482999	0.51674295
POLB	chr17	11356129	11388821	410074.1141	chr17	11410074	11411067	0.5170283
ABT1	chr7	21024635	21027794	996957.21000	chr7	20996957	21000957	0.51719872
KIAA0586	chr1	187504638	187642492	718846.18772	chr1	187718846	187720384	0.5175175
MORF4L2	chrX	84553774	84569030	173200.84177	chrX	84173200	84177200	0.5183264
GAR1	chr8	112400005	112414033	865664.11286	chr8	112865664	112869664	0.51833499
BAZ1A	chr7	64969640	65069889	238220.65242	chr7	65238220	65242220	0.51861066
ENSSSCG00000033293	chr12	4535650	4593526	798624.4802	chr12	4798624	4802624	0.51881771
ACTL6A	chr13	117598496	117628488	370272.1173	chr13	117370272	117371363	0.51882763
FFAR4	chr14	105011942	105037498	009942.1050	chr14	105009942	105013942	0.51884293
STX12	chr6	84918678	84960720	964791.84968	chr6	84964791	84968791	0.51928099
RHEB	chr18	5779212	5822257	242381.6244	chr18	6242381	6244253	0.51941386
CSGALNACT2	chr14	61372318	61415620	666240.6166	chr14	61666240	61668094	0.51960137
RHOT1	chr12	42711233	42783421	848632.4285	chr12	42848632	42852632	0.5203465
C5orf34	chr16	28058264	28094452	856157.2786	chr16	27856157	27860157	0.52063863
PARD6B	chr17	52195041	52213843	721967.5172	chr17	51721967	51724107	0.52066013
CRY1	chr5	13275545	13364593	365438.13369	chr5	13365438	13369438	0.52124197
GPR160	chr13	108748145	108793348	486808.1084	chr13	108486808	108487146	0.52144416
ENSSSCG00000016869	chr16	27803439	27805161	133077.2813	chr16	28133077	28134347	0.52168258
PARP14	chr13	137809510	137865106	453346.1374	chr13	137453346	137457346	0.52203945
PRKCI	chr13	108913173	108997931	486808.1084	chr13	108486808	108487146	0.52250181
TRMT5	chr1	189909660	189923234	471076.18947	chr1	189471076	189471705	0.52287273
STAMBP	chr3	69133978	69188192	545649.69549	chr3	69545649	69549649	0.52290808
DUSP11	chr3	69207475	69225729	020319.69021	chr3	69020319	69021858	0.52334054
FBXO4	chr16	26707470	26752024	124734.2712	chr16	27124734	27128734	0.52398815
CREG1	chr4	83458242	83469930	067748.83071	chr4	83067748	83071748	0.52402087
LACTB2	chr4	64710771	64748936	768402.64769	chr4	64768402	64769676	0.52416787
FLT3	chr11	5370496	5455358	793860.5797	chr11	5793860	5797121	0.52467386
TET3	chr3	68918933	69020986	570415.68574	chr3	68570415	68574415	0.52486412
SLC17A2	chr7	20644244	20663423	902846.20906	chr7	20902846	20906846	0.52509367
CENPW	chr1	36685265	36695955	022295.37026	chr1	37022295	37026295	0.52528366
RBM17	chr10	64885870	64908963	266094.6526	chr10	65266094	65267932	0.52539222
ZNF606	chr6	62695610	62719642	965994.62969	chr6	62965994	62969994	0.52555352
ENSSSCG00000004151	chr1	26043665	26044123	254832.26255	chr1	26254832	26255836	0.52595937
TMED5	chr4	124009816	124030773	834362.12383	chr4	123834362	123836334	0.52633368

STK38	chr7	32206541	32253624	924308.31928	chr7	31924308	31928308	0.52714871
PPP6C	chr1	265870477	265898124	934267.26593	chr1	265934267	265935360	0.52791739
FBXO33	chr1	169877197	169915798	725317.16972	chr1	169725317	169726241	0.52815686
SLC25A40	chr9	92481564	92528186	270443.92271	chr9	92270443	92271593	0.52826257
RHEB	chr18	5779212	5822257	690552.5691	chr18	5690552	5691835	0.52826568
POLE2	chr1	179495214	179529896	711299.17971	chr1	179711299	179715299	0.52851396
SHLD2	chr14	88005611	88121352	473596.8847	chr14	88473596	88474327	0.529699
SEPTIN2	chr15	140024049	140058289	540602.1395	chr15	139540602	139544602	0.53014808
MIER1	chr6	145609626	145680005	573985.14567	chr6	145673985	145674943	0.53030888
HHEX	chr14	104194343	104200219	835011.1038	chr14	103835011	103835799	0.53042563
DPP8	chr1	163446899	163517413	525770.16352	chr1	163525770	163526535	0.53097142
RNF216	chr3	4158450	4329378	089899.40938	chr3	4089899	4093899	0.53102358
TIGD7	chr3	38893694	38900327	128777.39132	chr3	39128777	39132777	0.53115907
CEP20	chr3	6977215	6996022	441088.74415	chr3	7441088	7441520	0.53159846
KLHL7	chr9	91924495	91971695	270443.92271	chr9	92270443	92271593	0.53184771
MTA3	chr3	97601412	97782863	199101.97202	chr3	97199101	97202900	0.53279023
DTL	chr9	131227705	131275451	631585.13163	chr9	131631585	131635585	0.53299363
TTC8	chr7	110731731	110784853	463371.11046	chr7	110463371	110463994	0.53302695
CNOT6	chr2	78132939	78202492	943841.77947	chr2	77943841	77947841	0.53378942
F2R	chr2	85641474	85656636	086481.86087	chr2	86086481	86087566	0.53405007
TNFSF12	chr12	52843026	52853325	399810.5240	chr12	52399810	52403810	0.53460197
TRAPPC6B	chr1	169709558	169722735	723649.16972	chr1	169723649	169727649	0.53517396
RHNO1	chr5	67239202	67246124	445777.67446	chr5	67445777	67446595	0.53530361
SNAPC3	chr1	207215738	207252396	173457.20717	chr1	207173457	207174369	0.53565477
WDR48	chr13	23735567	23786308	235699.2323	chr13	23235699	23239699	0.53580917
CDK17	chr5	87179615	87295167	567798.87571	chr5	87567798	87571798	0.53594851
LIN9	chr10	14109768	14184586	293023.1429	chr10	14293023	14297023	0.53607949
ENSSSCG00000008845	chr8	42024370	42044719	022370.42026	chr8	42022370	42026370	0.53620344
SGO2	chr15	104099384	104150720	523451.1045	chr15	104523451	104527451	0.5363233
CNBP	chr13	71630566	71646563	760305.7176	chr13	71760305	71762524	0.53700858
NRAS	chr4	105845872	105858227	056109.10606	chr4	106056109	106060109	0.5377546
DTL	chr9	131227705	131275451	784511.13078	chr9	130784511	130786914	0.53786428
SLC17A5	chr1	92286841	92342776	421305.92422	chr1	92421305	92422860	0.53804229
ENSSSCG00000040187	chr2	66650451	66674686	345269.66346	chr2	66345269	66346152	0.53818461
ENSSSCG00000008996	chr8	74614893	74633102	662598.74666	chr8	74662598	74666598	0.53922939
XPA	chr1	239532272	239568570	490721.23949	chr1	239490721	239494721	0.53999146
ST6GAL1	chr13	124791751	124837841	582327.1245	chr13	124582327	124586327	0.54059367
ENSSSCG00000000296	chr5	19672068	19673217	483096.19487	chr5	19483096	19487096	0.54063428
RHOT1	chr12	42711233	42783421	849222.4285	chr12	42849222	42850200	0.54127065
SLC25A20	chr13	31523821	31553286	989610.3199	chr13	31989610	31993610	0.54164196
CD47	chr13	151429288	151488544	427288.1514	chr13	151427288	151431288	0.54250926
ZNF140	chr14	22533916	22542728	650568.2265	chr14	22650568	22654568	0.54273829
ENSSSCG00000028423	chr6	88987099	89006811	530010.88531	chr6	88530010	88531426	0.54317893
PBX3	chr1	266382640	266610640	931340.26593	chr1	265931340	265935340	0.5432107
MYEF2	chr1	123600908	123638236	461438.12346	chr1	123461438	123462218	0.54543884
CDK17	chr5	87179615	87295167	485168.87489	chr5	87485168	87489478	0.54581902
PARP14	chr13	137809510	137865106	455216.1374	chr13	137455216	137457020	0.5466584
ZNF484	chr3	41893285	41918685	476270.41480	chr3	41476270	41480270	0.54666879
CD164	chr1	75441575	75458145	169857.75170	chr1	75169857	75170800	0.54720609
PPIL4	chr1	16400983	16446854	139115.16140	chr1	16139115	16140322	0.54760463
LONP2	chr6	36372599	36466190	340872.36344	chr6	36340872	36344872	0.54762582
PRDM10	chr9	56657270	56749482	748541.56750	chr9	56748541	56750127	0.54836367
KAT2A	chr12	20638497	20647461	256958.2025	chr12	20256958	20257834	0.54839773
KLHL11	chr12	20842393	20852980	937554.2094	chr12	20937554	20941554	0.54853451
EIF4G2	chr2	48666520	48678950	082751.49083	chr2	49082751	49083432	0.54908594
RPA3	chr9	77910881	77937060	215213.78219	chr9	78215213	78219213	0.54924098
SCAI	chr1	265714211	265867101	931340.26593	chr1	265931340	265935340	0.54924588
ENSSSCG00000059603	chr14	16624296	16629222	680792.1668	chr14	16680792	16682843	0.54979668
YAP1	chr9	32811416	32925603	437726.32439	chr9	32437726	32439106	0.551888
DCLRE1A	chr14	124144273	124162102	399551.1244	chr14	124399551	124401921	0.55229274
INTS14	chr1	163567777	163603184	525770.16352	chr1	163525770	163526535	0.55249305
RANBP17	chr16	52836297	53160162	821657.5282	chr16	52821657	52825657	0.55253747
SNAPC5	chr1	164467599	164475011	273811.16427	chr1	164273811	164275327	0.55256524

NUP133	chr14	60353474	60408828	528065.6052	chr14	60528065	60529075	0.55261551
TMCC1	chr13	69036428	69290098	402021.6940	chr13	69402021	69406021	0.55310246
EIF5A2	chr13	109510812	109530413	229745.1092	chr13	109229745	109232545	0.55339481
PTGR2	chr7	97174219	97195595	821130.96825	chr7	96821130	96825130	0.55379687
MELK	chr1	237317191	237431589	737586.23774	chr1	237737586	237740566	0.55381341
CD164	chr1	75441575	75458145	169061.75173	chr1	75169061	75173061	0.55445465
COG3	chr11	21759180	21826913	499492.2150	chr11	21499492	21503492	0.55542923
TOR1B	chr1	269967008	269973693	190175.27019	chr1	270190175	270190671	0.5572611
CTNNBL1	chr17	40774720	40949022	092032.4109	chr17	41092032	41096032	0.55737868
CD164	chr1	75441575	75458145	522280.75523	chr1	75522280	75523303	0.55765999
WDR47	chr4	111006968	111076918	006543.11100	chr4	111006543	111007819	0.55769063
MAPK8	chr14	88988949	89108874	192374.8919	chr14	89192374	89193566	0.55853329
SNX7	chr4	119035133	119255877	947829.11894	chr4	118947829	118949869	0.5585929
CEP135	chr8	55251005	55323350	727116.55731	chr8	55727116	55731116	0.55950743
ENSSSCG00000001769	chr7	48050045	48075795	837392.47841	chr7	47837392	47841392	0.55952271
SGO2	chr15	104099384	104150720	879439.1038	chr15	103879439	103880711	0.55971288
DTL	chr9	131227705	131275451	784592.13078	chr9	130784592	130788222	0.56034727
RNFT1	chr12	36162444	36174851	846540.3585	chr12	35846540	35850540	0.5603877
PPP1R12A	chr5	101490341	101639429	691246.10169	chr5	101691246	101695246	0.56132716
AZIN1	chr4	34234679	34257134	221012.34222	chr4	34221012	34222788	0.56162286
SEPTIN2	chr15	140024049	140058289	131918.1401	chr15	140131918	140133932	0.56180485
TMBIM4	chr5	30488328	30511533	186885.30190	chr5	30186885	30190885	0.56236104
POLE2	chr1	179495214	179529896	475992.17947	chr1	179475992	179479992	0.56252956
OMA1	chr6	153882451	153949468	859444.15386	chr6	153859444	153860669	0.56348625
TET3	chr3	68918933	69020986	519015.68520	chr3	68519015	68520573	0.56387796
CBFB	chr6	27684030	27750852	682030.27686	chr6	27682030	27686030	0.56482307
ENSSSCG00000004983	chr1	169777596	169870892	723649.16972	chr1	169723649	169727649	0.56583454
ACTL6A	chr13	117598496	117628488	368544.1173	chr13	117368544	117372544	0.5661877
ZNF567	chr6	45707074	45737962	741855.45745	chr6	45741855	45745855	0.56682419
ELK3	chr5	87285238	87354208	567798.87571	chr5	87567798	87571798	0.56686195
KDM1A	chr6	80903424	80971873	516429.80517	chr6	80516429	80517008	0.56885468
LIN9	chr10	14109768	14184586	939003.1394	chr10	13939003	13940930	0.56952503
NPM1	chr16	52765298	52781771	819079.5282	chr16	52819079	52825559	0.56991719
LONP2	chr6	36372599	36466190	342480.36343	chr6	36342480	36343795	0.57243952
ENSSSCG00000002877	chr6	44345463	44357772	933498.43934	chr6	43933498	43934468	0.57259566
PRKCI	chr13	108913173	108997931	229745.1092	chr13	109229745	109232545	0.57289011
NFYB	chr5	80334748	80353768	408372.80412	chr5	80408372	80412372	0.57312201
TOR1B	chr1	269967008	269973693	995271.26999	chr1	269995271	269995975	0.57315533
CNN3	chr4	122504433	122528348	824644.12282	chr4	122824644	122828644	0.57338829
MSX1	chr8	5628380	5632607	022066.60240	chr8	6022066	6024012	0.5734783
ABT1	chr7	21024635	21027794	997828.20999	chr7	20997828	20999197	0.57376178
TET3	chr3	68918933	69020986	496471.68497	chr3	68496471	68497933	0.57398683
DNAAF2	chr1	179482208	179491142	711299.17971	chr1	179711299	179715299	0.57411462
CREG1	chr4	83458242	83469930	068725.83070	chr4	83068725	83070535	0.57456547
NUDT19	chr6	42574071	42583926	558113.42562	chr6	42558113	42562113	0.57466513
SIKE1	chr4	105779814	105789389	057753.10605	chr4	106057753	106059218	0.57512503
AP3S1	chr2	119965539	120039947	803806.11980	chr2	119803806	119804690	0.57531405
POLN	chr8	1073889	1197086	053894.10572	chr8	1053894	1057212	0.57661166
RCN2	chr7	57041003	57059330	040612.57041	chr7	57040612	57041309	0.57691049
BANP	chr6	1421462	1495486	494442.14961	chr6	1494442	1496104	0.57776088
GALNT11	chr18	5289917	5348058	992746.4995	chr18	4992746	4995436	0.57840763
TMEM128	chr8	6115002	6125139	890392.58913	chr8	5890392	5891389	0.57870468
PSMA5	chr4	110662692	110717290	004968.11100	chr4	111004968	111008968	0.57884564
TOR1B	chr1	269967008	269973693	965008.26996	chr1	269965008	269969008	0.57971741
AURKB	chr12	53410177	53423225	925589.5292	chr12	52925589	52926771	0.58315633
MKNK1	chr6	164887748	164933521	132690.16513	chr6	165132690	165136690	0.58325068
CIAO2A	chr1	107951646	107969298	995293.10799	chr1	107995293	107999293	0.58462101
MAP3K20	chr15	79173033	79380993	773073.7877	chr15	78773073	78773732	0.58480105
SIRT4	chr14	40290288	40303879	561159.4056	chr14	40561159	40565159	0.58792532
LEKR1	chr13	96652441	96879731	651679.9665	chr13	96651679	96652236	0.58800498
TOR1B	chr1	269967008	269973693	993520.26999	chr1	269993520	269997520	0.58875147
ENSSSCG00000032353	chr8	55787215	55830694	192440.56196	chr8	56192440	56196440	0.58891815
SLAIN2	chr8	38241598	38322031	934847.37938	chr8	37934847	37938847	0.5890656

STX12	chr6	84918678	84960720	467181.84468	chr6	84467181	84468278	0.59128119
ENSSSCG00000026746	chr13	79305680	79386940	707967.7971	chr13	79707967	79710829	0.59145859
ALG6	chr6	149027980	149112665	160941.14916	chr6	149160941	149161931	0.59177529
ENSSSCG00000014071	chr2	82660495	82668374	535019.82537	chr2	82535019	82537507	0.59202955
TTC8	chr7	110731731	110784853	461517.11046	chr7	110461517	110465517	0.59249548
DCLRE1A	chr14	124144273	124162102	017164.1240	chr14	124017164	124021164	0.59295273
TMEM128	chr8	6115002	6125139	888765.58927	chr8	5888765	5892765	0.5935677
CNOT8	chr16	68329790	68350260	675963.6867	chr16	68675963	68679963	0.59388871
PDE8A	chr7	52410779	52557645	302472.52306	chr7	52302472	52306472	0.59389945
ENSSSCG00000011253	chr13	23020584	23116955	236881.2323	chr13	23236881	23238804	0.59508785
SLC41A2	chr5	79593915	79720895	591915.79595	chr5	79591915	79595915	0.59547787
ENSSSCG00000053231	chr14	61024449	61030097	677026.6067	chr14	60677026	60678251	0.59587208
NSL1	chr9	130622331	130668936	784592.13078	chr9	130784592	130788222	0.5964835
SRSF7	chr3	101603707	101613237	456930.10146	chr3	101456930	101460930	0.59700064
STAG1	chr13	77321872	77863491	749660.7775	chr13	77749660	77751338	0.59806475
F2R	chr2	85641474	85656636	642347.85643	chr2	85642347	85643055	0.59823178
AZIN1	chr4	34234679	34257134	429189.34431	chr4	34429189	34431968	0.59887249
RHNO1	chr5	67239202	67246124	237801.67238	chr5	67237801	67238687	0.59979188
SIKE1	chr4	105779814	105789389	056109.10606	chr4	106056109	106060109	0.59987244
IMPA1	chr4	54935860	54984238	281589.55282	chr4	55281589	55282904	0.59995662
SPPL2A	chr1	121067454	121134888	411506.12141	chr1	121411506	121415506	0.60020067
GALNT11	chr18	5289917	5348058	474457.5476	chr18	5474457	5476891	0.60195752
ENSSSCG00000059603	chr14	16624296	16629222	708864.1671	chr14	16708864	16710745	0.60401696
CNOT8	chr16	68329790	68350260	678219.6868	chr16	68678219	68680897	0.60431704
DNAL1	chr7	96999602	97047090	123873.97124	chr7	97123873	97124625	0.60694396
DTX3	chr5	22867711	22873393	044561.23048	chr5	23044561	23048561	0.60781905
ENSSSCG00000016869	chr16	27803439	27805161	639997.2764	chr16	27639997	27640867	0.60851947
NSL1	chr9	130622331	130668936	784511.13078	chr9	130784511	130786914	0.60882922
USP25	chr13	180571342	180712435	569342.1805	chr13	180569342	180573342	0.60934512
C12orf40	chr5	71185592	71270728	672760.71673	chr5	71672760	71673927	0.60944687
SNX7	chr4	119035133	119255877	945440.11894	chr4	118945440	118949440	0.60951009
INTS14	chr1	163567777	163603184	435340.16343	chr1	163435340	163436160	0.60991858
GNG10	chr1	252453107	252460315	323217.25232	chr1	252323217	252324977	0.60993059
RDX	chr9	38220447	38322097	580776.38582	chr9	38580776	38582849	0.61121691
ANKRD13C	chr6	142516426	142616328	666213.14267	chr6	142666213	142670213	0.61234725
CAMK4	chr2	116000791	116229758	285018.11628	chr2	116285018	116289018	0.61249143
SLC35G1	chr14	105335160	105345274	011963.1050	chr14	105011963	105012625	0.61273086
PRKCI	chr13	108913173	108997931	230698.1092	chr13	109230698	109231563	0.61376732
ITSN2	chr3	114363529	114508444	576199.11458	chr3	114576199	114580199	0.61446313
ENSSSCG00000013715	chr2	66829154	66853255	345269.66346	chr2	66345269	66346152	0.61534714
FFAR4	chr14	105011942	105037498	010519.1050	chr14	105010519	105014699	0.61542434
ENSSSCG00000033395	chr10	55709135	55820506	129857.5613	chr10	56129857	56130854	0.6170523
POLB	chr17	11356129	11388821	166763.1116	chr17	11166763	11167738	0.61815382
MOCS2	chr16	32436205	32462521	334292.3233	chr16	32334292	32338292	0.61854928
C12orf40	chr5	71185592	71270728	028825.71030	chr5	71028825	71030050	0.61874379
NPM1	chr16	52765298	52781771	821657.5282	chr16	52821657	52825657	0.62013788
STT3B	chr13	17534171	17635035	030794.1803	chr13	18030794	18031998	0.620307
TBC1D15	chr5	35827473	35908385	670415.35671	chr5	35670415	35671285	0.62129833
RANBP17	chr16	52836297	53160162	819079.5282	chr16	52819079	52825559	0.6244887
CRYZ	chr6	138435392	138460975	433392.13843	chr6	138433392	138437392	0.62608409
XRCC2	chr18	4808470	4835802	991227.4995	chr18	4991227	4995227	0.62656765
LDAF1	chr3	24876637	24910760	285886.25286	chr3	25285886	25286690	0.62674791
ENSSSCG00000033814	chr16	39954354	39958520	103948.4010	chr16	40103948	40107227	0.62687614
GPR160	chr13	108748145	108793348	229745.1092	chr13	109229745	109232545	0.62753431
TRAPPC6B	chr1	169709558	169722735	725317.16972	chr1	169725317	169726241	0.62871238
THOC6	chr3	39161434	39164707	128777.39132	chr3	39128777	39132777	0.62961313
CMAS	chr5	51438020	51458615	808943.51812	chr5	51808943	51812943	0.63083549
ENSSSCG00000013715	chr2	66829154	66853255	344600.66347	chr2	66344600	66347110	0.63127817
POLN	chr8	1073889	1197086	386266.13902	chr8	1386266	1390266	0.63131038
ENSSSCG00000026746	chr13	79305680	79386940	706693.7971	chr13	79706693	79710693	0.63247036
PSMA5	chr4	110662692	110717290	006543.11100	chr4	111006543	111007819	0.63331214
ENSSSCG00000063355	chr15	133206674	133208289	376576.1333	chr15	133376576	133378660	0.63529405
XRCC2	chr18	4808470	4835802	992746.4995	chr18	4992746	4995436	0.63625907

HHEX	chr14	104194343	104200219	549119.1045	chr14	104549119	104554279	0.63863861
SCAF11	chr5	76809514	76848660	126152.7712	chr5	77126152	77127879	0.63881303
ENSSSCG00000026746	chr13	79305680	79386940	610176.7961	chr13	79610176	79611044	0.63917338
TMBIM4	chr5	30488328	30511533	187944.3019	chr5	30187944	30190487	0.64236645
LIMA1	chr5	16074438	16177103	964706.1596	chr5	15964706	15965446	0.64320963
PPP6C	chr1	265870477	265898124	931340.2659	chr1	265931340	265935340	0.64539516
PDE8A	chr7	52410779	52557645	254159.5225	chr7	52254159	52258159	0.64746632
ANKLE2	chr14	22745641	22771727	650568.2265	chr14	22650568	22654568	0.6481573
ALG6	chr6	149027980	149112665	152033.1491	chr6	149152033	149156033	0.64868141
LRPPRC	chr3	96474549	96595143	472549.9647	chr3	96472549	96476549	0.65192153
SNX7	chr4	119035133	119255877	947397.1189	chr4	118947397	118948903	0.65267695
HHEX	chr14	104194343	104200219	549134.1045	chr14	104549134	104553134	0.65465189
DNM3	chr9	114262791	114797674	200484.1142	chr9	114200484	114204484	0.65731675
CCT8	chr13	192406281	192423413	247246.1922	chr13	192247246	192251246	0.65787705
USP25	chr13	180571342	180712435	570640.1805	chr13	180570640	180571603	0.65865279
SS18	chr6	110710569	110790379	147751.1111	chr6	111147751	111151751	0.6649037
TAF1A	chr10	11340642	11362432	334439.1133	chr10	11334439	11336379	0.66751822
ZNF567	chr6	45707074	45737962	304692.4530	chr6	45304692	45306421	0.66999751
COG3	chr11	21759180	21826913	927221.2193	chr11	21927221	21931221	0.67239613
ENSSSCG00000051290	chr11	17731530	17762314	945850.1794	chr11	17945850	17949850	0.67292346
ZNF606	chr6	62695610	62719642	690463.6269	chr6	62690463	62691254	0.67295123
TMEM216	chr2	10115607	10121649	733322.9737	chr2	9733322	9737322	0.67330523
CNBP	chr13	71630566	71646563	558012.7156	chr13	71558012	71562012	0.67397995
SLC35G1	chr14	105335160	105345274	009942.1050	chr14	105009942	105013942	0.68072485
GPR160	chr13	108748145	108793348	230698.1092	chr13	109230698	109231563	0.68473123
SCLT1	chr8	95949572	96199110	267859.9626	chr8	96267859	96269407	0.68664357
GNG10	chr1	252453107	252460315	323757.2523	chr1	252323757	252327757	0.6887167
STAMBP	chr3	69133978	69188192	020319.6902	chr3	69020319	69021858	0.69179529
ENSSSCG00000042498	chr16	18299051	18305299	537410.1853	chr16	18537410	18538073	0.69291809
ENSSSCG00000051290	chr11	17731530	17762314	947617.1794	chr11	17947617	17948835	0.69644294
ITCH	chr17	37810235	37918209	955507.3795	chr17	37955507	37959507	0.70575892
SLC35G1	chr14	105335160	105345274	010519.1050	chr14	105010519	105014699	0.71118119
HENMT1	chr4	111364966	111382572	004968.1110	chr4	111004968	111008968	0.71473632
CEP57L1	chr1	75171061	75227022	522280.7552	chr1	75522280	75523303	0.72679978
HENMT1	chr4	111364966	111382572	006543.1110	chr4	111006543	111007819	0.73024919
GOLT1B	chr5	51939485	51953315	808943.5181	chr5	51808943	51812943	0.75170073
