# Supplementary material for: Deciphering differences in DNA methylation and transcriptome profiles of oocytes from pigs with high and low developmental competence
Source: Environ Epigenet. 2025 Jun 3;11(1):dvaf018. doi: 10.1093/eep/dvaf018 (PMC12418950; doi:10.1093/eep/dvaf018)
Supplement: dvaf018_Supplemental_Files [file dvaf018_supplemental_files.zip › Sup table 18.pdf]

Gene	Gene-Chr	Gene-ini	Gene-end	Loci	Loci-Chr	Loci-ini	Loci-end	cor
EFR3A	chr4	9053086	9137265	051086.90550	chr4	9051086	9055086	-0.7608383
TEX12	chr9	39864148	39869979	158462.40161	chr9	40158462	40161372	-0.7431674
BLM	chr7	53317540	53412645	285609.53288	chr7	53285609	53288709	-0.7349497
TADA1	chr4	84174476	84187514	108767.84112	chr4	84108767	84112767	-0.7044881
TADA1	chr4	84174476	84187514	111766.84112	chr4	84111766	84112592	-0.6831932
ENSSSCG00000063355	chr15	133206674	133208289	473548.1334	chr15	133473548	133476628	-0.6716355
RARS2	chr1	55893480	55982068	376101.56380	chr1	56376101	56380101	-0.6667698
ZNF300	chr2	151842009	151856042	555380.15156	chr2	151555380	151563830	-0.6636798
VPS37A	chr17	4949644	4988074	4947644.4951	chr17	4947644	4951644	-0.6574041
TOR1B	chr1	269967008	269973693	598798.26960	chr1	269598798	269602798	-0.6548929
ZNF449	chrX	110928720	110948614	247183.11124	chrX	111247183	111248501	-0.6508967
VEZF1	chr12	34245884	34263538	896719.3390	chr12	33896719	33901049	-0.650731
ZFP1	chr6	12480278	12531530	550006.12551	chr6	12550006	12551126	-0.6494921
GALNT11	chr18	5289917	5348058	4980080.4981	chr18	4980080	4981148	-0.6392882
NCAPG2	chr18	653224	717200	1130327.1131	chr18	1130327	1131165	-0.637561
PTS	chr9	39920273	39928634	158462.40161	chr9	40158462	40161372	-0.6351548
CFAP57	chr6	167988092	168049263	986876.16798	chr6	167986876	167989686	-0.6326948
ENSSSCG00000001769	chr7	48050045	48075795	008589.48012	chr7	48008589	48012589	-0.6304671
SYCP2	chr17	59875993	59955209	0235822.6023	chr17	60235822	60239962	-0.6292017
ROCK2	chr3	125352920	125493362	103753.12510	chr3	125103753	125104644	-0.6241188
DSCC1	chr4	19161226	19178568	317810.19321	chr4	19317810	19321810	-0.6239561
POLN	chr8	1073889	1197086	702463.70552	chr8	702463	705529	-0.6227604
CNOT7	chr17	4933453	4950346	4947644.4951	chr17	4947644	4951644	-0.6224747
MPP7	chr10	39532370	39798356	9874289.3987	chr10	39874289	39876849	-0.6180078
CEP295	chr9	26103283	26155076	546594.26550	chr9	26546594	26550594	-0.6176766
ATP13A3	chr13	131391798	131493623	1383044.1313	chr13	131383044	131387044	-0.6171484
SEPTIN2	chr15	140024049	140058289	9858888.1398	chr15	139858888	139868418	-0.6171017
ZNF664	chr14	29031605	29068864	8802819.2880	chr14	28802819	28808389	-0.614801
APPBP2	chr12	37613770	37678708	8059927.3806	chr12	38059927	38063927	-0.6141951
RNF2	chr9	126172301	126287603	172009.12617	chr9	126172009	126172651	-0.6078482
KIF4A	chrX	56319529	56447553	132853.56136	chrX	56132853	56136853	-0.6065243
DONSON	chr13	197193658	197203932	7465015.1974	chr13	197465015	197468485	-0.6055793
ATP13A3	chr13	131391798	131493623	1341940.1313	chr13	131341940	131345940	-0.6050735
NXT2	chrX	89684671	89691805	994848.89998	chrX	89994848	89998308	-0.6020805
AFF4	chr2	135200076	135283675	137272.13514	chr2	135137272	135141272	-0.5992346
LAPTM4A	chr3	118152587	118170278	444356.11844	chr3	118444356	118447936	-0.5982496
ENSSSCG00000056411	chr13	205345690	205349487	5423454.2054	chr13	205423454	205427454	-0.5982
ENSSSCG00000060529	chr11	25541019	25595113	5903366.2590	chr11	25903366	25907366	-0.5978392
ZDHHC20	chr11	1328110	1405872	1836951.8409	chr11	836951	840951	-0.5976899
RHEB	chr18	5779212	5822257	5873195.5876	chr18	5873195	5876335	-0.5973598
FUNDC1	chrX	39724998	39737532	255608.39260	chrX	39255608	39260588	-0.5972778
MKLN1	chr18	17501857	17832503	7986022.1799	chr18	17986022	17990022	-0.5972483
MPP7	chr10	39532370	39798356	9875244.3987	chr10	39875244	39875701	-0.5950923
TAF1A	chr10	11340642	11362432	0870521.1087	chr10	10870521	10871478	-0.5926971
ENSSSCG00000002020	chr7	75618988	75629447	005976.76006	chr7	76005976	76006524	-0.5916653
MAPK6	chr1	119725029	119771312	597054.11959	chr1	119597054	119597567	-0.5895446
LACTB2	chr4	64710771	64748936	953940.64956	chr4	64953940	64956027	-0.5885036
EIF5A2	chr13	109510812	109530413	9529851.1095	chr13	109529851	109530793	-0.5869992
CNN3	chr4	122504433	122528348	771109.12277	chr4	122771109	122773489	-0.5867099
TOR1B	chr1	269967008	269973693	573594.26957	chr1	269573594	269577594	-0.5861427
HAUS4	chr7	76085608	76097953	005976.76006	chr7	76005976	76006524	-0.5855849
ATP13A3	chr13	131391798	131493623	1327565.1313	chr13	131327565	131331315	-0.585302
PITRM1	chr10	66996954	67047235	6811869.6681	chr10	66811869	66815749	-0.5831604
SEPTIN2	chr15	140024049	140058289	9865812.1398	chr15	139865812	139867602	-0.5821924
KHDRBS1	chr6	88530385	88572463	808587.88809	chr6	88808587	88809709	-0.580337
PITRM1	chr10	66996954	67047235	7435519.6744	chr10	67435519	67446299	-0.5796333
PTS	chr9	39920273	39928634	790010.39790	chr9	39790010	39790626	-0.5780028
RABGAP1	chr1	263857513	264030574	731927.26373	chr1	263731927	263735927	-0.5767059
WTAP	chr1	7622547	7651961	364266.73678	chr1	7364266	7367856	-0.5756975
HDAC3	chr2	143273192	143287399	317870.14332	chr2	143317870	143325360	-0.5733276
ATL2	chr3	101957209	102029667	193741.10219	chr3	102193741	102197741	-0.5726689
TOR1B	chr1	269967008	269973693	259836.27026	chr1	270259836	270264736	-0.5715756

PPFIA1	chr2	3073241	3164235	723860.27334	chr2	2723860	2733450	-0.5713659
PSMG1	chr13	202762512	202775642	561255.2025	chr13	202561255	202564075	-0.5699792
SMC3	chr14	120969631	121021497	347059.1213	chr14	121347059	121351059	-0.5685397
XRCC2	chr18	4808470	4835802	943185.4946	chr18	4943185	4946905	-0.5674818
PRDM10	chr9	56657270	56749482	098862.57101	chr9	57098862	57101522	-0.5674284
GRPEL2	chr2	150511178	150520462	981810.15098	chr2	150981810	150986810	-0.567368
ENSSSCG00000063355	chr15	133206674	133208289	933798.1329	chr15	132933798	132935628	-0.5670037
TBC1D23	chr13	158665077	158729274	326227.1583	chr13	158326227	158326985	-0.5661287
NXT2	chrX	89684671	89691805	739190.89743	chrX	89739190	89743190	-0.5658678
ENSSSCG00000005440	chr1	249898712	249905370	876176.24987	chr1	249876176	249878826	-0.5655663
SEC23IP	chr14	129758288	129810642	577544.1295	chr14	129577544	129578376	-0.5628973
SEPTIN2	chr15	140024049	140058289	764414.1397	chr15	139764414	139768414	-0.5625779
TFG	chr13	158288579	158326885	326227.1583	chr13	158326227	158326985	-0.5609832
ENSSSCG00000033293	chr12	4535650	4593526	005799.5007	chr12	5005799	5007699	-0.560497
STAG2	chrX	101478247	101617563	477610.10147	chrX	101477610	101479275	-0.5590943
TIGD7	chr3	38893694	38900327	260264.39264	chr3	39260264	39264264	-0.5573602
UBIAD1	chr6	71419602	71433210	017576.71021	chr6	71017576	71021076	-0.5572284
UBE2G1	chr12	50266696	50348689	736832.5073	chr12	50736832	50738032	-0.55527
ZCCHC10	chr2	135305852	135328527	147046.13515	chr2	135147046	135151046	-0.5546122
FERMT2	chr1	182519684	182606869	357377.18235	chr1	182357377	182358281	-0.552894
DTL	chr9	131227705	131275451	707002.13170	chr9	131707002	131709562	-0.5519138
ENSSSCG00000033293	chr12	4535650	4593526	908039.4909	chr12	4908039	4909329	-0.5507279
ENSSSCG00000012165	chrX	18013930	18054247	839324.17842	chrX	17839324	17842394	-0.549302
ZDHHC20	chr11	1328110	1405872	559030.1562	chr11	1559030	1562300	-0.5489007
WAC	chr10	39932599	40022338	875244.3987	chr10	39875244	39875701	-0.5486428
MORC3	chr13	199997156	200041236	128785.2001	chr13	200128785	200129482	-0.5468321
UBR2	chr7	37655926	37784359	097569.38102	chr7	38097569	38102239	-0.5465325
ADH5	chr8	121266764	121282310	794058.12079	chr8	120794058	120796588	-0.5455828
KHDRBS1	chr6	88530385	88572463	049896.88059	chr6	88049896	88059876	-0.5455723
ENSSSCG00000014071	chr2	82660495	82668374	713416.82717	chr2	82713416	82717416	-0.5445592
CCDC127	chr16	79862686	79870755	877864.7987	chr16	79877864	79878678	-0.5400646
GALNT11	chr18	5289917	5348058	943185.4946	chr18	4943185	4946905	-0.5400341
MEIOC	chr12	18651511	18671987	465382.1846	chr12	18465382	18469382	-0.5383009
ENSSSCG00000012152	chrX	14210164	14222047	247778.14250	chrX	14247778	14250728	-0.5377026
FNBP4	chr2	14896175	14932183	164306.15168	chr2	15164306	15168306	-0.5376022
SWT1	chr9	126347517	126481803	172009.12617	chr9	126172009	126172651	-0.5364748
ZNF606	chr6	62695610	62719642	745816.62746	chr6	62745816	62746568	-0.5355229
WDR47	chr4	111006968	111076918	584109.11058	chr4	110584109	110585339	-0.5329223
DONSON	chr13	197193658	197203932	955955.1969	chr13	196955955	196959185	-0.5325581
PSMG1	chr13	202762512	202775642	234243.2032	chr13	203234243	203238243	-0.5313164
CLK3	chr7	58895419	58909551	068728.59072	chr7	59068728	59072728	-0.5308227
F3	chr4	122826644	122837666	218039.12322	chr4	123218039	123221889	-0.529741
ATP13A3	chr13	131391798	131493623	343175.13134	chr13	131343175	131346375	-0.5295176
TFDP1	chr11	78755533	78778309	645567.7864	chr11	78645567	78646089	-0.5294319
ENSSSCG00000002020	chr7	75618988	75629447	003612.76007	chr7	76003612	76007612	-0.5284715
RNF216	chr3	4158450	4329378	044136.40488	chr3	4044136	4048850	-0.5264299
CDC123	chr10	59718340	59765621	587169.5959	chr10	59587169	59591069	-0.5254505
GALNT11	chr18	5289917	5348058	977436.4981	chr18	4977436	4981436	-0.5250574
WTAP	chr1	7622547	7651961	517526.75263	chr1	7517526	7526346	-0.5248272
MEST	chr18	18327536	18345843	817641.1882	chr18	18817641	18821641	-0.5237521
SCML2	chrX	14717606	14823413	086758.15090	chrX	15086758	15090018	-0.520721
ENSSSCG00000026719	chr5	40416335	40423016	197598.40200	chr5	40197598	40200348	-0.5202416
MAP2K1	chr1	164381845	164471226	734118.16473	chr1	164734118	164734722	-0.5201875
PTGR2	chr7	97174219	97195595	314711.97318	chr7	97314711	97318711	-0.5186577
CDC7	chr4	125432772	125459288	366989.12537	chr4	125366989	125370459	-0.5179807
HSD17B12	chr2	18456019	18611782	969090.17975	chr2	17969090	17975170	-0.5178353
ATAD2	chr4	16012561	16082638	790997.15794	chr4	15790997	15794997	-0.5170517
PDP2	chr6	27565450	27575455	825350.27829	chr6	27825350	27829350	-0.5164717
FLT3	chr11	5370496	5455358	241105.5241	chr11	5241105	5241689	-0.5158095
ARPIN	chr7	55468568	55483536	363709.55366	chr7	55363709	55366089	-0.5157043
MRPL15	chr4	77069092	77083723	888489.76894	chr4	76888489	76894389	-0.5142664
DLAT	chr9	39738564	39796806	696254.39697	chr9	39696254	39697000	-0.5121136
SEPSECS	chr8	19097456	19136222	797198.18803	chr8	18797198	18803368	-0.509522

ZNF606	chr6	62695610	62719642	054906.63058	chr6	63054906	63058226	-0.5085161
ZHX2	chr4	16374902	16553002	527039.16532	chr4	16527039	16532019	-0.5084073
SPTLC1	chr14	3081972	3138103	2802061.2806	chr14	2802061	2806061	-0.5081514
CFAP57	chr6	167988092	168049263	986092.16799	chr6	167986092	167990092	-0.5079123
ZC3H8	chr3	44161310	44184683	973665.43974	chr3	43973665	43974341	-0.5055722
ZDHHC20	chr11	1328110	1405872	1661120.1663	chr11	1661120	1663910	-0.5052623
GALNT11	chr18	5289917	5348058	4978355.4984	chr18	4978355	4984775	-0.5044738
CFAP57	chr6	167988092	168049263	801936.16780	chr6	167801936	167808346	-0.5042291
SRPX	chrX	34075579	34198467	296220.34296	chrX	34296220	34296757	-0.5040744
STX12	chr6	84918678	84960720	918888.84919	chr6	84918888	84919217	-0.5038682
NCAPG	chr8	12759641	12807163	689067.12689	chr8	12689067	12689731	-0.5037506
XRCC2	chr18	4808470	4835802	4977436.4981	chr18	4977436	4981436	-0.5036917
UBXN2B	chr4	74405902	74437615	619249.74623	chr4	74619249	74623839	-0.5029863
ZDHHC20	chr11	1328110	1405872	1606840.1609	chr11	1606840	1609800	-0.5012085
ODF2L	chr4	129721291	129758495	795209.12979	chr4	129795209	129797689	-0.5005479
SCML2	chrX	14717606	14823413	821461.14822	chrX	14821461	14822726	-0.500383
CYLD	chr6	34059081	34121264	740746.33747	chr6	33740746	33747206	-0.5003599
DGAT1	chr4	452662	466684	801299.80574	chr4	801299	805749	0.50000674
PSMA7	chr17	61566373	61572438	1331052.6133	chr17	61331052	61338302	0.50008182
LYRM4	chr7	3070470	3192922	614139.26162	chr7	2614139	2616239	0.50009639
NAPA	chr6	53246968	53275265	504410.53508	chr6	53504410	53508410	0.50009878
TMEM147	chr6	44979190	44981049	805436.44807	chr6	44805436	44807246	0.50028825
CSTB	chr13	206706063	206710646	6843554.2068	chr13	206843554	206847737	0.50045765
CLN8	chr15	33288301	33304631	3261878.3326	chr15	33261878	33268778	0.50054852
FBXO31	chr6	1949276	1995462	574996.15795	chr6	1574996	1579556	0.50056098
FAM50A	chrX	124967510	124973483	271488.12527	chrX	125271488	125274598	0.50077667
LAT2	chr3	11352579	11372092	052516.11053	chr3	11052516	11053085	0.50082614
PIP4K2B	chr12	23317208	23346424	3729110.2373	chr12	23729110	23733110	0.50083627
TUBB2A	chr7	1910269	1914761	230059.22352	chr7	2230059	2235289	0.50085304
ENSSSCG00000018046	chr12	59977028	60019814	9576119.5957	chr12	59576119	59578709	0.50092662
STX8	chr12	54296995	54544646	4052571.5405	chr12	54052571	54056571	0.50093964
MRPL36	chr16	79001549	79004358	9037029.7904	chr16	79037029	79040119	0.50105766
DBNL	chr18	48713340	48725697	9159885.4917	chr18	49159885	49170155	0.50112392
OSBPL2	chr17	61643377	61685819	1331052.6133	chr17	61331052	61338302	0.5012067
WDR25	chr7	121238268	121384292	895092.12089	chr7	120895092	120899092	0.5012257
RANGRF	chr12	53485265	53486713	3904511.5390	chr12	53904511	53908511	0.50142196
PKN1	chr2	64800721	64824957	716906.64720	chr2	64716906	64720906	0.5014298
ENSSSCG00000003253	chr6	56236311	56244185	389302.56390	chr6	56389302	56390042	0.50143371
MTM1	chrX	122286916	122379299	707368.12270	chrX	122707368	122709996	0.50143999
TMEM39B	chr6	88585291	88606906	808587.88809	chr6	88808587	88809709	0.50153383
SDHA	chr16	79834044	79862524	9891263.7989	chr16	79891263	79895263	0.50157517
BRD9	chr16	79473745	79492635	9511609.7951	chr16	79511609	79518859	0.50175323
GABARAP	chr12	52596543	52598194	3006559.5300	chr12	53006559	53008829	0.50182234
KCNAB3	chr12	53171319	53180823	3006559.5300	chr12	53006559	53008829	0.50193236
POLR2G	chr2	9004390	9017779	193765.91977	chr2	9193765	9197765	0.50199102
PPP1CA	chr2	5119939	5123617	099970.51007	chr2	5099970	5100709	0.50199432
BNIP3	chr14	140362127	140371907	0430596.1404	chr14	140430596	140434596	0.50200926
XRCC6	chr5	6903495	6929052	576101.65771	chr5	6576101	6577139	0.50207552
TMC4	chr6	55955991	55970280	781617.55782	chr6	55781617	55782322	0.50212511
RPS9	chr6	55920934	55927280	781617.55782	chr6	55781617	55782322	0.50217799
TSPAN7	chrX	34522933	34660212	296220.34296	chrX	34296220	34296757	0.5023116
PSENEN	chr6	45171825	45174102	805436.44807	chr6	44805436	44807246	0.50231956
FIGNL1	chr9	136484216	136488250	707472.13671	chr9	136707472	136712522	0.50250495
FTSJ3	chr12	15106719	15114398	5380779.1538	chr12	15380779	15384509	0.50253526
EBNA1BP2	chr6	168049758	168061013	524156.16853	chr6	168524156	168530576	0.50298445
WARS1	chr7	121212981	121238113	705122.12170	chr7	121705122	121709122	0.50305032
RPTOR	chr12	1709128	1991574	1783700.1786	chr12	1783700	1786995	0.50320053
RIOK1	chr7	4750752	4782448	273899.42749	chr7	4273899	4274969	0.50332105
ZNF212	chr18	55528138	55541841	5614429.5561	chr18	55614429	55616784	0.50337021
CARS2	chr11	77229971	77265272	6748420.7675	chr11	76748420	76752040	0.50350034
CFL1	chr2	6469254	6475035	895080.68997	chr2	6895080	6899700	0.50361707
RPP40	chr7	2987271	3005271	064599.30706	chr7	3064599	3070619	0.50366147
ENSSSCG00000038404	chr7	1752689	1776087	748321.17494	chr7	1748321	1749415	0.50367391

FSD1	chr2	74471744	74485786	040989.74044	chr2	74040989	74044989	0.50388422
EIF4E2	chr15	133060247	133103719	118093.1331	chr15	133118093	133122093	0.50409348
ENSSSCG00000012088	chr13	207801096	207818130	214622.2082	chr13	208214622	208222830	0.50421833
ENSSSCG00000027723	chr15	137567231	137621880	444508.1374	chr15	137444508	137445469	0.50427649
NCF1	chr3	11820266	11839354	038056.12042	chr3	12038056	12042056	0.5043675
CLDN10	chr11	65121607	65145072	200901.6520	chr11	65200901	65201681	0.50453632
POLM	chr18	51079503	51088781	951075.5095	chr18	50951075	50958745	0.50453741
GGA1	chr5	10190367	10213477	587800.10588	chr5	10587800	10588562	0.50456488
ENSSSCG00000013064	chr2	9163317	9174468	082256.90862	chr2	9082256	9086256	0.50461283
EEF1D	chr4	969527	983270	785393.79847	chr4	785393	798477	0.50467174
ENSSSCG00000013064	chr2	9163317	9174468	293700.92984	chr2	9293700	9298410	0.50468397
DOK5	chr17	55390802	55583858	527122.5553	chr17	55527122	55531472	0.50468574
RNF220	chr6	166666138	166910646	808576.16681	chr6	166808576	166811236	0.50492836
SEC61G	chr9	139128296	139135727	147222.13915	chr9	139147222	139153542	0.50500076
CFDP1	chr6	12223748	12354177	054571.12058	chr6	12054571	12058571	0.50500872
MRPL36	chr16	79001549	79004358	168279.7917	chr16	79168279	79175129	0.50504884
ADAM3A	chr17	9088860	9166340	596773.8597	chr17	8596773	8597366	0.50515698
C1orf174	chr6	65323756	65333886	005436.65012	chr6	65005436	65012316	0.50520894
TCP1	chr1	7590140	7601795	527865.75286	chr1	7527865	7528661	0.505368
GTF3A	chr11	4857023	4869272	241105.5241	chr11	5241105	5241689	0.50543436
NPC2	chr7	97730516	97740331	314711.97318	chr7	97314711	97318711	0.50556104
WARS1	chr7	121212981	121238113	706792.12171	chr7	121706792	121711117	0.5055859
ILK	chr9	3145608	3159858	486681.34906	chr9	3486681	3490681	0.50570011
NIPAL3	chr6	82111626	82167383	533691.82534	chr6	82533691	82534381	0.50570957
ANKRD40CL	chr12	27038840	27043741	745423.2674	chr12	26745423	26746618	0.50576719
G6PD	chrX	125029150	125041040	129837.12513	chrX	125129837	125130618	0.50582588
FBXO31	chr6	1949276	1995462	565705.15725	chr6	1565705	1572513	0.50584116
CYP2E1	chr14	141690426	141736817	189763.1411	chr14	141189763	141193763	0.50585596
PHRF1	chr2	340528	371692	212170.21617	chr2	212170	216170	0.50590849
ENSSSCG00000031249	chr13	207493659	207499867	375502.2073	chr13	207375502	207379502	0.50610465
PSENEN	chr6	45171825	45174102	344637.45348	chr6	45344637	45348637	0.50611296
TRAF3IP1	chr15	137863609	137912477	707322.1377	chr15	137707322	137711322	0.50628195
ATP6V0B	chr6	167316947	167320212	943409.16694	chr6	166943409	166947409	0.50629958
SKA3	chr11	1302886	1320981	836951.8409	chr11	836951	840951	0.50630869
TUBGCP2	chr14	141233856	141253359	396639.1414	chr14	141396639	141400649	0.50632115
RPP40	chr7	2987271	3005271	720009.27279	chr7	2720009	2727929	0.50634095
MVP	chr3	18057177	18081155	803416.17807	chr3	17803416	17807526	0.50639999
MYL6	chr5	21559029	21562413	663252.21667	chr5	21663252	21667252	0.50641257
TRAF3IP1	chr15	137863609	137912477	032918.1380	chr15	138032918	138036878	0.50642963
TUBB2A	chr7	1910269	1914761	325359.23277	chr7	2325359	2327729	0.50662191
ZCCHC17	chr6	87879830	87942014	049896.88059	chr6	88049896	88059876	0.50678635
ACO2	chr5	7008719	7071025	576101.65771	chr5	6576101	6577139	0.50679911
SRRT	chr3	8717785	8731201	492530.84965	chr3	8492530	8496530	0.50698296
ENSSSCG00000036988	chr7	1980292	1988727	618809.16227	chr7	1618809	1622759	0.5071619
AFAP1L1	chr2	150445126	150510002	88203.15059	chr2	150588203	150592203	0.50718958
CARS2	chr11	77229971	77265272	586457.7758	chr11	77586457	77587718	0.50733521
NAXD	chr11	77210228	77230680	710558.7671	chr11	76710558	76711935	0.50738322
ENSSSCG00000062841	chr15	115446825	115450516	349501.1153	chr15	115349501	115353501	0.50776866
RNF220	chr6	166666138	166910646	943423.16694	chr6	166943423	166947423	0.50803725
PSMG3	chr3	1052191	1054688	312596.13169	chr3	1312596	1316956	0.50809838
PDZD11	chrX	56312754	56319405	132853.56136	chrX	56132853	56136853	0.50830571
ENSSSCG00000061173	chrX	110774840	110792950	959577.11096	chrX	110959577	110960315	0.50838345
AP1S1	chr3	8881107	8887566	836970.88376	chr3	8836970	8837652	0.50839745
NDUFA7	chr2	70954896	70963322	110600.71114	chr2	71110600	71114230	0.5085734
GET1	chr13	202962664	202979902	234243.2032	chr13	203234243	203238243	0.50863114
HARS2	chr2	142401328	142409619	547516.14255	chr2	142547516	142551516	0.50873003
TIMM17B	chrX	42970108	42975955	680269.42684	chrX	42680269	42684269	0.50880048
CENPS	chr6	70701694	70711717	017576.71021	chr6	71017576	71021076	0.50882847
DDX56	chr18	50705632	50715033	750846.5075	chr18	50750846	50751473	0.50888834
GLRX3	chr14	139072575	139111249	121536.1391	chr14	139121536	139122737	0.50898985
LIG1	chr6	53620483	53686562	428926.53431	chr6	53428926	53431616	0.5092411
PTPRS	chr2	73574854	73680686	042210.74044	chr2	74042210	74044130	0.50928474
GLP2R	chr12	54680057	54738102	553579.5455	chr12	54553579	54559169	0.50936787

HIRIP3	chr3	18198807	18202089	809718.17813	chr3	17809718	17813718	0.50951693
SELENOF	chr4	129258944	129287720	841529.12885	chr4	128841529	128852539	0.50959297
MYBBP1A	chr12	50491044	50506050	0728138.5072	chr12	50728138	50729135	0.50970756
ENSSSCG00000008056	chr3	39532921	39538681	117229.39118	chr3	39117229	39118770	0.50974388
LYRM4	chr7	3070470	3192922	053192.30571	chr7	3053192	3057192	0.50978655
GAS7	chr12	54752507	54973515	676329.5467	chr12	54676329	54677869	0.50994078
LIG1	chr6	53620483	53686562	070265.54074	chr6	54070265	54074265	0.50998012
CMC4	chrX	125372941	125376999	903258.12490	chrX	124903258	124909988	0.5103378
NARF	chr12	632363	651817	145208.1492	chr12	145208	149208	0.51041531
ENSSSCG00000052263	chr4	98779348	98794830	803588.98807	chr4	98803588	98807588	0.51046384
PPP1R7	chr15	139917257	139941251	479553.1394	chr15	139479553	139483165	0.51050545
RAB5C	chr12	20613996	20636073	957709.2096	chr12	20957709	20961019	0.51065012
ENO1	chr6	69385879	69401151	723206.69728	chr6	69723206	69728846	0.5106923
NDUFA10	chr15	138986421	139031897	477888.1394	chr15	139477888	139482538	0.51093089
TUBA4A	chr15	121288957	121294853	560936.1215	chr15	121560936	121562466	0.51104698
PYGB	chr17	30940452	30995691	801572.3080	chr17	30801572	30807492	0.5114659
SEPTIN8	chr2	135077170	135104471	147046.13515	chr2	135147046	135151046	0.51155086
BCAP31	chrX	124457001	124484743	423872.12442	chrX	124423872	124427872	0.51158641
SYT5	chr6	59381642	59389298	950381.58954	chr6	58950381	58954381	0.51159494
ENSSSCG00000035728	chr12	1095096	1099180	162187.1166	chr12	1162187	1166187	0.51177015
ZNRD2	chr2	6683057	6684494	526990.65343	chr2	6526990	6534360	0.5118706
ENSSSCG00000057427	chr18	2568274	2595510	949925.2952	chr18	2949925	2952915	0.51198271
NDUFA10	chr15	138986421	139031897	497388.1385	chr15	138497388	138508728	0.5120235
MRPL36	chr16	79001549	79004358	436079.7944	chr16	79436079	79447039	0.51204008
EIPR1	chr3	131360883	131435579	304376.13131	chr3	131304376	131310136	0.51210564
ARL3	chr14	113665270	113707591	597569.1136	chr14	113597569	113600209	0.51211669
ENSSSCG00000032916	chr1	2541383	2552858	044406.20479	chr1	2044406	2047956	0.51225485
MRPL36	chr16	79001549	79004358	206448.7920	chr16	79206448	79209364	0.51226536
ABHD17A	chr2	76658068	76666552	276671.76278	chr2	76276671	76278423	0.51230706
BRD9	chr16	79473745	79492635	792309.7979	chr16	79792309	79794329	0.51235785
SCPEP1	chr12	33265562	33298142	816761.3281	chr12	32816761	32817407	0.51253477
RPP40	chr7	2987271	3005271	566999.25682	chr7	2566999	2568269	0.51268833
FANK1	chr14	135197450	135302516	863359.1348	chr14	134863359	134867349	0.51295349
ENSSSCG00000017913	chr12	52095888	52098231	975989.5197	chr12	51975989	51978009	0.51309724
TMEM176B	chr18	6342611	6348867	873195.5876	chr18	5873195	5876335	0.51310615
PIH1D1	chr6	54524172	54535249	346936.54350	chr6	54346936	54350546	0.51316769
RPS11	chr6	54577667	54580846	346936.54350	chr6	54346936	54350546	0.51319936
NRAP	chr14	123926927	124018440	871319.1238	chr14	123871319	123873859	0.5132491
FIGNL1	chr9	136484216	136488250	627192.13663	chr9	136627192	136633732	0.51335565
NLRP8	chr6	60350622	60370529	594958.60596	chr6	60594958	60596272	0.51335783
CTPS1	chr6	170201751	170233016	242316.17024	chr6	170242316	170248076	0.51336712
PFKP	chr10	67022655	67082449	844399.6684	chr10	66844399	66847999	0.51341766
STX5	chr2	8937778	8968932	293700.92984	chr2	9293700	9298410	0.51357003
RPS16	chr6	48085678	48088423	969275.47973	chr6	47969275	47973275	0.51367911
MED8	chr6	167861373	167869411	435586.16743	chr6	167435586	167437366	0.51370804
MPPED1	chr5	5417833	5486726	176438.51815	chr5	5176438	5181598	0.51373803
TBC1D10B	chr3	17944629	17956069	021096.18025	chr3	18021096	18025096	0.51374393
HADHA	chr3	112752865	112797733	698376.11270	chr3	112698376	112701986	0.51379208
DAD1	chr7	76432051	76457192	003612.76007	chr7	76003612	76007612	0.51382328
ZFTRAF1	chr4	335301	346534	377258.37834	chr4	377258	378347	0.51386505
ENSSSCG00000044567	chr17	31217462	31238052	516319.3151	chr17	31516319	31518091	0.51401734
TUBGCP2	chr14	141233856	141253359	391083.1413	chr14	141391083	141395083	0.51406757
MRPL28	chr3	41329928	41334451	248196.41252	chr3	41248196	41252396	0.5142642
GPR32	chr6	55519957	55525919	717729.55721	chr6	55717729	55721729	0.51435754
ENSSSCG00000035997	chrX	124926772	124929791	717923.12472	chrX	124717923	124727079	0.51436274
RPS7	chr3	131258722	131263256	623366.13162	chr3	131623366	131629856	0.51436863
TIMM44	chr2	71271805	71287817	951708.70955	chr2	70951708	70955708	0.51455845
PMF1	chr4	93810507	93837652	460308.93464	chr4	93460308	93464308	0.51458671
ENSSSCG00000047692	chr16	55617518	55623070	458699.5546	chr16	55458699	55460509	0.51468387
NEDD8	chr7	75070161	75085899	957429.74959	chr7	74957429	74959919	0.51475079
G6PD	chrX	125029150	125041040	148743.12515	chrX	125148743	125152743	0.51489119
ENSSSCG00000044567	chr17	31217462	31238052	801572.3080	chr17	30801572	30807492	0.51497821
PTPRS	chr2	73574854	73680686	485267.73486	chr2	73485267	73486251	0.51499488

ATP6AP1	chrX	124952500	124960343	123038.12512	chrX	125123038	125124988	0.51513724
ENSSSCG00000051526	chrX	34521562	34522768	296220.34296	chrX	34296220	34296757	0.51516774
ACTR1A	chr14	113480196	113498740	597569.1136	chr14	113597569	113600209	0.51523478
MRPL36	chr16	79001549	79004358	896319.7890	chr16	78896319	78901689	0.51526249
ENSSSCG00000049777	chr7	121082820	121091813	897229.12090	chr7	120897229	120900569	0.5152775
MRPS24	chr18	48825782	48830609	708347.4871	chr18	48708347	48712347	0.51540298
PSMC3	chr2	15166306	15193047	164306.15168	chr2	15164306	15168306	0.51541941
TRAF3IP1	chr15	137863609	137912477	7442855.1374	chr15	137442855	137446855	0.51566455
GPR32	chr6	55519957	55525919	781617.55782	chr6	55781617	55782322	0.51589277
MAF1	chr4	597472	600447	441311.44531	chr4	441311	445311	0.51589792
TCP1	chr1	7590140	7601795	525861.75298	chr1	7525861	7529861	0.51591619
STK25	chr15	140144336	140154366	9764414.1397	chr15	139764414	139768414	0.51615608
PCDH12	chr2	143550849	143565033	317870.14332	chr2	143317870	143325360	0.51616497
MEI1	chr5	6800961	6871625	576101.65771	chr5	6576101	6577139	0.51629113
STX8	chr12	54296995	54544646	3904511.5390	chr12	53904511	53908511	0.51632745
BRCC3	chrX	125383414	125439082	903258.12490	chrX	124903258	124909988	0.51660714
RPS20	chr4	75762209	75769955	739459.75740	chr4	75739459	75740629	0.51686651
ERI3	chr6	166957316	167082631	829006.16683	chr6	166829006	166831646	0.51697639
BCKDHA	chr6	49381967	49398743	708933.49712	chr6	49708933	49712933	0.51713243
MRPL36	chr16	79001549	79004358	9036641.7904	chr16	79036641	79043345	0.51716344
TK1	chr12	3786031	3795917	3800240.3804	chr12	3800240	3804240	0.51716758
ENSSSCG00000036812	chr12	61077631	61186002	0918169.6092	chr12	60918169	60920239	0.51716852
COA4	chr9	8278311	8281153	358382.83636	chr9	8358382	8363692	0.51719055
TUBA4A	chr15	121288957	121294853	1428658.1214	chr15	121428658	121429211	0.51721807
NAA10	chrX	124658158	124662702	148743.12515	chrX	125148743	125152743	0.51722388
NPAS1	chr6	52845207	52875240	634601.52635	chr6	52634601	52635934	0.51723848
SDHA	chr16	79834044	79862524	9552127.7955	chr16	79552127	79553615	0.51733153
SCAMP4	chr2	76615000	76634264	277430.76279	chr2	76277430	76279160	0.51739809
ENSSSCG00000035728	chr12	1095096	1099180	1396069.1402	chr12	1396069	1402069	0.5174014
IDH3G	chrX	124528585	124537575	826225.12483	chrX	124826225	124830225	0.51744946
HSPBP1	chr6	59452935	59464533	233436.59233	chr6	59233436	59233978	0.5174505
WRAP73	chr6	65155209	65167079	316926.65322	chr6	65316926	65322026	0.51747686
ORAI1	chr14	31011644	31027993	0522232.3052	chr14	30522232	30526232	0.51764001
CCNL2	chr6	63659054	63668047	991306.63993	chr6	63991306	63993056	0.5177015
RPS14	chr2	151430049	151433856	981810.15098	chr2	150981810	150986810	0.51771337
MTM1	chrX	122286916	122379299	392921.12239	chrX	122392921	122396921	0.51775255
WARS1	chr7	121212981	121238113	707640.12171	chr7	121707640	121711640	0.51813455
ENSSSCG00000042487	chr7	58998922	59008157	068728.59072	chr7	59068728	59072728	0.51817739
LRCH4	chr3	8499285	8511262	688988.86902	chr3	8688988	8690214	0.51837661
SPECC1	chr12	59466791	59654192	9895496.5989	chr12	59895496	59899496	0.51840878
GALNTL5	chr18	5353769	5433814	4977436.4981	chr18	4977436	4981436	0.5185821
ENSSSCG00000024070	chr18	6166918	6171153	6312474.6314	chr18	6312474	6314041	0.51863555
PDCD6	chr16	79818958	79835746	9638839.7964	chr16	79638839	79642899	0.51866894
CBX8	chr12	2562374	2565780	2095212.2099	chr12	2095212	2099139	0.51879534
RDH13	chr6	59274387	59292765	713636.59717	chr6	59713636	59717636	0.51881418
ENSSSCG00000052671	chr6	54564793	54568340	761096.54766	chr6	54761096	54766246	0.51885425
UBXN6	chr2	74357517	74378592	040989.74044	chr2	74040989	74044989	0.51889691
NCF2	chr9	124776534	124812923	492652.12449	chr9	124492652	124494632	0.51895452
RPS4X	chrX	58149318	58155157	182285.58184	chrX	58182285	58184738	0.51900865
POP7	chr3	8605018	8610218	492530.84965	chr3	8492530	8496530	0.51904072
BRD9	chr16	79473745	79492635	9898299.7990	chr16	79898299	79904919	0.51905163
STK10	chr16	52003887	52140367	1831879.5183	chr16	51831879	51834749	0.51907348
MAF1	chr4	597472	600447	785393.79847	chr4	785393	798477	0.519091
SDHA	chr16	79834044	79862524	9605069.7960	chr16	79605069	79606470	0.51910301
NOP14	chr8	1716826	1737902	223429.12274	chr8	1223429	1227429	0.5191605
IAH1	chr3	126849443	126865734	639236.12664	chr3	126639236	126644176	0.51923908
BIRC5	chr12	3747279	3755215	3867743.3868	chr12	3867743	3868767	0.51924806
MEPCE	chr3	8357289	8364661	492530.84965	chr3	8492530	8496530	0.51928547
C6orf52	chr7	7430542	7437789	795369.77985	chr7	7795369	7798579	0.51942831
DBNL	chr18	48713340	48725697	8737495.4874	chr18	48737495	48741355	0.51950556
RIMS3	chr6	170506426	170546933	401157.17040	chr6	170401157	170403038	0.51974957
AP1S1	chr3	8881107	8887566	832274.88362	chr3	8832274	8836274	0.51977185
GADD45GIP1	chr2	66087453	66090710	910372.65911	chr2	65910372	65911524	0.51977657

TIMP2	chr12	3254493	3300834	166999.3170	chr12	3166999	3170069	0.51981153
FANK1	chr14	135197450	135302516	822589.1348	chr14	134822589	134825479	0.51982803
ZMAT2	chr2	142411089	142419137	906041.1429	chr2	142906041	142910041	0.51989381
RPTOR	chr12	1709128	1991574	141178.2144	chr12	2141178	2144859	0.52002705
DUSP28	chr15	139537326	139539438	477888.1394	chr15	139477888	139482538	0.52002966
GMPPA	chr15	121507564	121515300	560936.1215	chr15	121560936	121562466	0.52014159
GLP2R	chr12	54680057	54738102	207189.5421	chr12	54207189	54212279	0.5203872
COPS9	chr15	139260478	139264211	705558.1397	chr15	139705558	139712278	0.52043602
ENSSSCG00000025928	chr6	53974309	53978544	346936.5435	chr6	54346936	54350546	0.52059584
ALG8	chr9	12497721	12532307	477907.1248	chr9	12477907	12481907	0.52059654
FTL	chr6	54231172	54232750	461759.5446	chr6	54461759	54465759	0.52062525
KXD1	chr2	59242310	59247713	406904.5941	chr2	59406904	59410904	0.52067443
DPCD	chr14	112617398	112637913	079981.1130	chr14	113079981	113083981	0.52071123
ENSSSCG00000038506	chr9	135025443	135078913	787102.1347	chr9	134787102	134794662	0.52072361
OXA1L	chr7	76230889	76266429	003612.7600	chr7	76003612	76007612	0.52073889
ENSSSCG00000056015	chr6	61202803	61213758	473531.6147	chr6	61473531	61474411	0.52080201
ENSSSCG00000052671	chr6	54564793	54568340	070265.5407	chr6	54070265	54074265	0.52113399
TUBGCP2	chr14	141233856	141253359	746869.1407	chr14	140746869	140750169	0.52120259
STX5	chr2	8937778	8968932	295283.9299	chr2	9295283	9299283	0.52127304
CD63	chr5	21172283	21176232	622762.2162	chr5	21622762	21626762	0.52136598
ENSSSCG00000032916	chr1	2541383	2552858	935566.2938	chr1	2935566	2938576	0.52147688
RDH13	chr6	59274387	59292765	733286.5973	chr6	59733286	59736796	0.52158185
PI4KA	chr14	50390410	50491944	810984.5081	chr14	50810984	50814984	0.52177215
TUBB2A	chr7	1910269	1914761	618809.1622	chr7	1618809	1622759	0.52197834
NHEJ1	chr15	121100628	121190062	923499.1209	chr15	120923499	120927499	0.52208267
ECHS1	chr14	141339364	141348994	278460.1412	chr14	141278460	141282460	0.52208666
PIH1D1	chr6	54524172	54535249	070265.5407	chr6	54070265	54074265	0.52208954
RECQL5	chr12	5732348	5770133	025019.6028	chr12	6025019	6028749	0.52217004
UBE2J2	chr6	63555488	63568017	487976.6350	chr6	63487976	63500036	0.52232789
C11orf98	chr2	9084256	9090037	293700.9298	chr2	9293700	9298410	0.52249004
C19orf54	chr6	48962838	48970959	219763.4922	chr6	49219763	49223763	0.52254731
PRPSAP1	chr12	5144069	5174382	914069.4923	chr12	4914069	4923019	0.52256184
MRPL36	chr16	79001549	79004358	729349.7873	chr16	78729349	78736299	0.52266332
RPL28	chr6	59556687	59562147	703779.5970	chr6	59703779	59705229	0.52275536
ENSSSCG00000013613	chr2	70193730	70199975	656247.7066	chr2	70656247	70660247	0.52287213
ZNRF1	chr6	12555664	12668804	550006.1255	chr6	12550006	12551126	0.5228789
NCLN	chr2	75369955	75390545	396091.7539	chr2	75396091	75397399	0.52304049
ENSSSCG00000015632	chr9	136893356	137056101	634302.1366	chr9	136634302	136640792	0.52317741
SAT2	chr12	52911725	52913363	006559.5300	chr12	53006559	53008829	0.5232029
WARS1	chr7	121212981	121238113	706089.1217	chr7	121706089	121709769	0.5232042
ARHGDIA	chr12	1113379	1119315	304812.1306	chr12	1304812	1306458	0.52333909
WARS1	chr7	121212981	121238113	684359.1216	chr7	121684359	121686349	0.5233768
NFYC	chr6	170413638	170485840	193186.1701	chr6	170193186	170197086	0.52342571
EBNA1BP2	chr6	168049758	168061013	922536.1679	chr6	167922536	167926936	0.52347481
ENSSSCG00000024070	chr18	6166918	6171153	310855.6315	chr18	6310855	6315595	0.52351695
NFYC	chr6	170413638	170485840	192822.1701	chr6	170192822	170196822	0.52354745
PRDX5	chr2	7798670	7802952	286187.8288	chr2	8286187	8288147	0.52365841
RPS15A	chr3	26776780	26784124	464566.2646	chr3	26464566	26466636	0.52371736
BRD9	chr16	79473745	79492635	206448.7920	chr16	79206448	79209364	0.52376007
PWP2	chr13	207015125	207031879	843554.2068	chr13	206843554	206847737	0.52380474
AURKAIP1	chr6	63656119	63657472	991306.6399	chr6	63991306	63993056	0.52389039
PRRC2B	chr1	271336818	271430916	678866.2716	chr1	271678866	271684276	0.52439351
MRPS24	chr18	48825782	48830609	989585.4899	chr18	48989585	48995365	0.52440963
LANCL2	chr18	48550944	48593455	989585.4899	chr18	48989585	48995365	0.52441312
DBNL	chr18	48713340	48725697	708347.4871	chr18	48708347	48712347	0.5245005
ATP6V0B	chr6	167316947	167320212	796056.1677	chr6	167796056	167799916	0.5248027
ENSSSCG00000035997	chrX	124926772	124929791	636523.1246	chrX	124636523	124640523	0.52501132
ZFTRAF1	chr4	335301	346534	785393.7984	chr4	785393	798477	0.52510368
EEF1AKMT1	chr11	1067061	1082591	826930.8289	chr11	826930	828910	0.52516538
BRD9	chr16	79473745	79492635	767469.7977	chr16	79767469	79770829	0.52544745
SERPINB1	chr7	1684215	1693299	727489.1732	chr7	1727489	1732119	0.52572626
ZNF212	chr18	55528138	55541841	612730.5561	chr18	55612730	55616730	0.52579488
NDUFA7	chr2	70954896	70963322	658392.7065	chr2	70658392	70659484	0.52583317

SLC16A5	chr12	6185528	6200001	336839.6340	chr12	6336839	6340259	0.52593189
ING1	chr11	77270320	77277068	898450.7690	chr11	76898450	76905200	0.52605818
UBE2J2	chr6	63555488	63568017	991306.6399	chr6	63991306	63993056	0.52640759
C11orf98	chr2	9084256	9090037	195750.9198	chr2	9195750	9198850	0.52642381
RPP40	chr7	2987271	3005271	616409.2622	chr7	2616409	2622659	0.52647051
RANGRF	chr12	53485265	53486713	006559.5300	chr12	53006559	53008829	0.52674876
LSM2	chr7	23900395	23908490	086873.2409	chr7	24086873	24090873	0.52676878
RPP40	chr7	2987271	3005271	053192.3057	chr7	3053192	3057192	0.52680743
SKI	chr6	64188236	64242560	637886.6464	chr6	64637886	64641676	0.52699707
NXF1	chr2	8969581	8981984	293700.9298	chr2	9293700	9298410	0.5270416
GUCA1A	chr7	37282700	37296717	828394.3683	chr7	36828394	36832394	0.5272499
COTL1	chr6	4099414	4147469	710866.3717	chr6	3710866	3717166	0.52741897
PTCH2	chr6	166495763	166512648	829006.1668	chr6	166829006	166831646	0.52752519
ZDHHC16	chr14	108842966	108852789	884753.1088	chr14	108884753	108888753	0.5276122
ATP5MC2	chr5	18871026	18879609	149084.1915	chr5	19149084	19153084	0.52761379
ENSSSCG00000036988	chr7	1980292	1988727	229293.2233	chr7	2229293	2233293	0.52767183
CA5A	chr6	1506042	1538777	409866.1413	chr6	1409866	1413016	0.52771303
LYRM4	chr7	3070470	3192922	102006.3103	chr7	3102006	3103105	0.52799365
THAP7	chr14	50591410	50594105	810984.5081	chr14	50810984	50814984	0.52807641
PSMD13	chr2	55108	74829	403950.4053	chr2	403950	405332	0.52808079
ENSSSCG00000061886	chr11	66802860	66809872	330220.6633	chr11	66330220	66335220	0.52826769
MRPL10	chr12	24075632	24083052	729110.2373	chr12	23729110	23733110	0.52848389
ATP6AP1	chrX	124952500	124960343	271488.1252	chrX	125271488	125274598	0.52868592
LRRC47	chr6	65260793	65271729	025442.6502	chr6	65025442	65029442	0.52870864
GLRX3	chr14	139072575	139111249	120929.1391	chr14	139120929	139123949	0.52882812
CCDC136	chr18	19793781	19824453	165695.2016	chr18	20165695	20169695	0.52884597
DGAT1	chr4	452662	466684	204188.2081	chr4	204188	208188	0.52897145
PFKP	chr10	67022655	67082449	819339.6682	chr10	66819339	66823389	0.52903392
KCTD2	chr12	6226376	6241318	214528.6215	chr12	6214528	6215144	0.52917708
NELFE	chr7	24040525	24047025	086873.2409	chr7	24086873	24090873	0.52967701
YJU2	chr2	74509739	74528300	040989.7404	chr2	74040989	74044989	0.52971301
PRR14	chr3	17745892	17751099	613617.1761	chr3	17613617	17614750	0.52983872
ZNF410	chr7	97195975	97243614	316238.9731	chr7	97316238	97316776	0.52992871
SUV39H1	chrX	42797828	42811461	680269.4268	chrX	42680269	42684269	0.52998238
ATG2A	chr2	7262031	7282347	895080.6899	chr2	6895080	6899700	0.53000416
PLIN5	chr2	74300619	74314315	042210.7404	chr2	74042210	74044130	0.53049926
ENSSSCG00000030908	chrX	110622132	110658827	832171.1108	chrX	110832171	110836171	0.53052897
SPECC1	chr12	59466791	59654192	454279.5945	chr12	59454279	59457869	0.53058304
ENSSSCG00000063021	chr12	10292427	10297310	353259.1035	chr12	10353259	10356689	0.53063389
OXA1L	chr7	76230889	76266429	005976.7600	chr7	76005976	76006524	0.53066505
BRD9	chr16	79473745	79492635	037029.7904	chr16	79037029	79040119	0.53089968
SLC38A5	chrX	42590607	42601502	955808.4295	chrX	42955808	42959908	0.53090434
MOSPD1	chrX	110693552	110720762	961228.1109	chrX	110961228	110965038	0.53097298
PDE6D	chr15	132375743	132426683	220868.1322	chr15	132220868	132223458	0.53112209
ENSSSCG00000018046	chr12	59977028	60019814	531479.5953	chr12	59531479	59536229	0.53124279
MED10	chr16	75517023	75524758	908265.7590	chr16	75908265	75908603	0.53128086
TRAF3IP1	chr15	137863609	137912477	398238.1374	chr15	137398238	137401498	0.53130852
POLM	chr18	51079503	51088781	728428.5072	chr18	50728428	50729797	0.53154847
PCDHGA4	chr2	142993554	143156556	906041.1429	chr2	142906041	142910041	0.53164704
ENSSSCG00000002995	chr6	49002854	49010040	659926.4866	chr6	48659926	48665966	0.53186809
RPS20	chr4	75762209	75769955	926549.7593	chr4	75926549	75930959	0.5318856
SLC35A4	chr2	142323341	142325335	804530.1428	chr2	142804530	142808530	0.5319672
DBNL	chr18	48713340	48725697	984125.4898	chr18	48984125	48986935	0.53205397
DAD1	chr7	76432051	76457192	012901.7601	chr7	76012901	76016901	0.53209338
DEGS2	chr7	121058105	121076328	897229.1209	chr7	120897229	120900569	0.53217571
HSPBP1	chr6	59452935	59464533	733286.5973	chr6	59733286	59736796	0.53232239
DBNL	chr18	48713340	48725697	189535.4919	chr18	49189535	49195785	0.53232668
UTP25	chr9	133105063	133141217	306452.1333	chr9	133306452	133308672	0.53236994
BRD9	chr16	79473745	79492635	436079.7944	chr16	79436079	79447039	0.5324678
HSD17B10	chrX	46218548	46220883	223748.4622	chrX	46223748	46227988	0.53258344
SMARCD2	chr12	15091729	15101899	380779.1538	chr12	15380779	15384509	0.53259797
HDLBP	chr15	139957715	140022302	858888.1398	chr15	139858888	139868418	0.53264763
HADHA	chr3	112752865	112797733	042836.1130	chr3	113042836	113046836	0.53265238

C8orf76	chr4	16137193	16158255	527039.16532	chr4	16527039	16532019	0.53287522
RAB4B	chr6	48989172	48999608	219763.49223	chr6	49219763	49223763	0.53292992
CARS2	chr11	77229971	77265272	880640.7688	chr11	76880640	76886850	0.53299832
PLEKHO1	chr4	98923996	98934189	803588.98807	chr4	98803588	98807588	0.53303962
RPS5	chr6	62967994	62974350	444151.63445	chr6	63444151	63445363	0.533063
HARS2	chr2	142401328	142409619	483896.14248	chr2	142483896	142487896	0.53309271
CYBC1	chr12	656347	662513	.448428.4524	chr12	448428	452428	0.53318831
CSNK1D	chr12	768865	796595	.453099.4609	chr12	453099	460989	0.53323847
COG1	chr12	7743274	7760504	178209.8181	chr12	8178209	8181929	0.53325537
ASB6	chr1	269828985	269834271	259836.27026	chr1	270259836	270264736	0.53332795
SDHA	chr16	79834044	79862524	538042.7953	chr16	79538042	79539074	0.53336396
SRM	chr6	71246876	71253508	997366.71001	chr6	70997366	71001106	0.53336484
HMG20B	chr2	75093075	75098536	729195.74730	chr2	74729195	74730347	0.53338253
RECQL5	chr12	5732348	5770133	214528.6215	chr12	6214528	6215144	0.53379775
CYC1	chr4	606516	608996	782159.79387	chr4	782159	793879	0.53391011
TBCD	chr12	329714	449556	.453099.4609	chr12	453099	460989	0.53399912
MRPL20	chr6	63670755	63675839	679085.63683	chr6	63679085	63683085	0.53406859
DEF8	chr6	140908	160692	179225.18322	chr6	179225	183225	0.53410557
NSDHL	chrX	123906199	123929117	025315.12402	chrX	124025315	124028273	0.5343479
VKORC1	chr3	17386245	17389905	809718.17813	chr3	17809718	17813718	0.53436439
CARS2	chr11	77229971	77265272	887510.7689	chr11	76887510	76890510	0.53450439
ENSSSCG00000036988	chr7	1980292	1988727	417517.24215	chr7	2417517	2421517	0.53452282
SDHA	chr16	79834044	79862524	495219.7949	chr16	79495219	79497289	0.53459167
SHANK2	chr2	2864331	3015314	705840.27134	chr2	2705840	2713480	0.53461656
XAB2	chr2	71521081	71530929	110600.71114	chr2	71110600	71114230	0.53500607
BRMS1	chr2	6046098	6053667	456140.64608	chr2	6456140	6460880	0.5350433
ENSSSCG00000061352	chr7	85598675	85602661	919594.85923	chr7	85919594	85923594	0.53511015
FIGNL1	chr9	136484216	136488250	649752.13665	chr9	136649752	136654312	0.53557732
MED8	chr6	167861373	167869411	796056.16779	chr6	167796056	167799916	0.53566685
ATP5ME	chr8	110605	113510	.79738.81698	chr8	79738	81698	0.535678
MRPL36	chr16	79001549	79004358	888489.7889	chr16	78888489	78894099	0.53568679
MMD2	chr3	3729531	3775205	231956.32449	chr3	3231956	3244946	0.53579904
MRPL36	chr16	79001549	79004358	539179.7854	chr16	78539179	78544609	0.535807
AURKAIP1	chr6	63656119	63657472	453866.63457	chr6	63453866	63457336	0.53589457
NSDHL	chrX	123906199	123929117	024145.12402	chrX	124024145	124028145	0.53605231
GPS1	chr12	951867	957080	.304812.1306	chr12	1304812	1306458	0.53628037
TUBA4A	chr15	121288957	121294853	.425642.1214	chr15	121425642	121429642	0.53632002
FCGR1A	chr4	99233611	99242586	803588.98807	chr4	98803588	98807588	0.53634347
JOSD1	chr5	9321640	9335041	545177.95491	chr5	9545177	9549177	0.53644404
CCNL2	chr6	63659054	63668047	453866.63457	chr6	63453866	63457336	0.53674096
GLRX3	chr14	139072575	139111249	444509.1394	chr14	139444509	139447079	0.53676653
PCCB	chr13	77217428	77316568	943395.7694	chr13	76943395	76947395	0.53676791
C1orf174	chr6	65323756	65333886	025442.65029	chr6	65025442	65029442	0.53680423
ENSSSCG00000051057	chr4	129797110	129860442	319459.12932	chr4	129319459	129323069	0.53685647
MYL6	chr5	21559029	21562413	622762.21626	chr5	21622762	21626762	0.53696559
ENSSSCG00000032573	chr4	75636278	75646153	737402.75741	chr4	75737402	75741402	0.53704743
BRD9	chr16	79473745	79492635	295297.7930	chr16	79295297	79302303	0.53705833
TUBGCP2	chr14	141233856	141253359	812715.1408	chr14	140812715	140816165	0.537164
FAM50A	chrX	124967510	124973483	148743.12515	chrX	125148743	125152743	0.53726157
NIT1	chr4	89320712	89327066	972183.88973	chr4	88972183	88973295	0.53737082
CYC1	chr4	606516	608996	441311.44531	chr4	441311	445311	0.53742293
BUD23	chr3	10960092	10973082	052516.11053	chr3	11052516	11053085	0.53745427
TUBGCP2	chr14	141233856	141253359	.151169.1411	chr14	141151169	141169699	0.53746361
DFFA	chr6	70724590	70736236	017576.71021	chr6	71017576	71021076	0.53748173
FXR2	chr12	52882870	52903450	006559.5300	chr12	53006559	53008829	0.53759659
VPS28	chr4	362912	370588	441311.44531	chr4	441311	445311	0.53771373
PSMA7	chr17	61566373	61572438	.208112.6121	chr17	61208112	61211942	0.53773369
MAPK3	chr3	18291445	18299567	812944.17813	chr3	17812944	17813910	0.53774549
POP4	chr6	39734559	39760953	129356.40134	chr6	40129356	40134356	0.53780331
SLC35B1	chr12	25881256	25888293	153561.2615	chr12	26153561	26157561	0.53781795
SLC41A3	chr7	53672130	53748727	285609.53288	chr7	53285609	53288709	0.53785134
ENSSSCG00000012886	chr2	4682315	4695942	256940.42596	chr2	4256940	4259620	0.53790284
SLC7A7	chr7	76198256	76235402	003612.76007	chr7	76003612	76007612	0.53797299

FUS	chr3	17314332	17326637	303416.17807	chr3	17803416	17807526	0.53799843
PUF60	chr4	812131	824318	377258.37834	chr4	377258	378347	0.5380596
OTUD5	chrX	43000231	43029468	651789.42655	chrX	42651789	42655789	0.53818186
JPT1	chr12	6139428	6158452	6337182.6338	chr12	6337182	6338139	0.53822821
STAT5A	chr12	20474227	20499138	0166659.2016	chr12	20166659	20168579	0.53829482
DPEP1	chr6	361197	376616	180690.18282	chr6	180690	182821	0.53830504
NDUFA10	chr15	138986421	139031897	0052628.1390	chr15	139052628	139057078	0.53836647
NDUFS6	chr16	78993913	79000006	3729349.7873	chr16	78729349	78736299	0.53846001
USP11	chrX	41851602	41866355	174600.42175	chrX	42174600	42175367	0.53872469
ZP3	chr3	9934761	9944941	460014.94610	chr3	9460014	9461065	0.53872605
TCL1B	chr7	116912976	116917934	469559.11647	chr7	116469559	116474519	0.53879973
PDCD6	chr16	79818958	79835746	9550266.7955	chr16	79550266	79551345	0.53884908
ATP6V0E2	chr18	55877504	55882204	5614429.5561	chr18	55614429	55616784	0.5388568
MRM1	chr12	38124400	38144083	3059927.3806	chr12	38059927	38063927	0.53891321
ENSSSCG00000056015	chr6	61202803	61213758	857817.60859	chr6	60857817	60859961	0.53913922
C1orf54	chr4	98805588	98814563	803588.98807	chr4	98803588	98807588	0.5392271
BRD9	chr16	79473745	79492635	9741769.7974	chr16	79741769	79744539	0.53929051
ENSSSCG00000062841	chr15	115446825	115450516	5351188.1153	chr15	115351188	115353598	0.53955518
ENSSSCG00000045223	chr6	166945409	166957165	435586.16743	chr6	167435586	167437366	0.53995638
PALLD	chr14	20678452	21020125	1009729.2101	chr14	21009729	21013389	0.54026638
ASB11	chrX	11842855	11868793	091242.12095	chrX	12091242	12095242	0.54063687
GLRX3	chr14	139072575	139111249	8976219.1389	chr14	138976219	138981529	0.54070636
ENSSSCG00000032959	chrX	125001675	125002920	180305.12518	chrX	125180305	125182027	0.54083612
FANCA	chr6	253207	300152	179225.18322	chr6	179225	183225	0.54086445
DNAJB6	chr18	1444739	1499887	1095905.1098	chr18	1095905	1098925	0.54099538
SHANK2	chr2	2864331	3015314	723860.27334	chr2	2723860	2733450	0.54110353
ATP5PO	chr13	197509331	197519588	7465015.1974	chr13	197465015	197468485	0.54134651
DUSP28	chr15	139537326	139539438	9044907.1390	chr15	139044907	139048907	0.54154236
NUDT9	chr8	131443997	131472696	031428.13103	chr8	131031428	131035218	0.54177871
BNIP3	chr14	140362127	140371907	0087869.1400	chr14	140087869	140090589	0.54193834
SYTL3	chr1	8453283	8546530	357656.83601	chr1	8357656	8360166	0.541971
ENSSSCG00000057427	chr18	2568274	2595510	2272575.2275	chr18	2272575	2275685	0.54210694
DHRS7B	chr12	61320730	61350219	1055733.6105	chr12	61055733	61059733	0.54247094
NAA10	chrX	124658158	124662702	407289.12441	chrX	124407289	124411289	0.54252078
SAL1	chr1	253638015	253642866	792913.25379	chr1	253792913	253796913	0.54265573
EEF1B2	chr15	109451514	109455525	9247828.1092	chr15	109247828	109253198	0.54271004
SDHA	chr16	79834044	79862524	9653319.7966	chr16	79653319	79662869	0.54271483
SRM	chr6	71246876	71253508	017576.71021	chr6	71017576	71021076	0.54275172
MAF1	chr4	597472	600447	377258.37834	chr4	377258	378347	0.54282148
RPS14	chr2	151430049	151433856	153756.15115	chr2	151153756	151157756	0.54284761
ARHGAP27	chr12	18037138	18082348	8036976.1804	chr12	18036976	18040754	0.5429086
C19orf67	chr2	65100755	65104628	716906.64720	chr2	64716906	64720906	0.54298512
ENSSSCG00000035997	chrX	124926772	124929791	427335.12442	chrX	124427335	124429654	0.54312973
VPS41	chr18	55301624	55466628	5271610.5527	chr18	55271610	55272585	0.54341333
SENP3	chr12	52765187	52867576	3006559.5300	chr12	53006559	53008829	0.54350578
NARF	chr12	632363	651817	740005.7440	chr12	740005	744005	0.54363308
ENSSSCG00000038506	chr9	135025443	135078913	368712.13537	chr9	135368712	135371122	0.54368106
SDHA	chr16	79834044	79862524	9898299.7990	chr16	79898299	79904919	0.54372731
ENSSSCG00000031249	chr13	207493659	207499867	7225755.2072	chr13	207225755	207228460	0.54379102
AP2A2	chr2	580182	628087	212170.21617	chr2	212170	216170	0.5441471
PEX19	chr4	90195852	90205215	288394.90292	chr4	90288394	90292394	0.54431172
EDC3	chr7	58828727	58897919	068728.59072	chr7	59068728	59072728	0.54445019
IDH3G	chrX	124528585	124537575	642648.12464	chrX	124642648	124648648	0.54446954
IPO9	chr10	24110064	24150291	4458712.2445	chr10	24458712	24459669	0.54449769
ENSSSCG00000038506	chr9	135025443	135078913	794722.13480	chr9	134794722	134800842	0.54452022
RPS3	chr9	9624990	9630401	835482.98416	chr9	9835482	9841672	0.54452419
RNF4	chr8	1353658	1380634	223429.12274	chr8	1223429	1227429	0.54459844
VAT1	chr12	19870312	19879230	0166659.2016	chr12	20166659	20168579	0.54462021
AJUBA	chr7	76063058	76076585	005976.76006	chr7	76005976	76006524	0.54468546
TUBGCP2	chr14	141233856	141253359	1189763.1411	chr14	141189763	141193763	0.54493513
PRR14	chr3	17745892	17751099	445606.17447	chr3	17445606	17447676	0.54497317
PPP1CA	chr2	5119939	5123617	070700.50728	chr2	5070700	5072860	0.54505907
GATD1	chr2	474296	480736	212170.21617	chr2	212170	216170	0.54530335

ZMIZ2	chr18	50579655	50598562	125275.5012	chr18	50125275	50128495	0.54538327
ENSSSCG00000033019	chr1	267961404	267964881	094326.2681	chr1	268094326	268100136	0.54541037
BRD9	chr16	79473745	79492635	653319.7966	chr16	79653319	79662869	0.54555733
UBE2J2	chr6	63555488	63568017	017978.6402	chr6	64017978	64020207	0.54558668
ENSSSCG00000052671	chr6	54564793	54568340	346936.5435	chr6	54346936	54350546	0.54560674
BRD9	chr16	79473745	79492635	808628.7980	chr16	79808628	79809073	0.54563902
CSTB	chr13	206706063	206710646	906685.2069	chr13	206906685	206910205	0.54571052
PRPF31	chr6	55995091	56011441	701622.5570	chr6	55701622	55705622	0.5457222
TIMM44	chr2	71271805	71287817	110600.7111	chr2	71110600	71114230	0.54573221
ENSSSCG00000015632	chr9	136893356	137056101	578362.1365	chr9	136578362	136582362	0.54578086
OXTR	chr13	65134057	65153487	365608.6536	chr13	65365608	65369608	0.5457842
NSDHL	chrX	123906199	123929117	232736.1242	chrX	124232736	124236736	0.54581341
ZNRD2	chr2	6683057	6684494	456140.6460	chr2	6456140	6460880	0.5458315
MED8	chr6	167861373	167869411	584752.1676	chr6	167684752	167687008	0.5458463
FIGNL1	chr9	136484216	136488250	578362.1365	chr9	136578362	136582362	0.545961
CNPY2	chr5	21707834	21711292	622762.2162	chr5	21622762	21626762	0.54601761
MEA1	chr7	38074439	38081897	097569.3810	chr7	38097569	38102239	0.54635327
CDK5RAP3	chr12	24224263	24234467	729110.2373	chr12	23729110	23733110	0.54650476
ENSSSCG00000012088	chr13	207801096	207818130	369155.2073	chr13	207369155	207370835	0.54660728
DUSP28	chr15	139537326	139539438	822668.1398	chr15	139822668	139834538	0.54666899
COQ4	chr1	268756685	268768343	689502.2686	chr1	268689502	268693502	0.5466948
ENSSSCG00000033019	chr1	267961404	267964881	741236.2677	chr1	267741236	267743686	0.54692572
KAT5	chr2	6560513	6572466	895080.6899	chr2	6895080	6899700	0.54722324
ECHS1	chr14	141339364	141348994	098189.1411	chr14	141098189	141104699	0.54736434
WDR55	chr2	142378462	142384677	804530.1428	chr2	142804530	142808530	0.54750039
ENSSSCG00000038506	chr9	135025443	135078913	847862.1348	chr9	134847862	134850842	0.54751135
VPS25	chr12	20082865	20088566	166659.2016	chr12	20166659	20168579	0.54756374
PSMA7	chr17	61566373	61572438	683622.6168	chr17	61683622	61687972	0.54759059
EIF3B	chr3	1645706	1666091	228872.1232	chr3	1228872	1232833	0.54777146
IPO9	chr10	24110064	24150291	179769.2418	chr10	24179769	24184649	0.5478604
AGFG2	chr3	8468044	8490831	836970.8837	chr3	8836970	8837652	0.54803353
RIMKLA	chr6	168916725	168950046	524156.1685	chr6	168524156	168530576	0.54815207
SLC38A8	chr6	4564533	4594520	640764.4644	chr6	4640764	4644764	0.54835215
ENSSSCG00000061173	chrX	110774840	110792950	247183.1112	chrX	111247183	111248501	0.5483628
TBRG4	chr18	50365988	50378084	977355.4998	chr18	49977355	49983615	0.54838003
DGAT1	chr4	452662	466684	441311.4453	chr4	441311	445311	0.54843792
PCDHGA4	chr2	142993554	143156556	004701.1430	chr2	143004701	143005341	0.54884774
MRPS6	chr13	197738890	197756785	465015.1974	chr13	197465015	197468485	0.5488949
PRPF4	chr1	253906717	253925046	792913.2537	chr1	253792913	253796913	0.54913215
RPS20	chr4	75762209	75769955	065169.7606	chr4	76065169	76068979	0.54913987
CCER2	chr6	47712765	47715620	969275.4797	chr6	47969275	47973275	0.54924195
UQCRQ	chr2	135186501	135188293	137272.1351	chr2	135137272	135141272	0.54930102
ENSSSCG00000051352	chr14	73350356	73357245	180892.7318	chr14	73180892	73184892	0.54931473
TUBB2A	chr7	1910269	1914761	470509.1472	chr7	1470509	1472579	0.54939831
ATP6V0B	chr6	167316947	167320212	190926.1672	chr6	167190926	167200676	0.54953565
ENSSSCG00000035997	chrX	124926772	124929791	271488.1252	chrX	125271488	125274598	0.54963476
RETREG2	chr15	121212881	121218812	428658.1214	chr15	121428658	121429211	0.54965147
PGS1	chr12	3589541	3628961	166999.3170	chr12	3166999	3170069	0.54971832
ENSSSCG00000038506	chr9	135025443	135078913	571202.1345	chr9	134571202	134575572	0.54971868
COX5A	chr7	58622964	58644021	068728.5907	chr7	59068728	59072728	0.54974281
GPR137	chr2	7826456	7830969	286187.8288	chr2	8286187	8288147	0.54976823
AIFM1	chrX	106670520	106708317	659892.1066	chrX	106659892	106661951	0.54980808
NAXD	chr11	77210228	77230680	887510.7689	chr11	76887510	76890510	0.54981995
BNIP3	chr14	140362127	140371907	746869.1407	chr14	140746869	140750169	0.54997229
AFAP1L1	chr2	150445126	150510002	286910.1502	chr2	150286910	150293060	0.54999166
CYC1	chr4	606516	608996	801299.8057	chr4	801299	805749	0.55001263
FBXO17	chr6	47732303	47759606	969275.4797	chr6	47969275	47973275	0.55016519
ENSSSCG00000033707	chr12	61514310	61563818	055733.6105	chr12	61055733	61059733	0.55030448
PDXP	chr5	10162643	10169512	587800.1058	chr5	10587800	10588562	0.55039504
ENSSSCG00000032959	chrX	125001675	125002920	903258.1249	chrX	124903258	124909988	0.55050598
ENSSSCG00000029830	chr4	136107	138652	441311.4453	chr4	441311	445311	0.55053187
NCCRP1	chr6	47915118	47929937	969275.4797	chr6	47969275	47973275	0.55055763
LIG1	chr6	53620483	53686562	761062.5376	chr6	53761062	53765062	0.55058254

SSR1	chr7	4652921	4685901	504349.45086	chr7	4504349	4508659	0.55067993
LYPLAL1	chr10	8946487	9062412	8917679.8921	chr10	8917679	8921219	0.55071555
POLD1	chr6	55247880	55272085	717729.55721	chr6	55717729	55721729	0.55076377
ENSSSCG00000038506	chr9	135025443	135078913	373142.13538	chr9	135373142	135380772	0.55096902
C11orf98	chr2	9084256	9090037	082256.90862	chr2	9082256	9086256	0.55098826
ENSSSCG00000025928	chr6	53974309	53978544	070265.54074	chr6	54070265	54074265	0.55110835
IDH3G	chrX	124528585	124537575	822947.12482	chrX	124822947	124824355	0.5512602
RPS14	chr2	151430049	151433856	555380.15156	chr2	151555380	151563830	0.55127291
SELENOF	chr4	129258944	129287720	319564.12932	chr4	129319564	129323564	0.55147144
YJU2	chr2	74509739	74528300	042210.74044	chr2	74042210	74044130	0.55149865
ILK	chr9	3145608	3159858	120522.31249	chr9	3120522	3124932	0.55158371
ENSSSCG00000003612	chr6	88695926	88708678	808587.88809	chr6	88808587	88809709	0.55171031
ATP5ME	chr8	110605	113510	444863.45075	chr8	444863	450757	0.55178795
TMEM147	chr6	44979190	44981049	344637.45348	chr6	45344637	45348637	0.55181077
ENSSSCG00000057427	chr18	2568274	2595510	2154505.2156	chr18	2154505	2156285	0.5521237
ENSSSCG00000027723	chr15	137567231	137621880	7673710.1376	chr15	137673710	137677710	0.55221869
ENSSSCG00000032959	chrX	125001675	125002920	271488.12527	chrX	125271488	125274598	0.55228562
POLA2	chr2	6922387	6950179	363246.73672	chr2	7363246	7367246	0.55228949
ENSSSCG00000035997	chrX	124926772	124929791	148743.12515	chrX	125148743	125152743	0.55245627
POLR3H	chr5	6997939	7014421	431892.74326	chr5	7431892	7432609	0.5525008
DRAP1	chr2	6409655	6412395	526990.65343	chr2	6526990	6534360	0.55255674
PRR14	chr3	17745892	17751099	803416.17807	chr3	17803416	17807526	0.55271988
NDUFA6	chr5	6562011	6567647	576101.65771	chr5	6576101	6577139	0.55294099
EEF1AKMT1	chr11	1067061	1082591	1559030.1562	chr11	1559030	1562300	0.55303463
SLC35A4	chr2	142323341	142325335	483896.14248	chr2	142483896	142487896	0.55308376
FIGNL1	chr9	136484216	136488250	285762.13628	chr9	136285762	136289922	0.55331543
NARF	chr12	632363	651817	1448428.4524	chr12	448428	452428	0.55334668
MRPL10	chr12	24075632	24083052	3579721.2358	chr12	23579721	23583721	0.55346414
TRAF3IP1	chr15	137863609	137912477	7555738.1375	chr15	137555738	137558808	0.55364174
PRR14	chr3	17745892	17751099	812944.17813	chr3	17812944	17813910	0.55369908
MRPL11	chr2	5978483	5981449	456140.64608	chr2	6456140	6460880	0.55393088
ENSSSCG00000021624	chr10	23767206	23778722	3626979.2363	chr10	23626979	23630979	0.55394607
CYBC1	chr12	656347	662513	1053218.1057	chr12	1053218	1057218	0.55449361
NLRP8	chr6	60350622	60370529	422807.60424	chr6	60422807	60424327	0.55476375
EHD1	chr2	7298208	7321576	895080.68997	chr2	6895080	6899700	0.55479646
YKT6	chr18	50960113	50971384	0750846.5075	chr18	50750846	50751473	0.55479802
SERPINB1	chr7	1684215	1693299	748321.17494	chr7	1748321	1749415	0.55486155
MRPL36	chr16	79001549	79004358	8911469.7891	chr16	78911469	78914430	0.55526387
BCAP31	chrX	124457001	124484743	822947.12482	chrX	124822947	124824355	0.55529231
MRPL45	chr12	23751926	23766459	3729110.2373	chr12	23729110	23733110	0.55535801
PDGFA	chr3	301584	321712	343167.35035	chr3	343167	350359	0.55564531
AAAS	chr5	18526076	18537830	586758.18590	chr5	18586758	18590758	0.55575749
CDIPT	chr3	18084549	18091798	021096.18025	chr3	18021096	18025096	0.55579009
LRPAP1	chr8	2125405	2145647	299768.23084	chr8	2299768	2308478	0.55593366
ENSSSCG00000032166	chr12	6240277	6247343	6214528.6215	chr12	6214528	6215144	0.55649769
MED8	chr6	167861373	167869411	922536.16792	chr6	167922536	167926936	0.55657829
MRPS23	chr12	34122020	34129548	3861199.3386	chr12	33861199	33867119	0.55665497
APOE	chr6	51372292	51375330	402558.51406	chr6	51402558	51406558	0.55665581
FAM104A	chr12	7727796	7749240	7581599.7584	chr12	7581599	7584879	0.55670805
ENSSSCG00000014540	chr2	9535148	9537974	195750.91988	chr2	9195750	9198850	0.5567856
RPL27	chr12	19892217	19894504	0166659.2016	chr12	20166659	20168579	0.55691018
DYNLL2	chr12	34349294	34356472	3861199.3386	chr12	33861199	33867119	0.55699282
DAD1	chr7	76432051	76457192	005976.76006	chr7	76005976	76006524	0.55707608
POLR2G	chr2	9004390	9017779	195750.91988	chr2	9195750	9198850	0.55708001
NARF	chr12	632363	651817	1448754.4508	chr12	448754	450833	0.55715847
SAP18	chr11	1291202	1298983	1656336.1660	chr11	1656336	1660336	0.55717074
RPS11	chr6	54577667	54580846	761096.54766	chr6	54761096	54766246	0.55719862
PRSS8	chr3	17357802	17362220	613617.17614	chr3	17613617	17614750	0.55733807
NR2C2AP	chr2	58675990	58678130	631062.58632	chr2	58631062	58632193	0.55751787
ENSSSCG00000012088	chr13	207801096	207818130	8293451.2082	chr13	208293451	208297451	0.55765363
HIRIP3	chr3	18198807	18202089	812944.17813	chr3	17812944	17813910	0.55765939
ABHD17A	chr2	76658068	76666552	277430.76279	chr2	76277430	76279160	0.55768875
MRPL36	chr16	79001549	79004358	9295297.7930	chr16	79295297	79302303	0.55775887

SDHA	chr16	79834044	79862524	797679.7980	chr16	79797679	79802279	0.55783532
EBNA1BP2	chr6	168049758	168061013	796056.1677	chr6	167796056	167799916	0.55792196
BNIP3	chr14	140362127	140371907	835389.1408	chr14	140835389	140842459	0.55792744
ENSSSCG00000044155	chr2	142321430	142324016	483896.1424	chr2	142483896	142487896	0.5580582
MRPL40	chr14	51102613	51105767	810984.5081	chr14	50810984	50814984	0.55808061
TUBB2A	chr7	1910269	1914761	748321.1749	chr7	1748321	1749415	0.55844079
TTLL13	chr7	55811855	55827077	363709.5536	chr7	55363709	55366089	0.55857924
RPP40	chr7	2987271	3005271	614139.2616	chr7	2614139	2616239	0.55874749
ENSSSCG00000060915	chr17	59172433	59175227	150116.5915	chr17	59150116	59154116	0.55884548
STMP1	chr18	13641541	13665565	502464.1350	chr18	13502464	13506464	0.55888532
PFDN2	chr4	89326798	89342325	972183.8897	chr4	88972183	88973295	0.55906371
OSBPL2	chr17	61643377	61685819	208112.6121	chr17	61208112	61211942	0.55914218
ZMAT2	chr2	142411089	142419137	473803.1424	chr2	142473803	142474940	0.55948621
PRR13	chr5	18660814	18665925	671981.1867	chr5	18671981	18675981	0.55948841
RECQL4	chr4	287214	293913	441311.4453	chr4	441311	445311	0.55966617
PKIG	chr17	47008035	47044049	638022.4664	chr17	46638022	46640212	0.55994523
DNAJC11	chr6	67463374	67529930	938806.6794	chr6	67938806	67942416	0.5600792
PIMREG	chr12	50898501	50903464	728138.5072	chr12	50728138	50729135	0.56014722
TBC1D10B	chr3	17944629	17956069	812944.1781	chr3	17812944	17813910	0.56022838
TCL1B	chr7	116912976	116917934	476479.1164	chr7	116476479	116479379	0.56029444
GMPPA	chr15	121507564	121515300	428658.1214	chr15	121428658	121429211	0.56033968
WRAP73	chr6	65155209	65167079	005436.6501	chr6	65005436	65012316	0.56039754
COX8A	chr2	8101613	8103285	286187.8288	chr2	8286187	8288147	0.56050985
TMED4	chr18	50699470	50702705	728428.5072	chr18	50728428	50729797	0.56073392
TUBGCP2	chr14	141233856	141253359	133839.1411	chr14	141133839	141138489	0.56081882
AARSD1	chr12	19918248	19926825	166659.2016	chr12	20166659	20168579	0.56093877
CYP2E1	chr14	141690426	141736817	450562.1414	chr14	141450562	141451766	0.56094546
ENSSSCG00000045223	chr6	166945409	166957165	907657.1669	chr6	166907657	166910521	0.56096581
ARHGEF7	chr11	77427368	77530993	854120.7785	chr11	77854120	77859120	0.56115705
DPEP1	chr6	361197	376616	251207.2552	chr6	251207	255207	0.56120157
ENSSSCG00000031249	chr13	207493659	207499867	369155.2073	chr13	207369155	207370835	0.56133248
CLN6	chr1	166107228	166126808	599696.1666	chr1	166599696	166602986	0.56139457
VPS28	chr4	362912	370588	785393.7984	chr4	785393	798477	0.56141534
NLRP8	chr6	60350622	60370529	420391.6042	chr6	60420391	60424391	0.5614315
VKORC1	chr3	17386245	17389905	445606.1744	chr3	17445606	17447676	0.56145055
DNPEP	chr15	121391746	121412864	428658.1214	chr15	121428658	121429211	0.56201278
ENSSSCG00000037652	chr3	65951	140492	341139.3451	chr3	341139	345139	0.56205899
TBC1D10B	chr3	17944629	17956069	809718.1781	chr3	17809718	17813718	0.56215758
CNPY2	chr5	21707834	21711292	663252.2166	chr5	21663252	21667252	0.56216945
ENSSSCG00000037652	chr3	65951	140492	343167.3503	chr3	343167	350359	0.56253788
TRMT2A	chr14	51489892	51494190	291092.5129	chr14	51291092	51297177	0.56256684
ZNF212	chr18	55528138	55541841	271610.5527	chr18	55271610	55272585	0.56265652
HDGFL2	chr2	74332106	74357040	042210.7404	chr2	74042210	74044130	0.56294116
NSDHL	chrX	123906199	123929117	165690.1241	chrX	124165690	124169690	0.56296957
DGAT1	chr4	452662	466684	785393.7984	chr4	785393	798477	0.5630065
GTPBP3	chr2	60352026	60357176	740996.6074	chr2	60740996	60744996	0.56308258
PSMC5	chr12	15101859	15106213	392487.1539	chr12	15392487	15396487	0.56316451
MRPL20	chr6	63670755	63675839	017978.6402	chr6	64017978	64020207	0.56338479
FTSJ3	chr12	15106719	15114398	360359.1536	chr12	15360359	15364249	0.56358682
ENSSSCG00000048787	chr15	137765933	137767998	701312.1377	chr15	137701312	137702888	0.56416953
TALDO1	chr2	464728	472835	709596.7135	chr2	709596	713596	0.56433856
RDH13	chr6	59274387	59292765	233436.5923	chr6	59233436	59233978	0.56442977
GPS1	chr12	951867	957080	453099.4609	chr12	453099	460989	0.56450912
VAT1	chr12	19870312	19879230	529603.1953	chr12	19529603	19533603	0.56453066
ENSSSCG00000038506	chr9	135025443	135078913	113078.1351	chr9	135113078	135117078	0.56459463
GINS2	chr6	3218702	3243899	710866.3717	chr6	3710866	3717166	0.56465036
OTUD5	chrX	43000231	43029468	680269.4268	chrX	42680269	42684269	0.56475683
FEM1A	chr2	74042989	74044983	042210.7404	chr2	74042210	74044130	0.56479159
TUBGCP2	chr14	141233856	141253359	098189.1411	chr14	141098189	141104699	0.56479376
C10orf143	chr14	139013253	139050019	346949.1393	chr14	139346949	139351589	0.56484406
COG1	chr12	7743274	7760504	170949.8173	chr12	8170949	8173959	0.56485153
CUL7	chr7	38097854	38115822	097569.3810	chr7	38097569	38102239	0.56499039
CA5A	chr6	1506042	1538777	020937.1024	chr6	1020937	1024937	0.56536382

SEZ6L2	chr3	18098102	18119558	312944.17813	chr3	17812944	17813910	0.56539839
SLC16A5	chr12	6185528	6200001	214528.6215	chr12	6214528	6215144	0.56541379
DGAT1	chr4	452662	466684	377258.37834	chr4	377258	378347	0.56544107
WRAP73	chr6	65155209	65167079	710876.64718	chr6	64710876	64718966	0.56548402
BRD9	chr16	79473745	79492635	438153.7943	chr16	79438153	79439277	0.56549666
MTMR1	chrX	122394921	122453905	002378.12200	chrX	122002378	122004278	0.56578428
MRPL54	chr2	74925070	74928167	729195.74730	chr2	74729195	74730347	0.56586628
PCID2	chr11	78541318	78556733	167005.7817	chr11	78167005	78170890	0.56588715
TXNL4A	chr6	127974543	127991177	712046.12771	chr6	127712046	127713246	0.56595289
SERPINB1	chr7	1684215	1693299	518809.16227	chr7	1618809	1622759	0.56614374
SELENOF	chr4	129258944	129287720	319459.12932	chr4	129319459	129323069	0.56622214
FIGNL1	chr9	136484216	136488250	240759.13624	chr9	136240759	136244759	0.56634823
TBCD	chr12	329714	449556	.145208.1492	chr12	145208	149208	0.5663549
MGMT	chr14	138499309	138771540	976219.1389	chr14	138976219	138981529	0.56644054
NDUFA13	chr2	58401551	58411981	531062.58632	chr2	58631062	58632193	0.56656624
NLRP5	chr6	60379623	60409465	857817.60859	chr6	60857817	60859961	0.5666867
PRSS8	chr3	17357802	17362220	809718.17813	chr3	17809718	17813718	0.5673088
ENSSSCG00000035728	chr12	1095096	1099180	.304812.1306	chr12	1304812	1306458	0.56731674
ENSSSCG00000046487	chr6	48052136	48059215	150486.48153	chr6	48150486	48153526	0.56732759
ENSSSCG00000060915	chr17	59172433	59175227	145902.5914	chr17	59145902	59148352	0.56754017
NSDHL	chrX	123906199	123929117	234696.12423	chrX	124234696	124236903	0.56754628
POLR2F	chr5	9855983	9917726	547161.95483	chr5	9547161	9548308	0.56756201
GAA	chr12	2314591	2336988	095212.2099	chr12	2095212	2099139	0.56756714
TNNT1	chr6	59347582	59365284	733286.59736	chr6	59733286	59736796	0.56756727
ENSSSCG00000016990	chr16	51225724	51260716	.049039.5105	chr16	51049039	51051709	0.56766568
TEPSIN	chr12	1519760	1529542	.304812.1306	chr12	1304812	1306458	0.56772734
ENKD1	chr6	28283117	28287306	851452.27855	chr6	27851452	27855452	0.56788634
HAUS7	chrX	124234736	124258098	423872.12442	chrX	124423872	124427872	0.56802394
ENSSSCG00000057427	chr18	2568274	2595510	415625.2424	chr18	2415625	2424205	0.56842325
SMARCD2	chr12	15091729	15101899	360359.1536	chr12	15360359	15364249	0.56865289
LYRM4	chr7	3070470	3192922	523009.26262	chr7	2623009	2626219	0.56875354
DGAT1	chr4	452662	466684	782159.79387	chr4	782159	793879	0.56888147
BRD9	chr16	79473745	79492635	538042.7953	chr16	79538042	79539074	0.56907898
STMP1	chr18	13641541	13665565	995535.1399	chr18	13995535	13998965	0.56914218
MRPL17	chr9	3075995	3083580	120522.31249	chr9	3120522	3124932	0.5693029
ENSSSCG00000032573	chr4	75636278	75646153	328169.75830	chr4	75828169	75830959	0.56930972
BCAP31	chrX	124457001	124484743	814014.12481	chrX	124814014	124818014	0.56937721
FTL	chr6	54231172	54232750	761062.53765	chr6	53761062	53765062	0.56960777
TRAF3IP1	chr15	137863609	137912477	673710.1376	chr15	137673710	137677710	0.56963996
RPS16	chr6	48085678	48088423	150486.48153	chr6	48150486	48153526	0.56966637
SERPINB1	chr7	1684215	1693299	470509.14725	chr7	1470509	1472579	0.56975322
TRAF3IP1	chr15	137863609	137912477	383458.1373	chr15	137383458	137390388	0.56975985
ZNHIT6	chr4	130298280	130386955	737319.13073	chr4	130737319	130739599	0.56977868
ENSSSCG00000021624	chr10	23767206	23778722	786279.2379	chr10	23786279	23790279	0.56998971
CAMTA1	chr6	67605790	68471920	759306.67762	chr6	67759306	67762556	0.57011172
C9orf78	chr1	269986391	269995395	259836.27026	chr1	270259836	270264736	0.57017726
TUBB2B	chr7	1951407	1956119	325359.23277	chr7	2325359	2327729	0.57021992
IDH3G	chrX	124528585	124537575	903258.12490	chrX	124903258	124909988	0.57042121
TMED4	chr18	50699470	50702705	750846.5075	chr18	50750846	50751473	0.57074481
FZR1	chr2	75121835	75146835	395470.75398	chr2	75395470	75398190	0.57080292
MRPS7	chr12	6052063	6055668	337182.6338	chr12	6337182	6338139	0.57084564
SYT2	chr10	24638704	24669846	684409.2468	chr10	24684409	24686519	0.57104889
TAF1C	chr6	4448778	4457088	540764.46447	chr6	4640764	4644764	0.57147479
PLIN3	chr2	73969862	73995529	485267.73486	chr2	73485267	73486251	0.57171739
TMEM9B	chr9	495693	521013	258992.26107	chr9	258992	261072	0.57180935
ENO1	chr6	69385879	69401151	585646.69696	chr6	69685646	69696186	0.57187461
PDGFA	chr3	301584	321712	345186.35211	chr3	345186	352116	0.5719748
FTL	chr6	54231172	54232750	070265.54074	chr6	54070265	54074265	0.57246413
SDHA	chr16	79834044	79862524	544639.7954	chr16	79544639	79547779	0.57253467
ENSSSCG00000014540	chr2	9535148	9537974	082256.90862	chr2	9082256	9086256	0.57272141
CSNK1D	chr12	768865	796595	.162187.1166	chr12	1162187	1166187	0.57285938
POLA2	chr2	6922387	6950179	526990.65343	chr2	6526990	6534360	0.57349107
DYNLL2	chr12	34349294	34356472	896719.3390	chr12	33896719	33901049	0.57357762

PDCD6	chr16	79818958	79835746	877864.7987	chr16	79877864	79878678	0.57361288
MRPL40	chr14	51102613	51105767	291092.5129	chr14	51291092	51297177	0.57361816
COMT	chr14	51385738	51403998	291092.5129	chr14	51291092	51297177	0.57444171
CDIPT	chr3	18084549	18091798	803416.17807	chr3	17803416	17807526	0.5745527
PHRF1	chr2	340528	371692	709596.71359	chr2	709596	713596	0.57459909
SLC3A2	chr2	8892089	8919072	293700.92984	chr2	9293700	9298410	0.57484705
HMG20B	chr2	75093075	75098536	395470.75398	chr2	75395470	75398190	0.57485258
ATP6V0B	chr6	167316947	167320212	584752.16768	chr6	167684752	167687008	0.57515746
ENSSSCG00000045735	chr18	50458411	50463097	056015.5005	chr18	50056015	50059125	0.5752046
UBE2J2	chr6	63555488	63568017	054906.63058	chr6	63054906	63058226	0.57547638
IDH3G	chrX	124528585	124537575	427335.12442	chrX	124427335	124429654	0.57549522
ZFPL1	chr2	7098766	7103536	895080.68997	chr2	6895080	6899700	0.57561321
FAM104A	chr12	7727796	7749240	170949.8173	chr12	8170949	8173959	0.57594963
EIF4E2	chr15	133060247	133103719	933798.1329	chr15	132933798	132935628	0.57641797
AKR1C8	chr10	65559508	65572895	314989.6532	chr10	65314989	65324449	0.57652357
TMED3	chr7	48431375	48442645	558753.48562	chr7	48558753	48562753	0.57656393
DTYMK	chr15	140248079	140255805	822668.1398	chr15	139822668	139834538	0.5773297
MEPCE	chr3	8357289	8364661	836970.88376	chr3	8836970	8837652	0.57749647
ENSSSCG00000038506	chr9	135025443	135078913	272762.13528	chr9	135272762	135281422	0.57764353
NAXD	chr11	77210228	77230680	710950.7671	chr11	76710950	76713460	0.57789813
ENSSSCG00000014569	chr9	707693	711219	258992.26107	chr9	258992	261072	0.57796599
ZNF75D	chrX	110890341	110904062	961228.11096	chrX	110961228	110965038	0.57829006
PCID2	chr11	78541318	78556733	891930.7889	chr11	78891930	78896300	0.57846477
LDLRAP1	chr6	83030702	83054377	550096.82554	chr6	82550096	82554946	0.57875547
SLC43A2	chr12	47791913	47839012	166900.4817	chr12	48166900	48170900	0.57910722
DUSP28	chr15	139537326	139539438	048928.1390	chr15	139048928	139052328	0.57911832
ZNF584	chr6	62992506	63005691	745816.62746	chr6	62745816	62746568	0.57941254
NAA10	chrX	124658158	124662702	424348.12442	chrX	124424348	124429148	0.57964139
HARS1	chr2	142385872	142401208	804530.14280	chr2	142804530	142808530	0.57969612
BCAP31	chrX	124457001	124484743	903258.12490	chrX	124903258	124909988	0.57981062
PRKCA	chr12	12882064	13263554	390875.1239	chr12	12390875	12394875	0.5798825
TIMM17B	chrX	42970108	42975955	357991.43361	chrX	43357991	43361991	0.57991301
ATP6AP1	chrX	124952500	124960343	903258.12490	chrX	124903258	124909988	0.58012472
MRPL36	chr16	79001549	79004358	603069.7860	chr16	78603069	78605119	0.5801346
CBR3	chr13	199830668	199840442	126477.2001	chr13	200126477	200130477	0.58040513
ARMC12	chr7	31586797	31603600	159320.31163	chr7	31159320	31163320	0.58043745
PLIN3	chr2	73969862	73995529	042210.74044	chr2	74042210	74044130	0.58053354
BNIP3	chr14	140362127	140371907	303169.1403	chr14	140303169	140305619	0.58054351
BRD9	chr16	79473745	79492635	036641.7904	chr16	79036641	79043345	0.58065802
MTM1	chrX	122286916	122379299	898508.12190	chrX	121898508	121904128	0.58099862
MRPL2	chr7	38116179	38121756	097569.38102	chr7	38097569	38102239	0.58153705
MRPS7	chr12	6052063	6055668	336839.6340	chr12	6336839	6340259	0.58180368
CERS4	chr2	71004420	71048248	110600.71114	chr2	71110600	71114230	0.58182598
VPS28	chr4	362912	370588	204188.20818	chr4	204188	208188	0.5818983
CCER2	chr6	47712765	47715620	150486.48153	chr6	48150486	48153526	0.58191951
VKORC1	chr3	17386245	17389905	613617.17614	chr3	17613617	17614750	0.58254931
BCAP31	chrX	124457001	124484743	234696.12423	chrX	124234696	124236903	0.58282769
UXT	chrX	42176145	42184079	174600.42175	chrX	42174600	42175367	0.58283111
RPS20	chr4	75762209	75769955	828169.75830	chr4	75828169	75830959	0.58288746
ENSSSCG00000061173	chrX	110774840	110792950	890031.11089	chrX	110890031	110890938	0.58313362
PSMA7	chr17	61566373	61572438	222882.6122	chr17	61222882	61224592	0.58336845
ECHS1	chr14	141339364	141348994	450562.1414	chr14	141450562	141451766	0.5833742
NDUFA10	chr15	138986421	139031897	044907.1390	chr15	139044907	139048907	0.58341362
COPS9	chr15	139260478	139264211	868278.1388	chr15	138868278	138870448	0.58343469
JPT1	chr12	6139428	6158452	336839.6340	chr12	6336839	6340259	0.58415541
LSM7	chr2	76247058	76252598	276671.76278	chr2	76276671	76278423	0.58430861
SELENOF	chr4	129258944	129287720	968789.12897	chr4	128968789	128973549	0.58434362
ENSSSCG00000038506	chr9	135025443	135078913	273944.13527	chr9	135273944	135274674	0.58455484
EEF1D	chr4	969527	983270	321556.13255	chr4	1321556	1325556	0.58476967
TMEM223	chr2	8981963	8983263	082256.90862	chr2	9082256	9086256	0.58493363
BNIP3	chr14	140362127	140371907	414459.1404	chr14	140414459	140419889	0.58497522
GFUS	chr4	949867	959744	321556.13255	chr4	1321556	1325556	0.58503387
BCAP31	chrX	124457001	124484743	232736.12423	chrX	124232736	124236736	0.58514475

ENSSSCG00000017971	chr12	53111256	53112484	493453.5349	chr12	53493453	53497453	0.58520561
TNNT1	chr6	59347582	59365284	703779.59705	chr6	59703779	59705229	0.58539601
CTPS1	chr6	170201751	170233016	194906.17019	chr6	170194906	170197814	0.58561752
TUFM	chr3	18521128	18524959	021096.18025	chr3	18021096	18025096	0.58565902
ATP6V0B	chr6	167316947	167320212	435586.16743	chr6	167435586	167437366	0.58585155
MRPL20	chr6	63670755	63675839	974346.63978	chr6	63974346	63978346	0.58586126
PSMG3	chr3	1052191	1054688	066576.10701	chr3	1066576	1070166	0.58587245
COPS9	chr15	139260478	139264211	477888.1394	chr15	139477888	139482538	0.58592289
SEZ6L2	chr3	18098102	18119558	803416.17807	chr3	17803416	17807526	0.58651537
PBDC1	chrX	60907404	61019857	424975.60428	chrX	60424975	60428975	0.58655731
FAM110A	chr17	34425617	34437721	911812.3491	chr17	34911812	34917302	0.58701873
TUBB2B	chr7	1951407	1956119	424299.24311	chr7	2424299	2431169	0.58724528
NUDCD3	chr18	50759221	50830332	890905.5089	chr18	50890905	50896275	0.587393
UBL7	chr7	59070728	59084112	068728.59072	chr7	59068728	59072728	0.58750767
MRPL36	chr16	79001549	79004358	854486.7885	chr16	78854486	78856078	0.5877441
ENSSSCG00000045735	chr18	50458411	50463097	890905.5089	chr18	50890905	50896275	0.58776359
TNNT1	chr6	59347582	59365284	713636.59717	chr6	59713636	59717636	0.58815467
CBX8	chr12	2562374	2565780	133160.2137	chr12	2133160	2137160	0.5881729
COX4I1	chr6	3131019	3137530	658189.26621	chr6	2658189	2662189	0.58843614
WARS1	chr7	121212981	121238113	665209.12166	chr7	121665209	121667959	0.58852723
NCLN	chr2	75369955	75390545	395470.75398	chr2	75395470	75398190	0.58872563
SYCN	chr6	47931042	47932708	150486.48153	chr6	48150486	48153526	0.5888219
ARID3B	chr7	58932461	58998711	068728.59072	chr7	59068728	59072728	0.58891294
MVP	chr3	18057177	18081155	613617.17614	chr3	17613617	17614750	0.5889809
ARHGEF7	chr11	77427368	77530993	854226.7785	chr11	77854226	77857606	0.58900305
METTL23	chr12	4804037	4808913	914069.4923	chr12	4914069	4923019	0.58917951
FTL	chr6	54231172	54232750	346936.54350	chr6	54346936	54350546	0.58922646
MRPL36	chr16	79001549	79004358	495219.7949	chr16	79495219	79497289	0.58926132
ENSSSCG00000002036	chr7	76014901	76021919	003612.76007	chr7	76003612	76007612	0.58952982
FSD1	chr2	74471744	74485786	729195.74730	chr2	74729195	74730347	0.58969367
ENSSSCG00000027491	chr18	51220948	51310989	750846.5075	chr18	50750846	50751473	0.58998893
TUBB2A	chr7	1910269	1914761	259389.22659	chr7	2259389	2265929	0.58999184
DUSP28	chr15	139537326	139539438	479553.1394	chr15	139479553	139483165	0.59015734
POLR2H	chr13	122250020	122256195	482235.1224	chr13	122482235	122484295	0.59020001
ENSSSCG00000013064	chr2	9163317	9174468	195750.91988	chr2	9195750	9198850	0.59021289
ENSSSCG00000056719	chr6	61903216	61907832	473531.61474	chr6	61473531	61474411	0.5903009
VPS28	chr4	362912	370588	377258.37834	chr4	377258	378347	0.59031429
ENSSSCG00000053570	chr6	62255210	62260695	138474.62142	chr6	62138474	62142474	0.59048538
SHD	chr2	74497351	74504659	729195.74730	chr2	74729195	74730347	0.59058514
NDUFS6	chr16	78993913	79000006	837739.7884	chr16	78837739	78842699	0.59066725
ATP6V0E2	chr18	55877504	55882204	612730.5561	chr18	55612730	55616730	0.59086354
MED8	chr6	167861373	167869411	986876.16798	chr6	167986876	167989686	0.5910029
PPP1R7	chr15	139917257	139941251	865812.1398	chr15	139865812	139867602	0.59112572
SHANK2	chr2	2864331	3015314	577970.25863	chr2	2577970	2586340	0.59113815
ENSSSCG00000017907	chr12	51961952	51965464	975989.5197	chr12	51975989	51978009	0.59120892
SARS2	chr6	47716679	47728310	969275.47973	chr6	47969275	47973275	0.59126492
CYC1	chr4	606516	608996	204188.20818	chr4	204188	208188	0.59126801
BRD9	chr16	79473745	79492635	048763.7905	chr16	79048763	79050221	0.59133039
ECHS1	chr14	141339364	141348994	145779.1411	chr14	141145779	141148769	0.59139196
CDPF1	chr5	3292022	3296307	395198.34075	chr5	3395198	3407528	0.59195367
KDM4A	chr6	167597956	167642333	801936.16780	chr6	167801936	167808346	0.59208203
SEC61G	chr9	139128296	139135727	932632.13893	chr9	138932632	138938622	0.59220119
TOP1MT	chr4	1148890	1188130	476009.14858	chr4	1476009	1485859	0.59222441
ATG4B	chr15	140223904	140246321	764414.1397	chr15	139764414	139768414	0.59232392
CIAO2B	chr6	27610470	27612592	825350.27829	chr6	27825350	27829350	0.59239624
MRPL52	chr7	76187446	76191901	005976.76006	chr7	76005976	76006524	0.59308625
TMEM219	chr3	18167923	18176563	021096.18025	chr3	18021096	18025096	0.59321038
COPS9	chr15	139260478	139264211	984421.1389	chr15	138984421	138988421	0.59344437
ENSSSCG00000015632	chr9	136893356	137056101	753582.13675	chr9	136753582	136756492	0.59370223
BCAP31	chrX	124457001	124484743	214324.12421	chrX	124214324	124218324	0.59378382
SHANK2	chr2	2864331	3015314	743030.27504	chr2	2743030	2750430	0.59403428
MEPCE	chr3	8357289	8364661	832274.88362	chr3	8832274	8836274	0.59409516
VKORC1	chr3	17386245	17389905	803416.17807	chr3	17803416	17807526	0.59429022

STK25	chr15	140144336	140154366	865812.1398	chr15	139865812	139867602	0.59430337
CCNL2	chr6	63659054	63668047	679085.63683	chr6	63679085	63683085	0.59431239
TCP1	chr1	7590140	7601795	364266.73678	chr1	7364266	7367856	0.59436885
CSNK1D	chr12	768865	796595	448754.4508	chr12	448754	450833	0.59456726
ENSSSCG00000048787	chr15	137765933	137767998	240648.1382	chr15	138240648	138243538	0.59478412
LRCH4	chr3	8499285	8511262	836970.88376	chr3	8836970	8837652	0.59525021
TRAF3IP1	chr15	137863609	137912477	692318.1376	chr15	137692318	137698258	0.59542482
ENSSSCG00000045735	chr18	50458411	50463097	750846.5075	chr18	50750846	50751473	0.59563294
ATP6V0E2	chr18	55877504	55882204	493159.5549	chr18	55493159	55497159	0.5957259
TUBGCP2	chr14	141233856	141253359	119109.1411	chr14	141119109	141125409	0.59594717
MGMT	chr14	138499309	138771540	182749.1381	chr14	138182749	138186599	0.59595627
ATP5MC2	chr5	18871026	18879609	586758.18590	chr5	18586758	18590758	0.59613086
ENSSSCG00000017971	chr12	53111256	53112484	006559.5300	chr12	53006559	53008829	0.59665652
IDH3G	chrX	124528585	124537575	214324.12421	chrX	124214324	124218324	0.59674
LIG1	chr6	53620483	53686562	423936.53426	chr6	53423936	53426996	0.59716377
ATG4B	chr15	140223904	140246321	865812.1398	chr15	139865812	139867602	0.59736715
SEZ6L2	chr3	18098102	18119558	613617.17614	chr3	17613617	17614750	0.59742771
GLRX3	chr14	139072575	139111249	003294.1390	chr14	139003294	139003884	0.59759271
ENSSSCG00000012088	chr13	207801096	207818130	285845.2082	chr13	208285845	208289845	0.59789176
IDH3G	chrX	124528585	124537575	232736.12423	chrX	124232736	124236736	0.59848227
SPDYC	chr2	7030733	7058967	395080.68997	chr2	6895080	6899700	0.59852107
DNAJC30	chr3	10955877	10959916	052516.11053	chr3	11052516	11053085	0.5989485
SYT2	chr10	24638704	24669846	179769.2418	chr10	24179769	24184649	0.59901834
ENSSSCG00000027491	chr18	51220948	51310989	890905.5089	chr18	50890905	50896275	0.59913056
THEM5	chr4	97332432	97359428	041069.97043	chr4	97041069	97043669	0.59922048
NDUFS6	chr16	78993913	79000006	168279.7917	chr16	79168279	79175129	0.59930833
DND1	chr2	142384532	142387199	473803.14247	chr2	142473803	142474940	0.59947044
RPS20	chr4	75762209	75769955	737402.75741	chr4	75737402	75741402	0.5995187
SNU13	chr5	6885067	6900022	576101.65771	chr5	6576101	6577139	0.59953926
COA3	chr12	20062826	20063980	166659.2016	chr12	20166659	20168579	0.59961866
HDGFL2	chr2	74332106	74357040	729195.74730	chr2	74729195	74730347	0.59980321
UBE2J2	chr6	63555488	63568017	679085.63683	chr6	63679085	63683085	0.60058703
ZNHIT6	chr4	130298280	130386955	781369.13078	chr4	130781369	130787289	0.60070945
PSMC4	chr6	48406376	48416215	659926.48665	chr6	48659926	48665966	0.60085288
COPS3	chr12	60994310	61024846	055733.6105	chr12	61055733	61059733	0.60093978
IK	chr2	142361155	142376333	473803.14247	chr2	142473803	142474940	0.60101538
NDUFA10	chr15	138986421	139031897	479553.1394	chr15	139479553	139483165	0.60107334
DUSP28	chr15	139537326	139539438	865812.1398	chr15	139865812	139867602	0.60112904
HAUS7	chrX	124234736	124258098	232736.12423	chrX	124232736	124236736	0.60121607
ENSSSCG00000031249	chr13	207493659	207499867	882785.2078	chr13	207882785	207886425	0.60141066
NDUFB11	chrX	41770941	41774245	174600.42175	chrX	42174600	42175367	0.60176322
NCCRP1	chr6	47915118	47929937	150486.48153	chr6	48150486	48153526	0.60177491
NHEJ1	chr15	121100628	121190062	560936.1215	chr15	121560936	121562466	0.60208018
BRD9	chr16	79473745	79492635	891263.7989	chr16	79891263	79895263	0.60208524
NELFE	chr7	24040525	24047025	907960.23911	chr7	23907960	23911960	0.60221231
HAUS7	chrX	124234736	124258098	234696.12423	chrX	124234696	124236903	0.6022447
ILK	chr9	3145608	3159858	293892.32964	chr9	3293892	3296422	0.60231479
PFKM	chr5	78476123	78526997	139236.78143	chr5	78139236	78143236	0.6024654
MGMT	chr14	138499309	138771540	075609.1380	chr14	138075609	138081359	0.60248039
VPS41	chr18	55301624	55466628	493159.5549	chr18	55493159	55497159	0.60250653
POLR1D	chr11	5044329	5089766	390050.5395	chr11	5390050	5395160	0.60257696
ENSSSCG00000036988	chr7	1980292	1988727	748321.17494	chr7	1748321	1749415	0.60280072
PRPF31	chr6	55995091	56011441	020256.56024	chr6	56020256	56024256	0.60327201
BRD9	chr16	79473745	79492635	537609.7954	chr16	79537609	79543449	0.60336703
UBE2J2	chr6	63555488	63568017	444151.63445	chr6	63444151	63445363	0.60394538
BNIP1	chr16	51126926	51140394	049039.5105	chr16	51049039	51051709	0.60404173
ENSSSCG00000027723	chr15	137567231	137621880	045088.1380	chr15	138045088	138051538	0.60423485
RPS5	chr6	62967994	62974350	054906.63058	chr6	63054906	63058226	0.60433242
EBNA1BP2	chr6	168049758	168061013	684752.16768	chr6	167684752	167687008	0.60448992
BNIP3	chr14	140362127	140371907	308619.1403	chr14	140308619	140313739	0.60449267
ENSSSCG00000045735	chr18	50458411	50463097	095885.5009	chr18	50095885	50097985	0.60451262
LYRM4	chr7	3070470	3192922	573209.25814	chr7	2573209	2581459	0.60480084
ENSSSCG00000013613	chr2	70193730	70199975	658392.70659	chr2	70658392	70659484	0.60488483

TUBGCP2	chr14	141233856	141253359	.114989.1411	chr14	141114989	141118819	0.60522832
MRPL20	chr6	63670755	63675839	444151.63445	chr6	63444151	63445363	0.60522996
FAU	chr2	7070906	7072809	395080.68997	chr2	6895080	6899700	0.60532661
CD63	chr5	21172283	21176232	189251.21193	chr5	21189251	21193251	0.60561382
DTYMK	chr15	140248079	140255805	858888.1398	chr15	139858888	139868418	0.60569098
NLRP5	chr6	60379623	60409465	855442.60859	chr6	60855442	60859442	0.60602808
LIN37	chr6	45175500	45181391	344637.45348	chr6	45344637	45348637	0.6061176
ENSSSCG00000002036	chr7	76014901	76021919	005976.76006	chr7	76005976	76006524	0.60658629
INO80E	chr3	18202134	18212186	809718.17813	chr3	17809718	17813718	0.60666152
FUT2	chr6	54034684	54047224	078981.54081	chr6	54078981	54081394	0.60679641
EIPR1	chr3	131360883	131435579	726366.13173	chr3	131726366	131731426	0.60685699
SEPTIN8	chr2	135077170	135104471	137272.13514	chr2	135137272	135141272	0.6069075
ENSSSCG00000035997	chrX	124926772	124929791	822947.12482	chrX	124822947	124824355	0.60692815
FZR1	chr2	75121835	75146835	729195.74730	chr2	74729195	74730347	0.60694698
TUBB2B	chr7	1951407	1956119	498649.15032	chr7	1498649	1503249	0.60699706
ELAC2	chr12	57206600	57238444	7589920.5759	chr12	57589920	57593920	0.60702292
ZNF212	chr18	55528138	55541841	5493159.5549	chr18	55493159	55497159	0.60704577
NUDCD3	chr18	50759221	50830332	0750846.5075	chr18	50750846	50751473	0.60704752
DDX39B	chr7	23658088	23670031	907960.23911	chr7	23907960	23911960	0.60755314
TMEM219	chr3	18167923	18176563	812944.17813	chr3	17812944	17813910	0.60778423
POLR3H	chr5	6997939	7014421	576101.65771	chr5	6576101	6577139	0.60789796
ZNF574	chr6	49834650	49840391	568032.49572	chr6	49568032	49572032	0.60793427
SAP18	chr11	1291202	1298983	.836951.8409	chr11	836951	840951	0.60795498
MTMR1	chrX	122394921	122453905	227078.12223	chrX	122227078	122232108	0.6088662
ENSSSCG00000012088	chr13	207801096	207818130	3267585.2082	chr13	208267585	208270895	0.60900349
MRPS24	chr18	48825782	48830609	3737495.4874	chr18	48737495	48741355	0.60905804
MED8	chr6	167861373	167869411	556186.16766	chr6	167656186	167665426	0.60906528
MRPS24	chr18	48825782	48830609	0159885.4917	chr18	49159885	49170155	0.60965293
TBRG4	chr18	50365988	50378084	0750846.5075	chr18	50750846	50751473	0.60986694
HDLBP	chr15	139957715	140022302	865812.1398	chr15	139865812	139867602	0.61007464
TBRG4	chr18	50365988	50378084	0064445.5006	chr18	50064445	50066235	0.6100771
PSMG3	chr3	1052191	1054688	228872.12328	chr3	1228872	1232833	0.61008443
TMED4	chr18	50699470	50702705	0479985.5048	chr18	50479985	50482895	0.61017668
BCAP31	chrX	124457001	124484743	427335.12442	chrX	124427335	124429654	0.61073077
ENOX2	chrX	107085503	107368473	659892.10666	chrX	106659892	106661951	0.61091157
RPL3	chr5	8922665	8929767	775965.87799	chr5	8775965	8779965	0.61138919
ENSSSCG00000027723	chr15	137567231	137621880	7442855.1374	chr15	137442855	137446855	0.61141285
GAA	chr12	2314591	2336988	2133160.2137	chr12	2133160	2137160	0.61152473
CYC1	chr4	606516	608996	785393.79847	chr4	785393	798477	0.61158116
PER1	chr12	53361889	53374248	3493453.5349	chr12	53493453	53497453	0.61162567
LSM7	chr2	76247058	76252598	277430.76279	chr2	76277430	76279160	0.6120501
MRPS24	chr18	48825782	48830609	0189535.4919	chr18	49189535	49195785	0.61270546
TMED4	chr18	50699470	50702705	0890905.5089	chr18	50890905	50896275	0.61294932
HAUS7	chrX	124234736	124258098	424348.12442	chrX	124424348	124429148	0.6131717
HAUS7	chrX	124234736	124258098	427335.12442	chrX	124427335	124429654	0.61321331
PHRF1	chr2	340528	371692	:.90704.93254	chr2	90704	93254	0.61334754
FIGNL1	chr9	136484216	136488250	386442.13639	chr9	136386442	136391772	0.61375906
UBXN6	chr2	74357517	74378592	414316.74414	chr2	74414316	74414825	0.61376143
ZMAT2	chr2	142411089	142419137	483896.14248	chr2	142483896	142487896	0.61379893
TSTD1	chr4	89401159	89403277	972183.88973	chr4	88972183	88973295	0.61380181
USP27X	chrX	43555005	43556321	230192.43234	chrX	43230192	43234192	0.61395846
ATP6V0B	chr6	167316947	167320212	801936.16780	chr6	167801936	167808346	0.61426986
GINS2	chr6	3218702	3243899	809516.28170	chr6	2809516	2817016	0.61460198
KAT5	chr2	6560513	6572466	456140.64608	chr2	6456140	6460880	0.61464641
OSBPL2	chr17	61643377	61685819	.683622.6168	chr17	61683622	61687972	0.61470004
CIAO2B	chr6	27610470	27612592	851452.27855	chr6	27851452	27855452	0.61480605
CFL1	chr2	6469254	6475035	456140.64608	chr2	6456140	6460880	0.61481097
OSBPL2	chr17	61643377	61685819	.321802.6132	chr17	61321802	61328422	0.61485534
WRAP73	chr6	65155209	65167079	025442.65029	chr6	65025442	65029442	0.61507068
ENSSSCG00000012088	chr13	207801096	207818130	7882785.2078	chr13	207882785	207886425	0.61511142
ENSSSCG00000057427	chr18	2568274	2595510	2235895.2238	chr18	2235895	2238315	0.6152794
POLA2	chr2	6922387	6950179	395080.68997	chr2	6895080	6899700	0.61528543
ENSSSCG00000035904	chr1	272959831	272965634	535826.27254	chr1	272535826	272540916	0.61530478

NFYC	chr6	170413638	170485840	194906.17019	chr6	170194906	170197814	0.61570665
RNF220	chr6	166666138	166910646	593803.16659	chr6	166593803	166595225	0.61589578
FTSJ3	chr12	15106719	15114398	5392487.1539	chr12	15392487	15396487	0.6161268
FSD1	chr2	74471744	74485786	042210.74044	chr2	74042210	74044130	0.61619759
MRPL20	chr6	63670755	63675839	991306.63993	chr6	63991306	63993056	0.61635627
ENSSSCG00000035997	chrX	124926772	124929791	814014.12481	chrX	124814014	124818014	0.61650738
GALNTL5	chr18	5353769	5433814	4980080.4981	chr18	4980080	4981148	0.61673354
ING1	chr11	77270320	77277068	6880640.7688	chr11	76880640	76886850	0.61686474
LENG1	chr6	55970668	55976735	389302.56390	chr6	56389302	56390042	0.61708113
MRPL36	chr16	79001549	79004358	9011667.7901	chr16	79011667	79012408	0.61739899
TUBGCP2	chr14	141233856	141253359	1450562.1414	chr14	141450562	141451766	0.6174596
TLE2	chr2	75510004	75536804	395470.75398	chr2	75395470	75398190	0.61747485
TNNT1	chr6	59347582	59365284	233436.59233	chr6	59233436	59233978	0.61753749
BRD9	chr16	79473745	79492635	9552127.7955	chr16	79552127	79553615	0.61757612
NAPA	chr6	53246968	53275265	431154.53431	chr6	53431154	53431639	0.61792426
MED8	chr6	167861373	167869411	658726.16766	chr6	167658726	167662326	0.61804788
TUBGCP2	chr14	141233856	141253359	1115481.1411	chr14	141115481	141118289	0.61805999
MED8	chr6	167861373	167869411	801936.16780	chr6	167801936	167808346	0.61829373
CKM	chr6	51700507	51714814	601726.51606	chr6	51601726	51606356	0.61889501
DNPEP	chr15	121391746	121412864	1560936.1215	chr15	121560936	121562466	0.61901668
ZNRF1	chr6	12555664	12668804	054571.12058	chr6	12054571	12058571	0.61908404
CYC1	chr4	606516	608996	377258.37834	chr4	377258	378347	0.61911379
GLRX3	chr14	139072575	139111249	9222899.1392	chr14	139222899	139228039	0.61923061
ATG4B	chr15	140223904	140246321	9858888.1398	chr15	139858888	139868418	0.61971078
MRPL54	chr2	74925070	74928167	395470.75398	chr2	75395470	75398190	0.62015638
NFYC	chr6	170413638	170485840	242316.17024	chr6	170242316	170248076	0.62037007
ENSSSCG00000027723	chr15	137567231	137621880	7692318.1376	chr15	137692318	137698258	0.62052314
PRKAG2	chr18	5475077	5744657	5873195.5876	chr18	5873195	5876335	0.62065909
IDH3G	chrX	124528585	124537575	234696.12423	chrX	124234696	124236903	0.62111631
BRD9	chr16	79473745	79492635	9168279.7917	chr16	79168279	79175129	0.6211819
ENSSSCG00000021624	chr10	23767206	23778722	4179769.2418	chr10	24179769	24184649	0.62143718
ENSSSCG00000029830	chr4	136107	138652	204188.20818	chr4	204188	208188	0.62150646
CDIPT	chr3	18084549	18091798	613617.17614	chr3	17613617	17614750	0.62203075
TALDO1	chr2	464728	472835	130230.13366	chr2	130230	133660	0.62203579
NDUFA10	chr15	138986421	139031897	8868278.1388	chr15	138868278	138870448	0.62276313
ENSSSCG00000032573	chr4	75636278	75646153	065169.76068	chr4	76065169	76068979	0.62289896
IDH3G	chrX	124528585	124537575	814014.12481	chrX	124814014	124818014	0.62299352
TCP11	chr7	31007474	31114734	159320.31163	chr7	31159320	31163320	0.62317185
BNIP3	chr14	140362127	140371907	0038389.1400	chr14	140038389	140042669	0.6231784
BRD9	chr16	79473745	79492635	9544639.7954	chr16	79544639	79547779	0.62324754
SNAPC2	chr2	71291257	71294006	110600.71114	chr2	71110600	71114230	0.62420458
VKORC1	chr3	17386245	17389905	812944.17813	chr3	17812944	17813910	0.62546863
MRPL58	chr12	6255342	6266163	6214528.6215	chr12	6214528	6215144	0.62555166
CCNF	chr3	39585607	39603079	260264.39264	chr3	39260264	39264264	0.62595017
ENSSSCG00000057427	chr18	2568274	2595510	3040845.3044	chr18	3040845	3044465	0.62669555
EBNA1BP2	chr6	168049758	168061013	801936.16780	chr6	167801936	167808346	0.62670863
COX17	chr13	140584363	140587621	0599515.1406	chr13	140599515	140603515	0.62675423
WRAP73	chr6	65155209	65167079	663816.64666	chr6	64663816	64666816	0.62684515
NKIRAS2	chr12	20717547	20723704	0957709.2096	chr12	20957709	20961019	0.62751063
CDIPT	chr3	18084549	18091798	809718.17813	chr3	17809718	17813718	0.628387
PRPSAP1	chr12	5144069	5174382	4908039.4909	chr12	4908039	4909329	0.62873277
ALG8	chr9	12497721	12532307	479472.12481	chr9	12479472	12481122	0.62899978
RPL10A	chr7	31327440	31333638	159320.31163	chr7	31159320	31163320	0.62912911
BRD9	chr16	79473745	79492635	9797679.7980	chr16	79797679	79802279	0.62946501
NAA10	chrX	124658158	124662702	427335.12442	chrX	124427335	124429654	0.62947083
STX8	chr12	54296995	54544646	4676329.5467	chr12	54676329	54677869	0.62955289
FIGNL1	chr9	136484216	136488250	457852.13646	chr9	136457852	136463142	0.62966831
TXN2	chr5	11264645	11283395	811288.10814	chr5	10811288	10814498	0.6296892
ENKD1	chr6	28283117	28287306	825350.27829	chr6	27825350	27829350	0.62990907
GLRX3	chr14	139072575	139111249	9000749.1390	chr14	139000749	139004889	0.63016751
RNF181	chr3	59171082	59174229	183735.59187	chr3	59183735	59187735	0.6304201
ZFTRAF1	chr4	335301	346534	441311.44531	chr4	441311	445311	0.63049005
NAA10	chrX	124658158	124662702	214324.12421	chrX	124214324	124218324	0.63089559

FIGNL1	chr9	136484216	136488250	753582.13675	chr9	136753582	136756492	0.63094928
ATP6AP1	chrX	124952500	124960343	148743.12515	chrX	125148743	125152743	0.63110078
PSMD13	chr2	55108	74829	.90704.93254	chr2	90704	93254	0.63164546
RNASEH1	chr3	131268926	131281930	726366.13173	chr3	131726366	131731426	0.63180074
HAUS7	chrX	124234736	124258098	407289.12441	chrX	124407289	124411289	0.63232911
IDH3G	chrX	124528585	124537575	423872.12442	chrX	124423872	124427872	0.63238889
POLR2F	chr5	9855983	9917726	545177.95491	chr5	9545177	9549177	0.63250637
FIGNL1	chr9	136484216	136488250	721192.13672	chr9	136721192	136724212	0.63308962
SAP18	chr11	1291202	1298983	.559030.1562	chr11	1559030	1562300	0.63364155
LSM2	chr7	23900395	23908490	907960.23911	chr7	23907960	23911960	0.63383771
MGMT	chr14	138499309	138771540	239999.1382	chr14	138239999	138242709	0.63414926
TMEM213	chr18	10862206	10868435	593024.1059	chr18	10593024	10594111	0.63498042
TMEM219	chr3	18167923	18176563	809718.17813	chr3	17809718	17813718	0.63565135
YKT6	chr18	50960113	50971384	890905.5089	chr18	50890905	50896275	0.6359835
SHD	chr2	74497351	74504659	414316.74414	chr2	74414316	74414825	0.63609098
ENSSSCG00000030849	chrX	98178155	98181598	565158.98569	chrX	98565158	98569158	0.63628892
PFDN1	chr2	142011063	142077925	483896.14248	chr2	142483896	142487896	0.63632779
WDR55	chr2	142378462	142384677	483896.14248	chr2	142483896	142487896	0.63650116
SLIT3	chr16	54553426	55220296	267269.5426	chr16	54267269	54269829	0.63752746
IPO9	chr10	24110064	24150291	786279.2379	chr10	23786279	23790279	0.6379153
POLA2	chr2	6922387	6950179	456140.64608	chr2	6456140	6460880	0.63814434
ENSSSCG00000035997	chrX	124926772	124929791	903258.12490	chrX	124903258	124909988	0.63865776
PRSS8	chr3	17357802	17362220	812944.17813	chr3	17812944	17813910	0.63873939
ENSSSCG00000024588	chr2	75538090	75542189	395470.75398	chr2	75395470	75398190	0.63888577
BRD9	chr16	79473745	79492635	011667.7901	chr16	79011667	79012408	0.63903109
SURF4	chr1	272975160	272988688	535826.27254	chr1	272535826	272540916	0.63913154
RALGDS	chr1	272776846	272824184	535826.27254	chr1	272535826	272540916	0.63916941
C9orf78	chr1	269986391	269995395	329336.27033	chr1	270329336	270333876	0.63923939
AP1S1	chr3	8881107	8887566	588988.86902	chr3	8688988	8690214	0.63925804
FBXO17	chr6	47732303	47759606	150486.48153	chr6	48150486	48153526	0.63972231
NDUFA10	chr15	138986421	139031897	984421.1389	chr15	138984421	138988421	0.6402958
ARV1	chr14	59396962	59408958	543459.5954	chr14	59543459	59546189	0.64068788
ENSSSCG00000015632	chr9	136893356	137056101	457852.13646	chr9	136457852	136463142	0.64132685
IDH3G	chrX	124528585	124537575	407289.12441	chrX	124407289	124411289	0.6413458
ENSSSCG00000045223	chr6	166945409	166957165	285396.16728	chr6	167285396	167289306	0.64135058
GAA	chr12	2314591	2336988	134182.2135	chr12	2134182	2135153	0.64165141
TOLLIP	chr2	851512	864483	709596.71359	chr2	709596	713596	0.64185865
RPS20	chr4	75762209	75769955	143499.76145	chr4	76143499	76145939	0.64219784
LRCH4	chr3	8499285	8511262	832274.88362	chr3	8832274	8836274	0.64236013
USP36	chr12	3309237	3348525	166999.3170	chr12	3166999	3170069	0.64243882
HSF1	chr4	463393	486473	295751.29975	chr4	295751	299751	0.64245398
ADRM1	chr17	61692945	61698144	222882.6122	chr17	61222882	61224592	0.64258802
TALDO1	chr2	464728	472835	212170.21617	chr2	212170	216170	0.64367032
ENSSSCG00000056719	chr6	61903216	61907832	977286.61979	chr6	61977286	61979696	0.64378879
MRPL36	chr16	79001549	79004358	971273.7897	chr16	78971273	78974922	0.64380668
MVP	chr3	18057177	18081155	809718.17813	chr3	17809718	17813718	0.64395202
SDHA	chr16	79834044	79862524	638839.7964	chr16	79638839	79642899	0.64411369
CBX8	chr12	2562374	2565780	134182.2135	chr12	2134182	2135153	0.64435511
BRD9	chr16	79473745	79492635	495219.7949	chr16	79495219	79497289	0.64452001
ENSSSCG00000012088	chr13	207801096	207818130	959056.2079	chr13	207959056	207961576	0.64454797
PFDN1	chr2	142011063	142077925	473803.14247	chr2	142473803	142474940	0.64494302
CYTH2	chr6	53868409	53878856	431154.53431	chr6	53431154	53431639	0.64508286
ENSSSCG00000047605	chr4	128894115	128943092	935039.12893	chr4	128935039	128939899	0.64536438
TMUB1	chr18	6142023	6144894	873195.5876	chr18	5873195	5876335	0.64599879
POLR1D	chr11	5044329	5089766	415520.5417	chr11	5415520	5417840	0.64635036
BCAP31	chrX	124457001	124484743	424348.12442	chrX	124424348	124429148	0.64641803
TIMM13	chr2	76146867	76155729	277430.76279	chr2	76277430	76279160	0.64672607
PSMD13	chr2	55108	74829	212170.21617	chr2	212170	216170	0.64704802
HAUS7	chrX	124234736	124258098	214324.12421	chrX	124214324	124218324	0.6470684
UBE2J2	chr6	63555488	63568017	974346.63978	chr6	63974346	63978346	0.64759216
KAZN	chr6	74236437	74378956	378056.74379	chr6	74378056	74379376	0.64792319
MVP	chr3	18057177	18081155	812944.17813	chr3	17812944	17813910	0.648515
NAA10	chrX	124658158	124662702	903258.12490	chrX	124903258	124909988	0.64856113

IDH3G	chrX	124528585	124537575	424348.12442	chrX	124424348	124429148	0.64877218
PDGFA	chr3	301584	321712	341139.34513	chr3	341139	345139	0.64948696
BNIP3	chr14	140362127	140371907	407549.1404	chr14	140407549	140410609	0.6494879
NAXD	chr11	77210228	77230680	880640.7688	chr11	76880640	76886850	0.64952754
RPTOR	chr12	1709128	1991574	304812.1306	chr12	1304812	1306458	0.64968944
ENSSSCG00000003286	chr6	59113039	59134353	233436.59233	chr6	59233436	59233978	0.65012544
OSBPL2	chr17	61643377	61685819	222882.6122	chr17	61222882	61224592	0.65049635
RIMKLA	chr6	168916725	168950046	469856.16847	chr6	168469856	168474916	0.65055991
ENSSSCG00000059201	chr4	90261918	90263262	288394.90292	chr4	90288394	90292394	0.6508391
RIMS3	chr6	170506426	170546933	187216.17018	chr6	170187216	170189606	0.65089203
ENSSSCG00000021624	chr10	23767206	23778722	966485.2396	chr10	23966485	23968979	0.65118455
ENSSSCG00000029830	chr4	136107	138652	295751.29975	chr4	295751	299751	0.65162601
METTL23	chr12	4804037	4808913	908039.4909	chr12	4908039	4909329	0.65164323
EBNA1BP2	chr6	168049758	168061013	469856.16847	chr6	168469856	168474916	0.65166356
GALNTL5	chr18	5353769	5433814	978355.4984	chr18	4978355	4984775	0.65212508
HNRNPUL2	chr2	9034667	9048768	082256.90862	chr2	9082256	9086256	0.65255724
EBNA1BP2	chr6	168049758	168061013	556186.16766	chr6	167656186	167665426	0.65259418
COPS9	chr15	139260478	139264211	479553.1394	chr15	139479553	139483165	0.65271633
IK	chr2	142361155	142376333	483896.14248	chr2	142483896	142487896	0.65291751
TUBB2B	chr7	1951407	1956119	470509.14725	chr7	1470509	1472579	0.6530349
SEZ6L2	chr3	18098102	18119558	021096.18025	chr3	18021096	18025096	0.6533819
WDR5	chr1	273560283	273578063	551376.27365	chr1	273651376	273656236	0.65345299
POLR1D	chr11	5044329	5089766	241105.5241	chr11	5241105	5241689	0.65368869
CBX8	chr12	2562374	2565780	141178.2144	chr12	2141178	2144859	0.6537205
SSR4	chrX	124537540	124541637	423872.12442	chrX	124423872	124427872	0.65411663
MRPS24	chr18	48825782	48830609	267135.4926	chr18	49267135	49269745	0.65421461
MRPL36	chr16	79001549	79004358	970059.7897	chr16	78970059	78976349	0.65431604
RPL7L1	chr7	37940834	37951324	097569.38102	chr7	38097569	38102239	0.65449679
BRD9	chr16	79473745	79492635	638839.7964	chr16	79638839	79642899	0.65453587
SSR4	chrX	124537540	124541637	407289.12441	chrX	124407289	124411289	0.65487882
ATG4B	chr15	140223904	140246321	822668.1398	chr15	139822668	139834538	0.65488313
STK25	chr15	140144336	140154366	858888.1398	chr15	139858888	139868418	0.65503532
ENSSSCG00000017955	chr12	52878764	52882863	006559.5300	chr12	53006559	53008829	0.6555723
MRPL52	chr7	76187446	76191901	003612.76007	chr7	76003612	76007612	0.6563334
LIG1	chr6	53620483	53686562	431154.53431	chr6	53431154	53431639	0.65650402
ENSSSCG00000045223	chr6	166945409	166957165	829006.16683	chr6	166829006	166831646	0.65676563
NDUFA10	chr15	138986421	139031897	787006.1387	chr15	138787006	138788821	0.65684593
ENSSSCG00000035997	chrX	124926772	124929791	180305.12518	chrX	125180305	125182027	0.65741725
MRPS23	chr12	34122020	34129548	896719.3390	chr12	33896719	33901049	0.65744412
ENSSSCG00000031249	chr13	207493659	207499867	959056.2079	chr13	207959056	207961576	0.65778265
RPP40	chr7	2987271	3005271	573209.25814	chr7	2573209	2581459	0.65899626
MAF1	chr4	597472	600447	295751.29975	chr4	295751	299751	0.65900329
BRD9	chr16	79473745	79492635	605069.7960	chr16	79605069	79606470	0.65948601
POP7	chr3	8605018	8610218	836970.88376	chr3	8836970	8837652	0.65959159
COX4I1	chr6	3131019	3137530	809516.28170	chr6	2809516	2817016	0.65982335
CBR3	chr13	199830668	199840442	126305.2001	chr13	200126305	200129795	0.66021308
DUSP28	chr15	139537326	139539438	858888.1398	chr15	139858888	139868418	0.66043559
PSMC3IP	chr12	20244304	20250159	166659.2016	chr12	20166659	20168579	0.66069029
MGMT	chr14	138499309	138771540	416319.1384	chr14	138416319	138425189	0.66172784
RPS7	chr3	131258722	131263256	728816.13173	chr3	131728816	131731070	0.66192542
ENSSSCG00000036812	chr12	61077631	61186002	055733.6105	chr12	61055733	61059733	0.66205794
TUBB2B	chr7	1951407	1956119	259389.22659	chr7	2259389	2265929	0.66243374
TBCC	chr7	37822767	37824953	097569.38102	chr7	38097569	38102239	0.66350949
VPS26C	chr13	200869167	200926108	197137.2012	chr13	201197137	201201137	0.66441309
P4HB	chr12	1121824	1131289	304812.1306	chr12	1304812	1306458	0.6647682
CTPS1	chr6	170201751	170233016	907106.16991	chr6	169907106	169912386	0.66560373
SSR1	chr7	4652921	4685901	273899.42749	chr7	4273899	4274969	0.66567638
ENSSSCG00000057427	chr18	2568274	2595510	176050.2180	chr18	2176050	2180050	0.66596772
RPL4	chr1	164475357	164481534	734118.16473	chr1	164734118	164734722	0.66598741
ENSSSCG00000047605	chr4	128894115	128943092	095199.12909	chr4	129095199	129098159	0.66602911
HEXIM2	chr12	18251428	18258295	036976.1804	chr12	18036976	18040754	0.66631135
ENSSSCG00000045735	chr18	50458411	50463097	246693.5025	chr18	50246693	50250693	0.66640116
JPT1	chr12	6139428	6158452	214528.6215	chr12	6214528	6215144	0.66693713

COPS9	chr15	139260478	139264211	048928.1390	chr15	139048928	139052328	0.66762411
RPS5	chr6	62967994	62974350	745816.6274	chr6	62745816	62746568	0.66786857
ENSSSCG00000045735	chr18	50458411	50463097	233045.5023	chr18	50233045	50234445	0.66838024
ENSSSCG00000008097	chr3	43977111	44022535	973665.4397	chr3	43973665	43974341	0.66870514
NFYC	chr6	170413638	170485840	187216.1701	chr6	170187216	170189606	0.66877654
MRPL36	chr16	79001549	79004358	715419.7872	chr16	78715419	78721319	0.66934382
FAM104A	chr12	7727796	7749240	934529.7938	chr12	7934529	7938329	0.6695177
ENSSSCG00000045735	chr18	50458411	50463097	064445.5006	chr18	50064445	50066235	0.6696349
RNASEH2A	chr2	66193197	66207697	910372.6591	chr2	65910372	65911524	0.67036194
VPS28	chr4	362912	370588	295751.2997	chr4	295751	299751	0.67290356
POP7	chr3	8605018	8610218	832274.8836	chr3	8832274	8836274	0.6729648
USP36	chr12	3309237	3348525	215499.3219	chr12	3215499	3219479	0.67319271
GATD1	chr2	474296	480736	130230.1336	chr2	130230	133660	0.67397093
SELENOF	chr4	129258944	129287720	935039.1289	chr4	128935039	128939899	0.67404559
CTDNEP1	chr12	52599226	52606059	006559.5300	chr12	53006559	53008829	0.67469679
MRPL36	chr16	79001549	79004358	837739.7884	chr16	78837739	78842699	0.67522116
DND1	chr2	142384532	142387199	483896.1424	chr2	142483896	142487896	0.67586187
ENSSSCG00000037652	chr3	65951	140492	345186.3521	chr3	345186	352116	0.67608237
CYC1	chr4	606516	608996	295751.2997	chr4	295751	299751	0.67660555
CYBC1	chr12	656347	662513	453099.4609	chr12	453099	460989	0.67677096
CDIPT	chr3	18084549	18091798	812944.1781	chr3	17812944	17813910	0.67697382
GAA	chr12	2314591	2336988	141178.2144	chr12	2141178	2144859	0.67830672
CHCHD5	chr3	43908122	43915709	973665.4397	chr3	43973665	43974341	0.67855284
CCNL2	chr6	63659054	63668047	444151.6344	chr6	63444151	63445363	0.67888401
ENSSSCG00000045735	chr18	50458411	50463097	977355.4998	chr18	49977355	49983615	0.6800394
USP11	chrX	41851602	41866355	488466.4148	chrX	41488466	41489630	0.68083023
FIGNL1	chr9	136484216	136488250	460695.1364	chr9	136460695	136461442	0.68088733
MED8	chr6	167861373	167869411	986092.1679	chr6	167986092	167990092	0.68159164
CTPS1	chr6	170201751	170233016	187216.1701	chr6	170187216	170189606	0.68172316
PCGF3	chr8	140446	182261	444863.4507	chr8	444863	450757	0.68188859
NARF	chr12	632363	651817	143994.1479	chr12	143994	147994	0.68219344
MRPL36	chr16	79001549	79004358	534549.7853	chr16	78534549	78538079	0.68234122
MAP2K3	chr12	61396369	61417321	055733.6105	chr12	61055733	61059733	0.68236055
NDUFA10	chr15	138986421	139031897	509228.1385	chr15	138509228	138517658	0.68250111
MRPL36	chr16	79001549	79004358	203589.7921	chr16	79203589	79211199	0.68271127
ENSSSCG00000024070	chr18	6166918	6171153	873195.5876	chr18	5873195	5876335	0.68388541
WDR91	chr18	14092322	14126475	995535.1399	chr18	13995535	13998965	0.68464662
ENSSSCG00000031299	chr2	64723435	64731124	716906.6472	chr2	64716906	64720906	0.68562524
ZNF250	chr4	66932	84170	295751.2997	chr4	295751	299751	0.68648929
DTYMK	chr15	140248079	140255805	865812.1398	chr15	139865812	139867602	0.6866551
ARAF	chrX	42078206	42090250	174600.4217	chrX	42174600	42175367	0.68752819
BRD9	chr16	79473745	79492635	203589.7921	chr16	79203589	79211199	0.68890032
ENSSSCG00000029830	chr4	136107	138652	377258.3783	chr4	377258	378347	0.68991788
TXNL4A	chr6	127974543	127991177	713256.1277	chr6	127713256	127715196	0.69087878
MRPL36	chr16	79001549	79004358	048763.7905	chr16	79048763	79050221	0.69121762
DGAT1	chr4	452662	466684	295751.2997	chr4	295751	299751	0.69189783
SSR4	chrX	124537540	124541637	424348.1244	chrX	124424348	124429148	0.69224072
MRPS24	chr18	48825782	48830609	225095.4923	chr18	49225095	49233665	0.69253218
ENSSSCG00000003930	chr6	166549949	166553230	829006.1668	chr6	166829006	166831646	0.6935188
ATP6V0B	chr6	167316947	167320212	658726.1676	chr6	167658726	167662326	0.69605677
DPEP1	chr6	361197	376616	179225.1832	chr6	179225	183225	0.69622281
BCAP31	chrX	124457001	124484743	407289.1244	chrX	124407289	124411289	0.69664106
EBNA1BP2	chr6	168049758	168061013	986876.1679	chr6	167986876	167989686	0.69691195
MTMR1	chrX	122394921	122453905	707368.1227	chrX	122707368	122709996	0.69733178
TXN2	chr5	11264645	11283395	980748.1098	chr5	10980748	10985038	0.69801193
UBXN6	chr2	74357517	74378592	729195.7473	chr2	74729195	74730347	0.70026247
TBCD	chr12	329714	449556	143994.1479	chr12	143994	147994	0.70064283
EBNA1BP2	chr6	168049758	168061013	658726.1676	chr6	167658726	167662326	0.70324623
ATP6V0B	chr6	167316947	167320212	656186.1676	chr6	167656186	167665426	0.7032465
DRAP1	chr2	6409655	6412395	895080.6899	chr2	6895080	6899700	0.7051387
SHD	chr2	74497351	74504659	042210.7404	chr2	74042210	74044130	0.70644813
COX4I1	chr6	3131019	3137530	669978.2673	chr6	2669978	2673391	0.70704147
NAA60	chr3	38763447	38781988	260264.3926	chr3	39260264	39264264	0.70853161

CPB2	chr11	21234447	21298241	0743220.2074	chr11	20743220	20746240	0.70914911
ENSSSCG00000045735	chr18	50458411	50463097	0125275.5012	chr18	50125275	50128495	0.70992137
ENSSSCG00000061760	chr7	31573753	31585356	159320.31163	chr7	31159320	31163320	0.71034255
NARF	chr12	632363	651817	.453099.4609	chr12	453099	460989	0.71049724
NDUFA10	chr15	138986421	139031897	0048928.1390	chr15	139048928	139052328	0.71732611
GATD1	chr2	474296	480736	2.90704.93254	chr2	90704	93254	0.72243002
ENSSSCG00000032573	chr4	75636278	75646153	739459.75740	chr4	75739459	75740629	0.72259276
WRAP73	chr6	65155209	65167079	662641.64666	chr6	64662641	64666641	0.72476121
ATP6V0B	chr6	167316947	167320212	829006.16683	chr6	166829006	166831646	0.72832481
UBXN6	chr2	74357517	74378592	042210.74044	chr2	74042210	74044130	0.72942238
WDFY2	chr11	16092698	16195134	6514780.1651	chr11	16514780	16518890	0.73000259
EBNA1BP2	chr6	168049758	168061013	986092.16799	chr6	167986092	167990092	0.73386015
OTUD5	chrX	43000231	43029468	357991.43361	chrX	43357991	43361991	0.74125253
DRAP1	chr2	6409655	6412395	456140.64608	chr2	6456140	6460880	0.74426475
GLP2R	chr12	54680057	54738102	4676329.5467	chr12	54676329	54677869	0.74778092
TALDO1	chr2	464728	472835	2.90704.93254	chr2	90704	93254	0.75405653
RNF220	chr6	166666138	166910646	829006.16683	chr6	166829006	166831646	0.7546161
STAT5A	chr12	20474227	20499138	0957709.2096	chr12	20957709	20961019	0.75871512
RPS7	chr3	131258722	131263256	726366.13173	chr3	131726366	131731426	0.76106648
CBR3	chr13	199830668	199840442	0128785.2001	chr13	200128785	200129482	0.76292937
TOMM22	chr5	9336130	9350015	545177.95491	chr5	9545177	9549177	0.77525101
EIF4E2	chr15	133060247	133103719	3473548.1334	chr15	133473548	133476628	0.79026245
WRAP73	chr6	65155209	65167079	664948.64665	chr6	64664948	64665830	0.7915506
