# Supplementary material for: Deciphering differences in DNA methylation and transcriptome profiles of oocytes from pigs with high and low developmental competence
Source: Environ Epigenet. 2025 Jun 3;11(1):dvaf018. doi: 10.1093/eep/dvaf018 (PMC12418950; doi:10.1093/eep/dvaf018)
Supplement: dvaf018_Supplemental_Files [file dvaf018_supplemental_files.zip › Sup table 19.pdf]

ID	Term	Ontology Source	Term PValue	Term P-Value Corrected with Bonferroni step down	Group PValue	Group P-Value Corrected with Bonferroni step down	GO Levels	GO Groups	% Associated Genes	Number of Genes	Associated Genes Found
GO:0006900	vesicle budding from membrane	GO_BiologicalProcess-EBI-UniProt-GOA-ACAP-ARAP_23.01.2024_00h00	0.00	0.00	0.00	0.00	[3, 5, 6]	Group0	12.24	6.00	[ANXA2, PEF1, SEC13, SEC24A, TFG, VAPA]
GO:0032480	negative regulation of type I interferon production	GO_BiologicalProcess-EBI-UniProt-GOA-ACAP-ARAP_23.01.2024_00h00	0.00	0.00	0.00	0.00	[4, 5, 6, 7, 8, 9, 10]	Group1	16.67	5.00	[ATG12, ATG5, CACTIN, MORC3, UFD1]
GO:0043954	cellular component maintenance	GO_BiologicalProcess-EBI-UniProt-GOA-ACAP-ARAP_23.01.2024_00h00	0.00	0.03	0.00	0.00	[4]	Group2	10.87	5.00	[ABHD17B, APPL1, CTTN, F2R, FERMT2]
GO:0098687	chromosomal region	GO_CellularComponent-EBI-UniProt-GOA-ACAP-ARAP_23.01.2024_00h00	0.00	0.01	0.00	0.00	[2, 6]	Group3	4.83	13.00	[AURKC, CENPW, CLASP2, KDM1A, MEAF6, NDC80, PPP1CC, SEC13, SMC5, THOC7, TP53BP1, ZNF618, ZWINT]
GO:0000775	chromosome, centromeric region	GO_CellularComponent-EBI-UniProt-GOA-ACAP-ARAP_23.01.2024_00h00	0.00	0.02	0.00	0.00	[3, 7]	Group3	5.56	10.00	[AURKC, CENPW, CLASP2, MEAF6, NDC80, PPP1CC, SEC13, TP53BP1, ZNF618, ZWINT]
GO:0000776	kinetochore	GO_CellularComponent-EBI-UniProt-GOA-ACAP-ARAP_23.01.2024_00h00	0.00	0.01	0.00	0.00	[3, 5, 8, 9]	Group3	6.92	9.00	[AURKC, CENPW, CLASP2, MEAF6, NDC80, PPP1CC, SEC13, TP53BP1, ZWINT]
