# Supplementary material for: Deciphering differences in DNA methylation and transcriptome profiles of oocytes from pigs with high and low developmental competence
Source: Environ Epigenet. 2025 Jun 3;11(1):dvaf018. doi: 10.1093/eep/dvaf018 (PMC12418950; doi:10.1093/eep/dvaf018)
Supplement: dvaf018_Supplemental_Files [file dvaf018_supplemental_files.zip › Sup table 2.pdf]

Gene name	P-value	avg_log2FC	pct.1	pct.2	p_value_adj
GSTM3	1.04E-86	-4.433006734	1	1	1.80E-82
TFDP2	2.75E-84	-3.177096336	1	1	4.78E-80
ZP3	2.38E-83	-6.015396062	1	1	4.13E-79
ATP5MC1	5.83E-81	-5.399416519	0.938	1	1.01E-76
SPC24	8.57E-78	-4.870920051	0.938	1	1.49E-73
ENSSSCG00000044567	5.89E-71	-5.010647244	0.812	1	1.02E-66
STX8	1.41E-70	-3.993366908	1	1	2.44E-66
VTI1B	6.98E-70	-3.022510367	1	1	1.21E-65
PSMA6	7.79E-67	-3.33267553	1	1	1.35E-62
ATP5IF1	6.91E-66	-5.134680245	0.875	1	1.20E-61
NUDT5	9.53E-66	-4.13922368	1	1	1.65E-61
POLR2G	2.20E-63	-5.191775723	0.938	1	3.82E-59
ENSSSCG00000013386	7.35E-63	-4.143414486	0.938	1	1.28E-58
ZAR1	1.21E-60	-4.289123635	0.938	1	2.10E-56
STAT5A	1.42E-59	-4.833612988	0.812	1	2.46E-55
PSMD13	4.61E-59	-2.947201482	0.938	1	8.00E-55
SKIC8	9.03E-58	-3.334381207	0.938	1	1.57E-53
CNIH4	9.73E-58	-3.272066477	1	1	1.69E-53
RPL7L1	6.73E-57	-3.737951715	1	1	1.17E-52
ENSSSCG00000054115	9.59E-57	-2.75729124	1	1	1.66E-52
ENSSSCG00000002036	1.51E-55	-4.154829138	1	1	2.62E-51
LSM2	7.07E-55	-4.993866456	1	1	1.23E-50
C6orf52	1.10E-54	-4.108141464	1	1	1.91E-50
PIGP	2.71E-53	-3.652805147	1	1	4.70E-49
BCAP31	6.92E-53	-4.23299078	0.875	1	1.20E-48
ENSSSCG00000048076	7.84E-53	-4.707819249	0.875	1	1.36E-48
RPL6	1.38E-51	-3.963033674	1	1	2.39E-47
EMC6	5.65E-51	-3.724084303	1	1	9.81E-47
HSBP1	2.19E-50	-3.109274224	1	1	3.80E-46
MRPL37	2.25E-50	-3.359639762	1	1	3.90E-46
TLE2	2.66E-50	-3.733296946	1	1	4.62E-46
PARK7	2.84E-50	-4.237632299	1	1	4.92E-46
CRNKL1	4.54E-49	-1.49424903	1	1	7.87E-45
TUBA1B	6.94E-49	-4.676940823	1	1	1.20E-44
RPL23A	9.12E-49	-4.981852653	0.875	1	1.58E-44
ENSSSCG00000024070	2.86E-48	-3.633975668	0.938	1	4.95E-44
COPZ1	3.32E-47	-3.028014376	1	1	5.76E-43
NDUFB9	3.76E-47	-4.719499041	1	1	6.52E-43
ENSSSCG00000015217	8.26E-47	-1.861187839	1	1	1.43E-42
ENSSSCG00000032166	1.75E-46	-6.263568638	0.812	1	3.04E-42
NSDHL	2.01E-46	-2.836175198	1	1	3.48E-42
GSDMB	2.93E-46	-3.485642171	1	1	5.09E-42
ZP4	6.97E-45	-3.388611949	1	1	1.21E-40
CCDC167	2.22E-44	-6.637898252	0.375	1	3.85E-40
CDC26	2.70E-44	-3.839590794	1	1	4.68E-40
ARMC2	1.70E-43	-2.013514806	1	1	2.96E-39
ENSSSCG00000023691	1.75E-43	-2.916550707	0.938	1	3.04E-39
STYXL1	6.49E-43	-3.058661912	0.938	1	1.13E-38
NDUFA11	1.46E-42	-5.048339534	0.875	1	2.53E-38
PRDX1	1.81E-42	-2.421547541	1	1	3.15E-38
SERF1A	2.81E-42	-5.027783157	0.75	1	4.87E-38
C11orf98	3.41E-42	-3.182719114	1	1	5.92E-38
ENSSSCG00000005528	4.73E-42	-3.751728947	1	1	8.21E-38
ENSSSCG00000024588	1.37E-41	-3.330148602	1	1	2.38E-37
WDR5	1.74E-41	-3.285333073	0.875	1	3.03E-37
TIMM22	1.85E-41	-3.358127286	0.938	1	3.20E-37
CHCHD4	3.26E-41	-2.13162464	1	1	5.66E-37
RPL7	3.38E-41	-3.57740515	1	1	5.87E-37
ENSSSCG00000061888	4.35E-41	4.03180478	1	1	7.54E-37
MRPL40	4.88E-41	-3.820266461	1	1	8.46E-37
NR2C2AP	5.52E-41	-3.742923049	0.938	1	9.58E-37
NDUFB8	7.29E-41	-3.946675592	1	1	1.26E-36
ENSSSCG00000028414	1.19E-40	-3.493621187	1	1	2.06E-36
COX5B	1.81E-40	-4.20701957	1	1	3.15E-36
ENSSSCG00000035909	2.97E-40	-3.31177874	0.938	1	5.15E-36
ENSSSCG00000039544	1.91E-39	-2.718025096	1	1	3.31E-35
COX7A2	2.14E-39	-4.894811864	1	1	3.72E-35
FTL	4.04E-39	-3.506178543	1	1	7.02E-35
REC114	6.77E-39	-2.635806178	1	1	1.17E-34

ENSSSCG00000032060	8.76E-39	-6.079688889	0.688	1	1.52E-34
IDI1	9.56E-39	-2.926917122	1	1	1.66E-34
MAP1LC3B	1.76E-38	-2.022026306	1	1	3.05E-34
GINS2	1.93E-38	-2.758285966	1	1	3.36E-34
NDUFA7	3.74E-38	-5.034557222	0.875	1	6.49E-34
PSMB7	4.23E-38	-3.308349922	1	1	7.35E-34
CDIPT	4.47E-38	-3.147741853	1	1	7.75E-34
RHOC	7.04E-38	-3.987706724	0.875	1	1.22E-33
CNPY2	1.18E-37	-3.541854344	1	1	2.05E-33
MRPS25	1.38E-37	-3.21897236	1	1	2.40E-33
GDF9	1.63E-37	-2.261016009	1	1	2.83E-33
DPPA5	2.51E-37	-3.463134425	1	1	4.35E-33
ENSSSCG00000039523	3.75E-37	-3.090659313	1	1	6.51E-33
ENSSSCG00000028167	5.41E-37	-4.862881144	0.812	1	9.38E-33
HAUS1	1.20E-36	-2.349847571	1	1	2.08E-32
ENSSSCG00000007493	1.61E-36	-2.177421518	1	1	2.79E-32
NEDD8	1.95E-36	-3.312857104	1	1	3.38E-32
TFB1M	3.24E-36	-2.182425236	1	1	5.62E-32
MYL6	3.60E-36	-4.504923003	0.812	1	6.25E-32
MRPL51	3.98E-36	-2.904937803	1	1	6.91E-32
ENSSSCG00000009610	4.17E-36	-3.116655702	1	1	7.23E-32
CBR3	4.82E-36	-2.833538854	1	1	8.35E-32
GEMIN6	7.36E-36	-2.451362189	1	1	1.28E-31
ENSSSCG00000032129	8.37E-36	-3.418551983	1	1	1.45E-31
PFDN1	9.79E-36	-2.36656808	1	1	1.70E-31
POP4	1.93E-35	-3.189970944	0.938	1	3.35E-31
ENSSSCG00000009303	2.84E-35	-4.620378184	0.938	1	4.92E-31
TNNT1	5.42E-35	-4.763357093	0.625	1	9.41E-31
LSM10	6.93E-35	-3.055742552	1	1	1.20E-30
TK1	7.53E-35	-4.49966875	0.688	1	1.31E-30
NDUFA9	9.69E-35	-3.241753486	1	1	1.68E-30
POLR3H	1.13E-34	-3.287064475	1	1	1.96E-30
PPIB	1.49E-34	-3.708211765	0.938	1	2.58E-30
POLR2F	1.73E-34	-4.927685795	0.625	1	3.00E-30
ENSSSCG00000057874	1.77E-34	-3.306103128	1	1	3.08E-30
PDCD5	2.53E-34	-2.444916142	1	1	4.40E-30
SYCN	2.98E-34	-4.093109404	0.812	1	5.16E-30
ATP6V1D	7.02E-34	-1.394783349	1	1	1.22E-29
ARHGAP22	1.41E-33	-2.109076153	1	1	2.45E-29
CD320	1.76E-33	-6.590779827	0.375	1	3.05E-29
ENSSSCG00000032916	1.84E-33	-3.085020584	1	1	3.19E-29
COX5A	1.98E-33	-2.844683085	1	1	3.43E-29
BUD31	2.27E-33	-2.264183048	1	1	3.95E-29
MTRES1	2.35E-33	-2.19291522	0.938	1	4.08E-29
GGH	2.93E-33	-2.920516485	1	1	5.09E-29
GPN1	4.72E-33	-1.127382596	1	1	8.19E-29
ENSSSCG00000027041	5.02E-33	-2.339220179	1	1	8.72E-29
MRPL30	9.01E-33	-4.191843358	0.688	1	1.56E-28
EIF3M	9.09E-33	-1.01368084	1	1	1.58E-28
EEF1B2	1.22E-32	-2.816700776	1	1	2.11E-28
MRPS15	2.47E-32	-3.836501268	0.938	1	4.28E-28
ENSSSCG00000002963	3.16E-32	-3.149940627	0.938	1	5.48E-28
ENSSSCG00000015337	3.76E-32	-3.937715722	0.875	1	6.52E-28
CYTH2	4.56E-32	-3.523840711	1	1	7.91E-28
TMEM219	4.76E-32	-4.427010141	0.625	1	8.27E-28
TPI1	5.88E-32	-3.851403508	0.875	1	1.02E-27
RABAC1	7.54E-32	-4.64200265	0.75	1	1.31E-27
CUL7	8.33E-32	-3.56125824	0.938	1	1.45E-27
POLR2D	9.85E-32	-1.857845832	1	1	1.71E-27
ENSSSCG00000005217	1.01E-31	-2.325255617	0.938	1	1.75E-27
FIGLA	1.24E-31	-4.372532771	0.75	1	2.16E-27
ENSSSCG00000001696	1.25E-31	-4.375425643	0.688	1	2.16E-27
RAB7A	1.81E-31	-1.667076512	1	1	3.15E-27
ENSSSCG00000000905	1.84E-31	-3.9492467	1	1	3.19E-27
NDUFV2	1.92E-31	-2.049388027	1	1	3.33E-27
H2BK1	2.14E-31	-7.584962501	0.125	1	3.72E-27
MRPL28	4.09E-31	-4.910732662	0.625	1	7.09E-27
UQCRQ	6.43E-31	-3.437593624	1	1	1.12E-26
SHD	6.93E-31	-4.824428435	0.5	1	1.20E-26
ENSSSCG00000006081	8.10E-31	-2.750199047	1	1	1.41E-26

POLR2H	1.14E-30	-2.49986834	1	1	1.99E-26
FSD2	1.60E-30	-2.62995331	1	1	2.77E-26
POLE4	1.84E-30	-2.800647975	1	1	3.19E-26
SMIM26	1.90E-30	-4.730160416	0.375	1	3.30E-26
DAD1	1.93E-30	-3.080472579	0.938	1	3.34E-26
PRDX3	2.76E-30	-1.867487004	1	1	4.80E-26
ENSSSCG00000024520	3.07E-30	-1.706520335	1	1	5.33E-26
COX4I1	3.08E-30	-3.69743723	1	1	5.34E-26
VKORC1	3.53E-30	-3.464926375	0.75	1	6.12E-26
HAX1	4.20E-30	-1.716123211	1	1	7.28E-26
MYL12A	4.74E-30	-2.429016083	1	1	8.23E-26
CHMP2A	4.78E-30	-5.568842835	0.438	1	8.29E-26
VDAC3	5.40E-30	-2.721494185	0.938	1	9.38E-26
MRPS10	6.05E-30	-1.385167992	1	1	1.05E-25
MARS1	6.10E-30	-2.440010028	1	1	1.06E-25
EIF6	6.33E-30	-3.256408929	0.875	1	1.10E-25
AP2S1	6.45E-30	-5.299309792	0.562	1	1.12E-25
SLC25A26	8.03E-30	-1.282001028	1	1	1.39E-25
GPX4	8.51E-30	-4.381507876	0.688	1	1.48E-25
ITGB5	1.14E-29	-3.323575948	0.938	1	1.97E-25
MVP	1.19E-29	-2.662130361	1	1	2.06E-25
ENSSSCG00000017509	1.20E-29	-4.387588775	0.938	1	2.08E-25
POLR2K	1.33E-29	-2.581325519	1	1	2.31E-25
AMZ2	1.51E-29	-2.380368483	0.938	1	2.62E-25
MGMT	2.30E-29	-4.186037006	0.75	1	3.99E-25
PAIP2	2.53E-29	-3.047305715	1	1	4.40E-25
RPS5	2.64E-29	-4.450661409	0.938	1	4.59E-25
HADHA	2.75E-29	-2.194422891	1	1	4.77E-25
TMEM128	3.34E-29	3.015103393	1	1	5.80E-25
DEGS2	3.75E-29	-3.737548384	0.875	1	6.51E-25
ROMO1	3.84E-29	-5.458181991	0.438	1	6.66E-25
TIMM17B	5.42E-29	-3.693022247	0.812	1	9.41E-25
TOMM20	5.88E-29	-2.134301092	1	1	1.02E-24
COA4	7.59E-29	-2.543142325	1	1	1.32E-24
TAF1A	8.06E-29	2.647720015	1	1	1.40E-24
ENSSSCG00000040854	9.01E-29	-4.499101764	0.688	1	1.56E-24
NUDC	9.59E-29	-2.976106794	0.938	1	1.66E-24
ENSSSCG00000010058	1.41E-28	-2.457319548	1	1	2.44E-24
ENSSSCG00000013064	1.65E-28	-3.364342109	0.938	1	2.86E-24
VPS29	1.75E-28	-1.61027192	1	1	3.03E-24
PPAN	1.91E-28	-2.36143811	1	1	3.31E-24
SELENOS	1.93E-28	-2.663099398	0.938	1	3.34E-24
ENSSSCG00000014071	2.43E-28	2.820801619	1	1	4.22E-24
LALBA	2.56E-28	-3.833141302	0.812	1	4.44E-24
TMEM125	3.74E-28	-7.070389328	0.062	1	6.49E-24
RNH1	4.12E-28	-6.180572246	0.062	1	7.15E-24
CLDN10	4.78E-28	-2.1498785	1	1	8.29E-24
IMP4	5.22E-28	-3.545369368	0.812	1	9.06E-24
SFT2D1	5.58E-28	3.120036725	1	1	9.69E-24
UXT	6.01E-28	-4.46634959	0.812	1	1.04E-23
BUD23	6.03E-28	-2.697880386	0.938	1	1.05E-23
POMP	8.30E-28	-2.70286109	1	1	1.44E-23
EMG1	8.56E-28	-4.410522018	0.688	1	1.48E-23
DYDC1	8.75E-28	-2.802060622	0.938	1	1.52E-23
COMMD9	9.11E-28	-3.814644958	0.812	1	1.58E-23
ENSSSCG00000056716	9.23E-28	-3.159198595	0.875	1	1.60E-23
ENSSSCG00000038035	1.13E-27	-3.163058282	0.938	1	1.97E-23
NAXE	1.16E-27	-3.332180567	0.75	1	2.01E-23
MRPL55	1.20E-27	-4.193090285	0.875	1	2.08E-23
TLCD1	1.51E-27	-2.229753584	0.875	1	2.62E-23
PHB1	1.66E-27	-2.307003265	1	1	2.88E-23
EMC7	1.90E-27	-2.695681305	0.812	1	3.30E-23
UBL7	2.15E-27	-2.729380999	1	1	3.74E-23
HAUS4	2.44E-27	2.453180215	1	1	4.24E-23
ENSSSCG00000007826	2.94E-27	-2.505740406	1	1	5.10E-23
ARG2	3.06E-27	-3.033608393	0.875	1	5.31E-23
DPCD	3.32E-27	-3.404832717	1	1	5.76E-23
C1orf105	3.62E-27	-3.343846451	0.875	1	6.27E-23
SAT2	4.96E-27	-3.600904045	0.875	1	8.60E-23
AJUBA	5.41E-27	-2.760065277	0.938	1	9.39E-23

FLOT1	6.04E-27	-2.252063288	0.938	1	1.05E-22
GUCA1A	6.23E-27	-2.830074999	1	1	1.08E-22
GTF2F1	7.88E-27	-2.641313355	1	1	1.37E-22
SNX22	8.80E-27	-3.823502345	0.75	1	1.53E-22
TALDO1	9.07E-27	-4.133855747	0.688	1	1.57E-22
NOP10	1.05E-26	-2.281381064	1	1	1.82E-22
RPS18	1.10E-26	-3.563041882	1	1	1.90E-22
ENSSSCG00000042862	1.17E-26	-5.993976212	0.75	0.933	2.02E-22
ENSSSCG00000010056	1.26E-26	-3.254997087	1	1	2.18E-22
ADA	1.54E-26	-2.3111759	1	1	2.67E-22
ENSSSCG00000014284	1.66E-26	-2.572968905	1	1	2.88E-22
ENSSSCG00000012842	2.09E-26	-6.290817562	0.125	1	3.62E-22
NDUFA13	2.68E-26	-4.503805356	0.625	1	4.64E-22
MIEN1	3.05E-26	-4.732837709	0.562	1	5.30E-22
PTPMT1	3.09E-26	-3.107815903	0.812	1	5.37E-22
COX6A1	3.40E-26	-3.538668848	0.875	1	5.90E-22
RPL38	4.10E-26	-6.42103624	0.125	1	7.11E-22
ASB11	4.27E-26	-2.270431399	1	1	7.40E-22
CZIB	4.76E-26	-4.302042786	0.812	1	8.26E-22
IMP3	5.48E-26	-1.525461489	1	1	9.51E-22
TSPAN31	6.10E-26	-3.098110085	0.812	1	1.06E-21
SSBP1	6.24E-26	-1.543400369	1	1	1.08E-21
DNAJC15	7.29E-26	-1.999860197	1	1	1.26E-21
PDZD11	7.66E-26	-3.053363226	0.938	1	1.33E-21
ENSSSCG00000009851	8.03E-26	-2.701658725	1	1	1.39E-21
PTS	8.50E-26	2.946358034	1	1	1.48E-21
SLC25A38	9.15E-26	-2.978279803	0.875	1	1.59E-21
ENSSSCG00000000296	1.15E-25	3.605214326	1	1	2.00E-21
COPS9	1.20E-25	-4.421580345	0.625	1	2.07E-21
PTPRT	1.25E-25	-1.823371458	1	1	2.18E-21
ATP5MC2	1.27E-25	-2.416655778	1	1	2.20E-21
ENSSSCG00000045485	1.46E-25	-4.54092194	0.562	1	2.53E-21
SELENOF	1.48E-25	-1.351053043	1	1	2.56E-21
SNU13	1.51E-25	-2.076890894	1	1	2.61E-21
TMEM126A	2.18E-25	-3.275471509	0.938	1	3.78E-21
ACAD9	2.27E-25	-2.965234582	0.812	1	3.94E-21
BCKDHA	2.40E-25	-3.452259963	0.938	1	4.17E-21
TIMM13	2.95E-25	-2.714597781	0.875	1	5.11E-21
CDK5RAP3	2.97E-25	-3.986672468	0.625	1	5.14E-21
DLGAP4	3.26E-25	-1.542240102	1	1	5.65E-21
WRAP53	3.59E-25	-5.162782932	0.312	0.933	6.22E-21
ENSSSCG00000017913	4.57E-25	-2.531814801	1	1	7.94E-21
ATP5PO	4.63E-25	-2.068333059	1	1	8.04E-21
ENSSSCG00000052263	5.25E-25	-2.573374526	1	1	9.10E-21
NDUFB11	5.33E-25	-4.478540442	0.562	1	9.25E-21
NDUFA10	5.47E-25	-3.57797607	0.688	1	9.50E-21
ENSSSCG00000061874	5.56E-25	-5.870993208	0.688	1	9.65E-21
ENSSSCG00000038628	6.86E-25	-1.321073299	1	1	1.19E-20
RPS14	8.58E-25	-4.619282574	0.688	1	1.49E-20
FAM162A	8.76E-25	-2.020008913	1	1	1.52E-20
ENSSSCG00000021644	9.67E-25	1.990075288	1	1	1.68E-20
ENSSSCG00000041579	1.11E-24	-3.245833665	0.875	1	1.93E-20
ENSSSCG00000055438	1.14E-24	-6.045850652	0.625	1	1.98E-20
ENSSSCG00000028397	1.21E-24	-1.930727165	1	1	2.11E-20
MRPS33	1.31E-24	-2	1	1	2.28E-20
COPS3	1.36E-24	-2.806991385	0.938	1	2.36E-20
GLRX3	1.44E-24	-2.717600269	0.938	1	2.51E-20
TDRD10	1.45E-24	-3.101440679	0.938	1	2.52E-20
DDT	1.52E-24	-5.571156701	0.25	1	2.64E-20
HSD17B7	1.67E-24	-1.58770869	1	1	2.90E-20
ENSSSCG00000018046	2.00E-24	-3.623946691	0.875	1	3.47E-20
EBNA1BP2	2.41E-24	-2.130103612	1	1	4.18E-20
ALDOC	2.71E-24	-2.715262464	0.875	1	4.70E-20
ACAT1	3.20E-24	-1.997719059	1	1	5.55E-20
TRAPPC4	3.20E-24	-2.068810808	1	1	5.56E-20
C19orf38	3.64E-24	-4.584962501	0.375	1	6.31E-20
MANBA	4.71E-24	-1.741369417	1	1	8.17E-20
MIX23	5.17E-24	-2.010940863	1	1	8.98E-20
SUCLG1	6.13E-24	-2.486181572	0.938	1	1.06E-19
ATP5F1A	7.27E-24	-1.598644656	1	1	1.26E-19

MAPK13	7.63E-24	-3.710204526	0.688	1	1.32E-19
BTG4	8.42E-24	2.767651617	1	1	1.46E-19
PDCD6	8.68E-24	-1.213925	1	1	1.51E-19
CUTA	8.91E-24	-4.365132593	0.562	1	1.55E-19
CNBP	8.99E-24	1.758101516	1	1	1.56E-19
MRPS28	9.31E-24	-2.276470545	0.938	1	1.62E-19
LAMTOR2	9.49E-24	-3.888045067	0.75	1	1.65E-19
SLC35G1	9.66E-24	2.529820947	1	1	1.68E-19
NSMCE4A	9.70E-24	2.367134588	1	1	1.68E-19
MICOS13	9.88E-24	-5.137503524	0.312	1	1.71E-19
NDUFS3	1.12E-23	-2.803482798	0.812	1	1.95E-19
UQCRB	1.18E-23	-2.375613674	1	1	2.05E-19
SEC11C	1.46E-23	-2.531682418	1	1	2.53E-19
TMEM147	1.49E-23	-4.119909464	0.562	1	2.58E-19
WDR74	1.52E-23	-2.777013755	0.812	1	2.64E-19
GFER	1.60E-23	-4.389042291	0.375	1	2.78E-19
RPS10	1.76E-23	-3.92372842	0.938	1	3.06E-19
RPS9	1.89E-23	-4.202678914	0.938	1	3.27E-19
MRPL44	2.36E-23	3.135383216	1	0.867	4.09E-19
EEF1D	2.42E-23	-3.703351314	0.875	1	4.21E-19
DERL3	2.70E-23	-5.758445322	0.188	1	4.69E-19
MRPL54	2.88E-23	-2.796286685	0.875	1	4.99E-19
RTF2	3.19E-23	-1.445300338	1	1	5.54E-19
UQCR10	3.67E-23	-3.520970944	0.875	1	6.37E-19
EFHC1	3.76E-23	-1.765323099	1	1	6.52E-19
NR0B1	3.98E-23	-2.360289747	1	1	6.91E-19
OOEP	4.23E-23	-2.496253737	1	1	7.35E-19
EXOSC4	4.24E-23	-5.895302621	0.188	0.933	7.36E-19
ENSSSCG00000027538	4.31E-23	-3.969626351	0.625	1	7.48E-19
CDK5RAP2	4.54E-23	-2.672487787	1	1	7.88E-19
RNF26	4.85E-23	-2.253756592	1	1	8.42E-19
FIBP	4.95E-23	-4.315501826	0.312	1	8.58E-19
POLR1D	5.37E-23	-2.054281661	1	1	9.32E-19
ENSSSCG00000033310	5.53E-23	-5.173856818	0.5	1	9.59E-19
ENSSSCG00000036499	5.58E-23	-1.361992689	1	1	9.68E-19
STMP1	5.59E-23	-1.666769256	1	1	9.70E-19
GMPS	5.89E-23	-1.501278506	1	1	1.02E-18
LUC7L2	5.96E-23	-1.636956051	1	1	1.03E-18
ENSSSCG00000025673	6.86E-23	-3.097667686	0.875	1	1.19E-18
CCDC12	9.34E-23	-1.969102764	1	1	1.62E-18
ENSSSCG00000002020	1.00E-22	2.049370351	1	1	1.74E-18
GALK1	1.00E-22	-4.415037499	0.625	0.933	1.74E-18
ENSSSCG00000024974	1.04E-22	-4.60968493	0.312	1	1.80E-18
CHRAC1	1.05E-22	2.747817929	1	1	1.82E-18
COMMD4	1.11E-22	-4.653824359	0.562	0.933	1.93E-18
ENSSSCG00000011272	1.32E-22	-2.396632919	1	1	2.29E-18
ENSSSCG00000037510	1.35E-22	-3.152890633	0.938	1	2.34E-18
C11orf80	1.38E-22	-1.502180062	1	1	2.39E-18
PHB2	1.41E-22	-1.998170329	1	1	2.45E-18
TPRKB	1.43E-22	-5.24961389	0.625	1	2.48E-18
AP1M2	1.44E-22	-2.966503088	0.812	1	2.50E-18
EIF3H	1.48E-22	-1.706572228	1	1	2.56E-18
NLRP5	1.51E-22	-1.92919812	1	1	2.61E-18
FZR1	1.55E-22	-2.333531241	0.938	1	2.68E-18
AP1S1	1.69E-22	-2.55468949	0.938	1	2.93E-18
TEX35	1.86E-22	-2.928050158	0.812	1	3.24E-18
MRPL20	1.97E-22	-2.004800986	1	1	3.42E-18
FAM98A	1.98E-22	-1.385160458	1	1	3.44E-18
MRPL45	2.04E-22	-2.141318497	0.938	1	3.54E-18
FAM32A	2.21E-22	-2.258772379	1	1	3.83E-18
MEA1	2.30E-22	-2.845181891	0.938	1	3.99E-18
MALSU1	2.42E-22	-4.973527789	0.438	1	4.19E-18
GABARAP	2.51E-22	-2.632747915	0.938	1	4.36E-18
FKBP4	2.57E-22	-2.092006005	1	1	4.46E-18
TPT1	2.62E-22	-3.057143907	0.875	1	4.55E-18
SYT2	2.62E-22	-3.671377253	0.625	1	4.55E-18
ENSSSCG00000052310	3.13E-22	-3.395408693	0.812	1	5.44E-18
ERP29	3.49E-22	-3.653824359	0.812	1	6.06E-18
HSPA8	4.33E-22	-1.646974879	1	1	7.52E-18
FDPS	4.57E-22	-3.160032957	0.875	1	7.92E-18

ATOX1	4.61E-22	-4.552541023	0.562	1	8.00E-18
MRPL36	5.17E-22	-3.268648012	0.75	1	8.96E-18
DCTN3	6.64E-22	-3.398917834	0.938	1	1.15E-17
ENSSSCG00000022401	6.71E-22	-2.454930226	0.875	1	1.16E-17
ECRG4	6.96E-22	-5.006853671	0.312	1	1.21E-17
GDPD5	7.63E-22	-2.949373927	0.75	1	1.32E-17
METTL23	8.60E-22	-2.712718048	0.875	1	1.49E-17
SCNM1	9.75E-22	-3.102173487	1	1	1.69E-17
PRPF4	1.00E-21	-1.93844619	1	1	1.74E-17
WDR91	1.01E-21	-1.308978702	1	1	1.76E-17
PUF60	1.08E-21	-2.51524796	1	1	1.88E-17
RPS25	1.13E-21	-3.271284293	1	1	1.97E-17
RTN4IP1	1.14E-21	-1.682666479	0.938	1	1.97E-17
TMEM248	1.16E-21	-1.884414833	1	1	2.01E-17
ARPC3	1.22E-21	-2.343222583	1	1	2.12E-17
ENSSSCG00000008012	1.25E-21	-2.203219161	1	1	2.18E-17
MRPL17	1.28E-21	-2.91707904	0.688	1	2.22E-17
ENSSSCG00000017955	1.33E-21	-3.064780681	0.688	1	2.31E-17
ENSSSCG00000034739	1.51E-21	-1.781165398	0.938	1	2.62E-17
RPP40	1.52E-21	-3.299363179	0.812	1	2.64E-17
CCDC32	1.63E-21	-3.082248412	0.75	0.933	2.83E-17
MRPL13	1.66E-21	-1.053300551	1	1	2.88E-17
TCP1	1.70E-21	-1.320024384	1	1	2.94E-17
ILF2	1.73E-21	-1.238172684	1	1	3.00E-17
ZMAT5	1.87E-21	-2.232085818	0.938	1	3.24E-17
UST	1.89E-21	-2.360331607	0.875	1	3.28E-17
SNX7	1.90E-21	2.868352343	1	1	3.30E-17
CLPP	2.11E-21	-3.338865819	0.688	1	3.66E-17
AMFR	2.20E-21	2.2644426	1	1	3.81E-17
CEP78	2.52E-21	2.692330741	1	1	4.37E-17
MDH1	3.00E-21	-1.813769426	1	1	5.20E-17
ENSSSCG00000040498	3.81E-21	3.214415625	1	0.933	6.61E-17
HNRNPC	4.48E-21	1.833240347	1	1	7.77E-17
HSPA4	5.41E-21	-1.356605547	1	1	9.38E-17
MACROD1	6.13E-21	-3.202733896	0.75	1	1.06E-16
ENSSSCG00000058091	6.23E-21	-3.494359952	0.812	1	1.08E-16
ENSSSCG00000017971	7.07E-21	-4.284250892	0.5	1	1.23E-16
ENSSSCG00000004703	7.99E-21	-3.589785373	1	1	1.39E-16
OXA1L	8.05E-21	-1.341373156	1	1	1.40E-16
BPNT1	8.10E-21	-1.101314327	1	1	1.41E-16
CD40	8.33E-21	-2.051325934	0.938	1	1.45E-16
RPL36	8.79E-21	-3.4639471	0.75	1	1.53E-16
TAGLN	9.42E-21	-4.017921908	0.562	0.933	1.63E-16
RNF121	9.49E-21	-2.499734664	0.938	1	1.65E-16
SNX31	1.02E-20	2.372787211	1	1	1.78E-16
FIS1	1.06E-20	-5.130198723	0.188	1	1.85E-16
TMBIM6	1.09E-20	-1.862974638	1	1	1.89E-16
RPL32	1.30E-20	-2.59946207	1	1	2.26E-16
POP7	1.36E-20	-2.12015628	1	1	2.36E-16
DTYMK	1.37E-20	-3.703973769	0.625	1	2.38E-16
MELK	1.38E-20	1.956840472	1	1	2.39E-16
LHFPL1	1.43E-20	-1.620728464	1	1	2.48E-16
ENSSSCG00000046592	1.54E-20	-2.753669524	0.938	1	2.67E-16
CTNS	1.56E-20	-2.435262527	1	1	2.71E-16
DMAP1	1.58E-20	-4.627087976	0.25	1	2.74E-16
COX6C	1.59E-20	-2.84820373	1	1	2.76E-16
KCNIP4	1.74E-20	-1.505626031	1	1	3.01E-16
ZNHIT6	1.77E-20	-1.050108251	1	1	3.07E-16
MED8	1.77E-20	-2.326599535	1	1	3.08E-16
IMMP2L	2.02E-20	-2.847996907	0.938	1	3.50E-16
PET100	2.36E-20	-4.773229138	0.375	1	4.09E-16
DUS4L	2.42E-20	3.065426492	1	1	4.20E-16
TMED3	2.50E-20	-3.020647151	0.625	1	4.34E-16
ENSSSCG00000053939	2.51E-20	-3.103678646	0.875	1	4.35E-16
NGLY1	2.68E-20	2.278596252	1	1	4.65E-16
NRBF2	3.10E-20	2.095657031	1	1	5.38E-16
YIPF2	3.33E-20	-2.73231495	0.875	1	5.78E-16
ARF5	3.40E-20	-2.291379032	0.875	1	5.89E-16
SWSAP1	3.59E-20	-2.852569636	0.938	0.933	6.24E-16
MRPS9	3.89E-20	-1.597948935	1	1	6.75E-16

ARPP19	4.16E-20	-1.666789194	1	1	7.22E-16
GRHPR	4.46E-20	-2.634891081	1	1	7.75E-16
ZPR1	5.55E-20	-2.25762091	0.938	1	9.64E-16
ENSSSCG00000016100	5.59E-20	-2.576431643	0.875	1	9.70E-16
ISCA2	6.28E-20	-2.882816461	0.75	1	1.09E-15
ENSSSCG00000038475	6.36E-20	-3.756074417	0.375	1	1.10E-15
ENSSSCG00000060915	6.42E-20	-3.678071905	0.812	1	1.11E-15
ENSSSCG00000003286	6.88E-20	-2.458385042	1	1	1.19E-15
COMMD7	7.60E-20	-2.411285364	0.812	1	1.32E-15
SDF2	8.30E-20	-1.116585889	1	1	1.44E-15
PRSS2	8.43E-20	-3.304854582	0.812	1	1.46E-15
ENSSSCG00000036716	8.69E-20	-4.038732394	0.75	1	1.51E-15
LMF1	8.90E-20	-4.099108145	0.562	1	1.54E-15
PCID2	9.28E-20	-3.093109404	0.812	1	1.61E-15
ELOB	1.00E-19	-4.450661409	0.375	1	1.74E-15
ZNF821	1.09E-19	-3.080170349	0.812	1	1.89E-15
SLC35B1	1.21E-19	-2.251371488	1	1	2.10E-15
TBRG4	1.21E-19	-2.58812978	0.938	1	2.10E-15
ENSSSCG00000035997	1.24E-19	-4.636043713	0.875	1	2.16E-15
ADCK5	1.47E-19	-4.650486073	0.375	0.933	2.55E-15
RPS15A	1.58E-19	-3.761619696	0.875	1	2.74E-15
EPHX1	1.72E-19	-3.222392421	0.688	0.933	2.99E-15
DERL1	1.82E-19	-1.498522485	1	1	3.16E-15
GTF2E2	2.02E-19	-1.534452666	1	1	3.51E-15
RMDN1	2.03E-19	-1.193590797	1	1	3.52E-15
PRDX2	2.13E-19	-1.719882611	1	1	3.69E-15
RALY	2.20E-19	-1.698731376	0.938	1	3.81E-15
RPS7	2.20E-19	-2.784423886	0.875	1	3.82E-15
DUT	2.42E-19	-1.527512229	1	1	4.21E-15
RPL35	2.46E-19	-3.910732662	0.625	1	4.27E-15
FETUB	2.57E-19	-3.333021925	0.688	1	4.45E-15
APOO	2.81E-19	-1.865698908	1	1	4.87E-15
ADH5	3.25E-19	2.095906726	1	1	5.64E-15
MOBKL3	3.45E-19	2.651588877	1	1	5.99E-15
ATP5MK	3.51E-19	-2.856099887	1	1	6.09E-15
CASQ2	3.59E-19	-3.796716402	0.625	1	6.22E-15
FSTL3	3.59E-19	-2.591015171	0.875	1	6.23E-15
ENSSSCG00000006694	3.74E-19	-0.636148296	1	1	6.49E-15
PFDN5	4.69E-19	-2.375887746	1	1	8.13E-15
EXOSC8	4.86E-19	-1.736965594	1	1	8.43E-15
CDH6	4.86E-19	-3.655351829	0.562	0.933	8.44E-15
IFT57	6.40E-19	-1.71086184	1	1	1.11E-14
GLIPR1	6.86E-19	-0.984560378	1	1	1.19E-14
CD247	7.28E-19	-2.292418213	0.938	1	1.26E-14
WDR53	7.37E-19	-1.298156199	0.938	1	1.28E-14
JAM3	7.40E-19	-2.02233633	0.938	1	1.28E-14
TUBB4B	7.57E-19	-3.375663261	0.625	1	1.31E-14
TAF9	7.91E-19	2.398175692	1	1	1.37E-14
LYPLAL1	8.99E-19	-1.795723494	0.938	1	1.56E-14
C19orf53	1.02E-18	-3.597072183	0.562	1	1.78E-14
FAT2	1.03E-18	-2.374570908	0.875	0.933	1.79E-14
TMEM42	1.09E-18	-2.785600369	0.938	1	1.89E-14
ENSSSCG00000014133	1.12E-18	-4.232527869	0.812	1	1.95E-14
CDC40	1.21E-18	2.043233601	1	1	2.10E-14
AARSD1	1.28E-18	-1.67363284	1	1	2.22E-14
RFC4	1.29E-18	2.227744324	1	1	2.24E-14
PIH1D1	1.30E-18	-2.334770159	0.812	1	2.25E-14
BNIP3	1.31E-18	-2.415037499	0.938	0.933	2.28E-14
EPHB6	1.45E-18	-4.568842835	0.25	1	2.52E-14
ENSSSCG00000046487	1.49E-18	-3.034765418	0.75	1	2.59E-14
GTF2H5	1.56E-18	-2.406848101	0.938	1	2.70E-14
APRT	1.58E-18	-6	0.062	1	2.74E-14
POC1A	1.60E-18	-2.133186844	0.875	1	2.78E-14
ZNF830	1.81E-18	-0.939968987	1	1	3.14E-14
NOP2	1.84E-18	-1.805648956	0.938	1	3.19E-14
PDE8A	1.92E-18	1.993544978	1	1	3.33E-14
IDH3G	2.03E-18	-3.552541023	0.75	1	3.52E-14
SSMEM1	2.22E-18	-2.793549123	0.938	1	3.85E-14
JPT1	2.26E-18	-2.623624121	0.938	1	3.92E-14
CCT3	2.36E-18	-1.443101418	1	1	4.09E-14

RPL10A	2.38E-18	-3.538586852	0.938	1	4.13E-14
ENSSSCG00000032573	2.47E-18	-1.917113455	1	1	4.28E-14
BNIP1	2.48E-18	-2.195624315	1	1	4.30E-14
ENSSSCG00000006559	2.51E-18	-2.065364414	0.938	1	4.36E-14
CDC6	2.61E-18	-1.110381486	1	1	4.53E-14
NDUFC1	2.70E-18	-2.113008962	0.875	1	4.69E-14
NSL1	2.79E-18	2.760265682	1	1	4.85E-14
PARL	2.80E-18	2.158125	1	1	4.86E-14
VPS26C	3.05E-18	-1.414353595	1	1	5.29E-14
GADD45GIP1	3.27E-18	-2.920928429	0.688	1	5.67E-14
UMPS	3.60E-18	-1.01790306	1	1	6.25E-14
ENSSSCG00000031249	3.91E-18	-3.018204595	0.812	1	6.78E-14
SLC25A6	3.91E-18	-2.045944296	0.938	1	6.79E-14
ENSSSCG00000055313	4.01E-18	-1.760320317	0.938	1	6.95E-14
CFAP298	4.60E-18	-1.688224621	1	1	7.98E-14
ENSSSCG00000030849	4.67E-18	-3.612794188	1	1	8.10E-14
YKT6	4.80E-18	-2.434402824	0.812	1	8.33E-14
EIF4E2	4.88E-18	-1.021556143	1	1	8.47E-14
HCRT	4.93E-18	-3.95491211	0.438	1	8.56E-14
ATP6V1F	5.53E-18	-4.712019237	0.312	0.933	9.59E-14
PSMC3IP	5.85E-18	-2.117275716	0.938	1	1.01E-13
IP6K2	5.88E-18	-2.471621028	1	1	1.02E-13
ENSSSCG00000057536	5.93E-18	-5.938599455	0.062	0.933	1.03E-13
TRIM77	6.12E-18	2.249337225	1	1	1.06E-13
MKS1	6.15E-18	-3.474303449	0.625	1	1.07E-13
FTSJ3	6.22E-18	-1.657978335	1	1	1.08E-13
SPTLC1	6.24E-18	2.016150344	1	1	1.08E-13
ENSA	6.35E-18	-1.908040197	0.938	1	1.10E-13
RPS6	6.65E-18	-2.094454575	1	1	1.15E-13
ENSSSCG00000035904	6.96E-18	-1.790597077	1	1	1.21E-13
NCF1	6.96E-18	-2.35033181	0.875	1	1.21E-13
STX18	7.08E-18	-1.39509009	1	1	1.23E-13
SNX11	7.20E-18	-1.82058355	0.938	1	1.25E-13
GNL3	7.45E-18	-1.53331335	1	1	1.29E-13
SRA1	8.53E-18	-5.028569152	0.25	0.867	1.48E-13
PSME3IP1	8.71E-18	-2.032446622	1	1	1.51E-13
PATL2	9.01E-18	-1.139485211	1	1	1.56E-13
ENSSSCG00000028677	9.05E-18	-1.064485396	1	1	1.57E-13
MAP3K13	9.10E-18	1.874358342	1	1	1.58E-13
DPEP1	9.44E-18	-3.638245925	0.812	1	1.64E-13
POLE3	9.70E-18	-1.971499957	0.938	1	1.68E-13
LSM4	1.03E-17	-2.192645078	0.938	1	1.78E-13
STARD3NL	1.04E-17	2.076598078	1	1	1.81E-13
ENSSSCG00000008170	1.10E-17	-3.742034964	0.938	1	1.91E-13
IDH3B	1.16E-17	-2.108706259	0.875	1	2.01E-13
ENSSSCG00000058502	1.19E-17	-4.502500341	0.25	1	2.06E-13
AIMP2	1.20E-17	-3.779609932	0.5	1	2.07E-13
UQCC2	1.28E-17	-3.526068812	0.5	1	2.22E-13
RPLP1	1.31E-17	-3.788134272	1	1	2.27E-13
ATP5PF	1.34E-17	-4.656045599	0.875	1	2.33E-13
MRPL58	1.40E-17	-2.088909416	1	1	2.42E-13
HILPDA	1.44E-17	2.60711463	1	1	2.49E-13
NEIL1	1.51E-17	3.005693006	1	1	2.62E-13
ATP6V1G1	1.53E-17	-1.292031628	1	1	2.65E-13
CRHR1	1.60E-17	-3.137870855	0.938	1	2.78E-13
FAU	1.76E-17	-3.943965965	0.5	1	3.05E-13
PAAF1	1.81E-17	-2.18001747	1	1	3.14E-13
THOC6	1.98E-17	2.430452552	1	0.933	3.44E-13
TYMS	2.00E-17	2.501766903	1	1	3.48E-13
C4orf33	2.10E-17	-1.319365406	1	1	3.64E-13
IDH2	2.32E-17	-2.386373741	0.938	1	4.03E-13
NRG4	2.34E-17	-0.891307387	1	1	4.05E-13
ENSSSCG00000014569	2.39E-17	-3.73392514	0.875	1	4.15E-13
VPS25	2.40E-17	-2.22606081	1	1	4.17E-13
TCIRG1	2.45E-17	-2.761487913	0.75	1	4.25E-13
B4GALT4	2.45E-17	-1.875263772	0.938	1	4.25E-13
WDR54	2.50E-17	-3.832957507	0.438	1	4.33E-13
IAH1	2.55E-17	-2.655634516	0.812	1	4.43E-13
RPL11	2.59E-17	-2.846162942	1	1	4.50E-13
COX7A1	2.60E-17	-4.473931188	0.25	1	4.50E-13

LSM6	2.62E-17	-1.475579041	1	1	4.54E-13
ETFB	2.71E-17	-4.154220919	0.375	1	4.71E-13
ENSSSCG00000012088	2.78E-17	-4.310340121	0.562	0.867	4.83E-13
SCRG1	2.85E-17	-3.273018494	0.688	0.933	4.94E-13
CCT6B	2.86E-17	-1.694489665	1	1	4.96E-13
PEX16	2.86E-17	-6.273018494	0.062	0.867	4.96E-13
AIP	2.95E-17	-4.512648296	0.25	1	5.12E-13
ENSSSCG00000023783	3.06E-17	-5.341036918	0.062	0.933	5.31E-13
TCL1B	3.19E-17	-2.928167255	1	1	5.53E-13
TMEM225B	3.22E-17	-3.296049463	0.75	0.933	5.58E-13
DNAJB13	3.54E-17	-1.849440323	1	1	6.14E-13
NELFE	3.56E-17	-2.927685795	0.688	1	6.17E-13
NPC2	3.62E-17	-2.273176416	1	1	6.28E-13
MIB2	3.65E-17	-4.24838763	0.25	1	6.33E-13
ENSSSCG00000015083	3.91E-17	-2.295245716	1	1	6.79E-13
DUS1L	4.09E-17	-4.736965594	0.25	0.933	7.09E-13
NDUFV1	4.10E-17	-4.714347292	0.188	1	7.11E-13
ETHE1	4.51E-17	-2.696735749	0.875	0.933	7.82E-13
ENSSSCG00000021624	4.85E-17	-3.560467939	0.625	1	8.41E-13
BAG2	4.86E-17	-2.508146904	0.875	1	8.43E-13
NUP35	5.30E-17	2.084742217	1	1	9.19E-13
RPS15	5.49E-17	-4.754174884	0.625	1	9.52E-13
WTAP	5.88E-17	1.757829777	1	1	1.02E-12
ELK3	6.17E-17	2.593203241	1	1	1.07E-12
ENSSSCG00000034019	6.27E-17	-2.968973104	1	1	1.09E-12
EMC3	6.36E-17	-2.957295549	0.812	1	1.10E-12
TSPAN1	6.45E-17	-2.543599989	0.938	1	1.12E-12
C15orf48	6.51E-17	-4.062735755	0.375	1	1.13E-12
PNLDC1	6.76E-17	-1.684330896	0.938	1	1.17E-12
SPACA3	7.40E-17	-5.126532406	0.125	0.933	1.28E-12
RPS19	7.58E-17	-3.917776875	0.875	1	1.31E-12
LAT2	7.70E-17	-1.132782712	1	1	1.34E-12
MTMR14	7.71E-17	-2.494039957	0.938	1	1.34E-12
ENSSSCG00000058371	8.03E-17	-1.668662061	0.938	1	1.39E-12
SLC25A17	9.35E-17	-1.087601426	1	1	1.62E-12
CA9	9.64E-17	2.749230138	1	1	1.67E-12
PLA2G12A	9.78E-17	-2.116760672	0.938	1	1.70E-12
CRB3	9.87E-17	-2.010235222	0.938	1	1.71E-12
ISOC1	1.01E-16	2.154508152	1	1	1.75E-12
NDUFC2	1.02E-16	-3.492640317	0.625	1	1.78E-12
CARMIL1	1.07E-16	1.99051427	1	1	1.86E-12
ENSSSCG00000007646	1.13E-16	-4.712019237	0.25	1	1.96E-12
YTHDF1	1.35E-16	-2.817706441	0.688	1	2.35E-12
TOMM34	1.40E-16	-1.774667485	1	1	2.43E-12
ENSSSCG00000013232	1.52E-16	-0.691034212	1	1	2.63E-12
PQBP1	1.52E-16	-3.21649182	0.75	1	2.63E-12
MAST2	1.54E-16	-2.915111102	0.812	1	2.68E-12
ENSSSCG00000011875	1.56E-16	-1.200731638	1	1	2.71E-12
ANKRD40CL	1.63E-16	-1.765534746	0.938	1	2.83E-12
POLR2I	1.65E-16	-4.987927168	0.188	1	2.86E-12
ENSSSCG00000005440	1.73E-16	1.853706272	1	1	3.00E-12
OXSM	1.74E-16	-2.003842066	0.875	1	3.02E-12
GPS1	1.82E-16	-2.887525271	0.75	1	3.15E-12
EIPR1	1.92E-16	-3.450661409	0.562	1	3.34E-12
METTL9	1.94E-16	2.35331022	1	1	3.37E-12
MAP7	1.95E-16	-1.481446976	1	1	3.38E-12
BCAS2	2.11E-16	-1.99342083	1	1	3.66E-12
PRR13	2.12E-16	-2.113287286	0.938	1	3.67E-12
RNF31	2.21E-16	-3.449802917	0.688	1	3.84E-12
MRPL22	2.27E-16	-1.754741673	1	1	3.94E-12
ALKBH7	2.33E-16	-2.647045009	0.75	1	4.05E-12
NUP42	2.42E-16	2.699171005	1	1	4.21E-12
TMA7	2.44E-16	-2.407982742	1	1	4.24E-12
BORCS5	2.46E-16	-1.652671688	0.938	1	4.26E-12
AMN1	2.47E-16	-1.458962642	1	1	4.28E-12
DDX39B	2.50E-16	-1.359725826	1	1	4.34E-12
MAD2L1	2.58E-16	2.690511219	1	1	4.47E-12
SQLE	2.58E-16	-1.545038281	1	1	4.48E-12
DYNLL1	2.84E-16	-2.064648741	1	1	4.93E-12
COA8	3.18E-16	-2.718429643	0.75	1	5.51E-12

LRRC47	3.27E-16	-3.007777351	0.625	1	5.67E-12
OTUB1	3.36E-16	-2.148001535	1	1	5.83E-12
RASL11A	3.38E-16	3.302713962	1	0.867	5.86E-12
SWI5	3.39E-16	-2.528566445	1	1	5.88E-12
SRPX	3.66E-16	2.397800932	1	1	6.35E-12
WDR20	4.23E-16	1.861195383	1	1	7.34E-12
DDX11	4.56E-16	-1.861645078	0.938	1	7.91E-12
TSPAN14	4.66E-16	-1.477232261	1	1	8.08E-12
ATP6V0B	5.16E-16	-1.637587842	1	1	8.95E-12
AIDA	5.19E-16	-1.193952163	1	1	9.01E-12
GSTK1	5.21E-16	-3.977632187	0.5	0.933	9.04E-12
TIMM17A	5.27E-16	-2.499101764	0.688	1	9.15E-12
SDF4	5.43E-16	-4.074962058	0.438	1	9.42E-12
MED31	5.49E-16	-2.09797516	1	1	9.52E-12
ATG7	5.66E-16	-0.878058958	1	1	9.82E-12
RPL18A	5.73E-16	-4.178449074	0.562	1	9.94E-12
MCRS1	5.99E-16	-2.463567393	0.938	1	1.04E-11
GLRX5	6.01E-16	-1.056467561	1	1	1.04E-11
SLC17A5	7.19E-16	2.258422404	1	1	1.25E-11
JOSD2	7.34E-16	-5.201633861	0.125	0.933	1.27E-11
NUDT2	7.53E-16	-2.915839552	0.75	0.933	1.31E-11
AVPI1	8.33E-16	-4.180572246	0.438	0.867	1.45E-11
DNAJC14	8.40E-16	-0.933766302	1	1	1.46E-11
CCT4	8.40E-16	-1.673513623	1	1	1.46E-11
TUBGCP2	8.77E-16	-3.195724471	0.688	0.933	1.52E-11
RPL34	8.78E-16	-3.9510904	0.312	1	1.52E-11
PPP2R2B	9.03E-16	-1.301552267	1	1	1.57E-11
THYN1	9.25E-16	-1.089464261	1	1	1.61E-11
MEPCE	9.54E-16	-2.019777666	0.938	1	1.65E-11
SNPH	9.57E-16	-1.649743182	0.875	1	1.66E-11
ENSSSCG00000053447	9.85E-16	-3.481806511	0.438	1	1.71E-11
FHIT	1.00E-15	-1.865241853	0.938	1	1.74E-11
RFC3	1.01E-15	-1.74080766	1	1	1.76E-11
GFI1	1.11E-15	2.99906044	1	0.867	1.92E-11
H2AJ	1.14E-15	-4.20461772	0.312	0.933	1.98E-11
ENSSSCG00000052759	1.22E-15	-4.351843673	0.188	1	2.12E-11
ENSSSCG00000040571	1.25E-15	-1.640434248	1	1	2.16E-11
ENSSSCG00000002995	1.28E-15	-3.865698908	0.625	1	2.23E-11
SBDS	1.30E-15	1.867298417	1	1	2.26E-11
GPATCH11	1.33E-15	-1.565817451	1	1	2.30E-11
RBX1	1.34E-15	-0.741466986	1	1	2.33E-11
TEX264	1.43E-15	-2.735787403	0.875	1	2.48E-11
LMNA	1.46E-15	-1.562344198	0.938	1	2.53E-11
AVEN	1.47E-15	-1.551344593	1	1	2.56E-11
ENSSSCG00000037514	1.51E-15	-1.286678991	0.938	1	2.63E-11
CDK10	1.53E-15	-3.973527789	0.375	0.933	2.66E-11
FXR2	1.56E-15	-1.988412026	0.938	1	2.71E-11
ENSSSCG00000049464	1.58E-15	-2.124587636	1	1	2.74E-11
BTF3L4	1.62E-15	-1.468400725	1	1	2.81E-11
GCA	1.63E-15	2.219736947	1	1	2.84E-11
NDUFS4	1.66E-15	-1.886810959	1	1	2.87E-11
PAQR7	1.68E-15	-2.349449158	0.938	0.933	2.91E-11
RPS4X	1.74E-15	-3.062348377	1	1	3.02E-11
ENSSSCG00000031117	1.80E-15	-2.726981506	0.938	1	3.12E-11
ZMAT2	1.81E-15	-1.19088462	1	1	3.14E-11
ENSSSCG00000002379	1.84E-15	-2.188266637	1	1	3.19E-11
ARMC12	1.91E-15	-2.685807554	0.688	1	3.32E-11
TMEM179B	1.97E-15	-3.298223834	0.438	1	3.41E-11
NDUFS5	2.00E-15	-2.674904626	1	1	3.47E-11
TULP3	2.01E-15	-1.672596775	1	1	3.49E-11
IER2	2.07E-15	-2.263034406	0.875	0.933	3.59E-11
ENSSSCG00000031730	2.07E-15	-2.27897595	1	1	3.60E-11
CRYBB3	2.15E-15	-4.121015401	0.5	0.933	3.73E-11
DTD1	2.20E-15	-1.443541016	1	1	3.82E-11
NLRP8	2.26E-15	-1.143542393	1	1	3.93E-11
ENSSSCG00000061145	2.40E-15	-7.201633861	0	0.733	4.17E-11
ENSSSCG00000025928	2.44E-15	-4.180572246	0.562	1	4.23E-11
RHOT1	2.53E-15	1.728831319	1	1	4.39E-11
RCN2	2.55E-15	2.125407063	1	1	4.43E-11
RPS13	2.62E-15	-4.119909464	0.938	1	4.55E-11

ESYT1	2.69E-15	-1.310138641	1	1	4.66E-11
ENSSSCG00000033926	2.81E-15	-3.532221039	0.438	1	4.88E-11
LENG1	2.84E-15	-3.431911318	0.5	1	4.93E-11
CALR	2.95E-15	-1.254413923	1	1	5.12E-11
CARS2	3.24E-15	-1.946719917	0.875	1	5.62E-11
PSENEN	3.53E-15	-3.135995217	0.875	1	6.13E-11
RCBTB2	3.69E-15	1.998683967	1	1	6.41E-11
RNASEH2C	3.72E-15	-5.751320887	0.062	0.933	6.45E-11
CCNB1	3.78E-15	2.140053101	1	1	6.56E-11
GET1	3.90E-15	-0.943336134	1	1	6.76E-11
ENSSSCG00000038364	3.92E-15	-5.568842835	0.062	0.933	6.80E-11
B3GNTL1	4.18E-15	-3.90902634	0.375	0.933	7.25E-11
FBL	4.19E-15	-3.793549123	0.375	1	7.27E-11
COPS6	4.19E-15	-1.959092056	1	1	7.27E-11
SYTL3	4.31E-15	-1.186424189	1	1	7.48E-11
ENSSSCG00000056015	4.35E-15	-3.204140717	0.75	1	7.56E-11
ENSSSCG00000053570	4.36E-15	-1.292801985	1	1	7.57E-11
SLC25A31	4.46E-15	2.339218765	1	1	7.73E-11
RBPMS2	4.46E-15	-1.834323334	1	1	7.74E-11
ZNHIT1	4.49E-15	-4.653824359	0.188	0.867	7.79E-11
TMEM186	4.75E-15	-1.528365183	0.938	1	8.24E-11
SMPD2	4.85E-15	-3.254101281	0.75	0.933	8.41E-11
CHST10	4.93E-15	-1.27551518	1	1	8.56E-11
RPL4	5.15E-15	-1.626541604	1	1	8.94E-11
ENSSSCG00000031756	5.20E-15	-2.312467766	0.875	1	9.02E-11
RARS2	5.21E-15	2.350834658	1	1	9.05E-11
COX14	5.33E-15	-3.124587636	0.688	1	9.25E-11
SIL1	5.45E-15	-1.880000224	1	1	9.45E-11
RPS11	5.46E-15	-3.850539101	0.938	1	9.47E-11
RTRAF	5.75E-15	2.84955432	1	1	9.97E-11
LONP1	5.86E-15	-2.147901877	0.938	1	1.02E-10
MKKS	6.19E-15	-1.580224582	1	1	1.07E-10
KLHL7	6.63E-15	2.105913653	1	1	1.15E-10
FAM168B	7.21E-15	1.836792626	1	1	1.25E-10
VPS28	7.67E-15	-2.443606651	0.875	1	1.33E-10
ENSSSCG00000035007	8.18E-15	-3.748106152	1	1	1.42E-10
ACYP2	8.21E-15	2.185044731	1	1	1.42E-10
GSR	8.34E-15	1.886583089	1	1	1.45E-10
LRPAP1	8.61E-15	-3.040641984	0.5	0.933	1.49E-10
PDIA3	8.77E-15	-1.09427569	1	1	1.52E-10
C11orf58	9.04E-15	-0.843417126	1	1	1.57E-10
ENSSSCG00000038027	9.19E-15	-2.603724564	0.562	1	1.59E-10
CMC4	9.23E-15	-2.563429339	0.812	1	1.60E-10
FTSJ1	9.45E-15	-1.256608137	1	1	1.64E-10
MRPS11	9.65E-15	-4.21649182	0.25	1	1.68E-10
ENSSSCG00000012100	9.90E-15	-3.075995384	0.688	1	1.72E-10
POP5	1.00E-14	-1.812888488	1	1	1.74E-10
SNRPC	1.03E-14	2.25801286	1	1	1.79E-10
RPS12	1.06E-14	-3.920321745	0.875	1	1.84E-10
GPD1	1.08E-14	-2.863351153	0.812	1	1.87E-10
LDHC	1.10E-14	-1.241591694	1	1	1.91E-10
SPATA16	1.12E-14	-4.606178987	0.25	1	1.95E-10
ENSSSCG00000047189	1.15E-14	-2.878984599	0.688	1	2.00E-10
TMEM165	1.16E-14	-0.878370556	0.938	1	2.01E-10
PRIM2	1.23E-14	-0.860505178	1	1	2.13E-10
TMCC1	1.23E-14	1.811684666	1	1	2.14E-10
VPS4A	1.28E-14	-1.604720163	1	1	2.21E-10
MRPL46	1.45E-14	-1.621604953	1	1	2.52E-10
RNF170	1.45E-14	-1.429987841	1	1	2.52E-10
ZBTB48	1.48E-14	-3.699133521	0.375	0.933	2.56E-10
DRG1	1.59E-14	-2.094757257	1	1	2.76E-10
COQ3	1.61E-14	-1.980256065	0.875	1	2.79E-10
COX17	1.63E-14	-2.935568127	0.688	1	2.83E-10
ENSSSCG00000027573	1.70E-14	-2.993113614	0.875	1	2.95E-10
ESD	1.79E-14	-1.565990978	1	1	3.10E-10
MT3	1.82E-14	-4.362570079	0.125	0.933	3.15E-10
NDUFB2	1.83E-14	-2.607348581	1	1	3.18E-10
TIMM10	1.85E-14	-4.561878888	0.188	0.933	3.22E-10
CTDNEP1	1.97E-14	-2.315501826	0.875	1	3.42E-10
ENSSSCG00000060974	2.01E-14	-1.526354522	1	1	3.49E-10

MAMDC4	2.07E-14	-5	0.062	1	3.59E-10
SRM	2.14E-14	-3.341036918	0.625	0.933	3.72E-10
DNAJC11	2.15E-14	-1.827819025	0.938	1	3.74E-10
NLRP9	2.19E-14	-1.389012091	1	1	3.80E-10
ELP2	2.28E-14	-0.872110218	1	1	3.96E-10
ENSSSCG00000016990	2.34E-14	-1.64548461	1	1	4.07E-10
ENSSSCG00000052208	2.45E-14	-2.441631709	0.562	1	4.24E-10
C8orf76	2.88E-14	-1.710986528	1	1	5.00E-10
ENSSSCG00000032082	3.07E-14	-1.781165398	0.875	1	5.33E-10
CHMP4A	3.23E-14	-1.978938384	0.812	1	5.61E-10
IQCG	3.37E-14	-1.484578433	1	1	5.85E-10
ENSSSCG00000059201	3.37E-14	-1.801454321	1	1	5.85E-10
LRSAM1	3.48E-14	-2.581395886	0.812	1	6.04E-10
PSMB4	3.49E-14	-1.913559984	0.938	1	6.05E-10
NUDT16L1	3.53E-14	-4.113008962	0.375	0.867	6.12E-10
HSD17B10	3.53E-14	-2.988772745	0.812	0.933	6.13E-10
USE1	3.82E-14	-4.394278939	0.375	0.933	6.64E-10
WDFY2	3.86E-14	-1.424998609	1	1	6.69E-10
LGALS8	3.92E-14	-1.276937169	0.938	1	6.80E-10
TNNC2	4.13E-14	-2.935869663	0.625	0.933	7.16E-10
EZR	4.31E-14	-0.497619192	1	1	7.48E-10
ENSSSCG00000059584	4.39E-14	-4.546567122	0.375	0.933	7.62E-10
NOTO	4.49E-14	-1.614346308	0.938	1	7.79E-10
GOLT1B	4.50E-14	2.570474352	1	1	7.80E-10
UPB1	4.50E-14	-2.087118967	0.938	1	7.81E-10
GON7	4.89E-14	-1.900464326	1	1	8.48E-10
PARD6B	4.99E-14	1.85297505	1	1	8.66E-10
MRTO4	5.24E-14	-2.294743266	0.812	1	9.10E-10
APIP	5.29E-14	2.391364415	1	1	9.17E-10
PEX19	5.35E-14	-2.717018185	0.875	0.933	9.29E-10
PSMC4	5.38E-14	-1.646923207	1	1	9.34E-10
C1orf54	5.53E-14	-2.584962501	0.812	1	9.60E-10
ATRAID	5.55E-14	-2.14286244	0.875	1	9.63E-10
STX7	5.74E-14	1.989783604	1	1	9.97E-10
TOMM22	5.90E-14	-1.365635259	1	1	1.02E-09
ENSSSCG00000005006	6.08E-14	-2.117183539	1	1	1.06E-09
DHDDS	6.14E-14	-1.362156187	0.938	1	1.07E-09
PNP	6.61E-14	2.379167825	1	1	1.15E-09
UBE2J2	6.64E-14	-1.917259569	0.875	1	1.15E-09
TCP11	6.92E-14	-1.526412235	1	1	1.20E-09
CNBD2	6.97E-14	-1.957846984	1	1	1.21E-09
PIP4P1	7.40E-14	-2.144421495	0.938	1	1.28E-09
ALAD	7.55E-14	-2.805827452	0.688	1	1.31E-09
AZIN2	7.60E-14	2.618820598	1	1	1.32E-09
PMF1	7.90E-14	-2.264589872	0.938	1	1.37E-09
FOXJ3	8.06E-14	-0.829294953	1	1	1.40E-09
DUSP16	8.09E-14	-0.601232924	1	1	1.40E-09
TRIP4	8.22E-14	-1.103173088	1	1	1.43E-09
MMAA	8.46E-14	-0.828089183	1	1	1.47E-09
RPL26	8.55E-14	-4.500073603	0.312	1	1.48E-09
ECHS1	8.66E-14	-2.760964913	0.75	0.933	1.50E-09
RAB4B	8.69E-14	-3.058005603	0.75	1	1.51E-09
NOP14	8.87E-14	-2.080170349	0.812	1	1.54E-09
ISCA1	9.52E-14	-1.523955925	1	1	1.65E-09
ENSSSCG00000061531	1.02E-13	-5.450661409	0.062	1	1.77E-09
P2RX5	1.04E-13	-1.870407093	0.938	1	1.80E-09
EIF1B	1.04E-13	-1.350302932	1	1	1.81E-09
ATPSCKMT	1.06E-13	-1.688294765	1	1	1.84E-09
ENSSSCG00000047605	1.10E-13	-0.871722696	1	1	1.90E-09
SLC47A1	1.11E-13	-2.925999419	0.562	0.933	1.92E-09
UBXN6	1.16E-13	-1.810641025	0.875	0.933	2.01E-09
ENSSSCG00000021161	1.16E-13	-1.588162104	1	1	2.02E-09
NDUFAF1	1.23E-13	-1.306218585	1	1	2.14E-09
AIMP1	1.25E-13	-0.859603526	1	1	2.16E-09
CSTB	1.25E-13	-3.137503524	0.75	0.933	2.17E-09
ABHD17A	1.28E-13	-2.704544116	0.688	0.933	2.22E-09
ENSSSCG00000033814	1.34E-13	2.358864381	1	1	2.33E-09
CDA	1.37E-13	-2.119104613	0.75	1	2.38E-09
MRPL2	1.38E-13	-2.875517969	0.688	0.867	2.40E-09
FHIP1A	1.42E-13	-0.742019857	1	1	2.46E-09

CNN3	1.50E-13	2.168456354	1	1	2.60E-09
ME2	1.50E-13	1.873282203	1	1	2.60E-09
CALM3	1.54E-13	-1.32443495	1	1	2.67E-09
CCDC172	1.55E-13	-2.449594721	0.75	1	2.69E-09
DONSON	1.58E-13	1.848430865	1	1	2.73E-09
TMEM138	1.58E-13	-0.915111102	1	1	2.75E-09
SMIM12	1.61E-13	-2.78181983	0.812	0.933	2.80E-09
SERPINB1	1.67E-13	-2.193303692	0.938	1	2.91E-09
COPA	1.70E-13	1.701163208	1	1	2.95E-09
ENSSSCG00000011294	1.72E-13	-2.51731855	0.75	1	2.98E-09
TRAK2	1.73E-13	-1.96437609	0.938	1	3.00E-09
ENSSSCG00000025940	1.76E-13	1.932878128	1	1	3.05E-09
BCS1L	1.77E-13	-3.635636639	0.562	1	3.07E-09
SCPEP1	1.81E-13	-2.252449655	0.875	1	3.14E-09
EPB41L5	1.83E-13	2.057190776	1	1	3.18E-09
TXNL4A	1.84E-13	-1.207106175	1	1	3.19E-09
GPC3	1.86E-13	-1.87175303	0.75	1	3.23E-09
SNRNP25	1.92E-13	-3.315501826	0.438	0.933	3.33E-09
CEP44	1.98E-13	1.830519326	1	1	3.43E-09
RIMBP2	2.01E-13	2.3474175	1	1	3.49E-09
SDHAF1	2.14E-13	-3.228268988	0.375	1	3.71E-09
SNRPD2	2.19E-13	-2.793549123	0.812	1	3.80E-09
COA3	2.24E-13	-2.493647334	0.625	1	3.89E-09
SAR1A	2.52E-13	-0.785820052	1	1	4.38E-09
STIMATE	2.63E-13	-0.831947413	1	1	4.56E-09
LYRM4	2.63E-13	-2.840343334	0.688	0.933	4.57E-09
LYRM1	2.70E-13	-3.943965965	0.312	0.867	4.68E-09
C12orf40	2.93E-13	2.6555913	1	1	5.09E-09
GSK3B	3.02E-13	-1.035667512	1	1	5.24E-09
KHDRBS1	3.08E-13	2.138454663	1	1	5.34E-09
COG3	3.15E-13	1.938844457	1	1	5.47E-09
EDRF1	3.19E-13	1.765957912	1	1	5.53E-09
MCM5	3.22E-13	-1.503608696	0.938	1	5.59E-09
ENSSSCG00000029003	3.25E-13	-2.769219793	0.812	1	5.64E-09
TBC1D7	3.29E-13	-1.541397278	0.938	1	5.71E-09
FAM110A	3.31E-13	-1.68953102	0.938	1	5.74E-09
IK	3.42E-13	-0.779834621	1	1	5.93E-09
UQCR11	3.54E-13	-4.701918647	0.125	0.8	6.14E-09
PDE6D	3.66E-13	-1.28769646	1	1	6.34E-09
NSMAF	3.70E-13	-1.553301871	1	1	6.42E-09
NECAP1	3.98E-13	-0.986301941	1	1	6.90E-09
PSMD14	4.16E-13	-0.747475954	1	1	7.22E-09
SIRT5	4.23E-13	-3.621488377	0.438	0.933	7.33E-09
ENSSSCG00000004151	4.34E-13	2.261529099	1	1	7.53E-09
CCNA2	4.53E-13	1.946110343	1	1	7.86E-09
DDX49	4.59E-13	-1.860089979	1	1	7.97E-09
JPT2	4.63E-13	-2.613773412	0.75	0.933	8.03E-09
HACE1	4.63E-13	2.273501405	1	1	8.03E-09
CTSF	4.80E-13	-3.335184192	0.375	0.933	8.33E-09
C5orf34	4.84E-13	2.024233184	1	1	8.40E-09
SGSM2	5.05E-13	-1.707100954	0.938	1	8.76E-09
DYNLL2	5.21E-13	-1.215750078	1	1	9.05E-09
ACTR1B	5.30E-13	-3.067114196	0.562	1	9.19E-09
DMWD	5.31E-13	-2.583075391	0.75	1	9.21E-09
GFUS	5.56E-13	-2.352976531	0.812	1	9.64E-09
KANK1	5.60E-13	1.714174324	1	1	9.72E-09
ENSSSCG00000039815	5.75E-13	-2.239950793	0.688	1	9.97E-09
FANK1	5.86E-13	-1.436272141	1	1	1.02E-08
EIF2AK4	5.93E-13	1.685963583	1	1	1.03E-08
FAM104A	5.96E-13	-1.116087968	1	1	1.03E-08
PPIF	6.06E-13	-1.094876331	1	1	1.05E-08
SLC8B1	6.32E-13	-1.538169387	1	1	1.10E-08
ALDH18A1	6.44E-13	2.657370996	1	1	1.12E-08
SECISBP2	6.79E-13	1.947391555	1	1	1.18E-08
CLDN1	6.82E-13	2.013764314	1	1	1.18E-08
RAD1	7.00E-13	2.588906089	1	1	1.21E-08
TMEM131L	7.02E-13	1.628625223	1	1	1.22E-08
PBDC1	7.03E-13	-1.191673239	1	1	1.22E-08
RPS26	7.27E-13	-4.990810829	0.375	1	1.26E-08
ENSSSCG00000039972	7.28E-13	-1.341929951	0.938	1	1.26E-08

SLC25A14	7.34E-13	-2.697971463	0.75	0.933	1.27E-08
ATP6AP1	7.37E-13	-2.016873819	1	0.933	1.28E-08
TK2	7.48E-13	-2.156351838	1	1	1.30E-08
VPS37A	7.53E-13	2.011686786	1	1	1.31E-08
EXOSC5	7.59E-13	-1.427375834	1	1	1.32E-08
PSMC3	7.80E-13	-1.071840788	1	1	1.35E-08
NUDCD3	7.80E-13	-2.333669948	0.875	1	1.35E-08
HIGD2A	7.92E-13	-2.398038496	1	1	1.37E-08
MESD	7.99E-13	-2.409684265	0.75	1	1.39E-08
TANGO2	8.01E-13	-2.269432177	0.938	1	1.39E-08
MED10	8.09E-13	-2.337034987	0.75	1	1.40E-08
SCGB3A2	8.09E-13	-4.263034406	0.125	0.933	1.40E-08
ENSSSCG00000008996	8.14E-13	2.081859803	1	1	1.41E-08
FCGR1A	8.37E-13	-0.903610119	1	1	1.45E-08
C17orf80	8.39E-13	-0.775515625	1	1	1.46E-08
MIF	8.42E-13	-3.300704824	0.625	1	1.46E-08
EIF3G	8.46E-13	-3.557481764	0.688	1	1.47E-08
CBX8	8.54E-13	-1.779517169	0.938	1	1.48E-08
KIF4A	8.66E-13	1.840404308	1	1	1.50E-08
CERS4	9.10E-13	-2.911414626	0.938	1	1.58E-08
COPB1	9.11E-13	2.214169759	1	1	1.58E-08
TIMM50	9.32E-13	-2.434146322	0.812	1	1.62E-08
ENSSSCG00000034853	9.52E-13	-3.242856524	0.625	1	1.65E-08
ENSSSCG00000052760	9.88E-13	-1.060102299	1	1	1.71E-08
WDR48	9.90E-13	1.382770612	1	1	1.72E-08
LMO2	1.01E-12	-3.310340121	0.375	1	1.75E-08
TMEM160	1.03E-12	-5.975752454	0	0.933	1.79E-08
C9orf40	1.11E-12	2.077886243	1	1	1.92E-08
TMEM251	1.13E-12	-1.568688446	0.938	1	1.97E-08
YWHAQ	1.15E-12	-0.751953103	1	1	2.00E-08
ENSSSCG00000060365	1.20E-12	-1.334313562	1	1	2.08E-08
NRBP1	1.22E-12	-0.623062958	1	1	2.12E-08
ENSSSCG00000052671	1.23E-12	-3.261271797	0.938	1	2.14E-08
NARF	1.24E-12	-1.584458854	0.938	1	2.15E-08
ZNF212	1.26E-12	-1.852496293	0.75	1	2.19E-08
NKIRAS1	1.27E-12	-0.886596597	1	1	2.20E-08
ELAC2	1.38E-12	-2.918275603	0.625	1	2.40E-08
RDH13	1.42E-12	-2.435215381	0.812	0.933	2.46E-08
NSFL1C	1.43E-12	-2.447337991	0.75	1	2.49E-08
RNFT1	1.45E-12	2.348841623	1	1	2.52E-08
ERCC1	1.47E-12	-3.169925001	0.438	1	2.55E-08
ATPAF2	1.52E-12	-5.30256277	0.062	0.867	2.65E-08
DRC7	1.53E-12	-3.137503524	0.438	1	2.65E-08
HMGB2	1.53E-12	1.647952264	1	1	2.66E-08
COX7C	1.57E-12	-1.795937409	1	1	2.72E-08
SVOPL	1.59E-12	-0.970810921	1	1	2.75E-08
PPM1G	1.62E-12	-1.310667052	0.938	1	2.82E-08
MTUS2	1.64E-12	-1.453211228	0.938	1	2.85E-08
ENSSSCG00000036197	1.65E-12	-1.608466438	1	1	2.86E-08
GPR108	1.68E-12	-2.451301043	0.562	1	2.91E-08
RNF181	1.69E-12	-2.13492958	0.875	1	2.94E-08
SUGT1	1.72E-12	-0.833994037	1	1	2.98E-08
CDC45	1.74E-12	2.605578832	1	1	3.01E-08
SGMS1	1.75E-12	1.862972371	1	1	3.03E-08
DNAJB6	1.76E-12	-0.709277773	1	1	3.06E-08
ENSSSCG00000057138	1.78E-12	-2.292090439	0.75	0.933	3.08E-08
MYCBP	1.80E-12	-1.269030734	1	1	3.13E-08
RPS27A	1.84E-12	-2.369440633	1	1	3.19E-08
ATG4A	1.87E-12	-0.549425205	1	1	3.24E-08
MOCS2	1.90E-12	2.104562594	1	1	3.29E-08
SLC25A19	1.92E-12	-4.263034406	0.312	0.8	3.32E-08
TUBB2A	1.93E-12	-1.636452816	1	1	3.35E-08
FUNDC2	1.96E-12	-2.485426827	0.812	1	3.41E-08
ENSSSCG00000021991	2.03E-12	-4.013674937	0.312	0.867	3.52E-08
CMTM8	2.04E-12	2.305542121	1	1	3.54E-08
GALNT6	2.05E-12	-2.297799824	0.812	1	3.56E-08
TAB2	2.06E-12	1.831676759	1	1	3.57E-08
AGA	2.08E-12	-2.831751576	0.562	1	3.61E-08
CRYZ	2.14E-12	2.094147155	1	1	3.72E-08
ANKRD39	2.18E-12	-1.929610672	0.938	1	3.78E-08

ENSSSCG00000034927	2.21E-12	-1.033109415	1	1	3.84E-08
DCAF11	2.24E-12	-3.194647431	0.75	0.933	3.88E-08
PGLS	2.24E-12	-4.565597176	0.25	0.867	3.89E-08
POC1B	2.28E-12	2.351408963	1	1	3.95E-08
HAUS7	2.38E-12	-2.678071905	0.688	0.933	4.13E-08
ENSSSCG00000003930	2.38E-12	-2.964510915	0.938	1	4.13E-08
LGI1	2.64E-12	-3.987927168	0.25	0.867	4.59E-08
ATG3	2.72E-12	-0.761903496	0.938	1	4.73E-08
CHST7	2.84E-12	-1.636099635	0.688	1	4.92E-08
ENSSSCG00000032216	2.92E-12	-1.047562811	1	1	5.07E-08
ENSSSCG00000059649	2.95E-12	-0.626933056	1	1	5.12E-08
PPCDC	2.97E-12	-1.227212826	1	1	5.15E-08
ENSSSCG00000001075	3.01E-12	2.009949005	1	1	5.22E-08
DUSP5	3.04E-12	-1.17533253	1	1	5.28E-08
ARRDC4	3.11E-12	-2.41130478	1	1	5.40E-08
ENSSSCG00000006996	3.29E-12	-1.08435693	1	1	5.70E-08
ENSSSCG00000005596	3.33E-12	-1.281150321	0.938	1	5.78E-08
ARFGAP2	3.56E-12	-1.205430429	1	1	6.18E-08
PRDX5	3.61E-12	-2.501691882	0.812	1	6.26E-08
SPPL2A	3.61E-12	1.941824386	1	1	6.27E-08
CCDC137	3.66E-12	-3.693022247	0.25	0.933	6.35E-08
SCAND1	3.74E-12	-1.900464326	0.875	0.933	6.50E-08
MOSPD1	3.78E-12	-1.281099122	1	1	6.56E-08
FBXO43	4.20E-12	2.108918286	1	1	7.29E-08
ENSSSCG00000010719	4.26E-12	-1.016488123	1	1	7.39E-08
MPP7	4.53E-12	1.98179201	1	1	7.87E-08
PIM1	4.59E-12	2.316732069	1	1	7.97E-08
MAP3K7	4.76E-12	1.989567618	1	1	8.26E-08
MOK	4.81E-12	-2.093109404	0.75	0.933	8.35E-08
GRPEL2	4.84E-12	1.613787476	1	1	8.41E-08
BPGM	4.91E-12	-2.044453598	0.938	1	8.51E-08
RNF8	5.01E-12	-0.556955188	1	1	8.69E-08
SGCD	5.08E-12	-1.067114196	1	1	8.82E-08
GHITM	5.14E-12	-2.154565608	1	1	8.91E-08
ENSSSCG00000037143	5.17E-12	-0.482950751	1	1	8.97E-08
PSMD7	5.17E-12	-0.682340237	1	1	8.98E-08
ZNF235	5.18E-12	-1.52554995	0.938	1	8.99E-08
MRPS7	5.21E-12	-2.236945177	0.625	1	9.05E-08
SSNA1	5.49E-12	-1.729734025	0.875	1	9.52E-08
TBC1D1	5.58E-12	-1.547466069	1	1	9.68E-08
CRLS1	5.59E-12	2.064678618	1	1	9.70E-08
CAPZB	5.67E-12	-1.650415562	1	1	9.83E-08
SLC25A20	5.68E-12	1.82527683	1	1	9.86E-08
NUDT21	5.69E-12	1.464466188	1	1	9.87E-08
PRDX4	5.76E-12	-2.251741236	1	1	9.99E-08
C19orf67	5.84E-12	-1.564180939	1	1	1.01E-07
GALNT11	5.89E-12	2.112237651	1	1	1.02E-07
TMEM213	5.98E-12	-2.138005942	0.875	1	1.04E-07
ENSSSCG00000063355	6.18E-12	3.181226319	1	0.867	1.07E-07
C19orf81	6.24E-12	-4.230612928	0.188	0.8	1.08E-07
ENSSSCG00000052241	6.25E-12	-1.25918208	0.938	1	1.08E-07
MRPL19	6.28E-12	-1.271647536	0.938	1	1.09E-07
ARR3	6.35E-12	-3.033730857	0.625	0.933	1.10E-07
EIF4G2	6.39E-12	1.935715115	1	1	1.11E-07
PRR5L	6.39E-12	-1.6405972	1	1	1.11E-07
CCDC152	6.47E-12	3.060365076	1	0.867	1.12E-07
OSBPL2	6.47E-12	-1.265062087	1	1	1.12E-07
NRAP	6.58E-12	-2.267883805	0.625	1	1.14E-07
TXNDC12	6.68E-12	-2.313157885	0.688	1	1.16E-07
NUDT14	6.94E-12	-4.552541023	0.188	0.933	1.20E-07
MRPL50	7.19E-12	-0.983069604	1	1	1.25E-07
ENSSSCG00000052096	7.38E-12	-3.915111102	0.312	0.933	1.28E-07
CACYBP	7.73E-12	-1.033166864	1	1	1.34E-07
CCDC124	7.78E-12	-3.074000581	0.625	0.933	1.35E-07
HMGCR	7.90E-12	-1.494816573	1	1	1.37E-07
ENSSSCG00000021591	8.01E-12	-1.43641115	1	1	1.39E-07
OAZ1	8.02E-12	2.063053848	1	1	1.39E-07
TNK1	8.24E-12	-3.793549123	0.188	1	1.43E-07
PLIN5	8.63E-12	-2.247437551	0.875	1	1.50E-07
TPCN1	8.66E-12	-2.557390423	0.625	1	1.50E-07

PFKM	8.73E-12	-2.009586049	0.938	1	1.52E-07
RPS17	8.76E-12	-3.150011796	1	1	1.52E-07
ETFA	8.85E-12	1.951779687	1	1	1.53E-07
IER3IP1	8.87E-12	2.431573399	1	1	1.54E-07
ENSSSCG00000011147	9.26E-12	-2.074493726	0.812	1	1.61E-07
NAPRT	9.33E-12	-4.693022247	0.062	0.867	1.62E-07
NR2C1	9.34E-12	2.051872623	1	1	1.62E-07
KBTBD13	9.60E-12	-0.094786469	1	1	1.67E-07
DR1	9.91E-12	1.810473113	1	1	1.72E-07
MAPKAPK2	1.04E-11	-2.545621609	0.625	1	1.81E-07
NAXD	1.06E-11	-1.635258822	0.875	1	1.84E-07
PDLIM2	1.11E-11	-3.385891154	0.312	0.933	1.92E-07
SLC25A39	1.13E-11	-2.802060622	0.688	0.933	1.97E-07
SSR1	1.14E-11	-0.519348645	1	1	1.99E-07
ENSSSCG00000044108	1.18E-11	-2.026346871	1	1	2.04E-07
LEPROT	1.21E-11	2.055395592	1	1	2.10E-07
HECTD2	1.22E-11	2.316742956	1	1	2.12E-07
ENSSSCG00000056174	1.23E-11	2.124121312	1	1	2.13E-07
TMEM11	1.24E-11	-3.802060622	0.312	0.933	2.15E-07
ERGIC3	1.26E-11	-1.738223471	1	1	2.19E-07
CDH22	1.34E-11	-3.192645078	0.5	0.933	2.32E-07
ENSSSCG00000056323	1.34E-11	-2.156219448	0.812	1	2.33E-07
ADIPOR2	1.36E-11	-0.566082469	1	1	2.35E-07
LSG1	1.40E-11	-1.391549146	1	1	2.43E-07
SRP14	1.41E-11	-0.698551297	1	1	2.44E-07
ATP5ME	1.44E-11	-3.013674937	0.938	1	2.49E-07
PRKCI	1.47E-11	1.805465923	1	1	2.55E-07
CHID1	1.50E-11	-3.989273593	0.375	0.867	2.60E-07
PSMA2	1.51E-11	1.984092766	1	1	2.62E-07
NUCB1	1.51E-11	-2.322355497	0.812	1	2.63E-07
VEZF1	1.57E-11	1.870521458	1	1	2.73E-07
SDHA	1.59E-11	-0.927627927	1	1	2.75E-07
RFC2	1.65E-11	-1.369838549	1	1	2.87E-07
STK16	1.67E-11	-1.743363365	0.875	0.933	2.89E-07
PPFIA1	1.67E-11	1.752353315	1	1	2.90E-07
DMGDH	1.68E-11	-2.818694771	0.875	1	2.91E-07
MLH1	1.70E-11	-1.342920115	1	1	2.95E-07
ENSSSCG00000034524	1.72E-11	-1.224706287	0.938	1	2.98E-07
NAT10	1.74E-11	-0.990372648	1	1	3.02E-07
PRKAR1A	1.78E-11	1.594419608	1	1	3.09E-07
SF3B6	1.81E-11	2.330102026	1	1	3.15E-07
BCAP29	1.91E-11	-0.919510082	1	1	3.32E-07
EIF4A1	1.91E-11	-0.905834422	1	1	3.32E-07
RAF1	1.94E-11	1.845181503	1	1	3.37E-07
C2orf76	2.04E-11	-1.64345815	1	1	3.54E-07
SORD	2.08E-11	-4.450661409	0.188	0.8	3.61E-07
CLN8	2.09E-11	-1.620151929	0.938	1	3.62E-07
ENSSSCG00000056896	2.13E-11	-1.571156701	0.875	1	3.69E-07
NOL4L	2.19E-11	-2.093109404	0.625	0.933	3.80E-07
TMUB1	2.21E-11	-1.926537838	0.938	1	3.83E-07
TRAFD1	2.24E-11	1.86773925	1	1	3.89E-07
ENSSSCG00000056114	2.25E-11	-2.601450624	0.75	0.933	3.90E-07
ENSSSCG00000052306	2.30E-11	-0.894780688	1	1	3.99E-07
XRCC6	2.31E-11	-0.634027572	1	1	4.01E-07
ENSSSCG00000030908	2.39E-11	-1.324735106	1	1	4.15E-07
GREB1L	2.40E-11	-1.004036441	1	1	4.16E-07
SLC38A8	2.46E-11	-2.168599604	0.875	0.933	4.28E-07
DUS3L	2.47E-11	-2.50589093	0.625	0.933	4.29E-07
PRXL2A	2.51E-11	2.245797026	1	1	4.36E-07
ZNF280D	2.57E-11	2.159583057	1	0.933	4.46E-07
PLEKHB1	2.63E-11	-2.039069565	0.875	0.933	4.56E-07
NECAP2	2.67E-11	-0.765136267	1	1	4.64E-07
ENSSSCG00000029160	2.69E-11	-1.438389371	1	1	4.66E-07
ENSSSCG00000045681	2.84E-11	-2.17625064	0.875	1	4.92E-07
TBCE	2.89E-11	-0.879645855	1	1	5.01E-07
GRIN3B	2.90E-11	-2.970426885	0.562	0.933	5.03E-07
SRP19	2.91E-11	-0.663309844	1	1	5.05E-07
SIVA1	2.93E-11	-3.169925001	0.438	1	5.08E-07
MVK	3.02E-11	-2.61667136	0.625	0.933	5.25E-07
NEIL3	3.06E-11	1.847480932	1	1	5.31E-07

GSTZ1	3.10E-11	-3.152003093	0.375	1	5.39E-07
NPAS1	3.20E-11	-3.275312736	0.5	0.933	5.56E-07
KAT5	3.23E-11	-3.166358386	0.5	0.867	5.61E-07
PLIN3	3.27E-11	-2.541569905	0.875	1	5.68E-07
CCER2	3.27E-11	-2.628072519	1	1	5.68E-07
CYC1	3.32E-11	-1.560888366	1	1	5.75E-07
LAPTM4A	3.37E-11	1.806840594	1	1	5.84E-07
MRPL16	3.38E-11	2.258610984	1	1	5.87E-07
FYB2	3.41E-11	1.827074642	1	1	5.93E-07
NDUFAB1	3.42E-11	-3.428093652	0.625	0.933	5.93E-07
ENSSSCG00000009015	3.42E-11	-1.38502493	0.875	0.933	5.94E-07
C5orf49	3.47E-11	-2.405387329	0.938	1	6.02E-07
BLMH	3.47E-11	-0.4012317	1	1	6.02E-07
FOXN2	3.54E-11	1.964627887	1	1	6.14E-07
MTO1	3.63E-11	-1.76087221	0.875	1	6.31E-07
ENSSSCG00000026248	3.72E-11	-3.30256277	0.562	0.8	6.46E-07
TUFM	3.82E-11	-1.767516686	0.938	1	6.63E-07
SPOUT1	3.82E-11	-3.707819249	0.312	0.933	6.63E-07
CTH	3.88E-11	-1.952065523	0.812	0.933	6.73E-07
IGFBP6	3.90E-11	-3.450661409	0.375	0.933	6.77E-07
UROD	3.96E-11	-2.774933444	0.688	1	6.87E-07
LAMTOR1	3.98E-11	-4.263034406	0.125	1	6.91E-07
NXF1	4.07E-11	-0.968382924	1	1	7.06E-07
ENSSSCG00000041052	4.14E-11	-5.647698256	0	0.933	7.19E-07
SLC25A11	4.15E-11	-1.833141302	0.875	1	7.21E-07
CCDC63	4.18E-11	-2.437937901	0.438	1	7.25E-07
ATXN10	4.41E-11	-1.797866418	1	1	7.66E-07
ATP5F1D	4.43E-11	-3.462343214	0.375	0.867	7.69E-07
NDUFA2	4.44E-11	-4.072931522	0.438	1	7.70E-07
B3GNT3	4.47E-11	-1.475966498	0.875	1	7.75E-07
SH3GL2	4.60E-11	-0.648841194	1	1	7.98E-07
SEH1L	4.62E-11	1.726191123	1	1	8.01E-07
SMAD1	4.63E-11	2.335374792	1	1	8.03E-07
TMEM200C	4.64E-11	-2.669458775	0.5	0.867	8.05E-07
ENSSSCG00000030478	4.68E-11	-1.974102224	1	1	8.12E-07
ARMC8	4.72E-11	1.716357809	1	1	8.19E-07
FGFR1OP2	4.84E-11	2.131716682	1	1	8.40E-07
CTBS	5.13E-11	1.870909175	1	1	8.90E-07
RAD51D	5.36E-11	-1.751418055	0.938	1	9.30E-07
PSMC5	5.37E-11	-1.466397921	0.938	1	9.32E-07
AAAS	5.44E-11	-1.57589151	1	1	9.43E-07
CASP6	5.45E-11	1.956892733	1	1	9.45E-07
PBK	5.45E-11	2.029968463	1	1	9.46E-07
OOSP3	5.47E-11	2.24828312	1	1	9.50E-07
AIFM1	5.51E-11	-1.443606651	0.938	1	9.56E-07
LUC7L	5.63E-11	-3.468148836	0.312	0.933	9.76E-07
STAT4	5.83E-11	-0.879899096	1	1	1.01E-06
ERAP2	5.85E-11	-1.788094692	0.938	1	1.02E-06
CEP131	5.88E-11	-4.201633861	0.188	0.933	1.02E-06
MAP2K1	5.89E-11	2.030832606	1	1	1.02E-06
RRM1	5.90E-11	1.850669584	1	1	1.02E-06
LIN37	6.05E-11	-2.584249883	0.75	1	1.05E-06
ENSSSCG00000022738	6.09E-11	-2.173674886	0.812	0.933	1.06E-06
EHD1	6.19E-11	-2.315501826	0.625	1	1.07E-06
TPST1	6.31E-11	2.195676406	1	1	1.10E-06
WNT7A	6.35E-11	-1.715449515	0.875	1	1.10E-06
ENSSSCG00000047619	6.38E-11	-1.271446646	1	1	1.11E-06
FBXO31	6.39E-11	-2.229375094	0.812	0.933	1.11E-06
PSMD6	6.42E-11	-0.666940898	1	1	1.11E-06
PPP1R11	6.46E-11	-1.690044547	1	1	1.12E-06
ENSSSCG00000049161	6.46E-11	-5.222392421	0.062	0.733	1.12E-06
HERPUD2	6.47E-11	1.786094079	1	1	1.12E-06
UNC13B	6.53E-11	1.93477371	1	1	1.13E-06
ENSSSCG00000008016	6.55E-11	-3.081036572	0.438	1	1.14E-06
MLYCD	6.65E-11	-2.72537762	0.438	1	1.15E-06
ENSSSCG00000059061	6.76E-11	-2.689476668	0.562	0.933	1.17E-06
TMEM50A	6.85E-11	-0.669411567	1	1	1.19E-06
DNPH1	6.90E-11	-3.660793914	0.25	0.933	1.20E-06
NID2	6.95E-11	1.627675754	1	1	1.21E-06
ENY2	6.96E-11	1.859062071	1	1	1.21E-06

CIB2	7.21E-11	-3.081036572	0.375	0.933	1.25E-06
KMT2E	7.24E-11	1.646338602	1	1	1.26E-06
RNF2	7.30E-11	1.480553423	1	1	1.27E-06
ZDHHC4	7.52E-11	-1.159488244	1	1	1.31E-06
LSM3	7.53E-11	-1.200750128	1	1	1.31E-06
BIRC5	7.79E-11	-0.996147675	1	1	1.35E-06
SHANK2	8.01E-11	-2.651104858	0.688	0.933	1.39E-06
FAM3A	8.03E-11	-4.485426827	0.125	0.867	1.39E-06
MRPL9	8.20E-11	-1.272507158	1	1	1.42E-06
ENSSSCG00000037399	8.20E-11	-4.468148836	0.188	0.867	1.42E-06
HDAC3	8.35E-11	2.492707015	1	1	1.45E-06
ENSSSCG00000028786	8.48E-11	-1.236067358	1	1	1.47E-06
YBX2	8.59E-11	-2.202231127	0.875	1	1.49E-06
EEF1AKMT1	8.60E-11	-1.888289613	0.812	0.933	1.49E-06
DSN1	8.61E-11	-0.722499371	1	1	1.49E-06
DHPS	8.79E-11	-2.198462405	0.688	0.933	1.52E-06
CIRBP	9.02E-11	-1.533093261	0.938	1	1.56E-06
TBL3	9.08E-11	-3.623624121	0.188	0.933	1.58E-06
ADCK2	9.27E-11	-1.66780357	0.875	0.933	1.61E-06
ARL2	9.27E-11	-3.401862111	0.25	0.933	1.61E-06
NKIRAS2	9.39E-11	-1.607682577	1	1	1.63E-06
PPM1B	9.48E-11	1.844984691	1	1	1.65E-06
CDC42	9.51E-11	-0.672497035	1	1	1.65E-06
CMAS	9.56E-11	1.797178592	1	1	1.66E-06
CNOT8	9.69E-11	1.983875399	1	1	1.68E-06
GPN2	9.73E-11	-2.380391356	0.812	0.933	1.69E-06
SLC25A1	9.77E-11	-4.108059746	0.25	0.867	1.70E-06
ENSSSCG00000053454	9.89E-11	2.494450208	1	1	1.72E-06
ASPA	1.01E-10	-1.835613182	0.812	1	1.76E-06
SLC25A12	1.01E-10	-1.399536406	0.875	1	1.76E-06
PRMT6	1.03E-10	-0.643876985	1	1	1.78E-06
HDGFL2	1.04E-10	-1.786682403	0.938	1	1.81E-06
CLTA	1.10E-10	-0.620554795	1	1	1.91E-06
MORN3	1.14E-10	-5.678071905	0	0.933	1.98E-06
CDKN2AIPNL	1.14E-10	-1.346865997	1	1	1.98E-06
NUP43	1.15E-10	2.099140144	1	1	2.00E-06
DNASE2	1.16E-10	-2.873328197	0.438	0.933	2.01E-06
PGS1	1.21E-10	-1.862728216	1	1	2.10E-06
AKR1A1	1.21E-10	-2.316912775	0.812	0.933	2.10E-06
TXNDC17	1.23E-10	-1.740473974	1	1	2.13E-06
MRPS6	1.25E-10	-1.879402711	0.875	0.933	2.17E-06
KDM8	1.27E-10	-2.58185659	0.688	0.933	2.21E-06
MBD3	1.29E-10	-3.728697978	0.25	0.867	2.24E-06
CCNB2	1.30E-10	-0.152153171	1	1	2.25E-06
NEGR1	1.30E-10	-0.814941695	1	1	2.26E-06
POLB	1.30E-10	2.278539969	1	1	2.26E-06
CENPS	1.31E-10	-1.014383918	1	1	2.28E-06
TM2D3	1.32E-10	-0.7314716	1	1	2.29E-06
ENSSSCG00000001064	1.33E-10	-1.114923766	1	1	2.30E-06
ENSSSCG00000052822	1.37E-10	-1.61667136	1	1	2.38E-06
STX6	1.37E-10	1.558227658	1	1	2.39E-06
PCOLCE2	1.39E-10	1.637581225	1	1	2.41E-06
SPRING1	1.45E-10	-0.763188519	1	1	2.52E-06
MAPK7	1.46E-10	-2.40780593	0.812	1	2.53E-06
UTS2	1.49E-10	-3.804604311	0.188	0.933	2.58E-06
SKA2	1.53E-10	-1.219987467	0.938	1	2.65E-06
UBTD2	1.55E-10	-1.167266339	0.938	1	2.69E-06
ENSSSCG00000033744	1.56E-10	-3.27897595	0.312	0.933	2.70E-06
FAM219B	1.59E-10	-1.847244131	0.938	1	2.75E-06
NDUFB7	1.63E-10	-3.900464326	0.25	0.867	2.82E-06
PIGH	1.63E-10	-1.969726989	0.812	0.933	2.83E-06
ENSSSCG00000061183	1.64E-10	-4.595609745	0.125	0.8	2.85E-06
H1-8	1.67E-10	2.061878384	1	1	2.89E-06
CDK5RAP1	1.71E-10	-1.19005496	0.938	1	2.97E-06
ENSSSCG00000052576	1.74E-10	-3.308122295	0.375	0.867	3.02E-06
ENSSSCG00000032953	1.79E-10	-2.051289229	0.812	1	3.10E-06
HEXIM2	1.79E-10	-1.832518174	0.75	0.933	3.10E-06
ENSSSCG00000039612	1.80E-10	-3.068306013	0.562	1	3.13E-06
TMED9	1.82E-10	-1.949745229	0.812	1	3.15E-06
DNAJB11	1.82E-10	-0.838816224	1	1	3.16E-06

HSF4	1.84E-10	-3.325770161	0.438	0.8	3.20E-06
SPOCK1	1.85E-10	-1.280581387	0.938	1	3.20E-06
MFF	1.85E-10	1.8252513	1	1	3.21E-06
MRPL15	1.87E-10	1.780585735	1	1	3.25E-06
PCGF6	1.90E-10	2.258577835	1	1	3.30E-06
LRCH4	1.93E-10	-2.054635257	0.75	0.933	3.35E-06
TOMM40L	1.94E-10	-2.330148602	0.5	1	3.36E-06
VPS41	1.96E-10	-0.928255392	1	1	3.40E-06
USP33	1.96E-10	1.897881896	1	1	3.40E-06
ENSSSCG00000052254	1.96E-10	-2.197446064	0.5	0.933	3.40E-06
VPS26B	1.96E-10	-1.541498852	1	1	3.41E-06
FCF1	1.97E-10	-1.825629094	1	1	3.42E-06
TXN2	2.06E-10	-1.933168492	0.938	0.933	3.58E-06
WNT2B	2.08E-10	-1.013674937	0.938	1	3.61E-06
PELO	2.08E-10	-0.58236383	1	1	3.61E-06
RAB43	2.12E-10	-1.345027778	0.875	1	3.67E-06
ENSSSCG00000002707	2.13E-10	-0.517339328	1	1	3.69E-06
RIDA	2.14E-10	1.872844123	1	1	3.71E-06
HYI	2.14E-10	-3.595609745	0.25	0.933	3.71E-06
DARS1	2.17E-10	1.98681924	1	1	3.76E-06
TMEM216	2.17E-10	2.540692234	1	0.933	3.76E-06
NACC2	2.22E-10	-3.941106311	0.188	0.8	3.86E-06
TRAPPC5	2.23E-10	-5.707819249	0	0.867	3.87E-06
RPS29	2.25E-10	-2.984960601	0.75	1	3.90E-06
AURKAIP1	2.28E-10	-2.111968432	0.75	1	3.95E-06
SAT1	2.31E-10	2.065794191	1	1	4.00E-06
NCOA3	2.35E-10	1.885400313	1	1	4.08E-06
UXS1	2.38E-10	1.553617912	1	1	4.12E-06
NENF	2.38E-10	-3.180572246	0.188	0.933	4.13E-06
ENSSSCG00000008147	2.39E-10	-2.185758692	0.812	0.933	4.15E-06
ENSSSCG00000029830	2.42E-10	-2.521203056	0.938	1	4.19E-06
BCAR3	2.42E-10	-0.961319532	0.938	1	4.19E-06
SNX25	2.48E-10	1.606274683	1	1	4.30E-06
CENPA	2.48E-10	2.102857731	1	1	4.30E-06
SIAE	2.50E-10	-1.256083616	1	1	4.34E-06
SMS	2.52E-10	-0.544063405	1	1	4.38E-06
ENSSSCG00000043434	2.52E-10	-2.728697978	0.438	1	4.38E-06
MAP4K1	2.53E-10	-2.768674454	0.625	0.933	4.40E-06
NME2	2.55E-10	-2.082248412	1	1	4.42E-06
EMC4	2.55E-10	-1.415037499	1	1	4.43E-06
ENSSSCG00000051057	2.58E-10	-1.368743847	0.938	1	4.47E-06
SCCPDH	2.63E-10	1.722895174	1	1	4.56E-06
POLM	2.69E-10	-2.435501602	0.75	0.867	4.67E-06
DPYS	2.73E-10	1.71334129	1	1	4.74E-06
UQCRC1	2.79E-10	-2.004001931	0.875	1	4.84E-06
ENSSSCG00000053185	2.81E-10	-1.509273569	0.938	1	4.88E-06
NDC1	2.85E-10	1.65327006	1	1	4.94E-06
ENSSSCG00000010531	2.94E-10	-1.639852583	0.875	1	5.10E-06
KIAA1143	2.95E-10	-1.640302862	1	1	5.12E-06
DYNC2I2	2.97E-10	-2.830074999	0.5	0.867	5.16E-06
ENSSSCG00000003612	2.99E-10	-1.142455458	1	1	5.19E-06
NDUFB10	3.01E-10	-2.790546634	0.75	0.933	5.22E-06
SNURF	3.01E-10	-0.971171787	1	1	5.22E-06
NAA10	3.03E-10	-2.415037499	0.75	1	5.25E-06
TIMM23B	3.10E-10	-1.24691474	1	1	5.37E-06
ABCF1	3.10E-10	-0.934593004	1	1	5.37E-06
MAF	3.11E-10	-2.212408333	0.562	1	5.40E-06
SNRPN	3.18E-10	-0.776925292	1	1	5.52E-06
IRAK2	3.24E-10	2.135768962	1	1	5.62E-06
RNF141	3.24E-10	-2.74080766	0.5	0.933	5.63E-06
ENSSSCG00000057224	3.28E-10	-2.32443495	0.75	1	5.69E-06
STK35	3.30E-10	1.655013158	1	1	5.73E-06
TRAF4	3.34E-10	2.377825621	1	1	5.79E-06
RAB5C	3.35E-10	-1.195359463	0.938	1	5.81E-06
NUP133	3.35E-10	1.747885009	1	1	5.82E-06
HSPE1	3.36E-10	-1.085000647	1	1	5.82E-06
DPP7	3.39E-10	-3.446746359	0.312	0.867	5.89E-06
POLE2	3.55E-10	2.208871282	1	1	6.17E-06
IQCC	3.56E-10	-1.942732799	0.938	1	6.18E-06
LRRC49	3.58E-10	2.341827652	1	0.933	6.21E-06

EIF3F	3.66E-10	-3.036525876	0.312	0.933	6.35E-06
ENSSSCG00000056638	3.67E-10	-0.983136562	0.875	1	6.37E-06
MFSD12	3.70E-10	-3.047305715	0.625	0.867	6.42E-06
POLA2	3.78E-10	-1.662805148	0.938	1	6.56E-06
ACTL7B	3.83E-10	-3.121306296	0.375	0.933	6.65E-06
DYNC1I2	3.84E-10	1.357296662	1	1	6.67E-06
GDF3	3.85E-10	-3.793549123	0.5	0.8	6.67E-06
ENSSSCG00000047706	3.87E-10	-2.117771459	0.75	0.933	6.72E-06
SAMD4A	4.16E-10	-5.208586622	0.125	0.933	7.22E-06
SLC35A1	4.17E-10	1.971502958	1	1	7.23E-06
PSMA5	4.23E-10	2.050838491	1	1	7.35E-06
CCDC59	4.27E-10	-1.285458818	1	1	7.41E-06
DERL2	4.30E-10	-0.590104436	1	1	7.46E-06
THOC3	4.30E-10	1.840075642	1	1	7.47E-06
ARHGEF28	4.31E-10	-2.300704824	0.562	1	7.47E-06
SAMM50	4.32E-10	-2.119909464	0.812	1	7.49E-06
ENSSSCG00000038404	4.46E-10	-2.815575429	0.875	1	7.74E-06
NDUFA4	4.53E-10	-2.391682103	1	1	7.86E-06
ENSSSCG00000004623	4.55E-10	2.1452581	1	1	7.89E-06
AMDHD2	4.61E-10	-1.982926487	0.812	1	7.99E-06
GNL3L	4.62E-10	-1.949518551	0.75	1	8.02E-06
ASGR2	4.65E-10	-3.294743266	0.312	0.867	8.07E-06
ENSSSCG00000034188	4.70E-10	-2.736965594	0.438	1	8.15E-06
CFAP44	4.75E-10	-0.327618112	1	1	8.24E-06
RPA3	4.82E-10	2.155821258	1	1	8.37E-06
MORC2	4.99E-10	-0.684243872	1	1	8.66E-06
ZNF584	5.10E-10	-1.524375365	0.875	1	8.85E-06
EIF2A	5.14E-10	1.683206594	1	1	8.91E-06
DNAJA3	5.15E-10	-0.968208933	1	1	8.93E-06
NDUFS2	5.21E-10	-1.334825089	1	1	9.04E-06
ENSSSCG00000026229	5.28E-10	-1.240815562	1	1	9.17E-06
THAP7	5.29E-10	-2.296981738	0.688	1	9.17E-06
ARHGAP27	5.29E-10	-1.143067563	0.938	1	9.19E-06
HSPA5	5.33E-10	-1.064144386	1	1	9.25E-06
ENSSSCG00000055905	5.57E-10	-2.154509949	0.625	1	9.66E-06
UCHL3	5.60E-10	-0.605922119	1	1	9.71E-06
PIGO	5.62E-10	-1.522725369	0.875	0.933	9.75E-06
STEAP1	5.75E-10	3.000194315	1	0.8	9.98E-06
CARD19	5.80E-10	-2.36159824	0.812	1	1.01E-05
ENSSSCG00000061274	5.91E-10	-2.719892081	0.5	0.933	1.02E-05
SLC25A32	6.05E-10	1.924195482	1	1	1.05E-05
PMM1	6.12E-10	-2.900464326	0.312	0.933	1.06E-05
PSTPIP1	6.20E-10	-2.449802917	0.688	0.933	1.08E-05
LIMS2	6.29E-10	-1.565659362	0.938	1	1.09E-05
ENSSSCG00000054196	6.33E-10	-1.31817596	1	1	1.10E-05
ASTL	6.42E-10	-1.568770273	1	1	1.11E-05
GNL2	6.51E-10	-1.132339179	1	1	1.13E-05
GDPGP1	6.54E-10	-2.354069482	0.688	1	1.14E-05
EIF2S2	6.64E-10	-0.466033103	1	1	1.15E-05
CCDC127	6.66E-10	1.975345898	1	1	1.16E-05
SNX3	6.73E-10	-1.052314178	1	1	1.17E-05
PEX3	6.74E-10	2.089246271	1	1	1.17E-05
ENSSSCG00000021236	6.79E-10	-1.427528443	0.938	1	1.18E-05
C15orf40	7.17E-10	-3.074962058	0.375	0.867	1.24E-05
GABARAPL1	7.28E-10	-2.783424905	0.438	0.933	1.26E-05
NUB1	7.32E-10	-0.237291677	1	1	1.27E-05
ALKBH3	7.46E-10	-0.911030203	1	1	1.30E-05
ACAA1	7.54E-10	-2.03284888	0.75	0.933	1.31E-05
MRGBP	7.61E-10	-2.975752454	0.438	0.8	1.32E-05
RNF4	7.76E-10	-1.214258891	0.938	1	1.35E-05
EDF1	7.78E-10	-2.923184403	0.438	1	1.35E-05
RAB6B	7.93E-10	-1.217367507	1	1	1.38E-05
NUBP2	7.95E-10	-3.504042505	0.375	0.867	1.38E-05
LETM2	7.95E-10	-0.952931746	1	1	1.38E-05
CHN2	8.02E-10	-0.336995358	1	1	1.39E-05
SNF8	8.05E-10	-2.322843801	0.812	1	1.40E-05
SPG7	8.35E-10	-4.707819249	0.062	0.867	1.45E-05
ZNF207	8.44E-10	1.261019582	1	1	1.46E-05
KLHDC2	8.47E-10	1.706456971	1	1	1.47E-05
PSMC1	8.49E-10	-0.980799188	1	1	1.47E-05

PLPP4	8.60E-10	-2.423258006	0.688	1	1.49E-05
LMBR1L	8.63E-10	-2.168397532	0.812	0.933	1.50E-05
SLC16A5	8.65E-10	-2.145197916	0.625	1	1.50E-05
MGST2	8.77E-10	-1.570099394	1	1	1.52E-05
PPOX	8.81E-10	-2.285754482	0.5	0.933	1.53E-05
BRCC3	8.97E-10	-1.093109404	0.938	1	1.56E-05
ENSSSCG00000035728	9.07E-10	-2.175571565	0.812	1	1.57E-05
MASTL	9.12E-10	1.783366188	1	1	1.58E-05
PPEF2	9.30E-10	-1.678071905	0.938	0.933	1.61E-05
SUGP1	9.41E-10	-3	0.5	0.933	1.63E-05
SLC12A8	9.46E-10	1.938439431	1	1	1.64E-05
CD164	9.63E-10	1.693993495	1	1	1.67E-05
GATD1	9.67E-10	-2.542416806	0.625	0.933	1.68E-05
TOP1MT	1.00E-09	-1.983880335	0.812	0.933	1.74E-05
PSME2	1.00E-09	-2.938599455	0.438	0.933	1.74E-05
TKT	1.01E-09	-1.683332422	1	1	1.75E-05
ATG4B	1.01E-09	-1.418375785	1	1	1.75E-05
ENSSSCG00000044205	1.02E-09	-6.251538767	0.125	0.8	1.77E-05
PAFAH1B3	1.03E-09	-4.975752454	0.062	0.733	1.79E-05
ENSSSCG00000001769	1.04E-09	1.634028348	1	1	1.80E-05
RNF220	1.04E-09	-0.895401246	1	1	1.80E-05
ENSSSCG00000027506	1.05E-09	-0.585589642	1	1	1.81E-05
NAA20	1.05E-09	-1.199202794	1	1	1.82E-05
PDHX	1.05E-09	1.840777924	1	1	1.82E-05
ENSSSCG00000033019	1.06E-09	-3.061400545	0.812	1	1.84E-05
ZNF621	1.06E-09	-2.670538232	0.562	0.867	1.84E-05
MCMBP	1.07E-09	1.667316889	1	1	1.86E-05
ENSSSCG00000013768	1.08E-09	-2.678071905	0.562	0.933	1.87E-05
KCTD20	1.08E-09	-1.126024027	1	1	1.88E-05
ZCCHC17	1.09E-09	-0.981765817	1	1	1.89E-05
GEMIN2	1.09E-09	-2.111394757	0.812	0.933	1.90E-05
AUP1	1.10E-09	-1.801220136	0.938	1	1.90E-05
ZNF606	1.11E-09	1.927610269	1	1	1.93E-05
ENSSSCG00000003903	1.14E-09	-1.828958361	1	1	1.97E-05
PHYHIPL	1.15E-09	2.058262372	1	1	2.00E-05
MYBPC3	1.19E-09	-3.310340121	0.375	0.867	2.06E-05
TTC5	1.19E-09	1.896446704	1	1	2.07E-05
SAL1	1.21E-09	-2.982926487	0.562	1	2.10E-05
NUDT9	1.22E-09	-0.657124967	1	1	2.12E-05
EMC2	1.23E-09	2.026801923	1	1	2.14E-05
ALG8	1.27E-09	-1.195093437	1	1	2.20E-05
RPGRIP1	1.27E-09	-0.743969791	1	1	2.21E-05
UBA52	1.28E-09	-2.72416832	0.875	1	2.21E-05
TBCA	1.28E-09	-1.640750279	1	1	2.22E-05
MYL6B	1.31E-09	-1.331681753	0.938	1	2.27E-05
ATP5PB	1.32E-09	-0.475626662	1	1	2.28E-05
MAD2L1BP	1.32E-09	-1.250961574	0.938	1	2.30E-05
EIF3B	1.33E-09	-1.90902634	0.938	0.933	2.30E-05
CIDEA	1.34E-09	-2.345113482	0.938	1	2.33E-05
ENSSSCG00000012452	1.37E-09	2.175952849	1	1	2.39E-05
OOSP2	1.39E-09	1.988350296	1	1	2.40E-05
ENSSSCG00000050347	1.39E-09	-2.717600269	0.375	0.933	2.40E-05
PSTK	1.40E-09	-1.234328822	1	1	2.43E-05
RPS16	1.41E-09	-2.962322797	0.938	1	2.45E-05
LNX1	1.45E-09	1.930084168	1	1	2.52E-05
GLT8D1	1.46E-09	-1.879705766	0.875	0.933	2.53E-05
TUBA4A	1.46E-09	-1.273468407	0.938	1	2.54E-05
DDRGK1	1.53E-09	-2.810966176	0.438	0.933	2.66E-05
FNTB	1.54E-09	-0.854471653	1	1	2.67E-05
ENSSSCG00000058768	1.61E-09	-4.093109404	0.188	0.733	2.79E-05
BUD13	1.63E-09	2.217581719	1	1	2.82E-05
ANKLE2	1.65E-09	1.600442284	1	1	2.87E-05
MPV17	1.65E-09	-2.20461772	0.938	0.933	2.87E-05
RTCB	1.66E-09	-1.233444519	1	1	2.89E-05
UPP1	1.69E-09	-4.180572246	0.188	0.667	2.93E-05
ICA1	1.74E-09	1.782374089	1	1	3.02E-05
TDP2	1.75E-09	1.688618558	1	1	3.03E-05
ENSSSCG00000038630	1.75E-09	1.715600797	1	1	3.04E-05
XRCC2	1.76E-09	1.800268176	1	1	3.05E-05
GLP2R	1.79E-09	-0.711334359	1	1	3.11E-05

ENSSSCG00000061349	1.85E-09	-2.291048782	0.688	0.867	3.22E-05
CFDP1	1.86E-09	-1.167877173	1	1	3.22E-05
MTM1	1.91E-09	-0.685293132	1	1	3.32E-05
COQ10B	1.93E-09	1.786147538	1	1	3.35E-05
ENSSSCG00000039222	1.99E-09	-0.792683211	1	1	3.45E-05
KCNN2	2.01E-09	-1.546700144	0.938	1	3.50E-05
LZIC	2.08E-09	-0.198772777	1	1	3.61E-05
EAF1	2.08E-09	-0.993573731	0.938	1	3.62E-05
OXLD1	2.09E-09	-3.847996907	0.125	0.867	3.63E-05
GSE1	2.11E-09	-1.774933444	0.875	1	3.66E-05
CD274	2.16E-09	-1.567949091	1	1	3.74E-05
ENSSSCG00000062187	2.20E-09	-4.595609745	0.125	0.8	3.83E-05
SMYD3	2.27E-09	-2.515066318	0.812	1	3.95E-05
TMEM205	2.31E-09	-2.783424905	0.688	0.867	4.00E-05
ENSSSCG00000007203	2.32E-09	1.54863596	1	1	4.03E-05
KLHDC4	2.33E-09	-2.336563441	0.875	0.867	4.05E-05
NLRP4	2.34E-09	1.649275169	1	1	4.05E-05
MCAT	2.40E-09	-2.815575429	0.625	0.867	4.17E-05
SAP30BP	2.43E-09	-1.524955191	0.938	1	4.22E-05
NDUFB4	2.43E-09	-2.441837559	0.812	1	4.22E-05
TNIP1	2.47E-09	-3.334117504	0.25	0.867	4.28E-05
BRF2	2.48E-09	-1.399471242	0.938	1	4.31E-05
TUT1	2.54E-09	-3.793549123	0.188	0.867	4.42E-05
MIER1	2.57E-09	2.027383612	1	1	4.45E-05
CCDC28B	2.65E-09	-3.629162305	0.25	0.867	4.60E-05
SEPTIN2	2.69E-09	2.029509882	1	1	4.66E-05
SLC24A3	2.72E-09	-1.697808123	0.875	1	4.72E-05
PSMG3	2.83E-09	-1.220390068	1	1	4.91E-05
GRIN3A	2.86E-09	-1.103717504	0.938	1	4.96E-05
GSPT1	2.87E-09	-0.303046827	1	1	4.98E-05
RNASEH2A	2.89E-09	-1.078210528	0.938	1	5.02E-05
SMIM11	2.92E-09	2.316614295	1	0.933	5.06E-05
NAALAD2	2.92E-09	-0.530542495	1	1	5.06E-05
TCTN2	2.92E-09	1.790823198	1	1	5.06E-05
RFK	2.93E-09	2.394409312	1	1	5.08E-05
HSCB	2.93E-09	2.185191758	1	1	5.09E-05
NMD3	2.95E-09	1.935699219	1	1	5.11E-05
RPP25L	3.03E-09	1.970249993	1	1	5.26E-05
SLC16A13	3.09E-09	-1.236528652	1	1	5.36E-05
NOP9	3.11E-09	-1.110964769	1	1	5.40E-05
CHST3	3.16E-09	-1.051993551	0.938	1	5.49E-05
MLST8	3.18E-09	-2.285754482	0.75	0.933	5.52E-05
ENSSSCG00000060912	3.23E-09	-2.604071324	0.438	0.933	5.61E-05
GTSF1	3.24E-09	-0.613139669	1	1	5.62E-05
MTMR2	3.27E-09	-0.521088936	1	1	5.68E-05
MTA3	3.30E-09	1.706193245	1	1	5.72E-05
LIG1	3.33E-09	-1.163498732	0.938	1	5.78E-05
CARHSP1	3.38E-09	-1.925381959	0.75	0.933	5.86E-05
FBXO25	3.38E-09	1.451249671	1	1	5.87E-05
KAT2A	3.45E-09	2.307467282	1	1	5.98E-05
NPM1	3.50E-09	1.827574231	1	1	6.06E-05
CDC20	3.53E-09	2.325557038	1	1	6.12E-05
GPR160	3.61E-09	1.929964619	1	1	6.27E-05
METTL3	3.72E-09	2.364976417	1	1	6.45E-05
NUS1	3.78E-09	1.951599199	1	1	6.56E-05
SHFL	3.86E-09	-3.415037499	0.25	0.867	6.70E-05
ENSSSCG00000027374	3.88E-09	-0.675180732	1	1	6.74E-05
ENSSSCG00000062406	3.92E-09	-0.510404482	1	1	6.81E-05
ARL6IP4	3.99E-09	-2.078609835	0.688	0.933	6.93E-05
UBR2	4.04E-09	1.601089374	1	1	7.01E-05
ARV1	4.04E-09	-0.835721562	1	1	7.01E-05
ARHGAP9	4.13E-09	-1.452190498	0.875	0.933	7.16E-05
RND2	4.14E-09	-3.180572246	0.25	0.933	7.18E-05
CDH7	4.27E-09	1.586386027	1	1	7.42E-05
PADI3	4.28E-09	-2.055495113	0.75	1	7.43E-05
SPAG8	4.38E-09	-2.511610759	0.625	0.933	7.59E-05
TEX10	4.46E-09	1.587813114	1	1	7.73E-05
CHCHD3	4.60E-09	-0.507036711	1	1	7.98E-05
ENSSSCG00000039658	4.61E-09	2.01283646	1	1	8.00E-05
CHCHD5	4.67E-09	-1.608530972	0.938	1	8.10E-05

PYCR2	4.71E-09	2.209207973	1	1	8.18E-05
MMAB	4.73E-09	-2.519374159	0.5	0.933	8.20E-05
PAPLN	4.84E-09	-2.393504338	0.562	1	8.39E-05
FBXO7	4.86E-09	-0.98579638	1	1	8.44E-05
CCNI	4.98E-09	-0.404282534	1	1	8.63E-05
PDIA2	5.09E-09	-3.499101764	0.25	0.867	8.84E-05
ENSSSCG00000031636	5.20E-09	-0.248545069	1	1	9.02E-05
PSMA7	5.29E-09	-1.231888803	1	1	9.18E-05
ENSSSCG00000053801	5.31E-09	-3.606178987	0.312	0.8	9.22E-05
FAM50A	5.35E-09	-1.99492901	0.812	1	9.28E-05
PADI6	5.51E-09	-1.104206143	1	1	9.55E-05
MRPL3	5.55E-09	-0.316356756	1	1	9.63E-05
MAF1	5.56E-09	-1.547675268	0.812	1	9.64E-05
CDKN3	5.57E-09	-1.27104622	1	1	9.66E-05
SUPT3H	5.58E-09	-0.718579629	1	1	9.68E-05
GPANK1	5.63E-09	-1.276555545	1	1	9.76E-05
PALLD	5.70E-09	-1.798584712	1	1	9.88E-05
ENSSSCG00000060926	5.81E-09	-1.033576081	0.875	1	0.000100881
CCNL2	5.82E-09	-1.747334568	0.875	1	0.000100907
TBCC	5.82E-09	-1.181532655	1	1	0.000100917
SLC23A2	5.82E-09	1.516889647	1	1	0.000100922
ENSSSCG00000029257	5.98E-09	-1.35614381	0.875	1	0.000103762
NDFIP2	6.08E-09	2.000421286	1	1	0.000105492
TUBB	6.16E-09	-1.160028996	1	1	0.000106846
COL4A6	6.25E-09	-1.327019669	1	1	0.000108376
STOML2	6.26E-09	2.118982423	1	1	0.000108682
MCEE	6.27E-09	-1.614346308	0.938	1	0.000108723
MRM3	6.39E-09	-1.276081476	1	1	0.00011083
THBS3	6.39E-09	-2.212848649	0.625	1	0.000110875
ENSSSCG00000013613	6.40E-09	-2.188266637	0.688	1	0.000111102
PRPF6	6.41E-09	-1.555900035	0.875	1	0.00011122
ABI1	6.42E-09	1.29785963	1	1	0.000111428
PIGC	6.51E-09	-0.846536245	1	1	0.000112946
RPL23	6.56E-09	-3.379266368	0.812	1	0.000113782
SPIRE1	6.69E-09	1.840365135	1	1	0.00011607
PADI4	6.70E-09	-1.511822562	0.938	1	0.000116325
ZP2	6.75E-09	-0.806185294	1	1	0.000117114
RBP2	6.76E-09	-2.639597757	0.562	0.867	0.000117304
CFAP276	6.76E-09	-3.143735477	0.25	0.933	0.000117337
UBC	6.82E-09	2.248487915	1	1	0.000118316
SIAH3	6.84E-09	-2.516320835	0.688	0.933	0.000118658
EIF4EBP1	6.88E-09	-2.443606651	0.562	1	0.000119369
CHMP1A	7.01E-09	-3.629162305	0.125	0.867	0.000121637
RND1	7.11E-09	-1.047828525	1	1	0.000123304
ZFAND3	7.12E-09	-0.816485934	1	1	0.00012346
ENSSSCG00000031299	7.12E-09	-1.705552642	0.875	1	0.000123493
PENK	7.19E-09	-7.415037499	0	0.8	0.000124726
ENSSSCG00000061194	7.30E-09	-1.200024608	0.938	1	0.000126578
MST1	7.39E-09	-2.847996907	0.812	0.867	0.000128151
U2AF1L4	7.50E-09	-3.113008962	0.375	0.933	0.000130079
DTX2	7.69E-09	-0.756455166	1	1	0.000133488
KLK15	7.81E-09	-3.475579041	0.125	0.867	0.000135582
MVD	7.93E-09	-5.415037499	0	0.8	0.00013762
BBS5	7.96E-09	2.191781311	1	1	0.000138141
RNF115	8.03E-09	1.307835866	1	1	0.000139284
TTC7A	8.26E-09	-1.664461474	0.812	1	0.000143268
MCOLN1	8.27E-09	2.305169577	1	0.933	0.000143576
CAAP1	8.28E-09	1.905272317	1	1	0.000143675
UPK1A	8.31E-09	-2.344070978	0.625	0.867	0.000144219
GGA1	8.32E-09	-1.881605299	0.688	0.933	0.000144286
TMEM156	8.33E-09	-3.761487913	0.25	0.733	0.000144606
PCCB	8.40E-09	-0.586723668	1	1	0.000145774
SMYD2	8.45E-09	-0.953810367	0.938	1	0.000146609
ENSSSCG00000012235	8.55E-09	2.069324598	1	1	0.00014832
FLAD1	8.56E-09	-2.709780765	0.438	0.933	0.000148536
NHEJ1	8.78E-09	-1.487793842	0.875	1	0.000152356
CCDC179	8.85E-09	2.688785533	1	0.867	0.000153589
PSMG1	8.86E-09	1.422742753	1	1	0.000153662
SKA1	8.98E-09	-1.11137418	1	1	0.000155895
CHEK1	9.02E-09	1.717060111	1	1	0.000156589

ERH	9.16E-09	-0.923184403	1	1	0.000158857
ATRIP	9.18E-09	-1.62203787	0.875	1	0.000159234
MLLT11	9.36E-09	1.841624824	1	1	0.00016234
PCYOX1	9.55E-09	-0.992832176	1	1	0.000165667
REX1BD	9.58E-09	-2.719892081	0.438	1	0.000166203
PITRM1	9.67E-09	2.466180149	1	1	0.000167698
NPEPL1	9.80E-09	-3.821029859	0.125	0.8	0.000170093
GLMP	9.88E-09	-1.753259402	0.938	0.933	0.000171479
GAPDH	9.91E-09	-2.591295462	0.938	1	0.000172017
TRAP1	1.01E-08	-0.82930944	0.938	1	0.000174444
ITPRID2	1.02E-08	1.555273235	1	1	0.000176242
AHCTF1	1.02E-08	1.805775963	1	1	0.000176586
LANCL2	1.03E-08	-1.328557979	1	1	0.000177994
PRMT2	1.04E-08	2.121224571	1	1	0.000180156
CORO6	1.04E-08	-2.678071905	0.438	1	0.000180574
PPHLN1	1.04E-08	1.664068197	1	1	0.000180661
MEST	1.04E-08	1.874618149	1	1	0.000181144
ADRM1	1.05E-08	-1.744035167	0.812	0.933	0.000181866
MROH7	1.05E-08	-1.276114233	0.875	1	0.000182888
SFN	1.08E-08	-1.981464049	0.75	1	0.000186784
TIMD4	1.10E-08	-1.351177557	0.938	1	0.00019051
STMN1	1.12E-08	-1.81232633	1	1	0.000194453
CEP57L1	1.13E-08	2.106650827	1	1	0.000196073
REV1	1.13E-08	1.482413868	1	1	0.00019628
RPL15	1.13E-08	-0.465519923	1	1	0.000196294
ENSSSCG00000033189	1.15E-08	-1.891475543	0.875	1	0.000199362
KNG1	1.17E-08	-0.581762098	1	1	0.000202447
PRPF19	1.18E-08	-1.200024608	0.938	0.933	0.000204877
KCNAB3	1.19E-08	-1.467304809	1	1	0.000205751
ANTKMT	1.19E-08	-3.180572246	0.188	0.933	0.000205969
UBXN1	1.19E-08	-3.093109404	0.312	0.733	0.000205971
UNC50	1.20E-08	-0.594022028	1	1	0.00020741
EFCAB7	1.20E-08	-0.841346111	1	1	0.000207425
MITD1	1.20E-08	2.631992368	1	0.933	0.000207638
MFSD13A	1.21E-08	-2.432959407	0.625	0.933	0.000210064
ENSSSCG00000053975	1.22E-08	-3.208586622	0.25	0.933	0.000211856
ENSSSCG00000059210	1.23E-08	-2.341036918	0.5	0.933	0.000213461
ENSSSCG00000052563	1.23E-08	-3.159198595	0.25	0.867	0.000213917
OGFOD1	1.25E-08	-0.512664405	1	1	0.000217009
RDX	1.26E-08	1.554478431	1	1	0.000219143
USP27X	1.28E-08	-0.917382117	1	1	0.000221728
ZNHIT3	1.28E-08	-0.639471852	1	1	0.000222035
EPB41L2	1.29E-08	1.40721507	1	1	0.000223191
PPP1CC	1.29E-08	1.806780231	1	1	0.000223787
MBOAT1	1.29E-08	-0.368743847	1	1	0.00022394
USP28	1.30E-08	1.623563571	1	1	0.000225147
ZNF567	1.30E-08	2.117845904	1	1	0.00022568
ARL6IP6	1.30E-08	1.881799615	1	1	0.000225803
SUPV3L1	1.30E-08	2.075816056	1	1	0.000226395
NOLC1	1.31E-08	-0.494649119	1	1	0.000227016
MMD2	1.31E-08	-2.03866162	0.562	0.933	0.00022718
SMARCE1	1.34E-08	-0.544858682	1	1	0.000232963
SAP30	1.38E-08	1.879861364	1	1	0.000238832
ENSSSCG00000016475	1.38E-08	-1.335470242	1	0.933	0.000239913
WDR89	1.41E-08	1.980186105	1	1	0.000244806
PCBP1	1.42E-08	1.818636586	1	1	0.000245923
FKBP9	1.43E-08	-0.634882948	1	1	0.000247606
ENSSSCG00000042361	1.43E-08	-1.947258538	0.75	0.933	0.000247769
ZNF410	1.43E-08	-1.439434154	0.875	1	0.00024809
TARBP2	1.43E-08	-2.823502345	0.5	0.8	0.000248448
MFSD2A	1.44E-08	-1.924354947	0.812	1	0.000249106
ADPGK	1.45E-08	1.785155183	1	1	0.000252168
CLPTM1L	1.49E-08	2.196498097	1	1	0.0002587
ENSSSCG00000062012	1.50E-08	-2.719892081	0.562	0.8	0.000259453
PPP1R7	1.51E-08	-1.495741883	0.812	1	0.000261312
OMA1	1.52E-08	1.859597987	1	1	0.000263857
GLIPR2	1.52E-08	-1.189111224	1	1	0.000264251
TADA1	1.54E-08	1.708423358	1	1	0.000267767
WBP2	1.56E-08	-1.462343214	1	1	0.000270333
NCLN	1.57E-08	-2.012832815	0.875	0.933	0.000271961

BLM	1.58E-08	1.952296575	1	1	0.000274322
POLG2	1.60E-08	2.043271108	1	1	0.000276763
ECSIT	1.62E-08	-1.411380476	0.938	1	0.00028069
ENSSSCG00000036988	1.62E-08	-1.252307999	0.875	1	0.000280732
CTSD	1.63E-08	-2.910732662	0.375	0.933	0.000282431
DDX23	1.65E-08	-1.222877279	0.938	1	0.0002869
ENSSSCG00000043578	1.66E-08	-4.208586622	0.25	0.8	0.000287211
MPHOSPH8	1.66E-08	-0.403505662	1	1	0.000288422
JOSD1	1.67E-08	-0.864400137	0.938	1	0.00028996
PPIE	1.67E-08	-0.593538395	1	1	0.000290113
TMEM41A	1.68E-08	-0.283197445	1	1	0.000291124
RAB2B	1.69E-08	-0.368091664	1	1	0.00029342
ENSSSCG00000047379	1.70E-08	-2.171111916	0.625	1	0.000294962
CRYM	1.72E-08	-1.002200908	1	1	0.000298507
AKTIP	1.73E-08	2.009272088	1	1	0.000299952
ENSSSCG00000051352	1.78E-08	-2.118644496	0.75	0.867	0.000308745
CYFIP1	1.78E-08	1.938169762	1	1	0.000309135
CAPNS1	1.79E-08	-1.734974296	0.688	0.933	0.000311229
EIF1	1.84E-08	2.397698817	1	1	0.000319391
NME7	1.85E-08	1.914351909	1	1	0.00032113
CBFB	1.85E-08	1.910510911	1	1	0.000321405
ENSSSCG00000060529	1.86E-08	1.882935863	1	1	0.000322913
CHMP6	1.89E-08	-4.180572246	0.125	0.667	0.00032737
EIF2B2	1.89E-08	-2.561878888	0.625	0.933	0.000328771
CAPN8	1.91E-08	-1.915111102	0.625	1	0.000331314
HJURP	1.92E-08	2.624119972	1	0.933	0.000333931
ATL1	1.96E-08	1.876654589	1	1	0.000340165
LRRC61	1.96E-08	-2.877380713	0.312	0.933	0.000340228
PSEN1	1.96E-08	-0.417905003	1	1	0.000340793
ATP6V0A1	1.99E-08	-0.364227248	1	1	0.000345582
KCNMB4	2.00E-08	-0.836991861	1	1	0.000347219
ENSSSCG00000008335	2.02E-08	1.8217351	1	1	0.000349837
ENSSSCG00000033421	2.05E-08	-1.522974321	0.875	0.933	0.000355958
ENSSSCG00000059232	2.06E-08	-2.001961516	0.812	1	0.000357801
MRPS18C	2.07E-08	-2.013256097	0.875	1	0.000359545
MRPL35	2.07E-08	-0.786341117	0.938	1	0.00035971
CENPN	2.10E-08	2.279793831	1	1	0.000364593
COPS4	2.13E-08	-0.408894306	1	1	0.000368808
BRD9	2.15E-08	-0.993810064	1	1	0.000372688
PLD6	2.15E-08	-3.396890153	0.25	0.867	0.00037365
THEM5	2.18E-08	-2.093109404	0.562	1	0.000377851
GNB4	2.19E-08	1.716366119	1	1	0.000379406
SLC4A4	2.20E-08	1.822708174	1	1	0.000381831
MKRN1	2.22E-08	-0.754669598	1	1	0.000385886
EIF2S3B	2.25E-08	-0.477925205	1	1	0.000390186
TCEANC	2.27E-08	-3.351843673	0.25	0.733	0.000393546
TTC1	2.31E-08	-0.822171113	1	1	0.000400683
ENSSSCG00000051162	2.31E-08	-1.04800915	0.938	1	0.000401598
ENSSSCG00000015390	2.32E-08	1.209560482	1	1	0.000401959
UBAC2	2.32E-08	1.563842443	1	1	0.000403121
TXNRD3	2.35E-08	-2.183307213	0.688	0.933	0.000408179
CCDC3	2.37E-08	-1.632930774	1	1	0.000411688
PTP4A2	2.38E-08	-0.458319747	1	1	0.00041285
ENSSSCG00000043116	2.38E-08	-0.675166174	1	1	0.000413171
DOC2B	2.39E-08	-0.915785126	1	1	0.00041399
SSX2IP	2.40E-08	1.686716974	1	1	0.000416763
THOP1	2.45E-08	-3.508146904	0.312	0.867	0.000425137
UBE2D1	2.46E-08	2.371476543	1	0.933	0.000427081
ANXA5	2.47E-08	-0.587953107	1	1	0.000428998
CTDSP2	2.52E-08	-1.686789122	0.812	1	0.00043688
HNF1B	2.53E-08	-0.665304609	1	1	0.000438138
OARD1	2.57E-08	-0.935568127	0.875	1	0.000445342
SMG1	2.57E-08	-0.171291378	1	1	0.000446598
HARS1	2.60E-08	-1.375748368	0.812	1	0.000451205
ENSSSCG00000049122	2.65E-08	-4.584962501	0.062	0.8	0.000460665
DERA	2.67E-08	2.11585031	1	0.933	0.000462818
YJU2B	2.69E-08	-1.697489359	0.875	1	0.00046631
RXYLT1	2.73E-08	2.173398388	1	1	0.00047284
RAB5IF	2.74E-08	-0.964254339	0.938	1	0.000475238
ENSSSCG00000048122	2.75E-08	-3.415037499	0.188	0.867	0.00047779

ENSSSCG00000041692	2.83E-08	-2.185031894	0.688	0.867	0.000491629
DPM1	2.85E-08	-0.677186001	1	1	0.000495332
CFL1	2.88E-08	-1.015328283	1	1	0.000500276
APH1A	2.91E-08	1.849088499	1	1	0.000504213
ACOT13	2.98E-08	-3.010647244	0.375	0.867	0.000517156
TAF5	2.99E-08	1.810392008	1	1	0.00051917
MFSD11	2.99E-08	-0.979627156	1	1	0.000519259
KANK3	3.00E-08	-2.805827452	0.438	0.867	0.000519988
CDT1	3.01E-08	-3.306103128	0.25	0.867	0.000522439
SNRNP40	3.01E-08	1.932165403	1	1	0.000522748
FBXL20	3.03E-08	1.420819564	1	1	0.000525735
CALML4	3.07E-08	-2.12963528	0.75	1	0.000533479
OSER1	3.08E-08	-1.024677679	1	1	0.000535115
ENSSSCG00000063358	3.10E-08	-3.35614381	0.125	0.867	0.000537218
DAPK3	3.17E-08	-1.610508621	0.75	0.933	0.000550771
ACBD7	3.19E-08	-0.850084959	0.938	1	0.000552688
QPRT	3.21E-08	-3.756074417	0.062	0.867	0.000557806
ENSSSCG00000031991	3.26E-08	-0.968446831	1	1	0.000565058
C9orf72	3.27E-08	1.766723691	1	1	0.000568012
ENSSSCG00000032073	3.28E-08	-3.874469118	0.125	0.8	0.000568608
PPIL2	3.30E-08	-0.985040403	1	1	0.000573051
FRA10AC1	3.40E-08	-0.763869778	1	1	0.000589952
NPLOC4	3.46E-08	-1.332779817	0.875	1	0.000600957
SELENON	3.47E-08	-1.343246816	1	1	0.000601253
FAM174C	3.47E-08	-2.900464326	0.375	0.867	0.000602641
COPE	3.55E-08	-1.085949613	0.938	1	0.000616151
MRPS18A	3.59E-08	-3.552541023	0.188	0.8	0.000622699
PLEKHO1	3.61E-08	-1.208586622	0.938	1	0.000625613
DNPEP	3.61E-08	-2.164975391	1	1	0.000626059
ENSSSCG00000005649	3.66E-08	-1.709780765	0.875	1	0.000634529
SYCP3	3.67E-08	-1.07189795	1	1	0.00063663
FBXO17	3.70E-08	-1.390495315	0.812	1	0.000641464
MAPK6	3.71E-08	1.776318141	1	1	0.000643971
TMED5	3.73E-08	1.737527032	1	1	0.000647763
KDELR2	3.75E-08	1.62028149	1	1	0.000651357
FUNDC1	3.78E-08	1.710902779	1	1	0.000655535
RPF2	3.78E-08	-1.287506773	1	1	0.000656317
ENSSSCG00000010489	3.78E-08	-1.809316438	0.625	1	0.000656601
ENSSSCG00000027349	3.89E-08	1.870043711	1	1	0.000674888
GAB1	3.90E-08	1.629387342	1	1	0.000677459
ENSSSCG00000062560	3.94E-08	-0.802251316	1	1	0.000684344
COPS7B	3.98E-08	-1.283905584	1	0.933	0.000690438
VWA7	3.99E-08	2.382848703	1	1	0.000692398
IFT88	4.06E-08	-0.746855887	1	1	0.000704714
CBR4	4.07E-08	1.851964385	1	1	0.000705637
ENSSSCG00000057916	4.10E-08	-1.265659875	1	1	0.000711876
SNRPE	4.13E-08	1.809716512	1	1	0.000717241
HSD17B1	4.19E-08	-5.222392421	0	0.8	0.000727491
SPATA24	4.34E-08	-3.394278939	0.188	0.733	0.000753104
ZNF75D	4.37E-08	-0.510918653	1	1	0.000757648
TPGS2	4.40E-08	-0.877849776	0.938	1	0.000763194
VIRMA	4.42E-08	1.533280993	1	1	0.00076639
SLC28A3	4.47E-08	-1.447291698	0.812	1	0.000775123
SOS2	4.48E-08	1.777892239	1	1	0.000777224
DRAP1	4.48E-08	-1.475883054	0.938	1	0.000777337
RAP2C	4.49E-08	1.888796802	1	1	0.000779408
PPP6C	4.50E-08	1.683483653	1	1	0.000781549
ENSSSCG00000054294	4.50E-08	-1.485426827	0.625	1	0.000781598
ZC3H13	4.60E-08	-0.297718674	1	1	0.000797904
ENSSSCG00000062784	4.67E-08	-2.277533976	0.562	1	0.000810684
DDX28	4.69E-08	-1.155231116	0.938	1	0.000813766
CAPN7	4.72E-08	1.511395335	1	1	0.000819713
SLC37A4	4.80E-08	-1.000850897	1	0.933	0.000832331
MRPS14	4.80E-08	-0.574340501	1	1	0.00083264
SF3B5	4.81E-08	-5.519374159	0	0.667	0.000834372
MFAP1	4.91E-08	-0.266278777	1	1	0.00085178
ENSSSCG00000041717	4.91E-08	-1.633677786	0.938	1	0.000852766
SSR3	4.93E-08	-0.89426806	1	1	0.00085567
PRRT3	4.95E-08	-2.765534746	0.375	0.867	0.000858689
ENSSSCG00000011447	4.95E-08	-0.780545526	1	1	0.000858761

ASPDH	4.99E-08	-3.236067358	0.312	0.8	0.000866098
SSR2	5.09E-08	-1.479658347	1	0.933	0.000883038
NFATC2	5.17E-08	-0.563875051	1	1	0.000897696
DNMT3B	5.21E-08	1.861357454	1	1	0.000903329
MYOM1	5.21E-08	-1.77663574	0.812	0.933	0.000903873
CIAPIN1	5.21E-08	-1.574978412	0.938	1	0.000904518
HAT1	5.23E-08	-0.306923251	1	1	0.000907967
RCHY1	5.24E-08	1.778530344	1	1	0.000909226
PRSS8	5.26E-08	-1.199560346	0.938	1	0.000912608
ISYNA1	5.28E-08	-2.22059914	0.688	0.867	0.000915432
PIP4K2B	5.39E-08	-0.838925916	1	0.933	0.000935456
RABGAP1	5.44E-08	1.336867759	1	1	0.000944688
ENSSSCG00000044054	5.55E-08	-2.118644496	0.688	1	0.000962625
ANKRA2	5.57E-08	2.588196965	1	0.933	0.00096612
ALG14	5.57E-08	-0.783488205	1	0.933	0.000966633
ACSL5	5.60E-08	-0.444460047	1	1	0.000971096
UBE2R2	5.60E-08	-0.659212878	1	1	0.000972239
PATE1	5.67E-08	-4.378511623	0.062	0.8	0.000983854
MTHFS	5.67E-08	-1.513012659	0.75	1	0.000984381
ENSSSCG00000017907	5.73E-08	-1.2264715	1	1	0.00099503
AP3S2	5.74E-08	-1.910732662	0.562	1	0.000995144
SLC38A5	5.83E-08	-2.489999557	0.625	0.8	0.001012363
TTC32	5.87E-08	-1.072850565	1	1	0.001018402
TM9SF1	5.94E-08	1.939411738	1	1	0.001030794
ENSSSCG00000026746	5.97E-08	2.142680165	1	1	0.001035336
TTLL3	6.01E-08	-0.737743118	1	1	0.001042393
PTPN13	6.02E-08	1.270783108	1	1	0.001045285
LDHA	6.08E-08	-1.084296335	0.938	1	0.001054667
TMEM178A	6.19E-08	-2.703162886	0.375	0.933	0.001074113
GFRA4	6.20E-08	-1.965648495	0.625	0.933	0.001075958
UBXN8	6.21E-08	-1.079116132	1	0.933	0.001077879
SSR4	6.27E-08	-1.613263609	0.938	1	0.001087508
DNAJC3	6.33E-08	-0.682758059	1	1	0.001098572
ENSSSCG00000050296	6.34E-08	-3.629162305	0.125	0.867	0.001099562
ENSSSCG00000009422	6.39E-08	-0.945175975	1	1	0.001108813
PRPSAP1	6.45E-08	-1.370748223	1	0.933	0.00111983
ATP6V0E2	6.47E-08	-1.712019237	0.875	1	0.001122959
TRMT11	6.63E-08	2.138802484	1	0.933	0.001149704
TRPT1	6.65E-08	-3.040641984	0.188	0.867	0.001154201
LACTB2	6.74E-08	2.026037861	1	0.867	0.001169959
ENSSSCG00000037808	6.77E-08	-2.202338474	0.938	1	0.001175196
ENSSSCG00000028962	6.78E-08	-2.239950793	0.438	0.933	0.001175709
ARSL	6.84E-08	-3.385891154	0.25	0.8	0.001186894
MKLN1	6.86E-08	1.373074128	1	1	0.001189545
DPH7	6.91E-08	-1.949373927	0.688	0.933	0.001198132
CENPF	6.99E-08	-0.527420916	1	1	0.001213647
PXMP2	7.15E-08	-1.423417165	1	0.933	0.001241326
PRPF31	7.30E-08	-1.878984599	0.562	1	0.001267487
SMARCAL1	7.34E-08	-1.279730695	0.875	1	0.001273135
FAAP100	7.40E-08	-3.208586622	0.25	0.733	0.001284215
WDR47	7.41E-08	1.628470444	1	1	0.00128495
NUP107	7.44E-08	1.658083293	1	1	0.001290833
ACTL8	7.46E-08	2.184703057	1	1	0.001294641
ORMDL1	7.47E-08	1.813531352	1	1	0.001296922
PRKAG1	7.52E-08	-1.910564916	0.812	0.933	0.00130444
NELFCD	7.66E-08	-2.469485283	0.562	0.867	0.00132971
TAOK3	7.71E-08	1.78495202	1	1	0.001337646
PPP1CB	7.77E-08	1.543635638	1	1	0.001347377
NDRG3	7.80E-08	1.677781191	1	1	0.001353322
TRAIP	7.82E-08	-1.305262641	0.938	1	0.001356035
PPM1J	8.05E-08	-2.552541023	0.438	0.867	0.001396809
LDLRAP1	8.11E-08	-0.594507188	0.938	1	0.001407053
CCT5	8.13E-08	-0.254544523	1	1	0.00141008
SLAIN2	8.18E-08	1.523380394	1	1	0.001419624
SKP2	8.22E-08	1.834397152	1	1	0.00142677
RBBP7	8.23E-08	1.462745729	1	1	0.001428216
INSL6	8.26E-08	-3.152003093	0.375	0.8	0.00143348
POLR3C	8.28E-08	-0.748259753	1	1	0.001437235
PPP4R4	8.28E-08	1.405882705	1	1	0.001437339
CIAO2A	8.32E-08	1.707494476	1	1	0.001444283

ADI1	8.36E-08	-3.070389328	0.312	0.867	0.001450794
MON1A	8.37E-08	-1.238251305	0.875	1	0.001451877
PDCD1LG2	8.43E-08	-1.190650339	0.938	1	0.001462317
CPO	8.62E-08	3.969900393	1	0.333	0.001495808
RPS19BP1	8.70E-08	-4	0.125	0.8	0.001510242
PALB2	8.70E-08	1.494613246	1	1	0.001510305
COPS7A	8.76E-08	-2.105933445	0.562	0.933	0.001520427
SPATA7	8.82E-08	1.643024426	1	1	0.001529632
KNSTRN	8.82E-08	1.650188891	1	1	0.001529992
GNAI1	8.83E-08	1.492281641	1	1	0.001532561
ENSSSCG00000000576	8.88E-08	1.995947086	1	1	0.001540106
MAP2K2	8.96E-08	-2.428712436	0.5	0.867	0.001554729
DVL1	9.03E-08	-4.222392421	0.062	0.8	0.001567021
INPP1	9.04E-08	-0.130005882	1	1	0.001569273
RPL3	9.12E-08	-0.988772745	1	1	0.001582244
TM4SF5	9.21E-08	-3.756074417	0.125	0.867	0.00159808
TENT4A	9.33E-08	1.764018514	1	1	0.001619618
SLC41A3	9.38E-08	-0.817851743	1	1	0.001627598
TMEM233	9.42E-08	-0.994194378	0.938	1	0.001634981
COMMD1	9.46E-08	-2.366127899	0.5	0.867	0.001642246
ENSSSCG00000058049	9.54E-08	-5.450661409	0	0.733	0.001655271
ATG101	9.63E-08	-1.342723294	0.938	1	0.001671144
PCDHGA4	9.65E-08	-0.772700736	1	1	0.001673657
RBM17	9.77E-08	1.885682937	1	1	0.001694563
TFR2	9.77E-08	-1.790546634	0.812	1	0.001695803
PWP1	9.79E-08	-0.130594414	1	1	0.00169889
INTS14	9.97E-08	1.526377673	1	1	0.001729272
UBE2B	1.00E-07	-1.005646563	0.938	1	0.001736186
NOL7	1.01E-07	-0.954441948	0.938	1	0.00174761
ENSSSCG00000042939	1.01E-07	-1.858644151	0.562	0.933	0.001757709
BAZ1A	1.01E-07	1.66310998	1	1	0.001760857
ENSSSCG00000045404	1.02E-07	-1.477773255	0.938	0.933	0.001770807
BMAL1	1.03E-07	2.088060354	1	1	0.001783321
DCXR	1.03E-07	-3.462343214	0.25	0.667	0.001784623
TRIM4	1.03E-07	-2.24961389	0.562	0.8	0.001788072
NOC4L	1.03E-07	-2.13492958	0.625	0.933	0.001793292
NAA35	1.05E-07	1.675133385	1	1	0.001825756
CLINT1	1.05E-07	1.304413648	1	1	0.001829781
WAPL	1.06E-07	1.456452254	1	1	0.001840963
ENSSSCG00000049957	1.07E-07	-3.847996907	0.062	0.733	0.001851898
SART3	1.07E-07	-0.45429693	1	1	0.001852775
YIF1A	1.07E-07	-3.010647244	0.5	0.867	0.001863935
FAAP24	1.11E-07	-0.744992503	1	1	0.001923506
ENSSSCG00000063521	1.11E-07	2.33229481	1	0.933	0.001934452
KANSL2	1.13E-07	-0.20932751	1	1	0.001954415
DHX30	1.14E-07	2.133710104	1	1	0.001976659
SPDYC	1.14E-07	-0.770822028	1	1	0.001984676
ENDOU	1.15E-07	-1.790546634	0.562	0.933	0.001992765
ATP5F1C	1.15E-07	1.669914851	1	1	0.002002283
POLD1	1.17E-07	-1.816591769	0.812	0.933	0.002029088
LRRFIP2	1.18E-07	-0.009335781	1	1	0.002043169
PSMA3	1.18E-07	-0.384846706	1	1	0.002054864
FFAR4	1.19E-07	2.364059163	1	0.933	0.002058847
ARL4A	1.20E-07	-0.111073154	1	1	0.002083142
FBXO16	1.20E-07	-1.248626982	0.875	1	0.002086164
SFT2D2	1.22E-07	-1.003354752	1	1	0.002113404
GARS1	1.22E-07	-1.072645302	0.938	1	0.002119738
PLPP1	1.23E-07	-0.889178519	0.938	1	0.002137081
CTNND1	1.24E-07	1.418153376	1	1	0.002158661
TMEM120A	1.24E-07	-4.30256277	0.062	0.733	0.002159744
PPM1D	1.24E-07	1.872215328	1	1	0.002159884
CNOT7	1.25E-07	1.763788602	1	1	0.002167964
TXNDC5	1.25E-07	-1.825413621	1	0.933	0.002174115
TMEM223	1.25E-07	-2.208586622	0.625	0.867	0.002176893
XKRX	1.26E-07	-2.552541023	0.562	0.933	0.002184646
TSEN54	1.26E-07	-2.485426827	0.5	0.8	0.002192351
NCBP3	1.28E-07	-0.480953941	1	1	0.002215081
SURF2	1.29E-07	-3.366127899	0.188	0.733	0.002230672
DDB2	1.32E-07	-1.05403984	0.875	1	0.002295406
SLC30A4	1.34E-07	1.640024328	1	1	0.002320256

CTNNB1	1.34E-07	1.277044708	1	1	0.002328767
NQO2	1.34E-07	-1.809316438	0.625	1	0.002329011
GMPPA	1.36E-07	-2.437937901	0.5	0.867	0.002354595
BUB3	1.37E-07	-0.531026191	1	1	0.002375438
BLOC1S1	1.37E-07	-2.398917834	0.375	0.933	0.002378694
RAP1A	1.37E-07	1.759994393	1	1	0.002380976
ENSSSCG00000039365	1.40E-07	-3.471621028	0.25	0.667	0.002424684
TJP3	1.42E-07	-1.961864871	0.625	0.933	0.002472323
ENSSSCG00000032413	1.43E-07	-2.093109404	0.688	0.933	0.002483484
PPP2R1B	1.43E-07	1.371955583	1	1	0.002488651
C2orf88	1.44E-07	-0.445647895	1	1	0.002499494
PSMD8	1.45E-07	1.758339245	1	1	0.002524324
HMOX2	1.46E-07	-1.396387442	1	1	0.002538015
ENSSSCG00000048488	1.47E-07	-2.929610672	0.25	0.8	0.002553335
ZKSCAN4	1.48E-07	-1.868755467	0.875	1	0.002562623
NMT1	1.49E-07	-0.882665826	1	1	0.002576639
STX5	1.49E-07	-1.607682577	0.812	0.933	0.002587367
HEMK1	1.49E-07	-2.458758877	0.688	0.867	0.002588197
EAPP	1.49E-07	-0.909863024	1	1	0.002588854
DCAF6	1.50E-07	1.597061978	1	1	0.002596012
HAUS2	1.50E-07	-0.922268697	0.875	1	0.002601125
PIWIL1	1.50E-07	1.390492381	1	1	0.002602272
C10orf90	1.51E-07	1.653462185	1	1	0.002612155
ASRGL1	1.51E-07	-1.334117504	0.812	1	0.002616998
CDCA7L	1.52E-07	-0.385795866	0.938	1	0.002635562
NOX4	1.52E-07	-0.526511671	1	1	0.002636304
ENSSSCG00000056486	1.52E-07	-0.974014347	0.875	1	0.00264599
EIF2AK2	1.53E-07	-0.631629957	1	1	0.002658911
ZNRD2	1.55E-07	-2.485426827	0.5	0.867	0.002693137
NDUFS8	1.59E-07	-2.803602787	0.25	0.8	0.002762909
SRRD	1.60E-07	-1.71871389	0.688	0.933	0.002776821
WARS1	1.61E-07	-0.921222887	1	1	0.002786989
B4GALT7	1.62E-07	-2.2255597	0.625	0.867	0.002812829
SLC9A3R1	1.64E-07	-1.723159795	0.75	0.933	0.002849015
PROM2	1.67E-07	-2.27897595	0.5	0.8	0.002892421
PCDH12	1.67E-07	-1.860663318	0.75	1	0.002901763
CENPW	1.70E-07	1.557784573	1	1	0.002948231
CLN6	1.72E-07	-1.756074417	0.875	0.933	0.002982613
NDUFS1	1.73E-07	-1.165865747	0.938	1	0.00300361
ZNF79	1.75E-07	-0.075746696	1	1	0.003041681
HENMT1	1.76E-07	1.722047403	1	1	0.00304964
ENSSSCG00000044533	1.79E-07	2.391276142	1	1	0.003108455
GLCE	1.79E-07	2.224849788	1	0.933	0.00310916
PIN1	1.81E-07	-3.152003093	0.25	0.867	0.003140481
KLF1	1.83E-07	-2.30461351	0.5	0.933	0.003179516
STOM	1.85E-07	2.095073819	1	1	0.003217219
TMED1	1.87E-07	-5.61667136	0	0.733	0.003244314
ADAD1	1.88E-07	-0.289374798	1	1	0.003253645
ERI3	1.92E-07	-1.821029859	0.625	1	0.003338236
GM2A	1.93E-07	-1.25624524	0.875	1	0.003349115
CRY2	1.99E-07	-1.335859539	0.938	0.933	0.003453865
TMEM69	2.01E-07	-1.093109404	0.938	1	0.003480418
PDE6C	2.01E-07	-0.650169631	1	1	0.003493691
NTPCR	2.02E-07	-0.883846255	1	1	0.003503376
ENSSSCG00000056719	2.06E-07	-1.48436952	0.938	0.933	0.003578
SDHAF2	2.07E-07	-1.299560282	0.875	1	0.003583779
VWA3A	2.07E-07	-1.358699515	0.938	1	0.003598868
SEZ6L2	2.14E-07	-0.824378268	1	1	0.00370762
RAE1	2.16E-07	1.769979241	1	1	0.003740694
HSD17B12	2.16E-07	1.427998763	1	1	0.003746467
SNAPC2	2.16E-07	-0.451802311	1	1	0.003754215
ENSSSCG00000057665	2.17E-07	-1.088448027	0.938	1	0.003767757
SLC6A12	2.19E-07	-2.989273593	0.25	0.8	0.003792175
YEATS4	2.22E-07	-0.53470773	1	1	0.003845501
ACVR1B	2.25E-07	1.958460944	1	1	0.003900831
ENSSSCG00000043568	2.26E-07	-0.680697375	1	1	0.003925795
CFI	2.27E-07	-1.493040011	0.812	1	0.00394466
PEX1	2.28E-07	1.518716759	1	1	0.003951587
PDXP	2.29E-07	-1.367402928	0.938	0.933	0.003965373
SWT1	2.29E-07	1.611131884	1	1	0.003969631

ECD	2.31E-07	-0.304877643	1	1	0.004010403
CABLES1	2.34E-07	-1.735952821	0.875	1	0.00406362
ENSSSCG00000058372	2.35E-07	-2.805827452	0.438	0.867	0.00408124
CTHRC1	2.35E-07	2.178695211	1	0.8	0.004084409
LONP2	2.36E-07	1.72055616	1	1	0.004087095
ENSSSCG00000060526	2.36E-07	-2.36159824	0.312	0.933	0.004099612
GFM2	2.38E-07	-0.774040964	1	1	0.00413364
PLEK2	2.39E-07	-0.340989311	1	1	0.004148814
LRRC42	2.39E-07	2.031924912	1	1	0.00415109
SMYD5	2.40E-07	-1.925999419	0.625	0.8	0.004155848
ENSSSCG00000051290	2.40E-07	1.730291251	1	1	0.004166719
ENSSSCG00000050177	2.46E-07	-3.933212909	0.562	0.867	0.004276993
POLL	2.48E-07	-2.728697978	0.438	0.867	0.004300999
XPO4	2.48E-07	-1.579807889	0.812	1	0.004310781
CHEK2	2.49E-07	-1.375622818	0.875	1	0.004323089
ENSSSCG00000003234	2.56E-07	-2.388565288	0.438	0.933	0.004444173
DDX25	2.56E-07	-1.634128558	0.875	0.933	0.004449233
PFDN6	2.58E-07	-0.779609932	1	1	0.004484278
ENSSSCG00000059558	2.60E-07	-1.527512229	0.75	0.933	0.00450815
ENSSSCG00000013395	2.60E-07	1.597238307	1	1	0.004512775
ENSSSCG00000052707	2.60E-07	-0.465420878	1	1	0.004513583
SLC35B4	2.60E-07	-1.089702803	0.938	1	0.004515083
ENSSSCG00000051526	2.64E-07	-0.987022146	1	1	0.004576071
SLC39A12	2.70E-07	1.751436077	1	1	0.004680292
FERMT2	2.71E-07	1.330585528	1	1	0.004698905
CDC7	2.71E-07	1.638829373	1	1	0.004707555
CIAO2B	2.71E-07	-1.602123052	1	1	0.004708391
AURKB	2.75E-07	2.103927443	1	1	0.004769222
NT5DC2	2.75E-07	-2.146976276	0.75	0.933	0.004778405
ARL8B	2.76E-07	1.482679972	1	1	0.004783589
ENSSSCG00000057727	2.81E-07	-0.562322044	1	1	0.004872946
GLS2	2.83E-07	1.613110664	1	1	0.004916252
DNA2	2.84E-07	2.159083794	1	1	0.004921645
HABP4	2.86E-07	-0.973439174	1	1	0.004955696
CFAP36	2.92E-07	-1.387556763	0.938	1	0.005060204
ENSSSCG00000040118	2.95E-07	-2.809316438	0.312	0.867	0.005123008
IFRD2	2.96E-07	1.906890596	1	1	0.005128258
TMEM134	2.99E-07	-2.9861942	0.188	0.867	0.005190446
INTS11	2.99E-07	-2.385891154	0.562	0.933	0.005193427
GTF2IRD1	3.03E-07	-2.388565288	0.5	0.933	0.005252371
BTBD3	3.04E-07	1.410824552	1	1	0.005273494
ZNF16	3.14E-07	2.156169942	1	1	0.005445552
KLF16	3.15E-07	-3.263034406	0.25	0.733	0.005461918
SARS1	3.15E-07	-1.683718468	0.875	1	0.005466171
ID3	3.15E-07	1.827864414	1	1	0.005471755
ARRDC1	3.17E-07	-1.836333989	0.5	1	0.005507499
PITPNA	3.22E-07	-1.081968847	1	1	0.005585122
GNB1L	3.23E-07	-2.777607579	0.312	0.8	0.005607337
CLPS	3.23E-07	-2.635163314	0.688	1	0.005611858
ENSSSCG00000012125	3.24E-07	-0.459840707	1	1	0.005624378
CCS	3.28E-07	-2.862496476	0.25	0.867	0.005691302
LIG3	3.30E-07	-0.506088674	1	1	0.005724372
ADAD2	3.32E-07	-2.934411658	0.25	0.867	0.005752004
ENSSSCG00000051497	3.32E-07	2.241175897	1	0.867	0.005765392
TRMT2B	3.36E-07	2.240314329	1	0.8	0.005827112
CYB561D2	3.42E-07	-2.582494245	0.438	0.8	0.005927883
RMC1	3.42E-07	2.306702555	1	1	0.005937686
FHIP2B	3.46E-07	-2.77118131	0.375	0.733	0.006008928
NDUFA6	3.47E-07	-1.485299835	1	1	0.00602515
SPATA19	3.48E-07	-2.9861942	0.375	0.733	0.006034942
ABHD3	3.49E-07	2.628589433	1	0.8	0.00605455
WEE2	3.53E-07	1.900622379	1	1	0.006120335
MATCAP1	3.59E-07	-2.600904045	0.562	0.667	0.00622084
ENSSSCG00000044091	3.60E-07	-3.485426827	0.125	0.8	0.006239949
ETF1	3.61E-07	-0.760534065	1	1	0.006256611
NCF2	3.64E-07	-1.027046765	0.938	1	0.006315
BAG5	3.65E-07	-1.938134744	0.688	0.933	0.006328532
LY6G6D	3.66E-07	-4.341036918	0.062	0.733	0.006347928
DNAJA4	3.71E-07	-1.389158867	0.938	1	0.006428797
TNN	3.75E-07	-0.649270382	1	1	0.00650922

SAP18	3.77E-07	-1.089337651	1	1	0.006533722
ENSSSCG00000058562	3.77E-07	-1.281554494	0.875	0.933	0.00654411
ENSSSCG00000058326	3.79E-07	-0.86097087	1	1	0.006583778
FN3KRP	3.79E-07	-2.647698256	0.312	1	0.0065843
ASCC1	3.81E-07	-0.924555135	1	0.933	0.006610226
KCNN3	3.87E-07	-0.783781346	0.938	1	0.006706767
TMEM266	3.94E-07	-1.703835001	0.812	0.867	0.006839773
NSMCE1	3.97E-07	-1.779801792	0.812	0.933	0.006896021
MYOF	4.02E-07	-0.421320541	1	1	0.006975427
ARL13B	4.06E-07	2.108876806	1	0.8	0.007045686
MORF4L2	4.10E-07	1.680893083	1	1	0.007108665
TMEM45A	4.12E-07	-0.610815467	1	1	0.007145467
RIC1	4.15E-07	1.595086011	1	1	0.007209207
FKBP2	4.19E-07	-3.471621028	0.188	0.667	0.007265236
CD164L2	4.21E-07	-5.093109404	0	0.733	0.007300304
ENSSSCG00000056768	4.22E-07	-1.15787966	1	1	0.007329621
ENSSSCG00000003303	4.25E-07	1.411726362	1	1	0.007366974
FAM98C	4.25E-07	-3.793549123	0.125	0.8	0.007376677
RPS20	4.31E-07	-1.12145403	1	1	0.007475784
RGS11	4.35E-07	-3.552541023	0.188	0.733	0.007553073
HMMR	4.37E-07	1.617624648	1	1	0.007576955
NDUFA5	4.37E-07	-1.835951565	0.875	0.933	0.007590397
PMVK	4.39E-07	-3.385891154	0.125	0.8	0.007621551
ENSSSCG00000016678	4.40E-07	-1.019688335	0.938	1	0.007642081
MAPKBP1	4.41E-07	1.983921081	1	1	0.0076442
ENSSSCG00000038506	4.41E-07	-1.229130199	1	1	0.007648256
TP63	4.52E-07	0.034871345	1	1	0.007847919
AQP6	4.56E-07	-2.255380833	0.625	0.867	0.007907673
DTWD1	4.64E-07	1.916617142	1	1	0.008054912
ENSSSCG00000054357	4.65E-07	-3.294743266	0.25	0.8	0.008070855
LETMD1	4.70E-07	-0.433733593	1	1	0.008147322
LYSMD2	4.70E-07	-1.347923303	0.75	0.933	0.008150097
FBXW8	4.73E-07	-0.282722741	1	1	0.008215459
ALG3	4.74E-07	-2.678071905	0.438	0.867	0.008221613
FAM151B	4.79E-07	1.5710144	1	1	0.008302828
USP25	4.80E-07	1.607903722	1	1	0.008321593
TSR3	4.82E-07	-3.230612928	0.25	0.8	0.00836956
GPN3	4.84E-07	-0.655055252	1	1	0.008402802
USP36	4.86E-07	-1.03441541	0.938	1	0.008425773
ENSSSCG00000022773	4.87E-07	-1.377562794	0.938	1	0.008454279
GLYR1	4.91E-07	-0.812026362	1	1	0.008523416
CRY1	4.93E-07	1.930658762	1	1	0.008546989
ENSSSCG00000031705	5.03E-07	1.649493895	1	1	0.008734038
MTSS1	5.05E-07	1.695794892	1	1	0.008755967
ENSSSCG00000003282	5.05E-07	-1.94458688	0.312	0.867	0.008766849
TMSB10	5.15E-07	-3.440877504	0.812	1	0.008934754
RNPEP	5.17E-07	2.536391492	1	1	0.008962419
FAN1	5.19E-07	-0.661448075	1	1	0.00900548
ENSSSCG00000056115	5.21E-07	-3.192645078	0.25	0.667	0.009036211
NCOA1	5.25E-07	1.389367417	1	1	0.009105661
SPR	5.26E-07	-1.130198723	1	1	0.009124608
ENSSSCG00000033451	5.26E-07	-3.471621028	0.188	0.733	0.009125773
SRSF7	5.30E-07	2.201166457	1	1	0.009187886
ASTN1	5.34E-07	-0.662662466	1	1	0.009257207
GTF2B	5.37E-07	2.005660936	1	1	0.00932055
ELP6	5.41E-07	-1.334117504	0.875	1	0.009393972
CHIC2	5.43E-07	-0.455254599	1	1	0.009421176
PIN4	5.45E-07	-0.780226449	0.938	1	0.009449064
ITM2B	5.51E-07	-0.464949214	1	1	0.009561531
SCAMP4	5.52E-07	-2.282143229	0.562	0.867	0.009579811
PHLPP2	5.58E-07	1.284378753	1	1	0.009679752
ENSSSCG00000004983	5.59E-07	1.871744033	1	1	0.009706062
ETFDH	5.60E-07	2.449998253	1	0.933	0.009710765
TMEM50B	5.60E-07	-0.417682221	1	1	0.009718761
DBF4B	5.61E-07	-1.589940995	0.812	0.933	0.009736986
ASIP	5.61E-07	-3.128733314	0.25	0.733	0.009738643
GLA	5.71E-07	1.585964555	1	1	0.009911142
CFAP69	5.87E-07	2.572665142	1	0.667	0.010193507
XAB2	5.98E-07	-1.222151349	0.938	0.933	0.010375571
MAT2A	6.04E-07	-0.059391289	1	1	0.010480238

ENSSSCG00000028479	6.12E-07	-0.524721128	1	1	0.010612105
IFT81	6.13E-07	1.629034865	1	1	0.010641767
LIMA1	6.18E-07	1.904321236	1	0.933	0.010714595
HAS3	6.22E-07	1.668601566	1	1	0.010797688
PSMD3	6.29E-07	2.102635153	1	1	0.010907434
CENPO	6.40E-07	1.590260181	1	1	0.011097784
SLIT3	6.49E-07	-0.783504588	1	1	0.011269044
GNPTG	6.55E-07	-1.360589715	0.938	1	0.011361162
ARL3	6.55E-07	-1.668611576	0.938	1	0.011368771
BARD1	6.58E-07	1.674551523	1	1	0.01141825
TOE1	6.63E-07	-1.403835152	0.875	1	0.011503437
ENSSSCG00000001934	6.69E-07	1.54760354	1	1	0.011604171
IQCD	6.71E-07	-2.656045599	0.25	0.867	0.011636202
ITGB7	6.72E-07	-4.047305715	0.062	0.8	0.011655077
CNOT11	6.72E-07	-0.291005617	1	1	0.011658104
TAB3	6.91E-07	1.574600749	1	1	0.011988563
TP53INP2	6.92E-07	-1.423417165	0.938	1	0.012001392
SIKE1	6.94E-07	2.120486069	1	1	0.012045213
SMU1	6.98E-07	1.547856577	1	1	0.012110939
BMPR2	7.16E-07	1.410657013	1	1	0.012429407
CSPP1	7.19E-07	1.664133138	1	1	0.012480951
INO80B	7.27E-07	-2.9510904	0.188	0.867	0.012614057
WASHC3	7.35E-07	-2.5981036	0.562	0.933	0.012745113
MSL1	7.39E-07	-0.765961138	0.938	1	0.012823961
RPS21	7.39E-07	-3.296049463	0.625	0.867	0.012830063
FKBP1B	7.44E-07	-2.415037499	0.438	0.867	0.012901357
RBM22	7.44E-07	-0.693738502	1	1	0.012913496
SDS	7.48E-07	-3	0.188	0.8	0.01298216
TANK	7.69E-07	1.9021989	1	1	0.013349352
CDCA7	7.72E-07	1.892909841	1	1	0.013395458
DIP2B	7.72E-07	1.420817834	1	1	0.013398722
ZFYVE1	7.86E-07	-0.613876145	1	1	0.01363043
STRAP	7.93E-07	1.728606527	1	1	0.013753482
ENSSSCG00000010791	8.00E-07	-4.900464326	0	0.8	0.013888609
TMEM245	8.08E-07	-0.355736901	1	1	0.014024073
CLK3	8.11E-07	2.164121796	1	1	0.014078249
WWC3	8.14E-07	-2.188266637	0.438	0.867	0.014117306
C1orf174	8.17E-07	-1.895428737	0.875	0.933	0.014173403
DUSP12	8.21E-07	2.365391743	1	0.933	0.014243074
MND1	8.22E-07	2.09025786	1	0.933	0.014268021
MFGE8	8.26E-07	-3.180572246	0.188	0.733	0.014334346
LRIG3	8.26E-07	1.689172706	1	1	0.014334579
ENSSSCG00000060068	8.27E-07	-2.958179824	0.125	0.933	0.014348589
RPL28	8.31E-07	-2.160016063	0.875	1	0.014415636
THOC7	8.37E-07	1.637647225	1	1	0.014525265
NOCT	8.37E-07	1.27941248	1	1	0.014526955
TROAP	8.43E-07	1.920986939	1	1	0.014626141
PKN1	8.46E-07	-2.24838763	0.5	0.933	0.014683238
ENSSSCG00000022295	8.48E-07	-3.678071905	0.062	0.8	0.014719965
ENSSSCG00000006581	8.50E-07	-2.9510904	0.188	0.867	0.014742939
ENSSSCG00000006344	8.51E-07	1.56233476	1	1	0.01476067
SLC17A2	8.57E-07	2.063111982	1	1	0.014876895
HESX1	8.70E-07	2.14112463	1	0.8	0.015101778
STRN	8.72E-07	1.366006146	1	1	0.015128942
AKAIN1	8.73E-07	2.045505167	1	1	0.015150217
NCAPG	8.73E-07	1.697768694	1	1	0.015154501
UHRF1	8.74E-07	1.641121222	1	1	0.015165766
DCLK1	8.86E-07	-0.271165307	1	1	0.015367144
RSL24D1	9.00E-07	-1.462866024	1	1	0.015620425
PRKAG2	9.03E-07	-1.093109404	0.938	1	0.015663092
RIOK3	9.06E-07	1.705759832	1	1	0.01571312
CREG1	9.10E-07	1.776561601	1	1	0.015786707
SYCP2L	9.11E-07	-1.027020214	0.938	1	0.015800017
UTRN	9.13E-07	1.524803518	1	1	0.015846826
TVP23A	9.18E-07	-2.315501826	0.438	0.8	0.015924332
EIF4B	9.19E-07	-0.151258284	1	1	0.01594539
CAMTA1	9.19E-07	-0.753903318	1	1	0.015951316
SLC19A2	9.21E-07	1.687488217	1	1	0.015977283
RHBG	9.29E-07	-1.9861942	0.812	0.867	0.016123459
NANS	9.31E-07	-3.093109404	0.5	0.733	0.016158842

PELP1	9.42E-07	-1.574236094	0.688	1	0.016336806
ENSSSCG00000050758	9.54E-07	-1.218640286	0.875	1	0.016548929
ENSSSCG00000042524	9.58E-07	-1.084794136	0.938	1	0.016616612
HOATZ	9.82E-07	-2.61667136	0.312	0.8	0.01704113
SRRT	9.85E-07	-1.086930829	1	1	0.017086339
RFXANK	9.87E-07	-3.298223834	0.125	0.867	0.017128917
ENSSSCG00000009178	1.01E-06	1.989057724	1	1	0.017542931
ENSSSCG00000001776	1.04E-06	-2.323407024	0.438	0.867	0.017974564
ENSSSCG00000046622	1.05E-06	-1.609359155	0.625	0.867	0.018222684
ACTL6A	1.07E-06	1.483581834	1	1	0.018487178
NUDT18	1.07E-06	-3.013674937	0.312	0.733	0.018549614
SLC17A1	1.07E-06	-0.741441355	1	1	0.018603451
MPV17L2	1.08E-06	-2.865698908	0.25	0.867	0.018653594
ANO4	1.09E-06	-0.572030268	1	1	0.018861652
PYGB	1.09E-06	-0.855529184	1	1	0.018861771
TSPAN13	1.09E-06	-0.994194378	0.938	1	0.018923548
BORA	1.09E-06	2.040817399	1	1	0.018928374
FIGNL1	1.11E-06	-0.649057923	1	1	0.019251517
CYP2E1	1.12E-06	-1.39176772	0.688	1	0.019403534
RESF1	1.13E-06	1.646133374	1	1	0.019551068
GMNN	1.13E-06	1.776971761	1	1	0.01956387
WSB2	1.13E-06	-1.472455197	0.875	0.933	0.019619436
YIPF5	1.13E-06	-0.296158366	1	1	0.019642152
ENSSSCG00000050595	1.15E-06	-2.830074999	0.312	0.733	0.019875044
PEDS1	1.16E-06	-0.69366876	1	1	0.020041297
UFC1	1.16E-06	-0.96848275	1	1	0.02018581
PHYKPL	1.16E-06	-1.756074417	0.562	0.867	0.020198252
ASH2L	1.17E-06	-0.218408789	1	1	0.020296565
SERHL2	1.18E-06	-1.050040682	0.938	1	0.020409495
TMED4	1.18E-06	-0.491032883	1	1	0.020439474
PDLIM7	1.18E-06	-2.862496476	0.375	0.8	0.020443019
TM2D2	1.19E-06	-1.186891551	0.938	0.933	0.020570959
ARL14EPL	1.19E-06	1.544011951	1	1	0.020597373
IPO9	1.19E-06	-1.701155518	0.938	1	0.020616435
ABLIM3	1.20E-06	-1.697971463	0.875	0.933	0.020760594
TMEM86A	1.20E-06	-3.126124462	0.312	0.933	0.020824999
DNAJC18	1.21E-06	-0.604025554	1	1	0.020936913
ENSSSCG00000061760	1.21E-06	-1.71871389	0.812	0.933	0.02094427
YIF1B	1.21E-06	-2.208586622	0.438	0.933	0.020981163
PRMT1	1.21E-06	2.066235664	1	1	0.021007243
ENSSSCG00000049777	1.21E-06	-1.745186101	0.75	0.933	0.021069645
ARF4	1.22E-06	1.496439937	1	1	0.021167942
TTC8	1.22E-06	2.557655155	1	0.667	0.0211717
ENTREP1	1.23E-06	-1.062735755	0.938	1	0.021405049
TFPT	1.24E-06	-2.712019237	0.25	0.867	0.021533377
GRTP1	1.25E-06	-5.180572246	0	0.667	0.021630765
MRM1	1.25E-06	-1.903070822	0.812	0.933	0.021698092
MIOS	1.25E-06	-0.363279087	1	1	0.021720935
WAC	1.28E-06	1.359306738	1	1	0.022181346
GMFG	1.30E-06	-5.180572246	0	0.733	0.022583802
DCLRE1A	1.31E-06	1.892656985	1	1	0.022664081
CDCP1	1.31E-06	1.923077642	1	1	0.022767816
KRR1	1.31E-06	1.476972397	1	1	0.022775628
DCAF13	1.32E-06	-0.551130074	1	1	0.022961175
SMURF2	1.32E-06	1.302431308	1	1	0.022966123
ENSSSCG00000027270	1.33E-06	-0.762960803	1	1	0.023066082
AHSA1	1.34E-06	1.726522106	1	1	0.023237526
MRPS12	1.34E-06	-2.803602787	0.312	0.867	0.023333024
SEPTIN10	1.35E-06	1.471618613	1	1	0.023341009
DCTD	1.37E-06	1.711179215	1	1	0.02370505
RMND5A	1.37E-06	1.322632364	1	1	0.023798091
ENSSSCG00000048663	1.37E-06	-3.137503524	0.188	0.8	0.023812538
ACTR2	1.38E-06	1.360790027	1	1	0.023965323
USP11	1.39E-06	-1.371316122	0.938	1	0.024160544
ENSSSCG00000026520	1.40E-06	-1.019860422	1	1	0.024257867
MAP4K4	1.41E-06	-0.364234398	1	1	0.024391068
LAMA3	1.41E-06	-0.788015748	0.938	1	0.024515866
ENSSSCG00000021041	1.42E-06	-2.783424905	0.312	0.733	0.024657452
MRPS23	1.42E-06	-0.455504091	1	1	0.02466459
ENSSSCG00000040146	1.42E-06	-3.044199804	0.438	0.933	0.02470248

RPSA	1.42E-06	-0.638310836	1	1	0.02471224
DIMT1	1.43E-06	1.802513979	1	1	0.024792884
PAFAH2	1.44E-06	-0.607188419	1	1	0.02502292
ENSSSCG00000009327	1.44E-06	-0.025250546	1	1	0.025030087
TOGARAM2	1.45E-06	2.266786541	1	0.867	0.025094473
CPSF3	1.45E-06	-0.444581775	1	1	0.025183898
PCNX4	1.45E-06	1.437220647	1	1	0.025211619
NARS1	1.47E-06	-0.542980385	1	1	0.025467526
NIT1	1.47E-06	-1.72914609	0.688	0.933	0.025556536
LGR4	1.48E-06	1.622604417	1	1	0.025713213
CDKN2AIP	1.49E-06	1.522488781	1	1	0.025850132
PM20D2	1.49E-06	1.638073837	1	1	0.025853483
ENSSSCG00000052480	1.49E-06	-3.03170886	0.25	0.8	0.0259386
PCIF1	1.50E-06	-2.054635257	0.5	0.867	0.02595232
WIPF2	1.53E-06	-0.338761732	1	1	0.026620242
MAPK3	1.54E-06	-2.093109404	0.5	0.867	0.02671252
C14orf93	1.55E-06	-2.315501826	0.375	0.933	0.026980752
ATG5	1.56E-06	1.398267139	1	1	0.027063631
SLC2A8	1.56E-06	-0.847439041	1	1	0.027151158
OCRL	1.58E-06	1.532661013	1	1	0.027397493
ENSSSCG00000017061	1.58E-06	-1.328325866	0.75	1	0.027448638
CKS1B	1.59E-06	1.62728767	1	1	0.027525321
LDAF1	1.60E-06	1.95473037	1	1	0.027709869
TOLLIP	1.60E-06	-1.135753742	0.812	1	0.027762462
VBP1	1.64E-06	-0.516320835	1	1	0.028502808
ENSSSCG00000059030	1.65E-06	-4	0.062	0.733	0.028547392
ENSSSCG00000063102	1.65E-06	-1.8372705	0.5	0.8	0.028550015
ADORA2A	1.66E-06	2.725052273	1	0.6	0.028768688
PKIG	1.67E-06	-0.588211293	1	1	0.028931735
SLC25A10	1.67E-06	-2.35614381	0.188	0.933	0.028966505
ENSSSCG00000025913	1.69E-06	-0.926459535	1	0.933	0.02929923
NSG1	1.69E-06	-0.803227036	1	1	0.029355248
ENSSSCG00000027124	1.70E-06	-0.978825977	0.938	1	0.029497892
ABR	1.71E-06	-0.851011574	0.938	1	0.029704378
SYPL1	1.72E-06	1.724134845	1	1	0.029860201
HYAL3	1.75E-06	-3.341036918	0.188	0.733	0.030282698
RHPN2	1.75E-06	1.715332294	1	1	0.030306698
TPM1	1.76E-06	-0.523097245	1	1	0.030591207
ZNRF2	1.78E-06	1.94035188	1	1	0.030954364
FAM114A2	1.79E-06	-0.748350601	1	1	0.030991266
TMEM181	1.80E-06	-0.040734909	1	1	0.031269265
UBR1	1.80E-06	1.476235981	1	1	0.031287808
F3	1.81E-06	1.600852475	1	1	0.031403994
ACAD8	1.81E-06	-0.491088162	1	1	0.031409223
GUSB	1.82E-06	-3.987927168	0.188	0.733	0.031602718
ATF4	1.88E-06	-0.472370674	1	1	0.032533271
LRRC36	1.88E-06	-2.338221902	0.5	0.933	0.032575999
TENM4	1.88E-06	-1.478763097	0.812	0.933	0.03260626
YARS1	1.89E-06	-0.829001964	0.938	1	0.032843893
TAX1BP3	1.94E-06	-1.222989687	1	1	0.03366211
HSD17B2	1.95E-06	-3.415037499	0.125	0.8	0.033765532
ENSSSCG00000053208	1.97E-06	-3	0.312	0.733	0.03417565
ENSSSCG00000050083	1.98E-06	-0.394032934	1	1	0.034271292
RACK1	1.98E-06	-2.902380256	0.938	1	0.034348173
ENSSSCG00000033181	1.99E-06	2.400136927	0.938	0.8	0.034503582
SAR1B	1.99E-06	-0.920175653	1	1	0.034578546
BBS4	2.00E-06	2.326666014	1	0.733	0.034729117
CCDC9	2.00E-06	-2.571156701	0.375	0.867	0.034738138
ANAPC1	2.01E-06	1.160299843	1	1	0.03489341
NPDC1	2.01E-06	-3.552541023	0.125	0.733	0.034958267
LRRC59	2.02E-06	-0.793549123	0.938	1	0.035010006
NEU1	2.02E-06	-2.742202243	0.375	0.8	0.03501214
ENSSSCG00000051717	2.03E-06	-1.034215715	0.938	1	0.035167065
C6orf120	2.05E-06	-0.151673297	1	1	0.035645692
SDHAF4	2.07E-06	2.288139781	1	0.8	0.035939686
TRMT5	2.07E-06	1.784555053	1	1	0.035940575
FBXO34	2.07E-06	-1.259467791	0.938	1	0.035977983
GBA2	2.11E-06	-2.247437551	0.625	0.933	0.036532621
TSTD1	2.11E-06	-1.498821111	1	1	0.03657423
GZMA	2.12E-06	1.627940593	1	1	0.036828202

PRXL2B	2.16E-06	-2.874469118	0.375	0.733	0.037449795
ADD3	2.16E-06	1.617232363	1	1	0.037454415
DHCR24	2.17E-06	-1.217845632	0.938	0.933	0.037678632
MTERF3	2.18E-06	1.643119933	1	1	0.037747134
TCP11L1	2.20E-06	-0.243563045	1	1	0.038227094
B4GAT1	2.21E-06	-1.35938947	0.875	0.867	0.038422858
ENSSSCG00000033697	2.22E-06	-1.110140485	1	1	0.038483298
MTAP	2.23E-06	1.390063936	1	1	0.038701872
ENSSSCG00000027723	2.23E-06	-1.840343334	0.75	1	0.038729951
PTPN2	2.23E-06	1.207960086	1	1	0.038778272
TRMT10A	2.27E-06	-0.280861804	1	1	0.03938029
ZNF526	2.28E-06	-1.216167801	0.75	1	0.039571689
SF3B3	2.31E-06	-0.653409851	1	1	0.040006968
ENSSSCG00000057721	2.32E-06	-2.777607579	0.25	0.733	0.040188179
RIMS3	2.32E-06	-1.666294737	0.75	0.867	0.040224855
SMARCD3	2.36E-06	-2.180572246	0.375	0.867	0.040942038
VAPA	2.36E-06	1.564283981	1	1	0.041020352
HBEGF	2.38E-06	1.568922489	1	1	0.04127448
SPAG16	2.41E-06	1.622184163	1	1	0.041876173
ENSSSCG00000050765	2.43E-06	3.19865672	1	0.667	0.04212399
SRI	2.43E-06	-0.935403952	1	1	0.042147976
UTP23	2.43E-06	-0.663848491	1	1	0.04214827
SVOP	2.44E-06	-2.228624375	0.688	0.867	0.042398324
GFPT2	2.45E-06	2.148524367	1	1	0.042474278
KATNB1	2.45E-06	-1.311944006	0.812	1	0.042491403
TRNT1	2.46E-06	-0.354954449	1	1	0.04275075
RHOD	2.50E-06	-6.137503524	0	0.667	0.043342858
ENSSSCG00000016385	2.50E-06	-1.259467791	0.812	0.933	0.043432992
ENKD1	2.51E-06	-2.234465254	0.5	0.867	0.043503533
ENSSSCG00000030671	2.51E-06	2.077654617	1	0.933	0.043528982
RALA	2.51E-06	-0.107867594	1	1	0.04356708
EDC3	2.52E-06	-0.729536548	1	1	0.043669749
TIMP2	2.56E-06	-2.323407024	0.5	0.8	0.04440117
REN	2.56E-06	-3.047305715	0.188	0.8	0.044487341
SEPTIN8	2.57E-06	-0.701127141	1	1	0.044575922
LRMDA	2.58E-06	-0.693258309	0.938	1	0.04483446
ENSSSCG00000015011	2.60E-06	-0.340729471	1	1	0.045064088
SKA3	2.60E-06	-0.944146765	0.938	1	0.045089369
CYTH3	2.60E-06	-0.766299088	1	0.933	0.045174604
SLC4A1AP	2.63E-06	-0.226102741	1	1	0.045708593
TCEA3	2.64E-06	-0.660720571	1	1	0.045859029
DNAJB2	2.66E-06	-1.187746279	0.688	1	0.046236516
RNF166	2.68E-06	-2.840343334	0.312	0.733	0.046450154
DBT	2.68E-06	1.485016912	1	1	0.046461175
CTNNBL1	2.68E-06	1.989957207	1	1	0.046509475
SNRPA1	2.68E-06	1.685968961	1	1	0.046522886
TMEM163	2.68E-06	-1.009693396	1	1	0.046536988
DNAI1	2.70E-06	-2.325770161	0.438	0.867	0.046933095
ENSSSCG00000006084	2.71E-06	1.410299263	1	1	0.046964512
DBNL	2.83E-06	-1.782769284	0.562	1	0.049186401
CCL22	2.84E-06	-0.686292412	0.938	1	0.049357611
GORASP1	2.85E-06	-1.687290435	0.812	0.933	0.049447247
ENSSSCG00000053039	2.85E-06	1.810366527	1	1	0.04952177
TMCO6	2.87E-06	1.771904551	1	1	0.049723937
ODC1	2.90E-06	1.624044755	1	1	0.050316624
ENSSSCG00000063054	2.94E-06	-1.941106311	0.562	0.8	0.05101326
ENSSSCG00000057488	2.98E-06	-3.35614381	0.188	0.6	0.051635479
UBE4A	2.98E-06	1.61609715	1	1	0.051705333
MTARC2	2.98E-06	2.553053253	1	0.867	0.051718237
MAP1LC3A	2.99E-06	-1.726570423	0.688	0.933	0.051954671
ZNF789	3.00E-06	-1.529973266	0.688	0.933	0.051982289
FAM227B	3.01E-06	-0.309984821	1	1	0.052221614
BNIP3L	3.02E-06	-0.286651402	1	1	0.052466515
USP5	3.03E-06	-0.598259323	1	1	0.052507171
PPP1R36	3.06E-06	-0.431431938	1	1	0.05307027
AMN	3.08E-06	-2.858644151	0.188	0.867	0.053447624
OPA1	3.10E-06	1.340475394	1	1	0.053834068
PHC1	3.11E-06	-0.966352262	0.938	1	0.054019741
PABPC1	3.12E-06	-1.906340893	0.75	0.867	0.054179985
RPL24	3.13E-06	-2.019108823	0.875	1	0.054222088

ELP4	3.14E-06	1.893428336	1	1	0.054503179
MRPL52	3.19E-06	-1.25180715	0.688	1	0.055274611
ENSSSCG00000055613	3.19E-06	-3.197446064	0.188	0.733	0.055355867
CHIC1	3.21E-06	-0.882027078	0.938	1	0.055718106
RRP1	3.22E-06	-3.137503524	0.188	0.8	0.055840625
DPY30	3.26E-06	1.47066258	1	1	0.056481462
ENSSSCG00000061799	3.26E-06	-1.839922785	0.812	0.933	0.056492193
GSTO2	3.27E-06	-2.066142357	0.625	0.733	0.056674187
SCAF4	3.28E-06	1.586035535	1	1	0.056923755
PLAGL1	3.30E-06	1.540433277	1	1	0.057251611
INO80E	3.30E-06	-1.300434378	0.938	0.933	0.057314188
LTF	3.32E-06	-1.282933963	0.75	0.933	0.057667967
LATS1	3.34E-06	1.65593296	1	1	0.057874737
SHMT1	3.34E-06	1.950579413	1	1	0.057903108
SPAG5	3.36E-06	-1.025266759	1	1	0.058216688
CXorf38	3.36E-06	-1.843131151	0.688	0.867	0.058244754
ENSSSCG00000025759	3.38E-06	-0.489919808	1	1	0.05861693
C1QTNF2	3.40E-06	-2.315501826	0.438	0.8	0.058917784
ENSSSCG00000009496	3.41E-06	2.760341932	1	0.4	0.059195868
MRPS24	3.42E-06	-0.733980646	1	1	0.059376638
WDR37	3.47E-06	-1.072548082	1	0.933	0.060278624
SESTD1	3.48E-06	1.400197946	1	1	0.060424219
CHP1	3.50E-06	-0.22732119	1	1	0.060721184
ENSSSCG00000039286	3.50E-06	-0.441159924	1	1	0.060732361
RPL27	3.50E-06	-1.45602944	1	1	0.060767789
MEX3C	3.51E-06	1.421272417	1	1	0.060880459
MSH2	3.52E-06	1.416000874	1	1	0.061014938
RAD9A	3.53E-06	-1.363963315	0.812	0.933	0.061187754
CRYBB1	3.55E-06	-2.093109404	0.562	0.867	0.061550612
VAMP8	3.57E-06	-2.595609745	0.312	0.867	0.061883655
CCDC180	3.59E-06	-0.714597781	0.938	1	0.062309603
B3GNT2	3.61E-06	1.519333833	1	1	0.062560737
IPO8	3.63E-06	1.218662285	1	1	0.062969515
WFDC1	3.64E-06	-3.847996907	0.062	0.733	0.063171705
PDHB	3.69E-06	-0.612219358	1	0.933	0.064015493
ENSSSCG00000014969	3.72E-06	1.636489703	1	1	0.064506468
PSMD9	3.76E-06	1.881866572	1	1	0.065275542
ENSSSCG00000039753	3.78E-06	1.600037821	1	1	0.065574189
DOCK9	3.79E-06	1.649977811	1	1	0.065706539
MRPL47	3.79E-06	-0.802123252	1	1	0.065820034
TRPC4AP	3.81E-06	1.546486712	1	1	0.066066187
TSPAN17	3.81E-06	-0.599359929	0.938	1	0.066082557
MAGEB3	3.81E-06	-0.099629504	1	1	0.066180283
GBX1	3.83E-06	-4.9510904	0	0.667	0.066480121
C1orf74	3.87E-06	-2.491658781	0.312	0.8	0.067101582
CCDC60	3.88E-06	-0.848424308	0.938	1	0.067341218
ENSSSCG00000016719	3.90E-06	-0.543336332	1	1	0.06770951
HMGA1	3.93E-06	-1.781165398	0.625	0.867	0.06818686
RIPOR1	3.94E-06	2.119632847	1	0.933	0.068437936
ENSSSCG00000024144	3.98E-06	-3.093109404	0.188	0.733	0.068999159
LAMC1	4.00E-06	1.3163563	1	1	0.069320646
GTF3A	4.02E-06	-2.044199804	0.562	0.8	0.069765629
TMEM59	4.03E-06	-0.297349958	1	1	0.069892645
USPL1	4.06E-06	1.662715217	1	0.933	0.070467694
PARVA	4.08E-06	-1.05994254	1	0.933	0.070712151
PUS3	4.08E-06	-0.888524713	0.938	1	0.070750191
SUV39H1	4.08E-06	-2.074000581	0.5	0.867	0.070822558
BRMS1	4.08E-06	-1.803602787	0.75	1	0.070840656
ORC4	4.12E-06	-0.756628554	1	1	0.071507495
ENSSSCG00000016543	4.13E-06	-0.123502444	1	1	0.07164778
ENSSSCG00000023467	4.15E-06	-4.180572246	0.062	0.667	0.072055746
EIF3L	4.20E-06	-0.30705589	1	1	0.072900798
NUP37	4.22E-06	1.739054833	1	1	0.073166715
PSKH1	4.22E-06	-0.729986674	1	1	0.07320503
SSH2	4.23E-06	1.4086114	1	1	0.073318661
G3BP2	4.24E-06	-0.385772905	1	1	0.073565777
PPP1R12A	4.24E-06	1.567635022	1	1	0.073579464
PRMT7	4.27E-06	-1.698391889	0.75	1	0.074003933
XRCC5	4.27E-06	-0.672718671	1	1	0.074020008
CDK17	4.30E-06	1.521139843	1	1	0.074540894

IQGAP2	4.31E-06	1.329618456	1	1	0.074860495
TMEM63A	4.32E-06	-0.897653698	0.938	1	0.074913545
NIPA1	4.32E-06	1.555319515	1	1	0.074946764
TAF12	4.34E-06	1.707909561	1	1	0.075373317
ENSSSCG00000058767	4.39E-06	-4.9510904	0	0.667	0.076158333
HIGD1C	4.39E-06	-1.047305715	1	1	0.076255797
PI4K2B	4.46E-06	1.501284322	1	1	0.077315752
YBEY	4.48E-06	-1.799378201	0.625	0.8	0.077660018
FUS	4.49E-06	-1.106284793	1	1	0.077846101
EOGT	4.50E-06	1.591845066	1	1	0.078110842
LMOD3	4.51E-06	-1.042836198	0.938	1	0.078277989
TSHZ1	4.52E-06	1.630645316	1	1	0.078440395
ENSSSCG00000034715	4.55E-06	-1.813001485	0.75	0.8	0.0789516
SHLD2	4.57E-06	1.50701664	1	1	0.079282147
SIRT4	4.59E-06	2.125425541	1	0.933	0.079687753
SMARCB1	4.60E-06	-0.635705874	1	1	0.079771429
ENSSSCG00000017446	4.62E-06	-2.036525876	0.375	0.8	0.080236047
TCEANC2	4.63E-06	-1.934411658	0.562	0.867	0.080362685
MCM6	4.64E-06	1.327981215	1	1	0.080458304
GTPBP8	4.65E-06	-1.789103218	0.438	0.933	0.080727494
RBFOX2	4.69E-06	-0.417978775	1	1	0.081297554
METTL1	4.72E-06	-1.123195131	1	1	0.08188664
MYO5B	4.74E-06	-0.245063342	1	1	0.082188924
NICN1	4.77E-06	-1.523097245	0.75	0.867	0.082682479
PIM3	4.79E-06	2.171773426	1	1	0.083140178
KCTD9	4.83E-06	1.585869588	1	1	0.083852803
C19orf47	4.94E-06	-1.660149997	0.812	0.867	0.085728539
MYBPC2	4.95E-06	-3.197446064	0.125	0.667	0.085958801
DDB1	5.01E-06	1.925299062	1	1	0.086995594
ATP23	5.02E-06	-0.345088622	1	1	0.087070302
PRELID1	5.02E-06	-0.179584228	1	1	0.087109387
SKI	5.03E-06	-0.984037705	1	1	0.087293648
RANBP17	5.04E-06	1.651299173	1	1	0.087500185
ANAPC13	5.11E-06	-1.50157425	0.938	1	0.088611212
PTPRK	5.11E-06	2.424166289	1	0.933	0.088745602
ENSSSCG00000054608	5.18E-06	-0.468930507	1	1	0.089951688
TEX261	5.23E-06	-2.023846742	0.5	0.867	0.090721101
ASF1A	5.26E-06	-0.344542861	1	1	0.091221257
MARK3	5.29E-06	-0.623279432	0.938	1	0.09185179
RABEP2	5.33E-06	-2.044199804	0.438	0.8	0.092476015
LAMB1	5.37E-06	1.587081366	1	1	0.093176526
MB21D2	5.38E-06	-0.563247159	1	1	0.093316361
TRIM32	5.40E-06	-0.567243775	0.938	1	0.093621556
RRP36	5.44E-06	-1.373217324	0.75	1	0.094459297
SLC9B1	5.48E-06	1.94862116	1	0.933	0.09505672
RPN2	5.49E-06	-0.690744122	1	1	0.09525848
TRMT10B	5.58E-06	-0.347210685	1	1	0.096765827
ENSSSCG00000050918	5.58E-06	-0.647624289	1	1	0.096844174
ADGRA2	5.58E-06	-1.931358334	0.688	0.933	0.096879079
COL23A1	5.60E-06	-1.152003093	0.812	1	0.09714056
ENSSSCG00000004415	5.60E-06	-0.181973391	1	1	0.097140785
ABCF3	5.60E-06	-1.383786565	0.75	0.933	0.097233154
RASSF9	5.62E-06	-1.130103612	0.812	0.933	0.097435029
ENSSSCG00000008892	5.63E-06	1.600097018	1	0.933	0.09764421
DOK4	5.63E-06	-0.280322375	1	1	0.097724259
RHNO1	5.69E-06	1.482701398	1	1	0.09878976
BRIX1	5.70E-06	-0.533159438	1	1	0.098943654
ATP11C	5.73E-06	1.437953047	1	1	0.099382803
ENSSSCG00000004760	5.75E-06	-3.152003093	0.188	0.733	0.099779512
UTP25	5.77E-06	-1.30461351	0.812	0.933	0.100169888
TFDP1	5.81E-06	2.512317199	1	0.8	0.10082446
RBM28	5.83E-06	-0.23405592	1	1	0.101133341
ENSSSCG00000059371	5.87E-06	-1.468618539	0.75	0.933	0.101843389
ENSSSCG00000033298	5.88E-06	1.885516945	1	0.867	0.101983606
SAMD12	5.89E-06	-0.220659454	1	1	0.102225934
MRPL12	5.91E-06	-3.093109404	0.188	0.733	0.102531251
RABEP1	5.92E-06	-0.157165578	1	1	0.102802981
MAN1A2	5.95E-06	1.415231815	1	1	0.103250363
PFDN2	5.96E-06	-1.0131411	1	1	0.103470404
ZCWPW1	6.00E-06	-0.693433958	1	1	0.104027733

ZC3H7A	6.02E-06	1.959185103	1	0.933	0.104436735
ENSSSCG00000040187	6.02E-06	1.683939748	1	1	0.104469182
CYB5R3	6.05E-06	-2.078002512	0.625	0.933	0.104932374
ENSSSCG00000009926	6.11E-06	-1.878984599	0.625	0.8	0.105932118
UBB	6.13E-06	-0.680652651	1	1	0.106339925
NAGA	6.26E-06	-3.308122295	0.25	0.6	0.108603732
MEAF6	6.26E-06	1.559577182	1	1	0.10865816
UBE2M	6.27E-06	-3.415037499	0.188	0.6	0.108767834
LTV1	6.28E-06	1.540651385	1	1	0.10904125
STX17	6.29E-06	-1.375405712	1	1	0.109138232
POLR3E	6.30E-06	-0.675525223	1	1	0.109375687
A1CF	6.35E-06	1.669905979	1	1	0.110244179
BOLA1	6.38E-06	-2.756074417	0.188	0.733	0.110654941
C5orf24	6.42E-06	1.995619514	1	0.8	0.111362488
CCDC88C	6.46E-06	-1.115305151	0.875	1	0.112164404
POLD2	6.47E-06	-1.36994961	0.75	0.933	0.112178855
ENSSSCG00000058574	6.47E-06	-0.982685415	0.938	1	0.112238653
C3orf49	6.48E-06	1.383671445	1	1	0.112460654
ENSSSCG00000020808	6.58E-06	-0.429672846	1	1	0.11412395
ING1	6.61E-06	-0.636141225	1	1	0.114629241
TTLL12	6.61E-06	-1.89219671	0.625	0.867	0.114684448
KIF18A	6.62E-06	1.796320221	1	1	0.114839599
NUP155	6.62E-06	0.047371819	1	1	0.114870676
ENSSSCG00000061886	6.62E-06	-1.870716983	0.625	0.933	0.114903783
MARCHF2	6.63E-06	-2.61667136	0.375	0.667	0.115008207
TBC1D32	6.63E-06	1.432401636	1	1	0.115086521
ENSSSCG00000060138	6.64E-06	-0.893171834	0.875	1	0.115253447
MCC	6.66E-06	1.641434544	1	1	0.115608831
POMGNT1	6.69E-06	-1.635003183	0.625	1	0.116102622
ENSSSCG00000042487	6.69E-06	-1.183874868	0.812	1	0.116164099
ENSSSCG00000047692	6.72E-06	-1.173032949	0.938	0.933	0.116680191
SMC3	6.74E-06	1.495782688	1	1	0.1169753
CDKN2B	6.82E-06	-0.77325266	0.938	1	0.118394095
SERP1	6.84E-06	1.339885816	1	1	0.118630724
SNAP23	6.87E-06	-0.113249727	1	1	0.119202655
USP13	6.88E-06	1.628615446	1	1	0.119302802
SCN5A	6.91E-06	2.040289721	1	0.867	0.119923276
ALG6	6.92E-06	2.510389048	1	0.933	0.120079177
ZDHHC5	6.93E-06	1.016229444	1	1	0.12015659
HMGXB4	6.97E-06	1.42477075	1	1	0.120973287
TFG	7.00E-06	1.500313216	1	1	0.121448024
C5orf15	7.00E-06	2.372705831	1	0.867	0.121461535
CTSB	7.03E-06	-0.502837101	1	1	0.122032622
WRAP73	7.06E-06	-0.722740954	1	1	0.122424534
ENSSSCG00000060885	7.08E-06	-1.741637034	0.75	0.933	0.12290054
RUVBL2	7.09E-06	-2.800062429	0.312	0.8	0.122984666
CHORDC1	7.10E-06	1.527411478	1	1	0.123154277
ENSSSCG00000039915	7.14E-06	-3	0.125	0.867	0.123859431
UQCRFS1	7.24E-06	-0.28825699	1	1	0.125646163
EPS8L3	7.36E-06	-1.691010961	0.688	0.867	0.127732893
PAPSS1	7.39E-06	1.620859443	1	1	0.128274129
EXOSC6	7.42E-06	-2.093109404	0.438	0.867	0.128701956
AKIRIN2	7.43E-06	1.18639133	1	1	0.128868583
STAMBP	7.48E-06	1.891569796	1	1	0.129706761
ELMOD3	7.51E-06	-1.642447995	0.75	0.867	0.130362085
ENSSSCG00000016075	7.51E-06	1.204339007	1	1	0.130371259
TAFA4	7.54E-06	1.393952205	1	1	0.130829822
CD47	7.58E-06	1.480723282	1	1	0.131484959
ADSL	7.63E-06	-0.825217435	1	1	0.132433935
TOB2	7.72E-06	-0.913288367	0.938	1	0.133928702
SNAPC3	7.81E-06	1.351654834	1	1	0.135580815
HMCES	7.82E-06	1.757656172	1	1	0.135638171
DUSP22	7.82E-06	-2.678071905	0.25	0.8	0.135668465
FOSL2	7.84E-06	-1.919080005	0.562	0.867	0.136005484
PDHA1	7.86E-06	-0.171700411	1	1	0.136330553
RAB36	7.99E-06	-2.552541023	0.25	0.733	0.138671353
WIPI2	8.03E-06	1.968577991	1	1	0.139327235
FAM210B	8.05E-06	-0.761076432	1	1	0.139717935
NUP50	8.07E-06	-0.024226929	1	1	0.139972574
ENSSSCG00000004811	8.15E-06	-3.047305715	0.188	0.733	0.141481272

MRPL38	8.16E-06	-1.567040593	0.812	0.867	0.141530143
FBXO33	8.17E-06	1.421076416	1	1	0.141744759
CEBPG	8.20E-06	-0.267560189	1	1	0.142243276
ENSSSCG00000011307	8.27E-06	-0.618428959	0.875	1	0.143530623
MYO3B	8.33E-06	-0.311110057	1	1	0.144605225
ENSSSCG00000000298	8.40E-06	-3.678071905	0.062	0.733	0.145728037
ENSSSCG00000041524	8.41E-06	-0.910245347	0.875	1	0.145872557
ENSSSCG00000032959	8.42E-06	-2.11417102	0.562	0.867	0.146081944
CGNL1	8.43E-06	-0.161434677	1	1	0.146329231
YPEL5	8.44E-06	1.258112997	1	1	0.14650352
FGGY	8.45E-06	-1.157239742	0.812	0.933	0.146633848
ENSSSCG00000007733	8.49E-06	1.430046314	1	1	0.147362298
TMEM126B	8.55E-06	-0.275634443	1	1	0.148377317
ENSSSCG00000031854	8.57E-06	2.088384986	1	0.933	0.148677091
ILRUN	8.58E-06	-0.470368869	1	1	0.148868144
CCP110	8.58E-06	1.516170892	1	1	0.14895208
ZHX1	8.63E-06	2.220430905	1	0.933	0.149753533
LSR	8.65E-06	1.678957933	1	1	0.150001115
MXD1	8.71E-06	-0.191417775	1	1	0.151195181
ABCB8	8.79E-06	-3.197446064	0.125	0.733	0.152497441
FNDC3B	8.81E-06	1.354180133	1	1	0.152848068
DIO1	8.83E-06	-1.505235308	0.75	0.867	0.153225704
NUDT15	8.85E-06	1.521664771	1	1	0.153495479
SHROOM1	8.87E-06	-1.955605881	0.625	0.867	0.153962842
UCHL1	8.89E-06	-0.460931198	1	1	0.154184087
MKNK1	8.90E-06	1.624241808	1	1	0.154478092
KDM4A	9.02E-06	-0.785785722	0.875	1	0.156488642
CDIP1	9.11E-06	-2.376043368	0.375	0.867	0.157996959
SESN2	9.12E-06	-1.062735755	0.938	1	0.158224336
TOMM40	9.18E-06	-2.637429921	0.25	0.8	0.159279996
CCDC42	9.24E-06	-4.047305715	0.062	0.667	0.160404928
IFT43	9.25E-06	2.23457796	0.938	0.867	0.160482249
ENSSSCG00000061796	9.32E-06	-1.697180728	0.562	1	0.161646616
ENSSSCG00000056538	9.34E-06	1.607260273	1	1	0.162005649
ENSSSCG00000053692	9.42E-06	-0.868403117	1	1	0.163502347
GSTO1	9.42E-06	-0.978339217	0.938	1	0.163532763
C9orf78	9.53E-06	-0.669361267	0.938	1	0.165404741
GTF3C6	9.59E-06	-0.51077716	1	1	0.166357984
HROB	9.64E-06	-0.938266359	1	0.933	0.167177469
FMNL3	9.74E-06	-0.948266103	1	1	0.169069999
LRATD2	9.75E-06	1.39143531	1	1	0.1691432
ENSSSCG00000001970	9.78E-06	1.541386482	1	1	0.169684094
CHST12	9.81E-06	-1.678071905	0.625	0.933	0.170127645
USP45	9.82E-06	1.695136695	1	1	0.170452705
AHSG	9.88E-06	-1.166571566	0.938	0.867	0.171348957
ENSSSCG00000033293	9.92E-06	1.557114516	1	1	0.172070709
SEMA4B	9.97E-06	2.056637715	1	1	0.172932521
TAF7	9.99E-06	-0.225439415	1	1	0.173338345
ARPIN	1.00E-05	2.388583826	1	1	0.173453804
AKIRIN1	1.00E-05	1.533783777	1	1	0.173717986
NMB	1.00E-05	-3.450661409	0.125	0.667	0.174281517
TCL1A	1.01E-05	-0.876900855	1	1	0.174462939
DGAT1	1.01E-05	-1.535113951	0.625	0.933	0.175796819
VCPKMT	1.02E-05	2.868822555	0.938	0.6	0.177724516
PCYT1A	1.04E-05	1.45876231	1	1	0.179613237
CELA2A	1.05E-05	-2.277533976	0.312	0.8	0.18266326
MARCHF9	1.05E-05	-3.697971463	0.125	0.667	0.182720291
PLCG2	1.06E-05	-0.969374036	0.938	1	0.184039029
WDR18	1.07E-05	-3.462343214	0.062	0.733	0.185697734
PABPC1L	1.07E-05	-0.56056782	1	1	0.185909271
ZNF3	1.08E-05	-2.062735755	0.75	0.867	0.186764075
RGP1	1.09E-05	-1.15787966	0.938	0.933	0.189218064
ENSSSCG00000050302	1.09E-05	-2.595609745	0.375	0.667	0.189398155
DLST	1.10E-05	-0.283384719	1	1	0.190193076
COX8A	1.11E-05	-1.215966152	1	1	0.19193035
OSBPL6	1.11E-05	-0.298091142	1	1	0.191957823
BAIAP2L1	1.11E-05	-0.604811205	0.938	1	0.19307945
TSN	1.12E-05	0.025162037	1	1	0.193810016
SLC26A3	1.12E-05	-0.390328512	1	1	0.194081438
TRAPPC2L	1.13E-05	-4.159198595	0.062	0.667	0.195245832

CENPI	1.14E-05	1.698391566	1	1	0.197143822
ENO1	1.15E-05	-2.146220741	1	1	0.198932444
ENSSSCG00000058414	1.15E-05	-2.062735755	0.562	0.8	0.199677779
ENSSSCG00000061173	1.15E-05	-1.576431643	0.5	1	0.200264183
ZFYVE21	1.16E-05	-0.311454993	1	1	0.200405593
ITPR3	1.16E-05	-0.923897021	0.938	1	0.200877868
CDK8	1.16E-05	-0.463520924	1	1	0.20187421
ENSSSCG00000030013	1.18E-05	-0.756975933	1	1	0.204092632
OSGEPL1	1.18E-05	1.836501268	1	0.933	0.205458389
PIGT	1.18E-05	-1.810966176	0.688	0.8	0.20555731
ENSSSCG00000041032	1.19E-05	-3.793549123	0.062	0.667	0.206930247
ADCY9	1.19E-05	-0.511213207	1	1	0.206960238
ST7L	1.20E-05	-1.172543872	0.938	0.933	0.207818482
ENSSSCG00000013150	1.21E-05	-2.858644151	0.125	0.733	0.209369617
UFM1	1.21E-05	-0.296788533	1	1	0.210156641
ENSSSCG00000055548	1.21E-05	-2.571156701	0.375	0.733	0.21050367
DACT1	1.22E-05	1.837285128	1	0.8	0.211674209
SIRT7	1.22E-05	-1.183307213	0.875	0.867	0.211744675
ASB3	1.22E-05	1.314421112	1	1	0.211768201
MAGT1	1.22E-05	1.567340582	1	1	0.211919388
FABP3	1.24E-05	-0.915430177	1	1	0.215506789
PSD2	1.24E-05	-3.137503524	0.125	0.733	0.215676068
DNAH1	1.24E-05	-2.32443495	0.562	0.8	0.215981067
TBC1D8	1.26E-05	-0.486024093	1	1	0.218174944
ENSSSCG00000055956	1.26E-05	-2.132637769	0.5	0.933	0.219182824
ENSSSCG00000045719	1.26E-05	-2.341036918	0.312	0.8	0.219421208
ATP2C2	1.27E-05	-1.100488935	1	1	0.220717493
RUVBL1	1.27E-05	-0.763884719	1	1	0.221140499
TMEM109	1.28E-05	1.838729518	1	1	0.22149184
SHE	1.28E-05	1.546806787	1	1	0.221500709
LRRC17	1.29E-05	-0.375675067	1	1	0.223575058
TEK	1.29E-05	-0.595609745	1	1	0.223709651
KPNA1	1.29E-05	-0.567206888	1	1	0.224058403
GPR107	1.29E-05	-0.940421108	0.938	0.933	0.224112018
GNAI3	1.31E-05	-0.077661405	1	1	0.226932088
USP48	1.31E-05	1.422590434	1	1	0.227022507
MYEF2	1.32E-05	1.489892913	1	1	0.22837116
QSOX1	1.32E-05	-0.658014253	1	1	0.228413602
FASTKD2	1.32E-05	1.780110086	1	1	0.228664061
NLRP13	1.32E-05	-0.506108911	1	1	0.229019579
ANKRD24	1.32E-05	-2.858644151	0.188	0.667	0.229372822
TWF1	1.32E-05	1.470362568	1	1	0.229780442
ANKRD27	1.33E-05	-0.557414449	0.938	1	0.230688807
RNF146	1.33E-05	1.308131097	1	1	0.230923506
SP3	1.33E-05	1.674752061	1	1	0.231133265
ENSSSCG00000060152	1.34E-05	-1.117771459	0.875	1	0.231708892
GATM	1.34E-05	3.661778098	0.938	0.2	0.231997314
HSPBP1	1.35E-05	-1.423754716	0.688	0.867	0.233514616
CCM2	1.35E-05	-3.462343214	0.125	0.667	0.234459311
ATP6V0D1	1.36E-05	-0.63076619	1	1	0.236515802
NUTM1	1.36E-05	-2.167109986	0.438	0.867	0.2367438
RPLP0	1.36E-05	-0.253807453	1	1	0.236773708
KIF20A	1.37E-05	1.7062503	1	1	0.237143844
ACP2	1.37E-05	-2.019108823	0.5	0.867	0.237265453
SDAD1	1.37E-05	-0.336502124	1	1	0.237817793
ENSSSCG00000002907	1.38E-05	-1.580491545	1	1	0.239584594
NOM1	1.39E-05	1.637069481	1	1	0.241602856
ENTPD6	1.40E-05	-1.432739501	0.875	1	0.242238128
UEVLD	1.40E-05	2.127848213	1	1	0.24258544
TRIP10	1.41E-05	-1.122856748	0.875	1	0.244557192
ENSSSCG00000002850	1.41E-05	-4.047305715	0.062	0.6	0.245142351
PLS3	1.42E-05	1.366619047	1	1	0.245556762
NME1	1.43E-05	-3.180572246	0.312	0.8	0.247614796
ENSSSCG00000053231	1.43E-05	2.677408749	1	0.533	0.247934937
HDLBP	1.43E-05	-1.023475217	1	0.933	0.248239889
ENSSSCG00000012202	1.45E-05	-0.733007581	0.938	1	0.251882491
F8	1.46E-05	-1.335470242	0.812	0.867	0.25332453
CD72	1.47E-05	-1.386555123	0.875	0.933	0.255131508
ROPN1L	1.47E-05	-1.625604485	0.5	0.867	0.255669235
CTPS1	1.47E-05	-0.460078464	1	1	0.255848997

ZDHHC1	1.48E-05	2.136624992	1	0.733	0.256441191
ZC3H8	1.48E-05	1.356165997	1	1	0.256719681
SEMA4D	1.48E-05	2.059773113	1	0.933	0.256862392
ENSSSCG00000013992	1.48E-05	-0.175075004	1	1	0.257447926
ARHGDIA	1.49E-05	-1.854949667	0.625	0.933	0.258414323
TSC2	1.49E-05	-0.9861942	0.938	1	0.258704692
PRKCH	1.50E-05	1.495874549	1	1	0.25999483
ENSSSCG00000059900	1.50E-05	-2.900464326	0.125	0.867	0.260529518
SEC13	1.51E-05	1.729238232	1	1	0.261650911
APMAP	1.52E-05	-0.249862897	1	1	0.263452331
AQP11	1.53E-05	-1.69214709	0.562	0.933	0.265570194
NFIL3	1.53E-05	-0.408959329	1	1	0.265576897
ENSSSCG00000006621	1.53E-05	-0.828896807	0.938	0.933	0.265602649
HMGN3	1.54E-05	2.003147611	1	1	0.266884815
MAD2L2	1.55E-05	-1.3910776	0.938	1	0.268145831
YIPF3	1.55E-05	-1.709780765	0.75	0.8	0.269019104
PSMB1	1.56E-05	-0.285075669	1	1	0.271540006
ASB6	1.57E-05	-1.26895924	0.938	1	0.272638145
DIABLO	1.58E-05	-1.002200908	1	0.933	0.27403966
KCNMA1	1.59E-05	-0.897189194	0.875	1	0.276036496
IZUMO4	1.59E-05	-2.941106311	0.125	0.667	0.276052857
GALE	1.59E-05	-2.446746359	0.375	0.667	0.276196214
RNASEH2B	1.60E-05	-1.053849974	0.875	1	0.278295075
SMUG1	1.61E-05	-1.678071905	0.5	0.933	0.278737568
MCOLN2	1.61E-05	3.994353437	0.938	0.4	0.278897384
NXN	1.61E-05	-1.184257292	0.75	1	0.278926894
YWHAH	1.61E-05	1.585927837	1	1	0.280195518
LOXL2	1.62E-05	-1.292418213	0.688	0.933	0.281479455
ENSSSCG00000009670	1.63E-05	1.506701959	1	1	0.282043995
BANP	1.63E-05	1.853600893	1	1	0.282255094
ROS1	1.66E-05	3.157117225	0.938	0.4	0.288345814
HELQ	1.68E-05	1.70555979	1	1	0.291285595
E2F2	1.69E-05	-1.77118131	0.688	0.867	0.292621772
STT3A	1.69E-05	-0.101800365	1	1	0.293786936
ALDOB	1.70E-05	-1.085804604	0.875	0.933	0.294502154
ENSSSCG00000016226	1.70E-05	-0.435094511	1	1	0.295382318
PTCD2	1.70E-05	-0.942775131	0.875	0.933	0.295460605
ENSSSCG00000055543	1.73E-05	2.501837185	1	0.933	0.299675214
ATP6V0A4	1.75E-05	1.877890724	1	1	0.303124562
FRMD5	1.75E-05	-0.345208333	1	1	0.304463075
PFKP	1.76E-05	-0.659264637	1	1	0.304917465
DESI2	1.76E-05	-0.51481712	1	1	0.305027174
SRSF5	1.77E-05	-0.895170027	1	1	0.306539471
BAP1	1.77E-05	-1.087716146	0.938	1	0.307752445
CSNK1D	1.77E-05	-1.021216486	0.75	1	0.30779613
ABCG2	1.78E-05	-2.349449158	0.438	0.8	0.308496819
POLR1H	1.80E-05	-1.140415119	0.812	1	0.313016643
ENSSSCG00000003253	1.82E-05	-1.189970944	0.688	0.933	0.315652473
SS18L2	1.83E-05	-0.195868979	1	1	0.318251194
PHF24	1.84E-05	-0.659073803	0.938	1	0.318484576
PI4KB	1.84E-05	1.885321298	1	1	0.318720697
STK38	1.84E-05	1.482529159	1	1	0.318876216
EMC10	1.84E-05	-2.678071905	0.25	0.8	0.320109266
CELA1	1.85E-05	-4.678071905	0	0.667	0.321216314
MVB12A	1.86E-05	-3.128733314	0.125	0.667	0.322480245
MTMR1	1.87E-05	-0.354576515	1	1	0.323684676
NOL12	1.87E-05	-1.77118131	0.625	0.933	0.324045517
GRID2	1.87E-05	1.757225744	1	0.867	0.324568596
ODF2	1.88E-05	-0.628659711	0.938	1	0.326233217
PNPT1	1.88E-05	1.560534387	1	1	0.327025232
STAG2	1.89E-05	1.874900481	1	1	0.327529593
ARL5B	1.89E-05	1.5129088	1	1	0.3280715
APLF	1.89E-05	-0.070918831	1	1	0.328488559
NDST2	1.89E-05	-0.815201722	0.875	1	0.328661877
AGAP3	1.92E-05	-1.482151695	0.438	0.933	0.333211916
NLK	1.93E-05	-0.419073013	1	1	0.335001044
ARHGEF7	1.93E-05	-0.251371488	1	1	0.33561975
GRAMD1A	1.95E-05	-1.941106311	0.5	0.933	0.337806456
DNAAF10	1.95E-05	1.536172482	1	1	0.337902933
AKR7A2	1.97E-05	-1.974464908	0.438	0.933	0.341663967

NFXL1	1.97E-05	1.423801928	1	1	0.342618772
CC2D1A	1.98E-05	-1.852101305	0.5	0.867	0.343912106
KLHDC3	2.02E-05	-0.806991385	1	1	0.350129039
ZNF146	2.02E-05	1.914190116	1	1	0.351110907
CHTOP	2.03E-05	-0.430931146	0.938	1	0.351545738
TRMU	2.04E-05	-2.315501826	0.25	0.8	0.354032759
LIN7B	2.04E-05	-3.366127899	0.125	0.6	0.354072342
LEPROTL1	2.05E-05	-0.744238829	1	1	0.354829537
ZNRF1	2.06E-05	-1.040641984	1	0.933	0.357439182
BRK1	2.06E-05	-0.500363097	1	1	0.357761039
EIF2B3	2.08E-05	-0.932644732	1	0.933	0.360080037
IQCB1	2.08E-05	1.545006115	1	1	0.360397673
CPSF4	2.08E-05	1.82572945	1	1	0.360783421
SYT14	2.08E-05	1.340932701	1	1	0.361414202
ENSSSCG00000062804	2.09E-05	-1.182746617	0.625	1	0.362921467
RBM7	2.09E-05	-2.135753742	0.562	1	0.362972688
TYRO3	2.09E-05	-1.888968688	0.688	0.8	0.363241435
TNKS2	2.10E-05	1.43272344	1	1	0.364501063
ENSSSCG00000041142	2.10E-05	-0.631074426	0.938	1	0.364556308
ENSSSCG00000033805	2.11E-05	2.886712714	0.875	0.467	0.365276351
GBX2	2.11E-05	-2.495207848	0.375	0.733	0.365708323
ELP5	2.11E-05	-1.528046461	0.688	0.867	0.366185552
HHAT	2.11E-05	-0.413590463	1	1	0.366254438
NUP54	2.11E-05	1.51971371	1	1	0.366815484
HHEX	2.12E-05	1.397667455	1	1	0.367839196
ENSSSCG00000000908	2.15E-05	-0.256739446	1	1	0.372378212
NFYC	2.17E-05	-0.649695421	1	1	0.377102771
NPRL3	2.20E-05	-3.047305715	0.188	0.667	0.380911257
ENSSSCG00000058051	2.20E-05	-0.555289887	1	1	0.381807355
MAP7D1	2.20E-05	-0.975053643	0.875	0.933	0.382283439
RNF111	2.22E-05	1.335716203	1	1	0.38491094
NUDCD1	2.22E-05	1.379378367	1	1	0.385022286
ENSSSCG00000057586	2.23E-05	-1.131583552	0.875	1	0.386382757
KHK	2.23E-05	-2.865698908	0.25	0.667	0.387456083
GNAZ	2.23E-05	-3	0.125	0.733	0.387503965
ENSSSCG00000062136	2.25E-05	-2.036525876	0.438	0.867	0.389739592
HMG20A	2.25E-05	1.407266468	1	1	0.390304178
NUP88	2.26E-05	1.546921748	1	1	0.391286648
ENSSSCG00000012703	2.28E-05	-0.459152982	1	1	0.394839141
ENSSSCG00000055095	2.28E-05	-1.750221691	0.562	0.867	0.395473368
CKM	2.29E-05	-1.5360529	0.812	0.933	0.397585142
SS18	2.30E-05	1.430233978	1	1	0.399010406
RNASEH1	2.31E-05	-0.46844678	1	1	0.400625093
TSACC	2.31E-05	-3.208586622	0.062	0.733	0.401279085
DLG5	2.32E-05	1.597457891	1	1	0.401990183
QPCTL	2.32E-05	-1.9510904	0.562	0.867	0.402477828
ADGRL2	2.32E-05	2.233784943	1	0.667	0.402946353
DNAJC22	2.33E-05	-0.769045565	0.938	0.933	0.403684658
RANGRF	2.33E-05	-1.9510904	0.75	0.867	0.404051025
EDARADD	2.33E-05	-1.175571565	0.875	0.933	0.404443265
HOXD9	2.34E-05	-0.957992473	0.938	1	0.405357919
GNPNAT1	2.35E-05	1.568729619	1	1	0.407838233
SLC1A2	2.37E-05	-0.731313926	1	1	0.410758969
ENSSSCG00000016902	2.38E-05	2.13941054	1	1	0.412115224
NR2F2	2.38E-05	-1.967578522	0.625	0.8	0.412164867
ENSSSCG00000027491	2.39E-05	-0.636849242	1	1	0.414435956
MBOAT7	2.40E-05	-1.793549123	0.562	0.867	0.417246429
NAT8L	2.42E-05	-3.263034406	0.125	0.667	0.419938708
FNDC5	2.44E-05	-0.941106311	0.812	1	0.422868605
HDAC1	2.45E-05	1.898556112	1	1	0.425092722
FHAD1	2.45E-05	-1.167109986	0.688	0.933	0.425250116
ENSSSCG00000004404	2.45E-05	1.740880644	1	0.867	0.425919757
ZFP36L2	2.46E-05	-1.050465067	1	1	0.426120398
TLK1	2.46E-05	-0.657607803	1	1	0.426576905
ENSSSCG00000060282	2.48E-05	-1.888491685	0.75	1	0.4299434
GGA2	2.48E-05	-0.657238402	0.938	1	0.429999473
PAICS	2.49E-05	-0.171647455	1	1	0.431583562
TXNL1	2.49E-05	1.416099775	1	1	0.43181051
MED14	2.49E-05	1.22661442	1	1	0.432158728
SCG3	2.50E-05	-0.503197688	1	1	0.434083234

H6PD	2.51E-05	-3.9510904	0.062	0.6	0.434871351
STAG1	2.51E-05	1.419873145	1	1	0.435894025
KXD1	2.52E-05	-1.360589715	0.75	0.933	0.436499317
JAZF1	2.55E-05	-0.415307895	1	1	0.442972565
DNAJC4	2.57E-05	-3.128733314	0.125	0.667	0.445062713
ENSSSCG00000054785	2.57E-05	-0.966104128	0.938	0.933	0.446785315
OGFOD2	2.58E-05	-1.298223834	0.75	0.933	0.448094226
GABRR3	2.58E-05	3.728892294	0.75	0.2	0.448187857
ENSSSCG00000040885	2.60E-05	-1.582494245	0.562	0.733	0.451181727
CPT2	2.61E-05	-0.41439473	1	1	0.452383815
SMCO4	2.61E-05	2.531201764	1	0.933	0.453096133
RRP8	2.61E-05	-2.815575429	0.188	0.667	0.453098364
DOCK6	2.62E-05	-1.062735755	0.938	1	0.454288753
CAMK1	2.64E-05	1.850028259	1	1	0.457348269
CCT8	2.67E-05	1.528097269	1	1	0.463202576
HOMER3	2.68E-05	-3.152003093	0.062	0.8	0.465041135
MTERF1	2.68E-05	2.28771238	1	0.6	0.465759783
KNTC1	2.69E-05	-0.34316109	1	1	0.467386294
YPEL1	2.71E-05	-0.642843417	1	1	0.470515814
ENSSSCG00000046461	2.72E-05	-2.678071905	0.125	0.8	0.472298726
RIMKLA	2.73E-05	-0.673570513	1	1	0.473577194
IPO5	2.74E-05	-0.855825993	0.938	0.933	0.474911912
ENSSSCG00000042921	2.74E-05	-3.9510904	0.062	0.6	0.475747195
ENSSSCG00000051835	2.74E-05	2.5980525	0.938	0.333	0.475978512
EIF3E	2.75E-05	1.528509488	1	1	0.476507584
GBF1	2.75E-05	1.357023763	1	1	0.476852874
CDC123	2.75E-05	1.42475451	1	1	0.477073016
STX3	2.75E-05	1.683558614	1	1	0.477750001
OST4	2.77E-05	-1.423754716	0.812	0.867	0.479847451
TMEM39A	2.78E-05	1.386033416	1	1	0.481818525
OGA	2.78E-05	-0.854007741	0.875	1	0.482609398
RIOK1	2.79E-05	-0.383099904	1	1	0.483842942
DLAT	2.79E-05	1.668358093	1	1	0.484199125
SH2B2	2.79E-05	-1.865698908	0.438	0.733	0.484632499
RPUSD2	2.80E-05	-1.526068812	0.75	0.933	0.485901193
SMARCD2	2.83E-05	-0.779951519	0.938	0.933	0.490244378
FAM3B	2.83E-05	-4.793549123	0	0.667	0.490330769
ACOT6	2.84E-05	-0.610026518	1	1	0.493077843
SCYL2	2.86E-05	1.594134911	1	1	0.49577656
ENSSSCG00000011569	2.86E-05	-1.1602236	0.688	1	0.496228548
ENSSSCG00000052460	2.87E-05	-1.972815171	0.75	0.867	0.498221891
TMEM104	2.88E-05	-0.953706348	0.938	1	0.498874227
FGF16	2.88E-05	1.666576266	1	1	0.499243784
FBXO3	2.89E-05	-0.772831047	1	1	0.500775768
ENSSSCG00000062912	2.89E-05	0.096487628	1	1	0.501318599
PYGO1	2.89E-05	1.397667455	1	1	0.502262564
ENSSSCG00000059772	2.90E-05	-0.723652939	0.938	1	0.502385916
NEDD1	2.94E-05	1.498315347	1	1	0.509792009
CCDC97	2.95E-05	-1.477773255	0.688	0.933	0.512567136
SCAMP5	2.96E-05	-0.784423886	0.938	1	0.51343221
UBALD2	2.96E-05	-2.678071905	0.25	0.733	0.514095737
TMEM259	2.97E-05	-1.714597781	0.688	0.8	0.515039147
EIF5A2	2.99E-05	1.952214586	1	0.933	0.518649928
RBM34	3.02E-05	-0.85025288	0.938	0.933	0.524758051
ENSSSCG00000044694	3.04E-05	-0.199657183	1	1	0.527783734
CLIP1	3.05E-05	1.353839336	1	1	0.529362766
MRPL48	3.07E-05	-1.601256308	0.75	0.933	0.532837335
SURF1	3.08E-05	-2.145576824	0.438	0.8	0.534950396
ENSSSCG00000038518	3.08E-05	-0.518716145	0.938	1	0.534974254
TPP2	3.10E-05	1.290219235	1	1	0.537289584
CAPN2	3.10E-05	-0.23336729	1	1	0.53783869
BCLAF3	3.11E-05	1.580878757	1	1	0.538910035
FAM184A	3.11E-05	-0.268476491	1	1	0.539328386
R3HDM4	3.12E-05	1.943274746	1	1	0.541184323
TMCO4	3.14E-05	-2.036525876	0.438	0.867	0.545404808
ANKIB1	3.17E-05	1.29351763	1	1	0.549237721
EXOC1	3.17E-05	1.287555495	1	1	0.549781107
N4BP2	3.17E-05	1.47255949	1	1	0.549872307
GEM	3.17E-05	3.637283536	0.75	0.4	0.550712375
GOLM1	3.18E-05	-0.341188469	1	1	0.551929529

CGGBP1	3.23E-05	1.423132121	1	1	0.560283559
PIGS	3.24E-05	-0.612483563	0.938	0.933	0.561341605
METTL26	3.24E-05	-2.815575429	0.188	0.667	0.561471445
FAM118B	3.24E-05	-0.338498004	1	1	0.562665232
MRPL11	3.28E-05	-0.442063982	1	1	0.569879232
MIS12	3.29E-05	-0.194670132	1	1	0.571073583
C2CD2	3.29E-05	-1.449253215	0.562	0.933	0.571262195
DDX51	3.31E-05	-2.351843673	0.438	0.6	0.573630491
SPCS3	3.31E-05	-1.368344264	0.875	1	0.574120442
ARPP21	3.32E-05	-0.481223619	1	1	0.576870398
LMBRD1	3.34E-05	1.729181788	1	1	0.579471589
SIGMAR1	3.35E-05	-0.451839463	1	1	0.581257657
SMARCD1	3.38E-05	1.673070554	1	1	0.585965376
ECPAS	3.38E-05	-0.104266116	1	1	0.586398491
PHF20L1	3.38E-05	1.381648306	1	1	0.586970451
TBC1D15	3.40E-05	1.574329721	1	1	0.589434339
RAB1B	3.40E-05	-1.320178313	0.688	0.933	0.590424547
POLN	3.42E-05	1.568786832	1	1	0.592681923
KANSL3	3.42E-05	-0.663043696	0.938	1	0.593524241
ARMC10	3.44E-05	1.308989039	1	1	0.596577232
NOP53	3.44E-05	-2.330148602	0.312	0.733	0.597164486
ZNF280C	3.45E-05	2.264047179	1	0.733	0.599261524
BIRC6	3.46E-05	1.187556432	1	1	0.600272388
CPN1	3.46E-05	-1.967578522	0.625	0.8	0.601099192
SPNS3	3.47E-05	-2.477773255	0.375	0.667	0.602273866
PARP14	3.48E-05	1.653886555	1	1	0.603041858
MCRIP2	3.48E-05	-3.678071905	0.062	0.733	0.603738221
ZNF106	3.48E-05	1.214756045	1	1	0.604495232
RECQL5	3.49E-05	-0.880888788	0.875	1	0.605745272
ATP6V0A2	3.50E-05	1.265874556	1	1	0.60712944
F12	3.50E-05	-2.840343334	0.188	0.667	0.607138967
ENSSSCG00000054645	3.51E-05	2.4247389	1	0.6	0.608464903
HEXA	3.51E-05	-2.310340121	0.312	0.8	0.608560428
RHOT2	3.53E-05	-2.571156701	0.312	0.667	0.612868985
CINP	3.55E-05	-2.485426827	0.188	0.8	0.616052547
CTTN	3.55E-05	2.028585249	1	1	0.616457097
ENSSSCG00000058443	3.56E-05	-3.793549123	0.312	0.667	0.617246091
ENSSSCG00000001057	3.56E-05	2.522067709	0.875	0.933	0.618212818
LINGO2	3.57E-05	1.885086225	1	0.867	0.619168484
RNF180	3.58E-05	1.665882496	1	1	0.621581357
SGK2	3.59E-05	-2.455679484	0.188	0.8	0.622512068
RAB3B	3.60E-05	-0.299850826	1	1	0.624757547
SPRY2	3.61E-05	-1.587218475	0.812	0.8	0.626432117
LNX2	3.61E-05	-0.315129084	1	1	0.62683197
ENSSSCG00000062132	3.66E-05	2.362955363	1	0.8	0.635855237
ENSSSCG00000016869	3.67E-05	2.155918143	1	0.867	0.636693384
HIF1AN	3.69E-05	-0.299846332	1	1	0.639766784
ENSSSCG00000015034	3.70E-05	-0.566614327	1	1	0.642190943
PER1	3.73E-05	-0.749456538	0.938	1	0.646747966
FZD6	3.73E-05	1.443943111	1	1	0.646812644
GPD2	3.73E-05	2.649956389	0.938	0.6	0.647209828
ENSSSCG00000042028	3.76E-05	-1.651104858	0.625	0.867	0.651835491
DNLZ	3.76E-05	-5.137503524	0	0.667	0.652444844
HNF4G	3.76E-05	2.184529414	1	0.933	0.652526394
ENSSSCG00000005959	3.78E-05	1.513431348	1	1	0.656265328
CCNDBP1	3.78E-05	-0.914772163	0.938	1	0.656537805
HSP90AB1	3.79E-05	1.535407009	1	1	0.657914966
COQ4	3.87E-05	-1.4639471	0.688	0.8	0.671485657
TRMT10C	3.87E-05	-0.894448911	0.938	1	0.671874203
CHODL	3.88E-05	-0.031490286	1	1	0.672798881
PDPN	3.88E-05	-3.124587636	0.375	0.8	0.673276335
RRP1B	3.90E-05	-1.05704015	0.812	1	0.676601635
ENSSSCG00000031170	3.90E-05	1.574058891	1	1	0.676713122
ENSSSCG00000031398	3.92E-05	-1.916231642	0.5	0.933	0.679426822
CFAP58	3.94E-05	-0.386678178	1	1	0.683010677
FKBPL	3.94E-05	-2.481379994	0.312	0.733	0.683105802
TRPC7	3.94E-05	-1.706086281	0.625	0.8	0.68347366
DHCR7	3.97E-05	-2.707819249	0.188	0.667	0.688364429
L3MBTL3	3.98E-05	-0.010030365	1	1	0.689716632
SLC6A20	3.98E-05	-0.296069882	1	1	0.690520821

SLC7A7	3.99E-05	-0.598504648	1	1	0.692144201
KDF1	4.00E-05	-2.443606651	0.312	0.733	0.69353746
SYAP1	4.02E-05	-0.205584134	1	1	0.697072022
TIMM8B	4.02E-05	-0.652780986	1	1	0.697563475
PPP1CA	4.02E-05	-0.718260995	0.938	1	0.697634069
RANGAP1	4.03E-05	-1.171111916	0.688	0.933	0.698710361
TRADD	4.03E-05	-3.047305715	0.125	0.667	0.698731881
ENSSSCG00000016215	4.03E-05	-1.830074999	0.375	0.867	0.699995639
FAM98B	4.05E-05	-0.581687369	1	1	0.702002551
ENSSSCG00000053662	4.07E-05	1.622732898	1	0.933	0.705901918
AZIN1	4.09E-05	1.5203258	1	1	0.709035039
ENSSSCG00000038304	4.10E-05	1.869864739	1	1	0.711053595
TECPR1	4.10E-05	-3.678071905	0.062	0.667	0.711779288
RRP12	4.13E-05	-1.467084247	1	0.867	0.716262642
MAG	4.14E-05	-2.900464326	0.188	0.6	0.719178295
UBE2E1	4.16E-05	1.40844769	1	1	0.72180288
CACNA2D3	4.17E-05	-1.756074417	0.625	0.867	0.723601988
DOK6	4.17E-05	-1.399770743	0.688	0.933	0.724133605
CHST14	4.18E-05	2.796707678	0.875	0.4	0.72523318
KCTD2	4.19E-05	-0.605444105	1	0.933	0.726618369
SLC36A3	4.20E-05	-0.796716402	0.938	0.933	0.727963141
ENSSSCG00000006556	4.21E-05	-2.309427311	0.312	0.867	0.730071529
FANCD2	4.24E-05	-0.330497874	1	1	0.735900804
PTCD3	4.24E-05	-0.418232838	0.938	0.933	0.736086627
DPH1	4.24E-05	-0.418566311	1	1	0.736109371
NPHP3	4.29E-05	1.408309456	1	1	0.744056099
MAP2K3	4.29E-05	-0.42365063	0.875	1	0.744388843
TRMT2A	4.32E-05	-1.093109404	0.688	1	0.749589405
ARRDC2	4.33E-05	-0.515174171	0.938	1	0.751439456
C19orf12	4.33E-05	-1.560235415	0.438	0.933	0.751589107
ZNF664	4.33E-05	1.275523783	1	1	0.75167213
C18orf21	4.36E-05	-2.745186101	0.312	0.6	0.756216771
ATL2	4.36E-05	1.373788448	1	1	0.756478072
ORAI1	4.40E-05	-0.960659108	0.812	0.933	0.762711892
ANKRD13C	4.40E-05	1.82336724	1	1	0.763140072
ESRRG	4.41E-05	1.510450904	1	1	0.765950669
PALS2	4.42E-05	-0.16871209	1	1	0.76756659
FBXW9	4.43E-05	-1.508146904	0.875	0.867	0.768754708
NEDD4L	4.49E-05	1.198456389	1	1	0.77846021
ENSSSCG00000057751	4.51E-05	-0.926212096	0.875	1	0.781779164
BRPF1	4.51E-05	-0.860549957	0.938	1	0.783146132
ATAD1	4.52E-05	1.602394804	1	1	0.783457849
GRK6	4.54E-05	-1.799378201	0.375	0.8	0.788492163
ENSSSCG00000005508	4.59E-05	1.587737531	1	1	0.796051599
SLC10A3	4.59E-05	-1.30256277	0.5	0.867	0.796562751
ZNF529	4.64E-05	-1.179009478	0.938	1	0.805865151
RHBDD2	4.66E-05	-2.093109404	0.312	0.8	0.808416751
YIPF1	4.72E-05	-0.868593811	1	1	0.818244495
RBIS	4.73E-05	-1.581047451	0.938	0.933	0.821555299
FBXW11	4.74E-05	1.49878359	1	1	0.822421045
TINF2	4.75E-05	-0.716931684	0.938	0.933	0.824831125
HEATR5B	4.76E-05	1.21802541	1	1	0.826324195
CHMP4B	4.78E-05	-0.519202092	1	0.933	0.829996563
SLC4A7	4.80E-05	1.504478579	1	1	0.833382314
LPIN2	4.83E-05	-0.036049216	1	1	0.83750145
HIP1R	4.83E-05	-0.72416832	1	1	0.838194973
ENSSSCG00000003261	4.83E-05	-2.415037499	0.188	0.733	0.838228413
CRLF3	4.83E-05	1.310377472	1	1	0.83831394
REEP2	4.83E-05	-2.230612928	0.438	0.733	0.838417625
AP1G2	4.83E-05	-1.536716056	0.75	0.867	0.838453046
CIART	4.84E-05	1.797883564	1	0.933	0.840398834
ENSSSCG00000011549	4.85E-05	2.27462238	1	0.8	0.840982701
NGRN	4.86E-05	-0.451254035	0.938	1	0.843309104
REEP4	4.86E-05	-0.207726082	1	1	0.843861489
SERBP1	4.87E-05	1.118702702	1	1	0.845581917
FNBP4	4.88E-05	1.667048415	1	0.933	0.846684536
RRP7A	4.90E-05	-2.382616022	0.375	0.733	0.85018905
TRIM41	4.92E-05	-0.589262638	1	1	0.853890734
ENSSSCG00000031818	4.96E-05	-1.678071905	0.625	0.933	0.860755978
RFC5	4.98E-05	-0.509487076	0.938	1	0.864883749

GNL1	5.00E-05	-0.956292715	0.812	1	0.867341974
PPP4R2	5.05E-05	1.375877194	1	1	0.87619451
ENSSSCG00000028501	5.05E-05	-3.30256277	0.188	0.533	0.876330863
CSE1L	5.07E-05	-0.4045705	1	1	0.880168077
OS9	5.08E-05	-0.440242839	1	1	0.882004682
GPR137B	5.09E-05	0.002328806	1	1	0.882648147
VWC2L	5.09E-05	-0.830074999	1	1	0.882732502
SINHCAF	5.09E-05	1.754566225	1	1	0.883260165
ENSSSCG00000003792	5.10E-05	1.982981097	1	1	0.885225281
CCBE1	5.12E-05	-0.487388344	1	1	0.887702756
ENSSSCG00000012879	5.12E-05	1.519867472	1	1	0.888195147
RASAL2	5.18E-05	1.752283694	1	1	0.897961776
CDV3	5.20E-05	-1.301508554	1	1	0.903067764
ENSSSCG00000048769	5.23E-05	-0.445094733	1	1	0.907569397
CMPK1	5.27E-05	1.400177116	1	1	0.914028765
MAPKAPK5	5.28E-05	1.829486092	1	1	0.915323501
ENSSSCG00000009578	5.28E-05	-1.117356951	0.812	0.933	0.915386192
NFKBIB	5.28E-05	-2.495207848	0.312	0.733	0.915543583
NCAPG2	5.29E-05	1.615789908	1	1	0.918704649
ENSSSCG00000000703	5.30E-05	-1.840343334	0.438	0.867	0.919040688
TTLL4	5.32E-05	-0.784837754	0.938	1	0.922828823
ARFIP2	5.35E-05	-2.934411658	0.125	0.6	0.927555152
HMG20B	5.36E-05	-1.04580369	0.875	1	0.930808579
RUFY1	5.39E-05	-0.580096746	0.938	1	0.93445473
DUS2	5.46E-05	-1.458758877	0.375	0.933	0.947929314
GFOD2	5.48E-05	-1.877380713	0.438	0.8	0.950866611
CKAP2	5.50E-05	1.487014153	1	1	0.955059829
SUCLG2	5.51E-05	1.497862546	1	1	0.955954601
SLCO6A1	5.52E-05	2.457087678	1	0.8	0.958052417
PPP1R8	5.54E-05	-0.198505182	1	1	0.961870178
PAOX	5.55E-05	-6	0	0.6	0.962383406
ENSSSCG00000060900	5.58E-05	-2.980634675	0.062	0.667	0.968106166
NOP16	5.58E-05	-1.125052297	0.938	0.933	0.968970544
ARHGAP10	5.65E-05	-0.178627476	1	1	0.980656936
TMEM70	5.66E-05	1.555071874	1	1	0.98197382
ARID5A	5.66E-05	-2.77118131	0.188	0.667	0.982632268
ARAP1	5.67E-05	-1.571156701	0.562	0.867	0.983825953
PCBD1	5.71E-05	-0.339561335	1	1	0.990596896
ENSSSCG00000009963	5.76E-05	-1.977632187	0.312	0.733	0.999038947
CLASP2	5.76E-05	1.226568581	1	1	1
NDUFA1	5.80E-05	-0.837372871	1	1	1
HSD17B4	5.82E-05	-0.276958222	1	1	1
HSDL1	5.85E-05	-0.283474743	1	1	1
RPS6KA6	5.85E-05	2.444547382	0.938	0.4	1
PIGV	5.87E-05	-0.52770917	1	1	1
ABHD17B	5.88E-05	1.723157762	1	1	1
CMTM4	5.88E-05	1.401029504	1	1	1
LIN9	5.89E-05	2.269987879	1	0.667	1
KCMF1	5.90E-05	-0.068127048	1	1	1
KLF8	5.97E-05	3.272540068	0.75	0.6	1
FAM13C	5.99E-05	1.440706477	1	1	1
ARF3	6.00E-05	-0.643306487	1	1	1
MFSD4A	6.02E-05	-0.191222442	1	1	1
TMPRSS7	6.07E-05	4.994353437	0.688	0.067	1
ZNF692	6.08E-05	-2.315501826	0.438	0.733	1
PDLIM5	6.13E-05	-0.153526646	1	1	1
MAP3K12	6.16E-05	-1.254860474	0.688	0.933	1
C8orf58	6.26E-05	-1.631529319	0.562	0.867	1
MVB12B	6.28E-05	-0.464707642	0.938	1	1
ALDH3A2	6.29E-05	-2.140415119	0.562	0.667	1
ENSSSCG00000007520	6.32E-05	-1.121964267	1	0.933	1
TIMMDC1	6.33E-05	-1.114642566	1	1	1
SEC61G	6.34E-05	-1.206671963	0.938	1	1
PPFIBP2	6.38E-05	-0.990175882	0.812	1	1
MGARP	6.42E-05	1.583000984	1	0.867	1
RANBP1	6.45E-05	-0.765223854	0.938	1	1
PANK2	6.47E-05	-0.197195037	0.938	1	1
DNAJC30	6.49E-05	-0.680801698	0.938	0.933	1
CYB5A	6.54E-05	1.413631237	1	1	1
ENSSSCG00000040577	6.55E-05	-1.693501946	0.75	0.867	1

USP54	6.56E-05	1.62324116	1	1	1
NUP93	6.56E-05	1.562409027	1	1	1
ENSSSCG00000036122	6.57E-05	-0.77685006	1	0.933	1
STARD3	6.57E-05	-2.900464326	0.125	0.667	1
SLC2A6	6.57E-05	-3.152003093	0.062	0.667	1
TRAPPC6B	6.58E-05	1.443184971	1	1	1
ENSSSCG00000032110	6.61E-05	-1.701918647	0.75	0.867	1
ENSSSCG00000048711	6.67E-05	-1.793549123	0.375	1	1
PINK1	6.68E-05	-0.83719441	1	1	1
CARS1	6.69E-05	-3.30256277	0.125	0.533	1
ENSSSCG00000063468	6.69E-05	-2.093109404	0.438	0.733	1
IRF6	6.72E-05	-0.604609743	1	1	1
MED19	6.75E-05	-1.034215715	0.875	0.933	1
MCM2	6.75E-05	1.68896422	1	1	1
CPA1	6.77E-05	-2.900464326	0.188	0.6	1
EZH2	6.77E-05	-0.047987937	1	1	1
ENSSSCG00000003348	6.79E-05	-0.73792176	0.938	1	1
RAB3A	6.81E-05	-1.279987539	0.875	0.867	1
ENSSSCG00000054958	6.83E-05	-1.536716056	0.5	0.867	1
PPT2	6.84E-05	1.834684375	1	1	1
PEPD	6.86E-05	1.806037994	1	1	1
TSPAN7	6.86E-05	-0.978938384	0.938	1	1
ORC1	6.90E-05	-0.943161996	1	1	1
PIBF1	6.94E-05	1.872264252	1	1	1
FAM83F	6.97E-05	-2.637429921	0.188	0.667	1
MAP4K3	6.97E-05	1.559619653	1	1	1
ATP13A3	6.99E-05	1.353508165	1	1	1
FREM2	7.01E-05	1.524441023	1	1	1
EEFSEC	7.04E-05	-1.427528443	0.562	0.867	1
ANXA7	7.05E-05	1.664830319	1	1	1
RIIAD1	7.05E-05	-0.411732355	1	1	1
COL14A1	7.06E-05	0.044947925	1	1	1
HNRNPA2B1	7.06E-05	0.064431873	1	1	1
ZNF652	7.10E-05	-1.454107064	0.875	1	1
TANC1	7.11E-05	1.235626162	1	1	1
ENSSSCG00000029285	7.13E-05	-0.569547448	0.938	1	1
EHD4	7.13E-05	-0.565460356	1	1	1
MRPL24	7.19E-05	-1.241501244	0.812	0.8	1
NFYB	7.19E-05	1.436083558	1	1	1
LHX6	7.24E-05	-1.145104042	0.875	0.933	1
DMAC2	7.25E-05	-1.159604816	0.875	0.867	1
CCR5	7.27E-05	-1.25214609	0.938	1	1
SMIM4	7.27E-05	-2.1740294	0.438	0.733	1
CDKL1	7.29E-05	-1.001686377	0.938	1	1
MARCHF5	7.31E-05	1.490295951	1	1	1
STPG4	7.32E-05	-2.446746359	0.188	0.8	1
TNFSF12	7.33E-05	1.846853013	1	0.933	1
ago-02	7.36E-05	1.290906069	1	1	1
RAVER1	7.38E-05	1.896203917	1	1	1
HLTF	7.39E-05	1.740260674	1	1	1
KRAS	7.41E-05	1.5212479	1	1	1
SEMA3A	7.43E-05	1.300598032	1	1	1
HMBOX1	7.44E-05	-0.363561282	1	1	1
SDCCAG8	7.45E-05	1.367562176	1	1	1
ALKBH2	7.50E-05	-0.392669686	0.938	1	1
VAPB	7.53E-05	-0.306590772	1	1	1
SMIM8	7.55E-05	-0.67189333	1	1	1
G6PD	7.56E-05	-1.169460291	0.875	0.867	1
UNG	7.58E-05	1.310224834	1	1	1
BOP1	7.59E-05	-1.750221691	0.438	0.8	1
TWSG1	7.60E-05	-1.99122979	0.562	0.8	1
ENSSSCG00000036877	7.61E-05	4.065319958	0.625	0.267	1
SEC23B	7.61E-05	-0.121203618	1	1	1
FBXO30	7.72E-05	1.273850163	1	1	1
ALPK2	7.82E-05	-0.740228381	0.938	1	1
CDK3	7.83E-05	-1.620356407	0.5	0.867	1
DKK3	7.85E-05	-1.078002512	1	1	1
MTG1	7.92E-05	-3.847996907	0.062	0.667	1
BRD2	7.98E-05	1.844785094	1	0.933	1
SNCA	7.98E-05	-0.284535475	1	1	1

TNRC6A	7.99E-05	1.357153097	1	1	1
ARAF	7.99E-05	-1.367731785	0.688	0.8	1
ENSSSCG00000059019	8.01E-05	-0.458564875	1	1	1
EXOSC9	8.10E-05	-0.273939182	1	1	1
TMEM39B	8.15E-05	-1.082617031	0.75	0.933	1
USP39	8.20E-05	-0.79406842	0.938	1	1
MACROD2	8.24E-05	-0.106915204	1	1	1
PLCD3	8.26E-05	-1.703162886	0.375	0.8	1
TMEM167A	8.27E-05	-1.221539345	0.938	1	1
CCDC136	8.30E-05	-0.53337288	0.938	1	1
ENSSSCG00000043562	8.36E-05	-0.386840607	1	1	1
POLR2E	8.38E-05	1.53843989	1	1	1
PPIL1	8.41E-05	-0.129384791	1	1	1
SPICE1	8.46E-05	1.302360391	1	1	1
SYTL5	8.46E-05	1.06536036	1	1	1
INVS	8.47E-05	-0.439667166	1	1	1
ENSSSCG00000035762	8.48E-05	-0.65846362	1	1	1
ENSSSCG00000040582	8.51E-05	-2.900464326	0.188	0.6	1
AP2M1	8.51E-05	-0.086871463	1	1	1
ENSSSCG00000005453	8.54E-05	1.425627752	1	1	1
PSMB10	8.54E-05	-2.607682577	0.25	0.6	1
CCDC107	8.55E-05	-2.72537762	0.25	0.667	1
ZNF638	8.58E-05	1.506769506	1	1	1
ENSSSCG00000042603	8.62E-05	-0.575556994	1	1	1
UBE3D	8.63E-05	-0.109621116	1	1	1
MBD6	8.66E-05	-3.462343214	0.125	0.6	1
NUBP1	8.69E-05	-0.346001385	1	1	1
TMEM38B	8.70E-05	2.323381692	0.938	0.667	1
ENSSSCG00000054639	8.73E-05	-1.495207848	0.562	0.867	1
ZNF329	8.74E-05	-0.786596362	1	1	1
EYA1	8.75E-05	1.271463028	1	1	1
DOHH	8.76E-05	-0.811927652	0.875	0.867	1
BMS1	8.78E-05	-0.094617711	1	1	1
CNDP2	8.79E-05	-0.891150575	0.938	1	1
ENSSSCG00000063338	8.83E-05	1.889042594	1	0.867	1
ENSSSCG00000057940	8.85E-05	-1.116193018	0.75	0.867	1
PPEF1	8.90E-05	-0.699109895	0.938	1	1
CABP1	8.92E-05	-1.916231642	0.375	0.8	1
NDUFS6	8.92E-05	-1.656045599	0.562	0.867	1
ENSSSCG00000046083	8.92E-05	-1.109788146	0.875	1	1
SLC35A4	8.98E-05	-1.604071324	0.5	0.733	1
PPCS	8.98E-05	-0.275973462	0.938	1	1
PSRC1	9.02E-05	-0.613941568	1	1	1
NUP160	9.05E-05	1.2242826	1	1	1
GPR39	9.14E-05	-0.490180357	1	0.933	1
NXT2	9.15E-05	1.155449679	1	1	1
RCN3	9.15E-05	-2.552541023	0.312	0.533	1
HDAC11	9.16E-05	-2.285754482	0.188	0.8	1
STUB1	9.16E-05	-1.538909157	0.562	0.8	1
OTUD5	9.21E-05	-0.301759513	1	1	1
ENSSSCG00000063033	9.22E-05	1.539434161	1	1	1
KIN	9.24E-05	-0.219781208	0.938	1	1
GLS	9.29E-05	1.32042607	1	1	1
ENSSSCG00000002790	9.31E-05	-2.1655179	0.562	0.933	1
SNX17	9.37E-05	-0.654688978	1	1	1
UPF1	9.37E-05	1.645416704	1	1	1
MCM4	9.41E-05	1.573073346	1	1	1
ENSSSCG00000004806	9.43E-05	1.25409107	1	1	1
PPARG	9.49E-05	-0.384029934	1	1	1
ERMP1	9.49E-05	1.370145426	1	1	1
LARS1	9.55E-05	-0.342993704	1	1	1
ENSSSCG00000057441	9.56E-05	4.579315938	0.75	0.067	1
ENSSSCG00000049247	9.60E-05	-1.093109404	0.875	0.933	1
GEMIN7	9.61E-05	-3.462343214	0.062	0.533	1
INTS12	9.66E-05	-0.250980726	1	1	1
SCYL1	9.69E-05	-0.738014446	0.875	0.933	1
CTIF	9.74E-05	-2.192645078	0.25	0.667	1
FAM76B	9.82E-05	1.603142517	1	1	1
DMXL1	9.84E-05	1.226802658	1	1	1
CCN6	9.86E-05	-1.701918647	0.5	0.8	1

MOV10	9.87E-05	-0.99034983	0.875	1	1
HNRNPA1	9.89E-05	1.312182931	1	1	1
PIGU	9.93E-05	-0.16923585	1	1	1
PNISR	9.98E-05	1.467346923	1	1	1
CNEP1R1	0.000100492	1.577304997	1	0.933	1
CTU2	0.000100947	-1.607682577	0.5	0.867	1
LRRC8C	0.000102045	1.321928095	1	1	1
MSANTD3	0.00010237	-1.048255169	0.875	1	1
ENSSSCG00000056164	0.000102457	-0.363910693	0.938	1	1
JMJD6	0.000102699	1.909969989	1	0.867	1
ENSSSCG00000041572	0.000102748	2.845490051	0.938	0.4	1
SNX27	0.000103047	1.212604914	1	1	1
ELOVL4	0.000103461	1.459021704	1	1	1
SIRT6	0.000103626	-1.334117504	0.562	0.933	1
ENSSSCG00000034966	0.00010405	-1.734655433	0.562	0.733	1
ROR1	0.000104176	3.154818109	0.875	0.267	1
C2orf74	0.00010456	-0.747218066	1	1	1
NOS2	0.000105138	-1.499101764	0.688	0.8	1
ATP6V1B2	0.000105199	0.003425934	1	1	1
VIPAS39	0.000105593	-0.425185455	1	1	1
LHFPL6	0.000105897	-0.119052672	1	1	1
PARD6G	0.000106085	1.742079726	1	0.933	1
SEC61B	0.000106327	1.597206096	1	1	1
NCCRP1	0.000106348	-0.589785373	0.938	1	1
ENSSSCG00000053304	0.000106665	-1.267138804	0.562	1	1
ENSSSCG00000059681	0.000106674	-0.508146904	1	1	1
DPY19L3	0.000106757	1.899937835	1	0.8	1
CLCN3	0.00010726	1.234796214	1	1	1
CEP76	0.000107371	1.844705764	1	1	1
IPPK	0.000107676	-0.242165214	1	1	1
SELENOW	0.000107677	-0.717600269	1	1	1
KDM1A	0.000107784	1.40284809	1	1	1
ASIC1	0.000107808	-1.394278939	0.562	0.8	1
NFIB	0.000108014	-0.379482452	1	1	1
TBC1D25	0.000108556	-1.762960803	0.562	0.867	1
ENSSSCG00000033395	0.000108665	2.115477217	1	0.867	1
RBM45	0.000108721	2.141355849	1	0.867	1
ENSSSCG00000057847	0.000109467	-1.175571565	0.625	1	1
FLT3	0.000109679	1.607330314	1	1	1
CD28	0.000109771	-0.87435556	1	0.933	1
POLR2B	0.000110199	-1.264068225	1	1	1
ENSSSCG00000061752	0.000110284	1.450226001	1	1	1
ZNF577	0.000111227	1.521680663	1	1	1
U2AF1	0.000111502	1.600519782	1	1	1
TRAF1	0.00011157	-2.208586622	0.25	0.733	1
ZFAND2B	0.000112298	-1.777607579	0.438	0.8	1
CDC25A	0.000112309	-0.313046174	1	1	1
GNA14	0.00011272	-0.705038953	0.812	1	1
RXRG	0.000112739	1.666981692	1	1	1
CYCS	0.000112817	0.022983534	1	1	1
SNAP29	0.00011318	-1.058343986	0.688	1	1
ABHD12	0.000113338	-1.200024608	0.75	0.933	1
ENSSSCG00000062032	0.000113954	-3.093109404	0.125	0.533	1
AP1AR	0.000115037	1.454168621	1	0.933	1
MED23	0.000115513	1.501754954	1	1	1
NDUFB6	0.000115926	-0.954403134	0.938	1	1
RSPO2	0.000116395	-0.156946143	1	1	1
CNIH1	0.000116433	1.453490852	1	1	1
FSD1	0.000116586	-0.941106311	0.812	0.867	1
TRIR	0.00011704	-2.05246742	0.438	0.733	1
SOCS5	0.000117047	-0.307428525	1	1	1
ENSSSCG00000047468	0.000117267	-1.093109404	0.75	1	1
KRT25	0.000117414	-3.595609745	0.062	0.6	1
NCKAP1L	0.000118419	-0.262109305	1	1	1
RPS3	0.000118778	-0.571308924	1	1	1
SEC23IP	0.000118836	1.526893444	1	1	1
GPT2	0.000118937	1.316994502	1	1	1
SIGIRR	0.00011913	-3.485426827	0.062	0.733	1
CSMD3	0.000119507	1.366371427	1	1	1
CEP295	0.000119764	1.321115538	1	1	1

CD302	0.00012093	-1.508146904	0.688	0.867	1
CDH3	0.000121388	1.636089307	1	1	1
SLC25A5	0.00012171	-0.188820604	1	1	1
ENSSSCG00000052597	0.000122773	3.22881869	0.812	0.2	1
METTL13	0.000122903	-1.326599535	0.688	0.933	1
CCDC61	0.000123081	-1.47498004	0.625	0.933	1
TIPARP	0.00012319	1.961429872	1	0.867	1
RPL22	0.000123545	-1.422173681	1	1	1
CHAC1	0.000123787	-0.626915392	1	1	1
MTX1	0.000123886	-2.315501826	0.312	0.733	1
ENSSSCG00000027428	0.000124	-4.678071905	0	0.533	1
ENSSSCG00000046886	0.000124037	-0.714100214	0.938	1	1
ITGA11	0.00012452	-0.659740082	1	1	1
ENSSSCG00000062439	0.00012455	-0.846194664	1	0.933	1
F2R	0.00012488	3.233183883	1	0.6	1
ENSSSCG00000008899	0.000125124	-1.155393683	0.75	0.8	1
HEPACAM2	0.000125369	2.861086906	0.812	0.533	1
AFF1	0.000125478	-0.389648718	1	1	1
ENSSSCG00000061331	0.000126219	-0.756826017	0.875	1	1
PARP12	0.000126862	1.492625045	1	1	1
MPPED1	0.000127003	-0.6405972	0.875	1	1
MRPL41	0.000127106	-1.235128409	0.562	1	1
AVL9	0.000127489	1.131583966	1	1	1
GSTCD	0.000127808	1.456630623	1	1	1
DPYSL2	0.000128498	-0.729673102	0.938	1	1
MYO1B	0.000129246	1.178727157	1	1	1
FAM210A	0.000129932	-0.267899297	1	1	1
ACO2	0.000130072	-0.72603723	1	1	1
NDUFB5	0.000130124	-0.582549294	1	1	1
APPL2	0.000130513	1.336100342	1	1	1
ASNS	0.000130694	-0.369871017	1	1	1
ENSSSCG00000058013	0.000131035	-4.61667136	0	0.6	1
REPS1	0.000131486	1.363073115	1	1	1
ACCS	0.000132065	-2.171111916	0.312	0.667	1
STOML3	0.000132217	-1.462343214	0.625	0.933	1
XDH	0.000132477	-1.330148602	0.688	0.933	1
COL19A1	0.000133089	2.691722501	1	0.733	1
ENSSSCG00000060497	0.000133118	2.60249716	0.875	0.333	1
DNAJB12	0.000133464	-1.188266637	0.812	0.867	1
ZNF140	0.00013348	1.352393376	1	1	1
ENSSSCG00000040389	0.000133702	1.584438931	1	1	1
ENSSSCG00000040707	0.00013373	-2.125530882	0.312	0.8	1
TP53RK	0.000135136	-0.347050662	1	1	1
CORIN	0.000135278	-0.42241703	1	1	1
LEF1	0.000136039	1.447265262	1	1	1
IGSF3	0.000136182	-0.921997488	0.75	0.933	1
PLIN2	0.00013622	1.472895197	1	1	1
COBLL1	0.000136475	1.384074944	1	1	1
ABCB7	0.000136505	0.06211615	1	1	1
POLR3K	0.000136928	1.416362897	1	1	1
TCTN3	0.000137277	-0.511899039	1	0.933	1
SLC46A2	0.00013728	-0.118077485	1	1	1
ING3	0.000137526	1.544320516	1	1	1
ENSSSCG00000062067	0.000137771	-2.578536232	0.25	0.6	1
GGT6	0.000139195	-0.688407922	1	1	1
GLIS3	0.000139764	1.440542744	1	1	1
ALKBH5	0.000140193	-0.66195224	1	1	1
LRRC34	0.000140642	2.607330314	1	0.667	1
MLX	0.000142625	1.484933682	1	1	1
CLDN4	0.000142671	-1.40053793	0.625	0.8	1
UIMC1	0.00014317	-0.519000729	0.938	1	1
PRPSAP2	0.000143323	1.452364079	1	1	1
MAP4K2	0.000143502	-1.502500341	0.75	0.8	1
NAT9	0.000143869	-2.77118131	0.25	0.6	1
MSMO1	0.000144187	1.564790165	1	1	1
LEO1	0.000144569	-0.093109404	1	1	1
RHCG	0.000144943	-1.813001485	0.438	0.867	1
PAFAH1B2	0.000145015	1.277214871	1	1	1
ENSSSCG00000032016	0.000145813	-0.405917121	1	1	1
BDH1	0.000146158	-1.161280907	0.688	0.867	1

IQGAP1	0.000146715	1.407788832	1	1	1
KIAA2012	0.000147109	-1.360589715	0.812	0.867	1
ENSSSCG00000042533	0.000147266	-2.019108823	0.312	0.8	1
CASTOR1	0.000148126	1.92270572	1	1	1
H2AC1	0.000149959	-1.032988412	0.812	1	1
XPNPEP3	0.00015088	2.009857175	1	0.933	1
ENSSSCG00000057950	0.00015096	-0.699766976	1	1	1
ALOX15	0.000151932	-2.651104858	0.312	0.533	1
YAP1	0.000152191	1.360699139	1	1	1
TERF1	0.000152254	1.381748181	1	1	1
ENSSSCG00000006679	0.000152538	-0.789055208	0.938	1	1
DCP1A	0.00015316	1.416525846	1	1	1
ADGB	0.000153897	5.299208018	0.625	0.067	1
CMTM7	0.000154415	2.147699083	1	0.933	1
LCA5	0.000154858	1.586690225	1	1	1
RASL10B	0.000154993	-2.629162305	0.188	0.667	1
AKAP8	0.00015508	-0.197639988	1	1	1
IL6ST	0.000155158	-0.906373504	1	1	1
CALU	0.00015537	-0.084108604	1	1	1
AP2A2	0.000155719	-1.732519689	0.625	0.867	1
JCHAIN	0.000156012	-0.490701769	1	1	1
FH	0.00015623	-0.126740182	1	1	1
DGKZ	0.000157049	-1.040641984	0.688	0.867	1
ENSSSCG00000010161	0.000157272	-1.462343214	0.812	1	1
KLHL11	0.000157458	1.391627963	1	1	1
SLC38A1	0.000157716	-2.171111916	0.625	0.867	1
ENSSSCG00000060134	0.000158563	2.000402482	1	0.867	1
CYBC1	0.000158823	-1.138913094	0.75	0.867	1
GPR137	0.000159104	-1.607682577	0.562	0.8	1
MGME1	0.000159454	-0.561364873	1	1	1
RNF7	0.000159665	1.724747642	1	1	1
INPP5B	0.000160237	-0.309872656	1	1	1
KIF5C	0.000160511	-0.303768197	0.938	1	1
DEDD	0.00016067	-0.076586496	1	1	1
XPOT	0.00016145	1.571798324	1	1	1
BZW1	0.000162194	1.275076137	1	1	1
ENSSSCG00000046452	0.00016284	-1.923184403	0.375	0.8	1
ENSSSCG00000048017	0.000163843	-0.537894247	1	1	1
TMEM106B	0.000164559	1.50463037	1	1	1
NDUFAF5	0.000165106	-1.073209847	0.812	1	1
CTSK	0.000165205	1.878667436	1	1	1
SCAF11	0.000165268	1.597423016	1	1	1
ENSSSCG00000053738	0.000165533	-1.476438044	0.625	0.867	1
KIF3C	0.000165732	-0.417460162	1	0.933	1
ENSSSCG00000027988	0.000166973	-2.125530882	0.375	0.733	1
C1D	0.000168664	1.865311492	1	1	1
RBMS3	0.000168773	1.45571931	1	1	1
AMPD3	0.000170467	1.26388081	1	1	1
CAMKK2	0.00017196	-1.058062457	0.875	1	1
PTDSS2	0.000172685	-1.629162305	0.375	0.8	1
UNC5B	0.000173062	2.427722759	1	0.533	1
SLC35C2	0.000173481	-1.020959619	0.75	0.867	1
ENSSSCG00000054543	0.000173554	-1.113358088	0.938	0.933	1
CLTRN	0.0001737	1.782008845	1	0.933	1
DDX19A	0.000174178	-0.208034394	1	1	1
ENSSSCG00000037376	0.000174347	-1.03170886	0.5	0.933	1
LAS1L	0.000174397	-0.418648753	1	1	1
TMEM115	0.000174398	-3.047305715	0.188	0.533	1
RPIA	0.000175297	1.381162862	1	1	1
MOGAT1	0.000175342	-0.496377127	1	1	1
S100A11	0.000176223	-0.932356049	1	1	1
TREH	0.000176362	-2.315501826	0.312	0.6	1
ABT1	0.00017685	1.431989969	1	1	1
MPZL2	0.000176914	-0.594462472	1	1	1
ENSSSCG00000039407	0.00017727	-4.61667136	0	0.6	1
LTO1	0.000177887	-1.886658527	0.5	0.733	1
POGLUT2	0.000178744	2.208306093	1	0.867	1
NR1H3	0.000179224	-0.695213038	0.938	0.933	1
VTCN1	0.00017931	1.688889943	1	1	1
ENSSSCG00000043136	0.000179678	-1.299560282	0.625	0.867	1

ARHGAP18	0.000179919	1.390112493	1	1	1
PSMD5	0.000180227	1.285948798	1	1	1
PIK3R3	0.000181703	0.103754312	1	1	1
ENSSSCG00000014043	0.000182126	-3.263034406	0.125	0.6	1
MSH3	0.000183093	1.305354197	1	1	1
ENSSSCG00000043245	0.000184512	-1.595609745	0.438	0.733	1
EPN2	0.000185551	-0.402793904	1	1	1
SPINDOC	0.000187013	2.054232312	1	0.933	1
CROCC	0.00018776	-2.074962058	0.562	0.867	1
ZNF827	0.0001878	2	1	0.667	1
MCU	0.000188399	1.352629134	1	1	1
PLEKHA8	0.000188808	1.335943827	1	1	1
CEND1	0.00018881	-2.61667136	0.125	0.667	1
PSME4	0.00019068	1.254241287	1	1	1
HEATR6	0.000191499	1.379289342	1	1	1
GTPBP3	0.000191706	-1.297467903	0.5	0.867	1
GNG10	0.000192024	1.513403774	1	1	1
ENSSSCG00000024791	0.000192895	-0.545331824	1	1	1
KIF15	0.00019403	1.396089526	1	1	1
PIGF	0.000194115	1.868168598	1	1	1
NT5E	0.000194386	1.938368827	1	0.933	1
SCO2	0.000194416	-4.61667136	0	0.533	1
MKRN2	0.000195079	-0.769954006	0.938	1	1
ENSSSCG00000033766	0.000195283	-1.375509135	0.75	0.8	1
MBD5	0.000195706	1.671221089	1	1	1
PDAP1	0.000196443	-0.678071905	1	0.933	1
ENSSSCG00000059543	0.000196893	-0.939758627	0.812	1	1
BACE1	0.000196953	-2.651104858	0.25	0.533	1
ENSSSCG00000059729	0.000197345	-1.704544116	0.562	0.667	1
ENSSSCG00000057663	0.000197539	-0.647845118	1	1	1
ENSSSCG00000010888	0.000197663	-0.844081857	1	0.933	1
PTDSS1	0.000198039	-0.585757155	0.938	1	1
HMGN1	0.000198428	1.167950059	1	1	1
TBCEL	0.000198552	-0.143685712	1	1	1
SCN1A	0.000198672	1.712604537	1	1	1
ENSSSCG00000059603	0.000198791	1.526846702	1	1	1
PDS5A	0.000199009	1.17658648	1	1	1
NDC80	0.000199749	1.335046187	1	1	1
HEY1	0.000201355	1.450032921	1	1	1
ZWINT	0.000201381	1.669521073	1	1	1
DHRS13	0.000201534	-3.03170886	0.125	0.533	1
TPD52	0.000202108	-1.325770161	0.75	0.867	1
RMI2	0.000202332	-1.479167837	0.812	0.867	1
PCLAF	0.000202605	-2.584962501	0.188	0.733	1
SCFD2	0.000202813	-0.267652301	1	1	1
CCDC34	0.000203161	1.275592872	1	1	1
L2HGDH	0.00020326	-2.132637769	0.375	0.733	1
ZNF436	0.000204118	-0.119391869	1	1	1
DNAJC24	0.000206071	1.705977902	1	0.867	1
HNRNPR	0.000206285	1.251665759	1	1	1
IKZF4	0.000206872	2.581490309	0.938	0.467	1
ZDHHC6	0.000207053	1.655897749	1	0.933	1
GPBP1	0.000208	-0.168581373	1	1	1
LCA5L	0.000208484	-2.285754482	0.25	0.667	1
LPAR2	0.000208754	-1.805827452	0.562	0.8	1
RNF216	0.00020914	1.506543739	1	1	1
CCNC	0.000209177	2.197109831	1	0.733	1
RWDD3	0.000209228	-1.389350856	0.812	1	1
SETD6	0.000209347	-0.674156855	0.938	1	1
C5orf63	0.000210984	3.116343961	0.812	0.333	1
ENSSSCG00000050380	0.000211134	1.445932764	1	1	1
PMS1	0.000211815	1.599593156	1	1	1
RPP21	0.000211841	-0.872609042	1	1	1
ENSSSCG00000038327	0.000212721	-0.525189388	0.938	1	1
TRNAU1AP	0.000212824	1.63610669	1	1	1
ZC3H14	0.000213595	1.190448172	1	1	1
SYCP2	0.000214069	1.444596971	1	1	1
MAPK14	0.000214924	1.396084726	1	1	1
F13B	0.000215791	-0.600995682	0.875	1	1
RYK	0.000216761	1.50902824	1	1	1

RNPC3	0.00021795	1.181421931	1	1	1
DAAM2	0.000218448	-1.330148602	0.75	0.867	1
SLC2A4	0.00021874	-2.678071905	0.188	0.667	1
BEX3	0.000219957	-1.163498732	0.75	1	1
TXNDC11	0.000220694	2.362342363	1	1	1
POGK	0.000220761	1.243925583	1	1	1
HAS1	0.000221501	2.321928095	1	0.867	1
LLGL1	0.000221691	2.266360653	1	1	1
NEO1	0.000221892	1.171405448	1	1	1
UBR5	0.000222538	1.176444195	1	1	1
PEF1	0.000222615	1.53466618	1	0.933	1
CDIN1	0.00022345	1.287487318	1	1	1
ENSSSCG00000061064	0.000223816	-1.15787966	0.812	0.933	1
NSRP1	0.000224956	0.053905625	1	1	1
ASTN2	0.000225324	2.032146548	1	1	1
IDE	0.000225538	1.323269335	1	1	1
ERN1	0.000225718	1.268445765	1	1	1
ZNF398	0.000226557	-0.011639916	1	1	1
ANKRD46	0.000227368	1.763449516	1	1	1
SH3GLB2	0.000227622	-1.180572246	0.688	0.933	1
GAB2	0.000227958	-0.941106311	0.875	1	1
MRPL10	0.000228643	-1.361361519	0.625	0.933	1
PGD	0.000229152	1.498021105	1	1	1
DGUOK	0.000229399	1.80743568	1	0.933	1
CDK9	0.000229845	1.904531323	1	1	1
C4orf3	0.000229979	1.692078629	1	1	1
USP31	0.000230126	-0.645650427	0.875	1	1
GPLD1	0.000230794	-0.531656588	1	1	1
FBXW5	0.000231456	-4.30256277	0.062	0.533	1
MYL3	0.000231608	-1.818934441	0.5	0.733	1
MBTD1	0.000232555	1.393277939	1	1	1
ASAH1	0.000232649	-0.192514393	1	1	1
DLEC1	0.000233631	-0.122809639	1	1	1
ACP7	0.000234735	-1.73947245	0.625	0.867	1
XIAP	0.000235501	1.191168157	1	1	1
FTO	0.000236453	2.906890596	0.875	0.6	1
TM7SF2	0.000236911	-2.9510904	0.062	0.6	1
ENSSSCG00000039635	0.000237551	-2.366127899	0.125	0.733	1
KHDC4	0.000238037	-0.381752908	1	1	1
ENSSSCG00000063116	0.000238186	-0.07221418	0.938	1	1
CLPX	0.000239289	1.154140542	1	1	1
CD2BP2	0.000241659	-0.848305736	1	1	1
ENSSSCG00000057760	0.000241823	1.544120406	1	1	1
SIK2	0.000243004	-0.197217808	1	1	1
TDRD7	0.000243336	1.120529181	1	1	1
YJU2	0.00024335	-0.853921741	0.938	0.933	1
WBP1L	0.000243351	-1.479167837	0.812	0.867	1
C19orf54	0.00024336	-0.592098682	0.938	1	1
IREB2	0.000244702	1.513375895	1	1	1
CAP1	0.000244995	-0.393011193	1	1	1
NR3C1	0.000245576	-0.107629822	1	1	1
RAB11FIP4	0.000246384	-1.843131151	0.25	0.8	1
DBX2	0.000246825	-0.09683249	1	1	1
AP3S1	0.000247361	1.631286094	1	1	1
SBK1	0.000248073	-2.61667136	0.188	0.6	1
ARHGEF1	0.000248911	-0.845799351	0.875	0.8	1
TCAIM	0.00025006	-0.160814264	1	1	1
ARID2	0.000250568	1.198586195	1	1	1
SCML2	0.000251565	1.390863713	1	1	1
ENSSSCG00000049125	0.000252136	-0.075298128	1	1	1
TXNL4B	0.000252509	-0.585223127	0.875	1	1
ENSSSCG00000016107	0.000252847	-0.752684966	0.938	1	1
WDR25	0.000253733	-1.334117504	0.625	0.933	1
FRZB	0.000254755	-0.399480218	1	1	1
KRTCAP2	0.000255551	-1.409442036	0.875	1	1
CFAP61	0.000255601	-1.006952761	0.938	0.867	1
ENSSSCG00000058505	0.000257219	-1.016147423	0.875	0.933	1
KDM4C	0.000257924	1.725147547	1	0.867	1
DXO	0.000258246	-4.415037499	0	0.6	1
EXT1	0.000259196	-0.133207473	1	1	1

ENSSSCG00000055752	0.000260001	-1.150442579	0.688	0.933	1
ARHGEF18	0.000260194	-1.263034406	0.625	0.867	1
ENSSSCG00000056225	0.000261168	-1.595609745	0.5	1	1
GIPC2	0.000261602	-0.248659384	1	1	1
NFKB1	0.000261642	1.534202449	1	1	1
AKT1S1	0.00026174	-1.730539325	0.312	0.867	1
ENSSSCG00000052841	0.000262114	-0.764160882	1	1	1
FEN1	0.000262486	-0.268476491	1	1	1
TMC4	0.000264726	-1.131583552	0.938	0.867	1
ZNF74	0.000266246	-1.346865997	0.688	0.867	1
LYPD6B	0.000266795	-2.135278859	0.625	0.8	1
OR8S1	0.000268853	-1.133186844	0.688	0.933	1
CCSAP	0.000269807	1.563614322	1	1	1
CMTR2	0.000270367	1.529671646	1	0.867	1
GPR32	0.000270381	-1.308377391	0.938	0.933	1
ESF1	0.000270519	1.360889987	1	1	1
ENSSSCG00000028717	0.000271781	-1.169058258	0.688	0.933	1
KIAA0586	0.00027196	1.252072263	1	1	1
PRRT1B	0.000272442	-0.196000819	1	1	1
ENSSSCG00000063078	0.000274314	-2.366127899	0.062	0.667	1
NOBOX	0.000274455	-0.58435747	1	1	1
CPLANE1	0.000274909	1.171804289	1	1	1
CISD3	0.000274926	-3.137503524	0.125	0.6	1
ENSSSCG00000059138	0.000275304	-0.967578522	0.75	0.933	1
NDUFAF3	0.000275482	-1.517607233	0.625	0.8	1
DAP3	0.000275903	-0.444908029	1	0.933	1
NCOA4	0.000276086	0.023297249	1	1	1
ANP32B	0.000277572	1.424558259	1	1	1
LMAN2L	0.000277862	-1.911271081	0.375	0.667	1
ACAA2	0.000279905	-1.497499659	0.5	0.8	1
VPS35	0.000280546	1.364321168	1	1	1
ENSSSCG00000042029	0.00028063	-1.169058258	0.75	1	1
ACADS	0.000282663	-1.626541604	0.375	0.667	1
GEMIN4	0.000283082	-0.044813847	1	1	1
ENSSSCG00000045343	0.00028365	-1.558772977	0.688	0.933	1
MED27	0.000284188	1.472774004	1	1	1
ENSSSCG00000008769	0.000284721	-4.341036918	0	0.6	1
PIK3C2A	0.000284763	1.445955047	1	1	1
RBM23	0.000286378	-0.705293374	0.938	1	1
RBBP9	0.000287207	-1.72782494	0.625	0.733	1
HORMAD1	0.000287396	1.459237591	1	1	1
HNRNPH1	0.000287536	1.641366638	1	1	1
RP9	0.000288198	-0.798302653	0.938	1	1
ADIPOR1	0.000288959	-0.213224688	1	1	1
FANCG	0.000289129	1.637530552	1	0.933	1
SNRNP35	0.000289455	-0.545046486	0.812	1	1
SLC30A6	0.000290525	1.872889924	1	1	1
ENSSSCG00000030015	0.000290733	1.558721664	1	0.933	1
ST6GALNAC4	0.000291467	-2.629162305	0.188	0.6	1
RNF122	0.00029179	-0.539388814	1	1	1
ENSSSCG00000062841	0.000292238	-1.298223834	0.812	0.933	1
FBXO21	0.000294864	1.074460176	1	1	1
BLOC1S4	0.000294955	-1.311014435	0.875	0.867	1
ELL	0.000296536	-0.163213643	1	1	1
ACOT8	0.000297106	-1.730539325	0.375	0.867	1
ENSSSCG00000053955	0.00029721	1.590917224	1	1	1
ANO5	0.000300394	2.346759948	1	0.733	1
CETN2	0.000300735	1.268115048	1	1	1
XXYLT1	0.000302717	-1.27368165	0.562	0.733	1
ENSSSCG00000028159	0.000305069	-0.600646933	0.875	1	1
STN1	0.000305186	-1.076989739	0.688	0.8	1
WDFY4	0.000306248	2.89112328	0.938	0.533	1
CD3E	0.000306569	-1.607682577	0.438	0.733	1
BCR	0.00030726	-0.809861778	0.938	0.867	1
EFCAB12	0.000308799	-1.601256308	0.75	0.933	1
ENSSSCG00000048408	0.000309219	-1.678071905	0.375	0.8	1
RBL2	0.000309822	1.254303662	1	1	1
DKK4	0.000310125	-1.644124573	0.625	0.733	1
GPAA1	0.000310702	-1.967578522	0.438	0.8	1
SLC44A1	0.000310999	2.440322796	0.875	0.467	1

RAB35	0.000313255	-0.199394198	1	1	1
LLPH	0.000313721	-0.294865533	1	1	1
HAUS8	0.000314327	-0.568237354	1	1	1
MIER3	0.000314501	1.294271752	1	1	1
CAMLG	0.00031501	-0.073209847	1	1	1
PPIP5K2	0.000316745	1.430521133	1	1	1
SELENOO	0.000318099	-4.485426827	0	0.533	1
ENSSSCG00000055918	0.000318587	-1.30256277	0.625	0.933	1
ENSSSCG00000047248	0.000321623	2.237807474	0.875	0.667	1
ENSSSCG00000063044	0.000321717	-3.152003093	0.062	0.667	1
PTPN5	0.000321826	1.679604432	1	1	1
ENSSSCG00000057278	0.000322263	-4.485426827	0	0.533	1
ENSSSCG00000031083	0.000322687	-0.983484913	0.688	0.867	1
ENSSSCG00000061676	0.000323174	-2.093109404	0.25	0.667	1
SMC4	0.000323841	1.540549201	1	1	1
C18orf54	0.000323955	-1.649159213	0.688	1	1
ENSSSCG00000054866	0.000324164	-1.438884241	0.562	0.867	1
ENSSSCG00000050152	0.000325005	-0.856409146	0.938	1	1
ZDHHC13	0.000325052	1.589025149	1	1	1
ENSSSCG00000053718	0.000325064	2.087956155	0.938	0.4	1
HRH1	0.000325435	-1.773229138	0.688	0.933	1
DGKK	0.00032663	-0.317750766	1	1	1
MYH11	0.000327135	-0.285196056	1	1	1
CHRNA3	0.000327787	0.033667713	1	1	1
AMPD2	0.000328004	2.162953041	1	0.867	1
RBM25	0.00032861	-0.074936658	1	1	1
TNPO1	0.000328992	1.288719693	1	1	1
JKAMP	0.000329965	-0.372041437	1	1	1
VPS51	0.000330785	-1.42281485	0.562	0.867	1
SIDT2	0.000332603	-0.720450507	1	0.867	1
CFAP97	0.000333337	1.410404895	1	1	1
CHI3L1	0.000334352	-1.629162305	0.438	0.8	1
ENSSSCG00000016810	0.000334888	1.130190658	1	1	1
DCLK3	0.000337034	2.888743249	0.875	0.4	1
RIOX2	0.000337126	-0.218229204	1	1	1
OXTR	0.000338464	-0.608874395	0.875	1	1
SMIM14	0.000338836	1.403136421	1	1	1
RLF	0.000340558	1.237349154	1	1	1
TMEM242	0.000341061	-0.149596596	1	1	1
ATL3	0.000341409	1.160464672	1	1	1
CDC42SE1	0.000342563	1.31585286	1	1	1
GCN1	0.000343661	1.714245518	1	0.933	1
KIF23	0.000343829	1.08448661	1	1	1
DDIT4	0.000345571	-1.830074999	0.5	0.667	1
GABRA3	0.00034611	-0.430260322	1	1	1
FYB1	0.000347067	3.491853096	0.75	0.2	1
VPS11	0.000348657	-0.617445546	0.938	1	1
ENSSSCG00000049664	0.000349292	-1.220864952	0.688	0.867	1
TARS1	0.000349307	1.12654562	1	1	1
PCM1	0.000349857	1.30168359	1	1	1
ITSN2	0.00035234	1.398066964	1	1	1
PLAUR	0.00035235	2.349408831	1	0.8	1
CNOT6	0.000353657	1.316751061	1	1	1
CPEB4	0.000354003	1.296194126	1	1	1
PRKX	0.00035485	-0.967578522	0.75	0.867	1
MACROH2A1	0.00035534	-0.352113933	1	1	1
ENSSSCG00000062072	0.000357302	-1.093109404	0.688	0.867	1
CWC25	0.00035741	-0.20744208	1	1	1
MED30	0.000357645	1.371215033	1	1	1
CAMK2N2	0.00035907	-4.341036918	0	0.667	1
OCEL1	0.000360794	-1.526068812	0.438	0.667	1
CHMP2B	0.000361744	1.397004038	1	1	1
ATG2B	0.000361881	1.217067205	1	1	1
SLC12A5	0.000362223	1.693275744	1	1	1
ISY1	0.000363018	1.781833116	1	1	1
ENSSSCG00000057519	0.000363805	-0.814573905	0.875	0.933	1
SRPRA	0.000366089	-0.577952263	1	1	1
ENSSSCG00000061564	0.000366537	-1.109169255	0.812	1	1
MFSD14A	0.000367865	1.49739128	1	1	1
PIGW	0.000370598	-1.119581616	0.938	0.933	1

GPKOW	0.000370642	-1.517607233	0.562	0.8	1
ENSSSCG00000043636	0.000371579	-0.803602787	0.5	1	1
PCMTD1	0.00037235	1.373912443	1	1	1
OTUB2	0.000372551	-1.489999557	0.562	0.8	1
NT5C3A	0.000372641	-0.724546335	0.938	1	1
UBIAD1	0.000373253	1.547268264	1	1	1
FAM13B	0.000373401	1.314575114	1	1	1
DNAL1	0.000373509	1.496702496	1	1	1
GET3	0.000373922	-2.736965594	0.188	0.533	1
EOMES	0.000373932	1.920965781	1	0.667	1
E2F6	0.000374073	-3.180572246	0.125	0.533	1
STEAP2	0.00037491	1.862496476	0.938	1	1
FGF9	0.000375278	-0.443711808	1	1	1
NFE2L2	0.000375971	1.471247465	1	1	1
ZNF45	0.000376517	-0.670997379	0.938	1	1
TBX18	0.000376647	1.682901389	1	1	1
GNG12	0.000376898	-0.028665696	1	1	1
ENSSSCG00000050581	0.000377726	-3.9510904	0.062	0.6	1
RALGDS	0.000377967	-1.053849974	0.875	1	1
ZFAND5	0.000379471	1.139399817	1	1	1
SYTL4	0.000382978	1.47972265	1	1	1
ENSSSCG00000039538	0.000383518	-1.074962058	0.5	0.867	1
HOMER2	0.000385387	-0.220148593	1	1	1
ANGPTL6	0.000385928	-4.341036918	0	0.6	1
SORBS1	0.000386136	-0.217500267	1	1	1
YBX1	0.000386727	1.698665506	1	1	1
ENSSSCG00000060173	0.000386945	-0.757060201	0.812	1	1
NES	0.000387117	-3.197446064	0.188	0.533	1
ACRV1	0.000387442	-2.526068812	0.188	0.6	1
STX4	0.000394128	-1.388565288	0.562	0.733	1
AGFG2	0.000394485	-1.241208043	0.688	0.8	1
BBS2	0.000397272	-0.088196007	1	1	1
DNAJC8	0.000398465	0.067717539	1	1	1
DEF6	0.000398568	-1.205002284	0.75	0.933	1
BCL2L14	0.000398604	0.006086772	1	1	1
ZNF268	0.000398705	-2.495207848	0.188	0.533	1
ENSSSCG00000006738	0.000398946	-1.341036918	0.75	0.8	1
DICER1	0.00039931	1.451875795	1	1	1
ALDH4A1	0.000399511	-0.919777802	0.688	1	1
CETN3	0.000399677	1.627112926	1	1	1
HOMEZ	0.000400312	-0.093109404	1	1	1
RNASEK	0.000400498	-2.05246742	0.25	0.733	1
RBM42	0.000401769	-0.503058027	1	1	1
PRKCQ	0.00040219	2.139551352	1	0.733	1
STRN3	0.000402251	1.52823603	1	1	1
SHARPIN	0.000404701	-1.277533976	0.625	0.8	1
TLE7	0.000404813	1.997719574	1	1	1
VPS52	0.000406673	-0.701392979	0.938	1	1
IL17D	0.000407036	-3.208586622	0.125	0.6	1
FRMD7	0.000409274	1.354564243	1	0.933	1
KIF18B	0.000409524	2.703357202	0.938	0.6	1
BCL7B	0.000413736	-0.238070339	1	1	1
BZW2	0.000415523	1.362586521	1	1	1
RPUSD3	0.000417486	1.915254069	1	0.8	1
GPX3	0.000417566	-1.002294241	1	1	1
PIWIL3	0.000418695	-0.19548154	1	1	1
ENSSSCG00000009374	0.000420465	-0.493349902	0.938	1	1
ENSSSCG00000022164	0.000420809	1.710908519	1	1	1
ENSSSCG00000027916	0.000421401	-1.399770743	0.688	0.733	1
MFN1	0.000421961	1.277285169	1	1	1
ENSSSCG00000063088	0.000422443	-1.396890153	0.5	0.867	1
HMGCS1	0.000422704	-0.146503708	1	1	1
ENSSSCG00000062491	0.000423786	-1.74080766	0.5	0.8	1
TMED7	0.000423865	1.539255966	1	1	1
KAZN	0.000424643	-0.453469811	0.938	1	1
TOMM7	0.000425189	1.41737488	1	1	1
CFL2	0.000427877	-0.634935402	1	1	1
TSPAN5	0.000427904	-0.238960271	1	0.933	1
MEI1	0.000428439	-1.13965199	0.625	0.867	1
DPPA2	0.000431484	-1.269186633	0.875	0.867	1

ENSSSCG00000036736	0.000432388	-1.249228606	0.562	0.933	1
STAT6	0.000434835	-3.03170886	0.062	0.6	1
ENSSSCG00000008097	0.000435644	-1.394278939	0.562	0.867	1
ENSSSCG00000054830	0.000436805	-1.415037499	0.562	0.933	1
GALT	0.000437214	-2.8372705	0.312	0.6	1
SRSF3	0.0004378	1.309480261	1	1	1
CDKN2D	0.000437819	-0.617677307	0.938	1	1
APBA2	0.000438272	-1.438884241	0.312	0.867	1
ENSSSCG00000058154	0.000438564	-2.830074999	0.25	0.533	1
ENSSSCG00000015691	0.000443444	-0.517862059	0.938	1	1
CCNJL	0.000444221	-1.768269714	0.75	0.867	1
UBE2J1	0.000444741	-1.523743759	0.688	0.8	1
MFSD5	0.000444924	-2.495207848	0.188	0.6	1
ENSSSCG00000050221	0.000445555	-1.929610672	0.375	0.667	1
SNX2	0.000447045	1.347057803	1	1	1
MPC1L	0.000450171	-3.485426827	0.062	0.533	1
MECP2	0.000450377	-1.870716983	0.25	0.733	1
NUFIP1	0.000451153	1.182174898	1	1	1
ENSSSCG00000043676	0.000452591	-0.59171162	1	1	1
NELL1	0.000453226	-0.145013783	1	1	1
PTGR2	0.000454166	1.623642969	1	1	1
HK1	0.000458667	-2.552541023	0.188	0.533	1
TBCD	0.000459934	-0.785927895	0.812	1	1
ZYG11B	0.000460226	1.27059603	1	1	1
PUSL1	0.000460826	-2.230612928	0.25	0.733	1
SNRPA	0.00046402	-1.247437551	0.75	0.867	1
SLC7A6OS	0.000464628	1.267804459	1	1	1
ST6GALNAC1	0.000465678	3.661778098	0.75	0.133	1
PCGF3	0.00046724	-0.849838253	0.688	1	1
UPF3B	0.000468171	0.141686099	1	1	1
LY75	0.000468479	1.410498401	1	1	1
AK6	0.000468876	1.34351632	1	1	1
NF2	0.000469113	1.317271469	1	1	1
ZMAT3	0.000469148	1.344864906	1	1	1
CHAF1A	0.000469222	0.106170317	1	1	1
CCDC158	0.000469303	3.025285296	0.812	0.333	1
ENSSSCG00000045223	0.000470539	-1.227758931	0.688	0.867	1
AKAP12	0.000471689	-1.407217995	0.625	0.867	1
GART	0.000472104	1.875914946	1	0.867	1
ENSSSCG00000005101	0.000472359	0.090962171	1	1	1
FANCA	0.000474326	-1.196202897	0.562	0.8	1
SMARCA5	0.000474576	1.375405822	1	1	1
SVIL	0.000475786	1.280348991	1	1	1
ENSSSCG00000032891	0.000476116	-2.900464326	0.062	0.667	1
VRK1	0.000476833	-0.393475256	1	1	1
TUBB2B	0.000477982	-1.065095028	0.75	0.8	1
GPAM	0.000479065	-0.813331735	0.938	1	1
CDPF1	0.000481198	-1.373217324	0.5	0.867	1
TAF6L	0.000481406	-1.20987647	0.875	1	1
RCCD1	0.000481905	-0.752072487	0.875	1	1
CABLES2	0.000483517	-0.315501826	1	1	1
GJA5	0.000484741	-0.936707847	0.75	0.867	1
MCF2	0.000486204	1.869949017	1	0.8	1
TIAL1	0.000486384	1.415786438	1	1	1
DEF8	0.000486992	-1.157239742	0.688	0.933	1
ENSSSCG00000059847	0.000488266	-1.728697978	0.688	0.733	1
MAPKAPK3	0.000489374	1.610497593	1	1	1
PRELID2	0.000489554	-0.758690365	1	1	1
ZNF770	0.000490056	1.90172889	1	0.933	1
PGAM5	0.000491219	1.804042188	0.938	0.933	1
RNF34	0.00049181	1.171105654	1	1	1
SLC25A4	0.000492923	-0.480995297	1	1	1
RHOV	0.000493004	-2.678071905	0.188	0.533	1
ENSSSCG00000013387	0.000493894	-1.415037499	0.625	0.8	1
TAB1	0.000498203	-0.645013617	0.938	0.933	1
SLK	0.000499098	-1.736965594	0.75	0.867	1
NAB1	0.000501197	1.464886049	1	1	1
SRARP	0.000504586	-1.272815427	0.812	0.8	1
TEAD4	0.000506683	-2.415037499	0.125	0.667	1
KCNH3	0.000506825	-0.496114348	1	0.933	1

ENSSSCG00000032501	0.000506883	-4.415037499	0	0.533	1
CNRIP1	0.000507484	2.318316841	0.938	0.6	1
ENSSSCG00000051302	0.000508731	-0.454538111	1	1	1
CCDC138	0.000510607	-1.99492901	0.5	0.8	1
MDP1	0.000511815	-1.373217324	0.5	0.933	1
ENSSSCG00000008942	0.000512223	-0.482958572	1	1	1
VTI1A	0.000514539	-1.378511623	0.5	0.8	1
GTF3C3	0.000515429	1.594721467	1	0.933	1
ENSSSCG00000061608	0.000515542	-2.141472426	0.375	0.867	1
ENSSSCG00000013556	0.000517069	-2.019108823	0.188	0.6	1
VCL	0.000517339	1.163530416	1	1	1
PITPNC1	0.000523012	-1.777607579	0.438	0.733	1
MYH15	0.000523701	-0.053436097	1	1	1
HSD17B8	0.000527059	-1.929610672	0.25	0.667	1
TSNAXIP1	0.000527978	-2.533681996	0.25	0.533	1
MAL2	0.00052804	3.6794801	0.812	0.133	1
LAMTOR3	0.000530842	1.220099308	1	1	1
SERPINA11	0.000531678	-2.294743266	0.25	0.6	1
ENSSSCG00000041453	0.000533875	-2.093109404	0.312	0.667	1
HDHD3	0.000533902	-2.023846742	0.375	0.733	1
PLEKHG3	0.000534176	-1.793549123	0.75	0.733	1
ST6GAL1	0.000534901	1.611647609	1	0.933	1
UCHL5	0.000535917	0.179212003	1	1	1
ENSSSCG00000011324	0.000536765	-2.010647244	0.312	0.6	1
EIF4E3	0.000537382	1.401956476	1	1	1
ENSSSCG00000027746	0.00053835	-0.01939047	1	1	1
ADPRM	0.000538948	1.454776925	1	1	1
CRACD	0.000539483	2.157627643	1	0.733	1
SLF1	0.000542239	1.358987152	1	1	1
SYNE3	0.000542283	-1.945552216	0.5	0.733	1
SLC25A40	0.000542514	1.252128533	1	1	1
STRIT1	0.000544452	-2.180572246	0.188	0.667	1
CRAT	0.000545229	-1.076621282	0.75	0.933	1
ENSSSCG00000053787	0.000548205	-2.315501826	0.188	0.6	1
ENSSSCG00000055413	0.000551048	-2.30256277	0.25	0.667	1
PDCL2	0.000552205	-1.427528443	0.438	0.867	1
SOD1	0.000552328	-0.156655221	1	1	1
TSPAN15	0.000552379	1.612218049	1	0.933	1
RAB9A	0.000554094	0.040500203	1	1	1
TBC1D23	0.000558067	1.489352758	1	1	1
DMXL2	0.000558259	-0.728046547	0.938	1	1
COPB2	0.000560905	-0.059444993	1	1	1
WDR26	0.000561165	0.973806823	1	1	1
PSIP1	0.000563245	1.440964386	1	1	1
RNF38	0.000564654	1.228011102	1	1	1
EXOSC2	0.000564693	-1.338865819	0.625	0.867	1
PHAX	0.000566721	1.173826859	1	1	1
SCYL3	0.000567572	1.363530857	1	1	1
ENSSSCG00000029043	0.000569805	-2.35614381	0.25	0.6	1
ADIRF	0.00057061	-3.263034406	0.062	0.6	1
CIT	0.000570644	-0.016561391	1	1	1
ENSSSCG00000056619	0.000570911	-1.263034406	0.688	0.867	1
ABI2	0.000571004	-0.953946657	1	0.933	1
ENSSSCG00000057571	0.000571379	-1.068018423	0.688	0.933	1
WDR55	0.000571482	-1.379990552	0.625	0.8	1
DPF2	0.000576062	1.592524566	1	1	1
RAB37	0.000578401	-3.552541023	0.062	0.6	1
TMEM150A	0.000581442	1.911979472	1	1	1
ENSSSCG00000006153	0.000581501	-1.117771459	0.625	0.933	1
CENPM	0.000584473	-0.696219252	0.938	0.933	1
ENSSSCG00000056500	0.000584645	-0.340082349	1	1	1
NFAT5	0.000585339	-0.900464326	0.812	0.8	1
ENSSSCG00000012165	0.000587751	1.126517643	1	1	1
TMEM218	0.000588584	-0.379080622	0.938	1	1
AKAP8L	0.000592001	-0.742202243	0.938	0.933	1
CHRNE	0.000594655	-0.188855031	0.938	1	1
ACTR1A	0.000594994	-0.166781316	1	1	1
ILVBL	0.000596073	-1.285754482	0.562	0.867	1
CDH8	0.000596465	-0.036340308	0.875	1	1
PXK	0.000597508	1.414286844	1	1	1

TEX2	0.00059795	0.094362579	1	1	1
FBXO42	0.000601359	1.244285492	1	1	1
TFPI2	0.000602127	2.609563012	1	1	1
TAF8	0.000604426	1.783048064	1	1	1
ENSSSCG00000050213	0.000604514	-0.04257667	1	1	1
C6orf136	0.000605255	-0.36518895	1	0.933	1
KICS2	0.000605311	-0.403177344	0.938	1	1
ELOVL1	0.000606538	-1.039867383	0.875	0.933	1
GLO1	0.000607928	-0.143735477	1	1	1
ARMH1	0.000611156	-2.143735477	0.25	0.667	1
ENSSSCG00000045152	0.000611983	-1.929610672	0.312	0.667	1
ZNF226	0.000612123	-0.909312228	0.938	1	1
DLL3	0.000612323	-4.415037499	0	0.533	1
C20orf204	0.000612661	-4.093109404	0	0.6	1
ZNF496	0.000614879	-0.285078905	1	1	1
ENSSSCG00000018027	0.000616294	-0.138150369	1	1	1
DCC	0.000617313	1.38051191	1	1	1
MFSD1	0.000620956	1.352233727	1	1	1
ENSSSCG00000058521	0.000622587	-1.157681406	0.938	1	1
SGO2	0.000623039	1.512149901	1	1	1
CDKL2	0.000623256	-0.753259402	0.75	0.867	1
PLGRKT	0.000625321	1.721858702	0.938	0.733	1
HSP90AA1	0.000625918	-0.142229271	1	1	1
METTL25B	0.00062593	-2.05246742	0.25	0.667	1
ENSSSCG00000046397	0.00062604	-2.03170886	0.188	0.667	1
ENSSSCG00000012219	0.000627472	1.983511877	0.938	0.8	1
ENSSSCG00000007481	0.000630042	-0.491018105	1	0.933	1
ENSSSCG00000061765	0.000631218	-3.595609745	0.125	0.533	1
ENSSSCG00000002349	0.00063223	-1.637429921	0.5	0.867	1
FOXRED1	0.000635787	-1.709780765	0.375	0.8	1
RAVER2	0.000636343	-0.533093261	0.938	1	1
TJAP1	0.000637848	-0.748461233	0.875	1	1
XPO1	0.000637928	-0.444396419	1	1	1
SNUPN	0.000639955	-1.275312736	0.812	0.867	1
SALL2	0.000642335	1.460062029	1	1	1
BRAP	0.000642807	-0.212925558	1	1	1
ENSSSCG00000007947	0.0006431	-1.56125824	0.75	0.867	1
ZNF688	0.000643242	-4.485426827	0	0.533	1
ENSSSCG00000045539	0.000643468	-4.263034406	0	0.6	1
UBE2V1	0.000644706	0.201118095	1	1	1
ENSSSCG00000007719	0.000646271	-0.805307093	0.75	0.867	1
SOX30	0.00064707	1.466318004	1	1	1
ENSSSCG00000050661	0.000648463	-1.415037499	0.5	0.8	1
FBXL4	0.000648596	1.447978287	1	1	1
IL10RB	0.000649494	2.129283017	1	0.8	1
SPECC1L	0.000657149	1.513729977	1	1	1
ULK2	0.00065723	-0.854949667	1	0.867	1
CDKL5	0.000659206	1.10567046	1	1	1
APOD	0.000660482	-1.784987109	0.562	0.667	1
TMEM192	0.000661475	-0.638105498	0.938	1	1
BIN2	0.000662012	2.14671761	1	0.933	1
SPAST	0.000663816	1.309740839	1	1	1
PLA2G15	0.000663842	-0.490344081	0.875	1	1
ENSSSCG00000041170	0.000666533	-0.888750906	0.75	0.867	1
ENSSSCG00000053691	0.000668068	-2.208586622	0.25	0.533	1
IFI35	0.000668231	-3.263034406	0.062	0.533	1
MAGI3	0.000668859	1.3084434	1	1	1
AFG3L2	0.000670213	-0.210292944	1	1	1
STK17A	0.000670708	-0.242753025	1	1	1
NEMP2	0.000670965	1.601627954	1	0.933	1
ATP10D	0.000671201	0.071014458	1	1	1
ENSSSCG00000007963	0.000671928	-2.147557188	0.312	0.6	1
BCL2L1	0.000672	0.039043341	1	1	1
FAM221A	0.000673083	-0.563251503	0.938	1	1
DPY19L4	0.000675752	1.609740832	1	0.867	1
BTRC	0.000677092	-0.152720419	1	1	1
CDS2	0.000678022	-0.453951944	1	1	1
ENSSSCG00000059437	0.000681487	-2.263034406	0.188	0.6	1
IRAK3	0.000681568	-0.0806018	1	1	1
CSNK1G3	0.000683759	1.337674759	1	1	1

PFDN4	0.000684789	1.243717567	1	1	1
TNFRSF11A	0.000685386	-1.562594688	0.25	0.667	1
EFR3A	0.000685913	1.273579676	1	1	1
STIL	0.000685928	1.406350482	1	1	1
ENSSSCG00000041827	0.000690233	-0.744444927	0.938	1	1
PROP1	0.000693949	-0.735952821	0.812	1	1
ZNF740	0.000694531	1.519246863	1	1	1
ENSSSCG00000061304	0.000694837	-2.571156701	0.188	0.6	1
NRDC	0.000695472	1.313807633	1	1	1
ENSSSCG00000041998	0.000696586	-2.239950793	0.25	0.667	1
TMTC1	0.000696607	1.888274917	0.938	0.933	1
JAKMIP2	0.000700114	-0.214813897	1	1	1
AKAP1	0.000704486	-0.349449158	0.938	1	1
DTL	0.00070453	1.382643347	1	1	1
CYP39A1	0.000705062	-2.143735477	0.25	0.533	1
TMEM9B	0.000708447	-0.175145173	0.938	1	1
VAT1	0.000711482	-0.411185174	1	0.933	1
ENSSSCG00000002270	0.000712078	-3.093109404	0.125	0.6	1
AEN	0.000713612	-1.164900087	0.625	0.8	1
SHCBP1	0.000715425	1.092087416	1	1	1
MINDY2	0.000715447	1.591248764	0.938	0.933	1
SIDT1	0.000716013	-1.638543541	0.5	0.8	1
PHF5A	0.00071891	1.293383219	1	1	1
NAPA	0.000720076	-0.774747925	0.812	0.933	1
TECPR2	0.000721799	-1.255380833	0.75	0.8	1
ENSSSCG00000015632	0.000723596	-0.716039755	0.812	0.933	1
TOP2B	0.000723644	1.371270096	1	1	1
ENSSSCG00000050259	0.000726572	-0.847996907	0.875	0.933	1
STK25	0.000728071	-1.415037499	0.938	0.8	1
ENSSSCG00000053510	0.000728241	-2.263034406	0.25	0.6	1
DDX56	0.000731212	-1.06185847	0.875	1	1
NRAS	0.000733025	1.235347341	1	1	1
P3H1	0.000737995	-1.415037499	0.625	0.8	1
ARPC1B	0.000738599	-0.513842509	0.938	1	1
ENSSSCG00000061352	0.000739964	-0.819733054	0.938	0.867	1
FBXO48	0.000743156	-1.196732035	0.562	0.933	1
SCRN1	0.000744113	-0.581210366	0.875	0.933	1
ENSSSCG00000059315	0.000744431	-1.607682577	0.812	0.667	1
ENSSSCG00000010084	0.000746283	-2.736965594	0.125	0.533	1
HASPIN	0.000752432	-0.482034063	0.938	1	1
ZNF599	0.000753604	1.27964295	1	1	1
CYB5R4	0.000755481	-0.617712942	0.938	1	1
MYG1	0.000755484	-2.578536232	0.062	0.6	1
ENSSSCG00000041889	0.000755928	-2.056583528	0.375	0.6	1
CLP1	0.00075641	1.93004156	1	0.933	1
ENSSSCG00000036102	0.000756927	-2.27368165	0.188	0.8	1
FEM1C	0.000757181	1.064054142	1	1	1
DNAJB4	0.000757435	1.310354129	1	1	1
NIPAL3	0.000758901	-0.821029859	0.75	0.933	1
ENSSSCG00000051117	0.000759684	-1.736965594	0.375	0.733	1
ACADM	0.000762174	1.231338054	1	1	1
RPF1	0.000762492	-0.272725322	1	1	1
ENOX2	0.000763435	-0.723271325	0.938	1	1
PRR7	0.000764214	1.87036472	1	1	1
ENSSSCG00000032422	0.000764857	-1.397963986	0.375	0.667	1
ENSSSCG00000009435	0.000765006	1.325843145	1	1	1
TAF1C	0.000766409	-1.471621028	0.5	0.667	1
KDM3B	0.00076681	1.742852437	1	0.933	1
RDH11	0.000769184	1.808470186	1	0.933	1
SLC10A7	0.00077137	-0.110024155	1	1	1
ACADVL	0.000773399	-0.261863862	1	1	1
GALNTL6	0.000773429	-0.176913161	1	1	1
TBPL1	0.000775009	1.423636628	1	1	1
LRPPRC	0.000776369	1.366484057	1	1	1
LIG4	0.00077647	1.40107198	1	1	1
FOXP1	0.000777659	-0.053893008	1	1	1
RPTOR	0.000779638	-0.919777802	0.875	0.933	1
ANKRD12	0.000779718	1.378233501	1	1	1
GCNT1	0.000781596	1.296613242	1	1	1
SRP9	0.000782156	1.277354528	1	1	1

RPAP3	0.000787091	1.334692046	1	1	1
TCFL5	0.000788593	-0.783424905	0.812	0.933	1
ZWILCH	0.000789522	1.76858325	1	1	1
SYCE1	0.000791982	-3.047305715	0.25	0.533	1
RHOF	0.000792614	1.697070374	1	1	1
ENSSSCG00000031622	0.000793271	-0.886658527	0.438	0.933	1
ZNF593	0.000794205	-0.210679001	1	1	1
RFWD3	0.000795153	0.103240202	1	1	1
ZC3H4	0.000796745	-0.31188151	0.938	1	1
LYPLA2	0.000800299	-0.45369912	1	0.867	1
JAG2	0.000800785	-2.315501826	0.25	0.6	1
KIF22	0.000808309	-0.519958102	0.938	1	1
CD2	0.000809844	-1.015704901	0.875	0.933	1
AFG1L	0.000810602	2.113341473	0.875	0.6	1
PEX2	0.000811276	-0.148250959	1	1	1
NUP62	0.00082122	-0.870716983	0.812	0.867	1
CPB2	0.000821569	-1.590609064	0.5	0.733	1
ENSSSCG00000028423	0.000822164	1.360323168	1	1	1
LYZL4	0.000822907	-2.157239742	0.312	0.667	1
RHOBTB1	0.000823608	2.123546647	1	0.867	1
RBM39	0.000829394	-0.125385807	0.938	1	1
ENSSSCG00000008383	0.000829713	-0.071765869	1	1	1
TTLL13	0.000834385	-1.204140717	0.625	0.867	1
PRR19	0.000835321	-0.923184403	0.812	0.933	1
ABRACL	0.000836095	-1.075407403	0.812	1	1
ENSSSCG00000025041	0.000838314	-1.717600269	0.5	0.733	1
TMEM167B	0.000845595	-0.996894089	0.625	0.933	1
HCN2	0.000849037	-0.998717034	0.938	1	1
RSU1	0.000849626	1.302140923	1	1	1
HADH	0.000851424	0.072871467	1	1	1
AOAH	0.000853442	-1.645650427	0.375	0.667	1
STT3B	0.000853846	1.252179633	1	1	1
ISCU	0.000854578	-0.516790998	1	1	1
ENSSSCG00000062306	0.000856644	-2.415037499	0.125	0.6	1
CCSER2	0.000856716	1.214709795	1	1	1
MORC3	0.000859241	1.162220942	1	1	1
ENSSSCG00000007284	0.000860106	1.383073102	1	1	1
DDX4	0.000863773	-1.370447348	0.938	0.933	1
TMEM176B	0.000864313	-1.867549704	0.5	0.8	1
PRADC1	0.00086473	-0.393941664	1	1	1
SPIDR	0.000864896	-0.174618347	1	1	1
SF3A3	0.000864965	1.337838245	1	1	1
HSPBAP1	0.000866219	1.343482238	1	1	1
CEP89	0.000867009	0.138121678	1	1	1
CEACAM20	0.000868305	-1.407217995	0.562	0.933	1
LDOC1	0.000869578	-0.493647334	1	1	1
MAN2B1	0.00087253	-1.187436787	0.625	0.933	1
ATP5MC3	0.000878126	-0.251417075	1	1	1
SLC10A4	0.000879286	-2.250650681	0.312	0.667	1
TLR10	0.000881658	3.285402219	0.625	0.467	1
IGFBP7	0.000881807	-0.650850436	1	1	1
PDGFA	0.000882517	-0.162151049	0.938	1	1
SETD1B	0.000882539	-0.269987166	1	1	1
KLHL13	0.000886791	2.306821202	0.938	0.533	1
ZNF239	0.000888499	-0.457550927	0.938	1	1
WDR13	0.000890113	-1.900464326	0.375	0.733	1
ZNF200	0.000890231	-2.351843673	0.375	0.6	1
ENSSSCG00000014015	0.000890676	-0.638758981	1	1	1
ENSSSCG00000053482	0.000892019	-0.480132528	0.938	1	1
ZNF367	0.000892944	1.580662363	1	1	1
GNG4	0.000893945	-0.418595319	0.938	1	1
BCAS4	0.000896041	-0.363237108	1	1	1
XIRP2	0.000896813	1.284775096	1	1	1
PALM	0.000896875	-0.858644151	0.5	0.867	1
WDR5B	0.00090326	-2.036525876	0.188	0.667	1
ENSSSCG00000011317	0.000904488	1.902738952	1	1	1
COLGALT2	0.00090607	0.034532261	1	1	1
EPSTI1	0.000907967	1.590517745	1	1	1
ENSSSCG00000043931	0.000910965	-1.222392421	0.5	0.933	1
PDIA4	0.000911184	1.183957906	1	1	1

UBE2G1	0.00091324	1.385858473	1	1	1
AKR1B1	0.000914455	-0.345651436	1	1	1
ENSSSCG00000039695	0.000915867	-0.162974211	1	1	1
ENSSSCG00000028892	0.000916405	-1.093109404	0.75	1	1
ALOX15B	0.000916792	-1.548788888	0.562	0.733	1
SLC44A2	0.000918781	3.607330314	0.75	0.2	1
GLYCTK	0.000919386	-1.415037499	0.625	0.733	1
ZNF689	0.000919592	-1.620356407	0.562	0.733	1
ENSSSCG00000043990	0.000919607	-2.678071905	0.125	0.533	1
ENSSSCG00000052039	0.00092359	-2.047305715	0.25	0.6	1
APOBEC2	0.00092707	-1.325770161	0.562	0.8	1
IFFO1	0.000928854	-1.840343334	0.438	0.8	1
NPFFR2	0.000929599	-0.441371647	0.938	1	1
ENSSSCG00000044936	0.000931438	-1.344648171	0.562	0.933	1
GHRH	0.000936865	-3.485426827	0.062	0.533	1
DMAC2L	0.000938632	-1.571156701	0.5	0.667	1
VARS1	0.000938637	-2.208586622	0.125	0.667	1
TTC26	0.000941119	-0.809316438	0.75	0.933	1
CSTF1	0.00094141	-0.002528025	1	1	1
USP7	0.000943811	0.204855401	1	1	1
DENND3	0.000945346	-1.13492958	0.625	0.867	1
ITCH	0.000945523	1.269086678	1	1	1
CPEB1	0.000949263	-0.037179899	1	1	1
GAS7	0.000953265	-0.538141644	0.938	1	1
TAF13	0.00095587	1.484630578	1	1	1
ENSSSCG00000061086	0.000960396	-1.395672174	0.562	0.8	1
ARSI	0.000960803	-3.093109404	0.062	0.6	1
GPX1	0.000961328	-0.794669662	0.812	0.867	1
AKAP10	0.000961384	-0.034765418	1	1	1
B3GNT4	0.000962447	-0.096018065	1	1	1
MED9	0.000962805	-1.070025791	0.812	0.8	1
CENPJ	0.00096292	1.250059838	1	1	1
ENSSSCG00000057960	0.000964759	-1.35614381	0.5	0.8	1
BHMT	0.000966039	-1.678071905	0.188	0.733	1
ITGA8	0.000966332	-1.592680414	0.688	0.667	1
ING4	0.000967208	-0.299560282	1	1	1
ARSJ	0.000967261	1.314248597	1	1	1
KIF5B	0.000970432	1.581699714	1	1	1
WNK1	0.000973348	1.277508183	1	1	1
PHRF1	0.000978639	-1.034215715	0.812	0.933	1
ENSSSCG00000051427	0.000982939	-1.847996907	0.312	0.867	1
ENSSSCG00000053205	0.000987893	-0.929610672	0.5	0.933	1
SETD7	0.000987954	-0.782321867	0.938	1	1
ZRANB1	0.00098806	0.05489651	1	1	1
FIG4	0.00098867	-0.168904755	1	1	1
FASTKD5	0.000990118	-0.347952475	1	1	1
ENSSSCG00000017324	0.000991067	-1.415037499	0.5	0.8	1
ABHD18	0.000992322	-0.251021888	0.938	1	1
PLEKHG5	0.000992586	-1.756074417	0.375	0.867	1
GATAD1	0.000993329	-1.392669686	0.688	0.8	1
PEA15	0.000998209	-2.35614381	0.25	0.6	1
FKTN	0.000998416	1.151829281	1	1	1
GHR	0.000998964	1.218932342	1	1	1
ZNF174	0.00099919	-1.328325866	0.75	0.733	1
NIF3L1	0.00100021	-0.079941532	1	1	1
FBXO4	0.001001872	1.641918386	1	1	1
EML3	0.001008736	-1.277533976	0.5	0.8	1
COL4A4	0.001012807	-0.863627558	0.75	0.867	1
FCHO2	0.001016431	1.249964499	1	1	1
WSCD1	0.001017597	-1.781165398	0.188	0.733	1
ATAD2	0.001019111	1.225563878	1	1	1
TMEFF1	0.001020792	1.224136132	1	1	1
DHRS4	0.001024092	-1.426533138	0.562	0.8	1
MED28	0.001025746	0.087034915	1	1	1
MPHOSPH9	0.001028571	1.255924756	1	1	1
PTAR1	0.001041682	-1.557777671	0.562	0.8	1
AFF4	0.001049292	1.310622915	1	1	1
CAMSAP2	0.001052328	-1.192645078	0.5	0.867	1
ENSSSCG00000052268	0.001054024	-0.576192291	0.938	1	1
LRRC8D	0.001055866	1.091922489	1	1	1

ENSSSCG00000046125	0.001056881	-0.426403754	1	0.933	1
CD37	0.001058254	-0.541867522	0.875	0.933	1
ENSSSCG00000035992	0.001058718	-0.44503686	1	1	1
ITGA6	0.001063845	1.041036586	1	1	1
PISD	0.001064151	-0.17989749	1	1	1
ZNF524	0.001066351	-1.721140627	0.375	0.667	1
RAB3GAP2	0.001067506	1.102762544	1	1	1
IYD	0.001068566	-1.471621028	0.438	0.6	1
ENSSSCG00000053414	0.001070328	-3.415037499	0.062	0.533	1
MTFMT	0.001073564	1.664776841	0.938	1	1
FRMD4A	0.00107809	-0.299013703	0.938	1	1
DSCC1	0.001078871	1.442862799	1	1	1
ENSSSCG00000051230	0.001091966	-2.61667136	0.125	0.533	1
ENSSSCG00000057212	0.001092492	-1.519374159	0.438	0.8	1
SCN9A	0.001092805	1.319643542	1	0.933	1
CCDC117	0.001093834	1.204813227	1	1	1
PGAP3	0.001095092	-0.847996907	0.812	0.867	1
UTP18	0.001097131	0.050423831	1	1	1
KMT5B	0.001102479	0.227800198	1	1	1
QARS1	0.001103429	-0.738576644	0.875	1	1
ENSSSCG00000055443	0.001103919	-1.678071905	0.438	0.667	1
ENSSSCG00000062695	0.001117417	-2.019108823	0.375	0.6	1
DUSP11	0.001119864	1.543596671	1	1	1
ENSSSCG00000031838	0.001124191	-1.891065628	0.562	0.933	1
HARS2	0.001125861	-1.033166864	0.812	0.867	1
ZAR1L	0.00112657	0.174378046	1	1	1
HMGN4	0.001127421	2.861086906	0.688	0.2	1
EIF2B5	0.001128106	-1.4012317	0.688	0.867	1
NIPSNAP1	0.00112895	-0.468320923	1	1	1
MNAT1	0.001131737	1.126870483	1	1	1
ESRRA	0.001133258	-3.263034406	0.062	0.6	1
ZNF202	0.00113352	-0.916231642	1	0.933	1
CLCN4	0.001134044	-0.234592302	1	1	1
LIPI	0.001143647	3.321928095	0.75	0.2	1
RPS28	0.001146096	-1.14303463	1	1	1
GTF2H1	0.001149189	-0.42281485	0.875	1	1
SCAP	0.001150327	-0.556554086	1	1	1
SDHB	0.001153414	-0.089593207	1	1	1
MAPK8	0.001159485	1.129956607	1	1	1
CDC25B	0.001164362	-0.21183794	0.938	1	1
HMGCL	0.001167503	-0.152305197	1	0.933	1
ENSSSCG00000034572	0.001168727	-0.317930197	1	1	1
ACP3	0.001171109	-1.923184403	0.25	0.6	1
PTBP2	0.001172393	-0.040933939	1	1	1
HSF1	0.001182156	-0.62814668	0.875	0.933	1
UFD1	0.00118232	1.315740382	1	1	1
ENSSSCG00000042257	0.001183216	-2.040641984	0.375	0.6	1
ENSSSCG00000016982	0.001187593	1.348724522	1	1	1
XPC	0.001190451	-0.078065524	1	1	1
NYX	0.001194312	-1.929610672	0.25	0.667	1
EFCAB14	0.001194396	1.399314279	1	1	1
ENSSSCG00000011862	0.001199183	2.269460675	0.938	0.467	1
ENSSSCG00000038264	0.001200015	-3.180572246	0.062	0.533	1
COG8	0.001200803	-0.682872891	1	0.933	1
CLMP	0.001202286	-1.604071324	0.562	0.8	1
TEX12	0.001202805	1.895208791	1	0.933	1
FSIP2	0.001207015	-0.402640473	1	1	1
UCKL1	0.001209479	-1.341036918	0.375	0.667	1
HRAS	0.001210593	-4.093109404	0	0.533	1
CASK	0.001212202	1.095690045	1	1	1
ENSSSCG00000031897	0.001214961	-0.699340522	1	1	1
DEUP1	0.001216337	1.292425345	1	1	1
LSM7	0.001220509	-0.703973769	1	1	1
TMEM237	0.001223788	1.416085589	1	1	1
GIT1	0.001224559	-0.455679484	1	1	1
ENSSSCG00000059385	0.001228973	-1.075621978	0.938	0.867	1
TRA2A	0.00122946	1.238900458	1	1	1
ENSSSCG00000049223	0.001230046	-0.564861295	0.938	0.933	1
COMMD10	0.001231038	1.62058641	1	1	1
ENSSSCG00000041310	0.001236521	-0.593802988	0.875	0.933	1

MAMDC2	0.001238297	1.945571064	0.938	0.933	1
DYNC2I1	0.001239803	-1.967578522	0.25	0.6	1
ENSSSCG00000008056	0.001241669	-0.660704773	0.938	0.933	1
IER3	0.001242559	-0.52554995	0.938	1	1
ENSSSCG00000056962	0.001251866	-1.30461351	0.375	0.8	1
GDA	0.001252569	1.445579244	1	0.933	1
RSBN1L	0.001254002	1.197633985	1	1	1
ENSSSCG00000026486	0.001254163	1.744044787	1	1	1
EMILIN1	0.001255365	-1.397963986	0.562	0.867	1
ABCF2	0.00125861	-0.112474729	1	1	1
TNPO3	0.001258848	-0.278236131	1	1	1
EEF1AKMT4	0.001260248	-1.30461351	0.625	0.733	1
PLXDC2	0.001268147	-1.781165398	0.375	0.6	1
ENSSSCG00000012602	0.001271501	1.576288245	1	1	1
ENSSSCG00000048413	0.001274372	-1.027521063	0.562	0.8	1
ART3	0.001280065	1.440376879	1	1	1
ECI1	0.001282018	-1.714597781	0.438	0.733	1
SUB1	0.001282201	-0.263034406	1	1	1
ARHGDIG	0.001286582	-2.294743266	0.188	0.6	1
ERO1A	0.001286612	1.291096229	1	1	1
CEP85	0.00128735	1.226839345	1	1	1
NEK9	0.001288442	-1.803602787	0.5	0.533	1
LGALS3	0.001290641	1.275102822	1	1	1
TBC1D9	0.001291417	1.237949702	1	1	1
POC5	0.001292994	1.172588556	1	1	1
ENSSSCG00000012152	0.001293503	1.59373271	1	1	1
ACCSL	0.001293634	1.732786937	1	1	1
DHX58	0.001295128	-0.248432856	1	1	1
ATP2B1	0.001299043	1.258192314	1	1	1
ATG12	0.001299151	0.970941831	1	1	1
PPME1	0.001299629	-0.122661152	1	1	1
APBA3	0.001303534	-1.035776229	0.938	1	1
MMS22L	0.001311599	1.110478616	1	1	1
GPR19	0.001312493	-0.582494245	0.812	1	1
UBL5	0.001315608	-0.943791758	1	1	1
SLU7	0.001316041	1.154745861	1	1	1
RAB31	0.001326514	1.574127099	1	0.8	1
GAA	0.001328528	-0.403628336	1	1	1
ENSSSCG00000042996	0.001331415	3.951284715	0.688	0.067	1
DDX59	0.001331566	1.32369849	1	1	1
BRF1	0.00133451	-3.137503524	0.125	0.533	1
ZFP1	0.001339492	1.234130317	1	1	1
KCTD21	0.001340112	-0.987756404	0.688	1	1
ENSSSCG00000042842	0.001353441	-0.493647334	0.812	1	1
TCTN1	0.001353928	-0.465078182	1	1	1
BCL7A	0.001355083	-0.40861123	0.875	1	1
TM7SF3	0.001355528	1.859584881	1	0.8	1
TTC21A	0.001355603	-1.214124805	0.688	0.8	1
DDX46	0.001359858	0.011074553	1	1	1
AASS	0.001361161	1.626127687	1	1	1
SLC25A13	0.001361405	1.225043807	1	1	1
ENSSSCG00000013471	0.001361491	-0.471389048	0.938	1	1
STK10	0.001368389	-0.148250959	0.938	1	1
ENSSSCG00000044253	0.001370903	-1.36159824	0.625	0.667	1
FLVCR2	0.001372361	2.11146174	0.938	0.933	1
NABP2	0.001374907	-1.578536232	0.438	0.8	1
RIT1	0.001377128	-1.678071905	0.5	0.733	1
RTEL1	0.001388983	-2.756074417	0.125	0.533	1
EIF4H	0.001396339	1.128722856	1	1	1
PPIL4	0.00139645	1.325119758	1	1	1
DYNC1H1	0.001407447	-0.604822923	0.938	1	1
MMS19	0.001408874	1.436308974	1	1	1
GLB1	0.001410176	2.880895387	0.812	0.2	1
STRADB	0.001414933	1.505660065	1	1	1
RRAGC	0.001419506	0.00590939	1	1	1
LPAR3	0.001419778	2.259050892	1	0.867	1
ENSSSCG00000036748	0.001420254	-1.170588157	0.875	1	1
DPH2	0.001420968	-1.843131151	0.312	0.667	1
CCAR1	0.001426439	1.256297308	1	1	1
CHADL	0.001431625	-1.239950793	0.438	0.8	1

MED11	0.001431889	-1.543770813	0.562	0.733	1
SCG5	0.001432532	-1.352496033	0.812	1	1
CATSPERD	0.001433181	-2.967578522	0.062	0.533	1
GYG2	0.001435761	-2.736965594	0.125	0.533	1
ENSSSCG00000040835	0.001437667	-0.158785579	1	1	1
CEP68	0.001441325	-0.250078751	1	1	1
ENSSSCG00000060817	0.001444297	-1.323407024	0.438	0.8	1
ENSSSCG00000051966	0.001445443	-1.858644151	0.438	0.667	1
EGLN1	0.001448621	-0.732519689	0.812	0.933	1
DAPL1	0.001449243	-0.415037499	0.938	1	1
ENSSSCG00000053444	0.001451966	-1.9861942	0.312	0.667	1
SMG9	0.001456373	-1.629162305	0.25	0.733	1
AFF2	0.001466947	-0.440460096	0.938	1	1
ZMIZ2	0.001469163	-0.554243318	0.938	0.933	1
LMCD1	0.001472075	-0.233157364	1	1	1
GOLGA5	0.001474193	1.169203834	1	1	1
PRR3	0.001476568	1.975413565	1	1	1
SCAF8	0.001476784	1.309172087	1	1	1
HPS3	0.001477131	1.320229807	1	1	1
ATG16L2	0.001479128	-2.03170886	0.188	0.533	1
NEXN	0.001484305	1.407460909	1	1	1
ENSSSCG00000058485	0.001486762	-1.167109986	0.625	0.733	1
ENSSSCG00000062490	0.001488796	-1.042483331	0.688	0.867	1
TIGD7	0.001493801	1.263290908	1	1	1
KANSL1L	0.001496343	1.452378813	1	1	1
ENSSSCG00000037714	0.001496805	1.867896464	1	0.6	1
CCDC22	0.001499299	-2.678071905	0.062	0.533	1
ENSSSCG00000015814	0.001500564	1.432978637	1	1	1
FAM240B	0.001511034	-1.368116452	0.938	0.867	1
C6orf62	0.001516186	-0.907097419	1	0.933	1
ANXA9	0.001519795	-0.690044547	0.688	0.933	1
NFKBIE	0.001522973	-1.282933963	0.625	1	1
ENSSSCG00000003825	0.001524351	-0.802519276	0.688	0.867	1
CTNNA1	0.001529383	1.486344518	1	1	1
VSTM5	0.001533132	-0.395916616	1	1	1
ANAPC10	0.001535155	1.503325881	1	1	1
IRF5	0.001535512	3.638694485	0.75	0.2	1
HERPUD1	0.001537907	-1.803602787	0.812	0.533	1
ENSSSCG00000043234	0.001538685	-0.993573731	0.688	0.8	1
DIRAS2	0.001538794	1.233281404	1	1	1
ENSSSCG00000006176	0.001541211	1.246377062	1	1	1
ITGAD	0.001546073	-0.324166687	1	1	1
CEP20	0.001548215	1.217947955	1	1	1
IFRD1	0.00155116	0.00061746	1	1	1
MOCOS	0.001553908	-1.224353938	0.5	0.733	1
PPP1R10	0.001555607	-0.647210941	1	1	1
ENSSSCG00000057427	0.001559171	-0.500109585	0.938	0.933	1
ENSSSCG00000013715	0.001563839	1.328508792	1	1	1
MX1	0.001564555	1.668494844	1	1	1
NUDT17	0.001565701	-0.343846451	0.938	1	1
ZC2HC1A	0.001567378	1.22424914	1	1	1
ZHX2	0.001567488	1.695866988	1	0.933	1
ATG2A	0.001568119	-0.990015911	0.75	0.8	1
ENSSSCG00000058514	0.001569467	-2.415037499	0.25	0.6	1
MYSM1	0.001570562	1.298461413	1	1	1
MCL1	0.001575231	-0.522893489	1	0.933	1
MRPS2	0.00157801	-1.870716983	0.25	0.533	1
ORC5	0.001578219	1.10617339	1	1	1
CPNE2	0.001585845	1.747987	1	1	1
THAP2	0.001586233	1.909200756	1	0.933	1
LAT	0.001592028	-1.633677786	0.312	0.667	1
FUT2	0.001593268	-0.734655433	0.875	0.933	1
CLYBL	0.001593946	-0.650798975	0.938	1	1
CCDC113	0.001596974	-0.279877916	0.938	1	1
ENSSSCG00000063039	0.001597824	-4.415037499	0	0.533	1
EHMT2	0.001598526	1.677626926	1	0.933	1
GNPDA2	0.001598602	-0.111031312	1	1	1
ENSSSCG00000009182	0.001599231	-1.591915261	0.688	0.6	1
ZKSCAN5	0.001603033	-0.411964665	0.875	1	1
HYCC2	0.001604589	1.108303962	1	1	1

GJC1	0.001605385	-0.133373275	1	1	1
ENSSSCG00000016414	0.001614891	-2.03170886	0.312	0.6	1
SUPT16H	0.00161667	0.161030546	1	1	1
NADK	0.001619834	1.737403239	1	0.933	1
ENSSSCG00000059593	0.00162146	-2	0.25	0.6	1
PLB1	0.0016293	-0.254698547	1	1	1
RPS6KA5	0.001633482	1.275397057	1	0.933	1
DIAPH2	0.001635332	1.340287913	1	1	1
ENSSSCG00000028345	0.00163747	-1.067114196	0.5	0.867	1
TSR2	0.001651765	1.59844573	1	0.733	1
EPHA7	0.001653762	1.55488256	1	0.933	1
SLC35F6	0.001654312	-1.047305715	0.688	0.8	1
ENSSSCG00000057787	0.001668203	-1.415037499	0.562	0.733	1
EEF2	0.001669633	-0.177603882	1	1	1
FBXO38	0.001673163	-0.567551191	0.875	1	1
MED20	0.001678784	1.611434712	1	1	1
TBPL2	0.001679404	-1.197446064	0.812	1	1
TTC4	0.001680144	-0.234045194	1	1	1
ENSSSCG00000011068	0.001680231	1.121719536	1	1	1
ND4	0.001681275	1.826567838	1	1	1
BCDIN3D	0.001681591	-0.842301301	0.75	0.933	1
DYNC1LI1	0.001683799	-1.603593689	0.812	0.8	1
ATP10B	0.001687702	1.10839565	1	1	1
BET1L	0.001690318	-0.204418988	1	1	1
ENSSSCG00000017202	0.001694324	1.092137738	1	1	1
ENSSSCG00000035776	0.001695405	-1.171111916	0.625	0.8	1
CA5A	0.001695941	-0.313708544	0.812	0.933	1
RABEPK	0.001697418	1.947047722	1	0.867	1
UBE2W	0.001702935	1.204659418	1	1	1
P4HA3	0.001721933	2.248581945	0.812	0.4	1
NSUN5	0.001722774	-3.493040011	0.188	0.667	1
SOCS4	0.001723395	1.329477193	1	1	1
SEC24A	0.001723403	1.245302805	1	1	1
ENSSSCG00000039009	0.001726796	-0.138706271	1	1	1
ZBTB38	0.001731007	-0.202943956	1	1	1
ENSSSCG00000055388	0.001753278	0.038941751	1	1	1
SLC45A4	0.001760879	-0.49119377	0.938	0.933	1
ENSSSCG00000057821	0.001760892	-3.847996907	0.062	0.533	1
ENSSSCG00000059229	0.001762165	-1.900464326	0.188	0.667	1
ENSSSCG00000056422	0.001765862	-3.263034406	0.062	0.533	1
ACY1	0.001768244	-1.518415239	0.625	0.733	1
IMPA2	0.001777827	-1.830074999	0.25	0.733	1
ENSSSCG00000054032	0.001779439	-1.263034406	0.562	0.8	1
OTX2	0.001781526	-0.513154043	1	1	1
ENSSSCG00000034393	0.001785265	-0.945883451	0.812	0.933	1
HERC5	0.001791577	-1.009277814	0.875	1	1
SGK3	0.001802584	1.226237137	1	1	1
POPDC3	0.00180351	3.011227255	0.812	0.133	1
FBXL7	0.001806236	2.478432581	0.812	0.667	1
SHMT2	0.001809686	1.251217449	1	1	1
ENSSSCG00000054611	0.001811748	-1.967578522	0.312	0.6	1
RBMS2	0.001816125	0.126030661	1	1	1
SPI1	0.00181777	-2.093109404	0.25	0.6	1
TRIM14	0.001819295	-0.400164223	1	0.933	1
CLCC1	0.001819782	1.082110504	1	1	1
PLAGL2	0.001821929	0.998464983	1	1	1
CDO1	0.001829161	1.12307213	1	1	1
LYAR	0.001833956	-0.123287553	1	1	1
EEF1AKMT2	0.001834159	-0.808132446	0.875	0.867	1
PPFIA4	0.001837937	-2.446746359	0.25	0.6	1
RAD23A	0.001842833	2.382624027	1	0.933	1
ENSSSCG00000055595	0.00184384	-2.03170886	0.188	0.667	1
LYPD3	0.001845424	-0.516320835	0.938	0.933	1
CSGALNACT2	0.001850122	1.332368516	1	1	1
FITM2	0.001851643	-0.163905183	1	1	1
TMEM9	0.001852398	-4.900464326	0	0.533	1
COG1	0.001853129	-0.792449926	0.875	0.867	1
MLXIPL	0.001854344	-1.900464326	0.312	0.6	1
STAT3	0.001854529	1.378751664	1	1	1
PITPNM3	0.001865102	1.678528767	1	1	1

YDJC	0.001865437	-1.438884241	0.688	0.733	1
PLA2G4A	0.001870123	1.260314543	1	1	1
REL	0.001878266	-0.024513358	1	1	1
PDK2	0.001879081	-0.894563725	0.875	0.8	1
SLC25A48	0.001883479	-1.934411658	0.375	0.667	1
RAB40C	0.001884507	-0.343087657	1	0.867	1
ENSSSCG00000012886	0.001891292	-0.565862402	1	0.933	1
DIRAS1	0.001893362	-1.552541023	0.562	0.667	1
RETREG2	0.001907241	-0.347292893	1	0.933	1
FSTL1	0.001912845	2.274866058	1	0.867	1
SLC35D1	0.001915407	1.322994783	1	1	1
C4orf19	0.001917169	-0.93363119	0.625	0.933	1
NSUN6	0.00191912	1.272762038	1	1	1
ENSSSCG00000033787	0.001926275	-0.809316438	0.688	0.933	1
VLDLR	0.001933589	-0.309445776	1	1	1
SLC45A2	0.001939175	-0.816591769	0.75	0.8	1
USP15	0.001939519	1.090455274	1	1	1
DCUN1D2	0.001950888	-2.263034406	0.125	0.6	1
CEP126	0.001953998	1.639951735	1	0.733	1
P4HB	0.001966428	-0.389502407	0.938	1	1
ADAMTS12	0.00197353	-0.411697926	1	1	1
DYRK1A	0.001976112	-0.216211595	1	1	1
UBA1	0.00197791	1.508780424	1	1	1
ENSSSCG00000062820	0.001981727	-0.445990428	0.938	1	1
DNAH12	0.001983799	-0.207117375	1	1	1
BAG1	0.00198551	-0.205292454	1	1	1
REV3L	0.001988957	1.514814245	1	1	1
GINS1	0.002001299	-0.63584367	0.875	1	1
NRP2	0.002001995	1.788431762	1	0.8	1
RNF6	0.002006593	-0.848872594	0.688	0.867	1
ENSSSCG00000050594	0.002016825	1.397722457	1	1	1
NT5C1A	0.002026701	-4.093109404	0	0.533	1
SBF1	0.002035947	-1.140415119	0.312	0.733	1
TRIML2	0.00203605	-0.440004415	1	1	1
TUBE1	0.002036332	1.412594235	1	0.933	1
FZD7	0.002038389	-0.949745229	0.688	0.867	1
ENSSSCG00000011253	0.002045363	1.178858126	1	1	1
ENSSSCG00000049691	0.002047606	-0.730539325	0.5	0.867	1
DDHD2	0.002048831	1.179596302	1	1	1
SERPINI1	0.002050299	-3.109411217	0.312	0.6	1
ZNF34	0.002053408	1.568831912	1	1	1
TRAPPC2	0.002055838	-0.965101792	0.938	0.933	1
ARHGEF26	0.002061725	0.02710523	1	1	1
HIKESHI	0.002069165	-0.116555669	1	1	1
MTCL1	0.002084426	-1.211290831	0.625	0.867	1
ENSSSCG00000027557	0.002086787	-1.415037499	0.438	0.733	1
ARMT1	0.002090044	1.406928999	1	1	1
EIF2D	0.002090542	-0.568688446	0.875	1	1
MAD1L1	0.002095055	-1.208586622	0.562	0.8	1
ENSSSCG00000057403	0.002095847	-0.382616022	1	1	1
NBEAL1	0.002098841	1.150143437	1	1	1
CFAP92	0.00210167	-1.730539325	0.312	0.667	1
DHRS7	0.002104998	-0.275312736	1	0.933	1
SMYD4	0.00211439	-1.217098122	0.562	0.933	1
ARPC1A	0.002114903	-0.00904514	1	1	1
SLC25A16	0.002122331	1.693675311	1	0.8	1
RAB3C	0.002127106	-0.228726524	1	1	1
DDX41	0.002138854	-0.996894089	0.812	0.933	1
RBM5	0.002144802	-0.616574408	0.938	1	1
CDH15	0.002146592	-1.254572827	0.562	0.8	1
ENSSSCG00000043700	0.002149391	-0.277533976	1	1	1
HNRNPUL2	0.002157124	-0.363523341	1	1	1
NOPCHAP1	0.002158227	-0.53562764	0.875	0.933	1
HHATL	0.002158671	-2.294743266	0.062	0.6	1
SSUH2	0.002159926	-1.323407024	0.625	0.667	1
VPS4B	0.002161997	1.266121329	1	1	1
BMPR1A	0.002164878	1.426826953	1	1	1
UBE2Z	0.002166291	-0.709780765	0.688	0.867	1
USP53	0.002168241	1.455806755	1	1	1
CSKMT	0.002169932	-1.445411148	0.5	0.867	1

DVL2	0.002171952	2.246740598	0.875	0.8	1
PTPN23	0.002173558	-0.196055706	1	1	1
SARS2	0.002174878	-1.128733314	0.688	0.867	1
ENSSSCG00000012264	0.002176474	-0.556416113	0.938	1	1
NODAL	0.002180269	-1.637429921	0.375	0.667	1
NPTX2	0.002188264	-1.349449158	0.625	0.667	1
COQ8A	0.002195842	1.660411615	1	1	1
HNRNPK	0.002198658	0.927338124	1	1	1
ENSSSCG00000049460	0.002199454	-1.717600269	0.438	0.667	1
INO80D	0.002207992	-0.282399731	0.938	1	1
POLR2C	0.002211863	1.609767299	1	1	1
ENSSSCG00000060368	0.002212085	2.020101206	0.875	0.867	1
NT5DC1	0.002212154	-0.67320615	1	0.933	1
EPB41L3	0.002219417	-0.267022114	0.938	1	1
SFXN4	0.002219726	-1.159198595	0.625	0.8	1
LNPK	0.002229954	1.554588852	1	1	1
ENSSSCG00000007527	0.002230423	-1.900464326	0.188	0.667	1
CUEDC2	0.002235075	-2.315501826	0.25	0.867	1
MINDY3	0.002241749	0.141389891	1	1	1
FANCL	0.002246509	1.449038671	1	1	1
NUAK2	0.002256887	-1.730539325	0.312	0.6	1
RAB8A	0.002257746	-0.46723371	0.938	1	1
ENSSSCG00000002536	0.002267231	-2.341036918	0.188	0.533	1
SLC37A2	0.002270608	-0.124270336	1	1	1
TCF19	0.002271228	-1.793549123	0.375	0.6	1
METTL14	0.002283478	-0.224905197	1	1	1
SPECC1	0.002283501	-0.443606651	0.625	0.933	1
NOX3	0.002284131	-1.500284786	0.562	0.667	1
ENSSSCG00000006682	0.002297812	-0.171465042	0.938	1	1
ENSSSCG00000052302	0.002301072	-1.214932333	0.938	1	1
RAD51AP1	0.002304006	1.173297099	1	1	1
ABHD1	0.002306841	-1.552541023	0.438	0.6	1
DUSP28	0.002309557	-1.299560282	0.562	0.8	1
MROH1	0.002309851	-2.093109404	0.25	0.533	1
ENSSSCG00000062959	0.002310533	-0.422057927	1	0.867	1
IKBKE	0.002313142	-0.71871389	0.688	0.933	1
ENSSSCG00000060307	0.002317974	-3	0.062	0.6	1
GPATCH2L	0.002318625	-0.306939812	1	0.933	1
IVNS1ABP	0.002321743	1.024806667	1	1	1
RASSF8	0.002328162	1.201982943	1	1	1
GOLGA3	0.002330213	1.246377062	1	1	1
VPS26A	0.002333087	-0.559462018	1	1	1
FGB	0.002336931	-3.093109404	0.062	0.533	1
ENSSSCG00000047092	0.002347602	-1.730539325	0.25	0.6	1
MEX3D	0.002347777	3.076815597	0.812	0.133	1
RPL26L1	0.002351347	1.188010989	1	1	1
RRAGA	0.002356005	1.046551306	1	1	1
CYB5D1	0.002361347	-0.091690127	1	1	1
EHF	0.002364524	-0.047987937	1	1	1
ENSSSCG00000037274	0.002368693	-0.661393164	1	1	1
EIF2B4	0.002370094	-0.707819249	0.875	0.933	1
ENSSSCG00000041961	0.002379678	-1.04580369	0.688	0.933	1
GALNT7	0.002384689	1.34129342	1	1	1
PTPN9	0.002385685	-0.229394264	0.938	1	1
MRPL33	0.002389548	-0.38036269	1	1	1
ENSSSCG00000013600	0.002394591	-1.431911318	0.438	0.733	1
ENSSSCG00000042498	0.002396951	1.637500695	0.875	0.933	1
SYT5	0.002397748	-0.559890479	0.875	0.933	1
ENSSSCG00000029600	0.002414902	1.447797916	1	1	1
ENSSSCG00000014037	0.002418527	-1.706086281	0.375	0.733	1
ACP4	0.002420275	-2.341036918	0.062	0.533	1
ASB7	0.002427989	-0.086836807	1	1	1
ENSSSCG00000056648	0.002429314	-2.595609745	0.125	0.6	1
ENSSSCG00000033340	0.002432919	1.799975392	0.938	0.6	1
HCFC2	0.002435678	0.955899398	1	1	1
TRIM36	0.002439732	1.245812975	1	1	1
ENSSSCG00000062045	0.002440071	-1.415037499	0.438	0.733	1
CCDC150	0.002441316	0.113267144	1	1	1
MTMR3	0.002443444	1.132584204	1	1	1
NUP210	0.002449481	1.948443085	1	0.867	1

IRF3	0.00245263	-2.61667136	0.062	0.6	1
HEATR1	0.002459427	-0.199061487	1	1	1
ZNF250	0.002460837	-1.050674138	0.938	0.933	1
LRRC72	0.002466301	-1.321928095	0.625	0.8	1
ENSSSCG00000001396	0.0024671	-1.540568381	0.25	0.667	1
SMAD3	0.002473678	-0.540568381	0.875	0.933	1
SPDYA	0.002475669	1.491853096	1	0.867	1
CNIH3	0.002477367	-2.341036918	0.188	0.533	1
FOXN3	0.002477589	-0.105207547	1	1	1
CHRNA5	0.002478111	-0.326500825	0.938	1	1
ENSSSCG00000002532	0.002479398	-0.489038081	0.688	0.933	1
PAK4	0.00248282	-0.9861942	0.625	0.8	1
SNX12	0.002483053	-0.082865373	1	1	1
MAPK8IP3	0.002488697	-1.415037499	0.312	0.667	1
ENSSSCG00000010077	0.002491907	-1.967578522	0.25	0.6	1
DOCK7	0.002491938	1.229004953	1	1	1
ENSSSCG00000008121	0.002495553	-0.57589151	0.812	1	1
TAF15	0.002496621	-1.060687927	0.812	0.933	1
HSD11B1L	0.002499533	-2.093109404	0.312	0.533	1
YPEL4	0.002504365	-1.263034406	0.562	0.733	1
FCHO1	0.00250606	2.094517599	0.938	0.867	1
ERG28	0.002516538	0.096551815	1	1	1
PAXIP1	0.002524492	1.823474587	1	0.933	1
UBE2C	0.002530474	1.301463992	1	1	1
ENSSSCG00000055583	0.002534577	-3	0.062	0.6	1
RARS1	0.002535846	-0.086725788	1	1	1
NOC2L	0.002548354	-1.285754482	0.562	0.8	1
CAMKMT	0.002554095	-0.583744523	0.938	0.933	1
RAD51C	0.002558778	1.160647188	1	1	1
TP53BP1	0.002561505	1.083410786	1	1	1
SKIC2	0.002567599	-1.847996907	0.25	0.6	1
ENSSSCG00000017754	0.002570983	-1.830074999	0.312	0.667	1
INTS1	0.002578974	-1.714597781	0.375	0.6	1
GSS	0.002579709	-0.949518551	0.938	1	1
AHSP	0.002589442	-5.047305715	0	0.533	1
ENSSSCG00000050627	0.002590688	0.23166937	1	1	1
RNF215	0.002597316	1.67688499	1	0.933	1
ENSSSCG00000009692	0.002602077	-0.051256437	1	1	1
TGM7	0.002602379	-0.559194509	0.938	0.867	1
ENTPD4	0.002605775	-0.406837018	1	0.933	1
ZNF618	0.002607059	1.845490051	0.938	0.8	1
SURF4	0.00261036	-0.532641466	1	1	1
PIGQ	0.002616868	-1.602123052	0.312	0.667	1
SPSB3	0.002618279	-1.543770813	0.5	0.733	1
ENSSSCG00000061525	0.002619694	-2.455679484	0.312	0.533	1
ENSSSCG00000035336	0.002629758	-2.093109404	0.25	0.6	1
NEK6	0.002644661	-0.465078182	1	1	1
TPRG1L	0.002655145	-1.578536232	0.375	0.6	1
ENSSSCG00000012382	0.002657053	0.182012284	1	1	1
EIF3D	0.002659462	-0.921997488	1	1	1
RAD50	0.002660182	0.049835239	1	1	1
POLDIP2	0.002668171	-0.467712039	1	1	1
PDZK1	0.002671628	0.009263536	1	1	1
RANBP9	0.002680659	1.026593327	1	1	1
RSRC2	0.00268927	0.155977884	1	1	1
ASF1B	0.002690298	1.69698539	1	1	1
KARS1	0.002695551	1.436431505	1	1	1
ENSSSCG00000014540	0.002696532	-1.23413826	0.938	1	1
MSX1	0.002703947	1.756750246	1	0.8	1
PHKA2	0.002706709	0.011552957	1	1	1
SNX1	0.002715843	-0.548191623	0.875	1	1
ENSSSCG00000023603	0.002720472	-1.143735477	0.562	0.8	1
DENND4C	0.002725606	1.115473298	1	1	1
CHURC1	0.002727202	-0.000109832	1	1	1
DPPA4	0.002728882	2.182168597	1	0.733	1
C8orf34	0.002729071	1.741347662	1	0.867	1
PRSS23	0.002730919	-1.029544276	0.938	0.867	1
CAST	0.002734537	-0.462148479	0.875	1	1
IGF1R	0.002741866	-0.070986593	0.938	1	1
ELAVL1	0.002747541	1.294342263	1	1	1

FRRS1	0.002748165	-2.208586622	0.438	0.533	1
ENSSSCG00000043543	0.002753399	-2.485426827	0.062	0.667	1
ENSSSCG00000040775	0.002754117	-1.578536232	0.375	0.733	1
ACSS2	0.002754475	-0.291544571	1	1	1
SIRT2	0.00275651	-1.437063806	0.25	0.733	1
TTC6	0.002760885	-0.10295719	0.938	1	1
PARD6A	0.002764164	-2.047305715	0.188	0.6	1
PLPPR3	0.00277029	-1.198462405	0.5	0.8	1
PNRC1	0.002774922	-0.845181891	0.875	0.933	1
COMMD5	0.002784343	-1.620356407	0.5	0.733	1
SPATA5	0.002787572	1.282359375	1	1	1
DCTN4	0.00278762	-0.003746289	1	1	1
EFNA5	0.002791778	0.011881623	1	1	1
NAA60	0.002793938	-0.360456803	1	1	1
RPL35A	0.002796017	-0.938300255	1	1	1
RRAGB	0.002796695	-1.587874096	0.5	0.733	1
PDE3A	0.002797504	1.059660876	1	1	1
SLITRK4	0.002806168	1.428006614	1	0.933	1
RNF17	0.00280644	1.101408628	1	1	1
SLC25A33	0.002808313	0.033475694	1	1	1
ZNF32	0.00281029	-1.540568381	0.562	0.8	1
DNAAF2	0.002815552	1.534719746	1	0.933	1
KHDRBS2	0.002816552	-0.065887936	1	1	1
SRSF4	0.002821385	2.418212334	0.938	0.933	1
NOA1	0.002836838	-0.298383292	0.875	1	1
ENSSSCG00000062919	0.002840953	2.392317423	0.938	0.267	1
NUCKS1	0.002841192	-0.823502345	0.938	0.933	1
PEAK1	0.002841621	1.260364405	1	1	1
ENSSSCG00000053385	0.002845029	2.062168821	1	0.667	1
APCS	0.002867139	-1.519374159	0.375	0.667	1
YES1	0.002869704	1.254164657	1	1	1
CACNA2D2	0.002872048	-0.29127571	0.875	1	1
AGBL5	0.002873488	-0.347155027	0.938	0.933	1
BTBD18	0.002874978	1.626186441	1	1	1
GOLGA7	0.002884253	0.042624455	1	1	1
ODF2L	0.002885353	1.677545414	1	1	1
ZNF133	0.002890556	1.591054774	1	1	1
MEIKIN	0.002894882	-0.284927012	0.938	1	1
TM2D1	0.002895143	1.124197728	1	1	1
CDYL	0.002899997	1.790566478	1	0.933	1
ZNF408	0.002909701	-1.900464326	0.375	0.533	1
RPAP2	0.002916288	-0.061454066	1	1	1
THTPA	0.002920492	-0.94308396	0.812	0.933	1
CDK4	0.002922825	-0.090310771	1	1	1
TIGD2	0.002922939	-1.208586622	0.438	0.733	1
RAD51AP2	0.002927644	-1.607682577	0.25	0.6	1
TOMM70	0.002931983	-0.166884688	1	1	1
FAM135A	0.002940007	1.431126866	1	1	1
ZNF383	0.002942501	1.811733363	0.938	0.733	1
FBLN5	0.002942605	-0.399139872	1	1	1
SUN2	0.002947978	1.586972292	1	1	1
BARX2	0.002950413	1.812031409	1	0.933	1
COPRS	0.002954027	1.594304535	1	1	1
BUB1B	0.002957314	1.009320407	1	1	1
PIERCE1	0.002960432	2.064431873	1	0.867	1
DDIT3	0.00296383	-0.732391546	1	1	1
C22orf23	0.002966131	-2.263034406	0.125	0.533	1
ENSSSCG00000038150	0.002969411	-1.327574658	0.312	0.733	1
SERTAD1	0.002982407	-2.208586622	0.312	0.667	1
CHCHD6	0.002994975	-1.809316438	0.25	0.6	1
ENSSSCG00000015293	0.003001345	1.335363609	1	1	1
PNKP	0.003006059	-1.587874096	0.312	0.6	1
CARM1	0.003013252	-0.27716869	1	1	1
COX15	0.00301432	-0.255006032	0.938	1	1
MAX	0.003021309	-0.0199747	1	1	1
CTNNA3	0.003025658	0.161011946	1	1	1
HINT1	0.003027103	-0.737525755	1	1	1
ENSSSCG00000061957	0.003027791	-0.012340458	1	1	1
HYLS1	0.003034895	1.085536142	1	1	1
MPPE1	0.003038397	-1.049388027	0.75	0.867	1

IGF2R	0.003047981	1.552560356	1	1	1
TONSL	0.003054519	-1.263034406	0.312	0.667	1
ENSSSCG00000047552	0.003054988	3.813781191	0.688	0.067	1
METTL17	0.003075504	-0.473988042	0.938	1	1
MAPRE2	0.003076712	0.003427941	1	1	1
CUX1	0.003078112	-0.782769284	0.562	0.867	1
DNTTIP1	0.003080449	-0.176940995	1	0.933	1
HACD4	0.003082424	-0.314773376	1	1	1
ANKRD40	0.003083817	-0.265132872	1	1	1
CCDC77	0.003088143	1.286215998	1	1	1
OXR1	0.003091919	1.463670329	1	1	1
PLOD1	0.003100194	-0.297283968	1	1	1
CERCAM	0.003104146	-1.941106311	0.188	0.533	1
ENSSSCG00000060272	0.003104791	-0.109503843	0.938	1	1
ENSSSCG00000036445	0.003106706	1.783809111	1	1	1
APOE	0.003124447	-0.535331733	0.812	0.867	1
UBE2O	0.003134833	-0.607682577	0.938	0.933	1
LRRCC1	0.003134947	1.411759505	1	1	1
ENSSSCG00000012218	0.003165645	-1.306103128	0.25	0.733	1
IST1	0.003181505	0.010344864	1	1	1
PPP2R5C	0.003183784	0.175197429	1	1	1
PAIP2B	0.003195947	-0.161055556	1	1	1
LMNB2	0.00320227	1.389910307	1	1	1
ABHD2	0.003206231	-0.778236067	0.812	0.867	1
KCNQ1	0.003213231	-0.529208519	0.625	0.933	1
COQ9	0.003214892	-0.051202453	1	0.933	1
ZMYM2	0.003222513	1.200824448	1	1	1
VPS50	0.003239198	1.215978489	1	1	1
CLPTM1	0.003252446	-0.607682577	0.875	0.933	1
PROSER1	0.003252519	1.477187553	1	1	1
ZCCHC9	0.003261717	1.38436867	1	1	1
TBC1D10B	0.003264119	-1.029915578	0.688	0.733	1
NFU1	0.003272404	1.527433076	1	1	1
ENSSSCG00000040260	0.003275881	1.553253641	1	0.933	1
MGAT5	0.003278352	1.048801221	1	1	1
PTCHD3	0.003280556	-0.433083955	0.875	1	1
ENSSSCG00000046754	0.003284252	-2.093109404	0.188	0.6	1
SMPD1	0.003286849	-2.208586622	0.125	0.533	1
PSMA8	0.003289207	1.165229713	1	1	1
EXOC7	0.00330562	-1.881605299	0.375	0.6	1
CAMK1D	0.003308974	-0.844430292	0.75	0.933	1
HTR2A	0.003314899	-1.870716983	0.188	0.533	1
OCLN	0.003315386	1.258982702	1	1	1
RAB32	0.003316061	-0.076558228	1	1	1
AHCYL1	0.003318696	-1.602123052	0.562	0.733	1
CNOT6L	0.003321023	1.086049704	1	1	1
ZC3H15	0.003332213	1.088827148	1	1	1
CYLD	0.003333682	1.263034406	1	1	1
RAB4A	0.00333707	1.593524514	1	1	1
BPIFB3	0.00334031	-3	0.062	0.533	1
RADIL	0.003340936	1.574710029	1	1	1
TAGAP	0.003345055	-2.040641984	0.25	0.6	1
PLCD1	0.003346755	-2	0.188	0.533	1
ENSSSCG00000035645	0.003355656	-1.830074999	0.25	0.667	1
DHRS1	0.00335568	-0.576192291	0.938	0.867	1
MINDY1	0.003362145	-0.846826363	0.938	0.867	1
SFSWAP	0.003364212	0.006937772	1	1	1
E2F4	0.003368995	1.723473358	1	1	1
UFSP2	0.003379566	1.615841814	1	1	1
ENSSSCG00000025565	0.003384383	-0.367825615	1	1	1
AMBRA1	0.003385814	-0.175571565	1	1	1
ARMH3	0.003387423	-0.093109404	1	1	1
DIRAS3	0.0033939	-1.075407403	0.812	0.667	1
FBXL3	0.003394151	1.098640222	1	1	1
SNX24	0.003395123	1.228145949	1	1	1
ENSSSCG00000052085	0.003403266	-0.876298015	0.875	0.8	1
C4orf36	0.003407158	-1.331269142	0.875	0.6	1
IGHMBP2	0.003411726	-1.346865997	0.25	0.667	1
RNF157	0.003423326	-0.36994961	1	1	1
ENSSSCG00000006899	0.0034338	-0.120014362	1	1	1

ENSSSCG00000018055	0.003440821	-3.093109404	0.062	0.533	1
CCDC70	0.003451056	-1.633677786	0.438	0.667	1
KCNN4	0.003455423	-0.952931746	0.688	0.8	1
MTHFD1	0.003456934	-0.425953277	1	1	1
ENSSSCG00000056241	0.003472451	1.745954377	1	0.933	1
CDC37	0.003472899	-0.462632244	1	1	1
GRHL2	0.003476762	0.190683562	1	1	1
RECQL4	0.003481945	-0.707366212	0.812	0.933	1
ARFRP1	0.003495641	-2.093109404	0.125	0.533	1
ZFPL1	0.003499934	-0.8372705	0.75	1	1
CIB1	0.003509375	-0.40019899	1	0.933	1
PTCH1	0.003509959	2.047864223	1	0.933	1
ATXN1	0.003511234	1.312405321	1	1	1
TMEM101	0.003512418	-1.200024608	0.562	0.8	1
MEN1	0.003524306	-1.721140627	0.438	0.667	1
NR1H4	0.00352516	1.549127902	1	1	1
SUPT6H	0.003526454	-0.182208451	1	1	1
RAPGEF6	0.003529486	1.152967673	1	1	1
ENSSSCG00000055037	0.003554471	-1.678071905	0.312	0.733	1
CYSTM1	0.003556037	-0.292004644	1	1	1
DDX52	0.003571542	-0.484447068	0.938	1	1
ARFGEF2	0.003575271	0.094554913	1	1	1
TAPT1	0.003577088	1.107071417	1	1	1
CCNYL1	0.003578541	1.268773408	1	1	1
SYT16	0.003601286	1.267513981	1	0.933	1
PRR14	0.003606817	-0.534481051	0.875	1	1
PDP1	0.003608342	-0.24307191	0.938	1	1
KDM3A	0.003608406	1.295292968	1	1	1
ENSSSCG00000036117	0.003612109	-2.35614381	0.188	0.533	1
FBRS	0.003614807	-1.941106311	0.25	0.6	1
ENSSSCG00000043719	0.003620154	-0.689212463	0.75	1	1
KAT14	0.003622771	-0.941106311	0.812	0.867	1
CCDC126	0.003638713	1.213470726	1	1	1
ENSSSCG00000053468	0.003643463	2.994353437	0.688	0.133	1
ND3	0.003647303	1.903446038	1	1	1
NRDE2	0.003655863	-0.867227997	0.812	1	1
FOXM1	0.00365656	-0.250078751	0.938	1	1
ENSSSCG00000051926	0.003658365	-1.540568381	0.25	0.667	1
LYZL6	0.00366004	-2.010647244	0.25	0.6	1
SPRTN	0.003672097	0.075741982	1	1	1
ENSSSCG00000038171	0.003675617	2.379826479	0.812	0.4	1
ABHD5	0.003676693	-0.344334508	0.938	0.933	1
THPO	0.003684568	4.461479447	0.562	0.067	1
DHX37	0.00369846	-0.77118131	0.75	0.733	1
ENSSSCG00000042380	0.003718659	-0.330148602	0.812	1	1
ENSSSCG00000060794	0.003719697	-0.528324786	1	0.933	1
DBN1	0.003722907	-0.063114664	0.938	0.933	1
BOC	0.003736522	0.165202591	1	1	1
GNA13	0.003750963	0.992938723	1	1	1
SYT6	0.003754827	-0.540568381	0.625	0.933	1
FRMD4B	0.003765099	1.56139403	1	0.933	1
AMMECR1	0.003778771	-0.699445556	0.875	0.933	1
ZW10	0.0037897	-0.034264746	1	1	1
CHFR	0.003794691	-0.334117504	0.938	0.933	1
NTHL1	0.003797173	-0.520530628	0.938	0.867	1
ZACN	0.003801953	-1.508146904	0.312	0.667	1
TET3	0.003807019	1.161034496	1	1	1
ENSSSCG00000025525	0.003809767	-2.471621028	0.125	0.533	1
SMIM1	0.003814297	-3.793549123	0	0.533	1
PRMT9	0.003815807	1.603498452	1	1	1
PDE7A	0.003816812	1.587822155	1	0.867	1
SYT17	0.003830005	-1.567040593	0.25	0.533	1
ETV6	0.00383129	-0.486930418	0.938	1	1
VAC14	0.003837938	-0.971803108	0.562	0.867	1
ENSSSCG00000049047	0.003845953	-1.382616022	0.688	0.8	1
ENOX1	0.003848627	1.416400868	1	1	1
TSKU	0.003854453	-2.415037499	0.125	0.6	1
SLC16A10	0.003864344	1.321071133	1	1	1
SH3BP5	0.003874812	1.695344306	1	1	1
ENSSSCG00000055400	0.003889725	-2.163498732	0.25	0.6	1

DDR1	0.003892094	1.75227865	1	0.933	1
GRK7	0.003895052	1.443892058	1	0.933	1
CCNF	0.003898147	-0.811696469	0.812	0.933	1
TSR1	0.00390173	0.095126006	1	1	1
RTTN	0.003906411	1.106919538	1	1	1
BPNT2	0.003908411	1.100648435	1	1	1
CD99L2	0.003913237	-1.756074417	0.25	0.667	1
ENSSSCG00000060667	0.003926691	-1.005646563	0.562	0.867	1
DCAF5	0.003930267	1.009919479	1	1	1
RGS16	0.003934752	1.392214266	1	1	1
TMOD1	0.003936761	-0.166065438	1	1	1
ENSSSCG00000037703	0.003949153	1.62309763	0.938	0.733	1
CYFIP2	0.003950291	-0.082001837	1	1	1
TOR1B	0.003958137	1.760501109	1	0.867	1
HFM1	0.003960092	1.487514161	1	0.733	1
CERS2	0.003962174	1.428048282	1	1	1
ARL10	0.003963497	-0.132667878	1	1	1
RWDD2A	0.003964215	0.111941817	1	1	1
U2SURP	0.003967972	1.203810637	1	1	1
ENSSSCG00000020830	0.003969552	-0.159656666	1	1	1
COTL1	0.003972061	-0.451156925	1	0.933	1
DEPTOR	0.003980075	1.159583057	1	1	1
ENSSSCG00000055009	0.003985808	-0.379291838	1	0.933	1
PRKCA	0.003986832	-0.470179053	0.938	1	1
VWA5A	0.003996502	-0.827172191	1	0.933	1
DCAF8	0.003997394	1.214390052	1	1	1
CYTH1	0.004002644	-1.237499314	0.625	0.733	1
PPP5C	0.004004554	1.827078014	1	0.867	1
USP44	0.004066362	1.065578002	1	1	1
BST1	0.004073574	-1.877380713	0.25	0.6	1
GALK2	0.004084168	0.160761248	1	1	1
PRIM1	0.004085167	-0.423074818	0.938	1	1
ENSSSCG00000036013	0.004108756	-1.491658781	0.312	0.667	1
ENSSSCG00000004392	0.004116138	1.186605232	1	1	1
ENSSSCG00000056738	0.004118914	1.674056428	1	0.8	1
SPATS2	0.00412589	1.111801256	1	1	1
ENSSSCG00000045298	0.004134115	-1.929610672	0.312	0.6	1
ENSSSCG00000031300	0.004137477	-0.638543541	0.625	0.867	1
WDSUB1	0.004138511	1.13524704	1	1	1
EPHB1	0.004142522	3.076815597	0.812	0.2	1
TRIM33	0.004143069	1.311194433	1	1	1
TLN2	0.004144264	-0.524000855	0.938	1	1
SFMBT1	0.004160949	1.105427033	1	1	1
TOR3A	0.004165347	-0.815575429	0.75	0.933	1
PAIP1	0.00416589	1.133607905	1	1	1
NKAPD1	0.004170086	0.008292628	1	1	1
SUSD4	0.004170691	-1.093109404	0.812	0.867	1
DSCAML1	0.004179731	-0.736965594	0.812	0.933	1
ENSSSCG00000031365	0.004179786	-0.442916532	0.812	1	1
ENSSSCG00000059837	0.00420086	-0.542094236	0.875	0.933	1
EPHB2	0.004208899	-0.693501946	0.688	0.867	1
CAV2	0.004210329	2.555983434	0.812	0.2	1
ENSSSCG00000056139	0.00421262	-1.700791982	0.5	0.667	1
ENSSSCG00000002855	0.004212799	1.42096961	1	1	1
PLK1	0.004213681	0.066533751	1	1	1
COX19	0.00423249	-1.30256277	0.375	0.667	1
COX16	0.00424075	-0.76747099	1	1	1
TMEM97	0.004245652	-0.416486716	1	1	1
ENSSSCG00000049366	0.004249699	-2.093109404	0.25	0.533	1
TUBGCP5	0.004250061	1.097744618	1	1	1
PRTG	0.004253135	1.240959253	1	0.933	1
ZMYND11	0.004257926	1.203106442	1	1	1
YBX3	0.004259547	-0.443866152	1	1	1
C7orf50	0.004263598	-2.263034406	0.062	0.6	1
CC2D1B	0.004281894	-0.551539499	0.875	0.867	1
MESP2	0.004282651	-0.329307625	0.938	0.933	1
PIP5K1C	0.00429615	-0.079125175	1	0.933	1
ANO10	0.004318358	-0.172082579	0.938	1	1
SIN3B	0.00432188	1.41564253	1	0.933	1
SCAMP2	0.004329533	-0.249305511	1	1	1

WDR12	0.004345062	0.123267602	1	1	1
ENSSSCG00000050702	0.004359009	-1.607682577	0.25	0.667	1
EVI5L	0.004359258	-0.540568381	0.438	0.8	1
EPHA6	0.004363273	-0.005236648	1	1	1
ACER3	0.004363713	-0.04449946	1	1	1
CTSZ	0.004375029	-1.629162305	0.188	0.733	1
PRMT5	0.004386024	-0.115248382	1	1	1
C21orf91	0.004387499	1.58176149	1	1	1
GRK4	0.004392869	-0.870716983	0.5	0.867	1
ENSSSCG00000049490	0.004399501	3.965784285	0.562	0.133	1
RMI1	0.00441071	1.483167368	1	1	1
DAZAP1	0.00442163	-0.688267672	0.812	0.933	1
SLC46A3	0.004424853	-0.481179856	0.812	1	1
TELO2	0.004428386	-4.552541023	0	0.533	1
ENSSSCG00000005908	0.004444505	-1.678071905	0.312	0.533	1
ENSSSCG00000024263	0.004446487	-0.602304397	1	1	1
ENSSSCG00000048448	0.004455114	-1.756074417	0.25	0.667	1
ENSSSCG00000032840	0.004468855	-1.533681996	0.375	0.6	1
DCN	0.004477309	-1.415037499	0.25	0.667	1
CFAP100	0.004488589	-1.137503524	0.438	0.867	1
ENSSSCG00000059579	0.004488653	-1.809316438	0.25	0.533	1
HNRNPH3	0.004495153	0.218284396	1	1	1
BIRC3	0.004509153	1.335189994	1	1	1
MTRFR	0.004515436	-0.576533879	0.938	1	1
AUH	0.004519207	1.213061505	1	1	1
ZMYM3	0.004520551	1.276124405	1	1	1
KIFC1	0.00454935	1.63462835	1	1	1
ENSSSCG00000008850	0.004558646	1.491853096	1	1	1
SYCP1	0.004561086	1.491853096	1	1	1
ENSSSCG00000061649	0.004561682	2.025285296	0.812	0.667	1
UBE2D4	0.00456437	-1.900464326	0.125	0.533	1
TMA16	0.004568599	-0.357351592	1	1	1
DPP8	0.004570584	1.430602966	1	1	1
TBL2	0.004585269	-0.252013	0.875	1	1
ENSSSCG00000050522	0.004596683	-0.958179824	0.562	0.867	1
HINT3	0.004611473	-0.124405535	1	1	1
ENSSSCG00000052071	0.004614814	-0.9510904	0.688	0.867	1
SLC8A1	0.004616304	0.124312201	1	1	1
DTX3	0.00462001	1.807354922	1	0.733	1
STRIP2	0.00462387	1.27293544	1	1	1
ENSSSCG00000012654	0.004645889	1.317433078	1	1	1
YOD1	0.004662139	1.291838261	1	1	1
DKC1	0.004668682	1.360566774	1	1	1
HSPA14	0.004674531	1.111267567	1	1	1
TXNDC15	0.004695706	-1.643306487	0.438	0.667	1
SEL1L	0.004701149	0.15259286	1	1	1
RPS6KB1	0.00470322	-0.222946842	0.938	1	1
MAP3K15	0.004704048	0.91847857	1	1	1
GUCD1	0.004713396	2.121903487	0.938	0.933	1
ENSSSCG00000007749	0.004742423	1.048959768	1	1	1
RMND5B	0.004747565	-0.643923683	0.875	0.933	1
CENPE	0.004771629	1.456258982	1	1	1
MRAP2	0.004783019	-1.093109404	0.812	1	1
SNX30	0.004806712	-0.348106491	0.938	0.933	1
FAIM	0.004808346	0.053224939	1	1	1
CALCOCO1	0.004814925	1.767487539	1	0.867	1
B4GALT3	0.004815501	1.681123447	1	1	1
TERF2IP	0.004821764	0.116206035	1	1	1
VPS33B	0.004835043	-0.701648842	0.938	0.867	1
THAP5	0.004835657	1.315395125	1	1	1
DOCK3	0.004837583	1.289557848	1	1	1
ENSSSCG00000044306	0.004838811	-1.192645078	0.375	0.667	1
CYP2D6	0.004849954	-2.809316438	0.125	0.533	1
GPAT4	0.004880329	-0.040160524	1	0.933	1
SAV1	0.004882146	1.176322773	1	1	1
ENSSSCG00000053483	0.004886867	-2.24961389	0.688	0.467	1
ECT2	0.004887427	1.273988395	1	1	1
CS	0.004891176	1.146013232	1	1	1
LIMD2	0.004906286	-0.623624121	0.875	1	1
ENSSSCG00000053875	0.004906928	2.87036472	0.75	0.2	1

ENSSSCG00000044627	0.004913522	-0.270936273	1	1	1
SERPINE3	0.004922378	-0.798366139	0.75	0.8	1
ENSSSCG00000058938	0.004926601	-1.00904514	0.812	0.8	1
CDK1	0.004927524	-0.566246126	0.938	1	1
DCAF10	0.004943984	-0.805532814	0.75	0.933	1
ENSSSCG00000055605	0.004944753	-1.093109404	0.562	0.867	1
LIFR	0.004944861	-0.018943386	1	1	1
H2BC1	0.004947739	-0.420962333	0.938	1	1
LHX1	0.00494933	1.557942287	1	0.867	1
CLOCK	0.004957714	1.014081575	1	1	1
IFT20	0.00497319	-0.247437551	1	1	1
PIP4K2A	0.004975443	-0.600713022	0.938	0.933	1
LPP	0.004977295	1.405696453	1	0.733	1
ENSSSCG00000035622	0.004983301	1.392791136	1	1	1
SCN4A	0.00499137	-0.617268626	0.938	1	1
ENSSSCG00000007847	0.004996897	1.506352666	0.938	0.8	1
KCNK13	0.005009519	-1.366127899	0.438	0.733	1
ZNF248	0.005034404	1.310740414	1	1	1
ND4L	0.005053496	1.859288913	1	1	1
HLX	0.005058039	-1.186218809	0.438	0.8	1
ENSSSCG00000060401	0.00506609	-1.736965594	0.188	0.533	1
ENSSSCG00000048380	0.00508099	-1.35614381	0.5	0.733	1
PSME1	0.005084239	-2.359895945	0.125	0.933	1
ENSSSCG00000045735	0.005085673	-1.010647244	0.688	0.867	1
UTP3	0.00508885	-0.238208298	1	1	1
ENSSSCG00000060030	0.005091424	-0.252013	0.938	1	1
CNOT2	0.005103703	1.216283055	1	1	1
ENSSSCG00000042822	0.005104484	-1.678071905	0.25	0.533	1
KBTBD8	0.005107408	1.190062647	1	0.933	1
SELENOT	0.005109147	0.065783906	1	1	1
ENSSSCG00000059519	0.005110852	-0.913288367	0.812	1	1
FAM177A1	0.005123195	1.114998791	1	1	1
PRICKLE2	0.005123249	0.977229813	1	1	1
SLC26A9	0.005127875	1.967772838	0.938	0.667	1
LRP11	0.005128565	2.321928095	0.812	0.4	1
RBMS1	0.005142862	1.554411409	1	1	1
ADGRA3	0.005153442	0.137472707	1	1	1
GBE1	0.005157857	1.270619001	1	1	1
MFSD14B	0.005171349	1.112345022	1	1	1
SLC7A5	0.005176572	-1.545621609	0.5	0.667	1
ENSSSCG00000011121	0.005217488	-0.013765973	1	1	1
EFNA1	0.005218028	1.490233001	1	0.933	1
ENSSSCG00000042788	0.005235956	-0.334117504	0.938	0.933	1
C10orf143	0.00523728	-0.144639705	1	1	1
PRTFDC1	0.005241011	3.491853096	0.562	0.133	1
ARL6	0.005241351	-0.451902793	1	1	1
DNAJA2	0.005242834	0.062202624	1	1	1
FKBP5	0.005256536	0.241541542	1	1	1
QDPR	0.005269388	-0.301597987	1	1	1
ENSSSCG00000014242	0.005278195	-0.595609745	0.812	0.933	1
DHRS11	0.005286664	-0.941106311	0.562	0.733	1
ARID3B	0.005289192	-0.043356369	0.938	1	1
ENSSSCG00000061655	0.005291905	-0.881605299	0.812	0.933	1
SYS1	0.005292468	-0.068115025	1	0.933	1
AP5S1	0.005304202	-1.578536232	0.312	0.667	1
ELL2	0.005311439	-0.406863568	1	1	1
TUBA3C	0.005320275	1.438215133	1	0.933	1
DYNC2LI1	0.00532711	1.539729451	1	1	1
SUMF1	0.005328766	-0.093109404	1	1	1
FAM178B	0.005336157	-1.508146904	0.312	0.533	1
BLCAP	0.00534171	1.924812504	0.938	0.8	1
PKP2	0.005344945	3.74819285	0.562	0.067	1
IKBKG	0.005358922	-1.552541023	0.438	0.667	1
RBM15B	0.005359852	-0.641730058	0.812	0.933	1
RETREG3	0.005361475	-1.00843908	1	0.933	1
RTN2	0.005379844	-0.652536813	0.938	0.867	1
POFUT1	0.005407976	1.259515488	1	1	1
CDCA3	0.005421415	1.717073072	1	1	1
FAM193A	0.005423346	-0.286989138	0.938	0.933	1
TRAPPC9	0.005430356	-0.351711564	1	1	1

PBLD	0.005432964	-0.599569931	1	1	1
C12orf43	0.005442949	-0.877380713	0.812	0.933	1
TMEM254	0.005470772	-0.716039755	0.875	0.867	1
ITIH2	0.005472225	-1.127056736	0.375	0.667	1
HERC4	0.0054773	1.136535133	1	1	1
PIP5K1B	0.005481814	1.329666605	1	1	1
BLVRA	0.005489233	-0.9861942	0.5	0.733	1
HPGDS	0.005495061	1.094053307	1	1	1
KAT8	0.005509312	-0.100359143	1	1	1
SEC14L2	0.005512227	-1.236067358	0.625	0.867	1
FDXR	0.005524215	-2.211290831	0.438	0.933	1
STK39	0.005532642	0.053947225	1	1	1
RELA	0.005539256	-1.05994254	0.5	0.8	1
MTHFD1L	0.005540365	-0.443606651	0.938	0.933	1
MAPK1IP1L	0.005580373	-0.304216018	1	1	1
PRRG1	0.005597382	1.151396925	1	1	1
ENSSSCG00000044155	0.005602329	-0.706086281	0.75	0.933	1
ENSSSCG00000057365	0.005616955	-0.053226889	1	1	1
TTLL11	0.005622202	-0.858644151	0.625	0.733	1
GINM1	0.005622399	1.364265041	1	1	1
FLOT2	0.005637594	-0.644519345	0.75	0.8	1
ZNF462	0.005639236	1.134502947	1	1	1
ENSSSCG00000033707	0.005640175	-0.761903496	0.875	1	1
ENSSSCG00000063070	0.005641162	-0.967578522	0.688	0.733	1
DROSHA	0.005671023	0.042829253	1	1	1
GCAT	0.005671757	-2.595609745	0.125	0.533	1
FAM8A1	0.005672375	-0.703162886	0.812	1	1
CDK6	0.0056762	-2.571156701	0.125	0.6	1
ENSSSCG00000050357	0.005683974	-1.455679484	0.375	0.533	1
SLCO3A1	0.00568573	-0.739800895	1	0.867	1
E2F8	0.005703716	1.283680626	1	1	1
ENSSSCG00000035091	0.005704734	-0.809316438	0.688	0.8	1
TFE3	0.005715325	-0.260051781	1	1	1
MTMR8	0.005727756	-0.545621609	0.875	1	1
USP46	0.005734949	0.099011343	1	1	1
ENSSSCG00000002709	0.005736097	-0.125450124	1	1	1
ATIC	0.005745059	1.126662749	1	1	1
NUDT22	0.005752809	-1.567040593	0.188	0.533	1
PDCD10	0.005770791	1.168410588	1	1	1
PDCD7	0.005775975	1.281127564	1	1	1
ENSSSCG00000059276	0.00577862	-0.62814668	0.625	0.867	1
TMEM107	0.005783607	1.32349539	1	1	1
DCTPP1	0.005784441	1.27330723	1	1	1
PSMD11	0.005793122	1.131586109	1	1	1
ILKAP	0.005804703	-0.10718459	0.938	1	1
FEM1A	0.005844526	-0.753622938	0.562	0.8	1
TASP1	0.005847952	-0.900464326	0.875	0.733	1
APLP2	0.005864213	0.10671416	1	1	1
WDR19	0.005892945	1.118286568	1	1	1
ENSSSCG00000039249	0.005912017	1.12026654	1	1	1
TRAF6	0.005944952	1.218492838	1	1	1
ENSSSCG00000047668	0.005945823	-1.595609745	0.188	0.733	1
CASTOR2	0.005948285	-0.391632895	0.875	0.933	1
HNRNPLL	0.005971441	1.791710933	1	0.8	1
KYAT3	0.005975677	-0.530730189	0.938	0.933	1
MCCC1	0.005976278	1.147065047	1	1	1
GOLGA1	0.005980579	0.103815206	1	1	1
ENSSSCG00000050056	0.005990304	1.411565307	1	1	1
ENSSSCG00000004814	0.005996519	0.072186495	1	1	1
LUZP1	0.00599683	1.072080505	1	1	1
CD63	0.006002124	-1.569324242	0.938	1	1
TTC19	0.006009942	-1.830074999	0.312	0.533	1
MRC1	0.006037231	2.039340892	0.812	0.4	1
FAM209B	0.006040547	-1.967578522	0.188	0.6	1
ENSSSCG00000005457	0.006079988	1.555000815	1	0.867	1
C14orf119	0.006099237	1.290854135	1	1	1
UGP2	0.006102108	1.044777809	1	1	1
ILDR2	0.006106369	1.315331398	1	1	1
RABGGTA	0.006119668	-0.580461108	1	1	1
TMEM131	0.006133844	1.060022654	1	1	1

REPS2	0.006134178	-0.116140373	1	0.933	1
ENSSSCG00000036792	0.006140201	-0.249699767	1	1	1
DGKB	0.006145983	1.328454029	1	0.933	1
F2RL1	0.00616401	0.085860737	1	1	1
INTS13	0.006172333	0.005396141	1	1	1
ENSSSCG00000051336	0.006229765	-1.530514717	0.438	0.733	1
RING1	0.006237745	-3.793549123	0	0.533	1
UBE3B	0.006255331	-0.276139393	1	1	1
MAP3K20	0.006258928	1.329833293	1	0.933	1
MBLAC1	0.00627138	-0.7355574	0.75	0.8	1
PLA2G4D	0.00627531	1.269121736	1	1	1
ENSSSCG00000012541	0.006283995	-0.9861942	0.312	0.8	1
ZNF691	0.006296909	-1.220864952	0.625	0.533	1
RNPS1	0.006297178	1.239770693	1	1	1
SLC9A6	0.006321817	-0.074342375	0.938	1	1
KREMEN1	0.006330265	-0.206003461	1	1	1
NEK11	0.0063524	1.266934063	1	0.867	1
DCLRE1C	0.006364363	-1.032567862	0.938	0.867	1
C17orf107	0.006365252	-1.415037499	0.312	0.6	1
TSEN34	0.006369861	-1.736965594	0.125	0.6	1
ENSSSCG00000014031	0.006376259	1.520493144	1	1	1
SASS6	0.00638171	1.241090493	1	1	1
ENSSSCG00000017032	0.006385684	-0.407893937	1	1	1
P2RX4	0.006389982	-1.257496222	0.5	0.733	1
SUGP2	0.006406737	1.58739743	1	1	1
FAM102B	0.006422957	1.227046389	1	1	1
MYH3	0.006423398	-1.382616022	0.312	0.6	1
ACBD5	0.006444325	1.220390068	1	1	1
ENSSSCG00000060966	0.006447721	-1.973527789	0.5	0.667	1
ADAM10	0.00647117	0.960543575	1	1	1
CPNE4	0.006472851	1.761039729	1	0.667	1
HELZ	0.006482316	1.148965383	1	1	1
MGAT1	0.006489943	-0.278921468	1	1	1
MYOCD	0.006496678	1.906890596	0.875	0.667	1
ENSSSCG00000054140	0.006562858	-0.615530439	0.812	0.933	1
ATP2A2	0.006593387	1.004811316	1	1	1
SETD5	0.006595634	1.564002882	1	1	1
MAP2K4	0.006608175	1.136304089	1	1	1
TGFBR3	0.006642223	-1.093109404	0.625	0.733	1
ARHGEF39	0.006654822	1.882643049	0.938	0.667	1
SEMA3C	0.006656298	1.3534546	0.938	0.933	1
RGL1	0.006657928	1.322363205	1	1	1
GABBR2	0.006680015	-0.426846802	0.812	1	1
ZNF510	0.006696715	1.631046383	1	0.867	1
GALNTL5	0.006731749	-0.492716863	0.75	0.867	1
ENSSSCG00000055570	0.006733857	1.878876219	0.875	0.4	1
TMEM33	0.006744906	1.133594438	1	1	1
ASIC3	0.006757782	-1.900464326	0.125	0.533	1
SLC41A2	0.006760532	1.245885688	1	1	1
POGZ	0.006764842	1.120863413	1	1	1
RASGRP1	0.006772245	-0.417460162	0.938	1	1
GTF2E1	0.006778353	0.863482585	1	1	1
ENSSSCG00000012746	0.006813925	0.147533861	1	1	1
NOL6	0.006821983	-0.730539325	0.5	0.867	1
LMO7	0.00682268	0.290594888	1	1	1
WHAMM	0.006823959	1.232575012	1	1	1
ENSSSCG00000021767	0.006834887	0.026876452	1	1	1
HIRIP3	0.006836261	0.067762349	0.938	1	1
COMMD3	0.006838131	-0.044310106	1	1	1
ENSSSCG00000044382	0.006843274	-0.813001485	0.75	0.8	1
C18orf25	0.006843589	0.162005338	1	1	1
SEC22C	0.006847258	-0.121678557	1	1	1
SLC35F5	0.006856356	1.491853096	1	0.733	1
GUCY1B1	0.006870875	3.845490051	0.5	0.067	1
THBS4	0.006887517	1.349574513	1	1	1
LXN	0.006896796	-0.561298382	1	1	1
SCFD1	0.00693904	0.147663911	1	1	1
TEPSIN	0.006950135	-0.852101305	0.688	0.733	1
ITGA9	0.006962833	1.45462019	1	1	1
ENSSSCG00000020870	0.006969622	1.17077892	1	1	1

SERPINE2	0.006985018	-0.214950527	0.938	1	1
TMEM63B	0.00699644	-0.350267244	0.938	1	1
EMILIN2	0.006998782	-0.058663258	1	1	1
RANBP10	0.007002369	-0.259985774	0.938	1	1
AAR2	0.007050229	-0.268427665	0.875	0.933	1
TACR1	0.007059722	-0.761700256	0.75	1	1
RHEB	0.0070691	1.298346245	1	1	1
ENSSSCG00000058759	0.007126074	-0.701341684	0.938	0.867	1
PSMD10	0.007152424	1.066128376	1	1	1
FDX2	0.007154693	-1.781165398	0.25	0.667	1
CLK4	0.007159509	-0.161127828	1	1	1
ATP6AP2	0.00716564	0.244100955	1	1	1
DCBLD2	0.007192128	1.340776292	1	1	1
MPI	0.007194023	-1.458758877	0.438	0.667	1
ENSSSCG00000024765	0.007202274	-0.483315051	0.938	1	1
CFTR	0.007239212	1.315187949	1	0.867	1
MED22	0.007275677	-1.040641984	0.625	0.6	1
WWOX	0.007290126	0.11914457	1	1	1
TKFC	0.00729195	-1.61667136	0.438	0.533	1
ENSSSCG00000063021	0.007296875	-0.332297068	0.875	1	1
TSPAN3	0.007304546	1.089898012	1	1	1
C8orf88	0.00730874	-1.533681996	0.562	0.467	1
MRTFB	0.007313886	0.08628835	1	1	1
TIMM44	0.00733504	-0.751418055	0.875	1	1
TBC1D31	0.007340612	0.129563779	1	1	1
CHSY3	0.007351416	-0.551539499	1	1	1
ATG16L1	0.007359026	1.310389596	1	1	1
C17orf49	0.007383154	1.519867472	1	0.867	1
ALS2	0.007383888	1.172522113	1	1	1
ENSSSCG00000045482	0.007405644	1.966294883	1	0.933	1
MIER2	0.007407082	1.417421372	1	1	1
ZBTB1	0.007423155	1.614297999	1	0.733	1
EIF2AK3	0.007425416	-0.874469118	0.312	0.8	1
ENSSSCG00000055921	0.007425654	-1.607682577	0.25	0.6	1
PPIL6	0.007425687	-1.392669686	0.375	0.667	1
ENSSSCG00000056286	0.007447523	-1.629162305	0.375	0.6	1
ENSSSCG00000062599	0.007456053	1.126244484	1	1	1
UBXN2A	0.007456584	1.509351687	1	1	1
DAZAP2	0.007470889	1.190522872	1	1	1
PCSK4	0.007485478	-0.68975371	0.938	0.733	1
ADAMTS7	0.00749284	-0.337416602	0.938	1	1
ENSSSCG00000003421	0.007496963	-1.941106311	0.188	0.533	1
LIN7C	0.007504088	1.10206983	1	1	1
CLASP1	0.007504358	-0.120721636	0.875	0.933	1
BLOC1S2	0.007518544	-0.317982815	1	0.933	1
FBXO28	0.007519606	-0.13517904	1	1	1
FCSK	0.007520955	-1.678071905	0.438	0.533	1
RNF13	0.007536222	1.141044568	1	1	1
SPATA22	0.007537142	-0.457293688	0.938	0.933	1
GTF3C1	0.007554823	1.35108881	1	1	1
PBRM1	0.007561767	1.157894664	1	1	1
ASPHD2	0.007564091	-0.32287434	1	1	1
PCNP	0.007591315	1.453472005	1	1	1
FHOD1	0.00762823	1.611647609	1	0.8	1
ENSSSCG00000057584	0.007640168	-1.446746359	0.312	0.533	1
RECK	0.007643923	1.360847084	1	1	1
DZANK1	0.00764772	-0.462343214	1	0.867	1
ENSSSCG00000061166	0.00765453	-1.552541023	0.25	0.733	1
WIF1	0.007663325	1.37627507	1	1	1
ENSSSCG00000051948	0.007666266	-1.61667136	0.312	0.533	1
P2RY14	0.007713201	-0.996894089	0.625	0.667	1
IFTAP	0.007718904	-0.775919229	0.875	0.867	1
DUSP23	0.007722562	-1.392669686	0.5	0.667	1
GHRHR	0.007723854	-1.334117504	0.25	0.667	1
TMEM243	0.007726216	1.322578984	1	1	1
VPS33A	0.007731314	-1.05994254	0.688	0.8	1
KIAA0825	0.007731594	0.963834392	1	1	1
CDK2AP1	0.007744263	1.260000346	1	1	1
SMDT1	0.007780534	-1.600069393	0.375	0.867	1
SRGN	0.007804	-0.415037499	0.875	0.933	1

ZNF784	0.007818807	-2.093109404	0.125	0.533	1
FAF1	0.007846159	-0.263536824	1	0.933	1
MID1	0.007846521	-0.158134564	1	1	1
SCAMP3	0.007847933	-0.309317954	1	0.933	1
UBXN2B	0.007849614	1.206590127	1	1	1
SH2D5	0.007859236	-1.325770161	0.5	0.667	1
PRPF4B	0.007861151	0.201057619	1	1	1
ENSSSCG00000007188	0.007894975	1.396241185	1	1	1
KDM6A	0.007905764	1.204206136	1	1	1
JMJD7	0.007926179	-1.127056736	0.125	0.733	1
KLC1	0.007945081	1.259824075	1	1	1
TRMT112	0.007948199	-0.248792483	1	1	1
ENSSSCG00000032311	0.007952043	-0.093109404	0.938	1	1
CLGN	0.007970298	1.220618209	1	1	1
ZFP2	0.007989201	-0.186580886	1	1	1
PTPRM	0.008019595	-0.527512229	0.875	0.933	1
FASTKD1	0.008020315	1.329779267	1	1	1
PI4KA	0.008040544	-0.139279586	1	0.933	1
VWA2	0.008042179	-2.900464326	0.062	0.533	1
PGM5	0.008074074	1.512701653	1	0.933	1
NUDT6	0.008107561	-0.527512229	0.812	0.933	1
ENSSSCG00000057830	0.008107682	-1.736965594	0.25	0.6	1
ENSSSCG00000061141	0.008125764	1.479469372	0.938	0.867	1
MYORG	0.008128682	-0.600478784	1	1	1
ENSSSCG00000014075	0.008149589	-0.509879871	1	1	1
THEG	0.008195424	-1.441032708	0.25	0.667	1
COX18	0.008214384	-0.396890153	0.688	0.933	1
ATRN	0.008223796	1.337555209	1	1	1
CFAP410	0.008229363	-1.900464326	0.188	0.733	1
OTUD1	0.008250976	1.497434129	1	0.933	1
ENSSSCG00000042022	0.008257761	-0.136502	1	1	1
SLC29A4	0.00826434	-0.977632187	0.375	0.533	1
ZNF696	0.008274044	-0.963048864	0.688	0.8	1
PTPA	0.008324418	-2.128733314	0.188	0.667	1
NSUN7	0.00833371	1.035840998	1	1	1
RBBP5	0.008342716	0.920939673	1	1	1
PIGK	0.00834454	1.420280747	1	1	1
CAGE1	0.008359536	-0.212498134	1	1	1
SNAPIN	0.008361958	-0.315501826	1	1	1
KNOP1	0.008374206	-1.415037499	0.5	0.6	1
STX16	0.008376227	-1.587874096	0.25	0.733	1
IP6K1	0.008382438	-0.109199107	1	1	1
CUL4A	0.008390774	-0.115220106	1	1	1
GSN	0.008391142	2.540762697	0.875	0.6	1
KIAA1191	0.008405816	-0.056274188	1	1	1
PSMB2	0.008409854	-0.109597527	1	1	1
TMPRSS11E	0.008411896	2.528378972	0.688	0.333	1
RRP9	0.008421105	-0.447642165	0.938	1	1
CNTN4	0.008422065	1.571641122	1	0.867	1
ENSSSCG00000010440	0.008422372	-0.08323916	1	1	1
CCDC50	0.008425082	1.074874207	1	1	1
ENSSSCG00000002811	0.008446167	-1.830074999	0.125	0.533	1
PHEX	0.008456296	1.024221383	1	1	1
MED12	0.008463695	-0.304983954	0.938	0.933	1
RALGPS1	0.008476109	-1.337034987	0.562	0.733	1
ACSL1	0.008493716	0.04342228	1	1	1
CYB5R1	0.008494978	-1.11417102	0.688	0.933	1
JUP	0.008498157	2.5987683	0.688	0.4	1
ENSSSCG00000062217	0.008499836	-0.707819249	0.875	0.867	1
HMGXB3	0.008527872	-0.140588115	1	0.933	1
CXCL9	0.008542785	-0.444581775	0.875	0.933	1
CCNO	0.00856579	-0.912537159	1	0.867	1
ENSSSCG00000049325	0.008568402	-1.137503524	0.312	0.867	1
LARP6	0.008574291	1.63895739	1	0.8	1
EHD2	0.008589437	-1.607682577	0.25	0.667	1
ENSSSCG00000034353	0.008594299	1.821160722	1	0.667	1
CHMP3	0.008604423	0.16817297	1	1	1
ENSSSCG00000009523	0.008607321	1.359202468	1	1	1
KAT7	0.008617584	-0.118898086	1	1	1
ENSSSCG00000033770	0.008620243	2.091765939	1	0.733	1

NMRAL1	0.008634753	3.076815597	0.75	0.067	1
MST1R	0.008654325	-1.155393683	0.562	0.8	1
ENSSSCG00000037652	0.008676467	-0.578536232	0.938	1	1
ORC3	0.008679872	-0.004434723	1	1	1
NIBAN1	0.008690764	-1.019108823	0.625	0.667	1
TLCD5	0.00873065	1.349834091	1	1	1
PAK1IP1	0.008735031	0.037954351	1	1	1
ENSSSCG00000039774	0.008744576	-0.957225976	0.812	0.933	1
ENSSSCG00000061987	0.008777565	1.884864289	0.938	0.733	1
SHTN1	0.008783507	-0.186744477	1	1	1
TAF1D	0.008794031	-0.284250892	1	0.933	1
ETV3	0.008802665	-0.127056736	1	1	1
ELAPOR1	0.008832487	-0.075674811	0.938	1	1
ENSSSCG00000060630	0.008835699	-1.093109404	0.438	0.733	1
LPCAT4	0.008863533	-2.315501826	0.125	0.533	1
SLC48A1	0.008871185	-0.595609745	0.75	0.933	1
MAP10	0.008893822	-1.18932472	0.5	0.8	1
PPIL3	0.008931824	-1.256608137	0.688	1	1
VAMP7	0.008933744	1.140974982	1	1	1
YAF2	0.008944984	1.421072741	1	0.867	1
MARCHF7	0.008951602	1.101400699	1	1	1
ARL4D	0.008965862	1.29687293	1	1	1
CKAP4	0.008971613	-0.141364573	0.875	0.933	1
ENSSSCG00000057252	0.008990667	2.594946589	0.75	0.2	1
RSRC1	0.008994867	1.026001807	1	1	1
CCDC191	0.009000426	-0.378086363	0.938	1	1
ERFL	0.009002402	-0.929610672	0.5	0.733	1
KIZ	0.00900671	1.700039025	1	1	1
SRGAP1	0.009015529	-0.099350344	0.938	1	1
KLHL25	0.009024724	1.464169538	1	1	1
ENSSSCG00000011243	0.009072507	0.042338789	1	1	1
CCDC141	0.009076424	-0.900464326	0.688	0.733	1
MRPS27	0.009111366	-0.181918671	1	1	1
MOCS1	0.009120395	-0.127874823	0.875	0.933	1
ENSSSCG00000044312	0.009123229	-1.180572246	0.438	0.667	1
SLC6A6	0.00913751	1.121939343	1	1	1
C15orf39	0.009141976	-0.343336034	0.938	0.867	1
LIPE	0.009199204	1.949959318	0.938	0.733	1
SENP1	0.00921318	-0.208586622	0.938	1	1
ARHGEF37	0.009215453	-1.341036918	0.25	0.533	1
NOB1	0.009280988	0.181897643	1	1	1
MIIP	0.009301737	-0.604362219	0.938	0.933	1
IFT122	0.009314346	-0.683289614	0.875	1	1
IRAK1BP1	0.009317523	-0.638105498	0.938	0.8	1
ASNSD1	0.009339908	-1.18932472	0.688	0.733	1
ENSSSCG00000035290	0.009345975	-0.678071905	0.812	0.8	1
ENSSSCG00000052712	0.009352073	-0.227868926	1	1	1
RTN4	0.009368772	-0.745186101	0.75	0.8	1
ENSSSCG00000047723	0.009393826	3.550746785	0.688	0.067	1
CREB1	0.009414355	-0.588434693	0.812	1	1
ENSSSCG00000007639	0.009468773	3.861086906	0.562	0.067	1
ALB	0.009474861	-0.65870658	0.812	0.867	1
SDR42E1	0.009476	-1.707819249	0.438	0.6	1
AGFG1	0.00948893	-0.09230185	1	1	1
ESRRB	0.009496742	-1.415037499	0.312	0.6	1
TM9SF2	0.009511636	1.097805666	1	1	1
SNX29	0.009520239	-0.230612928	0.812	0.933	1
B3GLCT	0.009537811	-0.116760672	1	1	1
NAA50	0.009565712	1.027701716	1	1	1
RCC1L	0.009586893	1.75406098	1	1	1
ENSSSCG00000057040	0.009644749	-2.629162305	0.375	0.6	1
APOB	0.009649302	-0.358162978	0.812	0.933	1
ZBTB37	0.00965654	1.127686232	1	1	1
KYAT1	0.009674193	1.538980491	1	0.933	1
KCNMB3	0.009696457	-1.678071905	0.188	0.533	1
AP1S2	0.009697095	1.303863379	1	1	1
ZNF449	0.009714157	1.120260014	1	1	1
ENSSSCG00000048494	0.009715503	-1.607682577	0.375	0.533	1
FBXL2	0.009727541	-0.212949174	1	1	1
FAM53C	0.009755217	0.160324671	1	1	1

TRPV6	0.009769658	-1.607682577	0.188	0.6	1
TTC21B	0.009792474	1.182895784	1	1	1
STIM1	0.009825555	1.861903141	1	1	1
GGT7	0.009829074	-1.341036918	0.5	0.667	1
ABL1	0.009851161	1.096551815	1	1	1
ZNF354A	0.009863822	-0.08901665	1	1	1
CASP3	0.009905165	-0.27690658	1	1	1
CCDC92	0.009909554	-0.852101305	0.562	0.8	1
NUDT3	0.009919234	0.164090376	1	1	1
KIFBP	0.009921398	-0.289506617	1	1	1
VDAC2	0.009963083	-0.858644151	0.938	0.8	1
GCDH	0.009975813	-0.228886183	0.812	0.933	1
RPAIN	0.009995982	1.182525038	1	1	1
FKBP8	0.009996734	-0.900464326	0.625	0.8	1
ZNF655	0.010011132	1.0172456	1	1	1
ZNF713	0.010035345	1.558450845	0.938	0.933	1
ENSSSCG00000009333	0.01005876	-1.230612928	0.375	0.667	1
ENSSSCG00000060591	0.010073103	-1.497499659	0.5	0.6	1
ENSSSCG00000062462	0.010080175	-1.277533976	0.312	0.6	1
TRMT61A	0.010095336	-1.77118131	0.188	0.533	1
C11orf54	0.010098715	1.346513733	1	1	1
CARF	0.010106734	2.072228328	0.812	0.4	1
SC5D	0.010107106	-0.514936069	0.812	0.733	1
PRPS1	0.010126225	0.291008456	1	1	1
DNAJC2	0.010140525	0.160384234	1	1	1
SMC1A	0.010143871	0.245782697	1	1	1
MPC2	0.010159635	1.193533629	1	1	1
ENSSSCG00000059490	0.010162469	-1.299560282	0.375	0.533	1
ENSSSCG00000059628	0.010182865	-0.09965225	1	1	1
GPRASP2	0.010185205	1.370123784	1	1	1
TRAPPC3	0.01022478	1.137465129	1	1	1
HNRNPU	0.01023144	0.204345133	1	1	1
RAB8B	0.010242291	-0.157862197	1	1	1
ENSSSCG00000059905	0.01027855	2.316281532	0.812	0.467	1
SH3BGRL2	0.010288232	0.969024031	1	1	1
ZNF483	0.010342221	-0.180307846	1	1	1
TYSND1	0.010345805	-1.540568381	0.312	0.733	1
PWP2	0.010347576	-0.793549123	0.75	0.933	1
RO60	0.010388587	-0.316580476	1	1	1
ZNF668	0.010399104	-1.230612928	0.375	0.6	1
TMEM47	0.010470909	1.448351458	1	1	1
DDX24	0.010492246	-0.063337505	1	1	1
INTS2	0.010517815	-0.04608407	1	1	1
ZSWIM8	0.010530459	-0.330860446	1	1	1
WWP1	0.01055146	1.186697011	1	1	1
ENSSSCG00000061694	0.010565786	-0.704544116	0.688	0.867	1
BLTP1	0.010594311	0.951586053	1	1	1
ENSSSCG00000046323	0.010595497	1.446049407	1	0.867	1
ENKUR	0.010605179	-0.076929477	0.938	1	1
SLC5A2	0.010609115	-0.53562764	0.875	0.867	1
EHD3	0.010624948	-1.858644151	0.062	0.6	1
CNNM2	0.010636661	0.134864893	1	1	1
CDH18	0.010650361	2.417852515	0.75	0.333	1
KLHDC10	0.010668548	0.905725252	1	1	1
RBM11	0.01069402	-1.299560282	0.5	0.6	1
ZGLP1	0.010716376	-1.137503524	0.5	0.6	1
EXOC2	0.010731129	-0.076989739	1	1	1
STK19	0.01076807	-0.611576493	0.625	0.867	1
ENSSSCG00000004572	0.010779353	-1.277533976	0.188	0.6	1
ENSSSCG00000018034	0.010796718	1.35720357	1	1	1
RALGPS2	0.010801694	1.004049506	1	1	1
TRAF3IP1	0.010805838	-0.967578522	0.5	0.867	1
MYBBP1A	0.010807099	-0.516917113	0.938	1	1
ENSSSCG00000009802	0.010809493	-1.18932472	0.625	0.733	1
ENSSSCG00000052522	0.01084831	-0.937458534	0.562	0.733	1
CENPT	0.010850042	-0.033196503	0.938	1	1
PTPN20	0.010871208	1.452631787	1	0.933	1
EHBP1	0.010903262	1.365858698	1	1	1
KCTD3	0.0109093	0.178052882	1	1	1
MBNL3	0.010911503	1.243362748	1	1	1

FANCM	0.010940286	-0.276615973	0.938	1	1
MAP3K4	0.010946288	1.099149048	1	1	1
EAF2	0.01094979	1.106572784	1	1	1
NCSTN	0.010950731	1.16458258	1	1	1
COPS5	0.01097474	-0.441288391	0.938	1	1
ENSSSCG00000036529	0.010987272	-0.28530877	1	1	1
MTMR7	0.011027648	1.217476004	1	1	1
ENSSSCG00000058915	0.011070854	-0.323889611	0.812	0.933	1
ENSSSCG00000059428	0.011083863	-1.721140627	0.312	0.667	1
PDCD6IP	0.011094472	0.945709844	1	1	1
ENSSSCG00000001917	0.011106772	-0.931358334	0.688	0.8	1
ENSSSCG00000056992	0.0111121	-1.441032708	0.438	0.6	1
HECTD3	0.011113634	-1.075407403	0.5	0.667	1
TUT4	0.011124939	-0.972815171	0.812	0.8	1
SPATA2	0.011125741	0.340072756	1	1	1
SREBF2	0.011128994	1.493979397	1	0.933	1
ENSSSCG00000039144	0.011131566	-1.552541023	0.375	0.533	1
F11R	0.011165284	1.484399699	1	1	1
SNRPD1	0.011168055	0.052445169	1	1	1
ENSSSCG00000056070	0.011173031	-0.723159795	0.875	0.8	1
ARRB2	0.01117823	-1.870716983	0.25	0.533	1
PRPF3	0.011202839	0.975630186	1	1	1
ENSSSCG00000051889	0.011211861	0.012907484	1	1	1
RUNX1	0.011272417	-1.044199804	0.438	0.667	1
RCAN3	0.01133349	1.118629221	1	1	1
BET1	0.011387088	-0.188707502	1	1	1
PRKN	0.011400256	-0.19363628	1	0.933	1
DFFA	0.011405652	-0.19363628	0.938	1	1
PC	0.011416011	-1.050040682	0.5	0.667	1
ANKS3	0.01142995	-0.678071905	0.375	0.667	1
BCL2L13	0.011459069	1.311537649	1	1	1
ENSSSCG00000058426	0.011478566	2.584962501	0.688	0.2	1
ENSSSCG00000038362	0.011481419	-0.312890612	0.875	1	1
KRT10	0.011488455	-1.245112498	0.312	0.6	1
ENSSSCG00000039731	0.011516984	-0.060596591	1	0.933	1
SLC35B3	0.011556873	-0.853158612	0.625	0.867	1
TBC1D22A	0.011583123	-0.65870658	0.562	0.733	1
BCAS1	0.011604549	0.250502517	1	1	1
SKOR1	0.011645253	-1.062735755	0.562	0.8	1
CEP63	0.011655665	0.032421478	1	1	1
UROC1	0.011659996	-1.093109404	0.562	0.667	1
ERMARD	0.011670405	-0.529208519	0.562	0.8	1
TASOR2	0.011696611	0.912725879	1	1	1
ENSSSCG00000005371	0.011705797	-0.556241192	1	1	1
ABCC6	0.011719887	-0.543630617	1	1	1
MYH14	0.011790242	-1.595609745	0.188	0.6	1
EXTL2	0.011798274	-0.550959987	0.938	0.867	1
SNRK	0.011807638	0.887019674	1	1	1
ENSSSCG00000053759	0.011833258	-1.434146322	0.312	0.6	1
AGPS	0.011838706	1.147336394	1	1	1
ENSSSCG00000041959	0.01187174	-1.508146904	0.25	0.6	1
ENSSSCG00000057344	0.01192454	-0.384875529	0.75	0.933	1
APEX1	0.011925524	1.032421478	1	1	1
PLA2G12B	0.011933174	-1.093109404	0.562	0.733	1
ENSSSCG00000013990	0.011962502	-0.16930903	1	1	1
ARMC1	0.011976172	1.086924934	1	1	1
ZSWIM3	0.011997334	1.555677641	1	1	1
SLC1A4	0.012010982	0.019200812	0.938	1	1
ZNF165	0.012011737	-0.176674121	0.938	1	1
TGFBR1	0.012012429	-0.227671653	1	0.933	1
ZNF514	0.012038223	1.211698057	1	1	1
DISP1	0.012056176	-0.315501826	0.875	0.867	1
SCAPER	0.012057978	0.961644554	1	1	1
ENSSSCG00000047974	0.012081627	-1.678071905	0.188	0.6	1
ENSSSCG00000046630	0.012115437	2.366322214	0.688	0.4	1
BTBD6	0.012130485	2.027905997	1	0.733	1
ARID5B	0.012227974	-0.326828243	1	0.933	1
HCCS	0.012235556	1.449727621	1	1	1
ENSSSCG00000063163	0.012253825	0.029747343	1	1	1
GAP43	0.01225872	-0.272079545	0.75	1	1

ENSSSCG00000029696	0.012275246	-0.59400764	0.75	0.8	1
ADAMTS9	0.012281162	-1.13058411	0.625	0.6	1
ENSSSCG00000016119	0.012346359	-0.044942122	1	1	1
CCDC62	0.012410298	1.276124405	1	0.733	1
TREX1	0.012456663	-1.455679484	0.125	0.6	1
DCLRE1B	0.012461246	-0.421945869	0.812	1	1
BRDT	0.012461438	1.137210464	1	1	1
LIMD1	0.012462778	-0.496174262	0.812	0.933	1
ABCG5	0.012469728	-0.011809302	1	1	1
ENSSSCG00000053393	0.012470409	-1.27368165	0.5	0.533	1
ENSSSCG00000059780	0.012486272	-0.870716983	0.438	0.733	1
EXOC3L1	0.012491994	-1.678071905	0.062	0.667	1
NR4A3	0.01251167	2.534921818	0.75	0.333	1
DLG2	0.012521815	0.054622012	1	1	1
ANKRD44	0.012530839	0.997533482	1	1	1
ZNF281	0.012535079	1.189483778	1	1	1
NTMT1	0.012550984	-1.533681996	0.312	0.533	1
GALM	0.012555968	2.314975334	0.625	0.467	1
ENSSSCG00000048417	0.012602228	-0.312226694	1	0.933	1
ETV5	0.012610702	-0.348870575	0.938	1	1
ENSSSCG00000062960	0.012635362	-1.595609745	0.312	0.533	1
CAMSAP1	0.012659165	-0.274716211	0.875	0.867	1
SSBP3	0.012687898	-0.257496222	0.875	0.933	1
DCTN1	0.012692453	-0.353636955	0.875	0.933	1
TMPRSS9	0.012697286	-1.793549123	0.188	0.533	1
ENSSSCG00000054992	0.012725431	-1.188266637	0.5	0.8	1
DNAJC27	0.012778923	1.305637111	1	1	1
DPP6	0.012811877	-0.52279368	0.75	0.733	1
GLB1L3	0.012823835	3.344295908	0.5	0.267	1
SEC61A2	0.012826556	1.269396451	1	1	1
CNKSR1	0.012863242	0.107287949	1	1	1
SANBR	0.012875343	1.24710838	1	1	1
KMT2C	0.012905324	1.063528195	1	1	1
TENT2	0.012914465	1.389529605	1	1	1
ENSSSCG00000057240	0.012939502	2.254813899	0.812	0.4	1
TRIP11	0.012940883	0.095755469	1	1	1
ZNF286A	0.012964043	1.16040769	1	1	1
ENSSSCG00000055852	0.012971061	-1.093109404	0.438	0.733	1
PIAS1	0.012993877	1.178543238	1	1	1
ASPSCR1	0.013025065	-1.533681996	0.25	0.533	1
KDM2A	0.013032504	1.436926886	1	0.933	1
EGR1	0.013042966	-0.19345993	1	1	1
TIMP3	0.013073995	1.843164998	0.938	0.933	1
RPAP1	0.013116739	1.337734191	1	1	1
WASHC4	0.013125395	1.045983731	1	1	1
FOCAD	0.01312834	0.174785712	1	1	1
MOB3A	0.013160386	-1.020353062	0.75	0.867	1
ENPP4	0.013183414	-1.13492958	0.5	0.6	1
PUS10	0.013211612	1.329313022	1	1	1
LMO1	0.013231246	-0.364411426	0.812	0.933	1
TGDS	0.013249263	1.426884172	1	1	1
ENSSSCG00000042047	0.013253102	-1.678071905	0.25	0.533	1
ILF3	0.013258097	1.293618337	1	1	1
WWP2	0.013258777	-0.078801568	1	1	1
ORC2	0.013285693	1.064178292	1	1	1
UBA5	0.013296286	-0.05900155	1	1	1
SIGLEC1	0.013309049	1.276515433	1	1	1
ITPRID1	0.013320709	-0.496074071	0.812	0.933	1
ENSSSCG00000056033	0.013332756	1.790866799	0.938	0.667	1
ENSSSCG00000057312	0.013349228	-0.582147485	0.688	0.867	1
TMEM117	0.013351665	0.097555309	1	1	1
CDON	0.013358256	1.369033199	1	0.933	1
CCNE2	0.013366184	-1.495207848	0.5	0.467	1
TBRG1	0.01338026	1.205382135	1	1	1
EIF4E	0.013386209	0.005996832	1	1	1
ENSSSCG00000018064	0.013393554	1.794070765	1	0.933	1
RASSF1	0.013438765	-1.093109404	0.188	0.6	1
VSIG1	0.013451987	4.906890596	0.5	0	1
KIF12	0.01347262	-0.717600269	0.688	0.667	1
MAGEF1	0.013487277	-2.019108823	0.125	0.533	1

TNFRSF1A	0.013547623	-1.158697746	0.688	0.8	1
ENSSSCG00000048914	0.013565235	-0.11417102	1	1	1
RBMX2	0.013571103	-0.37996243	1	1	1
BOLL	0.01357797	-0.811927652	0.75	0.8	1
MYO19	0.013584993	-0.445682817	1	0.933	1
PDCL3	0.013585894	1.058326225	1	1	1
NTN1	0.013590561	-1.341036918	0.312	0.6	1
ACACA	0.013594705	0.259041632	1	1	1
HYKK	0.013603371	-0.466233262	0.875	0.933	1
THOC2	0.013613892	1.254344562	1	1	1
ENSSSCG00000045522	0.013618164	-0.182746617	0.938	1	1
SSB	0.013628834	0.054801153	1	1	1
PER2	0.013644639	1.672425342	0.938	0.467	1
TLCD2	0.01367414	-1.35614381	0.312	0.6	1
FLNB	0.013708519	-0.075161771	1	1	1
CDC42EP4	0.013723093	-0.521952703	0.625	0.867	1
SYBU	0.013734949	1.965784285	0.938	0.6	1
INTU	0.0137433	1.483316414	0.938	0.867	1
ENSSSCG00000049063	0.013745856	-1.334117504	0.25	0.667	1
PSMA1	0.013762313	1.146436363	1	1	1
DELE1	0.013781254	-0.745186101	0.5	0.667	1
RANBP2	0.013829883	0.981803636	1	1	1
HACL1	0.013830716	-1.239950793	0.25	0.667	1
ENSSSCG00000032946	0.013833412	1.36757076	0.938	0.933	1
PECR	0.01383761	1.412589818	1	1	1
HYPK	0.013844405	-0.254321974	1	1	1
ARRDC3	0.01384768	-0.272877024	0.938	1	1
VPS13B	0.013848144	0.878328789	1	1	1
PPID	0.01388368	1.289235932	1	0.933	1
RSRP1	0.013913257	-0.350907162	0.875	0.933	1
CREB3	0.01391657	-0.020353062	1	1	1
ENSSSCG00000056777	0.013944345	1.131720002	1	1	1
CIITA	0.013947547	-1.036525876	0.25	0.733	1
MEMO1	0.013958083	0.977670951	1	1	1
PARP2	0.013962328	1.749482698	1	0.733	1
ENSSSCG00000023195	0.013964652	-0.179266048	0.875	1	1
TTC9C	0.013984371	-0.056939668	0.938	1	1
SARAF	0.013997222	0.090769363	1	1	1
ADAM12	0.014008563	0.051280505	1	1	1
SSRP1	0.014015572	1.44578564	1	1	1
CILK1	0.014020198	1.381617534	1	0.867	1
SYTL2	0.014029323	0.959278622	1	1	1
MAIP1	0.014060373	-0.177010662	1	1	1
DND1	0.014066194	-0.02193064	1	1	1
KLK5	0.014069416	-0.809316438	0.625	0.8	1
ENSSSCG00000055200	0.014075347	-0.752072487	0.688	0.733	1
ENSSSCG00000055973	0.014104393	2.491853096	0.75	0.267	1
ARHGAP44	0.01418232	-0.263034406	0.812	1	1
GTF3C2	0.014194882	0.057974482	1	1	1
COMT	0.014195324	-1.589535231	0.562	0.8	1
SMG6	0.014210764	-0.095946564	1	1	1
ENSSSCG00000000148	0.014211157	-0.341036918	0.562	0.933	1
TXNRD1	0.014238034	-0.157526133	1	1	1
MRPS18B	0.01426208	-0.136698673	1	1	1
LGMN	0.014300191	1.263149412	1	1	1
NBR1	0.014325307	1.140536545	1	1	1
SLBP	0.014335463	1.290288394	1	1	1
APPL1	0.014347101	1.169521507	1	1	1
ARHGAP42	0.014363791	0.911936753	1	1	1
QKI	0.01438018	1.09703623	1	1	1
ENSSSCG00000039259	0.014382828	-0.9510904	0.5	0.733	1
PLEKHB2	0.014392212	0.278412854	1	1	1
TRMT6	0.014407743	1.181594482	1	1	1
C1orf87	0.014420524	-0.712019237	0.688	0.667	1
BLZF1	0.014430276	-0.166945285	0.938	1	1
HNRNPD	0.014442274	-0.381090167	1	1	1
SERPING1	0.014496038	-2.35614381	0.062	0.533	1
ENSSSCG00000045125	0.014522997	-0.54830403	1	0.867	1
TPD52L2	0.014523865	0.292544288	1	1	1
ZNF622	0.014551116	-1.349449158	0.25	0.533	1

ADAM3A	0.014556352	-0.37064338	0.812	0.933	1
DPAGT1	0.014557072	-0.200912694	1	1	1
ERAP1	0.014577948	1.067725256	0.938	0.933	1
LIN28A	0.014584435	1.213137068	1	1	1
CDK5R2	0.01458586	-1.341036918	0.188	0.533	1
CCDC86	0.014589784	1.582268392	1	1	1
ERCC5	0.014590048	-0.37690237	1	0.867	1
ZCCHC8	0.014621425	1.197847303	1	1	1
ENSSSCG00000052755	0.014630291	-0.334867151	1	0.933	1
ZNF777	0.014645877	-0.578536232	0.812	0.867	1
NUP214	0.014651229	1.558534875	1	1	1
VGLL4	0.014715507	-0.175051018	1	1	1
CTDSPL2	0.014729975	1.120747433	1	1	1
TIMM29	0.014739147	-0.257228784	0.938	1	1
NLRC5	0.014743129	-0.923184403	0.688	0.8	1
ENSSSCG00000001979	0.014778436	-0.93363119	0.875	0.8	1
ENSSSCG00000058487	0.014789665	-1.455679484	0.312	0.533	1
KCNG3	0.014807359	1.18730708	1	0.933	1
MTRF1	0.014812582	-0.521443726	1	0.867	1
TOM1	0.014813802	-0.941106311	0.875	0.8	1
APEX2	0.014844439	1.2306385	1	1	1
SNX9	0.014852698	0.09847697	1	1	1
CAPN12	0.014885653	0.135966723	1	1	1
ZFAND6	0.014916606	1.000446309	1	1	1
ENSSSCG00000012709	0.014931041	1.906890596	0.938	0.4	1
ENSSSCG00000045128	0.01494854	-1.027020214	0.875	0.8	1
MGST1	0.014953091	2.977279923	0.625	0.267	1
PHKG2	0.015020072	-1.489999557	0.562	0.6	1
L3MBTL2	0.015025709	-0.086054647	0.875	1	1
ATP8B2	0.015026486	-1.208586622	0.25	0.6	1
PACSIN3	0.015039994	-0.712019237	0.75	0.667	1
SLC30A5	0.015056542	1.1864724	1	1	1
ZCCHC10	0.0150575	1.353776961	1	1	1
GPR22	0.015084472	0.985364654	1	0.933	1
ENSSSCG00000058007	0.015102014	1.060325645	1	1	1
LAPTM4B	0.015106796	-0.128237628	1	1	1
NCAPH2	0.015143258	-0.756074417	0.375	0.8	1
BAALC	0.015154916	-0.319617934	1	1	1
SMARCA1	0.015157505	-0.757242119	0.5	0.8	1
JADE3	0.015158101	1.207345326	1	1	1
DAG1	0.015160165	1.202190537	1	1	1
ZKSCAN1	0.015161726	-0.717600269	1	0.933	1
ZDHHC3	0.015167713	0.051409756	1	1	1
ENSSSCG00000058720	0.015201121	-0.583435031	0.75	0.8	1
CNKSR3	0.01524067	-2.208586622	0.125	0.533	1
RABIF	0.015297166	1.991424106	0.938	0.533	1
C2CD3	0.01530118	-0.136178126	1	1	1
ENSSSCG00000007692	0.015315899	0.192138027	1	1	1
NCOR1	0.015358676	1.266416885	1	1	1
GTDC1	0.015364319	2.587010329	0.812	0.467	1
TMEM209	0.015397453	1.093073479	1	1	1
METTL18	0.015416189	-0.049630768	1	1	1
DNAJC1	0.015418173	1.038360097	1	1	1
TSNAX	0.015497949	-0.17420783	1	1	1
TADA2A	0.015513539	1.102125521	1	1	1
RRM2B	0.01551439	1.041075159	1	1	1
ENSSSCG00000055703	0.015524324	-1.285754482	0.438	0.6	1
PIK3R1	0.01555057	-0.191388922	1	1	1
PGBD1	0.015591116	-0.596875822	0.938	0.933	1
NDUFAF2	0.015592984	-0.929610672	0.312	0.867	1
ROCK2	0.015657789	1.083338327	1	1	1
ENSSSCG00000059383	0.015669557	-0.35614381	1	1	1
SEPSECS	0.015672327	1.154652672	1	1	1
ENSSSCG00000012961	0.015685987	-0.143963313	1	1	1
SH3BGR	0.015689397	-0.664266106	0.688	0.867	1
GORAB	0.015721035	2.994353437	0.688	0.2	1
NFKBIA	0.015725702	1.236930442	1	1	1
ZNF703	0.015753655	-0.535113951	0.812	0.933	1
ANGEL1	0.015805528	1.21888747	1	1	1
PPP2R1A	0.015806927	1.590126845	1	0.933	1

SPTAN1	0.015814572	-0.041163189	1	1	1
ATP9A	0.015831745	-0.107323264	0.938	1	1
CDH24	0.015842695	-0.485426827	0.688	0.8	1
ENSSSCG00000007065	0.015857923	-1.093109404	0.312	0.733	1
ENSSSCG00000017577	0.015906335	-0.463477854	0.875	0.867	1
TMEM169	0.015908945	1.524274574	0.938	0.733	1
GDAP2	0.015958714	1.039315807	1	1	1
TARDBP	0.01603271	1.083008806	1	1	1
ENSSSCG00000006771	0.016117054	1.17228408	1	1	1
TOP2A	0.016117722	1.048864334	1	1	1
BEND7	0.016144048	-0.549967079	0.875	0.933	1
KIAA1522	0.016152459	-0.098712111	1	1	1
ELP1	0.016158775	-0.867613299	0.938	0.933	1
INTS7	0.016159169	1.215158141	1	1	1
MUTYH	0.016174778	1.470319935	1	0.8	1
PERP	0.016182829	-0.154509949	0.812	0.867	1
ENSSSCG00000040466	0.016188813	2.54630088	0.812	0.4	1
ENSSSCG00000014062	0.016200796	-0.159451899	0.875	0.933	1
MATCAP2	0.016217371	-0.394393189	1	1	1
UBLCP1	0.016251866	-0.152097538	1	1	1
ANGPT1	0.016260743	1.32074118	1	1	1
MAST4	0.016274776	0.092336182	0.938	1	1
CCDC47	0.016275733	1.358174661	1	1	1
ANKDD1A	0.016276387	-1.446746359	0.375	0.533	1
ENSSSCG00000033490	0.016287399	-0.703162886	0.625	0.8	1
BIN3	0.016313553	1.402486094	1	1	1
GPC2	0.016324713	-1.157239742	0.312	0.667	1
SLC37A3	0.016351423	-0.326308581	0.938	1	1
ENSSSCG00000047398	0.016364047	-1.368743847	0.438	0.667	1
ZNF283	0.016365222	1.30959364	1	1	1
TIMP4	0.016370141	-0.879211777	0.938	0.8	1
BTBD8	0.01637505	-0.182235295	0.938	1	1
GPR158	0.016380771	0.309302612	0.938	1	1
ENSSSCG00000032999	0.01641747	1.332899213	0.938	1	1
MAML3	0.01642058	-0.249349491	0.938	1	1
ENSSSCG00000048278	0.016455348	-0.977632187	0.5	0.667	1
GNB2	0.016481903	2.140009424	0.938	0.733	1
ACTN2	0.016527983	-1.025995209	0.625	0.667	1
ENSSSCG00000052892	0.016532478	2.62935662	0.688	0.267	1
FAM199X	0.016587122	-0.045214714	0.938	1	1
ENSSSCG00000030260	0.016604761	0.034260753	1	1	1
ZNF135	0.016618851	-0.874469118	0.5	0.667	1
KLHDC1	0.016657494	-0.592680414	0.938	1	1
SH3BP5L	0.016674286	-0.156955886	0.875	0.933	1
EBP	0.016733128	-0.226375935	0.938	1	1
BAX	0.016755719	-1.093109404	0.562	0.533	1
ADCYAP1	0.016769435	-1.508146904	0.25	0.6	1
MALRD1	0.016778693	1.107317685	1	1	1
ENSSSCG00000003965	0.016791533	-1.180572246	0.312	0.6	1
ENSSSCG00000013607	0.016821837	1.669851398	0.938	0.6	1
MCAM	0.016823088	2.491853096	0.625	0.2	1
H2AZ2	0.016823742	0.230394545	1	1	1
SPINT1	0.016831402	-0.89061654	0.688	0.733	1
GPRC5A	0.016866661	-0.961864871	0.375	0.733	1
SLAIN1	0.016877534	-0.150537684	1	0.933	1
TNRC6B	0.01697714	0.185985966	1	1	1
NMRK1	0.016990301	-0.549369566	0.938	1	1
MAEA	0.017016918	0.01282404	1	1	1
SENP3	0.017060946	-0.335679708	0.875	0.933	1
CWC22	0.017065429	1.130251431	1	1	1
ATP1A1	0.017067232	1.584336881	1	1	1
FLII	0.017104058	-0.213963799	1	1	1
UNC45A	0.017149014	-1.230612928	0.25	0.6	1
RALGAPA2	0.017210266	1.220129743	1	1	1
PABPC4	0.017231932	1.075048671	1	1	1
ENSSSCG00000045932	0.017234501	-1.137503524	0.312	0.533	1
SLC23A1	0.017244561	-1.291048782	0.562	0.467	1
TAFAZZIN	0.017345766	1.200086972	1	1	1
KIAA2013	0.017385771	-0.419337637	0.938	0.933	1
ENSSSCG00000061039	0.017397118	1.837627933	0.875	0.467	1

ZIC2	0.017410692	-0.974464908	0.438	0.867	1
VASP	0.017492813	-0.477773255	0.812	0.733	1
CALD1	0.017542924	-0.420199204	0.938	0.933	1
ENSSSCG00000060891	0.017558153	-0.781165398	0.438	0.867	1
OSBPL3	0.017575431	-0.121214742	1	1	1
ZNF131	0.017628475	-1.469485283	0.812	0.467	1
ZFP28	0.017675661	-0.170391325	0.938	1	1
RAD21	0.017766166	-0.009550793	1	1	1
DHX38	0.01778314	1.309579702	1	1	1
KIF2A	0.0177863	0.942133754	1	1	1
ENSSSCG00000059539	0.0178	-0.732519689	0.938	0.8	1
NRXN1	0.017822621	0.975743916	1	1	1
ENSSSCG00000052641	0.017823781	-0.299560282	0.75	0.933	1
ENSSSCG00000014884	0.017833517	-0.003332697	1	1	1
ENSSSCG00000062144	0.017838429	-0.941106311	0.562	0.8	1
CCDC106	0.017840343	-1.093109404	0.25	0.6	1
DYNLT2B	0.017848166	-0.319880266	0.875	0.933	1
CSPG5	0.017865976	-1.552541023	0.25	0.6	1
ENSSSCG00000011162	0.017881877	0.10712295	1	1	1
ENSSSCG00000061116	0.017905635	-0.912537159	0.5	0.667	1
C12orf29	0.017975151	-0.574236094	0.875	0.867	1
FAM124A	0.017977472	-0.941106311	0.5	0.533	1
MTRF1L	0.018017353	-0.183711953	0.875	1	1
LMBRD2	0.018035993	1.340689023	1	1	1
ADCK1	0.018059391	-0.193730219	1	1	1
TLK2	0.018061726	1.047632728	1	1	1
MARVELD2	0.018116617	1.07710579	1	1	1
ANKRD54	0.018157752	-0.323407024	0.688	0.933	1
KPNA7	0.018169403	1.666609228	1	1	1
ZNF384	0.018184646	-1.341036918	0.312	0.667	1
CLIC4	0.018190635	0.987119621	1	1	1
ENSSSCG00000051957	0.018191093	1.82336724	0.875	0.667	1
TEDC2	0.018256515	-2.341036918	0.125	0.8	1
DDX54	0.018266454	0.005380459	0.938	1	1
GYG1	0.018273756	-0.860132232	0.625	0.733	1
DNTTIP2	0.018313341	1.177413775	1	1	1
FGL1	0.018337909	-0.385891154	0.438	0.733	1
ENSSSCG00000016733	0.018338051	-0.471621028	0.938	0.867	1
PSEN2	0.018351014	1.474111514	1	0.933	1
STAM2	0.018393072	1.160009533	1	1	1
TACSTD2	0.018393508	1.195877697	1	1	1
STAU1	0.018429427	1.197111828	1	1	1
DHX9	0.018443451	-0.197320399	1	1	1
AARS1	0.01844604	-0.399212532	0.938	1	1
ARMC9	0.01849303	-0.47689597	0.875	0.933	1
ARL15	0.01849749	0.950825021	1	1	1
STEAP3	0.018533634	-0.315501826	1	1	1
COMTD1	0.018579096	1.982178723	0.875	0.4	1
MIPEP	0.01858398	-0.346228341	0.812	0.867	1
OBSCN	0.018604277	-0.818934441	0.75	0.733	1
DYM	0.018624467	0.998813085	1	1	1
METAP2	0.018644615	0.246224967	1	1	1
ENSSSCG00000033366	0.018647786	1.236961864	1	1	1
ENSSSCG00000042658	0.018648807	-0.275700818	1	1	1
ANGEL2	0.018686503	-0.010647244	1	1	1
ZMYND19	0.018698795	-1.143735477	0.375	0.667	1
ENSSSCG00000047302	0.018744033	-0.016147423	1	1	1
MSH6	0.018878232	1.148524367	1	1	1
PGAP2	0.018879838	1.935841969	0.938	0.667	1
ZSWIM6	0.018898169	-0.688719149	0.812	0.733	1
EPDR1	0.018968256	-1.415037499	0.625	0.6	1
BRMS1L	0.018974934	1.321928095	1	0.933	1
PIH1D2	0.018992002	-0.384158186	0.875	0.8	1
PAN3	0.019004057	1.013652101	1	1	1
ENSSSCG00000028572	0.0190137	-0.522502197	0.812	0.8	1
B3GALNT2	0.019023627	0.206297955	1	1	1
CCDC160	0.019041329	-0.41975991	0.938	1	1
DOK5	0.019105579	-0.704544116	0.875	1	1
FREM1	0.019178358	-0.313235219	0.875	0.933	1
ENSSSCG00000062937	0.01918945	1.994353437	0.812	0.467	1

ENSSSCG00000054552	0.019230765	1.353025392	0.938	0.933	1
BYSL	0.019273462	1.526410318	1	0.867	1
NPAS3	0.019306139	-0.001235713	1	1	1
PTRH2	0.019324866	-3.415037499	0	0.533	1
SUSD3	0.019332627	1.373300197	1	1	1
WDR1	0.01936322	-0.340490546	0.938	0.933	1
ENSSSCG00000021322	0.019374633	0.312956589	1	1	1
GOLIM4	0.019423828	1.625531532	1	1	1
MME	0.019516039	2.254813899	0.75	0.333	1
IRS1	0.019527208	-0.471621028	0.875	0.933	1
RAD51B	0.019533463	-0.01160662	1	1	1
CDCA8	0.019541786	1.107553428	1	1	1
ARID1A	0.019542556	1.217428052	1	1	1
GTF2F2	0.019549983	-0.240916	1	1	1
ENSSSCG00000062092	0.019552084	-1.192645078	0.375	0.667	1
DCAF17	0.019558283	-0.486342533	0.875	0.933	1
ENSSSCG00000032202	0.019579766	0.180795737	1	1	1
NFKB2	0.019582478	-0.806805219	0.375	0.667	1
POLR3A	0.019589998	-0.232173442	1	1	1
BLOC1S6	0.019616824	1.242493627	1	0.867	1
UBXN4	0.019627226	0.945990415	1	1	1
ENSSSCG00000036812	0.019634682	-0.601256308	0.75	0.8	1
ENSSSCG00000041031	0.019658732	1.130156055	1	0.933	1
KIF26B	0.019673128	-0.214671384	0.875	1	1
HTR1B	0.019796211	-1.029915578	0.75	0.8	1
LIN28B	0.019809087	1.025210563	1	1	1
GRM5	0.019843019	1.124970024	1	1	1
ENSSSCG00000043207	0.019843921	-0.955605881	0.312	0.533	1
CALM2	0.019943439	-0.023933828	1	1	1
ENSSSCG00000016704	0.019966803	-0.815575429	0.562	0.667	1
CTC1	0.019995338	-0.009693396	1	1	1
ENSSSCG00000005656	0.0200676	0.167306646	1	1	1
PHC3	0.020073943	0.127578711	1	1	1
ENSSSCG00000007066	0.020075457	1.274866058	0.938	1	1
MAP1A	0.020077122	-0.717600269	0.562	0.8	1
GINS3	0.020079862	1.220706045	1	1	1
RDH10	0.020132936	0.894553936	1	1	1
ENSSSCG00000037473	0.020134344	-1.533681996	0.125	0.667	1
SF3B4	0.020163363	-0.030373649	0.938	1	1
SLC17A3	0.020210388	1.330790664	1	0.933	1
ZDHHC20	0.020241758	1.152593444	1	1	1
MYO5A	0.020242336	1.252755356	1	1	1
SIRT1	0.020260139	1.051735037	1	1	1
ENSSSCG00000059059	0.020277997	-1.093109404	0.562	0.6	1
GCG	0.020288164	-0.736965594	0.875	0.933	1
GGA3	0.020302755	-0.398735687	0.812	1	1
GSK3A	0.020312693	-0.208586622	1	1	1
CAMKK1	0.020313852	-0.30565404	0.75	0.867	1
ZNF37A	0.020337668	-1.063362061	0.812	0.733	1
DZIP3	0.020352045	1.093285934	1	1	1
GLTP	0.020375724	1.416143437	1	1	1
XPA	0.020393155	1.218351691	1	0.933	1
ENSSSCG00000054646	0.020393292	2.104829973	0.75	0.2	1
ENSSSCG00000055001	0.020403279	1.972182056	0.875	0.667	1
SHB	0.020424974	-1.455679484	0.25	0.533	1
ENSSSCG00000053064	0.02044332	1.781359714	0.938	0.6	1
CEP135	0.020448494	1.259515488	1	1	1
NSUN4	0.020458967	0.089137826	0.938	1	1
ATP1A3	0.020501466	-0.784987109	0.438	0.867	1
SAE1	0.020508451	1.521710708	1	1	1
ENSSSCG00000032978	0.02054667	-0.525060287	0.812	0.933	1
SLC12A4	0.02054701	-0.297265052	1	1	1
SMARCC1	0.020551205	0.100379437	1	1	1
PSAT1	0.020602663	-0.23397194	0.938	1	1
ENSSSCG00000001931	0.020645526	0.049914521	1	1	1
ENSSSCG00000060436	0.020666294	-1.140415119	0.562	0.6	1
SCAI	0.020666752	1.169925001	1	1	1
ENSSSCG00000051978	0.020689842	-0.678071905	0.625	0.933	1
ENSSSCG00000040337	0.020698397	1.218768811	1	1	1
ENSSSCG00000038960	0.020713711	-0.372085354	1	1	1

TARBP1	0.020734282	1.282589947	0.938	0.867	1
ANKS1B	0.020756831	0.923946557	1	1	1
ZBTB17	0.020796194	-0.571763003	0.812	0.667	1
BMP2	0.020820589	1.615124471	0.938	0.933	1
ONECUT2	0.020850419	1.40598098	1	1	1
ZNF793	0.020872988	0.969927257	1	1	1
LHX2	0.020879729	-0.467504919	0.625	0.8	1
IRGQ	0.020891856	-0.963826387	0.625	0.667	1
SLC6A5	0.020928641	0.236359985	1	1	1
MORN4	0.020950131	-0.239950793	0.438	0.8	1
ARMCX5	0.020950342	1.423140346	1	1	1
SLC38A6	0.020970471	1.242713132	1	1	1
C12orf4	0.020975145	0.030598742	0.938	1	1
NT5C3B	0.02104992	-0.808379927	0.688	0.733	1
TRMT1	0.021088507	-0.858644151	0.5	0.867	1
CDH1	0.021113033	0.960646246	1	1	1
TCEA1	0.021132172	1.021883118	1	1	1
GRIN2C	0.021166149	-1.578536232	0.188	0.6	1
FUBP1	0.021191015	0.983953929	1	1	1
NCL	0.021244979	1.207607492	1	1	1
EZH1	0.02124766	0.105272043	0.938	1	1
CERS6	0.02124899	1.583775586	1	0.733	1
ACADL	0.021277202	-0.489038081	0.75	0.8	1
ING2	0.021303736	-0.874469118	0.688	0.733	1
ZC3H18	0.021305701	-0.476438044	0.375	0.867	1
TMEM196	0.021315177	-1.35614381	0.25	0.667	1
DBP	0.021350868	-1.578536232	0.25	0.533	1
EFHD2	0.021355534	-1.245112498	0.438	0.533	1
CXADR	0.021373682	1.457905764	1	1	1
ENSSSCG00000003990	0.021379891	-1.127056736	0.562	0.733	1
RORB	0.021402209	0.022113566	1	1	1
ENSSSCG00000038326	0.021417175	1.441667339	1	0.867	1
CLDND1	0.021444191	-0.117513205	1	1	1
SNTA1	0.021467762	-0.623624121	0.5	0.667	1
CDK18	0.021498363	-1.056583528	0.438	0.667	1
ENSSSCG00000061538	0.02152849	-1.140415119	0.5	0.467	1
ENSSSCG00000061401	0.021545362	-1.192645078	0.312	0.533	1
ENSSSCG00000063544	0.021545444	1.106631251	1	1	1
CCNG1	0.021545852	0.202721024	1	1	1
EEF1E1	0.021586545	-0.390577307	1	1	1
PDPR	0.021587179	1.253630446	1	1	1
WDR35	0.021620811	1.753345337	1	0.733	1
UCK1	0.021803824	-1.093109404	0.312	0.667	1
PLXNB2	0.021813103	-0.9861942	0.438	0.667	1
IVD	0.021826408	-0.357293826	1	1	1
ENSSSCG00000009348	0.021833341	-0.745186101	0.812	0.867	1
CFAP206	0.02192581	0.035405336	1	1	1
TAF4B	0.021925979	1.256447447	1	1	1
LACTB	0.021929059	0.100732104	1	1	1
RWDD1	0.021941096	0.024290979	1	1	1
ENSSSCG00000032364	0.021988107	-0.034970968	1	1	1
EI24	0.022007511	-0.005646563	1	1	1
APBB1IP	0.022027624	3.22881869	0.562	0.133	1
DOLPP1	0.022037026	-0.678071905	0.5	0.8	1
NEK5	0.022061599	-0.415037499	0.938	1	1
POLI	0.022128883	0.007960977	0.938	1	1
VPS36	0.022188066	0.051031571	1	1	1
SLC24A1	0.022192514	-0.020353062	0.938	1	1
ARHGAP12	0.02226128	1.044022179	1	1	1
NUDT19	0.022268494	1.504102993	1	1	1
ENSSSCG00000008845	0.022287783	1.259450309	1	1	1
ENSSSCG00000037475	0.022291329	-0.334117504	0.75	0.733	1
LLGL2	0.022311768	1.326882977	1	1	1
SPNS1	0.022332347	-0.266174113	0.875	1	1
NPTN	0.022335336	1.052794662	1	1	1
NAGPA	0.02233941	-0.070025791	0.938	0.867	1
UGT8	0.022365941	1.177571813	1	1	1
PRKCSH	0.022377159	-0.843131151	0.75	0.8	1
OPTN	0.022380429	-0.047594745	1	1	1
ENSSSCG00000022842	0.022413607	-0.854949667	0.562	0.8	1

SORCS1	0.022426471	0.946920374	1	1	1
FAR1	0.022455702	-0.107944446	0.938	1	1
ENSSSCG00000034914	0.022468099	4.430452552	0.562	0	1
CEP192	0.022508333	0.888210641	1	1	1
CTNND2	0.022559829	-1.508146904	0.312	0.6	1
TMBIM4	0.022562451	1.599912842	0.938	0.667	1
BLOC1S5	0.022628017	0.281379365	1	1	1
ENSSSCG00000048787	0.02263035	-0.155037154	0.938	1	1
TMEM231	0.022630493	-0.498365883	0.688	0.867	1
NOP56	0.022646939	-0.191513108	1	1	1
S100PBP	0.022671903	0.314316709	1	1	1
ENSSSCG00000005971	0.022683204	-0.402156208	0.938	1	1
THOC5	0.022708039	-0.166873459	1	1	1
CSK	0.022716466	-0.097926421	1	1	1
SNX16	0.022745152	1.844063758	1	0.667	1
SGO1	0.022825873	1.141977292	1	1	1
ENSSSCG00000021891	0.022835093	1.463283944	0.938	1	1
PLLP	0.022844721	1.18893539	1	1	1
ENSSSCG00000038487	0.022916995	1.62935662	1	0.667	1
KLF2	0.022925547	-0.487388344	0.875	0.8	1
DGKG	0.022953247	0.068543859	1	0.933	1
TCERG1	0.022984364	1.085408208	1	1	1
USF2	0.022994578	1.730012834	1	0.733	1
GLUD1	0.023009587	1.170782727	1	1	1
MLF1	0.02302587	0.882643049	1	1	1
INTS9	0.023029964	1.18924186	1	1	1
RASAL1	0.023053399	-1.450661409	0.375	0.6	1
FMC1	0.023069525	1.180392	1	0.933	1
ZBTB24	0.023075143	-0.297467903	0.812	1	1
ENSSSCG00000000104	0.023211311	-0.02923958	1	1	1
RFX5	0.023211484	1.179049462	1	1	1
GNAT2	0.023220817	1.738767837	0.875	0.533	1
RARB	0.023246226	-0.359624379	1	1	1
IQCE	0.02324818	-1.455679484	0.188	0.533	1
ENSSSCG00000057725	0.023259404	-0.032263849	1	1	1
DOP1B	0.0232776	0.031435694	1	1	1
PLA2G4E	0.023277981	1.180336049	1	1	1
DNAJA1	0.023280555	1.173775486	1	1	1
TMPRSS15	0.023290561	0.978165284	1	1	1
ENSSSCG00000048343	0.023296127	-0.027307346	1	1	1
ARHGAP19	0.023331104	-0.131841799	1	1	1
GPR182	0.023340269	0.968153891	1	1	1
LNPEP	0.023377874	1.134711263	1	1	1
TNFAIP8	0.023388345	0.334873673	1	1	1
RB1	0.023399055	1.283509332	1	1	1
SLC25A3	0.02344446	-0.509909512	1	1	1
PIGA	0.023515009	0.925873699	1	1	1
ENSSSCG00000003461	0.023527611	1.148316888	1	1	1
ITSN1	0.023531316	0.000576041	1	0.933	1
CYP26A1	0.023546975	1.346261242	1	0.933	1
ENSSSCG00000037476	0.02355105	-0.941106311	0.5	0.6	1
VPS13C	0.023576279	0.233918227	1	1	1
EEF2K	0.023609579	-0.269987166	0.938	1	1
ENSSSCG00000060951	0.023627364	2.089093927	0.812	0.4	1
CAT	0.023656365	0.924072558	1	1	1
VPS9D1	0.023725653	-1.678071905	0.125	0.533	1
ENSSSCG00000059863	0.023756654	-1.415037499	0.188	0.6	1
ENSSSCG00000035160	0.023758365	-1.142019005	0.812	0.8	1
ZSCAN26	0.023792611	0.933739224	1	1	1
ALG13	0.023816827	0.344933756	1	1	1
URI1	0.023825164	-2.11417102	0.5	0.4	1
ENSSSCG00000010221	0.023857608	1.314394422	1	0.867	1
FANCC	0.023871576	-0.026558211	1	0.933	1
DCPS	0.023894349	1.458362031	0.938	1	1
PTCH2	0.023931896	-0.30256277	0.812	0.867	1
IQSEC3	0.024018386	-1.010647244	0.562	0.667	1
RB1CC1	0.02414826	1.217952454	1	1	1
ACBD3	0.02416921	0.141666741	1	1	1
KATNBL1	0.024213874	0.012243596	1	1	1
NSD2	0.02422268	0.093149231	1	1	1

TRIM39	0.024275126	0.153456691	1	1	1
CALM1	0.024276455	0.216813049	1	1	1
TBC1D2	0.024290838	-0.466924241	0.875	0.8	1
SPTLC2	0.024315865	0.079001263	1	1	1
METAP1	0.024318544	-1.072931522	0.625	0.6	1
ENSSSCG00000042348	0.024337656	-1	0.562	0.667	1
SV2C	0.024480839	-0.568447414	0.812	0.8	1
HPRT1	0.024499583	0.276044577	1	1	1
SUSD1	0.024577759	-0.347292893	0.938	0.933	1
DHRS7B	0.024579096	-0.809316438	0.688	0.867	1
MDM2	0.024590078	-0.421163602	0.938	1	1
ZBTB10	0.024619221	-0.025404545	1	1	1
NCAPH	0.024655061	-0.324859536	1	1	1
DHX32	0.024713794	1.373342642	1	1	1
MTRR	0.024746635	-0.206003461	0.812	1	1
PARP11	0.024818197	-1.157239742	0.312	0.533	1
HNMT	0.024901534	2.137188215	0.75	0.267	1
KCNT2	0.024904795	1.461202883	1	0.933	1
CCDC39	0.024908331	1.446410126	0.875	0.933	1
NUTF2	0.024910952	-0.001415606	1	1	1
TMIGD1	0.025012166	-0.941106311	0.875	0.867	1
ERGIC2	0.02501359	1.071448575	1	1	1
SUPT5H	0.025017039	1.246395827	1	1	1
ANKEF1	0.025021919	-0.224353938	0.938	0.933	1
RPGRIP1L	0.025039463	0.954110877	1	1	1
MBOAT2	0.025074633	-0.159707153	1	1	1
ZYG11A	0.025133663	0.126090891	1	1	1
SNAPC5	0.025143502	1.665882496	1	0.933	1
ZNF574	0.025181826	-0.300648698	1	0.933	1
TMC1	0.025222512	-1.415037499	0.312	0.6	1
MBL2	0.025247975	-0.415037499	0.75	0.867	1
NPSR1	0.025251976	-1.03170886	0.188	0.533	1
ABCD3	0.025286899	0.408754967	1	1	1
SORCS3	0.025304202	-0.221842719	1	1	1
RNF14	0.025357724	0.23639191	1	1	1
PRRC2B	0.025372915	-0.35614381	0.938	0.933	1
CALHM6	0.025389813	-0.845181891	0.562	0.8	1
SHOX2	0.025414357	-0.565177849	0.812	0.867	1
EMD	0.0254659	-0.190806078	0.938	1	1
PPM1A	0.025486612	1.0887579	1	1	1
TGIF1	0.025493967	3.33315535	0.5	0.133	1
SERINC3	0.025511419	0.329041198	1	1	1
LRIG2	0.02556471	-0.195207592	1	1	1
OLFML3	0.025631851	-0.508146904	0.312	0.667	1
SEMA3D	0.025662059	1.059439028	1	1	1
ZCCHC7	0.02569299	1.168905789	1	1	1
GIGYF2	0.025723928	0.150725354	1	1	1
IPMK	0.025735402	1.531632998	1	1	1
SACM1L	0.02573724	1.22063633	1	1	1
NFX1	0.02576411	1.196397213	1	0.8	1
TMEM94	0.025776928	1.298256184	1	1	1
FPGS	0.025812825	-0.607682577	0.75	0.733	1
RHBDF2	0.025862538	1.935459748	0.938	0.667	1
TMEM183A	0.025947101	0.150406309	1	1	1
ENSSSCG00000060015	0.025966547	0.050085231	0.938	1	1
SLC35B2	0.025975147	1.192068904	1	1	1
SCN11A	0.025979584	-0.814573905	0.875	0.867	1
TRAF2	0.026087105	-0.35614381	0.75	0.867	1
COG5	0.026128531	0.296934313	1	1	1
CACTIN	0.026143402	1.941378972	0.938	0.533	1
ENSSSCG00000029449	0.026223386	1.285402219	0.875	0.4	1
SPACA9	0.026233627	-0.974464908	0.438	0.8	1
MYOZ1	0.026242449	-0.281181753	0.938	0.867	1
ENSSSCG00000057683	0.026283025	-0.485426827	0.625	0.733	1
UTP11	0.026297188	1.059028514	1	1	1
ENSSSCG00000052863	0.026339242	1.717620244	1	0.867	1
AFF3	0.026460563	1.287812737	1	1	1
ENSSSCG00000052562	0.026502642	-0.886658527	0.5	0.6	1
NXPH4	0.026560198	-1.010647244	0.375	0.533	1
ENSSSCG00000055609	0.026601156	-1.040641984	0.312	0.6	1

BSDC1	0.026658116	0.086685841	1	0.933	1
FRS3	0.026667736	-0.993573731	0.5	0.8	1
ENSSSCG00000041066	0.026712917	2.396275436	0.75	0.467	1
ENSSSCG00000007435	0.026734318	-0.442443657	0.875	0.867	1
NAF1	0.026735805	0.878221253	1	1	1
ENSSSCG00000044013	0.026759255	-0.052258167	1	1	1
CLVS2	0.02677932	0.026146152	1	1	1
PLEKHJ1	0.02684552	1.290856649	1	1	1
LSAMP	0.02691409	-0.337034987	0.812	0.867	1
PITHD1	0.026949073	1.26693143	1	1	1
S100Z	0.026953129	-0.861783858	0.688	0.733	1
ABRAXAS1	0.026972869	-2.03170886	0.438	0.6	1
ENSSSCG00000061680	0.026985755	-1.245112498	0.25	0.533	1
AGR2	0.027014233	-0.305479906	1	1	1
ENSSSCG00000038924	0.027077152	2.906890596	0.562	0.133	1
TTC28	0.027081792	1.180500203	1	0.933	1
MSL3	0.027088962	0.013199498	1	1	1
SFR1	0.027124498	-0.348542087	1	1	1
MYNN	0.027171937	1.18255029	1	1	1
GPR4	0.027225391	0.035405336	1	1	1
LARP1B	0.027237832	0.935947608	1	1	1
KPNA6	0.02728806	0.272508541	1	1	1
DHX40	0.027347372	0.299913182	1	1	1
MARK4	0.027359535	-0.863627558	0.625	0.6	1
WDR82	0.027435219	0.21673554	1	1	1
USP16	0.027470937	0.171504149	1	1	1
ENSSSCG00000051232	0.027484399	-0.752072487	0.5	0.8	1
WDHD1	0.027687355	1.19106151	1	1	1
PAK3	0.027710935	1.176872475	1	1	1
CEP95	0.027720231	1.020276069	1	1	1
AREL1	0.027737042	-0.033667713	1	1	1
SMURF1	0.027748773	-0.814393376	0.625	0.667	1
FARP2	0.027757241	-0.600069393	0.688	0.8	1
SLC35F2	0.027784752	1.272625175	1	1	1
ENSSSCG00000006395	0.027809439	1.533075759	1	1	1
PAGE2B	0.027840339	1.590961241	0.938	0.667	1
RRBP1	0.027841581	-0.329176763	0.875	0.867	1
C1orf112	0.027875371	-0.088216143	0.938	1	1
ENSSSCG00000021069	0.027929977	-0.452190498	0.688	0.8	1
TRHR	0.027950312	1.109198771	1	1	1
ENSSSCG00000002034	0.027981656	1.750489038	0.938	0.933	1
ENSSSCG00000050888	0.027997117	-1.192645078	0.25	0.533	1
CUL3	0.028004169	-0.015642748	1	1	1
PRR16	0.02804283	0.922183245	1	1	1
MAP4	0.028084185	1.346097018	1	1	1
ENSSSCG00000049849	0.028108694	-0.649502753	0.438	0.8	1
ENSSSCG00000021343	0.028108837	-1.598344713	0.438	0.667	1
SUV39H2	0.02815471	0.024837625	1	1	1
PLEKHM2	0.028161394	-0.054115273	1	0.933	1
ZNF451	0.028238218	0.997498752	1	1	1
PCSK5	0.028252749	1.267535798	1	1	1
KIFAP3	0.028258033	1.051562805	1	1	1
HPSE	0.028259943	3.461479447	0.5	0.133	1
RPS6KC1	0.028280677	0.932776056	1	1	1
SPATC1L	0.028306664	-3.263034406	0	0.533	1
FAM76A	0.028393649	-0.237499314	0.938	1	1
ENSSSCG00000051511	0.028423937	-0.834191107	0.75	0.8	1
SCP2D1	0.028446633	-1.521952703	0.25	0.533	1
HCN4	0.028472864	0.00507099	1	1	1
TAF1B	0.028482248	0.136580173	1	1	1
FNDC1	0.028490603	-0.319880266	0.875	1	1
PSPH	0.028534806	1.093057032	1	1	1
MRPL34	0.028563998	1.285402219	1	0.867	1
SUMO1	0.028613037	0.976326142	1	1	1
ENSSSCG00000035459	0.028664221	-1.068861858	0.5	0.733	1
SAYSD1	0.028690558	-1.77118131	0.125	0.533	1
PDIK1L	0.028702879	1.578267848	1	0.467	1
MANEA	0.028763458	1.211147664	1	0.933	1
NCAPD3	0.028775394	1.282940806	1	0.933	1
HEG1	0.028791445	-1.076235586	0.75	0.8	1

AATF	0.028826634	0.259241611	1	1	1
NCK2	0.028873734	0.226333427	1	1	1
GFRA3	0.028924753	-0.782769284	0.812	0.733	1
ILDR1	0.028936908	0.020402288	1	1	1
XRCC1	0.028980515	-0.466924241	0.812	0.733	1
SPART	0.029002135	1.210648936	1	1	1
IFNLR1	0.029138978	2.936637939	0.562	0.2	1
ST8SIA4	0.029152727	-0.289115059	1	1	1
ZNF397	0.02916413	-1.006397771	0.812	0.533	1
ENSSSCG00000045465	0.029168157	-0.65701029	0.625	0.867	1
BBX	0.029219628	1.408316303	1	1	1
MFSD6	0.029246824	1.042242449	1	1	1
ENSSSCG00000000563	0.029258298	1.016484057	1	1	1
STX12	0.029355286	1.11782289	1	1	1
ENSSSCG00000053769	0.029422199	2.63481105	0.625	0.333	1
HIBADH	0.029442759	1.350130729	1	1	1
ZFR	0.029474621	0.986787185	1	1	1
ENSSSCG00000060175	0.029502864	-1.093109404	0.188	0.6	1
ZFTRAF1	0.029521589	-0.945552216	0.688	0.867	1
ENSSSCG00000061948	0.029529414	-0.955605881	0.375	0.6	1
ZNF558	0.029568953	1.09978612	1	1	1
ENSSSCG00000054038	0.029681221	-1.093109404	0.312	0.533	1
SDR42E2	0.029688286	-0.678071905	0.5	0.467	1
GRB2	0.029715672	-0.57068837	0.812	0.867	1
ALG11	0.029779982	-0.281871048	0.938	0.933	1
NAALADL2	0.029824237	1.1205658	1	1	1
ADAMTSL3	0.029835564	-0.967578522	0.375	0.733	1
SLC26A4	0.029881097	1.128957131	1	1	1
SUMO2	0.029908953	1.040568166	1	1	1
ENSSSCG00000009240	0.029940743	-0.555452618	0.938	0.667	1
BIVM	0.0300522	1.394991557	0.938	0.933	1
ENSSSCG00000061723	0.030062196	0.979905393	1	0.933	1
ENSSSCG00000015193	0.030071012	-1.093109404	0.5	0.667	1
RSL1D1	0.030150788	0.125735596	1	1	1
HECA	0.030188104	-0.111927433	1	1	1
DKK1	0.030223897	-0.220221322	0.938	0.933	1
TRIO	0.030336736	-0.109692289	1	1	1
EXO5	0.030367451	1.397525714	1	0.933	1
CDHR3	0.030379083	-0.297727124	0.875	0.933	1
IL34	0.030408328	-0.601256308	0.812	0.667	1
ENSSSCG00000058307	0.03048722	-0.692571475	0.938	0.733	1
IFT22	0.030491741	-0.10718459	0.812	0.933	1
ENSSSCG00000052763	0.03051887	-0.654988292	0.438	0.733	1
SHC1	0.030591396	1.190820226	1	1	1
VGLL3	0.030622003	1.09427151	1	1	1
APPBP2	0.030629913	0.979540718	1	1	1
FAM169A	0.03071155	0.8498451	1	1	1
NR1I3	0.030745366	-1.315501826	0.188	0.533	1
TAP1	0.030748496	-0.9510904	0.625	0.667	1
PPP1R14B	0.030758345	1.475733431	1	0.667	1
DCUN1D3	0.030851825	-0.448590059	0.875	0.867	1
ZNF22	0.030851886	0.048458236	1	1	1
SLC38A2	0.031005857	-0.254915673	1	1	1
ENSSSCG00000047016	0.031008553	1.491853096	0.812	0.733	1
ENSSSCG00000058777	0.03101315	-0.752072487	0.625	0.667	1
EPS8	0.031028357	-0.651104858	0.875	0.733	1
C9orf85	0.031038125	-0.849838253	0.688	0.6	1
DNM3	0.03104416	1.144140976	1	1	1
NDST1	0.031121789	-0.613941568	0.438	0.6	1
AP3M1	0.031135634	0.916686375	1	1	1
ENSSSCG00000057928	0.031135731	1.190986617	0.938	1	1
MRM2	0.031235894	1.196268876	1	1	1
ELP3	0.03130955	0.302885792	1	1	1
SRSF12	0.031337517	-0.384875529	0.938	1	1
WDTC1	0.031384548	-0.639199013	0.875	0.867	1
RIBC1	0.031529674	-1.005646563	0.438	0.667	1
SPTY2D1	0.031570508	-0.678071905	0.875	0.867	1
DCLK2	0.031633894	1.083553032	1	1	1
ENSSSCG00000058221	0.031645597	-0.852101305	0.625	0.533	1
PLXNB1	0.031697262	-2	0.188	0.533	1

FAM168A	0.03170874	-0.279987539	1	1	1
SMC1B	0.031721722	0.299602036	1	1	1
ENSSSCG00000057350	0.031766091	2.607330314	0.688	0.2	1
SPC25	0.031777555	-0.023815499	1	1	1
VPS72	0.031834591	1.334237236	1	1	1
UNK	0.031859455	-0.10433666	1	1	1
ADARB1	0.031905639	-1.208586622	0.188	0.6	1
RAB2A	0.031918056	1.065738803	1	1	1
ALG5	0.031944519	1.654703572	1	0.733	1
PLXNC1	0.031956791	-0.31890441	0.938	0.933	1
EXOC3L4	0.032011687	-1.341036918	0.125	0.533	1
SH3BGRL3	0.032068046	-1.552541023	0.188	0.6	1
FAHD1	0.032095514	-0.578536232	0.688	0.8	1
EIF4EBP2	0.032167927	0.181397786	0.938	1	1
ATXN1L	0.032177137	1.144396156	1	1	1
ENSSSCG00000062485	0.032269609	1.484199524	1	0.933	1
NBAS	0.032300779	1.080431472	1	1	1
DSTN	0.032305942	0.999537014	1	1	1
CADPS2	0.032386364	0.981278854	1	1	1
PNRC2	0.032427817	1.091661719	1	1	1
ENSSSCG00000049230	0.032475589	-0.289506617	0.938	0.867	1
KIAA1958	0.032476897	1.027283303	1	1	1
ENSSSCG00000059101	0.032496867	-0.254390311	0.875	0.867	1
LIF	0.032539833	1.770829046	1	0.733	1
ENSSSCG00000059082	0.032544448	2.845490051	0.688	0.067	1
ACSBG1	0.032585224	-0.749155003	0.688	0.733	1
FARS2	0.032699651	-1.028979067	0.75	0.667	1
CMSS1	0.032705593	-0.982078092	0.438	0.667	1
PRKDC	0.032709021	0.123919832	1	1	1
AMOT	0.032732558	-0.087736232	1	1	1
PDK1	0.032762191	1.374158054	1	1	1
TUBGCP4	0.032799038	-0.286960245	0.938	0.933	1
RCBTB1	0.032809971	1.166777944	1	1	1
APAF1	0.032828785	-0.149526956	1	1	1
GRAMD2B	0.03287133	1.090838164	1	1	1
ANAPC4	0.032877752	1.426635437	1	1	1
ENSSSCG00000042690	0.03288025	-0.093109404	0.875	0.933	1
VWA3B	0.032923056	-0.529208519	0.75	0.8	1
SLC66A2	0.033127534	1.303586459	1	1	1
TPX2	0.033147481	0.977645579	1	1	1
SEC22A	0.03314977	0.355711971	1	1	1
SPG21	0.033167806	0.301132895	1	1	1
KLHL2	0.033267461	1.209453366	1	0.8	1
SEPHS1	0.033309726	1.837627933	1	0.6	1
CCDC91	0.033417377	1.217230716	1	0.933	1
CABIN1	0.033438121	1.272112201	1	0.8	1
OTULINL	0.033456822	-0.24691474	0.812	0.867	1
COPS8	0.033542125	0.359592168	1	1	1
ZNF326	0.033586792	1.544061574	1	1	1
DDX3X	0.033595246	1.015760038	1	1	1
FBXL16	0.033651284	-0.455679484	0.75	0.867	1
ENSSSCG00000052025	0.033775213	-1.00737953	0.75	0.667	1
PPP1R27	0.033806223	2.192292814	0.812	0.333	1
RBM18	0.033808562	1.216073462	1	1	1
ENSSSCG00000056205	0.033819025	-0.847996907	0.688	0.533	1
EPB41L4B	0.033838901	0.292274026	1	1	1
ENSSSCG00000060362	0.033857131	-0.595609745	0.375	0.6	1
DNAAF4	0.0339206	-0.912537159	0.5	0.667	1
MED1	0.0339719	0.85076664	1	1	1
COQ5	0.033997108	1.313845749	1	1	1
SAMHD1	0.034053004	1.572367702	1	1	1
INPP5F	0.034053032	1.042848283	1	1	1
ENSSSCG00000032119	0.034115759	1.054535913	1	1	1
GMPPB	0.034138429	-0.385891154	0.938	1	1
ENSSSCG00000038396	0.034333818	1.906890596	0.875	0.267	1
SENP7	0.034380632	1.237144623	1	1	1
ENSSSCG00000034843	0.034393031	-0.678071905	0.812	0.867	1
JUN	0.03441827	1.805010982	1	0.733	1
ARHGAP39	0.034477462	1.730012834	1	0.667	1
CDKN1A	0.034500102	-0.064540252	0.938	1	1

MTCH1	0.03451355	0.085060351	0.938	1	1
ETNK2	0.034527295	-0.299560282	0.875	0.8	1
LIPA	0.034570818	1.342372198	1	1	1
VMAC	0.034633615	-0.73947245	0.562	0.667	1
CPSF6	0.03463908	1.0408363	1	1	1
CALCOCO2	0.034685479	0.207030002	1	1	1
NFE2L1	0.034695482	0.109489148	1	1	1
OXNAD1	0.034720754	0.997711823	1	1	1
NCOA5	0.034737791	-0.112050968	0.938	1	1
ICE1	0.034897799	1.338606836	1	1	1
PLEKHA3	0.034948459	-0.617771395	0.75	0.8	1
TCP11L2	0.03503198	-0.647698256	0.5	0.733	1
ZNF541	0.035031985	1.984893108	0.812	0.4	1
CUX2	0.035041526	1.051172652	1	1	1
USP35	0.035076008	-0.88957601	0.5	0.733	1
OPN4	0.035143155	-1.044199804	0.438	0.733	1
NBN	0.035149894	0.027604461	1	1	1
ENSSSCG00000034174	0.03524116	1.638073837	0.875	0.8	1
CRYZL1	0.035261286	-0.123062626	0.938	1	1
MEAK7	0.035267945	-1.294743266	0.438	0.533	1
DALRD3	0.035275245	-0.79193987	0.688	0.933	1
HINFP	0.035314208	-0.73617486	0.812	0.8	1
SZT2	0.03531996	-0.633677786	0.375	0.8	1
ENSSSCG00000035376	0.035333853	0.227125768	1	1	1
TAOK2	0.035370512	-0.549487699	0.75	0.867	1
DGKE	0.035372801	1.027629237	1	1	1
SDC2	0.035382687	1.085961242	1	1	1
LIAS	0.035393673	-0.158165288	1	1	1
IDH3A	0.035470547	1.227524213	1	1	1
ENSSSCG00000046071	0.035476051	-1.471621028	0.125	0.533	1
PAXBP1	0.035482489	-0.410591594	1	1	1
KPNA4	0.035488299	-1.055438986	0.875	0.733	1
CCDC90B	0.035489516	-0.111056084	1	1	1
REEP1	0.03551577	-0.587874096	0.312	0.733	1
C1orf131	0.035530602	-0.721140627	0.625	0.733	1
SLC45A3	0.035556045	1.407650862	1	0.933	1
PGAP1	0.035633443	1.231392634	1	1	1
GTF2H4	0.035710065	2.476256241	0.875	0.6	1
ENSSSCG00000058744	0.035765519	-1.093109404	0.312	0.667	1
SMAGP	0.035850343	1.421463768	1	0.8	1
BICRA	0.035880463	-0.567040593	0.5	0.667	1
C18orf32	0.035931901	0.943141122	1	1	1
KCTD16	0.036036376	0.15368436	1	1	1
AP5M1	0.036111884	-0.091030593	1	1	1
ENSSSCG00000059840	0.036235085	1.33752495	1	0.867	1
ENSSSCG00000047186	0.036240881	-1.455679484	0.25	0.533	1
UBR7	0.036358415	0.943027924	1	1	1
PTPRG	0.03647229	0.039671709	1	1	1
ENSSSCG00000053618	0.036485966	-0.736965594	0.438	0.667	1
BCAS3	0.036528062	-0.259119356	1	0.933	1
CERK	0.036537715	1.501951544	1	1	1
IPO11	0.036552581	0.973659608	1	1	1
PDZRN4	0.036557597	-0.280736408	0.875	1	1
SMAD2	0.03658482	0.200921595	1	1	1
CAMTA2	0.036653837	-0.700791982	0.625	0.733	1
ERLIN2	0.036695281	0.994154677	1	1	1
ENSSSCG00000036022	0.036728179	1.805010982	1	0.6	1
SYCE2	0.036737586	-0.045412662	1	1	1
CDKL3	0.036771924	1.566815154	0.938	0.6	1
SLC6A4	0.036782732	-0.13076753	1	1	1
ENSSSCG00000054177	0.036786998	1.232507925	1	1	1
UVRAG	0.03683489	0.279877291	1	1	1
INTS3	0.036836396	1.397609295	1	1	1
ENSSSCG00000053387	0.036849578	-0.019647242	0.938	1	1
TXLNA	0.036859702	1.457279948	1	0.933	1
ERI2	0.036859961	1.534261244	1	1	1
SPCS2	0.03689336	1.07525667	1	1	1
AP1M1	0.036900088	-0.20475476	0.938	0.933	1
VOPP1	0.036909539	-0.877380713	0.5	0.467	1
PTGER2	0.036937984	3.661778098	0.5	0.067	1

GPR173	0.036966559	-0.9861942	0.375	0.533	1
MIPOL1	0.036967201	1.023808347	1	0.933	1
SHF	0.037029225	-1.093109404	0.188	0.6	1
AAMP	0.037065652	1.651794009	1	0.933	1
MEF2D	0.037089565	-0.339749372	0.812	0.867	1
GIT2	0.037150922	0.194871359	1	1	1
ENSSSCG00000058517	0.037205685	-0.186218809	1	0.933	1
ZNF354C	0.037208435	-0.909397451	0.688	0.733	1
ZNF471	0.037285105	-0.716546053	0.438	0.733	1
UBE2G2	0.037286352	1.352537521	1	0.933	1
SUPT20H	0.037307491	0.109295183	1	1	1
CELSR1	0.037390399	-0.706086281	0.375	0.667	1
FRYL	0.037478497	1.021066452	1	1	1
H1-0	0.037541727	-1.093109404	0.312	0.6	1
MGAT4A	0.037548048	0.845444956	1	1	1
ENSSSCG00000039545	0.037594895	-0.177998302	0.812	0.867	1
KLF11	0.037753418	0.088410736	1	1	1
GINS4	0.037785304	1.363480584	1	1	1
ENSSSCG00000002877	0.037788361	1.187733721	1	1	1
MMP1	0.037841609	-0.439759929	0.812	0.933	1
QSOX2	0.037896092	-1.415037499	0.25	0.533	1
MTERF4	0.037906075	-1.171111916	0.438	0.533	1
LRRC58	0.037909018	-0.230612928	1	0.733	1
ZSCAN12	0.037924956	0.067054717	0.938	1	1
CLCN2	0.037937673	1.393449392	1	0.933	1
ENSSSCG00000053345	0.037961601	-1.678071905	0.25	0.6	1
DDIT4L	0.037984569	1.158265411	1	1	1
MTHFSD	0.038144455	-0.218640286	0.938	0.8	1
ENSSSCG00000046327	0.038164111	-0.36104261	0.938	0.867	1
PDCD4	0.038207202	0.271660912	1	1	1
SRR	0.038227511	-0.205375637	0.875	0.867	1
SLC22A23	0.038248246	-0.186976328	1	1	1
ZBBX	0.038251224	1.067355268	1	1	1
NME3	0.038284029	1.796707678	0.938	0.6	1
FKBP6	0.038299463	0.313794237	1	1	1
EID1	0.038378964	-0.331269142	0.938	0.867	1
CPNE3	0.038396244	1.102578693	1	1	1
PRSS22	0.038434016	4.154818109	0.625	0	1
USP3	0.038479426	-0.12855824	1	1	1
KBTBD6	0.038528074	-0.242856524	0.688	0.867	1
ADNP	0.038536514	1.887154378	0.938	0.933	1
ZPBP2	0.038570798	1.519074565	1	1	1
MLLT1	0.0386133	1.339850003	1	0.867	1
ENSSSCG00000063245	0.03868756	-0.283212288	1	1	1
ENSSSCG00000031855	0.038747945	2	0.812	0.533	1
CCL17	0.038750312	-1	0.375	0.667	1
RBM27	0.038868685	0.997552319	1	1	1
MRPS36	0.038912115	0.065821678	1	1	1
ATP2C1	0.038983915	0.089543902	1	1	1
BCAT2	0.03899998	-0.035393907	0.875	0.933	1
ABHD12B	0.039000419	1.236717705	1	1	1
SLC26A6	0.039024629	1.32011998	1	1	1
NKAPL	0.03904782	-0.006694653	1	1	1
ENSSSCG00000056471	0.039052872	-1.015106892	0.5	0.667	1
TRAM2	0.039055604	0.235743745	1	1	1
CDR2L	0.039100123	-1.093109404	0.375	0.667	1
HIC2	0.039216932	1.95419631	0.75	0.533	1
TDRD9	0.039225133	-0.555452618	0.688	0.867	1
ENSSSCG00000056411	0.03926358	1.017692015	1	1	1
ACTR3	0.039290903	0.30934125	1	1	1
ENSSSCG00000059570	0.039302428	-0.654988292	0.625	0.667	1
UNC13D	0.03933527	-0.689212463	0.875	0.867	1
IFIT5	0.039352693	1.014950341	1	1	1
SLC37A1	0.039377941	-0.134136673	0.938	1	1
DYRK2	0.039436336	-1.093109404	0.688	0.733	1
ENSSSCG00000033894	0.039586426	0.013199498	1	1	1
FAM102A	0.0396821	-0.176767333	0.875	1	1
TM9SF4	0.039698279	1.249577251	1	1	1
CCNQ	0.039779764	2.22881869	0.688	0.267	1
TRPC3	0.039803374	0.40272621	1	1	1

PRRG4	0.039935978	1.441927871	1	0.933	1
KIAA1549	0.03993892	-0.018341636	1	1	1
TBXA2R	0.040022109	-0.040160524	1	1	1
CEP164	0.040143412	-0.063091259	1	1	1
ENSSSCG00000023865	0.040173757	-0.863627558	0.438	0.533	1
NAPB	0.040224892	-0.471621028	0.75	0.867	1
CCDC28A	0.040296503	1.638073837	1	0.867	1
ABHD10	0.040315157	1.254241287	1	0.933	1
RSPH3	0.040441647	0.022367813	0.875	0.933	1
TUBGCP3	0.040496916	1.375794967	1	1	1
PCF11	0.040500435	0.19661818	1	1	1
ATP6V1H	0.040531335	-0.03729219	1	1	1
ENSSSCG00000037445	0.040532421	1.043156286	1	1	1
SAG	0.04054574	-1.093109404	0.25	0.667	1
ATP9B	0.0405476	1.513069582	0.812	0.667	1
DSE	0.040570717	1.536641018	1	0.933	1
UBA3	0.040580233	0.848943246	1	1	1
ADNP2	0.040603054	0.163512263	1	1	1
COX7B2	0.04065802	-0.512485839	1	1	1
PACC1	0.040787212	-0.217736059	1	0.867	1
GSKIP	0.040835877	1.152284842	1	1	1
YTHDC1	0.040874154	0.242110187	1	1	1
CSTF3	0.040902039	1.164351157	1	1	1
INPP5E	0.040972668	1.999336844	0.875	0.733	1
GK5	0.041018731	-0.024803391	0.938	0.933	1
PPP2CB	0.041022106	0.976070623	1	1	1
KPNB1	0.041079488	0.066781004	1	1	1
ENSSSCG00000055817	0.041178292	-1.208586622	0.188	0.533	1
SPG11	0.04119017	0.981022325	1	1	1
AAGAB	0.041216439	0.128234318	1	1	1
MAP2K6	0.041245839	-1.678071905	0.25	0.533	1
MTREX	0.041317858	0.029323367	1	1	1
AKAP11	0.041355159	0.953898822	1	1	1
PHF11	0.041386291	1.15254972	1	1	1
ZFP91	0.041436049	0.890211854	1	1	1
TRIM11	0.041441311	1.459431619	0.875	0.6	1
PHF19	0.041492443	0.089794851	1	1	1
CNOT10	0.041496602	1.038223472	1	1	1
GAR1	0.041530763	1.312488399	1	1	1
ENSSSCG00000000746	0.041554072	-0.326599535	0.938	0.933	1
SLX4	0.041603243	-0.449253215	0.5	0.6	1
ENSSSCG00000026454	0.041617104	-0.118644496	0.75	0.933	1
ND5	0.041688592	1.671794797	1	1	1
CRELD1	0.04175462	-0.455679484	0.875	0.8	1
BVES	0.041757761	-0.050465067	1	1	1
POT1	0.041791472	-0.274174963	1	1	1
MTMR10	0.041877432	1.457254435	1	1	1
NUP188	0.041902285	0.978275229	1	1	1
COG4	0.041928056	-0.642882477	0.938	0.8	1
MLEC	0.041937656	0.29053196	1	1	1
EGFL6	0.042010135	1.091315167	1	1	1
BRI3	0.042024693	1.05166212	1	0.867	1
YPEL2	0.042043461	1.209307867	1	1	1
ANKRD11	0.042174586	-0.540568381	0.5	0.6	1
SEC62	0.042184853	0.265082234	1	1	1
INIP	0.042196702	0.166628373	1	1	1
CDC34	0.042236836	0.22743746	1	1	1
CLPB	0.042367913	-0.200912694	0.938	1	1
TFRC	0.042402459	-0.411606594	1	1	1
UMAD1	0.042438842	0.240677774	1	1	1
ACAT2	0.042467195	-0.723875595	0.812	0.733	1
TFIP11	0.042482226	0.068058979	1	1	1
TLE4	0.042511092	-0.121201711	1	1	1
GDI1	0.042601517	0.017921908	1	0.933	1
RPRD1A	0.0426483	1.225252075	1	1	1
SYNRG	0.042699904	0.28171874	1	1	1
ENSSSCG00000040973	0.042716332	1.36833856	1	0.8	1
SLC5A6	0.042766315	-0.910906073	0.812	0.933	1
GRIK2	0.042776482	-0.151605054	0.875	0.933	1
PLCB1	0.042856141	-0.445781023	0.812	0.933	1

ISL2	0.042877151	-0.912537159	0.625	0.6	1
NDEL1	0.042939118	0.304028822	1	1	1
NSD1	0.042987848	0.898515919	1	1	1
TMLHE	0.04302367	1.164021717	1	1	1
ARHGAP31	0.043077731	1.829722735	0.875	0.467	1
EML1	0.043122317	2.330102026	0.562	0.333	1
TRAPPC13	0.043134153	0.093871809	1	1	1
C16orf46	0.043146656	-0.545621609	0.5	0.733	1
SNAP47	0.043239207	0.107156901	1	1	1
GCNT3	0.043284895	0.010171805	1	1	1
EPS15L1	0.043296683	0.159612627	1	1	1
RNF149	0.043326989	0.010144557	1	1	1
COX1	0.043376122	1.736681473	1	1	1
ENSSSCG00000057034	0.043419411	-0.578536232	0.938	0.933	1
LIMK1	0.043485024	-0.431525622	0.938	0.933	1
TGFBI	0.043522634	-0.2255597	0.812	0.933	1
RBM6	0.043599715	0.299317583	1	1	1
BICC1	0.043750503	0.960636167	1	1	1
SLC3A2	0.043798902	-0.584095757	0.688	0.733	1
DENR	0.0438554	-1.093109404	0.5	0.533	1
RHOBTB3	0.043890564	-0.267607136	0.875	0.933	1
ENSSSCG00000026719	0.043932244	1.253121938	1	1	1
GRSF1	0.043950991	1.00476625	1	1	1
PPIG	0.043971732	1.082365823	1	1	1
ENSSSCG00000054218	0.044117122	-0.242487028	0.688	0.933	1
CDC16	0.044166311	1.218658824	1	1	1
PRKCD	0.044344735	1.312032059	1	1	1
ADAMTSL5	0.044366316	-0.531230517	0.812	0.667	1
SYF2	0.044374137	0.003566615	1	1	1
BSCL2	0.04437603	0.018638953	0.938	0.933	1
BRD8	0.044377458	0.787050826	1	1	1
GPAT3	0.044387011	1.637283536	0.812	0.667	1
PLEKHM1	0.044421405	1.159533396	1	1	1
ZNF227	0.044428341	1.208804901	1	1	1
METTL5	0.044433241	-0.396387442	1	0.933	1
ZC4H2	0.044510271	1.540159293	0.938	1	1
NT5DC3	0.044579544	-0.109983223	0.938	1	1
GEN1	0.044595483	-0.303876501	0.875	0.867	1
PDE5A	0.044614949	0.005970369	1	1	1
KLHL8	0.044686951	1.096715154	1	1	1
DDX55	0.044694485	1.250222542	0.938	1	1
KCNK9	0.044815527	-0.552541023	0.875	0.667	1
GRIN2A	0.044821034	1.370291116	0.938	0.867	1
SEC24B	0.044873665	-0.170242875	1	1	1
SMG7	0.044917339	0.187316801	1	1	1
ALDH5A1	0.044953015	1.951284715	0.688	0.267	1
CDC14A	0.045069625	2.154818109	0.688	0.533	1
NUP85	0.045082517	1.193585413	1	1	1
CMKLR1	0.045083473	1.56872186	0.875	0.8	1
SH2B1	0.045121314	1.600787468	1	0.8	1
WDR36	0.045144147	1.003675658	1	1	1
SRP72	0.045157948	-0.55468949	1	0.933	1
ENSSSCG00000062130	0.045177003	-1.35614381	0.188	0.533	1
DZIP1	0.045275784	1.170655665	1	1	1
ITK	0.045322767	-1.093109404	0.5	0.533	1
ACD	0.045451999	-0.213403638	1	0.8	1
DYRK3	0.045491002	1.140282016	1	1	1
PMP22	0.045523773	-0.384010604	0.875	0.933	1
KLRG2	0.045548638	-0.977632187	0.438	0.6	1
PKNOX1	0.045644561	0.225920742	1	1	1
ENSSSCG00000059754	0.045659117	1.321928095	1	0.933	1
ENSSSCG00000041267	0.045823678	2.22881869	0.688	0.267	1
SRP68	0.04583928	0.114344897	1	1	1
KIF16B	0.045940838	0.34307587	1	1	1
ZNF330	0.046034984	1.274930615	1	0.933	1
ENSSSCG00000007688	0.046070596	0.883558092	1	1	1
SPDL1	0.046100854	-0.064091304	1	1	1
EIF5B	0.046192783	0.447486904	1	1	1
PREX1	0.046196128	1.613159393	0.938	0.733	1
ENSSSCG00000021534	0.046207414	-1.230612928	0.125	0.6	1

SNRNP27	0.046270531	1.137422362	1	1	1
H2AC4	0.046271683	-0.458393868	0.75	0.733	1
RNF10	0.046290538	1.516804943	1	1	1
SLC40A1	0.046336986	-0.158327064	0.938	0.867	1
ENSSSCG00000061793	0.04635005	1.122396631	1	0.867	1
PHLDA1	0.0463882	0.071949842	0.938	0.933	1
TMEM150C	0.046434136	0.046963479	1	1	1
PIGN	0.046435274	1.019365325	1	1	1
C14orf39	0.046466188	1.534921818	0.812	0.667	1
FAM217B	0.046516056	-0.923184403	0.25	0.533	1
HEY2	0.046543887	0.91411699	1	1	1
WASF3	0.046569349	1.088546963	1	1	1
ACTG1	0.046582719	0.317806106	1	1	1
ENSSSCG00000042700	0.046686412	2.714245518	0.5	0.133	1
LDLRAD4	0.046696072	1.578657924	0.938	0.733	1
HPS1	0.046725972	-0.625983394	0.75	0.6	1
ENSSSCG00000061446	0.046775415	-0.435997118	0.812	0.8	1
EFCAB10	0.046800942	-0.295530405	0.938	1	1
RNF227	0.046903353	-0.760534065	0.562	0.667	1
TXLNG	0.046919942	0.362085221	1	1	1
NTAN1	0.0469341	1.095335685	1	0.8	1
PDE11A	0.046936096	-0.4639471	0.688	0.867	1
APBB2	0.046944995	0.211994068	0.938	1	1
PECAM1	0.046992337	1.301750213	0.938	0.667	1
PSMD4	0.047130311	1.234695257	1	1	1
LTN1	0.047130891	0.947001803	1	1	1
POMT2	0.047178394	-0.63673111	0.625	0.867	1
CCL25	0.047195093	-0.493647334	0.812	0.8	1
ENSSSCG00000008984	0.047207785	-0.471621028	0.875	0.867	1
PIKFYVE	0.047221969	-0.150140349	0.938	0.933	1
ARPC2	0.047251296	0.399207546	1	1	1
STK4	0.047258137	1.021308488	1	1	1
ETFRF1	0.047314811	-0.098110085	0.938	1	1
ELOVL6	0.047410704	0.29192645	1	1	1
NDE1	0.047474319	0.288253561	1	1	1
RYR3	0.047489651	0.196909443	1	1	1
FRMD6	0.047540474	0.945341437	1	1	1
ENSSSCG00000014314	0.047550226	-0.178276788	0.875	1	1
PPAT	0.047593162	1.029617552	0.938	1	1
TET1	0.047716667	2.070389328	0.75	0.667	1
CD151	0.047720493	-0.721140627	0.438	0.533	1
DDOST	0.047757641	0.201633861	1	1	1
UVSSA	0.047780765	-0.166358386	1	0.933	1
COQ8B	0.047809506	-1.066142357	0.5	0.733	1
ENSSSCG00000017507	0.047905541	0.263498078	1	1	1
STK38L	0.047906177	1.199283181	1	1	1
ENSSSCG00000003102	0.047942635	-0.967578522	0.375	0.533	1
ATP1B1	0.047948708	-0.027421501	0.875	1	1
ENSSSCG00000055321	0.047980653	1.369517553	0.875	0.867	1
M6PR	0.048023192	0.191458163	1	1	1
TRAF7	0.048101892	1.38466385	1	0.933	1
PTK7	0.048154875	2.430452552	0.625	0.267	1
PAN2	0.048176091	1.082252408	1	1	1
MYO9B	0.048183459	-0.455679484	0.938	0.867	1
DCTN6	0.048199093	0.852709557	1	1	1
XPR1	0.048288385	0.927749201	1	1	1
ENSSSCG00000045808	0.048297671	1.037227795	1	1	1
USP38	0.048343521	0.937717122	1	1	1
NKAIN2	0.048363453	-0.924986646	0.812	0.733	1
PHTF2	0.048397312	1.093626496	1	1	1
PREP	0.048398878	1.044712162	1	1	1
MAP2K5	0.048606473	-0.220488711	0.875	0.8	1
PPT1	0.048667446	0.338505964	1	1	1
ENSSSCG00000049958	0.048720064	-1.277533976	0.312	0.533	1
ITGB1BP1	0.048725898	-0.10137702	0.938	1	1
DOT1L	0.048732575	0.059593857	0.938	1	1
ENSSSCG00000061001	0.048854711	1.294494865	0.938	0.6	1
FAM163A	0.048984046	-0.587874096	0.688	0.8	1
ENSSSCG00000062471	0.049024065	-0.9861942	0.375	0.667	1
SPTB	0.049060227	1.383442484	1	1	1

GSX2	0.049076626	1.350725359	1	1	1
VPS16	0.049119582	1.323661062	1	1	1
SEZ6L	0.049124515	-1.388565288	0.562	0.467	1
R3HDM2	0.049137306	0.135019386	0.938	1	1
ENSSSCG00000036144	0.049305232	-0.256608137	0.812	0.933	1
STAT5B	0.04935411	1.391090119	0.938	0.933	1
MFN2	0.049355576	-0.062412607	1	0.867	1
METTL8	0.049431224	-0.211753901	0.562	0.667	1
ENSSSCG00000049427	0.04945828	-0.852101305	0.25	0.733	1
ADAR	0.049513065	0.941872792	1	1	1
ENSSSCG00000061041	0.049624469	-0.423258006	0.875	0.8	1
AMMECR1L	0.049686609	0.114556502	0.938	1	1
ARL14EP	0.049690895	1.105770994	1	1	1
ABRAXAS2	0.049776677	0.241406512	1	1	1
BCL2L2	0.049806585	-0.77118131	0.5	0.6	1
KDM5B	0.049834997	-1.749155003	0.625	0.533	1
ACOX1	0.049836478	1.055527091	1	1	1
SESN1	0.049850534	1.253776113	1	1	1
ENSSSCG00000044685	0.049873702	-1.230612928	0.188	0.533	1
CNOT3	0.049952273	1.743391863	1	0.933	1
ESPL1	0.049992993	1.076744948	1	1	1
ENSSSCG00000061301	0.049994377	-0.809316438	0.312	0.667	1
SQSTM1	0.050024742	1.201857503	1	1	1
TMEM201	0.050056228	-0.34836646	0.625	0.733	1
PCSK1	0.05006224	2.658963082	0.75	0.6	1
GABPA	0.050143386	0.941576824	1	1	1
TAF5L	0.050149719	0.098405773	1	1	1
ENSSSCG00000042411	0.05021929	2.321928095	0.625	0.2	1
MYO10	0.050269654	0.175222682	1	1	1
MTF2	0.05029783	0.94473488	1	1	1
KIF20B	0.050301479	1.13767323	1	1	1
RAB6A	0.050309173	0.891293741	1	1	1
C5	0.050320434	-0.779951519	0.688	0.6	1
E2F7	0.050323806	-0.093109404	0.875	1	1
NEPRO	0.050353471	-0.054635257	0.938	0.933	1
ENSSSCG00000041243	0.050358258	3.076815597	0.562	0.067	1
POLR1C	0.050392918	-1.096747981	1	0.733	1
ENSSSCG00000040773	0.050581634	1.217859479	0.938	0.933	1
GUF1	0.050643294	-0.026189812	0.938	1	1
NAP1L3	0.050690037	-0.112940576	1	1	1
ENSSSCG00000004246	0.050787972	1.369417021	0.938	0.867	1
ENSSSCG00000051194	0.050794418	-1	0.312	0.6	1
ENSSSCG00000048507	0.050826526	-0.607682577	0.75	0.8	1
MARCKS	0.051032683	1.516781493	1	0.867	1
ENSSSCG00000056774	0.051053445	-0.47498004	0.438	0.6	1
GREB1	0.051074094	-0.815575429	0.438	0.733	1
ZFP62	0.05112333	1.282399731	0.938	0.867	1
WDR72	0.051147878	1.100662339	1	1	1
APP	0.051149789	-0.4639471	0.625	0.8	1
SKIDA1	0.051265435	-0.77118131	0.5	0.533	1
OR51Q1	0.051274421	1.656912343	0.812	0.267	1
ENSSSCG00000062038	0.051304872	-0.883656039	0.625	0.467	1
GNPAT	0.051382311	1.067643951	1	1	1
AMIGO1	0.051488547	-0.065453061	0.875	0.933	1
ENSSSCG00000048831	0.051507794	-0.830074999	0.25	0.667	1
ENSSSCG00000055578	0.051513756	-0.585505786	0.875	0.933	1
WDCP	0.0515304	-0.088260005	1	1	1
ZNF473	0.051638893	-0.363963315	0.875	0.8	1
ENSSSCG00000007088	0.051642522	0.371164306	1	1	1
IPCEF1	0.051841153	-0.052082136	0.875	0.933	1
TUBG2	0.051930853	1.216575095	1	1	1
TUBAL3	0.052051178	0.897904923	1	1	1
TESC	0.05228381	1.153233766	1	1	1
SLC44A5	0.052300007	0.215322848	1	1	1
COX7A2L	0.052346008	0.323881021	1	1	1
ENSSSCG00000051466	0.052414724	-0.382616022	0.562	0.733	1
NPR3	0.052460616	0.067355268	1	1	1
TRIM44	0.052488514	0.026921299	0.938	0.933	1
PRKACB	0.052503927	1.189926954	1	1	1
FOXK1	0.052529832	1.491853096	0.938	0.533	1

CCIN	0.052547222	-0.075727326	0.875	0.933	1
AP3B1	0.052547491	0.81606443	1	1	1
ENSSSCG00000043595	0.052552205	-0.804604311	0.688	0.867	1
TMEM127	0.052557273	-0.204140717	0.75	0.8	1
PABPN1L	0.05257893	1.986375379	1	0.933	1
TNIK	0.052611317	1.183788482	1	1	1
BID	0.052655863	-2.341036918	0.188	0.667	1
IMPA1	0.052727857	1.081780535	1	1	1
ZSCAN23	0.052817609	0.098189248	0.875	1	1
ZSCAN16	0.052818505	-0.111256751	1	0.933	1
RBCK1	0.05282138	0.289610375	1	0.933	1
ENSSSCG00000052558	0.052940743	-0.35614381	0.625	0.933	1
VAMP4	0.053025157	-0.361857461	0.938	0.933	1
ENSSSCG00000053698	0.05312643	-0.14286244	0.938	0.933	1
RBPJ	0.053174403	1.103369232	1	1	1
ENSSSCG00000032337	0.053181365	-0.253574077	0.875	0.933	1
CMIP	0.053182484	-0.449253215	0.625	0.733	1
TTC14	0.053275831	1.633325522	0.938	0.933	1
ENSSSCG00000061435	0.053411562	-0.800928653	0.438	0.6	1
PRMT3	0.053504303	1.392317423	0.938	0.8	1
SLC39A5	0.053516202	-0.079812582	0.875	0.933	1
CAPRIN1	0.053558484	-0.120177648	1	0.933	1
ANGPTL1	0.053558861	-0.295926287	0.812	0.933	1
ENSSSCG00000032445	0.053642861	1.751526534	1	0.933	1
NUP98	0.053677943	0.974142842	1	1	1
LPAR6	0.053723569	1.567404129	0.875	0.8	1
ENSSSCG00000032456	0.05373808	-0.745186101	0.438	0.667	1
DYNLRB2	0.053857023	-0.877380713	0.5	0.6	1
ENSSSCG00000061311	0.053945311	-0.205584134	0.875	0.867	1
ADGRG1	0.054052292	0.206292121	1	1	1
ENSSSCG00000043560	0.054067552	1.845490051	0.812	0.467	1
DDHD1	0.054079846	0.151309323	1	1	1
WDR83OS	0.054188173	-0.152216022	1	1	1
TPR	0.054200283	1.176270513	1	1	1
SUN1	0.054216592	1.299901789	1	1	1
BAG4	0.054242741	0.26709366	1	1	1
COMMD2	0.05438539	0.951114596	1	1	1
CAP2	0.054460977	1.396037309	1	0.933	1
PAQR3	0.054499354	1.054989235	1	1	1
ENSSSCG00000051907	0.054609406	-0.967578522	0.625	0.6	1
ENSSSCG00000058908	0.054610608	1.701306462	0.688	0.533	1
RNF11	0.054634939	-0.048715285	1	1	1
PIMREG	0.054637434	-0.312890612	0.688	0.733	1
ATG4C	0.054666781	1.435269568	0.938	0.467	1
TJP1	0.054682366	0.985235486	1	1	1
GALNT2	0.054724939	-0.499101764	0.812	0.8	1
ENSSSCG00000011905	0.054873241	1.704397732	0.688	0.667	1
TBC1D2B	0.054944295	0.171718283	1	1	1
ENSSSCG00000004573	0.054945587	-0.706086281	0.5	0.733	1
THG1L	0.055057323	0.078501974	1	1	1
ENSSSCG00000052350	0.055073862	0.074639735	1	1	1
CYP20A1	0.05517065	1.142662047	1	1	1
CPEB2	0.055213505	1.080707412	1	1	1
ENSSSCG00000055258	0.055259269	-1.35614381	0.125	0.533	1
RIC8B	0.055323678	-0.162372067	0.875	1	1
MXI1	0.055366328	1.018783476	1	1	1
CEP43	0.055372106	0.416000874	1	1	1
NIN	0.055494541	1.038135129	1	1	1
MICU1	0.05573889	-0.300704824	0.938	0.867	1
CDR2	0.05580099	0.014950341	1	1	1
RASIP1	0.055811559	-0.495207848	0.625	0.667	1
SUDS3	0.055833361	0.290052753	1	1	1
TACO1	0.055841609	-0.062412607	1	0.867	1
WDR46	0.055931553	-0.578536232	0.562	0.6	1
ND1	0.055989398	1.596896499	1	1	1
SLC16A7	0.056104961	2.19865672	0.812	0.667	1
DUSP6	0.056126894	-0.497499659	0.562	0.933	1
HNRNPDL	0.056238512	0.999979205	1	1	1
AEBP2	0.056289459	-1.54830403	0.688	0.467	1
SLC27A6	0.056357448	2.621136113	0.562	0.467	1

SAFB	0.056633834	0.337219747	0.938	1	1
MYL12B	0.056637916	0.114503297	1	1	1
ENSSSCG00000062276	0.056796836	-0.578536232	0.875	0.733	1
KIF17	0.056813054	-1.378511623	0.438	0.6	1
HACD1	0.05683109	1.602884409	0.875	0.467	1
SARNP	0.056832071	1.084027222	1	1	1
YIPF6	0.056834228	1.180494243	1	0.867	1
RINT1	0.056858212	0.160838429	1	1	1
EWSR1	0.057058721	-0.021556143	1	1	1
UBE3A	0.057068788	1.289063461	1	1	1
STAG3	0.05714234	-0.061676897	0.875	1	1
RNF169	0.057214201	-0.113968009	1	1	1
USP37	0.057245388	0.240815036	1	1	1
ENSSSCG00000041164	0.057281314	1.302819272	0.875	0.733	1
TRAM1L1	0.057338023	-0.101146723	1	0.933	1
KRIT1	0.057365217	1.237807474	1	0.933	1
PTPRA	0.057380602	0.261692988	1	1	1
IFT172	0.057403917	-0.849838253	0.625	0.8	1
DOP1A	0.05745075	0.139946631	1	1	1
ARID4A	0.057452687	1.065327804	1	1	1
ENSSSCG00000027232	0.057546205	2.17990909	0.625	0.2	1
NETO2	0.057654598	1.024585638	1	1	1
ago-03	0.057655801	-0.147951475	1	1	1
RNF185	0.057703584	-0.027602298	0.938	1	1
CHKB	0.057814415	-0.623624121	0.625	0.733	1
RRS1	0.05782403	0.204434532	1	1	1
RAB23	0.057859488	1.164558492	1	1	1
TPMT	0.057884299	-0.253574077	0.5	0.733	1
ENSSSCG00000003989	0.057934428	1.617860981	0.938	0.933	1
ENSSSCG00000056123	0.058004475	-0.338221902	0.938	1	1
SNX21	0.058064803	-1.010647244	0.188	0.533	1
BTBD9	0.058086798	0.226926631	0.938	1	1
BBS9	0.058105352	1.558967292	0.875	0.4	1
ESRP1	0.058126029	0.358465299	1	1	1
MACO1	0.058148008	0.052549227	0.938	1	1
ZFP30	0.058149706	0.948158614	1	1	1
SCN10A	0.058195618	-0.065394026	1	1	1
ENSSSCG00000061639	0.058206395	-0.734655433	0.75	0.733	1
ORMDL3	0.05826311	0.213221835	1	0.933	1
HMCN1	0.058443404	0.188012474	1	1	1
ENSSSCG00000056598	0.058456599	-0.279730695	0.875	1	1
SPNS2	0.058487563	-0.707819249	0.438	0.6	1
SLC46A1	0.058493827	-0.415037499	0.875	0.733	1
KIF7	0.058498024	1.271084417	1	0.933	1
COPG1	0.058571776	1.240651054	1	1	1
TIMM10B	0.058753294	0.324714405	1	1	1
MTMR6	0.058782509	0.330744242	1	1	1
SCUBE3	0.058813618	2.044394119	0.688	0.333	1
ALDH1B1	0.058846164	-0.050040682	0.875	0.933	1
ENSSSCG00000038540	0.058864271	-0.437063806	0.75	0.933	1
ENSSSCG00000045852	0.058867806	1.029509882	0.938	0.733	1
CSNK1G1	0.058878331	0.932653692	1	1	1
ENSSSCG00000058423	0.058902081	-0.721140627	0.375	0.533	1
DNAJC9	0.058929926	0.441767865	1	1	1
MSTO1	0.05908921	-0.508146904	0.75	0.933	1
ENSSSCG00000003746	0.059136109	-0.923184403	0.375	0.6	1
ZKSCAN8	0.05919456	0.881093716	1	1	1
FECH	0.059239535	1.260037421	1	0.933	1
ENSSSCG00000044152	0.059298776	-0.678071905	0.5	0.667	1
ENSSSCG00000056615	0.059300978	-0.745186101	0.375	0.533	1
FGF20	0.059475363	1.934371332	0.812	0.333	1
SETD3	0.059544756	1.089176256	1	1	1
SULT4A1	0.059569285	1.725052273	0.938	0.667	1
ENTHD1	0.059649598	-0.318669105	0.75	0.667	1
SH3PXD2A	0.059674649	1.191134897	1	1	1
SHQ1	0.059708473	1.155719624	1	1	1
RFLNB	0.059763675	-0.590609064	0.5	0.8	1
NDUFAF7	0.059820701	0.019274012	1	1	1
PTGFRN	0.059853403	-0.546827372	0.812	0.667	1
ENSSSCG00000029203	0.059900398	0.12889556	1	1	1

KLHL41	0.059941014	-0.923184403	0.562	0.6	1
KLHL15	0.060038348	1.014429788	1	1	1
ENSSSCG00000022039	0.060043215	-0.556081381	0.625	0.667	1
ENSSSCG00000045442	0.060055052	-0.693501946	0.75	0.867	1
RHEBL1	0.06011643	0.321073164	1	1	1
ZDHHC18	0.060134998	1.337318403	1	1	1
RAB21	0.060201722	0.149500051	1	1	1
KHDRBS3	0.060216416	-0.05173897	1	1	1
MET	0.060427628	-0.347923303	0.812	0.867	1
ENSSSCG00000004252	0.060432569	-0.254611489	1	1	1
ENSSSCG00000041452	0.06071077	-0.900464326	0.312	0.667	1
FBXO9	0.060895069	0.279475953	1	1	1
RIC8A	0.060944781	-0.863627558	0.312	0.533	1
TNFRSF19	0.060967955	1.581795222	0.875	0.733	1
ENSSSCG00000027907	0.060992393	0.094823546	1	0.933	1
CAVIN4	0.061052881	1.401655287	1	0.8	1
SLC25A24	0.061093437	0.087217193	1	1	1
AGGF1	0.06116062	1.096846029	1	1	1
EPS15	0.061199777	1.280948203	1	1	1
RAD18	0.061346366	0.202875423	1	1	1
ZFP69B	0.061372485	0.820733952	1	1	1
ATP8A1	0.061406636	-0.303327112	0.938	0.933	1
ENSSSCG00000022044	0.061422197	0.069443608	0.688	0.933	1
SND1	0.061447688	0.272035193	1	1	1
BMP6	0.061575518	0.119298928	1	1	1
ACP1	0.061641711	0.901846202	1	1	1
DET1	0.061643493	0.391301798	1	1	1
AASDHPPT	0.061706132	0.906426731	1	1	1
TBCK	0.061776206	1.118594009	1	1	1
RFESD	0.061813427	0.963167526	1	1	1
MRPL57	0.06181725	-0.495207848	0.75	0.8	1
SLC8A2	0.061887224	-0.803602787	0.25	0.533	1
ENSSSCG00000062875	0.061932252	-0.395672174	0.562	0.8	1
PABPC5	0.061966914	2.845490051	0.5	0.133	1
GPA33	0.062160992	1.078671154	1	1	1
NIT2	0.062167959	0.401154786	1	1	1
FANCI	0.062210822	0.903984864	1	1	1
ABCC10	0.062292706	-0.697180728	0.5	0.733	1
TTC9	0.062320922	-0.533681996	0.688	0.667	1
CAND1	0.062526553	0.402046583	1	1	1
KIF21A	0.062530583	1.27462238	1	0.933	1
AKIP1	0.062542306	1.22881869	1	1	1
ENSSSCG00000032146	0.062564407	1.329248781	1	0.933	1
ENSSSCG00000037720	0.062632797	-0.803602787	0.375	0.6	1
PRPF40B	0.062651581	-0.22521294	0.75	0.867	1
ENSSSCG00000060990	0.062652333	-0.495207848	0.625	0.667	1
OLIG1	0.062667315	2.299208018	0.5	0.2	1
PXN	0.062702838	1.189648093	1	1	1
ACSL4	0.063154754	0.138279375	1	1	1
GBP5	0.063195017	-1.044199804	0.562	0.6	1
OSBPL7	0.063196318	-0.166172867	0.875	0.867	1
MEIS2	0.063254965	-0.475579041	0.875	0.867	1
MPHOSPH10	0.063288257	0.183661615	1	1	1
PRKAA1	0.063359114	-0.449253215	0.875	0.867	1
AKR1C8	0.063398628	-0.105182237	1	1	1
RBM4	0.063471341	0.352374773	1	1	1
ENSSSCG00000015151	0.063471583	-0.870716983	0.125	0.733	1
ENSSSCG00000054797	0.06349402	-0.693501946	0.688	0.667	1
TMEM74	0.063533072	0.336355166	1	1	1
ELOVL5	0.063759217	-0.090886453	1	1	1
NAA30	0.063824863	1.041395099	1	1	1
ENSSSCG00000055086	0.063961888	1.143929793	1	1	1
UFL1	0.064001561	0.952096792	1	1	1
EFNB1	0.064076269	0.057622032	1	1	1
ENSSSCG00000055357	0.06409534	1.672425342	0.625	0.267	1
ENSSSCG00000036416	0.064124584	-0.923184403	0.375	0.533	1
DECR1	0.064428566	0.999222476	1	1	1
DENND2A	0.064433131	-0.183874868	0.938	0.8	1
ENSSSCG00000061594	0.064630368	-0.405053411	0.812	0.933	1
EN2	0.06469455	-0.858644151	0.312	0.667	1

ENSSSCG00000010391	0.064741862	1.700439718	0.875	0.533	1
GOLGB1	0.064773442	0.05735809	1	1	1
ECH1	0.064777397	-0.518415239	1	0.867	1
AXIN2	0.064921089	-0.374880373	0.75	0.8	1
NAGLU	0.064946766	1.419703311	1	0.533	1
CD2AP	0.065101735	1.046790892	1	1	1
NADK2	0.065130831	1.115379178	1	1	1
ENSSSCG00000052804	0.065140812	2.64385619	0.5	0.133	1
ZNF18	0.06515961	-0.552541023	0.375	0.6	1
TMTC3	0.065164541	1.066522143	1	1	1
ACAP2	0.065384452	1.132229549	1	1	1
AFAP1L1	0.065531232	0.132657744	0.938	1	1
PLK2	0.065557144	1.866248611	0.75	0.467	1
ENSSSCG00000044479	0.065701569	1.926790153	0.688	0.533	1
SUOX	0.065753918	-0.26021939	0.875	0.933	1
ATP6	0.065770207	1.447202939	1	1	1
PBX3	0.066084808	1.194623771	0.938	0.933	1
ENSSSCG00000053554	0.066168582	-1.230612928	0.188	0.533	1
MAN1A1	0.066253887	1.166757722	1	0.867	1
CPNE1	0.066255883	0.247414373	1	1	1
LRP2BP	0.066261401	-0.462343214	0.625	0.667	1
PDP2	0.066307345	1.158429363	1	1	1
FMNL1	0.066337065	1.369322621	1	0.933	1
ENSSSCG00000041892	0.066427086	-0.385891154	0.812	0.8	1
PPP4R3B	0.066435939	1.054653583	1	1	1
DTD2	0.066479295	1.186464678	1	0.933	1
PAX5	0.066521894	0.016301812	0.938	1	1
ENSSSCG00000036659	0.066691128	1.094663598	1	1	1
CSNK1E	0.066798707	0.977880035	1	1	1
ACP6	0.066805033	1.141174138	1	1	1
ARF6	0.067024329	0.098983234	0.938	1	1
LRIG1	0.067045148	1.383643879	1	0.933	1
GOSR2	0.067117061	1.049676256	1	1	1
GRIA1	0.067124008	-0.491658781	0.5	0.8	1
MYLK	0.067143531	1.102810806	1	1	1
AMIGO3	0.067199638	-1.010647244	0.312	0.533	1
G3BP1	0.067213429	-0.199042849	1	1	1
TIMM8A	0.067266971	-1.093109404	0.375	0.533	1
ENSSSCG00000058942	0.067280288	0.929078921	1	1	1
SHROOM2	0.067356506	1.05572641	1	1	1
ENSSSCG00000015383	0.067396764	-0.313878083	1	0.933	1
STAM	0.067431842	0.773044694	1	1	1
GOLPH3L	0.067489624	1.394005773	1	1	1
DOCK4	0.067595128	1.170604077	1	0.933	1
ENSSSCG00000063011	0.067631192	-0.590609064	0.438	0.6	1
ZYX	0.067766593	-0.529208519	0.562	0.6	1
ZSCAN30	0.067792224	1.653134003	0.938	0.8	1
LARP7	0.06779352	-0.108706259	0.938	1	1
SLC12A2	0.067842774	-0.226965151	0.875	0.8	1
EDAR	0.067853591	-0.18473988	0.812	0.867	1
PKN2	0.067861994	0.124721132	1	1	1
DGKD	0.06794729	1.109141879	1	1	1
ENSSSCG00000055280	0.067973875	-0.290146251	0.875	0.933	1
ZNF883	0.068062351	1.465686434	1	1	1
RPRD2	0.068079327	0.297893163	1	1	1
TMEM184B	0.068095417	1.336574871	0.938	0.867	1
SMNDC1	0.068158316	1.126335733	1	1	1
SERINC5	0.068289747	-0.477932291	0.938	0.867	1
SMAD4	0.06831736	-0.415037499	0.75	0.8	1
SEC11A	0.06835519	0.400142859	1	1	1
ENSSSCG00000022206	0.068492751	1.041540122	1	0.933	1
SGSM3	0.068519208	0.062419376	1	1	1
CACHD1	0.068685377	0.360544296	1	1	1
ERO1B	0.068836551	0.854397021	1	1	1
ARHGAP5	0.068886308	1.078285485	1	1	1
VPS13A	0.068994631	0.9354255	1	1	1
STK11	0.069056289	-0.0596347	0.938	0.933	1
VKORC1L1	0.0690904	1.041331884	1	1	1
SRCAP	0.069184229	1.166534412	1	1	1
PTBP3	0.069192212	0.930779741	1	1	1

SLC25A30	0.069207613	0.475338621	1	1	1
LYRM9	0.069241847	1.33315535	1	0.933	1
TYR	0.0692984	1.991424106	0.688	0.733	1
ENSSSCG00000054996	0.069375291	0.102881701	1	1	1
CERS3	0.069423038	1.138414029	0.938	1	1
DDX21	0.069423715	0.987183941	1	1	1
PLOD3	0.06948533	-0.584962501	0.5	0.733	1
PLEKHD1	0.069544478	-0.208586622	0.875	0.933	1
CCDC85C	0.069664994	-0.385891154	0.75	0.733	1
ENSSSCG00000042319	0.069678483	-0.654988292	0.562	0.533	1
ENSSSCG00000053904	0.069714671	1.824428435	0.625	0.333	1
POLR1B	0.069746166	0.954837447	1	1	1
CNDP1	0.069829396	-0.048290901	1	0.933	1
FER	0.070079374	0.938599455	1	1	1
ENSSSCG00000052117	0.070203944	0.139775391	0.938	0.933	1
TOM1L1	0.070215281	0.128793387	1	0.933	1
SPRYD7	0.070258916	1.139867768	1	1	1
NPTXR	0.070308	-0.046815752	0.875	0.933	1
ENSSSCG00000026446	0.070325959	1.308023574	1	1	1
ENSSSCG00000056195	0.070331033	1.951284715	0.688	0.333	1
SNRNP200	0.070355552	0.073009379	1	1	1
WDR24	0.070369483	-0.376043368	0.812	0.867	1
ENSSSCG00000011567	0.070428435	0.965809473	1	1	1
SLMAP	0.070495942	0.968181522	1	1	1
EXOC6B	0.070583814	0.201390862	1	1	1
ENSSSCG00000032894	0.070598579	1.481584761	1	0.733	1
CCDC81	0.070694621	2.269460675	0.688	0.2	1
ARHGEF11	0.070769996	-0.188868387	0.938	0.933	1
ZNF389	0.070805522	1.009984089	0.938	0.4	1
TTC39A	0.070811019	1.447772028	0.938	0.933	1
PLEKHF2	0.070825112	1.200141366	1	1	1
PRPS2	0.070949302	1.103691303	1	1	1
LAMC3	0.071005548	-0.607682577	0.625	0.6	1
MFAP2	0.071011778	-0.487388344	0.812	0.667	1
ENSSSCG00000004963	0.071016021	0.207338963	1	1	1
TOP3B	0.071189005	-1.05246742	0.375	0.6	1
PPP1R15B	0.071492396	-0.287756835	0.688	0.667	1
SLC24A4	0.071516188	0.072669068	1	1	1
DAPK2	0.071551634	1.569855608	0.625	0.333	1
NEK3	0.071568733	-1.044199804	0.812	0.733	1
ENSSSCG00000014132	0.071626454	0.162572335	1	1	1
GPC6	0.071735125	-0.159451899	0.875	1	1
AGPAT4	0.07185345	-0.236700258	0.875	1	1
PLEKHH1	0.071865332	0.051280505	1	1	1
MUS81	0.07189278	-0.100845053	0.938	0.867	1
ZNF786	0.07195202	-1.192645078	0.438	0.667	1
ENSSSCG00000063225	0.072017599	2.036173613	0.625	0.2	1
SUZ12	0.072039635	-0.214610409	1	0.933	1
TMCC3	0.072085185	0.082980277	1	1	1
TTC24	0.072088191	0.040157126	0.875	0.933	1
SRD5A1	0.072146323	1.303549566	1	0.933	1
TMEM268	0.072281323	-0.116696225	0.875	0.933	1
CCDC115	0.072341416	0.333094013	1	1	1
IMPDH1	0.072415343	-0.977632187	0.5	0.467	1
ENSSSCG00000063446	0.072427393	-1.157239742	0.312	0.6	1
ENSSSCG00000025060	0.072436547	-0.351642418	1	1	1
MED18	0.07244769	0.038621294	1	1	1
ZDHHC7	0.072528037	-0.248760275	0.875	0.867	1
ENSSSCG00000035307	0.072734178	-0.56125824	0.75	0.867	1
SENP2	0.072844349	0.08367679	1	1	1
COG2	0.072948775	-0.12217187	1	1	1
TRPM4	0.073095024	-0.037766371	0.938	1	1
CST3	0.073105149	-0.289506617	1	0.933	1
ENSSSCG00000020777	0.073133154	1.74819285	0.938	0.867	1
CBX5	0.073374588	0.964261775	1	1	1
ENSSSCG00000034625	0.073630953	1.300628693	1	1	1
TDRD5	0.073639212	0.148747003	1	1	1
CCPG1	0.073782179	0.425550726	1	1	1
PLK3	0.073802653	1.513831465	1	0.933	1
PFKFB4	0.074014809	-0.02406776	0.875	1	1

LIN54	0.074086022	-0.023985044	0.938	0.933	1
CCNY	0.074272939	0.39243472	1	1	1
HPSE2	0.074342665	1.965784285	0.562	0.267	1
SLC39A6	0.074396236	0.948493951	1	1	1
REXO5	0.074419818	-0.143735477	0.938	1	1
ATM	0.074711717	-0.089478459	1	1	1
KCNK2	0.074723941	0.205156013	1	1	1
SOAT1	0.074727639	0.413145356	1	1	1
LSM11	0.074820494	1.096134517	1	1	1
MARCHF8	0.074825835	0.225959036	1	1	1
PCDH10	0.074909657	-0.830074999	0.438	0.533	1
PRR11	0.074975308	-0.678071905	0.625	0.667	1
SEMA3E	0.075126393	-0.550657199	0.938	0.933	1
ENSSSCG00000057777	0.075313186	-0.678071905	0.375	0.533	1
RAB14	0.075402587	1.207844561	1	0.933	1
PMFBP1	0.075415629	-0.071495591	1	1	1
ARHGEF3	0.075546289	0.019365325	1	1	1
ENSSSCG00000029803	0.075558888	-0.282933963	0.812	0.867	1
TAF9B	0.075561702	0.968818345	1	1	1
MOGS	0.075603484	1.736965594	0.75	0.333	1
ZNF346	0.075679646	0.282399731	0.688	0.933	1
ENSSSCG00000018085	0.075766291	-1.327574658	0.438	0.6	1
SLC43A1	0.075864468	-0.119340946	0.875	0.867	1
SMC6	0.075876661	1.06799945	1	1	1
ENSSSCG00000037885	0.075896931	-0.003472192	1	0.933	1
CERS5	0.075901252	0.415468205	1	1	1
XRRA1	0.075907787	-0.0717884	0.938	1	1
CSRNP1	0.076005544	-0.309920793	0.75	0.667	1
LYRM7	0.076037587	0.985415776	1	1	1
DNAJC7	0.076057587	0.474443697	1	1	1
KLF4	0.076199512	1.059175438	0.938	1	1
ENSSSCG00000001594	0.076229289	2.64385619	0.5	0.133	1
CHD7	0.076298221	0.19958285	1	1	1
RCC2	0.076327864	0.37997989	1	1	1
PPP1R12B	0.076530664	1.271164981	1	0.933	1
CERKL	0.076579409	-0.001428714	1	1	1
BRWD3	0.076606641	0.091466623	1	1	1
DNMT3A	0.076619643	0.894280547	1	1	1
ENSSSCG00000041063	0.076833241	0.918553513	1	1	1
FGFR2	0.077102476	1.064803079	1	1	1
ZNF454	0.077141926	-0.233878998	0.875	1	1
CDK19	0.077161919	-1.058343986	0.438	0.533	1
ENSSSCG00000003826	0.07722116	1.010900068	1	1	1
JMJD1C	0.077289518	1.168654464	1	1	1
ENSSSCG00000037359	0.077315238	1.308989039	0.938	0.8	1
FMO5	0.077575361	1.555816155	0.938	0.933	1
PAF1	0.077629755	0.008299323	1	1	1
TASL	0.077761178	-0.793549123	0.562	0.6	1
ANXA2	0.07776732	1.651585463	1	1	1
PEX11A	0.077813807	2.698303974	0.625	0.2	1
CUL1	0.077927589	1.088167065	1	1	1
PRKRA	0.078079093	1.326115892	1	1	1
ENSSSCG00000050494	0.078122157	-0.77118131	0.312	0.6	1
C18orf63	0.078128137	1.014079842	1	1	1
MICAL1	0.078255111	-0.959842874	0.375	0.533	1
ZBTB39	0.078327129	1.001862158	1	1	1
ENSSSCG00000056588	0.078330506	2.906890596	0.5	0.067	1
SGTB	0.078381191	1.185932878	0.938	1	1
TRERF1	0.078390516	-0.607682577	0.375	0.667	1
ENSSSCG00000061444	0.078422145	1.371558863	1	0.733	1
ARCN1	0.078470473	0.989209265	1	1	1
DDX20	0.078555778	1.18509388	1	1	1
C5orf58	0.078586602	-0.389502407	0.938	1	1
ENSSSCG00000041434	0.078660673	-0.929610672	0.375	0.6	1
MRE11	0.078665432	-0.046780353	1	1	1
ENSSSCG00000046164	0.078752093	1.321928095	0.75	0.4	1
SON	0.07875698	0.818563126	1	1	1
ENSSSCG00000039529	0.078892971	1.271463028	0.875	0.867	1
CYP51A1	0.078995835	0.146941683	1	1	1
GLE1	0.079054723	1.119084367	1	1	1

GRWD1	0.079062397	-0.041314789	1	1	1
ENSSSCG00000035105	0.079118236	0.16754235	0.875	1	1
ENSSSCG00000035856	0.079145154	-0.27368165	0.625	0.733	1
TTC23L	0.079161373	1.048343105	1	1	1
ZNF532	0.079247233	0.928980138	1	1	1
ENSSSCG00000000854	0.079325406	-0.05994254	0.562	0.667	1
USP21	0.079452408	1.243022554	1	1	1
TMX2	0.079579236	0.169183447	1	1	1
TMEM87B	0.079678596	1.018005788	1	1	1
ENSSSCG00000062377	0.0797576	-0.113768875	0.938	1	1
ENSSSCG00000056505	0.07978251	1.714245518	0.75	0.4	1
ENSSSCG00000063426	0.079822727	1.015247774	1	1	1
SPOCK3	0.079867814	1.501634117	1	0.733	1
ANKZF1	0.079920877	-0.912537159	0.438	0.533	1
PEX11B	0.079962975	0.807217333	1	1	1
MED24	0.079986339	1.1109822	1	1	1
ENSSSCG00000048962	0.080162772	2.392317423	0.562	0.267	1
ZFYVE27	0.080194636	0.319829629	1	0.933	1
PLOD2	0.080361516	1.05197597	1	1	1
FGD1	0.080475579	0.053560245	1	1	1
ENSSSCG00000018063	0.080481015	1.220141466	1	1	1
ENSSSCG00000023569	0.080520719	0.097221808	0.938	1	1
ENSSSCG00000052847	0.080541478	1.607330314	0.75	0.533	1
LDAH	0.080545937	-0.900464326	0.625	0.533	1
ENSSSCG00000002653	0.080631081	1.767123231	1	1	1
CFAP54	0.080688546	0.324371271	1	1	1
WDR11	0.080783876	1.084871814	1	1	1
ENSSSCG00000015182	0.080814968	-0.312422433	0.688	0.867	1
ISL1	0.080847551	0.880010931	1	1	1
TMCO1	0.081029763	0.83662826	1	1	1
FOXR2	0.081157725	2.321928095	0.625	0.2	1
NEK4	0.08117301	1.277605764	1	0.933	1
EP400	0.081305765	-0.721140627	0.625	0.4	1
ENSSSCG00000047440	0.081332546	2.169925001	0.562	0.2	1
KCNAB1	0.081370497	-0.015506586	1	1	1
TCF7L2	0.081414523	-0.37941359	0.938	0.867	1
OBI1	0.081557501	1.1109822	1	1	1
HAAO	0.081768246	-0.756074417	0.5	0.4	1
BCAN	0.081934618	-0.923184403	0.25	0.6	1
CECR2	0.081962947	1.181619719	1	1	1
STEEP1	0.081965085	0.90459392	1	1	1
ENSSSCG00000054384	0.082132855	0.006426269	0.625	0.933	1
FAM172A	0.082185017	1.040309387	1	1	1
PLPPR4	0.082240352	-0.621488377	0.625	0.867	1
ENSSSCG00000042623	0.08226403	1.876516947	0.75	0.533	1
CYB561D1	0.082338392	-0.765534746	0.562	0.8	1
ENSSSCG00000057171	0.082342093	-0.830074999	0.625	0.6	1
AHCY	0.08237323	0.248447497	1	1	1
LAMP1	0.082440805	0.007584595	0.938	0.867	1
IL1R1	0.082590498	1.312843901	1	1	1
ENSSSCG00000034866	0.082591604	1.618765209	0.812	0.933	1
ENSSSCG00000053746	0.082613738	-0.645650427	0.5	0.533	1
IL6	0.082708685	2.299208018	0.562	0.2	1
TAF2	0.082723283	0.921574641	1	0.933	1
ENSSSCG00000057642	0.082800447	0.208919133	0.938	1	1
AGL	0.083127629	0.971403357	1	1	1
GPX8	0.083194783	0.074490544	0.938	0.933	1
CENPP	0.083221001	-0.150065289	0.938	0.933	1
CSRNP2	0.083248831	0.272817883	0.938	1	1
ENSSSCG00000033601	0.083277003	0.227571224	1	1	1
GXYLT2	0.083328745	-0.400290914	1	0.867	1
RANBP3	0.083521666	0.201337954	1	1	1
DPP9	0.083541277	1.296661336	1	0.933	1
CACNA1E	0.083550129	-0.029684024	0.938	0.933	1
ZXDC	0.083568695	0.134301092	0.938	0.933	1
FAM117B	0.083617151	0.140539472	1	1	1
GPR63	0.083630575	0.146021815	1	1	1
ENSSSCG00000053851	0.083669239	-0.492379588	0.875	0.8	1
IL12B	0.083891536	1.018152205	1	1	1
PPP2R5B	0.08396385	1.771961016	0.625	0.533	1

DENND5B	0.084056258	-0.325770161	1	0.933	1
PAK5	0.084123301	0.951284715	1	1	1
LIN52	0.084286513	0.034513098	1	1	1
ENSSSCG00000049258	0.08442163	2.714245518	0.5	0.133	1
GPR161	0.08448126	1.111721905	1	1	1
TEKT1	0.084678817	-0.886658527	0.5	0.333	1
SALL4	0.08468827	1.029993645	1	1	1
PRORP	0.084738795	-0.176767333	0.875	0.8	1
PODN	0.084762736	0.048246445	0.875	0.933	1
FAM228B	0.084971968	-0.326308581	0.75	0.733	1
APC	0.085141434	0.942041051	1	1	1
CTSS	0.08515204	0.113935053	1	1	1
ENSSSCG00000007023	0.085358519	0.95298701	1	1	1
MPG	0.08537407	1.282757498	1	0.867	1
PCNX1	0.085692208	0.348764793	1	1	1
TIMM21	0.085748339	0.098095139	1	1	1
TMEM129	0.08581133	1.528198924	1	1	1
ENSSSCG00000059540	0.085827607	0.176588732	1	1	1
C3orf70	0.085998886	0.346979467	1	1	1
SYNPR	0.086050557	1.584962501	0.812	0.467	1
TAMM41	0.086064775	1.04615794	1	1	1
RPRM	0.086189986	-0.678071905	0.438	0.6	1
CRKL	0.086192682	0.846190728	1	1	1
MRPS5	0.086314722	1.192562805	1	0.933	1
CENATAC	0.086457737	1.518325308	0.938	0.4	1
SMAP2	0.086464472	-0.779743323	0.938	0.8	1
MAN2A2	0.086677798	1.302375297	1	0.933	1
ARAP3	0.086751987	-0.256608137	0.5	0.6	1
PTPRU	0.086847085	-0.067460817	1	1	1
API5	0.086866817	1.30232736	1	1	1
ENSSSCG00000055073	0.086878848	-0.629162305	0.375	0.533	1
ZNF318	0.087014869	0.90971664	1	1	1
ENSSSCG00000053065	0.087156679	-0.500284786	0.75	0.867	1
CIAO1	0.087313874	0.376272456	1	1	1
ENSSSCG00000007482	0.087327958	1.107140134	1	1	1
ENSSSCG00000053776	0.087358252	1.491853096	0.812	0.733	1
SULF2	0.087374686	0.059258882	1	1	1
CATSPER2	0.08737837	0.264784188	0.875	1	1
PIGBOS1	0.087431432	-0.678071905	0.75	0.8	1
PUM3	0.087569067	0.157157705	1	1	1
ENSSSCG00000003984	0.087571589	0.301422439	1	1	1
PARP1	0.087634385	0.29759211	1	1	1
LTBP1	0.087755063	0.876592736	1	1	1
PHF20	0.087768463	0.975993619	1	1	1
METTL6	0.087848533	1.230119428	1	1	1
ENSSSCG00000051414	0.087879616	2.64385619	0.5	0.133	1
H1-6	0.087957661	1.305439972	0.75	0.467	1
ENSSSCG00000008990	0.088196389	-0.455679484	0.562	0.733	1
SLC1A5	0.08820269	1.130677262	1	1	1
ASPM	0.088231129	0.280159813	1	1	1
PDE8B	0.088370905	0.220716518	1	1	1
TMEM263	0.088396999	1.049243519	1	1	1
ZBTB18	0.088420491	-0.618200449	0.812	0.8	1
VIT	0.088597609	0.911772817	1	1	1
ERBB4	0.088677323	1.247927513	1	0.867	1
PAPOLG	0.088769236	1.027184829	1	1	1
ENSSSCG00000003076	0.088946576	-0.703162886	0.812	0.8	1
ENSSSCG00000045269	0.089111997	-0.9861942	0.438	0.533	1
FAM120A	0.089161781	1.034152781	1	1	1
AAK1	0.089244755	0.298315538	1	1	1
CCDC15	0.089280614	0.937385714	1	1	1
TRPV3	0.089340197	1.112005026	1	0.867	1
GALNT17	0.089349585	-0.784987109	0.562	0.533	1
SPATA9	0.08935253	-0.645650427	0.625	0.6	1
ENSSSCG00000059452	0.089392684	1.866248611	0.75	0.333	1
ENSSSCG00000062636	0.089558941	1.510961919	0.812	0.533	1
PPM1H	0.089618844	-0.023312437	1	1	1
CA12	0.089694035	-0.745186101	0.625	0.533	1
NUFIP2	0.08973896	0.259340815	1	1	1
TOR2A	0.089774479	-0.239950793	0.875	0.667	1

EDIL3	0.089783646	0.894043716	1	1	1
ENSSSCG00000058178	0.089788075	0.007819504	1	1	1
ENSSSCG00000044640	0.089819464	1.539158811	1	0.933	1
GCLM	0.089871118	1.278012188	0.938	0.933	1
ZMPSTE24	0.089887696	1.086283687	1	1	1
CPQ	0.089923927	-0.385891154	0.688	0.6	1
ENSSSCG00000053788	0.090031296	-0.299560282	0.5	0.8	1
ENSSSCG00000023596	0.090071282	1.356693513	0.938	0.867	1
ENSSSCG00000000887	0.090073745	1.560894741	0.812	0.8	1
PGPEP1	0.090074184	-0.03500645	0.812	0.933	1
DMTF1	0.090124457	1.019899557	1	0.933	1
VEPH1	0.090188387	1.204571144	0.75	0.467	1
ZNF251	0.090199263	0.278859373	0.438	0.867	1
ENSSSCG00000021024	0.090220912	-0.250650681	0.562	0.8	1
ILK	0.090269167	-0.449253215	0.562	0.667	1
STMND1	0.090286413	-0.607682577	0.562	0.533	1
TBC1D12	0.090385101	-0.531230517	0.812	0.733	1
PKD1L2	0.090797753	-0.632268215	0.688	0.933	1
LYSMD3	0.090913005	-0.104080522	0.938	0.8	1
NCALD	0.091005617	-0.169058258	0.812	0.933	1
ANKMY2	0.091030774	0.957777106	1	1	1
ZNF484	0.091035382	1.714245518	0.75	0.6	1
KIAA0319	0.091048992	1.428993072	1	0.8	1
TRHDE	0.091075183	1.297805301	1	0.867	1
ITGAE	0.09113626	1.152674254	1	1	1
PEX5L	0.091202323	0.24391206	1	1	1
SLC1A1	0.091238555	-0.253574077	0.688	0.667	1
IWS1	0.09135124	0.59363488	1	1	1
ATF7IP2	0.091385376	-0.082011696	1	1	1
ENSSSCG00000006510	0.09172587	0.150106714	1	1	1
SLC35F4	0.091731348	1.053975621	1	1	1
TUBD1	0.091733956	-0.229926094	0.938	1	1
KIF2C	0.091942478	1.041837477	1	1	1
SEPTIN7	0.092135908	0.132657744	1	1	1
RER1	0.092282322	0.351736904	0.938	1	1
NDNF	0.092354361	1.148328773	0.938	0.8	1
USO1	0.092357127	0.994002922	1	1	1
ATP6V1C1	0.092357853	0.208217804	1	1	1
TOR1A	0.092408701	-0.03451475	1	1	1
ENSSSCG00000013052	0.092549046	0.246655376	1	1	1
CSTPP1	0.092557214	-0.392669686	0.562	0.733	1
CEP170	0.092597512	0.928734417	1	1	1
FXR1	0.092638171	0.913349997	1	1	1
ENSSSCG00000009497	0.092782141	1.564002882	0.75	0.6	1
DYNC1LI2	0.09284445	0.277957852	1	1	1
SHOC2	0.092909667	-1.484300162	0.875	0.533	1
ENSSSCG00000061630	0.092917949	-0.552541023	0.625	0.6	1
UBASH3B	0.092961909	0.254813899	1	1	1
ABHD14A	0.093100646	-0.363198568	0.688	0.667	1
ZFAND1	0.093126305	1.09085343	1	0.933	1
ESYT2	0.093234621	0.220198208	1	1	1
TRIM50	0.093421474	1.104829973	1	0.933	1
LYSMD1	0.093530482	0.109707479	0.688	0.867	1
ENSSSCG00000040413	0.093544672	-1.054635257	0.312	0.533	1
PPP2R3A	0.093570684	1.238096504	1	1	1
ENSSSCG00000055939	0.093594279	-1.143735477	0.5	0.667	1
CDC42SE2	0.093622531	1.089748087	1	1	1
DLGAP1	0.093704764	-1.093109404	0.125	0.533	1
SIM2	0.093774926	-0.446746359	0.5	0.667	1
ENSSSCG00000026817	0.093907634	-0.350907162	0.688	0.867	1
FAM204A	0.093962551	0.346526349	1	1	1
ENSSSCG00000011106	0.094022518	0.20407607	1	1	1
SGPL1	0.094060946	-0.219321767	1	1	1
BNC2	0.094083066	0.887185186	1	1	1
TSC22D2	0.094246777	0.868318475	1	1	1
ENSSSCG00000009499	0.09443057	-0.177998302	0.75	0.8	1
CREB3L4	0.094492386	1.906890596	0.812	0.533	1
SF3B2	0.094494526	1.411803005	1	1	1
SPINK5	0.094497579	0.313058562	1	1	1
MSRB2	0.094548857	-0.881605299	0.562	0.6	1

CCDC87	0.09458273	-0.721140627	0.312	0.533	1
TAFA1	0.094745757	-0.714597781	0.375	0.6	1
SYVN1	0.094813775	1.564279199	1	0.933	1
AFAP1	0.094816951	-0.465078182	0.438	0.533	1
PHACTR3	0.094850043	0.243498586	1	1	1
NFATC2IP	0.094903514	-0.058343986	0.625	0.8	1
KCTD13	0.09490543	1.34488208	1	1	1
TENM3	0.094921579	1.600787468	0.812	0.667	1
LCLAT1	0.094939278	0.03562391	1	1	1
MYO6	0.095069177	0.872789988	1	1	1
ARHGEF33	0.09516669	0.108524457	0.812	1	1
HIRA	0.095346481	1.432959407	1	0.8	1
ENSSSCG00000023791	0.095438684	0.00877021	0.938	0.867	1
TARS2	0.095617344	-0.90669628	0.562	0.467	1
SNRPF	0.096011003	0.964725784	1	1	1
ENSSSCG00000054135	0.096108516	0.0138058	0.875	0.933	1
PES1	0.096248603	-0.043204499	0.938	1	1
ENSSSCG00000059368	0.096481511	1.548997003	0.938	0.733	1
TESK2	0.096640731	1.416751641	1	0.933	1
ENSSSCG00000042179	0.096778386	1.544320516	0.75	0.4	1
ENSSSCG00000045166	0.096835788	1.442943496	0.938	0.733	1
SRSF1	0.096836305	0.227904338	1	1	1
PRICKLE1	0.096888017	1.016780296	1	1	1
IRF8	0.09694836	0.120210935	1	1	1
GARRE1	0.09700304	1.052088511	1	1	1
TOMM20L	0.097036929	-0.449253215	0.5	0.667	1
GFPT1	0.097157278	0.83351337	1	1	1
GALNT10	0.097191285	-0.135922488	0.875	0.933	1
GALNT3	0.09732238	1.415037499	0.938	0.733	1
SLC43A2	0.097373788	0.076815597	0.938	0.933	1
CLCN5	0.097619453	0.231617324	0.938	0.933	1
MMP13	0.09767939	0.913962644	1	1	1
NKAP	0.097717417	0.144354177	1	1	1
CDC25C	0.097737762	1.017591648	1	1	1
JADE1	0.097804485	0.973774274	1	1	1
ENSSSCG00000007003	0.097841827	-0.678071905	0.562	0.533	1
ENSSSCG00000037257	0.097916474	-0.852101305	0.312	0.533	1
EIF3A	0.098093396	0.127619968	1	1	1
RNF20	0.098121817	0.258499326	1	1	1
ENSSSCG00000061205	0.098262881	-0.533681996	0.25	0.533	1
DPP3	0.09831783	-0.4040385	0.688	0.8	1
CCAR2	0.09834675	-0.001961516	0.938	0.933	1
ASCC2	0.098425859	-0.154509949	0.938	0.867	1
COG7	0.098438562	1.667702932	0.625	0.467	1
RGS18	0.098557348	2.539158811	0.562	0.133	1
DPYD	0.098629215	1.058503719	1	0.933	1
ENSSSCG00000055749	0.098752737	-0.397963986	0.5	0.733	1
TC2N	0.098868025	1.096482631	1	1	1
ENSSSCG00000058193	0.098904872	1.52160044	0.688	0.267	1
THOC1	0.098987899	-0.033520608	1	1	1
IL1RAPL1	0.099161768	0.967586527	1	0.933	1
HTATIP2	0.099283461	0.040746342	1	1	1
ENSSSCG00000034311	0.099296748	1.906890596	0.625	0.267	1
ASXL3	0.099606266	1.146302303	1	1	1
GPM6A	0.09962406	2.327222395	0.5	0.267	1
CYP7A1	0.099707346	1.813781191	0.625	0.4	1
DNAJB1	0.099718795	0.165890676	1	1	1
SEC63	0.099732762	0.379459093	1	1	1
ZBTB22	0.099753351	2.764871591	0.562	0.2	1
ENSSSCG00000062914	0.099758872	-0.693501946	0.75	0.667	1
BNIP2	0.099785018	1.048246445	1	1	1
IQUB	0.099797417	1.136372442	0.938	0.8	1
CROT	0.099809563	0.975906582	1	1	1
ZDHHC2	0.099951057	-0.263034406	1	0.867	1
RFC1	0.100007724	0.788467248	1	1	1
RRP15	0.100193912	0.269365876	1	1	1
ENSSSCG00000061277	0.100217901	-0.508146904	0.5	0.667	1
CCDC93	0.100323869	0.287849539	1	1	1
COIL	0.100385218	-0.43824489	0.75	0.867	1
ENSSSCG00000010734	0.100441194	1.454231798	1	1	1

BORCS7	0.100618654	-0.830074999	0.688	0.867	1
SLC31A2	0.100699885	0.876231112	1	1	1
ENSSSCG00000041731	0.100740432	-0.533681996	0.562	1	1
PGM2	0.100907277	1.126884521	1	1	1
SLC49A4	0.100942349	1.026899925	1	1	1
SNAP91	0.101057095	0.241240249	1	1	1
IARS1	0.101154429	1.346932101	1	1	1
DPH6	0.101209369	0.829722735	1	1	1
ADCY7	0.101226191	1.491853096	0.562	0.4	1
HIBCH	0.101332676	1.004309089	1	1	1
CDS1	0.101416046	0.160745575	0.938	1	1
LRWD1	0.101658214	1.611647609	0.938	0.8	1
ERAL1	0.10167379	0.098114999	0.938	0.933	1
ATXN2L	0.101673792	1.022184065	1	1	1
EXOSC1	0.101744966	0.3447539	1	1	1
ZNF879	0.101913549	-0.35614381	0.75	0.8	1
LAMB4	0.102038459	1.288418833	0.938	0.8	1
NXPE3	0.102064971	0.253693359	1	1	1
PRCP	0.102195319	-0.540568381	0.812	0.933	1
ZC3H12C	0.102199146	-0.076868763	1	1	1
NCKAP5L	0.102469582	-0.315501826	0.688	0.733	1
ENSSSCG00000046222	0.10250396	-0.321378392	0.75	0.733	1
UQCC1	0.102653192	-0.025995209	0.938	0.933	1
UBA6	0.102694699	0.102928437	1	1	1
THUMPD1	0.102750221	1.089065057	1	1	1
DHX35	0.102838471	0.844847036	1	1	1
ENSSSCG00000042522	0.102875958	0.899710442	1	1	1
CUL4B	0.102884339	0.861276847	1	1	1
PCMT1	0.103035061	0.906890596	1	1	1
PANK1	0.103129744	0.108097687	0.938	1	1
MANF	0.103154739	0.163736055	1	1	1
ENSSSCG00000051934	0.103180197	3.076815597	0.562	0.067	1
NASP	0.103207106	1.339476948	1	1	1
PAFAH1B1	0.103387506	0.121385032	1	1	1
ENSSSCG00000059776	0.10352622	-0.709780765	0.438	0.667	1
SLC36A4	0.103614106	0.099535674	1	0.933	1
YWHAZ	0.103714509	0.303761196	1	1	1
C1orf43	0.103865033	0.157174299	0.938	1	1
ENSSSCG00000059823	0.103958125	0.926790153	1	1	1
ENSSSCG00000062865	0.104095874	0.998346752	1	1	1
KIAA1328	0.104272725	0.023535514	1	1	1
ENSSSCG00000000547	0.10443827	0.884039656	1	0.933	1
PMS2	0.104478466	-0.32443495	0.688	0.867	1
ZNF521	0.104540028	1.183837308	0.938	1	1
DCDC1	0.104600335	-0.529208519	0.625	0.667	1
ALKBH1	0.104623108	1.102020937	0.938	0.867	1
ANKHD1	0.104862031	0.782788478	1	1	1
ENSSSCG00000055582	0.10491072	1.666939803	0.812	0.733	1
ENSSSCG00000061549	0.105108365	2.076815597	0.562	0.2	1
SNX13	0.105125498	1.07753425	1	1	1
GPR151	0.105201234	0.119634591	1	1	1
MYO1D	0.105202873	0.34298971	1	1	1
MIB1	0.105242358	0.881714867	1	1	1
PI4K2A	0.105271394	1.165117862	0.938	1	1
RAB11FIP2	0.105428771	1.079592682	0.938	1	1
TXNDC16	0.105625952	1.299874852	1	0.933	1
ENSSSCG00000054995	0.105681684	-0.540568381	0.375	0.667	1
TTC3	0.105712206	1.030149825	1	1	1
DLG1	0.105719504	0.970722207	1	1	1
LANCL1	0.105827676	0.057740916	1	1	1
LRR1	0.105893414	0.252487596	1	1	1
LAMTOR5	0.105949237	0.003105911	1	1	1
CHPT1	0.10629476	1.121177967	1	1	1
PLA2G6	0.106403268	1.362570079	1	0.667	1
CCDC196	0.106421925	-0.434146322	0.312	0.533	1
FBXO22	0.106462057	0.151712198	0.938	1	1
BANK1	0.106464595	1.023214714	1	1	1
ENSSSCG00000062612	0.106531359	-0.607682577	0.5	0.533	1
RHOH	0.106557797	2.169925001	0.5	0.267	1
FHL1	0.106671703	1.156599434	1	1	1

NUMB	0.106856627	0.328431615	1	1	1
PPP1R13B	0.106941023	1.05254496	1	1	1
ATXN7L2	0.107001693	-0.338221902	0.75	0.733	1
CDCA2	0.107017788	1.111977125	1	1	1
OSGEP	0.107135292	-0.040958122	1	1	1
ENSSSCG00000058436	0.107139155	-0.313075088	0.875	0.867	1
FANCE	0.107148711	-0.228461257	0.688	0.733	1
B4GALT6	0.107177009	0.753515094	1	1	1
C7orf31	0.107222996	0.049848549	0.812	0.933	1
CORO2A	0.107234525	2.22881869	0.625	0.333	1
CDC27	0.107251743	0.766587484	1	1	1
ATRNL1	0.107426319	1.074000581	0.938	0.667	1
ALKBH8	0.107450249	1.329123596	0.938	0.8	1
SUFU	0.107450407	1.754887502	0.75	0.4	1
TMEM62	0.107542146	-0.042483331	0.938	0.933	1
PLXNA4	0.107552956	2.053731984	0.625	0.2	1
PIAS2	0.107571971	0.966398661	1	1	1
ENSSSCG00000046945	0.107616094	1.41550221	0.938	0.8	1
LHX4	0.107704226	-0.272079545	0.812	0.733	1
AXIN1	0.107760264	1.688889943	1	0.8	1
PMPCA	0.107935232	-0.030645317	0.938	1	1
ATG13	0.108250031	1.156000355	1	1	1
ENSSSCG00000057596	0.108338494	-0.557056504	0.625	0.667	1
ALDH1A2	0.108421437	1.194692907	0.875	0.867	1
ENSSSCG00000047655	0.108431022	-0.093109404	1	1	1
RAD17	0.108450292	0.957236212	1	1	1
GGCT	0.108450412	1.009847786	1	1	1
ENSSSCG00000008582	0.108520481	-0.567040593	0.5	0.533	1
UNC80	0.108550259	0.454668176	1	1	1
NPC1	0.108611035	0.750197985	1	1	1
RAB3IP	0.108619511	0.425021588	1	1	1
CPEB3	0.10862231	1.08813091	1	0.933	1
HSF5	0.108701584	-0.392669686	0.812	0.667	1
LAX1	0.108795149	1.316636569	1	0.867	1
ZNF804B	0.108843179	0.952453173	1	1	1
TAPBPL	0.108965575	-0.497499659	0.75	0.6	1
MYZAP	0.109115349	-0.093109404	0.375	0.667	1
KTI12	0.109134488	-0.134529332	0.875	0.867	1
MED13	0.109378929	0.825516931	1	1	1
RNF182	0.109417698	1.124569863	1	1	1
BEND3	0.109622198	-0.131583552	0.875	0.867	1
SMCR8	0.109632964	1.111657346	1	1	1
ENSSSCG00000063154	0.10963922	1.510961919	0.938	0.733	1
ENSSSCG00000036554	0.109696671	0.847474897	0.938	1	1
CSRP2	0.109815931	1.155918143	1	0.8	1
UNC5A	0.109851489	0.949156323	1	0.933	1
ENSSSCG00000053400	0.109877881	0.910226308	1	0.933	1
SLC36A1	0.110107081	0.086440016	1	0.867	1
DIS3	0.110118067	0.172702086	1	1	1
KDSR	0.110445065	0.419340597	1	0.933	1
ENSSSCG00000061303	0.110492103	0.108524457	0.875	0.8	1
ATRX	0.110606834	1.098531966	1	1	1
ENSSSCG00000058286	0.110682269	1.244760234	1	0.867	1
APOOL	0.110821103	1.120195144	1	0.867	1
USP49	0.111006569	1.094962944	0.875	0.733	1
SLC17A4	0.111089197	-0.297467903	0.688	0.733	1
ALG2	0.111259027	1.24046905	1	0.8	1
ENSSSCG00000055798	0.11131108	-0.583435031	0.438	0.667	1
TAF4	0.111345981	1.119501996	1	1	1
JRKL	0.111387755	-0.050465067	0.875	1	1
JAK2	0.111464382	0.297805301	0.938	0.933	1
DENND1A	0.111502253	0.154515562	1	1	1
CGAS	0.111552723	-0.13058411	0.875	0.8	1
BFAR	0.11158924	1.100770329	1	1	1
VRK3	0.111658396	-0.584962501	0.562	0.533	1
CHD2	0.111743749	0.404849055	1	1	1
ENSSSCG00000029430	0.111811889	-0.144508557	0.75	0.933	1
CCDC125	0.111920115	0.249880306	1	1	1
NIBAN2	0.111937941	0.204571144	0.938	1	1
ENSSSCG00000055828	0.111970692	0.886072124	1	1	1

ADAM17	0.112115246	0.333536229	1	1	1
BPHL	0.112363166	1.555983434	1	1	1
PTPN1	0.112441249	0.218351691	1	1	1
ENSSSCG00000028944	0.112452658	-0.080857144	1	1	1
HDAC8	0.112607949	1.074000581	0.938	0.867	1
PDE1A	0.112907471	1.558967292	0.875	0.6	1
ENSSSCG00000055365	0.112918334	1.61214733	0.875	0.733	1
SGMS2	0.113120238	-0.042777729	0.875	1	1
PPRC1	0.113211096	0.963815631	1	1	1
VPS53	0.113211744	0.210467664	1	0.933	1
MANSC4	0.113237254	-0.649502753	0.562	0.467	1
FURIN	0.113268044	0.993047239	1	0.933	1
ATAT1	0.113429763	2.339850003	0.5	0.4	1
RNF113B	0.113448685	0.12389647	1	1	1
CASC3	0.113617677	1.057597682	1	1	1
ENSSSCG00000055233	0.113704762	-0.086932386	1	1	1
REXO2	0.113887302	1.041181883	1	1	1
RAP2A	0.113889683	0.176730179	1	1	1
DBI	0.113973304	-0.16250047	1	1	1
ENSSSCG00000010203	0.113987564	0.360214195	1	1	1
ENSSSCG00000022634	0.1142109	0.971020933	1	0.8	1
SH2B3	0.114342778	0.361033886	1	1	1
CCDC43	0.114395705	0.865398467	1	1	1
MMUT	0.11440239	0.397852864	1	1	1
TM6SF1	0.114468835	1.317522916	0.875	0.733	1
MRGPRF	0.114475498	-0.182376742	0.688	0.8	1
STOX1	0.114541989	-0.523743759	0.875	0.8	1
ENSSSCG00000007000	0.114555035	0.782071172	1	1	1
INSIG2	0.114595285	0.96274383	1	1	1
IL10RA	0.114597454	-0.427528443	0.688	0.6	1
RAB28	0.114732406	1.000070774	1	1	1
SNX10	0.114753031	1.216854683	1	1	1
RNF126	0.114909824	1.558967292	0.938	0.933	1
JMY	0.114931959	0.967011588	1	1	1
PET117	0.115102379	-0.222392421	0.75	0.8	1
TMUB2	0.115413143	-0.093109404	0.625	0.667	1
ZNF277	0.115520498	0.178043082	1	1	1
AKAP6	0.11564896	-0.1740294	0.938	0.933	1
RIPK2	0.115698752	2.22881869	0.562	0.133	1
SLC31A1	0.115901601	0.918773587	1	1	1
MCM3	0.116161462	0.886880635	1	1	1
FAM133B	0.116291438	1.005755092	1	1	1
MEIOC	0.116334034	1.137934887	0.938	0.933	1
ENSSSCG00000041238	0.116347907	-0.245112498	0.562	0.533	1
BRCA2	0.116375234	0.897348091	1	1	1
TCF12	0.116382368	0.28273632	1	1	1
PHLDB3	0.116590855	-0.288365696	0.75	0.8	1
TANC2	0.116806652	0.308193069	1	1	1
ENSSSCG00000061887	0.116850723	1.714245518	0.688	0.533	1
MTURN	0.116981657	2.006426269	0.5	0.267	1
RNF183	0.117053329	0.073540465	0.938	1	1
THSD1	0.117177954	0.25883126	1	1	1
MCF2L2	0.117220316	1.117457582	0.938	0.733	1
TRPM7	0.117302807	0.877382291	1	1	1
ABCD4	0.117361507	1.060365076	0.938	0.867	1
ENSSSCG00000053298	0.117457313	-0.253574077	0.688	0.8	1
ENSSSCG00000029261	0.117472186	0.4147487	1	1	1
CHSY1	0.117545491	0.149068448	1	1	1
ENSSSCG00000061577	0.117616279	0.901273346	1	1	1
UACA	0.117905125	0.417743882	1	1	1
GSDME	0.117980354	0.459178403	1	1	1
ENSSSCG00000050482	0.117980412	2.22881869	0.562	0.2	1
TLCD3A	0.118078916	-0.803602787	0.562	0.533	1
ENSSSCG00000011729	0.118083373	1.326604582	1	0.867	1
ENSSSCG00000061482	0.118128823	1.482202926	0.938	0.867	1
SYMPK	0.118273302	-0.7355574	0.625	0.733	1
LYPD1	0.118444561	-0.366127899	0.5	0.8	1
ITGA4	0.118610869	0.778314729	1	1	1
TMEM67	0.118713158	1.117310375	0.938	0.867	1
USP24	0.118734692	0.842079186	1	1	1

ENSSSCG00000031893	0.118971709	-1.30461351	0.25	0.533	1
ENSSSCG00000012322	0.119013355	-0.230612928	0.688	0.667	1
ABCC2	0.119172251	0.81983067	1	1	1
CFAP20	0.119186334	1.650262316	1	0.867	1
RAP1B	0.119326667	0.900549069	1	1	1
IQCM	0.119704823	-0.042483331	1	1	1
SGF29	0.119744448	0.232826798	1	1	1
ENSSSCG00000063276	0.120210872	-0.678071905	0.438	0.6	1
ENSSSCG00000054327	0.120223584	1.374496146	0.938	0.867	1
GGCX	0.120232728	0.001306198	1	0.933	1
HBS1L	0.120266837	1.26052755	1	0.933	1
PELI1	0.120453748	0.89796708	1	1	1
MAEL	0.120467805	0.901730429	1	1	1
SEPTIN11	0.120545454	0.233131472	1	1	1
SAMD8	0.120687345	0.045986205	1	1	1
ENSSSCG00000031169	0.120874806	1.202088325	1	0.933	1
ZDHHC16	0.120906414	-0.179914232	0.938	1	1
MMADHC	0.120919685	0.884522783	1	1	1
ENSSSCG00000047735	0.121035671	-0.349449158	0.812	0.933	1
ENSSSCG00000049449	0.121241516	0.301169535	0.938	0.933	1
ENSSSCG00000056134	0.121389336	1.006426269	0.875	0.6	1
RNaseP-nuc.3	0.121394418	1.885516945	0.75	0.4	1
STRADA	0.1215471	0.158173996	1	1	1
QSER1	0.121724262	1.029194693	1	1	1
PHF10	0.121748784	1.11803826	1	1	1
ZNF445	0.121859528	0.768470427	1	1	1
PRKRIP1	0.1218632	0.419798159	1	1	1
ENSSSCG00000042151	0.121977932	-0.232272152	0.812	1	1
MITF	0.121985789	-0.492716863	0.812	0.667	1
PIK3CB	0.122013227	-0.281554494	0.688	0.733	1
GLCCI1	0.122038622	0.077849417	1	0.933	1
ENSSSCG00000005241	0.12237996	0.319107576	1	1	1
KLF3	0.122424268	0.836602152	1	1	1
CBFA2T2	0.122437135	0.690411087	1	1	1
PAX6	0.122470636	0.353706295	1	1	1
MISP	0.122551555	-0.858644151	0.25	0.6	1
IRAK4	0.122607302	1.289924778	0.938	1	1
SLC25A36	0.122610452	0.928293072	1	1	1
CCNT2	0.122624296	1.026604151	1	0.933	1
HSPA12B	0.122748723	-0.415037499	0.312	0.6	1
CISD2	0.122783261	1.037964668	1	1	1
CEBPZOS	0.122930373	0.941257255	1	1	1
ENSSSCG00000049042	0.122991127	-0.312675782	0.938	0.867	1
CCNT1	0.123096986	0.935683352	1	1	1
PBXIP1	0.123159116	1.217977131	1	1	1
ETV1	0.123307781	0.75445883	1	1	1
RAB15	0.123415572	1.278694597	1	1	1
SLC17A6	0.123749532	-0.707819249	0.688	0.533	1
CENPH	0.123823092	0.990836247	1	1	1
RELN	0.124093212	-0.958179824	0.562	0.4	1
CCDC69	0.124207665	-0.216257635	0.938	0.867	1
ZNF583	0.12440798	1.192977223	0.938	1	1
ENSSSCG00000054606	0.124532309	-0.562594688	0.312	0.667	1
ENSSSCG00000002259	0.12457517	0.996037327	1	1	1
ZNF76	0.124801413	1.194692907	1	0.733	1
SETDB2	0.12481778	0.231415802	0.938	1	1
ENSSSCG00000021560	0.124922666	0.989808272	1	1	1
PUS7L	0.12505572	1.225338122	1	0.733	1
ULK3	0.125232657	-0.042483331	0.438	0.6	1
KITLG	0.125341015	0.81563823	1	1	1
ZFAT	0.12544874	-0.480132528	0.75	0.867	1
TMTC4	0.125629179	0.2644426	1	1	1
ENSSSCG00000003240	0.125658532	-0.065275196	0.875	0.933	1
ENSSSCG00000054793	0.125699288	0.980600282	1	0.933	1
AGTR1	0.125729599	-0.242856524	0.875	0.8	1
CMTR1	0.125791972	0.807779008	1	1	1
STXBP5L	0.12657988	1.054232312	1	0.8	1
TICRR	0.126624794	0.20994098	1	1	1
WDR3	0.126641634	0.393241339	1	1	1
RBM15	0.126657485	-0.077427782	0.938	1	1

MON1B	0.126662447	1.02722737	1	1	1
USP9X	0.126867961	0.851741146	1	1	1
DDX27	0.127003182	0.299533573	1	1	1
SOCS2	0.127231026	-0.093109404	0.938	0.867	1
ENSSSCG00000041766	0.127256027	-0.441032708	0.5	0.667	1
ZNF502	0.127332196	-0.110596831	0.938	0.867	1
ENSSSCG00000030362	0.127412911	0.129876841	1	1	1
COA5	0.12750547	0.936006339	1	1	1
ZUP1	0.127835072	0.914075858	1	1	1
RBL1	0.127896439	1.142957954	1	1	1
NAMPT	0.127909091	0.291623423	1	1	1
SYN2	0.127983457	2.22881869	0.5	0.067	1
PDCD11	0.128090597	0.063796651	0.938	1	1
IGSF11	0.128244255	1.13138454	1	0.933	1
CLSTN1	0.128285556	1.224400019	1	0.933	1
BCL6	0.128288498	-0.607682577	0.438	0.6	1
ENSSSCG00000039370	0.128434177	0.910439663	1	1	1
ALAS1	0.128437401	1.639194812	0.938	0.6	1
ENSSSCG00000062273	0.128438398	-0.281554494	0.562	0.867	1
ELMOD2	0.128477368	0.956268962	1	0.933	1
MAP2K7	0.128730778	1.299997455	1	1	1
DUSP18	0.128867414	0.8881038	1	1	1
THUMPD3	0.128868684	0.058893689	0.938	0.867	1
MLXIP	0.128899349	1.487221648	1	0.867	1
ZIC1	0.12902575	0.071949842	0.938	0.933	1
ENSSSCG00000055045	0.12906513	-0.149692933	0.625	0.8	1
GADD45A	0.129131864	1.327222395	0.875	0.733	1
IRF1	0.129150949	1.002206045	1	1	1
UNC119B	0.129302308	-0.249228606	0.812	1	1
MTX3	0.129356727	0.407095478	0.938	1	1
SLCO4C1	0.12957691	0.855631089	1	1	1
ETAA1	0.129880806	0.849521227	1	1	1
ERBB3	0.129999433	-0.180572246	0.312	0.6	1
ACBD6	0.130090867	0.034269902	0.875	0.867	1
WBP4	0.130326179	0.11564334	0.938	1	1
ENSSSCG00000016828	0.130448499	0.311893078	1	1	1
PYROXD1	0.130450622	-0.335810808	0.938	0.933	1
CCNH	0.13065334	0.07945341	1	1	1
ZKSCAN7	0.130707025	0.878508794	1	0.933	1
CHM	0.130742168	1.110188785	1	1	1
ITGB8	0.131214832	1.553253641	0.688	0.667	1
PRELID3A	0.131362137	0.375443604	1	1	1
ENSSSCG00000034545	0.131427111	0.038135129	0.938	0.933	1
PTPRR	0.13181801	1.12701641	0.938	1	1
GRK5	0.131843956	-0.247437551	0.812	0.867	1
ERC1	0.131946115	0.522077018	1	1	1
CWC15	0.131951257	0.925702781	1	1	1
PPP3CB	0.131994422	-0.131244533	1	1	1
TRPC6	0.132138767	0.097113011	1	1	1
CBX1	0.132403633	0.269116644	1	1	1
PCDHAC2	0.132416217	0.20968683	1	1	1
DTNBP1	0.132460971	-0.043068722	0.875	0.933	1
LMBR1	0.132639211	0.040157126	0.938	0.867	1
STARD7	0.132692765	0.984543151	1	1	1
HCFC1R1	0.132798982	-0.529208519	0.625	0.6	1
ENSSSCG00000026564	0.132924268	1.491853096	0.812	0.6	1
ENSSSCG00000035901	0.132962627	0.347252251	1	1	1
TRIT1	0.133013556	1.568610295	0.938	0.733	1
ENSSSCG00000052027	0.133206883	2.044394119	0.5	0.2	1
KLHL21	0.133218159	0.717229376	1	0.867	1
HSD17B11	0.133268796	-0.239658068	1	1	1
ATP7A	0.133312257	-0.149692933	0.938	0.933	1
ENSSSCG00000059134	0.133489196	0.903332776	1	1	1
SFI1	0.133707142	0.032421478	0.875	1	1
PGBD5	0.133932861	0.306346554	1	1	1
DPH3	0.134043107	1.124764558	1	1	1
ENSSSCG00000011059	0.134095836	0.039645804	0.938	0.8	1
ENSSSCG00000013281	0.134122177	1.197853745	1	1	1
DENND2C	0.134201481	0.674943868	1	1	1
AFAP1L2	0.134233002	-0.195207592	0.938	0.867	1

ENSSSCG00000061235	0.134410303	0.904402475	1	1	1
RHOBTB2	0.134579441	1.269136146	1	1	1
ENSSSCG00000041888	0.134621971	-0.292418213	0.438	0.733	1
ENSSSCG00000026996	0.134870149	-0.489038081	0.438	0.6	1
HIGD1A	0.134965364	0.26216246	1	1	1
ENSSSCG00000054771	0.135023227	-0.562594688	0.375	0.6	1
DPF1	0.135063082	1.46982679	0.938	0.8	1
LRP8	0.135330552	0.006426269	0.875	0.867	1
NEK1	0.135426442	0.940423977	1	1	1
PDE4C	0.135514925	-0.784987109	0.312	0.533	1
FYN	0.13552643	0.857980995	1	1	1
MTFR1L	0.135608497	0.991659063	1	1	1
ZSWIM1	0.135740098	0.212516878	0.812	0.933	1
ICE2	0.135744328	0.85680062	1	1	1
TRIM59	0.135748689	1.11038918	1	1	1
LONRF1	0.135809138	0.870885642	1	1	1
ENSSSCG00000004687	0.135896125	-0.327574658	0.312	0.533	1
SLC25A46	0.13603294	1.028196892	0.938	0.933	1
ZBTB14	0.136165567	0.020547377	0.625	0.8	1
HSPA4L	0.136414163	0.841086772	1	1	1
SERTAD2	0.136436892	0.91034781	1	1	1
PTPRN	0.136507585	-0.623624121	0.125	0.533	1
RETSAT	0.13679959	-0.063536289	1	0.933	1
ENSSSCG00000015565	0.137298199	0.928023408	1	1	1
IFNAR1	0.137553043	0.864356552	1	1	1
HSPA12A	0.137566813	0.905564792	1	1	1
ENSSSCG00000046421	0.137692354	-0.470344871	0.938	0.933	1
DHTKD1	0.137896123	-0.259119356	0.688	0.667	1
MOSPD2	0.137923798	0.231538192	1	1	1
ENSSSCG00000028092	0.137976765	0.449551154	1	1	1
PCSK7	0.138087384	-0.587874096	0.5	0.533	1
MMD	0.138147356	1.093163591	0.938	0.933	1
TNRC18	0.138216681	0.058893689	0.75	0.867	1
OLA1	0.138290887	0.179093317	1	1	1
ENSSSCG00000008981	0.138405543	-0.736965594	0.375	0.533	1
GABPB2	0.138405626	1.178850231	0.938	1	1
ADGRG7	0.138482796	0.299473244	0.938	1	1
C12orf73	0.138509644	0.341827652	0.688	0.933	1
ZNF772	0.139147426	-0.101671418	0.938	0.933	1
SBNO2	0.139312982	0.293913719	0.875	1	1
ENSSSCG00000054839	0.139373539	2.108524457	0.5	0.2	1
BRCA1	0.13939757	0.206938671	1	1	1
BTBD7	0.139441199	0.715672751	1	1	1
KLK4	0.139478386	-0.714597781	0.5	0.467	1
CPS1	0.139519781	1.794415866	0.688	0.267	1
PAK1	0.139574443	0.411471107	1	1	1
CYTB	0.139748639	1.472024351	1	1	1
PDIA5	0.139762784	1.460826201	1	1	1
CA13	0.140090332	0.000503646	0.938	0.867	1
HDGF	0.140118544	1.442943496	0.938	0.467	1
FBXO41	0.140247462	-0.335679708	0.812	0.867	1
NLRP3	0.140327107	1.14964073	0.938	1	1
ENSSSCG00000032196	0.140404997	-0.048885404	0.938	0.933	1
DVL3	0.140522399	0.335964155	1	1	1
ZMAT1	0.140632222	1.444156354	0.812	0.667	1
RILPL1	0.140674669	-0.745186101	0.312	0.533	1
ENSSSCG00000036417	0.140707851	1.153051183	0.938	0.533	1
ASAP1	0.140861536	0.972186506	1	1	1
ENSSSCG00000046239	0.141012243	2.044394119	0.562	0.133	1
MNT	0.141399609	-0.154509949	0.625	0.667	1
ENSSSCG00000050485	0.141659047	-1.019108823	0.188	0.533	1
C4orf46	0.141662888	-0.299560282	0.562	0.6	1
RTCA	0.141665467	0.938194716	1	1	1
ZCCHC4	0.141672712	-0.099395667	0.938	0.933	1
MBNL1	0.141767262	-0.264720782	1	0.733	1
ACSS3	0.141826295	0.274195484	0.938	1	1
ATG14	0.141927395	0.116646862	1	1	1
ENSSSCG00000062271	0.141984139	-0.071735754	0.812	0.8	1
WDR31	0.141989634	1.293599372	1	0.8	1
ENSSSCG00000052396	0.142179796	1.182525038	0.688	0.267	1

KIF1B	0.14219709	0.888743249	1	1	1
UBE2Q2	0.142330537	0.903372815	1	1	1
ZNF566	0.14237909	-0.10433666	0.938	0.867	1
NUDCD2	0.142656649	0.95922206	1	1	1
POLDIP3	0.142661917	0.409069688	1	1	1
DPY19L1	0.14270741	1.152829008	1	1	1
NCOA2	0.142779749	0.946600305	1	1	1
GNGT1	0.142932414	-0.327574658	0.625	0.867	1
SPO11	0.142932997	-0.010647244	0.875	0.933	1
PCOLCE	0.142991706	-0.529208519	0.625	0.533	1
MCTP1	0.143114383	0.924623682	1	1	1
BTBD1	0.14326733	0.016863438	0.938	0.933	1
ENSSSCG00000054288	0.143323427	1.799975392	0.5	0.2	1
ENSSSCG00000032549	0.143348379	0.297680549	1	1	1
WNT5A	0.143386794	-0.552541023	0.438	0.6	1
TRIM13	0.14350416	1.136896201	1	1	1
ENSSSCG00000026365	0.143628688	1.672425342	0.812	0.267	1
MFHAS1	0.143698477	0.215965165	1	1	1
SF3A2	0.143704375	0.213551934	0.875	1	1
RTKN2	0.143707577	0.793813688	1	1	1
BTBD2	0.143746074	1.251225104	1	0.933	1
ENSSSCG00000036465	0.143827023	1.764871591	0.75	0.2	1
GJD2	0.1438851	1.054561444	1	1	1
RBM26	0.144207735	0.973192966	1	1	1
PHACTR1	0.14423576	0.17990909	0.938	0.933	1
ENSSSCG00000022331	0.144271768	-0.830074999	0.25	0.6	1
NDUFAF4	0.144435127	0.806286655	1	1	1
POLQ	0.144504739	0.993602229	1	1	1
RNF130	0.144571227	0.906890596	1	1	1
FAM118A	0.144583344	1.286550927	1	1	1
SORL1	0.144959148	0.792074104	1	1	1
LGALSL	0.145303274	1.541606132	0.812	0.533	1
APOF	0.145540629	-0.294743266	0.312	0.533	1
SMTNL2	0.145632627	0.389452089	0.938	1	1
SHROOM4	0.145641265	0.866701505	0.938	0.8	1
SCN3B	0.145898366	0.172053394	1	1	1
PDE1B	0.14600354	-0.847996907	0.375	0.533	1
PANX1	0.146064842	0.139383607	1	1	1
ENSSSCG00000059731	0.146072046	-0.900464326	0.375	0.533	1
SLC38A3	0.146963976	-0.638543541	0.625	0.733	1
ENSSSCG00000055296	0.147046703	-1.093109404	0.5	0.6	1
ENSSSCG00000059103	0.147123402	1.480075929	0.875	0.667	1
COL12A1	0.147141062	0.927249683	1	0.933	1
NOVA1	0.147288771	0.171485515	0.938	1	1
ENSSSCG00000038556	0.147335713	0.37895904	1	1	1
UBP1	0.147382669	1.081141522	1	1	1
ENSSSCG00000057625	0.147456677	1.861086906	0.688	0.2	1
CACNB1	0.147494784	-0.540568381	0.375	0.6	1
ENSSSCG00000056100	0.147629126	1.093163591	1	1	1
LAMA5	0.147635111	-0.245112498	0.438	0.6	1
LRRC57	0.147646832	-0.649502753	0.5	0.533	1
UCK2	0.147716933	0.032421478	1	1	1
ATP13A2	0.147728261	-0.550316358	0.75	0.733	1
MTBP	0.147752682	1.09592442	1	1	1
ATAD5	0.14776766	0.954135948	1	1	1
RC3H1	0.148128017	0.914534031	1	1	1
GCLC	0.148235447	0.937730215	1	1	1
RGS6	0.148261684	-0.285754482	0.438	0.667	1
TJP2	0.148283807	0.95155511	1	1	1
ATOSA	0.148300068	0.28101045	1	1	1
FADS1	0.14869998	-0.009819061	0.938	0.933	1
CTDP1	0.148756257	1.619314006	1	1	1
TTC29	0.148810329	0.02768874	1	0.933	1
UROS	0.148918534	0.111249094	0.688	0.8	1
ENSSSCG00000056001	0.148960815	-0.594272678	0.938	1	1
ENSSSCG00000014165	0.149166555	0.286687089	0.938	1	1
CCDC88A	0.149304763	0.910632772	1	1	1
PHLPP1	0.149334227	0.128569694	1	1	1
ENSSSCG00000061233	0.149594729	1.832890014	0.562	0.4	1
GPS2	0.14986372	-0.033403158	0.875	0.933	1

ENSSSCG00000012362	0.149888618	1.188460953	0.812	0.6	1
ACAD11	0.149904586	0.315289453	0.938	1	1
RAC3	0.14997162	1.64385619	0.75	0.467	1
FLT1	0.150004642	0.464516012	1	1	1
ERCC3	0.150106626	1.275612902	1	0.933	1
ADGRB3	0.15017488	1.114998791	1	0.733	1
NANP	0.150174949	-0.424952968	0.625	0.667	1
ENSSSCG00000049146	0.150371176	1.05166212	0.875	0.667	1
EFNA3	0.150582756	-0.30723421	0.438	0.733	1
SHLD3	0.150616173	1.164188632	1	1	1
ENSSSCG00000060358	0.150727435	0.067355268	0.875	0.533	1
ADARB2	0.15073226	-0.250650681	0.812	0.867	1
ENSSSCG00000052364	0.151201049	0.197708158	1	1	1
ENSSSCG00000048319	0.151491602	-0.279522529	0.688	0.6	1
DAGLB	0.151620752	0.370345224	1	0.933	1
ENSSSCG00000053028	0.151664611	2.108524457	0.5	0.267	1
UPRT	0.151757325	1.06161319	1	0.8	1
NOL4	0.151861762	0.727834582	1	1	1
LYST	0.152430524	0.898425198	1	1	1
ENSSSCG00000055282	0.15243186	-0.242856524	0.938	0.867	1
DDX18	0.15293913	0.828363791	1	1	1
SIN3A	0.15300743	0.855442585	1	1	1
MICU2	0.153380643	1.118508545	1	1	1
MCPH1	0.153467174	0.747692001	1	1	1
CSDE1	0.153551409	0.346345021	1	1	1
PTPDC1	0.153559293	1.134158638	1	0.933	1
CCDC120	0.153608313	-0.175571565	0.812	0.733	1
ALMS1	0.153768217	0.596036936	1	1	1
LHX8	0.153828625	0.863314717	1	1	1
KCNN1	0.153930532	-0.197446064	0.812	0.733	1
ENSSSCG00000005255	0.153935404	0.961664818	1	1	1
SLC27A3	0.15408703	1.62309763	0.812	0.533	1
ACTC1	0.154565599	2.409390936	0.5	0.333	1
NKAIN1	0.154678277	0.032421478	0.75	0.733	1
CMKLR2	0.154678331	1.714245518	0.5	0.2	1
ENSSSCG00000004170	0.154693716	0.271654888	0.938	0.933	1
ENSSSCG00000054874	0.154751232	-0.093109404	0.75	0.533	1
ENSSSCG00000052077	0.155290246	-0.745186101	0.125	0.533	1
IDH1	0.15559963	0.339779971	1	1	1
NLN	0.15561847	0.328779441	1	1	1
ENSSSCG00000050064	0.15562824	1.528378972	0.812	0.267	1
METTL4	0.155661499	0.894489129	1	1	1
RAB3D	0.155698485	-0.526068812	0.562	0.4	1
ENSSSCG00000027046	0.155729582	1.173677136	0.812	0.667	1
ENSSSCG00000059407	0.156076752	1.558967292	0.625	0.267	1
ENSSSCG00000000611	0.156086352	0.943321052	1	1	1
UTP6	0.156145295	0.39662852	1	1	1
ANLN	0.156240248	0.676203738	1	1	1
IMPACT	0.156242311	1.245059332	1	0.933	1
HHLA2	0.156252964	0.091984476	0.938	1	1
RCOR2	0.156708408	1.394828642	1	0.8	1
NEFH	0.156768335	-0.044900311	1	0.933	1
AXDND1	0.156782702	-0.113008962	0.875	0.8	1
ADD1	0.156897374	1.066130049	1	1	1
ENSSSCG00000007848	0.157395801	0.979791143	1	1	1
TAF10	0.157405345	-0.093109404	0.938	0.933	1
ENSSSCG00000062863	0.157436532	1.69538649	0.625	0.4	1
GNPTAB	0.157524764	0.441023152	1	1	1
PCK2	0.157668342	-0.545621609	0.375	0.8	1
ATF1	0.157791472	1.082332602	1	1	1
SRPK1	0.157879238	0.692705012	1	1	1
ABHD13	0.157976638	0.372828993	1	1	1
MOB3C	0.158265447	-0.35614381	0.375	0.6	1
MALT1	0.158303972	0.01995326	0.875	0.933	1
COASY	0.158459933	-0.2255597	0.688	0.667	1
THNSL1	0.15847327	0.030879313	0.875	0.667	1
ENSSSCG00000062141	0.158677262	1.039340892	0.938	0.8	1
ZFP82	0.158682301	-0.112671671	0.938	1	1
KDM7A	0.158729477	0.928089761	1	1	1
KLK7	0.15878457	1.584962501	0.5	0.467	1

ENSSSCG00000021579	0.158859737	1.421463768	0.812	0.533	1
COG6	0.158967085	0.385870992	1	1	1
PDE4B	0.159006388	1.409390936	0.938	0.8	1
GABRB2	0.15923696	0.863519743	1	1	1
GNAQ	0.159313355	1.06801621	1	1	1
RASSF3	0.159342043	0.858934552	1	1	1
ATE1	0.159668474	0.426897654	1	1	1
ZNF687	0.159967904	-0.455679484	0.562	0.333	1
RCAN1	0.160005031	0.434710974	1	1	1
SRRM2	0.160144987	0.923964109	1	1	1
KLHL14	0.160479248	0.877265426	0.938	0.8	1
ENSSSCG00000049984	0.160487278	-0.285754482	0.5	0.533	1
ENSSSCG00000056769	0.160631114	0.969069587	1	1	1
ABCA5	0.160649556	0.268856405	1	1	1
ENO3	0.160848647	-1.947258538	0.062	0.667	1
CCND3	0.161008167	1.122143157	1	0.933	1
HAUS6	0.16120839	-0.166732268	0.875	0.933	1
CAMKV	0.161279744	0.193470429	1	1	1
BCCIP	0.161314596	1.30448296	1	0.933	1
ENSSSCG00000050974	0.161497421	-0.186218809	1	0.933	1
ANKRD28	0.161506396	0.834078605	1	1	1
STK36	0.161531619	-0.434146322	0.562	0.867	1
CHERP	0.161684634	1.197492356	1	1	1
ZNF184	0.161863618	0.779056928	1	1	1
ZSCAN25	0.161956792	1.624747367	0.875	0.667	1
RTP1	0.162338188	0.063759444	0.875	0.867	1
NATD1	0.162363944	0.169925001	0.75	0.6	1
SP2	0.162412135	1.468769483	1	0.867	1
RIOX1	0.162501256	0.285735443	1	1	1
C16orf87	0.162538165	0.276124405	1	1	1
CNOT1	0.162629092	0.155495443	1	1	1
ENSSSCG00000006685	0.162637701	-0.386840607	0.688	0.6	1
ENSSSCG00000059696	0.162676278	-0.157239742	0.688	0.8	1
NALCN	0.162799775	-0.318401716	0.812	0.8	1
ATXN2	0.16310826	0.969418279	1	1	1
ZSWIM2	0.16313828	-0.331269142	0.75	0.667	1
CEPT1	0.163183963	0.844812507	1	1	1
ENSSSCG00000051458	0.163218316	-0.77118131	0.625	0.4	1
ENSSSCG00000010426	0.163292483	0.734945484	1	1	1
TCF20	0.163473132	0.913352846	1	1	1
RAPGEF1	0.163496166	0.230893019	0.938	1	1
MED12L	0.163496706	0.370788489	1	1	1
MED25	0.163544827	1.11461763	1	1	1
ENSSSCG00000044654	0.163550578	2.076815597	0.5	0.133	1
ENSSSCG00000054102	0.163641233	-0.285754482	0.688	0.733	1
DNER	0.163813221	1.399930607	0.938	0.733	1
FMO2	0.163957623	0.951838002	1	0.933	1
ZBED9	0.164070409	1.142518844	0.938	0.6	1
TMEM170B	0.1641406	1.025614516	1	1	1
PSMA4	0.164316961	0.448494566	1	1	1
KDM5A	0.164490328	0.474621927	1	1	1
BAG3	0.164579801	0.16307133	1	1	1
CEP19	0.164978712	-0.489038081	0.5	0.4	1
SLC7A11	0.165024124	1.005476815	1	1	1
ENSSSCG00000050495	0.165032294	-0.27368165	0.562	0.6	1
IGF2BP3	0.165099071	0.379367454	1	1	1
CAMK4	0.165405683	1.186998515	0.938	0.533	1
ARHGEF25	0.165431382	1.386883537	0.875	0.533	1
PLEKHG1	0.165451448	0.925278402	1	1	1
PPA2	0.165485129	1.348996338	0.875	0.667	1
STON1	0.165537074	0.288285251	1	1	1
ITPR2	0.165557752	1.473931188	0.938	0.933	1
IFIH1	0.166106585	1.106199404	0.938	0.533	1
HSP90B1	0.166139028	0.416738738	1	1	1
MYH9	0.166235828	-0.120330873	0.938	0.933	1
ERI1	0.166252538	0.788814276	1	1	1
CLTC	0.166266406	0.846678425	1	1	1
EGFLAM	0.166338556	0.265812898	1	1	1
PURA	0.166346661	0.036173613	0.875	0.933	1
RAB29	0.166709322	0.364827658	1	1	1

MAT2B	0.166874528	0.149707354	1	1	1
MYO1E	0.167049912	0.383520475	1	0.933	1
MAPRE1	0.167214574	0.845684064	1	1	1
CELF6	0.167374108	0.018207618	0.938	1	1
SPINT2	0.167411734	1.001465409	1	1	1
ENSSSCG00000063457	0.167577792	0.970750538	1	1	1
TOPBP1	0.167893969	0.761931926	1	1	1
FMNL2	0.167906729	0.944696468	1	1	1
ENSSSCG00000063474	0.16792142	-0.415037499	0.562	0.467	1
RHOQ	0.168030299	0.979040381	0.938	0.667	1
DNAJC28	0.168045342	1.209453366	0.625	0.267	1
ENSSSCG00000062434	0.168056934	-0.315501826	0.625	0.667	1
DLX4	0.168113522	-0.480132528	0.5	0.667	1
MAZ	0.168513391	1.423466121	0.938	0.867	1
ENSSSCG00000050531	0.16859401	0.316360768	1	1	1
ENSSSCG00000028693	0.168640662	1.252665432	0.938	0.667	1
LRCH3	0.168783389	0.869817163	1	1	1
AP4S1	0.168895323	1.311280851	0.688	0.533	1
PCBP2	0.168901478	0.683136177	1	1	1
C8B	0.168964117	1.564002882	0.625	0.2	1
EYA3	0.169034667	0.344505282	1	1	1
IL6R	0.169141326	-0.344648171	0.5	0.467	1
MYBL2	0.169749473	0.152515323	1	1	1
RNF19A	0.169854032	0.227699889	1	1	1
ENSSSCG00000058608	0.170028918	-0.441032708	0.375	0.533	1
CENPQ	0.170110053	0.210631901	1	1	1
YTHDF2	0.170128155	1.20330367	1	1	1
ENSSSCG00000005869	0.17047391	1.152003093	0.875	0.533	1
ENSSSCG00000062392	0.170524797	-0.4012317	0.438	0.733	1
TRIM24	0.170573071	-0.13112273	1	1	1
TSPYL5	0.170591347	-0.441032708	0.875	0.867	1
HUNK	0.170867286	0.022367813	0.438	0.533	1
ENSSSCG00000016486	0.171160163	-0.480132528	0.25	0.533	1
ESCO1	0.171250675	1.079875397	1	0.8	1
ADSS2	0.171313561	0.255344985	1	0.933	1
MED7	0.171687874	0.437323529	1	1	1
ENSSSCG00000005982	0.171791229	0.816233073	1	1	1
SYDE2	0.171812612	2.094517599	0.625	0.2	1
SLC4A2	0.172003841	1.491853096	0.625	0.4	1
AASDH	0.172194466	0.271046926	1	1	1
ENSSSCG00000035527	0.172367751	0.232290882	0.938	0.867	1
EXD2	0.172615238	1.374016606	0.875	0.867	1
SPRED2	0.172659997	0.90366349	1	1	1
RBM12B	0.172794694	0.860849848	1	1	1
DNM1L	0.172866896	0.849760488	1	1	1
ENSSSCG00000007355	0.172917253	0.859573387	1	1	1
ENSSSCG00000005424	0.172955038	-0.280736408	0.688	0.733	1
LTA4H	0.173125181	0.020547377	0.625	0.667	1
ANKRD37	0.173441965	0.944487382	1	1	1
ALG9	0.173464078	0.276536192	0.75	0.867	1
KCTD10	0.17349381	0.485426827	1	1	1
PEG10	0.173580792	0.155030786	1	1	1
ODAD3	0.17363725	-1.062735755	0.562	0.733	1
NRG3	0.173896106	1.188965044	1	1	1
TSC1	0.174043815	0.103713768	0.938	1	1
EPB42	0.174153297	-0.388565288	0.625	0.733	1
IL1RAPL2	0.174310594	1.246096682	1	0.933	1
CSNK1G2	0.174330926	0.154818109	0.875	0.933	1
NDUFAF6	0.174341494	0.0138058	0.875	0.933	1
CPPED1	0.174347319	-0.9861942	0.312	0.533	1
ENSSSCG00000002749	0.174499776	0.366322214	0.312	0.8	1
GTPBP1	0.174611532	1.051441385	1	1	1
SOWAHA	0.174689039	1.161074084	0.938	0.867	1
C1orf109	0.174998722	-0.023846742	0.875	0.8	1
ELF1	0.175085725	0.931409959	1	1	1
ENSSSCG00000048585	0.175113382	1.360256214	1	0.933	1
ENSSSCG00000042520	0.175158452	-0.415037499	0.375	0.533	1
ENSSSCG00000061821	0.175228795	1.906890596	0.625	0.267	1
ASZ1	0.175642602	0.665335917	1	0.867	1
RNF41	0.175723672	0.751392665	1	1	1

ENSSSCG00000057006	0.17576282	-0.055634699	0.875	0.8	1
ZNF614	0.175844571	1.539158811	0.875	0.867	1
WNT16	0.175967925	-0.397963986	0.625	0.733	1
ENSSSCG00000057680	0.175975445	-0.230612928	0.5	0.6	1
ENSSSCG00000062750	0.176158661	0.952453173	0.875	0.933	1
VCPIP1	0.176220908	0.917207887	1	1	1
EXOSC7	0.176246836	0.757747156	1	0.933	1
ZBTB43	0.176317378	0.024727086	0.875	0.867	1
HDAC2	0.176318172	0.199402203	1	1	1
ST6GALNAC3	0.17637609	0.59248018	1	1	1
ENSSSCG00000047687	0.176383074	1.491853096	0.688	0.333	1
GPX6	0.176836482	-0.805827452	0.438	0.6	1
RALB	0.177083834	-0.294743266	0.688	0.733	1
ZMYND8	0.177125082	0.85828457	1	1	1
WWC2	0.177141081	0.964223771	0.875	0.733	1
TUSC3	0.17732396	1.031691358	1	1	1
TPRG1	0.177428869	2.154818109	0.5	0.133	1
ANKRD42	0.177432124	0.9919565	1	1	1
NAT14	0.177461961	1.62309763	0.812	0.467	1
HYOU1	0.177499304	1.346028003	1	1	1
RWDD4	0.177511173	-0.024396654	0.812	0.733	1
TCERG1L	0.177543953	1.321928095	1	0.867	1
SPAG1	0.177548536	0.359476882	1	1	1
ENSSSCG00000032299	0.177607126	1.714245518	0.625	0.4	1
LEKR1	0.177980154	1.32439635	1	0.933	1
LRBA	0.178395729	0.833680749	1	1	1
TCF15	0.178454812	1.661778098	0.75	0.133	1
ENSSSCG00000050757	0.17846551	-0.093109404	0.688	0.867	1
NOL9	0.178475029	0.418643249	0.938	1	1
SIRT3	0.17859048	0.161311871	1	0.867	1
TANGO6	0.178708922	-0.17663276	0.938	0.933	1
ENSSSCG00000059556	0.178880965	-0.392669686	0.5	0.6	1
ENSSSCG00000051017	0.178908357	1.22881869	0.75	0.4	1
USP40	0.178948621	0.154018611	1	1	1
SEMA7A	0.179042198	2.22881869	0.5	0.4	1
DIS3L	0.179223703	1.027771172	1	0.933	1
MTFR1	0.17942505	0.873841924	1	1	1
DDX60	0.179578098	0.939495261	1	1	1
IMMT	0.179665093	0.095318055	1	1	1
CFAP70	0.179676125	-0.291048782	0.562	0.6	1
CCDC186	0.179862424	0.984781204	1	1	1
ENSSSCG00000014098	0.179874489	1.067355268	1	0.867	1
ARID1B	0.179910374	1.040414268	1	1	1
TATDN1	0.180138502	0.327076642	1	1	1
STRIP1	0.180164787	1.147939465	1	1	1
ENSSSCG00000048231	0.180229973	1.046615359	0.875	0.6	1
TCEA2	0.180310426	-0.327574658	0.5	0.467	1
ESR2	0.180376157	1.279755656	1	1	1
ENSSSCG00000050484	0.180418356	-0.415037499	0.312	0.6	1
CACNB2	0.180540566	0.324617182	0.938	1	1
ENSSSCG00000062745	0.180791545	-0.415037499	0.5	0.467	1
UTP15	0.180865845	0.208470045	0.938	1	1
APBA1	0.181220921	0.436827194	1	1	1
ENSSSCG00000011623	0.181265122	1.154157686	0.938	0.933	1
TGS1	0.181265411	0.958151611	1	1	1
CNNM4	0.18135723	0.070389328	0.812	0.867	1
IER5L	0.181398928	-0.334117504	0.125	0.533	1
UNC119	0.181429665	0.22881869	0.938	0.933	1
PPARD	0.181554785	1.089267338	1	0.933	1
MTERF2	0.181587292	-0.390789953	0.688	0.6	1
FSTL4	0.18191422	0.268214873	1	1	1
ENSSSCG00000047937	0.182092969	-0.213403638	0.5	0.667	1
SLC29A1	0.182502341	1.162545471	1	0.8	1
MAPKAP1	0.182520935	0.437603471	1	1	1
ENSSSCG00000046110	0.182521048	2.714245518	0.5	0.067	1
MINPP1	0.182559143	0.877295813	1	0.933	1
DNAJC19	0.182794978	0.873467594	0.938	0.933	1
FDFT1	0.182874899	0.974908581	1	1	1
ARHGAP24	0.182977956	1.301750213	0.938	0.733	1
SLC9A7	0.18321079	0.885597246	1	1	1

UGGT1	0.183215545	-0.034481674	1	1	1
BORCS8	0.183376023	-0.437063806	0.625	0.533	1
PIK3C2B	0.183396448	1.109982461	1	0.733	1
ENSSSCG00000035799	0.183738914	1.977279923	0.625	0.267	1
ENSSSCG00000060443	0.183934429	0.129283017	0.938	0.733	1
RBM19	0.184044013	-0.019108823	0.75	0.733	1
PATL1	0.184051628	1.199780986	1	1	1
PCCA	0.184404614	0.155803893	0.938	0.933	1
ALDH1A1	0.184418818	-0.415037499	0.312	0.533	1
STPG2	0.184536224	0.710999858	1	1	1
IKZF5	0.184754425	0.96829114	1	0.933	1
TRIM21	0.184761124	1.240791332	0.938	0.867	1
ENSSSCG00000009495	0.184843907	0.315586568	1	1	1
ENSSSCG00000063436	0.185031299	-0.1241363	0.875	0.733	1
PWWP3A	0.185162055	-0.529208519	0.875	0.867	1
GEMIN8	0.185193138	1.238433779	1	1	1
ENSSSCG00000023850	0.18526282	0.008674334	1	1	1
UNC79	0.18559125	1.994353437	0.562	0.4	1
COL11A1	0.185746163	0.250502517	1	1	1
DENND11	0.18574864	0.358457907	1	1	1
GXYLT1	0.185808948	0.375241868	1	1	1
SEC22B	0.18585631	0.804719341	1	1	1
ENSSSCG00000061915	0.186249626	0.027184829	0.312	0.667	1
PLPPR5	0.186380334	2.058893689	0.625	0.267	1
SLC39A13	0.186387197	0.343530349	1	0.867	1
LARS2	0.18650185	1.194467186	1	1	1
USP34	0.186507441	0.758306802	1	1	1
CNOT4	0.186610806	0.755297417	1	1	1
MLF2	0.186694739	1.343432935	1	1	1
HECW1	0.186746806	0.81998253	1	1	1
ENSSSCG00000059022	0.186873176	-0.093109404	0.812	0.6	1
TNFAIP1	0.187056086	1.842760258	0.688	0.533	1
ANAPC16	0.187060762	0.469571649	1	1	1
TNS2	0.187233323	1.746425923	0.812	0.667	1
ENSSSCG00000059416	0.187389541	1.617383978	0.688	0.533	1
PDRG1	0.187568283	0.805010982	1	1	1
BRAF	0.187604926	0.805059921	1	1	1
PLXNA1	0.187649543	0.079727192	1	0.933	1
SYNE2	0.187695131	0.310698708	1	1	1
IFT46	0.187974262	0.993146329	1	1	1
MARK2	0.188080407	1.324787749	1	1	1
ZNF518B	0.188118342	0.178521025	1	1	1
ENSSSCG00000036083	0.188222525	1.198302264	1	0.933	1
NARS2	0.188236862	0.311133779	1	1	1
DCTN2	0.18857274	-0.870716983	0.5	0.733	1
ENSSSCG00000009281	0.188686894	0.300711609	1	0.867	1
DDAH1	0.188691328	0.296302287	0.812	0.933	1
POU2F1	0.18870108	1.272540068	0.938	0.933	1
ENSSSCG00000050878	0.188810219	-0.810966176	0.812	0.667	1
ENSSSCG00000016191	0.188860752	0.020348645	1	0.933	1
CEP162	0.189172247	0.859362226	1	1	1
DHODH	0.189216548	0.897730596	1	1	1
VDR	0.189558728	-0.093109404	0.75	0.933	1
RBPMS	0.18959106	1.111482729	1	1	1
ZRANB2	0.189749592	0.918525296	1	1	1
SMPD4	0.189765539	-0.150824902	0.875	0.933	1
RAD51	0.189820972	0.877046085	1	1	1
SREK1IP1	0.189944072	0.860843228	1	1	1
HK2	0.190130762	0.922974319	1	1	1
COP1	0.190181486	0.190762464	1	1	1
RALBP1	0.190207661	0.338093682	1	1	1
GALC	0.190243214	1.114215569	1	0.933	1
NOL10	0.190364792	1.096507981	1	1	1
ERCC8	0.190375199	0.071949842	1	0.933	1
ENSSSCG00000060577	0.190950324	1.854423176	0.625	0.2	1
PRDM10	0.191169299	1.069162025	1	0.733	1
ENSSSCG00000055078	0.191210642	1.134696513	0.812	0.667	1
PCBP4	0.191331229	0.095335685	0.75	0.867	1
MAGI1	0.191333696	0.399930607	1	1	1
SCD	0.19144109	0.379911279	1	1	1

CAPS2	0.191699077	0.243416067	0.875	0.933	1
SGTA	0.191831265	0.351629217	1	1	1
ADAM9	0.191831275	0.176979759	0.688	0.867	1
GCH1	0.191926015	0.216079579	1	1	1
IPO7	0.1920306	0.998633164	1	1	1
SNX14	0.192267714	1.071991678	1	1	1
FBXO10	0.192313226	0.702837218	1	1	1
ENSSSCG00000048283	0.192421606	-0.397963986	0.625	0.467	1
ZBTB5	0.192598982	-0.339749372	1	0.933	1
ENSSSCG00000061997	0.192774725	-0.228764504	1	0.8	1
CNTLN	0.192789543	1.152135688	1	1	1
MAU2	0.192797855	0.892256201	1	1	1
XYLB	0.192948377	1.525800428	0.812	0.467	1
NAGK	0.193001696	0.276311295	1	1	1
ENSSSCG00000061607	0.193125401	1.070389328	0.688	0.4	1
EFHB	0.193219562	2.22881869	0.5	0.133	1
BICD2	0.193371787	0.892996154	1	1	1
TCN2	0.193427714	-0.63076619	0.562	0.6	1
SH3BP1	0.193538764	1.607330314	0.688	0.333	1
PHF14	0.194008491	0.465889077	1	1	1
NUDT13	0.194106658	1.038257158	1	0.933	1
GRIA4	0.194354663	0.304312552	1	1	1
PICALM	0.194601187	0.809700668	1	1	1
NEU3	0.194605582	-0.043068722	0.688	0.6	1
SLC35E3	0.194748831	0.91580532	1	0.933	1
PLAC9	0.194754049	-0.437063806	0.5	0.6	1
ATP13A4	0.194802572	1.568305157	0.812	0.733	1
FBXL12	0.194857188	0.445531528	1	1	1
NREP	0.195077249	0.852326235	1	1	1
CA14	0.195123351	1.743391863	0.562	0.267	1
UBE2H	0.195401281	0.512635903	1	1	1
SERPINB5	0.195695158	1.541606132	0.688	0.533	1
NUDT16	0.195853082	0.309437151	0.938	0.8	1
MUC15	0.196107969	0.980139578	0.938	0.867	1
TTI1	0.196338726	0.047488926	0.875	1	1
PDS5B	0.196464138	0.881193058	1	1	1
ZMYM5	0.196506011	0.35132188	1	1	1
ARHGAP17	0.196595682	0.120486069	1	0.867	1
CNGA3	0.196631233	-0.253574077	0.5	0.6	1
RPA1	0.196816107	0.938769896	1	1	1
EXD1	0.196888645	0.958658657	1	1	1
ENSSSCG00000062995	0.196923727	1.089093927	1	1	1
PDE3B	0.196935206	1.054320959	1	0.933	1
PRRC2A	0.197086308	-0.042923647	0.938	0.933	1
CD81	0.19723949	0.266886731	1	0.933	1
USP1	0.197576473	0.754887502	1	1	1
ENSSSCG00000056876	0.197628862	1.343407822	0.812	0.867	1
ME3	0.197668607	0.088337467	0.812	0.867	1
BMPER	0.197907856	0.861123695	1	1	1
PUS1	0.198197612	1.534921818	0.562	0.333	1
XKR8	0.198224865	-0.366127899	0.562	0.467	1
ENSSSCG00000055999	0.198673324	1.085227837	0.625	0.267	1
RIPPLY2	0.198931464	1.321928095	0.562	0.4	1
KLHL20	0.198937264	0.845810045	1	1	1
SMCHD1	0.199079493	0.879145605	1	1	1
RNF25	0.199416943	-0.246519319	0.938	0.933	1
ENSSSCG00000058314	0.199446284	0.091630475	0.875	1	1
MMRN1	0.199488925	-0.16525919	0.812	0.733	1
USB1	0.199545872	1.137503524	1	0.933	1
ENSSSCG00000058343	0.19961959	-0.1271904	1	1	1
MIS18BP1	0.199677019	0.946968035	1	0.933	1
WIPF3	0.199715262	0.265150903	1	0.933	1
ENSSSCG00000017038	0.199788153	0.438046653	1	1	1
BDNF	0.19992184	1.10938346	0.812	0.933	1
RBM41	0.200013435	0.101086125	1	1	1
CDK5R1	0.200193228	1.531381461	0.875	0.867	1
NEURL3	0.20021715	0.190683562	0.938	0.867	1
MAGOH	0.200454989	0.732794179	1	1	1
AP5B1	0.20089276	-0.388565288	0.5	0.467	1
FYTTD1	0.201232376	0.851894027	1	1	1

ENSSSCG00000059637	0.201273691	0.165237996	1	1	1
POMGNT2	0.201333006	-0.433449536	0.875	0.933	1
CEP152	0.201598316	0.950825021	1	1	1
ENSSSCG00000044105	0.201602385	1.845490051	0.5	0.267	1
GEMIN5	0.201816472	0.252387162	1	1	1
C1QBP	0.202086102	0.948994627	1	1	1
SCOC	0.202266365	0.138216142	0.938	1	1
TMEM222	0.202575854	-0.044199804	0.688	0.733	1
ENSSSCG00000060099	0.202590506	-0.186218809	0.688	0.733	1
OXCT1	0.202595342	0.20523187	0.938	0.933	1
PHLDB1	0.20280384	0.238872355	1	0.933	1
VSIG2	0.202942105	0.868416448	0.938	0.867	1
ENSSSCG00000051245	0.203081196	1.118729239	0.938	0.933	1
PTGIS	0.203316128	1.035505028	1	1	1
ENSSSCG00000057811	0.20342235	0.781359714	1	0.867	1
ENSSSCG00000022423	0.203500093	1.017534659	0.938	0.667	1
ENSSSCG00000052887	0.203508211	-0.508146904	0.312	0.6	1
TMEM232	0.2036905	0.074257302	1	1	1
TLE1	0.203795397	0.220999186	1	0.867	1
TBCB	0.203906422	0.886280316	1	1	1
ENSSSCG00000056414	0.204003132	1.441064986	1	0.867	1
RTBDN	0.204036801	0.0489096	0.75	0.667	1
MESP1	0.204088878	1.159656666	0.938	1	1
HERC1	0.204140377	0.365456136	1	1	1
RAP1GDS1	0.204179848	1.118532848	1	1	1
RBM44	0.204181196	1.030648371	1	1	1
KLHL36	0.204271718	1.441479574	1	0.867	1
ENSSSCG00000058222	0.204279791	-0.206320015	0.625	0.867	1
PML	0.204774173	-0.250650681	0.438	0.6	1
ADAMTS6	0.204921733	0.306034448	1	1	1
OSBP	0.205004531	0.311262686	1	1	1
LEMD3	0.205135157	0.927424604	1	1	1
PDE4DIP	0.205182546	0.388367537	1	1	1
SYNGR2	0.205216598	-0.392669686	0.5	0.467	1
KCNH1	0.205520206	0.452422952	1	1	1
CXXC4	0.205698181	1.430452552	0.625	0.2	1
USF1	0.205875762	-0.043356369	0.75	0.733	1
ENSSSCG00000060568	0.206088399	0.179262866	0.938	0.867	1
EFTUD2	0.206238852	0.304237619	1	1	1
PPP2R3B	0.206296088	1.231482948	1	1	1
SNX19	0.206553302	-0.076989739	0.812	0.933	1
ENSSSCG00000059277	0.206823995	1.401655287	0.75	0.2	1
SPHK2	0.206970948	-0.338221902	0.812	0.6	1
STK3	0.207789314	0.346247774	0.938	0.933	1
USP8	0.207920789	0.760230302	1	1	1
PAPOLA	0.208042787	0.953618076	1	1	1
JARID2	0.208281945	1.023517371	1	1	1
MRPS30	0.208289002	0.483140665	1	1	1
MLH3	0.208611483	0.003808739	1	0.933	1
ENSSSCG00000054702	0.208720604	1.201221132	0.938	0.933	1
ENSSSCG00000058499	0.208818943	-0.115135711	0.75	0.867	1
PRKCE	0.208920415	0.968951564	0.938	0.867	1
TMEM64	0.208946635	1.027642755	1	0.867	1
GDAP1	0.208949024	-0.37690237	0.5	0.6	1
EFCAB5	0.20955957	0.154328146	1	1	1
ENSSSCG00000063005	0.209698396	0.815410435	1	1	1
FKBP14	0.209702479	0.529327802	0.812	0.933	1
ENSSSCG00000012192	0.209936408	0.203640127	1	1	1
ATP13A1	0.209955685	1.327877361	1	1	1
ZNF711	0.2101833	1.277780991	1	1	1
HIPK1	0.210203238	0.857963805	1	1	1
ZFAND2A	0.210271797	1.262302389	1	1	1
ZC3H10	0.21030983	0.240016897	0.875	0.933	1
LRRC70	0.210382824	1.034002514	0.812	0.667	1
CHPF	0.210529997	0.254813899	0.812	0.867	1
STXBP3	0.210561085	0.060435854	1	1	1
RIF1	0.21064945	0.810909554	1	1	1
TM9SF3	0.211049913	0.330304517	1	1	1
AGTPBP1	0.211198568	0.840893037	1	1	1
ENSSSCG00000059564	0.211204248	0.225075826	1	1	1

ACLY	0.211224955	-0.007479333	1	1	1
FAM171B	0.211252023	0.94061747	1	1	1
LRP2	0.211291695	1.282757498	0.812	0.867	1
SLC7A1	0.21130234	1.265971689	0.938	0.933	1
NUP153	0.21149216	0.758020075	1	1	1
CRYBG2	0.211529031	0.233700912	1	1	1
RAB12	0.211547348	0.993544978	1	0.933	1
ENSSSCG00000061018	0.211551284	-0.1602236	0.625	0.533	1
RCC1	0.211708	1.125070766	1	0.933	1
NMT2	0.211769206	1.047753131	0.875	0.6	1
PKDCC	0.211785534	-0.093109404	0.625	0.333	1
ZNF365	0.211819836	1.352690349	0.875	0.6	1
ADM	0.211850349	-0.034215715	0.688	0.467	1
VEZT	0.211872472	0.978374325	0.938	1	1
RABGAP1L	0.212003036	0.674506403	1	1	1
PLBD1	0.212055654	-0.366127899	0.875	0.6	1
FBXW4	0.212080592	1.218476746	1	0.867	1
ZMYM6	0.212210606	0.311607289	1	1	1
NEK7	0.212423298	0.224860677	0.875	0.867	1
ENSSSCG00000040433	0.212494276	-0.137503524	0.938	0.667	1
ENSSSCG00000059817	0.212648634	-0.167109986	0.5	0.733	1
ENSSSCG00000015027	0.21265691	-0.134332067	0.875	0.933	1
POLR3B	0.212704003	0.192292814	0.938	0.933	1
PGP	0.212710535	0.1661826	0.938	0.933	1
NOD2	0.212734443	-0.218640286	0.25	0.533	1
OGT	0.21277482	0.332775477	1	1	1
C1orf56	0.212868879	-0.077679364	1	0.867	1
HCFC1	0.213045696	1.106114322	1	1	1
SULT1C3	0.213197601	0.091315167	0.688	0.8	1
SURF6	0.213401352	0.053731984	0.812	0.533	1
C1QTNF3	0.213438117	1.807354922	0.5	0.2	1
OSR2	0.213513988	0.286082896	1	0.933	1
POLR1G	0.213566074	0.085693748	0.875	0.933	1
ENSSSCG00000048082	0.214191974	-0.467504919	0.438	0.6	1
PRPF8	0.214239257	0.928529075	1	1	1
ELK1	0.214654366	0.481584761	0.875	0.867	1
SELENOI	0.214827301	0.824001489	1	1	1
NOP58	0.214832665	1.072305076	1	1	1
TBCCD1	0.215688267	0.982178723	0.75	0.533	1
ENSSSCG00000063326	0.215714821	1.534921818	0.688	0.333	1
ENSSSCG00000060264	0.215737952	-0.043356369	0.938	0.867	1
ENSSSCG00000018068	0.215894606	0.041191687	0.875	0.667	1
FAM180A	0.215896517	0.017921908	0.562	0.4	1
SP4	0.216030834	-0.382616022	0.875	0.867	1
EXOC3	0.216535944	-0.176940995	0.75	0.933	1
DARS2	0.216556896	-0.136611043	0.75	0.667	1
YRDC	0.216668398	0.339850003	0.938	0.933	1
ENSSSCG00000046312	0.216691588	0.032421478	0.625	0.533	1
ZNF300	0.217055602	1.54059743	0.938	0.867	1
LBH	0.217380437	0.240134004	1	0.933	1
TLDC2	0.217496413	1.335733894	0.75	0.4	1
NEMP1	0.217617288	0.321128153	1	1	1
ENSSSCG00000061203	0.21768749	0.886623197	1	1	1
ENSSSCG00000059967	0.217927306	1.34298971	0.812	0.467	1
CFAP20DC	0.21803128	0.966762052	1	1	1
ENSSSCG00000055335	0.218199384	-0.062082509	0.812	0.733	1
SF3A1	0.218451241	0.430452552	1	1	1
BPTF	0.21868868	0.837958932	1	1	1
ENSSSCG00000015988	0.21870024	0.343474534	1	1	1
ITPRIP	0.218707135	1.529820947	0.75	0.4	1
FNBP1	0.21876728	1.965784285	0.625	0.133	1
AP3D1	0.218857606	0.302819272	1	1	1
ITPR1	0.218886298	0.323392314	1	1	1
UPK1B	0.219076648	1.866248611	0.562	0.333	1
MTMR9	0.219117176	0.790499528	1	1	1
ENSSSCG00000033390	0.219182322	0.877546634	1	1	1
ENSSSCG00000029920	0.219303368	1.324125651	0.875	0.8	1
IFN-OMEGA-6	0.219410362	-0.03866162	0.5	0.533	1
FOXP2	0.219599636	1.321928095	0.625	0.4	1
GRAMD4	0.219636108	0.438935212	1	1	1

SLC38A7	0.219776094	1.491853096	0.625	0.533	1
SLC16A9	0.219895855	-0.128733314	0.438	0.533	1
ENSSSCG00000000377	0.219931137	0.544899331	1	1	1
ZDHHC17	0.220055136	1.303780748	0.812	0.467	1
PLD5	0.220100404	0.282589947	1	0.933	1
HDDC2	0.220305609	0.894917954	0.938	1	1
CNP	0.220314448	0.266746929	1	1	1
PSMD1	0.220316856	0.993256226	1	1	1
KLF6	0.220342798	-0.497499659	0.5	0.6	1
FAM161B	0.220482438	0.123701985	0.875	1	1
CFB	0.220589893	-0.180572246	0.438	0.533	1
PRKACA	0.220973157	1.558967292	0.875	0.6	1
LARGE1	0.22101771	0.262827583	1	0.933	1
ZNF512	0.221025229	-0.072645302	0.938	0.867	1
SPON1	0.221138343	0.131013905	1	1	1
CFAP65	0.221662227	-0.315501826	0.5	0.4	1
ENSSSCG00000051090	0.221674928	0.838719093	0.938	0.8	1
WLS	0.221737489	0.484131075	1	1	1
SLC29A3	0.221933091	0.183408231	1	0.933	1
PTPRS	0.221957438	-0.471621028	0.562	0.8	1
PPIH	0.221978239	0.774720516	1	0.933	1
DRD3	0.222028545	1.754887502	0.5	0.133	1
SOCS7	0.222042769	0.75130248	1	1	1
ZNF395	0.222348589	0.385135698	1	1	1
CD19	0.222394678	-0.044199804	0.875	0.867	1
NEURL1	0.222455515	0.478199034	1	1	1
DDX31	0.222599146	1.326604582	0.938	1	1
ENSSSCG00000009770	0.222750445	1.011720569	1	1	1
ELOVL2	0.222791383	0.07756657	1	1	1
GCC1	0.222904695	0.908215993	1	1	1
ENSSSCG00000034690	0.222929047	0.950833943	0.938	0.867	1
ENSSSCG00000058715	0.223196147	0.036049076	1	0.933	1
ENSSSCG00000052974	0.223311942	1.076815597	1	0.933	1
LHX5	0.223408589	1.951284715	0.562	0.333	1
GGPS1	0.223486227	0.156270447	1	1	1
MORC1	0.223734258	1.339850003	0.938	0.733	1
LSM12	0.223758202	-0.019108823	0.938	0.867	1
ENSSSCG00000016426	0.224188931	1.209389756	1	1	1
PPP2R5E	0.224260706	0.856264523	1	1	1
ZNF335	0.224384993	0.159871337	1	0.933	1
ENSSSCG00000008058	0.224398694	1.140120489	1	1	1
ENSSSCG00000038679	0.224509618	-0.022720077	0.562	0.667	1
FAM20B	0.224565984	0.318086029	0.812	0.867	1
MRRF	0.224677606	0.808262563	1	1	1
DIP2A	0.224713855	1.165624864	0.812	0.733	1
DDX5	0.224722119	0.452590854	1	1	1
RNF214	0.224765651	0.285225212	1	1	1
PPP4R3A	0.224779091	0.824905123	1	1	1
ADGRG6	0.224839315	2.464886049	0.625	0.133	1
ANKRD49	0.225223595	0.075881512	1	1	1
ENSSSCG00000040203	0.225236126	1.307428525	1	1	1
TRIM37	0.225316283	0.770289532	1	1	1
ENSSSCG00000027515	0.225468106	-0.055876498	0.812	0.867	1
STRC	0.225647265	1.190683562	0.75	0.533	1
TGIF2	0.225681648	0.17139041	1	0.867	1
SDE2	0.22573086	0.955823563	1	1	1
SRBD1	0.226108749	0.894421244	1	1	1
ENSSSCG00000043051	0.22623927	0.86466236	1	1	1
PIAS3	0.226259617	1.161512762	1	0.933	1
DCAF1	0.226364972	0.865408216	1	1	1
OPA3	0.226736629	0.174823801	0.875	0.8	1
LEAP2	0.226897432	0.945109918	1	1	1
SENP8	0.226910012	0.280204683	1	1	1
CRHBP	0.227183425	0.185650831	1	1	1
ENSSSCG00000046546	0.227284115	1.3594028	0.75	0.533	1
DGCR8	0.227563662	0.027584413	1	1	1
RFT1	0.227745768	1.280621121	0.875	0.8	1
FGD6	0.227759324	1.020648886	1	1	1
PALM3	0.227885804	0.427722759	0.5	0.667	1
FOXR1	0.227916171	0.404421629	1	1	1

GPC4	0.228154532	0.399675781	1	1	1
CDCA4	0.228344453	1.691161905	0.5	0.4	1
MYCBPAP	0.228697799	0.052321035	0.812	0.8	1
ENSSSCG00000059707	0.22893925	-0.063963059	0.875	0.8	1
GYS1	0.228999813	0.016768213	1	0.867	1
NR6A1	0.229262595	0.895674978	1	1	1
DHX16	0.229439155	0.230667115	1	1	1
KLHL42	0.229835539	0.295509747	1	1	1
PEX7	0.229855066	0.993783765	1	0.933	1
ENSSSCG00000003555	0.229914941	0.788507548	1	1	1
RP2	0.230081469	0.268609276	1	1	1
ENSSSCG00000000635	0.230099641	1.083388251	0.938	1	1
UTP20	0.230279398	0.777403871	0.938	1	1
POU6F1	0.230344948	-0.039670145	1	1	1
SPEF2	0.230352706	1.096715154	0.938	0.733	1
FAM237B	0.230371479	1.854423176	0.5	0.267	1
BCKDK	0.230664292	0.383643879	1	0.933	1
CEP97	0.231015718	1.034269902	1	1	1
ZNF366	0.231210079	1.573647187	0.938	0.733	1
ENSSSCG00000055760	0.231457999	0.625119627	0.938	0.867	1
MYO1H	0.231475703	-0.175571565	0.562	0.667	1
NPL	0.23151208	1.018311878	1	0.867	1
THRA	0.231536714	0.172624151	0.938	0.933	1
SLITRK6	0.231817573	1.047753131	0.938	0.533	1
IL18R1	0.231920533	-0.2255597	0.875	0.667	1
PROSER3	0.231999954	0.343954401	0.938	0.867	1
DCDC2C	0.232032845	1.994353437	0.562	0.133	1
GBGT1	0.23214006	-0.315501826	0.5	0.533	1
RFTN1	0.232141051	1.419960178	0.812	0.867	1
PCED1A	0.232624152	0	0.625	0.8	1
RACGAP1	0.232886684	0.165500992	1	1	1
HECW2	0.232970762	0.234055339	1	1	1
CNTROB	0.233299118	0.789320999	1	0.933	1
PNPO	0.233477246	0.025535092	0.5	0.8	1
PAQR8	0.233532859	-0.520530628	0.625	0.6	1
DNAI7	0.233546576	0.65150664	1	1	1
RNF123	0.233695812	0.059720212	0.938	0.933	1
ENSSSCG00000039073	0.233917093	1.661778098	0.625	0.333	1
CRYBG3	0.233982068	0.844201052	1	1	1
TMOD3	0.233999426	0.668662606	1	1	1
THAP11	0.23424815	0.886022848	1	1	1
PLCG1	0.234261718	0.845634907	1	1	1
ANKDD1B	0.234323084	0.96916374	1	1	1
MYO5C	0.23446579	0.296876133	1	1	1
COLEC12	0.234697266	-0.455679484	0.5	0.467	1
GALNT14	0.234732776	0.278314308	0.938	1	1
ENSSSCG00000053748	0.234816045	1.558967292	0.5	0.333	1
ENSSSCG00000052642	0.234893012	-0.051289229	0.75	0.8	1
DOCK5	0.234938264	0.945544027	1	1	1
ENSSSCG00000008677	0.235093513	1.319787788	1	1	1
NSUN3	0.235132736	-0.483055923	0.625	0.6	1
KCTD19	0.235178184	1.222392421	1	0.733	1
TCF7	0.235247763	1.047753131	1	1	1
SGCE	0.235620162	1.004378441	1	1	1
SCLT1	0.235641836	0.985245414	1	1	1
ARMH4	0.235801051	0.571974398	1	1	1
ZNF829	0.235972465	0.151073222	1	1	1
HYCC1	0.236409086	0.732519689	1	1	1
ENSSSCG00000055844	0.23642498	-0.32443495	0.5	0.6	1
IRF2BP2	0.236802705	1.015666942	0.938	0.667	1
ZNF581	0.237063482	-0.192645078	0.312	0.533	1
HCN1	0.237095725	1.252026082	0.875	0.867	1
CCDC18	0.237808735	1.012367996	1	1	1
THAP6	0.237833331	1.06667104	1	0.867	1
MAPK9	0.237923344	0.320484678	1	1	1
NPM3	0.238265256	1.16753157	1	1	1
DLG3	0.238372835	-0.781165398	0.375	0.667	1
SOS1	0.238399853	0.856114234	1	1	1
LIMK2	0.2387265	0.20968683	0.875	0.933	1
ENSSSCG00000051170	0.238911409	0.098189248	1	1	1

ENSSSCG00000052437	0.238937255	-0.163498732	0.312	0.6	1
ENSSSCG00000052253	0.23894299	1.569855608	0.625	0.2	1
ENSSSCG00000016224	0.239106608	1.080538683	0.875	0.8	1
MASP1	0.239233592	-0.346865997	0.875	0.667	1
SLC25A22	0.239234907	0.252026082	0.875	0.8	1
ENSSSCG00000050796	0.239406404	1.491853096	0.625	0.333	1
TRIM28	0.23941248	0.530585491	1	0.933	1
ENSSSCG00000062625	0.239594957	1.22881869	0.562	0.4	1
COX2	0.239731717	1.186670972	1	1	1
ENSSSCG00000037812	0.240185673	1.236596041	1	0.667	1
DNAJC25	0.240344703	-0.093109404	0.25	0.533	1
ENSSSCG00000058062	0.240742975	1.824428435	0.5	0.4	1
TSTD2	0.240756104	0.92346482	1	1	1
VPS54	0.240827805	-0.182192948	1	0.867	1
CUL5	0.240837582	0.919121609	1	1	1
KLHL32	0.241001262	0.629321601	1	1	1
ENSSSCG00000060550	0.241190334	0.991206882	1	0.933	1
METTL21A	0.241472657	1.029287227	1	1	1
ZNF24	0.24156702	0.903681039	1	1	1
CENPK	0.241615314	0.978876708	1	0.867	1
ENSSSCG00000052279	0.241731237	0.0138058	0.875	0.733	1
DMRT2	0.241762029	0.136587818	0.875	1	1
TMEM14A	0.242168668	0.767834952	1	1	1
ENSSSCG00000042369	0.24224415	-0.306103128	0.625	0.733	1
MICU3	0.242261912	1.791413378	0.625	0.333	1
ENSSSCG00000010432	0.242569416	0.546715031	1	1	1
ENSSSCG00000059054	0.24270276	1.447458977	0.562	0.333	1
ENSSSCG00000044869	0.242806451	1.315355441	0.875	0.8	1
KIAA1549L	0.243004585	0.16754235	0.938	0.933	1
ENSSSCG00000021676	0.243592056	0.239465935	1	0.867	1
RAB33B	0.243691566	0.979040381	0.938	0.8	1
TRMT61B	0.243698723	0.352301744	0.938	1	1
RNF103	0.244400915	0.19479297	1	1	1
ENSSSCG00000057932	0.244804756	-0.562594688	0.5	0.6	1
ENSSSCG00000063049	0.245022836	-0.263034406	0.438	0.533	1
KIT	0.245597534	0.755578779	1	1	1
CDKAL1	0.245604845	0.367599953	1	1	1
PNPLA2	0.24597869	0.078656944	1	0.8	1
RASGEF1A	0.246120479	0.205709577	0.938	1	1
FAM114A1	0.246166615	1.058562758	1	1	1
NEB	0.246265262	0.326917966	1	1	1
SLC9A8	0.24639597	0.112359714	0.938	0.933	1
EIF4E1B	0.246984902	1.022367813	1	1	1
MIGA2	0.247280559	0.217230716	0.438	0.667	1
DAAM1	0.247312694	0.788065299	1	1	1
ASXL1	0.247342423	0.010601945	0.938	0.867	1
NCK1	0.247427255	0.485870938	1	1	1
GCC2	0.247660119	0.898941842	1	1	1
LARP4	0.247777204	0.691328274	1	1	1
CFAP418	0.247857205	1.384507687	1	0.867	1
RNF114	0.248018912	1.220656602	1	1	1
ENSSSCG00000051861	0.248234062	0.894612266	0.938	1	1
MYDGF	0.248704834	1.011511558	1	1	1
MUL1	0.248754635	-0.102154544	0.75	0.733	1
ZDHHC12	0.249017123	0.200621799	0.938	0.933	1
CFAP95	0.249126785	0.906890596	0.875	0.733	1
KAT6B	0.249318762	0.853719937	1	1	1
PLCE1	0.249642144	0.947798539	1	0.933	1
TRANK1	0.249956554	1.158808969	1	0.933	1
PDSS2	0.250227194	0.226169112	0.938	0.933	1
KIF27	0.250596744	0.461479447	1	1	1
PNMA1	0.250752164	0.958504703	1	1	1
TENT5C	0.250860439	0.870030666	1	1	1
CCT7	0.251242129	0.888248189	1	1	1
ENSSSCG00000035836	0.251301216	1.531381461	0.562	0.333	1
ATXN7	0.251360326	0.26524996	1	1	1
MLLT10	0.251591345	0.879409859	1	0.8	1
ENSSSCG00000014779	0.251615399	0.09330372	0.875	0.8	1
GPD1L	0.252005406	0.854646728	1	1	1
ABCA1	0.252014251	0.394747877	1	1	1

ENSSSCG00000042120	0.252043789	-0.415037499	0.375	0.533	1
ENSSSCG00000035561	0.252157745	1.007893914	1	0.933	1
GSC	0.2522768	1.321928095	0.75	0.533	1
TMX3	0.252280255	0.342475472	1	1	1
STX11	0.252390718	0.872457099	0.938	0.867	1
OSBPL9	0.252538532	0.264634391	1	1	1
SEC16A	0.252538802	0.509743255	1	1	1
KIAA0513	0.25259866	0.087462841	1	0.733	1
SEC24C	0.25282545	0.386883537	1	0.933	1
ENSSSCG00000005475	0.252875931	1.564002882	0.5	0.333	1
GAS2	0.253010493	0.317678622	0.938	0.867	1
MARCKSL1	0.25307656	1.987810591	0.688	0.4	1
METTL16	0.253149082	0.659413211	1	1	1
FRAT2	0.253189613	1.299208018	0.812	0.533	1
GOT1	0.253463754	1.249122787	1	0.867	1
HACD2	0.25352632	0.716959902	1	1	1
ENSSSCG00000032896	0.253561488	1.271463028	0.875	0.733	1
EIF4G1	0.25358693	0.340400755	1	0.933	1
DDX19B	0.253724065	0.283453947	0.688	0.933	1
ADAMTS2	0.253753677	1.035720576	1	0.933	1
KMT5C	0.253845006	1.154818109	0.938	0.867	1
ERCC6L2	0.254031956	0.472319209	1	1	1
TTYH3	0.254042972	0.186998515	0.75	0.6	1
C5AR1	0.254119861	0.431157165	0.938	1	1
SIPA1L1	0.254432653	0.242904447	1	1	1
ENSSSCG00000045643	0.254502947	1.362955363	0.875	0.8	1
ENSSSCG00000041350	0.254657374	2.813781191	0.5	0.067	1
ZNF169	0.254806717	0.480625841	1	0.867	1
CLNS1A	0.25480999	0.955389113	1	1	1
SLC16A11	0.254895635	-0.285754482	0.562	0.6	1
GALNT13	0.254904032	0.885453425	1	1	1
ENSSSCG00000056346	0.25491165	-0.093109404	0.75	0.733	1
CHRNA1	0.254929255	-0.16663844	1	1	1
LIX1	0.254930653	0.906890596	0.812	0.4	1
KIF11	0.254945951	0.406611758	1	1	1
THEMIS	0.255051954	1.491853096	0.625	0.4	1
TRIM23	0.255082306	0.159755369	0.938	0.867	1
MIA3	0.255307934	0.249458457	1	1	1
PDE4A	0.255452462	1.409390936	0.562	0.333	1
LRP6	0.255540037	0.810859769	1	1	1
MCM8	0.255841833	0.131013905	1	1	1
ARFGEF1	0.255935723	0.780077508	1	1	1
GPC5	0.256018594	0.280348991	0.812	0.733	1
RIGI	0.256088616	0.632048459	1	1	1
ENSSSCG00000062580	0.256612387	1.417852515	0.688	0.533	1
ENSSSCG00000060507	0.256629895	1.398743692	0.875	0.6	1
POLR2A	0.256729038	1.309393741	1	1	1
AARD	0.25709506	-0.093109404	0.688	0.733	1
C4orf17	0.257643267	0.376375879	0.938	0.933	1
DYNC2H1	0.257753741	1.199071347	1	1	1
NOS1	0.2580049	0.138216142	0.438	0.533	1
THAP4	0.258152376	-0.093109404	0.625	0.6	1
PKD1	0.258374627	-0.049607766	0.812	0.867	1
ENSSSCG00000051679	0.258739246	-0.200024608	0.438	0.533	1
LMTK2	0.258978437	-0.222392421	0.625	0.667	1
PRR14L	0.259259163	0.852255339	1	1	1
C11orf87	0.259304751	1.252665432	0.75	0.667	1
SCAMP1	0.259311411	0.520151708	1	1	1
ENTREP3	0.259359996	-0.034215715	0.438	0.533	1
ZNF461	0.259461475	-0.545621609	0.625	0.6	1
ENSSSCG00000033232	0.259538649	-0.093109404	0.625	0.733	1
SYT11	0.25984394	0.0138058	0.75	0.867	1
PGRMC1	0.259846376	1.024542184	1	1	1
IGDCC4	0.260234941	1.607330314	0.5	0.4	1
TRAF3	0.260680924	0.423681594	1	1	1
ZNF513	0.260913502	1.083213368	1	0.533	1
THSD7A	0.260985786	0.499524576	1	1	1
ENSSSCG00000060492	0.261006015	0.906890596	1	1	1
ZBTB2	0.261055575	0.809866141	1	0.933	1
CNKSR2	0.261186942	1.047068254	0.938	0.667	1

LPCAT3	0.261531754	-0.451563375	0.625	0.667	1
SERTAD3	0.261581802	1.125900378	1	1	1
DIPK2A	0.261643532	1.053600883	1	1	1
ENSSSCG00000062726	0.261691006	0.136896201	1	0.933	1
ENSSSCG00000041277	0.261902402	-0.130723696	1	1	1
SETD2	0.262005631	-0.718069973	0.75	0.533	1
MSANTD7	0.262685962	0.803472836	1	1	1
ZIC3	0.262749964	0.082570272	1	1	1
FRMPD1	0.262767385	1.111249094	0.938	0.8	1
ZDHHC8	0.262806432	0.254813899	0.812	0.733	1
CIC	0.263097737	1.33315535	0.812	0.467	1
ENSSSCG00000018079	0.263133769	0.099535674	1	0.933	1
USP42	0.263174424	0.043603818	0.938	1	1
ANTXR2	0.263215404	0.445058885	1	0.933	1
TSPYL2	0.263315304	0.378196315	0.75	0.867	1
ENSSSCG00000061416	0.263424666	0.769387072	0.938	0.867	1
PPP3CC	0.263498426	0.405800652	1	0.933	1
DDX42	0.263516464	0.871073044	1	1	1
ENSSSCG00000055991	0.263776996	1.305439972	0.625	0.4	1
ATF2	0.264040348	1.079499682	1	1	1
ZSWIM5	0.264241005	-0.174409506	0.812	0.8	1
ADCY3	0.264347299	0.820682454	1	1	1
ENSSSCG00000062269	0.26443004	1.218092284	0.875	0.667	1
NPRL2	0.264636743	0.922093002	1	1	1
CYBRD1	0.264655073	1.723178642	0.688	0.467	1
LSM14A	0.264665496	0.79470045	1	1	1
ENSSSCG00000025711	0.264813444	0.34227674	1	1	1
RPUSD4	0.26486518	0.226827393	1	1	1
C15orf61	0.265283257	-0.128733314	0.812	0.867	1
VPS37B	0.265374188	-0.093109404	1	0.933	1
GNE	0.265436508	0.856280241	1	1	1
ENSSSCG00000063549	0.26560288	1.26052755	0.875	0.533	1
ACACB	0.265618655	1.076815597	1	0.667	1
ENSSSCG00000022957	0.265668019	0.219274918	0.875	0.933	1
RBAK	0.265818123	-0.480132528	0.5	0.4	1
PRPF39	0.266156638	0.472794761	0.875	0.933	1
ENSSSCG00000057934	0.266181237	-0.253574077	0.562	0.467	1
TIAM2	0.26646926	0.388423507	1	1	1
PTGDR	0.266644651	1.17990909	0.938	0.867	1
NINJ1	0.266900174	0.824428435	0.938	0.533	1
ENSSSCG00000057414	0.267029528	-0.163498732	0.75	0.667	1
BTG1	0.267260351	1.001749782	0.938	0.867	1
GPATCH4	0.267426923	0.062541466	0.812	0.933	1
RUBCNL	0.267440596	-0.017821277	0.75	0.8	1
DHX29	0.26766562	0.13289427	1	1	1
EPB41	0.267674594	0.956875345	1	1	1
ENSSSCG00000009341	0.267780601	0.293242352	1	1	1
COL1A2	0.268054818	1.030273011	1	0.933	1
INO80	0.268056091	0.890173201	1	1	1
ENSSSCG00000037609	0.268113333	0.33752495	0.5	0.6	1
PHOSPHO2	0.268138885	0.805305409	1	1	1
LARP1	0.268708042	0.147657771	1	1	1
CPSF2	0.268815675	0.750000112	1	1	1
NPHP1	0.268885262	1.380821784	0.625	0.267	1
NMI	0.268921508	-0.855417847	0.75	0.533	1
ENSSSCG00000006324	0.26894876	0.961052175	1	1	1
ZNF143	0.268981617	0.747442311	1	1	1
CCDC146	0.269060996	-0.035393907	0.875	1	1
ENSSSCG00000018077	0.269300584	-0.386840607	0.75	0.533	1
COLGALT1	0.269308928	0.419740118	1	0.933	1
TMEM87A	0.269934871	0.916481955	1	1	1
AGPAT5	0.269955513	0.429103831	1	1	1
CDK2AP2	0.269963971	0.473970391	1	1	1
TLR7	0.26996786	0.408717252	0.938	1	1
ARHGAP35	0.270091465	0.753846221	1	1	1
POLA1	0.270138414	0.719846724	1	1	1
ADAMTS3	0.270269252	1.17990909	0.625	0.267	1
SECISBP2L	0.270318836	0.744787649	1	1	1
XK	0.270328548	0.445866891	1	1	1
DHX8	0.270556809	0.268056934	1	1	1

ENSSSCG00000042928	0.270652161	-0.093109404	0.5	0.6	1
VXN	0.270684695	0.884170519	1	1	1
PIM2	0.270686322	0.415795018	1	1	1
GTPBP2	0.270786395	0.82799505	1	1	1
DDX1	0.271012196	0.287759733	1	1	1
ENSSSCG00000047889	0.271202977	0.174112798	1	1	1
CDC73	0.271339261	0.97549441	1	0.933	1
HM13	0.271478253	0.829928614	0.938	0.867	1
ZNF217	0.271511324	0.922386932	1	1	1
MGRN1	0.271531582	1.483651283	1	0.933	1
NIP7	0.271818024	0.700807143	1	1	1
MDFIC	0.271915434	0.273948066	1	0.867	1
UNC5C	0.271927317	0.076075264	1	1	1
ATF6	0.272482124	0.800517741	1	1	1
PPM1K	0.272671744	-0.35614381	0.5	0.6	1
C1orf35	0.27301546	0.89077093	1	1	1
LSM5	0.273150324	0.693445879	1	1	1
LMAN2	0.273253839	0.600009052	1	1	1
TEFM	0.273301943	1.097712938	0.875	0.8	1
BAZ1B	0.273602313	0.889179724	1	1	1
ENSSSCG00000056010	0.273642128	0.009984089	0.438	0.533	1
ENSSSCG00000062352	0.273747484	-0.042483331	0.375	0.6	1
AADAC	0.273813099	1.147898695	0.875	0.6	1
EMC1	0.273982263	0.885426086	1	1	1
CLUAP1	0.274055002	1.067674943	1	1	1
DNAI3	0.274211628	-0.025995209	0.5	0.733	1
TDRD6	0.274436666	0.810525498	1	1	1
LBR	0.27463775	0.751169214	1	1	1
HIPK3	0.27475068	0.390099598	1	1	1
TECTA	0.274917686	0.418940725	1	1	1
ENSSSCG00000045922	0.27502312	0.121015401	0.5	0.667	1
EMSY	0.275131109	0.818582177	1	1	1
ENSSSCG00000051999	0.275187527	-0.253574077	0.438	0.6	1
PLA2G10	0.275292261	1.409390936	0.5	0.2	1
SIK1	0.275428062	0.129283017	0.562	0.6	1
AKAP7	0.275626804	-0.093109404	0.562	0.533	1
PLP2	0.275723861	-0.455679484	0.25	0.533	1
MAP3K14	0.275774549	-0.327574658	0.438	0.533	1
RAI14	0.275783019	0.430551641	1	1	1
TDRD1	0.275801329	0.762880293	1	1	1
DISP2	0.275845586	0.15264701	0.938	0.933	1
PHKA1	0.275847084	0.91906531	1	0.667	1
YWHAE	0.276697255	0.297259747	1	1	1
RNF144B	0.276704824	0.426897654	1	1	1
ENSSSCG00000045636	0.276745756	-0.66247505	0.5	0.533	1
PJA2	0.276997838	0.293583469	1	1	1
ENSSSCG00000041432	0.277124506	1.087462841	0.75	0.333	1
IGF2BP2	0.27718135	0.27409957	0.938	0.8	1
ENSSSCG00000063045	0.27721485	-0.108791027	1	0.933	1
ENSSSCG00000015401	0.277228091	0.297266041	1	1	1
MLLT3	0.277262417	0.315793978	1	1	1
TMEM35B	0.277368439	1.076815597	0.812	0.8	1
TOP3A	0.277717289	-0.526068812	0.438	0.533	1
ENSSSCG00000007919	0.277872668	-0.042483331	0.562	0.6	1
IL1R2	0.278299645	0.624608305	0.938	0.933	1
AP3B2	0.278410894	0.428843299	1	0.933	1
ASAP2	0.278470324	1.171718283	1	0.933	1
MCUB	0.278516857	1.0878928	1	1	1
ENSSSCG00000053166	0.278779587	-0.35614381	0.5	0.6	1
MDH2	0.278842136	1.156527853	1	1	1
KCNQ5	0.278871311	1.331388424	0.75	0.333	1
MTR	0.278928534	0.201565081	1	1	1
KLHL28	0.278971172	1.256873421	1	1	1
RLIM	0.279172133	0.189577059	1	1	1
ITGA2	0.279190532	0.805403528	1	1	1
CASP7	0.279319208	0.270669975	1	1	1
ENSSSCG00000060901	0.279327681	1.299208018	0.625	0.333	1
FAM120B	0.279536985	0.860616087	1	1	1
DAXX	0.280165571	1.181253007	1	0.8	1
FDXACB1	0.280368428	-0.275312736	0.438	0.533	1

PLPP2	0.280447641	-0.204140717	0.5	0.667	1
LRP4	0.280453206	0.130946833	0.938	1	1
CHMP7	0.280745417	0.742287465	1	1	1
CHD6	0.280821552	0.428699368	1	1	1
ITPA	0.280830047	0.859193854	0.938	1	1
ENSSSCG00000039473	0.28095802	0.729366381	1	1	1
RC3H2	0.281076532	-0.104514168	0.938	0.933	1
STIP1	0.281100753	0.777625634	1	1	1
PCSK6	0.281164991	1.196397213	0.75	0.467	1
CHD1L	0.281202159	0.412189569	1	1	1
ENSSSCG00000052814	0.281323033	0.33149598	1	1	1
TMEM19	0.281323772	0.052321035	0.875	0.733	1
DOCK8	0.281472572	0.29281969	1	1	1
ME1	0.281545222	0.398205568	1	1	1
CDC42BPA	0.281937911	0.400655306	1	1	1
ENSSSCG00000048959	0.282201369	-0.489038081	0.5	0.667	1
ENSSSCG00000042915	0.282392073	-0.315501826	0.375	0.6	1
ENSSSCG00000018061	0.282392881	0.928451584	1	1	1
DCP2	0.282584302	0.45888605	1	1	1
HHIP	0.28263564	0.791898073	1	1	1
STAT1	0.282652483	0.370616458	1	1	1
MAP3K9	0.282742821	0.396695863	0.75	0.667	1
ST13	0.282859138	0.889995977	1	1	1
PTPN11	0.283278947	0.575896692	1	1	1
DTNA	0.28360545	0.782711184	1	0.933	1
LYRM2	0.283649096	-0.062521085	0.875	0.933	1
SS18L1	0.283770377	1.147898695	0.625	0.333	1
FNIP2	0.284416233	0.28128611	0.812	1	1
HOMER1	0.284557306	-0.018762063	0.938	0.933	1
MAP9	0.284642576	1.043786797	1	1	1
UHRF2	0.284679909	0.542275785	1	1	1
KDELR1	0.284819239	0.651188836	0.938	1	1
MSR1	0.285210057	1.049848549	0.938	0.933	1
CTCF	0.285355723	0.519499344	1	1	1
GNAI2	0.285379681	0.651893509	1	1	1
TTLL1	0.285509951	0.339586761	1	0.933	1
MT1X	0.285734279	0.196397213	1	1	1
ENSSSCG00000062174	0.285760516	0.748785122	1	1	1
ENSSSCG00000063055	0.285832527	0.781359714	0.938	0.467	1
GJB2	0.285991449	0.058893689	0.5	0.533	1
NHSL1	0.286024445	0.755966556	1	1	1
PHF3	0.286300431	0.707766705	1	1	1
ENSSSCG00000061382	0.28644733	-0.093109404	0.5	0.667	1
PHACTR4	0.286643641	0.185191758	1	0.933	1
COPG2	0.287082146	0.950239503	1	1	1
C1orf185	0.287094032	1.491853096	0.5	0.133	1
ARHGAP26	0.287183973	0.366009163	1	1	1
ENSSSCG00000051054	0.287188161	1.282181916	0.875	0.667	1
AKAP17A	0.287241533	0.334687326	1	1	1
ENSSSCG00000008459	0.287441269	0.913744267	0.938	0.867	1
PATZ1	0.287520541	0.269460675	0.562	0.733	1
CRTC3	0.287763655	0.016012318	0.938	0.867	1
LRRC41	0.288159957	1.576741994	0.562	0.333	1
CFAP300	0.288216252	0.857980995	0.938	1	1
ENSSSCG00000053107	0.288339993	0.845490051	0.688	0.267	1
ZNF619	0.288831396	-0.706086281	0.5	0.6	1
INPPL1	0.288838024	1.458300537	0.812	0.733	1
ENSSSCG00000037699	0.288868392	-0.245112498	0.5	0.4	1
SLC39A14	0.289196561	0.45538154	1	1	1
C2orf68	0.289361579	-0.093109404	0.5	0.4	1
TNPO2	0.28941721	0.921277701	0.938	1	1
AVPR1A	0.289459742	-0.817474962	0.562	0.267	1
ENSSSCG00000027301	0.289501684	0.924145626	1	1	1
HDGFL3	0.289607509	0.84395934	1	1	1
GRAMD1B	0.289886546	-0.150824902	0.625	0.8	1
RNF187	0.289950187	0.0138058	0.438	0.533	1
AGBL1	0.290022722	0.309523074	0.938	0.933	1
REXO1	0.290027181	1.176979759	1	0.867	1
GTPBP4	0.290031343	0.40092413	0.938	1	1
ENSSSCG00000058386	0.290062368	1.64385619	0.562	0.533	1

TSSK4	0.290078201	0.212993723	0.875	0.933	1
ENSSSCG00000052824	0.29007964	-0.093109404	0.688	0.667	1
CREBL2	0.290294367	1.198056437	0.938	0.933	1
MRPL4	0.290327844	0.451855028	1	1	1
MSL2	0.290442996	0.365661099	1	1	1
CD83	0.29044769	-0.590609064	0.188	0.533	1
SPATA17	0.291070525	-0.093109404	0.812	0.667	1
ENSSSCG00000045074	0.291084631	-0.12963528	0.562	0.6	1
SPPL3	0.291112838	0.631163776	1	1	1
ENSSSCG00000002937	0.29143356	-0.081925666	0.938	0.867	1
ZNF630	0.29159468	0.787949523	0.875	0.8	1
ENSSSCG00000044342	0.292155352	0.099535674	0.562	0.6	1
SUCO	0.292733165	0.915286517	1	1	1
SMG5	0.292887821	-0.215099929	0.875	1	1
FARP1	0.292909602	0.525349366	1	1	1
ENSSSCG00000042401	0.293098446	-0.623624121	0.625	0.667	1
STIM2	0.293196061	0.22881869	0.875	0.867	1
GTF2A1	0.293419143	0.32214024	1	1	1
ENSSSCG00000003741	0.293453897	1.021710998	0.938	0.867	1
ENSSSCG00000052854	0.29397635	-0.441032708	0.438	0.533	1
BECN1	0.29425058	0.868416448	1	1	1
ARSB	0.294445649	0.338606836	0.875	0.8	1
ATP6V1A	0.294723542	0.389518789	1	1	1
FGL2	0.294745809	0.867719998	0.938	0.933	1
CCNL1	0.294845234	0.367405725	1	1	1
SNRPG	0.294866862	0.737089959	1	1	1
SMOX	0.294922384	-0.363198568	0.5	0.533	1
ENSSSCG00000009399	0.294970021	-0.134332067	0.75	0.733	1
ENSSSCG00000055398	0.295112929	0.027184829	0.625	0.533	1
ENSSSCG00000005643	0.295125139	0.386331128	1	1	1
CDK2	0.295187455	0.254458512	1	1	1
MYD88	0.29522353	1.196397213	0.75	0.6	1
ADCY6	0.295466727	1.186250617	1	1	1
SAAL1	0.29578646	0.348596045	0.875	0.933	1
PITPNM2	0.295895121	-0.167109986	0.438	0.6	1
TADA2B	0.29609508	1.036173613	0.875	0.667	1
BRD4	0.296222053	0.956478605	1	1	1
SMAD5	0.296347389	0.827496454	1	1	1
SLITRK1	0.296575942	1.94641896	0.5	0.267	1
FZD5	0.297052865	0.372265744	1	1	1
FBXO45	0.297112314	0.022367813	1	0.867	1
MYLK3	0.297119427	0.022367813	0.812	0.6	1
PEX14	0.297404756	0.150816178	0.875	0.867	1
TTLL5	0.297479619	1.108297992	1	1	1
CASP2	0.297506333	0.513439959	1	0.933	1
ENSSSCG00000014219	0.297614427	1.442943496	0.562	0.333	1
ENSSSCG00000056190	0.297732599	1.366322214	0.5	0.4	1
PKP4	0.297961268	0.269460675	1	1	1
SCML4	0.297972239	0.780133453	1	1	1
ACADSB	0.298456891	-0.322591251	0.812	0.667	1
FEZ1	0.298495424	0.901199484	1	1	1
ENSSSCG00000046349	0.298701139	0.285402219	0.438	0.533	1
ENSSSCG00000059120	0.298719716	0.345774837	0.938	0.933	1
EPCAM	0.298764215	0.324623882	1	1	1
INPP5D	0.299159151	0.123208503	0.938	0.8	1
QTRT2	0.299431664	1.133166451	1	0.733	1
LRRIQ4	0.299573789	-0.405053411	0.688	0.6	1
PLK4	0.299709641	0.725994476	1	1	1
ENSSSCG00000053762	0.299710802	0.516944077	0.438	0.533	1
KATNAL1	0.29976718	0.31187443	1	0.933	1
ABHD17C	0.299839241	0.462105753	1	1	1
BLTP3B	0.300000167	0.898458689	1	1	1
MACC1	0.300077361	0.878719923	0.938	0.933	1
UBE2Q1	0.300276478	0.309969631	1	1	1
RECQL	0.300369388	0.18758637	0.938	1	1
MOSMO	0.300379373	-0.552541023	0.5	0.4	1
ENSSSCG00000056369	0.300414899	1.33752495	0.5	0.467	1
CMBL	0.300604163	0.890649955	1	1	1
PHETA2	0.300721466	0.062168821	0.75	0.733	1
MOS	0.300873027	0.539087595	1	1	1

ENSSSCG00000009672	0.301119367	0.41612577	1	0.933	1
DOLK	0.30113252	0.931373281	1	1	1
RRN3	0.301146327	0.636213495	1	1	1
ZNF112	0.301370957	0.076815597	0.625	0.6	1
BMP2K	0.301626678	0.852882493	1	1	1
TRIP13	0.301767293	1.074618041	1	1	1
RLBP1	0.302239216	-0.490444902	0.688	0.6	1
GFM1	0.30242208	0.923569337	1	1	1
KBTBD3	0.302777059	-0.165621904	0.938	0.867	1
SYNCRIP	0.30299733	0.90194399	1	1	1
PRPF40A	0.303139849	0.906407793	1	1	1
OTUD7B	0.303301153	0.0138058	0.938	0.867	1
ZNF667	0.30364198	0.459919098	1	1	1
ZNF648	0.303746032	-0.200024608	0.5	0.4	1
KCNH2	0.303872984	1.299208018	0.688	0.533	1
GNB5	0.304167617	0.921733932	1	1	1
ENSSSCG00000058262	0.304307346	1.072008212	1	0.867	1
TMEM214	0.304320122	0.163766209	1	0.933	1
GABRR1	0.304597062	0.967482001	1	1	1
SLITRK3	0.304905469	-0.341036918	0.75	0.867	1
LSM8	0.305022669	0.340075442	1	1	1
SLC33A1	0.305099749	0.993086247	0.938	0.867	1
COPS2	0.305310773	0.148057679	1	1	1
NPNT	0.305441491	-0.256608137	0.562	0.467	1
IARS2	0.305695484	0.954023615	1	1	1
IL13RA1	0.30576602	0.465275845	1	1	1
SAP30L	0.306077193	0.764033837	1	1	1
ENSSSCG00000063185	0.306895864	1.138216142	0.625	0.333	1
SPAG6	0.307054419	0.087462841	0.812	0.8	1
WDR59	0.307112658	0.11042399	1	1	1
BAG6	0.307507088	0.889417289	1	1	1
FHIP1B	0.307762577	0.279802619	1	1	1
ENSSSCG00000063467	0.307819845	0.696714124	1	1	1
CDH13	0.308427551	0.429763092	1	1	1
GDNF	0.308481647	-0.093109404	0.688	0.733	1
DENND1B	0.308858377	0.101063143	1	1	1
HSPB8	0.308928144	1.327466278	0.938	0.867	1
PDCL	0.309043576	0.420468463	1	1	1
ABHD11	0.309212199	0.142778859	1	0.933	1
ENSSSCG00000040688	0.309261843	0.775061943	1	1	1
THAP1	0.309305897	0.406637094	1	1	1
SLC30A1	0.309701621	0.79058171	1	1	1
PPP2R5D	0.309769083	0.436143664	1	1	1
TOR1AIP1	0.309985423	0.918712304	1	1	1
ENSSSCG00000053805	0.31010221	-0.186218809	0.5	0.533	1
AZI2	0.310459266	0.328717261	0.938	0.933	1
PDPK1	0.310495448	0.413953337	1	1	1
ENSSSCG00000044256	0.310566186	0.141355849	0.688	0.733	1
RTF1	0.310944288	0.25402403	1	1	1
MTF1	0.310951697	0.247304261	1	1	1
CREB3L2	0.311111953	0.878656915	1	0.933	1
EIF4ENIF1	0.31145534	0.873345396	1	1	1
DSP	0.31158528	1.150619479	0.938	0.933	1
GPATCH2	0.311683389	0.449683642	0.938	1	1
ENSSSCG00000055825	0.311895074	-0.397963986	0.625	0.533	1
TMF1	0.312066984	0.873925832	1	1	1
DLGAP5	0.31241498	0.528867939	1	1	1
PLEKHM3	0.312621215	0.407544877	1	1	1
WDR45	0.312673214	0.777677802	1	1	1
ENSSSCG00000050930	0.312749597	1.154818109	0.688	0.467	1
AGPAT1	0.312985764	1.0138058	1	0.733	1
MTPAP	0.313058339	0.750040936	1	1	1
HEBP1	0.313106186	1.22881869	0.5	0.267	1
FHOD3	0.313396889	1.113935053	0.938	0.733	1
WDFY1	0.313487425	0.243864985	1	1	1
MDN1	0.313508243	0.372924949	1	1	1
PHAF1	0.313563208	0.997887708	0.938	1	1
CRACDL	0.313602824	-0.493647334	0.5	0.533	1
HAPSTR1	0.314066285	0.284064532	1	1	1
SMC2	0.314141109	0.78696344	1	1	1

ENSSSCG00000052455	0.314225279	1.254813899	0.562	0.4	1
CLK2	0.314493315	0.978443857	1	1	1
SPPL2B	0.314503656	0.308730793	1	0.933	1
ENSSSCG00000058381	0.314752006	-0.032567862	0.625	0.867	1
ENSSSCG00000054974	0.315381845	-0.35614381	0.5	0.4	1
DHX33	0.315396035	-0.021674266	1	0.867	1
TMTC2	0.315705291	0.948274516	1	1	1
SH3KBP1	0.315776017	0.914204655	1	1	1
ZBED5	0.315879541	0.093511886	0.938	1	1
RANBP6	0.315921107	1.181065559	0.875	0.667	1
ABTB3	0.316032426	0.261650083	1	1	1
ENSSSCG00000042640	0.316201241	1.108524457	0.938	0.8	1
ENSSSCG00000035362	0.316260626	0.34298971	0.625	0.867	1
LDHB	0.316456117	1.26052755	1	0.667	1
ZNF791	0.316758601	-0.332297068	0.812	0.6	1
URB2	0.316893552	0.91675634	1	1	1
CAPN13	0.316981374	0.47217073	1	1	1
PIAS4	0.31707595	1.103963386	1	1	1
CDK13	0.317137458	0.347039873	1	1	1
FASTKD3	0.317191327	0.051280505	0.75	0.8	1
CAB39L	0.31792647	0.847389584	1	1	1
COL15A1	0.317946852	0.913616459	0.812	0.867	1
DSTYK	0.318016115	-0.32443495	0.562	0.533	1
SIX5	0.318366477	1.569855608	0.562	0.333	1
PSMF1	0.318481852	0.168430755	0.938	0.867	1
PDGFRA	0.318543624	-0.037614292	0.875	0.6	1
YARS2	0.319093258	1.041090867	1	0.8	1
THRAP3	0.319109678	0.923764414	1	1	1
ENSSSCG00000060599	0.320257036	-0.346865997	0.5	0.6	1
AP3M2	0.3203323	0.234944793	0.938	1	1
TIMELESS	0.320739774	0.803990688	0.938	1	1
BMT2	0.32074761	1.048551745	0.938	0.8	1
SLTM	0.320792569	0.455863239	1	1	1
POLR1F	0.321226553	0.25100131	1	1	1
PARD3	0.321253476	0.974209705	1	1	1
COL24A1	0.321838011	0.764150423	1	1	1
CADM2	0.321875025	1.401655287	0.688	0.333	1
BMF	0.321915653	0.334752135	1	1	1
CENPC	0.321970223	0.746865361	1	1	1
SLC9A9	0.322526223	0.418536419	0.938	1	1
VPS13D	0.323027347	0.881712033	1	1	1
INTS5	0.323096791	1.129283017	0.562	0.333	1
PIK3R4	0.323298143	0.748157717	1	1	1
ENSSSCG00000031883	0.323318485	1.183730801	0.812	0.6	1
AKT2	0.323592772	0.47436567	1	1	1
ENSSSCG00000061502	0.32366712	1.053731984	0.688	0.267	1
OTULIN	0.323697489	0.410028873	1	1	1
TOPORS	0.323786451	0.833796372	1	1	1
MTIF2	0.323872231	-0.180572246	0.75	0.6	1
FAM117A	0.323888783	0.284257677	0.938	1	1
SCMH1	0.324312871	1.100901216	0.938	0.933	1
ENSSSCG00000030335	0.324464559	0.767673657	1	1	1
ENSSSCG00000059464	0.324487124	0.070389328	0.688	0.667	1
ZFP64	0.324616906	0.038135129	0.875	0.667	1
KBTBD2	0.324704761	0.830718915	0.938	1	1
ENSSSCG00000010278	0.324814929	0.312147074	0.938	1	1
TP53BP2	0.324916037	1.043688817	1	0.933	1
SRSF2	0.325284457	1.189824559	0.938	0.733	1
ENSSSCG00000061969	0.325296095	1.347463187	0.562	0.467	1
TUFT1	0.32557816	0.395006093	1	1	1
EPC1	0.325754346	0.881279952	1	1	1
GZF1	0.325818197	-0.019108823	0.5	0.667	1
FAM120C	0.325838977	-0.15298086	0.875	0.933	1
IFFO2	0.325896042	0.121015401	0.812	0.867	1
LHFPL3	0.325944435	1.006426269	0.75	0.8	1
NAV2	0.326128306	0.884067825	1	1	1
LMLN	0.326295846	0.123701985	0.875	0.867	1
METTL22	0.326545421	0.168665556	1	0.933	1
RSF1	0.326727494	1.001012773	0.938	0.933	1
UQCRC2	0.326802273	-0.054635257	0.812	0.8	1

B4GALNT1	0.326917635	-0.064540252	0.688	0.733	1
STON2	0.327062453	0.443376023	1	1	1
FGFR1	0.327104801	0.906890596	1	0.733	1
ENSSSCG00000053970	0.327144052	0.413075983	0.938	1	1
PITPNB	0.327204092	0.438002699	1	1	1
ZBTB33	0.327205552	0.759167529	1	1	1
COQ7	0.327497209	0.215143754	1	1	1
STAU2	0.327511828	0.837181624	1	0.933	1
PDIA6	0.327638933	1.154464897	1	1	1
HRG	0.327803932	-0.072645302	0.75	0.867	1
KIF14	0.327817256	0.939123103	1	1	1
ENSSSCG00000039071	0.327868257	1.435819061	0.875	0.733	1
LETM1	0.327885866	0.344099476	1	0.933	1
CAPN14	0.328116515	-0.483055923	0.75	0.6	1
ENSSSCG00000024411	0.328145075	0.893215659	1	0.933	1
UBE3C	0.328241873	1.202346479	0.5	0.4	1
YTHDC2	0.328408733	0.984166309	1	1	1
SAMD4B	0.328765974	0.505242474	1	1	1
PATJ	0.329093237	0.759761164	1	1	1
ENSSSCG00000055520	0.329535245	0.030273011	0.75	0.733	1
DYNLT1	0.329571747	0.232405999	1	1	1
BMP4	0.329703022	0.5585134	1	1	1
C20orf27	0.329998607	0.412125904	1	0.933	1
TLCD4	0.330148505	0.343697996	1	1	1
N4BP2L1	0.330229164	0.193771743	1	1	1
DDIAS	0.330408787	0.522628475	1	1	1
ENSSSCG00000055646	0.33046609	1.321928095	0.625	0.533	1
ENSSSCG00000028810	0.330731924	0.091315167	0.688	0.533	1
PLPPR2	0.330822385	0.491853096	0.875	0.933	1
CWC27	0.331057682	0.704654371	1	1	1
ENSSSCG00000003791	0.331153215	0.79733067	1	1	1
ZNF662	0.331323024	0.342029301	1	1	1
ENSSSCG00000051810	0.331361582	0.850307067	0.938	0.8	1
KCTD6	0.331752179	-0.093109404	0.875	0.733	1
ANAPC5	0.331813056	1.271586976	1	0.933	1
ENSSSCG00000014186	0.332030086	0.032421478	0.625	0.667	1
ENSSSCG00000011489	0.332531776	1.046053343	0.938	0.733	1
ZKSCAN2	0.332664562	-0.321378392	0.75	0.733	1
ENSSSCG00000059700	0.332715776	1.186998515	0.812	0.8	1
ENSSSCG00000053210	0.332752084	0.141355849	0.562	0.733	1
USP43	0.332813137	1.115477217	0.938	0.867	1
NLE1	0.333261305	0.153530563	0.875	0.867	1
ENSSSCG00000032483	0.333464509	1.124695747	0.875	1	1
USP10	0.333713104	0.922312782	1	1	1
FUCA2	0.333852274	0.300035489	1	0.933	1
ENSSSCG00000055963	0.333934013	0.846769603	0.938	0.933	1
ENSSSCG00000049656	0.334567707	0.447023315	1	1	1
TSPAN2	0.334733431	0.932686596	1	1	1
ASTE1	0.334891979	0.360608563	0.812	0.933	1
ZNF177	0.335118984	-0.163498732	0.625	0.733	1
ENSSSCG00000035223	0.335225678	-0.180572246	0.5	0.4	1
CRELD2	0.335245953	1.146124006	1	1	1
EPRS1	0.335354828	0.419564236	1	1	1
ENSSSCG00000031706	0.335487769	0.289772857	1	1	1
ITGBL1	0.33556332	0.512611656	0.812	0.467	1
MBD1	0.335855659	1.099535674	1	0.933	1
LRP1B	0.335926932	0.976003767	1	0.933	1
RIMS2	0.335933447	0.455390646	1	1	1
FBXL5	0.336114056	0.617863222	1	1	1
ADAM29	0.336136374	-0.446746359	0.5	0.4	1
ITGB1	0.336277203	0.262071696	1	1	1
P4HA1	0.336828777	0.52968517	1	1	1
DUSP3	0.336895927	0.696967526	1	1	1
MTUS1	0.336904725	0.71052704	1	1	1
ACSL3	0.337127139	0.700798448	1	1	1
NME6	0.337490061	0.162147651	0.875	0.933	1
RMND1	0.337638824	1.134996668	1	1	1
ENSSSCG00000056149	0.337714418	1.106199404	0.875	0.867	1
ENSSSCG00000047137	0.337900003	-0.042483331	0.625	0.467	1
GPR180	0.338002856	0.922157352	1	0.867	1

UBE2T	0.338003099	0.4606772	1	1	1
TRPM3	0.338159665	0.675697456	1	1	1
ENC1	0.338229336	0.171075017	1	0.933	1
CRMP1	0.338255983	1.076815597	0.875	0.667	1
ENSSSCG00000043804	0.338341742	0.087462841	0.5	0.667	1
ENSSSCG00000056589	0.338674541	-0.526068812	0.438	0.6	1
GLYATL3	0.338785592	0.780537196	1	1	1
SLC15A4	0.338865451	1.023614202	1	0.933	1
TPGS1	0.33910096	-0.180572246	0.562	0.533	1
ENSSSCG00000026045	0.339320997	0.759628105	0.938	1	1
ENSSSCG00000062614	0.339336875	0.823232667	0.938	0.933	1
HEXB	0.33934963	0.445088629	1	0.933	1
ITGB6	0.339718569	0.906890596	0.562	0.2	1
FAM234B	0.340158884	-0.093109404	0.5	0.333	1
C2	0.34051464	0.172624151	1	0.933	1
ENSSSCG00000056226	0.340530528	0.25354112	0.938	0.933	1
FUBP3	0.340794248	0.984893108	0.875	0.6	1
PKMYT1	0.341048917	1.335733894	0.688	0.467	1
CHRDL1	0.341442252	0.238096504	0.75	0.733	1
PALM2AKAP2	0.341537091	0.285923141	0.938	1	1
RHOU	0.341728698	0.083768358	0.938	1	1
ENSSSCG00000045748	0.342194678	1.714245518	0.5	0.133	1
CEP104	0.342251914	0.983616386	1	0.8	1
ENSSSCG00000042530	0.342279823	0.34298971	0.625	0.667	1
ENSSSCG00000056152	0.342568488	1.398743692	0.688	0.533	1
ZBED6	0.342910057	-0.069650432	0.812	0.733	1
ENSSSCG00000007004	0.343156592	0.878350182	1	1	1
ZSWIM4	0.343324158	0.963474124	0.938	0.6	1
MTA1	0.343688196	-0.093109404	0.375	0.533	1
NONO	0.343762532	0.182806293	1	1	1
INTS6	0.344150424	0.909843913	1	0.933	1
SPTSSA	0.344476877	0.454953514	1	0.8	1
TDRD3	0.344733029	0.185426095	0.875	0.933	1
NFRKB	0.345082243	0.971407937	1	1	1
ATF7IP	0.345115733	0.842660061	1	1	1
KMO	0.345123978	1.206450877	0.688	0.6	1
GSDMA	0.345723854	-0.415037499	0.312	0.533	1
ZNF550	0.345898296	-0.192645078	0.562	0.533	1
ARHGEF10L	0.345990079	0.145050333	0.812	0.6	1
ZMYM4	0.346013792	0.187490874	0.938	1	1
ANKRD16	0.346349952	1.196397213	0.875	0.733	1
TRIM71	0.346422792	0.31046898	1	0.933	1
NEDD9	0.346544848	0.084982918	0.938	0.867	1
VDAC1	0.346740253	1.110685798	1	1	1
ENSSSCG00000038757	0.347136233	0.735812821	1	0.933	1
MINAR1	0.34716615	1.861086906	0.562	0.267	1
ENSSSCG00000048697	0.347405963	-0.140415119	0.562	0.733	1
DCT	0.347569653	0.900662485	1	1	1
ENSSSCG00000032353	0.347795473	1.273672926	0.812	0.533	1
KIF3B	0.348029949	-0.03196911	1	1	1
ENSSSCG00000036234	0.348163954	0.64385619	1	0.933	1
ENSSSCG00000062901	0.348550069	-0.373217324	0.5	0.333	1
CDC20B	0.348633298	0.445663463	0.812	0.867	1
PTH2R	0.348652708	-0.590609064	0.625	0.4	1
NTNG1	0.348857174	1.065588342	0.938	0.733	1
AP2B1	0.349542821	0.872412395	1	1	1
SLC35A3	0.349686922	0.758188865	1	0.933	1
TNFRSF1B	0.350008557	1.491853096	0.562	0.2	1
CPD	0.350049255	0.788702672	1	1	1
VASH2	0.350093336	0.569855608	0.938	0.8	1
LMF2	0.350490629	-0.196202897	0.5	0.733	1
TNNI3K	0.350702195	0.427722759	0.438	0.667	1
SLC34A1	0.350943917	0.051280505	0.5	0.533	1
RAD23B	0.351143921	1.100062878	1	1	1
TRMT1L	0.351153481	0.853064743	1	1	1
SUCLA2	0.351159824	0.526033943	1	1	1
UBAP1	0.351311458	0.445511081	1	1	1
HIF1A	0.351752601	0.846902594	1	1	1
LRRIQ1	0.35199449	1.106199404	0.938	0.533	1
ENSSSCG00000048161	0.352199555	0.409390936	0.688	0.8	1

CHST11	0.352306345	0.123701985	0.938	1	1
ENSSSCG00000059156	0.352344403	0.269460675	0.562	0.4	1
B3GALNT1	0.352461083	0.390017085	1	1	1
FCHSD2	0.35257296	0.190423312	1	0.933	1
TIPRL	0.352635576	0.357142787	1	1	1
ABHD4	0.352822414	0.466370049	1	1	1
DGKI	0.352838313	0.914100481	1	1	1
DYDC2	0.353131616	0.126456973	0.875	0.8	1
PELI2	0.353311561	0.193771743	0.875	0.933	1
ENSSSCG00000059909	0.353386353	1.033117054	1	1	1
OIP5	0.35357007	0.784998291	1	1	1
NET1	0.353650849	0.276399755	1	1	1
ACKR2	0.354167155	-0.08152143	0.938	0.933	1
ENSSSCG00000062401	0.354250831	0.83336156	1	0.933	1
BRI3BP	0.354285091	0.726248904	1	1	1
ENSSSCG00000061431	0.354590249	-0.167109986	0.5	0.4	1
ENSSSCG00000002935	0.354675776	0.705256734	1	1	1
EPG5	0.354916012	0.240846787	0.938	0.933	1
F2	0.355118254	1.046723532	1	1	1
FASLG	0.355132618	0.214072105	0.938	0.667	1
YEATS2	0.355965326	0.783407542	1	1	1
THAP12	0.356560843	0.150816178	0.75	0.733	1
FUT10	0.356706216	1.321928095	0.5	0.333	1
DCBLD1	0.356813837	1.018083583	1	1	1
POLH	0.356871543	0.424922088	0.938	0.933	1
XPO5	0.356893177	0.304515563	1	1	1
LRRK2	0.35711136	0.832890014	1	0.933	1
HSPA9	0.357214113	0.898614467	1	1	1
ENSSSCG00000041471	0.357767025	1.062168821	0.812	0.867	1
ENSSSCG00000054707	0.358064403	1.0138058	0.812	0.6	1
UBE2A	0.358090886	0.809775396	1	1	1
ABCE1	0.35813538	0.856791984	1	1	1
SYNJ2BP	0.358437264	1.039176795	1	1	1
HSPB11	0.358526541	1.321928095	0.75	0.533	1
ENSSSCG00000051601	0.35872325	0.810814624	1	1	1
MMGT1	0.358791298	0.935255487	1	1	1
TRPM6	0.358851735	0.655828831	0.938	1	1
PPP1R13L	0.359106978	1.202346479	0.75	0.4	1
CDK16	0.359256376	0.283631575	1	0.933	1
ENSSSCG00000015823	0.359503139	0.768156103	1	1	1
FBLN2	0.359528044	0.842760258	0.875	0.8	1
ANKRD52	0.359537451	0.800360675	1	1	1
HECTD4	0.359600397	0.443625984	1	1	1
IGFBP3	0.359602251	1.594946589	0.562	0.267	1
ALDH1L2	0.359651674	0.655351829	1	0.933	1
PPWD1	0.359736312	0.848278435	1	1	1
HAVCR2	0.359828559	-0.285754482	0.875	0.733	1
MYO18B	0.35992481	1.246740598	0.812	0.8	1
ENSSSCG00000047145	0.360078173	0.578468739	1	1	1
MAP3K11	0.360111837	0.022367813	0.5	0.6	1
CENPU	0.360167595	0.393413279	1	1	1
ENSSSCG00000057494	0.360397109	-0.208586622	0.688	0.667	1
CTBP2	0.360562027	1.057777052	1	1	1
FMN1	0.360601191	0.233239618	1	1	1
PHF8	0.360665934	0.799541259	1	1	1
PGGT1B	0.361018492	0.383643879	1	1	1
ACTB	0.361439063	0.421463768	1	0.933	1
COQ2	0.362008037	0.533212126	1	1	1
ZCRB1	0.362086317	0.865785392	0.875	0.8	1
PPA1	0.362117966	0.918374299	1	1	1
MRPS22	0.362190394	0.3819102	1	1	1
CCT2	0.3622233	0.416933361	1	1	1
FLT4	0.362323681	0.26052755	0.938	1	1
BBS12	0.3625601	-0.925381959	0.5	0.467	1
ENSSSCG00000030800	0.362928472	0.717066037	1	0.733	1
FUCA1	0.363029075	0.987858661	1	1	1
KIF24	0.363179993	0.439001214	1	1	1
ENSSSCG00000055922	0.363197611	0.129283017	0.688	0.8	1
CPSF7	0.363382236	-0.560235415	0.438	0.8	1
RPRD1B	0.363445552	0.114695481	0.688	0.867	1

DSCAM	0.363575292	0.762500686	0.688	0.4	1
MZT1	0.363650857	0.781849011	1	1	1
EHMT1	0.364063226	0.870862286	1	0.933	1
CYB561A3	0.364210479	0.941346669	1	1	1
OSBPL10	0.364697464	0.657883894	1	1	1
CCSER1	0.364763823	0.738480235	1	1	1
ATXN7L1	0.365099161	0.60225139	1	1	1
PTOV1	0.365122478	1.117945473	1	0.933	1
ENSSSCG00000042273	0.365170722	0.054989235	0.75	0.733	1
ENSSSCG00000036685	0.365342663	-0.022720077	0.562	0.6	1
TYK2	0.365677938	-0.042483331	0.75	0.867	1
POLE	0.365810833	-0.043121269	0.875	1	1
CHD8	0.366140852	0.754322957	0.938	0.933	1
BRINP2	0.366198067	0.468006354	1	1	1
TMEM108	0.366295702	0.774135387	1	1	1
RBM10	0.36647435	0.813781191	0.875	0.733	1
TRIM8	0.36647601	0.842760258	0.938	1	1
ENSSSCG00000049674	0.366580017	1.02888112	0.688	0.733	1
CHMP5	0.366677275	0.938344745	1	1	1
KLF7	0.367117436	0.332766075	1	1	1
TRPS1	0.367167367	0.758791957	0.875	0.6	1
NPHS1	0.367207266	-0.093109404	0.5	0.4	1
ENSSSCG00000062186	0.367284219	0.361456459	0.562	0.6	1
CDC5L	0.36729102	0.402047138	1	1	1
DLGAP2	0.367589343	-0.093109404	0.5	0.6	1
CELSR2	0.367645001	0.089093927	0.688	0.8	1
OSTC	0.367931845	0.152153617	1	1	1
CKAP2L	0.367972344	0.268603204	1	1	1
FAM219A	0.368032602	0.52160044	0.75	0.867	1
ENSSSCG00000044910	0.368134262	0.413236796	1	1	1
DDX50	0.368137114	0.442943496	1	1	1
DERPC	0.368368194	0.176979759	0.812	0.6	1
ENSSSCG00000059270	0.36839058	-0.9861942	0.5	0.467	1
TTBK2	0.368652768	1.162323279	1	0.933	1
ZCCHC3	0.368748908	-0.206766186	0.75	0.867	1
ENSSSCG00000037996	0.368877761	1.017921908	0.812	0.467	1
STRA6	0.368897015	0.208546295	0.812	0.8	1
H1-3	0.369044003	0.129283017	0.75	0.867	1
EDC4	0.369267006	0.81721855	1	1	1
ZNF10	0.369376634	0.240791332	0.938	0.933	1
MSH5	0.370078084	1.206876138	0.938	0.867	1
GRIPAP1	0.371027723	0.316003261	0.875	0.8	1
EPAS1	0.371183937	0.805961687	1	1	1
PPP1R35	0.371237926	0.289834465	1	1	1
RUNDC3B	0.371669227	0.824165673	1	1	1
ARSK	0.372295119	0.883806982	0.938	0.8	1
XRN1	0.37239821	0.96144133	1	1	1
ABCG8	0.372401267	-0.093109404	0.688	0.533	1
ENSSSCG00000055840	0.372576823	0.530922661	0.812	0.667	1
CISH	0.37296398	-0.285754482	0.562	0.533	1
ENSSSCG00000009125	0.373000434	0.461819211	1	1	1
ATP8B4	0.373150574	0.795607262	1	1	1
NR4A2	0.373171878	1.525800428	0.562	0.4	1
ENSSSCG00000045408	0.373389226	0.176077228	0.875	0.8	1
GFI1B	0.373432798	0.994353437	0.688	0.533	1
ENSSSCG00000059736	0.374375153	-0.397963986	0.562	0.533	1
FAM83C	0.37455346	0.87036472	0.75	0.333	1
ENSSSCG00000011676	0.374566922	0.901951063	1	1	1
COL2A1	0.374610453	0.301169535	1	0.867	1
FAM167A	0.374844874	0.360608563	0.75	0.867	1
GANC	0.374931585	0.882368276	0.938	0.867	1
TUBG1	0.374993039	1.031948075	1	0.933	1
YWHAB	0.375713006	0.885603745	1	1	1
DNAH11	0.375825039	0.383486207	1	1	1
EGLN3	0.376426349	0.355390854	1	1	1
ENSSSCG00000061675	0.377343316	1.069162025	0.875	0.667	1
ENSSSCG00000055262	0.377372051	-0.540568381	0.5	0.467	1
STAT2	0.377434797	0.218834602	1	1	1
INSIG1	0.377510078	0.977973694	1	1	1
ENSSSCG00000063364	0.377722528	1.118394701	0.938	0.867	1

TTI2	0.377879841	0.276671814	1	1	1
CCNJ	0.377892945	0.777782993	1	1	1
MRPS31	0.378006029	0.785430778	1	1	1
ENSSSCG00000052954	0.378207546	0.041191687	0.938	0.867	1
BIRC2	0.378331554	0.90456179	1	1	1
RPL37	0.378535286	-0.185969013	1	1	1
MNS1	0.378843756	0.929988423	1	1	1
URB1	0.37885876	1.672425342	0.625	0.467	1
FUT11	0.379191828	0.945364743	0.938	0.8	1
CENPL	0.379615922	0.484486127	1	1	1
IL12RB2	0.379725876	1.331388424	0.562	0.4	1
HGS	0.379752955	0.274261661	0.938	0.933	1
CHCHD2	0.38009765	0.440480995	1	1	1
TICAM1	0.380317396	1.392317423	0.938	0.6	1
TCAF1	0.380574392	0.673218249	1	0.933	1
HDDC3	0.380838949	1.491853096	0.625	0.467	1
PPP1R15A	0.381122342	1.179262866	1	1	1
PCMTD2	0.381214251	0.478410513	1	1	1
GAK	0.381391362	0.175142711	1	1	1
PAM	0.381476662	1.49833713	1	0.733	1
SOCS1	0.381506547	0.820475844	1	0.8	1
BLTP2	0.381649376	0.279510654	0.938	0.933	1
UBE2S	0.382077919	1.111822047	0.938	0.933	1
ATP8B1	0.382253754	0.948331398	0.938	1	1
ENSSSCG00000004287	0.382596826	0.779384183	1	1	1
PAPPA2	0.382708198	0.295730016	1	1	1
FLVCR1	0.382834845	1.19865672	0.812	0.6	1
ENSSSCG00000038187	0.382889236	-0.071083098	0.875	0.6	1
PAX9	0.382895092	1.202346479	0.875	0.867	1
ENSSSCG00000059653	0.383102427	0.301422439	0.938	1	1
ATXN3	0.383118301	0.819844497	1	1	1
OSBPL1A	0.383626191	0.791738859	1	1	1
DNAJB9	0.384001562	0.354545046	1	1	1
ENSSSCG00000037332	0.384031403	0.083768358	1	1	1
FAM133A	0.384279712	1.063009798	0.812	0.6	1
DYNC1I1	0.384805174	0.414752128	1	1	1
RIBC2	0.385130377	0.489133595	0.875	1	1
ENSSSCG00000041773	0.385159195	0.200621799	0.688	0.733	1
ZNF605	0.385182328	0.973110282	1	1	1
STXBP1	0.385202179	0.112671076	1	0.933	1
UBFD1	0.385536208	0.962017021	1	1	1
ENSSSCG00000048686	0.386076626	0.038994132	0.938	0.8	1
DIAPH3	0.38649005	0.823635092	1	1	1
NFYA	0.387303536	0.871719817	1	1	1
DNAJC17	0.387330783	0.928029363	1	1	1
ENSSSCG00000047853	0.38810005	1.139551352	0.75	0.667	1
UBN2	0.388902872	0.474880018	1	1	1
MRI1	0.38898151	0.955800196	0.938	0.6	1
ENSSSCG00000041180	0.389218423	0.696535264	1	1	1
BACH2	0.389604118	0.021061616	1	0.867	1
TRPC4	0.389631832	0.609599884	1	1	1
PUM1	0.390010284	1.012759231	1	1	1
KIAA0319L	0.390094577	0.829147612	1	1	1
ENSSSCG00000053589	0.390770845	-0.136178126	0.625	0.6	1
ALYREF	0.390882251	0.067355268	0.75	0.867	1
TMOD2	0.391253752	0.733775907	1	1	1
ENSSSCG00000044577	0.39143649	0.251106758	1	1	1
RELB	0.391493615	1.617383978	0.75	0.4	1
ENSSSCG00000038188	0.391527581	0.097412502	0.938	0.8	1
ACTN4	0.391636735	1.188303531	1	1	1
PTGES3L	0.391645917	0.125070766	0.75	0.867	1
CTBP1	0.391923639	1.354349573	0.625	0.267	1
TSEN15	0.392016236	0.756119187	1	1	1
FAM131B	0.392093864	-0.093109404	0.5	0.333	1
UAP1	0.392190533	0.652573407	0.938	0.8	1
HTATSF1	0.392574545	0.376375879	1	0.933	1
PSMB3	0.39261118	0.09330372	0.875	1	1
ENSSSCG00000059580	0.393085212	0.247927513	1	1	1
SMPDL3A	0.393502146	1.28771238	0.812	0.667	1
DST	0.393647914	0.475197933	1	1	1

HSD17B6	0.393703402	1.531381461	0.688	0.467	1
ENSSSCG00000055079	0.393821769	1.006426269	0.562	0.4	1
PRKAB1	0.394539837	1.036242656	1	1	1
ENSSSCG00000053714	0.395102749	0.868416448	0.875	0.733	1
ENSSSCG00000026529	0.395201443	-0.093109404	0.688	0.667	1
ENSSSCG00000061829	0.396122703	0.740351409	0.938	0.933	1
PRDX6	0.39614732	0.319934045	1	0.933	1
HVCN1	0.396317414	0.362401845	1	1	1
MYO1C	0.396752762	0.267888255	1	1	1
NIM1K	0.396870806	0.7508994	1	1	1
MDGA2	0.397061786	0.391469028	1	1	1
ENSSSCG00000061357	0.39727748	1.06608919	0.812	0.933	1
NT5C2	0.397443793	0.850118114	1	1	1
MATN2	0.397539214	0.009984089	0.5	0.467	1
ENSSSCG00000061672	0.39776516	0.152003093	0.5	0.4	1
TUB	0.397990183	0.299208018	0.625	0.467	1
GLRA2	0.398080861	-0.373217324	0.5	0.467	1
ENSSSCG00000061319	0.398189205	0.22881869	0.625	0.667	1
FAM91A1	0.398229585	0.380821784	1	0.667	1
IZUMO1R	0.398371384	1.154818109	0.938	0.867	1
OR10A4	0.398539777	1.169925001	0.75	0.2	1
RNASE4	0.398991248	1.335733894	0.688	0.333	1
EXOSC10	0.399049718	0.049848549	0.75	0.733	1
GATAD2A	0.399131617	0.502500341	1	1	1
FZD3	0.399182548	0.574468242	1	1	1
POP1	0.399319569	0.785584299	0.938	0.733	1
FANCF	0.399360523	0.888178335	1	1	1
ENSSSCG00000058981	0.399411667	0.080657663	0.938	0.733	1
ENSSSCG00000053175	0.399510011	-0.082138287	0.938	0.8	1
FAF2	0.399571567	0.857980995	1	1	1
RPN1	0.399598844	0.858372659	1	1	1
CSNK1A1	0.399878124	0.553998038	1	1	1
TBC1D30	0.399909223	0.108818914	0.938	1	1
ENSSSCG00000050566	0.400075413	0.083768358	0.562	0.6	1
ENSSSCG00000046897	0.400119015	1.003105911	0.688	0.467	1
PPP1R3D	0.400167653	1.144958086	0.938	0.933	1
BCL2L11	0.400637705	-0.138913094	0.625	0.4	1
PTK2	0.401455893	0.904484098	1	1	1
CCDC112	0.40155891	0.980891177	0.688	0.6	1
INTS15	0.401674215	1.233700912	1	1	1
ODAD2	0.401877958	0.302510641	1	0.933	1
ENSSSCG00000052080	0.401952566	-0.294743266	0.562	0.6	1
ENSSSCG00000012832	0.402107712	1.714245518	0.5	0.4	1
FST	0.40245818	1.049413649	1	1	1
ENSSSCG00000047367	0.402771042	0.584962501	0.938	0.667	1
SEC31A	0.402882167	0.90265609	1	1	1
ENSSSCG00000052019	0.402998235	1.22881869	0.562	0.333	1
FMR1	0.403198764	0.756731341	1	1	1
FARSA	0.404042295	0.87389232	1	0.933	1
ALOX12	0.404270727	-0.175571565	0.562	0.333	1
USP19	0.404289198	0.894447533	1	1	1
SNX4	0.404539492	0.945304479	1	1	1
CLN3	0.404544497	0.539158811	0.938	1	1
ENSSSCG00000060014	0.405008917	1.186998515	0.5	0.267	1
IKZF2	0.405233028	0.189824559	0.875	0.8	1
UPF3A	0.405469968	0.547532869	1	1	1
STYX	0.405561488	0.946857003	1	0.933	1
PDXDC1	0.405830723	1.273672926	0.812	0.8	1
RAB27A	0.405873061	0.520299784	1	1	1
ENSSSCG00000040690	0.405874717	0.64385619	0.375	0.6	1
ENSSSCG00000040742	0.406447178	1.036173613	0.688	0.467	1
MTX2	0.406466091	0.952199757	1	1	1
ARIH2	0.406870528	0.362763009	1	1	1
MYO18A	0.407043467	0.358269942	1	0.933	1
ENSSSCG00000043175	0.407722373	-0.1602236	0.562	0.533	1
GID4	0.408010678	1.252026082	0.938	0.8	1
ENSSSCG00000011531	0.408055929	-0.093109404	0.5	0.533	1
ENSSSCG00000041123	0.408091934	0.017921908	0.5	0.467	1
SH3RF1	0.408136379	0.906890596	0.875	1	1
HNRNPH2	0.408268488	0.289971328	1	1	1

DIAPH1	0.408468908	0.924612366	1	1	1
MIDEAS	0.408581272	0.854423176	0.938	0.933	1
BMAL2	0.408894631	0.39702955	0.938	1	1
RELCH	0.409077812	0.429412522	0.938	0.933	1
CEP85L	0.409275654	0.454739923	1	1	1
ENSSSCG00000032301	0.409405549	1.104829973	0.938	0.667	1
TRIM35	0.409426011	1.305439972	0.562	0.267	1
GREM1	0.409731113	0.308363367	0.938	0.933	1
ENSSSCG00000057851	0.409839619	0.518325308	0.625	0.8	1
ENAH	0.410219054	0.369990336	1	1	1
ENSSSCG00000007645	0.410250569	0.022367813	0.625	0.467	1
TRPV5	0.410510923	1.26052755	0.562	0.2	1
SNW1	0.410681271	0.455063066	1	1	1
TAT	0.410920682	-0.061948473	0.938	1	1
DHX15	0.411107439	1.071587082	1	0.933	1
ZFPM2	0.411237429	0.26689873	1	1	1
PTPN14	0.411628706	0.576655937	1	1	1
STK24	0.411719352	0.669076769	1	1	1
ST3GAL6	0.411748312	0.787151351	1	1	1
LFNG	0.411959799	0.22881869	0.812	0.6	1
H2BC18	0.412218432	-0.334117504	0.5	0.6	1
ENSSSCG00000062858	0.412441893	0.553300037	1	1	1
ZC3HC1	0.41254911	0.750378573	1	1	1
TMEM116	0.412598773	1.129283017	0.688	0.467	1
FNDC9	0.413000875	1.194467186	0.875	0.533	1
KCNH7	0.413124384	0.521471863	1	1	1
MED15	0.41339073	0.944770479	1	1	1
ENSSSCG00000038275	0.413393805	0.09330372	0.812	0.667	1
ENSSSCG00000024459	0.413581485	0.376375879	0.812	0.667	1
DNAAF11	0.413654443	-0.005646563	0.5	0.4	1
HIVEP1	0.413828455	0.434519921	0.938	0.8	1
MLKL	0.413881148	0.612386762	0.938	1	1
LYSMD4	0.413884092	0.346422657	1	1	1
IFT74	0.414465841	0.646898247	1	1	1
DEPDC7	0.414551663	0.321235969	1	1	1
ENSSSCG00000060818	0.414646918	0.950592302	1	1	1
LIX1L	0.41478612	0.776944423	1	1	1
CUBN	0.415153258	0.389042291	0.812	0.867	1
GAREM1	0.41531151	0.566815154	0.75	0.733	1
CCNP	0.415316661	0.914795794	1	0.867	1
THAP9	0.416402475	-0.329809662	0.875	0.733	1
MBTPS1	0.416525084	1.163230349	0.875	0.8	1
ENSSSCG00000034963	0.416555385	0.058893689	0.5	0.533	1
SEC61A1	0.417140302	0.761383617	1	1	1
NMNAT1	0.417159382	0.883633321	1	1	1
WASL	0.417527893	0.750976794	1	1	1
RBM12	0.417555497	0.630605166	1	0.8	1
BCL10	0.417559437	0.766429124	1	1	1
SSTR1	0.417875453	1.044394119	0.812	0.733	1
DENND6A	0.41793856	0.380701804	0.938	1	1
TRIQK	0.41816939	1.62935662	0.625	0.467	1
RNF145	0.418335026	0.730745807	1	1	1
SDHC	0.418385505	0.660196369	1	1	1
EBPL	0.418634378	0.566334927	1	1	1
KHSRP	0.419169419	0.328892043	1	1	1
ENSSSCG00000051049	0.419338465	0.260942894	0.938	1	1
CANT1	0.419493751	1.26237125	1	0.867	1
PARPBP	0.419697638	0.875181736	1	0.933	1
ARL5A	0.419860317	0.821921035	1	1	1
PACS1	0.419978209	1.083270619	1	0.933	1
ENSSSCG00000052587	0.420026934	0.215012891	0.5	0.667	1
PARD3B	0.420125526	0.590719771	1	1	1
CELF1	0.420170189	0.438758425	1	1	1
JAG1	0.420344622	0.816761002	1	1	1
ENSSSCG00000032067	0.420767733	0.497462774	1	1	1
CYP2C49	0.420901006	0.186998515	0.5	0.467	1
TIFA	0.421109944	1.347463187	0.5	0.4	1
PIWIL2	0.421332095	0.654596689	1	1	1
LYVE1	0.421448112	0.249778309	0.875	0.933	1
ENSSSCG00000057459	0.421749676	0.697172004	1	0.933	1

ENSSSCG00000063375	0.421757741	0	0.688	0.533	1
PIK3C3	0.422340442	0.680180879	0.938	1	1
VSTM1	0.422429735	0.121015401	0.75	0.6	1
ENSSSCG00000059862	0.422699101	2.022367813	0.5	0.133	1
PTPN4	0.422854924	-0.075621978	0.875	0.6	1
SBF2	0.422948789	0.934088737	0.938	1	1
ANXA6	0.422984696	0.094517599	0.688	0.733	1
CCNB1IP1	0.423145229	0.729431921	1	0.667	1
PSMD2	0.423578529	0.795972254	1	1	1
GRK2	0.423653601	0.186998515	0.562	0.467	1
OSMR	0.42371644	0.705163534	1	0.933	1
ENSSSCG00000062788	0.423816132	0.072476661	0.812	0.667	1
WDR75	0.423873624	0.721273808	1	1	1
RBM14	0.424071695	0.265344567	0.938	0.933	1
RAPGEF2	0.424256147	0.179724141	1	1	1
PEX26	0.424450095	1.00023685	1	1	1
ENSSSCG00000060636	0.424505793	0.735124247	0.938	0.8	1
GOT2	0.424676521	0.520162855	1	1	1
IFNGR1	0.424753345	0.080919995	0.875	0.867	1
CFAP46	0.42521589	-0.04580369	0.75	0.6	1
LZTFL1	0.425489919	1.129283017	1	1	1
PPP1R3C	0.425738346	0.664917805	1	1	1
WIZ	0.425761747	0.31676639	0.938	0.867	1
ENSSSCG00000027115	0.425981344	0.265885687	0.938	1	1
ENSSSCG00000001393	0.426373963	0.99305565	1	1	1
DENND5A	0.426560812	0.74130453	1	1	1
OSTM1	0.427391346	-0.516917113	0.562	0.667	1
HDHD5	0.427552481	1.392317423	0.5	0.267	1
RNF24	0.427589492	0.452196896	1	0.933	1
ENSSSCG00000040632	0.427593526	-0.218640286	0.5	0.4	1
AGK	0.427917567	0.783924704	1	1	1
SLC44A3	0.428169919	0.057307438	0.75	0.867	1
FGD5	0.428284478	0.055141554	0.938	0.933	1
WDR70	0.428292132	0.36496011	1	1	1
MBD4	0.428298998	0.827383149	1	1	1
CLSPN	0.428340213	0.867845863	1	1	1
FIGN	0.428610051	1.044394119	0.938	0.8	1
ENSSSCG00000059998	0.428617594	0.610497593	0.812	0.8	1
ENSSSCG00000044589	0.428663727	0.661778098	0.812	0.733	1
ENSSSCG00000059284	0.428829459	-0.093109404	0.5	0.533	1
ENPP1	0.429313469	0.356803136	0.938	1	1
PDGFRL	0.429380118	0.079357792	0.938	0.933	1
ENSSSCG00000059777	0.429398331	0.164048435	0.875	0.933	1
VMP1	0.429752931	0.941378972	1	1	1
SLC25A27	0.429912236	0.718033778	1	1	1
ENSSSCG00000058479	0.430018461	0.754887502	0.688	0.467	1
ZNF341	0.430135765	0.26052755	0.688	0.733	1
ENSSSCG00000002251	0.430230812	0.957516669	0.625	0.4	1
ABCC1	0.430231087	0.875366507	1	1	1
STK33	0.430719975	0.147898695	0.5	0.533	1
ENTPD7	0.431180766	0.647914406	1	1	1
SMARCA4	0.431263884	0.335733894	1	0.933	1
PARG	0.431319185	0.56175511	1	1	1
SDC3	0.431501573	0.96133838	0.812	0.6	1
ENSSSCG00000045095	0.431672504	-0.049388027	0.938	0.867	1
DCAF12	0.432752886	0.871615121	1	0.933	1
ENSSSCG00000057903	0.432902726	0.211745177	0.562	0.467	1
ZBTB6	0.432921971	-0.093109404	0.875	0.733	1
TRBV27	0.433276093	0.217678133	0.938	0.933	1
PRKD3	0.434225912	0.632836376	1	1	1
MTCP1	0.434339182	0.378642486	1	0.933	1
VPS8	0.43454555	0.365440401	1	1	1
PLAA	0.43509955	0.59017693	1	1	1
ITPRIPL1	0.435300347	0.880706664	1	1	1
ENSSSCG00000063016	0.435313568	1.516944077	0.625	0.6	1
CTDSPL	0.435352637	0.801280408	1	0.933	1
TASOR	0.435539807	0.633255885	1	1	1
SLC15A2	0.435567244	1.376375879	0.562	0.333	1
HNRNPM	0.435825884	0.973495994	1	1	1
ZSCAN20	0.436638897	0.198494154	1	0.8	1

ENSSSCG00000056610	0.436747981	0.376551764	1	1	1
TRAPPC14	0.436987213	-0.093109404	0.5	0.533	1
ENSSSCG00000044591	0.437045332	0.856617389	0.938	0.733	1
EXTL3	0.437125439	0.621509896	1	1	1
WDR43	0.437775918	0.921104455	1	1	1
NSD3	0.437878379	0.41043592	1	1	1
ENSSSCG00000060593	0.438106177	1.196397213	0.562	0.4	1
NUF2	0.438234108	0.871809944	1	1	1
ENSSSCG00000047048	0.43830258	1.730860232	0.75	0.533	1
WDR73	0.43880272	0.113341473	0.875	0.6	1
TXLNB	0.438922153	0.311280851	1	0.867	1
ENSSSCG00000037602	0.438946018	0.601758653	1	1	1
DUSP1	0.439117293	0.50907951	1	1	1
SEC24D	0.439319624	0.509274531	1	1	1
CHD9	0.439336504	-0.13965199	0.875	0.533	1
ENSSSCG00000048249	0.439584974	0.50084188	1	1	1
CYP2R1	0.440006478	0.196397213	1	0.933	1
ENSSSCG00000032352	0.440150357	0.235839118	0.688	0.667	1
ENSSSCG00000035650	0.440612295	0.194871359	1	0.8	1
PPP3CA	0.440750276	-0.03170886	0.562	0.533	1
ENSSSCG00000053249	0.441321911	1.032421478	0.625	0.467	1
HEXIM1	0.441616331	0.407319586	0.875	0.733	1
ENSSSCG00000062400	0.441722151	0.448476034	0.938	1	1
ACVR2B	0.442071006	0.603498452	1	1	1
ZNF546	0.44213655	0.885516945	0.938	0.733	1
TLR5	0.442213641	-0.040641984	0.5	0.667	1
HTD2	0.442277516	0.69619233	1	0.933	1
ENSSSCG00000056294	0.442282087	1.607330314	0.5	0.2	1
ARHGEF5	0.442289489	0.237901795	0.938	1	1
SSH1	0.442422491	1.019100099	1	0.933	1
SLC35E1	0.442837758	0.399594588	0.938	0.933	1
NCKAP5	0.442947154	0.698949331	1	1	1
TUBB3	0.442980033	0.070389328	0.812	0.4	1
CCDC38	0.443072154	0.984893108	0.562	0.267	1
PFKFB2	0.443082605	0.987509056	0.875	0.933	1
MED4	0.443123445	0.415732463	1	1	1
ENSSSCG00000008849	0.443407036	0.338606836	0.812	0.8	1
SLC18A2	0.443521263	0.349123022	1	1	1
MAN2A1	0.443594174	0.837181624	1	1	1
UBAP2	0.443706953	0.78508774	1	1	1
COX3	0.443870451	1.169709762	1	1	1
NFE2L3	0.444002925	0.356234217	1	1	1
TAFA2	0.444205602	0.47436567	1	0.933	1
MPDZ	0.444976817	0.528551396	1	1	1
CSNK2A2	0.445024989	0.383047889	1	1	1
CREB3L3	0.445144763	0.540644535	1	1	1
KDM1B	0.445514907	0.784935168	1	1	1
ENSSSCG00000014198	0.445792845	0.622813774	1	1	1
ZBTB44	0.445832367	0.639870842	1	1	1
ANKRD17	0.445879002	0.467523767	1	1	1
TTC17	0.446043136	0.809965545	1	1	1
LAMP2	0.446305005	0.774112576	1	1	1
BMP15	0.446413656	0.377368237	1	1	1
ENSSSCG00000003847	0.446454459	1.594946589	0.562	0.333	1
KIF13B	0.446579256	0.35638509	1	1	1
TBC1D24	0.446732749	0.542734265	1	1	1
NMNAT2	0.446805474	0.334061851	0.875	0.933	1
ENSSSCG00000009211	0.447216371	0.512582076	1	1	1
STRBP	0.447400412	0.901119808	1	1	1
ZFP36	0.447445805	0.497499659	0.938	0.933	1
RRAGD	0.447491411	0.432734881	0.938	1	1
ENSSSCG00000047947	0.447546308	0.129283017	0.875	0.867	1
MFAP3	0.447568882	0.734224688	1	1	1
ENSSSCG00000052153	0.447611347	1.076815597	0.5	0.267	1
RUSC2	0.447671214	0.189620803	0.938	1	1
LIPH	0.447757649	0.307428525	0.875	0.6	1
ENSSSCG00000009105	0.448077347	1.392317423	0.562	0.133	1
METTL15	0.448079533	1.00877021	0.875	0.6	1
CSTF2	0.448243019	0.743992861	1	0.8	1
ADAL	0.448738733	0.031070007	0.938	0.933	1

APTX	0.448772241	0.586695468	1	1	1
AUTS2	0.448908019	0.978443857	0.938	0.933	1
MRS2	0.44919284	0.188845863	1	1	1
ENSSSCG00000045959	0.449240557	1.569855608	0.5	0.2	1
MORC4	0.449538932	-0.003472192	0.812	0.867	1
POLR3D	0.449578459	0.917317249	1	1	1
C2CD5	0.449602545	0.692879983	1	1	1
PKLR	0.449618769	0.0138058	0.5	0.333	1
RITA1	0.449694789	0.951037699	1	1	1
CAMK2D	0.449916816	0.816125132	1	1	1
CYB561	0.450158655	0.427147407	0.938	1	1
ENSSSCG00000059573	0.45019858	0.239087026	0.938	1	1
ENSSSCG00000012517	0.450291771	0.837524651	1	0.933	1
SLC26A2	0.450332774	1.347463187	0.5	0.2	1
KLHL5	0.450540788	0.525327801	1	1	1
BBOX1	0.450752986	0.649941915	1	1	1
ENSSSCG00000032596	0.450898225	0.994353437	0.688	0.533	1
BRD1	0.451113391	0.909641207	1	1	1
CAVIN2	0.451215059	0.866248611	0.938	0.8	1
CHL1	0.451499004	0.402480665	1	1	1
AKAP9	0.451622169	0.816550218	1	1	1
BSPRY	0.451671142	-0.200024608	0.5	0.333	1
ENSSSCG00000060888	0.452000136	0.01268526	1	1	1
ND6	0.452121073	1.129150854	1	1	1
EYA2	0.452137713	0.054989235	0.688	0.867	1
SLC26A8	0.452189659	1.22881869	0.75	0.533	1
ENSSSCG00000054171	0.452254898	-0.093109404	0.5	0.467	1
KATNA1	0.45232848	0.904047846	1	1	1
USP4	0.452466982	0.188122109	1	0.933	1
PRKAR2A	0.452755042	0.491853096	0.938	0.8	1
RAB39A	0.452850596	0.856264523	0.938	1	1
ENSSSCG00000055276	0.45290027	0.838801606	1	0.933	1
FRMD8	0.453087142	1.096493082	1	1	1
PALS1	0.453189321	0.479833514	1	1	1
ENSSSCG00000055511	0.453492399	0.769387072	0.688	0.4	1
TAX1BP1	0.453549763	0.733714577	1	1	1
MYF6	0.453652205	0.491853096	0.875	0.733	1
SENP6	0.453680788	0.450153805	1	1	1
FAM186B	0.454205647	1.023404604	0.938	1	1
LYPD6	0.454617115	1.507283137	0.688	0.733	1
ARFGAP3	0.455359308	0.530979428	1	1	1
DEGS1	0.455436494	1.343661617	1	0.933	1
ENSSSCG00000002938	0.455592875	0.791988501	0.938	1	1
PMPCB	0.455688775	0.399717984	1	1	1
KIF9	0.455738964	0.269460675	0.812	0.733	1
RAI2	0.455779612	0.106199404	0.625	0.6	1
HDHD2	0.455863563	0.704778654	1	1	1
STRN4	0.455919143	1.109830654	1	1	1
BCL9	0.456467654	0.424166289	1	1	1
ENSSSCG00000052282	0.456512324	0.160647188	0.875	0.8	1
PABPN1	0.456645506	0.696703569	1	1	1
ENSSSCG00000059954	0.456882089	0.64385619	0.938	0.8	1
ENSSSCG00000045434	0.457280412	1.242493627	0.812	0.733	1
GNA12	0.457321598	0.500676947	0.938	0.933	1
ENSSSCG00000001038	0.457687289	1.001217978	1	1	1
PRDM5	0.457689868	0.529430554	1	1	1
PLPPR1	0.458619444	0.278859373	0.5	0.467	1
CFAP299	0.458643772	1.002047829	1	0.867	1
LMAN1	0.45929146	1.041615698	1	0.933	1
ATP11A	0.459460665	0.26052755	0.875	0.733	1
TBC1D8B	0.459563002	0.816203853	0.938	1	1
LSM14B	0.459696484	0.214072105	0.938	0.733	1
GIMAP8	0.460093782	0.380821784	0.5	0.6	1
USF3	0.460334398	0.203176807	1	1	1
ENSSSCG00000049992	0.460377553	1.594946589	0.562	0.133	1
DDX47	0.460386142	0.337034987	0.875	1	1
RASGRF2	0.46056112	1.243925583	0.875	0.733	1
ENSSSCG00000057066	0.461059079	-0.093109404	0.625	0.667	1
EPHA2	0.461092222	1.103691303	0.938	0.6	1
ENSSSCG00000061060	0.461288346	-0.127874823	0.812	0.467	1

DUOX2	0.461404226	1.392317423	0.562	0.333	1
ENSSSCG00000055040	0.461724284	0.415904243	0.812	0.8	1
FBXO15	0.463126485	0.844105321	1	1	1
AHI1	0.4634218	0.770132458	1	1	1
FBH1	0.463495666	0.934635586	1	0.933	1
TLR1	0.464031559	1.137188215	0.625	0.667	1
ENSSSCG00000046579	0.464066403	1.076815597	0.688	0.467	1
EBF2	0.464134388	0.027905997	0.875	0.8	1
IMMP1L	0.46429822	0.726101322	1	1	1
ODR4	0.464409023	0.485752897	0.75	0.933	1
ERCC2	0.464637923	0.252914366	1	0.933	1
MRPL18	0.465192601	0.881210049	1	1	1
CCDC134	0.465350734	0.401655287	0.938	0.8	1
CACNA2D1	0.465611744	0.955191928	1	1	1
RCOR3	0.465710226	0.243072674	1	1	1
ENSSSCG00000021380	0.465835015	1.136024595	0.812	0.6	1
AGBL3	0.465840114	0.423373358	0.938	0.933	1
ENSSSCG00000059042	0.465945686	1.05691604	1	0.933	1
GPR156	0.466033291	0.291808107	0.938	0.933	1
ASCC3	0.46616508	0.526110479	1	1	1
CEP128	0.467118404	0.549034757	1	1	1
MSN	0.467161334	1.321928095	0.875	0.667	1
ENOSF1	0.467667255	1.160647188	0.625	0.533	1
ZBTB34	0.468004459	0.807998839	1	1	1
SETD4	0.468267513	0.858635427	0.938	0.867	1
TBK1	0.46833407	0.828035175	0.938	0.733	1
ENSSSCG00000061302	0.468389814	-0.093109404	0.688	0.733	1
TBC1D20	0.468831583	0.785295758	1	1	1
ENSSSCG00000056401	0.468852772	0.206450877	0.625	0.6	1
ENSSSCG00000056542	0.468883604	0.495715736	1	1	1
GPATCH1	0.469018814	0.715591943	1	1	1
TNKS	0.469094083	0.641290746	1	1	1
TENT4B	0.469168011	0.8470812	1	0.933	1
ENSSSCG00000027421	0.469182065	1.354349573	0.5	0.4	1
SPATS2L	0.469335467	1.186998515	0.812	0.533	1
CRYBG1	0.469747024	0.321928095	1	1	1
DNM2	0.470241591	0.390099598	0.875	0.867	1
RSBN1	0.470436373	0.22336426	0.812	0.667	1
SNIP1	0.47071785	0.22881869	0.812	0.733	1
ENSSSCG00000013880	0.4709294	1.160647188	0.562	0.333	1
TCEAL9	0.471030095	0.716919652	1	0.867	1
WDR45B	0.471042215	0.88472327	1	1	1
YAE1	0.471055385	0.367731785	0.938	1	1
STX2	0.471072017	0.615427781	1	0.867	1
PTGES3	0.471433498	0.921587107	1	0.933	1
POR	0.471523308	0.906890596	0.938	1	1
B4GALT5	0.471631206	1.064122204	1	1	1
ARGLU1	0.471967289	0.89566334	1	0.933	1
ALDH16A1	0.472181313	1.00877021	0.938	0.8	1
ETS2	0.472258191	0.099535674	0.938	0.6	1
TBX3	0.472282616	0.906890596	0.688	0.6	1
GORASP2	0.472365978	0.631763897	1	1	1
N4BP1	0.472410655	0.189290326	1	0.867	1
ENSSSCG00000043817	0.472564835	0.491853096	0.938	0.8	1
ENSSSCG00000038066	0.472857781	0.526738614	1	1	1
TENM2	0.473197443	0.381027135	1	1	1
EPB41L4A	0.473767645	0.614337103	0.938	1	1
ARHGAP29	0.473897522	0.680585574	1	1	1
CHAF1B	0.475001291	0.297072906	1	1	1
DUSP19	0.4753741	0.902375114	1	1	1
CIP2A	0.475665192	0.472184936	0.938	1	1
C17orf58	0.475718187	0.468769483	0.688	0.8	1
NF1	0.475856975	0.571990204	1	1	1
SGPP2	0.476139595	0.174270472	1	1	1
BABAM2	0.476397952	0.079727192	0.812	0.733	1
ENSSSCG00000004583	0.4764599	0.62548294	1	1	1
SUPT4H1	0.476482753	0.137188215	0.688	0.667	1
ARPC5	0.47653955	0.494684687	1	1	1
SUSD6	0.476573633	0.682613909	1	1	1
NANOS1	0.477514598	0.293913719	0.562	0.6	1

RNFT2	0.477947505	0.353224225	1	1	1
TEC	0.477973669	0.328276061	1	1	1
B3GNT5	0.478051765	-0.194989018	0.562	0.667	1
ENSSSCG00000007525	0.478269659	0.906890596	0.562	0.267	1
CUL2	0.478339694	0.102441405	0.875	0.867	1
PTPRQ	0.478739259	0.814444347	0.938	1	1
PARP6	0.478746128	0.150816178	0.938	0.867	1
PSAP	0.478798331	0.033916034	1	1	1
ENSSSCG00000054758	0.478962265	0.103927443	0.75	0.533	1
ACAD10	0.47996241	0.951978485	0.875	0.733	1
ENSSSCG00000045708	0.480206429	1.154818109	0.562	0.4	1
UBE2V2	0.480632015	0.848156771	1	1	1
ENSSSCG00000062427	0.480644296	0.664771769	1	1	1
ENSSSCG00000035439	0.481123683	0.076815597	0.5	0.4	1
MYOG	0.481509712	0.129283017	0.625	0.667	1
XRN2	0.481677421	0.393532836	1	1	1
RAG1	0.481841805	1.044394119	0.625	0.267	1
BABAM1	0.482130766	0.812285395	1	1	1
TBC1D5	0.482477892	0.771163946	1	0.867	1
HS2ST1	0.482589818	0.385290156	1	1	1
HTR4	0.482669475	0.496425826	0.812	0.867	1
SCO1	0.482681476	0.361456459	1	0.933	1
C9orf64	0.482724364	0.925175949	1	0.933	1
KCTD12	0.482843238	0.583691963	1	0.933	1
ANKFY1	0.482914106	0.560170039	1	1	1
SLC35E2B	0.483029408	0.470791481	0.875	0.867	1
PTBP1	0.483379654	0.593543032	1	1	1
MRPS26	0.48359105	0.587915818	1	1	1
RMDN3	0.483723921	0.229700803	0.938	1	1
CBL	0.483921014	0.316145742	0.938	1	1
TMPPE	0.484373137	2.518325308	0.5	0.133	1
HERC2	0.484442603	0.757225744	1	1	1
SNRNP70	0.484574259	0.022367813	0.375	0.533	1
ADK	0.48457505	0.750771394	0.875	0.667	1
ENSSSCG00000043661	0.484669994	0.822409873	1	1	1
ENSSSCG00000058363	0.484889373	0.252026082	0.688	0.533	1
CEP120	0.484920868	0.65250806	1	1	1
RALGAPB	0.484931886	0.769431217	1	1	1
ZDHHC23	0.485024929	0.019899557	0.938	0.867	1
ZNF518A	0.485081966	0.234008247	1	0.933	1
ATG9A	0.485327772	0.142268659	0.812	0.933	1
CCNB3	0.486866039	0.269120055	1	1	1
SCEL	0.486902991	1.409390936	0.5	0.267	1
HPS4	0.487077551	1.038135129	0.875	0.8	1
BDP1	0.487129853	0.50846781	1	1	1
SMIM7	0.487242365	0.681330895	1	1	1
DOCK11	0.487307359	0.872125177	0.875	0.6	1
PHF6	0.487505899	0.35979628	1	1	1
ENSSSCG00000052578	0.487524116	1.0138058	0.562	0.333	1
ESYT3	0.487969464	0.423965793	1	1	1
SENP5	0.488045358	0.756995165	1	1	1
CBLL1	0.488250277	0.660422982	1	1	1
ENSSSCG00000060231	0.488547153	0.903817762	0.938	0.933	1
CEMIP2	0.488997682	0.40050415	1	1	1
RYBP	0.489180668	0.267621675	1	1	1
IFT52	0.489327828	0.331068749	1	1	1
UBQLN1	0.489331435	0.586153066	1	1	1
ENSSSCG00000048597	0.489336731	1.055753982	0.75	0.467	1
ENSSSCG00000039095	0.489357938	0.437405312	0.812	0.667	1
SLC1A7	0.489398569	0.154818109	0.562	0.467	1
RASGEF1B	0.489438718	0.375443604	0.875	0.933	1
SPTBN1	0.489519999	0.575890595	1	1	1
RNF40	0.49024897	0.79524524	1	1	1
NR3C2	0.490555332	0.835765011	1	1	1
ENSSSCG00000048640	0.490889179	0.418390934	0.75	0.867	1
SPAG7	0.490923835	0.392317423	0.938	0.8	1
SLC30A9	0.491658971	0.262567482	1	1	1
ENSSSCG00000012523	0.491933016	0.389973482	1	0.933	1
ZNF609	0.492006983	1.025285296	1	0.933	1
PPP6R2	0.492028239	0.380821784	0.938	0.8	1

TMEM168	0.492078759	0.90875334	1	1	1
KLHL18	0.492086552	0.762320572	1	1	1
CREBRF	0.492801457	-0.100971522	0.938	0.867	1
NELL2	0.492942506	0.732212064	0.812	0.667	1
EEF1AKMT3	0.493030189	0.182525038	0.625	0.333	1
WDR77	0.49376678	0.908926863	1	1	1
NUP58	0.494092162	0.781051083	1	1	1
DNAH9	0.494216336	0.540762697	1	0.8	1
ZNF644	0.494229803	0.36225255	1	1	1
PSMD12	0.494414823	0.367931667	1	1	1
XKR9	0.495015945	0.5980525	1	1	1
ZNF569	0.495165909	0.683914442	0.938	1	1
TAOK1	0.495588333	0.573280696	1	1	1
ENSSSCG00000045014	0.495741511	0.971020933	0.562	0.467	1
CNPPD1	0.49600904	1.066290417	1	0.933	1
ENSSSCG00000027890	0.496180412	1.491853096	0.5	0.2	1
ENSSSCG00000063156	0.496485066	0.147898695	0.938	0.8	1
SOCS6	0.496967876	0.67071565	1	1	1
PNPLA1	0.497089158	0.05626822	0.688	0.733	1
ENSSSCG00000031239	0.497097596	0.006426269	0.5	0.467	1
ZCCHC14	0.497272595	0.830269314	1	0.867	1
PTPRJ	0.497668369	0.841795567	1	1	1
DNAJC13	0.497698904	0.437949774	1	1	1
ENSSSCG00000053722	0.498383551	1.209453366	0.75	0.733	1
SLA2	0.49861774	0.036658471	0.812	0.8	1
RGS2	0.499110823	0.632802534	1	1	1
PPP2R5A	0.499376522	0.446723721	1	1	1
ENSSSCG00000016384	0.499410327	0.285402219	0.625	0.867	1
TMEM267	0.499501503	0.941067711	0.938	0.933	1
POLG	0.50010242	0.186998515	0.438	0.6	1
MED6	0.50020598	0.798329001	1	1	1
ARHGEF38	0.500401649	0.723726571	0.875	0.8	1
ENSSSCG00000057369	0.500698372	0.817623258	0.75	0.533	1
PIF1	0.500751359	0.003105911	0.562	0.6	1
TRMT13	0.500815746	0.906890596	0.938	0.8	1
TMSB4X	0.500849614	0.377279328	1	1	1
SNX18	0.501198077	0.616762529	1	1	1
NAA25	0.501280472	0.824428435	1	0.733	1
LAP3	0.501726699	0.678071905	1	1	1
ZC3H6	0.50181996	0.401087632	0.938	0.867	1
UBE2E2	0.502307324	0.830269314	0.875	0.667	1
CRK	0.502433745	0.733316212	1	1	1
SRSF6	0.502747977	0.323268267	1	1	1
AP4B1	0.502883797	0.22881869	0.938	0.933	1
UBN1	0.503118635	1.116790208	1	1	1
CASP8AP2	0.503471408	0.78722566	1	1	1
ZFYVE9	0.503528619	0.506972655	1	1	1
MORN2	0.503897152	0.692490965	1	1	1
ENSSSCG00000059507	0.504690198	0.754887502	0.812	0.333	1
ENSSSCG00000053580	0.504829036	0.276124405	0.938	0.933	1
EXOC8	0.505211265	0.107587946	0.875	0.667	1
LONRF2	0.505408984	0.577001215	1	1	1
PGM3	0.505935009	0.6794801	0.812	0.533	1
PTPRB	0.506412333	0.92347348	1	0.933	1
SNRPB	0.506977442	0.027184829	0.812	0.8	1
EIF1AX	0.507246587	0.700690208	1	1	1
ERLEC1	0.507379424	0.653499449	1	1	1
ENSSSCG00000060137	0.507931127	1.254813899	0.688	0.667	1
WDR33	0.508274472	0.671821198	1	1	1
MON2	0.50869214	0.717794585	1	1	1
SWAP70	0.508721058	0.855201119	1	1	1
ENSSSCG00000044572	0.508830565	0.254813899	0.625	0.667	1
ENSSSCG00000026233	0.508887725	0.482802282	1	1	1
CASR	0.509162946	0.652317769	0.938	0.867	1
ATP11B	0.509643804	0.775492706	1	1	1
B4GALT1	0.509805298	0.568634331	0.938	0.933	1
EGFL8	0.510195795	0.118394701	0.562	0.533	1
ENSSSCG00000056580	0.510716354	0.827323338	0.938	1	1
SCARB2	0.511221178	0.354211377	1	0.933	1
DNAH17	0.511305988	0.0489096	0.625	0.6	1

ENSSSCG00000056336	0.511487444	0.922157352	0.938	0.867	1
ANKRD50	0.51152759	0.425776064	1	1	1
PYCR3	0.511807481	1.169925001	0.625	0.467	1
TERF2	0.511967703	0.412046893	1	1	1
ENSSSCG00000027741	0.512734221	0.319740536	1	1	1
ENSSSCG00000036356	0.513030671	0.129283017	0.438	0.533	1
ATG4D	0.513456666	0.169925001	0.938	0.8	1
ENSSSCG00000024021	0.513741484	1.299208018	0.812	0.667	1
IRAG1	0.513844522	0.046615359	0.75	0.6	1
ENSSSCG00000044174	0.513870006	0.977279923	0.625	0.4	1
ZNF84	0.514734415	1.010513227	0.938	0.733	1
PTPRD	0.515313229	0.532824877	1	1	1
ZBTB21	0.515667116	0.468769483	0.625	0.667	1
PABIR2	0.515966161	0.595304445	1	1	1
ZC2HC1C	0.516227012	0.8758637	0.875	0.8	1
PLS1	0.516639243	0.925269125	0.688	0.667	1
HUS1	0.51720295	0.38283637	1	1	1
NEK10	0.517298618	0.563111779	0.938	0.933	1
SLC2A1	0.517317636	0.379643593	0.875	0.867	1
CPM	0.517506361	1.004933569	1	1	1
ATXN7L3B	0.51757809	0.356491482	1	1	1
RIPK4	0.517950569	1.130134416	1	0.933	1
SOGA1	0.518555408	0.918295359	1	0.867	1
ENSSSCG00000045070	0.518760014	0.607330314	0.562	0.467	1
CPNE8	0.518944536	0.775646062	0.625	0.667	1
ENSSSCG00000061161	0.519253122	1.154818109	0.562	0.267	1
RUBCN	0.519260703	0.776448407	1	1	1
ENSSSCG00000062358	0.519536807	0.951284715	0.688	0.6	1
CNTN5	0.519823319	0.467523767	1	1	1
SLC24A2	0.519934571	0.356197997	1	1	1
LCP1	0.519938305	0.202346479	0.438	0.533	1
TTC39B	0.520199983	0.649502753	1	0.867	1
U2AF2	0.520210602	0.928159212	1	0.867	1
DSG1	0.520262518	0.824428435	0.688	0.467	1
KSR2	0.520765564	0.437752887	1	1	1
ZNF12	0.520810135	-0.224353938	0.688	0.6	1
NAPEPLD	0.520923491	0.770255904	1	1	1
ENSSSCG00000055882	0.521149341	0.837627933	1	1	1
LINS1	0.521235549	0.372055978	1	1	1
S100A14	0.521322927	1.285402219	0.562	0.267	1
ZSCAN31	0.522126487	0.220048481	0.875	0.667	1
DPY19L2	0.522311865	0.752168001	0.875	0.667	1
TINAGL1	0.522370372	1.160647188	0.688	0.267	1
SLC66A1	0.52276555	0.215012891	0.625	0.533	1
BAIAP2	0.523163549	0.560326231	1	1	1
ENSSSCG00000052619	0.523164755	0.852028661	0.938	0.733	1
ATF6B	0.523176005	0.155711142	1	0.867	1
CLDN20	0.52317747	0.94295985	0.938	0.8	1
CCDC40	0.523362219	1.089862667	1	1	1
AP2A1	0.523367075	0.380821784	0.625	0.533	1
RPS6KL1	0.523415657	0.206450877	0.562	0.533	1
PALD1	0.524007217	1.217230716	0.688	0.667	1
MAN1C1	0.524126015	0.929258409	0.938	0.533	1
ZNF592	0.524139321	0.459838872	1	1	1
PEX12	0.524812015	0.61363636	1	1	1
PON2	0.524868071	0.233700912	0.812	0.867	1
RFX7	0.524918973	0.87734632	1	1	1
ZNF215	0.524995511	0.758765717	1	1	1
PORCN	0.525517203	1.305439972	0.625	0.333	1
ENSSSCG00000018060	0.525646902	0.769387072	1	0.6	1
AKT3	0.525707835	0.977312268	1	1	1
FNBP1L	0.526014971	0.733919888	1	1	1
MCFD2	0.526308981	0.633826835	1	1	1
RPH3AL	0.526673627	0.2644426	0.875	0.933	1
PLXND1	0.526774305	0.847777502	1	0.933	1
ENSSSCG00000059811	0.527525497	0.62309763	0.562	0.333	1
GID8	0.528310414	1.053208984	1	0.867	1
TEAD3	0.52833829	0.301169535	0.75	0.733	1
SLC4A8	0.528347722	0.702351839	0.875	0.733	1
SLC35A5	0.528480006	0.850307067	1	1	1

CAMK2G	0.528523254	0.778205092	1	1	1
MKNK2	0.528644666	0.906890596	1	0.933	1
TRRAP	0.528845491	0.864035117	1	1	1
PGK1	0.528964414	0.536438263	1	1	1
ENSSSCG00000062615	0.529124964	0.458132478	0.812	0.867	1
KDM6B	0.529139053	0.468769483	0.938	0.8	1
C8orf48	0.530079797	0.040157126	0.875	0.6	1
PUS7	0.530295089	0.534921818	1	1	1
PRC1	0.530361138	0.696427021	1	1	1
RIMOC1	0.530540027	0.744240258	1	1	1
CTTNBP2	0.530662899	0.618850763	1	1	1
PGAP4	0.530960239	1.206450877	0.5	0.133	1
ST3GAL5	0.531171481	0.513745702	1	0.933	1
GMDS	0.531580352	0.750771394	0.875	0.733	1
MGAT5B	0.531614291	0.973192966	1	1	1
ENSSSCG00000055759	0.532133543	0.006426269	0.375	0.533	1
SAP130	0.532567575	0.817474182	1	1	1
RAB5B	0.533099737	0.86476512	0.938	1	1
RASL12	0.533170639	0.705293917	1	1	1
MSX2	0.533338533	0.09330372	0.875	0.733	1
ANXA11	0.53407429	0.513795649	0.938	1	1
TMEM135	0.534212135	0.407674062	1	1	1
CD109	0.534298563	0.73470962	1	1	1
ENSSSCG00000010212	0.534312763	0.681598284	0.938	0.933	1
KMT2B	0.534652402	0.892293219	1	1	1
QRSL1	0.534688211	0.839427628	1	1	1
TTK	0.534746354	0.66264644	1	1	1
GATAD2B	0.534897788	0.420207612	1	1	1
SEC14L1	0.535082939	1.257387843	0.875	0.8	1
ZFAND4	0.535494543	1.26052755	0.562	0.333	1
PDZRN3	0.535834741	0.192292814	0.812	0.667	1
PACSIN2	0.536183301	0.841524316	1	1	1
ENSSSCG00000062318	0.536237572	-0.520530628	0.562	0.4	1
SCRN3	0.536322562	0.862768042	0.938	0.8	1
SNX15	0.536414137	0.3594028	0.625	0.467	1
ANO6	0.536509715	0.455750818	1	1	1
ZNF236	0.536667552	0.924099886	1	1	1
PON1	0.53730346	1.108524457	0.75	0.6	1
CDC42EP3	0.537370033	0.314065977	1	0.933	1
CLHC1	0.537379852	0.665882496	0.812	0.533	1
ANKRD10	0.537425864	0.887562335	1	1	1
UBR4	0.537443019	0.371154597	1	1	1
ENSSSCG00000035663	0.537891786	0.7426612	1	1	1
ENSSSCG00000040103	0.538058629	0.6036671	1	1	1
PAH	0.538270993	0.397069793	1	1	1
SLC7A8	0.538328105	0.403043829	1	1	1
ENSSSCG00000029199	0.538479614	0.818970747	1	1	1
ENSSSCG00000061287	0.538675136	0.083768358	0.875	0.667	1
ZNRF3	0.538770737	0.512611656	0.75	1	1
MCM7	0.53894917	1.03092031	1	1	1
ENSSSCG00000053596	0.538961482	0.713286598	1	1	1
ENSSSCG00000041876	0.539517931	0.169925001	0.938	0.667	1
ALDH7A1	0.539579627	0.640810922	1	1	1
SCN8A	0.540505495	0.377271129	1	1	1
PDE12	0.540658101	0.695850827	1	0.867	1
ENSSSCG00000034367	0.54106977	-0.006521719	0.938	0.933	1
PHF1	0.54179138	1.430452552	0.938	0.8	1
PJVK	0.54180117	0.236198221	0.812	0.733	1
NR2E3	0.541928671	0.211745177	0.562	0.533	1
NDUFA4L2	0.542123111	0.147898695	0.5	0.4	1
GFOD1	0.542313233	0.841795567	1	1	1
ENSSSCG00000056431	0.54232635	1.366322214	0.75	0.267	1
ENSSSCG00000012126	0.542581519	0.552459861	1	1	1
MAGI2	0.542746923	0.496885173	1	1	1
IL17RD	0.542813657	0.849405101	1	0.933	1
KPNA2	0.542949066	0.715784997	1	1	1
RBM8A	0.542986461	0.167481826	1	1	1
NOL11	0.544197302	0.690144737	1	1	1
ANKS4B	0.544254392	0.649633199	1	1	1
RAB10	0.544272804	0.629766785	1	1	1

SFPQ	0.544807901	0.631792371	1	1	1
LUC7L3	0.545029337	0.875752084	1	1	1
ENSSSCG00000055060	0.545248521	0.434628228	0.938	0.667	1
ARIH1	0.54528304	0.689188583	1	1	1
PTPN12	0.545697844	0.453172628	0.938	0.933	1
CLUL1	0.546519479	0.863821874	1	0.933	1
TFCP2	0.546777626	0.965784285	0.75	0.733	1
BCL7C	0.547762932	1.044394119	0.625	0.4	1
ZNFX1	0.548096303	0.905546678	1	1	1
ABHD16A	0.548147788	0.704073713	1	1	1
ENTPD5	0.548331886	0.824805771	1	1	1
ENSSSCG00000034696	0.548754796	0.261985554	0.75	0.8	1
ENSSSCG00000048793	0.548764435	0.88582898	1	1	1
AMER1	0.548948054	0.819427754	0.625	0.4	1
ENSSSCG00000056041	0.549537876	0.169925001	0.5	0.333	1
GIMAP4	0.549740286	0.022367813	0.375	0.533	1
PLCH1	0.549802547	0.665200425	1	1	1
NAV3	0.55029098	0.404601903	1	1	1
SLC12A9	0.550417296	1.206450877	0.75	0.733	1
ENSSSCG00000037928	0.550548433	0.906890596	0.812	0.667	1
ENSSSCG00000042741	0.550591229	0.104829973	0.688	0.6	1
ANKRD31	0.550721962	0.34639709	1	1	1
PPP6R3	0.550873128	0.552026651	1	1	1
ACTR6	0.550971081	0.677933239	1	1	1
SMAP1	0.550999582	0.797266104	1	0.933	1
CEP350	0.551320803	0.435831713	1	1	1
ITGAV	0.551528685	1.067836985	1	1	1
RICTOR	0.551549789	0.651496252	1	1	1
ENSSSCG00000053830	0.551781015	1.006426269	0.5	0.533	1
EIF3J	0.552347459	0.422439331	1	1	1
ENSSSCG00000056613	0.55262235	0.321928095	1	0.933	1
VPS39	0.552846961	0.400599871	1	1	1
HBP1	0.552851714	0.874415055	1	1	1
ENSSSCG00000033497	0.552858561	0.662505446	1	1	1
TAF6	0.55301546	0.72597481	1	1	1
ITFG1	0.553135518	0.733956844	1	1	1
SLC20A1	0.553400514	0.700085602	1	1	1
C9orf131	0.553833162	0.196397213	0.688	0.733	1
ENSSSCG00000023531	0.554268952	0.428427716	1	0.933	1
GRHL1	0.554345022	0.951284715	0.75	0.8	1
CLIP4	0.554391994	0.533478642	1	1	1
DDX39A	0.554568415	0.770241749	1	1	1
ARID4B	0.55463036	0.872443103	1	1	1
ENSSSCG00000033786	0.555235577	0.397216222	1	1	1
RAB11FIP5	0.555332958	0.321928095	0.75	0.6	1
KBTBD4	0.555375274	0.64542861	1	1	1
RBM38	0.55545798	1.073248982	1	0.867	1
ENSSSCG00000007705	0.555506944	0.739002746	1	1	1
AFDN	0.555582266	0.912515145	1	1	1
NAA11	0.555629448	0.984893108	0.75	0.667	1
LPGAT1	0.556465898	0.712791576	1	1	1
SPOP	0.556686804	0.606093965	1	1	1
ING5	0.556805815	0.903252019	1	1	1
FRY	0.556909798	0.539636868	1	1	1
HSPH1	0.557106199	0.494902449	1	1	1
TMEM174	0.557265009	0.738136138	0.875	0.867	1
ZMYM1	0.557653715	0.694995039	1	1	1
ENSSSCG00000054251	0.557751368	-0.015106892	0.438	0.533	1
ENSSSCG00000035428	0.557807648	0.875729664	0.938	1	1
DNAH14	0.558050907	0.6987824	1	1	1
CEP41	0.558396944	0.94753258	0.688	0.533	1
SLC39A8	0.558800267	0.35359601	0.938	1	1
ENSSSCG00000055775	0.559335408	-0.373217324	0.375	0.533	1
DNAJC16	0.559635061	0.755966556	1	1	1
ENSSSCG00000052335	0.559998399	0.67507492	0.938	0.733	1
PCP4L1	0.560041138	0.058893689	0.75	0.467	1
SP1	0.560182247	0.270638866	1	0.867	1
DCUN1D4	0.560378982	0.586651741	1	1	1
ENSSSCG00000053584	0.560650302	0.690398776	1	0.8	1
ZNF654	0.560774611	0.727003405	1	1	1

ENSSSCG00000045310	0.560897853	1.022367813	0.5	0.2	1
HSPD1	0.561000398	0.616634721	1	1	1
ZNF570	0.561029458	0.819040298	0.938	1	1
ENSSSCG00000037372	0.561077957	0.248996572	1	1	1
ENSSSCG00000015826	0.561407825	0.337130502	0.875	0.933	1
GNS	0.561525285	0.59101847	1	1	1
ROBO1	0.56153483	0.860383735	1	1	1
SIK3	0.561571943	0.733493402	1	1	1
CSNK2B	0.561769361	1.399758806	1	0.933	1
KATNAL2	0.561909445	0.536591411	1	1	1
MBD2	0.562089981	0.353989304	1	1	1
RUFY3	0.562206407	0.309971121	1	1	1
ERBIN	0.562242006	0.419929077	1	1	1
ENSSSCG00000058114	0.562585922	0.154818109	0.5	0.533	1
PRKG1	0.562886565	0.664320292	1	1	1
ENSSSCG00000055627	0.563201282	0.765714533	1	0.733	1
FAM222B	0.563265873	0.724026538	0.875	0.733	1
HPF1	0.563403905	0.681127885	1	1	1
ENSSSCG00000040442	0.563727494	0.661778098	0.688	0.467	1
SLC6A15	0.563775548	0.857397322	1	1	1
ENSSSCG00000008488	0.563857227	0.507534719	1	0.867	1
RPA2	0.564017406	0.573199051	1	1	1
ENSSSCG00000053892	0.564032345	0.906890596	0.562	0.4	1
IBTK	0.564081433	0.542262311	1	1	1
MCM3AP	0.564122619	0.093976148	0.938	1	1
ENSSSCG00000057567	0.56468102	0.22881869	0.75	0.8	1
MICAL3	0.564718789	0.809972452	1	1	1
ST3GAL3	0.565074956	0.994353437	0.562	0.333	1
RDH14	0.565123342	0.979040381	1	0.933	1
UBAP2L	0.565154069	0.622478677	1	1	1
NCOA7	0.565369125	0.215335461	1	1	1
DCAF7	0.566371734	0.657921911	1	1	1
ENSSSCG00000063104	0.566483093	0.827456128	0.625	0.267	1
BUB1	0.567381465	0.727471468	1	1	1
DMD	0.56779201	0.770829046	1	1	1
ALDH6A1	0.567811435	0.49842575	0.812	0.867	1
EDEM2	0.567848124	0.29620553	1	1	1
RTL6	0.567881807	0.437405312	0.625	0.533	1
TMEM184C	0.568320759	0.862240838	1	1	1
VPS18	0.568358332	0.332545202	1	1	1
NEIL2	0.568406287	-0.015106892	0.75	0.467	1
SRRM1	0.568589597	0.617383978	1	1	1
VPS35L	0.56869713	0.469904123	1	0.933	1
ENSSSCG00000054089	0.568944432	0.390212834	0.938	1	1
PRKAR2B	0.568964842	1.015294188	1	0.933	1
SBNO1	0.569125612	0.691331867	1	1	1
KANK2	0.569797064	0.512424706	1	1	1
ENSSSCG00000055266	0.569852879	0.276124405	0.938	0.8	1
ELMO1	0.569857184	0.743391863	0.75	0.4	1
CRTC1	0.569870104	0.788246099	0.75	0.533	1
ENSSSCG00000053847	0.570116124	0.57758297	0.812	0.867	1
WASF2	0.570357932	0.497897073	1	1	1
NAPG	0.57052315	0.534921818	1	1	1
ANKRD6	0.571011205	0.657001566	1	1	1
JPH1	0.571509492	0.590197038	1	1	1
ENSSSCG00000059296	0.571914085	1.067355268	0.75	0.6	1
DUSP7	0.572108436	0.757693523	1	1	1
WSB1	0.572265565	0.748888762	1	1	1
ENSSSCG00000059326	0.572433736	-0.27368165	0.688	0.467	1
ZNF280B	0.572705436	0.437613508	1	1	1
SLC25A15	0.572737226	0.382469637	0.875	0.8	1
PCDHB1	0.572808157	0.399671874	0.938	1	1
ENSSSCG00000057818	0.573127028	0.791413378	0.438	0.533	1
ENSSSCG00000017906	0.573275624	0.288761231	0.812	0.867	1
TMEM265	0.573723218	0.27462238	0.75	0.667	1
SLC25A35	0.573790848	0.364572432	0.812	0.867	1
FAM81A	0.574067633	0.100977648	0.938	0.933	1
PDF	0.57407776	0.283744901	0.812	0.8	1
EP300	0.574215042	0.510697633	1	1	1
MSH4	0.574237128	0.642928113	1	1	1

ENSSSCG00000007135	0.57425743	1.154515562	1	1	1
VANGL1	0.574391637	0.530585491	1	1	1
EXO1	0.574471725	0.377826955	1	1	1
SPRYD4	0.574868589	0.4559064	1	0.933	1
ELOVL7	0.575043944	0.254813899	0.562	0.8	1
AP4E1	0.575060743	0.451019379	1	0.933	1
ENSSSCG00000007589	0.575152002	0.788627714	0.938	0.867	1
ENSSSCG00000059288	0.575179447	0.540762697	0.938	0.867	1
MADD	0.575225714	0.377755129	1	1	1
ROCK1	0.57527551	0.935459748	1	1	1
CNTNAP5	0.575664207	0.719805043	0.875	0.8	1
TYW5	0.576175447	0.138972073	1	0.933	1
ADAMTS17	0.57643328	0.802193217	0.812	0.733	1
CTNNBIP1	0.576729345	0.321928095	0.375	0.667	1
ENSSSCG00000000405	0.576973201	0.706799333	1	0.933	1
LRRC8B	0.577073704	0.584962501	1	1	1
CNST	0.577640449	0.270379004	1	1	1
ENSSSCG00000062102	0.578245278	1.269460675	0.5	0.267	1
ZNF639	0.578706187	0.647478985	1	1	1
TATDN3	0.578720368	1.096715154	0.938	0.6	1
TTF2	0.579449978	0.329232896	0.938	1	1
KPNA5	0.579892792	0.848787641	1	0.8	1
KANTR	0.580163562	1.147898695	0.562	0.467	1
ENSSSCG00000053935	0.581586161	0.781359714	0.562	0.533	1
SLC39A7	0.581684667	0.966596842	0.938	0.933	1
GMEB1	0.581758079	0.402418013	0.938	0.867	1
YPEL3	0.581758619	0.452736966	1	0.933	1
ENSSSCG00000052555	0.581884138	0.785584299	0.938	0.8	1
DUSP10	0.581926853	0.186998515	0.75	0.8	1
CCDC181	0.582431488	0.387780642	0.938	0.8	1
GKAP1	0.582798639	0.723709497	1	1	1
ENSSSCG00000060864	0.583101496	0.206450877	0.5	0.533	1
TMCO3	0.583103048	0.967125703	0.938	1	1
ADCY10	0.583131639	0.002649578	0.875	0.733	1
COX10	0.58337544	0.217230716	0.812	0.667	1
ENSSSCG00000061843	0.584327156	0.628589433	1	0.933	1
MRAS	0.584374232	0.749534268	1	0.933	1
ENSSSCG00000041019	0.584522629	0.875181736	0.875	0.6	1
STK40	0.584630916	0.681123447	1	1	1
ENSSSCG00000037413	0.584695485	0.123701985	0.625	0.667	1
ARHGAP21	0.584963088	0.692482263	1	1	1
GALNT1	0.585154471	0.665882496	1	1	1
PDLIM1	0.585199165	0.819427754	0.938	0.8	1
PPP2CA	0.585483095	0.601953938	1	1	1
ENSSSCG00000008137	0.585684765	-0.491658781	0.562	0.667	1
REPIN1	0.585863333	1.312602303	1	1	1
ENSSSCG00000039046	0.586016726	0.12701641	0.875	0.867	1
THSD7B	0.586582	0.488844346	1	1	1
ENSSSCG00000035073	0.587023288	0.492938237	1	1	1
ZNF175	0.587024383	1.058893689	0.625	0.8	1
ENSSSCG00000054299	0.587427643	0.635588574	0.625	0.4	1
ENSSSCG00000062996	0.587443074	0.906890596	0.938	1	1
TMX1	0.587496863	0.614024966	1	0.933	1
GPR137C	0.58762996	0.185791407	1	1	1
SART1	0.587630324	0.771961016	0.938	0.933	1
ZRSR2	0.588742009	0.37185332	0.938	0.867	1
ST8SIA6	0.588816679	0.576741994	1	1	1
RBSN	0.588979699	0.259906368	1	1	1
CEP290	0.589189884	0.641574621	1	1	1
NCBP2	0.589321302	0.557504953	1	1	1
HIPK2	0.590087737	0.366322214	0.938	0.867	1
INSC	0.590202077	0.63907905	1	1	1
ENSSSCG00000056178	0.590597337	1.049848549	0.562	0.4	1
RIPK1	0.590671358	0.782776899	1	1	1
C10orf105	0.591093228	0.736965594	0.562	0.333	1
ENSSSCG00000054636	0.591152599	0.392317423	0.75	0.667	1
ENSSSCG00000034711	0.592055551	0.758040636	1	1	1
WDR7	0.592402153	0.082740431	0.938	0.867	1
PIEZO1	0.592451662	0.321928095	0.812	0.867	1
ANAPC7	0.59255778	0.615395425	1	1	1

MINK1	0.592572841	0.94641896	0.562	0.467	1
ENSSSCG00000048555	0.593228532	1.076815597	0.562	0.267	1
ENSSSCG00000043473	0.593298763	0.040157126	0.688	0.6	1
ENSSSCG00000054056	0.593465463	1.196397213	0.625	0.533	1
ENSSSCG00000031741	0.59356104	0.949451058	1	1	1
PREPL	0.593593932	0.679182345	1	1	1
MED21	0.593603269	0.59047402	1	1	1
MAP7D2	0.593793919	0.57766591	0.938	1	1
TEX9	0.594145195	0.314314911	0.938	0.933	1
RNF139	0.594646489	0.514706182	1	1	1
ENSSSCG00000050691	0.59477878	0.761039729	0.875	0.733	1
AIG1	0.595030345	0.26052755	0.5	0.4	1
MDM4	0.595044628	0.679810684	1	1	1
SNX8	0.595267106	0.760170723	1	1	1
PXYLP1	0.59539086	-0.147557188	0.812	0.733	1
ENSSSCG00000055510	0.595433083	1.169925001	0.562	0.4	1
UNC5D	0.595667038	0.798691855	1	0.933	1
RSPRY1	0.596006979	0.103287808	0.625	0.533	1
NAP1L2	0.596627095	0.206450877	0.75	0.733	1
PARN	0.597197241	0.669561904	1	1	1
MDH1B	0.597388041	0.169925001	0.562	0.4	1
SOX4	0.597782409	0.906890596	0.812	0.4	1
CHPF2	0.598111154	0.453633774	0.938	0.867	1
SLC17A7	0.598221368	0.984893108	0.562	0.267	1
GRM8	0.598340973	0.658277307	1	1	1
ZNF391	0.598777459	-0.042923647	0.875	0.867	1
MAP3K7CL	0.598840502	0.363128955	1	1	1
XPO7	0.598904384	0.650375193	1	1	1
ZNF596	0.599016857	0.534437489	1	1	1
ZNF580	0.59904807	0.070389328	0.75	0.867	1
SFT2D3	0.599121072	0.531381461	0.75	0.8	1
PHC2	0.599260372	0.52501996	1	0.867	1
MMACHC	0.599335282	-0.03866162	0.625	0.667	1
DEPDC1	0.600524141	0.752856967	0.938	0.933	1
RAN	0.601119387	0.557450587	1	1	1
NRIP1	0.601348359	0.710667107	1	0.8	1
MAPRE3	0.601399403	0.824428435	0.562	0.6	1
RNF19B	0.601907733	0.628558301	1	1	1
ABLIM1	0.602140514	0.744444968	0.938	1	1
ZNF613	0.60310775	-0.047305715	0.875	0.6	1
ENSSSCG00000005880	0.603153543	0.427722759	0.438	0.6	1
ENSSSCG00000045157	0.603245434	0.994353437	0.625	0.2	1
ACOT9	0.603543096	0.577042837	1	0.867	1
WNK3	0.603607565	0.459431619	1	1	1
TTLL7	0.603662141	0.403722186	0.938	0.867	1
ENSSSCG00000053756	0.603967807	0.906890596	0.625	0.333	1
MMP9	0.604124534	0.211745177	0.5	0.4	1
HOOK3	0.60505202	0.749490575	1	1	1
ZDHHC21	0.605187851	0.835099913	0.875	0.8	1
ENSSSCG00000047436	0.60561398	0.54630088	1	0.933	1
ENSSSCG00000048636	0.606081024	0.564784619	0.75	0.667	1
TLN1	0.606203636	0.78730898	0.938	0.867	1
PIGB	0.606256655	0.305986551	1	0.867	1
ADGRV1	0.606300461	0.668279664	1	1	1
TFAP2C	0.606450467	1.07534421	1	1	1
NFILZ	0.606708483	1.26052755	0.5	0.267	1
ENSSSCG00000046422	0.606755623	0.810214576	0.875	0.733	1
ENSSSCG00000057139	0.60683477	0.584962501	0.688	0.6	1
DGCR2	0.607021179	0.639194812	1	0.933	1
ST7	0.607470041	0.369183002	1	1	1
GNB1	0.607774925	0.783686016	1	1	1
LHFPL2	0.60832416	0.562349932	1	1	1
PITX2	0.608548179	0.269460675	0.562	0.6	1
PRRC2C	0.608728443	0.762450796	1	1	1
TCF3	0.608977717	0.636801432	0.938	1	1
ACTR10	0.610130682	0.457087678	1	1	1
DYNLT3	0.610392951	0.656912343	0.875	0.867	1
TMEM236	0.61046928	0.724026538	0.562	0.533	1
MSANTD4	0.61055229	0.166757722	0.875	0.733	1
UGGT2	0.610671736	0.257046981	1	1	1

DNAL4	0.61079047	0.67577137	1	1	1
TADA3	0.610863276	1.086514083	1	1	1
ENSSSCG00000020988	0.610912432	-0.4012317	0.5	0.4	1
ZFYVE16	0.610950618	0.235513343	0.625	0.4	1
ATP7B	0.611381642	0.551103697	1	1	1
C2orf69	0.611589679	0.582946155	1	0.933	1
JMJD4	0.611845474	0.802110258	1	1	1
ENSSSCG00000063532	0.612643102	0.619480615	0.938	0.933	1
ENSSSCG00000053530	0.61403575	0.708455429	1	1	1
SVIP	0.614800867	0.923569337	0.812	0.733	1
ENSSSCG00000062546	0.615078376	0.076815597	0.562	0.4	1
TAF11	0.615164197	0.502936595	1	1	1
EML6	0.615191582	0.509555098	1	1	1
ABI3BP	0.616020492	0.496537175	1	1	1
ENSSSCG00000056714	0.616487851	0.442943496	0.688	0.533	1
CNOT9	0.616510727	0.600253408	1	1	1
SERPINA6	0.616930915	0.555418225	0.875	0.667	1
TEX30	0.617111266	0.745252205	1	1	1
ELF5	0.617759433	1.193771743	0.875	0.6	1
ZSCAN29	0.618095215	0.539158811	0.5	0.533	1
ENSSSCG00000012074	0.61829248	0.993768046	1	1	1
SLC22A15	0.618303174	1.055753982	0.938	0.933	1
PLEKHA1	0.618377289	0.812613895	1	1	1
ENSSSCG00000055399	0.618568056	0.669496011	0.938	0.867	1
ENSSSCG00000028195	0.619076279	0.387516437	0.812	0.733	1
DCTN5	0.619187351	0.538957331	1	1	1
C14orf28	0.619312233	0.770569124	0.938	0.933	1
TMEM161B	0.619485374	0.730567823	0.875	0.867	1
C2orf42	0.620062271	0.411909462	1	1	1
SPRYD3	0.620104039	0.354349573	0.875	1	1
CLCN6	0.620165702	0.532495081	0.75	0.733	1
PRSS12	0.620289976	0.437405312	0.688	0.667	1
TRIM65	0.620451935	0.121015401	0.562	0.733	1
CLUH	0.62053959	0.894893063	1	1	1
MYLIP	0.620959878	0.823059005	1	1	1
PPFIA2	0.621294228	0.486624582	1	1	1
ENSSSCG00000056325	0.621372231	0.409390936	0.5	0.533	1
ASMT	0.62153154	0.701571688	0.938	0.733	1
PLEKHA2	0.621674208	0.760109258	1	1	1
FAM216A	0.621757454	0.372554168	0.688	0.533	1
FN1	0.622253576	0.505149919	0.688	0.733	1
IGSF9	0.622573503	1.285402219	0.562	0.267	1
ZNF385B	0.62319137	0.906890596	0.75	0.667	1
FADD	0.623200517	0.476256241	0.625	0.667	1
ZNF148	0.623243855	0.682184308	1	0.733	1
SYNPO2	0.623922409	0.735124247	0.812	0.933	1
BMERB1	0.623940233	0.483815777	0.938	0.867	1
NEK2	0.624049125	0.676827161	1	1	1
LTBP2	0.624129456	1.321928095	0.562	0.333	1
ITGB4	0.624226044	0.256224848	0.75	0.733	1
NUP205	0.625136272	0.489572155	1	1	1
POLK	0.625345371	0.685475279	1	0.8	1
CDK12	0.625668033	0.530087315	1	1	1
RPS6KA3	0.625974526	0.568177916	1	1	1
SKAP2	0.626247792	1.073787904	1	1	1
GABRR2	0.626273533	0.817005092	0.938	0.933	1
FBXW7	0.626350213	0.721535554	1	0.933	1
RGS22	0.626471043	0.562445064	1	1	1
GABPB1	0.626596532	0.71897567	1	1	1
COCH	0.627218264	0.568490066	1	1	1
SLC20A2	0.627935112	0.807354922	0.812	0.8	1
AGBL2	0.628378153	0.3594028	0.875	1	1
CCR1	0.62838867	0.65011518	0.938	1	1
ATP2B4	0.628610199	0.60694087	1	1	1
THSD4	0.628732013	1.006426269	0.625	0.4	1
ENSSSCG00000037963	0.628776172	0.685676365	1	1	1
NAA15	0.628789607	0.392964033	1	1	1
MRPL39	0.629263997	0.458193785	1	1	1
ENSSSCG00000035908	0.629319724	0.595935033	1	1	1
RBM48	0.629669362	0.314768303	1	1	1

MAP2	0.629772887	0.461479447	0.938	0.8	1
SLC43A3	0.630176404	0.881579507	1	1	1
QRFPR	0.630269601	0.293913719	0.5	0.333	1
ENSSSCG00000047090	0.630355821	0.965784285	0.875	0.6	1
ENSSSCG00000058385	0.630423441	0.576465324	1	1	1
SLC25A53	0.630746769	0.312882955	0.688	0.6	1
CORO1C	0.630751553	0.702598293	1	1	1
ENSSSCG00000061837	0.63125623	1.036173613	0.688	0.667	1
CDYL2	0.631723903	0.63481105	1	1	1
PRKD2	0.631762467	0.613159393	0.562	0.6	1
LMNB1	0.631948608	0.69930442	1	1	1
ICMT	0.631965543	0.954141942	1	1	1
ENSSSCG00000004489	0.632251862	0.520571407	1	1	1
EIF2S1	0.632498249	0.339385292	1	1	1
GRM7	0.632555555	0.033087388	1	1	1
STXBP5	0.63267688	0.916015832	1	1	1
ZNF292	0.632881779	0.166494096	1	1	1
FERD3L	0.632922143	0.984893108	0.562	0.2	1
MAML1	0.632957141	0.68061474	1	1	1
GLMN	0.633336235	0.392317423	1	0.867	1
PRDM6	0.633608883	0.574212891	1	1	1
GSAP	0.633652711	0.524274574	0.812	0.8	1
UBE2K	0.633825933	0.536849267	1	1	1
RAB18	0.633963601	0.57505611	1	1	1
HS6ST1	0.634342227	0.430452552	0.5	0.4	1
CACNA2D4	0.634372486	0.705274519	1	1	1
C3orf38	0.634612645	0.509887019	1	1	1
CNNM3	0.634725339	0.757302055	1	1	1
ADAMTS18	0.634904184	0.624082516	1	0.933	1
ABCC5	0.634990332	0.394684437	0.812	0.867	1
MECR	0.635169875	0.3594028	0.75	0.733	1
MRPL43	0.63523784	0.795036291	1	1	1
GRAMD1C	0.63579771	0.058893689	0.5	0.533	1
TIGD4	0.636267878	0.761285273	0.938	0.933	1
CXCR4	0.636665477	0.789054105	0.875	0.533	1
TDRKH	0.636916581	0.182455186	0.938	1	1
KASH5	0.636927439	0.440322796	0.812	0.6	1
DNAJC6	0.636928725	0.484393787	1	1	1
ENSSSCG00000009157	0.637082468	0.345846722	1	1	1
HARBI1	0.637203589	0.710140058	0.938	1	1
ENSSSCG00000055074	0.637350469	0.509555098	0.625	0.733	1
PPIP5K1	0.637716007	0.927952211	0.625	0.667	1
ENSSSCG00000010884	0.638060224	1.044394119	0.688	0.6	1
ENSSSCG00000059944	0.638360715	0.563359061	1	1	1
GTF2A1L	0.638509838	0.163104187	1	1	1
ZCWPW2	0.638607752	0.799281622	0.938	0.933	1
MFSD4B	0.638659102	0.680324357	1	1	1
ENSSSCG00000058576	0.638678839	0.769387072	0.5	0.267	1
ENSSSCG00000058300	0.638750572	0.653771659	0.875	0.8	1
TMEM35A	0.638979338	0.805010982	0.688	0.6	1
SLF2	0.639270791	0.539833125	1	1	1
ENSSSCG00000051837	0.639497406	0.411884792	1	0.933	1
HLF	0.639730796	0.825753833	0.938	1	1
E2F5	0.639800421	0.211745177	0.5	0.533	1
CNN2	0.639942778	0.357153757	1	1	1
CANX	0.640434965	0.679294694	1	1	1
PLEKHA5	0.640517502	0.807354922	0.875	0.533	1
ASXL2	0.640523415	0.897283268	1	1	1
GPSM2	0.640602959	0.540216118	1	1	1
RAD9B	0.640775692	1.055282436	0.875	0.8	1
TMEM51	0.64106893	0.762500686	0.938	1	1
DUSP14	0.64154995	0.773349745	1	1	1
PEX6	0.641555279	0.211745177	0.5	0.533	1
ENSSSCG00000055034	0.641565026	0.064431873	0.5	0.6	1
TMEM18	0.641983049	0.619314006	1	0.933	1
ENSSSCG00000062177	0.64215586	0.850307067	0.562	0.4	1
PRCC	0.642172138	0.648697453	1	1	1
RAB11A	0.642318895	0.712561703	1	1	1
ACO1	0.64262078	0.484435625	0.938	0.867	1
KTN1	0.642647571	0.623388711	1	1	1

SMARCAD1	0.6429263	0.579595991	1	1	1
ENSSSCG00000047287	0.642999974	0.321928095	1	1	1
TTF1	0.643116892	0.915352174	0.875	1	1
NFATC3	0.643699498	0.711166125	1	0.867	1
ENSSSCG00000063479	0.643875218	0.52931011	1	1	1
DNAH6	0.644509139	0.748805924	1	1	1
RNMT	0.644650049	0.688374229	1	1	1
ENSSSCG00000037401	0.644773354	0.837627933	0.75	0.6	1
CAD	0.644779652	0.466318004	0.625	0.6	1
DNAJB14	0.644924089	0.7570668	1	1	1
NID1	0.645037734	0.480075929	0.812	1	1
ORC6	0.645085941	0.573845582	1	1	1
ADAM19	0.645113587	0.674282491	1	1	1
ENSSSCG00000045838	0.645654305	0.250844997	0.625	0.467	1
ENSSSCG00000051325	0.64575708	0.526859546	0.938	1	1
POU3F2	0.645799032	0.421463768	0.688	0.467	1
ABAT	0.645846029	-0.093109404	0.562	0.733	1
ENSSSCG00000057133	0.645967641	0.867719998	0.812	0.733	1
ENSSSCG00000044626	0.646182527	0.929258409	0.875	0.6	1
ACTN1	0.64632393	0.984893108	0.875	0.733	1
BAZ2B	0.646517313	-0.005291526	0.938	0.733	1
OR10G3	0.64693414	0.965784285	0.625	0.4	1
CELF3	0.647129357	0.514573173	0.75	0.533	1
GCFC2	0.64724192	0.67507492	0.812	1	1
PGM2L1	0.647451792	0.539021882	1	1	1
ENSSSCG00000054374	0.647557933	0.347463187	0.625	0.667	1
ENSSSCG00000059141	0.647920355	0.72631835	0.438	0.533	1
CCDC174	0.648024082	0.47720632	0.938	0.867	1
PAWR	0.648398431	0.769049954	1	1	1
PCNX3	0.648555878	0.351192689	0.938	0.867	1
CYP2U1	0.64874598	0.184424571	0.688	0.6	1
COX11	0.648800238	0.209453366	0.625	0.533	1
IL17RB	0.649545187	0.508154909	0.875	0.933	1
GADL1	0.649557043	0.792431871	0.875	0.8	1
ENSSSCG00000054723	0.64990478	0.354349573	0.625	0.8	1
PIK3C2G	0.65012851	0.453633774	0.938	0.933	1
STK11IP	0.650290948	0.881445579	1	0.8	1
ENSSSCG00000055210	0.650419069	0.638862	0.938	1	1
KIAA2026	0.650552665	0.324448087	0.938	1	1
IFT80	0.650770576	0.517323784	1	1	1
RABL3	0.650923876	0.214522845	1	1	1
TWNK	0.651087684	0.636243006	0.938	1	1
NOC3L	0.651575246	0.998038484	0.875	0.933	1
TRIM66	0.651645888	0.321928095	0.688	0.6	1
FAM174A	0.653209025	1.491853096	0.5	0.467	1
ZBTB40	0.653276303	0.814133455	1	1	1
GPNMB	0.653362736	0.298023528	1	1	1
ENSSSCG00000043807	0.653663861	0.113341473	0.625	0.467	1
MCTP2	0.653817424	0.49816687	0.875	0.867	1
ENSSSCG00000054366	0.654496989	0.378196315	0.875	0.867	1
SOWAHB	0.654608026	0.293913719	0.75	0.6	1
LVRN	0.654705857	0.427722759	1	0.933	1
HERC3	0.654856902	0.757195445	1	1	1
ZNF527	0.654954516	0.468317582	0.938	1	1
GMPR2	0.655011438	0.797266104	0.875	0.667	1
MED17	0.655197918	0.8560152	1	1	1
GBA1	0.65541859	0.042242449	0.812	0.733	1
LDLR	0.655952459	0.639162314	1	1	1
ELAVL3	0.656075788	1.169925001	0.5	0.133	1
FANCB	0.656165384	0.643414255	1	1	1
COL17A1	0.656417081	0.245692509	0.75	0.8	1
ENSSSCG00000053550	0.656741728	1.09330372	0.812	0.733	1
SH3GLB1	0.656777059	0.620081839	1	1	1
SVEP1	0.65689782	0.541073171	1	1	1
RAPGEF4	0.656982393	0.519867472	0.875	0.933	1
MLIP	0.657404268	0.347463187	0.75	0.8	1
ENSSSCG00000058582	0.657971604	0.906890596	0.562	0.667	1
NABP1	0.658063325	0.880579318	1	1	1
SEMA6D	0.658255797	1.432352085	0.812	0.6	1
HADHB	0.658521456	0.649224364	1	1	1

NLGN1	0.658674512	1.169925001	0.75	0.667	1
TSPYL1	0.658992986	0.516944077	1	1	1
HCN3	0.659441398	0.727341175	0.938	0.867	1
WRNIP1	0.659497514	0.731119336	1	0.933	1
ZNF438	0.659665597	0.321928095	0.75	0.6	1
ENSSSCG00000044651	0.660324762	0.906890596	0.5	0.133	1
DEPDC5	0.660352498	0.641508435	1	0.867	1
ENSSSCG00000053141	0.660516911	0.38332864	0.75	0.867	1
ENSSSCG00000045328	0.660879908	0.285402219	0.375	0.6	1
PPM1E	0.66090447	0.498269838	1	1	1
PURG	0.661387384	0.099535674	0.812	0.533	1
UBAC1	0.661394433	0.669851398	0.875	0.6	1
TGFBRAP1	0.661579014	0.750634898	1	1	1
NRF1	0.661599416	0.727283041	1	1	1
RUNX1T1	0.661748801	0.5493026	1	1	1
SLC35A2	0.661782599	0.895575282	1	0.867	1
SCIN	0.662375918	0.6622055	0.938	1	1
SPAG9	0.662423562	0.822935073	1	1	1
ENSSSCG00000056189	0.662552415	0.64385619	0.188	0.533	1
GPR12	0.662855656	0.781359714	0.75	0.8	1
RAD54L2	0.662973103	0.732507724	1	1	1
CALB1	0.663236637	0.584962501	0.5	0.733	1
DAPP1	0.663429634	0.641500048	1	1	1
BRINP1	0.663634726	0.083768358	0.625	0.533	1
EIF4G3	0.663814198	0.475094183	1	1	1
MDM1	0.663893999	0.74624586	1	1	1
PSME3	0.6641385	0.429161318	1	1	1
SLC2A5	0.664214203	0.427722759	0.625	0.467	1
ENSSSCG00000051151	0.664300951	0.62935662	0.562	0.733	1
TRIM42	0.664447281	1.519867472	0.562	0.467	1
KLF5	0.66464179	0.167757165	1	1	1
GOSR1	0.664951403	0.529874941	1	1	1
ENSSSCG00000034192	0.664999383	0.655537995	1	1	1
ENSSSCG00000056202	0.665359338	0.421463768	0.5	0.667	1
ENSSSCG00000036124	0.665495695	0.096551815	1	1	1
ENSSSCG00000063471	0.665647326	0.510468774	0.875	0.8	1
SLC38A9	0.665848169	0.811706174	1	1	1
ENSSSCG00000063173	0.6658642	0.332816411	1	0.933	1
ZNF197	0.665969836	0.553253641	0.5	0.533	1
ITPK1	0.66670331	0.210282739	0.75	0.8	1
ENSSSCG00000032297	0.666705397	0.680254951	1	1	1
CHUK	0.666816821	0.679154853	1	1	1
TLNRD1	0.666912416	0.706865987	1	1	1
ENSSSCG00000059771	0.667170341	0.406461605	0.938	0.933	1
ENSSSCG00000052502	0.667785639	0.74819285	0.312	0.533	1
PBX2	0.667986452	1.022367813	0.5	0.333	1
COL21A1	0.668123596	0.276124405	0.562	0.6	1
TIGD3	0.668233407	0.169925001	0.5	0.333	1
ZHX3	0.668391412	0.242310927	1	1	1
ZFP90	0.668598009	0.299976268	1	1	1
SLC25A42	0.668879791	0.457905764	0.75	0.667	1
FAM3C	0.669248628	0.789342298	1	1	1
CRBN	0.669475841	0.480861081	1	1	1
SH3D19	0.669758294	0.392317423	0.75	0.533	1
HSDL2	0.669798034	0.588613355	1	1	1
RIPOR2	0.669804645	0.659975855	1	0.867	1
NOTCH2	0.669827019	0.811733363	1	0.8	1
ABL2	0.670046728	0.680832838	1	1	1
ENSSSCG00000055153	0.670212813	0.906890596	0.875	0.8	1
RALGAPA1	0.670441821	0.603498452	1	1	1
SLC10A6	0.670488906	0.743391863	0.625	0.533	1
NAP1L4	0.670561144	0.753195038	1	1	1
SLC2A13	0.670715343	0.670247689	1	1	1
DDR2	0.670934815	0.945025724	1	1	1
ZNF26	0.671274212	0.619743734	1	1	1
ENSSSCG00000045607	0.67154914	0.773624065	0.562	0.6	1
SDF2L1	0.671949294	0.595761902	1	0.933	1
FEM1B	0.671994918	0.381286158	1	1	1
ENSSSCG00000053861	0.672000305	0.1627295	1	0.933	1
DNAH10	0.672204059	0.824428435	0.812	0.733	1

BTG3	0.672381255	0.886712714	0.75	0.467	1
JAK1	0.673417755	0.646743376	1	1	1
SEC31B	0.673458879	0.646922493	0.875	1	1
RGS7	0.673634122	0.94641896	0.625	0.467	1
MARK1	0.673788887	0.609504685	1	1	1
ENSSSCG00000049364	0.674289085	0.321928095	0.625	0.533	1
CHRM2	0.674438194	0.349248701	1	0.933	1
MPZL3	0.674637185	0.957516669	0.938	0.867	1
CARNMT1	0.675430445	0.526986633	1	1	1
PLBD2	0.6759078	0.794997716	0.875	1	1
ENSSSCG00000046103	0.676128651	0.906890596	0.75	0.533	1
POGLUT3	0.676452024	1.136896201	1	0.733	1
ENSSSCG00000032439	0.676480452	0.254813899	0.562	0.333	1
NDFIP1	0.677119266	0.732743256	1	1	1
HTR7	0.677230403	0.305439972	0.625	0.467	1
FKBP15	0.677610254	0.126271126	1	0.933	1
ENSSSCG00000045184	0.678463074	0.099535674	0.688	0.8	1
ENSSSCG00000031911	0.678480754	1.064431873	0.625	0.467	1
NELFB	0.678491057	0.797266104	0.938	0.667	1
DNAAF5	0.678853469	0.899801171	0.938	0.8	1
MTTP	0.679027333	1.058893689	0.5	0.267	1
MCUR1	0.679627607	0.585961251	1	1	1
RUSF1	0.679773832	0.512611656	0.688	0.8	1
ENSSSCG00000052959	0.679828498	0.178192617	0.875	0.667	1
ENSSSCG00000021757	0.679881118	0.327176173	0.938	1	1
PRPF18	0.680580808	0.436759251	1	1	1
ENSSSCG00000058255	0.680703075	0.774618136	1	1	1
ZNF800	0.681156052	0.593474001	0.938	0.933	1
IDNK	0.681284371	0.888628389	0.938	1	1
TMEM164	0.681322715	0.632952444	1	1	1
LSM1	0.681567189	0.438337587	1	0.867	1
ZNF7	0.681818545	0.745455007	0.938	1	1
EEA1	0.681870635	0.74199799	1	1	1
MTPN	0.681893393	0.745491984	1	1	1
ENSSSCG00000002202	0.682007042	1.299208018	0.5	0.2	1
EXOG	0.682066322	0.336283388	0.875	0.8	1
ENSSSCG00000054944	0.682496378	0.380821784	0.562	0.4	1
DNAH7	0.682732169	0.602538083	1	1	1
DCAF15	0.683148082	0.588361077	1	0.867	1
ARL1	0.683738813	0.639410285	1	1	1
ENSSSCG00000060078	0.683915244	0.22881869	0.688	0.533	1
ANKS1A	0.684149826	0.847996907	1	0.933	1
RAB30	0.684523868	0.636969804	0.938	1	1
UNKL	0.68482678	0.957280876	1	1	1
TTC31	0.685134506	0.754887502	0.688	0.4	1
ABCB10	0.68530049	1.026800059	1	1	1
BNIPL	0.685336806	-0.005646563	0.5	0.533	1
CAPN15	0.685370437	1.22881869	0.5	0.2	1
PRICKLE4	0.685593792	0.756421352	1	1	1
FBLIM1	0.685887011	0.676770812	1	0.933	1
ENSSSCG00000023320	0.685975672	0.780653869	1	1	1
HYAL2	0.686092377	0.908506156	1	1	1
CBLB	0.686233972	0.369739575	1	1	1
GPATCH3	0.686523323	0.554588852	0.812	0.8	1
HOXA7	0.686713375	0.793233814	0.938	0.733	1
ENSSSCG00000038192	0.686883349	0.321928095	0.625	0.4	1
SLC5A3	0.686910793	-0.377676972	0.625	0.667	1
RNF44	0.687048817	0.432959407	0.938	0.933	1
ATR	0.687111401	0.547934648	1	1	1
NECAB1	0.687173136	0.509298231	0.938	0.867	1
RBBP6	0.68726885	0.637062457	1	1	1
THUMPD2	0.687298021	0.581795222	0.875	0.8	1
PRPF38A	0.687510673	0.528378972	1	1	1
CAPN10	0.687569621	1.030625964	0.938	0.8	1
FGF11	0.688181111	0.988684687	0.75	0.733	1
PACRGL	0.6885506	0.513795649	0.938	0.867	1
PLCL2	0.68861493	0.056699476	0.938	1	1
UBE2E3	0.688722005	0.703053993	1	1	1
RGMB	0.689049918	0.564498398	0.938	0.867	1
PTPRZ1	0.68950452	0.832350117	1	1	1

NEDD4	0.689780819	0.812344959	1	0.933	1
ENSSSCG00000002657	0.689960526	0.491853096	0.688	0.533	1
ENSSSCG00000039823	0.690652111	0.646922493	1	1	1
ENSSSCG00000004387	0.690731503	0.437977243	1	1	1
ENSSSCG00000032084	0.691138408	0.312882955	0.875	0.667	1
SLC41A1	0.691208385	0.22881869	0.75	0.533	1
ENSSSCG00000002520	0.692116989	1.058893689	0.688	0.467	1
ABTB2	0.69222528	0.904634622	1	1	1
LRP5	0.692976202	0.34298971	0.938	0.933	1
YTHDF3	0.693190475	0.827354555	1	1	1
F9	0.693461127	0.764871591	0.812	0.6	1
ENSSSCG00000016548	0.693540728	0.484330138	1	1	1
SASH1	0.693719228	0.583276124	0.938	0.867	1
ASPH	0.693790311	0.841302254	0.812	0.733	1
MTMR12	0.693880681	0.672834023	1	1	1
MDC1	0.693880908	0.044394119	0.75	0.533	1
PAQR5	0.69425312	0.451944375	1	0.933	1
ENSSSCG00000055221	0.694670521	0.575158627	1	1	1
BCKDHB	0.695463636	0.533766738	0.938	0.867	1
PPP3R1	0.69560038	0.458706828	1	1	1
OSGIN2	0.695823762	0.635468404	1	0.867	1
LSMEM2	0.696329096	0.421463768	0.5	0.4	1
FBXO6	0.696766158	0.633872101	0.688	0.8	1
TMEM80	0.697105977	0.361456459	0.75	0.6	1
ENSSSCG00000047109	0.697155743	0.417085328	0.812	0.8	1
WRN	0.697195159	0.290563924	1	1	1
GPBP1L1	0.697378523	0.714717064	1	1	1
PHIP	0.697568358	0.073669042	0.938	0.867	1
FAM83D	0.698057427	0.633740846	0.938	1	1
MEGF10	0.698059526	0.562674434	1	0.933	1
OTUD3	0.698355272	0.600705237	1	0.933	1
SOX5	0.698527848	0.64385619	0.625	0.467	1
CACUL1	0.699129036	0.313688809	1	1	1
ENSSSCG00000028674	0.699399229	-0.637429921	0.5	0.333	1
TMEM255A	0.69995396	0.767993745	1	1	1
KDM4B	0.70094424	0.979299091	1	0.8	1
FAM13A	0.701228391	0.649007478	1	1	1
SMARCC2	0.701378172	0.789054105	1	1	1
ENSSSCG00000055878	0.701394186	0.129283017	0.5	0.4	1
TXNDC9	0.701714038	0.926057569	1	1	1
ZNF624	0.702573289	0.631487632	0.938	1	1
EBF1	0.702592967	0.8518673	1	1	1
ENSSSCG00000057227	0.703426205	-0.03170886	0.688	0.533	1
TBL1XR1	0.703441707	0.577315042	1	1	1
AUNIP	0.70347692	0.354349573	1	0.8	1
KRI1	0.703805552	0.290219235	0.5	0.6	1
PYM1	0.70394556	0.497370101	1	0.8	1
EML4	0.703952226	0.668399579	1	1	1
CDC23	0.704473089	1.165624864	0.5	0.467	1
GRK3	0.704670657	0.454763778	1	1	1
RYR2	0.704712284	0.627402258	1	1	1
ZIC5	0.705016487	0.43198164	0.812	0.6	1
TRAPPC11	0.705156924	0.408798818	0.938	1	1
FBXO32	0.705583224	1.372554168	0.688	0.533	1
ENSSSCG00000026253	0.705842762	0.819427754	0.5	0.4	1
SDK1	0.705890342	0.62309763	0.812	0.667	1
MAP3K5	0.706100684	0.313515855	0.812	0.867	1
ENSSSCG00000050890	0.706159543	0.930737338	0.75	0.533	1
PHTF1	0.706451941	0.654283561	1	1	1
YIPF4	0.706704943	0.736356219	1	1	1
ENSSSCG00000052917	0.707230908	0.687880814	0.938	0.867	1
SNTB2	0.707339865	0.556393349	0.875	0.933	1
DMRT1	0.707536171	0.489133595	1	1	1
KCNV1	0.707784622	0.447458977	0.75	0.933	1
HCK	0.708251428	0.778069644	1	0.933	1
GAPVD1	0.708416712	0.430066325	1	1	1
HAUS5	0.708506916	0.784649342	1	1	1
SEPTIN3	0.708539826	0.712404171	0.938	1	1
FBXO8	0.708709472	0.518213683	1	1	1
MAST3	0.709351668	0.425855538	1	0.933	1

RPS27L	0.709760079	0.465924631	1	1	1
ENSSSCG00000061734	0.70983615	0.299208018	0.812	0.533	1
TMED10	0.709899238	0.623542425	1	1	1
PNPLA8	0.710536325	0.46001183	1	1	1
TSFM	0.71154598	0.452324732	0.938	0.733	1
LMX1A	0.711620133	0.765846714	0.875	0.8	1
ENSSSCG00000010401	0.711686397	0.55288253	1	1	1
PPTC7	0.711767969	1.219059724	0.875	0.933	1
GPI	0.711976443	0.520264709	1	1	1
ENSSSCG00000007143	0.712092123	0.402659367	1	0.933	1
CCDC6	0.712121553	0.546811741	1	1	1
EXOSC3	0.712166816	0.399930607	0.938	0.8	1
NRGN	0.712291006	0.762500686	0.938	0.667	1
ENSSSCG00000061246	0.7126964	0.794415866	0.875	0.867	1
RASSF5	0.712780083	0.269460675	0.5	0.4	1
ENSSSCG00000059615	0.712781157	0.684235651	1	1	1
ADAP1	0.713305301	0.74819285	1	1	1
ENSSSCG00000046060	0.714065065	0.209453366	0.5	0.6	1
GTSE1	0.714123813	0.693896872	0.938	0.733	1
MEGF11	0.71418488	0.509131088	1	0.8	1
MED26	0.714220585	0.108524457	0.438	0.533	1
LRRC8A	0.714272712	0.816125132	0.812	0.933	1
MOCS3	0.714702919	0.436670181	1	1	1
ANP32E	0.714875638	1.213551934	0.812	0.733	1
HES6	0.715106994	1.012542383	1	0.933	1
RASA1	0.715324211	0.494780288	1	1	1
SSBP2	0.715379506	0.711702532	1	1	1
RASGRP3	0.715706302	0.380000352	1	1	1
ECHDC1	0.715895075	0.473931188	0.812	0.733	1
BRD3	0.716228273	0.517848305	1	1	1
UBE2D2	0.716309012	0.769609008	1	1	1
SATB2	0.716977517	0.242493627	0.875	0.6	1
MATR3	0.71741442	0.59886779	1	1	1
RAB22A	0.717522287	0.99993932	1	1	1
SAXO2	0.717596484	0.645327577	1	1	1
NCBP1	0.718181729	0.629844099	1	1	1
GTF3C4	0.718256874	0.588068412	0.938	0.867	1
IQSEC2	0.718655659	0.854423176	0.688	0.4	1
CTSV	0.719419683	1.233784943	0.938	0.8	1
RXFP2	0.719493385	0.684498174	0.938	0.8	1
H3-3A	0.719609182	0.41603244	1	1	1
SERINC1	0.719727161	0.588334039	1	1	1
GABRA4	0.719846401	0.478242665	0.938	0.8	1
DCUN1D5	0.719867203	0.809711527	1	1	1
ENSSSCG00000052540	0.719913988	0.451886689	0.688	0.733	1
CMC1	0.720205176	0.311944006	1	1	1
ELAVL4	0.720456943	0.475174355	0.812	0.667	1
ENSSSCG00000032495	0.720993584	0.333705263	0.938	0.8	1
CACNG8	0.721012818	0.658963082	0.75	0.4	1
TAF3	0.721444538	0.860635005	1	1	1
ENSSSCG00000059577	0.722364492	0.872125177	0.812	0.733	1
IL27RA	0.722619028	0.906890596	0.875	0.6	1
GJA8	0.722814498	0.354349573	0.5	0.533	1
BICRAL	0.72300819	0.3594028	0.875	0.8	1
ENSSSCG00000054421	0.723456942	0.006426269	0.562	0.533	1
LINGO3	0.723504007	0.854423176	0.938	0.8	1
NIPAL2	0.723518067	0.321928095	0.5	0.4	1
PHKB	0.723720162	0.493694443	1	1	1
TMEM25	0.72410875	0.942895549	1	1	1
C1orf146	0.72425827	0.676211563	1	1	1
ENSSSCG00000052265	0.724544982	0.764871591	0.625	0.6	1
SF1	0.724648069	0.63907905	1	0.933	1
PDZD9	0.724649997	0.868416448	0.75	0.733	1
PTPN3	0.724980091	0.554472133	1	1	1
NISCH	0.725066236	0.794415866	1	1	1
PDE4D	0.725912598	0.576741994	0.812	0.6	1
FGD4	0.726698572	0.594128132	1	1	1
TCF4	0.726713636	0.824428435	0.5	0.333	1
TXNIP	0.726811145	0.138432454	0.812	0.8	1
CAPN3	0.72717625	0.289360232	0.812	0.733	1

EDNRB	0.727442576	0.123284276	0.875	1	1
KLHL3	0.727917412	0.781990124	1	1	1
ENSSSCG00000059602	0.728120675	0.491853096	0.562	0.267	1
C7orf25	0.729584944	0.716919652	0.938	1	1
KLC4	0.729760094	0.836501268	1	0.733	1
TRIM45	0.730312654	0.187292133	1	1	1
SELENOK	0.730791931	0.276753168	1	1	1
PDCD2L	0.730992751	0.500182081	1	1	1
SLC9A1	0.731916047	0.639410285	0.812	0.933	1
DENND10	0.731943985	0.981389718	1	1	1
MRTFA	0.732582213	0.569855608	0.938	1	1
OGFRL1	0.732728568	0.75802721	0.938	0.8	1
ENSSSCG00000054437	0.733211598	0.044394119	0.688	0.667	1
LDB1	0.73380596	0.539471117	1	1	1
MYO9A	0.733846605	0.358532513	0.938	1	1
ANKRD45	0.733966848	0.052968855	1	0.933	1
TERB1	0.734192235	0.743941357	1	1	1
SH2D3C	0.734285781	0.404390255	0.5	0.533	1
IPP	0.7342987	0.635260166	1	1	1
GALNT4	0.734393626	0.459431619	0.625	0.333	1
TRBV9	0.73467249	0.531381461	0.688	0.667	1
SORT1	0.734726508	0.643378595	1	1	1
ENSSSCG00000041810	0.734875681	0.452052088	0.938	0.8	1
ENSSSCG00000054816	0.735056334	0.222392421	0.938	0.733	1
ENSSSCG00000055326	0.735132631	0.584962501	0.75	0.467	1
ZNF182	0.736486937	0.467847633	1	0.933	1
FDX1	0.736574402	0.602803813	1	1	1
FOXK2	0.736805101	0.537244999	1	1	1
TXK	0.737312736	0.321928095	0.75	0.667	1
ENSSSCG00000046648	0.737961174	0.017921908	0.75	0.6	1
DHX57	0.73896282	0.710781662	1	1	1
KPNA3	0.739154724	0.697662633	1	1	1
E2F3	0.73932422	0.314065977	1	1	1
FLCN	0.739412664	1.052968855	0.938	0.8	1
OCIAD1	0.73952599	0.530528506	1	1	1
VCP	0.740203139	0.563124918	1	1	1
LIN7A	0.740537299	0.807354922	0.5	0.533	1
ENSSSCG00000010943	0.740600316	0.658606641	1	1	1
GATA4	0.740897475	0.716311923	1	1	1
CDC42BPG	0.741656558	0.321928095	0.688	0.667	1
LATS2	0.741915155	0.86561432	1	1	1
ENSSSCG00000014822	0.741949359	0.558967292	0.562	0.467	1
GRPR	0.742080328	0.0138058	0.5	0.533	1
DNAH5	0.742927213	0.141355849	0.812	0.667	1
SLC25A44	0.743029912	0.679394063	0.938	1	1
L3HYPDH	0.743373897	0.884864289	0.875	0.733	1
PPP2R2A	0.743945631	0.667982087	1	0.933	1
ASH1L	0.743977192	0.557898431	1	1	1
ENSSSCG00000042032	0.743985431	1.22881869	0.5	0.2	1
NNT	0.744106289	0.694049917	1	1	1
FGF2	0.744213317	0.758791957	0.938	0.867	1
NCAPD2	0.74424237	0.716990894	0.938	1	1
FAM161A	0.74427144	0.716538786	1	1	1
AMDHD1	0.744459007	0.684498174	0.688	0.333	1
PNN	0.744645602	0.746459125	1	1	1
NEBL	0.744888391	0.440587318	1	1	1
DDA1	0.744998332	0.473023028	1	1	1
GIGYF1	0.745041048	0.723178642	1	0.8	1
ENSSSCG00000063417	0.745305742	0.413850584	0.938	0.933	1
ZFYVE19	0.745917533	0.36289674	1	1	1
BROX	0.746205983	0.910532847	1	1	1
BAZ2A	0.746478198	0.777717895	0.938	1	1
LDLRAD3	0.747135705	0.463283944	0.938	0.533	1
MPZL1	0.74741741	0.635901805	1	1	1
ZNF287	0.747433962	0.549136506	0.938	1	1
DENND2B	0.747652888	0.189290326	0.75	0.867	1
ELK4	0.748438525	0.588730971	1	1	1
SGSM1	0.748520284	0.651051691	0.75	0.733	1
ENOPH1	0.748809045	0.632030754	0.875	1	1
ENSSSCG00000003352	0.748854607	0.767123231	1	1	1

ENSSSCG00000051454	0.748986725	1.076815597	0.562	0.4	1
HACD3	0.749226734	0.571922296	1	1	1
SLC15A1	0.749402872	0.44467116	1	1	1
TDP1	0.750015183	0.652250631	1	1	1
GAN	0.750242279	0.441729617	1	0.933	1
CSMD1	0.751032131	0.33752495	0.875	0.867	1
ENSSSCG00000041656	0.751226697	0.906890596	0.625	0.533	1
EXOC5	0.751374835	0.848303569	1	1	1
TMEM41B	0.751782358	0.609653665	1	1	1
ENSSSCG00000060025	0.752185713	0.470791481	0.75	0.6	1
FOXN1	0.752717665	0.86507042	0.812	0.867	1
ENSSSCG00000063030	0.75281997	0.514573173	1	1	1
MYO7A	0.753109756	0.488664819	0.875	1	1
COA7	0.753178856	0.452446056	1	1	1
TOPAZ1	0.753360693	0.723178642	1	1	1
CACNA1D	0.753434638	0.633325522	0.812	0.6	1
CERT1	0.753921045	0.597906604	1	1	1
TRIB1	0.754281394	1.169925001	0.5	0.267	1
ENSSSCG00000042386	0.754487425	0.550746785	0.938	0.733	1
FNIP1	0.75449222	0.638614105	1	1	1
BOD1L1	0.754636526	0.735124247	1	1	1
ENSSSCG00000059073	0.754857885	0.384937892	0.812	0.467	1
ENSSSCG00000036608	0.754989398	0.405159624	0.938	1	1
DAP	0.755143977	1.039085208	1	0.867	1
ATAD3A	0.755179372	0.540108265	0.75	0.667	1
ENSSSCG00000038151	0.755322482	0.786596362	0.75	0.733	1
BRIP1	0.755508387	0.518776381	0.938	1	1
TUBA1A	0.756560237	0.425855538	0.75	0.933	1
VPS37C	0.756947121	0.673691419	0.938	0.933	1
SIM1	0.757207333	0.247927513	0.625	0.6	1
CLK1	0.757291717	0.753878977	0.938	0.8	1
ARFGEF3	0.757830154	0.547706331	1	0.933	1
ATOSB	0.757959253	0.754887502	0.375	0.533	1
ENSSSCG00000035142	0.758161137	0.650399036	1	1	1
ENSSSCG00000050552	0.758432801	0.652135668	1	0.933	1
G2E3	0.75844302	0.446175522	1	1	1
ZNF507	0.758576013	0.54819108	1	1	1
NR1H2	0.758603649	0.906890596	1	0.933	1
NRIP3	0.7586988	0.878754806	1	1	1
ENSSSCG00000042371	0.758956328	-0.292418213	0.562	0.467	1
EFHC2	0.759045038	0.658813127	1	1	1
CCDC25	0.759763476	0.633452094	1	1	1
MEIS3	0.760876634	0.366322214	0.812	0.733	1
ENSSSCG00000038009	0.761087454	0.538578172	1	1	1
ZBTB41	0.761234695	0.777053158	0.938	0.933	1
ZRANB3	0.761388834	0.591585443	0.938	0.933	1
GNAL	0.761419032	0.906890596	0.5	0.333	1
MPV17L	0.762273514	0.500570313	0.812	0.8	1
EML2	0.762286399	1.32679385	0.875	0.8	1
HUWE1	0.762370975	0.376087956	1	1	1
TES	0.762769778	0.591828762	1	1	1
ENSSSCG00000018083	0.762950123	0.354349573	0.5	0.4	1
NUMA1	0.763017871	0.746036585	1	0.933	1
METTL24	0.763214839	0.726736462	1	1	1
NOL8	0.76355267	0.560652093	1	1	1
KCND2	0.763641504	0.491853096	0.562	0.4	1
ALCAM	0.763908902	0.710625202	0.875	0.8	1
SNTN	0.763923819	0.458631677	1	1	1
GNA11	0.764112801	0.547348209	0.875	0.667	1
PRUNE1	0.764152144	0.736072561	1	1	1
ENSSSCG00000024860	0.764315102	0.787050826	0.75	0.8	1
ENSSSCG00000008576	0.764537798	0.64385619	0.938	0.933	1
SLC39A10	0.764600851	0.687467702	1	1	1
DNMBP	0.765219196	0.705541449	1	1	1
WDPCP	0.765444147	0.547562529	1	1	1
HTT	0.765465652	0.757648642	1	1	1
EIF1AD	0.765529639	0.639530264	1	1	1
ENSSSCG00000059679	0.76559041	0.829722735	0.688	0.4	1
ADAMTSL4	0.76577751	0.509555098	0.875	0.933	1
ANGPTL2	0.766028747	1.058893689	0.75	0.667	1

CUTC	0.766222789	0.022367813	0.688	0.4	1
ZNF565	0.766766333	0.792855352	0.875	0.933	1
SLC6A11	0.766800058	0.874829386	1	1	1
ZNF773	0.76693679	0.611692866	1	1	1
GDI2	0.767004805	0.623263474	1	1	1
ENSSSCG00000052280	0.767105856	0.174948617	1	1	1
SETX	0.76713029	0.652387738	1	1	1
FOXA1	0.767131823	0.736965594	0.5	0.267	1
RAD54B	0.767179995	0.561678276	1	1	1
ENSSSCG00000063161	0.767279642	0.34129342	0.625	0.467	1
WDR62	0.768115612	0.566815154	0.812	0.733	1
MANEAL	0.768227807	0.538351558	0.938	0.933	1
PRKAB2	0.768261085	0.719805043	1	0.933	1
TIAM1	0.768345681	0.556523525	1	1	1
DENND4A	0.768361885	0.405103578	1	1	1
PRRC1	0.768380766	0.65905148	1	1	1
STK17B	0.768513071	-0.1602236	0.875	0.667	1
CFAP210	0.768745078	0.784555053	1	1	1
SIPA1L3	0.768754784	0.866248611	0.938	0.733	1
CHD4	0.768997293	0.79105381	1	1	1
UNC13C	0.769200352	0.510824853	1	1	1
CDADC1	0.76936314	0.676592976	1	1	1
ENSSSCG00000053778	0.769444562	0.206450877	0.875	0.533	1
ZNF511	0.769520589	0.362955363	1	0.733	1
ENSSSCG00000045377	0.769580201	0.410933101	0.938	0.8	1
HRH2	0.769856108	0.34298971	0.562	0.467	1
ENSSSCG00000059162	0.770124595	0.69743723	0.875	0.667	1
ENSSSCG00000048556	0.770565873	0.006426269	0.812	0.733	1
ENSSSCG00000025500	0.77083601	0.796600665	1	1	1
ENSSSCG00000062556	0.771033134	0.993651908	0.938	1	1
ANKRD33B	0.771454099	0.404009747	0.875	0.933	1
ACVR2A	0.771887733	0.411682748	1	1	1
NUSAP1	0.771916993	0.619199433	1	1	1
ENDOV	0.772642169	0.386883537	0.938	0.8	1
CCDC85A	0.772645899	0.458466928	1	1	1
RAB3GAP1	0.77275902	0.782594158	1	1	1
ZNF304	0.773037255	0.423949032	0.938	0.867	1
ENSSSCG00000061085	0.773279455	0.154818109	0.625	0.4	1
ENSSSCG00000032554	0.773580825	0.752760556	1	1	1
DNAJC10	0.773581752	0.546008866	1	1	1
ENSSSCG00000033241	0.773718347	0.637985602	1	1	1
RPUSD1	0.773838997	-0.167109986	0.438	0.6	1
CEBPZ	0.774045584	0.547735181	1	1	1
TTC39C	0.774476307	0.769808851	0.938	0.933	1
UBE4B	0.774599389	0.828562235	1	1	1
ENSSSCG00000056080	0.774760256	0.799975392	0.75	0.4	1
ENSSSCG00000013120	0.775150097	0.571706404	0.875	0.933	1
PTPN21	0.775481017	0.578457585	1	1	1
MEF2A	0.77551373	0.64385619	1	0.733	1
SEMA6A	0.775904015	0.52501996	0.812	0.733	1
ENSSSCG00000056025	0.776001812	0.356693513	0.812	0.733	1
CKAP5	0.776056534	0.673498479	1	1	1
ENSSSCG00000016093	0.776078137	0.759333407	0.812	0.8	1
RABGEF1	0.776738017	0.584340515	1	1	1
NVL	0.77700832	0.557655155	1	1	1
CCDC82	0.777321187	0.186998515	1	0.733	1
PIGL	0.778024072	0.607330314	0.5	0.4	1
IQSEC1	0.77810387	0.714245518	0.812	0.6	1
KLHL40	0.778731278	0.906890596	0.812	0.667	1
ENSSSCG00000031776	0.778793965	0.830787617	1	1	1
GAD2	0.778807084	0.531381461	0.562	0.4	1
SMIM29	0.779123769	0.392317423	1	0.733	1
ZNF81	0.779163615	0.687267263	1	1	1
ENSSSCG00000045006	0.779501776	0.449667866	1	1	1
GABRG2	0.78100983	0.675866585	1	1	1
NCKAP1	0.781230561	0.474575105	1	1	1
FBXO11	0.781526425	0.487434425	1	0.933	1
FBXO47	0.781745656	0.269460675	0.625	0.733	1
CCDC51	0.78177527	0.541373232	1	0.8	1
AMER3	0.781787269	0.781359714	0.625	0.533	1

ENSSSCG00000060228	0.782329496	1.058893689	0.5	0.333	1
ENSSSCG00000047466	0.782550004	0.761039729	1	0.867	1
ENSSSCG00000002260	0.783135215	0.531606829	1	1	1
FHIP2A	0.78326006	0.844606317	0.938	1	1
ARNT	0.783405427	0.497566748	1	0.867	1
LRRTM1	0.783579483	-0.093109404	0.5	0.533	1
ENSSSCG00000030140	0.783726151	0.451211112	0.625	0.667	1
ENSSSCG00000062147	0.784720462	0.710970386	1	1	1
NFS1	0.784791796	0.624747367	1	1	1
ENSSSCG00000042074	0.785680338	0.34298971	0.562	0.467	1
SLC27A1	0.785870091	0.472487771	0.688	0.6	1
KLHL26	0.785966012	0.292180751	0.688	0.6	1
ENSSSCG00000056845	0.787303786	0.536299824	1	1	1
ENSSSCG00000055269	0.787480356	0.714245518	0.5	0.467	1
TRIB3	0.787700286	0.597562538	0.688	0.867	1
SMIM35	0.787717637	0.743952024	0.875	0.8	1
MEOX2	0.787762512	0.312882955	0.875	0.533	1
ENSSSCG00000057514	0.788067242	0.087462841	0.375	0.533	1
ENSSSCG00000060154	0.788315524	0.689299161	0.875	0.6	1
SPATA6	0.788332913	0.664320292	0.875	0.867	1
WDR17	0.788641741	1.426264755	0.5	0.4	1
PHF13	0.788710441	0.682103659	1	1	1
MAPK1	0.788720037	0.611015361	1	1	1
GATC	0.789096928	0.609449328	1	1	1
HOXD8	0.789568853	0.739290647	1	1	1
NPAS2	0.789610597	0.87036472	0.688	0.467	1
TLR8	0.789727965	0.520937589	1	1	1
ENSSSCG00000055025	0.789964348	0.243925583	0.688	0.267	1
KLF12	0.789976723	0.632110982	1	0.933	1
ENSSSCG00000045742	0.790753782	0.440322796	0.938	0.8	1
ERRFI1	0.791048959	1.22881869	0.5	0.333	1
ZNF382	0.791175002	0.414685236	0.875	0.8	1
BARHL2	0.791343292	0.482750889	0.938	0.867	1
ENSSSCG00000022345	0.791576536	1.081977302	0.812	0.733	1
DHX34	0.793283553	0.530327244	0.688	0.6	1
SLC25A21	0.793326157	0.461264776	0.938	0.867	1
ENSSSCG00000055926	0.793352953	0.752168001	0.812	0.667	1
SOHLH2	0.794215646	0.669391282	1	0.6	1
LRRC1	0.794674634	0.867719998	1	1	1
ENSSSCG00000015489	0.794936449	0.631256153	0.625	0.467	1
ENSSSCG00000017834	0.795095846	0.129283017	0.562	0.333	1
RARRES1	0.795453768	0.870023942	1	1	1
GIN1	0.795613009	0.435269568	0.75	0.733	1
ENSSSCG00000034749	0.795617569	0.82130204	1	1	1
METTL25	0.796643834	0.288761231	0.812	0.667	1
TMEM185B	0.797075109	0.600105987	1	0.933	1
ENSSSCG00000062704	0.797513707	0.69538649	0.75	0.733	1
RBBP8	0.797647537	0.440743141	1	1	1
AKT1	0.797870837	0.660730008	0.938	0.867	1
UBQLN2	0.798102709	0.766960334	1	0.933	1
ENSSSCG00000047003	0.79858679	0.663221515	0.938	0.867	1
ENSSSCG00000038289	0.799085437	0.714725856	1	1	1
RFX1	0.799097285	0.668730858	0.75	0.533	1
ENSSSCG00000049335	0.799201347	0.375039431	0.938	0.8	1
ENSSSCG00000038337	0.799356855	0.714245518	0.812	0.533	1
CCDC122	0.799637634	0.730012834	0.625	0.467	1
SDCBP	0.799696198	0.794737317	1	1	1
ENSSSCG00000031787	0.799753369	0.666064139	1	1	1
EFCAB11	0.80002839	0.906890596	0.875	0.933	1
PRIMPOL	0.800088442	0.601335312	1	1	1
UHMK1	0.800157814	0.603022472	1	1	1
DRAM2	0.800216197	0.578836398	1	1	1
PDK3	0.800623972	0.703643224	1	1	1
PLD1	0.800861935	0.328717261	0.688	0.733	1
EPC2	0.801575286	0.484075746	1	0.933	1
P4HA2	0.802428518	0.658543633	0.938	0.933	1
HECTD1	0.80258547	0.524809129	1	1	1
INTS8	0.802602716	0.329581667	0.938	0.667	1
CCDC14	0.803220316	0.280348991	0.875	0.667	1
ERCC4	0.803427252	0.635818871	1	1	1

GOPC	0.80358512	0.613279876	1	1	1
ENSSSCG00000000327	0.803618105	0.206450877	0.875	0.667	1
ENSSSCG00000003322	0.804193373	0.399930607	0.938	0.933	1
ENSSSCG00000055908	0.804460834	0.8397764	0.75	0.533	1
GLYAT	0.804685911	1.046565218	1	1	1
C6orf89	0.805059112	0.620305524	1	1	1
ENSSSCG00000057246	0.805424297	0.534573971	0.938	0.933	1
HELB	0.805665323	0.514623602	1	1	1
FAR2	0.805675509	0.487265827	0.875	0.8	1
ZNF555	0.805737628	0.431775276	0.938	1	1
ENTR1	0.805986611	0.527301932	0.938	0.867	1
ENSSSCG00000046607	0.806032425	0.672425342	0.562	0.4	1
EXOC6	0.806166519	0.628749297	1	1	1
ZNF396	0.806474912	0.770829046	0.875	0.667	1
DHX36	0.806585603	0.55316365	1	1	1
ENSSSCG00000048605	0.806927934	0.539158811	0.688	0.467	1
ENSSSCG00000062678	0.806948762	0.755428953	1	1	1
TRAPPC10	0.807173757	0.820475844	0.938	0.933	1
DEPDC1B	0.807350788	0.439119815	1	1	1
ADGRG2	0.807570044	0.269460675	0.688	0.733	1
EME1	0.807857602	0.737264134	1	1	1
GTPBP10	0.807871236	0.439937626	1	1	1
PLCB2	0.807893247	0.491853096	0.812	0.933	1
ENSSSCG00000063050	0.808465927	0.478432581	1	1	1
ARAP2	0.80861427	0.493457874	1	1	1
ENSSSCG00000028004	0.808727587	0.269460675	1	1	1
OSTF1	0.808894928	0.631064636	1	1	1
ENSSSCG00000026271	0.809083213	0.554009572	1	0.867	1
RUNDC1	0.809237454	0.762500686	0.562	0.4	1
ENSSSCG00000040056	0.809334657	0.394019049	1	1	1
ENSSSCG00000015545	0.809692463	0.663793781	1	1	1
ENSSSCG00000060644	0.809730921	0.051625689	1	0.933	1
ZEB1	0.810238322	0.626782676	0.938	0.933	1
ENSSSCG00000038702	0.811654498	0.52501996	0.812	0.8	1
ENSSSCG00000046699	0.811718545	0.607330314	0.562	0.2	1
ENSSSCG00000053808	0.811972461	0.463283944	0.562	0.333	1
RFX3	0.812243004	0.219678037	0.875	0.8	1
FAM170A	0.812682028	0.74819285	0.438	0.6	1
MAK16	0.812684097	0.515716989	1	1	1
ENSSSCG00000057502	0.812809763	0.913082429	1	1	1
ENSSSCG00000015810	0.813138217	0.614108846	0.875	0.8	1
IL31RA	0.813362619	-0.32443495	0.625	0.4	1
ENSSSCG00000032113	0.814614059	0.852442812	0.75	0.6	1
MAPT	0.814732059	0.856797256	1	1	1
MGST3	0.81482354	0.599566914	1	1	1
FLNC	0.815245304	0.566757905	0.938	1	1
ENSSSCG00000008241	0.815392045	0.22881869	0.688	0.6	1
FNTA	0.815551322	0.526443025	1	1	1
PHF23	0.815606496	0.638872782	1	1	1
ACVR1	0.815615318	0.525853768	1	1	1
EHHADH	0.815850811	0.586961683	1	1	1
HDAC6	0.815960554	0.69276579	0.562	0.733	1
CCDC171	0.816065995	0.725216635	1	1	1
DNMT1	0.816542679	0.726406141	1	1	1
CFAP91	0.816676214	1.089093927	0.625	0.533	1
ENSSSCG00000055112	0.816806975	0.652317769	0.75	0.6	1
APH1B	0.817075112	0.861228042	1	1	1
FILIP1L	0.817129367	0.211745177	0.562	0.8	1
MIS18A	0.817265103	0.707882476	1	1	1
NFIC	0.817597801	0.602884409	0.875	0.867	1
SRSF10	0.817721554	0.565304352	1	1	1
CLIP3	0.818403916	1.120669887	0.875	0.867	1
LRRC4C	0.818681513	0.97593224	0.938	0.867	1
YLPM1	0.819653776	0.688250309	1	0.933	1
HP1BP3	0.819740657	0.527450369	1	1	1
ENSSSCG00000057417	0.819997558	0.635614812	1	1	1
TMEM260	0.820600904	0.668730858	0.812	0.733	1
S1PR2	0.820605887	0.459431619	0.812	0.733	1
RFFL	0.821178262	0.792530275	1	1	1
ENSSSCG00000033643	0.821180349	0.491853096	0.5	0.4	1

BRPF3	0.821508559	0.473237418	0.938	1	1
ENSSSCG00000023172	0.821701732	0.813781191	0.75	0.6	1
ADCY5	0.821802136	0.156868849	0.625	0.6	1
BCLAF1	0.821957267	0.617579452	1	1	1
GRIP2	0.822060114	0.765490729	1	1	1
ATPAF1	0.82250466	0.508455064	1	0.933	1
DNAAF9	0.822539006	0.801045508	1	1	1
TENT5B	0.822630236	0.96281601	1	1	1
RCOR1	0.823060505	0.678179372	1	1	1
ABCA2	0.823064066	0.5987683	0.625	0.467	1
SLN	0.823400019	0.22336426	0.875	0.8	1
NCEH1	0.82343434	0.714245518	0.688	0.667	1
ENSSSCG00000057038	0.823709982	0.287980763	0.812	0.667	1
ZCCHC2	0.823719279	0.601403519	1	1	1
ENSSSCG00000000540	0.824098092	0.399072811	1	1	1
BCORL1	0.824662219	0.73586556	0.938	1	1
CALCR	0.824703127	0.658160649	1	0.933	1
WDFY3	0.825301966	0.53162156	1	1	1
DPH5	0.825429593	0.638260727	1	1	1
LRIF1	0.826224233	0.35974956	1	1	1
NIPBL	0.826637762	0.571848583	1	1	1
POLR2M	0.826731876	0.820755755	1	1	1
ZNF260	0.826846373	0.213868349	0.938	0.8	1
FZD4	0.827004191	0.957516669	0.875	0.4	1
ENSSSCG00000052612	0.827223824	0.542479169	1	1	1
INPP4A	0.827282376	0.615427781	0.938	0.867	1
ZC3H12A	0.827309609	0.392317423	0.5	0.267	1
HSD11B2	0.827454287	0.671359705	1	1	1
RNGTT	0.82748998	0.459910204	1	1	1
MAP4K5	0.827524796	0.656673987	1	1	1
TMEM43	0.827748853	0.807303241	1	1	1
RAD52	0.828042934	0.516171727	0.938	1	1
ENSSSCG00000009498	0.828063439	0.714245518	0.75	0.667	1
ZNF658	0.828409571	-0.228461257	0.688	0.333	1
ENSSSCG00000062885	0.828564409	0.994353437	0.562	0.467	1
ERC2	0.828566684	0.651486631	1	1	1
CCDC65	0.828885087	0.684498174	0.938	1	1
ENSSSCG00000050278	0.829154889	0.451211112	0.688	0.467	1
OGDH	0.829618556	0.737390854	0.938	1	1
ENSSSCG00000000194	0.830212199	0.890666558	1	1	1
ENSSSCG00000034578	0.830326295	0.719805043	0.938	0.733	1
SRGAP3	0.830401549	0.749349319	0.562	0.8	1
CIPC	0.830456274	0.873930397	1	1	1
CPE	0.830481537	0.639786525	0.812	0.6	1
FEZ2	0.830529051	0.459041226	1	1	1
ENSSSCG00000042218	0.830636588	0.607330314	0.812	0.467	1
ENSSSCG00000041874	0.830812947	0.773624065	0.75	0.6	1
DTWD2	0.831887225	-0.183307213	0.562	0.6	1
EVI5	0.831989968	0.645710554	1	0.933	1
ENSSSCG00000055415	0.832135093	0.679975616	1	1	1
ALPK1	0.832391876	0.57758297	0.75	0.733	1
ZFC3H1	0.832696415	0.654310547	1	1	1
RAC1	0.832877464	0.601952811	1	1	1
CEP55	0.833159641	0.460193941	1	1	1
SRPK2	0.83365742	0.711034532	1	1	1
NR2F1	0.83373653	0.5360529	0.938	0.867	1
CTR9	0.833924899	0.535673374	1	1	1
ENSSSCG00000053016	0.834364923	0.537656786	0.875	0.6	1
ARHGAP32	0.834551146	0.475733431	0.938	0.933	1
ZNF71	0.834827538	0.555418225	0.938	0.733	1
ENSSSCG00000055301	0.835107014	1.016515087	0.688	0.667	1
ENSSSCG00000031890	0.835202718	0.46760555	1	0.933	1
GPCPD1	0.835521341	0.692181782	1	1	1
SLC13A2	0.835622146	0.575684687	0.625	0.667	1
FOXO1	0.836039108	0.589281107	1	1	1
C1orf52	0.836434025	0.525800428	0.75	0.667	1
SETDB1	0.836597746	0.453448557	1	1	1
CXCL10	0.836673127	0.087462841	0.625	0.467	1
PANK3	0.836864442	0.650157116	1	1	1
ENSSSCG00000039706	0.837259235	0.421463768	0.625	0.8	1

ND2	0.837406115	0.627053486	1	1	1
PAPSS2	0.837423297	0.384469561	1	1	1
C17orf75	0.837600489	0.22881869	0.812	0.467	1
ENSSSCG00000042026	0.837654344	0.584962501	0.875	0.733	1
EMP2	0.837705057	0.862947248	0.875	0.733	1
PJA1	0.837761381	0.854423176	0.562	0.467	1
ZNF2	0.838012515	0.651196199	0.938	1	1
ENSSSCG00000038455	0.83801781	0.866248611	0.75	0.333	1
NSMCE2	0.838093085	0.64385619	0.875	0.867	1
BLTP3A	0.838102065	0.476256241	0.625	0.6	1
DLD	0.838703926	0.572288671	1	0.933	1
OAZ2	0.83883902	0.587385533	1	1	1
SLC25A23	0.838919077	0.584962501	0.812	0.733	1
MEI4	0.839162922	0.22881869	0.75	0.467	1
GLT8D2	0.839263259	0.728399223	1	1	1
SETD1A	0.83936481	0.906890596	1	1	1
ARFIP1	0.839449736	0.692987026	1	1	1
UBTF	0.839827353	0.625403427	1	1	1
PLEKHG2	0.840016966	0.37895904	0.75	0.733	1
ENSSSCG00000044943	0.840061606	0.514573173	0.938	1	1
ZDHHC14	0.840622592	0.769387072	0.625	0.467	1
LACC1	0.840977004	0.663884969	1	1	1
KIF3A	0.841176593	0.540895719	1	1	1
ENSSSCG00000038144	0.841294382	0.796466606	0.938	0.867	1
POGLUT1	0.841362205	0.409390936	0.625	0.533	1
ZNF782	0.841453803	0.467191042	1	0.867	1
ENSSSCG00000059077	0.842013774	0.421463768	0.562	0.4	1
PEX5	0.842070283	0.529224526	1	1	1
MARF1	0.842113398	0.434137598	1	1	1
ZNF710	0.842405821	0.8397764	0.812	0.6	1
RBFOX1	0.842453891	0.652915529	1	1	1
GABRA1	0.842636085	0.454378391	1	0.867	1
MCCC2	0.842656457	0.637283536	1	1	1
PPL	0.842791035	0.725443724	0.938	0.933	1
HSPB1	0.843517473	0.661778098	0.562	0.533	1
ENSSSCG00000005121	0.843924053	0.459431619	0.562	0.533	1
RWDD2B	0.843949795	0.823232667	1	1	1
ENSSSCG00000061420	0.843953653	0.206450877	0.5	0.267	1
SCN2A	0.843988138	-0.125530882	0.688	0.533	1
ENSSSCG00000040367	0.844012367	0.514573173	0.75	0.467	1
STXBP6	0.844419425	0.798987718	1	1	1
MBIP	0.844550902	0.616300467	1	0.933	1
NR2C2	0.844708777	0.604058012	1	1	1
BACH1	0.844781856	0.588920514	1	1	1
XPO6	0.845236785	0.734779929	1	1	1
OSBP2	0.84552927	0.058893689	0.375	0.533	1
ENSSSCG00000043563	0.845987592	0.64385619	0.875	0.533	1
ELAPOR2	0.84643165	0.515552932	1	1	1
CCDC142	0.846689645	0.169925001	0.562	0.267	1
ENSSSCG00000047832	0.846805569	0.730012834	0.812	0.733	1
CLCN7	0.846815727	0.752242781	1	1	1
RAG2	0.847066842	0.26052755	0.5	0.533	1
ENSSSCG00000021941	0.847197556	0.613339763	1	1	1
ARHGAP20	0.847233557	0.776830055	0.688	0.467	1
ENSSSCG00000034709	0.847679711	0.566275457	1	1	1
SATB1	0.847873894	0.354349573	1	1	1
ENSSSCG00000041398	0.847963921	0.293913719	0.375	0.533	1
HTR3B	0.848343771	0.26052755	0.875	0.667	1
FOXJ2	0.848437018	0.661778098	0.75	0.533	1
ZFP36L1	0.848548446	0.475551284	1	1	1
ANKH	0.848924031	0.650325685	0.812	0.933	1
RTL4	0.848942341	0.235513343	0.938	0.8	1
ENSSSCG00000043724	0.849252129	0.380821784	0.562	0.4	1
ENSSSCG00000062411	0.849262195	0.607330314	0.688	0.4	1
PGR	0.849522042	0.680244846	1	1	1
EIF4A3	0.849776409	0.343084746	1	1	1
ENSSSCG00000052923	0.850029203	0.51793375	1	1	1
CAND2	0.850282693	0.603022472	1	1	1
KMT5A	0.850371431	0.621829883	1	1	1
SLC39A1	0.850578338	0.531851164	1	0.933	1

MS4A2	0.850678041	0.542734265	0.75	0.667	1
RHOA	0.850770231	0.679895738	1	1	1
BMPR1B	0.850934399	0.689546748	1	1	1
PDSS1	0.850988805	0.627273306	1	1	1
C5orf22	0.851223195	0.624015277	1	0.933	1
ENSSSCG00000025590	0.851499962	0.432959407	0.875	0.6	1
ENSSSCG00000054550	0.851506458	0.956359272	0.688	0.533	1
POU4F2	0.851632791	0.813781191	0.562	0.333	1
CCNK	0.852710443	0.516581898	1	1	1
PRPH	0.852839337	0.906890596	0.688	0.467	1
RAB5A	0.853148643	0.341518823	1	0.867	1
ENSSSCG00000061091	0.853220408	0.432959407	0.625	0.467	1
ENSSSCG00000054193	0.853439914	0.703357202	0.625	0.6	1
PPP1R9A	0.853567115	0.827089884	1	1	1
RAB27B	0.853651466	-0.215099929	0.75	0.4	1
CLDN7	0.854158535	0.625550035	1	1	1
CYP1A1	0.854531266	0.550746785	0.688	0.467	1
RGS14	0.855058509	0.805010982	0.75	0.667	1
TRIM52	0.855260953	0.617383978	0.938	0.8	1
ENSSSCG00000017285	0.855499511	0.804710201	1	0.933	1
TRIP12	0.855570236	0.716113181	1	1	1
TRIM38	0.855769942	0.65137304	1	0.933	1
GTF2H3	0.855987485	0.648156327	0.875	0.667	1
CASD1	0.855997187	0.485146774	1	1	1
ABTB1	0.856700307	0.425357685	0.812	0.867	1
ENSSSCG00000057242	0.857107705	0.732212064	0.938	0.933	1
ACTR5	0.857207871	0.601264313	0.875	0.8	1
TIPIN	0.857263076	0.642474959	1	1	1
ENSSSCG00000055690	0.8576402	0.574315257	0.625	0.6	1
SHISA2	0.858044041	0.547996174	1	1	1
ACTR8	0.858431415	0.525985781	1	1	1
ENSSSCG00000047157	0.858659207	1.0138058	0.875	0.8	1
MTOR	0.858870164	0.614877123	1	1	1
COQ10A	0.859640858	1.006426269	0.688	0.733	1
ENSSSCG00000057507	0.859817322	0.742259894	0.75	0.733	1
TBC1D22B	0.859861917	0.986117287	1	0.933	1
NLRP11	0.859873533	0.598113868	1	1	1
RBP1	0.860020199	0.047068254	0.375	0.733	1
C19orf44	0.860250654	0.736965594	0.5	0.267	1
KLC2	0.860418553	0.99095486	0.938	0.867	1
CLN5	0.860466453	0.702200176	1	1	1
ENSSSCG00000002427	0.860560649	0.706865987	0.938	0.933	1
PSD3	0.861440581	0.340349292	1	1	1
ENSSSCG00000052590	0.861563501	0.5987683	0.75	0.8	1
IL2RA	0.861718842	0.80786122	1	1	1
ENSSSCG00000026167	0.86197738	0.239465935	0.812	0.533	1
DIP2C	0.862013055	0.757512972	1	1	1
ENSSSCG00000021656	0.862031232	0.454378391	0.562	0.467	1
ARHGEF10	0.862041938	0.799975392	0.75	0.4	1
ELAVL2	0.862203744	0.669347851	1	1	1
SLC4A11	0.862217445	0.738013684	0.938	0.8	1
ENSSSCG00000053948	0.862429468	0.965784285	0.75	0.4	1
EDEM1	0.862439856	0.606877277	1	0.933	1
MAB21L3	0.862839522	0.762500686	0.562	0.533	1
MYCBP2	0.86285274	0.566769763	1	1	1
TMEM120B	0.863081213	0.872125177	0.938	0.867	1
HEATR3	0.863378538	0.396695863	0.688	0.533	1
SIAH2	0.863642775	0.707581787	0.812	0.8	1
DDX6	0.863684511	0.519995222	1	1	1
DNAJC12	0.863977322	0.616479751	1	1	1
TMC7	0.864101604	0.857980995	0.625	0.6	1
FRMD3	0.864116418	0.534124749	1	1	1
DESI1	0.864308999	0.794891297	1	1	1
ENSSSCG00000009419	0.864405869	0.732190487	1	1	1
ENSSSCG00000005232	0.864725862	0.462105753	0.938	0.6	1
ANKRD13A	0.865162687	0.693606679	1	1	1
BRWD1	0.865323531	0.633768535	1	1	1
PCDHA13	0.865404784	0.321928095	0.5	0.267	1
SPOPL	0.865424594	0.59373271	1	1	1
ENSSSCG00000058419	0.865611053	0.552225714	0.688	0.6	1

SYNJ1	0.865621475	0.798787988	0.938	1	1
MRPL49	0.86566591	0.710198685	1	0.933	1
UBOX5	0.865733381	0.533273024	0.938	1	1
PPFIBP1	0.865805901	0.417628511	1	0.8	1
ENSSSCG00000034769	0.865884484	0.544320516	0.625	0.333	1
ANAPC15	0.866004079	0.606231117	1	1	1
CYB5B	0.866013565	0.624219525	1	1	1
CXXC1	0.86676485	0.850981854	1	1	1
SLC26A5	0.866906881	0.760596824	1	1	1
PLEKHA7	0.867097877	0.656359722	1	1	1
CLASRP	0.867536612	0.727566896	1	1	1
NELFA	0.867828703	0.77131305	1	1	1
TEX14	0.867880669	0.906890596	0.812	0.4	1
RNF207	0.868151287	0.769387072	0.938	0.8	1
ENSSSCG00000061293	0.868241569	0.22881869	0.688	0.733	1
ENSSSCG00000049199	0.868417492	0.536940986	0.938	0.867	1
B3GAT2	0.868542216	0.671176796	1	1	1
ENSSSCG00000052142	0.868607729	0.64385619	1	1	1
ZNF385C	0.868692696	0.921537372	0.875	0.867	1
YY1	0.868693399	0.463643077	1	1	1
RASA2	0.868985314	0.5017959	1	1	1
TRDMT1	0.869030159	0.645775306	1	0.933	1
ENSSSCG00000038269	0.8694568	0.64385619	0.562	0.333	1
S1PR1	0.869563113	1.030879313	0.938	0.733	1
HDAC9	0.869797711	0.524643031	1	1	1
CAPZA1	0.869990935	0.465598456	1	1	1
ENSSSCG00000052972	0.870864524	0.5987683	0.562	0.333	1
ENSSSCG00000063274	0.871529747	0.665882496	0.5	0.4	1
LYPLA1	0.871541629	0.572195205	1	1	1
WDR44	0.871565935	0.680298186	1	1	1
ENSSSCG00000062082	0.872039277	0.468769483	0.875	0.8	1
CWF19L1	0.872555421	0.927952211	0.938	0.8	1
NSUN2	0.873399218	0.69538649	0.625	0.467	1
RHBDD1	0.873622054	0.668777565	1	1	1
ENSSSCG00000022176	0.873696393	0.746425923	0.812	0.4	1
ENSSSCG00000016737	0.873945525	0.50754662	1	1	1
ENSSSCG00000061941	0.873974522	0.722134789	1	1	1
ZZZ3	0.873976363	0.749198117	1	1	1
UPF2	0.874023975	0.655592298	1	1	1
LCOR	0.874503102	0.511613797	1	1	1
PIP5K1A	0.874599311	0.631256153	0.75	0.6	1
ENSSSCG00000013380	0.874995316	0.64385619	1	1	1
TMX4	0.875912245	0.597068129	1	1	1
ENSSSCG00000034743	0.876245248	0.458794808	1	0.933	1
NCKIPSD	0.87642152	0.766712938	1	1	1
ZGRF1	0.876519141	0.517658514	0.938	1	1
MTHFD2	0.876855992	0.541012709	1	1	1
PCGF2	0.877246155	0.611434712	0.875	0.667	1
IQGAP3	0.877396373	0.493300859	1	1	1
ZNF598	0.877497199	0.825147446	1	0.933	1
GTF2I	0.877565669	0.675489474	1	1	1
ENSSSCG00000060843	0.877930905	0.678138009	1	1	1
ATP8	0.87800678	0.659693116	1	1	1
COQ6	0.878216466	0.646706663	1	1	1
ENSSSCG00000028035	0.87826176	0.59980101	1	0.933	1
ENSSSCG00000009595	0.878320862	0.542734265	0.938	0.867	1
ENSSSCG00000047290	0.878823007	0.380821784	0.688	0.467	1
POMK	0.878982834	0.674229839	0.75	0.6	1
ATF7	0.879356534	0.677498115	1	1	1
ZZEF1	0.879486123	0.561655127	0.938	0.933	1
CRTAP	0.879699942	0.68061474	0.812	0.6	1
ENSSSCG00000045286	0.879790859	0.606082847	1	1	1
CCDC73	0.880005176	0.651923471	1	1	1
GPHN	0.880471948	0.541241123	0.875	0.733	1
ENSSSCG00000006205	0.880586198	0.637832402	0.938	0.8	1
GABRA2	0.880648794	0.672425342	1	0.933	1
CTTNBP2NL	0.880679303	0.312529397	0.938	0.8	1
ENSSSCG00000061109	0.881335684	0.417628511	0.812	0.933	1
ENSSSCG00000004410	0.881538069	0.514573173	0.625	0.6	1
ELOVL3	0.881597818	1.006426269	0.625	0.6	1

TEAD1	0.881609471	0.677975568	1	1	1
FRS2	0.88200597	0.990658953	0.875	0.8	1
CHCHD1	0.882058033	0.857980995	1	0.8	1
NMNAT3	0.882851458	0.50153565	1	0.867	1
OTUD4	0.882869425	0.7367343	1	1	1
AP1B1	0.882971342	0.733496245	1	1	1
ID1	0.882994028	0.658963082	0.75	0.467	1
EBF3	0.883088622	0.376375879	0.562	0.467	1
VPS45	0.883193531	0.701000097	1	1	1
ARHGEF12	0.883202879	0.597346225	0.938	0.867	1
ENSSSCG00000043429	0.883744171	0.202346479	0.5	0.333	1
UGDH	0.883849016	0.552549028	1	1	1
PTP4A1	0.883964606	0.731701007	1	1	1
MOB3B	0.884251663	0.761039729	0.75	0.533	1
SIAH1	0.884593278	0.636951781	1	1	1
CAMSAP3	0.884651845	0.424289813	1	0.867	1
TMEM185A	0.884717567	0.703357202	0.688	0.533	1
SEMA3F	0.88555638	0.457087678	0.625	0.467	1
ENSSSCG00000044144	0.885570974	0.689299161	0.625	0.6	1
DENND4B	0.886036125	0.677408749	0.625	0.6	1
SHISA5	0.886071642	0.731671921	1	1	1
MCM10	0.886268287	0.636376225	1	1	1
KLKB1	0.886386214	0.781359714	0.5	0.667	1
INCENP	0.886454829	0.765336451	1	1	1
ENSSSCG00000010893	0.886613778	0.432518957	0.938	0.733	1
HNRNPL	0.886700765	0.62639522	1	1	1
USP12	0.886787411	0.247230728	0.938	0.8	1
NHLRC3	0.886881617	0.818638126	1	1	1
CDK7	0.887280689	0.285874171	1	1	1
WFS1	0.887334966	0.895208791	0.75	0.867	1
HORMAD2	0.887955183	0.558967292	0.562	0.467	1
ENSSSCG00000047289	0.888040831	0.521339861	0.938	0.867	1
ADAT1	0.888165649	0.715557655	0.812	0.867	1
OTP	0.888326198	0.781359714	0.625	0.667	1
ENSSSCG00000052175	0.889533632	0.88582898	0.938	0.933	1
CAB39	0.889631945	0.386294016	1	1	1
NWD2	0.889917131	0.881799615	0.938	1	1
ENSSSCG00000061191	0.890161107	0.534921818	0.5	0.467	1
TRAM1	0.890345625	0.713780207	1	1	1
TRA2B	0.890365197	0.686634648	1	1	1
ENSSSCG00000057241	0.890425714	0.37895904	0.562	0.6	1
E2F1	0.890487137	0.662653785	1	1	1
NKX2-8	0.890817075	0.754887502	0.688	0.467	1
ENSSSCG00000048684	0.891270448	0.761039729	0.75	0.667	1
FBXL18	0.891697145	1.150161747	0.875	0.867	1
C2CD2L	0.891941726	0.409390936	0.5	0.333	1
NIPA2	0.892180115	0.589041015	1	1	1
NHLRC2	0.892191691	0.691067503	1	1	1
PKHD1	0.892676093	0.475174355	0.5	0.533	1
TNK2	0.892824456	0.613159393	0.562	0.267	1
ZNF333	0.892970256	0.489980682	1	1	1
ENSSSCG00000061077	0.893109709	0.196397213	0.562	0.267	1
SNAPC1	0.893289441	0.481496304	1	0.933	1
MAP1B	0.893577065	0.575821398	1	1	1
NAP1L1	0.893660515	0.7409819	1	1	1
CACNB4	0.895213427	0.153805336	0.938	0.8	1
LSS	0.895311358	0.647131322	0.938	0.933	1
ENSSSCG00000010952	0.89613669	0.636903429	0.938	1	1
CILP	0.896162558	0.426724285	0.938	0.933	1
C12orf76	0.89616845	0.923316144	1	1	1
SRFBP1	0.89645862	0.639870842	1	0.933	1
MGAM2	0.896688699	0.539102223	1	1	1
NDUFB1	0.896872807	0.317078695	1	0.933	1
ENSSSCG00000044127	0.897111236	0.654124525	0.875	0.867	1
SREK1	0.897218311	0.82536071	1	1	1
TRPV4	0.897443438	0.78754965	1	1	1
MELTF	0.897606147	0.464580166	1	0.933	1
ENSSSCG00000061869	0.897902111	0.546008866	1	0.933	1
ENSSSCG00000055563	0.897947153	0.512611656	0.875	0.733	1
GAS2L3	0.897998102	0.41550221	0.938	0.733	1

ENSSSCG00000045906	0.898271311	0.376375879	0.5	0.467	1
TRIM26	0.898275429	0.278859373	0.812	0.733	1
ROBO2	0.898427104	0.75987091	1	1	1
ENSSSCG00000045464	0.898612802	0.714245518	0.5	0.4	1
PRDM1	0.899613051	0.416564969	0.938	0.733	1
ENSSSCG00000054101	0.899954937	0.64385619	0.562	0.467	1
NAAA	0.900242287	0.673093411	0.938	0.867	1
ENSSSCG00000035000	0.90026724	0.569587275	1	1	1
ENSSSCG00000012006	0.900435588	0.398702217	0.75	1	1
ENSSSCG00000034540	0.901130226	0.378440116	0.938	0.933	1
NPEPPS	0.901418229	0.661210013	1	1	1
CHST4	0.901751779	0.708344916	0.688	0.667	1
PHF2	0.902152866	0.847184349	0.875	0.867	1
HIP1	0.90217591	0.547251335	1	1	1
TRIM68	0.902655669	0.452324732	0.875	0.6	1
OTUD6B	0.903369376	0.305439972	1	0.533	1
ARHGAP15	0.903940713	0.64385619	0.562	0.533	1
NRXN3	0.904033876	0.662552283	1	1	1
RNLS	0.904582811	0.476256241	0.812	0.6	1
KMT2A	0.904725163	0.631436931	1	1	1
NAA80	0.904930031	0.145050333	0.75	0.867	1
IGSF10	0.905066511	0.362707945	1	1	1
KIF13A	0.905483924	0.547476603	1	1	1
LRRIQ3	0.906848539	0.483162136	1	1	1
RIMS4	0.907380568	0.515699838	0.875	0.667	1
KEAP1	0.907617013	0.485063931	0.875	0.733	1
ENSSSCG00000061934	0.907687767	0.529626439	0.938	1	1
TPK1	0.908065374	0.655734946	1	1	1
ERCC6L	0.90819168	0.676032081	1	1	1
MYBL1	0.908570225	0.51737146	1	1	1
ESS2	0.908809943	0.691430888	1	1	1
ARL2BP	0.908818962	0.598266231	1	1	1
USP14	0.909110295	0.653288355	1	1	1
CDC37L1	0.909790246	0.524435919	1	1	1
SMIM19	0.910022284	0.556393349	1	1	1
HELLS	0.910376458	0.67507492	1	1	1
EGF	0.910452161	0.585689482	1	1	1
ADCYAP1R1	0.910604767	0.72744282	1	1	1
CIR1	0.911249372	0.709698728	1	1	1
ENSSSCG00000058555	0.911408241	0.186998515	0.938	0.733	1
PSPC1	0.911475914	0.655828831	0.938	1	1
ZNF354B	0.911898941	0.378341404	0.938	0.933	1
C11orf65	0.912136111	0.505336326	0.875	0.867	1
BLNK	0.912172751	0.781359714	0.625	0.4	1
ENSSSCG00000044841	0.9123598	0.719467004	1	1	1
AP1G1	0.913155265	0.719643261	1	0.933	1
AHR	0.913273396	0.546596061	1	1	1
ENSSSCG00000035189	0.913880899	0.72631835	0.625	0.267	1
TDRD12	0.914125169	0.673803573	1	1	1
MEGF9	0.914156207	0.531109309	1	1	1
SERAC1	0.914707313	0.483082887	0.938	0.933	1
ENSSSCG00000060381	0.915016413	0.321928095	0.812	0.6	1
PIK3CA	0.915369628	0.334553776	1	1	1
DAB2IP	0.915892995	0.557962892	1	1	1
KANSL1	0.915964435	0.452584453	1	1	1
CABCOCO1	0.916146847	0.736965594	0.812	0.667	1
ENSSSCG00000041329	0.917018406	0.491853096	0.875	0.867	1
ZFYVE26	0.917073109	0.567305325	0.938	0.867	1
MARS2	0.917274356	0.92347348	1	0.933	1
SLC12A6	0.917399292	0.546008866	1	1	1
ENSSSCG00000047646	0.917624267	0.339850003	0.5	0.467	1
ENSSSCG00000056848	0.918109612	0.38332864	0.562	0.467	1
CPOX	0.918628963	0.439385676	0.75	0.733	1
XPNPEP1	0.918644621	0.347463187	0.625	0.533	1
SLC39A9	0.918748869	0.573617527	1	1	1
HAPLN1	0.918937798	-0.005646563	0.125	0.533	1
PHF12	0.919042629	0.43650068	1	1	1
AGMO	0.919287078	0.574459468	1	1	1
TSGA10	0.919476545	0.582480292	1	1	1
TOGARAM1	0.91958071	0.542326466	1	1	1

ENSSSCG00000004520	0.91978427	0.698806786	0.938	1	1
ENSSSCG00000044331	0.919844368	0.670190338	1	0.933	1
ODAD4	0.920692944	0.632201348	0.938	1	1
AFTPH	0.920972415	0.594862268	1	1	1
ENSSSCG00000003654	0.921064984	0.493378951	1	1	1
SPRY1	0.921294806	0.60232269	1	1	1
ATAD2B	0.921522168	0.673492424	1	1	1
ENSSSCG00000060388	0.921983071	0.556648219	0.938	1	1
ZNF180	0.922240999	0.493378951	0.938	1	1
SACS	0.923171642	0.736271824	1	0.867	1
MIGA1	0.923211512	0.637667978	1	1	1
ELMO2	0.92372534	0.627153311	1	1	1
ENSSSCG00000057454	0.923967887	0.301608111	0.938	0.933	1
TMED8	0.924533372	0.47023452	1	1	1
PIP4P2	0.924571734	0.511589314	0.938	0.8	1
TMEM199	0.924649502	0.715359138	1	0.933	1
CREB5	0.924654995	0.309855263	0.938	0.867	1
BNC1	0.924658793	0.693304679	1	1	1
ENSSSCG00000056312	0.925030473	0.64385619	0.438	0.533	1
PYGL	0.925124971	0.491853096	1	0.8	1
PSMC2	0.925534184	0.496179022	1	1	1
NECTIN3	0.925669873	0.220551075	0.938	1	1
BRINP3	0.925728257	0.754887502	1	1	1
PPP2R3C	0.926420651	0.469666333	1	1	1
IGSF9B	0.926454559	0.285402219	0.75	0.467	1
ENSSSCG00000036656	0.926624742	0.479959418	0.812	1	1
ENSSSCG00000016085	0.926704482	0.504792152	0.938	0.933	1
NPAT	0.926710692	0.507158416	1	1	1
CTSH	0.92692105	0.863821874	1	1	1
ENSSSCG00000012627	0.92745233	0.633776984	1	1	1
ENSSSCG00000010669	0.927526827	0.461899874	1	1	1
ADAM22	0.927601085	-0.093109404	0.688	0.533	1
CRPPA	0.927854534	0.599256192	1	1	1
PLAG1	0.927912274	0.714245518	1	0.933	1
CDKN1C	0.928454231	0.447458977	0.5	0.467	1
MGA	0.928987194	0.627891636	1	1	1
FGF12	0.92899307	0.508531837	1	0.867	1
FBXO5	0.929315137	0.488253107	1	1	1
TMEM178B	0.929502285	0.299208018	0.625	0.467	1
WASHC5	0.930924374	0.616240978	1	1	1
IMPDH2	0.931290283	0.733028068	1	1	1
TBC1D10A	0.931364668	0.776529796	1	1	1
ZNF470	0.931582867	0.604327826	1	0.933	1
ENSSSCG00000060892	0.931693244	0.836501268	0.5	0.533	1
SUPT7L	0.931788004	0.620397217	0.938	1	1
OSBPL8	0.932188435	0.415732463	1	1	1
LENG8	0.932932399	0.441227023	0.938	0.867	1
MRO	0.933066518	0.681882226	0.938	1	1
CEP83	0.933070097	0.765622053	1	1	1
MFSD9	0.933177166	0.672425342	0.625	0.667	1
ENSSSCG00000060691	0.933395131	0.87036472	0.5	0.533	1
AFMID	0.933939884	0.616032508	0.938	1	1
CRIM1	0.934020185	0.472728441	1	1	1
CMYA5	0.93423218	0.671870566	1	0.933	1
NEMF	0.934260412	0.627623163	1	1	1
PPP4R1	0.934475234	0.584223226	1	1	1
MIF4GD	0.934692785	0.772113916	1	0.933	1
PPP1R21	0.934983138	0.5585134	1	1	1
ENSSSCG00000058394	0.934998218	0.582247201	1	1	1
KIDINS220	0.935383246	0.728355919	1	1	1
GLDC	0.935390633	0.62309763	0.938	0.733	1
JCAD	0.935889269	0.607330314	0.5	0.333	1
ZBTB26	0.936085015	-0.001478929	0.812	0.467	1
ULK4	0.936240726	0.726082307	1	1	1
EDEM3	0.936769925	0.584962501	0.938	0.667	1
CIZ1	0.936971436	0.564002882	0.75	0.8	1
PPP4C	0.937137488	0.340877198	1	1	1
ENSSSCG00000053348	0.937604177	0.196397213	0.625	0.533	1
NUBPL	0.937875152	0.593615812	1	1	1
ENSSSCG00000059926	0.938090956	0.633325522	1	0.8	1

DIPK1A	0.938561951	0.736965594	1	1	1
TECTB	0.938669285	0.304974962	0.875	0.933	1
LPIN1	0.938764231	0.330102026	0.938	0.733	1
MED16	0.938939161	0.626782676	1	0.933	1
ENSSSCG00000015876	0.939220091	0.432302293	1	0.8	1
C2orf49	0.939744957	0.438413837	1	1	1
PHF7	0.940082692	0.728553354	0.812	0.8	1
NIFK	0.940208361	0.496006578	1	1	1
ENSSSCG00000058823	0.940241775	0.673913424	0.688	0.8	1
PIGG	0.941273423	0.349408831	0.875	0.867	1
RBM20	0.941359727	0.733291273	1	0.933	1
KIAA1217	0.941430438	0.906890596	0.938	0.867	1
ENSSSCG00000040412	0.941559967	0.447458977	0.5	0.4	1
SYNJ2	0.941563858	0.609504685	1	1	1
AQR	0.941963988	0.491285218	1	1	1
ENSSSCG00000054931	0.942013839	0.64385619	0.562	0.467	1
FSIP1	0.942143669	0.940837928	1	1	1
GOLM2	0.942144108	0.747110152	0.875	0.867	1
PSMC6	0.94236755	0.432959407	1	0.867	1
TAPBP	0.942541818	0.427722759	0.75	0.733	1
MED13L	0.942620493	0.613125097	1	1	1
ZBTB11	0.943066412	0.531381461	0.938	0.933	1
PRDM2	0.943115667	0.823078608	1	1	1
ENSSSCG00000062978	0.943364782	0.591794534	1	0.933	1
KLHL9	0.943411495	0.139551352	0.5	0.6	1
POLR1A	0.943774424	0.430109747	1	0.933	1
FRMPD4	0.943895199	0.794232795	1	1	1
SLC50A1	0.945105904	0.302819272	0.562	0.4	1
CTPS2	0.945242672	0.611434712	0.688	0.6	1
DFFB	0.945245552	0.468769483	0.562	0.4	1
ENSSSCG00000059588	0.94561861	0.906890596	0.562	0.6	1
NUAK1	0.945643912	0.646299366	1	1	1
TBC1D9B	0.946012426	0.285402219	0.75	0.667	1
CRTC2	0.946772465	0.771235496	0.938	0.8	1
ORMDL2	0.946813198	0.665882496	1	0.933	1
ENSSSCG00000050165	0.947291699	0.64385619	0.688	0.467	1
Vault.2	0.947327102	0.491853096	0.5	0.4	1
ENSSSCG00000057557	0.947554151	0.475174355	0.688	0.467	1
MYBPC1	0.948008361	0.384469561	0.938	0.867	1
CAMK2N1	0.948255031	0.219274918	1	1	1
ZNF684	0.94841773	0.156868849	0.625	0.6	1
ENSSSCG00000057597	0.948870221	0.69276579	0.75	0.6	1
ENSSSCG00000053625	0.948917506	0.863821874	0.562	0.467	1
GALNT12	0.948927937	0.335127593	1	1	1
SPRED1	0.949267239	0.331925684	1	1	1
WNT3	0.949324834	0.776775776	1	1	1
CHD1	0.949330178	0.71104009	1	1	1
GRIP1	0.949355488	0.60118352	1	1	1
MEP1B	0.949593091	0.653471279	1	1	1
OGDHL	0.950530948	0.385751672	0.938	1	1
ENSSSCG00000057119	0.950701371	0.549374315	1	1	1
ENSSSCG00000039618	0.950909216	0.361005617	1	1	1
ENSSSCG00000033351	0.951276894	0.622051819	0.938	0.8	1
CLEC16A	0.951450872	0.797661526	1	1	1
MTMR4	0.951772693	0.300554444	0.875	0.867	1
GJA10	0.951809901	0.719377179	0.938	1	1
VRK2	0.951922934	0.674552987	1	1	1
CDK14	0.952052906	0.568872798	1	1	1
IDS	0.952058636	0.757460298	1	1	1
MFAP3L	0.952112595	0.701421477	0.938	0.933	1
ENSSSCG00000063513	0.952440453	0.202346479	0.625	0.467	1
ENSSSCG00000056036	0.952904661	0.366322214	0.562	0.333	1
ENSSSCG00000056261	0.95297842	0.944365301	0.812	0.867	1
GMFB	0.953076449	0.664264621	1	1	1
FBLN1	0.95309568	0.415037499	0.812	0.867	1
ENSSSCG00000055669	0.953334444	1.036173613	0.5	0.4	1
PDE1C	0.953787937	0.736490945	1	1	1
ENSSSCG00000053386	0.953845626	0.491853096	0.5	0.467	1
PREB	0.953863173	0.749534268	1	0.933	1
ENSSSCG00000011803	0.953867903	0.771815941	1	1	1

MAP3K3	0.954516751	0.870053827	1	1	1
SOD2	0.955402246	0.570173278	1	1	1
FUT8	0.955891839	0.495934273	0.938	1	1
ENSSSCG00000060561	0.956515391	0.534921818	0.562	0.333	1
TYW3	0.956536612	0.148476582	1	1	1
ENSSSCG00000053433	0.957088358	0.68195598	0.938	1	1
RAB1A	0.957370362	0.714511428	1	1	1
ENSSSCG00000060721	0.957472784	0.668730858	0.5	0.533	1
WARS2	0.957474039	0.49330523	1	1	1
ENSSSCG00000042615	0.957766382	0.531381461	1	0.867	1
POLR1E	0.95779739	0.520638226	1	1	1
LCORL	0.957860957	0.468440551	1	1	1
MTFR2	0.957898217	0.604543863	1	1	1
COMMD8	0.958199267	0.469510895	1	1	1
FBXL17	0.958211823	0.558967292	1	0.867	1
CWF19L2	0.958307693	0.654090904	1	1	1
MGAT4B	0.958313429	0.754887502	0.812	0.667	1
CDCA5	0.958321929	0.867362231	0.938	0.933	1
SPAG17	0.959104485	0.706953025	1	1	1
ENSSSCG00000057954	0.959123807	0.491853096	0.5	0.4	1
SHPRH	0.959672068	0.5987683	0.938	0.8	1
RNF5	0.959898008	0.836501268	0.75	0.8	1
ENSSSCG00000033117	0.960230012	0.455679484	1	1	1
USP2	0.960629115	0.648031866	1	1	1
HOXA9	0.960638	0.728892294	0.938	0.8	1
ENSSSCG00000053300	0.960777829	0.491853096	0.938	0.8	1
CBX3	0.96097963	0.497089745	1	1	1
ALX1	0.961133499	0.425528337	1	1	1
STK26	0.962156649	0.61381643	1	1	1
MFSD8	0.96226215	0.911203588	1	0.933	1
ABCA4	0.962302079	0.347463187	0.625	0.467	1
ZNF317	0.962399378	0.500525398	1	1	1
ZFHX2	0.962611928	0.676006713	1	1	1
RGS10	0.962860769	0.186998515	0.625	0.4	1
ENSSSCG00000055215	0.962911244	0.516944077	0.688	0.467	1
EFL1	0.962977889	0.622583672	1	1	1
KLHL12	0.963153657	0.770073906	0.875	0.933	1
HID1	0.963177918	0.81688043	1	0.867	1
RPP30	0.963399779	0.741552864	1	1	1
MEIS1	0.964051382	0.613159393	0.688	0.733	1
PIGX	0.964054576	0.420377885	1	1	1
NBEA	0.964338127	0.671735222	1	0.867	1
PRDM4	0.964369463	0.686017125	1	1	1
BICDL1	0.964477308	0.689299161	0.875	0.667	1
PUM2	0.964896941	0.619808732	1	1	1
TMEM30A	0.965043877	0.548360193	1	0.933	1
ZNF23	0.965079697	0.549734012	0.938	0.867	1
TNFSF18	0.965835464	0.581314724	1	1	1
MYPN	0.965905716	0.454378391	0.562	0.467	1
SAFB2	0.965995843	0.798514435	0.938	0.8	1
WDR76	0.966300978	0.564097981	1	1	1
BBOF1	0.966369935	0.951284715	0.5	0.467	1
ETNK1	0.966569078	0.550034167	0.938	0.933	1
HNRNPUL1	0.966855499	0.801793533	1	1	1
SLC7A3	0.967004332	0.404606211	1	1	1
UBR3	0.967176055	0.462370075	1	1	1
ENSSSCG00000041163	0.967496068	0.454378391	0.5	0.267	1
RCE1	0.967832436	0.836117521	1	0.867	1
CAPRIN2	0.967938317	0.634197046	1	1	1
ENSSSCG00000012520	0.968184736	0.578205135	1	1	1
SHISA9	0.968511072	0.673691419	0.938	0.933	1
ENSSSCG00000060472	0.969410112	0.64385619	0.625	0.4	1
TBC1D13	0.969904497	1.002649578	0.875	0.933	1
TRAF3IP2	0.97076885	0.5987683	0.5	0.333	1
GLG1	0.970947837	0.503441071	1	1	1
BBS7	0.971318749	0.461479447	1	0.8	1
FAM185A	0.972298556	0.371294172	0.875	0.8	1
LRATD1	0.972466494	0.661778098	0.562	0.467	1
PGRMC2	0.973044098	0.576319886	1	1	1
TP53INP1	0.973137093	0.600768001	1	1	1

ENSSSCG00000059586	0.973176458	0.564002882	0.625	0.533	1
SYT1	0.973658713	0.736965594	0.5	0.333	1
DPP10	0.973684385	0.523561956	0.562	0.6	1
EBAG9	0.973890907	0.692219211	1	1	1
DHRS9	0.974422692	0.656912343	0.562	0.467	1
USP6NL	0.974756401	0.321928095	1	1	1
CFAP43	0.975415668	0.491853096	0.625	0.467	1
SNX6	0.975570776	0.608558314	1	1	1
MARCHF6	0.975581117	0.727470816	1	1	1
KLHL22	0.97580569	0.588638159	1	1	1
TRIM9	0.97627211	0.69538649	0.5	0.333	1
MAFG	0.976448927	0.785328616	1	1	1
ENSSSCG00000058281	0.976783926	0.154818109	0.5	0.4	1
SLC5A8	0.977042585	0.769387072	0.75	0.533	1
SH3GL1	0.977373553	0.627273306	1	1	1
ITGAM	0.977475515	0.587272661	1	0.933	1
SAXO1	0.977583191	0.564654775	1	1	1
FAT4	0.977929577	0.267832351	1	0.933	1
MTIF3	0.977946167	0.650550842	0.875	0.667	1
DAB1	0.977956418	0.594946589	1	1	1
TOR1AIP2	0.978395547	0.369862572	0.938	0.933	1
ENSSSCG00000050726	0.979005602	0.617383978	0.625	0.4	1
ADAMTS14	0.979800484	0.743391863	0.75	0.667	1
HLCS	0.979897675	0.80580447	1	1	1
PAMR1	0.979914447	0.343954401	1	1	1
CCDC66	0.980233522	0.588059422	1	1	1
ENSSSCG00000025440	0.980383672	0.318817923	1	1	1
ENSSSCG00000009356	0.980754103	0.521434087	1	0.933	1
USP30	0.980857304	0.6036671	1	1	1
ENSSSCG00000003248	0.981013784	0.392317423	0.625	0.4	1
TFCP2L1	0.981856625	0.392317423	0.688	0.4	1
GGNBP2	0.982764364	0.792821887	1	1	1
ZNF544	0.982904557	0.700439718	1	1	1
ENSSSCG00000049625	0.983472309	0.594946589	0.75	0.4	1
TMEM106A	0.983966506	0.909129068	1	1	1
ENSSSCG00000013760	0.983978893	0.642771049	1	1	1
SLC11A2	0.984393529	0.623373929	1	1	1
MYOC	0.984527	0.491853096	0.625	0.533	1
ZBTB9	0.984556359	0.956643631	0.938	1	1
USP47	0.984869382	0.580662363	1	1	1
R3HDM1	0.985323775	0.544518675	1	1	1
EARS2	0.985389259	0.340306624	1	0.8	1
POLRMT	0.985498709	-0.028979067	0.562	0.267	1
ADAMTS19	0.98555962	0.564784619	0.875	0.667	1
CREBBP	0.985636884	0.596004672	1	1	1
ENSSSCG00000038359	0.986013408	0.661149335	1	1	1
GANAB	0.986188112	0.75276745	1	1	1
ENSSSCG00000053419	0.986445117	0.718128952	0.938	0.933	1
NSF	0.986583048	0.636283873	1	1	1
SRSF9	0.987248779	0.673815892	1	1	1
ZBTB47	0.987352322	0.769387072	0.812	0.8	1
SFXN2	0.987380232	0.933984256	1	1	1
SGCB	0.987711551	0.441227023	0.625	0.4	1
BTAF1	0.987857497	0.637077657	1	1	1
PIP4K2C	0.988373159	0.730899146	1	0.933	1
TDRP	0.989059141	0.738858647	1	1	1
OSBPL11	0.989075999	0.590389573	1	1	1
PCYT1B	0.989548294	0.633872101	1	0.933	1
ENSSSCG00000056449	0.989917424	0.64385619	0.5	0.4	1
PAK2	0.990126339	0.641977855	1	1	1
RTN1	0.990219874	0.34298971	0.875	0.867	1
UTP4	0.990981414	0.540594639	1	1	1
TERB2	0.991706536	0.596550475	0.938	0.8	1
ENSSSCG00000014896	0.992288255	0.832890014	0.5	0.4	1
ENSSSCG00000062223	0.992716475	0.722807531	0.875	0.933	1
OCA2	0.992782799	0.22881869	0.5	0.4	1
ENSSSCG00000031683	0.992799746	0.689299161	0.75	0.4	1
RAD54L	0.992860428	0.639118271	1	1	1
SLC26A11	0.992944293	0.829353995	1	1	1
SRP54	0.992947569	0.721198278	1	1	1

C4orf47	0.993002995	0.526204601	0.938	0.867	1
FBN1	0.993150706	0.496386039	1	1	1
ULK1	0.993235394	0.548997003	1	1	1
ENSSSCG00000026430	0.994277712	0.626338429	1	1	1
ADAMTSL1	0.994366355	0.402522205	1	0.933	1
GPATCH8	0.994440873	0.527765219	1	1	1
TRAPPC8	0.99477295	0.531845072	1	1	1
DIS3L2	0.995294971	0.583913649	0.938	1	1
ENSSSCG00000050748	0.995585647	0.392317423	0.688	0.467	1
ENSSSCG00000034356	0.995596654	0.544320516	0.625	0.4	1
ENSSSCG00000060962	0.995966054	0.553253641	0.625	0.4	1
ENSSSCG00000045200	0.996004633	0.437405312	0.562	0.267	1
ZNF189	0.996330671	0.549848648	1	1	1
SHKBP1	0.996444095	0.417085328	0.75	0.733	1
NAA40	0.99658086	0.806779111	1	1	1
FNDC3A	0.996595735	0.548825282	1	1	1
ABHD6	0.996873564	0.665882496	0.812	0.667	1
ARHGAP1	0.997071351	0.780793062	1	1	1
DSEL	0.997109775	0.523561956	0.5	0.267	1
ENSSSCG00000053448	0.997315713	0.62309763	0.688	0.533	1
GNPDA1	0.997779038	0.750771394	0.812	0.8	1
ICA1L	0.997812987	0.608466145	1	1	1
TTC27	0.997917571	0.743004511	1	0.867	1
NKTR	0.997996955	0.566217031	1	1	1
ENSSSCG00000033739	0.998016592	0.508341219	0.938	0.733	1
ATP8A2	0.998022064	0.545567552	1	1	1
ZFHX3	0.998208452	0.857027319	1	1	1
USP22	0.998292863	0.822847	1	1	1
EPHX2	0.998823691	0.64385619	0.75	0.6	1
VTA1	0.999118589	0.580061324	1	1	1
ENSSSCG00000059963	0.99935236	0.508341219	0.812	0.8	1
PHF21A	0.999991502	0.54630088	0.938	0.933	1
ENSSSCG00000036114	NA	-0.780321963	0.25	1	NA
ENSSSCG00000045657	NA	-0.694560028	0.25	0.733	NA
ENSSSCG00000044939	NA	-1.756074417	0.125	0.6	NA
ENSSSCG00000063504	NA	2.845490051	1	0.533	NA
RAB13	NA	-1.543770813	0.562	1	NA
SDCBP2	NA	0.565853678	0.562	0.133	NA
ENSSSCG00000018067	NA	2.714245518	0.688	0.267	NA
TMEM98	NA	0.296837114	0.25	0.667	NA
NR1D2	NA	-1.030825126	0.75	0.333	NA
ENSSSCG00000056354	NA	-0.802330008	0.75	0.333	NA
ENSSSCG00000061722	NA	-1.269432177	0.812	0.4	NA
GSTA4	NA	3.293913719	0.812	0.4	NA
INHBA	NA	-1.230612928	0.188	0.6	NA
GPRASP1	NA	3.192292814	0.875	0.467	NA
ENSSSCG00000054266	NA	3.154818109	0.5	0.133	NA
LRCH2	NA	2.086214295	0.5	0.133	NA
ENSSSCG00000013849	NA	0.249282793	0.438	0.8	NA
WWTR1	NA	-0.158697746	0.375	0.733	NA
EED	NA	-0.119104613	0.312	0.667	NA
PROS1	NA	0.651051691	0.75	0.4	NA
SBSN	NA	-2.714597781	0.25	0.6	NA
TEX47	NA	1.347463187	0.25	0.6	NA
SERPINE1	NA	-3.710270728	0.25	0.6	NA
ZBTB8OS	NA	-0.678071905	0.188	0.533	NA
BTG2	NA	-1.900464326	0.188	0.533	NA
CFAP52	NA	1.25334101	0.812	0.467	NA
TBC1D4	NA	0.025072022	0.875	0.533	NA
ZC3HAV1	NA	-0.165865747	0.875	0.533	NA
FBN2	NA	-0.595609745	0.938	0.6	NA
HSF2	NA	-1.425953277	1	0.667	NA
POF1B	NA	3.366322214	0.5	0.2	NA
PAG1	NA	1.781359714	0.562	0.267	NA
ENSSSCG00000018076	NA	1.186998515	0.75	0.467	NA
RPS6KA1	NA	-0.280194957	0.812	0.533	NA
ENSSSCG00000060412	NA	-1.286790529	0.938	0.667	NA
CAPZA2	NA	0.704530363	0.938	0.667	NA
ABCA7	NA	-1.563429339	0.562	0.8	NA
GNG11	NA	1.138972073	0.5	0.733	NA

ENSSSCG00000002623	NA	3.831483193	0.5	0.267	NA
GABRB1	NA	-0.208586622	0.562	0.333	NA
GRIA3	NA	0.054989235	0.438	0.667	NA
CCNG2	NA	0.803054785	0.625	0.4	NA
ENSSSCG00000032381	NA	-2.526068812	0.375	0.6	NA
TIMP1	NA	-0.66247505	0.375	0.6	NA
CHAC2	NA	1.506352666	0.688	0.467	NA
SKIL	NA	-0.03170886	0.812	0.6	NA
MOB1A	NA	-1.093109404	0.875	0.667	NA
RUFY2	NA	1.577322439	0.938	0.733	NA
SESN3	NA	0.266432982	0.562	0.733	NA
ALDOA	NA	-0.526458782	0.562	0.733	NA
RGS5	NA	0.760638423	0.5	0.667	NA
ENSSSCG00000057898	NA	-1.012189409	0.562	0.4	NA
VIM	NA	-0.232272152	0.375	0.533	NA
ENSSSCG00000048190	NA	-0.001961516	0.688	0.533	NA
SERPINF1	NA	1.939312073	0.688	0.533	NA
ENSSSCG00000009772	NA	-0.53006168	0.75	0.6	NA
THBS1	NA	-3.390657772	0.812	0.667	NA
ALDH2	NA	-0.191194404	0.812	0.933	NA
SRPRB	NA	-0.900464326	0.562	0.667	NA
ENSSSCG00000022925	NA	-2.370267629	0.5	0.6	NA
HSPG2	NA	-0.093109404	0.438	0.533	NA
BMP1	NA	-0.208586622	0.438	0.533	NA
ENSSSCG00000061178	NA	-2.575502171	0.562	0.467	NA
KIAA0930	NA	2.049848549	0.562	0.467	NA
PHLDB2	NA	-1.05246742	0.625	0.533	NA
ENSSSCG00000031640	NA	1.22881869	0.625	0.533	NA
ENTPD1	NA	1.457905764	0.625	0.533	NA
ZNF25	NA	0.836501268	0.688	0.6	NA
ENSSSCG00000048856	NA	-1.703351314	0.812	0.733	NA
CTSC	NA	1.705541449	0.812	0.733	NA
MAGED1	NA	0.311586151	0.875	0.8	NA
RPS3A	NA	0.893215659	1	0.933	NA
CFAP57	NA	2.851451609	0.938	1	NA
FGF1	NA	0.158429363	0.688	0.733	NA
PIK3IP1	NA	0.989352756	0.5	0.467	NA
SLC30A7	NA	-0.689212463	0.562	0.533	NA
ENSSSCG00000004081	NA	0.828888084	0.625	0.6	NA
ENSSSCG00000061243	NA	-0.392669686	0.625	0.6	NA
TIA1	NA	0.352301744	0.75	0.733	NA
RETREG1	NA	-0.747334568	0.812	0.8	NA
PKM	NA	-0.380596854	0.938	0.933	NA
FKBP3	NA	-1.285754482	1	1	NA
ERP44	NA	0.897688103	1	1	NA
RNF138	NA	2.14974712	1	1	NA
VCAN	NA	1.156385161	1	1	NA
TMEM60	NA	1.963673317	1	1	NA
SERPINH1	NA	0.842978832	1	1	NA
ENSSSCG00000016970	NA	1.686780978	1	1	NA
