# Supplementary material for: Deciphering differences in DNA methylation and transcriptome profiles of oocytes from pigs with high and low developmental competence
Source: Environ Epigenet. 2025 Jun 3;11(1):dvaf018. doi: 10.1093/eep/dvaf018 (PMC12418950; doi:10.1093/eep/dvaf018)
Supplement: dvaf018_Supplemental_Files [file dvaf018_supplemental_files.zip › Sup table 3.pdf]

| Gene | p_val | avg_log2FC | pct.1 | pct.2 | p_val_adj |
|---|---|---|---|---|---|
| ENSSSCG00000061888 | 4.35E-41 | 4.03180478 | 1 | 1 | 7.54E-37 |
| CPO | 8.62E-08 | 3.969900393 | 1 | 0.333 | 0.001495808 |
| ENSSSCG00000000296 | 1.15E-25 | 3.605214326 | 1 | 1 | 2.00E-21 |
| RASL11A | 3.38E-16 | 3.302713962 | 1 | 0.867 | 5.86E-12 |
| ENSSSCG00000040498 | 3.81E-21 | 3.214415625 | 1 | 0.933 | 6.61E-17 |
| ENSSSCG00000050765 | 2.43E-06 | 3.19865672 | 1 | 0.667 | 0.04212399 |
| ENSSSCG00000063355 | 6.18E-12 | 3.181226319 | 1 | 0.867 | 1.07E-07 |
| MRPL44 | 2.36E-23 | 3.135383216 | 1 | 0.867 | 4.09E-19 |
| SFT2D1 | 5.58E-28 | 3.120036725 | 1 | 1 | 9.69E-24 |
| DUS4L | 2.42E-20 | 3.065426492 | 1 | 1 | 4.20E-16 |
| CCDC152 | 6.47E-12 | 3.060365076 | 1 | 0.867 | 1.12E-07 |
| TMEM128 | 3.34E-29 | 3.015103393 | 1 | 1 | 5.80E-25 |
| NEIL1 | 1.51E-17 | 3.005693006 | 1 | 1 | 2.62E-13 |
| STEAP1 | 5.75E-10 | 3.000194315 | 1 | 0.8 | 9.98E-06 |
| GFI1 | 1.11E-15 | 2.99906044 | 1 | 0.867 | 1.92E-11 |
| PTS | 8.50E-26 | 2.946358034 | 1 | 1 | 1.48E-21 |
| SNX7 | 1.90E-21 | 2.868352343 | 1 | 1 | 3.30E-17 |
| RTRAF | 5.75E-15 | 2.84955432 | 1 | 1 | 9.97E-11 |
| ENSSSCG00000014071 | 2.43E-28 | 2.820801619 | 1 | 1 | 4.22E-24 |
| BTG4 | 8.42E-24 | 2.767651617 | 1 | 1 | 1.46E-19 |
| NSL1 | 2.79E-18 | 2.760265682 | 1 | 1 | 4.85E-14 |
| CA9 | 9.64E-17 | 2.749230138 | 1 | 1 | 1.67E-12 |
| CHRAC1 | 1.05E-22 | 2.747817929 | 1 | 1 | 1.82E-18 |
| ADORA2A | 1.66E-06 | 2.725052273 | 1 | 0.6 | 0.028768688 |
| NUP42 | 2.42E-16 | 2.699171005 | 1 | 1 | 4.21E-12 |
| CEP78 | 2.52E-21 | 2.692330741 | 1 | 1 | 4.37E-17 |
| MAD2L1 | 2.58E-16 | 2.690511219 | 1 | 1 | 4.47E-12 |
| CCDC179 | 8.85E-09 | 2.688785533 | 1 | 0.867 | 0.000153589 |
| ALDH18A1 | 6.44E-13 | 2.657370996 | 1 | 1 | 1.12E-08 |
| C12orf40 | 2.93E-13 | 2.6555913 | 1 | 1 | 5.09E-09 |
| MOBKL3 | 3.45E-19 | 2.651588877 | 1 | 1 | 5.99E-15 |
| TAF1A | 8.06E-29 | 2.647720015 | 1 | 1 | 1.40E-24 |
| MITD1 | 1.20E-08 | 2.631992368 | 1 | 0.933 | 0.000207638 |
| ABHD3 | 3.49E-07 | 2.628589433 | 1 | 0.8 | 0.00605455 |
| HJURP | 1.92E-08 | 2.624119972 | 1 | 0.933 | 0.000333931 |
| AZIN2 | 7.60E-14 | 2.618820598 | 1 | 1 | 1.32E-09 |
| HILPDA | 1.44E-17 | 2.60711463 | 1 | 1 | 2.49E-13 |
| CDC45 | 1.74E-12 | 2.605578832 | 1 | 1 | 3.01E-08 |
| ELK3 | 6.17E-17 | 2.593203241 | 1 | 1 | 1.07E-12 |
| RAD1 | 7.00E-13 | 2.588906089 | 1 | 1 | 1.21E-08 |
| ANKRA2 | 5.57E-08 | 2.588196965 | 1 | 0.933 | 0.00096612 |
| CFAP69 | 5.87E-07 | 2.572665142 | 1 | 0.667 | 0.010193507 |
| GOLT1B | 4.50E-14 | 2.570474352 | 1 | 1 | 7.80E-10 |
| TTC8 | 1.22E-06 | 2.557655155 | 1 | 0.667 | 0.0211717 |
| TMEM216 | 2.17E-10 | 2.540692234 | 1 | 0.933 | 3.76E-06 |
| RNPEP | 5.17E-07 | 2.536391492 | 1 | 1 | 0.008962419 |
| SLC35G1 | 9.66E-24 | 2.529820947 | 1 | 1 | 1.68E-19 |
| TYMS | 2.00E-17 | 2.501766903 | 1 | 1 | 3.48E-13 |
| ENSSSCG00000053454 | 9.89E-11 | 2.494450208 | 1 | 1 | 1.72E-06 |
| HDAC3 | 8.35E-11 | 2.492707015 | 1 | 1 | 1.45E-06 |
| PITRM1 | 9.67E-09 | 2.466180149 | 1 | 1 | 0.000167698 |
| HAUS4 | 2.44E-27 | 2.453180215 | 1 | 1 | 4.24E-23 |
| ETFDH | 5.60E-07 | 2.449998253 | 1 | 0.933 | 0.009710765 |
| IER3IP1 | 8.87E-12 | 2.431573399 | 1 | 1 | 1.54E-07 |
| THOC6 | 1.98E-17 | 2.430452552 | 1 | 0.933 | 3.44E-13 |
| ENSSSCG00000033181 | 1.99E-06 | 2.400136927 | 0.938 | 0.8 | 0.034503582 |
| TAF9 | 7.91E-19 | 2.398175692 | 1 | 1 | 1.37E-14 |
| SRPX | 3.66E-16 | 2.397800932 | 1 | 1 | 6.35E-12 |
| EIF1 | 1.84E-08 | 2.397698817 | 1 | 1 | 0.000319391 |
| RFK | 2.93E-09 | 2.394409312 | 1 | 1 | 5.08E-05 |
| APIP | 5.29E-14 | 2.391364415 | 1 | 1 | 9.17E-10 |
| ENSSSCG00000044533 | 1.79E-07 | 2.391276142 | 1 | 1 | 0.003108455 |
| VWA7 | 3.99E-08 | 2.382848703 | 1 | 1 | 0.000692398 |
| PNP | 6.61E-14 | 2.379167825 | 1 | 1 | 1.15E-09 |
| TRAF4 | 3.34E-10 | 2.377825621 | 1 | 1 | 5.79E-06 |

| | | | | | |
|---|---|---|---|---|---|
| SNX31 | 1.02E-20 | 2.372787211 | 1 | 1 | 1.78E-16 |
| UBE2D1 | 2.46E-08 | 2.371476543 | 1 | 0.933 | 0.000427081 |
| NSMCE4A | 9.70E-24 | 2.367134588 | 1 | 1 | 1.68E-19 |
| DUSP12 | 8.21E-07 | 2.365391743 | 1 | 0.933 | 0.014243074 |
| METTL3 | 3.72E-09 | 2.364976417 | 1 | 1 | 6.45E-05 |
| FFAR4 | 1.19E-07 | 2.364059163 | 1 | 0.933 | 0.002058847 |
| ENSSSCG00000033814 | 1.34E-13 | 2.358864381 | 1 | 1 | 2.33E-09 |
| METTL9 | 1.94E-16 | 2.35331022 | 1 | 1 | 3.37E-12 |
| POC1B | 2.28E-12 | 2.351408963 | 1 | 1 | 3.95E-08 |
| RARS2 | 5.21E-15 | 2.350834658 | 1 | 1 | 9.05E-11 |
| RNFT1 | 1.45E-12 | 2.348841623 | 1 | 1 | 2.52E-08 |
| RIMBP2 | 2.01E-13 | 2.3474175 | 1 | 1 | 3.49E-09 |
| LRRC49 | 3.58E-10 | 2.341827652 | 1 | 0.933 | 6.21E-06 |
| SLC25A31 | 4.46E-15 | 2.339218765 | 1 | 1 | 7.73E-11 |
| SMAD1 | 4.63E-11 | 2.335374792 | 1 | 1 | 8.03E-07 |
| ENSSSCG00000063521 | 1.11E-07 | 2.33229481 | 1 | 0.933 | 0.001934452 |
| SF3B6 | 1.81E-11 | 2.330102026 | 1 | 1 | 3.15E-07 |
| BBS4 | 2.00E-06 | 2.326666014 | 1 | 0.733 | 0.034729117 |
| CDC20 | 3.53E-09 | 2.325557038 | 1 | 1 | 6.12E-05 |
| HECTD2 | 1.22E-11 | 2.316742956 | 1 | 1 | 2.12E-07 |
| PIM1 | 4.59E-12 | 2.316732069 | 1 | 1 | 7.97E-08 |
| SMIM11 | 2.92E-09 | 2.316614295 | 1 | 0.933 | 5.06E-05 |
| KAT2A | 3.45E-09 | 2.307467282 | 1 | 1 | 5.98E-05 |
| RMC1 | 3.42E-07 | 2.306702555 | 1 | 1 | 0.005937686 |
| CMTM8 | 2.04E-12 | 2.305542121 | 1 | 1 | 3.54E-08 |
| MCOLN1 | 8.27E-09 | 2.305169577 | 1 | 0.933 | 0.000143576 |
| SDHAF4 | 2.07E-06 | 2.288139781 | 1 | 0.8 | 0.035939686 |
| CENPN | 2.10E-08 | 2.279793831 | 1 | 1 | 0.000364593 |
| NGLY1 | 2.68E-20 | 2.278596252 | 1 | 1 | 4.65E-16 |
| POLB | 1.30E-10 | 2.278539969 | 1 | 1 | 2.26E-06 |
| HACE1 | 4.63E-13 | 2.273501405 | 1 | 1 | 8.03E-09 |
| TOGARAM2 | 1.45E-06 | 2.266786541 | 1 | 0.867 | 0.025094473 |
| AMFR | 2.20E-21 | 2.2644426 | 1 | 1 | 3.81E-17 |
| ENSSSCG00000004151 | 4.34E-13 | 2.261529099 | 1 | 1 | 7.53E-09 |
| MRPL16 | 3.38E-11 | 2.258610984 | 1 | 1 | 5.87E-07 |
| PCGF6 | 1.90E-10 | 2.258577835 | 1 | 1 | 3.30E-06 |
| SLC17A5 | 7.19E-16 | 2.258422404 | 1 | 1 | 1.25E-11 |
| SNRPC | 1.03E-14 | 2.25801286 | 1 | 1 | 1.79E-10 |
| TRIM77 | 6.12E-18 | 2.249337225 | 1 | 1 | 1.06E-13 |
| UBC | 6.82E-09 | 2.248487915 | 1 | 1 | 0.000118316 |
| OOSP3 | 5.47E-11 | 2.24828312 | 1 | 1 | 9.50E-07 |
| PRXL2A | 2.51E-11 | 2.245797026 | 1 | 1 | 4.36E-07 |
| ENSSSCG00000051497 | 3.32E-07 | 2.241175897 | 1 | 0.867 | 0.005765392 |
| TRMT2B | 3.36E-07 | 2.240314329 | 1 | 0.8 | 0.005827112 |
| RFC4 | 1.29E-18 | 2.227744324 | 1 | 1 | 2.24E-14 |
| GLCE | 1.79E-07 | 2.224849788 | 1 | 0.933 | 0.00310916 |
| GCA | 1.63E-15 | 2.219736947 | 1 | 1 | 2.84E-11 |
| BUD13 | 1.63E-09 | 2.217581719 | 1 | 1 | 2.82E-05 |
| COPB1 | 9.11E-13 | 2.214169759 | 1 | 1 | 1.58E-08 |
| PYCR2 | 4.71E-09 | 2.209207973 | 1 | 1 | 8.18E-05 |
| POLE2 | 3.55E-10 | 2.208871282 | 1 | 1 | 6.17E-06 |
| SRSF7 | 5.30E-07 | 2.201166457 | 1 | 1 | 0.009187886 |
| CLPTM1L | 1.49E-08 | 2.196498097 | 1 | 1 | 0.0002587 |
| TPST1 | 6.31E-11 | 2.195676406 | 1 | 1 | 1.10E-06 |
| BBS5 | 7.96E-09 | 2.191781311 | 1 | 1 | 0.000138141 |
| HSCB | 2.93E-09 | 2.185191758 | 1 | 1 | 5.09E-05 |
| ACYP2 | 8.21E-15 | 2.185044731 | 1 | 1 | 1.42E-10 |
| ACTL8 | 7.46E-08 | 2.184703057 | 1 | 1 | 0.001294641 |
| CTHRC1 | 2.35E-07 | 2.178695211 | 1 | 0.8 | 0.004084409 |
| ENSSSCG00000012452 | 1.37E-09 | 2.175952849 | 1 | 1 | 2.39E-05 |
| RXYLT1 | 2.73E-08 | 2.173398388 | 1 | 1 | 0.00047284 |
| CNN3 | 1.50E-13 | 2.168456354 | 1 | 1 | 2.60E-09 |
| CLK3 | 8.11E-07 | 2.164121796 | 1 | 1 | 0.014078249 |
| ZNF280D | 2.57E-11 | 2.159583057 | 1 | 0.933 | 4.46E-07 |
| DNA2 | 2.84E-07 | 2.159083794 | 1 | 1 | 0.004921645 |
| PARL | 2.80E-18 | 2.158125 | 1 | 1 | 4.86E-14 |

| | | | | | |
|---|---|---|---|---|---|
| ZNF16 | 3.14E-07 | 2.156169942 | 1 | 1 | 0.005445552 |
| RPA3 | 4.82E-10 | 2.155821258 | 1 | 1 | 8.37E-06 |
| ISOC1 | 1.01E-16 | 2.154508152 | 1 | 1 | 1.75E-12 |
| GFPT2 | 2.45E-06 | 2.148524367 | 1 | 1 | 0.042474278 |
| ENSSSCG00000004623 | 4.55E-10 | 2.1452581 | 1 | 1 | 7.89E-06 |
| ENSSSCG00000026746 | 5.97E-08 | 2.142680165 | 1 | 1 | 0.001035336 |
| HESX1 | 8.70E-07 | 2.14112463 | 1 | 0.8 | 0.015101778 |
| CCNB1 | 3.78E-15 | 2.140053101 | 1 | 1 | 6.56E-11 |
| TRMT11 | 6.63E-08 | 2.138802484 | 1 | 0.933 | 0.001149704 |
| KHDRBS1 | 3.08E-13 | 2.138454663 | 1 | 1 | 5.34E-09 |
| IRAK2 | 3.24E-10 | 2.135768962 | 1 | 1 | 5.62E-06 |
| DHX30 | 1.14E-07 | 2.133710104 | 1 | 1 | 0.001976659 |
| FGFR1OP2 | 4.84E-11 | 2.131716682 | 1 | 1 | 8.40E-07 |
| RCN2 | 2.55E-15 | 2.125407063 | 1 | 1 | 4.43E-11 |
| ENSSSCG00000056174 | 1.23E-11 | 2.124121312 | 1 | 1 | 2.13E-07 |
| PRMT2 | 1.04E-08 | 2.121224571 | 1 | 1 | 0.000180156 |
| SIKE1 | 6.94E-07 | 2.120486069 | 1 | 1 | 0.012045213 |
| STOML2 | 6.26E-09 | 2.118982423 | 1 | 1 | 0.000108682 |
| ZNF567 | 1.30E-08 | 2.117845904 | 1 | 1 | 0.00022568 |
| DERA | 2.67E-08 | 2.11585031 | 1 | 0.933 | 0.000462818 |
| GALNT11 | 5.89E-12 | 2.112237651 | 1 | 1 | 1.02E-07 |
| FBXO43 | 4.20E-12 | 2.108918286 | 1 | 1 | 7.29E-08 |
| ARL13B | 4.06E-07 | 2.108876806 | 1 | 0.8 | 0.007045686 |
| CEP57L1 | 1.13E-08 | 2.106650827 | 1 | 1 | 0.000196073 |
| KLHL7 | 6.63E-15 | 2.105913653 | 1 | 1 | 1.15E-10 |
| MOCS2 | 1.90E-12 | 2.104562594 | 1 | 1 | 3.29E-08 |
| AURKB | 2.75E-07 | 2.103927443 | 1 | 1 | 0.004769222 |
| CENPA | 2.48E-10 | 2.102857731 | 1 | 1 | 4.30E-06 |
| PSMD3 | 6.29E-07 | 2.102635153 | 1 | 1 | 0.010907434 |
| NUP43 | 1.15E-10 | 2.099140144 | 1 | 1 | 2.00E-06 |
| ADH5 | 3.25E-19 | 2.095906726 | 1 | 1 | 5.64E-15 |
| NRBF2 | 3.10E-20 | 2.095657031 | 1 | 1 | 5.38E-16 |
| STOM | 1.85E-07 | 2.095073819 | 1 | 1 | 0.003217219 |
| CRYZ | 2.14E-12 | 2.094147155 | 1 | 1 | 3.72E-08 |
| MND1 | 8.22E-07 | 2.09025786 | 1 | 0.933 | 0.014268021 |
| PEX3 | 6.74E-10 | 2.089246271 | 1 | 1 | 1.17E-05 |
| BMAL1 | 1.03E-07 | 2.088060354 | 1 | 1 | 0.001783321 |
| NUP35 | 5.30E-17 | 2.084742217 | 1 | 1 | 9.19E-13 |
| ENSSSCG00000008996 | 8.14E-13 | 2.081859803 | 1 | 1 | 1.41E-08 |
| C9orf40 | 1.11E-12 | 2.077886243 | 1 | 1 | 1.92E-08 |
| ENSSSCG00000030671 | 2.51E-06 | 2.077654617 | 1 | 0.933 | 0.043528982 |
| STARD3NL | 1.04E-17 | 2.076598078 | 1 | 1 | 1.81E-13 |
| SUPV3L1 | 1.30E-08 | 2.075816056 | 1 | 1 | 0.000226395 |
| ENSSSCG00000012235 | 8.55E-09 | 2.069324598 | 1 | 1 | 0.00014832 |
| PRMT1 | 1.21E-06 | 2.066235664 | 1 | 1 | 0.021007243 |
| SAT1 | 2.31E-10 | 2.065794191 | 1 | 1 | 4.00E-06 |
| CRLS1 | 5.59E-12 | 2.064678618 | 1 | 1 | 9.70E-08 |
| SLC17A2 | 8.57E-07 | 2.063111982 | 1 | 1 | 0.014876895 |
| OAZ1 | 8.02E-12 | 2.063053848 | 1 | 1 | 1.39E-07 |
| H1-8 | 1.67E-10 | 2.061878384 | 1 | 1 | 2.89E-06 |
| PHYHIPL | 1.15E-09 | 2.058262372 | 1 | 1 | 2.00E-05 |
| EPB41L5 | 1.83E-13 | 2.057190776 | 1 | 1 | 3.18E-09 |
| LEPROT | 1.21E-11 | 2.055395592 | 1 | 1 | 2.10E-07 |
| NR2C1 | 9.34E-12 | 2.051872623 | 1 | 1 | 1.62E-07 |
| PSMA5 | 4.23E-10 | 2.050838491 | 1 | 1 | 7.35E-06 |
| ENSSSCG00000002020 | 1.00E-22 | 2.049370351 | 1 | 1 | 1.74E-18 |
| AKAIN1 | 8.73E-07 | 2.045505167 | 1 | 1 | 0.015150217 |
| POLG2 | 1.60E-08 | 2.043271108 | 1 | 1 | 0.000276763 |
| CDC40 | 1.21E-18 | 2.043233601 | 1 | 1 | 2.10E-14 |
| BORA | 1.09E-06 | 2.040817399 | 1 | 1 | 0.018928374 |
| LRRC42 | 2.39E-07 | 2.031924912 | 1 | 1 | 0.00415109 |
| MAP2K1 | 5.89E-11 | 2.030832606 | 1 | 1 | 1.02E-06 |
| PBK | 5.45E-11 | 2.029968463 | 1 | 1 | 9.46E-07 |
| SEPTIN2 | 2.69E-09 | 2.029509882 | 1 | 1 | 4.66E-05 |
| MIER1 | 2.57E-09 | 2.027383612 | 1 | 1 | 4.45E-05 |
| EMC2 | 1.23E-09 | 2.026801923 | 1 | 1 | 2.14E-05 |

| | | | | | |
|---|---|---|---|---|---|
| LACTB2 | 6.74E-08 | 2.026037861 | 1 | 0.867 | 0.001169959 |
| C5orf34 | 4.84E-13 | 2.024233184 | 1 | 1 | 8.40E-09 |
| SPTLC1 | 6.24E-18 | 2.016150344 | 1 | 1 | 1.08E-13 |
| CLDN1 | 6.82E-13 | 2.013764314 | 1 | 1 | 1.18E-08 |
| ENSSSCG00000039658 | 4.61E-09 | 2.01283646 | 1 | 1 | 8.00E-05 |
| VPS37A | 7.53E-13 | 2.011686786 | 1 | 1 | 1.31E-08 |
| ENSSSCG00000001075 | 3.01E-12 | 2.009949005 | 1 | 1 | 5.22E-08 |
| AKTIP | 1.73E-08 | 2.009272088 | 1 | 1 | 0.000299952 |
| GTF2B | 5.37E-07 | 2.005660936 | 1 | 1 | 0.00932055 |
| NDFIP2 | 6.08E-09 | 2.000421286 | 1 | 1 | 0.000105492 |
| RCBTB2 | 3.69E-15 | 1.998683967 | 1 | 1 | 6.41E-11 |
| ENSSSCG00000000576 | 8.88E-08 | 1.995947086 | 1 | 1 | 0.001540106 |
| PDE8A | 1.92E-18 | 1.993544978 | 1 | 1 | 3.33E-14 |
| CARMIL1 | 1.07E-16 | 1.99051427 | 1 | 1 | 1.86E-12 |
| ENSSSCG00000021644 | 9.67E-25 | 1.990075288 | 1 | 1 | 1.68E-20 |
| CTNNBL1 | 2.68E-06 | 1.989957207 | 1 | 1 | 0.046509475 |
| STX7 | 5.74E-14 | 1.989783604 | 1 | 1 | 9.97E-10 |
| MAP3K7 | 4.76E-12 | 1.989567618 | 1 | 1 | 8.26E-08 |
| ENSSSCG00000009178 | 1.01E-06 | 1.989057724 | 1 | 1 | 0.017542931 |
| OOSP2 | 1.39E-09 | 1.988350296 | 1 | 1 | 2.40E-05 |
| DARS1 | 2.17E-10 | 1.98681924 | 1 | 1 | 3.76E-06 |
| PSMA2 | 1.51E-11 | 1.984092766 | 1 | 1 | 2.62E-07 |
| MAPKBP1 | 4.41E-07 | 1.983921081 | 1 | 1 | 0.0076442 |
| CNOT8 | 9.69E-11 | 1.983875399 | 1 | 1 | 1.68E-06 |
| MPP7 | 4.53E-12 | 1.98179201 | 1 | 1 | 7.87E-08 |
| WDR89 | 1.41E-08 | 1.980186105 | 1 | 1 | 0.000244806 |
| CCDC127 | 6.66E-10 | 1.975345898 | 1 | 1 | 1.16E-05 |
| SLC35A1 | 4.17E-10 | 1.971502958 | 1 | 1 | 7.23E-06 |
| RPP25L | 3.03E-09 | 1.970249993 | 1 | 1 | 5.26E-05 |
| FOXN2 | 3.54E-11 | 1.964627887 | 1 | 1 | 6.14E-07 |
| ACVR1B | 2.25E-07 | 1.958460944 | 1 | 1 | 0.003900831 |
| CASP6 | 5.45E-11 | 1.956892733 | 1 | 1 | 9.45E-07 |
| MELK | 1.38E-20 | 1.956840472 | 1 | 1 | 2.39E-16 |
| LDAF1 | 1.60E-06 | 1.95473037 | 1 | 1 | 0.027709869 |
| BLM | 1.58E-08 | 1.952296575 | 1 | 1 | 0.000274322 |
| ETFA | 8.85E-12 | 1.951779687 | 1 | 1 | 1.53E-07 |
| NUS1 | 3.78E-09 | 1.951599199 | 1 | 1 | 6.56E-05 |
| SECISBP2 | 6.79E-13 | 1.947391555 | 1 | 1 | 1.18E-08 |
| CCNA2 | 4.53E-13 | 1.946110343 | 1 | 1 | 7.86E-09 |
| SPPL2A | 3.61E-12 | 1.941824386 | 1 | 1 | 6.27E-08 |
| ZNRF2 | 1.78E-06 | 1.94035188 | 1 | 1 | 0.030954364 |
| TM9SF1 | 5.94E-08 | 1.939411738 | 1 | 1 | 0.001030794 |
| COG3 | 3.15E-13 | 1.938844457 | 1 | 1 | 5.47E-09 |
| SLC12A8 | 9.46E-10 | 1.938439431 | 1 | 1 | 1.64E-05 |
| CYFIP1 | 1.78E-08 | 1.938169762 | 1 | 1 | 0.000309135 |
| EIF4G2 | 6.39E-12 | 1.935715115 | 1 | 1 | 1.11E-07 |
| NMD3 | 2.95E-09 | 1.935699219 | 1 | 1 | 5.11E-05 |
| UNC13B | 6.53E-11 | 1.93477371 | 1 | 1 | 1.13E-06 |
| ENSSSCG00000025940 | 1.76E-13 | 1.932878128 | 1 | 1 | 3.05E-09 |
| SNRNP40 | 3.01E-08 | 1.932165403 | 1 | 1 | 0.000522748 |
| CRY1 | 4.93E-07 | 1.930658762 | 1 | 1 | 0.008546989 |
| LNX1 | 1.45E-09 | 1.930084168 | 1 | 1 | 2.52E-05 |
| GPR160 | 3.61E-09 | 1.929964619 | 1 | 1 | 6.27E-05 |
| ZNF606 | 1.11E-09 | 1.927610269 | 1 | 1 | 1.93E-05 |
| SLC25A32 | 6.05E-10 | 1.924195482 | 1 | 1 | 1.05E-05 |
| CDCP1 | 1.31E-06 | 1.923077642 | 1 | 1 | 0.022767816 |
| TROAP | 8.43E-07 | 1.920986939 | 1 | 1 | 0.014626141 |
| DTWD1 | 4.64E-07 | 1.916617142 | 1 | 1 | 0.008054912 |
| NME7 | 1.85E-08 | 1.914351909 | 1 | 1 | 0.00032113 |
| CBFB | 1.85E-08 | 1.910510911 | 1 | 1 | 0.000321405 |
| IFRD2 | 2.96E-07 | 1.906890596 | 1 | 1 | 0.005128258 |
| CAAP1 | 8.28E-09 | 1.905272317 | 1 | 1 | 0.000143675 |
| LIMA1 | 6.18E-07 | 1.904321236 | 1 | 0.933 | 0.010714595 |
| TANK | 7.69E-07 | 1.9021989 | 1 | 1 | 0.013349352 |
| WEE2 | 3.53E-07 | 1.900622379 | 1 | 1 | 0.006120335 |
| USP33 | 1.96E-10 | 1.897881896 | 1 | 1 | 3.40E-06 |

| | | | | | |
|---|---|---|---|---|---|
| TTC5 | 1.19E-09 | 1.896446704 | 1 | 1 | 2.07E-05 |
| CDCA7 | 7.72E-07 | 1.892909841 | 1 | 1 | 0.013395458 |
| DCLRE1A | 1.31E-06 | 1.892656985 | 1 | 1 | 0.022664081 |
| RAP2C | 4.49E-08 | 1.888796802 | 1 | 1 | 0.000779408 |
| GSR | 8.34E-15 | 1.886583089 | 1 | 1 | 1.45E-10 |
| RBM17 | 9.77E-08 | 1.885682937 | 1 | 1 | 0.001694563 |
| NCOA3 | 2.35E-10 | 1.885400313 | 1 | 1 | 4.08E-06 |
| ENSSSCG00000060529 | 1.86E-08 | 1.882935863 | 1 | 1 | 0.000322913 |
| ARL6IP6 | 1.30E-08 | 1.881799615 | 1 | 1 | 0.000225803 |
| SAP30 | 1.38E-08 | 1.879861364 | 1 | 1 | 0.000238832 |
| ATL1 | 1.96E-08 | 1.876654589 | 1 | 1 | 0.000340165 |
| MEST | 1.04E-08 | 1.874618149 | 1 | 1 | 0.000181144 |
| MAP3K13 | 9.10E-18 | 1.874358342 | 1 | 1 | 1.58E-13 |
| ME2 | 1.50E-13 | 1.873282203 | 1 | 1 | 2.60E-09 |
| RIDA | 2.14E-10 | 1.872844123 | 1 | 1 | 3.71E-06 |
| PPM1D | 1.24E-07 | 1.872215328 | 1 | 1 | 0.002159884 |
| ENSSSCG00000004983 | 5.59E-07 | 1.871744033 | 1 | 1 | 0.009706062 |
| CTBS | 5.13E-11 | 1.870909175 | 1 | 1 | 8.90E-07 |
| VEZF1 | 1.57E-11 | 1.870521458 | 1 | 1 | 2.73E-07 |
| ENSSSCG00000027349 | 3.89E-08 | 1.870043711 | 1 | 1 | 0.000674888 |
| TRAFD1 | 2.24E-11 | 1.86773925 | 1 | 1 | 3.89E-07 |
| SBDS | 1.30E-15 | 1.867298417 | 1 | 1 | 2.26E-11 |
| SGMS1 | 1.75E-12 | 1.862972371 | 1 | 1 | 3.03E-08 |
| DNMT3B | 5.21E-08 | 1.861357454 | 1 | 1 | 0.000903329 |
| WDR20 | 4.23E-16 | 1.861195383 | 1 | 1 | 7.34E-12 |
| OMA1 | 1.52E-08 | 1.859597987 | 1 | 1 | 0.000263857 |
| ENY2 | 6.96E-11 | 1.859062071 | 1 | 1 | 1.21E-06 |
| ENSSSCG00000005440 | 1.73E-16 | 1.853706272 | 1 | 1 | 3.00E-12 |
| PARD6B | 4.99E-14 | 1.85297505 | 1 | 1 | 8.66E-10 |
| CBR4 | 4.07E-08 | 1.851964385 | 1 | 1 | 0.000705637 |
| RRM1 | 5.90E-11 | 1.850669584 | 1 | 1 | 1.02E-06 |
| APH1A | 2.91E-08 | 1.849088499 | 1 | 1 | 0.000504213 |
| DONSON | 1.58E-13 | 1.848430865 | 1 | 1 | 2.73E-09 |
| NEIL3 | 3.06E-11 | 1.847480932 | 1 | 1 | 5.31E-07 |
| RAF1 | 1.94E-11 | 1.845181503 | 1 | 1 | 3.37E-07 |
| PPM1B | 9.48E-11 | 1.844984691 | 1 | 1 | 1.65E-06 |
| MLLT11 | 9.36E-09 | 1.841624824 | 1 | 1 | 0.00016234 |
| PDHX | 1.05E-09 | 1.840777924 | 1 | 1 | 1.82E-05 |
| KIF4A | 8.66E-13 | 1.840404308 | 1 | 1 | 1.50E-08 |
| SPIRE1 | 6.69E-09 | 1.840365135 | 1 | 1 | 0.00011607 |
| THOC3 | 4.30E-10 | 1.840075642 | 1 | 1 | 7.47E-06 |
| FAM168B | 7.21E-15 | 1.836792626 | 1 | 1 | 1.25E-10 |
| SKP2 | 8.22E-08 | 1.834397152 | 1 | 1 | 0.00142677 |
| HNRNPC | 4.48E-21 | 1.833240347 | 1 | 1 | 7.77E-17 |
| TAB2 | 2.06E-12 | 1.831676759 | 1 | 1 | 3.57E-08 |
| CEP44 | 1.98E-13 | 1.830519326 | 1 | 1 | 3.43E-09 |
| ID3 | 3.15E-07 | 1.827864414 | 1 | 1 | 0.005471755 |
| NPM1 | 3.50E-09 | 1.827574231 | 1 | 1 | 6.06E-05 |
| FYB2 | 3.41E-11 | 1.827074642 | 1 | 1 | 5.93E-07 |
| SLC25A20 | 5.68E-12 | 1.82527683 | 1 | 1 | 9.86E-08 |
| MFF | 1.85E-10 | 1.8252513 | 1 | 1 | 3.21E-06 |
| SLC4A4 | 2.20E-08 | 1.822708174 | 1 | 1 | 0.000381831 |
| ENSSSCG00000008335 | 2.02E-08 | 1.8217351 | 1 | 1 | 0.000349837 |
| PCBP1 | 1.42E-08 | 1.818636586 | 1 | 1 | 0.000245923 |
| ORMDL1 | 7.47E-08 | 1.813531352 | 1 | 1 | 0.001296922 |
| TMCC1 | 1.23E-14 | 1.811684666 | 1 | 1 | 2.14E-10 |
| DR1 | 9.91E-12 | 1.810473113 | 1 | 1 | 1.72E-07 |
| TAF5 | 2.99E-08 | 1.810392008 | 1 | 1 | 0.00051917 |
| ENSSSCG00000053039 | 2.85E-06 | 1.810366527 | 1 | 1 | 0.04952177 |
| SNRPE | 4.13E-08 | 1.809716512 | 1 | 1 | 0.000717241 |
| LAPTM4A | 3.37E-11 | 1.806840594 | 1 | 1 | 5.84E-07 |
| PPP1CC | 1.29E-08 | 1.806780231 | 1 | 1 | 0.000223787 |
| AHCTF1 | 1.02E-08 | 1.805775963 | 1 | 1 | 0.000176586 |
| PRKCI | 1.47E-11 | 1.805465923 | 1 | 1 | 2.55E-07 |
| DIMT1 | 1.43E-06 | 1.802513979 | 1 | 1 | 0.024792884 |
| XRCC2 | 1.76E-09 | 1.800268176 | 1 | 1 | 3.05E-05 |

| | | | | | |
|---|---|---|---|---|---|
| CMAS | 9.56E-11 | 1.797178592 | 1 | 1 | 1.66E-06 |
| TCTN2 | 2.92E-09 | 1.790823198 | 1 | 1 | 5.06E-05 |
| COQ10B | 1.93E-09 | 1.786147538 | 1 | 1 | 3.35E-05 |
| HERPUD2 | 6.47E-11 | 1.786094079 | 1 | 1 | 1.12E-06 |
| ADPGK | 1.45E-08 | 1.785155183 | 1 | 1 | 0.000252168 |
| TAOK3 | 7.71E-08 | 1.78495202 | 1 | 1 | 0.001337646 |
| TRMT5 | 2.07E-06 | 1.784555053 | 1 | 1 | 0.035940575 |
| MASTL | 9.12E-10 | 1.783366188 | 1 | 1 | 1.58E-05 |
| ICA1 | 1.74E-09 | 1.782374089 | 1 | 1 | 3.02E-05 |
| MRPL15 | 1.87E-10 | 1.780585735 | 1 | 1 | 3.25E-06 |
| RCHY1 | 5.24E-08 | 1.778530344 | 1 | 1 | 0.000909226 |
| SOS2 | 4.48E-08 | 1.777892239 | 1 | 1 | 0.000777224 |
| GMNN | 1.13E-06 | 1.776971761 | 1 | 1 | 0.01956387 |
| CREG1 | 9.10E-07 | 1.776561601 | 1 | 1 | 0.015786707 |
| MAPK6 | 3.71E-08 | 1.776318141 | 1 | 1 | 0.000643971 |
| TMCO6 | 2.87E-06 | 1.771904551 | 1 | 1 | 0.049723937 |
| RAE1 | 2.16E-07 | 1.769979241 | 1 | 1 | 0.003740694 |
| C9orf72 | 3.27E-08 | 1.766723691 | 1 | 1 | 0.000568012 |
| EDRF1 | 3.19E-13 | 1.765957912 | 1 | 1 | 5.53E-09 |
| TENT4A | 9.33E-08 | 1.764018514 | 1 | 1 | 0.001619618 |
| CNOT7 | 1.25E-07 | 1.763788602 | 1 | 1 | 0.002167964 |
| RAP1A | 1.37E-07 | 1.759994393 | 1 | 1 | 0.002380976 |
| PSMD8 | 1.45E-07 | 1.758339245 | 1 | 1 | 0.002524324 |
| CNBP | 8.99E-24 | 1.758101516 | 1 | 1 | 1.56E-19 |
| WTAP | 5.88E-17 | 1.757829777 | 1 | 1 | 1.02E-12 |
| PPFIA1 | 1.67E-11 | 1.752353315 | 1 | 1 | 2.90E-07 |
| SLC39A12 | 2.70E-07 | 1.751436077 | 1 | 1 | 0.004680292 |
| NUP133 | 3.35E-10 | 1.747885009 | 1 | 1 | 5.82E-06 |
| TMED5 | 3.73E-08 | 1.737527032 | 1 | 1 | 0.000647763 |
| ENSSSCG00000051290 | 2.40E-07 | 1.730291251 | 1 | 1 | 0.004166719 |
| RHOT1 | 2.53E-15 | 1.728831319 | 1 | 1 | 4.39E-11 |
| STRAP | 7.93E-07 | 1.728606527 | 1 | 1 | 0.013753482 |
| AHSA1 | 1.34E-06 | 1.726522106 | 1 | 1 | 0.023237526 |
| SEH1L | 4.62E-11 | 1.726191123 | 1 | 1 | 8.01E-07 |
| SYPL1 | 1.72E-06 | 1.724134845 | 1 | 1 | 0.029860201 |
| SCCPDH | 2.63E-10 | 1.722895174 | 1 | 1 | 4.56E-06 |
| HENMT1 | 1.76E-07 | 1.722047403 | 1 | 1 | 0.00304964 |
| LONP2 | 2.36E-07 | 1.72055616 | 1 | 1 | 0.004087095 |
| CHEK1 | 9.02E-09 | 1.717060111 | 1 | 1 | 0.000156589 |
| GNB4 | 2.19E-08 | 1.716366119 | 1 | 1 | 0.000379406 |
| ARMC8 | 4.72E-11 | 1.716357809 | 1 | 1 | 8.19E-07 |
| ENSSSCG00000038630 | 1.75E-09 | 1.715600797 | 1 | 1 | 3.04E-05 |
| RHPN2 | 1.75E-06 | 1.715332294 | 1 | 1 | 0.030306698 |
| KANK1 | 5.60E-13 | 1.714174324 | 1 | 1 | 9.72E-09 |
| DPYS | 2.73E-10 | 1.71334129 | 1 | 1 | 4.74E-06 |
| DCTD | 1.37E-06 | 1.711179215 | 1 | 1 | 0.02370505 |
| FUNDC1 | 3.78E-08 | 1.710902779 | 1 | 1 | 0.000655535 |
| TADA1 | 1.54E-08 | 1.708423358 | 1 | 1 | 0.000267767 |
| CIAO2A | 8.32E-08 | 1.707494476 | 1 | 1 | 0.001444283 |
| KLHDC2 | 8.47E-10 | 1.706456971 | 1 | 1 | 1.47E-05 |
| MTA3 | 3.30E-09 | 1.706193245 | 1 | 1 | 5.72E-05 |
| RIOK3 | 9.06E-07 | 1.705759832 | 1 | 1 | 0.01571312 |
| COPA | 1.70E-13 | 1.701163208 | 1 | 1 | 2.95E-09 |
| NCAPG | 8.73E-07 | 1.697768694 | 1 | 1 | 0.015154501 |
| MTSS1 | 5.05E-07 | 1.695794892 | 1 | 1 | 0.008755967 |
| CD164 | 9.63E-10 | 1.693993495 | 1 | 1 | 1.67E-05 |
| LRIG3 | 8.26E-07 | 1.689172706 | 1 | 1 | 0.014334579 |
| TDP2 | 1.75E-09 | 1.688618558 | 1 | 1 | 3.03E-05 |
| SLC19A2 | 9.21E-07 | 1.687488217 | 1 | 1 | 0.015977283 |
| SSX2IP | 2.40E-08 | 1.686716974 | 1 | 1 | 0.000416763 |
| SNRPA1 | 2.68E-06 | 1.685968961 | 1 | 1 | 0.046522886 |
| EIF2AK4 | 5.93E-13 | 1.685963583 | 1 | 1 | 1.03E-08 |
| PPP6C | 4.50E-08 | 1.683483653 | 1 | 1 | 0.000781549 |
| EIF2A | 5.14E-10 | 1.683206594 | 1 | 1 | 8.91E-06 |
| MORF4L2 | 4.10E-07 | 1.680893083 | 1 | 1 | 0.007108665 |
| NDRG3 | 7.80E-08 | 1.677781191 | 1 | 1 | 0.001353322 |

| | | | | | |
|---|---|---|---|---|---|
| NAA35 | 1.05E-07 | 1.675133385 | 1 | 1 | 0.001825756 |
| BARD1 | 6.58E-07 | 1.674551523 | 1 | 1 | 0.01141825 |
| ATP5F1C | 1.15E-07 | 1.669914851 | 1 | 1 | 0.002002283 |
| HAS3 | 6.22E-07 | 1.668601566 | 1 | 1 | 0.010797688 |
| MCMBP | 1.07E-09 | 1.667316889 | 1 | 1 | 1.86E-05 |
| CSPP1 | 7.19E-07 | 1.664133138 | 1 | 1 | 0.012480951 |
| PPHLN1 | 1.04E-08 | 1.664068197 | 1 | 1 | 0.000180661 |
| BAZ1A | 1.01E-07 | 1.66310998 | 1 | 1 | 0.001760857 |
| NUP107 | 7.44E-08 | 1.658083293 | 1 | 1 | 0.001290833 |
| STK35 | 3.30E-10 | 1.655013158 | 1 | 1 | 5.73E-06 |
| C10orf90 | 1.51E-07 | 1.653462185 | 1 | 1 | 0.002612155 |
| NDC1 | 2.85E-10 | 1.65327006 | 1 | 1 | 4.94E-06 |
| KNSTRN | 8.82E-08 | 1.650188891 | 1 | 1 | 0.001529992 |
| ENSSSCG00000031705 | 5.03E-07 | 1.649493895 | 1 | 1 | 0.008734038 |
| NLRP4 | 2.34E-09 | 1.649275169 | 1 | 1 | 4.05E-05 |
| HMGB2 | 1.53E-12 | 1.647952264 | 1 | 1 | 2.66E-08 |
| KMT2E | 7.24E-11 | 1.646338602 | 1 | 1 | 1.26E-06 |
| RESF1 | 1.13E-06 | 1.646133374 | 1 | 1 | 0.019551068 |
| MTERF3 | 2.18E-06 | 1.643119933 | 1 | 1 | 0.037747134 |
| SPATA7 | 8.82E-08 | 1.643024426 | 1 | 1 | 0.001529632 |
| UHRF1 | 8.74E-07 | 1.641121222 | 1 | 1 | 0.015165766 |
| SLC30A4 | 1.34E-07 | 1.640024328 | 1 | 1 | 0.002320256 |
| CDC7 | 2.71E-07 | 1.638829373 | 1 | 1 | 0.004707555 |
| PM20D2 | 1.49E-06 | 1.638073837 | 1 | 1 | 0.025853483 |
| THOC7 | 8.37E-07 | 1.637647225 | 1 | 1 | 0.014525265 |
| PCOLCE2 | 1.39E-10 | 1.637581225 | 1 | 1 | 2.41E-06 |
| ENSSSCG00000001769 | 1.04E-09 | 1.634028348 | 1 | 1 | 1.80E-05 |
| GAB1 | 3.90E-08 | 1.629387342 | 1 | 1 | 0.000677459 |
| IFT81 | 6.13E-07 | 1.629034865 | 1 | 1 | 0.010641767 |
| TMEM131L | 7.02E-13 | 1.628625223 | 1 | 1 | 1.22E-08 |
| WDR47 | 7.41E-08 | 1.628470444 | 1 | 1 | 0.00128495 |
| GZMA | 2.12E-06 | 1.627940593 | 1 | 1 | 0.036828202 |
| NID2 | 6.95E-11 | 1.627675754 | 1 | 1 | 1.21E-06 |
| CKS1B | 1.59E-06 | 1.62728767 | 1 | 1 | 0.027525321 |
| USP28 | 1.30E-08 | 1.623563571 | 1 | 1 | 0.000225147 |
| LGR4 | 1.48E-06 | 1.622604417 | 1 | 1 | 0.025713213 |
| SPAG16 | 2.41E-06 | 1.622184163 | 1 | 1 | 0.041876173 |
| KDELR2 | 3.75E-08 | 1.62028149 | 1 | 1 | 0.000651357 |
| HMMR | 4.37E-07 | 1.617624648 | 1 | 1 | 0.007576955 |
| ADD3 | 2.16E-06 | 1.617232363 | 1 | 1 | 0.037454415 |
| GRPEL2 | 4.84E-12 | 1.613787476 | 1 | 1 | 8.41E-08 |
| GLS2 | 2.83E-07 | 1.613110664 | 1 | 1 | 0.004916252 |
| SWT1 | 2.29E-07 | 1.611131884 | 1 | 1 | 0.003969631 |
| USP25 | 4.80E-07 | 1.607903722 | 1 | 1 | 0.008321593 |
| SNX25 | 2.48E-10 | 1.606274683 | 1 | 1 | 4.30E-06 |
| UBR2 | 4.04E-09 | 1.601089374 | 1 | 1 | 7.01E-05 |
| F3 | 1.81E-06 | 1.600852475 | 1 | 1 | 0.031403994 |
| ANKLE2 | 1.65E-09 | 1.600442284 | 1 | 1 | 2.87E-05 |
| ENSSSCG00000013395 | 2.60E-07 | 1.597238307 | 1 | 1 | 0.004512775 |
| DCAF6 | 1.50E-07 | 1.597061978 | 1 | 1 | 0.002596012 |
| RIC1 | 4.15E-07 | 1.595086011 | 1 | 1 | 0.007209207 |
| PRKAR1A | 1.78E-11 | 1.594419608 | 1 | 1 | 3.09E-07 |
| CENPO | 6.40E-07 | 1.590260181 | 1 | 1 | 0.011097784 |
| TEX10 | 4.46E-09 | 1.587813114 | 1 | 1 | 7.73E-05 |
| CDH7 | 4.27E-09 | 1.586386027 | 1 | 1 | 7.42E-05 |
| GLA | 5.71E-07 | 1.585964555 | 1 | 1 | 0.009911142 |
| TAB3 | 6.91E-07 | 1.574600749 | 1 | 1 | 0.011988563 |
| FAM151B | 4.79E-07 | 1.5710144 | 1 | 1 | 0.008302828 |
| HBEGF | 2.38E-06 | 1.568922489 | 1 | 1 | 0.04127448 |
| VAPA | 2.36E-06 | 1.564283981 | 1 | 1 | 0.041020352 |
| UBAC2 | 2.32E-08 | 1.563842443 | 1 | 1 | 0.000403121 |
| ENSSSCG00000006344 | 8.51E-07 | 1.56233476 | 1 | 1 | 0.01476067 |
| STX6 | 1.37E-10 | 1.558227658 | 1 | 1 | 2.39E-06 |
| CENPW | 1.70E-07 | 1.557784573 | 1 | 1 | 0.002948231 |
| ITPRID2 | 1.02E-08 | 1.555273235 | 1 | 1 | 0.000176242 |
| RDX | 1.26E-08 | 1.554478431 | 1 | 1 | 0.000219143 |

| | | | | | |
|---|---|---|---|---|---|
| UXS1 | 2.38E-10 | 1.553617912 | 1 | 1 | 4.12E-06 |
| ENSSSCG00000007203 | 2.32E-09 | 1.54863596 | 1 | 1 | 4.03E-05 |
| SMU1 | 6.98E-07 | 1.547856577 | 1 | 1 | 0.012110939 |
| ENSSSCG00000001934 | 6.69E-07 | 1.54760354 | 1 | 1 | 0.011604171 |
| ARL14EPL | 1.19E-06 | 1.544011951 | 1 | 1 | 0.020597373 |
| PPP1CB | 7.77E-08 | 1.543635638 | 1 | 1 | 0.001347377 |
| VIRMA | 4.42E-08 | 1.533280993 | 1 | 1 | 0.00076639 |
| OCRL | 1.58E-06 | 1.532661013 | 1 | 1 | 0.027397493 |
| INTS14 | 9.97E-08 | 1.526377673 | 1 | 1 | 0.001729272 |
| UTRN | 9.13E-07 | 1.524803518 | 1 | 1 | 0.015846826 |
| SLAIN2 | 8.18E-08 | 1.523380394 | 1 | 1 | 0.001419624 |
| CDKN2AIP | 1.49E-06 | 1.522488781 | 1 | 1 | 0.025850132 |
| PEX1 | 2.28E-07 | 1.518716759 | 1 | 1 | 0.003951587 |
| SLC23A2 | 5.82E-09 | 1.516889647 | 1 | 1 | 0.000100922 |
| CAPN7 | 4.72E-08 | 1.511395335 | 1 | 1 | 0.000819713 |
| ARF4 | 1.22E-06 | 1.496439937 | 1 | 1 | 0.021167942 |
| PALB2 | 8.70E-08 | 1.494613246 | 1 | 1 | 0.001510305 |
| GNAI1 | 8.83E-08 | 1.492281641 | 1 | 1 | 0.001532561 |
| DBT | 2.68E-06 | 1.485016912 | 1 | 1 | 0.046461175 |
| ACTL6A | 1.07E-06 | 1.483581834 | 1 | 1 | 0.018487178 |
| ARL8B | 2.76E-07 | 1.482679972 | 1 | 1 | 0.004783589 |
| REV1 | 1.13E-08 | 1.482413868 | 1 | 1 | 0.00019628 |
| RNF2 | 7.30E-11 | 1.480553423 | 1 | 1 | 1.27E-06 |
| KRR1 | 1.31E-06 | 1.476972397 | 1 | 1 | 0.022775628 |
| UBR1 | 1.80E-06 | 1.476235981 | 1 | 1 | 0.031287808 |
| SEPTIN10 | 1.35E-06 | 1.471618613 | 1 | 1 | 0.023341009 |
| NUDT21 | 5.69E-12 | 1.464466188 | 1 | 1 | 9.87E-08 |
| RBBP7 | 8.23E-08 | 1.462745729 | 1 | 1 | 0.001428216 |
| WAPL | 1.06E-07 | 1.456452254 | 1 | 1 | 0.001840963 |
| FBXO25 | 3.38E-09 | 1.451249671 | 1 | 1 | 5.87E-05 |
| PCNX4 | 1.45E-06 | 1.437220647 | 1 | 1 | 0.025211619 |
| HSD17B12 | 2.16E-07 | 1.427998763 | 1 | 1 | 0.003746467 |
| PSMG1 | 8.86E-09 | 1.422742753 | 1 | 1 | 0.000153662 |
| FBXL20 | 3.03E-08 | 1.420819564 | 1 | 1 | 0.000525735 |
| DIP2B | 7.72E-07 | 1.420817834 | 1 | 1 | 0.013398722 |
| CTNND1 | 1.24E-07 | 1.418153376 | 1 | 1 | 0.002158661 |
| ENSSSCG00000003303 | 4.25E-07 | 1.411726362 | 1 | 1 | 0.007366974 |
| BTBD3 | 3.04E-07 | 1.410824552 | 1 | 1 | 0.005273494 |
| BMPR2 | 7.16E-07 | 1.410657013 | 1 | 1 | 0.012429407 |
| ENSSSCG00000006084 | 2.71E-06 | 1.410299263 | 1 | 1 | 0.046964512 |
| EPB41L2 | 1.29E-08 | 1.40721507 | 1 | 1 | 0.000223191 |
| PPP4R4 | 8.28E-08 | 1.405882705 | 1 | 1 | 0.001437339 |
| ATG5 | 1.56E-06 | 1.398267139 | 1 | 1 | 0.027063631 |
| PIWIL1 | 1.50E-07 | 1.390492381 | 1 | 1 | 0.002602272 |
| MTAP | 2.23E-06 | 1.390063936 | 1 | 1 | 0.038701872 |
| NCOA1 | 5.25E-07 | 1.389367417 | 1 | 1 | 0.009105661 |
| WDR48 | 9.90E-13 | 1.382770612 | 1 | 1 | 1.72E-08 |
| MKLN1 | 6.86E-08 | 1.373074128 | 1 | 1 | 0.001189545 |
| PPP2R1B | 1.43E-07 | 1.371955583 | 1 | 1 | 0.002488651 |
| STRN | 8.72E-07 | 1.366006146 | 1 | 1 | 0.015128942 |
| ACTR2 | 1.38E-06 | 1.360790027 | 1 | 1 | 0.023965323 |
| WAC | 1.28E-06 | 1.359306738 | 1 | 1 | 0.022181346 |
| DYNC1I2 | 3.84E-10 | 1.357296662 | 1 | 1 | 6.67E-06 |
| RABGAP1 | 5.44E-08 | 1.336867759 | 1 | 1 | 0.000944688 |
| FERMT2 | 2.71E-07 | 1.330585528 | 1 | 1 | 0.004698905 |
| RMND5A | 1.37E-06 | 1.322632364 | 1 | 1 | 0.023798091 |
| RNF115 | 8.03E-09 | 1.307835866 | 1 | 1 | 0.000139284 |
| CLINT1 | 1.05E-07 | 1.304413648 | 1 | 1 | 0.001829781 |
| SMURF2 | 1.32E-06 | 1.302431308 | 1 | 1 | 0.022966123 |
| ABI1 | 6.42E-09 | 1.29785963 | 1 | 1 | 0.000111428 |
| PHLPP2 | 5.58E-07 | 1.284378753 | 1 | 1 | 0.009679752 |
| NOCT | 8.37E-07 | 1.27941248 | 1 | 1 | 0.014526955 |
| CTNNB1 | 1.34E-07 | 1.277044708 | 1 | 1 | 0.002328767 |
| PTPN13 | 6.02E-08 | 1.270783108 | 1 | 1 | 0.001045285 |
| ZNF207 | 8.44E-10 | 1.261019582 | 1 | 1 | 1.46E-05 |
| ENSSSCG00000015390 | 2.32E-08 | 1.209560482 | 1 | 1 | 0.000401959 |

| | | | | | |
|---|---|---|---|---|---|
| PTPN2 | 2.23E-06 | 1.207960086 | 1 | 1 | 0.038778272 |
| ANAPC1 | 2.01E-06 | 1.160299843 | 1 | 1 | 0.03489341 |
| TP63 | 4.52E-07 | 0.034871345 | 1 | 1 | 0.007847919 |
| LRRFIP2 | 1.18E-07 | -0.009335781 | 1 | 1 | 0.002043169 |
| ENSSSCG00000009327 | 1.44E-06 | -0.025250546 | 1 | 1 | 0.025030087 |
| TMEM181 | 1.80E-06 | -0.040734909 | 1 | 1 | 0.031269265 |
| MAT2A | 6.04E-07 | -0.059391289 | 1 | 1 | 0.010480238 |
| ZNF79 | 1.75E-07 | -0.075746696 | 1 | 1 | 0.003041681 |
| KBTBD13 | 9.60E-12 | -0.094786469 | 1 | 1 | 1.67E-07 |
| RALA | 2.51E-06 | -0.107867594 | 1 | 1 | 0.04356708 |
| ARL4A | 1.20E-07 | -0.111073154 | 1 | 1 | 0.002083142 |
| INPP1 | 9.04E-08 | -0.130005882 | 1 | 1 | 0.001569273 |
| PWP1 | 9.79E-08 | -0.130594414 | 1 | 1 | 0.00169889 |
| EIF4B | 9.19E-07 | -0.151258284 | 1 | 1 | 0.01594539 |
| C6orf120 | 2.05E-06 | -0.151673297 | 1 | 1 | 0.035645692 |
| CCNB2 | 1.30E-10 | -0.152153171 | 1 | 1 | 2.25E-06 |
| SMG1 | 2.57E-08 | -0.171291378 | 1 | 1 | 0.000446598 |
| LZIC | 2.08E-09 | -0.198772777 | 1 | 1 | 3.61E-05 |
| KANSL2 | 1.13E-07 | -0.20932751 | 1 | 1 | 0.001954415 |
| ASH2L | 1.17E-06 | -0.218408789 | 1 | 1 | 0.020296565 |
| SLC4A1AP | 2.63E-06 | -0.226102741 | 1 | 1 | 0.045708593 |
| NUB1 | 7.32E-10 | -0.237291677 | 1 | 1 | 1.27E-05 |
| TCP11L1 | 2.20E-06 | -0.243563045 | 1 | 1 | 0.038227094 |
| ENSSSCG00000031636 | 5.20E-09 | -0.248545069 | 1 | 1 | 9.02E-05 |
| CCT5 | 8.13E-08 | -0.254544523 | 1 | 1 | 0.00141008 |
| MFAP1 | 4.91E-08 | -0.266278777 | 1 | 1 | 0.00085178 |
| DCLK1 | 8.86E-07 | -0.271165307 | 1 | 1 | 0.015367144 |
| TRMT10A | 2.27E-06 | -0.280861804 | 1 | 1 | 0.03938029 |
| FBXW8 | 4.73E-07 | -0.282722741 | 1 | 1 | 0.008215459 |
| TMEM41A | 1.68E-08 | -0.283197445 | 1 | 1 | 0.000291124 |
| ADAD1 | 1.88E-07 | -0.289374798 | 1 | 1 | 0.003253645 |
| CNOT11 | 6.72E-07 | -0.291005617 | 1 | 1 | 0.011658104 |
| YIPF5 | 1.13E-06 | -0.296158366 | 1 | 1 | 0.019642152 |
| ZC3H13 | 4.60E-08 | -0.297718674 | 1 | 1 | 0.000797904 |
| GSPT1 | 2.87E-09 | -0.303046827 | 1 | 1 | 4.98E-05 |
| ECD | 2.31E-07 | -0.304877643 | 1 | 1 | 0.004010403 |
| HAT1 | 5.23E-08 | -0.306923251 | 1 | 1 | 0.000907967 |
| MRPL3 | 5.55E-09 | -0.316356756 | 1 | 1 | 9.63E-05 |
| CFAP44 | 4.75E-10 | -0.327618112 | 1 | 1 | 8.24E-06 |
| CHN2 | 8.02E-10 | -0.336995358 | 1 | 1 | 1.39E-05 |
| WIPF2 | 1.53E-06 | -0.338761732 | 1 | 1 | 0.026620242 |
| ENSSSCG00000015011 | 2.60E-06 | -0.340729471 | 1 | 1 | 0.045064088 |
| PLEK2 | 2.39E-07 | -0.340989311 | 1 | 1 | 0.004148814 |
| TRNT1 | 2.46E-06 | -0.354954449 | 1 | 1 | 0.04275075 |
| TMEM245 | 8.08E-07 | -0.355736901 | 1 | 1 | 0.014024073 |
| MIOS | 1.25E-06 | -0.363279087 | 1 | 1 | 0.021720935 |
| ATP6V0A1 | 1.99E-08 | -0.364227248 | 1 | 1 | 0.000345582 |
| MAP4K4 | 1.41E-06 | -0.364234398 | 1 | 1 | 0.024391068 |
| RAB2B | 1.69E-08 | -0.368091664 | 1 | 1 | 0.00029342 |
| MBOAT1 | 1.29E-08 | -0.368743847 | 1 | 1 | 0.00022394 |
| PSMA3 | 1.18E-07 | -0.384846706 | 1 | 1 | 0.002054864 |
| CDCA7L | 1.52E-07 | -0.385795866 | 0.938 | 1 | 0.002635562 |
| ENSSSCG00000050083 | 1.98E-06 | -0.394032934 | 1 | 1 | 0.034271292 |
| BLMH | 3.47E-11 | -0.4012317 | 1 | 1 | 6.02E-07 |
| MPHOSPH8 | 1.66E-08 | -0.403505662 | 1 | 1 | 0.000288422 |
| CCNI | 4.98E-09 | -0.404282534 | 1 | 1 | 8.63E-05 |
| COPS4 | 2.13E-08 | -0.408894306 | 1 | 1 | 0.000368808 |
| TMEM50B | 5.60E-07 | -0.417682221 | 1 | 1 | 0.009718761 |
| PSEN1 | 1.96E-08 | -0.417905003 | 1 | 1 | 0.000340793 |
| MYOF | 4.02E-07 | -0.421320541 | 1 | 1 | 0.006975427 |
| LETMD1 | 4.70E-07 | -0.433733593 | 1 | 1 | 0.008147322 |
| ACSL5 | 5.60E-08 | -0.444460047 | 1 | 1 | 0.000971096 |
| CPSF3 | 1.45E-06 | -0.444581775 | 1 | 1 | 0.025183898 |
| C2orf88 | 1.44E-07 | -0.445647895 | 1 | 1 | 0.002499494 |
| SNAPC2 | 2.16E-07 | -0.451802311 | 1 | 1 | 0.003754215 |
| SART3 | 1.07E-07 | -0.45429693 | 1 | 1 | 0.001852775 |

| | | | | | |
|---|---|---|---|---|---|
| CHIC2 | 5.43E-07 | -0.455254599 | 1 | 1 | 0.009421176 |
| MRPS23 | 1.42E-06 | -0.455504091 | 1 | 1 | 0.02466459 |
| PTP4A2 | 2.38E-08 | -0.458319747 | 1 | 1 | 0.00041285 |
| ENSSSCG00000012125 | 3.24E-07 | -0.459840707 | 1 | 1 | 0.005624378 |
| ITM2B | 5.51E-07 | -0.464949214 | 1 | 1 | 0.009561531 |
| ENSSSCG00000052707 | 2.60E-07 | -0.465420878 | 1 | 1 | 0.004513583 |
| RPL15 | 1.13E-08 | -0.465519923 | 1 | 1 | 0.000196294 |
| EIF2S2 | 6.64E-10 | -0.466033103 | 1 | 1 | 1.15E-05 |
| ATF4 | 1.88E-06 | -0.472370674 | 1 | 1 | 0.032533271 |
| ATP5PB | 1.32E-09 | -0.475626662 | 1 | 1 | 2.28E-05 |
| EIF2S3B | 2.25E-08 | -0.477925205 | 1 | 1 | 0.000390186 |
| NCBP3 | 1.28E-07 | -0.480953941 | 1 | 1 | 0.002215081 |
| ENSSSCG00000037143 | 5.17E-12 | -0.482950751 | 1 | 1 | 8.97E-08 |
| TMED4 | 1.18E-06 | -0.491032883 | 1 | 1 | 0.020439474 |
| ACAD8 | 1.81E-06 | -0.491088162 | 1 | 1 | 0.031409223 |
| NOLC1 | 1.31E-08 | -0.494649119 | 1 | 1 | 0.000227016 |
| EZR | 4.31E-14 | -0.497619192 | 1 | 1 | 7.48E-10 |
| LIG3 | 3.30E-07 | -0.506088674 | 1 | 1 | 0.005724372 |
| CHCHD3 | 4.60E-09 | -0.507036711 | 1 | 1 | 7.98E-05 |
| ENSSSCG00000062406 | 3.92E-09 | -0.510404482 | 1 | 1 | 6.81E-05 |
| ZNF75D | 4.37E-08 | -0.510918653 | 1 | 1 | 0.000757648 |
| OGFOD1 | 1.25E-08 | -0.512664405 | 1 | 1 | 0.000217009 |
| VBP1 | 1.64E-06 | -0.516320835 | 1 | 1 | 0.028502808 |
| ENSSSCG00000002707 | 2.13E-10 | -0.517339328 | 1 | 1 | 3.69E-06 |
| SSR1 | 1.14E-11 | -0.519348645 | 1 | 1 | 1.99E-07 |
| MTMR2 | 3.27E-09 | -0.521088936 | 1 | 1 | 5.68E-05 |
| TPM1 | 1.76E-06 | -0.523097245 | 1 | 1 | 0.030591207 |
| ENSSSCG00000028479 | 6.12E-07 | -0.524721128 | 1 | 1 | 0.010612105 |
| NOX4 | 1.52E-07 | -0.526511671 | 1 | 1 | 0.002636304 |
| CENPF | 6.99E-08 | -0.527420916 | 1 | 1 | 0.001213647 |
| NAALAD2 | 2.92E-09 | -0.530542495 | 1 | 1 | 5.06E-05 |
| BUB3 | 1.37E-07 | -0.531026191 | 1 | 1 | 0.002375438 |
| YEATS4 | 2.22E-07 | -0.53470773 | 1 | 1 | 0.003845501 |
| NARS1 | 1.47E-06 | -0.542980385 | 1 | 1 | 0.025467526 |
| SMS | 2.52E-10 | -0.544063405 | 1 | 1 | 4.38E-06 |
| SMARCE1 | 1.34E-08 | -0.544858682 | 1 | 1 | 0.000232963 |
| ATG4A | 1.87E-12 | -0.549425205 | 1 | 1 | 3.24E-08 |
| DCAF13 | 1.32E-06 | -0.551130074 | 1 | 1 | 0.022961175 |
| RNF8 | 5.01E-12 | -0.556955188 | 1 | 1 | 8.69E-08 |
| ENSSSCG00000057727 | 2.81E-07 | -0.562322044 | 1 | 1 | 0.004872946 |
| NFATC2 | 5.17E-08 | -0.563875051 | 1 | 1 | 0.000897696 |
| ADIPOR2 | 1.36E-11 | -0.566082469 | 1 | 1 | 2.35E-07 |
| ANO4 | 1.09E-06 | -0.572030268 | 1 | 1 | 0.018861652 |
| MRPS14 | 4.80E-08 | -0.574340501 | 1 | 1 | 0.00083264 |
| KNG1 | 1.17E-08 | -0.581762098 | 1 | 1 | 0.000202447 |
| PELO | 2.08E-10 | -0.58236383 | 1 | 1 | 3.61E-06 |
| ENSSSCG00000027506 | 1.05E-09 | -0.585589642 | 1 | 1 | 1.81E-05 |
| PCCB | 8.40E-09 | -0.586723668 | 1 | 1 | 0.000145774 |
| ANXA5 | 2.47E-08 | -0.587953107 | 1 | 1 | 0.000428998 |
| PKIG | 1.67E-06 | -0.588211293 | 1 | 1 | 0.028931735 |
| DERL2 | 4.30E-10 | -0.590104436 | 1 | 1 | 7.46E-06 |
| PPIE | 1.67E-08 | -0.593538395 | 1 | 1 | 0.000290113 |
| UNC50 | 1.20E-08 | -0.594022028 | 1 | 1 | 0.00020741 |
| LDLRAP1 | 8.11E-08 | -0.594507188 | 0.938 | 1 | 0.001407053 |
| DUSP16 | 8.09E-14 | -0.601232924 | 1 | 1 | 1.40E-09 |
| DNAJC18 | 1.21E-06 | -0.604025554 | 1 | 1 | 0.020936913 |
| UCHL3 | 5.60E-10 | -0.605922119 | 1 | 1 | 9.71E-06 |
| PAFAH2 | 1.44E-06 | -0.607188419 | 1 | 1 | 0.02502292 |
| TMEM45A | 4.12E-07 | -0.610815467 | 1 | 1 | 0.007145467 |
| GTSF1 | 3.24E-09 | -0.613139669 | 1 | 1 | 5.62E-05 |
| ZFYVE1 | 7.86E-07 | -0.613876145 | 1 | 1 | 0.01363043 |
| CLTA | 1.10E-10 | -0.620554795 | 1 | 1 | 1.91E-06 |
| NRBP1 | 1.22E-12 | -0.623062958 | 1 | 1 | 2.12E-08 |
| ENSSSCG00000059649 | 2.95E-12 | -0.626933056 | 1 | 1 | 5.12E-08 |
| EIF2AK2 | 1.53E-07 | -0.631629957 | 1 | 1 | 0.002658911 |
| XRCC6 | 2.31E-11 | -0.634027572 | 1 | 1 | 4.01E-07 |

| | | | | | |
|---|---|---|---|---|---|
| FKBP9 | 1.43E-08 | -0.634882948 | 1 | 1 | 0.000247606 |
| ENSSSCG00000006694 | 3.74E-19 | -0.636148296 | 1 | 1 | 6.49E-15 |
| RPSA | 1.42E-06 | -0.638310836 | 1 | 1 | 0.02471224 |
| ZNHIT3 | 1.28E-08 | -0.639471852 | 1 | 1 | 0.000222035 |
| PRMT6 | 1.03E-10 | -0.643876985 | 1 | 1 | 1.78E-06 |
| SH3GL2 | 4.60E-11 | -0.648841194 | 1 | 1 | 7.98E-07 |
| FIGNL1 | 1.11E-06 | -0.649057923 | 1 | 1 | 0.019251517 |
| TNN | 3.75E-07 | -0.649270382 | 1 | 1 | 0.00650922 |
| PDE6C | 2.01E-07 | -0.650169631 | 1 | 1 | 0.003493691 |
| SF3B3 | 2.31E-06 | -0.653409851 | 1 | 1 | 0.040006968 |
| GPN3 | 4.84E-07 | -0.655055252 | 1 | 1 | 0.008402802 |
| NUDT9 | 1.22E-09 | -0.657124967 | 1 | 1 | 2.12E-05 |
| UBE2R2 | 5.60E-08 | -0.659212878 | 1 | 1 | 0.000972239 |
| TCEA3 | 2.64E-06 | -0.660720571 | 1 | 1 | 0.045859029 |
| FAN1 | 5.19E-07 | -0.661448075 | 1 | 1 | 0.00900548 |
| ASTN1 | 5.34E-07 | -0.662662466 | 1 | 1 | 0.009257207 |
| SRP19 | 2.91E-11 | -0.663309844 | 1 | 1 | 5.05E-07 |
| UTP23 | 2.43E-06 | -0.663848491 | 1 | 1 | 0.04214827 |
| HNF1B | 2.53E-08 | -0.665304609 | 1 | 1 | 0.000438138 |
| PSMD6 | 6.42E-11 | -0.666940898 | 1 | 1 | 1.11E-06 |
| TMEM50A | 6.85E-11 | -0.669411567 | 1 | 1 | 1.19E-06 |
| CDC42 | 9.51E-11 | -0.672497035 | 1 | 1 | 1.65E-06 |
| ENSSSCG00000043116 | 2.38E-08 | -0.675166174 | 1 | 1 | 0.000413171 |
| ENSSSCG00000027374 | 3.88E-09 | -0.675180732 | 1 | 1 | 6.74E-05 |
| DPM1 | 2.85E-08 | -0.677186001 | 1 | 1 | 0.000495332 |
| ENSSSCG00000043568 | 2.26E-07 | -0.680697375 | 1 | 1 | 0.003925795 |
| PSMD7 | 5.17E-12 | -0.682340237 | 1 | 1 | 8.98E-08 |
| DNAJC3 | 6.33E-08 | -0.682758059 | 1 | 1 | 0.001098572 |
| MORC2 | 4.99E-10 | -0.684243872 | 1 | 1 | 8.66E-06 |
| MTM1 | 1.91E-09 | -0.685293132 | 1 | 1 | 3.32E-05 |
| CCL22 | 2.84E-06 | -0.686292412 | 0.938 | 1 | 0.049357611 |
| ENSSSCG00000013232 | 1.52E-16 | -0.691034212 | 1 | 1 | 2.63E-12 |
| LRMDA | 2.58E-06 | -0.693258309 | 0.938 | 1 | 0.04483446 |
| PEDS1 | 1.16E-06 | -0.69366876 | 1 | 1 | 0.020041297 |
| RBM22 | 7.44E-07 | -0.693738502 | 1 | 1 | 0.012913496 |
| SRP14 | 1.41E-11 | -0.698551297 | 1 | 1 | 2.44E-07 |
| SEPTIN8 | 2.57E-06 | -0.701127141 | 1 | 1 | 0.044575922 |
| DNAJB6 | 1.76E-12 | -0.709277773 | 1 | 1 | 3.06E-08 |
| GLP2R | 1.79E-09 | -0.711334359 | 1 | 1 | 3.11E-05 |
| SUPT3H | 5.58E-09 | -0.718579629 | 1 | 1 | 9.68E-05 |
| DSN1 | 8.61E-11 | -0.722499371 | 1 | 1 | 1.49E-06 |
| EDC3 | 2.52E-06 | -0.729536548 | 1 | 1 | 0.043669749 |
| TM2D3 | 1.32E-10 | -0.7314716 | 1 | 1 | 2.29E-06 |
| TTLL3 | 6.01E-08 | -0.737743118 | 1 | 1 | 0.001042393 |
| SLC17A1 | 1.07E-06 | -0.741441355 | 1 | 1 | 0.018603451 |
| RBX1 | 1.34E-15 | -0.741466986 | 1 | 1 | 2.33E-11 |
| FHIP1A | 1.42E-13 | -0.742019857 | 1 | 1 | 2.46E-09 |
| RPGRIP1 | 1.27E-09 | -0.743969791 | 1 | 1 | 2.21E-05 |
| FAAP24 | 1.11E-07 | -0.744992503 | 1 | 1 | 0.001923506 |
| IFT88 | 4.06E-08 | -0.746855887 | 1 | 1 | 0.000704714 |
| PSMD14 | 4.16E-13 | -0.747475954 | 1 | 1 | 7.22E-09 |
| POLR3C | 8.28E-08 | -0.748259753 | 1 | 1 | 0.001437235 |
| FAM114A2 | 1.79E-06 | -0.748350601 | 1 | 1 | 0.030991266 |
| YWHAQ | 1.15E-12 | -0.751953103 | 1 | 1 | 2.00E-08 |
| CAMTA1 | 9.19E-07 | -0.753903318 | 1 | 1 | 0.015951316 |
| MKRN1 | 2.22E-08 | -0.754669598 | 1 | 1 | 0.000385886 |
| DTX2 | 7.69E-09 | -0.756455166 | 1 | 1 | 0.000133488 |
| ETF1 | 3.61E-07 | -0.760534065 | 1 | 1 | 0.006256611 |
| ATG3 | 2.72E-12 | -0.761903496 | 0.938 | 1 | 4.73E-08 |
| ENSSSCG00000027270 | 1.33E-06 | -0.762960803 | 1 | 1 | 0.023066082 |
| SPRING1 | 1.45E-10 | -0.763188519 | 1 | 1 | 2.52E-06 |
| FRA10AC1 | 3.40E-08 | -0.763869778 | 1 | 1 | 0.000589952 |
| NECAP2 | 2.67E-11 | -0.765136267 | 1 | 1 | 4.64E-07 |
| MSL1 | 7.39E-07 | -0.765961138 | 0.938 | 1 | 0.012823961 |
| CYTH3 | 2.60E-06 | -0.766299088 | 1 | 0.933 | 0.045174604 |
| SPDYC | 1.14E-07 | -0.770822028 | 1 | 1 | 0.001984676 |

| | | | | | |
|---|---|---|---|---|---|
| PCDHGA4 | 9.65E-08 | -0.772700736 | 1 | 1 | 0.001673657 |
| GFM2 | 2.38E-07 | -0.774040964 | 1 | 1 | 0.00413364 |
| C17orf80 | 8.39E-13 | -0.775515625 | 1 | 1 | 1.46E-08 |
| SNRPN | 3.18E-10 | -0.776925292 | 1 | 1 | 5.52E-06 |
| PFDN6 | 2.58E-07 | -0.779609932 | 1 | 1 | 0.004484278 |
| IK | 3.42E-13 | -0.779834621 | 1 | 1 | 5.93E-09 |
| PIN4 | 5.45E-07 | -0.780226449 | 0.938 | 1 | 0.009449064 |
| ENSSSCG00000011447 | 4.95E-08 | -0.780545526 | 1 | 1 | 0.000858761 |
| ALG14 | 5.57E-08 | -0.783488205 | 1 | 0.933 | 0.000966633 |
| SLIT3 | 6.49E-07 | -0.783504588 | 1 | 1 | 0.011269044 |
| KCNN3 | 3.87E-07 | -0.783781346 | 0.938 | 1 | 0.006706767 |
| SAR1A | 2.52E-13 | -0.785820052 | 1 | 1 | 4.38E-09 |
| MRPL35 | 2.07E-08 | -0.786341117 | 0.938 | 1 | 0.00035971 |
| LAMA3 | 1.41E-06 | -0.788015748 | 0.938 | 1 | 0.024515866 |
| ENSSSCG00000039222 | 1.99E-09 | -0.792683211 | 1 | 1 | 3.45E-05 |
| LRRC59 | 2.02E-06 | -0.793549123 | 0.938 | 1 | 0.035010006 |
| ENSSSCG00000062560 | 3.94E-08 | -0.802251316 | 1 | 1 | 0.000684344 |
| NSG1 | 1.69E-06 | -0.803227036 | 1 | 1 | 0.029355248 |
| ZP2 | 6.75E-09 | -0.806185294 | 1 | 1 | 0.000117114 |
| GLYR1 | 4.91E-07 | -0.812026362 | 1 | 1 | 0.008523416 |
| NEGR1 | 1.30E-10 | -0.814941695 | 1 | 1 | 2.26E-06 |
| ZFAND3 | 7.12E-09 | -0.816485934 | 1 | 1 | 0.00012346 |
| SLC41A3 | 9.38E-08 | -0.817851743 | 1 | 1 | 0.001627598 |
| TTC1 | 2.31E-08 | -0.822171113 | 1 | 1 | 0.000400683 |
| SEZ6L2 | 2.14E-07 | -0.824378268 | 1 | 1 | 0.00370762 |
| MMAA | 8.46E-14 | -0.828089183 | 1 | 1 | 1.47E-09 |
| YARS1 | 1.89E-06 | -0.829001964 | 0.938 | 1 | 0.032843893 |
| FOXJ3 | 8.06E-14 | -0.829294953 | 1 | 1 | 1.40E-09 |
| TRAP1 | 1.01E-08 | -0.82930944 | 0.938 | 1 | 0.000174444 |
| STIMATE | 2.63E-13 | -0.831947413 | 1 | 1 | 4.56E-09 |
| SUGT1 | 1.72E-12 | -0.833994037 | 1 | 1 | 2.98E-08 |
| ARV1 | 4.04E-09 | -0.835721562 | 1 | 1 | 7.01E-05 |
| KCNMB4 | 2.00E-08 | -0.836991861 | 1 | 1 | 0.000347219 |
| DNAJB11 | 1.82E-10 | -0.838816224 | 1 | 1 | 3.16E-06 |
| PIP4K2B | 5.39E-08 | -0.838925916 | 1 | 0.933 | 0.000935456 |
| EFCAB7 | 1.20E-08 | -0.841346111 | 1 | 1 | 0.000207425 |
| C11orf58 | 9.04E-15 | -0.843417126 | 1 | 1 | 1.57E-10 |
| PIGC | 6.51E-09 | -0.846536245 | 1 | 1 | 0.000112946 |
| SLC2A8 | 1.56E-06 | -0.847439041 | 1 | 1 | 0.027151158 |
| ACBD7 | 3.19E-08 | -0.850084959 | 0.938 | 1 | 0.000552688 |
| ABR | 1.71E-06 | -0.851011574 | 0.938 | 1 | 0.029704378 |
| FNTB | 1.54E-09 | -0.854471653 | 1 | 1 | 2.67E-05 |
| PYGB | 1.09E-06 | -0.855529184 | 1 | 1 | 0.018861771 |
| AIMP1 | 1.25E-13 | -0.859603526 | 1 | 1 | 2.16E-09 |
| PRIM2 | 1.23E-14 | -0.860505178 | 1 | 1 | 2.13E-10 |
| ENSSSCG00000058326 | 3.79E-07 | -0.86097087 | 1 | 1 | 0.006583778 |
| JOSD1 | 1.67E-08 | -0.864400137 | 0.938 | 1 | 0.00028996 |
| ENSSSCG00000047605 | 1.10E-13 | -0.871722696 | 1 | 1 | 1.90E-09 |
| ELP2 | 2.28E-14 | -0.872110218 | 1 | 1 | 3.96E-10 |
| TPGS2 | 4.40E-08 | -0.877849776 | 0.938 | 1 | 0.000763194 |
| ATG7 | 5.66E-16 | -0.878058958 | 1 | 1 | 9.82E-12 |
| TMEM165 | 1.16E-14 | -0.878370556 | 0.938 | 1 | 2.01E-10 |
| TBCE | 2.89E-11 | -0.879645855 | 1 | 1 | 5.01E-07 |
| STAT4 | 5.83E-11 | -0.879899096 | 1 | 1 | 1.01E-06 |
| NMT1 | 1.49E-07 | -0.882665826 | 1 | 1 | 0.002576639 |
| NTPCR | 2.02E-07 | -0.883846255 | 1 | 1 | 0.003503376 |
| NKIRAS1 | 1.27E-12 | -0.886596597 | 1 | 1 | 2.20E-08 |
| PLPP1 | 1.23E-07 | -0.889178519 | 0.938 | 1 | 0.002137081 |
| NRG4 | 2.34E-17 | -0.891307387 | 1 | 1 | 4.05E-13 |
| SSR3 | 4.93E-08 | -0.89426806 | 1 | 1 | 0.00085567 |
| ENSSSCG00000052306 | 2.30E-11 | -0.894780688 | 1 | 1 | 3.99E-07 |
| RNF220 | 1.04E-09 | -0.895401246 | 1 | 1 | 1.80E-05 |
| FCGR1A | 8.37E-13 | -0.903610119 | 1 | 1 | 1.45E-08 |
| EIF4A1 | 1.91E-11 | -0.905834422 | 1 | 1 | 3.32E-07 |
| EAPP | 1.49E-07 | -0.909863024 | 1 | 1 | 0.002588854 |
| ALKBH3 | 7.46E-10 | -0.911030203 | 1 | 1 | 1.30E-05 |

| | | | | | |
|---|---|---|---|---|---|
| TMEM138 | 1.58E-13 | -0.915111102 | 1 | 1 | 2.75E-09 |
| DOC2B | 2.39E-08 | -0.915785126 | 1 | 1 | 0.00041399 |
| USP27X | 1.28E-08 | -0.917382117 | 1 | 1 | 0.000221728 |
| BCAP29 | 1.91E-11 | -0.919510082 | 1 | 1 | 3.32E-07 |
| SAR1B | 1.99E-06 | -0.920175653 | 1 | 1 | 0.034578546 |
| WARS1 | 1.61E-07 | -0.921222887 | 1 | 1 | 0.002786989 |
| HAUS2 | 1.50E-07 | -0.922268697 | 0.875 | 1 | 0.002601125 |
| ERH | 9.16E-09 | -0.923184403 | 1 | 1 | 0.000158857 |
| ASCC1 | 3.81E-07 | -0.924555135 | 1 | 0.933 | 0.006610226 |
| ENSSSCG00000025913 | 1.69E-06 | -0.926459535 | 1 | 0.933 | 0.02929923 |
| SDHA | 1.59E-11 | -0.927627927 | 1 | 1 | 2.75E-07 |
| VPS41 | 1.96E-10 | -0.928255392 | 1 | 1 | 3.40E-06 |
| DNAJC14 | 8.40E-16 | -0.933766302 | 1 | 1 | 1.46E-11 |
| ABCF1 | 3.10E-10 | -0.934593004 | 1 | 1 | 5.37E-06 |
| SRI | 2.43E-06 | -0.935403952 | 1 | 1 | 0.042147976 |
| OARD1 | 2.57E-08 | -0.935568127 | 0.875 | 1 | 0.000445342 |
| ZNF830 | 1.81E-18 | -0.939968987 | 1 | 1 | 3.14E-14 |
| GET1 | 3.90E-15 | -0.943336134 | 1 | 1 | 6.76E-11 |
| SKA3 | 2.60E-06 | -0.944146765 | 0.938 | 1 | 0.045089369 |
| ENSSSCG00000009422 | 6.39E-08 | -0.945175975 | 1 | 1 | 0.001108813 |
| LETM2 | 7.95E-10 | -0.952931746 | 1 | 1 | 1.38E-05 |
| SMYD2 | 8.45E-09 | -0.953810367 | 0.938 | 1 | 0.000146609 |
| NOL7 | 1.01E-07 | -0.954441948 | 0.938 | 1 | 0.00174761 |
| BCAR3 | 2.42E-10 | -0.961319532 | 0.938 | 1 | 4.19E-06 |
| RAB5IF | 2.74E-08 | -0.964254339 | 0.938 | 1 | 0.000475238 |
| DNAJA3 | 5.15E-10 | -0.968208933 | 1 | 1 | 8.93E-06 |
| NXF1 | 4.07E-11 | -0.968382924 | 1 | 1 | 7.06E-07 |
| ENSSSCG00000031991 | 3.26E-08 | -0.968446831 | 1 | 1 | 0.000565058 |
| UFC1 | 1.16E-06 | -0.96848275 | 1 | 1 | 0.02018581 |
| SVOPL | 1.59E-12 | -0.970810921 | 1 | 1 | 2.75E-08 |
| SNURF | 3.01E-10 | -0.971171787 | 1 | 1 | 5.22E-06 |
| HABP4 | 2.86E-07 | -0.973439174 | 1 | 1 | 0.004955696 |
| ENSSSCG00000056486 | 1.52E-07 | -0.974014347 | 0.875 | 1 | 0.00264599 |
| ENSSSCG00000027124 | 1.70E-06 | -0.978825977 | 0.938 | 1 | 0.029497892 |
| MFSD11 | 2.99E-08 | -0.979627156 | 1 | 1 | 0.000519259 |
| PSMC1 | 8.49E-10 | -0.980799188 | 1 | 1 | 1.47E-05 |
| ZCCHC17 | 1.09E-09 | -0.981765817 | 1 | 1 | 1.89E-05 |
| MRPL50 | 7.19E-12 | -0.983069604 | 1 | 1 | 1.25E-07 |
| ENSSSCG00000056638 | 3.67E-10 | -0.983136562 | 0.875 | 1 | 6.37E-06 |
| GLIPR1 | 6.86E-19 | -0.984560378 | 1 | 1 | 1.19E-14 |
| PPIL2 | 3.30E-08 | -0.985040403 | 1 | 1 | 0.000573051 |
| FBXO7 | 4.86E-09 | -0.98579638 | 1 | 1 | 8.44E-05 |
| NECAP1 | 3.98E-13 | -0.986301941 | 1 | 1 | 6.90E-09 |
| ENSSSCG00000051526 | 2.64E-07 | -0.987022146 | 1 | 1 | 0.004576071 |
| RPL3 | 9.12E-08 | -0.988772745 | 1 | 1 | 0.001582244 |
| NAT10 | 1.74E-11 | -0.990372648 | 1 | 1 | 3.02E-07 |
| PCYOX1 | 9.55E-09 | -0.992832176 | 1 | 1 | 0.000165667 |
| EAF1 | 2.08E-09 | -0.993573731 | 0.938 | 1 | 3.62E-05 |
| BRD9 | 2.15E-08 | -0.993810064 | 1 | 1 | 0.000372688 |
| TMEM233 | 9.42E-08 | -0.994194378 | 0.938 | 1 | 0.001634981 |
| TSPAN13 | 1.09E-06 | -0.994194378 | 0.938 | 1 | 0.018923548 |
| BIRC5 | 7.79E-11 | -0.996147675 | 1 | 1 | 1.35E-06 |
| SLC37A4 | 4.80E-08 | -1.000850897 | 1 | 0.933 | 0.000832331 |
| CRYM | 1.72E-08 | -1.002200908 | 1 | 1 | 0.000298507 |
| SFT2D2 | 1.22E-07 | -1.003354752 | 1 | 1 | 0.002113404 |
| GREB1L | 2.40E-11 | -1.004036441 | 1 | 1 | 4.16E-07 |
| UBE2B | 1.00E-07 | -1.005646563 | 0.938 | 1 | 0.001736186 |
| TMEM163 | 2.68E-06 | -1.009693396 | 1 | 1 | 0.046536988 |
| WNT2B | 2.08E-10 | -1.013674937 | 0.938 | 1 | 3.61E-06 |
| EIF3M | 9.09E-33 | -1.01368084 | 1 | 1 | 1.58E-28 |
| CENPS | 1.31E-10 | -1.014383918 | 1 | 1 | 2.28E-06 |
| CFL1 | 2.88E-08 | -1.015328283 | 1 | 1 | 0.000500276 |
| ENSSSCG00000010719 | 4.26E-12 | -1.016488123 | 1 | 1 | 7.39E-08 |
| UMPS | 3.60E-18 | -1.01790306 | 1 | 1 | 6.25E-14 |
| ENSSSCG00000016678 | 4.40E-07 | -1.019688335 | 0.938 | 1 | 0.007642081 |
| ENSSSCG00000026520 | 1.40E-06 | -1.019860422 | 1 | 1 | 0.024257867 |

| | | | | | |
|---|---|---|---|---|---|
| EIF4E2 | 4.88E-18 | -1.021556143 | 1 | 1 | 8.47E-14 |
| OSER1 | 3.08E-08 | -1.024677679 | 1 | 1 | 0.000535115 |
| SYCP2L | 9.11E-07 | -1.027020214 | 0.938 | 1 | 0.015800017 |
| NCF2 | 3.64E-07 | -1.027046765 | 0.938 | 1 | 0.006315 |
| ENSSSCG00000034927 | 2.21E-12 | -1.033109415 | 1 | 1 | 3.84E-08 |
| CACYBP | 7.73E-12 | -1.033166864 | 1 | 1 | 1.34E-07 |
| ENSSSCG00000060926 | 5.81E-09 | -1.033576081 | 0.875 | 1 | 0.000100881 |
| ENSSSCG00000051717 | 2.03E-06 | -1.034215715 | 0.938 | 1 | 0.035167065 |
| USP36 | 4.86E-07 | -1.03441541 | 0.938 | 1 | 0.008425773 |
| GSK3B | 3.02E-13 | -1.035667512 | 1 | 1 | 5.24E-09 |
| ENSSSCG00000032216 | 2.92E-12 | -1.047562811 | 1 | 1 | 5.07E-08 |
| RND1 | 7.11E-09 | -1.047828525 | 1 | 1 | 0.000123304 |
| ENSSSCG00000051162 | 2.31E-08 | -1.04800915 | 0.938 | 1 | 0.000401598 |
| SERHL2 | 1.18E-06 | -1.050040682 | 0.938 | 1 | 0.020409495 |
| ZNHIT6 | 1.77E-20 | -1.050108251 | 1 | 1 | 3.07E-16 |
| CHST3 | 3.16E-09 | -1.051993551 | 0.938 | 1 | 5.49E-05 |
| SNX3 | 6.73E-10 | -1.052314178 | 1 | 1 | 1.17E-05 |
| MRPL13 | 1.66E-21 | -1.053300551 | 1 | 1 | 2.88E-17 |
| DDB2 | 1.32E-07 | -1.05403984 | 0.875 | 1 | 0.002295406 |
| GLRX5 | 6.01E-16 | -1.056467561 | 1 | 1 | 1.04E-11 |
| ENSSSCG00000052760 | 9.88E-13 | -1.060102299 | 1 | 1 | 1.71E-08 |
| ENTREP1 | 1.23E-06 | -1.062735755 | 0.938 | 1 | 0.021405049 |
| HSPA5 | 5.33E-10 | -1.064144386 | 1 | 1 | 9.25E-06 |
| ENSSSCG00000028677 | 9.05E-18 | -1.064485396 | 1 | 1 | 1.57E-13 |
| SGCD | 5.08E-12 | -1.067114196 | 1 | 1 | 8.82E-08 |
| PSMC3 | 7.80E-13 | -1.071840788 | 1 | 1 | 1.35E-08 |
| SYCP3 | 3.67E-08 | -1.07189795 | 1 | 1 | 0.00063663 |
| GARS1 | 1.22E-07 | -1.072645302 | 0.938 | 1 | 0.002119738 |
| TTC32 | 5.87E-08 | -1.072850565 | 1 | 1 | 0.001018402 |
| RNASEH2A | 2.89E-09 | -1.078210528 | 0.938 | 1 | 5.02E-05 |
| UBXN8 | 6.21E-08 | -1.079116132 | 1 | 0.933 | 0.001077879 |
| PITPNA | 3.22E-07 | -1.081968847 | 1 | 1 | 0.005585122 |
| LDHA | 6.08E-08 | -1.084296335 | 0.938 | 1 | 0.001054667 |
| ENSSSCG00000006996 | 3.29E-12 | -1.08435693 | 1 | 1 | 5.70E-08 |
| ENSSSCG00000042524 | 9.58E-07 | -1.084794136 | 0.938 | 1 | 0.016616612 |
| HSPE1 | 3.36E-10 | -1.085000647 | 1 | 1 | 5.82E-06 |
| COPE | 3.55E-08 | -1.085949613 | 0.938 | 1 | 0.000616151 |
| SRRT | 9.85E-07 | -1.086930829 | 1 | 1 | 0.017086339 |
| SLC25A17 | 9.35E-17 | -1.087601426 | 1 | 1 | 1.62E-12 |
| ENSSSCG00000057665 | 2.17E-07 | -1.088448027 | 0.938 | 1 | 0.003767757 |
| SAP18 | 3.77E-07 | -1.089337651 | 1 | 1 | 0.006533722 |
| THYN1 | 9.25E-16 | -1.089464261 | 1 | 1 | 1.61E-11 |
| SLC35B4 | 2.60E-07 | -1.089702803 | 0.938 | 1 | 0.004515083 |
| BRCC3 | 8.97E-10 | -1.093109404 | 0.938 | 1 | 1.56E-05 |
| TMEM69 | 2.01E-07 | -1.093109404 | 0.938 | 1 | 0.003480418 |
| PRKAG2 | 9.03E-07 | -1.093109404 | 0.938 | 1 | 0.015663092 |
| PDIA3 | 8.77E-15 | -1.09427569 | 1 | 1 | 1.52E-10 |
| PPIF | 6.06E-13 | -1.094876331 | 1 | 1 | 1.05E-08 |
| BPNT1 | 8.10E-21 | -1.101314327 | 1 | 1 | 1.41E-16 |
| TRIP4 | 8.22E-14 | -1.103173088 | 1 | 1 | 1.43E-09 |
| GRIN3A | 2.86E-09 | -1.103717504 | 0.938 | 1 | 4.96E-05 |
| PADI6 | 5.51E-09 | -1.104206143 | 1 | 1 | 9.55E-05 |
| ENSSSCG00000033697 | 2.22E-06 | -1.110140485 | 1 | 1 | 0.038483298 |
| CDC6 | 2.61E-18 | -1.110381486 | 1 | 1 | 4.53E-14 |
| NOP9 | 3.11E-09 | -1.110964769 | 1 | 1 | 5.40E-05 |
| SKA1 | 8.98E-09 | -1.11137418 | 1 | 1 | 0.000155895 |
| ENSSSCG00000001064 | 1.33E-10 | -1.114923766 | 1 | 1 | 2.30E-06 |
| FAM104A | 5.96E-13 | -1.116087968 | 1 | 1 | 1.03E-08 |
| SDF2 | 8.30E-20 | -1.116585889 | 1 | 1 | 1.44E-15 |
| RPS20 | 4.31E-07 | -1.12145403 | 1 | 1 | 0.007475784 |
| KCTD20 | 1.08E-09 | -1.126024027 | 1 | 1 | 1.88E-05 |
| GPN1 | 4.72E-33 | -1.127382596 | 1 | 1 | 8.19E-29 |
| SPR | 5.26E-07 | -1.130198723 | 1 | 1 | 0.009124608 |
| GNL2 | 6.51E-10 | -1.132339179 | 1 | 1 | 1.13E-05 |
| LAT2 | 7.70E-17 | -1.132782712 | 1 | 1 | 1.34E-12 |
| TOLLIP | 1.60E-06 | -1.135753742 | 0.812 | 1 | 0.027762462 |

| | | | | | |
|---|---|---|---|---|---|
| PATL2 | 9.01E-18 | -1.139485211 | 1 | 1 | 1.56E-13 |
| ENSSSCG00000003612 | 2.99E-10 | -1.142455458 | 1 | 1 | 5.19E-06 |
| ARHGAP27 | 5.29E-10 | -1.143067563 | 0.938 | 1 | 9.19E-06 |
| NLRP8 | 2.26E-15 | -1.143542393 | 1 | 1 | 3.93E-11 |
| DDX28 | 4.69E-08 | -1.155231116 | 0.938 | 1 | 0.000813766 |
| ENSSSCG00000056768 | 4.22E-07 | -1.15787966 | 1 | 1 | 0.007329621 |
| ZDHHC4 | 7.52E-11 | -1.159488244 | 1 | 1 | 1.31E-06 |
| TUBB | 6.16E-09 | -1.160028996 | 1 | 1 | 0.000106846 |
| LIG1 | 3.33E-09 | -1.163498732 | 0.938 | 1 | 5.78E-05 |
| NDUFS1 | 1.73E-07 | -1.165865747 | 0.938 | 1 | 0.00300361 |
| UBTD2 | 1.55E-10 | -1.167266339 | 0.938 | 1 | 2.69E-06 |
| CFDP1 | 1.86E-09 | -1.167877173 | 1 | 1 | 3.22E-05 |
| DUSP5 | 3.04E-12 | -1.17533253 | 1 | 1 | 5.28E-08 |
| TBCC | 5.82E-09 | -1.181532655 | 1 | 1 | 0.000100917 |
| SYTL3 | 4.31E-15 | -1.186424189 | 1 | 1 | 7.48E-11 |
| TM2D2 | 1.19E-06 | -1.186891551 | 0.938 | 0.933 | 0.020570959 |
| DNAJB2 | 2.66E-06 | -1.187746279 | 0.688 | 1 | 0.046236516 |
| GLIPR2 | 1.52E-08 | -1.189111224 | 1 | 1 | 0.000264251 |
| CDK5RAP1 | 1.71E-10 | -1.19005496 | 0.938 | 1 | 2.97E-06 |
| PDCD1LG2 | 8.43E-08 | -1.190650339 | 0.938 | 1 | 0.001462317 |
| ZMAT2 | 1.81E-15 | -1.19088462 | 1 | 1 | 3.14E-11 |
| PBDC1 | 7.03E-13 | -1.191673239 | 1 | 1 | 1.22E-08 |
| RMDN1 | 2.03E-19 | -1.193590797 | 1 | 1 | 3.52E-15 |
| AIDA | 5.19E-16 | -1.193952163 | 1 | 1 | 9.01E-12 |
| ALG8 | 1.27E-09 | -1.195093437 | 1 | 1 | 2.20E-05 |
| RAB5C | 3.35E-10 | -1.195359463 | 0.938 | 1 | 5.81E-06 |
| NAA20 | 1.05E-09 | -1.199202794 | 1 | 1 | 1.82E-05 |
| PRSS8 | 5.26E-08 | -1.199560346 | 0.938 | 1 | 0.000912608 |
| ENSSSCG00000061194 | 7.30E-09 | -1.200024608 | 0.938 | 1 | 0.000126578 |
| PRPF19 | 1.18E-08 | -1.200024608 | 0.938 | 0.933 | 0.000204877 |
| ENSSSCG00000011875 | 1.56E-16 | -1.200731638 | 1 | 1 | 2.71E-12 |
| LSM3 | 7.53E-11 | -1.200750128 | 1 | 1 | 1.31E-06 |
| ARFGAP2 | 3.56E-12 | -1.205430429 | 1 | 1 | 6.18E-08 |
| TXNL4A | 1.84E-13 | -1.207106175 | 1 | 1 | 3.19E-09 |
| PLEKHO1 | 3.61E-08 | -1.208586622 | 0.938 | 1 | 0.000625613 |
| PDCD6 | 8.68E-24 | -1.213925 | 1 | 1 | 1.51E-19 |
| RNF4 | 7.76E-10 | -1.214258891 | 0.938 | 1 | 1.35E-05 |
| DYNLL2 | 5.21E-13 | -1.215750078 | 1 | 1 | 9.05E-09 |
| ZNF526 | 2.28E-06 | -1.216167801 | 0.75 | 1 | 0.039571689 |
| RAB6B | 7.93E-10 | -1.217367507 | 1 | 1 | 1.38E-05 |
| DHCR24 | 2.17E-06 | -1.217845632 | 0.938 | 0.933 | 0.037678632 |
| ENSSSCG00000050758 | 9.54E-07 | -1.218640286 | 0.875 | 1 | 0.016548929 |
| SKA2 | 1.53E-10 | -1.219987467 | 0.938 | 1 | 2.65E-06 |
| PSMG3 | 2.83E-09 | -1.220390068 | 1 | 1 | 4.91E-05 |
| XAB2 | 5.98E-07 | -1.222151349 | 0.938 | 0.933 | 0.010375571 |
| DDX23 | 1.65E-08 | -1.222877279 | 0.938 | 1 | 0.0002869 |
| TAX1BP3 | 1.94E-06 | -1.222989687 | 1 | 1 | 0.03366211 |
| ENSSSCG00000034524 | 1.72E-11 | -1.224706287 | 0.938 | 1 | 2.98E-07 |
| ENSSSCG00000017907 | 5.73E-08 | -1.2264715 | 1 | 1 | 0.00099503 |
| PPCDC | 2.97E-12 | -1.227212826 | 1 | 1 | 5.15E-08 |
| ENSSSCG00000038506 | 4.41E-07 | -1.229130199 | 1 | 1 | 0.007648256 |
| PSMA7 | 5.29E-09 | -1.231888803 | 1 | 1 | 9.18E-05 |
| RTCB | 1.66E-09 | -1.233444519 | 1 | 1 | 2.89E-05 |
| PSTK | 1.40E-09 | -1.234328822 | 1 | 1 | 2.43E-05 |
| ENSSSCG00000028786 | 8.48E-11 | -1.236067358 | 1 | 1 | 1.47E-06 |
| SLC16A13 | 3.09E-09 | -1.236528652 | 1 | 1 | 5.36E-05 |
| ILF2 | 1.73E-21 | -1.238172684 | 1 | 1 | 3.00E-17 |
| MON1A | 8.37E-08 | -1.238251305 | 0.875 | 1 | 0.001451877 |
| ENSSSCG00000026229 | 5.28E-10 | -1.240815562 | 1 | 1 | 9.17E-06 |
| LDHC | 1.10E-14 | -1.241591694 | 1 | 1 | 1.91E-10 |
| TIMM23B | 3.10E-10 | -1.24691474 | 1 | 1 | 5.37E-06 |
| FBXO16 | 1.20E-07 | -1.248626982 | 0.875 | 1 | 0.002086164 |
| MAD2L1BP | 1.32E-09 | -1.250961574 | 0.938 | 1 | 2.30E-05 |
| ENSSSCG00000036988 | 1.62E-08 | -1.252307999 | 0.875 | 1 | 0.000280732 |
| CALR | 2.95E-15 | -1.254413923 | 1 | 1 | 5.12E-11 |
| SIAE | 2.50E-10 | -1.256083616 | 1 | 1 | 4.34E-06 |

| | | | | | |
|---|---|---|---|---|---|
| GM2A | 1.93E-07 | -1.25624524 | 0.875 | 1 | 0.003349115 |
| FTSJ1 | 9.45E-15 | -1.256608137 | 1 | 1 | 1.64E-10 |
| ENSSSCG00000052241 | 6.25E-12 | -1.25918208 | 0.938 | 1 | 1.08E-07 |
| FBXO34 | 2.07E-06 | -1.259467791 | 0.938 | 1 | 0.035977983 |
| ENSSSCG00000016385 | 2.50E-06 | -1.259467791 | 0.812 | 0.933 | 0.043432992 |
| OSBPL2 | 6.47E-12 | -1.265062087 | 1 | 1 | 1.12E-07 |
| ENSSSCG00000057916 | 4.10E-08 | -1.265659875 | 1 | 1 | 0.000711876 |
| MYCBP | 1.80E-12 | -1.269030734 | 1 | 1 | 3.13E-08 |
| CDKN3 | 5.57E-09 | -1.27104622 | 1 | 1 | 9.66E-05 |
| ENSSSCG00000047619 | 6.38E-11 | -1.271446646 | 1 | 1 | 1.11E-06 |
| MRPL19 | 6.28E-12 | -1.271647536 | 0.938 | 1 | 1.09E-07 |
| MRPL9 | 8.20E-11 | -1.272507158 | 1 | 1 | 1.42E-06 |
| TUBA4A | 1.46E-09 | -1.273468407 | 0.938 | 1 | 2.54E-05 |
| CHST10 | 4.93E-15 | -1.27551518 | 1 | 1 | 8.56E-11 |
| MRM3 | 6.39E-09 | -1.276081476 | 1 | 1 | 0.00011083 |
| MROH7 | 1.05E-08 | -1.276114233 | 0.875 | 1 | 0.000182888 |
| GPANK1 | 5.63E-09 | -1.276555545 | 1 | 1 | 9.76E-05 |
| LGALS8 | 3.92E-14 | -1.276937169 | 0.938 | 1 | 6.80E-10 |
| SMARCAL1 | 7.34E-08 | -1.279730695 | 0.875 | 1 | 0.001273135 |
| SPOCK1 | 1.85E-10 | -1.280581387 | 0.938 | 1 | 3.20E-06 |
| MOSPD1 | 3.78E-12 | -1.281099122 | 1 | 1 | 6.56E-08 |
| ENSSSCG00000005596 | 3.33E-12 | -1.281150321 | 0.938 | 1 | 5.78E-08 |
| ENSSSCG00000058562 | 3.77E-07 | -1.281554494 | 0.875 | 0.933 | 0.00654411 |
| SLC25A26 | 8.03E-30 | -1.282001028 | 1 | 1 | 1.39E-25 |
| COPS7B | 3.98E-08 | -1.283905584 | 1 | 0.933 | 0.000690438 |
| CCDC59 | 4.27E-10 | -1.285458818 | 1 | 1 | 7.41E-06 |
| ENSSSCG00000037514 | 1.51E-15 | -1.286678991 | 0.938 | 1 | 2.63E-11 |
| RPF2 | 3.78E-08 | -1.287506773 | 1 | 1 | 0.000656317 |
| PDE6D | 3.66E-13 | -1.28769646 | 1 | 1 | 6.34E-09 |
| ATP6V1G1 | 1.53E-17 | -1.292031628 | 1 | 1 | 2.65E-13 |
| ENSSSCG00000053570 | 4.36E-15 | -1.292801985 | 1 | 1 | 7.57E-11 |
| WDR53 | 7.37E-19 | -1.298156199 | 0.938 | 1 | 1.28E-14 |
| SDHAF2 | 2.07E-07 | -1.299560282 | 0.875 | 1 | 0.003583779 |
| PPP2R2B | 9.03E-16 | -1.301552267 | 1 | 1 | 1.57E-11 |
| TRAIP | 7.82E-08 | -1.305262641 | 0.938 | 1 | 0.001356035 |
| NDUFAF1 | 1.23E-13 | -1.306218585 | 1 | 1 | 2.14E-09 |
| WDR91 | 1.01E-21 | -1.308978702 | 1 | 1 | 1.76E-17 |
| ESYT1 | 2.69E-15 | -1.310138641 | 1 | 1 | 4.66E-11 |
| PPM1G | 1.62E-12 | -1.310667052 | 0.938 | 1 | 2.82E-08 |
| KATNB1 | 2.45E-06 | -1.311944006 | 0.812 | 1 | 0.042491403 |
| ENSSSCG00000054196 | 6.33E-10 | -1.31817596 | 1 | 1 | 1.10E-05 |
| C4orf33 | 2.10E-17 | -1.319365406 | 1 | 1 | 3.64E-13 |
| TCP1 | 1.70E-21 | -1.320024384 | 1 | 1 | 2.94E-17 |
| ENSSSCG00000038628 | 6.86E-25 | -1.321073299 | 1 | 1 | 1.19E-20 |
| CALM3 | 1.54E-13 | -1.32443495 | 1 | 1 | 2.67E-09 |
| ENSSSCG00000030908 | 2.39E-11 | -1.324735106 | 1 | 1 | 4.15E-07 |
| COL4A6 | 6.25E-09 | -1.327019669 | 1 | 1 | 0.000108376 |
| ENSSSCG00000017061 | 1.58E-06 | -1.328325866 | 0.75 | 1 | 0.027448638 |
| LANCL2 | 1.03E-08 | -1.328557979 | 1 | 1 | 0.000177994 |
| MYL6B | 1.31E-09 | -1.331681753 | 0.938 | 1 | 2.27E-05 |
| NPLOC4 | 3.46E-08 | -1.332779817 | 0.875 | 1 | 0.000600957 |
| ASRGL1 | 1.51E-07 | -1.334117504 | 0.812 | 1 | 0.002616998 |
| ELP6 | 5.41E-07 | -1.334117504 | 0.875 | 1 | 0.009393972 |
| ENSSSCG00000060365 | 1.20E-12 | -1.334313562 | 1 | 1 | 2.08E-08 |
| NDUFS2 | 5.21E-10 | -1.334825089 | 1 | 1 | 9.04E-06 |
| ENSSSCG00000016475 | 1.38E-08 | -1.335470242 | 1 | 0.933 | 0.000239913 |
| CRY2 | 1.99E-07 | -1.335859539 | 0.938 | 0.933 | 0.003453865 |
| OXA1L | 8.05E-21 | -1.341373156 | 1 | 1 | 1.40E-16 |
| ENSSSCG00000039972 | 7.28E-13 | -1.341929951 | 0.938 | 1 | 1.26E-08 |
| ATG101 | 9.63E-08 | -1.342723294 | 0.938 | 1 | 0.001671144 |
| MLH1 | 1.70E-11 | -1.342920115 | 1 | 1 | 2.95E-07 |
| SELENON | 3.47E-08 | -1.343246816 | 1 | 1 | 0.000601253 |
| RAB43 | 2.12E-10 | -1.345027778 | 0.875 | 1 | 3.67E-06 |
| CDKN2AIPNL | 1.14E-10 | -1.346865997 | 1 | 1 | 1.98E-06 |
| LYSMD2 | 4.70E-07 | -1.347923303 | 0.75 | 0.933 | 0.008150097 |
| EIF1B | 1.04E-13 | -1.350302932 | 1 | 1 | 1.81E-09 |

| | | | | | |
|---|---|---|---|---|---|
| SELENOF | 1.48E-25 | -1.351053043 | 1 | 1 | 2.56E-21 |
| TIMD4 | 1.10E-08 | -1.351177557 | 0.938 | 1 | 0.00019051 |
| ENSSSCG00000029257 | 5.98E-09 | -1.35614381 | 0.875 | 1 | 0.000103762 |
| HSPA4 | 5.41E-21 | -1.356605547 | 1 | 1 | 9.38E-17 |
| VWA3A | 2.07E-07 | -1.358699515 | 0.938 | 1 | 0.003598868 |
| B4GAT1 | 2.21E-06 | -1.35938947 | 0.875 | 0.867 | 0.038422858 |
| DDX39B | 2.50E-16 | -1.359725826 | 1 | 1 | 4.34E-12 |
| GNPTG | 6.55E-07 | -1.360589715 | 0.938 | 1 | 0.011361162 |
| ENSSSCG00000036499 | 5.58E-23 | -1.361992689 | 1 | 1 | 9.68E-19 |
| DHDDS | 6.14E-14 | -1.362156187 | 0.938 | 1 | 1.07E-09 |
| TOMM22 | 5.90E-14 | -1.365635259 | 1 | 1 | 1.02E-09 |
| PDXP | 2.29E-07 | -1.367402928 | 0.938 | 0.933 | 0.003965373 |
| ENSSSCG00000051057 | 2.58E-10 | -1.368743847 | 0.938 | 1 | 4.47E-06 |
| RFC2 | 1.65E-11 | -1.369838549 | 1 | 1 | 2.87E-07 |
| PRPSAP1 | 6.45E-08 | -1.370748223 | 1 | 0.933 | 0.00111983 |
| USP11 | 1.39E-06 | -1.371316122 | 0.938 | 1 | 0.024160544 |
| CHEK2 | 2.49E-07 | -1.375622818 | 0.875 | 1 | 0.004323089 |
| HARS1 | 2.60E-08 | -1.375748368 | 0.812 | 1 | 0.000451205 |
| ENSSSCG00000022773 | 4.87E-07 | -1.377562794 | 0.938 | 1 | 0.008454279 |
| ENSSSCG00000009015 | 3.42E-11 | -1.38502493 | 0.875 | 0.933 | 5.94E-07 |
| FAM98A | 1.98E-22 | -1.385160458 | 1 | 1 | 3.44E-18 |
| MRPS10 | 6.05E-30 | -1.385167992 | 1 | 1 | 1.05E-25 |
| CFAP36 | 2.92E-07 | -1.387556763 | 0.938 | 1 | 0.005060204 |
| NLRP9 | 2.19E-14 | -1.389012091 | 1 | 1 | 3.80E-10 |
| DNAJA4 | 3.71E-07 | -1.389158867 | 0.938 | 1 | 0.006428797 |
| FBXO17 | 3.70E-08 | -1.390495315 | 0.812 | 1 | 0.000641464 |
| LSG1 | 1.40E-11 | -1.391549146 | 1 | 1 | 2.43E-07 |
| CYP2E1 | 1.12E-06 | -1.39176772 | 0.688 | 1 | 0.019403534 |
| ATP6V1D | 7.02E-34 | -1.394783349 | 1 | 1 | 1.22E-29 |
| STX18 | 7.08E-18 | -1.39509009 | 1 | 1 | 1.23E-13 |
| HMOX2 | 1.46E-07 | -1.396387442 | 1 | 1 | 0.002538015 |
| BRF2 | 2.48E-09 | -1.399471242 | 0.938 | 1 | 4.31E-05 |
| SLC25A12 | 1.01E-10 | -1.399536406 | 0.875 | 1 | 1.76E-06 |
| TOE1 | 6.63E-07 | -1.403835152 | 0.875 | 1 | 0.011503437 |
| ECSIT | 1.62E-08 | -1.411380476 | 0.938 | 1 | 0.00028069 |
| VPS26C | 3.05E-18 | -1.414353595 | 1 | 1 | 5.29E-14 |
| EMC4 | 2.55E-10 | -1.415037499 | 1 | 1 | 4.43E-06 |
| ATG4B | 1.01E-09 | -1.418375785 | 1 | 1 | 1.75E-05 |
| PXMP2 | 7.15E-08 | -1.423417165 | 1 | 0.933 | 0.001241326 |
| TP53INP2 | 6.92E-07 | -1.423417165 | 0.938 | 1 | 0.012001392 |
| WDFY2 | 3.86E-14 | -1.424998609 | 1 | 1 | 6.69E-10 |
| EXOSC5 | 7.59E-13 | -1.427375834 | 1 | 1 | 1.32E-08 |
| ENSSSCG00000021236 | 6.79E-10 | -1.427528443 | 0.938 | 1 | 1.18E-05 |
| RNF170 | 1.45E-14 | -1.429987841 | 1 | 1 | 2.52E-10 |
| FANK1 | 5.86E-13 | -1.436272141 | 1 | 1 | 1.02E-08 |
| ENSSSCG00000021591 | 8.01E-12 | -1.43641115 | 1 | 1 | 1.39E-07 |
| ENSSSCG00000029160 | 2.69E-11 | -1.438389371 | 1 | 1 | 4.66E-07 |
| ZNF410 | 1.43E-08 | -1.439434154 | 0.875 | 1 | 0.00024809 |
| CCT3 | 2.36E-18 | -1.443101418 | 1 | 1 | 4.09E-14 |
| DTD1 | 2.20E-15 | -1.443541016 | 1 | 1 | 3.82E-11 |
| AIFM1 | 5.51E-11 | -1.443606651 | 0.938 | 1 | 9.56E-07 |
| RTF2 | 3.19E-23 | -1.445300338 | 1 | 1 | 5.54E-19 |
| SLC28A3 | 4.47E-08 | -1.447291698 | 0.812 | 1 | 0.000775123 |
| ARHGAP9 | 4.13E-09 | -1.452190498 | 0.875 | 0.933 | 7.16E-05 |
| MTUS2 | 1.64E-12 | -1.453211228 | 0.938 | 1 | 2.85E-08 |
| AMN1 | 2.47E-16 | -1.458962642 | 1 | 1 | 4.28E-12 |
| WBP2 | 1.56E-08 | -1.462343214 | 1 | 1 | 0.000270333 |
| RSL24D1 | 9.00E-07 | -1.462866024 | 1 | 1 | 0.015620425 |
| PSMC5 | 5.37E-11 | -1.466397921 | 0.938 | 1 | 9.32E-07 |
| KCNAB3 | 1.19E-08 | -1.467304809 | 1 | 1 | 0.000205751 |
| BTF3L4 | 1.62E-15 | -1.468400725 | 1 | 1 | 2.81E-11 |
| WSB2 | 1.13E-06 | -1.472455197 | 0.875 | 0.933 | 0.019619436 |
| LSM6 | 2.62E-17 | -1.475579041 | 1 | 1 | 4.54E-13 |
| DRAP1 | 4.48E-08 | -1.475883054 | 0.938 | 1 | 0.000777337 |
| B3GNT3 | 4.47E-11 | -1.475966498 | 0.875 | 1 | 7.75E-07 |
| TSPAN14 | 4.66E-16 | -1.477232261 | 1 | 1 | 8.08E-12 |

| | | | | | |
|---|---|---|---|---|---|
| ENSSSCG00000045404 | 1.02E-07 | -1.477773255 | 0.938 | 0.933 | 0.001770807 |
| TENM4 | 1.88E-06 | -1.478763097 | 0.812 | 0.933 | 0.03260626 |
| SSR2 | 5.09E-08 | -1.479658347 | 1 | 0.933 | 0.000883038 |
| MAP7 | 1.95E-16 | -1.481446976 | 1 | 1 | 3.38E-12 |
| ENSSSCG00000056719 | 2.06E-07 | -1.48436952 | 0.938 | 0.933 | 0.003578 |
| IQCG | 3.37E-14 | -1.484578433 | 1 | 1 | 5.85E-10 |
| NDUFA6 | 3.47E-07 | -1.485299835 | 1 | 1 | 0.00602515 |
| ENSSSCG00000054294 | 4.50E-08 | -1.485426827 | 0.625 | 1 | 0.000781598 |
| NHEJ1 | 8.78E-09 | -1.487793842 | 0.875 | 1 | 0.000152356 |
| CFI | 2.27E-07 | -1.493040011 | 0.812 | 1 | 0.00394466 |
| CRNKL1 | 4.54E-49 | -1.49424903 | 1 | 1 | 7.87E-45 |
| HMGCR | 7.90E-12 | -1.494816573 | 1 | 1 | 1.37E-07 |
| PPP1R7 | 1.51E-08 | -1.495741883 | 0.812 | 1 | 0.000261312 |
| DERL1 | 1.82E-19 | -1.498522485 | 1 | 1 | 3.16E-15 |
| TSTD1 | 2.11E-06 | -1.498821111 | 1 | 1 | 0.03657423 |
| GMPS | 5.89E-23 | -1.501278506 | 1 | 1 | 1.02E-18 |
| C11orf80 | 1.38E-22 | -1.502180062 | 1 | 1 | 2.39E-18 |
| MCM5 | 3.22E-13 | -1.503608696 | 0.938 | 1 | 5.59E-09 |
| KCNIP4 | 1.74E-20 | -1.505626031 | 1 | 1 | 3.01E-16 |
| ENSSSCG00000053185 | 2.81E-10 | -1.509273569 | 0.938 | 1 | 4.88E-06 |
| PADI4 | 6.70E-09 | -1.511822562 | 0.938 | 1 | 0.000116325 |
| MTHFS | 5.67E-08 | -1.513012659 | 0.75 | 1 | 0.000984381 |
| PIGO | 5.62E-10 | -1.522725369 | 0.875 | 0.933 | 9.75E-06 |
| ENSSSCG00000033421 | 2.05E-08 | -1.522974321 | 0.875 | 0.933 | 0.000355958 |
| ISCA1 | 9.52E-14 | -1.523955925 | 1 | 1 | 1.65E-09 |
| ZNF584 | 5.10E-10 | -1.524375365 | 0.875 | 1 | 8.85E-06 |
| SAP30BP | 2.43E-09 | -1.524955191 | 0.938 | 1 | 4.22E-05 |
| IMP3 | 5.48E-26 | -1.525461489 | 1 | 1 | 9.51E-22 |
| ZNF235 | 5.18E-12 | -1.52554995 | 0.938 | 1 | 8.99E-08 |
| ENSSSCG00000060974 | 2.01E-14 | -1.526354522 | 1 | 1 | 3.49E-10 |
| TCP11 | 6.92E-14 | -1.526412235 | 1 | 1 | 1.20E-09 |
| DUT | 2.42E-19 | -1.527512229 | 1 | 1 | 4.21E-15 |
| ENSSSCG00000059558 | 2.60E-07 | -1.527512229 | 0.75 | 0.933 | 0.00450815 |
| TMEM186 | 4.75E-15 | -1.528365183 | 0.938 | 1 | 8.24E-11 |
| CIRBP | 9.02E-11 | -1.533093261 | 0.938 | 1 | 1.56E-06 |
| GNL3 | 7.45E-18 | -1.53331335 | 1 | 1 | 1.29E-13 |
| GTF2E2 | 2.02E-19 | -1.534452666 | 1 | 1 | 3.51E-15 |
| SLC8B1 | 6.32E-13 | -1.538169387 | 1 | 1 | 1.10E-08 |
| TBC1D7 | 3.29E-13 | -1.541397278 | 0.938 | 1 | 5.71E-09 |
| VPS26B | 1.96E-10 | -1.541498852 | 1 | 1 | 3.41E-06 |
| DLGAP4 | 3.26E-25 | -1.542240102 | 1 | 1 | 5.65E-21 |
| SSBP1 | 6.24E-26 | -1.543400369 | 1 | 1 | 1.08E-21 |
| SQLE | 2.58E-16 | -1.545038281 | 1 | 1 | 4.48E-12 |
| KCNN2 | 2.01E-09 | -1.546700144 | 0.938 | 1 | 3.50E-05 |
| TBC1D1 | 5.58E-12 | -1.547466069 | 1 | 1 | 9.68E-08 |
| MAF1 | 5.56E-09 | -1.547675268 | 0.812 | 1 | 9.64E-05 |
| AVEN | 1.47E-15 | -1.551344593 | 1 | 1 | 2.56E-11 |
| NSMAF | 3.70E-13 | -1.553301871 | 1 | 1 | 6.42E-09 |
| PRPF6 | 6.41E-09 | -1.555900035 | 0.875 | 1 | 0.00011122 |
| CYC1 | 3.32E-11 | -1.560888366 | 1 | 1 | 5.75E-07 |
| LMNA | 1.46E-15 | -1.562344198 | 0.938 | 1 | 2.53E-11 |
| C19orf67 | 5.84E-12 | -1.564180939 | 1 | 1 | 1.01E-07 |
| LIMS2 | 6.29E-10 | -1.565659362 | 0.938 | 1 | 1.09E-05 |
| GPATCH11 | 1.33E-15 | -1.565817451 | 1 | 1 | 2.30E-11 |
| ESD | 1.79E-14 | -1.565990978 | 1 | 1 | 3.10E-10 |
| CD274 | 2.16E-09 | -1.567949091 | 1 | 1 | 3.74E-05 |
| TMEM251 | 1.13E-12 | -1.568688446 | 0.938 | 1 | 1.97E-08 |
| ASTL | 6.42E-10 | -1.568770273 | 1 | 1 | 1.11E-05 |
| MGST2 | 8.77E-10 | -1.570099394 | 1 | 1 | 1.52E-05 |
| ENSSSCG00000056896 | 2.13E-11 | -1.571156701 | 0.875 | 1 | 3.69E-07 |
| PELP1 | 9.42E-07 | -1.574236094 | 0.688 | 1 | 0.016336806 |
| CIAPIN1 | 5.21E-08 | -1.574978412 | 0.938 | 1 | 0.000904518 |
| AAAS | 5.44E-11 | -1.57589151 | 1 | 1 | 9.43E-07 |
| XPO4 | 2.48E-07 | -1.579807889 | 0.812 | 1 | 0.004310781 |
| MKKS | 6.19E-15 | -1.580224582 | 1 | 1 | 1.07E-10 |
| NARF | 1.24E-12 | -1.584458854 | 0.938 | 1 | 2.15E-08 |

| | | | | | |
|---|---|---|---|---|---|
| HSD17B7 | 1.67E-24 | -1.58770869 | 1 | 1 | 2.90E-20 |
| ENSSSCG00000021161 | 1.16E-13 | -1.588162104 | 1 | 1 | 2.02E-09 |
| DBF4B | 5.61E-07 | -1.589940995 | 0.812 | 0.933 | 0.009736986 |
| MRPS9 | 3.89E-20 | -1.597948935 | 1 | 1 | 6.75E-16 |
| ATP5F1A | 7.27E-24 | -1.598644656 | 1 | 1 | 1.26E-19 |
| CIAO2B | 2.71E-07 | -1.602123052 | 1 | 1 | 0.004708391 |
| VPS4A | 1.28E-14 | -1.604720163 | 1 | 1 | 2.21E-10 |
| NKIRAS2 | 9.39E-11 | -1.607682577 | 1 | 1 | 1.63E-06 |
| STX5 | 1.49E-07 | -1.607682577 | 0.812 | 0.933 | 0.002587367 |
| ENSSSCG00000036197 | 1.65E-12 | -1.608466438 | 1 | 1 | 2.86E-08 |
| CHCHD5 | 4.67E-09 | -1.608530972 | 0.938 | 1 | 8.10E-05 |
| ENSSSCG00000046622 | 1.05E-06 | -1.609359155 | 0.625 | 0.867 | 0.018222684 |
| VPS29 | 1.75E-28 | -1.61027192 | 1 | 1 | 3.03E-24 |
| DAPK3 | 3.17E-08 | -1.610508621 | 0.75 | 0.933 | 0.000550771 |
| SSR4 | 6.27E-08 | -1.613263609 | 0.938 | 1 | 0.001087508 |
| NOTO | 4.49E-14 | -1.614346308 | 0.938 | 1 | 7.79E-10 |
| MCEE | 6.27E-09 | -1.614346308 | 0.938 | 1 | 0.000108723 |
| ENSSSCG00000052822 | 1.37E-10 | -1.61667136 | 1 | 1 | 2.38E-06 |
| CLN8 | 2.09E-11 | -1.620151929 | 0.938 | 1 | 3.62E-07 |
| LHFPL1 | 1.43E-20 | -1.620728464 | 1 | 1 | 2.48E-16 |
| MRPL46 | 1.45E-14 | -1.621604953 | 1 | 1 | 2.52E-10 |
| ATRIP | 9.18E-09 | -1.62203787 | 0.875 | 1 | 0.000159234 |
| RPL4 | 5.15E-15 | -1.626541604 | 1 | 1 | 8.94E-11 |
| CCDC3 | 2.37E-08 | -1.632930774 | 1 | 1 | 0.000411688 |
| ENSSSCG00000041717 | 4.91E-08 | -1.633677786 | 0.938 | 1 | 0.000852766 |
| DDX25 | 2.56E-07 | -1.634128558 | 0.875 | 0.933 | 0.004449233 |
| NAXD | 1.06E-11 | -1.635258822 | 0.875 | 1 | 1.84E-07 |
| CHST7 | 2.84E-12 | -1.636099635 | 0.688 | 1 | 4.92E-08 |
| TUBB2A | 1.93E-12 | -1.636452816 | 1 | 1 | 3.35E-08 |
| LUC7L2 | 5.96E-23 | -1.636956051 | 1 | 1 | 1.03E-18 |
| ATP6V0B | 5.16E-16 | -1.637587842 | 1 | 1 | 8.95E-12 |
| ENSSSCG00000010531 | 2.94E-10 | -1.639852583 | 0.875 | 1 | 5.10E-06 |
| KIAA1143 | 2.95E-10 | -1.640302862 | 1 | 1 | 5.12E-06 |
| ENSSSCG00000040571 | 1.25E-15 | -1.640434248 | 1 | 1 | 2.16E-11 |
| PRR5L | 6.39E-12 | -1.6405972 | 1 | 1 | 1.11E-07 |
| TBCA | 1.28E-09 | -1.640750279 | 1 | 1 | 2.22E-05 |
| C2orf76 | 2.04E-11 | -1.64345815 | 1 | 1 | 3.54E-07 |
| ENSSSCG00000016990 | 2.34E-14 | -1.64548461 | 1 | 1 | 4.07E-10 |
| PSMC4 | 5.38E-14 | -1.646923207 | 1 | 1 | 9.34E-10 |
| HSPA8 | 4.33E-22 | -1.646974879 | 1 | 1 | 7.52E-18 |
| SNPH | 9.57E-16 | -1.649743182 | 0.875 | 1 | 1.66E-11 |
| CAPZB | 5.67E-12 | -1.650415562 | 1 | 1 | 9.83E-08 |
| BORCS5 | 2.46E-16 | -1.652671688 | 0.938 | 1 | 4.26E-12 |
| FTSJ3 | 6.22E-18 | -1.657978335 | 1 | 1 | 1.08E-13 |
| POLA2 | 3.78E-10 | -1.662805148 | 0.938 | 1 | 6.56E-06 |
| TTC7A | 8.26E-09 | -1.664461474 | 0.812 | 1 | 0.000143268 |
| RIMS3 | 2.32E-06 | -1.666294737 | 0.75 | 0.867 | 0.040224855 |
| STMP1 | 5.59E-23 | -1.666769256 | 1 | 1 | 9.70E-19 |
| ARPP19 | 4.16E-20 | -1.666789194 | 1 | 1 | 7.22E-16 |
| RAB7A | 1.81E-31 | -1.667076512 | 1 | 1 | 3.15E-27 |
| ADCK2 | 9.27E-11 | -1.66780357 | 0.875 | 0.933 | 1.61E-06 |
| ARL3 | 6.55E-07 | -1.668611576 | 0.938 | 1 | 0.011368771 |
| ENSSSCG00000058371 | 8.03E-17 | -1.668662061 | 0.938 | 1 | 1.39E-12 |
| TULP3 | 2.01E-15 | -1.672596775 | 1 | 1 | 3.49E-11 |
| CCT4 | 8.40E-16 | -1.673513623 | 1 | 1 | 1.46E-11 |
| AARSD1 | 1.28E-18 | -1.67363284 | 1 | 1 | 2.22E-14 |
| PPEF2 | 9.30E-10 | -1.678071905 | 0.938 | 0.933 | 1.61E-05 |
| RTN4IP1 | 1.14E-21 | -1.682666479 | 0.938 | 1 | 1.97E-17 |
| TKT | 1.01E-09 | -1.683332422 | 1 | 1 | 1.75E-05 |
| SARS1 | 3.15E-07 | -1.683718468 | 0.875 | 1 | 0.005466171 |
| PNLDC1 | 6.76E-17 | -1.684330896 | 0.938 | 1 | 1.17E-12 |
| CTDSP2 | 2.52E-08 | -1.686789122 | 0.812 | 1 | 0.00043688 |
| GORASP1 | 2.85E-06 | -1.687290435 | 0.812 | 0.933 | 0.049447247 |
| CFAP298 | 4.60E-18 | -1.688224621 | 1 | 1 | 7.98E-14 |
| ATPSCKMT | 1.06E-13 | -1.688294765 | 1 | 1 | 1.84E-09 |
| FAM110A | 3.31E-13 | -1.68953102 | 0.938 | 1 | 5.74E-09 |

| | | | | | |
|---|---|---|---|---|---|
| PPP1R11 | 6.46E-11 | -1.690044547 | 1 | 1 | 1.12E-06 |
| CCT6B | 2.86E-17 | -1.694489665 | 1 | 1 | 4.96E-13 |
| YJU2B | 2.69E-08 | -1.697489359 | 0.875 | 1 | 0.00046631 |
| SLC24A3 | 2.72E-09 | -1.697808123 | 0.875 | 1 | 4.72E-05 |
| ABLIM3 | 1.20E-06 | -1.697971463 | 0.875 | 0.933 | 0.020760594 |
| RALY | 2.20E-19 | -1.698731376 | 0.938 | 1 | 3.81E-15 |
| IPO9 | 1.19E-06 | -1.701155518 | 0.938 | 1 | 0.020616435 |
| TMEM266 | 3.94E-07 | -1.703835001 | 0.812 | 0.867 | 0.006839773 |
| ENSSSCG00000031299 | 7.12E-09 | -1.705552642 | 0.875 | 1 | 0.000123493 |
| ENSSSCG00000024520 | 3.07E-30 | -1.706520335 | 1 | 1 | 5.33E-26 |
| EIF3H | 1.48E-22 | -1.706572228 | 1 | 1 | 2.56E-18 |
| SGSM2 | 5.05E-13 | -1.707100954 | 0.938 | 1 | 8.76E-09 |
| ENSSSCG00000005649 | 3.66E-08 | -1.709780765 | 0.875 | 1 | 0.000634529 |
| IFT57 | 6.40E-19 | -1.71086184 | 1 | 1 | 1.11E-14 |
| C8orf76 | 2.88E-14 | -1.710986528 | 1 | 1 | 5.00E-10 |
| ATP6V0E2 | 6.47E-08 | -1.712019237 | 0.875 | 1 | 0.001122959 |
| WNT7A | 6.35E-11 | -1.715449515 | 0.875 | 1 | 1.10E-06 |
| HAX1 | 4.20E-30 | -1.716123211 | 1 | 1 | 7.28E-26 |
| SRRD | 1.60E-07 | -1.71871389 | 0.688 | 0.933 | 0.002776821 |
| ENSSSCG00000061760 | 1.21E-06 | -1.71871389 | 0.812 | 0.933 | 0.02094427 |
| PRDX2 | 2.13E-19 | -1.719882611 | 1 | 1 | 3.69E-15 |
| SLC9A3R1 | 1.64E-07 | -1.723159795 | 0.75 | 0.933 | 0.002849015 |
| NIT1 | 1.47E-06 | -1.72914609 | 0.688 | 0.933 | 0.025556536 |
| SSNA1 | 5.49E-12 | -1.729734025 | 0.875 | 1 | 9.52E-08 |
| CAPNS1 | 1.79E-08 | -1.734974296 | 0.688 | 0.933 | 0.000311229 |
| CABLES1 | 2.34E-07 | -1.735952821 | 0.875 | 1 | 0.00406362 |
| EXOSC8 | 4.86E-19 | -1.736965594 | 1 | 1 | 8.43E-15 |
| ERGIC3 | 1.26E-11 | -1.738223471 | 1 | 1 | 2.19E-07 |
| TXNDC17 | 1.23E-10 | -1.740473974 | 1 | 1 | 2.13E-06 |
| RFC3 | 1.01E-15 | -1.74080766 | 1 | 1 | 1.76E-11 |
| MANBA | 4.71E-24 | -1.741369417 | 1 | 1 | 8.17E-20 |
| STK16 | 1.67E-11 | -1.743363365 | 0.875 | 0.933 | 2.89E-07 |
| ADRM1 | 1.05E-08 | -1.744035167 | 0.812 | 0.933 | 0.000181866 |
| ENSSSCG00000049777 | 1.21E-06 | -1.745186101 | 0.75 | 0.933 | 0.021069645 |
| CCNL2 | 5.82E-09 | -1.747334568 | 0.875 | 1 | 0.000100907 |
| RAD51D | 5.36E-11 | -1.751418055 | 0.938 | 1 | 9.30E-07 |
| GLMP | 9.88E-09 | -1.753259402 | 0.938 | 0.933 | 0.000171479 |
| MRPL22 | 2.27E-16 | -1.754741673 | 1 | 1 | 3.94E-12 |
| CLN6 | 1.72E-07 | -1.756074417 | 0.875 | 0.933 | 0.002982613 |
| PHYKPL | 1.16E-06 | -1.756074417 | 0.562 | 0.867 | 0.020198252 |
| ENSSSCG00000055313 | 4.01E-18 | -1.760320317 | 0.938 | 1 | 6.95E-14 |
| MTO1 | 3.63E-11 | -1.76087221 | 0.875 | 1 | 6.31E-07 |
| EFHC1 | 3.76E-23 | -1.765323099 | 1 | 1 | 6.52E-19 |
| ANKRD40CL | 1.63E-16 | -1.765534746 | 0.938 | 1 | 2.83E-12 |
| TUFM | 3.82E-11 | -1.767516686 | 0.938 | 1 | 6.63E-07 |
| TOMM34 | 1.40E-16 | -1.774667485 | 1 | 1 | 2.43E-12 |
| GSE1 | 2.11E-09 | -1.774933444 | 0.875 | 1 | 3.66E-05 |
| MYOM1 | 5.21E-08 | -1.77663574 | 0.812 | 0.933 | 0.000903873 |
| CBX8 | 8.54E-13 | -1.779517169 | 0.938 | 1 | 1.48E-08 |
| NSMCE1 | 3.97E-07 | -1.779801792 | 0.812 | 0.933 | 0.006896021 |
| ENSSSCG00000034739 | 1.51E-21 | -1.781165398 | 0.938 | 1 | 2.62E-17 |
| ENSSSCG00000032082 | 3.07E-14 | -1.781165398 | 0.875 | 1 | 5.33E-10 |
| DBNL | 2.83E-06 | -1.782769284 | 0.562 | 1 | 0.049186401 |
| HDGFL2 | 1.04E-10 | -1.786682403 | 0.938 | 1 | 1.81E-06 |
| ERAP2 | 5.85E-11 | -1.788094692 | 0.938 | 1 | 1.02E-06 |
| TFR2 | 9.77E-08 | -1.790546634 | 0.812 | 1 | 0.001695803 |
| ENDOU | 1.15E-07 | -1.790546634 | 0.562 | 0.933 | 0.001992765 |
| ENSSSCG00000035904 | 6.96E-18 | -1.790597077 | 1 | 1 | 1.21E-13 |
| LYPLAL1 | 8.99E-19 | -1.795723494 | 0.938 | 1 | 1.56E-14 |
| COX7C | 1.57E-12 | -1.795937409 | 1 | 1 | 2.72E-08 |
| ATXN10 | 4.41E-11 | -1.797866418 | 1 | 1 | 7.66E-07 |
| PALLD | 5.70E-09 | -1.798584712 | 1 | 1 | 9.88E-05 |
| AUP1 | 1.10E-09 | -1.801220136 | 0.938 | 1 | 1.90E-05 |
| ENSSSCG00000059201 | 3.37E-14 | -1.801454321 | 1 | 1 | 5.85E-10 |
| NOP2 | 1.84E-18 | -1.805648956 | 0.938 | 1 | 3.19E-14 |
| ENSSSCG00000010489 | 3.78E-08 | -1.809316438 | 0.625 | 1 | 0.000656601 |

| | | | | | |
|---|---|---|---|---|---|
| NQO2 | 1.34E-07 | -1.809316438 | 0.625 | 1 | 0.002329011 |
| UBXN6 | 1.16E-13 | -1.810641025 | 0.875 | 0.933 | 2.01E-09 |
| STMN1 | 1.12E-08 | -1.81232633 | 1 | 1 | 0.000194453 |
| POP5 | 1.00E-14 | -1.812888488 | 1 | 1 | 1.74E-10 |
| MDH1 | 3.00E-21 | -1.813769426 | 1 | 1 | 5.20E-17 |
| POLD1 | 1.17E-07 | -1.816591769 | 0.812 | 0.933 | 0.002029088 |
| SNX11 | 7.20E-18 | -1.82058355 | 0.938 | 1 | 1.25E-13 |
| ERI3 | 1.92E-07 | -1.821029859 | 0.625 | 1 | 0.003338236 |
| PTPRT | 1.25E-25 | -1.823371458 | 1 | 1 | 2.18E-21 |
| TXNDC5 | 1.25E-07 | -1.825413621 | 1 | 0.933 | 0.002174115 |
| FCF1 | 1.97E-10 | -1.825629094 | 1 | 1 | 3.42E-06 |
| DNAJC11 | 2.15E-14 | -1.827819025 | 0.938 | 1 | 3.74E-10 |
| ENSSSCG00000003903 | 1.14E-09 | -1.828958361 | 1 | 1 | 1.97E-05 |
| HEXIM2 | 1.79E-10 | -1.832518174 | 0.75 | 0.933 | 3.10E-06 |
| SLC25A11 | 4.15E-11 | -1.833141302 | 0.875 | 1 | 7.21E-07 |
| RBPMS2 | 4.46E-15 | -1.834323334 | 1 | 1 | 7.74E-11 |
| ASPA | 1.01E-10 | -1.835613182 | 0.812 | 1 | 1.76E-06 |
| NDUFA5 | 4.37E-07 | -1.835951565 | 0.875 | 0.933 | 0.007590397 |
| ARRDC1 | 3.17E-07 | -1.836333989 | 0.5 | 1 | 0.005507499 |
| ENSSSCG00000063102 | 1.65E-06 | -1.8372705 | 0.5 | 0.8 | 0.028550015 |
| ENSSSCG00000027723 | 2.23E-06 | -1.840343334 | 0.75 | 1 | 0.038729951 |
| FAM219B | 1.59E-10 | -1.847244131 | 0.938 | 1 | 2.75E-06 |
| DNAJB13 | 3.54E-17 | -1.849440323 | 1 | 1 | 6.14E-13 |
| ZNF212 | 1.26E-12 | -1.852496293 | 0.75 | 1 | 2.19E-08 |
| POLR2D | 9.85E-32 | -1.857845832 | 1 | 1 | 1.71E-27 |
| ENSSSCG00000042939 | 1.01E-07 | -1.858644151 | 0.562 | 0.933 | 0.001757709 |
| DDX49 | 4.59E-13 | -1.860089979 | 1 | 1 | 7.97E-09 |
| PCDH12 | 1.67E-07 | -1.860663318 | 0.75 | 1 | 0.002901763 |
| ENSSSCG00000015217 | 8.26E-47 | -1.861187839 | 1 | 1 | 1.43E-42 |
| DDX11 | 4.56E-16 | -1.861645078 | 0.938 | 1 | 7.91E-12 |
| PGS1 | 1.21E-10 | -1.862728216 | 1 | 1 | 2.10E-06 |
| TMBIM6 | 1.09E-20 | -1.862974638 | 1 | 1 | 1.89E-16 |
| FHIT | 1.00E-15 | -1.865241853 | 0.938 | 1 | 1.74E-11 |
| APOO | 2.81E-19 | -1.865698908 | 1 | 1 | 4.87E-15 |
| PRDX3 | 2.76E-30 | -1.867487004 | 1 | 1 | 4.80E-26 |
| ZKSCAN4 | 1.48E-07 | -1.868755467 | 0.875 | 1 | 0.002562623 |
| P2RX5 | 1.04E-13 | -1.870407093 | 0.938 | 1 | 1.80E-09 |
| GPC3 | 1.86E-13 | -1.87175303 | 0.75 | 1 | 3.23E-09 |
| B4GALT4 | 2.45E-17 | -1.875263772 | 0.938 | 1 | 4.25E-13 |
| PRPF31 | 7.30E-08 | -1.878984599 | 0.562 | 1 | 0.001267487 |
| MRPS6 | 1.25E-10 | -1.879402711 | 0.875 | 0.933 | 2.17E-06 |
| GLT8D1 | 1.46E-09 | -1.879705766 | 0.875 | 0.933 | 2.53E-05 |
| SIL1 | 5.45E-15 | -1.880000224 | 1 | 1 | 9.45E-11 |
| GGA1 | 8.32E-09 | -1.881605299 | 0.688 | 0.933 | 0.000144286 |
| TMEM248 | 1.16E-21 | -1.884414833 | 1 | 1 | 2.01E-17 |
| NDUFS4 | 1.66E-15 | -1.886810959 | 1 | 1 | 2.87E-11 |
| EEF1AKMT1 | 8.60E-11 | -1.888289613 | 0.812 | 0.933 | 1.49E-06 |
| ENSSSCG00000033189 | 1.15E-08 | -1.891475543 | 0.875 | 1 | 0.000199362 |
| C1orf174 | 8.17E-07 | -1.895428737 | 0.875 | 0.933 | 0.014173403 |
| GON7 | 4.89E-14 | -1.900464326 | 1 | 1 | 8.48E-10 |
| SCAND1 | 3.74E-12 | -1.900464326 | 0.875 | 0.933 | 6.50E-08 |
| MRM1 | 1.25E-06 | -1.903070822 | 0.812 | 0.933 | 0.021698092 |
| ENSA | 6.35E-18 | -1.908040197 | 0.938 | 1 | 1.10E-13 |
| EIF3B | 1.33E-09 | -1.90902634 | 0.938 | 0.933 | 2.30E-05 |
| PRKAG1 | 7.52E-08 | -1.910564916 | 0.812 | 0.933 | 0.00130444 |
| AP3S2 | 5.74E-08 | -1.910732662 | 0.562 | 1 | 0.000995144 |
| PSMB4 | 3.49E-14 | -1.913559984 | 0.938 | 1 | 6.05E-10 |
| CAPN8 | 1.91E-08 | -1.915111102 | 0.625 | 1 | 0.000331314 |
| ENSSSCG00000032573 | 2.47E-18 | -1.917113455 | 1 | 1 | 4.28E-14 |
| UBE2J2 | 6.64E-14 | -1.917259569 | 0.875 | 1 | 1.15E-09 |
| MFSD2A | 1.44E-08 | -1.924354947 | 0.812 | 1 | 0.000249106 |
| CARHSP1 | 3.38E-09 | -1.925381959 | 0.75 | 0.933 | 5.86E-05 |
| SMYD5 | 2.40E-07 | -1.925999419 | 0.625 | 0.8 | 0.004155848 |
| TMUB1 | 2.21E-11 | -1.926537838 | 0.938 | 1 | 3.83E-07 |
| NLRP5 | 1.51E-22 | -1.92919812 | 1 | 1 | 2.61E-18 |
| ANKRD39 | 2.18E-12 | -1.929610672 | 0.938 | 1 | 3.78E-08 |

| | | | | | |
|---|---|---|---|---|---|
| ENSSSCG00000028397 | 1.21E-24 | -1.930727165 | 1 | 1 | 2.11E-20 |
| TXN2 | 2.06E-10 | -1.933168492 | 0.938 | 0.933 | 3.58E-06 |
| BAG5 | 3.65E-07 | -1.938134744 | 0.688 | 0.933 | 0.006328532 |
| PRPF4 | 1.00E-21 | -1.93844619 | 1 | 1 | 1.74E-17 |
| IQCC | 3.56E-10 | -1.942732799 | 0.938 | 1 | 6.18E-06 |
| ENSSSCG00000003282 | 5.05E-07 | -1.94458688 | 0.312 | 0.867 | 0.008766849 |
| CARS2 | 3.24E-15 | -1.946719917 | 0.875 | 1 | 5.62E-11 |
| ENSSSCG00000042361 | 1.43E-08 | -1.947258538 | 0.75 | 0.933 | 0.000247769 |
| DPH7 | 6.91E-08 | -1.949373927 | 0.688 | 0.933 | 0.001198132 |
| GNL3L | 4.62E-10 | -1.949518551 | 0.75 | 1 | 8.02E-06 |
| TMED9 | 1.82E-10 | -1.949745229 | 0.812 | 1 | 3.15E-06 |
| CTH | 3.88E-11 | -1.952065523 | 0.812 | 0.933 | 6.73E-07 |
| CNBD2 | 6.97E-14 | -1.957846984 | 1 | 1 | 1.21E-09 |
| COPS6 | 4.19E-15 | -1.959092056 | 1 | 1 | 7.27E-11 |
| TJP3 | 1.42E-07 | -1.961864871 | 0.625 | 0.933 | 0.002472323 |
| TRAK2 | 1.73E-13 | -1.96437609 | 0.938 | 1 | 3.00E-09 |
| GFRA4 | 6.20E-08 | -1.965648495 | 0.625 | 0.933 | 0.001075958 |
| CCDC12 | 9.34E-23 | -1.969102764 | 1 | 1 | 1.62E-18 |
| PIGH | 1.63E-10 | -1.969726989 | 0.812 | 0.933 | 2.83E-06 |
| POLE3 | 9.70E-18 | -1.971499957 | 0.938 | 1 | 1.68E-13 |
| ENSSSCG00000030478 | 4.68E-11 | -1.974102224 | 1 | 1 | 8.12E-07 |
| CHMP4A | 3.23E-14 | -1.978938384 | 0.812 | 1 | 5.61E-10 |
| COQ3 | 1.61E-14 | -1.980256065 | 0.875 | 1 | 2.79E-10 |
| SFN | 1.08E-08 | -1.981464049 | 0.75 | 1 | 0.000186784 |
| AMDHD2 | 4.61E-10 | -1.982926487 | 0.812 | 1 | 7.99E-06 |
| TOP1MT | 1.00E-09 | -1.983880335 | 0.812 | 0.933 | 1.74E-05 |
| RHBG | 9.29E-07 | -1.9861942 | 0.812 | 0.867 | 0.016123459 |
| FXR2 | 1.56E-15 | -1.988412026 | 0.938 | 1 | 2.71E-11 |
| BCAS2 | 2.11E-16 | -1.99342083 | 1 | 1 | 3.66E-12 |
| FAM50A | 5.35E-09 | -1.99492901 | 0.812 | 1 | 9.28E-05 |
| ACAT1 | 3.20E-24 | -1.997719059 | 1 | 1 | 5.55E-20 |
| PHB2 | 1.41E-22 | -1.998170329 | 1 | 1 | 2.45E-18 |
| DNAJC15 | 7.29E-26 | -1.999860197 | 1 | 1 | 1.26E-21 |
| MRPS33 | 1.31E-24 | -2 | 1 | 1 | 2.28E-20 |
| ENSSSCG00000059232 | 2.06E-08 | -2.001961516 | 0.812 | 1 | 0.000357801 |
| OXSM | 1.74E-16 | -2.003842066 | 0.875 | 1 | 3.02E-12 |
| UQCRC1 | 2.79E-10 | -2.004001931 | 0.875 | 1 | 4.84E-06 |
| MRPL20 | 1.97E-22 | -2.004800986 | 1 | 1 | 3.42E-18 |
| PFKM | 8.73E-12 | -2.009586049 | 0.938 | 1 | 1.52E-07 |
| CRB3 | 9.87E-17 | -2.010235222 | 0.938 | 1 | 1.71E-12 |
| MIX23 | 5.17E-24 | -2.010940863 | 1 | 1 | 8.98E-20 |
| NCLN | 1.57E-08 | -2.012832815 | 0.875 | 0.933 | 0.000271961 |
| MRPS18C | 2.07E-08 | -2.013256097 | 0.875 | 1 | 0.000359545 |
| ARMC2 | 1.70E-43 | -2.013514806 | 1 | 1 | 2.96E-39 |
| ATP6AP1 | 7.37E-13 | -2.016873819 | 1 | 0.933 | 1.28E-08 |
| MEPCE | 9.54E-16 | -2.019777666 | 0.938 | 1 | 1.65E-11 |
| FAM162A | 8.76E-25 | -2.020008913 | 1 | 1 | 1.52E-20 |
| MAP1LC3B | 1.76E-38 | -2.022026306 | 1 | 1 | 3.05E-34 |
| JAM3 | 7.40E-19 | -2.02233633 | 0.938 | 1 | 1.28E-14 |
| ENSSSCG00000044108 | 1.18E-11 | -2.026346871 | 1 | 1 | 2.04E-07 |
| PSME3IP1 | 8.71E-18 | -2.032446622 | 1 | 1 | 1.51E-13 |
| ACAA1 | 7.54E-10 | -2.03284888 | 0.75 | 0.933 | 1.31E-05 |
| MMD2 | 1.31E-08 | -2.03866162 | 0.562 | 0.933 | 0.00022718 |
| PLEKHB1 | 2.63E-11 | -2.039069565 | 0.875 | 0.933 | 4.56E-07 |
| BPGM | 4.91E-12 | -2.044453598 | 0.938 | 1 | 8.51E-08 |
| SLC25A6 | 3.91E-18 | -2.045944296 | 0.938 | 1 | 6.79E-14 |
| NDUFV2 | 1.92E-31 | -2.049388027 | 1 | 1 | 3.33E-27 |
| ENSSSCG00000032953 | 1.79E-10 | -2.051289229 | 0.812 | 1 | 3.10E-06 |
| CD40 | 8.33E-21 | -2.051325934 | 0.938 | 1 | 1.45E-16 |
| POLR1D | 5.37E-23 | -2.054281661 | 1 | 1 | 9.32E-19 |
| LRCH4 | 1.93E-10 | -2.054635257 | 0.75 | 0.933 | 3.35E-06 |
| PCIF1 | 1.50E-06 | -2.054635257 | 0.5 | 0.867 | 0.02595232 |
| PADI3 | 4.28E-09 | -2.055495113 | 0.75 | 1 | 7.43E-05 |
| DYNLL1 | 2.84E-16 | -2.064648741 | 1 | 1 | 4.93E-12 |
| ENSSSCG00000006559 | 2.51E-18 | -2.065364414 | 0.938 | 1 | 4.36E-14 |
| ATP5PO | 4.63E-25 | -2.068333059 | 1 | 1 | 8.04E-21 |

| | | | | | |
|---|---|---|---|---|---|
| TRAPPC4 | 3.20E-24 | -2.068810808 | 1 | 1 | 5.56E-20 |
| ENSSSCG00000011147 | 9.26E-12 | -2.074493726 | 0.812 | 1 | 1.61E-07 |
| SNU13 | 1.51E-25 | -2.076890894 | 1 | 1 | 2.61E-21 |
| ARL6IP4 | 3.99E-09 | -2.078609835 | 0.688 | 0.933 | 6.93E-05 |
| NOP14 | 8.87E-14 | -2.080170349 | 0.812 | 1 | 1.54E-09 |
| NME2 | 2.55E-10 | -2.082248412 | 1 | 1 | 4.42E-06 |
| UPB1 | 4.50E-14 | -2.087118967 | 0.938 | 1 | 7.81E-10 |
| MRPL58 | 1.40E-17 | -2.088909416 | 1 | 1 | 2.42E-13 |
| FKBP4 | 2.57E-22 | -2.092006005 | 1 | 1 | 4.46E-18 |
| MOK | 4.81E-12 | -2.093109404 | 0.75 | 0.933 | 8.35E-08 |
| NOL4L | 2.19E-11 | -2.093109404 | 0.625 | 0.933 | 3.80E-07 |
| THEM5 | 2.18E-08 | -2.093109404 | 0.562 | 1 | 0.000377851 |
| ENSSSCG00000032413 | 1.43E-07 | -2.093109404 | 0.688 | 0.933 | 0.002483484 |
| MAPK3 | 1.54E-06 | -2.093109404 | 0.5 | 0.867 | 0.02671252 |
| RPS6 | 6.65E-18 | -2.094454575 | 1 | 1 | 1.15E-13 |
| DRG1 | 1.59E-14 | -2.094757257 | 1 | 1 | 2.76E-10 |
| MED31 | 5.49E-16 | -2.09797516 | 1 | 1 | 9.52E-12 |
| COPS7A | 8.76E-08 | -2.105933445 | 0.562 | 0.933 | 0.001520427 |
| IDH3B | 1.16E-17 | -2.108706259 | 0.875 | 1 | 2.01E-13 |
| ARHGAP22 | 1.41E-33 | -2.109076153 | 1 | 1 | 2.45E-29 |
| GEMIN2 | 1.09E-09 | -2.111394757 | 0.812 | 0.933 | 1.90E-05 |
| AURKAIP1 | 2.28E-10 | -2.111968432 | 0.75 | 1 | 3.95E-06 |
| NDUFC1 | 2.70E-18 | -2.113008962 | 0.875 | 1 | 4.69E-14 |
| PRR13 | 2.12E-16 | -2.113287286 | 0.938 | 1 | 3.67E-12 |
| PLA2G12A | 9.78E-17 | -2.116760672 | 0.938 | 1 | 1.70E-12 |
| ENSSSCG00000005006 | 6.08E-14 | -2.117183539 | 1 | 1 | 1.06E-09 |
| PSMC3IP | 5.85E-18 | -2.117275716 | 0.938 | 1 | 1.01E-13 |
| ENSSSCG00000047706 | 3.87E-10 | -2.117771459 | 0.75 | 0.933 | 6.72E-06 |
| ENSSSCG00000051352 | 1.78E-08 | -2.118644496 | 0.75 | 0.867 | 0.000308745 |
| ENSSSCG00000044054 | 5.55E-08 | -2.118644496 | 0.688 | 1 | 0.000962625 |
| CDA | 1.37E-13 | -2.119104613 | 0.75 | 1 | 2.38E-09 |
| SAMM50 | 4.32E-10 | -2.119909464 | 0.812 | 1 | 7.49E-06 |
| POP7 | 1.36E-20 | -2.12015628 | 1 | 1 | 2.36E-16 |
| ENSSSCG00000049464 | 1.58E-15 | -2.124587636 | 1 | 1 | 2.74E-11 |
| CALML4 | 3.07E-08 | -2.12963528 | 0.75 | 1 | 0.000533479 |
| EBNA1BP2 | 2.41E-24 | -2.130103612 | 1 | 1 | 4.18E-20 |
| CHCHD4 | 3.26E-41 | -2.13162464 | 1 | 1 | 5.66E-37 |
| POC1A | 1.60E-18 | -2.133186844 | 0.875 | 1 | 2.78E-14 |
| TOMM20 | 5.88E-29 | -2.134301092 | 1 | 1 | 1.02E-24 |
| RNF181 | 1.69E-12 | -2.13492958 | 0.875 | 1 | 2.94E-08 |
| NOC4L | 1.03E-07 | -2.13492958 | 0.625 | 0.933 | 0.001793292 |
| TMEM213 | 5.98E-12 | -2.138005942 | 0.875 | 1 | 1.04E-07 |
| MRPL45 | 2.04E-22 | -2.141318497 | 0.938 | 1 | 3.54E-18 |
| ATRAID | 5.55E-14 | -2.14286244 | 0.875 | 1 | 9.63E-10 |
| PIP4P1 | 7.40E-14 | -2.144421495 | 0.938 | 1 | 1.28E-09 |
| SLC16A5 | 8.65E-10 | -2.145197916 | 0.625 | 1 | 1.50E-05 |
| NT5DC2 | 2.75E-07 | -2.146976276 | 0.75 | 0.933 | 0.004778405 |
| LONP1 | 5.86E-15 | -2.147901877 | 0.938 | 1 | 1.02E-10 |
| OTUB1 | 3.36E-16 | -2.148001535 | 1 | 1 | 5.83E-12 |
| CLDN10 | 4.78E-28 | -2.1498785 | 1 | 1 | 8.29E-24 |
| ENSSSCG00000055905 | 5.57E-10 | -2.154509949 | 0.625 | 1 | 9.66E-06 |
| GHITM | 5.14E-12 | -2.154565608 | 1 | 1 | 8.91E-08 |
| ENSSSCG00000056323 | 1.34E-11 | -2.156219448 | 0.812 | 1 | 2.33E-07 |
| TK2 | 7.48E-13 | -2.156351838 | 1 | 1 | 1.30E-08 |
| RPL28 | 8.31E-07 | -2.160016063 | 0.875 | 1 | 0.014415636 |
| DNPEP | 3.61E-08 | -2.164975391 | 1 | 1 | 0.000626059 |
| LMBR1L | 8.63E-10 | -2.168397532 | 0.812 | 0.933 | 1.50E-05 |
| SLC38A8 | 2.46E-11 | -2.168599604 | 0.875 | 0.933 | 4.28E-07 |
| ENSSSCG00000047379 | 1.70E-08 | -2.171111916 | 0.625 | 1 | 0.000294962 |
| ENSSSCG00000022738 | 6.09E-11 | -2.173674886 | 0.812 | 0.933 | 1.06E-06 |
| ENSSSCG00000035728 | 9.07E-10 | -2.175571565 | 0.812 | 1 | 1.57E-05 |
| ENSSSCG00000045681 | 2.84E-11 | -2.17625064 | 0.875 | 1 | 4.92E-07 |
| ENSSSCG00000007493 | 1.61E-36 | -2.177421518 | 1 | 1 | 2.79E-32 |
| PAAF1 | 1.81E-17 | -2.18001747 | 1 | 1 | 3.14E-13 |
| SMARCD3 | 2.36E-06 | -2.180572246 | 0.375 | 0.867 | 0.040942038 |
| TFB1M | 3.24E-36 | -2.182425236 | 1 | 1 | 5.62E-32 |

| | | | | | |
|---|---|---|---|---|---|
| TXNRD3 | 2.35E-08 | -2.183307213 | 0.688 | 0.933 | 0.000408179 |
| ENSSSCG00000041692 | 2.83E-08 | -2.185031894 | 0.688 | 0.867 | 0.000491629 |
| ENSSSCG00000008147 | 2.39E-10 | -2.185758692 | 0.812 | 0.933 | 4.15E-06 |
| ENSSSCG00000002379 | 1.84E-15 | -2.188266637 | 1 | 1 | 3.19E-11 |
| ENSSSCG00000013613 | 6.40E-09 | -2.188266637 | 0.688 | 1 | 0.000111102 |
| WWC3 | 8.14E-07 | -2.188266637 | 0.438 | 0.867 | 0.014117306 |
| LSM4 | 1.03E-17 | -2.192645078 | 0.938 | 1 | 1.78E-13 |
| MTRES1 | 2.35E-33 | -2.19291522 | 0.938 | 1 | 4.08E-29 |
| SERPINB1 | 1.67E-13 | -2.193303692 | 0.938 | 1 | 2.91E-09 |
| HADHA | 2.75E-29 | -2.194422891 | 1 | 1 | 4.77E-25 |
| BNIP1 | 2.48E-18 | -2.195624315 | 1 | 1 | 4.30E-14 |
| ENSSSCG00000052254 | 1.96E-10 | -2.197446064 | 0.5 | 0.933 | 3.40E-06 |
| DHPS | 8.79E-11 | -2.198462405 | 0.688 | 0.933 | 1.52E-06 |
| YBX2 | 8.59E-11 | -2.202231127 | 0.875 | 1 | 1.49E-06 |
| ENSSSCG00000037808 | 6.77E-08 | -2.202338474 | 0.938 | 1 | 0.001175196 |
| ENSSSCG00000008012 | 1.25E-21 | -2.203219161 | 1 | 1 | 2.18E-17 |
| MPV17 | 1.65E-09 | -2.20461772 | 0.938 | 0.933 | 2.87E-05 |
| TMEM223 | 1.25E-07 | -2.208586622 | 0.625 | 0.867 | 0.002176893 |
| YIF1B | 1.21E-06 | -2.208586622 | 0.438 | 0.933 | 0.020981163 |
| MAF | 3.11E-10 | -2.212408333 | 0.562 | 1 | 5.40E-06 |
| THBS3 | 6.39E-09 | -2.212848649 | 0.625 | 1 | 0.000110875 |
| ISYNA1 | 5.28E-08 | -2.22059914 | 0.688 | 0.867 | 0.000915432 |
| B4GALT7 | 1.62E-07 | -2.2255597 | 0.625 | 0.867 | 0.002812829 |
| VPS25 | 2.40E-17 | -2.22606081 | 1 | 1 | 4.17E-13 |
| SVOP | 2.44E-06 | -2.228624375 | 0.688 | 0.867 | 0.042398324 |
| FBXO31 | 6.39E-11 | -2.229375094 | 0.812 | 0.933 | 1.11E-06 |
| TLCD1 | 1.51E-27 | -2.229753584 | 0.875 | 1 | 2.62E-23 |
| ZMAT5 | 1.87E-21 | -2.232085818 | 0.938 | 1 | 3.24E-17 |
| ENKD1 | 2.51E-06 | -2.234465254 | 0.5 | 0.867 | 0.043503533 |
| MRPS7 | 5.21E-12 | -2.236945177 | 0.625 | 1 | 9.05E-08 |
| ENSSSCG00000039815 | 5.75E-13 | -2.239950793 | 0.688 | 1 | 9.97E-09 |
| ENSSSCG00000028962 | 6.78E-08 | -2.239950793 | 0.438 | 0.933 | 0.001175709 |
| PLIN5 | 8.63E-12 | -2.247437551 | 0.875 | 1 | 1.50E-07 |
| GBA2 | 2.11E-06 | -2.247437551 | 0.625 | 0.933 | 0.036532621 |
| PKN1 | 8.46E-07 | -2.24838763 | 0.5 | 0.933 | 0.014683238 |
| TRIM4 | 1.03E-07 | -2.24961389 | 0.562 | 0.8 | 0.001788072 |
| SLC35B1 | 1.21E-19 | -2.251371488 | 1 | 1 | 2.10E-15 |
| PRDX4 | 5.76E-12 | -2.251741236 | 1 | 1 | 9.99E-08 |
| FLOT1 | 6.04E-27 | -2.252063288 | 0.938 | 1 | 1.05E-22 |
| SCPEP1 | 1.81E-13 | -2.252449655 | 0.875 | 1 | 3.14E-09 |
| RNF26 | 4.85E-23 | -2.253756592 | 1 | 1 | 8.42E-19 |
| AQP6 | 4.56E-07 | -2.255380833 | 0.625 | 0.867 | 0.007907673 |
| ZPR1 | 5.55E-20 | -2.25762091 | 0.938 | 1 | 9.64E-16 |
| FAM32A | 2.21E-22 | -2.258772379 | 1 | 1 | 3.83E-18 |
| GDF9 | 1.63E-37 | -2.261016009 | 1 | 1 | 2.83E-33 |
| IER2 | 2.07E-15 | -2.263034406 | 0.875 | 0.933 | 3.59E-11 |
| BUD31 | 2.27E-33 | -2.264183048 | 1 | 1 | 3.95E-29 |
| PMF1 | 7.90E-14 | -2.264589872 | 0.938 | 1 | 1.37E-09 |
| NRAP | 6.58E-12 | -2.267883805 | 0.625 | 1 | 1.14E-07 |
| TANGO2 | 8.01E-13 | -2.269432177 | 0.938 | 1 | 1.39E-08 |
| ASB11 | 4.27E-26 | -2.270431399 | 1 | 1 | 7.40E-22 |
| NPC2 | 3.62E-17 | -2.273176416 | 1 | 1 | 6.28E-13 |
| MRPS28 | 9.31E-24 | -2.276470545 | 0.938 | 1 | 1.62E-19 |
| ENSSSCG00000062784 | 4.67E-08 | -2.277533976 | 0.562 | 1 | 0.000810684 |
| ENSSSCG00000031730 | 2.07E-15 | -2.27897595 | 1 | 1 | 3.60E-11 |
| PROM2 | 1.67E-07 | -2.27897595 | 0.5 | 0.8 | 0.002892421 |
| NOP10 | 1.05E-26 | -2.281381064 | 1 | 1 | 1.82E-22 |
| SCAMP4 | 5.52E-07 | -2.282143229 | 0.562 | 0.867 | 0.009579811 |
| PPOX | 8.81E-10 | -2.285754482 | 0.5 | 0.933 | 1.53E-05 |
| MLST8 | 3.18E-09 | -2.285754482 | 0.75 | 0.933 | 5.52E-05 |
| ENSSSCG00000061349 | 1.85E-09 | -2.291048782 | 0.688 | 0.867 | 3.22E-05 |
| ARF5 | 3.40E-20 | -2.291379032 | 0.875 | 1 | 5.89E-16 |
| ENSSSCG00000057138 | 1.78E-12 | -2.292090439 | 0.75 | 0.933 | 3.08E-08 |
| CD247 | 7.28E-19 | -2.292418213 | 0.938 | 1 | 1.26E-14 |
| MRTO4 | 5.24E-14 | -2.294743266 | 0.812 | 1 | 9.10E-10 |
| ENSSSCG00000015083 | 3.91E-17 | -2.295245716 | 1 | 1 | 6.79E-13 |

| | | | | | |
|---|---|---|---|---|---|
| THAP7 | 5.29E-10 | -2.296981738 | 0.688 | 1 | 9.17E-06 |
| GALNT6 | 2.05E-12 | -2.297799824 | 0.812 | 1 | 3.56E-08 |
| ARHGEF28 | 4.31E-10 | -2.300704824 | 0.562 | 1 | 7.47E-06 |
| KLF1 | 1.83E-07 | -2.30461351 | 0.5 | 0.933 | 0.003179516 |
| PHB1 | 1.66E-27 | -2.307003265 | 1 | 1 | 2.88E-23 |
| ADA | 1.54E-26 | -2.3111759 | 1 | 1 | 2.67E-22 |
| ENSSSCG00000031756 | 5.20E-15 | -2.312467766 | 0.875 | 1 | 9.02E-11 |
| TXNDC12 | 6.68E-12 | -2.313157885 | 0.688 | 1 | 1.16E-07 |
| CTDNEP1 | 1.97E-14 | -2.315501826 | 0.875 | 1 | 3.42E-10 |
| EHD1 | 6.19E-11 | -2.315501826 | 0.625 | 1 | 1.07E-06 |
| TVP23A | 9.18E-07 | -2.315501826 | 0.438 | 0.8 | 0.015924332 |
| C14orf93 | 1.55E-06 | -2.315501826 | 0.375 | 0.933 | 0.026980752 |
| AKR1A1 | 1.21E-10 | -2.316912775 | 0.812 | 0.933 | 2.10E-06 |
| NUCB1 | 1.51E-11 | -2.322355497 | 0.812 | 1 | 2.63E-07 |
| SNF8 | 8.05E-10 | -2.322843801 | 0.812 | 1 | 1.40E-05 |
| ENSSSCG00000001776 | 1.04E-06 | -2.323407024 | 0.438 | 0.867 | 0.017974564 |
| TIMP2 | 2.56E-06 | -2.323407024 | 0.5 | 0.8 | 0.04440117 |
| ENSSSCG00000057224 | 3.28E-10 | -2.32443495 | 0.75 | 1 | 5.69E-06 |
| ENSSSCG00000005217 | 1.01E-31 | -2.325255617 | 0.938 | 1 | 1.75E-27 |
| DNAI1 | 2.70E-06 | -2.325770161 | 0.438 | 0.867 | 0.046933095 |
| MED8 | 1.77E-20 | -2.326599535 | 1 | 1 | 3.08E-16 |
| TOMM40L | 1.94E-10 | -2.330148602 | 0.5 | 1 | 3.36E-06 |
| FZR1 | 1.55E-22 | -2.333531241 | 0.938 | 1 | 2.68E-18 |
| NUDCD3 | 7.80E-13 | -2.333669948 | 0.875 | 1 | 1.35E-08 |
| PIH1D1 | 1.30E-18 | -2.334770159 | 0.812 | 1 | 2.25E-14 |
| KLHDC4 | 2.33E-09 | -2.336563441 | 0.875 | 0.867 | 4.05E-05 |
| MED10 | 8.09E-13 | -2.337034987 | 0.75 | 1 | 1.40E-08 |
| LRRC36 | 1.88E-06 | -2.338221902 | 0.5 | 0.933 | 0.032575999 |
| ENSSSCG00000027041 | 5.02E-33 | -2.339220179 | 1 | 1 | 8.72E-29 |
| ENSSSCG00000059210 | 1.23E-08 | -2.341036918 | 0.5 | 0.933 | 0.000213461 |
| ARPC3 | 1.22E-21 | -2.343222583 | 1 | 1 | 2.12E-17 |
| UPK1A | 8.31E-09 | -2.344070978 | 0.625 | 0.867 | 0.000144219 |
| CIDEA | 1.34E-09 | -2.345113482 | 0.938 | 1 | 2.33E-05 |
| PAQR7 | 1.68E-15 | -2.349449158 | 0.938 | 0.933 | 2.91E-11 |
| HAUS1 | 1.20E-36 | -2.349847571 | 1 | 1 | 2.08E-32 |
| NCF1 | 6.96E-18 | -2.35033181 | 0.875 | 1 | 1.21E-13 |
| GFUS | 5.56E-13 | -2.352976531 | 0.812 | 1 | 9.64E-09 |
| GDPGP1 | 6.54E-10 | -2.354069482 | 0.688 | 1 | 1.14E-05 |
| SLC25A10 | 1.67E-06 | -2.35614381 | 0.188 | 0.933 | 0.028966505 |
| NR0B1 | 3.98E-23 | -2.360289747 | 1 | 1 | 6.91E-19 |
| UST | 1.89E-21 | -2.360331607 | 0.875 | 1 | 3.28E-17 |
| PPAN | 1.91E-28 | -2.36143811 | 1 | 1 | 3.31E-24 |
| CARD19 | 5.80E-10 | -2.36159824 | 0.812 | 1 | 1.01E-05 |
| ENSSSCG00000060526 | 2.36E-07 | -2.36159824 | 0.312 | 0.933 | 0.004099612 |
| COMMD1 | 9.46E-08 | -2.366127899 | 0.5 | 0.867 | 0.001642246 |
| PFDN1 | 9.79E-36 | -2.36656808 | 1 | 1 | 1.70E-31 |
| RPS27A | 1.84E-12 | -2.369440633 | 1 | 1 | 3.19E-08 |
| FAT2 | 1.03E-18 | -2.374570908 | 0.875 | 0.933 | 1.79E-14 |
| UQCRB | 1.18E-23 | -2.375613674 | 1 | 1 | 2.05E-19 |
| PFDN5 | 4.69E-19 | -2.375887746 | 1 | 1 | 8.13E-15 |
| AMZ2 | 1.51E-29 | -2.380368483 | 0.938 | 1 | 2.62E-25 |
| GPN2 | 9.73E-11 | -2.380391356 | 0.812 | 0.933 | 1.69E-06 |
| INTS11 | 2.99E-07 | -2.385891154 | 0.562 | 0.933 | 0.005193427 |
| IDH2 | 2.32E-17 | -2.386373741 | 0.938 | 1 | 4.03E-13 |
| ENSSSCG00000003234 | 2.56E-07 | -2.388565288 | 0.438 | 0.933 | 0.004444173 |
| GTF2IRD1 | 3.03E-07 | -2.388565288 | 0.5 | 0.933 | 0.005252371 |
| NDUFA4 | 4.53E-10 | -2.391682103 | 1 | 1 | 7.86E-06 |
| PAPLN | 4.84E-09 | -2.393504338 | 0.562 | 1 | 8.39E-05 |
| ENSSSCG00000011272 | 1.32E-22 | -2.396632919 | 1 | 1 | 2.29E-18 |
| HIGD2A | 7.92E-13 | -2.398038496 | 1 | 1 | 1.37E-08 |
| BLOC1S1 | 1.37E-07 | -2.398917834 | 0.375 | 0.933 | 0.002378694 |
| C5orf49 | 3.47E-11 | -2.405387329 | 0.938 | 1 | 6.02E-07 |
| GTF2H5 | 1.56E-18 | -2.406848101 | 0.938 | 1 | 2.70E-14 |
| MAPK7 | 1.46E-10 | -2.40780593 | 0.812 | 1 | 2.53E-06 |
| TMA7 | 2.44E-16 | -2.407982742 | 1 | 1 | 4.24E-12 |
| MESD | 7.99E-13 | -2.409684265 | 0.75 | 1 | 1.39E-08 |

| | | | | | |
|---|---|---|---|---|---|
| COMMD7 | 7.60E-20 | -2.411285364 | 0.812 | 1 | 1.32E-15 |
| ARRDC4 | 3.11E-12 | -2.41130478 | 1 | 1 | 5.40E-08 |
| BNIP3 | 1.31E-18 | -2.415037499 | 0.938 | 0.933 | 2.28E-14 |
| NAA10 | 3.03E-10 | -2.415037499 | 0.75 | 1 | 5.25E-06 |
| FKBP1B | 7.44E-07 | -2.415037499 | 0.438 | 0.867 | 0.012901357 |
| ATP5MC2 | 1.27E-25 | -2.416655778 | 1 | 1 | 2.20E-21 |
| PRDX1 | 1.81E-42 | -2.421547541 | 1 | 1 | 3.15E-38 |
| PLPP4 | 8.60E-10 | -2.423258006 | 0.688 | 1 | 1.49E-05 |
| MAP2K2 | 8.96E-08 | -2.428712436 | 0.5 | 0.867 | 0.001554729 |
| MYL12A | 4.74E-30 | -2.429016083 | 1 | 1 | 8.23E-26 |
| MFSD13A | 1.21E-08 | -2.432959407 | 0.625 | 0.933 | 0.000210064 |
| TIMM50 | 9.32E-13 | -2.434146322 | 0.812 | 1 | 1.62E-08 |
| YKT6 | 4.80E-18 | -2.434402824 | 0.812 | 1 | 8.33E-14 |
| RDH13 | 1.42E-12 | -2.435215381 | 0.812 | 0.933 | 2.46E-08 |
| CTNS | 1.56E-20 | -2.435262527 | 1 | 1 | 2.71E-16 |
| POLM | 2.69E-10 | -2.435501602 | 0.75 | 0.867 | 4.67E-06 |
| CCDC63 | 4.18E-11 | -2.437937901 | 0.438 | 1 | 7.25E-07 |
| GMPPA | 1.36E-07 | -2.437937901 | 0.5 | 0.867 | 0.002354595 |
| MARS1 | 6.10E-30 | -2.440010028 | 1 | 1 | 1.06E-25 |
| ENSSSCG00000052208 | 2.45E-14 | -2.441631709 | 0.562 | 1 | 4.24E-10 |
| NDUFB4 | 2.43E-09 | -2.441837559 | 0.812 | 1 | 4.22E-05 |
| VPS28 | 7.67E-15 | -2.443606651 | 0.875 | 1 | 1.33E-10 |
| EIF4EBP1 | 6.88E-09 | -2.443606651 | 0.562 | 1 | 0.000119369 |
| PDCD5 | 2.53E-34 | -2.444916142 | 1 | 1 | 4.40E-30 |
| NSFL1C | 1.43E-12 | -2.447337991 | 0.75 | 1 | 2.49E-08 |
| CCDC172 | 1.55E-13 | -2.449594721 | 0.75 | 1 | 2.69E-09 |
| PSTPIP1 | 6.20E-10 | -2.449802917 | 0.688 | 0.933 | 1.08E-05 |
| GPR108 | 1.68E-12 | -2.451301043 | 0.562 | 1 | 2.91E-08 |
| GEMIN6 | 7.36E-36 | -2.451362189 | 1 | 1 | 1.28E-31 |
| ENSSSCG00000022401 | 6.71E-22 | -2.454930226 | 0.875 | 1 | 1.16E-17 |
| ENSSSCG00000010058 | 1.41E-28 | -2.457319548 | 1 | 1 | 2.44E-24 |
| ENSSSCG00000003286 | 6.88E-20 | -2.458385042 | 1 | 1 | 1.19E-15 |
| HEMK1 | 1.49E-07 | -2.458758877 | 0.688 | 0.867 | 0.002588197 |
| MCRS1 | 5.99E-16 | -2.463567393 | 0.938 | 1 | 1.04E-11 |
| NELFCD | 7.66E-08 | -2.469485283 | 0.562 | 0.867 | 0.00132971 |
| IP6K2 | 5.88E-18 | -2.471621028 | 1 | 1 | 1.02E-13 |
| FUNDC2 | 1.96E-12 | -2.485426827 | 0.812 | 1 | 3.41E-08 |
| TSEN54 | 1.26E-07 | -2.485426827 | 0.5 | 0.8 | 0.002192351 |
| ZNRD2 | 1.55E-07 | -2.485426827 | 0.5 | 0.867 | 0.002693137 |
| SUCLG1 | 6.13E-24 | -2.486181572 | 0.938 | 1 | 1.06E-19 |
| SLC38A5 | 5.83E-08 | -2.489999557 | 0.625 | 0.8 | 0.001012363 |
| COA3 | 2.24E-13 | -2.493647334 | 0.625 | 1 | 3.89E-09 |
| MTMR14 | 7.71E-17 | -2.494039957 | 0.938 | 1 | 1.34E-12 |
| OOEP | 4.23E-23 | -2.496253737 | 1 | 1 | 7.35E-19 |
| TIMM17A | 5.27E-16 | -2.499101764 | 0.688 | 1 | 9.15E-12 |
| RNF121 | 9.49E-21 | -2.499734664 | 0.938 | 1 | 1.65E-16 |
| POLR2H | 1.14E-30 | -2.49986834 | 1 | 1 | 1.99E-26 |
| PRDX5 | 3.61E-12 | -2.501691882 | 0.812 | 1 | 6.26E-08 |
| ENSSSCG00000007826 | 2.94E-27 | -2.505740406 | 1 | 1 | 5.10E-23 |
| DUS3L | 2.47E-11 | -2.50589093 | 0.625 | 0.933 | 4.29E-07 |
| BAG2 | 4.86E-17 | -2.508146904 | 0.875 | 1 | 8.43E-13 |
| SPAG8 | 4.38E-09 | -2.511610759 | 0.625 | 0.933 | 7.59E-05 |
| SMYD3 | 2.27E-09 | -2.515066318 | 0.812 | 1 | 3.95E-05 |
| PUF60 | 1.08E-21 | -2.51524796 | 1 | 1 | 1.88E-17 |
| SIAH3 | 6.84E-09 | -2.516320835 | 0.688 | 0.933 | 0.000118658 |
| ENSSSCG00000011294 | 1.72E-13 | -2.51731855 | 0.75 | 1 | 2.98E-09 |
| MMAB | 4.73E-09 | -2.519374159 | 0.5 | 0.933 | 8.20E-05 |
| ENSSSCG00000029830 | 2.42E-10 | -2.521203056 | 0.938 | 1 | 4.19E-06 |
| SWI5 | 3.39E-16 | -2.528566445 | 1 | 1 | 5.88E-12 |
| SEC11C | 1.46E-23 | -2.531682418 | 1 | 1 | 2.53E-19 |
| ENSSSCG00000017913 | 4.57E-25 | -2.531814801 | 1 | 1 | 7.94E-21 |
| PLIN3 | 3.27E-11 | -2.541569905 | 0.875 | 1 | 5.68E-07 |
| GATD1 | 9.67E-10 | -2.542416806 | 0.625 | 0.933 | 1.68E-05 |
| COA4 | 7.59E-29 | -2.543142325 | 1 | 1 | 1.32E-24 |
| TSPAN1 | 6.45E-17 | -2.543599989 | 0.938 | 1 | 1.12E-12 |
| MAPKAPK2 | 1.04E-11 | -2.545621609 | 0.625 | 1 | 1.81E-07 |

| | | | | | |
|---|---|---|---|---|---|
| PPM1J | 8.05E-08 | -2.552541023 | 0.438 | 0.867 | 0.001396809 |
| XKRX | 1.26E-07 | -2.552541023 | 0.562 | 0.933 | 0.002184646 |
| AP1S1 | 1.69E-22 | -2.55468949 | 0.938 | 1 | 2.93E-18 |
| TPCN1 | 8.66E-12 | -2.557390423 | 0.625 | 1 | 1.50E-07 |
| EIF2B2 | 1.89E-08 | -2.561878888 | 0.625 | 0.933 | 0.000328771 |
| CMC4 | 9.23E-15 | -2.563429339 | 0.812 | 1 | 1.60E-10 |
| CCDC9 | 2.00E-06 | -2.571156701 | 0.375 | 0.867 | 0.034738138 |
| ENSSSCG00000014284 | 1.66E-26 | -2.572968905 | 1 | 1 | 2.88E-22 |
| ENSSSCG00000052263 | 5.25E-25 | -2.573374526 | 1 | 1 | 9.10E-21 |
| ENSSSCG00000016100 | 5.59E-20 | -2.576431643 | 0.875 | 1 | 9.70E-16 |
| POLR2K | 1.33E-29 | -2.581325519 | 1 | 1 | 2.31E-25 |
| LRSAM1 | 3.48E-14 | -2.581395886 | 0.812 | 1 | 6.04E-10 |
| KDM8 | 1.27E-10 | -2.58185659 | 0.688 | 0.933 | 2.21E-06 |
| CYB561D2 | 3.42E-07 | -2.582494245 | 0.438 | 0.8 | 0.005927883 |
| DMWD | 5.31E-13 | -2.583075391 | 0.75 | 1 | 9.21E-09 |
| LIN37 | 6.05E-11 | -2.584249883 | 0.75 | 1 | 1.05E-06 |
| C1orf54 | 5.53E-14 | -2.584962501 | 0.812 | 1 | 9.60E-10 |
| TBRG4 | 1.21E-19 | -2.58812978 | 0.938 | 1 | 2.10E-15 |
| FSTL3 | 3.59E-19 | -2.591015171 | 0.875 | 1 | 6.23E-15 |
| GAPDH | 9.91E-09 | -2.591295462 | 0.938 | 1 | 0.000172017 |
| WASHC3 | 7.35E-07 | -2.5981036 | 0.562 | 0.933 | 0.012745113 |
| RPL32 | 1.30E-20 | -2.59946207 | 1 | 1 | 2.26E-16 |
| MATCAP1 | 3.59E-07 | -2.600904045 | 0.562 | 0.667 | 0.00622084 |
| ENSSSCG00000056114 | 2.25E-11 | -2.601450624 | 0.75 | 0.933 | 3.90E-07 |
| ENSSSCG00000038027 | 9.19E-15 | -2.603724564 | 0.562 | 1 | 1.59E-10 |
| ENSSSCG00000060912 | 3.23E-09 | -2.604071324 | 0.438 | 0.933 | 5.61E-05 |
| NDUFB2 | 1.83E-14 | -2.607348581 | 1 | 1 | 3.18E-10 |
| JPT2 | 4.63E-13 | -2.613773412 | 0.75 | 0.933 | 8.03E-09 |
| MVK | 3.02E-11 | -2.61667136 | 0.625 | 0.933 | 5.25E-07 |
| HOATZ | 9.82E-07 | -2.61667136 | 0.312 | 0.8 | 0.01704113 |
| JPT1 | 2.26E-18 | -2.623624121 | 0.938 | 1 | 3.92E-14 |
| CCER2 | 3.27E-11 | -2.628072519 | 1 | 1 | 5.68E-07 |
| FSD2 | 1.60E-30 | -2.62995331 | 1 | 1 | 2.77E-26 |
| GABARAP | 2.51E-22 | -2.632747915 | 0.938 | 1 | 4.36E-18 |
| GRHPR | 4.46E-20 | -2.634891081 | 1 | 1 | 7.75E-16 |
| CLPS | 3.23E-07 | -2.635163314 | 0.688 | 1 | 0.005611858 |
| REC114 | 6.77E-39 | -2.635806178 | 1 | 1 | 1.17E-34 |
| RBP2 | 6.76E-09 | -2.639597757 | 0.562 | 0.867 | 0.000117304 |
| GTF2F1 | 7.88E-27 | -2.641313355 | 1 | 1 | 1.37E-22 |
| ALKBH7 | 2.33E-16 | -2.647045009 | 0.75 | 1 | 4.05E-12 |
| FN3KRP | 3.79E-07 | -2.647698256 | 0.312 | 1 | 0.0065843 |
| SHANK2 | 8.01E-11 | -2.651104858 | 0.688 | 0.933 | 1.39E-06 |
| IAH1 | 2.55E-17 | -2.655634516 | 0.812 | 1 | 4.43E-13 |
| IQCD | 6.71E-07 | -2.656045599 | 0.25 | 0.867 | 0.011636202 |
| MVP | 1.19E-29 | -2.662130361 | 1 | 1 | 2.06E-25 |
| SELENOS | 1.93E-28 | -2.663099398 | 0.938 | 1 | 3.34E-24 |
| TMEM200C | 4.64E-11 | -2.669458775 | 0.5 | 0.867 | 8.05E-07 |
| ZNF621 | 1.06E-09 | -2.670538232 | 0.562 | 0.867 | 1.84E-05 |
| CDK5RAP2 | 4.54E-23 | -2.672487787 | 1 | 1 | 7.88E-19 |
| NDUFS5 | 2.00E-15 | -2.674904626 | 1 | 1 | 3.47E-11 |
| HAUS7 | 2.38E-12 | -2.678071905 | 0.688 | 0.933 | 4.13E-08 |
| ENSSSCG00000013768 | 1.08E-09 | -2.678071905 | 0.562 | 0.933 | 1.87E-05 |
| CORO6 | 1.04E-08 | -2.678071905 | 0.438 | 1 | 0.000180574 |
| ALG3 | 4.74E-07 | -2.678071905 | 0.438 | 0.867 | 0.008221613 |
| ARMC12 | 1.91E-15 | -2.685807554 | 0.688 | 1 | 3.32E-11 |
| ENSSSCG00000059061 | 6.76E-11 | -2.689476668 | 0.562 | 0.933 | 1.17E-06 |
| EMC7 | 1.90E-27 | -2.695681305 | 0.812 | 1 | 3.30E-23 |
| ETHE1 | 4.51E-17 | -2.696735749 | 0.875 | 0.933 | 7.82E-13 |
| BUD23 | 6.03E-28 | -2.697880386 | 0.938 | 1 | 1.05E-23 |
| SLC25A14 | 7.34E-13 | -2.697971463 | 0.75 | 0.933 | 1.27E-08 |
| ENSSSCG00000009851 | 8.03E-26 | -2.701658725 | 1 | 1 | 1.39E-21 |
| POMP | 8.30E-28 | -2.70286109 | 1 | 1 | 1.44E-23 |
| TMEM178A | 6.19E-08 | -2.703162886 | 0.375 | 0.933 | 0.001074113 |
| ABHD17A | 1.28E-13 | -2.704544116 | 0.688 | 0.933 | 2.22E-09 |
| FLAD1 | 8.56E-09 | -2.709780765 | 0.438 | 0.933 | 0.000148536 |
| TFPT | 1.24E-06 | -2.712019237 | 0.25 | 0.867 | 0.021533377 |

| | | | | | |
|---|---|---|---|---|---|
| METTL23 | 8.60E-22 | -2.712718048 | 0.875 | 1 | 1.49E-17 |
| TIMM13 | 2.95E-25 | -2.714597781 | 0.875 | 1 | 5.11E-21 |
| ALDOC | 2.71E-24 | -2.715262464 | 0.875 | 1 | 4.70E-20 |
| PEX19 | 5.35E-14 | -2.717018185 | 0.875 | 0.933 | 9.29E-10 |
| GLRX3 | 1.44E-24 | -2.717600269 | 0.938 | 1 | 2.51E-20 |
| ENSSSCG00000050347 | 1.39E-09 | -2.717600269 | 0.375 | 0.933 | 2.40E-05 |
| ENSSSCG00000039544 | 1.91E-39 | -2.718025096 | 1 | 1 | 3.31E-35 |
| COA8 | 3.18E-16 | -2.718429643 | 0.75 | 1 | 5.51E-12 |
| ENSSSCG00000061274 | 5.91E-10 | -2.719892081 | 0.5 | 0.933 | 1.02E-05 |
| REX1BD | 9.58E-09 | -2.719892081 | 0.438 | 1 | 0.000166203 |
| ENSSSCG00000062012 | 1.50E-08 | -2.719892081 | 0.562 | 0.8 | 0.000259453 |
| VDAC3 | 5.40E-30 | -2.721494185 | 0.938 | 1 | 9.38E-26 |
| UBA52 | 1.28E-09 | -2.72416832 | 0.875 | 1 | 2.21E-05 |
| MLYCD | 6.65E-11 | -2.72537762 | 0.438 | 1 | 1.15E-06 |
| ENSSSCG00000031117 | 1.80E-15 | -2.726981506 | 0.938 | 1 | 3.12E-11 |
| ENSSSCG00000043434 | 2.52E-10 | -2.728697978 | 0.438 | 1 | 4.38E-06 |
| POLL | 2.48E-07 | -2.728697978 | 0.438 | 0.867 | 0.004300999 |
| UBL7 | 2.15E-27 | -2.729380999 | 1 | 1 | 3.74E-23 |
| YIPF2 | 3.33E-20 | -2.73231495 | 0.875 | 1 | 5.78E-16 |
| TEX264 | 1.43E-15 | -2.735787403 | 0.875 | 1 | 2.48E-11 |
| ENSSSCG00000034188 | 4.70E-10 | -2.736965594 | 0.438 | 1 | 8.15E-06 |
| RNF141 | 3.24E-10 | -2.74080766 | 0.5 | 0.933 | 5.63E-06 |
| NEU1 | 2.02E-06 | -2.742202243 | 0.375 | 0.8 | 0.03501214 |
| ENSSSCG00000006081 | 8.10E-31 | -2.750199047 | 1 | 1 | 1.41E-26 |
| ENSSSCG00000046592 | 1.54E-20 | -2.753669524 | 0.938 | 1 | 2.67E-16 |
| ENSSSCG00000054115 | 9.59E-57 | -2.75729124 | 1 | 1 | 1.66E-52 |
| GINS2 | 1.93E-38 | -2.758285966 | 1 | 1 | 3.36E-34 |
| AJUBA | 5.41E-27 | -2.760065277 | 0.938 | 1 | 9.39E-23 |
| ECHS1 | 8.66E-14 | -2.760964913 | 0.75 | 0.933 | 1.50E-09 |
| TCIRG1 | 2.45E-17 | -2.761487913 | 0.75 | 1 | 4.25E-13 |
| PRRT3 | 4.95E-08 | -2.765534746 | 0.375 | 0.867 | 0.000858689 |
| MAP4K1 | 2.53E-10 | -2.768674454 | 0.625 | 0.933 | 4.40E-06 |
| ENSSSCG00000029003 | 3.25E-13 | -2.769219793 | 0.812 | 1 | 5.64E-09 |
| FHIP2B | 3.46E-07 | -2.77118131 | 0.375 | 0.733 | 0.006008928 |
| UROD | 3.96E-11 | -2.774933444 | 0.688 | 1 | 6.87E-07 |
| WDR74 | 1.52E-23 | -2.777013755 | 0.812 | 1 | 2.64E-19 |
| GNB1L | 3.23E-07 | -2.777607579 | 0.312 | 0.8 | 0.005607337 |
| ENSSSCG00000057721 | 2.32E-06 | -2.777607579 | 0.25 | 0.733 | 0.040188179 |
| SMIM12 | 1.61E-13 | -2.78181983 | 0.812 | 0.933 | 2.80E-09 |
| GABARAPL1 | 7.28E-10 | -2.783424905 | 0.438 | 0.933 | 1.26E-05 |
| TMEM205 | 2.31E-09 | -2.783424905 | 0.688 | 0.867 | 4.00E-05 |
| ENSSSCG00000021041 | 1.42E-06 | -2.783424905 | 0.312 | 0.733 | 0.024657452 |
| RPS7 | 2.20E-19 | -2.784423886 | 0.875 | 1 | 3.82E-15 |
| TMEM42 | 1.09E-18 | -2.785600369 | 0.938 | 1 | 1.89E-14 |
| NDUFB10 | 3.01E-10 | -2.790546634 | 0.75 | 0.933 | 5.22E-06 |
| SSMEM1 | 2.22E-18 | -2.793549123 | 0.938 | 1 | 3.85E-14 |
| SNRPD2 | 2.19E-13 | -2.793549123 | 0.812 | 1 | 3.80E-09 |
| MRPL54 | 2.88E-23 | -2.796286685 | 0.875 | 1 | 4.99E-19 |
| POLE4 | 1.84E-30 | -2.800647975 | 1 | 1 | 3.19E-26 |
| DYDC1 | 8.75E-28 | -2.802060622 | 0.938 | 1 | 1.52E-23 |
| SLC25A39 | 1.13E-11 | -2.802060622 | 0.688 | 0.933 | 1.97E-07 |
| NDUFS3 | 1.12E-23 | -2.803482798 | 0.812 | 1 | 1.95E-19 |
| NDUFS8 | 1.59E-07 | -2.803602787 | 0.25 | 0.8 | 0.002762909 |
| MRPS12 | 1.34E-06 | -2.803602787 | 0.312 | 0.867 | 0.023333024 |
| ALAD | 7.55E-14 | -2.805827452 | 0.688 | 1 | 1.31E-09 |
| KANK3 | 3.00E-08 | -2.805827452 | 0.438 | 0.867 | 0.000519988 |
| ENSSSCG00000058372 | 2.35E-07 | -2.805827452 | 0.438 | 0.867 | 0.00408124 |
| COPS3 | 1.36E-24 | -2.806991385 | 0.938 | 1 | 2.36E-20 |
| ENSSSCG00000040118 | 2.95E-07 | -2.809316438 | 0.312 | 0.867 | 0.005123008 |
| DDRGK1 | 1.53E-09 | -2.810966176 | 0.438 | 0.933 | 2.66E-05 |
| ENSSSCG00000038404 | 4.46E-10 | -2.815575429 | 0.875 | 1 | 7.74E-06 |
| MCAT | 2.40E-09 | -2.815575429 | 0.625 | 0.867 | 4.17E-05 |
| EEF1B2 | 1.22E-32 | -2.816700776 | 1 | 1 | 2.11E-28 |
| YTHDF1 | 1.35E-16 | -2.817706441 | 0.688 | 1 | 2.35E-12 |
| DMGDH | 1.68E-11 | -2.818694771 | 0.875 | 1 | 2.91E-07 |
| TARBP2 | 1.43E-08 | -2.823502345 | 0.5 | 0.8 | 0.000248448 |

| | | | | | |
|---|---|---|---|---|---|
| GUCA1A | 6.23E-27 | -2.830074999 | 1 | 1 | 1.08E-22 |
| DYNC2I2 | 2.97E-10 | -2.830074999 | 0.5 | 0.867 | 5.16E-06 |
| ENSSSCG00000050595 | 1.15E-06 | -2.830074999 | 0.312 | 0.733 | 0.019875044 |
| AGA | 2.08E-12 | -2.831751576 | 0.562 | 1 | 3.61E-08 |
| CBR3 | 4.82E-36 | -2.833538854 | 1 | 1 | 8.35E-32 |
| NSDHL | 2.01E-46 | -2.836175198 | 1 | 1 | 3.48E-42 |
| LYRM4 | 2.63E-13 | -2.840343334 | 0.688 | 0.933 | 4.57E-09 |
| RNF166 | 2.68E-06 | -2.840343334 | 0.312 | 0.733 | 0.046450154 |
| COX5A | 1.98E-33 | -2.844683085 | 1 | 1 | 3.43E-29 |
| MEA1 | 2.30E-22 | -2.845181891 | 0.938 | 1 | 3.99E-18 |
| RPL11 | 2.59E-17 | -2.846162942 | 1 | 1 | 4.50E-13 |
| IMMP2L | 2.02E-20 | -2.847996907 | 0.938 | 1 | 3.50E-16 |
| MST1 | 7.39E-09 | -2.847996907 | 0.812 | 0.867 | 0.000128151 |
| COX6C | 1.59E-20 | -2.84820373 | 1 | 1 | 2.76E-16 |
| SWSAP1 | 3.59E-20 | -2.852569636 | 0.938 | 0.933 | 6.24E-16 |
| ATP5MK | 3.51E-19 | -2.856099887 | 1 | 1 | 6.09E-15 |
| CCS | 3.28E-07 | -2.862496476 | 0.25 | 0.867 | 0.005691302 |
| PDLIM7 | 1.18E-06 | -2.862496476 | 0.375 | 0.8 | 0.020443019 |
| GPD1 | 1.08E-14 | -2.863351153 | 0.812 | 1 | 1.87E-10 |
| MPV17L2 | 1.08E-06 | -2.865698908 | 0.25 | 0.867 | 0.018653594 |
| DNASE2 | 1.16E-10 | -2.873328197 | 0.438 | 0.933 | 2.01E-06 |
| PRXL2B | 2.16E-06 | -2.874469118 | 0.375 | 0.733 | 0.037449795 |
| MRPL2 | 1.38E-13 | -2.875517969 | 0.688 | 0.867 | 2.40E-09 |
| LRRC61 | 1.96E-08 | -2.877380713 | 0.312 | 0.933 | 0.000340228 |
| ENSSSCG00000047189 | 1.15E-14 | -2.878984599 | 0.688 | 1 | 2.00E-10 |
| ISCA2 | 6.28E-20 | -2.882816461 | 0.75 | 1 | 1.09E-15 |
| GPS1 | 1.82E-16 | -2.887525271 | 0.75 | 1 | 3.15E-12 |
| PMM1 | 6.12E-10 | -2.900464326 | 0.312 | 0.933 | 1.06E-05 |
| FAM174C | 3.47E-08 | -2.900464326 | 0.375 | 0.867 | 0.000602641 |
| RACK1 | 1.98E-06 | -2.902380256 | 0.938 | 1 | 0.034348173 |
| MRPL51 | 3.98E-36 | -2.904937803 | 1 | 1 | 6.91E-32 |
| CTSD | 1.63E-08 | -2.910732662 | 0.375 | 0.933 | 0.000282431 |
| CERS4 | 9.10E-13 | -2.911414626 | 0.938 | 1 | 1.58E-08 |
| MAST2 | 1.54E-16 | -2.915111102 | 0.812 | 1 | 2.68E-12 |
| NUDT2 | 7.53E-16 | -2.915839552 | 0.75 | 0.933 | 1.31E-11 |
| ENSSSCG00000023691 | 1.75E-43 | -2.916550707 | 0.938 | 1 | 3.04E-39 |
| MRPL17 | 1.28E-21 | -2.91707904 | 0.688 | 1 | 2.22E-17 |
| ELAC2 | 1.38E-12 | -2.918275603 | 0.625 | 1 | 2.40E-08 |
| GGH | 2.93E-33 | -2.920516485 | 1 | 1 | 5.09E-29 |
| GADD45GIP1 | 3.27E-18 | -2.920928429 | 0.688 | 1 | 5.67E-14 |
| EDF1 | 7.78E-10 | -2.923184403 | 0.438 | 1 | 1.35E-05 |
| SLC47A1 | 1.11E-13 | -2.925999419 | 0.562 | 0.933 | 1.92E-09 |
| IDI1 | 9.56E-39 | -2.926917122 | 1 | 1 | 1.66E-34 |
| NELFE | 3.56E-17 | -2.927685795 | 0.688 | 1 | 6.17E-13 |
| TEX35 | 1.86E-22 | -2.928050158 | 0.812 | 1 | 3.24E-18 |
| TCL1B | 3.19E-17 | -2.928167255 | 1 | 1 | 5.53E-13 |
| ENSSSCG00000048488 | 1.47E-07 | -2.929610672 | 0.25 | 0.8 | 0.002553335 |
| ADAD2 | 3.32E-07 | -2.934411658 | 0.25 | 0.867 | 0.005752004 |
| COX17 | 1.63E-14 | -2.935568127 | 0.688 | 1 | 2.83E-10 |
| TNNC2 | 4.13E-14 | -2.935869663 | 0.625 | 0.933 | 7.16E-10 |
| PSME2 | 1.00E-09 | -2.938599455 | 0.438 | 0.933 | 1.74E-05 |
| PSMD13 | 4.61E-59 | -2.947201482 | 0.938 | 1 | 8.00E-55 |
| GDPD5 | 7.63E-22 | -2.949373927 | 0.75 | 1 | 1.32E-17 |
| INO80B | 7.27E-07 | -2.9510904 | 0.188 | 0.867 | 0.012614057 |
| ENSSSCG00000006581 | 8.50E-07 | -2.9510904 | 0.188 | 0.867 | 0.014742939 |
| EMC3 | 6.36E-17 | -2.957295549 | 0.812 | 1 | 1.10E-12 |
| ENSSSCG00000060068 | 8.27E-07 | -2.958179824 | 0.125 | 0.933 | 0.014348589 |
| RPS16 | 1.41E-09 | -2.962322797 | 0.938 | 1 | 2.45E-05 |
| ENSSSCG00000003930 | 2.38E-12 | -2.964510915 | 0.938 | 1 | 4.13E-08 |
| ACAD9 | 2.27E-25 | -2.965234582 | 0.812 | 1 | 3.94E-21 |
| AP1M2 | 1.44E-22 | -2.966503088 | 0.812 | 1 | 2.50E-18 |
| ENSSSCG00000034019 | 6.27E-17 | -2.968973104 | 1 | 1 | 1.09E-12 |
| GRIN3B | 2.90E-11 | -2.970426885 | 0.562 | 0.933 | 5.03E-07 |
| MRGBP | 7.61E-10 | -2.975752454 | 0.438 | 0.8 | 1.32E-05 |
| NUDC | 9.59E-29 | -2.976106794 | 0.938 | 1 | 1.66E-24 |
| SLC25A38 | 9.15E-26 | -2.978279803 | 0.875 | 1 | 1.59E-21 |

| | | | | | |
|---|---|---|---|---|---|
| SAL1 | 1.21E-09 | -2.982926487 | 0.562 | 1 | 2.10E-05 |
| RPS29 | 2.25E-10 | -2.984960601 | 0.75 | 1 | 3.90E-06 |
| TMEM134 | 2.99E-07 | -2.9861942 | 0.188 | 0.867 | 0.005190446 |
| SPATA19 | 3.48E-07 | -2.9861942 | 0.375 | 0.733 | 0.006034942 |
| HSD17B10 | 3.53E-14 | -2.988772745 | 0.812 | 0.933 | 6.13E-10 |
| SLC6A12 | 2.19E-07 | -2.989273593 | 0.25 | 0.8 | 0.003792175 |
| ENSSSCG00000027573 | 1.70E-14 | -2.993113614 | 0.875 | 1 | 2.95E-10 |
| SUGP1 | 9.41E-10 | -3 | 0.5 | 0.933 | 1.63E-05 |
| SDS | 7.48E-07 | -3 | 0.188 | 0.8 | 0.01298216 |
| ENSSSCG00000053208 | 1.97E-06 | -3 | 0.312 | 0.733 | 0.03417565 |
| LRRC47 | 3.27E-16 | -3.007777351 | 0.625 | 1 | 5.67E-12 |
| ACOT13 | 2.98E-08 | -3.010647244 | 0.375 | 0.867 | 0.000517156 |
| YIF1A | 1.07E-07 | -3.010647244 | 0.5 | 0.867 | 0.001863935 |
| ATP5ME | 1.44E-11 | -3.013674937 | 0.938 | 1 | 2.49E-07 |
| NUDT18 | 1.07E-06 | -3.013674937 | 0.312 | 0.733 | 0.018549614 |
| ENSSSCG00000031249 | 3.91E-18 | -3.018204595 | 0.812 | 1 | 6.78E-14 |
| TMED3 | 2.50E-20 | -3.020647151 | 0.625 | 1 | 4.34E-16 |
| VTI1B | 6.98E-70 | -3.022510367 | 1 | 1 | 1.21E-65 |
| COPZ1 | 3.32E-47 | -3.028014376 | 1 | 1 | 5.76E-43 |
| ENSSSCG00000052480 | 1.49E-06 | -3.03170886 | 0.25 | 0.8 | 0.0259386 |
| ARG2 | 3.06E-27 | -3.033608393 | 0.875 | 1 | 5.31E-23 |
| ARR3 | 6.35E-12 | -3.033730857 | 0.625 | 0.933 | 1.10E-07 |
| ENSSSCG00000046487 | 1.49E-18 | -3.034765418 | 0.75 | 1 | 2.59E-14 |
| EIF3F | 3.66E-10 | -3.036525876 | 0.312 | 0.933 | 6.35E-06 |
| LRPAP1 | 8.61E-15 | -3.040641984 | 0.5 | 0.933 | 1.49E-10 |
| TRPT1 | 6.65E-08 | -3.040641984 | 0.188 | 0.867 | 0.001154201 |
| ENSSSCG00000040146 | 1.42E-06 | -3.044199804 | 0.438 | 0.933 | 0.02470248 |
| PAIP2 | 2.53E-29 | -3.047305715 | 1 | 1 | 4.40E-25 |
| MFSD12 | 3.70E-10 | -3.047305715 | 0.625 | 0.867 | 6.42E-06 |
| REN | 2.56E-06 | -3.047305715 | 0.188 | 0.8 | 0.044487341 |
| PDZD11 | 7.66E-26 | -3.053363226 | 0.938 | 1 | 1.33E-21 |
| LSM10 | 6.93E-35 | -3.055742552 | 1 | 1 | 1.20E-30 |
| TPT1 | 2.62E-22 | -3.057143907 | 0.875 | 1 | 4.55E-18 |
| RAB4B | 8.69E-14 | -3.058005603 | 0.75 | 1 | 1.51E-09 |
| STYXL1 | 6.49E-43 | -3.058661912 | 0.938 | 1 | 1.13E-38 |
| ENSSSCG00000033019 | 1.06E-09 | -3.061400545 | 0.812 | 1 | 1.84E-05 |
| RPS4X | 1.74E-15 | -3.062348377 | 1 | 1 | 3.02E-11 |
| ENSSSCG00000017955 | 1.33E-21 | -3.064780681 | 0.688 | 1 | 2.31E-17 |
| ACTR1B | 5.30E-13 | -3.067114196 | 0.562 | 1 | 9.19E-09 |
| ENSSSCG00000039612 | 1.80E-10 | -3.068306013 | 0.562 | 1 | 3.13E-06 |
| ADI1 | 8.36E-08 | -3.070389328 | 0.312 | 0.867 | 0.001450794 |
| CCDC124 | 7.78E-12 | -3.074000581 | 0.625 | 0.933 | 1.35E-07 |
| C15orf40 | 7.17E-10 | -3.074962058 | 0.375 | 0.867 | 1.24E-05 |
| ENSSSCG00000012100 | 9.90E-15 | -3.075995384 | 0.688 | 1 | 1.72E-10 |
| ZNF821 | 1.09E-19 | -3.080170349 | 0.812 | 1 | 1.89E-15 |
| DAD1 | 1.93E-30 | -3.080472579 | 0.938 | 1 | 3.34E-26 |
| ENSSSCG00000008016 | 6.55E-11 | -3.081036572 | 0.438 | 1 | 1.14E-06 |
| CIB2 | 7.21E-11 | -3.081036572 | 0.375 | 0.933 | 1.25E-06 |
| CCDC32 | 1.63E-21 | -3.082248412 | 0.75 | 0.933 | 2.83E-17 |
| ENSSSCG00000032916 | 1.84E-33 | -3.085020584 | 1 | 1 | 3.19E-29 |
| ENSSSCG00000039523 | 3.75E-37 | -3.090659313 | 1 | 1 | 6.51E-33 |
| PCID2 | 9.28E-20 | -3.093109404 | 0.812 | 1 | 1.61E-15 |
| UBXN1 | 1.19E-08 | -3.093109404 | 0.312 | 0.733 | 0.000205971 |
| NANS | 9.31E-07 | -3.093109404 | 0.5 | 0.733 | 0.016158842 |
| ENSSSCG00000025673 | 6.86E-23 | -3.097667686 | 0.875 | 1 | 1.19E-18 |
| TSPAN31 | 6.10E-26 | -3.098110085 | 0.812 | 1 | 1.06E-21 |
| TDRD10 | 1.45E-24 | -3.101440679 | 0.938 | 1 | 2.52E-20 |
| SCNM1 | 9.75E-22 | -3.102173487 | 1 | 1 | 1.69E-17 |
| ENSSSCG00000053939 | 2.51E-20 | -3.103678646 | 0.875 | 1 | 4.35E-16 |
| PTPMT1 | 3.09E-26 | -3.107815903 | 0.812 | 1 | 5.37E-22 |
| HSBP1 | 2.19E-50 | -3.109274224 | 1 | 1 | 3.80E-46 |
| U2AF1L4 | 7.50E-09 | -3.113008962 | 0.375 | 0.933 | 0.000130079 |
| ENSSSCG00000009610 | 4.17E-36 | -3.116655702 | 1 | 1 | 7.23E-32 |
| ACTL7B | 3.83E-10 | -3.121306296 | 0.375 | 0.933 | 6.65E-06 |
| COX14 | 5.33E-15 | -3.124587636 | 0.688 | 1 | 9.25E-11 |
| TMEM86A | 1.20E-06 | -3.126124462 | 0.312 | 0.933 | 0.020824999 |

| | | | | | |
|---|---|---|---|---|---|
| ASIP | 5.61E-07 | -3.128733314 | 0.25 | 0.733 | 0.009738643 |
| PSENEN | 3.53E-15 | -3.135995217 | 0.875 | 1 | 6.13E-11 |
| CSTB | 1.25E-13 | -3.137503524 | 0.75 | 0.933 | 2.17E-09 |
| DRC7 | 1.53E-12 | -3.137503524 | 0.438 | 1 | 2.65E-08 |
| ENSSSCG00000048663 | 1.37E-06 | -3.137503524 | 0.188 | 0.8 | 0.023812538 |
| CRHR1 | 1.60E-17 | -3.137870855 | 0.938 | 1 | 2.78E-13 |
| CFAP276 | 6.76E-09 | -3.143735477 | 0.25 | 0.933 | 0.000117337 |
| CDIPT | 4.47E-38 | -3.147741853 | 1 | 1 | 7.75E-34 |
| ENSSSCG00000002963 | 3.16E-32 | -3.149940627 | 0.938 | 1 | 5.48E-28 |
| RPS17 | 8.76E-12 | -3.150011796 | 1 | 1 | 1.52E-07 |
| GSTZ1 | 3.10E-11 | -3.152003093 | 0.375 | 1 | 5.39E-07 |
| INSL6 | 8.26E-08 | -3.152003093 | 0.375 | 0.8 | 0.00143348 |
| PIN1 | 1.81E-07 | -3.152003093 | 0.25 | 0.867 | 0.003140481 |
| ENSSSCG00000037510 | 1.35E-22 | -3.152890633 | 0.938 | 1 | 2.34E-18 |
| ENSSSCG00000056716 | 9.23E-28 | -3.159198595 | 0.875 | 1 | 1.60E-23 |
| ENSSSCG00000052563 | 1.23E-08 | -3.159198595 | 0.25 | 0.867 | 0.000213917 |
| FDPS | 4.57E-22 | -3.160032957 | 0.875 | 1 | 7.92E-18 |
| ENSSSCG00000038035 | 1.13E-27 | -3.163058282 | 0.938 | 1 | 1.97E-23 |
| KAT5 | 3.23E-11 | -3.166358386 | 0.5 | 0.867 | 5.61E-07 |
| ERCC1 | 1.47E-12 | -3.169925001 | 0.438 | 1 | 2.55E-08 |
| SIVA1 | 2.93E-11 | -3.169925001 | 0.438 | 1 | 5.08E-07 |
| TFDP2 | 2.75E-84 | -3.177096336 | 1 | 1 | 4.78E-80 |
| NENF | 2.38E-10 | -3.180572246 | 0.188 | 0.933 | 4.13E-06 |
| RND2 | 4.14E-09 | -3.180572246 | 0.25 | 0.933 | 7.18E-05 |
| ANTKMT | 1.19E-08 | -3.180572246 | 0.188 | 0.933 | 0.000205969 |
| MFGE8 | 8.26E-07 | -3.180572246 | 0.188 | 0.733 | 0.014334346 |
| C11orf98 | 3.41E-42 | -3.182719114 | 1 | 1 | 5.92E-38 |
| POP4 | 1.93E-35 | -3.189970944 | 0.938 | 1 | 3.35E-31 |
| CDH22 | 1.34E-11 | -3.192645078 | 0.5 | 0.933 | 2.32E-07 |
| ENSSSCG00000056115 | 5.21E-07 | -3.192645078 | 0.25 | 0.667 | 0.009036211 |
| DCAF11 | 2.24E-12 | -3.194647431 | 0.75 | 0.933 | 3.88E-08 |
| TUBGCP2 | 8.77E-16 | -3.195724471 | 0.688 | 0.933 | 1.52E-11 |
| MACROD1 | 6.13E-21 | -3.202733896 | 0.75 | 1 | 1.06E-16 |
| ENSSSCG00000056015 | 4.35E-15 | -3.204140717 | 0.75 | 1 | 7.56E-11 |
| ENSSSCG00000053975 | 1.22E-08 | -3.208586622 | 0.25 | 0.933 | 0.000211856 |
| FAAP100 | 7.40E-08 | -3.208586622 | 0.25 | 0.733 | 0.001284215 |
| PQBP1 | 1.52E-16 | -3.21649182 | 0.75 | 1 | 2.63E-12 |
| MRPS25 | 1.38E-37 | -3.21897236 | 1 | 1 | 2.40E-33 |
| EPHX1 | 1.72E-19 | -3.222392421 | 0.688 | 0.933 | 2.99E-15 |
| SDHAF1 | 2.14E-13 | -3.228268988 | 0.375 | 1 | 3.71E-09 |
| TSR3 | 4.82E-07 | -3.230612928 | 0.25 | 0.8 | 0.00836956 |
| ASPDH | 4.99E-08 | -3.236067358 | 0.312 | 0.8 | 0.000866098 |
| NDUFA9 | 9.69E-35 | -3.241753486 | 1 | 1 | 1.68E-30 |
| ENSSSCG00000034853 | 9.52E-13 | -3.242856524 | 0.625 | 1 | 1.65E-08 |
| ENSSSCG00000041579 | 1.11E-24 | -3.245833665 | 0.875 | 1 | 1.93E-20 |
| SMPD2 | 4.85E-15 | -3.254101281 | 0.75 | 0.933 | 8.41E-11 |
| ENSSSCG00000010056 | 1.26E-26 | -3.254997087 | 1 | 1 | 2.18E-22 |
| EIF6 | 6.33E-30 | -3.256408929 | 0.875 | 1 | 1.10E-25 |
| ENSSSCG00000052671 | 1.23E-12 | -3.261271797 | 0.938 | 1 | 2.14E-08 |
| KLF16 | 3.15E-07 | -3.263034406 | 0.25 | 0.733 | 0.005461918 |
| MRPL36 | 5.17E-22 | -3.268648012 | 0.75 | 1 | 8.96E-18 |
| RPS25 | 1.13E-21 | -3.271284293 | 1 | 1 | 1.97E-17 |
| CNIH4 | 9.73E-58 | -3.272066477 | 1 | 1 | 1.69E-53 |
| SCRG1 | 2.85E-17 | -3.273018494 | 0.688 | 0.933 | 4.94E-13 |
| NPAS1 | 3.20E-11 | -3.275312736 | 0.5 | 0.933 | 5.56E-07 |
| TMEM126A | 2.18E-25 | -3.275471509 | 0.938 | 1 | 3.78E-21 |
| ENSSSCG00000033744 | 1.56E-10 | -3.27897595 | 0.312 | 0.933 | 2.70E-06 |
| WDR5 | 1.74E-41 | -3.285333073 | 0.875 | 1 | 3.03E-37 |
| POLR3H | 1.13E-34 | -3.287064475 | 1 | 1 | 1.96E-30 |
| ASGR2 | 4.65E-10 | -3.294743266 | 0.312 | 0.867 | 8.07E-06 |
| ENSSSCG00000054357 | 4.65E-07 | -3.294743266 | 0.25 | 0.8 | 0.008070855 |
| TMEM225B | 3.22E-17 | -3.296049463 | 0.75 | 0.933 | 5.58E-13 |
| RPS21 | 7.39E-07 | -3.296049463 | 0.625 | 0.867 | 0.012830063 |
| TMEM179B | 1.97E-15 | -3.298223834 | 0.438 | 1 | 3.41E-11 |
| RFXANK | 9.87E-07 | -3.298223834 | 0.125 | 0.867 | 0.017128917 |
| RPP40 | 1.52E-21 | -3.299363179 | 0.812 | 1 | 2.64E-17 |

| | | | | | |
|---|---|---|---|---|---|
| MIF | 8.42E-13 | -3.300704824 | 0.625 | 1 | 1.46E-08 |
| ENSSSCG00000026248 | 3.72E-11 | -3.30256277 | 0.562 | 0.8 | 6.46E-07 |
| PRSS2 | 8.43E-20 | -3.304854582 | 0.812 | 1 | 1.46E-15 |
| ENSSSCG00000057874 | 1.77E-34 | -3.306103128 | 1 | 1 | 3.08E-30 |
| CDT1 | 3.01E-08 | -3.306103128 | 0.25 | 0.867 | 0.000522439 |
| ENSSSCG00000052576 | 1.74E-10 | -3.308122295 | 0.375 | 0.867 | 3.02E-06 |
| PSMB7 | 4.23E-38 | -3.308349922 | 1 | 1 | 7.35E-34 |
| LMO2 | 1.01E-12 | -3.310340121 | 0.375 | 1 | 1.75E-08 |
| MYBPC3 | 1.19E-09 | -3.310340121 | 0.375 | 0.867 | 2.06E-05 |
| ENSSSCG00000035909 | 2.97E-40 | -3.31177874 | 0.938 | 1 | 5.15E-36 |
| NEDD8 | 1.95E-36 | -3.312857104 | 1 | 1 | 3.38E-32 |
| SNRNP25 | 1.92E-13 | -3.315501826 | 0.438 | 0.933 | 3.33E-09 |
| ITGB5 | 1.14E-29 | -3.323575948 | 0.938 | 1 | 1.97E-25 |
| HSF4 | 1.84E-10 | -3.325770161 | 0.438 | 0.8 | 3.20E-06 |
| ENSSSCG00000024588 | 1.37E-41 | -3.330148602 | 1 | 1 | 2.38E-37 |
| NAXE | 1.16E-27 | -3.332180567 | 0.75 | 1 | 2.01E-23 |
| PSMA6 | 7.79E-67 | -3.33267553 | 1 | 1 | 1.35E-62 |
| FETUB | 2.57E-19 | -3.333021925 | 0.688 | 1 | 4.45E-15 |
| TNIP1 | 2.47E-09 | -3.334117504 | 0.25 | 0.867 | 4.28E-05 |
| SKIC8 | 9.03E-58 | -3.334381207 | 0.938 | 1 | 1.57E-53 |
| CTSF | 4.80E-13 | -3.335184192 | 0.375 | 0.933 | 8.33E-09 |
| CLPP | 2.11E-21 | -3.338865819 | 0.688 | 1 | 3.66E-17 |
| SRM | 2.14E-14 | -3.341036918 | 0.625 | 0.933 | 3.72E-10 |
| HYAL3 | 1.75E-06 | -3.341036918 | 0.188 | 0.733 | 0.030282698 |
| C1orf105 | 3.62E-27 | -3.343846451 | 0.875 | 1 | 6.27E-23 |
| TCEANC | 2.27E-08 | -3.351843673 | 0.25 | 0.733 | 0.000393546 |
| ENSSSCG00000063358 | 3.10E-08 | -3.35614381 | 0.125 | 0.867 | 0.000537218 |
| TIMM22 | 1.85E-41 | -3.358127286 | 0.938 | 1 | 3.20E-37 |
| MRPL37 | 2.25E-50 | -3.359639762 | 1 | 1 | 3.90E-46 |
| ENSSSCG00000013064 | 1.65E-28 | -3.364342109 | 0.938 | 1 | 2.86E-24 |
| SURF2 | 1.29E-07 | -3.366127899 | 0.188 | 0.733 | 0.002230672 |
| TUBB4B | 7.57E-19 | -3.375663261 | 0.625 | 1 | 1.31E-14 |
| RPL23 | 6.56E-09 | -3.379266368 | 0.812 | 1 | 0.000113782 |
| PDLIM2 | 1.11E-11 | -3.385891154 | 0.312 | 0.933 | 1.92E-07 |
| ARSL | 6.84E-08 | -3.385891154 | 0.25 | 0.8 | 0.001186894 |
| PMVK | 4.39E-07 | -3.385891154 | 0.125 | 0.8 | 0.007621551 |
| ZP4 | 6.97E-45 | -3.388611949 | 1 | 1 | 1.21E-40 |
| SPATA24 | 4.34E-08 | -3.394278939 | 0.188 | 0.733 | 0.000753104 |
| ENSSSCG00000052310 | 3.13E-22 | -3.395408693 | 0.812 | 1 | 5.44E-18 |
| PLD6 | 2.15E-08 | -3.396890153 | 0.25 | 0.867 | 0.00037365 |
| DCTN3 | 6.64E-22 | -3.398917834 | 0.938 | 1 | 1.15E-17 |
| ARL2 | 9.27E-11 | -3.401862111 | 0.25 | 0.933 | 1.61E-06 |
| DPCD | 3.32E-27 | -3.404832717 | 1 | 1 | 5.76E-23 |
| SHFL | 3.86E-09 | -3.415037499 | 0.25 | 0.867 | 6.70E-05 |
| ENSSSCG00000048122 | 2.75E-08 | -3.415037499 | 0.188 | 0.867 | 0.00047779 |
| HSD17B2 | 1.95E-06 | -3.415037499 | 0.125 | 0.8 | 0.033765532 |
| ENSSSCG00000032129 | 8.37E-36 | -3.418551983 | 1 | 1 | 1.45E-31 |
| NDUFAB1 | 3.42E-11 | -3.428093652 | 0.625 | 0.933 | 5.93E-07 |
| LENG1 | 2.84E-15 | -3.431911318 | 0.5 | 1 | 4.93E-11 |
| UQCRQ | 6.43E-31 | -3.437593624 | 1 | 1 | 1.12E-26 |
| TMSB10 | 5.15E-07 | -3.440877504 | 0.812 | 1 | 0.008934754 |
| DPP7 | 3.39E-10 | -3.446746359 | 0.312 | 0.867 | 5.89E-06 |
| RNF31 | 2.21E-16 | -3.449802917 | 0.688 | 1 | 3.84E-12 |
| EIPR1 | 1.92E-16 | -3.450661409 | 0.562 | 1 | 3.34E-12 |
| IGFBP6 | 3.90E-11 | -3.450661409 | 0.375 | 0.933 | 6.77E-07 |
| BCKDHA | 2.40E-25 | -3.452259963 | 0.938 | 1 | 4.17E-21 |
| ATP5F1D | 4.43E-11 | -3.462343214 | 0.375 | 0.867 | 7.69E-07 |
| DCXR | 1.03E-07 | -3.462343214 | 0.25 | 0.667 | 0.001784623 |
| DPPA5 | 2.51E-37 | -3.463134425 | 1 | 1 | 4.35E-33 |
| RPL36 | 8.79E-21 | -3.4639471 | 0.75 | 1 | 1.53E-16 |
| VKORC1 | 3.53E-30 | -3.464926375 | 0.75 | 1 | 6.12E-26 |
| LUC7L | 5.63E-11 | -3.468148836 | 0.312 | 0.933 | 9.76E-07 |
| ENSSSCG00000039365 | 1.40E-07 | -3.471621028 | 0.25 | 0.667 | 0.002424684 |
| FKBP2 | 4.19E-07 | -3.471621028 | 0.188 | 0.667 | 0.007265236 |
| ENSSSCG00000033451 | 5.26E-07 | -3.471621028 | 0.188 | 0.733 | 0.009125773 |
| MKS1 | 6.15E-18 | -3.474303449 | 0.625 | 1 | 1.07E-13 |

| | | | | | |
|---|---|---|---|---|---|
| KLK15 | 7.81E-09 | -3.475579041 | 0.125 | 0.867 | 0.000135582 |
| ENSSSCG00000053447 | 9.85E-16 | -3.481806511 | 0.438 | 1 | 1.71E-11 |
| ENSSSCG00000044091 | 3.60E-07 | -3.485426827 | 0.125 | 0.8 | 0.006239949 |
| GSDMB | 2.93E-46 | -3.485642171 | 1 | 1 | 5.09E-42 |
| NDUFC2 | 1.02E-16 | -3.492640317 | 0.625 | 1 | 1.78E-12 |
| ENSSSCG00000028414 | 1.19E-40 | -3.493621187 | 1 | 1 | 2.06E-36 |
| ENSSSCG00000058091 | 6.23E-21 | -3.494359952 | 0.812 | 1 | 1.08E-16 |
| PDIA2 | 5.09E-09 | -3.499101764 | 0.25 | 0.867 | 8.84E-05 |
| NUBP2 | 7.95E-10 | -3.504042505 | 0.375 | 0.867 | 1.38E-05 |
| FTL | 4.04E-39 | -3.506178543 | 1 | 1 | 7.02E-35 |
| THOP1 | 2.45E-08 | -3.508146904 | 0.312 | 0.867 | 0.000425137 |
| UQCR10 | 3.67E-23 | -3.520970944 | 0.875 | 1 | 6.37E-19 |
| CYTH2 | 4.56E-32 | -3.523840711 | 1 | 1 | 7.91E-28 |
| UQCC2 | 1.28E-17 | -3.526068812 | 0.5 | 1 | 2.22E-13 |
| ENSSSCG00000033926 | 2.81E-15 | -3.532221039 | 0.438 | 1 | 4.88E-11 |
| RPL10A | 2.38E-18 | -3.538586852 | 0.938 | 1 | 4.13E-14 |
| COX6A1 | 3.40E-26 | -3.538668848 | 0.875 | 1 | 5.90E-22 |
| CNPY2 | 1.18E-37 | -3.541854344 | 1 | 1 | 2.05E-33 |
| IMP4 | 5.22E-28 | -3.545369368 | 0.812 | 1 | 9.06E-24 |
| IDH3G | 2.03E-18 | -3.552541023 | 0.75 | 1 | 3.52E-14 |
| MRPS18A | 3.59E-08 | -3.552541023 | 0.188 | 0.8 | 0.000622699 |
| RGS11 | 4.35E-07 | -3.552541023 | 0.188 | 0.733 | 0.007553073 |
| NPDC1 | 2.01E-06 | -3.552541023 | 0.125 | 0.733 | 0.034958267 |
| EIF3G | 8.46E-13 | -3.557481764 | 0.688 | 1 | 1.47E-08 |
| ENSSSCG00000021624 | 4.85E-17 | -3.560467939 | 0.625 | 1 | 8.41E-13 |
| CUL7 | 8.33E-32 | -3.56125824 | 0.938 | 1 | 1.45E-27 |
| RPS18 | 1.10E-26 | -3.563041882 | 1 | 1 | 1.90E-22 |
| RPL7 | 3.38E-41 | -3.57740515 | 1 | 1 | 5.87E-37 |
| NDUFA10 | 5.47E-25 | -3.57797607 | 0.688 | 1 | 9.50E-21 |
| ENSSSCG00000004703 | 7.99E-21 | -3.589785373 | 1 | 1 | 1.39E-16 |
| HYI | 2.14E-10 | -3.595609745 | 0.25 | 0.933 | 3.71E-06 |
| C19orf53 | 1.02E-18 | -3.597072183 | 0.562 | 1 | 1.78E-14 |
| SAT2 | 4.96E-27 | -3.600904045 | 0.875 | 1 | 8.60E-23 |
| ENSSSCG00000053801 | 5.31E-09 | -3.606178987 | 0.312 | 0.8 | 9.22E-05 |
| ENSSSCG00000030849 | 4.67E-18 | -3.612794188 | 1 | 1 | 8.10E-14 |
| SIRT5 | 4.23E-13 | -3.621488377 | 0.438 | 0.933 | 7.33E-09 |
| TBL3 | 9.08E-11 | -3.623624121 | 0.188 | 0.933 | 1.58E-06 |
| ENSSSCG00000018046 | 2.00E-24 | -3.623946691 | 0.875 | 1 | 3.47E-20 |
| CCDC28B | 2.65E-09 | -3.629162305 | 0.25 | 0.867 | 4.60E-05 |
| CHMP1A | 7.01E-09 | -3.629162305 | 0.125 | 0.867 | 0.000121637 |
| ENSSSCG00000050296 | 6.34E-08 | -3.629162305 | 0.125 | 0.867 | 0.001099562 |
| ENSSSCG00000024070 | 2.86E-48 | -3.633975668 | 0.938 | 1 | 4.95E-44 |
| BCS1L | 1.77E-13 | -3.635636639 | 0.562 | 1 | 3.07E-09 |
| DPEP1 | 9.44E-18 | -3.638245925 | 0.812 | 1 | 1.64E-13 |
| PIGP | 2.71E-53 | -3.652805147 | 1 | 1 | 4.70E-49 |
| ERP29 | 3.49E-22 | -3.653824359 | 0.812 | 1 | 6.06E-18 |
| CDH6 | 4.86E-19 | -3.655351829 | 0.562 | 0.933 | 8.44E-15 |
| DNPH1 | 6.90E-11 | -3.660793914 | 0.25 | 0.933 | 1.20E-06 |
| SYT2 | 2.62E-22 | -3.671377253 | 0.625 | 1 | 4.55E-18 |
| ENSSSCG00000060915 | 6.42E-20 | -3.678071905 | 0.812 | 1 | 1.11E-15 |
| ENSSSCG00000022295 | 8.48E-07 | -3.678071905 | 0.062 | 0.8 | 0.014719965 |
| TIMM17B | 5.42E-29 | -3.693022247 | 0.812 | 1 | 9.41E-25 |
| CCDC137 | 3.66E-12 | -3.693022247 | 0.25 | 0.933 | 6.35E-08 |
| COX4I1 | 3.08E-30 | -3.69743723 | 1 | 1 | 5.34E-26 |
| ZBTB48 | 1.48E-14 | -3.699133521 | 0.375 | 0.933 | 2.56E-10 |
| EEF1D | 2.42E-23 | -3.703351314 | 0.875 | 1 | 4.21E-19 |
| DTYMK | 1.37E-20 | -3.703973769 | 0.625 | 1 | 2.38E-16 |
| SPOUT1 | 3.82E-11 | -3.707819249 | 0.312 | 0.933 | 6.63E-07 |
| PPIB | 1.49E-34 | -3.708211765 | 0.938 | 1 | 2.58E-30 |
| MAPK13 | 7.63E-24 | -3.710204526 | 0.688 | 1 | 1.32E-19 |
| EMC6 | 5.65E-51 | -3.724084303 | 1 | 1 | 9.81E-47 |
| MBD3 | 1.29E-10 | -3.728697978 | 0.25 | 0.867 | 2.24E-06 |
| TLE2 | 2.66E-50 | -3.733296946 | 1 | 1 | 4.62E-46 |
| ENSSSCG00000014569 | 2.39E-17 | -3.73392514 | 0.875 | 1 | 4.15E-13 |
| DEGS2 | 3.75E-29 | -3.737548384 | 0.875 | 1 | 6.51E-25 |
| RPL7L1 | 6.73E-57 | -3.737951715 | 1 | 1 | 1.17E-52 |

| | | | | | |
|---|---|---|---|---|---|
| ENSSSCG00000008170 | 1.10E-17 | -3.742034964 | 0.938 | 1 | 1.91E-13 |
| NR2C2AP | 5.52E-41 | -3.742923049 | 0.938 | 1 | 9.58E-37 |
| ENSSSCG00000035007 | 8.18E-15 | -3.748106152 | 1 | 1 | 1.42E-10 |
| ENSSSCG00000005528 | 4.73E-42 | -3.751728947 | 1 | 1 | 8.21E-38 |
| ENSSSCG00000038475 | 6.36E-20 | -3.756074417 | 0.375 | 1 | 1.10E-15 |
| QPRT | 3.21E-08 | -3.756074417 | 0.062 | 0.867 | 0.000557806 |
| TM4SF5 | 9.21E-08 | -3.756074417 | 0.125 | 0.867 | 0.00159808 |
| TMEM156 | 8.33E-09 | -3.761487913 | 0.25 | 0.733 | 0.000144606 |
| RPS15A | 1.58E-19 | -3.761619696 | 0.875 | 1 | 2.74E-15 |
| AIMP2 | 1.20E-17 | -3.779609932 | 0.5 | 1 | 2.07E-13 |
| RPLP1 | 1.31E-17 | -3.788134272 | 1 | 1 | 2.27E-13 |
| FBL | 4.19E-15 | -3.793549123 | 0.375 | 1 | 7.27E-11 |
| TNK1 | 8.24E-12 | -3.793549123 | 0.188 | 1 | 1.43E-07 |
| GDF3 | 3.85E-10 | -3.793549123 | 0.5 | 0.8 | 6.67E-06 |
| TUT1 | 2.54E-09 | -3.793549123 | 0.188 | 0.867 | 4.42E-05 |
| FAM98C | 4.25E-07 | -3.793549123 | 0.125 | 0.8 | 0.007376677 |
| CASQ2 | 3.59E-19 | -3.796716402 | 0.625 | 1 | 6.22E-15 |
| TMEM11 | 1.24E-11 | -3.802060622 | 0.312 | 0.933 | 2.15E-07 |
| UTS2 | 1.49E-10 | -3.804604311 | 0.188 | 0.933 | 2.58E-06 |
| COMMD9 | 9.11E-28 | -3.814644958 | 0.812 | 1 | 1.58E-23 |
| MRPL40 | 4.88E-41 | -3.820266461 | 1 | 1 | 8.46E-37 |
| NPEPL1 | 9.80E-09 | -3.821029859 | 0.125 | 0.8 | 0.000170093 |
| SNX22 | 8.80E-27 | -3.823502345 | 0.75 | 1 | 1.53E-22 |
| WDR54 | 2.50E-17 | -3.832957507 | 0.438 | 1 | 4.33E-13 |
| LALBA | 2.56E-28 | -3.833141302 | 0.812 | 1 | 4.44E-24 |
| MRPS15 | 2.47E-32 | -3.836501268 | 0.938 | 1 | 4.28E-28 |
| CDC26 | 2.70E-44 | -3.839590794 | 1 | 1 | 4.68E-40 |
| OXLD1 | 2.09E-09 | -3.847996907 | 0.125 | 0.867 | 3.63E-05 |
| ENSSSCG00000049957 | 1.07E-07 | -3.847996907 | 0.062 | 0.733 | 0.001851898 |
| RPS11 | 5.46E-15 | -3.850539101 | 0.938 | 1 | 9.47E-11 |
| TPI1 | 5.88E-32 | -3.851403508 | 0.875 | 1 | 1.02E-27 |
| ENSSSCG00000002995 | 1.28E-15 | -3.865698908 | 0.625 | 1 | 2.23E-11 |
| ENSSSCG00000032073 | 3.28E-08 | -3.874469118 | 0.125 | 0.8 | 0.000568608 |
| LAMTOR2 | 9.49E-24 | -3.888045067 | 0.75 | 1 | 1.65E-19 |
| NDUFB7 | 1.63E-10 | -3.900464326 | 0.25 | 0.867 | 2.82E-06 |
| B3GNTL1 | 4.18E-15 | -3.90902634 | 0.375 | 0.933 | 7.25E-11 |
| RPL35 | 2.46E-19 | -3.910732662 | 0.625 | 1 | 4.27E-15 |
| ENSSSCG00000052096 | 7.38E-12 | -3.915111102 | 0.312 | 0.933 | 1.28E-07 |
| RPS19 | 7.58E-17 | -3.917776875 | 0.875 | 1 | 1.31E-12 |
| RPS12 | 1.06E-14 | -3.920321745 | 0.875 | 1 | 1.84E-10 |
| RPS10 | 1.76E-23 | -3.92372842 | 0.938 | 1 | 3.06E-19 |
| ENSSSCG00000050177 | 2.46E-07 | -3.933212909 | 0.562 | 0.867 | 0.004276993 |
| ENSSSCG00000015337 | 3.76E-32 | -3.937715722 | 0.875 | 1 | 6.52E-28 |
| NACC2 | 2.22E-10 | -3.941106311 | 0.188 | 0.8 | 3.86E-06 |
| FAU | 1.76E-17 | -3.943965965 | 0.5 | 1 | 3.05E-13 |
| LYRM1 | 2.70E-13 | -3.943965965 | 0.312 | 0.867 | 4.68E-09 |
| NDUFB8 | 7.29E-41 | -3.946675592 | 1 | 1 | 1.26E-36 |
| ENSSSCG00000000905 | 1.84E-31 | -3.9492467 | 1 | 1 | 3.19E-27 |
| RPL34 | 8.78E-16 | -3.9510904 | 0.312 | 1 | 1.52E-11 |
| HCRT | 4.93E-18 | -3.95491211 | 0.438 | 1 | 8.56E-14 |
| RPL6 | 1.38E-51 | -3.963033674 | 1 | 1 | 2.39E-47 |
| ENSSSCG00000027538 | 4.31E-23 | -3.969626351 | 0.625 | 1 | 7.48E-19 |
| CDK10 | 1.53E-15 | -3.973527789 | 0.375 | 0.933 | 2.66E-11 |
| GSTK1 | 5.21E-16 | -3.977632187 | 0.5 | 0.933 | 9.04E-12 |
| CDK5RAP3 | 2.97E-25 | -3.986672468 | 0.625 | 1 | 5.14E-21 |
| RHOC | 7.04E-38 | -3.987706724 | 0.875 | 1 | 1.22E-33 |
| LGI1 | 2.64E-12 | -3.987927168 | 0.25 | 0.867 | 4.59E-08 |
| GUSB | 1.82E-06 | -3.987927168 | 0.188 | 0.733 | 0.031602718 |
| CHID1 | 1.50E-11 | -3.989273593 | 0.375 | 0.867 | 2.60E-07 |
| STX8 | 1.41E-70 | -3.993366908 | 1 | 1 | 2.44E-66 |
| RPS19BP1 | 8.70E-08 | -4 | 0.125 | 0.8 | 0.001510242 |
| ENSSSCG00000059030 | 1.65E-06 | -4 | 0.062 | 0.733 | 0.028547392 |
| ENSSSCG00000021991 | 2.03E-12 | -4.013674937 | 0.312 | 0.867 | 3.52E-08 |
| TAGLN | 9.42E-21 | -4.017921908 | 0.562 | 0.933 | 1.63E-16 |
| ENSSSCG00000036716 | 8.69E-20 | -4.038732394 | 0.75 | 1 | 1.51E-15 |
| ITGB7 | 6.72E-07 | -4.047305715 | 0.062 | 0.8 | 0.011655077 |

| | | | | | |
|---|---|---|---|---|---|
| C15orf48 | 6.51E-17 | -4.062735755 | 0.375 | 1 | 1.13E-12 |
| NDUFA2 | 4.44E-11 | -4.072931522 | 0.438 | 1 | 7.70E-07 |
| SDF4 | 5.43E-16 | -4.074962058 | 0.438 | 1 | 9.42E-12 |
| SYCN | 2.98E-34 | -4.093109404 | 0.812 | 1 | 5.16E-30 |
| ENSSSCG00000058768 | 1.61E-09 | -4.093109404 | 0.188 | 0.733 | 2.79E-05 |
| LMF1 | 8.90E-20 | -4.099108145 | 0.562 | 1 | 1.54E-15 |
| SLC25A1 | 9.77E-11 | -4.108059746 | 0.25 | 0.867 | 1.70E-06 |
| C6orf52 | 1.10E-54 | -4.108141464 | 1 | 1 | 1.91E-50 |
| NUDT16L1 | 3.53E-14 | -4.113008962 | 0.375 | 0.867 | 6.12E-10 |
| TMEM147 | 1.49E-23 | -4.119909464 | 0.562 | 1 | 2.58E-19 |
| RPS13 | 2.62E-15 | -4.119909464 | 0.938 | 1 | 4.55E-11 |
| CRYBB3 | 2.15E-15 | -4.121015401 | 0.5 | 0.933 | 3.73E-11 |
| TALDO1 | 9.07E-27 | -4.133855747 | 0.688 | 1 | 1.57E-22 |
| NUDT5 | 9.53E-66 | -4.13922368 | 1 | 1 | 1.65E-61 |
| ENSSSCG00000013386 | 7.35E-63 | -4.143414486 | 0.938 | 1 | 1.28E-58 |
| ETFB | 2.71E-17 | -4.154220919 | 0.375 | 1 | 4.71E-13 |
| ENSSSCG00000002036 | 1.51E-55 | -4.154829138 | 1 | 1 | 2.62E-51 |
| RPL18A | 5.73E-16 | -4.178449074 | 0.562 | 1 | 9.94E-12 |
| AVPI1 | 8.33E-16 | -4.180572246 | 0.438 | 0.867 | 1.45E-11 |
| ENSSSCG00000025928 | 2.44E-15 | -4.180572246 | 0.562 | 1 | 4.23E-11 |
| UPP1 | 1.69E-09 | -4.180572246 | 0.188 | 0.667 | 2.93E-05 |
| CHMP6 | 1.89E-08 | -4.180572246 | 0.125 | 0.667 | 0.00032737 |
| MGMT | 2.30E-29 | -4.186037006 | 0.75 | 1 | 3.99E-25 |
| MRPL30 | 9.01E-33 | -4.191843358 | 0.688 | 1 | 1.56E-28 |
| MRPL55 | 1.20E-27 | -4.193090285 | 0.875 | 1 | 2.08E-23 |
| CEP131 | 5.88E-11 | -4.201633861 | 0.188 | 0.933 | 1.02E-06 |
| RPS9 | 1.89E-23 | -4.202678914 | 0.938 | 1 | 3.27E-19 |
| H2AJ | 1.14E-15 | -4.20461772 | 0.312 | 0.933 | 1.98E-11 |
| COX5B | 1.81E-40 | -4.20701957 | 1 | 1 | 3.15E-36 |
| ENSSSCG00000043578 | 1.66E-08 | -4.208586622 | 0.25 | 0.8 | 0.000287211 |
| MRPS11 | 9.65E-15 | -4.21649182 | 0.25 | 1 | 1.68E-10 |
| DVL1 | 9.03E-08 | -4.222392421 | 0.062 | 0.8 | 0.001567021 |
| C19orf81 | 6.24E-12 | -4.230612928 | 0.188 | 0.8 | 1.08E-07 |
| ENSSSCG00000014133 | 1.12E-18 | -4.232527869 | 0.812 | 1 | 1.95E-14 |
| BCAP31 | 6.92E-53 | -4.23299078 | 0.875 | 1 | 1.20E-48 |
| PARK7 | 2.84E-50 | -4.237632299 | 1 | 1 | 4.92E-46 |
| MIB2 | 3.65E-17 | -4.24838763 | 0.25 | 1 | 6.33E-13 |
| SCGB3A2 | 8.09E-13 | -4.263034406 | 0.125 | 0.933 | 1.40E-08 |
| SLC25A19 | 1.92E-12 | -4.263034406 | 0.312 | 0.8 | 3.32E-08 |
| LAMTOR1 | 3.98E-11 | -4.263034406 | 0.125 | 1 | 6.91E-07 |
| ENSSSCG00000017971 | 7.07E-21 | -4.284250892 | 0.5 | 1 | 1.23E-16 |
| ZAR1 | 1.21E-60 | -4.289123635 | 0.938 | 1 | 2.10E-56 |
| CZIB | 4.76E-26 | -4.302042786 | 0.812 | 1 | 8.26E-22 |
| TMEM120A | 1.24E-07 | -4.30256277 | 0.062 | 0.733 | 0.002159744 |
| ENSSSCG00000012088 | 2.78E-17 | -4.310340121 | 0.562 | 0.867 | 4.83E-13 |
| FIBP | 4.95E-23 | -4.315501826 | 0.312 | 1 | 8.58E-19 |
| LY6G6D | 3.66E-07 | -4.341036918 | 0.062 | 0.733 | 0.006347928 |
| ENSSSCG00000052759 | 1.22E-15 | -4.351843673 | 0.188 | 1 | 2.12E-11 |
| MT3 | 1.82E-14 | -4.362570079 | 0.125 | 0.933 | 3.15E-10 |
| CUTA | 8.91E-24 | -4.365132593 | 0.562 | 1 | 1.55E-19 |
| FIGLA | 1.24E-31 | -4.372532771 | 0.75 | 1 | 2.16E-27 |
| ENSSSCG00000001696 | 1.25E-31 | -4.375425643 | 0.688 | 1 | 2.16E-27 |
| PATE1 | 5.67E-08 | -4.378511623 | 0.062 | 0.8 | 0.000983854 |
| GPX4 | 8.51E-30 | -4.381507876 | 0.688 | 1 | 1.48E-25 |
| ENSSSCG00000017509 | 1.20E-29 | -4.387588775 | 0.938 | 1 | 2.08E-25 |
| GFER | 1.60E-23 | -4.389042291 | 0.375 | 1 | 2.78E-19 |
| USE1 | 3.82E-14 | -4.394278939 | 0.375 | 0.933 | 6.64E-10 |
| EMG1 | 8.56E-28 | -4.410522018 | 0.688 | 1 | 1.48E-23 |
| GALK1 | 1.00E-22 | -4.415037499 | 0.625 | 0.933 | 1.74E-18 |
| COPS9 | 1.20E-25 | -4.421580345 | 0.625 | 1 | 2.07E-21 |
| TMEM219 | 4.76E-32 | -4.427010141 | 0.625 | 1 | 8.27E-28 |
| GSTM3 | 1.04E-86 | -4.433006734 | 1 | 1 | 1.80E-82 |
| RPS5 | 2.64E-29 | -4.450661409 | 0.938 | 1 | 4.59E-25 |
| ELOB | 1.00E-19 | -4.450661409 | 0.375 | 1 | 1.74E-15 |
| SORD | 2.08E-11 | -4.450661409 | 0.188 | 0.8 | 3.61E-07 |
| UXT | 6.01E-28 | -4.46634959 | 0.812 | 1 | 1.04E-23 |

| | | | | | |
|---|---|---|---|---|---|
| ENSSSCG00000037399 | 8.20E-11 | -4.468148836 | 0.188 | 0.867 | 1.42E-06 |
| COX7A1 | 2.60E-17 | -4.473931188 | 0.25 | 1 | 4.50E-13 |
| NDUFB11 | 5.33E-25 | -4.478540442 | 0.562 | 1 | 9.25E-21 |
| FAM3A | 8.03E-11 | -4.485426827 | 0.125 | 0.867 | 1.39E-06 |
| ENSSSCG00000040854 | 9.01E-29 | -4.499101764 | 0.688 | 1 | 1.56E-24 |
| TK1 | 7.53E-35 | -4.49966875 | 0.688 | 1 | 1.31E-30 |
| RPL26 | 8.55E-14 | -4.500073603 | 0.312 | 1 | 1.48E-09 |
| ENSSSCG00000058502 | 1.19E-17 | -4.502500341 | 0.25 | 1 | 2.06E-13 |
| NDUFA13 | 2.68E-26 | -4.503805356 | 0.625 | 1 | 4.64E-22 |
| MYL6 | 3.60E-36 | -4.504923003 | 0.812 | 1 | 6.25E-32 |
| AIP | 2.95E-17 | -4.512648296 | 0.25 | 1 | 5.12E-13 |
| ENSSSCG00000045485 | 1.46E-25 | -4.54092194 | 0.562 | 1 | 2.53E-21 |
| ENSSSCG00000059584 | 4.39E-14 | -4.546567122 | 0.375 | 0.933 | 7.62E-10 |
| ATOX1 | 4.61E-22 | -4.552541023 | 0.562 | 1 | 8.00E-18 |
| NUDT14 | 6.94E-12 | -4.552541023 | 0.188 | 0.933 | 1.20E-07 |
| TIMM10 | 1.85E-14 | -4.561878888 | 0.188 | 0.933 | 3.22E-10 |
| PGLS | 2.24E-12 | -4.565597176 | 0.25 | 0.867 | 3.89E-08 |
| EPHB6 | 1.45E-18 | -4.568842835 | 0.25 | 1 | 2.52E-14 |
| C19orf38 | 3.64E-24 | -4.584962501 | 0.375 | 1 | 6.31E-20 |
| ENSSSCG00000049122 | 2.65E-08 | -4.584962501 | 0.062 | 0.8 | 0.000460665 |
| ENSSSCG00000061183 | 1.64E-10 | -4.595609745 | 0.125 | 0.8 | 2.85E-06 |
| ENSSSCG00000062187 | 2.20E-09 | -4.595609745 | 0.125 | 0.8 | 3.83E-05 |
| SPATA16 | 1.12E-14 | -4.606178987 | 0.25 | 1 | 1.95E-10 |
| ENSSSCG00000024974 | 1.04E-22 | -4.60968493 | 0.312 | 1 | 1.80E-18 |
| RPS14 | 8.58E-25 | -4.619282574 | 0.688 | 1 | 1.49E-20 |
| ENSSSCG00000009303 | 2.84E-35 | -4.620378184 | 0.938 | 1 | 4.92E-31 |
| DMAP1 | 1.58E-20 | -4.627087976 | 0.25 | 1 | 2.74E-16 |
| ENSSSCG00000035997 | 1.24E-19 | -4.636043713 | 0.875 | 1 | 2.16E-15 |
| RABAC1 | 7.54E-32 | -4.64200265 | 0.75 | 1 | 1.31E-27 |
| ADCK5 | 1.47E-19 | -4.650486073 | 0.375 | 0.933 | 2.55E-15 |
| COMMD4 | 1.11E-22 | -4.653824359 | 0.562 | 0.933 | 1.93E-18 |
| ZNHIT1 | 4.49E-15 | -4.653824359 | 0.188 | 0.867 | 7.79E-11 |
| ATP5PF | 1.34E-17 | -4.656045599 | 0.875 | 1 | 2.33E-13 |
| TUBA1B | 6.94E-49 | -4.676940823 | 1 | 1 | 1.20E-44 |
| NAPRT | 9.33E-12 | -4.693022247 | 0.062 | 0.867 | 1.62E-07 |
| UQCR11 | 3.54E-13 | -4.701918647 | 0.125 | 0.8 | 6.14E-09 |
| ENSSSCG00000048076 | 7.84E-53 | -4.707819249 | 0.875 | 1 | 1.36E-48 |
| SPG7 | 8.35E-10 | -4.707819249 | 0.062 | 0.867 | 1.45E-05 |
| ATP6V1F | 5.53E-18 | -4.712019237 | 0.312 | 0.933 | 9.59E-14 |
| ENSSSCG00000007646 | 1.13E-16 | -4.712019237 | 0.25 | 1 | 1.96E-12 |
| NDUFV1 | 4.10E-17 | -4.714347292 | 0.188 | 1 | 7.11E-13 |
| NDUFB9 | 3.76E-47 | -4.719499041 | 1 | 1 | 6.52E-43 |
| SMIM26 | 1.90E-30 | -4.730160416 | 0.375 | 1 | 3.30E-26 |
| MIEN1 | 3.05E-26 | -4.732837709 | 0.562 | 1 | 5.30E-22 |
| DUS1L | 4.09E-17 | -4.736965594 | 0.25 | 0.933 | 7.09E-13 |
| RPS15 | 5.49E-17 | -4.754174884 | 0.625 | 1 | 9.52E-13 |
| TNNT1 | 5.42E-35 | -4.763357093 | 0.625 | 1 | 9.41E-31 |
| PET100 | 2.36E-20 | -4.773229138 | 0.375 | 1 | 4.09E-16 |
| SHD | 6.93E-31 | -4.824428435 | 0.5 | 1 | 1.20E-26 |
| STAT5A | 1.42E-59 | -4.833612988 | 0.812 | 1 | 2.46E-55 |
| ENSSSCG00000028167 | 5.41E-37 | -4.862881144 | 0.812 | 1 | 9.38E-33 |
| SPC24 | 8.57E-78 | -4.870920051 | 0.938 | 1 | 1.49E-73 |
| COX7A2 | 2.14E-39 | -4.894811864 | 1 | 1 | 3.72E-35 |
| ENSSSCG00000010791 | 8.00E-07 | -4.900464326 | 0 | 0.8 | 0.013888609 |
| MRPL28 | 4.09E-31 | -4.910732662 | 0.625 | 1 | 7.09E-27 |
| POLR2F | 1.73E-34 | -4.927685795 | 0.625 | 1 | 3.00E-30 |
| MALSU1 | 2.42E-22 | -4.973527789 | 0.438 | 1 | 4.19E-18 |
| PAFAH1B3 | 1.03E-09 | -4.975752454 | 0.062 | 0.733 | 1.79E-05 |
| RPL23A | 9.12E-49 | -4.981852653 | 0.875 | 1 | 1.58E-44 |
| POLR2I | 1.65E-16 | -4.987927168 | 0.188 | 1 | 2.86E-12 |
| RPS26 | 7.27E-13 | -4.990810829 | 0.375 | 1 | 1.26E-08 |
| LSM2 | 7.07E-55 | -4.993866456 | 1 | 1 | 1.23E-50 |
| MAMDC4 | 2.07E-14 | -5 | 0.062 | 1 | 3.59E-10 |
| ECRG4 | 6.96E-22 | -5.006853671 | 0.312 | 1 | 1.21E-17 |
| ENSSSCG00000044567 | 5.89E-71 | -5.010647244 | 0.812 | 1 | 1.02E-66 |
| SERF1A | 2.81E-42 | -5.027783157 | 0.75 | 1 | 4.87E-38 |

| | | | | | |
|---|---|---|---|---|---|
| SRA1 | 8.53E-18 | -5.028569152 | 0.25 | 0.867 | 1.48E-13 |
| NDUFA7 | 3.74E-38 | -5.034557222 | 0.875 | 1 | 6.49E-34 |
| NDUFA11 | 1.46E-42 | -5.048339534 | 0.875 | 1 | 2.53E-38 |
| CD164L2 | 4.21E-07 | -5.093109404 | 0 | 0.733 | 0.007300304 |
| SPACA3 | 7.40E-17 | -5.126532406 | 0.125 | 0.933 | 1.28E-12 |
| FIS1 | 1.06E-20 | -5.130198723 | 0.188 | 1 | 1.85E-16 |
| ATP5IF1 | 6.91E-66 | -5.134680245 | 0.875 | 1 | 1.20E-61 |
| MICOS13 | 9.88E-24 | -5.137503524 | 0.312 | 1 | 1.71E-19 |
| WRAP53 | 3.59E-25 | -5.162782932 | 0.312 | 0.933 | 6.22E-21 |
| ENSSSCG00000033310 | 5.53E-23 | -5.173856818 | 0.5 | 1 | 9.59E-19 |
| GRTP1 | 1.25E-06 | -5.180572246 | 0 | 0.667 | 0.021630765 |
| GMFG | 1.30E-06 | -5.180572246 | 0 | 0.733 | 0.022583802 |
| POLR2G | 2.20E-63 | -5.191775723 | 0.938 | 1 | 3.82E-59 |
| JOSD2 | 7.34E-16 | -5.201633861 | 0.125 | 0.933 | 1.27E-11 |
| SAMD4A | 4.16E-10 | -5.208586622 | 0.125 | 0.933 | 7.22E-06 |
| ENSSSCG00000049161 | 6.46E-11 | -5.222392421 | 0.062 | 0.733 | 1.12E-06 |
| HSD17B1 | 4.19E-08 | -5.222392421 | 0 | 0.8 | 0.000727491 |
| TPRKB | 1.43E-22 | -5.24961389 | 0.625 | 1 | 2.48E-18 |
| AP2S1 | 6.45E-30 | -5.299309792 | 0.562 | 1 | 1.12E-25 |
| ATPAF2 | 1.52E-12 | -5.30256277 | 0.062 | 0.867 | 2.65E-08 |
| ENSSSCG00000023783 | 3.06E-17 | -5.341036918 | 0.062 | 0.933 | 5.31E-13 |
| ATP5MC1 | 5.83E-81 | -5.399416519 | 0.938 | 1 | 1.01E-76 |
| MVD | 7.93E-09 | -5.415037499 | 0 | 0.8 | 0.00013762 |
| ENSSSCG00000061531 | 1.02E-13 | -5.450661409 | 0.062 | 1 | 1.77E-09 |
| ENSSSCG00000058049 | 9.54E-08 | -5.450661409 | 0 | 0.733 | 0.001655271 |
| ROMO1 | 3.84E-29 | -5.458181991 | 0.438 | 1 | 6.66E-25 |
| SF3B5 | 4.81E-08 | -5.519374159 | 0 | 0.667 | 0.000834372 |
| CHMP2A | 4.78E-30 | -5.568842835 | 0.438 | 1 | 8.29E-26 |
| ENSSSCG00000038364 | 3.92E-15 | -5.568842835 | 0.062 | 0.933 | 6.80E-11 |
| DDT | 1.52E-24 | -5.571156701 | 0.25 | 1 | 2.64E-20 |
| TMED1 | 1.87E-07 | -5.61667136 | 0 | 0.733 | 0.003244314 |
| ENSSSCG00000041052 | 4.14E-11 | -5.647698256 | 0 | 0.933 | 7.19E-07 |
| MORN3 | 1.14E-10 | -5.678071905 | 0 | 0.933 | 1.98E-06 |
| TRAPPC5 | 2.23E-10 | -5.707819249 | 0 | 0.867 | 3.87E-06 |
| RNASEH2C | 3.72E-15 | -5.751320887 | 0.062 | 0.933 | 6.45E-11 |
| DERL3 | 2.70E-23 | -5.758445322 | 0.188 | 1 | 4.69E-19 |
| ENSSSCG00000061874 | 5.56E-25 | -5.870993208 | 0.688 | 1 | 9.65E-21 |
| EXOSC4 | 4.24E-23 | -5.895302621 | 0.188 | 0.933 | 7.36E-19 |
| ENSSSCG00000057536 | 5.93E-18 | -5.938599455 | 0.062 | 0.933 | 1.03E-13 |
| TMEM160 | 1.03E-12 | -5.975752454 | 0 | 0.933 | 1.79E-08 |
| ENSSSCG00000042862 | 1.17E-26 | -5.993976212 | 0.75 | 0.933 | 2.02E-22 |
| APRT | 1.58E-18 | -6 | 0.062 | 1 | 2.74E-14 |
| ZP3 | 2.38E-83 | -6.015396062 | 1 | 1 | 4.13E-79 |
| ENSSSCG00000055438 | 1.14E-24 | -6.045850652 | 0.625 | 1 | 1.98E-20 |
| ENSSSCG00000032060 | 8.76E-39 | -6.079688889 | 0.688 | 1 | 1.52E-34 |
| RHOD | 2.50E-06 | -6.137503524 | 0 | 0.667 | 0.043342858 |
| RNH1 | 4.12E-28 | -6.180572246 | 0.062 | 1 | 7.15E-24 |
| ENSSSCG00000044205 | 1.02E-09 | -6.251538767 | 0.125 | 0.8 | 1.77E-05 |
| ENSSSCG00000032166 | 1.75E-46 | -6.263568638 | 0.812 | 1 | 3.04E-42 |
| PEX16 | 2.86E-17 | -6.273018494 | 0.062 | 0.867 | 4.96E-13 |
| ENSSSCG00000012842 | 2.09E-26 | -6.290817562 | 0.125 | 1 | 3.62E-22 |
| RPL38 | 4.10E-26 | -6.42103624 | 0.125 | 1 | 7.11E-22 |
| CD320 | 1.76E-33 | -6.590779827 | 0.375 | 1 | 3.05E-29 |
| CCDC167 | 2.22E-44 | -6.637898252 | 0.375 | 1 | 3.85E-40 |
| TMEM125 | 3.74E-28 | -7.070389328 | 0.062 | 1 | 6.49E-24 |
| ENSSSCG00000061145 | 2.40E-15 | -7.201633861 | 0 | 0.733 | 4.17E-11 |
| PENK | 7.19E-09 | -7.415037499 | 0 | 0.8 | 0.000124726 |
| H2BK1 | 2.14E-31 | -7.584962501 | 0.125 | 1 | 3.72E-27 |
