# Supplementary material for: Deciphering differences in DNA methylation and transcriptome profiles of oocytes from pigs with high and low developmental competence
Source: Environ Epigenet. 2025 Jun 3;11(1):dvaf018. doi: 10.1093/eep/dvaf018 (PMC12418950; doi:10.1093/eep/dvaf018)
Supplement: dvaf018_Supplemental_Files [file dvaf018_supplemental_files.zip › Sup table 4.pdf]

ID	Term	Ontology Source	Term P-Value	Term P-Value Corrected with Bonferroni step down	Group P-Value	Group P-Value Corrected with Bonferroni step down	GO Levels	GO Groups	% Associated Genes	Number of Genes	Associated Genes Found
GO:0002181	cytoplasmic translation	GO_BiologicalProcess-EBI-UniProt-GOA-ACAP-ARAP_23.01.2024_0	0.00	0.00	0.00	0.00	[5, 6, 7, 8]	Group00	29.76	25.00	[DRG1, EIF2B2, EIF3B, EIF3F, EIF3G, EIF3H, EIF3K, LOC100738684, RPL10A, RPL17, RPL26, RPL29, RPL30, RPL31, RPL32, RPL36, RPL38, RPL6, RPL8, RPLP1, RPLP2, RPS14, RPS21, RPS23, RPS29]
GO:0033617	mitochondrial cytochrome c oxidase assembly	GO_BiologicalProcess-EBI-UniProt-GOA-ACAP-ARAP_23.01.2024_0	0.00	0.03	0.00	0.00	[7, 8]	Group02	50.00	6.00	[BCS1L, COX17, PET100, SMIM20, STMP1, TMEM223]
GO:0045182	translation regulator activity	GO_BiologicalProcess-EBI-UniProt-GOA-ACAP-ARAP_23.01.2024_0	0.00	0.03	0.00	0.00	[6, 7, 8, 9, 10]	Group03	16.39	20.00	[CIRBP, EEF1B2, EEF1D, EEF1G, EIF2B2, EIF3B, EIF3F, EIF3G, EIF3H, EIF3K, EIF6, FXR2, MRPL58, NELFE, PAIP2, RACK1, RPS14, RPS9, SAMD4A, TUFM]
GO:1901657	glycosyl compound metabolic process	GO_BiologicalProcess-EBI-UniProt-GOA-ACAP-ARAP_23.01.2024_0	0.00	0.03	0.00	0.00	[4]	Group04	23.53	12.00	[ADA, AKR1A1, AKR1C3, CDA, DNPH1, DTYMK, GBA2, GUSB, MACROD1, TK1, UPB1, UPP1]
GO:0006081	cellular aldehyde metabolic process	GO_BiologicalProcess-EBI-UniProt-GOA-ACAP-ARAP_23.01.2024_0	0.00	0.03	0.00	0.00	[3]	Group05	22.41	13.00	[AKR1A1, ESD, GATD1, GRHPR, HAGH, HYI, IDH2, PARK7, RDH13, SLC25A10, TALDO1, TKT, TPI1]
GO:0031163	metallo-sulfur cluster assembly	GO_BiologicalProcess-EBI-UniProt-GOA-ACAP-ARAP_23.01.2024_0	0.00	0.05	0.00	0.00	[5]	Group06	33.33	8.00	[CIAO2B, CIAPIN1, GLRX3, ISCA1, ISCA2, LYRM4, NDUFAB1, NUBP2]
GO:0016226	iron-sulfur cluster assembly	GO_BiologicalProcess-EBI-UniProt-GOA-ACAP-ARAP_23.01.2024_0	0.00	0.05	0.00	0.00	[6]	Group06	33.33	8.00	[CIAO2B, CIAPIN1, GLRX3, ISCA1, ISCA2, LYRM4, NDUFAB1, NUBP2]
GO:0048518	positive regulation of biological process	GO_BiologicalProcess-EBI-UniProt-GOA-ACAP-ARAP_23.01.2024_0	0.00	0.00	0.00	0.00	[1, 2, 3]	Group07	4.66	226.00	[ABLIM3, ADA, AIMP2, AJUBA, ARG2, ARL2, ARMC12, ARPP19, ARRDC4, ASB11, ASIP, ATP5F1A, ATP5IF1, ATRAID, AURKA, AURKAIP1, AVPI1, BAG2, BCAP31, BNIP3, BORCS5, BUD23, BUD31, CCT4, CD
GO:0050794	regulation of cellular process	GO_BiologicalProcess-EBI-UniProt-GOA-ACAP-ARAP_23.01.2024_0	0.00	0.00	0.00	0.00	[2, 3]	Group07	4.44	418.00	[ABHD17A, ABLIM3, ADA, ADCK2, AGTRAP, AIMP2, AJUBA, ARG2, ARHGAP22, ARHGEF28, ARL2, ARL3, ARMC12, ARPC3, ARPP19, ARR3, ARRDC1, ARRDC4, ASB11, ASIP, ATP5F1A, ATP5IF1, ATRAID, A
GO:0048522	positive regulation of cellular process	GO_BiologicalProcess-EBI-UniProt-GOA-ACAP-ARAP_23.01.2024_0	0.00	0.00	0.00	0.00	[2, 3, 4]	Group07	4.58	203.00	[ABLIM3, ADA, AJUBA, ARG2, ARL2, ARMC12, ARPP19, ARRDC4, ASIP, ATP5F1A, ATP5IF1, ATRAID, AURKA, AVPI1, BAG2, BCAP31, BNIP3, BORCS5, BUD23, BUD31, CCT4, CD274, CD320, CD40, CDK10,
GO:0044248	cellular catabolic process	GO_BiologicalProcess-EBI-UniProt-GOA-ACAP-ARAP_23.01.2024_0	0.00	0.04	0.00	0.00	[3]	Group08	8.76	108.00	[ACAA1, ACAT1, ADA, AKR1A1, ALDOC, AMDHD2, ARG2, ARL2, ATP5IF1, AUP1, BCKDHA, BNIP3, BPGM, CARHSP1, CDA, CHMP1A, CHMP2A, CHMP4A, CHMP6, CIDEA, CTSD, DDRGK1, DDX49, DNASE2,
GO:1901575	organic substance catabolic process	GO_BiologicalProcess-EBI-UniProt-GOA-ACAP-ARAP_23.01.2024_0	0.00	0.00	0.00	0.00	[3]	Group08	8.71	144.00	[ACAA1, ACAT1, ADA, AKR1A1, ALAD, ALDOC, AMDHD2, ANAPC11, ARG2, ARL2, ARRDC1, ASB11, ATP5IF1, ATRAID, AUP1, BAG2, BAG5, BCAP31, BCKDHA, BNIP3, BPGM, CARHSP1, CDA, CDC26, CDK
GO:1901565	organonitrogen compound catabolic process	GO_BiologicalProcess-EBI-UniProt-GOA-ACAP-ARAP_23.01.2024_0	0.00	0.02	0.00	0.00	[4]	Group08	9.08	103.00	[ACAT1, ADA, ALAD, ALDOC, AMDHD2, ANAPC11, ARG2, ARL2, ARRDC1, ASB11, ATP5IF1, ATRAID, AUP1, BAG2, BAG5, BCAP31, BCKDHA, BNIP3, BPGM, CDA, CDC26, CDK5RAP3, CHMP1A, CHMP2A, C
GO:0042273	ribosomal large subunit biogenesis	GO_BiologicalProcess-EBI-UniProt-GOA-ACAP-ARAP_23.01.2024_0	0.00	0.00	0.00	0.00	[5, 6]	Group09	25.00	14.00	[EIF6, FTSJ3, LOC100739087, MALSU1, NOP2, RPL10A, RPL14, RPL26, RPL35, RPL7, RPL7A, RPL7L1, SNU13, WDR74]
GO:0016072	rRNA metabolic process	GO_BiologicalProcess-EBI-UniProt-GOA-ACAP-ARAP_23.01.2024_0	0.00	0.00	0.00	0.00	[7, 8]	Group09	20.00	39.00	[BUD23, DDX11, DDX49, EIF6, EMG1, ERI3, EXOSC4, FBL, FCF1, FTSJ3, IMP3, IMP4, MRM1, NOP10, NOP14, NOP2, PELP1, PIH1D1, POP7, PPAN, RPL10A, RPL26, RPL35, RPL7, RPL7A, RPL7L1, RPP40, R
GO:0006364	rRNA processing	GO_BiologicalProcess-EBI-UniProt-GOA-ACAP-ARAP_23.01.2024_0	0.00	0.00	0.00	0.00	[6, 8, 9]	Group09	23.31	38.00	[BUD23, DDX49, EIF6, EMG1, ERI3, EXOSC4, FBL, FCF1, FTSJ3, IMP3, IMP4, MRM1, NOP10, NOP14, NOP2, PELP1, PIH1D1, POP7, PPAN, RPL10A, RPL26, RPL35, RPL7, RPL7A, RPL7L1, RPP40, RPS16, R
GO:0000470	maturation of LSU-rRNA	GO_BiologicalProcess-EBI-UniProt-GOA-ACAP-ARAP_23.01.2024_0	0.00	0.01	0.00	0.00	[6, 7, 9, 10]	Group09	36.00	9.00	[EIF6, FTSJ3, NOP2, RPL10A, RPL35, RPL7, RPL7A, RPL7L1, SNU13]
GO:0006996	organelle organization	GO_BiologicalProcess-EBI-UniProt-GOA-ACAP-ARAP_23.01.2024_0	0.00	0.00	0.00	0.00	[4]	Group10	7.89	220.00	[AAAS, ABLIM3, ACAD9, ALKBH7, APOO, ARHGEF28, ARL2, ARL3, ARMC2, ARPC3, ASIP, ATP5F1D, ATP5IF1, ATP6AP1, ATP6V1F, ATPAF2, ATXN10, AUP1, AURKA, BAG5, BCS1L, BNIP1, BNIP3, CAPZB, (
GO:0007005	mitochondrion organization	GO_BiologicalProcess-EBI-UniProt-GOA-ACAP-ARAP_23.01.2024_0	0.00	0.00	0.00	0.00	[5]	Group10	18.87	70.00	[ACAD9, ALKBH7, APOO, ATP5F1D, ATP5IF1, ATPAF2, BCS1L, BNIP3, CHCHD7, CLN8, COX17, COX7A1, COX7A2, DNAJC11, DYNLL1, FAM162A, FIS1, GHITM, HIGD2A, HSD17B10, IMMP2L, LMNA, LOC10
GO:0010257	NADH dehydrogenase complex assembly	GO_BiologicalProcess-EBI-UniProt-GOA-ACAP-ARAP_23.01.2024_0	0.00	0.00	0.00	0.00	[6]	Group10	44.23	23.00	[ACAD9, BCS1L, NDUFA10, NDUFA11, NDUFA12, NDUFA5, NDUFA9, NDUFAB1, NDUFB10, NDUFB11, NDUFB2, NDUFB4, NDUFB7, NDUFB8, NDUFB9, NDUFC1, NDUFC2, NDUFS3, NDUFS4, NDUFS5, NDU
GO:0033108	mitochondrial respiratory chain complex assembly	GO_BiologicalProcess-EBI-UniProt-GOA-ACAP-ARAP_23.01.2024_0	0.00	0.00	0.00	0.00	[6]	Group10	42.67	32.00	[ACAD9, BCS1L, CHCHD7, COX17, IMMP2L, NDUFA10, NDUFA11, NDUFA12, NDUFA5, NDUFA9, NDUFAB1, NDUFB10, NDUFB11, NDUFB2, NDUFB4, NDUFB7, NDUFB8, NDUFB9, NDUFC1, NDUFC2, NDUF
GO:0032981	mitochondrial respiratory chain complex I assembly	GO_BiologicalProcess-EBI-UniProt-GOA-ACAP-ARAP_23.01.2024_0	0.00	0.00	0.00	0.00	[7]	Group10	44.23	23.00	[ACAD9, BCS1L, NDUFA10, NDUFA11, NDUFA12, NDUFA5, NDUFA9, NDUFAB1, NDUFB10, NDUFB11, NDUFB2, NDUFB4, NDUFB7, NDUFB8, NDUFB9, NDUFC1, NDUFC2, NDUFS3, NDUFS4, NDUFS5, NDU
GO:0006839	mitochondrial transport	GO_BiologicalProcess-EBI-UniProt-GOA-ACAP-ARAP_23.01.2024_0	0.00	0.01	0.00	0.00	[4]	Group11	17.19	22.00	[ALKBH7, ATP5IF1, BNIP3, CHCHD4, GHITM, MPC1, PDCD5, ROMO1, SAMM50, SLC25A1, SLC25A38, SLC25A39, SLC25A6, SLC8B1, SPG7, TIMM10, TIMM17A, TIMM17B, TIMM22, TIMM50, TOMM20, TOMM
GO:0007006	mitochondrial membrane organization	GO_BiologicalProcess-EBI-UniProt-GOA-ACAP-ARAP_23.01.2024_0	0.00	0.00	0.00	0.00	[5, 6]	Group11	23.68	18.00	[ALKBH7, APOO, ATP5IF1, BCS1L, BNIP3, CLN8, DNAJC11, GHITM, MICOS13, PDCD5, ROMO1, SAMM50, SLC25A6, SPG7, TIMM10, TIMM22, TIMM50, TMEM11]
GO:0070585	protein localization to mitochondrion	GO_BiologicalProcess-EBI-UniProt-GOA-ACAP-ARAP_23.01.2024_0	0.00	0.00	0.00	0.00	[6]	Group11	25.33	19.00	[AIP, ATP5IF1, BCAP31, BCS1L, CHCHD4, FIS1, IMMP2L, PDCD5, ROMO1, SAMM50, SIAH3, TIMM10, TIMM17A, TIMM17B, TIMM22, TIMM50, TOMM20, TOMM34, TOMM40L]
GO:0072655	establishment of protein localization to mitochondrion	GO_BiologicalProcess-EBI-UniProt-GOA-ACAP-ARAP_23.01.2024_0	0.00	0.00	0.00	0.00	[5, 7]	Group11	27.14	19.00	[AIP, ATP5IF1, BCAP31, BCS1L, CHCHD4, FIS1, IMMP2L, PDCD5, ROMO1, SAMM50, SIAH3, TIMM10, TIMM17A, TIMM17B, TIMM22, TIMM50, TOMM20, TOMM34, TOMM40L]
GO:0006626	protein targeting to mitochondrion	GO_BiologicalProcess-EBI-UniProt-GOA-ACAP-ARAP_23.01.2024_0	0.00	0.00	0.00	0.00	[5, 6, 7, 8]	Group11	31.03	18.00	[AIP, ATP5IF1, BCAP31, CHCHD4, FIS1, IMMP2L, PDCD5, ROMO1, SAMM50, SIAH3, TIMM10, TIMM17A, TIMM17B, TIMM22, TIMM50, TOMM20, TOMM34, TOMM40L]
GO:0007007	inner mitochondrial membrane organization	GO_BiologicalProcess-EBI-UniProt-GOA-ACAP-ARAP_23.01.2024_0	0.00	0.00	0.00	0.00	[6, 7]	Group11	34.48	10.00	[APOO, BCS1L, DNAJC11, GHITM, MICOS13, ROMO1, SAMM50, TIMM10, TIMM22, TMEM11]
GO:0044085	cellular component biogenesis	GO_BiologicalProcess-EBI-UniProt-GOA-ACAP-ARAP_23.01.2024_0	0.00	0.00	0.00	0.00	[3]	Group12	9.70	243.00	[AAAS, ABLIM3, ACAD9, ACOT13, ADRM1, AIMP2, AJUBA, ALAD, ARL2, ARL3, ARMC2, ARPC3, ARRDC1, ATP5F1D, ATPAF2, ATXN10, AUP1, BAG5, BCS1L, BUD23, CAPZB, CASQ2, CCDC28B, CD247, CDI
GO:0006996	organelle organization	GO_BiologicalProcess-EBI-UniProt-GOA-ACAP-ARAP_23.01.2024_0	0.00	0.00	0.00	0.00	[4]	Group12	7.89	220.00	[AAAS, ABLIM3, ACAD9, ALKBH7, APOO, ARHGEF28, ARL2, ARL3, ARMC2, ARPC3, ASIP, ATP5F1D, ATP5IF1, ATP6AP1, ATP6V1F, ATPAF2, ATXN10, AUP1, AURKA, BAG5, BCS1L, BNIP1, BNIP3, CAPZB, (
GO:0022607	cellular component assembly	GO_BiologicalProcess-EBI-UniProt-GOA-ACAP-ARAP_23.01.2024_0	0.00	0.00	0.00	0.00	[4]	Group12	8.29	188.00	[AAAS, ABLIM3, ACAD9, ACOT13, ADRM1, AIMP2, AJUBA, ALAD, ARL2, ARL3, ARMC2, ARPC3, ATP5F1D, ATPAF2, ATXN10, AUP1, BAG5, BCS1L, CAPZB, CASQ2, CCDC28B, CD247, CDH6, CDK10, CDK5F
GO:0022613	ribonucleoprotein complex biogenesis	GO_BiologicalProcess-EBI-UniProt-GOA-ACAP-ARAP_23.01.2024_0	0.00	0.00	0.00	0.00	[4]	Group12	21.24	79.00	[BUD23, DDX49, EBNA1BP2, EIF3B, EIF3F, EIF3G, EIF3H, EIF3K, EIF6, EMG1, ERI3, EXOSC4, FBL, FCF1, FTSJ3, GEMIN2, GEMIN6, IMP3, IMP4, LOC100738684, LOC100739087, LOC102162486, LSM2, LUC
GO:0043933	protein-containing complex organization	GO_BiologicalProcess-EBI-UniProt-GOA-ACAP-ARAP_23.01.2024_0	0.00	0.00	0.00	0.00	[4]	Group12	8.56	150.00	[ACAD9, ACOT13, ADRM1, AIMP2, AJUBA, ALAD, ARL2, ARPC3, ASIP, ATP5F1D, ATPAF2, BCS1L, BNIP3, CAPZB, CASQ2, CD247, CDK5RAP2, CDT1, CHCHD7, CHMP1A, CHMP2A, CHMP4A, CHMP6, COP
GO:0007005	mitochondrion organization	GO_BiologicalProcess-EBI-UniProt-GOA-ACAP-ARAP_23.01.2024_0	0.00	0.00	0.00	0.00	[5]	Group12	18.87	70.00	[ACAD9, ALKBH7, APOO, ATP5F1D, ATP5IF1, ATPAF2, BCS1L, BNIP3, CHCHD7, CLN8, COX17, COX7A1, COX7A2, DNAJC11, DYNLL1, FAM162A, FIS1, GHITM, HIGD2A, HSD17B10, IMMP2L, LMNA, LOC10
GO:0065003	protein-containing complex assembly	GO_BiologicalProcess-EBI-UniProt-GOA-ACAP-ARAP_23.01.2024_0	0.00	0.00	0.00	0.00	[5]	Group12	10.41	121.00	[ACAD9, ACOT13, ADRM1, AIMP2, AJUBA, ALAD, ARL2, ARPC3, ATP5F1D, ATPAF2, BCS1L, CAPZB, CASQ2, CD247, CDK5RAP2, CDT1, CHCHD7, CHMP2A, CHMP4A, COPS7A, COX17, COX7A1, COX7A2,
GO:0098754	detoxification	GO_BiologicalProcess-EBI-UniProt-GOA-ACAP-ARAP_23.01.2024_0	0.00	0.02	0.00	0.00	[1, 4]	Group13	18.37	18.00	[AKR1A1, CCS, DHFR, ESD, GPX4, GSTK1, GSTM3, MGST2, MT3, PARK7, PRDX1, PRDX2, PRDX3, PRDX4, PRDX5, SELENOS, TXNDC17, TXNRD3]
GO:0045454	cell redox homeostasis	GO_BiologicalProcess-EBI-UniProt-GOA-ACAP-ARAP_23.01.2024_0	0.00	0.03	0.00	0.00	[3]	Group13	34.78	8.00	[GLRX3, PRDX1, PRDX2, PRDX3, PRDX4, SELENOS, TXN2, TXNRD3]
GO:1990748	cellular detoxification	GO_BiologicalProcess-EBI-UniProt-GOA-ACAP-ARAP_23.01.2024_0	0.00	0.00	0.00	0.00	[2, 5]	Group13	20.22	18.00	[AKR1A1, CCS, DHFR, ESD, GPX4, GSTK1, GSTM3, MGST2, MT3, PARK7, PRDX1, PRDX2, PRDX3, PRDX4, PRDX5, SELENOS, TXNDC17, TXNRD3]
GO:0097237	cellular response to toxic substance	GO_BiologicalProcess-EBI-UniProt-GOA-ACAP-ARAP_23.01.2024_0	0.00	0.01	0.00	0.00	[4]	Group13	19.15	18.00	[AKR1A1, CCS, DHFR, ESD, GPX4, GSTK1, GSTM3, MGST2, MT3, PARK7, PRDX1, PRDX2, PRDX3, PRDX4, PRDX5, SELENOS, TXNDC17, TXNRD3]
GO:0098869	cellular oxidant detoxification	GO_BiologicalProcess-EBI-UniProt-GOA-ACAP-ARAP_23.01.2024_0	0.00	0.02	0.00	0.00	[3, 6]	Group13	20.55	15.00	[CCS, DHFR, GPX4, GSTK1, MGST2, MT3, PARK7, PRDX1, PRDX2, PRDX3, PRDX4, PRDX5, SELENOS, TXNDC17, TXNRD3]
GO:0051920	peroxiredoxin activity	GO_BiologicalProcess-EBI-UniProt-GOA-ACAP-ARAP_23.01.2024_0	0.00	0.00	0.00	0.00	[6, 9]	Group13	85.71	6.00	[PARK7, PRDX1, PRDX2, PRDX3, PRDX4, PRDX5]
GO:0140824	thioredoxin-dependent peroxiredoxin activity	GO_BiologicalProcess-EBI-UniProt-GOA-ACAP-ARAP_23.01.2024_0	0.00	0.00	0.00	0.00	[7, 10]	Group13	100.00	5.00	[PRDX1, PRDX2, PRDX3, PRDX4, PRDX5]
GO:0008379	thioredoxin peroxidase activity	GO_BiologicalProcess-EBI-UniProt-GOA-ACAP-ARAP_23.01.2024_0	0.00	0.00	0.00	0.00	[8, 11]	Group13	100.00	5.00	[PRDX1, PRDX2, PRDX3, PRDX4, PRDX5]
GO:0022900	electron transport chain	GO_BiologicalProcess-EBI-UniProt-GOA-ACAP-ARAP_23.01.2024_0	0.00	0.00	0.00	0.00	[4]	Group14	32.61	30.00	[CIAPIN1, COX5B, COX6C, COX7A1, COX7A2, COX7C, DNAJC15, ETFB, GPD1, IMMP2L, LOC100524873, NDUFA10, NDUFA5, NDUFA7, NDUFA8, NDUFB3, NDUFB7, NDUFB8, NDUFB9, NDUFC2, NDUFS3,
GO:0045333	cellular respiration	GO_BiologicalProcess-EBI-UniProt-GOA-ACAP-ARAP_23.01.2024_0	0.00	0.00	0.00	0.00	[5]	Group14	25.64	40.00	[ARL2, ATP5F1A, ATP5F1D, ATP5ME, ATP5MF, ATP5PF, ATP5PO, BLOC1S1, COX5B, COX6A1, COX6C, COX7A1, COX7A2, COX7C, DNAJC15, ETFB, GPD1, IDH2, IDH3B, IDH3G, IMMP2L, LOC100524873, M
GO:0022904	respiratory electron transport chain	GO_BiologicalProcess-EBI-UniProt-GOA-ACAP-ARAP_23.01.2024_0	0.00	0.00	0.00	0.00	[5, 6]	Group14	33.78	25.00	[COX5B, COX6C, COX7A1, COX7A2, COX7C, DNAJC15, ETFB, GPD1, IMMP2L, LOC100524873, NDUFA10, NDUFA5, NDUFA7, NDUFA8, NDUFB8, NDUFB9, NDUFC2, NDUFS3, NDUFS8, NDUFV1, NDUFV2,
GO:0009060	aerobic respiration	GO_BiologicalProcess-EBI-UniProt-GOA-ACAP-ARAP_23.01.2024_0	0.00	0.00	0.00	0.00	[6]	Group14	28.93	35.00	[ARL2, ATP5F1A, ATP5F1D, ATP5ME, ATP5MF, ATP5PF, ATP5PO, BLOC1S1, COX5B, COX6A1, COX6C, COX7A1, COX7A2, COX7C, DNAJC15, IDH2, IDH3B, IDH3G, LOC100524873, MDH1, NDUFA10, NDUF
GO:0006119	oxidative phosphorylation	GO_BiologicalProcess-EBI-UniProt-GOA-ACAP-ARAP_23.01.2024_0	0.00	0.00	0.00	0.00	[7]	Group14	34.57	28.00	[ATP5F1A, ATP5F1D, ATP5ME, ATP5MF, ATP5PF, ATP5PO, COX5B, COX6A1, COX6C, COX7A1, COX7A2, COX7C, DNAJC15, LOC100524873, NDUFA10, NDUFA7, NDUFA8, NDUFB8, NDUFB9, NDUFC2, ND
GO:0019646	aerobic electron transport chain	GO_BiologicalProcess-EBI-UniProt-GOA-ACAP-ARAP_23.01.2024_0	0.00	0.00	0.00	0.00	[6, 7, 8]	Group14	42.22	19.00	[COX5B, COX6C, COX7A1, COX7A2, COX7C, DNAJC15, LOC100524873, NDUFA10, NDUFA8, NDUFB8, NDUFB9, NDUFC2, NDUFS3, NDUFS8, NDUFV1, NDUFV2, PARK7, UQCR10, UQCRB]
GO:0042773	ATP synthesis coupled electron transport	GO_BiologicalProcess-EBI-UniProt-GOA-ACAP-ARAP_23.01.2024_0	0.00	0.00	0.00	0.00	[6, 7, 8]	Group14	37.50	21.00	[COX5B, COX6C, COX7A1, COX7A2, COX7C, DNAJC15, LOC100524873, NDUFA10, NDUFA7, NDUFA8, NDUFB8, NDUFB9, NDUFC2, NDUFS3, NDUFS8, NDUFV1, NDUFV2, NDUFV3, PARK7, UQCR10, UQC
GO:0008137	NADH dehydrogenase (ubiquinone) activity	GO_BiologicalProcess-EBI-UniProt-GOA-ACAP-ARAP_23.01.2024_0	0.00	0.03	0.00	0.00	[7, 9]	Group14	41.18	7.00	[NDUFA10, NDUFB7, NDUFS3, NDUFS4, NDUFS8, NDUFV1, NDUFV2]
GO:0042775	mitochondrial ATP synthesis coupled electron transport	GO_BiologicalProcess-EBI-UniProt-GOA-ACAP-ARAP_23.01.2024_0	0.00	0.00	0.00	0.00	[7, 8, 9]	Group14	38.46	20.00	[COX5B, COX6C, COX7A1, COX7A2, COX7C, DNAJC15, LOC100524873, NDUFA10, NDUFA8, NDUFB8, NDUFB9, NDUFC2, NDUFS3, NDUFS8, NDUFV1, NDUFV2, NDUFV3, PARK7, UQCR10, UQCRB]
GO:0006120	mitochondrial electron transport, NADH to ubiquinone	GO_BiologicalProcess-EBI-UniProt-GOA-ACAP-ARAP_23.01.2024_0	0.00	0.00	0.00	0.00	[7, 8, 9, 10]	Group14	45.83	11.00	[DNAJC15, NDUFA10, NDUFA8, NDUFB8, NDUFB9, NDUFC2, NDUFS3, NDUFS8, NDUFV1, NDUFV2, PARK7]
GO:0044085	cellular component biogenesis	GO_BiologicalProcess-EBI-UniProt-GOA-ACAP-ARAP_23.01.2024_0	0.00	0.00	0.00	0.00	[3]	Group15	9.70	243.00	[AAAS, ABLIM3, ACAD9, ACOT13, ADRM1, AIMP2, AJUBA, ALAD, ARL2, ARL3, ARMC2, ARPC3, ARRDC1, ATP5F1D, ATPAF2, ATXN10, AUP1, BAG5, BCS1L, BUD23, CAPZB, CASQ2, CCDC28B, CD247, CDI
GO:0022613	ribonucleoprotein complex biogenesis	GO_BiologicalProcess-EBI-UniProt-GOA-ACAP-ARAP_23.01.2024_0	0.00	0.00	0.00	0.00	[4]	Group15	21.24	79.00	[BUD23, DDX49, EBNA1BP2, EIF3B, EIF3F, EIF3G, EIF3H, EIF3K, EIF6, EMG1, ERI3, EXOSC4, FBL, FCF1, FTSJ3, GEMIN2, GEMIN6, IMP3, IMP4, LOC100738684, LOC100739087, LOC102162486, LSM2, LUC
GO:0042254	ribosome biogenesis	GO_BiologicalProcess-EBI-UniProt-GOA-ACAP-ARAP_23.01.2024_0	0.00	0.00	0.00	0.00	[5]	Group15	23.74	61.00	[BUD23, DDX49, EBNA1BP2, EIF6, EMG1, ERI3, EXOSC4, FBL, FCF1, FTSJ3, IMP3, IMP4, LOC100739087, MALSU1, MPV17L2, MRM1, MRPS7, MRTO4, NOC4L, NOP10, NOP14, NOP2, PELP1, PIH1D1, POP
GO:0071826	protein-RNA complex organization	GO_BiologicalProcess-EBI-UniProt-GOA-ACAP-ARAP_23.01.2024_0	0.00	0.00	0.00	0.00	[5]	Group15	17.69	26.00	[EIF3B, EIF3F, EIF3G, EIF3H, EIF3K, EIF6, GEMIN2, GEMIN6, LOC100738684, LOC102162486, LSM2, LUC7L, MRPS7, PIH1D1, POLR2D, PRPF31, PRPF6, PUF60, RPL38, RPS15, RPS19, RPS5, SNRPD2, SN
GO:0022618	protein-RNA complex assembly	GO_BiologicalProcess-EBI-UniProt-GOA-ACAP-ARAP_23.01.2024_0	0.00	0.00	0.00	0.00	[5, 6]	Group15	18.71	26.00	[EIF3B, EIF3F, EIF3G, EIF3H, EIF3K, EIF6, GEMIN2, GEMIN6, LOC100738684, LOC102162486, LSM2, LUC7L, MRPS7, PIH1D1, POLR2D, PRPF31, PRPF6, PUF60, RPL38, RPS15, RPS19, RPS5, SNRPD2, SN
GO:0042273	ribosomal large subunit biogenesis	GO_BiologicalProcess-EBI-UniProt-GOA-ACAP-ARAP_23.01.2024_0	0.00	0.00	0.00	0.00	[5, 6]	Group15	25.00	14.00	[EIF6, FTSJ3, LOC100739087, MALSU1, NOP2, RPL10A, RPL14, RPL26, RPL35, RPL7, RPL7A, RPL7L1, SNU13, WDR74]
GO:0042274	ribosomal small subunit biogenesis	GO_BiologicalProcess-EBI-UniProt-GOA-ACAP-ARAP_23.01.2024_0	0.00	0.00	0.00	0.00	[5, 6]	Group15	34.09	30.00	[EMG1, FBL, FCF1, IMP3, IMP4, MRPS7, NOP14, RPP40, RPS11, RPS12, RPS13, RPS14, RPS15, RPS15A, RPS16, RPS17, RPS19, RPS19BP1, RPS21, RPS23, RPS24, RPS4X, RPS5, RPS6, RPS7, RPS8, RP
GO:0006396	RNA processing	GO_BiologicalProcess-EBI-UniProt-GOA-ACAP-ARAP_23.01.2024_0	0.00	0.00	0.00	0.00	[6, 7]	Group15	11.89	88.00	[ADAD2, BCAS2, BUD23, BUD31, CCNL2, DDX49, DUS1L, DUS3L, EIF6, ELAC2, EMG1, ERI3, EXOSC4, EXOSC8, FAM50A, FBL, FCF1, FTSJ3, FXR2, GEMIN2, GEMIN6, GON7, HSD17B10, HSPA8, IMP3, IMP
GO:0034660	ncRNA metabolic process	GO_BiologicalProcess-EBI-UniProt-GOA-ACAP-ARAP_23.01.2024_0	0.00	0.00	0.00	0.00	[6, 7]	Group15	12.33	65.00	[AARSD1, BUD23, CARS2, DDX11, DDX49, DUS1L, DUS3L, EIF6, ELAC2, ELOB, EMG1, ERI3, EXOSC4, FBL, FCF1, FTSJ3, GNL3, GON7, HSD17B10, IMP3, IMP4, INTS11, LRRC47, MEPCE, METTL2A, MRM1
GO:0016072	rRNA metabolic process	GO_BiologicalProcess-EBI-UniProt-GOA-ACAP-ARAP_23.01.2024_0	0.00	0.00	0.00	0.00	[7, 8]	Group15	20.00	39.00	[BUD23, DDX11, DDX49, EIF6, EMG1, ERI3, EXOSC4, FBL, FCF1, FTSJ3, IMP3, IMP4, MRM1, NOP10, NOP14, NOP2, PELP1, PIH1D1, POP7, PPAN, RPL10A, RPL26, RPL35, RPL7, RPL7A, RPL7L1, RPP40, R
GO:0034470	ncRNA processing	GO_BiologicalProcess-EBI-UniProt-GOA-ACAP-ARAP_23.01.2024_0	0.00	0.00	0.00	0.00	[7, 8]	Group15	16.81	57.00	[BUD23, DDX49, DUS1L, DUS3L, EIF6, ELAC2, EMG1, ERI3, EXOSC4, FBL, FCF1, FTSJ3, GON7, HSD17B10, IMP3, IMP4, INTS11, METTL2A, MRM1, MTO1, NOP10, NOP14, NOP2, PELP1, PIH1D1, PLD6, PN
GO:0006364	rRNA processing	GO_BiologicalProcess-EBI-UniProt-GOA-ACAP-ARAP_23.01.2024_0	0.00	0.00	0.00	0.00	[6, 8, 9]	Group15	23.31	38.00	[BUD23, DDX49, EIF6, EMG1, ERI3, EXOSC4, FBL, FCF1, FTSJ3, IMP3, IMP4, MRM1, NOP10, NOP14, NOP2, PELP1, PIH1D1, POP7, PPAN, RPL10A, RPL26, RPL35, RPL7, RPL7A, RPL7L1, RPP40, RPS16, R
GO:0034641	cellular nitrogen compound metabolic process	GO_BiologicalProcess-EBI-UniProt-GOA-ACAP-ARAP_23.01.2024_0	0.00	0.00	0.00	0.00	[3]	Group16	8.28	401.00	[AARSD1, ABLIM3, ACAT1, ADA, ADAD2, ADRM1, AJUBA, ALAD, ALDOC, APRT, ARG2, ARL2, ASPDH, ATP5F1A, ATP5F1D, ATP5IF1, ATP5MC1, ATP5MC2, ATP5ME, ATP5MF, ATP5PD, ATP5PF, ATP5PO, A
GO:0043170	macromolecule metabolic process	GO_BiologicalProcess-EBI-UniProt-GOA-ACAP-ARAP_23.01.2024_0	0.00	0.00	0.00	0.00	[3]	Group16	7.38	504.00	[AARSD1, ABHD17A, ABLIM3, ADAD2, ADRM1, AGA, AIMP2, AIP, AJUBA, ALAD, ALG3, AMZ2, ANAPC11, ARG2, ARL2, ARPP19, ARR3, ARRDC1, ARRDC4, ASB11, ASGR2, ASIP, ASTL, ATP5IF1, ATRAID, AU
GO:0043603	amide metabolic process	GO_BiologicalProcess-EBI-UniProt-GOA-ACAP-ARAP_23.01.2024_0	0.00	0.00	0.00	0.00	[3]	Group16	16.48	150.00	[AARSD1, ACAT1, ADA, AJUBA, AMDHD2, ARG2, AURKA, B4GALT4, CARS2, CERS4, CIRBP, CLN6, CLN8, CTNS, DAPK3, DDX25, DHFR, DPEP1, DRG1, DUS3L, EEF1B2, EEF1D, EEF1G, EIF2B2, EIF3B, EIF
GO:0044249	cellular biosynthetic process	GO_BiologicalProcess-EBI-UniProt-GOA-ACAP-ARAP_23.01.2024_0	0.00	0.00	0.00	0.00	[3]	Group16	7.73	451.00	[AARSD1, ABLIM3, ACAT1, ADA, ADAD2, ADCK2, ADI1, ADRM1, AIP, AJUBA, AKR1A1, ALAD, ALG3, APRT, ARG2, ARL2, ARPP19, ARRDC4, ASIP, ASPDH, ATP5F1A, ATP5F1D, ATP5IF1, ATP5MC1, ATP5M
GO:1901564	organonitrogen compound metabolic process	GO_BiologicalProcess-EBI-UniProt-GOA-ACAP-ARAP_23.01.2024_0	0.00	0.00	0.00	0.00	[3]	Group16	9.46	377.00	[AARSD1, ACAT1, ADA, ADI1, AGA, AIMP2, AIP, AJUBA, ALAD, ALDOC, ALG3, AMDHD2, AMZ2, ANAPC11, APRT, ARG2, ARL2, ARPP19, ARR3, ARRDC1, ARRDC4, ASB11, ASGR2, ASPA, ASPDH, ASTL, AT
GO:1901576	organic substance biosynthetic process	GO_BiologicalProcess-EBI-UniProt-GOA-ACAP-ARAP_23.01.2024_0	0.00	0.00	0.00	0.00	[3]	Group16	7.81	461.00	[AARSD1, ABLIM3, ACAT1, ADA, ADAD2, ADCK2, ADI1, ADRM1, AIP, AJUBA, AKR1A1, ALAD, ALDOC, ALG3, APRT, ARG2, ARL2, ARPP19, ARRDC4, ASIP, ASPDH, ATP5F1A, ATP5F1D, ATP5IF1, ATP5MC1,
GO:0019538	protein metabolic process	GO_BiologicalProcess-EBI-UniProt-GOA-ACAP-ARAP_23.01.2024_0	0.00	0.00	0.00	0.00	[3, 4]	Group16	9.28	292.00	[AARSD1, AGA, AIMP2, AIP, AJUBA, ALAD, ALG3, AMZ2, ANAPC11, ARL2, ARPP19, ARR3, ARRDC1, ARRDC4, ASB11, ASGR2, ASTL, ATP5IF1, ATRAID, AUP1, AURKA, AURKAIP1, B4GALT4, B4GALT7, BA
GO:0006518	peptide metabolic process	GO_BiologicalProcess-EBI-UniProt-GOA-ACAP-ARAP_23.01.2024_0	0.00	0.00	0.00	0.00	[4]	Group16	18.16	126.00	[AARSD1, AJUBA, AURKA, CARS2, CIRBP, CTNS, DAPK3, DDX25, DPEP1, DRG1, DUS3L, EEF1B2, EEF1D, EEF1G, EIF2B2, EIF3B, EIF3F, EIF3G, EIF3H, EIF3K, EIF4EBP1, EIF6, ERAP2, ETHE1, FAU, FXR2
GO:0009059	macromolecule biosynthetic process	GO_BiologicalProcess-EBI-UniProt-GOA-ACAP-ARAP_23.01.2024_0	0.00	0.00	0.00	0.00	[4]	Group16	7.29	378.00	[AARSD1, ABLIM3, ADAD2, ADRM1, AIP, AJUBA, ALG3, ARG2, ARL2, ARRDC4, ASIP, ATRAID, AURKA, B4GALT4, B4GALT7, BAG2, BCAS2, BOLA3, BUD23, BUD31, CARHSP1, CARS2, CBX8, CCNL2, CCT4
GO:0044271	cellular nitrogen compound biosynthetic process	GO_BiologicalProcess-EBI-UniProt-GOA-ACAP-ARAP_23.01.2024_0	0.00	0.00	0.00	0.00	[4]	Group16	7.82	280.00	[AARSD1, ABLIM3, ACAT1, ADA, ADRM1, AJUBA, ALAD, APRT, ARG2, ASPDH, ATP5F1A, ATP5F1D, ATP5IF1, ATP5MC1, ATP5MC2, ATP5ME, ATP5MF, ATP5PD, ATP5PF, ATP5PO, AURKA, B4GALT4, BUD
GO:1901566	organonitrogen compound biosynthetic process	GO_BiologicalProcess-EBI-UniProt-GOA-ACAP-ARAP_23.01.2024_0	0.00	0.00	0.00	0.00	[4]	Group16	13.50	172.00	[AARSD1, ACAT1, ADA, ADI1, AJUBA, ALAD, ALG3, APRT, ARG2, ASPDH, ATP5F1A, ATP5F1D, ATP5IF1, ATP5MC1, ATP5MC2, ATP5ME, ATP5MF, ATP5PD, ATP5PF, ATP5PO, AURKA, B4GALT4, B4GALT7,
GO:0043604	amide biosynthetic process	GO_BiologicalProcess-EBI-UniProt-GOA-ACAP-ARAP_23.01.2024_0	0.00	0.00	0.00	0.00	[4, 5]	Group16	18.58	120.00	[AARSD1, ACAT1, AJUBA, ARG2, AURKA, B4GALT4, CARS2, CERS4, CIRBP, DAPK3, DDX25, DRG1, DUS3L, EEF1B2, EEF1D, EEF1G, EIF2B2, EIF3B, EIF3F, EIF3G, EIF3H, EIF3K, EIF4EBP1, EIF6, FAU, FX
GO:0010467	gene expression	GO_BiologicalProcess-EBI-UniProt-GOA-ACAP-ARAP_23.01.2024_0	0.00	0.00	0.00	0.00	[5]	Group16	7.39	356.00	[AARSD1, ABLIM3, ADAD2, ADRM1, AIP, AJUBA, ARG2, ARL2, ARRDC4, ASIP, ATRAID, AURKA, BAG2, BCAS2, BOLA3, BUD23, BUD31, CARHSP1, CARS2, CBX8, CCNL2, CCT4, CCT6B, CD274, CD40, CDK
GO:0006412	translation	GO_BiologicalProcess-EBI-UniProt-GOA-ACAP-ARAP_23.01.2024_0	0.00	0.00	0.00	0.00	[4, 5, 6, 7]	Group16	20.95	110.00	[AARSD1, AJUBA, AURKA, CARS2, CIRBP, DAPK3, DDX25, DRG1, DUS3L, EEF1B2, EEF1D, EEF1G, EIF2B2, EIF3B, EIF3F, EIF3G, EIF3H, EIF3K, EIF4EBP1, EIF6, FAU, FXR2, GAPDH, LOC100738684, LOC1
GO:0043043	peptide biosynthetic process	GO_BiologicalProcess-EBI-UniProt-GOA-ACAP-ARAP_23.01.2024_0	0.00	0.00	0.00	0.00	[5, 6]	Group16	20.55	112.00	[AARSD1, AJUBA, AURKA, CARS2, CIRBP, DAPK3, DDX25, DRG1, DUS3L, EEF1B2, EEF1D, EEF1G, EIF2B2, EIF3B, EIF3F, EIF3G, EIF3H, EIF3K, EIF4EBP1, EIF6, FAU, FXR2, GAPDH, GGT1, LOC10073868
GO:2001141	regulation of RNA biosynthetic process	GO_BiologicalProcess-EBI-UniProt-GOA-ACAP-ARAP_23.01.2024_0	0.00	0.01	0.00	0.00	[5, 6, 7, 8]	Group16	4.13	106.00	[ABLIM3, AJUBA, BUD31, CBX8, CCNL2, CD274, CD40, CDK5RAP2, CDK5RAP3, COMMD7, CTDSP2, CTH, DDRGK1, DDX11, DMAP1, DVL1, EDF1, ERCC1, FHL2, FIGLA, FSTL3, FZR1, GLMP, GNL3, GTF2F
GO:0006355	regulation of DNA-templated transcription	GO_BiologicalProcess-EBI-UniProt-GOA-ACAP-ARAP_23.01.2024_0	0.00	0.01	0.00	0.00	[6, 7, 8, 9]	Group16	4.14	106.00	[ABLIM3, AJUBA, BUD31, CBX8, CCNL2, CD274, CD40, CDK5RAP2, CDK5RAP3, COMMD7, CTDSP2, CTH, DDRGK1, DDX11, DMAP1, DVL1, EDF1, ERCC1, FHL2, FIGLA, FSTL3, FZR1, GLMP, GNL3, GTF2F
GO:0046933	proton-transporting ATP synthase activity, rotational mechanism	GO_BiologicalProcess-EBI-UniProt-GOA-ACAP-ARAP_23.01.2024_0	0.00	0.01	0.00	0.00	8, 9, 10, 11, 12, 13]	Group17	60.00	6.00	[ATP5F1A, ATP5F1D, ATP5ME, ATP5MF, ATP5PF, ATP5PO]
GO:0042776	proton motive force-driven mitochondrial ATP synthesis	GO_BiologicalProcess-EBI-UniProt-GOA-ACAP-ARAP_23.01.2024_0	0.00	0.01	0.00	0.00	[8, 10, 11, 12, 13]	Group17	60.00	6.00	[ATP5F1A, ATP5F1D, ATP5ME, ATP5MF, ATP5PF, ATP5PO]
GO:0019637	organophosphate metabolic process	GO_BiologicalProcess-EBI-UniProt-GOA-ACAP-ARAP_23.01.2024_0	0.00	0.00	0.00	0.00	[3, 4]	Group17	10.76	85.00	[ACAT1, ADA, AJUBA, AKR1A1, ALDOC, APRT, ARL2, ASPDH, ATP5F1A, ATP5F1D, ATP5IF1, ATP5MC1, ATP5MC2, ATP5ME, ATP5MF, ATP5PD, ATP5PF, ATP5PO, BPGM, CDA, CDIPT, CKMT1A, CLN8, CTN
GO:0055086	nucleobase-containing small molecule metabolic process	GO_BiologicalProcess-EBI-UniProt-GOA-ACAP-ARAP_23.01.2024_0	0.00	0.00	0.00	0.00	[3, 4, 5]	Group17	12.81	62.00	[ACAT1, ADA, ALDOC, APRT, ARL2, ASPDH, ATP5F1A, ATP5F1D, ATP5IF1, ATP5MC1, ATP5MC2, ATP5ME, ATP5MF, ATP5PD, ATP5PF, ATP5PO, BPGM, CDA, CTNS, DNPH1, DTYMK, DUT, EEF1D, EIF6, FI
GO:0072521	purine-containing compound metabolic process	GO_BiologicalProcess-EBI-UniProt-GOA-ACAP-ARAP_23.01.2024_0	0.00	0.01	0.00	0.00	[4]	Group17	11.88	48.00	[ACAT1, ADA, ALDOC, APRT, ARL2, ASPDH, ATP5F1A, ATP5F1D, ATP5IF1, ATP5MC1, ATP5MC2, ATP5ME, ATP5MF, ATP5PD, ATP5PF, ATP5PO, BPGM, CTNS, EEF1D, EIF6, FHIT, FIS1, GALK1, GAPDH, G
GO:0019693	ribose phosphate metabolic process	GO_BiologicalProcess-EBI-UniProt-GOA-ACAP-ARAP_23.01.2024_0	0.00	0.00	0.00	0.00	[4, 5]	Group17	12.65	43.00	[ACAT1, ADA, ALDOC, APRT, ARL2, ATP5F1A, ATP5F1D, ATP5IF1, ATP5MC1, ATP5MC2, ATP5ME, ATP5MF, ATP5PD, ATP5PF, ATP5PO, BPGM, CDA, CTNS, EIF6, FIS1, GALK1, GAPDH, GMPS, GPD1, HSF
GO:0090407	organophosphate biosynthetic process	GO_BiologicalProcess-EBI-UniProt-GOA-ACAP-ARAP_23.01.2024_0	0.00	0.00	0.00	0.00	[4, 5]	Group17	12.25	49.00	[ACAT1, ADA, AJUBA, AKR1A1, APRT, ASPDH, ATP5F1A, ATP5F1D, ATP5MC1, ATP5MC2, ATP5ME, ATP5MF, ATP5PD, ATP5PF, ATP5PO, CDA, CDIPT, CKMT1A, DTYMK, DUT, EEF1D, FDPS, FLAD1, GMP
GO:0006753	nucleoside phosphate metabolic process	GO_BiologicalProcess-EBI-UniProt-GOA-ACAP-ARAP_23.01.2024_0	0.00	0.00	0.00	0.00	[4, 5, 6]	Group17	13.76	60.00	[ACAT1, ADA, ALDOC, APRT, ARL2, ASPDH, ATP5F1A, ATP5F1D, ATP5IF1, ATP5MC1, ATP5MC2, ATP5ME, ATP5MF, ATP5PD, ATP5PF, ATP5PO, BPGM, CDA, CTNS, DNPH1, DTYMK, DUT, EEF1D, EIF6, FI
GO:0009117	nucleotide metabolic process	GO_BiologicalProcess-EBI-UniProt-GOA-ACAP-ARAP_23.01.2024_0	0.00	0.00	0.00	0.00	[5, 6, 7]	Group17	13.75	59.00	[ACAT1, ADA, ALDOC, APRT, ARL2, ASPDH, ATP5F1A, ATP5F1D, ATP5IF1, ATP5MC1, ATP5MC2, ATP5ME, ATP5MF, ATP5PD, ATP5PF, ATP5PO, BPGM, CDA, CTNS, DNPH1, DTYMK, DUT, EEF1D, EIF6, FI
GO:0009141	nucleoside triphosphate metabolic process	GO_BiologicalProcess-EBI-UniProt-GOA-ACAP-ARAP_23.01.2024_0	0.00	0.00	0.00	0.00	[5, 6, 7]	Group17	15.38	28.00	[ADA, ALDOC, ARL2, ATP5F1A, ATP5F1D, ATP5IF1, ATP5MC1, ATP5MC2, ATP5ME, ATP5MF, ATP5PD, ATP5PF, ATP5PO, BPGM, CTNS, DTYMK, DUT, EIF6, FIS1, GALK1, GAPDH, GPD1, HSPA8, NME2, NU
GO:1901293	nucleoside phosphate biosynthetic process	GO_BiologicalProcess-EBI-UniProt-GOA-ACAP-ARAP_23.01.2024_0	0.00	0.01	0.00	0.00	[5, 6, 7]	Group17	14.84	27.00	[ACAT1, ADA, APRT, ASPDH, ATP5F1A, ATP5F1D, ATP5MC1, ATP5MC2, ATP5ME, ATP5MF, ATP5PD, ATP5PF, ATP5PO, CDA, DTYMK, DUT, EEF1D, FLAD1, GMPS, MLYCD, NAPRT, NME2, NUDT2, QPRT,
GO:0006163	purine nucleotide metabolic process	GO_BiologicalProcess-EBI-UniProt-GOA-ACAP-ARAP_23.01.2024_0	0.00	0.00	0.00	0.00	[5, 6, 7, 8]	Group17	12.30	47.00	[ACAT1, ADA, ALDOC, APRT, ARL2, ASPDH, ATP5F1A, ATP5F1D, ATP5IF1, ATP5MC1, ATP5MC2, ATP5ME, ATP5MF, ATP5PD, ATP5PF, ATP5PO, BPGM, CTNS, EEF1D, EIF6, FHIT, FIS1, GALK1, GAPDH, G
GO:0009259	ribonucleotide metabolic process	GO_BiologicalProcess-EBI-UniProt-GOA-ACAP-ARAP_23.01.2024_0	0.00	0.02	0.00	0.00	[5, 6, 7, 8]	Group17	12.12	40.00	[ACAT1, ADA, ALDOC, APRT, ARL2, ATP5F1A, ATP5F1D, ATP5IF1, ATP5MC1, ATP5MC2, ATP5ME, ATP5MF, ATP5PD, ATP5PF, ATP5PO, BPGM, CDA, CTNS, EIF6, FIS1, GALK1, GAPDH, GMPS, GPD1, HSF
GO:0009144	purine nucleoside triphosphate metabolic process	GO_BiologicalProcess-EBI-UniProt-GOA-ACAP-ARAP_23.01.2024_0	0.00	0.01	0.00	0.00	[6, 7, 8]	Group17	15.66	26.00	[ADA, ALDOC, ARL2, ATP5F1A, ATP5F1D, ATP5IF1, ATP5MC1, ATP5MC2, ATP5ME, ATP5MF, ATP5PD, ATP5PF, ATP5PO, BPGM, CTNS, EIF6, FIS1, GALK1, GAPDH, GPD1, HSPA8, NME2, NUDT2, NUDT5,
GO:0009165	nucleotide biosynthetic process	GO_BiologicalProcess-EBI-UniProt-GOA-ACAP-ARAP_23.01.2024_0	0.00	0.01	0.00	0.00	[6, 7, 8]	Group17	14.92	27.00	[ACAT1, ADA, APRT, ASPDH, ATP5F1A, ATP5F1D, ATP5MC1, ATP5MC2, ATP5ME, ATP5MF, ATP5PD, ATP5PF, ATP5PO, CDA, DTYMK, DUT, EEF1D, FLAD1, GMPS, MLYCD, NAPRT, NME2, NUDT2, QPRT,
GO:0009199	ribonucleoside triphosphate metabolic process	GO_BiologicalProcess-EBI-UniProt-GOA-ACAP-ARAP_23.01.2024_0	0.00	0.02	0.00	0.00	[6, 7, 8]	Group17	14.88	25.00	[ALDOC, ARL2, ATP5F1A, ATP5F1D, ATP5IF1, ATP5MC1, ATP5MC2, ATP5ME, ATP5MF, ATP5PD, ATP5PF, ATP5PO, BPGM, CTNS, EIF6, FIS1, GALK1, GAPDH, GPD1, HSPA8, NME2, NUDT2, NUDT5, PFKM
GO:0015078	proton transmembrane transporter activity	GO_BiologicalProcess-EBI-UniProt-GOA-ACAP-ARAP_23.01.2024_0	0.00	0.04	0.00	0.00	[6, 8]	Group17	17.14	18.00	[ATP5F1A, ATP5F1D, ATP5MC1, ATP5MC2, ATP5ME, ATP5MF, ATP5PD, ATP5PF, ATP5PO, ATP6V0B, ATP6V0E1, ATP6V0E2, ATP6V1F, CTNS, GHITM, SLC25A14, SLC38A5, TCIRG1]
GO:0009145	purine nucleoside triphosphate biosynthetic process	GO_BiologicalProcess-EBI-UniProt-GOA-ACAP-ARAP_23.01.2024_0	0.00	0.04	0.00	0.00	[7, 8, 9]	Group17	25.00	11.00	[ATP5F1A, ATP5F1D, ATP5MC1, ATP5MC2, ATP5ME, ATP5MF, ATP5PD, ATP5PF, ATP5PO, NME2, NUDT2]
GO:0009205	purine ribonucleoside triphosphate metabolic process	GO_BiologicalProcess-EBI-UniProt-GOA-ACAP-ARAP_23.01.2024_0	0.00	0.01	0.00	0.00	[7, 8, 9]	Group17	15.53	25.00	[ALDOC, ARL2, ATP5F1A, ATP5F1D, ATP5IF1, ATP5MC1, ATP5MC2, ATP5ME, ATP5MF, ATP5PD, ATP5PF, ATP5PO, BPGM, CTNS, EIF6, FIS1, GALK1, GAPDH, GPD1, HSPA8, NME2, NUDT2, NUDT5, PFKM
GO:0046034	ATP metabolic process	GO_BiologicalProcess-EBI-UniProt-GOA-ACAP-ARAP_23.01.2024_0	0.00	0.00	0.00	0.00	[7, 8, 9, 10]	Group17	17.27	24.00	[ALDOC, ARL2, ATP5F1A, ATP5F1D, ATP5IF1, ATP5MC1, ATP5MC2, ATP5ME, ATP5MF, ATP5PD, ATP5PF, ATP5PO, BPGM, CTNS, EIF6, FIS1, GALK1, GAPDH, GPD1, HSPA8, NUDT2, NUDT5, PFKM, TPI1]
GO:0009206	purine ribonucleoside triphosphate biosynthetic process	GO_BiologicalProcess-EBI-UniProt-GOA-ACAP-ARAP_23.01.2024_0	0.00	0.03	0.00	0.00	[8, 9, 10]	Group17	25.58	11.00	[ATP5F1A, ATP5F1D, ATP5MC1, ATP5MC2, ATP5ME, ATP5MF, ATP5PD, ATP5PF, ATP5PO, NME2, NUDT2]
GO:0006754	ATP biosynthetic process	GO_BiologicalProcess-EBI-UniProt-GOA-ACAP-ARAP_23.01.2024_0	0.00	0.01	0.00	0.00	[8, 9, 10, 11]	Group17	30.30	10.00	[ATP5F1A, ATP5F1D, ATP5MC1, ATP5MC2, ATP5ME, ATP5MF, ATP5PD, ATP5PF, ATP5PO, NUDT2]

GO:0006091	generation of precursor metabolites and energy	GO_BiologicalProcess-EBI-UniProt-GOA-ACAP-ARAP_23.01.2024_0(	0.00	0.00	0.00	0.00	[3]	Group18	17.01	58.00	[ACAT1, ALDOC, ARL2, ASIP, ATP5F1A, ATP5F1D, ATP5ME, ATP5MF, ATP5PF, ATP5PO, BLOC1S1, BPGM, CIAPIN1, COX5B, COX6A1, COX6C, COX7A1, COX7A2, COX7C, DNAJC15, EIF6, ETFB, GALK1, G
GO:0015980	energy derivation by oxidation of organic compounds	GO_BiologicalProcess-EBI-UniProt-GOA-ACAP-ARAP_23.01.2024_0(	0.00	0.00	0.00	0.00	[4]	Group18	18.70	43.00	[ARL2, ATP5F1A, ATP5F1D, ATP5ME, ATP5MF, ATP5PF, ATP5PO, BLOC1S1, COX5B, COX6A1, COX6C, COX7A1, COX7A2, COX7C, DNAJC15, ETFB, GPD1, IDH2, IDH3B, IDH3G, IMMP2L, LOC100524873, M
GO:0022900	electron transport chain	GO_BiologicalProcess-EBI-UniProt-GOA-ACAP-ARAP_23.01.2024_0(	0.00	0.00	0.00	0.00	[4]	Group18	32.61	30.00	[CIAPIN1, COX5B, COX6C, COX7A1, COX7A2, COX7C, DNAJC15, ETFB, GPD1, IMMP2L, LOC100524873, NDUFA10, NDUFA5, NDUFA7, NDUFA8, NDUFB3, NDUFB7, NDUFB8, NDUFB9, NDUFC2, NDUFS3,
GO:0007005	mitochondrion organization	GO_BiologicalProcess-EBI-UniProt-GOA-ACAP-ARAP_23.01.2024_0(	0.00	0.00	0.00	0.00	[5]	Group18	18.87	70.00	[ACAD9, ALKBH7, APOO, ATP5F1D, ATP5IF1, ATPAF2, BCS1L, BNIP3, CHCHD7, CLN8, COX17, COX7A1, COX7A2, DNAJC11, DYNLL1, FAM162A, FIS1, GHITM, HIGD2A, HSD17B10, IMMP2L, LMNA, LOC10(
GO:0045333	cellular respiration	GO_BiologicalProcess-EBI-UniProt-GOA-ACAP-ARAP_23.01.2024_0(	0.00	0.00	0.00	0.00	[5]	Group18	25.64	40.00	[ARL2, ATP5F1A, ATP5F1D, ATP5ME, ATP5MF, ATP5PF, ATP5PO, BLOC1S1, COX5B, COX6A1, COX6C, COX7A1, COX7A2, COX7C, DNAJC15, ETFB, GPD1, IDH2, IDH3B, IDH3G, IMMP2L, LOC100524873, M
GO:0022904	respiratory electron transport chain	GO_BiologicalProcess-EBI-UniProt-GOA-ACAP-ARAP_23.01.2024_0(	0.00	0.00	0.00	0.00	[5, 6]	Group18	33.78	25.00	[COX5B, COX6C, COX7A1, COX7A2, COX7C, DNAJC15, ETFB, GPD1, IMMP2L, LOC100524873, NDUFA10, NDUFA5, NDUFA7, NDUFA8, NDUFB8, NDUFB9, NDUFC2, NDUFS3, NDUFS8, NDUFV1, NDUFV2,
GO:0009060	aerobic respiration	GO_BiologicalProcess-EBI-UniProt-GOA-ACAP-ARAP_23.01.2024_0(	0.00	0.00	0.00	0.00	[6]	Group18	28.93	35.00	[ARL2, ATP5F1A, ATP5F1D, ATP5ME, ATP5MF, ATP5PF, ATP5PO, BLOC1S1, COX5B, COX6A1, COX6C, COX7A1, COX7A2, COX7C, DNAJC15, IDH2, IDH3B, IDH3G, LOC100524873, MDH1, NDUFA10, NDUF
GO:0010257	NADH dehydrogenase complex assembly	GO_BiologicalProcess-EBI-UniProt-GOA-ACAP-ARAP_23.01.2024_0(	0.00	0.00	0.00	0.00	[6]	Group18	44.23	23.00	[ACAD9, BCS1L, NDUFA10, NDUFA11, NDUFA12, NDUFA5, NDUFA9, NDUFAB1, NDUFB10, NDUFB11, NDUFB2, NDUFB4, NDUFB7, NDUFB8, NDUFB9, NDUFC1, NDUFC2, NDUFS3, NDUFS4, NDUFS5, NDU
GO:0033108	mitochondrial respiratory chain complex assembly	GO_BiologicalProcess-EBI-UniProt-GOA-ACAP-ARAP_23.01.2024_0(	0.00	0.00	0.00	0.00	[6]	Group18	42.67	32.00	[ACAD9, BCS1L, CHCHD7, COX17, IMMP2L, NDUFA10, NDUFA11, NDUFA12, NDUFA5, NDUFA9, NDUFAB1, NDUFB10, NDUFB11, NDUFB2, NDUFB4, NDUFB7, NDUFB8, NDUFB9, NDUFC1, NDUFC2, NDUF
GO:0006119	oxidative phosphorylation	GO_BiologicalProcess-EBI-UniProt-GOA-ACAP-ARAP_23.01.2024_0(	0.00	0.00	0.00	0.00	[7]	Group18	34.57	28.00	[ATP5F1A, ATP5F1D, ATP5ME, ATP5MF, ATP5PF, ATP5PO, COX5B, COX6A1, COX6C, COX7A1, COX7A2, COX7C, DNAJC15, LOC100524873, NDUFA10, NDUFA7, NDUFA8, NDUFB8, NDUFB9, NDUFC2, ND
GO:0019646	aerobic electron transport chain	GO_BiologicalProcess-EBI-UniProt-GOA-ACAP-ARAP_23.01.2024_0(	0.00	0.00	0.00	0.00	[6, 7, 8]	Group18	42.22	19.00	[COX5B, COX6C, COX7A1, COX7A2, COX7C, DNAJC15, LOC100524873, NDUFA10, NDUFA8, NDUFB8, NDUFB9, NDUFC2, NDUFS3, NDUFS8, NDUFV1, NDUFV2, PARK7, UQCR10, UQCRB]
GO:0032981	mitochondrial respiratory chain complex I assembly	GO_BiologicalProcess-EBI-UniProt-GOA-ACAP-ARAP_23.01.2024_0(	0.00	0.00	0.00	0.00	[7]	Group18	44.23	23.00	[ACAD9, BCS1L, NDUFA10, NDUFA11, NDUFA12, NDUFA5, NDUFA9, NDUFAB1, NDUFB10, NDUFB11, NDUFB2, NDUFB4, NDUFB7, NDUFB8, NDUFB9, NDUFC1, NDUFC2, NDUFS3, NDUFS4, NDUFS5, NDU
GO:0042773	ATP synthesis coupled electron transport	GO_BiologicalProcess-EBI-UniProt-GOA-ACAP-ARAP_23.01.2024_0(	0.00	0.00	0.00	0.00	[6, 7, 8]	Group18	37.50	21.00	[COX5B, COX6C, COX7A1, COX7A2, COX7C, DNAJC15, LOC100524873, NDUFA10, NDUFA7, NDUFA8, NDUFB8, NDUFB9, NDUFC2, NDUFS3, NDUFS8, NDUFV1, NDUFV2, NDUFV3, PARK7, UQCR10, UQC
GO:0042775	mitochondrial ATP synthesis coupled electron transport	GO_BiologicalProcess-EBI-UniProt-GOA-ACAP-ARAP_23.01.2024_0(	0.00	0.00	0.00	0.00	[7, 8, 9]	Group18	38.46	20.00	[COX5B, COX6C, COX7A1, COX7A2, COX7C, DNAJC15, LOC100524873, NDUFA10, NDUFA8, NDUFB8, NDUFB9, NDUFC2, NDUFS3, NDUFS8, NDUFV1, NDUFV2, NDUFV3, PARK7, UQCR10, UQCRB]
GO:0006120	mitochondrial electron transport, NADH to ubiquinone	GO_BiologicalProcess-EBI-UniProt-GOA-ACAP-ARAP_23.01.2024_0(	0.00	0.00	0.00	0.00	[7, 8, 9, 10]	Group18	45.83	11.00	[DNAJC15, NDUFA10, NDUFA8, NDUFB8, NDUFB9, NDUFC2, NDUFS3, NDUFS8, NDUFV1, NDUFV2, PARK7]

247, CD274, CD320, CD40, CDK10, CDK5RAP2, CDK5RAP3, CDT1, CEP131, CHMP2A, CIDEA, CLN6, COMMD1, COPS9, COX17, CRB3, CTDNEP1, CTH, CTNS, CTSD, CUL7, DAPK3, DBF4B, DBNL, DDRGK1, DDT, DDX11, DDX49, DHPS, DMAP1, DRG1, DVL1, DYNLL1, EDF1, EHD1, EIF2B2, EIF6, EIPR1, EPHB6, ERCC1, ERP29, EXOSC4, FAM162A, FIS1, FKBP1B, FLOT1, FSTL3, FXR2, FZR1, GAPDH, GDF3, GDF9, GGA1, GLMP, GNL3, GNL3L, GPC3, GPD1, GPR108, GTF2F1, HAX1, HCRT, HDGFL2, HSPA8, HY
ATRIP, AURKA, AVEN, AVPI1, B4GALT7, BAG2, BAG5, BCAP31, BMP8A, BNIP1, BNIP3, BORCS5, BUD23, BUD31, CABLES1, CAPZB, CARHSP1, CASQ2, CBX8, CCDC32, CCNL2, CCT4, CD247, CD274, CD320, CD40, CDA, CDH6, CDK10, CDK5, CDK5RAP2, CDK5RAP3, CDT1, CEP131, CFAP298, CHID1, CHMP1A, CHMP2A, CHMP4A, CHMP6, CIAPIN1, CIDEA, CIRBP, CKMT1A, CLN8, CNPY2, COMMD1, COMMD7, COPS3, COPS9, COX17, COX7A1, COX7A2, CRB3, CRHR1, CRSP3, CTDNEP1, CTDSP2, CTH, CTN
CDK5RAP2, CDK5RAP3, CDT1, CEP131, CHMP2A, COPS9, COX17, CRB3, CTDNEP1, CTH, CTNS, CTSD, CUL7, DAPK3, DBF4B, DBNL, DDRGK1, DDX11, DDX49, DHPS, DMAP1, DRG1, DVL1, DYNLL1, EDF1, EHD1, EIF6, EIPR1, EPHB6, ERCC1, ERP29, EXOSC4, FAM162A, FIS1, FKBP1B, FLOT1, FSTL3, FXR2, FZR1, GAPDH, GDF3, GDF9, GLMP, GNL3, GNL3L, GPC3, GPD1, GTF2F1, HAX1, HCRT, HDGFL2, HSPA8, HYAL3, IER2, IGFBP6, INO80B, IP6K2, ITGB7, JAM3, KAT5, KDM8, KLF1, LAMTOR1, LAMTOR2
DNPH1, DPEP1, DUT, ECHS1, EIF6, ELOB, EMC6, EPHX1, ERAP2, ESD, ETFB, EXOSC4, EXOSC8, FHIT, FXR2, GABARAP, GABARAPL1, GALK1, GAPDH, GATD1, GBA2, GGT1, GPD1, GSTM3, GSTZ1, GUSB, HADHA, HAGH, HSD17B10, HSPA8, KAT5, LRSAM1, LSM2, LSM4, MAP1LC3B, MAPK3, MAPKAPK2, MFSD2A, MGST2, MLYCD, MTHFS, NEU1, NQO2, NSFL1C, NUDT16L1, NUDT18, NUDT5, PARK7, PCID2, PFKM, PHB2, PNLDC1, PNPLA4, POLR2G, PRDX1, PRDX2, PRDX3, PRDX4, PRR5L, QPRT, RAB7.
RAP3, CHMP1A, CHMP2A, CHMP4A, CHMP6, CIDEA, CLN6, CLN8, CLPP, CLPS, COMMD1, COPS3, CTSD, CTSF, DDRGK1, DDX49, DERL3, DMGDH, DNASE2, DNPH1, DPEP1, DUT, DVL1, ECHS1, EIF3H, EIF6, ELOB, ERAP2, ESD, ETFB, EXOSC4, EXOSC8, FBXO31, FHIT, FXR2, FZR1, GALK1, GAPDH, GATD1, GBA2, GGA1, GGT1, GPC3, GPD1, GSTZ1, GUSB, HADHA, HAGH, HSD17B10, HSPA8, HYAL3, KAT5, LONP1, LRSAM1, LSM2, LSM4, MANBA, MAPKAPK2, MFSD2A, MGST2, MLYCD, MTHFS, NEDD8, I
HMP4A, CHMP6, CLN8, CLPP, COMMD1, COPS3, CTSD, CTSF, DDRGK1, DERL3, DMGDH, DPEP1, DUT, DVL1, EIF3H, EIF6, ERAP2, ETFB, FBXO31, FHIT, FZR1, GALK1, GAPDH, GBA2, GGA1, GGT1, GPC3, GPD1, GSTZ1, GUSB, HSD17B10, HSPA8, HYAL3, KAT5, LONP1, LRSAM1, MANBA, MLYCD, MTHFS, NEDD8, NEU1, NSFL1C, NUDT18, PARK7, PDLIM2, PFKM, PIN1, PPP1R11, PSMA6, PSMB4, PSMB6, PSMB7, PSMD13, PSME2, QPRT, RAB7A, RACK1, RNF166, RNF26, RPL11, RPS27A, SDS, SELENOS,

RPS16, RPS17, RPS21, RPS24, RPS6, RPS7, RPS8, SNU13, TBL3, TFB1M, TSR3, WDR74]
RPS17, RPS21, RPS24, RPS6, RPS7, RPS8, SNU13, TBL3, TFB1M, TSR3, WDR74]

CCDC28B, CCT4, CDK10, CDK5RAP2, CDT1, CEP131, CFAP298, CHCHD7, CHMP1A, CHMP2A, CHMP4A, CHMP6, CIDEA, CIRBP, CLN6, CLN8, CORO6, COX17, COX7A1, COX7A2, CTDNEP1, CTSD, CUL7, DAPK3, DBNL, DDRGK1, DDX11, DNAI1, DNAJB13, DNAJC11, DRC7, DRG1, DVL1, DYNLL1, EFHC1, EHD1, EIF6, EMC3, EMC6, EMC7, EMG1, ENKD1, ERCC1, FAM107A, FAM110A, FAM162A, FIS1, FZR1, GABARAP, GAPDH, GBA2, GHITM, GINS2, GMFG, GNL3, GNL3L, GORASP1, HAUS1, HAUS7, HAX1, HI
0523672, LONP1, MICOS13, MPV17, NDUFA10, NDUFA11, NDUFA12, NDUFA5, NDUFA9, NDUFAB1, NDUFB10, NDUFB11, NDUFB2, NDUFB4, NDUFB7, NDUFB8, NDUFB9, NDUFC1, NDUFC2, NDUFS3, NDUFS4, NDUFS5, NDUFS8, PARK7, PDCD5, PET100, PHB2, PLD6, PRDX3, ROMO1, SAMM50, SDHAF1, SFN, SLC25A6, SLIRP, SMIM20, SPATA19, SPG7, SSBP1, STMP1, TIMM10, TIMM22, TIMM50, TMEM11, TMEM126A, TMEM186, TMEM223, TRAK2]
UFS8, TMEM126A, TMEM186]
FS3, NDUFS4, NDUFS5, NDUFS8, PET100, SAMM50, SDHAF1, SMIM20, STMP1, TMEM126A, TMEM186, TMEM223]
UFS8, TMEM126A, TMEM186]
M40L]

H6, CDK10, CDK5RAP2, CDT1, CEP131, CFAP298, CHCHD7, CHMP1A, CHMP2A, CHMP4A, CHMP6, CIAO2B, CIAPIN1, CIRBP, CLDN10, COPS7A, COX17, COX7A1, COX7A2, CRB3, CTH, CTSD, DAPK3, DBNL, DDX49, DNAI1, DNAJB13, DNAJC15, DRC7, DRG1, DVL1, DYNLL1, EBNA1BP2, EHD1, EIF3B, EIF3F, EIF3G, EIF3H, EIF3K, EIF6, EMC6, EMG1, ENKD1, ERCC1, ERI3, EXOSC4, FAM107A, FBL, FCF1, FLOT1, FTSJ3, FXR2, FZR1, GABARAP, GBA2, GEMIN2, GEMIN6, GLRX3, GNL3L, GPX4, GTF2H5, HAUS
CCDC28B, CCT4, CDK10, CDK5RAP2, CDT1, CEP131, CFAP298, CHCHD7, CHMP1A, CHMP2A, CHMP4A, CHMP6, CIDEA, CIRBP, CLN6, CLN8, CORO6, COX17, COX7A1, COX7A2, CTDNEP1, CTSD, CUL7, DAPK3, DBNL, DDRGK1, DDX11, DNAI1, DNAJB13, DNAJC11, DRC7, DRG1, DVL1, DYNLL1, EFHC1, EHD1, EIF6, EMC3, EMC6, EMC7, EMG1, ENKD1, ERCC1, FAM107A, FAM110A, FAM162A, FIS1, FZR1, GABARAP, GAPDH, GBA2, GHITM, GINS2, GMFG, GNL3, GNL3L, GORASP1, HAUS1, HAUS7, HAX1, HI
RAP2, CDT1, CEP131, CFAP298, CHCHD7, CHMP1A, CHMP2A, CHMP4A, CHMP6, CIAO2B, CIAPIN1, CIRBP, CLDN10, COPS7A, COX17, COX7A1, COX7A2, CRB3, CTH, CTSD, DAPK3, DBNL, DNAI1, DNAJB13, DNAJC15, DRC7, DRG1, DVL1, DYNLL1, EHD1, EIF3B, EIF3F, EIF3G, EIF3H, EIF3K, EIF6, EMC6, ENKD1, ERCC1, FAM107A, FLOT1, FXR2, FZR1, GABARAP, GBA2, GEMIN2, GEMIN6, GLRX3, GNL3L, GPX4, GTF2H5, HAUS1, HAUS7, HAX1, HIGD2A, HSD17B10, HSPA8, IFT57, IMMP2L, ISCA1, ISCA2, ITG
7L, MALSU1, MPV17L2, MRM1, MRPS7, MRTO4, NOC4L, NOP10, NOP14, NOP2, PELP1, PIH1D1, POLR2D, POP7, PPAN, PRPF31, PRPF6, PUF60, RCL1, RPL10A, RPL14, RPL26, RPL35, RPL38, RPL7, RPL7A, RPL7L1, RPP40, RPS11, RPS12, RPS13, RPS14, RPS15, RPS15A, RPS16, RPS17, RPS19, RPS19BP1, RPS21, RPS23, RPS24, RPS4X, RPS5, RPS6, RPS7, RPS8, RPS9, SNRPD2, SNRPD3, SNU13, TARBP2, TBL3, TFB1M, TNIP1, TSR3, WDR74]
S7A, COX17, COX7A1, COX7A2, CTH, DBNL, DDX11, DMAP1, DNAI1, DNAJC15, DRG1, EHD1, EIF3B, EIF3F, EIF3G, EIF3H, EIF3K, EIF6, FAM107A, FAM50A, FBL, GBA2, GEMIN2, GEMIN6, GNL3L, GPX4, GTF2H5, HAX1, HDGFL2, HIGD2A, HSD17B10, HSPA8, IMMP2L, INO80B, JAM3, KANK3, KAT5, KDM8, LMNA, LOC100738684, LOC102162486, LONP1, LSM2, LUC7L, MAP1LC3B, MBD3, MCRS1, METTL23, MFSD2A, MIF, MKKS, MLST8, MRPL58, MRPS7, NACC2, NCLN, NDUFA10, NDUFA11, NDUFA12, NDUFA
0523672, LONP1, MICOS13, MPV17, NDUFA10, NDUFA11, NDUFA12, NDUFA5, NDUFA9, NDUFAB1, NDUFB10, NDUFB11, NDUFB2, NDUFB4, NDUFB7, NDUFB8, NDUFB9, NDUFC1, NDUFC2, NDUFS3, NDUFS4, NDUFS5, NDUFS8, PARK7, PDCD5, PET100, PHB2, PLD6, PRDX3, ROMO1, SAMM50, SDHAF1, SFN, SLC25A6, SLIRP, SMIM20, SPATA19, SPG7, SSBP1, STMP1, TIMM10, TIMM22, TIMM50, TMEM11, TMEM126A, TMEM186, TMEM223, TRAK2]
CTH, DBNL, DNAI1, DNAJC15, DRG1, EHD1, EIF3B, EIF3F, EIF3G, EIF3H, EIF3K, EIF6, FAM107A, GBA2, GEMIN2, GEMIN6, GNL3L, GPX4, GTF2H5, HAX1, HIGD2A, HSD17B10, HSPA8, IMMP2L, JAM3, KANK3, LOC100738684, LOC102162486, LONP1, LSM2, LUC7L, MFSD2A, MIF, MKKS, MLST8, MRPS7, NACC2, NCLN, NDUFA10, NDUFA11, NDUFA12, NDUFA5, NDUFA9, NDUFAB1, NDUFB10, NDUFB11, NDUFB2, NDUFB4, NDUFB7, NDUFB8, NDUFB9, NDUFC1, NDUFC2, NDUFS3, NDUFS4, NDUFS5, NDUFS8,

NDUFS4, NDUFS8, NDUFV1, NDUFV2, NDUFV3, NQO2, PARK7, UQCR10, UQCRB]
MDH1, NDUFA10, NDUFA5, NDUFA7, NDUFA8, NDUFB8, NDUFB9, NDUFC2, NDUFS3, NDUFS4, NDUFS8, NDUFV1, NDUFV2, NDUFV3, PARK7, SUCLG1, UQCR10, UQCRB]
NDUFV3, PARK7, UQCR10, UQCRB]
FA7, NDUFA8, NDUFB8, NDUFB9, NDUFC2, NDUFS3, NDUFS8, NDUFV1, NDUFV2, NDUFV3, PARK7, SUCLG1, UQCR10, UQCRB]
UFS3, NDUFS8, NDUFV1, NDUFV2, NDUFV3, PARK7, UQCR10, UQCRB]

RB]

H6, CDK10, CDK5RAP2, CDT1, CEP131, CFAP298, CHCHD7, CHMP1A, CHMP2A, CHMP4A, CHMP6, CIAO2B, CIAPIN1, CIRBP, CLDN10, COPS7A, COX17, COX7A1, COX7A2, CRB3, CTH, CTSD, DAPK3, DBNL, DDX49, DNAI1, DNAJB13, DNAJC15, DRC7, DRG1, DVL1, DYNLL1, EBNA1BP2, EHD1, EIF3B, EIF3F, EIF3G, EIF3H, EIF3K, EIF6, EMC6, EMG1, ENKD1, ERCC1, ERI3, EXOSC4, FAM107A, FBL, FCF1, FLOT1, FTSJ3, FXR2, FZR1, GABARAP, GBA2, GEMIN2, GEMIN6, GLRX3, GNL3L, GPX4, GTF2H5, HAUS
7L, MALSU1, MPV17L2, MRM1, MRPS7, MRTO4, NOC4L, NOP10, NOP14, NOP2, PELP1, PIH1D1, POLR2D, POP7, PPAN, PRPF31, PRPF6, PUF60, RCL1, RPL10A, RPL14, RPL26, RPL35, RPL38, RPL7, RPL7A, RPL7L1, RPP40, RPS11, RPS12, RPS13, RPS14, RPS15, RPS15A, RPS16, RPS17, RPS19, RPS19BP1, RPS21, RPS23, RPS24, RPS4X, RPS5, RPS6, RPS7, RPS8, RPS9, SNRPD2, SNRPD3, SNU13, TARBP2, TBL3, TFB1M, TNIP1, TSR3, WDR74]
7, PPAN, RCL1, RPL10A, RPL14, RPL26, RPL35, RPL38, RPL7, RPL7A, RPL7L1, RPP40, RPS11, RPS12, RPS13, RPS14, RPS15, RPS15A, RPS16, RPS17, RPS19, RPS19BP1, RPS21, RPS23, RPS24, RPS4X, RPS5, RPS6, RPS7, RPS8, RPS9, SNU13, TBL3, TFB1M, TNIP1, TSR3, WDR74]
RPD3, TARBP2, TNIP1]
RPD3, TARBP2, TNIP1]

PS9, SNU13, TBL3, TSR3]
4, INTS11, LOC102162486, LSM2, LSM4, LUC7L, METTL2A, MRM1, MTO1, NOP10, NOP14, NOP2, PELP1, PIH1D1, PLD6, PNLDC1, POLR2D, POP4, POP5, POP7, PPAN, PQBP1, PRPF31, PRPF4, PRPF6, PUF60, RCL1, RPL10A, RPL26, RPL35, RPL7, RPL7A, RPL7L1, RPP40, RPS16, RPS17, RPS21, RPS24, RPS6, RPS7, RPS8, SCNM1, SF3B5, SNRNP25, SNRPD2, SNRPD3, SNU13, SPOUT1, SUGP1, TARBP2, TBL3, TBRG4, TFB1M, TMBIM6, TPRKB, TRPT1, TSEN54, TSR3, TUT1, U2AF1L4, URM1, WDR74, ZPR
, MTO1, NOP10, NOP14, NOP2, NUDT16L1, PELP1, PIH1D1, PLD6, PNLDC1, POP4, POP5, POP7, PPAN, RPL10A, RPL26, RPL35, RPL7, RPL7A, RPL7L1, RPP40, RPS16, RPS17, RPS21, RPS24, RPS6, RPS7, RPS8, SNU13, SPOUT1, TARBP2, TBL3, TFB1M, TPRKB, TRPT1, TSEN54, TSR3, TUT1, URM1, WDR74]
RPS16, RPS17, RPS21, RPS24, RPS6, RPS7, RPS8, SNU13, TBL3, TFB1M, TSR3, WDR74]
LDC1, POP4, POP5, POP7, PPAN, RPL10A, RPL26, RPL35, RPL7, RPL7A, RPL7L1, RPP40, RPS16, RPS17, RPS21, RPS24, RPS6, RPS7, RPS8, SNU13, SPOUT1, TARBP2, TBL3, TFB1M, TPRKB, TRPT1, TSEN54, TSR3, TUT1, URM1, WDR74]
PS17, RPS21, RPS24, RPS6, RPS7, RPS8, SNU13, TBL3, TFB1M, TSR3, WDR74]
AURKA, B4GALT4, BCAS2, BPGM, BUD23, BUD31, CARHSP1, CARS2, CBX8, CCNL2, CCT4, CD274, CD40, CDA, CDK5RAP2, CDK5RAP3, CDT1, CERS4, CIDEA, CIRBP, COMMD1, COMMD7, CTDSP2, CTH, CTNS, DAPK3, DBF4B, DDRGK1, DDX11, DDX25, DDX49, DHFR, DMAP1, DNASE2, DNPH1, DRG1, DTYMK, DUS1L, DUS3L, DUT, DVL1, EDF1, EEF1B2, EEF1D, EEF1G, EIF2B2, EIF3B, EIF3F, EIF3G, EIF3H, EIF3K, EIF4EBP1, EIF6, ELAC2, ELOB, ELOF1, EMG1, ERCC1, ERI3, EXOSC4, EXOSC8, FAAP100, F
IP1, AURKA, AURKAIP1, B4GALT4, B4GALT7, BAG2, BAG5, BCAP31, BCAS2, BNIP3, BOLA3, BUD23, BUD31, CALML4, CAPN8, CARHSP1, CARS2, CBX8, CCNL2, CCT4, CCT6B, CD274, CD40, CDC26, CDK5RAP2, CDK5RAP3, CDT1, CHCHD4, CHID1, CHMP1A, CHMP2A, CHMP4A, CHMP6, CHST7, CIAO2B, CIDEA, CIRBP, CLN6, CLN8, CLPP, CNPY2, COMMD1, COMMD7, COPS3, COPS6, COPS7A, CSTB, CTDSP2, CTH, CTSD, CTSF, CUL7, DAD1, DAPK3, DBF4B, DCAF11, DDRGK1, DDX11, DDX25, DDX49, D
3F, EIF3G, EIF3H, EIF3K, EIF4EBP1, EIF6, ERAP2, ETHE1, FAU, FXR2, GAPDH, GBA2, GGT1, GSTK1, GSTM3, HAGH, IMMP2L, LOC100519130, LOC100738684, LOC100739087, LRRC47, MALSU1, MCEE, MGST2, MLYCD, MPV17L2, MRPL14, MRPL17, MRPL2, MRPL20, MRPL22, MRPL23, MRPL27, MRPL30, MRPL36, MRPL37, MRPL51, MRPL58, MRPS11, MRPS12, MRPS15, MRPS18A, MRPS18C, MRPS28, MRPS34, MRPS6, MRPS7, MRPS9, MTHFS, MVD, MVK, NELFE, NEU1, NIT1, NSMAF, OXSM, PAIP2, PAF
C2, ATP5ME, ATP5MF, ATP5PD, ATP5PF, ATP5PO, ATRAID, AURKA, B4GALT4, B4GALT7, BAG2, BCAS2, BOLA3, BUD23, BUD31, CARHSP1, CARS2, CBX8, CCNL2, CCT4, CCT6B, CD274, CD40, CDA, CDIPT, CDK5RAP2, CDK5RAP3, CERS4, CHCHD4, CHID1, CHMP1A, CHST7, CIAO2B, CIDEA, CIRBP, CNPY2, COMMD7, COQ3, CTDNEP1, CTDSP2, CTH, CTNS, DAD1, DAPK3, DDRGK1, DDT, DDX11, DDX25, DDX49, DEGS2, DERL3, DHFR, DMAP1, DNAJB13, DNPH1, DPPA5, DRG1, DTYMK, DUS1L, DUS3L, DI
P5F1A, ATP5F1D, ATP5F1E, ATP5MC1, ATP5MC2, ATP5ME, ATP5MF, ATP5PD, ATP5PF, ATP5PO, ATRAID, AUP1, AURKA, AURKAIP1, B4GALT4, B4GALT7, BAG2, BAG5, BCAP31, BCKDHA, BNIP3, BOLA3, BPGM, CALML4, CAPN8, CARS2, CCT4, CCT6B, CD40, CDA, CDC26, CDK5RAP3, CERS4, CHCHD4, CHMP1A, CHMP2A, CHMP4A, CHMP6, CHST7, CIAO2B, CIRBP, CKMT1A, CLN6, CLN8, CLPP, COMMD1, COPS3, COPS6, COPS7A, CSTB, CTDSP2, CTH, CTNS, CTSD, CTSF, CUL7, DAD1, DAPK3, DCAF11
ATP5MC2, ATP5ME, ATP5MF, ATP5PD, ATP5PF, ATP5PO, ATRAID, AURKA, B4GALT4, B4GALT7, BAG2, BCAS2, BOLA3, BUD23, BUD31, CARHSP1, CARS2, CBX8, CCNL2, CCT4, CCT6B, CD274, CD40, CDA, CDIPT, CDK5RAP2, CDK5RAP3, CERS4, CHCHD4, CHID1, CHMP1A, CHST7, CIAO2B, CIDEA, CIRBP, CKMT1A, CNPY2, COMMD7, COQ3, CTDNEP1, CTDSP2, CTH, CTNS, DAD1, DAPK3, DDRGK1, DDT, DDX11, DDX25, DDX49, DEGS2, DERL3, DHFR, DMAP1, DNAJB13, DNPH1, DPPA5, DRG1, DTYMK,
G2, BAG5, BCAP31, BNIP3, BOLA3, CALML4, CAPN8, CARS2, CCT4, CCT6B, CD40, CDC26, CDK5RAP3, CHCHD4, CHMP1A, CHMP2A, CHMP4A, CHMP6, CHST7, CIAO2B, CIRBP, CLN6, CLN8, CLPP, COMMD1, COPS3, COPS6, COPS7A, CSTB, CTDSP2, CTH, CTSD, CTSF, CUL7, DAD1, DAPK3, DCAF11, DDRGK1, DDX25, DERL3, DNAJB13, DNPEP, DPEP1, DPH7, DPP7, DRG1, DUS3L, DVL1, EEF1B2, EEF1D, EEF1G, EIF2B2, EIF3B, EIF3F, EIF3G, EIF3H, EIF3K, EIF4EBP1, EIF6, EIPR1, ENDOU, ENSA, ERAP2,
, GAPDH, GGT1, GSTK1, GSTM3, HAGH, IMMP2L, LOC100738684, LOC100739087, LRRC47, MALSU1, MGST2, MPV17L2, MRPL14, MRPL17, MRPL2, MRPL20, MRPL22, MRPL23, MRPL27, MRPL30, MRPL36, MRPL37, MRPL51, MRPL58, MRPS11, MRPS12, MRPS15, MRPS18A, MRPS18C, MRPS28, MRPS34, MRPS6, MRPS7, MRPS9, NELFE, PAIP2, PARK7, PCIF1, PIN1, POLR2G, PSENEN, RACK1, REN, RPL10A, RPL11, RPL12, RPL14, RPL17, RPL18, RPL18A, RPL21, RPL23, RPL23A, RPL26, RPL28, RPL29, RF
1, CCT6B, CD274, CD40, CDK5RAP2, CDK5RAP3, CHCHD4, CHID1, CHMP1A, CHST7, CIAO2B, CIDEA, CIRBP, CNPY2, COMMD7, CTDSP2, CTH, DAD1, DAPK3, DDRGK1, DDX11, DDX25, DDX49, DERL3, DMAP1, DNAJB13, DPPA5, DRG1, DUS1L, DUS3L, DVL1, EDF1, EEF1B2, EEF1D, EEF1G, EIF2B2, EIF3B, EIF3F, EIF3G, EIF3H, EIF3K, EIF4EBP1, EIF6, ELAC2, ELOB, ELOF1, EMG1, ERCC1, ERI3, ERP29, EXOSC4, EXOSC8, FAM50A, FAU, FBL, FCF1, FHL2, FIGLA, FKBP2, FKBP4, FLOT1, FSTL3, FTSJ3, FXR2
31, CARS2, CBX8, CCNL2, CCT4, CD274, CD40, CDA, CDK5RAP2, CDK5RAP3, CERS4, CIRBP, COMMD7, CTDSP2, CTH, DAPK3, DDRGK1, DDX11, DDX25, DHFR, DMAP1, DNPH1, DRG1, DTYMK, DUS3L, DUT, DVL1, EDF1, EEF1B2, EEF1D, EEF1G, EIF2B2, EIF3B, EIF3F, EIF3G, EIF3H, EIF3K, EIF4EBP1, EIF6, ELOB, ELOF1, ERCC1, FAU, FHL2, FIGLA, FLAD1, FSTL3, FXR2, FZR1, GAPDH, GGT1, GLMP, GMPS, GNL3, GNL3L, GTF2E2, GTF2F1, GTF2H5, GTF2IRD1, HAX1, HEXIM2, HSBP1, HSPA8, IER2, INO80
CARS2, CDA, CERS4, CHST7, CIRBP, CKMT1A, CTH, DAD1, DAPK3, DDX25, DEGS2, DERL3, DHFR, DNPH1, DRG1, DTYMK, DUS3L, DUT, EEF1B2, EEF1D, EEF1G, EIF2B2, EIF3B, EIF3F, EIF3G, EIF3H, EIF3K, EIF4EBP1, EIF6, FAU, FLAD1, FXR2, GALNT6, GAPDH, GGT1, GMPS, GORASP1, LOC100738684, LOC100739087, LRRC47, MALSU1, MGST2, MLYCD, MPV17L2, MRPL14, MRPL17, MRPL2, MRPL20, MRPL22, MRPL23, MRPL30, MRPL36, MRPL37, MRPL51, MRPL58, MRPS11, MRPS12, MRPS
R2, GAPDH, GGT1, LOC100519130, LOC100738684, LOC100739087, LRRC47, MALSU1, MGST2, MLYCD, MPV17L2, MRPL14, MRPL17, MRPL2, MRPL20, MRPL22, MRPL23, MRPL27, MRPL30, MRPL36, MRPL37, MRPL51, MRPL58, MRPS11, MRPS12, MRPS15, MRPS18A, MRPS18C, MRPS28, MRPS34, MRPS6, MRPS7, MRPS9, NELFE, PAIP2, PCIF1, PM20D1, POLR2G, RACK1, RPL10A, RPL11, RPL12, RPL14, RPL17, RPL18, RPL18A, RPL21, RPL23, RPL23A, RPL26, RPL28, RPL29, RPL30, RPL31, RPL3
RAP2, CDK5RAP3, CHCHD4, CHID1, CHMP1A, CIAO2B, CIDEA, CIRBP, CNPY2, COMMD7, CTDSP2, CTH, DAPK3, DDRGK1, DDX11, DDX25, DDX49, DMAP1, DNAJB13, DPPA5, DRG1, DUS1L, DUS3L, DVL1, EDF1, EEF1B2, EEF1D, EEF1G, EIF2B2, EIF3B, EIF3F, EIF3G, EIF3H, EIF3K, EIF4EBP1, EIF6, ELAC2, ELOB, ELOF1, EMG1, ERCC1, ERI3, ERP29, EXOSC4, EXOSC8, FAM50A, FAU, FBL, FCF1, FHL2, FIGLA, FKBP2, FKBP4, FLOT1, FSTL3, FTSJ3, FXR2, FZR1, GAPDH, GEMIN2, GEMIN6, GGT1, GLMP, G
00739087, LRRC47, MALSU1, MPV17L2, MRPL14, MRPL17, MRPL2, MRPL20, MRPL22, MRPL23, MRPL27, MRPL30, MRPL36, MRPL37, MRPL51, MRPL58, MRPS11, MRPS12, MRPS15, MRPS18A, MRPS18C, MRPS28, MRPS34, MRPS6, MRPS7, MRPS9, NELFE, PAIP2, PCIF1, POLR2G, RACK1, RPL10A, RPL11, RPL12, RPL14, RPL17, RPL18, RPL18A, RPL21, RPL23, RPL23A, RPL26, RPL28, RPL29, RPL30, RPL31, RPL32, RPL34, RPL35, RPL36, RPL37A, RPL38, RPL39, RPL4, RPL6, RPL8, RPLP1, RPLP2, RPS1
, LOC100739087, LRRC47, MALSU1, MGST2, MPV17L2, MRPL14, MRPL17, MRPL2, MRPL20, MRPL22, MRPL23, MRPL27, MRPL30, MRPL36, MRPL37, MRPL51, MRPL58, MRPS11, MRPS12, MRPS15, MRPS18A, MRPS18C, MRPS28, MRPS34, MRPS6, MRPS7, MRPS9, NELFE, PAIP2, PCIF1, POLR2G, RACK1, RPL10A, RPL11, RPL12, RPL14, RPL17, RPL18, RPL18A, RPL21, RPL23, RPL23A, RPL26, RPL28, RPL29, RPL30, RPL31, RPL32, RPL34, RPL35, RPL36, RPL37A, RPL38, RPL39, RPL4, RPL6, RPL8, RPLP1
, GTF2IRD1, HAX1, HEXIM2, HSBP1, HSPA8, IER2, INO80B, ITGB7, KAT5, KDM8, KLF1, KLF16, LIN37, LMO2, LOC100523672, LOC100623639, MAF, MAF1, MAP2K2, MAPK3, MAPK7, MBD3, MCRS1, MED10, MED31, MED8, MEPCE, METTL23, MRGBP, MT3, NACC2, NCF1, NEDD8, NELFCD, NELFE, NME2, NPAS1, NR0B1, PARK7, PCID2, PELP1, PFDN5, PFKM, PHB2, PIH1D1, PIN1, PKN1, POLE3, PRDX5, PRPF6, PSMC3IP, RALY, RFXANK, RNF141, RPS14, SAP30BP, SCAND1, SFN, SMARCD3, SMYD3, SNF8,
, GTF2IRD1, HAX1, HEXIM2, HSBP1, HSPA8, IER2, INO80B, ITGB7, KAT5, KDM8, KLF1, KLF16, LIN37, LMO2, LOC100523672, LOC100623639, MAF, MAF1, MAP2K2, MAPK3, MAPK7, MBD3, MCRS1, MED10, MED31, MED8, MEPCE, METTL23, MRGBP, MT3, NACC2, NCF1, NEDD8, NELFCD, NELFE, NME2, NPAS1, NR0B1, PARK7, PCID2, PELP1, PFDN5, PFKM, PHB2, PIH1D1, PIN1, PKN1, POLE3, PRDX5, PRPF6, PSMC3IP, RALY, RFXANK, RNF141, RPS14, SAP30BP, SCAND1, SFN, SMARCD3, SMYD3, SNF8,

NS, DNPH1, DTYMK, DUT, EEF1D, EIF6, FDPS, FHIT, FIS1, FLAD1, GALK1, GAPDH, GMPS, GPD1, HADHA, HSPA8, IDH2, IP6K2, ISYNA1, LOC100625049, MCEE, MDH1, MFSD2A, MLYCD, MVD, MVK, NAPRT, NAXD, NAXE, NME2, NUDT14, NUDT18, NUDT2, NUDT5, OXSM, PARK7, PFKM, PGLS, PGS1, PHB2, PIGH, PIGO, PIGP, PIP4P1, PLA2G12A, PLPP4, PMVK, PTPMT1, QPRT, SLC25A10, SLC25A19, SMPD2, SORD, TALDO1, THEM5, TK1, TK2, TKT, TPI1, TTC7A, UPB1, UPP1]
HIT, FIS1, FLAD1, GALK1, GAPDH, GMPS, GPD1, HSPA8, IDH2, MACROD1, MCEE, MDH1, MLYCD, MVD, MVK, NAPRT, NAXD, NAXE, NME2, NUDT14, NUDT18, NUDT2, NUDT5, OXSM, PARK7, PFKM, PGLS, PMM1, PMVK, QPRT, TALDO1, THEM5, TK1, TK2, TKT, TPI1, UPB1, UPP1]
GMPS, GPD1, HSPA8, IDH2, MACROD1, MCEE, MDH1, MLYCD, MVD, MVK, NAPRT, NME2, NUDT18, NUDT2, NUDT5, OXSM, PFKM, PGLS, PMVK, QPRT, TALDO1, THEM5, TKT, TPI1]
PA8, MCEE, MLYCD, MVD, MVK, NME2, NUDT14, NUDT18, NUDT2, NUDT5, OXSM, PFKM, PMVK, TALDO1, THEM5, TKT, TPI1, UPB1, UPP1]
S, IP6K2, ISYNA1, LOC100625049, MLYCD, MVD, MVK, NAPRT, NME2, NUDT2, PGS1, PIGH, PIGO, PIGP, PMVK, PTPMT1, QPRT, SLC25A10, SLC25A19, SORD, TK1, TK2, TKT, TPI1, TTC7A, UPP1]
HIT, FIS1, FLAD1, GALK1, GAPDH, GMPS, GPD1, HSPA8, IDH2, MCEE, MDH1, MLYCD, MVD, MVK, NAPRT, NAXD, NAXE, NME2, NUDT14, NUDT18, NUDT2, NUDT5, OXSM, PARK7, PFKM, PGLS, PMVK, QPRT, TALDO1, THEM5, TK1, TK2, TKT, TPI1, UPB1, UPP1]
HIT, FIS1, FLAD1, GALK1, GAPDH, GMPS, GPD1, HSPA8, IDH2, MCEE, MDH1, MLYCD, MVD, MVK, NAPRT, NAXD, NAXE, NME2, NUDT18, NUDT2, NUDT5, OXSM, PARK7, PFKM, PGLS, PMVK, QPRT, TALDO1, THEM5, TK1, TK2, TKT, TPI1, UPB1, UPP1]
JDT2, NUDT5, PFKM, TPI1]
TK1, TK2, UPP1]
GMPS, GPD1, HSPA8, IDH2, MCEE, MDH1, MLYCD, MVD, MVK, NAPRT, NME2, NUDT18, NUDT2, NUDT5, OXSM, PFKM, PGLS, PMVK, QPRT, TALDO1, THEM5, TKT, TPI1]
PA8, MCEE, MLYCD, MVD, MVK, NME2, NUDT18, NUDT2, NUDT5, OXSM, PFKM, PMVK, THEM5, TPI1, UPB1, UPP1]
PFKM, TPI1]
TK1, TK2, UPP1]
, TPI1]

, TPI1]

;APDH, GPD1, IDH2, IDH3B, IDH3G, IMMP2L, LOC100524873, MDH1, MT3, NDUFA10, NDUFA5, NDUFA7, NDUFA8, NDUFB3, NDUFB7, NDUFB8, NDUFB9, NDUFC2, NDUFS3, NDUFS4, NDUFS8, NDUFV1, NDUFV2, NDUFV3, NQO2, PARK7, PCDH12, PFKM, PGLS, SUCLG1, TALDO1, TKT, TPI1, UQCR10, UQCRB]
MDH1, MT3, NDUFA10, NDUFA5, NDUFA7, NDUFA8, NDUFB8, NDUFB9, NDUFC2, NDUFS3, NDUFS4, NDUFS8, NDUFV1, NDUFV2, NDUFV3, PARK7, PCDH12, PFKM, SUCLG1, UQCR10, UQCRB]
NDUFS4, NDUFS8, NDUFV1, NDUFV2, NDUFV3, NQO2, PARK7, UQCR10, UQCRB]
0523672, LONP1, MICOS13, MPV17, NDUFA10, NDUFA11, NDUFA12, NDUFA5, NDUFA9, NDUFAB1, NDUFB10, NDUFB11, NDUFB2, NDUFB4, NDUFB7, NDUFB8, NDUFB9, NDUFC1, NDUFC2, NDUFS3, NDUFS4, NDUFS5, NDUFS8, PARK7, PDCD5, PET100, PHB2, PLD6, PRDX3, ROMO1, SAMM50, SDHAF1, SFN, SLC25A6, SLIRP, SMIM20, SPATA19, SPG7, SSBP1, STMP1, TIMM10, TIMM22, TIMM50, TMEM11, TMEM126A, TMEM186, TMEM223, TRAK2]
MDH1, NDUFA10, NDUFA5, NDUFA7, NDUFA8, NDUFB8, NDUFB9, NDUFC2, NDUFS3, NDUFS4, NDUFS8, NDUFV1, NDUFV2, NDUFV3, PARK7, SUCLG1, UQCR10, UQCRB]
NDUFV3, PARK7, UQCR10, UQCRB]
FA7, NDUFA8, NDUFB8, NDUFB9, NDUFC2, NDUFS3, NDUFS8, NDUFV1, NDUFV2, NDUFV3, PARK7, SUCLG1, UQCR10, UQCRB]
JFS8, TMEM126A, TMEM186]
FS3, NDUFS4, NDUFS5, NDUFS8, PET100, SAMM50, SDHAF1, SMIM20, STMP1, TMEM126A, TMEM186, TMEM223]
)UFS3, NDUFS8, NDUFV1, NDUFV2, NDUFV3, PARK7, UQCR10, UQCRB]
JFS8, TMEM126A, TMEM186]
RB]

AL3, IER2, IGFBP6, INO80B, IP6K2, ITGB7, JAM3, KAT5, KDM8, KLF1, LAMTOR1, LAMTOR2, LAMTOR4, LGI1, LIMS2, LMNA, LMO2, LRCH4, LRSAM1, LSM10, LYPLAL1, MAF, MAP2K2, MAP4K1, MAPK13, MAPK3, MAPK7, MAPKAPK2, MCRS1, MED10, MEPCE, MESD, METTL23, MFSD2A, MGMT, MIEN1, MIF, MKKS, MLST8, MLYCD, MMD2, MPV17L2, MRGBP, MRPS6, MST1, MT3, MVD, MYOM1, NACC2, NCF1, NDUFS4, NENF, NME2, NOP2, NPM2, NSFL1C, NSMAF, OTUB1, PAIP2

IS, CTSD, CUL7, CYTH2, DAD1, DAPK3, DBF4B, DBNL, DDRGK1, DDX11, DDX25, DDX49, DERL3, DHFR, DHPS, DMAP1, DNAI1, DNAJC15, DPEP1, DPPA5, DRG1, DUS3L, DVL1, DYNLL1, EDF1, EEF1B2, EEF1D, EEF1G, EFHC1, EHD1, EIF2B2, EIF3B, EIF3F, EIF3G, EIF3H, EIF3K, EIF4EBP1, EIF6, EIPR1, ELOB, ENSA, EPHB6, ERCC1, ERP29, EXOSC4, FAM107A, FAM162A, FAM50A, FBXO31, FHIT, FHL2, FIGLA, FIS1, FKBP1B, FLOT1, FSTL3, FUNDC2, FXR2, FZR1, GAPDH, GB

2, LAMTOR4, LGI1, LIMS2, LMNA, LMO2, LRCH4, LRSAM1, LSM10, MAF, MAP2K2, MAP4K1, MAPK13, MAPK3, MAPK7, MAPKAPK2, MCRS1, MED10, MEPCE, MESD, METTL23, MFSD2A, MGMT, MIEN1, MIF, MLST8, MLYCD, MMD2, MPV17L2, MRGBP, MRPS6, MST1, MT3, MVD, MYOM1, NACC2, NCF1, NDUFS4, NENF, NME2, NOP2, NPM2, NSFL1C, OTUB1, PAIP2, PARK7, PCID2, PCIF1, PDCD5, PELP1, PFKM, PHB2, PIH1D1, PIN1, PIP4P1, PKN1, PLD6, PNLDC1, POLR2G, PQ

A, RNASEH2C, RNF31, SAMD4A, SDS, SLIRP, SMPD2, SORD, TBRG4, TCIRG1, TEX264, TIMP2, TMBIM6, TNIP1, TPCN1, TPI1, TRAPPC4, UBXN6, UPB1, UPP1, UROD, VPS4A, YTHDF1, ZPR1]

NEU1, NQO2, NSFL1C, NUDT16L1, NUDT18, NUDT5, PARK7, PCID2, PDLIM2, PFKM, PIN1, PLA2G12A, PLD6, PNLDC1, PNPLA4, POLR2G, PPP1R11, PRR5L, PSMA6, PSMB4, PSMB6, PSMB7, PSMD13, PSME2, QPRT, RAB7A, RACK1, RNASEH2C, RNF166, RNF26, RPL11, RPS27A, SAMD4A, SDS, SELENOS, SIAH3, SLIRP, SMPD2, SNF8, SORD, STX5, TBRG4, TCIRG1, TIMP2, TNIP1, TPI1, UBE2J2, UBXN1, UBXN6, UPB1, UPP1, UROD, VPS25, VPS28, VPS4A, YTHDF1, ZPR1]

SIAH3, SMPD2, SNF8, STX5, TCIRG1, TIMP2, TPI1, UBE2J2, UBXN1, UBXN6, UPB1, UPP1, UROD, VPS25, VPS28, VPS4A]

GD2A, HSD17B1, HSD17B10, IFT57, IMMP2L, INO80B, ITGB5, JAM3, KANK3, KAT5, LAMTOR1, LMNA, LOC100523672, LONP1, LRSAM1, MAP1LC3B, MAP2K2, MAPK3, MAST2, MCM5, MCRS1, MICOS13, MKKS, MKS1, MLST8, MPV17, MPV17L2, MRPL58, MRPS7, NAA10, NCLN, NDUFA10, NDUFA11, NDUFA12, NDUFA5, NDUFA9, NDUFAB1, NDUFB10, NDUFB11, NDUFB2, NDUFB4, NDUFB7, NDUFB8, NDUFB9, NDUFC1, NDUFC2, NDUFS3, NDUFS4, NDUFS5, NDUFS8, NOP10, NOTO, NPM2, NSF

1, HAUS7, HAX1, HIGD2A, HSD17B10, HSPA8, IFT57, IMMP2L, IMP3, IMP4, ISCA1, ISCA2, ITGB5, JAM3, KANK3, LOC100738684, LOC100739087, LOC102162486, LONP1, LRCH4, LRSAM1, LSM2, LUC7L, LYRM4, MALSU1, MAP1LC3B, MFSD2A, MIEN1, MIF, MKKS, MKS1, MLST8, MPV17L2, MRM1, MRPS7, MRTO4, NACC2, NCLN, NDUFA10, NDUFA11, NDUFA12, NDUFA5, NDUFA9, NDUFAB1, NDUFB10, NDUFB11, NDUFB2, NDUFB4, NDUFB7, NDUFB8, NDUFB9, NDUFC1, NDUFC2,

GD2A, HSD17B1, HSD17B10, IFT57, IMMP2L, INO80B, ITGB5, JAM3, KANK3, KAT5, LAMTOR1, LMNA, LOC100523672, LONP1, LRSAM1, MAP1LC3B, MAP2K2, MAPK3, MAST2, MCM5, MCRS1, MICOS13, MKKS, MKS1, MLST8, MPV17, MPV17L2, MRPL58, MRPS7, NAA10, NCLN, NDUFA10, NDUFA11, NDUFA12, NDUFA5, NDUFA9, NDUFAB1, NDUFB10, NDUFB11, NDUFB2, NDUFB4, NDUFB7, NDUFB8, NDUFB9, NDUFC1, NDUFC2, NDUFS3, NDUFS4, NDUFS5, NDUFS8, NOP10, NOTO, NPM2, NSF

B5, JAM3, KANK3, LOC100738684, LOC102162486, LONP1, LRCH4, LRSAM1, LSM2, LUC7L, LYRM4, MAP1LC3B, MFSD2A, MIEN1, MIF, MKKS, MKS1, MLST8, MPV17L2, MRPS7, NACC2, NCLN, NDUFA10, NDUFA11, NDUFA12, NDUFA5, NDUFA9, NDUFAB1, NDUFB10, NDUFB11, NDUFB2, NDUFB4, NDUFB7, NDUFB8, NDUFB9, NDUFC1, NDUFC2, NDUFS3, NDUFS4, NDUFS5, NDUFS8, NOTO, NSFL1C, NUBP2, NUDCD3, PADI4, PARK7, PDZD11, PET100, PIH1D1, PIP4P1, PLD6, POL

5, NDUFA9, NDUFAB1, NDUFB10, NDUFB11, NDUFB2, NDUFB4, NDUFB7, NDUFB8, NDUFB9, NDUFC1, NDUFC2, NDUFS3, NDUFS4, NDUFS5, NDUFS8, NPM2, NUDT5, PADI4, PARK7, PCID2, PDZD11, PET100, PIH1D1, PIP4P1, PKN1, POLE3, POLR2D, POMP, PRPF31, PRPF6, PSMG2, PUF60, RACK1, RHOC, RPL38, RPS15, RPS19, RPS5, SAMM50, SDHAF1, SEM1, SMARCD3, SMIM20, SMYD3, SMYD5, SNRPD2, SNRPD3, SSBP1, SSNA1, STMN1, STMP1, TARBP2, TBCA, TFPT, THAP7, T

, PADI4, PARK7, PDZD11, PET100, PIH1D1, PIP4P1, POLE3, POLR2D, POMP, PRPF31, PRPF6, PSMG2, PUF60, RACK1, RHOC, RPL38, RPS15, RPS19, RPS5, SAMM50, SDHAF1, SEM1, SMIM20, SMYD3, SNRPD2, SNRPD3, SSBP1, SSNA1, STMN1, STMP1, TARBP2, TBCA, TK1, TMEM120A, TMEM126A, TMEM186, TMEM223, TMSB10, TNIP1, TUBGCP2, UPB1]

1, HAUS7, HAX1, HIGD2A, HSD17B10, HSPA8, IFT57, IMMP2L, IMP3, IMP4, ISCA1, ISCA2, ITGB5, JAM3, KANK3, LOC100738684, LOC100739087, LOC102162486, LONP1, LRCH4, LRSAM1, LSM2, LUC7L, LYRM4, MALSU1, MAP1LC3B, MFSD2A, MIEN1, MIF, MKKS, MKS1, MLST8, MPV17L2, MRM1, MRPS7, MRTO4, NACC2, NCLN, NDUFA10, NDUFA11, NDUFA12, NDUFA5, NDUFA9, NDUFAB1, NDUFB10, NDUFB11, NDUFB2, NDUFB4, NDUFB7, NDUFB8, NDUFB9, NDUFC1, NDUFC2,

1]

AM50A, FAU, FBL, FCF1, FHIT, FHL2, FIGLA, FIS1, FLAD1, FSTL3, FTSJ3, FXR2, FZR1, GALK1, GAPDH, GEMIN2, GEMIN6, GGT1, GINS2, GLMP, GMPS, GNL3, GNL3L, GON7, GPD1, GTF2E2, GTF2F1, GTF2H5, GTF2IRD1, HAX1, HDGFL2, HEMK1, HEXIM2, HSBP1, HSD17B10, HSPA8, IDH2, IER2, IMP3, IMP4, INO80B, INTS11, ITGB7, KAT5, KDM8, KLF1, KLF16, LIN37, LMO2, LOC100519130, LOC100523672, LOC100623639, LOC100738684, LOC100739087, LOC102162

ERL3, DMAP1, DNAJB13, DNASE2, DNPEP, DPEP1, DPH7, DPP7, DPPA5, DRG1, DUS1L, DUS3L, DVL1, EDF1, EEF1B2, EEF1D, EEF1G, EIF2B2, EIF3B, EIF3F, EIF3G, EIF3H, EIF3K, EIF4EBP1, EIF6, EIPR1, ELAC2, ELOB, ELOF1, EMG1, ENDOU, ENSA, ERAP2, ERCC1, ERI3, ERP29, EXOSC4, EXOSC8, FAAP100, FAM50A, FAU, FBL, FBXO31, FCF1, FHIT, FHL2, FIGLA, FKBP2, FKBP4, FLOT1, FSTL3, FTSJ3, FXR2, FZR1, GALNT6, GAPDH, GDF9, GEMIN2, GEMIN6, GGA1, G

RK7, PCIF1, PIN1, PM20D1, PMVK, POLR2G, PSENEN, RACK1, REN, RPL10A, RPL11, RPL12, RPL14, RPL17, RPL18, RPL18A, RPL21, RPL23, RPL23A, RPL26, RPL28, RPL29, RPL30, RPL31, RPL32, RPL34, RPL35, RPL36, RPL37A, RPL38, RPL39, RPL4, RPL6, RPL8, RPLP1, RPLP2, RPS11, RPS12, RPS13, RPS14, RPS15, RPS15A, RPS16, RPS17, RPS18, RPS19, RPS21, RPS23, RPS24, RPS26, RPS27A, RPS29, RPS4X, RPS5, RPS6, RPS7, RPS8, RPS9, SAMD4A, SEC11C

JT, DVL1, EDF1, EEF1B2, EEF1D, EEF1G, EIF2B2, EIF3B, EIF3F, EIF3G, EIF3H, EIF3K, EIF4EBP1, EIF6, ELAC2, ELOB, ELOF1, EMG1, ERCC1, ERI3, ERP29, EXOSC4, EXOSC8, FAM50A, FAU, FBL, FCF1, FDPS, FHL2, FIGLA, FKBP2, FKBP4, FLAD1, FLOT1, FSTL3, FTSJ3, FXR2, FZR1, GALNT6, GAPDH, GATD1, GEMIN2, GEMIN6, GGT1, GLMP, GLRX3, GMPS, GNL3, GNL3L, GON7, GORASP1, GTF2E2, GTF2F1, GTF2H5, GTF2IRD1, HAX1, HEXIM2, HSBP1, HSD17B1, HSD17B

, DDRGK1, DDX25, DEGS2, DERL3, DHFR, DHPS, DMGDH, DNAJB13, DNPEP, DNPH1, DPEP1, DPH7, DPP7, DRG1, DTYMK, DUS3L, DUT, DVL1, EEF1B2, EEF1D, EEF1G, EIF2B2, EIF3B, EIF3F, EIF3G, EIF3H, EIF3K, EIF4EBP1, EIF6, EIPR1, ENDOU, ENSA, ERAP2, ERP29, ETFB, ETHE1, FAU, FBXO31, FHIT, FIS1, FKBP2, FKBP4, FLAD1, FLOT1, FXR2, FZR1, GALK1, GALNT6, GAPDH, GATD1, GBA2, GDF9, GGA1, GGH, GGT1, GLRX3, GMPS, GNL3, GNL3L, GORASP1, GPC3, GPD1

DUS1L, DUS3L, DUT, DVL1, EDF1, EEF1B2, EEF1D, EEF1G, EIF2B2, EIF3B, EIF3F, EIF3G, EIF3H, EIF3K, EIF4EBP1, EIF6, ELAC2, ELOB, ELOF1, EMG1, ERCC1, ERI3, ERP29, EXOSC4, EXOSC8, FAM50A, FAU, FBL, FCF1, FDPS, FHL2, FIGLA, FKBP2, FKBP4, FLAD1, FLOT1, FSTL3, FTSJ3, FXR2, FZR1, GALNT6, GAPDH, GATD1, GEMIN2, GEMIN6, GGT1, GLMP, GLRX3, GMPS, GNL3, GNL3L, GON7, GORASP1, GPD1, GTF2E2, GTF2F1, GTF2H5, GTF2IRD1, HAX1, HEXIM2, H

, ERP29, FAU, FBXO31, FHIT, FKBP2, FKBP4, FLOT1, FXR2, FZR1, GALNT6, GAPDH, GDF9, GGA1, GGT1, GLRX3, GNL3, GNL3L, GORASP1, GPC3, GPS1, GUSB, HAX1, HSPA8, IMMP2L, IP6K2, ISCA2, JOSD2, KAT5, KLK15, LMF1, LOC100517502, LOC100519130, LOC100738684, LOC100739087, LONP1, LRRC47, LRSAM1, MALSU1, MANBA, MAPK7, MESD, MIB2, MIF, MKKS, MLST8, MPV17L2, MRPL14, MRPL17, MRPL2, MRPL20, MRPL22, MRPL23, MRPL27, MRPL30, MRPL

PL30, RPL31, RPL32, RPL34, RPL35, RPL36, RPL37A, RPL38, RPL39, RPL4, RPL6, RPL8, RPLP1, RPLP2, RPS11, RPS12, RPS13, RPS14, RPS15, RPS15A, RPS16, RPS17, RPS18, RPS19, RPS21, RPS23, RPS24, RPS26, RPS27A, RPS29, RPS4X, RPS5, RPS6, RPS7, RPS8, RPS9, SAMD4A, SEC11C, TARBP2, THOP1, TUFM, UBA52, YTHDF1, ZAR1]

, FZR1, GALNT6, GAPDH, GEMIN2, GEMIN6, GGT1, GLMP, GLRX3, GNL3, GNL3L, GON7, GORASP1, GTF2E2, GTF2F1, GTF2H5, GTF2IRD1, HAX1, HEXIM2, HSBP1, HSD17B1, HSD17B10, HSPA8, IER2, IMMP2L, IMP3, IMP4, INO80B, INTS11, ISCA2, ITGB7, KAT5, KDM8, KLF1, KLF16, LIN37, LMF1, LMNA, LMO2, LOC100510975, LOC100523672, LOC100623639, LOC100738684, LOC100739087, LOC102162486, LRRC47, LSM2, LSM4, LUC7L, MAF, MAF1, MALSU1, M

B, ITGB7, KAT5, KDM8, KLF1, KLF16, LIN37, LMO2, LOC100519130, LOC100523672, LOC100623639, LOC100738684, LOC100739087, LRRC47, MAF, MAF1, MALSU1, MAP2K2, MAPK3, MAPK7, MBD3, MCRS1, MED10, MED31, MED8, MEPCE, METTL23, MGST2, MLYCD, MPV17L2, MRGBP, MRPL14, MRPL17, MRPL2, MRPL20, MRPL22, MRPL23, MRPL27, MRPL30, MRPL36, MRPL37, MRPL51, MRPL58, MRPS11, MRPS12, MRPS15, MRPS18A, MRPS18C, MRPS28, MRPS34, MRPS6, M

15, MRPS18A, MRPS18C, MRPS28, MRPS34, MRPS6, MRPS7, MRPS9, MTHFS, NAPRT, NELFE, NME2, NUDT2, PAIP2, PARK7, PCIF1, PIGH, PIGO, PIGP, PMM1, POFUT2, POLR2G, PPOX, QPRT, RACK1, RPL10A, RPL11, RPL12, RPL14, RPL17, RPL18, RPL18A, RPL21, RPL23, RPL23A, RPL26, RPL28, RPL29, RPL30, RPL31, RPL32, RPL34, RPL35, RPL36, RPL37A, RPL38, RPL39, RPL4, RPL6, RPL8, RPLP1, RPLP2, RPS11, RPS12, RPS13, RPS14, RPS15, RPS15A, RPS16, R

4, RPL35, RPL36, RPL37A, RPL38, RPL39, RPL4, RPL6, RPL8, RPLP1, RPLP2, RPS11, RPS12, RPS13, RPS14, RPS15, RPS15A, RPS16, RPS17, RPS18, RPS19, RPS21, RPS23, RPS24, RPS26, RPS27A, RPS29, RPS4X, RPS5, RPS6, RPS7, RPS8, RPS9, SAMD4A, SMPD2, TARBP2, TUFM, UBA52, YTHDF1, ZAR1]

LRX3, GNL3, GON7, GTF2E2, GTF2F1, GTF2H5, GTF2IRD1, HAX1, HEXIM2, HSBP1, HSD17B1, HSD17B10, HSPA8, IER2, IMMP2L, IMP3, IMP4, INO80B, INTS11, ISCA2, ITGB7, KAT5, KDM8, KLF1, KLF16, LIN37, LMF1, LMNA, LMO2, LOC100510975, LOC100523672, LOC100623639, LOC100738684, LOC100739087, LOC102162486, LRRC47, LSM2, LSM4, LUC7L, MAF, MAF1, MALSU1, MAP2K2, MAPK13, MAPK3, MAPK7, MAPKAPK2, MBD3, MCRS1, MED10, MED31, ME

1, RPS12, RPS13, RPS14, RPS15, RPS15A, RPS16, RPS17, RPS18, RPS19, RPS21, RPS23, RPS24, RPS26, RPS27A, RPS29, RPS4X, RPS5, RPS6, RPS7, RPS8, RPS9, SAMD4A, TARBP2, TUFM, UBA52, YTHDF1, ZAR1]

1, RPLP2, RPS11, RPS12, RPS13, RPS14, RPS15, RPS15A, RPS16, RPS17, RPS18, RPS19, RPS21, RPS23, RPS24, RPS26, RPS27A, RPS29, RPS4X, RPS5, RPS6, RPS7, RPS8, RPS9, SAMD4A, TARBP2, TUFM, UBA52, YTHDF1, ZAR1]

SRA1, STAT5A, TFDP2, TFPT, TFR2, THAP7, TLE2, TLE6, TNIP1, UXT, WDR5, WNT7A, WWC3, ZBTB48, ZKSCAN4, ZNF212, ZNF235, ZNF584, ZNF821, ZP3]

SRA1, STAT5A, TFDP2, TFPT, TFR2, THAP7, TLE2, TLE6, TNIP1, UXT, WDR5, WNT7A, WWC3, ZBTB48, ZKSCAN4, ZNF212, ZNF235, ZNF584, ZNF821, ZP3]
