# Supplementary material for: Deciphering differences in DNA methylation and transcriptome profiles of oocytes from pigs with high and low developmental competence
Source: Environ Epigenet. 2025 Jun 3;11(1):dvaf018. doi: 10.1093/eep/dvaf018 (PMC12418950; doi:10.1093/eep/dvaf018)
Supplement: dvaf018_Supplemental_Files [file dvaf018_supplemental_files.zip › Sup table 5.pdf]

ID	Term	Ontology Source	Term PValue	Term P-Value Corrected with Bonferroni step down	Group P-Value	Group P-Value Corrected with Bonferroni step down	GO Levels	GO Groups	% Associated Genes	Number of Genes	Associated Genes Found
GO:0006996	organelle organization	GO_BiologicalProcess-EBI-UniProt-GOA-ACAP-ARAP_23.01.2024_00h00	0.00	0.00	0.00	0.00	[4]	Group00	4.20	117.00	[ADD3, AHCTF1, AKTIP, ANKLE2, ARL13B, ATL1, AURKB, BBS4, BBS5, BLM, B
GO:0010639	negative regulation of organelle organization	GO_BiologicalProcess-EBI-UniProt-GOA-ACAP-ARAP_23.01.2024_00h00	0.00	0.03	0.00	0.00	[4, 5, 6]	Group02	6.64	19.00	[BBS4, CARMIL1, CCNB1, CHEK1, FBXO43, KANK1, KAT2A, LIMA1, MAD2L1, N
GO:0043414	macromolecule methylation	GO_BiologicalProcess-EBI-UniProt-GOA-ACAP-ARAP_23.01.2024_00h00	0.00	0.01	0.00	0.00	[3, 5]	Group03	10.78	11.00	[DIMT1, DNMT3B, HENMT1, KMT2E, METTL3, PPM1D, RAMAC, TRMT2B, TRMT
GO:0051276	chromosome organization	GO_BiologicalProcess-EBI-UniProt-GOA-ACAP-ARAP_23.01.2024_00h00	0.00	0.00	0.00	0.00	[5]	Group04	6.51	30.00	[AURKB, BLM, CCNB1, CDC20, CDC45, CENPA, CENPN, CENPO, CENPW, DN
GO:0006413	translational initiation	GO_BiologicalProcess-EBI-UniProt-GOA-ACAP-ARAP_23.01.2024_00h00	0.00	0.01	0.00	0.00	[3, 5, 6, 7, 8]	Group05	10.43	12.00	[DAZL, EIF1, EIF2A, EIF2AK4, EIF4G2, GTF2B, KHDRBS1, METTL3, NPM1, STK
GO:0006446	regulation of translational initiation	GO_BiologicalProcess-EBI-UniProt-GOA-ACAP-ARAP_23.01.2024_00h00	0.00	0.03	0.00	0.00	[4, 6, 7, 8, 9, 10]	Group05	13.56	8.00	[DAZL, EIF1, EIF2AK4, EIF4G2, KHDRBS1, METTL3, NPM1, STK35]
GO:0006999	nuclear pore organization	GO_BiologicalProcess-EBI-UniProt-GOA-ACAP-ARAP_23.01.2024_00h00	0.00	0.01	0.00	0.00	[5, 6]	Group06	35.71	5.00	[AHCTF1, NDC1, NUP107, NUP133, NUP35]
GO:0006259	DNA metabolic process	GO_BiologicalProcess-EBI-UniProt-GOA-ACAP-ARAP_23.01.2024_00h00	0.00	0.01	0.00	0.00	[5, 6]	Group07	5.06	38.00	[AURKB, BARD1, BAZ1A, BLM, CCNA2, CDC45, CDC7, CHEK1, CHRAC1, DCLR
GO:0006260	DNA replication	GO_BiologicalProcess-EBI-UniProt-GOA-ACAP-ARAP_23.01.2024_00h00	0.00	0.02	0.00	0.00	[6, 7]	Group07	7.73	16.00	[BAZ1A, BLM, CCNA2, CDC45, CDC7, CHRAC1, DNA2, DONSON, GMNN, MCM
GO:0006261	DNA-templated DNA replication	GO_BiologicalProcess-EBI-UniProt-GOA-ACAP-ARAP_23.01.2024_00h00	0.00	0.02	0.00	0.00	[7, 8]	Group07	9.60	12.00	[BAZ1A, BLM, CDC45, CDC7, DNA2, DONSON, GMNN, MCMBP, POLE2, POLG2
GO:0000226	microtubule cytoskeleton organization	GO_BiologicalProcess-EBI-UniProt-GOA-ACAP-ARAP_23.01.2024_00h00	0.00	0.00	0.00	0.00	[3, 6]	Group08	6.09	33.00	[AURKB, BBS4, BORA, CCNB1, CDC20, CENPA, CEP44, CEP70, CFAP69, CHE
GO:0070925	organelle assembly	GO_BiologicalProcess-EBI-UniProt-GOA-ACAP-ARAP_23.01.2024_00h00	0.00	0.03	0.00	0.00	[5]	Group08	4.82	37.00	[ARL13B, AURKB, BBS4, BBS5, CDC20, CENPA, CENPW, CEP44, CEP70, CFA
GO:0140694	non-membrane-bounded organelle assembly	GO_BiologicalProcess-EBI-UniProt-GOA-ACAP-ARAP_23.01.2024_00h00	0.00	0.04	0.00	0.00	[6]	Group08	6.31	20.00	[AURKB, CDC20, CENPA, CENPW, CEP44, CNOT7, DHX30, EIF2A, GTF2B, HA
GO:0022402	cell cycle process	GO_BiologicalProcess-EBI-UniProt-GOA-ACAP-ARAP_23.01.2024_00h00	0.00	0.00	0.00	0.00	[2, 3]	Group09	6.61	64.00	[ACVR1B, AHCTF1, ANKLE2, AURKB, BBS4, BLM, BORA, BRD7, CCNA2, CCNB
GO:1903047	mitotic cell cycle process	GO_BiologicalProcess-EBI-UniProt-GOA-ACAP-ARAP_23.01.2024_00h00	0.00	0.00	0.00	0.00	[3, 4]	Group09	6.36	36.00	[ACVR1B, ANKLE2, AURKB, BBS4, BLM, BORA, BRD7, CCNA2, CCNB1, CDC20
GO:0048285	organelle fission	GO_BiologicalProcess-EBI-UniProt-GOA-ACAP-ARAP_23.01.2024_00h00	0.00	0.03	0.00	0.00	[5]	Group09	5.99	23.00	[ANKLE2, AURKB, BORA, CCNB1, CDC20, CHEK1, FBXO43, KIF4A, KNSTRN, M
GO:0000280	nuclear division	GO_BiologicalProcess-EBI-UniProt-GOA-ACAP-ARAP_23.01.2024_00h00	0.00	0.04	0.00	0.00	[6]	Group09	6.09	21.00	[ANKLE2, AURKB, BORA, CCNB1, CDC20, CHEK1, FBXO43, KIF4A, KNSTRN, M
GO:0043933	protein-containing complex organization	GO_BiologicalProcess-EBI-UniProt-GOA-ACAP-ARAP_23.01.2024_00h00	0.00	0.00	0.00	0.00	[4]	Group10	4.34	76.00	[AHCTF1, AKAIN1, ANKRA2, APIP, ATL1, AURKB, BAZ1A, BBS4, BLM, BRD7, C
GO:0065003	protein-containing complex assembly	GO_BiologicalProcess-EBI-UniProt-GOA-ACAP-ARAP_23.01.2024_00h00	0.00	0.03	0.00	0.00	[5]	Group10	4.30	50.00	[AHCTF1, AKAIN1, ANKRA2, APIP, ATL1, BAZ1A, BBS4, BLM, CARMIL1, CBR4,
GO:0071824	protein-DNA complex organization	GO_BiologicalProcess-EBI-UniProt-GOA-ACAP-ARAP_23.01.2024_00h00	0.00	0.00	0.00	0.00	[5]	Group10	6.08	37.00	[AURKB, BAZ1A, BRD7, CCNB1, CDC45, CENPA, CENPN, CENPO, CENPW, CH
GO:0006325	chromatin organization	GO_BiologicalProcess-EBI-UniProt-GOA-ACAP-ARAP_23.01.2024_00h00	0.00	0.00	0.00	0.00	[6]	Group10	5.75	32.00	[AURKB, BAZ1A, BRD7, CCNB1, CENPA, CENPN, CHEK1, CHRAC1, DEK, ENY
GO:0065004	protein-DNA complex assembly	GO_BiologicalProcess-EBI-UniProt-GOA-ACAP-ARAP_23.01.2024_00h00	0.00	0.00	0.00	0.00	[6]	Group10	12.17	14.00	[BAZ1A, CDC45, CENPA, CENPN, CENPO, CENPW, CHRAC1, GMNN, GTF2B,
GO:0006338	chromatin remodeling	GO_BiologicalProcess-EBI-UniProt-GOA-ACAP-ARAP_23.01.2024_00h00	0.00	0.02	0.00	0.00	[7]	Group10	5.67	27.00	[AURKB, BAZ1A, BRD7, CCNB1, CENPA, CENPN, CHEK1, CHRAC1, GTF2B, HD
GO:0022402	cell cycle process	GO_BiologicalProcess-EBI-UniProt-GOA-ACAP-ARAP_23.01.2024_00h00	0.00	0.00	0.00	0.00	[2, 3]	Group11	6.61	64.00	[ACVR1B, AHCTF1, ANKLE2, AURKB, BBS4, BLM, BORA, BRD7, CCNA2, CCNB
GO:0000278	mitotic cell cycle	GO_BiologicalProcess-EBI-UniProt-GOA-ACAP-ARAP_23.01.2024_00h00	0.00	0.00	0.00	0.00	[3]	Group11	6.09	42.00	[ACVR1B, ANKLE2, AURKB, BBS4, BLM, BORA, BRD7, BTG4, CCNA2, CCNB1,
GO:0044770	cell cycle phase transition	GO_BiologicalProcess-EBI-UniProt-GOA-ACAP-ARAP_23.01.2024_00h00	0.00	0.00	0.00	0.00	[3, 4]	Group11	6.70	26.00	[ACVR1B, AURKB, BLM, BRD7, CCNA2, CCNB1, CDC20, CDC7, CHEK1, CKS1B
GO:0051726	regulation of cell cycle	GO_BiologicalProcess-EBI-UniProt-GOA-ACAP-ARAP_23.01.2024_00h00	0.00	0.01	0.00	0.00	[3, 4]	Group11	4.87	40.00	[AHCTF1, AURKB, BBS4, BLM, BORA, BRD7, BTG4, CCNB1, CDC20, CDC7, CH
GO:1903047	mitotic cell cycle process	GO_BiologicalProcess-EBI-UniProt-GOA-ACAP-ARAP_23.01.2024_00h00	0.00	0.00	0.00	0.00	[3, 4]	Group11	6.36	36.00	[ACVR1B, ANKLE2, AURKB, BBS4, BLM, BORA, BRD7, CCNA2, CCNB1, CDC20
GO:0010564	regulation of cell cycle process	GO_BiologicalProcess-EBI-UniProt-GOA-ACAP-ARAP_23.01.2024_00h00	0.00	0.00	0.00	0.00	[3, 4, 5]	Group11	5.69	31.00	[AHCTF1, AURKB, BBS4, BLM, BORA, BRD7, CCNB1, CDC20, CDC7, CHEK1, C
GO:0044772	mitotic cell cycle phase transition	GO_BiologicalProcess-EBI-UniProt-GOA-ACAP-ARAP_23.01.2024_00h00	0.00	0.03	0.00	0.00	[4, 5]	Group11	6.49	20.00	[ACVR1B, AURKB, BLM, BRD7, CCNA2, CCNB1, CDC20, CDC7, CHEK1, CKS1B
GO:0044839	cell cycle G2/M phase transition	GO_BiologicalProcess-EBI-UniProt-GOA-ACAP-ARAP_23.01.2024_00h00	0.00	0.00	0.00	0.00	[4, 5]	Group11	11.57	14.00	[AURKB, BLM, CCNA2, CCNB1, CDC7, CHEK1, DONSON, MASTL, MTA3, NPM1
GO:0000086	G2/M transition of mitotic cell cycle	GO_BiologicalProcess-EBI-UniProt-GOA-ACAP-ARAP_23.01.2024_00h00	0.00	0.00	0.00	0.00	[5, 6]	Group11	12.04	13.00	[AURKB, BLM, CCNA2, CCNB1, CDC7, CHEK1, DONSON, MASTL, MTA3, PPM1
GO:0000280	nuclear division	GO_BiologicalProcess-EBI-UniProt-GOA-ACAP-ARAP_23.01.2024_00h00	0.00	0.04	0.00	0.00	[6]	Group11	6.09	21.00	[ANKLE2, AURKB, BORA, CCNB1, CDC20, CHEK1, FBXO43, KIF4A, KNSTRN, M
GO:1902749	regulation of cell cycle G2/M phase transition	GO_BiologicalProcess-EBI-UniProt-GOA-ACAP-ARAP_23.01.2024_00h00	0.00	0.03	0.00	0.00	[5, 6, 7]	Group11	10.75	10.00	[AURKB, BLM, CCNB1, CDC7, CHEK1, DONSON, MTA3, NPM1, RRM1, TAOK3]

RA, CARMIL1, CCNB1, CDC20, CDC45, CENPA, CENPN, CENPO, CENPW, CEP44, CEP70, CEP78, CFAP69, CHEK1, CNN3, CNOT7, COG3, CREG1, CYFIP1, DHX30, DNA2, EIF2A, EMC2, EPB41L5, FBXO43, GTF2B, HACE1, HAUS4, HDAC3, HJURP, HMGB2, IFT81, KANK1, KAT2A, KIF4A, KNSTRN, LIMA1, LOC110257335, LONP2, MAD2L1, MAP2K1, MASTL, MCMBP, MCOLN1, MFF, MLL
PM1, OMA1, PARL, PPFIA1, RAD1, RDX, RHPN2, SLC25A31, TOGARAM2, WDR47]
5, VIRMA, WTAP]
2, GTF2B, HJURP, HMGB2, KIF4A, KNSTRN, MAD2L1, MASTL, MCMBP, MND1, NCAPG, NDC1, NSL1, NUP107, NUP133, PPHLN1, RAE1, RESF1, RFC4, SUPV3L1, UHRF1]
35, TAF5, TAF9]

E1A, DEK, DNA2, DNMT3B, DONSON, GMNN, HMGB2, KMT2E, MCMBP, MND1, MORF4L1, MORF4L2, NEIL1, NEIL3, NPM1, NSMCE4A, POLB, POLE2, POLG2, PPM1D, RAD1, RFC4, RPA3, RRM1, SPIRE1, SUPV3L1, TDP2, UBR2, XRCC2]
P, POLB, POLE2, POLG2, RFC4, RPA3, RRM1]
, RFC4, RRM1]
1, GTF2B, HAUS4, HDAC3, KAT2A, KIF4A, KNSTRN, MAD2L1, MASTL, NME7, NPM1, POC1B, RAE1, SBDS, SLAIN2, SLC39A12, SPAG16, SPATA7, SPIRE1, SSX2IP, UHRF1, USP33, WDR47, XRCC2]
69, CNOT7, DHX30, EIF2A, GTF2B, HAUS4, HDAC3, IFT81, KAT2A, KIF4A, MASTL, MTERF3, NPM1, OCRL, POC1B, PRKAR1A, RDX, SBDS, SNX7, SPAG16, SRPX, SSX2IP, STX7, TCTN2, TMEM216, TTC8, UHRF1, WEE2]
S4, HDAC3, KAT2A, KIF4A, MASTL, MTERF3, NPM1, POC1B, PRKAR1A, SBDS, UHRF1]
1, CDC20, CDC45, CDC7, CENPA, CENPN, CENPW, CEP44, CHEK1, CIAO2A, CKS1B, CRY1, CSPP1, DNA2, DONSON, EIF2AK4, FBXO43, GMNN, GTF2B, HAUS4, HDAC3, HJURP, KAT2A, KIF4A, KMT2E, KNSTRN, LOC100621388, MAD2L1, MASTL, MCMBP, MITD1, MND1, MTA3, NCAPG, NDC1, NPM1, NSL1, POC1B, PPM1D, PPP6C, PRKAR1A, RAD1, RAE1, RRM1, SBDS, SKP2, SLC25
, CDC45, CDC7, CENPA, CHEK1, CKS1B, DNA2, DONSON, FBXO43, HDAC3, KIF4A, KMT2E, KNSTRN, MAD2L1, MASTL, MITD1, MTA3, NCAPG, NSL1, PPM1D, PPP6C, RAE1, RRM1, SBDS, SKP2, TAOK3, UHRF1]
AD2L1, MASTL, MFF, MND1, NCAPG, NDC1, NSL1, RAD1, SLC25A31, SPIRE1, TMCC1, UBR2, UHRF1, WEE2]
AD2L1, MASTL, MND1, NCAPG, NDC1, NSL1, RAD1, SLC25A31, SPIRE1, UBR2, UHRF1, WEE2]
RMIL1, CBR4, CCNB1, CDC45, CENPA, CENPN, CENPO, CENPW, CHEK1, CHRAC1, CLDN1, CRYZ, CYFIP1, DEK, DHX30, ENY2, GMNN, GTF2B, HDAC3, HJURP, HMGB2, HSCB, KANK1, KAT2A, KMT2E, LIMA1, MCOLN1, METTL3, MIER1, MORF4L1, MORF4L2, MPP7, MTA3, NCOA3, NDC1, NME7, NPM1, NUP107, NUP133, NUP35, OMA1, PARD6B, PCGF6, PPHLN1, PPM1D, PRMT1, PR
CDC45, CENPA, CENPN, CENPO, CENPW, CHRAC1, CLDN1, CRYZ, CYFIP1, DHX30, GMNN, GTF2B, HJURP, HMGB2, HSCB, KANK1, MCOLN1, MPP7, NDC1, NME7, NPM1, NUP107, OMA1, PARD6B, PRMT1, RAF1, RDX, RIOK3, RRM1, SDHAF4, SLAIN2, SLC39A12, SNRPC, SNRPE, SPIRE1, STOML2, STRAP, TAF5, TAF9, UNC13B]
EK1, CHRAC1, DEK, ENY2, GMNN, GTF2B, HDAC3, HJURP, HMGB2, KAT2A, KMT2E, METTL3, MIER1, MORF4L1, MORF4L2, MTA3, NCOA3, NPM1, PCGF6, PPHLN1, PPM1D, PRMT1, PRMT2, RESF1, TAF5, TAF9, UBR2, UHRF1]
, GTF2B, HDAC3, HJURP, HMGB2, KAT2A, KMT2E, METTL3, MIER1, MORF4L1, MORF4L2, MTA3, NCOA3, NPM1, PCGF6, PPHLN1, PPM1D, PRMT1, PRMT2, RESF1, TAF9, UBR2, UHRF1]
JURP, HMGB2, NPM1, TAF5, TAF9]
AC3, HJURP, HMGB2, KAT2A, METTL3, MIER1, MTA3, NCOA3, NPM1, PCGF6, PPHLN1, PPM1D, PRMT1, PRMT2, RESF1, TAF9, UBR2, UHRF1]
1, CDC20, CDC45, CDC7, CENPA, CENPN, CENPW, CEP44, CHEK1, CIAO2A, CKS1B, CRY1, CSPP1, DNA2, DONSON, EIF2AK4, FBXO43, GMNN, GTF2B, HAUS4, HDAC3, HJURP, KAT2A, KIF4A, KMT2E, KNSTRN, LOC100621388, MAD2L1, MASTL, MCMBP, MITD1, MND1, MTA3, NCAPG, NDC1, NPM1, NSL1, POC1B, PPM1D, PPP6C, PRKAR1A, RAD1, RAE1, RRM1, SBDS, SKP2, SLC25
CDC20, CDC45, CDC7, CENPA, CENPW, CHEK1, CKS1B, DNA2, DONSON, FBXO43, GMNN, HDAC3, KIF4A, KMT2E, KNSTRN, MAD2L1, MASTL, MITD1, MTA3, NCAPG, NSL1, PPM1D, PPP6C, RAE1, RPA3, RRM1, SBDS, SKP2, TAOK3, UHRF1, WEE2, XRCC2]
, CRY1, DNA2, DONSON, EIF2AK4, KMT2E, LOC100621388, MAD2L1, MASTL, MTA3, NPM1, PPM1D, PPP6C, RAD1, RRM1, SKP2, TAOK3]
EK1, CRY1, CSNK2A1, CSPP1, DNA2, DONSON, DR1, EIF2AK4, FBXO43, GMNN, HDAC3, KAT2A, KMT2E, KNSTRN, LOC100621388, MAD2L1, MASTL, MORF4L1, MORF4L2, MTA3, NCAPG, NPM1, PRKAR1A, RAD1, RAE1, RPA3, RRM1, SKP2, TAOK3, WEE2]
, CDC45, CDC7, CENPA, CHEK1, CKS1B, DNA2, DONSON, FBXO43, HDAC3, KIF4A, KMT2E, KNSTRN, MAD2L1, MASTL, MITD1, MTA3, NCAPG, NSL1, PPM1D, PPP6C, RAE1, RRM1, SBDS, SKP2, TAOK3, UHRF1]
RY1, CSPP1, DNA2, DONSON, EIF2AK4, FBXO43, KAT2A, KMT2E, KNSTRN, LOC100621388, MAD2L1, MASTL, MTA3, NCAPG, NPM1, PRKAR1A, RAD1, RAE1, RRM1, TAOK3, WEE2]
, DONSON, KMT2E, MAD2L1, MASTL, MTA3, PPM1D, PPP6C, RRM1, SKP2, TAOK3]
, PPM1D, RRM1, SKP2, TAOK3]
D, RRM1, SKP2, TAOK3]
AD2L1, MASTL, MND1, NCAPG, NDC1, NSL1, RAD1, SLC25A31, SPIRE1, UBR2, UHRF1, WEE2]

.T11, MND1, MTERF3, NCAPG, NDC1, NME7, NPM1, NSL1, NUP107, NUP133, NUP35, OCRL, OMA1, PARL, PEX1, PEX3, POC1B, POLG2, PPFIA1, PPHLN1, PRKAR1A, PRKCI, RAD1, RAE1, RAF1, RCHY1, RDX, RESF1, RFC4, RHOT1, RHPN2, RRM1, SBDS, SDHAF4, SEC23A, SLAIN2, SLC25A31, SLC39A12, SNX7, SPAG16, SPATA7, SPIRE1, SRPX, SSX2IP, STOML2, STX6, STX7, SUPV3

A31, SPIRE1, SSX2IP, TAOK3, UBR2, UHRF1, USP33, WEE2, XRCC2]

MT2, RAF1, RDX, RESF1, RIOK3, RRM1, SDHAF4, SLAIN2, SLC39A12, SNRPC, SNRPE, SPIRE1, STOML2, STRAP, TAF5, TAF9, UBR2, UHRF1, UNC13B, WDR47]

A31, SPIRE1, SSX2IP, TAOK3, UBR2, UHRF1, USP33, WEE2, XRCC2]

[illegible]L1, TCTN2, TMCC1, TMED5, TMEM216, TOGARAM2, TTC8, UBR2, UHRF1, UNC13B, USP33, VAPA, WDR47, WEE2, XRCC2]
