# Supplementary material for: Deciphering differences in DNA methylation and transcriptome profiles of oocytes from pigs with high and low developmental competence
Source: Environ Epigenet. 2025 Jun 3;11(1):dvaf018. doi: 10.1093/eep/dvaf018 (PMC12418950; doi:10.1093/eep/dvaf018)
Supplement: dvaf018_Supplemental_Files [file dvaf018_supplemental_files.zip › Sup table 6.pdf]

Rank	TF	Score	Library
1	THAP3	6.14E-04	ARCHS4 Coexpression,6.143E-4
2	NEUROG1	6.22E-04	GTEx Coexpression,6.223E-4
3	DRAP1	7.12E-04	Enrichr Queries,7.123E-4
4	BRF2	0.001229	ARCHS4 Coexpression,0.001229
5	THAP4	0.001245	GTEx Coexpression,0.001245
6	THAP7	0.001425	Enrichr Queries,0.001425
7	ZNF511	0.001843	ARCHS4 Coexpression,0.001843
8	GTF3A	0.001867	GTEx Coexpression,0.001867
9	ZNF706	0.002137	Enrichr Queries,0.002137
10	ZNF581	0.002457	ARCHS4 Coexpression,0.002457
11	NME2	0.003071	ARCHS4 Coexpression,0.003071
12	ELK1	0.003367	ReMap ChIP-seq,0.003367
13	YBX1	0.003561	Enrichr Queries,0.003561
14	ESRRA	0.003734	GTEx Coexpression,0.003734
15	ZNF428	0.004274	Enrichr Queries,0.004274
16	THAP8	0.0043	ARCHS4 Coexpression,0.0043
17	NRL	0.004356	GTEx Coexpression,0.004356
18	DNTTIP1	0.004914	ARCHS4 Coexpression,0.004914
19	ZNF414	0.004978	GTEx Coexpression,0.004978
20	CREB3	0.005528	ARCHS4 Coexpression,0.005528
21	REPIN1	0.0056	GTEx Coexpression,0.0056
22	THAP11	0.005698	Enrichr Queries,0.005698
23	MYC	0.006098	Literature ChIP-seq,0.006098
24	ALX1	0.006143	ARCHS4 Coexpression,0.006143
25	HMGN3	0.006223	GTEx Coexpression,0.006223
26	MBD3	0.00641	Enrichr Queries,0.00641
27	GABPA	0.006734	ReMap ChIP-seq,0.006734
28	CENPS	0.006757	ARCHS4 Coexpression,0.006757
29	SNAPC5	0.006845	GTEx Coexpression,0.006845
30	ZNF787	0.007123	Enrichr Queries,0.007123
31	CHCHD3	0.007371	ARCHS4 Coexpression,0.007371
32	ZNF32	0.007467	GTEx Coexpression,0.007467
33	ATF4	0.007835	Enrichr Queries,0.007835
34	ZNF672	0.00809	GTEx Coexpression,0.00809
35	SIX5	0.008475	ENCODE ChIP-seq,0.008475
36	ZNF576	0.0086	ARCHS4 Coexpression,0.0086
37	TCF24	0.008712	GTEx Coexpression,0.008712
38	ZNF232	0.009214	ARCHS4 Coexpression,0.009214
39	CENPX	0.009334	GTEx Coexpression,0.009334
40	PIN1	0.009828	ARCHS4 Coexpression,0.009828
41	TCF15	0.009956	GTEx Coexpression,0.009956
42	PREB	0.009972	Enrichr Queries,0.009972
43	SP2	0.0101	ReMap ChIP-seq,0.0101
44	THYN1	0.01044	ARCHS4 Coexpression,0.01044
45	ARGFX	0.01058	GTEx Coexpression,0.01058
46	ARNT	0.01106	ARCHS4 Coexpression,0.01106
47	ZNF524	0.0114	Enrichr Queries,0.0114
48	HOXC9	0.01167	ARCHS4 Coexpression,0.01167
49	NKX25	0.01182	GTEx Coexpression,0.01182
50	E2F1	0.0122	Literature ChIP-seq,0.0122
51	ZNF408	0.01229	ARCHS4 Coexpression,0.01229
52	ZNF837	0.01245	GTEx Coexpression,0.01245

53	CENPB	0.01282	Enrichr Queries,0.01282
54	NR2C2	0.01347	ReMap ChIP-seq,0.01347
55	ZNF574	0.01353	Enrichr Queries,0.01353
56	MLX	0.01369	GTEx Coexpression,0.01369
57	ZNF395	0.01431	GTEx Coexpression,0.01431
58	FOXB1	0.01474	ARCHS4 Coexpression,0.01474
59	ZNF598	0.01496	Enrichr Queries,0.01496
60	ZNF691	0.01536	ARCHS4 Coexpression,0.01536
61	ESRRB	0.01556	GTEx Coexpression,0.01556
62	IRF3	0.01567	Enrichr Queries,0.01567
63	HESX1	0.01597	ARCHS4 Coexpression,0.01597
64	ZNF768	0.01618	GTEx Coexpression,0.01618
65	ISL2	0.01658	ARCHS4 Coexpression,0.01658
66	SOX4	0.0168	GTEx Coexpression,0.0168
67	MXI1	0.01684	ReMap ChIP-seq,0.01684
68	JUND	0.01709	Enrichr Queries,0.01709
69	SMYD3	0.0172	ARCHS4 Coexpression,0.0172
70	ZBTB45	0.01742	GTEx Coexpression,0.01742
71	POU5F1B	0.01781	ARCHS4 Coexpression,0.01781
72	KLF1	0.01829	Literature ChIP-seq,0.01829
73	ZNF174	0.01843	ARCHS4 Coexpression,0.01843
74	HMG20B	0.01852	Enrichr Queries,0.01852
75	GATA4	0.01867	GTEx Coexpression,0.01867
76	NR1H2	0.01923	Enrichr Queries,0.01923
77	SNAPC2	0.01966	ARCHS4 Coexpression,0.01966
78	ZNF668	0.01994	Enrichr Queries,0.01994
79	PRDM2	0.02054	GTEx Coexpression,0.02054
80	HOXA7	0.02088	ARCHS4 Coexpression,0.02088
81	MTERF3	0.02116	GTEx Coexpression,0.02116
82	ZNF3	0.0215	ARCHS4 Coexpression,0.0215
83	RBPJ	0.02178	GTEx Coexpression,0.02178
84	ZNF749	0.02211	ARCHS4 Coexpression,0.02211
85	SPEN	0.0224	GTEx Coexpression,0.0224
86	REXO4	0.02273	ARCHS4 Coexpression,0.02273
87	HMGA1	0.02279	Enrichr Queries,0.02279
88	CXXC1	0.0235	Enrichr Queries,0.0235
89	ELF1	0.02357	ReMap ChIP-seq,0.02357
90	GLMP	0.02396	ARCHS4 Coexpression,0.02396
91	ZNF579	0.02422	Enrichr Queries,0.02422
92	ZSCAN5C	0.02427	GTEx Coexpression,0.02427
93	ETS1	0.02439	Literature ChIP-seq,0.02439
94	FAM200B	0.02457	ARCHS4 Coexpression,0.02457
95	TP53	0.02489	GTEx Coexpression,0.02489
96	TBP	0.02542	ENCODE ChIP-seq,0.02542
97	ZNF784	0.02551	GTEx Coexpression,0.02551
98	ZNF653	0.02564	Enrichr Queries,0.02564
99	ZNF830	0.0258	ARCHS4 Coexpression,0.0258
100	ESRRG	0.02614	GTEx Coexpression,0.02614
101	ZNF358	0.02635	Enrichr Queries,0.02635
102	TIGD5	0.02676	GTEx Coexpression,0.02676
103	HES4	0.02703	ARCHS4 Coexpression,0.02703
104	TBX5	0.02738	GTEx Coexpression,0.02738
105	HES1	0.02764	ARCHS4 Coexpression,0.02764

106	SLC2A4RG	0.02778	Enrichr Queries,0.02778
107	TFAP4	0.028	GTEx Coexpression,0.028
108	ZKSCAN8	0.02826	ARCHS4 Coexpression,0.02826
109	USF2	0.02849	Enrichr Queries,0.02849
110	ZNF580	0.02887	ARCHS4 Coexpression,0.02887
111	XBP1	0.0292	Enrichr Queries,0.0292
112	ZNF747	0.02925	GTEx Coexpression,0.02925
113	NPAS1	0.02948	ARCHS4 Coexpression,0.02948
114	ZNF865	0.02987	GTEx Coexpression,0.02987
115	E4F1	0.02991	Enrichr Queries,0.02991
116	MESP1	0.0301	ARCHS4 Coexpression,0.0301
117	NRF1	0.0303	ReMap ChIP-seq,0.0303
118	CEBPB	0.03049	Literature ChIP-seq,0.03049
119	NKX26	0.03049	GTEx Coexpression,0.03049
120	ZGPAT	0.03063	Enrichr Queries,0.03063
121	HOXC5	0.03133	ARCHS4 Coexpression,0.03133
122	MAZ	0.03134	Enrichr Queries,0.03134
123	TBX20	0.03174	GTEx Coexpression,0.03174
124	ZNF207	0.03205	Enrichr Queries,0.03205
125	ZNF771	0.03256	ARCHS4 Coexpression,0.03256
126	ZNF688	0.03276	Enrichr Queries,0.03276
127	BAZ2B	0.03298	GTEx Coexpression,0.03298
128	VAX2	0.03317	ARCHS4 Coexpression,0.03317
129	ZNF444	0.03348	Enrichr Queries,0.03348
130	ELK4	0.03367	ReMap ChIP-seq,0.03367
131	ZNF721	0.03419	Enrichr Queries,0.03419
132	ATF6B	0.03423	GTEx Coexpression,0.03423
133	PRDM13	0.0344	ARCHS4 Coexpression,0.0344
134	HSFX1	0.03485	GTEx Coexpression,0.03485
135	MXD3	0.03501	ARCHS4 Coexpression,0.03501
136	E2F4	0.03561	Enrichr Queries,0.03561
137	TTF1	0.03624	ARCHS4 Coexpression,0.03624
138	VDR	0.03659	Literature ChIP-seq,0.03659
139	ZNF670	0.03686	ARCHS4 Coexpression,0.03686
140	SMAD5	0.03704	ReMap ChIP-seq,0.03704
141	NR2F6	0.03704	Enrichr Queries,0.03704
142	DPF3	0.03734	GTEx Coexpression,0.03734
143	NFAT5	0.03747	ARCHS4 Coexpression,0.03747
144	KLF16	0.03775	Enrichr Queries,0.03775
145	CREB1	0.03808	ARCHS4 Coexpression,0.03808
146	FIZ1	0.03846	Enrichr Queries,0.03846
147	HOXB7	0.0387	ARCHS4 Coexpression,0.0387
148	GLI4	0.0392	GTEx Coexpression,0.0392
149	HMX1	0.03931	ARCHS4 Coexpression,0.03931
150	ZNF584	0.03983	GTEx Coexpression,0.03983
151	RXRB	0.03989	Enrichr Queries,0.03989
152	MYNN	0.0404	ReMap ChIP-seq,0.0404
153	CEBPZ	0.0406	Enrichr Queries,0.0406
154	ZNF692	0.04131	Enrichr Queries,0.04131
155	ZNF513	0.04231	GTEx Coexpression,0.04231
156	MAX	0.04237	ENCODE ChIP-seq,0.04237
157	TEAD4	0.04238	ARCHS4 Coexpression,0.04238
158	ZNF205	0.04274	Enrichr Queries,0.04274

159	LBX2	0.043	ARCHS4 Coexpression,0.043
160	ZNF446	0.04345	Enrichr Queries,0.04345
161	E2F8	0.04356	GTEx Coexpression,0.04356
162	NKX28	0.04361	ARCHS4 Coexpression,0.04361
163	ZNF76	0.04416	Enrichr Queries,0.04416
164	ZNF480	0.04418	GTEx Coexpression,0.04418
165	THAP10	0.04423	ARCHS4 Coexpression,0.04423
166	RBCK1	0.0448	GTEx Coexpression,0.0448
167	ZBED1	0.04543	GTEx Coexpression,0.04543
168	HIVEP1	0.04545	ARCHS4 Coexpression,0.04545
169	CENPT	0.04558	Enrichr Queries,0.04558
170	DMTF1	0.04605	GTEx Coexpression,0.04605
171	DLX4	0.04607	ARCHS4 Coexpression,0.04607
172	HES6	0.0463	Enrichr Queries,0.0463
173	GMEB2	0.04667	GTEx Coexpression,0.04667
174	ZNF623	0.04668	ARCHS4 Coexpression,0.04668
175	DEAF1	0.04701	Enrichr Queries,0.04701
176	POU2F2	0.04714	ReMap ChIP-seq,0.04714
177	ZFP3	0.04729	GTEx Coexpression,0.04729
178	ZNF212	0.0473	ARCHS4 Coexpression,0.0473
179	TSC22D1	0.04772	Enrichr Queries,0.04772
180	TIGD6	0.04791	ARCHS4 Coexpression,0.04791
181	CDC5L	0.04843	Enrichr Queries,0.04843
182	FOXJ2	0.04853	ARCHS4 Coexpression,0.04853
183	ZFPM1	0.04854	GTEx Coexpression,0.04854
184	CDX4	0.04914	ARCHS4 Coexpression,0.04914
185	ZBTB22	0.04915	Enrichr Queries,0.04915
186	AKAP8L	0.04986	Enrichr Queries,0.04986
187	ZBTB47	0.0504	GTEx Coexpression,0.0504
188	THAP1	0.05051	ReMap ChIP-seq,0.05051
189	GATA1	0.05085	ENCODE ChIP-seq,0.05085
190	ZBTB11	0.05098	ARCHS4 Coexpression,0.05098
191	NFE2L1	0.05128	Enrichr Queries,0.05128
192	ZBTB9	0.0516	ARCHS4 Coexpression,0.0516
193	SMAD4	0.05221	ARCHS4 Coexpression,0.05221
194	ZBTB42	0.05227	GTEx Coexpression,0.05227
195	PRMT3	0.05271	Enrichr Queries,0.05271
196	ZNF296	0.05283	ARCHS4 Coexpression,0.05283
197	ASH1L	0.05289	GTEx Coexpression,0.05289
198	ANKZF1	0.05342	Enrichr Queries,0.05342
199	NFYA	0.05387	ReMap ChIP-seq,0.05387
200	ZNF410	0.05413	Enrichr Queries,0.05413
201	MZF1	0.05414	GTEx Coexpression,0.05414
202	TMF1	0.05467	ARCHS4 Coexpression,0.05467
203	ZNF48	0.05484	Enrichr Queries,0.05484
204	ZFP42	0.05488	Literature ChIP-seq,0.05488
205	PA2G4	0.05528	ARCHS4 Coexpression,0.05528
206	SREBF1	0.05556	Enrichr Queries,0.05556
207	DMRTA1	0.0559	ARCHS4 Coexpression,0.0559
208	ZBTB17	0.056	GTEx Coexpression,0.056
209	ZNF547	0.05651	ARCHS4 Coexpression,0.05651
210	ZNF777	0.05663	GTEx Coexpression,0.05663
211	OTX1	0.05713	ARCHS4 Coexpression,0.05713

212	ZFX	0.05724	ReMap ChIP-seq,0.05724
213	ZNF558	0.05725	GTEx Coexpression,0.05725
214	SREBF2	0.05769	Enrichr Queries,0.05769
215	OLIG3	0.05787	GTEx Coexpression,0.05787
216	BARX1	0.05835	ARCHS4 Coexpression,0.05835
217	ZBTB48	0.0584	Enrichr Queries,0.0584
218	ZNF761	0.05849	GTEx Coexpression,0.05849
219	GBX1	0.05912	GTEx Coexpression,0.05912
220	NR5A1	0.05912	Enrichr Queries,0.05912
221	YY1	0.05932	ENCODE ChIP-seq,0.05932
222	ZNF57	0.05958	ARCHS4 Coexpression,0.05958
223	ZSCAN2	0.05974	GTEx Coexpression,0.05974
224	ZNF213	0.05983	Enrichr Queries,0.05983
225	NKX32	0.0602	ARCHS4 Coexpression,0.0602
226	PPARA	0.06036	GTEx Coexpression,0.06036
227	USF1	0.06054	Enrichr Queries,0.06054
228	ZNF629	0.06098	GTEx Coexpression,0.06098
229	NFYC	0.06125	Enrichr Queries,0.06125
230	ZNF148	0.06143	ARCHS4 Coexpression,0.06143
231	ZNF121	0.06161	GTEx Coexpression,0.06161
232	TFDP1	0.06197	Enrichr Queries,0.06197
233	GATA2	0.06204	ARCHS4 Coexpression,0.06204
234	RUNX1	0.06265	ARCHS4 Coexpression,0.06265
235	ZNF628	0.06268	Enrichr Queries,0.06268
236	ZNF117	0.06327	ARCHS4 Coexpression,0.06327
237	MECP2	0.06347	GTEx Coexpression,0.06347
238	ZNF282	0.06409	GTEx Coexpression,0.06409
239	ZNF674	0.0641	Enrichr Queries,0.0641
240	RLF	0.0645	ARCHS4 Coexpression,0.0645
241	ZNF335	0.06472	GTEx Coexpression,0.06472
242	CENPBD1	0.06511	ARCHS4 Coexpression,0.06511
243	ZNF707	0.06534	GTEx Coexpression,0.06534
244	NCOA3	0.06553	Enrichr Queries,0.06553
245	ZSCAN29	0.06572	ARCHS4 Coexpression,0.06572
246	FOXK2	0.06596	GTEx Coexpression,0.06596
247	ZNF22	0.06624	Enrichr Queries,0.06624
248	ZNF324	0.06634	ARCHS4 Coexpression,0.06634
249	ZNF202	0.06658	GTEx Coexpression,0.06658
250	ZNF160	0.06695	ARCHS4 Coexpression,0.06695
251	BAZ2A	0.06721	GTEx Coexpression,0.06721
252	GATAD2A	0.06766	Enrichr Queries,0.06766
253	FLI1	0.0678	ENCODE ChIP-seq,0.0678
254	GZF1	0.06783	GTEx Coexpression,0.06783
255	MYBL2	0.06818	ARCHS4 Coexpression,0.06818
256	KCMF1	0.06838	Enrichr Queries,0.06838
257	FIGLA	0.06845	GTEx Coexpression,0.06845
258	BBX	0.0688	ARCHS4 Coexpression,0.0688
259	ZNF700	0.06907	GTEx Coexpression,0.06907
260	HSF1	0.06941	ARCHS4 Coexpression,0.06941
261	HOXD13	0.07002	ARCHS4 Coexpression,0.07002
262	ZNF764	0.07032	GTEx Coexpression,0.07032
263	CREB3L4	0.07064	ARCHS4 Coexpression,0.07064
264	ZBTB33	0.07071	ReMap ChIP-seq,0.07071

265	ZNF705E	0.07094	GTEx Coexpression,0.07094
266	SNAPC4	0.07123	Enrichr Queries,0.07123
267	ZNF791	0.07156	GTEx Coexpression,0.07156
268	ZNF775	0.07194	Enrichr Queries,0.07194
269	TCF3	0.07265	Enrichr Queries,0.07265
270	POU3F1	0.0731	ARCHS4 Coexpression,0.0731
271	SPI1	0.07317	Literature ChIP-seq,0.07317
272	ZNF512	0.07336	Enrichr Queries,0.07336
273	NANOG	0.07343	GTEx Coexpression,0.07343
274	LMX1A	0.07371	ARCHS4 Coexpression,0.07371
275	NKRF	0.07407	Enrichr Queries,0.07407
276	ATF1	0.07407	ReMap ChIP-seq,0.07407
277	ZNF696	0.07432	ARCHS4 Coexpression,0.07432
278	ZNF79	0.07467	GTEx Coexpression,0.07467
279	RELA	0.07479	Enrichr Queries,0.07479
280	CEBPG	0.07494	ARCHS4 Coexpression,0.07494
281	ETV4	0.0753	GTEx Coexpression,0.0753
282	AKAP8	0.0755	Enrichr Queries,0.0755
283	ZNF175	0.07678	ARCHS4 Coexpression,0.07678
284	ZNF549	0.07692	Enrichr Queries,0.07692
285	RARA	0.07744	ReMap ChIP-seq,0.07744
286	DBP	0.07764	Enrichr Queries,0.07764
287	RORC	0.07778	GTEx Coexpression,0.07778
288	FOXN3	0.07801	ARCHS4 Coexpression,0.07801
289	MAFG	0.07835	Enrichr Queries,0.07835
290	HOXB2	0.07862	ARCHS4 Coexpression,0.07862
291	ZNF582	0.07924	ARCHS4 Coexpression,0.07924
292	ZNF208	0.07965	GTEx Coexpression,0.07965
293	ATF5	0.07977	Enrichr Queries,0.07977
294	MTERF2	0.08027	GTEx Coexpression,0.08027
295	VEZF1	0.08047	ARCHS4 Coexpression,0.08047
296	MXD4	0.08048	Enrichr Queries,0.08048
297	MYCN	0.08081	ReMap ChIP-seq,0.08081
298	ZNF211	0.0809	GTEx Coexpression,0.0809
299	FOXK1	0.08108	ARCHS4 Coexpression,0.08108
300	ZNF875	0.08152	GTEx Coexpression,0.08152
301	ZNF680	0.0817	ARCHS4 Coexpression,0.0817
302	ZNF689	0.08191	Enrichr Queries,0.08191
303	NKX23	0.08231	ARCHS4 Coexpression,0.08231
304	ZNF600	0.08276	GTEx Coexpression,0.08276
305	HOXA9	0.08292	ARCHS4 Coexpression,0.08292
306	ZNF655	0.08339	GTEx Coexpression,0.08339
307	ZNF346	0.08401	GTEx Coexpression,0.08401
308	ETV1	0.08418	ReMap ChIP-seq,0.08418
309	ZNF75D	0.08463	GTEx Coexpression,0.08463
310	NFE2L2	0.08476	Enrichr Queries,0.08476
311	ZNF841	0.08477	ARCHS4 Coexpression,0.08477
312	ZNF317	0.08525	GTEx Coexpression,0.08525
313	CREM	0.08537	Literature ChIP-seq,0.08537
314	E2F6	0.08538	ARCHS4 Coexpression,0.08538
315	TEAD2	0.08618	Enrichr Queries,0.08618
316	CREBZF	0.0865	GTEx Coexpression,0.0865
317	SRF	0.08689	Enrichr Queries,0.08689

318	ZNF142	0.08761	Enrichr Queries,0.08761
319	ZNF189	0.08784	ARCHS4 Coexpression,0.08784
320	HSF4	0.08836	GTEx Coexpression,0.08836
321	FOXJ3	0.08845	ARCHS4 Coexpression,0.08845
322	NR1H4	0.08907	ARCHS4 Coexpression,0.08907
323	SON	0.08961	GTEx Coexpression,0.08961
324	ZNF276	0.08974	Enrichr Queries,0.08974
325	HOXC13	0.09029	ARCHS4 Coexpression,0.09029
326	PLAGL1	0.09085	GTEx Coexpression,0.09085
327	KMT2B	0.09091	ReMap ChIP-seq,0.09091
328	ZNF473	0.09117	Enrichr Queries,0.09117
329	CTCF	0.09146	Literature ChIP-seq,0.09146
330	ZNF44	0.09147	GTEx Coexpression,0.09147
331	HOXC10	0.09152	ARCHS4 Coexpression,0.09152
332	ZNF263	0.0921	GTEx Coexpression,0.0921
333	ZFP57	0.09214	ARCHS4 Coexpression,0.09214
334	STAT3	0.09259	Enrichr Queries,0.09259
335	ATF7	0.09272	GTEx Coexpression,0.09272
336	RFX7	0.09275	ARCHS4 Coexpression,0.09275
337	ATF3	0.09322	ENCODE ChIP-seq,0.09322
338	ZNF16	0.0933	Enrichr Queries,0.0933
339	ZNF783	0.09402	Enrichr Queries,0.09402
340	CTCFL	0.09428	ReMap ChIP-seq,0.09428
341	ZNF333	0.09459	GTEx Coexpression,0.09459
342	NFKB2	0.09473	Enrichr Queries,0.09473
343	SP3	0.09521	ARCHS4 Coexpression,0.09521
344	ZNF7	0.09544	Enrichr Queries,0.09544
345	MEF2A	0.09582	ARCHS4 Coexpression,0.09582
346	NR1I3	0.09615	Enrichr Queries,0.09615
347	USF3	0.09644	ARCHS4 Coexpression,0.09644
348	ZSCAN5B	0.09687	Enrichr Queries,0.09687
349	TBX3	0.09708	GTEx Coexpression,0.09708
350	TET1	0.09756	Literature ChIP-seq,0.09756
351	PBX2	0.09758	Enrichr Queries,0.09758
352	SP1	0.09764	ReMap ChIP-seq,0.09764
353	DDIT3	0.09767	ARCHS4 Coexpression,0.09767
354	STAT2	0.0977	GTEx Coexpression,0.0977
355	ZBTB44	0.09889	ARCHS4 Coexpression,0.09889
356	FOXM1	0.099	Enrichr Queries,0.099
357	ZKSCAN2	0.09956	GTEx Coexpression,0.09956
358	ZBED5	0.1001	ARCHS4 Coexpression,0.1001
359	MSANTD3	0.1002	GTEx Coexpression,0.1002
360	ZNF526	0.1004	Enrichr Queries,0.1004
361	FOXA2	0.1007	ARCHS4 Coexpression,0.1007
362	IRX1	0.1008	GTEx Coexpression,0.1008
363	CEBPD	0.101	ReMap ChIP-seq,0.101
364	ZNF514	0.1014	ARCHS4 Coexpression,0.1014
365	RXRA	0.1014	GTEx Coexpression,0.1014
366	ZNF430	0.1019	Enrichr Queries,0.1019
367	TCF4	0.1021	GTEx Coexpression,0.1021
368	ZC3H8	0.1026	Enrichr Queries,0.1026
369	TCFL5	0.1033	Enrichr Queries,0.1033
370	SAFB	0.1033	GTEx Coexpression,0.1033

371	TAL1	0.1037	Literature ChIP-seq,0.1037
372	ZNF35	0.1038	ARCHS4 Coexpression,0.1038
373	ZNF146	0.104	Enrichr Queries,0.104
374	ZHX2	0.1044	ReMap ChIP-seq,0.1044
375	ZBTB10	0.1044	ARCHS4 Coexpression,0.1044
376	ZNF704	0.1045	GTEx Coexpression,0.1045
377	MEF2B	0.1047	Enrichr Queries,0.1047
378	HOXA10	0.105	ARCHS4 Coexpression,0.105
379	KLF11	0.1052	GTEx Coexpression,0.1052
380	ZNF251	0.1054	Enrichr Queries,0.1054
381	ZNF219	0.1061	Enrichr Queries,0.1061
382	MYSM1	0.107	GTEx Coexpression,0.107
383	HOXA1	0.1075	ARCHS4 Coexpression,0.1075
384	YBX2	0.1075	Enrichr Queries,0.1075
385	ZBTB40	0.1077	GTEx Coexpression,0.1077
386	STAT6	0.1083	Enrichr Queries,0.1083
387	TCF7L2	0.1089	GTEx Coexpression,0.1089
388	NFKB1	0.109	Enrichr Queries,0.109
389	FOXD2	0.1093	ARCHS4 Coexpression,0.1093
390	THAP9	0.1095	GTEx Coexpression,0.1095
391	DACH1	0.1098	Literature ChIP-seq,0.1098
392	ZNF451	0.11	ARCHS4 Coexpression,0.11
393	ZNF280A	0.1101	GTEx Coexpression,0.1101
394	SIX3	0.1106	ARCHS4 Coexpression,0.1106
395	HSF2	0.1108	GTEx Coexpression,0.1108
396	DPRX	0.1112	ARCHS4 Coexpression,0.1112
397	ZUP1	0.1118	ARCHS4 Coexpression,0.1118
398	E2F2	0.1118	Enrichr Queries,0.1118
399	ZNF740	0.112	GTEx Coexpression,0.112
400	TFE3	0.1125	Enrichr Queries,0.1125
401	CREBL2	0.1126	GTEx Coexpression,0.1126
402	ZSCAN21	0.113	ARCHS4 Coexpression,0.113
403	RFX5	0.1132	Enrichr Queries,0.1132
404	ATF2	0.1136	ARCHS4 Coexpression,0.1136
405	ERG	0.1145	ReMap ChIP-seq,0.1145
406	ZNF326	0.1147	Enrichr Queries,0.1147
407	ZHX1	0.1151	GTEx Coexpression,0.1151
408	ZNF324B	0.1155	ARCHS4 Coexpression,0.1155
409	ZNF592	0.1157	GTEx Coexpression,0.1157
410	HOXC4	0.1161	ARCHS4 Coexpression,0.1161
411	ZNF394	0.1161	Enrichr Queries,0.1161
412	NKX11	0.1164	GTEx Coexpression,0.1164
413	GATA3	0.1167	ARCHS4 Coexpression,0.1167
414	ZNF41	0.117	GTEx Coexpression,0.117
415	HES5	0.1173	ARCHS4 Coexpression,0.1173
416	FOXR1	0.1175	Enrichr Queries,0.1175
417	MAFK	0.1176	GTEx Coexpression,0.1176
418	CENPA	0.1179	ARCHS4 Coexpression,0.1179
419	BPTF	0.1186	ARCHS4 Coexpression,0.1186
420	UBP1	0.1189	GTEx Coexpression,0.1189
421	ZNF367	0.1189	Enrichr Queries,0.1189
422	MSX1	0.1204	ARCHS4 Coexpression,0.1204
423	PLAGL2	0.1207	GTEx Coexpression,0.1207

424	CBX2	0.121	ARCHS4 Coexpression,0.121
425	NR1D1	0.1211	Enrichr Queries,0.1211
426	TGIF2	0.1212	ReMap ChIP-seq,0.1212
427	CEBPA	0.1213	GTEx Coexpression,0.1213
428	ZBTB3	0.1218	Enrichr Queries,0.1218
429	FOXP3	0.122	Literature ChIP-seq,0.122
430	MBD6	0.1226	GTEx Coexpression,0.1226
431	ZNF33A	0.1229	ARCHS4 Coexpression,0.1229
432	ZNF33B	0.1235	ARCHS4 Coexpression,0.1235
433	ZBTB12	0.1239	Enrichr Queries,0.1239
434	HOXA5	0.1241	ARCHS4 Coexpression,0.1241
435	ZNF512B	0.1245	GTEx Coexpression,0.1245
436	CREB3L3	0.1246	Enrichr Queries,0.1246
437	GPBP1	0.1247	ARCHS4 Coexpression,0.1247
438	YBX3	0.1251	GTEx Coexpression,0.1251
439	ZNF500	0.1254	Enrichr Queries,0.1254
440	ZNF843	0.1263	GTEx Coexpression,0.1263
441	HMBOX1	0.1265	ARCHS4 Coexpression,0.1265
442	FOXN4	0.1269	GTEx Coexpression,0.1269
443	ZKSCAN5	0.1276	GTEx Coexpression,0.1276
444	FOXO3	0.1278	ARCHS4 Coexpression,0.1278
445	CREB3L1	0.1279	ReMap ChIP-seq,0.1279
446	HOXB4	0.128	Literature ChIP-seq,0.128
447	ZNF710	0.1282	GTEx Coexpression,0.1282
448	ZNF12	0.1288	GTEx Coexpression,0.1288
449	WIZ	0.1289	Enrichr Queries,0.1289
450	HOXA11	0.129	ARCHS4 Coexpression,0.129
451	SOX15	0.1296	ARCHS4 Coexpression,0.1296
452	ZNF385A	0.1296	Enrichr Queries,0.1296
453	ZXDB	0.1302	ARCHS4 Coexpression,0.1302
454	PHF1	0.1307	GTEx Coexpression,0.1307
455	ELF5	0.1308	ARCHS4 Coexpression,0.1308
456	TIGD3	0.1311	Enrichr Queries,0.1311
457	ZNF662	0.1313	GTEx Coexpression,0.1313
458	EGR1	0.1313	ReMap ChIP-seq,0.1313
459	CC2D1A	0.1314	ARCHS4 Coexpression,0.1314
460	PCGF6	0.1318	Enrichr Queries,0.1318
461	ZSCAN25	0.1319	GTEx Coexpression,0.1319
462	ZBED6	0.1321	ARCHS4 Coexpression,0.1321
463	ZNF131	0.1325	Enrichr Queries,0.1325
464	ELF2	0.1325	GTEx Coexpression,0.1325
465	ZNF138	0.1327	ARCHS4 Coexpression,0.1327
466	ZNF81	0.1333	ARCHS4 Coexpression,0.1333
467	ZNF550	0.1338	GTEx Coexpression,0.1338
468	ZFP62	0.1344	GTEx Coexpression,0.1344
469	LCOR	0.1345	ARCHS4 Coexpression,0.1345
470	ZNF266	0.1346	Enrichr Queries,0.1346
471	PBX3	0.1347	ReMap ChIP-seq,0.1347
472	REST	0.1356	ENCODE ChIP-seq,0.1356
473	ZNF560	0.1363	GTEx Coexpression,0.1363
474	EEA1	0.1364	ARCHS4 Coexpression,0.1364
475	RAX2	0.1368	Enrichr Queries,0.1368
476	TAL2	0.1369	GTEx Coexpression,0.1369

477	SCX	0.137	ARCHS4 Coexpression,0.137
478	ETV2	0.1375	Enrichr Queries,0.1375
479	NCOA2	0.1375	GTEx Coexpression,0.1375
480	JAZF1	0.1376	ARCHS4 Coexpression,0.1376
481	SP4	0.138	ReMap ChIP-seq,0.138
482	NFYB	0.1382	ARCHS4 Coexpression,0.1382
483	KLF9	0.1388	GTEx Coexpression,0.1388
484	ZNF746	0.1389	Enrichr Queries,0.1389
485	ZNF772	0.1394	GTEx Coexpression,0.1394
486	TEF	0.1396	Enrichr Queries,0.1396
487	NEUROG3	0.14	ARCHS4 Coexpression,0.14
488	KLF6	0.141	Enrichr Queries,0.141
489	NR0B1	0.1413	GTEx Coexpression,0.1413
490	NCOA1	0.1413	ARCHS4 Coexpression,0.1413
491	ZNF34	0.1417	Enrichr Queries,0.1417
492	ZNF518A	0.1419	GTEx Coexpression,0.1419
493	MNX1	0.1419	ARCHS4 Coexpression,0.1419
494	ZNF23	0.1425	Enrichr Queries,0.1425
495	ZNF133	0.1425	GTEx Coexpression,0.1425
496	ZNF300	0.1431	GTEx Coexpression,0.1431
497	ZSCAN10	0.1432	Enrichr Queries,0.1432
498	HBP1	0.1437	ARCHS4 Coexpression,0.1437
499	TBX1	0.1437	GTEx Coexpression,0.1437
500	HNF4A	0.1439	Enrichr Queries,0.1439
501	PITX1	0.1443	ARCHS4 Coexpression,0.1443
502	LHX3	0.1444	GTEx Coexpression,0.1444
503	ZNF789	0.1446	Enrichr Queries,0.1446
504	DMRTA2	0.145	ARCHS4 Coexpression,0.145
505	DBX1	0.145	GTEx Coexpression,0.145
506	HOXA4	0.1456	ARCHS4 Coexpression,0.1456
507	MYB	0.146	Enrichr Queries,0.146
508	ZNF503	0.1462	ARCHS4 Coexpression,0.1462
509	RFX1	0.1462	GTEx Coexpression,0.1462
510	POU5F1	0.1467	Enrichr Queries,0.1467
511	ZNF292	0.1468	ARCHS4 Coexpression,0.1468
512	HOXD11	0.1474	ARCHS4 Coexpression,0.1474
513	RARG	0.1474	Enrichr Queries,0.1474
514	ZXDC	0.1475	GTEx Coexpression,0.1475
515	ZNF83	0.1481	GTEx Coexpression,0.1481
516	ZNF143	0.1481	ReMap ChIP-seq,0.1481
517	HIF1A	0.1481	Enrichr Queries,0.1481
518	ZSCAN9	0.1487	GTEx Coexpression,0.1487
519	SNAI3	0.1489	Enrichr Queries,0.1489
520	ZZZ3	0.1493	ARCHS4 Coexpression,0.1493
521	ASCL5	0.1493	GTEx Coexpression,0.1493
522	ERF	0.1496	Enrichr Queries,0.1496
523	ZNF594	0.1499	ARCHS4 Coexpression,0.1499
524	ZNF541	0.15	GTEx Coexpression,0.15
525	MBD4	0.1503	Enrichr Queries,0.1503
526	ZBTB43	0.1505	ARCHS4 Coexpression,0.1505
527	ELF3	0.151	Enrichr Queries,0.151
528	MGA	0.1511	ARCHS4 Coexpression,0.1511
529	KLF13	0.1515	ReMap ChIP-seq,0.1515

530	MBD1	0.1517	Enrichr Queries,0.1517
531	RXRG	0.1518	GTEx Coexpression,0.1518
532	ZBTB49	0.1529	ARCHS4 Coexpression,0.1529
533	ZNF778	0.1531	Enrichr Queries,0.1531
534	FOSL1	0.1536	ARCHS4 Coexpression,0.1536
535	ZNF215	0.1542	ARCHS4 Coexpression,0.1542
536	ZNF496	0.1546	Enrichr Queries,0.1546
537	TFDP2	0.1548	ARCHS4 Coexpression,0.1548
538	ESR2	0.1549	GTEx Coexpression,0.1549
539	ZNF416	0.1554	ARCHS4 Coexpression,0.1554
540	SOHLH1	0.156	Enrichr Queries,0.156
541	ZNF575	0.1562	GTEx Coexpression,0.1562
542	MNT	0.1567	Enrichr Queries,0.1567
543	SAFB2	0.1568	GTEx Coexpression,0.1568
544	ZNF544	0.1572	ARCHS4 Coexpression,0.1572
545	MEF2D	0.1574	Enrichr Queries,0.1574
546	HOMEZ	0.1574	GTEx Coexpression,0.1574
547	KLF12	0.1579	ARCHS4 Coexpression,0.1579
548	STAT5B	0.1581	Enrichr Queries,0.1581
549	HOXC12	0.1585	ARCHS4 Coexpression,0.1585
550	RARB	0.1585	Literature ChIP-seq,0.1585
551	SOX6	0.1587	GTEx Coexpression,0.1587
552	ZNF776	0.1591	ARCHS4 Coexpression,0.1591
553	ZNF853	0.1593	GTEx Coexpression,0.1593
554	ZXDA	0.1597	ARCHS4 Coexpression,0.1597
555	SRCAP	0.1599	GTEx Coexpression,0.1599
556	ZBTB7B	0.1603	Enrichr Queries,0.1603
557	BHLHA15	0.1609	ARCHS4 Coexpression,0.1609
558	ZKSCAN3	0.1617	Enrichr Queries,0.1617
559	ZNF485	0.1618	GTEx Coexpression,0.1618
560	ZNF236	0.1628	ARCHS4 Coexpression,0.1628
561	SETDB1	0.163	GTEx Coexpression,0.163
562	ZNF341	0.1631	Enrichr Queries,0.1631
563	FOXD4L4	0.1634	ARCHS4 Coexpression,0.1634
564	LBX1	0.1643	GTEx Coexpression,0.1643
565	FOXE3	0.1652	ARCHS4 Coexpression,0.1652
566	AIRE	0.1652	Enrichr Queries,0.1652
567	ZNF384	0.1655	GTEx Coexpression,0.1655
568	TBPL1	0.1658	ARCHS4 Coexpression,0.1658
569	ZNF497	0.166	Enrichr Queries,0.166
570	ZNF354B	0.1661	GTEx Coexpression,0.1661
571	ZNF654	0.1665	ARCHS4 Coexpression,0.1665
572	NR1D2	0.1667	Enrichr Queries,0.1667
573	ZNF587	0.1668	GTEx Coexpression,0.1668
574	ZNF714	0.1671	ARCHS4 Coexpression,0.1671
575	ZNF362	0.1674	Enrichr Queries,0.1674
576	MAFA	0.1674	GTEx Coexpression,0.1674
577	LCORL	0.1683	ARCHS4 Coexpression,0.1683
578	ARID2	0.1689	ARCHS4 Coexpression,0.1689
579	PAX1	0.1693	GTEx Coexpression,0.1693
580	ZNF260	0.1695	ARCHS4 Coexpression,0.1695
581	ZNF695	0.1701	ARCHS4 Coexpression,0.1701
582	MLXIPL	0.1702	Enrichr Queries,0.1702

583	ZNF19	0.1705	GTEx Coexpression,0.1705
584	TERF2	0.1709	Enrichr Queries,0.1709
585	ZBTB4	0.1711	GTEx Coexpression,0.1711
586	ZNF280C	0.1714	ARCHS4 Coexpression,0.1714
587	FOXP2	0.1717	ReMap ChIP-seq,0.1717
588	ZNF565	0.1717	GTEx Coexpression,0.1717
589	MTF2	0.172	ARCHS4 Coexpression,0.172
590	JUNB	0.1731	Enrichr Queries,0.1731
591	SPDEF	0.1738	Enrichr Queries,0.1738
592	ZNF197	0.1738	ARCHS4 Coexpression,0.1738
593	RFX4	0.1742	GTEx Coexpression,0.1742
594	BCL11A	0.1749	GTEx Coexpression,0.1749
595	ZNF460	0.1751	ARCHS4 Coexpression,0.1751
596	IRF2	0.1751	ReMap ChIP-seq,0.1751
597	ZBTB7A	0.1752	Enrichr Queries,0.1752
598	ZNF66	0.1755	GTEx Coexpression,0.1755
599	CLOCK	0.1757	ARCHS4 Coexpression,0.1757
600	KLF2	0.1759	Enrichr Queries,0.1759
601	PDX1	0.1761	GTEx Coexpression,0.1761
602	ZNF10	0.1763	ARCHS4 Coexpression,0.1763
603	NOBOX	0.1767	GTEx Coexpression,0.1767
604	KLF4	0.1768	Literature ChIP-seq,0.1768
605	FOXD4L1	0.1769	ARCHS4 Coexpression,0.1769
606	FOXA3	0.1773	GTEx Coexpression,0.1773
607	ZNF277	0.1774	Enrichr Queries,0.1774
608	ZNF615	0.1775	ARCHS4 Coexpression,0.1775
609	JUN	0.178	ENCODE ChIP-seq,0.178
610	MITF	0.178	GTEx Coexpression,0.178
611	ZBTB37	0.1781	ARCHS4 Coexpression,0.1781
612	CIC	0.1786	GTEx Coexpression,0.1786
613	KLF10	0.1788	Enrichr Queries,0.1788
614	MYOG	0.1792	GTEx Coexpression,0.1792
615	TFEB	0.1795	Enrichr Queries,0.1795
616	MBD2	0.1802	Enrichr Queries,0.1802
617	ZNF639	0.1809	Enrichr Queries,0.1809
618	PITX2	0.1811	GTEx Coexpression,0.1811
619	ZFP90	0.1816	Enrichr Queries,0.1816
620	DMRTB1	0.1817	GTEx Coexpression,0.1817
621	GSC	0.1818	ARCHS4 Coexpression,0.1818
622	ZMAT1	0.1824	ARCHS4 Coexpression,0.1824
623	FOXP1	0.1829	Literature ChIP-seq,0.1829
624	CGGBP1	0.1829	GTEx Coexpression,0.1829
625	ZNF74	0.1837	ARCHS4 Coexpression,0.1837
626	ZNF274	0.1838	Enrichr Queries,0.1838
627	ZBTB32	0.1845	Enrichr Queries,0.1845
628	KIN	0.1852	Enrichr Queries,0.1852
629	PITX3	0.1854	GTEx Coexpression,0.1854
630	XPA	0.1855	ARCHS4 Coexpression,0.1855
631	ZNF780B	0.1861	GTEx Coexpression,0.1861
632	NR1H3	0.1861	ARCHS4 Coexpression,0.1861
633	CAMTA2	0.1866	Enrichr Queries,0.1866
634	FOXP4	0.1867	GTEx Coexpression,0.1867
635	ZNF233	0.1873	GTEx Coexpression,0.1873

636	ZNF821	0.1873	Enrichr Queries,0.1873
637	FOXD4	0.1873	ARCHS4 Coexpression,0.1873
638	ZNF275	0.1879	GTEx Coexpression,0.1879
639	HOXC11	0.188	ARCHS4 Coexpression,0.188
640	ZNF664	0.188	Enrichr Queries,0.188
641	ZFP91	0.1886	GTEx Coexpression,0.1886
642	SOX3	0.1886	ARCHS4 Coexpression,0.1886
643	ZNF687	0.1887	Enrichr Queries,0.1887
644	FEV	0.1892	GTEx Coexpression,0.1892
645	ZNF519	0.1895	Enrichr Queries,0.1895
646	LIN28B	0.1898	GTEx Coexpression,0.1898
647	SOX2	0.1898	ARCHS4 Coexpression,0.1898
648	ZNF217	0.1902	Enrichr Queries,0.1902
649	ZNF546	0.191	GTEx Coexpression,0.191
650	ZNF530	0.1916	ARCHS4 Coexpression,0.1916
651	ZNF852	0.1923	ARCHS4 Coexpression,0.1923
652	ZNF516	0.1923	GTEx Coexpression,0.1923
653	MTERF1	0.1929	ARCHS4 Coexpression,0.1929
654	ZNF467	0.193	Enrichr Queries,0.193
655	ZNF195	0.1935	ARCHS4 Coexpression,0.1935
656	GLI3	0.1935	GTEx Coexpression,0.1935
657	TBX10	0.1937	Enrichr Queries,0.1937
658	ZNF222	0.1941	ARCHS4 Coexpression,0.1941
659	KMT2A	0.1942	GTEx Coexpression,0.1942
660	ZNF551	0.1944	Enrichr Queries,0.1944
661	ZNF140	0.1947	ARCHS4 Coexpression,0.1947
662	ZFAT	0.1948	GTEx Coexpression,0.1948
663	STAT1	0.1952	Enrichr Queries,0.1952
664	ZKSCAN1	0.1953	ReMap ChIP-seq,0.1953
665	ZSCAN16	0.1953	ARCHS4 Coexpression,0.1953
666	MYF6	0.1954	GTEx Coexpression,0.1954
667	TEAD3	0.1959	Enrichr Queries,0.1959
668	ZNF573	0.1966	ARCHS4 Coexpression,0.1966
669	TERF1	0.1966	Enrichr Queries,0.1966
670	ZNF200	0.1966	GTEx Coexpression,0.1966
671	HHEX	0.1972	ARCHS4 Coexpression,0.1972
672	ZNF316	0.1973	GTEx Coexpression,0.1973
673	ZNF589	0.1973	Enrichr Queries,0.1973
674	NFIB	0.1979	GTEx Coexpression,0.1979
675	KLF15	0.198	Enrichr Queries,0.198
676	SMAD9	0.1984	ARCHS4 Coexpression,0.1984
677	GTF2IRD2B	0.1985	GTEx Coexpression,0.1985
678	NEUROD1	0.1987	ReMap ChIP-seq,0.1987
679	NFXL1	0.199	ARCHS4 Coexpression,0.199
680	HSFY1	0.1991	GTEx Coexpression,0.1991
681	ZNF669	0.1994	Enrichr Queries,0.1994
682	MYOD1	0.1998	GTEx Coexpression,0.1998
683	ZNF223	0.2001	Enrichr Queries,0.2001
684	ZNF391	0.2002	ARCHS4 Coexpression,0.2002
685	ARID3B	0.2004	GTEx Coexpression,0.2004
686	PPARD	0.2009	Enrichr Queries,0.2009
687	E2F5	0.2009	ARCHS4 Coexpression,0.2009
688	ZNF646	0.2016	Enrichr Queries,0.2016

689	PAX7	0.2022	GTEx Coexpression,0.2022
690	RBPJL	0.2027	ARCHS4 Coexpression,0.2027
691	KDM2A	0.2029	GTEx Coexpression,0.2029
692	HNF1A	0.203	Enrichr Queries,0.203
693	IRF1	0.2034	ENCODE ChIP-seq,0.2034
694	PATZ1	0.2037	Enrichr Queries,0.2037
695	ZNF703	0.2039	ARCHS4 Coexpression,0.2039
696	RHOXF2	0.2044	Enrichr Queries,0.2044
697	ZNF417	0.2045	ARCHS4 Coexpression,0.2045
698	HES7	0.2051	Enrichr Queries,0.2051
699	ZNF626	0.2052	ARCHS4 Coexpression,0.2052
700	MAF	0.2054	ReMap ChIP-seq,0.2054
701	ZNF432	0.206	GTEx Coexpression,0.206
702	FEZF1	0.207	ARCHS4 Coexpression,0.207
703	ZNF786	0.2072	GTEx Coexpression,0.2072
704	ATMIN	0.208	Enrichr Queries,0.208
705	DNMT1	0.2082	ARCHS4 Coexpression,0.2082
706	ZIM3	0.2085	GTEx Coexpression,0.2085
707	ZNF517	0.2087	Enrichr Queries,0.2087
708	NR2F1	0.2088	ReMap ChIP-seq,0.2088
709	BORCS8MEF2B	0.2088	ARCHS4 Coexpression,0.2088
710	LYL1	0.2091	GTEx Coexpression,0.2091
711	RELB	0.2094	Enrichr Queries,0.2094
712	ZNF71	0.2095	ARCHS4 Coexpression,0.2095
713	SKOR1	0.2101	ARCHS4 Coexpression,0.2101
714	HMX2	0.2107	ARCHS4 Coexpression,0.2107
715	ARID3A	0.2108	Enrichr Queries,0.2108
716	ZNF658	0.2113	ARCHS4 Coexpression,0.2113
717	GFI1B	0.2121	ReMap ChIP-seq,0.2121
718	SP5	0.2122	GTEx Coexpression,0.2122
719	ZNF552	0.2125	ARCHS4 Coexpression,0.2125
720	ZFP14	0.2128	GTEx Coexpression,0.2128
721	ZNF554	0.213	Enrichr Queries,0.213
722	ZNF718	0.2131	ARCHS4 Coexpression,0.2131
723	ZNF77	0.2137	Enrichr Queries,0.2137
724	ZNF433	0.2138	ARCHS4 Coexpression,0.2138
725	NR2E3	0.2144	ARCHS4 Coexpression,0.2144
726	SOX12	0.2144	Enrichr Queries,0.2144
727	GPBP1L1	0.2147	GTEx Coexpression,0.2147
728	ZNF2	0.2151	Enrichr Queries,0.2151
729	ZNF226	0.2156	ARCHS4 Coexpression,0.2156
730	SIX1	0.2159	GTEx Coexpression,0.2159
731	ZNF155	0.2162	ARCHS4 Coexpression,0.2162
732	DMRTC2	0.2172	GTEx Coexpression,0.2172
733	ZNF586	0.2172	Enrichr Queries,0.2172
734	ZNF69	0.2174	ARCHS4 Coexpression,0.2174
735	DOT1L	0.2184	GTEx Coexpression,0.2184
736	NR2C1	0.2187	ARCHS4 Coexpression,0.2187
737	FBXL19	0.2193	ARCHS4 Coexpression,0.2193
738	ZSCAN18	0.2194	Enrichr Queries,0.2194
739	SOX17	0.2195	Literature ChIP-seq,0.2195
740	ZBED9	0.2197	GTEx Coexpression,0.2197
741	IKZF5	0.2199	ARCHS4 Coexpression,0.2199

742	OVOL3	0.2203	GTEx Coexpression,0.2203
743	ZNF737	0.2205	ARCHS4 Coexpression,0.2205
744	RAG1	0.2209	GTEx Coexpression,0.2209
745	ZNF319	0.2215	Enrichr Queries,0.2215
746	HIC2	0.2215	GTEx Coexpression,0.2215
747	TGIF1	0.2217	ARCHS4 Coexpression,0.2217
748	BHLHE40	0.2222	ReMap ChIP-seq,0.2222
749	ZFP41	0.2222	Enrichr Queries,0.2222
750	ZNF625	0.223	ARCHS4 Coexpression,0.223
751	FOXD1	0.2236	ARCHS4 Coexpression,0.2236
752	MSANTD4	0.224	GTEx Coexpression,0.224
753	ZNF606	0.2242	ARCHS4 Coexpression,0.2242
754	ZNF354A	0.2244	Enrichr Queries,0.2244
755	FAM170A	0.2253	GTEx Coexpression,0.2253
756	MBNL2	0.2259	GTEx Coexpression,0.2259
757	OVOL2	0.2265	Enrichr Queries,0.2265
758	OTX2	0.2267	ARCHS4 Coexpression,0.2267
759	NFIC	0.2272	Enrichr Queries,0.2272
760	ZNF599	0.2278	GTEx Coexpression,0.2278
761	THRA	0.2279	Enrichr Queries,0.2279
762	ARHGAP35	0.2284	GTEx Coexpression,0.2284
763	E2F3	0.2286	Enrichr Queries,0.2286
764	ZNF808	0.2291	ARCHS4 Coexpression,0.2291
765	ZNF577	0.2296	GTEx Coexpression,0.2296
766	ZNF846	0.2297	ARCHS4 Coexpression,0.2297
767	ZNF90	0.2303	ARCHS4 Coexpression,0.2303
768	FOXO4	0.2308	Enrichr Queries,0.2308
769	DMRT1	0.2309	GTEx Coexpression,0.2309
770	ETS2	0.2315	Enrichr Queries,0.2315
771	NFATC3	0.2315	GTEx Coexpression,0.2315
772	ZNF75A	0.2316	ARCHS4 Coexpression,0.2316
773	E2F7	0.2322	Enrichr Queries,0.2322
774	SNAI2	0.2323	ReMap ChIP-seq,0.2323
775	GFI1	0.2328	ARCHS4 Coexpression,0.2328
776	GLIS2	0.2329	Enrichr Queries,0.2329
777	SIX2	0.2334	GTEx Coexpression,0.2334
778	POGK	0.2336	Enrichr Queries,0.2336
779	POU5F2	0.234	GTEx Coexpression,0.234
780	ASCL2	0.234	ARCHS4 Coexpression,0.234
781	TIGD2	0.2343	Enrichr Queries,0.2343
782	HOXC6	0.2346	ARCHS4 Coexpression,0.2346
783	PRR12	0.2352	GTEx Coexpression,0.2352
784	GATAD2B	0.2353	ARCHS4 Coexpression,0.2353
785	JDP2	0.2358	Enrichr Queries,0.2358
786	FOXF2	0.2359	ARCHS4 Coexpression,0.2359
787	EBF4	0.2365	GTEx Coexpression,0.2365
788	ETV5	0.2365	Enrichr Queries,0.2365
789	IRX5	0.2371	ARCHS4 Coexpression,0.2371
790	HINFP	0.2377	GTEx Coexpression,0.2377
791	SRY	0.2378	Literature ChIP-seq,0.2378
792	ZNF652	0.2383	ARCHS4 Coexpression,0.2383
793	POU1F1	0.2383	GTEx Coexpression,0.2383
794	NEUROD4	0.239	GTEx Coexpression,0.239

795	ZEB1	0.2391	ReMap ChIP-seq,0.2391
796	STAT5A	0.2393	Enrichr Queries,0.2393
797	TBX15	0.2396	GTEx Coexpression,0.2396
798	ZBTB2	0.24	Enrichr Queries,0.24
799	ZNF699	0.2402	ARCHS4 Coexpression,0.2402
800	MYF5	0.2402	GTEx Coexpression,0.2402
801	ZNF239	0.2407	Enrichr Queries,0.2407
802	ZNF345	0.2408	GTEx Coexpression,0.2408
803	ZFY	0.2414	ARCHS4 Coexpression,0.2414
804	ZNF20	0.2414	GTEx Coexpression,0.2414
805	ZNF716	0.2421	GTEx Coexpression,0.2421
806	ZNF564	0.2422	Enrichr Queries,0.2422
807	TLX2	0.2426	ARCHS4 Coexpression,0.2426
808	ZNF684	0.2427	GTEx Coexpression,0.2427
809	ADNP2	0.2429	Enrichr Queries,0.2429
810	NFE2L3	0.2433	GTEx Coexpression,0.2433
811	ZNF415	0.2439	ARCHS4 Coexpression,0.2439
812	SIX4	0.2439	GTEx Coexpression,0.2439
813	FOXH1	0.245	Enrichr Queries,0.245
814	KAT7	0.2452	GTEx Coexpression,0.2452
815	SPIC	0.2457	ARCHS4 Coexpression,0.2457
816	KDM5B	0.2458	ReMap ChIP-seq,0.2458
817	FOS	0.2463	ARCHS4 Coexpression,0.2463
818	ZSCAN22	0.2464	GTEx Coexpression,0.2464
819	ZNF180	0.2469	ARCHS4 Coexpression,0.2469
820	TFCP2	0.2472	Enrichr Queries,0.2472
821	SOX18	0.2475	ARCHS4 Coexpression,0.2475
822	MESP2	0.2477	GTEx Coexpression,0.2477
823	MAFF	0.2482	ARCHS4 Coexpression,0.2482
824	THAP5	0.2483	GTEx Coexpression,0.2483
825	ZNF141	0.2488	ARCHS4 Coexpression,0.2488
826	PRDM15	0.2489	GTEx Coexpression,0.2489
827	CXXC5	0.2494	ARCHS4 Coexpression,0.2494
828	ZNF587B	0.2495	GTEx Coexpression,0.2495
829	CEBPE	0.25	Enrichr Queries,0.25
830	ZNF256	0.25	ARCHS4 Coexpression,0.25
831	RFX3	0.2502	GTEx Coexpression,0.2502
832	ZNF254	0.2506	ARCHS4 Coexpression,0.2506
833	NR3C2	0.2508	GTEx Coexpression,0.2508
834	TOPORS	0.2512	ARCHS4 Coexpression,0.2512
835	ZNF486	0.2514	Enrichr Queries,0.2514
836	ZNF597	0.2521	Enrichr Queries,0.2521
837	ZNF813	0.2525	ARCHS4 Coexpression,0.2525
838	ZNF253	0.2531	ARCHS4 Coexpression,0.2531
839	FOXL1	0.2537	ARCHS4 Coexpression,0.2537
840	OLIG1	0.2543	Enrichr Queries,0.2543
841	ZGLP1	0.2543	ARCHS4 Coexpression,0.2543
842	ZNF644	0.2549	ARCHS4 Coexpression,0.2549
843	ZSCAN12	0.2555	ARCHS4 Coexpression,0.2555
844	ETV6	0.2558	GTEx Coexpression,0.2558
845	GTF2B	0.2559	ReMap ChIP-seq,0.2559
846	ZFP82	0.2561	ARCHS4 Coexpression,0.2561
847	ZNF627	0.2568	ARCHS4 Coexpression,0.2568

848	CRX	0.257	GTEx Coexpression,0.257
849	NFIX	0.2578	Enrichr Queries,0.2578
850	ZNF98	0.2582	GTEx Coexpression,0.2582
851	HOXA6	0.2586	ARCHS4 Coexpression,0.2586
852	ONECUT3	0.2589	GTEx Coexpression,0.2589
853	ZIK1	0.2593	Enrichr Queries,0.2593
854	KLF5	0.2593	ReMap ChIP-seq,0.2593
855	PTF1A	0.2595	GTEx Coexpression,0.2595
856	SALL2	0.26	Enrichr Queries,0.26
857	ZSCAN5A	0.2614	Enrichr Queries,0.2614
858	TBX6	0.262	GTEx Coexpression,0.262
859	FOXO1	0.2621	Enrichr Queries,0.2621
860	SMAD1	0.2626	ReMap ChIP-seq,0.2626
861	THAP6	0.2635	ARCHS4 Coexpression,0.2635
862	ZNF561	0.2641	ARCHS4 Coexpression,0.2641
863	NPAS4	0.2642	Enrichr Queries,0.2642
864	ZFP2	0.2647	ARCHS4 Coexpression,0.2647
865	ZNF250	0.265	Enrichr Queries,0.265
866	SOX30	0.2651	GTEx Coexpression,0.2651
867	ZFP1	0.2657	Enrichr Queries,0.2657
868	HOXA2	0.2663	GTEx Coexpression,0.2663
869	ASCL3	0.2664	Enrichr Queries,0.2664
870	ZNF566	0.2666	ARCHS4 Coexpression,0.2666
871	DMBX1	0.267	GTEx Coexpression,0.267
872	GRHL2	0.2671	Enrichr Queries,0.2671
873	ZNF165	0.2676	GTEx Coexpression,0.2676
874	IRF6	0.2678	Enrichr Queries,0.2678
875	ZNF620	0.2678	ARCHS4 Coexpression,0.2678
876	SCMH1	0.2682	GTEx Coexpression,0.2682
877	ZNF429	0.2684	ARCHS4 Coexpression,0.2684
878	RFX2	0.2685	Enrichr Queries,0.2685
879	SOHLH2	0.2688	GTEx Coexpression,0.2688
880	DLX5	0.269	ARCHS4 Coexpression,0.269
881	PAX5	0.2694	ReMap ChIP-seq,0.2694
882	PRDM4	0.2699	Enrichr Queries,0.2699
883	FOXL2	0.2707	GTEx Coexpression,0.2707
884	GSX2	0.2726	GTEx Coexpression,0.2726
885	MEIS3	0.2735	Enrichr Queries,0.2735
886	HSF5	0.2738	GTEx Coexpression,0.2738
887	SOX9	0.2744	Literature ChIP-seq,0.2744
888	TP73	0.2746	ARCHS4 Coexpression,0.2746
889	PKNOX1	0.2749	Enrichr Queries,0.2749
890	ZNF709	0.275	GTEx Coexpression,0.275
891	ZNF136	0.2752	ARCHS4 Coexpression,0.2752
892	ZNF641	0.2757	GTEx Coexpression,0.2757
893	ZEB2	0.2758	ARCHS4 Coexpression,0.2758
894	ADNP	0.2764	Enrichr Queries,0.2764
895	FOXC1	0.2764	ARCHS4 Coexpression,0.2764
896	ZKSCAN4	0.2769	GTEx Coexpression,0.2769
897	TLX1	0.277	ARCHS4 Coexpression,0.277
898	ZNF93	0.2771	Enrichr Queries,0.2771
899	KLF7	0.2776	ARCHS4 Coexpression,0.2776
900	FOXA1	0.2789	ARCHS4 Coexpression,0.2789

901	AHCTF1	0.2799	Enrichr Queries,0.2799
902	ZNF671	0.2801	ARCHS4 Coexpression,0.2801
903	TRAFD1	0.2806	Enrichr Queries,0.2806
904	TGIF2LX	0.2807	ARCHS4 Coexpression,0.2807
905	FOXI1	0.2813	ARCHS4 Coexpression,0.2813
906	ZNF385C	0.2813	Enrichr Queries,0.2813
907	ZNF630	0.2819	ARCHS4 Coexpression,0.2819
908	DMRT3	0.2825	GTEx Coexpression,0.2825
909	DR1	0.2828	Enrichr Queries,0.2828
910	TBX19	0.2831	GTEx Coexpression,0.2831
911	TFAP2D	0.2832	ARCHS4 Coexpression,0.2832
912	ZBTB1	0.2838	GTEx Coexpression,0.2838
913	FLYWCH1	0.2844	GTEx Coexpression,0.2844
914	SIX6	0.285	GTEx Coexpression,0.285
915	POU2F1	0.285	ARCHS4 Coexpression,0.285
916	ETV3	0.2856	Enrichr Queries,0.2856
917	ZNF548	0.2856	GTEx Coexpression,0.2856
918	TCF12	0.2862	ReMap ChIP-seq,0.2862
919	ZNF337	0.2863	Enrichr Queries,0.2863
920	ZNF681	0.2869	ARCHS4 Coexpression,0.2869
921	ZNF816	0.2875	ARCHS4 Coexpression,0.2875
922	BATF2	0.2875	GTEx Coexpression,0.2875
923	IRF9	0.2877	Enrichr Queries,0.2877
924	ZNF235	0.2881	ARCHS4 Coexpression,0.2881
925	ZNF569	0.2881	GTEx Coexpression,0.2881
926	ZNF484	0.2893	ARCHS4 Coexpression,0.2893
927	ZNF648	0.2894	GTEx Coexpression,0.2894
928	MSANTD1	0.2899	ARCHS4 Coexpression,0.2899
929	NR2E1	0.2905	ARCHS4 Coexpression,0.2905
930	FOSL2	0.2906	GTEx Coexpression,0.2906
931	ZNF799	0.2912	ARCHS4 Coexpression,0.2912
932	ZNF616	0.2913	Enrichr Queries,0.2913
933	ZBED3	0.2918	ARCHS4 Coexpression,0.2918
934	ZBTB14	0.2918	GTEx Coexpression,0.2918
935	ZNF177	0.292	Enrichr Queries,0.292
936	HDX	0.2924	ARCHS4 Coexpression,0.2924
937	ZNF91	0.2934	Enrichr Queries,0.2934
938	PHF20	0.2936	ARCHS4 Coexpression,0.2936
939	ARNTL	0.2942	Enrichr Queries,0.2942
940	ZNF708	0.2948	ARCHS4 Coexpression,0.2948
941	ZNF624	0.2955	ARCHS4 Coexpression,0.2955
942	ZNF107	0.2961	ARCHS4 Coexpression,0.2961
943	SALL4	0.2963	Enrichr Queries,0.2963
944	KLF17	0.297	Enrichr Queries,0.297
945	RAX	0.2979	ARCHS4 Coexpression,0.2979
946	ZNF181	0.2981	GTEx Coexpression,0.2981
947	ZBTB5	0.2984	Enrichr Queries,0.2984
948	PPARG	0.2988	Literature ChIP-seq,0.2988
949	ZNF556	0.2993	GTEx Coexpression,0.2993
950	CXXC4	0.2997	ReMap ChIP-seq,0.2997
951	ZNF850	0.2998	ARCHS4 Coexpression,0.2998
952	TERB1	0.2999	GTEx Coexpression,0.2999
953	ZNF765	0.3004	ARCHS4 Coexpression,0.3004

954	POU4F2	0.3006	GTEx Coexpression,0.3006
955	ZNF320	0.301	ARCHS4 Coexpression,0.301
956	HOXB1	0.3012	GTEx Coexpression,0.3012
957	GMEB1	0.3018	GTEx Coexpression,0.3018
958	CARF	0.3022	ARCHS4 Coexpression,0.3022
959	CCDC17	0.3024	GTEx Coexpression,0.3024
960	ZNF442	0.3027	Enrichr Queries,0.3027
961	ZNF880	0.3028	ARCHS4 Coexpression,0.3028
962	ZNF343	0.303	GTEx Coexpression,0.303
963	SATB1	0.3037	GTEx Coexpression,0.3037
964	PRDM10	0.3047	ARCHS4 Coexpression,0.3047
965	ZNF182	0.3049	GTEx Coexpression,0.3049
966	SETDB2	0.3053	ARCHS4 Coexpression,0.3053
967	NFATC1	0.3056	Enrichr Queries,0.3056
968	ZNF273	0.3065	ARCHS4 Coexpression,0.3065
969	ZBTB8A	0.3068	GTEx Coexpression,0.3068
970	CREB3L2	0.307	Enrichr Queries,0.307
971	SMAD3	0.3077	Enrichr Queries,0.3077
972	ZNF610	0.3077	ARCHS4 Coexpression,0.3077
973	TGIF2LY	0.308	GTEx Coexpression,0.308
974	SALL3	0.3099	GTEx Coexpression,0.3099
975	HOXD9	0.3105	GTEx Coexpression,0.3105
976	TCF7L1	0.3105	Enrichr Queries,0.3105
977	MEIS1	0.311	Literature ChIP-seq,0.311
978	MYPOP	0.3111	GTEx Coexpression,0.3111
979	GTF2IRD1	0.3118	GTEx Coexpression,0.3118
980	FOXJ1	0.312	Enrichr Queries,0.312
981	ZSCAN1	0.3127	ARCHS4 Coexpression,0.3127
982	ZBTB16	0.3131	ReMap ChIP-seq,0.3131
983	ZNF607	0.3136	GTEx Coexpression,0.3136
984	RREB1	0.3145	ARCHS4 Coexpression,0.3145
985	NR6A1	0.3149	GTEx Coexpression,0.3149
986	CDX1	0.3155	Enrichr Queries,0.3155
987	SOX13	0.3161	GTEx Coexpression,0.3161
988	ZNF487	0.3162	Enrichr Queries,0.3162
989	IRX3	0.3163	ARCHS4 Coexpression,0.3163
990	ZNF559	0.317	ARCHS4 Coexpression,0.317
991	MSC	0.3176	ARCHS4 Coexpression,0.3176
992	POU4F1	0.318	GTEx Coexpression,0.318
993	TBXT	0.3182	ARCHS4 Coexpression,0.3182
994	NR4A1	0.3184	Enrichr Queries,0.3184
995	LIN28A	0.3192	GTEx Coexpression,0.3192
996	ZNF491	0.3194	ARCHS4 Coexpression,0.3194
997	TFAP2A	0.3199	ReMap ChIP-seq,0.3199
998	ZNF287	0.3205	GTEx Coexpression,0.3205
999	HOXD1	0.3206	ARCHS4 Coexpression,0.3206
1000	ZNF334	0.3211	GTEx Coexpression,0.3211
1001	ZFHX3	0.3212	Enrichr Queries,0.3212
1002	ELF4	0.3234	Enrichr Queries,0.3234
1003	DMRT2	0.3236	GTEx Coexpression,0.3236
1004	ZNF790	0.3237	ARCHS4 Coexpression,0.3237
1005	SP140L	0.3243	ARCHS4 Coexpression,0.3243
1006	ZNF555	0.3249	ARCHS4 Coexpression,0.3249

1007	ZNF543	0.3256	ARCHS4 Coexpression,0.3256
1008	ZNF449	0.3261	GTEx Coexpression,0.3261
1009	ZNF814	0.3262	ARCHS4 Coexpression,0.3262
1010	CUX1	0.3266	ReMap ChIP-seq,0.3266
1011	ZNF418	0.3268	ARCHS4 Coexpression,0.3268
1012	ANHX	0.3286	GTEx Coexpression,0.3286
1013	MLXIP	0.3292	GTEx Coexpression,0.3292
1014	ZNF682	0.3292	ARCHS4 Coexpression,0.3292
1015	OVOL1	0.3298	Enrichr Queries,0.3298
1016	ZNF227	0.3299	ARCHS4 Coexpression,0.3299
1017	ZNF214	0.3305	ARCHS4 Coexpression,0.3305
1018	ZNF43	0.3311	ARCHS4 Coexpression,0.3311
1019	PAX8	0.3312	Enrichr Queries,0.3312
1020	ZNF727	0.3317	ARCHS4 Coexpression,0.3317
1021	ZNF532	0.3323	GTEx Coexpression,0.3323
1022	ZBTB25	0.3329	ARCHS4 Coexpression,0.3329
1023	PAX6	0.3333	ReMap ChIP-seq,0.3333
1024	ZNF280B	0.3333	Enrichr Queries,0.3333
1025	ZNF570	0.3335	ARCHS4 Coexpression,0.3335
1026	CSRNP2	0.3335	GTEx Coexpression,0.3335
1027	TBPL2	0.3354	GTEx Coexpression,0.3354
1028	ZNF100	0.336	ARCHS4 Coexpression,0.336
1029	GTF2IRD2	0.3362	Enrichr Queries,0.3362
1030	ZNF678	0.3366	ARCHS4 Coexpression,0.3366
1031	ZNF462	0.3367	GTEx Coexpression,0.3367
1032	ATOH8	0.3372	ARCHS4 Coexpression,0.3372
1033	ZNF518B	0.3378	ARCHS4 Coexpression,0.3378
1034	ZNF883	0.3385	ARCHS4 Coexpression,0.3385
1035	PRDM5	0.3391	ARCHS4 Coexpression,0.3391
1036	ZNF423	0.3391	GTEx Coexpression,0.3391
1037	BNC1	0.3398	GTEx Coexpression,0.3398
1038	FOXS1	0.3403	ARCHS4 Coexpression,0.3403
1039	GLYR1	0.341	GTEx Coexpression,0.341
1040	ZNF101	0.3412	Enrichr Queries,0.3412
1041	ZNF705A	0.3419	Enrichr Queries,0.3419
1042	FOXR2	0.3426	Enrichr Queries,0.3426
1043	ZNF829	0.3428	ARCHS4 Coexpression,0.3428
1044	ONECUT1	0.3429	GTEx Coexpression,0.3429
1045	ZNF14	0.3433	Enrichr Queries,0.3433
1046	NR1I2	0.3435	GTEx Coexpression,0.3435
1047	ZNF426	0.344	ARCHS4 Coexpression,0.344
1048	PRDM14	0.344	Enrichr Queries,0.344
1049	ZBTB39	0.3441	GTEx Coexpression,0.3441
1050	LHX8	0.3452	ARCHS4 Coexpression,0.3452
1051	ZFP28	0.3454	GTEx Coexpression,0.3454
1052	ZNF350	0.3454	Enrichr Queries,0.3454
1053	ZNF675	0.3464	ARCHS4 Coexpression,0.3464
1054	ZNF608	0.3466	GTEx Coexpression,0.3466
1055	PGR	0.3477	ARCHS4 Coexpression,0.3477
1056	ETV7	0.3479	GTEx Coexpression,0.3479
1057	ZNF383	0.3483	ARCHS4 Coexpression,0.3483
1058	ARID5B	0.3489	ARCHS4 Coexpression,0.3489
1059	ZNF304	0.349	Enrichr Queries,0.349

1060	SOX5	0.3491	GTEx Coexpression,0.3491
1061	ZNF585A	0.3495	ARCHS4 Coexpression,0.3495
1062	HNF4G	0.3502	ReMap ChIP-seq,0.3502
1063	OSR2	0.3503	GTEx Coexpression,0.3503
1064	ZMAT4	0.3504	Enrichr Queries,0.3504
1065	ZNF25	0.3511	Enrichr Queries,0.3511
1066	ZNF766	0.3514	ARCHS4 Coexpression,0.3514
1067	ZNF385B	0.3516	GTEx Coexpression,0.3516
1068	SOX10	0.3519	Enrichr Queries,0.3519
1069	ESR1	0.352	ARCHS4 Coexpression,0.352
1070	ZNF169	0.3526	Enrichr Queries,0.3526
1071	HLF	0.3533	Enrichr Queries,0.3533
1072	TCF7	0.3537	Literature ChIP-seq,0.3537
1073	ZNF431	0.3544	ARCHS4 Coexpression,0.3544
1074	ZNF436	0.3547	Enrichr Queries,0.3547
1075	ZNF676	0.3553	GTEx Coexpression,0.3553
1076	ZNF891	0.3557	ARCHS4 Coexpression,0.3557
1077	TCF23	0.3561	Enrichr Queries,0.3561
1078	HOXB5	0.3563	ARCHS4 Coexpression,0.3563
1079	ZNF184	0.3569	ARCHS4 Coexpression,0.3569
1080	ZNF736	0.3572	GTEx Coexpression,0.3572
1081	GLI1	0.3575	Enrichr Queries,0.3575
1082	ZNF836	0.3578	GTEx Coexpression,0.3578
1083	NPAS3	0.3584	GTEx Coexpression,0.3584
1084	NR3C1	0.359	Enrichr Queries,0.359
1085	ZNF679	0.3591	GTEx Coexpression,0.3591
1086	ZNF683	0.3597	GTEx Coexpression,0.3597
1087	NR5A2	0.3606	ARCHS4 Coexpression,0.3606
1088	NKX24	0.3609	GTEx Coexpression,0.3609
1089	EPAS1	0.3611	Enrichr Queries,0.3611
1090	ZFP30	0.3612	ARCHS4 Coexpression,0.3612
1091	PHOX2A	0.3624	ARCHS4 Coexpression,0.3624
1092	MYBL1	0.3625	Enrichr Queries,0.3625
1093	ZNF729	0.3628	GTEx Coexpression,0.3628
1094	ZNF461	0.363	ARCHS4 Coexpression,0.363
1095	KLF8	0.3632	Enrichr Queries,0.3632
1096	HIF3A	0.3634	GTEx Coexpression,0.3634
1097	NR2F2	0.3636	ReMap ChIP-seq,0.3636
1098	ZNF474	0.3636	ARCHS4 Coexpression,0.3636
1099	TFCP2L1	0.364	Enrichr Queries,0.364
1100	ALX4	0.364	GTEx Coexpression,0.364
1101	HMX3	0.3643	ARCHS4 Coexpression,0.3643
1102	ZNF281	0.3654	Enrichr Queries,0.3654
1103	SOX11	0.3659	Literature ChIP-seq,0.3659
1104	PRDM9	0.3659	GTEx Coexpression,0.3659
1105	DPF1	0.3661	Enrichr Queries,0.3661
1106	TIGD1	0.3665	GTEx Coexpression,0.3665
1107	TLX3	0.3667	ARCHS4 Coexpression,0.3667
1108	WT1	0.3668	Enrichr Queries,0.3668
1109	ZNF525	0.3671	GTEx Coexpression,0.3671
1110	FOXD3	0.3673	ARCHS4 Coexpression,0.3673
1111	CUX2	0.3675	Enrichr Queries,0.3675
1112	HAND1	0.3679	ARCHS4 Coexpression,0.3679

1113	TPRX1	0.3682	Enrichr Queries,0.3682
1114	ZNF774	0.3703	GTEx Coexpression,0.3703
1115	ZNF763	0.3704	ARCHS4 Coexpression,0.3704
1116	TSHZ1	0.3709	GTEx Coexpression,0.3709
1117	RBSN	0.371	ARCHS4 Coexpression,0.371
1118	ELK3	0.3711	Enrichr Queries,0.3711
1119	ZNF407	0.3715	GTEx Coexpression,0.3715
1120	ZNF347	0.3721	GTEx Coexpression,0.3721
1121	ZNF17	0.3732	Enrichr Queries,0.3732
1122	IRX2	0.374	GTEx Coexpression,0.374
1123	ZFPM2	0.3741	ARCHS4 Coexpression,0.3741
1124	ARID3C	0.3746	GTEx Coexpression,0.3746
1125	BCL6	0.3746	Enrichr Queries,0.3746
1126	ZNF438	0.3761	Enrichr Queries,0.3761
1127	PAX9	0.3765	GTEx Coexpression,0.3765
1128	ZNF697	0.3768	Enrichr Queries,0.3768
1129	ZNF613	0.3771	GTEx Coexpression,0.3771
1130	DBX2	0.3775	Enrichr Queries,0.3775
1131	ZNF827	0.3778	ARCHS4 Coexpression,0.3778
1132	TP63	0.378	Literature ChIP-seq,0.378
1133	HMG20A	0.3782	Enrichr Queries,0.3782
1134	RFX6	0.3789	Enrichr Queries,0.3789
1135	SOX8	0.379	ARCHS4 Coexpression,0.379
1136	ZNF583	0.3796	GTEx Coexpression,0.3796
1137	AHR	0.3796	Enrichr Queries,0.3796
1138	NKX63	0.3802	ARCHS4 Coexpression,0.3802
1139	NFE2	0.3805	ReMap ChIP-seq,0.3805
1140	TIGD4	0.3814	ARCHS4 Coexpression,0.3814
1141	NAIF1	0.3815	GTEx Coexpression,0.3815
1142	ZNF785	0.3827	ARCHS4 Coexpression,0.3827
1143	PAX2	0.3827	GTEx Coexpression,0.3827
1144	ZNF468	0.3833	ARCHS4 Coexpression,0.3833
1145	SOX21	0.3839	GTEx Coexpression,0.3839
1146	ZBTB7C	0.3846	GTEx Coexpression,0.3846
1147	ZNF28	0.3851	ARCHS4 Coexpression,0.3851
1148	ZNF18	0.386	Enrichr Queries,0.386
1149	LIN54	0.3864	ARCHS4 Coexpression,0.3864
1150	BACH2	0.3864	GTEx Coexpression,0.3864
1151	NFIL3	0.3868	Enrichr Queries,0.3868
1152	ZFP64	0.387	ARCHS4 Coexpression,0.387
1153	TRPS1	0.3882	ARCHS4 Coexpression,0.3882
1154	MYCL	0.3883	GTEx Coexpression,0.3883
1155	ZBTB41	0.3888	ARCHS4 Coexpression,0.3888
1156	MXD1	0.3896	Enrichr Queries,0.3896
1157	ZNF425	0.3902	GTEx Coexpression,0.3902
1158	HES2	0.3908	GTEx Coexpression,0.3908
1159	POU6F1	0.391	Enrichr Queries,0.391
1160	PAX4	0.3914	GTEx Coexpression,0.3914
1161	STAT4	0.3927	GTEx Coexpression,0.3927
1162	FOXN2	0.3931	ARCHS4 Coexpression,0.3931
1163	HIVEP3	0.3939	Enrichr Queries,0.3939
1164	ZNF490	0.3953	Enrichr Queries,0.3953
1165	NPAS2	0.3964	GTEx Coexpression,0.3964

1166	NKX21	0.3976	GTEx Coexpression,0.3976
1167	ZBTB46	0.3989	Enrichr Queries,0.3989
1168	ZNF24	0.3996	Enrichr Queries,0.3996
1169	ZNF157	0.401	Enrichr Queries,0.401
1170	SOX7	0.4011	ARCHS4 Coexpression,0.4011
1171	L3MBTL4	0.4014	GTEx Coexpression,0.4014
1172	SEBOX	0.4017	Enrichr Queries,0.4017
1173	DLX6	0.4023	ARCHS4 Coexpression,0.4023
1174	TFAP2E	0.4024	Enrichr Queries,0.4024
1175	SPIB	0.4029	ARCHS4 Coexpression,0.4029
1176	GATA6	0.4032	GTEx Coexpression,0.4032
1177	ZNF529	0.4036	ARCHS4 Coexpression,0.4036
1178	KLF3	0.4038	Enrichr Queries,0.4038
1179	ZNF419	0.4042	ARCHS4 Coexpression,0.4042
1180	POU3F2	0.4045	GTEx Coexpression,0.4045
1181	PROX2	0.4046	Enrichr Queries,0.4046
1182	ZNF92	0.4054	ARCHS4 Coexpression,0.4054
1183	ZBTB20	0.4057	GTEx Coexpression,0.4057
1184	ZNF398	0.4067	Enrichr Queries,0.4067
1185	ZNF311	0.407	GTEx Coexpression,0.407
1186	TFDP3	0.4081	Enrichr Queries,0.4081
1187	GATA5	0.4082	GTEx Coexpression,0.4082
1188	HOXD4	0.4085	ARCHS4 Coexpression,0.4085
1189	ZFHX4	0.4088	GTEx Coexpression,0.4088
1190	CBLL2	0.4095	GTEx Coexpression,0.4095
1191	ZNF563	0.4101	GTEx Coexpression,0.4101
1192	ZNF557	0.4103	Enrichr Queries,0.4103
1193	SHOX	0.4103	ARCHS4 Coexpression,0.4103
1194	LHX2	0.4107	GTEx Coexpression,0.4107
1195	SPZ1	0.4119	GTEx Coexpression,0.4119
1196	ZNF397	0.4122	ARCHS4 Coexpression,0.4122
1197	ZBED4	0.4124	Enrichr Queries,0.4124
1198	AR	0.4126	GTEx Coexpression,0.4126
1199	ZBTB8B	0.4134	ARCHS4 Coexpression,0.4134
1200	BATF	0.4138	Enrichr Queries,0.4138
1201	ZNF750	0.4141	ReMap ChIP-seq,0.4141
1202	SKIL	0.4144	GTEx Coexpression,0.4144
1203	IRF8	0.4146	Literature ChIP-seq,0.4146
1204	HOXD8	0.4151	GTEx Coexpression,0.4151
1205	ZNF280D	0.4157	GTEx Coexpression,0.4157
1206	ZNF8	0.4165	ARCHS4 Coexpression,0.4165
1207	IRF5	0.4167	Enrichr Queries,0.4167
1208	ZSCAN26	0.4171	ARCHS4 Coexpression,0.4171
1209	RUNX3	0.4174	Enrichr Queries,0.4174
1210	ZSCAN32	0.4177	ARCHS4 Coexpression,0.4177
1211	NFX1	0.4188	Enrichr Queries,0.4188
1212	MTERF4	0.4194	GTEx Coexpression,0.4194
1213	ISX	0.4195	Enrichr Queries,0.4195
1214	SETBP1	0.42	GTEx Coexpression,0.42
1215	ZIC5	0.4201	ARCHS4 Coexpression,0.4201
1216	ZNF705G	0.4207	GTEx Coexpression,0.4207
1217	EOMES	0.4207	Literature ChIP-seq,0.4207
1218	ZNF568	0.4214	ARCHS4 Coexpression,0.4214

1219	ZNF331	0.4217	Enrichr Queries,0.4217
1220	MEF2C	0.4219	GTEx Coexpression,0.4219
1221	HES3	0.4226	ARCHS4 Coexpression,0.4226
1222	ZNF595	0.4238	Enrichr Queries,0.4238
1223	ARID5A	0.4238	ARCHS4 Coexpression,0.4238
1224	EBF1	0.4242	ReMap ChIP-seq,0.4242
1225	ZNF711	0.425	GTEx Coexpression,0.425
1226	PROP1	0.4256	GTEx Coexpression,0.4256
1227	ETV3L	0.4257	ARCHS4 Coexpression,0.4257
1228	TBX22	0.4269	GTEx Coexpression,0.4269
1229	ZKSCAN7	0.4269	ARCHS4 Coexpression,0.4269
1230	ZNF728	0.4275	GTEx Coexpression,0.4275
1231	FOXO6	0.4281	GTEx Coexpression,0.4281
1232	VENTX	0.4295	Enrichr Queries,0.4295
1233	CREB5	0.4302	Enrichr Queries,0.4302
1234	ASCL1	0.4306	GTEx Coexpression,0.4306
1235	ZNF618	0.4309	Enrichr Queries,0.4309
1236	HOXB6	0.4312	ARCHS4 Coexpression,0.4312
1237	ZNF567	0.4318	ARCHS4 Coexpression,0.4318
1238	LHX6	0.4323	Enrichr Queries,0.4323
1239	NFIA	0.4337	GTEx Coexpression,0.4337
1240	ATOH1	0.4343	GTEx Coexpression,0.4343
1241	NFATC4	0.4356	GTEx Coexpression,0.4356
1242	AHRR	0.4367	ARCHS4 Coexpression,0.4367
1243	ZNF502	0.4387	Enrichr Queries,0.4387
1244	GRHL1	0.4393	GTEx Coexpression,0.4393
1245	ZNF649	0.4395	Enrichr Queries,0.4395
1246	HOXB9	0.44	GTEx Coexpression,0.44
1247	ARX	0.4402	Enrichr Queries,0.4402
1248	ZBED2	0.4409	Enrichr Queries,0.4409
1249	ZNF660	0.4416	ARCHS4 Coexpression,0.4416
1250	ZNF528	0.4423	ARCHS4 Coexpression,0.4423
1251	HIC1	0.443	Enrichr Queries,0.443
1252	ZNF701	0.4441	ARCHS4 Coexpression,0.4441
1253	IRX4	0.4443	GTEx Coexpression,0.4443
1254	ZNF443	0.4444	Enrichr Queries,0.4444
1255	ZNF614	0.4453	ARCHS4 Coexpression,0.4453
1256	FOXI3	0.4456	GTEx Coexpression,0.4456
1257	ZNF420	0.4459	ARCHS4 Coexpression,0.4459
1258	FOXF1	0.4466	ARCHS4 Coexpression,0.4466
1259	MAFB	0.4468	GTEx Coexpression,0.4468
1260	EGR4	0.4473	Enrichr Queries,0.4473
1261	ZNF804B	0.4474	GTEx Coexpression,0.4474
1262	SALL1	0.448	GTEx Coexpression,0.448
1263	IRX6	0.4484	ARCHS4 Coexpression,0.4484
1264	ZNF229	0.449	ARCHS4 Coexpression,0.449
1265	ONECUT2	0.4512	GTEx Coexpression,0.4512
1266	TFAP2C	0.4512	ReMap ChIP-seq,0.4512
1267	HOXD10	0.4515	ARCHS4 Coexpression,0.4515
1268	ZNF284	0.4527	ARCHS4 Coexpression,0.4527
1269	ZNF713	0.4533	ARCHS4 Coexpression,0.4533
1270	ZNF483	0.4536	GTEx Coexpression,0.4536
1271	ZNF224	0.4537	Enrichr Queries,0.4537

1272	ZNF823	0.4539	ARCHS4 Coexpression,0.4539
1273	ZNF793	0.4552	ARCHS4 Coexpression,0.4552
1274	NANOGP8	0.4574	GTEx Coexpression,0.4574
1275	BACH1	0.4579	ReMap ChIP-seq,0.4579
1276	EMX2	0.458	Enrichr Queries,0.458
1277	ZNF471	0.458	GTEx Coexpression,0.458
1278	ZNF501	0.4582	ARCHS4 Coexpression,0.4582
1279	EN1	0.4605	GTEx Coexpression,0.4605
1280	PBX4	0.4611	GTEx Coexpression,0.4611
1281	ZNF221	0.4619	ARCHS4 Coexpression,0.4619
1282	ZNF492	0.4638	ARCHS4 Coexpression,0.4638
1283	MSGN1	0.4642	GTEx Coexpression,0.4642
1284	FOXI2	0.4661	GTEx Coexpression,0.4661
1285	SIM2	0.4667	GTEx Coexpression,0.4667
1286	ZNF135	0.4679	Enrichr Queries,0.4679
1287	ZNF878	0.468	GTEx Coexpression,0.468
1288	UNCX	0.4693	ARCHS4 Coexpression,0.4693
1289	IRF7	0.4694	Enrichr Queries,0.4694
1290	ZNF264	0.4699	ARCHS4 Coexpression,0.4699
1291	ZNF234	0.4708	Enrichr Queries,0.4708
1292	GRHL3	0.4717	GTEx Coexpression,0.4717
1293	ZNF257	0.4717	ARCHS4 Coexpression,0.4717
1294	EHF	0.4729	GTEx Coexpression,0.4729
1295	CAMTA1	0.4729	Enrichr Queries,0.4729
1296	BARX2	0.4742	GTEx Coexpression,0.4742
1297	SP100	0.4742	ARCHS4 Coexpression,0.4742
1298	ZFP92	0.4744	Enrichr Queries,0.4744
1299	ZNF479	0.4748	GTEx Coexpression,0.4748
1300	NACC2	0.4754	GTEx Coexpression,0.4754
1301	ZNF844	0.4754	ARCHS4 Coexpression,0.4754
1302	HEY1	0.476	GTEx Coexpression,0.476
1303	EMX1	0.4773	ARCHS4 Coexpression,0.4773
1304	VAX1	0.4779	GTEx Coexpression,0.4779
1305	ZNF860	0.4785	ARCHS4 Coexpression,0.4785
1306	ZNF773	0.4793	Enrichr Queries,0.4793
1307	ZNF605	0.4804	GTEx Coexpression,0.4804
1308	ZNF677	0.4808	Enrichr Queries,0.4808
1309	ZNF84	0.481	ARCHS4 Coexpression,0.481
1310	RHOXF1	0.4822	Enrichr Queries,0.4822
1311	ZNF154	0.4834	ARCHS4 Coexpression,0.4834
1312	REL	0.4836	Enrichr Queries,0.4836
1313	TSHZ3	0.4841	GTEx Coexpression,0.4841
1314	IRF4	0.4843	Enrichr Queries,0.4843
1315	ZNF619	0.4846	ARCHS4 Coexpression,0.4846
1316	TSHZ2	0.4848	GTEx Coexpression,0.4848
1317	AEBP2	0.4854	GTEx Coexpression,0.4854
1318	CPXCR1	0.4858	Enrichr Queries,0.4858
1319	ZNF665	0.4859	ARCHS4 Coexpression,0.4859
1320	LTF	0.4866	GTEx Coexpression,0.4866
1321	TBX21	0.4871	ARCHS4 Coexpression,0.4871
1322	MECOM	0.4878	Literature ChIP-seq,0.4878
1323	ZFHX2	0.4879	GTEx Coexpression,0.4879
1324	ZNF85	0.4879	Enrichr Queries,0.4879

1325	ZNF506	0.4885	GTEx Coexpression,0.4885
1326	ZNF114	0.4902	ARCHS4 Coexpression,0.4902
1327	LEUTX	0.4908	ARCHS4 Coexpression,0.4908
1328	HOXB13	0.4915	Enrichr Queries,0.4915
1329	ESX1	0.4935	GTEx Coexpression,0.4935
1330	SKOR2	0.4939	ARCHS4 Coexpression,0.4939
1331	ZBTB24	0.4941	GTEx Coexpression,0.4941
1332	HOXA3	0.4947	GTEx Coexpression,0.4947
1333	DLX3	0.4957	ARCHS4 Coexpression,0.4957
1334	GLI2	0.4957	Enrichr Queries,0.4957
1335	NANOGNB	0.496	GTEx Coexpression,0.496
1336	FOXB2	0.4963	ARCHS4 Coexpression,0.4963
1337	ZNF788P	0.4964	Enrichr Queries,0.4964
1338	MEOX2	0.4972	Enrichr Queries,0.4972
1339	LMX1B	0.4988	ARCHS4 Coexpression,0.4988
1340	ZNF800	0.4991	GTEx Coexpression,0.4991
1341	ZNF782	0.4997	GTEx Coexpression,0.4997
1342	OTP	0.5006	ARCHS4 Coexpression,0.5006
1343	MIXL1	0.5007	Enrichr Queries,0.5007
1344	HNF1B	0.5014	Enrichr Queries,0.5014
1345	TRERF1	0.5047	GTEx Coexpression,0.5047
1346	ZNF609	0.5053	GTEx Coexpression,0.5053
1347	ZFP37	0.5057	Enrichr Queries,0.5057
1348	NHLH1	0.5065	GTEx Coexpression,0.5065
1349	ZSCAN30	0.5072	GTEx Coexpression,0.5072
1350	ZNF286A	0.5078	Enrichr Queries,0.5078
1351	CDX2	0.5084	GTEx Coexpression,0.5084
1352	ZNF45	0.5085	Enrichr Queries,0.5085
1353	MSX2	0.5092	ARCHS4 Coexpression,0.5092
1354	FOXG1	0.5093	Enrichr Queries,0.5093
1355	ZNF441	0.5098	ARCHS4 Coexpression,0.5098
1356	FOXE1	0.5103	GTEx Coexpression,0.5103
1357	ZNF724	0.5104	ARCHS4 Coexpression,0.5104
1358	ZNF302	0.5115	GTEx Coexpression,0.5115
1359	ZNF225	0.5117	ARCHS4 Coexpression,0.5117
1360	ZNF562	0.514	GTEx Coexpression,0.514
1361	ZNF454	0.516	ARCHS4 Coexpression,0.516
1362	ZNF124	0.5165	GTEx Coexpression,0.5165
1363	PROX1	0.5177	GTEx Coexpression,0.5177
1364	LHX5	0.5178	ARCHS4 Coexpression,0.5178
1365	HOXB3	0.5184	GTEx Coexpression,0.5184
1366	IKZF4	0.5184	ARCHS4 Coexpression,0.5184
1367	NOTO	0.519	ARCHS4 Coexpression,0.519
1368	ZNF527	0.5199	Enrichr Queries,0.5199
1369	ZNF540	0.5203	ARCHS4 Coexpression,0.5203
1370	NKX12	0.5208	GTEx Coexpression,0.5208
1371	PRDM8	0.5221	Enrichr Queries,0.5221
1372	GBX2	0.5221	ARCHS4 Coexpression,0.5221
1373	MEOX1	0.5233	ARCHS4 Coexpression,0.5233
1374	L3MBTL3	0.5246	ARCHS4 Coexpression,0.5246
1375	SATB2	0.5246	GTEx Coexpression,0.5246
1376	TEAD1	0.5249	Enrichr Queries,0.5249
1377	ZNF726	0.5252	GTEx Coexpression,0.5252

1378	EGR3	0.5258	GTEx Coexpression,0.5258
1379	FOXN1	0.5271	Enrichr Queries,0.5271
1380	ZNF879	0.5276	ARCHS4 Coexpression,0.5276
1381	PRRX2	0.5278	Enrichr Queries,0.5278
1382	ZNF283	0.5289	ARCHS4 Coexpression,0.5289
1383	NR4A3	0.5296	GTEx Coexpression,0.5296
1384	ZIC3	0.5321	Enrichr Queries,0.5321
1385	HELT	0.5327	GTEx Coexpression,0.5327
1386	ZNF322	0.5332	ARCHS4 Coexpression,0.5332
1387	ZNF99	0.535	ARCHS4 Coexpression,0.535
1388	ZNF404	0.5352	GTEx Coexpression,0.5352
1389	ZNF70	0.5356	ARCHS4 Coexpression,0.5356
1390	ZNF440	0.5358	GTEx Coexpression,0.5358
1391	ZNF717	0.5362	ARCHS4 Coexpression,0.5362
1392	SP7	0.5381	ARCHS4 Coexpression,0.5381
1393	EVX1	0.5383	GTEx Coexpression,0.5383
1394	POU2F3	0.5385	Enrichr Queries,0.5385
1395	HLX	0.5392	Enrichr Queries,0.5392
1396	ZSCAN23	0.5393	ARCHS4 Coexpression,0.5393
1397	FOXD4L5	0.5408	GTEx Coexpression,0.5408
1398	ZNF285	0.5412	ARCHS4 Coexpression,0.5412
1399	HMGA2	0.5413	Enrichr Queries,0.5413
1400	ZNF329	0.5418	ARCHS4 Coexpression,0.5418
1401	ZNF286B	0.5442	ARCHS4 Coexpression,0.5442
1402	FEZF2	0.5445	GTEx Coexpression,0.5445
1403	AHDC1	0.5463	Enrichr Queries,0.5463
1404	HOXB8	0.547	GTEx Coexpression,0.547
1405	ZNF585B	0.547	Enrichr Queries,0.547
1406	ARNTL2	0.5477	Enrichr Queries,0.5477
1407	POU3F3	0.5482	GTEx Coexpression,0.5482
1408	DLX1	0.5485	ARCHS4 Coexpression,0.5485
1409	ZNF705B	0.5488	GTEx Coexpression,0.5488
1410	ZNF667	0.5498	ARCHS4 Coexpression,0.5498
1411	GSC2	0.5513	GTEx Coexpression,0.5513
1412	FOSB	0.552	Enrichr Queries,0.552
1413	ZBTB21	0.5522	ARCHS4 Coexpression,0.5522
1414	RHOXF2B	0.5532	GTEx Coexpression,0.5532
1415	TCF21	0.5549	Literature ChIP-seq,0.5549
1416	MYRFL	0.5551	GTEx Coexpression,0.5551
1417	TBR1	0.5553	ARCHS4 Coexpression,0.5553
1418	BATF3	0.5556	Enrichr Queries,0.5556
1419	HOXA13	0.5588	GTEx Coexpression,0.5588
1420	THAP12	0.559	ARCHS4 Coexpression,0.559
1421	NEUROD2	0.561	Literature ChIP-seq,0.561
1422	HAND2	0.5619	GTEx Coexpression,0.5619
1423	ZNF382	0.562	ARCHS4 Coexpression,0.562
1424	ZNF792	0.5625	GTEx Coexpression,0.5625
1425	INSM1	0.5632	GTEx Coexpression,0.5632
1426	THRB	0.5644	GTEx Coexpression,0.5644
1427	EGR2	0.5681	GTEx Coexpression,0.5681
1428	TCF20	0.5726	Enrichr Queries,0.5726
1429	ZBTB26	0.5734	Enrichr Queries,0.5734
1430	BCL11B	0.5737	GTEx Coexpression,0.5737

1431	RFX8	0.5756	ARCHS4 Coexpression,0.5756
1432	POU3F4	0.578	ARCHS4 Coexpression,0.578
1433	ZNF572	0.5783	Enrichr Queries,0.5783
1434	BHLHE22	0.5791	ReMap ChIP-seq,0.5791
1435	HEY2	0.5805	ARCHS4 Coexpression,0.5805
1436	CASZ1	0.5805	Enrichr Queries,0.5805
1437	PRDM1	0.5812	GTEx Coexpression,0.5812
1438	KDM2B	0.5817	ARCHS4 Coexpression,0.5817
1439	DACH2	0.5826	Enrichr Queries,0.5826
1440	MYT1	0.5833	Enrichr Queries,0.5833
1441	HEYL	0.5835	ARCHS4 Coexpression,0.5835
1442	ZNF37A	0.5843	GTEx Coexpression,0.5843
1443	ZNF596	0.5848	ARCHS4 Coexpression,0.5848
1444	DLX2	0.5849	GTEx Coexpression,0.5849
1445	ATF6	0.5855	Enrichr Queries,0.5855
1446	TIGD7	0.5862	GTEx Coexpression,0.5862
1447	SCML4	0.5869	Enrichr Queries,0.5869
1448	ZSCAN20	0.5876	Enrichr Queries,0.5876
1449	TBX4	0.5878	ARCHS4 Coexpression,0.5878
1450	POU2AF1	0.5887	GTEx Coexpression,0.5887
1451	PLSCR1	0.5897	ARCHS4 Coexpression,0.5897
1452	ZNF488	0.5905	Enrichr Queries,0.5905
1453	SHOX2	0.5933	Enrichr Queries,0.5933
1454	OSR1	0.5977	ARCHS4 Coexpression,0.5977
1455	ZNF845	0.5983	ARCHS4 Coexpression,0.5983
1456	YY2	0.5989	ARCHS4 Coexpression,0.5989
1457	PAX3	0.5993	GTEx Coexpression,0.5993
1458	ZNF439	0.6004	Enrichr Queries,0.6004
1459	PKNOX2	0.6019	Enrichr Queries,0.6019
1460	THAP2	0.6032	ARCHS4 Coexpression,0.6032
1461	ZNF469	0.605	ARCHS4 Coexpression,0.605
1462	ZNF835	0.6057	ARCHS4 Coexpression,0.6057
1463	GLIS1	0.6075	ARCHS4 Coexpression,0.6075
1464	ZIM2	0.6086	GTEx Coexpression,0.6086
1465	IKZF1	0.6093	ARCHS4 Coexpression,0.6093
1466	EVX2	0.6098	GTEx Coexpression,0.6098
1467	ARNT2	0.6104	Enrichr Queries,0.6104
1468	IKZF3	0.6111	Enrichr Queries,0.6111
1469	ZNF735	0.6118	ARCHS4 Coexpression,0.6118
1470	ZNF26	0.613	ARCHS4 Coexpression,0.613
1471	GSX1	0.6143	ARCHS4 Coexpression,0.6143
1472	ZNF132	0.6161	ARCHS4 Coexpression,0.6161
1473	HOXC8	0.6161	Enrichr Queries,0.6161
1474	ZNF611	0.6173	ARCHS4 Coexpression,0.6173
1475	SNAI1	0.6175	Enrichr Queries,0.6175
1476	PRDM16	0.6192	GTEx Coexpression,0.6192
1477	VSX2	0.6198	GTEx Coexpression,0.6198
1478	MEIS2	0.6204	GTEx Coexpression,0.6204
1479	ZNF267	0.6218	Enrichr Queries,0.6218
1480	ZNF396	0.6222	ARCHS4 Coexpression,0.6222
1481	RUNX2	0.6225	Enrichr Queries,0.6225
1482	ZNF268	0.6229	ARCHS4 Coexpression,0.6229
1483	NKX31	0.6229	GTEx Coexpression,0.6229

1484	ZSCAN4	0.6235	ARCHS4 Coexpression,0.6235
1485	HOXD3	0.6241	GTEx Coexpression,0.6241
1486	ZNF831	0.6253	ARCHS4 Coexpression,0.6253
1487	HIVEP2	0.6254	Enrichr Queries,0.6254
1488	ZSCAN31	0.6259	ARCHS4 Coexpression,0.6259
1489	GCM2	0.626	GTEx Coexpression,0.626
1490	SP6	0.6265	ARCHS4 Coexpression,0.6265
1491	DUXA	0.6271	ARCHS4 Coexpression,0.6271
1492	NEUROD6	0.6296	ARCHS4 Coexpression,0.6296
1493	LEF1	0.6297	GTEx Coexpression,0.6297
1494	ZNF805	0.6311	Enrichr Queries,0.6311
1495	AEBP1	0.6314	ARCHS4 Coexpression,0.6314
1496	FOXQ1	0.6316	GTEx Coexpression,0.6316
1497	ZHX3	0.6329	GTEx Coexpression,0.6329
1498	ZBTB38	0.6333	ARCHS4 Coexpression,0.6333
1499	NR4A2	0.6341	GTEx Coexpression,0.6341
1500	CSRNP1	0.6345	ARCHS4 Coexpression,0.6345
1501	C11ORF95	0.6353	GTEx Coexpression,0.6353
1502	PRDM6	0.636	GTEx Coexpression,0.636
1503	ZNF534	0.6388	ARCHS4 Coexpression,0.6388
1504	BHLHE23	0.6407	ARCHS4 Coexpression,0.6407
1505	DZIP1	0.6432	Enrichr Queries,0.6432
1506	MTF1	0.6441	GTEx Coexpression,0.6441
1507	SP8	0.6447	GTEx Coexpression,0.6447
1508	POU6F2	0.645	ARCHS4 Coexpression,0.645
1509	TFAP2B	0.6486	ARCHS4 Coexpression,0.6486
1510	RORB	0.651	Enrichr Queries,0.651
1511	ZNF493	0.6515	GTEx Coexpression,0.6515
1512	RORA	0.6528	GTEx Coexpression,0.6528
1513	EN2	0.6529	ARCHS4 Coexpression,0.6529
1514	ZNF318	0.6532	ReMap ChIP-seq,0.6532
1515	ZNF536	0.6546	Enrichr Queries,0.6546
1516	POU4F3	0.6548	ARCHS4 Coexpression,0.6548
1517	ZNF705D	0.6567	Enrichr Queries,0.6567
1518	ST18	0.6579	ARCHS4 Coexpression,0.6579
1519	SCRT2	0.661	Enrichr Queries,0.661
1520	ZIC2	0.6667	Enrichr Queries,0.6667
1521	ZNF365	0.6674	Enrichr Queries,0.6674
1522	FOXC2	0.6677	ARCHS4 Coexpression,0.6677
1523	OLIG2	0.6683	ARCHS4 Coexpression,0.6683
1524	ASCL4	0.6696	GTEx Coexpression,0.6696
1525	ZNF134	0.6714	ARCHS4 Coexpression,0.6714
1526	ZNF730	0.672	ARCHS4 Coexpression,0.672
1527	INSM2	0.6752	Enrichr Queries,0.6752
1528	ZNF470	0.6763	ARCHS4 Coexpression,0.6763
1529	ZBTB34	0.6766	Enrichr Queries,0.6766
1530	ZNF571	0.6769	ARCHS4 Coexpression,0.6769
1531	GLIS3	0.6781	ARCHS4 Coexpression,0.6781
1532	ZNF445	0.6787	ARCHS4 Coexpression,0.6787
1533	MKX	0.6795	GTEx Coexpression,0.6795
1534	JRKL	0.6814	GTEx Coexpression,0.6814
1535	ZNF230	0.683	Enrichr Queries,0.683
1536	ZNF30	0.6843	ARCHS4 Coexpression,0.6843

1537	KCNIP3	0.6845	GTEx Coexpression,0.6845
1538	TBX18	0.6882	GTEx Coexpression,0.6882
1539	SCRT1	0.6902	Enrichr Queries,0.6902
1540	PLAG1	0.691	ARCHS4 Coexpression,0.691
1541	ZNF621	0.6923	Enrichr Queries,0.6923
1542	LHX1	0.6947	ARCHS4 Coexpression,0.6947
1543	PURB	0.699	ARCHS4 Coexpression,0.699
1544	BNC2	0.6994	Enrichr Queries,0.6994
1545	FERD3L	0.6996	ARCHS4 Coexpression,0.6996
1546	CPEB1	0.7002	ARCHS4 Coexpression,0.7002
1547	SP110	0.7023	Enrichr Queries,0.7023
1548	ZNF510	0.7051	Enrichr Queries,0.7051
1549	GTF2I	0.7064	ARCHS4 Coexpression,0.7064
1550	PHOX2B	0.707	ARCHS4 Coexpression,0.707
1551	ZNF521	0.7076	ARCHS4 Coexpression,0.7076
1552	GCM1	0.708	Enrichr Queries,0.708
1553	TBX2	0.7106	GTEx Coexpression,0.7106
1554	ZBTB6	0.7131	GTEx Coexpression,0.7131
1555	VSX1	0.7131	ARCHS4 Coexpression,0.7131
1556	TFEC	0.7144	ARCHS4 Coexpression,0.7144
1557	SKI	0.7156	GTEx Coexpression,0.7156
1558	ZNF507	0.7165	Enrichr Queries,0.7165
1559	ALX3	0.7181	GTEx Coexpression,0.7181
1560	EBF2	0.7211	ARCHS4 Coexpression,0.7211
1561	ATOH7	0.7222	Enrichr Queries,0.7222
1562	ZIC1	0.7229	Enrichr Queries,0.7229
1563	SIM1	0.7236	Enrichr Queries,0.7236
1564	ZNF770	0.7244	Enrichr Queries,0.7244
1565	NKX61	0.725	GTEx Coexpression,0.725
1566	ZNF780A	0.7251	Enrichr Queries,0.7251
1567	PRRX1	0.7272	Enrichr Queries,0.7272
1568	BCL6B	0.7291	ARCHS4 Coexpression,0.7291
1569	ZNF732	0.7293	GTEx Coexpression,0.7293
1570	TWIST2	0.7316	ARCHS4 Coexpression,0.7316
1571	PBX1	0.735	Enrichr Queries,0.735
1572	NEUROG2	0.7362	GTEx Coexpression,0.7362
1573	ZNF112	0.7371	ARCHS4 Coexpression,0.7371
1574	NKX62	0.7383	ARCHS4 Coexpression,0.7383
1575	ZFP69B	0.7439	ARCHS4 Coexpression,0.7439
1576	NFE4	0.7449	GTEx Coexpression,0.7449
1577	HOXD12	0.7461	GTEx Coexpression,0.7461
1578	ZNF248	0.7472	Enrichr Queries,0.7472
1579	TWIST1	0.7508	ReMap ChIP-seq,0.7508
1580	EBF3	0.7543	Enrichr Queries,0.7543
1581	NFATC2	0.7549	ARCHS4 Coexpression,0.7549
1582	BHLHA9	0.7567	GTEx Coexpression,0.7567
1583	HSFY2	0.7598	ARCHS4 Coexpression,0.7598
1584	SP9	0.7654	GTEx Coexpression,0.7654
1585	ZNF366	0.7666	GTEx Coexpression,0.7666
1586	NKX22	0.7685	GTEx Coexpression,0.7685
1587	PCGF2	0.7709	ARCHS4 Coexpression,0.7709
1588	SOX1	0.7758	ARCHS4 Coexpression,0.7758
1589	ZNF354C	0.7778	Enrichr Queries,0.7778

1590	CHAMP1	0.7785	GTEx Coexpression,0.7785
1591	JRK	0.7797	GTEx Coexpression,0.7797
1592	ZNF80	0.7801	ARCHS4 Coexpression,0.7801
1593	RBAK	0.7863	Enrichr Queries,0.7863
1594	ZNF578	0.7893	ARCHS4 Coexpression,0.7893
1595	BARHL2	0.7897	GTEx Coexpression,0.7897
1596	PRDM12	0.7903	GTEx Coexpression,0.7903
1597	PURG	0.7912	ARCHS4 Coexpression,0.7912
1598	MYT1L	0.797	Enrichr Queries,0.797
1599	PHF21A	0.7978	GTEx Coexpression,0.7978
1600	ZNF385D	0.7979	ARCHS4 Coexpression,0.7979
1601	L3MBTL1	0.8034	ARCHS4 Coexpression,0.8034
1602	ZNF781	0.8053	ARCHS4 Coexpression,0.8053
1603	SP140	0.8059	ARCHS4 Coexpression,0.8059
1604	IKZF2	0.8063	Enrichr Queries,0.8063
1605	AKNA	0.8077	Enrichr Queries,0.8077
1606	ZNF888	0.8139	GTEx Coexpression,0.8139
1607	DUX4	0.8151	ARCHS4 Coexpression,0.8151
1608	SOX14	0.8182	ARCHS4 Coexpression,0.8182
1609	FOXD4L3	0.8191	Enrichr Queries,0.8191
1610	KLF14	0.8213	ARCHS4 Coexpression,0.8213
1611	ZBTB18	0.8214	GTEx Coexpression,0.8214
1612	ZIC4	0.8219	Enrichr Queries,0.8219
1613	BARHL1	0.8264	GTEx Coexpression,0.8264
1614	TET3	0.8307	GTEx Coexpression,0.8307
1615	DRGX	0.8378	ARCHS4 Coexpression,0.8378
1616	ZFP69	0.8444	GTEx Coexpression,0.8444
1617	ISL1	0.8482	GTEx Coexpression,0.8482
1618	SGSM2	0.8507	ARCHS4 Coexpression,0.8507
1619	LHX4	0.8682	Enrichr Queries,0.8682
1620	NHLH2	0.8724	GTEx Coexpression,0.8724
1621	PURA	0.8787	GTEx Coexpression,0.8787
1622	CSRNP3	0.8805	GTEx Coexpression,0.8805
1623	ZNF804A	0.8808	ARCHS4 Coexpression,0.8808
1624	BHLHE41	0.89	ARCHS4 Coexpression,0.89
1625	MYRF	0.8998	GTEx Coexpression,0.8998
1626	LHX9	0.9042	GTEx Coexpression,0.9042
1627	BSX	0.9117	Enrichr Queries,0.9117
1628	PEG3	0.916	GTEx Coexpression,0.916
1629	HSFX2	0.9429	ARCHS4 Coexpression,0.9429
1630	CCDC169SOHLH2	0.9459	ARCHS4 Coexpression,0.9459
1631	FOXD4L6	0.9793	Enrichr Queries,0.9793
1632	TET2	0.9844	GTEx Coexpression,0.9844
