# Supplementary material for: Deciphering differences in DNA methylation and transcriptome profiles of oocytes from pigs with high and low developmental competence
Source: Environ Epigenet. 2025 Jun 3;11(1):dvaf018. doi: 10.1093/eep/dvaf018 (PMC12418950; doi:10.1093/eep/dvaf018)
Supplement: dvaf018_Supplemental_Files [file dvaf018_supplemental_files.zip › Sup table 7.pdf]

Rank	TF	Score	Library
1	CENPA	6.14E-04	ARCHS4 Coexpression,6.143E-4
2	ZNF367	6.22E-04	GTEx Coexpression,6.223E-4
3	ZNF695	7.12E-04	Enrichr Queries,7.123E-4
4	E2F8	0.001229	ARCHS4 Coexpression,0.001229
5	TFDP1	0.001245	GTEx Coexpression,0.001245
6	MTERF3	0.001843	ARCHS4 Coexpression,0.001843
7	FOXM1	0.001867	GTEx Coexpression,0.001867
8	ZNF689	0.002457	ARCHS4 Coexpression,0.002457
9	ZNF691	0.003071	ARCHS4 Coexpression,0.003071
10	ZNF492	0.003111	GTEx Coexpression,0.003111
11	GLYR1	0.003367	ReMap ChIP-seq,0.003367
12	E2F7	0.003561	Enrichr Queries,0.003561
13	PRMT3	0.003686	ARCHS4 Coexpression,0.003686
14	DNMT1	0.003734	GTEx Coexpression,0.003734
15	MYBL2	0.004274	Enrichr Queries,0.004274
16	ZNF227	0.0043	ARCHS4 Coexpression,0.0043
17	HMGA1	0.004356	GTEx Coexpression,0.004356
18	SOHLH2	0.004914	ARCHS4 Coexpression,0.004914
19	LCORL	0.004978	GTEx Coexpression,0.004978
20	PCGF6	0.004986	Enrichr Queries,0.004986
21	ZNF670	0.005528	ARCHS4 Coexpression,0.005528
22	PA2G4	0.0056	GTEx Coexpression,0.0056
23	ZNF519	0.005698	Enrichr Queries,0.005698
24	CREM	0.006098	Literature ChIP-seq,0.006098
25	LIN28B	0.006143	ARCHS4 Coexpression,0.006143
26	ZNF215	0.006223	GTEx Coexpression,0.006223
27	MYBL1	0.00641	Enrichr Queries,0.00641
28	KMT2A	0.006734	ReMap ChIP-seq,0.006734
29	E2F2	0.007123	Enrichr Queries,0.007123
30	ZNF146	0.007371	ARCHS4 Coexpression,0.007371
31	ZNF726	0.00809	GTEx Coexpression,0.00809
32	MAX	0.008475	ENCODE ChIP-seq,0.008475
33	MXD3	0.008547	Enrichr Queries,0.008547
34	TGIF2	0.0086	ARCHS4 Coexpression,0.0086
35	ZNF239	0.009214	ARCHS4 Coexpression,0.009214
36	CBX2	0.009259	Enrichr Queries,0.009259
37	ZNF124	0.009334	GTEx Coexpression,0.009334
38	ZNF732	0.009828	ARCHS4 Coexpression,0.009828
39	ZNF724	0.009956	GTEx Coexpression,0.009956
40	E2F1	0.009972	Enrichr Queries,0.009972
41	NCOA3	0.0101	ReMap ChIP-seq,0.0101
42	NR6A1	0.01044	ARCHS4 Coexpression,0.01044
43	ZNF93	0.01068	Enrichr Queries,0.01068
44	ZNF155	0.01106	ARCHS4 Coexpression,0.01106
45	ZNF625	0.0112	GTEx Coexpression,0.0112
46	OTX2	0.01167	ARCHS4 Coexpression,0.01167
47	ZFP69B	0.01182	GTEx Coexpression,0.01182
48	ZFX	0.0122	Literature ChIP-seq,0.0122
49	ZNF281	0.01229	ARCHS4 Coexpression,0.01229
50	AHRR	0.01245	GTEx Coexpression,0.01245
51	ZNF85	0.01282	Enrichr Queries,0.01282
52	ALX1	0.0129	ARCHS4 Coexpression,0.0129

53	ETV4	0.01307	GTEx Coexpression,0.01307
54	GATA2	0.01347	ReMap ChIP-seq,0.01347
55	ZNF878	0.01351	ARCHS4 Coexpression,0.01351
56	ZNF530	0.01353	Enrichr Queries,0.01353
57	MTERF1	0.01369	GTEx Coexpression,0.01369
58	THAP10	0.01425	Enrichr Queries,0.01425
59	ZNF850	0.01431	GTEx Coexpression,0.01431
60	ZIC2	0.01474	ARCHS4 Coexpression,0.01474
61	IKZF2	0.01493	GTEx Coexpression,0.01493
62	CENPT	0.01496	Enrichr Queries,0.01496
63	LEUTX	0.01556	GTEx Coexpression,0.01556
64	ZNF273	0.01567	Enrichr Queries,0.01567
65	ZNF888	0.01618	GTEx Coexpression,0.01618
66	ZNF788P	0.01638	Enrichr Queries,0.01638
67	ZNF286A	0.01658	ARCHS4 Coexpression,0.01658
68	ZNF92	0.0168	GTEx Coexpression,0.0168
69	HMBOX1	0.01684	ReMap ChIP-seq,0.01684
70	E2F4	0.01695	ENCODE ChIP-seq,0.01695
71	MYB	0.01709	Enrichr Queries,0.01709
72	STAT1	0.01742	GTEx Coexpression,0.01742
73	E2F3	0.01781	Enrichr Queries,0.01781
74	ZNF749	0.01781	ARCHS4 Coexpression,0.01781
75	ZNF257	0.01805	GTEx Coexpression,0.01805
76	KDM5B	0.01829	Literature ChIP-seq,0.01829
77	HOXA7	0.01843	ARCHS4 Coexpression,0.01843
78	ZNF473	0.01852	Enrichr Queries,0.01852
79	ZSCAN5C	0.01867	GTEx Coexpression,0.01867
80	ZNF560	0.01904	ARCHS4 Coexpression,0.01904
81	ZNF714	0.01923	Enrichr Queries,0.01923
82	MBD4	0.01994	Enrichr Queries,0.01994
83	CEBPG	0.0202	ReMap ChIP-seq,0.0202
84	CENPS	0.02027	ARCHS4 Coexpression,0.02027
85	ZNF100	0.02054	GTEx Coexpression,0.02054
86	ZNF511	0.02066	Enrichr Queries,0.02066
87	NKX12	0.02088	ARCHS4 Coexpression,0.02088
88	ZNF90	0.02116	GTEx Coexpression,0.02116
89	ZC3H8	0.02137	Enrichr Queries,0.02137
90	HESX1	0.0215	ARCHS4 Coexpression,0.0215
91	ZNF813	0.02178	GTEx Coexpression,0.02178
92	ZNF256	0.02211	ARCHS4 Coexpression,0.02211
93	ZNF26	0.0224	GTEx Coexpression,0.0224
94	SKIL	0.02273	ARCHS4 Coexpression,0.02273
95	DR1	0.02279	Enrichr Queries,0.02279
96	ZUP1	0.02302	GTEx Coexpression,0.02302
97	TERF1	0.0235	Enrichr Queries,0.0235
98	DUX4	0.02357	ReMap ChIP-seq,0.02357
99	FIGLA	0.02396	ARCHS4 Coexpression,0.02396
100	ZNF22	0.02422	Enrichr Queries,0.02422
101	ZNF717	0.02427	GTEx Coexpression,0.02427
102	CDX4	0.02457	ARCHS4 Coexpression,0.02457
103	HMX3	0.02489	GTEx Coexpression,0.02489
104	HOXA1	0.02518	ARCHS4 Coexpression,0.02518
105	GABPA	0.02542	ENCODE ChIP-seq,0.02542

106	IRF4	0.02551	GTEx Coexpression,0.02551
107	ZNF684	0.02564	Enrichr Queries,0.02564
108	CEBPZ	0.0258	ARCHS4 Coexpression,0.0258
109	ZBED4	0.02614	GTEx Coexpression,0.02614
110	KIN	0.02635	Enrichr Queries,0.02635
111	HMX2	0.02676	GTEx Coexpression,0.02676
112	HOXB13	0.02694	ReMap ChIP-seq,0.02694
113	CHCHD3	0.02703	ARCHS4 Coexpression,0.02703
114	ZNF232	0.02707	Enrichr Queries,0.02707
115	ZNF624	0.02738	GTEx Coexpression,0.02738
116	ZNF229	0.02764	ARCHS4 Coexpression,0.02764
117	ZIK1	0.02778	Enrichr Queries,0.02778
118	RFX8	0.028	GTEx Coexpression,0.028
119	ZNF214	0.02826	ARCHS4 Coexpression,0.02826
120	ZNF639	0.02849	Enrichr Queries,0.02849
121	ZNF277	0.02862	GTEx Coexpression,0.02862
122	ZNF280C	0.02887	ARCHS4 Coexpression,0.02887
123	ZNF77	0.02948	ARCHS4 Coexpression,0.02948
124	BHLHE23	0.02987	GTEx Coexpression,0.02987
125	THAP9	0.02991	Enrichr Queries,0.02991
126	ZNF35	0.0301	ARCHS4 Coexpression,0.0301
127	MAFG	0.0303	ReMap ChIP-seq,0.0303
128	GFI1B	0.03049	Literature ChIP-seq,0.03049
129	ZNF131	0.03049	GTEx Coexpression,0.03049
130	CREB3L4	0.03063	Enrichr Queries,0.03063
131	ZNF416	0.03071	ARCHS4 Coexpression,0.03071
132	ZNF891	0.03111	GTEx Coexpression,0.03111
133	TTF1	0.03133	ARCHS4 Coexpression,0.03133
134	ARNTL2	0.03134	Enrichr Queries,0.03134
135	PRDM13	0.03174	GTEx Coexpression,0.03174
136	HMGA2	0.03236	GTEx Coexpression,0.03236
137	E2F5	0.03276	Enrichr Queries,0.03276
138	TOPORS	0.03298	GTEx Coexpression,0.03298
139	ZNF449	0.03317	ARCHS4 Coexpression,0.03317
140	THAP1	0.03348	Enrichr Queries,0.03348
141	CREB1	0.0336	GTEx Coexpression,0.0336
142	POU2F1	0.03367	ReMap ChIP-seq,0.03367
143	ZBTB49	0.03378	ARCHS4 Coexpression,0.03378
144	MYC	0.0339	ENCODE ChIP-seq,0.0339
145	ZNF107	0.03419	Enrichr Queries,0.03419
146	ZNF567	0.03423	GTEx Coexpression,0.03423
147	ZBTB2	0.0344	ARCHS4 Coexpression,0.0344
148	MLX	0.03485	GTEx Coexpression,0.03485
149	YY2	0.0349	Enrichr Queries,0.0349
150	ZNF770	0.03501	ARCHS4 Coexpression,0.03501
151	TP53	0.03547	GTEx Coexpression,0.03547
152	ZSCAN21	0.03563	ARCHS4 Coexpression,0.03563
153	CHAMP1	0.03609	GTEx Coexpression,0.03609
154	NFYB	0.03624	ARCHS4 Coexpression,0.03624
155	MYCN	0.03659	Literature ChIP-seq,0.03659
156	ZNF543	0.03671	GTEx Coexpression,0.03671
157	ZNF597	0.03686	ARCHS4 Coexpression,0.03686
158	NFX1	0.03734	GTEx Coexpression,0.03734

159	TET1	0.03747	ARCHS4 Coexpression,0.03747
160	ZNF789	0.03775	Enrichr Queries,0.03775
161	MIXL1	0.03796	GTEx Coexpression,0.03796
162	LIN28A	0.03808	ARCHS4 Coexpression,0.03808
163	ADNP2	0.0387	ARCHS4 Coexpression,0.0387
164	ZNF410	0.03917	Enrichr Queries,0.03917
165	MSANTD4	0.03931	ARCHS4 Coexpression,0.03931
166	BACH1	0.03983	GTEx Coexpression,0.03983
167	ATF1	0.03989	Enrichr Queries,0.03989
168	ZNF304	0.03993	ARCHS4 Coexpression,0.03993
169	ONECUT1	0.0404	ReMap ChIP-seq,0.0404
170	HIF1A	0.04045	GTEx Coexpression,0.04045
171	TFAP2D	0.04054	ARCHS4 Coexpression,0.04054
172	ZNF654	0.04107	GTEx Coexpression,0.04107
173	ZFAT	0.04169	GTEx Coexpression,0.04169
174	HMG20A	0.04177	ARCHS4 Coexpression,0.04177
175	ZNF200	0.04202	Enrichr Queries,0.04202
176	ZNF69	0.04231	GTEx Coexpression,0.04231
177	HES6	0.04274	Enrichr Queries,0.04274
178	SETDB1	0.04294	GTEx Coexpression,0.04294
179	ZNF443	0.043	ARCHS4 Coexpression,0.043
180	SALL4	0.04345	Enrichr Queries,0.04345
181	ZNF468	0.04356	GTEx Coexpression,0.04356
182	TCF7L2	0.04361	ARCHS4 Coexpression,0.04361
183	FOXA1	0.04377	ReMap ChIP-seq,0.04377
184	ZBED2	0.04416	Enrichr Queries,0.04416
185	ZNF845	0.04418	GTEx Coexpression,0.04418
186	ZNF311	0.0448	GTEx Coexpression,0.0448
187	ADNP	0.04484	ARCHS4 Coexpression,0.04484
188	ZNF300	0.04487	Enrichr Queries,0.04487
189	ZNF28	0.04543	GTEx Coexpression,0.04543
190	LHX8	0.04545	ARCHS4 Coexpression,0.04545
191	TEAD4	0.04558	Enrichr Queries,0.04558
192	ZNF268	0.04605	GTEx Coexpression,0.04605
193	ZNF287	0.04607	ARCHS4 Coexpression,0.04607
194	E2F6	0.0463	Enrichr Queries,0.0463
195	ZNF572	0.04667	GTEx Coexpression,0.04667
196	ZNF57	0.04668	ARCHS4 Coexpression,0.04668
197	ZFP1	0.04701	Enrichr Queries,0.04701
198	HHEX	0.04714	ReMap ChIP-seq,0.04714
199	POU5F1B	0.04791	ARCHS4 Coexpression,0.04791
200	TIGD6	0.04792	GTEx Coexpression,0.04792
201	ZNF680	0.04843	Enrichr Queries,0.04843
202	ZNF578	0.04854	GTEx Coexpression,0.04854
203	ZFP69	0.04914	ARCHS4 Coexpression,0.04914
204	MBD2	0.04915	Enrichr Queries,0.04915
205	ZNF699	0.04916	GTEx Coexpression,0.04916
206	NANOGP8	0.04975	ARCHS4 Coexpression,0.04975
207	ZBED1	0.04978	GTEx Coexpression,0.04978
208	ZNF318	0.0504	GTEx Coexpression,0.0504
209	SOX2	0.05051	ReMap ChIP-seq,0.05051
210	GTF3A	0.05057	Enrichr Queries,0.05057
211	FLI1	0.05085	ENCODE ChIP-seq,0.05085

212	KLF1	0.05098	ARCHS4 Coexpression,0.05098
213	ZNF507	0.05103	GTEx Coexpression,0.05103
214	ZNF620	0.05128	Enrichr Queries,0.05128
215	FOXE3	0.0516	ARCHS4 Coexpression,0.0516
216	ZNF202	0.05165	GTEx Coexpression,0.05165
217	TFAP4	0.05199	Enrichr Queries,0.05199
218	FOXN4	0.05221	ARCHS4 Coexpression,0.05221
219	ARNTL	0.05227	GTEx Coexpression,0.05227
220	FOXN2	0.05271	Enrichr Queries,0.05271
221	ATF6B	0.05289	GTEx Coexpression,0.05289
222	HMGN3	0.05344	ARCHS4 Coexpression,0.05344
223	KLF3	0.05387	ReMap ChIP-seq,0.05387
224	NR2E1	0.05405	ARCHS4 Coexpression,0.05405
225	ZNF551	0.05413	Enrichr Queries,0.05413
226	ZNF649	0.05414	GTEx Coexpression,0.05414
227	AEBP2	0.05467	ARCHS4 Coexpression,0.05467
228	ZNF790	0.05476	GTEx Coexpression,0.05476
229	ZNF721	0.05484	Enrichr Queries,0.05484
230	ERG	0.05488	Literature ChIP-seq,0.05488
231	FOXO1	0.05538	GTEx Coexpression,0.05538
232	TEAD2	0.05556	Enrichr Queries,0.05556
233	ZNF823	0.0559	ARCHS4 Coexpression,0.0559
234	ZNF730	0.056	GTEx Coexpression,0.056
235	ZNF66	0.05663	GTEx Coexpression,0.05663
236	HINFP	0.05724	ReMap ChIP-seq,0.05724
237	KCMF1	0.05769	Enrichr Queries,0.05769
238	ZNF404	0.05774	ARCHS4 Coexpression,0.05774
239	ZBTB9	0.05835	ARCHS4 Coexpression,0.05835
240	TIGD3	0.0584	Enrichr Queries,0.0584
241	POU2AF1	0.05849	GTEx Coexpression,0.05849
242	ZFP42	0.05897	ARCHS4 Coexpression,0.05897
243	ZFP91	0.05912	Enrichr Queries,0.05912
244	NRF1	0.05932	ENCODE ChIP-seq,0.05932
245	ZIC5	0.05958	ARCHS4 Coexpression,0.05958
246	ZNF765	0.05974	GTEx Coexpression,0.05974
247	VEZF1	0.05983	Enrichr Queries,0.05983
248	NCOA1	0.0602	ARCHS4 Coexpression,0.0602
249	KLF10	0.06036	GTEx Coexpression,0.06036
250	PREB	0.06054	Enrichr Queries,0.06054
251	KLF11	0.06081	ARCHS4 Coexpression,0.06081
252	KLF4	0.06098	Literature ChIP-seq,0.06098
253	RAG1	0.06098	GTEx Coexpression,0.06098
254	THAP5	0.06125	Enrichr Queries,0.06125
255	JRKL	0.06161	GTEx Coexpression,0.06161
256	ZNF121	0.06197	Enrichr Queries,0.06197
257	ZFPM2	0.06204	ARCHS4 Coexpression,0.06204
258	SOX15	0.06265	ARCHS4 Coexpression,0.06265
259	SNAPC5	0.06285	GTEx Coexpression,0.06285
260	HIC2	0.06327	ARCHS4 Coexpression,0.06327
261	ZNF195	0.06339	Enrichr Queries,0.06339
262	ZNF573	0.06347	GTEx Coexpression,0.06347
263	ZBTB16	0.06409	GTEx Coexpression,0.06409
264	ZNF138	0.0641	Enrichr Queries,0.0641

265	CGGBP1	0.0645	ARCHS4 Coexpression,0.0645
266	ZKSCAN4	0.06472	GTEx Coexpression,0.06472
267	ZNF143	0.06481	Enrichr Queries,0.06481
268	TIGD7	0.06511	ARCHS4 Coexpression,0.06511
269	ZNF675	0.06534	GTEx Coexpression,0.06534
270	TCF3	0.06553	Enrichr Queries,0.06553
271	ZNF518B	0.06572	ARCHS4 Coexpression,0.06572
272	ZNF17	0.06596	GTEx Coexpression,0.06596
273	CDC5L	0.06624	Enrichr Queries,0.06624
274	ZNF544	0.06658	GTEx Coexpression,0.06658
275	SMYD3	0.06695	ARCHS4 Coexpression,0.06695
276	FOXO3	0.06707	Literature ChIP-seq,0.06707
277	ZNF354C	0.06721	GTEx Coexpression,0.06721
278	FOXP1	0.06734	ReMap ChIP-seq,0.06734
279	ZNF91	0.06757	ARCHS4 Coexpression,0.06757
280	CTCF	0.06766	Enrichr Queries,0.06766
281	ZFY	0.06783	GTEx Coexpression,0.06783
282	GSX2	0.06818	ARCHS4 Coexpression,0.06818
283	ZNF317	0.06838	Enrichr Queries,0.06838
284	GLIS3	0.06845	GTEx Coexpression,0.06845
285	PRDM14	0.0688	ARCHS4 Coexpression,0.0688
286	ZNF706	0.06909	Enrichr Queries,0.06909
287	ZNF581	0.06941	ARCHS4 Coexpression,0.06941
288	RBAK	0.0697	GTEx Coexpression,0.0697
289	ZNF45	0.07002	ARCHS4 Coexpression,0.07002
290	ZNF565	0.07032	GTEx Coexpression,0.07032
291	ZNF589	0.07064	ARCHS4 Coexpression,0.07064
292	ZNF398	0.07094	GTEx Coexpression,0.07094
293	UBP1	0.07123	Enrichr Queries,0.07123
294	ZBED5	0.07125	ARCHS4 Coexpression,0.07125
295	ZNF681	0.07156	GTEx Coexpression,0.07156
296	ZBTB39	0.07187	ARCHS4 Coexpression,0.07187
297	ZZZ3	0.07194	Enrichr Queries,0.07194
298	ARID5B	0.07218	GTEx Coexpression,0.07218
299	POU5F1	0.07248	ARCHS4 Coexpression,0.07248
300	ZNF800	0.07265	Enrichr Queries,0.07265
301	ZNF7	0.07281	GTEx Coexpression,0.07281
302	ZNF292	0.0731	ARCHS4 Coexpression,0.0731
303	SPI1	0.07317	Literature ChIP-seq,0.07317
304	ZNF705E	0.07343	GTEx Coexpression,0.07343
305	ZNF502	0.07371	ARCHS4 Coexpression,0.07371
306	BNC1	0.07405	GTEx Coexpression,0.07405
307	YY1	0.07407	Enrichr Queries,0.07407
308	MAFB	0.07407	ReMap ChIP-seq,0.07407
309	DMRTB1	0.07432	ARCHS4 Coexpression,0.07432
310	NEUROG2	0.07467	GTEx Coexpression,0.07467
311	ZNF207	0.07479	Enrichr Queries,0.07479
312	ZNF664	0.0755	Enrichr Queries,0.0755
313	ARID2	0.07617	ARCHS4 Coexpression,0.07617
314	ZNF682	0.07621	Enrichr Queries,0.07621
315	BHLHE40	0.07627	ENCODE ChIP-seq,0.07627
316	FOXJ2	0.07654	GTEx Coexpression,0.07654
317	TBP	0.07678	ARCHS4 Coexpression,0.07678

318	THAP6	0.07716	GTEx Coexpression,0.07716
319	ZNF181	0.0774	ARCHS4 Coexpression,0.0774
320	NFE2L1	0.07744	ReMap ChIP-seq,0.07744
321	TCFL5	0.07764	Enrichr Queries,0.07764
322	ZNF569	0.07801	ARCHS4 Coexpression,0.07801
323	SP3	0.07835	Enrichr Queries,0.07835
324	ZNF480	0.07841	GTEx Coexpression,0.07841
325	LIN54	0.07862	ARCHS4 Coexpression,0.07862
326	FOXN3	0.07903	GTEx Coexpression,0.07903
327	ZNF322	0.07924	ARCHS4 Coexpression,0.07924
328	FOXP3	0.07927	Literature ChIP-seq,0.07927
329	ZNF678	0.07985	ARCHS4 Coexpression,0.07985
330	KLF15	0.08027	GTEx Coexpression,0.08027
331	ZNF134	0.08047	ARCHS4 Coexpression,0.08047
332	YBX1	0.08048	Enrichr Queries,0.08048
333	FOXK1	0.08081	ReMap ChIP-seq,0.08081
334	NFYC	0.0812	Enrichr Queries,0.0812
335	ELK3	0.08152	GTEx Coexpression,0.08152
336	ZNF112	0.0817	ARCHS4 Coexpression,0.0817
337	TCF12	0.08191	Enrichr Queries,0.08191
338	PRRX1	0.08214	GTEx Coexpression,0.08214
339	DLX2	0.08231	ARCHS4 Coexpression,0.08231
340	ZSCAN12	0.08262	Enrichr Queries,0.08262
341	ZNF644	0.08276	GTEx Coexpression,0.08276
342	ISL2	0.08292	ARCHS4 Coexpression,0.08292
343	ZNF197	0.08333	Enrichr Queries,0.08333
344	ZNF830	0.08339	GTEx Coexpression,0.08339
345	TAL2	0.08354	ARCHS4 Coexpression,0.08354
346	ZNF12	0.08405	Enrichr Queries,0.08405
347	ZNF395	0.08415	ARCHS4 Coexpression,0.08415
348	ZKSCAN2	0.08463	GTEx Coexpression,0.08463
349	REST	0.08475	ENCODE ChIP-seq,0.08475
350	GTF2B	0.08477	ARCHS4 Coexpression,0.08477
351	TBX5	0.08537	Literature ChIP-seq,0.08537
352	ZNF30	0.08538	ARCHS4 Coexpression,0.08538
353	CLOCK	0.08587	GTEx Coexpression,0.08587
354	TPRX1	0.086	ARCHS4 Coexpression,0.086
355	ZBTB47	0.0865	GTEx Coexpression,0.0865
356	SOX6	0.08661	ARCHS4 Coexpression,0.08661
357	MAZ	0.08689	Enrichr Queries,0.08689
358	ZNF697	0.08712	GTEx Coexpression,0.08712
359	NFXL1	0.08722	ARCHS4 Coexpression,0.08722
360	CEBPA	0.08754	ReMap ChIP-seq,0.08754
361	FOXK2	0.08774	GTEx Coexpression,0.08774
362	PRDM9	0.08784	ARCHS4 Coexpression,0.08784
363	NR3C1	0.08836	GTEx Coexpression,0.08836
364	ZSCAN5B	0.08845	ARCHS4 Coexpression,0.08845
365	TIGD2	0.08903	Enrichr Queries,0.08903
366	MBD3	0.08974	Enrichr Queries,0.08974
367	SNAI1	0.09023	GTEx Coexpression,0.09023
368	ZNF177	0.09029	ARCHS4 Coexpression,0.09029
369	TFDP2	0.09046	Enrichr Queries,0.09046
370	ZNF485	0.09085	GTEx Coexpression,0.09085

371	GATA3	0.09091	ReMap ChIP-seq,0.09091
372	VDR	0.09146	Literature ChIP-seq,0.09146
373	ZFP64	0.09147	GTEx Coexpression,0.09147
374	TFAP2C	0.09152	ARCHS4 Coexpression,0.09152
375	ZNF512	0.09188	Enrichr Queries,0.09188
376	TFCP2	0.0921	GTEx Coexpression,0.0921
377	ARNT	0.09214	ARCHS4 Coexpression,0.09214
378	FOSL1	0.09259	Enrichr Queries,0.09259
379	ZBTB24	0.09272	GTEx Coexpression,0.09272
380	ZNF114	0.0933	Enrichr Queries,0.0933
381	ZNF117	0.09337	ARCHS4 Coexpression,0.09337
382	ZNF43	0.09396	GTEx Coexpression,0.09396
383	ZSCAN10	0.09398	ARCHS4 Coexpression,0.09398
384	PRDM4	0.09402	Enrichr Queries,0.09402
385	AHR	0.09428	ReMap ChIP-seq,0.09428
386	ZNF383	0.09459	GTEx Coexpression,0.09459
387	RFX6	0.09459	ARCHS4 Coexpression,0.09459
388	ZNF165	0.09473	Enrichr Queries,0.09473
389	ZNF837	0.09521	GTEx Coexpression,0.09521
390	ZNF574	0.09582	ARCHS4 Coexpression,0.09582
391	XPA	0.09583	GTEx Coexpression,0.09583
392	RBPJ	0.09615	Enrichr Queries,0.09615
393	TBXT	0.09644	ARCHS4 Coexpression,0.09644
394	ZNF407	0.09645	GTEx Coexpression,0.09645
395	ZNF217	0.09764	ReMap ChIP-seq,0.09764
396	ZNF71	0.09767	ARCHS4 Coexpression,0.09767
397	ZNF491	0.09828	ARCHS4 Coexpression,0.09828
398	NR2C1	0.09829	Enrichr Queries,0.09829
399	PRDM15	0.09832	GTEx Coexpression,0.09832
400	CXXC4	0.09889	ARCHS4 Coexpression,0.09889
401	ZNF669	0.09894	GTEx Coexpression,0.09894
402	RXRB	0.099	Enrichr Queries,0.099
403	NFIL3	0.09956	GTEx Coexpression,0.09956
404	SKOR1	0.1001	ARCHS4 Coexpression,0.1001
405	ZNF764	0.1002	GTEx Coexpression,0.1002
406	ZNF24	0.1004	Enrichr Queries,0.1004
407	PRDM1	0.101	ReMap ChIP-seq,0.101
408	SP4	0.1017	ENCODE ChIP-seq,0.1017
409	ZNF586	0.1026	Enrichr Queries,0.1026
410	ZNF852	0.1026	ARCHS4 Coexpression,0.1026
411	MITF	0.1027	GTEx Coexpression,0.1027
412	ZNF280B	0.1033	Enrichr Queries,0.1033
413	ZNF766	0.1033	GTEx Coexpression,0.1033
414	PRDM5	0.1037	Literature ChIP-seq,0.1037
415	NFKB1	0.1039	GTEx Coexpression,0.1039
416	ZNF562	0.104	Enrichr Queries,0.104
417	POU4F2	0.1044	ReMap ChIP-seq,0.1044
418	TIGD4	0.1044	ARCHS4 Coexpression,0.1044
419	ZNF211	0.105	ARCHS4 Coexpression,0.105
420	SMAD1	0.1052	GTEx Coexpression,0.1052
421	MYNN	0.1054	Enrichr Queries,0.1054
422	ZNF260	0.1057	ARCHS4 Coexpression,0.1057
423	SMAD4	0.1064	GTEx Coexpression,0.1064

424	ZNF222	0.1069	ARCHS4 Coexpression,0.1069
425	GZF1	0.1075	Enrichr Queries,0.1075
426	RFX7	0.1077	GTEx Coexpression,0.1077
427	ZNF384	0.1077	ReMap ChIP-seq,0.1077
428	ZFP62	0.1081	ARCHS4 Coexpression,0.1081
429	ZNF334	0.1087	ARCHS4 Coexpression,0.1087
430	ZNF883	0.1093	ARCHS4 Coexpression,0.1093
431	ZNF358	0.1095	GTEx Coexpression,0.1095
432	CREBZF	0.1097	Enrichr Queries,0.1097
433	THAP11	0.11	ARCHS4 Coexpression,0.11
434	ESR2	0.1101	GTEx Coexpression,0.1101
435	ZNF326	0.1104	Enrichr Queries,0.1104
436	ZNF136	0.1108	GTEx Coexpression,0.1108
437	ZNF83	0.1111	ReMap ChIP-seq,0.1111
438	SETDB2	0.1111	Enrichr Queries,0.1111
439	ZBTB26	0.1114	GTEx Coexpression,0.1114
440	NME2	0.112	GTEx Coexpression,0.112
441	ZNF337	0.1132	Enrichr Queries,0.1132
442	ZNF616	0.1133	GTEx Coexpression,0.1133
443	HOXD8	0.1139	GTEx Coexpression,0.1139
444	ZNF486	0.114	Enrichr Queries,0.114
445	EGR1	0.1143	ARCHS4 Coexpression,0.1143
446	LHX2	0.1145	ReMap ChIP-seq,0.1145
447	MSC	0.1145	GTEx Coexpression,0.1145
448	ZNF488	0.1147	Enrichr Queries,0.1147
449	DUXA	0.1149	ARCHS4 Coexpression,0.1149
450	HSF2	0.1155	ARCHS4 Coexpression,0.1155
451	KLF7	0.1157	GTEx Coexpression,0.1157
452	MSANTD3	0.117	GTEx Coexpression,0.117
453	ZNF160	0.1175	Enrichr Queries,0.1175
454	ZNF718	0.1176	GTEx Coexpression,0.1176
455	ELF5	0.1178	ReMap ChIP-seq,0.1178
456	PDX1	0.1179	ARCHS4 Coexpression,0.1179
457	IRF3	0.1182	GTEx Coexpression,0.1182
458	HES3	0.1186	ARCHS4 Coexpression,0.1186
459	ZNF799	0.1189	GTEx Coexpression,0.1189
460	SOX11	0.1189	Enrichr Queries,0.1189
461	ZNF534	0.1192	ARCHS4 Coexpression,0.1192
462	ZNF264	0.1195	GTEx Coexpression,0.1195
463	HBP1	0.1198	ARCHS4 Coexpression,0.1198
464	TSC22D1	0.1204	Enrichr Queries,0.1204
465	NOBOX	0.1204	ARCHS4 Coexpression,0.1204
466	ZSCAN20	0.1207	GTEx Coexpression,0.1207
467	ZBTB6	0.1211	Enrichr Queries,0.1211
468	HNF1B	0.1212	ReMap ChIP-seq,0.1212
469	ZNF84	0.1213	GTEx Coexpression,0.1213
470	SIX6	0.1216	ARCHS4 Coexpression,0.1216
471	POU3F2	0.122	Literature ChIP-seq,0.122
472	ZBTB1	0.122	GTEx Coexpression,0.122
473	ZNF223	0.1225	Enrichr Queries,0.1225
474	THYN1	0.1229	ARCHS4 Coexpression,0.1229
475	ZNF782	0.1232	GTEx Coexpression,0.1232
476	DPF1	0.1232	Enrichr Queries,0.1232

477	EBF2	0.1235	ARCHS4 Coexpression,0.1235
478	ZNF75D	0.1238	GTEx Coexpression,0.1238
479	ZBED3	0.1239	Enrichr Queries,0.1239
480	TEAD1	0.1245	GTEx Coexpression,0.1245
481	POGK	0.1246	Enrichr Queries,0.1246
482	ZNF354B	0.1251	GTEx Coexpression,0.1251
483	RHOXF2	0.1253	ARCHS4 Coexpression,0.1253
484	ZNF559	0.1254	Enrichr Queries,0.1254
485	ZBTB12	0.1261	Enrichr Queries,0.1261
486	ZNF484	0.1265	ARCHS4 Coexpression,0.1265
487	ATF4	0.1268	Enrichr Queries,0.1268
488	SP1	0.1271	ENCODE ChIP-seq,0.1271
489	ZNF594	0.1271	ARCHS4 Coexpression,0.1271
490	THAP4	0.1275	Enrichr Queries,0.1275
491	ZNF662	0.1276	GTEx Coexpression,0.1276
492	GATA1	0.1279	ReMap ChIP-seq,0.1279
493	MLXIP	0.1282	GTEx Coexpression,0.1282
494	ZNF3	0.1282	Enrichr Queries,0.1282
495	ZNF614	0.1284	ARCHS4 Coexpression,0.1284
496	ZNF584	0.1288	GTEx Coexpression,0.1288
497	ZNF420	0.129	ARCHS4 Coexpression,0.129
498	ZNF773	0.1294	GTEx Coexpression,0.1294
499	CREBL2	0.1296	ARCHS4 Coexpression,0.1296
500	ETV3L	0.1301	GTEx Coexpression,0.1301
501	ZNF711	0.1302	ARCHS4 Coexpression,0.1302
502	ZBTB11	0.1307	GTEx Coexpression,0.1307
503	ZNF254	0.1308	ARCHS4 Coexpression,0.1308
504	YBX2	0.1311	Enrichr Queries,0.1311
505	ZNF674	0.1313	GTEx Coexpression,0.1313
506	FOXA2	0.1313	ReMap ChIP-seq,0.1313
507	ZNF587B	0.1314	ARCHS4 Coexpression,0.1314
508	ZFP28	0.1318	Enrichr Queries,0.1318
509	NR2C2	0.1319	GTEx Coexpression,0.1319
510	MTF2	0.1321	ARCHS4 Coexpression,0.1321
511	IKZF4	0.1332	GTEx Coexpression,0.1332
512	ZNF490	0.1332	Enrichr Queries,0.1332
513	ELK4	0.1339	Enrichr Queries,0.1339
514	ZSCAN9	0.1339	ARCHS4 Coexpression,0.1339
515	TAL1	0.1341	Literature ChIP-seq,0.1341
516	PAX5	0.1344	GTEx Coexpression,0.1344
517	STAT2	0.1347	ReMap ChIP-seq,0.1347
518	ZNF570	0.135	GTEx Coexpression,0.135
519	ZNF708	0.1351	ARCHS4 Coexpression,0.1351
520	IRF1	0.1356	ENCODE ChIP-seq,0.1356
521	ZSCAN23	0.1357	ARCHS4 Coexpression,0.1357
522	MSGN1	0.1364	ARCHS4 Coexpression,0.1364
523	ZNF141	0.1368	Enrichr Queries,0.1368
524	ZSCAN16	0.137	ARCHS4 Coexpression,0.137
525	IKZF5	0.1375	Enrichr Queries,0.1375
526	ZNF772	0.1375	GTEx Coexpression,0.1375
527	ZNF529	0.1382	Enrichr Queries,0.1382
528	ZNF627	0.1382	ARCHS4 Coexpression,0.1382
529	SIM1	0.1394	GTEx Coexpression,0.1394

530	GLI4	0.14	GTEx Coexpression,0.14
531	PURG	0.14	ARCHS4 Coexpression,0.14
532	SRF	0.1402	Literature ChIP-seq,0.1402
533	AKAP8	0.1403	Enrichr Queries,0.1403
534	ZNF880	0.1406	GTEx Coexpression,0.1406
535	MTERF2	0.1407	ARCHS4 Coexpression,0.1407
536	SALL3	0.1413	ARCHS4 Coexpression,0.1413
537	ZNF394	0.1417	Enrichr Queries,0.1417
538	ZNF665	0.1419	GTEx Coexpression,0.1419
539	ATF3	0.1419	ARCHS4 Coexpression,0.1419
540	ZFP41	0.1425	Enrichr Queries,0.1425
541	ZNF14	0.1425	GTEx Coexpression,0.1425
542	ZNF10	0.1425	ARCHS4 Coexpression,0.1425
543	TGIF1	0.1431	ARCHS4 Coexpression,0.1431
544	ZBTB8A	0.1431	GTEx Coexpression,0.1431
545	SLC2A4RG	0.1432	Enrichr Queries,0.1432
546	ZNF442	0.1437	GTEx Coexpression,0.1437
547	ZNF606	0.1443	ARCHS4 Coexpression,0.1443
548	ZNF623	0.1444	GTEx Coexpression,0.1444
549	ZNF709	0.145	ARCHS4 Coexpression,0.145
550	MGA	0.145	GTEx Coexpression,0.145
551	ZNF585A	0.1456	ARCHS4 Coexpression,0.1456
552	DLX6	0.1462	ARCHS4 Coexpression,0.1462
553	SHOX2	0.1462	GTEx Coexpression,0.1462
554	ZNF354A	0.1467	Enrichr Queries,0.1467
555	JAZF1	0.1468	ARCHS4 Coexpression,0.1468
556	ZNF221	0.1469	GTEx Coexpression,0.1469
557	ZNF679	0.1474	ARCHS4 Coexpression,0.1474
558	NFATC3	0.1475	GTEx Coexpression,0.1475
559	KMT2B	0.1481	ReMap ChIP-seq,0.1481
560	RAX2	0.1481	Enrichr Queries,0.1481
561	MEF2A	0.1486	ARCHS4 Coexpression,0.1486
562	SNAI2	0.1489	Enrichr Queries,0.1489
563	PRDM10	0.1499	ARCHS4 Coexpression,0.1499
564	GTF2IRD2	0.15	GTEx Coexpression,0.15
565	ZNF44	0.1506	GTEx Coexpression,0.1506
566	ZFP57	0.151	Enrichr Queries,0.151
567	ZNF346	0.1511	ARCHS4 Coexpression,0.1511
568	BAZ2A	0.1512	GTEx Coexpression,0.1512
569	TCF7	0.1515	ReMap ChIP-seq,0.1515
570	ZNF280A	0.1517	Enrichr Queries,0.1517
571	ZNF184	0.1517	ARCHS4 Coexpression,0.1517
572	ZSCAN4	0.1523	ARCHS4 Coexpression,0.1523
573	RUNX1	0.1524	Literature ChIP-seq,0.1524
574	NFIC	0.1525	GTEx Coexpression,0.1525
575	CEBPB	0.1525	ENCODE ChIP-seq,0.1525
576	FOXR1	0.1529	ARCHS4 Coexpression,0.1529
577	ZNF496	0.1531	GTEx Coexpression,0.1531
578	THAP12	0.1536	ARCHS4 Coexpression,0.1536
579	DLX5	0.1542	ARCHS4 Coexpression,0.1542
580	ZNF180	0.1546	Enrichr Queries,0.1546
581	ZBTB44	0.1548	ARCHS4 Coexpression,0.1548
582	SOX10	0.1549	ReMap ChIP-seq,0.1549

583	NKX21	0.1554	ARCHS4 Coexpression,0.1554
584	ZNF829	0.156	Enrichr Queries,0.156
585	ZSCAN32	0.156	ARCHS4 Coexpression,0.156
586	ZNF816	0.1562	GTEx Coexpression,0.1562
587	GSC	0.1566	ARCHS4 Coexpression,0.1566
588	ZBTB33	0.1567	Enrichr Queries,0.1567
589	PPARA	0.1568	GTEx Coexpression,0.1568
590	SOX4	0.1574	Enrichr Queries,0.1574
591	CARF	0.1574	GTEx Coexpression,0.1574
592	GTF2IRD2B	0.1581	GTEx Coexpression,0.1581
593	ZNF576	0.1581	Enrichr Queries,0.1581
594	ZIC3	0.1585	ARCHS4 Coexpression,0.1585
595	ZHX3	0.1587	GTEx Coexpression,0.1587
596	ATMIN	0.1588	Enrichr Queries,0.1588
597	PAX6	0.1591	ARCHS4 Coexpression,0.1591
598	ZNF619	0.1595	Enrichr Queries,0.1595
599	ZNF525	0.1599	GTEx Coexpression,0.1599
600	ZNF692	0.1603	Enrichr Queries,0.1603
601	ZNF821	0.1603	ARCHS4 Coexpression,0.1603
602	XBP1	0.1609	ARCHS4 Coexpression,0.1609
603	RFX5	0.161	ENCODE ChIP-seq,0.161
604	ZNF700	0.1612	GTEx Coexpression,0.1612
605	TCF15	0.1615	ARCHS4 Coexpression,0.1615
606	NR1H3	0.1616	ReMap ChIP-seq,0.1616
607	ZXDB	0.1617	Enrichr Queries,0.1617
608	PHF20	0.1618	GTEx Coexpression,0.1618
609	TGIF2LX	0.1622	ARCHS4 Coexpression,0.1622
610	ZNF740	0.1624	Enrichr Queries,0.1624
611	DNTTIP1	0.1628	ARCHS4 Coexpression,0.1628
612	ZHX1	0.1631	Enrichr Queries,0.1631
613	ZNF582	0.1634	ARCHS4 Coexpression,0.1634
614	ZNF225	0.1637	GTEx Coexpression,0.1637
615	ZNF235	0.1638	Enrichr Queries,0.1638
616	NKRF	0.164	ARCHS4 Coexpression,0.164
617	HIVEP1	0.1643	GTEx Coexpression,0.1643
618	ZNF2	0.1645	Enrichr Queries,0.1645
619	EOMES	0.1646	Literature ChIP-seq,0.1646
620	ZSCAN25	0.1649	GTEx Coexpression,0.1649
621	ZBTB14	0.1655	GTEx Coexpression,0.1655
622	ZNF415	0.1658	ARCHS4 Coexpression,0.1658
623	GLMP	0.1661	GTEx Coexpression,0.1661
624	BAZ2B	0.1665	ARCHS4 Coexpression,0.1665
625	KLF16	0.1667	Enrichr Queries,0.1667
626	ZXDA	0.1668	GTEx Coexpression,0.1668
627	L3MBTL3	0.1671	ARCHS4 Coexpression,0.1671
628	AKAP8L	0.1674	Enrichr Queries,0.1674
629	CSRNP2	0.1674	GTEx Coexpression,0.1674
630	MSANTD1	0.1677	ARCHS4 Coexpression,0.1677
631	CEBPD	0.168	GTEx Coexpression,0.168
632	ATF2	0.1681	Enrichr Queries,0.1681
633	HOXC11	0.1684	ReMap ChIP-seq,0.1684
634	ZNF224	0.1686	GTEx Coexpression,0.1686
635	ZNF433	0.1689	ARCHS4 Coexpression,0.1689

636	ZNF226	0.1695	Enrichr Queries,0.1695
637	PPARG	0.1707	Literature ChIP-seq,0.1707
638	ZNF786	0.1709	Enrichr Queries,0.1709
639	ZNF501	0.172	ARCHS4 Coexpression,0.172
640	ZNF430	0.1731	Enrichr Queries,0.1731
641	ZNF253	0.1732	ARCHS4 Coexpression,0.1732
642	ZNF844	0.1736	GTEx Coexpression,0.1736
643	ZNF761	0.1738	Enrichr Queries,0.1738
644	ZBED6	0.1738	ARCHS4 Coexpression,0.1738
645	ZNF676	0.1744	ARCHS4 Coexpression,0.1744
646	ZBTB41	0.1751	ARCHS4 Coexpression,0.1751
647	NCOA2	0.1751	ReMap ChIP-seq,0.1751
648	ZNF879	0.1761	GTEx Coexpression,0.1761
649	GATA4	0.1768	Literature ChIP-seq,0.1768
650	JUN	0.1769	ARCHS4 Coexpression,0.1769
651	NR5A1	0.1774	Enrichr Queries,0.1774
652	ZNF737	0.1775	ARCHS4 Coexpression,0.1775
653	ESRRA	0.178	GTEx Coexpression,0.178
654	PAX8	0.1785	ReMap ChIP-seq,0.1785
655	ZNF561	0.1788	Enrichr Queries,0.1788
656	ZNF234	0.1792	GTEx Coexpression,0.1792
657	EEA1	0.1794	ARCHS4 Coexpression,0.1794
658	ZNF132	0.1805	GTEx Coexpression,0.1805
659	ZNF735	0.1806	ARCHS4 Coexpression,0.1806
660	ZFP82	0.1809	Enrichr Queries,0.1809
661	BBX	0.1816	Enrichr Queries,0.1816
662	MAFK	0.1818	ReMap ChIP-seq,0.1818
663	DLX1	0.1818	ARCHS4 Coexpression,0.1818
664	TMF1	0.1823	GTEx Coexpression,0.1823
665	HES1	0.1823	Enrichr Queries,0.1823
666	SOX17	0.1829	Literature ChIP-seq,0.1829
667	EMX1	0.183	ARCHS4 Coexpression,0.183
668	ZBTB5	0.183	Enrichr Queries,0.183
669	CREB3L2	0.1836	GTEx Coexpression,0.1836
670	TP73	0.1838	Enrichr Queries,0.1838
671	ZNF860	0.1843	ARCHS4 Coexpression,0.1843
672	ZNF33A	0.1848	GTEx Coexpression,0.1848
673	ZNF302	0.1852	Enrichr Queries,0.1852
674	STAT5B	0.1852	ReMap ChIP-seq,0.1852
675	MBNL2	0.1861	GTEx Coexpression,0.1861
676	REXO4	0.1867	GTEx Coexpression,0.1867
677	ZNF599	0.1873	GTEx Coexpression,0.1873
678	ZNF33B	0.1873	Enrichr Queries,0.1873
679	RARB	0.1879	GTEx Coexpression,0.1879
680	USF3	0.188	ARCHS4 Coexpression,0.188
681	ZNF781	0.1886	ARCHS4 Coexpression,0.1886
682	ZNF808	0.1887	Enrichr Queries,0.1887
683	DDIT3	0.1892	ARCHS4 Coexpression,0.1892
684	NFIA	0.1895	Enrichr Queries,0.1895
685	FOXD1	0.1898	GTEx Coexpression,0.1898
686	ZNF655	0.1902	Enrichr Queries,0.1902
687	ZBTB3	0.1909	Enrichr Queries,0.1909
688	HOXC6	0.191	GTEx Coexpression,0.191

689	MXI1	0.1916	ARCHS4 Coexpression,0.1916
690	DMTF1	0.1917	GTEx Coexpression,0.1917
691	ZNF23	0.1923	ARCHS4 Coexpression,0.1923
692	DRAP1	0.1923	Enrichr Queries,0.1923
693	ZNF577	0.1929	GTEx Coexpression,0.1929
694	ZFP30	0.1935	ARCHS4 Coexpression,0.1935
695	CREB3L1	0.1935	GTEx Coexpression,0.1935
696	REPIN1	0.1942	GTEx Coexpression,0.1942
697	ZNF34	0.1944	Enrichr Queries,0.1944
698	ATF6	0.1947	ARCHS4 Coexpression,0.1947
699	NR2F1	0.1948	GTEx Coexpression,0.1948
700	FOS	0.1949	ENCODE ChIP-seq,0.1949
701	NANOG	0.1951	Literature ChIP-seq,0.1951
702	ZNF174	0.1959	ARCHS4 Coexpression,0.1959
703	FOXD3	0.1966	ARCHS4 Coexpression,0.1966
704	THAP7	0.1972	ARCHS4 Coexpression,0.1972
705	ZKSCAN5	0.1973	GTEx Coexpression,0.1973
706	HEY2	0.1978	ARCHS4 Coexpression,0.1978
707	ZBTB21	0.1979	GTEx Coexpression,0.1979
708	ZNF563	0.198	Enrichr Queries,0.198
709	CREB3	0.1984	ARCHS4 Coexpression,0.1984
710	ZNF451	0.1985	GTEx Coexpression,0.1985
711	ZNF446	0.1987	Enrichr Queries,0.1987
712	ZNF747	0.1991	GTEx Coexpression,0.1991
713	ZBTB40	0.1994	Enrichr Queries,0.1994
714	ZKSCAN1	0.1996	ARCHS4 Coexpression,0.1996
715	ZNF250	0.2001	Enrichr Queries,0.2001
716	LHX6	0.2002	ARCHS4 Coexpression,0.2002
717	LCOR	0.2004	GTEx Coexpression,0.2004
718	TERF2	0.2009	Enrichr Queries,0.2009
719	ISL1	0.2009	ARCHS4 Coexpression,0.2009
720	HOXB4	0.2012	Literature ChIP-seq,0.2012
721	ZNF408	0.2015	ARCHS4 Coexpression,0.2015
722	ZNF140	0.2016	Enrichr Queries,0.2016
723	NRL	0.2016	GTEx Coexpression,0.2016
724	DEAF1	0.202	ReMap ChIP-seq,0.202
725	ZNF763	0.2021	ARCHS4 Coexpression,0.2021
726	ZBTB43	0.2023	Enrichr Queries,0.2023
727	ZNF652	0.2027	ARCHS4 Coexpression,0.2027
728	ZNF592	0.2029	GTEx Coexpression,0.2029
729	ZNF506	0.203	Enrichr Queries,0.203
730	MSX2	0.2033	ARCHS4 Coexpression,0.2033
731	ZNF585B	0.2037	Enrichr Queries,0.2037
732	ZNF441	0.2039	ARCHS4 Coexpression,0.2039
733	ZNF135	0.2041	GTEx Coexpression,0.2041
734	PITX1	0.2044	Enrichr Queries,0.2044
735	HOXB2	0.2045	ARCHS4 Coexpression,0.2045
736	ZEB2	0.2054	ReMap ChIP-seq,0.2054
737	SP9	0.2058	ARCHS4 Coexpression,0.2058
738	ZNF462	0.2058	Enrichr Queries,0.2058
739	WT1	0.206	GTEx Coexpression,0.206
740	NFE2L3	0.2073	Enrichr Queries,0.2073
741	ETS1	0.2073	Literature ChIP-seq,0.2073

742	ZNF607	0.2076	ARCHS4 Coexpression,0.2076
743	JUND	0.2078	GTEx Coexpression,0.2078
744	OTX1	0.2082	ARCHS4 Coexpression,0.2082
745	GATAD2A	0.2085	GTEx Coexpression,0.2085
746	NFYA	0.2087	Enrichr Queries,0.2087
747	ETV6	0.2088	ReMap ChIP-seq,0.2088
748	MEF2D	0.2091	GTEx Coexpression,0.2091
749	ZNF658	0.2095	ARCHS4 Coexpression,0.2095
750	RUNX2	0.2097	GTEx Coexpression,0.2097
751	RLF	0.2101	ARCHS4 Coexpression,0.2101
752	BCL6B	0.2107	ARCHS4 Coexpression,0.2107
753	IKZF3	0.2116	GTEx Coexpression,0.2116
754	HOXB7	0.2121	ReMap ChIP-seq,0.2121
755	ZNF778	0.2122	GTEx Coexpression,0.2122
756	ZNF320	0.2123	Enrichr Queries,0.2123
757	HDX	0.2125	ARCHS4 Coexpression,0.2125
758	SIX3	0.2131	ARCHS4 Coexpression,0.2131
759	HNF4A	0.2134	Literature ChIP-seq,0.2134
760	ZNF391	0.2134	GTEx Coexpression,0.2134
761	DPRX	0.2144	ARCHS4 Coexpression,0.2144
762	ZNF79	0.2144	Enrichr Queries,0.2144
763	ZNF426	0.2151	Enrichr Queries,0.2151
764	HSFX1	0.2153	GTEx Coexpression,0.2153
765	PLSCR1	0.2172	GTEx Coexpression,0.2172
766	RREB1	0.2172	Enrichr Queries,0.2172
767	POU1F1	0.2174	ARCHS4 Coexpression,0.2174
768	ZNF558	0.2178	GTEx Coexpression,0.2178
769	CUX1	0.2195	Literature ChIP-seq,0.2195
770	ZNF266	0.2197	GTEx Coexpression,0.2197
771	HOXA9	0.2199	ARCHS4 Coexpression,0.2199
772	ZNF16	0.2203	GTEx Coexpression,0.2203
773	MECP2	0.2205	ARCHS4 Coexpression,0.2205
774	ZHX2	0.2209	GTEx Coexpression,0.2209
775	ZNF596	0.2222	GTEx Coexpression,0.2222
776	RELA	0.2222	Enrichr Queries,0.2222
777	BACH2	0.2222	ReMap ChIP-seq,0.2222
778	GMEB2	0.2229	Enrichr Queries,0.2229
779	ZXDC	0.2236	Enrichr Queries,0.2236
780	TSHZ1	0.2242	ARCHS4 Coexpression,0.2242
781	ELF2	0.2251	Enrichr Queries,0.2251
782	ZNF703	0.2253	GTEx Coexpression,0.2253
783	ZNF280D	0.2254	ARCHS4 Coexpression,0.2254
784	TFEB	0.2256	Literature ChIP-seq,0.2256
785	HMG20B	0.2258	Enrichr Queries,0.2258
786	BARX1	0.226	ARCHS4 Coexpression,0.226
787	KLF9	0.2265	GTEx Coexpression,0.2265
788	STAT3	0.2271	GTEx Coexpression,0.2271
789	ASCL4	0.2297	ARCHS4 Coexpression,0.2297
790	ZKSCAN8	0.2302	GTEx Coexpression,0.2302
791	ZNF263	0.2308	Enrichr Queries,0.2308
792	TCF4	0.231	ARCHS4 Coexpression,0.231
793	PHF21A	0.2315	Enrichr Queries,0.2315
794	ZBTB37	0.2316	ARCHS4 Coexpression,0.2316

795	AR	0.2317	Literature ChIP-seq,0.2317
796	SATB1	0.2321	GTEx Coexpression,0.2321
797	AHCTF1	0.2322	Enrichr Queries,0.2322
798	MAFF	0.2323	ReMap ChIP-seq,0.2323
799	ZEB1	0.2334	GTEx Coexpression,0.2334
800	ESR1	0.2334	ARCHS4 Coexpression,0.2334
801	NR0B1	0.2352	GTEx Coexpression,0.2352
802	ZNF547	0.2353	ARCHS4 Coexpression,0.2353
803	LEF1	0.2357	ReMap ChIP-seq,0.2357
804	CXXC1	0.2358	Enrichr Queries,0.2358
805	TWIST1	0.2365	GTEx Coexpression,0.2365
806	HOXC5	0.2371	GTEx Coexpression,0.2371
807	TWIST2	0.2377	GTEx Coexpression,0.2377
808	PLAGL2	0.2379	Enrichr Queries,0.2379
809	ZSCAN29	0.2386	Enrichr Queries,0.2386
810	ZBTB38	0.239	GTEx Coexpression,0.239
811	MEIS2	0.2391	ReMap ChIP-seq,0.2391
812	ZSCAN26	0.2396	ARCHS4 Coexpression,0.2396
813	ZNF148	0.2396	GTEx Coexpression,0.2396
814	ZNF610	0.24	Enrichr Queries,0.24
815	NEUROG3	0.2402	ARCHS4 Coexpression,0.2402
816	SALL2	0.2408	ARCHS4 Coexpression,0.2408
817	ZNF248	0.2414	ARCHS4 Coexpression,0.2414
818	SCMH1	0.2414	GTEx Coexpression,0.2414
819	ANKZF1	0.2415	Enrichr Queries,0.2415
820	ANHX	0.242	ARCHS4 Coexpression,0.242
821	ZNF212	0.2422	Enrichr Queries,0.2422
822	TET3	0.2426	ARCHS4 Coexpression,0.2426
823	RFX4	0.2432	ARCHS4 Coexpression,0.2432
824	HES4	0.2436	Enrichr Queries,0.2436
825	ELK1	0.2439	Literature ChIP-seq,0.2439
826	ZNF736	0.2439	GTEx Coexpression,0.2439
827	ZNF836	0.2446	GTEx Coexpression,0.2446
828	HOXC9	0.2451	ARCHS4 Coexpression,0.2451
829	ZNF331	0.2457	Enrichr Queries,0.2457
830	HAND2	0.2463	ARCHS4 Coexpression,0.2463
831	PRDM2	0.2469	ARCHS4 Coexpression,0.2469
832	ZNF32	0.247	GTEx Coexpression,0.247
833	NFE2L2	0.2475	ARCHS4 Coexpression,0.2475
834	TSHZ2	0.2477	GTEx Coexpression,0.2477
835	ZNF641	0.2479	Enrichr Queries,0.2479
836	KLF14	0.2482	ARCHS4 Coexpression,0.2482
837	HOXA4	0.2483	GTEx Coexpression,0.2483
838	YBX3	0.2489	GTEx Coexpression,0.2489
839	MEIS1	0.2492	ReMap ChIP-seq,0.2492
840	ZNF189	0.2493	Enrichr Queries,0.2493
841	POU3F4	0.2494	ARCHS4 Coexpression,0.2494
842	THAP3	0.25	ARCHS4 Coexpression,0.25
843	ZNF514	0.25	Enrichr Queries,0.25
844	TBX3	0.25	Literature ChIP-seq,0.25
845	ZNF521	0.2507	Enrichr Queries,0.2507
846	HAND1	0.2512	ARCHS4 Coexpression,0.2512
847	ZNF696	0.2514	GTEx Coexpression,0.2514

848	ETV5	0.2518	ARCHS4 Coexpression,0.2518
849	TBX21	0.2525	ReMap ChIP-seq,0.2525
850	ZNF236	0.2526	GTEx Coexpression,0.2526
851	ZNF296	0.2528	Enrichr Queries,0.2528
852	PLAGL1	0.2531	ARCHS4 Coexpression,0.2531
853	ZNF608	0.2543	Enrichr Queries,0.2543
854	ZNF660	0.2545	GTEx Coexpression,0.2545
855	ZNF169	0.255	Enrichr Queries,0.255
856	ZNF843	0.2551	GTEx Coexpression,0.2551
857	ZNF805	0.2555	ARCHS4 Coexpression,0.2555
858	PBX1	0.2557	Enrichr Queries,0.2557
859	NR4A1	0.2559	ReMap ChIP-seq,0.2559
860	ARID3B	0.2561	ARCHS4 Coexpression,0.2561
861	ZIM2	0.2574	ARCHS4 Coexpression,0.2574
862	FOXO6	0.2576	GTEx Coexpression,0.2576
863	HOXB9	0.2578	Enrichr Queries,0.2578
864	EGR2	0.258	ARCHS4 Coexpression,0.258
865	ZNF600	0.2582	GTEx Coexpression,0.2582
866	PBX3	0.2585	Enrichr Queries,0.2585
867	TIGD5	0.2589	GTEx Coexpression,0.2589
868	ARX	0.2592	ARCHS4 Coexpression,0.2592
869	ZNF362	0.2593	Enrichr Queries,0.2593
870	ZNF791	0.26	Enrichr Queries,0.26
871	POU4F1	0.2601	GTEx Coexpression,0.2601
872	ZBTB20	0.2607	Enrichr Queries,0.2607
873	ARGFX	0.2607	GTEx Coexpression,0.2607
874	TCF7L1	0.2617	ARCHS4 Coexpression,0.2617
875	NFIB	0.2621	Enrichr Queries,0.2621
876	NKX28	0.2623	ARCHS4 Coexpression,0.2623
877	ZNF727	0.2632	GTEx Coexpression,0.2632
878	ZFP90	0.2638	GTEx Coexpression,0.2638
879	ZNF80	0.2641	ARCHS4 Coexpression,0.2641
880	NEUROD1	0.2647	ARCHS4 Coexpression,0.2647
881	HIC1	0.265	Enrichr Queries,0.265
882	ZNF283	0.2651	GTEx Coexpression,0.2651
883	ZNF571	0.2657	GTEx Coexpression,0.2657
884	FOSB	0.2663	GTEx Coexpression,0.2663
885	ZNF397	0.267	GTEx Coexpression,0.267
886	ZNF436	0.2672	ARCHS4 Coexpression,0.2672
887	FOXC2	0.2678	Enrichr Queries,0.2678
888	ZNF629	0.2682	GTEx Coexpression,0.2682
889	FEV	0.269	ARCHS4 Coexpression,0.269
890	SAFB	0.2694	GTEx Coexpression,0.2694
891	PROX2	0.2701	GTEx Coexpression,0.2701
892	ZFP37	0.2703	ARCHS4 Coexpression,0.2703
893	SIX4	0.2707	Enrichr Queries,0.2707
894	ZNF347	0.2713	GTEx Coexpression,0.2713
895	HIVEP2	0.2714	Enrichr Queries,0.2714
896	DBX2	0.2715	ARCHS4 Coexpression,0.2715
897	RAX	0.2719	GTEx Coexpression,0.2719
898	ZSCAN5A	0.2721	Enrichr Queries,0.2721
899	TCF24	0.2726	GTEx Coexpression,0.2726
900	ZBTB42	0.2738	GTEx Coexpression,0.2738

901	ZNF701	0.274	ARCHS4 Coexpression,0.274
902	FOXP2	0.2744	Literature ChIP-seq,0.2744
903	ASH1L	0.2744	GTEx Coexpression,0.2744
904	ZNF418	0.2746	ARCHS4 Coexpression,0.2746
905	TFCP2L1	0.275	GTEx Coexpression,0.275
906	TFAP2A	0.2756	Enrichr Queries,0.2756
907	GPBP1	0.2757	GTEx Coexpression,0.2757
908	ZNF438	0.2758	ARCHS4 Coexpression,0.2758
909	NFATC1	0.2763	GTEx Coexpression,0.2763
910	PATZ1	0.2785	Enrichr Queries,0.2785
911	NR2F6	0.2792	Enrichr Queries,0.2792
912	HEY1	0.2795	ARCHS4 Coexpression,0.2795
913	ARID3A	0.2797	ENCODE ChIP-seq,0.2797
914	ZBTB4	0.28	GTEx Coexpression,0.28
915	MSX1	0.2806	Enrichr Queries,0.2806
916	RBPJL	0.2806	GTEx Coexpression,0.2806
917	STAT6	0.2813	GTEx Coexpression,0.2813
918	ZNF324B	0.2819	GTEx Coexpression,0.2819
919	ZNF550	0.2825	GTEx Coexpression,0.2825
920	ZNF432	0.2828	Enrichr Queries,0.2828
921	ZNF487	0.2856	Enrichr Queries,0.2856
922	MYSM1	0.2856	GTEx Coexpression,0.2856
923	SNAPC4	0.2862	ReMap ChIP-seq,0.2862
924	ZBTB10	0.2863	Enrichr Queries,0.2863
925	PROP1	0.2869	ARCHS4 Coexpression,0.2869
926	FOXA3	0.2869	GTEx Coexpression,0.2869
927	RORA	0.2875	ARCHS4 Coexpression,0.2875
928	FOXJ3	0.2875	GTEx Coexpression,0.2875
929	FOXG1	0.2881	ARCHS4 Coexpression,0.2881
930	SOX9	0.2885	Enrichr Queries,0.2885
931	KLF17	0.2893	ARCHS4 Coexpression,0.2893
932	ZNF284	0.2894	GTEx Coexpression,0.2894
933	THAP2	0.2899	Enrichr Queries,0.2899
934	ZNF780B	0.2899	ARCHS4 Coexpression,0.2899
935	TIGD1	0.2905	ARCHS4 Coexpression,0.2905
936	ZNF566	0.2906	Enrichr Queries,0.2906
937	CTCFL	0.2912	ARCHS4 Coexpression,0.2912
938	NFAT5	0.2913	Enrichr Queries,0.2913
939	ZNF333	0.2918	GTEx Coexpression,0.2918
940	ZBTB25	0.292	Enrichr Queries,0.292
941	ZNF527	0.2925	GTEx Coexpression,0.2925
942	DACH1	0.2927	Literature ChIP-seq,0.2927
943	ZNF792	0.2927	Enrichr Queries,0.2927
944	ZNF471	0.2936	ARCHS4 Coexpression,0.2936
945	ZNF133	0.2942	Enrichr Queries,0.2942
946	ZNF615	0.2943	GTEx Coexpression,0.2943
947	USF2	0.2949	Enrichr Queries,0.2949
948	ZNF444	0.295	GTEx Coexpression,0.295
949	RORC	0.2962	GTEx Coexpression,0.2962
950	ZNF460	0.2963	Enrichr Queries,0.2963
951	PLAG1	0.2968	GTEx Coexpression,0.2968
952	MBD1	0.2977	Enrichr Queries,0.2977
953	DMRTA1	0.2979	ARCHS4 Coexpression,0.2979

954	GATA6	0.2988	Literature ChIP-seq,0.2988
955	TET2	0.2991	ARCHS4 Coexpression,0.2991
956	ZBTB7A	0.2991	Enrichr Queries,0.2991
957	MKX	0.2993	GTEx Coexpression,0.2993
958	SMAD9	0.2998	ARCHS4 Coexpression,0.2998
959	ZNF469	0.2999	GTEx Coexpression,0.2999
960	ZNF425	0.3006	Enrichr Queries,0.3006
961	ZNF728	0.301	ARCHS4 Coexpression,0.301
962	HOXC4	0.3012	GTEx Coexpression,0.3012
963	HOXD4	0.3016	ARCHS4 Coexpression,0.3016
964	PKNOX1	0.302	Enrichr Queries,0.302
965	RFX1	0.303	ReMap ChIP-seq,0.303
966	HOXA2	0.303	GTEx Coexpression,0.303
967	ZNF564	0.3041	Enrichr Queries,0.3041
968	ZNF729	0.3047	ARCHS4 Coexpression,0.3047
969	BCL11A	0.3053	ARCHS4 Coexpression,0.3053
970	SREBF1	0.3055	GTEx Coexpression,0.3055
971	ZNF335	0.3062	GTEx Coexpression,0.3062
972	ZSCAN30	0.3096	ARCHS4 Coexpression,0.3096
973	ZBTB46	0.3098	Enrichr Queries,0.3098
974	ZNF512B	0.3099	GTEx Coexpression,0.3099
975	ZNF37A	0.3102	ARCHS4 Coexpression,0.3102
976	NEUROD6	0.3108	ARCHS4 Coexpression,0.3108
977	BPTF	0.3118	GTEx Coexpression,0.3118
978	ZNF774	0.312	Enrichr Queries,0.312
979	ZNF182	0.312	ARCHS4 Coexpression,0.312
980	HLF	0.3124	GTEx Coexpression,0.3124
981	OVOL1	0.3127	Enrichr Queries,0.3127
982	MEF2B	0.3131	ReMap ChIP-seq,0.3131
983	KLF13	0.3134	Enrichr Queries,0.3134
984	USF1	0.3141	Enrichr Queries,0.3141
985	GSC2	0.3143	GTEx Coexpression,0.3143
986	ZNF251	0.3145	ARCHS4 Coexpression,0.3145
987	FOXD4L4	0.3149	GTEx Coexpression,0.3149
988	ZNF780A	0.3161	GTEx Coexpression,0.3161
989	ZNF20	0.3162	Enrichr Queries,0.3162
990	GCM2	0.317	ARCHS4 Coexpression,0.317
991	SALL1	0.3171	Literature ChIP-seq,0.3171
992	NR3C2	0.3176	ARCHS4 Coexpression,0.3176
993	ZNF552	0.3177	Enrichr Queries,0.3177
994	PHOX2A	0.3182	ARCHS4 Coexpression,0.3182
995	RXRA	0.3184	Enrichr Queries,0.3184
996	EBF1	0.3188	ARCHS4 Coexpression,0.3188
997	ZNF230	0.3198	Enrichr Queries,0.3198
998	ZNF516	0.3199	GTEx Coexpression,0.3199
999	TBX22	0.32	ARCHS4 Coexpression,0.32
1000	HIF3A	0.3205	GTEx Coexpression,0.3205
1001	TRERF1	0.3205	Enrichr Queries,0.3205
1002	SIX2	0.3211	GTEx Coexpression,0.3211
1003	ZFP3	0.3219	ARCHS4 Coexpression,0.3219
1004	RFX2	0.3225	ARCHS4 Coexpression,0.3225
1005	SMAD5	0.323	GTEx Coexpression,0.323
1006	ATOH7	0.3231	ARCHS4 Coexpression,0.3231

1007	ESX1	0.3236	GTEx Coexpression,0.3236
1008	CPEB1	0.3237	ARCHS4 Coexpression,0.3237
1009	ZNF316	0.3242	GTEx Coexpression,0.3242
1010	ZFHX4	0.3243	ARCHS4 Coexpression,0.3243
1011	ZNF503	0.3248	GTEx Coexpression,0.3248
1012	NEUROG1	0.3255	GTEx Coexpression,0.3255
1013	ZBTB32	0.3255	Enrichr Queries,0.3255
1014	NANOGNB	0.3261	GTEx Coexpression,0.3261
1015	BRF2	0.3266	ReMap ChIP-seq,0.3266
1016	FEZF1	0.3268	ARCHS4 Coexpression,0.3268
1017	TBX15	0.3279	GTEx Coexpression,0.3279
1018	NR4A2	0.328	ARCHS4 Coexpression,0.328
1019	ZNF526	0.3283	Enrichr Queries,0.3283
1020	SHOX	0.3286	GTEx Coexpression,0.3286
1021	SNAPC2	0.3286	ARCHS4 Coexpression,0.3286
1022	DOT1L	0.3291	Enrichr Queries,0.3291
1023	EGR4	0.3292	ARCHS4 Coexpression,0.3292
1024	ETV3	0.3298	Enrichr Queries,0.3298
1025	ZNF345	0.3298	GTEx Coexpression,0.3298
1026	ZNF274	0.3299	ARCHS4 Coexpression,0.3299
1027	SPIB	0.33	ReMap ChIP-seq,0.33
1028	POU2F2	0.3305	ENCODE ChIP-seq,0.3305
1029	JRK	0.3311	GTEx Coexpression,0.3311
1030	RFX3	0.3311	ARCHS4 Coexpression,0.3311
1031	ZNF341	0.3326	Enrichr Queries,0.3326
1032	ZNF555	0.3329	GTEx Coexpression,0.3329
1033	SRCAP	0.3333	Enrichr Queries,0.3333
1034	NR1H2	0.3335	GTEx Coexpression,0.3335
1035	NR1H4	0.3342	ARCHS4 Coexpression,0.3342
1036	ZNF536	0.3348	ARCHS4 Coexpression,0.3348
1037	THRB	0.3348	GTEx Coexpression,0.3348
1038	HOXC8	0.3354	ARCHS4 Coexpression,0.3354
1039	ZNF549	0.3355	Enrichr Queries,0.3355
1040	KLF6	0.336	ARCHS4 Coexpression,0.336
1041	NKX22	0.3366	ARCHS4 Coexpression,0.3366
1042	ZNF587	0.3367	GTEx Coexpression,0.3367
1043	MNX1	0.3376	Enrichr Queries,0.3376
1044	ZNF528	0.3378	ARCHS4 Coexpression,0.3378
1045	ZNF175	0.3379	GTEx Coexpression,0.3379
1046	OSR2	0.3383	Enrichr Queries,0.3383
1047	NR1D2	0.3385	ARCHS4 Coexpression,0.3385
1048	GCM1	0.339	Enrichr Queries,0.339
1049	REL	0.3404	GTEx Coexpression,0.3404
1050	ZNF814	0.3409	ARCHS4 Coexpression,0.3409
1051	FOXS1	0.3419	Enrichr Queries,0.3419
1052	MEOX2	0.3426	Enrichr Queries,0.3426
1053	ZNF439	0.3433	Enrichr Queries,0.3433
1054	ZKSCAN3	0.3441	GTEx Coexpression,0.3441
1055	NPAS2	0.3447	Enrichr Queries,0.3447
1056	ZNF350	0.3454	Enrichr Queries,0.3454
1057	TCF20	0.3466	GTEx Coexpression,0.3466
1058	SOX13	0.3468	ReMap ChIP-seq,0.3468
1059	CRX	0.3469	Enrichr Queries,0.3469

1060	SMAD3	0.3476	Literature ChIP-seq,0.3476
1061	PURA	0.3503	GTEx Coexpression,0.3503
1062	ZNF618	0.3511	Enrichr Queries,0.3511
1063	ELF1	0.3516	GTEx Coexpression,0.3516
1064	TRPS1	0.352	ARCHS4 Coexpression,0.352
1065	NR5A2	0.3522	GTEx Coexpression,0.3522
1066	ZNF382	0.3526	Enrichr Queries,0.3526
1067	ZSCAN31	0.3528	GTEx Coexpression,0.3528
1068	SP7	0.3532	ARCHS4 Coexpression,0.3532
1069	ZNF275	0.354	Enrichr Queries,0.354
1070	ZNF423	0.3541	GTEx Coexpression,0.3541
1071	ZNF81	0.355	ARCHS4 Coexpression,0.355
1072	ZNF428	0.3554	Enrichr Queries,0.3554
1073	ZNF285	0.3563	ARCHS4 Coexpression,0.3563
1074	EHF	0.3569	ReMap ChIP-seq,0.3569
1075	SKOR2	0.3572	GTEx Coexpression,0.3572
1076	PRRX2	0.3575	ARCHS4 Coexpression,0.3575
1077	GLIS1	0.3581	ARCHS4 Coexpression,0.3581
1078	ZNF583	0.3583	Enrichr Queries,0.3583
1079	ZNF98	0.3584	GTEx Coexpression,0.3584
1080	ZNF286B	0.3587	ARCHS4 Coexpression,0.3587
1081	ETV2	0.359	Enrichr Queries,0.359
1082	ZFPM1	0.3597	GTEx Coexpression,0.3597
1083	GLI1	0.3597	Enrichr Queries,0.3597
1084	MECOM	0.3598	Literature ChIP-seq,0.3598
1085	ZNF713	0.3603	GTEx Coexpression,0.3603
1086	ZNF841	0.3606	ARCHS4 Coexpression,0.3606
1087	ZFP14	0.3628	GTEx Coexpression,0.3628
1088	STAT5A	0.3634	GTEx Coexpression,0.3634
1089	PAX4	0.3636	ARCHS4 Coexpression,0.3636
1090	ZNF419	0.364	Enrichr Queries,0.364
1091	RUNX3	0.3644	ENCODE ChIP-seq,0.3644
1092	POU5F2	0.3661	ARCHS4 Coexpression,0.3661
1093	ZFP2	0.3668	Enrichr Queries,0.3668
1094	ZNF343	0.3678	GTEx Coexpression,0.3678
1095	ZNF282	0.369	GTEx Coexpression,0.369
1096	ZNF267	0.3697	Enrichr Queries,0.3697
1097	RELB	0.3703	GTEx Coexpression,0.3703
1098	BATF	0.3704	ReMap ChIP-seq,0.3704
1099	HOXA5	0.3715	GTEx Coexpression,0.3715
1100	SIM2	0.3722	ARCHS4 Coexpression,0.3722
1101	ZNF518A	0.3725	Enrichr Queries,0.3725
1102	KLF8	0.3732	Enrichr Queries,0.3732
1103	ELF3	0.3737	ReMap ChIP-seq,0.3737
1104	SOX7	0.3739	Enrichr Queries,0.3739
1105	ZNF787	0.3746	Enrichr Queries,0.3746
1106	RBSN	0.3752	GTEx Coexpression,0.3752
1107	ZNF532	0.3754	Enrichr Queries,0.3754
1108	HOXA10	0.3759	ARCHS4 Coexpression,0.3759
1109	HSF5	0.3768	Enrichr Queries,0.3768
1110	FOXP4	0.3777	GTEx Coexpression,0.3777
1111	ZNF429	0.3778	ARCHS4 Coexpression,0.3778
1112	OLIG2	0.378	Literature ChIP-seq,0.378

1113	SIX5	0.3783	GTEx Coexpression,0.3783
1114	RXRG	0.3796	Enrichr Queries,0.3796
1115	ZNF707	0.3802	GTEx Coexpression,0.3802
1116	PTF1A	0.3802	ARCHS4 Coexpression,0.3802
1117	HOXC12	0.3808	ARCHS4 Coexpression,0.3808
1118	ONECUT3	0.3833	ARCHS4 Coexpression,0.3833
1119	ZNF776	0.3839	ARCHS4 Coexpression,0.3839
1120	SETBP1	0.3839	GTEx Coexpression,0.3839
1121	CSRNP1	0.3845	ARCHS4 Coexpression,0.3845
1122	SPDEF	0.3846	GTEx Coexpression,0.3846
1123	ZSCAN22	0.3846	Enrichr Queries,0.3846
1124	PPARD	0.3853	Enrichr Queries,0.3853
1125	ZNF677	0.3864	ARCHS4 Coexpression,0.3864
1126	HIVEP3	0.387	ARCHS4 Coexpression,0.387
1127	GTF2IRD1	0.3871	GTEx Coexpression,0.3871
1128	FOXD4L5	0.3876	ARCHS4 Coexpression,0.3876
1129	ZMAT1	0.3882	ARCHS4 Coexpression,0.3882
1130	DLX4	0.3888	ARCHS4 Coexpression,0.3888
1131	IRX1	0.3889	GTEx Coexpression,0.3889
1132	TFDP3	0.3902	GTEx Coexpression,0.3902
1133	CDX2	0.3906	ReMap ChIP-seq,0.3906
1134	DMRTC2	0.3919	ARCHS4 Coexpression,0.3919
1135	ZNF579	0.392	GTEx Coexpression,0.392
1136	NR4A3	0.3925	ARCHS4 Coexpression,0.3925
1137	NKX31	0.3943	ARCHS4 Coexpression,0.3943
1138	DMRT2	0.3946	Enrichr Queries,0.3946
1139	FAM200B	0.395	ARCHS4 Coexpression,0.395
1140	HOXB1	0.3956	ARCHS4 Coexpression,0.3956
1141	TBX10	0.3958	GTEx Coexpression,0.3958
1142	POU3F1	0.3963	Literature ChIP-seq,0.3963
1143	ZNF557	0.3964	GTEx Coexpression,0.3964
1144	SP2	0.3967	Enrichr Queries,0.3967
1145	ZKSCAN7	0.3976	GTEx Coexpression,0.3976
1146	FOXH1	0.398	ARCHS4 Coexpression,0.398
1147	INSM2	0.3986	ARCHS4 Coexpression,0.3986
1148	MESP1	0.3989	GTEx Coexpression,0.3989
1149	MYF6	0.3995	GTEx Coexpression,0.3995
1150	KLF5	0.4003	Enrichr Queries,0.4003
1151	PHOX2B	0.4005	ARCHS4 Coexpression,0.4005
1152	ZNF827	0.4007	GTEx Coexpression,0.4007
1153	LMX1A	0.402	GTEx Coexpression,0.402
1154	ZNF846	0.4024	Enrichr Queries,0.4024
1155	ZNF454	0.4026	GTEx Coexpression,0.4026
1156	VAX1	0.4036	ARCHS4 Coexpression,0.4036
1157	ZSCAN2	0.4039	GTEx Coexpression,0.4039
1158	RARG	0.4046	Enrichr Queries,0.4046
1159	ZFP92	0.4051	GTEx Coexpression,0.4051
1160	ASCL3	0.406	Enrichr Queries,0.406
1161	LBX2	0.406	ARCHS4 Coexpression,0.406
1162	JUNB	0.4081	Enrichr Queries,0.4081
1163	OTP	0.4088	GTEx Coexpression,0.4088
1164	EBF3	0.4095	GTEx Coexpression,0.4095
1165	GRHL2	0.4095	Enrichr Queries,0.4095

1166	IRF6	0.4097	ARCHS4 Coexpression,0.4097
1167	BSX	0.4101	GTEx Coexpression,0.4101
1168	TFEC	0.4103	ARCHS4 Coexpression,0.4103
1169	ZNF671	0.4109	ARCHS4 Coexpression,0.4109
1170	ZNF548	0.4113	GTEx Coexpression,0.4113
1171	BHLHE41	0.4122	ARCHS4 Coexpression,0.4122
1172	TBPL1	0.4128	ARCHS4 Coexpression,0.4128
1173	HOXC13	0.4134	ARCHS4 Coexpression,0.4134
1174	TEAD3	0.4138	GTEx Coexpression,0.4138
1175	ZNF41	0.4144	GTEx Coexpression,0.4144
1176	FOXB1	0.4152	ARCHS4 Coexpression,0.4152
1177	ST18	0.4158	ARCHS4 Coexpression,0.4158
1178	SPEN	0.4169	GTEx Coexpression,0.4169
1179	TP63	0.4171	ARCHS4 Coexpression,0.4171
1180	MEF2C	0.4175	ReMap ChIP-seq,0.4175
1181	ZNF653	0.4181	Enrichr Queries,0.4181
1182	TLX2	0.4183	ARCHS4 Coexpression,0.4183
1183	NR2F2	0.4188	GTEx Coexpression,0.4188
1184	SIX1	0.4188	Enrichr Queries,0.4188
1185	PITX2	0.4189	ARCHS4 Coexpression,0.4189
1186	PEG3	0.4194	GTEx Coexpression,0.4194
1187	PRDM16	0.4195	ARCHS4 Coexpression,0.4195
1188	DPF3	0.42	GTEx Coexpression,0.42
1189	KLF12	0.4202	Enrichr Queries,0.4202
1190	NFATC4	0.4207	GTEx Coexpression,0.4207
1191	ESRRB	0.4207	Literature ChIP-seq,0.4207
1192	DBX1	0.422	ARCHS4 Coexpression,0.422
1193	ZNF609	0.4231	GTEx Coexpression,0.4231
1194	KDM2A	0.4238	GTEx Coexpression,0.4238
1195	ZNF598	0.4256	GTEx Coexpression,0.4256
1196	SPIC	0.4257	ARCHS4 Coexpression,0.4257
1197	ERF	0.4263	GTEx Coexpression,0.4263
1198	ZNF750	0.4266	Enrichr Queries,0.4266
1199	ZNF417	0.4269	GTEx Coexpression,0.4269
1200	GBX2	0.4275	ARCHS4 Coexpression,0.4275
1201	ZNF672	0.4295	Enrichr Queries,0.4295
1202	BHLHA15	0.4306	GTEx Coexpression,0.4306
1203	BCL6	0.4312	GTEx Coexpression,0.4312
1204	BATF3	0.4319	GTEx Coexpression,0.4319
1205	EPAS1	0.4331	GTEx Coexpression,0.4331
1206	FOSL2	0.4343	ReMap ChIP-seq,0.4343
1207	SREBF2	0.4359	Enrichr Queries,0.4359
1208	TBX20	0.4361	ARCHS4 Coexpression,0.4361
1209	NKX63	0.4367	ARCHS4 Coexpression,0.4367
1210	ZNF483	0.4368	GTEx Coexpression,0.4368
1211	FOXF1	0.4386	ARCHS4 Coexpression,0.4386
1212	PROX1	0.4393	GTEx Coexpression,0.4393
1213	HOXA6	0.44	GTEx Coexpression,0.44
1214	ZNF431	0.4409	Enrichr Queries,0.4409
1215	ZNF25	0.4418	GTEx Coexpression,0.4418
1216	MAF	0.4423	ARCHS4 Coexpression,0.4423
1217	HOMEZ	0.4423	Enrichr Queries,0.4423
1218	ZNF613	0.4429	ARCHS4 Coexpression,0.4429

1219	SP5	0.4449	GTEx Coexpression,0.4449
1220	TCF23	0.4453	ARCHS4 Coexpression,0.4453
1221	ZNF99	0.4459	ARCHS4 Coexpression,0.4459
1222	SATB2	0.4474	GTEx Coexpression,0.4474
1223	ZGLP1	0.4478	ARCHS4 Coexpression,0.4478
1224	L3MBTL4	0.4484	ARCHS4 Coexpression,0.4484
1225	DZIP1	0.4505	GTEx Coexpression,0.4505
1226	PAX3	0.4512	GTEx Coexpression,0.4512
1227	E4F1	0.4512	Literature ChIP-seq,0.4512
1228	TFE3	0.4523	Enrichr Queries,0.4523
1229	ZNF875	0.453	Enrichr Queries,0.453
1230	ATOH8	0.453	GTEx Coexpression,0.453
1231	ZNF621	0.4537	Enrichr Queries,0.4537
1232	GMEB1	0.4549	GTEx Coexpression,0.4549
1233	ZNF440	0.4555	GTEx Coexpression,0.4555
1234	ZNF324	0.4573	Enrichr Queries,0.4573
1235	DMRTA2	0.4574	GTEx Coexpression,0.4574
1236	FOXD4L1	0.4582	ARCHS4 Coexpression,0.4582
1237	ZNF385D	0.4588	ARCHS4 Coexpression,0.4588
1238	PRDM8	0.4592	GTEx Coexpression,0.4592
1239	ZNF445	0.4595	ARCHS4 Coexpression,0.4595
1240	EVX1	0.4599	GTEx Coexpression,0.4599
1241	NPAS1	0.4601	ARCHS4 Coexpression,0.4601
1242	CC2D1A	0.4611	GTEx Coexpression,0.4611
1243	ZBTB17	0.4615	Enrichr Queries,0.4615
1244	GATA5	0.4619	ARCHS4 Coexpression,0.4619
1245	ZBTB48	0.4623	Enrichr Queries,0.4623
1246	GATAD2B	0.463	Enrichr Queries,0.463
1247	ZNF784	0.4644	Enrichr Queries,0.4644
1248	DRGX	0.4655	GTEx Coexpression,0.4655
1249	FOXD4L6	0.4665	Enrichr Queries,0.4665
1250	THAP8	0.4667	GTEx Coexpression,0.4667
1251	ZNF667	0.4672	Enrichr Queries,0.4672
1252	ZNF611	0.4681	ARCHS4 Coexpression,0.4681
1253	MBD6	0.4686	GTEx Coexpression,0.4686
1254	FOXO4	0.4698	GTEx Coexpression,0.4698
1255	ZNF595	0.4708	Enrichr Queries,0.4708
1256	MEOX1	0.4722	Enrichr Queries,0.4722
1257	NKX25	0.4723	GTEx Coexpression,0.4723
1258	SON	0.4729	GTEx Coexpression,0.4729
1259	ZNF396	0.4748	ARCHS4 Coexpression,0.4748
1260	ESRRG	0.4754	GTEx Coexpression,0.4754
1261	ZNF233	0.476	GTEx Coexpression,0.476
1262	MYOD1	0.4772	Enrichr Queries,0.4772
1263	ZNF630	0.4773	GTEx Coexpression,0.4773
1264	FOXB2	0.4792	GTEx Coexpression,0.4792
1265	FIZ1	0.4803	ARCHS4 Coexpression,0.4803
1266	ARHGAP35	0.4804	GTEx Coexpression,0.4804
1267	AIRE	0.4822	Enrichr Queries,0.4822
1268	DMBX1	0.4828	ARCHS4 Coexpression,0.4828
1269	HOXD3	0.4829	GTEx Coexpression,0.4829
1270	ZNF497	0.4829	Enrichr Queries,0.4829
1271	ZNF768	0.4835	GTEx Coexpression,0.4835

1272	ZNF853	0.4848	GTEx Coexpression,0.4848
1273	OVOL2	0.4872	Enrichr Queries,0.4872
1274	TBX1	0.4879	GTEx Coexpression,0.4879
1275	MYF5	0.4879	Enrichr Queries,0.4879
1276	SOX5	0.4889	ARCHS4 Coexpression,0.4889
1277	NFIX	0.4893	Enrichr Queries,0.4893
1278	ZFHX3	0.491	GTEx Coexpression,0.491
1279	LHX3	0.4922	GTEx Coexpression,0.4922
1280	ZFHX2	0.4928	GTEx Coexpression,0.4928
1281	ZNF208	0.4939	ARCHS4 Coexpression,0.4939
1282	RHOXF2B	0.4941	GTEx Coexpression,0.4941
1283	MYRFL	0.4947	GTEx Coexpression,0.4947
1284	NFE2	0.4949	ReMap ChIP-seq,0.4949
1285	ZNF467	0.495	Enrichr Queries,0.495
1286	ZNF710	0.496	GTEx Coexpression,0.496
1287	GLI3	0.4966	GTEx Coexpression,0.4966
1288	ZSCAN1	0.4975	ARCHS4 Coexpression,0.4975
1289	NR1D1	0.4986	Enrichr Queries,0.4986
1290	LHX9	0.4988	ARCHS4 Coexpression,0.4988
1291	CENPBD1	0.4991	GTEx Coexpression,0.4991
1292	ZNF461	0.5	ARCHS4 Coexpression,0.5
1293	FOXQ1	0.5003	GTEx Coexpression,0.5003
1294	ZNF568	0.5018	ARCHS4 Coexpression,0.5018
1295	FOXF2	0.5021	Enrichr Queries,0.5021
1296	NEUROD4	0.5028	GTEx Coexpression,0.5028
1297	THRA	0.5028	Enrichr Queries,0.5028
1298	ZNF74	0.5031	ARCHS4 Coexpression,0.5031
1299	ZNF626	0.5037	ARCHS4 Coexpression,0.5037
1300	TBX2	0.504	GTEx Coexpression,0.504
1301	CREB5	0.5043	ARCHS4 Coexpression,0.5043
1302	HOXC10	0.5064	Enrichr Queries,0.5064
1303	GLI2	0.5074	ARCHS4 Coexpression,0.5074
1304	HNF4G	0.5078	GTEx Coexpression,0.5078
1305	ZNF746	0.5078	Enrichr Queries,0.5078
1306	ATOH1	0.5103	GTEx Coexpression,0.5103
1307	VSX2	0.5104	ARCHS4 Coexpression,0.5104
1308	ZNF75A	0.5107	Enrichr Queries,0.5107
1309	DACH2	0.5117	ARCHS4 Coexpression,0.5117
1310	MLXIPL	0.5121	Enrichr Queries,0.5121
1311	CREB3L3	0.5129	ARCHS4 Coexpression,0.5129
1312	BATF2	0.514	GTEx Coexpression,0.514
1313	SOX14	0.5152	GTEx Coexpression,0.5152
1314	ZNF804A	0.5166	ARCHS4 Coexpression,0.5166
1315	HOXD1	0.5171	GTEx Coexpression,0.5171
1316	ALX3	0.5177	GTEx Coexpression,0.5177
1317	ETS2	0.5178	Enrichr Queries,0.5178
1318	TCF21	0.5183	Literature ChIP-seq,0.5183
1319	GLIS2	0.5185	Enrichr Queries,0.5185
1320	ETV1	0.5196	GTEx Coexpression,0.5196
1321	MAFA	0.5199	Enrichr Queries,0.5199
1322	RARA	0.5207	Enrichr Queries,0.5207
1323	ISX	0.5208	GTEx Coexpression,0.5208
1324	HELT	0.5221	ARCHS4 Coexpression,0.5221

1325	GRHL1	0.5235	Enrichr Queries,0.5235
1326	ARID3C	0.5246	GTEx Coexpression,0.5246
1327	MYOG	0.5254	ENCODE ChIP-seq,0.5254
1328	OLIG3	0.5264	ARCHS4 Coexpression,0.5264
1329	SRY	0.5264	GTEx Coexpression,0.5264
1330	SP100	0.5277	GTEx Coexpression,0.5277
1331	ZNF205	0.5283	GTEx Coexpression,0.5283
1332	TBX19	0.5285	Enrichr Queries,0.5285
1333	NHLH2	0.5289	ARCHS4 Coexpression,0.5289
1334	ZNF19	0.5289	GTEx Coexpression,0.5289
1335	CDX1	0.5299	Enrichr Queries,0.5299
1336	LTF	0.5301	ARCHS4 Coexpression,0.5301
1337	FOXE1	0.5302	GTEx Coexpression,0.5302
1338	ONECUT2	0.5307	ARCHS4 Coexpression,0.5307
1339	NKX61	0.5308	GTEx Coexpression,0.5308
1340	TFAP2E	0.5314	GTEx Coexpression,0.5314
1341	ZNF804B	0.532	GTEx Coexpression,0.532
1342	NFKB2	0.5328	Enrichr Queries,0.5328
1343	ATF5	0.5335	Enrichr Queries,0.5335
1344	HOXD11	0.5338	ARCHS4 Coexpression,0.5338
1345	INSM1	0.5342	Enrichr Queries,0.5342
1346	HOXB8	0.535	ARCHS4 Coexpression,0.535
1347	SP6	0.5356	Enrichr Queries,0.5356
1348	PKNOX2	0.5362	ARCHS4 Coexpression,0.5362
1349	ZNF777	0.5383	GTEx Coexpression,0.5383
1350	ZNF385C	0.5389	GTEx Coexpression,0.5389
1351	CSRNP3	0.5405	ARCHS4 Coexpression,0.5405
1352	HES5	0.5406	Enrichr Queries,0.5406
1353	EGR3	0.5413	Enrichr Queries,0.5413
1354	NPAS4	0.542	GTEx Coexpression,0.542
1355	SOX21	0.543	ARCHS4 Coexpression,0.543
1356	GPBP1L1	0.5436	ARCHS4 Coexpression,0.5436
1357	TRAFD1	0.5445	GTEx Coexpression,0.5445
1358	AHDC1	0.5449	Enrichr Queries,0.5449
1359	PBX4	0.5461	ARCHS4 Coexpression,0.5461
1360	HOXB5	0.547	GTEx Coexpression,0.547
1361	ZNF605	0.547	Enrichr Queries,0.547
1362	ZNF540	0.5485	ARCHS4 Coexpression,0.5485
1363	ZBTB7B	0.5488	GTEx Coexpression,0.5488
1364	SNAI3	0.5499	Enrichr Queries,0.5499
1365	LHX4	0.5501	GTEx Coexpression,0.5501
1366	OLIG1	0.5516	ARCHS4 Coexpression,0.5516
1367	OSR1	0.5528	ARCHS4 Coexpression,0.5528
1368	GRHL3	0.5534	Enrichr Queries,0.5534
1369	ZNF556	0.5557	GTEx Coexpression,0.5557
1370	ZNF18	0.557	Enrichr Queries,0.557
1371	PRDM6	0.5571	ARCHS4 Coexpression,0.5571
1372	ZNF329	0.5577	Enrichr Queries,0.5577
1373	ZNF479	0.5577	ARCHS4 Coexpression,0.5577
1374	IRF9	0.5589	ReMap ChIP-seq,0.5589
1375	NR2E3	0.559	ARCHS4 Coexpression,0.559
1376	MXD1	0.5591	Enrichr Queries,0.5591
1377	ZNF785	0.5596	ARCHS4 Coexpression,0.5596

1378	ZNF793	0.562	ARCHS4 Coexpression,0.562
1379	NFE4	0.5625	GTEx Coexpression,0.5625
1380	ZNF783	0.5638	GTEx Coexpression,0.5638
1381	TBX18	0.5645	ARCHS4 Coexpression,0.5645
1382	ZNF365	0.5648	Enrichr Queries,0.5648
1383	CENPB	0.5657	ARCHS4 Coexpression,0.5657
1384	STAT4	0.5671	Literature ChIP-seq,0.5671
1385	FOXL1	0.5676	ARCHS4 Coexpression,0.5676
1386	ZNF668	0.5677	Enrichr Queries,0.5677
1387	ZNF76	0.5684	Enrichr Queries,0.5684
1388	ZNF142	0.5698	Enrichr Queries,0.5698
1389	ZNF835	0.5706	ARCHS4 Coexpression,0.5706
1390	ZNF500	0.5725	ARCHS4 Coexpression,0.5725
1391	CAMTA1	0.5726	Enrichr Queries,0.5726
1392	FBXL19	0.5731	ARCHS4 Coexpression,0.5731
1393	ZNF154	0.5734	Enrichr Queries,0.5734
1394	SOX12	0.5737	GTEx Coexpression,0.5737
1395	ZNF213	0.5741	Enrichr Queries,0.5741
1396	HOXA11	0.575	GTEx Coexpression,0.575
1397	ZNF157	0.5755	Enrichr Queries,0.5755
1398	RBCK1	0.5806	GTEx Coexpression,0.5806
1399	ZNF470	0.5811	ARCHS4 Coexpression,0.5811
1400	MNT	0.5812	Enrichr Queries,0.5812
1401	PGR	0.5817	ARCHS4 Coexpression,0.5817
1402	ATF7	0.5825	ReMap ChIP-seq,0.5825
1403	ASCL2	0.5826	Enrichr Queries,0.5826
1404	ZNF385B	0.5831	GTEx Coexpression,0.5831
1405	ZNF101	0.5842	ARCHS4 Coexpression,0.5842
1406	NOTO	0.5843	GTEx Coexpression,0.5843
1407	BNC2	0.5848	ARCHS4 Coexpression,0.5848
1408	FERD3L	0.5862	GTEx Coexpression,0.5862
1409	ZNF580	0.5881	GTEx Coexpression,0.5881
1410	MTERF4	0.5912	GTEx Coexpression,0.5912
1411	CASZ1	0.5912	Enrichr Queries,0.5912
1412	RHOXF1	0.5919	Enrichr Queries,0.5919
1413	WIZ	0.5921	ARCHS4 Coexpression,0.5921
1414	POU4F3	0.5924	GTEx Coexpression,0.5924
1415	ZSCAN18	0.5926	Enrichr Queries,0.5926
1416	ZNF493	0.5946	ARCHS4 Coexpression,0.5946
1417	DMRT1	0.5949	GTEx Coexpression,0.5949
1418	ZNF319	0.5962	Enrichr Queries,0.5962
1419	CEBPE	0.5969	Enrichr Queries,0.5969
1420	KAT7	0.5971	ARCHS4 Coexpression,0.5971
1421	NKX24	0.5974	GTEx Coexpression,0.5974
1422	GBX1	0.5993	GTEx Coexpression,0.5993
1423	ZNF546	0.5995	ARCHS4 Coexpression,0.5995
1424	SCML4	0.6004	Enrichr Queries,0.6004
1425	GFI1	0.6019	Enrichr Queries,0.6019
1426	SOX18	0.6024	GTEx Coexpression,0.6024
1427	L3MBTL1	0.6026	ARCHS4 Coexpression,0.6026
1428	IRF2	0.6027	ReMap ChIP-seq,0.6027
1429	IRF8	0.6037	Literature ChIP-seq,0.6037
1430	BCL11B	0.6038	ARCHS4 Coexpression,0.6038

1431	ZBTB8B	0.6044	ARCHS4 Coexpression,0.6044
1432	ASCL1	0.6055	GTEx Coexpression,0.6055
1433	PRR12	0.6061	GTEx Coexpression,0.6061
1434	POU3F3	0.6069	ARCHS4 Coexpression,0.6069
1435	ZNF705A	0.6075	Enrichr Queries,0.6075
1436	HOXA13	0.608	GTEx Coexpression,0.608
1437	ZNF688	0.609	Enrichr Queries,0.609
1438	HOXA3	0.6092	GTEx Coexpression,0.6092
1439	ZNF524	0.6123	GTEx Coexpression,0.6123
1440	FOXD4	0.6125	Enrichr Queries,0.6125
1441	DMRT3	0.6136	ARCHS4 Coexpression,0.6136
1442	ZNF510	0.6161	Enrichr Queries,0.6161
1443	ZNF716	0.6179	GTEx Coexpression,0.6179
1444	ZNF8	0.6179	ARCHS4 Coexpression,0.6179
1445	HSF1	0.6186	ARCHS4 Coexpression,0.6186
1446	ZNF474	0.6189	Enrichr Queries,0.6189
1447	NKX26	0.6192	GTEx Coexpression,0.6192
1448	C11ORF95	0.6204	GTEx Coexpression,0.6204
1449	FOXI3	0.621	ARCHS4 Coexpression,0.621
1450	FOXR2	0.6217	GTEx Coexpression,0.6217
1451	PAX7	0.6235	GTEx Coexpression,0.6235
1452	TSHZ3	0.6254	Enrichr Queries,0.6254
1453	ZGPAT	0.6266	GTEx Coexpression,0.6266
1454	EMX2	0.6278	ARCHS4 Coexpression,0.6278
1455	MESP2	0.6279	GTEx Coexpression,0.6279
1456	NKX23	0.6285	GTEx Coexpression,0.6285
1457	LHX1	0.6296	ARCHS4 Coexpression,0.6296
1458	LYL1	0.6296	ReMap ChIP-seq,0.6296
1459	VSX1	0.6304	GTEx Coexpression,0.6304
1460	ZNF705B	0.6321	ARCHS4 Coexpression,0.6321
1461	ZNF648	0.6322	GTEx Coexpression,0.6322
1462	IRX2	0.6325	Enrichr Queries,0.6325
1463	ETV7	0.6333	ARCHS4 Coexpression,0.6333
1464	LMX1B	0.6339	ARCHS4 Coexpression,0.6339
1465	BARHL2	0.6341	GTEx Coexpression,0.6341
1466	SP8	0.6347	GTEx Coexpression,0.6347
1467	FOXD4L3	0.6351	ARCHS4 Coexpression,0.6351
1468	UNCX	0.636	GTEx Coexpression,0.636
1469	ZIC1	0.6396	Enrichr Queries,0.6396
1470	ZNF366	0.6403	Enrichr Queries,0.6403
1471	POU2F3	0.6403	GTEx Coexpression,0.6403
1472	PCGF2	0.6407	ARCHS4 Coexpression,0.6407
1473	ZNF704	0.6409	GTEx Coexpression,0.6409
1474	ZNF70	0.6434	GTEx Coexpression,0.6434
1475	PAX9	0.6447	GTEx Coexpression,0.6447
1476	ARID5A	0.6459	GTEx Coexpression,0.6459
1477	KDM2B	0.6468	ARCHS4 Coexpression,0.6468
1478	FOXI1	0.6474	Enrichr Queries,0.6474
1479	NR1I3	0.6484	GTEx Coexpression,0.6484
1480	GTF2I	0.6493	ARCHS4 Coexpression,0.6493
1481	POU6F2	0.6499	ARCHS4 Coexpression,0.6499
1482	MYT1	0.6515	GTEx Coexpression,0.6515
1483	ARNT2	0.6524	Enrichr Queries,0.6524

1484	ZBTB45	0.6529	ARCHS4 Coexpression,0.6529
1485	IKZF1	0.6532	ReMap ChIP-seq,0.6532
1486	TLX1	0.6536	ARCHS4 Coexpression,0.6536
1487	JDP2	0.654	GTEx Coexpression,0.654
1488	HOXB6	0.6574	Enrichr Queries,0.6574
1489	ZBTB34	0.6602	GTEx Coexpression,0.6602
1490	ZNF48	0.6609	ARCHS4 Coexpression,0.6609
1491	SOX1	0.6634	ARCHS4 Coexpression,0.6634
1492	ZNF646	0.6646	GTEx Coexpression,0.6646
1493	NEUROD2	0.6658	GTEx Coexpression,0.6658
1494	HOXB3	0.6665	GTEx Coexpression,0.6665
1495	MYCL	0.6671	ARCHS4 Coexpression,0.6671
1496	DBP	0.6674	Enrichr Queries,0.6674
1497	PRDM12	0.6689	ARCHS4 Coexpression,0.6689
1498	NPAS3	0.6744	ARCHS4 Coexpression,0.6744
1499	HOXD13	0.6745	GTEx Coexpression,0.6745
1500	EN1	0.6777	GTEx Coexpression,0.6777
1501	ZNF775	0.6781	Enrichr Queries,0.6781
1502	ZBTB18	0.6801	GTEx Coexpression,0.6801
1503	ZBTB7C	0.6802	Enrichr Queries,0.6802
1504	ZNF541	0.6806	ARCHS4 Coexpression,0.6806
1505	TEF	0.6809	Enrichr Queries,0.6809
1506	NR1I2	0.6812	ARCHS4 Coexpression,0.6812
1507	CXXC5	0.6814	GTEx Coexpression,0.6814
1508	ZIC4	0.6845	GTEx Coexpression,0.6845
1509	NFATC2	0.6859	Enrichr Queries,0.6859
1510	PURB	0.6864	GTEx Coexpression,0.6864
1511	MZF1	0.688	Enrichr Queries,0.688
1512	IRX6	0.6886	ARCHS4 Coexpression,0.6886
1513	IRF5	0.6895	GTEx Coexpression,0.6895
1514	HOXD12	0.6907	GTEx Coexpression,0.6907
1515	ELF4	0.6926	GTEx Coexpression,0.6926
1516	ZNF575	0.6929	ARCHS4 Coexpression,0.6929
1517	OVOL3	0.6951	GTEx Coexpression,0.6951
1518	HLX	0.6973	Enrichr Queries,0.6973
1519	ZNF865	0.6994	GTEx Coexpression,0.6994
1520	TLX3	0.7007	GTEx Coexpression,0.7007
1521	TERB1	0.7009	ARCHS4 Coexpression,0.7009
1522	TBPL2	0.7015	ARCHS4 Coexpression,0.7015
1523	NACC2	0.7026	GTEx Coexpression,0.7026
1524	FOXC1	0.705	GTEx Coexpression,0.705
1525	RORB	0.7075	GTEx Coexpression,0.7075
1526	SOX3	0.7082	GTEx Coexpression,0.7082
1527	ALX4	0.7082	ARCHS4 Coexpression,0.7082
1528	SAFB2	0.71	GTEx Coexpression,0.71
1529	ZBTB22	0.7115	Enrichr Queries,0.7115
1530	PITX3	0.7119	GTEx Coexpression,0.7119
1531	GSX1	0.7144	ARCHS4 Coexpression,0.7144
1532	FOXD2	0.7144	Enrichr Queries,0.7144
1533	PAX1	0.7174	ARCHS4 Coexpression,0.7174
1534	FOXL2	0.7175	GTEx Coexpression,0.7175
1535	CUX2	0.7187	GTEx Coexpression,0.7187
1536	VENTX	0.7193	ARCHS4 Coexpression,0.7193

1537	ZNF414	0.7201	Enrichr Queries,0.7201
1538	HOXD9	0.7212	GTEx Coexpression,0.7212
1539	TBR1	0.7224	ARCHS4 Coexpression,0.7224
1540	ZNF705D	0.723	ARCHS4 Coexpression,0.723
1541	KLF2	0.7236	Enrichr Queries,0.7236
1542	IRX3	0.7265	Enrichr Queries,0.7265
1543	VAX2	0.7285	ARCHS4 Coexpression,0.7285
1544	HES2	0.7293	Enrichr Queries,0.7293
1545	SOX8	0.7322	Enrichr Queries,0.7322
1546	EBF4	0.7337	GTEx Coexpression,0.7337
1547	TGIF2LY	0.7353	ARCHS4 Coexpression,0.7353
1548	TBX4	0.7355	GTEx Coexpression,0.7355
1549	AEBP1	0.7371	ARCHS4 Coexpression,0.7371
1550	CBLL2	0.7372	Enrichr Queries,0.7372
1551	HSF4	0.74	Enrichr Queries,0.74
1552	LBX1	0.7405	GTEx Coexpression,0.7405
1553	PBX2	0.7429	Enrichr Queries,0.7429
1554	ZNF219	0.7445	ARCHS4 Coexpression,0.7445
1555	MXD4	0.7455	GTEx Coexpression,0.7455
1556	ZNF385A	0.7472	Enrichr Queries,0.7472
1557	SEBOX	0.7479	Enrichr Queries,0.7479
1558	MTF1	0.748	GTEx Coexpression,0.748
1559	NHLH1	0.75	ARCHS4 Coexpression,0.75
1560	PIN1	0.7537	ARCHS4 Coexpression,0.7537
1561	NKX32	0.7579	GTEx Coexpression,0.7579
1562	ZNF771	0.758	ARCHS4 Coexpression,0.758
1563	BHLHE22	0.7586	GTEx Coexpression,0.7586
1564	HNF1A	0.7604	GTEx Coexpression,0.7604
1565	SCRT2	0.761	GTEx Coexpression,0.761
1566	AKNA	0.7614	Enrichr Queries,0.7614
1567	CIC	0.7628	Enrichr Queries,0.7628
1568	ZIM3	0.7629	ARCHS4 Coexpression,0.7629
1569	CPXCR1	0.7635	ARCHS4 Coexpression,0.7635
1570	CAMTA2	0.7642	Enrichr Queries,0.7642
1571	PAX2	0.7666	GTEx Coexpression,0.7666
1572	ZNF687	0.7685	Enrichr Queries,0.7685
1573	EVX2	0.7707	Enrichr Queries,0.7707
1574	EN2	0.771	GTEx Coexpression,0.771
1575	HMX1	0.7714	Enrichr Queries,0.7714
1576	BARX2	0.7746	ARCHS4 Coexpression,0.7746
1577	IRX5	0.7758	ARCHS4 Coexpression,0.7758
1578	ZNF554	0.7772	GTEx Coexpression,0.7772
1579	CENPX	0.7778	GTEx Coexpression,0.7778
1580	ZNF831	0.7799	Enrichr Queries,0.7799
1581	IRX4	0.785	ARCHS4 Coexpression,0.785
1582	ZMAT4	0.7869	ARCHS4 Coexpression,0.7869
1583	SOX30	0.7885	Enrichr Queries,0.7885
1584	ZNF705G	0.7899	ARCHS4 Coexpression,0.7899
1585	FOXI2	0.7984	Enrichr Queries,0.7984
1586	BARHL1	0.799	GTEx Coexpression,0.799
1587	ZNF628	0.8016	ARCHS4 Coexpression,0.8016
1588	HES7	0.8052	GTEx Coexpression,0.8052
1589	ZBED9	0.8083	GTEx Coexpression,0.8083

1590	LHX5	0.8084	ARCHS4 Coexpression,0.8084
1591	NAIF1	0.8108	ARCHS4 Coexpression,0.8108
1592	IRF7	0.8114	GTEx Coexpression,0.8114
1593	TBX6	0.8152	GTEx Coexpression,0.8152
1594	MYT1L	0.8177	GTEx Coexpression,0.8177
1595	HOXD10	0.8194	ARCHS4 Coexpression,0.8194
1596	SGSM2	0.8219	ARCHS4 Coexpression,0.8219
1597	SKI	0.8237	ARCHS4 Coexpression,0.8237
1598	MEIS3	0.8239	GTEx Coexpression,0.8239
1599	ZNF513	0.8283	Enrichr Queries,0.8283
1600	BHLHA9	0.8289	GTEx Coexpression,0.8289
1601	FOXN1	0.8291	Enrichr Queries,0.8291
1602	MYPOP	0.8299	ARCHS4 Coexpression,0.8299
1603	NKX62	0.8307	GTEx Coexpression,0.8307
1604	HEYL	0.8376	GTEx Coexpression,0.8376
1605	FOXJ1	0.8376	Enrichr Queries,0.8376
1606	SP110	0.855	ARCHS4 Coexpression,0.855
1607	NKX11	0.8556	GTEx Coexpression,0.8556
1608	HSFY1	0.8563	GTEx Coexpression,0.8563
1609	DLX3	0.8568	Enrichr Queries,0.8568
1610	PHF1	0.8569	GTEx Coexpression,0.8569
1611	FLYWCH1	0.8575	GTEx Coexpression,0.8575
1612	SP140	0.86	ARCHS4 Coexpression,0.86
1613	POU6F1	0.8618	Enrichr Queries,0.8618
1614	FAM170A	0.8667	ARCHS4 Coexpression,0.8667
1615	HSFY2	0.8704	ARCHS4 Coexpression,0.8704
1616	ASCL5	0.8718	GTEx Coexpression,0.8718
1617	SPZ1	0.8821	ARCHS4 Coexpression,0.8821
1618	ZNF276	0.8889	Enrichr Queries,0.8889
1619	ZNF683	0.8939	Enrichr Queries,0.8939
1620	TFAP2B	0.8986	GTEx Coexpression,0.8986
1621	ORCS8MEF2	0.8993	ARCHS4 Coexpression,0.8993
1622	CCDC17	0.9115	ARCHS4 Coexpression,0.9115
1623	KCNIP3	0.9197	GTEx Coexpression,0.9197
1624	HSFX2	0.9201	ARCHS4 Coexpression,0.9201
1625	FEZF2	0.9203	GTEx Coexpression,0.9203
1626	MYRF	0.9247	GTEx Coexpression,0.9247
1627	DC169SOHL	0.9281	ARCHS4 Coexpression,0.9281
1628	ZNF517	0.9459	Enrichr Queries,0.9459
1629	SOHLH1	0.9544	Enrichr Queries,0.9544
1630	SP140L	0.9546	GTEx Coexpression,0.9546
1631	SCX	0.9693	ARCHS4 Coexpression,0.9693
1632	SCRT1	0.9939	ARCHS4 Coexpression,0.9939
