# Supplementary material for: Deciphering differences in DNA methylation and transcriptome profiles of oocytes from pigs with high and low developmental competence
Source: Environ Epigenet. 2025 Jun 3;11(1):dvaf018. doi: 10.1093/eep/dvaf018 (PMC12418950; doi:10.1093/eep/dvaf018)
Supplement: dvaf018_Supplemental_Files [file dvaf018_supplemental_files.zip › Sup table 9.pdf]

chr	start	end	group1	group2	n1	n2	estimate1	estimate2	estimate	statistic	p- value	adj.p.value	name
chr12	61029157	61033157	in_vitro	in_vivo	12	14	60.9133333	20.0107143	40.902619	c(t = 4.14691	0.000969	0.89488417	FLCN
chr16	17529952	17533952	in_vitro	in_vivo	10	12	72.582	23.3366667	49.2453333	c(t = 3.58581	0.00185	0.89488417	CDH6
chr8	53702714	53706714	in_vitro	in_vivo	9	9	25.1844444	7.88888889	17.2955556	c(t = 2.97538	0.00999	0.89488417	ENSSSCG00000031750
chr4	118945440	118949440	in_vitro	in_vivo	13	13	41.4138462	13.2407692	28.1730769	c(t = 2.78266	0.0118	0.89488417	PLPPR5
chr6	70876983	70880983	in_vitro	in_vivo	12	11	40.4825	15.9618182	24.5206818	c(t = 2.80488	0.0121	0.89488417	CASZ1
chr9	133385162	133389162	in_vitro	in_vivo	10	12	51.884	24.045	27.839	c(t = 2.73316	0.0128	0.89488417	ENSSSCG00000058599
chr2	66142777	66146777	in_vitro	in_vivo	14	10	33.0235714	8.251	24.7725714	c(t = 2.74656	0.0147	0.89488417	KLF1
chr3	39737308	39741308	in_vitro	in_vivo	11	10	34.0690909	9.933	24.1360909	c(t = 2.84646	0.015	0.89488417	RNPS1
chr1	236392306	236396306	in_vitro	in_vivo	10	12	34.652	13.6666667	20.9853333	c(t = 2.80653	0.0152	0.89488417	CA9
chr7	52254159	52258159	in_vitro	in_vivo	13	14	50.69	24.4114286	26.2785714	c(t = 2.65872	0.0165	0.89488417	AP3B2
chr6	54705237	54709237	in_vitro	in_vivo	10	9	37.887	12.4666667	25.4203333	c(t = 2.56571	0.0216	0.89488417	ENSSSCG00000003177
chr1	253973973	253977973	in_vitro	in_vivo	11	12	34.9545455	11.8408333	23.1137121	c(t = 2.60968	0.0226	0.89488417	BSPRY
chr17	41489534	41493534	in_vitro	in_vivo	14	10	38.2442857	18.329	19.9152857	c(t = 2.38348	0.0264	0.89488417	SNORA71
chr15	46085696	46089696	in_vitro	in_vivo	12	11	44.25	17.0327273	27.2172727	c(t = 2.40741	0.0266	0.89488417	HELT
chr6	54142020	54146020	in_vitro	in_vivo	9	10	30.6655556	12.838	17.8275556	c(t = 2.44178	0.0286	0.89488417	PLEKHA4
chr2	71471389	71475389	in_vitro	in_vivo	9	11	40.1355556	19.3827273	20.7528283	c(t = 2.51710	0.03	0.89488417	ENSSSCG00000033183
chr3	17619269	17623269	in_vitro	in_vivo	13	14	31.1723077	10.57	20.6023077	c(t = 2.38975	0.033	0.89488417	ENSSSCG00000058184
chr8	5888765	5892765	in_vitro	in_vivo	11	11	39.0654545	17.5409091	21.5245455	c(t = 2.31237	0.0339	0.89488417	STX18
chr14	49838305	49842305	in_vitro	in_vivo	11	14	41.4418182	18.3607143	23.0811039	c(t = 2.36780	0.0343	0.89488417	MIF
chr15	76989928	76993928	in_vitro	in_vivo	11	14	38.9490909	11.2442857	27.7048052	c(t = 2.34167	0.0363	0.89488417	GAD1
chr6	53078236	53082236	in_vitro	in_vivo	11	12	32.1781818	10.6233333	21.5548485	c(t = 2.32506	0.0365	0.89488417	ENSSSCG00000037986
chr1	268614034	268618034	in_vitro	in_vivo	11	12	37.9427273	15.4666667	22.4760606	c(t = 2.28174	0.0377	0.89488417	ENSSSCG00000022295
chr6	78239624	78243624	in_vitro	in_vivo	12	12	25.8816667	6.57916667	19.3025	c(t = 2.26160	0.0404	0.89488417	OTUD3
chrX	42076206	42080206	in_vitro	in_vivo	12	14	45.0608333	23.9371429	21.1236905	c(t = 2.15408	0.0423	0.89488417	ARAF
chr6	101814967	101818967	in_vitro	in_vivo	9	9	37.6511111	17.6522222	19.9988889	c(t = 2.19675	0.0439	0.89488417	AKAIN1
chr6	49347708	49351708	in_vitro	in_vivo	12	10	34.9966667	11.154	23.8426667	c(t = 2.19011	0.0458	0.89488417	B9D2
chr2	71468445	71472445	in_vitro	in_vivo	11	10	41.9654545	19.199	22.7664545	c(t = 2.13656	0.0472	0.89488417	TRAPPC5
chr4	134107	138107	in_vitro	in_vivo	10	14	44.266	22.2885714	21.9774286	c(t = 2.11452	0.0475	0.89488417	ENSSSCG00000029830
chr15	46191746	46195746	in_vitro	in_vivo	11	10	26.8654545	8.543	18.3224545	c(t = 2.18902	0.0486	0.89488417	SLC25A4
chr14	140327081	140331081	in_vitro	in_vivo	9	13	46.5466667	24.7969231	21.7497436	c(t = 2.11603	0.0493	0.89488417	ENSSSCG00000053237
chr2	7207377	7211377	in_vitro	in_vivo	9	9	35.25	11.8577778	23.3922222	c(t = 2.23730	0.0494	0.89488417	ENSSSCG00000040674
chr14	51401923	51405923	in_vitro	in_vivo	11	10	71.5827273	35.89	35.6927273	c(t = 4.68229	0.00019	0.58885667	ARVCF
chr5	6536907	6540907	in_vitro	in_vivo	10	13	30.391	71.8161538	-41.425154	c(t = -4.4874	0.000214	0.58885667	CYP2D6
chr6	45179443	45183443	in_vitro	in_vivo	11	12	27.5463636	61.8891667	-34.342803	c(t = -3.4780	0.00225	0.89488417	HSPB6
chr7	17693258	17697258	in_vitro	in_vivo	11	11	70.9645455	28.3190909	42.6454545	c(t = 3.41087	0.00306	0.89488417	ENSSSCG00000001083
chr1	268310767	268314767	in_vitro	in_vivo	10	9	35.819	74.0577778	-38.238778	c(t = -3.4148	0.00345	0.89488417	U2
chr3	39128777	39132777	in_vitro	in_vivo	10	12	67.187	28.8883333	38.2986667	c(t = 3.19013	0.00607	0.89488417	MMP25
chr2	59399246	59403246	in_vitro	in_vivo	9	10	59.3966667	27.916	31.4806667	c(t = 3.03742	0.00744	0.89488417	LRRC25
chr5	22880669	22884669	in_vitro	in_vivo	9	13	71.9922222	30.5076923	41.4845299	c(t = 3.00473	0.00902	0.89488417	ENSSSCG00000036029
chr2	71517392	71521392	in_vitro	in_vivo	9	12	70.6377778	37.0883333	33.5494444	c(t = 2.85172	0.0103	0.89488417	PET100
chr14	48822977	48826977	in_vitro	in_vivo	9	12	32.0044444	69.1933333	-37.188889	c(t = -2.95681	0.0105	0.89488417	ENSSSCG00000039180
chr7	30513925	30517925	in_vitro	in_vivo	11	10	58.53	28.702	29.828	c(t = 2.72474	0.0147	0.89488417	SPDEF
chrY	8587526	8591526	in_vitro	in_vivo	11	12	56.5254545	29.7758333	26.7496212	c(t = 2.72462	0.0168	0.89488417	ENSSSCG00000057434
chr6	3007259	3011259	in_vitro	in_vivo	10	11	42.576	70.4509091	-27.874909	c(t = -2.6420	0.0175	0.89488417	ENSSSCG00000054743
chr13	168788207	168792207	in_vitro	in_vivo	9	9	62.0066667	25.1933333	36.8133333	c(t = 2.64679	0.0193	0.89488417	VGLL3
chr3	40996504	41000504	in_vitro	in_vivo	10	9	51.458	28.5577778	22.9002222	c(t = 2.52145	0.0241	0.89488417	GNG13
chr5	36272165	36276165	in_vitro	in_vivo	11	13	59.7509091	27.9184615	31.8324476	c(t = 2.39840	0.0254	0.89488417	TRHDE
chr9	5762261	5766261	in_vitro	in_vivo	10	11	72.413	46.0372727	26.3757273	c(t = 2.43411	0.027	0.89488417	ENSSSCG00000014779
chr11	67650193	67654193	in_vitro	in_vivo	11	10	55.4590909	72.622	-17.162909	c(t = -2.3490	0.0298	0.89488417	SLC15A1
chr4	93430330	93434330	in_vitro	in_vivo	12	10	64.7908333	40.218	24.5728333	c(t = 2.31500	0.0315	0.89488417	BCAN
chr11	4192605	4196605	in_vitro	in_vivo	10	14	36.376	56.3871429	-20.011143	c(t = -2.2998	0.0324	0.89488417	ENSSSCG00000056808
chr12	1090329	1094329	in_vitro	in_vivo	10	12	67.118	42.0058333	25.1121667	c(t = 2.35614	0.0328	0.89488417	ALYREF
chrX	110757000	110761000	in_vitro	in_vivo	12	11	27.4733333	50.4309091	-22.957576	c(t = -2.2981	0.0346	0.89488417	ENSSSCG00000034882
chr2	51241294	51245294	in_vitro	in_vivo	9	13	53.5188889	28.9315385	24.5873504	c(t = 2.25226	0.0358	0.89488417	MRPL55
chrX	42720730	42724730	in_vitro	in_vivo	12	11	27.4891667	57.1918182	-29.702652	c(t = -2.2944	0.036	0.89488417	WDR13
chrX	110758028	110762028	in_vitro	in_vivo	12	11	25.09	46.3027273	-21.212727	c(t = -2.2042	0.0411	0.89488417	ENSSSCG00000061518
chr6	47140702	47144702	in_vitro	in_vivo	9	10	64.9733333	43.075	21.8983333	c(t = 2.18390	0.0457	0.89488417	DPF1
chr13	22872429	22876429	in_vitro	in_vivo	9	10	43.0166667	71.178	-28.161333	c(t = -2.1416	0.0474	0.89488417	ENSSSCG00000062897
chr4	96092147	96096147	in_vitro	in_vivo	9	10	28.0322222	48.792	-20.759778	c(t = -2.1319	0.0483	0.89488417	S100A5
chr1	2592117	2596117	in_vitro	in_vivo	9	12	48.1711111	67.2758333	-19.104722	c(t = -2.1186	0.0488	0.89488417	ENSSSCG00000062461
chr12	2074491	2078491	in_vitro	in_vivo	9	9	53.4955556	92.8877778	-39.392222	c(t = -4.2337	0.00206	0.89488417	ENDOV
chr3	664918	668918	in_vitro	in_vivo	9	12	58.9533333	82.5691667	-23.615833	c(t = -3.5051	0.00527	0.89488417	ssc-mir-339-1
chr8	52324545	52328545	in_vitro	in_vivo	9	11	38.4322222	84.2327273	-45.800505	c(t = -3.0894	0.0108	0.89488417	TKTL2
chr1	236946636	236950636	in_vitro	in_vivo	11	13	54.7272727	77.0061538	-22.278881	c(t = -2.6327	0.0186	0.89488417	CCIN
chr6	2991213	2995213	in_vitro	in_vivo	9	11	66.0933333	89.7536364	-23.660303	c(t = -2.7461	0.0206	0.89488417	ENSSSCG00000057541
chr6	72512079	72516079	in_vitro	in_vivo	9	10	48.1822222	78.086	-29.903778	c(t = -2.5143	0.0255	0.89488417	ENSSSCG00000059184
chr1	1554460	1558460	in_vitro	in_vivo	10	11	46.11	76.37	-30.26	c(t = -2.4212	0.0265	0.89488417	ENSSSCG00000054333
chr7	59479412	59483412	in_vitro	in_vivo	10	9	74.5	89.12	-14.62	c(t = -2.4068	0.0308	0.89488417	LOXL1
chr14	135099610	135103610	in_vitro	in_vivo	10	9	55.323	79.27	-23.947	c(t = -2.2210	0.0413	0.89488417	UROS
chr2	138371189	138375189	in_vitro	in_vivo	9	9	55.3844444	80.3866667	-25.002222	c(t = -2.2201	0.0486	0.89488417	TRPC7
chr1	169723649	169727649	in_vitro	in_vivo	9	10	20.2633333	5.025	15.2383333	c(t = 3.54190	0.00488	0.89488417	PNN
chr4	98105360	98109360	in_vitro	in_vivo	11	11	10.0481818	23.7590909	-13.710909	c(t = -3.0368	0.00725	0.89488417	SEMA6C
chr1	55406112	55410112	in_vitro	in_vivo	12	12	12.3425	2.26	10.0825	c(t = 3.19459	0.00763	0.89488417	HTR1E
chr2	15379707	15383707	in_vitro	in_vivo	10	10	14.457	4.073	10.384	c(t = 2.71251	0.0168	0.89488417	PACSIN3
chrX	14208164	14212164	in_vitro	in_vivo	12	10	6.32416667	19.873	-13.548833	c(t = -2.7864	0.0181	0.89488417	ENSSSCG00000012152
chr4	97366453	97370453	in_vitro	in_vivo	10	11	22.33	7.06363636	15.2663636	c(t = 2.75748	0.0195	0.89488417	C2CD4D
chr12	34921458	34925458	in_vitro	in_vivo	11	9	18.25	4.29777778	13.9522222	c(t = 2.62667	0.0197	0.89488417	U1
chr17	57582956	57586956	in_vitro	in_vivo	9	14	5.70555556	21.5507143	-15.845159	c(t = -2.4853	0.0244	0.89488417	BMP7
chr7	54653804	54657804	in_vitro	in_vivo	12	10	14.6841667	3.972	10.7121667	c(t = 2.51060	0.0258	0.89488417	ABHD2
chr7	48008589	48012589	in_vitro	in_vivo	15	14	5.728	19.5764286	-13.848429	c(t = -2.4257	0.027	0.89488417	ENSSSCG00000041217
chr3	37926096	37930096	in_vitro	in_vivo	10	11	4.114	14.4709091	-10.356909	c(t = -2.4722	0.0274	0.89488417	CORO7
chr14	23260776	23264776	in_vitro	in_vivo	10	11	14.875	4.04363636	10.8313636	c(t = 2.54343	0.0277	0.89488417	DDX51
chr2	65094877	65098877	in_vitro	in_vivo	12	14	21.8383333	7.06571429	14.772619	c(t = 2.44536	0.0277	0.89488417	ENSSSCG00000033298
chrX	89739190	89743190	in_vitro	in_vivo	13	13	3.94461538	20.54	-16.595385	c(t = -2.4553	0.0293	0.89488417	KCNE5
chr7	64505822	64509822	in_vitro	in_vivo	11	13	21.9754545	5.85153846	16.1239161	c(t = 2.42962	0.0337	0.89488417	NFKBIA
chr5	64104098	64108098	in_vitro	in_vivo	10	11	23.156	7.06545455	16.0905455	c(t = 2.39942	0.0344	0.89488417	NOP2
chr2	49227223	49231223	in_vitro	in_vivo	15	11	18.762	5.43272727	13.3292727	c(t = 2.24350	0.0377	0.89488417	ADM
chrX	124407289	124411289	in_vitro	in_vivo	9	14	3.91888889	23.6664286	-19.74754	c(t = -2.2865	0.0383	0.89488417	DUSP9
chr13	122103344	122107344	in_vitro	in_vivo	11	11	14.1836364	4.06454545	10.1190909	c(t = 2.31250	0.0384	0.89488417	ABCF3
chr1	273558283	273562283	in_vitro	in_vivo	10	15	19.746	5.606	14.14	c(t = 2.37736	0.0386	0.89488417	WDR5
chr6	168875249	168879249	in_vitro	in_vivo	14	16	17.2571429	5.08	12.1771429	c(t = 2.24589	0.0386	0.89488417	ZMYND12
chr13	66019325	66023325	in_vitro	in_vivo	14	12	14.2735714	4.00916667	10.2644048	c(t = 2.24209	0.0389	0.89488417	BRPF1
chr5	15493157	15497157	in_vitro	in_vivo	12	12	19.24	8.57833333	10.6616667	c(t = 2.18994	0.0402	0.89488417	KCNH3
chr6	43527806	43531806	in_vitro	in_vivo	10	14	23.715	8.46357143	15.2514286	c(t = 2.29412	0.041	0.89488417	KCTD15

chr2	1673360	1677360	in_vitro	in_vivo	9	10	7.40555556	24.856	-17.450444	c(t = -2.25452	0.0411	0.89488417	KCNQ1
chr9	120858104	120862104	in_vitro	in_vivo	12	13	12.5558333	1.77461538	10.7812179	c(t = 2.29390	0.0411	0.89488417	TOR3A
chr1	21740495	21744495	in_vitro	in_vivo	12	11	4.9575	15.2727273	-10.315227	c(t = -2.23047	0.0424	0.89488417	FUCA2
chr7	29671027	29675027	in_vitro	in_vivo	14	13	22.7792857	11.6553846	11.1239011	c(t = 2.15391	0.043	0.89488417	ZBTB22
chr1	194964334	194968334	in_vitro	in_vivo	14	11	13.4635714	2.79090909	10.6726623	c(t = 2.17188	0.0473	0.89488417	KCNH5
chr2	7140291	7144291	in_vitro	in_vivo	10	12	24.048	9.64416667	14.4038333	c(t = 2.17379	0.0478	0.89488417	SNX15
chr2	136650785	136654785	in_vitro	in_vivo	11	9	15.4163636	5.20222222	10.2141414	c(t = 2.11408	0.0496	0.89488417	UBE2B
chr1	181336525	181340525	in_vitro	in_vivo	14	14	3.16357143	0.30214286	2.86142857	c(t = 4.03254	0.00123	0.89488417	FRMD6
chr4	125520334	125524334	in_vitro	in_vivo	10	11	4.302	12.5363636	-8.2343636	c(t = -3.53434	0.00222	0.89488417	HFM1
chr2	88389167	88393167	in_vitro	in_vivo	13	13	10.5807692	3.41230769	7.16846154	c(t = 3.44760	0.00247	0.89488417	CMYA5
chr2	79464779	79468779	in_vitro	in_vivo	11	13	13.85	4.44846154	9.40153846	c(t = 3.55240	0.00318	0.89488417	GRM6
chr6	165132690	165136690	in_vitro	in_vivo	11	13	6.77636364	2.03384615	4.74251748	c(t = 3.27604	0.00425	0.89488417	LRRC41
chr3	8904591	8908591	in_vitro	in_vivo	9	13	13.9988889	5.33076923	8.66811966	c(t = 3.44340	0.00435	0.89488417	ENSSSCG00000030709
chr4	7812542	7816542	in_vitro	in_vivo	13	11	1.29692308	3.77363636	-2.4767133	c(t = -3.17395	0.00449	0.89488417	ENSSSCG00000059334
chr3	55675073	55679073	in_vitro	in_vivo	11	13	8.25090909	1.17461538	7.07629371	c(t = 3.38281	0.00588	0.89488417	COA5
chr9	57125442	57129442	in_vitro	in_vivo	12	16	9.2575	3.0475	6.21	c(t = 3.18291	0.00612	0.89488417	ADAMTS15
chr10	68619828	68623828	in_vitro	in_vivo	12	9	3.70916667	1.16333333	2.54583333	c(t = 3.06154	0.00667	0.89488417	ENSSSCG00000011162
chr16	22150469	22154469	in_vitro	in_vivo	9	12	2.40222222	0.12666667	2.27555556	c(t = 3.48211	0.00738	0.89488417	NIPBL
chr1	92419230	92423230	in_vitro	in_vivo	10	15	4.747	0.92866667	3.81833333	c(t = 3.18802	0.00846	0.89488417	ENSSSCG00000004489
chr18	45419663	45423663	in_vitro	in_vivo	12	13	7.28583333	1.93461538	5.35121795	c(t = 3.06757	0.0091	0.89488417	HOXA5
chr3	92988021	92992021	in_vitro	in_vivo	13	13	4.63230769	1.21615385	3.41615385	c(t = 2.90669	0.00933	0.89488417	KCNK12
chr12	20256373	20260373	in_vitro	in_vivo	12	13	17.9483333	8.81615385	9.13217949	c(t = 2.83175	0.00963	0.89488417	COASY
chr6	13293125	13297125	in_vitro	in_vivo	10	13	3.769	11.1053846	-7.3363846	c(t = -2.86504	0.0098	0.89488417	EXOSC6
chr17	22633284	22637284	in_vitro	in_vivo	13	11	3.39307692	0.07363636	3.31944056	c(t = 2.99546	0.0111	0.89488417	MACROD2
chr4	68283803	68287803	in_vitro	in_vivo	14	14	2.06214286	5.86357143	-3.8014286	c(t = -2.75469	0.0112	0.89488417	RRS1
chr14	16468323	16472323	in_vitro	in_vivo	12	14	6.34583333	1.37357143	4.9722619	c(t = 2.89684	0.0116	0.89488417	ENSSSCG00000058445
chr9	12583955	12587955	in_vitro	in_vivo	12	9	10.1083333	1.24555556	8.86277778	c(t = 2.96455	0.0121	0.89488417	USP35
chr1	75169061	75173061	in_vitro	in_vivo	14	10	3.00214286	0	3.00214286	c(t = 2.86606	0.0132	0.89488417	CEP57L1
chr2	59529154	59533154	in_vitro	in_vivo	12	15	7.5725	0.49266667	7.07983333	c(t = 2.87353	0.0148	0.89488417	ENSSSCG00000031657
chr2	396766	400766	in_vitro	in_vivo	13	14	8.07846154	3.02571429	5.05274725	c(t = 2.71542	0.0153	0.89488417	DRD4
chr14	9195293	9199293	in_vitro	in_vivo	12	14	2.865	0.89714286	1.96785714	c(t = 2.69392	0.0158	0.89488417	DOCK5
chr11	23235775	23239775	in_vitro	in_vivo	12	13	5.8775	1.74923077	4.12826923	c(t = 2.69494	0.0169	0.89488417	ENOX1
chr14	112214732	112218732	in_vitro	in_vivo	14	14	6.00571429	1.22714286	4.77857143	c(t = 2.69510	0.0172	0.89488417	ENSSSCG00000048585
chr2	108731357	108735357	in_vitro	in_vivo	10	12	5.253	1.535	3.718	c(t = 2.73085	0.0174	0.89488417	MACIR
chr4	110328028	110332028	in_vitro	in_vivo	10	13	1.132	9.09461538	-7.9626154	c(t = -2.71988	0.0174	0.89488417	GSTM3
chr18	4991227	4995227	in_vitro	in_vivo	13	11	1.75076923	0.35	1.40076923	c(t = 2.56181	0.0178	0.89488417	KMT2C
chr5	15024080	15028080	in_vitro	in_vivo	10	13	9.58	3.79076923	5.78923077	c(t = 2.59412	0.0186	0.89488417	DDN
chr3	117985557	117989557	in_vitro	in_vivo	10	15	7.38	1.36333333	6.01666667	c(t = 2.78604	0.0197	0.89488417	SDC1
chr7	93222130	93226130	in_vitro	in_vivo	13	13	12.9207692	5.48	7.44076923	c(t = 2.54446	0.0203	0.89488417	CCDC177
chr9	12276570	12280570	in_vitro	in_vivo	11	16	1.83727273	5.448125	-3.6108523	c(t = -2.48425	0.0208	0.89488417	ENSSSCG00000037251
chr3	111326750	111330750	in_vitro	in_vivo	12	15	3.925	1.118	2.807	c(t = 2.60456	0.0213	0.89488417	RBKS
chr10	63875586	63879586	in_vitro	in_vivo	11	11	0.31909091	5.49090909	-5.1718182	c(t = -2.69390	0.0216	0.89488417	SFMBT2
chr15	120983257	120987257	in_vitro	in_vivo	14	14	2.67	0.73785714	1.93214286	c(t = 2.50378	0.0219	0.89488417	CDK5R2
chr16	22597936	22601936	in_vitro	in_vivo	9	11	9.36444444	1.67545455	7.6889899	c(t = 2.67898	0.0223	0.89488417	WDR70
chr2	125810261	125814261	in_vitro	in_vivo	12	12	3.62166667	0.3025	3.31916667	c(t = 2.63911	0.0225	0.89488417	ENSSSCG00000014235
chr8	131470582	131474582	in_vitro	in_vivo	10	13	5.927	2.60846154	3.31853846	c(t = 2.47824	0.0225	0.89488417	HSD17B11
chr1	115939940	115943940	in_vitro	in_vivo	9	10	9.49222222	2.564	6.92822222	c(t = 2.64364	0.023	0.89488417	NEDD4
chr14	88986949	88990949	in_vitro	in_vivo	12	11	6.41333333	1.67272727	4.74060606	c(t = 2.55757	0.023	0.89488417	MAPK8
chr5	74915463	74919463	in_vitro	in_vivo	10	11	4.157	0.45636364	3.70063636	c(t = 2.66035	0.0235	0.89488417	TMEM117
chr6	27682030	27686030	in_vitro	in_vivo	13	11	3.75307692	0.95818182	2.7948951	c(t = 2.52540	0.0237	0.89488417	CBFB
chr4	111362966	111366966	in_vitro	in_vivo	9	10	2.87555556	11.447	-8.5714444	c(t = -2.59976	0.0239	0.89488417	HENMT1
chr13	122691760	122695760	in_vitro	in_vivo	10	10	4.769	0	4.769	c(t = 2.69993	0.0244	0.89488417	VPS8
chr2	16770262	16774262	in_vitro	in_vivo	10	13	4.299	13.0769231	-8.7779231	c(t = -2.43875	0.0253	0.89488417	CHST1
chr11	47826421	47830421	in_vitro	in_vivo	10	13	3.746	9.28923077	-5.5432308	c(t = -2.40418	0.0255	0.89488417	UCHL3
chr1	215606849	215610849	in_vitro	in_vivo	10	14	2.992	9.52357143	-6.5315714	c(t = -2.39477	0.0256	0.89488417	GLDC
chr16	49046686	49050686	in_vitro	in_vivo	11	14	0.95909091	0.11142857	0.84766234	c(t = 2.52682	0.0259	0.89488417	TNPO1
chr5	82323790	82327790	in_vitro	in_vivo	10	12	6.83	15.0808333	-8.2508333	c(t = -2.45618	0.026	0.89488417	ENSSSCG00000056574
chr16	44970859	44974859	in_vitro	in_vivo	13	13	6.10923077	1.64692308	4.46230769	c(t = 2.45337	0.0271	0.89488417	ENSSSCG00000063417
chr9	3073995	3077995	in_vitro	in_vivo	11	12	8.86272727	4.00166667	4.86106061	c(t = 2.42607	0.0271	0.89488417	MRPL17
chr11	21927221	21931221	in_vitro	in_vivo	9	11	9.01333333	3.06909091	5.94424242	c(t = 2.46033	0.0275	0.89488417	TPT1
chr13	13325220	13329220	in_vitro	in_vivo	9	13	3.32555556	0.57230769	2.75324786	c(t = 2.61816	0.0275	0.89488417	LRRC3B
chr4	106654809	106658809	in_vitro	in_vivo	12	14	3.12083333	7.08142857	-3.9605952	c(t = -2.34012	0.0282	0.89488417	AP4B1
chr1	65833995	65837995	in_vitro	in_vivo	16	16	1.598125	0.305	1.293125	c(t = 2.38820	0.0287	0.89488417	POU3F2
chr9	5500945	5504945	in_vitro	in_vivo	9	11	14.5655556	5.80363636	8.76191919	c(t = 2.43261	0.0287	0.89488417	TRIM68
chr6	145678052	145682052	in_vitro	in_vivo	12	12	5.07083333	0.82333333	4.2475	c(t = 2.44531	0.0288	0.89488417	DNAI4
chr7	96286789	96290789	in_vitro	in_vivo	9	14	8.56	17.9492857	-9.3892857	c(t = -2.34150	0.0294	0.89488417	ENSSSCG00000028159
chr6	107277316	107281316	in_vitro	in_vivo	15	10	1.48066667	4.454	-2.9733333	c(t = -2.42506	0.0295	0.89488417	ENSSSCG00000039279
chr2	77477273	77481273	in_vitro	in_vivo	11	10	6.54545455	1.087	5.45845455	c(t = 2.46488	0.0296	0.89488417	R3HDM4
chr6	42558113	42562113	in_vitro	in_vivo	16	11	6.2975	3.18272727	3.11477273	c(t = 2.31447	0.0296	0.89488417	RGS9BP
chr10	40762396	40766396	in_vitro	in_vivo	9	9	5.44333333	0.7	4.74333333	c(t = 2.56864	0.0297	0.89488417	MTPAP
chr1	236819414	236823414	in_vitro	in_vivo	13	11	3.79	0.87909091	2.91090909	c(t = 2.35306	0.03	0.89488417	RECK
chr5	19483096	19487096	in_vitro	in_vivo	12	12	8.0025	1.095	6.9075	c(t = 2.46859	0.03	0.89488417	HNRNPA1
chr5	7581048	7585048	in_vitro	in_vivo	12	11	0.70416667	3.23	-2.5258333	c(t = -2.45874	0.0303	0.89488417	ST13
chr14	30522232	30526232	in_vitro	in_vivo	10	12	2.52	9.03833333	-6.5183333	c(t = -2.32686	0.0316	0.89488417	LRRC43
chr4	98847196	98851196	in_vitro	in_vivo	9	10	9.99777778	2.364	7.63377778	c(t = 2.51488	0.0316	0.89488417	ANP32E
chr1	130110847	130114847	in_vitro	in_vivo	13	9	3.65230769	0.73888889	2.9134188	c(t = 2.33988	0.0318	0.89488417	EXD1
chr17	35707349	35711349	in_vitro	in_vivo	9	12	3.29777778	8.815	-5.5172222	c(t = -2.36657	0.0319	0.89488417	HCK
chr16	65640223	65644223	in_vitro	in_vivo	12	12	4.39583333	13.1933333	-8.7975	c(t = -2.40640	0.032	0.89488417	SOX30
chr6	17231858	17235858	in_vitro	in_vivo	10	11	8.936	2.10454545	6.83145455	c(t = 2.45606	0.0321	0.89488417	NOB1
chr13	34837384	34841384	in_vitro	in_vivo	12	10	4.94083333	1.108	3.83283333	c(t = 2.32764	0.0326	0.89488417	ENSSSCG00000011447
chr5	19165157	19169157	in_vitro	in_vivo	13	14	4.49538462	0.89142857	3.60395604	c(t = 2.36901	0.0327	0.89488417	HOXC12
chr18	44260081	44264081	in_vitro	in_vivo	11	12	2.25454545	0.47666667	1.77787879	c(t = 2.39165	0.033	0.89488417	ENSSSCG00000062314
chr6	101756014	101760014	in_vitro	in_vivo	11	14	2.49545455	0.91142857	1.58402597	c(t = 2.36065	0.0335	0.89488417	ZBTB14
chr14	124160521	124164521	in_vitro	in_vivo	12	11	4.07583333	0.64909091	3.42674242	c(t = 2.32264	0.0338	0.89488417	NHLRC2
chr16	52001887	52005887	in_vitro	in_vivo	12	14	8.01083333	1.25857143	6.7522619	c(t = 2.38944	0.0341	0.89488417	STK10
chr7	33376286	33380286	in_vitro	in_vivo	13	9	3.03384615	0.20222222	2.83162393	c(t = 2.37403	0.0341	0.89488417	ZFAND3
chr3	97828678	97832678	in_vitro	in_vivo	15	15	2.99266667	1.04333333	1.94933333	c(t = 2.28971	0.0348	0.89488417	KCNG3
chr5	68221729	68225729	in_vitro	in_vivo	12	10	1.21166667	2.784	-1.5723333	c(t = -2.26738	0.0349	0.89488417	ERC1
chr14	103394459	103398459	in_vitro	in_vivo	13	11	3.74769231	0.98363636	2.76405594	c(t = 2.30734	0.0351	0.89488417	TNKS2
chr9	135980227	135984227	in_vitro	in_vivo	10	15	10.126	5.808	4.318	c(t = 2.25987	0.0351	0.89488417	VWC2
chr11	1552763	1556763	in_vitro	in_vivo	13	16	1.85076923	0.673125	1.17764423	c(t = 2.29084	0.0353	0.89488417	FGF9
chr15	38788655	38792655	in_vitro	in_vivo	13	14	2.52846154	0.78214286	1.74631868	c(t = 2.26088	0.0358	0.89488417	SPCS3
chr2	58826251	58830251	in_vitro	in_vivo	12	12	5.4675	2.44	3.0275	c(t = 2.26225	0.0358	0.89488417	SUGP2
chr14	50810984	50814984	in_vitro	in_vivo	10	12	3.275	10.4741667	-7.1991667	c(t = -2.35347	0.0359	0.89488417	SCARF2
chr2	27184673	27188673	in_vitro	in_vivo	10	9	12.529	2.83	9.699	c(t = 2.40161	0.0359	0.89488417	ENSSSCG00000024791

chr9	52114030	52118030	in_vitro	in_vivo	11	10	11.1954545	3.879	7.31645455	c(t = 2.29346	0.0366	0.89488417	ENSSSCG00000050132
chr1	55614957	55618957	in_vitro	in_vivo	12	12	0.405	2.11083333	-1.7058333	c(t = -2.2861	0.0371	0.89488417	ZNF292
chr2	36701790	36705790	in_vitro	in_vivo	9	12	1.20111111	6.38083333	-5.1797222	c(t = -2.2919	0.0372	0.89488417	SVIP
chr17	18956165	18960165	in_vitro	in_vivo	9	10	12.4666667	5.612	6.85466667	c(t = 2.30372	0.0375	0.89488417	ANKEF1
chr17	38102200	38106200	in_vitro	in_vivo	9	13	15.6366667	5.98461538	9.65205128	c(t = 2.40131	0.0375	0.89488417	TP53INP2
chr4	112699088	112703088	in_vitro	in_vivo	12	14	7.1175	1.24714286	5.87035714	c(t = 2.28522	0.0379	0.89488417	PRMT6
chr8	137603600	137607600	in_vitro	in_vivo	14	14	6.47714286	2.98571429	3.49142857	c(t = 2.21245	0.0383	0.89488417	PRDM8
chr6	30514948	30518948	in_vitro	in_vivo	12	13	1.77416667	0.28076923	1.49339744	c(t = 2.30893	0.0392	0.89488417	ENSSSCG00000047270
chr9	68981827	68985827	in_vitro	in_vivo	11	15	7.10363636	0.73133333	6.37230303	c(t = 2.36378	0.0392	0.89488417	ZNF804B
chr18	50757221	50761221	in_vitro	in_vivo	12	13	7.06416667	2.53	4.53416667	c(t = 2.27673	0.0397	0.89488417	NUDCD3
chr1	255149863	255153863	in_vitro	in_vivo	9	10	4.00444444	11.575	-7.5705556	c(t = -2.24692	0.0401	0.89488417	ATP6V1G1
chr13	110585463	110589463	in_vitro	in_vivo	12	14	5.66416667	2.06285714	3.60130952	c(t = 2.21092	0.041	0.89488417	FNDC3B
chr13	189714190	189718190	in_vitro	in_vivo	13	13	6.47769231	1.19769231	5.28	c(t = 2.24934	0.041	0.89488417	ENSSSCG00000052162
chr15	78502078	78506078	in_vitro	in_vivo	12	13	5.32666667	2.07923077	3.2474359	c(t = 2.25160	0.041	0.89488417	ITGA6
chr2	59220367	59224367	in_vitro	in_vivo	11	12	4.60090909	2.03666667	2.56424242	c(t = 2.19959	0.0414	0.89488417	CRLF1
chr8	43958709	43962709	in_vitro	in_vivo	9	12	1.69	9.11166667	-7.4216667	c(t = -2.25473	0.0418	0.89488417	TMEM192
chr1	99309561	99313561	in_vitro	in_vivo	10	9	13.099	3.33666667	9.76233333	c(t = 2.26745	0.0423	0.89488417	ENSSSCG00000020045
chr17	11408472	11412472	in_vitro	in_vivo	14	10	3.16428571	0.629	2.53528571	c(t = 2.22337	0.0424	0.89488417	VDAC3
chr8	87852699	87856699	in_vitro	in_vivo	11	13	2.48727273	0.37615385	2.11111888	c(t = 2.28987	0.0427	0.89488417	ENSSSCG00000029920
chr9	67931220	67935220	in_vitro	in_vivo	13	9	9.07692308	1.18777778	7.8891453	c(t = 2.24143	0.0432	0.89488417	ENSSSCG00000015664
chr14	44071638	44075638	in_vitro	in_vivo	10	11	3.228	0.81454545	2.41345455	c(t = 2.22217	0.0437	0.89488417	SRRD
chr15	68983328	68987328	in_vitro	in_vivo	10	11	9.261	0.84272727	8.41827273	c(t = 2.33383	0.0437	0.89488417	GCA
chr17	47789175	47793175	in_vitro	in_vivo	9	13	9.42	3.88153846	5.53846154	c(t = 2.19272	0.0437	0.89488417	ENSSSCG00000051240
chr4	35766190	35770190	in_vitro	in_vivo	11	12	0.44090909	2.70916667	-2.2682576	c(t = -2.22962	0.044	0.89488417	ENSSSCG00000056174
chr4	77107594	77111594	in_vitro	in_vivo	12	10	6.87	1.253	5.617	c(t = 2.24033	0.0442	0.89488417	TCEA1
chr14	11062492	11066492	in_vitro	in_vivo	9	10	14.4	24.396	-9.996	c(t = -2.18532	0.0443	0.89488417	PTK2B
chr10	24108064	24112064	in_vitro	in_vivo	11	10	3.00909091	10.205	-7.1959091	c(t = -2.26729	0.0444	0.89488417	IPO9
chr7	52956106	52960106	in_vitro	in_vivo	12	12	9.99333333	2.80333333	7.19	c(t = 2.19939	0.0444	0.89488417	IQGAP1
chr1	113287209	113291209	in_vitro	in_vivo	13	11	3.66	0.66454545	2.99545455	c(t = 2.19933	0.0447	0.89488417	ADAM10
chr18	45386375	45390375	in_vitro	in_vivo	9	11	4.12555556	0.87363636	3.25191919	c(t = 2.32033	0.045	0.89488417	HOXA11
chrX	19505739	19509739	in_vitro	in_vivo	12	13	1.8875	8.64846154	-6.7609615	c(t = -2.20337	0.045	0.89488417	PTCHD1
chr1	201340788	201344788	in_vitro	in_vivo	10	10	5.764	0.286	5.478	c(t = 2.30426	0.0459	0.89488417	IFN-DELTA-6
chr2	117502129	117506129	in_vitro	in_vivo	14	9	11.0042857	1.27666667	9.72761905	c(t = 2.18851	0.046	0.89488417	ENSSSCG00000057006
chr12	47123236	47127236	in_vitro	in_vivo	9	15	8.04	1.294	6.746	c(t = 2.31540	0.0472	0.89488417	ENSSSCG00000057748
chr11	49805994	49809994	in_vitro	in_vivo	10	14	4.294	1.37071429	2.92328571	c(t = 2.17619	0.0475	0.89488417	SLAIN1
chr2	143286004	143290004	in_vitro	in_vivo	13	12	12.1838462	5.43916667	6.74467949	c(t = 2.09196	0.0477	0.89488417	RELL2
chr14	88472122	88476122	in_vitro	in_vivo	12	12	2.66166667	0.5175	2.14416667	c(t = 2.14284	0.0479	0.89488417	ZNF488
chr14	24339549	24343549	in_vitro	in_vivo	11	10	7.88818182	2.937	4.95118182	c(t = 2.12021	0.0486	0.89488417	STX2
chr15	83144002	83148002	in_vitro	in_vivo	12	12	3.31666667	0.73	2.58666667	c(t = 2.16690	0.0486	0.89488417	AGPS
chr7	55234348	55238348	in_vitro	in_vivo	11	12	16.8390909	7.69583333	9.14325758	c(t = 2.12823	0.0488	0.89488417	PLIN1
chr1	99309788	99313788	in_vitro	in_vivo	10	9	12.803	3.33666667	9.46633333	c(t = 2.18655	0.049	0.89488417	ENSSSCG00000020491
chr9	49903428	49907428	in_vitro	in_vivo	12	14	3.28166667	0.94714286	2.33452381	c(t = 2.14056	0.049	0.89488417	BSX
chr3	34081830	34085830	in_vitro	in_vivo	10	14	1.758	5.62071429	-3.8627143	c(t = -2.10381	0.0493	0.89488417	TMEM114
chr14	133851193	133855193	in_vitro	in_vivo	10	14	3.012	0.84285714	2.16914286	c(t = 2.18107	0.0499	0.89488417	NKX1-2
chr9	4099022	4103022	in_vitro	in_vivo	9	9	12.7011111	44.71	-32.008889	c(t = -3.83487	0.00149	0.89488417	ENSSSCG00000034983
chr8	120790258	120794258	in_vitro	in_vivo	9	14	15.0111111	53.6328571	-38.621746	c(t = -3.62224	0.00161	0.89488417	C4orf54
chr6	62138474	62142474	in_vitro	in_vivo	9	9	6.85222222	35.49	-28.637778	c(t = -3.62131	0.00238	0.89488417	ENSSSCG00000053530
chr9	3164485	3168485	in_vitro	in_vivo	11	11	17.8163636	49.7190909	-31.902727	c(t = -3.31897	0.00515	0.89488417	ENSSSCG00000057514
chr12	145208	149208	in_vitro	in_vivo	14	13	10.9364286	29.8276923	-18.891264	c(t = -2.96775	0.00846	0.89488417	ENSSSCG00000048182
chr15	121558008	121562008	in_vitro	in_vivo	9	10	20.74	45.052	-24.312	c(t = -2.99272	0.0093	0.89488417	OBSL1
chr13	200126477	200130477	in_vitro	in_vivo	10	12	6.005	38.7208333	-32.715833	c(t = -3.00425	0.0104	0.89488417	CLDN14
chr6	45055837	45059837	in_vitro	in_vivo	10	10	15.778	44.478	-28.7	c(t = -2.92978	0.011	0.89488417	HAUS5
chr14	141358608	141362608	in_vitro	in_vivo	10	13	8.491	29.52	-21.029	c(t = -2.89416	0.0112	0.89488417	PAOX
chr18	48950906	48954906	in_vitro	in_vivo	10	10	10.115	37.592	-27.477	c(t = -2.86513	0.0122	0.89488417	ENSSSCG00000052165
chr12	143994	147994	in_vitro	in_vivo	14	14	9.95357143	28.6357143	-18.682143	c(t = -2.73902	0.0129	0.89488417	ENSSSCG00000048083
chr5	78139236	78143236	in_vitro	in_vivo	10	9	3.906	30.1611111	-26.255111	c(t = -3.06029	0.013	0.89488417	SLC48A1
chr11	2955295	2959295	in_vitro	in_vivo	12	12	10.1641667	30.3291667	-20.165	c(t = -2.83228	0.0134	0.89488417	ENSSSCG00000062603
chr1	268689502	268693502	in_vitro	in_vivo	9	9	11.5933333	37.88	-26.286667	c(t = -2.89672	0.0159	0.89488417	MIR199B
chr1	267425147	267429147	in_vitro	in_vivo	9	9	5.35444444	30.92	-25.565556	c(t = -2.90660	0.0167	0.89488417	ENSSSCG00000063069
chr4	84108767	84112767	in_vitro	in_vivo	11	13	3.50909091	31.2638462	-27.754755	c(t = -2.75965	0.0167	0.89488417	ssc-mir-9825
chr5	495385	499385	in_vitro	in_vivo	10	13	17.927	47.7723077	-29.845308	c(t = -2.64418	0.0175	0.89488417	PANX2
chr12	53904511	53908511	in_vitro	in_vivo	9	12	5.19777778	32.1791667	-26.981389	c(t = -2.76424	0.0177	0.89488417	PIK3R6
chr6	27851452	27855452	in_vitro	in_vivo	10	10	11.379	44.411	-33.032	c(t = -2.82005	0.0177	0.89488417	ENSSSCG00000002790
chr6	45344637	45348637	in_vitro	in_vivo	11	10	15.9663636	48.943	-32.976636	c(t = -2.63659	0.0177	0.89488417	LRFN3
chr3	5412074	5416074	in_vitro	in_vivo	11	14	9.70909091	38.3878571	-28.678766	c(t = -2.64210	0.0184	0.89488417	TECPR1
chr12	60414463	60418463	in_vitro	in_vivo	10	9	12.681	45.2544444	-32.573444	c(t = -2.79416	0.0185	0.89488417	SHMT1
chr13	208285845	208289845	in_vitro	in_vivo	15	12	10.1066667	33.6216667	-23.515	c(t = -2.65881	0.019	0.89488417	ENSSSCG00000057439
chr1	2016608	2020608	in_vitro	in_vivo	11	13	4.47363636	25.75	-21.276364	c(t = -2.64979	0.0195	0.89488417	CCR6
chrX	42998231	43002231	in_vitro	in_vivo	11	11	15.5490909	36.5109091	-20.961818	c(t = -2.56524	0.0204	0.89488417	OTUD5
chr12	52108718	52112718	in_vitro	in_vivo	9	14	9.73222222	26.5478571	-16.815635	c(t = -2.49841	0.021	0.89488417	TM4SF5
chr1	166816037	166820037	in_vitro	in_vivo	13	10	8.25	31.343	-23.093	c(t = -2.68509	0.0216	0.89488417	SPESP1
chr18	55612730	55616730	in_vitro	in_vivo	13	15	12.46	33.834	-21.374	c(t = -2.50389	0.0224	0.89488417	ZNF777
chr14	140430596	140434596	in_vitro	in_vivo	9	9	16.8188889	50.5844444	-33.765556	c(t = -2.55861	0.0226	0.89488417	U6
chr5	848983	852983	in_vitro	in_vivo	12	12	11.5925	40.1033333	-28.510833	c(t = -2.43557	0.0243	0.89488417	ENSSSCG00000053256
chr3	113513116	113517116	in_vitro	in_vivo	10	14	10.688	36.535	-25.847	c(t = -2.48197	0.0251	0.89488417	ENSSSCG00000044418
chrX	34320298	34324298	in_vitro	in_vivo	13	10	10.2253846	38.137	-27.911615	c(t = -2.51234	0.0251	0.89488417	OTC
chr10	9516977	9520977	in_vitro	in_vivo	9	12	11.84	34.2966667	-22.456667	c(t = -2.43504	0.0281	0.89488417	ENSSSCG00000053979
chr12	3800240	3804240	in_vitro	in_vivo	11	13	14.2272727	33.4976923	-19.27042	c(t = -2.38066	0.0282	0.89488417	ENSSSCG00000041583
chr9	10445249	10449249	in_vitro	in_vivo	10	9	8.853	48.4744444	-39.621444	c(t = -2.64029	0.0287	0.89488417	ENSSSCG00000048879
chr2	9295283	9299283	in_vitro	in_vivo	10	11	9.034	40.2681818	-31.234182	c(t = -2.48287	0.0288	0.89488417	SCGB1A1
chr14	16483081	16487081	in_vitro	in_vivo	14	9	13.1857143	30.9711111	-17.785397	c(t = -2.35299	0.0311	0.89488417	ENSSSCG00000043271
chr2	150588203	150592203	in_vitro	in_vivo	9	10	7.87555556	35.604	-27.728444	c(t = -2.47489	0.0311	0.89488417	ENSSSCG00000055888
chr2	150588203	150592203	in_vitro	in_vivo	9	10	7.87555556	35.604	-27.728444	c(t = -2.47489	0.0311	0.89488417	ENSSSCG00000055888
chrX	124507526	124511526	in_vitro	in_vivo	9	15	8.15888889	30.8913333	-22.732444	c(t = -2.30493	0.0311	0.89488417	PLXNB3
chr5	3275506	3279506	in_vitro	in_vivo	12	14	9.73166667	28.0614286	-18.329762	c(t = -2.38052	0.0314	0.89488417	ENSSSCG00000063551
chr17	57997287	58001287	in_vitro	in_vivo	9	10	8.41666667	38.388	-29.971333	c(t = -2.44951	0.0322	0.89488417	PMEPA1
chr11	3130164	3134164	in_vitro	in_vivo	11	13	10.1045455	27.0523077	-16.947762	c(t = -2.34823	0.0324	0.89488417	ENSSSCG00000059877
chr14	141422468	141426468	in_vitro	in_vivo	12	12	7.1775	27.2266667	-20.049167	c(t = -2.37342	0.0347	0.89488417	ENSSSCG00000059266
chr3	59183735	59187735	in_vitro	in_vivo	9	14	12.2244444	32.9764286	-20.751984	c(t = -2.25144	0.0367	0.89488417	VAMP8
chr3	113515808	113519808	in_vitro	in_vivo	11	10	12.6681818	41.908	-29.239818	c(t = -2.36200	0.0374	0.89488417	ENSSSCG00000041111
chr14	133458167	133462167	in_vitro	in_vivo	13	13	5.73769231	29.6223077	-23.884615	c(t = -2.29997	0.0393	0.89488417	CHST15
chr4	1627425	1631425	in_vitro	in_vivo	9	10	12.5122222	44.363	-31.850778	c(t = -2.25956	0.0397	0.89488417	ENSSSCG00000050724
chr6	51402558	51406558	in_vitro	in_vivo	11	12	15.6463636	37.6658333	-22.01947	c(t = -2.25194	0.0412	0.89488417	ENSSSCG00000003086
chr2	71286242	71290242	in_vitro	in_vivo	10	12	13.504	35.1566667	-21.652667	c(t = -2.28306	0.0416	0.89488417	CTXN1
chr18	50246693	50250693	in_vitro	in_vivo	13	14	11.2076923	31.16	-19.952308	c(t = -2.15171	0.0422	0.89488417	ENSSSCG00000062187

chr15	115349501	115353501	in_vitro	in_vivo	9	14	16.9866667	44.4742857	-27.487619	c(t = -2.16386	0.0426	0.89488417	ENSSSCG00000037703
chr7	48558753	48562753	in_vitro	in_vivo	9	12	15.7433333	38.8558333	-23.1125	c(t = -2.21717	0.0434	0.89488417	ENSSSCG00000001776
chr6	88137498	88141498	in_vitro	in_vivo	15	12	15.114	36.8358333	-21.721833	c(t = -2.16222	0.0437	0.89488417	HCRTR1
chr5	4107938	4111938	in_vitro	in_vivo	10	9	6.727	37.0677778	-30.340778	c(t = -2.29241	0.0444	0.89488417	UPK3A
chr6	71745227	71749227	in_vitro	in_vivo	11	10	11.3045455	34.821	-23.516455	c(t = -2.26532	0.0452	0.89488417	ENSSSCG00000003421
chr7	121695837	121699837	in_vitro	in_vivo	11	14	13.21	29.4457143	-16.235714	c(t = -2.13706	0.0462	0.89488417	ssc-mir-493
chr9	65944483	65948483	in_vitro	in_vivo	11	11	15.3618182	45.6890909	-30.327273	c(t = -2.14363	0.0463	0.89488417	ENSSSCG00000047928
chr1	6730360	6734360	in_vitro	in_vivo	13	10	10.8253846	34.991	-24.165615	c(t = -2.18706	0.0464	0.89488417	ENSSSCG00000059587
chr6	59713636	59717636	in_vitro	in_vivo	11	11	22.1418182	47.7818182	-25.64	c(t = -2.14009	0.0465	0.89488417	FIZ1
chr5	1412144	1416144	in_vitro	in_vivo	10	10	16.048	45.886	-29.838	c(t = -2.16305	0.0466	0.89488417	TAFA5
chr11	3804302	3808302	in_vitro	in_vivo	10	11	20.918	42.5318182	-21.613818	c(t = -2.12027	0.048	0.89488417	SHISA2
chr7	74877260	74881260	in_vitro	in_vivo	10	11	23.414	47.9709091	-24.556909	c(t = -2.13574	0.048	0.89488417	NYNRIN
chr12	12364615	12368615	in_vitro	in_vivo	9	13	11.1133333	32.01	-20.896667	c(t = -2.15301	0.0485	0.89488417	ENSSSCG00000060709
chr2	41755356	41759356	in_vitro	in_vivo	12	13	85.4916667	53.6630769	31.8285897	c(t = 3.92018	0.00103	0.89488417	KCNJ11
chr1	253654010	253658010	in_vitro	in_vivo	11	15	92.7554545	73.0213333	19.7341212	c(t = 3.74011	0.00155	0.89488417	ENSSSCG00000043950
chr12	5424762	5428762	in_vitro	in_vivo	13	15	76.0376923	44.688	31.3496923	c(t = 3.00838	0.00585	0.89488417	CDK3
chr2	6295855	6299855	in_vitro	in_vivo	13	13	87.8746154	59.7953846	28.0792308	c(t = 3.04072	0.00733	0.89488417	GAL3ST3
chr18	18984830	18988830	in_vitro	in_vivo	12	14	90.0791667	66.7885714	23.2905952	c(t = 2.99919	0.00749	0.89488417	ssc-mir-182
chr9	37924858	37928858	in_vitro	in_vivo	9	9	81.95	58.0588889	23.8911111	c(t = 3.08616	0.00833	0.89488417	ENSSSCG00000052243
chr13	122139967	122143967	in_vitro	in_vivo	10	13	96.063	74.5646154	21.4983846	c(t = 2.89505	0.0126	0.89488417	ALG3
chr4	94498141	94502141	in_vitro	in_vivo	11	13	94.7790909	65.3607692	29.4183217	c(t = 2.82409	0.013	0.89488417	FDPS
chr13	133155321	133159321	in_vitro	in_vivo	11	10	97.7718182	59.302	38.4698182	c(t = 3.01243	0.0145	0.89488417	ENSSSCG00000029291
chr3	47836558	47840558	in_vitro	in_vivo	11	13	85.1563636	66.5615385	18.5948252	c(t = 2.60425	0.0165	0.89488417	SULT1C3
chr3	112798719	112802719	in_vitro	in_vivo	10	12	87.168	71.8525	15.3155	c(t = 2.62234	0.0174	0.89488417	GAREM2
chr6	54821668	54825668	in_vitro	in_vivo	9	11	80.2611111	58.2109091	22.050202	c(t = 2.46348	0.0241	0.89488417	FUZ
chr12	61269641	61273641	in_vitro	in_vivo	12	13	81.385	68.6792308	12.7057692	c(t = 2.17642	0.0402	0.89488417	USP22
chr7	58762493	58766493	in_vitro	in_vivo	9	9	79.48	62.7888889	16.6911111	c(t = 2.14231	0.0495	0.89488417	ENSSSCG00000062579
chr6	83316411	83320411	in_vitro	in_vivo	11	10	75.9918182	49.12	26.8718182	c(t = 2.10965	0.0496	0.89488417	PAQR7
chr2	134355485	134359485	in_vitro	in_vivo	10	10	95.79	79.029	16.761	c(t = 6.74436	3.44E-06	0.0283972	ENSSSCG00000055451
chr13	122139042	122143042	in_vitro	in_vivo	10	14	96.399	76.6492857	19.7497143	c(t = 3.25840	0.00539	0.89488417	ssc-mir-1224
chr9	5513403	5517403	in_vitro	in_vivo	9	9	92	81.9744444	10.0255556	c(t = 2.95987	0.0104	0.89488417	ENSSSCG00000014769
chr9	6289930	6293930	in_vitro	in_vivo	10	10	95.912	84.832	11.08	c(t = 3.03740	0.0108	0.89488417	PGAP2
chr13	31177291	31181291	in_vitro	in_vivo	11	12	92.9072727	76.9233333	15.9839394	c(t = 2.73537	0.0138	0.89488417	TREX1
chr2	151428049	151432049	in_vitro	in_vivo	9	10	97.2555556	80.848	16.4075556	c(t = 2.98121	0.014	0.89488417	RPS14
chr6	89161378	89165378	in_vitro	in_vivo	9	11	93.5244444	81.8372727	11.6871717	c(t = 2.73899	0.014	0.89488417	FNDC5
chr1	269079272	269083272	in_vitro	in_vivo	10	10	93.021	79.595	13.426	c(t = 2.84114	0.0141	0.89488417	ZDHHC12
chr2	10156188	10160188	in_vitro	in_vivo	9	12	93.5255556	82.6483333	10.8772222	c(t = 2.55438	0.0196	0.89488417	TKFC
chr5	63955064	63959064	in_vitro	in_vivo	9	11	94.8044444	82.7981818	12.0062626	c(t = 2.58620	0.0218	0.89488417	COPS7A
chr2	71529614	71533614	in_vitro	in_vivo	10	12	91.947	75.665	16.282	c(t = 2.53580	0.0227	0.89488417	CAMSAP3
chr2	151633672	151637672	in_vitro	in_vivo	12	10	94.35	78.683	15.667	c(t = 2.52423	0.0291	0.89488417	MYOZ3
chr2	24644871	24648871	in_vitro	in_vivo	9	9	94.7422222	81.2933333	13.4488889	c(t = 2.39699	0.0331	0.89488417	PRR5L
chr1	7557745	7561745	in_vitro	in_vivo	11	12	95.0054545	84.3216667	10.6837879	c(t = 2.19746	0.0436	0.89488417	PNLDC1
chr4	100458304	100462304	in_vitro	in_vivo	9	10	91.7633333	77.354	14.4093333	c(t = 2.20266	0.0439	0.89488417	ENSSSCG00000060248
chr17	48510803	48514803	in_vitro	in_vivo	11	10	94.0281818	83.437	10.5911818	c(t = 2.17469	0.049	0.89488417	SLC35C2
chr3	17131476	17135476	in_vitro	in_vivo	9	9	88.6388889	97.1555556	-8.5166667	c(t = -2.83172	0.0152	0.89488417	COX6A2
chr12	47114565	47118565	in_vitro	in_vivo	13	12	98.0861538	93.2475	4.83865385	c(t = 2.62857	0.0153	0.89488417	TLCD3A
chr16	71978475	71982475	in_vitro	in_vivo	12	13	93.0775	84.1115385	8.96596154	c(t = 2.63486	0.0167	0.89488417	GPX3
chr5	15306210	15310210	in_vitro	in_vivo	9	11	98.6844444	91.26	7.42444444	c(t = 2.71048	0.0191	0.89488417	C1QL4
chr9	3181690	3185690	in_vitro	in_vivo	11	11	97.4127273	89.1672727	8.24545455	c(t = 2.59637	0.0213	0.89488417	ENSSSCG00000057908
chr14	30575661	30579661	in_vitro	in_vivo	9	9	89.8788889	80.2166667	9.66222222	c(t = 2.47794	0.025	0.89488417	MLXIP
chr17	28406099	28410099	in_vitro	in_vivo	9	9	98.83	88.9966667	9.83333333	c(t = 2.62661	0.0259	0.89488417	RALGAPA2
chr13	26328233	26332233	in_vitro	in_vivo	9	10	97.4755556	90.269	7.20655556	c(t = 2.50493	0.0264	0.89488417	CYP8B1
chr6	3039645	3043645	in_vitro	in_vivo	9	10	98.4266667	94.192	4.23466667	c(t = 2.42860	0.0299	0.89488417	IRF8
chr2	66282462	66286462	in_vitro	in_vivo	9	12	97.6622222	90.6691667	6.99305556	c(t = 2.40628	0.0308	0.89488417	ENSSSCG00000019671
chr4	16167708	16171708	in_vitro	in_vivo	12	11	88.0441667	96.1590909	-8.1149242	c(t = -2.30896	0.0365	0.89488417	FAM83A
chr7	9733660	9737660	in_vitro	in_vivo	9	9	89.6544444	98.4655556	-8.8111111	c(t = -2.43423	0.0367	0.89488417	GFOD1
chr3	8228328	8232328	in_vitro	in_vivo	9	13	91.6766667	82.4715385	9.20512821	c(t = 2.23101	0.0373	0.89488417	PVRIG
chr9	1289661	1293661	in_vitro	in_vivo	11	11	93.4827273	85.3245455	8.15818182	c(t = 2.17745	0.0447	0.89488417	EIF3F
chr12	47716491	47720491	in_vitro	in_vivo	9	11	95.8966667	88.5827273	7.31393939	c(t = 2.21096	0.0454	0.89488417	INPP5K
