# Supplementary material for: Deciphering differences in DNA methylation and transcriptome profiles of oocytes from pigs with high and low developmental competence
Source: Environ Epigenet. 2025 Jun 3;11(1):dvaf018. doi: 10.1093/eep/dvaf018 (PMC12418950; doi:10.1093/eep/dvaf018)
Supplement: dvaf018_Supplemental_Files [file dvaf018_supplemental_files.zip › Suple Figure S1.pdf]

Supplementary Figure S1

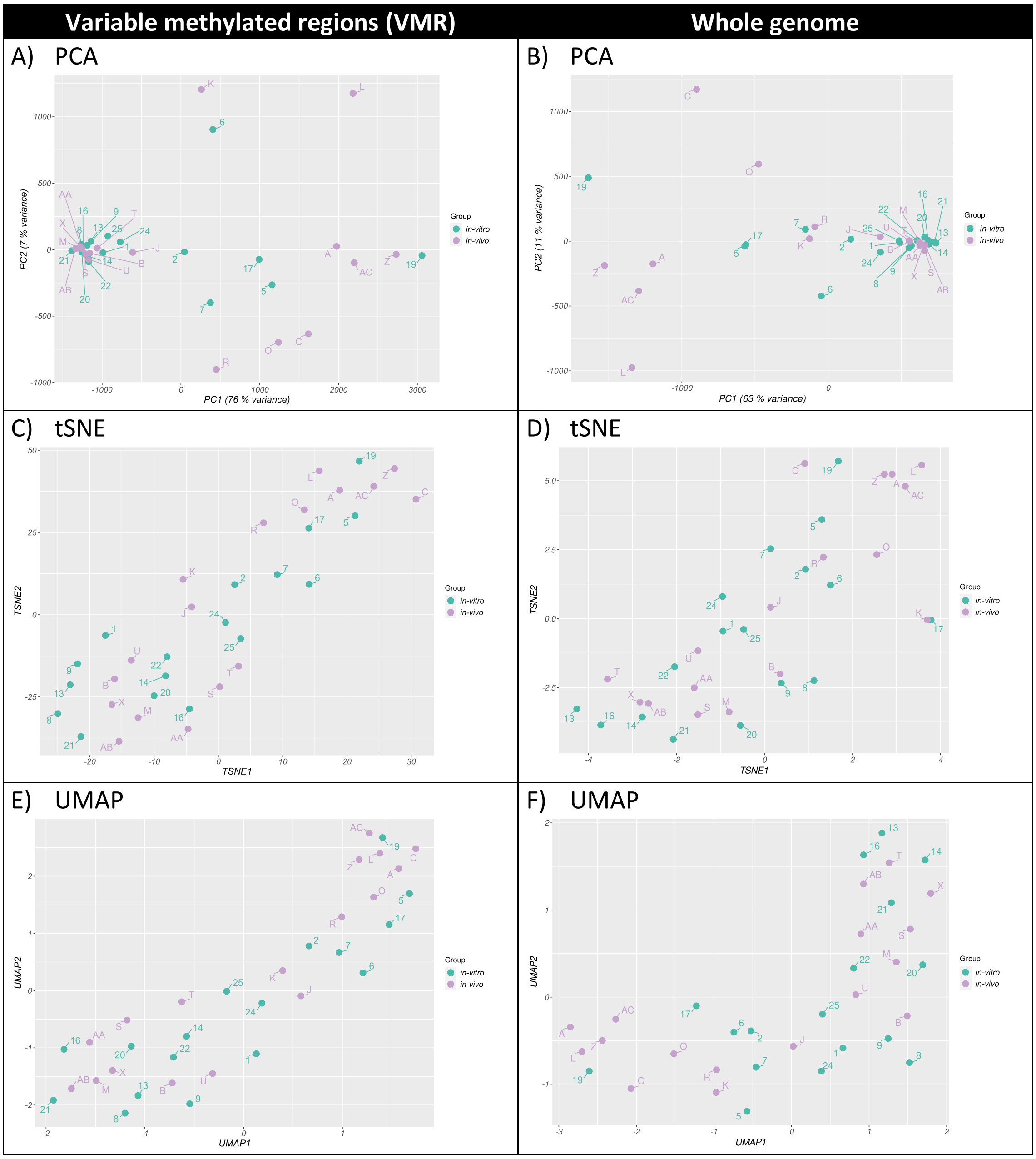

Figure S1. Clustering of individual porcine oocytes matured in vivo and in vitro using different algorithms. Clustering by Principal Components Analysis (PCA) of the samples for A) Variable methylated regions (VMR) regions and B) whole genome. Clustering by T-distributed Stochastic Neighbour Embedding (t-SNE) of the samples for C) VMR regions and D) whole genome. Uniform Manifold Approximation and Projection (UMAP) clustering of the samples for E) VMR regions and F) whole genome.
