# Supplementary material for: Deciphering differences in DNA methylation and transcriptome profiles of oocytes from pigs with high and low developmental competence
Source: Environ Epigenet. 2025 Jun 3;11(1):dvaf018. doi: 10.1093/eep/dvaf018 (PMC12418950; doi:10.1093/eep/dvaf018)
Supplement: dvaf018_Supplemental_Files [file Sup table 20.pdf › Sup table 20.pdf]

ID	Term	Ontology Source	Term PValue	Term P-Value Corrected with Bonferroni step down	Group P-Value	Group P-Value Corrected with Bonferroni step down	GO Levels	GO Groups	% Associated Genes	Number of Genes	Associated Genes Found
GO:0005737	cytoplasm	GO_CellularComponent-EBI-UniProt-GOA-ACAP-ARAP_23.01.2024_00h00	0.00	0.00	0.00	0.00	[2, 3]	Group0	4.15	368.00	[ABLIM3, ACAA1, ACAD9, ACY1, AFAP1L1, AFG3L2, AHSG, AKAP12, AKR1A1, ALAD, ALDH4A1, ALDOB, AP2S1, APOD, APOE, ARHGEF1, ARPC5L, ARPP19, ATG3, ATG7, ATP2C2, ATP5F1A, ATP5IF1, ATP5PF, ATP5PO, ATP6V0D1, ATP6V1G1, AURKAIP1, B4GALT4, BCKDHA, BDH1, BRPF1, CA5A, CABLES1, CALM3, CAMTA1, CAPN12, CAPNS1, CAPZB, CBR3, CC2D1A, CC2D1B, CCDC61, CCN6, CCNDBP1, CCR5, CD274, CDA, CDH15, CDK5RAP2, CDKN3, CHAC1, CHCHD4, CHSY3, CIAO2B, CIAPIN1, CIDEA, CKMT1A, CLN6, CLTA, CMC2, CNDP2, COG8, COMMD9, COQ3, COQ4, COQ8B, COQ9, COTL1, COX17, COX6B, COX7A2, COX7C, CPT2, CRAT, CROCC, CSTB, CTH, CTPS1, CTU2, CYTH2, DDX28, DELE1, DFFA, DHCR24, DIO1, DMAC2, DMAC2L, DMWD, DNAAF4, DNAJB11, DNAJC11, DND1, DOK6, DOLPP1, DYN
GO:0043232	intracellular non-membrane-bounded organelle	GO_CellularComponent-EBI-UniProt-GOA-ACAP-ARAP_23.01.2024_00h00	0.00	0.02	0.00	0.00	[4]	Group1	4.18	153.00	[ABLIM3, AEN, AFAP1L1, ALDOB, APOD, ARPC5L, ATP6V0D1, BRPF1, CALM3, CAMTA1, CAPZB, CC2D1A, CCDC113, CCDC61, CD274, CDK5RAP2, CENPT, CFDP1, CIAO2B, CIAPIN1, CIDEA, CLN6, CPT2, CROCC, CSTB, DDX28, DFFA, DNAAF4, DYNC1LI1, EBNA1BP2, ENKD1, ETF1, EXOSC2, EXOSC5, FAAP24, FAM107A, FAM32A, FHIT, GEMIN2, GINS2, GNL2, GNL3, GSK3B, HAUS1, HAUS2, IFT57, IK, IP6K1, IP6K2, IQCG, KATNB1, KDM4A, KLF1, KLF2, LDLRAP1, LIMD1, LMOD3, LOC100359361, LOC100739087, MAD2L2, MAP1A, MAP1LC3B, MAP7D1, MAST2, MFAP1, MLH1, MRPL37, MRPL46, MRPL47, MRPL50, MRPS15, MRPS25, MRPS6, MST1R, MTCL1, MYL3, MYOM1, NAT10, NLRC5, NOC2L, NUDC, NUDT16, NUP62, ODF2, PARVA, PIH1D1, PINK1, PNRC1, POC1A, POLE3, POLR2H, PPAN, PRMT7, PRI
GO:0005829	cytosol	GO_CellularComponent-EBI-UniProt-GOA-ACAP-ARAP_23.01.2024_00h00	0.00	0.00	0.00	0.00	[2, 3, 4]	Group2	4.71	145.00	[AFAP1L1, AKAP12, AKR1A1, ALAD, ALDH4A1, ALDOB, APOD, ARHGEF1, ATG3, BRPF1, CABLES1, CAMTA1, CAPNS1, CBR3, CC2D1A, CC2D1B, CDA, CDK5RAP2, CDKN3, CHAC1, CIAO2B, CNDP2, COMMD9, COTL1, CROCC, CSTB, DDX28, DELE1, DFFA, DNAAF4, EIF3G, ELP6, ENO1, ETF1, EXOSC2, EXOSC5, FHIT, GBA2, GEMIN2, GLYCTK, GRAMD1A, GRHPR, GSK3A, HAUS1, HINT1, HRH1, HSBP1, HSPA4, HSPA5, INTS11, IP6K1, IQCG, KATNB1, KAZN, KCNN4, KDM4A, KPNA1, LDHC, LDLRAP1, LOC100521256, LOC100739087, LRSAM1, LSAMP, LSG1, LYPLA2, MAP1A, MAP1LC3B, MRPL50, MTHFD1L, MTO1, MVB12B, NANS, NCCRP1, NDUFAF1, NFAT5, NKIRAS1, NLRC5, NOC2L, NUDC, OXSM, PADI3, PARVA, PDCD5, PINK1, PLCG2, PPIE, PRDX1, PRDX2, PRKN, PRMT7, PSKH1, PSMB7, PSMC4, PSMG2, PTPN
GO:0042175	nuclear outer membrane-endoplasmic reticulum membrane network	GO_CellularComponent-EBI-UniProt-GOA-ACAP-ARAP_23.01.2024_00h00	0.00	0.02	0.00	0.00	[3]	Group3	5.94	42.00	[DIO1, DOLPP1, ELOVL1, EMC3, ENO1, GBA2, GRAMD1A, HSPA5, ILVBL, PDIA3, PIGO, PIGP, PIGV, PLOD1, RASGRP1, RPL11, RPL14, RPL29, RPL32, RPL35, RPS23, RPS29, RPSA, SAR1B, SCAP, SEC11C, SELENON, SELENOS, SLC37A1, SPCS1, SRPRB, SSR3, STX17, SURF4, TECR, TEX264, TMEM147, TMEM208, TMEM39B, TYRO3, UBE2J1, XXYLT1]
GO:0031090	organelle membrane	GO_CellularComponent-EBI-UniProt-GOA-ACAP-ARAP_23.01.2024_00h00	0.00	0.00	0.00	0.00	[3, 4]	Group3	5.26	122.00	[ACAD9, AEN, AFG3L2, AP2S1, ATP2C2, ATP5F1A, ATP5PF, ATP5PO, ATP6V0D1, ATP6V1G1, B4GALT4, BDH1, CC2D1A, CC2D1B, CD274, CHSY3, CKMT1A, CLTA, COG8, COQ3, COQ4, COQ9, COX6B, COX7A2, COX7C, CPT2, DELE1, DIO1, DMAC2, DNAJC11, DOLPP1, DYNC1LI1, EHD4, ELOVL1, EMC3, ENO1, EXOSC2, GBA2, GLIPR2, GRAMD1A, HSPA5, ILVBL, LIG1, LOC100522551, LOC100524873, LOC100739087, MAP1LC3B, MPC1, MPPE1, MVB12B, NDUFA8, NDUFAF3, NDUFB4, NDUFS5, NDUFV2, NECAP2, NUP62, PDIA3, PEX10, PIGO, PIGP, PIGV, PINK1, PLEKHM2, PLOD1, POLR2H, POMGNT1, PSENEN, PSMC4, RAB6B, RAB7A, RAB8A, RASGRP1, RDH13, RER1, RPL11, RPL14, RPL29, RPL32, RPL35, RPS23, RPS29, RPSA, RTN4IP1, SAR1B, SCAP, SCG3, SEC11C, SELENON, SELENOS, SESN2, SLC25A26
GO:0005783	endoplasmic reticulum	GO_CellularComponent-EBI-UniProt-GOA-ACAP-ARAP_23.01.2024_00h00	0.00	0.01	0.00	0.00	[3, 4, 5]	Group3	5.29	68.00	[APOD, APOE, ATP2C2, CLN6, DHCR24, DIO1, DNAJB11, DOLPP1, EHD4, ELOVL1, EMC3, GBA2, GRAMD1A, HSPA5, HSPBP1, ILVBL, LAMA3, LSG1, MBOAT7, MRAP2, NKIRAS1, P3H1, PDIA3, PIGO, PIGP, PIGV, PINK1, PLOD1, PNLDC1, POFUT2, PPIB, PRKN, PSENEN, RASGRP1, RER1, RPL11, RPL14, RPL18, RPL24, RPL29, RPL32, RPL35, RPL4, RPS23, RPS29, RPSA, SAR1B, SCAP, SEC11C, SELENON, SELENOS, SIL1, SLC37A1, SMPD2, SPCS1, SRPRB, SSR3, STX17, SURF4, TECR, TEX264, TMEM147, TMEM208, TMEM39B, TXNDC12, TYRO3, UBE2J1, XXYLT1]
GO:0034641	cellular nitrogen compound metabolic process	GO_BiologicalProcess-EBI-UniProt-GOA-ACAP-ARAP_23.01.2024_00h00	0.00	0.01	0.00	0.00	[3]	Group4	4.00	194.00	[ABLIM3, AEN, ALAD, ALDOB, APOE, ATMIN, ATP5F1A, ATP5IF1, ATP5PF, ATP5PO, B4GALT4, BRPF1, CAMTA1, CC2D1A, CC2D1B, CD274, CDA, CDK5RAP2, CIDEA, CPT2, CRAT, CTH, CTPS1, CTU2, DALRD3, DFFA, DKK3, DNAJB11, DND1, DUT, E2F2, EAF1, EEFSEC, EIF1B, EIF2B3, EIF3G, EIF3I, EIF3K, ELOVL1, ELP6, ENO1, ERI3, ETF1, EXOSC2, EXOSC5, FAAP24, FHIT, FOXJ3, GEMIN2, GINS2, GMPPB, GNL3, GSK3A, GTF2H5, GTF3C5, HEMK1, HINT1, HSBP1, IK, INTS11, KDM4A, KLF1, KLF2, LDHC, LIG1, LIMD1, LIN37, LMCD1, LOC100359361, LOC100520273, LOC100521256, LOC100738134, LOC100739087, LRRC47, MAD2L2, MAF, MCEE, MED22, MED8, MFAP1, MLH1, MRPL37, MRPL47, MRPS15, MRPS6, MTHFD1L, MTO1, MTRF1L, MYCBP, NAT10, NFAT5, NFYC, NLRC5, NOC2L, NPAS1, NUDT16, NUP62
GO:0044249	cellular biosynthetic process	GO_BiologicalProcess-EBI-UniProt-GOA-ACAP-ARAP_23.01.2024_00h00	0.00	0.00	0.00	0.00	[3]	Group4	4.08	238.00	[ABLIM3, AFG3L2, AKR1A1, ALAD, ALPK2, APOD, APOE, ARPP19, ATMIN, ATP5F1A, ATP5IF1, ATP5PF, ATP5PO, B4GALT4, BRPF1, CAMTA1, CC2D1A, CC2D1B, CCBE1, CD274, CDA, CDK5RAP2, CHAC1, CHCHD4, CHSY3, CIAO2B, CIDEA, COQ3, COQ4, COQ8B, COQ9, CTH, CTPS1, CTU2, DALRD3, DFFA, DHCR24, DHDDS, DKK3, DNAJB11, DND1, DOLPP1, DUT, E2F2, EAF1, EEFSEC, EIF1B, EIF2B3, EIF3G, EIF3I, EIF3K, ELOVL1, ELP6, ENO1, EPHB2, ERI3, ETF1, EXOSC2, EXOSC5, FOXJ3, GEMIN2, GMPPB, GNL3, GORASP1, GSK3A, GSK3B, GTF2H5, GTF3C5, HEG1, HINT1, HMGCL, HSBP1, HSPA4, HSPA5, IK, ILVBL, INTS11, IP6K1, IP6K2, KDM4A, KLF1, KLF2, LDHC, LIG1, LIMD1, LIN37, LMCD1, LOC100359361, LOC100520273, LOC100521256, LOC100522551, LOC100738134, LOC100739087, LRRC47, LTF, MAI
GO:1901576	organic substance biosynthetic process	GO_BiologicalProcess-EBI-UniProt-GOA-ACAP-ARAP_23.01.2024_00h00	0.00	0.00	0.00	0.00	[3]	Group4	4.08	241.00	[ABLIM3, AFG3L2, AKR1A1, ALAD, ALPK2, APOD, APOE, ARPP19, ATMIN, ATP5F1A, ATP5IF1, ATP5PF, ATP5PO, B4GALT4, BRPF1, CAMTA1, CC2D1A, CC2D1B, CCBE1, CD274, CDA, CDK5RAP2, CHAC1, CHCHD4, CHSY3, CIAO2B, CIDEA, CKM, CKMT1A, COQ3, COQ4, COQ8B, COQ9, CTH, CTPS1, CTU2, DALRD3, DFFA, DHCR24, DHDDS, DKK3, DNAJB11, DND1, DOLPP1, DUT, E2F2, EAF1, EEFSEC, EIF1B, EIF2B3, EIF3G, EIF3I, EIF3K, ELOVL1, ELP6, ENO1, EPHB2, ERI3, ETF1, EXOSC2, EXOSC5, FOXJ3, GEMIN2, GMPPB, GNL3, GORASP1, GSK3A, GSK3B, GTF2H5, GTF3C5, HEG1, HINT1, HMGCL, HSBP1, HSPA4, HSPA5, IK, ILVBL, INTS11, IP6K1, IP6K2, KDM4A, KLF1, KLF2, LDHC, LIG1, LIMD1, LIN37, LMCD1, LOC100359361, LOC100520273, LOC100521256, LOC100522551, LOC100738134, LOC100739087, LI
GO:0022626	cytosolic ribosome	GO_CellularComponent-EBI-UniProt-GOA-ACAP-ARAP_23.01.2024_00h00	0.00	0.00	0.00	0.00	[3, 4, 5, 6]	Group5	25.71	27.00	[APOD, ETF1, LOC100739087, RPL11, RPL12, RPL14, RPL17, RPL24, RPL28, RPL29, RPL32, RPL35, RPL4, RPL7A, RPLP1, RPS12, RPS13, RPS14, RPS16, RPS23, RPS29, RPS5, RPS6, RPS8, RPS9, RPSA, ZCCHC17]
GO:0043043	peptide biosynthetic process	GO_BiologicalProcess-EBI-UniProt-GOA-ACAP-ARAP_23.01.2024_00h00	0.00	0.00	0.00	0.00	[5, 6]	Group5	9.36	51.00	[DALRD3, EEFSEC, EIF1B, EIF2B3, EIF3G, EIF3I, EIF3K, ETF1, GMPPB, LIMD1, LOC100521256, LOC100739087, LRRC47, MRPL37, MRPL47, MRPS15, MRPS6, MTRF1L, NAT10, PAIP2, PINK1, RPL11, RPL12, RPL14, RPL17, RPL18, RPL24, RPL28, RPL29, RPL32, RPL35, RPL4, RPLP1, RPS12, RPS13, RPS14, RPS16, RPS23, RPS29, RPS5, RPS6, RPS8, RPS9, RPSA, RSL24D1, SARS2, SESN2, SLC1A2, TCEANC2, TRIP4, TRMT10C]
GO:0002181	cytoplasmic translation	GO_BiologicalProcess-EBI-UniProt-GOA-ACAP-ARAP_23.01.2024_00h00	0.00	0.00	0.00	0.00	[5, 6, 7, 8]	Group5	16.67	14.00	[EIF2B3, EIF3G, EIF3I, EIF3K, ETF1, RPL17, RPL24, RPL29, RPL32, RPLP1, RPS14, RPS23, RPS29, RPSA]
GO:0030867	rough endoplasmic reticulum membrane	GO_CellularComponent-EBI-UniProt-GOA-ACAP-ARAP_23.01.2024_00h00	0.00	0.00	0.00	0.00	[5, 6, 7, 8]	Group5	27.03	10.00	[PLOD1, RPL11, RPL14, RPL29, RPL32, RPL35, RPS23, RPS29, RPSA, SRPRB]
GO:0098556	cytoplasmic side of rough endoplasmic reticulum membrane	GO_CellularComponent-EBI-UniProt-GOA-ACAP-ARAP_23.01.2024_00h00	0.00	0.00	0.00	0.00	[5, 6, 7, 8, 9]	Group5	36.36	8.00	[RPL11, RPL14, RPL29, RPL32, RPL35, RPS23, RPS29, RPSA]
GO:0045182	translation regulator activity	GO_BiologicalProcess-EBI-UniProt-GOA-ACAP-ARAP_23.01.2024_00h00	0.00	0.04	0.00	0.00	[6, 7, 8, 9, 10]	Group5	10.66	13.00	[EEFSEC, EIF1B, EIF2B3, EIF3G, EIF3I, EIF3K, ETF1, GMPPB, MTRF1L, PAIP2, RPS14, RPS9, TCEANC2]
KEGG:00190	Oxidative phosphorylation	KEGG_23.01.2024	0.00	0.04	0.00	0.00	[-1]	Group6	10.22	14.00	[ATP5F1A, ATP5PF, ATP5PO, ATP6V0D1, ATP6V1G1, COX17, COX6B, COX7A2, COX7C, LOC100524873, NDUFA8, NDUFB4, NDUFS5, NDUFV2]
GO:0005739	mitochondrion	GO_CellularComponent-EBI-UniProt-GOA-ACAP-ARAP_23.01.2024_00h00	0.00	0.00	0.00	0.00	[3, 4, 5]	Group6	6.31	82.00	[ACAD9, AFG3L2, ALDH4A1, ATP5F1A, ATP5IF1, ATP5PF, ATP5PO, AURKAIP1, BCKDHA, BDH1, CA5A, CCN6, CHCHD4, CIAPIN1, CIDEA, CKMT1A, CMC2, COQ3, COQ4, COQ8B, COQ9, COX17, COX6B, COX7A2, COX7C, CPT2, CRAT, DDX28, DELE1, DMAC2, DNAJC11, ECH1, ETHE1, GSK3A, HMGCL, HSPA4, HSPA5, LDHA, LDHC, LIG1, LOC100521256, LOC100524873, MPC1, MRPL37, MRPL46, MRPL47, MRPL50, MRPS15, MRPS25, MRPS6, MTHFD1L, MTO1, MTRF1L, MYCBP, NDUFA8, NDUFAF1, NDUFAF3, NDUFB4, NDUFS5, NDUFV2, NLRP5, OXSM, PCCB, PINK1, PMAIP1, PRELID2, PRKN, RAB7A, RDH13, RTN4IP1, SARS2, SESN2, SLC25A26, SLC25A38, STX17, TFB1M, TIMM50, TIMM9, TIMMDC1, TK2, TRMT10C, TXNRD3]
GO:0098798	mitochondrial protein-containing complex	GO_CellularComponent-EBI-UniProt-GOA-ACAP-ARAP_23.01.2024_00h00	0.00	0.00	0.00	0.00	[2, 4, 5, 6]	Group6	9.79	23.00	[AFG3L2, ATP5F1A, ATP5PF, ATP5PO, BCKDHA, COX6B, COX7A2, COX7C, DMAC2, DNAJC11, LOC100524873, MRPL37, MRPL46, MRPL47, MRPL50, MRPS15, MRPS6, NDUFA8, NDUFB4, NDUFS5, NDUFV2, TIMM50, TRMT10C]
GO:0005740	mitochondrial envelope	GO_CellularComponent-EBI-UniProt-GOA-ACAP-ARAP_23.01.2024_00h00	0.00	0.00	0.00	0.00	[4, 5, 6]	Group6	7.63	38.00	[ACAD9, AFG3L2, ATP5F1A, ATP5PF, ATP5PO, BDH1, CHCHD4, CIAPIN1, CIDEA, CKMT1A, COQ3, COQ4, COQ9, COX17, COX6B, COX7A2, COX7C, CPT2, DELE1, DMAC2, DNAJC11, LOC100524873, MPC1, NDUFA8, NDUFAF3, NDUFB4, NDUFS5, NDUFV2, PINK1, PRELID2, RAB7A, RDH13, RTN4IP1, SLC25A26, SLC25A38, TIMM50, TIMM9, TIMMDC1]
GO:0031966	mitochondrial membrane	GO_CellularComponent-EBI-UniProt-GOA-ACAP-ARAP_23.01.2024_00h00	0.00	0.00	0.00	0.00	[4, 5, 6, 7]	Group6	7.24	33.00	[ACAD9, AFG3L2, ATP5F1A, ATP5PF, ATP5PO, BDH1, CKMT1A, COQ3, COQ4, COQ9, COX6B, COX7A2, COX7C, CPT2, DELE1, DMAC2, DNAJC11, LOC100524873, MPC1, NDUFA8, NDUFAF3, NDUFB4, NDUFS5, NDUFV2, PINK1, RAB7A, RDH13, RTN4IP1, SLC25A26, SLC25A38, TIMM50, TIMM9, TIMMDC1]
GO:0098800	inner mitochondrial membrane protein complex	GO_CellularComponent-EBI-UniProt-GOA-ACAP-ARAP_23.01.2024_00h00	0.00	0.00	0.00	0.00	[3, 4, 5, 6, 7, 8, 9]	Group6	12.50	15.00	[AFG3L2, ATP5F1A, ATP5PF, ATP5PO, COX6B, COX7A2, COX7C, DMAC2, DNAJC11, LOC100524873, NDUFA8, NDUFB4, NDUFS5, NDUFV2, TIMM50]
GO:0005743	mitochondrial inner membrane	GO_CellularComponent-EBI-UniProt-GOA-ACAP-ARAP_23.01.2024_00h00	0.00	0.00	0.00	0.00	[5, 6, 7, 8]	Group6	10.11	28.00	[AFG3L2, ATP5F1A, ATP5PF, ATP5PO, BDH1, CKMT1A, COQ3, COQ4, COQ9, COX6B, COX7A2, COX7C, CPT2, DMAC2, DNAJC11, LOC100524873, MPC1, NDUFA8, NDUFAF3, NDUFB4, NDUFS5, NDUFV2, PINK1, RDH13, SLC25A26, SLC25A38, TIMM50, TIMM9]
GO:0030684	preribosome	GO_CellularComponent-EBI-UniProt-GOA-ACAP-ARAP_23.01.2024_00h00	0.00	0.04	0.00	0.00	[3]	Group7	12.36	11.00	[NAT10, NOC2L, PPAN, PWP2, RPS12, RPS13, RPS14, RPS23, RPS5, RPS9, RRP1B]
GO:0043603	amide metabolic process	GO_BiologicalProcess-EBI-UniProt-GOA-ACAP-ARAP_23.01.2024_00h00	0.00	0.00	0.00	0.00	[3]	Group7	7.36	67.00	[ALDOB, APOE, B4GALT4, CHAC1, CLN6, DALRD3, EEFSEC, EIF1B, EIF2B3, EIF3G, EIF3I, EIF3K, ELOVL1, ETF1, ETHE1, GBA2, GMPPB, GSK3A, LIMD1, LOC100521256, LOC100739087, LRRC47, MCEE, MRPL37, MRPL47, MRPS15, MRPS6, MTRF1L, NAT10, OXSM, PAIP2, PINK1, PSENEN, RPL11, RPL12, RPL14, RPL17, RPL18, RPL24, RPL28, RPL29, RPL32, RPL35, RPL4, RPLP1, RPS12, RPS13, RPS14, RPS16, RPS23, RPS29, RPS5, RPS6, RPS8, RPS9, RPSA, RSL24D1, SARS2, SCG5, SEC11C, SESN2, SLC1A2, SMPD2, SPCS1, TCEANC2, TRIP4, TRMT10C]
GO:0044085	cellular component biogenesis	GO_BiologicalProcess-EBI-UniProt-GOA-ACAP-ARAP_23.01.2024_00h00	0.00	0.00	0.00	0.00	[3]	Group7	4.71	118.00	[ABLIM3, ACAD9, ALAD, ALDOB, APOD, APOE, ARMC2, ARPC5L, ATG3, ATG7, CAPZB, CCDC113, CCDC61, CDH15, CDK5RAP2, CENPT, CFAP298, CIAO2B, CIAPIN1, CLTA, COX17, COX7A2, CROCC, CTH, DDX28, DEF8, DMAC2, DNAAF4, EBNA1BP2, EHD4, EIF3G, EIF3I, EIF3K, ENKD1, EPHB2, ERI3, EXOSC2, FAM107A, GAP43, GBA2, GEMIN2, GSK3B, GTF2H5, HAUS1, HAUS2, HSPA4, HSPA5, IFT57, IQCG, ITGB5, LIMD1, LMOD3, LOC100739087, LRSAM1, LSG1, MAP1LC3B, MLH1, NAPA, NAT10, NDUFAF1, NDUFAF3, NDUFB4, NDUFS5, NOC2L, NUP62, ODF2, PADI4, PARVA, PDIA3, PIH1D1, PINK1, PLCG2, POLE3, PPAN, PRKN, PRMT7, PSMG2, PTPN23, PWP2, RAB7A, RAB8A, RCL1, RPL14, RPL35, RPL7A, RPS12, RPS13, RPS14, RPS16, RPS23, RPS5, RPS6, RPS8, RPS9, RPSA, RRP1B, RSL24D1, RSRP1, RUVBI
GO:1901564	organonitrogen compound metabolic process	GO_BiologicalProcess-EBI-UniProt-GOA-ACAP-ARAP_23.01.2024_00h00	0.00	0.00	0.00	0.00	[3]	Group7	4.64	185.00	[AFG3L2, ALAD, ALDH4A1, ALDOB, ANAPC13, APOD, APOE, ARPP19, ASB6, ATG3, ATG7, ATP5F1A, ATP5IF1, ATP5PF, ATP5PO, AURKAIP1, B4GALT4, BCKDHA, CALM3, CAMTA1, CAPN12, CCBE1, CDA, CDC26, CHAC1, CHCHD4, CHSY3, CIAO2B, CKM, CKMT1A, CLN6, CNDP2, CPT2, CRAT, CSTB, CTH, CTPS1, CTU2, DALRD3, DFFA, DHCR24, DHDDS, DIO1, DNAAF4, DNAJB11, DOLPP1, DUT, EEFSEC, EIF1B, EIF2B3, EIF3G, EIF3I, EIF3K, ELOVL1, ENO1, EPHB2, ETF1, ETHE1, FBXO17, FBXO31, FBXO38, FHIT, GBA2, GMPPB, GNL3, GORASP1, GSK3A, GSK3B, HECTD3, HINT1, HMGCL, HSPA4, HSPA5, HSPBP1, ILVBL, IP6K2, KLF2, LDHC, LDLRAP1, LIMD1, LOC100521256, LOC100522551, LOC100739087, LRRC47, LRSAM1, LTF, MAD2L2, MBOAT7, MCEE, MKRN2, MPPE1, MRPL37, MRPL47, MRPS15, MRPS6, MTH
GO:0019538	protein metabolic process	GO_BiologicalProcess-EBI-UniProt-GOA-ACAP-ARAP_23.01.2024_00h00	0.00	0.00	0.00	0.00	[3, 4]	Group7	4.93	155.00	[AFG3L2, ALAD, ANAPC13, APOD, APOE, ARPP19, ASB6, ATG3, ATG7, ATP5IF1, AURKAIP1, B4GALT4, CALM3, CAMTA1, CAPN12, CCBE1, CDC26, CHAC1, CHCHD4, CHSY3, CIAO2B, CLN6, CNDP2, CSTB, CTH, CTU2, DALRD3, DFFA, DHCR24, DHDDS, DNAAF4, DNAJB11, DOLPP1, EEFSEC, EIF1B, EIF2B3, EIF3G, EIF3I, EIF3K, ENO1, EPHB2, ETF1, FBXO17, FBXO31, FBXO38, FHIT, GMPPB, GNL3, GORASP1, GSK3A, GSK3B, HECTD3, HINT1, HSPA4, HSPA5, HSPBP1, IP6K2, KLF2, LDLRAP1, LIMD1, LOC100521256, LOC100739087, LRRC47, LRSAM1, LTF, MAD2L2, MKRN2, MPPE1, MRPL37, MRPL47, MRPS15, MRPS6, MTRF1L, NAT10, NUDC, P3H1, PADI4, PAIP2, PDCD5, PDIA3, PFDN1, PIGO, PIGP, PIGV, PINK1, PKN1, PLCG2, POFUT2, POMGNT1, PPIB, PPIE, PPP1R8, PRKN, PRR5L, PSENEN, PSMB7, PSMD7, P
GO:0003723	RNA binding	GO_MolecularFunction-EBI-UniProt-GOA-ACAP-ARAP_23.01.2024_00h00	0.00	0.04	0.00	0.00	[4]	Group7	5.47	51.00	[CTU2, DALRD3, DDX28, DND1, EEFSEC, EIF1B, EIF3G, EIF3I, EIF3K, ETF1, EXOSC2, GNL3, LOC100521256, LOC100739087, LRRC47, LSM10, MRPS6, NAT10, NUDT16, OOEP, PAIP2, PNLDC1, PPAN, PPIE, PPP1R8, PRPF4, RBM15B, RBM22, RBM42, RBPMS2, RNASEH2A, RPL11, RPL12, RPL14, RPL35, RPL7A, RPS13, RPS14, RPS16, RPS29, RPS5, RPS9, RPUSD2, SARS2, SIDT1, SNRPA, SNRPD2, TFB1M, TOE1, TRIM32, TRMT10C]
GO:0006518	peptide metabolic process	GO_BiologicalProcess-EBI-UniProt-GOA-ACAP-ARAP_23.01.2024_00h00	0.00	0.00	0.00	0.00	[4]	Group7	8.50	59.00	[APOE, CHAC1, DALRD3, EEFSEC, EIF1B, EIF2B3, EIF3G, EIF3I, EIF3K, ETF1, ETHE1, GMPPB, GSK3A, LIMD1, LOC100521256, LOC100739087, LRRC47, MRPL37, MRPL47, MRPS15, MRPS6, MTRF1L, NAT10, PAIP2, PINK1, PSENEN, RPL11, RPL12, RPL14, RPL17, RPL18, RPL24, RPL28, RPL29, RPL32, RPL35, RPL4, RPLP1, RPS12, RPS13, RPS14, RPS16, RPS23, RPS29, RPS5, RPS6, RPS8, RPS9, RPSA, RSL24D1, SARS2, SCG5, SEC11C, SESN2, SLC1A2, SPCS1, TCEANC2, TRIP4, TRMT10C]
GO:0022613	ribonucleoprotein complex biogenesis	GO_BiologicalProcess-EBI-UniProt-GOA-ACAP-ARAP_23.01.2024_00h00	0.00	0.00	0.00	0.00	[4]	Group7	9.95	37.00	[DDX28, EBNA1BP2, EIF3G, EIF3I, EIF3K, ERI3, EXOSC2, GEMIN2, LOC100739087, LSG1, NAT10, NOC2L, PIH1D1, PPAN, PRMT7, PWP2, RCL1, RPL14, RPL35, RPL7A, RPS12, RPS13, RPS14, RPS16, RPS23, RPS5, RPS6, RPS8, RPS9, RPSA, RRP1B, RSL24D1, RSRP1, RUVBL1, SNRPD2, TFB1M, ZNF593]
GO:1901566	organonitrogen compound biosynthetic process	GO_BiologicalProcess-EBI-UniProt-GOA-ACAP-ARAP_23.01.2024_00h00	0.00	0.00	0.00	0.00	[4]	Group7	6.83	87.00	[ALAD, APOE, ATP5F1A, ATP5IF1, ATP5PF, ATP5PO, B4GALT4, CDA, CHSY3, CKM, CKMT1A, CTH, CTPS1, DALRD3, DHDDS, DOLPP1, DUT, EEFSEC, EIF1B, EIF2B3, EIF3G, EIF3I, EIF3K, ELOVL1, ENO1, ETF1, GMPPB, GORASP1, ILVBL, LDHC, LIMD1, LOC100521256, LOC100522551, LOC100739087, LRRC47, MPPE1, MRPL37, MRPL47, MRPS15, MRPS6, MTHFD1L, MTRF1L, NAT10, PAIP2, PCBD2, PIGO, PIGP, PIGV, PINK1, POFUT2, POMGNT1, RASGRP1, RPL11, RPL12, RPL14, RPL17, RPL18, RPL24, RPL28, RPL29, RPL32, RPL35, RPL4, RPLP1, RPS12, RPS13, RPS14, RPS16, RPS23, RPS29, RPS5, RPS6, RPS8, RPS9, RPSA, RSL24D1, SARS2, SESN2, SLC1A2, SMPD2, SRM, TCEANC2, TRIP4, TRMT10C, UBE2J1, UROD, XXYLT1]
GO:0022626	cytosolic ribosome	GO_CellularComponent-EBI-UniProt-GOA-ACAP-ARAP_23.01.2024_00h00	0.00	0.00	0.00	0.00	[3, 4, 5, 6]	Group7	25.71	27.00	[APOD, ETF1, LOC100739087, RPL11, RPL12, RPL14, RPL17, RPL24, RPL28, RPL29, RPL32, RPL35, RPL4, RPL7A, RPLP1, RPS12, RPS13, RPS14, RPS16, RPS23, RPS29, RPS5, RPS6, RPS8, RPS9, RPSA, ZCCHC17]
GO:0043604	amide biosynthetic process	GO_BiologicalProcess-EBI-UniProt-GOA-ACAP-ARAP_23.01.2024_00h00	0.00	0.00	0.00	0.00	[4, 5]	Group7	8.36	54.00	[B4GALT4, DALRD3, EEFSEC, EIF1B, EIF2B3, EIF3G, EIF3I, EIF3K, ELOVL1, ETF1, GMPPB, LIMD1, LOC100521256, LOC100739087, LRRC47, MRPL37, MRPL47, MRPS15, MRPS6, MTRF1L, NAT10, PAIP2, PINK1, RPL11, RPL12, RPL14, RPL17, RPL18, RPL24, RPL28, RPL29, RPL32, RPL35, RPL4, RPLP1, RPS12, RPS13, RPS14, RPS16, RPS23, RPS29, RPS5, RPS6, RPS8, RPS9, RPSA, RSL24D1, SARS2, SESN2, SLC1A2, SMPD2, TCEANC2, TRIP4, TRMT10C]
GO:0044391	ribosomal subunit	GO_CellularComponent-EBI-UniProt-GOA-ACAP-ARAP_23.01.2024_00h00	0.00	0.00	0.00	0.00	[3, 6]	Group7	19.25	31.00	[LOC100739087, MRPL37, MRPL46, MRPL47, MRPL50, MRPS15, MRPS6, RPL11, RPL12, RPL14, RPL17, RPL24, RPL28, RPL29, RPL32, RPL35, RPL4, RPL7A, RPLP1, RPS12, RPS13, RPS14, RPS16, RPS23, RPS29, RPS5, RPS6, RPS8, RPS9, RPSA, ZCCHC17]
GO:0005791	rough endoplasmic reticulum	GO_CellularComponent-EBI-UniProt-GOA-ACAP-ARAP_23.01.2024_00h00	0.00	0.00	0.00	0.00	[4, 5, 6]	Group7	20.34	12.00	[PLOD1, RPL11, RPL14, RPL18, RPL29, RPL32, RPL35, RPL4, RPS23, RPS29, RPSA, SRPRB]
GO:0005840	ribosome	GO_CellularComponent-EBI-UniProt-GOA-ACAP-ARAP_23.01.2024_00h00	0.00	0.00	0.00	0.00	[5]	Group7	16.74	36.00	[APOD, ETF1, LOC100739087, MRPL37, MRPL46, MRPL47, MRPL50, MRPS15, MRPS25, MRPS6, RPL11, RPL12, RPL14, RPL17, RPL18, RPL24, RPL28, RPL29, RPL32, RPL35, RPL4, RPL7A, RPLP1, RPS12, RPS13, RPS14, RPS16, RPS23, RPS29, RPS5, RPS6, RPS8, RPS9, RPSA, RSL24D1, ZCCHC17]
GO:0042254	ribosome biogenesis	GO_BiologicalProcess-EBI-UniProt-GOA-ACAP-ARAP_23.01.2024_00h00	0.00	0.00	0.00	0.00	[5]	Group7	11.28	29.00	[DDX28, EBNA1BP2, ERI3, EXOSC2, LOC100739087, LSG1, NAT10, NOC2L, PIH1D1, PPAN, PWP2, RCL1, RPL14, RPL35, RPL7A, RPS12, RPS13, RPS14, RPS16, RPS23, RPS5, RPS6, RPS8, RPS9, RPSA, RRP1B, RSL24D1, TFB1M, ZNF593]
GO:0015934	large ribosomal subunit	GO_CellularComponent-EBI-UniProt-GOA-ACAP-ARAP_23.01.2024_00h00	0.00	0.00	0.00	0.00	[4, 7]	Group7	18.00	18.00	[LOC100739087, MRPL37, MRPL46, MRPL47, MRPL50, RPL11, RPL12, RPL14, RPL17, RPL24, RPL28, RPL29, RPL32, RPL35, RPL4, RPL7A, RPLP1, ZCCHC17]
GO:0015935	small ribosomal subunit	GO_CellularComponent-EBI-UniProt-GOA-ACAP-ARAP_23.01.2024_00h00	0.00	0.00	0.00	0.00	[4, 7]	Group7	20.97	13.00	[MRPS15, MRPS6, RPS12, RPS13, RPS14, RPS16, RPS23, RPS29, RPS5, RPS6, RPS8, RPS9, RPSA]
GO:0042274	ribosomal small subunit biogenesis	GO_BiologicalProcess-EBI-UniProt-GOA-ACAP-ARAP_23.01.2024_00h00	0.00	0.01	0.00	0.00	[5, 6]	Group7	13.64	12.00	[NAT10, PWP2, RPS12, RPS13, RPS14, RPS16, RPS23, RPS5, RPS6, RPS8, RPS9, RPSA]
GO:0043043	peptide biosynthetic process	GO_BiologicalProcess-EBI-UniProt-GOA-ACAP-ARAP_23.01.2024_00h00	0.00	0.00	0.00	0.00	[5, 6]	Group7	9.36	51.00	[DALRD3, EEFSEC, EIF1B, EIF2B3, EIF3G, EIF3I, EIF3K, ETF1, GMPPB, LIMD1, LOC100521256, LOC100739087, LRRC47, MRPL37, MRPL47, MRPS15, MRPS6, MTRF1L, NAT10, PAIP2, PINK1, RPL11, RPL12, RPL14, RPL17, RPL18, RPL24, RPL28, RPL29, RPL32, RPL35, RPL4, RPLP1, RPS12, RPS13, RPS14, RPS16, RPS23, RPS29, RPS5, RPS6, RPS8, RPS9, RPSA, RSL24D1, SARS2, SESN2, SLC1A2, TCEANC2, TRIP4, TRMT10C]
GO:0022625	cytosolic large ribosomal subunit	GO_CellularComponent-EBI-UniProt-GOA-ACAP-ARAP_23.01.2024_00h00	0.00	0.00	0.00	0.00	[4, 5, 6, 7, 8]	Group7	27.45	14.00	[LOC100739087, RPL11, RPL12, RPL14, RPL17, RPL24, RPL28, RPL29, RPL32, RPL35, RPL4, RPL7A, RPLP1, ZCCHC17]
GO:0022627	cytosolic small ribosomal subunit	GO_CellularComponent-EBI-UniProt-GOA-ACAP-ARAP_23.01.2024_00h00	0.00	0.00	0.00	0.00	[4, 5, 6, 7, 8]	Group7	28.95	11.00	[RPS12, RPS13, RPS14, RPS16, RPS23, RPS29, RPS5, RPS6, RPS8, RPS9, RPSA]
GO:0002181	cytoplasmic translation	GO_BiologicalProcess-EBI-UniProt-GOA-ACAP-ARAP_23.01.2024_00h00	0.00	0.00	0.00	0.00	[5, 6, 7, 8]	Group7	16.67	14.00	[EIF2B3, EIF3G, EIF3I, EIF3K, ETF1, RPL17, RPL24, RPL29, RPL32, RPLP1, RPS14, RPS23, RPS29, RPSA]
GO:0030867	rough endoplasmic reticulum membrane	GO_CellularComponent-EBI-UniProt-GOA-ACAP-ARAP_23.01.2024_00h00	0.00	0.00	0.00	0.00	[5, 6, 7, 8]	Group7	27.03	10.00	[PLOD1, RPL11, RPL14, RPL29, RPL32, RPL35, RPS23, RPS29, RPSA, SRPRB]
GO:0098556	cytoplasmic side of rough endoplasmic reticulum membrane	GO_CellularComponent-EBI-UniProt-GOA-ACAP-ARAP_23.01.2024_00h00	0.00	0.00	0.00	0.00	[5, 6, 7, 8, 9]	Group7	36.36	8.00	[RPL11, RPL14, RPL29, RPL32, RPL35, RPS23, RPS29, RPSA]
