# Supplementary material for: Deciphering differences in DNA methylation and transcriptome profiles of oocytes from pigs with high and low developmental competence
Source: Environ Epigenet. 2025 Jun 3;11(1):dvaf018. doi: 10.1093/eep/dvaf018 (PMC12418950; doi:10.1093/eep/dvaf018)
Supplement: dvaf018_Supplemental_Files [file Sup table 8.pdf › Sup table 8.pdf]

seqnames	start	end	width	strand	chr	start	end	group1	group2	n1	n2	estimate1	estimate2	estimate	statistic	p-value	adj.p.value	annotation	ENSEMBL	SYMBOL	GENENAME
chr13	109230698	109231563	866	*	chr13	109230698	109231563	in_vitro	in_vivo	10	10	78.682	19.604	59.078	c(t = 4.93689	0.000115	0.83731181	Promoter (<=1kb)	ENSSSCG000	SLC7A14	solute carrier family 7 member 14 [Source:VGNC Symbol;Acc:VGNC:93173]
chr8	1721157	1729364	8208	*	chr8	1721157	1729364	in_vitro	in_vivo	10	15	96.623	91.1833333	5.43966667	c(t = 3.69487	0.00121	0.83731181	Distal Intergenic	ENSSSCG000	HTT	huntingtin [Source:VGNC Symbol;Acc:VGNC:89006]
chr15	140131918	140133932	2015	*	chr15	140131918	140133932	in_vitro	in_vivo	9	11	91.9411111	40.3609091	51.580202	c(t = 3.78604	0.00213	0.83731181	Distal Intergenic	ENSSSCG000	ATG4B	autophagy related 4B cysteine peptidase [Source:VGNC Symbol;Acc:VGNC:95947]
chr6	55340821	55341307	487	*	chr6	55340821	55341307	in_vitro	in_vivo	10	10	3.456	0	3.456	c(t = 4.15184	0.00248	0.83731181	Promoter (<=1kb)	ENSSSCG000	JOSD2	Josephin domain containing 2 [Source:VGNC Symbol;Acc:VGNC:111449]
chr1	95727119	95728266	1148	*	chr1	95727119	95728266	in_vitro	in_vivo	13	11	19.7646154	5.64	14.1246154	c(t = 3.58940	0.00265	0.83731181	Distal Intergenic	ENSSSCG000	ATP5F1A	ATP synthase F1 subunit alpha [Source:NCBI gene (formerly Entrezgene);Acc:100157880]
chr18	4992746	4995436	2691	*	chr18	4992746	4995436	in_vitro	in_vivo	13	11	1.95307692	0.10818182	1.8448951	c(t = 3.65079	0.00288	0.83731181	Distal Intergenic	ENSSSCG000	RHEB	Ras homolog, mTORC1 binding [Source:NCBI gene (formerly Entrezgene);Acc:100516050]
chr3	78901679	78906385	4707	*	chr3	78901679	78906385	in_vitro	in_vivo	15	16	6.44733333	1.128125	5.31920833	c(t = 3.38742	0.00323	0.83731181	Distal Intergenic	ENSSSCG000	TMEM17	transmembrane protein 17 [Source:NCBI gene (formerly Entrezgene);Acc:100627459]
chr14	54742312	54743052	741	*	chr14	54742312	54743052	in_vitro	in_vivo	12	14	8.88666667	1.085	7.80166667	c(t = 3.56339	0.00377	0.83731181	Promoter (<=1kb)	ENSSSCG000	ACTN2	actinin alpha 2 [Source:NCBI gene (formerly Entrezgene);Acc:100157406]
chr1	215806690	215808328	1639	*	chr1	215806690	215808328	in_vitro	in_vivo	12	14	1.69416667	0.06714286	1.62702381	c(t = 3.53981	0.00435	0.83731181	Distal Intergenic	ENSSSCG000	IL33	interleukin 33 [Source:VGNC Symbol;Acc:VGNC:89106]
chr1	181337919	181339414	1496	*	chr1	181337919	181339414	in_vitro	in_vivo	14	13	2.30428571	0.23923077	2.06505495	c(t = 3.37360	0.00445	0.83731181	Distal Intergenic	ENSSSCG000	RTRAF	RNA transcription, translation and transport factor [Source:NCBI gene (formerly Entrezgene);Acc:100154555]
chr2	66674117	66674817	701	*	chr2	66674117	66674817	in_vitro	in_vivo	11	10	5.42272727	0	5.42272727	c(t = 3.63378	0.00458	0.83731181	Distal Intergenic	NA	NA	NA
chr12	3893280	3894487	1208	*	chr12	3893280	3894487	in_vitro	in_vivo	12	9	30.2241667	69.9433333	-39.719167	c(t = -3.2262	0.0047	0.83731181	Distal Intergenic	ENSSSCG000	BIRC5	baculoviral IAP repeat containing 5 [Source:VGNC Symbol;Acc:VGNC:85827]
chr6	287312	290634	3323	*	chr6	287312	290634	in_vitro	in_vivo	11	9	99.2318182	93.9477778	5.2840404	c(t = 3.58188	0.00513	0.83731181	Distal Intergenic	ENSSSCG000	SPATA2L	spermatogenesis associated 2 like [Source:NCBI gene (formerly Entrezgene);Acc:100415811]
chr7	2130734	2133042	2309	*	chr7	2130734	2133042	in_vitro	in_vivo	11	12	2.33727273	0.615	1.72227273	c(t = 3.13515	0.00518	0.83731181	Distal Intergenic	ENSSSCG000	TUBB2B	tubulin, beta 2B class IIb [Source:NCBI gene (formerly Entrezgene);Acc:100153507]
chr6	14715131	14716191	1061	*	chr6	14715131	14716191	in_vitro	in_vivo	14	10	2.27357143	0.307	1.96657143	c(t = 3.21195	0.00535	0.83731181	Distal Intergenic	ENSSSCG000	MARVELD3	MARVEL domain containing 3 [Source:NCBI gene (formerly Entrezgene);Acc:100511453]
chr13	208214622	208222830	8209	*	chr13	208214622	208222830	in_vitro	in_vivo	12	16	11.955	34.85125	-22.89625	c(t = -3.0938	0.00559	0.83731181	Distal Intergenic	ENSSSCG000	ITGB2	integrin subunit beta 2 [Source:NCBI gene (formerly Entrezgene);Acc:396943]
chr5	2624003	2625906	1904	*	chr5	2624003	2625906	in_vitro	in_vivo	10	11	22.396	58.3554545	-35.959455	c(t = -3.1933	0.00625	0.83731181	Distal Intergenic	ENSSSCG000	PPARA	peroxisome proliferator activated receptor alpha [Source:NCBI gene (formerly Entrezgene);Acc:397239]
chr13	200128785	200129482	698	*	chr13	200128785	200129482	in_vitro	in_vivo	9	12	0.88222222	42.9241667	-42.041944	c(t = -3.3627	0.0063	0.83731181	Promoter (<=1kb)	ENSSSCG000	CLDN14	claudin 14 [Source:VGNC Symbol;Acc:VGNC:86731]
chr3	3610054	3619820	9767	*	chr3	3610054	3619820	in_vitro	in_vivo	12	13	64.9966667	85.11	-20.113333	c(t = -3.1238	0.00634	0.83731181	Distal Intergenic	ENSSSCG000	AP5Z1	adaptor related protein complex 5 subunit zeta 1 [Source:VGNC Symbol;Acc:VGNC:85397]
chr1	92421305	92422860	1556	*	chr1	92421305	92422860	in_vitro	in_vivo	10	14	3.948	0.90571429	3.04228571	c(t = 3.19933	0.00649	0.83731181	Promoter (<=1kb)	ENSSSCG00000004489		eukaryotic translation elongation factor 1 alpha 1 [Source:NCBI gene (formerly Entrezgene);Acc:574059]
chr11	15734905	15735621	717	*	chr11	15734905	15735621	in_vitro	in_vivo	13	12	2.15923077	0.25833333	1.90089744	c(t = 3.14482	0.00665	0.83731181	Distal Intergenic	ENSSSCG000	SLC25A15	solute carrier family 25 member 15 [Source:VGNC Symbol;Acc:VGNC:92994]
chr1	147781309	147782595	1287	*	chr1	147781309	147782595	in_vitro	in_vivo	13	10	3.13538462	0.529	2.60638462	c(t = 3.15888	0.00675	0.83731181	Distal Intergenic	ENSSSCG000	MBP	myelin basic protein [Source:NCBI gene (formerly Entrezgene);Acc:414286]
chr7	57702066	57703313	1248	*	chr7	57702066	57703313	in_vitro	in_vivo	9	12	4.00777778	0.26666667	3.74111111	c(t = 3.53339	0.00727	0.83731181	Distal Intergenic	ENSSSCG000	PEAK1	pseudopodium enriched atypical kinase 1 [Source:VGNC Symbol;Acc:VGNC:91300]
chr16	22151535	22153268	1734	*	chr16	22151535	22153268	in_vitro	in_vivo	9	12	2.40444444	0.1325	2.27194444	c(t = 3.47297	0.0074	0.83731181	Distal Intergenic	ENSSSCG000	SLC1A3	solute carrier family 1 member 3 [Source:VGNC Symbol;Acc:VGNC:92965]
chr8	45807958	45809344	1387	*	chr8	45807958	45809344	in_vitro	in_vivo	12	13	4.82916667	1.79769231	3.03147436	c(t = 2.94296	0.00768	0.83731181	Distal Intergenic	ENSSSCG000	GLRB	glycine receptor beta [Source:VGNC Symbol;Acc:VGNC:88494]
chr3	92988582	92990937	2356	*	chr3	92988582	92990937	in_vitro	in_vivo	13	13	3.85076923	0.40846154	3.44230769	c(t = 3.12833	0.0077	0.83731181	Distal Intergenic	ENSSSCG000	MSH2	mutS homolog 2 [Source:NCBI gene (formerly Entrezgene);Acc:100337661]
chr7	46185166	46186082	917	*	chr7	46185166	46186082	in_vitro	in_vivo	10	12	3.007	0	3.007	c(t = 3.40962	0.00775	0.83731181	Promoter (<=1kb)	ENSSSCG000	PAQR8	progestin and adipoQ receptor family member 8 [Source:NCBI gene (formerly Entrezgene);Acc:396559]
chr14	41233137	41234555	1419	*	chr14	41233137	41234555	in_vitro	in_vivo	13	15	3.88230769	0.436	3.44630769	c(t = 3.13477	0.00786	0.83731181	Distal Intergenic	ENSSSCG000	TRPV4	transient receptor potential cation channel subfamily V member 4 [Source:VGNC Symbol;Acc:VGNC:94476]
chr5	80409299	80410463	1165	*	chr5	80409299	80410463	in_vitro	in_vivo	11	12	1.26636364	0	1.26636364	c(t = 3.28517	0.00822	0.83731181	Distal Intergenic	ENSSSCG000	NFYB	nuclear transcription factor Y subunit beta [Source:VGNC Symbol;Acc:VGNC:90730]
chr1	25155426	25156648	1223	*	chr1	25155426	25156648	in_vitro	in_vivo	9	9	84.42	34.8288889	49.5911111	c(t = 3.14701	0.00836	0.83731181	Distal Intergenic	ENSSSCG000	CCDC28A	coiled-coil domain containing 28A [Source:NCBI gene (formerly Entrezgene);Acc:100512200]
chr3	56810364	56812121	1758	*	chr3	56810364	56812121	in_vitro	in_vivo	11	14	2.72	0.59214286	2.12785714	c(t = 3.09877	0.00862	0.83731181	Distal Intergenic	ENSSSCG000	ANKRD23	ankyrin repeat domain 23 [Source:NCBI gene (formerly Entrezgene);Acc:100525457]
chr1	268115922	268117045	1124	*	chr1	268115922	268117045	in_vitro	in_vivo	10	10	2.211	0.086	2.125	c(t = 3.30270	0.00876	0.83731181	Distal Intergenic	ENSSSCG000	PTRH1	peptidyl-tRNA hydrolase 1 homolog [Source:NCBI gene (formerly Entrezgene);Acc:100157969]
chr8	118202577	118204400	1824	*	chr8	118202577	118204400	in_vitro	in_vivo	11	16	2.21181818	0.415625	1.79619318	c(t = 2.96106	0.00882	0.83731181	Distal Intergenic	ENSSSCG000	UBE2D3	ubiquitin conjugating enzyme E2 D3 [Source:NCBI gene (formerly Entrezgene);Acc:780418]
chr14	55923940	55925554	1615	*	chr14	55923940	55925554	in_vitro	in_vivo	12	14	2.51666667	0.38	2.13666667	c(t = 3.06702	0.00889	0.83731181	Distal Intergenic	ENSSSCG000	EDARADD	EDAR associated death domain [Source:VGNC Symbol;Acc:VGNC:87542]
chr6	78241571	78242116	546	*	chr6	78241571	78242116	in_vitro	in_vivo	9	12	1.98666667	0.16	1.82666667	c(t = 3.27078	0.00908	0.83731181	Distal Intergenic	ENSSSCG000	PLA2G2D	phospholipase A2 group IID [Source:NCBI gene (formerly Entrezgene);Acc:100512662]
chr7	22637719	22638283	565	*	chr7	22637719	22638283	in_vitro	in_vivo	9	9	15.21	49.7622222	-34.552222	c(t = -2.9805	0.00919	0.83731181	Exon (NM_001128446/100141418, exon 4 of 5)	ENSSSCG000	PPP1R11	protein phosphatase 1 regulatory inhibitor subunit 11 [Source:VGNC Symbol;Acc:VGNC:91718]
chr2	16127932	16129218	1287	*	chr2	16127932	16129218	in_vitro	in_vivo	9	11	93.07	58.4809091	34.5890909	c(t = 3.13114	0.00927	0.83731181	Distal Intergenic	ENSSSCG000	MDK	midkine [Source:VGNC Symbol;Acc:VGNC:100167]
chr17	61552863	61572712	19850	*	chr17	61552863	61572712	in_vitro	in_vivo	15	15	51.1886667	29.0226667	22.166	c(t = 2.80783	0.00938	0.83731181	Promoter (<=1kb)	ENSSSCG000	PSMA7	proteasome 20S subunit alpha 7 [Source:NCBI gene (formerly Entrezgene);Acc:100626738]
chr2	143124047	143126730	2684	*	chr2	143124047	143126730	in_vitro	in_vivo	10	13	87.222	51.7246154	35.4973846	c(t = 2.86662	0.00992	0.83731181	Distal Intergenic	ENSSSCG000	DIAPH1	diaphanous related formin 1 [Source:VGNC Symbol;Acc:VGNC:99632]
chr1	142936355	142937652	1298	*	chr1	142936355	142937652	in_vitro	in_vivo	11	14	4.81181818	1.23714286	3.57467532	c(t = 2.96763	0.00997	0.83731181	Distal Intergenic	ENSSSCG000	NDN	necdin, MAGE family member [Source:NCBI gene (formerly Entrezgene);Acc:100144475]
chr6	2669978	2673391	3414	*	chr6	2669978	2673391	in_vitro	in_vivo	9	9	4.01	31.3711111	-27.361111	c(t = -3.3021	0.0102	0.83731181	Distal Intergenic	ENSSSCG000	IRF8	interferon regulatory factor 8 [Source:VGNC Symbol;Acc:VGNC:89211]
chr15	140119347	140127064	7718	*	chr15	140119347	140127064	in_vitro	in_vivo	13	12	94.2684615	81.2691667	12.9992949	c(t = 2.94776	0.0106	0.83731181	Distal Intergenic	ENSSSCG000	SEPTIN2	septin 2 [Source:VGNC Symbol;Acc:VGNC:104054]
chr6	64295076	64296992	1917	*	chr6	64295076	64296992	in_vitro	in_vivo	10	11	18.455	52.1227273	-33.667727	c(t = -2.9577	0.0108	0.83731181	Distal Intergenic	ENSSSCG000	PRXL2B	peroxiredoxin like 2B [Source:VGNC Symbol;Acc:VGNC:98543]
chr4	70961529	70962086	558	*	chr4	70961529	70962086	in_vitro	in_vivo	12	9	1.83083333	0	1.83083333	c(t = 3.04678	0.0111	0.83731181	Distal Intergenic	ENSSSCG000	ssc-mir-124a	ssc-mir-124a-1 [Source:miRBase;Acc:MI0010680]
chr10	68621049	68622278	1230	*	chr10	68621049	68622278	in_vitro	in_vivo	11	9	3.49090909	0.58111111	2.90979798	c(t = 2.97618	0.0113	0.83731181	Distal Intergenic	ENSSSCG000	WDR37	WD repeat domain 37 [Source:VGNC Symbol;Acc:VGNC:96258]
chr11	1552416	1558026	5611	*	chr11	1552416	1558026	in_vitro	in_vivo	13	16	2.01230769	0.67375	1.33855769	c(t = 2.83601	0.0114	0.83731181	Promoter (<=1kb)	ENSSSCG000	FGF9	fibroblast growth factor 9 [Source:NCBI gene (formerly Entrezgene);Acc:396717]
chr9	53178204	53178954	751	*	chr9	53178204	53178954	in_vitro	in_vivo	10	11	0	1.65	-1.65	c(t = -3.0759	0.0117	0.83731181	Distal Intergenic	ENSSSCG000	RPUSD4	RNA pseudouridine synthase D4 [Source:VGNC Symbol;Acc:VGNC:92453]
chr3	112250919	112253609	2691	*	chr3	112250919	112253609	in_vitro	in_vivo	12	10	4.85583333	1.611	3.24483333	c(t = 2.84110	0.0117	0.83731181	Distal Intergenic	ENSSSCG000	KCNK3	potassium two pore domain channel subfamily K member 3 [Source:NCBI gene (formerly Entrezgene);Acc:100737895]
chr17	48081366	48082939	1574	*	chr17	48081366	48082939	in_vitro	in_vivo	12	11	6.0575	2.12909091	3.92840909	c(t = 2.80696	0.012	0.83731181	Promoter (<=1kb)	ENSSSCG000	CTSA	cathepsin A [Source:NCBI gene (formerly Entrezgene);Acc:100156798]
chr8	40881417	40883397	1981	*	chr8	40881417	40883397	in_vitro	in_vivo	13	14	5.81076923	1.44285714	4.36791209	c(t = 2.79337	0.012	0.83731181	Distal Intergenic	ENSSSCG000	PDGFRA	platelet derived growth factor receptor alpha [Source:NCBI gene (formerly Entrezgene);Acc:100627123]
chr12	4808396	4809752	1357	*	chr12	4808396	4809752	in_vitro	in_vivo	10	14	2.922	0.28142857	2.64057143	c(t = 3.07332	0.0123	0.83731181	Distal Intergenic	ENSSSCG00000036592		serine and arginine rich splicing factor 2 [Source:VGNC Symbol;Acc:VGNC:99085]
chr6	60845868	60846733	866	*	chr6	60845868	60846733	in_vitro	in_vivo	9	9	3.02	0.71888889	2.30111111	c(t = 2.84091	0.0124	0.83731181	Distal Intergenic	ENSSSCG000	GALP	galanin like peptide [Source:NCBI gene (formerly Entrezgene);Acc:396772]
chr8	2732517	2734817	2301	*	chr8	2732517	2734817	in_vitro	in_vivo	11	12	7.62818182	35.6025	-27.974318	c(t = -2.9040	0.0125	0.83731181	Distal Intergenic	ENSSSCG000	HTRA3	HtrA serine peptidase 3 [Source:NCBI gene (formerly Entrezgene);Acc:100337673]
chr1	75169857	75170800	944	*	chr1	75169857	75170800	in_vitro	in_vivo	14	9	3.15	0	3.15	c(t = 2.89462	0.0125	0.83731181	Distal Intergenic	ENSSSCG000	PPIL6	peptidylprolyl isomerase like 6 [Source:VGNC Symbol;Acc:VGNC:98216]
chrX	10218003	10218798	796	*	chrX	10218003	10218798	in_vitro	in_vivo	9	11	99.4155556	62.1245455	37.2910101	c(t = 3.02488	0.0127	0.83731181	Distal Intergenic	ENSSSCG000	RAB9A	RAB9A, member RAS onco family [Source:VGNC Symbol;Acc:VGNC:98277]
chr8	56082833	56084870	2038	*	chr8	56082833	56084870	in_vitro	in_vivo	13	15	4.04846154	0.92133333	3.12712821	c(t = 2.84195	0.0127	0.83731181	Distal Intergenic	ENSSSCG00000008898		HOP homeobox [Source:VGNC Symbol;Acc:VGNC:88932]
chr17	59960804	59962397	1594	*	chr17	59960804	59962397	in_vitro	in_vivo	13	14	2.10076923	0.47357143	1.6271978	c(t = 2.75233	0.013	0.83731181	Distal Intergenic	ENSSSCG000	PHACTR3	phosphatase and actin regulator 3 [Source:VGNC Symbol;Acc:VGNC:95667]
chr6	34929284	34933833	4550	*	chr6	34929284	34933833	in_vitro	in_vivo	14	12	3.805	1.355	2.45	c(t = 2.74599	0.0131	0.83731181	Distal Intergenic	ENSSSCG000	CBLN1	cerebellin 1 precursor [Source:NCBI gene (formerly Entrezgene);Acc:100127148]
chr18	2236071	2237045	975	*	chr18	2236071	2237045	in_vitro	in_vivo	11	12	4.46727273	44.575	-40.107727	c(t = -2.9059	0.0132	0.83731181	Distal Intergenic	ENSSSCG000	SHH	sonic hedgehog signaling molecule [Source:NCBI gene (formerly Entrezgene);Acc:100512749]
chr1	255168342	255169051	710	*	chr1	255168342	255169051	in_vitro	in_vivo	10	9	2.737	0.16555556	2.57144444	c(t = 3.02323	0.0132	0.83731181	Distal Intergenic	ENSSSCG000	ATP6V1G1	ATPase H+ transporting V1 subunit G1 [Source:NCBI gene (formerly Entrezgene);Acc:100154379]
chr3	126510477	126511819	1343	*	chr3	126510477	126511819	in_vitro	in_vivo	9	12	2.79777778	0.82833333	1.96944444	c(t = 2.94781	0.0133	0.83731181	Distal Intergenic	ENSSSCG000	KLF11	KLF transcription factor 11 [Source:NCBI gene (formerly Entrezgene);Acc:100174954]
chr17	31194616	31195316	701	*	chr17	31194616	31195316	in_vitro	in_vivo	9	9	2.83777778	0.19888889	2.63888889	c(t = 3.07587	0.0134	0.83731181	Distal Intergenic	ENSSSCG000	ADRA1D	adrenoceptor alpha 1D [Source:NCBI gene (formerly Entrezgene);Acc:552898]
chr17	22634883	22636026	1144	*	chr17	22634883	22636026	in_vitro	in_vivo	13	10	3.64307692	0	3.64307692	c(t = 2.88433	0.0137	0.83731181	Distal Intergenic	ENSSSCG00000037602		small nuclear ribonucleoprotein polypeptide B2 [Source:VGNC Symbol;Acc:VGNC:108738]
chr12	27119120	27120471	1352	*	chr12	27119120	27120471	in_vitro	in_vivo	11	10	1.29545455	0	1.29545455	c(t = 2.96538	0.0142	0.83731181	Promoter (<=1kb)	ENSSSCG000	TOB1	transducer of ERBB2, 1 [Source:NCBI gene (formerly Entrezgene);Acc:100144440]
chr14	4019687	4020252	566	*	chr14	4019687	4020252	in_vitro	in_vivo	12	11	42.7691667	11.1018182	31.6673485	c(t = 2.76810	0.0143	0.83731181	Distal Intergenic	ENSSSCG000	LPL	lipoprotein lipase [Source:VGNC Symbol;Acc:VGNC:89795]
chr17	62754250	62755455	1206	*	chr17	62754250	62755455	in_vitro	in_vivo	12	9	7.75166667	2.73555556	5.01611111	c(t = 2.71768	0.0143	0.83731181	Distal Intergenic	ENSSSCG000	ssc-mir-1388	ssc-mir-1388 [Source:miRBase;Acc:MI0033392]
chr7	51730443	51731835	1393	*	chr7	51730443	51731835	in_vitro	in_vivo	14	14	2.54	0.30071429	2.23928571	c(t = 2.74859	0.0143	0.83731181	Distal Intergenic	ENSSSCG000	ssc-mir-1839	ssc-mir-1839 [Source:miRBase;Acc:MI0013162]
chr6	53044724	53046058	1335	*	chr6	53044724	53046058	in_vitro	in_vivo	9	9	0.08888889	3.08666667	-2.9977778	c(t = -3.1004	0.0144	0.83731181	Distal Intergenic	ENSSSCG000	C5AR1	complement C5a receptor 1 [Source:NCBI gene (formerly Entrezgene);Acc:100048955]
chr15	54724766	54726020	1255	*	chr15	54724766	54726020	in_vitro	in_vivo	12	11	3.6775	0.80272727	2.87477273	c(t = 2.70630	0.0145	0.83731181	Distal Intergenic	ENSSSCG000	DCTN6	dynactin subunit 6 [Source:NCBI gene (formerly Entrezgene);Acc:100312966]
chr14	66277597	66278796	1200	*	chr14	66277597	66278796	in_vitro	in_vivo	12	12	1.71083333	0.24583333	1.465	c(t = 2.80495	0.0145	0.83731181	Distal Intergenic	ENSSSCG000	EGR2	early growth response 2 [Source:VGNC Symbol;Acc:VGNC:103939]
chr13	206189636	206192636	3001	*	chr13	206189636	206192636	in_vitro	in_vivo	9	10	97.9444444	87.956	9.98844444	c(t = 2.94539	0.0149	0.83731181	Distal Intergenic	ENSSSCG000	PDXK	pyridoxal kinase [Source:NCBI gene (formerly Entrezgene);Acc:396983]
chr6	70879224	70881068	1845	*	chr6	70879224	70881068	in_vitro	in_vivo	12	10	43.6158333	18.869	24.7468333	c(t = 2.71850	0.0149	0.83731181	Distal Intergenic	ENSSSCG000	CORT	cortistatin [Source:NCBI gene (formerly Entrezgene);Acc:100526112]
chr17	17915689	17916830	1142	*	chr17	17915689	17916830	in_vitro	in_vivo	12	9	2.4825	0.27777778	2.20472222	c(t = 2.77839	0.0149	0.83731181	Distal Intergenic	ENSSSCG000	TMX4	thioredoxin related transmembrane protein 4 [Source:NCBI gene (formerly Entrezgene);Acc:100627887]
chr12	27097716	27099057	1342	*	chr12	27097716	27099057	in_vitro	in_vivo	11	12	7.76818182	39.1708333	-31.402652	c(t = -2.7762	0.0151	0.83731181	Distal Intergenic	ENSSSCG000	TOB1	transducer of ERBB2, 1 [Source:NCBI gene (formerly Entrezgene);Acc:100144440]
chr1	239918638	239919296	659	*	chr1	239918638	239919296	in_vitro	in_vivo	11	10	30.9636364	3.73	27.2336364	c(t = 2.86277	0.0153	0.83731181	Distal Intergenic	ENSSSCG000	NANS	N-acetylneuraminate synthase [Source:NCBI gene (formerly Entrezgene);Acc:100155883]
chr1	179610971	179611880	910	*	chr1	179610971	179611880	in_vitro	in_vivo	11	11	3.04	0.67272727	2.36727273	c(t = 2.80270	0.0153	0.83731181	Distal Intergenic	ENSSSCG000	Metazoa_SR	Metazoan signal recognition particle RNA [Source:RFAM;Acc:RF00017]
chr4	118947397	118948903	1507	*	chr4	118947397	118948903	in_vitro	in_vivo	13	13	42.5838462	14.1907692	28.3930769	c(t = 2.64348	0.0154	0.83731181	Promoter (<=1kb)	ENSSSCG000	PLPPR5	phospholipid phosphatase related 5 [Source:VGNC Symbol;Acc:VGNC:109449]
chr11	12576418	12577666	1249	*	chr11	12576418	12577666	in_vitro	in_vivo	11	13	4.22727273	1.52923077	2.69804196	c(t = 2.71317	0.0154	0.83731181	Distal Intergenic	ENSSSCG000	SMAD9	SMAD family member 9 [Source:NCBI gene (formerly Entrezgene);Acc:100153584]
chr11	5793860	5797121	3262	*	chr11	5793860	5797121	in_vitro	in_vivo	13	16	3.14538462	1.0025	2.14288462	c(t = 2.71611	0.0156	0.83731181	Distal Intergenic	ENSSSCG000	SLC46A3	solute carrier family 46 member 3 [Source:VGNC Symbol;Acc:VGNC:93128]
chr5	15494578	15495561	984	*	chr5	15494578	15495561	in_vitro	in_vivo	10	9	7.694	0.60555556	7.08844444	c(t = 2.93174	0.0157	0.83731181	Distal Intergenic	ENSSSCG000	TMBIM6	transmembrane BAX inhibitor motif containing 6 [Source:VGNC Symbol;Acc:VGNC:98374]
chr17	51721967	51724107	2141	*	chr17	51721967	51724107	in_vitro	in_vivo	13	15	2.57846154	0.63266667	1.94579487	c(t = 2.67706	0.0159	0.83731181	Promoter (<=1kb)	ENSSSCG000	CEBPB	CCAAT enhancer binding protein beta [Source:NCBI gene (formerly Entrezgene);Acc:397360]
chr14	141450562	141451766	1205	*	chr14	141450562	141451766	in_vitro	in_vivo	9	11	2.55888889	32.6045455	-30.045657	c(t = -2.8634	0.0165	0.83731181	Distal Intergenic	ENSSSCG000	ECHS1	enoyl-CoA hydratase, short chain 1 [Source:NCBI gene (formerly Entrezgene);Acc:100156927]
chr1	2017975	2020386	2412	*	chr1	2017975	2020386	in_vitro	in_vivo	11	13	4.00363636	25.9415385	-21.937902	c(t = -2.7354	0.0167	0.83731181	Distal Intergenic	ENSSSCG000	ssc-mir-9824	ssc-mir-9824 [Source:miRBase;Acc:MI0031591]
chr1	166817977	166818586	610	*	chr1	166817977	166818586	in_vitro	in_vivo	13	10	3.30384615	28.268	-24.964154	c(t = -2.8990	0.0168	0.83731181	Promoter (<=1kb)	ENSSSCG000	SPESP1	sperm equatorial segment protein 1 [Source:NCBI gene (formerly Entrezgene);Acc:100415935]
chr2	13275391	13276315	925	*	chr2	13275391	13276315	in_vitro	in_vivo	12	11	1.045	0.05636364	0.98863636	c(t = 2.78667	0.017	0.83731181	Distal Intergenic	ENSSSCG000	ssc-mir-130a	ssc-mir-130a [Source:miRBase;Acc:MI0008217]
chr6	51254570	51256407	1838	*	chr6	51254570	51256407	in_vitro	in_vivo	11	10	0.38909091	2.981	-2.5919091	c(t = -2.8515	0.0173	0.83731181	Promoter (<=1kb)	ENSSSCG000	BCL3	BCL3 transcription coactivator [Source:NCBI gene (formerly Entrezgene);Acc:100738193]
chr13	132269186	132270126	941	*	chr13	132269186	132270126	in_vitro	in_vivo	11	14	2.01636364	0.25571429	1.76064935	c(t = 2.77052	0.0173	0.83731181	Distal Intergenic	ENSSSCG000	HES1	hes family bHLH transcription factor 1 [Source:NCBI gene (formerly Entrezgene);Acc:100499567]
chr9	6627185	6628280	1096	*	chr9	6627185	6628280	in_vitro	in_vivo	15	13	12.2793333	4.74230769	7.53702564	c(t = 2.57046	0.0174	0.83731181	Distal Intergenic	ENSSSCG000	LRRC51	leucine-rich repeat-containing protein 51 [Source:NCBI gene (formerly Entrezgene);Acc:100520208]
chr2	103567539	103568117	579	*	chr2	103567539	103568117	in_vitro	in_vivo	12	10	2.54083333	0	2.54083333	c(t = 2.79469	0.0174	0.83731181	Promoter (<=1kb)	ENSSSCG000	LNPEP	leucyl and cystinyl aminopeptidase [Source:NCBI gene (formerly Entrezgene);Acc:100125826]
chr6	3738547	3742530	3984	*	chr6	3738547	3742530	in_vitro	in_vivo	12	10	80.7458333	94.215	-13.469167	c(t = -2.6047	0.0177	0.83731181	Distal Intergenic	ENSSSCG000	CIBAR2	CBY1 interacting BAR domain containing 2 [Source:VGNC Symbol;Acc:VGNC:86698]
chr9	7692907	7694323	1417	*	chr9	7692907	7694323	in_vitro	in_vivo	10	10	2.671	46.192	-43.521	c(t = -2.8933	0.0177	0.83731181	5' UTR	ENSSSCG000	P2RY2	purinergic receptor P2Y2 [Source:VGNC Symbol;Acc:VGNC:98173]
chr15	76991728	76993989	2262	*	chr15	76991728	76993989	in_vitro	in_vivo	9	12	55.0744444	14.0666667	41.0077778	c(t = 2.81698	0.0181	0.83731181	Promoter (<=1kb)	ENSSSCG000	GAD1	glutamate decarboxylase 1 [Source:VGNC Symbol;Acc:VGNC:96312]
chr6	60706305	60707327	1023	*	chr6	60706305	60707327	in_vitro	in_vivo	11	12	2.62454545	0.47	2.15454545	c(t = 2.74226	0.0181	0.83731181	Distal Intergenic	ENSSSCG000	GALP	galanin like peptide [Source:NCBI gene (formerly Entrezgene);Acc:396772]
chr5	3275226	3282696	7471	*	chr5	3275226	3282696	in_vitro	in_vivo	13	16	12.0853846	30.1225	-18.037115	c(t = -2.5804	0.0183	0.83731181	Distal Intergenic	ENSSSCG000	PPARA	peroxisome proliferator activated receptor alpha [Source:NCBI gene (formerly Entrezgene);Acc:397239]
chr6	94978926	94980105	1180	*	chr6	94978926	94980105	in_vitro	in_vivo	13	9	2.78076923	0.48666667	2.29410256	c(t = 2.62579	0.0187	0.83731181	Distal Intergenic	ENSSSCG000	NDUFS5	NADH:ubiquinone oxidoreductase subunit S5 [Source:NCBI gene (formerly Entrezgene);Acc:100514655]
chr14	134118957	134120680	1724	*	chr14	134118957	134120680	in_vitro	in_vivo	11	10	5.64636364	1.937	3.70936364	c(t = 2.59496	0.0189	0.83731181	Distal Intergenic	ENSSSCG000	LHPP	phospholysine phosphohistidine inorganic pyrophosphate phosphatase [Source:VGNC Symbol;Acc:VGNC:89710]
chr8	137606015	137607614	1600	*	chr8	137606015	137607614	in_vitro	in_vivo	13	14	3.40923077	1.23642857	2.1728022	c(t = 2.56273	0.0189	0.83731181	Distal Intergenic	ENSSSCG000	BMP3	bone morphogenetic protein 3 [Source:NCBI gene (formerly Entrezgene);Acc:100512974]
chr16	52212235	52213033	799	*	chr16	52212235	52213033	in_vitro	in_vivo	13	10	2.71076923	0.327	2.38376923	c(t = 2.66486	0.0189	0.83731181	Distal Intergenic	ENSSSCG000	DUSP1	dual specificity phosphatase 1 [Source:VGNC Symbol;Acc:VGNC:87476]
chr13	208171184	208172636	1453	*	chr13	208171184	208172636	in_vitro	in_vivo	14	13	20.715	50.1561538	-29.441154	c(t = -2.5226	0.0191	0.83731181	Distal Intergenic	ENSSSCG000	ITGB2	integrin subunit beta 2 [Source:NCBI gene (formerly Entrezgene);Acc:396943]
chr16	78971273	78974922	3650	*	chr16	78971273	78974922	in_vitro	in_vivo	11	12	7.14909091	38.8233333	-31.674242	c(t = -2.6995	0.0192	0.83731181	Distal Intergenic	ENSSSCG000	TERT	telomerase reverse transcriptase [Source:NCBI gene (formerly Entrezgene);Acc:492280]
chr2	80496241	80497919	1679	*	chr2	80496241	80497919	in_vitro	in_vivo	10	12	10.251	3.4725	6.7785	c(t = 2.64500	0.0192	0.83731181	Distal Intergenic	ENSSSCG000	F12	coagulation factor XII [Source:VGNC Symbol;Acc:VGNC:99647]
chr18	1795033	1796221	1189	*	chr18	1795033	1796221	in_vitro	in_vivo	11	13	2.52818182	0.49076923	2.03741259	c(t = 2.69768	0.0196	0.83731181	Distal Intergenic	ENSSSCG000	ssc-mir-153	ssc-mir-153 [Source:miRBase;Acc:MI0002454]
chr1	26254832	26255836	1005	*	chr1	26254832	26255836	in_vitro	in_vivo	12	12	2.46416667	0.425	2.03916667	c(t = 2.59556	0.0197	0.83731181	Distal Intergenic	ENSSSCG000	PERP	p53 apoptosis effector related to PMP22 [Source:NCBI gene (formerly Entrezgene);Acc:100513507]
chr2	105202132	105203988	1857	*	chr2	105202132	105203988	in_vitro	in_vivo	13	11	1.86769231	0.39	1.47769231	c(t = 2.57457	0.0197	0.83731181	Distal Intergenic	ENSSSCG000	LNPEP	leucyl and cystinyl aminopeptidase [Source:NCBI gene (formerly Entrezgene);Acc:100125826]
chr6	33743421	33745073	1653	*	chr6	33743421	33745073	in_vitro	in_vivo	11	13	8.68090909	41.0253846	-32.344476	c(t = -2.6056	0.0198	0.83731181	Distal Intergenic	ENSSSCG000	NOD2	nucleotide binding oligomerization domain containing 2 [Source:NCBI gene (formerly Entrezgene);Acc:100125838]
chr11	67018760	67019748	989	*	chr11	67018760	67019748	in_vitro	in_vivo	10	13	3.422	1.24	2.182	c(t = 2.51698	0.02	0.83731181	Promoter (<=1kb)	ENSSSCG000	IPO5	importin 5 [Source:NCBI gene (formerly Entrezgene);Acc:100152933]
chr7	63501337	63503611	2275	*	chr7	63501337	63503611	in_vitro	in_vivo	14	14	2.46428571	0.52	1.94428571	c(t = 2.58834	0.02	0.83731181	Distal Intergenic	ENSSSCG000	NKX2-1	NK2 homeobox 1 [Source:VGNC Symbol;Acc:VGNC:90767]
chr17	32031730	32034057	2328	*	chr17	32031730	32034057	in_vitro	in_vivo	10	12	90.229	58.5433333	31.6856667	c(t = 2.61708	0.0202	0.83731181	Distal Intergenic	ENSSSCG000	SIGLEC1	sialic acid binding Ig like lectin 1 [Source:VGNC Symbol;Acc:VGNC:108736]
chr17	10488716	10489921	1206	*	chr17	10488716	10489921	in_vitro	in_vivo	10	9	2.663	0.69	1.973	c(t = 2.65084	0.0204	0.83731181	Distal Intergenic	ENSSSCG000	GPAT4	glycerol-3-phosphate acyltransferase 4 [Source:NCBI gene (formerly Entrezgene);Acc:100152836]
chr6	52660294	52661512	1219	*	chr6	52660294	52661512	in_vitro	in_vivo	10	12	13.89	2.8325	11.0575	c(t = 2.73058	0.0205	0.83731181	Distal Intergenic	ENSSSCG000	AP2S1	adaptor related protein complex 2 subunit sigma 1 [Source:VGNC Symbol;Acc:VGNC:96909]
chr1	71635638	71636643	1006	*	chr1	71635638	71636643	in_vitro	in_vivo	11	9	2.80363636	0.62888889	2.17474747	c(t = 2.62283	0.0206	0.83731181	Promoter (<=1kb)	ENSSSCG000	PREP	prolyl endopeptidase [Source:VGNC Symbol;Acc:VGNC:103157]
chr3	132092659	132102628	9970	*	chr3	132092659	132102628	in_vitro	in_vivo	10	15	9.145	25.3766667	-16.231667	c(t = -2.5262	0.0208	0.83731181	Distal Intergenic	ENSSSCG000	TPO	thyroid peroxidase [Source:VGNC Symbol;Acc:VGNC:94341]
chr10	65471676	65472178	503	*	chr10	65471676	65472178	in_vitro	in_vivo	10	10	5.025	0.73	4.295	c(t = 2.67500	0.0212	0.83731181	Distal Intergenic	ENSSSCG000	AKR1C8	aldo-keto reductase family 1 member C4 [Source:NCBI gene (formerly Entrezgene);Acc:654406]
chr2	58296585	58297568	984	*	chr2	58296585	58297568	in_vitro	in_vivo	10	11	13.501	2.17181818	11.3291818	c(t = 2.69992	0.0213	0.83731181	Promoter (<=1kb)	ENSSSCG000	LPAR2	lysophosphatidic acid receptor 2 [Source:VGNC Symbol;Acc:VGNC:89784]
chr3	9460014	9461065	1052	*	chr3	9460014	9461065	in_vitro	in_vivo	9	11	13.1688889	47.0990909	-33.930202	c(t = -2.5688	0.0214	0.83731181	Distal Intergenic	ENSSSCG000	ZP3	zona pellucida glycoprotein 3 [Source:NCBI gene (formerly Entrezgene);Acc:396927]
chr6	44536486	44537168	683	*	chr6	44536486	44537168	in_vitro	in_vivo	10	9	9.379	1.65666667	7.72233333	c(t = 2.64823	0.0214	0.83731181	Distal Intergenic	ENSSSCG000	FXYD3	FXYD domain containing ion transport regulator 3 [Source:VGNC Symbol;Acc:VGNC:97060]
chr12	43091170	43091884	715	*	chr12	43091170	43091884	in_vitro	in_vivo	9	11	6.35666667	2.01181818	4.34484848	c(t = 2.57581	0.0216	0.83731181	Distal Intergenic	ENSSSCG000	CRLF3	cytokine receptor like factor 3 [Source:VGNC Symbol;Acc:VGNC:86999]
chr14	103835011	103835799	789	*	chr14	103835011	103835799	in_vitro	in_vivo	10	9	45.122	9.74777778	35.3742222	c(t = 2.54637	0.0217	0.83731181	Distal Intergenic	ENSSSCG000	FGFBP3	fibroblast growth factor binding protein 3 [Source:VGNC Symbol;Acc:VGNC:88118]
chr18	6109429	6111011	1583	*	chr18	6109429	6111011	in_vitro	in_vivo	11	11	5.49363636	22.5209091	-17.027273	c(t = -2.6154	0.022	0.83731181	Distal Intergenic	ENSSSCG000	TMUB1	transmembrane and ubiquitin like domain containing 1 [Source:NCBI gene (formerly Entrezgene);Acc:100625342]
chr1	271180563	271182055	1493	*	chr1	271180563	271182055	in_vitro	in_vivo	12	12	92.055	59.45	32.605	c(t = 2.61680	0.0222	0.83731181	Distal Intergenic	ENSSSCG000	FUBP3	far upstream element binding protein 3 [Source:VGNC Symbol;Acc:VGNC:103093]
chr7	68143330	68144149	820	*	chr7	68143330	68144149	in_vitro	in_vivo	11	10	5.71	0.575	5.135	c(t = 2.66967	0.0225	0.83731181	Distal Intergenic	ENSSSCG000	GPR33	G protein-coupled receptor 33 [Source:NCBI gene (formerly Entrezgene);Acc:100157823]
chr3	69020319	69021858	1540	*	chr3	69020319	69021858	in_vitro	in_vivo	10	11	4.7	0.75090909	3.94909091	c(t = 2.70565	0.0226	0.83731181	Distal Intergenic	ENSSSCG000	TPRKB	TP53RK binding protein [Source:VGNC Symbol;Acc:VGNC:94350]
chr1	230906557	230908239	1683	*	chr1	230906557	230908239	in_vitro	in_vivo	12	13	1.36416667	0.38	0.98416667	c(t = 2.51096	0.0227	0.83731181	Promoter (<=1kb)	ENSSSCG000	GNAQ	G protein subunit alpha q [Source:NCBI gene (formerly Entrezgene);Acc:654405]
chr7	65156263	65156988	726	*	chr7	65156263	65156988	in_vitro	in_vivo	9	11	1.86	0	1.86	c(t = 2.81496	0.0227	0.83731181	Promoter (<=1kb)	ENSSSCG000	SNX6	sorting nexin 6 [Source:NCBI gene (formerly Entrezgene);Acc:100154775]
chr16	65599888	65600792	905	*	chr16	65599888	65600792	in_vitro	in_vivo	10	9	2.071	0.26333333	1.80766667	c(t = 2.64969	0.0231	0.83731181	Distal Intergenic	ENSSSCG000	MED7	mediator complex subunit 7 [Source:VGNC Symbol;Acc:VGNC:90123]
chr5	45954512	45955449	938	*	chr5	45954512	45955449	in_vitro	in_vivo	11	10	2.27545455	0.359	1.91645455	c(t = 2.61591	0.0234	0.83731181	Distal Intergenic	ENSSSCG000	ssc-mir-9852	ssc-mir-9852 [Source:miRBase;Acc:MI0031628]
chr6	160653772	160654621	850	*	chr6	160653772	160654621	in_vitro	in_vivo	9	11	3.33666667	0.35	2.98666667	c(t = 2.68356	0.0234	0.83731181	Distal Intergenic	ENSSSCG000	COA7	cytochrome c oxidase assembly factor 7 [Source:NCBI gene (formerly Entrezgene);Acc:100627075]
chr2	143031725	143032458	734	*	chr2	143031725	143032458	in_vitro	in_vivo	9	9	10.6255556	42.6322222	-32.006667	c(t = -2.7124	0.0237	0.83731181	Distal Intergenic	ENSSSCG000	DIAPH1	diaphanous related formin 1 [Source:VGNC Symbol;Acc:VGNC:99632]
chr13	106894307	106895551	1245	*	chr13	106894307	106895551	in_vitro	in_vivo	14	12	2.59285714	0.605	1.98785714	c(t = 2.48738	0.0238	0.83731181	Distal Intergenic	ENSSSCG000	PDCD10	programmed cell death 10 [Source:VGNC Symbol;Acc:VGNC:91241]
chr1	130746387	130746999	613	*	chr1	130746387	130746999	in_vitro	in_vivo	10	10	4.222	0.93	3.292	c(t = 2.50971	0.024	0.83731181	Distal Intergenic	ENSSSCG000	RAD51	RAD51 recombinase [Source:VGNC Symbol;Acc:VGNC:92056]
chr13	6984630	6986375	1746	*	chr13	6984630	6986375	in_vitro	in_vivo	11	10	8.96	2.844	6.116	c(t = 2.53616	0.0241	0.83731181	Distal Intergenic	ENSSSCG000	RAB5A	RAB5A, member RAS onco family [Source:VGNC Symbol;Acc:VGNC:108687]
chr14	112069537	112070757	1221	*	chr14	112069537	112070757	in_vitro	in_vivo	9	12	14.0155556	3.8975	10.1180556	c(t = 2.58906	0.0244	0.83731181	Distal Intergenic	ENSSSCG000	ssc-mir-1038	ssc-mir-10382 [Source:miRBase;Acc:MI0033391]
chr1	108408470	108409594	1125	*	chr1	108408470	108409594	in_vitro	in_vivo	14	11	4.94071429	0.77545455	4.16525974	c(t = 2.52287	0.0245	0.83731181	Distal Intergenic	ENSSSCG000	DAPK2	death associated protein kinase 2 [Source:VGNC Symbol;Acc:VGNC:87157]
chr12	42782672	42783572	901	*	chr12	42782672	42783572	in_vitro	in_vivo	11	11	1.92818182	0.27818182	1.65	c(t = 2.57271	0.0245	0.83731181	Distal Intergenic	ENSSSCG000	ADAP2	ArfGAP with dual PH domains 2 [Source:VGNC Symbol;Acc:VGNC:85096]
chr9	130784511	130786914	2404	*	chr9	130784511	130786914	in_vitro	in_vivo	10	12	34.473	7.2975	27.1755	c(t = 2.60955	0.0246	0.83731181	Distal Intergenic	ENSSSCG000	FLVCR1	FLVCR heme transporter 1 [Source:NCBI gene (formerly Entrezgene);Acc:100217398]
chr2	59221175	59223270	2096	*	chr2	59221175	59223270	in_vitro	in_vivo	10	11	3.174	0.77363636	2.40036364	c(t = 2.56123	0.0246	0.83731181	Distal Intergenic	ENSSSCG000	TMEM59L	transmembrane protein 59 like [Source:VGNC Symbol;Acc:VGNC:94193]
chrX	84793439	84794215	777	*	chrX	84793439	84794215	in_vitro	in_vivo	10	9	24.861	61.1044444	-36.243444	c(t = -2.4740	0.0247	0.83731181	Distal Intergenic	ENSSSCG000	PLP1	proteolipid protein 1 [Source:NCBI gene (formerly Entrezgene);Acc:397029]
chr8	5890392	5891389	998	*	chr8	5890392	5891389	in_vitro	in_vivo	11	11	40.8727273	16.3181818	24.5545455	c(t = 2.48737	0.0247	0.83731181	Distal Intergenic	ENSSSCG000	MSX1	msh homeobox 1 [Source:NCBI gene (formerly Entrezgene);Acc:100302364]
chr7	1748321	1749415	1095	*	chr7	1748321	1749415	in_vitro	in_vivo	9	11	1.62444444	34.4372727	-32.812828	c(t = -2.6289	0.0248	0.83731181	Distal Intergenic	ENSSSCG000	SERPINB9	serpin peptidase inhibitor, clade B (ovalbumin), member 9 [Source:NCBI gene (formerly Entrezgene);Acc:100126851]
chr8	1038195	1039688	1494	*	chr8	1038195	1039688	in_vitro	in_vivo	13	12	2.66692308	0.37333333	2.29358974	c(t = 2.53618	0.0249	0.83731181	Distal Intergenic	ENSSSCG000	LETM1	leucine zipper and EF-hand containing transmembrane protein 1 [Source:VGNC Symbol;Acc:VGNC:89687]
chr2	94878601	94879832	1232	*	chr2	94878601	94879832	in_vitro	in_vivo	13	11	4.11769231	0	4.11769231	c(t = 2.55760	0.0251	0.83731181	Distal Intergenic	ENSSSCG000	CCNH	cyclin H [Source:VGNC Symbol;Acc:VGNC:86359]
chr4	76733723	76734353	631	*	chr4	76733723	76734353	in_vitro	in_vivo	12	10	3.64916667	40.45	-36.800833	c(t = -2.6547	0.0253	0.83731181	Distal Intergenic	ENSSSCG000	MRPL15	mitochondrial ribosomal protein L15 [Source:VGNC Symbol;Acc:VGNC:98820]
chr1	120398506	120399196	691	*	chr1	120398506	120399196	in_vitro	in_vivo	12	9	20.1641667	2.91444444	17.2497222	c(t = 2.55342	0.0256	0.83731181	Distal Intergenic	ENSSSCG00000030168		cytochrome P450 family 19 subfamily A member 1 [Source:VGNC Symbol;Acc:VGNC:103367]
chr6	63355058	63356735	1678	*	chr6	63355058	63356735	in_vitro	in_vivo	10	10	6.547	1.475	5.072	c(t = 2.60031	0.0256	0.83731181	Distal Intergenic	ENSSSCG000	ISG15	ISG15 ubiquitin like modifier [Source:NCBI gene (formerly Entrezgene);Acc:100145895]
chr1	58279845	58281931	2087	*	chr1	58279845	58281931	in_vitro	in_vivo	11	11	4.87636364	1.29727273	3.57909091	c(t = 2.54492	0.0258	0.83731181	Distal Intergenic	ENSSSCG000	MAP3K7	mitogen-activated protein kinase kinase kinase 7 [Source:VGNC Symbol;Acc:VGNC:89987]
chr7	52256466	52256966	501	*	chr7	52256466	52256966	in_vitro	in_vivo	11	14	23.34	2.21071429	21.1292857	c(t = 2.59785	0.026	0.83731181	Distal Intergenic	ENSSSCG000	CPEB1	cytoplasmic polyadenylation element binding protein 1 [Source:NCBI gene (formerly Entrezgene);Acc:100048944]
chr8	86088048	86089310	1263	*	chr8	86088048	86089310	in_vitro	in_vivo	12	15	3.03	0.69866667	2.33133333	c(t = 2.51242	0.0261	0.83731181	Distal Intergenic	ENSSSCG000	IL15	interleukin 15 [Source:VGNC Symbol;Acc:VGNC:89083]
chr5	74917239	74918065	827	*	chr5	74917239	74918065	in_vitro	in_vivo	10	11	3.602	0.45636364	3.14563636	c(t = 2.58379	0.0261	0.83731181	Distal Intergenic	ENSSSCG000	IRAK4	interleukin 1 receptor associated kinase 4 [Source:NCBI gene (formerly Entrezgene);Acc:100127166]
chr16	49048701	49049545	845	*	chr16	49048701	49049545	in_vitro	in_vivo	11	14	1.02454545	0.11142857	0.91311688	c(t = 2.53080	0.0262	0.83731181	Distal Intergenic	ENSSSCG000	DRD1	dopamine receptor D1 [Source:VGNC Symbol;Acc:VGNC:87443]
chr10	38889028	38889711	684	*	chr10	38889028	38889711	in_vitro	in_vivo	9	11	1.66111111	0.10454545	1.55656566	c(t = 2.68386	0.0262	0.83731181	Promoter (<=1kb)	ENSSSCG000	MFSD14B	major facilitator superfamily domain containing 14B [Source:NCBI gene (formerly Entrezgene);Acc:100627901]
chr1	147568066	147569382	1317	*	chr1	147568066	147569382	in_vitro	in_vivo	11	12	0.27363636	1.645	-1.3713636	c(t = -2.4861	0.0264	0.83731181	Distal Intergenic	ENSSSCG000	MBP	myelin basic protein [Source:NCBI gene (formerly Entrezgene);Acc:414286]
chr6	36121847	36122624	778	*	chr6	36121847	36122624	in_vitro	in_vivo	10	14	2.729	0.67642857	2.05257143	c(t = 2.47315	0.0265	0.83731181	Distal Intergenic	ENSSSCG000	CBLN1	cerebellin 1 precursor [Source:NCBI gene (formerly Entrezgene);Acc:100127148]
chr7	90955179	90956189	1011	*	chr7	90955179	90956189	in_vitro	in_vivo	12	12	3.3075	0.53833333	2.76916667	c(t = 2.45734	0.0266	0.83731181	Distal Intergenic	ENSSSCG000	VTI1B	vesicle transport through interaction with t-SNAREs 1B [Source:NCBI gene (formerly Entrezgene);Acc:404692]
chr1	8045564	8047844	2281	*	chr1	8045564	8047844	in_vitro	in_vivo	12	14	94.9516667	76.0685714	18.8830952	c(t = 2.46573	0.0267	0.83731181	Distal Intergenic	ENSSSCG000	TAGAP	T cell activation RhoGTPase activating protein [Source:NCBI gene (formerly Entrezgene);Acc:100155114]
chr14	9196934	9197987	1054	*	chr14	9196934	9197987	in_vitro	in_vivo	12	12	3.17083333	0.66	2.51083333	c(t = 2.47677	0.0271	0.83731181	Distal Intergenic	ENSSSCG000	NEFL	neurofilament light chain [Source:NCBI gene (formerly Entrezgene);Acc:100521224]
chr8	6022066	6024012	1947	*	chr8	6022066	6024012	in_vitro	in_vivo	13	9	18.9238462	3.09444444	15.8294017	c(t = 2.48418	0.0273	0.83731181	Distal Intergenic	ENSSSCG000	MSX1	msh homeobox 1 [Source:NCBI gene (formerly Entrezgene);Acc:100302364]
chr3	1663916	1670556	6641	*	chr3	1663916	1670556	in_vitro	in_vivo	12	14	55.2383333	32.69	22.5483333	c(t = 2.35910	0.0274	0.83731181	Distal Intergenic	ENSSSCG000	NUDT1	nudix hydrolase 1 [Source:NCBI gene (formerly Entrezgene);Acc:100624718]
chr11	71084133	71085216	1084	*	chr11	71084133	71085216	in_vitro	in_vivo	10	14	4.201	0.79428571	3.40671429	c(t = 2.57584	0.0274	0.83731181	Distal Intergenic	ENSSSCG000	BIVM	basic, immunoglobulin-like variable motif containing [Source:VGNC Symbol;Acc:VGNC:109180]
chr7	5235922	5237118	1197	*	chr7	5235922	5237118	in_vitro	in_vivo	10	10	2.352	0.689	1.663	c(t = 2.44033	0.0274	0.83731181	Distal Intergenic	ENSSSCG000	BLOC1S5	biogenesis of lysosomal organelles complex 1 subunit 5 [Source:NCBI gene (formerly Entrezgene);Acc:100049677]
chr9	60784990	60786456	1467	*	chr9	60784990	60786456	in_vitro	in_vivo	11	12	0.77090909	0	0.77090909	c(t = 2.58081	0.0274	0.83731181	Promoter (<=1kb)	ENSSSCG000	VPS26B	VPS26, retromer complex component B [Source:NCBI gene (formerly Entrezgene);Acc:100192441]
chr2	2124343	2126553	2211	*	chr2	2124343	2126553	in_vitro	in_vivo	10	11	21.99	57.0327273	-35.042727	c(t = -2.4101	0.0276	0.83731181	Distal Intergenic	ENSSSCG000	NAP1L4	nucleosome assembly protein 1 like 4 [Source:VGNC Symbol;Acc:VGNC:90577]
chr8	998450	1026433	27984	*	chr8	998450	1026433	in_vitro	in_vivo	16	17	96.174375	92.3188235	3.85555147	c(t = 2.36928	0.0278	0.83731181	Distal Intergenic	ENSSSCG000	LETM1	leucine zipper and EF-hand containing transmembrane protein 1 [Source:VGNC Symbol;Acc:VGNC:89687]
chrX	14244629	14245912	1284	*	chrX	14244629	14245912	in_vitro	in_vivo	10	12	4.16	30.72	-26.56	c(t = -2.4868	0.0284	0.83731181	Distal Intergenic	ENSSSCG000	SYAP1	synapse associated protein 1 [Source:VGNC Symbol;Acc:VGNC:93648]
chr6	59716607	59717786	1180	*	chr6	59716607	59717786	in_vitro	in_vivo	9	9	19.5555556	55.4466667	-35.891111	c(t = -2.4270	0.0286	0.83731181	Distal Intergenic	ENSSSCG000	U2AF2	U2 small nuclear RNA auxiliary factor 2 [Source:VGNC Symbol;Acc:VGNC:94623]
chr3	51522582	51523239	658	*	chr3	51522582	51523239	in_vitro	in_vivo	12	9	97.8116667	99.7355556	-1.9238889	c(t = -2.4447	0.0287	0.83731181	Distal Intergenic	ENSSSCG000	SLC9A2	solute carrier family 9 member A2 [Source:NCBI gene (formerly Entrezgene);Acc:100101921]
chr15	117617305	117617820	516	*	chr15	117617305	117617820	in_vitro	in_vivo	10	10	2.232	0	2.232	c(t = 2.59940	0.0288	0.83731181	Promoter (<=1kb)	ENSSSCG000	ATIC	5-aminoimidazole-4-carboxamide ribonucleotide formyltransferase/IMP cyclohydrolase [Source:NCBI gene (formerly Entrezgene);Acc:100170854
chr4	72780274	72781395	1122	*	chr4	72780274	72781395	in_vitro	in_vivo	9	11	3.06444444	0.37909091	2.68535354	c(t = 2.61478	0.029	0.83731181	Distal Intergenic	ENSSSCG000	SDCBP	syndecan binding protein [Source:VGNC Symbol;Acc:VGNC:92657]
chr6	96893194	96894015	822	*	chr6	96893194	96894015	in_vitro	in_vivo	15	10	2.664	0.208	2.456	c(t = 2.41065	0.029	0.83731181	Distal Intergenic	ENSSSCG000	CIDEA	cell death inducing DFFA like effector a [Source:NCBI gene (formerly Entrezgene);Acc:100127171]
chr11	18297273	18297949	677	*	chr11	18297273	18297949	in_vitro	in_vivo	10	11	2.588	0.16545455	2.42254545	c(t = 2.56492	0.0291	0.83731181	Distal Intergenic	ENSSSCG000	PHF11	PHD finger protein 11 [Source:VGNC Symbol;Acc:VGNC:91377]
chr6	167889784	167891016	1233	*	chr6	167889784	167891016	in_vitro	in_vivo	10	11	5.057	0.90090909	4.15609091	c(t = 2.48208	0.0292	0.83731181	Promoter (<=1kb)	ENSSSCG000	CDC20	cell division cycle 20 [Source:VGNC Symbol;Acc:VGNC:86444]
chr6	28464744	28466409	1666	*	chr6	28464744	28466409	in_vitro	in_vivo	11	13	8.17363636	1.94	6.23363636	c(t = 2.45151	0.0294	0.83731181	Promoter (<=1kb)	ENSSSCG000	THAP11	THAP domain containing 11 [Source:NCBI gene (formerly Entrezgene);Acc:100520959]
chr4	40472366	40476373	4008	*	chr4	40472366	40476373	in_vitro	in_vivo	14	14	3.87714286	1.45928571	2.41785714	c(t = 2.33839	0.0294	0.83731181	Distal Intergenic	ENSSSCG000	UQCRB	ubiquinol-cytochrome c reductase binding protein [Source:NCBI gene (formerly Entrezgene);Acc:100157621]
chr7	7537477	7538431	955	*	chr7	7537477	7538431	in_vitro	in_vivo	12	11	2.46166667	0	2.46166667	c(t = 2.49978	0.0295	0.83731181	Distal Intergenic	ENSSSCG000	C6orf52	chromosome 7 C6orf52 homolog [Source:VGNC Symbol;Acc:VGNC:86073]
chr4	130312787	130314301	1515	*	chr4	130312787	130314301	in_vitro	in_vivo	9	10	96.3855556	91.256	5.12955556	c(t = 2.38384	0.0297	0.83731181	Intron (NM_001243449/100153815, intron 5 of 9)	ENSSSCG000	ZNHIT6	zinc finger HIT-type containing 6 [Source:VGNC Symbol;Acc:VGNC:98909]
chr2	59530503	59533108	2606	*	chr2	59530503	59533108	in_vitro	in_vivo	11	15	5.93454545	0.33133333	5.60321212	c(t = 2.52800	0.0298	0.83731181	Distal Intergenic	ENSSSCG000	RAB3A	RAB3A, member RAS onco family [Source:VGNC Symbol;Acc:VGNC:98264]
chr6	68487103	68487955	853	*	chr6	68487103	68487955	in_vitro	in_vivo	10	9	2.415	0.62666667	1.78833333	c(t = 2.43366	0.0299	0.83731181	Distal Intergenic	ENSSSCG000	UTS2	urotensin 2 [Source:VGNC Symbol;Acc:VGNC:94788]
chr7	38388035	38389283	1249	*	chr7	38388035	38389283	in_vitro	in_vivo	13	11	3.38615385	1.13090909	2.25524476	c(t = 2.35530	0.03	0.83731181	Distal Intergenic	ENSSSCG000	SLC22A7	solute carrier family 22 member 7 [Source:VGNC Symbol;Acc:VGNC:92983]
chr1	217236861	217237767	907	*	chr1	217236861	217237767	in_vitro	in_vivo	13	10	2.62384615	0.345	2.27884615	c(t = 2.42007	0.0302	0.83731181	Distal Intergenic	ENSSSCG000	ssc-mir-101-1	ssc-mir-101-1 [Source:miRBase;Acc:MI0010678]
chr17	31055369	31056573	1205	*	chr17	31055369	31056573	in_vitro	in_vivo	12	9	1.61416667	0.06555556	1.54861111	c(t = 2.47816	0.0302	0.83731181	Distal Intergenic	ENSSSCG000	CST7	cystatin F [Source:NCBI gene (formerly Entrezgene);Acc:100152012]
chr7	120627082	120627764	683	*	chr7	120627082	120627764	in_vitro	in_vivo	10	9	2.188	0.37666667	1.81133333	c(t = 2.47225	0.0304	0.83731181	Distal Intergenic	ENSSSCG000	BCL11B	BCL11 transcription factor B [Source:NCBI gene (formerly Entrezgene);Acc:100154158]
chr3	46748129	46748614	486	*	chr3	46748129	46748614	in_vitro	in_vivo	10	13	0.943	7.98076923	-7.0377692	c(t = -2.4397	0.0305	0.83731181	Distal Intergenic	ENSSSCG000	MALL	mal, T cell differentiation protein like [Source:NCBI gene (formerly Entrezgene);Acc:100626835]
chr15	63482472	63482999	528	*	chr15	63482472	63482999	in_vitro	in_vivo	10	9	2.938	0.53333333	2.40466667	c(t = 2.46102	0.0305	0.83731181	Distal Intergenic	ENSSSCG000	NR4A2	nuclear receptor subfamily 4 group A member 2 [Source:VGNC Symbol;Acc:VGNC:96451]
chr14	140335405	140336916	1512	*	chr14	140335405	140336916	in_vitro	in_vivo	13	11	3.85461538	0.85818182	2.99643357	c(t = 2.37679	0.0307	0.83731181	Distal Intergenic	ENSSSCG000	ssc-mir-202	ssc-mir-202 [Source:miRBase;Acc:MI0013157]
chr13	206031271	206032506	1236	*	chr13	206031271	206032506	in_vitro	in_vivo	9	13	12.6033333	40.8184615	-28.215128	c(t = -2.3680	0.031	0.83731181	Distal Intergenic	ENSSSCG000	TFF3	trefoil factor 3 [Source:NCBI gene (formerly Entrezgene);Acc:100627480]
chr16	75473622	75475781	2160	*	chr16	75473622	75475781	in_vitro	in_vivo	11	14	1.80909091	0.435	1.37409091	c(t = 2.43629	0.031	0.83731181	Distal Intergenic	ENSSSCG000	CCT5	chaperonin containing TCP1 subunit 5 [Source:VGNC Symbol;Acc:VGNC:86383]
chr3	10991204	10991839	636	*	chr3	10991204	10991839	in_vitro	in_vivo	10	11	3.377	0.77727273	2.59972727	c(t = 2.38745	0.0315	0.83731181	Distal Intergenic	ENSSSCG000	ssc-mir-7137	ssc-mir-7137 [Source:miRBase;Acc:MI0023570]
chr14	139121536	139122737	1202	*	chr14	139121536	139122737	in_vitro	in_vivo	10	11	12.542	41.4045455	-28.862545	c(t = -2.3695	0.0319	0.83731181	Distal Intergenic	ENSSSCG000	GLRX3	glutaredoxin 3 [Source:VGNC Symbol;Acc:VGNC:88495]
chr15	139479553	139483165	3613	*	chr15	139479553	139483165	in_vitro	in_vivo	12	12	4.67833333	32.0675	-27.389167	c(t = -2.4369	0.0321	0.83731181	Distal Intergenic	ENSSSCG000	ssc-mir-149	ssc-mir-149 [Source:miRBase;Acc:MI0016620]
chr13	97001727	97002621	895	*	chr13	97001727	97002621	in_vitro	in_vivo	14	10	2.6	0.383	2.217	c(t = 2.36709	0.0322	0.83731181	Distal Intergenic	ENSSSCG000	PTX3	pentraxin 3 [Source:NCBI gene (formerly Entrezgene);Acc:100513178]
chrX	34296220	34296757	538	*	chrX	34296220	34296757	in_vitro	in_vivo	10	13	1.519	15.2292308	-13.710231	c(t = -2.4102	0.0324	0.83731181	Distal Intergenic	ENSSSCG000	OTC	ornithine transcarbamylase [Source:NCBI gene (formerly Entrezgene);Acc:397438]
chr3	97199101	97202900	3800	*	chr3	97199101	97202900	in_vitro	in_vivo	16	13	10.84	5.22923077	5.61076923	c(t = 2.25673	0.0325	0.83731181	Distal Intergenic	ENSSSCG000	ssc-mir-9855	ssc-mir-9855-1 [Source:miRBase;Acc:MI0031632]
chr17	3047616	3048263	648	*	chr17	3047616	3048263	in_vitro	in_vivo	11	10	17.2309091	6.613	10.6179091	c(t = 2.33529	0.0326	0.83731181	Distal Intergenic	ENSSSCG000	ssc-mir-383	ssc-mir-383 [Source:miRBase;Acc:MI0015927]
chr3	41489356	41492002	2647	*	chr3	41489356	41492002	in_vitro	in_vivo	12	12	2.62333333	8.72416667	-6.1008333	c(t = -2.3343	0.0327	0.83731181	Distal Intergenic	ENSSSCG000	NME4	NME/NM23 nucleoside diphosphate kinase 4 [Source:VGNC Symbol;Acc:VGNC:90792]
chr15	76997399	76998753	1355	*	chr15	76997399	76998753	in_vitro	in_vivo	9	11	34.7366667	7.09	27.6466667	c(t = 2.54796	0.0327	0.83731181	Intron (NM_213894/396928, intron 3 of 16)	ENSSSCG000	GAD1	glutamate decarboxylase 1 [Source:VGNC Symbol;Acc:VGNC:96312]
chr7	47674119	47675412	1294	*	chr7	47674119	47675412	in_vitro	in_vivo	10	11	6.036	1.87909091	4.15690909	c(t = 2.41364	0.0327	0.83731181	Promoter (<=1kb)	ENSSSCG000	DNAJA4	DnaJ heat shock protein family (Hsp40) member A4 [Source:NCBI gene (formerly Entrezgene);Acc:397613]
chr1	2594033	2595401	1369	*	chr1	2594033	2595401	in_vitro	in_vivo	9	11	45.4244444	66.6472727	-21.222828	c(t = -2.3148	0.0332	0.83731181	Distal Intergenic	ENSSSCG000	SFT2D1	SFT2 domain containing 1 [Source:NCBI gene (formerly Entrezgene);Acc:100513321]
chr14	7517560	7518083	524	*	chr14	7517560	7518083	in_vitro	in_vivo	11	12	4.20818182	0.79416667	3.41401515	c(t = 2.40503	0.0333	0.83731181	Distal Intergenic	ENSSSCG000	PEBP4	phosphatidylethanolamine binding protein 4 [Source:VGNC Symbol;Acc:VGNC:91303]
chr15	138151863	138153104	1242	*	chr15	138151863	138153104	in_vitro	in_vivo	9	9	12.1922222	49.7355556	-37.543333	c(t = -2.3383	0.0334	0.83731181	Distal Intergenic	ENSSSCG000	RAMP1	receptor activity modifying protein 1 [Source:NCBI gene (formerly Entrezgene);Acc:397401]
chr18	55614429	55616784	2356	*	chr18	55614429	55616784	in_vitro	in_vivo	12	14	12.8741667	34.2335714	-21.359405	c(t = -2.3140	0.0338	0.83731181	Distal Intergenic	ENSSSCG000	RARRES2	retinoic acid receptor responder 2 [Source:VGNC Symbol;Acc:VGNC:92098]
chr8	702463	705529	3067	*	chr8	702463	705529	in_vitro	in_vivo	10	11	12.147	38.6090909	-26.462091	c(t = -2.3774	0.034	0.83731181	Distal Intergenic	ENSSSCG000	SLBP	stem-loop binding protein [Source:NCBI gene (formerly Entrezgene);Acc:100513605]
chr15	84155847	84156647	801	*	chr15	84155847	84156647	in_vitro	in_vivo	10	11	4.192	0.23909091	3.95290909	c(t = 2.47421	0.0342	0.83731181	Distal Intergenic	ENSSSCG000	PLEKHA3	pleckstrin homology domain containing A3 [Source:NCBI gene (formerly Entrezgene);Acc:100154010]
chr1	240984394	240985684	1291	*	chr1	240984394	240985684	in_vitro	in_vivo	12	9	1.50833333	0.21111111	1.29722222	c(t = 2.37636	0.0342	0.83731181	Promoter (<=1kb)	ENSSSCG000	ALG2	ALG2 alpha-1,3/1,6-mannosyltransferase [Source:VGNC Symbol;Acc:VGNC:85250]
chr4	36653656	36655064	1409	*	chr4	36653656	36655064	in_vitro	in_vivo	10	14	3.809	0.50714286	3.30185714	c(t = 2.45177	0.0344	0.83731181	Distal Intergenic	ENSSSCG000	RNF19A	ring finger protein 19A, RBR E3 ubiquitin protein ligase [Source:VGNC Symbol;Acc:VGNC:92375]
chr1	27247922	27250919	2998	*	chr1	27247922	27250919	in_vitro	in_vivo	14	14	4.40142857	1.25785714	3.14357143	c(t = 2.29315	0.0345	0.83731181	Distal Intergenic	ENSSSCG000	IFNGR1	interferon gamma receptor 1 [Source:VGNC Symbol;Acc:VGNC:89041]
chr13	108340607	108341546	940	*	chr13	108340607	108341546	in_vitro	in_vivo	10	10	2.907	0.385	2.522	c(t = 2.39238	0.0345	0.83731181	Distal Intergenic	ENSSSCG000	SEC62	SEC62 homolog, preprotein translocation factor [Source:NCBI gene (formerly Entrezgene);Acc:100125961]
chr14	106793021	106793727	707	*	chr14	106793021	106793727	in_vitro	in_vivo	11	10	3.33909091	0.642	2.69709091	c(t = 2.37992	0.0346	0.83731181	Promoter (<=1kb)	ENSSSCG000	PDLIM1	PDZ and LIM domain 1 [Source:NCBI gene (formerly Entrezgene);Acc:100155592]
chr4	83068725	83070535	1811	*	chr4	83068725	83070535	in_vitro	in_vivo	14	14	1.74642857	0.31571429	1.43071429	c(t = 2.29372	0.0348	0.83731181	Promoter (<=1kb)	ENSSSCG000	ssc-mir-9810	ssc-mir-9810 [Source:miRBase;Acc:MI0031577]
chr9	33041216	33041867	652	*	chr9	33041216	33041867	in_vitro	in_vivo	12	13	2.34416667	0	2.34416667	c(t = 2.40333	0.035	0.83731181	Distal Intergenic	ENSSSCG000	MMP7	matrix metallopeptidase 7 [Source:NCBI gene (formerly Entrezgene);Acc:397411]
chrX	112110461	112113992	3532	*	chrX	112110461	112113992	in_vitro	in_vivo	11	15	5.29	16.512	-11.222	c(t = -2.2977	0.0352	0.83731181	Distal Intergenic	ENSSSCG00000012703		RNA binding motif protein X-linked [Source:NCBI gene (formerly Entrezgene);Acc:100158042]
chr11	22323921	22324836	916	*	chr11	22323921	22324836	in_vitro	in_vivo	11	10	3.70363636	0	3.70363636	c(t = 2.43337	0.0352	0.83731181	Distal Intergenic	ENSSSCG000	GTF2F2	general transcription factor IIF subunit 2 [Source:NCBI gene (formerly Entrezgene);Acc:100626869]
chr3	4454836	4455680	845	*	chr3	4454836	4455680	in_vitro	in_vivo	11	13	1.73363636	0.06846154	1.66517483	c(t = 2.42435	0.0353	0.83731181	Distal Intergenic	ENSSSCG000	FSCN1	fascin actin-bundling protein 1 [Source:NCBI gene (formerly Entrezgene);Acc:100286741]
chr14	23506208	23507324	1117	*	chr14	23506208	23507324	in_vitro	in_vivo	14	9	4.64	0.93555556	3.70444444	c(t = 2.30064	0.0354	0.83731181	Distal Intergenic	ENSSSCG000	PIWIL1	piwi like RNA-mediated silencing 1 [Source:VGNC Symbol;Acc:VGNC:98197]
chr12	59594416	59595578	1163	*	chr12	59594416	59595578	in_vitro	in_vivo	9	10	1.35888889	0.093	1.26588889	c(t = 2.49332	0.0355	0.83731181	Distal Intergenic	ENSSSCG000	AKAP10	A-kinase anchoring protein 10 [Source:NCBI gene (formerly Entrezgene);Acc:397262]
chr3	113502110	113504784	2675	*	chr3	113502110	113504784	in_vitro	in_vivo	11	11	6.39272727	2.01909091	4.37363636	c(t = 2.38085	0.0356	0.83731181	Distal Intergenic	ENSSSCG000	DNMT3A	DNA methyltransferase 3 alpha [Source:VGNC Symbol;Acc:VGNC:87384]
chr5	15964706	15965446	741	*	chr5	15964706	15965446	in_vitro	in_vivo	9	10	4.06666667	0.818	3.24866667	c(t = 2.45054	0.0359	0.83731181	Distal Intergenic	ENSSSCG000	SMARCD1	SWI/SNF related, matrix associated, actin dependent regulator of chromatin, subfamily d, member 1 [Source:VGNC Symbol;Acc:VGNC:93233]
chr14	52363010	52363920	911	*	chr14	52363010	52363920	in_vitro	in_vivo	10	14	2.939	0.69571429	2.24328571	c(t = 2.30819	0.0359	0.83731181	Distal Intergenic	ENSSSCG000	CHRM3	cholinergic receptor muscarinic 3 [Source:VGNC Symbol;Acc:VGNC:86664]
chr4	106809183	106809889	707	*	chr4	106809183	106809889	in_vitro	in_vivo	11	9	0.96363636	4.54333333	-3.579697	c(t = -2.4409	0.036	0.83731181	Distal Intergenic	ENSSSCG00000063479		solute carrier family 16 member 1 [Source:NCBI gene (formerly Entrezgene);Acc:100127159]
chr4	117874916	117875834	919	*	chr4	117874916	117875834	in_vitro	in_vivo	9	12	1.73888889	0.21333333	1.52555556	c(t = 2.41598	0.0361	0.83731181	Promoter (<=1kb)	ENSSSCG000	RTCA	RNA 3'-terminal phosphate cyclase [Source:NCBI gene (formerly Entrezgene);Acc:100154102]
chr18	19689063	19690697	1635	*	chr18	19689063	19690697	in_vitro	in_vivo	13	10	23.4338462	6.188	17.2458462	c(t = 2.28371	0.0362	0.83731181	Distal Intergenic	ENSSSCG000	OPN1SW	opsin 1, short wave sensitive [Source:NCBI gene (formerly Entrezgene);Acc:397164]
chr6	45346622	45347932	1311	*	chr6	45346622	45347932	in_vitro	in_vivo	11	10	16.9990909	48.832	-31.832909	c(t = -2.2760	0.0365	0.83731181	Distal Intergenic	ENSSSCG000	TYROBP	transmembrane immune signaling adaptor TYROBP [Source:VGNC Symbol;Acc:VGNC:94619]
chr8	76269629	76270726	1098	*	chr8	76269629	76270726	in_vitro	in_vivo	9	13	3.49888889	0.31384615	3.18504274	c(t = 2.45352	0.0365	0.83731181	Promoter (<=1kb)	ENSSSCG000	ARFIP1	ADP ribosylation factor interacting protein 1 [Source:VGNC Symbol;Acc:VGNC:85453]
chr14	30888852	30890087	1236	*	chr14	30888852	30890087	in_vitro	in_vivo	9	9	3.58111111	0.94666667	2.63444444	c(t = 2.35013	0.0367	0.83731181	Distal Intergenic	ENSSSCG000	RHOF	ras homolog family member F, filopodia associated [Source:VGNC Symbol;Acc:VGNC:92288]
chr14	51406309	51407289	981	*	chr14	51406309	51407289	in_vitro	in_vivo	9	10	52.1266667	24.791	27.3356667	c(t = 2.30893	0.0368	0.83731181	Distal Intergenic	ENSSSCG000	COMT	catechol-O-methyltransferase [Source:VGNC Symbol;Acc:VGNC:107378]
chr4	205764	207099	1336	*	chr4	205764	207099	in_vitro	in_vivo	12	12	1.7275	0.35083333	1.37666667	c(t = 2.30613	0.0368	0.83731181	Distal Intergenic	ENSSSCG000	SLC52A1	solute carrier family 52 member 2 [Source:NCBI gene (formerly Entrezgene);Acc:445519]
chr7	80287942	80288596	655	*	chr7	80287942	80288596	in_vitro	in_vivo	12	10	1.30333333	0.11	1.19333333	c(t = 2.34732	0.0368	0.83731181	Promoter (<=1kb)	ENSSSCG000	KATNBL1	katanin regulatory subunit B1 like 1 [Source:VGNC Symbol;Acc:VGNC:89313]
chr7	58908967	58910668	1702	*	chr7	58908967	58910668	in_vitro	in_vivo	10	9	7.244	1.78111111	5.46288889	c(t = 2.39499	0.0369	0.83731181	Distal Intergenic	ENSSSCG000	CYP1A1	cytochrome P450 family 1 subfamily A member 1 [Source:VGNC Symbol;Acc:VGNC:103347]
chr17	39683942	39684278	337	*	chr17	39683942	39684278	in_vitro	in_vivo	9	10	2.98222222	0	2.98222222	c(t = 2.49897	0.037	0.83731181	Distal Intergenic	ENSSSCG000	MYL9	myosin light chain 9 [Source:NCBI gene (formerly Entrezgene);Acc:100157760]
chr9	713731	714401	671	*	chr9	713731	714401	in_vitro	in_vivo	10	9	38.356	67.01	-28.654	c(t = -2.2907	0.0372	0.83731181	Distal Intergenic	NA	NA	NA
chr7	83093084	83094502	1419	*	chr7	83093084	83094502	in_vitro	in_vivo	12	12	6.58	1.245	5.335	c(t = 2.31029	0.0372	0.83731181	Distal Intergenic	ENSSSCG000	NR2F2	nuclear receptor subfamily 2 group F member 2 [Source:VGNC Symbol;Acc:VGNC:90881]
chr1	169725317	169726241	925	*	chr1	169725317	169726241	in_vitro	in_vivo	9	10	4.71666667	0.845	3.87166667	c(t = 2.40583	0.0373	0.83731181	Promoter (<=1kb)	ENSSSCG000	PNN	pinin, desmosome associated protein [Source:NCBI gene (formerly Entrezgene);Acc:100526149]
chr11	77817247	77824787	7541	*	chr11	77817247	77824787	in_vitro	in_vivo	12	13	10.1641667	35.3830769	-25.21891	c(t = -2.2369	0.0375	0.83731181	Distal Intergenic	ENSSSCG000	ANKRD10	ankyrin repeat domain 10 [Source:VGNC Symbol;Acc:VGNC:85322]
chr13	71760305	71762524	2220	*	chr13	71760305	71762524	in_vitro	in_vivo	13	12	4.61846154	1.81333333	2.80512821	c(t = 2.22354	0.0375	0.83731181	Distal Intergenic	ENSSSCG000	RPN1	ribophorin I [Source:NCBI gene (formerly Entrezgene);Acc:397606]
chr7	3102006	3103105	1100	*	chr7	3102006	3103105	in_vitro	in_vivo	10	10	13.384	40.306	-26.922	c(t = -2.3142	0.0377	0.83731181	Distal Intergenic	ENSSSCG000	NRN1	neuritin 1 [Source:VGNC Symbol;Acc:VGNC:90900]
chr3	8356207	8358016	1810	*	chr3	8356207	8358016	in_vitro	in_vivo	12	10	8.33	1.008	7.322	c(t = 2.34036	0.0378	0.83731181	Distal Intergenic	ENSSSCG000	PVRIG	PVR related immunoglobulin domain containing [Source:VGNC Symbol;Acc:VGNC:92008]
chr1	239285331	239286111	781	*	chr1	239285331	239286111	in_vitro	in_vivo	11	12	1.27727273	0	1.27727273	c(t = 2.39221	0.0378	0.83731181	Distal Intergenic	ENSSSCG000	TRMO	tRNA methyltransferase O [Source:NCBI gene (formerly Entrezgene);Acc:100156700]
chr4	41803316	41805611	2296	*	chr4	41803316	41805611	in_vitro	in_vivo	15	14	3.17866667	0.85357143	2.32509524	c(t = 2.22860	0.0379	0.83731181	Promoter (<=1kb)	ENSSSCG000	CCNE2	cyclin E2 [Source:VGNC Symbol;Acc:VGNC:86355]
chr4	84111766	84112592	827	*	chr4	84111766	84112592	in_vitro	in_vivo	10	13	1.336	26.3184615	-24.982462	c(t = -2.3280	0.038	0.83731181	Promoter (<=1kb)	ENSSSCG000	ssc-mir-9825	ssc-mir-9825 [Source:miRBase;Acc:MI0031592]
chr14	131960740	131961674	935	*	chr14	131960740	131961674	in_vitro	in_vivo	13	9	2.18538462	0.34333333	1.84205128	c(t = 2.28425	0.0383	0.83731181	Distal Intergenic	ENSSSCG000	TACC2	transforming acidic coiled-coil containing protein 2 [Source:NCBI gene (formerly Entrezgene);Acc:100152804]
chr18	45387546	45389319	1774	*	chr18	45387546	45389319	in_vitro	in_vivo	9	11	4.08444444	0.87545455	3.2089899	c(t = 2.42010	0.0384	0.83731181	Distal Intergenic	ENSSSCG000	HOXA10	homeobox A10 [Source:VGNC Symbol;Acc:VGNC:88934]
chr7	55237411	55238015	605	*	chr7	55237411	55238015	in_vitro	in_vivo	10	12	5.541	1.5725	3.9685	c(t = 2.33263	0.0385	0.83731181	3' UTR	ENSSSCG000	PLIN1	perilipin 1 [Source:NCBI gene (formerly Entrezgene);Acc:654411]
chr17	15137297	15137913	617	*	chr17	15137297	15137913	in_vitro	in_vivo	11	9	2.17727273	0	2.17727273	c(t = 2.37797	0.0387	0.83731181	Distal Intergenic	ENSSSCG000	CHGB	chromogranin B [Source:NCBI gene (formerly Entrezgene);Acc:397154]
chr9	130994762	130995869	1108	*	chr9	130994762	130995869	in_vitro	in_vivo	9	9	96.3855556	87.9533333	8.43222222	c(t = 2.39299	0.0388	0.83731181	Distal Intergenic	ENSSSCG000	PPP2R5A	protein phosphatase 2 regulatory subunit B'alpha [Source:NCBI gene (formerly Entrezgene);Acc:100101925]
chr2	150916235	150919215	2981	*	chr2	150916235	150919215	in_vitro	in_vivo	9	10	15.4488889	45.062	-29.613111	c(t = -2.3309	0.039	0.83731181	Distal Intergenic	ENSSSCG000	ssc-mir-378-1	ssc-mir-378-1 [Source:miRBase;Acc:MI0013088]

chr14	22654113	22655056	944	*	chr14	22654113	22655056	in_vitro	in_vivo	9	12	2.83555556	0.21416667	2.62138889	c(t = 2.44840	0.039	0.83731181	Distal Intergenic	ENSSSCG000	PIWIL1	piwi like RNA-mediated silencing 1 [Source:VGNC Symbol;Acc:VGNC:98197]
chr4	377258	378347	1090	*	chr4	377258	378347	in_vitro	in_vivo	9	12	4.78444444	16.99	-12.205556	c(t = -2.2832	0.0391	0.83731181	Distal Intergenic	ENSSSCG000	SLC52A1	solute carrier family 52 member 2 [Source:NCBI gene (formerly Entrezgene);Acc:445519]
chr2	8985672	8986851	1180	*	chr2	8985672	8986851	in_vitro	in_vivo	12	14	3.255	1.40357143	1.85142857	c(t = 2.20899	0.0391	0.83731181	Distal Intergenic	ENSSSCG000	NXF1	nuclear RNA export factor 1 [Source:VGNC Symbol;Acc:VGNC:90993]
chr9	74635301	74635903	603	*	chr9	74635301	74635903	in_vitro	in_vivo	11	9	2.30727273	0.37	1.93727273	c(t = 2.27061	0.0392	0.83731181	Distal Intergenic	ENSSSCG000	PEG10	paternally expressed 10 [Source:NCBI gene (formerly Entrezgene);Acc:654416]
chr4	125109312	125110933	1622	*	chr4	125109312	125110933	in_vitro	in_vivo	10	13	1.568	0.33153846	1.23646154	c(t = 2.32874	0.0392	0.83731181	Distal Intergenic	ENSSSCG000	TGFBR3	transforming growth factor beta receptor 3 [Source:VGNC Symbol;Acc:VGNC:93932]
chr2	8969768	8970328	561	*	chr2	8969768	8970328	in_vitro	in_vivo	9	11	0	1.25090909	-1.2509091	c(t = -2.3682	0.0394	0.83731181	Promoter (<=1kb)	ENSSSCG000	NXF1	nuclear RNA export factor 1 [Source:VGNC Symbol;Acc:VGNC:90993]
chr4	127136039	127137255	1217	*	chr4	127136039	127137255	in_vitro	in_vivo	11	11	2.78363636	0.72090909	2.06272727	c(t = 2.22096	0.0395	0.83731181	Distal Intergenic	ENSSSCG000	GBP2	guanylate binding protein 2, interferon-inducible [Source:NCBI gene (formerly Entrezgene);Acc:100153137]
chr12	61028808	61045523	16716	*	chr12	61028808	61045523	in_vitro	in_vivo	13	14	76.39	61.3128571	15.0771429	c(t = 2.18805	0.0398	0.83731181	Distal Intergenic	ENSSSCG000	COPS3	COP9 signalosome subunit 3 [Source:VGNC Symbol;Acc:VGNC:86900]
chr6	69693111	69694449	1339	*	chr6	69693111	69694449	in_vitro	in_vivo	10	9	15.498	48.0988889	-32.600889	c(t = -2.3039	0.0398	0.83731181	Distal Intergenic	ENSSSCG000	CA6	carbonic anhydrase 6 [Source:NCBI gene (formerly Entrezgene);Acc:100240717]
chr13	79723572	79724099	528	*	chr13	79723572	79724099	in_vitro	in_vivo	11	11	6.73454545	2.46545455	4.26909091	c(t = 2.23379	0.0398	0.83731181	Distal Intergenic	ENSSSCG000	FOXL2	forkhead box L2 [Source:NCBI gene (formerly Entrezgene);Acc:100622956]
chr15	56200224	56201602	1379	*	chr15	56200224	56201602	in_vitro	in_vivo	11	14	2.52090909	0.96857143	1.55233766	c(t = 2.21745	0.0398	0.83731181	Distal Intergenic	ENSSSCG000	OCA2	OCA2 melanosomal transmembrane protein [Source:NCBI gene (formerly Entrezgene);Acc:397171]
chr13	198204658	198205975	1318	*	chr13	198204658	198205975	in_vitro	in_vivo	13	14	9.19615385	0.93357143	8.26258242	c(t = 2.29936	0.0398	0.83731181	Distal Intergenic	ENSSSCG000	RCAN1	regulator of calcineurin 1 [Source:VGNC Symbol;Acc:VGNC:92170]
chr1	161187719	161188444	726	*	chr1	161187719	161188444	in_vitro	in_vivo	10	11	0.529	2.55272727	-2.0237273	c(t = -2.3003	0.0399	0.83731181	Promoter (<=1kb)	ENSSSCG000	PMAIP1	phorbol-12-myristate-13-acetate-induced protein 1 [Source:NCBI gene (formerly Entrezgene);Acc:397278]
chr12	51960595	51963596	3002	*	chr12	51960595	51963596	in_vitro	in_vivo	13	12	2.60461538	0.33583333	2.26878205	c(t = 2.27491	0.04	0.83731181	Promoter (<=1kb)	ENSSSCG000	PFN1	profilin 1 [Source:NCBI gene (formerly Entrezgene);Acc:100512494]
chr4	54940555	54941014	460	*	chr4	54940555	54941014	in_vitro	in_vivo	9	12	4.43	0	4.43	c(t = 2.44947	0.04	0.83731181	Distal Intergenic	ENSSSCG000	IMPA1	inositol monophosphatase 1 [Source:VGNC Symbol;Acc:VGNC:89125]
chr11	1771434	1772733	1300	*	chr11	1771434	1772733	in_vitro	in_vivo	10	9	93.367	97.6633333	-4.2963333	c(t = -2.2182	0.0406	0.83731181	Distal Intergenic	ENSSSCG000	FGF9	fibroblast growth factor 9 [Source:NCBI gene (formerly Entrezgene);Acc:396717]
chr2	80193189	80194780	1592	*	chr2	80193189	80194780	in_vitro	in_vivo	9	11	2.14222222	0.52727273	1.61494949	c(t = 2.32721	0.0406	0.83731181	Promoter (<=1kb)	ENSSSCG00000014031		heterous nuclear ribonucleoprotein A/B [Source:NCBI gene (formerly Entrezgene);Acc:100127170]
chr13	72578711	72579793	1083	*	chr13	72578711	72579793	in_vitro	in_vivo	12	12	5.11916667	1.72916667	3.39	c(t = 2.24487	0.0407	0.83731181	Distal Intergenic	ENSSSCG000	MGLL	monoglyceride lipase [Source:VGNC Symbol;Acc:VGNC:90199]
chr5	1412892	1415114	2223	*	chr5	1412892	1415114	in_vitro	in_vivo	9	10	10.9011111	46.543	-35.641889	c(t = -2.2811	0.0408	0.83731181	Distal Intergenic	ENSSSCG000	SELENOO	selenoprotein O [Source:NCBI gene (formerly Entrezgene);Acc:100518971]
chr15	120984962	120986538	1577	*	chr15	120984962	120986538	in_vitro	in_vivo	14	13	1.42357143	0.18461538	1.23895604	c(t = 2.24653	0.0408	0.83731181	Distal Intergenic	ENSSSCG000	ssc-mir-375	ssc-mir-375 [Source:miRBase;Acc:MI0033409]
chr8	101936783	101937261	479	*	chr8	101936783	101937261	in_vitro	in_vivo	11	9	1.36727273	0	1.36727273	c(t = 2.34815	0.0408	0.83731181	Distal Intergenic	ENSSSCG000	TRPC3	transient receptor potential cation channel subfamily C member 3 [Source:VGNC Symbol;Acc:VGNC:94461]
chr2	42081357	42082577	1221	*	chr2	42081357	42082577	in_vitro	in_vivo	11	11	6.08727273	1.90090909	4.18636364	c(t = 2.23873	0.0412	0.83731181	Distal Intergenic	ENSSSCG000	RPS13	ribosomal protein S13 [Source:NCBI gene (formerly Entrezgene);Acc:100513520]
chr3	8889825	8890734	910	*	chr3	8889825	8890734	in_vitro	in_vivo	9	12	18.5233333	4.49666667	14.0266667	c(t = 2.33025	0.0413	0.83731181	Distal Intergenic	ENSSSCG000	SERPINE1	serpin family E member 1 [Source:NCBI gene (formerly Entrezgene);Acc:396945]
chr2	134092700	134094111	1412	*	chr2	134092700	134094111	in_vitro	in_vivo	11	15	0.56	3.38	-2.82	c(t = -2.2300	0.0413	0.83731181	Distal Intergenic	ENSSSCG000	CSF2	colony stimulating factor 2 [Source:NCBI gene (formerly Entrezgene);Acc:397208]
chr9	62933183	62933665	483	*	chr9	62933183	62933665	in_vitro	in_vivo	10	10	26.072	3.886	22.186	c(t = 2.32958	0.0415	0.83731181	Distal Intergenic	ENSSSCG000	FMO1	flavin containing dimethylaniline monoxygenase 1 [Source:VGNC Symbol;Acc:VGNC:88170]
chr5	7978754	7982596	3843	*	chr5	7978754	7982596	in_vitro	in_vivo	11	13	96.2190909	88.3492308	7.86986014	c(t = 2.18825	0.042	0.83731181	Distal Intergenic	ENSSSCG000	ADSL	adenylosuccinate lyase [Source:NCBI gene (formerly Entrezgene);Acc:100170844]
chr15	48442185	48443666	1482	*	chr15	48442185	48443666	in_vitro	in_vivo	10	13	3.156	8.68230769	-5.5263077	c(t = -2.1838	0.042	0.83731181	Promoter (<=1kb)	ENSSSCG000	EIF4EBP1	eukaryotic translation initiation factor 4E binding protein 1 [Source:NCBI gene (formerly Entrezgene);Acc:100627508]
chr5	82795815	82796633	819	*	chr5	82795815	82796633	in_vitro	in_vivo	13	11	0.59153846	5.41727273	-4.8257343	c(t = -2.2831	0.042	0.83731181	Promoter (<=1kb)	ENSSSCG000	ARL1	ADP ribosylation factor like GTPase 1 [Source:VGNC Symbol;Acc:VGNC:85513]
chr1	221578260	221579399	1140	*	chr1	221578260	221579399	in_vitro	in_vivo	13	14	6.16461538	1.35642857	4.80818681	c(t = 2.20328	0.042	0.83731181	Distal Intergenic	ENSSSCG000	ssc-mir-9823	ssc-mir-9823 [Source:miRBase;Acc:MI0031590]
chr8	102129442	102130697	1256	*	chr8	102129442	102130697	in_vitro	in_vivo	12	13	0.67416667	2.40538462	-1.7312179	c(t = -2.2044	0.0421	0.83731181	Distal Intergenic	ENSSSCG000	TRPC3	transient receptor potential cation channel subfamily C member 3 [Source:VGNC Symbol;Acc:VGNC:94461]
chr14	127174214	127175160	947	*	chr14	127174214	127175160	in_vitro	in_vivo	11	12	2.82727273	1.05166667	1.77560606	c(t = 2.18173	0.0423	0.83731181	Distal Intergenic	NA	NA	NA
chr14	50346903	50348220	1318	*	chr14	50346903	50348220	in_vitro	in_vivo	11	12	3.21909091	0.71833333	2.50075758	c(t = 2.26925	0.0423	0.83731181	Distal Intergenic	ENSSSCG00000037143		ubiquitin conjugating enzyme E2 L3 [Source:NCBI gene (formerly Entrezgene);Acc:100154268]
chr6	97643080	97644518	1439	*	chr6	97643080	97644518	in_vitro	in_vivo	13	13	3.63769231	1.20230769	2.43538462	c(t = 2.17156	0.0424	0.83731181	Distal Intergenic	ENSSSCG000	CIDEA	cell death inducing DFFA like effector a [Source:NCBI gene (formerly Entrezgene);Acc:100127171]
chr1	31796141	31797056	916	*	chr1	31796141	31797056	in_vitro	in_vivo	9	9	2.22	0.51777778	1.70222222	c(t = 2.27178	0.0424	0.83731181	Distal Intergenic	ENSSSCG000	CCN2	cellular communication network factor 2 [Source:NCBI gene (formerly Entrezgene);Acc:100152141]
chr17	61527565	61532404	4840	*	chr17	61527565	61532404	in_vitro	in_vivo	14	13	4.75928571	2.33230769	2.42697802	c(t = 2.15759	0.0427	0.83731181	Distal Intergenic	ENSSSCG000	PSMA7	proteasome 20S subunit alpha 7 [Source:NCBI gene (formerly Entrezgene);Acc:100626738]
chr11	23237035	23238925	1891	*	chr11	23237035	23238925	in_vitro	in_vivo	12	13	4.37416667	1.01846154	3.35570513	c(t = 2.25150	0.0427	0.83731181	Distal Intergenic	ENSSSCG000	FAM216B	family with sequence similarity 216 member B [Source:VGNC Symbol;Acc:VGNC:87955]
chr2	142944617	142945445	829	*	chr2	142944617	142945445	in_vitro	in_vivo	11	12	13.5118182	29.9416667	-16.429848	c(t = -2.1558	0.0429	0.83731181	Distal Intergenic	ENSSSCG000	DIAPH1	diaphanous related formin 1 [Source:VGNC Symbol;Acc:VGNC:99632]
chr18	46249140	46250558	1419	*	chr18	46249140	46250558	in_vitro	in_vivo	14	12	2.02428571	0.41416667	1.61011905	c(t = 2.19242	0.0431	0.83731181	Distal Intergenic	NA	NA	NA
chr1	121067661	121068085	425	*	chr1	121067661	121068085	in_vitro	in_vivo	9	10	3.05666667	0.27	2.78666667	c(t = 2.35743	0.0431	0.83731181	Distal Intergenic	ENSSSCG00000054479		cytochrome P450 19A2 [Source:NCBI gene (formerly Entrezgene);Acc:403332]
chr11	68838249	68842592	4344	*	chr11	68838249	68842592	in_vitro	in_vivo	14	17	1.93285714	0.80588235	1.12697479	c(t = 2.16987	0.0434	0.83731181	Distal Intergenic	ENSSSCG000	TMTC4	transmembrane O-mannosyltransferase targeting cadherins 4 [Source:VGNC Symbol;Acc:VGNC:94244]
chr15	100498157	100499384	1228	*	chr15	100498157	100499384	in_vitro	in_vivo	11	12	2.59909091	0.565	2.03409091	c(t = 2.19033	0.0435	0.83731181	Distal Intergenic	ENSSSCG000	HECW2	HECT, C2 and WW domain containing E3 ubiquitin protein ligase 2 [Source:NCBI gene (formerly Entrezgene);Acc:100155879]
chr9	3440966	3442583	1618	*	chr9	3440966	3442583	in_vitro	in_vivo	10	11	2.997	0.73636364	2.26063636	c(t = 2.27595	0.0436	0.83731181	Distal Intergenic	ENSSSCG000	HPX	hemopexin [Source:VGNC Symbol;Acc:VGNC:88968]
chr17	37743961	37744581	621	*	chr17	37743961	37744581	in_vitro	in_vivo	11	11	0.89909091	0	0.89909091	c(t = 2.30811	0.0436	0.83731181	Promoter (<=1kb)	ENSSSCG000	AHCY	adenosylhomocysteinase [Source:NCBI gene (formerly Entrezgene);Acc:497050]
chr6	148009356	148011156	1801	*	chr6	148009356	148011156	in_vitro	in_vivo	12	11	11.61	2.06	9.55	c(t = 2.24308	0.0437	0.83731181	Distal Intergenic	ENSSSCG000	JAK1	Janus kinase 1 [Source:VGNC Symbol;Acc:VGNC:89270]
chr16	64205145	64205815	671	*	chr16	64205145	64205815	in_vitro	in_vivo	11	12	1.32454545	0.1575	1.16704545	c(t = 2.24894	0.044	0.83731181	Distal Intergenic	ENSSSCG000	IL12B	interleukin 12B [Source:NCBI gene (formerly Entrezgene);Acc:397076]
chrX	112473156	112475691	2536	*	chrX	112473156	112475691	in_vitro	in_vivo	13	13	2.30692308	7.09076923	-4.7838462	c(t = -2.1808	0.0441	0.83731181	Distal Intergenic	ENSSSCG00000012703		RNA binding motif protein X-linked [Source:NCBI gene (formerly Entrezgene);Acc:100158042]
chr12	14571199	14573341	2143	*	chr12	14571199	14573341	in_vitro	in_vivo	11	15	2.79	0.75333333	2.03666667	c(t = 2.23892	0.0441	0.83731181	Distal Intergenic	ENSSSCG000	PECAM1	platelet and endothelial cell adhesion molecule 1 [Source:VGNC Symbol;Acc:VGNC:91304]
chr6	101214882	101216684	1803	*	chr6	101214882	101216684	in_vitro	in_vivo	10	11	5.948	1.16	4.788	c(t = 2.25945	0.0443	0.83731181	Distal Intergenic	ENSSSCG000	TGIF1	TGFB induced factor homeobox 1 [Source:VGNC Symbol;Acc:VGNC:93935]
chr9	421276	423480	2205	*	chr9	421276	423480	in_vitro	in_vivo	9	10	72.1655556	95.906	-23.740444	c(t = -2.3549	0.0445	0.83731181	Distal Intergenic	NA	NA	NA
chr13	79610176	79611044	869	*	chr13	79610176	79611044	in_vitro	in_vivo	11	11	2.18909091	0.51454545	1.67454545	c(t = 2.20544	0.0447	0.83731181	Distal Intergenic	ENSSSCG000	FOXL2	forkhead box L2 [Source:NCBI gene (formerly Entrezgene);Acc:100622956]
chr10	59816556	59817109	554	*	chr10	59816556	59817109	in_vitro	in_vivo	9	9	1.06111111	0.11444444	0.94666667	c(t = 2.31402	0.0449	0.83731181	Distal Intergenic	ENSSSCG000	CDC123	cell division cycle 123 [Source:VGNC Symbol;Acc:VGNC:95819]
chr5	76634663	76635581	919	*	chr5	76634663	76635581	in_vitro	in_vivo	12	12	0.57833333	0	0.57833333	c(t = 2.26243	0.0449	0.83731181	Distal Intergenic	ENSSSCG000	SLC38A2	solute carrier family 38 member 2 [Source:VGNC Symbol;Acc:VGNC:93094]
chr12	24679720	24680359	640	*	chr12	24679720	24680359	in_vitro	in_vivo	9	12	3.84	0.35333333	3.48666667	c(t = 2.34684	0.0452	0.83731181	Distal Intergenic	NA	NA	NA
chr12	5116522	5120486	3965	*	chr12	5116522	5120486	in_vitro	in_vivo	14	11	3.90071429	1.50363636	2.39707792	c(t = 2.13603	0.0453	0.83731181	Distal Intergenic	ENSSSCG00000036592		serine and arginine rich splicing factor 2 [Source:VGNC Symbol;Acc:VGNC:99085]
chr15	3525008	3526746	1739	*	chr15	3525008	3526746	in_vitro	in_vivo	9	12	2.27777778	0.38333333	1.89444444	c(t = 2.31409	0.0453	0.83731181	Distal Intergenic	ENSSSCG000	ACVR2A	activin A receptor type 2A [Source:NCBI gene (formerly Entrezgene);Acc:397282]
chr2	150679035	150679341	307	*	chr2	150679035	150679341	in_vitro	in_vivo	10	9	2.331	0	2.331	c(t = 2.31774	0.0457	0.83731181	Distal Intergenic	ENSSSCG000	ssc-mir-145	ssc-mir-145 [Source:miRBase;Acc:MI0002417]
chr8	43960461	43961175	715	*	chr8	43960461	43961175	in_vitro	in_vivo	9	12	1.46444444	4.89916667	-3.4347222	c(t = -2.1546	0.0458	0.83731181	Distal Intergenic	ENSSSCG000	MSMO1	methylsterol monooxygenase 1 [Source:VGNC Symbol;Acc:VGNC:98941]
chr7	115099677	115101064	1388	*	chr7	115099677	115101064	in_vitro	in_vivo	9	10	4.87222222	1.222	3.65022222	c(t = 2.20365	0.0458	0.83731181	Distal Intergenic	ENSSSCG000	ISG12(A)	putative ISG12(a) protein [Source:NCBI gene (formerly Entrezgene);Acc:100153902]
chr11	4879361	4880102	742	*	chr11	4879361	4880102	in_vitro	in_vivo	11	12	4.44	0.34083333	4.09916667	c(t = 2.26909	0.0458	0.83731181	Distal Intergenic	ENSSSCG000	ssc-mir-9803	ssc-mir-9803 [Source:miRBase;Acc:MI0031567]
chr14	22379509	22380259	751	*	chr14	22379509	22380259	in_vitro	in_vivo	9	13	4.34333333	0.19230769	4.15102564	c(t = 2.35287	0.0458	0.83731181	Distal Intergenic	ENSSSCG000	PIWIL1	piwi like RNA-mediated silencing 1 [Source:VGNC Symbol;Acc:VGNC:98197]
chr7	48010546	48011792	1247	*	chr7	48010546	48011792	in_vitro	in_vivo	14	14	5.285	18.7871429	-13.502143	c(t = -2.1644	0.0462	0.83731181	Distal Intergenic	ENSSSCG000	CTSH	cathepsin H [Source:NCBI gene (formerly Entrezgene);Acc:396969]
chr2	137205574	137207668	2095	*	chr2	137205574	137207668	in_vitro	in_vivo	12	15	4.07166667	1.522	2.54966667	c(t = 2.17364	0.0464	0.83731181	Promoter (<=1kb)	ENSSSCG000	PITX1	paired like homeodomain 1 [Source:NCBI gene (formerly Entrezgene);Acc:100689266]
chr1	161320999	161322117	1119	*	chr1	161320999	161322117	in_vitro	in_vivo	12	10	3.799	0.2775	3.5215	c(t = 2.28241	0.0466	0.83731181	Distal Intergenic	ENSSSCG000	PMAIP1	phorbol-12-myristate-13-acetate-induced protein 1 [Source:NCBI gene (formerly Entrezgene);Acc:397278]
chr15	137400785	137401797	1013	*	chr15	137400785	137401797	in_vitro	in_vivo	9	10	9.28666667	35.879	-26.592333	c(t = -2.2428	0.0469	0.83731181	Distal Intergenic	ENSSSCG000	RAMP1	receptor activity modifying protein 1 [Source:NCBI gene (formerly Entrezgene);Acc:397401]
chr1	159530453	159532086	1634	*	chr1	159530453	159532086	in_vitro	in_vivo	10	11	2.542	0.52545455	2.01654545	c(t = 2.25848	0.0469	0.83731181	Promoter (<=1kb)	ENSSSCG000	RNF152	ring finger protein 152 [Source:VGNC Symbol;Acc:VGNC:92364]
chr6	1476941	1481053	4113	*	chr6	1476941	1481053	in_vitro	in_vivo	11	11	95.93	84.7036364	11.2263636	c(t = 2.21257	0.0471	0.83731181	Intron (NM_001244650/100622381, intron 1 of 12)	ENSSSCG000	BANP	BTG3 associated nuclear protein [Source:NCBI gene (formerly Entrezgene);Acc:100622381]
chr11	76710558	76711935	1378	*	chr11	76710558	76711935	in_vitro	in_vivo	13	15	6.36384615	21.8993333	-15.535487	c(t = -2.1096	0.0472	0.83731181	Distal Intergenic	ENSSSCG000	NAXD	NAD(P)HX dehydratase [Source:NCBI gene (formerly Entrezgene);Acc:100153456]
chr3	979624	1005727	26104	*	chr3	979624	1005727	in_vitro	in_vivo	11	15	85.7318182	67.9546667	17.7771515	c(t = 2.10196	0.0473	0.83731181	Distal Intergenic	ENSSSCG000	ssc-mir-9817	ssc-mir-9817 [Source:miRBase;Acc:MI0031584]
chr8	607842	613265	5424	*	chr8	607842	613265	in_vitro	in_vivo	14	14	4.30214286	1.11857143	3.18357143	c(t = 2.18006	0.0473	0.83731181	Distal Intergenic	ENSSSCG000	SLBP	stem-loop binding protein [Source:NCBI gene (formerly Entrezgene);Acc:100513605]
chr13	208211722	208213513	1792	*	chr13	208211722	208213513	in_vitro	in_vivo	9	11	8.69555556	29.2372727	-20.541717	c(t = -2.2179	0.0476	0.83731181	Distal Intergenic	ENSSSCG000	ITGB2	integrin subunit beta 2 [Source:NCBI gene (formerly Entrezgene);Acc:396943]
chr18	37724345	37725032	688	*	chr18	37724345	37725032	in_vitro	in_vivo	11	11	2.69636364	0.32	2.37636364	c(t = 2.22738	0.0477	0.83731181	Distal Intergenic	ENSSSCG000	FKBP9	FKBP prolyl isomerase 9 [Source:NCBI gene (formerly Entrezgene);Acc:100623924]
chr17	15752061	15752568	508	*	chr17	15752061	15752568	in_vitro	in_vivo	10	9	47.584	14.9033333	32.6806667	c(t = 2.15390	0.0478	0.83731181	Promoter (1-2kb)	ENSSSCG000	BMP2	bone morphotic protein 2 [Source:VGNC Symbol;Acc:VGNC:95488]
chr2	81125621	81126933	1313	*	chr2	81125621	81126933	in_vitro	in_vivo	14	9	3.415	1.10111111	2.31388889	c(t = 2.11855	0.0482	0.83731181	Distal Intergenic	ENSSSCG000	SNCB	synuclein beta [Source:VGNC Symbol;Acc:VGNC:93286]
chr13	131475772	131476583	812	*	chr13	131475772	131476583	in_vitro	in_vivo	10	12	2.636	0.45416667	2.18183333	c(t = 2.21910	0.0482	0.83731181	Distal Intergenic	ENSSSCG000	HES1	hes family bHLH transcription factor 1 [Source:NCBI gene (formerly Entrezgene);Acc:100499567]
chr13	32216849	32218331	1483	*	chr13	32216849	32218331	in_vitro	in_vivo	9	10	14.1988889	4.937	9.26188889	c(t = 2.27759	0.0483	0.83731181	Distal Intergenic	ENSSSCG000	APEH	acylaminoacyl-peptide hydrolase [Source:VGNC Symbol;Acc:VGNC:85408]
chr4	127568383	127569330	948	*	chr4	127568383	127569330	in_vitro	in_vivo	9	11	68.3711111	29.8036364	38.5674747	c(t = 2.10334	0.0498	0.83731181	Distal Intergenic	ENSSSCG000	GBP1	guanylate binding protein 1, interferon-inducible [Source:NCBI gene (formerly Entrezgene);Acc:100151938]
chr2	60378324	60379067	744	*	chr2	60378324	60379067	in_vitro	in_vivo	10	12	1.383	0	1.383	c(t = 2.26508	0.0498	0.83731181	Distal Intergenic	ENSSSCG000	BST2	bone marrow stromal cell antigen 2 [Source:NCBI gene (formerly Entrezgene);Acc:100302088]
chr10	16190362	16191160	799	*	chr10	16190362	16191160	in_vitro	in_vivo	10	11	3.657	0.93181818	2.72518182	c(t = 2.16898	0.0499	0.83731181	Distal Intergenic	ENSSSCG000	AKT3	AKT serine/threonine kinase 3 [Source:VGNC Symbol;Acc:VGNC:96306]
chr16	6383448	6384749	1302	*	chr16	6383448	6384749	in_vitro	in_vivo	12	12	2.3125	0.41666667	1.89583333	c(t = 2.11297	0.05	0.83731181	Distal Intergenic	ENSSSCG000	C1QTNF3	C1q and TNF related 3 [Source:NCBI gene (formerly Entrezgene);Acc:100270814]
chr3	45198701	45201575	2875	*	chr3	45198701	45201575	in_vitro	in_vivo	13	16	2.00076923	0.70875	1.29201923	c(t = 2.12412	0.0501	0.83731181	Promoter (<=1kb)	ENSSSCG000	BCL2L11	BCL2 like 11 [Source:NCBI gene (formerly Entrezgene);Acc:396632]
chr9	135981067	135983958	2892	*	chr9	135981067	135983958	in_vitro	in_vivo	10	15	9.819	5.61	4.209	c(t = 2.09655	0.0502	0.83731181	Distal Intergenic	ENSSSCG000	ZPBP	zona pellucida binding protein [Source:NCBI gene (formerly Entrezgene);Acc:397194]
chr6	83361071	83362136	1066	*	chr6	83361071	83362136	in_vitro	in_vivo	10	9	1.913	0	1.913	c(t = 2.25604	0.0505	0.83731181	Promoter (<=1kb)	ENSSSCG000	STMN1	stathmin 1 [Source:VGNC Symbol;Acc:VGNC:93560]
chr18	8842902	8845088	2187	*	chr18	8842902	8845088	in_vitro	in_vivo	13	13	2.83923077	0.74384615	2.09538462	c(t = 2.12956	0.0507	0.83731181	Distal Intergenic	ENSSSCG000	NDUFB2	NADH:ubiquinone oxidoreductase subunit B2 [Source:NCBI gene (formerly Entrezgene);Acc:100513895]
chr10	8305655	8307855	2201	*	chr10	8305655	8307855	in_vitro	in_vivo	12	12	33.3391667	8.68916667	24.65	c(t = 2.15423	0.0508	0.83731181	Distal Intergenic	ENSSSCG000	SPATA17	spermatosis associated 17 [Source:VGNC Symbol;Acc:VGNC:96065]
chr5	36273859	36275362	1504	*	chr5	36273859	36275362	in_vitro	in_vivo	10	12	62.218	32.5308333	29.6871667	c(t = 2.07603	0.0511	0.83731181	Distal Intergenic	ENSSSCG000	LGR5	leucine rich repeat containing G protein-coupled receptor 5 [Source:VGNC Symbol;Acc:VGNC:103296]
chr1	229954958	229956566	1609	*	chr1	229954958	229956566	in_vitro	in_vivo	11	12	2	0.1325	1.8675	c(t = 2.20003	0.0512	0.83731181	Distal Intergenic	ENSSSCG000	GNA14	G protein subunit alpha 14 [Source:VGNC Symbol;Acc:VGNC:88519]
chr13	31627106	31627958	853	*	chr13	31627106	31627958	in_vitro	in_vivo	11	9	6.82090909	1.49222222	5.32868687	c(t = 2.14371	0.0514	0.83731181	Distal Intergenic	ENSSSCG000	ssc-mir-425	ssc-mir-425 [Source:miRBase;Acc:MI0013133]
chr8	104270796	104272872	2077	*	chr8	104270796	104272872	in_vitro	in_vivo	13	17	1.98307692	0.38588235	1.59719457	c(t = 2.14136	0.0514	0.83731181	Distal Intergenic	ENSSSCG000	FABP2	fatty acid binding protein 2 [Source:NCBI gene (formerly Entrezgene);Acc:595106]
chr1	131745993	131746903	911	*	chr1	131745993	131746903	in_vitro	in_vivo	12	9	3.18916667	0.30888889	2.88027778	c(t = 2.15704	0.0516	0.83731181	Promoter (<=1kb)	ENSSSCG000	THBS1	thrombospondin 1 [Source:NCBI gene (formerly Entrezgene);Acc:492313]
chr10	49119792	49120632	841	*	chr10	49119792	49120632	in_vitro	in_vivo	14	9	2.06142857	0.42777778	1.63365079	c(t = 2.09224	0.0517	0.83731181	Distal Intergenic	ENSSSCG000	GAD2	glutamate decarboxylase 2 [Source:NCBI gene (formerly Entrezgene);Acc:396929]
chr5	41561475	41562280	806	*	chr5	41561475	41562280	in_vitro	in_vivo	12	12	2.75916667	0.28833333	2.47083333	c(t = 2.16563	0.0517	0.83731181	Distal Intergenic	ENSSSCG000	RESF1	retroelement silencing factor 1 [Source:VGNC Symbol;Acc:VGNC:92219]
chr14	6639221	6639907	687	*	chr14	6639221	6639907	in_vitro	in_vivo	10	11	3.127	0.92636364	2.20063636	c(t = 2.17282	0.0518	0.83731181	Distal Intergenic	ENSSSCG000	PIWIL2	piwi like RNA-mediated silencing 2 [Source:VGNC Symbol;Acc:VGNC:98198]
chr3	85810808	85811477	670	*	chr3	85810808	85811477	in_vitro	in_vivo	11	12	2.77545455	0.3475	2.42795455	c(t = 2.15704	0.0518	0.83731181	Distal Intergenic	ENSSSCG000	CFAP36	cilia and flagella associated protein 36 [Source:NCBI gene (formerly Entrezgene);Acc:100513146]
chr4	64953940	64956027	2088	*	chr4	64953940	64956027	in_vitro	in_vivo	11	14	0.28454545	1.49642857	-1.2118831	c(t = -2.0986	0.052	0.83731181	Promoter (<=1kb)	ENSSSCG000	NCOA2	nuclear receptor coactivator 2 [Source:VGNC Symbol;Acc:VGNC:90616]
chr2	135919072	135920821	1750	*	chr2	135919072	135920821	in_vitro	in_vivo	14	16	2.68928571	0.91875	1.77053571	c(t = 2.07137	0.0521	0.83731181	Distal Intergenic	ENSSSCG00000014296		voltage dependent anion channel 1 [Source:VGNC Symbol;Acc:VGNC:100881]
chr6	63724265	63728822	4558	*	chr6	63724265	63728822	in_vitro	in_vivo	15	12	10.2026667	24.5891667	-14.3865	c(t = -2.1342	0.0523	0.83731181	Distal Intergenic	ENSSSCG000	TAS1R3	taste 1 receptor member 3 [Source:NCBI gene (formerly Entrezgene);Acc:100127440]
chr15	25328709	25330385	1677	*	chr15	25328709	25330385	in_vitro	in_vivo	12	11	2.9225	0.33454545	2.58795455	c(t = 2.14993	0.0526	0.83731181	Promoter (<=1kb)	ENSSSCG000	BIN1	bridging integrator 1 [Source:VGNC Symbol;Acc:VGNC:95475]
chr11	79083807	79084575	769	*	chr11	79083807	79084575	in_vitro	in_vivo	10	11	3.23	0.26	2.97	c(t = 2.20510	0.0528	0.83731181	Promoter (<=1kb)	ENSSSCG000	CDC16	cell division cycle 16 [Source:NCBI gene (formerly Entrezgene);Acc:100621739]
chr4	94747476	94748744	1269	*	chr4	94747476	94748744	in_vitro	in_vivo	11	12	2.83272727	0.69	2.14272727	c(t = 2.10927	0.0531	0.83731181	Distal Intergenic	ENSSSCG000	CKS1B	CDC28 protein kinase regulatory subunit 1B [Source:NCBI gene (formerly Entrezgene);Acc:100155110]
chr7	121012195	121014850	2656	*	chr7	121012195	121014850	in_vitro	in_vivo	13	9	98.47	91.4888889	6.98111111	c(t = 2.20605	0.0532	0.83731181	Exon (NM_001244777/100513162, exon 3 of 14)	ENSSSCG000	ssc-mir-342	ssc-mir-342 [Source:miRBase;Acc:MI0013153]
chr9	136741863	136743267	1405	*	chr9	136741863	136743267	in_vitro	in_vivo	10	12	3.585	1.29	2.295	c(t = 2.14747	0.0532	0.83731181	Promoter (<=1kb)	ENSSSCG000	GRB10	growth factor receptor bound protein 10 [Source:VGNC Symbol;Acc:VGNC:88662]
chr7	117469021	117469789	769	*	chr7	117469021	117469789	in_vitro	in_vivo	11	12	10.9081818	40.3941667	-29.485985	c(t = -2.0554	0.0533	0.83731181	3' UTR	ENSSSCG000	BDKRB1	bradykinin receptor B1 [Source:NCBI gene (formerly Entrezgene);Acc:100127469]
chr8	87854548	87855663	1116	*	chr8	87854548	87855663	in_vitro	in_vivo	10	13	1.056	0.19923077	0.85676923	c(t = 2.15478	0.0534	0.83731181	Distal Intergenic	ENSSSCG000	MGARP	mitochondria localized glutamic acid rich protein [Source:NCBI gene (formerly Entrezgene);Acc:100359351]
chr3	125103753	125104644	892	*	chr3	125103753	125104644	in_vitro	in_vivo	9	10	7.35222222	39.089	-31.736778	c(t = -2.1229	0.0535	0.83731181	Distal Intergenic	ENSSSCG000	LPIN1	lipin 1 [Source:VGNC Symbol;Acc:VGNC:89793]
chr1	254671485	254672860	1376	*	chr1	254671485	254672860	in_vitro	in_vivo	10	11	97.432	93.5045455	3.92745455	c(t = 2.06504	0.054	0.83731181	Distal Intergenic	ENSSSCG000	AMBP	alpha-1-microglobulin/bikunin [Source:VGNC Symbol;Acc:VGNC:109523]
chr3	42711254	42712670	1417	*	chr3	42711254	42712670	in_vitro	in_vivo	12	13	16.1491667	2.10461538	14.0445513	c(t = 2.15244	0.054	0.83731181	Distal Intergenic	ENSSSCG000	CARD19	caspase recruitment domain family member 19 [Source:VGNC Symbol;Acc:VGNC:86188]
chr13	206034618	206037701	3084	*	chr13	206034618	206037701	in_vitro	in_vivo	11	13	11.9836364	33.1415385	-21.157902	c(t = -2.0470	0.0542	0.83731181	Distal Intergenic	ENSSSCG000	TFF3	trefoil factor 3 [Source:NCBI gene (formerly Entrezgene);Acc:100627480]
chr1	44056457	44058235	1779	*	chr1	44056457	44058235	in_vitro	in_vivo	12	10	1.51	5.058	-3.548	c(t = -2.1566	0.0542	0.83731181	Distal Intergenic	ENSSSCG000	PLN	phospholamban [Source:NCBI gene (formerly Entrezgene);Acc:397421]
chr17	31868517	31869375	859	*	chr17	31868517	31869375	in_vitro	in_vivo	11	10	10.43	3.205	7.225	c(t = 2.05773	0.0542	0.83731181	Distal Intergenic	ENSSSCG00000007143		mitochondrial antiviral signaling protein [Source:VGNC Symbol;Acc:VGNC:96208]
chr11	2602205	2603499	1295	*	chr11	2602205	2603499	in_vitro	in_vivo	11	12	10.8336364	36.0616667	-25.22803	c(t = -2.0439	0.0543	0.83731181	Distal Intergenic	ENSSSCG000	ssc-mir-9814	ssc-mir-9814 [Source:miRBase;Acc:MI0031581]
chr13	131662423	131664783	2361	*	chr13	131662423	131664783	in_vitro	in_vivo	12	15	3.69	11.5346667	-7.8446667	c(t = -2.0349	0.0543	0.83731181	Distal Intergenic	ENSSSCG000	HES1	hes family bHLH transcription factor 1 [Source:NCBI gene (formerly Entrezgene);Acc:100499567]
chr1	161720590	161721936	1347	*	chr1	161720590	161721936	in_vitro	in_vivo	11	14	2.51727273	0.91857143	1.5987013	c(t = 2.05459	0.0545	0.83731181	Distal Intergenic	ENSSSCG000	PMAIP1	phorbol-12-myristate-13-acetate-induced protein 1 [Source:NCBI gene (formerly Entrezgene);Acc:397278]
chr1	76998122	76999023	902	*	chr1	76998122	76999023	in_vitro	in_vivo	12	10	3.2375	0.308	2.9295	c(t = 2.13984	0.0546	0.83731181	Distal Intergenic	ENSSSCG000	DDO	D-aspartate oxidase [Source:NCBI gene (formerly Entrezgene);Acc:100037287]
chr1	181771496	181773290	1795	*	chr1	181771496	181773290	in_vitro	in_vivo	11	11	6.97454545	3.36090909	3.61363636	c(t = 2.04421	0.0547	0.83731181	Distal Intergenic	ENSSSCG000	RTRAF	RNA transcription, translation and transport factor [Source:NCBI gene (formerly Entrezgene);Acc:100154555]
chr1	60942998	60944419	1422	*	chr1	60942998	60944419	in_vitro	in_vivo	14	9	2.13428571	0.66111111	1.4731746	c(t = 2.04734	0.0547	0.83731181	Distal Intergenic	ENSSSCG000	MAP3K7	mitogen-activated protein kinase kinase kinase 7 [Source:VGNC Symbol;Acc:VGNC:89987]
chr7	47607575	47608982	1408	*	chr7	47607575	47608982	in_vitro	in_vivo	13	12	5.76692308	1.76916667	3.99775641	c(t = 2.08543	0.0548	0.83731181	Promoter (<=1kb)	ENSSSCG000	CRABP1	cellular retinoic acid binding protein 1 [Source:NCBI gene (formerly Entrezgene);Acc:100169745]
chr11	22517984	22521671	3688	*	chr11	22517984	22521671	in_vitro	in_vivo	13	15	19.5823077	8.62266667	10.959641	c(t = 2.11220	0.0549	0.83731181	Distal Intergenic	ENSSSCG000	GTF2F2	general transcription factor IIF subunit 2 [Source:NCBI gene (formerly Entrezgene);Acc:100626869]
chr1	7378251	7380208	1958	*	chr1	7378251	7380208	in_vitro	in_vivo	10	11	98.195	92.6136364	5.58136364	c(t = 2.11745	0.055	0.83731181	Exon (NM_001244473/397214, exon 42 of 48)	ENSSSCG000	SLC22A1	solute carrier family 22 member 1 [Source:VGNC Symbol;Acc:VGNC:103179]
chr1	141494263	141495332	1070	*	chr1	141494263	141495332	in_vitro	in_vivo	13	14	0.92538462	2.7	-1.7746154	c(t = -2.0634	0.055	0.83731181	Distal Intergenic	ENSSSCG000	UBE3A	ubiquitin protein ligase E3A [Source:VGNC Symbol;Acc:VGNC:94652]
chr3	72619059	72619800	742	*	chr3	72619059	72619800	in_vitro	in_vivo	13	14	2.08	0	2.08	c(t = 2.12496	0.055	0.83731181	Distal Intergenic	ENSSSCG000	ANXA4	annexin A4 [Source:NCBI gene (formerly Entrezgene);Acc:100312960]
chr7	7234684	7245005	10322	*	chr7	7234684	7245005	in_vitro	in_vivo	16	16	2.004375	3.346875	-1.3425	c(t = -1.9961	0.0551	0.83731181	Distal Intergenic	ENSSSCG000	C6orf52	chromosome 7 C6orf52 homolog [Source:VGNC Symbol;Acc:VGNC:86073]
chr6	153859444	153860669	1226	*	chr6	153859444	153860669	in_vitro	in_vivo	12	14	3.90083333	0.975	2.92583333	c(t = 2.11945	0.0551	0.83731181	Distal Intergenic	ENSSSCG000	JUN	Jun proto-oncogene, AP-1 transcription factor subunit [Source:NCBI gene (formerly Entrezgene);Acc:396913]
chr9	25545447	25546144	698	*	chr9	25545447	25546144	in_vitro	in_vivo	9	13	4.62666667	0.74769231	3.87897436	c(t = 2.19641	0.0554	0.83731181	Distal Intergenic	ENSSSCG000	C11orf54	chromosome 9 C11orf54 homolog [Source:VGNC Symbol;Acc:VGNC:86087]
chr18	1968728	1969685	958	*	chr18	1968728	1969685	in_vitro	in_vivo	9	12	0.80222222	4.68583333	-3.8836111	c(t = -2.1281	0.0557	0.83731181	Distal Intergenic	ENSSSCG000	SHH	sonic hedgehog signaling molecule [Source:NCBI gene (formerly Entrezgene);Acc:100512749]
chr1	156435049	156435996	948	*	chr1	156435049	156435996	in_vitro	in_vivo	12	12	3.17416667	0.48166667	2.6925	c(t = 2.11100	0.0561	0.83731181	Distal Intergenic	ENSSSCG000	SERPINB10	serpin family B member 10 [Source:NCBI gene (formerly Entrezgene);Acc:100154994]
chr11	65200901	65201681	781	*	chr11	65200901	65201681	in_vitro	in_vivo	12	13	1.21333333	6.74384615	-5.5305128	c(t = -2.0804	0.0562	0.83731181	Distal Intergenic	ENSSSCG000	DNAJC3	DnaJ heat shock protein family (Hsp40) member C3 [Source:NCBI gene (formerly Entrezgene);Acc:100154166]
chr8	81023772	81024870	1099	*	chr8	81023772	81024870	in_vitro	in_vivo	11	11	0.72909091	4.20909091	-3.48	c(t = -2.1273	0.0562	0.83731181	Distal Intergenic	ENSSSCG000	EDNRA	endothelin receptor type A [Source:VGNC Symbol;Acc:VGNC:87549]
chr1	187718846	187720384	1539	*	chr1	187718846	187720384	in_vitro	in_vivo	14	11	1.65142857	0.46363636	1.18779221	c(t = 2.03047	0.0564	0.83731181	Distal Intergenic	ENSSSCG000	ARID4A	AT-rich interaction domain 4A [Source:VGNC Symbol;Acc:VGNC:85509]
chr1	25363215	25364243	1029	*	chr1	25363215	25364243	in_vitro	in_vivo	14	13	0.77928571	4.43153846	-3.6522527	c(t = -2.0940	0.0566	0.83731181	Distal Intergenic	ENSSSCG000	CCDC28A	coiled-coil domain containing 28A [Source:NCBI gene (formerly Entrezgene);Acc:100512200]
chr7	64507971	64508850	880	*	chr7	64507971	64508850	in_vitro	in_vivo	10	13	11.253	4.16	7.093	c(t = 2.12228	0.0567	0.83731181	Promoter (<=1kb)	ENSSSCG000	NFKBIA	NFKB inhibitor alpha [Source:NCBI gene (formerly Entrezgene);Acc:406188]
chr5	66066979	66067842	864	*	chr5	66066979	66067842	in_vitro	in_vivo	10	13	0.322	2.99384615	-2.6718462	c(t = -2.0956	0.0567	0.83731181	Distal Intergenic	ENSSSCG000	CCND2	cyclin D2 [Source:NCBI gene (formerly Entrezgene);Acc:397162]
chr12	5423055	5426347	3293	*	chr12	5423055	5426347	in_vitro	in_vivo	12	13	72.3091667	49.3607692	22.9483974	c(t = 2.00438	0.0569	0.83731181	Distal Intergenic	ENSSSCG000	ACOX1	acyl-CoA oxidase 1 [Source:VGNC Symbol;Acc:VGNC:85020]
chr14	76864199	76864522	324	*	chr14	76864199	76864522	in_vitro	in_vivo	11	10	5.00363636	1.045	3.95863636	c(t = 2.12196	0.0569	0.83731181	Distal Intergenic	ENSSSCG000	AP3M1	adaptor related protein complex 3 subunit mu 1 [Source:VGNC Symbol;Acc:VGNC:85390]
chr13	78982541	78983412	872	*	chr13	78982541	78983412	in_vitro	in_vivo	9	10	3.84888889	0.747	3.10188889	c(t = 2.17467	0.0569	0.83731181	Distal Intergenic	ENSSSCG000	CLDN18	claudin 18 [Source:VGNC Symbol;Acc:VGNC:86735]
chr3	22654598	22655683	1086	*	chr3	22654598	22655683	in_vitro	in_vivo	10	12	2.586	0.10666667	2.47933333	c(t = 2.17770	0.0569	0.83731181	Distal Intergenic	ENSSSCG000	NDUFAB1	NADH:ubiquinone oxidoreductase subunit AB1 [Source:VGNC Symbol;Acc:VGNC:104004]
chr14	20621737	20622911	1175	*	chr14	20621737	20622911	in_vitro	in_vivo	11	14	0.81272727	2.93	-2.1172727	c(t = -2.0276	0.0572	0.83731181	Distal Intergenic	ENSSSCG000	HMGB2	high mobility group box 2 [Source:VGNC Symbol;Acc:VGNC:88902]
chr1	1026523	1027672	1150	*	chr1	1026523	1027672	in_vitro	in_vivo	9	9	3.3	31.8411111	-28.541111	c(t = -2.2125	0.0573	0.83731181	Distal Intergenic	ENSSSCG000	ssc-mir-9815	ssc-mir-9815 [Source:miRBase;Acc:MI0031582]
chr17	62340780	62341942	1163	*	chr17	62340780	62341942	in_vitro	in_vivo	11	11	3.42545455	1.49545455	1.93	c(t = 2.03007	0.0574	0.83731181	Distal Intergenic	ENSSSCG000	LIME1	Lck interacting transmembrane adaptor 1 [Source:NCBI gene (formerly Entrezgene);Acc:100625216]
chr2	44375307	44376119	813	*	chr2	44375307	44376119	in_vitro	in_vivo	11	12	2.68090909	1.065	1.61590909	c(t = 2.01668	0.0574	0.83731181	Distal Intergenic	ENSSSCG00000035649		calcitonin-related polypeptide beta [Source:NCBI gene (formerly Entrezgene);Acc:396563]
chr13	108334208	108334864	657	*	chr13	108334208	108334864	in_vitro	in_vivo	10	9	4.27	0	4.27	c(t = 2.17808	0.0574	0.83731181	Distal Intergenic	ENSSSCG000	SEC62	SEC62 homolog, preprotein translocation factor [Source:NCBI gene (formerly Entrezgene);Acc:100125961]
chr3	102306317	102307068	752	*	chr3	102306317	102307068	in_vitro	in_vivo	9	9	0.18222222	1.60111111	-1.4188889	c(t = -2.1642	0.0576	0.83731181	Distal Intergenic	ENSSSCG000	GALM	galactose mutarotase [Source:NCBI gene (formerly Entrezgene);Acc:399536]
chr1	270738916	270739959	1044	*	chr1	270738916	270739959	in_vitro	in_vivo	14	12	2.51571429	0.90333333	1.61238095	c(t = 2.01818	0.0576	0.83731181	Distal Intergenic	ENSSSCG000	FUBP3	far upstream element binding protein 3 [Source:VGNC Symbol;Acc:VGNC:103093]
chr18	45636790	45637705	916	*	chr18	45636790	45637705	in_vitro	in_vivo	11	13	0	0.98307692	-0.9830769	c(t = -2.0985	0.0577	0.83731181	Distal Intergenic	ENSSSCG000	HOXA5	homeobox A5 [Source:NCBI gene (formerly Entrezgene);Acc:100499568]
chr9	32126081	32128169	2089	*	chr9	32126081	32128169	in_vitro	in_vivo	12	16	3.2675	0.871875	2.395625	c(t = 2.06644	0.0578	0.83731181	Promoter (1-2kb)	ENSSSCG000	PGR	progesterone receptor [Source:VGNC Symbol;Acc:VGNC:91362]
chr8	56193369	56194969	1601	*	chr8	56193369	56194969	in_vitro	in_vivo	11	13	2.26636364	0.65615385	1.61020979	c(t = 2.08669	0.0579	0.83731181	Distal Intergenic	ENSSSCG000	IGFBP7	insulin like growth factor binding protein 7 [Source:NCBI gene (formerly Entrezgene);Acc:100302573]
chr2	60567234	60567972	739	*	chr2	60567234	60567972	in_vitro	in_vivo	9	10	4.64555556	1.119	3.52655556	c(t = 2.12517	0.0581	0.83731181	Distal Intergenic	ENSSSCG000	BST2	bone marrow stromal cell antigen 2 [Source:NCBI gene (formerly Entrezgene);Acc:100302088]
chr10	10156482	10157235	754	*	chr10	10156482	10157235	in_vitro	in_vivo	10	11	3.559	0.80181818	2.75718182	c(t = 2.11498	0.0581	0.83731181	Distal Intergenic	ENSSSCG000	ssc-mir-664	ssc-mir-664 [Source:miRBase;Acc:MI0013123]
chr1	240494463	240496777	2315	*	chr1	240494463	240496777	in_vitro	in_vivo	12	12	8.55833333	1.635	6.92333333	c(t = 2.09164	0.0583	0.83731181	Distal Intergenic	ENSSSCG000	TGFBR1	transforming growth factor beta receptor 1 [Source:VGNC Symbol;Acc:VGNC:98368]
chr14	51291092	51297177	6086	*	chr14	51291092	51297177	in_vitro	in_vivo	15	14	2.176	6.65142857	-4.4754286	c(t = -2.0506	0.0585	0.83731181	Distal Intergenic	ENSSSCG000	GP1BB	glycoprotein Ib platelet subunit beta [Source:NCBI gene (formerly Entrezgene);Acc:100155101]
chr4	100443981	100444218	238	*	chr4	100443981	100444218	in_vitro	in_vivo	10	9	7.05	1.80333333	5.24666667	c(t = 2.06430	0.0585	0.83731181	Distal Intergenic	ENSSSCG000	PRKAB2	protein kinase AMP-activated non-catalytic subunit beta 2 [Source:VGNC Symbol;Acc:VGNC:91799]
chr12	52286754	52289412	2659	*	chr12	52286754	52289412	in_vitro	in_vivo	13	11	14.4	35.7827273	-21.382727	c(t = -2.0415	0.0586	0.83731181	Promoter (<=1kb)	ENSSSCG000	ALOX15	arachidonate 15-lipoxygenase [Source:NCBI gene (formerly Entrezgene);Acc:396971]
chr2	81372325	81373010	686	*	chr2	81372325	81373010	in_vitro	in_vivo	9	10	4.75333333	1.114	3.63933333	c(t = 2.08663	0.0586	0.83731181	Distal Intergenic	ENSSSCG000	SNCB	synuclein beta [Source:VGNC Symbol;Acc:VGNC:93286]
chr14	111166854	111167480	627	*	chr14	111166854	111167480	in_vitro	in_vivo	12	9	2.64833333	0.45666667	2.19166667	c(t = 2.04317	0.0586	0.83731181	Distal Intergenic	ENSSSCG00000058016		
chr10	51619996	51621825	1830	*	chr10	51619996	51621825	in_vitro	in_vivo	13	14	4.92923077	2.3	2.62923077	c(t = 2.02574	0.0587	0.83731181	Distal Intergenic	ENSSSCG000	PIP4K2A	phosphatidylinositol-5-phosphate 4-kinase type 2 alpha [Source:VGNC Symbol;Acc:VGNC:96485]
chr11	50724406	50725888	1483	*	chr11	50724406	50725888	in_vitro	in_vivo	9	10	1.80333333	0.446	1.35733333	c(t = 2.12002	0.0588	0.83731181	Distal Intergenic	ENSSSCG000	EDNRB	endothelin receptor type B [Source:VGNC Symbol;Acc:VGNC:87550]
chr11	3807702	3808665	964	*	chr11	3807702	3808665	in_vitro	in_vivo	11	11	12.56	33.7281818	-21.168182	c(t = -2.0709	0.059	0.83731181	Distal Intergenic	ENSSSCG000	GPR12	G protein-coupled receptor 12 [Source:NCBI gene (formerly Entrezgene);Acc:100736641]
chr10	20635351	20636292	942	*	chr10	20635351	20636292	in_vitro	in_vivo	10	14	0.213	1.72357143	-1.5105714	c(t = -2.0402	0.059	0.83731181	Distal Intergenic	ENSSSCG000	CAPN2	calpain 2 [Source:NCBI gene (formerly Entrezgene);Acc:397393]
chr8	52162182	52163224	1043	*	chr8	52162182	52163224	in_vitro	in_vivo	11	11	3.04	0.71181818	2.32818182	c(t = 2.06362	0.059	0.83731181	Distal Intergenic	ENSSSCG000	NPY1R	neuropeptide Y receptor Y1 [Source:VGNC Symbol;Acc:VGNC:90865]
chr1	198992230	198993785	1556	*	chr1	198992230	198993785	in_vitro	in_vivo	11	13	51.5427273	24.2130769	27.3296503	c(t = 2.00796	0.0591	0.83731181	Distal Intergenic	ENSSSCG000	CDKN2B	cyclin dependent kinase inhibitor 2B [Source:NCBI gene (formerly Entrezgene);Acc:397227]
chr10	67735763	67737924	2162	*	chr10	67735763	67737924	in_vitro	in_vivo	9	9	13.6444444	42.9466667	-29.302222	c(t = -2.0315	0.0592	0.83731181	Distal Intergenic	ENSSSCG000	WDR37	WD repeat domain 37 [Source:VGNC Symbol;Acc:VGNC:96258]
chr7	86268771	86269496	726	*	chr7	86268771	86269496	in_vitro	in_vivo	9	14	0.12111111	1.03571429	-0.9146032	c(t = -2.0407	0.0593	0.83731181	Distal Intergenic	NA	NA	NA
chr14	39885763	39886534	772	*	chr14	39885763	39886534	in_vitro	in_vivo	12	10	4.03166667	0.465	3.56666667	c(t = 2.09308	0.0594	0.83731181	Promoter (<=1kb)	ENSSSCG000	MAPKAPK5	MAPK activated protein kinase 5 [Source:NCBI gene (formerly Entrezgene);Acc:100153404]
chr9	60520395	60521865	1471	*	chr9	60520395	60521865	in_vitro	in_vivo	9	11	2.19888889	0.44363636	1.75525253	c(t = 2.14200	0.0595	0.83731181	Distal Intergenic	ENSSSCG000	SPATA19	spermatogenesis associated 19 [Source:NCBI gene (formerly Entrezgene);Acc:100415923]
chr11	6762590	6763775	1186	*	chr11	6762590	6763775	in_vitro	in_vivo	11	13	1.20363636	0.28230769	0.92132867	c(t = 2.08515	0.0595	0.83731181	Distal Intergenic	ENSSSCG000	SLC7A1	solute carrier family 7 member 1 [Source:VGNC Symbol;Acc:VGNC:93169]
chr17	29984578	29985610	1033	*	chr17	29984578	29985610	in_vitro	in_vivo	10	12	2.483	0.60666667	1.87633333	c(t = 2.08209	0.0596	0.83731181	Distal Intergenic	ENSSSCG000	THBD	thrombomodulin [Source:NCBI gene (formerly Entrezgene);Acc:100157642]
chr18	45420847	45423475	2629	*	chr18	45420847	45423475	in_vitro	in_vivo	12	13	4.7125	1.07692308	3.63557692	c(t = 2.08568	0.0597	0.83731181	Distal Intergenic	ENSSSCG000	HOXA5	homeobox A5 [Source:NCBI gene (formerly Entrezgene);Acc:100499568]
chr12	52925589	52926771	1183	*	chr12	52925589	52926771	in_vitro	in_vivo	10	10	3.886	0.476	3.41	c(t = 2.10472	0.0598	0.83731181	Distal Intergenic	ENSSSCG000	SAT2	spermidine/spermine N1-acetyltransferase family member 2 [Source:NCBI gene (formerly Entrezgene);Acc:100170117]
chr1	1555066	1558772	3707	*	chr1	1555066	1558772	in_vitro	in_vivo	10	11	47.535	72.4163636	-24.881364	c(t = -2.0111	0.0601	0.83731181	Distal Intergenic	ENSSSCG000	ssc-mir-9824	ssc-mir-9824 [Source:miRBase;Acc:MI0031591]
chr13	74179911	74180817	907	*	chr13	74179911	74180817	in_vitro	in_vivo	13	12	2.12538462	0.3875	1.73788462	c(t = 2.00976	0.0601	0.83731181	Distal Intergenic	ENSSSCG000	ACKR4	atypical chemokine receptor 4 [Source:NCBI gene (formerly Entrezgene);Acc:100037292]
chr13	75507034	75508117	1084	*	chr13	75507034	75508117	in_vitro	in_vivo	10	11	3.151	0.83727273	2.31372727	c(t = 2.09260	0.0602	0.83731181	Distal Intergenic	ENSSSCG000	ANAPC13	anaphase promoting complex subunit 13 [Source:VGNC Symbol;Acc:VGNC:85294]
chr1	144787214	144788783	1570	*	chr1	144787214	144788783	in_vitro	in_vivo	12	14	2.2275	0.60142857	1.62607143	c(t = 2.05903	0.0602	0.83731181	Distal Intergenic	ENSSSCG000	MCEE	methylmalonyl-CoA epimerase [Source:VGNC Symbol;Acc:VGNC:90066]
chr4	83775141	83777373	2233	*	chr4	83775141	83777373	in_vitro	in_vivo	13	13	0.78923077	2.98230769	-2.1930769	c(t = -1.9970	0.0603	0.83731181	Distal Intergenic	ENSSSCG000	POU2F1	POU class 2 homeobox 1 [Source:VGNC Symbol;Acc:VGNC:91672]
chr11	68855883	68856700	818	*	chr11	68855883	68856700	in_vitro	in_vivo	9	12	1.16222222	3.78916667	-2.6269444	c(t = -2.0257	0.0604	0.83731181	Distal Intergenic	ENSSSCG000	TMTC4	transmembrane O-mannosyltransferase targeting cadherins 4 [Source:VGNC Symbol;Acc:VGNC:94244]
chr2	57849075	57849575	501	*	chr2	57849075	57849575	in_vitro	in_vivo	11	12	1.26727273	0	1.26727273	c(t = 2.11441	0.0606	0.83731181	Distal Intergenic	ENSSSCG000	MGAT1	alpha-1,3-mannosyl-glycoprotein 2-beta-N-acetylglucosaminyltransferase [Source:VGNC Symbol;Acc:VGNC:96738]
chr2	65593675	65594233	559	*	chr2	65593675	65594233	in_vitro	in_vivo	13	10	1.09461538	0	1.09461538	c(t = 2.07119	0.0606	0.83731181	Distal Intergenic	ENSSSCG000	ssc-mir-24-1	ssc-mir-24-1 [Source:miRBase;Acc:MI0002428]
chr3	6373763	6374740	978	*	chr3	6373763	6374740	in_vitro	in_vivo	11	14	4.35636364	0.90428571	3.45207792	c(t = 2.07948	0.0607	0.83731181	Distal Intergenic	ENSSSCG00000034019		ATP synthase membrane subunit f [Source:VGNC Symbol;Acc:VGNC:97036]
chr12	57205516	57209565	4050	*	chr12	57205516	57209565	in_vitro	in_vivo	12	13	94.4066667	89.3615385	5.04512821	c(t = 1.97609	0.0608	0.83731181	3' UTR	ENSSSCG000	ELAC2	elaC ribonuclease Z 2 [Source:NCBI gene (formerly Entrezgene);Acc:100520804]
chr1	66513715	66514116	402	*	chr1	66513715	66514116	in_vitro	in_vivo	9	10	4.72666667	0	4.72666667	c(t = 2.18059	0.0608	0.83731181	Distal Intergenic	ENSSSCG000	CCNC	cyclin C [Source:VGNC Symbol;Acc:VGNC:103046]
chr12	33141356	33142511	1156	*	chr12	33141356	33142511	in_vitro	in_vivo	12	12	0.64916667	3.33583333	-2.6866667	c(t = -2.0566	0.0609	0.83731181	Promoter (<=1kb)	ENSSSCG000	DGKE	diacylglycerol kinase epsilon [Source:VGNC Symbol;Acc:VGNC:87271]
chr7	62481722	62483191	1470	*	chr7	62481722	62483191	in_vitro	in_vivo	9	13	4.16111111	1.50076923	2.66034188	c(t = 2.00233	0.0609	0.83731181	Distal Intergenic	ENSSSCG000	SSTR1	somatostatin receptor 1 [Source:VGNC Symbol;Acc:VGNC:93496]
chr1	248607333	248610234	2902	*	chr1	248607333	248610234	in_vitro	in_vivo	15	15	22.7933333	9.832	12.9613333	c(t = 1.99132	0.0611	0.83731181	Promoter (<=1kb)	ENSSSCG000	KLF4	KLF transcription factor 4 [Source:VGNC Symbol;Acc:VGNC:98062]
chrX	44860542	44862581	2040	*	chrX	44860542	44862581	in_vitro	in_vivo	15	15	5.998	22.346	-16.348	c(t = -2.0027	0.0613	0.83731181	Distal Intergenic	ENSSSCG000	BMP15	bone morphogenetic protein 15 [Source:NCBI gene (formerly Entrezgene);Acc:448811]
chr1	182202638	182204120	1483	*	chr1	182202638	182204120	in_vitro	in_vivo	12	14	1.67916667	0.385	1.29416667	c(t = 2.01641	0.0615	0.83731181	Distal Intergenic	ENSSSCG000	ERO1A	endoplasmic reticulum oxidoreductase 1 alpha [Source:NCBI gene (formerly Entrezgene);Acc:100192435]
chr16	33776872	33778708	1837	*	chr16	33776872	33778708	in_vitro	in_vivo	12	15	8.15166667	3.21466667	4.937	c(t = 1.98432	0.0617	0.83731181	Distal Intergenic	ENSSSCG000	GZMK	granzyme K [Source:NCBI gene (formerly Entrezgene);Acc:100233185]
chr11	6541133	6542447	1315	*	chr11	6541133	6542447	in_vitro	in_vivo	9	10	97.2577778	91.448	5.80977778	c(t = 2.03389	0.0618	0.83731181	3' UTR	ENSSSCG000	SLC7A1	solute carrier family 7 member 1 [Source:VGNC Symbol;Acc:VGNC:93169]
chr4	32092828	32094332	1505	*	chr4	32092828	32094332	in_vitro	in_vivo	13	13	4.29769231	0.88230769	3.41538462	c(t = 2.04163	0.062	0.83731181	Promoter (<=1kb)	ENSSSCG000	ZFPM2	zinc finger protein, FOG family member 2 [Source:NCBI gene (formerly Entrezgene);Acc:100337657]
chr10	10190759	10191758	1000	*	chr10	10190759	10191758	in_vitro	in_vivo	10	14	0.318	2.30785714	-1.9898571	c(t = -2.0238	0.0621	0.83731181	Distal Intergenic	ENSSSCG000	ssc-mir-664	ssc-mir-664 [Source:miRBase;Acc:MI0013123]
chr8	4446979	4447902	924	*	chr8	4446979	4447902	in_vitro	in_vivo	9	13	2.02	6.68692308	-4.6669231	c(t = -1.9780	0.0622	0.83731181	Distal Intergenic	ENSSSCG000	MAN2B2	mannosidase alpha class 2B member 2 [Source:VGNC Symbol;Acc:VGNC:98100]
chr3	42367639	42368394	756	*	chr3	42367639	42368394	in_vitro	in_vivo	10	11	3.078	0.58	2.498	c(t = 2.06709	0.0625	0.83731181	Distal Intergenic	ENSSSCG000	ASPN	asporin [Source:VGNC Symbol;Acc:VGNC:85586]
chr8	67830747	67831964	1218	*	chr8	67830747	67831964	in_vitro	in_vivo	13	14	2.25615385	0.22285714	2.0332967	c(t = 2.04189	0.0625	0.83731181	Distal Intergenic	ENSSSCG000	SLC4A4	solute carrier family 4 member 4 [Source:VGNC Symbol;Acc:VGNC:93133]
chr6	62833127	62834033	907	*	chr6	62833127	62834033	in_vitro	in_vivo	9	9	1.87555556	0	1.87555556	c(t = 2.16308	0.0625	0.83731181	Distal Intergenic	ENSSSCG000	ISG15	ISG15 ubiquitin like modifier [Source:NCBI gene (formerly Entrezgene);Acc:100145895]
chr10	12557515	12558552	1038	*	chr10	12557515	12558552	in_vitro	in_vivo	11	13	2.74181818	0.50076923	2.24104895	c(t = 2.07116	0.0626	0.83731181	Promoter (<=1kb)	ENSSSCG000	DEGS1	delta 4-desaturase, sphingolipid 1 [Source:VGNC Symbol;Acc:VGNC:95590]
chr7	65805345	65806263	919	*	chr7	65805345	65806263	in_vitro	in_vivo	13	11	2.15153846	0.27363636	1.8779021	c(t = 2.03547	0.0626	0.83731181	Distal Intergenic	ENSSSCG000	SNX6	sorting nexin 6 [Source:NCBI gene (formerly Entrezgene);Acc:100154775]
chr18	43559542	43559992	451	*	chr18	43559542	43559992	in_vitro	in_vivo	10	11	4.183	0.44363636	3.73936364	c(t = 2.10368	0.0627	0.83731181	Distal Intergenic	ENSSSCG000	FKBP14	FKBP prolyl isomerase 14 [Source:NCBI gene (formerly Entrezgene);Acc:100514551]
chr2	28497037	28497821	785	*	chr2	28497037	28497821	in_vitro	in_vivo	12	11	0.78583333	0	0.78583333	c(t = 2.07079	0.0627	0.83731181	Distal Intergenic	ENSSSCG000	WT1	WT1 transcription factor [Source:VGNC Symbol;Acc:VGNC:100884]
chr3	54775204	54776418	1215	*	chr3	54775204	54776418	in_vitro	in_vivo	10	11	2.823	1.23636364	1.58663636	c(t = 2.02462	0.063	0.83731181	Distal Intergenic	ENSSSCG000	MGAT4A	alpha-1,3-mannosyl-glycoprotein 4-beta-N-acetylglucosaminyltransferase A [Source:VGNC Symbol;Acc:VGNC:108158]
chr15	44917351	44919630	2280	*	chr15	44917351	44919630	in_vitro	in_vivo	12	13	2.72333333	0.73230769	1.99102564	c(t = 1.97995	0.063	0.83731181	Distal Intergenic	ENSSSCG000	CLDN22	claudin 22 [Source:NCBI gene (formerly Entrezgene);Acc:100294683]
chr5	1425406	1428979	3574	*	chr5	1425406	1428979	in_vitro	in_vivo	9	12	12.3477778	36.3675	-24.019722	c(t = -1.9919	0.0631	0.83731181	Distal Intergenic	ENSSSCG000	SELENOO	selenoprotein O [Source:NCBI gene (formerly Entrezgene);Acc:100518971]
chr18	4968572	4969838	1267	*	chr18	4968572	4969838	in_vitro	in_vivo	12	13	2.10083333	0.74769231	1.35314103	c(t = 1.96083	0.0634	0.83731181	Distal Intergenic	ENSSSCG000	RHEB	Ras homolog, mTORC1 binding [Source:NCBI gene (formerly Entrezgene);Acc:100516050]
chr1	227962394	227962842	449	*	chr1	227962394	227962842	in_vitro	in_vivo	9	12	4.42666667	0.53166667	3.895	c(t = 2.10293	0.0634	0.83731181	Distal Intergenic	ENSSSCG000	OSTF1	osteoclast stimulating factor 1 [Source:NCBI gene (formerly Entrezgene);Acc:397066]
chr16	67480975	67482118	1144	*	chr16	67480975	67482118	in_vitro	in_vivo	12	11	1.83583333	0.14454545	1.69128788	c(t = 2.05055	0.0634	0.83731181	Distal Intergenic	ENSSSCG000	SGCD	sarcoglycan delta [Source:NCBI gene (formerly Entrezgene);Acc:100240724]
chr1	220952750	220953643	894	*	chr1	220952750	220953643	in_vitro	in_vivo	12	12	2.05166667	0.3675	1.68416667	c(t = 1.99453	0.0636	0.83731181	Promoter (<=1kb)	ENSSSCG000	DMRT1	doublesex and mab-3 related transcription factor 1 [Source:NCBI gene (formerly Entrezgene);Acc:397199]
chr14	48565981	48567279	1299	*	chr14	48565981	48567279	in_vitro	in_vivo	9	14	0.79444444	3.77714286	-2.9826984	c(t = -1.9877	0.0638	0.83731181	Distal Intergenic	ENSSSCG000	SLC5A1	solute carrier family 5 member 1 [Source:VGNC Symbol;Acc:VGNC:93140]
chr17	11166763	11167738	976	*	chr17	11166763	11167738	in_vitro	in_vivo	13	10	4.65538462	0	4.65538462	c(t = 2.04181	0.0638	0.83731181	Distal Intergenic	ENSSSCG000	PLAT	plasminogen activator, tissue type [Source:NCBI gene (formerly Entrezgene);Acc:397121]
chr14	3033556	3035784	2229	*	chr14	3033556	3035784	in_vitro	in_vivo	13	16	0.46153846	1.421875	-0.9603365	c(t = -1.9512	0.0639	0.83731181	Distal Intergenic	ENSSSCG000	SYK	spleen associated tyrosine kinase [Source:NCBI gene (formerly Entrezgene);Acc:100125540]
chr8	18800641	18801470	830	*	chr8	18800641	18801470	in_vitro	in_vivo	9	12	8.78444444	37.3583333	-28.573889	c(t = -1.9983	0.064	0.83731181	Promoter (<=1kb)	ENSSSCG000	SOD3	superoxide dismutase 3 [Source:VGNC Symbol;Acc:VGNC:93335]
chr13	177966838	177967654	817	*	chr13	177966838	177967654	in_vitro	in_vivo	9	10	0	1.839	-1.839	c(t = -2.1069	0.0644	0.83731181	Distal Intergenic	NA	NA	NA
chr2	90261901	90263826	1926	*	chr2	90261901	90263826	in_vitro	in_vivo	14	11	3.475	0.51	2.965	c(t = 2.00950	0.0647	0.83731181	Distal Intergenic	ENSSSCG000	ATG10	autophagy related 10 [Source:VGNC Symbol;Acc:VGNC:85613]
chr13	192655845	192656999	1155	*	chr13	192655845	192656999	in_vitro	in_vivo	9	13	2.13888889	0.20230769	1.9365812	c(t = 2.10948	0.0647	0.83731181	Distal Intergenic	ENSSSCG000	CLDN17	claudin 17 [Source:NCBI gene (formerly Entrezgene);Acc:100294681]
chr13	202895426	202897439	2014	*	chr13	202895426	202897439	in_vitro	in_vivo	14	14	3.48857143	1.13357143	2.355	c(t = 1.96838	0.0648	0.83731181	Distal Intergenic	ENSSSCG000	GET1	guided entry of tail-anchored proteins factor 1 [Source:VGNC Symbol;Acc:VGNC:103949]
chr12	19335968	19337026	1059	*	chr12	19335968	19337026	in_vitro	in_vivo	9	10	2.26111111	0.573	1.68811111	c(t = 2.07726	0.0648	0.83731181	Distal Intergenic	ENSSSCG00000025414		peptide YY [Source:VGNC Symbol;Acc:VGNC:99029]
chr3	2092348	2094552	2205	*	chr3	2092348	2094552	in_vitro	in_vivo	9	11	92.8677778	85.7381818	7.12959596	c(t = 1.96876	0.0649	0.83731181	Distal Intergenic	ENSSSCG000	AMZ1	archaelysin family metallopeptidase 1 [Source:NCBI gene (formerly Entrezgene);Acc:100337670]
chr10	18195378	18196288	911	*	chr10	18195378	18196288	in_vitro	in_vivo	9	10	1.81222222	21.874	-20.061778	c(t = -2.0959	0.0649	0.83731181	Distal Intergenic	ENSSSCG000	SMYD3	SET and MYND domain containing 3 [Source:NCBI gene (formerly Entrezgene);Acc:100294707]
chr6	51444462	51444930	469	*	chr6	51444462	51444930	in_vitro	in_vivo	11	10	1.98636364	0.189	1.79736364	c(t = 2.05116	0.0649	0.83731181	Distal Intergenic	ENSSSCG00000003086		apolipoprotein C2 [Source:VGNC Symbol;Acc:VGNC:110413]
chr2	142578003	142579756	1754	*	chr2	142578003	142579756	in_vitro	in_vivo	11	13	76.2009091	45.2223077	30.9786014	c(t = 1.94236	0.0652	0.83731181	Distal Intergenic	ENSSSCG000	CD14	CD14 molecule [Source:NCBI gene (formerly Entrezgene);Acc:100037938]
chr7	113599654	113600565	912	*	chr7	113599654	113600565	in_vitro	in_vivo	13	13	2.43153846	0.62615385	1.80538462	c(t = 1.99070	0.0652	0.83731181	Distal Intergenic	ENSSSCG00000002446		ataxin 3 [Source:NCBI gene (formerly Entrezgene);Acc:100144422]
chr1	259543849	259544959	1111	*	chr1	259543849	259544959	in_vitro	in_vivo	10	10	0.47	4.5	-4.03	c(t = -2.0832	0.0653	0.83731181	Distal Intergenic	ENSSSCG000	BRINP1	BMP/retinoic acid inducible neural specific 1 [Source:VGNC Symbol;Acc:VGNC:85881]
chr1	14892590	14893116	527	*	chr1	14892590	14893116	in_vitro	in_vivo	9	9	20.6533333	52.1544444	-31.501111	c(t = -1.9781	0.0654	0.83731181	Distal Intergenic	ENSSSCG000	ARMT1	acidic residue methyltransferase 1 [Source:NCBI gene (formerly Entrezgene);Acc:100157036]
chr13	180570640	180571603	964	*	chr13	180570640	180571603	in_vitro	in_vivo	11	12	2.03181818	0.5225	1.50931818	c(t = 2.01111	0.0654	0.83731181	Distal Intergenic	NA	NA	NA
chr13	137738702	137739377	676	*	chr13	137738702	137739377	in_vitro	in_vivo	12	13	2.1475	0.16538462	1.98211538	c(t = 2.03329	0.0654	0.83731181	Distal Intergenic	ENSSSCG000	KPNA1	karyopherin subunit alpha 1 [Source:NCBI gene (formerly Entrezgene);Acc:397235]
chr11	15408631	15411432	2802	*	chr11	15408631	15411432	in_vitro	in_vivo	16	14	2.309375	0.75785714	1.55151786	c(t = 1.96019	0.0655	0.83731181	Promoter (<=1kb)	ENSSSCG000	FOXO1	forkhead box O1 [Source:NCBI gene (formerly Entrezgene);Acc:397077]
chr7	64969685	64971342	1658	*	chr7	64969685	64971342	in_vitro	in_vivo	13	13	3.62923077	1.00461538	2.62461538	c(t = 1.98399	0.0656	0.83731181	Promoter (<=1kb)	ENSSSCG000	BAZ1A	bromodomain adjacent to zinc finger domain 1A [Source:VGNC Symbol;Acc:VGNC:85761]
chr1	230544669	230546091	1423	*	chr1	230544669	230546091	in_vitro	in_vivo	14	14	2.235	0.74285714	1.49214286	c(t = 1.92439	0.0656	0.83731181	Promoter (<=1kb)	ENSSSCG000	GNA14	G protein subunit alpha 14 [Source:VGNC Symbol;Acc:VGNC:88519]
chr17	30473845	30474530	686	*	chr17	30473845	30474530	in_vitro	in_vivo	11	14	6.24545455	0	6.24545455	c(t = 2.06710	0.0656	0.83731181	Promoter (<=1kb)	ENSSSCG000	CST3	cystatin C [Source:NCBI gene (formerly Entrezgene);Acc:733672]
chr15	88527575	88528771	1197	*	chr15	88527575	88528771	in_vitro	in_vivo	13	15	2.54076923	0.24933333	2.2914359	c(t = 2.01570	0.0657	0.83731181	Distal Intergenic	ENSSSCG000	FRZB	frizzled related protein [Source:VGNC Symbol;Acc:VGNC:96308]
chr12	4729034	4730767	1734	*	chr12	4729034	4730767	in_vitro	in_vivo	9	15	0.46444444	3.00266667	-2.5382222	c(t = -1.9776	0.0658	0.83731181	Distal Intergenic	ENSSSCG00000036592		serine and arginine rich splicing factor 2 [Source:VGNC Symbol;Acc:VGNC:99085]
chr1	189248441	189249849	1409	*	chr1	189248441	189249849	in_vitro	in_vivo	11	12	5.97272727	1.8625	4.11022727	c(t = 2.00888	0.0658	0.83731181	Distal Intergenic	ENSSSCG000	DHRS7	dehydrogenase/reductase 7 [Source:NCBI gene (formerly Entrezgene);Acc:100152785]
chrX	19506726	19509193	2468	*	chrX	19506726	19509193	in_vitro	in_vivo	11	12	1.84545455	8.95333333	-7.1078788	c(t = -2.0220	0.0659	0.83731181	Distal Intergenic	ENSSSCG000	SAT1	spermidine/spermine N1-acetyltransferase 1 [Source:NCBI gene (formerly Entrezgene);Acc:397645]
chr14	22542135	22542788	654	*	chr14	22542135	22542788	in_vitro	in_vivo	11	12	0.66181818	2.97333333	-2.3115152	c(t = -2.0049	0.0659	0.83731181	Distal Intergenic	ENSSSCG000	PIWIL1	piwi like RNA-mediated silencing 1 [Source:VGNC Symbol;Acc:VGNC:98197]
chr9	5257871	5259319	1449	*	chr9	5257871	5259319	in_vitro	in_vivo	11	14	6.06454545	25.1107143	-19.046169	c(t = -1.9961	0.0661	0.83731181	Distal Intergenic	ENSSSCG000	HBE1	hemoglobin subunit epsilon 1 [Source:NCBI gene (formerly Entrezgene);Acc:407067]
chr12	52104602	52105217	616	*	chr12	52104602	52105217	in_vitro	in_vivo	10	10	16.931	5.096	11.835	c(t = 2.00507	0.0661	0.83731181	Distal Intergenic	ENSSSCG000	VMO1	vitelline membrane outer layer 1 homolog [Source:NCBI gene (formerly Entrezgene);Acc:100512921]
chr10	57829168	57830499	1332	*	chr10	57829168	57830499	in_vitro	in_vivo	12	10	2.90833333	0.878	2.03033333	c(t = 1.98979	0.0661	0.83731181	Distal Intergenic	ENSSSCG000	CCNY	cyclin Y [Source:VGNC Symbol;Acc:VGNC:95998]
chr7	64808007	64808662	656	*	chr7	64808007	64808662	in_vitro	in_vivo	9	14	0.27777778	2.46428571	-2.1865079	c(t = -1.9845	0.0662	0.83731181	Promoter (<=1kb)	ENSSSCG000	FAM177A1	family with sequence similarity 177 member A1 [Source:VGNC Symbol;Acc:VGNC:87933]
chr18	50294414	50295043	630	*	chr18	50294414	50295043	in_vitro	in_vivo	10	10	4.275	1.696	2.579	c(t = 1.95641	0.0662	0.83731181	Distal Intergenic	ENSSSCG000	RAMP3	receptor activity modifying protein 3 [Source:VGNC Symbol;Acc:VGNC:92075]
chr3	34083531	34084719	1189	*	chr3	34083531	34084719	in_vitro	in_vivo	9	14	0.86666667	3.65571429	-2.7890476	c(t = -1.9734	0.0663	0.83731181	Distal Intergenic	ENSSSCG000	ABAT	4-aminobutyrate aminotransferase [Source:NCBI gene (formerly Entrezgene);Acc:397500]
chr13	48026295	48027741	1447	*	chr13	48026295	48027741	in_vitro	in_vivo	13	10	1.88230769	0.595	1.28730769	c(t = 1.94794	0.0664	0.83731181	Distal Intergenic	ENSSSCG000	EOGT	EGF domain specific O-linked N-acetylglucosamine transferase [Source:VGNC Symbol;Acc:VGNC:87718]
chr15	33478739	33479825	1087	*	chr15	33478739	33479825	in_vitro	in_vivo	9	11	4.95888889	2.08363636	2.87525253	c(t = 1.97885	0.0665	0.83731181	Distal Intergenic	ENSSSCG000	CLN8	CLN8 transmembrane ER and ERGIC protein [Source:VGNC Symbol;Acc:VGNC:103067]
chr17	29199471	29201230	1760	*	chr17	29199471	29201230	in_vitro	in_vivo	15	17	2.48066667	0.90235294	1.57831373	c(t = 1.95441	0.0666	0.83731181	Distal Intergenic	ENSSSCG000	THBD	thrombomodulin [Source:NCBI gene (formerly Entrezgene);Acc:100157642]
chr8	27960315	27960942	628	*	chr8	27960315	27960942	in_vitro	in_vivo	11	15	1.56272727	0.26666667	1.29606061	c(t = 1.98866	0.0666	0.83731181	Distal Intergenic	ENSSSCG000	ssc-mir-9840	ssc-mir-9840 [Source:miRBase;Acc:MI0031609]
chr7	94789576	94791047	1472	*	chr7	94789576	94791047	in_vitro	in_vivo	9	15	0	1.84	-1.84	c(t = -1.9874	0.0668	0.83731181	Distal Intergenic	ENSSSCG000	SYNJ2BP	synaptojanin 2 binding protein [Source:NCBI gene (formerly Entrezgene);Acc:100514718]
chr10	68475939	68478569	2631	*	chr10	68475939	68478569	in_vitro	in_vivo	10	11	93.958	98.1409091	-4.1829091	c(t = -1.9818	0.0669	0.83731181	Distal Intergenic	ENSSSCG000	WDR37	WD repeat domain 37 [Source:VGNC Symbol;Acc:VGNC:96258]
chr13	32329740	32330722	983	*	chr13	32329740	32330722	in_vitro	in_vivo	9	9	7.51777778	1.71111111	5.80666667	c(t = 2.04470	0.0671	0.83731181	Distal Intergenic	ENSSSCG000	GMPPB	GDP-mannose pyrophosphorylase B [Source:NCBI gene (formerly Entrezgene);Acc:100513376]
chr12	1196816	1214341	17526	*	chr12	1196816	1214341	in_vitro	in_vivo	12	14	44.6216667	25.2857143	19.3359524	c(t = 1.99139	0.0672	0.83731181	Distal Intergenic	ENSSSCG000	ARHGDIA	Rho GDP dissociation inhibitor alpha [Source:VGNC Symbol;Acc:VGNC:85483]
chr13	129502323	129504542	2220	*	chr13	129502323	129504542	in_vitro	in_vivo	12	12	31.0258333	12.1058333	18.92	c(t = 1.97518	0.0672	0.83731181	Distal Intergenic	ENSSSCG000	OSTN	osteocrin [Source:NCBI gene (formerly Entrezgene);Acc:100049691]

chr2	65062232	65063433	1202	*	chr2	65062232	65063433	in_vitro	in_vivo	9	12	0	1.7825	-1.7825	c(t = -2.0304	0.0672	0.83731181	Promoter (<=1kb)	ENSSSCG000	ASF1B	anti-silencing function 1B histone chaperone [Source:NCBI gene (formerly Entrezgene);Acc:100192434]
chr16	68709316	68710732	1417	*	chr16	68709316	68710732	in_vitro	in_vivo	9	12	2.77111111	0.55916667	2.21194444	c(t = 2.07110	0.0672	0.83731181	Distal Intergenic	ENSSSCG000	HAND1	heart and neural crest derivatives expressed 1 [Source:VGNC Symbol;Acc:VGNC:88777]
chr8	38696266	38697244	979	*	chr8	38696266	38697244	in_vitro	in_vivo	10	13	2.883	0.89230769	1.99069231	c(t = 2.00417	0.0673	0.83731181	Distal Intergenic	ENSSSCG000	ZAR1	zygote arrest 1 [Source:VGNC Symbol;Acc:VGNC:95051]
chr3	26382742	26383314	573	*	chr3	26382742	26383314	in_vitro	in_vivo	9	10	0.24666667	2.287	-2.0403333	c(t = -2.0454	0.0676	0.83731181	Distal Intergenic	ENSSSCG000	SMG1	SMG1 nonsense mediated mRNA decay associated PI3K related kinase [Source:VGNC Symbol;Acc:VGNC:93248]
chr1	270179344	270180174	831	*	chr1	270179344	270180174	in_vitro	in_vivo	11	9	2.31909091	0.42777778	1.89131313	c(t = 1.97533	0.0676	0.83731181	Distal Intergenic	ENSSSCG000	TOR1A	torsin family 1 member A [Source:NCBI gene (formerly Entrezgene);Acc:100125953]
chr11	79118460	79119509	1050	*	chr11	79118460	79119509	in_vitro	in_vivo	9	10	3.31555556	0.238	3.07755556	c(t = 2.09565	0.0676	0.83731181	Distal Intergenic	ENSSSCG000	CDC16	cell division cycle 16 [Source:NCBI gene (formerly Entrezgene);Acc:100621739]
chr17	30855515	30856217	703	*	chr17	30855515	30856217	in_vitro	in_vivo	12	9	2.93416667	0.54111111	2.39305556	c(t = 1.99171	0.0677	0.83731181	Distal Intergenic	ENSSSCG000	CST7	cystatin F [Source:NCBI gene (formerly Entrezgene);Acc:100152012]
chr6	161614842	161617460	2619	*	chr6	161614842	161617460	in_vitro	in_vivo	11	11	2.84272727	0.94363636	1.89909091	c(t = 1.93160	0.0678	0.83731181	Distal Intergenic	ENSSSCG000	SPATA6	spermatosis associated 6 [Source:VGNC Symbol;Acc:VGNC:93387]
chr6	118817339	118818238	900	*	chr6	118817339	118818238	in_vitro	in_vivo	11	9	3.31363636	0.32666667	2.9869697	c(t = 2.03621	0.0679	0.83731181	Distal Intergenic	ENSSSCG000	ZNF24	zinc finger protein 24 [Source:VGNC Symbol;Acc:VGNC:98422]
chr11	21928915	21930059	1145	*	chr11	21928915	21930059	in_vitro	in_vivo	9	11	4.92	1.43909091	3.48090909	c(t = 2.03093	0.068	0.83731181	Promoter (<=1kb)	ENSSSCG000	TPT1	tumor protein, translationally-controlled 1 [Source:NCBI gene (formerly Entrezgene);Acc:397664]
chr15	139865812	139867602	1791	*	chr15	139865812	139867602	in_vitro	in_vivo	9	13	16.0422222	44.2038462	-28.161624	c(t = -1.9288	0.0683	0.83731181	Distal Intergenic	ENSSSCG000	PPP1R7	protein phosphatase 1 regulatory subunit 7 [Source:NCBI gene (formerly Entrezgene);Acc:100511842]
chr10	68409572	68410930	1359	*	chr10	68409572	68410930	in_vitro	in_vivo	9	9	70.9477778	91.8544444	-20.906667	c(t = -2.0299	0.0687	0.83731181	Distal Intergenic	ENSSSCG000	WDR37	WD repeat domain 37 [Source:VGNC Symbol;Acc:VGNC:96258]
chr12	58964200	58965105	906	*	chr12	58964200	58965105	in_vitro	in_vivo	9	9	2.69111111	0.69222222	1.99888889	c(t = 1.96255	0.0687	0.83731181	Distal Intergenic	ENSSSCG00000018027		trans-golgi network vesicle B [Source:NCBI gene (formerly Entrezgene);Acc:100522572]
chr4	70928130	70929245	1116	*	chr4	70928130	70929245	in_vitro	in_vivo	11	13	3.95090909	0.58846154	3.36244755	c(t = 2.01821	0.0687	0.83731181	Distal Intergenic	ENSSSCG000	ssc-mir-124a	ssc-mir-124a-1 [Source:miRBase;Acc:MI0010680]
chr9	1290206	1295177	4972	*	chr9	1290206	1295177	in_vitro	in_vivo	12	15	93.5316667	88.1006667	5.431	c(t = 1.90441	0.0692	0.83731181	Distal Intergenic	NA	NA	NA
chr1	73548019	73549424	1406	*	chr1	73548019	73549424	in_vitro	in_vivo	9	10	3.11666667	0.301	2.81566667	c(t = 2.08147	0.0694	0.83731181	Distal Intergenic	ENSSSCG000	PDSS2	decaprenyl diphosphate synthase subunit 2 [Source:VGNC Symbol;Acc:VGNC:91291]
chr3	58436237	58437288	1052	*	chr3	58436237	58437288	in_vitro	in_vivo	12	11	2.37	0.97	1.4	c(t = 1.91858	0.0695	0.83731181	Distal Intergenic	ENSSSCG000	PTCD3	pentatricopeptide repeat domain 3 [Source:VGNC Symbol;Acc:VGNC:91940]
chr12	4446902	4451651	4750	*	chr12	4446902	4451651	in_vitro	in_vivo	9	11	97.6211111	89.7127273	7.90838384	c(t = 2.00107	0.0697	0.83731181	Distal Intergenic	ENSSSCG000	ssc-mir-6516	ssc-mir-6516 [Source:miRBase;Acc:MI0033411]
chr8	108314575	108315347	773	*	chr8	108314575	108315347	in_vitro	in_vivo	10	12	2.986	0.97583333	2.01016667	c(t = 1.94242	0.0698	0.83731181	Distal Intergenic	ENSSSCG000	UGT8	UDP glycosyltransferase 8 [Source:VGNC Symbol;Acc:VGNC:98401]
chr3	59806553	59807393	841	*	chr3	59806553	59807393	in_vitro	in_vivo	9	10	1.98444444	0.376	1.60844444	c(t = 2.01210	0.0698	0.83731181	Distal Intergenic	ENSSSCG000	TMSB10	thymosin beta 10 [Source:NCBI gene (formerly Entrezgene);Acc:100037998]
chrX	88049579	88050930	1352	*	chrX	88049579	88050930	in_vitro	in_vivo	12	13	12.7733333	34.9692308	-22.195897	c(t = -1.9399	0.0699	0.83731181	Distal Intergenic	ENSSSCG000	CLDN2	claudin 2 [Source:VGNC Symbol;Acc:VGNC:86737]
chr13	207225755	207228460	2706	*	chr13	207225755	207228460	in_vitro	in_vivo	11	9	11.7872727	37.4922222	-25.704949	c(t = -2.0167	0.07	0.83731181	Distal Intergenic	ENSSSCG000	ITGB2	integrin subunit beta 2 [Source:NCBI gene (formerly Entrezgene);Acc:396943]
chr12	26157977	26159662	1686	*	chr12	26157977	26159662	in_vitro	in_vivo	9	12	1.17333333	4.68083333	-3.5075	c(t = -1.9481	0.0702	0.83731181	Distal Intergenic	ENSSSCG000	TAC4	tachykinin 4 [Source:VGNC Symbol;Acc:VGNC:96765]
chr6	158905407	158907023	1617	*	chr6	158905407	158907023	in_vitro	in_vivo	11	10	7.54	3.018	4.522	c(t = 1.96135	0.0702	0.83731181	Promoter (<=1kb)	ENSSSCG000	LRP8	LDL receptor related protein 8 [Source:VGNC Symbol;Acc:VGNC:89822]
chr8	55763269	55764103	835	*	chr8	55763269	55764103	in_vitro	in_vivo	9	11	2.30333333	0.71727273	1.58606061	c(t = 1.97240	0.0703	0.83731181	Distal Intergenic	ENSSSCG00000008898		HOP homeobox [Source:VGNC Symbol;Acc:VGNC:88932]
chr12	6493324	6494050	727	*	chr12	6493324	6494050	in_vitro	in_vivo	9	9	2.08111111	0.19111111	1.89	c(t = 2.05945	0.0705	0.83731181	Distal Intergenic	ENSSSCG000	NHERF1	NHERF family PDZ scaffold protein 1 [Source:NCBI gene (formerly Entrezgene);Acc:100233201]
chr6	44038140	44038870	731	*	chr6	44038140	44038870	in_vitro	in_vivo	10	9	10.416	0.94888889	9.46711111	c(t = 2.04374	0.0706	0.83731181	Promoter (<=1kb)	ENSSSCG000	GPI	glucose-6-phosphate isomerase [Source:NCBI gene (formerly Entrezgene);Acc:397602]
chr1	127317598	127318399	802	*	chr1	127317598	127318399	in_vitro	in_vivo	11	10	2.36727273	0.705	1.66227273	c(t = 1.92264	0.0706	0.83731181	Distal Intergenic	ENSSSCG000	B2M	beta-2-microglobulin [Source:NCBI gene (formerly Entrezgene);Acc:397033]
chr1	272453457	272455247	1791	*	chr1	272453457	272455247	in_vitro	in_vivo	11	14	2.11818182	0.41428571	1.7038961	c(t = 1.98956	0.0707	0.83731181	Distal Intergenic	ENSSSCG00000035964		odorant binding protein 2B [Source:NCBI gene (formerly Entrezgene);Acc:396861]
chr1	51040040	51041845	1806	*	chr1	51040040	51041845	in_vitro	in_vivo	15	13	2.354	0.49307692	1.86092308	c(t = 1.93020	0.0708	0.83731181	Distal Intergenic	ENSSSCG000	ssc-mir-30c-2	ssc-mir-30c-2 [Source:miRBase;Acc:MI0002461]
chr8	1090785	1092173	1389	*	chr8	1090785	1092173	in_vitro	in_vivo	11	9	30.7490909	62.9144444	-32.165354	c(t = -1.9400	0.0709	0.83731181	Distal Intergenic	ENSSSCG000	LETM1	leucine zipper and EF-hand containing transmembrane protein 1 [Source:VGNC Symbol;Acc:VGNC:89687]
chr6	105505463	105506783	1321	*	chr6	105505463	105506783	in_vitro	in_vivo	11	10	2.95454545	0.951	2.00354545	c(t = 1.96107	0.0709	0.83731181	Distal Intergenic	ENSSSCG000	ADCYAP1	adenylate cyclase activating polypeptide 1 [Source:VGNC Symbol;Acc:VGNC:85114]
chr2	77478916	77479754	839	*	chr2	77478916	77479754	in_vitro	in_vivo	11	10	4.05181818	0.364	3.68781818	c(t = 2.01269	0.0709	0.83731181	Promoter (<=1kb)	ENSSSCG000	KISS1R	KISS1 receptor [Source:NCBI gene (formerly Entrezgene);Acc:733704]
chr12	1819704	1821601	1898	*	chr12	1819704	1821601	in_vitro	in_vivo	9	11	74.8033333	95.9727273	-21.169394	c(t = -2.0719	0.071	0.83731181	Distal Intergenic	NA	NA	NA
chr14	29399797	29400582	786	*	chr14	29399797	29400582	in_vitro	in_vivo	12	10	1.99666667	0.516	1.48066667	c(t = 1.93300	0.0711	0.83731181	Distal Intergenic	ENSSSCG000	SUMO4	SMT3 suppressor of mif two 3 homolog 4 (S. cerevisiae) [Source:NCBI gene (formerly Entrezgene);Acc:100127140]
chr13	50435577	50436446	870	*	chr13	50435577	50436446	in_vitro	in_vivo	12	13	1.78	0.10846154	1.67153846	c(t = 1.98913	0.0713	0.83731181	Distal Intergenic	ENSSSCG000	EOGT	EGF domain specific O-linked N-acetylglucosamine transferase [Source:VGNC Symbol;Acc:VGNC:87718]
chr7	1970754	1971917	1164	*	chr7	1970754	1971917	in_vitro	in_vivo	10	9	0.833	5.15222222	-4.3192222	c(t = -2.0170	0.0714	0.83731181	Distal Intergenic	ENSSSCG000	TUBB2B	tubulin, beta 2B class IIb [Source:NCBI gene (formerly Entrezgene);Acc:100153507]
chr8	43870528	43871480	953	*	chr8	43870528	43871480	in_vitro	in_vivo	9	16	0.39444444	2.953125	-2.5586806	c(t = -1.9294	0.0714	0.83731181	Distal Intergenic	ENSSSCG000	MSMO1	methylsterol monooxygenase 1 [Source:VGNC Symbol;Acc:VGNC:98941]
chr1	180635934	180636147	214	*	chr1	180635934	180636147	in_vitro	in_vivo	9	9	10.2911111	1.11666667	9.17444444	c(t = 2.06030	0.0714	0.83731181	Distal Intergenic	ENSSSCG000	PYGL	glycogen phosphorylase L [Source:NCBI gene (formerly Entrezgene);Acc:100141306]
chr12	53365897	53366923	1027	*	chr12	53365897	53366923	in_vitro	in_vivo	9	9	94.6855556	78.2211111	16.4644444	c(t = 2.01707	0.0715	0.83731181	Distal Intergenic	ENSSSCG000	AURKB	aurora kinase B [Source:VGNC Symbol;Acc:VGNC:85698]
chr14	139835458	139836943	1486	*	chr14	139835458	139836943	in_vitro	in_vivo	11	10	9.36909091	40.719	-31.349909	c(t = -1.9950	0.0715	0.83731181	Distal Intergenic	ENSSSCG000	GLRX3	glutaredoxin 3 [Source:VGNC Symbol;Acc:VGNC:88495]
chr9	494702	496526	1825	*	chr9	494702	496526	in_vitro	in_vivo	9	10	2.30444444	0.719	1.58544444	c(t = 2.01548	0.0715	0.83731181	Distal Intergenic	NA	NA	NA
chr7	31360867	31362660	1794	*	chr7	31360867	31362660	in_vitro	in_vivo	9	11	3.68555556	0.33545455	3.35010101	c(t = 2.06847	0.0715	0.83731181	Distal Intergenic	ENSSSCG000	TEAD3	TEA domain transcription factor 3 [Source:NCBI gene (formerly Entrezgene);Acc:654299]
chr1	200773084	200774782	1699	*	chr1	200773084	200774782	in_vitro	in_vivo	13	10	2.79384615	0.41	2.38384615	c(t = 1.95604	0.0717	0.83731181	Promoter (<=1kb)	NA	NA	NA
chr4	35767515	35768745	1231	*	chr4	35767515	35768745	in_vitro	in_vivo	11	12	0.48272727	2.105	-1.6222727	c(t = -1.9384	0.072	0.83731181	Distal Intergenic	ENSSSCG000	YWHAZ	tyrosine 3-monooxygenase/tryptophan 5-monooxygenase activation protein zeta [Source:VGNC Symbol;Acc:VGNC:95047]
chr1	272961037	272962500	1464	*	chr1	272961037	272962500	in_vitro	in_vivo	9	9	6.98111111	0.72777778	6.25333333	c(t = 2.05455	0.072	0.83731181	Distal Intergenic	NA	NA	NA
chr2	123313408	123313825	418	*	chr2	123313408	123313825	in_vitro	in_vivo	9	15	0	1.55	-1.55	c(t = -1.9453	0.0721	0.83731181	Promoter (<=1kb)	ENSSSCG000	HSD17B4	hydroxysteroid 17-beta dehydrogenase 4 [Source:VGNC Symbol;Acc:VGNC:99760]
chr10	10870521	10871478	958	*	chr10	10870521	10871478	in_vitro	in_vivo	11	9	13.2145455	41.64	-28.425455	c(t = -1.9399	0.0722	0.83731181	Distal Intergenic	ENSSSCG000	ssc-mir-664	ssc-mir-664 [Source:miRBase;Acc:MI0013123]
chr13	22706057	22708111	2055	*	chr13	22706057	22708111	in_vitro	in_vivo	11	13	3.21545455	1.28307692	1.93237762	c(t = 1.93771	0.0723	0.83731181	Distal Intergenic	ENSSSCG000	PLCD1	phospholipase C delta 1 [Source:NCBI gene (formerly Entrezgene);Acc:100627099]
chr13	63318004	63318792	789	*	chr13	63318004	63318792	in_vitro	in_vivo	9	16	2.36888889	0.6325	1.73638889	c(t = 1.97649	0.0723	0.83731181	Distal Intergenic	ENSSSCG000	LMCD1	LIM and cysteine rich domains 1 [Source:VGNC Symbol;Acc:VGNC:89756]
chr5	735223	741079	5857	*	chr5	735223	741079	in_vitro	in_vivo	11	15	87.8390909	93.8666667	-6.0275758	c(t = -1.9325	0.0724	0.83731181	Distal Intergenic	ENSSSCG000	SELENOO	selenoprotein O [Source:NCBI gene (formerly Entrezgene);Acc:100518971]
chr11	49807826	49808981	1156	*	chr11	49807826	49808981	in_vitro	in_vivo	10	14	3.677	0.84	2.837	c(t = 1.95034	0.0724	0.83731181	Distal Intergenic	ENSSSCG000	EDNRB	endothelin receptor type B [Source:VGNC Symbol;Acc:VGNC:87550]
chr2	150295741	150296850	1110	*	chr2	150295741	150296850	in_vitro	in_vivo	9	10	3.07777778	0.341	2.73677778	c(t = 2.03106	0.0725	0.83731181	Distal Intergenic	ENSSSCG000	AFAP1L1	actin filament associated protein 1 like 1 [Source:NCBI gene (formerly Entrezgene);Acc:100513463]
chrX	6814765	6816269	1505	*	chrX	6814765	6816269	in_vitro	in_vivo	13	12	2.80846154	12.4558333	-9.6473718	c(t = -1.9643	0.0726	0.83731181	Distal Intergenic	ENSSSCG000	AMELY	amelogenin X-linked [Source:NCBI gene (formerly Entrezgene);Acc:396940]
chr15	121025670	121027619	1950	*	chr15	121025670	121027619	in_vitro	in_vivo	12	9	5.985	2.27333333	3.71166667	c(t = 1.94598	0.0728	0.83731181	Promoter (<=1kb)	ENSSSCG000	ssc-mir-375	ssc-mir-375 [Source:miRBase;Acc:MI0033409]
chr1	215608623	215609505	883	*	chr1	215608623	215609505	in_vitro	in_vivo	9	14	0.73111111	3.33285714	-2.601746	c(t = -1.9092	0.073	0.83731181	Distal Intergenic	ENSSSCG000	IL33	interleukin 33 [Source:VGNC Symbol;Acc:VGNC:89106]
chr1	273559701	273560994	1294	*	chr1	273559701	273560994	in_vitro	in_vivo	10	15	6.718	0.434	6.284	c(t = 2.02734	0.073	0.83731181	Distal Intergenic	ENSSSCG000	ssc-mir-9812	ssc-mir-9812 [Source:miRBase;Acc:MI0031579]
chr2	150386554	150387326	773	*	chr2	150386554	150387326	in_vitro	in_vivo	10	9	97.075	87.5044444	9.57055556	c(t = 2.03386	0.0732	0.83731181	Distal Intergenic	ENSSSCG000	AFAP1L1	actin filament associated protein 1 like 1 [Source:NCBI gene (formerly Entrezgene);Acc:100513463]
chr12	13654362	13656200	1839	*	chr12	13654362	13656200	in_vitro	in_vivo	10	14	1.078	4.77571429	-3.6977143	c(t = -1.8968	0.0733	0.83731181	Distal Intergenic	ENSSSCG000	PITPNC1	phosphatidylinositol transfer protein cytoplasmic 1 [Source:NCBI gene (formerly Entrezgene);Acc:100233199]
chr2	7938601	7939439	839	*	chr2	7938601	7939439	in_vitro	in_vivo	10	12	0.255	1.71666667	-1.4616667	c(t = -1.9593	0.0733	0.83731181	Promoter (<=1kb)	ENSSSCG000	MACROD1	mono-ADP ribosylhydrolase 1 [Source:NCBI gene (formerly Entrezgene);Acc:100522538]
chr14	40217722	40218386	665	*	chr14	40217722	40218386	in_vitro	in_vivo	9	12	3.03555556	0.2875	2.74805556	c(t = 2.03439	0.0733	0.83731181	Distal Intergenic	ENSSSCG000	RPLP0	ribosomal protein lateral stalk subunit P0 [Source:NCBI gene (formerly Entrezgene);Acc:100049695]
chr17	25998123	25999465	1343	*	chr17	25998123	25999465	in_vitro	in_vivo	11	13	0.22909091	1.25692308	-1.0278322	c(t = -1.9259	0.0735	0.83731181	Promoter (<=1kb)	ENSSSCG000	DSTN	destrin, actin depolymerizing factor [Source:VGNC Symbol;Acc:VGNC:108715]
chr1	272006808	272009294	2487	*	chr1	272006808	272009294	in_vitro	in_vivo	9	11	5.52666667	0.54636364	4.98030303	c(t = 2.05239	0.0735	0.83731181	Distal Intergenic	ENSSSCG000	MED27	mediator complex subunit 27 [Source:VGNC Symbol;Acc:VGNC:90117]
chr1	1030583	1032651	2069	*	chr1	1030583	1032651	in_vitro	in_vivo	10	11	12.036	36.2963636	-24.260364	c(t = -1.9228	0.0736	0.83731181	Distal Intergenic	ENSSSCG000	ssc-mir-9815	ssc-mir-9815 [Source:miRBase;Acc:MI0031582]
chr1	163369769	163370776	1008	*	chr1	163369769	163370776	in_vitro	in_vivo	13	9	3.11230769	0.95777778	2.15452991	c(t = 1.89140	0.0736	0.83731181	Distal Intergenic	ENSSSCG000	CILP	cartilage intermediate layer protein [Source:VGNC Symbol;Acc:VGNC:86703]
chr13	207124039	207124752	714	*	chr13	207124039	207124752	in_vitro	in_vivo	9	9	9.68555556	34.88	-25.194444	c(t = -2.0107	0.0737	0.83731181	Distal Intergenic	ENSSSCG000	AGPAT3	1-acylglycerol-3-phosphate O-acyltransferase 3 [Source:VGNC Symbol;Acc:VGNC:85185]
chr10	68769934	68770936	1003	*	chr10	68769934	68770936	in_vitro	in_vivo	10	11	2.987	1.02818182	1.95881818	c(t = 1.93745	0.074	0.83731181	Distal Intergenic	ENSSSCG000	WDR37	WD repeat domain 37 [Source:VGNC Symbol;Acc:VGNC:96258]
chr4	124949857	124950545	689	*	chr4	124949857	124950545	in_vitro	in_vivo	9	13	3.13	0.50538462	2.62461538	c(t = 2.02514	0.074	0.83731181	Distal Intergenic	ENSSSCG000	TGFBR3	transforming growth factor beta receptor 3 [Source:VGNC Symbol;Acc:VGNC:93932]
chr6	159396297	159397375	1079	*	chr6	159396297	159397375	in_vitro	in_vivo	11	14	1.26090909	6.575	-5.3140909	c(t = -1.9172	0.0742	0.83731181	Distal Intergenic	NA	NA	NA
chr8	100860864	100862970	2107	*	chr8	100860864	100862970	in_vitro	in_vivo	11	13	2.15272727	0.47692308	1.6758042	c(t = 1.96581	0.075	0.83731181	Promoter (<=1kb)	ENSSSCG000	SPRY1	sprouty RTK signaling antagonist 1 [Source:VGNC Symbol;Acc:VGNC:93424]
chr10	43754214	43755645	1432	*	chr10	43754214	43755645	in_vitro	in_vivo	16	15	2.160625	0.80266667	1.35795833	c(t = 1.87304	0.0752	0.83731181	Distal Intergenic	ENSSSCG000	TRDMT1	tRNA aspartic acid methyltransferase 1 [Source:VGNC Symbol;Acc:VGNC:96227]
chr3	343167	350359	7193	*	chr3	343167	350359	in_vitro	in_vivo	10	14	13.833	35.6342857	-21.801286	c(t = -1.8687	0.0753	0.83731181	Distal Intergenic	ENSSSCG000	ADAP1	ArfGAP with dual PH domains 1 [Source:NCBI gene (formerly Entrezgene);Acc:397453]
chr14	40355984	40357105	1122	*	chr14	40355984	40357105	in_vitro	in_vivo	10	13	2.921	0.93692308	1.98407692	c(t = 1.92931	0.0753	0.83731181	Promoter (<=1kb)	ENSSSCG000	MSI1	musashi RNA binding protein 1 [Source:VGNC Symbol;Acc:VGNC:90421]
chr13	176082750	176083616	867	*	chr13	176082750	176083616	in_vitro	in_vivo	13	10	2.34615385	0.357	1.98915385	c(t = 1.91057	0.0753	0.83731181	Distal Intergenic	NA	NA	NA
chr13	207269952	207273071	3120	*	chr13	207269952	207273071	in_vitro	in_vivo	11	11	7.79545455	28.0390909	-20.243636	c(t = -1.9422	0.0754	0.83731181	Distal Intergenic	ENSSSCG000	ITGB2	integrin subunit beta 2 [Source:NCBI gene (formerly Entrezgene);Acc:396943]
chr14	111996448	111997329	882	*	chr14	111996448	111997329	in_vitro	in_vivo	9	11	2.15888889	0.21636364	1.94252525	c(t = 2.01365	0.0754	0.83731181	Distal Intergenic	ENSSSCG000	ssc-mir-1038	ssc-mir-10382 [Source:miRBase;Acc:MI0033391]
chr3	17742406	17744406	2001	*	chr3	17742406	17744406	in_vitro	in_vivo	10	10	3.451	0.747	2.704	c(t = 1.94989	0.0755	0.83731181	Distal Intergenic	ENSSSCG000	TMEM265	transmembrane protein 265 [Source:NCBI gene (formerly Entrezgene);Acc:100513980]
chr9	6064374	6065627	1254	*	chr9	6064374	6065627	in_vitro	in_vivo	10	13	3.124	1.09230769	2.03169231	c(t = 1.95331	0.0756	0.83731181	Distal Intergenic	ENSSSCG000	STIM1	stromal interaction molecule 1 [Source:VGNC Symbol;Acc:VGNC:93546]
chr1	190170203	190171392	1190	*	chr1	190170203	190171392	in_vitro	in_vivo	11	15	1.16727273	2.82466667	-1.6573939	c(t = -1.8809	0.076	0.83731181	Distal Intergenic	ENSSSCG000	SLC38A6	solute carrier family 38 member 6 [Source:VGNC Symbol;Acc:VGNC:93098]
chr2	51670936	51671748	813	*	chr2	51670936	51671748	in_vitro	in_vivo	13	14	5.46538462	1.18857143	4.27681319	c(t = 1.92067	0.0762	0.83731181	Distal Intergenic	NA	NA	NA
chr18	45372787	45374238	1452	*	chr18	45372787	45374238	in_vitro	in_vivo	11	11	0.68727273	2.36909091	-1.6818182	c(t = -1.8698	0.0764	0.83731181	Promoter (<=1kb)	ENSSSCG000	HOXA13	homeobox A13 [Source:NCBI gene (formerly Entrezgene);Acc:100359352]
chr12	7637681	7639269	1589	*	chr12	7637681	7639269	in_vitro	in_vivo	10	11	3.734	0.84454545	2.88945455	c(t = 1.95414	0.0765	0.83731181	Distal Intergenic	ENSSSCG000	SSTR2	somatostatin receptor 2 [Source:VGNC Symbol;Acc:VGNC:99086]
chr13	196329651	196330677	1027	*	chr13	196329651	196330677	in_vitro	in_vivo	14	12	1.79571429	0.43333333	1.36238095	c(t = 1.87151	0.0767	0.83731181	Distal Intergenic	ENSSSCG000	C21orf62	chromosome 13 C21orf62 homolog [Source:VGNC Symbol;Acc:VGNC:85929]
chr14	34558557	34559520	964	*	chr14	34558557	34559520	in_vitro	in_vivo	9	13	0.33888889	3.02923077	-2.6903419	c(t = -1.9263	0.0768	0.83731181	Distal Intergenic	ENSSSCG000	SUDS3	SDS3 homolog, SIN3A corepressor complex component [Source:VGNC Symbol;Acc:VGNC:93607]
chr8	29189941	29190831	891	*	chr8	29189941	29190831	in_vitro	in_vivo	10	13	3.682	1.16769231	2.51430769	c(t = 1.95380	0.0768	0.83731181	Distal Intergenic	ENSSSCG000	ssc-mir-9840	ssc-mir-9840 [Source:miRBase;Acc:MI0031609]
chr7	29638251	29639301	1051	*	chr7	29638251	29639301	in_vitro	in_vivo	10	11	0.278	3.00272727	-2.7247273	c(t = -1.9549	0.0769	0.83731181	Promoter (<=1kb)	ENSSSCG000	RPS18	ribosomal protein S18 [Source:NCBI gene (formerly Entrezgene);Acc:396980]
chr17	38939146	38939986	841	*	chr17	38939146	38939986	in_vitro	in_vivo	9	10	13.1044444	2.725	10.3794444	c(t = 2.00582	0.077	0.83731181	Distal Intergenic	ENSSSCG000	CPNE1	copine 1 [Source:NCBI gene (formerly Entrezgene);Acc:100233174]
chr16	79011667	79012408	742	*	chr16	79011667	79012408	in_vitro	in_vivo	9	11	11.9033333	33.4636364	-21.560303	c(t = -1.9370	0.0771	0.83731181	Distal Intergenic	ENSSSCG000	TERT	telomerase reverse transcriptase [Source:NCBI gene (formerly Entrezgene);Acc:492280]
chr7	15643353	15644140	788	*	chr7	15643353	15644140	in_vitro	in_vivo	11	13	4.36818182	1.15538462	3.2127972	c(t = 1.89866	0.0774	0.83731181	Distal Intergenic	ENSSSCG000	ID4	inhibitor of DNA binding 4 [Source:VGNC Symbol;Acc:VGNC:89024]
chr6	40292760	40296390	3631	*	chr6	40292760	40296390	in_vitro	in_vivo	10	13	2.842	1.18076923	1.66123077	c(t = 1.93327	0.0775	0.83731181	Distal Intergenic	ENSSSCG000	SLC7A9	solute carrier family 7 member 9 [Source:VGNC Symbol;Acc:VGNC:93177]
chr4	16551603	16554570	2968	*	chr4	16551603	16554570	in_vitro	in_vivo	10	11	3.518	1.35545455	2.16254545	c(t = 1.90671	0.0778	0.83731181	Distal Intergenic	ENSSSCG000	DERL1	derlin 1 [Source:NCBI gene (formerly Entrezgene);Acc:100626802]
chr8	33176147	33178159	2013	*	chr8	33176147	33178159	in_vitro	in_vivo	14	13	2.82142857	1.14230769	1.67912088	c(t = 1.86895	0.0778	0.83731181	Distal Intergenic	ENSSSCG000	SLC30A9	solute carrier family 30 member 9 [Source:NCBI gene (formerly Entrezgene);Acc:100192440]
chr9	3177660	3178660	1001	*	chr9	3177660	3178660	in_vitro	in_vivo	10	12	84.654	54.5791667	30.0748333	c(t = 1.86733	0.0779	0.83731181	Distal Intergenic	ENSSSCG000	MRPL17	mitochondrial ribosomal protein L17 [Source:NCBI gene (formerly Entrezgene);Acc:100512482]
chr13	39429201	39430494	1294	*	chr13	39429201	39430494	in_vitro	in_vivo	11	10	0.18909091	1.463	-1.2739091	c(t = -1.9505	0.0779	0.83731181	Promoter (<=1kb)	ENSSSCG000	ARF4	ADP ribosylation factor 4 [Source:VGNC Symbol;Acc:VGNC:85446]
chr9	56748541	56750127	1587	*	chr9	56748541	56750127	in_vitro	in_vivo	14	15	2.845	1.018	1.827	c(t = 1.86765	0.0782	0.83731181	Distal Intergenic	ENSSSCG000	KCNJ5	potassium inwardly rectifying channel subfamily J member 5 [Source:NCBI gene (formerly Entrezgene);Acc:397448]
chr11	4260856	4262050	1195	*	chr11	4260856	4262050	in_vitro	in_vivo	14	13	1.88285714	0.64692308	1.23593407	c(t = 1.86405	0.0783	0.83731181	Distal Intergenic	ENSSSCG000	GPR12	G protein-coupled receptor 12 [Source:NCBI gene (formerly Entrezgene);Acc:100736641]
chr2	125811919	125812616	698	*	chr2	125811919	125812616	in_vitro	in_vivo	12	12	1.74416667	0.08583333	1.65833333	c(t = 1.93748	0.0783	0.83731181	Distal Intergenic	NA	NA	NA
chr8	709518	712522	3005	*	chr8	709518	712522	in_vitro	in_vivo	11	11	8.56090909	28.6090909	-20.048182	c(t = -1.9095	0.0784	0.83731181	Distal Intergenic	ENSSSCG000	SLBP	stem-loop binding protein [Source:NCBI gene (formerly Entrezgene);Acc:100513605]
chr6	68415006	68417225	2220	*	chr6	68415006	68417225	in_vitro	in_vivo	9	9	1.09444444	3.76555556	-2.6711111	c(t = -1.9272	0.0785	0.83731181	Distal Intergenic	ENSSSCG000	UTS2	urotensin 2 [Source:VGNC Symbol;Acc:VGNC:94788]
chr7	2382314	2383183	870	*	chr7	2382314	2383183	in_vitro	in_vivo	11	13	2.68181818	0.57076923	2.11104895	c(t = 1.93273	0.0785	0.83731181	Distal Intergenic	ENSSSCG000	ECI2	enoyl-CoA delta isomerase 2 [Source:VGNC Symbol;Acc:VGNC:87532]
chr7	13845808	13847130	1323	*	chr7	13845808	13847130	in_vitro	in_vivo	11	15	1.29181818	0.28266667	1.00915152	c(t = 1.91040	0.0786	0.83731181	Promoter (<=1kb)	ENSSSCG000	TPMT	thiopurine S-methyltransferase [Source:NCBI gene (formerly Entrezgene);Acc:100157630]
chr6	40605226	40606633	1408	*	chr6	40605226	40606633	in_vitro	in_vivo	9	12	2.74444444	24.26	-21.515556	c(t = -1.9318	0.0787	0.83731181	Distal Intergenic	ENSSSCG000	SLC7A9	solute carrier family 7 member 9 [Source:VGNC Symbol;Acc:VGNC:93177]
chr12	59381441	59383672	2232	*	chr12	59381441	59383672	in_vitro	in_vivo	9	10	99	96.065	2.935	c(t = 1.91307	0.0794	0.83731181	Distal Intergenic	ENSSSCG000	UBB	ubiquitin B [Source:VGNC Symbol;Acc:VGNC:98389]
chr6	118333276	118334137	862	*	chr6	118333276	118334137	in_vitro	in_vivo	10	11	2.049	0.48	1.569	c(t = 1.93395	0.0794	0.83731181	Distal Intergenic	ENSSSCG000	ZNF24	zinc finger protein 24 [Source:VGNC Symbol;Acc:VGNC:98422]
chr14	75518432	75519036	605	*	chr14	75518432	75519036	in_vitro	in_vivo	14	9	1.75357143	0.28444444	1.46912698	c(t = 1.86684	0.0797	0.83731181	Distal Intergenic	ENSSSCG000	MICU1	mitochondrial calcium uptake 1 [Source:VGNC Symbol;Acc:VGNC:90211]
chr1	79900853	79901991	1139	*	chr1	79900853	79901991	in_vitro	in_vivo	12	11	1.385	0.17181818	1.21318182	c(t = 1.90263	0.08	0.83731181	Distal Intergenic	ENSSSCG000	COL10A1	collagen type X alpha 1 chain [Source:NCBI gene (formerly Entrezgene);Acc:448809]
chr5	359412	361343	1932	*	chr5	359412	361343	in_vitro	in_vivo	10	13	42.697	17.1353846	25.5616154	c(t = 1.85283	0.0801	0.83731181	Distal Intergenic	ENSSSCG000	PPP6R2	protein phosphatase 6 regulatory subunit 2 [Source:VGNC Symbol;Acc:VGNC:91761]
chr14	101254498	101255249	752	*	chr14	101254498	101255249	in_vitro	in_vivo	12	10	2.46666667	0.849	1.61766667	c(t = 1.85525	0.0801	0.83731181	Promoter (<=1kb)	NA	NA	NA
chr11	62438139	62440011	1873	*	chr11	62438139	62440011	in_vitro	in_vivo	12	15	1.63	0.61533333	1.01466667	c(t = 1.84634	0.0801	0.83731181	Distal Intergenic	ENSSSCG000	DCT	dopachrome tautomerase [Source:VGNC Symbol;Acc:VGNC:87192]
chr12	22466401	22466731	331	*	chr12	22466401	22466731	in_vitro	in_vivo	9	9	3.95666667	0.50555556	3.45111111	c(t = 1.96043	0.0801	0.83731181	Distal Intergenic	ENSSSCG000	ZPBP2	zona pellucida binding protein 2 [Source:VGNC Symbol;Acc:VGNC:95320]
chr11	52157608	52160831	3224	*	chr11	52157608	52160831	in_vitro	in_vivo	12	15	3.375	0.48866667	2.88633333	c(t = 1.91748	0.0801	0.83731181	Distal Intergenic	ENSSSCG000	EDNRB	endothelin receptor type B [Source:VGNC Symbol;Acc:VGNC:87550]
chr3	126218701	126221073	2373	*	chr3	126218701	126221073	in_vitro	in_vivo	10	11	3.522	0.85727273	2.66472727	c(t = 1.92852	0.0803	0.83731181	Distal Intergenic	ENSSSCG000	HPCAL1	hippocalcin like 1 [Source:VGNC Symbol;Acc:VGNC:88958]
chr1	461673	464321	2649	*	chr1	461673	464321	in_vitro	in_vivo	11	11	7.32	29.4936364	-22.173636	c(t = -1.8867	0.0808	0.83731181	Distal Intergenic	ENSSSCG000	PSMB1	proteasome 20S subunit beta 1 [Source:NCBI gene (formerly Entrezgene);Acc:100621969]
chr3	10212893	10214505	1613	*	chr3	10212893	10214505	in_vitro	in_vivo	10	10	90.044	79.667	10.377	c(t = 1.89861	0.081	0.83731181	3' UTR	ENSSSCG000	POR	cytochrome p450 oxidoreductase [Source:VGNC Symbol;Acc:VGNC:109477]
chr2	66345269	66346152	884	*	chr2	66345269	66346152	in_vitro	in_vivo	12	11	89.6375	59.8190909	29.8184091	c(t = 1.85633	0.0812	0.83731181	Distal Intergenic	NA	NA	NA
chr4	785393	798477	13085	*	chr4	785393	798477	in_vitro	in_vivo	10	15	17.478	36.992	-19.514	c(t = -1.8336	0.0812	0.83731181	Distal Intergenic	ENSSSCG000	HSF1	heat shock transcription factor 1 [Source:VGNC Symbol;Acc:VGNC:88993]
chr7	53889447	53891085	1639	*	chr7	53889447	53891085	in_vitro	in_vivo	10	10	2.398	0.786	1.612	c(t = 1.88044	0.0814	0.83731181	Promoter (<=1kb)	ENSSSCG000	KLF15	KLF transcription factor 15 [Source:VGNC Symbol;Acc:VGNC:89495]
chrX	60118739	60119931	1193	*	chrX	60118739	60119931	in_vitro	in_vivo	12	11	5.92416667	2.32727273	3.59689394	c(t = 1.86502	0.0815	0.83731181	Distal Intergenic	ENSSSCG000	UPRT	uracil phosphoribosyltransferase homolog [Source:VGNC Symbol;Acc:VGNC:98403]
chr18	39663091	39664836	1746	*	chr18	39663091	39664836	in_vitro	in_vivo	14	9	2.19285714	0.19111111	2.00174603	c(t = 1.87886	0.0815	0.83731181	Distal Intergenic	ENSSSCG000	FKBP9	FKBP prolyl isomerase 9 [Source:NCBI gene (formerly Entrezgene);Acc:100623924]
chr8	113820763	113822729	1967	*	chr8	113820763	113822729	in_vitro	in_vivo	9	14	29.8344444	10.7992857	19.0351587	c(t = 1.90001	0.0818	0.83731181	Distal Intergenic	ENSSSCG000	LEF1	lymphoid enhancer binding factor 1 [Source:VGNC Symbol;Acc:VGNC:89676]
chr1	213483568	213484017	450	*	chr1	213483568	213484017	in_vitro	in_vivo	11	12	0.26	1.95333333	-1.6933333	c(t = -1.9003	0.0819	0.83731181	Distal Intergenic	ENSSSCG000	IL33	interleukin 33 [Source:VGNC Symbol;Acc:VGNC:89106]
chr1	255596234	255597286	1053	*	chr1	255596234	255597286	in_vitro	in_vivo	14	12	4.28428571	22.2816667	-17.997381	c(t = -1.9061	0.0821	0.83731181	Exon (NM_214230/397460, exon 3 of 23)	ENSSSCG000	TNC	tenascin C [Source:VGNC Symbol;Acc:VGNC:103192]
chr4	56472959	56473695	737	*	chr4	56472959	56473695	in_vitro	in_vivo	13	12	2.91692308	0.89166667	2.02525641	c(t = 1.85543	0.0821	0.83731181	Promoter (<=1kb)	ENSSSCG000	TPD52	tumor protein D52 [Source:VGNC Symbol;Acc:VGNC:98383]
chr6	664926	672794	7869	*	chr6	664926	672794	in_vitro	in_vivo	12	12	43.37	61.8108333	-18.440833	c(t = -1.8350	0.0822	0.83731181	Distal Intergenic	ENSSSCG000	GALNS	galactosamine (N-acetyl)-6-sulfatase [Source:VGNC Symbol;Acc:VGNC:97061]
chr2	77776470	77777125	656	*	chr2	77776470	77777125	in_vitro	in_vivo	12	12	3.18166667	1.00083333	2.18083333	c(t = 1.86281	0.0822	0.83731181	Distal Intergenic	ENSSSCG000	MADCAM1	mucosal vascular addressin cell adhesion molecule 1 [Source:VGNC Symbol;Acc:VGNC:108754]
chr7	116435602	116436857	1256	*	chr7	116435602	116436857	in_vitro	in_vivo	9	12	2.74777778	0.35166667	2.39611111	c(t = 1.97497	0.0822	0.83731181	Distal Intergenic	ENSSSCG000	DICER1	dicer 1, ribonuclease III [Source:VGNC Symbol;Acc:VGNC:87302]
chr5	61337687	61338942	1256	*	chr5	61337687	61338942	in_vitro	in_vivo	14	14	4.33	0.51357143	3.81642857	c(t = 1.87558	0.0824	0.83731181	Distal Intergenic	ENSSSCG00000000636		killer cell lectin-like receptor subfamily A, member 1 [Source:NCBI gene (formerly Entrezgene);Acc:397612]
chr2	18758228	18758904	677	*	chr2	18758228	18758904	in_vitro	in_vivo	12	11	7.5525	3.03545455	4.51704545	c(t = 1.83495	0.0827	0.83731181	Distal Intergenic	ENSSSCG000	ssc-mir-670	ssc-mir-670 [Source:miRBase;Acc:MI0033398]
chr1	184256497	184257503	1007	*	chr1	184256497	184257503	in_vitro	in_vivo	10	13	0.169	1.55615385	-1.3871538	c(t = -1.8761	0.0827	0.83731181	Distal Intergenic	ENSSSCG000	SOCS4	suppressor of cytokine signaling 4 [Source:VGNC Symbol;Acc:VGNC:93331]
chr3	5074732	5075550	819	*	chr3	5074732	5075550	in_vitro	in_vivo	10	11	4.447	0.92090909	3.52609091	c(t = 1.91886	0.0827	0.83731181	Distal Intergenic	ENSSSCG000	AIMP2	aminoacyl tRNA synthetase complex interacting multifunctional protein 2 [Source:NCBI gene (formerly Entrezgene);Acc:100514707]
chr8	30758869	30759906	1038	*	chr8	30758869	30759906	in_vitro	in_vivo	12	15	2.83583333	0.356	2.47983333	c(t = 1.89623	0.0827	0.83731181	Distal Intergenic	ENSSSCG000	LIAS	lipoic acid synthetase [Source:VGNC Symbol;Acc:VGNC:89718]
chr10	65266094	65267932	1839	*	chr10	65266094	65267932	in_vitro	in_vivo	11	13	2.44454545	0.90769231	1.53685315	c(t = 1.86073	0.0828	0.83731181	Distal Intergenic	ENSSSCG000	GDI2	GDP dissociation inhibitor 2 [Source:NCBI gene (formerly Entrezgene);Acc:414427]
chr10	19888436	19889542	1107	*	chr10	19888436	19889542	in_vitro	in_vivo	10	11	3.443	0.96454545	2.47845455	c(t = 1.86125	0.083	0.83731181	Distal Intergenic	ENSSSCG000	CAPN2	calpain 2 [Source:NCBI gene (formerly Entrezgene);Acc:397393]
chr15	38790311	38791397	1087	*	chr15	38790311	38791397	in_vitro	in_vivo	13	14	2.25307692	0.795	1.45807692	c(t = 1.83278	0.083	0.83731181	Distal Intergenic	ENSSSCG000	SPATA4	spermatosis associated 4 [Source:VGNC Symbol;Acc:VGNC:95846]
chr12	51843603	51844550	948	*	chr12	51843603	51844550	in_vitro	in_vivo	9	14	1.51888889	3.90142857	-2.3825397	c(t = -1.8195	0.0832	0.83731181	Distal Intergenic	ENSSSCG000	ZNF232	zinc finger protein 232 [Source:VGNC Symbol;Acc:VGNC:95208]
chr5	2725267	2727222	1956	*	chr5	2725267	2727222	in_vitro	in_vivo	9	9	31.6944444	58.4777778	-26.783333	c(t = -1.8523	0.0833	0.83731181	Distal Intergenic	ENSSSCG000	PPARA	peroxisome proliferator activated receptor alpha [Source:NCBI gene (formerly Entrezgene);Acc:397239]
chr1	239492188	239493251	1064	*	chr1	239492188	239493251	in_vitro	in_vivo	14	10	2.415	0.233	2.182	c(t = 1.86353	0.0834	0.83731181	Distal Intergenic	ENSSSCG000	TRMO	tRNA methyltransferase O [Source:NCBI gene (formerly Entrezgene);Acc:100156700]
chr15	8058502	8059080	579	*	chr15	8058502	8059080	in_vitro	in_vivo	12	11	0	0.38636364	-0.3863636	c(t = -1.9222	0.0835	0.83731181	Distal Intergenic	ENSSSCG000	ACVR2A	activin A receptor type 2A [Source:NCBI gene (formerly Entrezgene);Acc:397282]
chr13	31308410	31309009	600	*	chr13	31308410	31309009	in_vitro	in_vivo	9	10	2.28666667	0.411	1.87566667	c(t = 1.93497	0.0836	0.83731181	Distal Intergenic	ENSSSCG000	SLC26A6	solute carrier family 26 member 6 [Source:VGNC Symbol;Acc:VGNC:93031]
chr17	14895814	14896617	804	*	chr17	14895814	14896617	in_vitro	in_vivo	9	10	5.07	14.113	-9.043	c(t = -1.9150	0.0837	0.83731181	Distal Intergenic	ENSSSCG000	CHGB	chromogranin B [Source:NCBI gene (formerly Entrezgene);Acc:397154]
chr2	147691322	147694729	3408	*	chr2	147691322	147694729	in_vitro	in_vivo	14	14	3.75	1.21071429	2.53928571	c(t = 1.85487	0.0837	0.83731181	Distal Intergenic	ENSSSCG000	PPP2R2B	protein phosphatase 2 regulatory subunit Bbeta [Source:VGNC Symbol;Acc:VGNC:100336]
chr4	98933489	98934588	1100	*	chr4	98933489	98934588	in_vitro	in_vivo	10	13	0	0.86307692	-0.8630769	c(t = -1.8860	0.0837	0.83731181	Distal Intergenic	ENSSSCG000	VPS45	vacuolar protein sorting 45 homolog [Source:NCBI gene (formerly Entrezgene);Acc:100514766]
chr16	52823014	52827774	4761	*	chr16	52823014	52827774	in_vitro	in_vivo	15	13	38.608	17.6930769	20.9149231	c(t = 1.79975	0.0838	0.83731181	Distal Intergenic	ENSSSCG000	KCNIP1	potassium voltage-gated channel interacting protein 1 [Source:VGNC Symbol;Acc:VGNC:89348]
chr13	141148425	141149278	854	*	chr13	141148425	141149278	in_vitro	in_vivo	13	13	2.22461538	0.66230769	1.56230769	c(t = 1.82487	0.0838	0.83731181	Distal Intergenic	ENSSSCG000	UPK1B	uroplakin 1B [Source:NCBI gene (formerly Entrezgene);Acc:100144488]
chr4	40487794	40489065	1272	*	chr4	40487794	40489065	in_vitro	in_vivo	10	9	1.603	3.37444444	-1.7714444	c(t = -1.8445	0.0839	0.83731181	Distal Intergenic	ENSSSCG000	UQCRB	ubiquinol-cytochrome c reductase binding protein [Source:NCBI gene (formerly Entrezgene);Acc:100157621]
chr13	14207614	14209199	1586	*	chr13	14207614	14209199	in_vitro	in_vivo	9	10	2.49444444	0.617	1.87744444	c(t = 1.89449	0.0839	0.83731181	Distal Intergenic	ENSSSCG000	TOP2B	DNA topoisomerase II beta [Source:NCBI gene (formerly Entrezgene);Acc:100156319]
chr12	369214	371816	2603	*	chr12	369214	371816	in_vitro	in_vivo	10	11	95.748	91.3863636	4.36163636	c(t = 1.85031	0.084	0.83731181	Exon (NM_001244105/100512127, exon 15 of 40)	ENSSSCG000	TBCD	tubulin folding cofactor D [Source:NCBI gene (formerly Entrezgene);Acc:100512127]
chr15	137444508	137445469	962	*	chr15	137444508	137445469	in_vitro	in_vivo	10	10	3.296	20.5	-17.204	c(t = -1.9267	0.0841	0.83731181	Distal Intergenic	ENSSSCG000	RAMP1	receptor activity modifying protein 1 [Source:NCBI gene (formerly Entrezgene);Acc:397401]
chr5	41635356	41636159	804	*	chr5	41635356	41636159	in_vitro	in_vivo	10	9	3.181	0.62666667	2.55433333	c(t = 1.89056	0.0843	0.83731181	Distal Intergenic	ENSSSCG000	RESF1	retroelement silencing factor 1 [Source:VGNC Symbol;Acc:VGNC:92219]
chr14	44073399	44073873	475	*	chr14	44073399	44073873	in_vitro	in_vivo	10	11	1.859	0.37090909	1.48809091	c(t = 1.88581	0.0844	0.83731181	Promoter (1-2kb)	ENSSSCG000	HPS4	HPS4 biosis of lysosomal organelles complex 3 subunit 2 [Source:VGNC Symbol;Acc:VGNC:88966]
chr11	6000360	6001074	715	*	chr11	6000360	6001074	in_vitro	in_vivo	11	15	3.89090909	1.332	2.55890909	c(t = 1.86309	0.0846	0.83731181	Promoter (<=1kb)	ENSSSCG000	SLC46A3	solute carrier family 46 member 3 [Source:VGNC Symbol;Acc:VGNC:93128]
chr9	121849916	121852846	2931	*	chr9	121849916	121852846	in_vitro	in_vivo	9	10	2.61	0.997	1.613	c(t = 1.86851	0.0846	0.83731181	Distal Intergenic	ENSSSCG000	TOR1AIP2	torsin 1A interacting protein 2 [Source:VGNC Symbol;Acc:VGNC:94318]
chr1	32418386	32419693	1308	*	chr1	32418386	32419693	in_vitro	in_vivo	9	15	2.85111111	0.94	1.91111111	c(t = 1.82865	0.0847	0.83731181	Distal Intergenic	ENSSSCG000	ARG1	arginase 1 [Source:VGNC Symbol;Acc:VGNC:85454]
chr10	63387055	63394037	6983	*	chr10	63387055	63394037	in_vitro	in_vivo	14	15	3.80642857	1.114	2.69242857	c(t = 1.85129	0.0848	0.83731181	Promoter (<=1kb)	ENSSSCG000	GATA3	GATA binding protein 3 [Source:VGNC Symbol;Acc:VGNC:96315]
chr1	253569435	253570326	892	*	chr1	253569435	253570326	in_vitro	in_vivo	11	9	76.2763636	49.9622222	26.3141414	c(t = 1.87206	0.085	0.83731181	Distal Intergenic	ENSSSCG000	SAL1	salivary lipocalin [Source:NCBI gene (formerly Entrezgene);Acc:396739]
chr17	38775062	38775462	401	*	chr17	38775062	38775462	in_vitro	in_vivo	11	10	3.70545455	0	3.70545455	c(t = 1.91153	0.085	0.83731181	Promoter (<=1kb)	ENSSSCG000	UQCC1	ubiquinol-cytochrome c reductase complex assembly factor 1 [Source:VGNC Symbol;Acc:VGNC:95694]
chr7	103768199	103768525	327	*	chr7	103768199	103768525	in_vitro	in_vivo	11	9	7.01909091	1.50777778	5.51131313	c(t = 1.86907	0.0851	0.83731181	Promoter (<=1kb)	ENSSSCG000	TSHR	thyroid stimulating hormone receptor [Source:VGNC Symbol;Acc:VGNC:94491]
chr5	63951223	63952157	935	*	chr5	63951223	63952157	in_vitro	in_vivo	11	12	1.92363636	0.63083333	1.29280303	c(t = 1.83688	0.0854	0.83731181	Distal Intergenic	ENSSSCG000	LAG3	lymphocyte activating 3 [Source:NCBI gene (formerly Entrezgene);Acc:100125962]
chr13	190065960	190066598	639	*	chr13	190065960	190066598	in_vitro	in_vivo	10	10	4.911	0.541	4.37	c(t = 1.90648	0.0855	0.83731181	Distal Intergenic	ENSSSCG000	ADAMTS1	ADAM metallopeptidase with thrombospondin type 1 motif 1 [Source:NCBI gene (formerly Entrezgene);Acc:497061]
chr1	4882178	4885075	2898	*	chr1	4882178	4885075	in_vitro	in_vivo	12	13	4.56416667	2.18769231	2.37647436	c(t = 1.82333	0.0857	0.83731181	Promoter (<=1kb)	ENSSSCG000	QKI	QKI, KH domain containing RNA binding [Source:VGNC Symbol;Acc:VGNC:92025]
chr13	90760706	90761674	969	*	chr13	90760706	90761674	in_vitro	in_vivo	15	10	3.47733333	0.135	3.34233333	c(t = 1.84695	0.0858	0.83731181	Promoter (<=1kb)	ENSSSCG000	SERP1	stress associated endoplasmic reticulum protein 1 [Source:NCBI gene (formerly Entrezgene);Acc:100156392]
chr5	19166882	19167840	959	*	chr5	19166882	19167840	in_vitro	in_vivo	12	11	3.76833333	0.64272727	3.12560606	c(t = 1.87879	0.0859	0.83731181	Distal Intergenic	ENSSSCG000	ssc-mir-196a	ssc-mir-196a-2 [Source:miRBase;Acc:MI0002457]
chr12	48167692	48171833	4142	*	chr12	48167692	48171833	in_vitro	in_vivo	13	15	9.85846154	20.6973333	-10.838872	c(t = -1.8059	0.086	0.83731181	Distal Intergenic	NA	NA	NA
chr16	44972535	44974039	1505	*	chr16	44972535	44974039	in_vitro	in_vivo	11	13	3.68454545	0.91615385	2.76839161	c(t = 1.88162	0.086	0.83731181	Distal Intergenic	ENSSSCG000	CD180	CD180 molecule [Source:NCBI gene (formerly Entrezgene);Acc:397644]
chr2	27146241	27147074	834	*	chr2	27146241	27147074	in_vitro	in_vivo	10	10	3.089	0.609	2.48	c(t = 1.88740	0.086	0.83731181	Distal Intergenic	ENSSSCG00000024791		CD59 molecule [Source:VGNC Symbol;Acc:VGNC:99615]
chr7	66800662	66802735	2074	*	chr7	66800662	66802735	in_vitro	in_vivo	11	15	4.49545455	1.276	3.21945455	c(t = 1.86701	0.0862	0.83731181	Distal Intergenic	ENSSSCG000	GPR33	G protein-coupled receptor 33 [Source:NCBI gene (formerly Entrezgene);Acc:100157823]
chr11	50730488	50732710	2223	*	chr11	50730488	50732710	in_vitro	in_vivo	16	15	3.106875	0.86	2.246875	c(t = 1.82662	0.0863	0.83731181	Distal Intergenic	ENSSSCG000	EDNRB	endothelin receptor type B [Source:VGNC Symbol;Acc:VGNC:87550]
chr13	157272010	157273158	1149	*	chr13	157272010	157273158	in_vitro	in_vivo	12	11	2.21	0.60636364	1.60363636	c(t = 1.85800	0.0863	0.83731181	Promoter (<=1kb)	ENSSSCG000	NFKBIZ	NFKB inhibitor zeta [Source:VGNC Symbol;Acc:VGNC:90726]
chr14	112215507	112215944	438	*	chr14	112215507	112215944	in_vitro	in_vivo	10	9	4.325	0.31777778	4.00722222	c(t = 1.91474	0.0863	0.83731181	Distal Intergenic	ENSSSCG000	LBX1	ladybird homeobox 1 [Source:VGNC Symbol;Acc:VGNC:89652]
chr15	138787006	138788821	1816	*	chr15	138787006	138788821	in_vitro	in_vivo	11	9	8.07727273	34.9988889	-26.921616	c(t = -1.8666	0.0864	0.83731181	Distal Intergenic	ENSSSCG000	ssc-mir-149	ssc-mir-149 [Source:miRBase;Acc:MI0016620]
chr6	79006917	79008162	1246	*	chr6	79006917	79008162	in_vitro	in_vivo	10	11	4.039	0.82636364	3.21263636	c(t = 1.89177	0.0866	0.83731181	Distal Intergenic	ENSSSCG000	DDOST	dolichyl-diphosphooligosaccharide--protein glycosyltransferase non-catalytic subunit [Source:NCBI gene (formerly Entrezgene);Acc:397385]
chr5	7000235	7000974	740	*	chr5	7000235	7000974	in_vitro	in_vivo	9	12	2.91666667	0.30916667	2.6075	c(t = 1.93779	0.0869	0.83731181	Distal Intergenic	ENSSSCG000	CSDC2	cold shock domain containing C2 [Source:VGNC Symbol;Acc:VGNC:87028]
chr5	89583700	89587150	3451	*	chr5	89583700	89587150	in_vitro	in_vivo	15	13	5.10133333	2.04230769	3.05902564	c(t = 1.79228	0.087	0.83731181	Promoter (<=1kb)	ENSSSCG000	SOCS2	suppressor of cytokine signaling 2 [Source:NCBI gene (formerly Entrezgene);Acc:100037966]
chr6	41347543	41349673	2131	*	chr6	41347543	41349673	in_vitro	in_vivo	12	14	13.2008333	34.4257143	-21.224881	c(t = -1.8024	0.0871	0.83731181	Distal Intergenic	ENSSSCG000	SLC7A9	solute carrier family 7 member 9 [Source:VGNC Symbol;Acc:VGNC:93177]
chr4	2577459	2578563	1105	*	chr4	2577459	2578563	in_vitro	in_vivo	9	9	4.81222222	1.23888889	3.57333333	c(t = 1.89588	0.0871	0.83731181	Distal Intergenic	NA	NA	NA
chr16	34383825	34384890	1066	*	chr16	34383825	34384890	in_vitro	in_vivo	9	14	3.36666667	0.99785714	2.36880952	c(t = 1.90849	0.0871	0.83731181	Distal Intergenic	ENSSSCG000	GZMA	granzyme A (granzyme 1, cytotoxic T-lymphocyte-associated serine esterase 3) [Source:NCBI gene (formerly Entrezgene);Acc:100526762]
chr13	22124847	22125444	598	*	chr13	22124847	22125444	in_vitro	in_vivo	12	13	1.7	0.21384615	1.48615385	c(t = 1.85444	0.0871	0.83731181	Distal Intergenic	ENSSSCG000	MLH1	mutL homolog 1 [Source:VGNC Symbol;Acc:VGNC:90252]
chr18	45379664	45381822	2159	*	chr18	45379664	45381822	in_vitro	in_vivo	10	13	1.159	3.62692308	-2.4679231	c(t = -1.8074	0.0872	0.83731181	Distal Intergenic	ENSSSCG000	HOXA13	homeobox A13 [Source:NCBI gene (formerly Entrezgene);Acc:100359352]
chr10	14183392	14184638	1247	*	chr10	14183392	14184638	in_vitro	in_vivo	14	12	1.88285714	0.14083333	1.74202381	c(t = 1.84476	0.0875	0.83731181	Distal Intergenic	ENSSSCG000	ACBD3	acyl-CoA binding domain containing 3 [Source:NCBI gene (formerly Entrezgene);Acc:494461]
chr2	65108939	65110643	1705	*	chr2	65108939	65110643	in_vitro	in_vivo	12	13	0.62083333	1.65076923	-1.0299359	c(t = -1.7878	0.0876	0.83731181	Promoter (<=1kb)	ENSSSCG00000013768		MISP family member 3 [Source:NCBI gene (formerly Entrezgene);Acc:100513647]
chr18	32928081	32928790	710	*	chr18	32928081	32928790	in_vitro	in_vivo	13	12	1.94076923	0.2375	1.70326923	c(t = 1.84980	0.0876	0.83731181	Distal Intergenic	ENSSSCG000	IFRD1	interferon related developmental regulator 1 [Source:NCBI gene (formerly Entrezgene);Acc:493185]
chr8	33012447	33012991	545	*	chr8	33012447	33012991	in_vitro	in_vivo	12	9	1.2475	0	1.2475	c(t = 1.87425	0.0877	0.83731181	Promoter (<=1kb)	ENSSSCG000	TMEM33	transmembrane protein 33 [Source:VGNC Symbol;Acc:VGNC:94171]
chr18	942780	944834	2055	*	chr18	942780	944834	in_vitro	in_vivo	11	14	14.3781818	37.6042857	-23.226104	c(t = -1.7881	0.0879	0.83731181	Distal Intergenic	ENSSSCG000	ssc-mir-153	ssc-mir-153 [Source:miRBase;Acc:MI0002454]
chr15	120589830	120591198	1369	*	chr15	120589830	120591198	in_vitro	in_vivo	14	13	2.105	0.50538462	1.59961538	c(t = 1.81890	0.0881	0.83731181	Distal Intergenic	ENSSSCG000	PLCD4	phospholipase C delta 4 [Source:NCBI gene (formerly Entrezgene);Acc:397119]
chr3	56276903	56278081	1179	*	chr3	56276903	56278081	in_vitro	in_vivo	15	13	1.368	0.50307692	0.86492308	c(t = 1.78321	0.0881	0.83731181	Distal Intergenic	ENSSSCG000	COX5B	mitochondrial cytochrome c oxidase subunit Vb [Source:NCBI gene (formerly Entrezgene);Acc:492822]
chr12	20796281	20797629	1349	*	chr12	20796281	20797629	in_vitro	in_vivo	9	12	1.44	3.655	-2.215	c(t = -1.7988	0.0882	0.83731181	Promoter (<=1kb)	ENSSSCG000	ACLY	ATP citrate lyase [Source:VGNC Symbol;Acc:VGNC:85014]
chr13	82230099	82231055	957	*	chr13	82230099	82231055	in_vitro	in_vivo	10	10	1.955	0.421	1.534	c(t = 1.84921	0.0882	0.83731181	Distal Intergenic	ENSSSCG000	ZBTB38	zinc finger and BTB domain containing 38 [Source:VGNC Symbol;Acc:VGNC:95072]
chr13	131133496	131134901	1406	*	chr13	131133496	131134901	in_vitro	in_vivo	9	12	25.6633333	5.75583333	19.9075	c(t = 1.91723	0.0883	0.83731181	Promoter (1-2kb)	ENSSSCG000	HES1	hes family bHLH transcription factor 1 [Source:NCBI gene (formerly Entrezgene);Acc:100499567]
chr5	68222967	68224233	1267	*	chr5	68222967	68224233	in_vitro	in_vivo	12	10	0.7225	1.753	-1.0305	c(t = -1.8108	0.0883	0.83731181	Distal Intergenic	ENSSSCG000	RAD52	RAD52 homolog, DNA repair protein [Source:VGNC Symbol;Acc:VGNC:92060]
chr10	13939003	13940930	1928	*	chr10	13939003	13940930	in_vitro	in_vivo	9	12	3.77777778	0.9575	2.82027778	c(t = 1.83837	0.0883	0.83731181	Promoter (<=1kb)	ENSSSCG000	H3-3A	H3.3 histone A [Source:NCBI gene (formerly Entrezgene);Acc:396970]
chr13	22883055	22883603	549	*	chr13	22883055	22883603	in_vitro	in_vivo	10	12	2.982	0.3625	2.6195	c(t = 1.88133	0.0884	0.83731181	Distal Intergenic	ENSSSCG000	PLCD1	phospholipase C delta 1 [Source:NCBI gene (formerly Entrezgene);Acc:100627099]
chr14	120050910	120051497	588	*	chr14	120050910	120051497	in_vitro	in_vivo	10	11	0.323	2.99545455	-2.6724545	c(t = -1.8678	0.0885	0.83731181	Distal Intergenic	ENSSSCG000	SMNDC1	survival motor neuron domain containing 1 [Source:VGNC Symbol;Acc:VGNC:93257]
chr18	4052778	4055572	2795	*	chr18	4052778	4055572	in_vitro	in_vivo	14	13	2.45	0.95769231	1.49230769	c(t = 1.80735	0.0885	0.83731181	Distal Intergenic	ENSSSCG000	INSIG1	insulin induced gene 1 [Source:NCBI gene (formerly Entrezgene);Acc:403162]
chrX	97394305	97394720	416	*	chrX	97394305	97394720	in_vitro	in_vivo	10	10	1.299	8.572	-7.273	c(t = -1.8931	0.0887	0.83731181	Distal Intergenic	ENSSSCG000	IL13RA1	interleukin 13 receptor subunit alpha 1 [Source:NCBI gene (formerly Entrezgene);Acc:397615]
chr12	56045287	56045889	603	*	chr12	56045287	56045889	in_vitro	in_vivo	13	12	0.31153846	1.28416667	-0.9726282	c(t = -1.8189	0.0888	0.83731181	Distal Intergenic	ENSSSCG000	ssc-mir-744	ssc-mir-744 [Source:miRBase;Acc:MI0014769]
chr6	53431154	53431639	486	*	chr6	53431154	53431639	in_vitro	in_vivo	10	11	2.56	25.9590909	-23.399091	c(t = -1.8762	0.089	0.83731181	Distal Intergenic	ENSSSCG000	SELENOW	selenoprotein W [Source:VGNC Symbol;Acc:VGNC:110393]
chr6	1494442	1496104	1663	*	chr6	1494442	1496104	in_vitro	in_vivo	10	13	5.286	1.65384615	3.63215385	c(t = 1.81487	0.089	0.83731181	Promoter (<=1kb)	ENSSSCG000	BANP	BTG3 associated nuclear protein [Source:NCBI gene (formerly Entrezgene);Acc:100622381]
chr9	8512578	8513484	907	*	chr9	8512578	8513484	in_vitro	in_vivo	12	12	1.36083333	0.18916667	1.17166667	c(t = 1.83486	0.0893	0.83731181	Distal Intergenic	ENSSSCG000	UCP3	uncoupling protein 3 [Source:NCBI gene (formerly Entrezgene);Acc:397116]
chr14	50822997	50823802	806	*	chr14	50822997	50823802	in_vitro	in_vivo	10	10	2.737	20.485	-17.748	c(t = -1.8842	0.0895	0.83731181	Distal Intergenic	ENSSSCG000	TSSK1B	testis specific serine kinase 1B [Source:NCBI gene (formerly Entrezgene);Acc:102159240]
chr9	710255	711662	1408	*	chr9	710255	711662	in_vitro	in_vivo	9	10	4.21444444	0.897	3.31744444	c(t = 1.89506	0.0895	0.83731181	Distal Intergenic	NA	NA	NA
chr2	96070275	96070742	468	*	chr2	96070275	96070742	in_vitro	in_vivo	9	10	2.41111111	0.314	2.09711111	c(t = 1.91075	0.0897	0.83731181	Distal Intergenic	NA	NA	NA
chrX	15699855	15700571	717	*	chrX	15699855	15700571	in_vitro	in_vivo	10	10	4.888	0.425	4.463	c(t = 1.89197	0.0898	0.83731181	Distal Intergenic	ENSSSCG000	EIF1AX	eukaryotic translation initiation factor 1A X-linked [Source:NCBI gene (formerly Entrezgene);Acc:100522912]
chr15	77649057	77650393	1337	*	chr15	77649057	77650393	in_vitro	in_vivo	9	13	5.35111111	1.36	3.99111111	c(t = 1.90130	0.0899	0.83731181	Promoter (<=1kb)	ENSSSCG000	CYBRD1	cytochrome b reductase 1 [Source:VGNC Symbol;Acc:VGNC:95948]
chr2	77977620	77978511	892	*	chr2	77977620	77978511	in_vitro	in_vivo	12	9	5.95833333	0.59666667	5.36166667	c(t = 1.85221	0.0899	0.83731181	Distal Intergenic	ENSSSCG000	PLPP2	phospholipid phosphatase 2 [Source:VGNC Symbol;Acc:VGNC:104028]
chr14	51705540	51706149	610	*	chr14	51705540	51706149	in_vitro	in_vivo	13	13	0.51307692	4.33153846	-3.8184615	c(t = -1.8258	0.09	0.83731181	Distal Intergenic	ENSSSCG000	CHRM3	cholinergic receptor muscarinic 3 [Source:VGNC Symbol;Acc:VGNC:86664]
chr7	16884227	16886077	1851	*	chr7	16884227	16886077	in_vitro	in_vivo	11	11	7.01818182	0.98090909	6.03727273	c(t = 1.86698	0.09	0.83731181	Distal Intergenic	ENSSSCG000	PRL	prolactin [Source:VGNC Symbol;Acc:VGNC:104037]
chr11	4856900	4857454	555	*	chr11	4856900	4857454	in_vitro	in_vivo	12	12	5.03583333	1.44583333	3.59	c(t = 1.83781	0.0901	0.83731181	Distal Intergenic	ENSSSCG000	ssc-mir-9803	ssc-mir-9803 [Source:miRBase;Acc:MI0031567]
chr9	85009387	85009638	252	*	chr9	85009387	85009638	in_vitro	in_vivo	9	9	4.48666667	0.61777778	3.86888889	c(t = 1.88341	0.0903	0.83731181	Promoter (<=1kb)	ENSSSCG000	MEOX2	mesenchyme homeobox 2 [Source:NCBI gene (formerly Entrezgene);Acc:397222]
chrX	110390440	110392443	2004	*	chrX	110390440	110392443	in_vitro	in_vivo	12	10	1.475	5.51	-4.035	c(t = -1.8688	0.0906	0.83731181	Distal Intergenic	ENSSSCG000	ssc-mir-424	ssc-mir-424 [Source:miRBase;Acc:MI0013135]
chr7	1655605	1658147	2543	*	chr7	1655605	1658147	in_vitro	in_vivo	11	13	3.06363636	1.21846154	1.84517483	c(t = 1.80532	0.0906	0.83731181	Distal Intergenic	ENSSSCG000	SERPINB9	serpin peptidase inhibitor, clade B (ovalbumin), member 9 [Source:NCBI gene (formerly Entrezgene);Acc:100126851]
chr1	144071402	144072799	1398	*	chr1	144071402	144072799	in_vitro	in_vivo	14	12	2.55214286	0.9	1.65214286	c(t = 1.76873	0.0906	0.83731181	Distal Intergenic	ENSSSCG000	MCEE	methylmalonyl-CoA epimerase [Source:VGNC Symbol;Acc:VGNC:90066]
chr5	10024456	10025231	776	*	chr5	10024456	10025231	in_vitro	in_vivo	11	11	2.66181818	0.54454545	2.11727273	c(t = 1.84857	0.0911	0.83731181	Distal Intergenic	ENSSSCG000	EIF3L	eukaryotic translation initiation factor 3 subunit L [Source:VGNC Symbol;Acc:VGNC:87623]
chr1	159133028	159133479	452	*	chr1	159133028	159133479	in_vitro	in_vivo	9	10	0.78666667	3.615	-2.8283333	c(t = -1.8443	0.0914	0.83731181	Distal Intergenic	ENSSSCG000	RNF152	ring finger protein 152 [Source:VGNC Symbol;Acc:VGNC:92364]
chr7	704023	705604	1582	*	chr7	704023	705604	in_vitro	in_vivo	10	12	5.423	0.71583333	4.70716667	c(t = 1.88254	0.0914	0.83731181	Distal Intergenic	ENSSSCG000	IRF4	interferon regulatory factor 4 [Source:VGNC Symbol;Acc:VGNC:89207]
chr14	49468536	49469708	1173	*	chr14	49468536	49469708	in_vitro	in_vivo	9	11	16.5333333	6.54181818	9.99151515	c(t = 1.83609	0.0916	0.83731181	Distal Intergenic	ENSSSCG000	GUCD1	guanylyl cyclase domain containing 1 [Source:VGNC Symbol;Acc:VGNC:88749]
chr9	8198088	8199446	1359	*	chr9	8198088	8199446	in_vitro	in_vivo	11	13	2.05545455	0.37076923	1.68468531	c(t = 1.84679	0.0918	0.83731181	Promoter (<=1kb)	ENSSSCG000	RAB6A	RAB6A, member RAS onco family [Source:VGNC Symbol;Acc:VGNC:108610]
chr2	17228084	17229058	975	*	chr2	17228084	17229058	in_vitro	in_vivo	9	11	1.60555556	0.36363636	1.24191919	c(t = 1.81052	0.092	0.83731181	Distal Intergenic	ENSSSCG000	SLC35C1	solute carrier family 35 member C1 [Source:VGNC Symbol;Acc:VGNC:93075]
chr5	6346283	6347246	964	*	chr5	6346283	6347246	in_vitro	in_vivo	9	10	5.96888889	0.483	5.48588889	c(t = 1.90626	0.0921	0.83731181	Distal Intergenic	ENSSSCG000	CYP2D6	cytochrome P450 family 2 subfamily D member 6 [Source:NCBI gene (formerly Entrezgene);Acc:397687]
chr9	51992222	51992940	719	*	chr9	51992222	51992940	in_vitro	in_vivo	9	11	12.0422222	27.25	-15.207778	c(t = -1.7965	0.0922	0.83731181	Distal Intergenic	ENSSSCG000	STT3A	STT3 oligosaccharyltransferase complex catalytic subunit A [Source:VGNC Symbol;Acc:VGNC:93581]
chr12	1990952	1991729	778	*	chr12	1990952	1991729	in_vitro	in_vivo	9	9	1.03	0	1.03	c(t = 1.91235	0.0922	0.83731181	Distal Intergenic	ENSSSCG000	SLC26A11	solute carrier family 26 member 11 [Source:NCBI gene (formerly Entrezgene);Acc:100191010]
chr2	70209412	70209772	361	*	chr2	70209412	70209772	in_vitro	in_vivo	9	10	38.4555556	19.178	19.2775556	c(t = 1.80175	0.0923	0.83731181	Promoter (<=1kb)	ENSSSCG000	ACP5	acid phosphatase 5, tartrate resistant [Source:VGNC Symbol;Acc:VGNC:85027]
chr14	112078473	112079282	810	*	chr14	112078473	112079282	in_vitro	in_vivo	9	11	4.64	0.53909091	4.10090909	c(t = 1.90256	0.0923	0.83731181	Distal Intergenic	ENSSSCG000	ssc-mir-1038	ssc-mir-10382 [Source:miRBase;Acc:MI0033391]
chr7	22498794	22499522	729	*	chr7	22498794	22499522	in_vitro	in_vivo	12	13	1.70916667	0.51692308	1.19224359	c(t = 1.80182	0.0925	0.83731181	Distal Intergenic	ENSSSCG000	UBD	ubiquitin D [Source:NCBI gene (formerly Entrezgene);Acc:100294688]
chr10	50778582	50779051	470	*	chr10	50778582	50779051	in_vitro	in_vivo	9	9	29.5588889	61.6744444	-32.115556	c(t = -1.7893	0.0926	0.83731181	Distal Intergenic	ENSSSCG000	GAD2	glutamate decarboxylase 2 [Source:NCBI gene (formerly Entrezgene);Acc:396929]
chr14	137242309	137242817	509	*	chr14	137242309	137242817	in_vitro	in_vivo	10	9	14.236	43.7166667	-29.480667	c(t = -1.8162	0.0927	0.83731181	Distal Intergenic	ENSSSCG000	GLRX3	glutaredoxin 3 [Source:VGNC Symbol;Acc:VGNC:88495]
chr13	207195553	207197440	1888	*	chr13	207195553	207197440	in_vitro	in_vivo	10	10	55.463	30.114	25.349	c(t = 1.77890	0.0927	0.83731181	Distal Intergenic	ENSSSCG000	AGPAT3	1-acylglycerol-3-phosphate O-acyltransferase 3 [Source:VGNC Symbol;Acc:VGNC:85185]
chr16	25783002	25783757	756	*	chr16	25783002	25783757	in_vitro	in_vivo	10	10	0.612	2.717	-2.105	c(t = -1.7947	0.093	0.83731181	Promoter (<=1kb)	ENSSSCG000	PRKAA1	protein kinase AMP-activated catalytic subunit alpha 1 [Source:VGNC Symbol;Acc:VGNC:91797]
chr9	46364840	46365875	1036	*	chr9	46364840	46365875	in_vitro	in_vivo	9	11	5.58	0.40363636	5.17636364	c(t = 1.89501	0.0931	0.83731181	Distal Intergenic	ENSSSCG000	NLRX1	NLR family member X1 [Source:NCBI gene (formerly Entrezgene);Acc:100518678]
chr14	127274751	127275985	1235	*	chr14	127274751	127275985	in_vitro	in_vivo	9	14	3.32222222	1.15285714	2.16936508	c(t = 1.86210	0.0933	0.83731181	Distal Intergenic	ENSSSCG000	SLC18A2	solute carrier family 18 member A2 [Source:NCBI gene (formerly Entrezgene);Acc:100154950]
chr1	219111651	219112615	965	*	chr1	219111651	219112615	in_vitro	in_vivo	10	10	60.435	86.815	-26.38	c(t = -1.7898	0.0934	0.83731181	Distal Intergenic	ENSSSCG000	VLDLR	very low density lipoprotein receptor [Source:VGNC Symbol;Acc:VGNC:94832]
chrX	36424419	36425127	709	*	chrX	36424419	36425127	in_vitro	in_vivo	11	9	2.01818182	0.26333333	1.75484848	c(t = 1.84113	0.0934	0.83731181	Distal Intergenic	ENSSSCG000	DDX3X	DEAD-box helicase 3 X-linked [Source:NCBI gene (formerly Entrezgene);Acc:100515940]
chr14	14576562	14577402	841	*	chr14	14576562	14577402	in_vitro	in_vivo	11	10	2.83181818	0	2.83181818	c(t = 1.85383	0.0935	0.83731181	Distal Intergenic	ENSSSCG00000021767		L-threonine dehydrogenase [Source:VGNC Symbol;Acc:VGNC:109162]
chr9	25784760	25785417	658	*	chr9	25784760	25785417	in_vitro	in_vivo	9	12	1.19666667	0	1.19666667	c(t = 1.90309	0.0935	0.83731181	Distal Intergenic	ENSSSCG000	C11orf54	chromosome 9 C11orf54 homolog [Source:VGNC Symbol;Acc:VGNC:86087]
chr12	51931617	51932898	1282	*	chr12	51931617	51932898	in_vitro	in_vivo	11	11	5.91727273	1.81545455	4.10181818	c(t = 1.80577	0.0937	0.83731181	Distal Intergenic	ENSSSCG000	SPAG7	sperm associated antigen 7 [Source:NCBI gene (formerly Entrezgene);Acc:100511958]
chr7	4542760	4543592	833	*	chr7	4542760	4543592	in_vitro	in_vivo	11	11	2.29909091	0.50454545	1.79454545	c(t = 1.82186	0.0938	0.83731181	Distal Intergenic	ENSSSCG000	SSR1	signal sequence receptor subunit 1 [Source:NCBI gene (formerly Entrezgene);Acc:100152689]
chr14	118008733	118009624	892	*	chr14	118008733	118009624	in_vitro	in_vivo	12	12	2.26666667	0.08583333	2.18083333	c(t = 1.83223	0.0938	0.83731181	Distal Intergenic	ENSSSCG000	SMNDC1	survival motor neuron domain containing 1 [Source:VGNC Symbol;Acc:VGNC:93257]
chr15	110873716	110874771	1056	*	chr15	110873716	110874771	in_vitro	in_vivo	13	13	3.10307692	0.61615385	2.48692308	c(t = 1.80226	0.0939	0.83731181	Distal Intergenic	NA	NA	NA
chr3	36547886	36550043	2158	*	chr3	36547886	36550043	in_vitro	in_vivo	11	12	6.54363636	1.585	4.95863636	c(t = 1.82759	0.094	0.83731181	Distal Intergenic	ENSSSCG000	NAGPA	N-acetylglucosamine-1-phosphodiester alpha-N-acetylglucosaminidase [Source:VGNC Symbol;Acc:VGNC:109427]
chr4	84204066	84204993	928	*	chr4	84204066	84204993	in_vitro	in_vivo	9	10	2.19111111	0.275	1.91611111	c(t = 1.88214	0.0942	0.83731181	Promoter (<=1kb)	ENSSSCG000	POGK	pogo transposable element derived with KRAB domain [Source:VGNC Symbol;Acc:VGNC:91622]
chr1	807820	809441	1622	*	chr1	807820	809441	in_vitro	in_vivo	11	12	17.8918182	38.5058333	-20.614015	c(t = -1.7522	0.0943	0.83731181	Distal Intergenic	ENSSSCG000	ssc-mir-9815	ssc-mir-9815 [Source:miRBase;Acc:MI0031582]
chr6	39373555	39374060	506	*	chr6	39373555	39374060	in_vitro	in_vivo	12	11	3.07083333	0.47818182	2.59265152	c(t = 1.79813	0.0943	0.83731181	Distal Intergenic	ENSSSCG000	SLC7A9	solute carrier family 7 member 9 [Source:VGNC Symbol;Acc:VGNC:93177]
chr3	98155512	98156023	512	*	chr3	98155512	98156023	in_vitro	in_vivo	11	11	2.34636364	0.33636364	2.01	c(t = 1.81683	0.0943	0.83731181	Distal Intergenic	ENSSSCG000	HAAO	3-hydroxyanthranilate 3,4-dioxygenase [Source:VGNC Symbol;Acc:VGNC:88765]
chr11	78735942	78737262	1321	*	chr11	78735942	78737262	in_vitro	in_vivo	9	10	96.6311111	91.843	4.78811111	c(t = 1.78140	0.0944	0.83731181	Exon (NM_001267851/100620378, exon 10 of 14)	ENSSSCG000	TMCO3	transmembrane and coiled-coil domains 3 [Source:VGNC Symbol;Acc:VGNC:94050]
chr6	115785969	115786941	973	*	chr6	115785969	115786941	in_vitro	in_vivo	14	12	0.56571429	0	0.56571429	c(t = 1.80201	0.0948	0.83731181	Distal Intergenic	ENSSSCG000	TTR	transthyretin [Source:NCBI gene (formerly Entrezgene);Acc:397419]

chr2	69095504	69097318	1815	*	chr2	69095504	69097318	in_vitro	in_vivo	9	13	4.76555556	0.80692308	3.95863248	c(t = 1.87983	0.0949	0.83731181	Promoter (<=1kb)	ENSSSCG000	ICAM1	intercellular adhesion molecule 1 [Source:NCBI gene (formerly Entrezgene);Acc:396750]
chr1	180433891	180434808	918	*	chr1	180433891	180434808	in_vitro	in_vivo	11	9	1.25727273	0	1.25727273	c(t = 1.84452	0.0949	0.83731181	Distal Intergenic	ENSSSCG000	PYGL	glycogen phosphorylase L [Source:NCBI gene (formerly Entrezgene);Acc:100141306]
chr6	95321439	95322907	1469	*	chr6	95321439	95322907	in_vitro	in_vivo	12	12	2.49083333	1.15833333	1.3325	c(t = 1.77132	0.0951	0.83731181	Distal Intergenic	ENSSSCG000	HPCAL4	hippocalcin like 4 [Source:VGNC Symbol;Acc:VGNC:88959]
chrX	111419723	111420322	600	*	chrX	111419723	111420322	in_vitro	in_vivo	11	10	14.5109091	24.644	-10.133091	c(t = -1.7954	0.0952	0.83731181	Distal Intergenic	ENSSSCG000	FHL1	four and a half LIM domains 1 [Source:VGNC Symbol;Acc:VGNC:88127]
chr9	7353388	7354452	1065	*	chr9	7353388	7354452	in_vitro	in_vivo	11	10	0.90727273	7.864	-6.9567273	c(t = -1.8592	0.0952	0.83731181	Distal Intergenic	ENSSSCG000	STARD10	StAR related lipid transfer domain containing 10 [Source:VGNC Symbol;Acc:VGNC:93530]
chr13	18371847	18373032	1186	*	chr13	18371847	18373032	in_vitro	in_vivo	10	10	4.262	1.119	3.143	c(t = 1.83489	0.0952	0.83731181	Promoter (<=1kb)	ENSSSCG000	CMTM7	CKLF like MARVEL transmembrane domain containing 7 [Source:VGNC Symbol;Acc:VGNC:103928]
chr5	27571456	27572855	1400	*	chr5	27571456	27572855	in_vitro	in_vivo	12	13	3.14666667	0.93538462	2.21128205	c(t = 1.79169	0.0952	0.83731181	Distal Intergenic	NA	NA	NA
chr1	44185474	44186334	861	*	chr1	44185474	44186334	in_vitro	in_vivo	12	11	1.5775	0.39636364	1.18113636	c(t = 1.79211	0.0952	0.83731181	Distal Intergenic	ENSSSCG000	PLN	phospholamban [Source:NCBI gene (formerly Entrezgene);Acc:397421]
chr8	2716786	2717374	589	*	chr8	2716786	2717374	in_vitro	in_vivo	9	11	0.41666667	2.15818182	-1.7415152	c(t = -1.8133	0.0953	0.83731181	Distal Intergenic	ENSSSCG000	HTRA3	HtrA serine peptidase 3 [Source:NCBI gene (formerly Entrezgene);Acc:100337673]
chr17	11410074	11411067	994	*	chr17	11410074	11411067	in_vitro	in_vivo	14	10	2.035	0.407	1.628	c(t = 1.78303	0.0954	0.83731181	Promoter (1-2kb)	ENSSSCG000	VDAC3	voltage dependent anion channel 3 [Source:VGNC Symbol;Acc:VGNC:95562]
chr7	41468134	41469623	1490	*	chr7	41468134	41469623	in_vitro	in_vivo	14	14	6.85214286	21.775	-14.922857	c(t = -1.7662	0.0955	0.83731181	Distal Intergenic	ENSSSCG000	SLC25A27	solute carrier family 25 member 27 [Source:VGNC Symbol;Acc:VGNC:93004]
chr9	34620728	34621250	523	*	chr9	34620728	34621250	in_vitro	in_vivo	10	11	2.451	0	2.451	c(t = 1.86063	0.0957	0.83731181	Distal Intergenic	ENSSSCG000	CASP1	caspase 1, apoptosis-related cysteine peptidase [Source:NCBI gene (formerly Entrezgene);Acc:397319]
chr5	76875793	76876934	1142	*	chr5	76875793	76876934	in_vitro	in_vivo	12	11	1.76583333	0.53909091	1.22674242	c(t = 1.75447	0.0958	0.83731181	Distal Intergenic	ENSSSCG000	SLC38A2	solute carrier family 38 member 2 [Source:VGNC Symbol;Acc:VGNC:93094]
chr8	83677759	83679223	1465	*	chr8	83677759	83679223	in_vitro	in_vivo	10	9	4.453	0.37888889	4.07411111	c(t = 1.85469	0.0958	0.83731181	Distal Intergenic	ENSSSCG000	ANAPC10	anaphase promoting complex subunit 10 [Source:VGNC Symbol;Acc:VGNC:98912]
chr6	62928534	62931535	3002	*	chr6	62928534	62931535	in_vitro	in_vivo	10	11	16.931	36.4672727	-19.536273	c(t = -1.7664	0.0959	0.83731181	Distal Intergenic	ENSSSCG000	ISG15	ISG15 ubiquitin like modifier [Source:NCBI gene (formerly Entrezgene);Acc:100145895]
chr3	44482670	44483422	753	*	chr3	44482670	44483422	in_vitro	in_vivo	11	10	4.72454545	0.5	4.22454545	c(t = 1.83034	0.0959	0.83731181	Distal Intergenic	ENSSSCG00000008097		tubulin tyrosine ligase [Source:NCBI gene (formerly Entrezgene);Acc:445530]
chr7	117242460	117243584	1125	*	chr7	117242460	117243584	in_vitro	in_vivo	13	14	2.65307692	1.17142857	1.48164835	c(t = 1.73917	0.0963	0.83731181	Distal Intergenic	ENSSSCG000	BDKRB2	bradykinin receptor B2 [Source:VGNC Symbol;Acc:VGNC:85794]
chr11	13959329	13960821	1493	*	chr11	13959329	13960821	in_vitro	in_vivo	14	16	4.55642857	1.741875	2.81455357	c(t = 1.73829	0.0964	0.83731181	Distal Intergenic	ENSSSCG000	TRPC4	transient receptor potential cation channel subfamily C member 4 [Source:VGNC Symbol;Acc:VGNC:94462]
chr5	13397169	13397904	736	*	chr5	13397169	13397904	in_vitro	in_vivo	10	11	1.548	0.23	1.318	c(t = 1.81609	0.0967	0.83731181	Distal Intergenic	ENSSSCG000	CKAP4	cytoskeleton associated protein 4 [Source:NCBI gene (formerly Entrezgene);Acc:100523493]
chr16	79877864	79878678	815	*	chr16	79877864	79878678	in_vitro	in_vivo	9	10	33.2555556	59.801	-26.545444	c(t = -1.7641	0.097	0.83731181	Distal Intergenic	ENSSSCG000	TPPP	tubulin polymerization promoting protein [Source:VGNC Symbol;Acc:VGNC:94344]
chr13	109529851	109530793	943	*	chr13	109529851	109530793	in_vitro	in_vivo	11	12	1.32090909	5.5225	-4.2015909	c(t = -1.7889	0.0971	0.83731181	Distal Intergenic	ENSSSCG000	SLC2A2	solute carrier family 2 member 2 [Source:NCBI gene (formerly Entrezgene);Acc:397429]
chr2	36703626	36704312	687	*	chr2	36703626	36704312	in_vitro	in_vivo	9	10	0.34666667	4.405	-4.0583333	c(t = -1.8403	0.0973	0.83731181	Distal Intergenic	ENSSSCG000	CSRP3	cysteine and glycine rich protein 3 [Source:NCBI gene (formerly Entrezgene);Acc:100337687]
chr3	97829681	97831549	1869	*	chr3	97829681	97831549	in_vitro	in_vivo	15	14	2.368	1.17785714	1.19014286	c(t = 1.72194	0.0973	0.83731181	Distal Intergenic	ENSSSCG000	HAAO	3-hydroxyanthranilate 3,4-dioxygenase [Source:VGNC Symbol;Acc:VGNC:88765]
chr7	23207467	23208121	655	*	chr7	23207467	23208121	in_vitro	in_vivo	11	11	1.68	0.27545455	1.40454545	c(t = 1.78909	0.0974	0.83731181	Promoter (<=1kb)	ENSSSCG000	DHX16	DEAH-box helicase 16 [Source:VGNC Symbol;Acc:VGNC:87287]
chr18	55271610	55272585	976	*	chr18	55271610	55272585	in_vitro	in_vivo	10	12	4.291	21.365	-17.074	c(t = -1.8001	0.0975	0.83731181	Distal Intergenic	ENSSSCG000	RARRES2	retinoic acid receptor responder 2 [Source:VGNC Symbol;Acc:VGNC:92098]
chrX	124610	126228	1619	*	chrX	124610	126228	in_vitro	in_vivo	12	9	4.1675	1.97	2.1975	c(t = 1.74289	0.0976	0.83731181	Distal Intergenic	ENSSSCG000	ARSL	arylsulfatase E (chondrodysplasia punctata 1) [Source:NCBI gene (formerly Entrezgene);Acc:100627778]
chr1	216115199	216116457	1259	*	chr1	216115199	216116457	in_vitro	in_vivo	10	12	3.606	1.2725	2.3335	c(t = 1.79533	0.0976	0.83731181	Distal Intergenic	ENSSSCG000	IL33	interleukin 33 [Source:VGNC Symbol;Acc:VGNC:89106]
chr15	31974112	31975637	1526	*	chr15	31974112	31975637	in_vitro	in_vivo	12	12	1.1175	0.23833333	0.87916667	c(t = 1.75334	0.0976	0.83731181	Distal Intergenic	ENSSSCG000	SCTR	secretin receptor [Source:VGNC Symbol;Acc:VGNC:95482]
chr17	29359959	29360197	239	*	chr17	29359959	29360197	in_vitro	in_vivo	9	9	3.16111111	22.0333333	-18.872222	c(t = -1.8655	0.0977	0.83731181	Distal Intergenic	ENSSSCG000	THBD	thrombomodulin [Source:NCBI gene (formerly Entrezgene);Acc:100157642]
chr2	5028938	5030058	1121	*	chr2	5028938	5030058	in_vitro	in_vivo	10	9	18.345	3.63444444	14.7105556	c(t = 1.82821	0.0978	0.83731181	Distal Intergenic	ENSSSCG000	PPP1CA	protein phosphatase 1 catalytic subunit alpha [Source:NCBI gene (formerly Entrezgene);Acc:733611]
chr14	110708953	110709949	997	*	chr14	110708953	110709949	in_vitro	in_vivo	12	11	5.74916667	1.16909091	4.58007576	c(t = 1.76759	0.0978	0.83731181	Distal Intergenic	ENSSSCG000	GOT1	glutamic-oxaloacetic transaminase 1 [Source:NCBI gene (formerly Entrezgene);Acc:396967]
chr14	2804901	2806267	1367	*	chr14	2804901	2806267	in_vitro	in_vivo	13	13	11.1146154	35.1723077	-24.057692	c(t = -1.7491	0.0979	0.83731181	Distal Intergenic	ENSSSCG000	SYK	spleen associated tyrosine kinase [Source:NCBI gene (formerly Entrezgene);Acc:100125540]
chr4	118751357	118752099	743	*	chr4	118751357	118752099	in_vitro	in_vivo	11	11	87.7409091	61.4481818	26.2927273	c(t = 1.75894	0.098	0.83731181	Distal Intergenic	ENSSSCG000	PLPPR5	phospholipid phosphatase related 5 [Source:VGNC Symbol;Acc:VGNC:109449]
chr1	265934267	265935360	1094	*	chr1	265934267	265935360	in_vitro	in_vivo	9	15	4.51777778	1.17266667	3.34511111	c(t = 1.84784	0.0981	0.83731181	Distal Intergenic	ENSSSCG000	RABEPK	Rab9 effector protein with kelch motifs [Source:VGNC Symbol;Acc:VGNC:92045]
chr8	87478129	87480437	2309	*	chr8	87478129	87480437	in_vitro	in_vivo	12	15	2.65666667	0.72933333	1.92733333	c(t = 1.76482	0.0981	0.83731181	Distal Intergenic	NA	NA	NA
chr14	50380670	50383399	2730	*	chr14	50380670	50383399	in_vitro	in_vivo	12	13	2.86083333	1.14307692	1.71775641	c(t = 1.77649	0.0982	0.83731181	Distal Intergenic	ENSSSCG00000037143		ubiquitin conjugating enzyme E2 L3 [Source:NCBI gene (formerly Entrezgene);Acc:100154268]
chr2	150660348	150662998	2651	*	chr2	150660348	150662998	in_vitro	in_vivo	12	12	1.89	0.66333333	1.22666667	c(t = 1.74413	0.0982	0.83731181	Distal Intergenic	ENSSSCG000	ssc-mir-145	ssc-mir-145 [Source:miRBase;Acc:MI0002417]
chr2	403950	405332	1383	*	chr2	403950	405332	in_vitro	in_vivo	10	9	5.374	29.36	-23.986	c(t = -1.8597	0.0983	0.83731181	Distal Intergenic	ENSSSCG000	TMEM80	transmembrane protein 80 [Source:NCBI gene (formerly Entrezgene);Acc:100514388]
chr3	75475892	75476432	541	*	chr3	75475892	75476432	in_vitro	in_vivo	10	10	2.919	0.625	2.294	c(t = 1.79898	0.0984	0.83731181	Distal Intergenic	ENSSSCG000	ACTR2	actin related protein 2 [Source:VGNC Symbol;Acc:VGNC:85051]
chr17	58889179	58890956	1778	*	chr17	58889179	58890956	in_vitro	in_vivo	11	10	87.9218182	94.774	-6.8521818	c(t = -1.7557	0.0985	0.83731181	3' UTR	ENSSSCG000	NPEPL1	aminopeptidase like 1 [Source:VGNC Symbol;Acc:VGNC:95798]
chr11	77808880	77809699	820	*	chr11	77808880	77809699	in_vitro	in_vivo	9	9	24.4888889	52.6611111	-28.172222	c(t = -1.7576	0.0985	0.83731181	Distal Intergenic	ENSSSCG000	ANKRD10	ankyrin repeat domain 10 [Source:VGNC Symbol;Acc:VGNC:85322]
chr4	4373624	4374318	695	*	chr4	4373624	4374318	in_vitro	in_vivo	11	11	4.07818182	1.01363636	3.06454545	c(t = 1.78588	0.0985	0.83731181	Distal Intergenic	ENSSSCG000	ago-02	argonaute RISC catalytic component 2 [Source:NCBI gene (formerly Entrezgene);Acc:100155245]
chr7	100638552	100639200	649	*	chr7	100638552	100639200	in_vitro	in_vivo	10	10	4.225	0.676	3.549	c(t = 1.80857	0.0986	0.83731181	Distal Intergenic	ENSSSCG000	SLIRP	SRA stem-loop interacting RNA binding protein [Source:NCBI gene (formerly Entrezgene);Acc:100037987]
chr6	1401764	1407176	5413	*	chr6	1401764	1407176	in_vitro	in_vivo	11	11	59.24	37.6854545	21.5545455	c(t = 1.73202	0.0987	0.83731181	Distal Intergenic	ENSSSCG000	BANP	BTG3 associated nuclear protein [Source:NCBI gene (formerly Entrezgene);Acc:100622381]
chrX	41488466	41489630	1165	*	chrX	41488466	41489630	in_vitro	in_vivo	10	12	1.997	10.18	-8.183	c(t = -1.7930	0.0987	0.83731181	Distal Intergenic	ENSSSCG000	RGN	regucalcin [Source:VGNC Symbol;Acc:VGNC:92256]
chr3	42456138	42457476	1339	*	chr3	42456138	42457476	in_vitro	in_vivo	9	14	4.14111111	1.72428571	2.4168254	c(t = 1.78584	0.0987	0.83731181	Distal Intergenic	ENSSSCG000	CARD19	caspase recruitment domain family member 19 [Source:VGNC Symbol;Acc:VGNC:86188]
chr12	15191801	15192748	948	*	chr12	15191801	15192748	in_vitro	in_vivo	10	10	1.244	0	1.244	c(t = 1.84150	0.0987	0.83731181	Distal Intergenic	ENSSSCG000	LIMD2	LIM domain containing 2 [Source:VGNC Symbol;Acc:VGNC:89726]
chr18	20653228	20653979	752	*	chr18	20653228	20653979	in_vitro	in_vivo	10	11	0.956	0	0.956	c(t = 1.84158	0.0987	0.83731181	Distal Intergenic	ENSSSCG000	FSCN3	fascin actin-bundling protein 3 [Source:NCBI gene (formerly Entrezgene);Acc:733598]
chr6	65009462	65011693	2232	*	chr6	65009462	65011693	in_vitro	in_vivo	9	10	10.8688889	39.214	-28.345111	c(t = -1.7487	0.0994	0.83731181	Distal Intergenic	ENSSSCG000	ssc-mir-551a	ssc-mir-551a [Source:miRBase;Acc:MI0022153]
chr11	1060139	1061186	1048	*	chr11	1060139	1061186	in_vitro	in_vivo	9	9	94.0255556	97.5955556	-3.57	c(t = -1.7483	0.0996	0.83731181	Distal Intergenic	ENSSSCG000	CRYL1	crystallin lambda 1 [Source:VGNC Symbol;Acc:VGNC:87023]
chr1	183914510	183917681	3172	*	chr1	183914510	183917681	in_vitro	in_vivo	15	13	6.01733333	2.68615385	3.33117949	c(t = 1.72852	0.0997	0.83731181	Distal Intergenic	ENSSSCG000	GMFB	glia maturation factor beta [Source:VGNC Symbol;Acc:VGNC:88509]
chr10	31928693	31929648	956	*	chr10	31928693	31929648	in_vitro	in_vivo	12	12	0.5725	1.84416667	-1.2716667	c(t = -1.7547	0.0997	0.83731181	Distal Intergenic	ENSSSCG000	CCL21	C-C motif chemokine ligand 21 [Source:NCBI gene (formerly Entrezgene);Acc:448797]
chr4	111006543	111007819	1277	*	chr4	111006543	111007819	in_vitro	in_vivo	12	14	7.38083333	2.35928571	5.02154762	c(t = 1.77194	0.0998	0.83731181	Distal Intergenic	ENSSSCG000	TAF13	TATA-box binding protein associated factor 13 [Source:NCBI gene (formerly Entrezgene);Acc:100157095]
chr16	79295297	79302303	7007	*	chr16	79295297	79302303	in_vitro	in_vivo	10	9	9.193	30.2433333	-21.050333	c(t = -1.8471	0.1	0.83731181	Distal Intergenic	ENSSSCG000	TERT	telomerase reverse transcriptase [Source:NCBI gene (formerly Entrezgene);Acc:492280]
chr3	52918642	52919666	1025	*	chr3	52918642	52919666	in_vitro	in_vivo	11	13	3.55727273	0.80076923	2.7565035	c(t = 1.79096	0.1	0.83731181	Distal Intergenic	ENSSSCG000	IL1R2	interleukin 1 receptor type 2 [Source:VGNC Symbol;Acc:VGNC:89093]
chrX	106246537	106246837	301	*	chrX	106246537	106246837	in_vitro	in_vivo	10	9	5.155	0.48333333	4.67166667	c(t = 1.81904	0.1	0.83731181	Distal Intergenic	ENSSSCG000	XPNPEP2	X-prolyl aminopeptidase 2 [Source:VGNC Symbol;Acc:VGNC:95001]
chr5	71672760	71673927	1168	*	chr5	71672760	71673927	in_vitro	in_vivo	13	12	1.23846154	0.42333333	0.81512821	c(t = 1.72525	0.1	0.83731181	Distal Intergenic	ENSSSCG000	LRRK2	leucine rich repeat kinase 2 [Source:NCBI gene (formerly Entrezgene);Acc:780403]
chr13	66421148	66422152	1005	*	chr13	66421148	66422152	in_vitro	in_vivo	10	12	1.473	0.30333333	1.16966667	c(t = 1.79581	0.1	0.83731181	Distal Intergenic	ENSSSCG000	GHRL	ghrelin and obestatin prepropeptide [Source:VGNC Symbol;Acc:VGNC:111192]
chr7	31160689	31161712	1024	*	chr7	31160689	31161712	in_vitro	in_vivo	13	12	0.74615385	0.1325	0.61365385	c(t = 1.74632	0.1	0.83731181	Distal Intergenic	ENSSSCG000	PPARD	peroxisome proliferator activated receptor delta [Source:VGNC Symbol;Acc:VGNC:91683]
chr12	32816761	32817407	647	*	chr12	32816761	32817407	in_vitro	in_vivo	11	10	13.11	38.366	-25.256	c(t = -1.7530	0.101	0.83731181	Distal Intergenic	ENSSSCG000	NOG	noggin [Source:NCBI gene (formerly Entrezgene);Acc:574073]
chr6	109998444	109999497	1054	*	chr6	109998444	109999497	in_vitro	in_vivo	12	14	10.9791667	35.56	-24.580833	c(t = -1.7120	0.101	0.83731181	Distal Intergenic	ENSSSCG000	HRH4	histamine receptor H4 [Source:NCBI gene (formerly Entrezgene);Acc:397224]
chr6	170194906	170197814	2909	*	chr6	170194906	170197814	in_vitro	in_vivo	14	10	8.20357143	33.425	-25.221429	c(t = -1.7585	0.101	0.83731181	Distal Intergenic	ENSSSCG000	ssc-mir-9829	ssc-mir-9829 [Source:miRBase;Acc:MI0031598]
chr1	100288868	100289605	738	*	chr1	100288868	100289605	in_vitro	in_vivo	11	10	4.52909091	18.329	-13.799909	c(t = -1.8119	0.101	0.83731181	Distal Intergenic	ENSSSCG000	MRO	maestro [Source:VGNC Symbol;Acc:VGNC:103127]
chr7	63422310	63422659	350	*	chr7	63422310	63422659	in_vitro	in_vivo	11	9	32.5727273	14.9411111	17.6316162	c(t = 1.72873	0.101	0.83731181	Distal Intergenic	ENSSSCG000	NKX2-1	NK2 homeobox 1 [Source:VGNC Symbol;Acc:VGNC:90767]
chr2	62435107	62436281	1175	*	chr2	62435107	62436281	in_vitro	in_vivo	13	10	9.60846154	2.529	7.07946154	c(t = 1.72867	0.101	0.83731181	Distal Intergenic	ENSSSCG000	BRD4	bromodomain containing 4 [Source:VGNC Symbol;Acc:VGNC:85873]
chr7	31549482	31550858	1377	*	chr7	31549482	31550858	in_vitro	in_vivo	10	13	2.341	1.24615385	1.09484615	c(t = 1.74205	0.101	0.83731181	Promoter (<=1kb)	ENSSSCG000	FKBP5	FKBP prolyl isomerase 5 [Source:VGNC Symbol;Acc:VGNC:88148]
chr14	66287640	66291506	3867	*	chr14	66287640	66291506	in_vitro	in_vivo	13	13	3.57153846	1.18076923	2.39076923	c(t = 1.74246	0.101	0.83731181	Promoter (<=1kb)	ENSSSCG000	EGR2	early growth response 2 [Source:VGNC Symbol;Acc:VGNC:103939]
chr7	112518546	112519247	702	*	chr7	112518546	112519247	in_vitro	in_vivo	10	11	2.565	0.72727273	1.83772727	c(t = 1.79420	0.101	0.83731181	Distal Intergenic	ENSSSCG000	CALM1	calmodulin 1 [Source:NCBI gene (formerly Entrezgene);Acc:100154056]
chr15	72341420	72342645	1226	*	chr15	72341420	72342645	in_vitro	in_vivo	13	13	2.31538462	0.49615385	1.81923077	c(t = 1.76182	0.101	0.83731181	Distal Intergenic	ENSSSCG000	IFIH1	interferon induced with helicase C domain 1 [Source:VGNC Symbol;Acc:VGNC:98042]
chr4	37867922	37868911	990	*	chr4	37867922	37868911	in_vitro	in_vivo	9	15	3.16777778	0.35133333	2.81644444	c(t = 1.84806	0.101	0.83731181	Distal Intergenic	ENSSSCG000	COX6C	COX6C protein [Source:NCBI gene (formerly Entrezgene);Acc:100037989]
chr15	65430945	65432760	1816	*	chr15	65430945	65432760	in_vitro	in_vivo	15	12	1.26866667	0.3025	0.96616667	c(t = 1.72409	0.101	0.83731181	Distal Intergenic	ENSSSCG000	DAPL1	death associated protein like 1 [Source:NCBI gene (formerly Entrezgene);Acc:100157537]
chr3	3114419	3117522	3104	*	chr3	3114419	3117522	in_vitro	in_vivo	11	11	51.8718182	72.6318182	-20.76	c(t = -1.7151	0.102	0.83731181	Distal Intergenic	ENSSSCG000	AP5Z1	adaptor related protein complex 5 subunit zeta 1 [Source:VGNC Symbol;Acc:VGNC:85397]
chrX	82149485	82153336	3852	*	chrX	82149485	82153336	in_vitro	in_vivo	14	17	7.08857143	16.9317647	-9.8431933	c(t = -1.7136	0.102	0.83731181	Distal Intergenic	ENSSSCG000	TNMD	tenomodulin [Source:NCBI gene (formerly Entrezgene);Acc:100048961]
chr2	77729691	77731119	1429	*	chr2	77729691	77731119	in_vitro	in_vivo	10	11	0.917	2.17181818	-1.2548182	c(t = -1.7381	0.102	0.83731181	Distal Intergenic	ENSSSCG00000027907		basigin [Source:VGNC Symbol;Acc:VGNC:99594]
chr14	58119117	58120295	1179	*	chr14	58119117	58120295	in_vitro	in_vivo	11	13	0.73363636	1.99692308	-1.2632867	c(t = -1.7211	0.102	0.83731181	Distal Intergenic	ENSSSCG000	TSNAX	translin associated factor X [Source:NCBI gene (formerly Entrezgene);Acc:100156036]
chr17	41507640	41508067	428	*	chr17	41507640	41508067	in_vitro	in_vivo	9	10	0.18222222	1.475	-1.2927778	c(t = -1.7985	0.102	0.83731181	Distal Intergenic	ENSSSCG000	ADIG	adipogenin [Source:VGNC Symbol;Acc:VGNC:96182]
chrX	35858147	35861047	2901	*	chrX	35858147	35861047	in_vitro	in_vivo	11	13	3.54	1.36692308	2.17307692	c(t = 1.73141	0.102	0.83731181	Distal Intergenic	ENSSSCG000	MID1IP1	MID1 interacting protein 1 [Source:VGNC Symbol;Acc:VGNC:90214]
chr1	158860711	158862808	2098	*	chr1	158860711	158862808	in_vitro	in_vivo	16	12	2.533125	1.08833333	1.44479167	c(t = 1.72709	0.102	0.83731181	Distal Intergenic	ENSSSCG000	RNF152	ring finger protein 152 [Source:VGNC Symbol;Acc:VGNC:92364]
chr5	68000040	68001488	1449	*	chr5	68000040	68001488	in_vitro	in_vivo	13	11	1.74923077	0.50454545	1.24468531	c(t = 1.73644	0.102	0.83731181	Distal Intergenic	ENSSSCG000	RAD52	RAD52 homolog, DNA repair protein [Source:VGNC Symbol;Acc:VGNC:92060]
chr1	186025525	186026828	1304	*	chr1	186025525	186026828	in_vitro	in_vivo	14	13	1.51428571	0.46615385	1.04813187	c(t = 1.71720	0.102	0.83731181	Distal Intergenic	ENSSSCG000	KTN1	kinectin 1 [Source:VGNC Symbol;Acc:VGNC:89608]
chr3	77740492	77741145	654	*	chr3	77740492	77741145	in_vitro	in_vivo	10	9	1.946	0.19888889	1.74711111	c(t = 1.80636	0.102	0.83731181	Distal Intergenic	ENSSSCG000	UGP2	UDP-glucose pyrophosphorylase 2 [Source:VGNC Symbol;Acc:VGNC:98400]
chr3	59401082	59401782	701	*	chr3	59401082	59401782	in_vitro	in_vivo	10	12	1.065	0.19833333	0.86666667	c(t = 1.76741	0.102	0.83731181	Distal Intergenic	ENSSSCG000	MAT2A	methionine adenosyltransferase 2A [Source:NCBI gene (formerly Entrezgene);Acc:100312974]
chr7	53317382	53317844	463	*	chr7	53317382	53317844	in_vitro	in_vivo	12	10	1.13333333	0	1.13333333	c(t = 1.78523	0.102	0.83731181	Distal Intergenic	ENSSSCG000	BLM	BLM RecQ like helicase [Source:VGNC Symbol;Acc:VGNC:96569]
chr3	1422795	1424243	1449	*	chr3	1422795	1424243	in_vitro	in_vivo	9	12	98.1477778	90.3183333	7.82944444	c(t = 1.73512	0.103	0.83731181	Distal Intergenic	ENSSSCG000	MRM2	mitochondrial rRNA methyltransferase 2 [Source:VGNC Symbol;Acc:VGNC:99722]
chr1	2035593	2036549	957	*	chr1	2035593	2036549	in_vitro	in_vivo	9	9	4.72333333	28.1133333	-23.39	c(t = -1.8198	0.103	0.83731181	Distal Intergenic	ENSSSCG000	ssc-mir-9824	ssc-mir-9824 [Source:miRBase;Acc:MI0031591]
chr14	71400887	71401470	584	*	chr14	71400887	71401470	in_vitro	in_vivo	11	10	11.9309091	2.344	9.58690909	c(t = 1.78415	0.103	0.83731181	Distal Intergenic	ENSSSCG000	HNRNPH3	heterous nuclear ribonucleoprotein H3 [Source:VGNC Symbol;Acc:VGNC:88922]
chr4	110694958	110695918	961	*	chr4	110694958	110695918	in_vitro	in_vivo	10	10	4.586	0.918	3.668	c(t = 1.78704	0.103	0.83731181	Promoter (<=1kb)	ENSSSCG000	PSMA5	proteasome 20S subunit alpha 5 [Source:VGNC Symbol;Acc:VGNC:98838]
chr14	125405677	125407409	1733	*	chr14	125405677	125407409	in_vitro	in_vivo	13	11	3.05153846	0.53272727	2.51881119	c(t = 1.74279	0.103	0.83731181	Distal Intergenic	ENSSSCG000	ADRB1	adrenoceptor beta 1 [Source:NCBI gene (formerly Entrezgene);Acc:397355]
chr14	66966202	66967086	885	*	chr14	66966202	66967086	in_vitro	in_vivo	12	13	1.99833333	0.51615385	1.48217949	c(t = 1.74595	0.103	0.83731181	Distal Intergenic	ENSSSCG000	ssc-mir-1296	ssc-mir-1296 [Source:miRBase;Acc:MI0015931]
chr13	30062491	30063365	875	*	chr13	30062491	30063365	in_vitro	in_vivo	10	12	1.004	0	1.004	c(t = 1.81576	0.103	0.83731181	Distal Intergenic	ENSSSCG000	SCAP	SREBF chaperone [Source:VGNC Symbol;Acc:VGNC:92610]
chr8	1915525	1922816	7292	*	chr8	1915525	1922816	in_vitro	in_vivo	9	13	96.8888889	92.9969231	3.89196581	c(t = 1.73126	0.104	0.83731181	Exon (NM_213964/397014, exon 52 of 67)	ENSSSCG000	HTT	huntingtin [Source:VGNC Symbol;Acc:VGNC:89006]
chr5	2531221	2539361	8141	*	chr5	2531221	2539361	in_vitro	in_vivo	13	13	16.0930769	35.7676923	-19.674615	c(t = -1.7032	0.104	0.83731181	Distal Intergenic	ENSSSCG000	PPARA	peroxisome proliferator activated receptor alpha [Source:NCBI gene (formerly Entrezgene);Acc:397239]
chr5	9547161	9548308	1148	*	chr5	9547161	9548308	in_vitro	in_vivo	11	10	4.67272727	23.292	-18.619273	c(t = -1.7906	0.104	0.83731181	Distal Intergenic	ENSSSCG000	KDELR3	KDEL endoplasmic reticulum protein retention receptor 3 [Source:NCBI gene (formerly Entrezgene);Acc:100514523]
chr2	71470469	71472755	2287	*	chr2	71470469	71472755	in_vitro	in_vivo	11	12	34.0536364	17	17.0536364	c(t = 1.73187	0.104	0.83731181	Distal Intergenic	ENSSSCG000	RETN	resistin [Source:NCBI gene (formerly Entrezgene);Acc:396674]
chr6	62817872	62818675	804	*	chr6	62817872	62818675	in_vitro	in_vivo	11	9	2.08454545	0.54666667	1.53787879	c(t = 1.74824	0.104	0.83731181	Distal Intergenic	ENSSSCG000	ISG15	ISG15 ubiquitin like modifier [Source:NCBI gene (formerly Entrezgene);Acc:100145895]
chr1	261586415	261587776	1362	*	chr1	261586415	261587776	in_vitro	in_vivo	10	11	2.853	0.41363636	2.43936364	c(t = 1.79019	0.104	0.83731181	Promoter (<=1kb)	ENSSSCG00000005518		glycoprotein alpha-galactosyltransferase 1 (inactive) [Source:NCBI gene (formerly Entrezgene);Acc:396733]
chr10	17495675	17497194	1520	*	chr10	17495675	17497194	in_vitro	in_vivo	12	13	3.15416667	0.31615385	2.83801282	c(t = 1.77200	0.104	0.83731181	Distal Intergenic	ENSSSCG000	DESI2	desumoylating isopeptidase 2 [Source:VGNC Symbol;Acc:VGNC:96037]
chr15	45632881	45634107	1227	*	chr15	45632881	45634107	in_vitro	in_vivo	13	10	1.28692308	0.308	0.97892308	c(t = 1.72790	0.104	0.83731181	Distal Intergenic	ENSSSCG000	CASP3	caspase 3 [Source:NCBI gene (formerly Entrezgene);Acc:397244]
chr13	189714642	189716452	1811	*	chr13	189714642	189716452	in_vitro	in_vivo	12	13	2.1225	0.17461538	1.94788462	c(t = 1.76175	0.104	0.83731181	Promoter (<=1kb)	ENSSSCG000	APP	amyloid beta protein [Source:VGNC Symbol;Acc:VGNC:103902]
chr8	86879182	86882738	3557	*	chr8	86879182	86882738	in_vitro	in_vivo	11	15	4.33454545	0.17133333	4.16321212	c(t = 1.78656	0.104	0.83731181	Distal Intergenic	ENSSSCG000	CLGN	calmegin [Source:VGNC Symbol;Acc:VGNC:86759]
chrX	35788444	35791349	2906	*	chrX	35788444	35791349	in_vitro	in_vivo	12	10	11.8475	26.714	-14.8665	c(t = -1.7191	0.105	0.83731181	Distal Intergenic	ENSSSCG000	MID1IP1	MID1 interacting protein 1 [Source:VGNC Symbol;Acc:VGNC:90214]
chr6	58307108	58307861	754	*	chr6	58307108	58307861	in_vitro	in_vivo	10	12	2.131	4.49916667	-2.3681667	c(t = -1.6977	0.105	0.83731181	Promoter (<=1kb)	ENSSSCG000	HAS1	hyaluronan synthase 1 [Source:NCBI gene (formerly Entrezgene);Acc:100191062]
chr9	10430489	10435279	4791	*	chr9	10430489	10435279	in_vitro	in_vivo	13	15	5.79615385	1.48933333	4.30682051	c(t = 1.74587	0.105	0.83731181	Distal Intergenic	ENSSSCG000	DGAT2	diacylglycerol O-acyltransferase 2 [Source:NCBI gene (formerly Entrezgene);Acc:100294675]
chr3	118767918	118768625	708	*	chr3	118767918	118768625	in_vitro	in_vivo	9	12	0.13333333	1.31916667	-1.1858333	c(t = -1.7529	0.105	0.83731181	Distal Intergenic	ENSSSCG000	SDC1	syndecan 1 [Source:VGNC Symbol;Acc:VGNC:92654]
chr15	43787167	43788743	1577	*	chr15	43787167	43788743	in_vitro	in_vivo	12	11	0.1175	0.76272727	-0.6452273	c(t = -1.7519	0.105	0.83731181	Distal Intergenic	ENSSSCG000	CLDN22	claudin 22 [Source:NCBI gene (formerly Entrezgene);Acc:100294683]
chr10	25471431	25472763	1333	*	chr10	25471431	25472763	in_vitro	in_vivo	13	12	1.74	0.53833333	1.20166667	c(t = 1.71930	0.105	0.83731181	Distal Intergenic	ENSSSCG000	HABP4	hyaluronan binding protein 4 [Source:NCBI gene (formerly Entrezgene);Acc:100512302]
chr15	78773073	78773732	660	*	chr15	78773073	78773732	in_vitro	in_vivo	11	9	3.23818182	0.52888889	2.70929293	c(t = 1.74778	0.105	0.83731181	Distal Intergenic	ENSSSCG000	PDK1	pyruvate dehydrogenase kinase 1 [Source:VGNC Symbol;Acc:VGNC:98180]
chr3	69235860	69236713	854	*	chr3	69235860	69236713	in_vitro	in_vivo	9	9	5.59333333	0.42333333	5.17	c(t = 1.82412	0.105	0.83731181	Promoter (1-2kb)	ENSSSCG000	TPRKB	TP53RK binding protein [Source:VGNC Symbol;Acc:VGNC:94350]
chr6	136019503	136020553	1051	*	chr6	136019503	136020553	in_vitro	in_vivo	11	14	2.05909091	0.26428571	1.79480519	c(t = 1.76116	0.105	0.83731181	Distal Intergenic	ENSSSCG000	PIGK	phosphatidylinositol glycan anchor biosynthesis class K [Source:NCBI gene (formerly Entrezgene);Acc:606750]
chr4	95380564	95381511	948	*	chr4	95380564	95381511	in_vitro	in_vivo	9	10	0.65888889	0	0.65888889	c(t = 1.82543	0.105	0.83731181	Promoter (<=1kb)	ENSSSCG000	IL6R	interleukin 6 receptor [Source:VGNC Symbol;Acc:VGNC:89112]
chr9	137902875	137903559	685	*	chr9	137902875	137903559	in_vitro	in_vivo	10	9	11	41.4833333	-30.483333	c(t = -1.7271	0.106	0.83731181	Distal Intergenic	ENSSSCG000	GRB10	growth factor receptor bound protein 10 [Source:VGNC Symbol;Acc:VGNC:88662]
chr2	40458273	40459582	1310	*	chr2	40458273	40459582	in_vitro	in_vivo	9	12	1.20555556	3.935	-2.7294444	c(t = -1.7011	0.106	0.83731181	Distal Intergenic	ENSSSCG000	CSRP3	cysteine and glycine rich protein 3 [Source:NCBI gene (formerly Entrezgene);Acc:100337687]
chr7	75720739	75722450	1712	*	chr7	75720739	75722450	in_vitro	in_vivo	10	11	6.405	2.8	3.605	c(t = 1.74569	0.106	0.83731181	Distal Intergenic	ENSSSCG000	CMTM5	CKLF like MARVEL transmembrane domain containing 5 [Source:NCBI gene (formerly Entrezgene);Acc:100514659]
chr11	68052033	68053789	1757	*	chr11	68052033	68053789	in_vitro	in_vivo	11	15	1.87454545	0.72666667	1.14787879	c(t = 1.73932	0.106	0.83731181	Distal Intergenic	ENSSSCG000	SLC15A1	solute carrier family 15 member 1 [Source:NCBI gene (formerly Entrezgene);Acc:397624]
chr1	249905208	249905824	617	*	chr1	249905208	249905824	in_vitro	in_vivo	9	10	3.37111111	0.577	2.79411111	c(t = 1.78878	0.106	0.83731181	Distal Intergenic	ENSSSCG000	ssc-mir-32	ssc-mir-32 [Source:miRBase;Acc:MI0002443]
chr13	134405918	134406641	724	*	chr13	134405918	134406641	in_vitro	in_vivo	13	12	3.35846154	0.20333333	3.15512821	c(t = 1.74346	0.106	0.83731181	Distal Intergenic	ENSSSCG000	RUBCN	rubicon autophagy regulator [Source:VGNC Symbol;Acc:VGNC:92509]
chr3	11052516	11053085	570	*	chr3	11052516	11053085	in_vitro	in_vivo	13	12	24.0769231	49.3866667	-25.309744	c(t = -1.6790	0.107	0.83731181	Promoter (<=1kb)	ENSSSCG000	CLDN4	claudin 4 [Source:VGNC Symbol;Acc:VGNC:100383]
chr13	203619487	203620128	642	*	chr13	203619487	203620128	in_vitro	in_vivo	10	9	15.954	42.6077778	-26.653778	c(t = -1.7730	0.107	0.83731181	Distal Intergenic	ENSSSCG000	ssc-mir-9805	ssc-mir-9805 [Source:miRBase;Acc:MI0031569]
chr12	1783700	1786995	3296	*	chr12	1783700	1786995	in_vitro	in_vivo	9	14	15.9855556	35.7721429	-19.786587	c(t = -1.6873	0.107	0.83731181	Distal Intergenic	NA	NA	NA
chr6	97308852	97310375	1524	*	chr6	97308852	97310375	in_vitro	in_vivo	10	14	0.655	2.15142857	-1.4964286	c(t = -1.7113	0.107	0.83731181	Distal Intergenic	ENSSSCG000	CIDEA	cell death inducing DFFA like effector a [Source:NCBI gene (formerly Entrezgene);Acc:100127171]
chr5	2947355	2948317	963	*	chr5	2947355	2948317	in_vitro	in_vivo	11	11	2.65727273	1.11363636	1.54363636	c(t = 1.72608	0.107	0.83731181	Distal Intergenic	ENSSSCG000	PPARA	peroxisome proliferator activated receptor alpha [Source:NCBI gene (formerly Entrezgene);Acc:397239]
chr3	8494167	8496577	2411	*	chr3	8494167	8496577	in_vitro	in_vivo	11	14	8.60636364	22.8692857	-14.262922	c(t = -1.7027	0.108	0.83731181	Distal Intergenic	ENSSSCG000	MOSPD3	motile sperm domain containing 3 [Source:NCBI gene (formerly Entrezgene);Acc:100337680]
chr7	2508208	2509226	1019	*	chr7	2508208	2509226	in_vitro	in_vivo	14	10	32.2092857	12.055	20.1542857	c(t = 1.67804	0.108	0.83731181	Distal Intergenic	ENSSSCG000	ECI2	enoyl-CoA delta isomerase 2 [Source:VGNC Symbol;Acc:VGNC:87532]
chr6	67445309	67447663	2355	*	chr6	67445309	67447663	in_vitro	in_vivo	10	10	4.884	0.945	3.939	c(t = 1.75128	0.108	0.83731181	Distal Intergenic	ENSSSCG000	ICMT	isoprenylcysteine carboxyl methyltransferase [Source:NCBI gene (formerly Entrezgene);Acc:100514410]
chr13	39571919	39572976	1058	*	chr13	39571919	39572976	in_vitro	in_vivo	10	14	2.422	0.68214286	1.73985714	c(t = 1.73027	0.108	0.83731181	Distal Intergenic	ENSSSCG000	ARF4	ADP ribosylation factor 4 [Source:VGNC Symbol;Acc:VGNC:85446]
chr13	109029108	109030588	1481	*	chr13	109029108	109030588	in_vitro	in_vivo	11	11	1.70636364	0.48	1.22636364	c(t = 1.70919	0.108	0.83731181	Distal Intergenic	ENSSSCG000	CLDN11	claudin 11 [Source:NCBI gene (formerly Entrezgene);Acc:100302016]
chr5	39743761	39744909	1149	*	chr5	39743761	39744909	in_vitro	in_vivo	13	12	2.28846154	0.43166667	1.85679487	c(t = 1.71935	0.108	0.83731181	Distal Intergenic	ENSSSCG000	NAP1L1	nucleosome assembly protein 1 like 1 [Source:VGNC Symbol;Acc:VGNC:90576]
chr4	76914027	76914510	484	*	chr4	76914027	76914510	in_vitro	in_vivo	9	9	1.37888889	0	1.37888889	c(t = 1.80980	0.108	0.83731181	Distal Intergenic	ENSSSCG000	MRPL15	mitochondrial ribosomal protein L15 [Source:VGNC Symbol;Acc:VGNC:98820]
chr8	878824	912677	33854	*	chr8	878824	912677	in_vitro	in_vivo	14	15	34.6121429	50.3406667	-15.728524	c(t = -1.6595	0.109	0.83731181	3' UTR	ENSSSCG000	LETM1	leucine zipper and EF-hand containing transmembrane protein 1 [Source:VGNC Symbol;Acc:VGNC:89687]
chr5	10744508	10745220	713	*	chr5	10744508	10745220	in_vitro	in_vivo	11	12	2.71090909	20.3433333	-17.632424	c(t = -1.7306	0.109	0.83731181	Distal Intergenic	ENSSSCG000	ssc-mir-9819	ssc-mir-9819 [Source:miRBase;Acc:MI0031586]
chr3	129798818	129801426	2609	*	chr3	129798818	129801426	in_vitro	in_vivo	13	15	0.79307692	2.10466667	-1.3115897	c(t = -1.6897	0.109	0.83731181	Distal Intergenic	ENSSSCG000	RSAD2	radical S-adenosyl methionine domain containing 2 [Source:NCBI gene (formerly Entrezgene);Acc:396752]
chr7	92811499	92813662	2164	*	chr7	92811499	92813662	in_vitro	in_vivo	12	13	2.52666667	0.83769231	1.68897436	c(t = 1.69676	0.109	0.83731181	Distal Intergenic	ENSSSCG000	GALNT16	polypeptide N-acetylgalactosaminyltransferase 16 [Source:VGNC Symbol;Acc:VGNC:88332]
chr7	8093395	8094830	1436	*	chr7	8093395	8094830	in_vitro	in_vivo	14	12	2.46214286	0.7425	1.71964286	c(t = 1.69448	0.109	0.83731181	Distal Intergenic	ENSSSCG000	ssc-mir-7136	ssc-mir-7136 [Source:miRBase;Acc:MI0023569]
chr13	71646123	71647004	882	*	chr13	71646123	71647004	in_vitro	in_vivo	11	15	1.77636364	0.648	1.12836364	c(t = 1.69724	0.109	0.83731181	Distal Intergenic	ENSSSCG000	GP9	glycoprotein IX platelet [Source:VGNC Symbol;Acc:VGNC:88567]
chr3	112923567	112924574	1008	*	chr3	112923567	112924574	in_vitro	in_vivo	10	13	1.668	0.29615385	1.37184615	c(t = 1.73260	0.109	0.83731181	Promoter (<=1kb)	ENSSSCG000	RAB10	RAB10, member RAS oncogene family [Source:NCBI gene (formerly Entrezgene);Acc:100526104]
chr2	75878830	75879514	685	*	chr2	75878830	75879514	in_vitro	in_vivo	10	9	5.052	0	5.052	c(t = 1.78142	0.109	0.83731181	Promoter (<=1kb)	ENSSSCG000	SGTA	small glutamine rich tetratricopeptide repeat co-chaperone alpha [Source:VGNC Symbol;Acc:VGNC:92803]
chr13	95723859	95724455	597	*	chr13	95723859	95724455	in_vitro	in_vivo	11	12	2.04	0	2.04	c(t = 1.75769	0.109	0.83731181	Promoter (<=1kb)	ENSSSCG000	SLC33A1	solute carrier family 33 member 1 [Source:VGNC Symbol;Acc:VGNC:103074]
chr12	1831832	1832939	1108	*	chr12	1831832	1832939	in_vitro	in_vivo	10	10	36.108	63.022	-26.914	c(t = -1.6843	0.11	0.83731181	Distal Intergenic	NA	NA	NA
chr11	76212570	76214054	1485	*	chr11	76212570	76214054	in_vitro	in_vivo	10	12	2.269	10.0575	-7.7885	c(t = -1.7351	0.11	0.83731181	Distal Intergenic	ENSSSCG000	TNFSF13B	TNF superfamily member 13b [Source:VGNC Symbol;Acc:VGNC:94269]
chr6	63656955	63658178	1224	*	chr6	63656955	63658178	in_vitro	in_vivo	9	11	9.47666667	1.68545455	7.79121212	c(t = 1.78254	0.11	0.83731181	Distal Intergenic	ENSSSCG000	TAS1R3	taste 1 receptor member 3 [Source:NCBI gene (formerly Entrezgene);Acc:100127440]
chr6	54230949	54232043	1095	*	chr6	54230949	54232043	in_vitro	in_vivo	11	10	5.83909091	1.193	4.64609091	c(t = 1.72465	0.11	0.83731181	Promoter (<=1kb)	ENSSSCG000	FTL	ferritin light chain [Source:NCBI gene (formerly Entrezgene);Acc:397035]
chr15	60053707	60055165	1459	*	chr15	60053707	60055165	in_vitro	in_vivo	12	9	3.2775	0.53111111	2.74638889	c(t = 1.72708	0.11	0.83731181	Distal Intergenic	ENSSSCG000	PRPF40A	pre-mRNA processing factor 40 homolog A [Source:VGNC Symbol;Acc:VGNC:98222]
chr1	182752958	182754479	1522	*	chr1	182752958	182754479	in_vitro	in_vivo	13	13	1.94153846	0.46846154	1.47307692	c(t = 1.71449	0.11	0.83731181	Distal Intergenic	ENSSSCG000	GNPNAT1	glucosamine-phosphate N-acetyltransferase 1 [Source:VGNC Symbol;Acc:VGNC:103102]
chr4	10182208	10183341	1134	*	chr4	10182208	10183341	in_vitro	in_vivo	9	9	1.81	0.33444444	1.47555556	c(t = 1.76998	0.11	0.83731181	Distal Intergenic	ENSSSCG00000005959		CYFIP related Rac1 interactor B [Source:VGNC Symbol;Acc:VGNC:97956]
chr11	21872178	21872950	773	*	chr11	21872178	21872950	in_vitro	in_vivo	10	12	1.64	0.2875	1.3525	c(t = 1.74755	0.11	0.83731181	Distal Intergenic	ENSSSCG000	TPT1	tumor protein, translationally-controlled 1 [Source:NCBI gene (formerly Entrezgene);Acc:397664]
chr6	170656549	170657433	885	*	chr6	170656549	170657433	in_vitro	in_vivo	12	10	4.09	0.274	3.816	c(t = 1.73352	0.11	0.83731181	Distal Intergenic	ENSSSCG000	SMAP2	small ArfGAP2 [Source:VGNC Symbol;Acc:VGNC:93224]
chr13	99785597	99786558	962	*	chr13	99785597	99786558	in_vitro	in_vivo	12	11	2.14666667	0.17181818	1.97484848	c(t = 1.73274	0.11	0.83731181	Distal Intergenic	ENSSSCG000	IL12A	interleukin 12A [Source:NCBI gene (formerly Entrezgene);Acc:397053]
chr6	60934919	60936218	1300	*	chr6	60934919	60936218	in_vitro	in_vivo	10	14	25.934	44.0671429	-18.133143	c(t = -1.6611	0.111	0.83731181	Distal Intergenic	ENSSSCG000	GALP	galanin like peptide [Source:NCBI gene (formerly Entrezgene);Acc:396772]
chr11	77828961	77832698	3738	*	chr11	77828961	77832698	in_vitro	in_vivo	14	12	11.8485714	32.1033333	-20.254762	c(t = -1.6895	0.111	0.83731181	Distal Intergenic	ENSSSCG000	ANKRD10	ankyrin repeat domain 10 [Source:VGNC Symbol;Acc:VGNC:85322]
chr14	112280113	112287673	7561	*	chr14	112280113	112287673	in_vitro	in_vivo	15	16	15.114	7.236875	7.877125	c(t = 1.65273	0.111	0.83731181	Promoter (<=1kb)	ENSSSCG000	LBX1	ladybird homeobox 1 [Source:VGNC Symbol;Acc:VGNC:89652]
chr7	828315	846360	18046	*	chr7	828315	846360	in_vitro	in_vivo	13	16	12.07	6.81125	5.25875	c(t = 1.66443	0.111	0.83731181	Distal Intergenic	ENSSSCG000	IRF4	interferon regulatory factor 4 [Source:VGNC Symbol;Acc:VGNC:89207]
chr6	63076530	63079307	2778	*	chr6	63076530	63079307	in_vitro	in_vivo	12	12	13.5925	3.8825	9.71	c(t = 1.72301	0.111	0.83731181	Distal Intergenic	ENSSSCG000	ISG15	ISG15 ubiquitin like modifier [Source:NCBI gene (formerly Entrezgene);Acc:100145895]
chr1	264899699	264901707	2009	*	chr1	264899699	264901707	in_vitro	in_vivo	10	11	1.282	2.85818182	-1.5761818	c(t = -1.6790	0.111	0.83731181	Promoter (<=1kb)	ENSSSCG000	LHX2	LIM homeobox 2 [Source:NCBI gene (formerly Entrezgene);Acc:100156063]
chr18	23530888	23532336	1449	*	chr18	23530888	23532336	in_vitro	in_vivo	12	14	0.475	2.43714286	-1.9621429	c(t = -1.6985	0.111	0.83731181	Distal Intergenic	ENSSSCG000	SPAM1	sperm adhesion molecule 1 [Source:NCBI gene (formerly Entrezgene);Acc:397074]
chr1	130036392	130036875	484	*	chr1	130036392	130036875	in_vitro	in_vivo	9	11	0	1.16	-1.16	c(t = -1.7505	0.111	0.83731181	Distal Intergenic	ENSSSCG000	DLL4	delta like canonical Notch ligand 4 [Source:NCBI gene (formerly Entrezgene);Acc:100152163]
chr6	33729523	33734724	5202	*	chr6	33729523	33734724	in_vitro	in_vivo	12	13	2.61583333	1.14846154	1.46737179	c(t = 1.68671	0.111	0.83731181	Distal Intergenic	ENSSSCG000	NOD2	nucleotide binding oligomerization domain containing 2 [Source:NCBI gene (formerly Entrezgene);Acc:100125838]
chr3	117987100	117989331	2232	*	chr3	117987100	117989331	in_vitro	in_vivo	9	15	3.31888889	1.01866667	2.30022222	c(t = 1.74968	0.111	0.83731181	Promoter (<=1kb)	ENSSSCG000	SDC1	syndecan 1 [Source:VGNC Symbol;Acc:VGNC:92654]
chr1	57825922	57826427	506	*	chr1	57825922	57826427	in_vitro	in_vivo	9	9	3.33888889	0.57	2.76888889	c(t = 1.74563	0.111	0.83731181	Distal Intergenic	NA	NA	NA
chr17	46135795	46136454	660	*	chr17	46135795	46136454	in_vitro	in_vivo	10	9	1.397	0.42555556	0.97144444	c(t = 1.69945	0.111	0.83731181	Distal Intergenic	ENSSSCG000	SRSF6	serine and arginine rich splicing factor 6 [Source:NCBI gene (formerly Entrezgene);Acc:768102]
chr1	186429429	186430242	814	*	chr1	186429429	186430242	in_vitro	in_vivo	14	12	1.42571429	0.15166667	1.27404762	c(t = 1.69820	0.111	0.83731181	Distal Intergenic	ENSSSCG000	ARMH4	armadillo like helical domain containing 4 [Source:VGNC Symbol;Acc:VGNC:85535]
chr2	96083763	96084601	839	*	chr2	96083763	96084601	in_vitro	in_vivo	11	9	0.77181818	3.51777778	-2.7459596	c(t = -1.7581	0.112	0.83731181	Distal Intergenic	NA	NA	NA
chr1	54400289	54401392	1104	*	chr1	54400289	54401392	in_vitro	in_vivo	12	9	10.5975	2.99666667	7.60083333	c(t = 1.70051	0.112	0.83731181	Distal Intergenic	ENSSSCG000	CGA	glycoprotein hormones, alpha polypeptide [Source:VGNC Symbol;Acc:VGNC:86613]
chr6	169221083	169222257	1175	*	chr6	169221083	169222257	in_vitro	in_vivo	11	9	0.51	2.94666667	-2.4366667	c(t = -1.7675	0.112	0.83731181	Distal Intergenic	ENSSSCG000	GUCA2B	guanylate cyclase activator 2B [Source:VGNC Symbol;Acc:VGNC:88748]
chr11	1207538	1208575	1038	*	chr11	1207538	1208575	in_vitro	in_vivo	11	16	1.12363636	2.825625	-1.7019886	c(t = -1.6605	0.112	0.83731181	Distal Intergenic	ENSSSCG000	LATS2	large tumor suppressor kinase 2 [Source:VGNC Symbol;Acc:VGNC:89649]
chr1	240717968	240718974	1007	*	chr1	240717968	240718974	in_vitro	in_vivo	10	9	5.152	1.86444444	3.28755556	c(t = 1.71668	0.112	0.83731181	Distal Intergenic	ENSSSCG000	TGFBR1	transforming growth factor beta receptor 1 [Source:VGNC Symbol;Acc:VGNC:98368]
chr14	108303791	108305408	1618	*	chr14	108303791	108305408	in_vitro	in_vivo	14	14	2.72642857	0.73714286	1.98928571	c(t = 1.69171	0.112	0.83731181	Distal Intergenic	NA	NA	NA
chr1	113288955	113290096	1142	*	chr1	113288955	113290096	in_vitro	in_vivo	12	10	3.23833333	0.693	2.54533333	c(t = 1.70509	0.112	0.83731181	Promoter (<=1kb)	ENSSSCG000	ADAM10	ADAM metallopeptidase domain 10 [Source:NCBI gene (formerly Entrezgene);Acc:397345]
chr7	112752700	112753589	890	*	chr7	112752700	112753589	in_vitro	in_vivo	12	11	2.48833333	0.10818182	2.38015152	c(t = 1.72781	0.112	0.83731181	Distal Intergenic	ENSSSCG000	CALM1	calmodulin 1 [Source:NCBI gene (formerly Entrezgene);Acc:100154056]
chr5	69771614	69772096	483	*	chr5	69771614	69772096	in_vitro	in_vivo	10	9	1.603	0	1.603	c(t = 1.75951	0.112	0.83731181	Distal Intergenic	ENSSSCG000	BID	BH3 interacting domain death agonist [Source:VGNC Symbol;Acc:VGNC:85824]
chr9	767448	771882	4435	*	chr9	767448	771882	in_vitro	in_vivo	11	15	30.9663636	48.5686667	-17.602303	c(t = -1.6450	0.113	0.83731181	Distal Intergenic	NA	NA	NA
chrX	86533552	86534969	1418	*	chrX	86533552	86534969	in_vitro	in_vivo	12	12	3.4325	15.43	-11.9975	c(t = -1.7091	0.113	0.83731181	Distal Intergenic	ENSSSCG000	SERPINA7	serpin family A member 7 [Source:VGNC Symbol;Acc:VGNC:109415]
chr1	261682797	261684641	1845	*	chr1	261682797	261684641	in_vitro	in_vivo	11	11	6.57363636	3.94545455	2.62818182	c(t = 1.66221	0.113	0.83731181	Distal Intergenic	ENSSSCG00000005518		glycoprotein alpha-galactosyltransferase 1 (inactive) [Source:NCBI gene (formerly Entrezgene);Acc:396733]
chr10	24669284	24670675	1392	*	chr10	24669284	24670675	in_vitro	in_vivo	9	12	4.69777778	1.4875	3.21027778	c(t = 1.71869	0.113	0.83731181	Distal Intergenic	ENSSSCG000	ADIPOR1	adiponectin receptor 1 [Source:NCBI gene (formerly Entrezgene);Acc:404695]
chr1	137855508	137856731	1224	*	chr1	137855508	137856731	in_vitro	in_vivo	12	12	2.87833333	1.43333333	1.445	c(t = 1.65684	0.113	0.83731181	Distal Intergenic	ENSSSCG000	IGF1R	insulin like growth factor 1 receptor [Source:VGNC Symbol;Acc:VGNC:109458]
chr15	91604314	91605456	1143	*	chr15	91604314	91605456	in_vitro	in_vivo	10	11	4.373	1.11090909	3.26209091	c(t = 1.71851	0.113	0.83731181	Promoter (<=1kb)	ENSSSCG000	ITGAV	integrin subunit alpha V [Source:VGNC Symbol;Acc:VGNC:96380]
chr14	108886269	108887967	1699	*	chr14	108886269	108887967	in_vitro	in_vivo	10	12	0.141	0.84083333	-0.6998333	c(t = -1.6945	0.113	0.83731181	Distal Intergenic	ENSSSCG000	HOGA1	4-hydroxy-2-oxoglutarate aldolase 1 [Source:NCBI gene (formerly Entrezgene);Acc:100153961]
chr12	38447112	38450960	3849	*	chr12	38447112	38450960	in_vitro	in_vivo	10	12	2.617	0.77916667	1.83783333	c(t = 1.72411	0.113	0.83731181	Distal Intergenic	ENSSSCG000	MRM1	mitochondrial rRNA methyltransferase 1 [Source:NCBI gene (formerly Entrezgene);Acc:100513493]
chr15	137739878	137746798	6921	*	chr15	137739878	137746798	in_vitro	in_vivo	13	9	97.8769231	94.2477778	3.6291453	c(t = 1.67894	0.114	0.83731181	Distal Intergenic	ENSSSCG000	RAMP1	receptor activity modifying protein 1 [Source:NCBI gene (formerly Entrezgene);Acc:397401]
chr1	270024487	270025424	938	*	chr1	270024487	270025424	in_vitro	in_vivo	10	10	94.775	89.526	5.249	c(t = 1.68683	0.114	0.83731181	Distal Intergenic	ENSSSCG000	TOR1A	torsin family 1 member A [Source:NCBI gene (formerly Entrezgene);Acc:100125953]
chr15	108977560	108979429	1870	*	chr15	108977560	108979429	in_vitro	in_vivo	10	13	4.367	2.07230769	2.29469231	c(t = 1.65963	0.114	0.83731181	Distal Intergenic	ENSSSCG000	EEF1B2	eukaryotic translation elongation factor 1 beta 2 [Source:NCBI gene (formerly Entrezgene);Acc:100154920]
chr15	139263540	139264259	720	*	chr15	139263540	139264259	in_vitro	in_vivo	10	11	3.552	1.16272727	2.38927273	c(t = 1.66494	0.114	0.83731181	Distal Intergenic	ENSSSCG000	ssc-mir-149	ssc-mir-149 [Source:miRBase;Acc:MI0016620]
chr12	39739482	39740013	532	*	chr12	39739482	39740013	in_vitro	in_vivo	9	9	2.74888889	1.04111111	1.70777778	c(t = 1.68468	0.114	0.83731181	Distal Intergenic	ENSSSCG000	TAF15	TATA-box binding protein associated factor 15 [Source:NCBI gene (formerly Entrezgene);Acc:100518332]
chr5	6436542	6438239	1698	*	chr5	6436542	6438239	in_vitro	in_vivo	11	10	3.72	1.026	2.694	c(t = 1.66435	0.114	0.83731181	Distal Intergenic	ENSSSCG000	CYP2D6	cytochrome P450 family 2 subfamily D member 6 [Source:NCBI gene (formerly Entrezgene);Acc:397687]
chr5	88770132	88771231	1100	*	chr5	88770132	88771231	in_vitro	in_vivo	13	12	2.10461538	0.47583333	1.62878205	c(t = 1.69287	0.114	0.83731181	Distal Intergenic	ENSSSCG000	ssc-mir-331	ssc-mir-331 [Source:miRBase;Acc:MI0013142]
chr18	6869541	6871020	1480	*	chr18	6869541	6871020	in_vitro	in_vivo	11	9	1.79	0.39666667	1.39333333	c(t = 1.67920	0.114	0.83731181	Distal Intergenic	ENSSSCG000	GSTK1	glutathione S-transferase kappa 1 [Source:NCBI gene (formerly Entrezgene);Acc:100521480]
chr1	77809767	77810781	1015	*	chr1	77809767	77810781	in_vitro	in_vivo	13	12	1.85461538	0.26666667	1.58794872	c(t = 1.69760	0.114	0.83731181	Distal Intergenic	ENSSSCG000	FYN	FYN proto-onco, Src family tyrosine kinase [Source:VGNC Symbol;Acc:VGNC:88276]
chr11	8342903	8343505	603	*	chr11	8342903	8343505	in_vitro	in_vivo	10	11	1.927	0.20636364	1.72063636	c(t = 1.73429	0.114	0.83731181	Distal Intergenic	ENSSSCG000	HSPH1	heat shock protein family H (Hsp110) member 1 [Source:NCBI gene (formerly Entrezgene);Acc:100048931]
chr8	43745452	43746152	701	*	chr8	43745452	43746152	in_vitro	in_vivo	12	14	1.03166667	0.16642857	0.8652381	c(t = 1.68774	0.114	0.83731181	Distal Intergenic	ENSSSCG000	MSMO1	methylsterol monooxygenase 1 [Source:VGNC Symbol;Acc:VGNC:98941]
chr18	14880634	14881184	551	*	chr18	14880634	14881184	in_vitro	in_vivo	10	11	1.334	0	1.334	c(t = 1.74752	0.114	0.83731181	Distal Intergenic	ENSSSCG000	AKR1B1	aldo-keto reductase family 1 member B [Source:VGNC Symbol;Acc:VGNC:109454]
chr18	52899435	52900656	1222	*	chr18	52899435	52900656	in_vitro	in_vivo	10	10	7.177	24.01	-16.833	c(t = -1.7102	0.115	0.83731181	3' UTR	ENSSSCG000	INHBA	inhibin subunit beta A [Source:NCBI gene (formerly Entrezgene);Acc:397093]
chr11	3270599	3271713	1115	*	chr11	3270599	3271713	in_vitro	in_vivo	10	14	35.635	19.0807143	16.5542857	c(t = 1.68938	0.115	0.83731181	Distal Intergenic	ENSSSCG000	ssc-mir-9814	ssc-mir-9814 [Source:miRBase;Acc:MI0031581]
chr18	51924072	51924784	713	*	chr18	51924072	51924784	in_vitro	in_vivo	10	9	4.016	1.47888889	2.53711111	c(t = 1.68622	0.115	0.83731181	Distal Intergenic	ENSSSCG000	POLD2	DNA polymerase delta 2, accessory subunit [Source:VGNC Symbol;Acc:VGNC:91628]
chr15	12952987	12954375	1389	*	chr15	12952987	12954375	in_vitro	in_vivo	11	12	2.14181818	0.71416667	1.42765152	c(t = 1.65742	0.115	0.83731181	Distal Intergenic	ENSSSCG000	HNMT	histamine N-methyltransferase [Source:NCBI gene (formerly Entrezgene);Acc:100624677]
chr9	103962980	103964626	1647	*	chr9	103962980	103964626	in_vitro	in_vivo	12	13	2.48833333	0.50846154	1.97987179	c(t = 1.70012	0.115	0.83731181	Distal Intergenic	ENSSSCG000	SLC26A5	solute carrier family 26 member 5 [Source:NCBI gene (formerly Entrezgene);Acc:100190993]
chr18	55494976	55495783	808	*	chr18	55494976	55495783	in_vitro	in_vivo	10	12	2.085	0.25666667	1.82833333	c(t = 1.73536	0.115	0.83731181	Distal Intergenic	ENSSSCG000	RARRES2	retinoic acid receptor responder 2 [Source:VGNC Symbol;Acc:VGNC:92098]
chr9	68983450	68983894	445	*	chr9	68983450	68983894	in_vitro	in_vivo	9	13	3.57111111	0.23307692	3.33803419	c(t = 1.75989	0.115	0.83731181	Distal Intergenic	ENSSSCG000	STEAP4	STEAP4 metalloreductase [Source:NCBI gene (formerly Entrezgene);Acc:100310802]
chr13	207025746	207030190	4445	*	chr13	207025746	207030190	in_vitro	in_vivo	11	11	93.8954545	96.9836364	-3.0881818	c(t = -1.6678	0.116	0.83731181	Distal Intergenic	ENSSSCG000	AGPAT3	1-acylglycerol-3-phosphate O-acyltransferase 3 [Source:VGNC Symbol;Acc:VGNC:85185]
chr1	164734118	164734722	605	*	chr1	164734118	164734722	in_vitro	in_vivo	10	10	10.84	32.698	-21.858	c(t = -1.6734	0.116	0.83731181	Distal Intergenic	ENSSSCG000	SNAPC5	small nuclear RNA activating complex polypeptide 5 [Source:VGNC Symbol;Acc:VGNC:93283]
chr2	135139150	135141215	2066	*	chr2	135139150	135141215	in_vitro	in_vivo	12	14	6.65583333	12.0185714	-5.3627381	c(t = -1.6487	0.116	0.83731181	Distal Intergenic	ENSSSCG000	GDF9	growth differentiation factor 9 [Source:NCBI gene (formerly Entrezgene);Acc:414285]
chr14	128618093	128618758	666	*	chr14	128618093	128618758	in_vitro	in_vivo	12	12	2.2875	6.52666667	-4.2391667	c(t = -1.6753	0.116	0.83731181	Promoter (<=1kb)	ENSSSCG000	PRLHR	prolactin releasing hormone receptor [Source:VGNC Symbol;Acc:VGNC:91818]
chr5	15316471	15318272	1802	*	chr5	15316471	15318272	in_vitro	in_vivo	10	11	1.233	3.25	-2.017	c(t = -1.6744	0.116	0.83731181	Distal Intergenic	ENSSSCG000	TUBA1A	tubulin alpha 1a [Source:NCBI gene (formerly Entrezgene);Acc:100135051]
chr6	84879723	84880671	949	*	chr6	84879723	84880671	in_vitro	in_vivo	13	12	3.17846154	0.90583333	2.27262821	c(t = 1.66952	0.116	0.83731181	Promoter (<=1kb)	ENSSSCG000	FAM76A	family with sequence similarity 76 member A [Source:VGNC Symbol;Acc:VGNC:87982]
chr4	82844627	82845774	1148	*	chr4	82844627	82845774	in_vitro	in_vivo	9	12	4.58777778	0.85666667	3.73111111	c(t = 1.74067	0.116	0.83731181	Distal Intergenic	ENSSSCG000	SFT2D2	SFT2 domain containing 2 [Source:NCBI gene (formerly Entrezgene);Acc:100157189]
chr2	58951371	58952756	1386	*	chr2	58951371	58952756	in_vitro	in_vivo	11	13	1.53545455	0.40076923	1.13468531	c(t = 1.69642	0.116	0.83731181	Distal Intergenic	ENSSSCG000	ARMC6	armadillo repeat containing 6 [Source:VGNC Symbol;Acc:VGNC:108748]
chr13	77217290	77217798	509	*	chr13	77217290	77217798	in_vitro	in_vivo	9	13	1.73333333	0.32692308	1.40641026	c(t = 1.73241	0.116	0.83731181	Promoter (<=1kb)	ENSSSCG000	PCCB	propionyl-CoA carboxylase subunit beta [Source:NCBI gene (formerly Entrezgene);Acc:100158147]
chr15	32359264	32359986	723	*	chr15	32359264	32359986	in_vitro	in_vivo	10	10	0.806	0	0.806	c(t = 1.73915	0.116	0.83731181	Distal Intergenic	NA	NA	NA
chr2	70066820	70068204	1385	*	chr2	70066820	70068204	in_vitro	in_vivo	12	14	3.10083333	8.63142857	-5.5305952	c(t = -1.6559	0.117	0.83731181	Promoter (<=1kb)	ENSSSCG000	EPOR	erythropoietin receptor [Source:NCBI gene (formerly Entrezgene);Acc:397554]
chr6	17854479	17856633	2155	*	chr6	17854479	17856633	in_vitro	in_vivo	12	13	16.1491667	8.17923077	7.9699359	c(t = 1.63909	0.117	0.83731181	Promoter (1-2kb)	ENSSSCG000	HAS3	hyaluronan synthase 3 [Source:VGNC Symbol;Acc:VGNC:110405]
chr2	8898604	8899470	867	*	chr2	8898604	8899470	in_vitro	in_vivo	9	14	10.0733333	1.98785714	8.08547619	c(t = 1.74741	0.117	0.83731181	Distal Intergenic	ENSSSCG000	WDR74	WD repeat domain 74 [Source:NCBI gene (formerly Entrezgene);Acc:100627951]
chr2	88390865	88391500	636	*	chr2	88390865	88391500	in_vitro	in_vivo	11	13	4.40545455	1.70538462	2.70006993	c(t = 1.64772	0.117	0.83731181	Promoter (<=1kb)	ENSSSCG000	CMYA5	cardiomyopathy associated 5 [Source:VGNC Symbol;Acc:VGNC:86809]
chr1	136616459	136618284	1826	*	chr1	136616459	136618284	in_vitro	in_vivo	14	13	4.19928571	1.56769231	2.63159341	c(t = 1.62740	0.117	0.83731181	Distal Intergenic	ENSSSCG000	SCG5	secretogranin V [Source:VGNC Symbol;Acc:VGNC:103171]
chr13	121378158	121378972	815	*	chr13	121378158	121378972	in_vitro	in_vivo	12	12	1.92083333	0.73416667	1.18666667	c(t = 1.63625	0.117	0.83731181	Distal Intergenic	ENSSSCG000	B3GNT5	UDP-GlcNAc:betaGal beta-1,3-N-acetylglucosaminyltransferase 5 [Source:VGNC Symbol;Acc:VGNC:85726]
chr7	53459588	53460730	1143	*	chr7	53459588	53460730	in_vitro	in_vivo	11	12	1.69545455	0.41583333	1.27962121	c(t = 1.69876	0.117	0.83731181	Distal Intergenic	ENSSSCG000	HDDC3	HD domain containing 3 [Source:VGNC Symbol;Acc:VGNC:88818]
chr15	65920153	65921474	1322	*	chr15	65920153	65921474	in_vitro	in_vivo	12	11	3.02333333	0.15363636	2.86969697	c(t = 1.69860	0.117	0.83731181	Distal Intergenic	ENSSSCG000	DAPL1	death associated protein like 1 [Source:NCBI gene (formerly Entrezgene);Acc:100157537]
chr3	69416558	69417246	689	*	chr3	69416558	69417246	in_vitro	in_vivo	10	11	0	3.97636364	-3.9763636	c(t = -1.7108	0.118	0.83731181	Distal Intergenic	ENSSSCG000	EGR4	early growth response 4 [Source:NCBI gene (formerly Entrezgene);Acc:100516906]
chrX	125208222	125209704	1483	*	chrX	125208222	125209704	in_vitro	in_vivo	9	14	19.1877778	3.815	15.3727778	c(t = 1.74266	0.118	0.83731181	Distal Intergenic	ENSSSCG000	DKC1	dyskerin pseudouridine synthase 1 [Source:NCBI gene (formerly Entrezgene);Acc:100216430]
chr1	126541347	126541988	642	*	chr1	126541347	126541988	in_vitro	in_vivo	11	9	0	3.61555556	-3.6155556	c(t = -1.7528	0.118	0.83731181	Distal Intergenic	ENSSSCG000	DUOX1	dual oxidase 1 [Source:NCBI gene (formerly Entrezgene);Acc:397177]
chr4	65271227	65274583	3357	*	chr4	65271227	65274583	in_vitro	in_vivo	11	14	3.89636364	1.35	2.54636364	c(t = 1.67338	0.118	0.83731181	Distal Intergenic	ENSSSCG000	NCOA2	nuclear receptor coactivator 2 [Source:VGNC Symbol;Acc:VGNC:90616]
chr2	77538465	77539792	1328	*	chr2	77538465	77539792	in_vitro	in_vivo	9	11	6.68888889	1.08090909	5.6079798	c(t = 1.73602	0.118	0.83731181	Distal Intergenic	ENSSSCG000	PTBP1	polypyrimidine tract binding protein 1 [Source:VGNC Symbol;Acc:VGNC:100330]
chr1	107896679	107897733	1055	*	chr1	107896679	107897733	in_vitro	in_vivo	13	11	3.08076923	1.02909091	2.05167832	c(t = 1.62607	0.118	0.83731181	Distal Intergenic	ENSSSCG000	DAPK2	death associated protein kinase 2 [Source:VGNC Symbol;Acc:VGNC:87157]
chr7	38568860	38569941	1082	*	chr7	38568860	38569941	in_vitro	in_vivo	10	12	2.545	0.77416667	1.77083333	c(t = 1.68272	0.118	0.83731181	Distal Intergenic	ENSSSCG000	YIPF3	Yip1 domain family member 3 [Source:VGNC Symbol;Acc:VGNC:95029]
chr14	103396276	103396980	705	*	chr14	103396276	103396980	in_vitro	in_vivo	13	11	2.52846154	0.62454545	1.90391608	c(t = 1.66635	0.118	0.83731181	Distal Intergenic	ENSSSCG000	FGFBP3	fibroblast growth factor binding protein 3 [Source:VGNC Symbol;Acc:VGNC:88118]
chr8	20455918	20457287	1370	*	chr8	20455918	20457287	in_vitro	in_vivo	12	12	3.13416667	0.39166667	2.7425	c(t = 1.68466	0.118	0.83731181	Distal Intergenic	ENSSSCG000	SLC34A2	solute carrier family 34 member 2 [Source:VGNC Symbol;Acc:VGNC:93065]
chr8	84811148	84812838	1691	*	chr8	84811148	84812838	in_vitro	in_vivo	11	11	1.43636364	0.36	1.07636364	c(t = 1.66477	0.118	0.83731181	Distal Intergenic	ENSSSCG000	IL15	interleukin 15 [Source:VGNC Symbol;Acc:VGNC:89083]
chr3	127502664	127503957	1294	*	chr3	127502664	127503957	in_vitro	in_vivo	10	13	1.612	0.28692308	1.32507692	c(t = 1.70499	0.118	0.83731181	Promoter (<=1kb)	ENSSSCG000	ID2	inhibitor of DNA binding 2 [Source:NCBI gene (formerly Entrezgene);Acc:654298]
chr6	71281864	71282494	631	*	chr6	71281864	71282494	in_vitro	in_vivo	10	11	5.049	0.14	4.909	c(t = 1.72953	0.118	0.83731181	Distal Intergenic	ENSSSCG000	MASP2	MBL associated serine protease 2 [Source:NCBI gene (formerly Entrezgene);Acc:100302537]
chr12	59875258	59877419	2162	*	chr12	59875258	59877419	in_vitro	in_vivo	9	11	98.1477778	92.63	5.51777778	c(t = 1.64471	0.119	0.83731181	Distal Intergenic	ENSSSCG000	AKAP10	A-kinase anchoring protein 10 [Source:NCBI gene (formerly Entrezgene);Acc:397262]
chr8	137728399	137729557	1159	*	chr8	137728399	137729557	in_vitro	in_vivo	9	10	64.3566667	32.136	32.2206667	c(t = 1.64747	0.119	0.83731181	Distal Intergenic	ENSSSCG000	BMP3	bone morphogenetic protein 3 [Source:NCBI gene (formerly Entrezgene);Acc:100512974]
chr7	3004452	3005235	784	*	chr7	3004452	3005235	in_vitro	in_vivo	10	11	4.721	17.4127273	-12.691727	c(t = -1.6721	0.119	0.83731181	Distal Intergenic	ENSSSCG000	ECI2	enoyl-CoA delta isomerase 2 [Source:VGNC Symbol;Acc:VGNC:87532]
chr8	73137478	73137764	287	*	chr8	73137478	73137764	in_vitro	in_vivo	14	17	3.62285714	9.95647059	-6.3336134	c(t = -1.6236	0.119	0.83731181	Distal Intergenic	ENSSSCG000	NPY2R	neuropeptide Y receptor Y2 [Source:VGNC Symbol;Acc:VGNC:109473]
chr1	123461438	123462218	781	*	chr1	123461438	123462218	in_vitro	in_vivo	13	11	4.78615385	1.87272727	2.91342657	c(t = 1.62622	0.119	0.83731181	Distal Intergenic	ENSSSCG000	FBN1	fibrillin 1 [Source:NCBI gene (formerly Entrezgene);Acc:414836]
chr11	32994496	32994967	472	*	chr11	32994496	32994967	in_vitro	in_vivo	10	13	0.217	1.64769231	-1.4306923	c(t = -1.6643	0.119	0.83731181	Distal Intergenic	ENSSSCG000	MTRF1	mitochondrial translation release factor 1 [Source:VGNC Symbol;Acc:VGNC:90471]
chr15	75753778	75754777	1000	*	chr15	75753778	75754777	in_vitro	in_vivo	11	11	2.60545455	1.29636364	1.30909091	c(t = 1.63837	0.119	0.83731181	Distal Intergenic	ENSSSCG000	PHOSPHO2	phosphatase, orphan 2 [Source:VGNC Symbol;Acc:VGNC:96172]
chr1	90299436	90300756	1321	*	chr1	90299436	90300756	in_vitro	in_vivo	13	14	1.89153846	0.855	1.03653846	c(t = 1.61998	0.119	0.83731181	Distal Intergenic	ENSSSCG000	MYO6	myosin VI [Source:VGNC Symbol;Acc:VGNC:103137]
chr5	33264154	33264918	765	*	chr5	33264154	33264918	in_vitro	in_vivo	12	10	2.02083333	0.111	1.90983333	c(t = 1.68655	0.119	0.83731181	Distal Intergenic	ENSSSCG000	MDM2	MDM2 proto-onco [Source:VGNC Symbol;Acc:VGNC:90093]
chr12	2141178	2144859	3682	*	chr12	2141178	2144859	in_vitro	in_vivo	12	16	11.185	24.455625	-13.270625	c(t = -1.6251	0.12	0.83731181	Distal Intergenic	ENSSSCG000	SLC26A11	solute carrier family 26 member 11 [Source:NCBI gene (formerly Entrezgene);Acc:100191010]
chr18	18350565	18351367	803	*	chr18	18350565	18351367	in_vitro	in_vivo	10	13	7.93	21.1061538	-13.176154	c(t = -1.6578	0.12	0.83731181	Distal Intergenic	ENSSSCG000	MEST	mesoderm specific transcript [Source:VGNC Symbol;Acc:VGNC:90150]
chr12	36831969	36832389	421	*	chr12	36831969	36832389	in_vitro	in_vivo	9	10	5.26	1.825	3.435	c(t = 1.65498	0.12	0.83731181	Promoter (1-2kb)	ENSSSCG000	TBX4	T-box transcription factor 4 [Source:NCBI gene (formerly Entrezgene);Acc:100620126]
chr14	35175543	35176998	1456	*	chr14	35175543	35176998	in_vitro	in_vivo	12	10	8.06833333	1.754	6.31433333	c(t = 1.67722	0.12	0.83731181	Distal Intergenic	ENSSSCG000	FBXO21	F-box protein 21 [Source:VGNC Symbol;Acc:VGNC:88035]
chr1	3118820	3122474	3655	*	chr1	3118820	3122474	in_vitro	in_vivo	15	13	3.43066667	1.75384615	1.67682051	c(t = 1.61135	0.12	0.83731181	Distal Intergenic	ENSSSCG000	ssc-mir-9831	ssc-mir-9831 [Source:miRBase;Acc:MI0031600]
chr13	196595651	196601071	5421	*	chr13	196595651	196601071	in_vitro	in_vivo	12	14	2.53333333	1.29571429	1.23761905	c(t = 1.61753	0.12	0.83731181	Distal Intergenic	ENSSSCG000	IFNAR2	interferon alpha and beta receptor subunit 2 [Source:NCBI gene (formerly Entrezgene);Acc:100533555]
chr16	37304535	37305366	832	*	chr16	37304535	37305366	in_vitro	in_vivo	13	13	5.53769231	1.01230769	4.52538462	c(t = 1.66341	0.12	0.83731181	Distal Intergenic	ENSSSCG000	ssc-mir-9799	ssc-mir-9799 [Source:miRBase;Acc:MI0031563]
chr2	39051753	39052290	538	*	chr2	39051753	39052290	in_vitro	in_vivo	10	10	4.122	0.9	3.222	c(t = 1.68387	0.12	0.83731181	Distal Intergenic	ENSSSCG000	CSRP3	cysteine and glycine rich protein 3 [Source:NCBI gene (formerly Entrezgene);Acc:100337687]
chr1	248447927	248448945	1019	*	chr1	248447927	248448945	in_vitro	in_vivo	12	15	3.85666667	0.75066667	3.106	c(t = 1.67885	0.12	0.83731181	Distal Intergenic	ENSSSCG000	KLF4	KLF transcription factor 4 [Source:VGNC Symbol;Acc:VGNC:98062]

chr5	479867	481076	1210	*	chr5	479867	481076	in_vitro	in_vivo	11	10	2.11090909	0.405	1.70590909	c(t = 1.67676	0.12	0.83731181	Promoter (<=1kb)	ENSSSCG000	SELENOO	selenoprotein O [Source:NCBI gene (formerly Entrezgene);Acc:100518971]
chr9	78217717	78218825	1109	*	chr9	78217717	78218825	in_vitro	in_vivo	13	12	4.23538462	1.87583333	2.35955128	c(t = 1.63215	0.121	0.83731181	Distal Intergenic	ENSSSCG000	MIOS	meiosis regulator for oocyte development [Source:VGNC Symbol;Acc:VGNC:90234]
chr14	23262363	23263437	1075	*	chr14	23262363	23263437	in_vitro	in_vivo	10	11	4.455	1.16090909	3.29409091	c(t = 1.68573	0.121	0.83731181	Distal Intergenic	ENSSSCG000	PIWIL1	piwi like RNA-mediated silencing 1 [Source:VGNC Symbol;Acc:VGNC:98197]
chr9	66837140	66840446	3307	*	chr9	66837140	66840446	in_vitro	in_vivo	12	11	2.70916667	1.06545455	1.64371212	c(t = 1.63383	0.121	0.83731181	Distal Intergenic	ENSSSCG00000021041		peptidase M20 domain containing 1 [Source:NCBI gene (formerly Entrezgene);Acc:100627595]
chr16	1521101	1522544	1444	*	chr16	1521101	1522544	in_vitro	in_vivo	14	12	2.19928571	0.52833333	1.67095238	c(t = 1.64651	0.121	0.83731181	Distal Intergenic	ENSSSCG000	C1QTNF3	C1q and TNF related 3 [Source:NCBI gene (formerly Entrezgene);Acc:100270814]
chr18	6965704	6966439	736	*	chr18	6965704	6966439	in_vitro	in_vivo	10	10	3.054	0.196	2.858	c(t = 1.70600	0.121	0.83731181	Distal Intergenic	ENSSSCG000	GSTK1	glutathione S-transferase kappa 1 [Source:NCBI gene (formerly Entrezgene);Acc:100521480]
chr10	24458712	24459669	958	*	chr10	24458712	24459669	in_vitro	in_vivo	9	9	1.14888889	4.64777778	-3.4988889	c(t = -1.6933	0.122	0.83731181	Distal Intergenic	ENSSSCG000	ADIPOR1	adiponectin receptor 1 [Source:NCBI gene (formerly Entrezgene);Acc:404695]
chr9	20721112	20722039	928	*	chr9	20721112	20722039	in_vitro	in_vivo	9	11	0.10666667	4.13	-4.0233333	c(t = -1.6874	0.122	0.83731181	Distal Intergenic	ENSSSCG000	ME3	malic enzyme 3 [Source:VGNC Symbol;Acc:VGNC:90097]
chr9	9596861	9598014	1154	*	chr9	9596861	9598014	in_vitro	in_vivo	11	13	4.73545455	1.77769231	2.95776224	c(t = 1.64262	0.122	0.83731181	Distal Intergenic	ENSSSCG000	ssc-mir-326	ssc-mir-326 [Source:miRBase;Acc:MI0002434]
chr1	147987193	147988527	1335	*	chr1	147987193	147988527	in_vitro	in_vivo	9	9	0	1.37	-1.37	c(t = -1.7304	0.122	0.83731181	Distal Intergenic	ENSSSCG000	MBP	myelin basic protein [Source:NCBI gene (formerly Entrezgene);Acc:414286]
chr7	24059962	24060711	750	*	chr7	24059962	24060711	in_vitro	in_vivo	10	13	2.903	1.04615385	1.85684615	c(t = 1.63869	0.122	0.83731181	Promoter (<=1kb)	ENSSSCG000	DXO	decapping exoribonuclease [Source:VGNC Symbol;Acc:VGNC:87493]
chr17	58052838	58054470	1633	*	chr17	58052838	58054470	in_vitro	in_vivo	11	11	2.94454545	0.64272727	2.30181818	c(t = 1.68063	0.122	0.83731181	Promoter (<=1kb)	ENSSSCG000	PMEPA1	prostate transmembrane protein, androgen induced 1 [Source:VGNC Symbol;Acc:VGNC:95606]
chr3	25285886	25286690	805	*	chr3	25285886	25286690	in_vitro	in_vivo	12	12	1.61	0.3475	1.2625	c(t = 1.63245	0.122	0.83731181	Promoter (<=1kb)	ENSSSCG000	THUMPD1	THUMP domain containing 1 [Source:VGNC Symbol;Acc:VGNC:93970]
chr6	106917497	106918880	1384	*	chr6	106917497	106918880	in_vitro	in_vivo	11	11	1.30636364	0.30090909	1.00545455	c(t = 1.65911	0.122	0.83731181	Distal Intergenic	NA	NA	NA
chr3	132355803	132362996	7194	*	chr3	132355803	132362996	in_vitro	in_vivo	9	11	11.6544444	35.6418182	-23.987374	c(t = -1.6288	0.123	0.83731181	Distal Intergenic	ENSSSCG000	TPO	thyroid peroxidase [Source:VGNC Symbol;Acc:VGNC:94341]
chr15	87182412	87183487	1076	*	chr15	87182412	87183487	in_vitro	in_vivo	9	10	0.5	1.957	-1.457	c(t = -1.6602	0.123	0.83731181	Distal Intergenic	ENSSSCG000	FRZB	frizzled related protein [Source:VGNC Symbol;Acc:VGNC:96308]
chr8	37808265	37809591	1327	*	chr8	37808265	37809591	in_vitro	in_vivo	12	13	3.09666667	0.99461538	2.10205128	c(t = 1.62825	0.123	0.83731181	Distal Intergenic	ENSSSCG000	ZAR1	zygote arrest 1 [Source:VGNC Symbol;Acc:VGNC:95051]
chr15	131787057	131787895	839	*	chr15	131787057	131787895	in_vitro	in_vivo	13	11	2.22846154	0.81818182	1.41027972	c(t = 1.62074	0.123	0.83731181	Distal Intergenic	ENSSSCG000	SPATA3	spermatosis associated 3 [Source:VGNC Symbol;Acc:VGNC:95863]
chr13	119668240	119669409	1170	*	chr13	119668240	119669409	in_vitro	in_vivo	12	14	3.47916667	0.71071429	2.76845238	c(t = 1.65309	0.123	0.83731181	Promoter (<=1kb)	ENSSSCG000	SOX2	SRY-box transcription factor 2 [Source:NCBI gene (formerly Entrezgene);Acc:407739]
chr14	55748571	55750084	1514	*	chr14	55748571	55750084	in_vitro	in_vivo	11	14	2.28909091	0.67571429	1.61337662	c(t = 1.64870	0.123	0.83731181	Distal Intergenic	ENSSSCG000	EDARADD	EDAR associated death domain [Source:VGNC Symbol;Acc:VGNC:87542]
chr15	61440321	61441513	1193	*	chr15	61440321	61441513	in_vitro	in_vivo	11	10	4.38636364	0.474	3.91236364	c(t = 1.67708	0.123	0.83731181	Distal Intergenic	ENSSSCG000	PRPF40A	pre-mRNA processing factor 40 homolog A [Source:VGNC Symbol;Acc:VGNC:98222]
chr7	64374809	64376853	2045	*	chr7	64374809	64376853	in_vitro	in_vivo	13	14	78.3976923	53.8614286	24.5362637	c(t = 1.59378	0.124	0.83731181	Distal Intergenic	ENSSSCG000	NFKBIA	NFKB inhibitor alpha [Source:NCBI gene (formerly Entrezgene);Acc:406188]
chr14	22857213	22858723	1511	*	chr14	22857213	22858723	in_vitro	in_vivo	10	14	13.71	4.60285714	9.10714286	c(t = 1.67483	0.124	0.83731181	Distal Intergenic	ENSSSCG000	PIWIL1	piwi like RNA-mediated silencing 1 [Source:VGNC Symbol;Acc:VGNC:98197]
chr1	50044935	50046041	1107	*	chr1	50044935	50046041	in_vitro	in_vivo	11	10	5.90636364	2.471	3.43536364	c(t = 1.64041	0.124	0.83731181	Distal Intergenic	ENSSSCG000	ssc-mir-30c-2	ssc-mir-30c-2 [Source:miRBase;Acc:MI0002461]
chr6	144942651	144944013	1363	*	chr6	144942651	144944013	in_vitro	in_vivo	13	11	16.3038462	1.40090909	14.9029371	c(t = 1.65242	0.124	0.83731181	Promoter (<=1kb)	ENSSSCG000	GADD45A	growth arrest and DNA damage inducible alpha [Source:NCBI gene (formerly Entrezgene);Acc:733669]
chr7	58172158	58177104	4947	*	chr7	58172158	58177104	in_vitro	in_vivo	13	14	2.58076923	0.975	1.60576923	c(t = 1.62798	0.124	0.83731181	Distal Intergenic	ENSSSCG000	IMP3	IMP U3 small nucleolar ribonucleoprotein 3 [Source:NCBI gene (formerly Entrezgene);Acc:100151831]
chr6	115592318	115593437	1120	*	chr6	115592318	115593437	in_vitro	in_vivo	14	10	2.98357143	0.959	2.02457143	c(t = 1.61533	0.124	0.83731181	Distal Intergenic	ENSSSCG000	TTR	transthyretin [Source:NCBI gene (formerly Entrezgene);Acc:397419]
chr6	62113466	62114049	584	*	chr6	62113466	62114049	in_vitro	in_vivo	9	11	2.93666667	0.71727273	2.21939394	c(t = 1.66649	0.124	0.83731181	Distal Intergenic	ENSSSCG000	ISG15	ISG15 ubiquitin like modifier [Source:NCBI gene (formerly Entrezgene);Acc:100145895]
chr14	60444708	60446071	1364	*	chr14	60444708	60446071	in_vitro	in_vivo	11	12	1.51909091	0.51583333	1.00325758	c(t = 1.64151	0.124	0.83731181	Distal Intergenic	ENSSSCG000	ACTA1	actin alpha 1, skeletal muscle [Source:NCBI gene (formerly Entrezgene);Acc:100154254]
chr2	3286110	3287711	1602	*	chr2	3286110	3287711	in_vitro	in_vivo	9	11	1.91111111	0.42909091	1.4820202	c(t = 1.69609	0.124	0.83731181	Distal Intergenic	ENSSSCG000	FADD	Fas associated via death domain [Source:NCBI gene (formerly Entrezgene);Acc:595129]
chr18	17443861	17445151	1291	*	chr18	17443861	17445151	in_vitro	in_vivo	11	9	2.00181818	0.36444444	1.63737374	c(t = 1.66027	0.124	0.83731181	Distal Intergenic	ENSSSCG000	ssc-mir-29b-	ssc-mir-29b-1 [Source:miRBase;Acc:MI0002431]
chr17	60982000	60983255	1256	*	chr17	60982000	60983255	in_vitro	in_vivo	10	11	2.99	17.2081818	-14.218182	c(t = -1.6712	0.125	0.83731181	Distal Intergenic	ENSSSCG000	PSMA7	proteasome 20S subunit alpha 7 [Source:NCBI gene (formerly Entrezgene);Acc:100626738]
chrX	35810108	35826549	16442	*	chrX	35810108	35826549	in_vitro	in_vivo	13	15	2.72615385	5.69666667	-2.9705128	c(t = -1.6096	0.125	0.83731181	Distal Intergenic	ENSSSCG000	MID1IP1	MID1 interacting protein 1 [Source:VGNC Symbol;Acc:VGNC:90214]
chr12	52627516	52629243	1728	*	chr12	52627516	52629243	in_vitro	in_vivo	10	15	4.419	1.542	2.877	c(t = 1.65877	0.125	0.83731181	Promoter (<=1kb)	ENSSSCG000	SLC2A4	solute carrier family 2 member 4 [Source:NCBI gene (formerly Entrezgene);Acc:396754]
chr1	181955320	181956450	1131	*	chr1	181955320	181956450	in_vitro	in_vivo	12	13	2.48666667	1.10230769	1.38435897	c(t = 1.60036	0.125	0.83731181	Distal Intergenic	ENSSSCG000	RTRAF	RNA transcription, translation and transport factor [Source:NCBI gene (formerly Entrezgene);Acc:100154555]
chr3	13563373	13566045	2673	*	chr3	13563373	13566045	in_vitro	in_vivo	12	15	2.35666667	0.70266667	1.654	c(t = 1.64593	0.125	0.83731181	Promoter (<=1kb)	ENSSSCG000	AUTS2	activator of transcription and developmental regulator AUTS2 [Source:NCBI gene (formerly Entrezgene);Acc:100515476]
chr7	19244417	19245134	718	*	chr7	19244417	19245134	in_vitro	in_vivo	11	13	2.93	0.68076923	2.24923077	c(t = 1.65183	0.125	0.83731181	Distal Intergenic	ENSSSCG000	ALDH5A1	aldehyde dehydrogenase 5 family member A1 [Source:NCBI gene (formerly Entrezgene);Acc:100157072]
chr11	67143886	67145549	1664	*	chr11	67143886	67145549	in_vitro	in_vivo	12	14	2.90666667	0.56428571	2.34238095	c(t = 1.65671	0.125	0.83731181	Distal Intergenic	ENSSSCG000	IPO5	importin 5 [Source:NCBI gene (formerly Entrezgene);Acc:100152933]
chr9	49904270	49905676	1407	*	chr9	49904270	49905676	in_vitro	in_vivo	10	11	2.712	0.53454545	2.17745455	c(t = 1.64631	0.125	0.83731181	Distal Intergenic	ENSSSCG000	HSPA8	heat shock protein family A (Hsp70) member 8 [Source:NCBI gene (formerly Entrezgene);Acc:100511890]
chr3	101752943	101754405	1463	*	chr3	101752943	101754405	in_vitro	in_vivo	11	12	1.35272727	0.4925	0.86022727	c(t = 1.62047	0.125	0.83731181	Distal Intergenic	ENSSSCG000	GALM	galactose mutarotase [Source:NCBI gene (formerly Entrezgene);Acc:399536]
chr2	4522311	4523724	1414	*	chr2	4522311	4523724	in_vitro	in_vivo	9	11	2.15333333	0.41363636	1.73969697	c(t = 1.65950	0.125	0.83731181	Distal Intergenic	ENSSSCG000	GAL	galanin and GMAP prepropeptide [Source:NCBI gene (formerly Entrezgene);Acc:397465]
chr4	123834362	123836334	1973	*	chr4	123834362	123836334	in_vitro	in_vivo	14	15	1.47071429	0.33	1.14071429	c(t = 1.62426	0.125	0.83731181	Distal Intergenic	ENSSSCG000	TMED5	transmembrane p24 trafficking protein 5 [Source:NCBI gene (formerly Entrezgene);Acc:100157999]
chr18	6312474	6314041	1568	*	chr18	6312474	6314041	in_vitro	in_vivo	12	12	20.5	41.0858333	-20.585833	c(t = -1.5887	0.126	0.83731181	Distal Intergenic	ENSSSCG000	NOS3	nitric oxide synthase 3 [Source:NCBI gene (formerly Entrezgene);Acc:397557]
chrX	43115832	43116730	899	*	chrX	43115832	43116730	in_vitro	in_vivo	9	11	14.3666667	7.53363636	6.8330303	c(t = 1.63505	0.126	0.83731181	Distal Intergenic	NA	NA	NA
chr12	18036976	18040754	3779	*	chr12	18036976	18040754	in_vitro	in_vivo	12	11	2.66166667	6.02833333	-3.3666667	c(t = -1.6272	0.126	0.83731181	Distal Intergenic	ENSSSCG000	C1QL1	complement C1q like 1 [Source:NCBI gene (formerly Entrezgene);Acc:780424]
chr13	23236881	23238804	1924	*	chr13	23236881	23238804	in_vitro	in_vivo	10	13	9.548	3.12615385	6.42184615	c(t = 1.66567	0.126	0.83731181	Promoter (<=1kb)	ENSSSCG000	ACVR2B	activin A receptor type 2B [Source:VGNC Symbol;Acc:VGNC:108629]
chr1	161682525	161684418	1894	*	chr1	161682525	161684418	in_vitro	in_vivo	12	12	3.695	1.42833333	2.26666667	c(t = 1.62656	0.126	0.83731181	Distal Intergenic	ENSSSCG000	PMAIP1	phorbol-12-myristate-13-acetate-induced protein 1 [Source:NCBI gene (formerly Entrezgene);Acc:397278]
chr12	14284911	14285657	747	*	chr12	14284911	14285657	in_vitro	in_vivo	9	10	0	1.29	-1.29	c(t = -1.6863	0.126	0.83731181	Promoter (<=1kb)	ENSSSCG000	KPNA2	karyopherin subunit alpha 2 [Source:VGNC Symbol;Acc:VGNC:89561]
chr2	57052957	57053769	813	*	chr2	57052957	57053769	in_vitro	in_vivo	11	11	6.88181818	1.27727273	5.60454545	c(t = 1.64456	0.126	0.83731181	Distal Intergenic	ENSSSCG000	NLRP3	NLR family pyrin domain containing 3 [Source:VGNC Symbol;Acc:VGNC:90785]
chr2	5682532	5683558	1027	*	chr2	5682532	5683558	in_vitro	in_vivo	9	10	8.96666667	1.074	7.89266667	c(t = 1.70651	0.126	0.83731181	Distal Intergenic	ENSSSCG00000038304		RNA binding motif protein 4B [Source:NCBI gene (formerly Entrezgene);Acc:100144418]
chr1	109010167	109010965	799	*	chr1	109010167	109010965	in_vitro	in_vivo	11	12	2.37916667	0.81	1.56916667	c(t = 1.61169	0.126	0.83731181	Intron (NM_001097483/100037999, intron 2 of 9)	ENSSSCG000	TPM1	tropomyosin 1 [Source:VGNC Symbol;Acc:VGNC:103194]
chr6	699443	702166	2724	*	chr6	699443	702166	in_vitro	in_vivo	11	9	58.9809091	79.8866667	-20.905758	c(t = -1.6022	0.127	0.83731181	Distal Intergenic	ENSSSCG000	GALNS	galactosamine (N-acetyl)-6-sulfatase [Source:VGNC Symbol;Acc:VGNC:97061]
chr4	967166	970490	3325	*	chr4	967166	970490	in_vitro	in_vivo	9	9	5.48	12.5544444	-7.0744444	c(t = -1.6196	0.127	0.83731181	Distal Intergenic	ENSSSCG000	GPIHBP1	glycosylphosphatidylinositol anchored high density lipoprotein binding protein 1 [Source:VGNC Symbol;Acc:VGNC:88588]
chr18	47117249	47117983	735	*	chr18	47117249	47117983	in_vitro	in_vivo	10	10	1.853	7.392	-5.539	c(t = -1.6575	0.127	0.83731181	Distal Intergenic	ENSSSCG000	CYCS	cytochrome c, somatic [Source:NCBI gene (formerly Entrezgene);Acc:100170131]
chr2	135345414	135346416	1003	*	chr2	135345414	135346416	in_vitro	in_vivo	10	12	1.851	0.17333333	1.67766667	c(t = 1.67615	0.127	0.83731181	Distal Intergenic	ENSSSCG000	LEAP2	liver enriched antimicrobial peptide 2 [Source:NCBI gene (formerly Entrezgene);Acc:396687]
chr18	19255276	19255774	499	*	chr18	19255276	19255774	in_vitro	in_vivo	11	10	1.63272727	0	1.63272727	c(t = 1.66612	0.127	0.83731181	Distal Intergenic	ENSSSCG000	ssc-mir-182	ssc-mir-182 [Source:miRBase;Acc:MI0022135]
chr14	31165490	31166856	1367	*	chr14	31165490	31166856	in_vitro	in_vivo	13	13	4.36	1.76692308	2.59307692	c(t = 1.61641	0.128	0.83731181	Distal Intergenic	ENSSSCG000	ORAI1	ORAI calcium release-activated calcium modulator 1 [Source:NCBI gene (formerly Entrezgene);Acc:100154923]
chr14	109007942	109008784	843	*	chr14	109007942	109008784	in_vitro	in_vivo	14	13	2.82714286	1.10076923	1.72637363	c(t = 1.58302	0.128	0.83731181	Distal Intergenic	ENSSSCG000	HOGA1	4-hydroxy-2-oxoglutarate aldolase 1 [Source:NCBI gene (formerly Entrezgene);Acc:100153961]
chr1	252323217	252324977	1761	*	chr1	252323217	252324977	in_vitro	in_vivo	15	13	2.21866667	0.98384615	1.23482051	c(t = 1.59266	0.128	0.83731181	Distal Intergenic	ENSSSCG000	PTGR1	prostaglandin reductase 1 [Source:VGNC Symbol;Acc:VGNC:91957]
chr1	189622401	189624931	2531	*	chr1	189622401	189624931	in_vitro	in_vivo	12	12	2.6	0.8725	1.7275	c(t = 1.61781	0.128	0.83731181	Promoter (<=1kb)	ENSSSCG000	SIX1	SIX homeobox 1 [Source:NCBI gene (formerly Entrezgene);Acc:100156847]
chr9	39368199	39369388	1190	*	chr9	39368199	39369388	in_vitro	in_vivo	14	14	1.94857143	0.74428571	1.20428571	c(t = 1.57859	0.128	0.83731181	Distal Intergenic	ENSSSCG000	LAYN	layilin [Source:NCBI gene (formerly Entrezgene);Acc:100513941]
chr7	13934571	13935536	966	*	chr7	13934571	13935536	in_vitro	in_vivo	12	13	2.44083333	0.70230769	1.73852564	c(t = 1.62311	0.128	0.83731181	Distal Intergenic	ENSSSCG000	TPMT	thiopurine S-methyltransferase [Source:NCBI gene (formerly Entrezgene);Acc:100157630]
chr11	5042384	5044586	2203	*	chr11	5042384	5044586	in_vitro	in_vivo	11	15	3.12545455	0.636	2.48945455	c(t = 1.64910	0.128	0.83731181	Distal Intergenic	ENSSSCG000	ssc-mir-9803	ssc-mir-9803 [Source:miRBase;Acc:MI0031567]
chr1	182604861	182606142	1282	*	chr1	182604861	182606142	in_vitro	in_vivo	12	11	1.09166667	0.30272727	0.78893939	c(t = 1.60918	0.128	0.83731181	Distal Intergenic	ENSSSCG000	GNPNAT1	glucosamine-phosphate N-acetyltransferase 1 [Source:VGNC Symbol;Acc:VGNC:103102]
chr7	1987462	1992334	4873	*	chr7	1987462	1992334	in_vitro	in_vivo	12	11	87.935	95.5172727	-7.5822727	c(t = -1.6125	0.129	0.83731181	Distal Intergenic	ENSSSCG000	TUBB2B	tubulin, beta 2B class IIb [Source:NCBI gene (formerly Entrezgene);Acc:100153507]
chr7	35766578	35767645	1068	*	chr7	35766578	35767645	in_vitro	in_vivo	9	9	10.43	34.6911111	-24.261111	c(t = -1.6334	0.129	0.83731181	Distal Intergenic	ENSSSCG000	NFYA	nuclear transcription factor Y subunit alpha [Source:VGNC Symbol;Acc:VGNC:90729]
chr2	90704	93254	2551	*	chr2	90704	93254	in_vitro	in_vivo	11	13	3.39909091	10.9446154	-7.5455245	c(t = -1.6174	0.129	0.83731181	Distal Intergenic	ENSSSCG000	COX8H	COX8H protein [Source:NCBI gene (formerly Entrezgene);Acc:100038031]
chr12	2709570	2711455	1886	*	chr12	2709570	2711455	in_vitro	in_vivo	10	14	10.551	4.91571429	5.63528571	c(t = 1.64692	0.129	0.83731181	Distal Intergenic	ENSSSCG000	CBX8	chromobox 8 [Source:NCBI gene (formerly Entrezgene);Acc:100514536]
chr1	130752923	130753394	472	*	chr1	130752923	130753394	in_vitro	in_vivo	10	9	0.476	2.44333333	-1.9673333	c(t = -1.6439	0.129	0.83731181	Distal Intergenic	ENSSSCG000	RAD51	RAD51 recombinase [Source:VGNC Symbol;Acc:VGNC:92056]
chr14	121062448	121063593	1146	*	chr14	121062448	121063593	in_vitro	in_vivo	11	12	2.77363636	1.265	1.50863636	c(t = 1.61321	0.129	0.83731181	Distal Intergenic	ENSSSCG000	SMNDC1	survival motor neuron domain containing 1 [Source:VGNC Symbol;Acc:VGNC:93257]
chr4	104856268	104858169	1902	*	chr4	104856268	104858169	in_vitro	in_vivo	12	14	2.94666667	1.18571429	1.76095238	c(t = 1.60611	0.129	0.83731181	Distal Intergenic	ENSSSCG000	ATP1A1	ATPase Na+/K+ transporting subunit alpha 1 [Source:VGNC Symbol;Acc:VGNC:98735]
chr12	49758420	49759697	1278	*	chr12	49758420	49759697	in_vitro	in_vivo	10	13	3.624	1.04076923	2.58323077	c(t = 1.64606	0.129	0.83731181	Distal Intergenic	ENSSSCG000	ASPA	aspartoacylase [Source:NCBI gene (formerly Entrezgene);Acc:100142661]
chr18	47709839	47711059	1221	*	chr18	47709839	47711059	in_vitro	in_vivo	10	11	4.057	0.80363636	3.25336364	c(t = 1.66424	0.129	0.83731181	Distal Intergenic	ENSSSCG000	NPY	neuropeptide Y [Source:NCBI gene (formerly Entrezgene);Acc:397304]
chr17	3312637	3313325	689	*	chr17	3312637	3313325	in_vitro	in_vivo	9	13	2.11444444	0.34769231	1.76675214	c(t = 1.67202	0.129	0.83731181	Distal Intergenic	ENSSSCG000	ssc-mir-383	ssc-mir-383 [Source:miRBase;Acc:MI0015927]
chr5	42143346	42144111	766	*	chr5	42143346	42144111	in_vitro	in_vivo	12	10	1.53083333	0.227	1.30383333	c(t = 1.62293	0.129	0.83731181	Distal Intergenic	ENSSSCG000	RESF1	retroelement silencing factor 1 [Source:VGNC Symbol;Acc:VGNC:92219]
chr12	2134182	2135153	972	*	chr12	2134182	2135153	in_vitro	in_vivo	9	11	2.57555556	18.0281818	-15.452626	c(t = -1.6416	0.13	0.83731181	Distal Intergenic	ENSSSCG000	SLC26A11	solute carrier family 26 member 11 [Source:NCBI gene (formerly Entrezgene);Acc:100191010]
chr7	99910131	99911945	1815	*	chr7	99910131	99911945	in_vitro	in_vivo	10	13	3.155	1.14230769	2.01269231	c(t = 1.63428	0.13	0.83731181	Distal Intergenic	ENSSSCG000	ssc-mir-9860	ssc-mir-9860 [Source:miRBase;Acc:MI0031638]
chr6	169999441	170000630	1190	*	chr6	169999441	170000630	in_vitro	in_vivo	12	9	2.8875	1.09222222	1.79527778	c(t = 1.58934	0.13	0.83731181	Distal Intergenic	ENSSSCG000	ssc-mir-9829	ssc-mir-9829 [Source:miRBase;Acc:MI0031598]
chr16	34377975	34378559	585	*	chr16	34377975	34378559	in_vitro	in_vivo	11	10	5.48272727	1.05	4.43272727	c(t = 1.62597	0.13	0.83731181	Distal Intergenic	ENSSSCG000	GZMA	granzyme A (granzyme 1, cytotoxic T-lymphocyte-associated serine esterase 3) [Source:NCBI gene (formerly Entrezgene);Acc:100526762]
chr14	124017787	124018756	970	*	chr14	124017787	124018756	in_vitro	in_vivo	10	12	1.621	0.64333333	0.97766667	c(t = 1.59397	0.13	0.83731181	Distal Intergenic	ENSSSCG000	NRAP	nebulin related anchoring protein [Source:VGNC Symbol;Acc:VGNC:90888]
chr3	23796274	23797371	1098	*	chr3	23796274	23797371	in_vitro	in_vivo	12	16	2.51166667	0.468125	2.04354167	c(t = 1.61986	0.13	0.83731181	Distal Intergenic	ENSSSCG000	METTL9	methyltransferase like 9 [Source:VGNC Symbol;Acc:VGNC:103984]
chr6	88987010	88987840	831	*	chr6	88987010	88987840	in_vitro	in_vivo	9	12	1.78111111	0.35583333	1.42527778	c(t = 1.66137	0.13	0.83731181	Distal Intergenic	ENSSSCG000	TSSK3	testis specific serine kinase 3 [Source:NCBI gene (formerly Entrezgene);Acc:100628162]
chr11	78677137	78678566	1430	*	chr11	78677137	78678566	in_vitro	in_vivo	11	10	4.44363636	17.245	-12.801364	c(t = -1.6487	0.131	0.83731181	Distal Intergenic	ENSSSCG000	TMCO3	transmembrane and coiled-coil domains 3 [Source:VGNC Symbol;Acc:VGNC:94050]
chr8	39633704	39634411	708	*	chr8	39633704	39634411	in_vitro	in_vivo	12	12	2.105	8.275	-6.17	c(t = -1.6032	0.131	0.83731181	Distal Intergenic	ENSSSCG000	SPATA18	spermatosis associated 18 [Source:VGNC Symbol;Acc:VGNC:98966]
chr2	58505416	58506571	1156	*	chr2	58505416	58506571	in_vitro	in_vivo	12	9	3.91333333	1.73777778	2.17555556	c(t = 1.58762	0.131	0.83731181	Distal Intergenic	ENSSSCG000	TSSK6	testis specific serine kinase 6 [Source:NCBI gene (formerly Entrezgene);Acc:100518240]
chr6	17651971	17653242	1272	*	chr6	17651971	17653242	in_vitro	in_vivo	9	11	4.66222222	1.44181818	3.22040404	c(t = 1.66746	0.131	0.83731181	Distal Intergenic	ENSSSCG000	NIP7	nucleolar pre-rRNA processing protein NIP7 [Source:NCBI gene (formerly Entrezgene);Acc:595107]
chr6	85545905	85546681	777	*	chr6	85545905	85546681	in_vitro	in_vivo	10	10	4.387	1.135	3.252	c(t = 1.64648	0.131	0.83731181	Distal Intergenic	ENSSSCG000	ATP5IF1	ATP synthase inhibitory factor subunit 1 [Source:NCBI gene (formerly Entrezgene);Acc:100038002]
chr16	50987679	50990847	3169	*	chr16	50987679	50990847	in_vitro	in_vivo	14	14	2.41428571	1.04928571	1.365	c(t = 1.58714	0.131	0.83731181	Promoter (<=1kb)	ENSSSCG000	STC2	stanniocalcin 2 [Source:NCBI gene (formerly Entrezgene);Acc:100126236]
chr7	29661762	29663591	1830	*	chr7	29661762	29663591	in_vitro	in_vivo	9	12	3.41444444	0.99166667	2.42277778	c(t = 1.64096	0.131	0.83731181	Promoter (<=1kb)	ENSSSCG000	RGL2	ral guanine nucleotide dissociation stimulator like 2 [Source:NCBI gene (formerly Entrezgene);Acc:100151794]
chr2	73294501	73295458	958	*	chr2	73294501	73295458	in_vitro	in_vivo	9	13	2.86111111	0.84538462	2.0157265	c(t = 1.63442	0.131	0.83731181	Promoter (<=1kb)	ENSSSCG000	MICOS13	mitochondrial contact site and cristae organizing system subunit 13 [Source:NCBI gene (formerly Entrezgene);Acc:100037978]
chr4	21215363	21215667	305	*	chr4	21215363	21215667	in_vitro	in_vivo	13	9	3.73230769	0.79333333	2.93897436	c(t = 1.58693	0.131	0.83731181	Distal Intergenic	NA	NA	NA
chr6	77761125	77762456	1332	*	chr6	77761125	77762456	in_vitro	in_vivo	9	11	2.01444444	0.66090909	1.35353535	c(t = 1.60697	0.131	0.83731181	Promoter (<=1kb)	ENSSSCG000	AKR7A2	aldo-keto reductase family 7 member A2 [Source:NCBI gene (formerly Entrezgene);Acc:100511331]
chr13	112850490	112851294	805	*	chr13	112850490	112851294	in_vitro	in_vivo	10	11	1.742	0.36636364	1.37563636	c(t = 1.62928	0.131	0.83731181	Distal Intergenic	ENSSSCG000	SPATA16	spermatogenesis associated 16 [Source:NCBI gene (formerly Entrezgene);Acc:100415806]
chr6	82308652	82310066	1415	*	chr6	82308652	82310066	in_vitro	in_vivo	12	10	2.15166667	0.359	1.79266667	c(t = 1.61965	0.131	0.83731181	Distal Intergenic	ENSSSCG000	RSRP1	arginine and serine rich protein 1 [Source:VGNC Symbol;Acc:VGNC:92489]
chr11	359409	360448	1040	*	chr11	359409	360448	in_vitro	in_vivo	10	11	2.515	0.35	2.165	c(t = 1.64724	0.131	0.83731181	Distal Intergenic	ENSSSCG000	CRYL1	crystallin lambda 1 [Source:VGNC Symbol;Acc:VGNC:87023]
chr5	773588	807293	33706	*	chr5	773588	807293	in_vitro	in_vivo	16	15	64.889375	78.5253333	-13.635958	c(t = -1.5628	0.132	0.83731181	Distal Intergenic	ENSSSCG000	SELENOO	selenoprotein O [Source:NCBI gene (formerly Entrezgene);Acc:100518971]
chr4	19513664	19514811	1148	*	chr4	19513664	19514811	in_vitro	in_vivo	10	14	0.637	1.94142857	-1.3044286	c(t = -1.5679	0.132	0.83731181	Distal Intergenic	ENSSSCG000	MAL2	mal, T cell differentiation protein 2 [Source:VGNC Symbol;Acc:VGNC:98818]
chr6	95701570	95703104	1535	*	chr6	95701570	95703104	in_vitro	in_vivo	10	13	3.76	1.88846154	1.87153846	c(t = 1.58256	0.132	0.83731181	Distal Intergenic	ENSSSCG000	HPCAL4	hippocalcin like 4 [Source:VGNC Symbol;Acc:VGNC:88959]
chr13	35179148	35181193	2046	*	chr13	35179148	35181193	in_vitro	in_vivo	12	15	3.69083333	1.138	2.55283333	c(t = 1.61045	0.132	0.83731181	Distal Intergenic	ENSSSCG000	TKT	transketolase [Source:VGNC Symbol;Acc:VGNC:94008]
chr13	123648246	123651419	3174	*	chr13	123648246	123651419	in_vitro	in_vivo	15	13	1.92266667	0.86	1.06266667	c(t = 1.55758	0.132	0.83731181	Distal Intergenic	ENSSSCG000	TRA2B	transformer 2 beta homolog [Source:VGNC Symbol;Acc:VGNC:94354]
chr5	38530309	38531273	965	*	chr5	38530309	38531273	in_vitro	in_vivo	13	12	1.97230769	0.61333333	1.35897436	c(t = 1.59419	0.132	0.83731181	Distal Intergenic	ENSSSCG000	NAP1L1	nucleosome assembly protein 1 like 1 [Source:VGNC Symbol;Acc:VGNC:90576]
chr2	12236783	12237730	948	*	chr2	12236783	12237730	in_vitro	in_vivo	11	10	1.38545455	0.596	0.78945455	c(t = 1.58513	0.132	0.83731181	Distal Intergenic	ENSSSCG000	MPEG1	macrophage expressed 1 [Source:NCBI gene (formerly Entrezgene);Acc:100516953]
chr3	110364513	110365147	635	*	chr3	110364513	110365147	in_vitro	in_vivo	9	11	2.80888889	0.42545455	2.38343434	c(t = 1.66383	0.132	0.83731181	Distal Intergenic	ENSSSCG000	SPDYA	speedy/RINGO cell cycle regulator family member A [Source:VGNC Symbol;Acc:VGNC:93396]
chr1	14750483	14751213	731	*	chr1	14750483	14751213	in_vitro	in_vivo	12	12	2.11833333	0.40583333	1.7125	c(t = 1.61284	0.132	0.83731181	Distal Intergenic	ENSSSCG000	ARMT1	acidic residue methyltransferase 1 [Source:NCBI gene (formerly Entrezgene);Acc:100157036]
chr18	42351599	42352432	834	*	chr18	42351599	42352432	in_vitro	in_vivo	9	11	0.95777778	0.11545455	0.84232323	c(t = 1.65671	0.132	0.83731181	Distal Intergenic	ENSSSCG000	CRHR2	corticotropin releasing hormone receptor 2 [Source:VGNC Symbol;Acc:VGNC:86994]
chr14	37581797	37584215	2419	*	chr14	37581797	37584215	in_vitro	in_vivo	11	12	2.54	0	2.54	c(t = 1.64241	0.132	0.83731181	Distal Intergenic	ENSSSCG000	OAS2	2'-5'-oligoadenylate synthetase 2 [Source:VGNC Symbol;Acc:VGNC:91007]
chr16	75217339	75224081	6743	*	chr16	75217339	75224081	in_vitro	in_vivo	11	13	95.5881818	91.8961538	3.69202797	c(t = 1.56404	0.133	0.83731181	Distal Intergenic	ENSSSCG000	CCT5	chaperonin containing TCP1 subunit 5 [Source:VGNC Symbol;Acc:VGNC:86383]
chr9	72761788	72765866	4079	*	chr9	72761788	72765866	in_vitro	in_vivo	14	15	1.50857143	2.974	-1.4654286	c(t = -1.5588	0.133	0.83731181	Distal Intergenic	ENSSSCG000	ssc-mir-489	ssc-mir-489 [Source:miRBase;Acc:MI0022150]
chr1	86954375	86955571	1197	*	chr1	86954375	86955571	in_vitro	in_vivo	9	11	0.81111111	2.25818182	-1.4470707	c(t = -1.5863	0.133	0.83731181	Promoter (<=1kb)	ENSSSCG000	HMGN3	high mobility group nucleosomal binding domain 3 [Source:VGNC Symbol;Acc:VGNC:88908]
chr3	108223220	108224622	1403	*	chr3	108223220	108224622	in_vitro	in_vivo	9	11	11.0163636	2.00818182	9.00818182	c(t = 1.62807	0.133	0.83731181	Distal Intergenic	ENSSSCG000	XDH	xanthine dehydrogenase [Source:VGNC Symbol;Acc:VGNC:94995]
chr10	22737587	22741951	4365	*	chr10	22737587	22741951	in_vitro	in_vivo	9	13	2.88333333	1.25153846	1.63179487	c(t = 1.64379	0.133	0.83731181	Intron (NM_001267893/100523688, intron 1 of 6)	ENSSSCG000	NR5A2	nuclear receptor subfamily 5 group A member 2 [Source:VGNC Symbol;Acc:VGNC:96452]
chr9	52117244	52117625	382	*	chr9	52117244	52117625	in_vitro	in_vivo	10	10	3.736	1.023	2.713	c(t = 1.58757	0.133	0.83731181	Distal Intergenic	ENSSSCG000	STT3A	STT3 oligosaccharyltransferase complex catalytic subunit A [Source:VGNC Symbol;Acc:VGNC:93581]
chr6	65223922	65225393	1472	*	chr6	65223922	65225393	in_vitro	in_vivo	9	12	5.00111111	0.9275	4.07361111	c(t = 1.65875	0.133	0.83731181	Distal Intergenic	ENSSSCG000	WRAP73	WD repeat containing, antisense to TP73 [Source:VGNC Symbol;Acc:VGNC:94980]
chr1	45219317	45220259	943	*	chr1	45219317	45220259	in_vitro	in_vivo	13	12	1.77153846	0.48166667	1.28987179	c(t = 1.59311	0.133	0.83731181	Distal Intergenic	ENSSSCG000	ZUP1	zinc finger containing ubiquitin peptidase 1 [Source:VGNC Symbol;Acc:VGNC:95339]
chr4	63960959	63962197	1239	*	chr4	63960959	63962197	in_vitro	in_vivo	10	11	1.716	0.47272727	1.24327273	c(t = 1.61663	0.133	0.83731181	Distal Intergenic	ENSSSCG000	LACTB2	lactamase beta 2 [Source:NCBI gene (formerly Entrezgene);Acc:100153212]
chr2	88254244	88255514	1271	*	chr2	88254244	88255514	in_vitro	in_vivo	13	10	0.74307692	0.179	0.56407692	c(t = 1.57054	0.133	0.83731181	Promoter (<=1kb)	ENSSSCG000	HOMER1	homer scaffold protein 1 [Source:VGNC Symbol;Acc:VGNC:88928]
chr12	16810575	16811355	781	*	chr12	16810575	16811355	in_vitro	in_vivo	12	11	2.17333333	0	2.17333333	c(t = 1.62044	0.133	0.83731181	Distal Intergenic	ENSSSCG000	ITGB3	integrin subunit beta 3 [Source:NCBI gene (formerly Entrezgene);Acc:397063]
chr15	136380048	136381950	1903	*	chr15	136380048	136381950	in_vitro	in_vivo	10	11	15.876	38.7036364	-22.827636	c(t = -1.5733	0.134	0.83731181	Distal Intergenic	ENSSSCG000	ssc-mir-7141	ssc-mir-7141 [Source:miRBase;Acc:MI0023574]
chrX	124839501	124841054	1554	*	chrX	124839501	124841054	in_vitro	in_vivo	11	11	10.07	26.9790909	-16.909091	c(t = -1.5896	0.134	0.83731181	Distal Intergenic	ENSSSCG00000029523		
chr11	77974252	77977295	3044	*	chr11	77974252	77977295	in_vitro	in_vivo	15	12	3.31133333	1.80916667	1.50216667	c(t = 1.55212	0.134	0.83731181	Distal Intergenic	ENSSSCG000	F7	coagulation factor VII [Source:NCBI gene (formerly Entrezgene);Acc:733661]
chrX	55770857	55771986	1130	*	chrX	55770857	55771986	in_vitro	in_vivo	11	14	3.47363636	1.125	2.34863636	c(t = 1.61294	0.134	0.83731181	Distal Intergenic	ENSSSCG000	ssc-mir-676-	ssc-mir-676-1 [Source:miRBase;Acc:MI0013152]
chr15	4259706	4260283	578	*	chr15	4259706	4260283	in_vitro	in_vivo	11	10	2.93818182	0.804	2.13418182	c(t = 1.60211	0.134	0.83731181	Promoter (<=1kb)	ENSSSCG000	ACVR2A	activin A receptor type 2A [Source:NCBI gene (formerly Entrezgene);Acc:397282]
chr1	128641100	128642036	937	*	chr1	128641100	128642036	in_vitro	in_vivo	11	10	2.88727273	0.647	2.24027273	c(t = 1.62508	0.134	0.83731181	Distal Intergenic	ENSSSCG000	HAUS2	HAUS augmin like complex subunit 2 [Source:VGNC Symbol;Acc:VGNC:103104]
chr2	77086573	77087448	876	*	chr2	77086573	77087448	in_vitro	in_vivo	10	10	2.15	0.471	1.679	c(t = 1.61816	0.134	0.83731181	Distal Intergenic	ENSSSCG000	DAZAP1	DAZ associated protein 1 [Source:NCBI gene (formerly Entrezgene);Acc:100625005]
chr7	11719560	11720406	847	*	chr7	11719560	11720406	in_vitro	in_vivo	9	11	1.09555556	0.25272727	0.84282828	c(t = 1.60344	0.134	0.83731181	Promoter (<=1kb)	ENSSSCG000	DTNBP1	dystrobrevin binding protein 1 [Source:NCBI gene (formerly Entrezgene);Acc:100049697]
chr16	77565586	77566195	610	*	chr16	77565586	77566195	in_vitro	in_vivo	9	9	8.88	33.4411111	-24.561111	c(t = -1.6130	0.135	0.83731181	Distal Intergenic	ENSSSCG000	TERT	telomerase reverse transcriptase [Source:NCBI gene (formerly Entrezgene);Acc:492280]
chrX	111309587	111311206	1620	*	chrX	111309587	111311206	in_vitro	in_vivo	12	9	4.84166667	18.9966667	-14.155	c(t = -1.6472	0.135	0.83731181	Distal Intergenic	ENSSSCG000	FHL1	four and a half LIM domains 1 [Source:VGNC Symbol;Acc:VGNC:88127]
chrX	108611633	108612981	1349	*	chrX	108611633	108612981	in_vitro	in_vivo	14	10	9.935	3.801	6.134	c(t = 1.58001	0.135	0.83731181	Distal Intergenic	ENSSSCG000	MBNL3	muscleblind like splicing regulator 3 [Source:VGNC Symbol;Acc:VGNC:90056]
chr11	2790234	2791422	1189	*	chr11	2790234	2791422	in_vitro	in_vivo	9	13	5.94666667	2.36846154	3.57820513	c(t = 1.56854	0.135	0.83731181	Distal Intergenic	ENSSSCG000	ssc-mir-9814	ssc-mir-9814 [Source:miRBase;Acc:MI0031581]
chr13	21877378	21878749	1372	*	chr13	21877378	21878749	in_vitro	in_vivo	10	12	3.719	0.99583333	2.72316667	c(t = 1.62310	0.135	0.83731181	Promoter (<=1kb)	ENSSSCG000	MLH1	mutL homolog 1 [Source:VGNC Symbol;Acc:VGNC:90252]
chr1	222038464	222039632	1169	*	chr1	222038464	222039632	in_vitro	in_vivo	11	13	2.86818182	0.99538462	1.8727972	c(t = 1.60451	0.135	0.83731181	Distal Intergenic	ENSSSCG000	TJP2	tight junction protein 2 [Source:VGNC Symbol;Acc:VGNC:103188]
chr9	38298778	38299617	840	*	chr9	38298778	38299617	in_vitro	in_vivo	11	13	2.30181818	0.46230769	1.83951049	c(t = 1.60452	0.135	0.83731181	Promoter (<=1kb)	ENSSSCG000	RDX	radixin [Source:NCBI gene (formerly Entrezgene);Acc:494457]
chr6	51807925	51808802	878	*	chr6	51807925	51808802	in_vitro	in_vivo	9	10	10.3111111	0.426	9.88511111	c(t = 1.66095	0.135	0.83731181	Distal Intergenic	ENSSSCG000	ERCC1	ERCC excision repair 1, endonuclease non-catalytic subunit [Source:NCBI gene (formerly Entrezgene);Acc:100286808]
chr12	1313313	1314443	1131	*	chr12	1313313	1314443	in_vitro	in_vivo	10	9	1.056	0	1.056	c(t = 1.64196	0.135	0.83731181	Distal Intergenic	ENSSSCG000	ARHGDIA	Rho GDP dissociation inhibitor alpha [Source:VGNC Symbol;Acc:VGNC:85483]
chr6	690420	691630	1211	*	chr6	690420	691630	in_vitro	in_vivo	9	10	99.7777778	94.342	5.43577778	c(t = 1.63443	0.136	0.83731181	Distal Intergenic	ENSSSCG000	GALNS	galactosamine (N-acetyl)-6-sulfatase [Source:VGNC Symbol;Acc:VGNC:97061]
chr15	59066115	59067613	1499	*	chr15	59066115	59067613	in_vitro	in_vivo	16	13	3.525625	8.93307692	-5.4074519	c(t = -1.5684	0.136	0.83731181	Distal Intergenic	ENSSSCG000	PRPF40A	pre-mRNA processing factor 40 homolog A [Source:VGNC Symbol;Acc:VGNC:98222]
chr2	78515563	78516593	1031	*	chr2	78515563	78516593	in_vitro	in_vivo	12	13	4.985	1.82615385	3.15884615	c(t = 1.57970	0.136	0.83731181	Distal Intergenic	NA	NA	NA
chr13	122104940	122106057	1118	*	chr13	122104940	122106057	in_vitro	in_vivo	10	11	6.071	1.68909091	4.38190909	c(t = 1.61165	0.136	0.83731181	Distal Intergenic	ENSSSCG000	AP2M1	adaptor related protein complex 2 subunit mu 1 [Source:VGNC Symbol;Acc:VGNC:85386]
chr8	112650474	112651762	1289	*	chr8	112650474	112651762	in_vitro	in_vivo	10	12	2.701	1.11	1.591	c(t = 1.56884	0.136	0.83731181	Distal Intergenic	ENSSSCG000	EGF	epidermal growth factor [Source:VGNC Symbol;Acc:VGNC:98921]
chr13	122187264	122187964	701	*	chr13	122187264	122187964	in_vitro	in_vivo	9	13	2.47888889	0.61307692	1.86581197	c(t = 1.63004	0.136	0.83731181	Promoter (<=1kb)	ENSSSCG000	PSMD2	proteasome 26S subunit ubiquitin receptor, non-ATPase 2 [Source:NCBI gene (formerly Entrezgene);Acc:100627568]
chr15	54210540	54211319	780	*	chr15	54210540	54211319	in_vitro	in_vivo	12	9	1.30166667	3.11333333	-1.8116667	c(t = -1.5555	0.137	0.83731181	Distal Intergenic	ENSSSCG000	UBXN8	UBX domain protein 8 [Source:VGNC Symbol;Acc:VGNC:96239]
chr12	49732011	49733007	997	*	chr12	49732011	49733007	in_vitro	in_vivo	9	12	0.52111111	2.18166667	-1.6605556	c(t = -1.5777	0.137	0.83731181	Distal Intergenic	ENSSSCG000	ASPA	aspartoacylase [Source:NCBI gene (formerly Entrezgene);Acc:100142661]
chr7	53498666	53499679	1014	*	chr7	53498666	53499679	in_vitro	in_vivo	12	10	3.3925	1.348	2.0445	c(t = 1.55134	0.137	0.83731181	Distal Intergenic	ENSSSCG000	HDDC3	HD domain containing 3 [Source:VGNC Symbol;Acc:VGNC:88818]
chr1	271075049	271075933	885	*	chr1	271075049	271075933	in_vitro	in_vivo	9	10	2.62444444	1.052	1.57244444	c(t = 1.58836	0.137	0.83731181	Distal Intergenic	ENSSSCG000	FUBP3	far upstream element binding protein 3 [Source:VGNC Symbol;Acc:VGNC:103093]
chr2	101919328	101920678	1351	*	chr2	101919328	101920678	in_vitro	in_vivo	14	13	1.98785714	0.87846154	1.1093956	c(t = 1.54547	0.137	0.83731181	Distal Intergenic	ENSSSCG000	SPATA9	spermatogenesis associated 9 [Source:NCBI gene (formerly Entrezgene);Acc:100415927]
chr9	125290220	125291393	1174	*	chr9	125290220	125291393	in_vitro	in_vivo	12	12	2.51916667	0.83538462	1.68378205	c(t = 1.58534	0.137	0.83731181	Distal Intergenic	ENSSSCG000	ARPC5	actin related protein 2/3 complex subunit 5 [Source:NCBI gene (formerly Entrezgene);Acc:100174965]
chr14	32770703	32771764	1062	*	chr14	32770703	32771764	in_vitro	in_vivo	12	11	2.10916667	0.76909091	1.34007576	c(t = 1.55644	0.137	0.83731181	Distal Intergenic	ENSSSCG000	PRKAB1	protein kinase AMP-activated non-catalytic subunit beta 1 [Source:VGNC Symbol;Acc:VGNC:109479]
chr1	184478519	184479240	722	*	chr1	184478519	184479240	in_vitro	in_vivo	9	12	1.34777778	0.32583333	1.02194444	c(t = 1.61551	0.137	0.83731181	Distal Intergenic	ENSSSCG000	LGALS3	galectin 3 [Source:VGNC Symbol;Acc:VGNC:89694]
chr14	141115481	141118289	2809	*	chr14	141115481	141118289	in_vitro	in_vivo	13	15	18.2776923	37.1206667	-18.842974	c(t = -1.5334	0.138	0.83731181	Distal Intergenic	ENSSSCG000	ssc-mir-202	ssc-mir-202 [Source:miRBase;Acc:MI0013157]
chr13	206884378	206890774	6397	*	chr13	206884378	206890774	in_vitro	in_vivo	13	15	11.4530769	24.1813333	-12.728256	c(t = -1.5486	0.138	0.83731181	Promoter (<=1kb)	ENSSSCG000	AGPAT3	1-acylglycerol-3-phosphate O-acyltransferase 3 [Source:VGNC Symbol;Acc:VGNC:85185]
chr12	1317683	1322503	4821	*	chr12	1317683	1322503	in_vitro	in_vivo	13	13	27.8276923	15.92	11.9076923	c(t = 1.56205	0.138	0.83731181	Distal Intergenic	ENSSSCG000	TEPSIN	TEPSIN adaptor related protein complex 4 accessory protein [Source:NCBI gene (formerly Entrezgene);Acc:100626379]
chr8	4107677	4108455	779	*	chr8	4107677	4108455	in_vitro	in_vivo	10	9	4.001	1.78	2.221	c(t = 1.58837	0.138	0.83731181	Distal Intergenic	ENSSSCG000	MAN2B2	mannosidase alpha class 2B member 2 [Source:VGNC Symbol;Acc:VGNC:98100]
chr18	38182246	38183657	1412	*	chr18	38182246	38183657	in_vitro	in_vivo	14	12	2.13571429	0.77416667	1.36154762	c(t = 1.54653	0.138	0.83731181	Distal Intergenic	ENSSSCG000	FKBP9	FKBP prolyl isomerase 9 [Source:NCBI gene (formerly Entrezgene);Acc:100623924]
chr17	30941728	30942375	648	*	chr17	30941728	30942375	in_vitro	in_vivo	9	13	2.92777778	0.62692308	2.3008547	c(t = 1.62708	0.138	0.83731181	Distal Intergenic	ENSSSCG000	CST7	cystatin F [Source:NCBI gene (formerly Entrezgene);Acc:100152012]
chr8	81276441	81276952	512	*	chr8	81276441	81276952	in_vitro	in_vivo	9	12	0.84555556	0.23666667	0.60888889	c(t = 1.59552	0.138	0.83731181	Distal Intergenic	ENSSSCG000	EDNRA	endothelin receptor type A [Source:VGNC Symbol;Acc:VGNC:87549]
chr1	690192	693228	3037	*	chr1	690192	693228	in_vitro	in_vivo	14	15	16.3385714	31.48	-15.141429	c(t = -1.5398	0.139	0.83731181	Distal Intergenic	ENSSSCG000	ssc-mir-9815	ssc-mir-9815 [Source:miRBase;Acc:MI0031582]
chrX	124167411	124168787	1377	*	chrX	124167411	124168787	in_vitro	in_vivo	14	13	10.1228571	28.3430769	-18.22022	c(t = -1.5424	0.139	0.83731181	Distal Intergenic	ENSSSCG000	NSDHL	NAD(P) dependent steroid dehydrogenase-like [Source:NCBI gene (formerly Entrezgene);Acc:100157894]
chr16	78999772	79002446	2675	*	chr16	78999772	79002446	in_vitro	in_vivo	11	13	15.6981818	6.36923077	9.32895105	c(t = 1.57995	0.139	0.83731181	Distal Intergenic	ENSSSCG000	TERT	telomerase reverse transcriptase [Source:NCBI gene (formerly Entrezgene);Acc:492280]
chr8	119114552	119114881	330	*	chr8	119114552	119114881	in_vitro	in_vivo	11	11	19.8145455	5.08	14.7345455	c(t = 1.58666	0.139	0.83731181	Distal Intergenic	ENSSSCG000	PPP3CA	protein phosphatase 3 catalytic subunit alpha [Source:VGNC Symbol;Acc:VGNC:98218]
chr3	40992621	40993631	1011	*	chr3	40992621	40993631	in_vitro	in_vivo	9	11	8.05	2.57818182	5.47181818	c(t = 1.62003	0.139	0.83731181	Distal Intergenic	ENSSSCG000	RHOT2	ras homolog family member T2 [Source:NCBI gene (formerly Entrezgene);Acc:397596]
chr10	11465908	11467200	1293	*	chr10	11465908	11467200	in_vitro	in_vivo	10	12	2.863	0.67	2.193	c(t = 1.60651	0.139	0.83731181	Distal Intergenic	ENSSSCG000	FH	fumarate hydratase [Source:VGNC Symbol;Acc:VGNC:96300]
chr15	137636504	137637619	1116	*	chr15	137636504	137637619	in_vitro	in_vivo	11	12	1.72727273	0.62333333	1.10393939	c(t = 1.55488	0.139	0.83731181	Distal Intergenic	ENSSSCG000	RAMP1	receptor activity modifying protein 1 [Source:NCBI gene (formerly Entrezgene);Acc:397401]
chr7	38213081	38214323	1243	*	chr7	38213081	38214323	in_vitro	in_vivo	11	12	2.40727273	0.54083333	1.86643939	c(t = 1.59076	0.139	0.83731181	Distal Intergenic	ENSSSCG000	MRPL2	mitochondrial ribosomal protein L2 [Source:VGNC Symbol;Acc:VGNC:103072]
chr13	13327129	13327837	709	*	chr13	13327129	13327837	in_vitro	in_vivo	9	11	1.58444444	0.43181818	1.15262626	c(t = 1.61109	0.139	0.83731181	Distal Intergenic	ENSSSCG000	TOP2B	DNA topoisomerase II beta [Source:NCBI gene (formerly Entrezgene);Acc:100156319]
chr2	124199377	124199620	244	*	chr2	124199377	124199620	in_vitro	in_vivo	9	11	2.26777778	0.31909091	1.94868687	c(t = 1.61747	0.139	0.83731181	Distal Intergenic	ENSSSCG000	HSD17B4	hydroxysteroid 17-beta dehydrogenase 4 [Source:VGNC Symbol;Acc:VGNC:99760]
chr10	65206990	65209297	2308	*	chr10	65206990	65209297	in_vitro	in_vivo	11	9	90.6772727	98.1855556	-7.5082828	c(t = -1.5975	0.14	0.83731181	Distal Intergenic	ENSSSCG000	GDI2	GDP dissociation inhibitor 2 [Source:NCBI gene (formerly Entrezgene);Acc:414427]
chrX	60426861	60427355	495	*	chrX	60426861	60427355	in_vitro	in_vivo	9	10	0.38333333	6.703	-6.3196667	c(t = -1.6165	0.14	0.83731181	Promoter (<=1kb)	ENSSSCG000	UPRT	uracil phosphoribosyltransferase homolog [Source:VGNC Symbol;Acc:VGNC:98403]
chr12	4937974	4939628	1655	*	chr12	4937974	4939628	in_vitro	in_vivo	10	12	1.673	4.57833333	-2.9053333	c(t = -1.5485	0.14	0.83731181	Distal Intergenic	ENSSSCG00000036592		serine and arginine rich splicing factor 2 [Source:VGNC Symbol;Acc:VGNC:99085]
chr1	250680302	250681256	955	*	chr1	250680302	250681256	in_vitro	in_vivo	9	12	4.45555556	0.8775	3.57805556	c(t = 1.60952	0.14	0.83731181	Distal Intergenic	ENSSSCG000	PALM2AKAP	PALM2-AKAP2 fusion [Source:NCBI gene (formerly Entrezgene);Acc:100152369]
chr11	5343358	5346519	3162	*	chr11	5343358	5346519	in_vitro	in_vivo	9	16	2.04111111	0.740625	1.30048611	c(t = 1.59170	0.14	0.83731181	Promoter (<=1kb)	ENSSSCG000	CDX2	caudal type homeobox 2 [Source:NCBI gene (formerly Entrezgene);Acc:100127132]
chr3	87231203	87232172	970	*	chr3	87231203	87232172	in_vitro	in_vivo	13	12	1.46615385	0.4875	0.97865385	c(t = 1.53785	0.14	0.83731181	Distal Intergenic	ENSSSCG000	GPR75	G protein-coupled receptor 75 [Source:VGNC Symbol;Acc:VGNC:88636]
chr2	935264	976497	41234	*	chr2	935264	976497	in_vitro	in_vivo	16	13	30.881875	44.9576923	-14.075817	c(t = -1.5287	0.141	0.83731181	Distal Intergenic	ENSSSCG000	TOLLIP	toll interacting protein [Source:NCBI gene (formerly Entrezgene);Acc:100625123]
chr3	70574089	70579203	5115	*	chr3	70574089	70579203	in_vitro	in_vivo	10	16	2.739	6.323125	-3.584125	c(t = -1.5223	0.141	0.83731181	Distal Intergenic	ENSSSCG000	CCT7	chaperonin containing TCP1 subunit 7 [Source:NCBI gene (formerly Entrezgene);Acc:100322871]
chr15	86946825	86948112	1288	*	chr15	86946825	86948112	in_vitro	in_vivo	12	10	15.2891667	4.967	10.3221667	c(t = 1.57102	0.141	0.83731181	Distal Intergenic	ENSSSCG000	FRZB	frizzled related protein [Source:VGNC Symbol;Acc:VGNC:96308]
chr2	77812280	77812937	658	*	chr2	77812280	77812937	in_vitro	in_vivo	12	11	12.5166667	2.17818182	10.3384848	c(t = 1.58091	0.141	0.83731181	Distal Intergenic	ENSSSCG000	MADCAM1	mucosal vascular addressin cell adhesion molecule 1 [Source:VGNC Symbol;Acc:VGNC:108754]
chr8	43091412	43092300	889	*	chr8	43091412	43092300	in_vitro	in_vivo	9	13	2.20888889	0.86692308	1.34196581	c(t = 1.59625	0.141	0.83731181	Distal Intergenic	ENSSSCG000	CPE	carboxypeptidase E [Source:NCBI gene (formerly Entrezgene);Acc:100037304]
chr16	5459114	5460528	1415	*	chr16	5459114	5460528	in_vitro	in_vivo	14	15	1.95428571	0.67866667	1.27561905	c(t = 1.54030	0.141	0.83731181	Distal Intergenic	ENSSSCG000	C1QTNF3	C1q and TNF related 3 [Source:NCBI gene (formerly Entrezgene);Acc:100270814]
chr12	22874333	22875345	1013	*	chr12	22874333	22875345	in_vitro	in_vivo	14	12	1.64428571	0.64666667	0.99761905	c(t = 1.53456	0.141	0.83731181	Distal Intergenic	ENSSSCG000	STARD3	StAR related lipid transfer domain containing 3 [Source:VGNC Symbol;Acc:VGNC:93532]
chr7	117586302	117587042	741	*	chr7	117586302	117587042	in_vitro	in_vivo	11	12	2.29090909	0.64166667	1.64924242	c(t = 1.57140	0.141	0.83731181	Promoter (<=1kb)	ENSSSCG000	GSKIP	GSK3B interacting protein [Source:VGNC Symbol;Acc:VGNC:103956]
chr9	87086263	87086975	713	*	chr9	87086263	87086975	in_vitro	in_vivo	13	16	1.77692308	0.629375	1.14754808	c(t = 1.54834	0.141	0.83731181	Distal Intergenic	ENSSSCG000	AHR	aryl hydrocarbon receptor [Source:NCBI gene (formerly Entrezgene);Acc:396654]
chr14	132042371	132043853	1483	*	chr14	132042371	132043853	in_vitro	in_vivo	10	12	2.042	0.5475	1.4945	c(t = 1.59359	0.141	0.83731181	Distal Intergenic	ENSSSCG000	AWN	sperm associated AWN protein [Source:NCBI gene (formerly Entrezgene);Acc:396783]
chr9	105388425	105389010	586	*	chr9	105388425	105389010	in_vitro	in_vivo	9	14	2.93777778	0.45357143	2.48420635	c(t = 1.60356	0.141	0.83731181	Distal Intergenic	ENSSSCG000	NAMPT	nicotinamide phosphoribosyltransferase [Source:VGNC Symbol;Acc:VGNC:90573]
chr1	166309549	166310588	1040	*	chr1	166309549	166310588	in_vitro	in_vivo	14	9	2.02571429	0.44444444	1.58126984	c(t = 1.53943	0.141	0.83731181	Distal Intergenic	ENSSSCG000	CLN6	CLN6 transmembrane ER protein [Source:VGNC Symbol;Acc:VGNC:86771]
chr10	68565826	68566865	1040	*	chr10	68565826	68566865	in_vitro	in_vivo	11	11	1.32	0.10454545	1.21545455	c(t = 1.59529	0.141	0.83731181	Distal Intergenic	ENSSSCG000	WDR37	WD repeat domain 37 [Source:VGNC Symbol;Acc:VGNC:96258]
chr3	18677091	18678207	1117	*	chr3	18677091	18678207	in_vitro	in_vivo	12	13	14.595	24.9407692	-10.345769	c(t = -1.5224	0.142	0.83731181	Distal Intergenic	ENSSSCG000	SPNS1	SPNS lysolipid transporter 1, lysophospholipid [Source:NCBI gene (formerly Entrezgene);Acc:100517643]
chr4	125061954	125062657	704	*	chr4	125061954	125062657	in_vitro	in_vivo	10	10	0.988	21.158	-20.17	c(t = -1.6100	0.142	0.83731181	Distal Intergenic	ENSSSCG000	TGFBR3	transforming growth factor beta receptor 3 [Source:VGNC Symbol;Acc:VGNC:93932]
chr5	39315271	39315937	667	*	chr5	39315271	39315937	in_vitro	in_vivo	10	11	17.382	6.96454545	10.4174545	c(t = 1.53987	0.142	0.83731181	Distal Intergenic	ENSSSCG000	NAP1L1	nucleosome assembly protein 1 like 1 [Source:VGNC Symbol;Acc:VGNC:90576]
chr6	103639512	103640575	1064	*	chr6	103639512	103640575	in_vitro	in_vivo	11	11	2.20545455	4.40272727	-2.1972727	c(t = -1.5389	0.142	0.83731181	Distal Intergenic	ENSSSCG000	LPIN2	lipin 2 [Source:VGNC Symbol;Acc:VGNC:89794]
chr1	15949440	15950476	1037	*	chr1	15949440	15950476	in_vitro	in_vivo	11	12	1.42	3.23	-1.81	c(t = -1.5390	0.142	0.83731181	Distal Intergenic	ENSSSCG000	ULBP1	UL16 binding protein 1 [Source:NCBI gene (formerly Entrezgene);Acc:445523]
chr7	62810855	62811490	636	*	chr7	62810855	62811490	in_vitro	in_vivo	10	9	0.792	2.55444444	-1.7624444	c(t = -1.5678	0.142	0.83731181	Distal Intergenic	ENSSSCG000	NKX2-1	NK2 homeobox 1 [Source:VGNC Symbol;Acc:VGNC:90767]
chr1	198227	200910	2684	*	chr1	198227	200910	in_vitro	in_vivo	11	12	0.95545455	2.3375	-1.3820455	c(t = -1.5482	0.142	0.83731181	Distal Intergenic	ENSSSCG000	PSMB1	proteasome 20S subunit beta 1 [Source:NCBI gene (formerly Entrezgene);Acc:100621969]
chr7	22888685	22890090	1406	*	chr7	22888685	22890090	in_vitro	in_vivo	11	11	3.93363636	1.04454545	2.88909091	c(t = 1.55696	0.142	0.83731181	Intron (NM_001246242/100513601, intron 3 of 7)	NA	NA	NA
chr6	88530010	88531426	1417	*	chr6	88530010	88531426	in_vitro	in_vivo	11	11	2.84727273	0.44272727	2.40454545	c(t = 1.58625	0.142	0.83731181	Promoter (<=1kb)	ENSSSCG000	KHDRBS1	KH RNA binding domain containing, signal transduction associated 1 [Source:VGNC Symbol;Acc:VGNC:89422]
chr4	107821702	107822770	1069	*	chr4	107821702	107822770	in_vitro	in_vivo	12	11	1.74833333	0.37909091	1.36924242	c(t = 1.54455	0.142	0.83731181	Promoter (<=1kb)	ENSSSCG000	CAPZA1	capping actin protein of muscle Z-line subunit alpha 1 [Source:NCBI gene (formerly Entrezgene);Acc:100037957]
chr1	126236649	126237621	973	*	chr1	126236649	126237621	in_vitro	in_vivo	13	10	1.29538462	0.343	0.95238462	c(t = 1.54351	0.142	0.83731181	Promoter (<=1kb)	ENSSSCG000	SLC30A4	solute carrier family 30 member 4 [Source:VGNC Symbol;Acc:VGNC:93058]
chr13	108486808	108487146	339	*	chr13	108486808	108487146	in_vitro	in_vivo	9	11	1.39333333	0.30272727	1.09060606	c(t = 1.57488	0.142	0.83731181	Distal Intergenic	ENSSSCG000	SEC62	SEC62 homolog, preprotein translocation factor [Source:NCBI gene (formerly Entrezgene);Acc:100125961]
chr4	104057228	104058340	1113	*	chr4	104057228	104058340	in_vitro	in_vivo	10	12	3.451	0.24916667	3.20183333	c(t = 1.60444	0.142	0.83731181	Distal Intergenic	ENSSSCG000	CD2	CD2 molecule [Source:NCBI gene (formerly Entrezgene);Acc:396662]
chr14	108158705	108159205	501	*	chr14	108158705	108159205	in_vitro	in_vivo	9	11	3.54222222	0	3.54222222	c(t = 1.62744	0.142	0.83731181	Promoter (<=1kb)	ENSSSCG000	PIK3AP1	phosphoinositide-3-kinase adaptor protein 1 [Source:VGNC Symbol;Acc:VGNC:91435]
chr12	559571	564852	5282	*	chr12	559571	564852	in_vitro	in_vivo	12	12	91.9333333	94.9766667	-3.0433333	c(t = -1.5210	0.143	0.83731181	Distal Intergenic	ENSSSCG000	WDR45B	WD repeat domain 45B [Source:NCBI gene (formerly Entrezgene);Acc:100462760]
chr7	8200349	8201369	1021	*	chr7	8200349	8201369	in_vitro	in_vivo	10	11	4.375	19.4027273	-15.027727	c(t = -1.5736	0.143	0.83731181	Distal Intergenic	ENSSSCG000	ssc-mir-7136	ssc-mir-7136 [Source:miRBase;Acc:MI0023569]
chr6	28347790	28348330	541	*	chr6	28347790	28348330	in_vitro	in_vivo	9	11	0	3.42818182	-3.4281818	c(t = -1.5879	0.143	0.83731181	Distal Intergenic	ENSSSCG000	CTCF	CCCTC-binding factor [Source:VGNC Symbol;Acc:VGNC:87057]
chr4	57685090	57685794	705	*	chr4	57685090	57685794	in_vitro	in_vivo	13	12	0.31230769	1.4425	-1.1301923	c(t = -1.5543	0.143	0.83731181	Promoter (<=1kb)	ENSSSCG000	IL7	interleukin 7 [Source:VGNC Symbol;Acc:VGNC:98812]
chr8	111717460	111720037	2578	*	chr8	111717460	111720037	in_vitro	in_vivo	12	16	0.4975	1.3875	-0.89	c(t = -1.5131	0.143	0.83731181	Promoter (<=1kb)	ENSSSCG000	PITX2	paired like homeodomain 2 [Source:VGNC Symbol;Acc:VGNC:91467]
chr4	77593770	77595169	1400	*	chr4	77593770	77595169	in_vitro	in_vivo	9	14	2.20888889	0.80357143	1.40531746	c(t = 1.57936	0.143	0.83731181	Distal Intergenic	ENSSSCG000	ATP6V1H	ATPase H+ transporting V1 subunit H [Source:VGNC Symbol;Acc:VGNC:85677]
chr1	40179564	40180454	891	*	chr1	40179564	40180454	in_vitro	in_vivo	10	12	2.308	0.7325	1.5755	c(t = 1.56291	0.143	0.83731181	Distal Intergenic	ENSSSCG000	FABP7	fatty acid binding protein 7 [Source:NCBI gene (formerly Entrezgene);Acc:574075]
chr17	61992822	61994952	2131	*	chr17	61992822	61994952	in_vitro	in_vivo	10	9	15.792	32.93	-17.138	c(t = -1.5480	0.144	0.83731181	Distal Intergenic	ENSSSCG000	ssc-mir-133a	ssc-mir-133a-1 [Source:miRBase;Acc:MI0010681]
chr12	49820108	49821044	937	*	chr12	49820108	49821044	in_vitro	in_vivo	11	14	1.14181818	4.42785714	-3.286039	c(t = -1.5429	0.144	0.83731181	Distal Intergenic	ENSSSCG000	ASPA	aspartoacylase [Source:NCBI gene (formerly Entrezgene);Acc:100142661]
chr15	118273197	118273642	446	*	chr15	118273197	118273642	in_vitro	in_vivo	11	11	0.40454545	2.70090909	-2.2963636	c(t = -1.5748	0.144	0.83731181	Distal Intergenic	ENSSSCG000	IGFBP2	insulin like growth factor binding protein 2 [Source:NCBI gene (formerly Entrezgene);Acc:397064]
chr7	48245154	48246906	1753	*	chr7	48245154	48246906	in_vitro	in_vivo	13	11	1.18076923	2.24454545	-1.0637762	c(t = -1.5208	0.144	0.83731181	Distal Intergenic	ENSSSCG000	ssc-mir-184	ssc-mir-184 [Source:miRBase;Acc:MI0002421]
chr5	15027205	15029851	2647	*	chr5	15027205	15029851	in_vitro	in_vivo	12	13	7.19916667	1.69153846	5.50762821	c(t = 1.56519	0.144	0.83731181	Distal Intergenic	ENSSSCG000	PRKAG1	protein kinase AMP-activated non-catalytic subunit gamma 1 [Source:VGNC Symbol;Acc:VGNC:103314]
chr11	77984833	77992369	7537	*	chr11	77984833	77992369	in_vitro	in_vivo	15	14	2.97733333	1.46357143	1.5137619	c(t = 1.51979	0.144	0.83731181	Distal Intergenic	ENSSSCG000	F7	coagulation factor VII [Source:NCBI gene (formerly Entrezgene);Acc:733661]
chr15	74926822	74928028	1207	*	chr15	74926822	74928028	in_vitro	in_vivo	12	15	3.16833333	1.35	1.81833333	c(t = 1.54347	0.144	0.83731181	Distal Intergenic	ENSSSCG000	PHOSPHO2	phosphatase, orphan 2 [Source:VGNC Symbol;Acc:VGNC:96172]
chr10	65107984	65109509	1526	*	chr10	65107984	65109509	in_vitro	in_vivo	10	11	3.42	1.27181818	2.14818182	c(t = 1.55305	0.144	0.83731181	Distal Intergenic	ENSSSCG000	GDI2	GDP dissociation inhibitor 2 [Source:NCBI gene (formerly Entrezgene);Acc:414427]
chr2	27684518	27686080	1563	*	chr2	27684518	27686080	in_vitro	in_vivo	14	15	3.53642857	1.23333333	2.30309524	c(t = 1.53910	0.144	0.83731181	Distal Intergenic	ENSSSCG000	CSTF3	cleavage stimulation factor subunit 3 [Source:NCBI gene (formerly Entrezgene);Acc:100622040]
chr8	130852700	130853243	544	*	chr8	130852700	130853243	in_vitro	in_vivo	12	13	2.405	0.51	1.895	c(t = 1.55233	0.144	0.83731181	Promoter (<=1kb)	ENSSSCG000	ABCG2	ATP-binding cassette, sub-family G (WHITE), member 2 [Source:NCBI gene (formerly Entrezgene);Acc:397073]
chr14	129113246	129114560	1315	*	chr14	129113246	129114560	in_vitro	in_vivo	12	12	1.47583333	0.50666667	0.96916667	c(t = 1.53309	0.144	0.83731181	Distal Intergenic	ENSSSCG000	PRDX3	peroxiredoxin 3 [Source:NCBI gene (formerly Entrezgene);Acc:100154143]
chr11	19317016	19318088	1073	*	chr11	19317016	19318088	in_vitro	in_vivo	12	14	1.68916667	0.5	1.18916667	c(t = 1.54745	0.144	0.83731181	Promoter (<=1kb)	ENSSSCG000	RB1	RB transcriptional corepressor 1 [Source:VGNC Symbol;Acc:VGNC:92132]
chr13	127933331	127933840	510	*	chr13	127933331	127933840	in_vitro	in_vivo	11	9	2.95909091	0	2.95909091	c(t = 1.58635	0.144	0.83731181	Distal Intergenic	ENSSSCG000	CLDN16	claudin 16 [Source:VGNC Symbol;Acc:VGNC:86733]
chr11	77854226	77857606	3381	*	chr11	77854226	77857606	in_vitro	in_vivo	12	12	13.2891667	30.4558333	-17.166667	c(t = -1.5150	0.145	0.83731181	Distal Intergenic	ENSSSCG000	ANKRD10	ankyrin repeat domain 10 [Source:VGNC Symbol;Acc:VGNC:85322]
chr11	17731253	17732521	1269	*	chr11	17731253	17732521	in_vitro	in_vivo	14	13	0.70357143	3.17923077	-2.4756593	c(t = -1.5421	0.145	0.83731181	Distal Intergenic	NA	NA	NA
chr4	77538186	77540808	2623	*	chr4	77538186	77540808	in_vitro	in_vivo	15	14	0.76066667	2.16428571	-1.403619	c(t = -1.5199	0.145	0.83731181	Distal Intergenic	ENSSSCG000	ATP6V1H	ATPase H+ transporting V1 subunit H [Source:VGNC Symbol;Acc:VGNC:85677]
chr17	32507940	32509303	1364	*	chr17	32507940	32509303	in_vitro	in_vivo	10	10	5.007	1.662	3.345	c(t = 1.54493	0.145	0.83731181	Distal Intergenic	ENSSSCG000	DDRGK1	DDRGK domain containing 1 [Source:NCBI gene (formerly Entrezgene);Acc:100155894]
chr14	48876565	48877265	701	*	chr14	48876565	48877265	in_vitro	in_vivo	13	11	2.27769231	0.63727273	1.64041958	c(t = 1.53983	0.145	0.83731181	Intron (NM_001243319/100152327, intron 2 of 4)	NA	NA	NA
chr8	96692176	96693464	1289	*	chr8	96692176	96693464	in_vitro	in_vivo	11	12	3.25090909	0.62083333	2.63007576	c(t = 1.56627	0.145	0.83731181	Promoter (<=1kb)	ENSSSCG000	PGRMC2	progesterone receptor membrane component 2 [Source:NCBI gene (formerly Entrezgene);Acc:100048965]
chr16	52003268	52004540	1273	*	chr16	52003268	52004540	in_vitro	in_vivo	12	14	3.20166667	0.42	2.78166667	c(t = 1.55836	0.145	0.83731181	Distal Intergenic	ENSSSCG000	DUSP1	dual specificity phosphatase 1 [Source:VGNC Symbol;Acc:VGNC:87476]
chr7	104007861	104009792	1932	*	chr7	104007861	104009792	in_vitro	in_vivo	14	13	1.48	0.34384615	1.13615385	c(t = 1.53258	0.145	0.83731181	Distal Intergenic	ENSSSCG000	TSHR	thyroid stimulating hormone receptor [Source:VGNC Symbol;Acc:VGNC:94491]
chr4	45923071	45923695	625	*	chr4	45923071	45923695	in_vitro	in_vivo	10	11	2.35	0	2.35	c(t = 1.59797	0.145	0.83731181	Distal Intergenic	ENSSSCG000	CALB1	calbindin 1 [Source:NCBI gene (formerly Entrezgene);Acc:100153645]
chr1	1179834	1183305	3472	*	chr1	1179834	1183305	in_vitro	in_vivo	9	10	14.9933333	37.329	-22.335667	c(t = -1.5331	0.146	0.83731181	Distal Intergenic	NA	NA	NA
chr7	37370107	37370513	407	*	chr7	37370107	37370513	in_vitro	in_vivo	9	9	2.80666667	21.3411111	-18.534444	c(t = -1.6062	0.146	0.83731181	Distal Intergenic	ENSSSCG000	CCND3	cyclin D3 [Source:VGNC Symbol;Acc:VGNC:86353]
chrX	18118201	18119393	1193	*	chrX	18118201	18119393	in_vitro	in_vivo	10	10	3.4	9.387	-5.987	c(t = -1.5418	0.146	0.83731181	Distal Intergenic	ENSSSCG000	PHEX	phosphate regulating endopeptidase X-linked [Source:NCBI gene (formerly Entrezgene);Acc:100154258]
chr12	18922849	18923907	1059	*	chr12	18922849	18923907	in_vitro	in_vivo	9	11	1.87777778	5.68909091	-3.8113131	c(t = -1.5415	0.146	0.83731181	Distal Intergenic	ENSSSCG000	SLC25A39	solute carrier family 25 member 39 [Source:VGNC Symbol;Acc:VGNC:93014]
chr15	131738358	131739243	886	*	chr15	131738358	131739243	in_vitro	in_vivo	13	14	1.34076923	4.85785714	-3.5170879	c(t = -1.5438	0.146	0.83731181	Distal Intergenic	ENSSSCG000	SPATA3	spermatosis associated 3 [Source:VGNC Symbol;Acc:VGNC:95863]
chr1	189616999	189618324	1326	*	chr1	189616999	189618324	in_vitro	in_vivo	9	12	3.80111111	1.71833333	2.08277778	c(t = 1.55579	0.146	0.83731181	Distal Intergenic	ENSSSCG000	SIX1	SIX homeobox 1 [Source:NCBI gene (formerly Entrezgene);Acc:100156847]
chr14	77928087	77928990	904	*	chr14	77928087	77928990	in_vitro	in_vivo	9	12	6.19	1.7025	4.4875	c(t = 1.58106	0.146	0.83731181	Distal Intergenic	ENSSSCG000	VDAC2	voltage dependent anion channel 2 [Source:VGNC Symbol;Acc:VGNC:111154]
chrX	36759922	36762228	2307	*	chrX	36759922	36762228	in_vitro	in_vivo	13	15	2.48615385	1.216	1.27015385	c(t = 1.51724	0.146	0.83731181	Distal Intergenic	ENSSSCG000	DDX3X	DEAD-box helicase 3 X-linked [Source:NCBI gene (formerly Entrezgene);Acc:100515940]
chr2	84379954	84380871	918	*	chr2	84379954	84380871	in_vitro	in_vivo	13	13	2.50846154	1.02363636	1.48482517	c(t = 1.51436	0.146	0.83731181	Promoter (<=1kb)	ENSSSCG000	HMGCR	3-hydroxy-3-methylglutaryl-CoA reductase [Source:NCBI gene (formerly Entrezgene);Acc:100144446]
chr1	272954630	272955607	978	*	chr1	272954630	272955607	in_vitro	in_vivo	11	11	3.46727273	0.81818182	2.64909091	c(t = 1.54748	0.146	0.83731181	Distal Intergenic	NA	NA	NA
chr4	119785458	119786378	921	*	chr4	119785458	119786378	in_vitro	in_vivo	13	15	2.26153846	0.81333333	1.44820513	c(t = 1.51821	0.146	0.83731181	Distal Intergenic	NA	NA	NA
chr1	270190175	270190671	497	*	chr1	270190175	270190671	in_vitro	in_vivo	9	13	3.48777778	0	3.48777778	c(t = 1.61013	0.146	0.83731181	Distal Intergenic	ENSSSCG000	TOR1A	torsin family 1 member A [Source:NCBI gene (formerly Entrezgene);Acc:100125953]
chr11	78397415	78399809	2395	*	chr11	78397415	78399809	in_vitro	in_vivo	11	13	46.1027273	23.9476923	22.155035	c(t = 1.51338	0.147	0.83731181	Distal Intergenic	ENSSSCG000	F7	coagulation factor VII [Source:NCBI gene (formerly Entrezgene);Acc:733661]
chrX	54616447	54617062	616	*	chrX	54616447	54617062	in_vitro	in_vivo	9	9	3.34444444	21.8822222	-18.537778	c(t = -1.5978	0.147	0.83731181	Distal Intergenic	ENSSSCG000	AR	androgen receptor [Source:VGNC Symbol;Acc:VGNC:103903]
chr6	64469327	64470745	1419	*	chr6	64469327	64470745	in_vitro	in_vivo	11	10	3.28818182	7.136	-3.8478182	c(t = -1.5518	0.147	0.83731181	Distal Intergenic	ENSSSCG000	PRXL2B	peroxiredoxin like 2B [Source:VGNC Symbol;Acc:VGNC:98543]
chr3	7982060	7983017	958	*	chr3	7982060	7983017	in_vitro	in_vivo	11	10	5.21545455	1.226	3.98945455	c(t = 1.55713	0.147	0.83731181	Distal Intergenic	ENSSSCG000	COPS6	COP9 signalosome subunit 6 [Source:NCBI gene (formerly Entrezgene);Acc:100125954]
chr5	30187944	30190487	2544	*	chr5	30187944	30190487	in_vitro	in_vivo	14	15	2.20285714	1.12133333	1.08152381	c(t = 1.49262	0.147	0.83731181	Distal Intergenic	ENSSSCG000	LEMD3	LEM domain containing 3 [Source:NCBI gene (formerly Entrezgene);Acc:100511508]

chr4	77211372	77212101	730	*	chr4	77211372	77212101	in_vitro	in_vivo	11	13	2.41454545	0.92	1.49454545	c(t = 1.52779	0.147	0.83731181	Promoter (<=1kb)	ENSSSCG000	ATP6V1H	ATPase H+ transporting V1 subunit H [Source:VGNC Symbol;Acc:VGNC:85677]
chr1	31184928	31185414	487	*	chr1	31184928	31185414	in_vitro	in_vivo	11	12	1.91090909	0.75083333	1.16007576	c(t = 1.53273	0.147	0.83731181	Distal Intergenic	ENSSSCG000	VNN1	vanin 1 [Source:NCBI gene (formerly Entrezgene);Acc:397246]
chr15	139537091	139538272	1182	*	chr15	139537091	139538272	in_vitro	in_vivo	9	12	3.99444444	0.67416667	3.32027778	c(t = 1.58224	0.147	0.83731181	Distal Intergenic	ENSSSCG000	CAPN10	calpain 10 [Source:VGNC Symbol;Acc:VGNC:95629]
chr8	71301759	71302949	1191	*	chr8	71301759	71302949	in_vitro	in_vivo	12	13	1.97833333	0.33461538	1.64371795	c(t = 1.54136	0.147	0.83731181	Distal Intergenic	ENSSSCG000	RCHY1	ring finger and CHY zinc finger domain containing 1 [Source:VGNC Symbol;Acc:VGNC:92179]
chr2	28080646	28081455	810	*	chr2	28080646	28081455	in_vitro	in_vivo	11	14	0.65363636	0.08285714	0.57077922	c(t = 1.55806	0.147	0.83731181	Promoter (<=1kb)	ENSSSCG000	PRRG4	proline rich and Gla domain 4 [Source:VGNC Symbol;Acc:VGNC:91870]
chr3	696614	736591	39978	*	chr3	696614	736591	in_vitro	in_vivo	15	16	65.1413333	74.270625	-9.1292917	c(t = -1.4898	0.148	0.83731181	Distal Intergenic	ENSSSCG000	ssc-mir-339-	ssc-mir-339-1 [Source:miRBase;Acc:MI0013149]
chr1	239374932	239376108	1177	*	chr1	239374932	239376108	in_vitro	in_vivo	10	9	1.329	3.03666667	-1.7076667	c(t = -1.5385	0.148	0.83731181	Distal Intergenic	ENSSSCG000	TRMO	tRNA methyltransferase O [Source:NCBI gene (formerly Entrezgene);Acc:100156700]
chr5	3434795	3437155	2361	*	chr5	3434795	3437155	in_vitro	in_vivo	11	13	1.31818182	2.84153846	-1.5233566	c(t = -1.5018	0.148	0.83731181	Distal Intergenic	ENSSSCG000	PPARA	peroxisome proliferator activated receptor alpha [Source:NCBI gene (formerly Entrezgene);Acc:397239]
chr15	94261981	94262374	394	*	chr15	94261981	94262374	in_vitro	in_vivo	9	11	6.79222222	1.99545455	4.79676768	c(t = 1.58459	0.148	0.83731181	Distal Intergenic	ENSSSCG000	MSTN	myostatin [Source:NCBI gene (formerly Entrezgene);Acc:399534]
chr2	9665941	9667507	1567	*	chr2	9665941	9667507	in_vitro	in_vivo	9	12	7.66222222	1.88166667	5.78055556	c(t = 1.58395	0.148	0.83731181	Promoter (<=1kb)	ENSSSCG000	FADS2	fatty acid desaturase 2 [Source:NCBI gene (formerly Entrezgene);Acc:444997]
chr1	235868779	235869966	1188	*	chr1	235868779	235869966	in_vitro	in_vivo	12	12	4.24833333	1.685	2.56333333	c(t = 1.51771	0.148	0.83731181	Promoter (<=1kb)	ENSSSCG000	VCP	valosin containing protein [Source:VGNC Symbol;Acc:VGNC:109420]
chr4	110662367	110663370	1004	*	chr4	110662367	110663370	in_vitro	in_vivo	11	12	4.89636364	1.4975	3.39886364	c(t = 1.53796	0.148	0.83731181	Distal Intergenic	ENSSSCG000	PSMA5	proteasome 20S subunit alpha 5 [Source:VGNC Symbol;Acc:VGNC:98838]
chr15	94376918	94377734	817	*	chr15	94376918	94377734	in_vitro	in_vivo	11	13	4.11272727	1.26923077	2.8434965	c(t = 1.54911	0.148	0.83731181	Distal Intergenic	ENSSSCG000	MSTN	myostatin [Source:NCBI gene (formerly Entrezgene);Acc:399534]
chr15	110788737	110790236	1500	*	chr15	110788737	110790236	in_vitro	in_vivo	9	12	2.24333333	0.80416667	1.43916667	c(t = 1.56415	0.148	0.83731181	Distal Intergenic	NA	NA	NA
chr5	21517601	21518414	814	*	chr5	21517601	21518414	in_vitro	in_vivo	11	10	3.31909091	0.759	2.56009091	c(t = 1.54391	0.148	0.83731181	Distal Intergenic	ENSSSCG000	MYL6	myosin light chain 6 [Source:NCBI gene (formerly Entrezgene);Acc:396807]
chr8	52342818	52343671	854	*	chr8	52342818	52343671	in_vitro	in_vivo	10	11	1.86	0.67	1.19	c(t = 1.53110	0.148	0.83731181	Distal Intergenic	ENSSSCG000	NPY1R	neuropeptide Y receptor Y1 [Source:VGNC Symbol;Acc:VGNC:90865]
chr1	67656599	67657325	727	*	chr1	67656599	67657325	in_vitro	in_vivo	12	9	2.46416667	0.26444444	2.19972222	c(t = 1.54781	0.148	0.83731181	Distal Intergenic	ENSSSCG000	SIM1	SIM bHLH transcription factor 1 [Source:VGNC Symbol;Acc:VGNC:92876]
chr15	68107137	68107746	610	*	chr15	68107137	68107746	in_vitro	in_vivo	10	11	1.336	0.25272727	1.08327273	c(t = 1.54916	0.148	0.83731181	Distal Intergenic	ENSSSCG000	DPP4	dipeptidyl peptidase 4 [Source:NCBI gene (formerly Entrezgene);Acc:397492]
chr2	134277890	134278684	795	*	chr2	134277890	134278684	in_vitro	in_vivo	11	9	18.3018182	5.34444444	12.9573737	c(t = 1.54922	0.149	0.83731181	Distal Intergenic	ENSSSCG000	CSF2	colony stimulating factor 2 [Source:NCBI gene (formerly Entrezgene);Acc:397208]
chrX	42078002	42078498	497	*	chrX	42078002	42078498	in_vitro	in_vivo	10	10	17.507	4.561	12.946	c(t = 1.55488	0.149	0.83731181	Promoter (<=1kb)	ENSSSCG000	ARAF	A-Raf proto-onco, serine/threonine kinase [Source:VGNC Symbol;Acc:VGNC:85438]
chr13	89888803	89890502	1700	*	chr13	89888803	89890502	in_vitro	in_vivo	10	13	0.748	1.78153846	-1.0335385	c(t = -1.5072	0.149	0.83731181	Distal Intergenic	ENSSSCG000	CPHL1	ceruloplasmin and hephaestin like 1 [Source:NCBI gene (formerly Entrezgene);Acc:100158001]
chr10	57928686	57930079	1394	*	chr10	57928686	57930079	in_vitro	in_vivo	13	14	2.12769231	0.89071429	1.23697802	c(t = 1.49771	0.149	0.83731181	Distal Intergenic	ENSSSCG000	CCNY	cyclin Y [Source:VGNC Symbol;Acc:VGNC:95998]
chr10	59052595	59053398	804	*	chr10	59052595	59053398	in_vitro	in_vivo	14	12	1.91785714	0.625	1.29285714	c(t = 1.51406	0.149	0.83731181	Distal Intergenic	ENSSSCG000	CAMK1D	calcium/calmodulin dependent protein kinase ID [Source:VGNC Symbol;Acc:VGNC:95909]
chr3	55676724	55677353	630	*	chr3	55676724	55677353	in_vitro	in_vivo	11	12	1.57636364	0.3225	1.25386364	c(t = 1.54509	0.149	0.83731181	Distal Intergenic	ENSSSCG000	INPP4A	inositol polyphosphate-4-phosphatase type I A [Source:VGNC Symbol;Acc:VGNC:89140]
chrX	101477610	101479275	1666	*	chrX	101477610	101479275	in_vitro	in_vivo	10	12	1.05	7.99	-6.94	c(t = -1.5442	0.15	0.83731181	Distal Intergenic	ENSSSCG000	XIAP	X-linked inhibitor of apoptosis [Source:NCBI gene (formerly Entrezgene);Acc:100037300]
chr15	105643390	105646686	3297	*	chr15	105643390	105646686	in_vitro	in_vivo	13	15	2.12076923	4.15266667	-2.0318974	c(t = -1.4951	0.15	0.83731181	Distal Intergenic	ENSSSCG000	SUMO1	small ubiquitin like modifier 1 [Source:NCBI gene (formerly Entrezgene);Acc:100127139]
chr2	10087466	10088176	711	*	chr2	10087466	10088176	in_vitro	in_vivo	9	10	1.32222222	3.751	-2.4287778	c(t = -1.5283	0.15	0.83731181	Distal Intergenic	ENSSSCG000	PGA5	pepsinogen 5, group I (pepsinogen A) [Source:NCBI gene (formerly Entrezgene);Acc:396892]
chr18	45397630	45401007	3378	*	chr18	45397630	45401007	in_vitro	in_vivo	14	14	3.28714286	1.93928571	1.34785714	c(t = 1.49134	0.15	0.83731181	Promoter (<=1kb)	ENSSSCG000	HOXA10	homeobox A10 [Source:VGNC Symbol;Acc:VGNC:88934]
chr1	55618248	55619402	1155	*	chr1	55618248	55619402	in_vitro	in_vivo	12	10	0.20333333	1.648	-1.4446667	c(t = -1.5614	0.15	0.83731181	Distal Intergenic	ENSSSCG000	CGA	glycoprotein hormones, alpha polypeptide [Source:VGNC Symbol;Acc:VGNC:86613]
chr4	34268359	34269548	1190	*	chr4	34268359	34269548	in_vitro	in_vivo	11	10	5.57727273	1.624	3.95327273	c(t = 1.54140	0.15	0.83731181	Distal Intergenic	ENSSSCG000	AZIN1	antizyme inhibitor 1 [Source:VGNC Symbol;Acc:VGNC:85712]
chr18	51022041	51023494	1454	*	chr18	51022041	51023494	in_vitro	in_vivo	9	12	3.65555556	1.55916667	2.09638889	c(t = 1.53830	0.15	0.83731181	Distal Intergenic	ENSSSCG000	POLD2	DNA polymerase delta 2, accessory subunit [Source:VGNC Symbol;Acc:VGNC:91628]
chr11	13515986	13516702	717	*	chr11	13515986	13516702	in_vitro	in_vivo	9	11	0.24111111	1.30818182	-1.0670707	c(t = -1.5310	0.15	0.83731181	Distal Intergenic	ENSSSCG000	TRPC4	transient receptor potential cation channel subfamily C member 4 [Source:VGNC Symbol;Acc:VGNC:94462]
chr6	161928273	161929161	889	*	chr6	161928273	161929161	in_vitro	in_vivo	12	14	3.51	1.16	2.35	c(t = 1.51512	0.15	0.83731181	Distal Intergenic	ENSSSCG000	SPATA6	spermatosis associated 6 [Source:VGNC Symbol;Acc:VGNC:93387]
chr18	44263282	44264082	801	*	chr18	44263282	44264082	in_vitro	in_vivo	11	12	1.21	0.3475	0.8625	c(t = 1.51843	0.15	0.83731181	Distal Intergenic	ENSSSCG000	JAZF1	JAZF zinc finger 1 [Source:VGNC Symbol;Acc:VGNC:89280]
chr3	26187661	26188222	562	*	chr3	26187661	26188222	in_vitro	in_vivo	11	9	1.38818182	0.24666667	1.14151515	c(t = 1.53274	0.15	0.83731181	Distal Intergenic	ENSSSCG000	SMG1	SMG1 nonsense mediated mRNA decay associated PI3K related kinase [Source:VGNC Symbol;Acc:VGNC:93248]
chrX	74465476	74465941	466	*	chrX	74465476	74465941	in_vitro	in_vivo	9	10	1.77555556	0	1.77555556	c(t = 1.59217	0.15	0.83731181	Distal Intergenic	ENSSSCG000	ssc-mir-361	ssc-mir-361 [Source:miRBase;Acc:MI0013145]
chr13	54660688	54662007	1320	*	chr13	54660688	54662007	in_vitro	in_vivo	9	11	90.0322222	96.3572727	-6.3250505	c(t = -1.5288	0.151	0.83731181	Distal Intergenic	ENSSSCG000	MITF	melanocyte inducing transcription factor [Source:VGNC Symbol;Acc:VGNC:90243]
chr10	66741888	66743377	1490	*	chr10	66741888	66743377	in_vitro	in_vivo	12	10	17.5875	43.732	-26.1445	c(t = -1.5044	0.151	0.83731181	Distal Intergenic	ENSSSCG000	KLF6	KLF transcription factor 6 [Source:NCBI gene (formerly Entrezgene);Acc:100174961]
chrX	11408637	11409673	1037	*	chrX	11408637	11409673	in_vitro	in_vivo	10	10	8.307	24.445	-16.138	c(t = -1.5147	0.151	0.83731181	Promoter (<=1kb)	ENSSSCG000	MOSPD2	motile sperm domain containing 2 [Source:VGNC Symbol;Acc:VGNC:90314]
chr13	207881641	207883728	2088	*	chr13	207881641	207883728	in_vitro	in_vivo	12	12	3.9475	10.4308333	-6.4833333	c(t = -1.5279	0.151	0.83731181	Distal Intergenic	ENSSSCG000	ITGB2	integrin subunit beta 2 [Source:NCBI gene (formerly Entrezgene);Acc:396943]
chr6	63320368	63322648	2281	*	chr6	63320368	63322648	in_vitro	in_vivo	9	11	0.76111111	3.69272727	-2.9316162	c(t = -1.5369	0.151	0.83731181	Distal Intergenic	ENSSSCG000	ISG15	ISG15 ubiquitin like modifier [Source:NCBI gene (formerly Entrezgene);Acc:100145895]
chrX	51795	53603	1809	*	chrX	51795	53603	in_vitro	in_vivo	9	12	17.3788889	3.3625	14.0163889	c(t = 1.57997	0.151	0.83731181	Distal Intergenic	ENSSSCG000	ARSL	arylsulfatase E (chondrodysplasia punctata 1) [Source:NCBI gene (formerly Entrezgene);Acc:100627778]
chr8	72356029	72356670	642	*	chr8	72356029	72356670	in_vitro	in_vivo	9	9	4.14	1.46555556	2.67444444	c(t = 1.52161	0.151	0.83731181	Distal Intergenic	ENSSSCG000	SCARB2	scavenger receptor class B member 2 [Source:VGNC Symbol;Acc:VGNC:92614]
chr1	225712962	225714198	1237	*	chr1	225712962	225714198	in_vitro	in_vivo	14	14	2.56571429	1.24428571	1.32142857	c(t = 1.49097	0.151	0.83731181	Distal Intergenic	ENSSSCG000	ZFAND5	zinc finger AN1-type containing 5 [Source:VGNC Symbol;Acc:VGNC:95137]
chr7	88575038	88577237	2200	*	chr7	88575038	88577237	in_vitro	in_vivo	14	15	2.435	1.186	1.249	c(t = 1.48547	0.151	0.83731181	Distal Intergenic	ENSSSCG000	GPX2	glutathione peroxidase 2 [Source:NCBI gene (formerly Entrezgene);Acc:767625]
chr11	16911110	16912081	972	*	chr11	16911110	16912081	in_vitro	in_vivo	13	15	3.27384615	1.07733333	2.19651282	c(t = 1.50880	0.151	0.83731181	Promoter (<=1kb)	ENSSSCG000	RNASEH2B	ribonuclease H2 subunit B [Source:VGNC Symbol;Acc:VGNC:92339]
chr4	36208364	36210329	1966	*	chr4	36208364	36210329	in_vitro	in_vivo	11	13	3.30272727	0.65692308	2.6458042	c(t = 1.54761	0.151	0.83731181	Distal Intergenic	ENSSSCG000	ANKRD46	ankyrin repeat domain 46 [Source:VGNC Symbol;Acc:VGNC:85339]
chr2	8361712	8362686	975	*	chr2	8361712	8362686	in_vitro	in_vivo	10	14	1.688	0.49	1.198	c(t = 1.53979	0.151	0.83731181	Distal Intergenic	ENSSSCG000	PLAAT3	phospholipase A and acyltransferase 3 [Source:VGNC Symbol;Acc:VGNC:91504]
chr16	79861949	79863041	1093	*	chr16	79861949	79863041	in_vitro	in_vivo	11	12	1.40454545	0.4875	0.91704545	c(t = 1.50888	0.151	0.83731181	Distal Intergenic	ENSSSCG000	TPPP	tubulin polymerization promoting protein [Source:VGNC Symbol;Acc:VGNC:94344]
chr8	42733606	42734094	489	*	chr8	42733606	42734094	in_vitro	in_vivo	11	9	1.91363636	0.42777778	1.48585859	c(t = 1.51864	0.151	0.83731181	Distal Intergenic	ENSSSCG000	CPE	carboxypeptidase E [Source:NCBI gene (formerly Entrezgene);Acc:100037304]
chr14	114166769	114167584	816	*	chr14	114166769	114167584	in_vitro	in_vivo	12	10	1.41833333	0.294	1.12433333	c(t = 1.52739	0.151	0.83731181	Distal Intergenic	ENSSSCG000	ssc-mir-1307	ssc-mir-1307 [Source:miRBase;Acc:MI0013147]
chr5	67237801	67238687	887	*	chr5	67237801	67238687	in_vitro	in_vivo	9	10	1.24111111	0.24	1.00111111	c(t = 1.54964	0.151	0.83731181	Distal Intergenic	ENSSSCG000	TEAD4	TEA domain transcription factor 4 [Source:NCBI gene (formerly Entrezgene);Acc:100152085]
chr11	67710536	67711157	622	*	chr11	67710536	67711157	in_vitro	in_vivo	10	11	4.035	1.35090909	2.68409091	c(t = 1.52427	0.152	0.83731181	Distal Intergenic	ENSSSCG000	SLC15A1	solute carrier family 15 member 1 [Source:NCBI gene (formerly Entrezgene);Acc:397624]
chr7	38051598	38052427	830	*	chr7	38051598	38052427	in_vitro	in_vivo	9	12	3.41111111	1.2125	2.19861111	c(t = 1.54648	0.152	0.83731181	Promoter (<=1kb)	ENSSSCG000	PEX6	peroxisomal biosis factor 6 [Source:VGNC Symbol;Acc:VGNC:91330]
chr7	113147752	113149302	1551	*	chr7	113147752	113149302	in_vitro	in_vivo	16	14	2.15	1.00071429	1.14928571	c(t = 1.48688	0.152	0.83731181	Distal Intergenic	ENSSSCG00000002446		ataxin 3 [Source:NCBI gene (formerly Entrezgene);Acc:100144422]
chr2	77076323	77077221	899	*	chr2	77076323	77077221	in_vitro	in_vivo	9	11	3.68555556	0.96545455	2.72010101	c(t = 1.53083	0.152	0.83731181	Distal Intergenic	ENSSSCG000	DAZAP1	DAZ associated protein 1 [Source:NCBI gene (formerly Entrezgene);Acc:100625005]
chr12	53908064	53908884	821	*	chr12	53908064	53908884	in_vitro	in_vivo	9	9	4.67666667	0.84333333	3.83333333	c(t = 1.56970	0.152	0.83731181	Distal Intergenic	ENSSSCG000	CCDC42	coiled-coil domain containing 42 [Source:NCBI gene (formerly Entrezgene);Acc:100514007]
chr14	25143636	25144744	1109	*	chr14	25143636	25144744	in_vitro	in_vivo	11	14	2.19545455	0.83857143	1.35688312	c(t = 1.49757	0.152	0.83731181	Distal Intergenic	ENSSSCG000	PIWIL1	piwi like RNA-mediated silencing 1 [Source:VGNC Symbol;Acc:VGNC:98197]
chr10	52595427	52597337	1911	*	chr10	52595427	52597337	in_vitro	in_vivo	12	10	2.8425	0.416	2.4265	c(t = 1.53465	0.152	0.83731181	Promoter (<=1kb)	ENSSSCG000	BMI1	BMI1 proto-onco, polycomb ring finger [Source:VGNC Symbol;Acc:VGNC:108265]
chr2	59523126	59524377	1252	*	chr2	59523126	59524377	in_vitro	in_vivo	10	10	2.018	0.385	1.633	c(t = 1.53435	0.152	0.83731181	Distal Intergenic	ENSSSCG000	RAB3A	RAB3A, member RAS onco family [Source:VGNC Symbol;Acc:VGNC:98264]
chr2	116286854	116287543	690	*	chr2	116286854	116287543	in_vitro	in_vivo	9	12	1.90444444	0.37333333	1.53111111	c(t = 1.56892	0.152	0.83731181	Distal Intergenic	ENSSSCG000	STARD4	StAR related lipid transfer domain containing 4 [Source:VGNC Symbol;Acc:VGNC:93534]
chrX	42174600	42175367	768	*	chrX	42174600	42175367	in_vitro	in_vivo	11	13	5.52	18.8023077	-13.282308	c(t = -1.5040	0.153	0.83731181	Distal Intergenic	ENSSSCG000	SYN1	synapsin I [Source:NCBI gene (formerly Entrezgene);Acc:100216310]
chr9	51975395	51976859	1465	*	chr9	51975395	51976859	in_vitro	in_vivo	9	11	2.81	6.84090909	-4.0309091	c(t = -1.4948	0.153	0.83731181	Distal Intergenic	ENSSSCG000	STT3A	STT3 oligosaccharyltransferase complex catalytic subunit A [Source:VGNC Symbol;Acc:VGNC:93581]
chr7	98822145	98822731	587	*	chr7	98822145	98822731	in_vitro	in_vivo	10	9	3.694	0.79333333	2.90066667	c(t = 1.52280	0.153	0.83731181	Distal Intergenic	ENSSSCG000	ERG28	ergosterol biosynthesis 28 homolog [Source:VGNC Symbol;Acc:VGNC:87769]
chr16	3969719	3970754	1036	*	chr16	3969719	3970754	in_vitro	in_vivo	9	13	1.89555556	0.60769231	1.28786325	c(t = 1.53461	0.153	0.83731181	Distal Intergenic	ENSSSCG000	C1QTNF3	C1q and TNF related 3 [Source:NCBI gene (formerly Entrezgene);Acc:100270814]
chr17	33410673	33412403	1731	*	chr17	33410673	33412403	in_vitro	in_vivo	11	14	18.8718182	7.54285714	11.328961	c(t = 1.50614	0.154	0.83731181	Distal Intergenic	ENSSSCG000	PDYN	prodynorphin [Source:VGNC Symbol;Acc:VGNC:96477]
chr14	3137776	3138192	417	*	chr14	3137776	3138192	in_vitro	in_vivo	9	11	0	2.38636364	-2.3863636	c(t = -1.5418	0.154	0.83731181	Distal Intergenic	ENSSSCG000	LPL	lipoprotein lipase [Source:VGNC Symbol;Acc:VGNC:89795]
chr13	96643128	96644106	979	*	chr13	96643128	96644106	in_vitro	in_vivo	13	12	2.53153846	1.31583333	1.21570513	c(t = 1.47816	0.154	0.83731181	Distal Intergenic	ENSSSCG000	SSR3	signal sequence receptor subunit 3 [Source:VGNC Symbol;Acc:VGNC:93492]
chr14	76699802	76700523	722	*	chr14	76699802	76700523	in_vitro	in_vivo	12	13	2.74083333	0.72076923	2.0200641	c(t = 1.50440	0.154	0.83731181	Promoter (<=1kb)	ENSSSCG000	VCL	vinculin [Source:VGNC Symbol;Acc:VGNC:94812]
chr4	69802240	69803647	1408	*	chr4	69802240	69803647	in_vitro	in_vivo	15	15	2.07533333	0.66333333	1.412	c(t = 1.49196	0.154	0.83731181	Distal Intergenic	ENSSSCG000	ssc-mir-124a	ssc-mir-124a-1 [Source:miRBase;Acc:MI0010680]
chr4	104589235	104589468	234	*	chr4	104589235	104589468	in_vitro	in_vivo	9	9	8.22444444	3.95333333	4.27111111	c(t = 1.49498	0.155	0.83731181	Distal Intergenic	ENSSSCG000	ATP1A1	ATPase Na+/K+ transporting subunit alpha 1 [Source:VGNC Symbol;Acc:VGNC:98735]
chr6	53809477	53810078	602	*	chr6	53809477	53810078	in_vitro	in_vivo	11	10	3.05272727	1.446	1.60672727	c(t = 1.49414	0.155	0.83731181	Distal Intergenic	ENSSSCG000	EMP3	epithelial membrane protein 3 [Source:VGNC Symbol;Acc:VGNC:87690]
chr10	19045922	19046994	1073	*	chr10	19045922	19046994	in_vitro	in_vivo	12	11	3.79333333	1.33909091	2.45424242	c(t = 1.48354	0.155	0.83731181	Distal Intergenic	ENSSSCG000	SMYD3	SET and MYND domain containing 3 [Source:NCBI gene (formerly Entrezgene);Acc:100294707]
chr1	237785173	237786908	1736	*	chr1	237785173	237786908	in_vitro	in_vivo	11	14	3.54090909	1.31428571	2.22662338	c(t = 1.48892	0.155	0.83731181	Distal Intergenic	ENSSSCG000	GNE	glucosamine (UDP-N-acetyl)-2-epimerase/N-acetylmannosamine kinase [Source:VGNC Symbol;Acc:VGNC:103101]
chr12	20256958	20257834	877	*	chr12	20256958	20257834	in_vitro	in_vivo	9	10	4.21666667	1.312	2.90466667	c(t = 1.53302	0.155	0.83731181	Distal Intergenic	ENSSSCG000	COASY	Coenzyme A synthase [Source:VGNC Symbol;Acc:VGNC:97935]
chr14	104105614	104106696	1083	*	chr14	104105614	104106696	in_vitro	in_vivo	15	13	2.49066667	1.03307692	1.45758974	c(t = 1.47115	0.155	0.83731181	Distal Intergenic	ENSSSCG000	HHEX	hematopoietically expressed homeobox [Source:NCBI gene (formerly Entrezgene);Acc:397232]
chr4	9643619	9646361	2743	*	chr4	9643619	9646361	in_vitro	in_vivo	13	15	1.67461538	0.61133333	1.06328205	c(t = 1.48954	0.155	0.83731181	Distal Intergenic	ENSSSCG00000005959		CYFIP related Rac1 interactor B [Source:VGNC Symbol;Acc:VGNC:97956]
chr11	17685097	17686093	997	*	chr11	17685097	17686093	in_vitro	in_vivo	10	13	2.654	0.58846154	2.06553846	c(t = 1.53378	0.155	0.83731181	Distal Intergenic	NA	NA	NA
chr6	28882914	28884051	1138	*	chr6	28882914	28884051	in_vitro	in_vivo	12	12	1.89666667	0.50166667	1.395	c(t = 1.50046	0.155	0.83731181	Distal Intergenic	ENSSSCG000	SLC12A4	solute carrier family 12 member 4 [Source:NCBI gene (formerly Entrezgene);Acc:396992]
chr5	21495512	21496740	1229	*	chr5	21495512	21496740	in_vitro	in_vivo	9	10	2.95666667	0.192	2.76466667	c(t = 1.56695	0.155	0.83731181	Distal Intergenic	ENSSSCG000	MYL6	myosin light chain 6 [Source:NCBI gene (formerly Entrezgene);Acc:396807]
chr3	48688936	48689760	825	*	chr3	48688936	48689760	in_vitro	in_vivo	9	10	17.3488889	37.027	-19.678111	c(t = -1.5094	0.156	0.83731181	Distal Intergenic	ENSSSCG000	UXS1	UDP-glucuronate decarboxylase 1 [Source:VGNC Symbol;Acc:VGNC:94791]
chr14	140616506	140618794	2289	*	chr14	140616506	140618794	in_vitro	in_vivo	9	9	11.8855556	30.8766667	-18.991111	c(t = -1.5300	0.156	0.83731181	Distal Intergenic	ENSSSCG000	ssc-mir-202	ssc-mir-202 [Source:miRBase;Acc:MI0013157]
chrX	68475481	68476646	1166	*	chrX	68475481	68476646	in_vitro	in_vivo	12	11	2.7375	11	-8.2625	c(t = -1.5103	0.156	0.83731181	Distal Intergenic	ENSSSCG000	ssc-mir-361	ssc-mir-361 [Source:miRBase;Acc:MI0013145]
chr9	1958181	1958677	497	*	chr9	1958181	1958677	in_vitro	in_vivo	9	11	17.07	5.70545455	11.3645455	c(t = 1.54050	0.156	0.83731181	Distal Intergenic	NA	NA	NA
chr3	634490	635426	937	*	chr3	634490	635426	in_vitro	in_vivo	9	12	0.76	3.42333333	-2.6633333	c(t = -1.5058	0.156	0.83731181	Distal Intergenic	ENSSSCG000	ADAP1	ArfGAP with dual PH domains 1 [Source:NCBI gene (formerly Entrezgene);Acc:397453]
chr3	8906543	8908021	1479	*	chr3	8906543	8908021	in_vitro	in_vivo	9	13	5.49777778	1.51153846	3.98623932	c(t = 1.55198	0.156	0.83731181	Distal Intergenic	ENSSSCG000	CLDN15	claudin 15 [Source:VGNC Symbol;Acc:VGNC:86732]
chr1	116292642	116293870	1229	*	chr1	116292642	116293870	in_vitro	in_vivo	15	12	2.99133333	1.2175	1.77383333	c(t = 1.46520	0.156	0.83731181	Distal Intergenic	ENSSSCG000	CCPG1	cell cycle progression 1 [Source:VGNC Symbol;Acc:VGNC:86368]
chr9	118113587	118114735	1149	*	chr9	118113587	118114735	in_vitro	in_vivo	12	13	2.14416667	0.97307692	1.17108974	c(t = 1.46754	0.156	0.83731181	Distal Intergenic	ENSSSCG000	SERPINC1	serpin family C member 1 [Source:NCBI gene (formerly Entrezgene);Acc:100125972]
chr2	5592836	5593606	771	*	chr2	5592836	5593606	in_vitro	in_vivo	11	11	3.24909091	0.91083333	2.33825758	c(t = 1.51579	0.156	0.83731181	Distal Intergenic	ENSSSCG000	PC	pyruvate carboxylase [Source:NCBI gene (formerly Entrezgene);Acc:397630]
chr14	61666240	61668094	1855	*	chr14	61666240	61668094	in_vitro	in_vivo	11	10	1.13454545	0.137	0.99754545	c(t = 1.52475	0.156	0.83731181	Distal Intergenic	ENSSSCG000	PHYHIPL	phytanoyl-CoA 2-hydroxylase interacting protein like [Source:VGNC Symbol;Acc:VGNC:91407]
chr12	59373799	59377144	3346	*	chr12	59373799	59377144	in_vitro	in_vivo	10	13	98.078	94.9384615	3.13953846	c(t = 1.49213	0.157	0.83731181	Distal Intergenic	ENSSSCG000	UBB	ubiquitin B [Source:VGNC Symbol;Acc:VGNC:98389]
chr12	23581861	23582956	1096	*	chr12	23581861	23582956	in_vitro	in_vivo	11	11	6.59	23.5954545	-17.005455	c(t = -1.5115	0.157	0.83731181	Distal Intergenic	ENSSSCG000	SOCS7	suppressor of cytokine signaling 7 [Source:VGNC Symbol;Acc:VGNC:93334]
chr1	140464192	140465203	1012	*	chr1	140464192	140465203	in_vitro	in_vivo	12	13	0.84083333	1.81923077	-0.9783974	c(t = -1.4674	0.157	0.83731181	Distal Intergenic	ENSSSCG000	SELENOS	selenoprotein S [Source:NCBI gene (formerly Entrezgene);Acc:100151836]
chr1	7589177	7590735	1559	*	chr1	7589177	7590735	in_vitro	in_vivo	13	12	0.59538462	1.7375	-1.1421154	c(t = -1.4881	0.157	0.83731181	Promoter (<=1kb)	ENSSSCG000	TCP1	t-complex 1 [Source:NCBI gene (formerly Entrezgene);Acc:100153485]
chr3	8363272	8363956	685	*	chr3	8363272	8363956	in_vitro	in_vivo	11	12	3.91363636	1.405	2.50863636	c(t = 1.50628	0.157	0.83731181	Distal Intergenic	ENSSSCG000	PVRIG	PVR related immunoglobulin domain containing [Source:VGNC Symbol;Acc:VGNC:92008]
chr7	38504395	38505802	1408	*	chr7	38504395	38505802	in_vitro	in_vivo	10	11	3.334	1.30363636	2.03036364	c(t = 1.49246	0.157	0.83731181	Distal Intergenic	ENSSSCG000	YIPF3	Yip1 domain family member 3 [Source:VGNC Symbol;Acc:VGNC:95029]
chr16	75258943	75261280	2338	*	chr16	75258943	75261280	in_vitro	in_vivo	12	10	2.73	1.131	1.599	c(t = 1.49947	0.157	0.83731181	Distal Intergenic	ENSSSCG000	CCT5	chaperonin containing TCP1 subunit 5 [Source:VGNC Symbol;Acc:VGNC:86383]
chr9	65154175	65154865	691	*	chr9	65154175	65154865	in_vitro	in_vivo	9	12	2.65111111	1.0825	1.56861111	c(t = 1.50329	0.157	0.83731181	Distal Intergenic	NA	NA	NA
chr5	10040803	10041588	786	*	chr5	10040803	10041588	in_vitro	in_vivo	9	9	3.53111111	0.83888889	2.69222222	c(t = 1.53611	0.157	0.83731181	Distal Intergenic	ENSSSCG000	EIF3L	eukaryotic translation initiation factor 3 subunit L [Source:VGNC Symbol;Acc:VGNC:87623]
chr1	121413109	121414480	1372	*	chr1	121413109	121414480	in_vitro	in_vivo	13	11	2.09692308	0.77909091	1.31783217	c(t = 1.47987	0.157	0.83731181	Distal Intergenic	ENSSSCG000	SLC27A2	solute carrier family 27 member 2 [Source:NCBI gene (formerly Entrezgene);Acc:494011]
chr6	120036547	120037803	1257	*	chr6	120036547	120037803	in_vitro	in_vivo	13	11	1.95230769	0.74272727	1.20958042	c(t = 1.46408	0.157	0.83731181	Distal Intergenic	NA	NA	NA
chr5	33049126	33049500	375	*	chr5	33049126	33049500	in_vitro	in_vivo	10	10	1.804	0.286	1.518	c(t = 1.52302	0.157	0.83731181	Distal Intergenic	ENSSSCG000	MDM2	MDM2 proto-onco [Source:VGNC Symbol;Acc:VGNC:90093]
chr6	47308509	47309252	744	*	chr6	47308509	47309252	in_vitro	in_vivo	9	10	7.65111111	20.951	-13.299889	c(t = -1.5251	0.158	0.83731181	Distal Intergenic	ENSSSCG000	RYR1	ryanodine receptor 1 [Source:NCBI gene (formerly Entrezgene);Acc:396718]
chrX	26728507	26729489	983	*	chrX	26728507	26729489	in_vitro	in_vivo	10	12	1.88	8.605	-6.725	c(t = -1.5098	0.158	0.83731181	Distal Intergenic	ENSSSCG00000012202		glycerol kinase [Source:NCBI gene (formerly Entrezgene);Acc:100233182]
chr11	12254653	12255670	1018	*	chr11	12254653	12255670	in_vitro	in_vivo	11	13	1.79636364	5.29615385	-3.4997902	c(t = -1.4944	0.158	0.83731181	Distal Intergenic	ENSSSCG000	SMAD9	SMAD family member 9 [Source:NCBI gene (formerly Entrezgene);Acc:100153584]
chr4	39362847	39363559	713	*	chr4	39362847	39363559	in_vitro	in_vivo	10	13	0.919	3.26769231	-2.3486923	c(t = -1.4782	0.158	0.83731181	Distal Intergenic	ENSSSCG000	MTDH	metadherin [Source:VGNC Symbol;Acc:VGNC:90439]
chr16	29111991	29112564	574	*	chr16	29111991	29112564	in_vitro	in_vivo	13	12	0.22615385	1.04666667	-0.8205128	c(t = -1.4847	0.158	0.83731181	Distal Intergenic	ENSSSCG000	CCL28	C-C motif chemokine ligand 28 [Source:NCBI gene (formerly Entrezgene);Acc:554303]
chr1	239109878	239110333	456	*	chr1	239109878	239110333	in_vitro	in_vivo	9	10	7.62222222	0.952	6.67022222	c(t = 1.54089	0.158	0.83731181	Distal Intergenic	ENSSSCG00000032554		solute carrier family 25 member 51 [Source:NCBI gene (formerly Entrezgene);Acc:100157218]
chr5	54015703	54017153	1451	*	chr5	54015703	54017153	in_vitro	in_vivo	12	12	3.025	0.93083333	2.09416667	c(t = 1.49594	0.158	0.83731181	Distal Intergenic	ENSSSCG000	PLCZ1	phospholipase C zeta 1 [Source:VGNC Symbol;Acc:VGNC:103310]
chr15	130938726	130940523	1798	*	chr15	130938726	130940523	in_vitro	in_vivo	10	12	2.489	0.46333333	2.02566667	c(t = 1.52454	0.158	0.83731181	Distal Intergenic	ENSSSCG000	PID1	phosphotyrosine interaction domain containing 1 [Source:NCBI gene (formerly Entrezgene);Acc:100302508]
chr3	113667494	113668510	1017	*	chr3	113667494	113668510	in_vitro	in_vivo	10	9	8.937	2.59777778	6.33922222	c(t = 1.51206	0.159	0.83731181	3' UTR	ENSSSCG00000021155		proopiomelanocortin [Source:VGNC Symbol;Acc:VGNC:110699]
chr6	128860057	128860790	734	*	chr6	128860057	128860790	in_vitro	in_vivo	9	13	0.77666667	2.38769231	-1.6110256	c(t = -1.4695	0.159	0.83731181	Distal Intergenic	ENSSSCG000	LPAR3	lysophosphatidic acid receptor 3 [Source:VGNC Symbol;Acc:VGNC:89785]
chr4	105779701	105780255	555	*	chr4	105779701	105780255	in_vitro	in_vivo	9	11	0.48	1.94181818	-1.4618182	c(t = -1.4973	0.159	0.83731181	Distal Intergenic	ENSSSCG000	NRAS	NRAS proto-oncogene, GTPase [Source:NCBI gene (formerly Entrezgene);Acc:100739349]
chr1	92472111	92472497	387	*	chr1	92472111	92472497	in_vitro	in_vivo	9	10	0.25888889	1.443	-1.1841111	c(t = -1.5110	0.159	0.83731181	Distal Intergenic	ENSSSCG00000004489		eukaryotic translation elongation factor 1 alpha 1 [Source:NCBI gene (formerly Entrezgene);Acc:574059]
chr3	46546187	46548494	2308	*	chr3	46546187	46548494	in_vitro	in_vivo	14	16	5.915	1.43625	4.47875	c(t = 1.48932	0.159	0.83731181	Distal Intergenic	ENSSSCG000	MALL	mal, T cell differentiation protein like [Source:NCBI gene (formerly Entrezgene);Acc:100626835]
chr7	65240198	65240605	408	*	chr7	65240198	65240605	in_vitro	in_vivo	9	11	2.80333333	1.14	1.66333333	c(t = 1.47763	0.159	0.83731181	Distal Intergenic	ENSSSCG000	SNX6	sorting nexin 6 [Source:NCBI gene (formerly Entrezgene);Acc:100154775]
chr1	268857050	268858138	1089	*	chr1	268857050	268858138	in_vitro	in_vivo	10	12	2.804	1.11333333	1.69066667	c(t = 1.49430	0.159	0.83731181	Promoter (<=1kb)	ENSSSCG000	ODF2	outer dense fiber of sperm tails 2 [Source:VGNC Symbol;Acc:VGNC:91017]
chr18	31888358	31890464	2107	*	chr18	31888358	31890464	in_vitro	in_vivo	13	14	2.90538462	0.81428571	2.0910989	c(t = 1.48318	0.159	0.83731181	Distal Intergenic	ENSSSCG000	FOXP2	forkhead box P2 [Source:VGNC Symbol;Acc:VGNC:98014]
chr13	56018700	56019145	446	*	chr13	56018700	56019145	in_vitro	in_vivo	11	12	1.03272727	0.16666667	0.86606061	c(t = 1.50374	0.159	0.83731181	Distal Intergenic	ENSSSCG000	MITF	melanocyte inducing transcription factor [Source:VGNC Symbol;Acc:VGNC:90243]
chr14	32311154	32312445	1292	*	chr14	32311154	32312445	in_vitro	in_vivo	11	9	3.68454545	1.61777778	2.06676768	c(t = 1.48026	0.16	0.83731181	Distal Intergenic	ENSSSCG000	MYL2	myosin light chain 2 [Source:VGNC Symbol;Acc:VGNC:90514]
chr14	82305943	82306949	1007	*	chr14	82305943	82306949	in_vitro	in_vivo	9	10	3.01	1.575	1.435	c(t = 1.49361	0.16	0.83731181	Distal Intergenic	ENSSSCG000	PRXL2A	peroxiredoxin like 2A [Source:VGNC Symbol;Acc:VGNC:91892]
chr4	129721167	129722142	976	*	chr4	129721167	129722142	in_vitro	in_vivo	13	11	2.04461538	0.79545455	1.24916084	c(t = 1.47140	0.16	0.83731181	Distal Intergenic	ENSSSCG000	CLCA1	chloride channel accessory 1 [Source:NCBI gene (formerly Entrezgene);Acc:397284]
chr11	19616364	19616989	626	*	chr11	19616364	19616989	in_vitro	in_vivo	12	13	2.34166667	0.69230769	1.64935897	c(t = 1.47647	0.16	0.83731181	Distal Intergenic	ENSSSCG000	ITM2B	integral membrane protein 2B [Source:VGNC Symbol;Acc:VGNC:103971]
chr13	90970844	90971971	1128	*	chr13	90970844	90971971	in_vitro	in_vivo	14	10	1.54357143	0.585	0.95857143	c(t = 1.46088	0.16	0.83731181	Distal Intergenic	ENSSSCG000	SELENOT	selenoprotein T [Source:NCBI gene (formerly Entrezgene);Acc:100302502]
chr4	84056469	84056948	480	*	chr4	84056469	84056948	in_vitro	in_vivo	10	12	1.424	0.4075	1.0165	c(t = 1.48453	0.16	0.83731181	Distal Intergenic	ENSSSCG000	MAEL	maelstrom spermatogenic transposon silencer [Source:NCBI gene (formerly Entrezgene);Acc:100048945]
chr10	46132765	46133420	656	*	chr10	46132765	46133420	in_vitro	in_vivo	12	12	2.99	0.31916667	2.67083333	c(t = 1.50277	0.16	0.83731181	Distal Intergenic	ENSSSCG000	RPP38	ribonuclease P/MRP subunit p38 [Source:VGNC Symbol;Acc:VGNC:96240]
chr11	77453549	77454446	898	*	chr11	77453549	77454446	in_vitro	in_vivo	9	13	2.75111111	0.30307692	2.44803419	c(t = 1.54702	0.16	0.83731181	Distal Intergenic	ENSSSCG000	ANKRD10	ankyrin repeat domain 10 [Source:VGNC Symbol;Acc:VGNC:85322]
chr3	79952642	79953227	586	*	chr3	79952642	79953227	in_vitro	in_vivo	12	11	1.8525	0	1.8525	c(t = 1.50648	0.16	0.83731181	Distal Intergenic	ENSSSCG000	TMEM17	transmembrane protein 17 [Source:NCBI gene (formerly Entrezgene);Acc:100627459]
chr11	7852607	7853387	781	*	chr11	7852607	7853387	in_vitro	in_vivo	9	12	93.6644444	97.4925	-3.8280556	c(t = -1.5029	0.161	0.83731181	Distal Intergenic	ENSSSCG000	HSPH1	heat shock protein family H (Hsp110) member 1 [Source:NCBI gene (formerly Entrezgene);Acc:100048931]
chr4	2510427	2518859	8433	*	chr4	2510427	2518859	in_vitro	in_vivo	14	14	81.065	89.36	-8.295	c(t = -1.4488	0.161	0.83731181	Distal Intergenic	NA	NA	NA
chrX	35050628	35051067	440	*	chrX	35050628	35051067	in_vitro	in_vivo	9	10	3.56888889	13.048	-9.4791111	c(t = -1.5023	0.161	0.83731181	Distal Intergenic	ENSSSCG000	MID1IP1	MID1 interacting protein 1 [Source:VGNC Symbol;Acc:VGNC:90214]
chr9	78652628	78653835	1208	*	chr9	78652628	78653835	in_vitro	in_vivo	10	12	1.791	5.33666667	-3.5456667	c(t = -1.4890	0.161	0.83731181	Distal Intergenic	ENSSSCG000	MIOS	meiosis regulator for oocyte development [Source:VGNC Symbol;Acc:VGNC:90234]
chr5	14806318	14807207	890	*	chr5	14806318	14807207	in_vitro	in_vivo	9	12	0.69444444	3.07583333	-2.3813889	c(t = -1.4740	0.161	0.83731181	Distal Intergenic	ENSSSCG000	ARF3	ADP ribosylation factor 3 [Source:NCBI gene (formerly Entrezgene);Acc:100301570]
chr6	166532945	166534118	1174	*	chr6	166532945	166534118	in_vitro	in_vivo	10	10	5.654	1.946	3.708	c(t = 1.49159	0.161	0.83731181	Distal Intergenic	ENSSSCG000	DYNLT4	dynein light chain Tctex-type 4 [Source:NCBI gene (formerly Entrezgene);Acc:606747]
chr2	51153535	51155710	2176	*	chr2	51153535	51155710	in_vitro	in_vivo	10	10	4.12	1.157	2.963	c(t = 1.50366	0.161	0.83731181	Distal Intergenic	ENSSSCG000	ARF1	ADP ribosylation factor 1 [Source:VGNC Symbol;Acc:VGNC:96793]
chr6	83542724	83544395	1672	*	chr6	83542724	83544395	in_vitro	in_vivo	14	13	2.16785714	0.99769231	1.17016484	c(t = 1.45766	0.161	0.83731181	Distal Intergenic	ENSSSCG000	TRIM63	tripartite motif containing 63 [Source:NCBI gene (formerly Entrezgene);Acc:100431101]
chr18	52691894	52694149	2256	*	chr18	52691894	52694149	in_vitro	in_vivo	12	15	10.2075	25.7833333	-15.575833	c(t = -1.4473	0.162	0.83731181	Distal Intergenic	ENSSSCG000	INHBA	inhibin subunit beta A [Source:NCBI gene (formerly Entrezgene);Acc:397093]
chr14	38632444	38633477	1034	*	chr14	38632444	38633477	in_vitro	in_vivo	9	13	3.04888889	1.06230769	1.9865812	c(t = 1.48807	0.162	0.83731181	Distal Intergenic	ENSSSCG000	OAS2	2'-5'-oligoadenylate synthetase 2 [Source:VGNC Symbol;Acc:VGNC:91007]
chr6	27390952	27391834	883	*	chr6	27390952	27391834	in_vitro	in_vivo	11	12	2.09636364	1.03583333	1.0605303	c(t = 1.45673	0.162	0.83731181	Distal Intergenic	ENSSSCG000	DYNC1LI2	dynein cytoplasmic 1 light intermediate chain 2 [Source:VGNC Symbol;Acc:VGNC:87498]
chr4	108393278	108395576	2299	*	chr4	108393278	108395576	in_vitro	in_vivo	12	13	4.48916667	1.00923077	3.4799359	c(t = 1.49527	0.162	0.83731181	Distal Intergenic	ENSSSCG000	ADORA3	adenosine A3 receptor [Source:NCBI gene (formerly Entrezgene);Acc:606753]
chr4	100377959	100378756	798	*	chr4	100377959	100378756	in_vitro	in_vivo	10	9	2.839	0.78555556	2.05344444	c(t = 1.49889	0.162	0.83731181	Promoter (<=1kb)	ENSSSCG000	PRKAB2	protein kinase AMP-activated non-catalytic subunit beta 2 [Source:VGNC Symbol;Acc:VGNC:91799]
chr13	80208262	80208721	460	*	chr13	80208262	80208721	in_vitro	in_vivo	9	9	2.15222222	0.55555556	1.59666667	c(t = 1.48096	0.162	0.83731181	Distal Intergenic	ENSSSCG000	RBP2	retinol binding protein 2 [Source:NCBI gene (formerly Entrezgene);Acc:407611]
chr1	230069115	230070221	1107	*	chr1	230069115	230070221	in_vitro	in_vivo	13	10	1.33615385	0.282	1.05415385	c(t = 1.47607	0.162	0.83731181	Distal Intergenic	ENSSSCG000	GNA14	G protein subunit alpha 14 [Source:VGNC Symbol;Acc:VGNC:88519]
chr8	124960816	124961492	677	*	chr8	124960816	124961492	in_vitro	in_vivo	9	12	65.3444444	38.3866667	26.9577778	c(t = 1.45255	0.163	0.83731181	Promoter (<=1kb)	ENSSSCG000	BMPR1B	bone morphotic protein receptor type 1B [Source:VGNC Symbol;Acc:VGNC:85847]
chr1	220838663	220840001	1339	*	chr1	220838663	220840001	in_vitro	in_vivo	11	9	1.03909091	3.52444444	-2.4853535	c(t = -1.4723	0.163	0.83731181	Distal Intergenic	ENSSSCG000	DMRT1	doublesex and mab-3 related transcription factor 1 [Source:NCBI gene (formerly Entrezgene);Acc:397199]
chr12	58703400	58706399	3000	*	chr12	58703400	58706399	in_vitro	in_vivo	10	15	6.415	3.30333333	3.11166667	c(t = 1.45335	0.163	0.83731181	Distal Intergenic	ENSSSCG000	ssc-mir-9828	ssc-mir-9828-1 [Source:miRBase;Acc:MI0031595]
chr6	43527098	43530021	2924	*	chr6	43527098	43530021	in_vitro	in_vivo	10	14	6.721	1.82285714	4.89814286	c(t = 1.51046	0.163	0.83731181	Distal Intergenic	ENSSSCG000	LSM14A	LSM14A mRNA processing body assembly factor [Source:NCBI gene (formerly Entrezgene);Acc:100511455]
chr7	32787351	32789591	2241	*	chr7	32787351	32789591	in_vitro	in_vivo	13	15	3.82538462	1.69933333	2.12605128	c(t = 1.44953	0.163	0.83731181	Distal Intergenic	ENSSSCG000	MTCH1	mitochondrial carrier 1 [Source:VGNC Symbol;Acc:VGNC:103996]
chr3	4328355	4329664	1310	*	chr3	4328355	4329664	in_vitro	in_vivo	11	15	0.78727273	1.59866667	-0.8113939	c(t = -1.4429	0.163	0.83731181	Distal Intergenic	ENSSSCG000	FSCN1	fascin actin-bundling protein 1 [Source:NCBI gene (formerly Entrezgene);Acc:100286741]
chr14	55450244	55451147	904	*	chr14	55450244	55451147	in_vitro	in_vivo	9	12	0.3	1.34583333	-1.0458333	c(t = -1.4647	0.163	0.83731181	Distal Intergenic	ENSSSCG000	EDARADD	EDAR associated death domain [Source:VGNC Symbol;Acc:VGNC:87542]
chr18	42431923	42432565	643	*	chr18	42431923	42432565	in_vitro	in_vivo	11	11	3.82272727	1.32181818	2.50090909	c(t = 1.47432	0.163	0.83731181	Distal Intergenic	ENSSSCG000	NOD1	nucleotide binding oligomerization domain containing 1 [Source:NCBI gene (formerly Entrezgene);Acc:100135660]
chr11	571169	572244	1076	*	chr11	571169	572244	in_vitro	in_vivo	10	12	2.405	1.0875	1.3175	c(t = 1.48519	0.163	0.83731181	Distal Intergenic	ENSSSCG000	CRYL1	crystallin lambda 1 [Source:VGNC Symbol;Acc:VGNC:87023]
chr1	37023575	37024368	794	*	chr1	37023575	37024368	in_vitro	in_vivo	9	12	1.74777778	0.3875	1.36027778	c(t = 1.51206	0.163	0.83731181	Distal Intergenic	ENSSSCG000	HEY2	hes related family bHLH transcription factor with YRPW motif 2 [Source:VGNC Symbol;Acc:VGNC:88864]
chr13	208182102	208185667	3566	*	chr13	208182102	208185667	in_vitro	in_vivo	14	13	12.4685714	28.4076923	-15.939121	c(t = -1.4359	0.164	0.83731181	Distal Intergenic	ENSSSCG000	ITGB2	integrin subunit beta 2 [Source:NCBI gene (formerly Entrezgene);Acc:396943]
chr5	678017	681372	3356	*	chr5	678017	681372	in_vitro	in_vivo	12	14	26.8383333	12.9278571	13.9104762	c(t = 1.45052	0.164	0.83731181	Distal Intergenic	ENSSSCG000	SELENOO	selenoprotein O [Source:NCBI gene (formerly Entrezgene);Acc:100518971]
chr1	237787543	237789267	1725	*	chr1	237787543	237789267	in_vitro	in_vivo	13	13	1.48153846	3.15846154	-1.6769231	c(t = -1.4516	0.164	0.83731181	Distal Intergenic	ENSSSCG000	GNE	glucosamine (UDP-N-acetyl)-2-epimerase/N-acetylmannosamine kinase [Source:VGNC Symbol;Acc:VGNC:103101]
chr4	101521421	101522406	986	*	chr4	101521421	101522406	in_vitro	in_vivo	10	14	0	1.30357143	-1.3035714	c(t = -1.4762	0.164	0.83731181	Distal Intergenic	ENSSSCG000	PHGDH	phosphoglycerate dehydrogenase [Source:NCBI gene (formerly Entrezgene);Acc:100144529]
chr3	67680786	67681060	275	*	chr3	67680786	67681060	in_vitro	in_vivo	9	9	5.12666667	0.85444444	4.27222222	c(t = 1.50767	0.164	0.83731181	Distal Intergenic	ENSSSCG000	HK2	hexokinase 2 [Source:NCBI gene (formerly Entrezgene);Acc:494561]
chr4	107510828	107512301	1474	*	chr4	107510828	107512301	in_vitro	in_vivo	12	14	1.77083333	0.66	1.11083333	c(t = 1.47128	0.164	0.83731181	Distal Intergenic	ENSSSCG00000063479		solute carrier family 16 member 1 [Source:NCBI gene (formerly Entrezgene);Acc:100127159]
chr14	131288053	131289691	1639	*	chr14	131288053	131289691	in_vitro	in_vivo	11	14	0.14545455	0.56214286	-0.4166883	c(t = -1.4567	0.164	0.83731181	Distal Intergenic	ENSSSCG000	FGFR2	fibroblast growth factor receptor 2 [Source:NCBI gene (formerly Entrezgene);Acc:396762]
chr4	106626390	106627405	1016	*	chr4	106626390	106627405	in_vitro	in_vivo	11	9	1.11272727	0.23111111	0.88161616	c(t = 1.47216	0.164	0.83731181	Distal Intergenic	ENSSSCG000	BCAS2	BCAS2 pre-mRNA processing factor [Source:NCBI gene (formerly Entrezgene);Acc:100154006]
chr11	78633632	78637487	3856	*	chr11	78633632	78637487	in_vitro	in_vivo	11	14	27.7390909	43.0178571	-15.278766	c(t = -1.4339	0.165	0.83731181	Distal Intergenic	ENSSSCG000	LAMP1	lysosomal associated membrane protein 1 [Source:NCBI gene (formerly Entrezgene);Acc:494565]
chr3	131354139	131355312	1174	*	chr3	131354139	131355312	in_vitro	in_vivo	11	11	53.0681818	41.6954545	11.3727273	c(t = 1.45985	0.165	0.83731181	Distal Intergenic	ENSSSCG000	EIPR1	EARP complex and GARP complex interacting protein 1 [Source:VGNC Symbol;Acc:VGNC:87635]
chr9	136460695	136461442	748	*	chr9	136460695	136461442	in_vitro	in_vivo	10	11	14.044	33.2045455	-19.160545	c(t = -1.4743	0.165	0.83731181	Distal Intergenic	ENSSSCG000	DDC	dopa decarboxylase [Source:VGNC Symbol;Acc:VGNC:87204]
chr14	50812609	50814603	1995	*	chr14	50812609	50814603	in_vitro	in_vivo	10	12	1.191	4.00916667	-2.8181667	c(t = -1.4747	0.165	0.83731181	Distal Intergenic	ENSSSCG000	TSSK1B	testis specific serine kinase 1B [Source:NCBI gene (formerly Entrezgene);Acc:102159240]
chr9	17333734	17334929	1196	*	chr9	17333734	17334929	in_vitro	in_vivo	15	14	0.76866667	2.70357143	-1.9349048	c(t = -1.4571	0.165	0.83731181	Distal Intergenic	ENSSSCG000	TMEM126B	transmembrane protein 126B [Source:NCBI gene (formerly Entrezgene);Acc:100626990]
chr15	31965211	31966188	978	*	chr15	31965211	31966188	in_vitro	in_vivo	10	12	0.639	2.12416667	-1.4851667	c(t = -1.4562	0.165	0.83731181	Distal Intergenic	ENSSSCG000	SCTR	secretin receptor [Source:VGNC Symbol;Acc:VGNC:95482]
chr1	166697611	166701753	4143	*	chr1	166697611	166701753	in_vitro	in_vivo	11	12	4.39272727	1.985	2.40772727	c(t = 1.46542	0.165	0.83731181	Distal Intergenic	ENSSSCG000	SPESP1	sperm equatorial segment protein 1 [Source:NCBI gene (formerly Entrezgene);Acc:100415935]
chr1	188729585	188729940	356	*	chr1	188729585	188729940	in_vitro	in_vivo	11	11	5.73727273	1.73363636	4.00363636	c(t = 1.48514	0.165	0.83731181	Promoter (<=1kb)	ENSSSCG000	RTN1	reticulon 1 [Source:NCBI gene (formerly Entrezgene);Acc:100152394]
chr11	25495412	25497295	1884	*	chr11	25495412	25497295	in_vitro	in_vivo	13	14	3.7	1.25	2.45	c(t = 1.44689	0.165	0.83731181	Promoter (<=1kb)	ENSSSCG000	RGCC	regulator of cell cycle [Source:VGNC Symbol;Acc:VGNC:92251]
chr6	165619464	165620284	821	*	chr6	165619464	165620284	in_vitro	in_vivo	13	11	2.55307692	1.13181818	1.42125874	c(t = 1.44365	0.165	0.83731181	Distal Intergenic	ENSSSCG000	AKR1A1	aldo-keto reductase family 1 member A1 [Source:VGNC Symbol;Acc:VGNC:85229]
chr4	126029104	126031784	2681	*	chr4	126029104	126031784	in_vitro	in_vivo	10	13	3.718	1.11461538	2.60338462	c(t = 1.49584	0.165	0.83731181	Distal Intergenic	ENSSSCG000	TGFBR3	transforming growth factor beta receptor 3 [Source:VGNC Symbol;Acc:VGNC:93932]
chr7	15793632	15796289	2658	*	chr7	15793632	15796289	in_vitro	in_vivo	12	14	2.69583333	0.90071429	1.79511905	c(t = 1.45941	0.165	0.83731181	Distal Intergenic	ENSSSCG000	ID4	inhibitor of DNA binding 4 [Source:VGNC Symbol;Acc:VGNC:89024]
chr1	163103130	163103731	602	*	chr1	163103130	163103731	in_vitro	in_vivo	11	9	1.4	0.47111111	0.92888889	c(t = 1.45532	0.165	0.83731181	Distal Intergenic	ENSSSCG000	CILP	cartilage intermediate layer protein [Source:VGNC Symbol;Acc:VGNC:86703]
chr13	140134838	140136710	1873	*	chr13	140134838	140136710	in_vitro	in_vivo	9	12	1.57333333	0.46916667	1.10416667	c(t = 1.47280	0.165	0.83731181	Promoter (<=1kb)	ENSSSCG000	GSK3B	glycogen synthase kinase 3 beta [Source:VGNC Symbol;Acc:VGNC:88723]
chr15	80461678	80462348	671	*	chr15	80461678	80462348	in_vitro	in_vivo	10	10	2.072	0.417	1.655	c(t = 1.48029	0.165	0.83731181	Distal Intergenic	ENSSSCG000	CIR1	corepressor interacting with RBPJ, CIR1 [Source:NCBI gene (formerly Entrezgene);Acc:100511415]
chr10	46934776	46936137	1362	*	chr10	46934776	46936137	in_vitro	in_vivo	13	11	1.85692308	0.35818182	1.49874126	c(t = 1.46865	0.165	0.83731181	Promoter (<=1kb)	ENSSSCG000	HSPA14	heat shock protein family A (Hsp70) member 14 [Source:VGNC Symbol;Acc:VGNC:96675]
chr1	185397819	185398991	1173	*	chr1	185397819	185398991	in_vitro	in_vivo	14	12	1.65285714	0.3075	1.34535714	c(t = 1.46338	0.165	0.83731181	Distal Intergenic	ENSSSCG000	KTN1	kinectin 1 [Source:VGNC Symbol;Acc:VGNC:89608]
chr15	186326	187602	1277	*	chr15	186326	187602	in_vitro	in_vivo	9	10	1.41111111	0	1.41111111	c(t = 1.52624	0.165	0.83731181	Promoter (<=1kb)	ENSSSCG000	CACNB4	calcium voltage-gated channel auxiliary subunit beta 4 [Source:NCBI gene (formerly Entrezgene);Acc:396585]
chr14	134361942	134363613	1672	*	chr14	134361942	134363613	in_vitro	in_vivo	9	10	96.71	93.113	3.597	c(t = 1.45091	0.166	0.83731181	Distal Intergenic	ENSSSCG000	LHPP	phospholysine phosphohistidine inorganic pyrophosphate phosphatase [Source:VGNC Symbol;Acc:VGNC:89710]
chr6	63112994	63114518	1525	*	chr6	63112994	63114518	in_vitro	in_vivo	9	10	49.0022222	29.108	19.8942222	c(t = 1.46313	0.166	0.83731181	Distal Intergenic	ENSSSCG000	ISG15	ISG15 ubiquitin like modifier [Source:NCBI gene (formerly Entrezgene);Acc:100145895]
chrX	125522485	125523146	662	*	chrX	125522485	125523146	in_vitro	in_vivo	12	11	3.87166667	16.82	-12.948333	c(t = -1.4810	0.166	0.83731181	Distal Intergenic	ENSSSCG000	CLIC2	chloride intracellular channel 2 [Source:VGNC Symbol;Acc:VGNC:86761]
chr6	48729426	48730140	715	*	chr6	48729426	48730140	in_vitro	in_vivo	10	11	1.913	9.55727273	-7.6442727	c(t = -1.4862	0.166	0.83731181	Distal Intergenic	ENSSSCG000	SERTAD3	SERTA domain containing 3 [Source:VGNC Symbol;Acc:VGNC:92751]
chr4	95283764	95285421	1658	*	chr4	95283764	95285421	in_vitro	in_vivo	11	15	1.42727273	4.524	-3.0967273	c(t = -1.4467	0.166	0.83731181	Promoter (<=1kb)	ENSSSCG000	TDRD10	tudor domain containing 10 [Source:NCBI gene (formerly Entrezgene);Acc:100157928]
chr17	47621149	47621853	705	*	chr17	47621149	47621853	in_vitro	in_vivo	11	9	9.60272727	2.74444444	6.85828283	c(t = 1.47875	0.166	0.83731181	Distal Intergenic	ENSSSCG000	SLPI	secretory leukocyte peptidase inhibitor [Source:NCBI gene (formerly Entrezgene);Acc:396886]
chr5	15090013	15091658	1646	*	chr5	15090013	15091658	in_vitro	in_vivo	9	12	2.74111111	1.15833333	1.58277778	c(t = 1.47971	0.166	0.83731181	Distal Intergenic	ENSSSCG000	PRKAG1	protein kinase AMP-activated non-catalytic subunit gamma 1 [Source:NCBI gene (formerly Entrezgene);Acc:[illegible]]
chr1	251276476	251277955	1480	*	chr1	251276476	251277955	in_vitro	in_vivo	9	14	2.08888889	0.73785714	1.35103175	c(t = 1.49717	0.166	0.83731181	Promoter (<=1kb)	ENSSSCG00000005453		thioredoxin [Source:NCBI gene (formerly Entrezgene);Acc:397581]
chr14	22976604	22977448	845	*	chr14	22976604	22977448	in_vitro	in_vivo	9	10	1.45888889	0.471	0.98788889	c(t = 1.48235	0.166	0.83731181	Distal Intergenic	ENSSSCG000	PIWIL1	piwi like RNA-mediated silencing 1 [Source:VGNC Symbol;Acc:VGNC:98197]
chr6	149748054	149749141	1088	*	chr6	149748054	149749141	in_vitro	in_vivo	10	11	1.928	0.25181818	1.67618182	c(t = 1.50067	0.166	0.83731181	Distal Intergenic	ENSSSCG000	ATG4C	autophagy related 4C cysteine peptidase [Source:VGNC Symbol;Acc:VGNC:85623]
chr12	61320499	61321054	556	*	chr12	61320499	61321054	in_vitro	in_vivo	12	9	4.25083333	0.17666667	4.07416667	c(t = 1.48157	0.166	0.83731181	Distal Intergenic	ENSSSCG000	COPS3	COP9 signalosome subunit 3 [Source:VGNC Symbol;Acc:VGNC:86900]
chr11	11084596	11085638	1043	*	chr11	11084596	11085638	in_vitro	in_vivo	11	11	2.76909091	0.14	2.62909091	c(t = 1.49450	0.166	0.83731181	Distal Intergenic	ENSSSCG000	RFC3	replication factor C subunit 3 [Source:VGNC Symbol;Acc:VGNC:92233]
chr6	85307093	85307507	415	*	chr6	85307093	85307507	in_vitro	in_vivo	10	10	1.78666667	0	1.78666667	c(t = 1.48339	0.166	0.83731181	Promoter (<=1kb)	ENSSSCG000	ATP5IF1	ATP synthase inhibitory factor subunit 1 [Source:NCBI gene (formerly Entrezgene);Acc:100038002]
chr5	955503	956407	905	*	chr5	955503	956407	in_vitro	in_vivo	9	9	20.3222222	44.8588889	-24.536667	c(t = -1.4602	0.167	0.83731181	Distal Intergenic	ENSSSCG000	SELENOO	selenoprotein O [Source:NCBI gene (formerly Entrezgene);Acc:100518971]
chr3	796214	797300	1087	*	chr3	796214	797300	in_vitro	in_vivo	9	9	10.4722222	35.0222222	-24.55	c(t = -1.5043	0.167	0.83731181	Distal Intergenic	ENSSSCG000	ssc-mir-339-	ssc-mir-339-1 [Source:miRBase;Acc:MI0013149]
chr14	141137294	141139749	2456	*	chr14	141137294	141139749	in_vitro	in_vivo	12	9	8.54333333	25.7322222	-17.188889	c(t = -1.4523	0.167	0.83731181	Distal Intergenic	ENSSSCG000	ssc-mir-202	ssc-mir-202 [Source:miRBase;Acc:MI0013157]
chrX	16574350	16576908	2559	*	chrX	16574350	16576908	in_vitro	in_vivo	14	14	5.015	12.4007143	-7.3857143	c(t = -1.4270	0.167	0.83731181	Distal Intergenic	ENSSSCG000	EIF1AX	eukaryotic translation initiation factor 1A X-linked [Source:NCBI gene (formerly Entrezgene);Acc:100522912]
chr2	9352422	9353463	1042	*	chr2	9352422	9353463	in_vitro	in_vivo	9	13	1.96333333	7.98153846	-6.0182051	c(t = -1.4556	0.167	0.83731181	Distal Intergenic	ENSSSCG000	PHEROC	pheromaxein C subunit [Source:NCBI gene (formerly Entrezgene);Acc:100144470]
chr14	10019641	10020958	1318	*	chr14	10019641	10020958	in_vitro	in_vivo	9	12	1.07444444	3.34833333	-2.2738889	c(t = -1.4565	0.167	0.83731181	Distal Intergenic	ENSSSCG000	PPP2R2A	protein phosphatase 2 regulatory subunit Balpha [Source:VGNC Symbol;Acc:VGNC:91748]
chr9	131821926	131822538	613	*	chr9	131821926	131822538	in_vitro	in_vivo	9	11	4.44222222	1.84909091	2.59313131	c(t = 1.45655	0.167	0.83731181	Distal Intergenic	ENSSSCG000	SLC30A1	solute carrier family 30 member 1 [Source:NCBI gene (formerly Entrezgene);Acc:503660]
chr1	130384819	130388088	3270	*	chr1	130384819	130388088	in_vitro	in_vivo	10	12	3.516	1.0175	2.4985	c(t = 1.48684	0.167	0.83731181	Promoter (<=1kb)	ENSSSCG000	DLL4	delta like canonical Notch ligand 4 [Source:NCBI gene (formerly Entrezgene);Acc:100152163]
chr13	54903880	54904991	1112	*	chr13	54903880	54904991	in_vitro	in_vivo	12	15	2.43916667	0.96733333	1.47183333	c(t = 1.46714	0.167	0.83731181	Distal Intergenic	ENSSSCG000	MITF	melanocyte inducing transcription factor [Source:VGNC Symbol;Acc:VGNC:90243]
chr3	57386491	57387138	648	*	chr3	57386491	57387138	in_vitro	in_vivo	10	9	0.149	0.89666667	-0.7476667	c(t = -1.4964	0.167	0.83731181	Promoter (<=1kb)	ENSSSCG000	RPIA	ribose 5-phosphate isomerase A [Source:VGNC Symbol;Acc:VGNC:109408]
chr6	165134359	165136122	1764	*	chr6	165134359	165136122	in_vitro	in_vivo	10	12	2.477	0.86	1.617	c(t = 1.47376	0.167	0.83731181	Distal Intergenic	ENSSSCG000	RAD54L	RAD54 like [Source:NCBI gene (formerly Entrezgene);Acc:100141305]
chr6	98597618	98598950	1333	*	chr6	98597618	98598950	in_vitro	in_vivo	9	10	2.52555556	0.813	1.71255556	c(t = 1.47433	0.167	0.83731181	Distal Intergenic	ENSSSCG000	NDUFV2	NADH:ubiquinone oxidoreductase core subunit V2 [Source:NCBI gene (formerly Entrezgene);Acc:100037991]
chr7	9853060	9854696	1637	*	chr7	9853060	9854696	in_vitro	in_vivo	14	12	1.78	0.7075	1.0725	c(t = 1.43631	0.167	0.83731181	Distal Intergenic	ENSSSCG000	SIRT5	sirtuin 5 [Source:NCBI gene (formerly Entrezgene);Acc:100125966]
chr5	78561157	78562099	943	*	chr5	78561157	78562099	in_vitro	in_vivo	11	13	0.78181818	2.29846154	-1.5166434	c(t = -1.4466	0.168	0.83731181	Distal Intergenic	ENSSSCG000	PFKM	phosphofructokinase, muscle [Source:VGNC Symbol;Acc:VGNC:91338]
chr1	75078471	75079199	729	*	chr1	75078471	75079199	in_vitro	in_vivo	14	11	0.48071429	1.71636364	-1.2356494	c(t = -1.4596	0.168	0.83731181	Distal Intergenic	ENSSSCG000	PPIL6	peptidylprolyl isomerase like 6 [Source:VGNC Symbol;Acc:VGNC:98216]
chr18	54428246	54431282	3037	*	chr18	54428246	54431282	in_vitro	in_vivo	16	15	1.73125	0.92533333	0.80591667	c(t = 1.42696	0.168	0.83731181	Distal Intergenic	ENSSSCG000	RARRES2	retinoic acid receptor responder 2 [Source:VGNC Symbol;Acc:VGNC:92098]
chr13	117598140	117599111	972	*	chr13	117598140	117599111	in_vitro	in_vivo	13	9	2.83769231	0.77888889	2.05880342	c(t = 1.45596	0.168	0.83731181	Distal Intergenic	ENSSSCG000	MFN1	mitofusin 1 [Source:VGNC Symbol;Acc:VGNC:90178]
chr2	70181160	70181415	256	*	chr2	70181160	70181415	in_vitro	in_vivo	11	9	2.76818182	0.61777778	2.15040404	c(t = 1.45586	0.168	0.83731181	Promoter (1-2kb)	ENSSSCG000	CNN1	calponin 1 [Source:VGNC Symbol;Acc:VGNC:86825]
chr2	73333659	73335052	1394	*	chr2	73333659	73335052	in_vitro	in_vivo	10	15	2.788	0.354	2.434	c(t = 1.49492	0.168	0.83731181	Distal Intergenic	ENSSSCG000	MICOS13	mitochondrial contact site and cristae organizing system subunit 13 [Source:NCBI gene (formerly Entrezgene);Acc:100037978]
chr8	114251850	114252900	1051	*	chr8	114251850	114252900	in_vitro	in_vivo	10	13	1.683	0	1.683	c(t = 1.49781	0.168	0.83731181	Distal Intergenic	ENSSSCG000	ssc-mir-9843	ssc-mir-9843 [Source:miRBase;Acc:MI0031612]
chr2	70658392	70659484	1093	*	chr2	70658392	70659484	in_vitro	in_vivo	12	14	15.5483333	31.9928571	-16.444524	c(t = -1.4182	0.169	0.83731181	Distal Intergenic	ENSSSCG000	ADAMTS10	ADAM metallopeptidase with thrombospondin type 1 motif 10 [Source:NCBI gene (formerly Entrezgene);Acc:100624519]
chr10	50310079	50310672	594	*	chr10	50310079	50310672	in_vitro	in_vivo	9	10	0.93555556	2.679	-1.7434444	c(t = -1.4441	0.169	0.83731181	Distal Intergenic	ENSSSCG000	GAD2	glutamate decarboxylase 2 [Source:NCBI gene (formerly Entrezgene);Acc:396929]
chr6	145673985	145674943	959	*	chr6	145673985	145674943	in_vitro	in_vivo	10	10	3.43	1.744	1.686	c(t = 1.44997	0.169	0.83731181	Distal Intergenic	ENSSSCG000	DYNLT5	dynein light chain Tctex-type family member 5 [Source:VGNC Symbol;Acc:VGNC:93838]
chr10	12854946	12856928	1983	*	chr10	12854946	12856928	in_vitro	in_vivo	13	12	0.45230769	1.62166667	-1.169359	c(t = -1.4541	0.169	0.83731181	Distal Intergenic	ENSSSCG000	DEGS1	delta 4-desaturase, sphingolipid 1 [Source:VGNC Symbol;Acc:VGNC:95590]
chr14	11367109	11367839	731	*	chr14	11367109	11367839	in_vitro	in_vivo	9	9	3.56888889	1.50555556	2.06333333	c(t = 1.44269	0.169	0.83731181	Distal Intergenic	ENSSSCG000	CLU	clusterin [Source:VGNC Symbol;Acc:VGNC:86790]
chr6	7170998	7171799	802	*	chr6	7170998	7171799	in_vitro	in_vivo	9	10	0	1.181	-1.181	c(t = -1.4968	0.169	0.83731181	Distal Intergenic	ENSSSCG000	BCO1	beta-carotene oxygenase 1 [Source:VGNC Symbol;Acc:VGNC:85789]
chr7	120486030	120487788	1759	*	chr7	120486030	120487788	in_vitro	in_vivo	13	13	2.12230769	0.91846154	1.20384615	c(t = 1.43685	0.169	0.83731181	Distal Intergenic	ENSSSCG000	BCL11B	BCL11 transcription factor B [Source:NCBI gene (formerly Entrezgene);Acc:100154158]
chr18	50862225	50863073	849	*	chr18	50862225	50863073	in_vitro	in_vivo	12	11	2.33083333	0.81636364	1.5144697	c(t = 1.43170	0.169	0.83731181	Distal Intergenic	ENSSSCG000	YKT6	YKT6 v-SNARE homolog [Source:NCBI gene (formerly Entrezgene);Acc:100513964]
chr5	23028369	23030272	1904	*	chr5	23028369	23030272	in_vitro	in_vivo	13	12	1.69615385	0.6	1.09615385	c(t = 1.43465	0.169	0.83731181	Distal Intergenic	ENSSSCG000	CDK4	cyclin dependent kinase 4 [Source:NCBI gene (formerly Entrezgene);Acc:100144492]
chr12	31207320	31208090	771	*	chr12	31207320	31208090	in_vitro	in_vivo	9	13	3.03555556	0.58923077	2.44632479	c(t = 1.49417	0.169	0.83731181	Distal Intergenic	ENSSSCG000	MMD	monocyte to macrophage differentiation associated [Source:VGNC Symbol;Acc:VGNC:90264]
chr7	38456387	38457636	1250	*	chr7	38456387	38457636	in_vitro	in_vivo	10	10	1.995	0.463	1.532	c(t = 1.46908	0.169	0.83731181	Promoter (<=1kb)	ENSSSCG000	DLK2	delta like non-canonical Notch ligand 2 [Source:VGNC Symbol;Acc:VGNC:87334]
chr4	61595843	61596236	394	*	chr4	61595843	61596236	in_vitro	in_vivo	9	12	1.38888889	0	1.38888889	c(t = 1.51185	0.169	0.83731181	Distal Intergenic	ENSSSCG000	LY96	lymphocyte antigen 96 [Source:VGNC Symbol;Acc:VGNC:89907]
chr4	36400524	36401066	543	*	chr4	36400524	36401066	in_vitro	in_vivo	10	11	0.935	0	0.935	c(t = 1.49548	0.169	0.83731181	Distal Intergenic	ENSSSCG000	ANKRD46	ankyrin repeat domain 46 [Source:VGNC Symbol;Acc:VGNC:85339]
chr3	39873222	39880541	7320	*	chr3	39873222	39880541	in_vitro	in_vivo	12	11	96.6766667	92.0445455	4.63212121	c(t = 1.45847	0.17	0.83731181	Exon (NM_001246202/100516429, exon 15 of 46)	ENSSSCG000	PKD1	polycystin 1, transient receptor potential channel interacting [Source:VGNC Symbol;Acc:VGNC:91471]
chr17	265698	267580	1883	*	chr17	265698	267580	in_vitro	in_vivo	12	11	3.30416667	11.0836364	-7.7794697	c(t = -1.4715	0.17	0.83731181	Distal Intergenic	ENSSSCG000	CLDN23	claudin 23 [Source:NCBI gene (formerly Entrezgene);Acc:100156235]
chrX	37535277	37536618	1342	*	chrX	37535277	37536618	in_vitro	in_vivo	11	14	1.69727273	4.52285714	-2.8255844	c(t = -1.4266	0.17	0.83731181	Distal Intergenic	ENSSSCG000	GPR82	G protein-coupled receptor 82 [Source:VGNC Symbol;Acc:VGNC:103955]
chr9	90806351	90807209	859	*	chr9	90806351	90807209	in_vitro	in_vivo	11	12	19.5518182	4.23083333	15.3209848	c(t = 1.47254	0.17	0.83731181	Distal Intergenic	ENSSSCG000	IL6	interleukin 6 [Source:NCBI gene (formerly Entrezgene);Acc:399500]
chr9	45902447	45905362	2916	*	chr9	45902447	45905362	in_vitro	in_vivo	13	15	6.36692308	3.06666667	3.30025641	c(t = 1.43287	0.17	0.83731181	Distal Intergenic	ENSSSCG000	ATP5MG	ATP synthase membrane subunit g [Source:NCBI gene (formerly Entrezgene);Acc:396956]
chr7	84414930	84415521	592	*	chr7	84414930	84415521	in_vitro	in_vivo	11	10	0.99636364	2.381	-1.3846364	c(t = -1.4591	0.17	0.83731181	Distal Intergenic	ENSSSCG000	NR2F2	nuclear receptor subfamily 2 group F member 2 [Source:VGNC Symbol;Acc:VGNC:90881]
chr12	48156254	48156978	725	*	chr12	48156254	48156978	in_vitro	in_vivo	9	9	0.83444444	2.13666667	-1.3022222	c(t = -1.4560	0.17	0.83731181	Distal Intergenic	NA	NA	NA
chr14	60528065	60529075	1011	*	chr14	60528065	60529075	in_vitro	in_vivo	10	11	8.927	1.64181818	7.28518182	c(t = 1.48556	0.17	0.83731181	Distal Intergenic	ENSSSCG000	ACTA1	actin alpha 1, skeletal muscle [Source:NCBI gene (formerly Entrezgene);Acc:100154254]
chr3	53489757	53491327	1571	*	chr3	53489757	53491327	in_vitro	in_vivo	13	13	2.19153846	0.68	1.51153846	c(t = 1.44329	0.17	0.83731181	Distal Intergenic	ENSSSCG000	IL1R2	interleukin 1 receptor type 2 [Source:VGNC Symbol;Acc:VGNC:89093]
chr12	20497596	20498373	778	*	chr12	20497596	20498373	in_vitro	in_vivo	12	10	1.59916667	0.381	1.21816667	c(t = 1.45229	0.17	0.83731181	Promoter (<=1kb)	ENSSSCG000	STAT5A	signal transducer and activator of transcription 5A [Source:VGNC Symbol;Acc:VGNC:99087]
chr6	108450217	108450740	524	*	chr6	108450217	108450740	in_vitro	in_vivo	9	11	1.58444444	0.33454545	1.24989899	c(t = 1.48688	0.17	0.83731181	Promoter (<=1kb)	ENSSSCG000	NPC1	NPC intracellular cholesterol transporter 1 [Source:NCBI gene (formerly Entrezgene);Acc:397591]
chr3	18903945	18905665	1721	*	chr3	18903945	18905665	in_vitro	in_vivo	11	11	1.07454545	0.31454545	0.76	c(t = 1.45725	0.17	0.83731181	Distal Intergenic	ENSSSCG000	SPNS1	SPNS lysolipid transporter 1, lysophospholipid [Source:NCBI gene (formerly Entrezgene);Acc:100517643]
chr9	122115320	122116176	857	*	chr9	122115320	122116176	in_vitro	in_vivo	9	12	2.50555556	0.2875	2.21805556	c(t = 1.49889	0.17	0.83731181	Distal Intergenic	ENSSSCG000	STX6	syntaxin 6 [Source:VGNC Symbol;Acc:VGNC:93594]
chr14	22502169	22502994	826	*	chr14	22502169	22502994	in_vitro	in_vivo	9	12	1.49222222	0.21416667	1.27805556	c(t = 1.49945	0.17	0.83731181	Distal Intergenic	ENSSSCG000	PIWIL1	piwi like RNA-mediated silencing 1 [Source:VGNC Symbol;Acc:VGNC:98197]
chr1	261847252	261848150	899	*	chr1	261847252	261848150	in_vitro	in_vivo	9	12	64.9488889	87.6025	-22.653611	c(t = -1.4450	0.171	0.83731181	Distal Intergenic	ENSSSCG00000005518		glycoprotein alpha-galactosyltransferase 1 (inactive) [Source:NCBI gene (formerly Entrezgene);Acc:396733]
chr1	96302626	96303169	544	*	chr1	96302626	96303169	in_vitro	in_vivo	11	10	19.8672727	43.628	-23.760727	c(t = -1.4425	0.171	0.83731181	Distal Intergenic	ENSSSCG000	HAUS1	HAUS augmin like complex subunit 1 [Source:NCBI gene (formerly Entrezgene);Acc:100152640]
chrX	89219731	89222218	2488	*	chrX	89219731	89222218	in_vitro	in_vivo	15	14	4.31933333	9.62071429	-5.301381	c(t = -1.4175	0.171	0.83731181	Distal Intergenic	ENSSSCG000	ACSL4	acyl-CoA synthetase long chain family member 4 [Source:VGNC Symbol;Acc:VGNC:85033]
chr1	272835449	272836800	1352	*	chr1	272835449	272836800	in_vitro	in_vivo	11	10	13.5636364	4.584	8.97963636	c(t = 1.45894	0.171	0.83731181	Intron (NM_213799/396713, intron 7 of 8)	ENSSSCG00000035964		odorant binding protein 2B [Source:NCBI gene (formerly Entrezgene);Acc:396861]
chr11	7545065	7546023	959	*	chr11	7545065	7546023	in_vitro	in_vivo	13	14	5.26615385	2.68	2.58615385	c(t = 1.41832	0.171	0.83731181	Distal Intergenic	ENSSSCG000	ALOX5AP	arachidonate 5-lipoxygenase activating protein [Source:VGNC Symbol;Acc:VGNC:109456]

chr12	40533057	40536192	3136	*	chr12	40533057	40536192	in_vitro	in_vivo	11	12	0.96636364	2.365	-1.3986364	c(t = -1.4367	0.171	0.83731181	Distal Intergenic	ENSSSCG000	CCL1	C-C motif chemokine ligand 1 [Source:NCBI gene (formerly Entrezgene);Acc:100310829]
chr4	117618519	117619958	1440	*	chr4	117618519	117619958	in_vitro	in_vivo	13	16	1.28	2.3025	-1.0225	c(t = -1.4126	0.171	0.83731181	Distal Intergenic	ENSSSCG000	VCAM1	vascular cell adhesion molecule 1 [Source:NCBI gene (formerly Entrezgene);Acc:396925]
chr11	69441722	69443247	1526	*	chr11	69441722	69443247	in_vitro	in_vivo	11	15	0.74636364	1.93466667	-1.188303	c(t = -1.4215	0.171	0.83731181	Promoter (<=1kb)	ENSSSCG000	TMTC4	transmembrane O-mannosyltransferase targeting cadherins 4 [Source:VGNC Symbol;Acc:VGNC:94244]
chr1	30531908	30533448	1541	*	chr1	30531908	30533448	in_vitro	in_vivo	13	9	2.29230769	0.86444444	1.42786325	c(t = 1.41964	0.171	0.83731181	Distal Intergenic	ENSSSCG000	RPS12	ribosomal protein S12 [Source:NCBI gene (formerly Entrezgene);Acc:397650]
chr10	27953468	27954613	1146	*	chr10	27953468	27954613	in_vitro	in_vivo	10	9	1.965	0.67333333	1.29166667	c(t = 1.43802	0.171	0.83731181	Distal Intergenic	ENSSSCG000	FBP2	fructose-bisphosphatase 2 [Source:NCBI gene (formerly Entrezgene);Acc:100134828]
chr8	131472484	131473084	601	*	chr8	131472484	131473084	in_vitro	in_vivo	10	13	1.793	0.52153846	1.27146154	c(t = 1.45169	0.171	0.83731181	Distal Intergenic	ENSSSCG000	HSD17B13	hydroxysteroid 17-beta dehydrogenase 13 [Source:NCBI gene (formerly Entrezgene);Acc:100626886]
chr8	102259975	102260625	651	*	chr8	102259975	102260625	in_vitro	in_vivo	12	14	1.95083333	0.495	1.45583333	c(t = 1.44537	0.171	0.83731181	Promoter (<=1kb)	ENSSSCG000	CCNA2	cyclin A2 [Source:NCBI gene (formerly Entrezgene);Acc:100415929]
chr12	16308018	16308769	752	*	chr12	16308018	16308769	in_vitro	in_vivo	9	9	2.89444444	0	2.89444444	c(t = 1.50277	0.171	0.83731181	Distal Intergenic	ENSSSCG000	ITGB3	integrin subunit beta 3 [Source:NCBI gene (formerly Entrezgene);Acc:397063]
chr1	9799623	9800706	1084	*	chr1	9799623	9800706	in_vitro	in_vivo	11	9	100	98.9522222	1.04777778	c(t = 1.49903	0.172	0.83731181	Distal Intergenic	NA	NA	NA
chr14	140515870	140524178	8309	*	chr14	140515870	140524178	in_vitro	in_vivo	11	9	26.9327273	50.0622222	-23.129495	c(t = -1.4325	0.172	0.83731181	Distal Intergenic	ENSSSCG000	ssc-mir-202	ssc-mir-202 [Source:miRBase;Acc:MI0013157]
chr15	135079939	135082163	2225	*	chr15	135079939	135082163	in_vitro	in_vivo	10	11	16.248	35.5427273	-19.294727	c(t = -1.4460	0.172	0.83731181	Distal Intergenic	ENSSSCG000	ARL4C	ADP ribosylation factor like GTPase 4C [Source:NCBI gene (formerly Entrezgene);Acc:100514255]
chr1	7527865	7528661	797	*	chr1	7527865	7528661	in_vitro	in_vivo	10	13	17.491	35.0015385	-17.510538	c(t = -1.4215	0.172	0.83731181	Distal Intergenic	ENSSSCG000	IGF2R	insulin like growth factor 2 receptor [Source:NCBI gene (formerly Entrezgene);Acc:397214]
chr7	3686450	3689176	2727	*	chr7	3686450	3689176	in_vitro	in_vivo	14	12	3.44142857	1.64583333	1.79559524	c(t = 1.42093	0.172	0.83731181	Exon (NM_001243601/100155948, exon 2 of 3)	ENSSSCG000	NRN1	neuritin 1 [Source:VGNC Symbol;Acc:VGNC:90900]
chr6	168667425	168669012	1588	*	chr6	168667425	168669012	in_vitro	in_vivo	9	14	3.27333333	1.48571429	1.78761905	c(t = 1.41658	0.172	0.83731181	Distal Intergenic	ENSSSCG000	CLDN19	claudin 19 [Source:NCBI gene (formerly Entrezgene);Acc:100294682]
chr2	69601362	69602633	1272	*	chr2	69601362	69602633	in_vitro	in_vivo	10	14	2.521	0.80857143	1.71242857	c(t = 1.46772	0.172	0.83731181	Distal Intergenic	ENSSSCG000	ssc-mir-199a	ssc-mir-199a-2 [Source:miRBase;Acc:MI0013093]
chr9	38580776	38582849	2074	*	chr9	38580776	38582849	in_vitro	in_vivo	13	15	1.37076923	0.734	0.63676923	c(t = 1.40598	0.172	0.83731181	Distal Intergenic	ENSSSCG000	FDX1	ferredoxin 1 [Source:NCBI gene (formerly Entrezgene);Acc:397133]
chr6	51651716	51652512	797	*	chr6	51651716	51652512	in_vitro	in_vivo	13	10	5.11	0.404	4.706	c(t = 1.45015	0.172	0.83731181	Distal Intergenic	ENSSSCG000	BLOC1S3	biogenesis of lysosomal organelles complex 1 subunit 3 [Source:NCBI gene (formerly Entrezgene);Acc:100049671]
chr9	12850636	12851763	1128	*	chr9	12850636	12851763	in_vitro	in_vivo	9	11	1.29666667	0.37909091	0.91757576	c(t = 1.43239	0.172	0.83731181	Distal Intergenic	ENSSSCG000	NDUFC2	NADH:ubiquinone oxidoreductase subunit C2 [Source:NCBI gene (formerly Entrezgene);Acc:100513030]
chr13	132820671	132821412	742	*	chr13	132820671	132821412	in_vitro	in_vivo	9	10	0.73777778	0	0.73777778	c(t = 1.49928	0.172	0.83731181	Distal Intergenic	ENSSSCG000	SLC51A	solute carrier family 51 subunit alpha [Source:VGNC Symbol;Acc:VGNC:93138]
chr16	71589175	71589734	560	*	chr16	71589175	71589734	in_vitro	in_vivo	10	11	0.463	0	0.463	c(t = 1.48293	0.172	0.83731181	Distal Intergenic	ENSSSCG000	SPARC	secreted protein acidic and cysteine rich [Source:NCBI gene (formerly Entrezgene);Acc:595124]
chr3	26465330	26465891	562	*	chr3	26465330	26465891	in_vitro	in_vivo	10	9	4.86	26.9922222	-22.132222	c(t = -1.4781	0.173	0.83731181	Distal Intergenic	ENSSSCG000	SMG1	SMG1 nonsense mediated mRNA decay associated PI3K related kinase [Source:VGNC Symbol;Acc:VGNC:93248]
chr4	63305579	63307211	1633	*	chr4	63305579	63307211	in_vitro	in_vivo	15	12	1.26466667	2.86416667	-1.5995	c(t = -1.4238	0.173	0.83731181	Distal Intergenic	ENSSSCG000	RPL7	ribosomal protein L7 [Source:NCBI gene (formerly Entrezgene);Acc:414418]
chr1	148991156	148992298	1143	*	chr1	148991156	148992298	in_vitro	in_vivo	11	11	4.88909091	2.76363636	2.12545455	c(t = 1.43324	0.173	0.83731181	Distal Intergenic	ENSSSCG000	CNDP1	carnosine dipeptidase 1 [Source:VGNC Symbol;Acc:VGNC:86811]
chr7	22851795	22853045	1251	*	chr7	22851795	22853045	in_vitro	in_vivo	13	14	5.33307692	2.06142857	3.27164835	c(t = 1.41433	0.173	0.83731181	Intron (NM_001246242/100513601, intron 3 of 7)	ENSSSCG00000062469		
chr13	30601700	30602411	712	*	chr13	30601700	30602411	in_vitro	in_vivo	9	13	2.76777778	0.99230769	1.77547009	c(t = 1.42910	0.173	0.83731181	Distal Intergenic	ENSSSCG000	SCAP	SREBF chaperone [Source:VGNC Symbol;Acc:VGNC:92610]
chr8	824805	826406	1602	*	chr8	824805	826406	in_vitro	in_vivo	10	13	3.844	0.98230769	2.86169231	c(t = 1.46597	0.173	0.83731181	Distal Intergenic	ENSSSCG000	SLBP	stem-loop binding protein [Source:NCBI gene (formerly Entrezgene);Acc:100513605]
chr7	1840959	1842348	1390	*	chr7	1840959	1842348	in_vitro	in_vivo	13	14	3.49461538	0.89285714	2.60175824	c(t = 1.43723	0.173	0.83731181	Distal Intergenic	ENSSSCG000	BPHL	biphenyl hydrolase like [Source:NCBI gene (formerly Entrezgene);Acc:100154272]
chr8	35004006	35006109	2104	*	chr8	35004006	35006109	in_vitro	in_vivo	15	15	1.75466667	0.78733333	0.96733333	c(t = 1.41652	0.173	0.83731181	Distal Intergenic	ENSSSCG000	SLC30A9	solute carrier family 30 member 9 [Source:NCBI gene (formerly Entrezgene);Acc:100192440]
chr1	76792411	76793529	1119	*	chr1	76792411	76793529	in_vitro	in_vivo	12	11	3.19666667	0.67545455	2.52121212	c(t = 1.45190	0.173	0.83731181	Distal Intergenic	ENSSSCG000	DDO	D-aspartate oxidase [Source:NCBI gene (formerly Entrezgene);Acc:100037287]
chr7	15281263	15282883	1621	*	chr7	15281263	15282883	in_vitro	in_vivo	10	13	2.578	0.39307692	2.18492308	c(t = 1.47679	0.173	0.83731181	Promoter (<=1kb)	ENSSSCG000	ID4	inhibitor of DNA binding 4 [Source:VGNC Symbol;Acc:VGNC:89024]
chr10	666756	668013	1258	*	chr10	666756	668013	in_vitro	in_vivo	10	15	2.961	0.09133333	2.86966667	c(t = 1.47770	0.173	0.83731181	Distal Intergenic	ENSSSCG00000031991		glutaredoxin 2 [Source:VGNC Symbol;Acc:VGNC:95730]
chr13	133800398	133801152	755	*	chr13	133800398	133801152	in_vitro	in_vivo	9	10	1.27222222	0	1.27222222	c(t = 1.49650	0.173	0.83731181	Distal Intergenic	ENSSSCG000	SLC51A	solute carrier family 51 subunit alpha [Source:VGNC Symbol;Acc:VGNC:93138]
chr12	7532439	7533276	838	*	chr12	7532439	7533276	in_vitro	in_vivo	10	10	1.833	6.647	-4.814	c(t = -1.4484	0.174	0.83731181	Distal Intergenic	ENSSSCG000	SSTR2	somatostatin receptor 2 [Source:VGNC Symbol;Acc:VGNC:99086]
chr14	28074604	28076493	1890	*	chr14	28074604	28076493	in_vitro	in_vivo	9	14	0.99333333	2.55928571	-1.5659524	c(t = -1.4075	0.174	0.83731181	Distal Intergenic	ENSSSCG000	SCARB1	scavenger receptor class B member 1 [Source:NCBI gene (formerly Entrezgene);Acc:397018]
chr6	36465701	36466269	569	*	chr6	36465701	36466269	in_vitro	in_vivo	12	10	0.51	1.579	-1.069	c(t = -1.4461	0.174	0.83731181	Distal Intergenic	ENSSSCG000	CBLN1	cerebellin 1 precursor [Source:NCBI gene (formerly Entrezgene);Acc:100127148]
chr10	11362153	11362717	565	*	chr10	11362153	11362717	in_vitro	in_vivo	9	12	0	1.56666667	-1.5666667	c(t = -1.4546	0.174	0.83731181	Distal Intergenic	ENSSSCG000	FH	fumarate hydratase [Source:VGNC Symbol;Acc:VGNC:96300]
chr15	81930782	81932098	1317	*	chr15	81930782	81932098	in_vitro	in_vivo	11	12	0.15363636	1.49	-1.3363636	c(t = -1.4469	0.174	0.83731181	Distal Intergenic	NA	NA	NA
chr11	16374841	16377014	2174	*	chr11	16374841	16377014	in_vitro	in_vivo	14	14	3.18357143	1.46571429	1.71785714	c(t = 1.39929	0.174	0.83731181	Distal Intergenic	ENSSSCG000	RNASEH2B	ribonuclease H2 subunit B [Source:VGNC Symbol;Acc:VGNC:92339]
chr12	22710244	22712260	2017	*	chr12	22710244	22712260	in_vitro	in_vivo	11	13	2.96	1.30076923	1.65923077	c(t = 1.43669	0.174	0.83731181	Distal Intergenic	ENSSSCG000	STARD3	StAR related lipid transfer domain containing 3 [Source:VGNC Symbol;Acc:VGNC:93532]
chr6	27548573	27549343	771	*	chr6	27548573	27549343	in_vitro	in_vivo	10	13	2.756	1.20538462	1.55061538	c(t = 1.43327	0.174	0.83731181	Distal Intergenic	ENSSSCG000	CES3	carboxylesterase 3 [Source:NCBI gene (formerly Entrezgene);Acc:100626873]
chr1	90137321	90138186	866	*	chr1	90137321	90138186	in_vitro	in_vivo	12	11	2.32833333	1.15454545	1.17378788	c(t = 1.41090	0.174	0.83731181	Distal Intergenic	ENSSSCG000	MYO6	myosin VI [Source:VGNC Symbol;Acc:VGNC:103137]
chr6	65309930	65311522	1593	*	chr6	65309930	65311522	in_vitro	in_vivo	9	13	2.17222222	0.62846154	1.54376068	c(t = 1.46070	0.174	0.83731181	Distal Intergenic	ENSSSCG000	ssc-mir-2320	ssc-mir-2320 [Source:miRBase;Acc:MI0017997]
chr17	12585276	12586058	783	*	chr17	12585276	12586058	in_vitro	in_vivo	11	9	2.92636364	0.29222222	2.63414141	c(t = 1.45770	0.174	0.83731181	Distal Intergenic	ENSSSCG000	SMIM19	small integral membrane protein 19 [Source:NCBI gene (formerly Entrezgene);Acc:100152445]
chr6	163414463	163420344	5882	*	chr6	163414463	163420344	in_vitro	in_vivo	10	10	34.917	56.326	-21.409	c(t = -1.4155	0.175	0.83731181	Distal Intergenic	ENSSSCG000	SPATA6	spermatosis associated 6 [Source:VGNC Symbol;Acc:VGNC:93387]
chr5	20238859	20239683	825	*	chr5	20238859	20239683	in_vitro	in_vivo	10	9	9.869	30.88	-21.011	c(t = -1.4249	0.175	0.83731181	Distal Intergenic	ENSSSCG000	GPR84	G protein-coupled receptor 84 [Source:NCBI gene (formerly Entrezgene);Acc:100152279]
chrX	97492997	97496435	3439	*	chrX	97492997	97496435	in_vitro	in_vivo	14	14	3.175	11.2428571	-8.0678571	c(t = -1.4307	0.175	0.83731181	Distal Intergenic	ENSSSCG000	IL13RA1	interleukin 13 receptor subunit alpha 1 [Source:NCBI gene (formerly Entrezgene);Acc:397615]
chr6	83089454	83091355	1902	*	chr6	83089454	83091355	in_vitro	in_vivo	11	11	14.8681818	6.77363636	8.09454545	c(t = 1.43446	0.175	0.83731181	Distal Intergenic	ENSSSCG000	MACO1	macoilin 1 [Source:NCBI gene (formerly Entrezgene);Acc:654414]
chr7	93223959	93226158	2200	*	chr7	93223959	93226158	in_vitro	in_vivo	13	13	8.50692308	4.29	4.21692308	c(t = 1.41856	0.175	0.83731181	Distal Intergenic	ENSSSCG000	PLEKHD1	pleckstrin homology and coiled-coil domain containing D1 [Source:VGNC Symbol;Acc:VGNC:91539]
chr3	47470392	47471564	1173	*	chr3	47470392	47471564	in_vitro	in_vivo	11	12	0.32727273	2.50916667	-2.1818939	c(t = -1.4463	0.175	0.83731181	Distal Intergenic	ENSSSCG00000008137		LIM zinc finger domain containing 1 [Source:VGNC Symbol;Acc:VGNC:110285]
chr4	7813784	7816376	2593	*	chr4	7813784	7816376	in_vitro	in_vivo	13	11	1.03076923	1.96545455	-0.9346853	c(t = -1.4011	0.175	0.83731181	Distal Intergenic	ENSSSCG000	ST3GAL1	ST3 beta-galactoside alpha-2,3-sialyltransferase 1 [Source:VGNC Symbol;Acc:VGNC:98863]
chr1	179670703	179671099	397	*	chr1	179670703	179671099	in_vitro	in_vivo	10	10	0.312	1.88	-1.568	c(t = -1.4525	0.175	0.83731181	Distal Intergenic	ENSSSCG000	Metazoa_SR	Metazoan signal recognition particle RNA [Source:RFAM;Acc:RF00017]
chr14	131718208	131719642	1435	*	chr14	131718208	131719642	in_vitro	in_vivo	11	13	3.76818182	1.81384615	1.95433566	c(t = 1.43079	0.175	0.83731181	Distal Intergenic	ENSSSCG000	TACC2	transforming acidic coiled-coil containing protein 2 [Source:NCBI gene (formerly Entrezgene);Acc:100152804]
chr2	140440615	140442507	1893	*	chr2	140440615	140442507	in_vitro	in_vivo	11	14	4.46454545	1.62428571	2.84025974	c(t = 1.44161	0.175	0.83731181	Distal Intergenic	ENSSSCG000	CDC25C	cell division cycle 25C [Source:NCBI gene (formerly Entrezgene);Acc:100144474]
chr2	81658651	81659068	418	*	chr2	81658651	81659068	in_vitro	in_vivo	10	11	0	1.60636364	-1.6063636	c(t = -1.4582	0.175	0.83731181	Distal Intergenic	ENSSSCG000	KIAA1191	KIAA1191 [Source:VGNC Symbol;Acc:VGNC:99783]
chr14	107220834	107221438	605	*	chr14	107220834	107221438	in_vitro	in_vivo	11	13	3.31545455	1.43615385	1.8793007	c(t = 1.44102	0.175	0.83731181	Distal Intergenic	ENSSSCG000	ENTPD1	ectonucleoside triphosphate diphosphohydrolase 1 [Source:VGNC Symbol;Acc:VGNC:97977]
chr4	98588798	98589676	879	*	chr4	98588798	98589676	in_vitro	in_vivo	9	10	3.66888889	1.218	2.45088889	c(t = 1.42727	0.175	0.83731181	Distal Intergenic	ENSSSCG000	MCL1	MCL1 apoptosis regulator, BCL2 family member [Source:NCBI gene (formerly Entrezgene);Acc:397648]
chr6	84048750	84051016	2267	*	chr6	84048750	84051016	in_vitro	in_vivo	13	12	5.82461538	1.11166667	4.71294872	c(t = 1.43916	0.175	0.83731181	Distal Intergenic	ENSSSCG000	SFN	stratifin [Source:NCBI gene (formerly Entrezgene);Acc:733625]
chr3	2464598	2466531	1934	*	chr3	2464598	2466531	in_vitro	in_vivo	12	14	2.09166667	0.97714286	1.11452381	c(t = 1.41863	0.175	0.83731181	Distal Intergenic	ENSSSCG000	AMZ1	archaelysin family metallopeptidase 1 [Source:NCBI gene (formerly Entrezgene);Acc:100337670]
chr14	60254453	60255837	1385	*	chr14	60254453	60255837	in_vitro	in_vivo	9	13	1.73555556	0.93384615	0.8017094	c(t = 1.40605	0.175	0.83731181	Distal Intergenic	ENSSSCG000	ACTA1	actin alpha 1, skeletal muscle [Source:NCBI gene (formerly Entrezgene);Acc:100154254]
chr9	45742104	45744257	2154	*	chr9	45742104	45744257	in_vitro	in_vivo	9	13	1.67	0.60769231	1.06230769	c(t = 1.44992	0.175	0.83731181	Distal Intergenic	ENSSSCG000	ATP5MG	ATP synthase membrane subunit g [Source:NCBI gene (formerly Entrezgene);Acc:396956]
chr2	78961546	78963266	1721	*	chr2	78961546	78963266	in_vitro	in_vivo	10	13	2.936	0.58230769	2.35369231	c(t = 1.46268	0.175	0.83731181	Distal Intergenic	ENSSSCG000	CANX	calnexin [Source:VGNC Symbol;Acc:VGNC:86167]
chr17	39028793	39029628	836	*	chr17	39028793	39029628	in_vitro	in_vivo	9	9	0	0.35555556	-0.3555556	c(t = -1.4886	0.175	0.83731181	Promoter (<=1kb)	ENSSSCG000	NFS1	NFS1 cysteine desulfurase [Source:VGNC Symbol;Acc:VGNC:99724]
chr17	61101019	61103598	2580	*	chr17	61101019	61103598	in_vitro	in_vivo	12	12	47.8666667	64.6475	-16.780833	c(t = -1.4016	0.176	0.83731181	Distal Intergenic	ENSSSCG000	PSMA7	proteasome 20S subunit alpha 7 [Source:NCBI gene (formerly Entrezgene);Acc:100626738]
chr12	5306850	5308582	1733	*	chr12	5306850	5308582	in_vitro	in_vivo	10	10	24.969	9.33	15.639	c(t = 1.43069	0.176	0.83731181	Distal Intergenic	ENSSSCG000	ACOX1	acyl-CoA oxidase 1 [Source:VGNC Symbol;Acc:VGNC:85020]
chr2	27044155	27045856	1702	*	chr2	27044155	27045856	in_vitro	in_vivo	14	10	16.1928571	5.446	10.7468571	c(t = 1.42403	0.176	0.83731181	Distal Intergenic	ENSSSCG00000024791		CD59 molecule [Source:VGNC Symbol;Acc:VGNC:99615]
chr13	122156603	122159576	2974	*	chr13	122156603	122159576	in_vitro	in_vivo	11	12	4.12454545	1.755	2.36954545	c(t = 1.42605	0.176	0.83731181	Distal Intergenic	ENSSSCG000	ssc-mir-1224	ssc-mir-1224 [Source:miRBase;Acc:MI0015920]
chr7	97850959	97853029	2071	*	chr7	97850959	97853029	in_vitro	in_vivo	10	13	3.19	1.37461538	1.81538462	c(t = 1.41414	0.176	0.83731181	Distal Intergenic	ENSSSCG000	NPC2	NPC intracellular cholesterol transporter 2 [Source:NCBI gene (formerly Entrezgene);Acc:397410]
chr3	68549928	68551940	2013	*	chr3	68549928	68551940	in_vitro	in_vivo	13	14	2.65461538	1.22857143	1.42604396	c(t = 1.40446	0.176	0.83731181	Distal Intergenic	ENSSSCG000	DOK1	docking protein 1 [Source:NCBI gene (formerly Entrezgene);Acc:100233179]
chr14	29067492	29069187	1696	*	chr14	29067492	29069187	in_vitro	in_vivo	9	9	2.60555556	1.01666667	1.58888889	c(t = 1.45596	0.176	0.83731181	Distal Intergenic	ENSSSCG000	SUMO4	SMT3 suppressor of mif two 3 homolog 4 (S. cerevisiae) [Source:NCBI gene (formerly Entrezgene);Acc:100127140]
chr1	65832488	65837351	4864	*	chr1	65832488	65837351	in_vitro	in_vivo	16	16	1.38125	0.588125	0.793125	c(t = 1.40089	0.176	0.83731181	Distal Intergenic	ENSSSCG000	FBXL4	F-box and leucine rich repeat protein 4 [Source:VGNC Symbol;Acc:VGNC:98007]
chr2	28282340	28282936	597	*	chr2	28282340	28282936	in_vitro	in_vivo	10	10	5.275	0.4	4.875	c(t = 1.46664	0.176	0.83731181	Distal Intergenic	ENSSSCG000	WT1	WT1 transcription factor [Source:VGNC Symbol;Acc:VGNC:100884]
chr7	25071326	25072425	1100	*	chr7	25071326	25072425	in_vitro	in_vivo	10	13	2.028	0.39307692	1.63492308	c(t = 1.45603	0.176	0.83731181	Promoter (<=1kb)	ENSSSCG000	TAP1	transporter 1, ATP binding cassette subfamily B member [Source:VGNC Symbol;Acc:VGNC:98358]
chr14	85544086	85544665	580	*	chr14	85544086	85544665	in_vitro	in_vivo	10	10	3.024	0.356	2.668	c(t = 1.46574	0.176	0.83731181	Distal Intergenic	ENSSSCG000	GPR15LG	G protein-coupled receptor 15 ligand [Source:NCBI gene (formerly Entrezgene);Acc:100511838]
chr6	42483464	42484143	680	*	chr6	42483464	42484143	in_vitro	in_vivo	12	11	1.3725	0.32	1.0525	c(t = 1.43013	0.176	0.83731181	Distal Intergenic	ENSSSCG000	SLC7A9	solute carrier family 7 member 9 [Source:VGNC Symbol;Acc:VGNC:93177]
chrX	115018100	115026468	8369	*	chrX	115018100	115026468	in_vitro	in_vivo	14	15	2.70285714	6.76733333	-4.0644762	c(t = -1.4013	0.177	0.83731181	Distal Intergenic	ENSSSCG000	ssc-mir-505	ssc-mir-505 [Source:miRBase;Acc:MI0013171]
chr4	69969711	69970489	779	*	chr4	69969711	69970489	in_vitro	in_vivo	10	11	1.8	3.57545455	-1.7754545	c(t = -1.4114	0.177	0.83731181	Promoter (<=1kb)	ENSSSCG000	ssc-mir-124a	ssc-mir-124a-1 [Source:miRBase;Acc:MI0010680]
chr15	137931342	137932530	1189	*	chr15	137931342	137932530	in_vitro	in_vivo	9	11	1.28777778	3.13636364	-1.8485859	c(t = -1.4063	0.177	0.83731181	Distal Intergenic	ENSSSCG000	RAMP1	receptor activity modifying protein 1 [Source:NCBI gene (formerly Entrezgene);Acc:397401]
chr2	1558660	1561185	2526	*	chr2	1558660	1561185	in_vitro	in_vivo	13	14	1.45769231	2.82714286	-1.3694505	c(t = -1.3961	0.177	0.83731181	Promoter (<=1kb)	ENSSSCG000	ASCL2	achaete-scute family bHLH transcription factor 2 [Source:NCBI gene (formerly Entrezgene);Acc:100144467]
chr14	63108129	63109534	1406	*	chr14	63108129	63109534	in_vitro	in_vivo	10	14	0.813	2.08642857	-1.2734286	c(t = -1.4042	0.177	0.83731181	Distal Intergenic	ENSSSCG00000032953		myoregulin [Source:NCBI gene (formerly Entrezgene);Acc:105667210]
chr7	63336782	63337665	884	*	chr7	63336782	63337665	in_vitro	in_vivo	12	14	3.635	1.80714286	1.82785714	c(t = 1.40226	0.177	0.83731181	Distal Intergenic	ENSSSCG000	NKX2-1	NK2 homeobox 1 [Source:VGNC Symbol;Acc:VGNC:90767]
chr15	124318397	124319396	1000	*	chr15	124318397	124319396	in_vitro	in_vivo	10	12	3.478	1.35833333	2.11966667	c(t = 1.42705	0.177	0.83731181	Distal Intergenic	ENSSSCG000	ACSL3	acyl-CoA synthetase long chain family member 3 [Source:VGNC Symbol;Acc:VGNC:96295]
chr17	47686035	47686889	855	*	chr17	47686035	47686889	in_vitro	in_vivo	9	10	2.29777778	0.812	1.48577778	c(t = 1.42590	0.177	0.83731181	Promoter (<=1kb)	ENSSSCG000	DBNDD2	dysbindin domain containing 2 [Source:VGNC Symbol;Acc:VGNC:107134]
chr13	23279037	23279516	480	*	chr13	23279037	23279516	in_vitro	in_vivo	9	11	3.24111111	0.67727273	2.56383838	c(t = 1.46287	0.177	0.83731181	Distal Intergenic	ENSSSCG000	ACVR2B	activin A receptor type 2B [Source:VGNC Symbol;Acc:VGNC:108629]
chr11	15472071	15472645	575	*	chr11	15472071	15472645	in_vitro	in_vivo	10	11	2.18	0.35	1.83	c(t = 1.44592	0.177	0.83731181	Distal Intergenic	ENSSSCG000	SLC25A15	solute carrier family 25 member 15 [Source:VGNC Symbol;Acc:VGNC:92994]
chrX	121749504	121753574	4071	*	chrX	121749504	121753574	in_vitro	in_vivo	11	14	9.34	23.5307143	-14.190714	c(t = -1.4172	0.178	0.83731181	Distal Intergenic	ENSSSCG000	FATE1	fetal and adult testis expressed 1 [Source:NCBI gene (formerly Entrezgene);Acc:396705]
chr14	141424471	141425338	868	*	chr14	141424471	141425338	in_vitro	in_vivo	10	9	5.223	18.66	-13.437	c(t = -1.4657	0.178	0.83731181	Distal Intergenic	ENSSSCG000	ECHS1	enoyl-CoA hydratase, short chain 1 [Source:NCBI gene (formerly Entrezgene);Acc:100156927]
chr1	2600859	2602515	1657	*	chr1	2600859	2602515	in_vitro	in_vivo	12	13	2.19583333	3.76923077	-1.5733974	c(t = -1.4028	0.178	0.83731181	Distal Intergenic	ENSSSCG000	SFT2D1	SFT2 domain containing 1 [Source:NCBI gene (formerly Entrezgene);Acc:100513321]
chr17	1213859	1215526	1668	*	chr17	1213859	1215526	in_vitro	in_vivo	12	13	0.84333333	3.03	-2.1866667	c(t = -1.4146	0.178	0.83731181	Distal Intergenic	ENSSSCG000	CLDN23	claudin 23 [Source:NCBI gene (formerly Entrezgene);Acc:100156235]
chr3	23365448	23367496	2049	*	chr3	23365448	23367496	in_vitro	in_vivo	10	16	1.428	2.431875	-1.003875	c(t = -1.3900	0.178	0.83731181	Distal Intergenic	ENSSSCG000	IGSF6	immunoglobulin superfamily member 6 [Source:NCBI gene (formerly Entrezgene);Acc:100627993]
chr2	144928471	144931857	3387	*	chr2	144928471	144931857	in_vitro	in_vivo	10	13	4.872	1.77153846	3.10046154	c(t = 1.44390	0.178	0.83731181	Promoter (<=1kb)	ENSSSCG000	NR3C1	nuclear receptor subfamily 3 group C member 1 [Source:VGNC Symbol;Acc:VGNC:90883]
chr4	56173277	56175322	2046	*	chr4	56173277	56175322	in_vitro	in_vivo	14	15	0.87071429	1.70866667	-0.8379524	c(t = -1.3896	0.178	0.83731181	Distal Intergenic	ENSSSCG000	TPD52	tumor protein D52 [Source:VGNC Symbol;Acc:VGNC:98383]
chr7	12116919	12117991	1073	*	chr7	12116919	12117991	in_vitro	in_vivo	10	11	2.421	0.94181818	1.47918182	c(t = 1.41926	0.178	0.83731181	Distal Intergenic	ENSSSCG000	DTNBP1	dystrobrevin binding protein 1 [Source:NCBI gene (formerly Entrezgene);Acc:100049697]
chr6	93987384	93987908	525	*	chr6	93987384	93987908	in_vitro	in_vivo	11	9	2.17727273	0.78666667	1.39060606	c(t = 1.40914	0.178	0.83731181	Promoter (<=1kb)	ENSSSCG000	FHL3	four and a half LIM domains 3 [Source:VGNC Symbol;Acc:VGNC:88129]
chr2	36884524	36885418	895	*	chr2	36884524	36885418	in_vitro	in_vivo	12	10	2.63166667	0.69	1.94166667	c(t = 1.40384	0.178	0.83731181	Distal Intergenic	ENSSSCG000	CSRP3	cysteine and glycine rich protein 3 [Source:NCBI gene (formerly Entrezgene);Acc:100337687]
chr3	71632987	71633473	487	*	chr3	71632987	71633473	in_vitro	in_vivo	10	11	1.558	0.42	1.138	c(t = 1.41329	0.178	0.83731181	Distal Intergenic	ENSSSCG000	CD207	CD207 molecule [Source:NCBI gene (formerly Entrezgene);Acc:100037935]
chr6	167823077	167825819	2743	*	chr6	167823077	167825819	in_vitro	in_vivo	9	10	94.5088889	97.624	-3.1151111	c(t = -1.4182	0.179	0.83731181	Distal Intergenic	ENSSSCG000	ELOVL1	ELOVL fatty acid elongase 1 [Source:VGNC Symbol;Acc:VGNC:87657]
chr1	270955829	270957567	1739	*	chr1	270955829	270957567	in_vitro	in_vivo	11	11	4.67454545	1.60090909	3.07363636	c(t = 1.42860	0.179	0.83731181	Distal Intergenic	ENSSSCG000	FUBP3	far upstream element binding protein 3 [Source:VGNC Symbol;Acc:VGNC:103093]
chr12	57728868	57730868	2001	*	chr12	57728868	57730868	in_vitro	in_vivo	12	15	2.49833333	1.19933333	1.299	c(t = 1.41764	0.179	0.83731181	Distal Intergenic	ENSSSCG000	COX10	cytochrome c oxidase assembly factor heme A:farnesyltransferase COX10 [Source:NCBI gene (formerly Entrezgene);Acc:100521863]
chr17	37447277	37448934	1658	*	chr17	37447277	37448934	in_vitro	in_vivo	14	13	1.95357143	0.92230769	1.03126374	c(t = 1.38975	0.179	0.83731181	Distal Intergenic	ENSSSCG000	ASIP	agouti signaling protein [Source:NCBI gene (formerly Entrezgene);Acc:414439]
chr1	12297263	12298975	1713	*	chr1	12297263	12298975	in_vitro	in_vivo	13	15	2	0.90866667	1.09133333	c(t = 1.38585	0.179	0.83731181	Distal Intergenic	ENSSSCG000	OPRM1	opioid receptor mu 1 [Source:NCBI gene (formerly Entrezgene);Acc:396802]
chr2	100446731	100448607	1877	*	chr2	100446731	100448607	in_vitro	in_vivo	12	10	2.46333333	0.906	1.55733333	c(t = 1.39320	0.179	0.83731181	Distal Intergenic	ENSSSCG000	SPATA9	spermatogenesis associated 9 [Source:NCBI gene (formerly Entrezgene);Acc:100415927]
chr4	124773441	124774402	962	*	chr4	124773441	124774402	in_vitro	in_vivo	9	12	0.96111111	0.09916667	0.86194444	c(t = 1.46373	0.179	0.83731181	Distal Intergenic	ENSSSCG000	TGFBR3	transforming growth factor beta receptor 3 [Source:VGNC Symbol;Acc:VGNC:93932]
chr2	69183279	69183764	486	*	chr2	69183279	69183764	in_vitro	in_vivo	10	10	0.913	0	0.913	c(t = 1.45735	0.179	0.83731181	Promoter (<=1kb)	ENSSSCG000	TYK2	tyrosine kinase 2 [Source:VGNC Symbol;Acc:VGNC:94615]
chr2	150346920	150347423	504	*	chr2	150346920	150347423	in_vitro	in_vivo	9	10	96.8077778	93.055	3.75277778	c(t = 1.41823	0.18	0.83731181	Distal Intergenic	ENSSSCG000	AFAP1L1	actin filament associated protein 1 like 1 [Source:NCBI gene (formerly Entrezgene);Acc:100513463]
chr11	6155334	6156225	892	*	chr11	6155334	6156225	in_vitro	in_vivo	9	10	58.8977778	39.335	19.5627778	c(t = 1.39979	0.18	0.83731181	Distal Intergenic	ENSSSCG000	SLC46A3	solute carrier family 46 member 3 [Source:VGNC Symbol;Acc:VGNC:93128]
chr6	51589227	51591098	1872	*	chr6	51589227	51591098	in_vitro	in_vivo	11	13	17.1027273	35.4953846	-18.392657	c(t = -1.3877	0.18	0.83731181	Promoter (<=1kb)	ENSSSCG000	BLOC1S3	biogenesis of lysosomal organelles complex 1 subunit 3 [Source:NCBI gene (formerly Entrezgene);Acc:100049671]
chr16	49770556	49771794	1239	*	chr16	49770556	49771794	in_vitro	in_vivo	9	11	13.35	5.55363636	7.79636364	c(t = 1.42820	0.18	0.83731181	Distal Intergenic	ENSSSCG000	DRD1	dopamine receptor D1 [Source:VGNC Symbol;Acc:VGNC:87443]
chr9	9052615	9053598	984	*	chr9	9052615	9053598	in_vitro	in_vivo	9	10	13.8244444	4.179	9.64544444	c(t = 1.43492	0.18	0.83731181	Distal Intergenic	ENSSSCG000	POLD3	DNA polymerase delta 3, accessory subunit [Source:NCBI gene (formerly Entrezgene);Acc:100626052]
chr2	130443253	130444090	838	*	chr2	130443253	130444090	in_vitro	in_vivo	11	12	8.07181818	2.4675	5.60431818	c(t = 1.41773	0.18	0.83731181	Distal Intergenic	ENSSSCG000	MARCHF3	membrane associated ring-CH-type finger 3 [Source:NCBI gene (formerly Entrezgene);Acc:100513526]
chr9	46276544	46277497	954	*	chr9	46276544	46277497	in_vitro	in_vivo	9	14	0.77555556	2.23428571	-1.4587302	c(t = -1.3895	0.18	0.83731181	Distal Intergenic	ENSSSCG000	HMBS	hydroxymethylbilane synthase [Source:VGNC Symbol;Acc:VGNC:88899]
chr13	38438611	38438951	341	*	chr13	38438611	38438951	in_vitro	in_vivo	12	13	5.75166667	1.74923077	4.0024359	c(t = 1.40419	0.18	0.83731181	Distal Intergenic	ENSSSCG000	APPL1	adaptor protein, phosphotyrosine interacting with PH domain and leucine zipper 1 [Source:NCBI gene (formerly Entrezgene);Acc:100188978]
chr1	271257212	271258895	1684	*	chr1	271257212	271258895	in_vitro	in_vivo	10	12	3.46	1.73083333	1.72916667	c(t = 1.39555	0.18	0.83731181	Distal Intergenic	ENSSSCG000	FUBP3	far upstream element binding protein 3 [Source:VGNC Symbol;Acc:VGNC:103093]
chr14	112217181	112217863	683	*	chr14	112217181	112217863	in_vitro	in_vivo	9	9	3.99555556	1.15222222	2.84333333	c(t = 1.45379	0.18	0.83731181	Distal Intergenic	ENSSSCG000	LBX1	ladybird homeobox 1 [Source:VGNC Symbol;Acc:VGNC:89652]
chr9	60841983	60842630	648	*	chr9	60841983	60842630	in_vitro	in_vivo	10	11	4.451	0.53454545	3.91645455	c(t = 1.44533	0.18	0.83731181	Distal Intergenic	ENSSSCG000	ACAD8	acyl-CoA dehydrogenase family member 8 [Source:NCBI gene (formerly Entrezgene);Acc:100627341]
chr4	97620195	97620824	630	*	chr4	97620195	97620824	in_vitro	in_vivo	9	11	0	0.43272727	-0.4327273	c(t = -1.4426	0.18	0.83731181	Distal Intergenic	ENSSSCG000	RIIAD1	regulatory subunit of type II PKA R-subunit domain containing 1 [Source:NCBI gene (formerly Entrezgene);Acc:100294636]
chr15	91493396	91494113	718	*	chr15	91493396	91494113	in_vitro	in_vivo	11	10	2.12636364	0.422	1.70436364	c(t = 1.43138	0.18	0.83731181	Distal Intergenic	ENSSSCG000	ITGAV	integrin subunit alpha V [Source:VGNC Symbol;Acc:VGNC:96380]
chr14	62525645	62526625	981	*	chr14	62525645	62526625	in_vitro	in_vivo	10	12	5.954	0.24666667	5.70733333	c(t = 1.45447	0.18	0.83731181	Promoter (<=1kb)	ENSSSCG000	PHYHIPL	phytanoyl-CoA 2-hydroxylase interacting protein like [Source:VGNC Symbol;Acc:VGNC:91407]
chr2	11689334	11689859	526	*	chr2	11689334	11689859	in_vitro	in_vivo	9	10	0.49444444	0	0.49444444	c(t = 1.46941	0.18	0.83731181	Promoter (<=1kb)	ENSSSCG000	MRPL16	mitochondrial ribosomal protein L16 [Source:NCBI gene (formerly Entrezgene);Acc:100514446]
chr6	63846909	63852030	5122	*	chr6	63846909	63852030	in_vitro	in_vivo	13	12	94.7676923	88.8941667	5.87352564	c(t = 1.40334	0.181	0.83731181	Distal Intergenic	ENSSSCG000	PRKCZ	protein kinase C zeta [Source:NCBI gene (formerly Entrezgene);Acc:100533200]
chr5	2548697	2550183	1487	*	chr5	2548697	2550183	in_vitro	in_vivo	10	10	7.013	24.644	-17.631	c(t = -1.4415	0.181	0.83731181	Distal Intergenic	ENSSSCG000	PPARA	peroxisome proliferator activated receptor alpha [Source:NCBI gene (formerly Entrezgene);Acc:397239]
chr11	77315503	77317573	2071	*	chr11	77315503	77317573	in_vitro	in_vivo	12	14	7.86083333	18.8071429	-10.94631	c(t = -1.3960	0.181	0.83731181	Distal Intergenic	ENSSSCG000	ANKRD10	ankyrin repeat domain 10 [Source:VGNC Symbol;Acc:VGNC:85322]
chrX	54661770	54662213	444	*	chrX	54661770	54662213	in_vitro	in_vivo	11	9	2.50727273	18.6966667	-16.189394	c(t = -1.4572	0.181	0.83731181	Distal Intergenic	ENSSSCG000	AR	androgen receptor [Source:VGNC Symbol;Acc:VGNC:103903]
chr16	51457610	51458746	1137	*	chr16	51457610	51458746	in_vitro	in_vivo	12	11	2.19416667	5.33727273	-3.1431061	c(t = -1.3871	0.181	0.83731181	Promoter (<=1kb)	ENSSSCG000	DUSP1	dual specificity phosphatase 1 [Source:VGNC Symbol;Acc:VGNC:87476]
chr6	31044413	31048622	4210	*	chr6	31044413	31048622	in_vitro	in_vivo	10	12	1.877	3.31583333	-1.4388333	c(t = -1.3855	0.181	0.83731181	Distal Intergenic	ENSSSCG000	FTO	FTO alpha-ketoglutarate dependent dioxygenase [Source:NCBI gene (formerly Entrezgene);Acc:100127165]
chr12	50013258	50014383	1126	*	chr12	50013258	50014383	in_vitro	in_vivo	9	9	6.39444444	3.16111111	3.23333333	c(t = 1.40805	0.181	0.83731181	Distal Intergenic	ENSSSCG000	ASPA	aspartoacylase [Source:NCBI gene (formerly Entrezgene);Acc:100142661]
chr13	160223888	160224761	874	*	chr13	160223888	160224761	in_vitro	in_vivo	11	9	6.58363636	2.50333333	4.08030303	c(t = 1.42009	0.181	0.83731181	Distal Intergenic	ENSSSCG000	CPOX	coproporphyrinogen oxidase [Source:NCBI gene (formerly Entrezgene);Acc:100511474]
chr14	6945493	6951286	5794	*	chr14	6945493	6951286	in_vitro	in_vivo	15	16	7.10466667	2.356875	4.74779167	c(t = 1.40166	0.181	0.83731181	Distal Intergenic	ENSSSCG000	CCAR2	cell cycle and apoptosis regulator 2 [Source:NCBI gene (formerly Entrezgene);Acc:100155718]
chr6	42559293	42561711	2419	*	chr6	42559293	42561711	in_vitro	in_vivo	16	11	3.4925	2.05727273	1.43522727	c(t = 1.37992	0.181	0.83731181	Distal Intergenic	ENSSSCG000	SLC7A9	solute carrier family 7 member 9 [Source:VGNC Symbol;Acc:VGNC:93177]
chr5	846439	847380	942	*	chr5	846439	847380	in_vitro	in_vivo	10	10	10.146	1.396	8.75	c(t = 1.44810	0.181	0.83731181	Distal Intergenic	ENSSSCG000	SELENOO	selenoprotein O [Source:NCBI gene (formerly Entrezgene);Acc:100518971]
chr7	22974495	22975595	1101	*	chr7	22974495	22975595	in_vitro	in_vivo	10	12	3.263	1.24166667	2.02133333	c(t = 1.41763	0.181	0.83731181	Distal Intergenic	ENSSSCG00000052432		MHC class I antigen 2 [Source:NCBI gene (formerly Entrezgene);Acc:100135031]
chr13	182226693	182228277	1585	*	chr13	182226693	182228277	in_vitro	in_vivo	11	12	8.74454545	0.80083333	7.94371212	c(t = 1.43801	0.181	0.83731181	Promoter (<=1kb)	ENSSSCG000	BTG3	BTG anti-proliferation factor 3 [Source:VGNC Symbol;Acc:VGNC:103908]
chr13	196370526	196371512	987	*	chr13	196370526	196371512	in_vitro	in_vivo	12	10	2.255	0.76	1.495	c(t = 1.39043	0.181	0.83731181	Distal Intergenic	ENSSSCG000	C21orf62	chromosome 13 C21orf62 homolog [Source:VGNC Symbol;Acc:VGNC:85929]
chr6	97428356	97429611	1256	*	chr6	97428356	97429611	in_vitro	in_vivo	9	12	2.04888889	0.58583333	1.46305556	c(t = 1.44444	0.181	0.83731181	Distal Intergenic	ENSSSCG000	CIDEA	cell death inducing DFFA like effector a [Source:NCBI gene (formerly Entrezgene);Acc:100127171]
chr12	37693741	37694252	512	*	chr12	37693741	37694252	in_vitro	in_vivo	11	9	2.16	0.49333333	1.66666667	c(t = 1.42165	0.181	0.83731181	Distal Intergenic	ENSSSCG000	CA4	carbonic anhydrase 4 [Source:NCBI gene (formerly Entrezgene);Acc:100511956]
chr11	74266261	74267612	1352	*	chr11	74266261	74267612	in_vitro	in_vivo	12	14	1.52416667	0.47714286	1.04702381	c(t = 1.40849	0.181	0.83731181	Promoter (<=1kb)	ENSSSCG000	ssc-mir-7144	ssc-mir-7144 [Source:miRBase;Acc:MI0023577]
chr13	984906	985330	425	*	chr13	984906	985330	in_vitro	in_vivo	9	10	1.98333333	0.333	1.65033333	c(t = 1.44537	0.181	0.83731181	Distal Intergenic	ENSSSCG000	CAPN7	calpain 7 [Source:NCBI gene (formerly Entrezgene);Acc:100037936]
chr7	57011483	57012115	633	*	chr7	57011483	57012115	in_vitro	in_vivo	12	9	1.02416667	0.19888889	0.82527778	c(t = 1.40828	0.181	0.83731181	Distal Intergenic	ENSSSCG000	RCN2	reticulocalbin 2 [Source:VGNC Symbol;Acc:VGNC:92181]
chr15	139935235	139937381	2147	*	chr15	139935235	139937381	in_vitro	in_vivo	9	11	91.5188889	95.3727273	-3.8538384	c(t = -1.3985	0.182	0.83731181	3' UTR	ENSSSCG000	PPP1R7	protein phosphatase 1 regulatory subunit 7 [Source:NCBI gene (formerly Entrezgene);Acc:100511842]
chr13	207038179	207039328	1150	*	chr13	207038179	207039328	in_vitro	in_vivo	10	9	21.65	41.3444444	-19.694444	c(t = -1.4084	0.182	0.83731181	Distal Intergenic	ENSSSCG000	AGPAT3	1-acylglycerol-3-phosphate O-acyltransferase 3 [Source:VGNC Symbol;Acc:VGNC:85185]
chr4	32682364	32682741	378	*	chr4	32682364	32682741	in_vitro	in_vivo	9	10	10.0788889	31.794	-21.715111	c(t = -1.3933	0.182	0.83731181	Distal Intergenic	ENSSSCG000	LRP12	LDL receptor related protein 12 [Source:VGNC Symbol;Acc:VGNC:89818]
chr13	205364060	205365651	1592	*	chr13	205364060	205365651	in_vitro	in_vivo	11	11	5.55181818	3.23636364	2.31545455	c(t = 1.39314	0.182	0.83731181	Distal Intergenic	ENSSSCG000	TFF3	trefoil factor 3 [Source:NCBI gene (formerly Entrezgene);Acc:100627480]
chr3	77191970	77192761	792	*	chr3	77191970	77192761	in_vitro	in_vivo	9	11	0.27777778	2.89636364	-2.6185859	c(t = -1.4303	0.182	0.83731181	Distal Intergenic	ENSSSCG000	RAB1A	RAB1A, member RAS oncogene family [Source:NCBI gene (formerly Entrezgene);Acc:595116]
chr6	80516429	80517008	580	*	chr6	80516429	80517008	in_vitro	in_vivo	9	9	4.32	1.89444444	2.42555556	c(t = 1.41626	0.182	0.83731181	Distal Intergenic	ENSSSCG000	C1QA	complement C1q A chain [Source:VGNC Symbol;Acc:VGNC:96926]
chr13	5415652	5417586	1935	*	chr13	5415652	5417586	in_vitro	in_vivo	11	13	3.28545455	1.47769231	1.80776224	c(t = 1.38983	0.182	0.83731181	Distal Intergenic	ENSSSCG000	RAB5A	RAB5A, member RAS onco family [Source:VGNC Symbol;Acc:VGNC:108687]
chr14	111817359	111819621	2263	*	chr14	111817359	111819621	in_vitro	in_vivo	11	13	3.26272727	1.47384615	1.78888112	c(t = 1.39132	0.182	0.83731181	Distal Intergenic	ENSSSCG000	NDUFB8	NADH:ubiquinone oxidoreductase subunit B8 [Source:NCBI gene (formerly Entrezgene);Acc:448979]
chr6	1545455	1546935	1481	*	chr6	1545455	1546935	in_vitro	in_vivo	11	10	4.21818182	0.825	3.39318182	c(t = 1.42593	0.182	0.83731181	Distal Intergenic	ENSSSCG000	BANP	BTG3 associated nuclear protein [Source:NCBI gene (formerly Entrezgene);Acc:100622381]
chr1	269374436	269375311	876	*	chr1	269374436	269375311	in_vitro	in_vivo	10	10	2.485	0.579	1.906	c(t = 1.42632	0.182	0.83731181	Distal Intergenic	ENSSSCG000	CRAT	carnitine O-acetyltransferase [Source:VGNC Symbol;Acc:VGNC:86976]
chr11	5454656	5455821	1166	*	chr11	5454656	5455821	in_vitro	in_vivo	12	12	5.15583333	0.52916667	4.62666667	c(t = 1.42414	0.182	0.83731181	Distal Intergenic	ENSSSCG000	CDX2	caudal type homeobox 2 [Source:NCBI gene (formerly Entrezgene);Acc:100127132]
chr6	60721048	60721771	724	*	chr6	60721048	60721771	in_vitro	in_vivo	11	11	2.60636364	0.28	2.32636364	c(t = 1.42836	0.182	0.83731181	Distal Intergenic	ENSSSCG000	GALP	galanin like peptide [Source:NCBI gene (formerly Entrezgene);Acc:396772]
chr14	59703631	59704143	513	*	chr14	59703631	59704143	in_vitro	in_vivo	9	10	1.15333333	0	1.15333333	c(t = 1.46321	0.182	0.83731181	Distal Intergenic	ENSSSCG000	ARV1	ARV1 homolog, fatty acid homeostasis modulator [Source:VGNC Symbol;Acc:VGNC:85554]
chr18	52599703	52600485	783	*	chr18	52599703	52600485	in_vitro	in_vivo	9	11	1.71666667	10.3054545	-8.5887879	c(t = -1.4254	0.183	0.83731181	Distal Intergenic	ENSSSCG000	INHBA	inhibin subunit beta A [Source:NCBI gene (formerly Entrezgene);Acc:397093]
chr7	7731725	7732699	975	*	chr7	7731725	7732699	in_vitro	in_vivo	12	12	0.85	2.14583333	-1.2958333	c(t = -1.4005	0.183	0.83731181	Distal Intergenic	ENSSSCG000	C6orf52	chromosome 7 C6orf52 homolog [Source:VGNC Symbol;Acc:VGNC:86073]
chr1	139804149	139805547	1399	*	chr1	139804149	139805547	in_vitro	in_vivo	14	13	0.79928571	1.81538462	-1.0160989	c(t = -1.3855	0.183	0.83731181	Promoter (<=1kb)	ENSSSCG000	CHSY1	chondroitin sulfate synthase 1 [Source:NCBI gene (formerly Entrezgene);Acc:100520288]
chr2	132145003	132146842	1840	*	chr2	132145003	132146842	in_vitro	in_vivo	14	14	3.06785714	1.42285714	1.645	c(t = 1.37111	0.183	0.83731181	Distal Intergenic	ENSSSCG000	MARCHF3	membrane associated ring-CH-type finger 3 [Source:NCBI gene (formerly Entrezgene);Acc:100513526]
chr17	34885011	34885919	909	*	chr17	34885011	34885919	in_vitro	in_vivo	9	11	2.23777778	0.94272727	1.29505051	c(t = 1.41268	0.183	0.83731181	Distal Intergenic	ENSSSCG000	DEFB129	defensin beta 129 [Source:NCBI gene (formerly Entrezgene);Acc:100170137]
chr12	59705031	59705770	740	*	chr12	59705031	59705770	in_vitro	in_vivo	11	13	2.15727273	0.85846154	1.29881119	c(t = 1.37711	0.183	0.83731181	Distal Intergenic	ENSSSCG000	AKAP10	A-kinase anchoring protein 10 [Source:NCBI gene (formerly Entrezgene);Acc:397262]
chr4	75902729	75903972	1244	*	chr4	75902729	75903972	in_vitro	in_vivo	11	11	2.47363636	0.77363636	1.7	c(t = 1.41016	0.183	0.83731181	Distal Intergenic	ENSSSCG000	RPS20	ribosomal protein S20 [Source:NCBI gene (formerly Entrezgene);Acc:414395]
chr1	128083556	128084394	839	*	chr1	128083556	128084394	in_vitro	in_vivo	11	11	2.12545455	0.56090909	1.56454545	c(t = 1.40529	0.183	0.83731181	Distal Intergenic	ENSSSCG000	ssc-mir-2366	ssc-mir-2366-1 [Source:miRBase;Acc:MI0022162]
chr3	39759888	39763675	3788	*	chr3	39759888	39763675	in_vitro	in_vivo	10	12	95.724	92.1116667	3.61233333	c(t = 1.38428	0.184	0.83731181	Distal Intergenic	ENSSSCG000	ECI1	enoyl-CoA delta isomerase 1 [Source:NCBI gene (formerly Entrezgene);Acc:100738177]
chr17	58996009	59001882	5874	*	chr17	58996009	59001882	in_vitro	in_vivo	13	16	92.0192308	80.249375	11.7698558	c(t = 1.36744	0.184	0.83731181	Intron (NM_001130215/100049657, intron 1 of 12)	ENSSSCG00000007520		GNAS complex locus [Source:VGNC Symbol;Acc:VGNC:110580]
chr18	50750846	50751473	628	*	chr18	50750846	50751473	in_vitro	in_vivo	9	13	6.38444444	19.9653846	-13.58094	c(t = -1.3820	0.184	0.83731181	Distal Intergenic	ENSSSCG000	DDX56	DEAD-box helicase 56 [Source:NCBI gene (formerly Entrezgene);Acc:100513388]
chr14	48092881	48095284	2404	*	chr14	48092881	48095284	in_vitro	in_vivo	13	14	3.86076923	1.80714286	2.05362637	c(t = 1.37135	0.184	0.83731181	Distal Intergenic	ENSSSCG000	SELENOM	selenoprotein M [Source:NCBI gene (formerly Entrezgene);Acc:100302025]
chr17	32796545	32797361	817	*	chr17	32796545	32797361	in_vitro	in_vivo	12	10	2.2975	1.009	1.2885	c(t = 1.38558	0.184	0.83731181	Promoter (<=1kb)	ENSSSCG000	PTPRA	protein tyrosine phosphatase receptor type A [Source:VGNC Symbol;Acc:VGNC:96522]
chr18	9580913	9582949	2037	*	chr18	9580913	9582949	in_vitro	in_vivo	13	15	1.66153846	0.78866667	0.87287179	c(t = 1.36910	0.184	0.83731181	Distal Intergenic	ENSSSCG000	SLC37A3	solute carrier family 37 member 3 [Source:VGNC Symbol;Acc:VGNC:98324]
chr2	14723938	14725343	1406	*	chr2	14723938	14725343	in_vitro	in_vivo	12	13	1.55333333	0.64384615	0.90948718	c(t = 1.37150	0.184	0.83731181	Distal Intergenic	ENSSSCG000	NDUFS3	NADH:ubiquinone oxidoreductase core subunit S3 [Source:NCBI gene (formerly Entrezgene);Acc:100510948]
chr3	69485823	69488494	2672	*	chr3	69485823	69488494	in_vitro	in_vivo	14	13	1.28785714	0.51692308	0.77093407	c(t = 1.38051	0.184	0.83731181	Promoter (<=1kb)	ENSSSCG000	EGR4	early growth response 4 [Source:NCBI gene (formerly Entrezgene);Acc:100516906]
chr17	33305567	33306649	1083	*	chr17	33305567	33306649	in_vitro	in_vivo	9	11	2.32222222	0.41363636	1.90858586	c(t = 1.43155	0.184	0.83731181	Distal Intergenic	ENSSSCG000	PDYN	prodynorphin [Source:VGNC Symbol;Acc:VGNC:96477]
chr5	19483902	19485653	1752	*	chr5	19483902	19485653	in_vitro	in_vivo	12	12	4.49083333	0.37	4.12083333	c(t = 1.41654	0.184	0.83731181	Promoter (<=1kb)	ENSSSCG00000000288		heterous nuclear ribonucleoprotein A1 [Source:VGNC Symbol;Acc:VGNC:88918]
chr12	36881939	36891306	9368	*	chr12	36881939	36891306	in_vitro	in_vivo	14	16	8.925	5.43125	3.49375	c(t = 1.35972	0.185	0.83731181	Promoter (<=1kb)	ENSSSCG000	TBX2	T-box transcription factor 2 [Source:NCBI gene (formerly Entrezgene);Acc:100622658]
chr3	16806689	16807315	627	*	chr3	16806689	16807315	in_vitro	in_vivo	9	13	4.06777778	1.51769231	2.55008547	c(t = 1.42449	0.185	0.83731181	Distal Intergenic	ENSSSCG000	PHKG1	phosphorylase kinase catalytic subunit gamma 1 [Source:NCBI gene (formerly Entrezgene);Acc:397548]
chr3	43973665	43974341	677	*	chr3	43973665	43974341	in_vitro	in_vivo	9	10	0.15888889	0.734	-0.5751111	c(t = -1.4061	0.185	0.83731181	Distal Intergenic	ENSSSCG00000008097		tubulin tyrosine ligase [Source:NCBI gene (formerly Entrezgene);Acc:445530]
chr11	12689286	12690041	756	*	chr11	12689286	12690041	in_vitro	in_vivo	11	13	2.01727273	0.67076923	1.3465035	c(t = 1.37553	0.185	0.83731181	Distal Intergenic	ENSSSCG000	SMAD9	SMAD family member 9 [Source:NCBI gene (formerly Entrezgene);Acc:100153584]
chrX	43215174	43216324	1151	*	chrX	43215174	43216324	in_vitro	in_vivo	13	10	3.06307692	0.622	2.44107692	c(t = 1.39515	0.185	0.83731181	Promoter (<=1kb)	ENSSSCG000	PLP2	proteolipid protein 2 [Source:NCBI gene (formerly Entrezgene);Acc:100512511]
chr5	63884199	63885126	928	*	chr5	63884199	63885126	in_vitro	in_vivo	9	9	0.99111111	0.34111111	0.65	c(t = 1.40193	0.185	0.83731181	Distal Intergenic	ENSSSCG000	GNB3	G protein subunit beta 3 [Source:VGNC Symbol;Acc:VGNC:88532]
chrX	98758069	98758634	566	*	chrX	98758069	98758634	in_vitro	in_vivo	9	9	2.96222222	0.32666667	2.63555556	c(t = 1.44035	0.185	0.83731181	Distal Intergenic	ENSSSCG000	LAMP2	lysosomal associated membrane protein 2 [Source:NCBI gene (formerly Entrezgene);Acc:100154598]
chr2	5056908	5057662	755	*	chr2	5056908	5057662	in_vitro	in_vivo	10	9	1.255	0.21222222	1.04277778	c(t = 1.42598	0.185	0.83731181	Distal Intergenic	ENSSSCG000	PPP1CA	protein phosphatase 1 catalytic subunit alpha [Source:NCBI gene (formerly Entrezgene);Acc:733611]
chr1	239786657	239787276	620	*	chr1	239786657	239787276	in_vitro	in_vivo	10	10	1.964	0	1.964	c(t = 1.43469	0.185	0.83731181	Promoter (<=1kb)	ENSSSCG000	TRMO	tRNA methyltransferase O [Source:NCBI gene (formerly Entrezgene);Acc:100156700]
chr4	69807627	69808568	942	*	chr4	69807627	69808568	in_vitro	in_vivo	9	10	1.91222222	0	1.91222222	c(t = 1.45193	0.185	0.83731181	Distal Intergenic	ENSSSCG000	ssc-mir-124a	ssc-mir-124a-1 [Source:miRBase;Acc:MI0010680]
chr6	63536716	63541364	4649	*	chr6	63536716	63541364	in_vitro	in_vivo	11	12	28.9209091	44.835	-15.914091	c(t = -1.3661	0.186	0.83731181	Distal Intergenic	ENSSSCG000	ssc-mir-429	ssc-mir-429 [Source:miRBase;Acc:MI0017991]
chr12	60141337	60149549	8213	*	chr12	60141337	60149549	in_vitro	in_vivo	13	13	39.7438462	27.6807692	12.0630769	c(t = 1.37048	0.186	0.83731181	Distal Intergenic	ENSSSCG000	SLC5A10	solute carrier family 5 member 10 [Source:NCBI gene (formerly Entrezgene);Acc:497235]
chr6	53078557	53080862	2306	*	chr6	53078557	53080862	in_vitro	in_vivo	10	11	19.161	8.78818182	10.3728182	c(t = 1.38841	0.186	0.83731181	Distal Intergenic	ENSSSCG000	C5AR1	complement C5a receptor 1 [Source:NCBI gene (formerly Entrezgene);Acc:100048955]
chr8	72542051	72543973	1923	*	chr8	72542051	72543973	in_vitro	in_vivo	12	15	1.70166667	6.43866667	-4.737	c(t = -1.3880	0.186	0.83731181	Distal Intergenic	ENSSSCG000	SCARB2	scavenger receptor class B member 2 [Source:VGNC Symbol;Acc:VGNC:92614]
chr2	86086481	86087566	1086	*	chr2	86086481	86087566	in_vitro	in_vivo	11	10	6.11	2.133	3.977	c(t = 1.40573	0.186	0.83731181	Distal Intergenic	ENSSSCG000	CRHBP	corticotropin releasing hormone binding protein [Source:VGNC Symbol;Acc:VGNC:86993]
chr12	50728138	50729135	998	*	chr12	50728138	50729135	in_vitro	in_vivo	10	14	0.701	2.07285714	-1.3718571	c(t = -1.3835	0.186	0.83731181	Distal Intergenic	ENSSSCG000	ZNF232	zinc finger protein 232 [Source:VGNC Symbol;Acc:VGNC:95208]
chr7	1732217	1733189	973	*	chr7	1732217	1733189	in_vitro	in_vivo	9	11	4.56333333	1.94545455	2.61787879	c(t = 1.40808	0.186	0.83731181	Promoter (<=1kb)	ENSSSCG000	SERPINB9	serpin peptidase inhibitor, clade B (ovalbumin), member 9 [Source:NCBI gene (formerly Entrezgene);Acc:100126851]
chr17	32512641	32513178	538	*	chr17	32512641	32513178	in_vitro	in_vivo	9	9	0	1.16888889	-1.1688889	c(t = -1.4465	0.186	0.83731181	Distal Intergenic	ENSSSCG000	DDRGK1	DDRGK domain containing 1 [Source:NCBI gene (formerly Entrezgene);Acc:100155894]
chr15	23470533	23471819	1287	*	chr15	23470533	23471819	in_vitro	in_vivo	11	10	2.68454545	1.127	1.55754545	c(t = 1.38648	0.186	0.83731181	Distal Intergenic	ENSSSCG000	INSIG2	insulin induced 2 [Source:VGNC Symbol;Acc:VGNC:103970]
chr8	16786527	16787338	812	*	chr8	16786527	16787338	in_vitro	in_vivo	11	11	1.37636364	0.51090909	0.86545455	c(t = 1.37468	0.186	0.83731181	Distal Intergenic	ENSSSCG000	PPARGC1A	PPARG coactivator 1 alpha [Source:VGNC Symbol;Acc:VGNC:91685]
chr13	24574798	24575650	853	*	chr13	24574798	24575650	in_vitro	in_vivo	9	9	1.46333333	0	1.46333333	c(t = 1.44695	0.186	0.83731181	Promoter (<=1kb)	ENSSSCG000	EIF1B	eukaryotic translation initiation factor 1B [Source:NCBI gene (formerly Entrezgene);Acc:414417]
chr17	61721309	61724447	3139	*	chr17	61721309	61724447	in_vitro	in_vivo	9	12	87.4766667	97.1075	-9.6308333	c(t = -1.4377	0.187	0.83731181	Distal Intergenic	ENSSSCG000	RPS21	ribosomal protein S21 [Source:NCBI gene (formerly Entrezgene);Acc:100037996]
chr14	140857075	140902248	45174	*	chr14	140857075	140902248	in_vitro	in_vivo	15	16	19.74	10.976875	8.763125	c(t = 1.35822	0.187	0.83731181	Distal Intergenic	ENSSSCG000	ssc-mir-202	ssc-mir-202 [Source:miRBase;Acc:MI0013157]
chr8	52174104	52174975	872	*	chr8	52174104	52174975	in_vitro	in_vivo	11	10	1.49909091	3.805	-2.3059091	c(t = -1.3764	0.187	0.83731181	Distal Intergenic	ENSSSCG000	NPY1R	neuropeptide Y receptor Y1 [Source:VGNC Symbol;Acc:VGNC:90865]
chr1	146279425	146280473	1049	*	chr1	146279425	146280473	in_vitro	in_vivo	10	9	1.649	3.64222222	-1.9932222	c(t = -1.3868	0.187	0.83731181	Distal Intergenic	ENSSSCG000	NFATC1	nuclear factor of activated T cells 1 [Source:NCBI gene (formerly Entrezgene);Acc:397318]
chr7	21220163	21221105	943	*	chr7	21220163	21221105	in_vitro	in_vivo	9	12	0.46333333	1.80666667	-1.3433333	c(t = -1.3783	0.187	0.83731181	Distal Intergenic	ENSSSCG000	ssc-mir-7857	ssc-mir-7857 [Source:miRBase;Acc:MI0031552]
chr2	79466602	79467529	928	*	chr2	79466602	79467529	in_vitro	in_vivo	11	13	2.12	0.91692308	1.20307692	c(t = 1.39028	0.187	0.83731181	Distal Intergenic	ENSSSCG000	PROP1	PROP paired-like homeobox 1 [Source:VGNC Symbol;Acc:VGNC:91834]
chr1	194351577	194352766	1190	*	chr1	194351577	194352766	in_vitro	in_vivo	12	12	4.21583333	0.88166667	3.33416667	c(t = 1.40461	0.187	0.83731181	Distal Intergenic	ENSSSCG000	PPP2R5E	protein phosphatase 2 regulatory subunit B'epsilon [Source:NCBI gene (formerly Entrezgene);Acc:100525377]
chr2	69073801	69074784	984	*	chr2	69073801	69074784	in_vitro	in_vivo	11	12	2.07636364	0.63333333	1.4430303	c(t = 1.40111	0.187	0.83731181	Distal Intergenic	ENSSSCG000	S1PR2	sphingosine-1-phosphate receptor 2 [Source:NCBI gene (formerly Entrezgene);Acc:100511551]
chr16	51209620	51211026	1407	*	chr16	51209620	51211026	in_vitro	in_vivo	11	13	1.36363636	0.48846154	0.87517483	c(t = 1.37312	0.187	0.83731181	Distal Intergenic	ENSSSCG00000016990		ATPase H+ transporting V0 subunit e1 [Source:NCBI gene (formerly Entrezgene);Acc:733646]
chr6	88451854	88453012	1159	*	chr6	88451854	88453012	in_vitro	in_vivo	10	12	1.643	0.2125	1.4305	c(t = 1.42120	0.187	0.83731181	Distal Intergenic	ENSSSCG000	PTP4A2	protein tyrosine phosphatase 4A2 [Source:VGNC Symbol;Acc:VGNC:91967]
chrX	46903510	46904358	849	*	chrX	46903510	46904358	in_vitro	in_vivo	11	11	0.77090909	11.8109091	-11.04	c(t = -1.4117	0.188	0.83731181	Distal Intergenic	ENSSSCG000	ssc-mir-1038	ssc-mir-10388 [Source:miRBase;Acc:MI0033401]
chr9	6269326	6270765	1440	*	chr9	6269326	6270765	in_vitro	in_vivo	12	15	1.40166667	6.48266667	-5.081	c(t = -1.3807	0.188	0.83731181	Promoter (<=1kb)	ENSSSCG000	STIM1	stromal interaction molecule 1 [Source:VGNC Symbol;Acc:VGNC:93546]
chr17	48227673	48229191	1519	*	chr17	48227673	48229191	in_vitro	in_vivo	11	12	1.26272727	3.95	-2.6872727	c(t = -1.3834	0.188	0.83731181	Distal Intergenic	ENSSSCG000	MMP9	matrix metallopeptidase 9 [Source:NCBI gene (formerly Entrezgene);Acc:654325]
chr5	7431892	7432609	718	*	chr5	7431892	7432609	in_vitro	in_vivo	9	10	0.77666667	2.711	-1.9343333	c(t = -1.3948	0.188	0.83731181	Distal Intergenic	ENSSSCG000	MCHR1	melanin concentrating hormone receptor 1 [Source:VGNC Symbol;Acc:VGNC:103300]
chr1	246548130	246549094	965	*	chr1	246548130	246549094	in_vitro	in_vivo	13	12	0.79923077	2.18583333	-1.3866026	c(t = -1.3680	0.188	0.83731181	Distal Intergenic	ENSSSCG000	ABCA1	ATP binding cassette subfamily A member 1 [Source:NCBI gene (formerly Entrezgene);Acc:100152112]
chr4	119930931	119931790	860	*	chr4	119930931	119931790	in_vitro	in_vivo	11	10	2.83181818	0.91	1.92181818	c(t = 1.37187	0.188	0.83731181	Promoter (<=1kb)	ENSSSCG000	DPYD	dihydropyrimidine dehydrogenase [Source:VGNC Symbol;Acc:VGNC:98787]
chr14	59841883	59842988	1106	*	chr14	59841883	59842988	in_vitro	in_vivo	11	12	1.88090909	0.81416667	1.06674242	c(t = 1.38573	0.188	0.83731181	Distal Intergenic	ENSSSCG000	ARV1	ARV1 homolog, fatty acid homeostasis modulator [Source:VGNC Symbol;Acc:VGNC:85554]
chr6	102332977	102334827	1851	*	chr6	102332977	102334827	in_vitro	in_vivo	10	14	1.961	0.715	1.246	c(t = 1.39677	0.188	0.83731181	Distal Intergenic	ENSSSCG000	TGIF1	TGFB induced factor homeobox 1 [Source:VGNC Symbol;Acc:VGNC:93935]
chr4	98250735	98252078	1344	*	chr4	98250735	98252078	in_vitro	in_vivo	9	10	1.97555556	0.6	1.37555556	c(t = 1.41478	0.188	0.83731181	Distal Intergenic	ENSSSCG000	ANXA9	annexin A9 [Source:VGNC Symbol;Acc:VGNC:85373]
chr1	238176747	238177655	909	*	chr1	238176747	238177655	in_vitro	in_vivo	10	10	1.809	0.554	1.255	c(t = 1.39681	0.188	0.83731181	Distal Intergenic	ENSSSCG00000032554		solute carrier family 25 member 51 [Source:NCBI gene (formerly Entrezgene);Acc:100157218]
chr7	32058236	32058987	752	*	chr7	32058236	32058987	in_vitro	in_vivo	9	11	0.52444444	0	0.52444444	c(t = 1.43912	0.188	0.83731181	Distal Intergenic	ENSSSCG000	SRPK1	SRSF protein kinase 1 [Source:VGNC Symbol;Acc:VGNC:93462]
chr5	17579653	17580342	690	*	chr5	17579653	17580342	in_vitro	in_vivo	10	10	0.737	5.867	-5.13	c(t = -1.4189	0.189	0.83731181	Distal Intergenic	ENSSSCG000	ACVR1B	activin A receptor type 1B [Source:VGNC Symbol;Acc:VGNC:85056]
chr2	66213257	66217787	4531	*	chr2	66213257	66217787	in_vitro	in_vivo	12	15	0.73083333	3.74533333	-3.0145	c(t = -1.3803	0.189	0.83731181	Distal Intergenic	ENSSSCG000	PRDX2	peroxiredoxin 2 [Source:NCBI gene (formerly Entrezgene);Acc:100512521]
chr7	75056630	75057310	681	*	chr7	75056630	75057310	in_vitro	in_vivo	9	10	0.55555556	2.733	-2.1774444	c(t = -1.3944	0.189	0.83731181	Distal Intergenic	ENSSSCG000	RABGGTA	Rab geranylgeranyltransferase subunit alpha [Source:VGNC Symbol;Acc:VGNC:92046]
chr7	55462095	55462628	534	*	chr7	55462095	55462628	in_vitro	in_vivo	10	11	3.209	1.01272727	2.19627273	c(t = 1.38326	0.189	0.83731181	Promoter (<=1kb)	ENSSSCG000	AP3S2	adaptor-related protein complex 3, sigma 2 subunit [Source:NCBI gene (formerly Entrezgene);Acc:100153862]
chr11	71109960	71112057	2098	*	chr11	71109960	71112057	in_vitro	in_vivo	16	15	0.336875	0.90066667	-0.5637917	c(t = -1.3538	0.189	0.83731181	Promoter (<=1kb)	ENSSSCG000	BIVM	basic, immunoglobulin-like variable motif containing [Source:VGNC Symbol;Acc:VGNC:109180]
chr1	144473288	144474112	825	*	chr1	144473288	144474112	in_vitro	in_vivo	13	10	2.11230769	0.823	1.28930769	c(t = 1.37285	0.189	0.83731181	Distal Intergenic	ENSSSCG000	MCEE	methylmalonyl-CoA epimerase [Source:VGNC Symbol;Acc:VGNC:90066]
chr5	3477101	3477726	626	*	chr5	3477101	3477726	in_vitro	in_vivo	12	10	1.53666667	0.72	0.81666667	c(t = 1.36555	0.189	0.83731181	Distal Intergenic	ENSSSCG000	PPARA	peroxisome proliferator activated receptor alpha [Source:NCBI gene (formerly Entrezgene);Acc:397239]
chr13	162477992	162478926	935	*	chr13	162477992	162478926	in_vitro	in_vivo	10	11	2.275	0.55363636	1.72136364	c(t = 1.39894	0.189	0.83731181	Distal Intergenic	ENSSSCG000	CLDND1	claudin domain containing 1 [Source:VGNC Symbol;Acc:VGNC:86742]
chr14	64399234	64400196	963	*	chr14	64399234	64400196	in_vitro	in_vivo	13	12	1.56	0.54583333	1.01416667	c(t = 1.36668	0.189	0.83731181	Distal Intergenic	ENSSSCG000	CDK1	cyclin dependent kinase 1 [Source:VGNC Symbol;Acc:VGNC:103917]
chr14	139049572	139050812	1241	*	chr14	139049572	139050812	in_vitro	in_vivo	13	12	1.73461538	0.37333333	1.36128205	c(t = 1.37308	0.189	0.83731181	Distal Intergenic	ENSSSCG000	GLRX3	glutaredoxin 3 [Source:VGNC Symbol;Acc:VGNC:88495]
chr6	84966637	84967274	638	*	chr6	84966637	84967274	in_vitro	in_vivo	11	10	0.70454545	0.145	0.55954545	c(t = 1.38795	0.189	0.83731181	Promoter (<=1kb)	ENSSSCG000	PPP1R8	protein phosphatase 1 regulatory subunit 8 [Source:VGNC Symbol;Acc:VGNC:91742]
chr6	47494021	47494228	208	*	chr6	47494021	47494228	in_vitro	in_vivo	9	9	1.07777778	0	1.07777778	c(t = 1.43684	0.189	0.83731181	Distal Intergenic	ENSSSCG00000024281		galectin 7 [Source:NCBI gene (formerly Entrezgene);Acc:100217394]
chr15	88198452	88199076	625	*	chr15	88198452	88199076	in_vitro	in_vivo	9	12	0.46555556	0	0.46555556	c(t = 1.43679	0.189	0.83731181	Distal Intergenic	ENSSSCG000	FRZB	frizzled related protein [Source:VGNC Symbol;Acc:VGNC:96308]
chr3	1991916	1999884	7969	*	chr3	1991916	1999884	in_vitro	in_vivo	13	15	84.9269231	93.2686667	-8.3417436	c(t = -1.3785	0.19	0.83731181	Exon (NM_001195325/100337670, exon 7 of 7)	ENSSSCG000	AMZ1	archaelysin family metallopeptidase 1 [Source:NCBI gene (formerly Entrezgene);Acc:100337670]
chr1	163435340	163436160	821	*	chr1	163435340	163436160	in_vitro	in_vivo	11	11	23.8872727	12.5254545	11.3618182	c(t = 1.37440	0.19	0.83731181	Distal Intergenic	ENSSSCG000	CILP	cartilage intermediate layer protein [Source:VGNC Symbol;Acc:VGNC:86703]
chr12	7331169	7333396	2228	*	chr12	7331169	7333396	in_vitro	in_vivo	11	14	2.17454545	10.3142857	-8.1397403	c(t = -1.3803	0.19	0.83731181	Distal Intergenic	ENSSSCG000	SSTR2	somatostatin receptor 2 [Source:VGNC Symbol;Acc:VGNC:99086]
chr14	59367234	59368759	1526	*	chr14	59367234	59368759	in_vitro	in_vivo	10	10	4.481	1.87	2.611	c(t = 1.37643	0.19	0.83731181	Distal Intergenic	ENSSSCG000	ARV1	ARV1 homolog, fatty acid homeostasis modulator [Source:VGNC Symbol;Acc:VGNC:85554]
chr13	1579511	1580617	1107	*	chr13	1579511	1580617	in_vitro	in_vivo	13	15	0.67076923	1.376	-0.7052308	c(t = -1.3545	0.19	0.83731181	Distal Intergenic	ENSSSCG000	CAPN7	calpain 7 [Source:NCBI gene (formerly Entrezgene);Acc:100037936]
chr2	76767827	76770247	2421	*	chr2	76767827	76770247	in_vitro	in_vivo	9	12	2.53	1.00166667	1.52833333	c(t = 1.41029	0.19	0.83731181	Distal Intergenic	ENSSSCG000	KLF16	KLF transcription factor 16 [Source:NCBI gene (formerly Entrezgene);Acc:100302638]
chr5	12525580	12526379	800	*	chr5	12525580	12526379	in_vitro	in_vivo	14	14	4.57428571	0.58571429	3.98857143	c(t = 1.37740	0.19	0.83731181	Promoter (<=1kb)	ENSSSCG000	FBXO7	F-box protein 7 [Source:VGNC Symbol;Acc:VGNC:88053]
chr14	76836194	76836627	434	*	chr14	76836194	76836627	in_vitro	in_vivo	13	9	1.16615385	0.3	0.86615385	c(t = 1.36308	0.19	0.83731181	Promoter (<=1kb)	ENSSSCG000	AP3M1	adaptor related protein complex 3 subunit mu 1 [Source:VGNC Symbol;Acc:VGNC:85390]
chr12	26181315	26183225	1911	*	chr12	26181315	26183225	in_vitro	in_vivo	10	13	0.707	5.10538462	-4.3983846	c(t = -1.3833	0.191	0.83731181	Distal Intergenic	ENSSSCG000	TAC4	tachykinin 4 [Source:VGNC Symbol;Acc:VGNC:96765]
chr6	62718915	62720246	1332	*	chr6	62718915	62720246	in_vitro	in_vivo	14	12	1.20142857	4.055	-2.8535714	c(t = -1.3838	0.191	0.83731181	Distal Intergenic	ENSSSCG000	ISG15	ISG15 ubiquitin like modifier [Source:NCBI gene (formerly Entrezgene);Acc:100145895]
chr1	9108367	9109453	1087	*	chr1	9108367	9109453	in_vitro	in_vivo	12	14	7.0975	2.00071429	5.09678571	c(t = 1.38857	0.191	0.83731181	Distal Intergenic	ENSSSCG000	DYNLT1	dynein light chain Tctex-type 1 [Source:NCBI gene (formerly Entrezgene);Acc:100158053]
chr6	69400407	69401729	1323	*	chr6	69400407	69401729	in_vitro	in_vivo	11	14	2.40363636	1.05285714	1.35077922	c(t = 1.36239	0.191	0.83731181	Distal Intergenic	ENSSSCG000	CA6	carbonic anhydrase 6 [Source:NCBI gene (formerly Entrezgene);Acc:100240717]
chr11	4688897	4690153	1257	*	chr11	4688897	4690153	in_vitro	in_vivo	11	12	2.63727273	0.84666667	1.79060606	c(t = 1.38242	0.191	0.83731181	Distal Intergenic	ENSSSCG000	GPR12	G protein-coupled receptor 12 [Source:NCBI gene (formerly Entrezgene);Acc:100736641]
chr3	10868468	10869352	885	*	chr3	10868468	10869352	in_vitro	in_vivo	9	12	6.44777778	0.40083333	6.04694444	c(t = 1.42884	0.191	0.83731181	Distal Intergenic	ENSSSCG000	ssc-mir-7137	ssc-mir-7137 [Source:miRBase;Acc:MI0023570]
chr2	133474679	133475628	950	*	chr2	133474679	133475628	in_vitro	in_vivo	12	11	1.02583333	0.33363636	0.69219697	c(t = 1.36189	0.191	0.83731181	Distal Intergenic	ENSSSCG000	CSF2	colony stimulating factor 2 [Source:NCBI gene (formerly Entrezgene);Acc:397208]
chr16	17848039	17848667	629	*	chr16	17848039	17848667	in_vitro	in_vivo	9	10	1.29333333	0.27	1.02333333	c(t = 1.39908	0.191	0.83731181	Distal Intergenic	ENSSSCG000	C1QTNF3	C1q and TNF related 3 [Source:NCBI gene (formerly Entrezgene);Acc:100270814]
chr16	21462521	21463346	826	*	chr16	21462521	21463346	in_vitro	in_vivo	9	10	1.98222222	0.263	1.71922222	c(t = 1.41814	0.191	0.83731181	Promoter (<=1kb)	ENSSSCG000	SKP2	S-phase kinase associated protein 2 [Source:NCBI gene (formerly Entrezgene);Acc:100329128]
chr9	122827798	122828224	427	*	chr9	122827798	122828224	in_vitro	in_vivo	10	9	0.948	0	0.948	c(t = 1.41304	0.191	0.83731181	Distal Intergenic	ENSSSCG000	IER5	immediate early response 5 [Source:NCBI gene (formerly Entrezgene);Acc:100511095]
chrX	125028816	125033740	4925	*	chrX	125028816	125033740	in_vitro	in_vivo	10	10	95.519	87.199	8.32	c(t = 1.39748	0.192	0.83731181	Distal Intergenic	ENSSSCG000	IKBKG	inhibitor of nuclear factor kappa B kinase regulatory subunit gamma [Source:VGNC Symbol;Acc:VGNC:89072]
chr6	111146191	111150565	4375	*	chr6	111146191	111150565	in_vitro	in_vivo	17	15	6.24470588	3.17333333	3.07137255	c(t = 1.35592	0.192	0.83731181	Promoter (<=1kb)	ENSSSCG000	KCTD1	potassium channel tetramerization domain containing 1 [Source:VGNC Symbol;Acc:VGNC:98482]
chr6	52322021	52322525	505	*	chr6	52322021	52322525	in_vitro	in_vivo	12	9	0.67666667	2.85777778	-2.1811111	c(t = -1.4037	0.192	0.83731181	Promoter (<=1kb)	ENSSSCG000	PGLYRP1	peptidoglycan recognition protein 1 [Source:NCBI gene (formerly Entrezgene);Acc:397213]

chr6	131916673	131919531	2859	*	chr6	131916673	131919531	in_vitro	in_vivo	13	16	2.63615385	1.203125	1.43302885	c(t = 1.36727	0.192	0.83731181	Distal Intergenic	ENSSSCG000	UOX	urate oxidase [Source:NCBI gene (formerly Entrezgene);Acc:397510]
chr7	58014743	58015645	903	*	chr7	58014743	58015645	in_vitro	in_vivo	9	9	1.53888889	0	1.53888889	c(t = 1.42497	0.192	0.83731181	Distal Intergenic	ENSSSCG000	IMP3	IMP U3 small nucleolar ribonucleoprotein 3 [Source:NCBI gene (formerly Entrezgene);Acc:100151831]
chr2	7979606	7981607	2002	*	chr2	7979606	7981607	in_vitro	in_vivo	10	12	84.381	94.0616667	-9.6806667	c(t = -1.4004	0.193	0.83731181	Intron (NM_001204770/100522538, intron 3 of 10)	ENSSSCG000	MACROD1	mono-ADP ribosylhydrolase 1 [Source:NCBI gene (formerly Entrezgene);Acc:100522538]
chr1	130412726	130413766	1041	*	chr1	130412726	130413766	in_vitro	in_vivo	9	11	96.95	84.5436364	12.4063636	c(t = 1.38317	0.193	0.83731181	Distal Intergenic	ENSSSCG000	DLL4	delta like canonical Notch ligand 4 [Source:NCBI gene (formerly Entrezgene);Acc:100152163]
chr11	70022260	70023229	970	*	chr11	70022260	70023229	in_vitro	in_vivo	10	9	78.213	54.84	23.373	c(t = 1.36179	0.193	0.83731181	Distal Intergenic	ENSSSCG000	TMTC4	transmembrane O-mannosyltransferase targeting cadherins 4 [Source:VGNC Symbol;Acc:VGNC:94244]
chr2	16786953	16788209	1257	*	chr2	16786953	16788209	in_vitro	in_vivo	13	10	19.1007692	41.187	-22.086231	c(t = -1.3559	0.193	0.83731181	Distal Intergenic	ENSSSCG000	SLC35C1	solute carrier family 35 member C1 [Source:VGNC Symbol;Acc:VGNC:93075]
chr6	169668576	169673083	4508	*	chr6	169668576	169673083	in_vitro	in_vivo	11	13	34.6872727	18.8715385	15.8157343	c(t = 1.35525	0.193	0.83731181	Distal Intergenic	ENSSSCG000	ssc-mir-4338	ssc-mir-4338 [Source:miRBase;Acc:MI0015933]
chr13	79796622	79797563	942	*	chr13	79796622	79797563	in_vitro	in_vivo	13	14	2.97923077	5.92785714	-2.9486264	c(t = -1.3430	0.193	0.83731181	Distal Intergenic	ENSSSCG000	FOXL2	forkhead box L2 [Source:NCBI gene (formerly Entrezgene);Acc:100622956]
chrX	55629781	55632028	2248	*	chrX	55629781	55632028	in_vitro	in_vivo	12	15	8.70416667	4.08533333	4.61883333	c(t = 1.36350	0.193	0.83731181	Distal Intergenic	ENSSSCG000	ssc-mir-676-	ssc-mir-676-1 [Source:miRBase;Acc:MI0013152]
chr5	32047507	32049078	1572	*	chr5	32047507	32049078	in_vitro	in_vivo	12	14	2.05916667	4.07571429	-2.0165476	c(t = -1.3450	0.193	0.83731181	Distal Intergenic	ENSSSCG000	IFNG	interferon gamma [Source:NCBI gene (formerly Entrezgene);Acc:396991]
chr14	108753807	108755517	1711	*	chr14	108753807	108755517	in_vitro	in_vivo	15	13	1.43466667	2.65076923	-1.2161026	c(t = -1.3489	0.193	0.83731181	Distal Intergenic	ENSSSCG000	HOGA1	4-hydroxy-2-oxoglutarate aldolase 1 [Source:NCBI gene (formerly Entrezgene);Acc:100153961]
chr1	238825022	238826808	1787	*	chr1	238825022	238826808	in_vitro	in_vivo	11	10	3.76909091	1.543	2.22609091	c(t = 1.35165	0.193	0.83731181	Distal Intergenic	ENSSSCG00000032554		solute carrier family 25 member 51 [Source:NCBI gene (formerly Entrezgene);Acc:100157218]
chr11	4053732	4054854	1123	*	chr11	4053732	4054854	in_vitro	in_vivo	9	12	3.40444444	1.14333333	2.26111111	c(t = 1.40728	0.193	0.83731181	Distal Intergenic	ENSSSCG000	GPR12	G protein-coupled receptor 12 [Source:NCBI gene (formerly Entrezgene);Acc:100736641]
chr15	81893059	81894671	1613	*	chr15	81893059	81894671	in_vitro	in_vivo	11	11	6.06181818	1	5.06181818	c(t = 1.39218	0.193	0.83731181	Distal Intergenic	NA	NA	NA
chr16	79534349	79535686	1338	*	chr16	79534349	79535686	in_vitro	in_vivo	9	9	2.19	0.85333333	1.33666667	c(t = 1.37218	0.193	0.83731181	Promoter (<=1kb)	ENSSSCG000	TPPP	tubulin polymerization promoting protein [Source:VGNC Symbol;Acc:VGNC:94344]
chr9	120859860	120860593	734	*	chr9	120859860	120860593	in_vitro	in_vivo	10	13	1.922	0.76384615	1.15815385	c(t = 1.36567	0.193	0.83731181	Distal Intergenic	ENSSSCG000	ANGPTL1	angiopoietin like 1 [Source:VGNC Symbol;Acc:VGNC:85303]
chr3	67547623	67548127	505	*	chr3	67547623	67548127	in_vitro	in_vivo	10	10	2.26	0.76	1.5	c(t = 1.36392	0.193	0.83731181	Distal Intergenic	ENSSSCG000	HK2	hexokinase 2 [Source:NCBI gene (formerly Entrezgene);Acc:494561]
chr1	7471833	7473008	1176	*	chr1	7471833	7473008	in_vitro	in_vivo	12	9	1.8725	0.55555556	1.31694444	c(t = 1.35273	0.193	0.83731181	Promoter (<=1kb)	ENSSSCG000	IGF2R	insulin like growth factor 2 receptor [Source:NCBI gene (formerly Entrezgene);Acc:397214]
chr14	2103850	2104226	377	*	chr14	2103850	2104226	in_vitro	in_vivo	9	10	38.2355556	15.309	22.9265556	c(t = 1.35980	0.194	0.83731181	Promoter (<=1kb)	ENSSSCG000	SYK	spleen associated tyrosine kinase [Source:NCBI gene (formerly Entrezgene);Acc:100125540]
chr12	52440268	52442734	2467	*	chr12	52440268	52442734	in_vitro	in_vivo	9	13	17.5233333	5.72615385	11.7971795	c(t = 1.40360	0.194	0.83731181	Distal Intergenic	ENSSSCG000	ssc-mir-497	ssc-mir-497 [Source:miRBase;Acc:MI0013139]
chr13	190284359	190286683	2325	*	chr13	190284359	190286683	in_vitro	in_vivo	11	13	0.60636364	1.75461538	-1.1482517	c(t = -1.3610	0.194	0.83731181	Promoter (<=1kb)	ENSSSCG000	ADAMTS1	ADAM metallopeptidase with thrombospondin type 1 motif 1 [Source:NCBI gene (formerly Entrezgene);Acc:497061]
chr7	57888355	57890245	1891	*	chr7	57888355	57890245	in_vitro	in_vivo	9	11	3.28	1.22181818	2.05818182	c(t = 1.38651	0.194	0.83731181	Distal Intergenic	ENSSSCG000	IMP3	IMP U3 small nucleolar ribonucleoprotein 3 [Source:NCBI gene (formerly Entrezgene);Acc:100151831]
chr2	58828025	58828910	886	*	chr2	58828025	58828910	in_vitro	in_vivo	12	12	2.12416667	1.05166667	1.0725	c(t = 1.34225	0.194	0.83731181	Promoter (<=1kb)	ENSSSCG000	ARMC6	armadillo repeat containing 6 [Source:VGNC Symbol;Acc:VGNC:108748]
chr9	138990014	138990729	716	*	chr9	138990014	138990729	in_vitro	in_vivo	12	11	2.45909091	0.93083333	1.52825758	c(t = 1.35734	0.194	0.83731181	Distal Intergenic	ENSSSCG000	EGFR	epidermal growth factor receptor [Source:VGNC Symbol;Acc:397070]
chr3	77292683	77294324	1642	*	chr3	77292683	77294324	in_vitro	in_vivo	14	12	2.10714286	0.71583333	1.39130952	c(t = 1.34274	0.194	0.83731181	Distal Intergenic	ENSSSCG000	RAB1A	RAB1A, member RAS oncogene family [Source:NCBI gene (formerly Entrezgene);Acc:595116]
chr2	4652994	4654255	1262	*	chr2	4652994	4654255	in_vitro	in_vivo	12	13	1.35416667	0.54769231	0.80647436	c(t = 1.34508	0.194	0.83731181	Distal Intergenic	ENSSSCG00000028501		aldehyde dehydrogenase 3 family member B1 [Source:NCBI gene (formerly Entrezgene);Acc:100739347]
chr4	467537	468316	780	*	chr4	467537	468316	in_vitro	in_vivo	9	9	98.4111111	96.0766667	2.33444444	c(t = 1.36809	0.195	0.83731181	Exon (NM_001243819/100511321, exon 2 of 13)	ENSSSCG000	DGAT1	diacylglycerol O-acyltransferase 1 [Source:VGNC Symbol;Acc:VGNC:87267]
chr3	39039427	39040455	1029	*	chr3	39039427	39040455	in_vitro	in_vivo	9	11	0.70666667	3.13	-2.4233333	c(t = -1.3746	0.195	0.83731181	Distal Intergenic	ENSSSCG000	ZNF205	zinc finger protein 205 [Source:VGNC Symbol;Acc:VGNC:95204]
chr5	7582694	7583956	1263	*	chr5	7582694	7583956	in_vitro	in_vivo	11	9	0.51181818	3.04	-2.5281818	c(t = -1.3830	0.195	0.83731181	Distal Intergenic	ENSSSCG000	MCHR1	melanin concentrating hormone receptor 1 [Source:VGNC Symbol;Acc:VGNC:103300]
chr2	25603683	25606037	2355	*	chr2	25603683	25606037	in_vitro	in_vivo	11	10	0.88909091	2.998	-2.1089091	c(t = -1.3694	0.195	0.83731181	Distal Intergenic	ENSSSCG000	PAMR1	peptidase domain containing associated with muscle reration 1 [Source:VGNC Symbol;Acc:VGNC:91164]
chr6	6254135	6254764	630	*	chr6	6254135	6254764	in_vitro	in_vivo	11	13	0.60636364	2.95692308	-2.3505594	c(t = -1.3557	0.195	0.83731181	Distal Intergenic	ENSSSCG000	HSD17B2	hydroxysteroid 17-beta dehydrogenase 2 [Source:NCBI gene (formerly Entrezgene);Acc:100312973]
chr14	41963583	41964327	745	*	chr14	41963583	41964327	in_vitro	in_vivo	10	13	0.918	2.28076923	-1.3627692	c(t = -1.3546	0.195	0.83731181	Distal Intergenic	ENSSSCG000	DAO	D-amino acid oxidase [Source:NCBI gene (formerly Entrezgene);Acc:397134]
chr13	135268389	135269219	831	*	chr13	135268389	135269219	in_vitro	in_vivo	11	9	4.57909091	0.99888889	3.58020202	c(t = 1.37471	0.195	0.83731181	Intron (NM_001105293/100125829, intron 1 of 10)	ENSSSCG000	MUC13	mucin 13, cell surface associated [Source:NCBI gene (formerly Entrezgene);Acc:100125829]
chr11	16271763	16272913	1151	*	chr11	16271763	16272913	in_vitro	in_vivo	11	13	1.86272727	0.70384615	1.15888112	c(t = 1.35440	0.195	0.83731181	Distal Intergenic	ENSSSCG000	RNASEH2B	ribonuclease H2 subunit B [Source:VGNC Symbol;Acc:VGNC:92339]
chr7	20997828	20999197	1370	*	chr7	20997828	20999197	in_vitro	in_vivo	15	12	2.84066667	0.6975	2.14316667	c(t = 1.35085	0.195	0.83731181	Distal Intergenic	NA	NA	NA
chr9	133040271	133041303	1033	*	chr9	133040271	133041303	in_vitro	in_vivo	12	11	3.47416667	0.64909091	2.82507576	c(t = 1.37290	0.195	0.83731181	Distal Intergenic	ENSSSCG000	IRF6	interferon regulatory factor 6 [Source:VGNC Symbol;Acc:VGNC:89209]
chr7	96868186	96868771	586	*	chr7	96868186	96868771	in_vitro	in_vivo	9	11	1.38888889	0	1.38888889	c(t = 1.41449	0.195	0.83731181	Distal Intergenic	ENSSSCG000	PSEN1	presenilin 1 [Source:VGNC Symbol;Acc:VGNC:91897]
chr9	7758444	7759399	956	*	chr9	7758444	7759399	in_vitro	in_vivo	9	10	29.6333333	55.078	-25.444667	c(t = -1.3469	0.196	0.83731181	5' UTR	ENSSSCG000	P2RY6	pyrimidinergic receptor P2Y6 [Source:VGNC Symbol;Acc:VGNC:91128]
chr17	58985369	58988683	3315	*	chr17	58985369	58988683	in_vitro	in_vivo	13	13	13.3438462	3.19	10.1538462	c(t = 1.36333	0.196	0.83731181	Promoter (<=1kb)	ENSSSCG00000007520		GNAS complex locus [Source:VGNC Symbol;Acc:VGNC:110580]
chr5	8017238	8017977	740	*	chr5	8017238	8017977	in_vitro	in_vivo	11	11	0.97888889	2.63545455	-1.6565657	c(t = -1.3544	0.196	0.83731181	Distal Intergenic	ENSSSCG000	ADSL	adenylosuccinate lyase [Source:NCBI gene (formerly Entrezgene);Acc:100170844]
chr13	77863462	77864279	818	*	chr13	77863462	77864279	in_vitro	in_vivo	11	11	0.85888889	2.16181818	-1.3029293	c(t = -1.3541	0.196	0.83731181	Distal Intergenic	ENSSSCG000	NCK1	NCK adaptor protein 1 [Source:VGNC Symbol;Acc:VGNC:90609]
chr10	5753626	5754856	1231	*	chr10	5753626	5754856	in_vitro	in_vivo	12	9	0.50916667	1.65888889	-1.1497222	c(t = -1.3688	0.196	0.83731181	Distal Intergenic	ENSSSCG000	SPATA17	spermatosis associated 17 [Source:VGNC Symbol;Acc:VGNC:96065]
chrX	36988912	36991150	2239	*	chrX	36988912	36991150	in_vitro	in_vivo	9	16	2.45444444	0.905625	1.54881944	c(t = 1.38724	0.196	0.83731181	Promoter (<=1kb)	ENSSSCG000	DDX3X	DEAD-box helicase 3 X-linked [Source:NCBI gene (formerly Entrezgene);Acc:100515940]
chr4	94831787	94832742	956	*	chr4	94831787	94832742	in_vitro	in_vivo	9	12	2.94555556	0.76583333	2.17972222	c(t = 1.40234	0.196	0.83731181	Promoter (<=1kb)	NA	NA	NA
chr6	46907965	46909187	1223	*	chr6	46907965	46909187	in_vitro	in_vivo	10	12	2.844	0.3975	2.4465	c(t = 1.39224	0.196	0.83731181	Distal Intergenic	ENSSSCG000	PPP1R14A	protein phosphatase 1 regulatory inhibitor subunit 14A [Source:VGNC Symbol;Acc:VGNC:98539]
chr13	157490683	157491344	662	*	chr13	157490683	157491344	in_vitro	in_vivo	10	12	2.806	0.345	2.461	c(t = 1.38859	0.196	0.83731181	Distal Intergenic	ENSSSCG000	NFKBIZ	NFKB inhibitor zeta [Source:VGNC Symbol;Acc:VGNC:90726]
chr7	29694166	29695202	1037	*	chr7	29694166	29695202	in_vitro	in_vivo	10	10	1.892	0.132	1.76	c(t = 1.39315	0.196	0.83731181	Distal Intergenic	ENSSSCG000	RGL2	ral guanine nucleotide dissociation stimulator like 2 [Source:NCBI gene (formerly Entrezgene);Acc:100151794]
chr12	52017237	52022230	4994	*	chr12	52017237	52022230	in_vitro	in_vivo	10	14	79.145	83.7864286	-4.6414286	c(t = -1.3322	0.197	0.83731181	Distal Intergenic	ENSSSCG000	PFN1	profilin 1 [Source:NCBI gene (formerly Entrezgene);Acc:100512494]
chr1	2003255	2004766	1512	*	chr1	2003255	2004766	in_vitro	in_vivo	10	11	8.68	24.7281818	-16.048182	c(t = -1.3615	0.197	0.83731181	Distal Intergenic	ENSSSCG000	ssc-mir-9824	ssc-mir-9824 [Source:miRBase;Acc:MI0031591]
chr7	3680566	3681745	1180	*	chr7	3680566	3681745	in_vitro	in_vivo	9	10	1.54333333	3.71	-2.1666667	c(t = -1.3497	0.197	0.83731181	Distal Intergenic	ENSSSCG000	NRN1	neuritin 1 [Source:VGNC Symbol;Acc:VGNC:90900]
chr9	66025241	66026632	1392	*	chr9	66025241	66026632	in_vitro	in_vivo	10	10	6.59	3.444	3.146	c(t = 1.34968	0.197	0.83731181	Distal Intergenic	ENSSSCG000	NUCKS1	nuclear casein kinase and cyclin dependent kinase substrate 1 [Source:VGNC Symbol;Acc:VGNC:90947]
chr15	31946340	31947196	857	*	chr15	31946340	31947196	in_vitro	in_vivo	9	12	1.01222222	3.12583333	-2.1136111	c(t = -1.3561	0.197	0.83731181	Distal Intergenic	ENSSSCG000	SCTR	secretin receptor [Source:VGNC Symbol;Acc:VGNC:95482]
chr9	2390868	2392382	1515	*	chr9	2390868	2392382	in_vitro	in_vivo	13	12	1.17	2.13916667	-0.9691667	c(t = -1.3317	0.197	0.83731181	Distal Intergenic	ENSSSCG000	MRPL17	mitochondrial ribosomal protein L17 [Source:NCBI gene (formerly Entrezgene);Acc:100512482]
chr10	25202239	25203052	814	*	chr10	25202239	25203052	in_vitro	in_vivo	10	11	6.452	1.71909091	4.73290909	c(t = 1.36807	0.197	0.83731181	Promoter (<=1kb)	ENSSSCG000	CTSV	cathepsin V [Source:NCBI gene (formerly Entrezgene);Acc:396926]
chr9	36142120	36143121	1002	*	chr9	36142120	36143121	in_vitro	in_vivo	13	13	1.78538462	0.75615385	1.02923077	c(t = 1.34572	0.197	0.83731181	Distal Intergenic	ENSSSCG000	CWF19L2	CWF19 like cell cycle control factor 2 [Source:VGNC Symbol;Acc:VGNC:96726]
chr12	18467636	18468623	988	*	chr12	18467636	18468623	in_vitro	in_vivo	13	13	9.88230769	22.6207692	-12.738462	c(t = -1.3350	0.198	0.83731181	Distal Intergenic	ENSSSCG000	GFAP	glial fibrillary acidic protein [Source:VGNC Symbol;Acc:VGNC:88419]
chr17	28957428	28958961	1534	*	chr17	28957428	28958961	in_vitro	in_vivo	12	11	7.01916667	4.63545455	2.38371212	c(t = 1.33012	0.198	0.83731181	Distal Intergenic	ENSSSCG000	THBD	thrombomodulin [Source:NCBI gene (formerly Entrezgene);Acc:100157642]
chr11	9426799	9428088	1290	*	chr11	9426799	9428088	in_vitro	in_vivo	14	14	1.86142857	3.65214286	-1.7907143	c(t = -1.3247	0.198	0.83731181	Distal Intergenic	ENSSSCG000	RFC3	replication factor C subunit 3 [Source:VGNC Symbol;Acc:VGNC:92233]
chr18	45476554	45478047	1494	*	chr18	45476554	45478047	in_vitro	in_vivo	9	10	1.19	3.163	-1.973	c(t = -1.3427	0.198	0.83731181	Distal Intergenic	ENSSSCG000	HOXA5	homeobox A5 [Source:NCBI gene (formerly Entrezgene);Acc:100499568]
chr1	57423069	57424350	1282	*	chr1	57423069	57424350	in_vitro	in_vivo	11	11	3.58454545	0.86363636	2.72090909	c(t = 1.36914	0.198	0.83731181	Promoter (<=1kb)	ENSSSCG000	RRAGD	Ras related GTP binding D [Source:VGNC Symbol;Acc:VGNC:92457]
chr14	111912057	111913431	1375	*	chr14	111912057	111913431	in_vitro	in_vivo	11	15	2.76727273	0.85866667	1.90860606	c(t = 1.36343	0.198	0.83731181	Distal Intergenic	ENSSSCG000	ssc-mir-1038	ssc-mir-10382 [Source:miRBase;Acc:MI0033391]
chr3	162680	164810	2131	*	chr3	162680	164810	in_vitro	in_vivo	10	9	2.031	0.79444444	1.23655556	c(t = 1.34032	0.198	0.83731181	Distal Intergenic	ENSSSCG000	ADAP1	ArfGAP with dual PH domains 1 [Source:NCBI gene (formerly Entrezgene);Acc:397453]
chr14	31027541	31028466	926	*	chr14	31027541	31028466	in_vitro	in_vivo	10	10	3.767	0.749	3.018	c(t = 1.38201	0.198	0.83731181	Promoter (<=1kb)	ENSSSCG000	ORAI1	ORAI calcium release-activated calcium modulator 1 [Source:NCBI gene (formerly Entrezgene);Acc:100154923]
chr7	20744142	20744659	518	*	chr7	20744142	20744659	in_vitro	in_vivo	10	12	2.735	0.70416667	2.03083333	c(t = 1.36765	0.198	0.83731181	Promoter (<=1kb)	NA	NA	NA
chr18	9390954	9391588	635	*	chr18	9390954	9391588	in_vitro	in_vivo	11	9	1.38545455	0.66333333	0.72212121	c(t = 1.33508	0.198	0.83731181	Intron (NM_001244154/100626074, intron 3 of 9)	ENSSSCG000	SLC37A3	solute carrier family 37 member 3 [Source:VGNC Symbol;Acc:VGNC:98324]
chr9	41639370	41641354	1985	*	chr9	41639370	41641354	in_vitro	in_vivo	9	9	1.89777778	0.52222222	1.37555556	c(t = 1.38480	0.198	0.83731181	Distal Intergenic	ENSSSCG000	NNMT	nicotinamide N-methyltransferase [Source:NCBI gene (formerly Entrezgene);Acc:100144485]
chr9	3410001	3410858	858	*	chr9	3410001	3410858	in_vitro	in_vivo	10	13	13.804	29.3692308	-15.565231	c(t = -1.3422	0.199	0.83731181	Distal Intergenic	ENSSSCG000	HPX	hemopexin [Source:VGNC Symbol;Acc:VGNC:88968]
chr2	7084418	7085678	1261	*	chr2	7084418	7085678	in_vitro	in_vivo	9	11	46.3566667	25.6818182	20.6748485	c(t = 1.33514	0.199	0.83731181	Distal Intergenic	ENSSSCG000	MRPL49	mitochondrial ribosomal protein L49 [Source:VGNC Symbol;Acc:VGNC:90373]
chr14	111460788	111462103	1316	*	chr14	111460788	111462103	in_vitro	in_vivo	9	10	5.32666667	11.51	-6.1833333	c(t = -1.3448	0.199	0.83731181	Promoter (<=1kb)	ENSSSCG000	SCD	stearoyl-CoA desaturase [Source:VGNC Symbol;Acc:VGNC:111140]
chrX	94885057	94886300	1244	*	chrX	94885057	94886300	in_vitro	in_vivo	9	10	2.50555556	7.174	-4.6684444	c(t = -1.3580	0.199	0.83731181	Distal Intergenic	ENSSSCG000	IL13RA2	interleukin 13 receptor subunit alpha 2 [Source:VGNC Symbol;Acc:VGNC:89082]
chr4	124229482	124230635	1154	*	chr4	124229482	124230635	in_vitro	in_vivo	10	12	1.064	2.4125	-1.3485	c(t = -1.3402	0.199	0.83731181	Distal Intergenic	ENSSSCG000	RPL5	ribosomal protein L5 [Source:NCBI gene (formerly Entrezgene);Acc:100135671]
chrX	106478675	106479241	567	*	chrX	106478675	106479241	in_vitro	in_vivo	9	9	0.46333333	2.09222222	-1.6288889	c(t = -1.3673	0.199	0.83731181	Distal Intergenic	ENSSSCG000	XPNPEP2	X-prolyl aminopeptidase 2 [Source:VGNC Symbol;Acc:VGNC:95001]
chr3	1618497	1618973	477	*	chr3	1618497	1618973	in_vitro	in_vivo	9	10	2.23222222	0.675	1.55722222	c(t = 1.36000	0.199	0.83731181	Distal Intergenic	ENSSSCG000	NUDT1	nudix hydrolase 1 [Source:NCBI gene (formerly Entrezgene);Acc:100624718]
chr1	115734123	115734746	624	*	chr1	115734123	115734746	in_vitro	in_vivo	9	10	1.43555556	0.484	0.95155556	c(t = 1.35342	0.199	0.83731181	Distal Intergenic	ENSSSCG000	TEX9	testis expressed 9 [Source:VGNC Symbol;Acc:VGNC:93907]
chr13	72250160	72250879	720	*	chr13	72250160	72250879	in_vitro	in_vivo	11	14	21.36	7.33142857	14.0285714	c(t = 1.36166	0.2	0.83731181	Distal Intergenic	ENSSSCG000	SEC61A1	SEC61 translocon subunit alpha 1 [Source:VGNC Symbol;Acc:VGNC:92682]
chr6	166520682	166521885	1204	*	chr6	166520682	166521885	in_vitro	in_vivo	11	11	2.47818182	7.05272727	-4.5745455	c(t = -1.3621	0.2	0.83731181	Distal Intergenic	ENSSSCG000	DYNLT4	dynein light chain Tctex-type 4 [Source:NCBI gene (formerly Entrezgene);Acc:606747]
chr6	39918980	39920248	1269	*	chr6	39918980	39920248	in_vitro	in_vivo	10	9	0.973	4.71333333	-3.7403333	c(t = -1.3917	0.2	0.83731181	Distal Intergenic	ENSSSCG000	SLC7A9	solute carrier family 7 member 9 [Source:VGNC Symbol;Acc:VGNC:93177]
chr6	28811894	28813180	1287	*	chr6	28811894	28813180	in_vitro	in_vivo	10	13	12.532	4.54538462	7.98661538	c(t = 1.36682	0.2	0.83731181	Distal Intergenic	ENSSSCG000	SLC12A4	solute carrier family 12 member 4 [Source:NCBI gene (formerly Entrezgene);Acc:396992]
chr6	152708957	152709909	953	*	chr6	152708957	152709909	in_vitro	in_vivo	11	10	0.76181818	2.539	-1.7771818	c(t = -1.3544	0.2	0.83731181	Distal Intergenic	ENSSSCG000	CYP2J34	cytochrome P450 family 2 subfamily J member 34 [Source:NCBI gene (formerly Entrezgene);Acc:100524750]
chr7	87889176	87890322	1147	*	chr7	87889176	87890322	in_vitro	in_vivo	14	10	4.59714286	1.721	2.87614286	c(t = 1.32304	0.2	0.83731181	Distal Intergenic	ENSSSCG000	GPX2	glutathione peroxidase 2 [Source:NCBI gene (formerly Entrezgene);Acc:767625]
chr2	108733271	108734465	1195	*	chr2	108733271	108734465	in_vitro	in_vivo	10	12	3.275	1.3975	1.8775	c(t = 1.35229	0.2	0.83731181	Distal Intergenic	ENSSSCG000	ST8SIA4	ST8 alpha-N-acetyl-neuraminide alpha-2,8-sialyltransferase 4 [Source:NCBI gene (formerly Entrezgene);Acc:641356]
chr16	51075321	51077044	1724	*	chr16	51075321	51077044	in_vitro	in_vivo	9	11	8.08111111	1.12545455	6.95565657	c(t = 1.38995	0.2	0.83731181	Distal Intergenic	ENSSSCG000	STC2	stanniocalcin 2 [Source:NCBI gene (formerly Entrezgene);Acc:100126236]
chr14	71665879	71666615	737	*	chr14	71665879	71666615	in_vitro	in_vivo	11	12	2.654	0.76833333	1.88566667	c(t = 1.36679	0.2	0.83731181	Distal Intergenic	ENSSSCG000	TET1	tet methylcytosine dioxygenase 1 [Source:NCBI gene (formerly Entrezgene);Acc:100625859]
chr2	5099970	5100709	740	*	chr2	5099970	5100709	in_vitro	in_vivo	9	10	3.58666667	7.203	-3.6163333	c(t = -1.3466	0.201	0.83731181	Distal Intergenic	ENSSSCG000	PPP1CA	protein phosphatase 1 catalytic subunit alpha [Source:NCBI gene (formerly Entrezgene);Acc:733611]
chrX	111623912	111624458	547	*	chrX	111623912	111624458	in_vitro	in_vivo	9	12	1.19555556	3.94833333	-2.7527778	c(t = -1.3464	0.201	0.83731181	Distal Intergenic	ENSSSCG000	CD40LG	CD40 ligand [Source:NCBI gene (formerly Entrezgene);Acc:397231]
chr7	22867809	22869220	1412	*	chr7	22867809	22869220	in_vitro	in_vivo	14	12	7.22857143	3.73833333	3.4902381	c(t = 1.32383	0.201	0.83731181	Intron (NM_001246242/100513601, intron 3 of 7)	ENSSSCG00000062469		
chr12	20375405	20377186	1782	*	chr12	20375405	20377186	in_vitro	in_vivo	13	11	1.03615385	3.63636364	-2.6002098	c(t = -1.3440	0.201	0.83731181	Distal Intergenic	ENSSSCG000	STAT3	signal transducer and activator of transcription 3 [Source:NCBI gene (formerly Entrezgene);Acc:733648]
chr14	125158444	125159376	933	*	chr14	125158444	125159376	in_vitro	in_vivo	11	12	0.74090909	2.7575	-2.0165909	c(t = -1.3444	0.201	0.83731181	Distal Intergenic	ENSSSCG000	ADRB1	adrenoceptor beta 1 [Source:NCBI gene (formerly Entrezgene);Acc:397355]
chr2	26873472	26875475	2004	*	chr2	26873472	26875475	in_vitro	in_vivo	12	12	7.51166667	2.54416667	4.9675	c(t = 1.34236	0.201	0.83731181	Distal Intergenic	ENSSSCG00000024791		CD59 molecule [Source:VGNC Symbol;Acc:VGNC:99615]
chrX	110159614	110160268	655	*	chrX	110159614	110160268	in_vitro	in_vivo	14	12	6.01357143	1.96416667	4.04940476	c(t = 1.33691	0.201	0.83731181	Distal Intergenic	ENSSSCG000	ssc-mir-106a	ssc-mir-106a [Source:miRBase;Acc:MI0002412]
chr13	145996370	145997471	1102	*	chr13	145996370	145997471	in_vitro	in_vivo	9	15	3.26111111	1.60666667	1.65444444	c(t = 1.32437	0.201	0.83731181	Distal Intergenic	ENSSSCG000	ATP6V1A	ATPase H+ transporting V1 subunit A [Source:NCBI gene (formerly Entrezgene);Acc:445531]
chr5	10038007	10038818	812	*	chr5	10038007	10038818	in_vitro	in_vivo	9	9	3.46222222	1.02666667	2.43555556	c(t = 1.35884	0.201	0.83731181	Distal Intergenic	ENSSSCG000	EIF3L	eukaryotic translation initiation factor 3 subunit L [Source:VGNC Symbol;Acc:VGNC:87623]
chr14	128961827	128963279	1453	*	chr14	128961827	128963279	in_vitro	in_vivo	13	14	2.77230769	0.78785714	1.98445055	c(t = 1.33868	0.201	0.83731181	Distal Intergenic	ENSSSCG000	PRDX3	peroxiredoxin 3 [Source:NCBI gene (formerly Entrezgene);Acc:100154143]
chr4	109182867	109183875	1009	*	chr4	109182867	109183875	in_vitro	in_vivo	9	15	1.72333333	0.45466667	1.26866667	c(t = 1.36234	0.201	0.83731181	Promoter (<=1kb)	ENSSSCG000	DRAM2	DNA damage regulated autophagy modulator 2 [Source:VGNC Symbol;Acc:VGNC:87437]
chr2	78876002	78876887	886	*	chr2	78876002	78876887	in_vitro	in_vivo	12	13	1.13	0.35	0.78	c(t = 1.33406	0.201	0.83731181	Distal Intergenic	ENSSSCG000	CANX	calnexin [Source:VGNC Symbol;Acc:VGNC:86167]
chr1	272949767	272951275	1509	*	chr1	272949767	272951275	in_vitro	in_vivo	9	9	95.3022222	97.9988889	-2.6966667	c(t = -1.3424	0.202	0.83731181	Distal Intergenic	NA	NA	NA
chr2	2134007	2142911	8905	*	chr2	2134007	2142911	in_vitro	in_vivo	10	12	67.123	78.5266667	-11.403667	c(t = -1.3344	0.202	0.83731181	Distal Intergenic	ENSSSCG000	NAP1L4	nucleosome assembly protein 1 like 4 [Source:VGNC Symbol;Acc:VGNC:90577]
chr1	270273979	270275413	1435	*	chr1	270273979	270275413	in_vitro	in_vivo	9	9	7.68333333	1.93	5.75333333	c(t = 1.37637	0.202	0.83731181	Distal Intergenic	ENSSSCG000	TOR1A	torsin family 1 member A [Source:NCBI gene (formerly Entrezgene);Acc:100125953]
chr7	28810995	28813559	2565	*	chr7	28810995	28813559	in_vitro	in_vivo	12	14	3.65333333	1.78142857	1.87190476	c(t = 1.33483	0.202	0.83731181	Distal Intergenic	ENSSSCG000	PRIM2	DNA primase subunit 2 [Source:VGNC Symbol;Acc:VGNC:91796]
chr5	11632774	11634069	1296	*	chr5	11632774	11634069	in_vitro	in_vivo	9	12	0	1.6775	-1.6775	c(t = -1.3581	0.202	0.83731181	Distal Intergenic	ENSSSCG000	RBFOX2	RNA binding fox-1 homolog 2 [Source:VGNC Symbol;Acc:VGNC:92140]
chr17	17007033	17008228	1196	*	chr17	17007033	17008228	in_vitro	in_vivo	10	14	3.353	1.54357143	1.80942857	c(t = 1.32330	0.202	0.83731181	Distal Intergenic	ENSSSCG000	TMX4	thioredoxin related transmembrane protein 4 [Source:NCBI gene (formerly Entrezgene);Acc:100627887]
chr1	152689344	152689882	539	*	chr1	152689344	152689882	in_vitro	in_vivo	9	9	3.12888889	0.44444444	2.68444444	c(t = 1.37632	0.202	0.83731181	Distal Intergenic	ENSSSCG000	SOCS6	suppressor of cytokine signaling 6 [Source:VGNC Symbol;Acc:VGNC:93333]
chr9	127539492	127540523	1032	*	chr9	127539492	127540523	in_vitro	in_vivo	12	10	2.08833333	0.443	1.64533333	c(t = 1.34472	0.202	0.83731181	Distal Intergenic	ENSSSCG000	PTGS2	prostaglandin-endoperoxide synthase 2 [Source:NCBI gene (formerly Entrezgene);Acc:397590]
chr13	108426112	108426607	496	*	chr13	108426112	108426607	in_vitro	in_vivo	10	10	1.821	0.4	1.421	c(t = 1.35019	0.202	0.83731181	Distal Intergenic	ENSSSCG000	SEC62	SEC62 homolog, preprotein translocation factor [Source:NCBI gene (formerly Entrezgene);Acc:100125961]
chr3	40272672	40274279	1608	*	chr3	40272672	40274279	in_vitro	in_vivo	10	11	98.8	96.1254545	2.67454545	c(t = 1.32744	0.203	0.83731181	Distal Intergenic	ENSSSCG000	JPT2	Jupiter microtubule associated homolog 2 [Source:NCBI gene (formerly Entrezgene);Acc:474165]
chr12	3201404	3202679	1276	*	chr12	3201404	3202679	in_vitro	in_vivo	10	10	97.713	94.706	3.007	c(t = 1.34626	0.203	0.83731181	Distal Intergenic	ENSSSCG00000017164		TIMP metallopeptidase inhibitor 2 [Source:VGNC Symbol;Acc:VGNC:93995]
chr1	970705	972819	2115	*	chr1	970705	972819	in_vitro	in_vivo	12	12	10.9833333	27.3841667	-16.400833	c(t = -1.3165	0.203	0.83731181	Distal Intergenic	ENSSSCG000	ssc-mir-9815	ssc-mir-9815 [Source:miRBase;Acc:MI0031582]
chr4	74612842	74614573	1732	*	chr4	74612842	74614573	in_vitro	in_vivo	10	15	6.302	17.252	-10.95	c(t = -1.3145	0.203	0.83731181	Distal Intergenic	ENSSSCG000	CYP7A1	cytochrome P450 family 7 subfamily A member 1 [Source:VGNC Symbol;Acc:VGNC:98774]
chr11	70421519	70422728	1210	*	chr11	70421519	70422728	in_vitro	in_vivo	10	13	0.661	1.50153846	-0.8405385	c(t = -1.3141	0.203	0.83731181	Distal Intergenic	ENSSSCG000	BIVM	basic, immunoglobulin-like variable motif containing [Source:VGNC Symbol;Acc:VGNC:109180]
chr13	199600809	199601305	497	*	chr13	199600809	199601305	in_vitro	in_vivo	9	10	4.28222222	0.909	3.37322222	c(t = 1.37960	0.203	0.83731181	Promoter (<=1kb)	ENSSSCG00000020706		
chr3	68230165	68232029	1865	*	chr3	68230165	68232029	in_vitro	in_vivo	11	12	2.23272727	0.80166667	1.43106061	c(t = 1.32970	0.203	0.83731181	Promoter (<=1kb)	ENSSSCG000	HK2	hexokinase 2 [Source:NCBI gene (formerly Entrezgene);Acc:494561]
chr8	9185231	9186610	1380	*	chr8	9185231	9186610	in_vitro	in_vivo	11	13	1.97818182	0.75	1.22818182	c(t = 1.34377	0.203	0.83731181	Distal Intergenic	ENSSSCG000	RAB28	RAB28, member RAS onco family [Source:VGNC Symbol;Acc:VGNC:98955]
chr12	50154886	50156103	1218	*	chr12	50154886	50156103	in_vitro	in_vivo	12	12	1.535	0.725	0.81	c(t = 1.33062	0.203	0.83731181	Distal Intergenic	ENSSSCG000	ASPA	aspartoacylase [Source:NCBI gene (formerly Entrezgene);Acc:100142661]
chr14	32841412	32842072	661	*	chr14	32841412	32842072	in_vitro	in_vivo	9	10	2.71555556	0.565	2.15055556	c(t = 1.37747	0.203	0.83731181	Distal Intergenic	ENSSSCG000	PRKAB1	protein kinase AMP-activated non-catalytic subunit beta 1 [Source:VGNC Symbol;Acc:VGNC:109479]
chr6	46121263	46121821	559	*	chr6	46121263	46121821	in_vitro	in_vivo	9	10	1.35888889	0.323	1.03588889	c(t = 1.35107	0.203	0.83731181	Distal Intergenic	ENSSSCG000	COX7A1	cytochrome c oxidase subunit 7A1 [Source:NCBI gene (formerly Entrezgene);Acc:399685]
chr14	113831542	113831950	409	*	chr14	113831542	113831950	in_vitro	in_vivo	10	9	2.054	0	2.054	c(t = 1.37416	0.203	0.83731181	Promoter (<=1kb)	ENSSSCG000	BORCS7	BLOC-1 related complex subunit 7 [Source:NCBI gene (formerly Entrezgene);Acc:100157047]
chrX	36325607	36326341	735	*	chrX	36325607	36326341	in_vitro	in_vivo	9	12	18.5455556	37.2625	-18.716944	c(t = -1.3162	0.204	0.83731181	Distal Intergenic	ENSSSCG000	DDX3X	DEAD-box helicase 3 X-linked [Source:NCBI gene (formerly Entrezgene);Acc:100515940]
chr3	39117229	39118770	1542	*	chr3	39117229	39118770	in_vitro	in_vivo	11	10	6.033	20.4236364	-14.390636	c(t = -1.3447	0.204	0.83731181	Distal Intergenic	ENSSSCG000	ZNF205	zinc finger protein 205 [Source:VGNC Symbol;Acc:VGNC:95204]
chr2	783574	786525	2952	*	chr2	783574	786525	in_vitro	in_vivo	10	10	38.215	20.053	18.162	c(t = 1.32169	0.204	0.83731181	Distal Intergenic	ENSSSCG000	TOLLIP	toll interacting protein [Source:NCBI gene (formerly Entrezgene);Acc:100625123]
chr3	132760729	132761249	521	*	chr3	132760729	132761249	in_vitro	in_vivo	9	11	3.12333333	18.2272727	-15.103939	c(t = -1.3520	0.204	0.83731181	Distal Intergenic	ENSSSCG000	SH3YL1	SH3 and SYLF domain containing 1 [Source:VGNC Symbol;Acc:VGNC:92834]
chr14	129423407	129424627	1221	*	chr14	129423407	129424627	in_vitro	in_vivo	11	11	35.3681818	17.9890909	17.3790909	c(t = 1.31624	0.204	0.83731181	Distal Intergenic	ENSSSCG000	MCMBP	minichromosome maintenance complex binding protein [Source:VGNC Symbol;Acc:VGNC:90077]
chr14	56669576	56670569	994	*	chr14	56669576	56670569	in_vitro	in_vivo	11	10	6.10454545	3.558	2.54654545	c(t = 1.32443	0.204	0.83731181	Distal Intergenic	ENSSSCG000	EDARADD	EDAR associated death domain [Source:VGNC Symbol;Acc:VGNC:87542]
chr7	97316238	97316776	539	*	chr7	97316238	97316776	in_vitro	in_vivo	10	13	0.555	3.28307692	-2.7280769	c(t = -1.3383	0.204	0.83731181	Distal Intergenic	ENSSSCG000	LIN52	lin-52 DREAM MuvB core complex component [Source:NCBI gene (formerly Entrezgene);Acc:100157325]
chr13	200402464	200403586	1123	*	chr13	200402464	200403586	in_vitro	in_vivo	9	11	5.64888889	2.66090909	2.9879798	c(t = 1.36238	0.204	0.83731181	Distal Intergenic	ENSSSCG000	CLDN14	claudin 14 [Source:VGNC Symbol;Acc:VGNC:86731]
chr11	25594473	25595178	706	*	chr11	25594473	25595178	in_vitro	in_vivo	9	11	0.92222222	2.21909091	-1.2968687	c(t = -1.3189	0.204	0.83731181	Distal Intergenic	ENSSSCG000	MTRF1	mitochondrial translation release factor 1 [Source:VGNC Symbol;Acc:VGNC:90471]
chr13	12574830	12575751	922	*	chr13	12574830	12575751	in_vitro	in_vivo	10	9	3.338	1.50666667	1.83133333	c(t = 1.32677	0.204	0.83731181	Promoter (<=1kb)	ENSSSCG000	TOP2B	DNA topoisomerase II beta [Source:NCBI gene (formerly Entrezgene);Acc:100156319]
chr11	77269829	77273481	3653	*	chr11	77269829	77273481	in_vitro	in_vivo	12	15	1.84583333	0.80866667	1.03716667	c(t = 1.31469	0.204	0.83731181	Distal Intergenic	ENSSSCG000	NAXD	NAD(P)HX dehydratase [Source:NCBI gene (formerly Entrezgene);Acc:100153456]
chr12	39198390	39201071	2682	*	chr12	39198390	39201071	in_vitro	in_vivo	12	12	10.5533333	21.6741667	-11.120833	c(t = -1.3309	0.205	0.83731181	Promoter (<=1kb)	ENSSSCG000	HNF1B	HNF1 homeobox B [Source:NCBI gene (formerly Entrezgene);Acc:397002]
chr2	74042733	74045574	2842	*	chr2	74042733	74045574	in_vitro	in_vivo	15	14	5.89933333	14.1128571	-8.2135238	c(t = -1.3203	0.205	0.83731181	Distal Intergenic	ENSSSCG000	TICAM1	TIR domain containing adaptor molecule 1 [Source:VGNC Symbol;Acc:VGNC:93976]
chr11	15751503	15752381	879	*	chr11	15751503	15752381	in_vitro	in_vivo	12	13	1.13416667	3.98	-2.8458333	c(t = -1.3300	0.205	0.83731181	Distal Intergenic	ENSSSCG000	SLC25A15	solute carrier family 25 member 15 [Source:VGNC Symbol;Acc:VGNC:92994]
chr1	139450801	139452477	1677	*	chr1	139450801	139452477	in_vitro	in_vivo	13	10	1.25615385	2.696	-1.4398462	c(t = -1.3199	0.205	0.83731181	Distal Intergenic	ENSSSCG000	CHSY1	chondroitin sulfate synthase 1 [Source:NCBI gene (formerly Entrezgene);Acc:100520288]
chr9	57127509	57129177	1669	*	chr9	57127509	57129177	in_vitro	in_vivo	12	16	4.24333333	1.845	2.39833333	c(t = 1.32859	0.205	0.83731181	Distal Intergenic	ENSSSCG000	KCNJ5	potassium inwardly rectifying channel subfamily J member 5 [Source:NCBI gene (formerly Entrezgene);Acc:397448]
chr12	59953179	59954298	1120	*	chr12	59953179	59954298	in_vitro	in_vivo	10	10	2.326	0.847	1.479	c(t = 1.32909	0.205	0.83731181	Distal Intergenic	ENSSSCG000	AKAP10	A-kinase anchoring protein 10 [Source:NCBI gene (formerly Entrezgene);Acc:397262]
chr13	24072275	24072883	609	*	chr13	24072275	24072883	in_vitro	in_vivo	10	10	2.69	0.417	2.273	c(t = 1.34566	0.205	0.83731181	Promoter (<=1kb)	ENSSSCG000	RPSA	ribosomal protein SA [Source:NCBI gene (formerly Entrezgene);Acc:641351]
chr6	162380879	162381936	1058	*	chr6	162380879	162381936	in_vitro	in_vivo	11	10	16.7872727	38.116	-21.328727	c(t = -1.3178	0.206	0.83731181	Distal Intergenic	ENSSSCG000	SPATA6	spermatogenesis associated 6 [Source:VGNC Symbol;Acc:VGNC:93387]
chrX	14209862	14210598	737	*	chrX	14209862	14210598	in_vitro	in_vivo	10	9	4.889	13.9288889	-9.0398889	c(t = -1.3693	0.206	0.83731181	Distal Intergenic	ENSSSCG000	SYAP1	synapse associated protein 1 [Source:VGNC Symbol;Acc:VGNC:93648]
chr16	51543970	51544842	873	*	chr16	51543970	51544842	in_vitro	in_vivo	9	10	2.05111111	9.64	-7.5888889	c(t = -1.3602	0.206	0.83731181	Distal Intergenic	ENSSSCG000	DUSP1	dual specificity phosphatase 1 [Source:VGNC Symbol;Acc:VGNC:87476]
chr3	112816323	112817925	1603	*	chr3	112816323	112817925	in_vitro	in_vivo	11	11	2.17	4.79181818	-2.6218182	c(t = -1.3066	0.206	0.83731181	Distal Intergenic	ENSSSCG000	HADHA	hydroxyacyl-CoA dehydrogenase trifunctional multienzyme complex subunit alpha [Source:NCBI gene (formerly Entrezgene);Acc:397012]
chr6	60784368	60785293	926	*	chr6	60784368	60785293	in_vitro	in_vivo	11	12	13.0018182	3.65	9.35181818	c(t = 1.34885	0.206	0.83731181	Distal Intergenic	ENSSSCG000	GALP	galanin like peptide [Source:NCBI gene (formerly Entrezgene);Acc:396772]
chr17	48180302	48182321	2020	*	chr17	48180302	48182321	in_vitro	in_vivo	10	11	6.305	2.30818182	3.99681818	c(t = 1.32208	0.206	0.83731181	Promoter (<=1kb)	ENSSSCG000	MMP9	matrix metallopeptidase 9 [Source:NCBI gene (formerly Entrezgene);Acc:654325]
chr13	135467337	135468789	1453	*	chr13	135467337	135468789	in_vitro	in_vivo	13	14	2.62307692	1.40857143	1.21450549	c(t = 1.30536	0.206	0.83731181	Promoter (<=1kb)	ENSSSCG000	ITGB5	integrin subunit beta 5 [Source:NCBI gene (formerly Entrezgene);Acc:100134977]
chr14	126936598	126937907	1310	*	chr14	126936598	126937907	in_vitro	in_vivo	10	12	2.487	1.33916667	1.14783333	c(t = 1.32233	0.206	0.83731181	Distal Intergenic	NA	NA	NA
chr16	51907074	51907933	860	*	chr16	51907074	51907933	in_vitro	in_vivo	11	10	2.78090909	1.013	1.76790909	c(t = 1.33488	0.206	0.83731181	Distal Intergenic	ENSSSCG000	DUSP1	dual specificity phosphatase 1 [Source:VGNC Symbol;Acc:VGNC:87476]
chr10	48359101	48360613	1513	*	chr10	48359101	48360613	in_vitro	in_vivo	10	12	2.495	1.01083333	1.48416667	c(t = 1.32793	0.206	0.83731181	Distal Intergenic	ENSSSCG000	PHYH	phytanoyl-CoA 2-hydroxylase [Source:VGNC Symbol;Acc:VGNC:96483]
chr11	39454285	39455234	950	*	chr11	39454285	39455234	in_vitro	in_vivo	13	12	3.05692308	0.96916667	2.08775641	c(t = 1.31092	0.206	0.83731181	Distal Intergenic	ENSSSCG000	KLF5	KLF transcription factor 5 [Source:NCBI gene (formerly Entrezgene);Acc:100038005]
chr4	20644421	20645576	1156	*	chr4	20644421	20645576	in_vitro	in_vivo	14	11	2.08357143	0.92363636	1.15993506	c(t = 1.30462	0.206	0.83731181	Distal Intergenic	NA	NA	NA
chr3	98267696	98269155	1460	*	chr3	98267696	98269155	in_vitro	in_vivo	11	13	2.15636364	0.91307692	1.24328671	c(t = 1.32233	0.206	0.83731181	Distal Intergenic	ENSSSCG000	HAAO	3-hydroxyanthranilate 3,4-dioxygenase [Source:VGNC Symbol;Acc:VGNC:88765]
chr14	911780	912340	561	*	chr14	911780	912340	in_vitro	in_vivo	9	11	2.64555556	0.69909091	1.94646465	c(t = 1.33252	0.206	0.83731181	Distal Intergenic	ENSSSCG000	GADD45G	growth arrest and DNA damage inducible gamma [Source:NCBI gene (formerly Entrezgene);Acc:100152997]
chr12	52868173	52869670	1498	*	chr12	52868173	52869670	in_vitro	in_vivo	11	12	4.23	0.43916667	3.79083333	c(t = 1.34954	0.206	0.83731181	Promoter (<=1kb)	ENSSSCG00000017957		eukaryotic translation initiation factor 4A1 [Source:VGNC Symbol;Acc:VGNC:98989]
chr11	4031196	4032447	1252	*	chr11	4031196	4032447	in_vitro	in_vivo	10	13	1.671	0.29692308	1.37407692	c(t = 1.35361	0.206	0.83731181	Distal Intergenic	ENSSSCG000	GPR12	G protein-coupled receptor 12 [Source:NCBI gene (formerly Entrezgene);Acc:100736641]
chr3	131294877	131295864	988	*	chr3	131294877	131295864	in_vitro	in_vivo	9	10	0.77333333	0	0.77333333	c(t = 1.37550	0.206	0.83731181	Promoter (<=1kb)	ENSSSCG000	ADI1	acireductone dioxygenase 1 [Source:NCBI gene (formerly Entrezgene);Acc:100626434]
chr13	110988947	110989813	867	*	chr13	110988947	110989813	in_vitro	in_vivo	9	13	0.77	2.56461538	-1.7946154	c(t = -1.3160	0.207	0.83731181	Promoter (<=1kb)	ENSSSCG000	GHSR	TNF superfamily member 10 [Source:NCBI gene (formerly Entrezgene);Acc:406191]
chr1	260868495	260869259	765	*	chr1	260868495	260869259	in_vitro	in_vivo	9	10	0	2.017	-2.017	c(t = -1.3595	0.207	0.83731181	Distal Intergenic	ENSSSCG000	C5	complement C5 [Source:VGNC Symbol;Acc:VGNC:103041]
chr10	13812753	13813888	1136	*	chr10	13812753	13813888	in_vitro	in_vivo	11	10	2.79363636	1.288	1.50563636	c(t = 1.31057	0.207	0.83731181	Promoter (<=1kb)	ENSSSCG000	TMEM63A	transmembrane protein 63A [Source:VGNC Symbol;Acc:VGNC:96198]
chr15	135441243	135443081	1839	*	chr15	135441243	135443081	in_vitro	in_vivo	11	13	2.19333333	1.06461538	1.12871795	c(t = 1.32248	0.207	0.83731181	Distal Intergenic	ENSSSCG000	ARL4C	ADP ribosylation factor like GTPase 4C [Source:NCBI gene (formerly Entrezgene);Acc:100514255]
chr5	43913148	43914564	1417	*	chr5	43913148	43914564	in_vitro	in_vivo	14	17	1.88285714	1.05588235	0.82697479	c(t = 1.29395	0.207	0.83731181	Distal Intergenic	ENSSSCG000	ERGIC2	ERGIC and golgi 2 [Source:VGNC Symbol;Acc:VGNC:87771]
chr1	90650386	90651370	985	*	chr1	90650386	90651370	in_vitro	in_vivo	12	13	2.33416667	0.99461538	1.33955128	c(t = 1.31498	0.207	0.83731181	Distal Intergenic	ENSSSCG000	COX7A2	cytochrome c oxidase subunit 7A2 [Source:NCBI gene (formerly Entrezgene);Acc:100038000]
chr1	166465252	166466453	1202	*	chr1	166465252	166466453	in_vitro	in_vivo	10	11	3.187	0.89545455	2.29154545	c(t = 1.35237	0.207	0.83731181	Distal Intergenic	ENSSSCG000	CLN6	CLN6 transmembrane ER protein [Source:VGNC Symbol;Acc:VGNC:86771]
chr12	24940950	24941545	596	*	chr12	24940950	24941545	in_vitro	in_vivo	12	10	4.18583333	0.714	3.47183333	c(t = 1.32956	0.207	0.83731181	Distal Intergenic	NA	NA	NA
chr5	5993898	5994690	793	*	chr5	5993898	5994690	in_vitro	in_vivo	10	12	2.082	0.65	1.432	c(t = 1.33802	0.207	0.83731181	Distal Intergenic	ENSSSCG000	TSPO	translocator protein [Source:NCBI gene (formerly Entrezgene);Acc:396592]
chr6	14868939	14870041	1103	*	chr6	14868939	14870041	in_vitro	in_vivo	13	13	1.41307692	0.41538462	0.99769231	c(t = 1.31657	0.207	0.83731181	Distal Intergenic	ENSSSCG00000002749		haptoglobin [Source:NCBI gene (formerly Entrezgene);Acc:397061]
chr1	246973300	246973971	672	*	chr1	246973300	246973971	in_vitro	in_vivo	9	10	2.49888889	0	2.49888889	c(t = 1.37238	0.207	0.83731181	Promoter (<=1kb)	ENSSSCG000	TMEM38B	transmembrane protein 38B [Source:NCBI gene (formerly Entrezgene);Acc:100153669]
chr14	41178551	41179836	1286	*	chr14	41178551	41179836	in_vitro	in_vivo	9	11	94.1811111	97.1690909	-2.9879798	c(t = -1.3292	0.208	0.83731181	Distal Intergenic	ENSSSCG000	TRPV4	transient receptor potential cation channel subfamily V member 4 [Source:VGNC Symbol;Acc:VGNC:94476]
chr2	151095242	151107853	12612	*	chr2	151095242	151107853	in_vitro	in_vivo	15	13	81.5593333	93.0992308	-11.539897	c(t = -1.3158	0.208	0.83731181	Distal Intergenic	ENSSSCG000	ssc-mir-378-	ssc-mir-378-1 [Source:miRBase;Acc:MI0013088]
chr14	129006408	129007344	937	*	chr14	129006408	129007344	in_vitro	in_vivo	12	11	0.8075	2.25363636	-1.4461364	c(t = -1.3190	0.208	0.83731181	Distal Intergenic	ENSSSCG000	PRDX3	peroxiredoxin 3 [Source:NCBI gene (formerly Entrezgene);Acc:100154143]
chr6	161165461	161167220	1760	*	chr6	161165461	161167220	in_vitro	in_vivo	13	12	1.92076923	0.89916667	1.02160256	c(t = 1.30333	0.208	0.83731181	Distal Intergenic	ENSSSCG000	COA7	cytochrome c oxidase assembly factor 7 [Source:NCBI gene (formerly Entrezgene);Acc:100627075]
chr5	51210476	51212313	1838	*	chr5	51210476	51212313	in_vitro	in_vivo	10	14	1.615	0.57857143	1.03642857	c(t = 1.32667	0.208	0.83731181	Distal Intergenic	ENSSSCG000	LDHB	lactate dehydrogenase B [Source:NCBI gene (formerly Entrezgene);Acc:100621540]
chr15	115095219	115096651	1433	*	chr15	115095219	115096651	in_vitro	in_vivo	11	14	2.2	0.45642857	1.74357143	c(t = 1.33890	0.208	0.83731181	Distal Intergenic	ENSSSCG000	MYL1	myosin light chain 1 [Source:NCBI gene (formerly Entrezgene);Acc:397666]
chr15	7501685	7503366	1682	*	chr15	7501685	7503366	in_vitro	in_vivo	12	11	2.01583333	0.32181818	1.69401515	c(t = 1.33365	0.208	0.83731181	Distal Intergenic	ENSSSCG000	ACVR2A	activin A receptor type 2A [Source:NCBI gene (formerly Entrezgene);Acc:397282]
chr3	114250160	114251490	1331	*	chr3	114250160	114251490	in_vitro	in_vivo	14	12	1.32142857	0.16666667	1.1547619	c(t = 1.32115	0.208	0.83731181	Distal Intergenic	ENSSSCG000	NCOA1	nuclear receptor coactivator 1 [Source:VGNC Symbol;Acc:VGNC:90615]
chr2	151521455	151526673	5219	*	chr2	151521455	151526673	in_vitro	in_vivo	14	13	94.5807143	91.7638462	2.81686813	c(t = 1.29580	0.209	0.83731181	Distal Intergenic	ENSSSCG000	MYOZ3	myozenin 3 [Source:VGNC Symbol;Acc:VGNC:90544]
chr10	53602092	53603913	1822	*	chr10	53602092	53603913	in_vitro	in_vivo	13	12	5.46166667	2.55538462	2.90628205	c(t = 1.31585	0.209	0.83731181	Distal Intergenic	ENSSSCG000	BMI1	BMI1 proto-onco, polycomb ring finger [Source:VGNC Symbol;Acc:VGNC:108265]
chr14	51161282	51162909	1628	*	chr14	51161282	51162909	in_vitro	in_vivo	15	13	5.326	2.12538462	3.20061538	c(t = 1.31114	0.209	0.83731181	Promoter (<=1kb)	ENSSSCG000	CLDN5	claudin 5 [Source:NCBI gene (formerly Entrezgene);Acc:396997]
chr17	31060473	31061251	779	*	chr17	31060473	31061251	in_vitro	in_vivo	12	12	3.22833333	1.03333333	2.195	c(t = 1.32227	0.209	0.83731181	Distal Intergenic	ENSSSCG000	CST7	cystatin F [Source:NCBI gene (formerly Entrezgene);Acc:100152012]
chr13	11724170	11724657	488	*	chr13	11724170	11724657	in_vitro	in_vivo	12	9	1.17666667	0.31777778	0.85888889	c(t = 1.30693	0.209	0.83731181	Distal Intergenic	ENSSSCG000	TOP2B	DNA topoisomerase II beta [Source:NCBI gene (formerly Entrezgene);Acc:100156319]
chr2	151020405	151025909	5505	*	chr2	151020405	151025909	in_vitro	in_vivo	9	14	92.7333333	86.6871429	6.04619048	c(t = 1.29859	0.21	0.83731181	Distal Intergenic	ENSSSCG000	ssc-mir-378-	ssc-mir-378-1 [Source:miRBase;Acc:MI0013088]
chr1	499641	502750	3110	*	chr1	499641	502750	in_vitro	in_vivo	13	9	14.6961538	30.4484615	-15.752308	c(t = -1.2913	0.21	0.83731181	Distal Intergenic	ENSSSCG000	PSMB1	proteasome 20S subunit beta 1 [Source:NCBI gene (formerly Entrezgene);Acc:100621969]
chr10	44982948	44984121	1174	*	chr10	44982948	44984121	in_vitro	in_vivo	9	11	23.5988889	15.2490909	8.34979798	c(t = 1.30110	0.21	0.83731181	Distal Intergenic	ENSSSCG000	PTER	phosphotriesterase related [Source:VGNC Symbol;Acc:VGNC:96514]
chr11	750318	752006	1689	*	chr11	750318	752006	in_vitro	in_vivo	12	12	4.1025	10.6791667	-6.5766667	c(t = -1.3204	0.21	0.83731181	Distal Intergenic	ENSSSCG000	CRYL1	crystallin lambda 1 [Source:VGNC Symbol;Acc:VGNC:87023]
chr1	27605703	27606825	1123	*	chr1	27605703	27606825	in_vitro	in_vivo	14	10	1.93428571	4.184	-2.2497143	c(t = -1.3204	0.21	0.83731181	Distal Intergenic	ENSSSCG000	BCLAF1	BCL2 associated transcription factor 1 [Source:VGNC Symbol;Acc:VGNC:85787]
chr14	88151959	88152413	455	*	chr14	88151959	88152413	in_vitro	in_vivo	9	10	8.73111111	2.576	6.15511111	c(t = 1.34338	0.21	0.83731181	Distal Intergenic	ENSSSCG000	GPRIN2	G protein regulated inducer of neurite outgrowth 2 [Source:NCBI gene (formerly Entrezgene);Acc:100568453]
chr2	15052066	15052940	875	*	chr2	15052066	15052940	in_vitro	in_vivo	9	9	0.80111111	1.91333333	-1.1122222	c(t = -1.3321	0.21	0.83731181	Promoter (<=1kb)	ENSSSCG000	PTPMT1	protein tyrosine phosphatase mitochondrial 1 [Source:NCBI gene (formerly Entrezgene);Acc:100514336]
chr15	37800617	37801941	1325	*	chr15	37800617	37801941	in_vitro	in_vivo	9	11	0.52666667	1.38636364	-0.859697	c(t = -1.3061	0.21	0.83731181	Promoter (<=1kb)	ENSSSCG000	AGPAT5	1-acylglycerol-3-phosphate O-acyltransferase 5 [Source:VGNC Symbol;Acc:VGNC:95954]
chr18	9186114	9187135	1022	*	chr18	9186114	9187135	in_vitro	in_vivo	11	13	3.45909091	1.21538462	2.24370629	c(t = 1.32447	0.21	0.83731181	Distal Intergenic	ENSSSCG000	NDUFB2	NADH:ubiquinone oxidoreductase subunit B2 [Source:NCBI gene (formerly Entrezgene);Acc:100513895]
chr14	4289139	4289518	380	*	chr14	4289139	4289518	in_vitro	in_vivo	11	10	2.47909091	0.769	1.71009091	c(t = 1.32525	0.21	0.83731181	Distal Intergenic	ENSSSCG000	LPL	lipoprotein lipase [Source:VGNC Symbol;Acc:VGNC:89795]
chr14	135679418	135680975	1558	*	chr14	135679418	135680975	in_vitro	in_vivo	12	10	5.0925	2.537	2.5555	c(t = 1.29981	0.211	0.83731181	Distal Intergenic	ENSSSCG000	UROS	uroporphyrinogen III synthase [Source:VGNC Symbol;Acc:VGNC:94735]
chr13	79707967	79710829	2863	*	chr13	79707967	79710829	in_vitro	in_vivo	14	13	2.18785714	1.08153846	1.10631868	c(t = 1.28999	0.211	0.83731181	Promoter (<=1kb)	ENSSSCG000	FOXL2	forkhead box L2 [Source:NCBI gene (formerly Entrezgene);Acc:100622956]
chr1	207173457	207174369	913	*	chr1	207173457	207174369	in_vitro	in_vivo	10	12	3.208	0.76666667	2.44133333	c(t = 1.33550	0.211	0.83731181	Promoter (<=1kb)	ENSSSCG000	PSIP1	PC4 and SFRS1 interacting protein 1 [Source:VGNC Symbol;Acc:VGNC:91898]
chr5	28300726	28301749	1024	*	chr5	28300726	28301749	in_vitro	in_vivo	9	12	1.31444444	0.49666667	0.81777778	c(t = 1.33957	0.211	0.83731181	Distal Intergenic	ENSSSCG000	TBK1	TANK binding kinase 1 [Source:VGNC Symbol;Acc:VGNC:93787]
chr4	77109293	77110223	931	*	chr4	77109293	77110223	in_vitro	in_vivo	10	10	2.016	0.484	1.532	c(t = 1.33603	0.211	0.83731181	Promoter (<=1kb)	ENSSSCG000	TCEA1	transcription elongation factor A1 [Source:VGNC Symbol;Acc:VGNC:93811]
chr6	60422807	60424327	1521	*	chr6	60422807	60424327	in_vitro	in_vivo	12	12	15.2183333	32.5025	-17.284167	c(t = -1.2906	0.212	0.83731181	Distal Intergenic	ENSSSCG000	GALP	galanin like peptide [Source:NCBI gene (formerly Entrezgene);Acc:396772]
chr18	48710523	48711236	714	*	chr18	48710523	48711236	in_vitro	in_vivo	9	11	6.04333333	16.91	-10.866667	c(t = -1.2998	0.212	0.83731181	Promoter (<=1kb)	ENSSSCG000	PGAM2	phosphoglycerate mutase 2 [Source:VGNC Symbol;Acc:VGNC:91341]
chr2	84621360	84621683	324	*	chr2	84621360	84621683	in_vitro	in_vivo	9	10	10.2611111	2.291	7.97011111	c(t = 1.34082	0.212	0.83731181	Distal Intergenic	ENSSSCG000	HMGCR	3-hydroxy-3-methylglutaryl-CoA reductase [Source:NCBI gene (formerly Entrezgene);Acc:100144446]
chr2	74455488	74455991	504	*	chr2	74455488	74455991	in_vitro	in_vivo	10	10	0.703	1.746	-1.043	c(t = -1.2970	0.212	0.83731181	Distal Intergenic	ENSSSCG000	EBI3	Epstein-Barr virus induced 3 [Source:NCBI gene (formerly Entrezgene);Acc:100522599]
chr5	27831439	27832672	1234	*	chr5	27831439	27832672	in_vitro	in_vivo	12	15	2.52333333	1.39066667	1.13266667	c(t = 1.29206	0.212	0.83731181	Distal Intergenic	NA	NA	NA
chr2	6563089	6563809	721	*	chr2	6563089	6563809	in_vitro	in_vivo	10	13	2.454	0.99076923	1.46323077	c(t = 1.30559	0.212	0.83731181	Distal Intergenic	ENSSSCG000	KAT5	lysine acetyltransferase 5 [Source:VGNC Symbol;Acc:VGNC:89305]
chr8	56035216	56036552	1337	*	chr8	56035216	56036552	in_vitro	in_vivo	13	14	1.96384615	0.94857143	1.01527473	c(t = 1.29021	0.212	0.83731181	Distal Intergenic	ENSSSCG00000008898		HOP homeobox [Source:VGNC Symbol;Acc:VGNC:88932]
chr16	44225307	44226069	763	*	chr16	44225307	44226069	in_vitro	in_vivo	12	15	1.52583333	0.634	0.89183333	c(t = 1.29935	0.212	0.83731181	Promoter (<=1kb)	ENSSSCG000	NLN	neurolysin [Source:VGNC Symbol;Acc:VGNC:90780]
chr9	126478588	126479935	1348	*	chr9	126478588	126479935	in_vitro	in_vivo	9	12	1.62333333	0.5775	1.04583333	c(t = 1.33076	0.212	0.83731181	Promoter (<=1kb)	ENSSSCG000	IVNS1ABP	influenza virus NS1A binding protein [Source:NCBI gene (formerly Entrezgene);Acc:100302027]
chr9	31720653	31721332	680	*	chr9	31720653	31721332	in_vitro	in_vivo	9	11	1.67333333	0.53272727	1.14060606	c(t = 1.32261	0.212	0.83731181	Distal Intergenic	ENSSSCG000	PGR	progesterone receptor [Source:VGNC Symbol;Acc:VGNC:91362]
chr13	99925168	99926563	1396	*	chr13	99925168	99926563	in_vitro	in_vivo	13	12	1.03692308	0.45916667	0.57775641	c(t = 1.29425	0.212	0.83731181	Distal Intergenic	ENSSSCG000	ssc-mir-15b	ssc-mir-15b [Source:miRBase;Acc:MI0002419]
chr13	71851975	71852765	791	*	chr13	71851975	71852765	in_vitro	in_vivo	13	10	0.88615385	0.401	0.48515385	c(t = 1.28831	0.212	0.83731181	Distal Intergenic	ENSSSCG000	RPN1	ribophorin I [Source:NCBI gene (formerly Entrezgene);Acc:397606]
chr3	125249016	125249813	798	*	chr3	125249016	125249813	in_vitro	in_vivo	11	12	1.90909091	0.38333333	1.57075758	c(t = 1.32751	0.212	0.83731181	Distal Intergenic	ENSSSCG000	LPIN1	lipin 1 [Source:VGNC Symbol;Acc:VGNC:89793]
chr13	25750718	25751503	786	*	chr13	25750718	25751503	in_vitro	in_vivo	10	9	0.923	0	0.923	c(t = 1.34279	0.212	0.83731181	Distal Intergenic	ENSSSCG000	CCK	cholecystokinin [Source:VGNC Symbol;Acc:VGNC:86334]
chr18	2794939	2797198	2260	*	chr18	2794939	2797198	in_vitro	in_vivo	10	10	3.434	1.383	2.051	c(t = 1.31437	0.213	0.83731181	Distal Intergenic	ENSSSCG000	INSIG1	insulin induced gene 1 [Source:NCBI gene (formerly Entrezgene);Acc:403162]
chr8	33373406	33375056	1651	*	chr8	33373406	33375056	in_vitro	in_vivo	12	13	2.57	1.23846154	1.33153846	c(t = 1.31165	0.213	0.83731181	Distal Intergenic	ENSSSCG000	SLC30A9	solute carrier family 30 member 9 [Source:NCBI gene (formerly Entrezgene);Acc:100192440]
chr7	47839232	47839834	603	*	chr7	47839232	47839834	in_vitro	in_vivo	10	9	0	0.99444444	-0.9944444	c(t = -1.3520	0.213	0.83731181	Distal Intergenic	ENSSSCG000	DNAJA4	DnaJ heat shock protein family (Hsp40) member A4 [Source:NCBI gene (formerly Entrezgene);Acc:397613]
chr11	78199005	78204915	5911	*	chr11	78199005	78204915	in_vitro	in_vivo	10	14	92.201	97.8578571	-5.6568571	c(t = -1.3349	0.214	0.83731181	Distal Intergenic	ENSSSCG000	F7	coagulation factor VII [Source:NCBI gene (formerly Entrezgene);Acc:733661]
chr5	2673850	2676183	2334	*	chr5	2673850	2676183	in_vitro	in_vivo	9	10	44.5044444	60.964	-16.459556	c(t = -1.2923	0.214	0.83731181	Distal Intergenic	ENSSSCG000	PPARA	peroxisome proliferator activated receptor alpha [Source:NCBI gene (formerly Entrezgene);Acc:397239]
chr15	81971924	81973015	1092	*	chr15	81971924	81973015	in_vitro	in_vivo	12	11	25.145	45.03	-19.885	c(t = -1.2800	0.214	0.83731181	Distal Intergenic	NA	NA	NA
chrX	45195355	45195972	618	*	chrX	45195355	45195972	in_vitro	in_vivo	9	10	3.10444444	11.778	-8.6735556	c(t = -1.3327	0.214	0.83731181	Distal Intergenic	ENSSSCG000	MAGED1	MAGE family member D1 [Source:VGNC Symbol;Acc:VGNC:89952]
chr13	100077810	100079784	1975	*	chr13	100077810	100079784	in_vitro	in_vivo	10	13	1.738	3.09307692	-1.3550769	c(t = -1.2803	0.214	0.83731181	Distal Intergenic	ENSSSCG000	ssc-mir-15b	ssc-mir-15b [Source:miRBase;Acc:MI0002419]
chr14	50324721	50326857	2137	*	chr14	50324721	50326857	in_vitro	in_vivo	12	11	3.44666667	1.76363636	1.6830303	c(t = 1.29235	0.214	0.83731181	Distal Intergenic	ENSSSCG00000037143		ubiquitin conjugating enzyme E2 L3 [Source:NCBI gene (formerly Entrezgene);Acc:100154268]
chr16	78300764	78309423	8660	*	chr16	78300764	78309423	in_vitro	in_vivo	16	16	2.4375	1.665	0.7725	c(t = 1.27499	0.214	0.83731181	Distal Intergenic	ENSSSCG000	TERT	telomerase reverse transcriptase [Source:NCBI gene (formerly Entrezgene);Acc:492280]
chr6	45182275	45184089	1815	*	chr6	45182275	45184089	in_vitro	in_vivo	13	12	2.44	1.27833333	1.16166667	c(t = 1.28095	0.214	0.83731181	Distal Intergenic	ENSSSCG000	LIN37	lin-37 DREAM MuvB core complex component [Source:VGNC Symbol;Acc:VGNC:98071]
chr8	93044478	93046326	1849	*	chr8	93044478	93046326	in_vitro	in_vivo	16	16	0.435625	0.941875	-0.50625	c(t = -1.2731	0.214	0.83731181	Distal Intergenic	ENSSSCG000	PGRMC2	progesterone receptor membrane component 2 [Source:NCBI gene (formerly Entrezgene);Acc:100048965]
chr15	131492473	131493547	1075	*	chr15	131492473	131493547	in_vitro	in_vivo	11	11	2.31909091	0.88363636	1.43545455	c(t = 1.30912	0.214	0.83731181	Distal Intergenic	ENSSSCG000	ITM2C	integral membrane protein 2C [Source:NCBI gene (formerly Entrezgene);Acc:100144481]
chr13	34088194	34089168	975	*	chr13	34088194	34089168	in_vitro	in_vivo	9	13	7.57222222	17.9384615	-10.366239	c(t = -1.2919	0.215	0.83731181	Distal Intergenic	ENSSSCG000	ABHD14A	abhydrolase domain containing 14A [Source:VGNC Symbol;Acc:VGNC:84973]
chr8	182041	185168	3128	*	chr8	182041	185168	in_vitro	in_vivo	11	11	31.6090909	15.7463636	15.8627273	c(t = 1.29433	0.215	0.83731181	Distal Intergenic	ENSSSCG000	ATP5ME	ATP synthase membrane subunit e [Source:NCBI gene (formerly Entrezgene);Acc:396950]
chr17	32445937	32447962	2026	*	chr17	32445937	32447962	in_vitro	in_vivo	9	15	4.17333333	7.102	-2.9286667	c(t = -1.2827	0.215	0.83731181	Distal Intergenic	ENSSSCG000	DDRGK1	DDRGK domain containing 1 [Source:NCBI gene (formerly Entrezgene);Acc:100155894]
chr14	64193315	64194152	838	*	chr14	64193315	64194152	in_vitro	in_vivo	9	9	0.61888889	1.59111111	-0.9722222	c(t = -1.2951	0.215	0.83731181	Distal Intergenic	ENSSSCG000	CDK1	cyclin dependent kinase 1 [Source:VGNC Symbol;Acc:VGNC:103917]
chr13	18557416	18558353	938	*	chr13	18557416	18558353	in_vitro	in_vivo	13	14	2.63230769	1.08214286	1.55016484	c(t = 1.29657	0.215	0.83731181	Distal Intergenic	ENSSSCG000	CMTM7	CKLF like MARVEL transmembrane domain containing 7 [Source:VGNC Symbol;Acc:VGNC:103928]
chr4	41915525	41916166	642	*	chr4	41915525	41916166	in_vitro	in_vivo	9	12	2.98636364	0.97	2.01636364	c(t = 1.30046	0.215	0.83731181	Distal Intergenic	ENSSSCG000	CCNE2	cyclin E2 [Source:VGNC Symbol;Acc:VGNC:86355]
chr16	71275601	71276563	963	*	chr16	71275601	71276563	in_vitro	in_vivo	13	12	1.70307692	0.59666667	1.10641026	c(t = 1.29193	0.215	0.83731181	Promoter (<=1kb)	ENSSSCG000	G3BP1	G3BP stress granule assembly factor 1 [Source:NCBI gene (formerly Entrezgene);Acc:100158288]
chr9	40470	40892	423	*	chr9	40470	40892	in_vitro	in_vivo	9	9	54.8677778	72.5033333	-17.635556	c(t = -1.2921	0.216	0.83731181	Distal Intergenic	NA	NA	NA
chr2	120117869	120118589	721	*	chr2	120117869	120118589	in_vitro	in_vivo	9	12	68.7466667	45.6566667	23.09	c(t = 1.29329	0.216	0.83731181	Distal Intergenic	ENSSSCG000	COMMD10	COMM domain containing 10 [Source:VGNC Symbol;Acc:VGNC:86886]
chr1	222836729	222838037	1309	*	chr1	222836729	222838037	in_vitro	in_vivo	10	11	8.211	22.8636364	-14.652636	c(t = -1.2917	0.216	0.83731181	Distal Intergenic	ENSSSCG000	TJP2	tight junction protein 2 [Source:VGNC Symbol;Acc:VGNC:103188]
chr2	79474154	79474808	655	*	chr2	79474154	79474808	in_vitro	in_vivo	9	9	4.57777778	19.3211111	-14.743333	c(t = -1.3385	0.216	0.83731181	Distal Intergenic	ENSSSCG000	PROP1	PROP paired-like homeobox 1 [Source:VGNC Symbol;Acc:VGNC:91834]
chr7	48523317	48523892	576	*	chr7	48523317	48523892	in_vitro	in_vivo	10	12	1.453	12.4325	-10.9795	c(t = -1.3115	0.216	0.83731181	Distal Intergenic	ENSSSCG000	TMED3	transmembrane p24 trafficking protein 3 [Source:VGNC Symbol;Acc:VGNC:94055]
chrX	122119871	122124979	5109	*	chrX	122119871	122124979	in_vitro	in_vivo	12	11	11.555	5.51454545	6.04045455	c(t = 1.29985	0.216	0.83731181	Distal Intergenic	ENSSSCG000	FATE1	fetal and adult testis expressed 1 [Source:NCBI gene (formerly Entrezgene);Acc:396705]
chr2	49229848	49231177	1330	*	chr2	49229848	49231177	in_vitro	in_vivo	13	11	7.65384615	4.13909091	3.51475524	c(t = 1.27413	0.216	0.83731181	Promoter (<=1kb)	ENSSSCG000	ADM	adrenomedullin [Source:NCBI gene (formerly Entrezgene);Acc:397195]
chr15	32599539	32601532	1994	*	chr15	32599539	32601532	in_vitro	in_vivo	13	13	2.06	3.99230769	-1.9323077	c(t = -1.2859	0.216	0.83731181	Distal Intergenic	NA	NA	NA
chr1	239135749	239136348	600	*	chr1	239135749	239136348	in_vitro	in_vivo	15	12	1.49733333	3.6875	-2.1901667	c(t = -1.2941	0.216	0.83731181	Distal Intergenic	ENSSSCG00000032554		solute carrier family 25 member 51 [Source:NCBI gene (formerly Entrezgene);Acc:100157218]
chr2	61271040	61273371	2332	*	chr2	61271040	61273371	in_vitro	in_vivo	17	14	5.26647059	3.49071429	1.7757563	c(t = 1.26494	0.216	0.83731181	Promoter (<=1kb)	ENSSSCG000	KLF2	KLF transcription factor 2 [Source:NCBI gene (formerly Entrezgene);Acc:100174959]
chr4	41373506	41374314	809	*	chr4	41373506	41374314	in_vitro	in_vivo	10	10	0.42777778	2.974	-2.5462222	c(t = -1.3224	0.216	0.83731181	Distal Intergenic	ENSSSCG000	CCNE2	cyclin E2 [Source:VGNC Symbol;Acc:VGNC:86355]
chr1	189679686	189682282	2597	*	chr1	189679686	189682282	in_vitro	in_vivo	14	13	0.92285714	2.97	-2.0471429	c(t = -1.2968	0.216	0.83731181	Promoter (<=1kb)	ENSSSCG000	SIX4	SIX homeobox 4 [Source:NCBI gene (formerly Entrezgene);Acc:100153985]
chr4	68778881	68779936	1056	*	chr4	68778881	68779936	in_vitro	in_vivo	12	15	1.08333333	2.0775	-0.9941667	c(t = -1.2764	0.216	0.83731181	Distal Intergenic	ENSSSCG000	DNAJC5B	DnaJ heat shock protein family (Hsp40) member C5 beta [Source:NCBI gene (formerly Entrezgene);Acc:100157247]
chr12	60993881	60994741	861	*	chr12	60993881	60994741	in_vitro	in_vivo	9	10	5.35555556	2.051	3.30455556	c(t = 1.32356	0.216	0.83731181	Distal Intergenic	ENSSSCG000	COPS3	COP9 signalosome subunit 3 [Source:VGNC Symbol;Acc:VGNC:86900]
chr2	73889403	73890442	1040	*	chr2	73889403	73890442	in_vitro	in_vivo	12	9	1.67583333	0.68555556	0.99027778	c(t = 1.28787	0.216	0.83731181	Distal Intergenic	ENSSSCG000	PLIN3	perilipin 3 [Source:VGNC Symbol;Acc:VGNC:91559]
chr3	33654223	33656529	2307	*	chr3	33654223	33656529	in_vitro	in_vivo	11	15	2.34181818	0.57733333	1.76448485	c(t = 1.31687	0.216	0.83731181	Distal Intergenic	ENSSSCG000	USP7	ubiquitin specific peptidase 7 [Source:VGNC Symbol;Acc:VGNC:94777]
chr6	12924722	12925418	697	*	chr6	12924722	12925418	in_vitro	in_vivo	9	10	5.88	0.52888889	5.35111111	c(t = 1.32767	0.216	0.83731181	Distal Intergenic	ENSSSCG000	COG4	component of oligomeric golgi complex 4 [Source:NCBI gene (formerly Entrezgene);Acc:100625346]
chrX	13181706	13182421	716	*	chrX	13181706	13182421	in_vitro	in_vivo	9	11	1.44166667	0.25222222	1.18944444	c(t = 1.30189	0.216	0.83731181	Promoter (<=1kb)	ENSSSCG000	SYAP1	synapse associated protein 1 [Source:VGNC Symbol;Acc:VGNC:93648]
chr13	81867610	81868480	871	*	chr13	81867610	81868480	in_vitro	in_vivo	11	10	11.0581818	30.685	-19.626818	c(t = -1.2955	0.217	0.83731181	Distal Intergenic	ENSSSCG000	SLC25A36	solute carrier family 25 member 36 [Source:VGNC Symbol;Acc:VGNC:93012]
chr3	356263	359237	2975	*	chr3	356263	359237	in_vitro	in_vivo	13	11	14.3866667	29.02	-14.633333	c(t = -1.2839	0.217	0.83731181	Distal Intergenic	ENSSSCG000	ADAP1	ArfGAP with dual PH domains 1 [Source:NCBI gene (formerly Entrezgene);Acc:397453]

chrX	113931507	113934661	3155	*	chrX	113931507	113934661	in_vitro	in_vivo	13	14	5.95769231	10.31	-4.3523077	c(t = -1.2747	0.217	0.83731181	Distal Intergenic	ENSSSCG000	F9	coagulation factor IX [Source:VGNC Symbol;Acc:VGNC:109453]
chr16	30969067	30969505	439	*	chr16	30969067	30969505	in_vitro	in_vivo	10	12	5.467	9.7275	-4.2605	c(t = -1.2761	0.217	0.83731181	Distal Intergenic	ENSSSCG000	ITGA2	integrin subunit alpha 2 [Source:VGNC Symbol;Acc:VGNC:89234]
chr9	6791923	6794162	2240	*	chr9	6791923	6794162	in_vitro	in_vivo	11	11	1.24545455	4.30090909	-3.0554545	c(t = -1.3043	0.217	0.83731181	Promoter (<=1kb)	ENSSSCG000	INPPL1	inositol polyphosphate phosphatase like 1 [Source:NCBI gene (formerly Entrezgene);Acc:100462718]
chr4	63970625	63971565	941	*	chr4	63970625	63971565	in_vitro	in_vivo	9	11	0.90333333	3.84636364	-2.9430303	c(t = -1.3097	0.217	0.83731181	Distal Intergenic	ENSSSCG000	LACTB2	lactamase beta 2 [Source:NCBI gene (formerly Entrezgene);Acc:100153212]
chr6	152321680	152322124	445	*	chr6	152321680	152322124	in_vitro	in_vivo	10	11	0.286	2.06727273	-1.7812727	c(t = -1.3109	0.217	0.83731181	Distal Intergenic	ENSSSCG000	CYP2J34	cytochrome P450 family 2 subfamily J member 34 [Source:NCBI gene (formerly Entrezgene);Acc:100524750]
chr9	76639910	76641296	1387	*	chr9	76639910	76641296	in_vitro	in_vivo	11	13	3.55181818	1.79307692	1.75874126	c(t = 1.29571	0.217	0.83731181	Exon (NM_001159660/100286863, exon 2 of 3)	ENSSSCG000	DLX5	distal-less homeobox 5 [Source:NCBI gene (formerly Entrezgene);Acc:100286863]
chr9	52758970	52759644	675	*	chr9	52758970	52759644	in_vitro	in_vivo	9	11	0	1.44818182	-1.4481818	c(t = -1.3189	0.217	0.83731181	Distal Intergenic	ENSSSCG000	STT3A	STT3 oligosaccharyltransferase complex catalytic subunit A [Source:VGNC Symbol;Acc:VGNC:93581]
chr15	130752250	130754118	1869	*	chr15	130752250	130754118	in_vitro	in_vivo	10	12	3.978	0.975	3.003	c(t = 1.31783	0.217	0.83731181	Distal Intergenic	ENSSSCG000	PID1	phosphotyrosine interaction domain containing 1 [Source:NCBI gene (formerly Entrezgene);Acc:100302508]
chr18	24875735	24876340	606	*	chr18	24875735	24876340	in_vitro	in_vivo	9	11	2.72777778	0.94727273	1.78050505	c(t = 1.29965	0.217	0.83731181	Distal Intergenic	ENSSSCG000	AASS	aminoadipate-semialdehyde synthase [Source:VGNC Symbol;Acc:VGNC:84944]
chr14	100798094	100798617	524	*	chr14	100798094	100798617	in_vitro	in_vivo	9	10	0	0.878	-0.878	c(t = -1.3282	0.217	0.83731181	Promoter (<=1kb)	ENSSSCG000	STAMBPL1	STAM binding protein like 1 [Source:VGNC Symbol;Acc:VGNC:93527]
chr11	7792203	7793236	1034	*	chr11	7792203	7793236	in_vitro	in_vivo	11	12	1.81181818	0.54583333	1.26598485	c(t = 1.29236	0.217	0.83731181	Distal Intergenic	ENSSSCG000	HSPH1	heat shock protein family H (Hsp110) member 1 [Source:NCBI gene (formerly Entrezgene);Acc:100048931]
chr6	3136991	3138436	1446	*	chr6	3136991	3138436	in_vitro	in_vivo	10	13	2.334	0.46153846	1.87246154	c(t = 1.32291	0.217	0.83731181	Distal Intergenic	ENSSSCG000	IRF8	interferon regulatory factor 8 [Source:VGNC Symbol;Acc:VGNC:89211]
chr3	1186699	1191376	4678	*	chr3	1186699	1191376	in_vitro	in_vivo	10	10	93.847	87.517	6.33	c(t = 1.28064	0.218	0.83731181	Distal Intergenic	ENSSSCG000	ssc-mir-9817	ssc-mir-9817 [Source:miRBase;Acc:MI0031584]
chr1	194966148	194966434	287	*	chr1	194966148	194966434	in_vitro	in_vivo	10	9	13.338	2.56	10.778	c(t = 1.31935	0.218	0.83731181	Distal Intergenic	ENSSSCG000	RHOJ	ras homolog family member J [Source:NCBI gene (formerly Entrezgene);Acc:100155474]
chr9	70284418	70284833	416	*	chr9	70284418	70284833	in_vitro	in_vivo	12	11	4.13083333	1.49272727	2.63810606	c(t = 1.26987	0.218	0.83731181	Promoter (<=1kb)	ENSSSCG000	STEAP1	STEAP family member 1 [Source:NCBI gene (formerly Entrezgene);Acc:397573]
chr5	62545747	62546793	1047	*	chr5	62545747	62546793	in_vitro	in_vivo	10	13	3.402	1.26538462	2.13661538	c(t = 1.31276	0.218	0.83731181	Distal Intergenic	ENSSSCG000	PHC1	polyhomeotic homolog 1 [Source:NCBI gene (formerly Entrezgene);Acc:100155344]
chr2	62251254	62251894	641	*	chr2	62251254	62251894	in_vitro	in_vivo	12	11	2.71916667	0.94909091	1.77007576	c(t = 1.27915	0.218	0.83731181	Distal Intergenic	ENSSSCG000	BRD4	bromodomain containing 4 [Source:VGNC Symbol;Acc:VGNC:85873]
chr1	119165528	119166193	666	*	chr1	119165528	119166193	in_vitro	in_vivo	11	13	1.92636364	0.85538462	1.07097902	c(t = 1.29177	0.218	0.83731181	Distal Intergenic	ENSSSCG000	ARPP19	cAMP regulated phosphoprotein 19 [Source:NCBI gene (formerly Entrezgene);Acc:397362]
chr12	14112323	14113140	818	*	chr12	14112323	14113140	in_vitro	in_vivo	9	11	2.25555556	0.78363636	1.47191919	c(t = 1.30546	0.218	0.83731181	Distal Intergenic	ENSSSCG000	KPNA2	karyopherin subunit alpha 2 [Source:VGNC Symbol;Acc:VGNC:89561]
chr1	25735449	25736948	1500	*	chr1	25735449	25736948	in_vitro	in_vivo	10	13	1.188	0.56846154	0.61953846	c(t = 1.27311	0.218	0.83731181	Distal Intergenic	ENSSSCG000	CCDC28A	coiled-coil domain containing 28A [Source:NCBI gene (formerly Entrezgene);Acc:100512200]
chr5	16618323	16619439	1117	*	chr5	16618323	16619439	in_vitro	in_vivo	9	9	3.01	0.29222222	2.71777778	c(t = 1.33393	0.218	0.83731181	Distal Intergenic	ENSSSCG000	LIMA1	LIM domain and actin binding 1 [Source:VGNC Symbol;Acc:VGNC:98070]
chr11	862370	863216	847	*	chr11	862370	863216	in_vitro	in_vivo	10	11	30.759	10.4236364	20.3353636	c(t = 1.28275	0.219	0.83731181	Intron (NM_213881/396914, intron 5 of 7)	ENSSSCG000	CRYL1	crystallin lambda 1 [Source:VGNC Symbol;Acc:VGNC:87023]
chr6	63840769	63842237	1469	*	chr6	63840769	63842237	in_vitro	in_vivo	12	11	18.1366667	6.14727273	11.9893939	c(t = 1.28802	0.219	0.83731181	Distal Intergenic	ENSSSCG000	TAS1R3	taste 1 receptor member 3 [Source:NCBI gene (formerly Entrezgene);Acc:100127440]
chr9	139302031	139303201	1171	*	chr9	139302031	139303201	in_vitro	in_vivo	11	12	1.43090909	3.87416667	-2.4432576	c(t = -1.2865	0.219	0.83731181	Promoter (<=1kb)	ENSSSCG000	EGFR	epidermal growth factor receptor [Source:NCBI gene (formerly Entrezgene);Acc:397070]
chr6	57029817	57030446	630	*	chr6	57029817	57030446	in_vitro	in_vivo	9	9	0.70444444	2.60555556	-1.9011111	c(t = -1.3112	0.219	0.83731181	Distal Intergenic	ENSSSCG00000058942		
chr4	35975871	35978365	2495	*	chr4	35975871	35978365	in_vitro	in_vivo	14	15	1.13071429	1.86533333	-0.734619	c(t = -1.2588	0.219	0.83731181	Promoter (1-2kb)	ENSSSCG000	YWHAZ	tyrosine 3-monooxygenase/tryptophan 5-monooxygenase activation protein zeta [Source:VGNC Symbol;Acc:VGNC:95047]
chr17	46432739	46434630	1892	*	chr17	46432739	46434630	in_vitro	in_vivo	15	15	3.74666667	1.80466667	1.942	c(t = 1.27179	0.219	0.83731181	Distal Intergenic	ENSSSCG000	GTSF1L	gametocyte specific factor 1 like [Source:NCBI gene (formerly Entrezgene);Acc:100156402]
chr13	158326227	158326985	759	*	chr13	158326227	158326985	in_vitro	in_vivo	10	11	0.323	1.60272727	-1.2797273	c(t = -1.2941	0.219	0.83731181	Distal Intergenic	ENSSSCG000	NIT2	nitrilase family member 2 [Source:VGNC Symbol;Acc:VGNC:108678]
chr10	36742477	36743225	749	*	chr10	36742477	36743225	in_vitro	in_vivo	10	9	3.733	1.55333333	2.17966667	c(t = 1.27821	0.219	0.83731181	Distal Intergenic	ENSSSCG000	IFNK	interferon kappa [Source:NCBI gene (formerly Entrezgene);Acc:100303725]
chr9	117063554	117064752	1199	*	chr9	117063554	117064752	in_vitro	in_vivo	13	13	3.97461538	1.27	2.70461538	c(t = 1.28683	0.219	0.83731181	Distal Intergenic	ENSSSCG000	SERPINC1	serpin family C member 1 [Source:NCBI gene (formerly Entrezgene);Acc:100125972]
chr10	25706694	25708890	2197	*	chr10	25706694	25708890	in_vitro	in_vivo	13	14	2.59692308	1.27	1.32692308	c(t = 1.26092	0.219	0.83731181	Distal Intergenic	ENSSSCG000	SLC35D2	solute carrier family 35 member D2 [Source:NCBI gene (formerly Entrezgene);Acc:100514312]
chr9	130510033	130511548	1516	*	chr9	130510033	130511548	in_vitro	in_vivo	10	10	2.372	1.171	1.201	c(t = 1.27395	0.219	0.83731181	Distal Intergenic	ENSSSCG000	FLVCR1	FLVCR heme transporter 1 [Source:NCBI gene (formerly Entrezgene);Acc:100217398]
chr14	32922499	32923565	1067	*	chr14	32922499	32923565	in_vitro	in_vivo	9	11	1.83666667	1.01090909	0.82575758	c(t = 1.27715	0.219	0.83731181	Distal Intergenic	ENSSSCG000	PRKAB1	protein kinase AMP-activated non-catalytic subunit beta 1 [Source:VGNC Symbol;Acc:VGNC:109479]
chr1	262203524	262204368	845	*	chr1	262203524	262204368	in_vitro	in_vivo	12	11	1.88166667	0.97909091	0.90257576	c(t = 1.26818	0.219	0.83731181	Distal Intergenic	ENSSSCG000	NDUFA8	NADH:ubiquinone oxidoreductase subunit A8 [Source:NCBI gene (formerly Entrezgene);Acc:100154443]
chr18	47169503	47170320	818	*	chr18	47169503	47170320	in_vitro	in_vivo	9	11	0.31555556	0.96727273	-0.6517172	c(t = -1.2875	0.219	0.83731181	Promoter (<=1kb)	ENSSSCG000	CYCS	cytochrome c, somatic [Source:NCBI gene (formerly Entrezgene);Acc:100170131]
chr9	46230195	46230898	704	*	chr9	46230195	46230898	in_vitro	in_vivo	9	14	3.33222222	0.85785714	2.47436508	c(t = 1.32098	0.219	0.83731181	Promoter (<=1kb)	ENSSSCG000	CENATAC	centrosomal AT-AC splicing factor [Source:VGNC Symbol;Acc:VGNC:86319]
chr13	196224458	196225516	1059	*	chr13	196224458	196225516	in_vitro	in_vivo	12	12	3.04083333	0.72916667	2.31166667	c(t = 1.28681	0.219	0.83731181	Distal Intergenic	ENSSSCG000	C21orf62	chromosome 13 C21orf62 homolog [Source:VGNC Symbol;Acc:VGNC:85929]
chr3	40240561	40241581	1021	*	chr3	40240561	40241581	in_vitro	in_vivo	12	10	1.72416667	0.721	1.00316667	c(t = 1.27709	0.219	0.83731181	Distal Intergenic	ENSSSCG000	JPT2	Jupiter microtubule associated homolog 2 [Source:NCBI gene (formerly Entrezgene);Acc:474165]
chr13	132488368	132489111	744	*	chr13	132488368	132489111	in_vitro	in_vivo	12	14	2.475	0.71785714	1.75714286	c(t = 1.29573	0.219	0.83731181	Distal Intergenic	ENSSSCG000	HES1	hes family bHLH transcription factor 1 [Source:NCBI gene (formerly Entrezgene);Acc:100499567]
chr13	18995013	18995748	736	*	chr13	18995013	18995748	in_vitro	in_vivo	12	12	1.645	0.56727273	1.07772727	c(t = 1.29117	0.219	0.83731181	Distal Intergenic	ENSSSCG000	CMTM7	CKLF like MARVEL transmembrane domain containing 7 [Source:VGNC Symbol;Acc:VGNC:103928]
chr17	58974072	58976064	1993	*	chr17	58974072	58976064	in_vitro	in_vivo	11	9	34.3672727	53.9488889	-19.581616	c(t = -1.2726	0.22	0.83731181	Distal Intergenic	ENSSSCG000	ssc-mir-296	ssc-mir-296 [Source:miRBase;Acc:MI0015906]
chr7	120295102	120299749	4648	*	chr7	120295102	120299749	in_vitro	in_vivo	13	12	4.81538462	2.2775	2.53788462	c(t = 1.27751	0.22	0.83731181	Promoter (<=1kb)	ENSSSCG000	BCL11B	BCL11 transcription factor B [Source:NCBI gene (formerly Entrezgene);Acc:100154158]
chr14	133542311	133544176	1866	*	chr14	133542311	133544176	in_vitro	in_vivo	14	10	2.80571429	1.148	1.65771429	c(t = 1.27623	0.22	0.83731181	Distal Intergenic	ENSSSCG00000010734		ornithine aminotransferase [Source:NCBI gene (formerly Entrezgene);Acc:100156865]
chr1	236380510	236381004	495	*	chr1	236380510	236381004	in_vitro	in_vivo	9	12	2.80666667	0.47333333	2.33333333	c(t = 1.32393	0.22	0.83731181	Distal Intergenic	ENSSSCG000	ARHGEF39	Rho guanine nucleotide exchange factor 39 [Source:VGNC Symbol;Acc:VGNC:85500]
chr1	179461838	179462603	766	*	chr1	179461838	179462603	in_vitro	in_vivo	10	11	1.713	0.35	1.363	c(t = 1.29947	0.22	0.83731181	Distal Intergenic	ENSSSCG000	Metazoa_SR	Metazoan signal recognition particle RNA [Source:RFAM;Acc:RF00017]
chr9	113973156	113973942	787	*	chr9	113973156	113973942	in_vitro	in_vivo	10	11	1.09	0.27545455	0.81454545	c(t = 1.28773	0.22	0.83731181	Distal Intergenic	ENSSSCG000	CHI3L1	chitinase 3 like 1 [Source:NCBI gene (formerly Entrezgene);Acc:396865]
chr12	61345329	61347750	2422	*	chr12	61345329	61347750	in_vitro	in_vivo	10	10	94.86	91.213	3.647	c(t = 1.27866	0.221	0.83731181	Distal Intergenic	ENSSSCG000	COPS3	COP9 signalosome subunit 3 [Source:VGNC Symbol;Acc:VGNC:86900]
chr12	18409698	18411236	1539	*	chr12	18409698	18411236	in_vitro	in_vivo	11	13	6.34181818	2.24923077	4.09258741	c(t = 1.28729	0.221	0.83731181	Promoter (<=1kb)	ENSSSCG000	C1QL1	complement C1q like 1 [Source:NCBI gene (formerly Entrezgene);Acc:780424]
chr1	262318935	262322380	3446	*	chr1	262318935	262322380	in_vitro	in_vivo	12	11	1.66333333	0.78727273	0.87606061	c(t = 1.27179	0.221	0.83731181	Distal Intergenic	ENSSSCG000	NDUFA8	NADH:ubiquinone oxidoreductase subunit A8 [Source:NCBI gene (formerly Entrezgene);Acc:100154443]
chr1	108648986	108649443	458	*	chr1	108648986	108649443	in_vitro	in_vivo	10	9	7.754	3.11222222	4.64177778	c(t = 1.29157	0.222	0.83731181	Distal Intergenic	ENSSSCG000	LACTB	lactamase beta [Source:VGNC Symbol;Acc:VGNC:89614]
chr3	31880460	31882752	2293	*	chr3	31880460	31882752	in_vitro	in_vivo	11	13	6.54636364	2.75923077	3.78713287	c(t = 1.27763	0.222	0.83731181	Promoter (<=1kb)	ENSSSCG000	SOCS1	suppressor of cytokine signaling 1 [Source:VGNC Symbol;Acc:VGNC:93330]
chr6	27308333	27309073	741	*	chr6	27308333	27309073	in_vitro	in_vivo	11	9	5.58818182	1.42222222	4.1659596	c(t = 1.29117	0.222	0.83731181	Distal Intergenic	ENSSSCG000	DYNC1LI2	dynein cytoplasmic 1 light intermediate chain 2 [Source:VGNC Symbol;Acc:VGNC:87498]
chr1	42729315	42730882	1568	*	chr1	42729315	42730882	in_vitro	in_vivo	12	9	0.58916667	1.39666667	-0.8075	c(t = -1.2750	0.222	0.83731181	Promoter (<=1kb)	ENSSSCG000	MAN1A1	mannosidase alpha class 1A member 1 [Source:VGNC Symbol;Acc:VGNC:89966]
chr13	91271610	91273774	2165	*	chr13	91271610	91273774	in_vitro	in_vivo	14	15	0.53285714	1.11933333	-0.5864762	c(t = -1.2583	0.222	0.83731181	Distal Intergenic	ENSSSCG000	P2RY12	purinergic receptor P2Y12 [Source:VGNC Symbol;Acc:VGNC:91124]
chr16	64250862	64251539	678	*	chr16	64250862	64251539	in_vitro	in_vivo	10	12	2.093	0.92833333	1.16466667	c(t = 1.27841	0.222	0.83731181	Distal Intergenic	ENSSSCG000	IL12B	interleukin 12B [Source:NCBI gene (formerly Entrezgene);Acc:397076]
chr12	38073488	38074719	1232	*	chr12	38073488	38074719	in_vitro	in_vivo	11	12	3.21454545	0.73833333	2.47621212	c(t = 1.29453	0.222	0.83731181	Distal Intergenic	ENSSSCG000	DHRS11	dehydrogenase/reductase 11 [Source:NCBI gene (formerly Entrezgene);Acc:100513307]
chr13	138681241	138682131	891	*	chr13	138681241	138682131	in_vitro	in_vivo	10	16	1.605	0.6525	0.9525	c(t = 1.26167	0.222	0.83731181	Distal Intergenic	ENSSSCG000	SLC15A2	solute carrier family 15 member 2 [Source:VGNC Symbol;Acc:VGNC:92935]
chrX	51903084	51903543	460	*	chrX	51903084	51903543	in_vitro	in_vivo	12	11	4.15083333	12.2036364	-8.052803	c(t = -1.2869	0.223	0.83731181	Promoter (<=1kb)	ENSSSCG000	MSN	moesin [Source:VGNC Symbol;Acc:VGNC:90426]
chr10	52654338	52656551	2214	*	chr10	52654338	52656551	in_vitro	in_vivo	12	15	2.225	8.10333333	-5.8783333	c(t = -1.2709	0.223	0.83731181	Distal Intergenic	ENSSSCG000	BMI1	BMI1 proto-onco, polycomb ring finger [Source:VGNC Symbol;Acc:VGNC:108265]
chr16	5314628	5315163	536	*	chr16	5314628	5315163	in_vitro	in_vivo	11	9	1.97545455	6.14444444	-4.1689899	c(t = -1.2948	0.223	0.83731181	Distal Intergenic	ENSSSCG000	C1QTNF3	C1q and TNF related 3 [Source:NCBI gene (formerly Entrezgene);Acc:100270814]
chr14	113081703	113082500	798	*	chr14	113081703	113082500	in_vitro	in_vivo	12	11	0.77666667	2.87636364	-2.099697	c(t = -1.2943	0.223	0.83731181	Promoter (<=1kb)	ENSSSCG000	HPS6	HPS6 biogenesis of lysosomal organelles complex 2 subunit 3 [Source:NCBI gene (formerly Entrezgene);Acc:100049675]
chr17	62611790	62612669	880	*	chr17	62611790	62612669	in_vitro	in_vivo	11	9	3.42727273	1.67555556	1.75171717	c(t = 1.26368	0.223	0.83731181	Distal Intergenic	ENSSSCG000	LIME1	Lck interacting transmembrane adaptor 1 [Source:NCBI gene (formerly Entrezgene);Acc:100625216]
chr14	112813312	112814912	1601	*	chr14	112813312	112814912	in_vitro	in_vivo	9	11	3.97	1.45545455	2.51454545	c(t = 1.30363	0.223	0.83731181	Distal Intergenic	ENSSSCG000	HPS6	HPS6 biogenesis of lysosomal organelles complex 2 subunit 3 [Source:NCBI gene (formerly Entrezgene);Acc:100049675]
chr3	49790017	49791469	1453	*	chr3	49790017	49791469	in_vitro	in_vivo	12	10	2.2575	0.989	1.2685	c(t = 1.26067	0.223	0.83731181	Distal Intergenic	ENSSSCG000	MRPS9	mitochondrial ribosomal protein S9 [Source:VGNC Symbol;Acc:VGNC:90401]
chr6	110122171	110123062	892	*	chr6	110122171	110123062	in_vitro	in_vivo	11	11	2.52181818	0.73363636	1.78818182	c(t = 1.28716	0.223	0.83731181	Distal Intergenic	ENSSSCG000	SS18	SS18 subunit of BAF chromatin remodeling complex [Source:NCBI gene (formerly Entrezgene);Acc:100513410]
chr5	16842666	16843234	569	*	chr5	16842666	16843234	in_vitro	in_vivo	9	11	2.05777778	0.69545455	1.36232323	c(t = 1.29218	0.223	0.83731181	Distal Intergenic	ENSSSCG000	ACVR1B	activin A receptor type 1B [Source:VGNC Symbol;Acc:VGNC:85056]
chr14	99454309	99456466	2158	*	chr14	99454309	99456466	in_vitro	in_vivo	12	14	1.92666667	0.64142857	1.2852381	c(t = 1.28305	0.223	0.83731181	Distal Intergenic	ENSSSCG000	SGMS1	sphingomyelin synthase 1 [Source:VGNC Symbol;Acc:VGNC:92794]
chr12	18670978	18672265	1288	*	chr12	18670978	18672265	in_vitro	in_vivo	12	11	3.6275	6.98272727	-3.3552273	c(t = -1.2686	0.224	0.83731181	Distal Intergenic	NA	NA	NA
chr3	41283502	41285294	1793	*	chr3	41283502	41285294	in_vitro	in_vivo	12	13	6.93666667	2.53230769	4.40435897	c(t = 1.27906	0.224	0.83731181	Distal Intergenic	ENSSSCG000	NME4	NME/NM23 nucleoside diphosphate kinase 4 [Source:VGNC Symbol;Acc:VGNC:90792]
chr15	78503725	78505060	1336	*	chr15	78503725	78505060	in_vitro	in_vivo	12	12	3.3575	1.8525	1.505	c(t = 1.26796	0.224	0.83731181	Distal Intergenic	ENSSSCG000	PDK1	pyruvate dehydrogenase kinase 1 [Source:VGNC Symbol;Acc:VGNC:98180]
chr1	97734395	97735500	1106	*	chr1	97734395	97735500	in_vitro	in_vivo	10	12	3.275	1.34833333	1.92666667	c(t = 1.27993	0.224	0.83731181	Distal Intergenic	ENSSSCG000	SMAD2	SMAD family member 2 [Source:VGNC Symbol;Acc:VGNC:98329]
chr2	13406817	13407883	1067	*	chr2	13406817	13407883	in_vitro	in_vivo	11	10	3.15545455	1.221	1.93445455	c(t = 1.27899	0.224	0.83731181	Distal Intergenic	ENSSSCG000	UBE2L6	ubiquitin conjugating enzyme E2 L6 [Source:NCBI gene (formerly Entrezgene);Acc:100514753]
chr7	23705101	23705806	706	*	chr7	23705101	23705806	in_vitro	in_vivo	10	13	0	1.00923077	-1.0092308	c(t = -1.2832	0.224	0.83731181	Promoter (<=1kb)	ENSSSCG000	LTB	lymphotoxin beta [Source:NCBI gene (formerly Entrezgene);Acc:100155581]
chr10	12670314	12670764	451	*	chr10	12670314	12670764	in_vitro	in_vivo	10	10	3.191	0.526	2.665	c(t = 1.29456	0.224	0.83731181	Distal Intergenic	ENSSSCG000	DEGS1	delta 4-desaturase, sphingolipid 1 [Source:VGNC Symbol;Acc:VGNC:95590]
chr13	82548375	82548979	605	*	chr13	82548375	82548979	in_vitro	in_vivo	11	11	1.58636364	0.39727273	1.18909091	c(t = 1.28076	0.224	0.83731181	Promoter (<=1kb)	ENSSSCG000	RNF7	ring finger protein 7 [Source:VGNC Symbol;Acc:VGNC:92398]
chr1	168552699	168553195	497	*	chr1	168552699	168553195	in_vitro	in_vivo	11	10	1.20272727	0	1.20272727	c(t = 1.29747	0.224	0.83731181	Distal Intergenic	ENSSSCG00000026486		
chr11	2256814	2257470	657	*	chr11	2256814	2257470	in_vitro	in_vivo	9	10	98.1744444	94.975	3.19944444	c(t = 1.27652	0.225	0.83731181	Distal Intergenic	ENSSSCG000	ssc-mir-9814	ssc-mir-9814 [Source:miRBase;Acc:MI0031581]
chr13	207836074	207837600	1527	*	chr13	207836074	207837600	in_vitro	in_vivo	12	12	14.3475	31.7241667	-17.376667	c(t = -1.2533	0.225	0.83731181	Distal Intergenic	ENSSSCG000	ITGB2	integrin subunit beta 2 [Source:NCBI gene (formerly Entrezgene);Acc:396943]
chr12	15444113	15446029	1917	*	chr12	15444113	15446029	in_vitro	in_vivo	11	10	10.41	23.568	-13.158	c(t = -1.2584	0.225	0.83731181	Promoter (<=1kb)	ENSSSCG000	CYB561	cytochrome b561 [Source:VGNC Symbol;Acc:VGNC:87119]
chrX	17839324	17842394	3071	*	chrX	17839324	17842394	in_vitro	in_vivo	14	14	7.20142857	12.385	-5.1835714	c(t = -1.2448	0.225	0.83731181	Distal Intergenic	ENSSSCG000	SMPX	small muscle protein X-linked [Source:NCBI gene (formerly Entrezgene);Acc:780438]
chr2	91700562	91701409	848	*	chr2	91700562	91701409	in_vitro	in_vivo	11	10	0.71636364	2.359	-1.6426364	c(t = -1.2785	0.225	0.83731181	Promoter (<=1kb)	ENSSSCG000	VCAN	versican [Source:VGNC Symbol;Acc:VGNC:108163]
chr3	75632881	75633942	1062	*	chr3	75632881	75633942	in_vitro	in_vivo	10	12	2.329	1.04416667	1.28483333	c(t = 1.27104	0.225	0.83731181	Distal Intergenic	ENSSSCG000	ACTR2	actin related protein 2 [Source:VGNC Symbol;Acc:VGNC:85051]
chr14	92383161	92384239	1079	*	chr14	92383161	92384239	in_vitro	in_vivo	10	12	2.187	0.77666667	1.41033333	c(t = 1.28377	0.225	0.83731181	Distal Intergenic	ENSSSCG000	UBE2D1	ubiquitin conjugating enzyme E2 D1 [Source:VGNC Symbol;Acc:VGNC:94641]
chr2	140531519	140532395	877	*	chr2	140531519	140532395	in_vitro	in_vivo	9	12	0.94555556	0.2525	0.69305556	c(t = 1.27826	0.225	0.83731181	Distal Intergenic	ENSSSCG000	CDC25C	cell division cycle 25C [Source:NCBI gene (formerly Entrezgene);Acc:100144474]
chr17	61733085	61734113	1029	*	chr17	61733085	61734113	in_vitro	in_vivo	9	12	27.6666667	11.495	16.1716667	c(t = 1.28091	0.226	0.83731181	Distal Intergenic	ENSSSCG000	RPS21	ribosomal protein S21 [Source:NCBI gene (formerly Entrezgene);Acc:100037996]
chr6	81303556	81304885	1330	*	chr6	81303556	81304885	in_vitro	in_vivo	9	9	17.6977778	3.38888889	14.3088889	c(t = 1.30633	0.226	0.83731181	Distal Intergenic	ENSSSCG000	ID3	inhibitor of DNA binding 3 [Source:NCBI gene (formerly Entrezgene);Acc:100626978]
chr15	30577526	30579857	2332	*	chr15	30577526	30579857	in_vitro	in_vivo	13	12	7.21384615	2.345	4.86884615	c(t = 1.26812	0.226	0.83731181	Promoter (<=1kb)	ENSSSCG000	GLI2	GLI family zinc finger 2 [Source:VGNC Symbol;Acc:VGNC:96324]
chr8	122006773	122008610	1838	*	chr8	122006773	122008610	in_vitro	in_vivo	13	14	4.04615385	2.185	1.86115385	c(t = 1.24293	0.226	0.83731181	Distal Intergenic	ENSSSCG000	ADH5	alcohol dehydrogenase 5 (class III), chi polypeptide [Source:NCBI gene (formerly Entrezgene);Acc:100513555]
chr7	88954232	88954707	476	*	chr7	88954232	88954707	in_vitro	in_vivo	9	12	0.61777778	2.04583333	-1.4280556	c(t = -1.2552	0.226	0.83731181	Distal Intergenic	ENSSSCG000	GPX2	glutathione peroxidase 2 [Source:NCBI gene (formerly Entrezgene);Acc:767625]
chr6	28616791	28617644	854	*	chr6	28616791	28617644	in_vitro	in_vivo	9	11	4.24888889	1.30181818	2.94707071	c(t = 1.30032	0.226	0.83731181	Distal Intergenic	ENSSSCG000	SLC12A4	solute carrier family 12 member 4 [Source:NCBI gene (formerly Entrezgene);Acc:396992]
chr3	10916330	10916834	505	*	chr3	10916330	10916834	in_vitro	in_vivo	9	11	2.84111111	1.18454545	1.65656566	c(t = 1.28490	0.226	0.83731181	Distal Intergenic	ENSSSCG000	ssc-mir-7137	ssc-mir-7137 [Source:miRBase;Acc:MI0023570]
chr6	43933498	43934468	971	*	chr6	43933498	43934468	in_vitro	in_vivo	9	11	3.99444444	1.03545455	2.9589899	c(t = 1.30124	0.226	0.83731181	Distal Intergenic	ENSSSCG000	LSM14A	LSM14A mRNA processing body assembly factor [Source:NCBI gene (formerly Entrezgene);Acc:100511455]
chr1	194263491	194264490	1000	*	chr1	194263491	194264490	in_vitro	in_vivo	11	11	1.78272727	0.46636364	1.31636364	c(t = 1.26421	0.226	0.83731181	Distal Intergenic	ENSSSCG000	PPP2R5E	protein phosphatase 2 regulatory subunit B'epsilon [Source:NCBI gene (formerly Entrezgene);Acc:100525377]
chr1	99916897	99917581	685	*	chr1	99916897	99917581	in_vitro	in_vivo	9	12	2.00666667	0.4225	1.58416667	c(t = 1.30086	0.226	0.83731181	Distal Intergenic	ENSSSCG000	MRO	maestro [Source:VGNC Symbol;Acc:VGNC:103127]
chr3	1506701	1509896	3196	*	chr3	1506701	1509896	in_vitro	in_vivo	11	12	96.7172727	94.6158333	2.10143939	c(t = 1.24910	0.227	0.83731181	Distal Intergenic	ENSSSCG000	MRM2	mitochondrial rRNA methyltransferase 2 [Source:VGNC Symbol;Acc:VGNC:99722]
chr15	30316072	30317018	947	*	chr15	30316072	30317018	in_vitro	in_vivo	13	11	10.46	27.8827273	-17.422727	c(t = -1.2521	0.227	0.83731181	Exon (NM_001315712/100520334, exon 14 of 14)	ENSSSCG000	GLI2	GLI family zinc finger 2 [Source:VGNC Symbol;Acc:VGNC:96324]
chr17	10656619	10657029	411	*	chr17	10656619	10657029	in_vitro	in_vivo	10	9	0.385	1.85777778	-1.4727778	c(t = -1.2878	0.227	0.83731181	Distal Intergenic	ENSSSCG000	GPAT4	glycerol-3-phosphate acyltransferase 4 [Source:NCBI gene (formerly Entrezgene);Acc:100152836]
chr8	12763506	12764638	1133	*	chr8	12763506	12764638	in_vitro	in_vivo	10	12	3.393	1.04166667	2.35133333	c(t = 1.27769	0.227	0.83731181	Distal Intergenic	ENSSSCG000	MED28	mediator complex subunit 28 [Source:VGNC Symbol;Acc:VGNC:90118]
chr17	43709928	43711256	1329	*	chr17	43709928	43711256	in_vitro	in_vivo	13	12	2.04846154	0.7075	1.34096154	c(t = 1.25669	0.227	0.83731181	Distal Intergenic	ENSSSCG000	ZHX3	zinc fingers and homeoboxes 3 [Source:VGNC Symbol;Acc:VGNC:95717]
chr9	114916639	114917283	645	*	chr9	114916639	114917283	in_vitro	in_vivo	10	10	1.737	0.4	1.337	c(t = 1.27259	0.227	0.83731181	Distal Intergenic	ENSSSCG000	PIGC	phosphatidylinositol glycan anchor biosynthesis class C [Source:VGNC Symbol;Acc:VGNC:91421]
chr3	4133904	4136379	2476	*	chr3	4133904	4136379	in_vitro	in_vivo	10	16	4.979	1.650625	3.328375	c(t = 1.28897	0.228	0.83731181	Promoter (<=1kb)	ENSSSCG000	FSCN1	fascin actin-bundling protein 1 [Source:NCBI gene (formerly Entrezgene);Acc:100286741]
chr9	22775846	22776456	611	*	chr9	22775846	22776456	in_vitro	in_vivo	13	11	2.41307692	0.99454545	1.41853147	c(t = 1.24165	0.228	0.83731181	Distal Intergenic	ENSSSCG000	FOLH1B	folate hydrolase 1B [Source:NCBI gene (formerly Entrezgene);Acc:397677]
chr6	81566634	81567383	750	*	chr6	81566634	81567383	in_vitro	in_vivo	9	9	1.83888889	0.68888889	1.15	c(t = 1.27533	0.228	0.83731181	Distal Intergenic	ENSSSCG000	RPL11	ribosomal protein L11 [Source:NCBI gene (formerly Entrezgene);Acc:414422]
chr3	111328291	111328798	508	*	chr3	111328291	111328798	in_vitro	in_vivo	11	15	1.67272727	0.658	1.01472727	c(t = 1.26004	0.228	0.83731181	Promoter (<=1kb)	ENSSSCG000	RBKS	ribokinase [Source:VGNC Symbol;Acc:VGNC:92142]
chr4	27898975	27899501	527	*	chr4	27898975	27899501	in_vitro	in_vivo	9	9	1.94111111	0.58666667	1.35444444	c(t = 1.27356	0.228	0.83731181	Distal Intergenic	ENSSSCG000	ENY2	ENY2 transcription and export complex 2 subunit [Source:VGNC Symbol;Acc:VGNC:98792]
chr7	80447519	80448268	750	*	chr7	80447519	80448268	in_vitro	in_vivo	10	12	2.297	0.5225	1.7745	c(t = 1.28469	0.228	0.83731181	Distal Intergenic	ENSSSCG000	EMC7	ER membrane protein complex subunit 7 [Source:NCBI gene (formerly Entrezgene);Acc:100626410]
chr6	13295973	13297027	1055	*	chr6	13295973	13297027	in_vitro	in_vivo	10	13	1.864	4.86307692	-2.9990769	c(t = -1.2566	0.229	0.83731181	Distal Intergenic	ENSSSCG000	COG4	component of oligomeric golgi complex 4 [Source:NCBI gene (formerly Entrezgene);Acc:100625346]
chr13	139927387	139928462	1076	*	chr13	139927387	139928462	in_vitro	in_vivo	11	14	1.35090909	4.39071429	-3.0398052	c(t = -1.2591	0.229	0.83731181	Distal Intergenic	ENSSSCG000	GSK3B	glycogen synthase kinase 3 beta [Source:VGNC Symbol;Acc:VGNC:88723]
chr5	35757137	35757884	748	*	chr5	35757137	35757884	in_vitro	in_vivo	12	12	0.62833333	2.325	-1.6966667	c(t = -1.2606	0.229	0.83731181	Distal Intergenic	ENSSSCG000	LGR5	leucine rich repeat containing G protein-coupled receptor 5 [Source:VGNC Symbol;Acc:VGNC:103296]
chr6	89249426	89250482	1057	*	chr6	89249426	89250482	in_vitro	in_vivo	13	11	0.69692308	1.32272727	-0.6258042	c(t = -1.2442	0.229	0.83731181	Distal Intergenic	ENSSSCG000	HPCA	hippocalcin [Source:VGNC Symbol;Acc:VGNC:88957]
chr10	54675617	54677199	1583	*	chr10	54675617	54677199	in_vitro	in_vivo	13	15	1.49538462	0.76066667	0.73471795	c(t = 1.23256	0.229	0.83731181	Distal Intergenic	ENSSSCG000	ITGB1	integrin subunit beta 1 [Source:VGNC Symbol;Acc:VGNC:96381]
chr1	52041127	52042104	978	*	chr1	52041127	52042104	in_vitro	in_vivo	14	13	1.52642857	0.69384615	0.83258242	c(t = 1.23645	0.229	0.83731181	Distal Intergenic	ENSSSCG000	ssc-mir-30a	ssc-mir-30a [Source:miRBase;Acc:MI0010688]
chr2	411934	416417	4484	*	chr2	411934	416417	in_vitro	in_vivo	12	13	98.2333333	96.7784615	1.45487179	c(t = 1.23833	0.23	0.83731181	Distal Intergenic	ENSSSCG000	TMEM80	transmembrane protein 80 [Source:NCBI gene (formerly Entrezgene);Acc:100514388]
chr9	57149152	57149993	842	*	chr9	57149152	57149993	in_vitro	in_vivo	12	9	27.9333333	12.6244444	15.3088889	c(t = 1.25262	0.23	0.83731181	Distal Intergenic	ENSSSCG000	KCNJ5	potassium inwardly rectifying channel subfamily J member 5 [Source:NCBI gene (formerly Entrezgene);Acc:397448]
chr3	68578108	68578777	670	*	chr3	68578108	68578777	in_vitro	in_vivo	9	10	2.89777778	9.257	-6.3592222	c(t = -1.2778	0.23	0.83731181	Distal Intergenic	ENSSSCG000	C2orf81	chromosome 3 C2orf81 homolog [Source:VGNC Symbol;Acc:VGNC:109179]
chr12	54053872	54054830	959	*	chr12	54053872	54054830	in_vitro	in_vivo	9	10	2.42777778	6.78	-4.3522222	c(t = -1.2726	0.23	0.83731181	Distal Intergenic	ENSSSCG000	PIK3R5	phosphoinositide-3-kinase regulatory subunit 5 [Source:NCBI gene (formerly Entrezgene);Acc:396851]
chr12	40204765	40205490	726	*	chr12	40204765	40205490	in_vitro	in_vivo	10	11	0.64	2.76727273	-2.1272727	c(t = -1.2657	0.23	0.83731181	Distal Intergenic	ENSSSCG000	LIG3	DNA ligase 3 [Source:VGNC Symbol;Acc:VGNC:89721]
chr12	54088759	54091265	2507	*	chr12	54088759	54091265	in_vitro	in_vivo	9	13	4.71222222	2.58307692	2.1291453	c(t = 1.27793	0.23	0.83731181	Promoter (<=1kb)	ENSSSCG000	NTN1	netrin 1 [Source:VGNC Symbol;Acc:VGNC:90931]
chr4	56844127	56844673	547	*	chr4	56844127	56844673	in_vitro	in_vivo	11	10	0.23272727	2.083	-1.8502727	c(t = -1.2831	0.23	0.83731181	Distal Intergenic	ENSSSCG000	MRPS28	mitochondrial ribosomal protein S28 [Source:NCBI gene (formerly Entrezgene);Acc:100152709]
chr3	11419176	11420477	1302	*	chr3	11419176	11420477	in_vitro	in_vivo	10	10	2.771	1.573	1.198	c(t = 1.24171	0.23	0.83731181	Distal Intergenic	ENSSSCG000	LAT2	linker for activation of T cells family member 2 [Source:VGNC Symbol;Acc:VGNC:89647]
chr14	32090912	32091778	867	*	chr14	32090912	32091778	in_vitro	in_vivo	11	9	2.13727273	0.58333333	1.55393939	c(t = 1.26480	0.23	0.83731181	Promoter (<=1kb)	ENSSSCG000	PPP1CC	protein phosphatase 1 catalytic subunit gamma [Source:NCBI gene (formerly Entrezgene);Acc:733612]
chr3	59000000	59001231	1232	*	chr3	59000000	59001231	in_vitro	in_vivo	9	11	1.18111111	0.50454545	0.67656566	c(t = 1.24855	0.23	0.83731181	Distal Intergenic	ENSSSCG000	GNLY	granulysin [Source:NCBI gene (formerly Entrezgene);Acc:396869]
chr1	253123899	253124858	960	*	chr1	253123899	253124858	in_vitro	in_vivo	10	9	3.46	0.46555556	2.99444444	c(t = 1.28529	0.23	0.83731181	Promoter (<=1kb)	ENSSSCG000	HSDL2	hydroxysteroid dehydrogenase like 2 [Source:VGNC Symbol;Acc:VGNC:88992]
chr10	42576661	42577922	1262	*	chr10	42576661	42577922	in_vitro	in_vivo	11	12	1.87818182	0.43833333	1.43984848	c(t = 1.25843	0.23	0.83731181	Distal Intergenic	ENSSSCG000	TRDMT1	tRNA aspartic acid methyltransferase 1 [Source:VGNC Symbol;Acc:VGNC:96227]
chr2	16343220	16344155	936	*	chr2	16343220	16344155	in_vitro	in_vivo	9	10	1.04	0	1.04	c(t = 1.29868	0.23	0.83731181	Distal Intergenic	ENSSSCG000	MDK	midkine [Source:VGNC Symbol;Acc:VGNC:100167]
chr8	136611699	136612503	805	*	chr8	136611699	136612503	in_vitro	in_vivo	9	11	9.86666667	24.5154545	-14.648788	c(t = -1.2507	0.231	0.83731181	Distal Intergenic	ENSSSCG000	RASGEF1B	RasGEF domain family member 1B [Source:VGNC Symbol;Acc:VGNC:92109]
chr9	130569903	130570921	1019	*	chr9	130569903	130570921	in_vitro	in_vivo	11	12	2.92545455	5.66666667	-2.7412121	c(t = -1.2450	0.231	0.83731181	Promoter (<=1kb)	ENSSSCG000	FLVCR1	FLVCR heme transporter 1 [Source:NCBI gene (formerly Entrezgene);Acc:100217398]
chr12	56249480	56250543	1064	*	chr12	56249480	56250543	in_vitro	in_vivo	9	10	1.65	4.962	-3.312	c(t = -1.2715	0.231	0.83731181	Distal Intergenic	ENSSSCG000	ssc-mir-744	ssc-mir-744 [Source:miRBase;Acc:MI0014769]
chr18	11150813	11151886	1074	*	chr18	11150813	11151886	in_vitro	in_vivo	11	13	1.44636364	3.41846154	-1.9720979	c(t = -1.2330	0.231	0.83731181	Distal Intergenic	ENSSSCG000	ZC3HAV1	zinc finger CCCH-type containing, antiviral 1 [Source:VGNC Symbol;Acc:VGNC:95102]
chr6	41423142	41426619	3478	*	chr6	41423142	41426619	in_vitro	in_vivo	14	14	1.85857143	1.14857143	0.71	c(t = 1.22951	0.231	0.83731181	Distal Intergenic	ENSSSCG000	SLC7A9	solute carrier family 7 member 9 [Source:VGNC Symbol;Acc:VGNC:93177]
chr8	1508427	1511191	2765	*	chr8	1508427	1511191	in_vitro	in_vivo	10	13	96.973	94.1530769	2.81992308	c(t = 1.23667	0.232	0.83731181	Distal Intergenic	ENSSSCG000	RNF4	ring finger protein 4 [Source:VGNC Symbol;Acc:VGNC:92392]
chr14	140246448	140247638	1191	*	chr14	140246448	140247638	in_vitro	in_vivo	9	10	21.2122222	41.948	-20.735778	c(t = -1.2402	0.232	0.83731181	Distal Intergenic	ENSSSCG000	ssc-mir-202	ssc-mir-202 [Source:miRBase;Acc:MI0013157]
chr18	18146926	18148322	1397	*	chr18	18146926	18148322	in_vitro	in_vivo	16	15	37.265	23.298	13.967	c(t = 1.22226	0.232	0.83731181	Promoter (<=1kb)	ENSSSCG000	KLF14	KLF transcription factor 14 [Source:NCBI gene (formerly Entrezgene);Acc:100174956]
chr8	1553686	1554898	1213	*	chr8	1553686	1554898	in_vitro	in_vivo	11	12	24.1772727	14.5225	9.65477273	c(t = 1.23728	0.232	0.83731181	Distal Intergenic	ENSSSCG000	RNF4	ring finger protein 4 [Source:VGNC Symbol;Acc:VGNC:92392]
chr7	23403895	23404762	868	*	chr7	23403895	23404762	in_vitro	in_vivo	11	9	2.69727273	4.20555556	-1.5082828	c(t = -1.2430	0.232	0.83731181	Promoter (<=1kb)	ENSSSCG000	VARS2	valyl-tRNA synthetase 2, mitochondrial [Source:VGNC Symbol;Acc:VGNC:109436]
chr2	136333708	136335199	1492	*	chr2	136333708	136335199	in_vitro	in_vivo	13	15	5.88230769	3.49533333	2.38697436	c(t = 1.23247	0.232	0.83731181	Distal Intergenic	ENSSSCG00000014296		voltage dependent anion channel 1 [Source:VGNC Symbol;Acc:VGNC:100881]
chr13	83289485	83290065	581	*	chr13	83289485	83290065	in_vitro	in_vivo	10	13	0	1.60230769	-1.6023077	c(t = -1.2576	0.232	0.83731181	Distal Intergenic	ENSSSCG000	TRPC1	transient receptor potential cation channel subfamily C member 1 [Source:VGNC Symbol;Acc:VGNC:94460]
chr3	38892964	38894098	1135	*	chr3	38892964	38894098	in_vitro	in_vivo	12	13	2.63833333	1.34538462	1.29294872	c(t = 1.23601	0.232	0.83731181	Distal Intergenic	ENSSSCG000	CLUAP1	clusterin associated protein 1 [Source:VGNC Symbol;Acc:VGNC:86791]
chr8	82048045	82048588	544	*	chr8	82048045	82048588	in_vitro	in_vivo	10	9	6.17	0.92	5.25	c(t = 1.27708	0.232	0.83731181	Distal Intergenic	ENSSSCG000	SLC10A7	solute carrier family 10 member 7 [Source:VGNC Symbol;Acc:VGNC:92919]
chr13	34205766	34207751	1986	*	chr13	34205766	34207751	in_vitro	in_vivo	14	14	1.23214286	0.57642857	0.65571429	c(t = 1.22324	0.232	0.83731181	Distal Intergenic	ENSSSCG000	RPL29	ribosomal protein L29 [Source:VGNC Symbol;Acc:VGNC:104048]
chr6	168949550	168950486	937	*	chr6	168949550	168950486	in_vitro	in_vivo	11	14	1.94545455	0.48285714	1.4625974	c(t = 1.26356	0.232	0.83731181	Distal Intergenic	ENSSSCG000	GUCA2A	guanylate cyclase activator 2A [Source:NCBI gene (formerly Entrezgene);Acc:100301560]
chr16	22599835	22600214	380	*	chr16	22599835	22600214	in_vitro	in_vivo	9	10	4.14111111	0.455	3.68611111	c(t = 1.28689	0.232	0.83731181	Distal Intergenic	ENSSSCG000	SLC1A3	solute carrier family 1 member 3 [Source:VGNC Symbol;Acc:VGNC:92965]
chr9	8840117	8840675	559	*	chr9	8840117	8840675	in_vitro	in_vivo	10	13	2.323	0.43769231	1.88530769	c(t = 1.27007	0.232	0.83731181	Distal Intergenic	ENSSSCG000	KCNE3	potassium voltage-gated channel subfamily E regulatory subunit 3 [Source:VGNC Symbol;Acc:VGNC:89337]
chrX	107520714	107521381	668	*	chrX	107520714	107521381	in_vitro	in_vivo	11	10	4.75363636	10.956	-6.2023636	c(t = -1.2629	0.233	0.83731181	Distal Intergenic	ENSSSCG000	RBMX2	RNA binding motif protein X-linked 2 [Source:NCBI gene (formerly Entrezgene);Acc:100626622]
chr12	21677092	21677592	501	*	chr12	21677092	21677592	in_vitro	in_vivo	11	9	20.22	6.84333333	13.3766667	c(t = 1.25797	0.233	0.83731181	Promoter (<=1kb)	ENSSSCG000	KRT27	keratin 27 [Source:NCBI gene (formerly Entrezgene);Acc:100521040]
chr18	5887933	5888881	949	*	chr18	5887933	5888881	in_vitro	in_vivo	11	10	4.67181818	2.029	2.64281818	c(t = 1.24008	0.233	0.83731181	Distal Intergenic	NA	NA	NA
chr2	136685721	136686259	539	*	chr2	136685721	136686259	in_vitro	in_vivo	12	10	3.00916667	1.376	1.63316667	c(t = 1.23641	0.233	0.83731181	Promoter (<=1kb)	ENSSSCG000	CDKN2AIPNL	CDKN2A interacting protein N-terminal like [Source:NCBI gene (formerly Entrezgene);Acc:100513343]
chr9	71895398	71895954	557	*	chr9	71895398	71895954	in_vitro	in_vivo	11	11	0	0.73090909	-0.7309091	c(t = -1.2707	0.233	0.83731181	Promoter (<=1kb)	ENSSSCG000	AKAP9	A-kinase anchoring protein 9 [Source:VGNC Symbol;Acc:VGNC:85225]
chr5	87179611	87181018	1408	*	chr5	87179611	87181018	in_vitro	in_vivo	13	13	1.61615385	0.60538462	1.01076923	c(t = 1.23348	0.233	0.83731181	Distal Intergenic	ENSSSCG000	LTA4H	leukotriene A4 hydrolase [Source:NCBI gene (formerly Entrezgene);Acc:100154219]
chr7	109961201	109961605	405	*	chr7	109961201	109961605	in_vitro	in_vivo	9	11	1.65888889	0.38	1.27888889	c(t = 1.27748	0.233	0.83731181	Promoter (<=1kb)	ENSSSCG000	GALC	galactosylceramidase [Source:VGNC Symbol;Acc:VGNC:88324]
chr4	83315929	83316452	524	*	chr4	83315929	83316452	in_vitro	in_vivo	9	10	2.24666667	0.37	1.87666667	c(t = 1.27738	0.233	0.83731181	Distal Intergenic	ENSSSCG000	RCSD1	RCSD domain containing 1 [Source:VGNC Symbol;Acc:VGNC:92186]
chr1	44889595	44890393	799	*	chr1	44889595	44890393	in_vitro	in_vivo	10	12	0.688	0.08833333	0.59966667	c(t = 1.27296	0.233	0.83731181	Distal Intergenic	ENSSSCG000	ZUP1	zinc finger containing ubiquitin peptidase 1 [Source:VGNC Symbol;Acc:VGNC:95339]
chrX	43148244	43149066	823	*	chrX	43148244	43149066	in_vitro	in_vivo	9	14	12.91	6.71857143	6.19142857	c(t = 1.24826	0.234	0.83731181	Promoter (1-2kb)	NA	NA	NA
chr10	16740096	16741797	1702	*	chr10	16740096	16741797	in_vitro	in_vivo	11	12	0.72181818	3.59166667	-2.8698485	c(t = -1.2526	0.234	0.83731181	Distal Intergenic	ENSSSCG000	AKT3	AKT serine/threonine kinase 3 [Source:VGNC Symbol;Acc:VGNC:96306]
chr15	137566785	137568095	1311	*	chr15	137566785	137568095	in_vitro	in_vivo	11	14	7.26909091	3.22214286	4.04694805	c(t = 1.23767	0.234	0.83731181	Distal Intergenic	ENSSSCG000	RAMP1	receptor activity modifying protein 1 [Source:NCBI gene (formerly Entrezgene);Acc:397401]
chr7	94218817	94219859	1043	*	chr7	94218817	94219859	in_vitro	in_vivo	10	11	0.811	2.86	-2.049	c(t = -1.2522	0.234	0.83731181	Distal Intergenic	ENSSSCG000	SYNJ2BP	synaptojanin 2 binding protein [Source:NCBI gene (formerly Entrezgene);Acc:100514718]
chr4	3061009	3061827	819	*	chr4	3061009	3061827	in_vitro	in_vivo	10	10	0.213	1.569	-1.356	c(t = -1.2699	0.234	0.83731181	Distal Intergenic	ENSSSCG000	ago-02	argonaute RISC catalytic component 2 [Source:NCBI gene (formerly Entrezgene);Acc:100155245]
chr14	92279283	92279892	610	*	chr14	92279283	92279892	in_vitro	in_vivo	10	11	2.857	1.02	1.837	c(t = 1.24796	0.234	0.83731181	Promoter (<=1kb)	ENSSSCG000	TFAM	transcription factor A, mitochondrial [Source:NCBI gene (formerly Entrezgene);Acc:397279]
chr4	34429189	34431968	2780	*	chr4	34429189	34431968	in_vitro	in_vivo	13	14	1.62	0.84928571	0.77071429	c(t = 1.23217	0.234	0.83731181	Promoter (<=1kb)	ENSSSCG000	KLF10	KLF transcription factor 10 [Source:NCBI gene (formerly Entrezgene);Acc:100153665]
chr13	195326182	195327233	1052	*	chr13	195326182	195327233	in_vitro	in_vivo	9	12	4.59333333	0.82583333	3.7675	c(t = 1.28174	0.234	0.83731181	Promoter (<=1kb)	ENSSSCG000	SOD1	superoxide dismutase 1 [Source:NCBI gene (formerly Entrezgene);Acc:397036]
chr2	46023941	46025544	1604	*	chr2	46023941	46025544	in_vitro	in_vivo	11	12	2.19272727	0.79916667	1.39356061	c(t = 1.24159	0.234	0.83731181	Distal Intergenic	ENSSSCG000	BMAL1	aryl hydrocarbon receptor nuclear translocator like [Source:VGNC Symbol;Acc:VGNC:96796]
chr13	117370272	117371363	1092	*	chr13	117370272	117371363	in_vitro	in_vivo	12	14	2.8025	0.68142857	2.12107143	c(t = 1.24558	0.234	0.83731181	Promoter (<=1kb)	ENSSSCG000	ZNF639	zinc finger protein 639 [Source:VGNC Symbol;Acc:VGNC:95282]
chr2	103568493	103569461	969	*	chr2	103568493	103569461	in_vitro	in_vivo	14	10	1.60785714	0.633	0.97485714	c(t = 1.22450	0.234	0.83731181	Promoter (<=1kb)	ENSSSCG000	LNPEP	leucyl and cystinyl aminopeptidase [Source:NCBI gene (formerly Entrezgene);Acc:100125826]
chr13	20934299	20935428	1130	*	chr13	20934299	20935428	in_vitro	in_vivo	14	10	1.55071429	0.615	0.93571429	c(t = 1.22982	0.234	0.83731181	Distal Intergenic	ENSSSCG000	ssc-mir-128-	ssc-mir-128-2 [Source:miRBase;Acc:MI0013094]
chr16	79652681	79653184	504	*	chr16	79652681	79653184	in_vitro	in_vivo	10	10	2.313	5.631	-3.318	c(t = -1.2502	0.235	0.83731181	Distal Intergenic	ENSSSCG000	TPPP	tubulin polymerization promoting protein [Source:VGNC Symbol;Acc:VGNC:94344]
chr3	18505886	18507873	1988	*	chr3	18505886	18507873	in_vitro	in_vivo	10	14	11.17	4.76357143	6.40642857	c(t = 1.25594	0.235	0.83731181	Distal Intergenic	ENSSSCG000	ATP2A1	ATPase sarcoplasmic/endoplasmic reticulum Ca2+ transporting 1 [Source:VGNC Symbol;Acc:VGNC:85646]
chr13	198250754	198252497	1744	*	chr13	198250754	198252497	in_vitro	in_vivo	14	13	1.435	3.5	-2.065	c(t = -1.2374	0.235	0.83731181	Distal Intergenic	ENSSSCG000	RCAN1	regulator of calcineurin 1 [Source:VGNC Symbol;Acc:VGNC:92170]
chr3	53941234	53942431	1198	*	chr3	53941234	53942431	in_vitro	in_vivo	9	15	0.69888889	1.55066667	-0.8517778	c(t = -1.2265	0.235	0.83731181	Distal Intergenic	ENSSSCG000	IL1R2	interleukin 1 receptor type 2 [Source:VGNC Symbol;Acc:VGNC:89093]
chr6	62745816	62746568	753	*	chr6	62745816	62746568	in_vitro	in_vivo	12	12	0.37666667	1.34454545	-0.9678788	c(t = -1.2489	0.235	0.83731181	Distal Intergenic	ENSSSCG000	ISG15	ISG15 ubiquitin like modifier [Source:NCBI gene (formerly Entrezgene);Acc:100145895]
chr2	60161828	60162696	869	*	chr2	60161828	60162696	in_vitro	in_vivo	13	10	5.02615385	1.225	3.80115385	c(t = 1.24729	0.235	0.83731181	Distal Intergenic	ENSSSCG000	SLC27A1	solute carrier family 27 member 1 [Source:VGNC Symbol;Acc:VGNC:93035]
chr6	170037125	170038071	947	*	chr6	170037125	170038071	in_vitro	in_vivo	11	11	92.45	96.7027273	-4.2527273	c(t = -1.2311	0.236	0.83731181	Distal Intergenic	ENSSSCG000	ssc-mir-9829	ssc-mir-9829 [Source:miRBase;Acc:MI0031598]
chr13	206843554	206847737	4184	*	chr13	206843554	206847737	in_vitro	in_vivo	9	13	8.01777778	18.28	-10.262222	c(t = -1.2215	0.236	0.83731181	Distal Intergenic	ENSSSCG000	AGPAT3	1-acylglycerol-3-phosphate O-acyltransferase 3 [Source:VGNC Symbol;Acc:VGNC:85185]
chr9	124696278	124697989	1712	*	chr9	124696278	124697989	in_vitro	in_vivo	11	10	1.03272727	3.028	-1.9952727	c(t = -1.2347	0.236	0.83731181	Distal Intergenic	ENSSSCG000	NCF2	neutrophil cytosolic factor 2 [Source:VGNC Symbol;Acc:VGNC:90607]
chr17	29421927	29425824	3898	*	chr17	29421927	29425824	in_vitro	in_vivo	13	16	3.43230769	2.075625	1.35668269	c(t = 1.21971	0.236	0.83731181	Distal Intergenic	ENSSSCG000	THBD	thrombomodulin [Source:NCBI gene (formerly Entrezgene);Acc:100157642]
chr5	3519278	3523072	3795	*	chr5	3519278	3523072	in_vitro	in_vivo	9	10	2.99555556	1.551	1.44455556	c(t = 1.26209	0.236	0.83731181	Distal Intergenic	ENSSSCG000	PPARA	peroxisome proliferator activated receptor alpha [Source:NCBI gene (formerly Entrezgene);Acc:397239]
chr8	87773476	87775718	2243	*	chr8	87773476	87775718	in_vitro	in_vivo	11	15	2.28636364	1.10733333	1.1790303	c(t = 1.23860	0.236	0.83731181	Distal Intergenic	ENSSSCG000	MGARP	mitochondria localized glutamic acid rich protein [Source:NCBI gene (formerly Entrezgene);Acc:100359351]
chr12	22236120	22238053	1934	*	chr12	22236120	22238053	in_vitro	in_vivo	11	11	2.09	1.04909091	1.04090909	c(t = 1.22198	0.236	0.83731181	Distal Intergenic	ENSSSCG000	THRA	thyroid hormone receptor alpha [Source:VGNC Symbol;Acc:VGNC:93963]
chr1	140566769	140568093	1325	*	chr1	140566769	140568093	in_vitro	in_vivo	12	14	1.8575	0.94214286	0.91535714	c(t = 1.23331	0.236	0.83731181	Distal Intergenic	ENSSSCG000	SELENOS	selenoprotein S [Source:NCBI gene (formerly Entrezgene);Acc:100151836]
chr18	24879054	24882419	3366	*	chr18	24879054	24882419	in_vitro	in_vivo	15	13	1.68133333	0.88153846	0.79979487	c(t = 1.21625	0.236	0.83731181	Distal Intergenic	ENSSSCG000	AASS	aminoadipate-semialdehyde synthase [Source:VGNC Symbol;Acc:VGNC:84944]
chr2	2386103	2387655	1553	*	chr2	2386103	2387655	in_vitro	in_vivo	9	9	90.8444444	86.5588889	4.28555556	c(t = 1.24094	0.237	0.83731181	Distal Intergenic	ENSSSCG000	NAP1L4	nucleosome assembly protein 1 like 4 [Source:VGNC Symbol;Acc:VGNC:90577]
chr3	125825047	125825821	775	*	chr3	125825047	125825821	in_vitro	in_vivo	12	11	3.76416667	1.70909091	2.05507576	c(t = 1.22024	0.237	0.83731181	Distal Intergenic	ENSSSCG000	PDIA6	protein disulfide isomerase family A member 6 [Source:NCBI gene (formerly Entrezgene);Acc:100499564]
chr2	57310618	57311496	879	*	chr2	57310618	57311496	in_vitro	in_vivo	13	11	2.85538462	1.57545455	1.27993007	c(t = 1.21813	0.237	0.83731181	Distal Intergenic	ENSSSCG000	RACK1	receptor for activated C kinase 1 [Source:NCBI gene (formerly Entrezgene);Acc:397605]
chr11	42418898	42419304	407	*	chr11	42418898	42419304	in_vitro	in_vivo	11	9	3.40909091	1.11111111	2.2979798	c(t = 1.22364	0.237	0.83731181	Distal Intergenic	ENSSSCG000	KLF5	KLF transcription factor 5 [Source:NCBI gene (formerly Entrezgene);Acc:100038005]
chr1	8685589	8686577	989	*	chr1	8685589	8686577	in_vitro	in_vivo	10	9	85.404	93.5333333	-8.1293333	c(t = -1.2467	0.238	0.83731181	Distal Intergenic	ENSSSCG000	DYNLT1	dynein light chain Tctex-type 1 [Source:NCBI gene (formerly Entrezgene);Acc:100158053]
chr1	859663	860994	1332	*	chr1	859663	860994	in_vitro	in_vivo	9	11	27.4888889	11.4609091	16.0279798	c(t = 1.25021	0.238	0.83731181	Distal Intergenic	ENSSSCG000	ssc-mir-9815	ssc-mir-9815 [Source:miRBase;Acc:MI0031582]
chr14	139115060	139116670	1611	*	chr14	139115060	139116670	in_vitro	in_vivo	10	12	1.98	3.7975	-1.8175	c(t = -1.2217	0.238	0.83731181	Distal Intergenic	ENSSSCG000	GLRX3	glutaredoxin 3 [Source:VGNC Symbol;Acc:VGNC:88495]
chr15	3178768	3179485	718	*	chr15	3178768	3179485	in_vitro	in_vivo	9	13	1.02888889	2.5	-1.4711111	c(t = -1.2260	0.238	0.83731181	Distal Intergenic	ENSSSCG000	ACVR2A	activin A receptor type 2A [Source:NCBI gene (formerly Entrezgene);Acc:397282]
chr18	45485129	45485713	585	*	chr18	45485129	45485713	in_vitro	in_vivo	9	12	4.66888889	2.08416667	2.58472222	c(t = 1.25413	0.238	0.83731181	Distal Intergenic	ENSSSCG000	HOXA5	homeobox A5 [Source:NCBI gene (formerly Entrezgene);Acc:100499568]
chr6	48461008	48461382	375	*	chr6	48461008	48461382	in_vitro	in_vivo	9	9	0	2.03222222	-2.0322222	c(t = -1.2745	0.238	0.83731181	Distal Intergenic	ENSSSCG000	AKT2	AKT serine/threonine kinase 2 [Source:VGNC Symbol;Acc:VGNC:85232]
chr13	87744854	87746832	1979	*	chr13	87744854	87746832	in_vitro	in_vivo	14	11	2.065	1.03454545	1.03045455	c(t = 1.21561	0.238	0.83731181	Distal Intergenic	ENSSSCG000	PLSCR1	phospholipid scramblase 1 [Source:NCBI gene (formerly Entrezgene);Acc:100620055]
chr3	125351698	125353660	1963	*	chr3	125351698	125353660	in_vitro	in_vivo	13	15	2.78923077	0.958	1.83123077	c(t = 1.23298	0.238	0.83731181	Distal Intergenic	ENSSSCG000	LPIN1	lipin 1 [Source:VGNC Symbol;Acc:VGNC:89793]
chr1	79791072	79792885	1814	*	chr1	79791072	79792885	in_vitro	in_vivo	13	11	3.34923077	0.94363636	2.40559441	c(t = 1.23221	0.238	0.83731181	Distal Intergenic	ENSSSCG000	COL10A1	collagen type X alpha 1 chain [Source:NCBI gene (formerly Entrezgene);Acc:448809]
chr4	61627920	61629427	1508	*	chr4	61627920	61629427	in_vitro	in_vivo	13	13	1.87692308	0.66692308	1.21	c(t = 1.23254	0.238	0.83731181	Distal Intergenic	ENSSSCG000	LY96	lymphocyte antigen 96 [Source:VGNC Symbol;Acc:VGNC:89907]
chr3	16671477	16672520	1044	*	chr3	16671477	16672520	in_vitro	in_vivo	9	10	1.81555556	0.607	1.20855556	c(t = 1.24461	0.238	0.83731181	Promoter (<=1kb)	ENSSSCG000	GUSB	glucuronidase beta [Source:NCBI gene (formerly Entrezgene);Acc:100144519]
chr18	40441060	40441729	670	*	chr18	40441060	40441729	in_vitro	in_vivo	9	9	3.73111111	0.50555556	3.22555556	c(t = 1.26868	0.238	0.83731181	Distal Intergenic	ENSSSCG000	FKBP9	FKBP prolyl isomerase 9 [Source:NCBI gene (formerly Entrezgene);Acc:100623924]
chrX	122376672	122377910	1239	*	chrX	122376672	122377910	in_vitro	in_vivo	9	9	72.6711111	86.0722222	-13.401111	c(t = -1.2277	0.239	0.83731181	Distal Intergenic	ENSSSCG000	FATE1	fetal and adult testis expressed 1 [Source:NCBI gene (formerly Entrezgene);Acc:396705]
chr1	1001116	1005693	4578	*	chr1	1001116	1005693	in_vitro	in_vivo	11	13	32.8809091	49.1146154	-16.233706	c(t = -1.2118	0.239	0.83731181	Distal Intergenic	ENSSSCG000	ssc-mir-9815	ssc-mir-9815 [Source:miRBase;Acc:MI0031582]
chr6	64145415	64146917	1503	*	chr6	64145415	64146917	in_vitro	in_vivo	9	9	28.5066667	46.1777778	-17.671111	c(t = -1.2256	0.239	0.83731181	Intron (NM_001204374/100533200, intron 10 of 17)	ENSSSCG000	PRKCZ	protein kinase C zeta [Source:NCBI gene (formerly Entrezgene);Acc:100533200]
chr13	208236198	208259432	23235	*	chr13	208236198	208259432	in_vitro	in_vivo	14	16	18.7307143	29.408125	-10.677411	c(t = -1.2030	0.239	0.83731181	Distal Intergenic	ENSSSCG000	ITGB2	integrin subunit beta 2 [Source:NCBI gene (formerly Entrezgene);Acc:396943]
chrX	98219382	98220365	984	*	chrX	98219382	98220365	in_vitro	in_vivo	9	11	4.15777778	10.0945455	-5.9367677	c(t = -1.2341	0.239	0.83731181	Distal Intergenic	ENSSSCG000	NKAP	NFKB activating protein [Source:VGNC Symbol;Acc:VGNC:90756]
chr12	53334923	53337620	2698	*	chr12	53334923	53337620	in_vitro	in_vivo	12	14	12.2816667	6.20857143	6.07309524	c(t = 1.23270	0.239	0.83731181	Distal Intergenic	ENSSSCG000	AURKB	aurora kinase B [Source:VGNC Symbol;Acc:VGNC:85698]
chrX	57520941	57522253	1313	*	chrX	57520941	57522253	in_vitro	in_vivo	9	11	2.01666667	4.20818182	-2.1915152	c(t = -1.2248	0.239	0.83731181	Promoter (<=1kb)	ENSSSCG000	OGT	O-linked N-acetylglucosamine (GlcNAc) transferase [Source:NCBI gene (formerly Entrezgene);Acc:664652]
chr14	35612661	35615512	2852	*	chr14	35612661	35615512	in_vitro	in_vivo	11	14	1.50454545	2.74428571	-1.2397403	c(t = -1.2083	0.239	0.83731181	Distal Intergenic	ENSSSCG000	FBXO21	F-box protein 21 [Source:VGNC Symbol;Acc:VGNC:88035]
chr5	104393596	104394739	1144	*	chr5	104393596	104394739	in_vitro	in_vivo	10	14	1.305	2.19857143	-0.8935714	c(t = -1.2121	0.239	0.83731181	Distal Intergenic	ENSSSCG000	ssc-mir-7139	ssc-mir-7139 [Source:miRBase;Acc:MI0023572]
chr15	44491989	44492797	809	*	chr15	44491989	44492797	in_vitro	in_vivo	10	9	3.004	1.52111111	1.48288889	c(t = 1.22288	0.239	0.83731181	Distal Intergenic	ENSSSCG000	CLDN22	claudin 22 [Source:NCBI gene (formerly Entrezgene);Acc:100294683]
chr17	50549764	50551067	1304	*	chr17	50549764	50551067	in_vitro	in_vivo	11	16	2.23909091	1.31625	0.92284091	c(t = 1.21538	0.239	0.83731181	Distal Intergenic	ENSSSCG000	CSE1L	chromosome segregation 1 like [Source:VGNC Symbol;Acc:VGNC:96039]
chr7	81668889	81669955	1067	*	chr7	81668889	81669955	in_vitro	in_vivo	14	14	3.91142857	1.01428571	2.89714286	c(t = 1.22871	0.239	0.83731181	Distal Intergenic	ENSSSCG000	EMC7	ER membrane protein complex subunit 7 [Source:NCBI gene (formerly Entrezgene);Acc:100626410]
chr18	14128278	14128750	473	*	chr18	14128278	14128750	in_vitro	in_vivo	10	10	3.894	0.755	3.139	c(t = 1.25120	0.239	0.83731181	Distal Intergenic	ENSSSCG000	STRA8	stimulated by retinoic acid 8 [Source:VGNC Symbol;Acc:VGNC:93574]
chr1	267964175	267965046	872	*	chr1	267964175	267965046	in_vitro	in_vivo	10	9	2.122	0.50555556	1.61644444	c(t = 1.23966	0.239	0.83731181	Distal Intergenic	ENSSSCG000	PTRH1	peptidyl-tRNA hydrolase 1 homolog [Source:NCBI gene (formerly Entrezgene);Acc:100157969]
chr14	104550563	104553460	2898	*	chr14	104550563	104553460	in_vitro	in_vivo	12	14	72.675	52.1157143	20.5592857	c(t = 1.20468	0.24	0.83731181	Distal Intergenic	ENSSSCG000	CYP26A1	cytochrome P450 family 26 subfamily A member 1 [Source:VGNC Symbol;Acc:VGNC:103370]
chr9	130958570	130959424	855	*	chr9	130958570	130959424	in_vitro	in_vivo	11	11	2.18363636	4.40636364	-2.2227273	c(t = -1.2193	0.24	0.83731181	Distal Intergenic	ENSSSCG000	PPP2R5A	protein phosphatase 2 regulatory subunit B'alpha [Source:NCBI gene (formerly Entrezgene);Acc:100101925]
chr15	25189558	25190048	491	*	chr15	25189558	25190048	in_vitro	in_vivo	10	10	0.688	2.248	-1.56	c(t = -1.2352	0.24	0.83731181	Distal Intergenic	ENSSSCG000	PROC	protein C, inactivator of coagulation factors Va and VIIIa [Source:VGNC Symbol;Acc:VGNC:109492]
chr8	115859547	115860986	1440	*	chr8	115859547	115860986	in_vitro	in_vivo	10	11	0.77	2.18454545	-1.4145455	c(t = -1.2339	0.24	0.83731181	Distal Intergenic	ENSSSCG000	AIMP1	aminoacyl tRNA synthetase complex interacting multifunctional protein 1 [Source:VGNC Symbol;Acc:VGNC:85202]
chr16	34113029	34113824	796	*	chr16	34113029	34113824	in_vitro	in_vivo	9	9	5.09333333	1.97666667	3.11666667	c(t = 1.25059	0.24	0.83731181	Distal Intergenic	ENSSSCG000	GZMK	granzyme K [Source:NCBI gene (formerly Entrezgene);Acc:100233185]
chr13	139444945	139445449	505	*	chr13	139444945	139445449	in_vitro	in_vivo	11	10	0.27545455	1.905	-1.6295455	c(t = -1.2512	0.24	0.83731181	Distal Intergenic	ENSSSCG000	GSK3B	glycogen synthase kinase 3 beta [Source:VGNC Symbol;Acc:VGNC:88723]
chr1	51428007	51428951	945	*	chr1	51428007	51428951	in_vitro	in_vivo	12	14	2.48166667	1.39214286	1.08952381	c(t = 1.22332	0.24	0.83731181	Distal Intergenic	ENSSSCG000	ssc-mir-30a	ssc-mir-30a [Source:miRBase;Acc:MI0010688]
chr3	48021702	48022538	837	*	chr3	48021702	48022538	in_vitro	in_vivo	11	11	2.48181818	1.22	1.26181818	c(t = 1.22465	0.24	0.83731181	Distal Intergenic	ENSSSCG00000008137		LIM zinc finger domain containing 1 [Source:VGNC Symbol;Acc:VGNC:110285]
chr12	41725597	41727813	2217	*	chr12	41725597	41727813	in_vitro	in_vivo	13	16	2.93923077	1.171875	1.76735577	c(t = 1.23022	0.24	0.83731181	Distal Intergenic	ENSSSCG000	SPACA3	sperm acrosome associated 3 [Source:NCBI gene (formerly Entrezgene);Acc:100415932]
chr7	3192430	3193675	1246	*	chr7	3192430	3193675	in_vitro	in_vivo	9	11	2.16777778	0.94454545	1.22323232	c(t = 1.22059	0.24	0.83731181	Distal Intergenic	ENSSSCG000	NRN1	neuritin 1 [Source:VGNC Symbol;Acc:VGNC:90900]
chr9	6282814	6283651	838	*	chr9	6282814	6283651	in_vitro	in_vivo	9	9	2.47	0.84888889	1.62111111	c(t = 1.23326	0.24	0.83731181	Distal Intergenic	ENSSSCG00000039656		ras homolog family member G [Source:VGNC Symbol;Acc:VGNC:92289]
chr10	29117644	29118971	1328	*	chr10	29117644	29118971	in_vitro	in_vivo	9	12	1.99888889	0.8325	1.16638889	c(t = 1.24587	0.24	0.83731181	Distal Intergenic	ENSSSCG000	FBP2	fructose-bisphosphatase 2 [Source:NCBI gene (formerly Entrezgene);Acc:100134828]
chr6	20371484	20372553	1070	*	chr6	20371484	20372553	in_vitro	in_vivo	12	11	1.64583333	0.29272727	1.35310606	c(t = 1.23776	0.24	0.83731181	Distal Intergenic	ENSSSCG000	GOT2	glutamic-oxaloacetic transaminase 2 [Source:NCBI gene (formerly Entrezgene);Acc:396968]
chr6	119885030	119886301	1272	*	chr6	119885030	119886301	in_vitro	in_vivo	9	14	0.98777778	0.26285714	0.72492063	c(t = 1.24578	0.24	0.83731181	Distal Intergenic	NA	NA	NA
chr1	73162197	73162659	463	*	chr1	73162197	73162659	in_vitro	in_vivo	9	9	2.38111111	0.20222222	2.17888889	c(t = 1.26792	0.24	0.83731181	Distal Intergenic	ENSSSCG000	PDSS2	decaprenyl diphosphate synthase subunit 2 [Source:VGNC Symbol;Acc:VGNC:91291]
chr11	8803477	8804155	679	*	chr11	8803477	8804155	in_vitro	in_vivo	11	13	31.6818182	14.7061538	16.9756643	c(t = 1.22124	0.241	0.83731181	Distal Intergenic	ENSSSCG000	HSPH1	heat shock protein family H (Hsp110) member 1 [Source:NCBI gene (formerly Entrezgene);Acc:100048931]
chr14	113240435	113241954	1520	*	chr14	113240435	113241954	in_vitro	in_vivo	10	13	2.289	4.59076923	-2.3017692	c(t = -1.2102	0.241	0.83731181	Distal Intergenic	ENSSSCG000	ELOVL3	ELOVL fatty acid elongase 3 [Source:VGNC Symbol;Acc:VGNC:87659]
chr2	7141062	7141836	775	*	chr2	7141062	7141836	in_vitro	in_vivo	10	12	9.933	3.70833333	6.22466667	c(t = 1.24626	0.241	0.83731181	Distal Intergenic	ENSSSCG000	MRPL49	mitochondrial ribosomal protein L49 [Source:VGNC Symbol;Acc:VGNC:90373]
chr14	103095221	103096176	956	*	chr14	103095221	103096176	in_vitro	in_vivo	10	11	1.48	2.81181818	-1.3318182	c(t = -1.2180	0.241	0.83731181	Distal Intergenic	ENSSSCG000	FGFBP3	fibroblast growth factor binding protein 3 [Source:VGNC Symbol;Acc:VGNC:88118]
chr6	166907657	166910521	2865	*	chr6	166907657	166910521	in_vitro	in_vivo	13	13	1.28538462	2.12076923	-0.8353846	c(t = -1.2042	0.241	0.83731181	Distal Intergenic	ENSSSCG000	DMAP1	DNA methyltransferase 1 associated protein 1 [Source:VGNC Symbol;Acc:VGNC:87344]
chr7	118059614	118060320	707	*	chr7	118059614	118060320	in_vitro	in_vivo	9	10	4.66444444	2.016	2.64844444	c(t = 1.23421	0.241	0.83731181	Distal Intergenic	ENSSSCG000	VRK1	VRK serine/threonine kinase 1 [Source:VGNC Symbol;Acc:VGNC:98408]
chr1	119134527	119135103	577	*	chr1	119134527	119135103	in_vitro	in_vivo	11	9	0.60363636	1.98555556	-1.3819192	c(t = -1.2429	0.241	0.83731181	Promoter (<=1kb)	ENSSSCG000	ARPP19	cAMP regulated phosphoprotein 19 [Source:NCBI gene (formerly Entrezgene);Acc:397362]
chr6	70164659	70165619	961	*	chr6	70164659	70165619	in_vitro	in_vivo	13	11	6.36307692	1.62181818	4.74125874	c(t = 1.22840	0.241	0.83731181	Distal Intergenic	ENSSSCG000	RBP7	retinol binding protein 7 [Source:NCBI gene (formerly Entrezgene);Acc:100270723]
chr16	68684437	68685509	1073	*	chr16	68684437	68685509	in_vitro	in_vivo	10	10	8.158	1.531	6.627	c(t = 1.25096	0.241	0.83731181	Distal Intergenic	ENSSSCG000	HAND1	heart and neural crest derivatives expressed 1 [Source:VGNC Symbol;Acc:VGNC:88777]
chr14	7539253	7540650	1398	*	chr14	7539253	7540650	in_vitro	in_vivo	11	13	2.40090909	1.24846154	1.15244755	c(t = 1.20707	0.241	0.83731181	Distal Intergenic	ENSSSCG000	PEBP4	phosphatidylethanolamine binding protein 4 [Source:VGNC Symbol;Acc:VGNC:91303]
chr15	39132727	39133879	1153	*	chr15	39132727	39133879	in_vitro	in_vivo	11	12	2.40727273	1.215	1.19227273	c(t = 1.20743	0.241	0.83731181	Distal Intergenic	ENSSSCG000	SPATA4	spermatosis associated 4 [Source:VGNC Symbol;Acc:VGNC:95846]
chr13	134081373	134082387	1015	*	chr13	134081373	134082387	in_vitro	in_vivo	11	11	2.58636364	1.18909091	1.39727273	c(t = 1.22746	0.241	0.83731181	Distal Intergenic	ENSSSCG000	TFRC	transferrin receptor [Source:NCBI gene (formerly Entrezgene);Acc:397062]
chr3	120096483	120097884	1402	*	chr3	120096483	120097884	in_vitro	in_vivo	10	12	4.023	1.09666667	2.92633333	c(t = 1.24053	0.241	0.83731181	Distal Intergenic	ENSSSCG000	KCNS3	potassium voltage-gated channel modifier subfamily S member 3 [Source:VGNC Symbol;Acc:VGNC:89387]
chr9	139135123	139136087	965	*	chr9	139135123	139136087	in_vitro	in_vivo	10	12	1.261	0.6275	0.6335	c(t = 1.21527	0.241	0.83731181	Distal Intergenic	ENSSSCG000	EGFR	epidermal growth factor receptor [Source:NCBI gene (formerly Entrezgene);Acc:397070]
chr4	39014316	39015331	1016	*	chr4	39014316	39015331	in_vitro	in_vivo	11	11	1.56272727	0.60272727	0.96	c(t = 1.22550	0.241	0.83731181	Distal Intergenic	ENSSSCG000	MTDH	metadherin [Source:VGNC Symbol;Acc:VGNC:90439]
chr7	2748128	2749282	1155	*	chr7	2748128	2749282	in_vitro	in_vivo	12	13	12.8425	29.9930769	-17.150577	c(t = -1.2075	0.242	0.83731181	Distal Intergenic	ENSSSCG000	ECI2	enoyl-CoA delta isomerase 2 [Source:VGNC Symbol;Acc:VGNC:87532]
chr14	13940064	13941293	1230	*	chr14	13940064	13941293	in_vitro	in_vivo	13	9	3.02076923	12.5377778	-9.5170085	c(t = -1.2598	0.242	0.83731181	Distal Intergenic	ENSSSCG00000021767		L-threonine dehydrogenase [Source:VGNC Symbol;Acc:VGNC:109162]
chr15	132354132	132358935	4804	*	chr15	132354132	132358935	in_vitro	in_vivo	11	12	7.02363636	2.975	4.04863636	c(t = 1.23347	0.242	0.83731181	Promoter (<=1kb)	ENSSSCG000	PTMA	prothymosin alpha [Source:VGNC Symbol;Acc:VGNC:106455]
chr8	131633916	131635050	1135	*	chr8	131633916	131635050	in_vitro	in_vivo	10	14	2.628	1.24714286	1.38085714	c(t = 1.22883	0.242	0.83731181	Distal Intergenic	ENSSSCG000	HSD17B13	hydroxysteroid 17-beta dehydrogenase 13 [Source:NCBI gene (formerly Entrezgene);Acc:100626886]
chr9	3075377	3076546	1170	*	chr9	3075377	3076546	in_vitro	in_vivo	10	12	2.023	0.75583333	1.26716667	c(t = 1.22576	0.242	0.83731181	Promoter (<=1kb)	ENSSSCG000	MRPL17	mitochondrial ribosomal protein L17 [Source:NCBI gene (formerly Entrezgene);Acc:100512482]

chr12	17241696	17242670	975	*	chr12	17241696	17242670	in_vitro	in_vivo	12	9	91.3691667	78.8066667	12.5625	c(t = 1.24165	0.243	0.83731181	Distal Intergenic	ENSSSCG000	CRHR1	corticotropin releasing hormone receptor 1 [Source:VGNC Symbol;Acc:VGNC:98983]
chr2	5577979	5580695	2717	*	chr2	5577979	5580695	in_vitro	in_vivo	11	12	47.1245455	30.9816667	16.1428788	c(t = 1.20055	0.243	0.83731181	Intron (NM_214349/397630, intron 9 of 19)	ENSSSCG000	PC	pyruvate carboxylase [Source:NCBI gene (formerly Entrezgene);Acc:397630]
chrX	111247183	111248501	1319	*	chrX	111247183	111248501	in_vitro	in_vivo	11	10	10.0836364	22.092	-12.008364	c(t = -1.2228	0.243	0.83731181	Distal Intergenic	ENSSSCG000	MMGT1	membrane magnesium transporter 1 [Source:NCBI gene (formerly Entrezgene);Acc:100520118]
chr12	51501426	51502992	1567	*	chr12	51501426	51502992	in_vitro	in_vivo	10	15	7.078	12.9673333	-5.8893333	c(t = -1.1993	0.243	0.83731181	Distal Intergenic	ENSSSCG000	ZNF232	zinc finger protein 232 [Source:VGNC Symbol;Acc:VGNC:95208]
chr13	71706180	71707384	1205	*	chr13	71706180	71707384	in_vitro	in_vivo	10	9	1.139	3.20111111	-2.0621111	c(t = -1.2242	0.243	0.83731181	Distal Intergenic	ENSSSCG000	GP9	glycoprotein IX platelet [Source:VGNC Symbol;Acc:VGNC:88567]
chr14	129510493	129511378	886	*	chr14	129510493	129511378	in_vitro	in_vivo	12	13	5.57916667	2.54153846	3.03762821	c(t = 1.20989	0.243	0.83731181	Distal Intergenic	ENSSSCG000	MCMBP	minichromosome maintenance complex binding protein [Source:VGNC Symbol;Acc:VGNC:90077]
chr13	66021047	66021983	937	*	chr13	66021047	66021983	in_vitro	in_vivo	13	11	5.97692308	2.26545455	3.71146853	c(t = 1.20509	0.243	0.83731181	Distal Intergenic	ENSSSCG000	CAMK1	calcium/calmodulin dependent protein kinase I [Source:VGNC Symbol;Acc:VGNC:86153]
chr1	242169220	242169827	608	*	chr1	242169220	242169827	in_vitro	in_vivo	9	10	4.03333333	2.126	1.90733333	c(t = 1.21074	0.243	0.83731181	Distal Intergenic	ENSSSCG000	ERP44	endoplasmic reticulum protein 44 [Source:NCBI gene (formerly Entrezgene);Acc:100192437]
chr3	58206755	58207622	868	*	chr3	58206755	58207622	in_vitro	in_vivo	10	10	3.479	1.853	1.626	c(t = 1.20870	0.243	0.83731181	Distal Intergenic	ENSSSCG000	CD8A	CD8 subunit alpha [Source:VGNC Symbol;Acc:VGNC:86436]
chr14	132683567	132686904	3338	*	chr14	132683567	132686904	in_vitro	in_vivo	14	14	3.53571429	1.42142857	2.11428571	c(t = 1.21372	0.243	0.83731181	Distal Intergenic	ENSSSCG000	ssc-mir-4331	ssc-mir-4331-2 [Source:miRBase;Acc:MI0033415]
chr1	36065071	36067575	2505	*	chr1	36065071	36067575	in_vitro	in_vivo	16	13	3.20125	1.08846154	2.11278846	c(t = 1.20998	0.243	0.83731181	Promoter (<=1kb)	ENSSSCG000	RSPO3	R-spondin 3 [Source:NCBI gene (formerly Entrezgene);Acc:100155208]
chr8	44449234	44449995	762	*	chr8	44449234	44449995	in_vitro	in_vivo	11	12	5.45363636	0.95666667	4.4969697	c(t = 1.23597	0.243	0.83731181	Promoter (<=1kb)	ENSSSCG000	GUCY1A1	guanylate cyclase 1 soluble subunit alpha 1 [Source:VGNC Symbol;Acc:VGNC:88750]
chr5	101490602	101491452	851	*	chr5	101490602	101491452	in_vitro	in_vivo	11	11	2.22727273	0.53454545	1.69272727	c(t = 1.22092	0.243	0.83731181	Distal Intergenic	ENSSSCG000	MYF6	myogenic factor 6 [Source:NCBI gene (formerly Entrezgene);Acc:397005]
chr5	38532277	38532844	568	*	chr5	38532277	38532844	in_vitro	in_vivo	12	10	1.58583333	0.495	1.09083333	c(t = 1.22158	0.243	0.83731181	Distal Intergenic	ENSSSCG000	NAP1L1	nucleosome assembly protein 1 like 1 [Source:VGNC Symbol;Acc:VGNC:90576]
chr3	58574855	58575743	889	*	chr3	58574855	58575743	in_vitro	in_vivo	10	11	1.671	0.31363636	1.35736364	c(t = 1.24219	0.243	0.83731181	Distal Intergenic	ENSSSCG000	PTCD3	pentatricopeptide repeat domain 3 [Source:VGNC Symbol;Acc:VGNC:91940]
chr14	65774520	65775439	920	*	chr14	65774520	65775439	in_vitro	in_vivo	9	11	0.96777778	0.23909091	0.72868687	c(t = 1.23381	0.243	0.83731181	Distal Intergenic	ENSSSCG000	ssc-mir-9836	ssc-mir-9836 [Source:miRBase;Acc:MI0031605]
chr10	65194847	65197405	2559	*	chr10	65194847	65197405	in_vitro	in_vivo	13	11	86.6961538	93.2072727	-6.5111189	c(t = -1.2003	0.244	0.83731181	3' UTR	ENSSSCG000	GDI2	GDP dissociation inhibitor 2 [Source:NCBI gene (formerly Entrezgene);Acc:414427]
chr5	2687562	2691348	3787	*	chr5	2687562	2691348	in_vitro	in_vivo	11	14	38.0636364	54.47	-16.406364	c(t = -1.1950	0.244	0.83731181	Distal Intergenic	ENSSSCG000	PPARA	peroxisome proliferator activated receptor alpha [Source:NCBI gene (formerly Entrezgene);Acc:397239]
chr1	259350947	259351845	899	*	chr1	259350947	259351845	in_vitro	in_vivo	11	11	48.1954545	33.6754545	14.52	c(t = 1.20256	0.244	0.83731181	Exon (NM_001315664/100625235, exon 7 of 7)	ENSSSCG000	BRINP1	BMP/retinoic acid inducible neural specific 1 [Source:VGNC Symbol;Acc:VGNC:85881]
chr14	127641787	127643488	1702	*	chr14	127641787	127643488	in_vitro	in_vivo	10	10	7.376	22.884	-15.508	c(t = -1.2317	0.244	0.83731181	Distal Intergenic	ENSSSCG000	SLC18A2	solute carrier family 18 member A2 [Source:NCBI gene (formerly Entrezgene);Acc:100154950]
chrX	43230208	43230523	316	*	chrX	43230208	43230523	in_vitro	in_vivo	13	9	1.81846154	9.12555556	-7.307094	c(t = -1.2508	0.244	0.83731181	Distal Intergenic	ENSSSCG000	PLP2	proteolipid protein 2 [Source:NCBI gene (formerly Entrezgene);Acc:100512511]
chr7	100540880	100541964	1085	*	chr7	100540880	100541964	in_vitro	in_vivo	12	12	2.5275	7.68333333	-5.1558333	c(t = -1.2253	0.244	0.83731181	Distal Intergenic	ENSSSCG000	GSTZ1	glutathione S-transferase zeta 1 [Source:VGNC Symbol;Acc:VGNC:88727]
chr6	99177342	99179159	1818	*	chr6	99177342	99179159	in_vitro	in_vivo	15	14	8.34866667	4.94785714	3.40080952	c(t = 1.19288	0.244	0.83731181	Distal Intergenic	ENSSSCG000	NDUFV2	NADH:ubiquinone oxidoreductase core subunit V2 [Source:NCBI gene (formerly Entrezgene);Acc:100037991]
chr6	52210875	52212498	1624	*	chr6	52210875	52212498	in_vitro	in_vivo	13	12	9.91461538	3.2475	6.66711538	c(t = 1.21983	0.244	0.83731181	Distal Intergenic	ENSSSCG000	ssc-mir-769	ssc-mir-769 [Source:miRBase;Acc:MI0013168]
chrX	103326090	103327810	1721	*	chrX	103326090	103327810	in_vitro	in_vivo	14	12	5.93285714	2.7825	3.15035714	c(t = 1.19975	0.244	0.83731181	Distal Intergenic	ENSSSCG000	ssc-mir-2483	ssc-mir-2483 [Source:miRBase;Acc:MI0022167]
chr1	72520150	72520489	340	*	chr1	72520150	72520489	in_vitro	in_vivo	9	10	0	2.5	-2.5	c(t = -1.2456	0.244	0.83731181	Promoter (<=1kb)	ENSSSCG000	ATG5	autophagy related 5 [Source:VGNC Symbol;Acc:VGNC:103029]
chr13	195395272	195396709	1438	*	chr13	195395272	195396709	in_vitro	in_vivo	13	13	3.39076923	1.31538462	2.07538462	c(t = 1.20855	0.244	0.83731181	Distal Intergenic	ENSSSCG000	SOD1	superoxide dismutase 1 [Source:NCBI gene (formerly Entrezgene);Acc:397036]
chr6	55456421	55458300	1880	*	chr6	55456421	55458300	in_vitro	in_vivo	9	12	5.47555556	1.28666667	4.18888889	c(t = 1.25192	0.244	0.83731181	Distal Intergenic	ENSSSCG000	ASPDH	aspartate dehydrogenase domain containing [Source:NCBI gene (formerly Entrezgene);Acc:100511638]
chr7	56581109	56582415	1307	*	chr7	56581109	56582415	in_vitro	in_vivo	11	10	0	0.834	-0.834	c(t = -1.2459	0.244	0.83731181	Distal Intergenic	ENSSSCG000	ETFA	electron transfer flavoprotein subunit alpha [Source:VGNC Symbol;Acc:VGNC:87801]
chr6	83590689	83591265	577	*	chr6	83590689	83591265	in_vitro	in_vivo	9	10	3.64555556	0.744	2.90155556	c(t = 1.25045	0.244	0.83731181	Distal Intergenic	ENSSSCG000	FAM110D	family with sequence similarity 110 member D [Source:VGNC Symbol;Acc:VGNC:87887]
chr16	4784256	4785188	933	*	chr16	4784256	4785188	in_vitro	in_vivo	12	14	2.81	0.51357143	2.29642857	c(t = 1.22776	0.244	0.83731181	Distal Intergenic	ENSSSCG000	C1QTNF3	C1q and TNF related 3 [Source:NCBI gene (formerly Entrezgene);Acc:100270814]
chr14	66919634	66920757	1124	*	chr14	66919634	66920757	in_vitro	in_vivo	12	11	1.72333333	0.46454545	1.25878788	c(t = 1.21831	0.244	0.83731181	Distal Intergenic	ENSSSCG000	ssc-mir-1296	ssc-mir-1296 [Source:miRBase;Acc:MI0015931]
chr5	100755180	100755881	702	*	chr5	100755180	100755881	in_vitro	in_vivo	10	11	81.168	65.1072727	16.0607273	c(t = 1.20509	0.245	0.83731181	Promoter (<=1kb)	ENSSSCG000	MYF5	myogenic factor 5 [Source:NCBI gene (formerly Entrezgene);Acc:100153269]
chr11	5301716	5302568	853	*	chr11	5301716	5302568	in_vitro	in_vivo	9	12	3.94333333	8.34416667	-4.4008333	c(t = -1.2047	0.245	0.83731181	Promoter (1-2kb)	ENSSSCG000	PDX1	pancreatic and duodenal homeobox 1 [Source:NCBI gene (formerly Entrezgene);Acc:397288]
chr1	255093812	255095359	1548	*	chr1	255093812	255095359	in_vitro	in_vivo	13	12	3.97923077	7.38833333	-3.4091026	c(t = -1.2013	0.245	0.83731181	Distal Intergenic	ENSSSCG000	ATP6V1G1	ATPase H+ transporting V1 subunit G1 [Source:NCBI gene (formerly Entrezgene);Acc:100154379]
chr1	92286637	92287355	719	*	chr1	92286637	92287355	in_vitro	in_vivo	10	12	1.039	4.41666667	-3.3776667	c(t = -1.2204	0.245	0.83731181	Distal Intergenic	ENSSSCG00000004489		eukaryotic translation elongation factor 1 alpha 1 [Source:NCBI gene (formerly Entrezgene);Acc:574059]
chr1	21801434	21801835	402	*	chr1	21801434	21801835	in_vitro	in_vivo	9	11	0.72	2.18181818	-1.4618182	c(t = -1.2141	0.245	0.83731181	Promoter (<=1kb)	ENSSSCG000	PEX3	peroxisomal biosis factor 3 [Source:VGNC Symbol;Acc:VGNC:91327]
chr12	11856832	11857872	1041	*	chr12	11856832	11857872	in_vitro	in_vivo	12	13	2.11166667	0.73846154	1.37320513	c(t = 1.21345	0.245	0.83731181	Distal Intergenic	ENSSSCG000	AMZ2	archaelysin family metallopeptidase 2 [Source:VGNC Symbol;Acc:VGNC:85292]
chr14	31387655	31388058	404	*	chr14	31387655	31388058	in_vitro	in_vivo	9	9	1.76111111	0.30888889	1.45222222	c(t = 1.24236	0.245	0.83731181	Distal Intergenic	ENSSSCG000	ATP2A2	ATPase sarcoplasmic/endoplasmic reticulum Ca2+ transporting 2 [Source:VGNC Symbol;Acc:VGNC:85647]
chr6	27757181	27757954	774	*	chr6	27757181	27757954	in_vitro	in_vivo	11	9	1.72090909	0.18222222	1.53868687	c(t = 1.23204	0.245	0.83731181	Distal Intergenic	ENSSSCG000	ssc-mir-328	ssc-mir-328 [Source:miRBase;Acc:MI0013158]
chr14	40563033	40564257	1225	*	chr14	40563033	40564257	in_vitro	in_vivo	12	13	9.97916667	4.11153846	5.86762821	c(t = 1.21735	0.246	0.83731181	Distal Intergenic	ENSSSCG000	ACADS	acyl-CoA dehydrogenase short chain [Source:VGNC Symbol;Acc:VGNC:97862]
chr7	28919287	28920841	1555	*	chr7	28919287	28920841	in_vitro	in_vivo	12	13	4.61307692	2.345	2.26807692	c(t = 1.19727	0.246	0.83731181	Distal Intergenic	ENSSSCG000	PRIM2	DNA primase subunit 2 [Source:VGNC Symbol;Acc:VGNC:91796]
chr17	10863659	10864925	1267	*	chr17	10863659	10864925	in_vitro	in_vivo	11	11	2.14909091	1.30181818	0.84727273	c(t = 1.19418	0.246	0.83731181	Distal Intergenic	NA	NA	NA
chr13	208059987	208062438	2452	*	chr13	208059987	208062438	in_vitro	in_vivo	11	12	7.79454545	1.26583333	6.52871212	c(t = 1.23039	0.246	0.83731181	Distal Intergenic	ENSSSCG000	ITGB2	integrin subunit beta 2 [Source:NCBI gene (formerly Entrezgene);Acc:396943]
chr10	40052626	40054149	1524	*	chr10	40052626	40054149	in_vitro	in_vivo	9	15	2.52111111	0.98933333	1.53177778	c(t = 1.23912	0.246	0.83731181	Promoter (<=1kb)	ENSSSCG000	BAMBI	BMP and activin membrane bound inhibitor [Source:NCBI gene (formerly Entrezgene);Acc:449001]
chr1	109930936	109931762	827	*	chr1	109930936	109931762	in_vitro	in_vivo	12	10	2.1675	0.717	1.4505	c(t = 1.21012	0.246	0.83731181	Distal Intergenic	ENSSSCG000	ssc-mir-190a	ssc-mir-190a [Source:miRBase;Acc:MI0022136]
chr9	130968046	130969046	1001	*	chr9	130968046	130969046	in_vitro	in_vivo	12	13	1.79166667	0.40615385	1.38551282	c(t = 1.21664	0.246	0.83731181	Distal Intergenic	ENSSSCG000	PPP2R5A	protein phosphatase 2 regulatory subunit B'alpha [Source:NCBI gene (formerly Entrezgene);Acc:100101925]
chr18	48522821	48523661	841	*	chr18	48522821	48523661	in_vitro	in_vivo	11	14	2.19909091	0.29357143	1.90551948	c(t = 1.22993	0.246	0.83731181	Promoter (<=1kb)	ENSSSCG000	HUS1	HUS1 checkpoint clamp component [Source:NCBI gene (formerly Entrezgene);Acc:100192318]
chr8	964756	966264	1509	*	chr8	964756	966264	in_vitro	in_vivo	11	10	71.3645455	87.811	-16.446455	c(t = -1.1958	0.247	0.83731181	Distal Intergenic	ENSSSCG000	LETM1	leucine zipper and EF-hand containing transmembrane protein 1 [Source:VGNC Symbol;Acc:VGNC:89687]
chr7	444903	445788	886	*	chr7	444903	445788	in_vitro	in_vivo	11	12	19.3227273	39.9633333	-20.640606	c(t = -1.1898	0.247	0.83731181	Distal Intergenic	ENSSSCG000	IRF4	interferon regulatory factor 4 [Source:VGNC Symbol;Acc:VGNC:89207]
chr12	60749059	60765232	16174	*	chr12	60749059	60765232	in_vitro	in_vivo	10	12	54.684	39.3783333	15.3056667	c(t = 1.20114	0.247	0.83731181	Promoter (<=1kb)	ENSSSCG000	ssc-mir-33b	ssc-mir-33b [Source:miRBase;Acc:MI0036557]
chr6	48526169	48527648	1480	*	chr6	48526169	48527648	in_vitro	in_vivo	11	11	12.0981818	23.51	-11.411818	c(t = -1.2104	0.247	0.83731181	Distal Intergenic	ENSSSCG000	AKT2	AKT serine/threonine kinase 2 [Source:VGNC Symbol;Acc:VGNC:85232]
chrX	110959577	110960315	739	*	chrX	110959577	110960315	in_vitro	in_vivo	11	9	3.50181818	8.99888889	-5.4970707	c(t = -1.2325	0.247	0.83731181	Distal Intergenic	ENSSSCG000	MMGT1	membrane magnesium transporter 1 [Source:NCBI gene (formerly Entrezgene);Acc:100520118]
chr9	40629144	40631040	1897	*	chr9	40629144	40631040	in_vitro	in_vivo	11	13	3.86	1.61	2.25	c(t = 1.19302	0.247	0.83731181	Distal Intergenic	ENSSSCG000	DRD2	dopamine receptor D2 [Source:VGNC Symbol;Acc:VGNC:87444]
chr1	200760765	200761552	788	*	chr1	200760765	200761552	in_vitro	in_vivo	11	10	3.19090909	1.417	1.77390909	c(t = 1.20343	0.247	0.83731181	Promoter (<=1kb)	ENSSSCG000	CDKN2B	cyclin dependent kinase inhibitor 2B [Source:NCBI gene (formerly Entrezgene);Acc:397227]
chr6	100091472	100093717	2246	*	chr6	100091472	100093717	in_vitro	in_vivo	14	14	1.92785714	1.11357143	0.81428571	c(t = 1.18928	0.247	0.83731181	Distal Intergenic	ENSSSCG000	NDUFV2	NADH:ubiquinone oxidoreductase core subunit V2 [Source:NCBI gene (formerly Entrezgene);Acc:100037991]
chr6	52626918	52628086	1169	*	chr6	52626918	52628086	in_vitro	in_vivo	9	12	1.95333333	0.62166667	1.33166667	c(t = 1.22585	0.247	0.83731181	Distal Intergenic	ENSSSCG000	AP2S1	adaptor related protein complex 2 subunit sigma 1 [Source:VGNC Symbol;Acc:VGNC:96909]
chr9	68071252	68071632	381	*	chr9	68071252	68071632	in_vitro	in_vivo	9	9	2.77777778	0	2.77777778	c(t = 1.25)	0.247	0.83731181	Distal Intergenic	ENSSSCG00000015664		CD55 molecule (Cromer blood group) [Source:NCBI gene (formerly Entrezgene);Acc:396743]
chr5	24712974	24713877	904	*	chr5	24712974	24713877	in_vitro	in_vivo	9	9	1.43777778	0	1.43777778	c(t = 1.24995	0.247	0.83731181	Distal Intergenic	ENSSSCG000	CTDSP2	CTD small phosphatase 2 [Source:NCBI gene (formerly Entrezgene);Acc:100624883]
chr17	60596806	60597587	782	*	chr17	60596806	60597587	in_vitro	in_vivo	9	12	12.3677778	28.09	-15.722222	c(t = -1.1951	0.248	0.83731181	Distal Intergenic	ENSSSCG000	PHACTR3	phosphatase and actin regulator 3 [Source:VGNC Symbol;Acc:VGNC:95667]
chr13	31355582	31357684	2103	*	chr13	31355582	31357684	in_vitro	in_vivo	15	11	13.004	22.6254545	-9.6214545	c(t = -1.2008	0.248	0.83731181	Distal Intergenic	ENSSSCG000	SLC26A6	solute carrier family 26 member 6 [Source:VGNC Symbol;Acc:VGNC:93031]
chr15	62262903	62264956	2054	*	chr15	62262903	62264956	in_vitro	in_vivo	13	14	4.97153846	10.9785714	-6.007033	c(t = -1.1901	0.248	0.83731181	Distal Intergenic	ENSSSCG000	NR4A2	nuclear receptor subfamily 4 group A member 2 [Source:VGNC Symbol;Acc:VGNC:96451]
chr15	107882458	107883229	772	*	chr15	107882458	107883229	in_vitro	in_vivo	9	12	0.68111111	1.97083333	-1.2897222	c(t = -1.2014	0.248	0.83731181	Distal Intergenic	ENSSSCG000	ICOS	inducible T cell costimulator [Source:NCBI gene (formerly Entrezgene);Acc:733597]
chr3	1720735	1721682	948	*	chr3	1720735	1721682	in_vitro	in_vivo	10	12	2.21	0.95833333	1.25166667	c(t = 1.20626	0.248	0.83731181	Distal Intergenic	ENSSSCG000	GRIFIN	galectin-related inter-fiber protein [Source:VGNC Symbol;Acc:VGNC:88676]
chr7	10142005	10143097	1093	*	chr7	10142005	10143097	in_vitro	in_vivo	10	14	1.303	0.61071429	0.69228571	c(t = 1.20528	0.248	0.83731181	Distal Intergenic	ENSSSCG000	ssc-mir-9837	ssc-mir-9837 [Source:miRBase;Acc:MI0031606]
chr1	229952611	229953398	788	*	chr1	229952611	229953398	in_vitro	in_vivo	9	12	2.44444444	0	2.44444444	c(t = 1.24700	0.248	0.83731181	Distal Intergenic	ENSSSCG000	GNA14	G protein subunit alpha 14 [Source:VGNC Symbol;Acc:VGNC:88519]
chr12	4813514	4816711	3198	*	chr12	4813514	4816711	in_vitro	in_vivo	9	11	44.4444444	59.8972727	-15.452828	c(t = -1.2112	0.249	0.83731181	Distal Intergenic	ENSSSCG00000036592		serine and arginine rich splicing factor 2 [Source:VGNC Symbol;Acc:VGNC:99085]
chr12	48484503	48487884	3382	*	chr12	48484503	48487884	in_vitro	in_vivo	10	12	7.482	3.62333333	3.85866667	c(t = 1.21721	0.249	0.83731181	Distal Intergenic	ENSSSCG000	PAFAH1B1	platelet activating factor acetylhydrolase 1b regulatory subunit 1 [Source:VGNC Symbol;Acc:VGNC:99026]
chr4	59573720	59574389	670	*	chr4	59573720	59574389	in_vitro	in_vivo	10	10	0.881	2.935	-2.054	c(t = -1.2135	0.249	0.83731181	Distal Intergenic	ENSSSCG000	PEX2	peroxisomal biosis factor 2 [Source:VGNC Symbol;Acc:VGNC:98182]
chr13	32252853	32253713	861	*	chr13	32252853	32253713	in_vitro	in_vivo	10	10	0.755	1.85	-1.095	c(t = -1.2084	0.249	0.83731181	Promoter (<=1kb)	ENSSSCG000	GMPPB	GDP-mannose pyrophosphorylase B [Source:NCBI gene (formerly Entrezgene);Acc:100513376]
chr4	42059127	42062904	3778	*	chr4	42059127	42062904	in_vitro	in_vivo	14	15	3.01071429	1.49733333	1.51338095	c(t = 1.19144	0.249	0.83731181	Distal Intergenic	ENSSSCG000	CCNE2	cyclin E2 [Source:VGNC Symbol;Acc:VGNC:86355]
chr2	18993678	18994315	638	*	chr2	18993678	18994315	in_vitro	in_vivo	9	13	1.63	0.51076923	1.11923077	c(t = 1.21845	0.249	0.83731181	Distal Intergenic	ENSSSCG000	ssc-mir-670	ssc-mir-670 [Source:miRBase;Acc:MI0033398]
chr3	16375939	16376821	883	*	chr3	16375939	16376821	in_vitro	in_vivo	11	11	1.16363636	0.29181818	0.87181818	c(t = 1.21295	0.249	0.83731181	Distal Intergenic	ENSSSCG000	TPST1	tyrosylprotein sulfotransferase 1 [Source:VGNC Symbol;Acc:VGNC:94352]
chr7	28679637	28680513	877	*	chr7	28679637	28680513	in_vitro	in_vivo	13	15	1.91615385	0.224	1.69215385	c(t = 1.21055	0.249	0.83731181	Distal Intergenic	ENSSSCG000	PRIM2	DNA primase subunit 2 [Source:VGNC Symbol;Acc:VGNC:91796]
chr2	151508399	151511016	2618	*	chr2	151508399	151511016	in_vitro	in_vivo	9	10	94.6355556	91.477	3.15855556	c(t = 1.20002	0.25	0.83731181	Distal Intergenic	ENSSSCG000	CD74	CD74 molecule [Source:VGNC Symbol;Acc:VGNC:86428]
chr5	2874849	2880389	5541	*	chr5	2874849	2880389	in_vitro	in_vivo	9	10	92.2566667	83.909	8.34766667	c(t = 1.19834	0.25	0.83731181	Distal Intergenic	ENSSSCG000	PPARA	peroxisome proliferator activated receptor alpha [Source:NCBI gene (formerly Entrezgene);Acc:397239]
chr14	114227695	114229208	1514	*	chr14	114227695	114229208	in_vitro	in_vivo	13	14	1.90384615	3.21928571	-1.3154396	c(t = -1.1817	0.25	0.83731181	Distal Intergenic	ENSSSCG000	ssc-mir-1307	ssc-mir-1307 [Source:miRBase;Acc:MI0013147]
chr6	63387	64608	1222	*	chr6	63387	64608	in_vitro	in_vivo	10	9	5.199	2.78	2.419	c(t = 1.20155	0.25	0.83731181	Distal Intergenic	ENSSSCG000	MC1R	melanocortin 1 receptor [Source:NCBI gene (formerly Entrezgene);Acc:494018]
chr8	9180178	9180933	756	*	chr8	9180178	9180933	in_vitro	in_vivo	14	9	1.01857143	1.99222222	-0.9736508	c(t = -1.1900	0.25	0.83731181	Distal Intergenic	ENSSSCG000	RAB28	RAB28, member RAS onco family [Source:VGNC Symbol;Acc:VGNC:98955]
chr11	47758928	47760953	2026	*	chr11	47758928	47760953	in_vitro	in_vivo	14	14	0.755	1.48428571	-0.7292857	c(t = -1.1904	0.25	0.83731181	Distal Intergenic	NA	NA	NA
chr3	68496471	68497933	1463	*	chr3	68496471	68497933	in_vitro	in_vivo	10	14	2.352	1.18357143	1.16842857	c(t = 1.19383	0.25	0.83731181	Promoter (<=1kb)	ENSSSCG000	DOK1	docking protein 1 [Source:NCBI gene (formerly Entrezgene);Acc:100233179]
chr3	71423713	71424316	604	*	chr3	71423713	71424316	in_vitro	in_vivo	10	10	1.764	0.526	1.238	c(t = 1.19819	0.25	0.83731181	Distal Intergenic	ENSSSCG000	CD207	CD207 molecule [Source:NCBI gene (formerly Entrezgene);Acc:100037935]
chr1	98014040	98016630	2591	*	chr1	98014040	98016630	in_vitro	in_vivo	12	11	1.61666667	5.37545455	-3.7587879	c(t = -1.2170	0.251	0.83731181	Distal Intergenic	ENSSSCG000	SMAD7	SMAD family member 7 [Source:VGNC Symbol;Acc:VGNC:93221]
chr17	62114550	62116202	1653	*	chr17	62114550	62116202	in_vitro	in_vivo	9	12	7.48666667	5.08333333	2.40333333	c(t = 1.18646	0.251	0.83731181	Distal Intergenic	ENSSSCG000	ssc-mir-133a	ssc-mir-133a-1 [Source:miRBase;Acc:MI0010681]
chr3	59224460	59225768	1309	*	chr3	59224460	59225768	in_vitro	in_vivo	9	14	8.53111111	4.12928571	4.4018254	c(t = 1.21888	0.251	0.83731181	Promoter (<=1kb)	ENSSSCG000	MAT2A	methionine adenosyltransferase 2A [Source:NCBI gene (formerly Entrezgene);Acc:100312974]
chr1	130362967	130364060	1094	*	chr1	130362967	130364060	in_vitro	in_vivo	10	10	5.546	3.091	2.455	c(t = 1.19734	0.251	0.83731181	Distal Intergenic	ENSSSCG000	DLL4	delta like canonical Notch ligand 4 [Source:NCBI gene (formerly Entrezgene);Acc:100152163]
chr6	161173607	161174290	684	*	chr6	161173607	161174290	in_vitro	in_vivo	11	11	0.68181818	2.6	-1.9181818	c(t = -1.2136	0.251	0.83731181	Distal Intergenic	ENSSSCG000	COA7	cytochrome c oxidase assembly factor 7 [Source:NCBI gene (formerly Entrezgene);Acc:100627075]
chr6	166897344	166898421	1078	*	chr6	166897344	166898421	in_vitro	in_vivo	10	10	0.536	1.272	-0.736	c(t = -1.1910	0.251	0.83731181	Distal Intergenic	ENSSSCG000	DMAP1	DNA methyltransferase 1 associated protein 1 [Source:VGNC Symbol;Acc:VGNC:87344]
chr2	7298937	7300054	1118	*	chr2	7298937	7300054	in_vitro	in_vivo	9	10	2.89222222	0.915	1.97722222	c(t = 1.21987	0.251	0.83731181	Distal Intergenic	ENSSSCG000	ssc-mir-192	ssc-mir-192 [Source:miRBase;Acc:MI0013127]
chr12	324320	345731	21412	*	chr12	324320	345731	in_vitro	in_vivo	12	15	89.73	93.142	-3.412	c(t = -1.1856	0.252	0.83731181	3' UTR	ENSSSCG000	TBCD	tubulin folding cofactor D [Source:NCBI gene (formerly Entrezgene);Acc:100512127]
chr10	69127062	69128175	1114	*	chr10	69127062	69128175	in_vitro	in_vivo	9	13	95.55	91.4238462	4.12615385	c(t = 1.18209	0.252	0.83731181	Distal Intergenic	ENSSSCG000	WDR37	WD repeat domain 37 [Source:VGNC Symbol;Acc:VGNC:96258]
chr12	60168337	60175100	6764	*	chr12	60168337	60175100	in_vitro	in_vivo	9	12	93.6811111	88.0241667	5.65694444	c(t = 1.18762	0.252	0.83731181	Distal Intergenic	ENSSSCG000	SLC5A10	solute carrier family 5 member 10 [Source:NCBI gene (formerly Entrezgene);Acc:497235]
chr8	572317	596562	24246	*	chr8	572317	596562	in_vitro	in_vivo	16	16	82.1525	73.38625	8.76625	c(t = 1.16877	0.252	0.83731181	Distal Intergenic	ENSSSCG000	SLBP	stem-loop binding protein [Source:NCBI gene (formerly Entrezgene);Acc:100513605]
chr2	64761429	64762414	986	*	chr2	64761429	64762414	in_vitro	in_vivo	10	12	13.799	4.20583333	9.59316667	c(t = 1.21805	0.252	0.83731181	Promoter (<=1kb)	ENSSSCG000	DNAJB1	DnaJ heat shock protein family (Hsp40) member B1 [Source:NCBI gene (formerly Entrezgene);Acc:396741]
chr3	102963849	102964578	730	*	chr3	102963849	102964578	in_vitro	in_vivo	12	12	0.4125	1.50833333	-1.0958333	c(t = -1.1965	0.252	0.83731181	Distal Intergenic	ENSSSCG000	EIF2AK2	eukaryotic translation initiation factor 2 alpha kinase 2 [Source:VGNC Symbol;Acc:VGNC:87607]
chr6	36342480	36343795	1316	*	chr6	36342480	36343795	in_vitro	in_vivo	12	13	1.56083333	0.91615385	0.64467949	c(t = 1.17597	0.252	0.83731181	Distal Intergenic	ENSSSCG000	CBLN1	cerebellin 1 precursor [Source:NCBI gene (formerly Entrezgene);Acc:100127148]
chr5	62714288	62715872	1585	*	chr5	62714288	62715872	in_vitro	in_vivo	12	10	2.685	0.673	2.012	c(t = 1.20388	0.252	0.83731181	Distal Intergenic	ENSSSCG000	MFAP5	microfibril associated protein 5 [Source:VGNC Symbol;Acc:VGNC:90177]
chr12	42493809	42495657	1849	*	chr12	42493809	42495657	in_vitro	in_vivo	14	14	11.3421429	3.05571429	8.28642857	c(t = 1.19335	0.253	0.83731181	Promoter (<=1kb)	ENSSSCG000	CDK5R1	cyclin dependent kinase 5 regulatory subunit 1 [Source:VGNC Symbol;Acc:VGNC:98980]
chr4	89082213	89086179	3967	*	chr4	89082213	89086179	in_vitro	in_vivo	9	12	1.06777778	3.05416667	-1.9863889	c(t = -1.1876	0.253	0.83731181	Distal Intergenic	ENSSSCG000	HSPA6	heat shock protein family A (Hsp70) member 6 [Source:NCBI gene (formerly Entrezgene);Acc:396906]
chr13	93307475	93308987	1513	*	chr13	93307475	93308987	in_vitro	in_vivo	13	13	1.87	0.93461538	0.93538462	c(t = 1.18813	0.253	0.83731181	Promoter (<=1kb)	ENSSSCG000	RAP2B	RAP2B, member of RAS oncogene family [Source:NCBI gene (formerly Entrezgene);Acc:100144507]
chr7	37094132	37095163	1032	*	chr7	37094132	37095163	in_vitro	in_vivo	12	13	5.795	0.71307692	5.08192308	c(t = 1.20440	0.253	0.83731181	Promoter (<=1kb)	ENSSSCG000	CCND3	cyclin D3 [Source:VGNC Symbol;Acc:VGNC:86353]
chr1	64044336	64045112	777	*	chr1	64044336	64045112	in_vitro	in_vivo	9	9	2.08111111	0.38333333	1.69777778	c(t = 1.21953	0.253	0.83731181	Distal Intergenic	ENSSSCG000	FHL5	four and a half LIM domains 5 [Source:VGNC Symbol;Acc:VGNC:88130]
chr13	35377003	35377727	725	*	chr13	35377003	35377727	in_vitro	in_vivo	12	12	0.91583333	0	0.91583333	c(t = 1.20559	0.253	0.83731181	Promoter (<=1kb)	ENSSSCG000	TKT	transketolase [Source:VGNC Symbol;Acc:VGNC:94008]
chr12	5104344	5106412	2069	*	chr12	5104344	5106412	in_vitro	in_vivo	10	11	92.438	80.99	11.448	c(t = 1.20001	0.254	0.83731181	Distal Intergenic	ENSSSCG00000036592		serine and arginine rich splicing factor 2 [Source:VGNC Symbol;Acc:VGNC:99085]
chrX	121644666	121648614	3949	*	chrX	121644666	121648614	in_vitro	in_vivo	9	11	17.87	32.0118182	-14.141818	c(t = -1.1782	0.254	0.83731181	Distal Intergenic	ENSSSCG000	FATE1	fetal and adult testis expressed 1 [Source:NCBI gene (formerly Entrezgene);Acc:396705]
chr12	448754	450833	2080	*	chr12	448754	450833	in_vitro	in_vivo	11	12	6.05090909	17.2933333	-11.242424	c(t = -1.1858	0.254	0.83731181	Promoter (<=1kb)	ENSSSCG000	TBCD	tubulin folding cofactor D [Source:NCBI gene (formerly Entrezgene);Acc:100512127]
chrX	124156514	124157355	842	*	chrX	124156514	124157355	in_vitro	in_vivo	11	9	4.24818182	15.99	-11.741818	c(t = -1.2251	0.254	0.83731181	Distal Intergenic	ENSSSCG000	NSDHL	NAD(P) dependent steroid dehydrogenase-like [Source:NCBI gene (formerly Entrezgene);Acc:100157894]
chr9	138992721	138994081	1361	*	chr9	138992721	138994081	in_vitro	in_vivo	11	15	2.37181818	4.456	-2.0841818	c(t = -1.1681	0.254	0.83731181	Distal Intergenic	ENSSSCG000	EGFR	epidermal growth factor receptor [Source:NCBI gene (formerly Entrezgene);Acc:397070]
chr11	4746266	4747069	804	*	chr11	4746266	4747069	in_vitro	in_vivo	11	10	0.74545455	1.657	-0.9115455	c(t = -1.1765	0.254	0.83731181	Distal Intergenic	ENSSSCG000	GPR12	G protein-coupled receptor 12 [Source:NCBI gene (formerly Entrezgene);Acc:100736641]
chr15	19521903	19524191	2289	*	chr15	19521903	19524191	in_vitro	in_vivo	12	13	2.83833333	1.63692308	1.20141026	c(t = 1.17013	0.254	0.83731181	Promoter (<=1kb)	ENSSSCG000	LYPD1	LY6/PLAUR domain containing 1 [Source:VGNC Symbol;Acc:VGNC:96176]
chr13	50805923	50806515	593	*	chr13	50805923	50806515	in_vitro	in_vivo	11	10	2.46090909	0.9	1.56090909	c(t = 1.18264	0.254	0.83731181	Distal Intergenic	ENSSSCG000	ARL6IP5	ADP ribosylation factor like GTPase 6 interacting protein 5 [Source:NCBI gene (formerly Entrezgene);Acc:595127]
chr1	140675581	140677235	1655	*	chr1	140675581	140677235	in_vitro	in_vivo	14	13	1.58571429	0.87461538	0.7110989	c(t = 1.16866	0.254	0.83731181	Distal Intergenic	ENSSSCG000	SELENOS	selenoprotein S [Source:NCBI gene (formerly Entrezgene);Acc:100151836]
chr14	87742769	87744183	1415	*	chr14	87742769	87744183	in_vitro	in_vivo	9	9	1.98333333	0.74333333	1.24	c(t = 1.19183	0.254	0.83731181	Distal Intergenic	ENSSSCG000	BMPR1A	bone morphogenetic protein receptor type 1A [Source:VGNC Symbol;Acc:VGNC:85846]
chr3	6480970	6481599	630	*	chr3	6480970	6481599	in_vitro	in_vivo	9	9	2.71222222	0.74111111	1.97111111	c(t = 1.20015	0.254	0.83731181	Distal Intergenic	ENSSSCG000	ssc-mir-9796	ssc-mir-9796 [Source:miRBase;Acc:MI0031560]
chr11	64308939	64309682	744	*	chr11	64308939	64309682	in_vitro	in_vivo	9	9	1.04222222	0.33222222	0.71	c(t = 1.20549	0.254	0.83731181	Distal Intergenic	ENSSSCG000	DCT	dopachrome tautomerase [Source:VGNC Symbol;Acc:VGNC:87192]
chr6	63628489	63634158	5670	*	chr6	63628489	63634158	in_vitro	in_vivo	12	12	15.7866667	9.49333333	6.29333333	c(t = 1.18451	0.255	0.83731181	Distal Intergenic	ENSSSCG000	TAS1R3	taste 1 receptor member 3 [Source:NCBI gene (formerly Entrezgene);Acc:100127440]
chr9	65065467	65067071	1605	*	chr9	65065467	65067071	in_vitro	in_vivo	11	13	3.95363636	2.27230769	1.68132867	c(t = 1.18739	0.255	0.83731181	Distal Intergenic	NA	NA	NA
chr14	54990279	54991202	924	*	chr14	54990279	54991202	in_vitro	in_vivo	9	14	0.26444444	1.36928571	-1.1048413	c(t = -1.1841	0.255	0.83731181	Promoter (<=1kb)	ENSSSCG000	EDARADD	EDAR associated death domain [Source:VGNC Symbol;Acc:VGNC:87542]
chr2	98441446	98441773	328	*	chr2	98441446	98441773	in_vitro	in_vivo	10	11	2.733	1.22454545	1.50845455	c(t = 1.17902	0.255	0.83731181	Distal Intergenic	ENSSSCG000	MEF2C	myocyte enhancer factor 2C [Source:VGNC Symbol;Acc:VGNC:90127]
chr13	72269277	72270454	1178	*	chr13	72269277	72270454	in_vitro	in_vivo	12	12	1.85333333	0.6725	1.18083333	c(t = 1.18824	0.255	0.83731181	Promoter (<=1kb)	ENSSSCG000	SEC61A1	SEC61 translocon subunit alpha 1 [Source:VGNC Symbol;Acc:VGNC:92682]
chr10	56428160	56430917	2758	*	chr10	56428160	56430917	in_vitro	in_vivo	11	13	1.81727273	0.60769231	1.20958042	c(t = 1.19740	0.255	0.83731181	Distal Intergenic	ENSSSCG000	ITGB1	integrin subunit beta 1 [Source:VGNC Symbol;Acc:VGNC:96381]
chr2	86528848	86530164	1317	*	chr2	86528848	86530164	in_vitro	in_vivo	9	14	15.3266667	6.80142857	8.5252381	c(t = 1.20914	0.256	0.83731181	Distal Intergenic	ENSSSCG000	AP3B1	adaptor related protein complex 3 subunit beta 1 [Source:VGNC Symbol;Acc:VGNC:85387]
chr2	120684711	120687392	2682	*	chr2	120684711	120687392	in_vitro	in_vivo	12	13	1.5825	3.47153846	-1.8890385	c(t = -1.1802	0.256	0.83731181	Distal Intergenic	ENSSSCG000	COMMD10	COMM domain containing 10 [Source:VGNC Symbol;Acc:VGNC:86886]
chr6	32012805	32014497	1693	*	chr6	32012805	32014497	in_vitro	in_vivo	10	10	0.955	2.004	-1.049	c(t = -1.1897	0.256	0.83731181	Distal Intergenic	ENSSSCG000	AKTIP	AKT interacting protein [Source:VGNC Symbol;Acc:VGNC:85233]
chr11	60587424	60587727	304	*	chr11	60587424	60587727	in_vitro	in_vivo	9	10	4.28444444	1.72	2.56444444	c(t = 1.18676	0.256	0.83731181	Distal Intergenic	ENSSSCG000	ssc-mir-17	ssc-mir-17 [Source:miRBase;Acc:MI0008214]
chr7	111866249	111868211	1963	*	chr7	111866249	111868211	in_vitro	in_vivo	14	14	3.78714286	1.65714286	2.13	c(t = 1.17950	0.256	0.83731181	Promoter (<=1kb)	ENSSSCG000	KCNK13	potassium two pore domain channel subfamily K member 13 [Source:VGNC Symbol;Acc:VGNC:89366]
chr1	250180819	250181855	1037	*	chr1	250180819	250181855	in_vitro	in_vivo	12	14	1.80666667	0.96642857	0.8402381	c(t = 1.16555	0.256	0.83731181	Distal Intergenic	ENSSSCG000	FRRS1L	ferric chelate reductase 1 like [Source:VGNC Symbol;Acc:VGNC:88245]
chr14	8995819	8997075	1257	*	chr14	8995819	8997075	in_vitro	in_vivo	13	12	1.67538462	0.86916667	0.80621795	c(t = 1.16896	0.256	0.83731181	Promoter (<=1kb)	ENSSSCG000	NEFL	neurofilament light chain [Source:NCBI gene (formerly Entrezgene);Acc:100521224]
chr1	8403375	8404452	1078	*	chr1	8403375	8404452	in_vitro	in_vivo	9	12	2.68333333	0.8275	1.85583333	c(t = 1.20810	0.256	0.83731181	Distal Intergenic	ENSSSCG000	DYNLT1	dynein light chain Tctex-type 1 [Source:NCBI gene (formerly Entrezgene);Acc:100158053]
chr14	51111847	51112805	959	*	chr14	51111847	51112805	in_vitro	in_vivo	9	13	2.19777778	0.65384615	1.54393162	c(t = 1.20041	0.256	0.83731181	Distal Intergenic	ENSSSCG000	MRPL40	mitochondrial ribosomal protein L40 [Source:NCBI gene (formerly Entrezgene);Acc:100155568]
chr12	14035466	14036044	579	*	chr12	14035466	14036044	in_vitro	in_vivo	10	12	1.227	0.43833333	0.78866667	c(t = 1.17099	0.256	0.83731181	Distal Intergenic	ENSSSCG000	KPNA2	karyopherin subunit alpha 2 [Source:VGNC Symbol;Acc:VGNC:89561]
chr14	107137035	107137559	525	*	chr14	107137035	107137559	in_vitro	in_vivo	13	11	0.62307692	0.15909091	0.46398601	c(t = 1.17828	0.256	0.83731181	Distal Intergenic	ENSSSCG000	ENTPD1	ectonucleoside triphosphate diphosphohydrolase 1 [Source:VGNC Symbol;Acc:VGNC:97977]
chr1	773861	779689	5829	*	chr1	773861	779689	in_vitro	in_vivo	14	15	25.685	40.218	-14.533	c(t = -1.1585	0.257	0.83731181	Distal Intergenic	ENSSSCG000	ssc-mir-9815	ssc-mir-9815 [Source:miRBase;Acc:MI0031582]
chr3	95462684	95463297	614	*	chr3	95462684	95463297	in_vitro	in_vivo	9	11	7.80666667	3.34818182	4.45848485	c(t = 1.19397	0.257	0.83731181	Distal Intergenic	ENSSSCG000	SLC3A1	solute carrier family 3 member 1 [Source:NCBI gene (formerly Entrezgene);Acc:100144588]
chr6	327613	328789	1177	*	chr6	327613	328789	in_vitro	in_vivo	11	11	6.07272727	2.28	3.79272727	c(t = 1.19613	0.257	0.83731181	Promoter (<=1kb)	ENSSSCG000	SPATA2L	spermatogenesis associated 2 like [Source:NCBI gene (formerly Entrezgene);Acc:100415811]
chr6	95598598	95600338	1741	*	chr6	95598598	95600338	in_vitro	in_vivo	17	14	3.17	1.86928571	1.30071429	c(t = 1.15704	0.257	0.83731181	Distal Intergenic	ENSSSCG000	HPCAL4	hippocalcin like 4 [Source:VGNC Symbol;Acc:VGNC:88959]
chr1	98541853	98544852	3000	*	chr1	98541853	98544852	in_vitro	in_vivo	15	12	1.83333333	0.935	0.89833333	c(t = 1.16425	0.257	0.83731181	Promoter (<=1kb)	ENSSSCG000	SMAD7	SMAD family member 7 [Source:VGNC Symbol;Acc:VGNC:93221]
chr1	267436593	267437611	1019	*	chr1	267436593	267437611	in_vitro	in_vivo	14	9	2.12428571	0.86888889	1.25539683	c(t = 1.16568	0.257	0.83731181	Distal Intergenic	ENSSSCG000	ANGPTL2	angiopoietin like 2 [Source:VGNC Symbol;Acc:VGNC:85304]
chr9	12532090	12532558	469	*	chr9	12532090	12532558	in_vitro	in_vivo	9	10	3.17444444	0.778	2.39644444	c(t = 1.20522	0.257	0.83731181	Distal Intergenic	ENSSSCG000	NDUFC2	NADH:ubiquinone oxidoreductase subunit C2 [Source:NCBI gene (formerly Entrezgene);Acc:100513030]
chr2	117505401	117505975	575	*	chr2	117505401	117505975	in_vitro	in_vivo	13	9	2.15461538	0.63888889	1.5157265	c(t = 1.17905	0.257	0.83731181	Distal Intergenic	ENSSSCG000	APC	APC regulator of WNT signaling pathway [Source:NCBI gene (formerly Entrezgene);Acc:100517932]
chr12	402889	405708	2820	*	chr12	402889	405708	in_vitro	in_vivo	10	14	90.393	96.8392857	-6.4462857	c(t = -1.1995	0.258	0.83731181	Intron (NM_001244105/100512127, intron 13 of 38)	ENSSSCG000	TBCD	tubulin folding cofactor D [Source:NCBI gene (formerly Entrezgene);Acc:100512127]
chr11	70948540	70949270	731	*	chr11	70948540	70949270	in_vitro	in_vivo	10	9	80.795	60.5177778	20.2772222	c(t = 1.17905	0.258	0.83731181	Distal Intergenic	ENSSSCG000	BIVM	basic, immunoglobulin-like variable motif containing [Source:VGNC Symbol;Acc:VGNC:109180]
chr3	68519015	68520573	1559	*	chr3	68519015	68520573	in_vitro	in_vivo	9	15	11.8222222	5.26666667	6.55555556	c(t = 1.19022	0.258	0.83731181	Distal Intergenic	ENSSSCG000	DOK1	docking protein 1 [Source:NCBI gene (formerly Entrezgene);Acc:100233179]
chr16	20523677	20524414	738	*	chr16	20523677	20524414	in_vitro	in_vivo	11	11	0.96818182	2.09636364	-1.1281818	c(t = -1.1793	0.258	0.83731181	Distal Intergenic	ENSSSCG000	BRIX1	biogenesis of ribosomes BRX1 [Source:NCBI gene (formerly Entrezgene);Acc:100513502]
chr14	60412032	60416075	4044	*	chr14	60412032	60416075	in_vitro	in_vivo	13	15	2.51692308	1.73466667	0.78225641	c(t = 1.15961	0.258	0.83731181	Promoter (<=1kb)	ENSSSCG000	ACTA1	actin alpha 1, skeletal muscle [Source:NCBI gene (formerly Entrezgene);Acc:100154254]
chr2	8281049	8282602	1554	*	chr2	8281049	8282602	in_vitro	in_vivo	11	13	2.84363636	1.63846154	1.20517483	c(t = 1.16843	0.258	0.83731181	Distal Intergenic	ENSSSCG000	ssc-mir-7142	ssc-mir-7142 [Source:miRBase;Acc:MI0023575]
chr1	114598500	114599062	563	*	chr1	114598500	114599062	in_vitro	in_vivo	10	10	2.174	1.085	1.089	c(t = 1.18183	0.258	0.83731181	Distal Intergenic	ENSSSCG000	AQP9	aquaporin 9 [Source:NCBI gene (formerly Entrezgene);Acc:100127153]
chr8	113825006	113827669	2664	*	chr8	113825006	113827669	in_vitro	in_vivo	12	13	3.7725	0.98846154	2.78403846	c(t = 1.18808	0.258	0.83731181	Promoter (<=1kb)	ENSSSCG000	LEF1	lymphoid enhancer binding factor 1 [Source:VGNC Symbol;Acc:VGNC:89676]
chr12	12881783	12882978	1196	*	chr12	12881783	12882978	in_vitro	in_vivo	10	13	2.271	0.80769231	1.46330769	c(t = 1.19548	0.258	0.83731181	Distal Intergenic	ENSSSCG000	CACNG1	calcium voltage-gated channel auxiliary subunit gamma 1 [Source:VGNC Symbol;Acc:VGNC:86124]
chr1	187384590	187386567	1978	*	chr1	187384590	187386567	in_vitro	in_vivo	12	13	1.48833333	0.62	0.86833333	c(t = 1.16866	0.258	0.83731181	Promoter (<=1kb)	ENSSSCG000	ARID4A	AT-rich interaction domain 4A [Source:VGNC Symbol;Acc:VGNC:85509]
chr14	50491311	50492732	1422	*	chr14	50491311	50492732	in_vitro	in_vivo	11	11	1.33727273	0.47181818	0.86545455	c(t = 1.18990	0.258	0.83731181	Distal Intergenic	ENSSSCG000	SLC7A4	solute carrier family 7 member 4 [Source:VGNC Symbol;Acc:VGNC:93174]
chr13	208271708	208273991	2284	*	chr13	208271708	208273991	in_vitro	in_vivo	14	13	14.835	26.3815385	-11.546538	c(t = -1.1578	0.259	0.83731181	Distal Intergenic	ENSSSCG000	ITGB2	integrin subunit beta 2 [Source:NCBI gene (formerly Entrezgene);Acc:396943]
chr14	137248593	137249342	750	*	chr14	137248593	137249342	in_vitro	in_vivo	10	9	27.333	10.2255556	17.1074444	c(t = 1.18079	0.259	0.83731181	Distal Intergenic	ENSSSCG000	GLRX3	glutaredoxin 3 [Source:VGNC Symbol;Acc:VGNC:88495]
chr1	67287133	67288316	1184	*	chr1	67287133	67288316	in_vitro	in_vivo	13	14	2.80230769	4.905	-2.1026923	c(t = -1.1577	0.259	0.83731181	Intron (NM_001172585/100154026, intron 1 of 10)	ENSSSCG000	SIM1	SIM bHLH transcription factor 1 [Source:VGNC Symbol;Acc:VGNC:92876]
chr7	55263901	55264474	574	*	chr7	55263901	55264474	in_vitro	in_vivo	13	12	0.50230769	1.22833333	-0.7260256	c(t = -1.1705	0.259	0.83731181	Distal Intergenic	ENSSSCG000	PLIN1	perilipin 1 [Source:NCBI gene (formerly Entrezgene);Acc:654411]
chr7	114706368	114707258	891	*	chr7	114706368	114707258	in_vitro	in_vivo	10	13	1.515	0.74461538	0.77038462	c(t = 1.16378	0.259	0.83731181	Distal Intergenic	ENSSSCG000	GON7	GON7 subunit of KEOPS complex [Source:NCBI gene (formerly Entrezgene);Acc:100155082]
chr14	20126658	20127364	707	*	chr14	20126658	20127364	in_vitro	in_vivo	13	9	1.50076923	0.68	0.82076923	c(t = 1.16180	0.259	0.83731181	Distal Intergenic	ENSSSCG000	HMGB2	high mobility group box 2 [Source:VGNC Symbol;Acc:VGNC:88902]
chr1	111588398	111589710	1313	*	chr1	111588398	111589710	in_vitro	in_vivo	10	11	7.576	3.39727273	4.17872727	c(t = 1.18146	0.26	0.83731181	Promoter (<=1kb)	ENSSSCG000	ANXA2	annexin A2 [Source:VGNC Symbol;Acc:VGNC:103027]
chr15	60439163	60440950	1788	*	chr15	60439163	60440950	in_vitro	in_vivo	12	15	3.38833333	1.61333333	1.775	c(t = 1.16927	0.26	0.83731181	Promoter (<=1kb)	ENSSSCG000	PRPF40A	pre-mRNA processing factor 40 homolog A [Source:VGNC Symbol;Acc:VGNC:98222]
chr3	10843172	10844430	1259	*	chr3	10843172	10844430	in_vitro	in_vivo	11	12	0.61727273	1.61166667	-0.9943939	c(t = -1.1739	0.26	0.83731181	Distal Intergenic	ENSSSCG000	ssc-mir-7137	ssc-mir-7137 [Source:miRBase;Acc:MI0023570]
chr13	87726882	87728725	1844	*	chr13	87726882	87728725	in_vitro	in_vivo	11	10	3.76	1.566	2.194	c(t = 1.16649	0.26	0.83731181	Distal Intergenic	ENSSSCG000	PLSCR1	phospholipid scramblase 1 [Source:NCBI gene (formerly Entrezgene);Acc:100620055]
chr3	121546774	121550025	3252	*	chr3	121546774	121550025	in_vitro	in_vivo	15	13	2.30133333	1.39307692	0.90825641	c(t = 1.15514	0.26	0.83731181	Distal Intergenic	ENSSSCG000	KCNS3	potassium voltage-gated channel modifier subfamily S member 3 [Source:VGNC Symbol;Acc:VGNC:89387]
chr15	120942295	120943824	1530	*	chr15	120942295	120943824	in_vitro	in_vivo	9	9	3.82555556	1.39222222	2.43333333	c(t = 1.20034	0.26	0.83731181	Distal Intergenic	ENSSSCG000	PRKAG3	protein kinase AMP-activated non-catalytic subunit gamma 3 [Source:NCBI gene (formerly Entrezgene);Acc:397149]
chr7	120910885	120912167	1283	*	chr7	120910885	120912167	in_vitro	in_vivo	10	13	2.082	1.11	0.972	c(t = 1.17683	0.26	0.83731181	Distal Intergenic	ENSSSCG000	EVL	Enah/Vasp-like [Source:VGNC Symbol;Acc:VGNC:87820]
chr15	83146070	83146555	486	*	chr15	83146070	83146555	in_vitro	in_vivo	12	12	1.73666667	0.67166667	1.065	c(t = 1.16601	0.26	0.83731181	Distal Intergenic	ENSSSCG000	PLEKHA3	pleckstrin homology domain containing A3 [Source:NCBI gene (formerly Entrezgene);Acc:100154010]
chr14	31945950	31946621	672	*	chr14	31945950	31946621	in_vitro	in_vivo	10	13	1.682	0.50461538	1.17738462	c(t = 1.18541	0.26	0.83731181	Distal Intergenic	ENSSSCG000	FAM216A	family with sequence similarity 216 member A [Source:NCBI gene (formerly Entrezgene);Acc:100513180]
chr17	48120133	48121156	1024	*	chr17	48120133	48121156	in_vitro	in_vivo	11	10	1.38727273	0.481	0.90627273	c(t = 1.17139	0.26	0.83731181	Distal Intergenic	ENSSSCG00000007435		phospholipid transfer protein [Source:VGNC Symbol;Acc:VGNC:104029]
chr15	83792726	83793555	830	*	chr15	83792726	83793555	in_vitro	in_vivo	14	13	6.995	0.35076923	6.64423077	c(t = 1.17725	0.26	0.83731181	Distal Intergenic	ENSSSCG000	PLEKHA3	pleckstrin homology domain containing A3 [Source:NCBI gene (formerly Entrezgene);Acc:100154010]
chr1	273567544	273569759	2216	*	chr1	273567544	273569759	in_vitro	in_vivo	9	12	84.0933333	89.3583333	-5.265	c(t = -1.1626	0.261	0.83731181	Distal Intergenic	ENSSSCG000	ssc-mir-9812	ssc-mir-9812 [Source:miRBase;Acc:MI0031579]
chr16	79605069	79606470	1402	*	chr16	79605069	79606470	in_vitro	in_vivo	9	12	17.4444444	32.1858333	-14.741389	c(t = -1.1590	0.261	0.83731181	Distal Intergenic	ENSSSCG000	TPPP	tubulin polymerization promoting protein [Source:VGNC Symbol;Acc:VGNC:94344]
chr17	46144449	46144699	251	*	chr17	46144449	46144699	in_vitro	in_vivo	9	11	5.45	11.0563636	-5.6063636	c(t = -1.1710	0.261	0.83731181	Distal Intergenic	ENSSSCG000	SRSF6	serine and arginine rich splicing factor 6 [Source:NCBI gene (formerly Entrezgene);Acc:768102]
chr14	14860858	14862136	1279	*	chr14	14860858	14862136	in_vitro	in_vivo	9	12	6.10777778	3.23333333	2.87444444	c(t = 1.18598	0.261	0.83731181	Distal Intergenic	ENSSSCG000	GATA4	GATA binding protein 4 [Source:VGNC Symbol;Acc:VGNC:88365]
chr3	78899688	78900082	395	*	chr3	78899688	78900082	in_vitro	in_vivo	12	12	10.5516667	2.59083333	7.96083333	c(t = 1.18047	0.261	0.83731181	Distal Intergenic	ENSSSCG000	TMEM17	transmembrane protein 17 [Source:NCBI gene (formerly Entrezgene);Acc:100627459]
chr9	131274898	131275967	1070	*	chr9	131274898	131275967	in_vitro	in_vivo	9	12	0.78777778	2.095	-1.3072222	c(t = -1.1675	0.261	0.83731181	Distal Intergenic	ENSSSCG000	PPP2R5A	protein phosphatase 2 regulatory subunit B'alpha [Source:NCBI gene (formerly Entrezgene);Acc:100101925]
chr8	38378748	38379815	1068	*	chr8	38378748	38379815	in_vitro	in_vivo	12	13	2.06166667	1.16384615	0.89782051	c(t = 1.16424	0.261	0.83731181	Distal Intergenic	ENSSSCG000	ZAR1	zygote arrest 1 [Source:VGNC Symbol;Acc:VGNC:95051]
chr12	47307290	47308427	1138	*	chr12	47307290	47308427	in_vitro	in_vivo	10	10	2.108	1.045	1.063	c(t = 1.16600	0.261	0.83731181	Distal Intergenic	ENSSSCG000	CRK	CRK proto-onco, adaptor protein [Source:NCBI gene (formerly Entrezgene);Acc:100192444]
chr14	27230324	27231093	770	*	chr14	27230324	27231093	in_vitro	in_vivo	11	13	1.99909091	0.93384615	1.06524476	c(t = 1.15447	0.261	0.83731181	Distal Intergenic	ENSSSCG000	SCARB1	scavenger receptor class B member 1 [Source:NCBI gene (formerly Entrezgene);Acc:397018]
chr7	70658807	70659804	998	*	chr7	70658807	70659804	in_vitro	in_vivo	11	13	2.44909091	0.90692308	1.54216783	c(t = 1.18169	0.261	0.83731181	Distal Intergenic	ENSSSCG000	AP4S1	adaptor related protein complex 4 subunit sigma 1 [Source:VGNC Symbol;Acc:VGNC:85394]
chr14	103871223	103871909	687	*	chr14	103871223	103871909	in_vitro	in_vivo	11	13	1.47909091	0.57307692	0.90601399	c(t = 1.17136	0.261	0.83731181	Distal Intergenic	ENSSSCG000	HHEX	hematopoietically expressed homeobox [Source:NCBI gene (formerly Entrezgene);Acc:397232]
chr15	100065480	100066240	761	*	chr15	100065480	100066240	in_vitro	in_vivo	11	9	1.18181818	0.47	0.71181818	c(t = 1.16427	0.261	0.83731181	Distal Intergenic	ENSSSCG000	HECW2	HECT, C2 and WW domain containing E3 ubiquitin protein ligase 2 [Source:NCBI gene (formerly Entrezgene);Acc:100155879]
chr14	102828990	102830596	1607	*	chr14	102828990	102830596	in_vitro	in_vivo	11	11	1.52727273	0.39181818	1.13545455	c(t = 1.18627	0.261	0.83731181	Distal Intergenic	ENSSSCG000	ANKRD1	ankyrin repeat domain 1 [Source:VGNC Symbol;Acc:VGNC:85321]
chr3	131430052	131435822	5771	*	chr3	131430052	131435822	in_vitro	in_vivo	13	12	92.5	95.8941667	-3.3941667	c(t = -1.1583	0.262	0.83731181	3' UTR	ENSSSCG000	EIPR1	EARP complex and GARP complex interacting protein 1 [Source:VGNC Symbol;Acc:VGNC:87635]
chr2	984059	988533	4475	*	chr2	984059	988533	in_vitro	in_vivo	9	10	96.0777778	92.425	3.65277778	c(t = 1.16304	0.262	0.83731181	Distal Intergenic	ENSSSCG000	TOLLIP	toll interacting protein [Source:NCBI gene (formerly Entrezgene);Acc:100625123]
chr2	28989743	28991063	1321	*	chr2	28989743	28991063	in_vitro	in_vivo	10	12	76.997	57.93	19.067	c(t = 1.15364	0.262	0.83731181	Intron (NM_001244172/100514152, intron 7 of 12)	ENSSSCG000	PAX6	paired box 6 [Source:VGNC Symbol;Acc:VGNC:91195]
chr4	76775033	76775589	557	*	chr4	76775033	76775589	in_vitro	in_vivo	11	11	14.0409091	30.05	-16.009091	c(t = -1.1555	0.262	0.83731181	Distal Intergenic	ENSSSCG000	MRPL15	mitochondrial ribosomal protein L15 [Source:VGNC Symbol;Acc:VGNC:98820]
chr8	110199849	110201826	1978	*	chr8	110199849	110201826	in_vitro	in_vivo	13	14	11.3023077	6.19214286	5.11016484	c(t = 1.16030	0.262	0.83731181	Distal Intergenic	ENSSSCG000	LARP7	La ribonucleoprotein 7, transcriptional regulator [Source:NCBI gene (formerly Entrezgene);Acc:100511273]
chr6	71026292	71029518	3227	*	chr6	71026292	71029518	in_vitro	in_vivo	11	15	1.83818182	3.636	-1.7978182	c(t = -1.1513	0.262	0.83731181	Distal Intergenic	ENSSSCG000	ssc-mir-9861	ssc-mir-9861 [Source:miRBase;Acc:MI0031639]
chr9	108413565	108414469	905	*	chr9	108413565	108414469	in_vitro	in_vivo	13	12	1.53692308	3.39916667	-1.8622436	c(t = -1.1610	0.262	0.83731181	Promoter (<=1kb)	ENSSSCG000	SFRP4	secreted frizzled related protein 4 [Source:NCBI gene (formerly Entrezgene);Acc:100145890]
chr6	69973176	69973989	814	*	chr6	69973176	69973989	in_vitro	in_vivo	11	12	1.10090909	3.11583333	-2.0149242	c(t = -1.1732	0.262	0.83731181	Distal Intergenic	ENSSSCG000	RBP7	retinol binding protein 7 [Source:NCBI gene (formerly Entrezgene);Acc:100270723]
chr7	112160511	112162269	1759	*	chr7	112160511	112162269	in_vitro	in_vivo	12	14	6.2225	3.08785714	3.13464286	c(t = 1.16025	0.262	0.83731181	Promoter (<=1kb)	ENSSSCG000	CALM1	calmodulin 1 [Source:NCBI gene (formerly Entrezgene);Acc:100154056]
chr14	124399551	124401921	2371	*	chr14	124399551	124401921	in_vitro	in_vivo	15	13	4.30266667	2.77461538	1.52805128	c(t = 1.15094	0.262	0.83731181	Promoter (<=1kb)	ENSSSCG000	ADRB1	adrenoceptor beta 1 [Source:NCBI gene (formerly Entrezgene);Acc:397355]
chr12	51507904	51508722	819	*	chr12	51507904	51508722	in_vitro	in_vivo	9	10	0.95666667	2.095	-1.1383333	c(t = -1.1617	0.262	0.83731181	Distal Intergenic	ENSSSCG000	ZNF232	zinc finger protein 232 [Source:VGNC Symbol;Acc:VGNC:95208]
chr7	33267685	33270693	3009	*	chr7	33267685	33270693	in_vitro	in_vivo	14	13	3.61571429	1.97	1.64571429	c(t = 1.15169	0.262	0.83731181	Distal Intergenic	ENSSSCG000	CCDC167	coiled-coil domain containing 167 [Source:VGNC Symbol;Acc:VGNC:86270]
chr13	3991914	3993286	1373	*	chr13	3991914	3993286	in_vitro	in_vivo	11	11	0.80909091	1.56454545	-0.7554545	c(t = -1.1641	0.262	0.83731181	Distal Intergenic	ENSSSCG000	DPH3	diphthamide biosynthesis 3 [Source:NCBI gene (formerly Entrezgene);Acc:100156362]
chr1	116448647	116449497	851	*	chr1	116448647	116449497	in_vitro	in_vivo	10	9	1.463	0.37	1.093	c(t = 1.17553	0.262	0.83731181	Promoter (<=1kb)	ENSSSCG000	CCPG1	cell cycle progression 1 [Source:VGNC Symbol;Acc:VGNC:86368]
chr14	50758442	50759388	947	*	chr14	50758442	50759388	in_vitro	in_vivo	12	11	1.22083333	4.73818182	-3.5173485	c(t = -1.1818	0.263	0.83731181	Distal Intergenic	ENSSSCG000	SLC7A4	solute carrier family 7 member 4 [Source:VGNC Symbol;Acc:VGNC:93174]
chr13	38556683	38557447	765	*	chr13	38556683	38557447	in_vitro	in_vivo	13	9	0.59461538	3.86666667	-3.2720513	c(t = -1.2008	0.263	0.83731181	Distal Intergenic	ENSSSCG000	APPL1	adaptor protein, phosphotyrosine interacting with PH domain and leucine zipper 1 [Source:NCBI gene (formerly Entrezgene);Acc:100188978]
chr5	103862401	103863532	1132	*	chr5	103862401	103863532	in_vitro	in_vivo	11	12	1.73636364	2.89833333	-1.1619697	c(t = -1.1533	0.263	0.83731181	Distal Intergenic	ENSSSCG000	ssc-mir-7139	ssc-mir-7139 [Source:miRBase;Acc:MI0023572]
chr1	231081044	231081611	568	*	chr1	231081044	231081611	in_vitro	in_vivo	9	12	0.16888889	2.13	-1.9611111	c(t = -1.1793	0.263	0.83731181	Distal Intergenic	ENSSSCG000	GNAQ	G protein subunit alpha q [Source:NCBI gene (formerly Entrezgene);Acc:654405]
chr6	51008033	51008599	567	*	chr6	51008033	51008599	in_vitro	in_vivo	9	9	0.49333333	1.82777778	-1.3344444	c(t = -1.1761	0.263	0.83731181	Distal Intergenic	ENSSSCG000	BCL3	BCL3 transcription coactivator [Source:NCBI gene (formerly Entrezgene);Acc:100738193]
chr14	51706840	51732873	26034	*	chr14	51706840	51732873	in_vitro	in_vivo	13	13	3.59538462	1.09307692	2.50230769	c(t = 1.16933	0.263	0.83731181	Distal Intergenic	ENSSSCG000	CHRM3	cholinergic receptor muscarinic 3 [Source:VGNC Symbol;Acc:VGNC:86664]
chr2	61037274	61038399	1126	*	chr2	61037274	61038399	in_vitro	in_vivo	11	15	2.03090909	1.05666667	0.97424242	c(t = 1.16053	0.263	0.83731181	Distal Intergenic	ENSSSCG000	SMIM7	small integral membrane protein 7 [Source:NCBI gene (formerly Entrezgene);Acc:100512765]
chr13	163442895	163444905	2011	*	chr13	163442895	163444905	in_vitro	in_vivo	14	13	2.36928571	0.94769231	1.42159341	c(t = 1.16520	0.263	0.83731181	Distal Intergenic	ENSSSCG000	CLDND1	claudin domain containing 1 [Source:VGNC Symbol;Acc:VGNC:86742]
chr14	16708864	16710745	1882	*	chr14	16708864	16710745	in_vitro	in_vivo	12	14	1.79333333	0.87	0.92333333	c(t = 1.15008	0.263	0.83731181	Promoter (<=1kb)	ENSSSCG000	HMGB2	high mobility group box 2 [Source:VGNC Symbol;Acc:VGNC:88902]
chr12	1112655	1114219	1565	*	chr12	1112655	1114219	in_vitro	in_vivo	9	9	1.32888889	0.65333333	0.67555556	c(t = 1.16134	0.263	0.83731181	Promoter (<=1kb)	ENSSSCG000	ARHGDIA	Rho GDP dissociation inhibitor alpha [Source:VGNC Symbol;Acc:VGNC:85483]
chr6	10488089	10488860	772	*	chr6	10488089	10488860	in_vitro	in_vivo	10	11	1.14	0.30090909	0.83909091	c(t = 1.17057	0.263	0.83731181	Distal Intergenic	ENSSSCG000	COG4	component of oligomeric golgi complex 4 [Source:NCBI gene (formerly Entrezgene);Acc:100625346]
chr13	192249136	192249547	412	*	chr13	192249136	192249547	in_vitro	in_vivo	10	10	2.163	0	2.163	c(t = 1.19344	0.263	0.83731181	Distal Intergenic	ENSSSCG000	CLDN17	claudin 17 [Source:NCBI gene (formerly Entrezgene);Acc:100294681]
chr15	128303902	128304964	1063	*	chr15	128303902	128304964	in_vitro	in_vivo	9	12	51.3966667	63.7258333	-12.329167	c(t = -1.1537	0.264	0.83731181	Exon (NM_001244489/100512686, exon 1 of 2)	ENSSSCG000	IRS1	insulin receptor substrate 1 [Source:VGNC Symbol;Acc:VGNC:96376]
chrX	125193685	125195164	1480	*	chrX	125193685	125195164	in_vitro	in_vivo	10	12	8.641	19.4441667	-10.803167	c(t = -1.1543	0.264	0.83731181	Distal Intergenic	ENSSSCG000	DKC1	dyskerin pseudouridine synthase 1 [Source:NCBI gene (formerly Entrezgene);Acc:100216430]
chr4	5600992	5602090	1099	*	chr4	5600992	5602090	in_vitro	in_vivo	10	10	2.9	13.495	-10.595	c(t = -1.1873	0.264	0.83731181	Distal Intergenic	ENSSSCG000	ssc-mir-30d	ssc-mir-30d [Source:miRBase;Acc:MI0013091]
chr6	44773642	44775397	1756	*	chr6	44773642	44775397	in_vitro	in_vivo	11	13	17.8390909	9.93846154	7.90062937	c(t = 1.16086	0.264	0.83731181	Distal Intergenic	ENSSSCG000	HAMP	hepcidin antimicrobial peptide [Source:NCBI gene (formerly Entrezgene);Acc:397207]
chr2	4709299	4712137	2839	*	chr2	4709299	4712137	in_vitro	in_vivo	15	16	3.884	7.46625	-3.58225	c(t = -1.1432	0.264	0.83731181	Distal Intergenic	ENSSSCG00000028501		aldehyde dehydrogenase 3 family member B1 [Source:NCBI gene (formerly Entrezgene);Acc:100739347]
chr5	64058625	64059702	1078	*	chr5	64058625	64059702	in_vitro	in_vivo	12	13	9.50833333	4.93923077	4.56910256	c(t = 1.14598	0.264	0.83731181	Distal Intergenic	ENSSSCG000	NOP2	NOP2 nucleolar protein [Source:VGNC Symbol;Acc:VGNC:103306]
chr3	118757512	118760544	3033	*	chr3	118757512	118760544	in_vitro	in_vivo	12	14	1.67	3.22928571	-1.5592857	c(t = -1.1512	0.264	0.83731181	Distal Intergenic	ENSSSCG000	SDC1	syndecan 1 [Source:VGNC Symbol;Acc:VGNC:92654]
chr7	75103845	75104691	847	*	chr7	75103845	75104691	in_vitro	in_vivo	10	13	0.781	2.32461538	-1.5436154	c(t = -1.1583	0.264	0.83731181	Distal Intergenic	ENSSSCG000	TSSK4	testis specific serine kinase 4 [Source:VGNC Symbol;Acc:VGNC:94527]
chr4	69977316	69978976	1661	*	chr4	69977316	69978976	in_vitro	in_vivo	11	13	4.13727273	2.18846154	1.94881119	c(t = 1.14740	0.264	0.83731181	Distal Intergenic	ENSSSCG000	ssc-mir-124a	ssc-mir-124a-1 [Source:miRBase;Acc:MI0010680]
chr7	91224180	91225237	1058	*	chr7	91224180	91225237	in_vitro	in_vivo	11	12	3.60363636	1.87333333	1.73030303	c(t = 1.15280	0.264	0.83731181	Distal Intergenic	ENSSSCG000	VTI1B	vesicle transport through interaction with t-SNAREs 1B [Source:NCBI gene (formerly Entrezgene);Acc:404692]
chr1	114266327	114266968	642	*	chr1	114266327	114266968	in_vitro	in_vivo	11	11	3.44454545	1.23909091	2.20545455	c(t = 1.16635	0.264	0.83731181	Distal Intergenic	ENSSSCG000	AQP9	aquaporin 9 [Source:NCBI gene (formerly Entrezgene);Acc:100127153]
chr13	32319453	32320311	859	*	chr13	32319453	32320311	in_vitro	in_vivo	12	11	2.37416667	0.40363636	1.9705303	c(t = 1.17116	0.264	0.83731181	Distal Intergenic	ENSSSCG000	GMPPB	GDP-mannose pyrophosphorylase B [Source:NCBI gene (formerly Entrezgene);Acc:100513376]
chr18	4576826	4577470	645	*	chr18	4576826	4577470	in_vitro	in_vivo	9	9	3.77888889	15.8522222	-12.073333	c(t = -1.1897	0.265	0.83731181	Distal Intergenic	ENSSSCG000	RHEB	Ras homolog, mTORC1 binding [Source:NCBI gene (formerly Entrezgene);Acc:100516050]
chr11	78645567	78646089	523	*	chr11	78645567	78646089	in_vitro	in_vivo	9	12	7.00444444	14.435	-7.4305556	c(t = -1.1538	0.265	0.83731181	Distal Intergenic	ENSSSCG000	LAMP1	lysosomal associated membrane protein 1 [Source:NCBI gene (formerly Entrezgene);Acc:494565]
chr2	81287760	81288900	1141	*	chr2	81287760	81288900	in_vitro	in_vivo	9	11	21.4211111	9.18181818	12.2392929	c(t = 1.17291	0.265	0.83731181	Promoter (<=1kb)	ENSSSCG000	SNCB	synuclein beta [Source:VGNC Symbol;Acc:VGNC:93286]
chrX	107530262	107530841	580	*	chrX	107530262	107530841	in_vitro	in_vivo	9	10	0.41888889	8.629	-8.2101111	c(t = -1.1882	0.265	0.83731181	Distal Intergenic	ENSSSCG000	STK26	serine/threonine kinase 26 [Source:NCBI gene (formerly Entrezgene);Acc:100233205]
chr13	77749660	77751338	1679	*	chr13	77749660	77751338	in_vitro	in_vivo	11	13	0.79636364	1.48538462	-0.689021	c(t = -1.1497	0.265	0.83731181	Distal Intergenic	ENSSSCG000	NCK1	NCK adaptor protein 1 [Source:VGNC Symbol;Acc:VGNC:90609]
chr1	147895498	147896110	613	*	chr1	147895498	147896110	in_vitro	in_vivo	11	11	2.83181818	1.38818182	1.44363636	c(t = 1.14921	0.265	0.83731181	Distal Intergenic	ENSSSCG000	MBP	myelin basic protein [Source:NCBI gene (formerly Entrezgene);Acc:414286]
chr5	29695884	29696861	978	*	chr5	29695884	29696861	in_vitro	in_vivo	12	14	2.66916667	1.215	1.45416667	c(t = 1.15329	0.265	0.83731181	Distal Intergenic	ENSSSCG000	LEMD3	LEM domain containing 3 [Source:NCBI gene (formerly Entrezgene);Acc:100511508]
chr6	29591756	29593220	1465	*	chr6	29591756	29593220	in_vitro	in_vivo	11	12	0.43636364	1.16333333	-0.7269697	c(t = -1.1520	0.265	0.83731181	Distal Intergenic	ENSSSCG000	NUDT21	nudix hydrolase 21 [Source:NCBI gene (formerly Entrezgene);Acc:100625681]
chr5	78266409	78267140	732	*	chr5	78266409	78267140	in_vitro	in_vivo	11	9	2.32909091	0.92555556	1.40353535	c(t = 1.15196	0.265	0.83731181	Promoter (<=1kb)	ENSSSCG000	VDR	vitamin D receptor [Source:VGNC Symbol;Acc:VGNC:94814]
chr18	50002300	50003906	1607	*	chr18	50002300	50003906	in_vitro	in_vivo	10	13	3.789	0.48461538	3.30438462	c(t = 1.18641	0.265	0.83731181	Promoter (<=1kb)	ENSSSCG000	IGFBP3	insulin like growth factor binding protein 3 [Source:NCBI gene (formerly Entrezgene);Acc:448812]
chr7	58023148	58024141	994	*	chr7	58023148	58024141	in_vitro	in_vivo	11	11	0.87090909	0.14454545	0.72636364	c(t = 1.17245	0.265	0.83731181	Promoter (<=1kb)	ENSSSCG000	IMP3	IMP U3 small nucleolar ribonucleoprotein 3 [Source:NCBI gene (formerly Entrezgene);Acc:100151831]
chr12	20842212	20843050	839	*	chr12	20842212	20843050	in_vitro	in_vivo	11	10	0.49727273	0.081	0.41627273	c(t = 1.17440	0.265	0.83731181	Distal Intergenic	ENSSSCG000	FKBP10	FKBP prolyl isomerase 10 [Source:NCBI gene (formerly Entrezgene);Acc:100511038]
chr6	101761404	101762389	986	*	chr6	101761404	101762389	in_vitro	in_vivo	10	9	57.004	44.0177778	12.9862222	c(t = 1.15047	0.266	0.83731181	Distal Intergenic	ENSSSCG000	TGIF1	TGFB induced factor homeobox 1 [Source:VGNC Symbol;Acc:VGNC:93935]
chr1	11885774	11887322	1549	*	chr1	11885774	11887322	in_vitro	in_vivo	10	11	1.503	2.73636364	-1.2333636	c(t = -1.1490	0.266	0.83731181	Distal Intergenic	ENSSSCG000	CLDN20	claudin 20 [Source:NCBI gene (formerly Entrezgene);Acc:100153817]
chr1	180828650	180830838	2189	*	chr1	180828650	180830838	in_vitro	in_vivo	15	13	1.62066667	0.71538462	0.90528205	c(t = 1.14408	0.266	0.83731181	Distal Intergenic	ENSSSCG000	PYGL	glycogen phosphorylase L [Source:NCBI gene (formerly Entrezgene);Acc:100141306]
chr1	16274723	16275409	687	*	chr1	16274723	16275409	in_vitro	in_vivo	10	9	1.212	0.36666667	0.84533333	c(t = 1.16729	0.266	0.83731181	Distal Intergenic	ENSSSCG000	PCMT1	protein-L-isoaspartate (D-aspartate) O-methyltransferase [Source:VGNC Symbol;Acc:VGNC:103146]
chr7	1060184	1062517	2334	*	chr7	1060184	1062517	in_vitro	in_vivo	11	11	56.9363636	72.1181818	-15.181818	c(t = -1.1439	0.267	0.83731181	Distal Intergenic	ENSSSCG000	SERPINB9	serpin peptidase inhibitor, clade B (ovalbumin), member 9 [Source:NCBI gene (formerly Entrezgene);Acc:100126851]
chr1	2320226	2323147	2922	*	chr1	2320226	2323147	in_vitro	in_vivo	10	9	14.145	30.9966667	-16.851667	c(t = -1.1537	0.267	0.83731181	Distal Intergenic	ENSSSCG00000032916		mitochondrial pyruvate carrier 1 [Source:VGNC Symbol;Acc:VGNC:90318]
chr1	143634080	143634652	573	*	chr1	143634080	143634652	in_vitro	in_vivo	10	9	1.288	2.76111111	-1.4731111	c(t = -1.1657	0.267	0.83731181	Distal Intergenic	ENSSSCG000	KLF13	KLF transcription factor 13 [Source:NCBI gene (formerly Entrezgene);Acc:494563]
chr1	108205774	108206782	1009	*	chr1	108205774	108206782	in_vitro	in_vivo	10	12	0.741	2.54166667	-1.8006667	c(t = -1.1626	0.267	0.83731181	Distal Intergenic	ENSSSCG000	DAPK2	death associated protein kinase 2 [Source:VGNC Symbol;Acc:VGNC:87157]
chr10	17710012	17713172	3161	*	chr10	17710012	17713172	in_vitro	in_vivo	14	17	3.34857143	1.82411765	1.52445378	c(t = 1.13911	0.267	0.83731181	Distal Intergenic	ENSSSCG000	DESI2	desumoylating isopeptidase 2 [Source:VGNC Symbol;Acc:VGNC:96037]
chr6	73117964	73119295	1332	*	chr6	73117964	73119295	in_vitro	in_vivo	13	12	7.59307692	1.7225	5.87057692	c(t = 1.16336	0.267	0.83731181	Distal Intergenic	ENSSSCG000	TNFRSF1B	TNF receptor superfamily member 1B [Source:VGNC Symbol;Acc:VGNC:94264]
chr3	58022649	58024475	1827	*	chr3	58022649	58024475	in_vitro	in_vivo	12	13	2.73416667	1.32461538	1.40955128	c(t = 1.14715	0.267	0.83731181	Promoter (<=1kb)	ENSSSCG000	CD8A	CD8 subunit alpha [Source:VGNC Symbol;Acc:VGNC:86436]
chr2	46669493	46671088	1596	*	chr2	46669493	46671088	in_vitro	in_vivo	10	10	1.894	1.11	0.784	c(t = 1.14504	0.267	0.83731181	Promoter (<=1kb)	ENSSSCG000	TEAD1	TEA domain transcription factor 1 [Source:VGNC Symbol;Acc:VGNC:93853]
chr18	50532582	50534287	1706	*	chr18	50532582	50534287	in_vitro	in_vivo	13	12	2.32307692	1.05083333	1.27224359	c(t = 1.15530	0.267	0.83731181	Distal Intergenic	ENSSSCG00000016737		peptidylprolyl isomerase A [Source:NCBI gene (formerly Entrezgene);Acc:397637]

chr8	75643575	75645530	1956	*	chr8	75643575	75645530	in_vitro	in_vivo	13	13	1.72307692	0.74615385	0.97692308	c(t = 1.15358	0.267	0.83731181	Distal Intergenic	ENSSSCG000	TLR2	toll like receptor 2 [Source:VGNC Symbol;Acc:VGNC:94024]
chr7	30331582	30332250	669	*	chr7	30331582	30332250	in_vitro	in_vivo	9	9	2.79222222	0.55555556	2.23666667	c(t = 1.17930	0.267	0.83731181	Promoter (<=1kb)	ENSSSCG000	SMIM29	small integral membrane protein 29 [Source:HGNC Symbol;Acc:HGNC:1340]
chrX	124544980	124548857	3878	*	chrX	124544980	124548857	in_vitro	in_vivo	9	11	70.8311111	54.4463636	16.3847475	c(t = 1.14391	0.268	0.83731181	Distal Intergenic	ENSSSCG00000024676		SRSF protein kinase 3 [Source:VGNC Symbol;Acc:VGNC:93464]
chr17	35657362	35658270	909	*	chr17	35657362	35658270	in_vitro	in_vivo	10	13	67.261	50.7107692	16.5502308	c(t = 1.13950	0.268	0.83731181	Distal Intergenic	ENSSSCG000	POFUT1	protein O-fucosyltransferase 1 [Source:VGNC Symbol;Acc:VGNC:95637]
chrX	125008695	125014215	5521	*	chrX	125008695	125014215	in_vitro	in_vivo	13	14	33.6092308	46.4035714	-12.794341	c(t = -1.1334	0.268	0.83731181	Distal Intergenic	ENSSSCG000	IKBKG	inhibitor of nuclear factor kappa B kinase regulatory subunit gamma [Source:VGNC Symbol;Acc:VGNC:89072]
chr1	56378061	56378402	342	*	chr1	56378061	56378402	in_vitro	in_vivo	9	9	17.2366667	39.0088889	-21.772222	c(t = -1.1532	0.268	0.83731181	Promoter (<=1kb)	ENSSSCG000	SPACA1	sperm acrosome associated 1 [Source:NCBI gene (formerly Entrezgene);Acc:100156797]
chr13	66204370	66205896	1527	*	chr13	66204370	66205896	in_vitro	in_vivo	14	14	18.5742857	11.4907143	7.08357143	c(t = 1.13850	0.268	0.83731181	Distal Intergenic	ENSSSCG000	CIDEC	cell death inducing DFFA like effector c [Source:VGNC Symbol;Acc:VGNC:86700]
chr4	137620	139244	1625	*	chr4	137620	139244	in_vitro	in_vivo	9	12	19.2733333	9.61333333	9.66	c(t = 1.15775	0.268	0.83731181	Distal Intergenic	ENSSSCG000	SLC52A1	solute carrier family 52 member 2 [Source:NCBI gene (formerly Entrezgene);Acc:445519]
chr7	93253509	93254384	876	*	chr7	93253509	93254384	in_vitro	in_vivo	11	11	1.20454545	3.41090909	-2.2063636	c(t = -1.1683	0.268	0.83731181	Distal Intergenic	ENSSSCG000	PLEKHD1	pleckstrin homology and coiled-coil domain containing D1 [Source:VGNC Symbol;Acc:VGNC:91539]
chr4	106656071	106656917	847	*	chr4	106656071	106656917	in_vitro	in_vivo	12	14	0.64166667	2.12	-1.4783333	c(t = -1.1508	0.268	0.83731181	Distal Intergenic	ENSSSCG000	BCAS2	BCAS2 pre-mRNA processing factor [Source:NCBI gene (formerly Entrezgene);Acc:100154006]
chr6	17605768	17606431	664	*	chr6	17605768	17606431	in_vitro	in_vivo	11	9	0.24909091	1.18333333	-0.9342424	c(t = -1.1749	0.268	0.83731181	Distal Intergenic	ENSSSCG000	NIP7	nucleolar pre-rRNA processing protein NIP7 [Source:NCBI gene (formerly Entrezgene);Acc:595107]
chr3	9672138	9673694	1557	*	chr3	9672138	9673694	in_vitro	in_vivo	9	10	4.79666667	1.138	3.65866667	c(t = 1.18330	0.268	0.83731181	Distal Intergenic	ENSSSCG000	ZP3	zona pellucida glycoprotein 3 [Source:NCBI gene (formerly Entrezgene);Acc:396927]
chr2	134700543	134701416	874	*	chr2	134700543	134701416	in_vitro	in_vivo	11	13	1.75181818	0.85	0.90181818	c(t = 1.13797	0.268	0.83731181	Distal Intergenic	ENSSSCG000	IRF1	interferon regulatory factor 1 [Source:VGNC Symbol;Acc:VGNC:89203]
chr14	35662639	35662978	340	*	chr14	35662639	35662978	in_vitro	in_vivo	9	10	0.85	1.952	-1.102	c(t = -1.1540	0.269	0.83731181	Distal Intergenic	ENSSSCG000	FBXO21	F-box protein 21 [Source:VGNC Symbol;Acc:VGNC:88035]
chr8	23457642	23460872	3231	*	chr8	23457642	23460872	in_vitro	in_vivo	14	15	2.66571429	1.744	0.92171429	c(t = 1.13795	0.269	0.83731181	Promoter (<=1kb)	ENSSSCG000	PCDH7	protocadherin 7 [Source:NCBI gene (formerly Entrezgene);Acc:100520035]
chr5	90093	91599	1507	*	chr5	90093	91599	in_vitro	in_vivo	11	12	3.58909091	1.73416667	1.85492424	c(t = 1.15606	0.269	0.83731181	Distal Intergenic	ENSSSCG000	ARSA	arylsulfatase A [Source:NCBI gene (formerly Entrezgene);Acc:396973]
chr14	139072367	139073598	1232	*	chr14	139072367	139073598	in_vitro	in_vivo	10	13	0.653	1.39307692	-0.7400769	c(t = -1.1375	0.269	0.83731181	Promoter (<=1kb)	ENSSSCG000	GLRX3	glutaredoxin 3 [Source:VGNC Symbol;Acc:VGNC:88495]
chr9	95280480	95282175	1696	*	chr9	95280480	95282175	in_vitro	in_vivo	12	15	3.12666667	1.188	1.93866667	c(t = 1.15595	0.269	0.83731181	Distal Intergenic	ENSSSCG000	CROT	carnitine O-octanoyltransferase [Source:NCBI gene (formerly Entrezgene);Acc:100521142]
chr6	166137705	166139053	1349	*	chr6	166137705	166139053	in_vitro	in_vivo	12	13	2.11416667	1.00076923	1.11339744	c(t = 1.15454	0.269	0.83731181	Distal Intergenic	ENSSSCG000	UROD	uroporphyrinogen decarboxylase [Source:NCBI gene (formerly Entrezgene);Acc:100514239]
chr11	66678135	66679652	1518	*	chr11	66678135	66679652	in_vitro	in_vivo	15	15	1.67	0.81866667	0.85133333	c(t = 1.12880	0.269	0.83731181	Distal Intergenic	ENSSSCG000	IPO5	importin 5 [Source:NCBI gene (formerly Entrezgene);Acc:100152933]
chr9	23207972	23209049	1078	*	chr9	23207972	23209049	in_vitro	in_vivo	13	14	2.08692308	0.72071429	1.36620879	c(t = 1.14814	0.269	0.83731181	Promoter (<=1kb)	ENSSSCG000	CHORDC1	cysteine and histidine rich domain containing 1 [Source:NCBI gene (formerly Entrezgene);Acc:100134961]
chr17	63028957	63029743	787	*	chr17	63028957	63029743	in_vitro	in_vivo	9	12	0.18222222	0.60666667	-0.4244444	c(t = -1.1436	0.269	0.83731181	Distal Intergenic	NA	NA	NA
chr11	33257361	33259093	1733	*	chr11	33257361	33259093	in_vitro	in_vivo	11	16	1.94363636	0.571875	1.37176136	c(t = 1.15900	0.269	0.83731181	Distal Intergenic	ENSSSCG000	MTRF1	mitochondrial translation release factor 1 [Source:VGNC Symbol;Acc:VGNC:90471]
chr8	71363372	71364228	857	*	chr8	71363372	71364228	in_vitro	in_vivo	9	11	1.18888889	0.48272727	0.70616162	c(t = 1.14710	0.269	0.83731181	Distal Intergenic	ENSSSCG000	RCHY1	ring finger and CHY zinc finger domain containing 1 [Source:VGNC Symbol;Acc:VGNC:92179]
chr9	130434921	130435588	668	*	chr9	130434921	130435588	in_vitro	in_vivo	12	12	1.29916667	0.42833333	0.87083333	c(t = 1.14883	0.269	0.83731181	Distal Intergenic	ENSSSCG000	FLVCR1	FLVCR heme transporter 1 [Source:NCBI gene (formerly Entrezgene);Acc:100217398]
chr16	78962485	78966170	3686	*	chr16	78962485	78966170	in_vitro	in_vivo	11	11	14.4463636	29.7190909	-15.272727	c(t = -1.1392	0.27	0.83731181	Distal Intergenic	ENSSSCG000	TERT	telomerase reverse transcriptase [Source:NCBI gene (formerly Entrezgene);Acc:492280]
chr2	38824408	38825157	750	*	chr2	38824408	38825157	in_vitro	in_vivo	9	9	9.61222222	26.7244444	-17.112222	c(t = -1.1564	0.27	0.83731181	Distal Intergenic	ENSSSCG000	CSRP3	cysteine and glycine rich protein 3 [Source:NCBI gene (formerly Entrezgene);Acc:100337687]
chr3	75613986	75615048	1063	*	chr3	75613986	75615048	in_vitro	in_vivo	9	12	24.56	9.99833333	14.5616667	c(t = 1.16351	0.27	0.83731181	Distal Intergenic	ENSSSCG000	ACTR2	actin related protein 2 [Source:VGNC Symbol;Acc:VGNC:85051]
chr2	69296943	69297623	681	*	chr2	69296943	69297623	in_vitro	in_vivo	12	11	7.92416667	3.27454545	4.64962121	c(t = 1.14526	0.27	0.83731181	Promoter (1-2kb)	ENSSSCG000	S1PR5	sphingosine-1-phosphate receptor 5 [Source:VGNC Symbol;Acc:VGNC:92555]
chr14	73074548	73074866	319	*	chr14	73074548	73074866	in_vitro	in_vivo	10	9	2.79	1.21444444	1.57555556	c(t = 1.15009	0.27	0.83731181	Distal Intergenic	ENSSSCG000	SAR1A	secretion associated Ras related GTPase 1A [Source:VGNC Symbol;Acc:VGNC:92579]
chr1	118913210	118914612	1403	*	chr1	118913210	118914612	in_vitro	in_vivo	10	11	1.893	1.13727273	0.75572727	c(t = 1.13583	0.27	0.83731181	Distal Intergenic	ENSSSCG000	ARPP19	cAMP regulated phosphoprotein 19 [Source:NCBI gene (formerly Entrezgene);Acc:397362]
chr5	12242109	12242865	757	*	chr5	12242109	12242865	in_vitro	in_vivo	9	10	3.20111111	1.02	2.18111111	c(t = 1.17120	0.27	0.83731181	Distal Intergenic	ENSSSCG000	ssc-mir-9790	ssc-mir-9790 [Source:miRBase;Acc:MI0031553]
chr9	33159532	33160468	937	*	chr9	33159532	33160468	in_vitro	in_vivo	9	10	2.10222222	0.68	1.42222222	c(t = 1.16313	0.27	0.83731181	Distal Intergenic	ENSSSCG000	MMP7	matrix metallopeptidase 7 [Source:NCBI gene (formerly Entrezgene);Acc:397411]
chr15	55680835	55681669	835	*	chr15	55680835	55681669	in_vitro	in_vivo	10	11	1.048	0.32454545	0.72345455	c(t = 1.14595	0.27	0.83731181	Distal Intergenic	ENSSSCG000	MBOAT4	membrane bound O-acyltransferase domain containing 4 [Source:NCBI gene (formerly Entrezgene);Acc:100153109]
chr4	110836126	110836847	722	*	chr4	110836126	110836847	in_vitro	in_vivo	9	11	14.1255556	31.7072727	-17.581717	c(t = -1.1352	0.271	0.83731181	Distal Intergenic	ENSSSCG000	PSMA5	proteasome 20S subunit alpha 5 [Source:VGNC Symbol;Acc:VGNC:98838]
chr9	89764171	89765975	1805	*	chr9	89764171	89765975	in_vitro	in_vivo	13	16	14.1646154	8.449375	5.71524038	c(t = 1.13656	0.271	0.83731181	Distal Intergenic	ENSSSCG000	ITGB8	integrin subunit beta 8 [Source:VGNC Symbol;Acc:VGNC:89246]
chrX	35881410	35882958	1549	*	chrX	35881410	35882958	in_vitro	in_vivo	11	9	1.21	7.91	-6.7	c(t = -1.1793	0.271	0.83731181	Distal Intergenic	ENSSSCG000	DDX3X	DEAD-box helicase 3 X-linked [Source:NCBI gene (formerly Entrezgene);Acc:100515940]
chr6	91229803	91230849	1047	*	chr6	91229803	91230849	in_vitro	in_vivo	14	13	5.48928571	3.49846154	1.99082418	c(t = 1.12856	0.271	0.83731181	Distal Intergenic	ENSSSCG000	GJA4	gap junction protein alpha 4 [Source:NCBI gene (formerly Entrezgene);Acc:100620145]
chr12	47528938	47531026	2089	*	chr12	47528938	47531026	in_vitro	in_vivo	11	11	5.18636364	2.89454545	2.29181818	c(t = 1.13452	0.271	0.83731181	Distal Intergenic	ENSSSCG000	CRK	CRK proto-onco, adaptor protein [Source:NCBI gene (formerly Entrezgene);Acc:100192444]
chr1	120945694	120946683	990	*	chr1	120945694	120946683	in_vitro	in_vivo	9	12	4.14666667	2.13916667	2.0075	c(t = 1.13918	0.271	0.83731181	Distal Intergenic	ENSSSCG00000054479		cytochrome P450 19A2 [Source:NCBI gene (formerly Entrezgene);Acc:403332]
chr1	236919501	236920390	890	*	chr1	236919501	236920390	in_vitro	in_vivo	9	12	4.99555556	1.31666667	3.67888889	c(t = 1.17708	0.271	0.83731181	Distal Intergenic	ENSSSCG000	GNE	glucosamine (UDP-N-acetyl)-2-epimerase/N-acetylmannosamine kinase [Source:VGNC Symbol;Acc:VGNC:103101]
chr1	7650678	7652010	1333	*	chr1	7650678	7652010	in_vitro	in_vivo	10	13	5.79	1.21923077	4.57076923	c(t = 1.16860	0.271	0.83731181	Promoter (<=1kb)	ENSSSCG00000004050		WT1 associated protein [Source:VGNC Symbol;Acc:VGNC:94984]
chr6	12855028	12855669	642	*	chr6	12855028	12855669	in_vitro	in_vivo	14	10	2.82785714	1.06	1.76785714	c(t = 1.13587	0.271	0.83731181	Distal Intergenic	ENSSSCG000	COG4	component of oligomeric golgi complex 4 [Source:NCBI gene (formerly Entrezgene);Acc:100625346]
chr18	4147656	4149260	1605	*	chr18	4147656	4149260	in_vitro	in_vivo	9	14	2.23777778	1.03571429	1.20206349	c(t = 1.14910	0.271	0.83731181	Distal Intergenic	ENSSSCG000	INSIG1	insulin induced gene 1 [Source:NCBI gene (formerly Entrezgene);Acc:403162]
chr6	74236274	74237206	933	*	chr6	74236274	74237206	in_vitro	in_vivo	13	12	2.11076923	0.9975	1.11326923	c(t = 1.12821	0.271	0.83731181	Distal Intergenic	ENSSSCG000	TMEM51	transmembrane protein 51 [Source:VGNC Symbol;Acc:VGNC:94187]
chr13	82937097	82937797	701	*	chr13	82937097	82937797	in_vitro	in_vivo	9	11	2.05666667	0.61909091	1.43757576	c(t = 1.17162	0.271	0.83731181	Distal Intergenic	ENSSSCG000	GRK7	G protein-coupled receptor kinase 7 [Source:VGNC Symbol;Acc:VGNC:88699]
chr1	271408923	271410200	1278	*	chr1	271408923	271410200	in_vitro	in_vivo	9	10	97.21	94.81	2.4	c(t = 1.13774	0.272	0.83731181	Distal Intergenic	ENSSSCG000	MED27	mediator complex subunit 27 [Source:VGNC Symbol;Acc:VGNC:90117]
chr13	31516383	31517443	1061	*	chr13	31516383	31517443	in_vitro	in_vivo	13	13	1.11384615	2.44846154	-1.3346154	c(t = -1.1422	0.272	0.83731181	Promoter (<=1kb)	ENSSSCG000	PRKAR2A	protein kinase cAMP-dependent type II regulatory subunit alpha [Source:NCBI gene (formerly Entrezgene);Acc:397493]
chr8	37874885	37875606	722	*	chr8	37874885	37875606	in_vitro	in_vivo	9	12	0.44333333	1.3	-0.8566667	c(t = -1.1415	0.272	0.83731181	Distal Intergenic	ENSSSCG000	ZAR1	zygote arrest 1 [Source:VGNC Symbol;Acc:VGNC:95051]
chr8	86546273	86546957	685	*	chr8	86546273	86546957	in_vitro	in_vivo	9	11	2.20111111	0.88454545	1.31656566	c(t = 1.14377	0.272	0.83731181	Distal Intergenic	ENSSSCG000	CLGN	calmegin [Source:VGNC Symbol;Acc:VGNC:86759]
chr10	31000652	31001558	907	*	chr10	31000652	31001558	in_vitro	in_vivo	12	11	2.01166667	0.58454545	1.42712121	c(t = 1.14615	0.272	0.83731181	Distal Intergenic	ENSSSCG000	ssc-mir-7-2	ssc-mir-7-2 [Source:miRBase;Acc:MI0002435]
chr6	81422970	81424316	1347	*	chr6	81422970	81424316	in_vitro	in_vivo	11	10	0.96272727	0.357	0.60572727	c(t = 1.13748	0.272	0.83731181	Promoter (<=1kb)	ENSSSCG000	ID3	inhibitor of DNA binding 3 [Source:NCBI gene (formerly Entrezgene);Acc:100626978]
chr1	253350595	253351048	454	*	chr1	253350595	253351048	in_vitro	in_vivo	10	9	12.631	32.2222222	-19.591222	c(t = -1.1638	0.273	0.83731181	Distal Intergenic	ENSSSCG000	INIP	INTS3 and NABP interacting protein [Source:VGNC Symbol;Acc:VGNC:89136]
chr3	131534166	131535301	1136	*	chr3	131534166	131535301	in_vitro	in_vivo	9	10	9.33111111	23.687	-14.355889	c(t = -1.1589	0.273	0.83731181	Distal Intergenic	ENSSSCG000	EIPR1	EARP complex and GARP complex interacting protein 1 [Source:VGNC Symbol;Acc:VGNC:87635]
chr6	53338166	53340485	2320	*	chr6	53338166	53340485	in_vitro	in_vivo	10	10	2.647	5.621	-2.974	c(t = -1.1332	0.273	0.83731181	Distal Intergenic	ENSSSCG000	NAPA	NSF attachment protein alpha [Source:VGNC Symbol;Acc:VGNC:90578]
chrX	122535268	122536981	1714	*	chrX	122535268	122536981	in_vitro	in_vivo	9	11	1.37888889	4.67454545	-3.2956566	c(t = -1.1493	0.273	0.83731181	Distal Intergenic	ENSSSCG000	FATE1	fetal and adult testis expressed 1 [Source:NCBI gene (formerly Entrezgene);Acc:396705]
chr4	99493239	99493892	654	*	chr4	99493239	99493892	in_vitro	in_vivo	12	10	0.30083333	3.031	-2.7301667	c(t = -1.1675	0.273	0.83731181	Distal Intergenic	ENSSSCG00000006685		gonadotropin releasing hormone receptor 2 [Source:VGNC Symbol;Acc:VGNC:109150]
chr9	39790010	39790626	617	*	chr9	39790010	39790626	in_vitro	in_vivo	9	11	0.90444444	2.73	-1.8255556	c(t = -1.1392	0.273	0.83731181	Promoter (<=1kb)	ENSSSCG000	SDHD	succinate dehydrogenase complex subunit D [Source:NCBI gene (formerly Entrezgene);Acc:100048954]
chr8	1809442	1810835	1394	*	chr8	1809442	1810835	in_vitro	in_vivo	11	11	0.94090909	2.41	-1.4690909	c(t = -1.1517	0.273	0.83731181	Promoter (<=1kb)	ENSSSCG000	HTT	huntingtin [Source:VGNC Symbol;Acc:VGNC:89006]
chr7	53020233	53021085	853	*	chr7	53020233	53021085	in_vitro	in_vivo	10	11	4.092	1.92909091	2.16290909	c(t = 1.14396	0.273	0.83731181	Distal Intergenic	ENSSSCG000	NMB	neuromedin B [Source:VGNC Symbol;Acc:VGNC:109471]
chr1	209181322	209182320	999	*	chr1	209181322	209182320	in_vitro	in_vivo	11	11	2.55272727	1.22727273	1.32545455	c(t = 1.13889	0.273	0.83731181	Distal Intergenic	ENSSSCG000	TYRP1	tyrosinase related protein 1 [Source:VGNC Symbol;Acc:VGNC:94620]
chr1	269966619	269967269	651	*	chr1	269966619	269967269	in_vitro	in_vivo	9	11	1.97555556	0.90181818	1.07373737	c(t = 1.13235	0.273	0.83731181	Distal Intergenic	ENSSSCG000	TOR1A	torsin family 1 member A [Source:NCBI gene (formerly Entrezgene);Acc:100125953]
chr11	22267040	22267799	760	*	chr11	22267040	22267799	in_vitro	in_vivo	10	13	1.68	0.40461538	1.27538462	c(t = 1.14937	0.273	0.83731181	Distal Intergenic	ENSSSCG000	GTF2F2	general transcription factor IIF subunit 2 [Source:NCBI gene (formerly Entrezgene);Acc:100626869]
chr10	28995319	28996127	809	*	chr10	28995319	28996127	in_vitro	in_vivo	9	13	2.08333333	0.32076923	1.7625641	c(t = 1.16907	0.273	0.83731181	Distal Intergenic	ENSSSCG000	FBP2	fructose-bisphosphatase 2 [Source:NCBI gene (formerly Entrezgene);Acc:100134828]
chr7	76005976	76006524	549	*	chr7	76005976	76006524	in_vitro	in_vivo	9	9	20.8155556	37.7466667	-16.931111	c(t = -1.1386	0.274	0.83731181	Distal Intergenic	ENSSSCG000	CDH24	cadherin 24 [Source:VGNC Symbol;Acc:VGNC:86485]
chr7	812368	814268	1901	*	chr7	812368	814268	in_vitro	in_vivo	9	13	15.21	32.9561538	-17.746154	c(t = -1.1243	0.274	0.83731181	Distal Intergenic	ENSSSCG000	IRF4	interferon regulatory factor 4 [Source:VGNC Symbol;Acc:VGNC:89207]
chr6	167188190	167188577	388	*	chr6	167188190	167188577	in_vitro	in_vivo	10	9	37	17.4777778	19.5222222	c(t = 1.13547	0.274	0.83731181	Promoter (<=1kb)	ENSSSCG000	KLF17	KLF transcription factor 17 [Source:NCBI gene (formerly Entrezgene);Acc:100302639]
chr13	140793256	140793917	662	*	chr13	140793256	140793917	in_vitro	in_vivo	9	12	5.83555556	2.79333333	3.04222222	c(t = 1.14562	0.274	0.83731181	Distal Intergenic	ENSSSCG000	CD80	CD80 molecule [Source:VGNC Symbol;Acc:VGNC:97920]
chr8	40967143	40971021	3879	*	chr8	40967143	40971021	in_vitro	in_vivo	12	14	2.87333333	1.60714286	1.26619048	c(t = 1.12449	0.274	0.83731181	Promoter (<=1kb)	ENSSSCG000	PDGFRA	platelet derived growth factor receptor alpha [Source:NCBI gene (formerly Entrezgene);Acc:100627123]
chr1	158336787	158338004	1218	*	chr1	158336787	158338004	in_vitro	in_vivo	9	10	0.46555556	1.296	-0.8304444	c(t = -1.1508	0.274	0.83731181	Distal Intergenic	ENSSSCG000	SERPINB10	serpin family B member 10 [Source:NCBI gene (formerly Entrezgene);Acc:100154994]
chr2	126605699	126607462	1764	*	chr2	126605699	126607462	in_vitro	in_vivo	10	12	2.231	1.08166667	1.14933333	c(t = 1.15501	0.274	0.83731181	Distal Intergenic	NA	NA	NA
chr7	50976716	50977512	797	*	chr7	50976716	50977512	in_vitro	in_vivo	13	13	2.26307692	0.87615385	1.38692308	c(t = 1.13796	0.274	0.83731181	Distal Intergenic	ENSSSCG000	IL16	interleukin 16 [Source:VGNC Symbol;Acc:VGNC:89084]
chr10	31416365	31417437	1073	*	chr10	31416365	31417437	in_vitro	in_vivo	11	12	3.22909091	0.82666667	2.40242424	c(t = 1.15041	0.274	0.83731181	Distal Intergenic	ENSSSCG000	ssc-mir-7-2	ssc-mir-7-2 [Source:miRBase;Acc:MI0002435]
chr3	2955907	2957696	1790	*	chr3	2955907	2957696	in_vitro	in_vivo	11	9	85.3663636	92.4711111	-7.1047475	c(t = -1.1256	0.275	0.83731181	Distal Intergenic	ENSSSCG000	AP5Z1	adaptor related protein complex 5 subunit zeta 1 [Source:VGNC Symbol;Acc:VGNC:85397]
chr3	131723982	131724647	666	*	chr3	131723982	131724647	in_vitro	in_vivo	9	11	25.5755556	10.4154545	15.160101	c(t = 1.14672	0.275	0.83731181	Distal Intergenic	ENSSSCG000	EIPR1	EARP complex and GARP complex interacting protein 1 [Source:VGNC Symbol;Acc:VGNC:87635]
chr14	127271456	127272220	765	*	chr14	127271456	127272220	in_vitro	in_vivo	10	13	7.244	4.74307692	2.50092308	c(t = 1.14051	0.275	0.83731181	Distal Intergenic	ENSSSCG000	SLC18A2	solute carrier family 18 member A2 [Source:NCBI gene (formerly Entrezgene);Acc:100154950]
chr11	76650550	76656366	5817	*	chr11	76650550	76656366	in_vitro	in_vivo	16	17	9.29	4.01352941	5.27647059	c(t = 1.12918	0.275	0.83731181	Distal Intergenic	ENSSSCG000	NAXD	NAD(P)HX dehydratase [Source:NCBI gene (formerly Entrezgene);Acc:100153456]
chr2	69649249	69650675	1427	*	chr2	69649249	69650675	in_vitro	in_vivo	13	13	1.42230769	2.84769231	-1.4253846	c(t = -1.1249	0.275	0.83731181	Distal Intergenic	ENSSSCG000	ssc-mir-199a	ssc-mir-199a-2 [Source:miRBase;Acc:MI0013093]
chr6	157910342	157912709	2368	*	chr6	157910342	157912709	in_vitro	in_vivo	13	12	1.49615385	2.59083333	-1.0946795	c(t = -1.1228	0.275	0.83731181	Distal Intergenic	ENSSSCG000	TMEM59	transmembrane protein 59 [Source:VGNC Symbol;Acc:VGNC:94192]
chr8	1215929	1217422	1494	*	chr8	1215929	1217422	in_vitro	in_vivo	9	11	8.30555556	1.98636364	6.31919192	c(t = 1.16778	0.275	0.83731181	Distal Intergenic	ENSSSCG000	RNF4	ring finger protein 4 [Source:VGNC Symbol;Acc:VGNC:92392]
chr15	106515974	106516776	803	*	chr15	106515974	106516776	in_vitro	in_vivo	10	9	4.103	1.83111111	2.27188889	c(t = 1.13143	0.275	0.83731181	Distal Intergenic	ENSSSCG000	CYP20A1	cytochrome P450 family 20 subfamily A member 1 [Source:NCBI gene (formerly Entrezgene);Acc:100124408]
chr4	85405225	85407586	2362	*	chr4	85405225	85407586	in_vitro	in_vivo	10	14	3.815	1.63785714	2.17714286	c(t = 1.13984	0.275	0.83731181	Distal Intergenic	ENSSSCG000	ssc-mir-9849	ssc-mir-9849 [Source:miRBase;Acc:MI0031625]
chr13	94155553	94157043	1491	*	chr13	94155553	94157043	in_vitro	in_vivo	12	13	2.5325	1.36615385	1.16634615	c(t = 1.11872	0.275	0.83731181	Distal Intergenic	ENSSSCG000	GPR149	G protein-coupled receptor 149 [Source:VGNC Symbol;Acc:VGNC:88605]
chr2	71047365	71048366	1002	*	chr2	71047365	71048366	in_vitro	in_vivo	10	9	2.335	0.94555556	1.38944444	c(t = 1.14880	0.275	0.83731181	Distal Intergenic	ENSSSCG000	CERS4	ceramide synthase 4 [Source:VGNC Symbol;Acc:VGNC:86589]
chr17	47297813	47298653	841	*	chr17	47297813	47298653	in_vitro	in_vivo	10	9	3.206	0.74222222	2.46377778	c(t = 1.15434	0.275	0.83731181	Distal Intergenic	ENSSSCG000	TOMM34	translocase of outer mitochondrial membrane 34 [Source:VGNC Symbol;Acc:VGNC:95647]
chr4	3558933	3562944	4012	*	chr4	3558933	3562944	in_vitro	in_vivo	13	15	5.44461538	3.518	1.92661538	c(t = 1.12288	0.276	0.83916667	Distal Intergenic	ENSSSCG000	ago-02	argonaute RISC catalytic component 2 [Source:NCBI gene (formerly Entrezgene);Acc:100155245]
chr17	31591390	31592201	812	*	chr17	31591390	31592201	in_vitro	in_vivo	10	13	5.954	1.12	4.834	c(t = 1.15630	0.276	0.83916667	Distal Intergenic	ENSSSCG000	SMOX	spermine oxidase [Source:VGNC Symbol;Acc:VGNC:95679]
chr1	270902900	270904959	2060	*	chr1	270902900	270904959	in_vitro	in_vivo	9	10	79.6433333	92.302	-12.658667	c(t = -1.1600	0.277	0.83916667	Distal Intergenic	ENSSSCG000	FUBP3	far upstream element binding protein 3 [Source:VGNC Symbol;Acc:VGNC:103093]
chr14	50976586	50978141	1556	*	chr14	50976586	50978141	in_vitro	in_vivo	11	12	4.28090909	9.16416667	-4.8832576	c(t = -1.1327	0.277	0.83916667	Promoter (<=1kb)	ENSSSCG000	SLC25A1	solute carrier family 25 member 1 [Source:VGNC Symbol;Acc:VGNC:92990]
chr18	6242381	6244253	1873	*	chr18	6242381	6244253	in_vitro	in_vivo	10	12	10.752	6.08833333	4.66366667	c(t = 1.12329	0.277	0.83916667	Distal Intergenic	ENSSSCG000	NOS3	nitric oxide synthase 3 [Source:NCBI gene (formerly Entrezgene);Acc:397557]
chr6	108115800	108117776	1977	*	chr6	108115800	108117776	in_vitro	in_vivo	14	11	8.58071429	2.86272727	5.71798701	c(t = 1.13355	0.277	0.83916667	Distal Intergenic	ENSSSCG000	NPC1	NPC intracellular cholesterol transporter 1 [Source:NCBI gene (formerly Entrezgene);Acc:397591]
chr18	26623809	26624680	872	*	chr18	26623809	26624680	in_vitro	in_vivo	12	13	4.06583333	1.77769231	2.28814103	c(t = 1.12210	0.277	0.83916667	Distal Intergenic	ENSSSCG000	TSPAN12	tetraspanin 12 [Source:VGNC Symbol;Acc:VGNC:94501]
chr6	103407862	103408758	897	*	chr6	103407862	103408758	in_vitro	in_vivo	11	11	1.68	0.87545455	0.80454545	c(t = 1.12293	0.277	0.83916667	Distal Intergenic	ENSSSCG000	MYL12A	myosin light chain 12A [Source:HGNC Symbol;Acc:HGNC:16701]
chr15	137658104	137658578	475	*	chr15	137658104	137658578	in_vitro	in_vivo	9	9	16.5933333	33.8255556	-17.232222	c(t = -1.1263	0.278	0.83916667	Distal Intergenic	ENSSSCG000	RAMP1	receptor activity modifying protein 1 [Source:NCBI gene (formerly Entrezgene);Acc:397401]
chr3	113874773	113877121	2349	*	chr3	113874773	113877121	in_vitro	in_vivo	10	12	6.482	11.65	-5.168	c(t = -1.1181	0.278	0.83916667	Distal Intergenic	ENSSSCG00000021155		proopiomelanocortin [Source:VGNC Symbol;Acc:VGNC:110699]
chr4	129257242	129259255	2014	*	chr4	129257242	129259255	in_vitro	in_vivo	12	13	0.6425	2.80153846	-2.1590385	c(t = -1.1335	0.278	0.83916667	Promoter (<=1kb)	ENSSSCG000	SELENOF	selenoprotein F [Source:NCBI gene (formerly Entrezgene);Acc:100038009]
chr12	60528556	60530790	2235	*	chr12	60528556	60530790	in_vitro	in_vivo	13	12	1.16	2.33	-1.17	c(t = -1.1243	0.278	0.83916667	Distal Intergenic	ENSSSCG000	SLC5A10	solute carrier family 5 member 10 [Source:NCBI gene (formerly Entrezgene);Acc:497235]
chrX	112131385	112132050	666	*	chrX	112131385	112132050	in_vitro	in_vivo	10	9	9.749	2.32	7.429	c(t = 1.15149	0.278	0.83916667	Distal Intergenic	ENSSSCG00000012703		RNA binding motif protein X-linked [Source:NCBI gene (formerly Entrezgene);Acc:100158042]
chr6	46347872	46348261	390	*	chr6	46347872	46348261	in_vitro	in_vivo	10	9	3.666	1.71888889	1.94711111	c(t = 1.12000	0.278	0.83916667	Distal Intergenic	ENSSSCG000	PPP1R14A	protein phosphatase 1 regulatory inhibitor subunit 14A [Source:VGNC Symbol;Acc:VGNC:98539]
chr1	143108915	143109914	1000	*	chr1	143108915	143109914	in_vitro	in_vivo	13	10	1.18538462	0.647	0.53838462	c(t = 1.11478	0.278	0.83916667	Distal Intergenic	ENSSSCG000	KLF13	KLF transcription factor 13 [Source:NCBI gene (formerly Entrezgene);Acc:494563]
chr9	19594328	19595127	800	*	chr9	19594328	19595127	in_vitro	in_vivo	12	12	1.77333333	0.62833333	1.145	c(t = 1.12835	0.278	0.83916667	Distal Intergenic	ENSSSCG000	TMEM126A	transmembrane protein 126A [Source:VGNC Symbol;Acc:VGNC:104077]
chr9	130835184	130835872	689	*	chr9	130835184	130835872	in_vitro	in_vivo	12	10	2.7625	0.385	2.3775	c(t = 1.13768	0.278	0.83916667	Distal Intergenic	ENSSSCG000	PPP2R5A	protein phosphatase 2 regulatory subunit B'alpha [Source:NCBI gene (formerly Entrezgene);Acc:100101925]
chr4	100481065	100481435	371	*	chr4	100481065	100481435	in_vitro	in_vivo	11	11	5.05	11.4381818	-6.3881818	c(t = -1.1152	0.279	0.83916667	Distal Intergenic	ENSSSCG000	PRKAB2	protein kinase AMP-activated non-catalytic subunit beta 2 [Source:VGNC Symbol;Acc:VGNC:91799]
chr2	51321094	51321956	863	*	chr2	51321094	51321956	in_vitro	in_vivo	13	11	10.6869231	4.95272727	5.7341958	c(t = 1.12056	0.279	0.83916667	Distal Intergenic	NA	NA	NA
chr9	6679608	6680398	791	*	chr9	6679608	6680398	in_vitro	in_vivo	10	11	2.175	3.81	-1.635	c(t = -1.1180	0.279	0.83916667	Distal Intergenic	ENSSSCG000	LRRC51	leucine-rich repeat-containing protein 51 [Source:NCBI gene (formerly Entrezgene);Acc:100520208]
chr6	86137854	86139444	1591	*	chr6	86137854	86139444	in_vitro	in_vivo	13	15	4.01692308	1.80066667	2.21625641	c(t = 1.12327	0.279	0.83916667	Distal Intergenic	ENSSSCG000	MECR	mitochondrial trans-2-enoyl-CoA reductase [Source:NCBI gene (formerly Entrezgene);Acc:100515782]
chr1	57128007	57129548	1542	*	chr1	57128007	57129548	in_vitro	in_vivo	13	11	1.25692308	0.54636364	0.71055944	c(t = 1.11494	0.279	0.83916667	Distal Intergenic	ENSSSCG000	SRSF12	serine and arginine rich splicing factor 12 [Source:VGNC Symbol;Acc:VGNC:93474]
chrX	58182285	58184738	2454	*	chrX	58182285	58184738	in_vitro	in_vivo	9	14	5.17777778	13.8221429	-8.6443651	c(t = -1.1169	0.28	0.83916667	Promoter (<=1kb)	ENSSSCG000	CITED1	Cbp/p300 interacting transactivator with Glu/Asp rich carboxy-terminal domain 1 [Source:VGNC Symbol;Acc:VGNC:86711]
chr15	136027774	136033785	6012	*	chr15	136027774	136033785	in_vitro	in_vivo	13	12	16.7938462	5.62833333	11.1655128	c(t = 1.12831	0.28	0.83916667	Distal Intergenic	ENSSSCG000	ssc-mir-7141	ssc-mir-7141 [Source:miRBase;Acc:MI0023574]
chr14	904075	905415	1341	*	chr14	904075	905415	in_vitro	in_vivo	9	12	0.70777778	1.705	-0.9972222	c(t = -1.1127	0.28	0.83916667	Distal Intergenic	ENSSSCG000	GADD45G	growth arrest and DNA damage inducible gamma [Source:NCBI gene (formerly Entrezgene);Acc:100152997]
chr4	18288718	18289961	1244	*	chr4	18288718	18289961	in_vitro	in_vivo	12	11	2.37	1.26090909	1.10909091	c(t = 1.11027	0.28	0.83916667	Distal Intergenic	ENSSSCG000	MRPL13	mitochondrial ribosomal protein L13 [Source:NCBI gene (formerly Entrezgene);Acc:100153348]
chr14	109051654	109052562	909	*	chr14	109051654	109052562	in_vitro	in_vivo	11	10	2.70818182	1.07	1.63818182	c(t = 1.12298	0.28	0.83916667	Distal Intergenic	ENSSSCG000	SFRP5	secreted frizzled related protein 5 [Source:NCBI gene (formerly Entrezgene);Acc:100153176]
chr3	86783397	86784793	1397	*	chr3	86783397	86784793	in_vitro	in_vivo	12	12	2.1775	0.87583333	1.30166667	c(t = 1.12590	0.28	0.83916667	Distal Intergenic	ENSSSCG000	GPR75	G protein-coupled receptor 75 [Source:VGNC Symbol;Acc:VGNC:88636]
chr1	241567559	241568284	726	*	chr1	241567559	241568284	in_vitro	in_vivo	11	9	6.72090909	0.53555556	6.18535354	c(t = 1.14121	0.28	0.83916667	Distal Intergenic	ENSSSCG000	NR4A3	nuclear receptor subfamily 4 group A member 3 [Source:VGNC Symbol;Acc:VGNC:90885]
chr6	4067472	4071617	4146	*	chr6	4067472	4071617	in_vitro	in_vivo	10	12	95.024	96.8833333	-1.8593333	c(t = -1.1095	0.281	0.83916667	Distal Intergenic	ENSSSCG000	WFDC1	WAP four-disulfide core domain 1 [Source:VGNC Symbol;Acc:VGNC:94953]
chr1	237754070	237754508	439	*	chr1	237754070	237754508	in_vitro	in_vivo	9	10	51.8466667	33.787	18.0596667	c(t = 1.11792	0.281	0.83916667	Distal Intergenic	ENSSSCG000	GNE	glucosamine (UDP-N-acetyl)-2-epimerase/N-acetylmannosamine kinase [Source:VGNC Symbol;Acc:VGNC:103101]
chrX	124717923	124727079	9157	*	chrX	124717923	124727079	in_vitro	in_vivo	16	15	14.545625	20.734	-6.188375	c(t = -1.0996	0.281	0.83916667	Distal Intergenic	ENSSSCG000	RENBP	renin binding protein [Source:NCBI gene (formerly Entrezgene);Acc:396934]
chr4	67465117	67466463	1347	*	chr4	67465117	67466463	in_vitro	in_vivo	13	12	0.71153846	6.09833333	-5.3867949	c(t = -1.1318	0.281	0.83916667	Distal Intergenic	ENSSSCG000	COPS5	COP9 signalosome subunit 5 [Source:VGNC Symbol;Acc:VGNC:86901]
chr6	93541004	93541990	987	*	chr6	93541004	93541990	in_vitro	in_vivo	9	11	12.8688889	3.52909091	9.33979798	c(t = 1.15085	0.281	0.83916667	Distal Intergenic	ENSSSCG000	MEAF6	MYST/Esa1 associated factor 6 [Source:VGNC Symbol;Acc:VGNC:90098]
chr7	4750322	4751281	960	*	chr7	4750322	4751281	in_vitro	in_vivo	11	14	1.44	3.25785714	-1.8178571	c(t = -1.1118	0.281	0.83916667	Promoter (<=1kb)	ENSSSCG000	RIOK1	RIO kinase 1 [Source:VGNC Symbol;Acc:VGNC:92315]
chr1	106683761	106685558	1798	*	chr1	106683761	106685558	in_vitro	in_vivo	10	10	0.351	2.892	-2.541	c(t = -1.1427	0.281	0.83916667	Distal Intergenic	ENSSSCG000	FECH	ferrochelatase [Source:VGNC Symbol;Acc:VGNC:88081]
chr1	57437731	57438760	1030	*	chr1	57437731	57438760	in_vitro	in_vivo	11	10	0.21090909	1.528	-1.3170909	c(t = -1.1440	0.281	0.83916667	Distal Intergenic	ENSSSCG000	RRAGD	Ras related GTP binding D [Source:VGNC Symbol;Acc:VGNC:92457]
chr6	106314117	106315302	1186	*	chr6	106314117	106315302	in_vitro	in_vivo	11	15	2.34181818	1.19	1.15181818	c(t = 1.11655	0.281	0.83916667	Distal Intergenic	ENSSSCG000	TYMS	thymidylate synthetase [Source:NCBI gene (formerly Entrezgene);Acc:100627209]
chr6	6881085	6882520	1436	*	chr6	6881085	6882520	in_vitro	in_vivo	11	10	3.11454545	1.105	2.00954545	c(t = 1.13142	0.281	0.83916667	Distal Intergenic	ENSSSCG000	BCO1	beta-carotene oxygenase 1 [Source:VGNC Symbol;Acc:VGNC:85789]
chr6	164196534	164197580	1047	*	chr6	164196534	164197580	in_vitro	in_vivo	10	13	2.466	1.07307692	1.39292308	c(t = 1.12832	0.281	0.83916667	Distal Intergenic	ENSSSCG000	CYP4A24	cytochrome P450 family 4 subfamily A member 24 [Source:NCBI gene (formerly Entrezgene);Acc:403326]
chr8	110027682	110028462	781	*	chr8	110027682	110028462	in_vitro	in_vivo	10	13	1.689	0.59153846	1.09746154	c(t = 1.11172	0.281	0.83916667	Distal Intergenic	ENSSSCG000	LARP7	La ribonucleoprotein 7, transcriptional regulator [Source:NCBI gene (formerly Entrezgene);Acc:100511273]
chr4	19180143	19180543	401	*	chr4	19180143	19180543	in_vitro	in_vivo	9	11	0.99666667	7.76636364	-6.769697	c(t = -1.1368	0.282	0.83916667	Distal Intergenic	ENSSSCG000	MAL2	mal, T cell differentiation protein 2 [Source:VGNC Symbol;Acc:VGNC:98818]
chrX	114354778	114356721	1944	*	chrX	114354778	114356721	in_vitro	in_vivo	13	14	9.93153846	4.12428571	5.80725275	c(t = 1.11673	0.282	0.83916667	Distal Intergenic	ENSSSCG000	F9	coagulation factor IX [Source:VGNC Symbol;Acc:VGNC:109453]
chr11	50102502	50102801	300	*	chr11	50102502	50102801	in_vitro	in_vivo	9	9	0.42777778	2.44222222	-2.0144444	c(t = -1.1450	0.282	0.83916667	Promoter (<=1kb)	ENSSSCG000	EDNRB	endothelin receptor type B [Source:VGNC Symbol;Acc:VGNC:87550]
chr8	4733628	4734848	1221	*	chr8	4733628	4734848	in_vitro	in_vivo	9	14	2.70444444	1.71642857	0.98801587	c(t = 1.12469	0.282	0.83916667	Distal Intergenic	ENSSSCG000	MAN2B2	mannosidase alpha class 2B member 2 [Source:VGNC Symbol;Acc:VGNC:98100]
chr14	55652524	55653975	1452	*	chr14	55652524	55653975	in_vitro	in_vivo	12	14	1.53083333	0.91071429	0.62011905	c(t = 1.10163	0.282	0.83916667	Distal Intergenic	ENSSSCG000	EDARADD	EDAR associated death domain [Source:VGNC Symbol;Acc:VGNC:87542]
chr10	47803760	47804441	682	*	chr10	47803760	47804441	in_vitro	in_vivo	11	12	0.22181818	0.6475	-0.4256818	c(t = -1.1072	0.282	0.83916667	Distal Intergenic	ENSSSCG000	PRPF18	pre-mRNA processing factor 18 [Source:VGNC Symbol;Acc:VGNC:108278]
chr1	187258629	187259377	749	*	chr1	187258629	187259377	in_vitro	in_vivo	11	10	1.95272727	0.545	1.40772727	c(t = 1.12710	0.282	0.83916667	Promoter (<=1kb)	ENSSSCG000	ARMH4	armadillo like helical domain containing 4 [Source:VGNC Symbol;Acc:VGNC:85535]
chr1	188334830	188335840	1011	*	chr1	188334830	188335840	in_vitro	in_vivo	10	10	1.87	0.385	1.485	c(t = 1.13419	0.282	0.83916667	Distal Intergenic	ENSSSCG000	RTN1	reticulon 1 [Source:NCBI gene (formerly Entrezgene);Acc:100152394]
chr2	5946290	5946872	583	*	chr2	5946290	5946872	in_vitro	in_vivo	11	9	1.68272727	0.19888889	1.48383838	c(t = 1.13544	0.282	0.83916667	Distal Intergenic	ENSSSCG000	B4GAT1	beta-1,4-glucuronyltransferase 1 [Source:NCBI gene (formerly Entrezgene);Acc:100518412]
chr6	62690463	62691254	792	*	chr6	62690463	62691254	in_vitro	in_vivo	9	11	76.6277778	60.4845455	16.1432323	c(t = 1.10561	0.283	0.83916667	Distal Intergenic	ENSSSCG000	ISG15	ISG15 ubiquitin like modifier [Source:NCBI gene (formerly Entrezgene);Acc:100145895]
chr4	108635683	108636579	897	*	chr4	108635683	108636579	in_vitro	in_vivo	9	10	1.26444444	3.445	-2.1805556	c(t = -1.1241	0.283	0.83916667	Distal Intergenic	ENSSSCG000	ADORA3	adenosine A3 receptor [Source:NCBI gene (formerly Entrezgene);Acc:606753]
chr17	33085940	33086507	568	*	chr17	33085940	33086507	in_vitro	in_vivo	9	10	1	2.732	-1.732	c(t = -1.1212	0.283	0.83916667	Distal Intergenic	ENSSSCG000	NOP56	NOP56 ribonucleoprotein [Source:VGNC Symbol;Acc:VGNC:95703]
chr14	89140815	89141391	577	*	chr14	89140815	89141391	in_vitro	in_vivo	10	10	1.474	2.639	-1.165	c(t = -1.1083	0.283	0.83916667	Distal Intergenic	ENSSSCG000	ssc-mir-9835	ssc-mir-9835 [Source:miRBase;Acc:MI0031604]
chr15	123500601	123503456	2856	*	chr15	123500601	123503456	in_vitro	in_vivo	13	13	0.82923077	2.42230769	-1.5930769	c(t = -1.1123	0.283	0.83916667	Promoter (<=1kb)	ENSSSCG000	EPHA4	EPH receptor A4 [Source:VGNC Symbol;Acc:VGNC:96280]
chr16	148928	150715	1788	*	chr16	148928	150715	in_vitro	in_vivo	11	12	4.10909091	2.3525	1.75659091	c(t = 1.10582	0.283	0.83916667	Distal Intergenic	ENSSSCG000	C1QTNF3	C1q and TNF related 3 [Source:NCBI gene (formerly Entrezgene);Acc:100270814]
chr18	46507283	46508187	905	*	chr18	46507283	46508187	in_vitro	in_vivo	10	10	5.212	2.289	2.923	c(t = 1.12658	0.283	0.83916667	Distal Intergenic	NA	NA	NA
chr18	6246349	6246886	538	*	chr18	6246349	6246886	in_vitro	in_vivo	9	9	3.93222222	1.54333333	2.38888889	c(t = 1.11599	0.283	0.83916667	Distal Intergenic	ENSSSCG000	NOS3	nitric oxide synthase 3 [Source:NCBI gene (formerly Entrezgene);Acc:397557]
chr10	17558598	17560252	1655	*	chr10	17558598	17560252	in_vitro	in_vivo	9	13	0.58	1.43076923	-0.8507692	c(t = -1.1046	0.283	0.83916667	Distal Intergenic	ENSSSCG000	DESI2	desumoylating isopeptidase 2 [Source:VGNC Symbol;Acc:VGNC:96037]
chr2	28961937	28964698	2762	*	chr2	28961937	28964698	in_vitro	in_vivo	13	11	2.96923077	1.18	1.78923077	c(t = 1.11924	0.283	0.83916667	Distal Intergenic	ENSSSCG000	PAX6	paired box 6 [Source:VGNC Symbol;Acc:VGNC:91195]
chr14	89192374	89193566	1193	*	chr14	89192374	89193566	in_vitro	in_vivo	10	13	2.041	1.12923077	0.91176923	c(t = 1.11043	0.283	0.83916667	Distal Intergenic	ENSSSCG000	ssc-mir-9835	ssc-mir-9835 [Source:miRBase;Acc:MI0031604]
chr4	87993510	87994473	964	*	chr4	87993510	87994473	in_vitro	in_vivo	14	12	1.70928571	0.72166667	0.98761905	c(t = 1.10642	0.283	0.83916667	Distal Intergenic	ENSSSCG000	HSD17B7	hydroxysteroid 17-beta dehydrogenase 7 [Source:VGNC Symbol;Acc:VGNC:88988]
chr9	133424572	133425979	1408	*	chr9	133424572	133425979	in_vitro	in_vivo	12	10	24.1741667	43.766	-19.591833	c(t = -1.1071	0.284	0.83916667	Distal Intergenic	ENSSSCG000	CAMK1G	calcium/calmodulin dependent protein kinase IG [Source:VGNC Symbol;Acc:VGNC:86154]
chr6	164879015	164879663	649	*	chr6	164879015	164879663	in_vitro	in_vivo	11	10	31.4227273	15.257	16.1657273	c(t = 1.10347	0.284	0.83916667	Distal Intergenic	ENSSSCG000	MKNK1	MAPK interacting serine/threonine kinase 1 [Source:VGNC Symbol;Acc:VGNC:90246]
chr14	51132181	51133805	1625	*	chr14	51132181	51133805	in_vitro	in_vivo	10	11	2.227	7.98636364	-5.7593636	c(t = -1.1271	0.284	0.83916667	Distal Intergenic	ENSSSCG000	CLDN5	claudin 5 [Source:NCBI gene (formerly Entrezgene);Acc:396997]
chr1	246085972	246086314	343	*	chr1	246085972	246086314	in_vitro	in_vivo	9	11	6.07777778	3.28272727	2.79505051	c(t = 1.10550	0.284	0.83916667	Distal Intergenic	ENSSSCG000	ABCA1	ATP binding cassette subfamily A member 1 [Source:NCBI gene (formerly Entrezgene);Acc:100152112]
chr10	33227139	33228075	937	*	chr10	33227139	33228075	in_vitro	in_vivo	9	11	4.38222222	2.79	1.59222222	c(t = 1.10385	0.284	0.83916667	Promoter (<=1kb)	ENSSSCG000	CHMP5	charged multivesicular body protein 5 [Source:NCBI gene (formerly Entrezgene);Acc:100513035]
chr15	124212832	124214286	1455	*	chr15	124212832	124214286	in_vitro	in_vivo	11	13	1.41272727	2.69692308	-1.2841958	c(t = -1.1040	0.284	0.83916667	Distal Intergenic	ENSSSCG000	ACSL3	acyl-CoA synthetase long chain family member 3 [Source:VGNC Symbol;Acc:VGNC:96295]
chr1	15587072	15587782	711	*	chr1	15587072	15587782	in_vitro	in_vivo	11	11	0.98181818	1.97363636	-0.9918182	c(t = -1.1081	0.284	0.83916667	Distal Intergenic	ENSSSCG000	IYD	iodotyrosine deiodinase [Source:VGNC Symbol;Acc:VGNC:89260]
chr1	167239843	167240597	755	*	chr1	167239843	167240597	in_vitro	in_vivo	16	11	2.186875	1.01181818	1.17505682	c(t = 1.09490	0.284	0.83916667	Distal Intergenic	ENSSSCG000	RPLP1	ribosomal protein lateral stalk subunit P1 [Source:NCBI gene (formerly Entrezgene);Acc:100170119]
chr18	5474457	5476891	2435	*	chr18	5474457	5476891	in_vitro	in_vivo	12	9	1.8025	0.97888889	0.82361111	c(t = 1.10481	0.284	0.83916667	Distal Intergenic	ENSSSCG000	RHEB	Ras homolog, mTORC1 binding [Source:NCBI gene (formerly Entrezgene);Acc:100516050]
chr1	263821450	263822086	637	*	chr1	263821450	263822086	in_vitro	in_vivo	11	11	2.39090909	0.91818182	1.47272727	c(t = 1.10944	0.284	0.83916667	Distal Intergenic	ENSSSCG000	LHX2	LIM homeobox 2 [Source:NCBI gene (formerly Entrezgene);Acc:100156063]
chr1	168529756	168530496	741	*	chr1	168529756	168530496	in_vitro	in_vivo	10	12	1.168	0.55083333	0.61716667	c(t = 1.12147	0.284	0.83916667	Distal Intergenic	ENSSSCG00000026486		
chr15	50801603	50803043	1441	*	chr15	50801603	50803043	in_vitro	in_vivo	13	12	1.37153846	0.545	0.82653846	c(t = 1.09984	0.284	0.83916667	Distal Intergenic	NA	NA	NA
chr11	70853955	70854539	585	*	chr11	70853955	70854539	in_vitro	in_vivo	12	11	1.26166667	0.46727273	0.79439394	c(t = 1.11501	0.284	0.83916667	Distal Intergenic	ENSSSCG000	BIVM	basic, immunoglobulin-like variable motif containing [Source:VGNC Symbol;Acc:VGNC:109180]
chr16	69121324	69121970	647	*	chr16	69121324	69121970	in_vitro	in_vivo	12	9	2.42666667	0.32666667	2.1	c(t = 1.12373	0.284	0.83916667	Distal Intergenic	ENSSSCG000	HAND1	heart and neural crest derivatives expressed 1 [Source:VGNC Symbol;Acc:VGNC:88777]
chr12	61012872	61018438	5567	*	chr12	61012872	61018438	in_vitro	in_vivo	11	13	92.8209091	95.3269231	-2.506014	c(t = -1.1002	0.285	0.83916667	Exon (NM_001244149/100512312, exon 7 of 12)	ENSSSCG000	COPS3	COP9 signalosome subunit 3 [Source:VGNC Symbol;Acc:VGNC:86900]
chr18	1082876	1083597	722	*	chr18	1082876	1083597	in_vitro	in_vivo	9	10	13.7344444	28.316	-14.581556	c(t = -1.1097	0.285	0.83916667	Distal Intergenic	ENSSSCG000	ssc-mir-153	ssc-mir-153 [Source:miRBase;Acc:MI0002454]
chr16	42482861	42483714	854	*	chr16	42482861	42483714	in_vitro	in_vivo	10	14	2.933	4.87214286	-1.9391429	c(t = -1.0966	0.285	0.83916667	Distal Intergenic	ENSSSCG000	RGS7BP	regulator of G protein signaling 7 binding protein [Source:VGNC Symbol;Acc:VGNC:92268]
chr14	47638106	47638634	529	*	chr14	47638106	47638634	in_vitro	in_vivo	9	10	9.51444444	4	5.51444444	c(t = 1.10460	0.285	0.83916667	Distal Intergenic	ENSSSCG000	TCN2	transcobalamin 2 [Source:VGNC Symbol;Acc:VGNC:93830]
chr14	112206912	112208732	1821	*	chr14	112206912	112208732	in_vitro	in_vivo	12	12	4.74166667	2.47916667	2.2625	c(t = 1.09633	0.285	0.83916667	Distal Intergenic	ENSSSCG000	KAZALD1	Kazal type serine peptidase inhibitor domain 1 [Source:NCBI gene (formerly Entrezgene);Acc:100624902]
chr12	1405645	1413313	7669	*	chr12	1405645	1413313	in_vitro	in_vivo	11	15	1.24272727	2.01266667	-0.7699394	c(t = -1.0934	0.285	0.83916667	Distal Intergenic	ENSSSCG000	TEPSIN	TEPSIN adaptor related protein complex 4 accessory protein [Source:NCBI gene (formerly Entrezgene);Acc:100626379]
chr6	112626524	112629611	3088	*	chr6	112626524	112629611	in_vitro	in_vivo	14	15	2.08214286	1.12333333	0.95880952	c(t = 1.09709	0.285	0.83916667	Distal Intergenic	ENSSSCG000	AQP4	aquaporin 4 [Source:VGNC Symbol;Acc:VGNC:85432]
chr1	113258121	113258999	879	*	chr1	113258121	113258999	in_vitro	in_vivo	10	10	2.141	0.941	1.2	c(t = 1.10118	0.285	0.83916667	Distal Intergenic	ENSSSCG000	ADAM10	ADAM metallopeptidase domain 10 [Source:NCBI gene (formerly Entrezgene);Acc:397345]
chr17	52194571	52195453	883	*	chr17	52194571	52195453	in_vitro	in_vivo	9	10	1.30888889	0.556	0.75288889	c(t = 1.12327	0.285	0.83916667	Promoter (<=1kb)	ENSSSCG000	PARD6B	par-6 family cell polarity regulator beta [Source:NCBI gene (formerly Entrezgene);Acc:100151957]
chrX	5361314	5363568	2255	*	chrX	5361314	5363568	in_vitro	in_vivo	10	11	1.884	0	1.884	c(t = 1.13700	0.285	0.83916667	Distal Intergenic	ENSSSCG00000038027		patatin like phospholipase domain containing 4 [Source:NCBI gene (formerly Entrezgene);Acc:100294680]
chr11	77502925	77508952	6028	*	chr11	77502925	77508952	in_vitro	in_vivo	10	12	98.531	89.3658333	9.16516667	c(t = 1.12056	0.286	0.83985862	Distal Intergenic	ENSSSCG000	ANKRD10	ankyrin repeat domain 10 [Source:VGNC Symbol;Acc:VGNC:85322]
chr18	1751604	1752965	1362	*	chr18	1751604	1752965	in_vitro	in_vivo	9	12	93.7388889	87.7891667	5.94972222	c(t = 1.10410	0.286	0.83985862	Distal Intergenic	ENSSSCG000	ssc-mir-153	ssc-mir-153 [Source:miRBase;Acc:MI0002454]
chr12	59442946	59444676	1731	*	chr12	59442946	59444676	in_vitro	in_vivo	10	10	94.013	86.527	7.486	c(t = 1.12434	0.286	0.83985862	Distal Intergenic	ENSSSCG000	UBB	ubiquitin B [Source:VGNC Symbol;Acc:VGNC:98389]
chr13	33663957	33666213	2257	*	chr13	33663957	33666213	in_vitro	in_vivo	11	12	37.4290909	24.2391667	13.1899242	c(t = 1.09591	0.286	0.83985862	Distal Intergenic	ENSSSCG000	MANF	mesencephalic astrocyte derived neurotrophic factor [Source:NCBI gene (formerly Entrezgene);Acc:100505460]
chr3	25881898	25882629	732	*	chr3	25881898	25882629	in_vitro	in_vivo	9	11	35.4844444	15.8509091	19.6335354	c(t = 1.10887	0.286	0.83985862	Distal Intergenic	ENSSSCG000	ACSM4	acyl-CoA synthetase medium-chain family member 4 [Source:NCBI gene (formerly Entrezgene);Acc:100512595]
chr2	28708831	28709715	885	*	chr2	28708831	28709715	in_vitro	in_vivo	11	10	3.94727273	1.513	2.43427273	c(t = 1.11658	0.286	0.83985862	Distal Intergenic	ENSSSCG000	PAX6	paired box 6 [Source:VGNC Symbol;Acc:VGNC:91195]
chr3	103249530	103250042	513	*	chr3	103249530	103250042	in_vitro	in_vivo	11	11	2.12363636	0.37909091	1.74454545	c(t = 1.12482	0.286	0.83985862	Distal Intergenic	ENSSSCG000	EIF2AK2	eukaryotic translation initiation factor 2 alpha kinase 2 [Source:VGNC Symbol;Acc:VGNC:87607]
chr17	34429947	34430745	799	*	chr17	34429947	34430745	in_vitro	in_vivo	9	10	2.42111111	4.5	-2.0788889	c(t = -1.0999	0.287	0.8404115	Distal Intergenic	ENSSSCG000	ssc-mir-9798	ssc-mir-9798 [Source:miRBase;Acc:MI0031562]
chr6	77707720	77708305	586	*	chr6	77707720	77708305	in_vitro	in_vivo	9	9	0.82666667	2.29222222	-1.4655556	c(t = -1.1148	0.287	0.8404115	Distal Intergenic	ENSSSCG000	MRTO4	MRT4 homolog, ribosome maturation factor [Source:VGNC Symbol;Acc:VGNC:90405]
chr14	50189939	50191300	1362	*	chr14	50189939	50191300	in_vitro	in_vivo	9	11	2.95888889	1.81181818	1.14707071	c(t = 1.11831	0.287	0.8404115	Distal Intergenic	ENSSSCG000	ssc-mir-130b	ssc-mir-130b [Source:miRBase;Acc:MI0013136]
chr1	76367408	76367899	492	*	chr1	76367408	76367899	in_vitro	in_vivo	12	9	2.56166667	1.38222222	1.17944444	c(t = 1.09628	0.287	0.8404115	Distal Intergenic	ENSSSCG000	DDO	D-aspartate oxidase [Source:NCBI gene (formerly Entrezgene);Acc:100037287]
chr13	16784118	16785020	903	*	chr13	16784118	16785020	in_vitro	in_vivo	12	11	2.48333333	1.28181818	1.20151515	c(t = 1.10351	0.287	0.8404115	Distal Intergenic	ENSSSCG000	CMTM7	CKLF like MARVEL transmembrane domain containing 7 [Source:VGNC Symbol;Acc:VGNC:103928]
chr7	91343485	91344178	694	*	chr7	91343485	91344178	in_vitro	in_vivo	11	10	0.35	1.194	-0.844	c(t = -1.1071	0.287	0.8404115	Distal Intergenic	ENSSSCG000	VTI1B	vesicle transport through interaction with t-SNAREs 1B [Source:NCBI gene (formerly Entrezgene);Acc:404692]
chr3	4992827	4994602	1776	*	chr3	4992827	4994602	in_vitro	in_vivo	11	10	78.9690909	58.585	20.3840909	c(t = 1.09530	0.288	0.8404115	Distal Intergenic	ENSSSCG000	AIMP2	aminoacyl tRNA synthetase complex interacting multifunctional protein 2 [Source:NCBI gene (formerly Entrezgene);Acc:100514707]
chr3	4044136	4048850	4715	*	chr3	4044136	4048850	in_vitro	in_vivo	9	14	30.4622222	44.7385714	-14.276349	c(t = -1.0945	0.288	0.8404115	Distal Intergenic	ENSSSCG000	FSCN1	fascin actin-bundling protein 1 [Source:NCBI gene (formerly Entrezgene);Acc:100286741]
chr6	95496289	95497668	1380	*	chr6	95496289	95497668	in_vitro	in_vivo	13	11	6.57692308	11.1245455	-4.5476224	c(t = -1.0963	0.288	0.8404115	Distal Intergenic	ENSSSCG000	HPCAL4	hippocalcin like 4 [Source:VGNC Symbol;Acc:VGNC:88959]
chr14	92329852	92330413	562	*	chr14	92329852	92330413	in_vitro	in_vivo	10	10	1.965	4.519	-2.554	c(t = -1.1140	0.288	0.8404115	Promoter (<=1kb)	ENSSSCG000	UBE2D1	ubiquitin conjugating enzyme E2 D1 [Source:VGNC Symbol;Acc:VGNC:94641]
chr13	27320290	27324941	4652	*	chr13	27320290	27324941	in_vitro	in_vivo	13	14	3.72153846	2.10857143	1.61296703	c(t = 1.08932	0.288	0.8404115	Distal Intergenic	ENSSSCG000	ssc-mir-138	ssc-mir-138 [Source:miRBase;Acc:MI0022131]
chr13	61526742	61529065	2324	*	chr13	61526742	61529065	in_vitro	in_vivo	10	16	3.725	1.830625	1.894375	c(t = 1.09373	0.288	0.8404115	Promoter (<=1kb)	ENSSSCG000	BHLHE40	basic helix-loop-helix family member e40 [Source:VGNC Symbol;Acc:VGNC:85814]
chr3	117774463	117776331	1869	*	chr3	117774463	117776331	in_vitro	in_vivo	10	13	0.616	1.28230769	-0.6663077	c(t = -1.0920	0.288	0.8404115	Promoter (<=1kb)	ENSSSCG000	RHOB	ras homolog family member B [Source:VGNC Symbol;Acc:VGNC:110709]
chr8	76481189	76483078	1890	*	chr8	76481189	76483078	in_vitro	in_vivo	10	14	1.808	0.92428571	0.88371429	c(t = 1.10704	0.288	0.8404115	Distal Intergenic	ENSSSCG000	TIGD4	tigger transposable element derived 4 [Source:NCBI gene (formerly Entrezgene);Acc:397327]
chr15	54929256	54929883	628	*	chr15	54929256	54929883	in_vitro	in_vivo	9	11	0.78333333	0	0.78333333	c(t = 1.13691	0.288	0.8404115	Distal Intergenic	ENSSSCG000	MBOAT4	membrane bound O-acyltransferase domain containing 4 [Source:NCBI gene (formerly Entrezgene);Acc:100153109]
chr15	101623623	101624643	1021	*	chr15	101623623	101624643	in_vitro	in_vivo	11	9	23.5190909	41.1944444	-17.675354	c(t = -1.0984	0.289	0.8404115	Distal Intergenic	ENSSSCG000	MOBKL3	MOB family member 4, phocein [Source:NCBI gene (formerly Entrezgene);Acc:613127]
chr12	2095212	2099139	3928	*	chr12	2095212	2099139	in_vitro	in_vivo	11	10	23.78	37.661	-13.881	c(t = -1.1106	0.289	0.8404115	Distal Intergenic	ENSSSCG000	SLC26A11	solute carrier family 26 member 11 [Source:NCBI gene (formerly Entrezgene);Acc:100191010]
chr8	83240039	83241417	1379	*	chr8	83240039	83241417	in_vitro	in_vivo	14	12	10.0764286	15.5416667	-5.4652381	c(t = -1.0930	0.289	0.8404115	Distal Intergenic	ENSSSCG000	ANAPC10	anaphase promoting complex subunit 10 [Source:VGNC Symbol;Acc:VGNC:98912]
chr17	14256515	14257554	1040	*	chr17	14256515	14257554	in_vitro	in_vivo	9	10	1.63777778	3.639	-2.0012222	c(t = -1.0995	0.289	0.8404115	Distal Intergenic	ENSSSCG000	CDS2	CDP-diacylglycerol synthase 2 [Source:VGNC Symbol;Acc:VGNC:95869]
chr13	168569249	168569755	507	*	chr13	168569249	168569755	in_vitro	in_vivo	9	11	0.69444444	2.51090909	-1.8164646	c(t = -1.1017	0.289	0.8404115	Promoter (<=1kb)	ENSSSCG000	CHMP2B	charged multivesicular body protein 2B [Source:VGNC Symbol;Acc:VGNC:86650]
chr13	200351881	200355931	4051	*	chr13	200351881	200355931	in_vitro	in_vivo	11	13	2.78090909	1.82538462	0.95552448	c(t = 1.09345	0.289	0.8404115	Distal Intergenic	ENSSSCG000	CLDN14	claudin 14 [Source:VGNC Symbol;Acc:VGNC:86731]
chr6	35989614	35990401	788	*	chr6	35989614	35990401	in_vitro	in_vivo	10	11	0.909	1.77545455	-0.8664545	c(t = -1.0919	0.289	0.8404115	Distal Intergenic	ENSSSCG000	CBLN1	cerebellin 1 precursor [Source:NCBI gene (formerly Entrezgene);Acc:100127148]
chr4	78098575	78099700	1126	*	chr4	78098575	78099700	in_vitro	in_vivo	11	12	2.89090909	0.81583333	2.07507576	c(t = 1.11183	0.289	0.8404115	Distal Intergenic	ENSSSCG000	ATP6V1H	ATPase H+ transporting V1 subunit H [Source:VGNC Symbol;Acc:VGNC:85677]
chr15	19971237	19971985	749	*	chr15	19971237	19971985	in_vitro	in_vivo	12	12	1.11083333	0.40833333	0.7025	c(t = 1.09724	0.289	0.8404115	Promoter (<=1kb)	ENSSSCG000	SLC35F5	solute carrier family 35 member F5 [Source:VGNC Symbol;Acc:VGNC:96015]
chr11	2407437	2408909	1473	*	chr11	2407437	2408909	in_vitro	in_vivo	9	11	93.2422222	95.5990909	-2.3568687	c(t = -1.0936	0.29	0.8404115	Distal Intergenic	ENSSSCG000	ssc-mir-9814	ssc-mir-9814 [Source:miRBase;Acc:MI0031581]
chr11	4363402	4364807	1406	*	chr11	4363402	4364807	in_vitro	in_vivo	9	10	93.9844444	82.517	11.4674444	c(t = 1.10176	0.29	0.8404115	Distal Intergenic	ENSSSCG000	GPR12	G protein-coupled receptor 12 [Source:NCBI gene (formerly Entrezgene);Acc:100736641]
chr11	26529327	26531483	2157	*	chr11	26529327	26531483	in_vitro	in_vivo	12	16	10.9366667	5.81875	5.11791667	c(t = 1.09769	0.29	0.8404115	Distal Intergenic	ENSSSCG000	MTRF1	mitochondrial translation release factor 1 [Source:VGNC Symbol;Acc:VGNC:90471]
chr6	177889	178679	791	*	chr6	177889	178679	in_vitro	in_vivo	9	9	1.11111111	2.48555556	-1.3744444	c(t = -1.1017	0.29	0.8404115	Distal Intergenic	ENSSSCG000	MC1R	melanocortin 1 receptor [Source:NCBI gene (formerly Entrezgene);Acc:494018]
chr5	68755479	68756479	1001	*	chr5	68755479	68756479	in_vitro	in_vivo	10	14	0.256	2.39785714	-2.1418571	c(t = -1.1022	0.29	0.8404115	Distal Intergenic	ENSSSCG000	ADIPOR2	adiponectin receptor 2 [Source:VGNC Symbol;Acc:VGNC:85141]
chr2	7390628	7392111	1484	*	chr2	7390628	7392111	in_vitro	in_vivo	9	11	0.80222222	2.18272727	-1.3805051	c(t = -1.1027	0.29	0.8404115	Distal Intergenic	ENSSSCG000	RASGRP2	RAS guanyl releasing protein 2 [Source:NCBI gene (formerly Entrezgene);Acc:100626093]
chr2	13458305	13460171	1867	*	chr2	13458305	13460171	in_vitro	in_vivo	12	9	3.89333333	1.84333333	2.05	c(t = 1.09279	0.29	0.8404115	Distal Intergenic	ENSSSCG000	UBE2L6	ubiquitin conjugating enzyme E2 L6 [Source:NCBI gene (formerly Entrezgene);Acc:100514753]
chr18	1729787	1733044	3258	*	chr18	1729787	1733044	in_vitro	in_vivo	11	15	0.97545455	1.61666667	-0.6412121	c(t = -1.0833	0.29	0.8404115	Distal Intergenic	ENSSSCG000	ssc-mir-153	ssc-mir-153 [Source:miRBase;Acc:MI0002454]
chr5	104126377	104127484	1108	*	chr5	104126377	104127484	in_vitro	in_vivo	15	10	2.95066667	1.482	1.46866667	c(t = 1.08875	0.29	0.8404115	Distal Intergenic	ENSSSCG000	ssc-mir-7139	ssc-mir-7139 [Source:miRBase;Acc:MI0023572]
chr7	51362686	51364275	1590	*	chr7	51362686	51364275	in_vitro	in_vivo	14	16	1.99142857	1.14	0.85142857	c(t = 1.08685	0.29	0.8404115	Distal Intergenic	ENSSSCG000	ssc-mir-1839	ssc-mir-1839 [Source:miRBase;Acc:MI0013162]
chr3	3008963	3010080	1118	*	chr3	3008963	3010080	in_vitro	in_vivo	10	13	22.853	37.1715385	-14.318538	c(t = -1.0825	0.291	0.8404115	Distal Intergenic	ENSSSCG000	AP5Z1	adaptor related protein complex 5 subunit zeta 1 [Source:VGNC Symbol;Acc:VGNC:85397]
chr7	23220795	23222248	1454	*	chr7	23220795	23222248	in_vitro	in_vivo	9	14	9.92666667	19.8742857	-9.947619	c(t = -1.0845	0.291	0.8404115	Promoter (<=1kb)	ENSSSCG000	PPP1R18	protein phosphatase 1 regulatory subunit 18 [Source:VGNC Symbol;Acc:VGNC:104034]
chr7	25234564	25235973	1410	*	chr7	25234564	25235973	in_vitro	in_vivo	9	11	2.81333333	6.07454545	-3.2612121	c(t = -1.0984	0.291	0.8404115	Distal Intergenic	ENSSSCG000	RXRB	retinoid X receptor beta [Source:VGNC Symbol;Acc:VGNC:92529]
chr14	81674564	81674952	389	*	chr14	81674564	81674952	in_vitro	in_vivo	9	10	5.08666667	2.406	2.68066667	c(t = 1.11040	0.291	0.8404115	Distal Intergenic	ENSSSCG000	PLAC9	placenta associated 9 [Source:NCBI gene (formerly Entrezgene);Acc:100155282]
chr13	60393352	60394382	1031	*	chr13	60393352	60394382	in_vitro	in_vivo	9	14	4.99444444	1.24928571	3.74515873	c(t = 1.12327	0.291	0.8404115	Distal Intergenic	ENSSSCG000	SUMF1	sulfatase modifying factor 1 [Source:VGNC Symbol;Acc:VGNC:93617]
chrX	11886002	11886904	903	*	chrX	11886002	11886904	in_vitro	in_vivo	10	11	10.903	1.18545455	9.71754545	c(t = 1.12010	0.291	0.8404115	Distal Intergenic	ENSSSCG000	ASB11	ankyrin repeat and SOCS box containing 11 [Source:VGNC Symbol;Acc:VGNC:85560]
chr9	120561923	120563303	1381	*	chr9	120561923	120563303	in_vitro	in_vivo	11	11	1.84818182	0.91818182	0.93	c(t = 1.08637	0.291	0.8404115	Distal Intergenic	ENSSSCG000	ANGPTL1	angiopoietin like 1 [Source:VGNC Symbol;Acc:VGNC:85303]
chr18	42900486	42901285	800	*	chr18	42900486	42901285	in_vitro	in_vivo	11	12	1.68545455	0.81583333	0.86962121	c(t = 1.08973	0.291	0.8404115	Promoter (1-2kb)	ENSSSCG000	FKBP14	FKBP prolyl isomerase 14 [Source:NCBI gene (formerly Entrezgene);Acc:100514551]
chr8	131924355	131925093	739	*	chr8	131924355	131925093	in_vitro	in_vivo	10	12	1.82	0.565	1.255	c(t = 1.10618	0.291	0.8404115	Distal Intergenic	ENSSSCG000	HSD17B13	hydroxysteroid 17-beta dehydrogenase 13 [Source:NCBI gene (formerly Entrezgene);Acc:100626886]
chr5	9484812	9486365	1554	*	chr5	9484812	9486365	in_vitro	in_vivo	11	13	1.07545455	0.47692308	0.59853147	c(t = 1.09252	0.291	0.8404115	Distal Intergenic	ENSSSCG000	KDELR3	KDEL endoplasmic reticulum protein retention receptor 3 [Source:NCBI gene (formerly Entrezgene);Acc:100514523]
chrX	107505780	107506648	869	*	chrX	107505780	107506648	in_vitro	in_vivo	12	9	5.03333333	13.36	-8.3266667	c(t = -1.1072	0.292	0.84050205	Distal Intergenic	ENSSSCG000	RBMX2	RNA binding motif protein X-linked 2 [Source:NCBI gene (formerly Entrezgene);Acc:100626622]
chr1	129868323	129869285	963	*	chr1	129868323	129869285	in_vitro	in_vivo	9	9	5.32444444	13.0966667	-7.7722222	c(t = -1.1213	0.292	0.84050205	Distal Intergenic	ENSSSCG000	JMJD7	jumonji domain containing 7 [Source:NCBI gene (formerly Entrezgene);Acc:100533119]
chr14	47301143	47301990	848	*	chr14	47301143	47301990	in_vitro	in_vivo	9	12	1.99111111	4.26333333	-2.2722222	c(t = -1.0891	0.292	0.84050205	Distal Intergenic	ENSSSCG000	LIF	LIF interleukin 6 family cytokine [Source:VGNC Symbol;Acc:VGNC:89719]
chr2	398245	401419	3175	*	chr2	398245	401419	in_vitro	in_vivo	11	13	5.09090909	3.38923077	1.70167832	c(t = 1.09279	0.292	0.84050205	Distal Intergenic	ENSSSCG000	IRF7	interferon regulatory factor 7 [Source:VGNC Symbol;Acc:VGNC:89210]
chrX	14821461	14822726	1266	*	chrX	14821461	14822726	in_vitro	in_vivo	12	12	1.15083333	2.58916667	-1.4383333	c(t = -1.0788	0.292	0.84050205	Distal Intergenic	ENSSSCG000	EIF1AX	eukaryotic translation initiation factor 1A X-linked [Source:NCBI gene (formerly Entrezgene);Acc:100522912]
chr11	15772434	15772911	478	*	chr11	15772434	15772911	in_vitro	in_vivo	9	11	0.65333333	1.89363636	-1.240303	c(t = -1.0866	0.292	0.84050205	Distal Intergenic	ENSSSCG000	SLC25A15	solute carrier family 25 member 15 [Source:VGNC Symbol;Acc:VGNC:92994]
chr17	15749629	15751232	1604	*	chr17	15749629	15751232	in_vitro	in_vivo	12	14	2.04666667	1.03571429	1.01095238	c(t = 1.09212	0.292	0.84050205	Promoter (<=1kb)	ENSSSCG000	BMP2	bone morphotic protein 2 [Source:VGNC Symbol;Acc:VGNC:95488]
chr4	34822900	34824189	1290	*	chr4	34822900	34824189	in_vitro	in_vivo	11	11	0.23909091	0.8	-0.5609091	c(t = -1.0926	0.292	0.84050205	Distal Intergenic	ENSSSCG000	ODF1	outer dense fiber of sperm tails 1 [Source:NCBI gene (formerly Entrezgene);Acc:397489]
chr11	4515155	4516404	1250	*	chr11	4515155	4516404	in_vitro	in_vivo	11	11	31.8118182	46.3818182	-14.57	c(t = -1.0848	0.293	0.84050205	Distal Intergenic	ENSSSCG000	GPR12	G protein-coupled receptor 12 [Source:NCBI gene (formerly Entrezgene);Acc:100736641]
chr10	23966485	23968979	2495	*	chr10	23966485	23968979	in_vitro	in_vivo	9	11	4.96555556	9.62909091	-4.6635354	c(t = -1.0857	0.293	0.84050205	Distal Intergenic	ENSSSCG000	TNNI1	troponin I1, slow skeletal type [Source:VGNC Symbol;Acc:VGNC:95548]
chr14	49840224	49841233	1010	*	chr14	49840224	49841233	in_vitro	in_vivo	9	14	16.3266667	9.27642857	7.0502381	c(t = 1.09924	0.293	0.84050205	Promoter (<=1kb)	ENSSSCG000	MIF	macrophage migration inhibitory factor [Source:NCBI gene (formerly Entrezgene);Acc:397412]
chr1	253428117	253429209	1093	*	chr1	253428117	253429209	in_vitro	in_vivo	11	12	1.43363636	3.09	-1.6563636	c(t = -1.0961	0.293	0.84050205	Distal Intergenic	ENSSSCG000	INIP	INTS3 and NABP interacting protein [Source:VGNC Symbol;Acc:VGNC:89136]
chr14	132454008	132454402	395	*	chr14	132454008	132454402	in_vitro	in_vivo	9	11	6.74777778	2.42090909	4.32686869	c(t = 1.11913	0.293	0.84050205	Distal Intergenic	ENSSSCG000	C14H10orf12	chromosome 14 C10orf120 homolog [Source:NCBI gene (formerly Entrezgene);Acc:100125841]
chr10	53351633	53354971	3339	*	chr10	53351633	53354971	in_vitro	in_vivo	12	12	2.84416667	1.785	1.05916667	c(t = 1.08174	0.293	0.84050205	Distal Intergenic	ENSSSCG000	BMI1	BMI1 proto-onco, polycomb ring finger [Source:VGNC Symbol;Acc:VGNC:108265]
chr2	77069584	77071011	1428	*	chr2	77069584	77071011	in_vitro	in_vivo	11	11	4.14090909	1.67	2.47090909	c(t = 1.08356	0.293	0.84050205	Promoter (<=1kb)	ENSSSCG000	DAZAP1	DAZ associated protein 1 [Source:NCBI gene (formerly Entrezgene);Acc:100625005]
chr1	128125656	128126657	1002	*	chr1	128125656	128126657	in_vitro	in_vivo	11	11	6.45272727	1.50545455	4.94727273	c(t = 1.10676	0.293	0.84050205	Distal Intergenic	ENSSSCG000	ssc-mir-9838	ssc-mir-9838 [Source:miRBase;Acc:MI0031607]
chr14	61517322	61518146	825	*	chr14	61517322	61518146	in_vitro	in_vivo	13	9	2.42384615	0.65888889	1.76495726	c(t = 1.08959	0.293	0.84050205	Distal Intergenic	ENSSSCG000	PHYHIPL	phytanoyl-CoA 2-hydroxylase interacting protein like [Source:VGNC Symbol;Acc:VGNC:91407]
chr17	28679078	28679600	523	*	chr17	28679078	28679600	in_vitro	in_vivo	12	10	0.3975	0.088	0.3095	c(t = 1.09585	0.293	0.84050205	Distal Intergenic	ENSSSCG000	THBD	thrombomodulin [Source:NCBI gene (formerly Entrezgene);Acc:100157642]
chr18	12598522	12600248	1727	*	chr18	12598522	12600248	in_vitro	in_vivo	13	13	4.85769231	7.45538462	-2.5976923	c(t = -1.0751	0.294	0.84076656	Promoter (<=1kb)	ENSSSCG000	CHRM2	cholinergic receptor muscarinic 2 [Source:VGNC Symbol;Acc:VGNC:103922]
chr18	20027545	20028961	1417	*	chr18	20027545	20028961	in_vitro	in_vivo	12	14	3.37166667	5.96142857	-2.5897619	c(t = -1.0766	0.294	0.84076656	Distal Intergenic	ENSSSCG000	HILPDA	hypoxia inducible lipid droplet associated [Source:NCBI gene (formerly Entrezgene);Acc:449525]
chr13	182134733	182135637	905	*	chr13	182134733	182135637	in_vitro	in_vivo	9	9	0.53777778	2.62333333	-2.0855556	c(t = -1.1162	0.294	0.84076656	Distal Intergenic	ENSSSCG000	BTG3	BTG anti-proliferation factor 3 [Source:VGNC Symbol;Acc:VGNC:103908]
chr14	55056675	55057801	1127	*	chr14	55056675	55057801	in_vitro	in_vivo	12	14	3.66	2.12071429	1.53928571	c(t = 1.08136	0.294	0.84076656	Distal Intergenic	ENSSSCG000	EDARADD	EDAR associated death domain [Source:VGNC Symbol;Acc:VGNC:87542]

chr4	99010527	99011374	848	*	chr4	99010527	99011374	in_vitro	in_vivo	9	9	4.03777778	1.20444444	2.83333333	c(t = 1.10777	0.294	0.84076656	Promoter (<=1kb)	ENSSSCG000	VPS45	vacuolar protein sorting 45 homolog [Source:NCBI gene (formerly Entrezgene);Acc:100514766]
chr13	167895365	167896638	1274	*	chr13	167895365	167896638	in_vitro	in_vivo	15	13	1.82533333	0.97538462	0.84994872	c(t = 1.07500	0.294	0.84076656	Distal Intergenic	ENSSSCG000	HTR1F	5-hydroxytryptamine receptor 1F [Source:VGNC Symbol;Acc:VGNC:88999]
chr3	106723882	106725048	1167	*	chr3	106723882	106725048	in_vitro	in_vivo	11	12	19.4227273	11.4416667	7.98106061	c(t = 1.08082	0.295	0.84076656	Distal Intergenic	ENSSSCG000	SLC30A6	solute carrier family 30 member 6 [Source:VGNC Symbol;Acc:VGNC:93060]
chrX	82508462	82509041	580	*	chrX	82508462	82509041	in_vitro	in_vivo	12	12	6.04666667	2.40083333	3.64583333	c(t = 1.08422	0.295	0.84076656	Distal Intergenic	ENSSSCG000	TSPAN6	tetraspanin 6 [Source:NCBI gene (formerly Entrezgene);Acc:100157741]
chr1	268465007	268466088	1082	*	chr1	268465007	268466088	in_vitro	in_vivo	11	11	5.93181818	2.29090909	3.64090909	c(t = 1.09693	0.295	0.84076656	Distal Intergenic	ENSSSCG000	DPM2	dolichyl-phosphate mannosyltransferase subunit 2, regulatory [Source:NCBI gene (formerly Entrezgene);Acc:100522057]
chr7	42944	43909	966	*	chr7	42944	43909	in_vitro	in_vivo	11	11	1.76272727	1.04727273	0.71545455	c(t = 1.08126	0.295	0.84076656	Distal Intergenic	ENSSSCG000	IRF4	interferon regulatory factor 4 [Source:VGNC Symbol;Acc:VGNC:89207]
chr16	78978851	78983932	5082	*	chr16	78978851	78983932	in_vitro	in_vivo	13	12	10.3730769	19.8666667	-9.4935897	c(t = -1.0709	0.296	0.84076656	Distal Intergenic	ENSSSCG000	TERT	telomerase reverse transcriptase [Source:NCBI gene (formerly Entrezgene);Acc:492280]
chr6	46619705	46620366	662	*	chr6	46619705	46620366	in_vitro	in_vivo	11	10	1.67181818	7.186	-5.5141818	c(t = -1.1057	0.296	0.84076656	Distal Intergenic	ENSSSCG000	PPP1R14A	protein phosphatase 1 regulatory inhibitor subunit 14A [Source:VGNC Symbol;Acc:VGNC:98539]
chr15	103151112	103155397	4286	*	chr15	103151112	103155397	in_vitro	in_vivo	12	13	1.75333333	2.70153846	-0.9482051	c(t = -1.0751	0.296	0.84076656	Distal Intergenic	ENSSSCG000	SPATS2L	spermatosis associated serine rich 2 like [Source:VGNC Symbol;Acc:VGNC:96047]
chr15	133329539	133330741	1203	*	chr15	133329539	133330741	in_vitro	in_vivo	9	11	5.57888889	2.53818182	3.04070707	c(t = 1.11016	0.296	0.84076656	3' UTR	ENSSSCG000	SNORC	secondary ossification center associated regulator of chondrocyte maturation [Source:NCBI gene (formerly Entrezgene);Acc:100157430]
chr6	59313979	59317055	3077	*	chr6	59313979	59317055	in_vitro	in_vivo	11	10	4.05272727	2.534	1.51872727	c(t = 1.07988	0.296	0.84076656	Distal Intergenic	ENSSSCG000	TNNT1	troponin T1, slow skeletal type [Source:VGNC Symbol;Acc:VGNC:94284]
chr8	71832490	71833346	857	*	chr8	71832490	71833346	in_vitro	in_vivo	10	9	0.327	1.19111111	-0.8641111	c(t = -1.1063	0.296	0.84076656	Distal Intergenic	ENSSSCG000	SCARB2	scavenger receptor class B member 2 [Source:VGNC Symbol;Acc:VGNC:92614]
chr1	40022514	40022951	438	*	chr1	40022514	40022951	in_vitro	in_vivo	12	11	2.57	1.06545455	1.50454545	c(t = 1.08253	0.296	0.84076656	Distal Intergenic	ENSSSCG000	FABP7	fatty acid binding protein 7 [Source:NCBI gene (formerly Entrezgene);Acc:574075]
chr4	127036253	127037904	1652	*	chr4	127036253	127037904	in_vitro	in_vivo	11	13	1.90636364	1.03769231	0.86867133	c(t = 1.07774	0.296	0.84076656	Distal Intergenic	ENSSSCG000	GBP2	guanylate binding protein 2, interferon-inducible [Source:NCBI gene (formerly Entrezgene);Acc:100153137]
chr8	55189270	55189977	708	*	chr8	55189270	55189977	in_vitro	in_vivo	11	12	0.44727273	1.02166667	-0.5743939	c(t = -1.0743	0.296	0.84076656	Distal Intergenic	ENSSSCG00000008898		HOP homeobox [Source:VGNC Symbol;Acc:VGNC:88932]
chr8	77969547	77970589	1043	*	chr8	77969547	77970589	in_vitro	in_vivo	13	11	1.70153846	0.7	1.00153846	c(t = 1.07924	0.296	0.84076656	Distal Intergenic	ENSSSCG000	RPS3A	ribosomal protein S3A [Source:NCBI gene (formerly Entrezgene);Acc:414390]
chr13	127571489	127572200	712	*	chr13	127571489	127572200	in_vitro	in_vivo	14	12	1.40857143	0.545	0.86357143	c(t = 1.08137	0.296	0.84076656	Distal Intergenic	NA	NA	NA
chr6	28489383	28490023	641	*	chr6	28489383	28490023	in_vitro	in_vivo	10	9	1.041	0.45666667	0.58433333	c(t = 1.08140	0.296	0.84076656	Distal Intergenic	ENSSSCG000	THAP11	THAP domain containing 11 [Source:NCBI gene (formerly Entrezgene);Acc:100520959]
chr1	169914979	169916174	1196	*	chr1	169914979	169916174	in_vitro	in_vivo	15	13	1.11533333	0.41230769	0.70302564	c(t = 1.07384	0.296	0.84076656	Distal Intergenic	ENSSSCG000	PNN	pinin, desmosome associated protein [Source:NCBI gene (formerly Entrezgene);Acc:100526149]
chr1	189631970	189632975	1006	*	chr1	189631970	189632975	in_vitro	in_vivo	12	9	0.9	0.25222222	0.64777778	c(t = 1.08196	0.296	0.84076656	Distal Intergenic	ENSSSCG000	SIX1	SIX homeobox 1 [Source:NCBI gene (formerly Entrezgene);Acc:100156847]
chr15	137612549	137614017	1469	*	chr15	137612549	137614017	in_vitro	in_vivo	12	11	91.75	83.6036364	8.14636364	c(t = 1.07927	0.297	0.84076656	Distal Intergenic	ENSSSCG000	RAMP1	receptor activity modifying protein 1 [Source:NCBI gene (formerly Entrezgene);Acc:397401]
chr14	140812715	140816165	3451	*	chr14	140812715	140816165	in_vitro	in_vivo	11	9	23.2991667	41.5922222	-18.293056	c(t = -1.0804	0.297	0.84076656	Distal Intergenic	ENSSSCG000	ssc-mir-202	ssc-mir-202 [Source:miRBase;Acc:MI0013157]
chr13	24347193	24348684	1492	*	chr13	24347193	24348684	in_vitro	in_vivo	10	12	8.79	5.10333333	3.68666667	c(t = 1.08493	0.297	0.84076656	Distal Intergenic	ENSSSCG000	MYRIP	myosin VIIA and Rab interacting protein [Source:VGNC Symbol;Acc:VGNC:109577]
chr1	264195399	264196413	1015	*	chr1	264195399	264196413	in_vitro	in_vivo	14	14	3.40357143	1.86928571	1.53428571	c(t = 1.06881	0.297	0.84076656	Distal Intergenic	ENSSSCG000	LHX2	LIM homeobox 2 [Source:NCBI gene (formerly Entrezgene);Acc:100156063]
chr9	106544100	106545296	1197	*	chr9	106544100	106545296	in_vitro	in_vivo	15	13	2.68866667	1.50923077	1.1794359	c(t = 1.06911	0.297	0.84076656	Distal Intergenic	ENSSSCG000	PIK3CG	phosphatidylinositol-4,5-bisphosphate 3-kinase catalytic subunit gamma [Source:VGNC Symbol;Acc:VGNC:91443]
chr9	9504111	9506051	1941	*	chr9	9504111	9506051	in_vitro	in_vivo	12	15	3.72666667	1.48466667	2.242	c(t = 1.08824	0.297	0.84076656	Distal Intergenic	ENSSSCG000	ssc-mir-326	ssc-mir-326 [Source:miRBase;Acc:MI0002434]
chr7	24975207	24975912	706	*	chr7	24975207	24975912	in_vitro	in_vivo	9	13	6.01555556	1.34076923	4.67478632	c(t = 1.11217	0.297	0.84076656	Promoter (1-2kb)	ENSSSCG000	SLA-DQB1	SLA-DQ beta1 domain [Source:NCBI gene (formerly Entrezgene);Acc:100037921]
chr14	28201278	28202176	899	*	chr14	28201278	28202176	in_vitro	in_vivo	9	15	2.21444444	1.16666667	1.04777778	c(t = 1.07515	0.297	0.84076656	Distal Intergenic	ENSSSCG000	SCARB1	scavenger receptor class B member 1 [Source:NCBI gene (formerly Entrezgene);Acc:397018]
chr8	86620961	86621998	1038	*	chr8	86620961	86621998	in_vitro	in_vivo	12	10	3.39833333	0.37	3.02833333	c(t = 1.09387	0.297	0.84076656	Promoter (<=1kb)	ENSSSCG000	CLGN	calmegin [Source:VGNC Symbol;Acc:VGNC:86759]
chr14	110728234	110728793	560	*	chr14	110728234	110728793	in_vitro	in_vivo	11	9	1.15454545	0.21	0.94454545	c(t = 1.09480	0.297	0.84076656	Distal Intergenic	ENSSSCG000	GOT1	glutamic-oxaloacetic transaminase 1 [Source:NCBI gene (formerly Entrezgene);Acc:396967]
chr2	36848454	36848802	349	*	chr2	36848454	36848802	in_vitro	in_vivo	9	9	1.17	2.66555556	-1.4955556	c(t = -1.0781	0.298	0.84076656	Distal Intergenic	ENSSSCG000	CSRP3	cysteine and glycine rich protein 3 [Source:NCBI gene (formerly Entrezgene);Acc:100337687]
chr2	97734899	97735415	517	*	chr2	97734899	97735415	in_vitro	in_vivo	10	11	0.625	2.38636364	-1.7613636	c(t = -1.0828	0.298	0.84076656	Distal Intergenic	ENSSSCG000	MEF2C	myocyte enhancer factor 2C [Source:VGNC Symbol;Acc:VGNC:90127]
chr6	161618670	161623312	4643	*	chr6	161618670	161623312	in_vitro	in_vivo	15	14	2.57066667	1.55	1.02066667	c(t = 1.07012	0.298	0.84076656	Distal Intergenic	ENSSSCG000	SPATA6	spermatosis associated 6 [Source:VGNC Symbol;Acc:VGNC:93387]
chr2	76510082	76510829	748	*	chr2	76510082	76510829	in_vitro	in_vivo	11	11	3.85272727	1.50909091	2.34363636	c(t = 1.07792	0.298	0.84076656	Distal Intergenic	ENSSSCG000	AMH	anti-Mullerian hormone [Source:VGNC Symbol;Acc:VGNC:110572]
chr8	11315618	11317517	1900	*	chr8	11315618	11317517	in_vitro	in_vivo	11	11	2.23909091	0.90909091	1.33	c(t = 1.07625	0.298	0.84076656	Distal Intergenic	ENSSSCG000	CD38	CD38 molecule [Source:NCBI gene (formerly Entrezgene);Acc:100511702]
chr10	14940468	14941416	949	*	chr10	14940468	14941416	in_vitro	in_vivo	11	9	0.23909091	0.79555556	-0.5564646	c(t = -1.0981	0.298	0.84076656	Distal Intergenic	ENSSSCG000	SCCPDH	saccharopine dehydrogenase (putative) [Source:NCBI gene (formerly Entrezgene);Acc:100512485]
chr6	7661083	7661822	740	*	chr6	7661083	7661822	in_vitro	in_vivo	9	11	0.19444444	0.79181818	-0.5973737	c(t = -1.0859	0.298	0.84076656	Distal Intergenic	ENSSSCG000	BCO1	beta-carotene oxygenase 1 [Source:VGNC Symbol;Acc:VGNC:85789]
chr7	25096227	25096930	704	*	chr7	25096227	25096930	in_vitro	in_vivo	10	11	1.618	0.64090909	0.97709091	c(t = 1.07676	0.298	0.84076656	Distal Intergenic	ENSSSCG000	PSMB9	proteasome 20S subunit beta 9 [Source:VGNC Symbol;Acc:VGNC:91911]
chr6	295000	301927	6928	*	chr6	295000	301927	in_vitro	in_vivo	13	13	86.8823077	91.7784615	-4.8961538	c(t = -1.0688	0.299	0.84076656	Distal Intergenic	ENSSSCG000	SPATA2L	spermatogenesis associated 2 like [Source:NCBI gene (formerly Entrezgene);Acc:100415811]
chr6	91747270	91748508	1239	*	chr6	91747270	91748508	in_vitro	in_vivo	10	10	2.755	5.256	-2.501	c(t = -1.0745	0.299	0.84076656	Distal Intergenic	NA	NA	NA
chrX	97997773	97998712	940	*	chrX	97997773	97998712	in_vitro	in_vivo	9	11	0.99666667	4.08181818	-3.0851515	c(t = -1.0901	0.299	0.84076656	Distal Intergenic	ENSSSCG000	PGRMC1	progesterone receptor membrane component 1 [Source:NCBI gene (formerly Entrezgene);Acc:396946]
chr2	134660379	134661263	885	*	chr2	134660379	134661263	in_vitro	in_vivo	12	10	1.02916667	1.916	-0.8868333	c(t = -1.0796	0.299	0.84076656	Distal Intergenic	ENSSSCG000	SLC22A4	solute carrier family 22 member 4 [Source:VGNC Symbol;Acc:VGNC:92981]
chr9	10627609	10629037	1429	*	chr9	10627609	10629037	in_vitro	in_vivo	13	11	4.51615385	1.77727273	2.73888112	c(t = 1.06821	0.299	0.84076656	Distal Intergenic	ENSSSCG000	DGAT2	diacylglycerol O-acyltransferase 2 [Source:NCBI gene (formerly Entrezgene);Acc:100294675]
chr7	94379628	94380577	950	*	chr7	94379628	94380577	in_vitro	in_vivo	12	14	2.1425	1.06714286	1.07535714	c(t = 1.06959	0.299	0.84076656	Distal Intergenic	ENSSSCG000	SYNJ2BP	synaptojanin 2 binding protein [Source:NCBI gene (formerly Entrezgene);Acc:100514718]
chr14	6187510	6188917	1408	*	chr14	6187510	6188917	in_vitro	in_vivo	12	15	2.28166667	0.886	1.39566667	c(t = 1.07499	0.299	0.84076656	Distal Intergenic	ENSSSCG000	DOK2	docking protein 2 [Source:NCBI gene (formerly Entrezgene);Acc:100152025]
chr14	46874912	46875862	951	*	chr14	46874912	46875862	in_vitro	in_vivo	9	13	2.1	0.87615385	1.22384615	c(t = 1.08791	0.299	0.84076656	Distal Intergenic	ENSSSCG000	UQCR10	ubiquinol-cytochrome c reductase, complex III subunit X [Source:NCBI gene (formerly Entrezgene);Acc:100155687]
chr1	37230785	37233378	2594	*	chr1	37230785	37233378	in_vitro	in_vivo	14	12	1.91714286	0.775	1.14214286	c(t = 1.07158	0.299	0.84076656	Promoter (<=1kb)	ENSSSCG000	HEY2	hes related family bHLH transcription factor with YRPW motif 2 [Source:VGNC Symbol;Acc:VGNC:88864]
chr10	23274482	23275709	1228	*	chr10	23274482	23275709	in_vitro	in_vivo	10	12	1.451	0.72416667	0.72683333	c(t = 1.08120	0.299	0.84076656	Distal Intergenic	ENSSSCG000	TNNT2	troponin T2, cardiac type [Source:NCBI gene (formerly Entrezgene);Acc:100622450]
chr6	27683973	27685294	1322	*	chr6	27683973	27685294	in_vitro	in_vivo	12	11	1.41916667	0.69818182	0.72098485	c(t = 1.07707	0.299	0.84076656	Distal Intergenic	ENSSSCG000	CES3	carboxylesterase 3 [Source:NCBI gene (formerly Entrezgene);Acc:100626873]
chr13	110375508	110376395	888	*	chr13	110375508	110376395	in_vitro	in_vivo	11	12	1.84636364	0.65083333	1.1955303	c(t = 1.08844	0.299	0.84076656	Promoter (<=1kb)	ENSSSCG000	PLD1	phospholipase D1 [Source:VGNC Symbol;Acc:VGNC:91525]
chr2	2999296	3006288	6993	*	chr2	2999296	3006288	in_vitro	in_vivo	10	13	95.207	92.8784615	2.32853846	c(t = 1.06467	0.3	0.84080963	Distal Intergenic	ENSSSCG000	FADD	Fas associated via death domain [Source:NCBI gene (formerly Entrezgene);Acc:595129]
chr3	131300600	131303395	2796	*	chr3	131300600	131303395	in_vitro	in_vivo	9	13	93.0755556	88.8915385	4.18401709	c(t = 1.06905	0.3	0.84080963	3' UTR	ENSSSCG000	ADI1	acireductone dioxygenase 1 [Source:NCBI gene (formerly Entrezgene);Acc:100626434]
chr3	38108772	38109727	956	*	chr3	38108772	38109727	in_vitro	in_vivo	9	10	7.06111111	18.799	-11.737889	c(t = -1.0762	0.3	0.84080963	Distal Intergenic	ENSSSCG000	HMOX2	heme oxygenase 2 [Source:VGNC Symbol;Acc:VGNC:88912]
chr7	114519635	114520899	1265	*	chr7	114519635	114520899	in_vitro	in_vivo	12	12	3.745	2.2825	1.4625	c(t = 1.06319	0.3	0.84080963	Promoter (<=1kb)	ENSSSCG000	ITPK1	inositol-tetrakisphosphate 1-kinase [Source:VGNC Symbol;Acc:VGNC:89251]
chr13	179913276	179914641	1366	*	chr13	179913276	179914641	in_vitro	in_vivo	12	13	0.79153846	1.97833333	-1.1867949	c(t = -1.0757	0.3	0.84080963	Distal Intergenic	NA	NA	NA
chr4	8468018	8468470	453	*	chr4	8468018	8468470	in_vitro	in_vivo	10	11	2.271	1.19818182	1.07281818	c(t = 1.07339	0.3	0.84080963	Distal Intergenic	ENSSSCG000	DNAAF11	dynein axonemal assembly factor 11 [Source:VGNC Symbol;Acc:VGNC:98083]
chr5	7210649	7211666	1018	*	chr5	7210649	7211666	in_vitro	in_vivo	9	10	3.94	1.197	2.743	c(t = 1.10036	0.3	0.84080963	Distal Intergenic	ENSSSCG000	ACO2	aconitase 2 [Source:NCBI gene (formerly Entrezgene);Acc:396999]
chr3	101957064	101958022	959	*	chr3	101957064	101958022	in_vitro	in_vivo	12	10	1.89	0.751	1.139	c(t = 1.07551	0.3	0.84080963	Distal Intergenic	ENSSSCG000	GALM	galactose mutarotase [Source:NCBI gene (formerly Entrezgene);Acc:399536]
chr12	46461997	46463264	1268	*	chr12	46461997	46463264	in_vitro	in_vivo	11	15	1.64636364	0.70066667	0.94569697	c(t = 1.08593	0.3	0.84080963	Distal Intergenic	ENSSSCG000	TRARG1	trafficking regulator of GLUT4 (SLC2A4) 1 [Source:NCBI gene (formerly Entrezgene);Acc:100126201]
chr16	78854486	78856078	1593	*	chr16	78854486	78856078	in_vitro	in_vivo	9	13	17.9133333	33.6869231	-15.77359	c(t = -1.0625	0.301	0.84085242	Distal Intergenic	ENSSSCG000	TERT	telomerase reverse transcriptase [Source:NCBI gene (formerly Entrezgene);Acc:492280]
chr10	33865976	33868184	2209	*	chr10	33865976	33868184	in_vitro	in_vivo	9	13	9.63222222	4.17076923	5.46145299	c(t = 1.08442	0.301	0.84085242	Distal Intergenic	ENSSSCG000	NDUFB6	NADH:ubiquinone oxidoreductase subunit B6 [Source:NCBI gene (formerly Entrezgene);Acc:733605]
chr4	56071475	56072547	1073	*	chr4	56071475	56072547	in_vitro	in_vivo	10	9	4.816	2.29222222	2.52377778	c(t = 1.07016	0.301	0.84085242	Distal Intergenic	ENSSSCG000	TPD52	tumor protein D52 [Source:VGNC Symbol;Acc:VGNC:98383]
chr13	72200329	72201343	1015	*	chr13	72200329	72201343	in_vitro	in_vivo	10	13	3.07846154	2.138	0.94046154	c(t = 1.06141	0.301	0.84085242	Distal Intergenic	ENSSSCG000	SEC61A1	SEC61 translocon subunit alpha 1 [Source:VGNC Symbol;Acc:VGNC:92682]
chr6	60638641	60640148	1508	*	chr6	60638641	60640148	in_vitro	in_vivo	9	11	0.77777778	1.94181818	-1.1640404	c(t = -1.0819	0.301	0.84085242	Distal Intergenic	ENSSSCG000	GALP	galanin like peptide [Source:NCBI gene (formerly Entrezgene);Acc:396772]
chr18	3032916	3034362	1447	*	chr18	3032916	3034362	in_vitro	in_vivo	10	10	6.143	1.851	4.292	c(t = 1.09163	0.301	0.84085242	Distal Intergenic	ENSSSCG000	INSIG1	insulin induced gene 1 [Source:NCBI gene (formerly Entrezgene);Acc:403162]
chr18	20123299	20123778	480	*	chr18	20123299	20123778	in_vitro	in_vivo	9	10	3.36555556	1.759	1.60655556	c(t = 1.06706	0.301	0.84085242	Distal Intergenic	ENSSSCG000	LEP	leptin [Source:VGNC Symbol;Acc:VGNC:89684]
chr4	17584459	17584932	474	*	chr4	17584459	17584932	in_vitro	in_vivo	11	9	2.53	1.04111111	1.48888889	c(t = 1.06795	0.301	0.84085242	Promoter (1-2kb)	ENSSSCG000	SHAS2	hyaluronan synthase 2 [Source:NCBI gene (formerly Entrezgene);Acc:397120]
chr12	16934181	16935468	1288	*	chr12	16934181	16935468	in_vitro	in_vivo	13	11	1.29692308	0.46363636	0.83328671	c(t = 1.07618	0.301	0.84085242	Distal Intergenic	ENSSSCG000	ITGB3	integrin subunit beta 3 [Source:NCBI gene (formerly Entrezgene);Acc:397063]
chr8	135769246	135769814	569	*	chr8	135769246	135769814	in_vitro	in_vivo	10	10	47.598	29.293	18.305	c(t = 1.06382	0.302	0.84089493	Distal Intergenic	ENSSSCG000	ENOPH1	enolase-phosphatase 1 [Source:VGNC Symbol;Acc:VGNC:98922]
chr3	132146786	132149102	2317	*	chr3	132146786	132149102	in_vitro	in_vivo	10	10	13.548	26.282	-12.734	c(t = -1.0648	0.302	0.84089493	Distal Intergenic	ENSSSCG000	TPO	thyroid peroxidase [Source:VGNC Symbol;Acc:VGNC:94341]
chr1	186022060	186023278	1219	*	chr1	186022060	186023278	in_vitro	in_vivo	14	11	4.10357143	10.3954545	-6.2918831	c(t = -1.0724	0.302	0.84089493	Distal Intergenic	ENSSSCG000	KTN1	kinectin 1 [Source:VGNC Symbol;Acc:VGNC:89608]
chr12	50602089	50603064	976	*	chr12	50602089	50603064	in_vitro	in_vivo	9	12	0.86111111	2.44916667	-1.5880556	c(t = -1.0749	0.302	0.84089493	Distal Intergenic	ENSSSCG000	ASPA	aspartoacylase [Source:NCBI gene (formerly Entrezgene);Acc:100142661]
chr15	83008082	83009848	1767	*	chr15	83008082	83009848	in_vitro	in_vivo	10	14	1.048	1.875	-0.827	c(t = -1.0584	0.302	0.84089493	Distal Intergenic	ENSSSCG000	MTX2	metaxin 2 [Source:VGNC Symbol;Acc:VGNC:96427]
chr8	10400557	10404013	3457	*	chr8	10400557	10404013	in_vitro	in_vivo	12	16	2.19	1.226875	0.963125	c(t = 1.07173	0.302	0.84089493	Promoter (<=1kb)	ENSSSCG000	CPEB2	cytoplasmic polyadenylation element binding protein 2 [Source:VGNC Symbol;Acc:VGNC:86937]
chr13	122268290	122269218	929	*	chr13	122268290	122269218	in_vitro	in_vivo	11	11	2.74272727	1.16727273	1.57545455	c(t = 1.06976	0.302	0.84089493	Distal Intergenic	ENSSSCG000	POLR2H	RNA polymerase II, I and III subunit H [Source:VGNC Symbol;Acc:VGNC:91652]
chr6	6988803	6989873	1071	*	chr6	6988803	6989873	in_vitro	in_vivo	9	13	2.17777778	1.06615385	1.11162393	c(t = 1.08320	0.302	0.84089493	Distal Intergenic	ENSSSCG000	BCO1	beta-carotene oxygenase 1 [Source:VGNC Symbol;Acc:VGNC:85789]
chr14	51806420	51806929	510	*	chr14	51806420	51806929	in_vitro	in_vivo	10	10	1.279	0.586	0.693	c(t = 1.07232	0.302	0.84089493	Distal Intergenic	ENSSSCG000	CHRM3	cholinergic receptor muscarinic 3 [Source:VGNC Symbol;Acc:VGNC:86664]
chr9	6806460	6810275	3816	*	chr9	6806460	6810275	in_vitro	in_vivo	9	12	31.3244444	46.6108333	-15.286389	c(t = -1.0630	0.303	0.84165302	Exon (NM_001190279/100462718, exon 30 of 32)	ENSSSCG000	INPPL1	inositol polyphosphate phosphatase like 1 [Source:NCBI gene (formerly Entrezgene);Acc:100462718]
chr12	2560207	2562838	2632	*	chr12	2560207	2562838	in_vitro	in_vivo	9	11	1.92444444	3.15363636	-1.2291919	c(t = -1.0617	0.303	0.84165302	Promoter (<=1kb)	ENSSSCG000	CBX8	chromobox 8 [Source:NCBI gene (formerly Entrezgene);Acc:100514536]
chr5	73825887	73826793	907	*	chr5	73825887	73826793	in_vitro	in_vivo	13	11	2.59461538	1.47818182	1.11643357	c(t = 1.05464	0.303	0.84165302	Distal Intergenic	ENSSSCG000	ADAMTS20	ADAM metallopeptidase with thrombospondin type 1 motif 20 [Source:VGNC Symbol;Acc:VGNC:85082]
chr6	119192553	119193411	859	*	chr6	119192553	119193411	in_vitro	in_vivo	11	9	1.78454545	0.74666667	1.03787879	c(t = 1.06179	0.303	0.84165302	Distal Intergenic	ENSSSCG000	ZNF24	zinc finger protein 24 [Source:VGNC Symbol;Acc:VGNC:98422]
chr11	26134218	26135404	1187	*	chr11	26134218	26135404	in_vitro	in_vivo	12	10	3.335	0.728	2.607	c(t = 1.07715	0.303	0.84165302	Distal Intergenic	ENSSSCG000	MTRF1	mitochondrial translation release factor 1 [Source:VGNC Symbol;Acc:VGNC:90471]
chr6	61227807	61229042	1236	*	chr6	61227807	61229042	in_vitro	in_vivo	9	10	1.26666667	0.294	0.97266667	c(t = 1.08549	0.303	0.84165302	Distal Intergenic	ENSSSCG000	GALP	galanin like peptide [Source:NCBI gene (formerly Entrezgene);Acc:396772]
chr7	133356	135131	1776	*	chr7	133356	135131	in_vitro	in_vivo	9	15	1.52222222	2.91	-1.3877778	c(t = -1.0535	0.304	0.84165302	Promoter (<=1kb)	ENSSSCG000	IRF4	interferon regulatory factor 4 [Source:VGNC Symbol;Acc:VGNC:89207]
chr7	29754354	29755343	990	*	chr7	29754354	29755343	in_vitro	in_vivo	10	12	6.857	2.10166667	4.75533333	c(t = 1.08231	0.304	0.84165302	Distal Intergenic	ENSSSCG000	RGL2	ral guanine nucleotide dissociation stimulator like 2 [Source:NCBI gene (formerly Entrezgene);Acc:100151794]
chr7	100263404	100264119	716	*	chr7	100263404	100264119	in_vitro	in_vivo	11	12	5.06363636	1.6775	3.38613636	c(t = 1.07144	0.304	0.84165302	Distal Intergenic	ENSSSCG000	NGB	neuroglobin [Source:NCBI gene (formerly Entrezgene);Acc:414438]
chr14	138934534	138945172	10639	*	chr14	138934534	138945172	in_vitro	in_vivo	15	16	2.05466667	1.445	0.60966667	c(t = 1.05034	0.304	0.84165302	Distal Intergenic	ENSSSCG000	GLRX3	glutaredoxin 3 [Source:VGNC Symbol;Acc:VGNC:88495]
chr14	39291315	39292295	981	*	chr14	39291315	39292295	in_vitro	in_vivo	10	10	1.486	0.526	0.96	c(t = 1.06087	0.304	0.84165302	Distal Intergenic	ENSSSCG000	RPL6	ribosomal protein L6 [Source:NCBI gene (formerly Entrezgene);Acc:733592]
chr18	50758794	50759735	942	*	chr18	50758794	50759735	in_vitro	in_vivo	11	13	1.49909091	0.47230769	1.02678322	c(t = 1.07923	0.304	0.84165302	Distal Intergenic	ENSSSCG000	DDX56	DEAD-box helicase 56 [Source:NCBI gene (formerly Entrezgene);Acc:100513388]
chr15	120756143	120756560	418	*	chr15	120756143	120756560	in_vitro	in_vivo	9	11	0.55444444	0.12818182	0.42626263	c(t = 1.08402	0.304	0.84165302	Distal Intergenic	ENSSSCG000	BCS1L	BCS1 homolog, ubiquinol-cytochrome c reductase complex chaperone [Source:VGNC Symbol;Acc:VGNC:96722]
chr3	1175818	1180351	4534	*	chr3	1175818	1180351	in_vitro	in_vivo	10	15	94.318	91.3346667	2.98333333	c(t = 1.04829	0.305	0.84165302	Distal Intergenic	ENSSSCG000	ssc-mir-9817	ssc-mir-9817 [Source:miRBase;Acc:MI0031584]
chr5	78717866	78718572	707	*	chr5	78717866	78718572	in_vitro	in_vivo	10	9	64.862	42.3866667	22.4753333	c(t = 1.06022	0.305	0.84165302	Distal Intergenic	ENSSSCG000	PFKM	phosphofructokinase, muscle [Source:VGNC Symbol;Acc:VGNC:91338]
chr2	9136891	9138911	2021	*	chr2	9136891	9138911	in_vitro	in_vivo	10	11	3.326	8.60818182	-5.2821818	c(t = -1.0750	0.305	0.84165302	Distal Intergenic	ENSSSCG000	GANAB	glucosidase II alpha subunit [Source:VGNC Symbol;Acc:VGNC:88344]
chr14	111831930	111834311	2382	*	chr14	111831930	111834311	in_vitro	in_vivo	13	11	2.06461538	4.01181818	-1.9472028	c(t = -1.0712	0.305	0.84165302	Distal Intergenic	ENSSSCG000	NDUFB8	NADH:ubiquinone oxidoreductase subunit B8 [Source:NCBI gene (formerly Entrezgene);Acc:448979]
chr14	136985821	136988516	2696	*	chr14	136985821	136988516	in_vitro	in_vivo	15	15	4.062	2.66266667	1.39933333	c(t = 1.04668	0.305	0.84165302	Distal Intergenic	ENSSSCG000	UROS	uroporphyrinogen III synthase [Source:VGNC Symbol;Acc:VGNC:94735]
chr1	217287626	217288039	414	*	chr1	217287626	217288039	in_vitro	in_vivo	9	9	1.06	2.65444444	-1.5944444	c(t = -1.0792	0.305	0.84165302	Distal Intergenic	ENSSSCG000	ssc-mir-101-	ssc-mir-101-1 [Source:miRBase;Acc:MI0010678]
chr8	134136848	134140941	4094	*	chr8	134136848	134140941	in_vitro	in_vivo	14	16	5.38928571	1.90375	3.48553571	c(t = 1.06376	0.305	0.84165302	Distal Intergenic	ENSSSCG000	CDS1	CDP-diacylglycerol synthase 1 [Source:VGNC Symbol;Acc:VGNC:86523]
chr11	60731373	60733345	1973	*	chr11	60731373	60733345	in_vitro	in_vivo	11	16	2.73909091	1.86125	0.87784091	c(t = 1.05976	0.305	0.84165302	Promoter (1-2kb)	ENSSSCG000	ssc-mir-17	ssc-mir-17 [Source:miRBase;Acc:MI0008214]
chr6	68473723	68474364	642	*	chr6	68473723	68474364	in_vitro	in_vivo	9	9	2.46666667	0.69777778	1.76888889	c(t = 1.08107	0.305	0.84165302	Distal Intergenic	ENSSSCG000	UTS2	urotensin 2 [Source:VGNC Symbol;Acc:VGNC:94788]
chr6	116324443	116325457	1015	*	chr6	116324443	116325457	in_vitro	in_vivo	12	12	1.10916667	0.48	0.62916667	c(t = 1.06039	0.305	0.84165302	Distal Intergenic	ENSSSCG000	TTR	transthyretin [Source:NCBI gene (formerly Entrezgene);Acc:397419]
chr14	10467108	10467964	857	*	chr14	10467108	10467964	in_vitro	in_vivo	10	12	1.344	0.255	1.089	c(t = 1.08332	0.305	0.84165302	Distal Intergenic	ENSSSCG000	ADRA1A	adrenoceptor alpha 1A [Source:VGNC Symbol;Acc:VGNC:85154]
chrX	21076176	21077766	1591	*	chrX	21076176	21077766	in_vitro	in_vivo	14	11	8.28428571	14.3881818	-6.1038961	c(t = -1.0501	0.306	0.84165302	Distal Intergenic	ENSSSCG000	SAT1	spermidine/spermine N1-acetyltransferase 1 [Source:NCBI gene (formerly Entrezgene);Acc:397645]
chr16	39679353	39680403	1051	*	chr16	39679353	39680403	in_vitro	in_vivo	11	13	0.41363636	1.39153846	-0.9779021	c(t = -1.0548	0.306	0.84165302	Distal Intergenic	ENSSSCG000	ssc-mir-9800	ssc-mir-9800 [Source:miRBase;Acc:MI0031564]
chr5	3365506	3367310	1805	*	chr5	3365506	3367310	in_vitro	in_vivo	9	13	0.74111111	1.38384615	-0.642735	c(t = -1.0508	0.306	0.84165302	Distal Intergenic	ENSSSCG000	PPARA	peroxisome proliferator activated receptor alpha [Source:NCBI gene (formerly Entrezgene);Acc:397239]
chr1	126361630	126362391	762	*	chr1	126361630	126362391	in_vitro	in_vivo	9	13	0.59555556	1.37076923	-0.7752137	c(t = -1.0516	0.306	0.84165302	Promoter (<=1kb)	ENSSSCG000	GATM	glycine amidinotransferase [Source:NCBI gene (formerly Entrezgene);Acc:100126844]
chr14	133829743	133830949	1207	*	chr14	133829743	133830949	in_vitro	in_vivo	10	11	2.827	1.18272727	1.64427273	c(t = 1.06531	0.306	0.84165302	Distal Intergenic	ENSSSCG00000010734		ornithine aminotransferase [Source:NCBI gene (formerly Entrezgene);Acc:100156865]
chr18	13821277	13821887	611	*	chr18	13821277	13821887	in_vitro	in_vivo	9	10	2.98777778	0.802	2.18577778	c(t = 1.08207	0.306	0.84165302	Distal Intergenic	ENSSSCG000	STRA8	stimulated by retinoic acid 8 [Source:VGNC Symbol;Acc:VGNC:93574]
chr2	40900154	40900741	588	*	chr2	40900154	40900741	in_vitro	in_vivo	14	11	1.43428571	0.50545455	0.92883117	c(t = 1.04908	0.306	0.84165302	Distal Intergenic	ENSSSCG000	HPS5	HPS5 biogenesis of lysosomal organelles complex 2 subunit 2 [Source:NCBI gene (formerly Entrezgene);Acc:100049701]
chr15	139973169	139977611	4443	*	chr15	139973169	139977611	in_vitro	in_vivo	11	10	82.2327273	93.143	-10.910273	c(t = -1.0708	0.307	0.84165302	Distal Intergenic	ENSSSCG000	SEPTIN2	septin 2 [Source:VGNC Symbol;Acc:VGNC:104054]
chr12	59883372	59885807	2436	*	chr12	59883372	59885807	in_vitro	in_vivo	11	12	95.2463636	91.83	3.41636364	c(t = 1.05046	0.307	0.84165302	Distal Intergenic	ENSSSCG000	AKAP10	A-kinase anchoring protein 10 [Source:NCBI gene (formerly Entrezgene);Acc:397262]
chr12	59835725	59837265	1541	*	chr12	59835725	59837265	in_vitro	in_vivo	11	11	92	86.8690909	5.13090909	c(t = 1.06363	0.307	0.84165302	Distal Intergenic	ENSSSCG000	AKAP10	A-kinase anchoring protein 10 [Source:NCBI gene (formerly Entrezgene);Acc:397262]
chr5	58925141	58926819	1679	*	chr5	58925141	58926819	in_vitro	in_vivo	11	13	10.6027273	22.6846154	-12.081888	c(t = -1.0450	0.307	0.84165302	Distal Intergenic	ENSSSCG000	EMP1	epithelial membrane protein 1 [Source:VGNC Symbol;Acc:VGNC:87688]
chrX	41397905	41399411	1507	*	chrX	41397905	41399411	in_vitro	in_vivo	10	12	5.73	10.815	-5.085	c(t = -1.0543	0.307	0.84165302	Distal Intergenic	ENSSSCG000	RGN	regucalcin [Source:VGNC Symbol;Acc:VGNC:92256]
chr7	54958637	54959826	1190	*	chr7	54958637	54959826	in_vitro	in_vivo	9	12	2.42666667	8.4175	-5.9908333	c(t = -1.0675	0.307	0.84165302	Distal Intergenic	ENSSSCG000	ssc-mir-9-3	ssc-mir-9-3 [Source:miRBase;Acc:MI0022123]
chr12	7788447	7789479	1033	*	chr12	7788447	7789479	in_vitro	in_vivo	12	14	6.945	1.58428571	5.36071429	c(t = 1.06894	0.307	0.84165302	Distal Intergenic	ENSSSCG000	SSTR2	somatostatin receptor 2 [Source:VGNC Symbol;Acc:VGNC:99086]
chr1	162935970	162937095	1126	*	chr1	162935970	162937095	in_vitro	in_vivo	11	9	2.80545455	1.27666667	1.52878788	c(t = 1.06406	0.307	0.84165302	Distal Intergenic	ENSSSCG000	CILP	cartilage intermediate layer protein [Source:VGNC Symbol;Acc:VGNC:86703]
chr8	111727887	111729068	1182	*	chr8	111727887	111729068	in_vitro	in_vivo	11	15	3.30727273	1.04733333	2.25993939	c(t = 1.06922	0.307	0.84165302	Distal Intergenic	ENSSSCG000	PITX2	paired like homeodomain 2 [Source:VGNC Symbol;Acc:VGNC:91467]
chr16	27813912	27814941	1030	*	chr16	27813912	27814941	in_vitro	in_vivo	11	12	1.23636364	0.62916667	0.60719697	c(t = 1.05173	0.307	0.84165302	Distal Intergenic	ENSSSCG000	HMGCS1	3-hydroxy-3-methylglutaryl-CoA synthase 1 [Source:VGNC Symbol;Acc:VGNC:88907]
chr11	24116885	24117332	448	*	chr11	24116885	24117332	in_vitro	in_vivo	9	11	4.01222222	0	4.01222222	c(t = 1.09101	0.307	0.84165302	Distal Intergenic	ENSSSCG000	FAM216B	family with sequence similarity 216 member B [Source:VGNC Symbol;Acc:VGNC:87955]
chr10	65400476	65401646	1171	*	chr10	65400476	65401646	in_vitro	in_vivo	9	9	91.6266667	95.4577778	-3.8311111	c(t = -1.0632	0.308	0.84165302	Distal Intergenic	ENSSSCG000	AKR1C8	aldo-keto reductase family 1 member C4 [Source:NCBI gene (formerly Entrezgene);Acc:654406]
chr18	50617184	50619543	2360	*	chr18	50617184	50619543	in_vitro	in_vivo	14	12	90.4664286	95.35	-4.8835714	c(t = -1.0513	0.308	0.84165302	Distal Intergenic	ENSSSCG00000016737		peptidylprolyl isomerase A [Source:NCBI gene (formerly Entrezgene);Acc:397637]
chr7	2915307	2917205	1899	*	chr7	2915307	2917205	in_vitro	in_vivo	11	9	91.8372727	94.9777778	-3.1405051	c(t = -1.0504	0.308	0.84165302	Distal Intergenic	ENSSSCG000	ECI2	enoyl-CoA delta isomerase 2 [Source:VGNC Symbol;Acc:VGNC:87532]
chr15	81901118	81902014	897	*	chr15	81901118	81902014	in_vitro	in_vivo	10	11	12.79	5.40545455	7.38454545	c(t = 1.06643	0.308	0.84165302	Distal Intergenic	NA	NA	NA
chr15	52838380	52839903	1524	*	chr15	52838380	52839903	in_vitro	in_vivo	12	16	1.11166667	1.953125	-0.8414583	c(t = -1.0401	0.308	0.84165302	Distal Intergenic	ENSSSCG000	UBXN8	UBX domain protein 8 [Source:VGNC Symbol;Acc:VGNC:96239]
chr13	25773473	25774601	1129	*	chr13	25773473	25774601	in_vitro	in_vivo	11	11	4.39	1.73545455	2.65454545	c(t = 1.06434	0.308	0.84165302	Distal Intergenic	ENSSSCG000	CCK	cholecystokinin [Source:VGNC Symbol;Acc:VGNC:86334]
chr9	267851	269384	1534	*	chr9	267851	269384	in_vitro	in_vivo	14	14	1.98357143	1.02214286	0.96142857	c(t = 1.04336	0.308	0.84165302	Distal Intergenic	NA	NA	NA
chr2	85642347	85643055	709	*	chr2	85642347	85643055	in_vitro	in_vivo	10	11	2.02	0.89909091	1.12090909	c(t = 1.05637	0.308	0.84165302	Promoter (<=1kb)	ENSSSCG000	F2R	coagulation factor II thrombin receptor [Source:NCBI gene (formerly Entrezgene);Acc:100521609]
chrX	98288615	98289975	1361	*	chrX	98288615	98289975	in_vitro	in_vivo	9	14	6.11777778	12.8457143	-6.7279365	c(t = -1.0481	0.309	0.84165302	Distal Intergenic	ENSSSCG000	NKAP	NFKB activating protein [Source:VGNC Symbol;Acc:VGNC:90756]
chr17	36199191	36201944	2754	*	chr17	36199191	36201944	in_vitro	in_vivo	12	13	1.89416667	3.31846154	-1.4242949	c(t = -1.0475	0.309	0.84165302	Distal Intergenic	ENSSSCG000	DNMT3B	DNA methyltransferase 3 beta [Source:VGNC Symbol;Acc:VGNC:96228]
chr6	119356359	119357822	1464	*	chr6	119356359	119357822	in_vitro	in_vivo	14	13	2.08214286	1.37384615	0.7082967	c(t = 1.04293	0.309	0.84165302	Distal Intergenic	ENSSSCG000	ZNF24	zinc finger protein 24 [Source:VGNC Symbol;Acc:VGNC:98422]
chr2	150259952	150260724	773	*	chr2	150259952	150260724	in_vitro	in_vivo	10	11	1.321	0.63916667	0.68183333	c(t = 1.05267	0.309	0.84165302	Distal Intergenic	ENSSSCG000	AFAP1L1	actin filament associated protein 1 like 1 [Source:NCBI gene (formerly Entrezgene);Acc:100513463]
chr9	41480496	41481252	757	*	chr9	41480496	41481252	in_vitro	in_vivo	9	11	1.48444444	0.54636364	0.93808081	c(t = 1.05606	0.309	0.84165302	Distal Intergenic	ENSSSCG000	NNMT	nicotinamide N-methyltransferase [Source:NCBI gene (formerly Entrezgene);Acc:100144485]
chr8	103047472	103049236	1765	*	chr8	103047472	103049236	in_vitro	in_vivo	12	13	16.3883333	9.81307692	6.57525641	c(t = 1.03859	0.31	0.84165302	Distal Intergenic	ENSSSCG000	SMIM43	small integral membrane protein 43 [Source:NCBI gene (formerly Entrezgene);Acc:110262163]
chr14	114898781	114899487	707	*	chr14	114898781	114899487	in_vitro	in_vivo	13	10	0.95153846	1.829	-0.8774615	c(t = -1.0497	0.31	0.84165302	Distal Intergenic	ENSSSCG000	STN1	STN1 subunit of CST complex [Source:VGNC Symbol;Acc:VGNC:93564]
chr12	48645255	48646633	1379	*	chr12	48645255	48646633	in_vitro	in_vivo	9	14	0.66222222	1.79357143	-1.1313492	c(t = -1.0469	0.31	0.84165302	Promoter (<=1kb)	ENSSSCG000	PAFAH1B1	platelet activating factor acetylhydrolase 1b regulatory subunit 1 [Source:VGNC Symbol;Acc:VGNC:99026]
chr14	113635977	113639044	3068	*	chr14	113635977	113639044	in_vitro	in_vivo	10	11	0.865	1.60545455	-0.7404545	c(t = -1.0512	0.31	0.84165302	Distal Intergenic	ENSSSCG000	ARL3	ADP ribosylation factor like GTPase 3 [Source:NCBI gene (formerly Entrezgene);Acc:595114]
chr14	39326528	39327468	941	*	chr14	39326528	39327468	in_vitro	in_vivo	10	11	0.337	1.38363636	-1.0466364	c(t = -1.0638	0.31	0.84165302	Distal Intergenic	ENSSSCG000	RPL6	ribosomal protein L6 [Source:NCBI gene (formerly Entrezgene);Acc:733592]
chr14	35575323	35575791	469	*	chr14	35575323	35575791	in_vitro	in_vivo	12	10	2.21083333	1.283	0.92783333	c(t = 1.04091	0.31	0.84165302	Distal Intergenic	ENSSSCG000	FBXO21	F-box protein 21 [Source:VGNC Symbol;Acc:VGNC:88035]
chr15	44065675	44066812	1138	*	chr15	44065675	44066812	in_vitro	in_vivo	10	10	3.522	0.851	2.671	c(t = 1.07368	0.31	0.84165302	Distal Intergenic	ENSSSCG000	CLDN22	claudin 22 [Source:NCBI gene (formerly Entrezgene);Acc:100294683]
chr5	33626689	33627307	619	*	chr5	33626689	33627307	in_vitro	in_vivo	10	13	1.54	0.40538462	1.13461538	c(t = 1.06809	0.31	0.84165302	Distal Intergenic	ENSSSCG000	LYZ	lysozyme [Source:NCBI gene (formerly Entrezgene);Acc:100157211]
chr2	7752679	7755259	2581	*	chr2	7752679	7755259	in_vitro	in_vivo	10	11	7.56	15.92	-8.36	c(t = -1.0495	0.311	0.84165302	Distal Intergenic	ENSSSCG000	PRDX5	peroxiredoxin 5 [Source:NCBI gene (formerly Entrezgene);Acc:397273]
chr14	50818150	50820813	2664	*	chr14	50818150	50820813	in_vitro	in_vivo	13	14	7.24	4.535	2.705	c(t = 1.04325	0.311	0.84165302	Distal Intergenic	ENSSSCG000	TSSK1B	testis specific serine kinase 1B [Source:NCBI gene (formerly Entrezgene);Acc:102159240]
chr12	18881300	18882124	825	*	chr12	18881300	18882124	in_vitro	in_vivo	9	13	1.53777778	3.87	-2.3322222	c(t = -1.0500	0.311	0.84165302	Distal Intergenic	ENSSSCG000	GRN	granulin precursor [Source:NCBI gene (formerly Entrezgene);Acc:733645]
chr17	27806619	27808167	1549	*	chr17	27806619	27808167	in_vitro	in_vivo	9	13	4.27888889	1.91461538	2.3642735	c(t = 1.05852	0.311	0.84165302	Distal Intergenic	ENSSSCG000	SEC23B	SEC23 homolog B, COPII coat complex component [Source:VGNC Symbol;Acc:VGNC:95490]
chr6	147394356	147395256	901	*	chr6	147394356	147395256	in_vitro	in_vivo	10	9	2.482	1.24777778	1.23422222	c(t = 1.04715	0.311	0.84165302	Distal Intergenic	ENSSSCG000	JAK1	Janus kinase 1 [Source:VGNC Symbol;Acc:VGNC:89270]
chr7	58622943	58623686	744	*	chr7	58622943	58623686	in_vitro	in_vivo	13	14	2.71642857	1.22615385	1.49027473	c(t = 1.04343	0.311	0.84165302	Distal Intergenic	ENSSSCG000	FAM219B	family with sequence similarity 219 member B [Source:VGNC Symbol;Acc:VGNC:87956]
chr3	126491367	126493529	2163	*	chr3	126491367	126493529	in_vitro	in_vivo	12	9	71.31	83.8688889	-12.558889	c(t = -1.0410	0.312	0.84165302	Distal Intergenic	ENSSSCG000	KLF11	KLF transcription factor 11 [Source:NCBI gene (formerly Entrezgene);Acc:100174954]
chr12	18581484	18582760	1277	*	chr12	18581484	18582760	in_vitro	in_vivo	11	10	2.09272727	6.136	-4.0432727	c(t = -1.0615	0.312	0.84165302	Distal Intergenic	ENSSSCG000	GJC1	gap junction protein gamma 1 [Source:VGNC Symbol;Acc:VGNC:88468]
chr7	8518417	8520340	1924	*	chr7	8518417	8520340	in_vitro	in_vivo	11	13	5.70272727	3.12076923	2.58195804	c(t = 1.04218	0.312	0.84165302	Distal Intergenic	ENSSSCG000	EDN1	endothelin 1 [Source:NCBI gene (formerly Entrezgene);Acc:396915]
chr5	4770397	4771659	1263	*	chr5	4770397	4771659	in_vitro	in_vivo	11	12	3.81	2.34333333	1.46666667	c(t = 1.04033	0.312	0.84165302	Distal Intergenic	ENSSSCG000	PARVG	parvin gamma [Source:VGNC Symbol;Acc:VGNC:91187]
chr15	111361388	111362917	1530	*	chr15	111361388	111362917	in_vitro	in_vivo	9	11	3.95666667	1.84727273	2.10939394	c(t = 1.06585	0.312	0.84165302	Distal Intergenic	NA	NA	NA
chr18	38635282	38636179	898	*	chr18	38635282	38636179	in_vitro	in_vivo	10	12	0.672	1.7125	-1.0405	c(t = -1.0423	0.312	0.84165302	Distal Intergenic	ENSSSCG000	FKBP9	FKBP prolyl isomerase 9 [Source:NCBI gene (formerly Entrezgene);Acc:100623924]
chr11	74231938	74234408	2471	*	chr11	74231938	74234408	in_vitro	in_vivo	11	13	2.74181818	1.55153846	1.19027972	c(t = 1.05465	0.312	0.84165302	Promoter (<=1kb)	ENSSSCG000	EFNB2	ephrin B2 [Source:NCBI gene (formerly Entrezgene);Acc:100135673]
chr5	16977229	16978245	1017	*	chr5	16977229	16978245	in_vitro	in_vivo	12	10	2.20666667	1.223	0.98366667	c(t = 1.03934	0.312	0.84165302	Distal Intergenic	ENSSSCG000	ACVR1B	activin A receptor type 1B [Source:VGNC Symbol;Acc:VGNC:85056]
chr11	9912809	9913316	508	*	chr11	9912809	9913316	in_vitro	in_vivo	9	11	2.96333333	1.17363636	1.78969697	c(t = 1.06157	0.312	0.84165302	Distal Intergenic	ENSSSCG000	RFC3	replication factor C subunit 3 [Source:VGNC Symbol;Acc:VGNC:92233]
chr16	18415572	18416759	1188	*	chr16	18415572	18416759	in_vitro	in_vivo	13	11	1.69454545	0.99384615	0.7006993	c(t = 1.04371	0.312	0.84165302	Distal Intergenic	ENSSSCG000	C1QTNF3	C1q and TNF related 3 [Source:NCBI gene (formerly Entrezgene);Acc:100270814]
chr14	101367425	101368332	908	*	chr14	101367425	101368332	in_vitro	in_vivo	15	13	1.706	0.93307692	0.77292308	c(t = 1.03321	0.312	0.84165302	Distal Intergenic	ENSSSCG000	ssc-mir-107	ssc-mir-107 [Source:miRBase;Acc:MI0002449]
chr8	36474080	36474606	527	*	chr8	36474080	36474606	in_vitro	in_vivo	10	9	2.097	0.57333333	1.52366667	c(t = 1.06251	0.312	0.84165302	Distal Intergenic	ENSSSCG000	ZAR1	zygote arrest 1 [Source:VGNC Symbol;Acc:VGNC:95051]
chr13	10715768	10717122	1355	*	chr13	10715768	10717122	in_vitro	in_vivo	11	11	1.50818182	0.49636364	1.01181818	c(t = 1.05191	0.312	0.84165302	Promoter (<=1kb)	ENSSSCG00000037030		ribosomal protein L15 [Source:NCBI gene (formerly Entrezgene);Acc:100620094]
chr15	95248034	95249335	1302	*	chr15	95248034	95249335	in_vitro	in_vivo	10	9	0.529	0.10111111	0.42788889	c(t = 1.06398	0.312	0.84165302	Distal Intergenic	ENSSSCG000	MFSD6	major facilitator superfamily domain containing 6 [Source:VGNC Symbol;Acc:VGNC:96052]
chrX	106795244	106795931	688	*	chrX	106795244	106795931	in_vitro	in_vivo	9	11	0.90777778	3.6	-2.6922222	c(t = -1.0571	0.313	0.84165302	Distal Intergenic	ENSSSCG000	GPR119	G protein-coupled receptor 119 [Source:NCBI gene (formerly Entrezgene);Acc:100524541]
chr14	137100970	137102284	1315	*	chr14	137100970	137102284	in_vitro	in_vivo	10	11	1.152	2.23636364	-1.0843636	c(t = -1.0454	0.313	0.84165302	Distal Intergenic	ENSSSCG000	UROS	uroporphyrinogen III synthase [Source:VGNC Symbol;Acc:VGNC:94735]
chr3	51622364	51623672	1309	*	chr3	51622364	51623672	in_vitro	in_vivo	13	14	1.99153846	1.27071429	0.72082418	c(t = 1.03157	0.313	0.84165302	Promoter (<=1kb)	ENSSSCG000	SLC9A2	solute carrier family 9 member A2 [Source:NCBI gene (formerly Entrezgene);Acc:100101921]
chr4	106057753	106059218	1466	*	chr4	106057753	106059218	in_vitro	in_vivo	12	16	1.90916667	1.05625	0.85291667	c(t = 1.03920	0.313	0.84165302	Distal Intergenic	ENSSSCG000	BCAS2	BCAS2 pre-mRNA processing factor [Source:NCBI gene (formerly Entrezgene);Acc:100154006]
chr15	63504468	63505671	1204	*	chr15	63504468	63505671	in_vitro	in_vivo	10	10	2.435	0.895	1.54	c(t = 1.05620	0.313	0.84165302	Distal Intergenic	ENSSSCG000	NR4A2	nuclear receptor subfamily 4 group A member 2 [Source:VGNC Symbol;Acc:VGNC:96451]
chr13	51178094	51179025	932	*	chr13	51178094	51179025	in_vitro	in_vivo	14	10	1.85	0.774	1.076	c(t = 1.03481	0.313	0.84165302	Distal Intergenic	ENSSSCG000	MITF	melanocyte inducing transcription factor [Source:VGNC Symbol;Acc:VGNC:90243]
chr2	6159157	6161564	2408	*	chr2	6159157	6161564	in_vitro	in_vivo	10	9	96.291	97.9744444	-1.6834444	c(t = -1.0376	0.314	0.84165302	Distal Intergenic	ENSSSCG000	RAB1B	RAB1B, member RAS oncogene family [Source:NCBI gene (formerly Entrezgene);Acc:100144502]
chr12	24846068	24848193	2126	*	chr12	24846068	24848193	in_vitro	in_vivo	10	10	5.214	3.202	2.012	c(t = 1.03724	0.314	0.84165302	Distal Intergenic	NA	NA	NA
chr1	267924478	267926016	1539	*	chr1	267924478	267926016	in_vitro	in_vivo	14	12	5.835	3.09916667	2.73583333	c(t = 1.03927	0.314	0.84165302	Distal Intergenic	ENSSSCG000	ANGPTL2	angiopoietin like 2 [Source:VGNC Symbol;Acc:VGNC:85304]
chr14	51493348	51494920	1573	*	chr14	51493348	51494920	in_vitro	in_vivo	10	12	5.492	2.61166667	2.88033333	c(t = 1.04494	0.314	0.84165302	Promoter (<=1kb)	ENSSSCG000	RANBP1	RAN binding protein 1 [Source:VGNC Symbol;Acc:VGNC:92076]
chr7	13126204	13127804	1601	*	chr7	13126204	13127804	in_vitro	in_vivo	14	14	1.90642857	1.14785714	0.75857143	c(t = 1.03220	0.314	0.84165302	Distal Intergenic	ENSSSCG000	NHLRC1	NHL repeat containing E3 ubiquitin protein ligase 1 [Source:NCBI gene (formerly Entrezgene);Acc:100152396]
chr18	29540788	29541659	872	*	chr18	29540788	29541659	in_vitro	in_vivo	12	11	2.3575	1.10727273	1.25022727	c(t = 1.04322	0.314	0.84165302	Distal Intergenic	ENSSSCG000	MET	MET proto-onco, receptor tyrosine kinase [Source:VGNC Symbol;Acc:VGNC:109462]
chr5	16285032	16286011	980	*	chr5	16285032	16286011	in_vitro	in_vivo	10	12	0.137	1.03166667	-0.8946667	c(t = -1.0525	0.314	0.84165302	Distal Intergenic	ENSSSCG000	LIMA1	LIM domain and actin binding 1 [Source:VGNC Symbol;Acc:VGNC:98070]
chr16	52759071	52760857	1787	*	chr16	52759071	52760857	in_vitro	in_vivo	10	13	2.393	0.89	1.503	c(t = 1.05992	0.314	0.84165302	Distal Intergenic	ENSSSCG000	KCNIP1	potassium voltage-gated channel interacting protein 1 [Source:VGNC Symbol;Acc:VGNC:89348]
chr4	33612583	33614901	2319	*	chr4	33612583	33614901	in_vitro	in_vivo	13	14	2.72538462	0.71285714	2.01252747	c(t = 1.04879	0.314	0.84165302	Distal Intergenic	ENSSSCG000	FZD6	frizzled class receptor 6 [Source:NCBI gene (formerly Entrezgene);Acc:100157360]
chr12	60073092	60074235	1144	*	chr12	60073092	60074235	in_vitro	in_vivo	9	13	32.0711111	20.7115385	11.3595726	c(t = 1.03328	0.315	0.84165302	Distal Intergenic	ENSSSCG000	SLC5A10	solute carrier family 5 member 10 [Source:NCBI gene (formerly Entrezgene);Acc:497235]
chrX	106502454	106504105	1652	*	chrX	106502454	106504105	in_vitro	in_vivo	12	10	2.59833333	4.479	-1.8806667	c(t = -1.0417	0.315	0.84165302	Distal Intergenic	ENSSSCG000	XPNPEP2	X-prolyl aminopeptidase 2 [Source:VGNC Symbol;Acc:VGNC:95001]
chr6	166333254	166334162	909	*	chr6	166333254	166334162	in_vitro	in_vivo	9	12	9.09777778	3.77916667	5.31861111	c(t = 1.06491	0.315	0.84165302	Distal Intergenic	ENSSSCG000	UROD	uroporphyrinogen decarboxylase [Source:NCBI gene (formerly Entrezgene);Acc:100514239]
chr9	71349560	71351710	2151	*	chr9	71349560	71351710	in_vitro	in_vivo	14	11	2.21214286	1.38454545	0.8275974	c(t = 1.02976	0.315	0.84165302	Distal Intergenic	ENSSSCG000	AKAP9	A-kinase anchoring protein 9 [Source:VGNC Symbol;Acc:VGNC:85225]
chr2	18725856	18726589	734	*	chr2	18725856	18726589	in_vitro	in_vivo	10	11	3.649	1.35272727	2.29627273	c(t = 1.05599	0.315	0.84165302	Promoter (1-2kb)	NA	NA	NA
chr6	2552607	2556130	3524	*	chr6	2552607	2556130	in_vitro	in_vivo	10	12	1.88545455	1.18416667	0.70128788	c(t = 1.03662	0.315	0.84165302	Distal Intergenic	ENSSSCG000	IRF8	interferon regulatory factor 8 [Source:VGNC Symbol;Acc:VGNC:89211]
chr14	113136901	113137814	914	*	chr14	113136901	113137814	in_vitro	in_vivo	12	10	2.68583333	0.986	1.69983333	c(t = 1.04123	0.315	0.84165302	Distal Intergenic	ENSSSCG000	LDB1	LIM domain binding 1 [Source:NCBI gene (formerly Entrezgene);Acc:780436]
chr2	136475272	136476355	1084	*	chr2	136475272	136476355	in_vitro	in_vivo	10	15	1.898	0.48666667	1.41133333	c(t = 1.05832	0.315	0.84165302	Distal Intergenic	ENSSSCG000	PPP2CA	protein phosphatase 2 catalytic subunit alpha [Source:NCBI gene (formerly Entrezgene);Acc:397656]
chr5	21665186	21665987	802	*	chr5	21665186	21665987	in_vitro	in_vivo	10	9	0.796	0.17333333	0.62266667	c(t = 1.05542	0.315	0.84165302	Distal Intergenic	ENSSSCG000	CS	citrate synthase [Source:VGNC Symbol;Acc:VGNC:103225]
chr6	107278317	107285577	7261	*	chr6	107278317	107285577	in_vitro	in_vivo	17	15	1.57823529	2.32333333	-0.745098	c(t = -1.0224	0.316	0.84165302	Promoter (<=1kb)	ENSSSCG000	GATA6	GATA binding protein 6 [Source:VGNC Symbol;Acc:VGNC:88366]
chr8	14756248	14760025	3778	*	chr8	14756248	14760025	in_vitro	in_vivo	14	17	0.99714286	1.83294118	-0.8357983	c(t = -1.0230	0.316	0.84165302	Distal Intergenic	ENSSSCG000	ssc-mir-218-	ssc-mir-218-1 [Source:miRBase;Acc:MI0015924]
chr2	105042235	105042857	623	*	chr2	105042235	105042857	in_vitro	in_vivo	10	12	0.5	1.49	-0.99	c(t = -1.0321	0.316	0.84165302	Distal Intergenic	ENSSSCG000	LNPEP	leucyl and cystinyl aminopeptidase [Source:NCBI gene (formerly Entrezgene);Acc:100125826]
chr2	89045219	89046280	1062	*	chr2	89045219	89046280	in_vitro	in_vivo	12	12	2.68083333	1.4625	1.21833333	c(t = 1.02731	0.316	0.84165302	Distal Intergenic	ENSSSCG00000031117		dihydrofolate reductase [Source:NCBI gene (formerly Entrezgene);Acc:100525912]
chr6	17433947	17435295	1349	*	chr6	17433947	17435295	in_vitro	in_vivo	10	10	0.244	0.703	-0.459	c(t = -1.0436	0.316	0.84165302	Distal Intergenic	ENSSSCG000	CYB5B	cytochrome b5 type B [Source:NCBI gene (formerly Entrezgene);Acc:100286856]
chr9	90336366	90337530	1165	*	chr9	90336366	90337530	in_vitro	in_vivo	12	11	3.63416667	0.53636364	3.09780303	c(t = 1.04803	0.316	0.84165302	Distal Intergenic	ENSSSCG000	ITGB8	integrin subunit beta 8 [Source:VGNC Symbol;Acc:VGNC:89246]
chr5	46574438	46574905	468	*	chr5	46574438	46574905	in_vitro	in_vivo	12	9	1.36833333	0.46111111	0.90722222	c(t = 1.04028	0.316	0.84165302	Distal Intergenic	ENSSSCG000	MED21	mediator complex subunit 21 [Source:VGNC Symbol;Acc:VGNC:109556]
chr6	69391289	69392780	1492	*	chr6	69391289	69392780	in_vitro	in_vivo	9	12	83.5455556	70.4683333	13.0772222	c(t = 1.02910	0.317	0.84165302	Distal Intergenic	ENSSSCG000	CA6	carbonic anhydrase 6 [Source:NCBI gene (formerly Entrezgene);Acc:100240717]
chr6	64017978	64020207	2230	*	chr6	64017978	64020207	in_vitro	in_vivo	11	10	31.4709091	47.866	-16.395091	c(t = -1.0287	0.317	0.84165302	Distal Intergenic	ENSSSCG000	PRKCZ	protein kinase C zeta [Source:NCBI gene (formerly Entrezgene);Acc:100533200]
chr8	1353171	1354590	1420	*	chr8	1353171	1354590	in_vitro	in_vivo	10	12	1.322	4.19166667	-2.8696667	c(t = -1.0439	0.317	0.84165302	Distal Intergenic	ENSSSCG000	RNF4	ring finger protein 4 [Source:VGNC Symbol;Acc:VGNC:92392]
chr4	14932985	14934360	1376	*	chr4	14932985	14934360	in_vitro	in_vivo	11	13	4.85454545	3.02307692	1.83146853	c(t = 1.04110	0.317	0.84165302	Distal Intergenic	ENSSSCG000	TATDN1	TatD DNase domain containing 1 [Source:VGNC Symbol;Acc:VGNC:98359]
chr14	28590596	28592578	1983	*	chr14	28590596	28592578	in_vitro	in_vivo	13	10	1.58923077	2.978	-1.3887692	c(t = -1.0473	0.317	0.84165302	Distal Intergenic	ENSSSCG000	NCOR2	nuclear receptor corepressor 2 [Source:NCBI gene (formerly Entrezgene);Acc:733643]
chr2	97647213	97648359	1147	*	chr2	97647213	97648359	in_vitro	in_vivo	11	14	3.10363636	1.635	1.46863636	c(t = 1.03125	0.317	0.84165302	Distal Intergenic	ENSSSCG000	MEF2C	myocyte enhancer factor 2C [Source:VGNC Symbol;Acc:VGNC:90127]
chr8	542680	543882	1203	*	chr8	542680	543882	in_vitro	in_vivo	9	15	4.42888889	1.09133333	3.33755556	c(t = 1.06340	0.317	0.84165302	Distal Intergenic	ENSSSCG000	SLBP	stem-loop binding protein [Source:NCBI gene (formerly Entrezgene);Acc:100513605]
chr1	236265869	236266351	483	*	chr1	236265869	236266351	in_vitro	in_vivo	11	10	2.40090909	0.788	1.61290909	c(t = 1.04138	0.317	0.84165302	Distal Intergenic	ENSSSCG000	CIMIP2B	ciliary microtubule inner protein 2B [Source:VGNC Symbol;Acc:VGNC:87922]
chr8	38241250	38242381	1132	*	chr8	38241250	38242381	in_vitro	in_vivo	13	14	1.08769231	0.50928571	0.57840659	c(t = 1.03138	0.317	0.84165302	Distal Intergenic	ENSSSCG000	ZAR1	zygote arrest 1 [Source:VGNC Symbol;Acc:VGNC:95051]
chr5	76636583	76638562	1980	*	chr5	76636583	76638562	in_vitro	in_vivo	12	12	0.83416667	0.3625	0.47166667	c(t = 1.03349	0.317	0.84165302	Distal Intergenic	ENSSSCG000	SLC38A2	solute carrier family 38 member 2 [Source:VGNC Symbol;Acc:VGNC:93094]
chr2	66617436	66617855	420	*	chr2	66617436	66617855	in_vitro	in_vivo	11	11	1.07818182	0.35636364	0.72181818	c(t = 1.03337	0.317	0.84165302	Distal Intergenic	NA	NA	NA
chr1	268995409	268997851	2443	*	chr1	268995409	268997851	in_vitro	in_vivo	11	10	95.3745455	97.458	-2.0834545	c(t = -1.0263	0.318	0.84165302	Distal Intergenic	ENSSSCG00000005656		SET nuclear proto-onco [Source:NCBI gene (formerly Entrezgene);Acc:100514028]
chr8	1481386	1484934	3549	*	chr8	1481386	1484934	in_vitro	in_vivo	10	13	97.53	96.2207692	1.30923077	c(t = 1.03026	0.318	0.84165302	Distal Intergenic	ENSSSCG000	RNF4	ring finger protein 4 [Source:VGNC Symbol;Acc:VGNC:92392]
chr15	133854591	133856068	1478	*	chr15	133854591	133856068	in_vitro	in_vivo	10	9	87.335	91.4711111	-4.1361111	c(t = -1.0339	0.318	0.84165302	Distal Intergenic	ENSSSCG000	SAG	S-antigen visual arrestin [Source:VGNC Symbol;Acc:VGNC:95476]
chr12	632579	638089	5511	*	chr12	632579	638089	in_vitro	in_vivo	11	12	94.3054545	86.8258333	7.47962121	c(t = 1.04057	0.318	0.84165302	Distal Intergenic	ENSSSCG000	WDR45B	WD repeat domain 45B [Source:NCBI gene (formerly Entrezgene);Acc:100462760]
chr3	39132088	39133142	1055	*	chr3	39132088	39133142	in_vitro	in_vivo	10	11	25.974	14.05	11.924	c(t = 1.03076	0.318	0.84165302	Distal Intergenic	ENSSSCG000	ZNF205	zinc finger protein 205 [Source:VGNC Symbol;Acc:VGNC:95204]
chr13	207075413	207076467	1055	*	chr13	207075413	207076467	in_vitro	in_vivo	12	9	14.05	6.12666667	7.92333333	c(t = 1.03199	0.318	0.84165302	Distal Intergenic	ENSSSCG000	AGPAT3	1-acylglycerol-3-phosphate O-acyltransferase 3 [Source:VGNC Symbol;Acc:VGNC:85185]
chrX	97868701	97869444	744	*	chrX	97868701	97869444	in_vitro	in_vivo	12	13	8.22916667	2.57307692	5.65608974	c(t = 1.04510	0.318	0.84165302	Distal Intergenic	ENSSSCG000	PGRMC1	progesterone receptor membrane component 1 [Source:NCBI gene (formerly Entrezgene);Acc:396946]
chr12	22185262	22185794	533	*	chr12	22185262	22185794	in_vitro	in_vivo	9	13	3.92666667	1.75076923	2.17589744	c(t = 1.04344	0.318	0.84165302	Distal Intergenic	ENSSSCG000	WIPF2	WAS/WASL interacting protein family member 2 [Source:VGNC Symbol;Acc:VGNC:99106]
chr17	48263013	48264102	1090	*	chr17	48263013	48264102	in_vitro	in_vivo	15	13	2.772	1.71	1.062	c(t = 1.02308	0.318	0.84165302	Distal Intergenic	ENSSSCG000	CD40	CD40 molecule [Source:NCBI gene (formerly Entrezgene);Acc:397395]
chr13	110587603	110588876	1274	*	chr13	110587603	110588876	in_vitro	in_vivo	11	14	2.11545455	1.30642857	0.80902597	c(t = 1.02033	0.318	0.84165302	Distal Intergenic	ENSSSCG000	PLD1	phospholipase D1 [Source:VGNC Symbol;Acc:VGNC:91525]
chr2	18037616	18038874	1259	*	chr2	18037616	18038874	in_vitro	in_vivo	9	10	3.03	1.287	1.743	c(t = 1.04739	0.318	0.84165302	Distal Intergenic	ENSSSCG000	CD82	CD82 molecule [Source:VGNC Symbol;Acc:VGNC:86432]
chr1	242214808	242215904	1097	*	chr1	242214808	242215904	in_vitro	in_vivo	12	14	2.04083333	1.12285714	0.91797619	c(t = 1.02624	0.318	0.84165302	Distal Intergenic	ENSSSCG000	ERP44	endoplasmic reticulum protein 44 [Source:NCBI gene (formerly Entrezgene);Acc:100192437]
chr9	129584961	129586234	1274	*	chr9	129584961	129586234	in_vitro	in_vivo	11	11	3.19272727	1.12272727	2.07	c(t = 1.03990	0.318	0.84165302	Distal Intergenic	ENSSSCG000	PROX1	prospero homeobox 1 [Source:VGNC Symbol;Acc:VGNC:91837]
chr4	77182992	77186016	3025	*	chr4	77182992	77186016	in_vitro	in_vivo	11	15	2.01636364	1.116	0.90036364	c(t = 1.02910	0.318	0.84165302	Distal Intergenic	ENSSSCG000	ATP6V1H	ATPase H+ transporting V1 subunit H [Source:VGNC Symbol;Acc:VGNC:85677]
chr6	81398094	81399002	909	*	chr6	81398094	81399002	in_vitro	in_vivo	12	9	2.2125	0.93	1.2825	c(t = 1.03663	0.318	0.84165302	Distal Intergenic	ENSSSCG000	ID3	inhibitor of DNA binding 3 [Source:NCBI gene (formerly Entrezgene);Acc:100626978]
chr8	54751364	54752255	892	*	chr8	54751364	54752255	in_vitro	in_vivo	9	12	1.13777778	0.58083333	0.55694444	c(t = 1.02834	0.318	0.84165302	Distal Intergenic	ENSSSCG00000008898		HOP homeobox [Source:VGNC Symbol;Acc:VGNC:88932]
chr7	7788699	7789382	684	*	chr7	7788699	7789382	in_vitro	in_vivo	10	9	64.044	76.0477778	-12.003778	c(t = -1.0268	0.319	0.84165302	Distal Intergenic	ENSSSCG000	ssc-mir-7136	ssc-mir-7136 [Source:miRBase;Acc:MI0023569]
chrX	57742179	57743168	990	*	chrX	57742179	57743168	in_vitro	in_vivo	11	11	3.44363636	7.91818182	-4.4745455	c(t = -1.0311	0.319	0.84165302	Distal Intergenic	ENSSSCG000	OGT	O-linked N-acetylglucosamine (GlcNAc) transferase [Source:NCBI gene (formerly Entrezgene);Acc:664652]
chrX	98349413	98351052	1640	*	chrX	98349413	98351052	in_vitro	in_vivo	12	11	6.1275	2.35363636	3.77386364	c(t = 1.02982	0.319	0.84165302	Distal Intergenic	ENSSSCG000	NKAP	NFKB activating protein [Source:VGNC Symbol;Acc:VGNC:90756]
chr1	164310254	164311025	772	*	chr1	164310254	164311025	in_vitro	in_vivo	10	11	4.437	1.78818182	2.64881818	c(t = 1.04000	0.319	0.84165302	Distal Intergenic	ENSSSCG000	MAP2K1	mitogen-activated protein kinase kinase 1 [Source:NCBI gene (formerly Entrezgene);Acc:100233191]
chr1	44372907	44374030	1124	*	chr1	44372907	44374030	in_vitro	in_vivo	14	13	2.07357143	1.06384615	1.00972527	c(t = 1.02571	0.319	0.84165302	Distal Intergenic	ENSSSCG000	ZUP1	zinc finger containing ubiquitin peptidase 1 [Source:VGNC Symbol;Acc:VGNC:95339]
chr8	124148846	124150881	2036	*	chr8	124148846	124150881	in_vitro	in_vivo	15	14	1.712	0.87714286	0.83485714	c(t = 1.01544	0.319	0.84165302	Distal Intergenic	ENSSSCG000	BMPR1B	bone morphogenetic protein receptor type 1B [Source:VGNC Symbol;Acc:VGNC:85847]
chr6	1449315	1455196	5882	*	chr6	1449315	1455196	in_vitro	in_vivo	13	11	96.8146154	94.7527273	2.06188811	c(t = 1.01982	0.32	0.84165302	Exon (NM_001244650/100622381, exon 5 of 14)	ENSSSCG000	BANP	BTG3 associated nuclear protein [Source:NCBI gene (formerly Entrezgene);Acc:100622381]
chr2	2349770	2351068	1299	*	chr2	2349770	2351068	in_vitro	in_vivo	12	10	45.3008333	29.255	16.0458333	c(t = 1.02057	0.32	0.84165302	Distal Intergenic	ENSSSCG000	NAP1L4	nucleosome assembly protein 1 like 4 [Source:VGNC Symbol;Acc:VGNC:90577]
chr15	121560936	121562466	1531	*	chr15	121560936	121562466	in_vitro	in_vivo	9	11	2.68222222	6.24090909	-3.5586869	c(t = -1.0371	0.32	0.84165302	Distal Intergenic	ENSSSCG000	INHA	inhibin subunit alpha [Source:VGNC Symbol;Acc:VGNC:96372]
chr2	83926092	83927200	1109	*	chr2	83926092	83927200	in_vitro	in_vivo	12	11	1.14	2.04636364	-0.9063636	c(t = -1.0283	0.32	0.84165302	Distal Intergenic	ENSSSCG000	HEXB	hexosaminidase subunit beta [Source:NCBI gene (formerly Entrezgene);Acc:396958]
chr1	19097298	19098191	894	*	chr1	19097298	19098191	in_vitro	in_vivo	12	13	0.78666667	1.66	-0.8733333	c(t = -1.0232	0.32	0.84165302	Promoter (<=1kb)	ENSSSCG000	RAB32	RAB32, member RAS oncogene family [Source:NCBI gene (formerly Entrezgene);Acc:100144496]
chr4	126873729	126875618	1890	*	chr4	126873729	126875618	in_vitro	in_vivo	14	13	0.82285714	1.55307692	-0.7302198	c(t = -1.0174	0.32	0.84165302	Distal Intergenic	ENSSSCG000	GBP2	guanylate binding protein 2, interferon-inducible [Source:NCBI gene (formerly Entrezgene);Acc:100153137]
chr4	90461394	90462415	1022	*	chr4	90461394	90462415	in_vitro	in_vivo	10	9	3.745	1.23777778	2.50722222	c(t = 1.03802	0.32	0.84165302	Distal Intergenic	ENSSSCG000	ATP1A2	ATPase Na+/K+ transporting subunit alpha 2 [Source:NCBI gene (formerly Entrezgene);Acc:396828]
chr12	43032439	43033268	830	*	chr12	43032439	43033268	in_vitro	in_vivo	11	9	3.17454545	0.79111111	2.38343434	c(t = 1.04133	0.32	0.84165302	Distal Intergenic	ENSSSCG000	CRLF3	cytokine receptor like factor 3 [Source:VGNC Symbol;Acc:VGNC:86999]
chr18	18345562	18347188	1627	*	chr18	18345562	18347188	in_vitro	in_vivo	13	10	81.5515385	66.358	15.1935385	c(t = 1.02345	0.321	0.84165302	Distal Intergenic	ENSSSCG000	MEST	mesoderm specific transcript [Source:VGNC Symbol;Acc:VGNC:90150]
chr2	6304558	6305906	1349	*	chr2	6304558	6305906	in_vitro	in_vivo	13	12	15.3823077	10.1833333	5.19897436	c(t = 1.01586	0.321	0.84165302	Distal Intergenic	ENSSSCG000	CATSPER1	cation channel sperm associated 1 [Source:NCBI gene (formerly Entrezgene);Acc:100689223]
chrX	98567091	98567717	627	*	chrX	98567091	98567717	in_vitro	in_vivo	10	10	1.991	9.937	-7.946	c(t = -1.0491	0.321	0.84165302	Distal Intergenic	ENSSSCG000	ATP1B4	ATPase Na+/K+ transporting family member beta 4 [Source:VGNC Symbol;Acc:VGNC:85644]
chr18	6164526	6165716	1191	*	chr18	6164526	6165716	in_vitro	in_vivo	9	11	11.4844444	6.29727273	5.18717172	c(t = 1.03178	0.321	0.84165302	Promoter (1-2kb)	ENSSSCG00000024070		cyclin dependent kinase 5 [Source:VGNC Symbol;Acc:VGNC:110291]
chr14	50245676	50246747	1072	*	chr14	50245676	50246747	in_vitro	in_vivo	11	10	8.61454545	3.498	5.11654545	c(t = 1.04002	0.321	0.84165302	Promoter (<=1kb)	ENSSSCG000	ssc-mir-130b	ssc-mir-130b [Source:miRBase;Acc:MI0013136]
chr4	57004125	57004743	619	*	chr4	57004125	57004743	in_vitro	in_vivo	12	10	1.32916667	2.999	-1.6698333	c(t = -1.0298	0.321	0.84165302	Distal Intergenic	ENSSSCG000	MRPS28	mitochondrial ribosomal protein S28 [Source:NCBI gene (formerly Entrezgene);Acc:100152709]
chr3	101603488	101604608	1121	*	chr3	101603488	101604608	in_vitro	in_vivo	10	10	0.714	2.858	-2.144	c(t = -1.0412	0.321	0.84165302	Distal Intergenic	ENSSSCG000	GALM	galactose mutarotase [Source:NCBI gene (formerly Entrezgene);Acc:399536]
chr8	84224237	84225226	990	*	chr8	84224237	84225226	in_vitro	in_vivo	9	12	0.69111111	2.10166667	-1.4105556	c(t = -1.0287	0.321	0.84165302	Distal Intergenic	ENSSSCG000	ANAPC10	anaphase promoting complex subunit 10 [Source:VGNC Symbol;Acc:VGNC:98912]
chr4	123418676	123420115	1440	*	chr4	123418676	123420115	in_vitro	in_vivo	13	14	1.00923077	1.89142857	-0.8821978	c(t = -1.0168	0.321	0.84165302	Distal Intergenic	ENSSSCG000	DNTTIP2	deoxynucleotidyltransferase terminal interacting protein 2 [Source:NCBI gene (formerly Entrezgene);Acc:100157622]
chr1	7206455	7207961	1507	*	chr1	7206455	7207961	in_vitro	in_vivo	12	14	2.69916667	1.47642857	1.2227381	c(t = 1.02951	0.321	0.84165302	Distal Intergenic	ENSSSCG000	SLC22A2	solute carrier family 22 member 2 [Source:VGNC Symbol;Acc:VGNC:92979]
chr8	29370077	29371263	1187	*	chr8	29370077	29371263	in_vitro	in_vivo	11	10	0.54454545	1.357	-0.8124545	c(t = -1.0291	0.321	0.84165302	Distal Intergenic	ENSSSCG000	ssc-mir-9840	ssc-mir-9840 [Source:miRBase;Acc:MI0031609]
chr13	50476425	50477456	1032	*	chr13	50476425	50477456	in_vitro	in_vivo	12	11	0.42916667	1.34545455	-0.9162879	c(t = -1.0297	0.321	0.84165302	Distal Intergenic	ENSSSCG000	ARL6IP5	ADP ribosylation factor like GTPase 6 interacting protein 5 [Source:NCBI gene (formerly Entrezgene);Acc:595127]

chr9	50092762	50093765	1004	*	chr9	50092762	50093765	in_vitro	in_vivo	11	9	2.01272727	1.01	1.00272727	c(t = 1.02754	0.321	0.84165302	Distal Intergenic	ENSSSCG000	HSPA8	heat shock protein family A (Hsp70) member 8 [Source:NCBI gene (formerly Entrezgene);Acc:100511890]
chr4	122928984	122929823	840	*	chr4	122928984	122929823	in_vitro	in_vivo	10	10	1.467	0.911	0.556	c(t = 1.02257	0.321	0.84165302	Promoter (<=1kb)	ENSSSCG000	ABCD3	ATP binding cassette subfamily D member 3 [Source:VGNC Symbol;Acc:VGNC:84963]
chr5	89727708	89728684	977	*	chr5	89727708	89728684	in_vitro	in_vivo	11	12	1.73363636	0.69166667	1.0419697	c(t = 1.03668	0.321	0.84165302	Distal Intergenic	ENSSSCG000	SOCS2	suppressor of cytokine signaling 2 [Source:NCBI gene (formerly Entrezgene);Acc:100037966]
chr4	64768402	64769676	1275	*	chr4	64768402	64769676	in_vitro	in_vivo	9	12	1.54	0.35916667	1.18083333	c(t = 1.05023	0.321	0.84165302	Distal Intergenic	ENSSSCG000	LACTB2	lactamase beta 2 [Source:NCBI gene (formerly Entrezgene);Acc:100153212]
chr14	50250758	50251859	1102	*	chr14	50250758	50251859	in_vitro	in_vivo	9	11	6.41777778	2.62454545	3.79323232	c(t = 1.04595	0.322	0.84255022	Distal Intergenic	ENSSSCG000	ssc-mir-130b	ssc-mir-130b [Source:miRBase;Acc:MI0013136]
chr3	75481009	75482055	1047	*	chr3	75481009	75482055	in_vitro	in_vivo	9	10	0.97777778	2.212	-1.2342222	c(t = -1.0302	0.322	0.84255022	Distal Intergenic	ENSSSCG000	ACTR2	actin related protein 2 [Source:VGNC Symbol;Acc:VGNC:85051]
chr3	78894161	78895245	1085	*	chr3	78894161	78895245	in_vitro	in_vivo	11	12	3.95727273	1.8875	2.06977273	c(t = 1.01726	0.322	0.84255022	Distal Intergenic	ENSSSCG000	TMEM17	transmembrane protein 17 [Source:NCBI gene (formerly Entrezgene);Acc:100627459]
chr1	236821237	236822030	794	*	chr1	236821237	236822030	in_vitro	in_vivo	13	11	1.45923077	0.65727273	0.80195804	c(t = 1.01284	0.322	0.84255022	Distal Intergenic	ENSSSCG000	GNE	glucosamine (UDP-N-acetyl)-2-epimerase/N-acetylmannosamine kinase [Source:VGNC Symbol;Acc:VGNC:103101]
chr14	88473596	88474327	732	*	chr14	88473596	88474327	in_vitro	in_vivo	12	12	1.18	0.43833333	0.74166667	c(t = 1.01482	0.322	0.84255022	Distal Intergenic	ENSSSCG00000010370		annexin A8 [Source:NCBI gene (formerly Entrezgene);Acc:100155930]
chr11	7320623	7321414	792	*	chr11	7320623	7321414	in_vitro	in_vivo	10	13	0.946	0.14538462	0.80061538	c(t = 1.04285	0.322	0.84255022	Distal Intergenic	ENSSSCG000	ALOX5AP	arachidonate 5-lipoxygenase activating protein [Source:VGNC Symbol;Acc:VGNC:109456]
chr5	486165	506835	20671	*	chr5	486165	506835	in_vitro	in_vivo	15	16	26.476	33.511875	-7.035875	c(t = -1.0067	0.323	0.84258486	Distal Intergenic	ENSSSCG000	SELENOO	selenoprotein O [Source:NCBI gene (formerly Entrezgene);Acc:100518971]
chr1	8348484	8349079	596	*	chr1	8348484	8349079	in_vitro	in_vivo	10	9	2.626	14.22	-11.594	c(t = -1.0514	0.323	0.84258486	Distal Intergenic	ENSSSCG000	TAGAP	T cell activation RhoGTPase activating protein [Source:NCBI gene (formerly Entrezgene);Acc:100155114]
chr9	36340793	36341465	673	*	chr9	36340793	36341465	in_vitro	in_vivo	10	10	0.918	3.03	-2.112	c(t = -1.0372	0.323	0.84258486	Distal Intergenic	ENSSSCG000	SLN	sarcolipin [Source:NCBI gene (formerly Entrezgene);Acc:733627]
chr6	97408064	97409697	1634	*	chr6	97408064	97409697	in_vitro	in_vivo	13	11	5.27076923	2.67090909	2.59986014	c(t = 1.02088	0.323	0.84258486	Distal Intergenic	ENSSSCG000	CIDEA	cell death inducing DFFA like effector a [Source:NCBI gene (formerly Entrezgene);Acc:100127171]
chr17	47234366	47235738	1373	*	chr17	47234366	47235738	in_vitro	in_vivo	9	12	4.99777778	2.66166667	2.33611111	c(t = 1.02906	0.323	0.84258486	Distal Intergenic	ENSSSCG000	TOMM34	translocase of outer mitochondrial membrane 34 [Source:VGNC Symbol;Acc:VGNC:95647]
chr12	3355852	3357321	1470	*	chr12	3355852	3357321	in_vitro	in_vivo	11	14	2.98818182	1.72642857	1.26175325	c(t = 1.01689	0.323	0.84258486	Distal Intergenic	ENSSSCG00000017164		TIMP metallopeptidase inhibitor 2 [Source:VGNC Symbol;Acc:VGNC:93995]
chr6	85716668	85717756	1089	*	chr6	85716668	85717756	in_vitro	in_vivo	13	11	2.11846154	1.12727273	0.99118881	c(t = 1.01476	0.323	0.84258486	Distal Intergenic	ENSSSCG000	SRSF4	serine and arginine rich splicing factor 4 [Source:NCBI gene (formerly Entrezgene);Acc:768101]
chr6	44103084	44103671	588	*	chr6	44103084	44103671	in_vitro	in_vivo	10	11	1.601	0.68272727	0.91827273	c(t = 1.02902	0.323	0.84258486	Distal Intergenic	ENSSSCG000	GPI	glucose-6-phosphate isomerase [Source:NCBI gene (formerly Entrezgene);Acc:397602]
chr13	90818284	90819115	832	*	chr13	90818284	90819115	in_vitro	in_vivo	9	12	2.58666667	0	2.58666667	c(t = 1.05279	0.323	0.84258486	Promoter (<=1kb)	ENSSSCG000	SELENOT	selenoprotein T [Source:NCBI gene (formerly Entrezgene);Acc:100302502]
chr1	65848864	65849529	666	*	chr1	65848864	65849529	in_vitro	in_vivo	11	10	1.71	4.188	-2.478	c(t = -1.0321	0.324	0.84261929	Distal Intergenic	ENSSSCG000	FBXL4	F-box and leucine rich repeat protein 4 [Source:VGNC Symbol;Acc:VGNC:98007]
chr6	2595642	2599489	3848	*	chr6	2595642	2599489	in_vitro	in_vivo	9	11	2.14111111	3.16	-1.0188889	c(t = -1.0177	0.324	0.84261929	Distal Intergenic	ENSSSCG000	IRF8	interferon regulatory factor 8 [Source:VGNC Symbol;Acc:VGNC:89211]
chr1	44557212	44558034	823	*	chr1	44557212	44558034	in_vitro	in_vivo	9	9	0.61111111	1.66	-1.0488889	c(t = -1.0334	0.324	0.84261929	Distal Intergenic	ENSSSCG000	ZUP1	zinc finger containing ubiquitin peptidase 1 [Source:VGNC Symbol;Acc:VGNC:95339]
chr14	71546552	71547596	1045	*	chr14	71546552	71547596	in_vitro	in_vivo	12	9	2.35	1.26333333	1.08666667	c(t = 1.01357	0.324	0.84261929	Distal Intergenic	ENSSSCG000	HNRNPH3	heterous nuclear ribonucleoprotein H3 [Source:VGNC Symbol;Acc:VGNC:88922]
chr5	18409267	18410275	1009	*	chr5	18409267	18410275	in_vitro	in_vivo	12	11	1.98	1.13272727	0.84727273	c(t = 1.00994	0.324	0.84261929	Distal Intergenic	ENSSSCG000	IGFBP6	insulin like growth factor binding protein 6 [Source:VGNC Symbol;Acc:VGNC:89059]
chr1	164273811	164275327	1517	*	chr1	164273811	164275327	in_vitro	in_vivo	10	12	1.969	1.00083333	0.96816667	c(t = 1.01133	0.324	0.84261929	Distal Intergenic	ENSSSCG000	MAP2K1	mitogen-activated protein kinase kinase 1 [Source:NCBI gene (formerly Entrezgene);Acc:100233191]
chr4	42946808	42947209	402	*	chr4	42946808	42947209	in_vitro	in_vivo	10	9	2.833	0.91222222	1.92077778	c(t = 1.03171	0.324	0.84261929	Distal Intergenic	ENSSSCG000	CCNE2	cyclin E2 [Source:VGNC Symbol;Acc:VGNC:86355]
chr7	32289298	32289759	462	*	chr7	32289298	32289759	in_vitro	in_vivo	10	9	2.398	0.74111111	1.65688889	c(t = 1.02503	0.324	0.84261929	Distal Intergenic	ENSSSCG000	PI16	peptidase inhibitor 16 [Source:NCBI gene (formerly Entrezgene);Acc:100155010]
chr8	70008184	70008453	270	*	chr8	70008184	70008453	in_vitro	in_vivo	10	10	1.435	0.345	1.09	c(t = 1.03192	0.324	0.84261929	Promoter (<=1kb)	ENSSSCG000	AMCF-II	alveolar macrophage-derived chemotactic factor-II [Source:NCBI gene (formerly Entrezgene);Acc:396900]
chr11	64042445	64043710	1266	*	chr11	64042445	64043710	in_vitro	in_vivo	10	11	3.793	2.19909091	1.59390909	c(t = 1.01815	0.325	0.84333076	Distal Intergenic	ENSSSCG000	DCT	dopachrome tautomerase [Source:VGNC Symbol;Acc:VGNC:87192]
chr3	132684271	132685559	1289	*	chr3	132684271	132685559	in_vitro	in_vivo	10	13	3.334	1.27615385	2.05784615	c(t = 1.02915	0.325	0.84333076	Promoter (<=1kb)	ENSSSCG000	SH3YL1	SH3 and SYLF domain containing 1 [Source:VGNC Symbol;Acc:VGNC:92834]
chr13	196066598	196068261	1664	*	chr13	196066598	196068261	in_vitro	in_vivo	11	13	1.94363636	0.92769231	1.01594406	c(t = 1.01991	0.325	0.84333076	Distal Intergenic	ENSSSCG000	C21orf62	chromosome 13 C21orf62 homolog [Source:VGNC Symbol;Acc:VGNC:85929]
chr2	80646149	80647103	955	*	chr2	80646149	80647103	in_vitro	in_vivo	9	10	1.40777778	0.466	0.94177778	c(t = 1.03438	0.325	0.84333076	Promoter (<=1kb)	ENSSSCG000	PRELID1	PRELI domain containing 1 [Source:NCBI gene (formerly Entrezgene);Acc:100512335]
chr12	59607694	59608259	566	*	chr12	59607694	59608259	in_vitro	in_vivo	10	10	3.744	6.822	-3.078	c(t = -1.0099	0.326	0.84333076	Distal Intergenic	ENSSSCG000	AKAP10	A-kinase anchoring protein 10 [Source:NCBI gene (formerly Entrezgene);Acc:397262]
chr9	109226400	109227212	813	*	chr9	109226400	109227212	in_vitro	in_vivo	11	10	3.4	6.69	-3.29	c(t = -1.0153	0.326	0.84333076	Distal Intergenic	ENSSSCG000	PDIA4	protein disulfide isomerase family A member 4 [Source:NCBI gene (formerly Entrezgene);Acc:100522203]
chr4	34645594	34646938	1345	*	chr4	34645594	34646938	in_vitro	in_vivo	11	9	1.63818182	2.89444444	-1.2562626	c(t = -1.0126	0.326	0.84333076	Distal Intergenic	ENSSSCG000	ODF1	outer dense fiber of sperm tails 1 [Source:NCBI gene (formerly Entrezgene);Acc:397489]
chr12	52426498	52428609	2112	*	chr12	52426498	52428609	in_vitro	in_vivo	9	10	5.29444444	2.544	2.75044444	c(t = 1.03352	0.326	0.84333076	Distal Intergenic	ENSSSCG000	ssc-mir-497	ssc-mir-497 [Source:miRBase;Acc:MI0013139]
chr2	78675736	78676481	746	*	chr2	78675736	78676481	in_vitro	in_vivo	10	12	1.265	2.21	-0.945	c(t = -1.0084	0.326	0.84333076	Distal Intergenic	NA	NA	NA
chr1	272216728	272217373	646	*	chr1	272216728	272217373	in_vitro	in_vivo	10	10	1.101	2.13	-1.029	c(t = -1.0116	0.326	0.84333076	Distal Intergenic	ENSSSCG000	MED27	mediator complex subunit 27 [Source:VGNC Symbol;Acc:VGNC:90117]
chr6	87686825	87687816	992	*	chr6	87686825	87687816	in_vitro	in_vivo	12	11	0.88916667	2.00454545	-1.1153788	c(t = -1.0105	0.326	0.84333076	Distal Intergenic	ENSSSCG000	FABP3	fatty acid binding protein 3 [Source:NCBI gene (formerly Entrezgene);Acc:399532]
chr13	200627175	200627596	422	*	chr13	200627175	200627596	in_vitro	in_vivo	9	11	0.95111111	1.99909091	-1.0479798	c(t = -1.0107	0.326	0.84333076	Distal Intergenic	ENSSSCG000	PIGP	phosphatidylinositol glycan anchor biosynthesis class P [Source:VGNC Symbol;Acc:VGNC:91427]
chr18	42410404	42411105	702	*	chr18	42410404	42411105	in_vitro	in_vivo	12	12	2.18333333	0.92583333	1.2575	c(t = 1.00544	0.326	0.84333076	Distal Intergenic	ENSSSCG000	NOD1	nucleotide binding oligomerization domain containing 1 [Source:NCBI gene (formerly Entrezgene);Acc:100135660]
chr3	23719283	23720348	1066	*	chr3	23719283	23720348	in_vitro	in_vivo	10	11	4.591	0.89727273	3.69372727	c(t = 1.03300	0.326	0.84333076	Promoter (<=1kb)	ENSSSCG000	METTL9	methyltransferase like 9 [Source:VGNC Symbol;Acc:VGNC:103984]
chr9	109277677	109278261	585	*	chr9	109277677	109278261	in_vitro	in_vivo	10	12	76.187	89.7416667	-13.554667	c(t = -1.0297	0.327	0.84333076	Exon (NM_001267834/100522203, exon 8 of 10)	ENSSSCG000	PDIA4	protein disulfide isomerase family A member 4 [Source:NCBI gene (formerly Entrezgene);Acc:100522203]
chr7	63344842	63345817	976	*	chr7	63344842	63345817	in_vitro	in_vivo	12	12	4.70583333	2.08166667	2.62416667	c(t = 1.00859	0.327	0.84333076	Distal Intergenic	ENSSSCG000	NKX2-1	NK2 homeobox 1 [Source:VGNC Symbol;Acc:VGNC:90767]
chr12	53405080	53406354	1275	*	chr12	53405080	53406354	in_vitro	in_vivo	12	12	2.97583333	1.66083333	1.315	c(t = 1.00763	0.327	0.84333076	Distal Intergenic	ENSSSCG000	AURKB	aurora kinase B [Source:VGNC Symbol;Acc:VGNC:85698]
chr10	19936340	19936876	537	*	chr10	19936340	19936876	in_vitro	in_vivo	9	9	2.66555556	1.27333333	1.39222222	c(t = 1.01912	0.327	0.84333076	Distal Intergenic	ENSSSCG000	CAPN2	calpain 2 [Source:NCBI gene (formerly Entrezgene);Acc:397393]
chr6	42890455	42891817	1363	*	chr6	42890455	42891817	in_vitro	in_vivo	11	13	2.94818182	1.03615385	1.91202797	c(t = 1.02634	0.327	0.84333076	Distal Intergenic	ENSSSCG000	SLC7A9	solute carrier family 7 member 9 [Source:VGNC Symbol;Acc:VGNC:93177]
chr9	124282702	124283947	1246	*	chr9	124282702	124283947	in_vitro	in_vivo	13	14	1.63384615	0.995	0.63884615	c(t = 1.00154	0.327	0.84333076	Promoter (<=1kb)	ENSSSCG000	LAMC1	laminin subunit gamma 1 [Source:NCBI gene (formerly Entrezgene);Acc:100514785]
chr12	61350028	61352309	2282	*	chr12	61350028	61352309	in_vitro	in_vivo	9	10	95.2244444	91.122	4.10244444	c(t = 1.01065	0.328	0.84333076	Distal Intergenic	ENSSSCG000	COPS3	COP9 signalosome subunit 3 [Source:VGNC Symbol;Acc:VGNC:86900]
chrX	125180305	125182027	1723	*	chrX	125180305	125182027	in_vitro	in_vivo	9	9	14.52	27.5222222	-13.002222	c(t = -1.0080	0.328	0.84333076	Distal Intergenic	ENSSSCG000	DKC1	dyskerin pseudouridine synthase 1 [Source:NCBI gene (formerly Entrezgene);Acc:100216430]
chr6	60594958	60596272	1315	*	chr6	60594958	60596272	in_vitro	in_vivo	13	9	14.4315385	25.4277778	-10.996239	c(t = -1.0072	0.328	0.84333076	Distal Intergenic	ENSSSCG000	GALP	galanin like peptide [Source:NCBI gene (formerly Entrezgene);Acc:396772]
chr6	85823506	85824050	545	*	chr6	85823506	85824050	in_vitro	in_vivo	10	10	31.502	18.218	13.284	c(t = 1.00705	0.328	0.84333076	Distal Intergenic	ENSSSCG000	SRSF4	serine and arginine rich splicing factor 4 [Source:NCBI gene (formerly Entrezgene);Acc:768101]
chr6	103785756	103787359	1604	*	chr6	103785756	103787359	in_vitro	in_vivo	11	12	2.27727273	4.90083333	-2.6235606	c(t = -1.0082	0.328	0.84333076	Distal Intergenic	ENSSSCG000	LPIN2	lipin 2 [Source:VGNC Symbol;Acc:VGNC:89794]
chr4	46911682	46912826	1145	*	chr4	46911682	46912826	in_vitro	in_vivo	14	12	6.71642857	3.76416667	2.9522619	c(t = 1.00067	0.328	0.84333076	Distal Intergenic	ENSSSCG000	DECR1	2,4-dienoyl-CoA reductase 1 [Source:NCBI gene (formerly Entrezgene);Acc:503544]
chr14	109069291	109070450	1160	*	chr14	109069291	109070450	in_vitro	in_vivo	10	10	1.52777778	3.122	-1.5942222	c(t = -1.0186	0.328	0.84333076	Distal Intergenic	ENSSSCG000	SFRP5	secreted frizzled related protein 5 [Source:NCBI gene (formerly Entrezgene);Acc:100153176]
chr6	351711	352349	639	*	chr6	351711	352349	in_vitro	in_vivo	9	10	2.75777778	1.394	1.36377778	c(t = 1.01633	0.328	0.84333076	Promoter (<=1kb)	ENSSSCG000	CHMP1A	charged multivesicular body protein 1A [Source:NCBI gene (formerly Entrezgene);Acc:100628127]
chr3	111992210	111992900	691	*	chr3	111992210	111992900	in_vitro	in_vivo	10	11	2.034	0.80818182	1.22581818	c(t = 1.01324	0.328	0.84333076	Distal Intergenic	ENSSSCG000	SLC30A3	solute carrier family 30 member 3 [Source:NCBI gene (formerly Entrezgene);Acc:100169931]
chr2	87142353	87142904	552	*	chr2	87142353	87142904	in_vitro	in_vivo	9	10	1.48	0.578	0.902	c(t = 1.01995	0.328	0.84333076	Promoter (<=1kb)	ENSSSCG000	AP3B1	adaptor related protein complex 3 subunit beta 1 [Source:VGNC Symbol;Acc:VGNC:85387]
chr13	197203025	197204121	1097	*	chr13	197203025	197204121	in_vitro	in_vivo	13	10	0.58153846	0.22	0.36153846	c(t = 1.00079	0.328	0.84333076	Distal Intergenic	ENSSSCG000	CRYZL1	crystallin zeta like 1 [Source:VGNC Symbol;Acc:VGNC:87026]
chr6	65290904	65292604	1701	*	chr6	65290904	65292604	in_vitro	in_vivo	9	10	94.67	91.093	3.577	c(t = 1.00526	0.329	0.84333076	Distal Intergenic	ENSSSCG000	ssc-mir-2320	ssc-mir-2320 [Source:miRBase;Acc:MI0017997]
chr14	6833996	6835311	1316	*	chr14	6833996	6835311	in_vitro	in_vivo	9	10	11.1166667	7.516	3.60066667	c(t = 1.01106	0.329	0.84333076	Distal Intergenic	ENSSSCG000	CCAR2	cell cycle and apoptosis regulator 2 [Source:NCBI gene (formerly Entrezgene);Acc:100155718]
chr7	84962321	84962853	533	*	chr7	84962321	84962853	in_vitro	in_vivo	10	12	0.726	2.51916667	-1.7931667	c(t = -1.0142	0.329	0.84333076	Distal Intergenic	NA	NA	NA
chr11	78231640	78232389	750	*	chr11	78231640	78232389	in_vitro	in_vivo	9	12	2.35111111	1.06916667	1.28194444	c(t = 1.00996	0.329	0.84333076	Distal Intergenic	ENSSSCG000	F7	coagulation factor VII [Source:NCBI gene (formerly Entrezgene);Acc:733661]
chr7	31925961	31927012	1052	*	chr7	31925961	31927012	in_vitro	in_vivo	10	9	1.377	0.55888889	0.81811111	c(t = 1.01214	0.329	0.84333076	Distal Intergenic	ENSSSCG000	SRPK1	SRSF protein kinase 1 [Source:VGNC Symbol;Acc:VGNC:93462]
chr17	61441055	61445357	4303	*	chr17	61441055	61445357	in_vitro	in_vivo	12	14	54.2441667	68.0657143	-13.821548	c(t = -0.9955	0.33	0.84333076	Distal Intergenic	ENSSSCG000	PSMA7	proteasome 20S subunit alpha 7 [Source:NCBI gene (formerly Entrezgene);Acc:100626738]
chr14	73087523	73088841	1319	*	chr14	73087523	73088841	in_vitro	in_vivo	10	12	3.34	7.105	-3.765	c(t = -1.0064	0.33	0.84333076	Distal Intergenic	ENSSSCG000	SAR1A	secretion associated Ras related GTPase 1A [Source:VGNC Symbol;Acc:VGNC:92579]
chr14	77934691	77936435	1745	*	chr14	77934691	77936435	in_vitro	in_vivo	10	12	2.337	3.98583333	-1.6488333	c(t = -0.9995	0.33	0.84333076	Distal Intergenic	ENSSSCG000	VDAC2	voltage dependent anion channel 2 [Source:VGNC Symbol;Acc:VGNC:111154]
chr1	191533757	191536473	2717	*	chr1	191533757	191536473	in_vitro	in_vivo	10	12	5.537	3.2475	2.2895	c(t = 1.00149	0.33	0.84333076	Promoter (<=1kb)	ENSSSCG000	NTRK3	neurotrophic receptor tyrosine kinase 3 [Source:VGNC Symbol;Acc:VGNC:103142]
chr10	12321666	12322822	1157	*	chr10	12321666	12322822	in_vitro	in_vivo	9	14	1.10777778	3.22	-2.1122222	c(t = -1.0088	0.33	0.84333076	Distal Intergenic	ENSSSCG000	FH	fumarate hydratase [Source:VGNC Symbol;Acc:VGNC:96300]
chr5	74671353	74672815	1463	*	chr5	74671353	74672815	in_vitro	in_vivo	14	11	2.03285714	1.08090909	0.95194805	c(t = 0.99869	0.33	0.84333076	Promoter (<=1kb)	ENSSSCG000	ADAMTS20	ADAM metallopeptidase with thrombospondin type 1 motif 20 [Source:VGNC Symbol;Acc:VGNC:85082]
chr3	6329169	6330628	1460	*	chr3	6329169	6330628	in_vitro	in_vivo	9	11	4.06888889	0.71272727	3.35616162	c(t = 1.03353	0.33	0.84333076	Distal Intergenic	ENSSSCG000	ARPC1B	actin related protein 2/3 complex subunit 1B [Source:VGNC Symbol;Acc:VGNC:85537]
chr9	82224923	82225957	1035	*	chr9	82224923	82225957	in_vitro	in_vivo	12	11	0.7675	0.26818182	0.49931818	c(t = 1.00944	0.33	0.84333076	Promoter (<=1kb)	ENSSSCG000	ARL4A	ADP ribosylation factor like GTPase 4A [Source:VGNC Symbol;Acc:VGNC:85522]
chr2	3694583	3695075	493	*	chr2	3694583	3695075	in_vitro	in_vivo	9	9	16.0388889	33.3633333	-17.324444	c(t = -1.0087	0.331	0.84333076	Distal Intergenic	ENSSSCG000	FADD	Fas associated via death domain [Source:NCBI gene (formerly Entrezgene);Acc:595129]
chr5	2543554	2544155	602	*	chr5	2543554	2544155	in_vitro	in_vivo	10	11	16.448	27.2518182	-10.803818	c(t = -1.0007	0.331	0.84333076	Distal Intergenic	ENSSSCG000	PPARA	peroxisome proliferator activated receptor alpha [Source:NCBI gene (formerly Entrezgene);Acc:397239]
chr4	75739459	75740629	1171	*	chr4	75739459	75740629	in_vitro	in_vivo	13	12	10.7123077	21.0133333	-10.301026	c(t = -0.9992	0.331	0.84333076	Promoter (<=1kb)	ENSSSCG000	MOS	MOS proto-oncogene, serine/threonine kinase [Source:NCBI gene (formerly Entrezgene);Acc:100127484]
chr17	35537066	35537697	632	*	chr17	35537066	35537697	in_vitro	in_vivo	9	10	3.85	13.224	-9.374	c(t = -1.0264	0.331	0.84333076	Distal Intergenic	ENSSSCG000	BCL2L1	BCL2 like 1 [Source:VGNC Symbol;Acc:VGNC:96535]
chr1	83973258	83974025	768	*	chr1	83973258	83974025	in_vitro	in_vivo	10	11	2.261	0.91636364	1.34463636	c(t = 1.00887	0.331	0.84333076	Distal Intergenic	ENSSSCG000	CALHM6	calcium homeostasis modulator family member 6 [Source:VGNC Symbol;Acc:VGNC:86147]
chr6	78731680	78735111	3432	*	chr6	78731680	78735111	in_vitro	in_vivo	11	11	2.21636364	3.69272727	-1.4763636	c(t = -0.9978	0.332	0.84333076	Distal Intergenic	ENSSSCG000	CDA	cytidine deaminase [Source:NCBI gene (formerly Entrezgene);Acc:100515954]
chr13	83436121	83437344	1224	*	chr13	83436121	83437344	in_vitro	in_vivo	12	10	1.055	1.901	-0.846	c(t = -1.0060	0.332	0.84333076	Promoter (<=1kb)	ENSSSCG000	TRPC1	transient receptor potential cation channel subfamily C member 1 [Source:VGNC Symbol;Acc:VGNC:94460]
chr2	141823949	141825196	1248	*	chr2	141823949	141825196	in_vitro	in_vivo	11	13	3.20727273	1.55384615	1.65342657	c(t = 1.00400	0.332	0.84333076	Distal Intergenic	ENSSSCG000	HBEGF	heparin binding EGF like growth factor [Source:VGNC Symbol;Acc:VGNC:88792]
chr6	94751193	94751754	562	*	chr6	94751193	94751754	in_vitro	in_vivo	10	12	3.315	1.065	2.25	c(t = 1.01693	0.332	0.84333076	Distal Intergenic	ENSSSCG000	MYCBP	MYC binding protein [Source:VGNC Symbol;Acc:VGNC:110209]
chr1	143993066	143993996	931	*	chr1	143993066	143993996	in_vitro	in_vivo	13	13	1.60846154	0.67692308	0.93153846	c(t = 1.00158	0.332	0.84333076	Promoter (<=1kb)	ENSSSCG000	MCEE	methylmalonyl-CoA epimerase [Source:VGNC Symbol;Acc:VGNC:90066]
chr13	207959056	207961576	2521	*	chr13	207959056	207961576	in_vitro	in_vivo	9	10	5.97666667	12.417	-6.4403333	c(t = -1.0076	0.333	0.84333076	Distal Intergenic	ENSSSCG000	ITGB2	integrin subunit beta 2 [Source:NCBI gene (formerly Entrezgene);Acc:396943]
chr16	48812716	48813157	442	*	chr16	48812716	48813157	in_vitro	in_vivo	9	10	8.65333333	5.137	3.51633333	c(t = 1.00575	0.333	0.84333076	Distal Intergenic	ENSSSCG000	DRD1	dopamine receptor D1 [Source:VGNC Symbol;Acc:VGNC:87443]
chr1	115219826	115220413	588	*	chr1	115219826	115220413	in_vitro	in_vivo	13	10	2.83307692	1.684	1.14907692	c(t = 0.99265	0.333	0.84333076	Distal Intergenic	ENSSSCG000	TEX9	testis expressed 9 [Source:VGNC Symbol;Acc:VGNC:93907]
chr13	139847057	139848117	1061	*	chr13	139847057	139848117	in_vitro	in_vivo	9	10	6.19	1.4	4.79	c(t = 1.02626	0.333	0.84333076	Distal Intergenic	ENSSSCG000	GSK3B	glycogen synthase kinase 3 beta [Source:VGNC Symbol;Acc:VGNC:88723]
chr3	4362584	4363091	508	*	chr3	4362584	4363091	in_vitro	in_vivo	11	9	69.6045455	86.1333333	-16.528788	c(t = -0.9938	0.334	0.84333076	Distal Intergenic	ENSSSCG000	FSCN1	fascin actin-bundling protein 1 [Source:NCBI gene (formerly Entrezgene);Acc:100286741]
chr14	105011963	105012625	663	*	chr14	105011963	105012625	in_vitro	in_vivo	9	9	58.2944444	36.4233333	21.8711111	c(t = 0.99612	0.334	0.84333076	Promoter (<=1kb)	ENSSSCG000	FFAR4	free fatty acid receptor 4 [Source:NCBI gene (formerly Entrezgene);Acc:116183081]
chr12	18740594	18742790	2197	*	chr12	18740594	18742790	in_vitro	in_vivo	13	15	5.65538462	3.02466667	2.63071795	c(t = 1.00077	0.334	0.84333076	Promoter (<=1kb)	NA	NA	NA
chr4	99297856	99300112	2257	*	chr4	99297856	99300112	in_vitro	in_vivo	12	15	3.56333333	1.41933333	2.144	c(t = 1.00739	0.334	0.84333076	Distal Intergenic	ENSSSCG000	TXNIP	thioredoxin interacting protein [Source:NCBI gene (formerly Entrezgene);Acc:733688]
chr8	135130373	135131010	638	*	chr8	135130373	135131010	in_vitro	in_vivo	9	12	0.49222222	1.2525	-0.7602778	c(t = -0.9961	0.334	0.84333076	Distal Intergenic	ENSSSCG000	HPSE	heparanase [Source:NCBI gene (formerly Entrezgene);Acc:100271932]
chr2	76095407	76096202	796	*	chr2	76095407	76096202	in_vitro	in_vivo	9	12	2.39888889	1.15666667	1.24222222	c(t = 1.00794	0.334	0.84333076	Distal Intergenic	ENSSSCG000	OAZ1	ornithine decarboxylase antizyme 1 [Source:NCBI gene (formerly Entrezgene);Acc:397034]
chr1	26800775	26801611	837	*	chr1	26800775	26801611	in_vitro	in_vivo	14	13	2.08	1.10846154	0.97153846	c(t = 0.98881	0.334	0.84333076	Distal Intergenic	ENSSSCG000	IFNGR1	interferon gamma receptor 1 [Source:VGNC Symbol;Acc:VGNC:89041]
chr11	45075638	45076165	528	*	chr11	45075638	45076165	in_vitro	in_vivo	10	9	8.385	0.24666667	8.13833333	c(t = 1.02109	0.334	0.84333076	Distal Intergenic	ENSSSCG000	KLF5	KLF transcription factor 5 [Source:NCBI gene (formerly Entrezgene);Acc:100038005]
chr2	90436512	90437261	750	*	chr2	90436512	90437261	in_vitro	in_vivo	9	10	0.91222222	0.244	0.66822222	c(t = 1.01255	0.334	0.84333076	Promoter (<=1kb)	ENSSSCG000	ATG10	autophagy related 10 [Source:VGNC Symbol;Acc:VGNC:85613]
chr5	1451424	1453785	2362	*	chr5	1451424	1453785	in_vitro	in_vivo	9	13	24.7566667	41.9407692	-17.184103	c(t = -0.9906	0.335	0.84333076	Distal Intergenic	ENSSSCG000	SELENOO	selenoprotein O [Source:NCBI gene (formerly Entrezgene);Acc:100518971]
chr17	483829	485146	1318	*	chr17	483829	485146	in_vitro	in_vivo	13	16	3.16923077	5.2675	-2.0982692	c(t = -0.9825	0.335	0.84333076	Promoter (<=1kb)	ENSSSCG000	CLDN23	claudin 23 [Source:NCBI gene (formerly Entrezgene);Acc:100156235]
chrX	54948029	54949706	1678	*	chrX	54948029	54949706	in_vitro	in_vivo	16	11	8.426875	4.04545455	4.38142045	c(t = 0.98314	0.335	0.84333076	Distal Intergenic	ENSSSCG000	ssc-mir-676-	ssc-mir-676-1 [Source:miRBase;Acc:MI0013152]
chr5	43454755	43455582	828	*	chr5	43454755	43455582	in_vitro	in_vivo	10	11	0.575	1.63	-1.055	c(t = -1.0004	0.335	0.84333076	Distal Intergenic	ENSSSCG000	ERGIC2	ERGIC and golgi 2 [Source:VGNC Symbol;Acc:VGNC:87771]
chr14	104342134	104343046	913	*	chr14	104342134	104343046	in_vitro	in_vivo	9	13	2.04111111	1.1	0.94111111	c(t = 0.99615	0.335	0.84333076	Distal Intergenic	ENSSSCG000	HHEX	hematopoietically expressed homeobox [Source:NCBI gene (formerly Entrezgene);Acc:397232]
chr2	113773770	113775051	1282	*	chr2	113773770	113775051	in_vitro	in_vivo	14	13	1.1	0.53923077	0.56076923	c(t = 0.98310	0.335	0.84333076	Distal Intergenic	ENSSSCG000	STARD4	StAR related lipid transfer domain containing 4 [Source:VGNC Symbol;Acc:VGNC:93534]
chr1	216312702	216313484	783	*	chr1	216312702	216313484	in_vitro	in_vivo	12	11	2.04583333	0.34363636	1.70219697	c(t = 1.00537	0.335	0.84333076	Distal Intergenic	ENSSSCG000	PDCD1LG2	programmed cell death 1 ligand 2 [Source:NCBI gene (formerly Entrezgene);Acc:574057]
chr11	1126982	1128616	1635	*	chr11	1126982	1128616	in_vitro	in_vivo	9	11	93.8533333	97.2309091	-3.3775758	c(t = -1.0028	0.336	0.84333076	Distal Intergenic	ENSSSCG000	LATS2	large tumor suppressor kinase 2 [Source:VGNC Symbol;Acc:VGNC:89649]
chr11	75386814	75388163	1350	*	chr11	75386814	75388163	in_vitro	in_vivo	13	12	5.70384615	11.8916667	-6.1878205	c(t = -0.9905	0.336	0.84333076	Distal Intergenic	ENSSSCG000	TNFSF13B	TNF superfamily member 13b [Source:VGNC Symbol;Acc:VGNC:94269]
chr14	121467726	121471128	3403	*	chr14	121467726	121471128	in_vitro	in_vivo	15	13	4.09333333	2.48692308	1.60641026	c(t = 0.99036	0.336	0.84333076	Promoter (<=1kb)	ENSSSCG000	ADRA2A	adrenoceptor alpha 2A [Source:NCBI gene (formerly Entrezgene);Acc:399501]
chr8	138789272	138790882	1611	*	chr8	138789272	138790882	in_vitro	in_vivo	12	14	1.18833333	2.08857143	-0.9002381	c(t = -0.9878	0.336	0.84333076	Distal Intergenic	ENSSSCG000	BMP3	bone morphogenetic protein 3 [Source:NCBI gene (formerly Entrezgene);Acc:100512974]
chr3	96474066	96475010	945	*	chr3	96474066	96475010	in_vitro	in_vivo	9	9	0.50555556	1.46777778	-0.9622222	c(t = -0.9980	0.336	0.84333076	Distal Intergenic	ENSSSCG000	DYNC2LI1	dynein cytoplasmic 2 light intermediate chain 1 [Source:VGNC Symbol;Acc:VGNC:87499]
chr9	27133595	27134629	1035	*	chr9	27133595	27134629	in_vitro	in_vivo	13	13	2.8	1.21769231	1.58230769	c(t = 0.98842	0.336	0.84333076	Distal Intergenic	ENSSSCG000	ssc-mir-7143	ssc-mir-7143 [Source:miRBase;Acc:MI0023576]
chr8	11415013	11416655	1643	*	chr8	11415013	11416655	in_vitro	in_vivo	11	13	1.99727273	1.20615385	0.79111888	c(t = 0.99010	0.336	0.84333076	Distal Intergenic	ENSSSCG000	CD38	CD38 molecule [Source:NCBI gene (formerly Entrezgene);Acc:100511702]
chr11	45369449	45370446	998	*	chr11	45369449	45370446	in_vitro	in_vivo	12	14	1.9075	1.07285714	0.83464286	c(t = 0.98677	0.336	0.84333076	Promoter (<=1kb)	ENSSSCG000	KLF5	KLF transcription factor 5 [Source:NCBI gene (formerly Entrezgene);Acc:100038005]
chr9	93393509	93394550	1042	*	chr9	93393509	93394550	in_vitro	in_vivo	10	12	1.949	0.88666667	1.06233333	c(t = 0.99212	0.336	0.84333076	Distal Intergenic	ENSSSCG000	CROT	carnitine O-octanoyltransferase [Source:NCBI gene (formerly Entrezgene);Acc:100521142]
chr12	36333086	36334175	1090	*	chr12	36333086	36334175	in_vitro	in_vivo	9	11	0.15888889	0.66181818	-0.5029293	c(t = -1.0025	0.336	0.84333076	Distal Intergenic	ENSSSCG000	ssc-mir-21	ssc-mir-21 [Source:miRBase;Acc:MI0002459]
chr10	65202229	65203747	1519	*	chr10	65202229	65203747	in_vitro	in_vivo	14	9	97.2457143	94.8544444	2.39126984	c(t = 1.00500	0.337	0.84333076	Distal Intergenic	ENSSSCG000	GDI2	GDP dissociation inhibitor 2 [Source:NCBI gene (formerly Entrezgene);Acc:414427]
chr6	13681590	13682859	1270	*	chr6	13681590	13682859	in_vitro	in_vivo	12	9	3.44416667	14.6466667	-11.2025	c(t = -1.0197	0.337	0.84333076	Distal Intergenic	ENSSSCG000	IL34	interleukin 34 [Source:VGNC Symbol;Acc:VGNC:89107]
chr3	76527411	76529741	2331	*	chr3	76527411	76529741	in_vitro	in_vivo	9	14	15.7722222	8.73714286	7.03507937	c(t = 1.00445	0.337	0.84333076	Distal Intergenic	ENSSSCG000	ACTR2	actin related protein 2 [Source:VGNC Symbol;Acc:VGNC:85051]
chr14	49208040	49210064	2025	*	chr14	49208040	49210064	in_vitro	in_vivo	9	12	2.56666667	4.68083333	-2.1141667	c(t = -0.9880	0.337	0.84333076	Distal Intergenic	ENSSSCG000	GUCD1	guanylyl cyclase domain containing 1 [Source:VGNC Symbol;Acc:VGNC:88749]
chr16	79698634	79701729	3096	*	chr16	79698634	79701729	in_vitro	in_vivo	11	13	7.82818182	3.99230769	3.83587413	c(t = 0.99692	0.337	0.84333076	Distal Intergenic	ENSSSCG000	TPPP	tubulin polymerization promoting protein [Source:VGNC Symbol;Acc:VGNC:94344]
chr12	18989915	18992990	3076	*	chr12	18989915	18992990	in_vitro	in_vivo	14	11	0.89642857	2.36454545	-1.4681169	c(t = -1.0013	0.337	0.84333076	Distal Intergenic	ENSSSCG000	SLC25A39	solute carrier family 25 member 39 [Source:VGNC Symbol;Acc:VGNC:93014]
chr15	81881154	81884936	3783	*	chr15	81881154	81884936	in_vitro	in_vivo	14	14	3.67	2.335	1.335	c(t = 0.98049	0.337	0.84333076	Distal Intergenic	NA	NA	NA
chr18	48550707	48551707	1001	*	chr18	48550707	48551707	in_vitro	in_vivo	10	10	1.881	1.105	0.776	c(t = 0.99155	0.337	0.84333076	Distal Intergenic	ENSSSCG000	HUS1	HUS1 checkpoint clamp component [Source:NCBI gene (formerly Entrezgene);Acc:100192318]
chr1	142566853	142567341	489	*	chr1	142566853	142567341	in_vitro	in_vivo	10	10	2.34	0.758	1.582	c(t = 1.00271	0.337	0.84333076	Distal Intergenic	ENSSSCG000	NDN	necdin, MAGE family member [Source:NCBI gene (formerly Entrezgene);Acc:100144475]
chr1	130112109	130112914	806	*	chr1	130112109	130112914	in_vitro	in_vivo	13	9	0.76923077	0	0.76923077	c(t = 1)	0.337	0.84333076	Distal Intergenic	ENSSSCG000	DLL4	delta like canonical Notch ligand 4 [Source:NCBI gene (formerly Entrezgene);Acc:100152163]
chr5	35691870	35692360	491	*	chr5	35691870	35692360	in_vitro	in_vivo	13	11	0.16384615	0	0.16384615	c(t = 1)	0.337	0.84333076	Distal Intergenic	ENSSSCG000	LGR5	leucine rich repeat containing G protein-coupled receptor 5 [Source:VGNC Symbol;Acc:VGNC:103296]
chr17	39868621	39869364	744	*	chr17	39868621	39869364	in_vitro	in_vivo	11	13	0.82363636	3.89307692	-3.0694406	c(t = -0.9964	0.338	0.84333076	Distal Intergenic	ENSSSCG000	MYL9	myosin light chain 9 [Source:NCBI gene (formerly Entrezgene);Acc:100157760]
chr18	43508604	43509990	1387	*	chr18	43508604	43509990	in_vitro	in_vivo	10	9	3.489	0.97555556	2.51344444	c(t = 1.00733	0.338	0.84333076	Distal Intergenic	ENSSSCG000	FKBP14	FKBP prolyl isomerase 14 [Source:NCBI gene (formerly Entrezgene);Acc:100514551]
chr9	131425819	131426842	1024	*	chr9	131425819	131426842	in_vitro	in_vivo	12	12	2.91833333	0.84416667	2.07416667	c(t = 0.99897	0.338	0.84333076	Distal Intergenic	ENSSSCG000	SLC30A1	solute carrier family 30 member 1 [Source:NCBI gene (formerly Entrezgene);Acc:503660]
chr1	76839128	76841495	2368	*	chr1	76839128	76841495	in_vitro	in_vivo	14	11	1.39714286	0.8	0.59714286	c(t = 0.97946	0.338	0.84333076	Distal Intergenic	ENSSSCG000	DDO	D-aspartate oxidase [Source:NCBI gene (formerly Entrezgene);Acc:100037287]
chr5	57951000	57951652	653	*	chr5	57951000	57951652	in_vitro	in_vivo	12	13	1.57916667	0.67076923	0.90839744	c(t = 0.98753	0.338	0.84333076	Promoter (<=1kb)	ENSSSCG000	PLBD1	phospholipase B domain containing 1 [Source:NCBI gene (formerly Entrezgene);Acc:100155493]
chr14	43684030	43684920	891	*	chr14	43684030	43684920	in_vitro	in_vivo	10	9	96.084	98.0277778	-1.9437778	c(t = -0.9881	0.339	0.84333076	Distal Intergenic	ENSSSCG000	HPS4	HPS4 biosis of lysosomal organelles complex 3 subunit 2 [Source:VGNC Symbol;Acc:VGNC:88966]
chr3	39288018	39289235	1218	*	chr3	39288018	39289235	in_vitro	in_vivo	10	10	87.233	94.577	-7.344	c(t = -1.0039	0.339	0.84333076	Exon (NM_001244545/100511011, exon 11 of 15)	ENSSSCG000	SRRM2	serine/arginine repetitive matrix 2 [Source:VGNC Symbol;Acc:VGNC:93468]
chr8	96267859	96269407	1549	*	chr8	96267859	96269407	in_vitro	in_vivo	9	14	3.53222222	5.67928571	-2.1470635	c(t = -0.9819	0.339	0.84333076	Distal Intergenic	ENSSSCG000	PGRMC2	progesterone receptor membrane component 2 [Source:NCBI gene (formerly Entrezgene);Acc:100048965]
chr10	34001454	34002137	684	*	chr10	34001454	34002137	in_vitro	in_vivo	11	11	5.49818182	3.31454545	2.18363636	c(t = 0.98083	0.339	0.84333076	Distal Intergenic	ENSSSCG000	RIGI	RNA sensor RIG-I [Source:VGNC Symbol;Acc:VGNC:95905]
chr7	98447522	98449816	2295	*	chr7	98447522	98449816	in_vitro	in_vivo	11	13	0.85636364	1.65615385	-0.7997902	c(t = -0.9773	0.339	0.84333076	Promoter (<=1kb)	ENSSSCG000	FOS	Fos proto-oncogene, AP-1 transcription factor subunit [Source:NCBI gene (formerly Entrezgene);Acc:100144486]
chr1	122139006	122139578	573	*	chr1	122139006	122139578	in_vitro	in_vivo	12	10	3.02083333	1.084	1.93683333	c(t = 0.98894	0.339	0.84333076	Distal Intergenic	ENSSSCG000	COPS2	COP9 signalosome subunit 2 [Source:VGNC Symbol;Acc:VGNC:86899]
chr4	125740158	125741117	960	*	chr4	125740158	125741117	in_vitro	in_vivo	12	14	1.90083333	0.97642857	0.92440476	c(t = 0.98116	0.339	0.84333076	Distal Intergenic	ENSSSCG000	TGFBR3	transforming growth factor beta receptor 3 [Source:VGNC Symbol;Acc:VGNC:93932]
chr15	63592661	63593639	979	*	chr15	63592661	63593639	in_vitro	in_vivo	12	11	0.26	0.96363636	-0.7036364	c(t = -0.9932	0.339	0.84333076	Distal Intergenic	ENSSSCG000	NR4A2	nuclear receptor subfamily 4 group A member 2 [Source:VGNC Symbol;Acc:VGNC:96451]
chr6	40043225	40044002	778	*	chr6	40043225	40044002	in_vitro	in_vivo	10	10	1.69	0.888	0.802	c(t = 0.98489	0.339	0.84333076	Distal Intergenic	ENSSSCG000	SLC7A9	solute carrier family 7 member 9 [Source:VGNC Symbol;Acc:VGNC:93177]
chr1	231142443	231143232	790	*	chr1	231142443	231143232	in_vitro	in_vivo	9	9	1.23444444	2.66222222	-1.4277778	c(t = -0.9837	0.34	0.84333076	Distal Intergenic	ENSSSCG000	GNAQ	G protein subunit alpha q [Source:NCBI gene (formerly Entrezgene);Acc:654405]
chr4	33704412	33705132	721	*	chr4	33704412	33705132	in_vitro	in_vivo	10	9	1.444	2.58444444	-1.1404444	c(t = -0.9868	0.34	0.84333076	Distal Intergenic	ENSSSCG000	FZD6	frizzled class receptor 6 [Source:NCBI gene (formerly Entrezgene);Acc:100157360]
chr8	116249397	116250335	939	*	chr8	116249397	116250335	in_vitro	in_vivo	13	16	2.72384615	1.345	1.37884615	c(t = 0.97765	0.34	0.84333076	Distal Intergenic	ENSSSCG000	AIMP1	aminoacyl tRNA synthetase complex interacting multifunctional protein 1 [Source:VGNC Symbol;Acc:VGNC:85202]
chr11	660207	662274	2068	*	chr11	660207	662274	in_vitro	in_vivo	13	13	1.69923077	0.83846154	0.86076923	c(t = 0.97755	0.34	0.84333076	Distal Intergenic	ENSSSCG000	CRYL1	crystallin lambda 1 [Source:VGNC Symbol;Acc:VGNC:87023]
chr18	55527984	55528662	679	*	chr18	55527984	55528662	in_vitro	in_vivo	9	9	2.32	0.82555556	1.49444444	c(t = 1.00245	0.34	0.84333076	Distal Intergenic	ENSSSCG000	RARRES2	retinoic acid receptor responder 2 [Source:VGNC Symbol;Acc:VGNC:92098]
chr5	19212370	19213667	1298	*	chr5	19212370	19213667	in_vitro	in_vivo	9	11	2.08333333	0.82272727	1.26060606	c(t = 0.99146	0.34	0.84333076	Distal Intergenic	ENSSSCG000	ssc-mir-196a	ssc-mir-196a-2 [Source:miRBase;Acc:MI0002457]
chr12	15813188	15813966	779	*	chr12	15813188	15813966	in_vitro	in_vivo	10	13	1.689	0.62846154	1.06053846	c(t = 0.99678	0.34	0.84333076	Distal Intergenic	ENSSSCG000	CYB561	cytochrome b561 [Source:VGNC Symbol;Acc:VGNC:87119]
chr7	10057140	10058482	1343	*	chr7	10057140	10058482	in_vitro	in_vivo	13	14	2.24230769	1.17571429	1.06659341	c(t = 0.98002	0.341	0.84333076	Distal Intergenic	ENSSSCG000	ssc-mir-9837	ssc-mir-9837 [Source:miRBase;Acc:MI0031606]
chr5	12623634	12624218	585	*	chr5	12623634	12624218	in_vitro	in_vivo	13	10	2.87384615	1.137	1.73684615	c(t = 0.98370	0.341	0.84333076	Distal Intergenic	ENSSSCG000	RTCB	RNA 2',3'-cyclic phosphate and 5'-OH ligase [Source:NCBI gene (formerly Entrezgene);Acc:733658]
chr15	139916778	139918537	1760	*	chr15	139916778	139918537	in_vitro	in_vivo	11	11	2.31727273	1.03909091	1.27818182	c(t = 0.99067	0.341	0.84333076	Promoter (<=1kb)	ENSSSCG000	PPP1R7	protein phosphatase 1 regulatory subunit 7 [Source:NCBI gene (formerly Entrezgene);Acc:100511842]
chr13	192376739	192376958	220	*	chr13	192376739	192376958	in_vitro	in_vivo	10	11	0	0.72727273	-0.7272727	c(t = -1)	0.341	0.84333076	Distal Intergenic	ENSSSCG000	CLDN17	claudin 17 [Source:NCBI gene (formerly Entrezgene);Acc:100294681]
chr15	48348339	48349236	898	*	chr15	48348339	48349236	in_vitro	in_vivo	10	9	2.047	0.57333333	1.47366667	c(t = 1.00034	0.341	0.84333076	Distal Intergenic	ENSSSCG000	STAR	steroidogenic acute regulatory protein [Source:NCBI gene (formerly Entrezgene);Acc:396597]
chr1	149737310	149737784	475	*	chr1	149737310	149737784	in_vitro	in_vivo	11	11	0	0.34272727	-0.3427273	c(t = -1)	0.341	0.84333076	Promoter (<=1kb)	ENSSSCG000	CYB5A	cytochrome b5 type A [Source:VGNC Symbol;Acc:VGNC:96727]
chr6	137200951	137201311	361	*	chr6	137200951	137201311	in_vitro	in_vivo	12	11	0	0.29363636	-0.2936364	c(t = -1)	0.341	0.84333076	Distal Intergenic	ENSSSCG000	MSH4	Rab geranylgeranyltransferase subunit beta [Source:NCBI gene (formerly Entrezgene);Acc:733698]
chr14	29894682	29895577	896	*	chr14	29894682	29895577	in_vitro	in_vivo	9	11	0	0.10727273	-0.1072727	c(t = -1)	0.341	0.84333076	Distal Intergenic	ENSSSCG000	ARL6IP4	ADP ribosylation factor like GTPase 6 interacting protein 4 [Source:VGNC Symbol;Acc:VGNC:107367]
chr11	51420135	51420621	487	*	chr11	51420135	51420621	in_vitro	in_vivo	11	10	1.29909091	0	1.29909091	c(t = 1)	0.341	0.84333076	Distal Intergenic	ENSSSCG000	EDNRB	endothelin receptor type B [Source:VGNC Symbol;Acc:VGNC:87550]
chr2	71871536	71871973	438	*	chr2	71871536	71871973	in_vitro	in_vivo	11	12	0.82636364	0	0.82636364	c(t = 1)	0.341	0.84333076	Distal Intergenic	ENSSSCG000	PET100	PET100 cytochrome c oxidase chaperone [Source:NCBI gene (formerly Entrezgene);Acc:100037981]
chr7	56146768	56147352	585	*	chr7	56146768	56147352	in_vitro	in_vivo	11	9	0.47818182	0	0.47818182	c(t = 1)	0.341	0.84333076	Distal Intergenic	ENSSSCG000	ETFA	electron transfer flavoprotein subunit alpha [Source:VGNC Symbol;Acc:VGNC:87801]
chr6	90423679	90425091	1413	*	chr6	90423679	90425091	in_vitro	in_vivo	11	10	0.38727273	0	0.38727273	c(t = 1)	0.341	0.84333076	Distal Intergenic	ENSSSCG000	GJA4	gap junction protein alpha 4 [Source:NCBI gene (formerly Entrezgene);Acc:100620145]
chr2	71616106	71617009	904	*	chr2	71616106	71617009	in_vitro	in_vivo	9	9	57.5211111	73.0511111	-15.53	c(t = -0.9797	0.342	0.84333076	Distal Intergenic	ENSSSCG000	PET100	PET100 cytochrome c oxidase chaperone [Source:NCBI gene (formerly Entrezgene);Acc:100037981]
chr8	1792641	1793782	1142	*	chr8	1792641	1793782	in_vitro	in_vivo	11	10	6.53272727	15.291	-8.7582727	c(t = -0.9976	0.342	0.84333076	Distal Intergenic	ENSSSCG000	HTT	huntingtin [Source:VGNC Symbol;Acc:VGNC:89006]
chr2	83690583	83692250	1668	*	chr2	83690583	83692250	in_vitro	in_vivo	11	12	4.57	12.6975	-8.1275	c(t = -0.9900	0.342	0.84333076	Distal Intergenic	ENSSSCG000	HEXB	hexosaminidase subunit beta [Source:NCBI gene (formerly Entrezgene);Acc:396958]
chr18	55775284	55776572	1289	*	chr18	55775284	55776572	in_vitro	in_vivo	10	11	6.883	4.54545455	2.33754545	c(t = 0.98962	0.342	0.84333076	Distal Intergenic	ENSSSCG000	RARRES2	retinoic acid receptor responder 2 [Source:VGNC Symbol;Acc:VGNC:92098]
chr13	75774229	75774905	677	*	chr13	75774229	75774905	in_vitro	in_vivo	13	13	1.97461538	3.15384615	-1.1792308	c(t = -0.9700	0.342	0.84333076	Distal Intergenic	ENSSSCG000	ANAPC13	anaphase promoting complex subunit 13 [Source:VGNC Symbol;Acc:VGNC:85294]
chr15	82958064	82958953	890	*	chr15	82958064	82958953	in_vitro	in_vivo	9	12	13.86	2.73833333	11.1216667	c(t = 1.00822	0.342	0.84333076	Distal Intergenic	ENSSSCG000	MTX2	metaxin 2 [Source:VGNC Symbol;Acc:VGNC:96427]
chr8	131058025	131059599	1575	*	chr8	131058025	131059599	in_vitro	in_vivo	13	13	1.15384615	2.42846154	-1.2746154	c(t = -0.9816	0.342	0.84333076	Promoter (<=1kb)	ENSSSCG000	PKD2	polycystin 2, transient receptor potential cation channel [Source:VGNC Symbol;Acc:VGNC:91472]
chrX	106245640	106246242	603	*	chrX	106245640	106246242	in_vitro	in_vivo	12	9	2.96333333	0.79777778	2.16555556	c(t = 0.98695	0.342	0.84333076	Distal Intergenic	ENSSSCG000	XPNPEP2	X-prolyl aminopeptidase 2 [Source:VGNC Symbol;Acc:VGNC:95001]
chr13	58642560	58643428	869	*	chr13	58642560	58643428	in_vitro	in_vivo	11	13	4.22272727	0.57	3.65272727	c(t = 0.99542	0.342	0.84333076	Distal Intergenic	ENSSSCG000	SUMF1	sulfatase modifying factor 1 [Source:VGNC Symbol;Acc:VGNC:93617]
chr17	14151397	14152143	747	*	chr17	14151397	14152143	in_vitro	in_vivo	9	11	1.14111111	0.48818182	0.65292929	c(t = 0.98393	0.342	0.84333076	Promoter (<=1kb)	ENSSSCG000	CDS2	CDP-diacylglycerol synthase 2 [Source:VGNC Symbol;Acc:VGNC:95869]
chr14	51020197	51020913	717	*	chr14	51020197	51020913	in_vitro	in_vivo	10	12	0.753	0.3	0.453	c(t = 0.98509	0.342	0.84333076	Distal Intergenic	ENSSSCG000	SLC25A1	solute carrier family 25 member 1 [Source:VGNC Symbol;Acc:VGNC:92990]
chr13	207494126	207502037	7912	*	chr13	207494126	207502037	in_vitro	in_vivo	10	13	97.215	94.9353846	2.27961538	c(t = 0.97156	0.343	0.84333076	Distal Intergenic	ENSSSCG000	ITGB2	integrin subunit beta 2 [Source:NCBI gene (formerly Entrezgene);Acc:396943]
chr12	1836929	1840298	3370	*	chr12	1836929	1840298	in_vitro	in_vivo	12	12	80.9775	89.0233333	-8.0458333	c(t = -0.9704	0.343	0.84333076	Distal Intergenic	NA	NA	NA
chr6	52348958	52349451	494	*	chr6	52348958	52349451	in_vitro	in_vivo	9	11	1.92444444	11.0018182	-9.0773737	c(t = -0.9936	0.343	0.84333076	Distal Intergenic	ENSSSCG000	PGLYRP1	peptidoglycan recognition protein 1 [Source:NCBI gene (formerly Entrezgene);Acc:397213]
chr7	43669188	43670032	845	*	chr7	43669188	43670032	in_vitro	in_vivo	10	12	4.571	2.29416667	2.27683333	c(t = 0.98958	0.343	0.84333076	Distal Intergenic	ENSSSCG000	MMUT	methylmalonyl-CoA mutase [Source:NCBI gene (formerly Entrezgene);Acc:399535]
chr1	127766732	127767898	1167	*	chr1	127766732	127767898	in_vitro	in_vivo	11	9	4.99454545	2.19111111	2.80343434	c(t = 0.98172	0.343	0.84333076	Promoter (<=1kb)	ENSSSCG000	SERF2	small EDRK-rich factor 2 [Source:NCBI gene (formerly Entrezgene);Acc:100154963]
chr8	118164265	118164837	573	*	chr8	118164265	118164837	in_vitro	in_vivo	12	11	0.60666667	1.63909091	-1.0324242	c(t = -0.9832	0.343	0.84333076	Distal Intergenic	ENSSSCG000	UBE2D3	ubiquitin conjugating enzyme E2 D3 [Source:NCBI gene (formerly Entrezgene);Acc:780418]
chr4	16012165	16012887	723	*	chr4	16012165	16012887	in_vitro	in_vivo	12	9	0.405	1.59444444	-1.1894444	c(t = -1.0015	0.343	0.84333076	Distal Intergenic	ENSSSCG000	FBXO32	F-box protein 32 [Source:NCBI gene (formerly Entrezgene);Acc:733657]
chr14	81405269	81409045	3777	*	chr14	81405269	81409045	in_vitro	in_vivo	12	12	3.175	1.46833333	1.70666667	c(t = 0.98599	0.343	0.84333076	Distal Intergenic	ENSSSCG000	PLAC9	placenta associated 9 [Source:NCBI gene (formerly Entrezgene);Acc:100155282]
chr11	19106367	19107200	834	*	chr11	19106367	19107200	in_vitro	in_vivo	9	12	2.29444444	1.16416667	1.13027778	c(t = 0.99039	0.343	0.84333076	Distal Intergenic	ENSSSCG000	CYSLTR2	cysteinyl leukotriene receptor 2 [Source:NCBI gene (formerly Entrezgene);Acc:397243]
chr6	91767719	91768312	594	*	chr6	91767719	91768312	in_vitro	in_vivo	9	10	2.58444444	1.032	1.55244444	c(t = 0.99301	0.343	0.84333076	Distal Intergenic	NA	NA	NA
chr13	121564660	121566208	1549	*	chr13	121564660	121566208	in_vitro	in_vivo	11	13	1.84363636	0.91384615	0.92979021	c(t = 0.98254	0.343	0.84333076	Distal Intergenic	ENSSSCG000	B3GNT5	UDP-GlcNAc:betaGal beta-1,3-N-acetylglucosaminyltransferase 5 [Source:VGNC Symbol;Acc:VGNC:85726]
chr18	20834172	20835541	1370	*	chr18	20834172	20835541	in_vitro	in_vivo	14	13	1.41714286	0.79846154	0.61868132	c(t = 0.96761	0.343	0.84333076	Distal Intergenic	ENSSSCG000	ARF5	ADP ribosylation factor 5 [Source:NCBI gene (formerly Entrezgene);Acc:100270722]
chr4	107920643	107921899	1257	*	chr4	107920643	107921899	in_vitro	in_vivo	10	16	1.799	0.771875	1.027125	c(t = 0.98977	0.343	0.84333076	Distal Intergenic	ENSSSCG000	CAPZA1	capping actin protein of muscle Z-line subunit alpha 1 [Source:NCBI gene (formerly Entrezgene);Acc:100037957]
chr14	61047832	61048430	599	*	chr14	61047832	61048430	in_vitro	in_vivo	12	11	1.33333333	0.58090909	0.75242424	c(t = 0.97371	0.343	0.84333076	Distal Intergenic	ENSSSCG000	ACTA1	actin alpha 1, skeletal muscle [Source:NCBI gene (formerly Entrezgene);Acc:100154254]
chr11	18755946	18757295	1350	*	chr11	18755946	18757295	in_vitro	in_vivo	14	16	0.985	0.558125	0.426875	c(t = 0.96883	0.343	0.84333076	Distal Intergenic	ENSSSCG000	CYSLTR2	cysteinyl leukotriene receptor 2 [Source:NCBI gene (formerly Entrezgene);Acc:397243]
chr14	100604477	100604991	515	*	chr14	100604477	100604991	in_vitro	in_vivo	9	10	0	0.5	-0.5	c(t = -1)	0.343	0.84333076	Distal Intergenic	ENSSSCG000	STAMBPL1	STAM binding protein like 1 [Source:VGNC Symbol;Acc:VGNC:93527]
chr3	15025520	15027394	1875	*	chr3	15025520	15027394	in_vitro	in_vivo	10	14	1.033	0.48928571	0.54371429	c(t = 0.98076	0.343	0.84333076	Distal Intergenic	ENSSSCG000	SBDS	SBDS ribosome maturation factor [Source:NCBI gene (formerly Entrezgene);Acc:100516003]
chr6	31564623	31565042	420	*	chr6	31564623	31565042	in_vitro	in_vivo	9	10	0	0.417	-0.417	c(t = -1)	0.343	0.84333076	Promoter (<=1kb)	ENSSSCG000	FTO	FTO alpha-ketoglutarate dependent dioxygenase [Source:NCBI gene (formerly Entrezgene);Acc:100127165]
chr7	100748752	100749429	678	*	chr7	100748752	100749429	in_vitro	in_vivo	10	10	0	0.227	-0.227	c(t = -1)	0.343	0.84333076	Distal Intergenic	ENSSSCG000	SLIRP	SRA stem-loop interacting RNA binding protein [Source:NCBI gene (formerly Entrezgene);Acc:100037987]
chr14	71486147	71487188	1042	*	chr14	71486147	71487188	in_vitro	in_vivo	10	13	0.588	0	0.588	c(t = 1)	0.343	0.84333076	Promoter (<=1kb)	ENSSSCG000	HNRNPH3	heterous nuclear ribonucleoprotein H3 [Source:VGNC Symbol;Acc:VGNC:88922]
chr4	21940489	21941088	600	*	chr4	21940489	21941088	in_vitro	in_vivo	10	9	0.435	0	0.435	c(t = 1)	0.343	0.84333076	Distal Intergenic	NA	NA	NA
chr6	83982319	83983320	1002	*	chr6	83982319	83983320	in_vitro	in_vivo	10	9	0.323	0	0.323	c(t = 1)	0.343	0.84333076	Distal Intergenic	ENSSSCG000	RPS6KA1	ribosomal protein S6 kinase A1 [Source:VGNC Symbol;Acc:VGNC:92440]
chr12	393763	395554	1792	*	chr12	393763	395554	in_vitro	in_vivo	9	9	96.3066667	92.8866667	3.42	c(t = 0.98282	0.344	0.84333076	Intron (NM_001244105/100512127, intron 13 of 38)	ENSSSCG000	TBCD	tubulin folding cofactor D [Source:NCBI gene (formerly Entrezgene);Acc:100512127]
chr9	135273944	135274674	731	*	chr9	135273944	135274674	in_vitro	in_vivo	9	10	12.6422222	26.649	-14.006778	c(t = -0.9741	0.344	0.84333076	Distal Intergenic	ENSSSCG00000038506		CD46 molecule, complement regulatory protein [Source:NCBI gene (formerly Entrezgene);Acc:396922]
chr6	63862113	63863570	1458	*	chr6	63862113	63863570	in_vitro	in_vivo	9	11	3.96444444	2.47818182	1.48626263	c(t = 0.97220	0.344	0.84333076	Distal Intergenic	ENSSSCG000	PRKCZ	protein kinase C zeta [Source:NCBI gene (formerly Entrezgene);Acc:100533200]
chr1	152635838	152636631	794	*	chr1	152635838	152636631	in_vitro	in_vivo	11	10	1.28090909	2.192	-0.9110909	c(t = -0.9808	0.344	0.84333076	Promoter (<=1kb)	ENSSSCG000	SOCS6	suppressor of cytokine signaling 6 [Source:VGNC Symbol;Acc:VGNC:93333]
chr9	52237232	52237795	564	*	chr9	52237232	52237795	in_vitro	in_vivo	11	9	3.54363636	1.24111111	2.30252525	c(t = 0.97956	0.344	0.84333076	Distal Intergenic	ENSSSCG000	STT3A	STT3 oligosaccharyltransferase complex catalytic subunit A [Source:VGNC Symbol;Acc:VGNC:93581]
chr2	10205755	10207262	1508	*	chr2	10205755	10207262	in_vitro	in_vivo	14	12	2.32142857	1.23583333	1.08559524	c(t = 0.96644	0.344	0.84333076	Distal Intergenic	ENSSSCG000	PGA5	pepsinogen 5, group I (pepsinogen A) [Source:NCBI gene (formerly Entrezgene);Acc:396892]
chr1	143867416	143868265	850	*	chr1	143867416	143868265	in_vitro	in_vivo	11	11	2.73545455	1.15272727	1.58272727	c(t = 0.98510	0.344	0.84333076	Distal Intergenic	ENSSSCG000	MCEE	methylmalonyl-CoA epimerase [Source:VGNC Symbol;Acc:VGNC:90066]
chr11	1131994	1133348	1355	*	chr11	1131994	1133348	in_vitro	in_vivo	9	11	98.1977778	96.2781818	1.91959596	c(t = 0.97107	0.345	0.84333076	Distal Intergenic	ENSSSCG000	LATS2	large tumor suppressor kinase 2 [Source:VGNC Symbol;Acc:VGNC:89649]
chr9	137054120	137056005	1886	*	chr9	137054120	137056005	in_vitro	in_vivo	14	13	27.0807143	16.9746154	10.1060989	c(t = 0.96228	0.345	0.84333076	Distal Intergenic	ENSSSCG000	GRB10	growth factor receptor bound protein 10 [Source:VGNC Symbol;Acc:VGNC:88662]
chr8	2319016	2321774	2759	*	chr8	2319016	2321774	in_vitro	in_vivo	9	11	4.58	2.62363636	1.95636364	c(t = 0.97999	0.345	0.84333076	Distal Intergenic	ENSSSCG000	LRPAP1	LDL receptor related protein associated protein 1 [Source:NCBI gene (formerly Entrezgene);Acc:399654]
chr6	78076877	78078416	1540	*	chr6	78076877	78078416	in_vitro	in_vivo	11	9	7.25909091	2.04555556	5.21353535	c(t = 0.98905	0.345	0.84333076	Distal Intergenic	ENSSSCG000	CAPZB	capping actin protein of muscle Z-line subunit beta [Source:VGNC Symbol;Acc:VGNC:86184]
chr15	106182884	106184304	1421	*	chr15	106182884	106184304	in_vitro	in_vivo	13	13	0.73461538	1.88153846	-1.1469231	c(t = -0.9760	0.345	0.84333076	Distal Intergenic	ENSSSCG000	BMPR2	bone morphogenetic protein receptor type 2 [Source:NCBI gene (formerly Entrezgene);Acc:100127483]
chr7	92928745	92930664	1920	*	chr7	92928745	92930664	in_vitro	in_vivo	12	14	2.14	1.39642857	0.74357143	c(t = 0.96519	0.345	0.84333076	Promoter (<=1kb)	ENSSSCG000	GALNT16	polypeptide N-acetylgalactosaminyltransferase 16 [Source:VGNC Symbol;Acc:VGNC:88332]
chr3	71947822	71948937	1116	*	chr3	71947822	71948937	in_vitro	in_vivo	10	11	2.888	5.43090909	-2.5429091	c(t = -0.9712	0.346	0.84333076	Promoter (<=1kb)	ENSSSCG000	TGFA	transforming growth factor alpha [Source:VGNC Symbol;Acc:VGNC:93928]
chrX	125382777	125383825	1049	*	chrX	125382777	125383825	in_vitro	in_vivo	10	10	1.994	4.928	-2.934	c(t = -0.9715	0.346	0.84333076	Promoter (<=1kb)	ENSSSCG000	BRCC3	BRCA1/BRCA2-containing complex subunit 3 [Source:VGNC Symbol;Acc:VGNC:85870]
chr3	47337926	47339720	1795	*	chr3	47337926	47339720	in_vitro	in_vivo	14	13	1.68142857	2.95846154	-1.277033	c(t = -0.9683	0.346	0.84333076	Distal Intergenic	ENSSSCG00000008137		LIM zinc finger domain containing 1 [Source:VGNC Symbol;Acc:VGNC:110285]
chr18	18722757	18723433	677	*	chr18	18722757	18723433	in_vitro	in_vivo	9	11	2.36888889	1.27727273	1.09161616	c(t = 0.97121	0.346	0.84333076	Distal Intergenic	ENSSSCG000	CPA1	carboxypeptidase A1 [Source:VGNC Symbol;Acc:VGNC:86930]
chr11	77426533	77428310	1778	*	chr11	77426533	77428310	in_vitro	in_vivo	11	10	0.68090909	1.23	-0.5490909	c(t = -0.9681	0.346	0.84333076	Distal Intergenic	ENSSSCG000	ANKRD10	ankyrin repeat domain 10 [Source:VGNC Symbol;Acc:VGNC:85322]
chr16	34702052	34703055	1004	*	chr16	34702052	34703055	in_vitro	in_vivo	11	11	0.52727273	1.14636364	-0.6190909	c(t = -0.9696	0.346	0.84333076	Distal Intergenic	ENSSSCG000	DDX4	DEAD-box helicase 4 [Source:VGNC Symbol;Acc:VGNC:87220]
chr4	124009024	124010421	1398	*	chr4	124009024	124010421	in_vitro	in_vivo	12	13	1.20583333	0.52153846	0.68429487	c(t = 0.96429	0.346	0.84333076	Promoter (<=1kb)	ENSSSCG000	TMED5	transmembrane p24 trafficking protein 5 [Source:NCBI gene (formerly Entrezgene);Acc:100157999]
chr6	48365196	48365837	642	*	chr6	48365196	48365837	in_vitro	in_vivo	10	9	25.502	37.8188889	-12.316889	c(t = -0.9755	0.347	0.84333076	Distal Intergenic	ENSSSCG000	LGALS13	lectin, galactoside-binding, soluble, 13 [Source:NCBI gene (formerly Entrezgene);Acc:100217392]
chr2	142385957	142387651	1695	*	chr2	142385957	142387651	in_vitro	in_vivo	15	14	9.134	13.9057143	-4.7717143	c(t = -0.9610	0.347	0.84333076	Distal Intergenic	ENSSSCG000	CD14	CD14 molecule [Source:NCBI gene (formerly Entrezgene);Acc:100037938]
chrX	124025315	124028273	2959	*	chrX	124025315	124028273	in_vitro	in_vivo	10	9	3.782	13.7177778	-9.9357778	c(t = -0.9953	0.347	0.84333076	Distal Intergenic	ENSSSCG000	NSDHL	NAD(P) dependent steroid dehydrogenase-like [Source:NCBI gene (formerly Entrezgene);Acc:100157894]
chr17	39360232	39362024	1793	*	chr17	39360232	39362024	in_vitro	in_vivo	12	13	2.51166667	3.80384615	-1.2921795	c(t = -0.9595	0.347	0.84333076	Distal Intergenic	ENSSSCG000	ROMO1	reactive oxygen species modulator 1 [Source:NCBI gene (formerly Entrezgene);Acc:100154394]
chr17	34626469	34627406	938	*	chr17	34626469	34627406	in_vitro	in_vivo	10	11	1.965	3.50181818	-1.5368182	c(t = -0.9716	0.347	0.84333076	Distal Intergenic	ENSSSCG000	TBC1D20	TBC1 domain family member 20 [Source:NCBI gene (formerly Entrezgene);Acc:100519805]
chr4	33770993	33771856	864	*	chr4	33770993	33771856	in_vitro	in_vivo	11	11	10.8009091	3.36	7.44090909	c(t = 0.98460	0.347	0.84333076	Distal Intergenic	ENSSSCG000	FZD6	frizzled class receptor 6 [Source:NCBI gene (formerly Entrezgene);Acc:100157360]
chr2	77178463	77179553	1091	*	chr2	77178463	77179553	in_vitro	in_vivo	9	10	4.43666667	2.334	2.10266667	c(t = 0.96776	0.347	0.84333076	Distal Intergenic	ENSSSCG000	CIRBP	cold inducible RNA binding protein [Source:NCBI gene (formerly Entrezgene);Acc:100522851]
chr2	114675500	114677370	1871	*	chr2	114675500	114677370	in_vitro	in_vivo	12	15	0.95166667	2.31666667	-1.365	c(t = -0.9651	0.347	0.84333076	Distal Intergenic	ENSSSCG000	STARD4	StAR related lipid transfer domain containing 4 [Source:VGNC Symbol;Acc:VGNC:93534]
chr13	106558592	106558988	397	*	chr13	106558592	106558988	in_vitro	in_vivo	10	9	0	2.22222222	-2.2222222	c(t = -1)	0.347	0.84333076	Promoter (<=1kb)	ENSSSCG000	PDCD10	programmed cell death 10 [Source:VGNC Symbol;Acc:VGNC:91241]
chr12	24899781	24900547	767	*	chr12	24899781	24900547	in_vitro	in_vivo	9	11	1.21444444	2.19454545	-0.980101	c(t = -0.9721	0.347	0.84333076	Distal Intergenic	NA	NA	NA
chr6	85785172	85785954	783	*	chr6	85785172	85785954	in_vitro	in_vivo	9	10	3.08888889	1.617	1.47188889	c(t = 0.96861	0.347	0.84333076	Distal Intergenic	ENSSSCG000	SRSF4	serine and arginine rich splicing factor 4 [Source:NCBI gene (formerly Entrezgene);Acc:768101]
chr7	116786532	116787890	1359	*	chr7	116786532	116787890	in_vitro	in_vivo	14	10	2.21928571	1.148	1.07128571	c(t = 0.96838	0.347	0.84333076	Distal Intergenic	ENSSSCG000	CLMN	calmin [Source:NCBI gene (formerly Entrezgene);Acc:100511700]
chr1	134538029	134538601	573	*	chr1	134538029	134538601	in_vitro	in_vivo	9	9	0	0.85444444	-0.8544444	c(t = -1)	0.347	0.84333076	Distal Intergenic	ENSSSCG000	ACTC1	actin alpha cardiac muscle 1 [Source:NCBI gene (formerly Entrezgene);Acc:100152267]
chr1	129587952	129588179	228	*	chr1	129587952	129588179	in_vitro	in_vivo	9	9	0	0.48333333	-0.4833333	c(t = -1)	0.347	0.84333076	Distal Intergenic	ENSSSCG000	JMJD7	jumonji domain containing 7 [Source:NCBI gene (formerly Entrezgene);Acc:100533119]
chr1	184599488	184600199	712	*	chr1	184599488	184600199	in_vitro	in_vivo	12	14	1.74666667	0.445	1.30166667	c(t = 0.97708	0.347	0.84333076	Distal Intergenic	ENSSSCG000	FBXO34	F-box protein 34 [Source:VGNC Symbol;Acc:VGNC:88041]
chr7	78067372	78067841	470	*	chr7	78067372	78067841	in_vitro	in_vivo	9	9	0	0.20555556	-0.2055556	c(t = -1)	0.347	0.84333076	Distal Intergenic	ENSSSCG000	NDRG2	NDRG family member 2 [Source:VGNC Symbol;Acc:VGNC:90632]
chr8	9166857	9167165	309	*	chr8	9166857	9167165	in_vitro	in_vivo	9	9	3.03	0	3.03	c(t = 1)	0.347	0.84333076	Distal Intergenic	ENSSSCG000	RAB28	RAB28, member RAS onco family [Source:VGNC Symbol;Acc:VGNC:98955]
chr6	19153969	19154904	936	*	chr6	19153969	19154904	in_vitro	in_vivo	9	10	1.58777778	0	1.58777778	c(t = 1)	0.347	0.84333076	Distal Intergenic	ENSSSCG000	CCL22	C-C motif chemokine ligand 22 [Source:NCBI gene (formerly Entrezgene);Acc:100516016]
chr6	145679982	145680346	365	*	chr6	145679982	145680346	in_vitro	in_vivo	9	9	1.01	0	1.01	c(t = 1)	0.347	0.84333076	Distal Intergenic	ENSSSCG000	DYNLT5	dynein light chain Tctex-type family member 5 [Source:VGNC Symbol;Acc:VGNC:93838]
chr7	38120348	38121141	794	*	chr7	38120348	38121141	in_vitro	in_vivo	9	9	0.88888889	0	0.88888889	c(t = 1)	0.347	0.84333076	Promoter (<=1kb)	ENSSSCG000	MRPL2	mitochondrial ribosomal protein L2 [Source:VGNC Symbol;Acc:VGNC:103072]
chrX	20146384	20147184	801	*	chrX	20146384	20147184	in_vitro	in_vivo	9	10	0.57	0	0.57	c(t = 1)	0.347	0.84333076	Distal Intergenic	ENSSSCG000	SAT1	spermidine/spermine N1-acetyltransferase 1 [Source:NCBI gene (formerly Entrezgene);Acc:397645]
chr14	14041383	14041923	541	*	chr14	14041383	14041923	in_vitro	in_vivo	9	10	0.47333333	0	0.47333333	c(t = 1)	0.347	0.84333076	Distal Intergenic	ENSSSCG00000021767		L-threonine dehydrogenase [Source:VGNC Symbol;Acc:VGNC:109162]
chr7	92848042	92848378	337	*	chr7	92848042	92848378	in_vitro	in_vivo	9	9	0.46333333	0	0.46333333	c(t = 1)	0.347	0.84333076	Distal Intergenic	ENSSSCG000	GALNT16	polypeptide N-acetylgalactosaminyltransferase 16 [Source:VGNC Symbol;Acc:VGNC:88332]
chr1	186039681	186039939	259	*	chr1	186039681	186039939	in_vitro	in_vivo	9	9	0.38333333	0	0.38333333	c(t = 1)	0.347	0.84333076	Distal Intergenic	ENSSSCG000	KTN1	kinectin 1 [Source:VGNC Symbol;Acc:VGNC:89608]
chr13	127730389	127730825	437	*	chr13	127730389	127730825	in_vitro	in_vivo	9	9	0.22666667	0	0.22666667	c(t = 1)	0.347	0.84333076	Promoter (<=1kb)	NA	NA	NA
chr12	27004717	27005308	592	*	chr12	27004717	27005308	in_vitro	in_vivo	9	10	0.22222222	0	0.22222222	c(t = 1)	0.347	0.84333076	Distal Intergenic	ENSSSCG000	TOB1	transducer of ERBB2, 1 [Source:NCBI gene (formerly Entrezgene);Acc:100144440]
chr11	129270	131178	1909	*	chr11	129270	131178	in_vitro	in_vivo	9	9	19.6277778	8.71666667	10.9111111	c(t = 0.97913	0.348	0.84333076	Distal Intergenic	ENSSSCG000	CRYL1	crystallin lambda 1 [Source:VGNC Symbol;Acc:VGNC:87023]
chr7	96823012	96824334	1323	*	chr7	96823012	96824334	in_vitro	in_vivo	13	15	5.84923077	3.44666667	2.4025641	c(t = 0.96339	0.348	0.84333076	Distal Intergenic	ENSSSCG000	PSEN1	presenilin 1 [Source:VGNC Symbol;Acc:VGNC:91897]
chr1	143416803	143418360	1558	*	chr1	143416803	143418360	in_vitro	in_vivo	12	11	0.8225	2.77818182	-1.9556818	c(t = -0.9816	0.348	0.84333076	Distal Intergenic	ENSSSCG000	KLF13	KLF transcription factor 13 [Source:NCBI gene (formerly Entrezgene);Acc:494563]
chr2	76913698	76914662	965	*	chr2	76913698	76914662	in_vitro	in_vivo	9	10	8.53444444	1.222	7.31244444	c(t = 0.99474	0.348	0.84333076	Distal Intergenic	ENSSSCG000	APC2	APC regulator of WNT signaling pathway 2 [Source:NCBI gene (formerly Entrezgene);Acc:100579172]
chr9	68252808	68254139	1332	*	chr9	68252808	68254139	in_vitro	in_vivo	14	14	0.55571429	1.06571429	-0.51	c(t = -0.9606	0.348	0.84333076	Distal Intergenic	ENSSSCG000	SRI	sorcin [Source:VGNC Symbol;Acc:VGNC:93457]
chr1	272370861	272373401	2541	*	chr1	272370861	272373401	in_vitro	in_vivo	12	9	2.07666667	0.95333333	1.12333333	c(t = 0.96579	0.348	0.84333076	Distal Intergenic	ENSSSCG000	MED27	mediator complex subunit 27 [Source:VGNC Symbol;Acc:VGNC:90117]
chr2	10142111	10142786	676	*	chr2	10142111	10142786	in_vitro	in_vivo	9	11	2.41888889	0.88727273	1.53161616	c(t = 0.97840	0.348	0.84333076	Distal Intergenic	ENSSSCG000	PGA5	pepsinogen 5, group I (pepsinogen A) [Source:NCBI gene (formerly Entrezgene);Acc:396892]
chr2	148726222	148727425	1204	*	chr2	148726222	148727425	in_vitro	in_vivo	11	11	1.34727273	0.61090909	0.73636364	c(t = 0.96411	0.348	0.84333076	Distal Intergenic	ENSSSCG000	PPP2R2B	protein phosphatase 2 regulatory subunit Bbeta [Source:VGNC Symbol;Acc:VGNC:100336]
chr6	91031458	91032433	976	*	chr6	91031458	91032433	in_vitro	in_vivo	11	12	6.75818182	2.3025	4.45568182	c(t = 0.97909	0.349	0.84333076	Distal Intergenic	ENSSSCG000	GJA4	gap junction protein alpha 4 [Source:NCBI gene (formerly Entrezgene);Acc:100620145]

chr8	55335999	55337356	1358	*	chr8	55335999	55337356	in_vitro	in_vivo	10	14	1.362	0.85214286	0.50985714	c(t = 0.96196	0.349	0.84333076	Distal Intergenic	ENSSSCG00000008898		HOP homeobox [Source:VGNC Symbol;Acc:VGNC:88932]
chr15	44886083	44887199	1117	*	chr15	44886083	44887199	in_vitro	in_vivo	13	13	1.31	0.68461538	0.62538462	c(t = 0.95752	0.349	0.84333076	Distal Intergenic	ENSSSCG000	CLDN22	claudin 22 [Source:NCBI gene (formerly Entrezgene);Acc:100294683]
chr6	101756543	101758489	1947	*	chr6	101756543	101758489	in_vitro	in_vivo	11	14	1.38909091	0.67	0.71909091	c(t = 0.97400	0.349	0.84333076	Distal Intergenic	ENSSSCG000	TGIF1	TGFB induced factor homeobox 1 [Source:VGNC Symbol;Acc:VGNC:93935]
chr6	1063980	1066754	2775	*	chr6	1063980	1066754	in_vitro	in_vivo	9	11	95.2877778	97.0054545	-1.7176768	c(t = -0.96658	0.35	0.84333076	Distal Intergenic	ENSSSCG000	CYBA	cytochrome b-245 alpha chain [Source:VGNC Symbol;Acc:VGNC:87124]
chr8	1926840	1933538	6699	*	chr8	1926840	1933538	in_vitro	in_vivo	10	14	95.821	93.3642857	2.45671429	c(t = 0.95445	0.35	0.84333076	3' UTR	ENSSSCG000	HTT	huntingtin [Source:VGNC Symbol;Acc:VGNC:89006]
chr12	1006990	1013460	6471	*	chr12	1006990	1013460	in_vitro	in_vivo	9	12	95.4888889	91.2758333	4.21305556	c(t = 0.96805	0.35	0.84333076	Distal Intergenic	ENSSSCG000	CBR2	carbonyl reductase 2 [Source:NCBI gene (formerly Entrezgene);Acc:396780]
chr8	74664147	74666704	2558	*	chr8	74664147	74666704	in_vitro	in_vivo	10	16	2.079	5.688125	-3.609125	c(t = -0.96328	0.35	0.84333076	Distal Intergenic	ENSSSCG000	PLRG1	pleiotropic regulator 1 [Source:NCBI gene (formerly Entrezgene);Acc:100514171]
chrX	85000219	85001227	1009	*	chrX	85000219	85001227	in_vitro	in_vivo	10	9	1.141	3.96	-2.819	c(t = -0.98659	0.35	0.84333076	Distal Intergenic	ENSSSCG000	PLP1	proteolipid protein 1 [Source:NCBI gene (formerly Entrezgene);Acc:397029]
chr6	45087857	45089567	1711	*	chr6	45087857	45089567	in_vitro	in_vivo	12	13	6.185	3.15769231	3.02730769	c(t = 0.95723	0.35	0.84333076	Distal Intergenic	ENSSSCG000	COX6B1	COX6B protein [Source:NCBI gene (formerly Entrezgene);Acc:100038022]
chr14	4013242	4013863	622	*	chr14	4013242	4013863	in_vitro	in_vivo	11	10	6.07181818	2.751	3.32081818	c(t = 0.96332	0.35	0.84333076	Distal Intergenic	ENSSSCG000	LPL	lipoprotein lipase [Source:VGNC Symbol;Acc:VGNC:89795]
chr14	118629070	118629611	542	*	chr14	118629070	118629611	in_vitro	in_vivo	10	9	3.192	1.33222222	1.85977778	c(t = 0.97015	0.35	0.84333076	Distal Intergenic	ENSSSCG000	SMNDC1	survival motor neuron domain containing 1 [Source:VGNC Symbol;Acc:VGNC:93257]
chr9	39920180	39920924	745	*	chr9	39920180	39920924	in_vitro	in_vivo	10	12	2.678	1.1975	1.4805	c(t = 0.96807	0.35	0.84333076	Distal Intergenic	ENSSSCG000	PLET1	placenta expressed transcript 1 [Source:NCBI gene (formerly Entrezgene);Acc:396570]
chr2	113479093	113480973	1881	*	chr2	113479093	113480973	in_vitro	in_vivo	14	15	1.49285714	0.48666667	1.00619048	c(t = 0.96805	0.35	0.84333076	Distal Intergenic	ENSSSCG000	STARD4	StAR related lipid transfer domain containing 4 [Source:VGNC Symbol;Acc:VGNC:93534]
chr6	17047199	17047921	723	*	chr6	17047199	17047921	in_vitro	in_vivo	14	9	0.885	0.35888889	0.52611111	c(t = 0.95964	0.35	0.84333076	Distal Intergenic	NA	NA	NA
chr15	30573168	30573535	368	*	chr15	30573168	30573535	in_vitro	in_vivo	9	10	1.84	0.26	1.58	c(t = 0.98902	0.35	0.84333076	Intron (NM_001315712/100520334, intron 1 of 13)	ENSSSCG000	GLI2	GLI family zinc finger 2 [Source:VGNC Symbol;Acc:VGNC:96324]
chr5	465511	468521	3011	*	chr5	465511	468521	in_vitro	in_vivo	14	10	90.6692857	94.343	-3.6737143	c(t = -0.95639	0.351	0.84333076	3' UTR	ENSSSCG000	SELENOO	selenoprotein O [Source:NCBI gene (formerly Entrezgene);Acc:100518971]
chr17	61426006	61428733	2728	*	chr17	61426006	61428733	in_vitro	in_vivo	10	11	76.332	86.5054545	-10.173455	c(t = -0.95765	0.351	0.84333076	Distal Intergenic	ENSSSCG000	PSMA7	proteasome 20S subunit alpha 7 [Source:NCBI gene (formerly Entrezgene);Acc:100626738]
chr7	1350734	1353234	2501	*	chr7	1350734	1353234	in_vitro	in_vivo	12	12	17.9433333	30.5758333	-12.6325	c(t = -0.95469	0.351	0.84333076	Distal Intergenic	ENSSSCG000	SERPINB9	serpin peptidase inhibitor, clade B (ovalbumin), member 9 [Source:NCBI gene (formerly Entrezgene);Acc:100126851]
chrX	122592332	122594426	2095	*	chrX	122592332	122594426	in_vitro	in_vivo	10	16	8.686	14.194375	-5.508375	c(t = -0.95548	0.351	0.84333076	Distal Intergenic	ENSSSCG000	FATE1	fetal and adult testis expressed 1 [Source:NCBI gene (formerly Entrezgene);Acc:396705]
chr15	101600509	101602423	1915	*	chr15	101600509	101602423	in_vitro	in_vivo	13	15	14.1438462	6.71066667	7.43317949	c(t = 0.95631	0.351	0.84333076	Distal Intergenic	ENSSSCG000	MOBKL3	MOB family member 4, phocein [Source:NCBI gene (formerly Entrezgene);Acc:613127]
chr5	6653516	6654369	854	*	chr5	6653516	6654369	in_vitro	in_vivo	12	10	1.29166667	6.153	-4.8613333	c(t = -0.98243	0.351	0.84333076	Distal Intergenic	ENSSSCG000	CENPM	centromere protein M [Source:VGNC Symbol;Acc:VGNC:86553]
chr7	55322206	55323605	1400	*	chr7	55322206	55323605	in_vitro	in_vivo	13	12	3.91846154	2.04083333	1.87762821	c(t = 0.95799	0.351	0.84333076	Distal Intergenic	ENSSSCG000	ANPEP	alanyl aminopeptidase, membrane [Source:VGNC Symbol;Acc:VGNC:85362]
chr11	12345072	12346297	1226	*	chr11	12345072	12346297	in_vitro	in_vivo	12	14	0.78083333	1.76071429	-0.979881	c(t = -0.95231	0.351	0.84333076	Distal Intergenic	ENSSSCG000	SMAD9	SMAD family member 9 [Source:NCBI gene (formerly Entrezgene);Acc:100153584]
chr13	69441026	69441812	787	*	chr13	69441026	69441812	in_vitro	in_vivo	9	11	2.31	1.14818182	1.16181818	c(t = 0.95653	0.351	0.84333076	Distal Intergenic	ENSSSCG000	TRH	thyrotropin releasing hormone [Source:NCBI gene (formerly Entrezgene);Acc:100513309]
chr8	9225744	9228128	2385	*	chr8	9225744	9228128	in_vitro	in_vivo	10	13	94.6	97.6892308	-3.0892308	c(t = -0.97734	0.352	0.84333076	Distal Intergenic	ENSSSCG000	RAB28	RAB28, member RAS onco family [Source:VGNC Symbol;Acc:VGNC:98955]
chr15	138920656	138922786	2131	*	chr15	138920656	138922786	in_vitro	in_vivo	10	12	12.875	25.755	-12.88	c(t = -0.95239	0.352	0.84333076	Distal Intergenic	ENSSSCG000	ssc-mir-149	ssc-mir-149 [Source:miRBase;Acc:MI0016620]
chr6	54265169	54267874	2706	*	chr6	54265169	54267874	in_vitro	in_vivo	10	12	6.561	9.18	-2.619	c(t = -0.95897	0.352	0.84333076	Promoter (<=1kb)	ENSSSCG000	SAXO3	stabilizer of axonemal microtubules 3 [Source:HGNC Symbol;Acc:HGNC:56771]
chr2	51732894	51733843	950	*	chr2	51732894	51733843	in_vitro	in_vivo	12	12	10.1475	7.03166667	3.11583333	c(t = 0.95070	0.352	0.84333076	Distal Intergenic	NA	NA	NA
chr6	27175664	27177023	1360	*	chr6	27175664	27177023	in_vitro	in_vivo	10	11	1.7	3.93090909	-2.2309091	c(t = -0.96963	0.352	0.84333076	Distal Intergenic	ENSSSCG000	CDH5	cadherin 5 [Source:NCBI gene (formerly Entrezgene);Acc:414737]
chr14	131608503	131609330	828	*	chr14	131608503	131609330	in_vitro	in_vivo	10	10	1.086	2.005	-0.919	c(t = -0.95715	0.352	0.84333076	Distal Intergenic	ENSSSCG000	TACC2	transforming acidic coiled-coil containing protein 2 [Source:NCBI gene (formerly Entrezgene);Acc:100152804]
chr2	69473825	69474563	739	*	chr2	69473825	69474563	in_vitro	in_vivo	9	14	3.09555556	1.17214286	1.9234127	c(t = 0.97405	0.352	0.84333076	Distal Intergenic	ENSSSCG000	ssc-mir-199a	ssc-mir-199a-2 [Source:miRBase;Acc:MI0013093]
chr15	59311788	59313408	1621	*	chr15	59311788	59313408	in_vitro	in_vivo	11	12	2.11909091	1.10583333	1.01325758	c(t = 0.95978	0.352	0.84333076	Distal Intergenic	ENSSSCG000	PRPF40A	pre-mRNA processing factor 40 homolog A [Source:VGNC Symbol;Acc:VGNC:98222]
chr9	92371926	92372657	732	*	chr9	92371926	92372657	in_vitro	in_vivo	10	12	1.73	0.80333333	0.92666667	c(t = 0.95433	0.352	0.84333076	Distal Intergenic	ENSSSCG000	TRA2A	transformer 2 alpha homolog [Source:VGNC Symbol;Acc:VGNC:94353]
chr1	76466882	76467897	1016	*	chr1	76466882	76467897	in_vitro	in_vivo	12	11	1.95833333	0.63181818	1.32651515	c(t = 0.96337	0.352	0.84333076	Distal Intergenic	ENSSSCG000	DDO	D-aspartate oxidase [Source:NCBI gene (formerly Entrezgene);Acc:100037287]
chr2	13229439	13230302	864	*	chr2	13229439	13230302	in_vitro	in_vivo	10	9	2.526	0.50555556	2.02044444	c(t = 0.97451	0.352	0.84333076	Promoter (<=1kb)	ENSSSCG000	SELENOH	selenoprotein H [Source:NCBI gene (formerly Entrezgene);Acc:100462651]
chr6	64491261	64492148	888	*	chr6	64491261	64492148	in_vitro	in_vivo	10	10	10.468	23.093	-12.625	c(t = -0.96125	0.353	0.84333076	Distal Intergenic	ENSSSCG000	PRXL2B	peroxiredoxin like 2B [Source:VGNC Symbol;Acc:VGNC:98543]
chr7	55343165	55343984	820	*	chr7	55343165	55343984	in_vitro	in_vivo	15	9	4.69533333	8.27333333	-3.578	c(t = -0.97511	0.353	0.84333076	Distal Intergenic	ENSSSCG000	ANPEP	alanyl aminopeptidase, membrane [Source:VGNC Symbol;Acc:VGNC:85362]
chr13	195232889	195235351	2463	*	chr13	195232889	195235351	in_vitro	in_vivo	13	15	6.30307692	3.35066667	2.95241026	c(t = 0.95725	0.353	0.84333076	Distal Intergenic	ENSSSCG000	SOD1	superoxide dismutase 1 [Source:NCBI gene (formerly Entrezgene);Acc:397036]
chr3	101206350	101206748	399	*	chr3	101206350	101206748	in_vitro	in_vivo	11	13	0.96363636	2.49692308	-1.5332867	c(t = -0.95279	0.353	0.84333076	Distal Intergenic	ENSSSCG000	GALM	galactose mutarotase [Source:NCBI gene (formerly Entrezgene);Acc:399536]
chr7	48105617	48106169	553	*	chr7	48105617	48106169	in_vitro	in_vivo	9	9	0.92777778	2.17222222	-1.2444444	c(t = -0.96523	0.353	0.84333076	Promoter (<=1kb)	ENSSSCG000	CTSH	cathepsin H [Source:NCBI gene (formerly Entrezgene);Acc:396969]
chr5	593573	594954	1382	*	chr5	593573	594954	in_vitro	in_vivo	11	11	3.63454545	2.07818182	1.55636364	c(t = 0.96109	0.353	0.84333076	Distal Intergenic	ENSSSCG000	SELENOO	selenoprotein O [Source:NCBI gene (formerly Entrezgene);Acc:100518971]
chr3	4716475	4717662	1188	*	chr3	4716475	4717662	in_vitro	in_vivo	12	14	0.84833333	1.60642857	-0.7580952	c(t = -0.94874	0.353	0.84333076	Distal Intergenic	ENSSSCG000	RAC1	Rac family small GTPase 1 [Source:VGNC Symbol;Acc:VGNC:92047]
chr1	18289279	18290726	1448	*	chr1	18289279	18290726	in_vitro	in_vivo	11	14	1.96	1.135	0.825	c(t = 0.95591	0.353	0.84333076	Distal Intergenic	ENSSSCG000	RAB32	RAB32, member RAS oncogene family [Source:NCBI gene (formerly Entrezgene);Acc:100144496]
chr6	17233736	17234273	538	*	chr6	17233736	17234273	in_vitro	in_vivo	9	11	2.50444444	0.97454545	1.52989899	c(t = 0.96516	0.353	0.84333076	Distal Intergenic	ENSSSCG000	NQO1	NAD(P)H quinone dehydrogenase 1 [Source:NCBI gene (formerly Entrezgene);Acc:100286873]
chr4	110841728	110843187	1460	*	chr4	110841728	110843187	in_vitro	in_vivo	10	11	48.357	30.9427273	17.4142727	c(t = 0.95096	0.354	0.84333076	Distal Intergenic	ENSSSCG000	TAF13	TATA-box binding protein associated factor 13 [Source:NCBI gene (formerly Entrezgene);Acc:100157095]
chr11	78534516	78538856	4341	*	chr11	78534516	78538856	in_vitro	in_vivo	11	13	17.4254545	25.8823077	-8.4568531	c(t = -0.94609	0.354	0.84333076	3' UTR	ENSSSCG000	F10	coagulation factor X [Source:NCBI gene (formerly Entrezgene);Acc:733662]
chr18	1256192	1257085	894	*	chr18	1256192	1257085	in_vitro	in_vivo	9	10	20.55	8.829	11.721	c(t = 0.95686	0.354	0.84333076	Distal Intergenic	ENSSSCG000	ssc-mir-153	ssc-mir-153 [Source:miRBase;Acc:MI0002454]
chr4	109907653	109909896	2244	*	chr4	109907653	109909896	in_vitro	in_vivo	10	11	2.927	1.53818182	1.38881818	c(t = 0.96831	0.354	0.84333076	Distal Intergenic	ENSSSCG000	PROK1	prokineticin 1 [Source:NCBI gene (formerly Entrezgene);Acc:100359362]
chr11	5310593	5312103	1511	*	chr11	5310593	5312103	in_vitro	in_vivo	11	15	2.15909091	1.09133333	1.06775758	c(t = 0.96233	0.354	0.84333076	Distal Intergenic	ENSSSCG000	PDX1	pancreatic and duodenal homeobox 1 [Source:NCBI gene (formerly Entrezgene);Acc:397288]
chr13	28729867	28730624	758	*	chr13	28729867	28730624	in_vitro	in_vivo	12	11	1.97416667	0.85545455	1.11871212	c(t = 0.94952	0.354	0.84333076	Distal Intergenic	ENSSSCG000	CCR9	C-C motif chemokine receptor 9 [Source:NCBI gene (formerly Entrezgene);Acc:414377]
chr2	59499309	59499726	418	*	chr2	59499309	59499726	in_vitro	in_vivo	9	9	1.47888889	0.52888889	0.95	c(t = 0.95997	0.354	0.84333076	Distal Intergenic	ENSSSCG000	GDF15	growth differentiation factor 15 [Source:NCBI gene (formerly Entrezgene);Acc:100379630]
chr5	748515	750493	1979	*	chr5	748515	750493	in_vitro	in_vivo	10	12	95.702	89.8008333	5.90116667	c(t = 0.95669	0.355	0.84333076	Distal Intergenic	ENSSSCG000	SELENOO	selenoprotein O [Source:NCBI gene (formerly Entrezgene);Acc:100518971]
chr2	60396052	60396781	730	*	chr2	60396052	60396781	in_vitro	in_vivo	9	13	8.83	2.69461538	6.13538462	c(t = 0.97819	0.355	0.84333076	Distal Intergenic	ENSSSCG000	BST2	bone marrow stromal cell antigen 2 [Source:NCBI gene (formerly Entrezgene);Acc:100302088]
chr14	5836469	5838940	2472	*	chr14	5836469	5838940	in_vitro	in_vivo	9	13	3.80777778	2.36846154	1.43931624	c(t = 0.95355	0.355	0.84333076	Distal Intergenic	ENSSSCG000	DOK2	docking protein 2 [Source:NCBI gene (formerly Entrezgene);Acc:100152025]
chr1	63973255	63974337	1083	*	chr1	63973255	63974337	in_vitro	in_vivo	11	9	6.06272727	2.35777778	3.70494949	c(t = 0.96150	0.355	0.84333076	Distal Intergenic	ENSSSCG000	FHL5	four and a half LIM domains 5 [Source:VGNC Symbol;Acc:VGNC:88130]
chr1	126570422	126571333	912	*	chr1	126570422	126571333	in_vitro	in_vivo	10	9	3.974	1.93222222	2.04177778	c(t = 0.95851	0.355	0.84333076	Distal Intergenic	ENSSSCG000	DUOX1	dual oxidase 1 [Source:NCBI gene (formerly Entrezgene);Acc:397177]
chr18	55933960	55935054	1095	*	chr18	55933960	55935054	in_vitro	in_vivo	10	12	0.765	1.69833333	-0.9333333	c(t = -0.95544	0.355	0.84333076	Distal Intergenic	ENSSSCG000	RARRES2	retinoic acid receptor responder 2 [Source:VGNC Symbol;Acc:VGNC:92098]
chr13	72622377	72623144	768	*	chr13	72622377	72623144	in_vitro	in_vivo	10	10	2.14	1.316	0.824	c(t = 0.95088	0.355	0.84333076	Distal Intergenic	ENSSSCG000	MGLL	monoglyceride lipase [Source:VGNC Symbol;Acc:VGNC:90199]
chr5	97092968	97094522	1555	*	chr5	97092968	97094522	in_vitro	in_vivo	11	10	2.05363636	1.008	1.04563636	c(t = 0.94818	0.355	0.84333076	Distal Intergenic	ENSSSCG000	MGAT4C	MGAT4 family member C [Source:NCBI gene (formerly Entrezgene);Acc:414851]
chr1	273474996	273476851	1856	*	chr1	273474996	273476851	in_vitro	in_vivo	12	14	2.14666667	1.00214286	1.14452381	c(t = 0.95947	0.355	0.84333076	Distal Intergenic	ENSSSCG000	ssc-mir-9812	ssc-mir-9812 [Source:miRBase;Acc:MI0031579]
chr8	125258948	125260092	1145	*	chr8	125258948	125260092	in_vitro	in_vivo	9	12	3.06	0.90583333	2.15416667	c(t = 0.97912	0.355	0.84333076	Distal Intergenic	ENSSSCG000	BMPR1B	bone morphotic protein receptor type 1B [Source:VGNC Symbol;Acc:VGNC:85847]
chr14	24341556	24342032	477	*	chr14	24341556	24342032	in_vitro	in_vivo	10	10	1.772	0.711	1.061	c(t = 0.95817	0.355	0.84333076	Distal Intergenic	ENSSSCG000	PIWIL1	piwi like RNA-mediated silencing 1 [Source:VGNC Symbol;Acc:VGNC:98197]
chrX	45802192	45802989	798	*	chrX	45802192	45802989	in_vitro	in_vivo	14	13	1.25571429	0.60384615	0.65186813	c(t = 0.94334	0.355	0.84333076	Distal Intergenic	ENSSSCG00000012322		lysine demethylase 5C [Source:NCBI gene (formerly Entrezgene);Acc:100037295]
chr6	134776157	134776932	776	*	chr6	134776157	134776932	in_vitro	in_vivo	12	11	0.88	0.32454545	0.55545455	c(t = 0.94818	0.355	0.84333076	Promoter (1-2kb)	ENSSSCG000	PTGFR	prostaglandin F receptor [Source:VGNC Symbol;Acc:VGNC:91954]
chr5	65440602	65442107	1506	*	chr5	65440602	65442107	in_vitro	in_vivo	10	10	5.772	14.114	-8.342	c(t = -0.96410	0.356	0.84388032	Promoter (<=1kb)	ENSSSCG000	KCNA5	potassium voltage-gated channel subfamily A member 5 [Source:NCBI gene (formerly Entrezgene);Acc:474166]
chr16	49090293	49091230	938	*	chr16	49090293	49091230	in_vitro	in_vivo	11	13	3.94545455	5.63923077	-1.6937762	c(t = -0.94437	0.356	0.84388032	Distal Intergenic	ENSSSCG000	DRD1	dopamine receptor D1 [Source:VGNC Symbol;Acc:VGNC:87443]
chr12	6047251	6047873	623	*	chr12	6047251	6047873	in_vitro	in_vivo	10	10	1.802	3.043	-1.241	c(t = -0.95112	0.356	0.84388032	Distal Intergenic	ENSSSCG000	SLC25A19	solute carrier family 25 member 19 [Source:VGNC Symbol;Acc:VGNC:92997]
chr14	101473488	101474161	674	*	chr14	101473488	101474161	in_vitro	in_vivo	10	14	5.231	2.32928571	2.90171429	c(t = 0.96388	0.356	0.84388032	Distal Intergenic	ENSSSCG000	ssc-mir-107	ssc-mir-107 [Source:miRBase;Acc:MI0002449]
chr12	50736832	50738032	1201	*	chr12	50736832	50738032	in_vitro	in_vivo	10	12	4.839	2.27416667	2.56483333	c(t = 0.96045	0.356	0.84388032	Distal Intergenic	ENSSSCG000	ZNF232	zinc finger protein 232 [Source:VGNC Symbol;Acc:VGNC:95208]
chr9	55620340	55622696	2357	*	chr9	55620340	55622696	in_vitro	in_vivo	14	16	1.73142857	0.994375	0.73705357	c(t = 0.93952	0.356	0.84388032	Distal Intergenic	ENSSSCG000	ETS1	ETS proto-onco 1, transcription factor [Source:VGNC Symbol;Acc:VGNC:87808]
chr3	59776579	59777730	1152	*	chr3	59776579	59777730	in_vitro	in_vivo	10	13	1.394	0.72076923	0.67323077	c(t = 0.94495	0.356	0.84388032	Promoter (<=1kb)	ENSSSCG000	TMSB10	thymosin beta 10 [Source:NCBI gene (formerly Entrezgene);Acc:100037998]
chr15	128305912	128308735	2824	*	chr15	128305912	128308735	in_vitro	in_vivo	11	13	21.9745455	16.6976923	5.27685315	c(t = 0.94079	0.357	0.84416769	Promoter (<=1kb)	ENSSSCG000	IRS1	insulin receptor substrate 1 [Source:VGNC Symbol;Acc:VGNC:96376]
chr9	129315436	129316506	1071	*	chr9	129315436	129316506	in_vitro	in_vivo	9	11	0.84777778	3.77636364	-2.9285859	c(t = -0.96190	0.357	0.84416769	Promoter (<=1kb)	ENSSSCG000	SMYD2	SET and MYND domain containing 2 [Source:VGNC Symbol;Acc:VGNC:93273]
chr5	99085090	99086357	1268	*	chr5	99085090	99086357	in_vitro	in_vivo	9	10	0.89555556	1.949	-1.0534444	c(t = -0.94947	0.357	0.84416769	Distal Intergenic	ENSSSCG000	CCDC59	coiled-coil domain containing 59 [Source:NCBI gene (formerly Entrezgene);Acc:100511567]
chr12	54105436	54108348	2913	*	chr12	54105436	54108348	in_vitro	in_vivo	16	12	2.26	1.61	0.65	c(t = 0.93939	0.357	0.84416769	5' UTR	ENSSSCG000	NTN1	netrin 1 [Source:VGNC Symbol;Acc:VGNC:90931]
chr13	65758915	65760402	1488	*	chr13	65758915	65760402	in_vitro	in_vivo	10	10	0.76	1.567	-0.807	c(t = -0.95264	0.357	0.84416769	Distal Intergenic	ENSSSCG000	CAMK1	calcium/calmodulin dependent protein kinase I [Source:VGNC Symbol;Acc:VGNC:86153]
chr3	76965704	76967022	1319	*	chr3	76965704	76967022	in_vitro	in_vivo	12	15	0.69583333	1.56533333	-0.8695	c(t = -0.94580	0.357	0.84416769	Distal Intergenic	ENSSSCG000	RAB1A	RAB1A, member RAS oncogene family [Source:NCBI gene (formerly Entrezgene);Acc:595116]
chr10	24158165	24159158	994	*	chr10	24158165	24159158	in_vitro	in_vivo	11	12	2.05818182	0.8425	1.21568182	c(t = 0.94936	0.357	0.84416769	Distal Intergenic	ENSSSCG000	TNNI1	troponin I1, slow skeletal type [Source:VGNC Symbol;Acc:VGNC:95548]
chr8	102213257	102213942	686	*	chr8	102213257	102213942	in_vitro	in_vivo	9	10	2.21222222	0.692	1.52022222	c(t = 0.97146	0.357	0.84416769	Distal Intergenic	ENSSSCG000	CCNA2	cyclin A2 [Source:NCBI gene (formerly Entrezgene);Acc:100415929]
chr12	1287861	1295777	7917	*	chr12	1287861	1295777	in_vitro	in_vivo	9	13	60.3811111	70.8561538	-10.475043	c(t = -0.95138	0.358	0.84445365	Distal Intergenic	ENSSSCG000	ARHGDIA	Rho GDP dissociation inhibitor alpha [Source:VGNC Symbol;Acc:VGNC:85483]
chr16	79881741	79882955	1215	*	chr16	79881741	79882955	in_vitro	in_vivo	12	11	15.3833333	26.6518182	-11.268485	c(t = -0.94035	0.358	0.84445365	Distal Intergenic	ENSSSCG000	TPPP	tubulin polymerization promoting protein [Source:VGNC Symbol;Acc:VGNC:94344]
chr1	270531147	270532477	1331	*	chr1	270531147	270532477	in_vitro	in_vivo	10	14	1.185	2.82928571	-1.6442857	c(t = -0.94535	0.358	0.84445365	Distal Intergenic	ENSSSCG000	FUBP3	far upstream element binding protein 3 [Source:VGNC Symbol;Acc:VGNC:103093]
chr2	78935084	78935819	736	*	chr2	78935084	78935819	in_vitro	in_vivo	11	13	4.28636364	2.74076923	1.54559441	c(t = 0.94247	0.358	0.84445365	Distal Intergenic	ENSSSCG000	CANX	calnexin [Source:VGNC Symbol;Acc:VGNC:86167]
chr14	2056512	2057196	685	*	chr14	2056512	2057196	in_vitro	in_vivo	10	10	1.294	2.67	-1.376	c(t = -0.94533	0.358	0.84445365	Distal Intergenic	ENSSSCG000	SYK	spleen associated tyrosine kinase [Source:NCBI gene (formerly Entrezgene);Acc:100125540]
chr15	83872448	83873390	943	*	chr15	83872448	83873390	in_vitro	in_vivo	15	14	1.56	0.75714286	0.80285714	c(t = 0.94350	0.358	0.84445365	Distal Intergenic	ENSSSCG000	PLEKHA3	pleckstrin homology domain containing A3 [Source:NCBI gene (formerly Entrezgene);Acc:100154010]
chr7	100180326	100181083	758	*	chr7	100180326	100181083	in_vitro	in_vivo	9	10	1.03333333	0.335	0.69833333	c(t = 0.96496	0.358	0.84445365	Distal Intergenic	ENSSSCG000	ssc-mir-9860	ssc-mir-9860 [Source:miRBase;Acc:MI0031638]
chr6	105807695	105809152	1458	*	chr6	105807695	105809152	in_vitro	in_vivo	10	10	0.592	0.26	0.332	c(t = 0.94946	0.358	0.84445365	Distal Intergenic	ENSSSCG000	TYMS	thymidylate synthetase [Source:NCBI gene (formerly Entrezgene);Acc:100627209]
chr12	51822475	51823097	623	*	chr12	51822475	51823097	in_vitro	in_vivo	9	10	28.0677778	14.847	13.2207778	c(t = 0.94410	0.359	0.84473821	Distal Intergenic	ENSSSCG000	ZNF232	zinc finger protein 232 [Source:VGNC Symbol;Acc:VGNC:95208]
chrX	15866692	15867342	651	*	chrX	15866692	15867342	in_vitro	in_vivo	13	10	3.72	7.972	-4.252	c(t = -0.94890	0.359	0.84473821	Distal Intergenic	ENSSSCG000	EIF1AX	eukaryotic translation initiation factor 1A X-linked [Source:NCBI gene (formerly Entrezgene);Acc:100522912]
chr5	27125895	27126407	513	*	chr5	27125895	27126407	in_vitro	in_vivo	12	10	0.86	2.032	-1.172	c(t = -0.94250	0.359	0.84473821	Distal Intergenic	NA	NA	NA
chr13	17533436	17535302	1867	*	chr13	17533436	17535302	in_vitro	in_vivo	13	14	1.02692308	1.68214286	-0.6552198	c(t = -0.93621	0.359	0.84473821	Distal Intergenic	ENSSSCG000	CMTM7	CKLF like MARVEL transmembrane domain containing 7 [Source:VGNC Symbol;Acc:VGNC:103928]
chr16	5713850	5715073	1224	*	chr16	5713850	5715073	in_vitro	in_vivo	14	15	0.92714286	1.46666667	-0.5395238	c(t = -0.93408	0.359	0.84473821	Distal Intergenic	ENSSSCG000	C1QTNF3	C1q and TNF related 3 [Source:NCBI gene (formerly Entrezgene);Acc:100270814]
chr7	23046988	23048084	1097	*	chr7	23046988	23048084	in_vitro	in_vivo	13	12	0.70538462	1.36583333	-0.6604487	c(t = -0.93668	0.359	0.84473821	Distal Intergenic	ENSSSCG000	TRIM39	tripartite motif containing 39 [Source:NCBI gene (formerly Entrezgene);Acc:100151742]
chr1	37193281	37194311	1031	*	chr1	37193281	37194311	in_vitro	in_vivo	11	10	1.53454545	0.942	0.59254545	c(t = 0.94090	0.359	0.84473821	Distal Intergenic	ENSSSCG000	HEY2	hes related family bHLH transcription factor with YRPW motif 2 [Source:VGNC Symbol;Acc:VGNC:88864]
chr2	61104912	61105587	676	*	chr2	61104912	61105587	in_vitro	in_vivo	9	14	1.02222222	0.33285714	0.68936508	c(t = 0.96238	0.359	0.84473821	Distal Intergenic	ENSSSCG000	SMIM7	small integral membrane protein 7 [Source:NCBI gene (formerly Entrezgene);Acc:100512765]
chr13	138363751	138364495	745	*	chr13	138363751	138364495	in_vitro	in_vivo	11	10	2.54181818	1.188	1.35381818	c(t = 0.94510	0.36	0.84605505	Distal Intergenic	ENSSSCG000	CASR	calcium sensing receptor [Source:VGNC Symbol;Acc:VGNC:108635]
chr7	57186638	57187460	823	*	chr7	57186638	57187460	in_vitro	in_vivo	10	13	1.767	0.70538462	1.06161538	c(t = 0.94973	0.36	0.84605505	Promoter (<=1kb)	ENSSSCG000	TSPAN3	tetraspanin 3 [Source:NCBI gene (formerly Entrezgene);Acc:100157865]
chr17	39092651	39093181	531	*	chr17	39092651	39093181	in_vitro	in_vivo	11	10	1.56545455	0.651	0.91445455	c(t = 0.94776	0.36	0.84605505	Distal Intergenic	ENSSSCG000	ROMO1	reactive oxygen species modulator 1 [Source:NCBI gene (formerly Entrezgene);Acc:100154394]
chr17	26480500	26481350	851	*	chr17	26480500	26481350	in_vitro	in_vivo	11	12	1.84272727	0.425	1.41772727	c(t = 0.95819	0.36	0.84605505	Distal Intergenic	ENSSSCG000	SEC23B	SEC23 homolog B, COPII coat complex component [Source:VGNC Symbol;Acc:VGNC:95490]
chrX	111352942	111353571	630	*	chrX	111352942	111353571	in_vitro	in_vivo	10	10	97.112	90.181	6.931	c(t = 0.95298	0.361	0.84625829	Distal Intergenic	ENSSSCG000	FHL1	four and a half LIM domains 1 [Source:VGNC Symbol;Acc:VGNC:88127]
chr6	47298652	47300548	1897	*	chr6	47298652	47300548	in_vitro	in_vivo	11	11	14.1654545	22.7190909	-8.5536364	c(t = -0.93607	0.361	0.84625829	Distal Intergenic	ENSSSCG000	RYR1	ryanodine receptor 1 [Source:NCBI gene (formerly Entrezgene);Acc:396718]
chr3	16518368	16519471	1104	*	chr3	16518368	16519471	in_vitro	in_vivo	9	13	1.66111111	0.72384615	0.93726496	c(t = 0.95617	0.361	0.84625829	Distal Intergenic	ENSSSCG000	TPST1	tyrosylprotein sulfotransferase 1 [Source:VGNC Symbol;Acc:VGNC:94352]
chr1	205923840	205925753	1914	*	chr1	205923840	205925753	in_vitro	in_vivo	13	12	1.68076923	0.69416667	0.98660256	c(t = 0.93139	0.361	0.84625829	Distal Intergenic	ENSSSCG000	PSIP1	PC4 and SFRS1 interacting protein 1 [Source:VGNC Symbol;Acc:VGNC:91898]
chr15	104525526	104526656	1131	*	chr15	104525526	104526656	in_vitro	in_vivo	13	13	1.59384615	0.52615385	1.06769231	c(t = 0.94504	0.361	0.84625829	Promoter (<=1kb)	ENSSSCG000	BZW1	basic leucine zipper and W2 domains 1 [Source:VGNC Symbol;Acc:VGNC:95885]
chr14	46016083	46016765	683	*	chr14	46016083	46016765	in_vitro	in_vivo	9	11	1.36111111	0.45454545	0.90656566	c(t = 0.94829	0.361	0.84625829	Promoter (<=1kb)	ENSSSCG000	XBP1	X-box binding protein 1 [Source:NCBI gene (formerly Entrezgene);Acc:100217386]
chr12	5111257	5113113	1857	*	chr12	5111257	5113113	in_vitro	in_vivo	11	9	94.6545455	89.6055556	5.0489899	c(t = 0.95417	0.362	0.84625829	Distal Intergenic	ENSSSCG00000036592		serine and arginine rich splicing factor 2 [Source:VGNC Symbol;Acc:VGNC:99085]
chr11	59864039	59864576	538	*	chr11	59864039	59864576	in_vitro	in_vivo	10	15	11.417	24.7353333	-13.318333	c(t = -0.93138	0.362	0.84625829	Distal Intergenic	ENSSSCG000	ssc-mir-17	ssc-mir-17 [Source:miRBase;Acc:MI0008214]
chr18	19875181	19876063	883	*	chr18	19875181	19876063	in_vitro	in_vivo	11	12	1.34636364	2.65083333	-1.3044697	c(t = -0.93761	0.362	0.84625829	Distal Intergenic	ENSSSCG000	OPN1SW	opsin 1, short wave sensitive [Source:NCBI gene (formerly Entrezgene);Acc:397164]
chr17	55129294	55130825	1532	*	chr17	55129294	55130825	in_vitro	in_vivo	13	13	3.74153846	1.90923077	1.83230769	c(t = 0.93296	0.362	0.84625829	Promoter (<=1kb)	ENSSSCG000	CYP24A1	cytochrome P450 family 24 subfamily A member 1 [Source:NCBI gene (formerly Entrezgene);Acc:397145]
chr1	252453043	252453703	661	*	chr1	252453043	252453703	in_vitro	in_vivo	10	14	2.524	1.49785714	1.02614286	c(t = 0.93492	0.362	0.84625829	Distal Intergenic	ENSSSCG000	PTGR1	prostaglandin reductase 1 [Source:VGNC Symbol;Acc:VGNC:91957]
chr3	42860905	42862141	1237	*	chr3	42860905	42862141	in_vitro	in_vivo	12	13	1.69166667	0.95	0.74166667	c(t = 0.93179	0.362	0.84625829	Distal Intergenic	ENSSSCG000	CARD19	caspase recruitment domain family member 19 [Source:VGNC Symbol;Acc:VGNC:86188]
chr5	28828176	28829273	1098	*	chr5	28828176	28829273	in_vitro	in_vivo	11	11	1.30363636	0.80727273	0.49636364	c(t = 0.93292	0.362	0.84625829	Distal Intergenic	ENSSSCG000	TBK1	TANK binding kinase 1 [Source:VGNC Symbol;Acc:VGNC:93787]
chr7	110453586	110454204	619	*	chr7	110453586	110454204	in_vitro	in_vivo	10	9	1.371	0.67777778	0.69322222	c(t = 0.93781	0.362	0.84625829	Distal Intergenic	ENSSSCG000	SPATA7	spermatosis associated 7 [Source:VGNC Symbol;Acc:VGNC:93389]
chr13	151428768	151429764	997	*	chr13	151428768	151429764	in_vitro	in_vivo	12	9	0.99666667	0.54333333	0.45333333	c(t = 0.93509	0.362	0.84625829	Promoter (<=1kb)	ENSSSCG000	CD47	CD47 molecule [Source:VGNC Symbol;Acc:VGNC:108637]
chr14	9517221	9518683	1463	*	chr14	9517221	9518683	in_vitro	in_vivo	10	11	1.677	0.46	1.217	c(t = 0.95354	0.362	0.84625829	Distal Intergenic	ENSSSCG000	GNRH1	gonadotropin releasing hormone 1 [Source:NCBI gene (formerly Entrezgene);Acc:397516]
chr12	35848537	35849273	737	*	chr12	35848537	35849273	in_vitro	in_vivo	10	13	1.389	0.43615385	0.95284615	c(t = 0.95061	0.362	0.84625829	Promoter (<=1kb)	ENSSSCG000	CLTC	clathrin heavy chain [Source:VGNC Symbol;Acc:VGNC:86789]
chr6	59233436	59233978	543	*	chr6	59233436	59233978	in_vitro	in_vivo	10	9	14.256	26.3533333	-12.097333	c(t = -0.95204	0.363	0.84625829	Distal Intergenic	ENSSSCG00000003278		
chr7	97095019	97096610	1592	*	chr7	97095019	97096610	in_vitro	in_vivo	11	14	11.2345455	5.41714286	5.8174026	c(t = 0.93357	0.363	0.84625829	Distal Intergenic	ENSSSCG000	LIN52	lin-52 DREAM MuvB core complex component [Source:NCBI gene (formerly Entrezgene);Acc:100157325]
chr16	28157496	28158158	663	*	chr16	28157496	28158158	in_vitro	in_vivo	9	12	1.92777778	3.30166667	-1.3738889	c(t = -0.93413	0.363	0.84625829	Distal Intergenic	ENSSSCG000	CCL28	C-C motif chemokine ligand 28 [Source:NCBI gene (formerly Entrezgene);Acc:554303]
chr11	49029946	49031917	1972	*	chr11	49029946	49031917	in_vitro	in_vivo	16	14	3.319375	2.305	1.014375	c(t = 0.92557	0.363	0.84625829	Distal Intergenic	ENSSSCG000	EDNRB	endothelin receptor type B [Source:VGNC Symbol;Acc:VGNC:87550]
chr3	49817420	49820219	2800	*	chr3	49817420	49820219	in_vitro	in_vivo	11	13	2.38090909	1.42384615	0.95706294	c(t = 0.93216	0.363	0.84625829	Distal Intergenic	ENSSSCG000	MRPS9	mitochondrial ribosomal protein S9 [Source:VGNC Symbol;Acc:VGNC:90401]
chr15	132209285	132209996	712	*	chr15	132209285	132209996	in_vitro	in_vivo	10	9	1.952	0.94555556	1.00644444	c(t = 0.94089	0.363	0.84625829	Distal Intergenic	ENSSSCG000	NMUR1	neuromedin U receptor 1 [Source:VGNC Symbol;Acc:VGNC:96335]
chr14	141239174	141248124	8951	*	chr14	141239174	141248124	in_vitro	in_vivo	12	13	90.335	95.7876923	-5.4526923	c(t = -0.94258	0.364	0.84625829	Distal Intergenic	ENSSSCG000	ssc-mir-202	ssc-mir-202 [Source:miRBase;Acc:MI0013157]
chr2	143068279	143070061	1783	*	chr2	143068279	143070061	in_vitro	in_vivo	11	13	11.1972727	18.5092308	-7.311958	c(t = -0.92800	0.364	0.84625829	Distal Intergenic	ENSSSCG000	DIAPH1	diaphanous related formin 1 [Source:VGNC Symbol;Acc:VGNC:99632]
chr7	58801699	58802390	692	*	chr7	58801699	58802390	in_vitro	in_vivo	11	11	6.71272727	10.7245455	-4.0118182	c(t = -0.93103	0.364	0.84625829	Promoter (<=1kb)	ENSSSCG000	CYP1A1	cytochrome P450 family 1 subfamily A member 1 [Source:VGNC Symbol;Acc:VGNC:103347]
chr3	71237334	71238306	973	*	chr3	71237334	71238306	in_vitro	in_vivo	9	13	11.1444444	5.15923077	5.98521368	c(t = 0.94440	0.364	0.84625829	Distal Intergenic	ENSSSCG000	CD207	CD207 molecule [Source:NCBI gene (formerly Entrezgene);Acc:100037935]
chr2	73574550	73576064	1515	*	chr2	73574550	73576064	in_vitro	in_vivo	11	14	4.31090909	2.82857143	1.48233766	c(t = 0.92662	0.364	0.84625829	Distal Intergenic	ENSSSCG000	MICOS13	mitochondrial contact site and cristae organizing system subunit 13 [Source:NCBI gene (formerly Entrezgene);Acc:100037978]
chr13	19275602	19276664	1063	*	chr13	19275602	19276664	in_vitro	in_vivo	13	12	1.66384615	2.66666667	-1.0028205	c(t = -0.93549	0.364	0.84625829	Distal Intergenic	ENSSSCG000	CMTM7	CKLF like MARVEL transmembrane domain containing 7 [Source:VGNC Symbol;Acc:VGNC:103928]
chr1	146272620	146275516	2897	*	chr1	146272620	146275516	in_vitro	in_vivo	14	13	2.51214286	1.63	0.88214286	c(t = 0.92518	0.364	0.84625829	Distal Intergenic	ENSSSCG000	NFATC1	nuclear factor of activated T cells 1 [Source:NCBI gene (formerly Entrezgene);Acc:397318]
chr2	131015687	131017282	1596	*	chr2	131015687	131017282	in_vitro	in_vivo	13	14	2.22538462	1.375	0.85038462	c(t = 0.93174	0.364	0.84625829	Distal Intergenic	ENSSSCG000	MARCHF3	membrane associated ring-CH-type finger 3 [Source:NCBI gene (formerly Entrezgene);Acc:100513526]
chr8	67706504	67707399	896	*	chr8	67706504	67707399	in_vitro	in_vivo	10	12	0.578	1.27166667	-0.6936667	c(t = -0.93399	0.364	0.84625829	Distal Intergenic	ENSSSCG000	UTP3	UTP3 small subunit processome component [Source:NCBI gene (formerly Entrezgene);Acc:100217385]
chr2	62221484	62222951	1468	*	chr2	62221484	62222951	in_vitro	in_vivo	13	15	1.83923077	0.97666667	0.8625641	c(t = 0.92466	0.364	0.84625829	Distal Intergenic	ENSSSCG000	BRD4	bromodomain containing 4 [Source:VGNC Symbol;Acc:VGNC:85873]
chr3	40756304	40758474	2171	*	chr3	40756304	40758474	in_vitro	in_vivo	9	9	26.5988889	41.8822222	-15.283333	c(t = -0.93490	0.365	0.84625829	Promoter (<=1kb)	NA	NA	NA
chr5	19246053	19247769	1717	*	chr5	19246053	19247769	in_vitro	in_vivo	11	12	6.36363636	14.0683333	-7.704697	c(t = -0.93421	0.365	0.84625829	Promoter (<=1kb)	ENSSSCG000	ssc-mir-615	ssc-mir-615 [Source:miRBase;Acc:MI0017995]
chr7	2308992	2310300	1309	*	chr7	2308992	2310300	in_vitro	in_vivo	11	12	24.7272727	11.7683333	12.9589394	c(t = 0.93112	0.365	0.84625829	Distal Intergenic	ENSSSCG000	ECI2	enoyl-CoA delta isomerase 2 [Source:VGNC Symbol;Acc:VGNC:87532]
chrX	20518326	20519242	917	*	chrX	20518326	20519242	in_vitro	in_vivo	13	9	5.26769231	9.45666667	-4.1889744	c(t = -0.93947	0.365	0.84625829	Distal Intergenic	ENSSSCG000	SAT1	spermidine/spermine N1-acetyltransferase 1 [Source:NCBI gene (formerly Entrezgene);Acc:397645]
chr17	58514058	58515327	1270	*	chr17	58514058	58515327	in_vitro	in_vivo	10	12	2.954	1.56166667	1.39233333	c(t = 0.93867	0.365	0.84625829	Promoter (<=1kb)	ENSSSCG000	RAB22A	RAB22A, member RAS oncogene family [Source:NCBI gene (formerly Entrezgene);Acc:100144535]
chr16	44578590	44579407	818	*	chr16	44578590	44579407	in_vitro	in_vivo	10	10	0.462	1.309	-0.847	c(t = -0.94337	0.365	0.84625829	Distal Intergenic	ENSSSCG000	NLN	neurolysin [Source:VGNC Symbol;Acc:VGNC:90780]
chr14	48210079	48211012	934	*	chr14	48210079	48211012	in_vitro	in_vivo	12	10	1.58	0.827	0.753	c(t = 0.92914	0.365	0.84625829	Distal Intergenic	ENSSSCG000	PISD	phosphatidylserine decarboxylase [Source:VGNC Symbol;Acc:VGNC:91462]
chr2	142360610	142361554	945	*	chr2	142360610	142361554	in_vitro	in_vivo	11	11	1.86	0.77636364	1.08363636	c(t = 0.93481	0.365	0.84625829	Distal Intergenic	ENSSSCG000	CD14	CD14 molecule [Source:NCBI gene (formerly Entrezgene);Acc:100037938]
chr3	10754663	10755905	1243	*	chr3	10754663	10755905	in_vitro	in_vivo	10	14	42.978	58.6042857	-15.626286	c(t = -0.92369	0.366	0.84625829	Distal Intergenic	ENSSSCG000	TRIM50	tripartite motif containing 50 [Source:NCBI gene (formerly Entrezgene);Acc:397383]
chr6	53299078	53300840	1763	*	chr6	53299078	53300840	in_vitro	in_vivo	12	11	34.1025	21.8363636	12.2661364	c(t = 0.92498	0.366	0.84625829	Distal Intergenic	ENSSSCG000	NAPA	NSF attachment protein alpha [Source:VGNC Symbol;Acc:VGNC:90578]
chr15	135592706	135595095	2390	*	chr15	135592706	135595095	in_vitro	in_vivo	9	14	17.6255556	8.93571429	8.68984127	c(t = 0.95165	0.366	0.84625829	Distal Intergenic	ENSSSCG000	ARL4C	ADP ribosylation factor like GTPase 4C [Source:NCBI gene (formerly Entrezgene);Acc:100514255]
chr18	52402677	52404651	1975	*	chr18	52402677	52404651	in_vitro	in_vivo	12	13	2.13666667	4.32538462	-2.1887179	c(t = -0.93394	0.366	0.84625829	Distal Intergenic	ENSSSCG000	INHBA	inhibin subunit beta A [Source:NCBI gene (formerly Entrezgene);Acc:397093]
chr6	105418165	105421189	3025	*	chr6	105418165	105421189	in_vitro	in_vivo	14	12	3.48571429	2.565	0.92071429	c(t = 0.92292	0.366	0.84625829	Promoter (<=1kb)	ENSSSCG000	ADCYAP1	adenylate cyclase activating polypeptide 1 [Source:VGNC Symbol;Acc:VGNC:85114]
chr1	82799678	82800487	810	*	chr1	82799678	82800487	in_vitro	in_vivo	13	12	2.93846154	1.43333333	1.50512821	c(t = 0.92925	0.366	0.84625829	Distal Intergenic	ENSSSCG000	CALHM6	calcium homeostasis modulator family member 6 [Source:VGNC Symbol;Acc:VGNC:86147]
chr4	73047741	73048575	835	*	chr4	73047741	73048575	in_vitro	in_vivo	9	12	2.47555556	1.22333333	1.25222222	c(t = 0.94182	0.366	0.84625829	Distal Intergenic	ENSSSCG000	SDCBP	syndecan binding protein [Source:VGNC Symbol;Acc:VGNC:92657]
chr13	29995480	29996773	1294	*	chr13	29995480	29996773	in_vitro	in_vivo	13	9	1.98769231	1.11333333	0.87435897	c(t = 0.92576	0.366	0.84625829	Distal Intergenic	ENSSSCG000	PTH1R	parathyroid hormone 1 receptor [Source:NCBI gene (formerly Entrezgene);Acc:397675]
chr12	18891635	18893275	1641	*	chr12	18891635	18893275	in_vitro	in_vivo	11	14	7.33	12.5921429	-5.2621429	c(t = -0.92057	0.367	0.84625829	Distal Intergenic	ENSSSCG000	GRN	granulin precursor [Source:NCBI gene (formerly Entrezgene);Acc:733645]
chr16	77146644	77147358	715	*	chr16	77146644	77147358	in_vitro	in_vivo	9	9	4.34222222	11.7377778	-7.3955556	c(t = -0.95128	0.367	0.84625829	Distal Intergenic	ENSSSCG000	TERT	telomerase reverse transcriptase [Source:NCBI gene (formerly Entrezgene);Acc:492280]
chr8	6179570	6181534	1965	*	chr8	6179570	6181534	in_vitro	in_vivo	11	10	6.98363636	10.39	-3.4063636	c(t = -0.92794	0.367	0.84625829	Distal Intergenic	ENSSSCG000	MSX1	msh homeobox 1 [Source:NCBI gene (formerly Entrezgene);Acc:100302364]
chr2	74729195	74730347	1153	*	chr2	74729195	74730347	in_vitro	in_vivo	11	10	1.56909091	3.507	-1.9379091	c(t = -0.93935	0.367	0.84625829	Distal Intergenic	ENSSSCG000	MAP2K2	mitogen-activated protein kinase kinase 2 [Source:NCBI gene (formerly Entrezgene);Acc:100525505]
chr14	50082950	50083924	975	*	chr14	50082950	50083924	in_vitro	in_vivo	10	11	2.438	1.03363636	1.40436364	c(t = 0.93547	0.367	0.84625829	Promoter (<=1kb)	ENSSSCG000	MAPK1	mitogen-activated protein kinase 1 [Source:VGNC Symbol;Acc:VGNC:89996]
chr9	138805882	138807228	1347	*	chr9	138805882	138807228	in_vitro	in_vivo	11	13	23.3009091	12.8569231	10.443986	c(t = 0.92480	0.368	0.84625829	Distal Intergenic	ENSSSCG000	EGFR	epidermal growth factor receptor [Source:NCBI gene (formerly Entrezgene);Acc:397070]
chr18	50684352	50685184	833	*	chr18	50684352	50685184	in_vitro	in_vivo	11	14	8.11545455	5.53928571	2.57616883	c(t = 0.92304	0.368	0.84625829	Distal Intergenic	ENSSSCG000	DDX56	DEAD-box helicase 56 [Source:NCBI gene (formerly Entrezgene);Acc:100513388]
chr14	71989045	71989965	921	*	chr14	71989045	71989965	in_vitro	in_vivo	10	10	1.676	3.889	-2.213	c(t = -0.92775	0.368	0.84625829	Distal Intergenic	ENSSSCG000	TET1	tet methylcytosine dioxygenase 1 [Source:NCBI gene (formerly Entrezgene);Acc:100625859]
chr2	60057798	60059511	1714	*	chr2	60057798	60059511	in_vitro	in_vivo	10	11	1.365	2.48181818	-1.1168182	c(t = -0.92297	0.368	0.84625829	Distal Intergenic	ENSSSCG000	MAP1S	microtubule associated protein 1S [Source:VGNC Symbol;Acc:VGNC:89980]
chr5	90131460	90132556	1097	*	chr5	90131460	90132556	in_vitro	in_vivo	13	10	1.07384615	2.457	-1.3831538	c(t = -0.93662	0.368	0.84625829	Distal Intergenic	ENSSSCG000	SOCS2	suppressor of cytokine signaling 2 [Source:NCBI gene (formerly Entrezgene);Acc:100037966]
chr9	130698445	130699824	1380	*	chr9	130698445	130699824	in_vitro	in_vivo	13	12	3.57615385	1.87666667	1.69948718	c(t = 0.92324	0.368	0.84625829	Distal Intergenic	ENSSSCG000	FLVCR1	FLVCR heme transporter 1 [Source:NCBI gene (formerly Entrezgene);Acc:100217398]
chr6	51162410	51163140	731	*	chr6	51162410	51163140	in_vitro	in_vivo	10	10	0.81	1.769	-0.959	c(t = -0.93344	0.368	0.84625829	Distal Intergenic	ENSSSCG000	BCL3	BCL3 transcription coactivator [Source:NCBI gene (formerly Entrezgene);Acc:100738193]
chr13	6905163	6906095	933	*	chr13	6905163	6906095	in_vitro	in_vivo	9	12	1.71333333	0.89666667	0.81666667	c(t = 0.92545	0.368	0.84625829	Distal Intergenic	ENSSSCG000	RAB5A	RAB5A, member RAS onco family [Source:VGNC Symbol;Acc:VGNC:108687]
chr12	52789892	52791046	1155	*	chr12	52789892	52791046	in_vitro	in_vivo	10	9	86.1	95.5655556	-9.4655556	c(t = -0.94172	0.369	0.84625829	Distal Intergenic	ENSSSCG000	TNFSF12	TNF superfamily member 12 [Source:VGNC Symbol;Acc:VGNC:99097]
chr13	207443907	207455579	11673	*	chr13	207443907	207455579	in_vitro	in_vivo	12	13	95.555	93.1146154	2.44038462	c(t = 0.91645	0.369	0.84625829	Distal Intergenic	ENSSSCG000	ITGB2	integrin subunit beta 2 [Source:NCBI gene (formerly Entrezgene);Acc:396943]
chr3	6048371	6055806	7436	*	chr3	6048371	6055806	in_vitro	in_vivo	14	14	89.1964286	90.9964286	-1.8	c(t = -0.91749	0.369	0.84625829	Distal Intergenic	ENSSSCG000	KPNA7	karyopherin subunit alpha 7 [Source:VGNC Symbol;Acc:VGNC:89566]
chr2	27095276	27096335	1060	*	chr2	27095276	27096335	in_vitro	in_vivo	11	12	13.5554545	18.6033333	-5.0478788	c(t = -0.91954	0.369	0.84625829	Distal Intergenic	ENSSSCG00000024791		CD59 molecule [Source:VGNC Symbol;Acc:VGNC:99615]
chr6	92413296	92414105	810	*	chr6	92413296	92414105	in_vitro	in_vivo	9	10	16.5311111	10.211	6.32011111	c(t = 0.92324	0.369	0.84625829	Distal Intergenic	NA	NA	NA
chr6	59569920	59571629	1710	*	chr6	59569920	59571629	in_vitro	in_vivo	10	10	3.964	7.808	-3.844	c(t = -0.93330	0.369	0.84625829	Distal Intergenic	ENSSSCG00000003302		transmembrane protein 86B [Source:VGNC Symbol;Acc:VGNC:94211]
chr7	13465585	13466712	1128	*	chr7	13465585	13466712	in_vitro	in_vivo	11	12	6.79545455	3.66666667	3.12878788	c(t = 0.92534	0.369	0.84625829	Distal Intergenic	ENSSSCG000	NHLRC1	NHL repeat containing E3 ubiquitin protein ligase 1 [Source:NCBI gene (formerly Entrezgene);Acc:100152396]
chr1	84543819	84546355	2537	*	chr1	84543819	84546355	in_vitro	in_vivo	15	14	2.504	1.59714286	0.90685714	c(t = 0.91391	0.369	0.84625829	Distal Intergenic	ENSSSCG000	BCKDHB	branched chain keto acid dehydrogenase E1 subunit beta [Source:VGNC Symbol;Acc:VGNC:85777]
chr8	42024285	42024928	644	*	chr8	42024285	42024928	in_vitro	in_vivo	11	13	2.98636364	1.59461538	1.39174825	c(t = 0.93003	0.369	0.84625829	Distal Intergenic	ENSSSCG000	KIT	KIT proto-oncogene, receptor tyrosine kinase [Source:NCBI gene (formerly Entrezgene);Acc:396810]
chr6	79809679	79810818	1140	*	chr6	79809679	79810818	in_vitro	in_vivo	11	11	2.21909091	0.93181818	1.28727273	c(t = 0.92954	0.369	0.84625829	Distal Intergenic	ENSSSCG00000030015		
chr13	136346063	136347663	1601	*	chr13	136346063	136347663	in_vitro	in_vivo	12	10	2.06833333	0.675	1.39333333	c(t = 0.93200	0.369	0.84625829	Distal Intergenic	ENSSSCG00000028717		rhophilin associated tail protein 1 [Source:NCBI gene (formerly Entrezgene);Acc:100359353]
chr6	81111669	81112470	802	*	chr6	81111669	81112470	in_vitro	in_vivo	9	9	1.70111111	0.61777778	1.08333333	c(t = 0.92916	0.369	0.84625829	Distal Intergenic	ENSSSCG00000025500		5-hydroxytryptamine receptor 1D [Source:VGNC Symbol;Acc:VGNC:97100]
chr5	45525194	45525723	530	*	chr5	45525194	45525723	in_vitro	in_vivo	12	10	1.3975	0.471	0.9265	c(t = 0.92916	0.369	0.84625829	Promoter (<=1kb)	ENSSSCG000	CCDC91	coiled-coil domain containing 91 [Source:VGNC Symbol;Acc:VGNC:86329]
chr15	138949988	138951159	1172	*	chr15	138949988	138951159	in_vitro	in_vivo	9	9	10.23	19.6777778	-9.4477778	c(t = -0.92319	0.37	0.84625829	Distal Intergenic	ENSSSCG000	ssc-mir-149	ssc-mir-149 [Source:miRBase;Acc:MI0016620]
chr3	73028493	73029142	650	*	chr3	73028493	73029142	in_vitro	in_vivo	10	12	1.96	3.4075	-1.4475	c(t = -0.91729	0.37	0.84625829	Promoter (<=1kb)	ENSSSCG000	GFPT1	glutamine--fructose-6-phosphate transaminase 1 [Source:VGNC Symbol;Acc:VGNC:88426]
chr7	29414721	29415659	939	*	chr7	29414721	29415659	in_vitro	in_vivo	11	10	1.54454545	2.707	-1.1624545	c(t = -0.92095	0.37	0.84625829	Distal Intergenic	ENSSSCG000	RPS18	ribosomal protein S18 [Source:NCBI gene (formerly Entrezgene);Acc:396980]
chr14	105422191	105422851	661	*	chr14	105422191	105422851	in_vitro	in_vivo	10	14	0.958	1.89357143	-0.9355714	c(t = -0.91795	0.37	0.84625829	Distal Intergenic	ENSSSCG000	FRA10AC1	FRA10A associated CGG repeat 1 [Source:VGNC Symbol;Acc:VGNC:96732]
chr15	61141093	61142260	1168	*	chr15	61141093	61142260	in_vitro	in_vivo	11	12	2.88818182	1.44583333	1.44234848	c(t = 0.92150	0.37	0.84625829	Distal Intergenic	ENSSSCG000	PRPF40A	pre-mRNA processing factor 40 homolog A [Source:VGNC Symbol;Acc:VGNC:98222]
chr4	100981485	100982325	841	*	chr4	100981485	100982325	in_vitro	in_vivo	10	11	2.146	1.14545455	1.00054545	c(t = 0.92046	0.37	0.84625829	Distal Intergenic	ENSSSCG000	REG4	regenerating family member 4 [Source:NCBI gene (formerly Entrezgene);Acc:100154580]
chr18	19513860	19514416	557	*	chr18	19513860	19514416	in_vitro	in_vivo	10	13	4.64	0.98307692	3.65692308	c(t = 0.94151	0.37	0.84625829	Distal Intergenic	ENSSSCG000	OPN1SW	opsin 1, short wave sensitive [Source:NCBI gene (formerly Entrezgene);Acc:397164]
chr1	163319162	163320156	995	*	chr1	163319162	163320156	in_vitro	in_vivo	9	11	1.46888889	0.71090909	0.7579798	c(t = 0.93126	0.37	0.84625829	Distal Intergenic	ENSSSCG000	CILP	cartilage intermediate layer protein [Source:VGNC Symbol;Acc:VGNC:86703]
chr5	516391	519494	3104	*	chr5	516391	519494	in_vitro	in_vivo	10	10	87.749	81.819	5.93	c(t = 0.91741	0.371	0.84625829	Distal Intergenic	ENSSSCG000	SELENOO	selenoprotein O [Source:NCBI gene (formerly Entrezgene);Acc:100518971]
chr12	3867743	3868767	1025	*	chr12	3867743	3868767	in_vitro	in_vivo	11	11	12.0509091	25.5390909	-13.488182	c(t = -0.91691	0.371	0.84625829	Distal Intergenic	ENSSSCG000	BIRC5	baculoviral IAP repeat containing 5 [Source:VGNC Symbol;Acc:VGNC:85827]
chr14	112734718	112735934	1217	*	chr14	112734718	112735934	in_vitro	in_vivo	10	13	39.475	24.9846154	14.4903846	c(t = 0.92130	0.371	0.84625829	Distal Intergenic	ENSSSCG000	HPS6	HPS6 biogenesis of lysosomal organelles complex 2 subunit 3 [Source:NCBI gene (formerly Entrezgene);Acc:100049675]
chr5	15010384	15014036	3653	*	chr5	15010384	15014036	in_vitro	in_vivo	13	13	5.59384615	7.79769231	-2.2038462	c(t = -0.91510	0.371	0.84625829	Distal Intergenic	ENSSSCG000	WNT10B	Wnt family member 10B [Source:VGNC Symbol;Acc:VGNC:94965]
chr12	19011019	19012882	1864	*	chr12	19011019	19012882	in_vitro	in_vivo	9	12	5.27222222	1.92333333	3.34888889	c(t = 0.93814	0.371	0.84625829	Distal Intergenic	ENSSSCG000	SLC25A39	solute carrier family 25 member 39 [Source:VGNC Symbol;Acc:VGNC:93014]
chr10	69293841	69295333	1493	*	chr10	69293841	69295333	in_vitro	in_vivo	11	14	1.90545455	1.29214286	0.61331169	c(t = 0.91244	0.371	0.84625829	Distal Intergenic	ENSSSCG000	WDR37	WD repeat domain 37 [Source:VGNC Symbol;Acc:VGNC:96258]
chr3	117857605	117858678	1074	*	chr3	117857605	117858678	in_vitro	in_vivo	10	10	0.303	1.119	-0.816	c(t = -0.93040	0.371	0.84625829	Distal Intergenic	ENSSSCG000	RHOB	ras homolog family member B [Source:VGNC Symbol;Acc:VGNC:110709]
chr13	61703225	61704329	1105	*	chr13	61703225	61704329	in_vitro	in_vivo	12	11	0.53916667	1.07363636	-0.5344697	c(t = -0.91617	0.371	0.84625829	Distal Intergenic	ENSSSCG000	ARL8B	ADP ribosylation factor like GTPase 8B [Source:VGNC Symbol;Acc:VGNC:103905]
chr4	118089579	118090559	981	*	chr4	118089579	118090559	in_vitro	in_vivo	13	12	1.87230769	1.0725	0.79980769	c(t = 0.91360	0.371	0.84625829	Distal Intergenic	ENSSSCG000	SLC35A3	solute carrier family 35 member A3 [Source:VGNC Symbol;Acc:VGNC:98860]
chr2	127016844	127017801	958	*	chr2	127016844	127017801	in_vitro	in_vivo	10	10	1.954	0.953	1.001	c(t = 0.91800	0.371	0.84625829	Distal Intergenic	NA	NA	NA
chr5	3286798	3287461	664	*	chr5	3286798	3287461	in_vitro	in_vivo	10	9	1.386	0.64333333	0.74266667	c(t = 0.91950	0.371	0.84625829	Distal Intergenic	ENSSSCG000	PPARA	peroxisome proliferator activated receptor alpha [Source:NCBI gene (formerly Entrezgene);Acc:397239]
chr14	132562556	132563534	979	*	chr14	132562556	132563534	in_vitro	in_vivo	9	13	1.14666667	0.46153846	0.68512821	c(t = 0.92620	0.371	0.84625829	Distal Intergenic	ENSSSCG000	ssc-mir-4331	ssc-mir-4331-2 [Source:miRBase;Acc:MI0033415]
chr5	51040211	51040872	662	*	chr5	51040211	51040872	in_vitro	in_vivo	11	13	1.27818182	0.42538462	0.8527972	c(t = 0.92852	0.371	0.84625829	Distal Intergenic	ENSSSCG000	LDHB	lactate dehydrogenase B [Source:NCBI gene (formerly Entrezgene);Acc:100621540]
chr16	46433989	46435608	1620	*	chr16	46433989	46435608	in_vitro	in_vivo	15	13	4.776	2.99461538	1.78138462	c(t = 0.90844	0.372	0.84625829	Distal Intergenic	ENSSSCG000	CD180	CD180 molecule [Source:NCBI gene (formerly Entrezgene);Acc:397644]
chr14	55239279	55240565	1287	*	chr14	55239279	55240565	in_vitro	in_vivo	12	11	4.365	2.38545455	1.97954545	c(t = 0.91271	0.372	0.84625829	Distal Intergenic	ENSSSCG000	EDARADD	EDAR associated death domain [Source:VGNC Symbol;Acc:VGNC:87542]
chr17	744836	745555	720	*	chr17	744836	745555	in_vitro	in_vivo	12	12	1.205	2.08916667	-0.8841667	c(t = -0.91087	0.372	0.84625829	Distal Intergenic	ENSSSCG000	CLDN23	claudin 23 [Source:NCBI gene (formerly Entrezgene);Acc:100156235]
chr3	15864849	15866695	1847	*	chr3	15864849	15866695	in_vitro	in_vivo	13	12	3.17538462	1.6575	1.51788462	c(t = 0.92019	0.372	0.84625829	Distal Intergenic	ENSSSCG000	SBDS	SBDS ribosome maturation factor [Source:NCBI gene (formerly Entrezgene);Acc:100516003]
chr6	55504057	55505135	1079	*	chr6	55504057	55505135	in_vitro	in_vivo	13	12	0.58076923	1.205	-0.6242308	c(t = -0.91500	0.372	0.84625829	Distal Intergenic	ENSSSCG000	KLK1	kallikrein 1 [Source:NCBI gene (formerly Entrezgene);Acc:431673]
chr14	41091452	41091872	421	*	chr14	41091452	41091872	in_vitro	in_vivo	9	10	1.87888889	0.778	1.10088889	c(t = 0.92575	0.372	0.84625829	Promoter (<=1kb)	ENSSSCG000	GLTP	glycolipid transfer protein [Source:NCBI gene (formerly Entrezgene);Acc:396765]
chr14	104556212	104556898	687	*	chr14	104556212	104556898	in_vitro	in_vivo	9	10	28.3122222	16.331	11.9812222	c(t = 0.91606	0.373	0.84625829	Distal Intergenic	ENSSSCG000	CYP26A1	cytochrome P450 family 26 subfamily A member 1 [Source:VGNC Symbol;Acc:VGNC:103370]
chr8	2423121	2425282	2162	*	chr8	2423121	2425282	in_vitro	in_vivo	9	10	2.58333333	5.985	-3.4016667	c(t = -0.92404	0.373	0.84625829	Distal Intergenic	ENSSSCG000	LRPAP1	LDL receptor related protein associated protein 1 [Source:NCBI gene (formerly Entrezgene);Acc:399654]
chr9	131960281	131961286	1006	*	chr9	131960281	131961286	in_vitro	in_vivo	14	14	4.76142857	3.01642857	1.745	c(t = 0.91095	0.373	0.84625829	Distal Intergenic	ENSSSCG000	SLC30A1	solute carrier family 30 member 1 [Source:NCBI gene (formerly Entrezgene);Acc:503660]
chr5	65658296	65659816	1521	*	chr5	65658296	65659816	in_vitro	in_vivo	9	13	2.96444444	1.95692308	1.00752137	c(t = 0.91191	0.373	0.84625829	Distal Intergenic	ENSSSCG000	AKAP3	A-kinase anchoring protein 3 [Source:VGNC Symbol;Acc:VGNC:85218]
chr18	2787104	2789169	2066	*	chr18	2787104	2789169	in_vitro	in_vivo	13	10	1.00769231	1.853	-0.8453077	c(t = -0.92391	0.373	0.84625829	Distal Intergenic	ENSSSCG000	INSIG1	insulin induced gene 1 [Source:NCBI gene (formerly Entrezgene);Acc:403162]
chr6	149112298	149112931	634	*	chr6	149112298	149112931	in_vitro	in_vivo	12	12	1.80583333	0.96583333	0.84	c(t = 0.91070	0.373	0.84625829	Distal Intergenic	ENSSSCG000	EFCAB7	phosphoglucomutase 1 [Source:VGNC Symbol;Acc:VGNC:91355]
chr12	25203385	25203880	496	*	chr12	25203385	25203880	in_vitro	in_vivo	9	11	2.82222222	0.95181818	1.87040404	c(t = 0.93188	0.373	0.84625829	Distal Intergenic	ENSSSCG000	GIP	gastric inhibitory polypeptide [Source:VGNC Symbol;Acc:VGNC:98995]
chr11	24504441	24505513	1073	*	chr11	24504441	24505513	in_vitro	in_vivo	11	11	1.62636364	0.86818182	0.75818182	c(t = 0.91645	0.373	0.84625829	Distal Intergenic	ENSSSCG000	FAM216B	family with sequence similarity 216 member B [Source:VGNC Symbol;Acc:VGNC:87955]
chr13	83667956	83669702	1747	*	chr13	83667956	83669702	in_vitro	in_vivo	14	12	5.10142857	10.9816667	-5.8802381	c(t = -0.91941	0.374	0.84625829	Distal Intergenic	ENSSSCG000	TRPC1	transient receptor potential cation channel subfamily C member 1 [Source:VGNC Symbol;Acc:VGNC:94460]
chr14	90582366	90583045	680	*	chr14	90582366	90583045	in_vitro	in_vivo	10	10	1.95	4.5	-2.55	c(t = -0.91961	0.374	0.84625829	Distal Intergenic	ENSSSCG000	MSMB	microseminoprotein beta [Source:NCBI gene (formerly Entrezgene);Acc:396852]
chr6	58396476	58397318	843	*	chr6	58396476	58397318	in_vitro	in_vivo	10	11	1.06	3.57727273	-2.5172727	c(t = -0.92362	0.374	0.84625829	Distal Intergenic	NA	NA	NA
chr4	77083505	77083940	436	*	chr4	77083505	77083940	in_vitro	in_vivo	10	9	1.267	2.76666667	-1.4996667	c(t = -0.92967	0.374	0.84625829	Distal Intergenic	ENSSSCG000	MRPL15	mitochondrial ribosomal protein L15 [Source:VGNC Symbol;Acc:VGNC:98820]
chr13	197007072	197008149	1078	*	chr13	197007072	197008149	in_vitro	in_vivo	11	12	1.23272727	2.07083333	-0.8381061	c(t = -0.91060	0.374	0.84625829	Promoter (<=1kb)	ENSSSCG000	IFNGR2	interferon gamma receptor 2 [Source:NCBI gene (formerly Entrezgene);Acc:100126864]
chr5	59671096	59671607	512	*	chr5	59671096	59671607	in_vitro	in_vivo	13	10	2.84923077	1.308	1.54123077	c(t = 0.91260	0.374	0.84625829	Distal Intergenic	ENSSSCG000	CDKN1B	cyclin dependent kinase inhibitor 1B [Source:NCBI gene (formerly Entrezgene);Acc:397584]
chr5	6799693	6800215	523	*	chr5	6799693	6800215	in_vitro	in_vivo	10	10	0.371	1.279	-0.908	c(t = -0.93032	0.374	0.84625829	Distal Intergenic	ENSSSCG000	SNU13	small nuclear ribonucleoprotein 13 [Source:NCBI gene (formerly Entrezgene);Acc:100155420]
chr12	20269412	20269983	572	*	chr12	20269412	20269983	in_vitro	in_vivo	9	10	0.24666667	1.204	-0.9573333	c(t = -0.93110	0.374	0.84625829	Promoter (1-2kb)	ENSSSCG000	HSD17B1	hydroxysteroid 17-beta dehydrogenase 1 [Source:NCBI gene (formerly Entrezgene);Acc:100147712]
chr13	207851085	207852185	1101	*	chr13	207851085	207852185	in_vitro	in_vivo	10	9	23.971	41.3566667	-17.385667	c(t = -0.91268	0.375	0.84625829	Distal Intergenic	ENSSSCG000	ITGB2	integrin subunit beta 2 [Source:NCBI gene (formerly Entrezgene);Acc:396943]
chr16	79808628	79809073	446	*	chr16	79808628	79809073	in_vitro	in_vivo	9	9	18.1744444	33.2966667	-15.122222	c(t = -0.91414	0.375	0.84625829	Distal Intergenic	ENSSSCG000	TPPP	tubulin polymerization promoting protein [Source:VGNC Symbol;Acc:VGNC:94344]
chrX	87657565	87658323	759	*	chrX	87657565	87658323	in_vitro	in_vivo	11	11	5.29545455	10.6709091	-5.3754545	c(t = -0.90875	0.375	0.84625829	Distal Intergenic	ENSSSCG000	CLDN2	claudin 2 [Source:VGNC Symbol;Acc:VGNC:86737]
chr17	32582379	32583215	837	*	chr17	32582379	32583215	in_vitro	in_vivo	10	10	1.683	3.375	-1.692	c(t = -0.92128	0.375	0.84625829	Promoter (1-2kb)	ENSSSCG000	AVP	vasopressin [Source:NCBI gene (formerly Entrezgene);Acc:396995]
chr17	58434458	58435050	593	*	chr17	58434458	58435050	in_vitro	in_vivo	10	11	1.22	2.75181818	-1.5318182	c(t = -0.91607	0.375	0.84625829	Promoter (<=1kb)	ENSSSCG000	ANKRD60	ankyrin repeat domain 60 [Source:NCBI gene (formerly Entrezgene);Acc:100144514]
chr8	67606202	67607569	1368	*	chr8	67606202	67607569	in_vitro	in_vivo	11	13	1.75090909	2.55076923	-0.7998601	c(t = -0.90654	0.375	0.84625829	Distal Intergenic	ENSSSCG000	UTP3	UTP3 small subunit processome component [Source:NCBI gene (formerly Entrezgene);Acc:100217385]
chr12	15164386	15165689	1304	*	chr12	15164386	15165689	in_vitro	in_vivo	9	12	0.88777778	2.4475	-1.5597222	c(t = -0.91737	0.375	0.84625829	Distal Intergenic	ENSSSCG000	PSMC5	proteasome 26S subunit, ATPase 5 [Source:VGNC Symbol;Acc:VGNC:91916]
chr14	24317210	24318261	1052	*	chr14	24317210	24318261	in_vitro	in_vivo	12	14	2.07333333	1.36714286	0.70619048	c(t = 0.90755	0.375	0.84625829	Distal Intergenic	ENSSSCG000	PIWIL1	piwi like RNA-mediated silencing 1 [Source:VGNC Symbol;Acc:VGNC:98197]
chr13	61640533	61641249	717	*	chr13	61640533	61641249	in_vitro	in_vivo	12	11	2.25083333	1.09272727	1.15810606	c(t = 0.91522	0.375	0.84625829	Promoter (<=1kb)	ENSSSCG000	ARL8B	ADP ribosylation factor like GTPase 8B [Source:VGNC Symbol;Acc:VGNC:103905]
chr7	42217962	42218901	940	*	chr7	42217962	42218901	in_vitro	in_vivo	13	10	1.49461538	0.728	0.76661538	c(t = 0.90945	0.375	0.84625829	Distal Intergenic	ENSSSCG000	CD2AP	CD2 associated protein [Source:VGNC Symbol;Acc:VGNC:86406]
chr2	73485267	73486251	985	*	chr2	73485267	73486251	in_vitro	in_vivo	11	12	14.3645455	24.2808333	-9.9162879	c(t = -0.90370	0.376	0.84625829	Distal Intergenic	ENSSSCG000	MICOS13	mitochondrial contact site and cristae organizing system subunit 13 [Source:NCBI gene (formerly Entrezgene);Acc:100037978]
chr9	120337047	120337872	826	*	chr9	120337047	120337872	in_vitro	in_vivo	10	9	3.848	8.43888889	-4.5908889	c(t = -0.91169	0.376	0.84625829	Distal Intergenic	ENSSSCG000	ANGPTL1	angiopoietin like 1 [Source:VGNC Symbol;Acc:VGNC:85303]
chr6	48943159	48943738	580	*	chr6	48943159	48943738	in_vitro	in_vivo	10	10	1.206	3.359	-2.153	c(t = -0.92440	0.376	0.84625829	Distal Intergenic	ENSSSCG000	SNRPA	small nuclear ribonucleoprotein polypeptide A [Source:NCBI gene (formerly Entrezgene);Acc:768109]
chr3	2105287	2106219	933	*	chr3	2105287	2106219	in_vitro	in_vivo	10	13	1.043	1.94230769	-0.8993077	c(t = -0.90689	0.376	0.84625829	Distal Intergenic	ENSSSCG000	AMZ1	archaelysin family metallopeptidase 1 [Source:NCBI gene (formerly Entrezgene);Acc:100337670]
chr3	58095813	58097552	1740	*	chr3	58095813	58097552	in_vitro	in_vivo	10	13	1.052	1.86307692	-0.8110769	c(t = -0.90642	0.376	0.84625829	Distal Intergenic	ENSSSCG000	CD8A	CD8 subunit alpha [Source:VGNC Symbol;Acc:VGNC:86436]
chr4	75524720	75526532	1813	*	chr4	75524720	75526532	in_vitro	in_vivo	15	14	2.688	1.73357143	0.95442857	c(t = 0.90065	0.376	0.84625829	Distal Intergenic	ENSSSCG000	SDR16C5	short chain dehydrogenase/reductase family 16C member 5 [Source:VGNC Symbol;Acc:VGNC:98853]
chr6	151247868	151248299	432	*	chr6	151247868	151248299	in_vitro	in_vivo	11	9	3.5	1.63666667	1.86333333	c(t = 0.90991	0.376	0.84625829	Distal Intergenic	ENSSSCG000	CYP2J34	cytochrome P450 family 2 subfamily J member 34 [Source:NCBI gene (formerly Entrezgene);Acc:100524750]
chr6	7254556	7255531	976	*	chr6	7254556	7255531	in_vitro	in_vivo	10	13	2.402	1.20076923	1.20123077	c(t = 0.90550	0.376	0.84625829	Distal Intergenic	ENSSSCG000	BCO1	beta-carotene oxygenase 1 [Source:VGNC Symbol;Acc:VGNC:85789]
chr18	38800616	38802120	1505	*	chr18	38800616	38802120	in_vitro	in_vivo	13	13	1.71692308	1.11153846	0.60538462	c(t = 0.90204	0.376	0.84625829	Distal Intergenic	ENSSSCG000	FKBP9	FKBP prolyl isomerase 9 [Source:NCBI gene (formerly Entrezgene);Acc:100623924]
chr16	28133077	28134347	1271	*	chr16	28133077	28134347	in_vitro	in_vivo	12	9	2.3575	0.82666667	1.53083333	c(t = 0.91263	0.376	0.84625829	Distal Intergenic	ENSSSCG000	CCL28	C-C motif chemokine ligand 28 [Source:NCBI gene (formerly Entrezgene);Acc:554303]
chr2	74414316	74414825	510	*	chr2	74414316	74414825	in_vitro	in_vivo	9	11	1.49555556	0.61545455	0.88010101	c(t = 0.92448	0.376	0.84625829	Distal Intergenic	ENSSSCG000	PLIN5	perilipin 5 [Source:VGNC Symbol;Acc:VGNC:91561]
chrX	97780970	97781705	736	*	chrX	97780970	97781705	in_vitro	in_vivo	11	11	2.11727273	0.36363636	1.75363636	c(t = 0.92369	0.376	0.84625829	Distal Intergenic	ENSSSCG000	PGRMC1	progesterone receptor membrane component 1 [Source:NCBI gene (formerly Entrezgene);Acc:396946]
chr12	60651612	60653996	2385	*	chr12	60651612	60653996	in_vitro	in_vivo	10	9	24.115	13.4888889	10.6261111	c(t = 0.91073	0.377	0.84625829	Distal Intergenic	ENSSSCG000	SREBF1	sterol regulatory element binding transcription factor 1 [Source:VGNC Symbol;Acc:VGNC:99083]
chr4	27861253	27862070	818	*	chr4	27861253	27862070	in_vitro	in_vivo	10	11	0.891	1.76	-0.869	c(t = -0.91060	0.377	0.84625829	Distal Intergenic	ENSSSCG000	ENY2	ENY2 transcription and export complex 2 subunit [Source:VGNC Symbol;Acc:VGNC:98792]
chr1	242682071	242683303	1233	*	chr1	242682071	242683303	in_vitro	in_vivo	10	13	3.089	1.56	1.529	c(t = 0.90298	0.377	0.84625829	Distal Intergenic	ENSSSCG000	ERP44	endoplasmic reticulum protein 44 [Source:NCBI gene (formerly Entrezgene);Acc:100192437]
chr3	107339474	107340030	557	*	chr3	107339474	107340030	in_vitro	in_vivo	9	10	0.53	1.23	-0.7	c(t = -0.91425	0.377	0.84625829	Distal Intergenic	ENSSSCG000	SLC30A6	solute carrier family 30 member 6 [Source:VGNC Symbol;Acc:VGNC:93060]
chr3	37554515	37555933	1419	*	chr3	37554515	37555933	in_vitro	in_vivo	12	14	1.16	0.70928571	0.45071429	c(t = 0.90027	0.377	0.84625829	Distal Intergenic	ENSSSCG000	SEPTIN12	septin 12 [Source:NCBI gene (formerly Entrezgene);Acc:100624981]

chr10	24464782	24465771	990	*	chr10	24464782	24465771	in_vitro	in_vivo	12	13	1.24	0.63461538	0.60538462	c(t = 0.90481	0.377	0.84625829	Distal Intergenic	ENSSSCG000	ADIPOR1	adiponectin receptor 1 [Source:NCBI gene (formerly Entrezgene);Acc:404695]
chr5	57349309	57349815	507	*	chr5	57349309	57349815	in_vitro	in_vivo	10	11	1.627	0.56818182	1.05881818	c(t = 0.91137	0.377	0.84625829	Distal Intergenic	ENSSSCG000	PDE6H	phosphodiesterase 6H [Source:NCBI gene (formerly Entrezgene);Acc:100156675]
chr3	1221728	1222695	968	*	chr3	1221728	1222695	in_vitro	in_vivo	9	9	89.8611111	76.7544444	13.1066667	c(t = 0.91313	0.378	0.84625829	Distal Intergenic	ENSSSCG000	ssc-mir-9817	ssc-mir-9817 [Source:miRBase;Acc:MI0031584]
chr3	40193520	40202614	9095	*	chr3	40193520	40202614	in_vitro	in_vivo	10	15	24.254	33.3766667	-9.1226667	c(t = -0.9083	0.378	0.84625829	Promoter (<=1kb)	ENSSSCG000	MRPS34	mitochondrial ribosomal protein S34 [Source:NCBI gene (formerly Entrezgene);Acc:100513531]
chr2	76276671	76278423	1753	*	chr2	76276671	76278423	in_vitro	in_vivo	13	15	20.9546154	29.97	-9.0153846	c(t = -0.8984	0.378	0.84625829	Distal Intergenic	ENSSSCG000	OAZ1	ornithine decarboxylase antizyme 1 [Source:NCBI gene (formerly Entrezgene);Acc:397034]
chr17	62500862	62506830	5969	*	chr17	62500862	62506830	in_vitro	in_vivo	9	9	6.74333333	10.0077778	-3.2644444	c(t = -0.9133	0.378	0.84625829	Distal Intergenic	ENSSSCG000	LIME1	Lck interacting transmembrane adaptor 1 [Source:NCBI gene (formerly Entrezgene);Acc:100625216]
chr2	59236197	59237084	888	*	chr2	59236197	59237084	in_vitro	in_vivo	10	12	2.294	5.45666667	-3.1626667	c(t = -0.9108	0.378	0.84625829	Distal Intergenic	ENSSSCG000	UBA52	ubiquitin A-52 residue ribosomal protein fusion product 1 [Source:NCBI gene (formerly Entrezgene);Acc:397418]
chr13	207411909	207412831	923	*	chr13	207411909	207412831	in_vitro	in_vivo	9	9	1.29888889	2.64888889	-1.35	c(t = -0.9081	0.378	0.84625829	Distal Intergenic	ENSSSCG000	ITGB2	integrin subunit beta 2 [Source:NCBI gene (formerly Entrezgene);Acc:396943]
chr13	196853454	196853933	480	*	chr13	196853454	196853933	in_vitro	in_vivo	11	11	4.90454545	2.29454545	2.61	c(t = 0.90826	0.378	0.84625829	Promoter (<=1kb)	ENSSSCG000	IL10RB	interleukin 10 receptor subunit beta [Source:NCBI gene (formerly Entrezgene);Acc:396657]
chr13	38925015	38926089	1075	*	chr13	38925015	38926089	in_vitro	in_vivo	11	12	3.52090909	1.92333333	1.59757576	c(t = 0.90728	0.378	0.84625829	Distal Intergenic	ENSSSCG000	APPL1	adaptor protein, phosphotyrosine interacting with PH domain and leucine zipper 1 [Source:NCBI gene (formerly Entrezgene);Acc:100188978]
chr1	128816811	128817663	853	*	chr1	128816811	128817663	in_vitro	in_vivo	12	11	3.32583333	1.49181818	1.83401515	c(t = 0.90721	0.378	0.84625829	Distal Intergenic	ENSSSCG000	HAUS2	HAUS augmin like complex subunit 2 [Source:VGNC Symbol;Acc:VGNC:103104]
chr9	136371429	136373047	1619	*	chr9	136371429	136373047	in_vitro	in_vivo	10	15	2.441	1.428	1.013	c(t = 0.90930	0.378	0.84625829	Distal Intergenic	ENSSSCG000	DDC	dopa decarboxylase [Source:VGNC Symbol;Acc:VGNC:87204]
chr12	61362051	61363522	1472	*	chr12	61362051	61363522	in_vitro	in_vivo	9	13	5.89888889	1.11615385	4.78273504	c(t = 0.93172	0.378	0.84625829	Distal Intergenic	ENSSSCG000	COPS3	COP9 signalosome subunit 3 [Source:VGNC Symbol;Acc:VGNC:86900]
chr14	72210313	72210949	637	*	chr14	72210313	72210949	in_vitro	in_vivo	10	10	2.5	0.879	1.621	c(t = 0.91753	0.378	0.84625829	Distal Intergenic	ENSSSCG000	HK1	hexokinase 1 [Source:NCBI gene (formerly Entrezgene);Acc:100152344]
chr4	104170286	104171473	1188	*	chr4	104170286	104171473	in_vitro	in_vivo	10	11	1.519	0.76545455	0.75354545	c(t = 0.90635	0.378	0.84625829	Promoter (<=1kb)	ENSSSCG000	CD58	CD58 molecule [Source:NCBI gene (formerly Entrezgene);Acc:396702]
chr17	40278698	40279433	736	*	chr17	40278698	40279433	in_vitro	in_vivo	9	11	2.75444444	0.68090909	2.07353535	c(t = 0.92781	0.378	0.84625829	Distal Intergenic	ENSSSCG000	RPN2	ribophorin II [Source:VGNC Symbol;Acc:VGNC:95471]
chr13	135105278	135106114	837	*	chr13	135105278	135106114	in_vitro	in_vivo	10	9	1.245	0.58555556	0.65944444	c(t = 0.90848	0.378	0.84625829	Distal Intergenic	ENSSSCG000	MUC13	mucin 13, cell surface associated [Source:NCBI gene (formerly Entrezgene);Acc:100125829]
chr3	18879839	18880377	539	*	chr3	18879839	18880377	in_vitro	in_vivo	9	9	85.2088889	95.3622222	-10.153333	c(t = -0.9273	0.379	0.84625829	Distal Intergenic	ENSSSCG000	SPNS1	SPNS lysolipid transporter 1, lysophospholipid [Source:NCBI gene (formerly Entrezgene);Acc:100517643]
chrX	110873257	110873955	699	*	chrX	110873257	110873955	in_vitro	in_vivo	10	12	1.011	3.39833333	-2.3873333	c(t = -0.9125	0.379	0.84625829	Distal Intergenic	ENSSSCG000	MOSPD1	motile sperm domain containing 1 [Source:VGNC Symbol;Acc:VGNC:103990]
chr7	78434300	78434853	554	*	chr7	78434300	78434853	in_vitro	in_vivo	10	12	5.776	3.13916667	2.63683333	c(t = 0.90436	0.379	0.84625829	Distal Intergenic	ENSSSCG000	APEX1	apurinic/apyrimidinic endodeoxyribonuclease 1 [Source:VGNC Symbol;Acc:VGNC:103896]
chr5	87671793	87673171	1379	*	chr5	87671793	87673171	in_vitro	in_vivo	13	10	1.24461538	3.115	-1.8703846	c(t = -0.9193	0.379	0.84625829	Distal Intergenic	ENSSSCG000	SNRPF	small nuclear ribonucleoprotein polypeptide F [Source:NCBI gene (formerly Entrezgene);Acc:100155855]
chrX	91111121	91111898	778	*	chrX	91111121	91111898	in_vitro	in_vivo	9	11	8.70666667	2.56454545	6.14212121	c(t = 0.92453	0.379	0.84625829	Distal Intergenic	ENSSSCG000	SERTM2	serine rich and transmembrane domain containing 2 [Source:NCBI gene (formerly Entrezgene);Acc:106507011]
chr7	97051208	97051998	791	*	chr7	97051208	97051998	in_vitro	in_vivo	10	12	3.053	1.63083333	1.42216667	c(t = 0.90183	0.379	0.84625829	Distal Intergenic	ENSSSCG000	LIN52	lin-52 DREAM MuvB core complex component [Source:NCBI gene (formerly Entrezgene);Acc:100157325]
chr7	114573438	114574442	1005	*	chr7	114573438	114574442	in_vitro	in_vivo	14	13	1.99357143	1.22769231	0.76587912	c(t = 0.90085	0.379	0.84625829	Promoter (<=1kb)	ENSSSCG000	LYSET	lysosomal enzyme trafficking factor [Source:NCBI gene (formerly Entrezgene);Acc:414384]
chr14	138930347	138931730	1384	*	chr14	138930347	138931730	in_vitro	in_vivo	10	13	1.568	0.94538462	0.62261538	c(t = 0.90215	0.379	0.84625829	Distal Intergenic	ENSSSCG000	GLRX3	glutaredoxin 3 [Source:VGNC Symbol;Acc:VGNC:88495]
chr5	1517572	1518794	1223	*	chr5	1517572	1518794	in_vitro	in_vivo	9	12	22.4733333	34.2141667	-11.740833	c(t = -0.9010	0.38	0.84625829	Distal Intergenic	ENSSSCG000	SELENOO	selenoprotein O [Source:NCBI gene (formerly Entrezgene);Acc:100518971]
chr7	23637458	23638380	923	*	chr7	23637458	23638380	in_vitro	in_vivo	11	12	5.14909091	7.97916667	-2.8300758	c(t = -0.9002	0.38	0.84625829	Promoter (<=1kb)	ENSSSCG00000001398		MHC class I antigen 7 [Source:NCBI gene (formerly Entrezgene);Acc:396650]
chr12	53381248	53382614	1367	*	chr12	53381248	53382614	in_vitro	in_vivo	9	9	4.05111111	2.10444444	1.94666667	c(t = 0.90443	0.38	0.84625829	Distal Intergenic	ENSSSCG000	AURKB	aurora kinase B [Source:VGNC Symbol;Acc:VGNC:85698]
chr17	20844702	20845613	912	*	chr17	20844702	20845613	in_vitro	in_vivo	11	11	1.91636364	0.79454545	1.12181818	c(t = 0.90520	0.38	0.84625829	Distal Intergenic	ENSSSCG000	TMX4	thioredoxin related transmembrane protein 4 [Source:NCBI gene (formerly Entrezgene);Acc:100627887]
chr1	181681232	181681657	426	*	chr1	181681232	181681657	in_vitro	in_vivo	10	9	1.424	0.58444444	0.83955556	c(t = 0.90158	0.38	0.84625829	Promoter (<=1kb)	ENSSSCG000	RTRAF	RNA transcription, translation and transport factor [Source:NCBI gene (formerly Entrezgene);Acc:100154555]
chr5	41878503	41879239	737	*	chr5	41878503	41879239	in_vitro	in_vivo	11	10	1.03	0.554	0.476	c(t = 0.90137	0.38	0.84625829	Distal Intergenic	ENSSSCG000	RESF1	retroelement silencing factor 1 [Source:VGNC Symbol;Acc:VGNC:92219]
chr5	6929051	6930053	1003	*	chr5	6929051	6930053	in_vitro	in_vivo	11	9	2.00818182	0.49777778	1.51040404	c(t = 0.91595	0.38	0.84625829	Promoter (<=1kb)	ENSSSCG000	XRCC6	X-ray repair cross complementing 6 [Source:VGNC Symbol;Acc:VGNC:95011]
chr11	78755186	78762110	6925	*	chr11	78755186	78762110	in_vitro	in_vivo	10	15	54.966	43.3326667	11.6333333	c(t = 0.89708	0.381	0.84625829	Distal Intergenic	ENSSSCG000	ATP4B	ATPase H+/K+ transporting subunit beta [Source:VGNC Symbol;Acc:VGNC:85655]
chr6	59732581	59735950	3370	*	chr6	59732581	59735950	in_vitro	in_vivo	12	12	25.3091667	37.6783333	-12.369167	c(t = -0.8945	0.381	0.84625829	Distal Intergenic	ENSSSCG000	U2AF2	U2 small nuclear RNA auxiliary factor 2 [Source:VGNC Symbol;Acc:VGNC:94623]
chr1	83849134	83850970	1837	*	chr1	83849134	83850970	in_vitro	in_vivo	12	12	10.7125	6.09916667	4.61333333	c(t = 0.89699	0.381	0.84625829	Distal Intergenic	ENSSSCG000	CALHM6	calcium homeostasis modulator family member 6 [Source:VGNC Symbol;Acc:VGNC:86147]
chr1	14866348	14867856	1509	*	chr1	14866348	14867856	in_vitro	in_vivo	10	12	0.988	2.325	-1.337	c(t = -0.9064	0.381	0.84625829	Distal Intergenic	ENSSSCG000	ARMT1	acidic residue methyltransferase 1 [Source:NCBI gene (formerly Entrezgene);Acc:100157036]
chr2	78915412	78915880	469	*	chr2	78915412	78915880	in_vitro	in_vivo	10	14	3.679	1.865	1.814	c(t = 0.91300	0.381	0.84625829	Promoter (<=1kb)	ENSSSCG000	CANX	calnexin [Source:VGNC Symbol;Acc:VGNC:86167]
chr15	23592877	23593880	1004	*	chr15	23592877	23593880	in_vitro	in_vivo	11	12	4.28090909	1.62583333	2.65507576	c(t = 0.90739	0.381	0.84625829	Distal Intergenic	ENSSSCG000	INSIG2	insulin induced 2 [Source:VGNC Symbol;Acc:VGNC:103970]
chr7	26988558	26990306	1749	*	chr7	26988558	26990306	in_vitro	in_vivo	10	15	2.809	1.448	1.361	c(t = 0.90980	0.381	0.84625829	Distal Intergenic	ENSSSCG000	HCRTR2	hypocretin receptor 2 [Source:NCBI gene (formerly Entrezgene);Acc:397428]
chr2	119803806	119804690	885	*	chr2	119803806	119804690	in_vitro	in_vivo	10	14	2.352	1.32214286	1.02985714	c(t = 0.90394	0.381	0.84625829	Promoter (<=1kb)	ENSSSCG000	TMED7	transmembrane p24 trafficking protein 7 [Source:NCBI gene (formerly Entrezgene);Acc:100522183]
chr11	75739283	75741016	1734	*	chr11	75739283	75741016	in_vitro	in_vivo	11	12	1.39727273	0.68666667	0.71060606	c(t = 0.90688	0.381	0.84625829	Distal Intergenic	ENSSSCG000	TNFSF13B	TNF superfamily member 13b [Source:VGNC Symbol;Acc:VGNC:94269]
chr13	89360623	89361267	645	*	chr13	89360623	89361267	in_vitro	in_vivo	9	11	1.14	0.43272727	0.70727273	c(t = 0.90357	0.381	0.84625829	Promoter (<=1kb)	ENSSSCG000	HPS3	HPS3 biosis of lysosomal organelles complex 2 subunit 1 [Source:VGNC Symbol;Acc:VGNC:88965]
chrX	120776682	120778680	1999	*	chrX	120776682	120778680	in_vitro	in_vivo	13	15	8.31461538	15.5	-7.1853846	c(t = -0.8938	0.382	0.84625829	Distal Intergenic	ENSSSCG000	FATE1	fetal and adult testis expressed 1 [Source:NCBI gene (formerly Entrezgene);Acc:396705]
chrX	40681259	40682392	1134	*	chrX	40681259	40682392	in_vitro	in_vivo	11	11	6.46909091	11.0218182	-4.5527273	c(t = -0.8964	0.382	0.84625829	Distal Intergenic	ENSSSCG000	ssc-mir-9859	ssc-mir-9859 [Source:miRBase;Acc:MI0031637]
chr13	206676149	206677317	1169	*	chr13	206676149	206677317	in_vitro	in_vivo	9	9	3.16444444	6.13666667	-2.9722222	c(t = -0.9183	0.382	0.84625829	Promoter (<=1kb)	ENSSSCG000	PDXK	pyridoxal kinase [Source:NCBI gene (formerly Entrezgene);Acc:396983]
chr2	82535019	82537507	2489	*	chr2	82535019	82537507	in_vitro	in_vivo	12	11	6.5025	4.25727273	2.24522727	c(t = 0.89377	0.382	0.84625829	Distal Intergenic	ENSSSCG000	BTF3	basic transcription factor 3 [Source:VGNC Symbol;Acc:VGNC:99597]
chr18	55605451	55606321	871	*	chr18	55605451	55606321	in_vitro	in_vivo	12	12	2.29833333	4.15333333	-1.855	c(t = -0.9045	0.382	0.84625829	Distal Intergenic	ENSSSCG000	RARRES2	retinoic acid receptor responder 2 [Source:VGNC Symbol;Acc:VGNC:92098]
chr5	9357809	9358824	1016	*	chr5	9357809	9358824	in_vitro	in_vivo	9	12	1.06333333	3.24	-2.1766667	c(t = -0.9063	0.382	0.84625829	Distal Intergenic	ENSSSCG00000043051		
chr4	40127960	40129740	1781	*	chr4	40127960	40129740	in_vitro	in_vivo	12	13	3.4725	1.54923077	1.92326923	c(t = 0.90511	0.382	0.84625829	Promoter (<=1kb)	ENSSSCG000	SDC2	syndecan 2 [Source:NCBI gene (formerly Entrezgene);Acc:100152754]
chr13	146193142	146193841	700	*	chr13	146193142	146193841	in_vitro	in_vivo	9	10	2.55666667	1.408	1.14866667	c(t = 0.90215	0.382	0.84625829	Promoter (<=1kb)	ENSSSCG000	NAA50	N-alpha-acetyltransferase 50, NatE catalytic subunit [Source:VGNC Symbol;Acc:VGNC:104002]
chr5	42642735	42644116	1382	*	chr5	42642735	42644116	in_vitro	in_vivo	14	12	4.59428571	1.39583333	3.19845238	c(t = 0.90422	0.382	0.84625829	Distal Intergenic	ENSSSCG000	RESF1	retroelement silencing factor 1 [Source:VGNC Symbol;Acc:VGNC:92219]
chr3	92335781	92337448	1668	*	chr3	92335781	92337448	in_vitro	in_vivo	11	11	3.37727273	1.36272727	2.01454545	c(t = 0.90752	0.382	0.84625829	Distal Intergenic	NA	NA	NA
chr2	125606214	125607275	1062	*	chr2	125606214	125607275	in_vitro	in_vivo	15	14	0.72266667	1.26428571	-0.541619	c(t = -0.8904	0.382	0.84625829	Promoter (<=1kb)	ENSSSCG000	LOX	lysyl oxidase [Source:NCBI gene (formerly Entrezgene);Acc:100525278]
chr5	49159642	49161415	1774	*	chr5	49159642	49161415	in_vitro	in_vivo	9	9	1.76555556	0.75888889	1.00666667	c(t = 0.91361	0.382	0.84625829	Distal Intergenic	ENSSSCG000	ssc-mir-9827	ssc-mir-9827 [Source:miRBase;Acc:MI0031594]
chr15	139956368	139962811	6444	*	chr15	139956368	139962811	in_vitro	in_vivo	13	11	95.5753846	87.4245455	8.15083916	c(t = 0.90994	0.383	0.84676496	Distal Intergenic	ENSSSCG000	PPP1R7	protein phosphatase 1 regulatory subunit 7 [Source:NCBI gene (formerly Entrezgene);Acc:100511842]
chr3	131713458	131715001	1544	*	chr3	131713458	131715001	in_vitro	in_vivo	11	12	13.6336364	24.0808333	-10.447197	c(t = -0.8921	0.383	0.84676496	Distal Intergenic	ENSSSCG000	EIPR1	EARP complex and GARP complex interacting protein 1 [Source:VGNC Symbol;Acc:VGNC:87635]
chr6	180690	182821	2132	*	chr6	180690	182821	in_vitro	in_vivo	11	13	11.3390909	21.5538462	-10.214755	c(t = -0.8896	0.383	0.84676496	Promoter (<=1kb)	ENSSSCG000	MC1R	melanocortin 1 receptor [Source:NCBI gene (formerly Entrezgene);Acc:494018]
chr3	108134333	108135076	744	*	chr3	108134333	108135076	in_vitro	in_vivo	9	10	16.2011111	10.534	5.66711111	c(t = 0.89616	0.383	0.84676496	Distal Intergenic	ENSSSCG000	XDH	xanthine dehydrogenase [Source:VGNC Symbol;Acc:VGNC:94995]
chr3	4554825	4557715	2891	*	chr3	4554825	4557715	in_vitro	in_vivo	12	13	19.0416667	9.49846154	9.54320513	c(t = 0.89579	0.383	0.84676496	Distal Intergenic	ENSSSCG000	RAC1	Rac family small GTPase 1 [Source:VGNC Symbol;Acc:VGNC:92047]
chr7	36919236	36921012	1777	*	chr7	36919236	36921012	in_vitro	in_vivo	13	10	1.99692308	3.849	-1.8520769	c(t = -0.8950	0.383	0.84676496	Distal Intergenic	ENSSSCG000	MED20	mediator complex subunit 20 [Source:NCBI gene (formerly Entrezgene);Acc:100157502]
chr6	69357243	69358360	1118	*	chr6	69357243	69358360	in_vitro	in_vivo	10	12	4.258	2.51166667	1.74633333	c(t = 0.89567	0.383	0.84676496	Distal Intergenic	ENSSSCG000	CA6	carbonic anhydrase 6 [Source:NCBI gene (formerly Entrezgene);Acc:100240717]
chrX	46201365	46202322	958	*	chrX	46201365	46202322	in_vitro	in_vivo	11	9	3.84727273	10.7933333	-6.9460606	c(t = -0.9133	0.384	0.84702641	Distal Intergenic	ENSSSCG000	ssc-mir-98	ssc-mir-98 [Source:miRBase;Acc:MI0013122]
chr7	21972253	21972998	746	*	chr7	21972253	21972998	in_vitro	in_vivo	10	9	1.612	3.75666667	-2.1446667	c(t = -0.9074	0.384	0.84702641	Distal Intergenic	ENSSSCG000	ZNF389	zinc finger protein 389 [Source:NCBI gene (formerly Entrezgene);Acc:100270748]
chr3	39035814	39037430	1617	*	chr3	39035814	39037430	in_vitro	in_vivo	10	13	1.455	2.97384615	-1.5188462	c(t = -0.8938	0.384	0.84702641	Distal Intergenic	ENSSSCG000	ZNF205	zinc finger protein 205 [Source:VGNC Symbol;Acc:VGNC:95204]
chr9	78661561	78662148	588	*	chr9	78661561	78662148	in_vitro	in_vivo	9	9	0.39666667	1.71555556	-1.3188889	c(t = -0.9145	0.384	0.84702641	Distal Intergenic	ENSSSCG000	MIOS	meiosis regulator for oocyte development [Source:VGNC Symbol;Acc:VGNC:90234]
chr5	34560999	34562850	1852	*	chr5	34560999	34562850	in_vitro	in_vivo	11	11	0.60363636	1.70727273	-1.1036364	c(t = -0.9048	0.384	0.84702641	Distal Intergenic	ENSSSCG000	CNOT2	CCR4-NOT transcription complex subunit 2 [Source:NCBI gene (formerly Entrezgene);Acc:100153354]
chr1	39039877	39040605	729	*	chr1	39039877	39040605	in_vitro	in_vivo	11	11	0.85181818	1.55909091	-0.7072727	c(t = -0.8911	0.384	0.84702641	Distal Intergenic	ENSSSCG000	FABP7	fatty acid binding protein 7 [Source:NCBI gene (formerly Entrezgene);Acc:574075]
chr9	20613443	20614159	717	*	chr9	20613443	20614159	in_vitro	in_vivo	10	11	2.003	1.13363636	0.86936364	c(t = 0.90235	0.384	0.84702641	Distal Intergenic	ENSSSCG000	ME3	malic enzyme 3 [Source:VGNC Symbol;Acc:VGNC:90097]
chr9	67268306	67269005	700	*	chr9	67268306	67269005	in_vitro	in_vivo	10	9	1.981	0.74111111	1.23988889	c(t = 0.89608	0.384	0.84702641	Distal Intergenic	ENSSSCG000	IL10	interleukin 10 [Source:VGNC Symbol;Acc:VGNC:108593]
chr10	68064769	68066841	2073	*	chr10	68064769	68066841	in_vitro	in_vivo	10	10	95.276	92.579	2.697	c(t = 0.89090	0.385	0.84754571	Distal Intergenic	ENSSSCG000	WDR37	WD repeat domain 37 [Source:VGNC Symbol;Acc:VGNC:96258]
chr8	96855265	96856418	1154	*	chr8	96855265	96856418	in_vitro	in_vivo	12	14	1.16083333	1.83571429	-0.674881	c(t = -0.8858	0.385	0.84754571	Distal Intergenic	ENSSSCG000	PGRMC2	progesterone receptor membrane component 2 [Source:NCBI gene (formerly Entrezgene);Acc:100048965]
chr5	6783880	6785296	1417	*	chr5	6783880	6785296	in_vitro	in_vivo	11	11	4.27636364	1.56	2.71636364	c(t = 0.90498	0.385	0.84754571	Distal Intergenic	ENSSSCG000	SNU13	small nuclear ribonucleoprotein 13 [Source:NCBI gene (formerly Entrezgene);Acc:100155420]
chr8	32432898	32434219	1322	*	chr8	32432898	32434219	in_vitro	in_vivo	11	11	2.15909091	1.14454545	1.01454545	c(t = 0.89222	0.385	0.84754571	Distal Intergenic	ENSSSCG000	UCHL1	ubiquitin C-terminal hydrolase L1 [Source:NCBI gene (formerly Entrezgene);Acc:396637]
chr6	65846598	65849370	2773	*	chr6	65846598	65849370	in_vitro	in_vivo	11	10	1.81545455	1.073	0.74245455	c(t = 0.89743	0.385	0.84754571	Distal Intergenic	ENSSSCG000	ssc-mir-4331	ssc-mir-4331-1 [Source:miRBase;Acc:MI0015910]
chr6	54650223	54652380	2158	*	chr6	54650223	54652380	in_vitro	in_vivo	11	13	85.5327273	93.9453846	-8.4126573	c(t = -0.8989	0.386	0.84754571	Distal Intergenic	ENSSSCG000	IRF3	interferon regulatory factor 3 [Source:NCBI gene (formerly Entrezgene);Acc:396656]
chr6	169924351	169925320	970	*	chr6	169924351	169925320	in_vitro	in_vivo	12	9	24.9025	11.5955556	13.3069444	c(t = 0.88888	0.386	0.84754571	Distal Intergenic	ENSSSCG000	ssc-mir-4338	ssc-mir-4338 [Source:miRBase;Acc:MI0015933]
chrX	16223259	16224564	1306	*	chrX	16223259	16224564	in_vitro	in_vivo	13	11	5.11076923	10.2490909	-5.1383217	c(t = -0.8899	0.386	0.84754571	Distal Intergenic	ENSSSCG000	EIF1AX	eukaryotic translation initiation factor 1A X-linked [Source:NCBI gene (formerly Entrezgene);Acc:100522912]
chrX	78602291	78603056	766	*	chrX	78602291	78603056	in_vitro	in_vivo	9	9	3.33333333	8.26111111	-4.9277778	c(t = -0.9057	0.386	0.84754571	Distal Intergenic	ENSSSCG000	TNMD	tenomodulin [Source:NCBI gene (formerly Entrezgene);Acc:100048961]
chr17	32964961	32965903	943	*	chr17	32964961	32965903	in_vitro	in_vivo	9	15	1.54444444	2.274	-0.7295556	c(t = -0.8904	0.386	0.84754571	Promoter (<=1kb)	ENSSSCG000	NOP56	NOP56 ribonucleoprotein [Source:VGNC Symbol;Acc:VGNC:95703]
chr6	167595211	167596409	1199	*	chr6	167595211	167596409	in_vitro	in_vivo	10	12	1.072	2.26583333	-1.1938333	c(t = -0.8900	0.386	0.84754571	Distal Intergenic	ENSSSCG000	ST3GAL3	ST3 beta-galactoside alpha-2,3-sialyltransferase 3 [Source:VGNC Symbol;Acc:VGNC:93503]
chr6	1948832	1950057	1226	*	chr6	1948832	1950057	in_vitro	in_vivo	10	11	7.197	1.79727273	5.39972727	c(t = 0.90875	0.386	0.84754571	Distal Intergenic	ENSSSCG000	MAP1LC3B	microtubule associated protein 1 light chain 3 beta [Source:NCBI gene (formerly Entrezgene);Acc:100462754]
chr17	34800600	34801198	599	*	chr17	34800600	34801198	in_vitro	in_vivo	10	9	3.183	1.74888889	1.43411111	c(t = 0.88980	0.386	0.84754571	Distal Intergenic	ENSSSCG000	TBC1D20	TBC1 domain family member 20 [Source:NCBI gene (formerly Entrezgene);Acc:100519805]
chr12	5207517	5208841	1325	*	chr12	5207517	5208841	in_vitro	in_vivo	13	11	2.75615385	1.71	1.04615385	c(t = 0.88605	0.386	0.84754571	Distal Intergenic	ENSSSCG000	ACOX1	acyl-CoA oxidase 1 [Source:VGNC Symbol;Acc:VGNC:85020]
chr15	132540518	132541145	628	*	chr15	132540518	132541145	in_vitro	in_vivo	11	13	2.27454545	1.18923077	1.08531469	c(t = 0.88436	0.386	0.84754571	Promoter (<=1kb)	ENSSSCG000	DIS3L2	DIS3 like 3'-5' exoribonuclease 2 [Source:VGNC Symbol;Acc:VGNC:96183]
chr2	123003130	123003892	763	*	chr2	123003130	123003892	in_vitro	in_vivo	12	12	2.3575	1	1.3575	c(t = 0.89720	0.386	0.84754571	Distal Intergenic	ENSSSCG000	TNFAIP8	TNF alpha induced protein 8 [Source:VGNC Symbol;Acc:VGNC:94253]
chr9	66249424	66250117	694	*	chr9	66249424	66250117	in_vitro	in_vivo	10	10	3.982	2.278	1.704	c(t = 0.89162	0.387	0.84839994	Distal Intergenic	ENSSSCG000	NUCKS1	nuclear casein kinase and cyclin dependent kinase substrate 1 [Source:VGNC Symbol;Acc:VGNC:90947]
chr8	87708757	87709223	467	*	chr8	87708757	87709223	in_vitro	in_vivo	11	12	0.50545455	1.71166667	-1.2062121	c(t = -0.8911	0.387	0.84839994	Promoter (<=1kb)	ENSSSCG000	MGARP	mitochondria localized glutamic acid rich protein [Source:NCBI gene (formerly Entrezgene);Acc:100359351]
chr11	70859146	70860493	1348	*	chr11	70859146	70860493	in_vitro	in_vivo	13	14	1.88153846	0.76	1.12153846	c(t = 0.89383	0.387	0.84839994	Distal Intergenic	ENSSSCG000	BIVM	basic, immunoglobulin-like variable motif containing [Source:VGNC Symbol;Acc:VGNC:109180]
chr15	75940381	75940896	516	*	chr15	75940381	75940896	in_vitro	in_vivo	9	9	2.70555556	0.61777778	2.08777778	c(t = 0.90815	0.387	0.84839994	Distal Intergenic	ENSSSCG000	PHOSPHO2	phosphatase, orphan 2 [Source:VGNC Symbol;Acc:VGNC:96172]
chr7	9140181	9140727	547	*	chr7	9140181	9140727	in_vitro	in_vivo	9	9	1.14	2.47444444	-1.3344444	c(t = -0.8910	0.388	0.84839994	Distal Intergenic	ENSSSCG000	EDN1	endothelin 1 [Source:NCBI gene (formerly Entrezgene);Acc:396915]
chr14	50773993	50774542	550	*	chr14	50773993	50774542	in_vitro	in_vivo	9	9	4.20555556	2.10333333	2.10222222	c(t = 0.89376	0.388	0.84839994	Distal Intergenic	ENSSSCG000	SLC7A4	solute carrier family 7 member 4 [Source:VGNC Symbol;Acc:VGNC:93174]
chr14	29423592	29424938	1347	*	chr14	29423592	29424938	in_vitro	in_vivo	13	13	2.75846154	1.75615385	1.00230769	c(t = 0.87964	0.388	0.84839994	Distal Intergenic	ENSSSCG000	SUMO4	SMT3 suppressor of mif two 3 homolog 4 (S. cerevisiae) [Source:NCBI gene (formerly Entrezgene);Acc:100127140]
chr7	117082702	117084111	1410	*	chr7	117082702	117084111	in_vitro	in_vivo	9	11	2.83666667	1.22545455	1.61121212	c(t = 0.90400	0.388	0.84839994	Distal Intergenic	ENSSSCG000	BDKRB2	bradykinin receptor B2 [Source:VGNC Symbol;Acc:VGNC:85794]
chr18	1653689	1654559	871	*	chr18	1653689	1654559	in_vitro	in_vivo	10	10	1.327	0.528	0.799	c(t = 0.89598	0.388	0.84839994	Distal Intergenic	ENSSSCG000	ssc-mir-153	ssc-mir-153 [Source:miRBase;Acc:MI0002454]
chr3	54930207	54930936	730	*	chr3	54930207	54930936	in_vitro	in_vivo	10	13	0.842	0.34615385	0.49584615	c(t = 0.89364	0.388	0.84839994	Distal Intergenic	ENSSSCG000	MGAT4A	alpha-1,3-mannosyl-glycoprotein 4-beta-N-acetylglucosaminyltransferase A [Source:VGNC Symbol;Acc:VGNC:108158]
chr10	939877	940319	443	*	chr10	939877	940319	in_vitro	in_vivo	12	10	0.8475	0.312	0.5355	c(t = 0.88442	0.388	0.84839994	Promoter (<=1kb)	ENSSSCG000	RGS2	regulator of G protein signaling 2 [Source:NCBI gene (formerly Entrezgene);Acc:733670]
chrX	124234696	124236903	2208	*	chrX	124234696	124236903	in_vitro	in_vivo	13	11	21.5838462	33.0354545	-11.451608	c(t = -0.8881	0.389	0.84839994	Distal Intergenic	ENSSSCG000	PNCK	pregnancy up-regulated nonubiquitous CaM kinase [Source:NCBI gene (formerly Entrezgene);Acc:100337678]
chr18	29935814	29936443	630	*	chr18	29935814	29936443	in_vitro	in_vivo	12	13	2.41666667	8.00615385	-5.5894872	c(t = -0.8889	0.389	0.84839994	Promoter (<=1kb)	ENSSSCG000	TES	testin LIM domain protein [Source:NCBI gene (formerly Entrezgene);Acc:100125376]
chr2	142131139	142132715	1577	*	chr2	142131139	142132715	in_vitro	in_vivo	11	15	2.58090909	3.73333333	-1.1524242	c(t = -0.8802	0.389	0.84839994	Distal Intergenic	ENSSSCG000	HBEGF	heparin binding EGF like growth factor [Source:VGNC Symbol;Acc:VGNC:88792]
chr6	94891069	94892194	1126	*	chr6	94891069	94892194	in_vitro	in_vivo	9	12	0.98333333	1.76666667	-0.7833333	c(t = -0.8812	0.389	0.84839994	Promoter (<=1kb)	ENSSSCG000	AKIRIN1	akirin 1 [Source:NCBI gene (formerly Entrezgene);Acc:100144621]
chr2	108255487	108256428	942	*	chr2	108255487	108256428	in_vitro	in_vivo	10	13	0.832	1.68	-0.848	c(t = -0.8845	0.389	0.84839994	Distal Intergenic	ENSSSCG000	ST8SIA4	ST8 alpha-N-acetyl-neuraminide alpha-2,8-sialyltransferase 4 [Source:NCBI gene (formerly Entrezgene);Acc:641356]
chr5	29205887	29206784	898	*	chr5	29205887	29206784	in_vitro	in_vivo	12	11	2.86	1.56818182	1.29181818	c(t = 0.88931	0.389	0.84839994	Distal Intergenic	ENSSSCG000	TBK1	TANK binding kinase 1 [Source:VGNC Symbol;Acc:VGNC:93787]
chr13	71886211	71887120	910	*	chr13	71886211	71887120	in_vitro	in_vivo	11	12	0.45454545	1.07916667	-0.6246212	c(t = -0.8791	0.389	0.84839994	Distal Intergenic	ENSSSCG000	RPN1	ribophorin I [Source:NCBI gene (formerly Entrezgene);Acc:397606]
chr10	68528038	68529249	1212	*	chr10	68528038	68529249	in_vitro	in_vivo	9	14	1.44888889	0.855	0.59388889	c(t = 0.89358	0.389	0.84839994	Promoter (<=1kb)	ENSSSCG000	WDR37	WD repeat domain 37 [Source:VGNC Symbol;Acc:VGNC:96258]
chr12	45760921	45763958	3038	*	chr12	45760921	45763958	in_vitro	in_vivo	10	11	54.77	37.5363636	17.2336364	c(t = 0.88058	0.39	0.84839994	Distal Intergenic	NA	NA	NA
chr13	206456277	206466338	10062	*	chr13	206456277	206466338	in_vitro	in_vivo	15	12	45.4886667	35.1258333	10.3628333	c(t = 0.87624	0.39	0.84839994	Distal Intergenic	ENSSSCG000	PDXK	pyridoxal kinase [Source:NCBI gene (formerly Entrezgene);Acc:396983]
chr14	23417781	23418601	821	*	chr14	23417781	23418601	in_vitro	in_vivo	10	10	6.118	11.18	-5.062	c(t = -0.8894	0.39	0.84839994	Distal Intergenic	ENSSSCG000	PIWIL1	piwi like RNA-mediated silencing 1 [Source:VGNC Symbol;Acc:VGNC:98197]
chr11	30512883	30515576	2694	*	chr11	30512883	30515576	in_vitro	in_vivo	14	17	4.94142857	7.57352941	-2.6321008	c(t = -0.8719	0.39	0.84839994	Distal Intergenic	ENSSSCG000	MTRF1	mitochondrial translation release factor 1 [Source:VGNC Symbol;Acc:VGNC:90471]
chr6	30506691	30508939	2249	*	chr6	30506691	30508939	in_vitro	in_vivo	11	12	11.8318182	6.9325	4.89931818	c(t = 0.88688	0.39	0.84839994	Distal Intergenic	ENSSSCG000	MMP2	matrix metallopeptidase 2 [Source:NCBI gene (formerly Entrezgene);Acc:397391]
chrX	123834682	123835457	776	*	chrX	123834682	123835457	in_vitro	in_vivo	10	10	8.733	4.116	4.617	c(t = 0.88345	0.39	0.84839994	Distal Intergenic	ENSSSCG000	NSDHL	NAD(P) dependent steroid dehydrogenase-like [Source:NCBI gene (formerly Entrezgene);Acc:100157894]
chr5	10290704	10291623	920	*	chr5	10290704	10291623	in_vitro	in_vivo	11	15	2.83909091	1.96266667	0.87642424	c(t = 0.87923	0.39	0.84839994	Distal Intergenic	ENSSSCG000	LGALS2	galectin 2 [Source:NCBI gene (formerly Entrezgene);Acc:100217393]
chr8	97087731	97088911	1181	*	chr8	97087731	97088911	in_vitro	in_vivo	11	13	3.20363636	1.29692308	1.90671329	c(t = 0.88712	0.39	0.84839994	Distal Intergenic	ENSSSCG000	PGRMC2	progesterone receptor membrane component 2 [Source:NCBI gene (formerly Entrezgene);Acc:100048965]
chr2	119965428	119966453	1026	*	chr2	119965428	119966453	in_vitro	in_vivo	15	10	1.89733333	0.95	0.94733333	c(t = 0.87769	0.39	0.84839994	Promoter (<=1kb)	ENSSSCG000	ATG12	autophagy related 12 [Source:NCBI gene (formerly Entrezgene);Acc:100462745]
chr3	48971115	48971563	449	*	chr3	48971115	48971563	in_vitro	in_vivo	9	13	9.47666667	0.84846154	8.62820513	c(t = 0.90828	0.39	0.84839994	Promoter (<=1kb)	ENSSSCG000	ECRG4	ECRG4 augurin [Source:VGNC Symbol;Acc:VGNC:87536]
chr8	12740885	12741821	937	*	chr8	12740885	12741821	in_vitro	in_vivo	12	14	1.34166667	0.63142857	0.7102381	c(t = 0.87784	0.39	0.84839994	Distal Intergenic	ENSSSCG000	MED28	mediator complex subunit 28 [Source:VGNC Symbol;Acc:VGNC:90118]
chrX	85348701	85349474	774	*	chrX	85348701	85349474	in_vitro	in_vivo	12	12	34.905	25.8291667	9.07583333	c(t = 0.87529	0.391	0.84839994	Distal Intergenic	ENSSSCG000	PLP1	proteolipid protein 1 [Source:NCBI gene (formerly Entrezgene);Acc:397029]
chr17	46596348	46597002	655	*	chr17	46596348	46597002	in_vitro	in_vivo	10	10	1.697	3.333	-1.636	c(t = -0.8880	0.391	0.84839994	Distal Intergenic	ENSSSCG000	FITM2	fat storage inducing transmembrane protein 2 [Source:NCBI gene (formerly Entrezgene);Acc:100158013]
chr12	43347469	43348573	1105	*	chr12	43347469	43348573	in_vitro	in_vivo	9	10	1.23888889	2.635	-1.3961111	c(t = -0.8814	0.391	0.84839994	Distal Intergenic	ENSSSCG000	ssc-mir-193a	ssc-mir-193a [Source:miRBase;Acc:MI0013113]
chr14	38667558	38668285	728	*	chr14	38667558	38668285	in_vitro	in_vivo	10	11	0.47	2.28727273	-1.8172727	c(t = -0.8951	0.391	0.84839994	Distal Intergenic	ENSSSCG000	OAS2	2'-5'-oligoadenylate synthetase 2 [Source:VGNC Symbol;Acc:VGNC:91007]
chr11	2421219	2422371	1153	*	chr11	2421219	2422371	in_vitro	in_vivo	12	11	0.565	1.06909091	-0.5040909	c(t = -0.8818	0.391	0.84839994	Distal Intergenic	ENSSSCG000	ssc-mir-9814	ssc-mir-9814 [Source:miRBase;Acc:MI0031581]
chr11	3835568	3836152	585	*	chr11	3835568	3836152	in_vitro	in_vivo	9	12	2.12	1.04166667	1.07833333	c(t = 0.88548	0.391	0.84839994	Distal Intergenic	ENSSSCG000	GPR12	G protein-coupled receptor 12 [Source:NCBI gene (formerly Entrezgene);Acc:100736641]
chr5	61162612	61163303	692	*	chr5	61162612	61163303	in_vitro	in_vivo	10	15	2.19	0.94733333	1.24266667	c(t = 0.88601	0.391	0.84839994	Distal Intergenic	ENSSSCG000	TP23	basic proline-rich protein [Source:NCBI gene (formerly Entrezgene);Acc:100049648]
chr1	54546699	54547818	1120	*	chr1	54546699	54547818	in_vitro	in_vivo	12	12	1.6525	0.87583333	0.77666667	c(t = 0.88276	0.391	0.84839994	Distal Intergenic	ENSSSCG000	CGA	glycoprotein hormones, alpha polypeptide [Source:VGNC Symbol;Acc:VGNC:86613]
chr2	60176106	60176795	690	*	chr2	60176106	60176795	in_vitro	in_vivo	10	10	1.621	0.66	0.961	c(t = 0.88630	0.391	0.84839994	Distal Intergenic	ENSSSCG000	SLC27A1	solute carrier family 27 member 1 [Source:VGNC Symbol;Acc:VGNC:93035]
chr14	28301155	28301821	667	*	chr14	28301155	28301821	in_vitro	in_vivo	11	10	1.33545455	0.556	0.77945455	c(t = 0.88836	0.391	0.84839994	Promoter (<=1kb)	ENSSSCG000	SCARB1	scavenger receptor class B member 1 [Source:NCBI gene (formerly Entrezgene);Acc:397018]
chr17	22480202	22480679	478	*	chr17	22480202	22480679	in_vitro	in_vivo	9	9	1.02333333	0.32666667	0.69666667	c(t = 0.89197	0.391	0.84839994	Distal Intergenic	ENSSSCG00000037602		small nuclear ribonucleoprotein polypeptide B2 [Source:VGNC Symbol;Acc:VGNC:108738]
chrX	125183346	125185313	1968	*	chrX	125183346	125185313	in_vitro	in_vivo	12	15	12.7791667	22.1893333	-9.4101667	c(t = -0.8705	0.392	0.84839994	Distal Intergenic	ENSSSCG000	DKC1	dyskerin pseudouridine synthase 1 [Source:NCBI gene (formerly Entrezgene);Acc:100216430]
chr8	1053894	1057212	3319	*	chr8	1053894	1057212	in_vitro	in_vivo	11	9	4.95545455	3.52555556	1.42989899	c(t = 0.87768	0.392	0.84839994	Distal Intergenic	ENSSSCG000	LETM1	leucine zipper and EF-hand containing transmembrane protein 1 [Source:VGNC Symbol;Acc:VGNC:89687]
chr17	51333333	51334502	1170	*	chr17	51333333	51334502	in_vitro	in_vivo	11	11	1.33272727	2.78272727	-1.45	c(t = -0.8878	0.392	0.84839994	Distal Intergenic	ENSSSCG000	IL29	interleukin 29 (interferon, lambda 1) [Source:NCBI gene (formerly Entrezgene);Acc:100217388]
chr6	81722201	81723288	1088	*	chr6	81722201	81723288	in_vitro	in_vivo	10	11	0.52	1.29454545	-0.7745455	c(t = -0.8850	0.392	0.84839994	Promoter (<=1kb)	ENSSSCG000	PNRC2	proline rich nuclear receptor coactivator 2 [Source:NCBI gene (formerly Entrezgene);Acc:100625012]
chr2	89839883	89840519	637	*	chr2	89839883	89840519	in_vitro	in_vivo	9	10	2.54777778	1.204	1.34377778	c(t = 0.88369	0.392	0.84839994	Promoter (<=1kb)	ENSSSCG000	ZCCHC9	zinc finger CCHC-type containing 9 [Source:NCBI gene (formerly Entrezgene);Acc:100514154]
chr4	110034682	110035810	1129	*	chr4	110034682	110035810	in_vitro	in_vivo	9	10	1.30333333	0.556	0.74733333	c(t = 0.87843	0.392	0.84839994	Distal Intergenic	ENSSSCG000	AHCYL1	adenosylhomocysteinase like 1 [Source:VGNC Symbol;Acc:VGNC:97873]
chr14	35313747	35314197	451	*	chr14	35313747	35314197	in_vitro	in_vivo	10	15	2.222	0.21533333	2.00666667	c(t = 0.89887	0.392	0.84839994	Promoter (<=1kb)	ENSSSCG000	FBXO21	F-box protein 21 [Source:VGNC Symbol;Acc:VGNC:88035]
chr17	30221721	30223844	2124	*	chr17	30221721	30223844	in_vitro	in_vivo	9	12	4.57666667	7.74666667	-3.17	c(t = -0.8755	0.393	0.84839994	Promoter (<=1kb)	ENSSSCG000	THBD	thrombomodulin [Source:NCBI gene (formerly Entrezgene);Acc:100157642]
chr13	18978028	18978900	873	*	chr13	18978028	18978900	in_vitro	in_vivo	10	10	0.444	1.359	-0.915	c(t = -0.8844	0.393	0.84839994	Distal Intergenic	ENSSSCG000	CMTM7	CKLF like MARVEL transmembrane domain containing 7 [Source:VGNC Symbol;Acc:VGNC:103928]
chr2	3581902	3583851	1950	*	chr2	3581902	3583851	in_vitro	in_vivo	11	14	1.78	1.12214286	0.65785714	c(t = 0.87173	0.393	0.84839994	Distal Intergenic	ENSSSCG000	FADD	Fas associated via death domain [Source:NCBI gene (formerly Entrezgene);Acc:595129]
chr15	3360580	3361510	931	*	chr15	3360580	3361510	in_vitro	in_vivo	10	11	1.781	0.92727273	0.85372727	c(t = 0.88071	0.393	0.84839994	Distal Intergenic	ENSSSCG000	ACVR2A	activin A receptor type 2A [Source:NCBI gene (formerly Entrezgene);Acc:397282]
chr15	59642046	59643076	1031	*	chr15	59642046	59643076	in_vitro	in_vivo	10	13	1.668	0.58	1.088	c(t = 0.89279	0.393	0.84839994	Distal Intergenic	ENSSSCG000	PRPF40A	pre-mRNA processing factor 40 homolog A [Source:VGNC Symbol;Acc:VGNC:98222]
chr3	40510127	40512022	1896	*	chr3	40510127	40512022	in_vitro	in_vivo	10	9	76.382	63.0655556	13.3164444	c(t = 0.87872	0.394	0.84839994	Distal Intergenic	ENSSSCG00000021560		ubiquitin conjugating enzyme E2 I [Source:NCBI gene (formerly Entrezgene);Acc:100533196]
chr2	65953836	65956067	2232	*	chr2	65953836	65956067	in_vitro	in_vivo	10	11	7.797	14.9763636	-7.1793636	c(t = -0.8758	0.394	0.84839994	Distal Intergenic	ENSSSCG000	TRMT1	tRNA methyltransferase 1 [Source:VGNC Symbol;Acc:VGNC:94441]
chr7	2548653	2549635	983	*	chr7	2548653	2549635	in_vitro	in_vivo	10	13	2.432	8.52230769	-6.0903077	c(t = -0.8816	0.394	0.84839994	Distal Intergenic	ENSSSCG000	ECI2	enoyl-CoA delta isomerase 2 [Source:VGNC Symbol;Acc:VGNC:87532]
chr9	100845795	100846904	1110	*	chr9	100845795	100846904	in_vitro	in_vivo	12	10	7.295	4.988	2.307	c(t = 0.87485	0.394	0.84839994	Distal Intergenic	ENSSSCG000	GNAI1	G protein subunit alpha i1 [Source:VGNC Symbol;Acc:VGNC:88521]
chr6	27881752	27883305	1554	*	chr6	27881752	27883305	in_vitro	in_vivo	9	11	2.47888889	4.70545455	-2.2265657	c(t = -0.8727	0.394	0.84839994	Distal Intergenic	ENSSSCG000	ssc-mir-328	ssc-mir-328 [Source:miRBase;Acc:MI0013158]
chr17	37217727	37218146	420	*	chr17	37217727	37218146	in_vitro	in_vivo	11	9	1.76181818	2.95	-1.1881818	c(t = -0.8746	0.394	0.84839994	Distal Intergenic	ENSSSCG00000007262		long palate lung and nasal epithelium protein 5 [Source:NCBI gene (formerly Entrezgene);Acc:100113428]
chr11	9014525	9016012	1488	*	chr11	9014525	9016012	in_vitro	in_vivo	10	13	0.926	1.96615385	-1.0401538	c(t = -0.8749	0.394	0.84839994	Distal Intergenic	ENSSSCG000	RFC3	replication factor C subunit 3 [Source:VGNC Symbol;Acc:VGNC:92233]
chr2	150510703	150511768	1066	*	chr2	150510703	150511768	in_vitro	in_vivo	11	11	3.63272727	1.87	1.76272727	c(t = 0.88448	0.394	0.84839994	Promoter (<=1kb)	ENSSSCG000	GRPEL2	GrpE like 2, mitochondrial [Source:VGNC Symbol;Acc:VGNC:88708]
chr5	67193294	67195014	1721	*	chr5	67193294	67195014	in_vitro	in_vivo	10	12	2.064	1.07833333	0.98566667	c(t = 0.88230	0.394	0.84839994	Distal Intergenic	ENSSSCG000	TEAD4	TEA domain transcription factor 4 [Source:NCBI gene (formerly Entrezgene);Acc:100152085]
chr16	41016412	41017473	1062	*	chr16	41016412	41017473	in_vitro	in_vivo	10	15	1.885	0.97	0.915	c(t = 0.87778	0.394	0.84839994	Promoter (<=1kb)	ENSSSCG000	KIF2A	kinesin family member 2A [Source:VGNC Symbol;Acc:VGNC:89467]
chr14	6859613	6860251	639	*	chr14	6859613	6860251	in_vitro	in_vivo	10	13	2.081	0.96153846	1.11946154	c(t = 0.86985	0.394	0.84839994	Promoter (<=1kb)	ENSSSCG000	CCAR2	cell cycle and apoptosis regulator 2 [Source:NCBI gene (formerly Entrezgene);Acc:100155718]
chr7	98321594	98322205	612	*	chr7	98321594	98322205	in_vitro	in_vivo	11	12	1.84636364	0.89166667	0.95469697	c(t = 0.87112	0.394	0.84839994	Distal Intergenic	ENSSSCG000	MLH3	mutL homolog 3 [Source:HGNC Symbol;Acc:HGNC:7128]
chr14	39606763	39607188	426	*	chr14	39606763	39607188	in_vitro	in_vivo	10	11	0.263	0.83454545	-0.5715455	c(t = -0.8799	0.394	0.84839994	Distal Intergenic	ENSSSCG000	MAPKAPK5	MAPK activated protein kinase 5 [Source:NCBI gene (formerly Entrezgene);Acc:100153404]
chr9	106161419	106162390	972	*	chr9	106161419	106162390	in_vitro	in_vivo	13	10	1.02538462	0.44	0.58538462	c(t = 0.87042	0.394	0.84839994	Promoter (<=1kb)	ENSSSCG000	NAMPT	nicotinamide phosphoribosyltransferase [Source:VGNC Symbol;Acc:VGNC:90573]
chr2	9119562	9120045	484	*	chr2	9119562	9120045	in_vitro	in_vivo	9	11	1.16888889	0.37909091	0.78979798	c(t = 0.88485	0.394	0.84839994	Distal Intergenic	ENSSSCG000	GANAB	glucosidase II alpha subunit [Source:VGNC Symbol;Acc:VGNC:88344]
chr1	14794865	14795890	1026	*	chr1	14794865	14795890	in_vitro	in_vivo	9	11	1.58	2.93727273	-1.3572727	c(t = -0.8724	0.395	0.84839994	Promoter (<=1kb)	ENSSSCG000	ARMT1	acidic residue methyltransferase 1 [Source:NCBI gene (formerly Entrezgene);Acc:100157036]
chrX	17560394	17562268	1875	*	chrX	17560394	17562268	in_vitro	in_vivo	12	11	5.375	2.83909091	2.53590909	c(t = 0.87035	0.395	0.84839994	Distal Intergenic	ENSSSCG000	SMPX	small muscle protein X-linked [Source:NCBI gene (formerly Entrezgene);Acc:780438]
chr3	33753250	33755159	1910	*	chr3	33753250	33755159	in_vitro	in_vivo	11	12	1.88909091	2.70166667	-0.8125758	c(t = -0.8679	0.395	0.84839994	Promoter (<=1kb)	ENSSSCG000	USP7	ubiquitin specific peptidase 7 [Source:VGNC Symbol;Acc:VGNC:94777]
chr11	5332263	5334043	1781	*	chr11	5332263	5334043	in_vitro	in_vivo	11	15	4.33	2.564	1.766	c(t = 0.88272	0.395	0.84839994	Distal Intergenic	ENSSSCG000	CDX2	caudal type homeobox 2 [Source:NCBI gene (formerly Entrezgene);Acc:100127132]
chr8	135801713	135804498	2786	*	chr8	135801713	135804498	in_vitro	in_vivo	13	14	2.68461538	1.56857143	1.11604396	c(t = 0.87270	0.395	0.84839994	Promoter (<=1kb)	ENSSSCG000	ENOPH1	enolase-phosphatase 1 [Source:VGNC Symbol;Acc:VGNC:98922]
chr6	103231795	103235011	3217	*	chr6	103231795	103235011	in_vitro	in_vivo	9	9	3.20888889	1.46777778	1.74111111	c(t = 0.88971	0.395	0.84839994	Promoter (<=1kb)	ENSSSCG000	TGIF1	TGFB induced factor homeobox 1 [Source:VGNC Symbol;Acc:VGNC:93935]
chr10	14294698	14296364	1667	*	chr10	14294698	14296364	in_vitro	in_vivo	12	12	0.75	1.41083333	-0.6608333	c(t = -0.8698	0.395	0.84839994	Distal Intergenic	ENSSSCG000	ACBD3	acyl-CoA binding domain containing 3 [Source:NCBI gene (formerly Entrezgene);Acc:494461]
chr14	107590758	107591563	806	*	chr14	107590758	107591563	in_vitro	in_vivo	13	10	1.78923077	0.747	1.04223077	c(t = 0.87506	0.395	0.84839994	Distal Intergenic	ENSSSCG000	OPALIN	oligodendrocytic myelin paranodal and inner loop protein [Source:VGNC Symbol;Acc:VGNC:91046]
chr8	105121278	105122468	1191	*	chr8	105121278	105122468	in_vitro	in_vivo	10	10	1.74	0.685	1.055	c(t = 0.87849	0.395	0.84839994	Distal Intergenic	ENSSSCG000	MYOZ2	myozenin 2 [Source:NCBI gene (formerly Entrezgene);Acc:733663]
chr2	95018311	95018788	478	*	chr2	95018311	95018788	in_vitro	in_vivo	9	13	1.74	0.55230769	1.18769231	c(t = 0.89145	0.395	0.84839994	Promoter (<=1kb)	ENSSSCG000	CCNH	cyclin H [Source:VGNC Symbol;Acc:VGNC:86359]
chr14	140969552	140980853	11302	*	chr14	140969552	140980853	in_vitro	in_vivo	11	11	40.7054545	49.6272727	-8.9218182	c(t = -0.8665	0.396	0.84936087	Distal Intergenic	ENSSSCG000	ssc-mir-202	ssc-mir-202 [Source:miRBase;Acc:MI0013157]
chr13	95019032	95019677	646	*	chr13	95019032	95019677	in_vitro	in_vivo	9	11	1.33444444	2.54636364	-1.2119192	c(t = -0.8767	0.396	0.84936087	Distal Intergenic	ENSSSCG000	GPR149	G protein-coupled receptor 149 [Source:VGNC Symbol;Acc:VGNC:88605]
chr4	101900654	101902737	2084	*	chr4	101900654	101902737	in_vitro	in_vivo	11	13	2.00363636	1.26461538	0.73902098	c(t = 0.86674	0.396	0.84936087	Distal Intergenic	ENSSSCG000	WARS2	tryptophanyl tRNA synthetase 2, mitochondrial [Source:NCBI gene (formerly Entrezgene);Acc:100737714]
chr5	23062627	23063817	1191	*	chr5	23062627	23063817	in_vitro	in_vivo	9	11	2.10666667	1.02090909	1.08575758	c(t = 0.87395	0.396	0.84936087	Distal Intergenic	ENSSSCG000	CYP27B1	cytochrome P450 family 27 subfamily B member 1 [Source:VGNC Symbol;Acc:VGNC:103376]
chr13	10756612	10758021	1410	*	chr13	10756612	10758021	in_vitro	in_vivo	10	16	1.988	0.9025	1.0855	c(t = 0.88543	0.396	0.84936087	Distal Intergenic	ENSSSCG00000037030		ribosomal protein L15 [Source:NCBI gene (formerly Entrezgene);Acc:100620094]
chrX	14308138	14309019	882	*	chrX	14308138	14309019	in_vitro	in_vivo	13	10	1.54615385	4.862	-3.3158462	c(t = -0.8871	0.397	0.84939474	Distal Intergenic	ENSSSCG000	SYAP1	synapse associated protein 1 [Source:VGNC Symbol;Acc:VGNC:93648]
chr6	93821264	93822248	985	*	chr6	93821264	93822248	in_vitro	in_vivo	9	9	6.85555556	2.69	4.16555556	c(t = 0.89126	0.397	0.84939474	Distal Intergenic	ENSSSCG000	MTF1	metal regulatory transcription factor 1 [Source:VGNC Symbol;Acc:VGNC:90443]
chr9	56453965	56454507	543	*	chr9	56453965	56454507	in_vitro	in_vivo	10	12	0.788	1.9275	-1.1395	c(t = -0.8719	0.397	0.84939474	Distal Intergenic	ENSSSCG000	KCNJ5	potassium inwardly rectifying channel subfamily J member 5 [Source:NCBI gene (formerly Entrezgene);Acc:397448]
chr11	25771414	25773436	2023	*	chr11	25771414	25773436	in_vitro	in_vivo	15	13	1.918	1.30538462	0.61261538	c(t = 0.86156	0.397	0.84939474	Distal Intergenic	ENSSSCG000	MTRF1	mitochondrial translation release factor 1 [Source:VGNC Symbol;Acc:VGNC:90471]
chr14	136478665	136480359	1695	*	chr14	136478665	136480359	in_vitro	in_vivo	12	12	0.78833333	1.21166667	-0.4233333	c(t = -0.8640	0.397	0.84939474	Distal Intergenic	ENSSSCG000	UROS	uroporphyrinogen III synthase [Source:VGNC Symbol;Acc:VGNC:94735]
chr5	100537877	100539441	1565	*	chr5	100537877	100539441	in_vitro	in_vivo	11	15	0.46181818	1.08866667	-0.6268485	c(t = -0.8651	0.397	0.84939474	Distal Intergenic	ENSSSCG000	MYF5	myogenic factor 5 [Source:NCBI gene (formerly Entrezgene);Acc:100153269]
chr14	132702929	132703932	1004	*	chr14	132702929	132703932	in_vitro	in_vivo	9	9	1.62888889	0.72555556	0.90333333	c(t = 0.88383	0.397	0.84939474	Distal Intergenic	ENSSSCG000	ssc-mir-4331	ssc-mir-4331-2 [Source:miRBase;Acc:MI0033415]
chr2	148775751	148776915	1165	*	chr2	148775751	148776915	in_vitro	in_vivo	13	11	1.09538462	0.63181818	0.46356643	c(t = 0.86641	0.397	0.84939474	Distal Intergenic	ENSSSCG000	PPP2R2B	protein phosphatase 2 regulatory subunit Bbeta [Source:VGNC Symbol;Acc:VGNC:100336]
chrX	97650133	97650587	455	*	chrX	97650133	97650587	in_vitro	in_vivo	10	11	3.809	8.93909091	-5.1300909	c(t = -0.8729	0.398	0.84939474	Distal Intergenic	ENSSSCG000	PGRMC1	progesterone receptor membrane component 1 [Source:NCBI gene (formerly Entrezgene);Acc:396946]
chr5	18709999	18710572	574	*	chr5	18709999	18710572	in_vitro	in_vivo	9	9	2.99888889	5.65555556	-2.6566667	c(t = -0.8706	0.398	0.84939474	Distal Intergenic	ENSSSCG000	PCBP2	poly(rC) binding protein 2 [Source:VGNC Symbol;Acc:VGNC:103309]
chr1	130472785	130474080	1296	*	chr1	130472785	130474080	in_vitro	in_vivo	12	15	6.21916667	3.61	2.60916667	c(t = 0.86555	0.398	0.84939474	Promoter (<=1kb)	ENSSSCG000	SPINT1	serine peptidase inhibitor, Kunitz type 1 [Source:VGNC Symbol;Acc:VGNC:93410]
chr16	51087883	51089061	1179	*	chr16	51087883	51089061	in_vitro	in_vivo	10	11	3.2	2.07454545	1.12545455	c(t = 0.87005	0.398	0.84939474	Distal Intergenic	ENSSSCG000	STC2	stanniocalcin 2 [Source:NCBI gene (formerly Entrezgene);Acc:100126236]
chr17	62708240	62710591	2352	*	chr17	62708240	62710591	in_vitro	in_vivo	11	12	2.01818182	1.31583333	0.70234848	c(t = 0.86752	0.398	0.84939474	Distal Intergenic	ENSSSCG000	LIME1	Lck interacting transmembrane adaptor 1 [Source:NCBI gene (formerly Entrezgene);Acc:100625216]
chr11	23159158	23159980	823	*	chr11	23159158	23159980	in_vitro	in_vivo	12	15	2.1825	1.19066667	0.99183333	c(t = 0.86516	0.398	0.84939474	Distal Intergenic	ENSSSCG000	GTF2F2	general transcription factor IIF subunit 2 [Source:NCBI gene (formerly Entrezgene);Acc:100626869]
chr10	26626765	26628311	1547	*	chr10	26626765	26628311	in_vitro	in_vivo	10	11	0.555	1.00727273	-0.4522727	c(t = -0.8678	0.398	0.84939474	Distal Intergenic	ENSSSCG000	ssc-mir-24-2	ssc-mir-24-2 [Source:miRBase;Acc:MI0013111]
chr5	7393150	7395020	1871	*	chr5	7393150	7395020	in_vitro	in_vivo	9	10	1.85666667	0.386	1.47066667	c(t = 0.88850	0.398	0.84939474	Distal Intergenic	ENSSSCG000	ACO2	aconitase 2 [Source:NCBI gene (formerly Entrezgene);Acc:396999]
chr3	5394749	5398377	3629	*	chr3	5394749	5398377	in_vitro	in_vivo	10	10	93.762	95.917	-2.155	c(t = -0.8713	0.399	0.84939474	Distal Intergenic	ENSSSCG000	AIMP2	aminoacyl tRNA synthetase complex interacting multifunctional protein 2 [Source:NCBI gene (formerly Entrezgene);Acc:100514707]
chr1	8215314	8218708	3395	*	chr1	8215314	8218708	in_vitro	in_vivo	13	11	44.3830769	33.5909091	10.7921678	c(t = 0.86039	0.399	0.84939474	3' UTR	ENSSSCG000	TAGAP	T cell activation RhoGTPase activating protein [Source:NCBI gene (formerly Entrezgene);Acc:100155114]
chr1	129873353	129876189	2837	*	chr1	129873353	129876189	in_vitro	in_vivo	14	13	5.49571429	3.58076923	1.91494505	c(t = 0.85939	0.399	0.84939474	Distal Intergenic	ENSSSCG000	JMJD7	jumonji domain containing 7 [Source:NCBI gene (formerly Entrezgene);Acc:100533119]
chr1	237777497	237779049	1553	*	chr1	237777497	237779049	in_vitro	in_vivo	12	13	4.695	3.05769231	1.63730769	c(t = 0.86230	0.399	0.84939474	Distal Intergenic	ENSSSCG000	GNE	glucosamine (UDP-N-acetyl)-2-epimerase/N-acetylmannosamine kinase [Source:VGNC Symbol;Acc:VGNC:103101]
chr6	65167928	65170313	2386	*	chr6	65167928	65170313	in_vitro	in_vivo	11	12	7.12909091	2.9025	4.22659091	c(t = 0.87925	0.399	0.84939474	Promoter (<=1kb)	ENSSSCG000	WRAP73	WD repeat containing, antisense to TP73 [Source:VGNC Symbol;Acc:VGNC:94980]
chr13	66997844	66998852	1009	*	chr13	66997844	66998852	in_vitro	in_vivo	12	13	1.2875	2.23538462	-0.9478846	c(t = -0.8604	0.399	0.84939474	Distal Intergenic	ENSSSCG000	ssc-mir-885	ssc-mir-885 [Source:miRBase;Acc:MI0013119]
chr1	577679	578628	950	*	chr1	577679	578628	in_vitro	in_vivo	10	9	3.285	2.09666667	1.18833333	c(t = 0.86573	0.399	0.84939474	Distal Intergenic	ENSSSCG000	ssc-mir-9815	ssc-mir-9815 [Source:miRBase;Acc:MI0031582]
chr6	110540	112050	1511	*	chr6	110540	112050	in_vitro	in_vivo	10	9	2.394	0.97777778	1.41622222	c(t = 0.87079	0.399	0.84939474	Distal Intergenic	ENSSSCG000	MC1R	melanocortin 1 receptor [Source:NCBI gene (formerly Entrezgene);Acc:494018]
chr13	67871394	67872720	1327	*	chr13	67871394	67872720	in_vitro	in_vivo	12	12	1.80916667	0.96916667	0.84	c(t = 0.86033	0.399	0.84939474	Distal Intergenic	ENSSSCG000	ATG7	autophagy related 7 [Source:VGNC Symbol;Acc:VGNC:85625]
chr16	47284471	47285239	769	*	chr16	47284471	47285239	in_vitro	in_vivo	13	11	1.53692308	0.81454545	0.72237762	c(t = 0.86010	0.399	0.84939474	Promoter (<=1kb)	ENSSSCG000	SLC30A5	solute carrier family 30 member 5 [Source:NCBI gene (formerly Entrezgene);Acc:100171388]
chr2	85294295	85295472	1178	*	chr2	85294295	85295472	in_vitro	in_vivo	9	12	1.76333333	0.70666667	1.05666667	c(t = 0.88100	0.399	0.84939474	Distal Intergenic	ENSSSCG000	F2R	coagulation factor II thrombin receptor [Source:NCBI gene (formerly Entrezgene);Acc:100521609]
chr6	1420275	1427018	6744	*	chr6	1420275	1427018	in_vitro	in_vivo	12	16	93.3241667	89.609375	3.71479167	c(t = 0.85848	0.4	0.84990532	3' UTR	ENSSSCG000	BANP	BTG3 associated nuclear protein [Source:NCBI gene (formerly Entrezgene);Acc:100622381]
chr6	1488806	1493127	4322	*	chr6	1488806	1493127	in_vitro	in_vivo	11	11	88.21	82.5827273	5.62727273	c(t = 0.86087	0.4	0.84990532	Intron (NM_001244650/100622381, intron 1 of 12)	ENSSSCG000	BANP	BTG3 associated nuclear protein [Source:NCBI gene (formerly Entrezgene);Acc:100622381]
chr6	56389302	56390042	741	*	chr6	56389302	56390042	in_vitro	in_vivo	11	12	16.4654545	23.8041667	-7.3387121	c(t = -0.8604	0.4	0.84990532	Distal Intergenic	ENSSSCG000	ssc-mir-371	ssc-mir-371 [Source:miRBase;Acc:MI0031640]
chr1	54633330	54634346	1017	*	chr1	54633330	54634346	in_vitro	in_vivo	9	11	1.05111111	2.32727273	-1.2761616	c(t = -0.8661	0.4	0.84990532	Distal Intergenic	ENSSSCG000	CGA	glycoprotein hormones, alpha polypeptide [Source:VGNC Symbol;Acc:VGNC:86613]
chr7	36385619	36387633	2015	*	chr7	36385619	36387633	in_vitro	in_vivo	13	11	2.13538462	1.03636364	1.09902098	c(t = 0.86174	0.4	0.84990532	Promoter (<=1kb)	ENSSSCG000	NFYA	nuclear transcription factor Y subunit alpha [Source:VGNC Symbol;Acc:VGNC:90729]
chr12	945459	961496	16038	*	chr12	945459	961496	in_vitro	in_vivo	11	14	43.3	34.4592857	8.84071429	c(t = 0.86094	0.401	0.84990532	Distal Intergenic	ENSSSCG000	CBR2	carbonyl reductase 2 [Source:NCBI gene (formerly Entrezgene);Acc:396780]
chr7	883887	888656	4770	*	chr7	883887	888656	in_vitro	in_vivo	9	15	17.0411111	27.354	-10.312889	c(t = -0.8591	0.401	0.84990532	Distal Intergenic	ENSSSCG000	IRF4	interferon regulatory factor 4 [Source:VGNC Symbol;Acc:VGNC:89207]
chr9	46548754	46550029	1276	*	chr9	46548754	46550029	in_vitro	in_vivo	9	13	6.42444444	3.06615385	3.3582906	c(t = 0.87824	0.401	0.84990532	Distal Intergenic	ENSSSCG00000032330		Thy-1 cell surface antigen [Source:VGNC Symbol;Acc:VGNC:93973]
chr9	8821161	8821930	770	*	chr9	8821161	8821930	in_vitro	in_vivo	9	11	4.33555556	2.13727273	2.19828283	c(t = 0.87147	0.401	0.84990532	Promoter (<=1kb)	ENSSSCG000	KCNE3	potassium voltage-gated channel subfamily E regulatory subunit 3 [Source:VGNC Symbol;Acc:VGNC:89337]
chr15	20104715	20105577	863	*	chr15	20104715	20105577	in_vitro	in_vivo	9	13	0.69444444	1.94615385	-1.2517094	c(t = -0.8602	0.401	0.84990532	Promoter (<=1kb)	ENSSSCG000	ACTR3	actin related protein 3 [Source:VGNC Symbol;Acc:VGNC:103894]
chr18	10675970	10677102	1133	*	chr18	10675970	10677102	in_vitro	in_vivo	12	15	0.98083333	1.80066667	-0.8198333	c(t = -0.8581	0.401	0.84990532	Distal Intergenic	ENSSSCG000	ZC3HAV1	zinc finger CCCH-type containing, antiviral 1 [Source:VGNC Symbol;Acc:VGNC:95102]
chr10	11377577	11378836	1260	*	chr10	11377577	11378836	in_vitro	in_vivo	11	14	2.32363636	1.38928571	0.93435065	c(t = 0.85562	0.401	0.84990532	Distal Intergenic	ENSSSCG000	FH	fumarate hydratase [Source:VGNC Symbol;Acc:VGNC:96300]
chr12	21115617	21116652	1036	*	chr12	21115617	21116652	in_vitro	in_vivo	10	10	1.937	0.873	1.064	c(t = 0.87156	0.401	0.84990532	Distal Intergenic	ENSSSCG000	KRT31	keratin 31 [Source:NCBI gene (formerly Entrezgene);Acc:100513946]
chr1	219812837	219815967	3131	*	chr1	219812837	219815967	in_vitro	in_vivo	14	12	1.25714286	0.80166667	0.45547619	c(t = 0.85612	0.401	0.84990532	Distal Intergenic	ENSSSCG000	VLDLR	very low density lipoprotein receptor [Source:VGNC Symbol;Acc:VGNC:94832]
chr5	67445777	67446595	819	*	chr5	67445777	67446595	in_vitro	in_vivo	10	12	49.62	34.6841667	14.9358333	c(t = 0.85757	0.402	0.84990532	Distal Intergenic	ENSSSCG000	WASHC1	WASH complex subunit 1 [Source:NCBI gene (formerly Entrezgene);Acc:100512536]
chr17	61684393	61685667	1275	*	chr17	61684393	61685667	in_vitro	in_vivo	9	11	18.3377778	31.3190909	-12.981313	c(t = -0.8576	0.402	0.84990532	Distal Intergenic	ENSSSCG000	RPS21	ribosomal protein S21 [Source:NCBI gene (formerly Entrezgene);Acc:100037996]
chr4	38366280	38367746	1467	*	chr4	38366280	38367746	in_vitro	in_vivo	10	9	4.544	3.08	1.464	c(t = 0.86221	0.402	0.84990532	Distal Intergenic	ENSSSCG000	RPL30	ribosomal protein L30 [Source:VGNC Symbol;Acc:VGNC:98849]
chr13	81749689	81750896	1208	*	chr13	81749689	81750896	in_vitro	in_vivo	13	10	3.19692308	1.753	1.44392308	c(t = 0.85730	0.402	0.84990532	Promoter (<=1kb)	ENSSSCG000	SLC25A36	solute carrier family 25 member 36 [Source:VGNC Symbol;Acc:VGNC:93012]
chr12	614851	616418	1568	*	chr12	614851	616418	in_vitro	in_vivo	11	11	1.32454545	0.77272727	0.55181818	c(t = 0.85682	0.402	0.84990532	Distal Intergenic	ENSSSCG000	WDR45B	WD repeat domain 45B [Source:NCBI gene (formerly Entrezgene);Acc:100462760]
chr6	106138870	106139585	716	*	chr6	106138870	106139585	in_vitro	in_vivo	11	10	1.75818182	0.714	1.04418182	c(t = 0.85898	0.402	0.84990532	Distal Intergenic	ENSSSCG000	TYMS	thymidylate synthetase [Source:NCBI gene (formerly Entrezgene);Acc:100627209]
chr3	40141520	40142616	1097	*	chr3	40141520	40142616	in_vitro	in_vivo	12	10	1.44333333	0.702	0.74133333	c(t = 0.86417	0.402	0.84990532	Distal Intergenic	ENSSSCG000	IGFALS	insulin like growth factor binding protein acid labile subunit [Source:VGNC Symbol;Acc:VGNC:110281]
chr7	80140642	80141126	485	*	chr7	80140642	80141126	in_vitro	in_vivo	9	10	1.42888889	0.417	1.01188889	c(t = 0.87857	0.402	0.84990532	Promoter (<=1kb)	ENSSSCG000	LPCAT4	lysophosphatidylcholine acyltransferase 4 [Source:VGNC Symbol;Acc:VGNC:89791]
chr1	1617308	1618835	1528	*	chr1	1617308	1618835	in_vitro	in_vivo	10	9	95.933	93.8688889	2.06411111	c(t = 0.86394	0.403	0.84990532	Distal Intergenic	ENSSSCG000	ssc-mir-9824	ssc-mir-9824 [Source:miRBase;Acc:MI0031591]
chrX	31903409	31903728	320	*	chrX	31903409	31903728	in_vitro	in_vivo	10	13	43.85	54.6530769	-10.803077	c(t = -0.8551	0.403	0.84990532	Distal Intergenic	ENSSSCG000	GP91-PHOX	NADPH oxidase heavy chain subunit [Source:NCBI gene (formerly Entrezgene);Acc:397108]
chr10	27726009	27727161	1153	*	chr10	27726009	27727161	in_vitro	in_vivo	10	9	11.428	22.2988889	-10.870889	c(t = -0.8673	0.403	0.84990532	Distal Intergenic	ENSSSCG000	FBP2	fructose-bisphosphatase 2 [Source:NCBI gene (formerly Entrezgene);Acc:100134828]
chr8	444863	450757	5895	*	chr8	444863	450757	in_vitro	in_vivo	12	16	11.77	17.74375	-5.97375	c(t = -0.8503	0.403	0.84990532	Distal Intergenic	ENSSSCG000	ATP5ME	ATP synthase membrane subunit e [Source:NCBI gene (formerly Entrezgene);Acc:396950]
chr14	141268828	141270936	2109	*	chr14	141268828	141270936	in_vitro	in_vivo	11	12	2.51363636	3.72916667	-1.2155303	c(t = -0.8541	0.403	0.84990532	Distal Intergenic	ENSSSCG000	ssc-mir-202	ssc-mir-202 [Source:miRBase;Acc:MI0013157]
chr4	77692667	77693053	387	*	chr4	77692667	77693053	in_vitro	in_vivo	9	9	1.62	3.63222222	-2.0122222	c(t = -0.8612	0.403	0.84990532	Distal Intergenic	ENSSSCG000	ATP6V1H	ATPase H+ transporting V1 subunit H [Source:VGNC Symbol;Acc:VGNC:85677]
chr2	102222002	102223412	1411	*	chr2	102222002	102223412	in_vitro	in_vivo	12	15	1.695	2.63666667	-0.9416667	c(t = -0.8509	0.403	0.84990532	Distal Intergenic	ENSSSCG000	SPATA9	spermatogenesis associated 9 [Source:NCBI gene (formerly Entrezgene);Acc:100415927]
chr18	1268587	1274242	5656	*	chr18	1268587	1274242	in_vitro	in_vivo	14	13	3.07785714	2.09769231	0.98016484	c(t = 0.85241	0.403	0.84990532	Distal Intergenic	ENSSSCG000	ssc-mir-153	ssc-mir-153 [Source:miRBase;Acc:MI0002454]
chr9	107292262	107292707	446	*	chr9	107292262	107292707	in_vitro	in_vivo	11	11	2.73	1.58	1.15	c(t = 0.85746	0.403	0.84990532	Distal Intergenic	ENSSSCG000	SLC26A3	solute carrier family 26 member 3 [Source:VGNC Symbol;Acc:VGNC:93028]
chrX	59919948	59920502	555	*	chrX	59919948	59920502	in_vitro	in_vivo	11	10	5.22363636	1.234	3.98963636	c(t = 0.87063	0.403	0.84990532	Distal Intergenic	ENSSSCG000	ssc-mir-374a	ssc-mir-374a [Source:miRBase;Acc:MI0013130]
chr6	153684681	153687894	3214	*	chr6	153684681	153687894	in_vitro	in_vivo	15	13	1.45266667	0.91076923	0.54189744	c(t = 0.85285	0.403	0.84990532	Promoter (<=1kb)	ENSSSCG000	JUN	Jun proto-oncogene, AP-1 transcription factor subunit [Source:NCBI gene (formerly Entrezgene);Acc:396913]
chr1	14490481	14491428	948	*	chr1	14490481	14491428	in_vitro	in_vivo	11	13	1.47909091	0.87153846	0.60755245	c(t = 0.85317	0.403	0.84990532	Promoter (<=1kb)	ENSSSCG000	ESR1	estrogen receptor 1 [Source:VGNC Symbol;Acc:VGNC:103089]
chr4	128996261	128997287	1027	*	chr4	128996261	128997287	in_vitro	in_vivo	10	10	32.49	19.159	13.331	c(t = 0.85551	0.404	0.84991514	Distal Intergenic	ENSSSCG00000047605		LIM domain only 4 [Source:NCBI gene (formerly Entrezgene);Acc:100127155]
chr12	1304812	1306458	1647	*	chr12	1304812	1306458	in_vitro	in_vivo	12	12	5.11166667	11.135	-6.0233333	c(t = -0.8629	0.404	0.84991514	Distal Intergenic	ENSSSCG000	ARHGDIA	Rho GDP dissociation inhibitor alpha [Source:VGNC Symbol;Acc:VGNC:85483]
chr2	6547216	6548856	1641	*	chr2	6547216	6548856	in_vitro	in_vivo	12	13	5.07083333	7.76923077	-2.6983974	c(t = -0.8506	0.404	0.84991514	Distal Intergenic	ENSSSCG000	KAT5	lysine acetyltransferase 5 [Source:VGNC Symbol;Acc:VGNC:89305]
chr1	236467711	236468689	979	*	chr1	236467711	236468689	in_vitro	in_vivo	10	9	1.142	1.93666667	-0.7946667	c(t = -0.8615	0.404	0.84991514	Promoter (<=1kb)	ENSSSCG000	GBA2	glucosylceramidase beta 2 [Source:NCBI gene (formerly Entrezgene);Acc:100155655]
chr7	95365145	95366014	870	*	chr7	95365145	95366014	in_vitro	in_vivo	12	12	0.89916667	1.72166667	-0.8225	c(t = -0.8547	0.404	0.84991514	Distal Intergenic	ENSSSCG000	RBM25	RNA binding motif protein 25 [Source:NCBI gene (formerly Entrezgene);Acc:100511390]
chr2	89255001	89256047	1047	*	chr2	89255001	89256047	in_vitro	in_vivo	12	16	1.84833333	1.125625	0.72270833	c(t = 0.85285	0.404	0.84991514	Promoter (<=1kb)	ENSSSCG00000031117		dihydrofolate reductase [Source:NCBI gene (formerly Entrezgene);Acc:100525912]
chr14	71827434	71828362	929	*	chr14	71827434	71828362	in_vitro	in_vivo	10	11	2.039	1.08727273	0.95172727	c(t = 0.85847	0.404	0.84991514	Distal Intergenic	ENSSSCG000	TET1	tet methylcytosine dioxygenase 1 [Source:NCBI gene (formerly Entrezgene);Acc:100625859]
chr7	83049237	83050408	1172	*	chr7	83049237	83050408	in_vitro	in_vivo	11	12	2.33454545	0.83333333	1.50121212	c(t = 0.85749	0.404	0.84991514	Distal Intergenic	ENSSSCG000	NR2F2	nuclear receptor subfamily 2 group F member 2 [Source:VGNC Symbol;Acc:VGNC:90881]
chr11	20652031	20652831	801	*	chr11	20652031	20652831	in_vitro	in_vivo	14	12	0.83857143	0.35416667	0.48440476	c(t = 0.85355	0.404	0.84991514	Promoter (<=1kb)	ENSSSCG000	ESD	esterase D [Source:VGNC Symbol;Acc:VGNC:87788]
chr8	31523164	31524414	1251	*	chr8	31523164	31524414	in_vitro	in_vivo	14	10	6.31642857	15.266	-8.9495714	c(t = -0.8624	0.405	0.8499249	Distal Intergenic	ENSSSCG000	CHRNA9	cholinergic receptor nicotinic alpha 9 subunit [Source:VGNC Symbol;Acc:VGNC:86671]
chr7	44640862	44641902	1041	*	chr7	44640862	44641902	in_vitro	in_vivo	9	9	1.30444444	6.43222222	-5.1277778	c(t = -0.8771	0.405	0.8499249	Distal Intergenic	ENSSSCG000	TFAP2B	transcription factor AP-2 beta [Source:VGNC Symbol;Acc:VGNC:93909]
chr15	136068923	136069597	675	*	chr15	136068923	136069597	in_vitro	in_vivo	9	11	11.2444444	5.44909091	5.79535354	c(t = 0.86813	0.405	0.8499249	Distal Intergenic	ENSSSCG000	ssc-mir-7141	ssc-mir-7141 [Source:miRBase;Acc:MI0023574]
chr6	70675763	70676314	552	*	chr6	70675763	70676314	in_vitro	in_vivo	13	13	2.1	4.06923077	-1.9692308	c(t = -0.8571	0.405	0.8499249	Distal Intergenic	ENSSSCG000	CENPS	apoptosis-inducing, TAF9-like domain 1 [Source:NCBI gene (formerly Entrezgene);Acc:100522376]
chr14	141191504	141193845	2342	*	chr14	141191504	141193845	in_vitro	in_vivo	13	11	1.88692308	3.50818182	-1.6212587	c(t = -0.8604	0.405	0.8499249	Distal Intergenic	ENSSSCG000	ssc-mir-202	ssc-mir-202 [Source:miRBase;Acc:MI0013157]
chr5	44558088	44559008	921	*	chr5	44558088	44559008	in_vitro	in_vivo	10	12	3.25	1.78916667	1.46083333	c(t = 0.86095	0.405	0.8499249	Distal Intergenic	ENSSSCG000	ERGIC2	ERGIC and golgi 2 [Source:VGNC Symbol;Acc:VGNC:87771]

chr9	92952842	92953673	832	*	chr9	92952842	92953673	in_vitro	in_vivo	13	11	0.75769231	1.71909091	-0.9613986	c(t = -0.85259	0.405	0.8499249	Distal Intergenic	ENSSSCG00000015390		ATP-binding cassette, sub-family B (MDR/TAP), member 1 [Source:NCBI gene (formerly Entrezgene);Acc:396910]
chr3	102195711	102199008	3298	*	chr3	102195711	102199008	in_vitro	in_vivo	15	14	2.092	1.465	0.627	c(t = -0.84886	0.405	0.8499249	Distal Intergenic	ENSSSCG000	GALM	galactose mutarotase [Source:NCBI gene (formerly Entrezgene);Acc:399536]
chr2	137082520	137083490	971	*	chr2	137082520	137083490	in_vitro	in_vivo	10	13	2.189	0.83461538	1.35438462	c(t = -0.86786	0.405	0.8499249	Distal Intergenic	ENSSSCG000	TXNDC15	thioredoxin domain containing 15 [Source:NCBI gene (formerly Entrezgene);Acc:100626939]
chrX	16449215	16449802	588	*	chrX	16449215	16449802	in_vitro	in_vivo	10	10	5.372	13.311	-7.939	c(t = -0.85852	0.406	0.85039793	Promoter (<=1kb)	ENSSSCG000	EIF1AX	eukaryotic translation initiation factor 1A X-linked [Source:NCBI gene (formerly Entrezgene);Acc:100522912]
chr9	36878970	36879685	716	*	chr9	36878970	36879685	in_vitro	in_vivo	11	9	0.63363636	3.05444444	-2.4208081	c(t = -0.87569	0.406	0.85039793	Distal Intergenic	ENSSSCG000	ATM	ATM serine/threonine kinase [Source:VGNC Symbol;Acc:VGNC:108572]
chr13	200925608	200926879	1272	*	chr13	200925608	200926879	in_vitro	in_vivo	12	10	1.42	2.488	-1.068	c(t = -0.85719	0.406	0.85039793	Distal Intergenic	ENSSSCG000	PIGP	phosphatidylinositol glycan anchor biosynthesis class P [Source:VGNC Symbol;Acc:VGNC:91427]
chr17	14142061	14142968	908	*	chr17	14142061	14142968	in_vitro	in_vivo	11	10	0.87545455	1.839	-0.9635455	c(t = -0.86590	0.406	0.85039793	Promoter (<=1kb)	ENSSSCG000	PCNA	proliferating cell nuclear antigen [Source:NCBI gene (formerly Entrezgene);Acc:692192]
chr2	76146669	76147753	1085	*	chr2	76146669	76147753	in_vitro	in_vivo	9	11	3.27444444	1.72363636	1.55080808	c(t = -0.86527	0.406	0.85039793	Distal Intergenic	ENSSSCG000	OAZ1	ornithine decarboxylase antizyme 1 [Source:NCBI gene (formerly Entrezgene);Acc:397034]
chr9	11193905	11195090	1186	*	chr9	11193905	11195090	in_vitro	in_vivo	12	10	2.01916667	1.159	0.86016667	c(t = -0.85031	0.406	0.85039793	Distal Intergenic	ENSSSCG000	MYO7A	myosin VIIA [Source:NCBI gene (formerly Entrezgene);Acc:397373]
chr2	52311759	52312351	593	*	chr2	52311759	52312351	in_vitro	in_vivo	9	9	1.43666667	0.65333333	0.78333333	c(t = -0.85373	0.406	0.85039793	Distal Intergenic	NA	NA	NA
chr9	6839530	6840473	944	*	chr9	6839530	6840473	in_vitro	in_vivo	9	9	85.73	93.4233333	-7.6933333	c(t = -0.86594	0.407	0.85110068	Distal Intergenic	ENSSSCG000	INPPL1	inositol polyphosphate phosphatase like 1 [Source:NCBI gene (formerly Entrezgene);Acc:100462718]
chr3	41211944	41213443	1500	*	chr3	41211944	41213443	in_vitro	in_vivo	10	10	15.993	28.563	-12.57	c(t = -0.86281	0.407	0.85110068	Distal Intergenic	ENSSSCG000	NME4	NME/NM23 nucleoside diphosphate kinase 4 [Source:VGNC Symbol;Acc:VGNC:90792]
chr6	40505210	40507364	2155	*	chr6	40505210	40507364	in_vitro	in_vivo	13	14	12.1492308	19.8214286	-7.6721978	c(t = -0.84325	0.407	0.85110068	Distal Intergenic	ENSSSCG000	SLC7A9	solute carrier family 7 member 9 [Source:VGNC Symbol;Acc:VGNC:93177]
chrX	26122119	26123080	962	*	chrX	26122119	26123080	in_vitro	in_vivo	10	13	11.098	5.87	5.228	c(t = -0.85808	0.407	0.85110068	Promoter (<=1kb)	ENSSSCG000	NR0B1	nuclear receptor subfamily 0 group B member 1 [Source:NCBI gene (formerly Entrezgene);Acc:397680]
chr2	70132892	70134521	1630	*	chr2	70132892	70134521	in_vitro	in_vivo	12	12	3.22	5.38583333	-2.1658333	c(t = -0.85450	0.407	0.85110068	Distal Intergenic	ENSSSCG000	PRKCSH	protein kinase C substrate 80K-H [Source:VGNC Symbol;Acc:VGNC:91810]
chr7	80164721	80165429	709	*	chr7	80164721	80165429	in_vitro	in_vivo	10	11	0.747	2.34090909	-1.5939091	c(t = -0.86153	0.407	0.85110068	Distal Intergenic	ENSSSCG000	LPCAT4	lysophosphatidylcholine acyltransferase 4 [Source:VGNC Symbol;Acc:VGNC:89791]
chr2	76750045	76751426	1382	*	chr2	76750045	76751426	in_vitro	in_vivo	9	12	4.71	2.70166667	2.00833333	c(t = -0.86055	0.408	0.8515698	Distal Intergenic	ENSSSCG000	KLF16	KLF transcription factor 16 [Source:NCBI gene (formerly Entrezgene);Acc:100302638]
chr2	9734823	9736008	1186	*	chr2	9734823	9736008	in_vitro	in_vivo	10	12	0.747	1.37	-0.623	c(t = -0.84521	0.408	0.8515698	Promoter (<=1kb)	ENSSSCG000	FADS1	fatty acid desaturase 1 [Source:NCBI gene (formerly Entrezgene);Acc:444995]
chr3	46137304	46137932	629	*	chr3	46137304	46137932	in_vitro	in_vivo	9	11	0.3	1.20363636	-0.9036364	c(t = -0.85828	0.408	0.8515698	Distal Intergenic	ENSSSCG000	MALL	mal, T cell differentiation protein like [Source:NCBI gene (formerly Entrezgene);Acc:100626835]
chr13	162555880	162556556	677	*	chr13	162555880	162556556	in_vitro	in_vivo	13	13	0.37846154	1.15384615	-0.7753846	c(t = -0.84927	0.408	0.8515698	Distal Intergenic	ENSSSCG000	CLDND1	claudin domain containing 1 [Source:VGNC Symbol;Acc:VGNC:86742]
chr18	553698	554416	719	*	chr18	553698	554416	in_vitro	in_vivo	9	11	1.74222222	0.97545455	0.76676768	c(t = -0.85122	0.408	0.8515698	Distal Intergenic	ENSSSCG000	VIPR2	vasoactive intestinal peptide receptor 2 [Source:NCBI gene (formerly Entrezgene);Acc:100499553]
chr18	6632339	6633452	1114	*	chr18	6632339	6633452	in_vitro	in_vivo	11	14	1.50545455	0.73142857	0.77402597	c(t = -0.84619	0.408	0.8515698	Distal Intergenic	ENSSSCG000	GSTK1	glutathione S-transferase kappa 1 [Source:NCBI gene (formerly Entrezgene);Acc:100521480]
chr7	64123140	64123902	763	*	chr7	64123140	64123902	in_vitro	in_vivo	10	13	0.741	0.22615385	0.51484615	c(t = -0.85833	0.408	0.8515698	Distal Intergenic	ENSSSCG000	NFKBIA	NFKB inhibitor alpha [Source:NCBI gene (formerly Entrezgene);Acc:406188]
chr11	4157836	4159079	1244	*	chr11	4157836	4159079	in_vitro	in_vivo	9	14	89.2622222	94.6607143	-5.3984921	c(t = -0.86570	0.409	0.85157545	Distal Intergenic	ENSSSCG000	GPR12	G protein-coupled receptor 12 [Source:NCBI gene (formerly Entrezgene);Acc:100736641]
chr5	94017005	94018206	1202	*	chr5	94017005	94018206	in_vitro	in_vivo	11	10	4.66181818	2.638	2.02381818	c(t = -0.84419	0.409	0.85157545	Promoter (<=1kb)	ENSSSCG000	KITLG	KIT ligand [Source:VGNC Symbol;Acc:VGNC:98061]
chr9	112931726	112932450	725	*	chr9	112931726	112932450	in_vitro	in_vivo	10	10	3.159	1.666	1.493	c(t = -0.85763	0.409	0.85157545	Distal Intergenic	ENSSSCG000	NOBOX	NOBOX oogenesis homeobox [Source:NCBI gene (formerly Entrezgene);Acc:100499552]
chr3	129795467	129796917	1451	*	chr3	129795467	129796917	in_vitro	in_vivo	10	11	2.619	1.22090909	1.39809091	c(t = -0.85405	0.409	0.85157545	Distal Intergenic	ENSSSCG000	RSAD2	radical S-adenosyl methionine domain containing 2 [Source:NCBI gene (formerly Entrezgene);Acc:396752]
chr14	107564824	107565403	580	*	chr14	107564824	107565403	in_vitro	in_vivo	10	12	3.017	1.15666667	1.86033333	c(t = -0.85568	0.409	0.85157545	Distal Intergenic	ENSSSCG000	OPALIN	oligodendrocytic myelin paranodal and inner loop protein [Source:VGNC Symbol;Acc:VGNC:91046]
chr14	140635542	140637683	2142	*	chr14	140635542	140637683	in_vitro	in_vivo	9	9	1.78444444	1.13777778	0.64666667	c(t = -0.85643	0.409	0.85157545	Distal Intergenic	ENSSSCG000	ssc-mir-202	ssc-mir-202 [Source:miRBase;Acc:MI0013157]
chr7	27101421	27102217	797	*	chr7	27101421	27102217	in_vitro	in_vivo	9	13	1.34222222	0.82153846	0.52068376	c(t = -0.84727	0.409	0.85157545	Distal Intergenic	ENSSSCG000	HCRTR2	hypocretin receptor 2 [Source:NCBI gene (formerly Entrezgene);Acc:397428]
chr4	76382096	76384193	2098	*	chr4	76382096	76384193	in_vitro	in_vivo	14	16	1.22214286	0.760625	0.46151786	c(t = -0.83931	0.409	0.85157545	Distal Intergenic	ENSSSCG000	RPS20	ribosomal protein S20 [Source:NCBI gene (formerly Entrezgene);Acc:414395]
chr8	33058542	33058959	418	*	chr8	33058542	33058959	in_vitro	in_vivo	11	13	0.77909091	0.31923077	0.45986014	c(t = -0.85157	0.409	0.85157545	Promoter (<=1kb)	ENSSSCG000	SLC30A9	solute carrier family 30 member 9 [Source:NCBI gene (formerly Entrezgene);Acc:100192440]
chr3	4975266	4977701	2436	*	chr3	4975266	4977701	in_vitro	in_vivo	11	12	93.8190909	96.3458333	-2.5267424	c(t = -0.84954	0.41	0.85181634	Distal Intergenic	ENSSSCG000	AIMP2	aminoacyl tRNA synthetase complex interacting multifunctional protein 2 [Source:NCBI gene (formerly Entrezgene);Acc:100514707]
chr14	138823848	138826635	2788	*	chr14	138823848	138826635	in_vitro	in_vivo	14	10	92.8828571	87.835	5.04785714	c(t = -0.84021	0.41	0.85181634	Distal Intergenic	ENSSSCG000	GLRX3	glutaredoxin 3 [Source:VGNC Symbol;Acc:VGNC:88495]
chr9	12592659	12593676	1018	*	chr9	12592659	12593676	in_vitro	in_vivo	9	9	32.12	18.5744444	13.5455556	c(t = -0.84608	0.41	0.85181634	Distal Intergenic	ENSSSCG000	NDUFC2	NADH:ubiquinone oxidoreductase subunit C2 [Source:NCBI gene (formerly Entrezgene);Acc:100513030]
chr7	60299428	60300812	1385	*	chr7	60299428	60300812	in_vitro	in_vivo	12	16	1.83833333	1.07625	0.76208333	c(t = -0.84404	0.41	0.85181634	Distal Intergenic	ENSSSCG000	BBS4	Bardet-Biedl syndrome 4 [Source:NCBI gene (formerly Entrezgene);Acc:100513212]
chr8	71602701	71603297	597	*	chr8	71602701	71603297	in_vitro	in_vivo	11	11	1.97727273	0.86545455	1.11181818	c(t = -0.84275	0.41	0.85181634	Distal Intergenic	ENSSSCG000	CXCL9	C-X-C motif chemokine ligand 9 [Source:NCBI gene (formerly Entrezgene);Acc:100135681]
chr15	89842307	89842879	573	*	chr15	89842307	89842879	in_vitro	in_vivo	10	13	0.988	0.31384615	0.67415385	c(t = -0.85286	0.41	0.85181634	Distal Intergenic	ENSSSCG000	FRZB	frizzled related protein [Source:VGNC Symbol;Acc:VGNC:96308]
chrX	121779410	121782510	3101	*	chrX	121779410	121782510	in_vitro	in_vivo	9	11	15.3555556	26.8690909	-11.513535	c(t = -0.84144	0.411	0.85181634	Distal Intergenic	ENSSSCG000	FATE1	fetal and adult testis expressed 1 [Source:NCBI gene (formerly Entrezgene);Acc:396705]
chr15	120937871	120938485	615	*	chr15	120937871	120938485	in_vitro	in_vivo	10	12	25.349	16.4866667	8.86233333	c(t = -0.84731	0.411	0.85181634	Distal Intergenic	ENSSSCG000	PRKAG3	protein kinase AMP-activated non-catalytic subunit gamma 3 [Source:NCBI gene (formerly Entrezgene);Acc:397149]
chrX	87150968	87151696	729	*	chrX	87150968	87151696	in_vitro	in_vivo	11	12	5.24363636	11.715	-6.4713636	c(t = -0.84393	0.411	0.85181634	Distal Intergenic	ENSSSCG000	CLDN2	claudin 2 [Source:VGNC Symbol;Acc:VGNC:86737]
chr12	8644313	8647506	3194	*	chr12	8644313	8647506	in_vitro	in_vivo	12	13	5.5225	8.73153846	-3.2090385	c(t = -0.83719	0.411	0.85181634	Promoter (<=1kb)	ENSSSCG000	SOX9	SRY-box transcription factor 9 [Source:NCBI gene (formerly Entrezgene);Acc:396840]
chr2	100450652	100451647	996	*	chr2	100450652	100451647	in_vitro	in_vivo	15	12	3.88466667	7.16083333	-3.2761667	c(t = -0.84994	0.411	0.85181634	Distal Intergenic	ENSSSCG000	SPATA9	spermatogenesis associated 9 [Source:NCBI gene (formerly Entrezgene);Acc:100415927]
chr3	4643448	4644121	674	*	chr3	4643448	4644121	in_vitro	in_vivo	11	9	0.88818182	2.34555556	-1.4573737	c(t = -0.85380	0.411	0.85181634	Distal Intergenic	ENSSSCG000	RAC1	Rac family small GTPase 1 [Source:VGNC Symbol;Acc:VGNC:92047]
chr17	8596773	8597366	594	*	chr17	8596773	8597366	in_vitro	in_vivo	10	10	0.87	1.851	-0.981	c(t = -0.84211	0.411	0.85181634	Distal Intergenic	ENSSSCG000	FAT1	FAT atypical cadherin 1 [Source:NCBI gene (formerly Entrezgene);Acc:100154043]
chr15	24403283	24403850	568	*	chr15	24403283	24403850	in_vitro	in_vivo	9	11	2.76	1.51636364	1.24363636	c(t = -0.84724	0.411	0.85181634	Distal Intergenic	ENSSSCG000	MARCO	macrophage receptor with collagenous structure [Source:NCBI gene (formerly Entrezgene);Acc:100516298]
chr13	70538158	70540781	2624	*	chr13	70538158	70540781	in_vitro	in_vivo	11	13	2.12818182	1.27307692	0.8551049	c(t = -0.84062	0.411	0.85181634	Distal Intergenic	ENSSSCG000	CHCHD4	coiled-coil-helix-coiled-coil-helix domain containing 4 [Source:NCBI gene (formerly Entrezgene);Acc:100154663]
chr5	24117060	24118111	1052	*	chr5	24117060	24118111	in_vitro	in_vivo	12	12	2.23583333	0.7875	1.44833333	c(t = -0.85117	0.411	0.85181634	Distal Intergenic	ENSSSCG000	CTDSP2	CTD small phosphatase 2 [Source:NCBI gene (formerly Entrezgene);Acc:100624883]
chr17	48817380	48818153	774	*	chr17	48817380	48818153	in_vitro	in_vivo	12	10	0.935	0.537	0.398	c(t = -0.84043	0.411	0.85181634	Distal Intergenic	ENSSSCG000	SLC35C2	solute carrier family 35 member C2 [Source:VGNC Symbol;Acc:VGNC:95831]
chr3	40165280	40166562	1283	*	chr3	40165280	40166562	in_vitro	in_vivo	9	11	94.2233333	90.0972727	4.12606061	c(t = -0.84089	0.412	0.85185582	Distal Intergenic	ENSSSCG000	IGFALS	insulin like growth factor binding protein acid labile subunit [Source:VGNC Symbol;Acc:VGNC:110281]
chr4	54895076	54895425	350	*	chr4	54895076	54895425	in_vitro	in_vivo	10	9	0.286	2.22222222	-1.9362222	c(t = -0.86417	0.412	0.85185582	Promoter (<=1kb)	ENSSSCG000	CHMP4C	charged multivesicular body protein 4C [Source:VGNC Symbol;Acc:VGNC:86652]
chr5	31462255	31463317	1063	*	chr5	31462255	31463317	in_vitro	in_vivo	9	9	2.3	1.30777778	0.99222222	c(t = -0.84855	0.412	0.85185582	Distal Intergenic	ENSSSCG000	ssc-mir-9808	ssc-mir-9808 [Source:miRBase;Acc:MI0031575]
chr14	22910053	22911037	985	*	chr14	22910053	22911037	in_vitro	in_vivo	9	9	2.00111111	1.02333333	0.97777778	c(t = -0.84843	0.412	0.85185582	Distal Intergenic	ENSSSCG000	PIWIL1	piwi like RNA-mediated silencing 1 [Source:VGNC Symbol;Acc:VGNC:98197]
chr18	52412029	52412961	933	*	chr18	52412029	52412961	in_vitro	in_vivo	9	10	3.97777778	7.384	-3.4062222	c(t = -0.84402	0.413	0.85185582	Distal Intergenic	ENSSSCG000	INHBA	inhibin subunit beta A [Source:NCBI gene (formerly Entrezgene);Acc:397093]
chr17	5191753	5192497	745	*	chr17	5191753	5192497	in_vitro	in_vivo	10	10	1.428	2.343	-0.915	c(t = -0.83739	0.413	0.85185582	Promoter (<=1kb)	ENSSSCG000	SLC7A2	solute carrier family 7 member 2 [Source:VGNC Symbol;Acc:VGNC:95506]
chr2	9183621	9185548	1928	*	chr2	9183621	9185548	in_vitro	in_vivo	11	11	3.73818182	1.61454545	2.12363636	c(t = -0.85060	0.413	0.85185582	Distal Intergenic	ENSSSCG000	GANAB	glucosidase II alpha subunit [Source:VGNC Symbol;Acc:VGNC:88344]
chr17	48152167	48153569	1403	*	chr17	48152167	48153569	in_vitro	in_vivo	12	12	2.50583333	1.5125	0.99333333	c(t = -0.83598	0.413	0.85185582	Distal Intergenic	ENSSSCG000	MMP9	matrix metallopeptidase 9 [Source:NCBI gene (formerly Entrezgene);Acc:654325]
chr1	164654720	164658000	3281	*	chr1	164654720	164658000	in_vitro	in_vivo	14	16	2.01571429	1.304375	0.71133929	c(t = -0.83612	0.413	0.85185582	Distal Intergenic	ENSSSCG000	SNAPC5	small nuclear RNA activating complex polypeptide 5 [Source:VGNC Symbol;Acc:VGNC:93283]
chr12	56329633	56330718	1086	*	chr12	56329633	56330718	in_vitro	in_vivo	9	12	1.12777778	0.51083333	0.61694444	c(t = -0.85160	0.413	0.85185582	Distal Intergenic	ENSSSCG000	ssc-mir-744	ssc-mir-744 [Source:miRBase;Acc:MI0014769]
chr17	44050377	44050884	508	*	chr17	44050377	44050884	in_vitro	in_vivo	10	9	94.284	89.6455556	4.63844444	c(t = -0.84923	0.414	0.85185582	Distal Intergenic	ENSSSCG000	LPIN3	lipin 3 [Source:NCBI gene (formerly Entrezgene);Acc:100170771]
chr13	206907169	206909236	2068	*	chr13	206907169	206909236	in_vitro	in_vivo	9	11	21.5055556	35.2163636	-13.710808	c(t = -0.83654	0.414	0.85185582	Distal Intergenic	ENSSSCG000	AGPAT3	1-acylglycerol-3-phosphate O-acyltransferase 3 [Source:VGNC Symbol;Acc:VGNC:85185]
chr14	51260885	51262051	1167	*	chr14	51260885	51262051	in_vitro	in_vivo	10	12	11.976	6.66083333	5.31516667	c(t = -0.84814	0.414	0.85185582	Promoter (<=1kb)	ENSSSCG000	GP1BB	glycoprotein Ib platelet subunit beta [Source:NCBI gene (formerly Entrezgene);Acc:100155101]
chr7	58608425	58609337	913	*	chr7	58608425	58609337	in_vitro	in_vivo	14	15	3.63285714	5.14733333	-1.5144762	c(t = -0.83142	0.414	0.85185582	Distal Intergenic	ENSSSCG000	FAM219B	family with sequence similarity 219 member B [Source:VGNC Symbol;Acc:VGNC:87956]
chr12	5225422	5226602	1181	*	chr12	5225422	5226602	in_vitro	in_vivo	11	9	1.81272727	2.58222222	-0.7694949	c(t = -0.83590	0.414	0.85185582	Distal Intergenic	ENSSSCG000	ACOX1	acyl-CoA oxidase 1 [Source:VGNC Symbol;Acc:VGNC:85020]
chr13	122129181	122130812	1632	*	chr13	122129181	122130812	in_vitro	in_vivo	13	13	3.26076923	1.96076923	1.3	c(t = -0.83113	0.414	0.85185582	Distal Intergenic	ENSSSCG000	ssc-mir-1224	ssc-mir-1224 [Source:miRBase;Acc:MI0015920]
chr6	155800991	155802258	1268	*	chr6	155800991	155802258	in_vitro	in_vivo	11	12	3.19909091	1.87416667	1.32492424	c(t = -0.83881	0.414	0.85185582	Distal Intergenic	ENSSSCG000	PRKAA2	protein kinase AMP-activated catalytic subunit alpha 2 [Source:VGNC Symbol;Acc:VGNC:91798]
chr13	206935891	206937242	1352	*	chr13	206935891	206937242	in_vitro	in_vivo	9	11	2.40555556	1.39181818	1.01373737	c(t = -0.84043	0.414	0.85185582	Distal Intergenic	ENSSSCG000	AGPAT3	1-acylglycerol-3-phosphate O-acyltransferase 3 [Source:VGNC Symbol;Acc:VGNC:85185]
chr6	82559858	82563452	3595	*	chr6	82559858	82563452	in_vitro	in_vivo	13	12	2.21153846	1.3725	0.83903846	c(t = -0.83625	0.414	0.85185582	Distal Intergenic	ENSSSCG000	RSRP1	arginine and serine rich protein 1 [Source:VGNC Symbol;Acc:VGNC:92489]
chr11	77979282	77981624	2343	*	chr11	77979282	77981624	in_vitro	in_vivo	11	11	2.60454545	1.37	1.23454545	c(t = -0.83897	0.414	0.85185582	Distal Intergenic	ENSSSCG000	F7	coagulation factor VII [Source:NCBI gene (formerly Entrezgene);Acc:733661]
chr1	45169939	45171310	1372	*	chr1	45169939	45171310	in_vitro	in_vivo	15	14	2.16866667	1.35714286	0.81152381	c(t = -0.83050	0.414	0.85185582	Distal Intergenic	ENSSSCG000	ZUP1	zinc finger containing ubiquitin peptidase 1 [Source:VGNC Symbol;Acc:VGNC:95339]
chr15	32474744	32476195	1452	*	chr15	32474744	32476195	in_vitro	in_vivo	13	11	1.25846154	0.51272727	0.74573427	c(t = -0.84049	0.414	0.85185582	Promoter (<=1kb)	NA	NA	NA
chr15	121428658	121429211	554	*	chr15	121428658	121429211	in_vitro	in_vivo	11	10	1.26818182	4.69	-3.4218182	c(t = -0.85290	0.415	0.85185582	Promoter (<=1kb)	ENSSSCG000	DES	desmin [Source:VGNC Symbol;Acc:VGNC:96159]
chrX	106815869	106817165	1297	*	chrX	106815869	106817165	in_vitro	in_vivo	13	13	6.86307692	4.23307692	2.63	c(t = -0.83009	0.415	0.85185582	Distal Intergenic	ENSSSCG000	GPR119	G protein-coupled receptor 119 [Source:NCBI gene (formerly Entrezgene);Acc:100524541]
chrX	38928740	38930757	2018	*	chrX	38928740	38930757	in_vitro	in_vivo	11	12	10.1381818	4.2275	5.91068182	c(t = -0.84358	0.415	0.85185582	Promoter (<=1kb)	ENSSSCG00000012257		monoamine oxidase A [Source:VGNC Symbol;Acc:VGNC:89975]
chr12	22041114	22041816	703	*	chr12	22041114	22041816	in_vitro	in_vivo	9	12	2.30888889	1.04666667	1.26222222	c(t = -0.84263	0.415	0.85185582	Distal Intergenic	ENSSSCG000	TOP2A	DNA topoisomerase II alpha [Source:NCBI gene (formerly Entrezgene);Acc:396917]
chr13	73573764	73574330	567	*	chr13	73573764	73574330	in_vitro	in_vivo	13	9	0.29615385	0.87666667	-0.5805128	c(t = -0.84493	0.415	0.85185582	Distal Intergenic	ENSSSCG000	ACKR4	atypical chemokine receptor 4 [Source:NCBI gene (formerly Entrezgene);Acc:100037292]
chr2	42165145	42166100	956	*	chr2	42165145	42166100	in_vitro	in_vivo	10	10	1.585	0.816	0.769	c(t = -0.83457	0.415	0.85185582	Distal Intergenic	ENSSSCG000	RPS13	ribosomal protein S13 [Source:NCBI gene (formerly Entrezgene);Acc:100513520]
chr5	10426797	10429585	2789	*	chr5	10426797	10429585	in_vitro	in_vivo	12	12	20.3925	32.8683333	-12.475833	c(t = -0.82921	0.416	0.85185582	Distal Intergenic	ENSSSCG000	CYTH4	cytohesin 4 [Source:NCBI gene (formerly Entrezgene);Acc:100240722]
chr2	1106981	1110406	3426	*	chr2	1106981	1110406	in_vitro	in_vivo	12	13	24.6175	15.0261538	9.59134615	c(t = -0.83264	0.416	0.85185582	Distal Intergenic	ENSSSCG000	CTSD	cathepsin D [Source:NCBI gene (formerly Entrezgene);Acc:494568]
chr2	136522024	136523709	1686	*	chr2	136522024	136523709	in_vitro	in_vivo	11	10	4.08181818	1.93	2.15181818	c(t = -0.84022	0.416	0.85185582	Promoter (<=1kb)	ENSSSCG000	PPP2CA	protein phosphatase 2 catalytic subunit alpha [Source:NCBI gene (formerly Entrezgene);Acc:397656]
chr1	10311010	10311738	729	*	chr1	10311010	10311738	in_vitro	in_vivo	10	12	3.192	1.65333333	1.53866667	c(t = -0.83994	0.416	0.85185582	Distal Intergenic	ENSSSCG000	ssc-mir-9857	ssc-mir-9857 [Source:miRBase;Acc:MI0031635]
chr5	69779093	69780033	941	*	chr5	69779093	69780033	in_vitro	in_vivo	11	11	2.60181818	1.36272727	1.23909091	c(t = -0.83626	0.416	0.85185582	Distal Intergenic	ENSSSCG000	BID	BH3 interacting domain death agonist [Source:VGNC Symbol;Acc:VGNC:85824]
chr1	133015727	133016951	1225	*	chr1	133015727	133016951	in_vitro	in_vivo	13	10	1.20153846	0.751	0.45053846	c(t = -0.83085	0.416	0.85185582	Distal Intergenic	ENSSSCG000	THBS1	thrombospondin 1 [Source:NCBI gene (formerly Entrezgene);Acc:492313]
chr8	401177	404465	3289	*	chr8	401177	404465	in_vitro	in_vivo	10	12	9.874	18.3958333	-8.5218333	c(t = -0.82841	0.417	0.85185582	Distal Intergenic	ENSSSCG000	ATP5ME	ATP synthase membrane subunit e [Source:NCBI gene (formerly Entrezgene);Acc:396950]
chr18	28944910	28946849	1940	*	chr18	28944910	28946849	in_vitro	in_vivo	10	14	15.221	9.56714286	5.65385714	c(t = -0.83988	0.417	0.85185582	Distal Intergenic	ENSSSCG000	CFTR	CF transmembrane conductance regulator [Source:VGNC Symbol;Acc:VGNC:97927]
chr11	16657653	16658609	957	*	chr11	16657653	16658609	in_vitro	in_vivo	12	11	1.13666667	2.04090909	-0.9042424	c(t = -0.83607	0.417	0.85185582	Distal Intergenic	ENSSSCG000	RNASEH2B	ribonuclease H2 subunit B [Source:VGNC Symbol;Acc:VGNC:92339]
chr2	107886727	107887527	801	*	chr2	107886727	107887527	in_vitro	in_vivo	11	12	0.75090909	2.0325	-1.2815909	c(t = -0.83577	0.417	0.85185582	Distal Intergenic	ENSSSCG000	ST8SIA4	ST8 alpha-N-acetyl-neuraminide alpha-2,8-sialyltransferase 4 [Source:NCBI gene (formerly Entrezgene);Acc:641356]
chr1	165753169	165758120	4952	*	chr1	165753169	165758120	in_vitro	in_vivo	15	12	2.44266667	1.79333333	0.64933333	c(t = -0.82609	0.417	0.85185582	Distal Intergenic	ENSSSCG000	CALML4	calmodulin like 4 [Source:NCBI gene (formerly Entrezgene);Acc:100519528]
chr10	64443110	64443785	676	*	chr10	64443110	64443785	in_vitro	in_vivo	10	9	2.2	0.88333333	1.31666667	c(t = -0.84640	0.417	0.85185582	Distal Intergenic	ENSSSCG000	IL2RA	interleukin 2 receptor subunit alpha [Source:VGNC Symbol;Acc:VGNC:108275]
chr11	19551676	19552448	773	*	chr11	19551676	19552448	in_vitro	in_vivo	11	10	1.86090909	0.855	1.00590909	c(t = -0.83431	0.417	0.85185582	Distal Intergenic	ENSSSCG000	ITM2B	integral membrane protein 2B [Source:VGNC Symbol;Acc:VGNC:103971]
chr7	68317948	68319341	1394	*	chr7	68317948	68319341	in_vitro	in_vivo	10	12	1.638	0.80666667	0.83133333	c(t = -0.84251	0.417	0.85185582	Distal Intergenic	ENSSSCG000	AP4S1	adaptor related protein complex 4 subunit sigma 1 [Source:VGNC Symbol;Acc:VGNC:85394]
chr10	40741656	40743514	1859	*	chr10	40741656	40743514	in_vitro	in_vivo	12	12	1.28333333	0.7725	0.51083333	c(t = -0.83023	0.417	0.85185582	Distal Intergenic	ENSSSCG000	MTPAP	mitochondrial poly(A) polymerase [Source:VGNC Symbol;Acc:VGNC:96676]
chr11	1022152	1024480	2329	*	chr11	1022152	1024480	in_vitro	in_vivo	11	13	93.5709091	95.4707692	-1.8998601	c(t = -0.82460	0.418	0.85185582	Distal Intergenic	ENSSSCG000	CRYL1	crystallin lambda 1 [Source:VGNC Symbol;Acc:VGNC:87023]
chr16	79487778	79492341	4564	*	chr16	79487778	79492341	in_vitro	in_vivo	10	10	91.386	95.157	-3.771	c(t = -0.83471	0.418	0.85185582	Distal Intergenic	ENSSSCG000	TRIP13	thyroid hormone receptor interactor 13 [Source:NCBI gene (formerly Entrezgene);Acc:100337674]
chr5	625127	628833	3707	*	chr5	625127	628833	in_vitro	in_vivo	12	13	21.2783333	31.17	-9.8916667	c(t = -0.82488	0.418	0.85185582	Distal Intergenic	ENSSSCG000	SELENOO	selenoprotein O [Source:NCBI gene (formerly Entrezgene);Acc:100518971]
chr9	25501071	25501328	258	*	chr9	25501071	25501328	in_vitro	in_vivo	10	10	17.565	31.069	-13.504	c(t = -0.82997	0.418	0.85185582	Distal Intergenic	ENSSSCG000	C11orf54	chromosome 9 C11orf54 homolog [Source:VGNC Symbol;Acc:VGNC:86087]
chr6	35145591	35148413	2823	*	chr6	35145591	35148413	in_vitro	in_vivo	10	10	36.552	22.749	13.803	c(t = -0.83642	0.418	0.85185582	Distal Intergenic	ENSSSCG000	CBLN1	cerebellin 1 precursor [Source:NCBI gene (formerly Entrezgene);Acc:100127148]
chr17	59965732	59967382	1651	*	chr17	59965732	59967382	in_vitro	in_vivo	14	11	8.67928571	6.14	2.53928571	c(t = -0.82611	0.418	0.85185582	Distal Intergenic	ENSSSCG000	PHACTR3	phosphatase and actin regulator 3 [Source:VGNC Symbol;Acc:VGNC:95667]
chrX	51231086	51231577	492	*	chrX	51231086	51231577	in_vitro	in_vivo	10	9	10.766	6.04777778	4.71822222	c(t = -0.83321	0.418	0.85185582	Distal Intergenic	ENSSSCG000	ZC4H2	zinc finger C4H2-type containing [Source:VGNC Symbol;Acc:VGNC:95105]
chr18	45427994	45431643	3650	*	chr18	45427994	45431643	in_vitro	in_vivo	12	16	7.06833333	4.314375	2.75395833	c(t = -0.83551	0.418	0.85185582	Promoter (<=1kb)	ENSSSCG000	HOXA5	homeobox A5 [Source:NCBI gene (formerly Entrezgene);Acc:100499568]
chr13	190410761	190413254	2494	*	chr13	190410761	190413254	in_vitro	in_vivo	14	13	2.085	3.05846154	-0.9734615	c(t = -0.83319	0.418	0.85185582	Distal Intergenic	ENSSSCG000	ADAMTS1	ADAM metallopeptidase with thrombospondin type 1 motif 1 [Source:NCBI gene (formerly Entrezgene);Acc:497061]
chr2	70604950	70605602	653	*	chr2	70604950	70605602	in_vitro	in_vivo	10	11	1.033	2.52818182	-1.4951818	c(t = -0.83197	0.418	0.85185582	Distal Intergenic	ENSSSCG000	ADAMTS10	ADAM metallopeptidase with thrombospondin type 1 motif 10 [Source:NCBI gene (formerly Entrezgene);Acc:100624519]
chr16	56914975	56915274	300	*	chr16	56914975	56915274	in_vitro	in_vivo	9	11	3.92888889	1.81818182	2.11070707	c(t = -0.83710	0.418	0.85185582	Distal Intergenic	ENSSSCG000	RARS1	arginyl-tRNA synthetase 1 [Source:NCBI gene (formerly Entrezgene);Acc:414410]
chr3	124194226	124196624	2399	*	chr3	124194226	124196624	in_vitro	in_vivo	9	12	2.81888889	1.5325	1.28638889	c(t = -0.84335	0.418	0.85185582	Distal Intergenic	ENSSSCG000	LPIN1	lipin 1 [Source:VGNC Symbol;Acc:VGNC:89793]
chr14	21754443	21756165	1723	*	chr14	21754443	21756165	in_vitro	in_vivo	12	14	1.95416667	1.34285714	0.61130952	c(t = -0.82547	0.418	0.85185582	Distal Intergenic	ENSSSCG000	PIWIL1	piwi like RNA-mediated silencing 1 [Source:VGNC Symbol;Acc:VGNC:98197]
chr14	30320690	30321567	878	*	chr14	30320690	30321567	in_vitro	in_vivo	9	12	2.15777778	1.0425	1.11527778	c(t = -0.84222	0.418	0.85185582	Distal Intergenic	ENSSSCG000	ZCCHC8	zinc finger CCHC-type containing 8 [Source:VGNC Symbol;Acc:VGNC:95112]
chr13	66334311	66335052	742	*	chr13	66334311	66335052	in_vitro	in_vivo	9	12	2.70666667	0.99916667	1.7075	c(t = -0.84770	0.418	0.85185582	Distal Intergenic	ENSSSCG000	GHRL	ghrelin and obestatin prepropeptide [Source:VGNC Symbol;Acc:VGNC:111192]
chr6	84747576	84751909	4334	*	chr6	84747576	84751909	in_vitro	in_vivo	9	12	88.5066667	94.5175	-6.0108333	c(t = -0.84550	0.419	0.85185582	Distal Intergenic	ENSSSCG000	WASF2	WASP family member 2 [Source:VGNC Symbol;Acc:VGNC:94893]
chr3	40376443	40382977	6535	*	chr3	40376443	40382977	in_vitro	in_vivo	10	10	75.684	64.108	11.576	c(t = -0.82761	0.419	0.85185582	Distal Intergenic	ENSSSCG000	JPT2	Jupiter microtubule associated homolog 2 [Source:NCBI gene (formerly Entrezgene);Acc:474165]
chr12	20167739	20168953	1215	*	chr12	20167739	20168953	in_vitro	in_vivo	9	12	11.1022222	18.3775	-7.2752778	c(t = -0.82827	0.419	0.85185582	Distal Intergenic	ENSSSCG000	ssc-mir-9788	ssc-mir-9788-1 [Source:miRBase;Acc:MI0031549]
chr4	90409094	90410075	982	*	chr4	90409094	90410075	in_vitro	in_vivo	10	13	3.765	6.62538462	-2.8603846	c(t = -0.82409	0.419	0.85185582	Distal Intergenic	ENSSSCG000	ATP1A2	ATPase Na+/K+ transporting subunit alpha 2 [Source:NCBI gene (formerly Entrezgene);Acc:396828]
chr14	30523460	30524341	882	*	chr14	30523460	30524341	in_vitro	in_vivo	9	9	2.10111111	3.73888889	-1.6377778	c(t = -0.83016	0.419	0.85185582	Distal Intergenic	ENSSSCG000	DIABLO	diablo IAP-binding mitochondrial protein [Source:NCBI gene (formerly Entrezgene);Acc:100155744]
chr6	62597760	62598431	672	*	chr6	62597760	62598431	in_vitro	in_vivo	9	11	3.91666667	2.46272727	1.45393939	c(t = -0.82842	0.419	0.85185582	Distal Intergenic	ENSSSCG000	ISG15	ISG15 ubiquitin like modifier [Source:NCBI gene (formerly Entrezgene);Acc:100145895]
chr7	48500852	48502007	1156	*	chr7	48500852	48502007	in_vitro	in_vivo	11	11	1.25727273	2.27	-1.0127273	c(t = -0.82798	0.419	0.85185582	Distal Intergenic	ENSSSCG000	TMED3	transmembrane p24 trafficking protein 3 [Source:VGNC Symbol;Acc:VGNC:94055]
chr13	23020018	23021153	1136	*	chr13	23020018	23021153	in_vitro	in_vivo	12	10	0.55166667	1.629	-1.0773333	c(t = -0.84211	0.419	0.85185582	Promoter (<=1kb)	NA	NA	NA
chr5	10168774	10169737	964	*	chr5	10168774	10169737	in_vitro	in_vivo	10	11	0.928	1.52909091	-0.6010909	c(t = -0.82574	0.419	0.85185582	Distal Intergenic	ENSSSCG000	LGALS1	galectin 1 [Source:NCBI gene (formerly Entrezgene);Acc:414915]
chr2	59583809	59585933	2125	*	chr2	59583809	59585933	in_vitro	in_vivo	12	9	2.17416667	1.16222222	1.01194444	c(t = -0.82697	0.419	0.85185582	Distal Intergenic	ENSSSCG000	RAB3A	RAB3A, member RAS onco family [Source:VGNC Symbol;Acc:VGNC:98264]
chr14	104193348	104195147	1800	*	chr14	104193348	104195147	in_vitro	in_vivo	11	15	1.48181818	0.808	0.67381818	c(t = -0.82579	0.419	0.85185582	Promoter (<=1kb)	ENSSSCG000	HHEX	hematopoietically expressed homeobox [Source:NCBI gene (formerly Entrezgene);Acc:397232]
chr15	108974202	108975206	1005	*	chr15	108974202	108975206	in_vitro	in_vivo	10	9	1.103	0.44444444	0.65855556	c(t = -0.83081	0.419	0.85185582	Distal Intergenic	ENSSSCG000	EEF1B2	eukaryotic translation elongation factor 1 beta 2 [Source:NCBI gene (formerly Entrezgene);Acc:100154920]
chr15	106997042	106998028	987	*	chr15	106997042	106998028	in_vitro	in_vivo	11	9	0.78545455	0.40444444	0.3810101	c(t = -0.82855	0.419	0.85185582	Distal Intergenic	ENSSSCG000	CD28	CD28 molecule [Source:NCBI gene (formerly Entrezgene);Acc:100515419]
chrX	20220883	20222377	1495	*	chrX	20220883	20222377	in_vitro	in_vivo	13	10	0.91461538	0.292	0.62261538	c(t = -0.83161	0.419	0.85185582	Distal Intergenic	ENSSSCG000	SAT1	spermidine/spermine N1-acetyltransferase 1 [Source:NCBI gene (formerly Entrezgene);Acc:397645]
chr16	77870528	77870974	447	*	chr16	77870528	77870974	in_vitro	in_vivo	9	10	24.9388889	39.505	-14.566111	c(t = -0.82697	0.42	0.85208553	Distal Intergenic	ENSSSCG000	TERT	telomerase reverse transcriptase [Source:NCBI gene (formerly Entrezgene);Acc:492280]
chr1	848481	851787	3307	*	chr1	848481	851787	in_vitro	in_vivo	13	12	16.7192308	26.34	-9.6207692	c(t = -0.82147	0.42	0.85208553	Distal Intergenic	ENSSSCG000	ssc-mir-9815	ssc-mir-9815 [Source:miRBase;Acc:MI0031582]
chr17	28375380	28381649	6270	*	chr17	28375380	28381649	in_vitro	in_vivo	16	15	4.940625	2.992	1.948625	c(t = -0.82182	0.42	0.85208553	Distal Intergenic	ENSSSCG000	SEC23B	SEC23 homolog B, COPII coat complex component [Source:VGNC Symbol;Acc:VGNC:95490]
chr1	243189046	243190242	1197	*	chr1	243189046	243190242	in_vitro	in_vivo	15	13	1.21466667	2.37153846	-1.1568718	c(t = -0.82852	0.42	0.85208553	Distal Intergenic	ENSSSCG000	ERP44	endoplasmic reticulum protein 44 [Source:NCBI gene (formerly Entrezgene);Acc:100192437]
chr14	63298261	63299790	1530	*	chr14	63298261	63299790	in_vitro	in_vivo	11	12	1.42636364	2.21583333	-0.7894697	c(t = -0.82234	0.42	0.85208553	Distal Intergenic	ENSSSCG00000032953		myoregulin [Source:NCBI gene (formerly Entrezgene);Acc:105667210]
chr14	110722361	110724969	2609	*	chr14	110722361	110724969	in_vitro	in_vivo	13	13	2.99153846	1.92692308	1.06461538	c(t = -0.82100	0.42	0.85208553	Distal Intergenic	ENSSSCG000	GOT1	glutamic-oxaloacetic transaminase 1 [Source:NCBI gene (formerly Entrezgene);Acc:396967]
chr3	46992189	46993592	1404	*	chr3	46992189	46993592	in_vitro	in_vivo	11	12	0.69454545	1.72583333	-1.0312879	c(t = -0.83335	0.42	0.85208553	Distal Intergenic	ENSSSCG00000008137		LIM zinc finger domain containing 1 [Source:VGNC Symbol;Acc:VGNC:110285]
chr17	37569698	37570461	764	*	chr17	37569698	37570461	in_vitro	in_vivo	9	13	1.84333333	0.71230769	1.13102564	c(t = -0.84195	0.42	0.85208553	Distal Intergenic	ENSSSCG000	ASIP	agouti signaling protein [Source:NCBI gene (formerly Entrezgene);Acc:414439]
chrX	20756277	20756841	565	*	chrX	20756277	20756841	in_vitro	in_vivo	11	9	1.79363636	5.68666667	-3.8930303	c(t = -0.84335	0.421	0.85279472	Distal Intergenic	ENSSSCG000	SAT1	spermidine/spermine N1-acetyltransferase 1 [Source:NCBI gene (formerly Entrezgene);Acc:397645]
chr1	222989516	222990649	1134	*	chr1	222989516	222990649	in_vitro	in_vivo	13	13	3.53230769	1.96923077	1.56307692	c(t = -0.82335	0.421	0.85279472	Distal Intergenic	ENSSSCG000	TJP2	tight junction protein 2 [Source:VGNC Symbol;Acc:VGNC:103188]
chr14	56555011	56555645	635	*	chr14	56555011	56555645	in_vitro	in_vivo	10	9	1.105	1.78333333	-0.6783333	c(t = -0.82781	0.421	0.85279472	Distal Intergenic	ENSSSCG000	EDARADD	EDAR associated death domain [Source:VGNC Symbol;Acc:VGNC:87542]
chr18	38631183	38632325	1143	*	chr18	38631183	38632325	in_vitro	in_vivo	10	11	3.333	1.64454545	1.68845455	c(t = -0.82655	0.421	0.85279472	Distal Intergenic	ENSSSCG000	FKBP9	FKBP prolyl isomerase 9 [Source:NCBI gene (formerly Entrezgene);Acc:100623924]
chr1	164474270	164475121	852	*	chr1	164474270	164475121	in_vitro	in_vivo	9	10	0.88	0.323	0.557	c(t = -0.83277	0.421	0.85279472	Promoter (<=1kb)	ENSSSCG000	SNAPC5	small nuclear RNA activating complex polypeptide 5 [Source:VGNC Symbol;Acc:VGNC:93283]
chr12	59976544	59979679	3136	*	chr12	59976544	59979679	in_vitro	in_vivo	9	14	97.2466667	95.8507143	1.39595238	c(t = -0.82277	0.422	0.85279472	Distal Intergenic	ENSSSCG000	AKAP10	A-kinase anchoring protein 10 [Source:NCBI gene (formerly Entrezgene);Acc:397262]
chr4	1723936	1727158	3223	*	chr4	1723936	1727158	in_vitro	in_vivo	11	11	94.6181818	92.0218182	2.59636364	c(t = -0.82016	0.422	0.85279472	Distal Intergenic	ENSSSCG000	GPIHBP1	glycosylphosphatidylinositol anchored high density lipoprotein binding protein 1 [Source:VGNC Symbol;Acc:VGNC:88588]
chr10	16911943	16913728	1786	*	chr10	16911943	16913728	in_vitro	in_vivo	11	12	2.72727273	3.92833333	-1.2010606	c(t = -0.81970	0.422	0.85279472	Distal Intergenic	ENSSSCG000	AKT3	AKT serine/threonine kinase 3 [Source:VGNC Symbol;Acc:VGNC:96306]
chr4	6446000	6447540	1541	*	chr4	6446000	6447540	in_vitro	in_vivo	10	13	4.126	2.63384615	1.49215385	c(t = -0.82435	0.422	0.85279472	Distal Intergenic	ENSSSCG000	ssc-mir-30d	ssc-mir-30d [Source:miRBase;Acc:MI0013091]
chr6	29366528	29367574	1047	*	chr6	29366528	29367574	in_vitro	in_vivo	13	12	1.65769231	1.115	0.54269231	c(t = -0.81728	0.422	0.85279472	Distal Intergenic	ENSSSCG000	NUDT21	nudix hydrolase 21 [Source:NCBI gene (formerly Entrezgene);Acc:100625681]
chr6	37193404	37194100	697	*	chr6	37193404	37194100	in_vitro	in_vivo	10	11	1.799	0.97090909	0.82809091	c(t = -0.82408	0.422	0.85279472	Distal Intergenic	ENSSSCG000	CBLN1	cerebellin 1 precursor [Source:NCBI gene (formerly Entrezgene);Acc:100127148]
chr13	68657307	68658064	758	*	chr13	68657307	68658064	in_vitro	in_vivo	12	11	0.31083333	0.81363636	-0.502803	c(t = -0.82970	0.422	0.85279472	Distal Intergenic	ENSSSCG000	RPL32	ribosomal protein L32 [Source:NCBI gene (formerly Entrezgene);Acc:414419]
chr3	131728816	131731070	2255	*	chr3	131728816	131731070	in_vitro	in_vivo	9	10	15.3666667	26.364	-10.997333	c(t = -0.82158	0.423	0.85279472	Distal Intergenic	ENSSSCG000	EIPR1	EARP complex and GARP complex interacting protein 1 [Source:VGNC Symbol;Acc:VGNC:87635]
chr1	82086200	82086744	545	*	chr1	82086200	82086744	in_vitro	in_vivo	12	11	1.55583333	3.73	-2.1741667	c(t = -0.83339	0.423	0.85279472	Promoter (<=1kb)	ENSSSCG000	CALHM6	calcium homeostasis modulator family member 6 [Source:VGNC Symbol;Acc:VGNC:86147]
chrX	20128994	20130229	1236	*	chrX	20128994	20130229	in_vitro	in_vivo	14	12	2.16571429	1.35166667	0.81404762	c(t = -0.81526	0.423	0.85279472	Distal Intergenic	ENSSSCG000	SAT1	spermidine/spermine N1-acetyltransferase 1 [Source:NCBI gene (formerly Entrezgene);Acc:397645]
chr18	54282923	54284268	1346	*	chr18	54282923	54284268	in_vitro	in_vivo	13	12	1.97692308	1.19333333	0.78358974	c(t = -0.81679	0.423	0.85279472	Distal Intergenic	ENSSSCG000	INHBA	inhibin subunit beta A [Source:NCBI gene (formerly Entrezgene);Acc:397093]
chr5	15319858	15321674	1817	*	chr5	15319858	15321674	in_vitro	in_vivo	9	10	2.14	1.071	1.069	c(t = -0.83318	0.423	0.85279472	Distal Intergenic	ENSSSCG000	TUBA1A	tubulin alpha 1a [Source:NCBI gene (formerly Entrezgene);Acc:100135051]
chr11	2753618	2764369	10752	*	chr11	2753618	2764369	in_vitro	in_vivo	13	17	92.4515385	95.3582353	-2.9066968	c(t = -0.82470	0.424	0.85279472	Distal Intergenic	ENSSSCG000	ssc-mir-9814	ssc-mir-9814 [Source:miRBase;Acc:MI0031581]
chr10	42986973	42987628	656	*	chr10	42986973	42987628	in_vitro	in_vivo	10	12	1.682	2.94	-1.258	c(t = -0.81693	0.424	0.85279472	Distal Intergenic	ENSSSCG000	TRDMT1	tRNA aspartic acid methyltransferase 1 [Source:VGNC Symbol;Acc:VGNC:96227]
chr4	34924106	34924924	819	*	chr4	34924106	34924924	in_vitro	in_vivo	11	14	4.42727273	2.41285714	2.01441558	c(t = -0.81684	0.424	0.85279472	Distal Intergenic	ENSSSCG000	NCALD	neurocalcin delta [Source:VGNC Symbol;Acc:VGNC:90594]
chr10	39462563	39463088	526	*	chr10	39462563	39463088	in_vitro	in_vivo	10	12	3.7	2.15583333	1.54416667	c(t = -0.81758	0.424	0.85279472	Distal Intergenic	ENSSSCG000	MFSD14B	major facilitator superfamily domain containing 14B [Source:NCBI gene (formerly Entrezgene);Acc:100627901]
chr9	56177002	56178365	1364	*	chr9	56177002	56178365	in_vitro	in_vivo	10	11	2.783	1.75363636	1.02936364	c(t = -0.82123	0.424	0.85279472	Distal Intergenic	ENSSSCG000	KCNJ5	potassium inwardly rectifying channel subfamily J member 5 [Source:NCBI gene (formerly Entrezgene);Acc:397448]
chr2	15066297	15066976	680	*	chr2	15066297	15066976	in_vitro	in_vivo	9	11	0.63888889	1.66090909	-1.0220202	c(t = -0.82487	0.424	0.85279472	Distal Intergenic	ENSSSCG000	PTPMT1	protein tyrosine phosphatase mitochondrial 1 [Source:NCBI gene (formerly Entrezgene);Acc:100514336]
chr4	50508684	50509357	674	*	chr4	50508684	50509357	in_vitro	in_vivo	9	13	0.48888889	1.05076923	-0.5618803	c(t = -0.81591	0.424	0.85279472	Distal Intergenic	ENSSSCG000	ssc-mir-9858	ssc-mir-9858 [Source:miRBase;Acc:MI0031636]
chr15	15923514	15924392	879	*	chr15	15923514	15924392	in_vitro	in_vivo	9	9	2.06555556	0.70555556	1.36	c(t = -0.83490	0.424	0.85279472	Distal Intergenic	ENSSSCG000	CXCR4	C-X-C motif chemokine receptor 4 [Source:NCBI gene (formerly Entrezgene);Acc:396659]
chr13	46206479	46207287	809	*	chr13	46206479	46207287	in_vitro	in_vivo	9	9	0.21777778	0.65444444	-0.4366667	c(t = -0.83044	0.424	0.85279472	Distal Intergenic	ENSSSCG000	EOGT	EGF domain specific O-linked N-acetylglucosamine transferase [Source:VGNC Symbol;Acc:VGNC:87718]
chr9	138444873	138445837	965	*	chr9	138444873	138445837	in_vitro	in_vivo	11	10	18.5027273	30.494	-11.991273	c(t = -0.81700	0.425	0.85279472	Distal Intergenic	ENSSSCG000	EGFR	epidermal growth factor receptor [Source:NCBI gene (formerly Entrezgene);Acc:397070]
chr3	1352308	1353299	992	*	chr3	1352308	1353299	in_vitro	in_vivo	9	10	35.4666667	25.76	9.70666667	c(t = -0.81716	0.425	0.85279472	Distal Intergenic	ENSSSCG000	MRM2	mitochondrial rRNA methyltransferase 2 [Source:VGNC Symbol;Acc:VGNC:99722]
chr1	149627080	149627573	494	*	chr1	149627080	149627573	in_vitro	in_vivo	10	9	12.711	22.0488889	-9.3378889	c(t = -0.81804	0.425	0.85279472	Distal Intergenic	ENSSSCG000	CNDP2	carnosine dipeptidase 2 [Source:VGNC Symbol;Acc:VGNC:86812]
chr6	58857000	58859772	2773	*	chr6	58857000	58859772	in_vitro	in_vivo	14	12	6.81285714	5.21	1.60285714	c(t = -0.81125	0.425	0.85279472	Distal Intergenic	ENSSSCG00000025687		leukocyte immunoglobulin-like receptor-like [Source:NCBI gene (formerly Entrezgene);Acc:100144538]
chr6	40438655	40439503	849	*	chr6	40438655	40439503	in_vitro	in_vivo	11	11	7.94727273	4.49818182	3.44909091	c(t = -0.82056	0.425	0.85279472	Distal Intergenic	ENSSSCG000	SLC7A9	solute carrier family 7 member 9 [Source:VGNC Symbol;Acc:VGNC:93177]
chr2	96101505	96101995	491	*	chr2	96101505	96101995	in_vitro	in_vivo	9	9	2.86222222	1.84555556	1.01666667	c(t = -0.82007	0.425	0.85279472	Distal Intergenic	NA	NA	NA
chr15	29782078	29783808	1731	*	chr15	29782078	29783808	in_vitro	in_vivo	9	10	3.81666667	1.786	2.03066667	c(t = -0.81825	0.425	0.85279472	Distal Intergenic	ENSSSCG000	NIFK	nucleolar protein interacting with the FHA domain of MKI67 [Source:VGNC Symbol;Acc:VGNC:99725]
chr7	110463371	110463994	624	*	chr7	110463371	110463994	in_vitro	in_vivo	10	11	2.82	1.58727273	1.23272727	c(t = -0.82426	0.425	0.85279472	Distal Intergenic	ENSSSCG000	SPATA7	spermatosis associated 7 [Source:VGNC Symbol;Acc:VGNC:93389]
chr10	15042026	15043019	994	*	chr10	15042026	15043019	in_vitro	in_vivo	11	12	0.11818182	0.725	-0.6068182	c(t = -0.82608	0.425	0.85279472	Distal Intergenic	ENSSSCG000	SCCPDH	saccharopine dehydrogenase (putative) [Source:NCBI gene (formerly Entrezgene);Acc:100512485]
chr12	42675440	42677045	1606	*	chr12	42675440	42677045	in_vitro	in_vivo	11	11	0.38545455	0.66272727	-0.2772727	c(t = -0.81530	0.425	0.85279472	Distal Intergenic	ENSSSCG000	C17orf75	chromosome 12 C17orf75 homolog [Source:VGNC Symbol;Acc:VGNC:85925]
chrX	8518864	8520159	1296	*	chrX	8518864	8520159	in_vitro	in_vivo	9	11	4.04222222	10.1018182	-6.059596	c(t = -0.81951	0.426	0.85279472	Promoter (<=1kb)	ENSSSCG000	MSL3	MSL complex subunit 3 [Source:NCBI gene (formerly Entrezgene);Acc:100526025]
chr17	52444271	52445580	1310	*	chr17	52444271	52445580	in_vitro	in_vivo	9	13	7.38888889	3.78307692	3.60581197	c(t = -0.83269	0.426	0.85279472	Promoter (<=1kb)	ENSSSCG000	KCNG1	potassium voltage-gated channel modifier subfamily G member 1 [Source:VGNC Symbol;Acc:VGNC:96388]
chr1	17136658	17138462	1805	*	chr1	17136658	17138462	in_vitro	in_vivo	13	10	5.69384615	2.532	3.16184615	c(t = -0.81538	0.426	0.85279472	Distal Intergenic	ENSSSCG000	PCMT1	protein-L-isoaspartate (D-aspartate) O-methyltransferase [Source:VGNC Symbol;Acc:VGNC:103146]
chr4	68119711	68120794	1084	*	chr4	68119711	68120794	in_vitro	in_vivo	12	11	1.2875	0.77636364	0.51113636	c(t = -0.81351	0.426	0.85279472	Distal Intergenic	ENSSSCG000	CRH	corticotropin releasing hormone [Source:VGNC Symbol;Acc:VGNC:86992]
chr6	74508020	74508306	287	*	chr6	74508020	74508306	in_vitro	in_vivo	9	9	1.42111111	0.61777778	0.80333333	c(t = -0.81851	0.426	0.85279472	Distal Intergenic	ENSSSCG000	TMEM51	transmembrane protein 51 [Source:VGNC Symbol;Acc:VGNC:94187]
chr13	26523973	26525508	1536	*	chr13	26523973	26525508	in_vitro	in_vivo	15	13	91.1306667	94.4615385	-3.3308718	c(t = -0.81189	0.427	0.85279472	Distal Intergenic	ENSSSCG000	CYP8B1	cytochrome P450 family 8 subfamily B member 1 [Source:NCBI gene (formerly Entrezgene);Acc:403328]
chr11	78131972	78133054	1083	*	chr11	78131972	78133054	in_vitro	in_vivo	11	14	26.0781818	37.82	-11.741818	c(t = -0.80959	0.427	0.85279472	Distal Intergenic	ENSSSCG000	F7	coagulation factor VII [Source:NCBI gene (formerly Entrezgene);Acc:733661]
chr7	895301	896197	897	*	chr7	895301	896197	in_vitro	in_vivo	9	9	6.55333333	14.0077778	-7.4544444	c(t = -0.82047	0.427	0.85279472	Distal Intergenic	ENSSSCG000	IRF4	interferon regulatory factor 4 [Source:VGNC Symbol;Acc:VGNC:89207]
chr1	171905754	171906217	464	*	chr1	171905754	171906217	in_vitro	in_vivo	10	10	1.433	3.264	-1.831	c(t = -0.82050	0.427	0.85279472	Distal Intergenic	ENSSSCG000	PNN	pinin, desmosome associated protein [Source:NCBI gene (formerly Entrezgene);Acc:100526149]
chr7	40204999	40207686	2688	*	chr7	40204999	40207686	in_vitro	in_vivo	13	17	1.50692308	2.44823529	-0.9413122	c(t = -0.80728	0.427	0.85279472	Distal Intergenic	ENSSSCG000	CLIC5	chloride intracellular channel 5 [Source:NCBI gene (formerly Entrezgene);Acc:100154589]
chr11	3125303	3127418	2116	*	chr11	3125303	3127418	in_vitro	in_vivo	12	15	3.4325	2.26866667	1.16383333	c(t = -0.80826	0.427	0.85279472	Distal Intergenic	ENSSSCG000	ssc-mir-9814	ssc-mir-9814 [Source:miRBase;Acc:MI0031581]
chr15	81987533	81988987	1455	*	chr15	81987533	81988987	in_vitro	in_vivo	9	11	3.73111111	2.19909091	1.5320202	c(t = -0.81337	0.427	0.85279472	Distal Intergenic	NA	NA	NA
chr1	129374179	129375207	1029	*	chr1	129374179	129375207	in_vitro	in_vivo	11	14	2.84636364	1.71214286	1.13422078	c(t = -0.81503	0.427	0.85279472	Distal Intergenic	ENSSSCG000	PLA2G4B	phospholipase A2 group IVB [Source:NCBI gene (formerly Entrezgene);Acc:100152927]
chr1	6996235	6997358	1124	*	chr1	6996235	6997358	in_vitro	in_vivo	13	9	2.39461538	1.54222222	0.85239316	c(t = -0.81168	0.427	0.85279472	Distal Intergenic	ENSSSCG000	PLG	plasminogen [Source:NCBI gene (formerly Entrezgene);Acc:733660]
chr11	46459363	46461173	1811	*	chr11	46459363	46461173	in_vitro	in_vivo	9	14	0.70666667	1.46142857	-0.7547619	c(t = -0.81600	0.427	0.85279472	Distal Intergenic	ENSSSCG000	KLF12	KLF transcription factor 12 [Source:VGNC Symbol;Acc:VGNC:89492]
chr2	141878543	141879691	1149	*	chr2	141878543	141879691	in_vitro	in_vivo	14	10	0.61785714	1.167	-0.5491429	c(t = -0.81159	0.427	0.85279472	Distal Intergenic	ENSSSCG000	HBEGF	heparin binding EGF like growth factor [Source:VGNC Symbol;Acc:VGNC:88792]
chr6	146719142	146720293	1152	*	chr6	146719142	146720293	in_vitro	in_vivo	12	12	1.89	1.10666667	0.78333333	c(t = -0.81681	0.427	0.85279472	Promoter (<=1kb)	ENSSSCG000	PDE4B	phosphodiesterase 4B [Source:VGNC Symbol;Acc:VGNC:91255]
chr6	98677640	98678700	1061	*	chr6	98677640	98678700	in_vitro	in_vivo	11	12	2.24636364	1.10583333	1.1405303	c(t = -0.81125	0.427	0.85279472	Distal Intergenic	ENSSSCG000	NDUFV2	NADH:ubiquinone oxidoreductase core subunit V2 [Source:NCBI gene (formerly Entrezgene);Acc:100037991]
chrX	84936868	84937815	948	*	chrX	84936868	84937815	in_vitro	in_vivo	12	14	1.50083333	0.68428571	0.81654762	c(t = -0.81249	0.427	0.85279472	Distal Intergenic	ENSSSCG000	PLP1	proteolipid protein 1 [Source:NCBI gene (formerly Entrezgene);Acc:397029]
chr4	3027848	3029148	1301	*	chr4	3027848	3029148	in_vitro	in_vivo	9	11	0.78666667	0.30272727	0.48393939	c(t = -0.81849	0.427	0.85279472	Distal Intergenic	ENSSSCG000	ago-02	argonaute RISC catalytic component 2 [Source:NCBI gene (formerly Entrezgene);Acc:100155245]
[illegible]	[illegible]	[illegible]	[illegible]	[illegible]	[illegible]	[illegible]	[illegible]	[illegible]	[illegible]	[illegible]	[illegible]	[illegible]	[illegible]	[illegible]	[illegible]	[illegible]	[illegible]	[illegible]	[illegible]	[illegible]	[illegible]

chr17	62127378	62154038	26661	*	chr17	62127378	62154038	in_vitro	in_vivo	13	17	64.3330769	70.3935294	-6.0604525	c(t = -0.7969	0.434	0.85367034	Distal Intergenic	ENSSSCG000	ssc-mir-133a	ssc-mir-133a-1 [Source:miRBase;Acc:MI0010681]
chr18	2166427	2166811	385	*	chr18	2166427	2166811	in_vitro	in_vivo	9	9	25.1588889	12.1066667	13.0522222	c(t = 0.80364	0.434	0.85367034	Distal Intergenic	ENSSSCG000	SHH	sonic hedgehog signaling molecule [Source:NCBI gene (formerly Entrezgene);Acc:100512749]
chr9	121515403	121516867	1465	*	chr9	121515403	121516867	in_vitro	in_vivo	12	14	1.68083333	1.22428571	0.45654762	c(t = 0.79684	0.434	0.85367034	Distal Intergenic	ENSSSCG000	TOR1AIP2	torsin 1A interacting protein 2 [Source:VGNC Symbol;Acc:VGNC:94318]
chr14	88988666	88989762	1097	*	chr14	88988666	88989762	in_vitro	in_vivo	11	11	1.54727273	0.78090909	0.76636364	c(t = 0.80243	0.434	0.85367034	Distal Intergenic	ENSSSCG000	ssc-mir-9835	ssc-mir-9835 [Source:miRBase;Acc:MI0031604]
chr2	151557220	151559103	1884	*	chr2	151557220	151559103	in_vitro	in_vivo	11	9	23.6654545	38.8988889	-15.233434	c(t = -0.8019	0.435	0.85410506	Distal Intergenic	ENSSSCG000	MYOZ3	myozenin 3 [Source:VGNC Symbol;Acc:VGNC:90544]
chr17	48687957	48689165	1209	*	chr17	48687957	48689165	in_vitro	in_vivo	9	13	22.1977778	15.7530769	6.44470085	c(t = 0.80653	0.435	0.85410506	Distal Intergenic	ENSSSCG000	SLC35C2	solute carrier family 35 member C2 [Source:VGNC Symbol;Acc:VGNC:95831]
chr5	23041803	23042333	531	*	chr5	23041803	23042333	in_vitro	in_vivo	9	9	3.67222222	6.65555556	-2.9833333	c(t = -0.8028	0.435	0.85410506	Promoter (<=1kb)	ENSSSCG000	CDK4	cyclin dependent kinase 4 [Source:NCBI gene (formerly Entrezgene);Acc:100144492]
chr15	81975100	81976025	926	*	chr15	81975100	81976025	in_vitro	in_vivo	9	12	0.91222222	1.89833333	-0.9861111	c(t = -0.7979	0.435	0.85410506	Distal Intergenic	NA	NA	NA
chr17	8451767	8452610	844	*	chr17	8451767	8452610	in_vitro	in_vivo	12	10	3.36	1.553	1.807	c(t = 0.80231	0.435	0.85410506	Distal Intergenic	ENSSSCG000	FAT1	FAT atypical cadherin 1 [Source:NCBI gene (formerly Entrezgene);Acc:100154043]
chr1	238567466	238568395	930	*	chr1	238567466	238568395	in_vitro	in_vivo	9	9	2.25333333	1.31	0.94333333	c(t = 0.80154	0.435	0.85410506	Distal Intergenic	ENSSSCG00000032554		solute carrier family 25 member 51 [Source:NCBI gene (formerly Entrezgene);Acc:100157218]
chr14	15895423	15896278	856	*	chr14	15895423	15896278	in_vitro	in_vivo	13	13	0.87538462	0.40384615	0.47153846	c(t = 0.79837	0.435	0.85410506	Distal Intergenic	ENSSSCG000	HPGD	15-hydroxyprostaglandin dehydrogenase [Source:VGNC Symbol;Acc:VGNC:88961]
chr6	59593670	59595403	1734	*	chr6	59593670	59595403	in_vitro	in_vivo	11	9	14.9572727	25.31	-10.352727	c(t = -0.7994	0.436	0.85410506	Distal Intergenic	ENSSSCG00000003302		transmembrane protein 86B [Source:VGNC Symbol;Acc:VGNC:94211]
chr14	74294456	74296247	1792	*	chr14	74294456	74296247	in_vitro	in_vivo	13	12	3.57846154	8.90166667	-5.3232051	c(t = -0.8062	0.436	0.85410506	Distal Intergenic	ENSSSCG000	UNC5B	unc-5 netrin receptor B [Source:VGNC Symbol;Acc:VGNC:94708]
chr17	29998421	30001686	3266	*	chr17	29998421	30001686	in_vitro	in_vivo	13	16	3.86615385	2.386875	1.47927885	c(t = 0.79846	0.436	0.85410506	Distal Intergenic	ENSSSCG000	THBD	thrombomodulin [Source:NCBI gene (formerly Entrezgene);Acc:100157642]
chr5	28880208	28880881	674	*	chr5	28880208	28880881	in_vitro	in_vivo	12	9	0.7575	1.66777778	-0.9102778	c(t = -0.7975	0.436	0.85410506	Distal Intergenic	ENSSSCG000	TBK1	TANK binding kinase 1 [Source:VGNC Symbol;Acc:VGNC:93787]
chr14	40528136	40528605	470	*	chr14	40528136	40528605	in_vitro	in_vivo	12	9	0.95416667	1.59555556	-0.6413889	c(t = -0.8008	0.436	0.85410506	Distal Intergenic	ENSSSCG000	DYNLL1	dynein light chain LC8-type 1 [Source:NCBI gene (formerly Entrezgene);Acc:100157773]
chr1	74348925	74349539	615	*	chr1	74348925	74349539	in_vitro	in_vivo	11	9	0.58363636	1.38333333	-0.799697	c(t = -0.8136	0.436	0.85410506	Distal Intergenic	ENSSSCG000	FOXO3	forkhead box O3 [Source:VGNC Symbol;Acc:VGNC:99715]
chr17	14860029	14860880	852	*	chr17	14860029	14860880	in_vitro	in_vivo	9	13	0.72111111	1.21076923	-0.4896581	c(t = -0.7953	0.436	0.85410506	Distal Intergenic	ENSSSCG000	CHGB	chromogranin B [Source:NCBI gene (formerly Entrezgene);Acc:397154]
chr3	40264788	40268457	3670	*	chr3	40264788	40268457	in_vitro	in_vivo	9	14	96.8155556	95.3892857	1.42626984	c(t = 0.79769	0.437	0.85410506	Distal Intergenic	ENSSSCG000	JPT2	Jupiter microtubule associated homolog 2 [Source:NCBI gene (formerly Entrezgene);Acc:474165]
chr7	4620600	4623278	2679	*	chr7	4620600	4623278	in_vitro	in_vivo	11	10	94.7236364	91.43	3.29363636	c(t = 0.80087	0.437	0.85410506	Distal Intergenic	ENSSSCG000	SSR1	signal sequence receptor subunit 1 [Source:NCBI gene (formerly Entrezgene);Acc:100152689]
chr5	19172985	19174124	1140	*	chr5	19172985	19174124	in_vitro	in_vivo	11	13	5.82090909	12.1646154	-6.3437063	c(t = -0.7952	0.437	0.85410506	Distal Intergenic	ENSSSCG000	ssc-mir-196a	ssc-mir-196a-2 [Source:miRBase;Acc:MI0002457]
chr2	69294622	69295889	1268	*	chr2	69294622	69295889	in_vitro	in_vivo	10	14	14.483	9.57928571	4.90371429	c(t = 0.79746	0.437	0.85410506	Promoter (<=1kb)	ENSSSCG000	S1PR5	sphingosine-1-phosphate receptor 5 [Source:VGNC Symbol;Acc:VGNC:92555]
chr5	75562	76727	1166	*	chr5	75562	76727	in_vitro	in_vivo	10	10	11.315	6.325	4.99	c(t = 0.80056	0.437	0.85410506	Distal Intergenic	ENSSSCG000	ARSA	arylsulfatase A [Source:NCBI gene (formerly Entrezgene);Acc:396973]
chr14	141092799	141093839	1041	*	chr14	141092799	141093839	in_vitro	in_vivo	9	9	1.57888889	3.58111111	-2.0022222	c(t = -0.8131	0.437	0.85410506	Distal Intergenic	ENSSSCG000	ssc-mir-202	ssc-mir-202 [Source:miRBase;Acc:MI0013157]
chr1	229336456	229337159	704	*	chr1	229336456	229337159	in_vitro	in_vivo	10	11	3.259	1.89272727	1.36627273	c(t = 0.80219	0.437	0.85410506	Distal Intergenic	ENSSSCG000	OSTF1	osteoclast stimulating factor 1 [Source:NCBI gene (formerly Entrezgene);Acc:397066]
chr12	38061163	38062529	1367	*	chr12	38061163	38062529	in_vitro	in_vivo	12	12	2.58666667	1.595	0.99166667	c(t = 0.79222	0.437	0.85410506	Distal Intergenic	ENSSSCG000	DHRS11	dehydrogenase/reductase 11 [Source:NCBI gene (formerly Entrezgene);Acc:100513307]
chr8	136903696	136905013	1318	*	chr8	136903696	136905013	in_vitro	in_vivo	13	16	2.14230769	1.54	0.60230769	c(t = 0.79350	0.437	0.85410506	Promoter (<=1kb)	ENSSSCG000	BMP3	bone morphogenetic protein 3 [Source:NCBI gene (formerly Entrezgene);Acc:100512974]
chr17	11772010	11772885	876	*	chr17	11772010	11772885	in_vitro	in_vivo	10	10	1.761	0.769	0.992	c(t = 0.79581	0.437	0.85410506	Distal Intergenic	ENSSSCG000	SMIM19	small integral membrane protein 19 [Source:NCBI gene (formerly Entrezgene);Acc:100152445]
chr16	79452841	79464661	11821	*	chr16	79452841	79464661	in_vitro	in_vivo	11	14	90.8045455	85.9071429	4.8974026	c(t = 0.79144	0.438	0.85410506	3' UTR	ENSSSCG000	TRIP13	thyroid hormone receptor interactor 13 [Source:NCBI gene (formerly Entrezgene);Acc:100337674]
chrX	122847042	122847447	406	*	chrX	122847042	122847447	in_vitro	in_vivo	10	10	3.389	10.476	-7.087	c(t = -0.8034	0.438	0.85410506	Distal Intergenic	ENSSSCG000	FATE1	fetal and adult testis expressed 1 [Source:NCBI gene (formerly Entrezgene);Acc:396705]
chr7	1952987	1958047	5061	*	chr7	1952987	1958047	in_vitro	in_vivo	11	13	12.9472727	5.77076923	7.1765035	c(t = 0.80652	0.438	0.85410506	Promoter (<=1kb)	ENSSSCG000	TUBB2B	tubulin, beta 2B class IIb [Source:NCBI gene (formerly Entrezgene);Acc:100153507]
chr2	14982871	14983485	615	*	chr2	14982871	14983485	in_vitro	in_vivo	10	12	1.755	2.71416667	-0.9591667	c(t = -0.7958	0.438	0.85410506	Distal Intergenic	ENSSSCG000	NDUFS3	NADH:ubiquinone oxidoreductase core subunit S3 [Source:NCBI gene (formerly Entrezgene);Acc:100510948]
chr5	21473488	21474885	1398	*	chr5	21473488	21474885	in_vitro	in_vivo	10	10	4.678	2.5	2.178	c(t = 0.80330	0.438	0.85410506	Distal Intergenic	ENSSSCG000	RPS26	ribosomal protein S26 [Source:NCBI gene (formerly Entrezgene);Acc:100037997]
chr1	148984369	148988473	4105	*	chr1	148984369	148988473	in_vitro	in_vivo	15	12	2.23	1.52583333	0.70416667	c(t = 0.78824	0.438	0.85410506	Distal Intergenic	ENSSSCG000	CNDP1	carnosine dipeptidase 1 [Source:VGNC Symbol;Acc:VGNC:86811]
chr9	28406788	28407470	683	*	chr9	28406788	28407470	in_vitro	in_vivo	9	10	0.48333333	1.255	-0.7716667	c(t = -0.7971	0.438	0.85410506	Distal Intergenic	ENSSSCG000	ssc-mir-7143	ssc-mir-7143 [Source:miRBase;Acc:MI0023576]
chr5	4151673	4153194	1522	*	chr5	4151673	4153194	in_vitro	in_vivo	13	12	1.83307692	0.95833333	0.87474359	c(t = 0.79917	0.438	0.85410506	Distal Intergenic	ENSSSCG000	ssc-mir-1249	ssc-mir-1249-1 [Source:miRBase;Acc:MI0022158]
chr13	198469387	198472806	3420	*	chr13	198469387	198472806	in_vitro	in_vivo	14	13	1.34785714	0.80230769	0.54554945	c(t = 0.78872	0.438	0.85410506	Intron (NM_001246252/100512633, intron 2 of 7)	ENSSSCG000	RUNX1	RUNX family transcription factor 1 [Source:VGNC Symbol;Acc:VGNC:92516]
chr11	1880559	1883247	2689	*	chr11	1880559	1883247	in_vitro	in_vivo	9	13	97.1511111	95.0130769	2.13803419	c(t = 0.79583	0.439	0.85410506	Distal Intergenic	ENSSSCG000	FGF9	fibroblast growth factor 9 [Source:NCBI gene (formerly Entrezgene);Acc:396717]
chr3	1450022	1461223	11202	*	chr3	1450022	1461223	in_vitro	in_vivo	13	12	88.6315385	92.5316667	-3.9001282	c(t = -0.7926	0.439	0.85410506	Distal Intergenic	ENSSSCG000	MRM2	mitochondrial rRNA methyltransferase 2 [Source:VGNC Symbol;Acc:VGNC:99722]
chr6	12185842	12187163	1322	*	chr6	12185842	12187163	in_vitro	in_vivo	11	10	81.3063636	70.119	11.1873636	c(t = 0.79301	0.439	0.85410506	Distal Intergenic	ENSSSCG000	COG4	component of oligomeric golgi complex 4 [Source:NCBI gene (formerly Entrezgene);Acc:100625346]
chr6	1565705	1572513	6809	*	chr6	1565705	1572513	in_vitro	in_vivo	9	15	25.9222222	35.9773333	-10.055111	c(t = -0.7883	0.439	0.85410506	Distal Intergenic	ENSSSCG000	KLHDC4	kelch domain containing 4 [Source:NCBI gene (formerly Entrezgene);Acc:100511452]
chr17	31516319	31518091	1773	*	chr17	31516319	31518091	in_vitro	in_vivo	13	15	7.74769231	13.418	-5.6703077	c(t = -0.7893	0.439	0.85410506	Promoter (<=1kb)	ENSSSCG000	ADRA1D	adrenoceptor alpha 1D [Source:NCBI gene (formerly Entrezgene);Acc:552898]
chr17	35632205	35632821	617	*	chr17	35632205	35632821	in_vitro	in_vivo	9	11	3.84444444	7.68	-3.8355556	c(t = -0.7995	0.439	0.85410506	Distal Intergenic	ENSSSCG000	BCL2L1	BCL2 like 1 [Source:VGNC Symbol;Acc:VGNC:96535]
chr4	186242	187013	772	*	chr4	186242	187013	in_vitro	in_vivo	11	11	2.33909091	5.44454545	-3.1054545	c(t = -0.8030	0.439	0.85410506	Distal Intergenic	ENSSSCG000	SLC52A1	solute carrier family 52 member 2 [Source:NCBI gene (formerly Entrezgene);Acc:445519]
chr9	3377762	3379626	1865	*	chr9	3377762	3379626	in_vitro	in_vivo	13	14	2.12230769	3.68071429	-1.5584066	c(t = -0.7886	0.439	0.85410506	Distal Intergenic	ENSSSCG000	HPX	hemopexin [Source:VGNC Symbol;Acc:VGNC:88968]
chr14	88132044	88132607	564	*	chr14	88132044	88132607	in_vitro	in_vivo	10	13	3.595	2.12846154	1.46653846	c(t = 0.79221	0.439	0.85410506	Distal Intergenic	ENSSSCG000	GPRIN2	G protein regulated inducer of neurite outgrowth 2 [Source:NCBI gene (formerly Entrezgene);Acc:100568453]
chr10	23826826	23827767	942	*	chr10	23826826	23827767	in_vitro	in_vivo	10	12	3.297	1.0725	2.2245	c(t = 0.80664	0.439	0.85410506	Distal Intergenic	ENSSSCG000	TNNI1	troponin I1, slow skeletal type [Source:VGNC Symbol;Acc:VGNC:95548]
chr14	135154849	135156948	2100	*	chr14	135154849	135156948	in_vitro	in_vivo	10	11	97.83	96.0545455	1.77545455	c(t = 0.78840	0.44	0.85432036	Distal Intergenic	ENSSSCG000	UROS	uroporphyrinogen III synthase [Source:VGNC Symbol;Acc:VGNC:94735]
chr3	1413680	1421880	8201	*	chr3	1413680	1421880	in_vitro	in_vivo	13	15	97.2815385	95.8786667	1.40287179	c(t = 0.78417	0.44	0.85432036	Distal Intergenic	ENSSSCG000	MRM2	mitochondrial rRNA methyltransferase 2 [Source:VGNC Symbol;Acc:VGNC:99722]
chr15	33173133	33173971	839	*	chr15	33173133	33173971	in_vitro	in_vivo	10	9	13.987	24.4466667	-10.459667	c(t = -0.7961	0.44	0.85432036	Promoter (<=1kb)	ENSSSCG000	DLGAP2	DLG associated protein 2 [Source:VGNC Symbol;Acc:VGNC:99711]
chr7	114209890	114210327	438	*	chr7	114209890	114210327	in_vitro	in_vivo	11	10	4.22727273	2.391	1.83627273	c(t = 0.78996	0.44	0.85432036	Distal Intergenic	ENSSSCG000	CHGA	chromogranin A [Source:NCBI gene (formerly Entrezgene);Acc:397540]
chr18	40820631	40821207	577	*	chr18	40820631	40821207	in_vitro	in_vivo	10	12	3.546	2.11	1.436	c(t = 0.79822	0.44	0.85432036	Distal Intergenic	ENSSSCG000	FKBP9	FKBP prolyl isomerase 9 [Source:NCBI gene (formerly Entrezgene);Acc:100623924]
chr6	81073651	81074198	548	*	chr6	81073651	81074198	in_vitro	in_vivo	9	11	3.05333333	1.38454545	1.66878788	c(t = 0.80447	0.44	0.85432036	Distal Intergenic	ENSSSCG00000025500		5-hydroxytryptamine receptor 1D [Source:VGNC Symbol;Acc:VGNC:97100]
chr17	37189264	37190070	807	*	chr17	37189264	37190070	in_vitro	in_vivo	9	11	2.18333333	1.32818182	0.85515152	c(t = 0.79943	0.44	0.85432036	Distal Intergenic	ENSSSCG00000007262		long palate lung and nasal epithelium protein 5 [Source:NCBI gene (formerly Entrezgene);Acc:100113428]
chr6	108338834	108339363	530	*	chr6	108338834	108339363	in_vitro	in_vivo	10	12	1.36	0.61833333	0.74166667	c(t = 0.79647	0.44	0.85432036	Distal Intergenic	ENSSSCG000	NPC1	NPC intracellular cholesterol transporter 1 [Source:NCBI gene (formerly Entrezgene);Acc:397591]
chr1	188580862	188582675	1814	*	chr1	188580862	188582675	in_vitro	in_vivo	11	15	7.20454545	4.53866667	2.66587879	c(t = 0.79464	0.441	0.8545348	Distal Intergenic	ENSSSCG000	RTN1	reticulon 1 [Source:NCBI gene (formerly Entrezgene);Acc:100152394]
chr11	12765176	12766128	953	*	chr11	12765176	12766128	in_vitro	in_vivo	13	13	0.81615385	1.69636364	-0.8802098	c(t = -0.7926	0.441	0.8545348	Distal Intergenic	ENSSSCG000	SMAD9	SMAD family member 9 [Source:NCBI gene (formerly Entrezgene);Acc:100153584]
chr16	4062684	4063416	733	*	chr16	4062684	4063416	in_vitro	in_vivo	10	12	0.969	1.6175	-0.6485	c(t = -0.7857	0.441	0.8545348	Distal Intergenic	ENSSSCG000	C1QTNF3	C1q and TNF related 3 [Source:NCBI gene (formerly Entrezgene);Acc:100270814]
chr13	70917126	70918268	1143	*	chr13	70917126	70918268	in_vitro	in_vivo	9	13	2.66444444	1.57769231	1.08675214	c(t = 0.79865	0.441	0.8545348	Distal Intergenic	ENSSSCG000	CHCHD4	coiled-coil-helix-coiled-coil-helix domain containing 4 [Source:NCBI gene (formerly Entrezgene);Acc:100154663]
chr8	82987121	82988025	905	*	chr8	82987121	82988025	in_vitro	in_vivo	9	9	2.28888889	1.12222222	1.16666667	c(t = 0.79080	0.441	0.8545348	Promoter (<=1kb)	ENSSSCG000	SMAD1	SMAD family member 1 [Source:VGNC Symbol;Acc:VGNC:93216]
chr15	133119928	133121274	1347	*	chr15	133119928	133121274	in_vitro	in_vivo	9	11	1.49222222	0.78727273	0.70494949	c(t = 0.79883	0.441	0.8545348	Distal Intergenic	ENSSSCG000	EIF4E2	eukaryotic translation initiation factor 4E family member 2 [Source:VGNC Symbol;Acc:VGNC:96269]
chr12	38461640	38462155	516	*	chr12	38461640	38462155	in_vitro	in_vivo	9	11	2.05	0.66272727	1.38727273	c(t = 0.80474	0.441	0.8545348	Distal Intergenic	ENSSSCG000	MRM1	mitochondrial rRNA methyltransferase 1 [Source:NCBI gene (formerly Entrezgene);Acc:100513493]
chr7	80391036	80391560	525	*	chr7	80391036	80391560	in_vitro	in_vivo	10	11	1.08	0.41363636	0.66636364	c(t = 0.79331	0.441	0.8545348	Promoter (<=1kb)	ENSSSCG000	EMC7	ER membrane protein complex subunit 7 [Source:NCBI gene (formerly Entrezgene);Acc:100626410]
chr11	64474262	64474908	647	*	chr11	64474262	64474908	in_vitro	in_vivo	10	9	59.732	42.0233333	17.7086667	c(t = 0.78643	0.442	0.85474836	Distal Intergenic	ENSSSCG000	CLDN10	claudin 10 [Source:NCBI gene (formerly Entrezgene);Acc:100153752]
chr2	68734538	68735138	601	*	chr2	68734538	68735138	in_vitro	in_vivo	10	11	34.352	22.0818182	12.2701818	c(t = 0.78616	0.442	0.85474836	Distal Intergenic	ENSSSCG000	PIN1	peptidylprolyl cis/trans isomerase, NIMA-interacting 1 [Source:NCBI gene (formerly Entrezgene);Acc:100512827]
chr10	11903586	11903986	401	*	chr10	11903586	11903986	in_vitro	in_vivo	9	9	10.0844444	5.34444444	4.74	c(t = 0.78896	0.442	0.85474836	Distal Intergenic	ENSSSCG000	FH	fumarate hydratase [Source:VGNC Symbol;Acc:VGNC:96300]
chr8	54978145	54979041	897	*	chr8	54978145	54979041	in_vitro	in_vivo	10	15	2.122	4.43533333	-2.3133333	c(t = -0.7849	0.442	0.85474836	Distal Intergenic	ENSSSCG00000008898		HOP homeobox [Source:VGNC Symbol;Acc:VGNC:88932]
chr2	65910372	65911524	1153	*	chr2	65910372	65911524	in_vitro	in_vivo	12	13	1.61666667	2.79	-1.1733333	c(t = -0.7891	0.442	0.85474836	Distal Intergenic	ENSSSCG000	TRMT1	tRNA methyltransferase 1 [Source:VGNC Symbol;Acc:VGNC:94441]
chr9	56584874	56585161	288	*	chr9	56584874	56585161	in_vitro	in_vivo	11	10	3.36272727	1.801	1.56172727	c(t = 0.78737	0.442	0.85474836	Distal Intergenic	ENSSSCG000	KCNJ5	potassium inwardly rectifying channel subfamily J member 5 [Source:NCBI gene (formerly Entrezgene);Acc:397448]
chr14	22780188	22780988	801	*	chr14	22780188	22780988	in_vitro	in_vivo	9	9	3.26444444	1.46111111	1.80333333	c(t = 0.80308	0.442	0.85474836	Distal Intergenic	ENSSSCG000	PIWIL1	piwi like RNA-mediated silencing 1 [Source:VGNC Symbol;Acc:VGNC:98197]
chr9	135097389	135097826	438	*	chr9	135097389	135097826	in_vitro	in_vivo	9	9	2.39888889	0.92555556	1.47333333	c(t = 0.79263	0.442	0.85474836	Distal Intergenic	ENSSSCG00000038506		CD46 molecule, complement regulatory protein [Source:NCBI gene (formerly Entrezgene);Acc:396922]
chr9	139040732	139042225	1494	*	chr9	139040732	139042225	in_vitro	in_vivo	9	16	14.9555556	9.136875	5.81868056	c(t = 0.78085	0.443	0.85517293	Distal Intergenic	ENSSSCG000	EGFR	epidermal growth factor receptor [Source:NCBI gene (formerly Entrezgene);Acc:397070]
chr4	33983451	33984334	884	*	chr4	33983451	33984334	in_vitro	in_vivo	9	13	1.25222222	2.66076923	-1.408547	c(t = -0.7849	0.443	0.85517293	Promoter (<=1kb)	ENSSSCG000	BAALC	BAALC binder of MAP3K1 and KLF4 [Source:NCBI gene (formerly Entrezgene);Acc:100170128]
chr1	246933168	246933653	486	*	chr1	246933168	246933653	in_vitro	in_vivo	11	10	1.09363636	2.154	-1.0603636	c(t = -0.7840	0.443	0.85517293	Distal Intergenic	ENSSSCG000	TMEM38B	transmembrane protein 38B [Source:NCBI gene (formerly Entrezgene);Acc:100153669]
chr7	12720374	12723110	2737	*	chr7	12720374	12723110	in_vitro	in_vivo	14	15	2.27571429	1.34866667	0.92704762	c(t = 0.77857	0.443	0.85517293	Distal Intergenic	ENSSSCG000	DTNBP1	dystrobrevin binding protein 1 [Source:NCBI gene (formerly Entrezgene);Acc:100049697]
chr4	102878291	102878904	614	*	chr4	102878291	102878904	in_vitro	in_vivo	9	9	1.98333333	0.77555556	1.20777778	c(t = 0.79085	0.443	0.85517293	Distal Intergenic	ENSSSCG000	TENT5C	terminal nucleotidyltransferase 5C [Source:VGNC Symbol;Acc:VGNC:93875]
chr16	76253911	76255002	1092	*	chr16	76253911	76255002	in_vitro	in_vivo	12	12	1.47333333	0.50916667	0.96416667	c(t = 0.79346	0.443	0.85517293	Distal Intergenic	ENSSSCG000	TERT	telomerase reverse transcriptase [Source:NCBI gene (formerly Entrezgene);Acc:492280]
chr12	1502356	1510950	8595	*	chr12	1502356	1510950	in_vitro	in_vivo	11	15	88.7763636	91.6593333	-2.8829697	c(t = -0.7795	0.444	0.85517293	Distal Intergenic	ENSSSCG000	TEPSIN	TEPSIN adaptor related protein complex 4 accessory protein [Source:NCBI gene (formerly Entrezgene);Acc:100626379]
chr4	93954444	93955531	1088	*	chr4	93954444	93955531	in_vitro	in_vivo	10	10	13.338	23.18	-9.842	c(t = -0.7863	0.444	0.85517293	Distal Intergenic	ENSSSCG000	LAMTOR2	late endosomal/lysosomal adaptor, MAPK and MTOR activator 2 [Source:NCBI gene (formerly Entrezgene);Acc:100156749]
chr18	2151680	2152679	1000	*	chr18	2151680	2152679	in_vitro	in_vivo	9	9	7.65888889	13.5022222	-5.8433333	c(t = -0.7888	0.444	0.85517293	Distal Intergenic	ENSSSCG000	SHH	sonic hedgehog signaling molecule [Source:NCBI gene (formerly Entrezgene);Acc:100512749]
chrX	87426352	87427374	1023	*	chrX	87426352	87427374	in_vitro	in_vivo	9	14	6.27111111	11.4778571	-5.206746	c(t = -0.7844	0.444	0.85517293	Distal Intergenic	ENSSSCG000	CLDN2	claudin 2 [Source:VGNC Symbol;Acc:VGNC:86737]
chr15	17917503	17919232	1730	*	chr15	17917503	17919232	in_vitro	in_vivo	12	12	5.49083333	3.5825	1.90833333	c(t = 0.78256	0.444	0.85517293	Distal Intergenic	ENSSSCG000	LYPD1	LY6/PLAUR domain containing 1 [Source:VGNC Symbol;Acc:VGNC:96176]
chrX	13173689	13174694	1006	*	chrX	13173689	13174694	in_vitro	in_vivo	13	11	1.43692308	2.89454545	-1.4576224	c(t = -0.7932	0.444	0.85517293	Distal Intergenic	ENSSSCG000	SYAP1	synapse associated protein 1 [Source:VGNC Symbol;Acc:VGNC:93648]
chr14	38579773	38580465	693	*	chr14	38579773	38580465	in_vitro	in_vivo	9	11	1.14666667	2.05	-0.9033333	c(t = -0.7860	0.444	0.85517293	Distal Intergenic	ENSSSCG000	OAS2	2'-5'-oligoadenylate synthetase 2 [Source:VGNC Symbol;Acc:VGNC:91007]
chr18	32797973	32798765	793	*	chr18	32797973	32798765	in_vitro	in_vivo	12	12	3.07	1.59083333	1.47916667	c(t = 0.78728	0.444	0.85517293	Distal Intergenic	ENSSSCG000	IFRD1	interferon related developmental regulator 1 [Source:NCBI gene (formerly Entrezgene);Acc:493185]
chr1	43392407	43393576	1170	*	chr1	43392407	43393576	in_vitro	in_vivo	12	13	1.92583333	1.27923077	0.64660256	c(t = 0.77932	0.444	0.85517293	Distal Intergenic	ENSSSCG000	PLN	phospholamban [Source:NCBI gene (formerly Entrezgene);Acc:397421]
chr4	29414793	29416100	1308	*	chr4	29414793	29416100	in_vitro	in_vivo	13	12	1.08461538	0.49916667	0.58544872	c(t = 0.78007	0.444	0.85517293	Promoter (<=1kb)	ENSSSCG000	RSPO2	R-spondin 2 [Source:NCBI gene (formerly Entrezgene);Acc:100154008]
chr2	9607023	9608275	1253	*	chr2	9607023	9608275	in_vitro	in_vivo	10	12	7.512	13.9725	-6.4605	c(t = -0.7896	0.445	0.85602628	Promoter (<=1kb)	ENSSSCG000	FADS3	fatty acid desaturase 3 [Source:VGNC Symbol;Acc:VGNC:87877]
chr11	57798430	57798837	408	*	chr11	57798430	57798837	in_vitro	in_vivo	9	13	4.29666667	7.36384615	-3.0671795	c(t = -0.7835	0.445	0.85602628	Distal Intergenic	ENSSSCG000	SLITRK1	SLIT and NTRK like family member 1 [Source:NCBI gene (formerly Entrezgene);Acc:100152470]
chr14	110714572	110715482	911	*	chr14	110714572	110715482	in_vitro	in_vivo	10	10	1.227	2.53	-1.303	c(t = -0.7880	0.445	0.85602628	Distal Intergenic	ENSSSCG000	GOT1	glutamic-oxaloacetic transaminase 1 [Source:NCBI gene (formerly Entrezgene);Acc:396967]
chr15	68742889	68743859	971	*	chr15	68742889	68743859	in_vitro	in_vivo	11	10	0.70272727	1.458	-0.7552727	c(t = -0.7882	0.445	0.85602628	Promoter (<=1kb)	ENSSSCG000	DPP4	dipeptidyl peptidase 4 [Source:NCBI gene (formerly Entrezgene);Acc:397492]
chr7	48873079	48873612	534	*	chr7	48873079	48873612	in_vitro	in_vivo	10	15	4.599	1.412	3.187	c(t = 0.79690	0.445	0.85602628	Distal Intergenic	ENSSSCG000	BCL2A1	BCL2 related protein A1 [Source:NCBI gene (formerly Entrezgene);Acc:100156860]
chr14	113230827	113232552	1726	*	chr14	113230827	113232552	in_vitro	in_vivo	12	12	3.0625	1.91833333	1.14416667	c(t = 0.77568	0.446	0.85685746	Distal Intergenic	ENSSSCG000	ELOVL3	ELOVL fatty acid elongase 3 [Source:VGNC Symbol;Acc:VGNC:87659]
chr15	67017802	67018429	628	*	chr15	67017802	67018429	in_vitro	in_vivo	10	12	2.475	1.5325	0.9425	c(t = 0.77919	0.446	0.85685746	Distal Intergenic	ENSSSCG000	ITGB6	integrin subunit beta 6 [Source:VGNC Symbol;Acc:VGNC:98054]
chr13	111852819	111853305	487	*	chr13	111852819	111853305	in_vitro	in_vivo	10	12	0.658	1.25833333	-0.6003333	c(t = -0.7802	0.446	0.85685746	Distal Intergenic	ENSSSCG000	SPATA16	spermatogenesis associated 16 [Source:NCBI gene (formerly Entrezgene);Acc:100415806]
chr6	37943241	37944533	1293	*	chr6	37943241	37944533	in_vitro	in_vivo	12	12	0.86583333	0.31166667	0.55416667	c(t = 0.78577	0.446	0.85685746	Distal Intergenic	ENSSSCG000	CBLN1	cerebellin 1 precursor [Source:NCBI gene (formerly Entrezgene);Acc:100127148]
chr14	60284626	60285135	510	*	chr14	60284626	60285135	in_vitro	in_vivo	10	11	94.582	92.0345455	2.54745455	c(t = 0.77638	0.447	0.85685746	Distal Intergenic	ENSSSCG000	ACTA1	actin alpha 1, skeletal muscle [Source:NCBI gene (formerly Entrezgene);Acc:100154254]
chr12	309421	311949	2529	*	chr12	309421	311949	in_vitro	in_vivo	9	12	86.8255556	91.4425	-4.6169444	c(t = -0.7766	0.447	0.85685746	Distal Intergenic	ENSSSCG000	TBCD	tubulin folding cofactor D [Source:NCBI gene (formerly Entrezgene);Acc:100512127]
chr10	68358331	68362636	4306	*	chr10	68358331	68362636	in_vitro	in_vivo	11	12	51.3690909	61.8108333	-10.441742	c(t = -0.7773	0.447	0.85685746	Distal Intergenic	ENSSSCG000	WDR37	WD repeat domain 37 [Source:VGNC Symbol;Acc:VGNC:96258]
chr6	63687831	63688978	1148	*	chr6	63687831	63688978	in_vitro	in_vivo	9	10	2.62666667	5.466	-2.8393333	c(t = -0.7885	0.447	0.85685746	Distal Intergenic	ENSSSCG000	TAS1R3	taste 1 receptor member 3 [Source:NCBI gene (formerly Entrezgene);Acc:100127440]
chr16	65642136	65643147	1012	*	chr16	65642136	65643147	in_vitro	in_vivo	12	12	2.83	4.75166667	-1.9216667	c(t = -0.7815	0.447	0.85685746	Distal Intergenic	ENSSSCG000	MED7	mediator complex subunit 7 [Source:VGNC Symbol;Acc:VGNC:90123]
chr2	6026791	6027941	1151	*	chr2	6026791	6027941	in_vitro	in_vivo	10	10	1.545	3.03	-1.485	c(t = -0.7832	0.447	0.85685746	Distal Intergenic	ENSSSCG000	B4GAT1	beta-1,4-glucuronyltransferase 1 [Source:NCBI gene (formerly Entrezgene);Acc:100518412]
chr17	11990147	11991047	901	*	chr17	11990147	11991047	in_vitro	in_vivo	12	12	2.355	1.5925	0.7625	c(t = 0.77766	0.447	0.85685746	Distal Intergenic	ENSSSCG000	SMIM19	small integral membrane protein 19 [Source:NCBI gene (formerly Entrezgene);Acc:100152445]
chr13	83035031	83035781	751	*	chr13	83035031	83035781	in_vitro	in_vivo	11	12	2.41818182	1.15166667	1.26651515	c(t = 0.78423	0.447	0.85685746	Distal Intergenic	ENSSSCG000	TRPC1	transient receptor potential cation channel subfamily C member 1 [Source:VGNC Symbol;Acc:VGNC:94460]
chr3	5404074	5405985	1912	*	chr3	5404074	5405985	in_vitro	in_vivo	9	10	86.3111111	93.704	-7.3928889	c(t = -0.7907	0.448	0.85685746	Distal Intergenic	ENSSSCG000	AIMP2	aminoacyl tRNA synthetase complex interacting multifunctional protein 2 [Source:NCBI gene (formerly Entrezgene);Acc:100514707]
chr7	56572389	56578001	5613	*	chr7	56572389	56578001	in_vitro	in_vivo	15	17	7.82533333	5.36058824	2.4647451	c(t = 0.77387	0.448	0.85685746	Distal Intergenic	ENSSSCG000	ETFA	electron transfer flavoprotein subunit alpha [Source:VGNC Symbol;Acc:VGNC:87801]
chr7	19218656	19219263	608	*	chr7	19218656	19219263	in_vitro	in_vivo	12	12	3.20083333	1.95833333	1.2425	c(t = 0.77236	0.448	0.85685746	Distal Intergenic	ENSSSCG000	ALDH5A1	aldehyde dehydrogenase 5 family member A1 [Source:NCBI gene (formerly Entrezgene);Acc:100157072]
chr6	34669034	34670243	1210	*	chr6	34669034	34670243	in_vitro	in_vivo	11	11	3.91909091	1.62090909	2.29818182	c(t = 0.78374	0.448	0.85685746	Distal Intergenic	ENSSSCG000	NOD2	nucleotide binding oligomerization domain containing 2 [Source:NCBI gene (formerly Entrezgene);Acc:100125838]
chr12	6116429	6117388	960	*	chr12	6116429	6117388	in_vitro	in_vivo	9	12	1.30333333	0.74166667	0.56166667	c(t = 0.77501	0.448	0.85685746	Promoter (<=1kb)	ENSSSCG000	SUMO2	small ubiquitin like modifier 2 [Source:NCBI gene (formerly Entrezgene);Acc:397044]
chr1	240583849	240584642	794	*	chr1	240583849	240584642	in_vitro	in_vivo	9	12	1.33444444	0.7375	0.59694444	c(t = 0.77894	0.448	0.85685746	Distal Intergenic	ENSSSCG000	TGFBR1	transforming growth factor beta receptor 1 [Source:VGNC Symbol;Acc:VGNC:98368]
chr2	32871899	32872989	1091	*	chr2	32871899	32872989	in_vitro	in_vivo	14	12	1.13142857	0.62666667	0.5047619	c(t = 0.77151	0.448	0.85685746	Distal Intergenic	ENSSSCG000	BDNF	brain derived neurotrophic factor [Source:VGNC Symbol;Acc:VGNC:85795]
chr1	6883621	6884602	982	*	chr1	6883621	6884602	in_vitro	in_vivo	9	9	98.4411111	96.9466667	1.49444444	c(t = 0.77981	0.449	0.85685746	Distal Intergenic	ENSSSCG000	AGPAT4	1-acylglycerol-3-phosphate O-acyltransferase 4 [Source:VGNC Symbol;Acc:VGNC:85186]
chrX	97919088	97920280	1193	*	chrX	97919088	97920280	in_vitro	in_vivo	12	12	12.5	20.08	-7.58	c(t = -0.7722	0.449	0.85685746	Distal Intergenic	ENSSSCG000	PGRMC1	progesterone receptor membrane component 1 [Source:NCBI gene (formerly Entrezgene);Acc:396946]
chr7	52303677	52305483	1807	*	chr7	52303677	52305483	in_vitro	in_vivo	11	16	6.58363636	9.10375	-2.5201136	c(t = -0.7740	0.449	0.85685746	Promoter (<=1kb)	ENSSSCG000	CPEB1	cytoplasmic polyadenylation element binding protein 1 [Source:NCBI gene (formerly Entrezgene);Acc:100048944]
chr11	7200074	7201910	1837	*	chr11	7200074	7201910	in_vitro	in_vivo	11	11	5.00727273	7.65545455	-2.6481818	c(t = -0.7785	0.449	0.85685746	Promoter (<=1kb)	ENSSSCG00000009327		high mobility group box 1 [Source:NCBI gene (formerly Entrezgene);Acc:445521]
chr5	68676805	68679332	2528	*	chr5	68676805	68679332	in_vitro	in_vivo	11	11	5.14363636	6.30333333	-1.159697	c(t = -0.7726	0.449	0.85685746	Distal Intergenic	ENSSSCG000	ADIPOR2	adiponectin receptor 2 [Source:VGNC Symbol;Acc:VGNC:85141]
chr3	8578968	8581511	2544	*	chr3	8578968	8581511	in_vitro	in_vivo	9	13	5.92	3.51769231	2.40230769	c(t = 0.78957	0.449	0.85685746	Distal Intergenic	ENSSSCG000	EPO	erythropoietin [Source:NCBI gene (formerly Entrezgene);Acc:397249]
chr2	66145769	66146410	642	*	chr2	66145769	66146410	in_vitro	in_vivo	11	9	4.31454545	2.88111111	1.43343434	c(t = 0.77426	0.449	0.85685746	Promoter (1-2kb)	ENSSSCG000	KLF1	KLF transcription factor 1 [Source:NCBI gene (formerly Entrezgene);Acc:100174958]
chr6	67131546	67136085	4540	*	chr6	67131546	67136085	in_vitro	in_vivo	9	13	3.03444444	2.21769231	0.81675214	c(t = 0.77191	0.449	0.85685746	Distal Intergenic	ENSSSCG000	ICMT	isoprenylcysteine carboxyl methyltransferase [Source:NCBI gene (formerly Entrezgene);Acc:100514410]
chr1	77399579	77401381	1803	*	chr1	77399579	77401381	in_vitro	in_vivo	13	12	3.09538462	1.89833333	1.19705128	c(t = 0.77136	0.449	0.85685746	Distal Intergenic	ENSSSCG000	FYN	FYN proto-onco, Src family tyrosine kinase [Source:VGNC Symbol;Acc:VGNC:88276]
chr1	145707133	145707900	768	*	chr1	145707133	145707900	in_vitro	in_vivo	13	9	0.74769231	1.52555556	-0.7778632	c(t = -0.7811	0.449	0.85685746	Distal Intergenic	ENSSSCG000	NFATC1	nuclear factor of activated T cells 1 [Source:NCBI gene (formerly Entrezgene);Acc:397318]
chr2	74677309	74679890	2582	*	chr2	74677309	74679890	in_vitro	in_vivo	12	9	3.8775	1.41555556	2.46194444	c(t = 0.77930	0.449	0.85685746	Distal Intergenic	ENSSSCG000	MAP2K2	mitogen-activated protein kinase kinase 2 [Source:NCBI gene (formerly Entrezgene);Acc:100525505]
chr9	105263058	105264699	1642	*	chr9	105263058	105264699	in_vitro	in_vivo	14	13	1.78857143	1.23307692	0.55549451	c(t = 0.76988	0.449	0.85685746	Distal Intergenic	ENSSSCG000	NAMPT	nicotinamide phosphoribosyltransferase [Source:VGNC Symbol;Acc:VGNC:90573]
chr5	45750476	45751175	700	*	chr5	45750476	45751175	in_vitro	in_vivo	9	15	1.45888889	0.86066667	0.59822222	c(t = 0.77749	0.449	0.85685746	Promoter (<=1kb)	ENSSSCG000	PTHLH	parathyroid hormone like hormone [Source:VGNC Symbol;Acc:VGNC:91963]
chr12	3423254	3425584	2331	*	chr12	3423254	3425584	in_vitro	in_vivo	10	9	96.665	94.5855556	2.07944444	c(t = 0.77679	0.45	0.8579047	Distal Intergenic	ENSSSCG00000017164		TIMP metallopeptidase inhibitor 2 [Source:VGNC Symbol;Acc:VGNC:93995]
chr16	48727943	48729301	1359	*	chr16	48727943	48729301	in_vitro	in_vivo	10	9	28.944	43.0122222	-14.068222	c(t = -0.7746	0.45	0.8579047	Distal Intergenic	ENSSSCG000	DRD1	dopamine receptor D1 [Source:VGNC Symbol;Acc:VGNC:87443]
chr14	90480493	90481457	965	*	chr14	90480493	90481457	in_vitro	in_vivo	11	13	0.86181818	1.46769231	-0.6058741	c(t = -0.7730	0.45	0.8579047	Distal Intergenic	ENSSSCG000	NCOA4	nuclear receptor coactivator 4 [Source:VGNC Symbol;Acc:VGNC:90617]
chr4	37900031	37902660	2630	*	chr4	37900031	37902660	in_vitro	in_vivo	10	15	0.833	1.28133333	-0.4483333	c(t = -0.7699	0.45	0.8579047	Distal Intergenic	ENSSSCG000	RPL30	ribosomal protein L30 [Source:VGNC Symbol;Acc:VGNC:98849]
chr16	77696414	77702600	6187	*	chr16	77696414	77702600	in_vitro	in_vivo	13	11	18.86	11.18	7.68	c(t = 0.76892	0.451	0.8579047	Distal Intergenic	ENSSSCG000	TERT	telomerase reverse transcriptase [Source:NCBI gene (formerly Entrezgene);Acc:492280]
chr3	47052333	47053125	793	*	chr3	47052333	47053125	in_vitro	in_vivo	11	14	14.4872727	8.87571429	5.61155844	c(t = 0.77112	0.451	0.8579047	Distal Intergenic	ENSSSCG00000008137		LIM zinc finger domain containing 1 [Source:VGNC Symbol;Acc:VGNC:110285]
chr1	113084842	113085832	991	*	chr1	113084842	113085832	in_vitro	in_vivo	13	10	3.28769231	7.14	-3.8523077	c(t = -0.7853	0.451	0.8579047	Distal Intergenic	ENSSSCG000	CCNB2	cyclin B2 [Source:VGNC Symbol;Acc:VGNC:86350]
chr18	45407067	45409470	2404	*	chr18	45407067	45409470	in_vitro	in_vivo	12	13	1.99166667	3.28384615	-1.2921795	c(t = -0.7708	0.451	0.8579047	Distal Intergenic	ENSSSCG000	ssc-mir-196b	ssc-mir-196b-1 [Source:miRBase;Acc:MI0013137]
chr4	13445200	13447361	2162	*	chr4	13445200	13447361	in_vitro	in_vivo	13	15	4.07384615	2.958	1.11584615	c(t = 0.76569	0.451	0.8579047	Distal Intergenic	ENSSSCG000	MYC	MYC proto-oncogene, bHLH transcription factor [Source:NCBI gene (formerly Entrezgene);Acc:448810]
chr8	117996716	117997403	688	*	chr8	117996716	117997403	in_vitro	in_vivo	11	13	4.89090909	2.16	2.73090909	c(t = 0.78176	0.451	0.8579047	Distal Intergenic	ENSSSCG000	UBE2D3	ubiquitin conjugating enzyme E2 D3 [Source:NCBI gene (formerly Entrezgene);Acc:780418]
chr2	59012611	59013741	1131	*	chr2	59012611	59013741	in_vitro	in_vivo	10	11	1.946	1.29545455	0.65054545	c(t = 0.77338	0.451	0.8579047	Distal Intergenic	ENSSSCG000	ARMC6	armadillo repeat containing 6 [Source:VGNC Symbol;Acc:VGNC:108748]
chr6	19206931	19207669	739	*	chr6	19206931	19207669	in_vitro	in_vivo	13	12	2.12076923	1.06583333	1.0549359	c(t = 0.77381	0.451	0.8579047	Distal Intergenic	ENSSSCG000	CCL22	C-C motif chemokine ligand 22 [Source:NCBI gene (formerly Entrezgene);Acc:100516016]
chr10	17471051	17472173	1123	*	chr10	17471051	17472173	in_vitro	in_vivo	9	10	2.01777778	1.02	0.99777778	c(t = 0.77899	0.451	0.8579047	Distal Intergenic	ENSSSCG000	DESI2	desumoylating isopeptidase 2 [Source:VGNC Symbol;Acc:VGNC:96037]
chr18	958004	959411	1408	*	chr18	958004	959411	in_vitro	in_vivo	12	10	14.9675	25.011	-10.0435	c(t = -0.7714	0.452	0.85810771	Distal Intergenic	ENSSSCG000	ssc-mir-153	ssc-mir-153 [Source:miRBase;Acc:MI0002454]
chrX	91644516	91645602	1087	*	chrX	91644516	91645602	in_vitro	in_vivo	11	13	1.33363636	3.02	-1.6863636	c(t = -0.7681	0.452	0.85810771	Distal Intergenic	ENSSSCG000	SERTM2	serine rich and transmembrane domain containing 2 [Source:NCBI gene (formerly Entrezgene);Acc:106507011]
chr4	117985350	117986145	796	*	chr4	117985350	117986145	in_vitro	in_vivo	12	12	1.31083333	2.51583333	-1.205	c(t = -0.7681	0.452	0.85810771	Distal Intergenic	ENSSSCG000	RTCA	RNA 3'-terminal phosphate cyclase [Source:NCBI gene (formerly Entrezgene);Acc:100154102]
chr8	136070721	136071642	922	*	chr8	136070721	136071642	in_vitro	in_vivo	9	12	1.10666667	2.20833333	-1.1016667	c(t = -0.7739	0.452	0.85810771	Distal Intergenic	ENSSSCG000	ENOPH1	enolase-phosphatase 1 [Source:VGNC Symbol;Acc:VGNC:98922]
chr4	122621129	122621793	665	*	chr4	122621129	122621793	in_vitro	in_vivo	13	12	3.42846154	2.19333333	1.23512821	c(t = 0.76658	0.452	0.85810771	Distal Intergenic	ENSSSCG000	F3	coagulation factor III, tissue factor [Source:VGNC Symbol;Acc:VGNC:87864]
chr14	99574135	99575136	1002	*	chr14	99574135	99575136	in_vitro	in_vivo	13	12	1.63384615	0.8975	0.73634615	c(t = 0.76550	0.452	0.85810771	Distal Intergenic	ENSSSCG00000010440		phosphatase and tensin homolog [Source:VGNC Symbol;Acc:VGNC:111132]
chr10	42914853	42916329	1477	*	chr10	42914853	42916329	in_vitro	in_vivo	13	15	1.38384615	0.86	0.52384615	c(t = 0.77015	0.452	0.85810771	Distal Intergenic	ENSSSCG000	TRDMT1	tRNA aspartic acid methyltransferase 1 [Source:VGNC Symbol;Acc:VGNC:96227]
chr6	34383113	34383570	458	*	chr6	34383113	34383570	in_vitro	in_vivo	9	9	1.53888889	0.58444444	0.95444444	c(t = 0.77726	0.452	0.85810771	Distal Intergenic	ENSSSCG000	NOD2	nucleotide binding oligomerization domain containing 2 [Source:NCBI gene (formerly Entrezgene);Acc:100125838]
chr2	69289089	69290282	1194	*	chr2	69289089	69290282	in_vitro	in_vivo	10	11	3.138	4.52181818	-1.3838182	c(t = -0.7677	0.453	0.85936929	Promoter (<=1kb)	ENSSSCG000	KEAP1	kelch like ECH associated protein 1 [Source:VGNC Symbol;Acc:VGNC:89419]
chr13	70879333	70880152	820	*	chr13	70879333	70880152	in_vitro	in_vivo	10	11	1.126	2.19181818	-1.0658182	c(t = -0.7738	0.453	0.85936929	Distal Intergenic	ENSSSCG000	CHCHD4	coiled-coil-helix-coiled-coil-helix domain containing 4 [Source:NCBI gene (formerly Entrezgene);Acc:100154663]
chr7	32635083	32636001	919	*	chr7	32635083	32636001	in_vitro	in_vivo	12	11	1.96333333	1.09636364	0.8669697	c(t = 0.77167	0.453	0.85936929	Promoter (<=1kb)	ENSSSCG000	MTCH1	mitochondrial carrier 1 [Source:VGNC Symbol;Acc:VGNC:103996]
chr5	2588186	2592661	4476	*	chr5	2588186	2592661	in_vitro	in_vivo	10	9	46.405	57.6777778	-11.272778	c(t = -0.7666	0.454	0.86020464	Distal Intergenic	ENSSSCG000	PPARA	peroxisome proliferator activated receptor alpha [Source:NCBI gene (formerly Entrezgene);Acc:397239]
chr2	5696154	5697681	1528	*	chr2	5696154	5697681	in_vitro	in_vivo	10	10	2.527	4.731	-2.204	c(t = -0.7730	0.454	0.86020464	Distal Intergenic	ENSSSCG00000038304		RNA binding motif protein 4B [Source:NCBI gene (formerly Entrezgene);Acc:100144418]
chr6	88710561	88712484	1924	*	chr6	88710561	88712484	in_vitro	in_vivo	12	12	7.53583333	3.52333333	4.0125	c(t = 0.77110	0.454	0.86020464	Distal Intergenic	ENSSSCG000	EIF3I	eukaryotic translation initiation factor 3 subunit I [Source:NCBI gene (formerly Entrezgene);Acc:100521499]
chr8	1387130	1388825	1696	*	chr8	1387130	1388825	in_vitro	in_vivo	13	12	1.76230769	1.11916667	0.64314103	c(t = 0.76191	0.454	0.86020464	Distal Intergenic	ENSSSCG000	RNF4	ring finger protein 4 [Source:VGNC Symbol;Acc:VGNC:92392]
chr3	11897471	11898076	606	*	chr3	11897471	11898076	in_vitro	in_vivo	10	12	1.393	0.54583333	0.84716667	c(t = 0.77447	0.454	0.86020464	Distal Intergenic	ENSSSCG000	NCF1	neutrophil cytosolic factor 1 [Source:NCBI gene (formerly Entrezgene);Acc:100134857]
chr7	20036267	20038074	1808	*	chr7	20036267	20038074	in_vitro	in_vivo	14	12	4.29285714	5.70333333	-1.4104762	c(t = -0.7605	0.455	0.86102211	Distal Intergenic	ENSSSCG000	CMAH	cytidine monophospho-N-acetylneuraminic acid hydroxylase [Source:NCBI gene (formerly Entrezgene);Acc:396918]
chr14	24921529	24924029	2501	*	chr14	24921529	24924029	in_vitro	in_vivo	12	13	4.06416667	5.53230769	-1.468141	c(t = -0.7595	0.455	0.86102211	Distal Intergenic	ENSSSCG000	PIWIL1	piwi like RNA-mediated silencing 1 [Source:VGNC Symbol;Acc:VGNC:98197]
chr1	2714623	2718074	3452	*	chr1	2714623	2718074	in_vitro	in_vivo	12	12	4.00916667	2.65416667	1.355	c(t = 0.76774	0.455	0.86102211	Distal Intergenic	ENSSSCG000	SFT2D1	SFT2 domain containing 1 [Source:NCBI gene (formerly Entrezgene);Acc:100513321]
chr15	133376576	133378660	2085	*	chr15	133376576	133378660	in_vitro	in_vivo	14	12	1.07357143	1.775	-0.7014286	c(t = -0.7635	0.455	0.86102211	Distal Intergenic	ENSSSCG000	SNORC	secondary ossification center associated regulator of chondrocyte maturation [Source:NCBI gene (formerly Entrezgene);Acc:100157430]
chr1	137689212	137692570	3359	*	chr1	137689212	137692570	in_vitro	in_vivo	16	11	1.644375	1.01363636	0.63073864	c(t = 0.75838	0.455	0.86102211	Promoter (<=1kb)	ENSSSCG000	IGF1R	insulin like growth factor 1 receptor [Source:VGNC Symbol;Acc:VGNC:109458]
chr3	86622979	86623438	460	*	chr3	86622979	86623438	in_vitro	in_vivo	9	9	87.4077778	93.7744444	-6.3666667	c(t = -0.7705	0.456	0.86102211	Distal Intergenic	ENSSSCG000	RTN4	reticulon 4 [Source:VGNC Symbol;Acc:VGNC:92502]
chr13	207573298	207577703	4406	*	chr13	207573298	207577703	in_vitro	in_vivo	9	11	95.3688889	92.5063636	2.86252525	c(t = 0.76682	0.456	0.86102211	Distal Intergenic	ENSSSCG000	ITGB2	integrin subunit beta 2 [Source:NCBI gene (formerly Entrezgene);Acc:396943]
chr15	48693951	48697396	3446	*	chr15	48693951	48697396	in_vitro	in_vivo	13	14	26.0538462	18.4378571	7.61598901	c(t = 0.75781	0.456	0.86102211	Distal Intergenic	ENSSSCG000	ERLIN2	ER lipid raft associated 2 [Source:VGNC Symbol;Acc:VGNC:95586]
chr3	69858146	69861226	3081	*	chr3	69858146	69861226	in_vitro	in_vivo	15	12	4.34266667	2.62	1.72266667	c(t = 0.76316	0.456	0.86102211	Distal Intergenic	ENSSSCG000	CCT7	chaperonin containing TCP1 subunit 7 [Source:NCBI gene (formerly Entrezgene);Acc:100322871]
chr6	37442940	37444502	1563	*	chr6	37442940	37444502	in_vitro	in_vivo	12	12	1.41416667	1.96083333	-0.5466667	c(t = -0.7594	0.456	0.86102211	Distal Intergenic	ENSSSCG000	CBLN1	cerebellin 1 precursor [Source:NCBI gene (formerly Entrezgene);Acc:100127148]
chr10	38389067	38390031	965	*	chr10	38389067	38390031	in_vitro	in_vivo	9	12	0.78666667	1.81666667	-1.03	c(t = -0.7651	0.456	0.86102211	Distal Intergenic	ENSSSCG000	IFNK	interferon kappa [Source:NCBI gene (formerly Entrezgene);Acc:100303725]
chr7	32145903	32146432	530	*	chr7	32145903	32146432	in_vitro	in_vivo	10	14	1.011	1.65571429	-0.6447143	c(t = -0.7613	0.456	0.86102211	Distal Intergenic	ENSSSCG000	SRPK1	SRSF protein kinase 1 [Source:VGNC Symbol;Acc:VGNC:93462]
chr3	55180428	55180966	539	*	chr3	55180428	55180966	in_vitro	in_vivo	11	13	1.93090909	1.14	0.79090909	c(t = 0.76413	0.456	0.86102211	Distal Intergenic	ENSSSCG000	MGAT4A	alpha-1,3-mannosyl-glycoprotein 4-beta-N-acetylglucosaminyltransferase A [Source:VGNC Symbol;Acc:VGNC:108158]
chr8	2916047	2917531	1485	*	chr8	2916047	2917531	in_vitro	in_vivo	9	9	2.25777778	1.11	1.14777778	c(t = 0.77362	0.456	0.86102211	Distal Intergenic	ENSSSCG000	HTRA3	HtrA serine peptidase 3 [Source:NCBI gene (formerly Entrezgene);Acc:100337673]
chr6	85842391	85843385	995	*	chr6	85842391	85843385	in_vitro	in_vivo	11	9	9.26181818	6.41222222	2.84959596	c(t = 0.76065	0.457	0.86134643	Distal Intergenic	ENSSSCG000	SRSF4	serine and arginine rich splicing factor 4 [Source:NCBI gene (formerly Entrezgene);Acc:768101]
chr9	9899863	9901539	1677	*	chr9	9899863	9901539	in_vitro	in_vivo	13	14	1.42076923	2.23857143	-0.8178022	c(t = -0.7564	0.457	0.86134643	Distal Intergenic	ENSSSCG00000014861		monoacylglycerol O-acyltransferase 2 [Source:NCBI gene (formerly Entrezgene);Acc:100312975]
chr16	63602631	63603226	596	*	chr16	63602631	63603226	in_vitro	in_vivo	11	11	2.78727273	1.84818182	0.93909091	c(t = 0.76020	0.457	0.86134643	Distal Intergenic	ENSSSCG000	FABP6	fatty acid binding protein 6 [Source:NCBI gene (formerly Entrezgene);Acc:397423]
chr6	84467181	84468278	1098	*	chr6	84467181	84468278	in_vitro	in_vivo	11	10	2.14636364	1.185	0.96136364	c(t = 0.76116	0.457	0.86134643	Distal Intergenic	ENSSSCG000	WDTC1	WD and tetratricopeptide repeats 1 [Source:VGNC Symbol;Acc:VGNC:94952]
chr10	68558368	68560753	2386	*	chr10	68558368	68560753	in_vitro	in_vivo	10	11	98.719	97.9745455	0.74445455	c(t = 0.75776	0.458	0.86134643	Distal Intergenic	ENSSSCG000	WDR37	WD repeat domain 37 [Source:VGNC Symbol;Acc:VGNC:96258]
chr11	15905559	15906721	1163	*	chr11	15905559	15906721	in_vitro	in_vivo	10	11	97.729	96.5536364	1.17536364	c(t = 0.75686	0.458	0.86134643	Distal Intergenic	ENSSSCG000	SLC25A15	solute carrier family 25 member 15 [Source:VGNC Symbol;Acc:VGNC:92994]
chr12	1120907	1129651	8745	*	chr12	1120907	1129651	in_vitro	in_vivo	9	13	41.7466667	52.2484615	-10.501795	c(t = -0.7598	0.458	0.86134643	Distal Intergenic	ENSSSCG000	ARHGDIA	Rho GDP dissociation inhibitor alpha [Source:VGNC Symbol;Acc:VGNC:85483]
chr14	49792613	49793506	894	*	chr14	49792613	49793506	in_vitro	in_vivo	10	13	2.712	5.25307692	-2.5410769	c(t = -0.7628	0.458	0.86134643	Promoter (<=1kb)	ENSSSCG000	DDT	D-dopachrome tautomerase [Source:NCBI gene (formerly Entrezgene);Acc:100155535]
chr4	864589	865478	890	*	chr4	864589	865478	in_vitro	in_vivo	10	11	1.67	4.51090909	-2.8409091	c(t = -0.7679	0.458	0.86134643	Distal Intergenic	ENSSSCG000	HSF1	heat shock transcription factor 1 [Source:VGNC Symbol;Acc:VGNC:88993]
chr3	92813000	92814492	1493	*	chr3	92813000	92814492	in_vitro	in_vivo	10	13	1.057	1.78692308	-0.7299231	c(t = -0.7567	0.458	0.86134643	Distal Intergenic	ENSSSCG000	MSH2	mutS homolog 2 [Source:NCBI gene (formerly Entrezgene);Acc:100337661]
chr1	15328656	15330141	1486	*	chr1	15328656	15330141	in_vitro	in_vivo	10	14	2.861	1.68214286	1.17885714	c(t = 0.76421	0.458	0.86134643	Distal Intergenic	ENSSSCG000	IYD	iodotyrosine deiodinase [Source:VGNC Symbol;Acc:VGNC:89260]
chr15	68220477	68221315	839	*	chr15	68220477	68221315	in_vitro	in_vivo	14	12	0.79714286	1.225	-0.4278571	c(t = -0.7557	0.458	0.86134643	Distal Intergenic	ENSSSCG000	DPP4	dipeptidyl peptidase 4 [Source:NCBI gene (formerly Entrezgene);Acc:397492]
chr11	78205489	78207669	2181	*	chr11	78205489	78207669	in_vitro	in_vivo	10	11	94.626	96.1109091	-1.4849091	c(t = -0.7556	0.459	0.86134643	Distal Intergenic	ENSSSCG000	F7	coagulation factor VII [Source:NCBI gene (formerly Entrezgene);Acc:733661]
chr4	547919	549723	1805	*	chr4	547919	549723	in_vitro	in_vivo	9	11	93.1811111	95.3345455	-2.1534343	c(t = -0.7674	0.459	0.86134643	Distal Intergenic	ENSSSCG000	HSF1	heat shock transcription factor 1 [Source:VGNC Symbol;Acc:VGNC:88993]
chr18	5865088	5866728	1641	*	chr18	5865088	5866728	in_vitro	in_vivo	10	9	8.522	5.85111111	2.67088889	c(t = 0.75863	0.459	0.86134643	Distal Intergenic	ENSSSCG000	RHEB	Ras homolog, mTORC1 binding [Source:NCBI gene (formerly Entrezgene);Acc:100516050]
chrX	113530387	113531817	1431	*	chrX	113530387	113531817	in_vitro	in_vivo	9	12	8.33888889	5.05083333	3.28805556	c(t = 0.75607	0.459	0.86134643	Distal Intergenic	ENSSSCG000	ssc-mir-504	ssc-mir-504 [Source:miRBase;Acc:MI0013143]
chr13	207907304	207909223	1920	*	chr13	207907304	207909223	in_vitro	in_vivo	11	13	2.64272727	4.51076923	-1.868042	c(t = -0.7591	0.459	0.86134643	Distal Intergenic	ENSSSCG000	ITGB2	integrin subunit beta 2 [Source:NCBI gene (formerly Entrezgene);Acc:396943]
chr6	72960288	72961643	1356	*	chr6	72960288	72961643	in_vitro	in_vivo	12	14	1.32666667	2.25928571	-0.932619	c(t = -0.7567	0.459	0.86134643	Distal Intergenic	ENSSSCG000	TNFRSF1B	TNF receptor superfamily member 1B [Source:VGNC Symbol;Acc:VGNC:94264]
chr9	90422871	90423728	858	*	chr9	90422871	90423728	in_vitro	in_vivo	12	12	0.69166667	1.87333333	-1.1816667	c(t = -0.7635	0.459	0.86134643	Distal Intergenic	ENSSSCG000	ITGB8	integrin subunit beta 8 [Source:VGNC Symbol;Acc:VGNC:89246]
chr2	101291929	101292505	577	*	chr2	101291929	101292505	in_vitro	in_vivo	10	11	2.218	1.43545455	0.78254545	c(t = 0.75632	0.459	0.86134643	Distal Intergenic	ENSSSCG000	SPATA9	spermatogenesis associated 9 [Source:NCBI gene (formerly Entrezgene);Acc:100415927]
chr1	241569484	241573110	3627	*	chr1	241569484	241573110	in_vitro	in_vivo	14	13	1.53928571	0.93230769	0.60697802	c(t = 0.75660	0.459	0.86134643	Promoter (<=1kb)	ENSSSCG000	NR4A3	nuclear receptor subfamily 4 group A member 3 [Source:VGNC Symbol;Acc:VGNC:90885]
chr14	29355331	29355705	375	*	chr14	29355331	29355705	in_vitro	in_vivo	12	11	1.82083333	0.74818182	1.07265152	c(t = 0.76034	0.459	0.86134643	Distal Intergenic	ENSSSCG000	SUMO4	SMT3 suppressor of mif two 3 homolog 4 (S. cerevisiae) [Source:NCBI gene (formerly Entrezgene);Acc:100127140]
chr1	5465142	5465952	811	*	chr1	5465142	5465952	in_vitro	in_vivo	13	16	1.01153846	0.64375	0.36778846	c(t = 0.75401	0.459	0.86134643	Distal Intergenic	ENSSSCG000	PRKN	parkin RBR E3 ubiquitin protein ligase [Source:NCBI gene (formerly Entrezgene);Acc:733673]
chr9	92822888	92823677	790	*	chr9	92822888	92823677	in_vitro	in_vivo	11	9	1.39818182	0.52888889	0.86929293	c(t = 0.76041	0.459	0.86134643	Distal Intergenic	ENSSSCG00000015390		ATP-binding cassette, sub-family B (MDR/TAP), member 1 [Source:NCBI gene (formerly Entrezgene);Acc:396910]
chr4	128641117	128641723	607	*	chr4	128641117	128641723	in_vitro	in_vivo	9	10	20.27	33.483	-13.213	c(t = -0.7565	0.46	0.86134643	Distal Intergenic	ENSSSCG00000047605		LIM domain only 4 [Source:NCBI gene (formerly Entrezgene);Acc:100127155]
chr9	46174212	46175155	944	*	chr9	46174212	46175155	in_vitro	in_vivo	9	10	1.10111111	2.201	-1.0998889	c(t = -0.7596	0.46	0.86134643	Distal Intergenic	ENSSSCG000	UPK2	uroplakin 2 [Source:VGNC Symbol;Acc:VGNC:94723]
chr3	71621062	71622408	1347	*	chr3	71621062	71622408	in_vitro	in_vivo	11	13	2.68363636	1.77846154	0.90517483	c(t = 0.75554	0.46	0.86134643	Distal Intergenic	ENSSSCG000	CD207	CD207 molecule [Source:NCBI gene (formerly Entrezgene);Acc:100037935]
chr12	25194965	25198370	3406	*	chr12	25194965	25198370	in_vitro	in_vivo	14	13	2.195	1.48	0.715	c(t = 0.75373	0.46	0.86134643	Distal Intergenic	ENSSSCG000	GIP	gastric inhibitory polypeptide [Source:VGNC Symbol;Acc:VGNC:98995]
chr16	24041161	24041905	745	*	chr16	24041161	24041905	in_vitro	in_vivo	9	14	1.90555556	1.11642857	0.78912698	c(t = 0.75973	0.46	0.86134643	Distal Intergenic	ENSSSCG000	C9	complement C9 [Source:NCBI gene (formerly Entrezgene);Acc:100037951]
chr13	110051617	110053129	1513	*	chr13	110051617	110053129	in_vitro	in_vivo	10	11	1.791	1.06363636	0.72736364	c(t = 0.75528	0.46	0.86134643	Distal Intergenic	ENSSSCG000	PLD1	phospholipase D1 [Source:VGNC Symbol;Acc:VGNC:91525]
chr6	63592979	63594604	1626	*	chr6	63592979	63594604	in_vitro	in_vivo	9	9	1.65	0.79666667	0.85333333	c(t = 0.76686	0.46	0.86134643	Distal Intergenic	ENSSSCG000	TAS1R3	taste 1 receptor member 3 [Source:NCBI gene (formerly Entrezgene);Acc:100127440]
chr9	1179075	1189335	10261	*	chr9	1179075	1189335	in_vitro	in_vivo	13	14	93.2738462	90.4164286	2.85741758	c(t = 0.75265	0.461	0.86134643	Distal Intergenic	NA	NA	NA
chr13	207730222	207735189	4968	*	chr13	207730222	207735189	in_vitro	in_vivo	9	13	79.9388889	86.8153846	-6.8764957	c(t = -0.7514	0.461	0.86134643	Distal Intergenic	ENSSSCG000	ITGB2	integrin subunit beta 2 [Source:NCBI gene (formerly Entrezgene);Acc:396943]
chr18	10593024	10594111	1088	*	chr18	10593024	10594111	in_vitro	in_vivo	11	14	1.37454545	3.32428571	-1.9497403	c(t = -0.7555	0.461	0.86134643	Distal Intergenic	ENSSSCG000	ZC3HAV1	zinc finger CCCH-type containing, antiviral 1 [Source:VGNC Symbol;Acc:VGNC:95102]
chr13	18649606	18650091	486	*	chr13	18649606	18650091	in_vitro	in_vivo	10	9	1.827	3.17777778	-1.3507778	c(t = -0.7606	0.461	0.86134643	Distal Intergenic	ENSSSCG000	CMTM7	CKLF like MARVEL transmembrane domain containing 7 [Source:VGNC Symbol;Acc:VGNC:103928]
chr10	43516434	43518340	1907	*	chr10	43516434	43518340	in_vitro	in_vivo	13	13	4.34846154	2.55769231	1.79076923	c(t = 0.75023	0.461	0.86134643	Distal Intergenic	ENSSSCG000	TRDMT1	tRNA aspartic acid methyltransferase 1 [Source:VGNC Symbol;Acc:VGNC:96227]
chr7	120596297	120598769	2473	*	chr7	120596297	120598769	in_vitro	in_vivo	13	9	3.45384615	2.23222222	1.22162393	c(t = 0.75116	0.461	0.86134643	Distal Intergenic	ENSSSCG000	BCL11B	BCL11 transcription factor B [Source:NCBI gene (formerly Entrezgene);Acc:100154158]
chr1	180591892	180593286	1395	*	chr1	180591892	180593286	in_vitro	in_vivo	11	14	2.37636364	1.56928571	0.80707792	c(t = 0.75377	0.461	0.86134643	Distal Intergenic	ENSSSCG000	PYGL	glycogen phosphorylase L [Source:NCBI gene (formerly Entrezgene);Acc:100141306]
chr16	47386266	47386800	535	*	chr16	47386266	47386800	in_vitro	in_vivo	11	9	0.71727273	1.50777778	-0.7905051	c(t = -0.7531	0.461	0.86134643	Distal Intergenic	ENSSSCG000	CDK7	cyclin dependent kinase 7 [Source:VGNC Symbol;Acc:VGNC:86506]
chr8	70787973	70789248	1276	*	chr8	70787973	70789248	in_vitro	in_vivo	13	15	1.57384615	0.95333333	0.62051282	c(t = 0.74809	0.461	0.86134643	Promoter (<=1kb)	ENSSSCG000	PARM1	prostate androgen-regulated mucin-like protein 1 [Source:NCBI gene (formerly Entrezgene);Acc:100511208]
chr2	113754365	113754953	589	*	chr2	113754365	113754953	in_vitro	in_vivo	9	9	0.38222222	0.89222222	-0.51	c(t = -0.7635	0.461	0.86134643	Distal Intergenic	ENSSSCG000	STARD4	StAR related lipid transfer domain containing 4 [Source:VGNC Symbol;Acc:VGNC:93534]
chr4	12457340	12461605	4266	*	chr4	12457340	12461605	in_vitro	in_vivo	13	15	1.35076923	0.75066667	0.60010256	c(t = 0.75078	0.461	0.86134643	Promoter (<=1kb)	ENSSSCG000	MYC	MYC proto-oncogene, bHLH transcription factor [Source:NCBI gene (formerly Entrezgene);Acc:448810]
chr12	1708838	1725965	17128	*	chr12	1708838	1725965	in_vitro	in_vivo	10	15	32.091	41.29	-9.199	c(t = -0.7494	0.462	0.86134643	Distal Intergenic	NA	NA	NA
chr12	186338	187465	1128	*	chr12	186338	187465	in_vitro	in_vivo	11	10	23.9954545	15.965	8.03045455	c(t = 0.75602	0.462	0.86134643	Distal Intergenic	ENSSSCG000	TBCD	tubulin folding cofactor D [Source:NCBI gene (formerly Entrezgene);Acc:100512127]
chr6	3655139	3657919	2781	*	chr6	3655139	3657919	in_vitro	in_vivo	9	13	20.3522222	12.8430769	7.5091453	c(t = 0.75273	0.462	0.86134643	Distal Intergenic	ENSSSCG000	CIBAR2	CBY1 interacting BAR domain containing 2 [Source:VGNC Symbol;Acc:VGNC:86698]
chr10	9542647	9543884	1238	*	chr10	9542647	9543884	in_vitro	in_vivo	14	14	4.65	9.21357143	-4.5635714	c(t = -0.7541	0.462	0.86134643	Distal Intergenic	ENSSSCG000	ssc-mir-215	ssc-mir-215 [Source:miRBase;Acc:MI0010687]
chr6	72195427	72195964	538	*	chr6	72195427	72195964	in_vitro	in_vivo	9	9	2.25666667	3.64222222	-1.3855556	c(t = -0.7612	0.462	0.86134643	Promoter (<=1kb)	ENSSSCG000	TNFRSF1B	TNF receptor superfamily member 1B [Source:VGNC Symbol;Acc:VGNC:94264]
chr3	112030847	112032670	1824	*	chr3	112030847	112032670	in_vitro	in_vivo	10	11	9.22	3.12363636	6.09636364	c(t = 0.76782	0.462	0.86134643	Distal Intergenic	ENSSSCG000	SLC30A3	solute carrier family 30 member 3 [Source:NCBI gene (formerly Entrezgene);Acc:100169931]
chr13	207557367	207561462	4096	*	chr13	207557367	207561462	in_vitro	in_vivo	10	12	1.491	2.6625	-1.1715	c(t = -0.7565	0.462	0.86134643	Distal Intergenic	ENSSSCG000	ITGB2	integrin subunit beta 2 [Source:NCBI gene (formerly Entrezgene);Acc:396943]
chr14	29729548	29730544	997	*	chr14	29729548	29730544	in_vitro	in_vivo	14	12	1.46357143	2.12916667	-0.6655952	c(t = -0.7484	0.462	0.86134643	Distal Intergenic	ENSSSCG000	CDK2AP1	cyclin dependent kinase 2 associated protein 1 [Source:VGNC Symbol;Acc:VGNC:103918]
chr17	39244558	39245553	996	*	chr17	39244558	39245553	in_vitro	in_vivo	9	12	1.58777778	0.79916667	0.78861111	c(t = 0.76423	0.462	0.86134643	Distal Intergenic	ENSSSCG000	ROMO1	reactive oxygen species modulator 1 [Source:NCBI gene (formerly Entrezgene);Acc:100154394]
chr9	93462604	93463174	571	*	chr9	93462604	93463174	in_vitro	in_vivo	11	10	1.22090909	0.642	0.57890909	c(t = 0.75158	0.462	0.86134643	Distal Intergenic	ENSSSCG000	CROT	carnitine O-octanoyltransferase [Source:NCBI gene (formerly Entrezgene);Acc:100521142]
chr11	5701363	5702038	676	*	chr11	5701363	5702038	in_vitro	in_vivo	10	10	71.627	79.304	-7.677	c(t = -0.7561	0.463	0.8617474	Distal Intergenic	ENSSSCG000	SLC46A3	solute carrier family 46 member 3 [Source:VGNC Symbol;Acc:VGNC:93128]
chr1	831845	834558	2714	*	chr1	831845	834558	in_vitro	in_vivo	11	10	31.11	43.74	-12.63	c(t = -0.7516	0.463	0.8617474	Distal Intergenic	ENSSSCG000	ssc-mir-9815	ssc-mir-9815 [Source:miRBase;Acc:MI0031582]
chr8	110504278	110504853	576	*	chr8	110504278	110504853	in_vitro	in_vivo	10	10	1.76	3.511	-1.751	c(t = -0.7611	0.463	0.8617474	Distal Intergenic	ENSSSCG000	LARP7	La ribonucleoprotein 7, transcriptional regulator [Source:NCBI gene (formerly Entrezgene);Acc:100511273]
chr6	82489644	82490390	747	*	chr6	82489644	82490390	in_vitro	in_vivo	10	10	1.323	2.303	-0.98	c(t = -0.7528	0.463	0.8617474	Distal Intergenic	ENSSSCG000	RSRP1	arginine and serine rich protein 1 [Source:VGNC Symbol;Acc:VGNC:92489]
chr14	55180753	55182094	1342	*	chr14	55180753	55182094	in_vitro	in_vivo	12	11	0.8525	1.68636364	-0.8338636	c(t = -0.7505	0.463	0.8617474	Distal Intergenic	ENSSSCG000	EDARADD	EDAR associated death domain [Source:VGNC Symbol;Acc:VGNC:87542]
chr7	51513508	51514484	977	*	chr7	51513508	51514484	in_vitro	in_vivo	13	12	2.61538462	1.4225	1.19288462	c(t = 0.74753	0.463	0.8617474	Distal Intergenic	ENSSSCG000	ssc-mir-1839	ssc-mir-1839 [Source:miRBase;Acc:MI0013162]
chr3	53032225	53033358	1134	*	chr3	53032225	53033358	in_vitro	in_vivo	10	13	1.318	0.67769231	0.64030769	c(t = 0.76166	0.463	0.8617474	Distal Intergenic	ENSSSCG000	IL1R2	interleukin 1 receptor type 2 [Source:VGNC Symbol;Acc:VGNC:89093]
chr16	79206448	79209364	2917	*	chr16	79206448	79209364	in_vitro	in_vivo	11	11	23.19	32.4981818	-9.3081818	c(t = -0.7486	0.464	0.86269609	Distal Intergenic	ENSSSCG000	TERT	telomerase reverse transcriptase [Source:NCBI gene (formerly Entrezgene);Acc:492280]
chr18	48403370	48403927	558	*	chr18	48403370	48403927	in_vitro	in_vivo	10	9	1.4	2.26111111	-0.8611111	c(t = -0.7512	0.464	0.86269609	Distal Intergenic	ENSSSCG000	HUS1	HUS1 checkpoint clamp component [Source:NCBI gene (formerly Entrezgene);Acc:100192318]
chr4	123548576	123549463	888	*	chr4	123548576	123549463	in_vitro	in_vivo	10	13	1.62	0.81384615	0.80615385	c(t = 0.75188	0.464	0.86269609	Distal Intergenic	ENSSSCG000	DNTTIP2	deoxynucleotidyltransferase terminal interacting protein 2 [Source:NCBI gene (formerly Entrezgene);Acc:100157622]
chr11	889016	890287	1272	*	chr11	889016	890287	in_vitro	in_vivo	9	10	96.0677778	93.192	2.87577778	c(t = 0.75175	0.465	0.86269609	Intron (NM_213881/396914, intron 3 of 7)	ENSSSCG000	CRYL1	crystallin lambda 1 [Source:VGNC Symbol;Acc:VGNC:87023]
chr4	1181019	1181629	611	*	chr4	1181019	1181629	in_vitro	in_vivo	9	9	83.31	91.0466667	-7.7366667	c(t = -0.7621	0.465	0.86269609	Distal Intergenic	ENSSSCG000	GPIHBP1	glycosylphosphatidylinositol anchored high density lipoprotein binding protein 1 [Source:VGNC Symbol;Acc:VGNC:88588]
chr7	88263152	88264687	1536	*	chr7	88263152	88264687	in_vitro	in_vivo	11	12	82.4409091	90.2175	-7.7765909	c(t = -0.7509	0.465	0.86269609	Distal Intergenic	ENSSSCG000	GPX2	glutathione peroxidase 2 [Source:NCBI gene (formerly Entrezgene);Acc:767625]
chr1	188925567	188926225	659	*	chr1	188925567	188926225	in_vitro	in_vivo	11	11	1.42363636	2.41	-0.9863636	c(t = -0.7472	0.465	0.86269609	Promoter (<=1kb)	ENSSSCG000	RTN1	reticulon 1 [Source:NCBI gene (formerly Entrezgene);Acc:100152394]
chr14	47557624	47558238	615	*	chr14	47557624	47558238	in_vitro	in_vivo	9	13	3.14888889	2.07692308	1.07196581	c(t = 0.74532	0.465	0.86269609	Distal Intergenic	ENSSSCG000	TCN2	transcobalamin 2 [Source:VGNC Symbol;Acc:VGNC:93830]
chr3	77855780	77856906	1127	*	chr3	77855780	77856906	in_vitro	in_vivo	10	13	3.23	1.89461538	1.33538462	c(t = 0.74826	0.465	0.86269609	Distal Intergenic	ENSSSCG000	UGP2	UDP-glucose pyrophosphorylase 2 [Source:VGNC Symbol;Acc:VGNC:98400]
chr3	11321023	11322131	1109	*	chr3	11321023	11322131	in_vitro	in_vivo	12	15	1	1.47933333	-0.4793333	c(t = -0.7415	0.465	0.86269609	Promoter (<=1kb)	ENSSSCG000	EIF4H	eukaryotic translation initiation factor 4H [Source:NCBI gene (formerly Entrezgene);Acc:100627717]
chr12	56401810	56402881	1072	*	chr12	56401810	56402881	in_vitro	in_vivo	14	16	1.62285714	1.116875	0.50598214	c(t = 0.74516	0.465	0.86269609	Distal Intergenic	ENSSSCG000	ssc-mir-744	ssc-mir-744 [Source:miRBase;Acc:MI0014769]
chr7	92497942	92498895	954	*	chr7	92497942	92498895	in_vitro	in_vivo	11	10	2.27	1.103	1.167	c(t = 0.74825	0.465	0.86269609	Distal Intergenic	ENSSSCG000	ACTN1	actinin alpha 1 [Source:NCBI gene (formerly Entrezgene);Acc:100513412]
chr10	40952722	40953660	939	*	chr10	40952722	40953660	in_vitro	in_vivo	9	11	1.60111111	0.56181818	1.03929293	c(t = 0.76106	0.465	0.86269609	Distal Intergenic	ENSSSCG000	MTPAP	mitochondrial poly(A) polymerase [Source:VGNC Symbol;Acc:VGNC:96676]
chr7	116099004	116102844	3841	*	chr7	116099004	116102844	in_vitro	in_vivo	13	13	6.38230769	10.8630769	-4.4807692	c(t = -0.7438	0.466	0.86269609	Distal Intergenic	ENSSSCG000	SERPINA3-2	alpha-1-antichymotrypsin 2 [Source:NCBI gene (formerly Entrezgene);Acc:396686]
chr12	60853240	60855192	1953	*	chr12	60853240	60855192	in_vitro	in_vivo	9	9	3.03111111	5.48111111	-2.45	c(t = -0.7554	0.466	0.86269609	Distal Intergenic	ENSSSCG00000018055		phosphatidylethanolamine N-methyltransferase [Source:VGNC Symbol;Acc:VGNC:91311]
chr2	28417596	28419538	1943	*	chr2	28417596	28419538	in_vitro	in_vivo	13	12	2.57846154	3.83916667	-1.2607051	c(t = -0.7429	0.466	0.86269609	Exon (NM_001001264/397338, exon 2 of 11)	ENSSSCG000	WT1	WT1 transcription factor [Source:VGNC Symbol;Acc:VGNC:100884]
chr16	63227310	63228360	1051	*	chr16	63227310	63228360	in_vitro	in_vivo	11	12	4.92454545	2.77333333	2.15121212	c(t = 0.74544	0.466	0.86269609	Distal Intergenic	ENSSSCG000	C1QTNF2	C1q and TNF related 2 [Source:VGNC Symbol;Acc:VGNC:85983]
chr4	99415106	99415898	793	*	chr4	99415106	99415898	in_vitro	in_vivo	10	10	4.396	2.351	2.045	c(t = 0.75177	0.466	0.86269609	Promoter (<=1kb)	ENSSSCG00000006685		gonadotropin releasing hormone receptor 2 [Source:VGNC Symbol;Acc:VGNC:109150]

chr5	42930098	42932877	2780	*	chr5	42930098	42932877	in_vitro	in_vivo	15	15	2.736	1.76933333	0.96666667	c(t = 0.73872	0.466	0.86269609	Distal Intergenic	ENSSSCG000	RESF1	retroelement silencing factor 1 [Source:VGNC Symbol;Acc:VGNC:92219]
chr8	68948215	68948869	655	*	chr8	68948215	68948869	in_vitro	in_vivo	10	9	1.874	1.06888889	0.80511111	c(t = 0.74599	0.466	0.86269609	Distal Intergenic	ENSSSCG000	ANKRD17	ankyrin repeat domain 17 [Source:VGNC Symbol;Acc:VGNC:85328]
chr7	1000404	1003515	3112	*	chr7	1000404	1003515	in_vitro	in_vivo	10	12	59.704	49.1383333	10.5656667	c(t = 0.74113	0.467	0.86269609	Distal Intergenic	ENSSSCG000	SERPINB9	serpin peptidase inhibitor, clade B (ovalbumin), member 9 [Source:NCBI gene (formerly Entrezgene);Acc:100126851]
chr10	7368435	7372085	3651	*	chr10	7368435	7372085	in_vitro	in_vivo	13	13	2.32538462	3.80307692	-1.4776923	c(t = -0.7417	0.467	0.86269609	Distal Intergenic	ENSSSCG000	SPATA17	spermatosis associated 17 [Source:VGNC Symbol;Acc:VGNC:96065]
chr1	269153781	269154816	1036	*	chr1	269153781	269154816	in_vitro	in_vivo	10	9	1.528	2.45777778	-0.9297778	c(t = -0.7455	0.467	0.86269609	Distal Intergenic	ENSSSCG000	ZER1	zyg-11 related cell cycle regulator [Source:VGNC Symbol;Acc:VGNC:95132]
chr1	235887622	235888107	486	*	chr1	235887622	235888107	in_vitro	in_vivo	11	10	0.68636364	1.833	-1.1466364	c(t = -0.7480	0.467	0.86269609	Distal Intergenic	ENSSSCG000	VCP	valosin containing protein [Source:VGNC Symbol;Acc:VGNC:109420]
chr8	12590064	12590695	632	*	chr8	12590064	12590695	in_vitro	in_vivo	9	11	2.71111111	1.51363636	1.19747475	c(t = 0.74754	0.467	0.86269609	Distal Intergenic	ENSSSCG000	MED28	mediator complex subunit 28 [Source:VGNC Symbol;Acc:VGNC:90118]
chr8	130747926	130749110	1185	*	chr8	130747926	130749110	in_vitro	in_vivo	9	13	0.81777778	1.36	-0.5422222	c(t = -0.7446	0.467	0.86269609	Distal Intergenic	ENSSSCG000	ABCG2	ATP-binding cassette, sub-family G (WHITE), member 2 [Source:NCBI gene (formerly Entrezgene);Acc:397073]
chr2	133950872	133951990	1119	*	chr2	133950872	133951990	in_vitro	in_vivo	10	12	0.441	1.04166667	-0.6006667	c(t = -0.7475	0.467	0.86269609	Distal Intergenic	ENSSSCG000	CSF2	colony stimulating factor 2 [Source:NCBI gene (formerly Entrezgene);Acc:397208]
chr12	48416580	48418425	1846	*	chr12	48416580	48418425	in_vitro	in_vivo	9	11	1.37777778	0.87636364	0.50141414	c(t = 0.74343	0.467	0.86269609	Distal Intergenic	ENSSSCG000	PAFAH1B1	platelet activating factor acetylhydrolase 1b regulatory subunit 1 [Source:VGNC Symbol;Acc:VGNC:99026]
chr6	95899989	95900651	663	*	chr6	95899989	95900651	in_vitro	in_vivo	11	10	1.51909091	0.854	0.66509091	c(t = 0.74366	0.467	0.86269609	Distal Intergenic	ENSSSCG000	HPCAL4	hippocalcin like 4 [Source:VGNC Symbol;Acc:VGNC:88959]
chr3	42632846	42633792	947	*	chr3	42632846	42633792	in_vitro	in_vivo	10	11	1.45	0.61090909	0.83909091	c(t = 0.74943	0.467	0.86269609	Promoter (<=1kb)	ENSSSCG000	CARD19	caspase recruitment domain family member 19 [Source:VGNC Symbol;Acc:VGNC:86188]
chr7	120971705	120972444	740	*	chr7	120971705	120972444	in_vitro	in_vivo	9	9	94.0711111	96.04	-1.9688889	c(t = -0.7463	0.468	0.86269609	Distal Intergenic	ENSSSCG000	EVL	Enah/Vasp-like [Source:VGNC Symbol;Acc:VGNC:87820]
chr18	48381360	48382157	798	*	chr18	48381360	48382157	in_vitro	in_vivo	11	12	12.2463636	20.3366667	-8.090303	c(t = -0.7397	0.468	0.86269609	Distal Intergenic	ENSSSCG000	HUS1	HUS1 checkpoint clamp component [Source:NCBI gene (formerly Entrezgene);Acc:100192318]
chr9	35442196	35442534	339	*	chr9	35442196	35442534	in_vitro	in_vivo	11	9	1.83363636	5.55555556	-3.7219192	c(t = -0.7566	0.468	0.86269609	Distal Intergenic	ENSSSCG000	CASP1	caspase 1, apoptosis-related cysteine peptidase [Source:NCBI gene (formerly Entrezgene);Acc:397319]
chr5	5131062	5131744	683	*	chr5	5131062	5131744	in_vitro	in_vivo	10	13	1.138	2.35692308	-1.2189231	c(t = -0.7441	0.468	0.86269609	Distal Intergenic	ENSSSCG000	PNPLA3	patatin like phospholipase domain containing 3 [Source:VGNC Symbol;Acc:VGNC:103312]
chr14	54405545	54406918	1374	*	chr14	54405545	54406918	in_vitro	in_vivo	11	12	1.57454545	2.17	-0.5954545	c(t = -0.7383	0.468	0.86269609	Distal Intergenic	ENSSSCG000	ACTN2	actinin alpha 2 [Source:NCBI gene (formerly Entrezgene);Acc:100157406]
chr2	11739329	11739906	578	*	chr2	11739329	11739906	in_vitro	in_vivo	9	11	1.07555556	2.09545455	-1.019899	c(t = -0.7434	0.468	0.86269609	Distal Intergenic	ENSSSCG000	MRPL16	mitochondrial ribosomal protein L16 [Source:NCBI gene (formerly Entrezgene);Acc:100514446]
chr1	8934635	8935543	909	*	chr1	8934635	8935543	in_vitro	in_vivo	12	12	2.7375	1.38083333	1.35666667	c(t = 0.74539	0.468	0.86269609	Distal Intergenic	ENSSSCG000	DYNLT1	dynein light chain Tctex-type 1 [Source:NCBI gene (formerly Entrezgene);Acc:100158053]
chr1	130991659	130993051	1393	*	chr1	130991659	130993051	in_vitro	in_vivo	12	11	0.7675	1.37727273	-0.6097727	c(t = -0.7436	0.468	0.86269609	Distal Intergenic	ENSSSCG000	IVD	isovaleryl-CoA dehydrogenase [Source:NCBI gene (formerly Entrezgene);Acc:100156047]
chr13	117147139	117147778	640	*	chr13	117147139	117147778	in_vitro	in_vivo	12	12	0.69	1.19416667	-0.5041667	c(t = -0.7393	0.468	0.86269609	Distal Intergenic	ENSSSCG000	ZNF639	zinc finger protein 639 [Source:VGNC Symbol;Acc:VGNC:95282]
chr1	238540755	238541406	652	*	chr1	238540755	238541406	in_vitro	in_vivo	11	9	0.34272727	0.82222222	-0.4794949	c(t = -0.7459	0.468	0.86269609	Distal Intergenic	ENSSSCG00000032554		solute carrier family 25 member 51 [Source:NCBI gene (formerly Entrezgene);Acc:100157218]
chr16	79538042	79539074	1033	*	chr16	79538042	79539074	in_vitro	in_vivo	9	9	21.1144444	34.4277778	-13.313333	c(t = -0.7421	0.469	0.86329701	Intron (NM_001123206/100144410, intron 1 of 3)	ENSSSCG000	TPPP	tubulin polymerization promoting protein [Source:VGNC Symbol;Acc:VGNC:94344]
chr7	121706792	121711117	4326	*	chr7	121706792	121711117	in_vitro	in_vivo	13	15	19.8807692	28.3713333	-8.4905641	c(t = -0.7345	0.469	0.86329701	Promoter (<=1kb)	ENSSSCG000	MIR127	microRNA mir-127 [Source:NCBI gene (formerly Entrezgene);Acc:100498735]
chr14	7358433	7358772	340	*	chr14	7358433	7358772	in_vitro	in_vivo	15	16	0.56133333	1.51625	-0.9549167	c(t = -0.7422	0.469	0.86329701	Distal Intergenic	ENSSSCG000	PEBP4	phosphatidylethanolamine binding protein 4 [Source:VGNC Symbol;Acc:VGNC:91303]
chr14	88004673	88005957	1285	*	chr14	88004673	88005957	in_vitro	in_vivo	10	11	2.096	1.29545455	0.80054545	c(t = 0.74677	0.469	0.86329701	Promoter (<=1kb)	ENSSSCG000	GLUD1	glutamate dehydrogenase 1 [Source:NCBI gene (formerly Entrezgene);Acc:100157162]
chr1	53865432	53867665	2234	*	chr1	53865432	53867665	in_vitro	in_vivo	11	13	0.47818182	0.96	-0.4818182	c(t = -0.7367	0.469	0.86329701	Distal Intergenic	ENSSSCG000	OOEP	oocyte expressed protein [Source:NCBI gene (formerly Entrezgene);Acc:100152355]
chr10	68534317	68535403	1087	*	chr10	68534317	68535403	in_vitro	in_vivo	9	13	0.61666667	0.36230769	0.25435897	c(t = 0.74115	0.469	0.86329701	Distal Intergenic	ENSSSCG000	WDR37	WD repeat domain 37 [Source:VGNC Symbol;Acc:VGNC:96258]
chr6	95508812	95509246	435	*	chr6	95508812	95509246	in_vitro	in_vivo	9	9	2.02222222	4.23333333	-2.2111111	c(t = -0.7528	0.47	0.86348315	Promoter (<=1kb)	ENSSSCG000	HPCAL4	hippocalcin like 4 [Source:VGNC Symbol;Acc:VGNC:88959]
chr14	23117919	23118857	939	*	chr14	23117919	23118857	in_vitro	in_vivo	11	14	2.49545455	3.64285714	-1.1474026	c(t = -0.7361	0.47	0.86348315	Distal Intergenic	ENSSSCG000	PIWIL1	piwi like RNA-mediated silencing 1 [Source:VGNC Symbol;Acc:VGNC:98197]
chr9	126172009	126172651	643	*	chr9	126172009	126172651	in_vitro	in_vivo	9	12	1.50111111	2.7875	-1.2863889	c(t = -0.7401	0.47	0.86348315	Promoter (<=1kb)	ENSSSCG000	NIBAN1	niban apoptosis regulator 1 [Source:NCBI gene (formerly Entrezgene);Acc:100516994]
chrX	15364910	15366034	1125	*	chrX	15364910	15366034	in_vitro	in_vivo	10	11	1.049	2.48272727	-1.4337273	c(t = -0.7461	0.47	0.86348315	Distal Intergenic	ENSSSCG000	EIF1AX	eukaryotic translation initiation factor 1A X-linked [Source:NCBI gene (formerly Entrezgene);Acc:100522912]
chr5	42517678	42518736	1059	*	chr5	42517678	42518736	in_vitro	in_vivo	11	12	4.01909091	2.08333333	1.93575758	c(t = 0.73653	0.47	0.86348315	Distal Intergenic	ENSSSCG000	RESF1	retroelement silencing factor 1 [Source:VGNC Symbol;Acc:VGNC:92219]
chr2	86664295	86665152	858	*	chr2	86664295	86665152	in_vitro	in_vivo	11	11	1.05636364	1.83545455	-0.7790909	c(t = -0.7407	0.47	0.86348315	Distal Intergenic	ENSSSCG000	AP3B1	adaptor related protein complex 3 subunit beta 1 [Source:VGNC Symbol;Acc:VGNC:85387]
chr6	127829778	127831259	1482	*	chr6	127829778	127831259	in_vitro	in_vivo	13	11	2.11615385	1.34454545	0.77160839	c(t = 0.73688	0.47	0.86348315	Distal Intergenic	ENSSSCG000	HSBP1L1	heat shock factor binding protein 1 like 1 [Source:NCBI gene (formerly Entrezgene);Acc:100192206]
chr1	252426235	252426865	631	*	chr1	252426235	252426865	in_vitro	in_vivo	10	11	2.219	1.12909091	1.08990909	c(t = 0.74939	0.47	0.86348315	Distal Intergenic	ENSSSCG000	PTGR1	prostaglandin reductase 1 [Source:VGNC Symbol;Acc:VGNC:91957]
chr6	60444766	60446432	1667	*	chr6	60444766	60446432	in_vitro	in_vivo	12	12	7.97833333	5.37916667	2.59916667	c(t = 0.73620	0.471	0.86408093	Distal Intergenic	ENSSSCG000	GALP	galanin like peptide [Source:NCBI gene (formerly Entrezgene);Acc:396772]
chr16	75908265	75908603	339	*	chr16	75908265	75908603	in_vitro	in_vivo	9	9	1.44777778	3.18555556	-1.7377778	c(t = -0.7434	0.471	0.86408093	Distal Intergenic	ENSSSCG000	TERT	telomerase reverse transcriptase [Source:NCBI gene (formerly Entrezgene);Acc:492280]
chr16	18655240	18656449	1210	*	chr16	18655240	18656449	in_vitro	in_vivo	10	9	4.099	2.51222222	1.58677778	c(t = 0.74202	0.471	0.86408093	Distal Intergenic	ENSSSCG000	C1QTNF3	C1q and TNF related 3 [Source:NCBI gene (formerly Entrezgene);Acc:100270814]
chr13	83818149	83821137	2989	*	chr13	83818149	83821137	in_vitro	in_vivo	14	15	4.45571429	2.39733333	2.05838095	c(t = 0.73858	0.471	0.86408093	Distal Intergenic	ENSSSCG000	TRPC1	transient receptor potential cation channel subfamily C member 1 [Source:VGNC Symbol;Acc:VGNC:94460]
chr18	3066008	3067401	1394	*	chr18	3066008	3067401	in_vitro	in_vivo	12	11	2.0275	1.29272727	0.73477273	c(t = 0.73505	0.471	0.86408093	Distal Intergenic	ENSSSCG000	INSIG1	insulin induced gene 1 [Source:NCBI gene (formerly Entrezgene);Acc:403162]
chr18	38284463	38285460	998	*	chr18	38284463	38285460	in_vitro	in_vivo	12	11	0.7225	0.36363636	0.35886364	c(t = 0.73522	0.471	0.86408093	Distal Intergenic	ENSSSCG000	FKBP9	FKBP prolyl isomerase 9 [Source:NCBI gene (formerly Entrezgene);Acc:100623924]
chr4	2529454	2533789	4336	*	chr4	2529454	2533789	in_vitro	in_vivo	11	13	95.1854545	92.3876923	2.79776224	c(t = 0.73519	0.472	0.86444828	Distal Intergenic	NA	NA	NA
chr12	42849222	42850200	979	*	chr12	42849222	42850200	in_vitro	in_vivo	10	14	6.709	3.81214286	2.89685714	c(t = 0.73276	0.472	0.86444828	Promoter (<=1kb)	ENSSSCG000	ADAP2	ArfGAP with dual PH domains 2 [Source:VGNC Symbol;Acc:VGNC:85096]
chr12	60921799	60924094	2296	*	chr12	60921799	60924094	in_vitro	in_vivo	13	13	1.41153846	2.23846154	-0.8269231	c(t = -0.7373	0.472	0.86444828	Distal Intergenic	ENSSSCG00000018055		phosphatidylethanolamine N-methyltransferase [Source:VGNC Symbol;Acc:VGNC:91311]
chr17	34856339	34859727	3389	*	chr17	34856339	34859727	in_vitro	in_vivo	12	15	1.73166667	1.17466667	0.557	c(t = 0.73447	0.472	0.86444828	Distal Intergenic	ENSSSCG000	DEFB129	defensin beta 129 [Source:NCBI gene (formerly Entrezgene);Acc:100170137]
chr6	3774677	3775199	523	*	chr6	3774677	3775199	in_vitro	in_vivo	11	9	1.09636364	0.46333333	0.6330303	c(t = 0.73609	0.472	0.86444828	Distal Intergenic	ENSSSCG000	CIBAR2	CBY1 interacting BAR domain containing 2 [Source:VGNC Symbol;Acc:VGNC:86698]
chr2	1844317	1849106	4790	*	chr2	1844317	1849106	in_vitro	in_vivo	10	11	88.417	91.9018182	-3.4848182	c(t = -0.7339	0.473	0.86444828	Distal Intergenic	ENSSSCG000	PHLDA2	pleckstrin homology like domain family A member 2 [Source:NCBI gene (formerly Entrezgene);Acc:100379631]
chr12	3145066	3146627	1562	*	chr12	3145066	3146627	in_vitro	in_vivo	9	10	59.6455556	49.938	9.70755556	c(t = 0.73378	0.473	0.86444828	Distal Intergenic	ENSSSCG00000017164		TIMP metallopeptidase inhibitor 2 [Source:VGNC Symbol;Acc:VGNC:93995]
chr11	2309684	2310874	1191	*	chr11	2309684	2310874	in_vitro	in_vivo	9	12	2.48833333	4.24583333	-1.7575	c(t = -0.7351	0.473	0.86444828	Distal Intergenic	ENSSSCG000	ssc-mir-9814	ssc-mir-9814 [Source:miRBase;Acc:MI0031581]
chr3	18609756	18610451	696	*	chr3	18609756	18610451	in_vitro	in_vivo	9	11	2.25888889	3.65181818	-1.3929293	c(t = -0.7338	0.473	0.86444828	Promoter (<=1kb)	ENSSSCG000	NFATC2IP	nuclear factor of activated T cells 2 interacting protein [Source:VGNC Symbol;Acc:VGNC:90709]
chr6	159655301	159656547	1247	*	chr6	159655301	159656547	in_vitro	in_vivo	10	9	0.664	2.77777778	-2.1137778	c(t = -0.7513	0.473	0.86444828	Distal Intergenic	ENSSSCG000	COA7	cytochrome c oxidase assembly factor 7 [Source:NCBI gene (formerly Entrezgene);Acc:100627075]
chr7	23112211	23112936	726	*	chr7	23112211	23112936	in_vitro	in_vivo	10	11	3.863	1.54818182	2.31481818	c(t = 0.74469	0.473	0.86444828	Promoter (<=1kb)	ENSSSCG000	GNL1	G protein nucleolar 1 (putative) [Source:NCBI gene (formerly Entrezgene);Acc:100151741]
chr7	47361535	47362322	788	*	chr7	47361535	47362322	in_vitro	in_vivo	9	11	2.07555556	1.46272727	0.61282828	c(t = 0.73284	0.473	0.86444828	Promoter (<=1kb)	ENSSSCG000	PSMA4	proteasome 20S subunit alpha 4 [Source:VGNC Symbol;Acc:VGNC:91901]
chr8	30887463	30888257	795	*	chr8	30887463	30888257	in_vitro	in_vivo	9	15	1.78666667	1.268	0.51866667	c(t = 0.73582	0.473	0.86444828	Promoter (<=1kb)	ENSSSCG000	UBE2K	ubiquitin conjugating enzyme E2 K [Source:VGNC Symbol;Acc:VGNC:94646]
chr7	57040612	57041309	698	*	chr7	57040612	57041309	in_vitro	in_vivo	9	12	1.79777778	1.065	0.73277778	c(t = 0.73535	0.473	0.86444828	Promoter (<=1kb)	ENSSSCG000	RCN2	reticulocalbin 2 [Source:VGNC Symbol;Acc:VGNC:92181]
chr12	11680417	11681477	1061	*	chr12	11680417	11681477	in_vitro	in_vivo	11	9	1.74636364	0.89888889	0.84747475	c(t = 0.74063	0.473	0.86444828	Promoter (<=1kb)	ENSSSCG000	PRKAR1A	protein kinase cAMP-dependent type I regulatory subunit alpha [Source:VGNC Symbol;Acc:VGNC:91802]
chr5	28247437	28247909	473	*	chr5	28247437	28247909	in_vitro	in_vivo	10	10	1.441	0.753	0.688	c(t = 0.73585	0.473	0.86444828	Distal Intergenic	ENSSSCG000	TBK1	TANK binding kinase 1 [Source:VGNC Symbol;Acc:VGNC:93787]
chr14	51664816	51667258	2443	*	chr14	51664816	51667258	in_vitro	in_vivo	11	12	95.5436364	93.355	2.18863636	c(t = 0.72987	0.474	0.86524703	Distal Intergenic	ENSSSCG000	CHRM3	cholinergic receptor muscarinic 3 [Source:VGNC Symbol;Acc:VGNC:86664]
chr9	2534139	2535508	1370	*	chr9	2534139	2535508	in_vitro	in_vivo	11	15	3.55363636	5.084	-1.5303636	c(t = -0.7281	0.474	0.86524703	Distal Intergenic	ENSSSCG000	MRPL17	mitochondrial ribosomal protein L17 [Source:NCBI gene (formerly Entrezgene);Acc:100512482]
chr12	4799653	4801709	2057	*	chr12	4799653	4801709	in_vitro	in_vivo	13	13	2.69769231	1.75538462	0.94230769	c(t = 0.73464	0.474	0.86524703	Promoter (<=1kb)	ENSSSCG00000036592		serine and arginine rich splicing factor 2 [Source:VGNC Symbol;Acc:VGNC:99085]
chr16	23402165	23403799	1635	*	chr16	23402165	23403799	in_vitro	in_vivo	12	12	1.94666667	1.17	0.77666667	c(t = 0.73530	0.474	0.86524703	Distal Intergenic	ENSSSCG000	C9	complement C9 [Source:NCBI gene (formerly Entrezgene);Acc:100037951]
chr1	223747244	223748493	1250	*	chr1	223747244	223748493	in_vitro	in_vivo	9	14	1.46222222	0.75357143	0.70865079	c(t = 0.74018	0.474	0.86524703	Promoter (<=1kb)	ENSSSCG000	KLF9	KLF transcription factor 9 [Source:NCBI gene (formerly Entrezgene);Acc:494562]
chr6	52054376	52055638	1263	*	chr6	52054376	52055638	in_vitro	in_vivo	10	10	86.449	93.295	-6.846	c(t = -0.7427	0.475	0.86604389	Distal Intergenic	ENSSSCG000	QPCTL	glutaminyl-peptide cyclotransferase like [Source:VGNC Symbol;Acc:VGNC:92027]
chr12	57233590	57235441	1852	*	chr12	57233590	57235441	in_vitro	in_vivo	9	9	87.8533333	81.5133333	6.34	c(t = 0.74648	0.475	0.86604389	Exon (NM_001243216/100520804, exon 6 of 25)	ENSSSCG000	ELAC2	elaC ribonuclease Z 2 [Source:NCBI gene (formerly Entrezgene);Acc:100520804]
chr2	16132039	16133561	1523	*	chr2	16132039	16133561	in_vitro	in_vivo	9	10	13.2755556	21.059	-7.7834444	c(t = -0.7366	0.475	0.86604389	Promoter (<=1kb)	ENSSSCG000	MDK	midkine [Source:VGNC Symbol;Acc:VGNC:100167]
chr12	51832445	51833136	692	*	chr12	51832445	51833136	in_vitro	in_vivo	10	10	1.895	1.088	0.807	c(t = 0.72954	0.475	0.86604389	Promoter (<=1kb)	ENSSSCG000	ZNF232	zinc finger protein 232 [Source:VGNC Symbol;Acc:VGNC:95208]
chr15	96855535	96856287	753	*	chr15	96855535	96856287	in_vitro	in_vivo	10	10	1.068	0.603	0.465	c(t = 0.72978	0.475	0.86604389	Distal Intergenic	ENSSSCG000	NABP1	nucleic acid binding protein 1 [Source:NCBI gene (formerly Entrezgene);Acc:100155066]
chr7	12320824	12322851	2028	*	chr7	12320824	12322851	in_vitro	in_vivo	12	11	97.3708333	95.7472727	1.62356061	c(t = 0.72955	0.476	0.86622306	Distal Intergenic	ENSSSCG000	DTNBP1	dystrobrevin binding protein 1 [Source:NCBI gene (formerly Entrezgene);Acc:100049697]
chr17	62329481	62338269	8789	*	chr17	62329481	62338269	in_vitro	in_vivo	13	12	88.5015385	79.8366667	8.66487179	c(t = 0.72551	0.476	0.86622306	Distal Intergenic	ENSSSCG000	LIME1	Lck interacting transmembrane adaptor 1 [Source:NCBI gene (formerly Entrezgene);Acc:100625216]
chr17	61692409	61715895	23487	*	chr17	61692409	61715895	in_vitro	in_vivo	16	17	69.0775	75.7794118	-6.7019118	c(t = -0.7227	0.476	0.86622306	Distal Intergenic	ENSSSCG000	RPS21	ribosomal protein S21 [Source:NCBI gene (formerly Entrezgene);Acc:100037996]
chr6	169677851	169678747	897	*	chr6	169677851	169678747	in_vitro	in_vivo	9	9	14.8511111	25.0655556	-10.214444	c(t = -0.7303	0.476	0.86622306	Distal Intergenic	ENSSSCG000	ssc-mir-4338	ssc-mir-4338 [Source:miRBase;Acc:MI0015933]
chr6	89558577	89559044	468	*	chr6	89558577	89559044	in_vitro	in_vivo	13	9	17.2092308	8.35222222	8.85700855	c(t = 0.72697	0.476	0.86622306	Distal Intergenic	ENSSSCG000	AZIN2	antizyme inhibitor 2 [Source:NCBI gene (formerly Entrezgene);Acc:100144439]
chr15	78196248	78198944	2697	*	chr15	78196248	78198944	in_vitro	in_vivo	10	13	2.511	1.65615385	0.85484615	c(t = 0.73176	0.476	0.86622306	Distal Intergenic	ENSSSCG000	DYNC1I2	dynein cytoplasmic 1 intermediate chain 2 [Source:NCBI gene (formerly Entrezgene);Acc:100154566]
chr11	3810286	3813165	2880	*	chr11	3810286	3813165	in_vitro	in_vivo	13	16	1.72	1.28875	0.43125	c(t = 0.72619	0.476	0.86622306	Distal Intergenic	ENSSSCG000	GPR12	G protein-coupled receptor 12 [Source:NCBI gene (formerly Entrezgene);Acc:100736641]
chr12	6240740	6241488	749	*	chr12	6240740	6241488	in_vitro	in_vivo	9	9	0.44	0.95444444	-0.5144444	c(t = -0.7381	0.476	0.86622306	Distal Intergenic	ENSSSCG00000032166		ATP synthase peripheral stalk subunit d [Source:NCBI gene (formerly Entrezgene);Acc:100512624]
chr6	166324614	166325615	1002	*	chr6	166324614	166325615	in_vitro	in_vivo	9	9	35.2188889	47.8166667	-12.597778	c(t = -0.7299	0.477	0.8668113	Distal Intergenic	ENSSSCG000	UROD	uroporphyrinogen decarboxylase [Source:NCBI gene (formerly Entrezgene);Acc:100514239]
chr11	63816099	63817302	1204	*	chr11	63816099	63817302	in_vitro	in_vivo	10	12	2.212	4.03416667	-1.8221667	c(t = -0.7309	0.477	0.8668113	Distal Intergenic	ENSSSCG000	DCT	dopachrome tautomerase [Source:VGNC Symbol;Acc:VGNC:87192]
chr12	5514268	5515060	793	*	chr12	5514268	5515060	in_vitro	in_vivo	9	12	0.98333333	1.76333333	-0.78	c(t = -0.7283	0.477	0.8668113	Distal Intergenic	ENSSSCG000	ACOX1	acyl-CoA oxidase 1 [Source:VGNC Symbol;Acc:VGNC:85020]
chr14	61793199	61794083	885	*	chr14	61793199	61794083	in_vitro	in_vivo	10	11	0.995	1.50272727	-0.5077273	c(t = -0.7257	0.477	0.8668113	Distal Intergenic	ENSSSCG000	PHYHIPL	phytanoyl-CoA 2-hydroxylase interacting protein like [Source:VGNC Symbol;Acc:VGNC:91407]
chr2	11827378	11828175	798	*	chr2	11827378	11828175	in_vitro	in_vivo	10	11	0.721	1.24090909	-0.5199091	c(t = -0.7252	0.477	0.8668113	Distal Intergenic	ENSSSCG000	MRPL16	mitochondrial ribosomal protein L16 [Source:NCBI gene (formerly Entrezgene);Acc:100514446]
chr2	88328154	88328673	520	*	chr2	88328154	88328673	in_vitro	in_vivo	10	12	1.712	0.81583333	0.89616667	c(t = 0.73019	0.477	0.8668113	Distal Intergenic	ENSSSCG000	CMYA5	cardiomyopathy associated 5 [Source:VGNC Symbol;Acc:VGNC:86809]
chr7	54084876	54085576	701	*	chr7	54084876	54085576	in_vitro	in_vivo	10	9	96.498	94.4944444	2.00355556	c(t = 0.72870	0.478	0.86690549	Distal Intergenic	ENSSSCG000	KLF15	KLF transcription factor 15 [Source:VGNC Symbol;Acc:VGNC:89495]
chr15	140291379	140293355	1977	*	chr15	140291379	140293355	in_vitro	in_vivo	9	11	95.5677778	93.4836364	2.08414141	c(t = 0.72696	0.478	0.86690549	Distal Intergenic	ENSSSCG000	GAL3ST2	galactose-3-O-sulfotransferase 2 [Source:NCBI gene (formerly Entrezgene);Acc:396666]
chr3	55469483	55470884	1402	*	chr3	55469483	55470884	in_vitro	in_vivo	13	12	10.6038462	6.25833333	4.34551282	c(t = 0.72663	0.478	0.86690549	Distal Intergenic	ENSSSCG000	MGAT4A	alpha-1,3-mannosyl-glycoprotein 4-beta-N-acetylglucosaminyltransferase A [Source:VGNC Symbol;Acc:VGNC:108158]
chr14	46457470	46458564	1095	*	chr14	46457470	46458564	in_vitro	in_vivo	11	11	1.60818182	2.32363636	-0.7154545	c(t = -0.7231	0.478	0.86690549	Distal Intergenic	ENSSSCG000	GAS2L1	growth arrest specific 2 like 1 [Source:NCBI gene (formerly Entrezgene);Acc:100513569]
chr1	265641668	265642480	813	*	chr1	265641668	265642480	in_vitro	in_vivo	10	12	0.98	1.92416667	-0.9441667	c(t = -0.7243	0.478	0.86690549	Promoter (<=1kb)	ENSSSCG000	RPL35	ribosomal protein L35 [Source:NCBI gene (formerly Entrezgene);Acc:397598]
chr15	54382942	54383631	690	*	chr15	54382942	54383631	in_vitro	in_vivo	9	9	0.97333333	1.8	-0.8266667	c(t = -0.7283	0.478	0.86690549	Distal Intergenic	ENSSSCG000	UBXN8	UBX domain protein 8 [Source:VGNC Symbol;Acc:VGNC:96239]
chr7	93479868	93480505	638	*	chr7	93479868	93480505	in_vitro	in_vivo	11	12	1.75181818	1.12583333	0.62598485	c(t = 0.72411	0.478	0.86690549	Distal Intergenic	ENSSSCG000	PLEKHD1	pleckstrin homology and coiled-coil domain containing D1 [Source:VGNC Symbol;Acc:VGNC:91539]
chr17	61597881	61598692	812	*	chr17	61597881	61598692	in_vitro	in_vivo	12	11	1.61916667	1.02181818	0.59734848	c(t = 0.72527	0.478	0.86690549	Distal Intergenic	ENSSSCG000	PSMA7	proteasome 20S subunit alpha 7 [Source:NCBI gene (formerly Entrezgene);Acc:100626738]
chr14	73183628	73184545	918	*	chr14	73183628	73184545	in_vitro	in_vivo	9	9	24.1322222	36.1688889	-12.036667	c(t = -0.7280	0.479	0.86690549	Exon (NM_001206457/100154351, exon 3 of 3)	ENSSSCG000	NPFFR1	neuropeptide FF receptor 1 [Source:NCBI gene (formerly Entrezgene);Acc:100154351]
chr16	79552127	79553615	1489	*	chr16	79552127	79553615	in_vitro	in_vivo	12	13	22.1016667	29.7369231	-7.6352564	c(t = -0.7195	0.479	0.86690549	3' UTR	ENSSSCG000	TPPP	tubulin polymerization promoting protein [Source:VGNC Symbol;Acc:VGNC:94344]
chr7	39272006	39274070	2065	*	chr7	39272006	39274070	in_vitro	in_vivo	13	13	8.88076923	16.0130769	-7.1323077	c(t = -0.7186	0.479	0.86690549	Distal Intergenic	ENSSSCG000	HSP90AB1	heat shock protein 90 alpha family class B member 1 [Source:NCBI gene (formerly Entrezgene);Acc:396742]
chr12	12341435	12343255	1821	*	chr12	12341435	12343255	in_vitro	in_vivo	10	11	1.042	1.78636364	-0.7443636	c(t = -0.7270	0.479	0.86690549	Distal Intergenic	ENSSSCG000	AMZ2	archaelysin family metallopeptidase 2 [Source:VGNC Symbol;Acc:VGNC:85292]
chr13	68301249	68302190	942	*	chr13	68301249	68302190	in_vitro	in_vivo	13	11	1.90692308	1.21153846	0.69538462	c(t = 0.72160	0.479	0.86690549	Distal Intergenic	ENSSSCG000	PPARG	peroxisome proliferator activated receptor gamma [Source:VGNC Symbol;Acc:VGNC:91684]
chr13	170752344	170752904	561	*	chr13	170752344	170752904	in_vitro	in_vivo	12	9	1.22333333	0.68666667	0.53666667	c(t = 0.72151	0.479	0.86690549	Distal Intergenic	ENSSSCG000	VGLL3	vestigial like family member 3 [Source:VGNC Symbol;Acc:VGNC:94823]
chr11	78583642	78588767	5126	*	chr11	78583642	78588767	in_vitro	in_vivo	11	13	87.9963636	94.5684615	-6.5720979	c(t = -0.7325	0.48	0.86690549	Distal Intergenic	ENSSSCG000	LAMP1	lysosomal associated membrane protein 1 [Source:NCBI gene (formerly Entrezgene);Acc:494565]
chr4	91175	92076	902	*	chr4	91175	92076	in_vitro	in_vivo	10	9	93.77	85.4955556	8.27444444	c(t = 0.73541	0.48	0.86690549	Distal Intergenic	ENSSSCG000	SLC52A1	solute carrier family 52 member 1 [Source:NCBI gene (formerly Entrezgene);Acc:445519]
chr5	2641788	2643715	1928	*	chr5	2641788	2643715	in_vitro	in_vivo	9	10	38.15	49.318	-11.168	c(t = -0.7215	0.48	0.86690549	Distal Intergenic	ENSSSCG000	PPARA	peroxisome proliferator activated receptor alpha [Source:NCBI gene (formerly Entrezgene);Acc:397239]
chr1	271448464	271453916	5453	*	chr1	271448464	271453916	in_vitro	in_vivo	13	9	51.3330769	44.3033333	7.02974359	c(t = 0.72181	0.48	0.86690549	Distal Intergenic	ENSSSCG000	MED27	mediator complex subunit 27 [Source:VGNC Symbol;Acc:VGNC:90117]
chr12	1792769	1793446	678	*	chr12	1792769	1793446	in_vitro	in_vivo	9	10	21.6422222	33.694	-12.051778	c(t = -0.7229	0.48	0.86690549	Distal Intergenic	NA	NA	NA
chr18	653081	653462	382	*	chr18	653081	653462	in_vitro	in_vivo	10	10	1.608	2.734	-1.126	c(t = -0.7237	0.48	0.86690549	Distal Intergenic	ENSSSCG000	VIPR2	vasoactive intestinal peptide receptor 2 [Source:NCBI gene (formerly Entrezgene);Acc:100499553]
chr14	7389728	7390623	896	*	chr14	7389728	7390623	in_vitro	in_vivo	10	10	3.997	2.394	1.603	c(t = 0.72442	0.48	0.86690549	Distal Intergenic	ENSSSCG000	PEBP4	phosphatidylethanolamine binding protein 4 [Source:VGNC Symbol;Acc:VGNC:91303]
chr17	37810051	37810770	720	*	chr17	37810051	37810770	in_vitro	in_vivo	13	9	1.40846154	2.08	-0.6715385	c(t = -0.7220	0.48	0.86690549	Distal Intergenic	ENSSSCG000	AHCY	adenosylhomocysteinase [Source:NCBI gene (formerly Entrezgene);Acc:497050]
chr17	13694260	13695226	967	*	chr17	13694260	13695226	in_vitro	in_vivo	12	11	1.85083333	0.90909091	0.94174242	c(t = 0.71967	0.48	0.86690549	Distal Intergenic	ENSSSCG000	PRNP	prion protein [Source:NCBI gene (formerly Entrezgene);Acc:494014]
chr9	21918647	21919408	762	*	chr9	21918647	21919408	in_vitro	in_vivo	9	12	1.65333333	0.8925	0.76083333	c(t = 0.72502	0.48	0.86690549	Distal Intergenic	ENSSSCG000	TYR	tyrosinase [Source:NCBI gene (formerly Entrezgene);Acc:407745]
chr15	81076505	81076865	361	*	chr15	81076505	81076865	in_vitro	in_vivo	10	9	2.667	0.65333333	2.01366667	c(t = 0.73334	0.48	0.86690549	Distal Intergenic	ENSSSCG000	ATP5MC3	ATP synthase membrane subunit c locus 3 [Source:NCBI gene (formerly Entrezgene);Acc:100513629]
chr14	131579213	131580337	1125	*	chr14	131579213	131580337	in_vitro	in_vivo	11	9	1.00454545	0.48888889	0.51565657	c(t = 0.72297	0.48	0.86690549	Distal Intergenic	ENSSSCG000	TACC2	transforming acidic coiled-coil containing protein 2 [Source:NCBI gene (formerly Entrezgene);Acc:100152804]
chr6	3253145	3258253	5109	*	chr6	3253145	3258253	in_vitro	in_vivo	11	12	91.8454545	94.635	-2.7895455	c(t = -0.7254	0.481	0.86690549	Distal Intergenic	ENSSSCG000	IRF8	interferon regulatory factor 8 [Source:VGNC Symbol;Acc:VGNC:89211]
chr1	895513	918409	22897	*	chr1	895513	918409	in_vitro	in_vivo	13	14	16.9846154	24.7014286	-7.7168132	c(t = -0.7165	0.481	0.86690549	Distal Intergenic	ENSSSCG000	ssc-mir-9815	ssc-mir-9815 [Source:miRBase;Acc:MI0031582]
chr7	29996081	29997233	1153	*	chr7	29996081	29997233	in_vitro	in_vivo	10	10	5.95	9.038	-3.088	c(t = -0.7279	0.481	0.86690549	Distal Intergenic	ENSSSCG000	MLN	motilin [Source:VGNC Symbol;Acc:VGNC:90258]
chr1	162735690	162737107	1418	*	chr1	162735690	162737107	in_vitro	in_vivo	10	12	1.6275	4.22416667	-2.5966667	c(t = -0.7280	0.481	0.86690549	Distal Intergenic	ENSSSCG000	ssc-mir-122	ssc-mir-122 [Source:miRBase;Acc:MI0002413]
chr3	38172773	38174424	1652	*	chr3	38172773	38174424	in_vitro	in_vivo	10	13	4.008	2.83153846	1.17646154	c(t = 0.71679	0.481	0.86690549	Distal Intergenic	ENSSSCG000	HMOX2	heme oxygenase 2 [Source:VGNC Symbol;Acc:VGNC:88912]
chr11	651580	652535	956	*	chr11	651580	652535	in_vitro	in_vivo	14	11	3.35857143	1.85181818	1.50675325	c(t = 0.72110	0.481	0.86690549	Distal Intergenic	ENSSSCG000	CRYL1	crystallin lambda 1 [Source:VGNC Symbol;Acc:VGNC:87023]
chr17	5506275	5507107	833	*	chr17	5506275	5507107	in_vitro	in_vivo	11	12	2.13909091	1.38916667	0.74992424	c(t = 0.72088	0.481	0.86690549	Distal Intergenic	ENSSSCG000	SLC7A2	solute carrier family 7 member 2 [Source:VGNC Symbol;Acc:VGNC:95506]
chr12	42933340	42933759	420	*	chr12	42933340	42933759	in_vitro	in_vivo	9	12	2.11	1.25916667	0.85083333	c(t = 0.71830	0.481	0.86690549	Promoter (<=1kb)	ENSSSCG000	CRLF3	cytokine receptor like factor 3 [Source:VGNC Symbol;Acc:VGNC:86999]
chr6	37591787	37592637	851	*	chr6	37591787	37592637	in_vitro	in_vivo	10	11	1.429	0.76818182	0.66081818	c(t = 0.72048	0.481	0.86690549	Distal Intergenic	ENSSSCG000	CBLN1	cerebellin 1 precursor [Source:NCBI gene (formerly Entrezgene);Acc:100127148]
chr16	79438153	79439277	1125	*	chr16	79438153	79439277	in_vitro	in_vivo	11	12	19.1181818	30.0166667	-10.898485	c(t = -0.7164	0.482	0.86708099	Distal Intergenic	ENSSSCG000	TRIP13	thyroid hormone receptor interactor 13 [Source:NCBI gene (formerly Entrezgene);Acc:100337674]
chr4	872957	873542	586	*	chr4	872957	873542	in_vitro	in_vivo	9	9	9.23222222	16.1933333	-6.9611111	c(t = -0.7249	0.482	0.86708099	Distal Intergenic	ENSSSCG000	GPIHBP1	glycosylphosphatidylinositol anchored high density lipoprotein binding protein 1 [Source:VGNC Symbol;Acc:VGNC:88588]
chr14	103516821	103517552	732	*	chr14	103516821	103517552	in_vitro	in_vivo	13	12	14.2246154	7.92083333	6.30378205	c(t = 0.71460	0.482	0.86708099	Promoter (<=1kb)	ENSSSCG000	FGFBP3	fibroblast growth factor binding protein 3 [Source:VGNC Symbol;Acc:VGNC:88118]
chr15	87378476	87379749	1274	*	chr15	87378476	87379749	in_vitro	in_vivo	11	10	7	4.596	2.404	c(t = 0.72027	0.482	0.86708099	Distal Intergenic	ENSSSCG000	FRZB	frizzled related protein [Source:VGNC Symbol;Acc:VGNC:96308]
chr7	32354605	32356516	1912	*	chr7	32354605	32356516	in_vitro	in_vivo	12	13	1.19416667	2.25384615	-1.0596795	c(t = -0.7193	0.482	0.86708099	Distal Intergenic	ENSSSCG000	PI16	peptidase inhibitor 16 [Source:NCBI gene (formerly Entrezgene);Acc:100155010]
chr1	207252052	207252703	652	*	chr1	207252052	207252703	in_vitro	in_vivo	12	9	0.34	1.38888889	-1.0488889	c(t = -0.7335	0.482	0.86708099	Distal Intergenic	ENSSSCG000	PSIP1	PC4 and SFRS1 interacting protein 1 [Source:VGNC Symbol;Acc:VGNC:91898]
chr16	55595054	55595869	816	*	chr16	55595054	55595869	in_vitro	in_vivo	10	12	1.856	1.14166667	0.71433333	c(t = 0.72456	0.482	0.86708099	Distal Intergenic	ENSSSCG000	RARS1	arginyl-tRNA synthetase 1 [Source:NCBI gene (formerly Entrezgene);Acc:414410]
chr10	38970355	38971153	799	*	chr10	38970355	38971153	in_vitro	in_vivo	12	13	1.2925	0.80846154	0.48403846	c(t = 0.71604	0.482	0.86708099	Distal Intergenic	ENSSSCG000	MFSD14B	major facilitator superfamily domain containing 14B [Source:NCBI gene (formerly Entrezgene);Acc:100627901]
chr17	43078566	43078980	415	*	chr17	43078566	43078980	in_vitro	in_vivo	9	10	23.0155556	11.897	11.1185556	c(t = 0.72940	0.483	0.86766129	Distal Intergenic	ENSSSCG000	ZHX3	zinc fingers and homeoboxes 3 [Source:VGNC Symbol;Acc:VGNC:95717]
chr2	59383762	59384683	922	*	chr2	59383762	59384683	in_vitro	in_vivo	9	11	5.43888889	2.9	2.53888889	c(t = 0.73175	0.483	0.86766129	Distal Intergenic	ENSSSCG000	GDF15	growth differentiation factor 15 [Source:NCBI gene (formerly Entrezgene);Acc:100379630]
chr11	24751560	24752453	894	*	chr11	24751560	24752453	in_vitro	in_vivo	11	11	1.18727273	2.32	-1.1327273	c(t = -0.7206	0.483	0.86766129	Distal Intergenic	ENSSSCG000	FAM216B	family with sequence similarity 216 member B [Source:VGNC Symbol;Acc:VGNC:87955]
chr6	28279781	28281182	1402	*	chr6	28279781	28281182	in_vitro	in_vivo	11	11	3.27909091	2.21272727	1.06636364	c(t = 0.71823	0.483	0.86766129	Distal Intergenic	ENSSSCG000	CTCF	CCCTC-binding factor [Source:VGNC Symbol;Acc:VGNC:87057]
chr14	40413720	40414385	666	*	chr14	40413720	40414385	in_vitro	in_vivo	11	11	0.70454545	1.34727273	-0.6427273	c(t = -0.7154	0.483	0.86766129	Distal Intergenic	ENSSSCG000	COX6A1	cytochrome c oxidase subunit 6A1 [Source:NCBI gene (formerly Entrezgene);Acc:100152232]
chr6	20431104	20432105	1002	*	chr6	20431104	20432105	in_vitro	in_vivo	9	10	0.55222222	0.924	-0.3717778	c(t = -0.7181	0.483	0.86766129	Promoter (<=1kb)	ENSSSCG000	GOT2	glutamic-oxaloacetic transaminase 2 [Source:NCBI gene (formerly Entrezgene);Acc:396968]
chr11	77586457	77587718	1262	*	chr11	77586457	77587718	in_vitro	in_vivo	9	9	33.5966667	21.1355556	12.4611111	c(t = 0.71926	0.484	0.86841216	Distal Intergenic	ENSSSCG000	ANKRD10	ankyrin repeat domain 10 [Source:VGNC Symbol;Acc:VGNC:85322]
chrX	21084215	21085098	884	*	chrX	21084215	21085098	in_vitro	in_vivo	11	10	2.12	3.377	-1.257	c(t = -0.7163	0.484	0.86841216	Distal Intergenic	ENSSSCG000	SAT1	spermidine/spermine N1-acetyltransferase 1 [Source:VGNC Symbol;Acc:VGNC:397645]
chr3	7441088	7441520	433	*	chr3	7441088	7441520	in_vitro	in_vivo	9	10	4.18222222	2.826	1.35622222	c(t = 0.72173	0.484	0.86841216	Distal Intergenic	ENSSSCG000	NTAN1	N-terminal asparagine amidase [Source:NCBI gene (formerly Entrezgene);Acc:397107]
chr5	65563030	65566190	3161	*	chr5	65563030	65566190	in_vitro	in_vivo	11	13	4.34727273	2.52307692	1.8241958	c(t = 0.72213	0.484	0.86841216	Distal Intergenic	ENSSSCG000	ssc-mir-9813	ssc-mir-9813 [Source:miRBase;Acc:MI0031580]
chr13	92991062	92992160	1099	*	chr13	92991062	92992160	in_vitro	in_vivo	14	10	2.03571429	1.292	0.74371429	c(t = 0.71113	0.484	0.86841216	Distal Intergenic	ENSSSCG000	RAP2B	RAP2B, member of RAS oncogene family [Source:NCBI gene (formerly Entrezgene);Acc:100144507]
chr17	61520397	61524632	4236	*	chr17	61520397	61524632	in_vitro	in_vivo	10	13	90.408	94.4430769	-4.0350769	c(t = -0.7224	0.485	0.86841216	Distal Intergenic	ENSSSCG000	PSMA7	proteasome 20S subunit alpha 7 [Source:NCBI gene (formerly Entrezgene);Acc:100626738]
chr6	45480521	45482766	2246	*	chr6	45480521	45482766	in_vitro	in_vivo	9	12	25.1811111	18.2058333	6.97527778	c(t = 0.71773	0.485	0.86841216	Promoter (<=1kb)	ENSSSCG000	POLR2I	RNA polymerase II subunit I [Source:NCBI gene (formerly Entrezgene);Acc:414403]
chr3	59319131	59319759	629	*	chr3	59319131	59319759	in_vitro	in_vivo	10	10	1.968	3.591	-1.623	c(t = -0.7178	0.485	0.86841216	Distal Intergenic	ENSSSCG000	MAT2A	methionine adenosyltransferase 2A [Source:NCBI gene (formerly Entrezgene);Acc:100312974]
chr6	46785621	46786657	1037	*	chr6	46785621	46786657	in_vitro	in_vivo	10	10	3.324	2.085	1.239	c(t = 0.71540	0.485	0.86841216	Distal Intergenic	ENSSSCG000	PPP1R14A	protein phosphatase 1 regulatory inhibitor subunit 14A [Source:VGNC Symbol;Acc:VGNC:98539]
chr1	112677813	112678800	988	*	chr1	112677813	112678800	in_vitro	in_vivo	9	12	3.02333333	1.82666667	1.19666667	c(t = 0.71191	0.485	0.86841216	Distal Intergenic	ENSSSCG000	CCNB2	cyclin B2 [Source:VGNC Symbol;Acc:VGNC:86350]
chr7	36571063	36574204	3142	*	chr7	36571063	36574204	in_vitro	in_vivo	13	14	2.10461538	1.36857143	0.73604396	c(t = 0.71443	0.485	0.86841216	Distal Intergenic	ENSSSCG000	TREM1	triggering receptor expressed on myeloid cells 1 [Source:NCBI gene (formerly Entrezgene);Acc:396601]
chr18	5928644	5929667	1024	*	chr18	5928644	5929667	in_vitro	in_vivo	11	11	0.625	1.29818182	-0.6731818	c(t = -0.7171	0.485	0.86841216	Distal Intergenic	NA	NA	NA
chr6	45811855	45812773	919	*	chr6	45811855	45812773	in_vitro	in_vivo	10	14	4.102	1.16285714	2.93914286	c(t = 0.72663	0.485	0.86841216	Distal Intergenic	ENSSSCG000	COX7A1	cytochrome c oxidase subunit 7A1 [Source:NCBI gene (formerly Entrezgene);Acc:399685]
chr8	45912877	45913259	383	*	chr8	45912877	45913259	in_vitro	in_vivo	11	10	1.31272727	0.53	0.78272727	c(t = 0.71988	0.485	0.86841216	Promoter (1-2kb)	ENSSSCG000	GLRB	glycine receptor beta [Source:VGNC Symbol;Acc:VGNC:88494]
chr15	31140421	31141534	1114	*	chr15	31140421	31141534	in_vitro	in_vivo	11	10	7.84909091	5.784	2.06509091	c(t = 0.71205	0.486	0.86895635	Distal Intergenic	ENSSSCG000	INHBB	inhibin subunit beta B [Source:NCBI gene (formerly Entrezgene);Acc:397490]
chrX	112592282	112596423	4142	*	chrX	112592282	112596423	in_vitro	in_vivo	13	14	7.14846154	4.75142857	2.39703297	c(t = 0.71054	0.486	0.86895635	Distal Intergenic	ENSSSCG00000012703		RNA binding motif protein X-linked [Source:NCBI gene (formerly Entrezgene);Acc:100158042]
chr3	10049396	10050986	1591	*	chr3	10049396	10050986	in_vitro	in_vivo	10	12	5.473	2.97916667	2.49383333	c(t = 0.71862	0.486	0.86895635	Distal Intergenic	ENSSSCG000	HSPB1	heat shock protein family B (small) member 1 [Source:NCBI gene (formerly Entrezgene);Acc:493184]
chr4	103612738	103613708	971	*	chr4	103612738	103613708	in_vitro	in_vivo	9	9	0.44444444	1.08777778	-0.6433333	c(t = -0.7180	0.486	0.86895635	Distal Intergenic	ENSSSCG000	CD2	CD2 molecule [Source:NCBI gene (formerly Entrezgene);Acc:396662]
chr10	56129857	56130854	998	*	chr10	56129857	56130854	in_vitro	in_vivo	11	11	1.39272727	0.89454545	0.49818182	c(t = 0.70938	0.486	0.86895635	Promoter (<=1kb)	ENSSSCG000	ITGB1	integrin subunit beta 1 [Source:VGNC Symbol;Acc:VGNC:96381]
chr4	62769695	62769940	246	*	chr4	62769695	62769940	in_vitro	in_vivo	9	10	4.81333333	10.433	-5.6196667	c(t = -0.7132	0.487	0.86895635	Distal Intergenic	ENSSSCG000	RPL7	ribosomal protein L7 [Source:NCBI gene (formerly Entrezgene);Acc:414418]
chr1	72815404	72815846	443	*	chr1	72815404	72815846	in_vitro	in_vivo	9	10	3.92666667	6.298	-2.3713333	c(t = -0.7131	0.487	0.86895635	Distal Intergenic	ENSSSCG000	ATG5	autophagy related 5 [Source:VGNC Symbol;Acc:VGNC:103029]
chr15	137534942	137536444	1503	*	chr15	137534942	137536444	in_vitro	in_vivo	9	12	9.55555556	6.26666667	3.28888889	c(t = 0.71415	0.487	0.86895635	3' UTR	ENSSSCG000	RAMP1	receptor activity modifying protein 1 [Source:NCBI gene (formerly Entrezgene);Acc:397401]
chr7	38223971	38224682	712	*	chr7	38223971	38224682	in_vitro	in_vivo	10	9	2.682	4.25222222	-1.5702222	c(t = -0.7186	0.487	0.86895635	Distal Intergenic	ENSSSCG000	MRPL2	mitochondrial ribosomal protein L2 [Source:VGNC Symbol;Acc:VGNC:103072]
chr3	80330641	80332276	1636	*	chr3	80330641	80332276	in_vitro	in_vivo	11	13	2.05181818	2.98461538	-0.9327972	c(t = -0.7070	0.487	0.86895635	Distal Intergenic	ENSSSCG000	ssc-mir-9811	ssc-mir-9811 [Source:miRBase;Acc:MI0031578]
chr18	19786327	19787975	1649	*	chr18	19786327	19787975	in_vitro	in_vivo	11	10	3.64090909	2.352	1.28890909	c(t = 0.70939	0.487	0.86895635	Distal Intergenic	ENSSSCG000	OPN1SW	opsin 1, short wave sensitive [Source:NCBI gene (formerly Entrezgene);Acc:397164]
chr10	28508350	28510600	2251	*	chr10	28508350	28510600	in_vitro	in_vivo	15	16	1.94466667	1.480625	0.46404167	c(t = 0.70581	0.487	0.86895635	Distal Intergenic	ENSSSCG000	FBP2	fructose-bisphosphatase 2 [Source:NCBI gene (formerly Entrezgene);Acc:100134828]
chr12	16751683	16752602	920	*	chr12	16751683	16752602	in_vitro	in_vivo	9	10	0.68222222	1.217	-0.5347778	c(t = -0.7115	0.487	0.86895635	Promoter (<=1kb)	ENSSSCG000	ITGB3	integrin subunit beta 3 [Source:NCBI gene (formerly Entrezgene);Acc:397063]
chr1	179477408	179478738	1331	*	chr1	179477408	179478738	in_vitro	in_vivo	14	12	1.385	0.87333333	0.51166667	c(t = 0.70910	0.487	0.86895635	Promoter (<=1kb)	ENSSSCG000	RPL36AL	ribosomal protein L36a like [Source:NCBI gene (formerly Entrezgene);Acc:396952]
chr9	134953631	134954670	1040	*	chr9	134953631	134954670	in_vitro	in_vivo	9	9	0.76888889	0.41666667	0.35222222	c(t = 0.71203	0.488	0.86895635	Distal Intergenic	ENSSSCG000	CD34	CD34 molecule [Source:VGNC Symbol;Acc:VGNC:86410]
chr5	57780634	57781132	499	*	chr5	57780634	57781132	in_vitro	in_vivo	10	13	2.236	7.32615385	-5.0901538	c(t = -0.7135	0.488	0.86932777	Distal Intergenic	ENSSSCG000	GUCY2C	guanylate cyclase 2C [Source:NCBI gene (formerly Entrezgene);Acc:397193]
chr7	46723026	46724479	1454	*	chr7	46723026	46724479	in_vitro	in_vivo	11	13	4.42909091	2.87384615	1.55524476	c(t = 0.70872	0.488	0.86932777	Promoter (<=1kb)	ENSSSCG000	FBXO9	F-box protein 9 [Source:NCBI gene (formerly Entrezgene);Acc:100152568]
chr7	86513136	86514793	1658	*	chr7	86513136	86514793	in_vitro	in_vivo	11	14	1.67909091	2.48785714	-0.8087662	c(t = -0.7060	0.488	0.86932777	Promoter (<=1kb)	ENSSSCG000	ST8SIA2	ST8 alpha-N-acetyl-neuraminide alpha-2,8-sialyltransferase 2 [Source:NCBI gene (formerly Entrezgene);Acc:100158046]
chr1	146007016	146010364	3349	*	chr1	146007016	146010364	in_vitro	in_vivo	16	11	2.918125	2.03272727	0.88539773	c(t = 0.70414	0.488	0.86932777	Distal Intergenic	ENSSSCG000	NFATC1	nuclear factor of activated T cells 1 [Source:NCBI gene (formerly Entrezgene);Acc:397318]
chr8	78389636	78390292	657	*	chr8	78389636	78390292	in_vitro	in_vivo	9	12	1.05111111	1.745	-0.6938889	c(t = -0.7102	0.488	0.86932777	Distal Intergenic	ENSSSCG000	RPS3A	ribosomal protein S3A [Source:NCBI gene (formerly Entrezgene);Acc:414390]
chr14	57317851	57319270	1420	*	chr14	57317851	57319270	in_vitro	in_vivo	12	13	1.055	1.67615385	-0.6211538	c(t = -0.7058	0.488	0.86932777	Distal Intergenic	ENSSSCG000	TSNAX	translin associated factor X [Source:NCBI gene (formerly Entrezgene);Acc:100156036]
chr15	37455116	37456014	899	*	chr15	37455116	37456014	in_vitro	in_vivo	9	14	2.03333333	0.93714286	1.09619048	c(t = 0.71602	0.488	0.86932777	Distal Intergenic	ENSSSCG000	ANGPT2	angiopoietin 2 [Source:NCBI gene (formerly Entrezgene);Acc:396730]
chr3	7873283	7874355	1073	*	chr3	7873283	7874355	in_vitro	in_vivo	11	12	18.7090909	13.1616667	5.54742424	c(t = 0.70639	0.489	0.86949676	Distal Intergenic	ENSSSCG000	COPS6	COP9 signalosome subunit 6 [Source:NCBI gene (formerly Entrezgene);Acc:100125954]
chr6	164951918	164952926	1009	*	chr6	164951918	164952926	in_vitro	in_vivo	9	9	1.73	3.25555556	-1.5255556	c(t = -0.7148	0.489	0.86949676	Distal Intergenic	ENSSSCG000	MKNK1	MAPK interacting serine/threonine kinase 1 [Source:VGNC Symbol;Acc:VGNC:90246]
chr10	19305835	19306725	891	*	chr10	19305835	19306725	in_vitro	in_vivo	12	13	3.97166667	2.67384615	1.29782051	c(t = 0.70497	0.489	0.86949676	Distal Intergenic	ENSSSCG000	TLR5	toll like receptor 5 [Source:NCBI gene (formerly Entrezgene);Acc:100144476]
chr14	120906003	120907343	1341	*	chr14	120906003	120907343	in_vitro	in_vivo	13	12	1.79923077	2.405	-0.6057692	c(t = -0.7045	0.489	0.86949676	Distal Intergenic	ENSSSCG000	SMNDC1	survival motor neuron domain containing 1 [Source:VGNC Symbol;Acc:VGNC:93257]
chr14	226335	227529	1195	*	chr14	226335	227529	in_vitro	in_vivo	15	14	2.248	1.50357143	0.74442857	c(t = 0.70359	0.489	0.86949676	Promoter (<=1kb)	ENSSSCG000	SPIN1	spindlin 1 [Source:NCBI gene (formerly Entrezgene);Acc:100337684]
chr9	38137909	38138833	925	*	chr9	38137909	38138833	in_vitro	in_vivo	10	12	2.228	1.47666667	0.75133333	c(t = 0.70642	0.489	0.86949676	Distal Intergenic	ENSSSCG000	RDX	radixin [Source:NCBI gene (formerly Entrezgene);Acc:494457]
chr13	3674186	3675154	969	*	chr13	3674186	3675154	in_vitro	in_vivo	12	11	0.82916667	1.37818182	-0.5490152	c(t = -0.7064	0.489	0.86949676	Distal Intergenic	ENSSSCG000	DPH3	diphthamide biosynthesis 3 [Source:NCBI gene (formerly Entrezgene);Acc:100156362]
chr7	77780741	77781816	1076	*	chr7	77780741	77781816	in_vitro	in_vivo	12	13	2.33307692	1.253	1.08007692	c(t = 0.70710	0.489	0.86949676	Distal Intergenic	ENSSSCG00000038947		heterous nuclear ribonucleoprotein C [Source:NCBI gene (formerly Entrezgene);Acc:100626496]
chr6	63055058	63056396	1339	*	chr6	63055058	63056396	in_vitro	in_vivo	10	9	17.186	28.5511111	-11.365111	c(t = -0.7069	0.49	0.87026808	Distal Intergenic	ENSSSCG000	ISG15	ISG15 ubiquitin like modifier [Source:NCBI gene (formerly Entrezgene);Acc:100145895]
chr5	587585	588182	598	*	chr5	587585	588182	in_vitro	in_vivo	10	10	16.025	27.356	-11.331	c(t = -0.7063	0.49	0.87026808	Distal Intergenic	ENSSSCG000	SELENOO	selenoprotein O [Source:NCBI gene (formerly Entrezgene);Acc:100518971]
chr12	53106369	53113327	6959	*	chr12	53106369	53113327	in_vitro	in_vivo	13	13	11.0984615	14.6884615	-3.59	c(t = -0.7006	0.49	0.87026808	Distal Intergenic	ENSSSCG000	EFNB3	ephrin B3 [Source:VGNC Symbol;Acc:VGNC:87578]
chr6	55937887	55940228	2342	*	chr6	55937887	55940228	in_vitro	in_vivo	13	14	2.49230769	4.30714286	-1.8148352	c(t = -0.7076	0.49	0.87026808	Distal Intergenic	ENSSSCG000	ssc-mir-935	ssc-mir-935 [Source:miRBase;Acc:MI0013156]
chr13	157422054	157423167	1114	*	chr13	157422054	157423167	in_vitro	in_vivo	13	12	1.12615385	0.68916667	0.43698718	c(t = 0.70185	0.49	0.87026808	Distal Intergenic	ENSSSCG000	NFKBIZ	NFKB inhibitor zeta [Source:VGNC Symbol;Acc:VGNC:90726]
chr11	2403850	2404785	936	*	chr11	2403850	2404785	in_vitro	in_vivo	11	10	97.5945455	95.586	2.00854545	c(t = 0.70557	0.491	0.87080147	Distal Intergenic	ENSSSCG000	ssc-mir-9814	ssc-mir-9814 [Source:miRBase;Acc:MI0031581]
chr6	49019958	49020957	1000	*	chr6	49019958	49020957	in_vitro	in_vivo	10	9	5.844	8.68	-2.836	c(t = -0.7083	0.491	0.87080147	Distal Intergenic	ENSSSCG000	CYP2A19	cytochrome P450 family 2 subfamily A member 19 [Source:NCBI gene (formerly Entrezgene);Acc:403149]
chr1	189471076	189471705	630	*	chr1	189471076	189471705	in_vitro	in_vivo	10	9	1.776	3.64333333	-1.8673333	c(t = -0.7094	0.491	0.87080147	Distal Intergenic	ENSSSCG000	SIX1	SIX homeobox 1 [Source:NCBI gene (formerly Entrezgene);Acc:100156847]
chr9	89340841	89342259	1419	*	chr9	89340841	89342259	in_vitro	in_vivo	14	14	0.80428571	1.28714286	-0.4828571	c(t = -0.7010	0.491	0.87080147	Promoter (<=1kb)	ENSSSCG000	ITGB8	integrin subunit beta 8 [Source:VGNC Symbol;Acc:VGNC:89246]
chr18	36583690	36584827	1138	*	chr18	36583690	36584827	in_vitro	in_vivo	12	10	0.43	0.845	-0.415	c(t = -0.7023	0.491	0.87080147	Distal Intergenic	ENSSSCG000	IFRD1	interferon related developmental regulator 1 [Source:NCBI gene (formerly Entrezgene);Acc:493185]
chr1	146221235	146222439	1205	*	chr1	146221235	146222439	in_vitro	in_vivo	13	11	1.07538462	0.58923077	0.48615385	c(t = 0.70297	0.491	0.87080147	Distal Intergenic	ENSSSCG000	NFATC1	nuclear factor of activated T cells 1 [Source:NCBI gene (formerly Entrezgene);Acc:397318]
chrX	122463683	122464789	1107	*	chrX	122463683	122464789	in_vitro	in_vivo	10	11	92.373	95.8109091	-3.4379091	c(t = -0.7069	0.492	0.87080147	Distal Intergenic	ENSSSCG000	FATE1	fetal and adult testis expressed 1 [Source:NCBI gene (formerly Entrezgene);Acc:396705]
chr4	55281589	55282904	1316	*	chr4	55281589	55282904	in_vitro	in_vivo	13	11	10.0646154	7.14545455	2.91916084	c(t = 0.69912	0.492	0.87080147	Promoter (<=1kb)	ENSSSCG000	FABP5	fatty acid binding protein 5 [Source:NCBI gene (formerly Entrezgene);Acc:574074]
chr3	87341437	87341979	543	*	chr3	87341437	87341979	in_vitro	in_vivo	9	9	3.59111111	5.79555556	-2.2044444	c(t = -0.7037	0.492	0.87080147	Promoter (<=1kb)	ENSSSCG000	GPR75	G protein-coupled receptor 75 [Source:VGNC Symbol;Acc:VGNC:88636]
chr7	1792741	1793388	648	*	chr7	1792741	1793388	in_vitro	in_vivo	9	12	1.55111111	2.51666667	-0.9655556	c(t = -0.7021	0.492	0.87080147	Distal Intergenic	ENSSSCG000	SERPINB9	serpin peptidase inhibitor, clade B (ovalbumin), member 9 [Source:NCBI gene (formerly Entrezgene);Acc:100126851]
chr17	32630745	32631847	1103	*	chr17	32630745	32631847	in_vitro	in_vivo	10	11	3.803	2.23272727	1.57027273	c(t = 0.70169	0.492	0.87080147	Distal Intergenic	ENSSSCG000	OXT	oxytocin/neurophysin I prepropeptide [Source:NCBI gene (formerly Entrezgene);Acc:100152272]
chr7	49540185	49541628	1444	*	chr7	49540185	49541628	in_vitro	in_vivo	12	12	1.1125	1.835	-0.7225	c(t = -0.7003	0.492	0.87080147	Distal Intergenic	ENSSSCG000	TLNRD1	talin rod domain containing 1 [Source:NCBI gene (formerly Entrezgene);Acc:100152002]
chr18	46203683	46206601	2919	*	chr18	46203683	46206601	in_vitro	in_vivo	12	14	1.91166667	1.32071429	0.59095238	c(t = 0.69710	0.492	0.87080147	Distal Intergenic	NA	NA	NA
chr15	124741648	124742637	990	*	chr15	124741648	124742637	in_vitro	in_vivo	9	13	2.01666667	1.23538462	0.78128205	c(t = 0.70490	0.492	0.87080147	Promoter (<=1kb)	ENSSSCG000	ACSL3	acyl-CoA synthetase long chain family member 3 [Source:VGNC Symbol;Acc:VGNC:96295]
chr2	51276343	51276760	418	*	chr2	51276343	51276760	in_vitro	in_vivo	9	9	1.05777778	0.49666667	0.56111111	c(t = 0.70911	0.492	0.87080147	Promoter (<=1kb)	NA	NA	NA
chr6	61473531	61474411	881	*	chr6	61473531	61474411	in_vitro	in_vivo	9	9	6.92777778	15.1855556	-8.2577778	c(t = -0.7118	0.493	0.87149485	Distal Intergenic	ENSSSCG000	GALP	galanin like peptide [Source:NCBI gene (formerly Entrezgene);Acc:396772]
chr12	15413941	15415068	1128	*	chr12	15413941	15415068	in_vitro	in_vivo	12	11	3.6275	5.63818182	-2.0106818	c(t = -0.7000	0.493	0.87149485	Promoter (<=1kb)	ENSSSCG000	ACE	angiotensin I converting enzyme (peptidyl-dipeptidase A) 1 [Source:NCBI gene (formerly Entrezgene);Acc:613133]
chr14	20014012	20014830	819	*	chr14	20014012	20014830	in_vitro	in_vivo	9	11	0.79222222	1.76181818	-0.969596	c(t = -0.7074	0.493	0.87149485	Distal Intergenic	ENSSSCG000	HMGB2	high mobility group box 2 [Source:VGNC Symbol;Acc:VGNC:88902]
chr15	66238648	66239234	587	*	chr15	66238648	66239234	in_vitro	in_vivo	9	9	2.47	1.15222222	1.31777778	c(t = 0.70733	0.493	0.87149485	Distal Intergenic	ENSSSCG000	DAPL1	death associated protein like 1 [Source:NCBI gene (formerly Entrezgene);Acc:100157537]
chr6	1337343	1339331	1989	*	chr6	1337343	1339331	in_vitro	in_vivo	11	12	96.5181818	94.6391667	1.87901515	c(t = 0.69673	0.494	0.87149485	Distal Intergenic	ENSSSCG000	BANP	BTG3 associated nuclear protein [Source:NCBI gene (formerly Entrezgene);Acc:100622381]
chr4	884100	885141	1042	*	chr4	884100	885141	in_vitro	in_vivo	11	10	30.1809091	43.303	-13.122091	c(t = -0.6967	0.494	0.87149485	Distal Intergenic	ENSSSCG000	GPIHBP1	glycosylphosphatidylinositol anchored high density lipoprotein binding protein 1 [Source:VGNC Symbol;Acc:VGNC:88588]
chr1	181995066	181995661	596	*	chr1	181995066	181995661	in_vitro	in_vivo	9	9	9.22666667	6.42333333	2.80333333	c(t = 0.70091	0.494	0.87149485	Distal Intergenic	ENSSSCG000	RTRAF	RNA transcription, translation and transport factor [Source:NCBI gene (formerly Entrezgene);Acc:100154555]
chr1	161678684	161680046	1363	*	chr1	161678684	161680046	in_vitro	in_vivo	13	13	1.96076923	3.08615385	-1.1253846	c(t = -0.7014	0.494	0.87149485	Distal Intergenic	ENSSSCG000	PMAIP1	phorbol-12-myristate-13-acetate-induced protein 1 [Source:NCBI gene (formerly Entrezgene);Acc:397278]
chr4	125458764	125459499	736	*	chr4	125458764	125459499	in_vitro	in_vivo	9	14	1.96111111	1.16785714	0.79325397	c(t = 0.70423	0.494	0.87149485	Distal Intergenic	ENSSSCG000	TGFBR3	transforming growth factor beta receptor 3 [Source:VGNC Symbol;Acc:VGNC:93932]
chr3	109104302	109106383	2082	*	chr3	109104302	109106383	in_vitro	in_vivo	13	11	2.19153846	1.11636364	1.07517483	c(t = 0.70135	0.494	0.87149485	Distal Intergenic	ENSSSCG000	LCLAT1	lysocardiolipin acyltransferase 1 [Source:NCBI gene (formerly Entrezgene);Acc:100217396]
chr7	58078043	58079195	1153	*	chr7	58078043	58079195	in_vitro	in_vivo	11	11	2.05272727	1.11090909	0.94181818	c(t = 0.70001	0.494	0.87149485	Distal Intergenic	ENSSSCG000	IMP3	IMP U3 small nucleolar ribonucleoprotein 3 [Source:NCBI gene (formerly Entrezgene);Acc:100151831]
chr14	30821806	30822160	355	*	chr14	30821806	30822160	in_vitro	in_vivo	10	11	0.833	0.22181818	0.61118182	c(t = 0.70900	0.494	0.87149485	Distal Intergenic	ENSSSCG000	HPD	4-hydroxyphenylpyruvate dioxygenase [Source:VGNC Symbol;Acc:VGNC:88960]
chr11	77226361	77234632	8272	*	chr11	77226361	77234632	in_vitro	in_vivo	10	15	86.131	89.0926667	-2.9616667	c(t = -0.6943	0.495	0.87149485	3' UTR	ENSSSCG000	NAXD	NAD(P)HX dehydratase [Source:NCBI gene (formerly Entrezgene);Acc:100153456]
chr1	130845975	130847552	1578	*	chr1	130845975	130847552	in_vitro	in_vivo	11	9	26.65	34.4077778	-7.7577778	c(t = -0.6959	0.495	0.87149485	Distal Intergenic	ENSSSCG000	IVD	isovaleryl-CoA dehydrogenase [Source:NCBI gene (formerly Entrezgene);Acc:100156047]
chr16	79043908	79044500	593	*	chr16	79043908	79044500	in_vitro	in_vivo	9	9	18.2088889	29.6444444	-11.435556	c(t = -0.7006	0.495	0.87149485	Distal Intergenic	ENSSSCG000	TERT	telomerase reverse transcriptase [Source:NCBI gene (formerly Entrezgene);Acc:492280]
chr16	5183814	5184354	541	*	chr16	5183814	5184354	in_vitro	in_vivo	12	10	35.7625	24.391	11.3715	c(t = 0.69641	0.495	0.87149485	Distal Intergenic	ENSSSCG000	C1QTNF3	C1q and TNF related 3 [Source:NCBI gene (formerly Entrezgene);Acc:100270814]
chr17	26118966	26120801	1836	*	chr17	26118966	26120801	in_vitro	in_vivo	10	13	8.01	4.38416667	3.62583333	c(t = 0.69720	0.495	0.87149485	Distal Intergenic	ENSSSCG000	DSTN	destrin, actin depolymerizing factor [Source:VGNC Symbol;Acc:VGNC:108715]
chr10	10263457	10265089	1633	*	chr10	10263457	10265089	in_vitro	in_vivo	11	15	1.21181818	1.98933333	-0.7775152	c(t = -0.6938	0.495	0.87149485	Distal Intergenic	ENSSSCG000	ssc-mir-664	ssc-mir-664 [Source:miRBase;Acc:MI0013123]
chr18	42801202	42802457	1256	*	chr18	42801202	42802457	in_vitro	in_vivo	10	9	2.57	1.65777778	0.91222222	c(t = 0.69674	0.495	0.87149485	Promoter (<=1kb)	ENSSSCG000	MTURN	maturin, neural progenitor differentiation regulator homolog [Source:VGNC Symbol;Acc:VGNC:90476]
chr14	18143901	18145432	1532	*	chr14	18143901	18145432	in_vitro	in_vivo	13	16	1.88307692	1.300625	0.58245192	c(t = 0.69392	0.495	0.87149485	Distal Intergenic	ENSSSCG000	HMGB2	high mobility group box 2 [Source:VGNC Symbol;Acc:VGNC:88902]
chr4	72752549	72754990	2442	*	chr4	72752549	72754990	in_vitro	in_vivo	11	14	1.92545455	1.22571429	0.69974026	c(t = 0.69964	0.495	0.87149485	Distal Intergenic	ENSSSCG000	SDCBP	syndecan binding protein [Source:VGNC Symbol;Acc:VGNC:92657]
chr17	38221275	38222076	802	*	chr17	38221275	38222076	in_vitro	in_vivo	9	12	1.29555556	0.69833333	0.59722222	c(t = 0.69719	0.495	0.87149485	Distal Intergenic	ENSSSCG000	ACSS2	acyl-CoA synthetase short chain family member 2 [Source:VGNC Symbol;Acc:VGNC:95665]
chr14	46408018	46409538	1521	*	chr14	46408018	46409538	in_vitro	in_vivo	10	9	0.872	0.41777778	0.45422222	c(t = 0.70301	0.495	0.87149485	Promoter (<=1kb)	ENSSSCG000	EWSR1	EWS RNA binding protein 1 [Source:VGNC Symbol;Acc:VGNC:87822]
chr3	10123368	10124803	1436	*	chr3	10123368	10124803	in_vitro	in_vivo	11	9	90.4909091	93.8433333	-3.3524242	c(t = -0.6996	0.496	0.87225629	Exon (NM_001244153/397039, exon 6 of 9)	ENSSSCG000	MDH2	malate dehydrogenase 2 [Source:NCBI gene (formerly Entrezgene);Acc:397039]
chr11	17689461	17691697	2237	*	chr11	17689461	17691697	in_vitro	in_vivo	16	15	2.770625	5.764	-2.993375	c(t = -0.6975	0.496	0.87225629	Distal Intergenic	NA	NA	NA
chr4	73809408	73811709	2302	*	chr4	73809408	73811709	in_vitro	in_vivo	12	15	1.9125	2.81133333	-0.8988333	c(t = -0.6918	0.496	0.87225629	Distal Intergenic	ENSSSCG000	SDCBP	syndecan binding protein [Source:VGNC Symbol;Acc:VGNC:92657]
chr14	29558914	29560120	1207	*	chr14	29558914	29560120	in_vitro	in_vivo	12	11	1.56583333	2.71363636	-1.147803	c(t = -0.7010	0.496	0.87225629	Distal Intergenic	ENSSSCG000	CDK2AP1	cyclin dependent kinase 2 associated protein 1 [Source:VGNC Symbol;Acc:VGNC:103918]
chr2	65172640	65174196	1557	*	chr2	65172640	65174196	in_vitro	in_vivo	10	9	0.977	2.18888889	-1.2118889	c(t = -0.7065	0.496	0.87225629	Distal Intergenic	ENSSSCG000	RLN3	relaxin 3 [Source:VGNC Symbol;Acc:VGNC:92329]
chr6	168493315	168494605	1291	*	chr6	168493315	168494605	in_vitro	in_vivo	11	12	10.9554545	6.88833333	4.06712121	c(t = 0.69801	0.497	0.87381492	Distal Intergenic	ENSSSCG000	CLDN19	claudin 19 [Source:NCBI gene (formerly Entrezgene);Acc:100294682]
chr3	444577	447973	3397	*	chr3	444577	447973	in_vitro	in_vivo	11	10	87.6709091	92.683	-5.0120909	c(t = -0.7003	0.498	0.87389129	Distal Intergenic	ENSSSCG000	ADAP1	ArfGAP with dual PH domains 1 [Source:NCBI gene (formerly Entrezgene);Acc:397453]
chr14	114391510	114391970	461	*	chr14	114391510	114391970	in_vitro	in_vivo	9	11	17.9855556	28.22	-10.234444	c(t = -0.6916	0.498	0.87389129	Distal Intergenic	ENSSSCG000	ssc-mir-1307	ssc-mir-1307 [Source:miRBase;Acc:MI0013147]
chr18	1208707	1209105	399	*	chr18	1208707	1209105	in_vitro	in_vivo	9	9	14.0344444	7.27444444	6.76	c(t = 0.69873	0.498	0.87389129	Distal Intergenic	ENSSSCG000	ssc-mir-153	ssc-mir-153 [Source:miRBase;Acc:MI0002454]
chrX	7420618	7421356	739	*	chrX	7420618	7421356	in_vitro	in_vivo	13	10	2.12	6.608	-4.488	c(t = -0.7045	0.498	0.87389129	Distal Intergenic	ENSSSCG000	AMELY	amelogenin Y-linked [Source:NCBI gene (formerly Entrezgene);Acc:396940]
chr9	132666107	132666735	629	*	chr9	132666107	132666735	in_vitro	in_vivo	11	9	1.78818182	3.00777778	-1.219596	c(t = -0.6968	0.498	0.87389129	Distal Intergenic	ENSSSCG000	IRF6	interferon regulatory factor 6 [Source:VGNC Symbol;Acc:VGNC:89209]
chr6	135382089	135382819	731	*	chr6	135382089	135382819	in_vitro	in_vivo	10	9	1.649	2.33555556	-0.6865556	c(t = -0.6925	0.498	0.87389129	Distal Intergenic	ENSSSCG000	PTGFR	prostaglandin F receptor [Source:VGNC Symbol;Acc:VGNC:91954]
chr3	73710713	73711538	826	*	chr3	73710713	73711538	in_vitro	in_vivo	9	15	1.65555556	0.96533333	0.69022222	c(t = 0.69961	0.498	0.87389129	Distal Intergenic	ENSSSCG000	GKN3	gastrokine 3 [Source:NCBI gene (formerly Entrezgene);Acc:100329220]
chr4	62014052	62014884	833	*	chr4	62014052	62014884	in_vitro	in_vivo	9	10	1.16444444	0.639	0.52544444	c(t = 0.69446	0.498	0.87389129	Promoter (<=1kb)	ENSSSCG000	UBE2W	ubiquitin conjugating enzyme E2 W [Source:NCBI gene (formerly Entrezgene);Acc:100152196]
chr12	907503	910006	2504	*	chr12	907503	910006	in_vitro	in_vivo	10	10	94.913	92.871	2.042	c(t = 0.69027	0.499	0.87389129	Distal Intergenic	ENSSSCG000	FASN	fatty acid synthase [Source:NCBI gene (formerly Entrezgene);Acc:397561]
chr12	59548031	59550169	2139	*	chr12	59548031	59550169	in_vitro	in_vivo	10	9	27.405	17.4711111	9.93388889	c(t = 0.69373	0.499	0.87389129	Distal Intergenic	ENSSSCG000	AKAP10	A-kinase anchoring protein 10 [Source:NCBI gene (formerly Entrezgene);Acc:397262]
chr14	50528419	50530015	1597	*	chr14	50528419	50530015	in_vitro	in_vivo	10	13	1.32	1.96923077	-0.6492308	c(t = -0.6914	0.499	0.87389129	Distal Intergenic	ENSSSCG000	SLC7A4	solute carrier family 7 member 4 [Source:VGNC Symbol;Acc:VGNC:93174]
chr12	54583845	54585106	1262	*	chr12	54583845	54585106	in_vitro	in_vivo	10	13	2.632	1.37307692	1.25892308	c(t = 0.69784	0.499	0.87389129	Distal Intergenic	ENSSSCG000	GLP2R	glucagon like peptide 2 receptor [Source:VGNC Symbol;Acc:VGNC:88490]
chr7	46406302	46407477	1176	*	chr7	46406302	46407477	in_vitro	in_vivo	13	14	1.47923077	0.93214286	0.54708791	c(t = 0.68747	0.499	0.87389129	Distal Intergenic	ENSSSCG000	GSTA1	glutathione S-transferase alpha 1 [Source:NCBI gene (formerly Entrezgene);Acc:397682]
chr2	11876330	11877002	673	*	chr2	11876330	11877002	in_vitro	in_vivo	12	10	1.08666667	0.645	0.44166667	c(t = 0.68898	0.499	0.87389129	Distal Intergenic	ENSSSCG000	MRPL16	mitochondrial ribosomal protein L16 [Source:NCBI gene (formerly Entrezgene);Acc:100514446]
chr12	59947208	59950800	3593	*	chr12	59947208	59950800	in_vitro	in_vivo	11	12	86.3672727	81.68	4.68727273	c(t = 0.68709	0.5	0.87389129	Distal Intergenic	ENSSSCG000	AKAP10	A-kinase anchoring protein 10 [Source:NCBI gene (formerly Entrezgene);Acc:397262]
chr17	62775484	62782650	7167	*	chr17	62775484	62782650	in_vitro	in_vivo	11	12	71.5463636	64.1875	7.35886364	c(t = 0.68727	0.5	0.87389129	Promoter (<=1kb)	ENSSSCG000	ssc-mir-1388	ssc-mir-1388 [Source:miRBase;Acc:MI0033392]
chr2	85948793	85949644	852	*	chr2	85948793	85949644	in_vitro	in_vivo	12	12	4.5175	2.95083333	1.56666667	c(t = 0.68591	0.5	0.87389129	Distal Intergenic	ENSSSCG000	CRHBP	corticotropin releasing hormone binding protein [Source:VGNC Symbol;Acc:VGNC:86993]
chr15	68211579	68212154	576	*	chr15	68211579	68212154	in_vitro	in_vivo	11	10	1.37181818	2.263	-0.8911818	c(t = -0.6948	0.5	0.87389129	Distal Intergenic	ENSSSCG000	DPP4	dipeptidyl peptidase 4 [Source:NCBI gene (formerly Entrezgene);Acc:397492]
chr5	101692178	101693841	1664	*	chr5	101692178	101693841	in_vitro	in_vivo	14	13	2.12142857	1.68846154	0.43296703	c(t = 0.68588	0.5	0.87389129	Distal Intergenic	ENSSSCG000	MYF6	myogenic factor 6 [Source:NCBI gene (formerly Entrezgene);Acc:397005]
chr5	68626785	68628151	1367	*	chr5	68626785	68628151	in_vitro	in_vivo	10	13	2.028	1.24615385	0.78184615	c(t = 0.69750	0.5	0.87389129	Distal Intergenic	ENSSSCG000	ADIPOR2	adiponectin receptor 2 [Source:VGNC Symbol;Acc:VGNC:85141]

chr18	45330214	45331086	873	*	chr18	45330214	45331086	in_vitro	in_vivo	12	11	2.11166667	1.11909091	0.99257576	c(t = 0.69256	0.5	0.87389129	Distal Intergenic	ENSSSCG000	HOXA13	homeobox A13 [Source:NCBI gene (formerly Entrezgene);Acc:100359352]
chr15	110704559	110706310	1752	*	chr15	110704559	110706310	in_vitro	in_vivo	14	13	1.49357143	1.08461538	0.40895604	c(t = 0.68519	0.5	0.87389129	Promoter (<=1kb)	ENSSSCG000	CREB1	cAMP responsive element binding protein 1 [Source:VGNC Symbol;Acc:VGNC:96004]
chr14	45924478	45925259	782	*	chr14	45924478	45925259	in_vitro	in_vivo	10	13	1.493	0.94384615	0.54915385	c(t = 0.68805	0.5	0.87389129	Distal Intergenic	ENSSSCG000	CHEK2	checkpoint kinase 2 [Source:VGNC Symbol;Acc:VGNC:86638]
chr2	140503774	140504956	1183	*	chr2	140503774	140504956	in_vitro	in_vivo	10	13	1.7	0.78076923	0.91923077	c(t = 0.69744	0.5	0.87389129	Distal Intergenic	ENSSSCG000	CDC25C	cell division cycle 25C [Source:NCBI gene (formerly Entrezgene);Acc:100144474]
chr10	17365200	17366363	1164	*	chr10	17365200	17366363	in_vitro	in_vivo	11	13	0.43636364	0.21692308	0.21944056	c(t = 0.69135	0.5	0.87389129	Promoter (<=1kb)	ENSSSCG000	DESI2	desumoylating isopeptidase 2 [Source:VGNC Symbol;Acc:VGNC:96037]
chr9	26103107	26103873	767	*	chr9	26103107	26103873	in_vitro	in_vivo	11	12	0.41636364	0.18083333	0.2355303	c(t = 0.68970	0.5	0.87389129	Distal Intergenic	ENSSSCG000	C11orf54	chromosome 9 C11orf54 homolog [Source:VGNC Symbol;Acc:VGNC:86087]
chr6	51773576	51774277	702	*	chr6	51773576	51774277	in_vitro	in_vivo	9	12	35.1211111	26.9125	8.20861111	c(t = 0.68899	0.501	0.8740077	Distal Intergenic	ENSSSCG000	ERCC1	ERCC excision repair 1, endonuclease non-catalytic subunit [Source:NCBI gene (formerly Entrezgene);Acc:100286808]
chr12	18875277	18875949	673	*	chr12	18875277	18875949	in_vitro	in_vivo	9	10	15.0522222	24.104	-9.0517778	c(t = -0.69089	0.501	0.8740077	Exon (NM_213998/397059, exon 27 of 30)	ENSSSCG000	ITGA2B	integrin subunit alpha 2b [Source:NCBI gene (formerly Entrezgene);Acc:397059]
chr3	39570647	39575423	4777	*	chr3	39570647	39575423	in_vitro	in_vivo	13	10	4.62769231	6.053	-1.4253077	c(t = -0.68410	0.501	0.8740077	Distal Intergenic	ENSSSCG000	ECI1	enoyl-CoA delta isomerase 1 [Source:NCBI gene (formerly Entrezgene);Acc:100738177]
chr2	40616963	40617536	574	*	chr2	40616963	40617536	in_vitro	in_vivo	9	10	1.39	2.251	-0.861	c(t = -0.68863	0.501	0.8740077	Distal Intergenic	ENSSSCG000	LDHC	lactate dehydrogenase C [Source:VGNC Symbol;Acc:VGNC:89669]
chr2	130025707	130027309	1603	*	chr2	130025707	130027309	in_vitro	in_vivo	12	15	2.75416667	1.80533333	0.94883333	c(t = 0.68597	0.501	0.8740077	Promoter (<=1kb)	ENSSSCG000	MARCHF3	membrane associated ring-CH-type finger 3 [Source:NCBI gene (formerly Entrezgene);Acc:100513526]
chr1	16611132	16612078	947	*	chr1	16611132	16612078	in_vitro	in_vivo	12	9	2.36	1.34222222	1.01777778	c(t = 0.68773	0.501	0.8740077	Distal Intergenic	ENSSSCG000	PCMT1	protein-L-isoaspartate (D-aspartate) O-methyltransferase [Source:VGNC Symbol;Acc:VGNC:103146]
chr9	131075103	131076502	1400	*	chr9	131075103	131076502	in_vitro	in_vivo	12	12	1.23166667	0.7425	0.48916667	c(t = 0.68460	0.501	0.8740077	Promoter (<=1kb)	ENSSSCG000	PPP2R5A	protein phosphatase 2 regulatory subunit B'alpha [Source:NCBI gene (formerly Entrezgene);Acc:100101925]
chr17	61642951	61649084	6134	*	chr17	61642951	61649084	in_vitro	in_vivo	12	12	53.0391667	44.8983333	8.14083333	c(t = 0.68285	0.502	0.8740077	Distal Intergenic	ENSSSCG000	PSMA7	proteasome 20S subunit alpha 7 [Source:NCBI gene (formerly Entrezgene);Acc:100626738]
chr7	1638610	1639630	1021	*	chr7	1638610	1639630	in_vitro	in_vivo	9	12	13.5777778	24.1066667	-10.528889	c(t = -0.68425	0.502	0.8740077	Distal Intergenic	ENSSSCG000	SERPINB9	serpin peptidase inhibitor, clade B (ovalbumin), member 9 [Source:NCBI gene (formerly Entrezgene);Acc:100126851]
chr2	6091603	6093068	1466	*	chr2	6091603	6093068	in_vitro	in_vivo	10	10	13.99	23.296	-9.306	c(t = -0.68548	0.502	0.8740077	Distal Intergenic	ENSSSCG000	RAB1B	RAB1B, member RAS oncogene family [Source:NCBI gene (formerly Entrezgene);Acc:100144502]
chr6	51866263	51867004	742	*	chr6	51866263	51867004	in_vitro	in_vivo	10	11	2.399	3.38636364	-0.9873636	c(t = -0.68574	0.502	0.8740077	Promoter (1-2kb)	ENSSSCG000	RTN2	reticulon 2 [Source:NCBI gene (formerly Entrezgene);Acc:100170115]
chr13	40768236	40768750	515	*	chr13	40768236	40768750	in_vitro	in_vivo	9	13	4.19444444	2.04461538	2.14982906	c(t = 0.69689	0.502	0.8740077	Distal Intergenic	ENSSSCG00000033189		family with sequence similarity 107 member A [Source:NCBI gene (formerly Entrezgene);Acc:100516512]
chr11	7758472	7759642	1171	*	chr11	7758472	7759642	in_vitro	in_vivo	12	11	0.66	1.14181818	-0.4818182	c(t = -0.68869	0.502	0.8740077	Promoter (<=1kb)	ENSSSCG000	HSPH1	heat shock protein family H (Hsp110) member 1 [Source:NCBI gene (formerly Entrezgene);Acc:100048931]
chr13	197161122	197162350	1229	*	chr13	197161122	197162350	in_vitro	in_vivo	14	14	0.61285714	1.14071429	-0.5278571	c(t = -0.68142	0.502	0.8740077	Distal Intergenic	ENSSSCG000	CRYZL1	crystallin zeta like 1 [Source:VGNC Symbol;Acc:VGNC:87026]
chr1	127018544	127019768	1225	*	chr1	127018544	127019768	in_vitro	in_vivo	14	11	1.79571429	1.10818182	0.68753247	c(t = 0.68258	0.502	0.8740077	Distal Intergenic	ENSSSCG000	B2M	beta-2-microglobulin [Source:NCBI gene (formerly Entrezgene);Acc:397033]
chr6	159326529	159327153	625	*	chr6	159326529	159327153	in_vitro	in_vivo	11	14	1.22454545	0.74285714	0.48168831	c(t = 0.68722	0.502	0.8740077	Distal Intergenic	NA	NA	NA
chr4	128909928	128911389	1462	*	chr4	128909928	128911389	in_vitro	in_vivo	10	11	0.961	0.43636364	0.52463636	c(t = 0.69272	0.502	0.8740077	Promoter (1-2kb)	ENSSSCG00000047605		LIM domain only 4 [Source:NCBI gene (formerly Entrezgene);Acc:100127155]
chr2	1831069	1837767	6699	*	chr2	1831069	1837767	in_vitro	in_vivo	10	11	92.795	94.9745455	-2.1795455	c(t = -0.69016	0.503	0.87455995	Distal Intergenic	ENSSSCG000	CD81	CD81 molecule [Source:VGNC Symbol;Acc:VGNC:86431]
chr7	1394281	1395440	1160	*	chr7	1394281	1395440	in_vitro	in_vivo	10	10	24.953	35.982	-11.029	c(t = -0.68407	0.503	0.87455995	Distal Intergenic	ENSSSCG000	SERPINB9	serpin peptidase inhibitor, clade B (ovalbumin), member 9 [Source:NCBI gene (formerly Entrezgene);Acc:100126851]
chr2	64798383	64799235	853	*	chr2	64798383	64799235	in_vitro	in_vivo	12	14	14.4691667	20.9971429	-6.5279762	c(t = -0.67973	0.503	0.87455995	Distal Intergenic	ENSSSCG000	DNAJB1	DnaJ heat shock protein family (Hsp40) member B1 [Source:NCBI gene (formerly Entrezgene);Acc:396741]
chr3	131260306	131263514	3209	*	chr3	131260306	131263514	in_vitro	in_vivo	11	12	12.2654545	18.3333333	-6.0678788	c(t = -0.68585	0.503	0.87455995	Distal Intergenic	ENSSSCG000	RNASEH1	ribonuclease H1 [Source:NCBI gene (formerly Entrezgene);Acc:100626539]
chr7	50749167	50753710	4544	*	chr7	50749167	50753710	in_vitro	in_vivo	12	13	2.28583333	3.13307692	-0.8472436	c(t = -0.68320	0.503	0.87455995	Distal Intergenic	ENSSSCG000	IL16	interleukin 16 [Source:VGNC Symbol;Acc:VGNC:89084]
chr6	89598921	89599866	946	*	chr6	89598921	89599866	in_vitro	in_vivo	11	9	1.89818182	1.30666667	0.59151515	c(t = 0.68582	0.503	0.87455995	Distal Intergenic	ENSSSCG000	AZIN2	antizyme inhibitor 2 [Source:NCBI gene (formerly Entrezgene);Acc:100144439]
chr14	139768742	139769312	571	*	chr14	139768742	139769312	in_vitro	in_vivo	9	10	17.1866667	9.556	7.63066667	c(t = 0.68989	0.504	0.87530847	Distal Intergenic	ENSSSCG000	GLRX3	glutaredoxin 3 [Source:VGNC Symbol;Acc:VGNC:88495]
chr14	73107773	73108369	597	*	chr14	73107773	73108369	in_vitro	in_vivo	10	13	3.55	1.97461538	1.57538462	c(t = 0.68916	0.504	0.87530847	Distal Intergenic	ENSSSCG000	SAR1A	secretion associated Ras related GTPase 1A [Source:VGNC Symbol;Acc:VGNC:92579]
chr5	70659216	70660242	1027	*	chr5	70659216	70660242	in_vitro	in_vivo	13	14	2.01923077	1.03357143	0.98565934	c(t = 0.68355	0.504	0.87530847	Distal Intergenic	ENSSSCG000	USP18	ubiquitin specific peptidase 18 [Source:NCBI gene (formerly Entrezgene);Acc:396777]
chr6	96933770	96935095	1326	*	chr6	96933770	96935095	in_vitro	in_vivo	14	13	0.79928571	0.45307692	0.34620879	c(t = 0.68084	0.504	0.87530847	Distal Intergenic	ENSSSCG000	CIDEA	cell death inducing DFFA like effector a [Source:NCBI gene (formerly Entrezgene);Acc:100127171]
chr6	46530116	46530624	509	*	chr6	46530116	46530624	in_vitro	in_vivo	10	10	0.691	0.303	0.388	c(t = 0.68394	0.504	0.87530847	Distal Intergenic	ENSSSCG000	PPP1R14A	protein phosphatase 1 regulatory inhibitor subunit 14A [Source:VGNC Symbol;Acc:VGNC:98539]
chr11	78167005	78170890	3886	*	chr11	78167005	78170890	in_vitro	in_vivo	12	12	32.61	41.525	-8.915	c(t = -0.67738	0.505	0.8760553	Distal Intergenic	ENSSSCG000	F7	coagulation factor VII [Source:NCBI gene (formerly Entrezgene);Acc:733661]
chr4	7082337	7083943	1607	*	chr4	7082337	7083943	in_vitro	in_vivo	11	13	42.77	32.5761538	10.1938462	c(t = 0.67856	0.505	0.8760553	Distal Intergenic	ENSSSCG000	ssc-mir-30b	ssc-mir-30b [Source:miRBase;Acc:MI0008215]
chr4	36886209	36887042	834	*	chr4	36886209	36887042	in_vitro	in_vivo	10	9	2.959	1.83333333	1.12566667	c(t = 0.68705	0.505	0.8760553	Distal Intergenic	ENSSSCG000	COX6C	COX6C protein [Source:NCBI gene (formerly Entrezgene);Acc:100037989]
chr2	65252152	65252696	545	*	chr2	65252152	65252696	in_vitro	in_vivo	10	11	0.667	1.29909091	-0.6320909	c(t = -0.67990	0.505	0.8760553	Promoter (<=1kb)	ENSSSCG000	CC2D1A	coiled-coil and C2 domain containing 1A [Source:VGNC Symbol;Acc:VGNC:86235]
chr1	186530326	186530920	595	*	chr1	186530326	186530920	in_vitro	in_vivo	10	12	1.752	1.15333333	0.59866667	c(t = 0.68091	0.505	0.8760553	Distal Intergenic	ENSSSCG000	ARMH4	armadillo like helical domain containing 4 [Source:VGNC Symbol;Acc:VGNC:85535]
chr3	45670293	45671169	877	*	chr3	45670293	45671169	in_vitro	in_vivo	10	11	6.498	4.22	2.278	c(t = 0.68678	0.506	0.87680045	Distal Intergenic	ENSSSCG000	ADRA2B	adrenoceptor alpha 2B [Source:NCBI gene (formerly Entrezgene);Acc:641353]
chr7	113835639	113837052	1414	*	chr7	113835639	113837052	in_vitro	in_vivo	9	13	1.61	2.35076923	-0.7407692	c(t = -0.67734	0.506	0.87680045	Distal Intergenic	ENSSSCG00000002446		ataxin 3 [Source:NCBI gene (formerly Entrezgene);Acc:100144422]
chr14	2602393	2604122	1730	*	chr14	2602393	2604122	in_vitro	in_vivo	11	15	1.21090909	1.86866667	-0.6577576	c(t = -0.67604	0.506	0.87680045	Distal Intergenic	ENSSSCG000	SYK	spleen associated tyrosine kinase [Source:NCBI gene (formerly Entrezgene);Acc:100125540]
chr7	13716361	13717374	1014	*	chr7	13716361	13717374	in_vitro	in_vivo	13	13	2.29923077	1.38769231	0.91153846	c(t = 0.68022	0.506	0.87680045	Distal Intergenic	ENSSSCG000	NHLRC1	NHL repeat containing E3 ubiquitin protein ligase 1 [Source:NCBI gene (formerly Entrezgene);Acc:100152396]
chr18	28415071	28415696	626	*	chr18	28415071	28415696	in_vitro	in_vivo	10	10	1.383	0.714	0.669	c(t = 0.67799	0.506	0.87680045	Distal Intergenic	ENSSSCG000	CFTR	CF transmembrane conductance regulator [Source:VGNC Symbol;Acc:VGNC:97927]
chr14	30645780	30646582	803	*	chr14	30645780	30646582	in_vitro	in_vivo	9	9	1.28222222	2.64333333	-1.3611111	c(t = -0.68425	0.507	0.87710177	Distal Intergenic	ENSSSCG000	DIABLO	diablo IAP-binding mitochondrial protein [Source:NCBI gene (formerly Entrezgene);Acc:100155744]
chr1	119758703	119759757	1055	*	chr1	119758703	119759757	in_vitro	in_vivo	12	13	1.89916667	1.25230769	0.64685897	c(t = 0.67728	0.507	0.87710177	Distal Intergenic	ENSSSCG000	TMOD3	tropomodulin 3 [Source:NCBI gene (formerly Entrezgene);Acc:397556]
chr18	564438	566239	1802	*	chr18	564438	566239	in_vitro	in_vivo	12	14	1.3175	0.87285714	0.44464286	c(t = 0.67704	0.507	0.87710177	Distal Intergenic	ENSSSCG000	VIPR2	vasoactive intestinal peptide receptor 2 [Source:NCBI gene (formerly Entrezgene);Acc:100499553]
chr6	88808587	88809709	1123	*	chr6	88808587	88809709	in_vitro	in_vivo	9	10	25.7566667	36.901	-11.144333	c(t = -0.67677	0.508	0.87710177	Promoter (<=1kb)	ENSSSCG000	TSSK3	testis specific serine kinase 3 [Source:NCBI gene (formerly Entrezgene);Acc:100628162]
chr3	56531107	56531723	617	*	chr3	56531107	56531723	in_vitro	in_vivo	9	14	5.15777778	6.88142857	-1.7236508	c(t = -0.67391	0.508	0.87710177	Distal Intergenic	ENSSSCG000	COX5B	mitochondrial cytochrome c oxidase subunit Vb [Source:NCBI gene (formerly Entrezgene);Acc:492822]
chr2	82386230	82386693	464	*	chr2	82386230	82386693	in_vitro	in_vivo	9	9	8.25222222	5.69111111	2.56111111	c(t = 0.67804	0.508	0.87710177	Distal Intergenic	ENSSSCG000	TMEM171	transmembrane protein 171 [Source:VGNC Symbol;Acc:VGNC:94109]
chrX	88232411	88234118	1708	*	chrX	88232411	88234118	in_vitro	in_vivo	11	13	2.86454545	5.38153846	-2.516993	c(t = -0.67952	0.508	0.87710177	Distal Intergenic	ENSSSCG000	CLDN2	claudin 2 [Source:VGNC Symbol;Acc:VGNC:86737]
chr10	27160110	27161504	1395	*	chr10	27160110	27161504	in_vitro	in_vivo	9	14	1.73555556	2.84642857	-1.110873	c(t = -0.67600	0.508	0.87710177	Distal Intergenic	ENSSSCG000	ssc-mir-23b	ssc-mir-23b [Source:miRBase;Acc:MI0013112]
chr14	29630876	29632175	1300	*	chr14	29630876	29632175	in_vitro	in_vivo	13	12	3.65153846	2.65583333	0.99570513	c(t = 0.67362	0.508	0.87710177	Promoter (<=1kb)	ENSSSCG000	CDK2AP1	cyclin dependent kinase 2 associated protein 1 [Source:VGNC Symbol;Acc:VGNC:103918]
chr14	30498253	30499310	1058	*	chr14	30498253	30499310	in_vitro	in_vivo	10	10	1.317	2.601	-1.284	c(t = -0.68379	0.508	0.87710177	Promoter (<=1kb)	ENSSSCG000	DIABLO	diablo IAP-binding mitochondrial protein [Source:NCBI gene (formerly Entrezgene);Acc:100155744]
chr3	17107888	17108843	956	*	chr3	17107888	17108843	in_vitro	in_vivo	9	11	3.53111111	2.14727273	1.38383838	c(t = 0.67786	0.508	0.87710177	Distal Intergenic	ENSSSCG000	FUS	FUS RNA binding protein [Source:VGNC Symbol;Acc:VGNC:88267]
chr4	126716871	126718247	1377	*	chr4	126716871	126718247	in_vitro	in_vivo	12	14	1.3825	2.01142857	-0.6289286	c(t = -0.67214	0.508	0.87710177	Distal Intergenic	ENSSSCG000	GBP2	guanylate binding protein 2, interferon-inducible [Source:NCBI gene (formerly Entrezgene);Acc:100153137]
chr17	59184459	59185350	892	*	chr17	59184459	59185350	in_vitro	in_vivo	13	10	1.19615385	1.766	-0.5698462	c(t = -0.68108	0.508	0.87710177	Promoter (<=1kb)	ENSSSCG00000007525		PRELI domain containing 3B [Source:NCBI gene (formerly Entrezgene);Acc:100134984]
chr7	24231962	24233054	1093	*	chr7	24231962	24233054	in_vitro	in_vivo	11	13	2.16636364	1.29846154	0.8679021	c(t = 0.67232	0.508	0.87710177	Exon (NM_001123147/100144522, exon 29 of 30)	ENSSSCG000	GPSM3	G protein signaling modulator 3 [Source:VGNC Symbol;Acc:VGNC:88650]
chr8	134142763	134143678	916	*	chr8	134142763	134143678	in_vitro	in_vivo	11	9	1.86272727	1.03	0.83272727	c(t = 0.67893	0.508	0.87710177	Distal Intergenic	ENSSSCG000	CDS1	CDP-diacylglycerol synthase 1 [Source:VGNC Symbol;Acc:VGNC:86523]
chr6	137594869	137595622	754	*	chr6	137594869	137595622	in_vitro	in_vivo	9	10	2.39333333	1.022	1.37133333	c(t = 0.68430	0.508	0.87710177	Distal Intergenic	ENSSSCG000	ACADM	acyl-CoA dehydrogenase medium chain [Source:NCBI gene (formerly Entrezgene);Acc:397104]
chr3	81286075	81287245	1171	*	chr3	81286075	81287245	in_vitro	in_vivo	9	13	26.8166667	17.9369231	8.87974359	c(t = 0.67682	0.509	0.87784224	Exon (NM_001297634/100523730, exon 4 of 5)	ENSSSCG000	BCL11A	BCL11 transcription factor A [Source:VGNC Symbol;Acc:VGNC:96562]
chr10	50201046	50202756	1711	*	chr10	50201046	50202756	in_vitro	in_vivo	12	11	2.93666667	4.07454545	-1.1378788	c(t = -0.67395	0.509	0.87784224	Distal Intergenic	ENSSSCG000	GAD2	glutamate decarboxylase 2 [Source:NCBI gene (formerly Entrezgene);Acc:396929]
chr13	69362783	69363954	1172	*	chr13	69362783	69363954	in_vitro	in_vivo	9	12	5.25666667	3.19	2.06666667	c(t = 0.68107	0.509	0.87784224	Promoter (<=1kb)	ENSSSCG000	TRH	thyrotropin releasing hormone [Source:NCBI gene (formerly Entrezgene);Acc:100513309]
chr14	23593267	23594441	1175	*	chr14	23593267	23594441	in_vitro	in_vivo	12	11	1.975	0.97545455	0.99954545	c(t = 0.67789	0.509	0.87784224	Distal Intergenic	ENSSSCG000	PIWIL1	piwi like RNA-mediated silencing 1 [Source:VGNC Symbol;Acc:VGNC:98197]
chr15	103542667	103543358	692	*	chr15	103542667	103543358	in_vitro	in_vivo	10	12	0.655	0.33333333	0.32166667	c(t = 0.67261	0.509	0.87784224	Distal Intergenic	ENSSSCG000	SPATS2L	spermatogenesis associated serine rich 2 like [Source:VGNC Symbol;Acc:VGNC:96047]
chr14	38454014	38455659	1646	*	chr14	38454014	38455659	in_vitro	in_vivo	9	11	2.85444444	4.23818182	-1.3837374	c(t = -0.67685	0.51	0.87917227	Distal Intergenic	ENSSSCG000	OAS2	2'-5'-oligoadenylate synthetase 2 [Source:VGNC Symbol;Acc:VGNC:91007]
chr9	134863586	134864477	892	*	chr9	134863586	134864477	in_vitro	in_vivo	9	12	2.42	1.39333333	1.02666667	c(t = 0.68310	0.51	0.87917227	Distal Intergenic	ENSSSCG000	CD34	CD34 molecule [Source:VGNC Symbol;Acc:VGNC:86410]
chr1	1530866	1535259	4394	*	chr1	1530866	1535259	in_vitro	in_vivo	9	9	28.3588889	39.4155556	-11.056667	c(t = -0.67270	0.511	0.87931818	Distal Intergenic	ENSSSCG000	ssc-mir-9824	ssc-mir-9824 [Source:miRBase;Acc:MI0031591]
chr2	5081209	5083819	2611	*	chr2	5081209	5083819	in_vitro	in_vivo	13	13	13.5284615	8.05461538	5.47384615	c(t = 0.67001	0.511	0.87931818	Distal Intergenic	ENSSSCG000	PPP1CA	protein phosphatase 1 catalytic subunit alpha [Source:NCBI gene (formerly Entrezgene);Acc:733611]
chr3	72379480	72381717	2238	*	chr3	72379480	72381717	in_vitro	in_vivo	11	11	4.82727273	2.83454545	1.99272727	c(t = 0.67372	0.511	0.87931818	Distal Intergenic	ENSSSCG000	C2orf42	chromosome 3 C2orf42 homolog [Source:VGNC Symbol;Acc:VGNC:86021]
chr14	8961742	8963206	1465	*	chr14	8961742	8963206	in_vitro	in_vivo	12	11	1.88166667	2.72733333	-0.8456667	c(t = -0.67044	0.511	0.87931818	Distal Intergenic	ENSSSCG000	NEFL	neurofilament light chain [Source:NCBI gene (formerly Entrezgene);Acc:100521224]
chr7	54941790	54943476	1687	*	chr7	54941790	54943476	in_vitro	in_vivo	12	10	2.84416667	2.033	0.81116667	c(t = 0.67126	0.511	0.87931818	Distal Intergenic	ENSSSCG000	ssc-mir-9-3	ssc-mir-9-3 [Source:miRBase;Acc:MI0022123]
chr6	84918888	84919217	330	*	chr6	84918888	84919217	in_vitro	in_vivo	11	9	0.53454545	1.85222222	-1.3176768	c(t = -0.68350	0.511	0.87931818	Promoter (<=1kb)	ENSSSCG000	STX12	syntaxin 12 [Source:NCBI gene (formerly Entrezgene);Acc:100627750]
chr2	61450085	61451069	985	*	chr2	61450085	61451069	in_vitro	in_vivo	12	13	3.21416667	1.84076923	1.37339744	c(t = 0.67116	0.511	0.87931818	Promoter (<=1kb)	ENSSSCG000	RAB8A	RAB8A, member RAS oncogene family [Source:NCBI gene (formerly Entrezgene);Acc:100511006]
chr13	43132225	43134615	2391	*	chr13	43132225	43134615	in_vitro	in_vivo	12	13	0.6225	1.00846154	-0.3859615	c(t = -0.66811	0.511	0.87931818	Distal Intergenic	ENSSSCG00000033189		family with sequence similarity 107 member A [Source:NCBI gene (formerly Entrezgene);Acc:100516512]
chr3	132345252	132353708	8457	*	chr3	132345252	132353708	in_vitro	in_vivo	9	12	14.3288889	21.5658333	-7.2369444	c(t = -0.66975	0.512	0.87966018	Distal Intergenic	ENSSSCG000	TPO	thyroid peroxidase [Source:VGNC Symbol;Acc:VGNC:94341]
chr17	36945660	36946509	850	*	chr17	36945660	36946509	in_vitro	in_vivo	11	11	5.61636364	7.90181818	-2.2854545	c(t = -0.66717	0.512	0.87966018	Distal Intergenic	ENSSSCG00000007262		long palate lung and nasal epithelium protein 5 [Source:NCBI gene (formerly Entrezgene);Acc:100113428]
chr17	32239068	32239912	845	*	chr17	32239068	32239912	in_vitro	in_vivo	10	11	1.042	1.77727273	-0.7352727	c(t = -0.66905	0.512	0.87966018	Distal Intergenic	ENSSSCG000	SIGLEC1	sialic acid binding Ig like lectin 1 [Source:VGNC Symbol;Acc:VGNC:108736]
chr6	83650906	83651688	783	*	chr6	83650906	83651688	in_vitro	in_vivo	10	10	1.63	0.909	0.721	c(t = 0.67200	0.512	0.87966018	Distal Intergenic	ENSSSCG000	CATSPER4	cation channel sperm associated 4 [Source:VGNC Symbol;Acc:VGNC:86212]
chr17	52349846	52351234	1389	*	chr17	52349846	52351234	in_vitro	in_vivo	10	10	1.317	0.893	0.424	c(t = 0.66923	0.512	0.87966018	Promoter (<=1kb)	ENSSSCG000	ADNP	activity dependent neuroprotector homeobox [Source:VGNC Symbol;Acc:VGNC:95657]
chr1	83136818	83137613	796	*	chr1	83136818	83137613	in_vitro	in_vivo	11	11	1.30545455	0.84363636	0.46181818	c(t = 0.66705	0.512	0.87966018	Promoter (<=1kb)	ENSSSCG000	CALHM6	calcium homeostasis modulator family member 6 [Source:VGNC Symbol;Acc:VGNC:86147]
chr1	180703594	180704362	769	*	chr1	180703594	180704362	in_vitro	in_vivo	9	11	1.53888889	0.76909091	0.76979798	c(t = 0.68022	0.512	0.87966018	Promoter (<=1kb)	ENSSSCG000	PYGL	glycogen phosphorylase L [Source:NCBI gene (formerly Entrezgene);Acc:100141306]
chr14	57105807	57107057	1251	*	chr14	57105807	57107057	in_vitro	in_vivo	11	13	2.40090909	1.34538462	1.05552448	c(t = 0.67383	0.513	0.88078753	Distal Intergenic	ENSSSCG000	TSNAX	translin associated factor X [Source:NCBI gene (formerly Entrezgene);Acc:100156036]
chr5	59735661	59736646	986	*	chr5	59735661	59736646	in_vitro	in_vivo	11	12	0.52090909	0.94818182	-0.4272727	c(t = -0.66770	0.513	0.88078753	Promoter (<=1kb)	ENSSSCG000	CDKN1B	cyclin dependent kinase inhibitor 1B [Source:NCBI gene (formerly Entrezgene);Acc:397584]
chr14	94094630	94095098	469	*	chr14	94094630	94095098	in_vitro	in_vivo	12	12	1.68333333	0.83	0.85333333	c(t = 0.66861	0.513	0.88078753	Distal Intergenic	ENSSSCG000	UBE2D1	ubiquitin conjugating enzyme E2 D1 [Source:VGNC Symbol;Acc:VGNC:94641]
chr2	140867932	140870751	2820	*	chr2	140867932	140870751	in_vitro	in_vivo	12	13	96.0441667	94.4538462	1.59032051	c(t = 0.66227	0.514	0.88112648	Distal Intergenic	ENSSSCG000	ssc-mir-9820	ssc-mir-9820 [Source:miRBase;Acc:MI0031587]
chr2	77848633	77850680	2048	*	chr2	77848633	77850680	in_vitro	in_vivo	11	12	4.23272727	6.19583333	-1.9631061	c(t = -0.66548	0.514	0.88112648	Distal Intergenic	ENSSSCG000	MADCAM1	mucosal vascular addressin cell adhesion molecule 1 [Source:VGNC Symbol;Acc:VGNC:108754]
chr17	29430108	29431152	1045	*	chr17	29430108	29431152	in_vitro	in_vivo	9	13	1.95666667	3.41692308	-1.4602564	c(t = -0.66745	0.514	0.88112648	Distal Intergenic	ENSSSCG000	THBD	thrombomodulin [Source:NCBI gene (formerly Entrezgene);Acc:100157642]
chr14	134253331	134254417	1087	*	chr14	134253331	134254417	in_vitro	in_vivo	11	10	2.13	3.183	-1.053	c(t = -0.66830	0.514	0.88112648	Distal Intergenic	ENSSSCG000	LHPP	phospholysine phosphohistidine inorganic pyrophosphate phosphatase [Source:VGNC Symbol;Acc:VGNC:89710]
chr6	1849069	1851141	2073	*	chr6	1849069	1851141	in_vitro	in_vivo	12	13	2.44333333	1.61230769	0.83102564	c(t = 0.66879	0.514	0.88112648	Distal Intergenic	ENSSSCG000	MAP1LC3B	microtubule associated protein 1 light chain 3 beta [Source:NCBI gene (formerly Entrezgene);Acc:100462754]
chr1	127125064	127125934	871	*	chr1	127125064	127125934	in_vitro	in_vivo	10	11	1.605	1	0.605	c(t = 0.66549	0.514	0.88112648	Distal Intergenic	ENSSSCG000	B2M	beta-2-microglobulin [Source:NCBI gene (formerly Entrezgene);Acc:397033]
chr2	73062646	73063742	1097	*	chr2	73062646	73063742	in_vitro	in_vivo	11	12	1.63090909	0.96916667	0.66174242	c(t = 0.66672	0.514	0.88112648	Distal Intergenic	ENSSSCG000	CAPS	calcyphosine [Source:VGNC Symbol;Acc:VGNC:86180]
chr7	100345866	100346672	807	*	chr7	100345866	100346672	in_vitro	in_vivo	10	10	12.416	5.572	6.844	c(t = 0.67380	0.515	0.88264384	Promoter (<=1kb)	ENSSSCG000	NGB	neuroglobin [Source:NCBI gene (formerly Entrezgene);Acc:414438]
chr5	87486464	87487005	542	*	chr5	87486464	87487005	in_vitro	in_vivo	9	10	47.4388889	33.681	13.7578889	c(t = 0.66593	0.516	0.88337269	Promoter (<=1kb)	ENSSSCG000	LTA4H	leukotriene A4 hydrolase [Source:NCBI gene (formerly Entrezgene);Acc:100154219]
chr16	27639997	27640867	871	*	chr16	27639997	27640867	in_vitro	in_vivo	9	12	16.2533333	10.9658333	5.2875	c(t = 0.66524	0.516	0.88337269	Distal Intergenic	NA	NA	NA
chr7	22916827	22918002	1176	*	chr7	22916827	22918002	in_vitro	in_vivo	10	14	1.519	3.62214286	-2.1031429	c(t = -0.66480	0.516	0.88337269	Promoter (<=1kb)	NA	NA	NA
chr16	72222647	72223804	1158	*	chr16	72222647	72223804	in_vitro	in_vivo	10	12	1.978	3.28583333	-1.3078333	c(t = -0.66183	0.516	0.88337269	Promoter (<=1kb)	ENSSSCG000	CCT5	chaperonin containing TCP1 subunit 5 [Source:VGNC Symbol;Acc:VGNC:86383]
chr1	37760791	37761845	1055	*	chr1	37760791	37761845	in_vitro	in_vivo	10	11	2.677	1.58727273	1.08972727	c(t = 0.66382	0.516	0.88337269	Distal Intergenic	ENSSSCG000	HEY2	hes related family bHLH transcription factor with YRPW motif 2 [Source:VGNC Symbol;Acc:VGNC:88864]
chr15	137819157	137820483	1327	*	chr15	137819157	137820483	in_vitro	in_vivo	11	10	35.05	44.494	-9.444	c(t = -0.66127	0.517	0.88395145	Distal Intergenic	ENSSSCG000	RAMP1	receptor activity modifying protein 1 [Source:NCBI gene (formerly Entrezgene);Acc:397401]
chr3	109411064	109412924	1861	*	chr3	109411064	109412924	in_vitro	in_vivo	14	14	1.32785714	1.87928571	-0.5514286	c(t = -0.65763	0.517	0.88395145	Distal Intergenic	ENSSSCG000	LCLAT1	lysocardiolipin acyltransferase 1 [Source:NCBI gene (formerly Entrezgene);Acc:100217396]
chr12	15298698	15299444	747	*	chr12	15298698	15299444	in_vitro	in_vivo	9	11	4.11	1.62545455	2.48454545	c(t = 0.67160	0.517	0.88395145	Distal Intergenic	ENSSSCG000	TACO1	translational activator of cytochrome c oxidase I [Source:VGNC Symbol;Acc:VGNC:93700]
chr12	18596965	18598152	1188	*	chr12	18596965	18598152	in_vitro	in_vivo	9	11	2.48444444	1.28545455	1.1989899	c(t = 0.66870	0.517	0.88395145	Distal Intergenic	ENSSSCG000	GJC1	gap junction protein gamma 1 [Source:VGNC Symbol;Acc:VGNC:88468]
chr1	116131788	116132927	1140	*	chr1	116131788	116132927	in_vitro	in_vivo	13	9	1.28538462	0.68111111	0.6042735	c(t = 0.66154	0.517	0.88395145	Distal Intergenic	ENSSSCG000	CCPG1	cell cycle progression 1 [Source:VGNC Symbol;Acc:VGNC:86368]
chr5	5234599	5235291	693	*	chr5	5234599	5235291	in_vitro	in_vivo	9	11	14.7966667	25.2036364	-10.40697	c(t = -0.65957	0.518	0.88395145	Distal Intergenic	ENSSSCG000	PNPLA3	patatin like phospholipase domain containing 3 [Source:VGNC Symbol;Acc:VGNC:103312]
chr14	47458141	47459148	1008	*	chr14	47458141	47459148	in_vitro	in_vivo	10	12	9.623	13.3866667	-3.7636667	c(t = -0.65959	0.518	0.88395145	Exon (NM_001244429/100155265, exon 3 of 3)	ENSSSCG000	GAL3ST1	galactose-3-O-sulfotransferase 1 [Source:VGNC Symbol;Acc:VGNC:88321]
chr10	13415633	13417080	1448	*	chr10	13415633	13417080	in_vitro	in_vivo	10	13	3.499	1.76076923	1.73823077	c(t = 0.66602	0.518	0.88395145	Distal Intergenic	ENSSSCG000	EPHX1	epoxide hydrolase 1 [Source:NCBI gene (formerly Entrezgene);Acc:397639]
chr6	1940258	1941162	905	*	chr6	1940258	1941162	in_vitro	in_vivo	9	12	2.94777778	1.41666667	1.53111111	c(t = 0.67172	0.518	0.88395145	Promoter (<=1kb)	ENSSSCG000	MAP1LC3B	microtubule associated protein 1 light chain 3 beta [Source:NCBI gene (formerly Entrezgene);Acc:100462754]
chr1	124042130	124043490	1361	*	chr1	124042130	124043490	in_vitro	in_vivo	13	10	2.14615385	1.333	0.81315385	c(t = 0.65839	0.518	0.88395145	Distal Intergenic	ENSSSCG000	FBN1	fibrillin 1 [Source:NCBI gene (formerly Entrezgene);Acc:414836]
chr7	7444211	7445022	812	*	chr7	7444211	7445022	in_vitro	in_vivo	9	11	0.31777778	0.69818182	-0.380404	c(t = -0.65991	0.518	0.88395145	Distal Intergenic	ENSSSCG000	C6orf52	chromosome 7 C6orf52 homolog [Source:VGNC Symbol;Acc:VGNC:86073]
chr14	120969166	120970151	986	*	chr14	120969166	120970151	in_vitro	in_vivo	10	12	1.085	0.595	0.49	c(t = 0.65819	0.518	0.88395145	Distal Intergenic	ENSSSCG000	SMNDC1	survival motor neuron domain containing 1 [Source:VGNC Symbol;Acc:VGNC:93257]
chr2	4844712	4846491	1780	*	chr2	4844712	4846491	in_vitro	in_vivo	11	9	90.4254545	93.2288889	-2.8034343	c(t = -0.65855	0.519	0.88395145	Distal Intergenic	ENSSSCG00000028501		aldehyde dehydrogenase 3 family member B1 [Source:NCBI gene (formerly Entrezgene);Acc:100739347]
chr17	62568789	62572832	4044	*	chr17	62568789	62572832	in_vitro	in_vivo	13	11	88.3853846	91.4936364	-3.1082517	c(t = -0.66274	0.519	0.88395145	Distal Intergenic	ENSSSCG000	LIME1	Lck interacting transmembrane adaptor 1 [Source:NCBI gene (formerly Entrezgene);Acc:100625216]
chrX	110928046	110929359	1314	*	chrX	110928046	110929359	in_vitro	in_vivo	9	9	2.85111111	4.73666667	-1.8855556	c(t = -0.66198	0.519	0.88395145	Distal Intergenic	ENSSSCG000	MOSPD1	motile sperm domain containing 1 [Source:VGNC Symbol;Acc:VGNC:103990]
chr1	90743992	90744888	897	*	chr1	90743992	90744888	in_vitro	in_vivo	9	10	2.71666667	1.42	1.29666667	c(t = 0.66958	0.519	0.88395145	Distal Intergenic	ENSSSCG000	COX7A2	cytochrome c oxidase subunit 7A2 [Source:NCBI gene (formerly Entrezgene);Acc:100038000]
chr5	14874397	14875373	977	*	chr5	14874397	14875373	in_vitro	in_vivo	10	10	2.299	1.228	1.071	c(t = 0.66075	0.519	0.88395145	Distal Intergenic	ENSSSCG000	ARF3	ADP ribosylation factor 3 [Source:NCBI gene (formerly Entrezgene);Acc:100301570]
chr11	67564513	67566112	1600	*	chr11	67564513	67566112	in_vitro	in_vivo	12	13	1.45916667	0.92846154	0.53070513	c(t = 0.65783	0.519	0.88395145	Distal Intergenic	ENSSSCG000	SLC15A1	solute carrier family 15 member 1 [Source:NCBI gene (formerly Entrezgene);Acc:397624]
chr14	7405745	7406457	713	*	chr14	7405745	7406457	in_vitro	in_vivo	9	10	7.27666667	14.397	-7.1203333	c(t = -0.66514	0.52	0.88395145	Distal Intergenic	ENSSSCG000	PEBP4	phosphatidylethanolamine binding protein 4 [Source:VGNC Symbol;Acc:VGNC:91303]
chr2	74294519	74295830	1312	*	chr2	74294519	74295830	in_vitro	in_vivo	11	13	6.63454545	10.6269231	-3.9923776	c(t = -0.65971	0.52	0.88395145	Distal Intergenic	ENSSSCG000	PLIN5	perilipin 5 [Source:VGNC Symbol;Acc:VGNC:91561]
chr6	45304692	45306421	1730	*	chr6	45304692	45306421	in_vitro	in_vivo	10	11	5.344	7.90727273	-2.5632727	c(t = -0.66261	0.52	0.88395145	Promoter (1-2kb)	ENSSSCG00000002918		hematopoietic cell signal transducer [Source:VGNC Symbol;Acc:VGNC:88809]
chr10	45358905	45360792	1888	*	chr10	45358905	45360792	in_vitro	in_vivo	13	15	1.39384615	2.06133333	-0.6674872	c(t = -0.65452	0.52	0.88395145	Distal Intergenic	ENSSSCG000	PTER	phosphotriesterase related [Source:VGNC Symbol;Acc:VGNC:96514]
chr3	114713659	114714276	618	*	chr3	114713659	114714276	in_vitro	in_vivo	10	12	2.438	1.56166667	0.87633333	c(t = 0.65746	0.52	0.88395145	Distal Intergenic	ENSSSCG000	NCOA1	nuclear receptor coactivator 1 [Source:VGNC Symbol;Acc:VGNC:90615]
chr11	75544552	75545320	769	*	chr11	75544552	75545320	in_vitro	in_vivo	11	16	0.65636364	1.301875	-0.6455114	c(t = -0.65515	0.52	0.88395145	Distal Intergenic	ENSSSCG000	TNFSF13B	TNF superfamily member 13b [Source:VGNC Symbol;Acc:VGNC:94269]
chr9	49630854	49631866	1013	*	chr9	49630854	49631866	in_vitro	in_vivo	11	11	1.59454545	1.02454545	0.57	c(t = 0.65858	0.52	0.88395145	Distal Intergenic	ENSSSCG000	HSPA8	heat shock protein family A (Hsp70) member 8 [Source:NCBI gene (formerly Entrezgene);Acc:100511890]
chr9	17297295	17298234	940	*	chr9	17297295	17298234	in_vitro	in_vivo	12	9	0.3475	0.78111111	-0.4336111	c(t = -0.66067	0.52	0.88395145	Distal Intergenic	ENSSSCG000	TMEM126B	transmembrane protein 126B [Source:NCBI gene (formerly Entrezgene);Acc:100626990]
chr12	52330931	52331649	719	*	chr12	52330931	52331649	in_vitro	in_vivo	10	12	84.268	78.0133333	6.25466667	c(t = 0.65327	0.521	0.88395145	Distal Intergenic	ENSSSCG000	ALOX15	arachidonate 15-lipoxygenase [Source:NCBI gene (formerly Entrezgene);Acc:396971]
chr18	20318218	20320557	2340	*	chr18	20318218	20320557	in_vitro	in_vivo	11	9	14.6041667	18.5966667	-3.9925	c(t = -0.65627	0.521	0.88395145	Distal Intergenic	ENSSSCG000	ssc-mir-129b	ssc-mir-129b [Source:miRBase;Acc:MI0017986]
chr12	2515047	2526675	11629	*	chr12	2515047	2526675	in_vitro	in_vivo	9	12	10.3377778	7.64833333	2.68944444	c(t = 0.65746	0.521	0.88395145	Distal Intergenic	ENSSSCG000	CBX8	chromobox 8 [Source:NCBI gene (formerly Entrezgene);Acc:100514536]
chr17	41900503	41900993	491	*	chr17	41900503	41900993	in_vitro	in_vivo	9	11	1.73222222	3.15181818	-1.419596	c(t = -0.65670	0.521	0.88395145	Distal Intergenic	ENSSSCG000	ADIG	adipogenin [Source:VGNC Symbol;Acc:VGNC:96182]
chr3	45585129	45585442	314	*	chr3	45585129	45585442	in_vitro	in_vivo	10	9	0.909	2.04222222	-1.1332222	c(t = -0.65919	0.521	0.88395145	Distal Intergenic	ENSSSCG000	ADRA2B	adrenoceptor alpha 2B [Source:NCBI gene (formerly Entrezgene);Acc:641353]
chr1	28968671	28969059	389	*	chr1	28968671	28969059	in_vitro	in_vivo	9	12	0.71666667	1.65416667	-0.9375	c(t = -0.65540	0.521	0.88395145	Distal Intergenic	ENSSSCG000	SGK1	serum/glucocorticoid regulated kinase 1 [Source:VGNC Symbol;Acc:VGNC:92793]
chr3	38952271	38952873	603	*	chr3	38952271	38952873	in_vitro	in_vivo	9	12	1.03333333	1.5475	-0.5141667	c(t = -0.65425	0.521	0.88395145	Distal Intergenic	ENSSSCG000	ZNF205	zinc finger protein 205 [Source:VGNC Symbol;Acc:VGNC:95204]
chr7	96984647	96985242	596	*	chr7	96984647	96985242	in_vitro	in_vivo	9	13	0.72	1.15615385	-0.4361538	c(t = -0.65287	0.521	0.88395145	Distal Intergenic	ENSSSCG000	LIN52	lin-52 DREAM MuvB core complex component [Source:NCBI gene (formerly Entrezgene);Acc:100157325]
chr13	124390684	124391253	570	*	chr13	124390684	124391253	in_vitro	in_vivo	12	9	0.49	1.14666667	-0.6566667	c(t = -0.65892	0.521	0.88395145	Promoter (<=1kb)	ENSSSCG000	DNAJB11	DnaJ heat shock protein family (Hsp40) member B11 [Source:VGNC Symbol;Acc:VGNC:108648]
chr1	194003	194875	873	*	chr1	194003	194875	in_vitro	in_vivo	11	10	1.27090909	0.75	0.52090909	c(t = 0.65407	0.521	0.88395145	Distal Intergenic	ENSSSCG000	PSMB1	proteasome 20S subunit beta 1 [Source:NCBI gene (formerly Entrezgene);Acc:100621969]
chr4	62139499	62140609	1111	*	chr4	62139499	62140609	in_vitro	in_vivo	11	12	0.80181818	0.47333333	0.32848485	c(t = 0.65276	0.521	0.88395145	Distal Intergenic	ENSSSCG000	UBE2W	ubiquitin conjugating enzyme E2 W [Source:NCBI gene (formerly Entrezgene);Acc:100152196]
chr4	98118813	98121204	2392	*	chr4	98118813	98121204	in_vitro	in_vivo	11	12	21.6609091	15.735	5.92590909	c(t = 0.65659	0.522	0.88395145	Distal Intergenic	ENSSSCG000	TNFAIP8L2	TNF alpha induced protein 8 like 2 [Source:VGNC Symbol;Acc:VGNC:94255]
chr13	207299458	207304981	5524	*	chr13	207299458	207304981	in_vitro	in_vivo	12	12	8.3525	12.58	-4.2275	c(t = -0.65199	0.522	0.88395145	Distal Intergenic	ENSSSCG000	ITGB2	integrin subunit beta 2 [Source:NCBI gene (formerly Entrezgene);Acc:396943]
chr3	63587671	63589717	2047	*	chr3	63587671	63589717	in_vitro	in_vivo	13	15	1.92153846	2.78466667	-0.8631282	c(t = -0.64925	0.522	0.88395145	Distal Intergenic	ENSSSCG00000037620		
chr6	119104749	119105286	538	*	chr6	119104749	119105286	in_vitro	in_vivo	9	9	3.25888889	2.01222222	1.24666667	c(t = 0.65700	0.522	0.88395145	Distal Intergenic	ENSSSCG000	ZNF24	zinc finger protein 24 [Source:VGNC Symbol;Acc:VGNC:98422]
chrX	42046222	42046809	588	*	chrX	42046222	42046809	in_vitro	in_vivo	11	11	2.61181818	1.47545455	1.13636364	c(t = 0.65352	0.522	0.88395145	Distal Intergenic	ENSSSCG000	ARAF	A-Raf proto-onco, serine/threonine kinase [Source:VGNC Symbol;Acc:VGNC:85438]
chr13	77180238	77182151	1914	*	chr13	77180238	77182151	in_vitro	in_vivo	11	11	1.3	0.82909091	0.47090909	c(t = 0.65227	0.522	0.88395145	Distal Intergenic	ENSSSCG000	PCCB	propionyl-CoA carboxylase subunit beta [Source:NCBI gene (formerly Entrezgene);Acc:100158147]
chr4	95972142	95973649	1508	*	chr4	95972142	95973649	in_vitro	in_vivo	9	13	0.95111111	0.48076923	0.47034188	c(t = 0.66583	0.522	0.88395145	Distal Intergenic	ENSSSCG000	SNAPIN	SNAP associated protein [Source:VGNC Symbol;Acc:VGNC:93284]
chr17	38467875	38468473	599	*	chr17	38467875	38468473	in_vitro	in_vivo	11	10	0.84818182	0.417	0.43118182	c(t = 0.65536	0.522	0.88395145	Distal Intergenic	ENSSSCG000	PROCR	protein C receptor [Source:VGNC Symbol;Acc:VGNC:96506]
chr4	27642620	27643168	549	*	chr4	27642620	27643168	in_vitro	in_vivo	11	11	8.85545455	6.02181818	2.83363636	c(t = 0.65174	0.523	0.88395145	Distal Intergenic	ENSSSCG000	ENY2	ENY2 transcription and export complex 2 subunit [Source:VGNC Symbol;Acc:VGNC:98792]
chr3	93039634	93040615	982	*	chr3	93039634	93040615	in_vitro	in_vivo	10	14	2.883	4.47285714	-1.5898571	c(t = -0.65060	0.523	0.88395145	Distal Intergenic	ENSSSCG000	MSH2	mutS homolog 2 [Source:NCBI gene (formerly Entrezgene);Acc:100337661]
chr2	78763343	78764182	840	*	chr2	78763343	78764182	in_vitro	in_vivo	9	9	4.98111111	3.49444444	1.48666667	c(t = 0.65314	0.523	0.88395145	Distal Intergenic	ENSSSCG000	CANX	calnexin [Source:VGNC Symbol;Acc:VGNC:86167]
chr7	3050184	3052610	2427	*	chr7	3050184	3052610	in_vitro	in_vivo	16	13	3.616875	2.49230769	1.12456731	c(t = 0.65058	0.523	0.88395145	Distal Intergenic	ENSSSCG000	ECI2	enoyl-CoA delta isomerase 2 [Source:VGNC Symbol;Acc:VGNC:87532]
chr9	104910297	104912327	2031	*	chr9	104910297	104912327	in_vitro	in_vivo	14	11	3.27285714	1.82636364	1.44649351	c(t = 0.65221	0.523	0.88395145	Distal Intergenic	ENSSSCG000	NAMPT	nicotinamide phosphoribosyltransferase [Source:VGNC Symbol;Acc:VGNC:90573]
chr6	84546751	84547362	612	*	chr6	84546751	84547362	in_vitro	in_vivo	11	11	1.81454545	0.97	0.84454545	c(t = 0.65519	0.523	0.88395145	Distal Intergenic	ENSSSCG000	WDTC1	WD and tetratricopeptide repeats 1 [Source:VGNC Symbol;Acc:VGNC:94952]
chr2	106845623	106846322	700	*	chr2	106845623	106846322	in_vitro	in_vivo	9	10	0.63	0.278	0.352	c(t = 0.65519	0.523	0.88395145	Promoter (<=1kb)	ENSSSCG000	ST8SIA4	ST8 alpha-N-acetyl-neuraminide alpha-2,8-sialyltransferase 4 [Source:NCBI gene (formerly Entrezgene);Acc:641356]
chr12	1481132	1495350	14219	*	chr12	1481132	1495350	in_vitro	in_vivo	11	15	76.6554545	71.4406667	5.21478788	c(t = 0.64633	0.524	0.88395145	Distal Intergenic	ENSSSCG000	TEPSIN	TEPSIN adaptor related protein complex 4 accessory protein [Source:NCBI gene (formerly Entrezgene);Acc:100626379]
chr3	126463389	126464544	1156	*	chr3	126463389	126464544	in_vitro	in_vivo	9	10	16.7633333	24.229	-7.4656667	c(t = -0.65397	0.524	0.88395145	Distal Intergenic	ENSSSCG000	KLF11	KLF transcription factor 11 [Source:NCBI gene (formerly Entrezgene);Acc:100174954]
chr7	936081	938202	2122	*	chr7	936081	938202	in_vitro	in_vivo	11	9	28.0354545	19.7633333	8.27212121	c(t = 0.65051	0.524	0.88395145	Distal Intergenic	ENSSSCG000	SERPINB9	serpin peptidase inhibitor, clade B (ovalbumin), member 9 [Source:NCBI gene (formerly Entrezgene);Acc:100126851]
chr7	100448913	100449594	682	*	chr7	100448913	100449594	in_vitro	in_vivo	9	11	1.87111111	2.67	-0.7988889	c(t = -0.65098	0.524	0.88395145	Distal Intergenic	ENSSSCG000	GSTZ1	glutathione S-transferase zeta 1 [Source:VGNC Symbol;Acc:VGNC:88727]
chr18	44488517	44489743	1227	*	chr18	44488517	44489743	in_vitro	in_vivo	11	10	3.76909091	2.494	1.27509091	c(t = 0.65225	0.524	0.88395145	Promoter (<=1kb)	ENSSSCG000	JAZF1	JAZF zinc finger 1 [Source:VGNC Symbol;Acc:VGNC:89280]
chr12	48762736	48763600	865	*	chr12	48762736	48763600	in_vitro	in_vivo	9	11	1.47	2.32	-0.85	c(t = -0.65151	0.524	0.88395145	Distal Intergenic	ENSSSCG000	PAFAH1B1	platelet activating factor acetylhydrolase 1b regulatory subunit 1 [Source:VGNC Symbol;Acc:VGNC:99026]
chr12	58125027	58129731	4705	*	chr12	58125027	58129731	in_vitro	in_vivo	12	15	1.41916667	2.17866667	-0.7595	c(t = -0.64624	0.524	0.88395145	Distal Intergenic	ENSSSCG000	COX10	cytochrome c oxidase assembly factor heme A:farnesyltransferase COX10 [Source:NCBI gene (formerly Entrezgene);Acc:100521863]
chr3	24228266	24228863	598	*	chr3	24228266	24228863	in_vitro	in_vivo	9	11	2.40333333	1.16090909	1.24242424	c(t = 0.66024	0.524	0.88395145	Distal Intergenic	ENSSSCG000	METTL9	methyltransferase like 9 [Source:VGNC Symbol;Acc:VGNC:103984]
chr2	38753823	38754955	1133	*	chr2	38753823	38754955	in_vitro	in_vivo	10	12	2.198	1.0225	1.1755	c(t = 0.65311	0.524	0.88395145	Distal Intergenic	ENSSSCG000	CSRP3	cysteine and glycine rich protein 3 [Source:NCBI gene (formerly Entrezgene);Acc:100337687]
chr4	61965698	61966186	489	*	chr4	61965698	61966186	in_vitro	in_vivo	9	9	0.61777778	0.28888889	0.32888889	c(t = 0.65304	0.524	0.88395145	Distal Intergenic	ENSSSCG000	LY96	lymphocyte antigen 96 [Source:VGNC Symbol;Acc:VGNC:89907]
chr2	66153750	66154740	991	*	chr2	66153750	66154740	in_vitro	in_vivo	11	9	12.4081818	19.9344444	-7.5262626	c(t = -0.65667	0.525	0.88395145	Distal Intergenic	ENSSSCG000	DNASE2	deoxyribonuclease 2, lysosomal [Source:NCBI gene (formerly Entrezgene);Acc:397398]
chr12	26745423	26746618	1196	*	chr12	26745423	26746618	in_vitro	in_vivo	9	12	12.6311111	18.7775	-6.1463889	c(t = -0.65290	0.525	0.88395145	Distal Intergenic	ENSSSCG000	LRRC59	leucine rich repeat containing 59 [Source:VGNC Symbol;Acc:VGNC:98082]
chr7	24203010	24203829	820	*	chr7	24203010	24203829	in_vitro	in_vivo	10	11	6.79	9.50181818	-2.7118182	c(t = -0.64700	0.525	0.88395145	Promoter (1-2kb)	ENSSSCG000	EGFL8	EGF like domain multiple 8 [Source:VGNC Symbol;Acc:VGNC:87585]
chr6	166543412	166543892	481	*	chr6	166543412	166543892	in_vitro	in_vivo	10	9	3.52	5.73444444	-2.2144444	c(t = -0.65094	0.525	0.88395145	Distal Intergenic	ENSSSCG000	DYNLT4	dynein light chain Tctex-type 4 [Source:NCBI gene (formerly Entrezgene);Acc:606747]
chr7	36661785	36662446	662	*	chr7	36661785	36662446	in_vitro	in_vivo	12	10	8.6825	4.489	4.1935	c(t = 0.65428	0.525	0.88395145	Distal Intergenic	ENSSSCG000	TREM1	triggering receptor expressed on myeloid cells 1 [Source:NCBI gene (formerly Entrezgene);Acc:396601]
chr10	19584279	19584992	714	*	chr10	19584279	19584992	in_vitro	in_vivo	9	9	5.48222222	3.02555556	2.45666667	c(t = 0.65588	0.525	0.88395145	Distal Intergenic	ENSSSCG000	CAPN2	calpain 2 [Source:NCBI gene (formerly Entrezgene);Acc:397393]
chr13	14042072	14043344	1273	*	chr13	14042072	14043344	in_vitro	in_vivo	12	12	1.63333333	1.08	0.55333333	c(t = 0.64641	0.525	0.88395145	Distal Intergenic	ENSSSCG000	TOP2B	DNA topoisomerase II beta [Source:NCBI gene (formerly Entrezgene);Acc:100156319]
chr3	21055224	21056845	1622	*	chr3	21055224	21056845	in_vitro	in_vivo	13	11	0.53692308	0.86636364	-0.3294406	c(t = -0.64904	0.525	0.88395145	Distal Intergenic	ENSSSCG000	AQP8	aquaporin 8 [Source:NCBI gene (formerly Entrezgene);Acc:100127152]
chr9	60954177	60954867	691	*	chr9	60954177	60954867	in_vitro	in_vivo	9	9	1.34888889	0.70444444	0.64444444	c(t = 0.65798	0.525	0.88395145	Distal Intergenic	ENSSSCG000	ACAD8	acyl-CoA dehydrogenase family member 8 [Source:NCBI gene (formerly Entrezgene);Acc:100627341]
chr14	34529347	34530281	935	*	chr14	34529347	34530281	in_vitro	in_vivo	12	15	1.03583333	0.664	0.37183333	c(t = 0.64632	0.525	0.88395145	Distal Intergenic	ENSSSCG000	SUDS3	SDS3 homolog, SIN3A corepressor complex component [Source:VGNC Symbol;Acc:VGNC:93607]
chr8	1962604	1963894	1291	*	chr8	1962604	1963894	in_vitro	in_vivo	11	11	2.64888889	3.74181818	-1.0929293	c(t = -0.64887	0.526	0.88395145	Distal Intergenic	ENSSSCG000	HTT	huntingtin [Source:VGNC Symbol;Acc:VGNC:89006]
chr3	54172419	54174055	1637	*	chr3	54172419	54174055	in_vitro	in_vivo	12	11	4.88833333	3.54272727	1.34560606	c(t = 0.64499	0.526	0.88395145	Distal Intergenic	ENSSSCG000	MGAT4A	alpha-1,3-mannosyl-glycoprotein 4-beta-N-acetylglucosaminyltransferase A [Source:VGNC Symbol;Acc:VGNC:108158]
chr7	41282330	41283115	786	*	chr7	41282330	41283115	in_vitro	in_vivo	9	9	2.55181818	3.52222222	-0.970404	c(t = -0.64985	0.526	0.88395145	Distal Intergenic	ENSSSCG000	CYP39A1	cytochrome P450 family 39 subfamily A member 1 [Source:NCBI gene (formerly Entrezgene);Acc:100113410]
chr4	44599383	44601148	1766	*	chr4	44599383	44601148	in_vitro	in_vivo	12	13	1.21833333	1.91461538	-0.6962821	c(t = -0.64551	0.526	0.88395145	Distal Intergenic	ENSSSCG000	CALB1	calbindin 1 [Source:NCBI gene (formerly Entrezgene);Acc:100153645]
chr7	120754580	120755756	1177	*	chr7	120754580	120755756	in_vitro	in_vivo	10	12	2.213	1.605	0.608	c(t = 0.64645	0.526	0.88395145	Distal Intergenic	ENSSSCG000	EVL	Enah/Vasp-like [Source:VGNC Symbol;Acc:VGNC:87820]
chr1	165436626	165437500	875	*	chr1	165436626	165437500	in_vitro	in_vivo	12	11	0.80333333	1.49636364	-0.6930303	c(t = -0.64652	0.526	0.88395145	Distal Intergenic	ENSSSCG000	SMAD3	SMAD family member 3 [Source:VGNC Symbol;Acc:VGNC:93217]
chr1	163857885	163858439	555	*	chr1	163857885	163858439	in_vitro	in_vivo	9	9	2.07333333	1.13777778	0.93555556	c(t = 0.64991	0.526	0.88395145	Promoter (<=1kb)	ENSSSCG000	RAB11A	RAB11A, member RAS oncogene family [Source:NCBI gene (formerly Entrezgene);Acc:595117]
chr3	49594640	49595315	676	*	chr3	49594640	49595315	in_vitro	in_vivo	9	11	1.89	1.13181818	0.75818182	c(t = 0.64684	0.526	0.88395145	Distal Intergenic	ENSSSCG000	MRPS9	mitochondrial ribosomal protein S9 [Source:VGNC Symbol;Acc:VGNC:90401]
chr10	19758509	19759380	872	*	chr10	19758509	19759380	in_vitro	in_vivo	9	14	1.39333333	0.95571429	0.43761905	c(t = 0.64927	0.526	0.88395145	Promoter (<=1kb)	ENSSSCG000	CAPN2	calpain 2 [Source:NCBI gene (formerly Entrezgene);Acc:397393]
chr13	123780185	123781190	1006	*	chr13	123780185	123781190	in_vitro	in_vivo	11	13	0.45454545	0.88615385	-0.4316084	c(t = -0.64462	0.526	0.88395145	Promoter (<=1kb)	ENSSSCG000	TRA2B	transformer 2 beta homolog [Source:VGNC Symbol;Acc:VGNC:94354]
chr1	16381110	16382007	898	*	chr1	16381110	16382007	in_vitro	in_vivo	9	9	0.85555556	0.42777778	0.42777778	c(t = 0.64896	0.526	0.88395145	Distal Intergenic	ENSSSCG000	PCMT1	protein-L-isoaspartate (D-aspartate) O-methyltransferase [Source:VGNC Symbol;Acc:VGNC:103146]
chr5	51927287	51927783	497	*	chr5	51927287	51927783	in_vitro	in_vivo	10	10	0.578	0.303	0.275	c(t = 0.64640	0.526	0.88395145	Distal Intergenic	ENSSSCG000	GOLT1B	golgi transport 1B [Source:NCBI gene (formerly Entrezgene);Acc:100154676]
chr1	146260568	146261762	1195	*	chr1	146260568	146261762	in_vitro	in_vivo	13	9	40.9946154	30.6388889	10.3557265	c(t = 0.64458	0.527	0.88427838	Distal Intergenic	ENSSSCG000	NFATC1	nuclear factor of activated T cells 1 [Source:NCBI gene (formerly Entrezgene);Acc:397318]
chr10	66532856	66534574	1719	*	chr10	66532856	66534574	in_vitro	in_vivo	11	12	33.9418182	23.16	10.7818182	c(t = 0.64398	0.527	0.88427838	Distal Intergenic	ENSSSCG000	KLF6	KLF transcription factor 6 [Source:NCBI gene (formerly Entrezgene);Acc:100174961]
chr9	65351646	65352805	1160	*	chr9	65351646	65352805	in_vitro	in_vivo	11	11	2.23909091	4.30363636	-2.0645455	c(t = -0.64497	0.527	0.88427838	Distal Intergenic	NA	NA	NA
chr15	75851978	75852553	576	*	chr15	75851978	75852553	in_vitro	in_vivo	10	11	2.164	1.32363636	0.84036364	c(t = 0.64753	0.527	0.88427838	Distal Intergenic	ENSSSCG000	PHOSPHO2	phosphatase, orphan 2 [Source:VGNC Symbol;Acc:VGNC:96172]
chr13	82679999	82681422	1424	*	chr13	82679999	82681422	in_vitro	in_vivo	9	13	1.56666667	0.96846154	0.59820513	c(t = 0.64935	0.527	0.88427838	Distal Intergenic	ENSSSCG000	GRK7	G protein-coupled receptor kinase 7 [Source:VGNC Symbol;Acc:VGNC:88699]
chr3	27492145	27492611	467	*	chr3	27492145	27492611	in_vitro	in_vivo	9	9	1.63111111	0.92555556	0.70555556	c(t = 0.64893	0.527	0.88427838	Distal Intergenic	ENSSSCG000	SMG1	SMG1 nonsense mediated mRNA decay associated PI3K related kinase [Source:VGNC Symbol;Acc:VGNC:93248]
chr11	57792515	57794442	1928	*	chr11	57792515	57794442	in_vitro	in_vivo	11	14	1.28909091	0.79928571	0.48980519	c(t = 0.64629	0.527	0.88427838	Distal Intergenic	ENSSSCG000	SLITRK1	SLIT and NTRK like family member 1 [Source:NCBI gene (formerly Entrezgene);Acc:100152470]
chr4	36779022	36779931	910	*	chr4	36779022	36779931	in_vitro	in_vivo	10	10	64.495	50.793	13.702	c(t = 0.64443	0.528	0.88460432	Distal Intergenic	ENSSSCG000	RNF19A	ring finger protein 19A, RBR E3 ubiquitin protein ligase [Source:VGNC Symbol;Acc:VGNC:92375]
chr2	729859	730616	758	*	chr2	729859	730616	in_vitro	in_vivo	10	9	13.89	23.6655556	-9.7755556	c(t = -0.64710	0.528	0.88460432	Distal Intergenic	ENSSSCG000	TOLLIP	toll interacting protein [Source:NCBI gene (formerly Entrezgene);Acc:100625123]
chr11	77868980	77871272	2293	*	chr11	77868980	77871272	in_vitro	in_vivo	12	13	16.3125	11.0938462	5.21865385	c(t = 0.64552	0.528	0.88460432	Distal Intergenic	ENSSSCG000	ANKRD10	ankyrin repeat domain 10 [Source:VGNC Symbol;Acc:VGNC:85322]
chr9	41977865	41978425	561	*	chr9	41977865	41978425	in_vitro	in_vivo	9	9	1.56888889	3.06888889	-1.5	c(t = -0.65440	0.528	0.88460432	Distal Intergenic	ENSSSCG000	NNMT	nicotinamide N-methyltransferase [Source:NCBI gene (formerly Entrezgene);Acc:100144485]
chr14	30468900	30469394	495	*	chr14	30468900	30469394	in_vitro	in_vivo	9	11	3.11444444	1.25	1.86444444	c(t = 0.65654	0.528	0.88460432	Promoter (<=1kb)	ENSSSCG000	VPS33A	VPS33A core subunit of CORVET and HOPS complexes [Source:VGNC Symbol;Acc:VGNC:110225]
chr14	115064521	115065266	746	*	chr14	115064521	115065266	in_vitro	in_vivo	10	9	1.628	0.90444444	0.72355556	c(t = 0.64808	0.528	0.88460432	Distal Intergenic	ENSSSCG000	GSTO1	glutathione S-transferase omega 1 [Source:NCBI gene (formerly Entrezgene);Acc:397117]
chr18	55842956	55843480	525	*	chr18	55842956	55843480	in_vitro	in_vivo	11	12	1.10090909	0.615	0.48590909	c(t = 0.64293	0.528	0.88460432	Distal Intergenic	ENSSSCG000	RARRES2	retinoic acid receptor responder 2 [Source:VGNC Symbol;Acc:VGNC:92098]
chr7	97123873	97124625	753	*	chr7	97123873	97124625	in_vitro	in_vivo	12	12	1.78416667	3.3525	-1.5683333	c(t = -0.64791	0.529	0.88531468	Distal Intergenic	ENSSSCG000	LIN52	lin-52 DREAM MuvB core complex component [Source:NCBI gene (formerly Entrezgene);Acc:100157325]
chr17	13821361	13822698	1338	*	chr17	13821361	13822698	in_vitro	in_vivo	12	9	2.25	3.28444444	-1.0344444	c(t = -0.64509	0.529	0.88531468	Distal Intergenic	ENSSSCG000	PRNP	prion protein [Source:NCBI gene (formerly Entrezgene);Acc:494014]
chr18	18819539	18820033	495	*	chr18	18819539	18820033	in_vitro	in_vivo	10	9	1.002	2.29666667	-1.2946667	c(t = -0.65157	0.529	0.88531468	Distal Intergenic	ENSSSCG000	ssc-mir-183	ssc-mir-183 [Source:miRBase;Acc:MI0002439]
chr2	131369532	131371871	2340	*	chr2	131369532	131371871	in_vitro	in_vivo	14	15	1.90214286	1.472	0.43014286	c(t = 0.63816	0.529	0.88531468	Distal Intergenic	ENSSSCG000	MARCHF3	membrane associated ring-CH-type finger 3 [Source:NCBI gene (formerly Entrezgene);Acc:100513526]
chr4	63709823	63710668	846	*	chr4	63709823	63710668	in_vitro	in_vivo	11	10	0.66545455	1.11	-0.4445455	c(t = -0.64535	0.529	0.88531468	Distal Intergenic	ENSSSCG000	LACTB2	lactamase beta 2 [Source:NCBI gene (formerly Entrezgene);Acc:100153212]
chr2	100433489	100434512	1024	*	chr2	100433489	100434512	in_vitro	in_vivo	10	12	10.736	7.5275	3.2085	c(t = 0.63850	0.53	0.88563818	Distal Intergenic	ENSSSCG000	SPATA9	spermatogenesis associated 9 [Source:NCBI gene (formerly Entrezgene);Acc:100415927]
chr17	60959084	60960870	1787	*	chr17	60959084	60960870	in_vitro	in_vivo	10	14	10.05	5.57142857	4.47857143	c(t = 0.64210	0.53	0.88563818	Distal Intergenic	ENSSSCG000	PSMA7	proteasome 20S subunit alpha 7 [Source:NCBI gene (formerly Entrezgene);Acc:100626738]
chr14	56544114	56546981	2868	*	chr14	56544114	56546981	in_vitro	in_vivo	15	15	6.672	4.374	2.298	c(t = 0.63887	0.53	0.88563818	Distal Intergenic	ENSSSCG000	EDARADD	EDAR associated death domain [Source:VGNC Symbol;Acc:VGNC:87542]
chr1	265308202	265309283	1082	*	chr1	265308202	265309283	in_vitro	in_vivo	9	9	3.87111111	2.64777778	1.22333333	c(t = 0.64491	0.53	0.88563818	Promoter (<=1kb)	ENSSSCG000	NR5A1	nuclear receptor subfamily 5 group A member 1 [Source:VGNC Symbol;Acc:VGNC:90886]
chr6	42335003	42336252	1250	*	chr6	42335003	42336252	in_vitro	in_vivo	10	11	1.118	1.79272727	-0.6747273	c(t = -0.64250	0.53	0.88563818	Distal Intergenic	ENSSSCG000	SLC7A9	solute carrier family 7 member 9 [Source:VGNC Symbol;Acc:VGNC:93177]
chr15	57293815	57294905	1091	*	chr15	57293815	57294905	in_vitro	in_vivo	12	16	1.50583333	0.8725	0.63333333	c(t = 0.64289	0.53	0.88563818	Distal Intergenic	ENSSSCG000	OCA2	OCA2 melanosomal transmembrane protein [Source:NCBI gene (formerly Entrezgene);Acc:397171]
chr9	125982302	125983364	1063	*	chr9	125982302	125983364	in_vitro	in_vivo	12	13	1.36	0.83307692	0.52692308	c(t = 0.64336	0.53	0.88563818	Distal Intergenic	ENSSSCG000	NIBAN1	niban apoptosis regulator 1 [Source:NCBI gene (formerly Entrezgene);Acc:100516994]
chr5	3426806	3428577	1772	*	chr5	3426806	3428577	in_vitro	in_vivo	9	13	17.33	23.68	-6.35	c(t = -0.63877	0.531	0.88653813	Distal Intergenic	ENSSSCG000	PPARA	peroxisome proliferator activated receptor alpha [Source:NCBI gene (formerly Entrezgene);Acc:397239]
chr12	6214528	6215144	617	*	chr12	6214528	6215144	in_vitro	in_vivo	10	12	10.042	16.0991667	-6.0571667	c(t = -0.63887	0.531	0.88653813	Distal Intergenic	ENSSSCG00000032166		ATP synthase peripheral stalk subunit d [Source:NCBI gene (formerly Entrezgene);Acc:100512624]
chr8	12689067	12689731	665	*	chr8	12689067	12689731	in_vitro	in_vivo	10	10	8.128	15.479	-7.351	c(t = -0.64151	0.531	0.88653813	Distal Intergenic	ENSSSCG000	MED28	mediator complex subunit 28 [Source:VGNC Symbol;Acc:VGNC:90118]
chr15	121193277	121194156	880	*	chr15	121193277	121194156	in_vitro	in_vivo	10	13	2.524	1.54636364	0.97763636	c(t = 0.64282	0.531	0.88653813	Distal Intergenic	ENSSSCG000	ATG9A	autophagy related 9A [Source:VGNC Symbol;Acc:VGNC:99703]
chr4	126962132	126964004	1873	*	chr4	126962132	126964004	in_vitro	in_vivo	10	13	97.465	96.4192308	1.04576923	c(t = 0.63519	0.532	0.886859	Distal Intergenic	ENSSSCG000	GBP2	guanylate binding protein 2, interferon-inducible [Source:NCBI gene (formerly Entrezgene);Acc:100153137]
chr2	151044454	151046552	2099	*	chr2	151044454	151046552	in_vitro	in_vivo	10	9	94.234	92.4455556	1.78844444	c(t = 0.63750	0.532	0.886859	Distal Intergenic	ENSSSCG000	ssc-mir-378-1	ssc-mir-378-1 [Source:miRBase;Acc:MI0013088]
chr11	78793520	78796717	3198	*	chr11	78793520	78796717	in_vitro	in_vivo	11	13	20.8709091	30.1992308	-9.3283217	c(t = -0.63543	0.532	0.886859	Distal Intergenic	ENSSSCG000	ATP4B	ATPase H+/K+ transporting subunit beta [Source:VGNC Symbol;Acc:VGNC:85655]
chr9	56928283	56929677	1395	*	chr9	56928283	56929677	in_vitro	in_vivo	11	12	17.9472727	25.8033333	-7.8560606	c(t = -0.63513	0.532	0.886859	Distal Intergenic	ENSSSCG000	KCNJ5	potassium inwardly rectifying channel subfamily J member 5 [Source:NCBI gene (formerly Entrezgene);Acc:397448]
chr13	23844245	23845421	1177	*	chr13	23844245	23845421	in_vitro	in_vivo	9	11	14.4577778	9.54363636	4.91414141	c(t = 0.64147	0.532	0.886859	Distal Intergenic	ENSSSCG000	XIRP1	xin actin binding repeat containing 1 [Source:NCBI gene (formerly Entrezgene);Acc:100134978]
chr1	22801435	22802399	965	*	chr1	22801435	22802399	in_vitro	in_vivo	9	13	2.67666667	3.94076923	-1.2641026	c(t = -0.63652	0.532	0.886859	Distal Intergenic	ENSSSCG000	NMBR	neuromedin B receptor [Source:VGNC Symbol;Acc:VGNC:90789]
chr17	62813261	62816171	2911	*	chr17	62813261	62816171	in_vitro	in_vivo	10	14	2.532	1.53214286	0.99985714	c(t = 0.63506	0.532	0.886859	Distal Intergenic	ENSSSCG000	ssc-mir-1388	ssc-mir-1388 [Source:miRBase;Acc:MI0033392]
chr11	5241105	5241689	585	*	chr11	5241105	5241689	in_vitro	in_vivo	12	12	10.6716667	15.2275	-4.5558333	c(t = -0.63430	0.533	0.88756338	Distal Intergenic	ENSSSCG000	ssc-mir-9803	ssc-mir-9803 [Source:miRBase;Acc:MI0031567]
chr8	76107128	76108367	1240	*	chr8	76107128	76108367	in_vitro	in_vivo	10	12	5.292	8.5575	-3.2655	c(t = -0.63517	0.533	0.88756338	Distal Intergenic	ENSSSCG000	ARFIP1	ADP ribosylation factor interacting protein 1 [Source:VGNC Symbol;Acc:VGNC:85453]
chr9	47344910	47345844	935	*	chr9	47344910	47345844	in_vitro	in_vivo	9	16	1.15555556	1.859375	-0.7038194	c(t = -0.63319	0.533	0.88756338	Distal Intergenic	ENSSSCG000	NECTIN1	nectin cell adhesion molecule 1 [Source:VGNC Symbol;Acc:VGNC:108604]
chr4	19146030	19146830	801	*	chr4	19146030	19146830	in_vitro	in_vivo	10	11	0.659	1.27181818	-0.6128182	c(t = -0.63680	0.533	0.88756338	Distal Intergenic	ENSSSCG000	MAL2	mal, T cell differentiation protein 2 [Source:VGNC Symbol;Acc:VGNC:98818]
chr12	3986934	3989222	2289	*	chr12	3986934	3989222	in_vitro	in_vivo	14	14	1.72071429	1.14928571	0.57142857	c(t = 0.63688	0.533	0.88756338	Distal Intergenic	ENSSSCG000	BIRC5	baculoviral IAP repeat containing 5 [Source:VGNC Symbol;Acc:VGNC:85827]
chr8	32955787	32956849	1063	*	chr8	32955787	32956849	in_vitro	in_vivo	11	11	1.479	2.46727273	-0.9882727	c(t = -0.63408	0.534	0.88768981	Distal Intergenic	ENSSSCG000	TMEM33	transmembrane protein 33 [Source:VGNC Symbol;Acc:VGNC:94171]
chr1	179540449	179541146	698	*	chr1	179540449	179541146	in_vitro	in_vivo	9	11	5.74333333	2.39272727	3.35060606	c(t = 0.64579	0.534	0.88768981	Distal Intergenic	NA	NA	NA
chr6	56234640	56235154	515	*	chr6	56234640	56235154	in_vitro	in_vivo	10	10	0.838	2.179	-1.341	c(t = -0.64230	0.534	0.88768981	Distal Intergenic	ENSSSCG000	ssc-mir-935	ssc-mir-935 [Source:miRBase;Acc:MI0013156]
chr9	120809501	120810627	1127	*	chr9	120809501	120810627	in_vitro	in_vivo	10	9	1.323	2.17444444	-0.8514444	c(t = -0.63571	0.534	0.88768981	Distal Intergenic	ENSSSCG000	ANGPTL1	angiopoietin like 1 [Source:VGNC Symbol;Acc:VGNC:85303]
chr10	14102193	14102788	596	*	chr10	14102193	14102788	in_vitro	in_vivo	9	12	1.34	2.025	-0.685	c(t = -0.63509	0.534	0.88768981	Distal Intergenic	ENSSSCG000	ACBD3	acyl-CoA binding domain containing 3 [Source:NCBI gene (formerly Entrezgene);Acc:494461]
chr1	108923714	108924471	758	*	chr1	108923714	108924471	in_vitro	in_vivo	15	11	3.23533333	1.97545455	1.25987879	c(t = 0.63146	0.534	0.88768981	Promoter (<=1kb)	ENSSSCG000	LACTB	lactamase beta [Source:VGNC Symbol;Acc:VGNC:89614]
chr8	33636867	33637932	1066	*	chr8	33636867	33637932	in_vitro	in_vivo	10	12	1.4	0.74583333	0.65416667	c(t = 0.63967	0.534	0.88768981	Distal Intergenic	ENSSSCG000	SLC30A9	solute carrier family 30 member 9 [Source:NCBI gene (formerly Entrezgene);Acc:100192440]
chr6	96706842	96707578	737	*	chr6	96706842	96707578	in_vitro	in_vivo	10	13	0.402	0.23153846	0.17046154	c(t = 0.63328	0.534	0.88768981	Distal Intergenic	ENSSSCG000	CIDEA	cell death inducing DFFA like effector a [Source:NCBI gene (formerly Entrezgene);Acc:100127171]
chr8	78385939	78386948	1010	*	chr8	78385939	78386948	in_vitro	in_vivo	13	9	6.26923077	8.42230769	-2.1530769	c(t = -0.62967	0.535	0.88819119	Distal Intergenic	ENSSSCG000	RPS3A	ribosomal protein S3A [Source:NCBI gene (formerly Entrezgene);Acc:414390]
chr14	7664863	7665988	1126	*	chr14	7664863	7665988	in_vitro	in_vivo	13	15	3.12461538	4.982	-1.8573846	c(t = -0.63172	0.535	0.88819119	Distal Intergenic	ENSSSCG000	STC1	stanniocalcin 1 [Source:NCBI gene (formerly Entrezgene);Acc:100125345]
chr15	48053034	48054419	1386	*	chr15	48053034	48054419	in_vitro	in_vivo	13	12	2.68846154	1.79666667	0.89179487	c(t = 0.63237	0.535	0.88819119	Distal Intergenic	ENSSSCG000	STAR	steroidogenic acute regulatory protein [Source:NCBI gene (formerly Entrezgene);Acc:396597]
chr3	8737293	8739730	2438	*	chr3	8737293	8739730	in_vitro	in_vivo	13	11	0.92	1.48181818	-0.5618182	c(t = -0.63811	0.535	0.88819119	Distal Intergenic	ENSSSCG000	ZAN	zonadhesin [Source:NCBI gene (formerly Entrezgene);Acc:397676]
chr2	51265727	51266890	1164	*	chr2	51265727	51266890	in_vitro	in_vivo	9	10	16.6655556	22.029	-5.3634444	c(t = -0.63574	0.536	0.88819119	Distal Intergenic	NA	NA	NA
chr2	16554423	16555522	1100	*	chr2	16554423	16555522	in_vitro	in_vivo	9	9	9.11555556	6.74444444	2.37111111	c(t = 0.63192	0.536	0.88819119	Distal Intergenic	ENSSSCG000	SLC35C1	solute carrier family 35 member C1 [Source:VGNC Symbol;Acc:VGNC:93075]
chr13	53151371	53153073	1703	*	chr13	53151371	53153073	in_vitro	in_vivo	13	15	3.00153846	3.844	-0.8424615	c(t = -0.62833	0.536	0.88819119	Distal Intergenic	ENSSSCG000	MITF	melanocyte inducing transcription factor [Source:VGNC Symbol;Acc:VGNC:90243]
chr18	43181856	43182754	899	*	chr18	43181856	43182754	in_vitro	in_vivo	11	12	5.8	3.605	2.195	c(t = 0.63469	0.536	0.88819119	Distal Intergenic	ENSSSCG000	FKBP14	FKBP prolyl isomerase 14 [Source:NCBI gene (formerly Entrezgene);Acc:100514551]
chr12	4404992	4406382	1391	*	chr12	4404992	4406382	in_vitro	in_vivo	9	10	1.17	2.236	-1.066	c(t = -0.63283	0.536	0.88819119	Distal Intergenic	ENSSSCG000	ssc-mir-6516	ssc-mir-6516 [Source:miRBase;Acc:MI0033411]
chr2	69336666	69337349	684	*	chr2	69336666	69337349	in_vitro	in_vivo	12	10	3.265	2.2125	1.0525	c(t = 0.63249	0.536	0.88819119	Distal Intergenic	ENSSSCG000	ATG4D	autophagy related 4D cysteine peptidase [Source:VGNC Symbol;Acc:VGNC:85624]
chr15	109695084	109695881	798	*	chr15	109695084	109695881	in_vitro	in_vivo	11	10	2.63818182	1.703	0.93518182	c(t = 0.63212	0.536	0.88819119	Distal Intergenic	ENSSSCG000	CMKLR2	chemerin chemokine-like receptor 2 [Source:VGNC Symbol;Acc:VGNC:96331]
chr1	119948447	119948891	445	*	chr1	119948447	119948891	in_vitro	in_vivo	13	10	0.94692308	1.629	-0.6820769	c(t = -0.63102	0.536	0.88819119	Promoter (<=1kb)	ENSSSCG000	TMOD3	tropomodulin 3 [Source:NCBI gene (formerly Entrezgene);Acc:397556]

chr8	9182044	9183480	1437	*	chr8	9182044	9183480	in_vitro	in_vivo	10	9	1.198	0.64555556	0.55244444	c(t = 0.63926	0.536	0.88819119	Distal Intergenic	ENSSSCG000	RAB28	RAB28, member RAS onco family [Source:VGNC Symbol;Acc:VGNC:98955]
chr6	70087359	70088739	1381	*	chr6	70087359	70088739	in_vitro	in_vivo	10	9	92.246	94.23	-1.984	c(t = -0.6305	0.537	0.88819119	Distal Intergenic	ENSSSCG000	RBP7	retinol binding protein 7 [Source:NCBI gene (formerly Entrezgene);Acc:100270723]
chr4	35563031	35564026	996	*	chr4	35563031	35564026	in_vitro	in_vivo	10	10	12.448	16.708	-4.26	c(t = -0.6302	0.537	0.88819119	Distal Intergenic	ENSSSCG000	NCALD	neurocalcin delta [Source:VGNC Symbol;Acc:VGNC:90594]
chr3	17613617	17614750	1134	*	chr3	17613617	17614750	in_vitro	in_vivo	10	13	24.674	15.25	9.424	c(t = 0.63140	0.537	0.88819119	Distal Intergenic	ENSSSCG000	PHKG2	phosphorylase kinase catalytic subunit gamma 2 [Source:VGNC Symbol;Acc:VGNC:91395]
chr5	15152611	15153926	1316	*	chr5	15152611	15153926	in_vitro	in_vivo	10	10	7.551	13.979	-6.428	c(t = -0.6296	0.537	0.88819119	Promoter (<=1kb)	ENSSSCG000	TUBA1B	tubulin alpha 1b [Source:NCBI gene (formerly Entrezgene);Acc:733594]
chr3	39807250	39810186	2937	*	chr3	39807250	39810186	in_vitro	in_vivo	9	12	5.63111111	8.0675	-2.4363889	c(t = -0.6301	0.537	0.88819119	Distal Intergenic	ENSSSCG000	ssc-mir-1842	ssc-mir-1842 [Source:miRBase;Acc:MI0033396]
chr1	9619043	9621873	2831	*	chr1	9619043	9621873	in_vitro	in_vivo	12	15	2.14916667	1.59733333	0.55183333	c(t = 0.62667	0.537	0.88819119	Distal Intergenic	NA	NA	NA
chr1	215507051	215508170	1120	*	chr1	215507051	215508170	in_vitro	in_vivo	10	10	0.687	1.536	-0.849	c(t = -0.6370	0.537	0.88819119	Distal Intergenic	ENSSSCG000	IL33	interleukin 33 [Source:VGNC Symbol;Acc:VGNC:89106]
chr8	84600237	84601280	1044	*	chr8	84600237	84601280	in_vitro	in_vivo	11	13	1.83909091	1.23230769	0.60678322	c(t = 0.62685	0.537	0.88819119	Distal Intergenic	ENSSSCG000	IL15	interleukin 15 [Source:VGNC Symbol;Acc:VGNC:89083]
chr18	40397513	40398252	740	*	chr18	40397513	40398252	in_vitro	in_vivo	12	13	1.46166667	1.04923077	0.4124359	c(t = 0.62685	0.537	0.88819119	Distal Intergenic	ENSSSCG000	FKBP9	FKBP prolyl isomerase 9 [Source:NCBI gene (formerly Entrezgene);Acc:100623924]
chrX	102641909	102643008	1100	*	chrX	102641909	102643008	in_vitro	in_vivo	9	12	5.27777778	9.08166667	-3.8038889	c(t = -0.6282	0.538	0.88819119	Distal Intergenic	ENSSSCG000	ssc-mir-2483	ssc-mir-2483 [Source:miRBase;Acc:MI0022167]
chr6	59634507	59636452	1946	*	chr6	59634507	59636452	in_vitro	in_vivo	14	12	6.21714286	4.59416667	1.62297619	c(t = 0.62479	0.538	0.88819119	Distal Intergenic	ENSSSCG000	U2AF2	U2 small nuclear RNA auxiliary factor 2 [Source:VGNC Symbol;Acc:VGNC:94623]
chr10	58094641	58098606	3966	*	chr10	58094641	58098606	in_vitro	in_vivo	16	14	3.66625	2.65142857	1.01482143	c(t = 0.62342	0.538	0.88819119	Distal Intergenic	ENSSSCG000	CCNY	cyclin Y [Source:VGNC Symbol;Acc:VGNC:95998]
chr15	44614625	44616544	1920	*	chr15	44614625	44616544	in_vitro	in_vivo	12	11	3.6325	2.52454545	1.10795455	c(t = 0.62820	0.538	0.88819119	Distal Intergenic	ENSSSCG000	CLDN22	claudin 22 [Source:NCBI gene (formerly Entrezgene);Acc:100294683]
chr11	7186906	7187595	690	*	chr11	7186906	7187595	in_vitro	in_vivo	12	10	0.9625	1.904	-0.9415	c(t = -0.6349	0.538	0.88819119	Distal Intergenic	ENSSSCG00000009327		high mobility group box 1 [Source:NCBI gene (formerly Entrezgene);Acc:445521]
chr1	50781392	50782140	749	*	chr1	50781392	50782140	in_vitro	in_vivo	13	9	2.40307692	1.69444444	0.70863248	c(t = 0.62728	0.538	0.88819119	Distal Intergenic	ENSSSCG000	ssc-mir-30c-2	ssc-mir-30c-2 [Source:miRBase;Acc:MI0002461]
chr2	112815113	112817489	2377	*	chr2	112815113	112817489	in_vitro	in_vivo	13	16	2.45692308	1.634375	0.82254808	c(t = 0.62765	0.538	0.88819119	Distal Intergenic	ENSSSCG000	STARD4	StAR related lipid transfer domain containing 4 [Source:VGNC Symbol;Acc:VGNC:93534]
chr14	124734796	124736117	1322	*	chr14	124734796	124736117	in_vitro	in_vivo	11	12	1.88181818	1.39166667	0.49015152	c(t = 0.62784	0.538	0.88819119	Distal Intergenic	ENSSSCG000	ADRB1	adrenoceptor beta 1 [Source:NCBI gene (formerly Entrezgene);Acc:397355]
chr14	126497670	126500377	2708	*	chr14	126497670	126500377	in_vitro	in_vivo	14	15	1.92285714	1.38866667	0.53419048	c(t = 0.62543	0.538	0.88819119	Distal Intergenic	ENSSSCG000	PNLIP	pancreatic lipase [Source:NCBI gene (formerly Entrezgene);Acc:100157809]
chr16	20022909	20023380	472	*	chr16	20022909	20023380	in_vitro	in_vivo	9	11	1.66222222	0.92454545	0.73767677	c(t = 0.62860	0.538	0.88819119	Distal Intergenic	ENSSSCG000	C1QTNF3	C1q and TNF related 3 [Source:NCBI gene (formerly Entrezgene);Acc:100270814]
chr1	239726870	239729114	2245	*	chr1	239726870	239729114	in_vitro	in_vivo	10	13	3.179	4.71153846	-1.5325385	c(t = -0.6267	0.539	0.88824914	Distal Intergenic	ENSSSCG000	TRMO	tRNA methyltransferase O [Source:NCBI gene (formerly Entrezgene);Acc:100156700]
chr6	39984449	39985277	829	*	chr6	39984449	39985277	in_vitro	in_vivo	9	9	5.92111111	3.02444444	2.89666667	c(t = 0.63570	0.539	0.88824914	Distal Intergenic	ENSSSCG000	SLC7A9	solute carrier family 7 member 9 [Source:VGNC Symbol;Acc:VGNC:93177]
chr10	51818738	51820749	2012	*	chr10	51818738	51820749	in_vitro	in_vivo	10	14	2.698	1.675	1.023	c(t = 0.63144	0.539	0.88824914	Distal Intergenic	ENSSSCG000	PIP4K2A	phosphatidylinositol-5-phosphate 4-kinase type 2 alpha [Source:VGNC Symbol;Acc:VGNC:96485]
chr8	4030302	4031725	1424	*	chr8	4030302	4031725	in_vitro	in_vivo	9	11	0.94888889	1.48272727	-0.5338384	c(t = -0.6281	0.539	0.88824914	Distal Intergenic	ENSSSCG000	MAN2B2	mannosidase alpha class 2B member 2 [Source:VGNC Symbol;Acc:VGNC:98100]
chr2	131856735	131857792	1058	*	chr2	131856735	131857792	in_vitro	in_vivo	11	13	1.67272727	1.02307692	0.64965035	c(t = 0.62776	0.539	0.88824914	Distal Intergenic	ENSSSCG000	MARCHF3	membrane associated ring-CH-type finger 3 [Source:NCBI gene (formerly Entrezgene);Acc:100513526]
chr3	1426325	1428379	2055	*	chr3	1426325	1428379	in_vitro	in_vivo	9	11	95.2944444	93.5163636	1.77808081	c(t = 0.62489	0.54	0.88824914	Distal Intergenic	ENSSSCG000	MRM2	mitochondrial rRNA methyltransferase 2 [Source:VGNC Symbol;Acc:VGNC:99722]
chr2	6070886	6071950	1065	*	chr2	6070886	6071950	in_vitro	in_vivo	9	10	19.93	27.909	-7.979	c(t = -0.6262	0.54	0.88824914	Distal Intergenic	ENSSSCG000	B4GAT1	beta-1,4-glucuronyltransferase 1 [Source:NCBI gene (formerly Entrezgene);Acc:100518412]
chr6	68949569	68950421	853	*	chr6	68949569	68950421	in_vitro	in_vivo	11	11	21.3363636	14.9636364	6.37272727	c(t = 0.62376	0.54	0.88824914	Distal Intergenic	ENSSSCG000	PARK7	Parkinsonism associated deglycase [Source:NCBI gene (formerly Entrezgene);Acc:780404]
chr11	4003012	4003919	908	*	chr11	4003012	4003919	in_vitro	in_vivo	11	13	3.71545455	7.81307692	-4.0976224	c(t = -0.6270	0.54	0.88824914	Distal Intergenic	ENSSSCG000	GPR12	G protein-coupled receptor 12 [Source:NCBI gene (formerly Entrezgene);Acc:100736641]
chr3	17621301	17621764	464	*	chr3	17621301	17621764	in_vitro	in_vivo	10	12	7.832	5.645	2.187	c(t = 0.62454	0.54	0.88824914	Distal Intergenic	ENSSSCG000	PHKG2	phosphorylase kinase catalytic subunit gamma 2 [Source:VGNC Symbol;Acc:VGNC:91395]
chr15	36240244	36243876	3633	*	chr15	36240244	36243876	in_vitro	in_vivo	13	15	5.37076923	4.128	1.24276923	c(t = 0.62147	0.54	0.88824914	Distal Intergenic	ENSSSCG000	ANGPT2	angiopoietin 2 [Source:NCBI gene (formerly Entrezgene);Acc:396730]
chr1	224680126	224680479	354	*	chr1	224680126	224680479	in_vitro	in_vivo	9	12	1.79	2.88	-1.09	c(t = -0.6265	0.54	0.88824914	Distal Intergenic	ENSSSCG000	ABHD17B	abhydrolase domain containing 17B, depalmitoylase [Source:VGNC Symbol;Acc:VGNC:84977]
chr2	88729306	88729896	591	*	chr2	88729306	88729896	in_vitro	in_vivo	10	12	1.208	1.74333333	-0.5353333	c(t = -0.6245	0.54	0.88824914	Distal Intergenic	ENSSSCG000	CMYA5	cardiomyopathy associated 5 [Source:VGNC Symbol;Acc:VGNC:86809]
chr6	158536200	158538129	1930	*	chr6	158536200	158538129	in_vitro	in_vivo	13	14	2.14692308	1.48714286	0.65978022	c(t = 0.62214	0.54	0.88824914	Distal Intergenic	ENSSSCG000	DIO1	iodothyronine deiodinase 1 [Source:NCBI gene (formerly Entrezgene);Acc:414380]
chr17	32943516	32945215	1700	*	chr17	32943516	32945215	in_vitro	in_vivo	10	10	2.141	1.377	0.764	c(t = 0.62857	0.54	0.88824914	Distal Intergenic	ENSSSCG000	IDH3B	isocitrate dehydrogenase (NAD(+)) 3 non-catalytic subunit beta [Source:VGNC Symbol;Acc:VGNC:108724]
chr6	13889391	13889910	520	*	chr6	13889391	13889910	in_vitro	in_vivo	9	9	1.39666667	0.94333333	0.45333333	c(t = 0.62629	0.54	0.88824914	Distal Intergenic	ENSSSCG000	IL34	interleukin 34 [Source:VGNC Symbol;Acc:VGNC:89107]
chr13	133182591	133183192	602	*	chr13	133182591	133183192	in_vitro	in_vivo	10	10	1.088	0.69	0.398	c(t = 0.62534	0.54	0.88824914	Distal Intergenic	ENSSSCG000	SLC51A	solute carrier family 51 subunit alpha [Source:VGNC Symbol;Acc:VGNC:93138]
chr4	297787	299732	1946	*	chr4	297787	299732	in_vitro	in_vivo	12	11	6.73	4.48545455	2.24454545	c(t = 0.62326	0.541	0.88932299	Distal Intergenic	ENSSSCG000	SLC52A1	solute carrier family 52 member 2 [Source:NCBI gene (formerly Entrezgene);Acc:445519]
chr5	71028825	71030050	1226	*	chr5	71028825	71030050	in_vitro	in_vivo	14	9	2.38428571	1.58777778	0.79650794	c(t = 0.62196	0.541	0.88932299	Distal Intergenic	ENSSSCG000	LRRK2	leucine rich repeat kinase 2 [Source:NCBI gene (formerly Entrezgene);Acc:780403]
chr6	106788196	106789255	1060	*	chr6	106788196	106789255	in_vitro	in_vivo	14	13	1.64428571	0.95923077	0.68505495	c(t = 0.62416	0.541	0.88932299	Distal Intergenic	NA	NA	NA
chr5	2978884	2984274	5391	*	chr5	2978884	2984274	in_vitro	in_vivo	12	14	7.48833333	10.4892857	-3.0009524	c(t = -0.6183	0.542	0.88963477	Distal Intergenic	ENSSSCG000	PPARA	peroxisome proliferator activated receptor alpha [Source:NCBI gene (formerly Entrezgene);Acc:397239]
chr16	64360870	64362137	1268	*	chr16	64360870	64362137	in_vitro	in_vivo	12	12	2.22916667	4.0625	-1.8333333	c(t = -0.6239	0.542	0.88963477	Distal Intergenic	ENSSSCG000	IL12B	interleukin 12B [Source:NCBI gene (formerly Entrezgene);Acc:397076]
chr1	106970567	106971054	488	*	chr1	106970567	106971054	in_vitro	in_vivo	9	11	1.11111111	2.11363636	-1.0025253	c(t = -0.6216	0.542	0.88963477	Promoter (<=1kb)	ENSSSCG000	MTFMT	mitochondrial methionyl-tRNA formyltransferase [Source:VGNC Symbol;Acc:VGNC:90445]
chr14	127831194	127831423	230	*	chr14	127831194	127831423	in_vitro	in_vivo	9	9	3.04555556	1.48111111	1.56444444	c(t = 0.62852	0.542	0.88963477	Distal Intergenic	ENSSSCG000	SLC18A2	solute carrier family 18 member A2 [Source:NCBI gene (formerly Entrezgene);Acc:100154950]
chr9	106850635	106851920	1286	*	chr9	106850635	106851920	in_vitro	in_vivo	10	12	0.957	1.46083333	-0.5038333	c(t = -0.6203	0.542	0.88963477	Distal Intergenic	ENSSSCG000	HBP1	HMG-box transcription factor 1 [Source:VGNC Symbol;Acc:VGNC:88794]
chr1	238666872	238667694	823	*	chr1	238666872	238667694	in_vitro	in_vivo	9	10	1.60777778	0.97	0.63777778	c(t = 0.62266	0.542	0.88963477	Promoter (<=1kb)	ENSSSCG00000032554		solute carrier family 25 member 51 [Source:NCBI gene (formerly Entrezgene);Acc:100157218]
chr1	177593153	177595171	2019	*	chr1	177593153	177595171	in_vitro	in_vivo	14	14	1.13	0.79	0.34	c(t = 0.61938	0.542	0.88963477	Distal Intergenic	ENSSSCG000	RPS29	ribosomal protein S29 [Source:NCBI gene (formerly Entrezgene);Acc:414391]
chrX	111158055	111158612	558	*	chrX	111158055	111158612	in_vitro	in_vivo	9	9	11.9255556	6.27111111	5.65444444	c(t = 0.62862	0.543	0.89032537	Promoter (<=1kb)	ENSSSCG000	MMGT1	membrane magnesium transporter 1 [Source:NCBI gene (formerly Entrezgene);Acc:100520118]
chr1	107997195	107997615	421	*	chr1	107997195	107997615	in_vitro	in_vivo	12	10	5.24333333	3.764	1.47933333	c(t = 0.61936	0.543	0.89032537	Promoter (<=1kb)	ENSSSCG000	DAPK2	death associated protein kinase 2 [Source:VGNC Symbol;Acc:VGNC:87157]
chr6	116609774	116611392	1619	*	chr6	116609774	116611392	in_vitro	in_vivo	14	13	0.99285714	1.56923077	-0.5763736	c(t = -0.6216	0.543	0.89032537	Distal Intergenic	ENSSSCG000	TTR	transthyretin [Source:NCBI gene (formerly Entrezgene);Acc:397419]
chr16	68349902	68350958	1057	*	chr16	68349902	68350958	in_vitro	in_vivo	9	12	0.71777778	1.2	-0.4822222	c(t = -0.6190	0.543	0.89032537	Distal Intergenic	ENSSSCG000	HAND1	heart and neural crest derivatives expressed 1 [Source:VGNC Symbol;Acc:VGNC:88777]
chr13	76945367	76946162	796	*	chr13	76945367	76946162	in_vitro	in_vivo	12	9	0.76416667	0.37	0.39416667	c(t = 0.61941	0.543	0.89032537	Distal Intergenic	ENSSSCG000	PCCB	propionyl-CoA carboxylase subunit beta [Source:NCBI gene (formerly Entrezgene);Acc:100158147]
chr10	68084996	68086970	1975	*	chr10	68084996	68086970	in_vitro	in_vivo	11	11	96.6718182	95.1281818	1.54363636	c(t = 0.62197	0.544	0.89056453	Distal Intergenic	ENSSSCG000	WDR37	WD repeat domain 37 [Source:VGNC Symbol;Acc:VGNC:96258]
chr9	47283223	47284119	897	*	chr9	47283223	47284119	in_vitro	in_vivo	9	10	12.4044444	20.359	-7.9545556	c(t = -0.6199	0.544	0.89056453	Distal Intergenic	ENSSSCG000	NECTIN1	nectin cell adhesion molecule 1 [Source:VGNC Symbol;Acc:VGNC:108604]
chr3	111678644	111679171	528	*	chr3	111678644	111679171	in_vitro	in_vivo	10	10	1.331	2.273	-0.942	c(t = -0.6204	0.544	0.89056453	Distal Intergenic	ENSSSCG000	NRBP1	nuclear receptor binding protein 1 [Source:VGNC Symbol;Acc:VGNC:90891]
chr18	51361444	51362193	750	*	chr18	51361444	51362193	in_vitro	in_vivo	11	9	2.20636364	1.36	0.84636364	c(t = 0.61969	0.544	0.89056453	Distal Intergenic	ENSSSCG000	POLD2	DNA polymerase delta 2, accessory subunit [Source:VGNC Symbol;Acc:VGNC:91628]
chr2	145668245	145668876	632	*	chr2	145668245	145668876	in_vitro	in_vivo	11	9	0.77181818	1.24666667	-0.4748485	c(t = -0.6185	0.544	0.89056453	Promoter (<=1kb)	ENSSSCG000	YIPF5	Yip1 domain family member 5 [Source:VGNC Symbol;Acc:VGNC:95031]
chr12	23404436	23408372	3937	*	chr12	23404436	23408372	in_vitro	in_vivo	10	13	0.574	0.99076923	-0.4167692	c(t = -0.6188	0.544	0.89056453	Distal Intergenic	ENSSSCG000	PSMB3	proteasome 20S subunit beta 3 [Source:NCBI gene (formerly Entrezgene);Acc:497064]
chr10	67012544	67021381	8838	*	chr10	67012544	67021381	in_vitro	in_vivo	11	14	88.1436364	93.7321429	-5.5885065	c(t = -0.6266	0.545	0.89056453	Distal Intergenic	ENSSSCG000	KLF6	KLF transcription factor 6 [Source:NCBI gene (formerly Entrezgene);Acc:100174961]
chr3	68563246	68564725	1480	*	chr3	68563246	68564725	in_vitro	in_vivo	12	13	6.98416667	9.23538462	-2.2512179	c(t = -0.6144	0.545	0.89056453	Distal Intergenic	ENSSSCG000	C2orf81	chromosome 3 C2orf81 homolog [Source:VGNC Symbol;Acc:VGNC:109179]
chr5	15311217	15312591	1375	*	chr5	15311217	15312591	in_vitro	in_vivo	10	12	4.475	6.09	-1.615	c(t = -0.6169	0.545	0.89056453	Distal Intergenic	ENSSSCG000	TUBA1A	tubulin alpha 1a [Source:NCBI gene (formerly Entrezgene);Acc:100135051]
chr18	20949226	20951804	2579	*	chr18	20949226	20951804	in_vitro	in_vivo	16	15	2.57375	1.87933333	0.69441667	c(t = 0.61204	0.545	0.89056453	Distal Intergenic	ENSSSCG000	ARF5	ADP ribosylation factor 5 [Source:NCBI gene (formerly Entrezgene);Acc:100270722]
chr8	38982176	38984473	2298	*	chr8	38982176	38984473	in_vitro	in_vivo	13	13	1.79846154	1.29923077	0.49923077	c(t = 0.61520	0.545	0.89056453	Distal Intergenic	ENSSSCG000	SGCB	sarcoglycan beta [Source:NCBI gene (formerly Entrezgene);Acc:100135674]
chr8	119428570	119430992	2423	*	chr8	119428570	119430992	in_vitro	in_vivo	12	15	1.84666667	1.218	0.62866667	c(t = 0.61716	0.545	0.89056453	Promoter (<=1kb)	ENSSSCG000	PPP3CA	protein phosphatase 3 catalytic subunit alpha [Source:VGNC Symbol;Acc:VGNC:98218]
chr7	30666632	30667390	759	*	chr7	30666632	30667390	in_vitro	in_vivo	11	11	1.71181818	1.08090909	0.63090909	c(t = 0.61886	0.545	0.89056453	Promoter (<=1kb)	ENSSSCG000	ILRUN	inflammation and lipid regulator with UBA-like and NBR1-like domains [Source:NCBI gene (formerly Entrezgene);Acc:100512239]
chr10	67080533	67083401	2869	*	chr10	67080533	67083401	in_vitro	in_vivo	12	13	1.39666667	0.98846154	0.40820513	c(t = 0.61495	0.545	0.89056453	Distal Intergenic	ENSSSCG000	KLF6	KLF transcription factor 6 [Source:NCBI gene (formerly Entrezgene);Acc:100174961]
chr14	128748468	128749516	1049	*	chr14	128748468	128749516	in_vitro	in_vivo	10	11	1.667	0.56818182	1.09881818	c(t = 0.62391	0.545	0.89056453	Distal Intergenic	ENSSSCG000	PRLHR	prolactin releasing hormone receptor [Source:VGNC Symbol;Acc:VGNC:91818]
chr15	71497007	71498364	1358	*	chr15	71497007	71498364	in_vitro	in_vivo	12	13	0.89666667	0.49153846	0.40512821	c(t = 0.61893	0.545	0.89056453	Distal Intergenic	ENSSSCG000	IFIH1	interferon induced with helicase C domain 1 [Source:VGNC Symbol;Acc:VGNC:98042]
chr4	4092876	4094036	1161	*	chr4	4092876	4094036	in_vitro	in_vivo	11	9	12.3454545	19.8344444	-7.4889899	c(t = -0.6162	0.546	0.8910618	Distal Intergenic	ENSSSCG000	ago-02	argonaute RISC catalytic component 2 [Source:NCBI gene (formerly Entrezgene);Acc:100155245]
chr18	5690552	5691835	1284	*	chr18	5690552	5691835	in_vitro	in_vivo	10	10	3.958	2.491	1.467	c(t = 0.62041	0.546	0.8910618	Distal Intergenic	ENSSSCG000	RHEB	Ras homolog, mTORC1 binding [Source:NCBI gene (formerly Entrezgene);Acc:100516050]
chr9	47639413	47640635	1223	*	chr9	47639413	47640635	in_vitro	in_vivo	14	12	3.12071429	1.84666667	1.27404762	c(t = 0.61375	0.546	0.8910618	Distal Intergenic	ENSSSCG000	ssc-mir-9818	ssc-mir-9818 [Source:miRBase;Acc:MI0031585]
chr11	65515301	65517102	1802	*	chr11	65515301	65517102	in_vitro	in_vivo	13	16	2.13846154	1.5825	0.55596154	c(t = 0.61187	0.546	0.8910618	Distal Intergenic	ENSSSCG000	DNAJC3	DnaJ heat shock protein family (Hsp40) member C3 [Source:NCBI gene (formerly Entrezgene);Acc:100154166]
chr7	47993389	47994787	1399	*	chr7	47993389	47994787	in_vitro	in_vivo	12	11	1.53666667	1.07181818	0.46484848	c(t = 0.61505	0.546	0.8910618	Distal Intergenic	ENSSSCG000	CTSH	cathepsin H [Source:NCBI gene (formerly Entrezgene);Acc:396969]
chr1	189503609	189504791	1183	*	chr1	189503609	189504791	in_vitro	in_vivo	10	9	0.391	0.73777778	-0.3467778	c(t = -0.6211	0.546	0.8910618	Distal Intergenic	ENSSSCG000	SIX1	SIX homeobox 1 [Source:NCBI gene (formerly Entrezgene);Acc:100156847]
chr6	61285230	61290563	5334	*	chr6	61285230	61290563	in_vitro	in_vivo	15	13	92.6693333	95.5738462	-2.9045128	c(t = -0.6139	0.547	0.89155779	Distal Intergenic	ENSSSCG000	GALP	galanin like peptide [Source:NCBI gene (formerly Entrezgene);Acc:396772]
chr15	138395839	138398445	2607	*	chr15	138395839	138398445	in_vitro	in_vivo	9	9	35.65	47.7677778	-12.117778	c(t = -0.6154	0.547	0.89155779	Distal Intergenic	ENSSSCG000	RAMP1	receptor activity modifying protein 1 [Source:NCBI gene (formerly Entrezgene);Acc:397401]
chr7	16891149	16893084	1936	*	chr7	16891149	16893084	in_vitro	in_vivo	13	15	1.81846154	2.37266667	-0.5542051	c(t = -0.6106	0.547	0.89155779	Distal Intergenic	ENSSSCG000	PRL	prolactin [Source:VGNC Symbol;Acc:VGNC:104037]
chr2	79021123	79021480	358	*	chr2	79021123	79021480	in_vitro	in_vivo	9	11	2.07111111	1.25181818	0.81929293	c(t = 0.61734	0.547	0.89155779	Distal Intergenic	ENSSSCG000	CANX	calnexin [Source:VGNC Symbol;Acc:VGNC:86167]
chr4	109973101	109974354	1254	*	chr4	109973101	109974354	in_vitro	in_vivo	9	15	1.59555556	1.096	0.49955556	c(t = 0.61439	0.547	0.89155779	Distal Intergenic	ENSSSCG000	AHCYL1	adenosylhomocysteinase like 1 [Source:VGNC Symbol;Acc:VGNC:97873]
chr1	34692972	34695030	2059	*	chr1	34692972	34695030	in_vitro	in_vivo	14	14	1.26857143	0.87142857	0.39714286	c(t = 0.61072	0.547	0.89155779	Distal Intergenic	ENSSSCG000	RSPO3	R-spondin 3 [Source:NCBI gene (formerly Entrezgene);Acc:100155208]
chr14	13946964	13948123	1160	*	chr14	13946964	13948123	in_vitro	in_vivo	12	9	5.4775	7.35444444	-1.8769444	c(t = -0.6139	0.548	0.89180771	Distal Intergenic	ENSSSCG00000021767		L-threonine dehydrogenase [Source:VGNC Symbol;Acc:VGNC:109162]
chr14	28256258	28257573	1316	*	chr14	28256258	28257573	in_vitro	in_vivo	9	10	3.68111111	2.404	1.27711111	c(t = 0.61409	0.548	0.89180771	Distal Intergenic	ENSSSCG000	SCARB1	scavenger receptor class B member 1 [Source:NCBI gene (formerly Entrezgene);Acc:397018]
chr15	45083639	45085067	1429	*	chr15	45083639	45085067	in_vitro	in_vivo	12	14	2.81916667	1.85642857	0.9627381	c(t = 0.60991	0.548	0.89180771	Distal Intergenic	ENSSSCG000	CLDN22	claudin 22 [Source:NCBI gene (formerly Entrezgene);Acc:100294683]
chr14	136157619	136158838	1220	*	chr14	136157619	136158838	in_vitro	in_vivo	10	9	2.233	1.32222222	0.91077778	c(t = 0.61441	0.548	0.89180771	Distal Intergenic	ENSSSCG000	UROS	uroporphyrinogen III synthase [Source:VGNC Symbol;Acc:VGNC:94735]
chr1	1245729	1247943	2215	*	chr1	1245729	1247943	in_vitro	in_vivo	9	10	18.4755556	25.674	-7.1984444	c(t = -0.6113	0.549	0.89180771	Distal Intergenic	NA	NA	NA
chr7	93152294	93153444	1151	*	chr7	93152294	93153444	in_vitro	in_vivo	10	12	10.896	15.0825	-4.1865	c(t = -0.6103	0.549	0.89180771	Promoter (<=1kb)	ENSSSCG000	PLEKHD1	pleckstrin homology and coiled-coil domain containing D1 [Source:VGNC Symbol;Acc:VGNC:91539]
chr14	90113008	90115354	2347	*	chr14	90113008	90115354	in_vitro	in_vivo	13	12	6.44076923	5.11583333	1.3249359	c(t = 0.61294	0.549	0.89180771	Distal Intergenic	ENSSSCG000	CHAT	choline O-acetyltransferase [Source:NCBI gene (formerly Entrezgene);Acc:396896]
chr17	53101373	53103056	1684	*	chr17	53101373	53103056	in_vitro	in_vivo	9	10	1.65555556	3.44	-1.7844444	c(t = -0.6203	0.549	0.89180771	Promoter (<=1kb)	ENSSSCG000	SALL4	spalt like transcription factor 4 [Source:NCBI gene (formerly Entrezgene);Acc:100136902]
chr11	3225197	3226084	888	*	chr11	3225197	3226084	in_vitro	in_vivo	12	12	2.87416667	1.88416667	0.99	c(t = 0.60919	0.549	0.89180771	Distal Intergenic	ENSSSCG000	ssc-mir-9814	ssc-mir-9814 [Source:miRBase;Acc:MI0031581]
chr15	80325240	80328355	3116	*	chr15	80325240	80328355	in_vitro	in_vivo	15	16	1.778	1.315625	0.462375	c(t = 0.60865	0.549	0.89180771	Distal Intergenic	ENSSSCG000	CIR1	corepressor interacting with RBPJ, CIR1 [Source:NCBI gene (formerly Entrezgene);Acc:100511415]
chr13	19509986	19510761	776	*	chr13	19509986	19510761	in_vitro	in_vivo	11	14	2.11909091	1.31285714	0.80623377	c(t = 0.61362	0.549	0.89180771	Distal Intergenic	ENSSSCG000	CMTM7	CKLF like MARVEL transmembrane domain containing 7 [Source:VGNC Symbol;Acc:VGNC:103928]
chr8	31132844	31134575	1732	*	chr8	31132844	31134575	in_vitro	in_vivo	12	16	0.81166667	1.18875	-0.3770833	c(t = -0.6075	0.549	0.89180771	Distal Intergenic	ENSSSCG000	RHOH	ras homolog family member H [Source:VGNC Symbol;Acc:VGNC:92290]
chr16	35871709	35873851	2143	*	chr16	35871709	35873851	in_vitro	in_vivo	14	14	1.32571429	0.92857143	0.39714286	c(t = 0.60875	0.549	0.89180771	Distal Intergenic	ENSSSCG000	IL6ST	interleukin 6 cytokine family signal transducer [Source:VGNC Symbol;Acc:VGNC:89113]
chr1	265897381	265898247	867	*	chr1	265897381	265898247	in_vitro	in_vivo	10	11	0.443	0.21090909	0.23209091	c(t = 0.61664	0.549	0.89180771	Promoter (<=1kb)	ENSSSCG000	PPP6C	protein phosphatase 6 catalytic subunit [Source:NCBI gene (formerly Entrezgene);Acc:100153256]
chr3	1310525	1331120	20596	*	chr3	1310525	1331120	in_vitro	in_vivo	10	15	28.593	36.2353333	-7.6423333	c(t = -0.6096	0.55	0.89180771	Distal Intergenic	ENSSSCG000	MRM2	mitochondrial rRNA methyltransferase 2 [Source:VGNC Symbol;Acc:VGNC:99722]
chr14	113432069	113433519	1451	*	chr14	113432069	113433519	in_vitro	in_vivo	9	12	5.47222222	7.37916667	-1.9069444	c(t = -0.6088	0.55	0.89180771	Distal Intergenic	NA	NA	NA
chr9	705335	706779	1445	*	chr9	705335	706779	in_vitro	in_vivo	12	10	10.6466667	7.115	3.53166667	c(t = 0.61084	0.55	0.89180771	Distal Intergenic	NA	NA	NA
chr17	29285155	29287617	2463	*	chr17	29285155	29287617	in_vitro	in_vivo	11	12	2.23	2.93916667	-0.7091667	c(t = -0.6073	0.55	0.89180771	Distal Intergenic	ENSSSCG000	THBD	thrombomodulin [Source:NCBI gene (formerly Entrezgene);Acc:100157642]
chr15	70393941	70394792	852	*	chr15	70393941	70394792	in_vitro	in_vivo	9	11	4.15	2.30363636	1.84636364	c(t = 0.61020	0.55	0.89180771	Distal Intergenic	ENSSSCG000	IFIH1	interferon induced with helicase C domain 1 [Source:VGNC Symbol;Acc:VGNC:98042]
chr13	90182764	90184187	1424	*	chr13	90182764	90184187	in_vitro	in_vivo	13	11	1.30153846	1.99363636	-0.6920979	c(t = -0.6107	0.55	0.89180771	Distal Intergenic	ENSSSCG000	SERP1	stress associated endoplasmic reticulum protein 1 [Source:NCBI gene (formerly Entrezgene);Acc:100156392]
chr10	42692652	42693706	1055	*	chr10	42692652	42693706	in_vitro	in_vivo	11	13	1.99090909	1.45615385	0.53475524	c(t = 0.60785	0.55	0.89180771	Distal Intergenic	ENSSSCG000	TRDMT1	tRNA aspartic acid methyltransferase 1 [Source:VGNC Symbol;Acc:VGNC:96227]
chr7	88470788	88471751	964	*	chr7	88470788	88471751	in_vitro	in_vivo	10	13	2.091	1.41769231	0.67330769	c(t = 0.61057	0.55	0.89180771	Distal Intergenic	ENSSSCG000	GPX2	glutathione peroxidase 2 [Source:NCBI gene (formerly Entrezgene);Acc:767625]
chr17	43809184	43810639	1456	*	chr17	43809184	43810639	in_vitro	in_vivo	10	14	2.985	2.01285714	0.97214286	c(t = 0.61146	0.551	0.89180771	Distal Intergenic	ENSSSCG000	ZHX3	zinc fingers and homeoboxes 3 [Source:VGNC Symbol;Acc:VGNC:95717]
chr1	129942078	129942716	639	*	chr1	129942078	129942716	in_vitro	in_vivo	12	9	2.75416667	1.88222222	0.87194444	c(t = 0.60774	0.551	0.89180771	Distal Intergenic	ENSSSCG000	JMJD7	jumonji domain containing 7 [Source:NCBI gene (formerly Entrezgene);Acc:100533119]
chr14	60140085	60141733	1649	*	chr14	60140085	60141733	in_vitro	in_vivo	12	13	2.30583333	1.75307692	0.55275641	c(t = 0.60589	0.551	0.89180771	Distal Intergenic	ENSSSCG000	ACTA1	actin alpha 1, skeletal muscle [Source:NCBI gene (formerly Entrezgene);Acc:100154254]
chr14	82377770	82378393	624	*	chr14	82377770	82378393	in_vitro	in_vivo	9	11	2.06222222	1.28272727	0.77949495	c(t = 0.61375	0.551	0.89180771	Distal Intergenic	ENSSSCG000	PRXL2A	peroxiredoxin like 2A [Source:VGNC Symbol;Acc:VGNC:91892]
chr7	33125288	33126225	938	*	chr7	33125288	33126225	in_vitro	in_vivo	9	10	14.0311111	21.136	-7.1048889	c(t = -0.6086	0.552	0.89180771	Distal Intergenic	ENSSSCG000	CCDC167	coiled-coil domain containing 167 [Source:VGNC Symbol;Acc:VGNC:86270]
chr5	77136423	77137554	1132	*	chr5	77136423	77137554	in_vitro	in_vivo	9	11	2.22222222	3.95	-1.7277778	c(t = -0.6069	0.552	0.89180771	Distal Intergenic	ENSSSCG000	SLC38A2	solute carrier family 38 member 2 [Source:VGNC Symbol;Acc:VGNC:93094]
chr1	270718603	270724414	5812	*	chr1	270718603	270724414	in_vitro	in_vivo	14	16	2.71714286	3.350625	-0.6334821	c(t = -0.6039	0.552	0.89180771	Distal Intergenic	ENSSSCG000	FUBP3	far upstream element binding protein 3 [Source:VGNC Symbol;Acc:VGNC:103093]
chr2	40550286	40550856	571	*	chr2	40550286	40550856	in_vitro	in_vivo	9	10	3.18888889	1.614	1.57488889	c(t = 0.61302	0.552	0.89180771	Distal Intergenic	ENSSSCG000	LDHC	lactate dehydrogenase C [Source:VGNC Symbol;Acc:VGNC:89669]
chr15	140222493	140224432	1940	*	chr15	140222493	140224432	in_vitro	in_vivo	10	14	2.013	1.54	0.473	c(t = 0.60483	0.552	0.89180771	Promoter (<=1kb)	ENSSSCG000	ATG4B	autophagy related 4B cysteine peptidase [Source:VGNC Symbol;Acc:VGNC:95947]
chr15	106696529	106697190	662	*	chr15	106696529	106697190	in_vitro	in_vivo	11	10	2.10090909	1.193	0.90790909	c(t = 0.61070	0.552	0.89180771	Promoter (<=1kb)	ENSSSCG000	CYP20A1	cytochrome P450 family 20 subfamily A member 1 [Source:NCBI gene (formerly Entrezgene);Acc:100124408]
chr15	56123148	56123647	500	*	chr15	56123148	56123647	in_vitro	in_vivo	12	10	1.40333333	0.821	0.58233333	c(t = 0.60617	0.552	0.89180771	Distal Intergenic	ENSSSCG000	OCA2	OCA2 melanosomal transmembrane protein [Source:NCBI gene (formerly Entrezgene);Acc:397171]
chr1	237239815	237240799	985	*	chr1	237239815	237240799	in_vitro	in_vivo	10	11	1.132	0.56727273	0.56472727	c(t = 0.61242	0.552	0.89180771	Distal Intergenic	ENSSSCG000	GNE	glucosamine (UDP-N-acetyl)-2-epimerase/N-acetylmannosamine kinase [Source:VGNC Symbol;Acc:VGNC:103101]
chr14	12182930	12184289	1360	*	chr14	12182930	12184289	in_vitro	in_vivo	11	12	0.79090909	0.45583333	0.33507576	c(t = 0.60611	0.552	0.89180771	Promoter (<=1kb)	ENSSSCG000	ZNF395	zinc finger protein 395 [Source:VGNC Symbol;Acc:VGNC:95235]
chr5	47686334	47688019	1686	*	chr5	47686334	47688019	in_vitro	in_vivo	12	12	0.72083333	0.33333333	0.3875	c(t = 0.60912	0.552	0.89180771	Distal Intergenic	ENSSSCG000	INTS13	integrator complex subunit 13 [Source:VGNC Symbol;Acc:VGNC:89160]
chr1	736416	739206	2791	*	chr1	736416	739206	in_vitro	in_vivo	12	14	25.8066667	34.1757143	-8.3690476	c(t = -0.6019	0.553	0.89180771	Distal Intergenic	ENSSSCG000	ssc-mir-9815	ssc-mir-9815 [Source:miRBase;Acc:MI0031582]
chrX	21079311	21080886	1576	*	chrX	21079311	21080886	in_vitro	in_vivo	13	11	7.31307692	11.1209091	-3.8078322	c(t = -0.6024	0.553	0.89180771	Distal Intergenic	ENSSSCG000	SAT1	spermidine/spermine N1-acetyltransferase 1 [Source:NCBI gene (formerly Entrezgene);Acc:397645]
chr9	49986069	49987812	1744	*	chr9	49986069	49987812	in_vitro	in_vivo	10	13	2.075	3.17769231	-1.1026923	c(t = -0.6032	0.553	0.89180771	Promoter (<=1kb)	ENSSSCG000	HSPA8	heat shock protein family A (Hsp70) member 8 [Source:NCBI gene (formerly Entrezgene);Acc:100511890]
chr8	9124777	9125814	1038	*	chr8	9124777	9125814	in_vitro	in_vivo	12	14	1.845	3.05714286	-1.2121429	c(t = -0.6043	0.553	0.89180771	Promoter (<=1kb)	ENSSSCG000	RAB28	RAB28, member RAS onco family [Source:VGNC Symbol;Acc:VGNC:98955]
chr11	26129227	26132219	2993	*	chr11	26129227	26132219	in_vitro	in_vivo	16	16	2.35375	2.83125	-0.4775	c(t = -0.5996	0.553	0.89180771	Distal Intergenic	ENSSSCG000	MTRF1	mitochondrial translation release factor 1 [Source:VGNC Symbol;Acc:VGNC:90471]
chr1	164997476	164999127	1652	*	chr1	164997476	164999127	in_vitro	in_vivo	11	12	4.31	2.67416667	1.63583333	c(t = 0.60663	0.553	0.89180771	Promoter (<=1kb)	ENSSSCG000	SMAD3	SMAD family member 3 [Source:VGNC Symbol;Acc:VGNC:93217]
chr2	79765833	79766991	1159	*	chr2	79765833	79766991	in_vitro	in_vivo	11	13	1.67090909	2.30307692	-0.6321678	c(t = -0.6022	0.553	0.89180771	Distal Intergenic	ENSSSCG000	PROP1	PROP paired-like homeobox 1 [Source:VGNC Symbol;Acc:VGNC:91834]
chr2	91357979	91358341	363	*	chr2	91357979	91358341	in_vitro	in_vivo	10	9	0.964	1.94444444	-0.9804444	c(t = -0.6107	0.553	0.89180771	Distal Intergenic	ENSSSCG000	VCAN	versican [Source:VGNC Symbol;Acc:VGNC:108163]
chr7	60583903	60584567	665	*	chr7	60583903	60584567	in_vitro	in_vivo	12	10	2.545	1.652	0.893	c(t = 0.60370	0.553	0.89180771	Distal Intergenic	ENSSSCG000	BBS4	Bardet-Biedl syndrome 4 [Source:NCBI gene (formerly Entrezgene);Acc:100513212]
chr17	56681623	56683243	1621	*	chr17	56681623	56683243	in_vitro	in_vivo	12	12	2.33583333	1.64166667	0.69416667	c(t = 0.60338	0.553	0.89180771	Promoter (<=1kb)	ENSSSCG000	CBLN4	cerebellin 4 precursor [Source:NCBI gene (formerly Entrezgene);Acc:100141408]
chr8	111750493	111751343	851	*	chr8	111750493	111751343	in_vitro	in_vivo	11	11	1.97	1.39181818	0.57818182	c(t = 0.60316	0.553	0.89180771	Distal Intergenic	ENSSSCG000	PITX2	paired like homeodomain 2 [Source:VGNC Symbol;Acc:VGNC:91467]
chr9	27811724	27813342	1619	*	chr9	27811724	27813342	in_vitro	in_vivo	15	13	0.62133333	0.95692308	-0.3355897	c(t = -0.6034	0.553	0.89180771	Distal Intergenic	ENSSSCG000	ssc-mir-7143	ssc-mir-7143 [Source:miRBase;Acc:MI0023576]
chr6	4797905	4798309	405	*	chr6	4797905	4798309	in_vitro	in_vivo	10	10	1.645	0.849	0.796	c(t = 0.60699	0.553	0.89180771	Promoter (<=1kb)	ENSSSCG000	HSBP1	heat shock factor binding protein 1 [Source:NCBI gene (formerly Entrezgene);Acc:100579143]
chr1	826036	828929	2894	*	chr1	826036	828929	in_vitro	in_vivo	9	11	30.6422222	40.3981818	-9.7559596	c(t = -0.6044	0.554	0.89180771	Distal Intergenic	ENSSSCG000	ssc-mir-9815	ssc-mir-9815 [Source:miRBase;Acc:MI0031582]
chr6	43491742	43492705	964	*	chr6	43491742	43492705	in_vitro	in_vivo	13	9	16.2615385	25.05	-8.7884615	c(t = -0.6063	0.554	0.89180771	Distal Intergenic	ENSSSCG000	LSM14A	LSM14A mRNA processing body assembly factor [Source:NCBI gene (formerly Entrezgene);Acc:100511455]
chr9	123761369	123762873	1505	*	chr9	123761369	123762873	in_vitro	in_vivo	14	14	2.855	3.765	-0.91	c(t = -0.6012	0.554	0.89180771	Promoter (<=1kb)	ENSSSCG00000015545		glutamate-ammonia ligase [Source:VGNC Symbol;Acc:VGNC:108587]
chr15	24394889	24396681	1793	*	chr15	24394889	24396681	in_vitro	in_vivo	12	12	4.5625	3.35583333	1.20666667	c(t = 0.60194	0.554	0.89180771	Distal Intergenic	ENSSSCG000	MARCO	macrophage receptor with collagenous structure [Source:NCBI gene (formerly Entrezgene);Acc:100516298]
chr4	110844773	110845639	867	*	chr4	110844773	110845639	in_vitro	in_vivo	9	9	1.25333333	1.91666667	-0.6633333	c(t = -0.6073	0.554	0.89180771	Distal Intergenic	ENSSSCG000	TAF13	TATA-box binding protein associated factor 13 [Source:NCBI gene (formerly Entrezgene);Acc:100157095]
chr5	34426160	34427295	1136	*	chr5	34426160	34427295	in_vitro	in_vivo	11	10	1.07181818	1.899	-0.8271818	c(t = -0.6027	0.554	0.89180771	Promoter (<=1kb)	ENSSSCG000	CNOT2	CCR4-NOT transcription complex subunit 2 [Source:NCBI gene (formerly Entrezgene);Acc:100153354]
chr8	74418892	74420781	1890	*	chr8	74418892	74420781	in_vitro	in_vivo	13	14	1.14461538	1.58642857	-0.4418132	c(t = -0.6007	0.554	0.89180771	5' UTR	ENSSSCG000	LRAT	lecithin retinol acyltransferase [Source:VGNC Symbol;Acc:VGNC:89798]
chr8	3796693	3798311	1619	*	chr8	3796693	3798311	in_vitro	in_vivo	9	11	2.18111111	1.47363636	0.70747475	c(t = 0.60538	0.554	0.89180771	Distal Intergenic	ENSSSCG000	MAN2B2	mannosidase alpha class 2B member 2 [Source:VGNC Symbol;Acc:VGNC:98100]
chr6	94960282	94961469	1188	*	chr6	94960282	94961469	in_vitro	in_vivo	12	11	1.67083333	1.18454545	0.48628788	c(t = 0.60161	0.554	0.89180771	Distal Intergenic	ENSSSCG000	NDUFS5	NADH:ubiquinone oxidoreductase subunit S5 [Source:NCBI gene (formerly Entrezgene);Acc:100514655]
chr14	7786247	7787261	1015	*	chr14	7786247	7787261	in_vitro	in_vivo	10	9	1.853	1.04111111	0.81188889	c(t = 0.60927	0.554	0.89180771	Distal Intergenic	ENSSSCG000	STC1	stanniocalcin 1 [Source:NCBI gene (formerly Entrezgene);Acc:100125345]
chr2	66271286	66272770	1485	*	chr2	66271286	66272770	in_vitro	in_vivo	10	11	10.793	15.4936364	-4.7006364	c(t = -0.6036	0.555	0.89203758	Distal Intergenic	NA	NA	NA
chr5	93131764	93133715	1952	*	chr5	93131764	93133715	in_vitro	in_vivo	10	14	7.605	5.95571429	1.64928571	c(t = 0.59994	0.555	0.89203758	Distal Intergenic	ENSSSCG000	DUSP6	dual specificity phosphatase 6 [Source:NCBI gene (formerly Entrezgene);Acc:100622968]
chr7	59663531	59664706	1176	*	chr7	59663531	59664706	in_vitro	in_vivo	9	10	8.70333333	5.151	3.55233333	c(t = 0.60282	0.555	0.89203758	Distal Intergenic	ENSSSCG000	CYP11A1	cytochrome P450 family 11 subfamily A member 1 [Source:VGNC Symbol;Acc:VGNC:103363]
chr4	441119	448198	7080	*	chr4	441119	448198	in_vitro	in_vivo	11	16	6.01545455	4.688125	1.32732955	c(t = 0.60013	0.555	0.89203758	Distal Intergenic	ENSSSCG000	DGAT1	diacylglycerol O-acyltransferase 1 [Source:VGNC Symbol;Acc:VGNC:87267]
chr4	56859211	56860420	1210	*	chr4	56859211	56860420	in_vitro	in_vivo	9	12	2.67666667	1.70583333	0.97083333	c(t = 0.60785	0.555	0.89203758	Distal Intergenic	ENSSSCG000	MRPS28	mitochondrial ribosomal protein S28 [Source:NCBI gene (formerly Entrezgene);Acc:100152709]
chr3	6141962	6142972	1011	*	chr3	6141962	6142972	in_vitro	in_vivo	10	16	1.916	1.18125	0.73475	c(t = 0.60496	0.555	0.89203758	Distal Intergenic	ENSSSCG000	KPNA7	karyopherin subunit alpha 7 [Source:VGNC Symbol;Acc:VGNC:89566]
chr6	70047661	70048758	1098	*	chr6	70047661	70048758	in_vitro	in_vivo	9	12	1.41444444	0.77083333	0.64361111	c(t = 0.60805	0.555	0.89203758	Distal Intergenic	ENSSSCG000	RBP7	retinol binding protein 7 [Source:NCBI gene (formerly Entrezgene);Acc:100270723]
chr17	62558809	62559642	834	*	chr17	62558809	62559642	in_vitro	in_vivo	12	10	34.9241667	29.0116667	5.9125	c(t = 0.59737	0.556	0.89203758	Distal Intergenic	ENSSSCG000	LIME1	Lck interacting transmembrane adaptor 1 [Source:NCBI gene (formerly Entrezgene);Acc:100625216]
chr3	42988524	42989398	875	*	chr3	42988524	42989398	in_vitro	in_vivo	9	12	1.97	3.05	-1.08	c(t = -0.5989	0.556	0.89203758	Distal Intergenic	ENSSSCG000	CARD19	caspase recruitment domain family member 19 [Source:VGNC Symbol;Acc:VGNC:86188]
chr6	75445780	75446327	548	*	chr6	75445780	75446327	in_vitro	in_vivo	12	11	1.70833333	2.90909091	-1.2007576	c(t = -0.6026	0.556	0.89203758	Distal Intergenic	ENSSSCG000	SDHB	succinate dehydrogenase complex iron sulfur subunit B [Source:NCBI gene (formerly Entrezgene);Acc:414412]
chr14	6957175	6957690	516	*	chr14	6957175	6957690	in_vitro	in_vivo	10	9	3.973	2.43777778	1.53522222	c(t = 0.60140	0.556	0.89203758	Distal Intergenic	ENSSSCG000	CCAR2	cell cycle and apoptosis regulator 2 [Source:NCBI gene (formerly Entrezgene);Acc:100155718]
chr1	253092108	253093556	1449	*	chr1	253092108	253093556	in_vitro	in_vivo	12	9	2.26083333	1.64444444	0.61638889	c(t = 0.59926	0.556	0.89203758	Distal Intergenic	ENSSSCG000	HSDL2	hydroxysteroid dehydrogenase like 2 [Source:VGNC Symbol;Acc:VGNC:88992]
chr13	119651539	119652628	1090	*	chr13	119651539	119652628	in_vitro	in_vivo	11	12	1.08363636	1.6275	-0.5438636	c(t = -0.5990	0.556	0.89203758	Distal Intergenic	ENSSSCG000	SOX2	SRY-box transcription factor 2 [Source:NCBI gene (formerly Entrezgene);Acc:407739]
chr2	119722125	119723245	1121	*	chr2	119722125	119723245	in_vitro	in_vivo	10	12	2.723	1.52333333	1.19966667	c(t = 0.60728	0.556	0.89203758	Distal Intergenic	ENSSSCG00000038141		toll like receptor adaptor molecule 2 [Source:NCBI gene (formerly Entrezgene);Acc:100520421]
chr1	97510272	97511929	1658	*	chr1	97510272	97511929	in_vitro	in_vivo	13	10	1.78153846	1.217	0.56453846	c(t = 0.59964	0.556	0.89203758	Distal Intergenic	ENSSSCG000	SMAD2	SMAD family member 2 [Source:VGNC Symbol;Acc:VGNC:98329]
chr1	27037435	27037872	438	*	chr1	27037435	27037872	in_vitro	in_vivo	10	11	0.811	0.42272727	0.38827273	c(t = 0.60374	0.556	0.89203758	Promoter (<=1kb)	ENSSSCG000	IFNGR1	interferon gamma receptor 1 [Source:VGNC Symbol;Acc:VGNC:89041]
chr2	1732655	1735985	3331	*	chr2	1732655	1735985	in_vitro	in_vivo	10	9	92.66	94.8522222	-2.1922222	c(t = -0.5999	0.557	0.89219418	Distal Intergenic	ENSSSCG000	CD81	CD81 molecule [Source:VGNC Symbol;Acc:VGNC:86431]
chr2	28415959	28416524	566	*	chr2	28415959	28416524	in_vitro	in_vivo	9	12	2.06111111	3.14	-1.0788889	c(t = -0.5979	0.557	0.89219418	Intron (NM_001001264/397338, intron 1 of 10)	ENSSSCG000	WT1	WT1 transcription factor [Source:VGNC Symbol;Acc:VGNC:100884]
chr10	32355353	32356987	1635	*	chr10	32355353	32356987	in_vitro	in_vivo	11	11	4.00727273	2.77363636	1.23363636	c(t = 0.60387	0.557	0.89219418	Distal Intergenic	ENSSSCG000	ENHO	energy homeostasis associated [Source:NCBI gene (formerly Entrezgene);Acc:100303726]
chr14	77403658	77405964	2307	*	chr14	77403658	77405964	in_vitro	in_vivo	12	12	3.11416667	2.18416667	0.93	c(t = 0.59740	0.557	0.89219418	Distal Intergenic	ENSSSCG00000035105		dual specificity phosphatase 13B [Source:VGNC Symbol;Acc:VGNC:107137]
chr3	93371546	93372929	1384	*	chr3	93371546	93372929	in_vitro	in_vivo	11	12	1.10636364	1.55333333	-0.4469697	c(t = -0.5980	0.557	0.89219418	Distal Intergenic	ENSSSCG000	EPCAM	epithelial cell adhesion molecule [Source:NCBI gene (formerly Entrezgene);Acc:403163]
chr1	28854258	28856687	2430	*	chr1	28854258	28856687	in_vitro	in_vivo	12	11	1.96333333	1.41363636	0.54969697	c(t = 0.59759	0.557	0.89219418	Distal Intergenic	ENSSSCG000	SGK1	serum/glucocorticoid regulated kinase 1 [Source:VGNC Symbol;Acc:VGNC:92793]
chr5	16069509	16070218	710	*	chr5	16069509	16070218	in_vitro	in_vivo	10	11	0.455	0.16272727	0.29227273	c(t = 0.60483	0.557	0.89219418	Distal Intergenic	ENSSSCG000	GPD1	glycerol-3-phosphate dehydrogenase 1 [Source:VGNC Symbol;Acc:VGNC:88583]
chr5	87568937	87569981	1045	*	chr5	87568937	87569981	in_vitro	in_vivo	9	10	71.4177778	59.87	11.5477778	c(t = 0.59723	0.558	0.89219418	Distal Intergenic	ENSSSCG000	SNRPF	small nuclear ribonucleoprotein polypeptide F [Source:NCBI gene (formerly Entrezgene);Acc:100155855]
chr12	59556135	59557812	1678	*	chr12	59556135	59557812	in_vitro	in_vivo	11	11	21.3463636	30.2472727	-8.9009091	c(t = -0.5966	0.558	0.89219418	Distal Intergenic	ENSSSCG000	AKAP10	A-kinase anchoring protein 10 [Source:NCBI gene (formerly Entrezgene);Acc:397262]
chr6	70894799	70895905	1107	*	chr6	70894799	70895905	in_vitro	in_vivo	11	10	18.4581818	27.362	-8.9038182	c(t = -0.5956	0.558	0.89219418	Distal Intergenic	ENSSSCG000	ssc-mir-9861	ssc-mir-9861 [Source:miRBase;Acc:MI0031639]
chr13	52971879	52973855	1977	*	chr13	52971879	52973855	in_vitro	in_vivo	9	11	3.73333333	2.22363636	1.50969697	c(t = 0.60482	0.558	0.89219418	Distal Intergenic	ENSSSCG000	MITF	melanocyte inducing transcription factor [Source:VGNC Symbol;Acc:VGNC:90243]
chr11	1405234	1406222	989	*	chr11	1405234	1406222	in_vitro	in_vivo	10	13	2.938	2.08230769	0.85569231	c(t = 0.60056	0.558	0.89219418	Distal Intergenic	ENSSSCG000	FGF9	fibroblast growth factor 9 [Source:NCBI gene (formerly Entrezgene);Acc:396717]
chr2	44613008	44614393	1386	*	chr2	44613008	44614393	in_vitro	in_vivo	13	15	2.60615385	1.952	0.65415385	c(t = 0.59297	0.558	0.89219418	Distal Intergenic	ENSSSCG000	COPB1	COPI coat complex subunit beta 1 [Source:NCBI gene (formerly Entrezgene);Acc:100337660]
chr17	30621224	30622586	1363	*	chr17	30621224	30622586	in_vitro	in_vivo	11	14	1.12545455	1.77928571	-0.6538312	c(t = -0.5959	0.558	0.89219418	Distal Intergenic	ENSSSCG000	CST7	cystatin F [Source:NCBI gene (formerly Entrezgene);Acc:100152012]
chr8	48020222	48021982	1761	*	chr8	48020222	48021982	in_vitro	in_vivo	13	15	0.93230769	1.43933333	-0.5070256	c(t = -0.5962	0.558	0.89219418	Distal Intergenic	ENSSSCG000	PPID	peptidylprolyl isomerase D [Source:VGNC Symbol;Acc:VGNC:98212]
chr6	160073675	160074672	998	*	chr6	160073675	160074672	in_vitro	in_vivo	11	11	0.88909091	0.59181818	0.29727273	c(t = 0.59851	0.558	0.89219418	Distal Intergenic	ENSSSCG000	COA7	cytochrome c oxidase assembly factor 7 [Source:NCBI gene (formerly Entrezgene);Acc:100627075]
chr10	67001447	67006088	4642	*	chr10	67001447	67006088	in_vitro	in_vivo	11	11	95.9972727	94.5745455	1.42272727	c(t = 0.59425	0.559	0.89219418	Distal Intergenic	ENSSSCG000	KLF6	KLF transcription factor 6 [Source:NCBI gene (formerly Entrezgene);Acc:100174961]
chr12	60911758	60915853	4096	*	chr12	60911758	60915853	in_vitro	in_vivo	10	13	32.232	38.6807692	-6.4487692	c(t = -0.5945	0.559	0.89219418	Exon (NM_214365/397654, exon 2 of 6)	ENSSSCG00000018055		phosphatidylethanolamine N-methyltransferase [Source:VGNC Symbol;Acc:VGNC:91311]
chr6	62738433	62740469	2037	*	chr6	62738433	62740469	in_vitro	in_vivo	16	12	6.828125	10.8716667	-4.0435417	c(t = -0.5973	0.559	0.89219418	Distal Intergenic	ENSSSCG000	ISG15	ISG15 ubiquitin like modifier [Source:NCBI gene (formerly Entrezgene);Acc:100145895]
chr9	139293670	139294528	859	*	chr9	139293670	139294528	in_vitro	in_vivo	10	10	5.64	2.533	3.107	c(t = 0.60309	0.559	0.89219418	Distal Intergenic	ENSSSCG000	EGFR	epidermal growth factor receptor [Source:NCBI gene (formerly Entrezgene);Acc:397070]
chr7	34122134	34122978	845	*	chr7	34122134	34122978	in_vitro	in_vivo	14	12	1.65857143	1.10583333	0.5527381	c(t = 0.59271	0.559	0.89219418	Promoter (<=1kb)	ENSSSCG000	BTBD9	BTB domain containing 9 [Source:VGNC Symbol;Acc:VGNC:85909]
chr6	98253620	98254689	1070	*	chr6	98253620	98254689	in_vitro	in_vivo	12	15	0.55583333	0.96066667	-0.4048333	c(t = -0.5942	0.559	0.89219418	Distal Intergenic	ENSSSCG000	NDUFV2	NADH:ubiquinone oxidoreductase core subunit V2 [Source:NCBI gene (formerly Entrezgene);Acc:100037991]
chr1	188597843	188598626	784	*	chr1	188597843	188598626	in_vitro	in_vivo	12	11	1.46	0.96	0.5	c(t = 0.59362	0.559	0.89219418	Distal Intergenic	ENSSSCG000	RTN1	reticulon 1 [Source:NCBI gene (formerly Entrezgene);Acc:100152394]
chr4	122306219	122307146	928	*	chr4	122306219	122307146	in_vitro	in_vivo	12	14	1.21666667	0.88928571	0.32738095	c(t = 0.59276	0.559	0.89219418	Distal Intergenic	ENSSSCG000	F3	coagulation factor III, tissue factor [Source:VGNC Symbol;Acc:VGNC:87864]
chr3	103814575	103816710	2136	*	chr3	103814575	103816710	in_vitro	in_vivo	12	14	1.12	0.66214286	0.45785714	c(t = 0.59863	0.559	0.89219418	Distal Intergenic	ENSSSCG000	EIF2AK2	eukaryotic translation initiation factor 2 alpha kinase 2 [Source:VGNC Symbol;Acc:VGNC:87607]
chr17	38793140	38793653	514	*	chr17	38793140	38793653	in_vitro	in_vivo	10	10	41.194	50.456	-9.262	c(t = -0.5936	0.56	0.89316813	Exon (NM_001244297/100520988, exon 2 of 2)	ENSSSCG000	GDF5	growth differentiation factor 5 [Source:NCBI gene (formerly Entrezgene);Acc:100520988]
chr6	166002934	166003747	814	*	chr6	166002934	166003747	in_vitro	in_vivo	9	10	1.42222222	0.988	0.43422222	c(t = 0.59925	0.56	0.89316813	Distal Intergenic	ENSSSCG000	MMACHC	metabolism of cobalamin associated C [Source:NCBI gene (formerly Entrezgene);Acc:100513159]
chr14	140997280	141003278	5999	*	chr14	140997280	141003278	in_vitro	in_vivo	12	12	72.9091667	66.0525	6.85666667	c(t = 0.59086	0.561	0.89316813	Distal Intergenic	ENSSSCG000	ssc-mir-202	ssc-mir-202 [Source:miRBase;Acc:MI0013157]
chr18	48811585	48814283	2699	*	chr18	48811585	48814283	in_vitro	in_vivo	15	13	19.8466667	26.8369231	-6.9902564	c(t = -0.5892	0.561	0.89316813	Distal Intergenic	ENSSSCG000	PGAM2	phosphoglycerate mutase 2 [Source:VGNC Symbol;Acc:VGNC:91341]
chr13	136971199	136974043	2845	*	chr13	136971199	136974043	in_vitro	in_vivo	12	16	14.0441667	17.5175	-3.4733333	c(t = -0.5890	0.561	0.89316813	Distal Intergenic	ENSSSCG00000028717		rhophilin associated tail protein 1 [Source:NCBI gene (formerly Entrezgene);Acc:100359353]
chrX	125218429	125219506	1078	*	chrX	125218429	125219506	in_vitro	in_vivo	11	12	6.20727273	9.43083333	-3.2235606	c(t = -0.5924	0.561	0.89316813	Promoter (<=1kb)	ENSSSCG000	DKC1	dyskerin pseudouridine synthase 1 [Source:NCBI gene (formerly Entrezgene);Acc:100216430]
chr7	85921224	85922401	1178	*	chr7	85921224	85922401	in_vitro	in_vivo	9	11	3.39777778	2.57	0.82777778	c(t = 0.59378	0.561	0.89316813	Distal Intergenic	NA	NA	NA
chr7	110270936	110271473	538	*	chr7	110270936	110271473	in_vitro	in_vivo	9	9	2.93666667	1.93333333	1.00333333	c(t = 0.59466	0.561	0.89316813	Distal Intergenic	ENSSSCG000	SPATA7	spermatosis associated 7 [Source:VGNC Symbol;Acc:VGNC:93389]
chr13	69575686	69577372	1687	*	chr13	69575686	69577372	in_vitro	in_vivo	14	14	1.69285714	1.24142857	0.45142857	c(t = 0.58882	0.561	0.89316813	Distal Intergenic	ENSSSCG000	TRH	thyrotropin releasing hormone [Source:NCBI gene (formerly Entrezgene);Acc:100513309]
chr3	49197475	49199024	1550	*	chr3	49197475	49199024	in_vitro	in_vivo	10	14	1.765	1.19571429	0.56928571	c(t = 0.59136	0.561	0.89316813	Distal Intergenic	ENSSSCG000	NCK2	NCK adaptor protein 2 [Source:VGNC Symbol;Acc:VGNC:109469]
chr9	3272186	3274610	2425	*	chr9	3272186	3274610	in_vitro	in_vivo	12	9	32.065	41.29	-9.225	c(t = -0.5982	0.562	0.89316813	Distal Intergenic	ENSSSCG000	HPX	hemopexin [Source:VGNC Symbol;Acc:VGNC:88968]
chr7	114035035	114036505	1471	*	chr7	114035035	114036505	in_vitro	in_vivo	10	14	22.531	28.5728571	-6.0418571	c(t = -0.5882	0.562	0.89316813	Distal Intergenic	ENSSSCG000	CHGA	chromogranin A [Source:NCBI gene (formerly Entrezgene);Acc:397540]
chr7	614078	616016	1939	*	chr7	614078	616016	in_vitro	in_vivo	13	12	26.3292308	18.73	7.59923077	c(t = 0.58881	0.562	0.89316813	Distal Intergenic	ENSSSCG000	IRF4	interferon regulatory factor 4 [Source:VGNC Symbol;Acc:VGNC:89207]
chr6	49710756	49712045	1290	*	chr6	49710756	49712045	in_vitro	in_vivo	11	11	5.12454545	3.65909091	1.46545455	c(t = 0.59220	0.562	0.89316813	Distal Intergenic	ENSSSCG000	ZNF526	zinc finger protein 526 [Source:VGNC Symbol;Acc:VGNC:95260]
chr14	27477593	27478141	549	*	chr14	27477593	27478141	in_vitro	in_vivo	10	10	4.116	2.827	1.289	c(t = 0.59162	0.562	0.89316813	Distal Intergenic	ENSSSCG000	SCARB1	scavenger receptor class B member 1 [Source:NCBI gene (formerly Entrezgene);Acc:397018]
chr1	271103816	271104402	587	*	chr1	271103816	271104402	in_vitro	in_vivo	10	10	2.824	1.595	1.229	c(t = 0.59230	0.562	0.89316813	Distal Intergenic	ENSSSCG000	FUBP3	far upstream element binding protein 3 [Source:VGNC Symbol;Acc:VGNC:103093]
chr9	39696254	39697000	747	*	chr9	39696254	39697000	in_vitro	in_vivo	10	9	0.892	1.33111111	-0.4391111	c(t = -0.5919	0.562	0.89316813	Distal Intergenic	ENSSSCG000	DLAT	dihydrolipoamide S-acetyltransferase [Source:NCBI gene (formerly Entrezgene);Acc:397054]
chr2	126929526	126930167	642	*	chr2	126929526	126930167	in_vitro	in_vivo	9	10	1.98222222	1.156	0.82622222	c(t = 0.59734	0.562	0.89316813	Distal Intergenic	NA	NA	NA
chr10	59629331	59631335	2005	*	chr10	59629331	59631335	in_vitro	in_vivo	10	14	1.573	1.01785714	0.55514286	c(t = 0.59327	0.562	0.89316813	Distal Intergenic	ENSSSCG000	CDC123	cell division cycle 123 [Source:VGNC Symbol;Acc:VGNC:95819]
chr2	100483262	100484415	1154	*	chr2	100483262	100484415	in_vitro	in_vivo	10	12	1.192	0.755	0.437	c(t = 0.59222	0.562	0.89316813	Distal Intergenic	ENSSSCG000	SPATA9	spermatogenesis associated 9 [Source:NCBI gene (formerly Entrezgene);Acc:100415927]
chr4	129315584	129316427	844	*	chr4	129315584	129316427	in_vitro	in_vivo	12	9	22.3766667	18.4211111	3.95555556	c(t = 0.58915	0.563	0.89316813	Distal Intergenic	ENSSSCG000	SELENOF	selenoprotein F [Source:NCBI gene (formerly Entrezgene);Acc:100038009]
chrX	42947761	42948854	1094	*	chrX	42947761	42948854	in_vitro	in_vivo	12	13	10.7983333	14.7861538	-3.9878205	c(t = -0.5876	0.563	0.89316813	Distal Intergenic	ENSSSCG000	PQBP1	polyglutamine binding protein 1 [Source:VGNC Symbol;Acc:VGNC:91767]
chr1	186951229	186952409	1181	*	chr1	186951229	186952409	in_vitro	in_vivo	13	12	3.92076923	3.1	0.82076923	c(t = 0.58702	0.563	0.89316813	Distal Intergenic	ENSSSCG000	ARMH4	armadillo like helical domain containing 4 [Source:VGNC Symbol;Acc:VGNC:85535]
chr3	94252035	94254714	2680	*	chr3	94252035	94254714	in_vitro	in_vivo	11	14	1.66636364	2.33	-0.6636364	c(t = -0.5876	0.563	0.89316813	Promoter (<=1kb)	ENSSSCG000	EPAS1	endothelial PAS domain protein 1 [Source:NCBI gene (formerly Entrezgene);Acc:100037272]
chr13	40210314	40211131	818	*	chr13	40210314	40211131	in_vitro	in_vivo	10	14	1.524	2.06714286	-0.5431429	c(t = -0.5889	0.563	0.89316813	Distal Intergenic	ENSSSCG000	PDHB	pyruvate dehydrogenase E1 subunit beta [Source:NCBI gene (formerly Entrezgene);Acc:100516042]
chr9	72360159	72360905	747	*	chr9	72360159	72360905	in_vitro	in_vivo	11	10	0.88454545	1.363	-0.4784545	c(t = -0.5884	0.563	0.89316813	Distal Intergenic	ENSSSCG000	CYP51A1	cytochrome P450 family 51 subfamily A member 1 [Source:VGNC Symbol;Acc:VGNC:103377]
chr1	179489467	179491166	1700	*	chr1	179489467	179491166	in_vitro	in_vivo	14	12	1.39	1.10583333	0.28416667	c(t = 0.58647	0.563	0.89316813	Distal Intergenic	NA	NA	NA
chr14	103530627	103531174	548	*	chr14	103530627	103531174	in_vitro	in_vivo	12	11	1.33916667	0.84545455	0.49371212	c(t = 0.58851	0.563	0.89316813	Distal Intergenic	ENSSSCG000	FGFBP3	fibroblast growth factor binding protein 3 [Source:VGNC Symbol;Acc:VGNC:88118]
chr4	117396324	117397377	1054	*	chr4	117396324	117397377	in_vitro	in_vivo	11	12	0.84272727	0.46333333	0.37939394	c(t = 0.58748	0.563	0.89316813	Promoter (<=1kb)	ENSSSCG000	SLC30A7	solute carrier family 30 member 7 [Source:NCBI gene (formerly Entrezgene);Acc:100156498]
chr2	82607657	82610024	2368	*	chr2	82607657	82610024	in_vitro	in_vivo	10	14	4.791	3.235	1.556	c(t = 0.59090	0.564	0.89316813	Distal Intergenic	ENSSSCG000	BTF3	basic transcription factor 3 [Source:VGNC Symbol;Acc:VGNC:99597]
chr17	18428363	18429820	1458	*	chr17	18428363	18429820	in_vitro	in_vivo	10	12	1.513	2.48916667	-0.9761667	c(t = -0.5874	0.564	0.89316813	Distal Intergenic	ENSSSCG000	TMX4	thioredoxin related transmembrane protein 4 [Source:NCBI gene (formerly Entrezgene);Acc:100627887]
chr12	54972468	54974045	1578	*	chr12	54972468	54974045	in_vitro	in_vivo	14	13	1.37857143	1.93538462	-0.5568132	c(t = -0.5847	0.564	0.89316813	Distal Intergenic	ENSSSCG000	ssc-mir-9853	ssc-mir-9853-1 [Source:miRBase;Acc:MI0031629]
chr8	114034621	114035686	1066	*	chr8	114034621	114035686	in_vitro	in_vivo	11	14	0.85545455	1.34	-0.4845455	c(t = -0.5859	0.564	0.89316813	Distal Intergenic	ENSSSCG000	HADH	hydroxyacyl-CoA dehydrogenase [Source:NCBI gene (formerly Entrezgene);Acc:397604]
chr9	102434271	102435540	1270	*	chr9	102434271	102435540	in_vitro	in_vivo	13	13	1.85307692	1.18461538	0.66846154	c(t = 0.58611	0.564	0.89316813	Distal Intergenic	ENSSSCG000	FGL2	fibrinogen like 2 [Source:NCBI gene (formerly Entrezgene);Acc:448808]
chr4	71607874	71609235	1362	*	chr4	71607874	71609235	in_vitro	in_vivo	12	12	0.93916667	0.62583333	0.31333333	c(t = 0.58872	0.564	0.89316813	Distal Intergenic	ENSSSCG000	ssc-mir-124a	ssc-mir-124a-1 [Source:miRBase;Acc:MI0010680]
chr2	137597247	137598176	930	*	chr2	137597247	137598176	in_vitro	in_vivo	9	12	22.6955556	31.645	-8.9494444	c(t = -0.5860	0.565	0.89316813	Intron (NM_001244562/100512769, intron 2 of 8)	ENSSSCG000	MACROH2A1	macroH2A.1 histone [Source:VGNC Symbol;Acc:VGNC:109808]
chr8	75332838	75333684	847	*	chr8	75332838	75333684	in_vitro	in_vivo	9	12	2.04333333	3.33833333	-1.295	c(t = -0.5898	0.565	0.89316813	Distal Intergenic	ENSSSCG000	SFRP2	secreted frizzled related protein 2 [Source:NCBI gene (formerly Entrezgene);Acc:100516027]
chr2	37254536	37255236	701	*	chr2	37254536	37255236	in_vitro	in_vivo	11	13	0.53454545	1.07384615	-0.5393007	c(t = -0.5842	0.565	0.89316813	Distal Intergenic	ENSSSCG000	CSRP3	cysteine and glycine rich protein 3 [Source:NCBI gene (formerly Entrezgene);Acc:100337687]
chr1	218676437	218677117	681	*	chr1	218676437	218677117	in_vitro	in_vivo	11	9	1.52909091	1.02777778	0.50131313	c(t = 0.58616	0.565	0.89316813	Distal Intergenic	ENSSSCG000	VLDLR	very low density lipoprotein receptor [Source:VGNC Symbol;Acc:VGNC:94832]
chr2	116256658	116257309	652	*	chr2	116256658	116257309	in_vitro	in_vivo	9	12	1.30222222	0.635	0.66722222	c(t = 0.59280	0.565	0.89316813	Promoter (<=1kb)	ENSSSCG000	STARD4	StAR related lipid transfer domain containing 4 [Source:VGNC Symbol;Acc:VGNC:93534]
chr9	55907731	55908731	1001	*	chr9	55907731	55908731	in_vitro	in_vivo	10	14	32.593	41.1164286	-8.5234286	c(t = -0.5839	0.566	0.89316813	Distal Intergenic	ENSSSCG000	KCNJ5	potassium inwardly rectifying channel subfamily J member 5 [Source:NCBI gene (formerly Entrezgene);Acc:397448]
chr8	30023767	30026140	2374	*	chr8	30023767	30026140	in_vitro	in_vivo	16	14	7.250625	5.23857143	2.01205357	c(t = 0.58144	0.566	0.89316813	Promoter (<=1kb)	ENSSSCG000	KLF3	KLF transcription factor 3 [Source:VGNC Symbol;Acc:VGNC:89498]
chr13	207676405	207678025	1621	*	chr13	207676405	207678025	in_vitro	in_vivo	12	10	3.4225	4.744	-1.3215	c(t = -0.5839	0.566	0.89316813	Distal Intergenic	ENSSSCG000	ITGB2	integrin subunit beta 2 [Source:NCBI gene (formerly Entrezgene);Acc:396943]
chr7	35913782	35915497	1716	*	chr7	35913782	35915497	in_vitro	in_vivo	14	11	2.78428571	2.12909091	0.65519481	c(t = 0.58323	0.566	0.89316813	Distal Intergenic	ENSSSCG000	NFYA	nuclear transcription factor Y subunit alpha [Source:VGNC Symbol;Acc:VGNC:90729]
chr15	132995267	132997607	2341	*	chr15	132995267	132997607	in_vitro	in_vivo	11	15	2.30090909	1.646	0.65490909	c(t = 0.58262	0.566	0.89316813	Distal Intergenic	ENSSSCG000	EIF4E2	eukaryotic translation initiation factor 4E family member 2 [Source:VGNC Symbol;Acc:VGNC:96269]
chr4	123778378	123779905	1528	*	chr4	123778378	123779905	in_vitro	in_vivo	13	14	1.48307692	1.09642857	0.38664835	c(t = 0.58216	0.566	0.89316813	Distal Intergenic	ENSSSCG000	TMED5	transmembrane p24 trafficking protein 5 [Source:NCBI gene (formerly Entrezgene);Acc:100157999]
chr15	86470767	86471759	993	*	chr15	86470767	86471759	in_vitro	in_vivo	11	12	1.54454545	0.96833333	0.57621212	c(t = 0.58343	0.566	0.89316813	Distal Intergenic	ENSSSCG000	FRZB	frizzled related protein [Source:VGNC Symbol;Acc:VGNC:96308]
chr1	190625006	190626173	1168	*	chr1	190625006	190626173	in_vitro	in_vivo	12	11	1.2325	0.85545455	0.37704545	c(t = 0.58374	0.566	0.89316813	Promoter (<=1kb)	ENSSSCG000	HIF1A	hypoxia inducible factor 1 subunit alpha [Source:NCBI gene (formerly Entrezgene);Acc:396696]
chr3	10415880	10416930	1051	*	chr3	10415880	10416930	in_vitro	in_vivo	11	10	1.18272727	0.678	0.50472727	c(t = 0.58559	0.566	0.89316813	Distal Intergenic	ENSSSCG000	CCL26	C-C motif chemokine ligand 26 [Source:NCBI gene (formerly Entrezgene);Acc:780409]
chr9	128588096	128588875	780	*	chr9	128588096	128588875	in_vitro	in_vivo	9	9	0.97555556	0.58666667	0.38888889	c(t = 0.59005	0.566	0.89316813	Promoter (<=1kb)	ENSSSCG000	KCNK2	potassium two pore domain channel subfamily K member 2 [Source:VGNC Symbol;Acc:VGNC:89369]
chr12	662579	667984	5406	*	chr12	662579	667984	in_vitro	in_vivo	11	15	16.7836364	23.0046667	-6.2210303	c(t = -0.5813	0.567	0.89316813	Distal Intergenic	ENSSSCG000	WDR45B	WD repeat domain 45B [Source:NCBI gene (formerly Entrezgene);Acc:100462760]
chr12	10395315	10396418	1104	*	chr12	10395315	10396418	in_vitro	in_vivo	9	11	24.3977778	15.5209091	8.87686869	c(t = 0.58590	0.567	0.89316813	Distal Intergenic	ENSSSCG000	KCNJ2	potassium inwardly rectifying channel subfamily J member 2 [Source:VGNC Symbol;Acc:VGNC:89357]
chr2	6471205	6472742	1538	*	chr2	6471205	6472742	in_vitro	in_vivo	10	10	6.496	3.743	2.753	c(t = 0.59117	0.567	0.89316813	Promoter (<=1kb)	ENSSSCG000	CFL1	cofilin 1 [Source:NCBI gene (formerly Entrezgene);Acc:445532]
chr6	60758737	60759740	1004	*	chr6	60758737	60759740	in_vitro	in_vivo	9	12	1.35777778	2.435	-1.0772222	c(t = -0.5840	0.567	0.89316813	Distal Intergenic	ENSSSCG000	GALP	galanin like peptide [Source:NCBI gene (formerly Entrezgene);Acc:396772]
chr1	227575167	227576174	1008	*	chr1	227575167	227576174	in_vitro	in_vivo	11	11	2.71272727	1.87363636	0.83909091	c(t = 0.58341	0.567	0.89316813	Distal Intergenic	ENSSSCG000	OSTF1	osteoclast stimulating factor 1 [Source:NCBI gene (formerly Entrezgene);Acc:397066]
chr1	208174086	208174753	668	*	chr1	208174086	208174753	in_vitro	in_vivo	11	11	2.49818182	1.65818182	0.84	c(t = 0.58480	0.567	0.89316813	Distal Intergenic	ENSSSCG000	PSIP1	PC4 and SFRS1 interacting protein 1 [Source:VGNC Symbol;Acc:VGNC:91898]
chr9	1277505	1279332	1828	*	chr9	1277505	1279332	in_vitro	in_vivo	11	10	0.83636364	1.393	-0.5566364	c(t = -0.5866	0.567	0.89316813	Distal Intergenic	NA	NA	NA
chr8	83352587	83353272	686	*	chr8	83352587	83353272	in_vitro	in_vivo	10	12	1.937	1.225	0.712	c(t = 0.58442	0.567	0.89316813	Promoter (<=1kb)	ENSSSCG000	ANAPC10	anaphase promoting complex subunit 10 [Source:VGNC Symbol;Acc:VGNC:98912]
chr7	19501808	19502711	904	*	chr7	19501808	19502711	in_vitro	in_vivo	9	9	0.48222222	0.86888889	-0.3866667	c(t = -0.5874	0.567	0.89316813	Promoter (<=1kb)	ENSSSCG000	TDP2	tyrosyl-DNA phosphodiesterase 2 [Source:NCBI gene (formerly Entrezgene);Acc:100157860]
chr4	733980	756160	22181	*	chr4	733980	756160	in_vitro	in_vivo	15	15	75.9753333	80.232	-4.2566667	c(t = -0.5783	0.568	0.89316813	Distal Intergenic	ENSSSCG000	HSF1	heat shock transcription factor 1 [Source:VGNC Symbol;Acc:VGNC:88993]
chr14	77921855	77924026	2172	*	chr14	77921855	77924026	in_vitro	in_vivo	11	13	11.1690909	8.31846154	2.85062937	c(t = 0.57950	0.568	0.89316813	Distal Intergenic	ENSSSCG000	VDAC2	voltage dependent anion channel 2 [Source:VGNC Symbol;Acc:VGNC:111154]
chrX	121681949	121682973	1025	*	chrX	121681949	121682973	in_vitro	in_vivo	11	9	11.1327273	6.19888889	4.93383838	c(t = 0.58522	0.568	0.89316813	Distal Intergenic	ENSSSCG000	FATE1	fetal and adult testis expressed 1 [Source:NCBI gene (formerly Entrezgene);Acc:396705]
chr14	127303730	127304711	982	*	chr14	127303730	127304711	in_vitro	in_vivo	9	10	5.63666667	4.126	1.51066667	c(t = 0.58636	0.568	0.89316813	Distal Intergenic	ENSSSCG000	SLC18A2	solute carrier family 18 member A2 [Source:NCBI gene (formerly Entrezgene);Acc:100154950]
chr6	115926717	115928284	1568	*	chr6	115926717	115928284	in_vitro	in_vivo	13	11	2.64461538	1.87818182	0.76643357	c(t = 0.58142	0.568	0.89316813	Distal Intergenic	ENSSSCG000	TTR	transthyretin [Source:NCBI gene (formerly Entrezgene);Acc:397419]

chr8	125769159	125770621	1463	*	chr8	125769159	125770621	in_vitro	in_vivo	9	14	2.53444444	1.81857143	0.71587302	c(t = 0.58558	0.568	0.89316813	Distal Intergenic	ENSSSCG000	BMPR1B	bone morphotic protein receptor type 1B [Source:VGNC Symbol;Acc:VGNC:85847]
chr3	107268510	107269840	1331	*	chr3	107268510	107269840	in_vitro	in_vivo	12	14	1.885	1.20714286	0.67785714	c(t = 0.58188	0.568	0.89316813	Distal Intergenic	ENSSSCG000	SLC30A6	solute carrier family 30 member 6 [Source:VGNC Symbol;Acc:VGNC:93060]
chrX	124884776	124912187	27412	*	chrX	124884776	124912187	in_vitro	in_vivo	15	15	22.7866667	26.9593333	-4.1726667	c(t = -0.5768	0.569	0.89316813	Distal Intergenic	ENSSSCG000	RPL10	ribosomal protein L10 [Source:NCBI gene (formerly Entrezgene);Acc:733593]
chrX	106435699	106436534	836	*	chrX	106435699	106436534	in_vitro	in_vivo	9	12	8.19333333	13.7525	-5.5591667	c(t = -0.5791	0.569	0.89316813	Distal Intergenic	ENSSSCG000	XPNPEP2	X-prolyl aminopeptidase 2 [Source:VGNC Symbol;Acc:VGNC:95001]
chrX	36351475	36351972	498	*	chrX	36351475	36351972	in_vitro	in_vivo	10	10	4.702	7.53	-2.828	c(t = -0.5843	0.569	0.89316813	Distal Intergenic	ENSSSCG000	DDX3X	DEAD-box helicase 3 X-linked [Source:NCBI gene (formerly Entrezgene);Acc:100515940]
chr6	7420066	7421566	1501	*	chr6	7420066	7421566	in_vitro	in_vivo	13	11	1.90076923	2.79454545	-0.8937762	c(t = -0.5818	0.569	0.89316813	Distal Intergenic	ENSSSCG000	BCO1	beta-carotene oxygenase 1 [Source:VGNC Symbol;Acc:VGNC:85789]
chr9	39305908	39307227	1320	*	chr9	39305908	39307227	in_vitro	in_vivo	12	15	1.47833333	2.062	-0.5836667	c(t = -0.5782	0.569	0.89316813	Promoter (<=1kb)	ENSSSCG000	LAYN	layilin [Source:NCBI gene (formerly Entrezgene);Acc:100513941]
chr9	72078586	72079698	1113	*	chr9	72078586	72079698	in_vitro	in_vivo	11	14	1.46818182	1.95785714	-0.4896753	c(t = -0.5782	0.569	0.89316813	Promoter (<=1kb)	ENSSSCG000	CYP51A1	cytochrome P450 family 51 subfamily A member 1 [Source:VGNC Symbol;Acc:VGNC:103377]
chr6	108859688	108861054	1367	*	chr6	108859688	108861054	in_vitro	in_vivo	11	12	2.76818182	1.89	0.87818182	c(t = 0.58074	0.569	0.89316813	Distal Intergenic	ENSSSCG000	CABYR	calcium binding tyrosine phosphorylation regulated [Source:NCBI gene (formerly Entrezgene);Acc:100511266]
chr2	58267573	58268127	555	*	chr2	58267573	58268127	in_vitro	in_vivo	11	12	2.79727273	1.65083333	1.14643939	c(t = 0.58545	0.569	0.89316813	Distal Intergenic	ENSSSCG000	LPAR2	lysophosphatidic acid receptor 2 [Source:VGNC Symbol;Acc:VGNC:89784]
chr5	47847146	47848180	1035	*	chr5	47847146	47848180	in_vitro	in_vivo	11	11	1.87545455	1.25636364	0.61909091	c(t = 0.58043	0.569	0.89316813	Distal Intergenic	ENSSSCG000	INTS13	integrator complex subunit 13 [Source:VGNC Symbol;Acc:VGNC:89160]
chr11	12395186	12396097	912	*	chr11	12395186	12396097	in_vitro	in_vivo	11	15	1.68272727	1.15266667	0.53006061	c(t = 0.57827	0.569	0.89316813	Distal Intergenic	ENSSSCG000	SMAD9	SMAD family member 9 [Source:NCBI gene (formerly Entrezgene);Acc:100153584]
chr2	1766772	1771959	5188	*	chr2	1766772	1771959	in_vitro	in_vivo	10	12	93.383	95.8858333	-2.5028333	c(t = -0.5866	0.57	0.89316813	Distal Intergenic	ENSSSCG000	CD81	CD81 molecule [Source:VGNC Symbol;Acc:VGNC:86431]
chr3	1239539	1244072	4534	*	chr3	1239539	1244072	in_vitro	in_vivo	10	12	25.636	19.6983333	5.93766667	c(t = 0.58033	0.57	0.89316813	Distal Intergenic	ENSSSCG000	ssc-mir-9817	ssc-mir-9817 [Source:miRBase;Acc:MI0031584]
chr4	101547405	101548610	1206	*	chr4	101547405	101548610	in_vitro	in_vivo	12	12	11.3433333	19.2508333	-7.9075	c(t = -0.5771	0.57	0.89316813	Distal Intergenic	ENSSSCG00000006719		hydroxy-delta-5-steroid dehydrogenase, 3 beta- and steroid delta-isomerase 1 [Source:NCBI gene (formerly Entrezgene);Acc:445539]
chr9	92838011	92838510	500	*	chr9	92838011	92838510	in_vitro	in_vivo	9	9	4.72444444	6.53333333	-1.8088889	c(t = -0.5799	0.57	0.89316813	Distal Intergenic	ENSSSCG00000015390		ATP-binding cassette, sub-family B (MDR/TAP), member 1 [Source:NCBI gene (formerly Entrezgene);Acc:396910]
chr7	63423912	63425691	1780	*	chr7	63423912	63425691	in_vitro	in_vivo	13	13	7.93153846	5.69076923	2.24076923	c(t = 0.57735	0.57	0.89316813	Distal Intergenic	ENSSSCG000	NKX2-1	NK2 homeobox 1 [Source:VGNC Symbol;Acc:VGNC:90767]
chr7	52556264	52558714	2451	*	chr7	52556264	52558714	in_vitro	in_vivo	16	17	2.6925	4.09882353	-1.4063235	c(t = -0.5768	0.57	0.89316813	Promoter (<=1kb)	ENSSSCG000	ssc-mir-9848	ssc-mir-9848 [Source:miRBase;Acc:MI0031624]
chr9	57557505	57558553	1049	*	chr9	57557505	57558553	in_vitro	in_vivo	12	14	6.27666667	3.89285714	2.38380952	c(t = 0.58000	0.57	0.89316813	Distal Intergenic	ENSSSCG000	KCNJ5	potassium inwardly rectifying channel subfamily J member 5 [Source:NCBI gene (formerly Entrezgene);Acc:397448]
chr1	161606942	161607485	544	*	chr1	161606942	161607485	in_vitro	in_vivo	10	9	1.221	2.24444444	-1.0234444	c(t = -0.5819	0.57	0.89316813	Distal Intergenic	ENSSSCG000	PMAIP1	phorbol-12-myristate-13-acetate-induced protein 1 [Source:NCBI gene (formerly Entrezgene);Acc:397278]
chr12	14881441	14882551	1111	*	chr12	14881441	14882551	in_vitro	in_vivo	9	11	1.22222222	2.01545455	-0.7932323	c(t = -0.5788	0.57	0.89316813	Distal Intergenic	ENSSSCG000	ICAM2	intercellular adhesion molecule 2 [Source:NCBI gene (formerly Entrezgene);Acc:414387]
chr10	41177870	41179742	1873	*	chr10	41177870	41179742	in_vitro	in_vivo	11	14	1.16272727	1.535	-0.3722727	c(t = -0.5783	0.57	0.89316813	Distal Intergenic	ENSSSCG000	MTPAP	mitochondrial poly(A) polymerase [Source:VGNC Symbol;Acc:VGNC:96676]
chr12	819581	820484	904	*	chr12	819581	820484	in_vitro	in_vivo	9	11	1.03333333	1.48818182	-0.4548485	c(t = -0.5796	0.57	0.89316813	Distal Intergenic	ENSSSCG000	FASN	fatty acid synthase [Source:NCBI gene (formerly Entrezgene);Acc:397561]
chr13	206645888	206648752	2865	*	chr13	206645888	206648752	in_vitro	in_vivo	11	12	96.8927273	97.7083333	-0.8156061	c(t = -0.5775	0.571	0.89316813	Distal Intergenic	ENSSSCG000	PDXK	pyridoxal kinase [Source:NCBI gene (formerly Entrezgene);Acc:396983]
chr15	139505895	139508283	2389	*	chr15	139505895	139508283	in_vitro	in_vivo	9	11	25.9777778	36.4090909	-10.431313	c(t = -0.5781	0.571	0.89316813	Distal Intergenic	ENSSSCG000	ssc-mir-149	ssc-mir-149 [Source:miRBase;Acc:MI0016620]
chr9	121380126	121381873	1748	*	chr9	121380126	121381873	in_vitro	in_vivo	12	12	5.25833333	4.195	1.06333333	c(t = 0.57579	0.571	0.89316813	Distal Intergenic	ENSSSCG000	TOR1AIP2	torsin 1A interacting protein 2 [Source:VGNC Symbol;Acc:VGNC:94318]
chr2	80263032	80263940	909	*	chr2	80263032	80263940	in_vitro	in_vivo	13	11	4.83153846	3.41636364	1.41517483	c(t = 0.57592	0.571	0.89316813	Distal Intergenic	ENSSSCG000	RMND5B	required for meiotic nuclear division 5 homolog B [Source:VGNC Symbol;Acc:VGNC:92337]
chr1	229848835	229849214	380	*	chr1	229848835	229849214	in_vitro	in_vivo	9	9	1.85222222	3.31888889	-1.4666667	c(t = -0.5782	0.571	0.89316813	Distal Intergenic	ENSSSCG000	GNA14	G protein subunit alpha 14 [Source:VGNC Symbol;Acc:VGNC:88519]
chr4	694878	699924	5047	*	chr4	694878	699924	in_vitro	in_vivo	10	11	4.242	3.22181818	1.02018182	c(t = 0.57924	0.571	0.89316813	Distal Intergenic	ENSSSCG000	HSF1	heat shock transcription factor 1 [Source:VGNC Symbol;Acc:VGNC:88993]
chr5	21700217	21700842	626	*	chr5	21700217	21700842	in_vitro	in_vivo	10	11	1.6	2.73	-1.13	c(t = -0.5769	0.571	0.89316813	Promoter (<=1kb)	ENSSSCG000	CS	citrate synthase [Source:VGNC Symbol;Acc:VGNC:103225]
chr17	49803158	49804883	1726	*	chr17	49803158	49804883	in_vitro	in_vivo	9	11	1.60666667	2.25454545	-0.6478788	c(t = -0.5796	0.571	0.89316813	Distal Intergenic	ENSSSCG000	NCOA3	nuclear receptor coactivator 3 [Source:NCBI gene (formerly Entrezgene);Acc:100135659]
chr1	9238155	9239563	1409	*	chr1	9238155	9239563	in_vitro	in_vivo	11	11	3.10636364	2.00272727	1.10363636	c(t = 0.57828	0.571	0.89316813	Distal Intergenic	NA	NA	NA
chr6	115020348	115021382	1035	*	chr6	115020348	115021382	in_vitro	in_vivo	10	10	1.343	1.886	-0.543	c(t = -0.5781	0.571	0.89316813	Distal Intergenic	ENSSSCG000	DSG1	desmoglein 1 [Source:NCBI gene (formerly Entrezgene);Acc:641355]
chr17	11108877	11110535	1659	*	chr17	11108877	11110535	in_vitro	in_vivo	14	12	2.11071429	1.46916667	0.64154762	c(t = 0.57523	0.571	0.89316813	Distal Intergenic	ENSSSCG000	PLAT	plasminogen activator, tissue type [Source:NCBI gene (formerly Entrezgene);Acc:397121]
chr5	90814519	90816470	1952	*	chr5	90814519	90816470	in_vitro	in_vivo	12	11	15.2541667	10.9527273	4.30143939	c(t = 0.57350	0.572	0.89345935	Promoter (<=1kb)	ENSSSCG000	BTG1	BTG anti-proliferation factor 1 [Source:NCBI gene (formerly Entrezgene);Acc:100101473]
chr6	242773	244492	1720	*	chr6	242773	244492	in_vitro	in_vivo	14	9	4.24	6.37666667	-2.1366667	c(t = -0.5785	0.572	0.89345935	Distal Intergenic	ENSSSCG000	MC1R	melanocortin 1 receptor [Source:NCBI gene (formerly Entrezgene);Acc:494018]
chrX	125442218	125443183	966	*	chrX	125442218	125443183	in_vitro	in_vivo	12	12	9.75333333	5.32083333	4.4325	c(t = 0.57638	0.572	0.89345935	Distal Intergenic	ENSSSCG000	VBP1	VHL binding protein 1 [Source:VGNC Symbol;Acc:VGNC:104090]
chr6	80650281	80651647	1367	*	chr6	80650281	80651647	in_vitro	in_vivo	12	13	3.03583333	2.31615385	0.71967949	c(t = 0.57417	0.572	0.89345935	Distal Intergenic	ENSSSCG000	C1QB	complement C1q B chain [Source:VGNC Symbol;Acc:VGNC:85977]
chr6	82853333	82854421	1089	*	chr6	82853333	82854421	in_vitro	in_vivo	11	14	1.23181818	1.99	-0.7581818	c(t = -0.5773	0.572	0.89345935	Promoter (<=1kb)	ENSSSCG000	RSRP1	arginine and serine rich protein 1 [Source:VGNC Symbol;Acc:VGNC:92489]
chr4	118152304	118153146	843	*	chr4	118152304	118153146	in_vitro	in_vivo	9	12	1.98666667	1.26083333	0.72583333	c(t = 0.57923	0.572	0.89345935	Distal Intergenic	ENSSSCG000	SLC35A3	solute carrier family 35 member A3 [Source:VGNC Symbol;Acc:VGNC:98860]
chr7	110309305	110309759	455	*	chr7	110309305	110309759	in_vitro	in_vivo	9	12	1.51555556	1.02166667	0.49388889	c(t = 0.57730	0.572	0.89345935	Promoter (<=1kb)	ENSSSCG000	SPATA7	spermatosis associated 7 [Source:VGNC Symbol;Acc:VGNC:93389]
chr11	1697358	1698303	946	*	chr11	1697358	1698303	in_vitro	in_vivo	11	13	23.7818182	16.7576923	7.02412587	c(t = 0.57373	0.573	0.89447593	Distal Intergenic	ENSSSCG000	FGF9	fibroblast growth factor 9 [Source:NCBI gene (formerly Entrezgene);Acc:396717]
chr9	87142788	87143234	447	*	chr9	87142788	87143234	in_vitro	in_vivo	11	11	17.84	11.7309091	6.10909091	c(t = 0.57347	0.573	0.89447593	Distal Intergenic	ENSSSCG000	AHR	aryl hydrocarbon receptor [Source:NCBI gene (formerly Entrezgene);Acc:396654]
chr6	106667200	106668015	816	*	chr6	106667200	106668015	in_vitro	in_vivo	10	9	17.846	11.5611111	6.28488889	c(t = 0.58048	0.573	0.89447593	Distal Intergenic	NA	NA	NA
chr6	1674910	1680819	5910	*	chr6	1674910	1680819	in_vitro	in_vivo	11	11	87.2536364	83.8090909	3.44454545	c(t = 0.57169	0.574	0.89494624	Distal Intergenic	ENSSSCG000	KLHDC4	kelch domain containing 4 [Source:NCBI gene (formerly Entrezgene);Acc:100511452]
chr4	128389583	128390068	486	*	chr4	128389583	128390068	in_vitro	in_vivo	10	9	15.24	24.1044444	-8.8644444	c(t = -0.5740	0.574	0.89494624	Distal Intergenic	ENSSSCG00000047605		LIM domain only 4 [Source:NCBI gene (formerly Entrezgene);Acc:100127155]
chr17	40098087	40099585	1499	*	chr17	40098087	40099585	in_vitro	in_vivo	10	15	3.779	2.64	1.139	c(t = 0.57199	0.574	0.89494624	Distal Intergenic	ENSSSCG000	SAMHD1	SAM and HD domain containing deoxynucleoside triphosphate triphosphohydrolase 1 [Source:VGNC Symbol;Acc:VGNC:95712]
chr11	78024030	78025925	1896	*	chr11	78024030	78025925	in_vitro	in_vivo	11	11	1.55818182	2.29454545	-0.7363636	c(t = -0.5750	0.574	0.89494624	Distal Intergenic	ENSSSCG000	F7	coagulation factor VII [Source:NCBI gene (formerly Entrezgene);Acc:733661]
chr1	225192532	225193021	490	*	chr1	225192532	225193021	in_vitro	in_vivo	11	10	2.33909091	1.507	0.83209091	c(t = 0.57288	0.574	0.89494624	Promoter (<=1kb)	ENSSSCG000	ABHD17B	abhydrolase domain containing 17B, depalmitoylase [Source:VGNC Symbol;Acc:VGNC:84977]
chr1	21047004	21049404	2401	*	chr1	21047004	21049404	in_vitro	in_vivo	12	14	1.98416667	1.375	0.60916667	c(t = 0.57395	0.574	0.89494624	Distal Intergenic	ENSSSCG000	PLAGL1	PLAG1 like zinc finger 1 [Source:VGNC Symbol;Acc:VGNC:91510]
chr1	183419666	183422280	2615	*	chr1	183419666	183422280	in_vitro	in_vivo	11	10	6.38818182	4.505	1.88318182	c(t = 0.57340	0.575	0.8954154	Promoter (<=1kb)	ENSSSCG000	BMP4	bone morphotic protein 4 [Source:VGNC Symbol;Acc:VGNC:85843]
chr6	63094012	63095299	1288	*	chr6	63094012	63095299	in_vitro	in_vivo	11	12	2.24636364	2.99666667	-0.750303	c(t = -0.5700	0.575	0.8954154	Distal Intergenic	ENSSSCG000	ISG15	ISG15 ubiquitin like modifier [Source:NCBI gene (formerly Entrezgene);Acc:100145895]
chr14	87226434	87228341	1908	*	chr14	87226434	87228341	in_vitro	in_vivo	16	15	2.055625	2.64333333	-0.5877083	c(t = -0.5671	0.575	0.8954154	Distal Intergenic	ENSSSCG000	BMPR1A	bone morphotic protein receptor type 1A [Source:VGNC Symbol;Acc:VGNC:85846]
chr13	160063728	160064451	724	*	chr13	160063728	160064451	in_vitro	in_vivo	11	11	1.17818182	1.85454545	-0.6763636	c(t = -0.5719	0.575	0.8954154	Distal Intergenic	ENSSSCG000	CPOX	coproporphyrinogen oxidase [Source:NCBI gene (formerly Entrezgene);Acc:100511474]
chr13	134718312	134718892	581	*	chr13	134718312	134718892	in_vitro	in_vivo	11	11	0.82818182	1.38727273	-0.5590909	c(t = -0.5708	0.575	0.8954154	Distal Intergenic	ENSSSCG000	RUBCN	rubicon autophagy regulator [Source:VGNC Symbol;Acc:VGNC:92509]
chr4	112612659	112614381	1723	*	chr4	112612659	112614381	in_vitro	in_vivo	11	13	1.64818182	1.12230769	0.52587413	c(t = 0.56982	0.575	0.8954154	Distal Intergenic	ENSSSCG000	PRMT6	protein arginine methyltransferase 6 [Source:VGNC Symbol;Acc:VGNC:91823]
chr12	786510	807454	20945	*	chr12	786510	807454	in_vitro	in_vivo	11	13	36.7645455	43.0069231	-6.2423776	c(t = -0.5710	0.576	0.89588342	Distal Intergenic	ENSSSCG000	FASN	fatty acid synthase [Source:NCBI gene (formerly Entrezgene);Acc:397561]
chr7	77895028	77895744	717	*	chr7	77895028	77895744	in_vitro	in_vivo	9	11	8.26888889	4.02545455	4.24343434	c(t = 0.57766	0.576	0.89588342	Distal Intergenic	ENSSSCG00000038947		heteroous nuclear ribonucleoprotein C [Source:NCBI gene (formerly Entrezgene);Acc:100626496]
chr7	115412153	115413208	1056	*	chr7	115412153	115413208	in_vitro	in_vivo	9	13	2.62333333	1.65384615	0.96948718	c(t = 0.57436	0.576	0.89588342	Distal Intergenic	ENSSSCG000	ISG12(A)	putative ISG12(a) protein [Source:NCBI gene (formerly Entrezgene);Acc:100153902]
chr11	5194593	5198471	3879	*	chr11	5194593	5198471	in_vitro	in_vivo	14	17	1.985	1.44941176	0.53558824	c(t = 0.56845	0.576	0.89588342	Distal Intergenic	ENSSSCG000	ssc-mir-9803	ssc-mir-9803 [Source:miRBase;Acc:MI0031567]
chr3	59340584	59341190	607	*	chr3	59340584	59341190	in_vitro	in_vivo	9	10	1.62222222	1.071	0.55122222	c(t = 0.57026	0.576	0.89588342	Distal Intergenic	ENSSSCG000	MAT2A	methionine adenosyltransferase 2A [Source:NCBI gene (formerly Entrezgene);Acc:100312974]
chr1	120807195	120808316	1122	*	chr1	120807195	120808316	in_vitro	in_vivo	11	11	1.28090909	0.80363636	0.47727273	c(t = 0.57133	0.576	0.89588342	Distal Intergenic	ENSSSCG00000054479		cytochrome P450 19A2 [Source:NCBI gene (formerly Entrezgene);Acc:403332]
chr12	61280425	61285071	4647	*	chr12	61280425	61285071	in_vitro	in_vivo	10	13	94.038	92.03	2.008	c(t = 0.56747	0.577	0.89598808	Distal Intergenic	ENSSSCG000	COPS3	COP9 signalosome subunit 3 [Source:VGNC Symbol;Acc:VGNC:86900]
chr13	208200212	208202940	2729	*	chr13	208200212	208202940	in_vitro	in_vivo	12	13	12.1283333	17.9215385	-5.7932051	c(t = -0.5661	0.577	0.89598808	Distal Intergenic	ENSSSCG000	ITGB2	integrin subunit beta 2 [Source:NCBI gene (formerly Entrezgene);Acc:396943]
chr6	101794092	101794983	892	*	chr6	101794092	101794983	in_vitro	in_vivo	10	13	1.483	2.65923077	-1.1762308	c(t = -0.5678	0.577	0.89598808	Distal Intergenic	ENSSSCG000	TGIF1	TGFB induced factor homeobox 1 [Source:VGNC Symbol;Acc:VGNC:93935]
chr11	799443	800391	949	*	chr11	799443	800391	in_vitro	in_vivo	9	10	3.32555556	2.617	0.70855556	c(t = 0.56882	0.577	0.89598808	Distal Intergenic	ENSSSCG000	CRYL1	crystallin lambda 1 [Source:VGNC Symbol;Acc:VGNC:87023]
chr18	50449417	50450737	1321	*	chr18	50449417	50450737	in_vitro	in_vivo	9	11	3.21666667	2.13454545	1.08212121	c(t = 0.57098	0.577	0.89598808	Distal Intergenic	ENSSSCG000	TBRG4	transforming growth factor beta regulator 4 [Source:VGNC Symbol;Acc:VGNC:104068]
chr1	48885203	48886377	1175	*	chr1	48885203	48886377	in_vitro	in_vivo	12	12	1.24833333	1.91666667	-0.6683333	c(t = -0.5677	0.577	0.89598808	Distal Intergenic	ENSSSCG000	ssc-mir-30c-2	ssc-mir-30c-2 [Source:miRBase;Acc:MI0002461]
chr6	4479510	4480297	788	*	chr6	4479510	4480297	in_vitro	in_vivo	11	9	2.24	1.31222222	0.92777778	c(t = 0.56968	0.577	0.89598808	Distal Intergenic	ENSSSCG000	WFDC1	WAP four-disulfide core domain 1 [Source:VGNC Symbol;Acc:VGNC:94953]
chr12	25970898	25971607	710	*	chr12	25970898	25971607	in_vitro	in_vivo	9	11	1.37	0.66272727	0.70727273	c(t = 0.57402	0.577	0.89598808	Promoter (<=1kb)	ENSSSCG000	KAT7	lysine acetyltransferase 7 [Source:VGNC Symbol;Acc:VGNC:89307]
chr7	52718236	52719053	818	*	chr7	52718236	52719053	in_vitro	in_vivo	12	11	2.84666667	4.29545455	-1.4487879	c(t = -0.5691	0.578	0.89645409	Distal Intergenic	ENSSSCG000	SLC28A1	solute carrier family 28 member 1 [Source:VGNC Symbol;Acc:VGNC:93041]
chr15	126286220	126287520	1301	*	chr15	126286220	126287520	in_vitro	in_vivo	10	12	3.009	1.80916667	1.19983333	c(t = 0.56708	0.578	0.89645409	Distal Intergenic	ENSSSCG000	SERPINE2	serpin family E member 2 [Source:NCBI gene (formerly Entrezgene);Acc:397542]
chr14	65152748	65153272	525	*	chr14	65152748	65153272	in_vitro	in_vivo	13	13	1.90307692	1.22692308	0.67615385	c(t = 0.56352	0.578	0.89645409	Distal Intergenic	ENSSSCG000	TMEM26	transmembrane protein 26 [Source:NCBI gene (formerly Entrezgene);Acc:100157089]
chr2	89528771	89529921	1151	*	chr2	89528771	89529921	in_vitro	in_vivo	12	15	0.77083333	1.118	-0.3471667	c(t = -0.5631	0.578	0.89645409	Distal Intergenic	ENSSSCG00000031117		dihydrofolate reductase [Source:NCBI gene (formerly Entrezgene);Acc:100525912]
chr6	145186374	145188049	1676	*	chr6	145186374	145188049	in_vitro	in_vivo	14	13	1.52357143	1.02846154	0.49510989	c(t = 0.56383	0.578	0.89645409	Distal Intergenic	ENSSSCG000	IL12RB2	interleukin 12 receptor subunit beta 2 [Source:VGNC Symbol;Acc:VGNC:98476]
chr1	52965806	52966674	869	*	chr1	52965806	52966674	in_vitro	in_vivo	11	10	0.96636364	0.476	0.49036364	c(t = 0.56646	0.578	0.89645409	Distal Intergenic	ENSSSCG000	DPPA5	developmental pluripotency associated 5 [Source:NCBI gene (formerly Entrezgene);Acc:100301558]
chr4	124618791	124625711	6921	*	chr4	124618791	124625711	in_vitro	in_vivo	14	16	4.67142857	4.055625	0.61580357	c(t = 0.56224	0.579	0.89666063	Distal Intergenic	ENSSSCG000	RPL5	ribosomal protein L5 [Source:NCBI gene (formerly Entrezgene);Acc:100135671]
chr14	59408985	59409978	994	*	chr14	59408985	59409978	in_vitro	in_vivo	10	12	3.155	2.32416667	0.83083333	c(t = 0.56439	0.579	0.89666063	Promoter (<=1kb)	ENSSSCG000	ARV1	ARV1 homolog, fatty acid homeostasis modulator [Source:VGNC Symbol;Acc:VGNC:85554]
chr2	77562227	77563880	1654	*	chr2	77562227	77563880	in_vitro	in_vivo	10	14	1.038	1.55214286	-0.5141429	c(t = -0.5632	0.579	0.89666063	Promoter (<=1kb)	ENSSSCG000	PTBP1	polypyrimidine tract binding protein 1 [Source:VGNC Symbol;Acc:VGNC:100330]
chr8	101342803	101344080	1278	*	chr8	101342803	101344080	in_vitro	in_vivo	10	13	1.883	1.26923077	0.61376923	c(t = 0.56778	0.579	0.89666063	Distal Intergenic	ENSSSCG000	AFG2A	AFG2 AAA ATPase homolog A [Source:NCBI gene (formerly Entrezgene);Acc:100415805]
chr3	38912927	38914056	1130	*	chr3	38912927	38914056	in_vitro	in_vivo	11	11	0.48727273	1.09363636	-0.6063636	c(t = -0.5700	0.579	0.89666063	Distal Intergenic	ENSSSCG000	CLUAP1	clusterin associated protein 1 [Source:VGNC Symbol;Acc:VGNC:86791]
chr7	872648	874205	1558	*	chr7	872648	874205	in_vitro	in_vivo	9	14	18.7044444	26.5228571	-7.8184127	c(t = -0.5641	0.58	0.89666063	Distal Intergenic	ENSSSCG000	IRF4	interferon regulatory factor 4 [Source:VGNC Symbol;Acc:VGNC:89207]
chrX	103640088	103641347	1260	*	chrX	103640088	103641347	in_vitro	in_vivo	10	12	8.542	14.1516667	-5.6096667	c(t = -0.5628	0.58	0.89666063	Distal Intergenic	ENSSSCG000	ssc-mir-2483	ssc-mir-2483 [Source:miRBase;Acc:MI0022167]
chr18	54614757	54616168	1412	*	chr18	54614757	54616168	in_vitro	in_vivo	12	13	13.2666667	9.73384615	3.53282051	c(t = 0.56217	0.58	0.89666063	Distal Intergenic	ENSSSCG000	RARRES2	retinoic acid receptor responder 2 [Source:VGNC Symbol;Acc:VGNC:92098]
chr5	3223835	3224850	1016	*	chr5	3223835	3224850	in_vitro	in_vivo	9	10	4.63777778	3.456	1.18177778	c(t = 0.57255	0.58	0.89666063	Distal Intergenic	ENSSSCG000	PPARA	peroxisome proliferator activated receptor alpha [Source:NCBI gene (formerly Entrezgene);Acc:397239]
chr5	80504162	80504845	684	*	chr5	80504162	80504845	in_vitro	in_vivo	11	12	2.10454545	3.29	-1.1854545	c(t = -0.5627	0.58	0.89666063	Distal Intergenic	ENSSSCG000	HSP90B1	heat shock protein 90 beta family member 1 [Source:VGNC Symbol;Acc:VGNC:103290]
chr11	1293666	1295160	1495	*	chr11	1293666	1295160	in_vitro	in_vivo	10	12	2.112	2.86416667	-0.7521667	c(t = -0.5660	0.58	0.89666063	Distal Intergenic	ENSSSCG000	LATS2	large tumor suppressor kinase 2 [Source:VGNC Symbol;Acc:VGNC:89649]
chr2	52210501	52211190	690	*	chr2	52210501	52211190	in_vitro	in_vivo	11	9	3.75818182	2.76888889	0.98929293	c(t = 0.56367	0.58	0.89666063	Distal Intergenic	NA	NA	NA
chr1	80243876	80245210	1335	*	chr1	80243876	80245210	in_vitro	in_vivo	15	11	1.406	1.93818182	-0.5321818	c(t = -0.5623	0.58	0.89666063	Distal Intergenic	ENSSSCG000	COL10A1	collagen type X alpha 1 chain [Source:NCBI gene (formerly Entrezgene);Acc:448809]
chr6	154936041	154937199	1159	*	chr6	154936041	154937199	in_vitro	in_vivo	14	14	2.15214286	1.56571429	0.58642857	c(t = 0.56155	0.58	0.89666063	Distal Intergenic	ENSSSCG000	DAB1	DAB adaptor protein 1 [Source:VGNC Symbol;Acc:VGNC:87143]
chr4	112702475	112703740	1266	*	chr4	112702475	112703740	in_vitro	in_vivo	15	15	1.60533333	1.03133333	0.574	c(t = 0.56187	0.58	0.89666063	Promoter (<=1kb)	ENSSSCG000	PRMT6	protein arginine methyltransferase 6 [Source:VGNC Symbol;Acc:VGNC:91823]
chr15	56437853	56438328	476	*	chr15	56437853	56438328	in_vitro	in_vivo	10	10	1.414	0.625	0.789	c(t = 0.56727	0.58	0.89666063	Distal Intergenic	ENSSSCG000	OCA2	OCA2 melanosomal transmembrane protein [Source:NCBI gene (formerly Entrezgene);Acc:397171]
chr15	137701312	137702888	1577	*	chr15	137701312	137702888	in_vitro	in_vivo	10	9	20.995	30.3922222	-9.3972222	c(t = -0.5634	0.581	0.89712377	Distal Intergenic	ENSSSCG000	RAMP1	receptor activity modifying protein 1 [Source:NCBI gene (formerly Entrezgene);Acc:397401]
chr4	1090168	1094742	4575	*	chr4	1090168	1094742	in_vitro	in_vivo	13	11	11.8546154	8.11818182	3.73643357	c(t = 0.56068	0.581	0.89712377	Distal Intergenic	ENSSSCG000	GPIHBP1	glycosylphosphatidylinositol anchored high density lipoprotein binding protein 1 [Source:VGNC Symbol;Acc:VGNC:88588]
chr8	32842837	32845039	2203	*	chr8	32842837	32845039	in_vitro	in_vivo	9	15	3.02333333	2.20866667	0.81466667	c(t = 0.56710	0.581	0.89712377	Distal Intergenic	ENSSSCG000	TMEM33	transmembrane protein 33 [Source:VGNC Symbol;Acc:VGNC:94171]
chr15	111509348	111509979	632	*	chr15	111509348	111509979	in_vitro	in_vivo	9	11	2.11777778	1.40636364	0.71141414	c(t = 0.56286	0.581	0.89712377	Distal Intergenic	NA	NA	NA
chr15	137344638	137346428	1791	*	chr15	137344638	137346428	in_vitro	in_vivo	9	10	1.62555556	1.087	0.53855556	c(t = 0.56552	0.581	0.89712377	Distal Intergenic	ENSSSCG000	RAB17	RAB17, member RAS onco family [Source:VGNC Symbol;Acc:VGNC:98240]
chr14	107521412	107522496	1085	*	chr14	107521412	107522496	in_vitro	in_vivo	14	12	0.74214286	0.51	0.23214286	c(t = 0.55963	0.581	0.89712377	Distal Intergenic	ENSSSCG000	ENTPD1	ectonucleoside triphosphate diphosphohydrolase 1 [Source:VGNC Symbol;Acc:VGNC:97977]
chr9	74481192	74482294	1103	*	chr9	74481192	74482294	in_vitro	in_vivo	11	10	75.7527273	66.644	9.10872727	c(t = 0.55984	0.582	0.89776596	3' UTR	ENSSSCG000	PEG10	paternally expressed 10 [Source:NCBI gene (formerly Entrezgene);Acc:654416]
chr9	39279112	39279990	879	*	chr9	39279112	39279990	in_vitro	in_vivo	9	14	1.11555556	1.57357143	-0.4580159	c(t = -0.5628	0.582	0.89776596	Promoter (<=1kb)	ENSSSCG000	ssc-mir-34c-1	ssc-mir-34c-1 [Source:miRBase;Acc:MI0013132]
chr1	201164783	201165513	731	*	chr1	201164783	201165513	in_vitro	in_vivo	12	10	0.56833333	1.006	-0.4376667	c(t = -0.5647	0.582	0.89776596	Distal Intergenic	ENSSSCG000	ssc-mir-31	ssc-mir-31 [Source:miRBase;Acc:MI0022127]
chr8	108914694	108916509	1816	*	chr8	108914694	108916509	in_vitro	in_vivo	11	16	1.25090909	0.79625	0.45465909	c(t = 0.55739	0.582	0.89776596	Promoter (<=1kb)	ENSSSCG000	CAMK2D	calcium/calmodulin dependent protein kinase II delta [Source:NCBI gene (formerly Entrezgene);Acc:397674]
chr12	8243844	8245010	1167	*	chr12	8243844	8245010	in_vitro	in_vivo	11	11	1.00636364	0.68545455	0.32090909	c(t = 0.56018	0.582	0.89776596	Distal Intergenic	ENSSSCG000	SOX9	SRY-box transcription factor 9 [Source:NCBI gene (formerly Entrezgene);Acc:396840]
chr8	552291	567692	15402	*	chr8	552291	567692	in_vitro	in_vivo	13	16	86.8938462	83.21	3.68384615	c(t = 0.55776	0.583	0.89822674	Distal Intergenic	ENSSSCG000	SLBP	stem-loop binding protein [Source:NCBI gene (formerly Entrezgene);Acc:100513605]
chr3	112322653	112323668	1016	*	chr3	112322653	112323668	in_vitro	in_vivo	9	10	10.3877778	16.073	-5.6852222	c(t = -0.5614	0.583	0.89822674	3' UTR	ENSSSCG000	KCNK3	potassium two pore domain channel subfamily K member 3 [Source:NCBI gene (formerly Entrezgene);Acc:100737895]
chr5	23016730	23018137	1408	*	chr5	23016730	23018137	in_vitro	in_vivo	9	13	2.99666667	1.83461538	1.16205128	c(t = 0.55821	0.583	0.89822674	Distal Intergenic	ENSSSCG000	CDK4	cyclin dependent kinase 4 [Source:NCBI gene (formerly Entrezgene);Acc:100144492]
chr17	10195982	10196266	285	*	chr17	10195982	10196266	in_vitro	in_vivo	9	10	1.88777778	0.919	0.96877778	c(t = 0.56614	0.583	0.89822674	Distal Intergenic	ENSSSCG000	GPAT4	glycerol-3-phosphate acyltransferase 4 [Source:NCBI gene (formerly Entrezgene);Acc:100152836]
chr4	84918264	84919304	1041	*	chr4	84918264	84919304	in_vitro	in_vivo	11	14	0.47	0.73214286	-0.2621429	c(t = -0.5581	0.583	0.89822674	Distal Intergenic	ENSSSCG000	TMCO1	transmembrane and coiled-coil domains 1 [Source:NCBI gene (formerly Entrezgene);Acc:100151809]
chr6	119166501	119167277	777	*	chr6	119166501	119167277	in_vitro	in_vivo	9	9	1.23444444	0.48333333	0.75111111	c(t = 0.56657	0.583	0.89822674	Promoter (<=1kb)	ENSSSCG000	ZNF24	zinc finger protein 24 [Source:VGNC Symbol;Acc:VGNC:98422]
chr6	51262900	51264294	1395	*	chr6	51262900	51264294	in_vitro	in_vivo	9	12	7.52	11.9216667	-4.4016667	c(t = -0.5612	0.584	0.89868642	Exon (NM_001315716/100738193, exon 5 of 10)	ENSSSCG000	BCL3	BCL3 transcription coactivator [Source:NCBI gene (formerly Entrezgene);Acc:100738193]
chr2	66055483	66056550	1068	*	chr2	66055483	66056550	in_vitro	in_vivo	10	10	6.096	7.443	-1.347	c(t = -0.5572	0.584	0.89868642	Distal Intergenic	ENSSSCG000	CALR	calreticulin [Source:NCBI gene (formerly Entrezgene);Acc:100381266]
chr2	143890145	143892568	2424	*	chr2	143890145	143892568	in_vitro	in_vivo	10	15	2.116	3.306	-1.19	c(t = -0.5632	0.584	0.89868642	Distal Intergenic	ENSSSCG000	NDFIP1	Nedd4 family interacting protein 1 [Source:NCBI gene (formerly Entrezgene);Acc:100628119]
chr4	3065202	3065955	754	*	chr4	3065202	3065955	in_vitro	in_vivo	10	11	0.973	1.69818182	-0.7251818	c(t = -0.5592	0.584	0.89868642	Distal Intergenic	ENSSSCG000	ago-02	argonaute RISC catalytic component 2 [Source:NCBI gene (formerly Entrezgene);Acc:100155245]
chr5	29596193	29597621	1429	*	chr5	29596193	29597621	in_vitro	in_vivo	12	12	1.99333333	1.45583333	0.5375	c(t = 0.55780	0.584	0.89868642	Promoter (<=1kb)	ENSSSCG000	LEMD3	LEM domain containing 3 [Source:NCBI gene (formerly Entrezgene);Acc:100511508]
chr3	68545677	68546069	393	*	chr3	68545677	68546069	in_vitro	in_vivo	11	10	2.38909091	1.375	1.01409091	c(t = 0.55878	0.584	0.89868642	Distal Intergenic	ENSSSCG000	DOK1	docking protein 1 [Source:NCBI gene (formerly Entrezgene);Acc:100233179]
chr12	27439282	27440471	1190	*	chr12	27439282	27440471	in_vitro	in_vivo	11	9	2.42272727	4.53888889	-2.1161616	c(t = -0.5663	0.585	0.89932486	Promoter (<=1kb)	ENSSSCG000	NME2	NME/NM23 nucleoside diphosphate kinase 2 [Source:NCBI gene (formerly Entrezgene);Acc:733683]
chr4	76859645	76861161	1517	*	chr4	76859645	76861161	in_vitro	in_vivo	9	14	2.28444444	3.165	-0.8805556	c(t = -0.5579	0.585	0.89932486	Distal Intergenic	ENSSSCG000	MRPL15	mitochondrial ribosomal protein L15 [Source:VGNC Symbol;Acc:VGNC:98820]
chr17	34818426	34819643	1218	*	chr17	34818426	34819643	in_vitro	in_vivo	13	13	3.58153846	2.56615385	1.01538462	c(t = 0.55496	0.585	0.89932486	Distal Intergenic	ENSSSCG000	TBC1D20	TBC1 domain family member 20 [Source:NCBI gene (formerly Entrezgene);Acc:100519805]
chr1	149611159	149611973	815	*	chr1	149611159	149611973	in_vitro	in_vivo	12	10	1.85916667	1.267	0.59216667	c(t = 0.55670	0.585	0.89932486	Distal Intergenic	ENSSSCG000	CNDP2	carnosine dipeptidase 2 [Source:VGNC Symbol;Acc:VGNC:86812]
chr13	75905729	75907626	1898	*	chr13	75905729	75907626	in_vitro	in_vivo	10	9	1.255	0.87111111	0.38388889	c(t = 0.55724	0.585	0.89932486	Distal Intergenic	ENSSSCG000	ANAPC13	anaphase promoting complex subunit 13 [Source:VGNC Symbol;Acc:VGNC:85294]
chr17	62582457	62583490	1034	*	chr17	62582457	62583490	in_vitro	in_vivo	10	9	93.56	90.3777778	3.18222222	c(t = 0.55599	0.586	0.89996203	Distal Intergenic	ENSSSCG000	LIME1	Lck interacting transmembrane adaptor 1 [Source:NCBI gene (formerly Entrezgene);Acc:100625216]
chr3	3873693	3876124	2432	*	chr3	3873693	3876124	in_vitro	in_vivo	10	13	20.376	13.59	6.786	c(t = 0.55700	0.586	0.89996203	Distal Intergenic	ENSSSCG000	WIPI2	WD repeat domain, phosphoinositide interacting 2 [Source:VGNC Symbol;Acc:VGNC:94960]
chr1	25480793	25481989	1197	*	chr1	25480793	25481989	in_vitro	in_vivo	12	13	1.67916667	2.60384615	-0.9246795	c(t = -0.5557	0.586	0.89996203	Distal Intergenic	ENSSSCG000	CCDC28A	coiled-coil domain containing 28A [Source:NCBI gene (formerly Entrezgene);Acc:100512200]
chr15	68215948	68216416	469	*	chr15	68215948	68216416	in_vitro	in_vivo	10	9	1.312	2.00222222	-0.6902222	c(t = -0.5567	0.586	0.89996203	Distal Intergenic	ENSSSCG000	DPP4	dipeptidyl peptidase 4 [Source:NCBI gene (formerly Entrezgene);Acc:397492]
chr8	73502715	73503641	927	*	chr8	73502715	73503641	in_vitro	in_vivo	11	14	1.70454545	1.01928571	0.68525974	c(t = 0.55371	0.586	0.89996203	Distal Intergenic	ENSSSCG000	NPY2R	neuropeptide Y receptor Y2 [Source:VGNC Symbol;Acc:VGNC:109473]
chr2	65202871	65204549	1679	*	chr2	65202871	65204549	in_vitro	in_vivo	9	10	60.23	52.296	7.934	c(t = 0.55469	0.587	0.90059792	Distal Intergenic	ENSSSCG000	CC2D1A	coiled-coil and C2 domain containing 1A [Source:VGNC Symbol;Acc:VGNC:86235]
chr6	52475105	52476162	1058	*	chr6	52475105	52476162	in_vitro	in_vivo	9	9	58.3722222	46.1255556	12.2466667	c(t = 0.55484	0.587	0.90059792	Distal Intergenic	ENSSSCG000	CALM3	calmodulin 3 [Source:NCBI gene (formerly Entrezgene);Acc:396838]
chr1	107011631	107012062	432	*	chr1	107011631	107012062	in_vitro	in_vivo	11	11	1.45272727	2.38636364	-0.9336364	c(t = -0.5529	0.587	0.90059792	Promoter (<=1kb)	ENSSSCG000	SPG21	SPG21 abhydrolase domain containing, maspardin [Source:VGNC Symbol;Acc:VGNC:93403]
chr6	166552520	166553784	1265	*	chr6	166552520	166553784	in_vitro	in_vivo	10	12	1.245	1.88666667	-0.6416667	c(t = -0.5518	0.587	0.90059792	Distal Intergenic	ENSSSCG000	DYNLT4	dynein light chain Tctex-type 4 [Source:NCBI gene (formerly Entrezgene);Acc:606747]
chr1	95484205	95484708	504	*	chr1	95484205	95484708	in_vitro	in_vivo	10	11	2.108	1.43454545	0.67345455	c(t = 0.55293	0.587	0.90059792	Distal Intergenic	ENSSSCG000	ATP5F1A	ATP synthase F1 subunit alpha [Source:NCBI gene (formerly Entrezgene);Acc:100157880]
chr4	127904391	127904789	399	*	chr4	127904391	127904789	in_vitro	in_vivo	9	10	25.6511111	16.621	9.03011111	c(t = 0.55528	0.588	0.90123255	Distal Intergenic	ENSSSCG000	GBP1	guanylate binding protein 1, interferon-inducible [Source:NCBI gene (formerly Entrezgene);Acc:100151938]
chr1	29746821	29747893	1073	*	chr1	29746821	29747893	in_vitro	in_vivo	10	13	1.448	2.18769231	-0.7396923	c(t = -0.5504	0.588	0.90123255	Promoter (<=1kb)	ENSSSCG000	SGK1	serum/glucocorticoid regulated kinase 1 [Source:VGNC Symbol;Acc:VGNC:92793]
chr14	48049624	48050222	599	*	chr14	48049624	48050222	in_vitro	in_vivo	10	9	2.557	1.60777778	0.94922222	c(t = 0.55502	0.588	0.90123255	Distal Intergenic	ENSSSCG000	SELENOM	selenoprotein M [Source:NCBI gene (formerly Entrezgene);Acc:100302025]
chr6	89441584	89442891	1308	*	chr6	89441584	89442891	in_vitro	in_vivo	13	11	2.48692308	1.57727273	0.90965035	c(t = 0.55238	0.588	0.90123255	Distal Intergenic	ENSSSCG000	AZIN2	antizyme inhibitor 2 [Source:NCBI gene (formerly Entrezgene);Acc:100144439]
chr5	81359016	81360619	1604	*	chr5	81359016	81360619	in_vitro	in_vivo	10	12	1.629	1.2	0.429	c(t = 0.55254	0.588	0.90123255	Distal Intergenic	ENSSSCG000	IGF1	insulin like growth factor 1 [Source:VGNC Symbol;Acc:VGNC:98044]
chr11	4477886	4478787	902	*	chr11	4477886	4478787	in_vitro	in_vivo	9	11	49.2455556	43.0090909	6.23646465	c(t = 0.54984	0.589	0.90132716	Distal Intergenic	ENSSSCG000	GPR12	G protein-coupled receptor 12 [Source:NCBI gene (formerly Entrezgene);Acc:100736641]
chr6	91123301	91123845	545	*	chr6	91123301	91123845	in_vitro	in_vivo	10	9	42.881	33.4	9.481	c(t = 0.55015	0.589	0.90132716	Distal Intergenic	ENSSSCG000	GJA4	gap junction protein alpha 4 [Source:NCBI gene (formerly Entrezgene);Acc:100620145]
chr5	15285135	15287701	2567	*	chr5	15285135	15287701	in_vitro	in_vivo	12	15	6.15416667	7.58533333	-1.4311667	c(t = -0.5468	0.589	0.90132716	Distal Intergenic	ENSSSCG000	TUBA1A	tubulin alpha 1a [Source:NCBI gene (formerly Entrezgene);Acc:100135051]
chrX	110890031	110890938	908	*	chrX	110890031	110890938	in_vitro	in_vivo	10	9	1.779	3.99333333	-2.2143333	c(t = -0.5596	0.589	0.90132716	Distal Intergenic	ENSSSCG000	MOSPD1	motile sperm domain containing 1 [Source:VGNC Symbol;Acc:VGNC:103990]
chr16	20268422	20269544	1123	*	chr16	20268422	20269544	in_vitro	in_vivo	11	13	2.45181818	3.67	-1.2181818	c(t = -0.5490	0.589	0.90132716	Promoter (<=1kb)	ENSSSCG000	RAI14	retinoic acid induced 14 [Source:NCBI gene (formerly Entrezgene);Acc:100145886]
chr7	64762791	64763478	688	*	chr7	64762791	64763478	in_vitro	in_vivo	10	11	0.244	0.60636364	-0.3623636	c(t = -0.5543	0.589	0.90132716	Distal Intergenic	ENSSSCG000	FAM177A1	family with sequence similarity 177 member A1 [Source:VGNC Symbol;Acc:VGNC:87933]
chr10	12688790	12689709	920	*	chr10	12688790	12689709	in_vitro	in_vivo	10	12	0.925	0.49	0.435	c(t = 0.55024	0.589	0.90132716	Distal Intergenic	ENSSSCG000	DEGS1	delta 4-desaturase, sphingolipid 1 [Source:VGNC Symbol;Acc:VGNC:95590]
chr2	81523428	81524025	598	*	chr2	81523428	81524025	in_vitro	in_vivo	12	11	0.83333333	0.33636364	0.4969697	c(t = 0.55301	0.589	0.90132716	Promoter (<=1kb)	ENSSSCG000	KIAA1191	KIAA1191 [Source:VGNC Symbol;Acc:VGNC:99783]
chr14	50260679	50262240	1562	*	chr14	50260679	50262240	in_vitro	in_vivo	9	11	17.9944444	22.9072727	-4.9128283	c(t = -0.5488	0.59	0.90231841	Promoter (<=1kb)	ENSSSCG000	YDJC	YdjC chitooligosaccharide deacetylase homolog [Source:NCBI gene (formerly Entrezgene);Acc:100155573]
chr9	107365757	107366991	1235	*	chr9	107365757	107366991	in_vitro	in_vivo	14	13	1.55	2.29846154	-0.7484615	c(t = -0.5459	0.59	0.90231841	Distal Intergenic	ENSSSCG000	SLC26A3	solute carrier family 26 member 3 [Source:VGNC Symbol;Acc:VGNC:93028]
chr15	129460430	129460971	542	*	chr15	129460430	129460971	in_vitro	in_vivo	9	11	1.47333333	0.75727273	0.71606061	c(t = 0.55061	0.59	0.90231841	Distal Intergenic	ENSSSCG00000016254		C-C motif chemokine ligand 20 [Source:VGNC Symbol;Acc:VGNC:95484]
chr6	170204855	170206973	2119	*	chr6	170204855	170206973	in_vitro	in_vivo	10	9	83.697	89.0255556	-5.3285556	c(t = -0.5470	0.591	0.90276983	Distal Intergenic	ENSSSCG000	ssc-mir-9829	ssc-mir-9829 [Source:miRBase;Acc:MI0031598]
chr4	850220	854120	3901	*	chr4	850220	854120	in_vitro	in_vivo	10	14	55.728	50.0171429	5.71085714	c(t = 0.54635	0.591	0.90276983	Distal Intergenic	ENSSSCG000	HSF1	heat shock transcription factor 1 [Source:VGNC Symbol;Acc:VGNC:88993]
chr7	59503573	59504772	1200	*	chr7	59503573	59504772	in_vitro	in_vivo	10	13	3.805	4.79	-0.985	c(t = -0.5466	0.591	0.90276983	Distal Intergenic	ENSSSCG000	CYP11A1	cytochrome P450 family 11 subfamily A member 1 [Source:VGNC Symbol;Acc:VGNC:103363]
chr2	1021742	1025823	4082	*	chr2	1021742	1025823	in_vitro	in_vivo	12	12	3.35166667	4.155	-0.8033333	c(t = -0.5474	0.591	0.90276983	Distal Intergenic	ENSSSCG000	TOLLIP	toll interacting protein [Source:NCBI gene (formerly Entrezgene);Acc:100625123]
chr16	47499264	47499847	584	*	chr16	47499264	47499847	in_vitro	in_vivo	10	11	2.173	3.29727273	-1.1242727	c(t = -0.5491	0.591	0.90276983	Distal Intergenic	ENSSSCG000	MARVELD2	MARVEL domain containing 2 [Source:VGNC Symbol;Acc:VGNC:90029]
chr14	31674168	31675679	1512	*	chr14	31674168	31675679	in_vitro	in_vivo	14	13	1.14	1.49	-0.35	c(t = -0.5448	0.591	0.90276983	Promoter (<=1kb)	ENSSSCG000	ATP2A2	ATPase sarcoplasmic/endoplasmic reticulum Ca2+ transporting 2 [Source:VGNC Symbol;Acc:VGNC:85647]
chr13	207706981	207709144	2164	*	chr13	207706981	207709144	in_vitro	in_vivo	9	9	94.0833333	91.8644444	2.21888889	c(t = 0.54720	0.592	0.90304089	Distal Intergenic	ENSSSCG000	ITGB2	integrin subunit beta 2 [Source:NCBI gene (formerly Entrezgene);Acc:396943]
chr2	66170809	66171607	799	*	chr2	66170809	66171607	in_vitro	in_vivo	9	13	10.2888889	7.36769231	2.92119658	c(t = 0.54784	0.592	0.90304089	Distal Intergenic	ENSSSCG000	DNASE2	deoxyribonuclease 2, lysosomal [Source:NCBI gene (formerly Entrezgene);Acc:397398]
chr9	64732269	64733851	1583	*	chr9	64732269	64733851	in_vitro	in_vivo	11	11	1.78090909	2.75272727	-0.9718182	c(t = -0.5468	0.592	0.90304089	Distal Intergenic	NA	NA	NA
chr6	63951497	63952855	1359	*	chr6	63951497	63952855	in_vitro	in_vivo	11	12	2.79181818	1.79083333	1.00098485	c(t = 0.55115	0.592	0.90304089	Distal Intergenic	ENSSSCG000	PRKCZ	protein kinase C zeta [Source:NCBI gene (formerly Entrezgene);Acc:100533200]
chr3	46966382	46967768	1387	*	chr3	46966382	46967768	in_vitro	in_vivo	10	11	1.118	1.68090909	-0.5629091	c(t = -0.5457	0.592	0.90304089	Distal Intergenic	ENSSSCG000	MALL	mal, T cell differentiation protein like [Source:NCBI gene (formerly Entrezgene);Acc:100626835]
chr4	14296690	14299005	2316	*	chr4	14296690	14299005	in_vitro	in_vivo	13	15	1.01	1.40666667	-0.3966667	c(t = -0.5454	0.592	0.90304089	Distal Intergenic	ENSSSCG000	SQLE	squalene epoxidase [Source:VGNC Symbol;Acc:VGNC:93444]
chr13	31569924	31570779	856	*	chr13	31569924	31570779	in_vitro	in_vivo	10	13	1.176	0.77769231	0.39830769	c(t = 0.54752	0.592	0.90304089	Distal Intergenic	ENSSSCG000	PRKAR2A	protein kinase cAMP-dependent type II regulatory subunit alpha [Source:NCBI gene (formerly Entrezgene);Acc:397493]
chr12	60457839	60467799	9961	*	chr12	60457839	60467799	in_vitro	in_vivo	10	11	89.421	91.8145455	-2.3935455	c(t = -0.5494	0.593	0.90366944	Distal Intergenic	ENSSSCG000	SLC5A10	solute carrier family 5 member 10 [Source:NCBI gene (formerly Entrezgene);Acc:497235]
chr6	63694711	63695543	833	*	chr6	63694711	63695543	in_vitro	in_vivo	9	11	2.46	1.61727273	0.84272727	c(t = 0.54921	0.593	0.90366944	Distal Intergenic	ENSSSCG000	TAS1R3	taste 1 receptor member 3 [Source:NCBI gene (formerly Entrezgene);Acc:100127440]
chr1	33032019	33033299	1281	*	chr1	33032019	33033299	in_vitro	in_vivo	10	10	0.715	1.203	-0.488	c(t = -0.5452	0.593	0.90366944	Distal Intergenic	ENSSSCG000	ARG1	arginase 1 [Source:VGNC Symbol;Acc:VGNC:85454]
chr7	37181018	37182626	1609	*	chr7	37181018	37182626	in_vitro	in_vivo	12	11	1.7575	1.01090909	0.74659091	c(t = 0.54587	0.593	0.90366944	Distal Intergenic	ENSSSCG000	CCND3	cyclin D3 [Source:VGNC Symbol;Acc:VGNC:86353]
chr6	115399830	115400776	947	*	chr6	115399830	115400776	in_vitro	in_vivo	9	11	1.12666667	0.65636364	0.47030303	c(t = 0.54940	0.593	0.90366944	Distal Intergenic	ENSSSCG000	TTR	transthyretin [Source:NCBI gene (formerly Entrezgene);Acc:397419]
chr11	2674428	2675937	1510	*	chr11	2674428	2675937	in_vitro	in_vivo	9	9	98.1811111	97.0044444	1.17666667	c(t = 0.54433	0.594	0.90411765	Distal Intergenic	ENSSSCG000	ssc-mir-9814	ssc-mir-9814 [Source:miRBase;Acc:MI0031581]
chr18	2002664	2004087	1424	*	chr18	2002664	2004087	in_vitro	in_vivo	11	11	30.6354545	21.98	8.65545455	c(t = 0.54201	0.594	0.90411765	Distal Intergenic	ENSSSCG000	SHH	sonic hedgehog signaling molecule [Source:NCBI gene (formerly Entrezgene);Acc:100512749]
chr4	78167642	78168256	615	*	chr4	78167642	78168256	in_vitro	in_vivo	11	9	2.95090909	6.08444444	-3.1335354	c(t = -0.5523	0.594	0.90411765	Distal Intergenic	ENSSSCG000	ATP6V1H	ATPase H+ transporting V1 subunit H [Source:VGNC Symbol;Acc:VGNC:85677]
chr18	45321588	45322617	1030	*	chr18	45321588	45322617	in_vitro	in_vivo	13	11	1.37615385	2.47181818	-1.0956643	c(t = -0.5449	0.594	0.90411765	Distal Intergenic	ENSSSCG000	HOXA13	homeobox A13 [Source:NCBI gene (formerly Entrezgene);Acc:100359352]
chr17	4949168	4949942	775	*	chr17	4949168	4949942	in_vitro	in_vivo	10	10	1.344	1.956	-0.612	c(t = -0.5422	0.594	0.90411765	Distal Intergenic	ENSSSCG000	SLC7A2	solute carrier family 7 member 2 [Source:VGNC Symbol;Acc:VGNC:95506]
chr13	68828290	68828965	676	*	chr13	68828290	68828965	in_vitro	in_vivo	12	10	0.8625	1.328	-0.4655	c(t = -0.5434	0.594	0.90411765	Distal Intergenic	ENSSSCG000	EFCAB12	EF-hand calcium binding domain 12 [Source:VGNC Symbol;Acc:VGNC:87562]
chr1	1623171	1627625	4455	*	chr1	1623171	1627625	in_vitro	in_vivo	11	11	94.6654545	96.5718182	-1.9063636	c(t = -0.5443	0.595	0.90438588	Distal Intergenic	ENSSSCG000	ssc-mir-9824	ssc-mir-9824 [Source:miRBase;Acc:MI0031591]
chr7	541358	544217	2860	*	chr7	541358	544217	in_vitro	in_vivo	13	11	24.4130769	31.5336364	-7.1205594	c(t = -0.5407	0.595	0.90438588	Distal Intergenic	ENSSSCG000	IRF4	interferon regulatory factor 4 [Source:VGNC Symbol;Acc:VGNC:89207]
chr13	1127397	1128336	940	*	chr13	1127397	1128336	in_vitro	in_vivo	13	14	4.11615385	5.40857143	-1.2924176	c(t = -0.5392	0.595	0.90438588	Distal Intergenic	ENSSSCG000	CAPN7	calpain 7 [Source:NCBI gene (formerly Entrezgene);Acc:100037936]
chr17	18958080	18958384	305	*	chr17	18958080	18958384	in_vitro	in_vivo	9	10	7.16444444	5.316	1.84844444	c(t = 0.54594	0.595	0.90438588	Distal Intergenic	ENSSSCG000	TMX4	thioredoxin related transmembrane protein 4 [Source:NCBI gene (formerly Entrezgene);Acc:100627887]
chr15	138226873	138230252	3380	*	chr15	138226873	138230252	in_vitro	in_vivo	11	12	3.95363636	3.275	0.67863636	c(t = 0.53981	0.595	0.90438588	Distal Intergenic	ENSSSCG000	RAMP1	receptor activity modifying protein 1 [Source:NCBI gene (formerly Entrezgene);Acc:397401]
chr4	99225345	99226081	737	*	chr4	99225345	99226081	in_vitro	in_vivo	11	12	1.47909091	2.3025	-0.8234091	c(t = -0.5412	0.595	0.90438588	Distal Intergenic	ENSSSCG000	FCGR1A	Fc fragment of IgG, high affinity Ia, receptor (CD64) [Source:NCBI gene (formerly Entrezgene);Acc:613130]
chr8	110205073	110205818	746	*	chr8	110205073	110205818	in_vitro	in_vivo	9	11	1.58777778	0.64909091	0.93868687	c(t = 0.54723	0.595	0.90438588	Distal Intergenic	ENSSSCG000	LARP7	La ribonucleoprotein 7, transcriptional regulator [Source:NCBI gene (formerly Entrezgene);Acc:100511273]
chr11	16972382	16973152	771	*	chr11	16972382	16973152	in_vitro	in_vivo	15	10	8.48466667	14.307	-5.8223333	c(t = -0.5399	0.596	0.90447472	Distal Intergenic	ENSSSCG000	RNASEH2B	ribonuclease H2 subunit B [Source:VGNC Symbol;Acc:VGNC:92339]
chr2	38828787	38829040	254	*	chr2	38828787	38829040	in_vitro	in_vivo	10	10	1.65	2.842	-1.192	c(t = -0.5414	0.596	0.90447472	Distal Intergenic	ENSSSCG000	CSRP3	cysteine and glycine rich protein 3 [Source:NCBI gene (formerly Entrezgene);Acc:100337687]
chr13	90640551	90643277	2727	*	chr13	90640551	90643277	in_vitro	in_vivo	15	15	2.686	2.218	0.468	c(t = 0.53663	0.596	0.90447472	Distal Intergenic	ENSSSCG000	SERP1	stress associated endoplasmic reticulum protein 1 [Source:NCBI gene (formerly Entrezgene);Acc:100156392]
chr6	28666388	28667243	856	*	chr6	28666388	28667243	in_vitro	in_vivo	10	9	1.253	2.03555556	-0.7825556	c(t = -0.5457	0.596	0.90447472	Distal Intergenic	ENSSSCG000	SLC12A4	solute carrier family 12 member 4 [Source:NCBI gene (formerly Entrezgene);Acc:396992]
chr1	147983496	147984710	1215	*	chr1	147983496	147984710	in_vitro	in_vivo	11	12	2.05727273	1.505	0.55227273	c(t = 0.53861	0.596	0.90447472	Distal Intergenic	ENSSSCG000	MBP	myelin basic protein [Source:NCBI gene (formerly Entrezgene);Acc:414286]
chrX	12295201	12295641	441	*	chrX	12295201	12295641	in_vitro	in_vivo	9	11	2.25777778	1.38909091	0.86868687	c(t = 0.54050	0.596	0.90447472	Distal Intergenic	ENSSSCG000	ACE2	angiotensin converting enzyme 2 [Source:VGNC Symbol;Acc:VGNC:85008]
chr4	16310206	16310817	612	*	chr4	16310206	16310817	in_vitro	in_vivo	9	9	1.23444444	0.50555556	0.72888889	c(t = 0.54641	0.596	0.90447472	Promoter (<=1kb)	ENSSSCG000	DERL1	derlin 1 [Source:NCBI gene (formerly Entrezgene);Acc:100626802]
chr6	115857800	115858532	733	*	chr6	115857800	115858532	in_vitro	in_vivo	11	13	1.04909091	0.45153846	0.59755245	c(t = 0.54513	0.596	0.90447472	Distal Intergenic	ENSSSCG000	TTR	transthyretin [Source:NCBI gene (formerly Entrezgene);Acc:397419]
chr6	270867	272033	1167	*	chr6	270867	272033	in_vitro	in_vivo	10	9	95.905	96.7422222	-0.8372222	c(t = -0.5383	0.597	0.90509864	Distal Intergenic	ENSSSCG000	SPATA2L	spermatogenesis associated 2 like [Source:NCBI gene (formerly Entrezgene);Acc:100415811]
chr10	68303867	68305905	2039	*	chr10	68303867	68305905	in_vitro	in_vivo	10	9	69.434	75.9577778	-6.5237778	c(t = -0.5390	0.597	0.90509864	Distal Intergenic	ENSSSCG000	WDR37	WD repeat domain 37 [Source:VGNC Symbol;Acc:VGNC:96258]
chr14	7107031	7108202	1172	*	chr14	7107031	7108202	in_vitro	in_vivo	10	11	3.181	2.54272727	0.63827273	c(t = 0.53788	0.597	0.90509864	Intron (NM_001162888/100302065, intron 2 of 5)	ENSSSCG000	PEBP4	phosphatidylethanolamine binding protein 4 [Source:VGNC Symbol;Acc:VGNC:91303]
chr6	52856377	52858587	2211	*	chr6	52856377	52858587	in_vitro	in_vivo	11	11	1.13363636	0.83727273	0.29636364	c(t = 0.53909	0.597	0.90509864	Distal Intergenic	ENSSSCG000	AP2S1	adaptor related protein complex 2 subunit sigma 1 [Source:VGNC Symbol;Acc:VGNC:96909]
chr7	112146476	112147817	1342	*	chr7	112146476	112147817	in_vitro	in_vivo	11	12	0.92545455	0.48916667	0.43628788	c(t = 0.54338	0.597	0.90509864	Distal Intergenic	ENSSSCG000	CALM1	calmodulin 1 [Source:NCBI gene (formerly Entrezgene);Acc:100154056]
chr15	132934568	132935243	676	*	chr15	132934568	132935243	in_vitro	in_vivo	10	10	15.687	24.554	-8.867	c(t = -0.5374	0.598	0.90554286	Distal Intergenic	ENSSSCG000	ssc-mir-1039	ssc-mir-10391 [Source:miRBase;Acc:MI0033405]
chr7	120204887	120207257	2371	*	chr7	120204887	120207257	in_vitro	in_vivo	13	11	17.9415385	12.0581818	5.88335664	c(t = 0.53493	0.598	0.90554286	Exon (NM_001297633/100154158, exon 4 of 5)	ENSSSCG000	BCL11B	BCL11 transcription factor B [Source:NCBI gene (formerly Entrezgene);Acc:100154158]
chr16	68678219	68680897	2679	*	chr16	68678219	68680897	in_vitro	in_vivo	14	12	6.83642857	5.20916667	1.6272619	c(t = 0.53451	0.598	0.90554286	Promoter (<=1kb)	ENSSSCG000	HAND1	heart and neural crest derivatives expressed 1 [Source:VGNC Symbol;Acc:VGNC:88777]
chr7	20789095	20789420	326	*	chr7	20789095	20789420	in_vitro	in_vivo	10	9	2.166	3.61666667	-1.4506667	c(t = -0.5390	0.598	0.90554286	Distal Intergenic	ENSSSCG000	H2BC5	H2B clustered histone 5 [Source:NCBI gene (formerly Entrezgene);Acc:100157327]
chr18	55561015	55561797	783	*	chr18	55561015	55561797	in_vitro	in_vivo	10	12	1.516	2.21583333	-0.6998333	c(t = -0.5359	0.598	0.90554286	Distal Intergenic	ENSSSCG000	RARRES2	retinoic acid receptor responder 2 [Source:VGNC Symbol;Acc:VGNC:92098]
chr9	47475496	47476606	1111	*	chr9	47475496	47476606	in_vitro	in_vivo	10	14	1.363	0.92642857	0.43657143	c(t = 0.54102	0.598	0.90554286	Distal Intergenic	ENSSSCG000	NECTIN1	nectin cell adhesion molecule 1 [Source:VGNC Symbol;Acc:VGNC:108604]
chr6	41427479	41428563	1085	*	chr6	41427479	41428563	in_vitro	in_vivo	12	10	8.56416667	11.89	-3.3258333	c(t = -0.5359	0.599	0.90598603	Distal Intergenic	ENSSSCG000	SLC7A9	solute carrier family 7 member 9 [Source:VGNC Symbol;Acc:VGNC:93177]
chr6	78787896	78789781	1886	*	chr6	78787896	78789781	in_vitro	in_vivo	15	14	1.588	2.00642857	-0.4184286	c(t = -0.5318	0.599	0.90598603	Distal Intergenic	ENSSSCG000	CDA	cytidine deaminase [Source:NCBI gene (formerly Entrezgene);Acc:100515954]
chr5	10003865	10004444	580	*	chr5	10003865	10004444	in_vitro	in_vivo	12	12	2.0925	1.43666667	0.65583333	c(t = 0.53596	0.599	0.90598603	Distal Intergenic	ENSSSCG000	EIF3L	eukaryotic translation initiation factor 3 subunit L [Source:VGNC Symbol;Acc:VGNC:87623]
chr1	26796784	26797440	657	*	chr1	26796784	26797440	in_vitro	in_vivo	9	11	1.77222222	0.93272727	0.83949495	c(t = 0.54101	0.599	0.90598603	Distal Intergenic	ENSSSCG000	IFNGR1	interferon gamma receptor 1 [Source:VGNC Symbol;Acc:VGNC:89041]
chr4	123371271	123371942	672	*	chr4	123371271	123371942	in_vitro	in_vivo	10	13	1.562	0.67923077	0.88276923	c(t = 0.54147	0.599	0.90598603	Distal Intergenic	ENSSSCG000	DNTTIP2	deoxynucleotidyltransferase terminal interacting protein 2 [Source:NCBI gene (formerly Entrezgene);Acc:100157622]
chr15	47012835	47013442	608	*	chr15	47012835	47013442	in_vitro	in_vivo	9	10	0.89333333	0.508	0.38533333	c(t = 0.53610	0.599	0.90598603	Distal Intergenic	ENSSSCG000	TLR3	toll like receptor 3 [Source:NCBI gene (formerly Entrezgene);Acc:100037937]
chr9	16921718	16923225	1508	*	chr9	16921718	16923225	in_vitro	in_vivo	10	10	52.262	40.381	11.881	c(t = 0.53438	0.6	0.90633536	Distal Intergenic	ENSSSCG000	TMEM126B	transmembrane protein 126B [Source:NCBI gene (formerly Entrezgene);Acc:100626990]
chr7	1140966	1142567	1602	*	chr7	1140966	1142567	in_vitro	in_vivo	11	14	32.83	39.4271429	-6.5971429	c(t = -0.5315	0.6	0.90633536	Distal Intergenic	ENSSSCG000	SERPINB9	serpin peptidase inhibitor, clade B (ovalbumin), member 9 [Source:NCBI gene (formerly Entrezgene);Acc:100126851]
chr6	55781617	55782322	706	*	chr6	55781617	55782322	in_vitro	in_vivo	9	9	11.6044444	19.3611111	-7.7566667	c(t = -0.5352	0.6	0.90633536	Distal Intergenic	ENSSSCG000	KLK7	kallikrein related peptidase 7 [Source:VGNC Symbol;Acc:VGNC:89546]
chr8	84122209	84123216	1008	*	chr8	84122209	84123216	in_vitro	in_vivo	11	14	11.2272727	8.59785714	2.62941558	c(t = 0.53277	0.6	0.90633536	Distal Intergenic	ENSSSCG000	ANAPC10	anaphase promoting complex subunit 10 [Source:VGNC Symbol;Acc:VGNC:98912]
chr8	137601725	137602573	849	*	chr8	137601725	137602573	in_vitro	in_vivo	12	10	2.99416667	1.955	1.03916667	c(t = 0.53358	0.6	0.90633536	Distal Intergenic	ENSSSCG000	BMP3	bone morphogenetic protein 3 [Source:NCBI gene (formerly Entrezgene);Acc:100512974]
chr15	46193363	46194177	815	*	chr15	46193363	46194177	in_vitro	in_vivo	9	10	2.16888889	1.319	0.84988889	c(t = 0.53783	0.6	0.90633536	Distal Intergenic	ENSSSCG000	LRP2BP	LRP2 binding protein [Source:VGNC Symbol;Acc:VGNC:96080]
chr10	68550474	68553286	2813	*	chr10	68550474	68553286	in_vitro	in_vivo	9	10	96.0966667	97.123	-1.0263333	c(t = -0.5326	0.601	0.90633536	Distal Intergenic	ENSSSCG000	WDR37	WD repeat domain 37 [Source:VGNC Symbol;Acc:VGNC:96258]
chr17	61484947	61497827	12881	*	chr17	61484947	61497827	in_vitro	in_vivo	14	15	95.4385714	92.7546667	2.68390476	c(t = 0.53120	0.601	0.90633536	Distal Intergenic	ENSSSCG000	PSMA7	proteasome 20S subunit alpha 7 [Source:NCBI gene (formerly Entrezgene);Acc:100626738]
chr13	47083221	47083855	635	*	chr13	47083221	47083855	in_vitro	in_vivo	9	13	13.7133333	20.6361538	-6.9228205	c(t = -0.5324	0.601	0.90633536	Distal Intergenic	ENSSSCG000	EOGT	EGF domain specific O-linked N-acetylglucosamine transferase [Source:VGNC Symbol;Acc:VGNC:87718]
chr9	113774648	113775299	652	*	chr9	113774648	113775299	in_vitro	in_vivo	10	10	23.418	18.668	4.75	c(t = 0.53289	0.601	0.90633536	Distal Intergenic	ENSSSCG000	MYOG	myogenin [Source:NCBI gene (formerly Entrezgene);Acc:497618]
chr13	121755014	121755621	608	*	chr13	121755014	121755621	in_vitro	in_vivo	10	12	13.627	10.0316667	3.59533333	c(t = 0.53275	0.601	0.90633536	Distal Intergenic	ENSSSCG000	AP2M1	adaptor related protein complex 2 subunit mu 1 [Source:VGNC Symbol;Acc:VGNC:85386]
chr7	65928021	65929427	1407	*	chr7	65928021	65929427	in_vitro	in_vivo	16	14	5.74625	6.79214286	-1.0458929	c(t = -0.5295	0.601	0.90633536	Distal Intergenic	ENSSSCG000	SNX6	sorting nexin 6 [Source:NCBI gene (formerly Entrezgene);Acc:100154775]
chr7	22938051	22939486	1436	*	chr7	22938051	22939486	in_vitro	in_vivo	12	10	1.54	2.583	-1.043	c(t = -0.5353	0.601	0.90633536	Promoter (<=1kb)	ENSSSCG00000001229		MHC class I antigen 3 [Source:NCBI gene (formerly Entrezgene);Acc:100037288]
chr10	24901644	24902251	608	*	chr10	24901644	24902251	in_vitro	in_vivo	9	12	2.15777778	1.36083333	0.79694444	c(t = 0.53423	0.601	0.90633536	Distal Intergenic	ENSSSCG000	ADIPOR1	adiponectin receptor 1 [Source:NCBI gene (formerly Entrezgene);Acc:404695]
chr8	86402752	86403898	1147	*	chr8	86402752	86403898	in_vitro	in_vivo	10	11	1.843	0.97636364	0.86663636	c(t = 0.53967	0.601	0.90633536	Distal Intergenic	ENSSSCG000	CLGN	calmegin [Source:VGNC Symbol;Acc:VGNC:86759]
chr6	47977077	47978408	1332	*	chr6	47977077	47978408	in_vitro	in_vivo	9	12	89.0911111	91.7566667	-2.6655556	c(t = -0.5317	0.602	0.90695354	Distal Intergenic	ENSSSCG000	IL28B	interleukin 28B (interferon, lambda 3) [Source:NCBI gene (formerly Entrezgene);Acc:100310828]
chr14	59301313	59304492	3180	*	chr14	59301313	59304492	in_vitro	in_vivo	13	13	2.68538462	4.35230769	-1.6669231	c(t = -0.5340	0.602	0.90695354	Distal Intergenic	ENSSSCG000	GNPAT	glyceronephosphate O-acyltransferase [Source:NCBI gene (formerly Entrezgene);Acc:100156253]
chr2	80433190	80433910	721	*	chr2	80433190	80433910	in_vitro	in_vivo	9	12	5.93777778	3.87833333	2.05944444	c(t = 0.53176	0.602	0.90695354	Distal Intergenic	ENSSSCG000	FAM193B	family with sequence similarity 193 member B [Source:NCBI gene (formerly Entrezgene);Acc:100511495]
chr5	151428	152527	1100	*	chr5	151428	152527	in_vitro	in_vivo	13	14	2.46769231	3.52785714	-1.0601648	c(t = -0.5306	0.602	0.90695354	Promoter (<=1kb)	ENSSSCG000	CHKB	choline kinase beta [Source:NCBI gene (formerly Entrezgene);Acc:100505461]
chr5	69621455	69622605	1151	*	chr5	69621455	69622605	in_vitro	in_vivo	12	11	1.85533333	1.27272727	0.58060606	c(t = 0.53182	0.602	0.90695354	Distal Intergenic	ENSSSCG000	ADA2	adenosine deaminase 2 [Source:NCBI gene (formerly Entrezgene);Acc:397079]
chr1	134018257	134018949	693	*	chr1	134018257	134018949	in_vitro	in_vivo	11	9	11.8190909	19.0122222	-7.1931313	c(t = -0.5298	0.603	0.90810406	Distal Intergenic	ENSSSCG000	ACTC1	actin alpha cardiac muscle 1 [Source:NCBI gene (formerly Entrezgene);Acc:100152267]
chr16	49760482	49760997	516	*	chr16	49760482	49760997	in_vitro	in_vivo	10	9	2.324	3.47222222	-1.1482222	c(t = -0.5322	0.603	0.90810406	Distal Intergenic	ENSSSCG000	DRD1	dopamine receptor D1 [Source:VGNC Symbol;Acc:VGNC:87443]
chr1	88596541	88598222	1682	*	chr1	88596541	88598222	in_vitro	in_vivo	14	13	1.27785714	1.74923077	-0.4713736	c(t = -0.5267	0.604	0.90933405	Promoter (<=1kb)	ENSSSCG000	HTR1B	5-hydroxytryptamine receptor 1B [Source:NCBI gene (formerly Entrezgene);Acc:397563]
chr7	2566991	2567375	385	*	chr7	2566991	2567375	in_vitro	in_vivo	9	9	21.5388889	30.6622222	-9.1233333	c(t = -0.5275	0.605	0.90933405	Distal Intergenic	ENSSSCG000	ECI2	enoyl-CoA delta isomerase 2 [Source:VGNC Symbol;Acc:VGNC:87532]
chr9	26679547	26681465	1919	*	chr9	26679547	26681465	in_vitro	in_vivo	14	12	13.0621429	16.5383333	-3.4761905	c(t = -0.5243	0.605	0.90933405	Distal Intergenic	ENSSSCG000	PIWIL4	piwi like RNA-mediated silencing 4 [Source:VGNC Symbol;Acc:VGNC:98200]
chr2	8982070	8982593	524	*	chr2	8982070	8982593	in_vitro	in_vivo	9	11	6.94	5.05090909	1.88909091	c(t = 0.53316	0.605	0.90933405	Downstream (<=300bp)	ENSSSCG000	NXF1	nuclear RNA export factor 1 [Source:VGNC Symbol;Acc:VGNC:90993]
chr5	23045947	23047008	1062	*	chr5	23045947	23047008	in_vitro	in_vivo	9	9	4.03666667	2.65	1.38666667	c(t = 0.52796	0.605	0.90933405	Distal Intergenic	ENSSSCG000	CDK4	cyclin dependent kinase 4 [Source:NCBI gene (formerly Entrezgene);Acc:100144492]
chr14	105043612	105044659	1048	*	chr14	105043612	105044659	in_vitro	in_vivo	12	12	1.0825	1.56333333	-0.4808333	c(t = -0.5247	0.605	0.90933405	Promoter (<=1kb)	ENSSSCG000	RBP4	retinol binding protein 4 [Source:VGNC Symbol;Acc:VGNC:92164]
chr6	62622907	62623504	598	*	chr6	62622907	62623504	in_vitro	in_vivo	10	10	0.714	1.212	-0.498	c(t = -0.5261	0.605	0.90933405	Distal Intergenic	ENSSSCG000	ISG15	ISG15 ubiquitin like modifier [Source:NCBI gene (formerly Entrezgene);Acc:100145895]
chrX	35452070	35453049	980	*	chrX	35452070	35453049	in_vitro	in_vivo	10	10	1.662	1.117	0.545	c(t = 0.52825	0.605	0.90933405	Distal Intergenic	ENSSSCG000	MID1IP1	MID1 interacting protein 1 [Source:VGNC Symbol;Acc:VGNC:90214]
chr15	99654372	99655817	1446	*	chr15	99654372	99655817	in_vitro	in_vivo	12	14	1.12833333	0.80071429	0.32761905	c(t = 0.52518	0.605	0.90933405	Distal Intergenic	ENSSSCG000	HECW2	HECT, C2 and WW domain containing E3 ubiquitin protein ligase 2 [Source:NCBI gene (formerly Entrezgene);Acc:100155879]
chr11	19377254	19378010	757	*	chr11	19377254	19378010	in_vitro	in_vivo	11	12	0.43090909	0.67416667	-0.2432576	c(t = -0.5251	0.605	0.90933405	Promoter (<=1kb)	ENSSSCG000	ITM2B	integral membrane protein 2B [Source:VGNC Symbol;Acc:VGNC:103971]
chrX	10581398	10582620	1223	*	chrX	10581398	10582620	in_vitro	in_vivo	9	10	7.83555556	11.413	-3.5774444	c(t = -0.5319	0.606	0.90949405	Distal Intergenic	ENSSSCG000	GEMIN8	gem nuclear organelle associated protein 8 [Source:VGNC Symbol;Acc:VGNC:88416]
chr8	99309155	99312124	2970	*	chr8	99309155	99312124	in_vitro	in_vivo	12	12	8.84666667	7.49416667	1.3525	c(t = 0.52472	0.606	0.90949405	Distal Intergenic	ENSSSCG000	SPRY1	sprouty RTK signaling antagonist 1 [Source:VGNC Symbol;Acc:VGNC:93424]
chr11	68849154	68849805	652	*	chr11	68849154	68849805	in_vitro	in_vivo	11	9	5.70181818	3.65777778	2.0440404	c(t = 0.52699	0.606	0.90949405	Distal Intergenic	ENSSSCG000	TMTC4	transmembrane O-mannosyltransferase targeting cadherins 4 [Source:VGNC Symbol;Acc:VGNC:94244]
chr2	82596978	82598732	1755	*	chr2	82596978	82598732	in_vitro	in_vivo	14	11	2.10071429	2.73454545	-0.6338312	c(t = -0.5246	0.606	0.90949405	Distal Intergenic	ENSSSCG000	BTF3	basic transcription factor 3 [Source:VGNC Symbol;Acc:VGNC:99597]
chr12	58010985	58012177	1193	*	chr12	58010985	58012177	in_vitro	in_vivo	13	14	1.71307692	2.32285714	-0.6097802	c(t = -0.5219	0.606	0.90949405	Promoter (<=1kb)	ENSSSCG000	COX10	cytochrome c oxidase assembly factor heme A:farnesyltransferase COX10 [Source:NCBI gene (formerly Entrezgene);Acc:100521863]
chr11	7063786	7064817	1032	*	chr11	7063786	7064817	in_vitro	in_vivo	12	12	2.59833333	1.81416667	0.78416667	c(t = 0.52570	0.606	0.90949405	Distal Intergenic	ENSSSCG00000009327		high mobility group box 1 [Source:NCBI gene (formerly Entrezgene);Acc:445521]
chr11	75547505	75548350	846	*	chr11	75547505	75548350	in_vitro	in_vivo	12	15	1.12083333	1.582	-0.4611667	c(t = -0.5217	0.606	0.90949405	Distal Intergenic	ENSSSCG000	TNFSF13B	TNF superfamily member 13b [Source:VGNC Symbol;Acc:VGNC:94269]
chrX	21066138	21066937	800	*	chrX	21066138	21066937	in_vitro	in_vivo	9	10	10.4322222	15.581	-5.1487778	c(t = -0.5236	0.607	0.90949405	Distal Intergenic	ENSSSCG000	SAT1	spermidine/spermine N1-acetyltransferase 1 [Source:NCBI gene (formerly Entrezgene);Acc:397645]
chr7	36603706	36604403	698	*	chr7	36603706	36604403	in_vitro	in_vivo	12	9	12.1983333	8.27222222	3.92611111	c(t = 0.52269	0.607	0.90949405	Distal Intergenic	ENSSSCG000	TREM1	triggering receptor expressed on myeloid cells 1 [Source:NCBI gene (formerly Entrezgene);Acc:396601]
chr4	129323239	129323634	396	*	chr4	129323239	129323634	in_vitro	in_vivo	9	10	12.2333333	6.018	6.21533333	c(t = 0.53159	0.607	0.90949405	Distal Intergenic	ENSSSCG000	SELENOF	selenoprotein F [Source:NCBI gene (formerly Entrezgene);Acc:100038009]

chr4	130392805	130399179	6375	*	chr4	130392805	130399179	in_vitro	in_vivo	13	17	7.56769231	5.65529412	1.91239819	c(t = 0.52390	0.607	0.90949405	Distal Intergenic	ENSSSCG000	ZNHIT6	zinc finger HIT-type containing 6 [Source:VGNC Symbol;Acc:VGNC:98909]
chr14	134486594	134489993	3400	*	chr14	134486594	134489993	in_vitro	in_vivo	11	12	3.16363636	2.11416667	1.0494697	c(t = 0.52805	0.607	0.90949405	Distal Intergenic	ENSSSCG000	LHPP	phospholysine phosphohistidine inorganic pyrophosphate phosphatase [Source:VGNC Symbol;Acc:VGNC:89710]
chr14	59113079	59114840	1762	*	chr14	59113079	59114840	in_vitro	in_vivo	14	14	1.54142857	2.02142857	-0.48	c(t = -0.5212	0.607	0.90949405	Distal Intergenic	ENSSSCG000	TSNAX	translin associated factor X [Source:NCBI gene (formerly Entrezgene);Acc:100156036]
chr18	10532151	10533086	936	*	chr18	10532151	10533086	in_vitro	in_vivo	13	13	1.91846154	1.41846154	0.5	c(t = 0.52114	0.607	0.90949405	Promoter (<=1kb)	ENSSSCG000	ZC3HAV1	zinc finger CCCH-type containing, antiviral 1 [Source:VGNC Symbol;Acc:VGNC:95102]
chr4	15181844	15182924	1081	*	chr4	15181844	15182924	in_vitro	in_vivo	10	12	0.794	1.29833333	-0.5043333	c(t = -0.5238	0.607	0.90949405	Distal Intergenic	ENSSSCG000	TATDN1	TatD DNase domain containing 1 [Source:VGNC Symbol;Acc:VGNC:98359]
chr1	147461608	147463377	1770	*	chr1	147461608	147463377	in_vitro	in_vivo	12	10	1.49666667	1.186	0.31066667	c(t = 0.52367	0.607	0.90949405	Distal Intergenic	ENSSSCG000	MBP	myelin basic protein [Source:NCBI gene (formerly Entrezgene);Acc:414286]
chrX	125072528	125075584	3057	*	chrX	125072528	125075584	in_vitro	in_vivo	9	11	13.3066667	20.6427273	-7.3360606	c(t = -0.5220	0.608	0.91010518	Distal Intergenic	ENSSSCG000	IKBKG	inhibitor of nuclear factor kappa B kinase regulatory subunit gamma [Source:VGNC Symbol;Acc:VGNC:89072]
chr6	60857817	60859961	2145	*	chr6	60857817	60859961	in_vitro	in_vivo	11	11	9	13.86	-4.86	c(t = -0.5217	0.608	0.91010518	Distal Intergenic	ENSSSCG000	GALP	galanin like peptide [Source:NCBI gene (formerly Entrezgene);Acc:396772]
chr13	71983803	71990542	6740	*	chr13	71983803	71990542	in_vitro	in_vivo	14	15	3.50714286	4.92933333	-1.4221905	c(t = -0.5226	0.608	0.91010518	Promoter (<=1kb)	ENSSSCG000	GATA2	GATA binding protein 2 [Source:VGNC Symbol;Acc:VGNC:88364]
chr2	139522238	139523465	1228	*	chr2	139522238	139523465	in_vitro	in_vivo	13	11	1.19153846	1.67	-0.4784615	c(t = -0.5227	0.608	0.91010518	Distal Intergenic	ENSSSCG000	ssc-mir-874	ssc-mir-874 [Source:miRBase;Acc:MI0022157]
chr2	13353694	13354123	430	*	chr2	13353694	13354123	in_vitro	in_vivo	9	9	0.68777778	1.06555556	-0.3777778	c(t = -0.5236	0.608	0.91010518	Promoter (<=1kb)	ENSSSCG000	UBE2L6	ubiquitin conjugating enzyme E2 L6 [Source:NCBI gene (formerly Entrezgene);Acc:100514753]
chr7	22955936	22957206	1271	*	chr7	22955936	22957206	in_vitro	in_vivo	12	11	2.24333333	3.18727273	-0.9439394	c(t = -0.5195	0.609	0.91074889	Promoter (<=1kb)	ENSSSCG00000052432		MHC class I antigen 2 [Source:NCBI gene (formerly Entrezgene);Acc:100135031]
chr4	129904638	129907181	2544	*	chr4	129904638	129907181	in_vitro	in_vivo	13	13	1.33	0.97846154	0.35153846	c(t = 0.51965	0.609	0.91074889	Distal Intergenic	ENSSSCG000	CLCA1	chloride channel accessory 1 [Source:NCBI gene (formerly Entrezgene);Acc:397284]
chr13	152099917	152100490	574	*	chr13	152099917	152100490	in_vitro	in_vivo	10	10	1.046	0.65	0.396	c(t = 0.52178	0.609	0.91074889	Distal Intergenic	ENSSSCG000	CD47	CD47 molecule [Source:VGNC Symbol;Acc:VGNC:108637]
chr2	58631062	58632193	1132	*	chr2	58631062	58632193	in_vitro	in_vivo	12	14	11.1433333	16.0428571	-4.8995238	c(t = -0.5161	0.61	0.91074889	Distal Intergenic	ENSSSCG000	RFXANK	regulatory factor X associated ankyrin containing protein [Source:VGNC Symbol;Acc:VGNC:92250]
chr8	44764446	44764933	488	*	chr8	44764446	44764933	in_vitro	in_vivo	9	9	2.94444444	2.02	0.92444444	c(t = 0.52029	0.61	0.91074889	Distal Intergenic	ENSSSCG000	GUCY1B1	guanylate cyclase 1 soluble subunit beta 1 [Source:VGNC Symbol;Acc:VGNC:88752]
chr2	60983602	60985160	1559	*	chr2	60983602	60985160	in_vitro	in_vivo	11	11	2.38090909	1.63818182	0.74272727	c(t = 0.51830	0.61	0.91074889	Distal Intergenic	ENSSSCG000	SMIM7	small integral membrane protein 7 [Source:NCBI gene (formerly Entrezgene);Acc:100512765]
chr12	3427268	3432122	4855	*	chr12	3427268	3432122	in_vitro	in_vivo	12	11	72.14	76.2945455	-4.1545455	c(t = -0.5165	0.611	0.91074889	Distal Intergenic	ENSSSCG00000017164		TIMP metallopeptidase inhibitor 2 [Source:VGNC Symbol;Acc:VGNC:93995]
chr5	17347588	17348705	1118	*	chr5	17347588	17348705	in_vitro	in_vivo	9	11	71.9544444	62.6	9.35444444	c(t = 0.51838	0.611	0.91074889	Exon (NM_001195322/100152462, exon 9 of 9)	ENSSSCG000	ACVR1B	activin A receptor type 1B [Source:VGNC Symbol;Acc:VGNC:85056]
chr18	42200141	42200584	444	*	chr18	42200141	42200584	in_vitro	in_vivo	11	9	8.49727273	12.5	-4.0027273	c(t = -0.5229	0.611	0.91074889	Distal Intergenic	ENSSSCG000	INMT	indolethylamine N-methyltransferase [Source:NCBI gene (formerly Entrezgene);Acc:100512997]
chr4	79872407	79873798	1392	*	chr4	79872407	79873798	in_vitro	in_vivo	13	15	5.83230769	7.57466667	-1.742359	c(t = -0.5160	0.611	0.91074889	Distal Intergenic	ENSSSCG000	UBE2V2	ubiquitin conjugating enzyme E2 V2 [Source:NCBI gene (formerly Entrezgene);Acc:100627960]
chr3	95458776	95460727	1952	*	chr3	95458776	95460727	in_vitro	in_vivo	10	14	1.981	2.70642857	-0.7254286	c(t = -0.5155	0.611	0.91074889	Distal Intergenic	ENSSSCG000	SLC3A1	solute carrier family 3 member 1 [Source:NCBI gene (formerly Entrezgene);Acc:100144588]
chr15	101372800	101374319	1520	*	chr15	101372800	101374319	in_vitro	in_vivo	11	12	1.61727273	2.27666667	-0.6593939	c(t = -0.5163	0.611	0.91074889	Promoter (<=1kb)	ENSSSCG000	HSPD1	heat shock protein family D (Hsp60) member 1 [Source:NCBI gene (formerly Entrezgene);Acc:492279]
chr12	2034808	2037621	2814	*	chr12	2034808	2037621	in_vitro	in_vivo	9	12	1.70111111	2.145	-0.4438889	c(t = -0.5177	0.611	0.91074889	Distal Intergenic	ENSSSCG000	SLC26A11	solute carrier family 26 member 11 [Source:NCBI gene (formerly Entrezgene);Acc:100191010]
chr3	31236814	31238380	1567	*	chr3	31236814	31238380	in_vitro	in_vivo	12	13	2.80333333	2.06769231	0.73564103	c(t = 0.51842	0.611	0.91074889	Distal Intergenic	ENSSSCG000	PRM1	protamine 1 [Source:NCBI gene (formerly Entrezgene);Acc:397487]
chr14	10730013	10732884	2872	*	chr14	10730013	10732884	in_vitro	in_vivo	15	15	2.20333333	1.73533333	0.468	c(t = 0.51678	0.611	0.91074889	Promoter (<=1kb)	ENSSSCG000	ADRA1A	adrenoceptor alpha 1A [Source:VGNC Symbol;Acc:VGNC:85154]
chr17	30908625	30909114	490	*	chr17	30908625	30909114	in_vitro	in_vivo	9	10	0.77444444	1.225	-0.4505556	c(t = -0.5194	0.611	0.91074889	Distal Intergenic	ENSSSCG000	CST7	cystatin F [Source:NCBI gene (formerly Entrezgene);Acc:100152012]
chr14	29297082	29297881	800	*	chr14	29297082	29297881	in_vitro	in_vivo	11	13	1.09909091	0.69692308	0.40216783	c(t = 0.51533	0.611	0.91074889	Distal Intergenic	ENSSSCG000	SUMO4	SMT3 suppressor of mif two 3 homolog 4 (S. cerevisiae) [Source:NCBI gene (formerly Entrezgene);Acc:100127140]
chr6	599029	614668	15640	*	chr6	599029	614668	in_vitro	in_vivo	14	14	96.2214286	96.9307143	-0.7092857	c(t = -0.5140	0.612	0.91074889	Distal Intergenic	ENSSSCG00000024974		ribosomal protein L13 [Source:NCBI gene (formerly Entrezgene);Acc:100628232]
chr17	32515986	32516624	639	*	chr17	32515986	32516624	in_vitro	in_vivo	10	10	11.334	18.288	-6.954	c(t = -0.5159	0.612	0.91074889	Distal Intergenic	ENSSSCG000	DDRGK1	DDRGK domain containing 1 [Source:NCBI gene (formerly Entrezgene);Acc:100155894]
chr2	82462988	82463502	515	*	chr2	82462988	82463502	in_vitro	in_vivo	9	10	12.8355556	9.086	3.74955556	c(t = 0.51812	0.612	0.91074889	Distal Intergenic	ENSSSCG000	TMEM171	transmembrane protein 171 [Source:VGNC Symbol;Acc:VGNC:94109]
chr6	170401157	170403038	1882	*	chr6	170401157	170403038	in_vitro	in_vivo	11	9	1.08272727	1.75888889	-0.6761616	c(t = -0.5237	0.612	0.91074889	Distal Intergenic	ENSSSCG000	ssc-mir-30c-1	ssc-mir-30c-1 [Source:miRBase;Acc:MI0016618]
chr12	39069418	39069923	506	*	chr12	39069418	39069923	in_vitro	in_vivo	9	9	0.88444444	1.46111111	-0.5766667	c(t = -0.5209	0.612	0.91074889	Distal Intergenic	ENSSSCG000	HNF1B	HNF1 homeobox B [Source:NCBI gene (formerly Entrezgene);Acc:397002]
chr2	44890049	44891037	989	*	chr2	44890049	44891037	in_vitro	in_vivo	15	13	2.11133333	1.37846154	0.73287179	c(t = 0.51622	0.612	0.91074889	Distal Intergenic	ENSSSCG000	COPB1	COPI coat complex subunit beta 1 [Source:NCBI gene (formerly Entrezgene);Acc:100337660]
chr14	140735848	140737091	1244	*	chr14	140735848	140737091	in_vitro	in_vivo	10	9	1.937	1.34777778	0.58922222	c(t = 0.51774	0.612	0.91074889	Distal Intergenic	ENSSSCG000	ssc-mir-202	ssc-mir-202 [Source:miRBase;Acc:MI0013157]
chr17	25363018	25363724	707	*	chr17	25363018	25363724	in_vitro	in_vivo	10	10	0.708	0.421	0.287	c(t = 0.51852	0.612	0.91074889	Promoter (1-2kb)	ENSSSCG00000037602		small nuclear ribonucleoprotein polypeptide B2 [Source:VGNC Symbol;Acc:VGNC:108738]
chr3	1378383	1388667	10285	*	chr3	1378383	1388667	in_vitro	in_vivo	11	15	94.7218182	93.4766667	1.24515152	c(t = 0.51281	0.613	0.91074889	Distal Intergenic	ENSSSCG000	MRM2	mitochondrial rRNA methyltransferase 2 [Source:VGNC Symbol;Acc:VGNC:99722]
chr12	317530	320924	3395	*	chr12	317530	320924	in_vitro	in_vivo	11	9	91.8763636	89.9022222	1.97414141	c(t = 0.51741	0.613	0.91074889	Distal Intergenic	ENSSSCG000	TBCD	tubulin folding cofactor D [Source:NCBI gene (formerly Entrezgene);Acc:100512127]
chr5	82973332	82973912	581	*	chr5	82973332	82973912	in_vitro	in_vivo	11	10	5.88545455	7.691	-1.8055455	c(t = -0.5181	0.613	0.91074889	Promoter (<=1kb)	ENSSSCG000	SLC5A8	solute carrier family 5 member 8 [Source:VGNC Symbol;Acc:VGNC:93148]
chr3	69700881	69702283	1403	*	chr3	69700881	69702283	in_vitro	in_vivo	10	12	3.739	4.73666667	-0.9976667	c(t = -0.5143	0.613	0.91074889	Distal Intergenic	ENSSSCG000	CCT7	chaperonin containing TCP1 subunit 7 [Source:NCBI gene (formerly Entrezgene);Acc:100322871]
chr18	45416788	45418928	2141	*	chr18	45416788	45418928	in_vitro	in_vivo	11	14	2.53181818	3.59214286	-1.0603247	c(t = -0.5128	0.613	0.91074889	Distal Intergenic	ENSSSCG000	HOXA5	homeobox A5 [Source:NCBI gene (formerly Entrezgene);Acc:100499568]
chr3	7174099	7174919	821	*	chr3	7174099	7174919	in_vitro	in_vivo	11	9	2.88909091	2	0.88909091	c(t = 0.51534	0.613	0.91074889	Distal Intergenic	ENSSSCG000	NTAN1	N-terminal asparagine amidase [Source:NCBI gene (formerly Entrezgene);Acc:397107]
chr2	26094674	26095683	1010	*	chr2	26094674	26095683	in_vitro	in_vivo	11	10	1.27545455	1.905	-0.6295455	c(t = -0.5149	0.613	0.91074889	Distal Intergenic	ENSSSCG000	ssc-mir-1343	ssc-mir-1343 [Source:miRBase;Acc:MI0017996]
chr14	129465934	129467679	1746	*	chr14	129465934	129467679	in_vitro	in_vivo	11	12	2.44363636	1.38916667	1.0544697	c(t = 0.51298	0.613	0.91074889	Distal Intergenic	ENSSSCG000	MCMBP	minichromosome maintenance complex binding protein [Source:VGNC Symbol;Acc:VGNC:90077]
chr6	42589290	42590345	1056	*	chr6	42589290	42590345	in_vitro	in_vivo	9	9	2.21666667	1.19777778	1.01888889	c(t = 0.52138	0.613	0.91074889	Distal Intergenic	ENSSSCG000	SLC7A9	solute carrier family 7 member 9 [Source:VGNC Symbol;Acc:VGNC:93177]
chr5	63751252	63752014	763	*	chr5	63751252	63752014	in_vitro	in_vivo	9	10	2.07333333	1.111	0.96233333	c(t = 0.51926	0.613	0.91074889	Promoter (<=1kb)	ENSSSCG000	EMG1	EMG1 N1-specific pseudouridine methyltransferase [Source:NCBI gene (formerly Entrezgene);Acc:100627558]
chr14	83315940	83317599	1660	*	chr14	83315940	83317599	in_vitro	in_vivo	13	13	1.44153846	0.94923077	0.49230769	c(t = 0.51624	0.613	0.91074889	Distal Intergenic	ENSSSCG000	PRXL2A	peroxiredoxin like 2A [Source:VGNC Symbol;Acc:VGNC:91892]
chr6	93833196	93834193	998	*	chr6	93833196	93834193	in_vitro	in_vivo	12	10	1.18833333	0.782	0.40633333	c(t = 0.51534	0.613	0.91074889	Distal Intergenic	ENSSSCG000	MTF1	metal regulatory transcription factor 1 [Source:VGNC Symbol;Acc:VGNC:90443]
chr13	24046101	24046910	810	*	chr13	24046101	24046910	in_vitro	in_vivo	13	12	0.88076923	0.51	0.37076923	c(t = 0.51639	0.613	0.91074889	Promoter (<=1kb)	ENSSSCG00000022773		
chr13	206074986	206078631	3646	*	chr13	206074986	206078631	in_vitro	in_vivo	10	11	92.897	89.7081818	3.18881818	c(t = 0.51263	0.614	0.91074889	Distal Intergenic	ENSSSCG000	TFF3	trefoil factor 3 [Source:NCBI gene (formerly Entrezgene);Acc:100627480]
chrX	124822947	124824355	1409	*	chrX	124822947	124824355	in_vitro	in_vivo	13	11	15.3723077	21.2854545	-5.9131469	c(t = -0.5118	0.614	0.91074889	Exon (NM_001011506/494564, exon 3 of 6)	ENSSSCG00000029523		
chrX	122872514	122873239	726	*	chrX	122872514	122873239	in_vitro	in_vivo	14	11	2.66571429	4.05636364	-1.3906494	c(t = -0.5176	0.614	0.91074889	Distal Intergenic	ENSSSCG000	FATE1	fetal and adult testis expressed 1 [Source:NCBI gene (formerly Entrezgene);Acc:396705]
chr1	143538747	143541809	3063	*	chr1	143538747	143541809	in_vitro	in_vivo	13	15	3.25846154	2.564	0.69446154	c(t = 0.51126	0.614	0.91074889	Promoter (<=1kb)	ENSSSCG000	KLF13	KLF transcription factor 13 [Source:NCBI gene (formerly Entrezgene);Acc:494563]
chr2	136972266	136973098	833	*	chr2	136972266	136973098	in_vitro	in_vivo	10	11	1.02	1.99818182	-0.9781818	c(t = -0.5173	0.614	0.91074889	Promoter (<=1kb)	ENSSSCG000	DDX46	DEAD-box helicase 46 [Source:VGNC Symbol;Acc:VGNC:87223]
chr9	126347033	126347900	868	*	chr9	126347033	126347900	in_vitro	in_vivo	12	15	1.19333333	1.77533333	-0.582	c(t = -0.5110	0.614	0.91074889	Distal Intergenic	ENSSSCG000	IVNS1ABP	influenza virus NS1A binding protein [Source:NCBI gene (formerly Entrezgene);Acc:100302027]
chr1	26798749	26799476	728	*	chr1	26798749	26799476	in_vitro	in_vivo	10	12	2.009	1.39833333	0.61066667	c(t = 0.51580	0.614	0.91074889	Distal Intergenic	ENSSSCG000	IFNGR1	interferon gamma receptor 1 [Source:VGNC Symbol;Acc:VGNC:89041]
chr14	134154295	134154917	623	*	chr14	134154295	134154917	in_vitro	in_vivo	10	10	0.758	1.25636364	-0.4983636	c(t = -0.5168	0.614	0.91074889	Distal Intergenic	ENSSSCG000	LHPP	phospholysine phosphohistidine inorganic pyrophosphate phosphatase [Source:VGNC Symbol;Acc:VGNC:89710]
chr5	88104146	88105318	1173	*	chr5	88104146	88105318	in_vitro	in_vivo	11	10	0.99363636	0.556	0.43763636	c(t = 0.51247	0.614	0.91074889	Distal Intergenic	ENSSSCG000	ssc-mir-331	ssc-mir-331 [Source:miRBase;Acc:MI0013142]
chr3	39776250	39777782	1533	*	chr3	39776250	39777782	in_vitro	in_vivo	11	12	10.0536364	7.11666667	2.9369697	c(t = 0.51126	0.615	0.91115392	Promoter (1-2kb)	ENSSSCG000	ssc-mir-1842	ssc-mir-1842 [Source:miRBase;Acc:MI0033396]
chr1	251067197	251067923	727	*	chr1	251067197	251067923	in_vitro	in_vivo	11	11	2.83454545	1.90909091	0.92545455	c(t = 0.51305	0.615	0.91115392	Promoter (<=1kb)	ENSSSCG000	PALM2AKAP;	PALM2-AKAP2 fusion [Source:NCBI gene (formerly Entrezgene);Acc:100152369]
chr6	93588711	93589107	397	*	chr6	93588711	93589107	in_vitro	in_vivo	13	11	0.98384615	1.72909091	-0.7452448	c(t = -0.5130	0.615	0.91115392	Promoter (<=1kb)	ENSSSCG000	MEAF6	MYST/Esa1 associated factor 6 [Source:VGNC Symbol;Acc:VGNC:90098]
chr5	92935043	92937351	2309	*	chr5	92935043	92937351	in_vitro	in_vivo	13	13	1.15	0.87	0.28	c(t = 0.50938	0.615	0.91115392	Promoter (<=1kb)	ENSSSCG000	ATP2B1	ATPase plasma membrane Ca2+ transporting 1 [Source:VGNC Symbol;Acc:VGNC:103219]
chr2	18611376	18612040	665	*	chr2	18611376	18612040	in_vitro	in_vivo	10	12	1.111	0.50916667	0.60183333	c(t = 0.51734	0.615	0.91115392	Distal Intergenic	NA	NA	NA
chr3	41225386	41228731	3346	*	chr3	41225386	41228731	in_vitro	in_vivo	12	11	50.3433333	57.9372727	-7.5939394	c(t = -0.5085	0.616	0.91115392	Distal Intergenic	ENSSSCG000	NME4	NME/NM23 nucleoside diphosphate kinase 4 [Source:VGNC Symbol;Acc:VGNC:90792]
chr10	11706226	11708540	2315	*	chr10	11706226	11708540	in_vitro	in_vivo	10	11	15.865	10.3845455	5.48045455	c(t = 0.51318	0.616	0.91115392	Distal Intergenic	ENSSSCG000	FH	fumarate hydratase [Source:VGNC Symbol;Acc:VGNC:96300]
chr13	137455216	137457020	1805	*	chr13	137455216	137457020	in_vitro	in_vivo	11	13	3.48727273	2.74615385	0.74111888	c(t = 0.50818	0.616	0.91115392	Distal Intergenic	ENSSSCG000	KPNA1	karyopherin subunit alpha 1 [Source:NCBI gene (formerly Entrezgene);Acc:397235]
chr15	132508587	132510578	1992	*	chr15	132508587	132510578	in_vitro	in_vivo	12	13	1.4525	1.89153846	-0.4390385	c(t = -0.5091	0.616	0.91115392	Promoter (<=1kb)	ENSSSCG000	NPPC	natriuretic peptide C [Source:VGNC Symbol;Acc:VGNC:96450]
chr13	124181513	124182896	1384	*	chr13	124181513	124182896	in_vitro	in_vivo	11	13	1.91090909	1.45769231	0.45321678	c(t = 0.50900	0.616	0.91115392	Distal Intergenic	ENSSSCG000	TBCCD1	TBCC domain containing 1 [Source:VGNC Symbol;Acc:VGNC:93784]
chr7	39221325	39222273	949	*	chr7	39221325	39222273	in_vitro	in_vivo	9	9	1.86555556	1.18888889	0.67666667	c(t = 0.51198	0.616	0.91115392	Distal Intergenic	ENSSSCG000	HSP90AB1	heat shock protein 90 alpha family class B member 1 [Source:NCBI gene (formerly Entrezgene);Acc:396742]
chr7	340874	341747	874	*	chr7	340874	341747	in_vitro	in_vivo	10	12	1.469	0.94666667	0.52233333	c(t = 0.51035	0.616	0.91115392	Distal Intergenic	ENSSSCG000	IRF4	interferon regulatory factor 4 [Source:VGNC Symbol;Acc:VGNC:89207]
chr7	1775647	1776106	460	*	chr7	1775647	1776106	in_vitro	in_vivo	9	11	1.02888889	0.63	0.39888889	c(t = 0.51526	0.616	0.91115392	Distal Intergenic	ENSSSCG000	SERPINB9	serpin peptidase inhibitor, clade B (ovalbumin), member 9 [Source:NCBI gene (formerly Entrezgene);Acc:100126851]
chr4	124367013	124368027	1015	*	chr4	124367013	124368027	in_vitro	in_vivo	9	14	0.94555556	0.50785714	0.43769841	c(t = 0.51565	0.616	0.91115392	Promoter (<=1kb)	ENSSSCG000	RPL5	ribosomal protein L5 [Source:NCBI gene (formerly Entrezgene);Acc:100135671]
chr13	207710740	207725706	14967	*	chr13	207710740	207725706	in_vitro	in_vivo	15	15	90.802	89.276	1.526	c(t = 0.50551	0.617	0.91115392	Distal Intergenic	ENSSSCG000	ITGB2	integrin subunit beta 2 [Source:NCBI gene (formerly Entrezgene);Acc:396943]
chrX	124194635	124195446	812	*	chrX	124194635	124195446	in_vitro	in_vivo	11	9	15.1445455	19.8111111	-4.6665657	c(t = -0.5092	0.617	0.91115392	Distal Intergenic	ENSSSCG000	PNCK	pregnancy up-regulated nonubiquitous CaM kinase [Source:NCBI gene (formerly Entrezgene);Acc:100337678]
chr7	59386220	59388505	2286	*	chr7	59386220	59388505	in_vitro	in_vivo	13	14	6.10153846	4.84357143	1.25796703	c(t = 0.50755	0.617	0.91115392	Distal Intergenic	ENSSSCG000	CYP11A1	cytochrome P450 family 11 subfamily A member 1 [Source:VGNC Symbol;Acc:VGNC:103363]
chr13	82144978	82145839	862	*	chr13	82144978	82145839	in_vitro	in_vivo	12	10	2.90833333	4.334	-1.4256667	c(t = -0.5126	0.617	0.91115392	Distal Intergenic	ENSSSCG000	ZBTB38	zinc finger and BTB domain containing 38 [Source:VGNC Symbol;Acc:VGNC:95072]
chr1	268652871	268653576	706	*	chr1	268652871	268653576	in_vitro	in_vivo	9	9	3.42333333	2.2	1.22333333	c(t = 0.51294	0.617	0.91115392	Distal Intergenic	NA	NA	NA
chr6	147428927	147429746	820	*	chr6	147428927	147429746	in_vitro	in_vivo	9	10	2.07888889	1.447	0.63188889	c(t = 0.51033	0.617	0.91115392	Promoter (<=1kb)	ENSSSCG000	JAK1	Janus kinase 1 [Source:VGNC Symbol;Acc:VGNC:89270]
chr6	108516701	108517275	575	*	chr6	108516701	108517275	in_vitro	in_vivo	12	11	0.77	1.25	-0.48	c(t = -0.5113	0.617	0.91115392	Distal Intergenic	ENSSSCG000	NPC1	NPC intracellular cholesterol transporter 1 [Source:NCBI gene (formerly Entrezgene);Acc:397591]
chr6	14808938	14809826	889	*	chr6	14808938	14809826	in_vitro	in_vivo	13	10	0.24846154	0.448	-0.1995385	c(t = -0.5079	0.617	0.91115392	Distal Intergenic	ENSSSCG00000002749		haptoglobin [Source:NCBI gene (formerly Entrezgene);Acc:397061]
chr4	83458199	83458885	687	*	chr4	83458199	83458885	in_vitro	in_vivo	9	11	0.71444444	0.41363636	0.30080808	c(t = 0.51277	0.617	0.91115392	Distal Intergenic	ENSSSCG000	CD247	CD247 molecule [Source:VGNC Symbol;Acc:VGNC:86401]
chr7	8660035	8661131	1097	*	chr7	8660035	8661131	in_vitro	in_vivo	11	12	92.6345455	94.8291667	-2.1946212	c(t = -0.5076	0.618	0.91175466	Distal Intergenic	ENSSSCG000	EDN1	endothelin 1 [Source:NCBI gene (formerly Entrezgene);Acc:396915]
chr14	102891463	102892205	743	*	chr14	102891463	102892205	in_vitro	in_vivo	11	12	7.09545455	8.96583333	-1.8703788	c(t = -0.5058	0.618	0.91175466	Distal Intergenic	ENSSSCG000	ANKRD1	ankyrin repeat domain 1 [Source:VGNC Symbol;Acc:VGNC:85321]
chr7	992703	995178	2476	*	chr7	992703	995178	in_vitro	in_vivo	9	13	11.7644444	8.49538462	3.26905983	c(t = 0.50798	0.618	0.91175466	Distal Intergenic	ENSSSCG000	SERPINB9	serpin peptidase inhibitor, clade B (ovalbumin), member 9 [Source:NCBI gene (formerly Entrezgene);Acc:100126851]
chr3	11018829	11020759	1931	*	chr3	11018829	11020759	in_vitro	in_vivo	14	15	2.27142857	2.90866667	-0.6372381	c(t = -0.5044	0.618	0.91175466	Promoter (<=1kb)	ENSSSCG000	CLDN3	claudin 3 [Source:NCBI gene (formerly Entrezgene);Acc:431781]
chr6	47741333	47741841	509	*	chr6	47741333	47741841	in_vitro	in_vivo	13	9	2.28846154	1.68666667	0.60179487	c(t = 0.50680	0.618	0.91175466	Distal Intergenic	ENSSSCG000	SIRT2	sirtuin 2 [Source:VGNC Symbol;Acc:VGNC:92885]
chr8	55569011	55571703	2693	*	chr8	55569011	55571703	in_vitro	in_vivo	13	13	13.7115385	18.6646154	-4.9530769	c(t = -0.5044	0.619	0.91200441	Distal Intergenic	ENSSSCG00000008898		HOP homeobox [Source:VGNC Symbol;Acc:VGNC:88932]
chr16	79733176	79734436	1261	*	chr16	79733176	79734436	in_vitro	in_vivo	9	11	5.68666667	7.64454545	-1.9578788	c(t = -0.5077	0.619	0.91200441	Distal Intergenic	ENSSSCG000	TPPP	tubulin polymerization promoting protein [Source:VGNC Symbol;Acc:VGNC:94344]
chr2	105046095	105047780	1686	*	chr2	105046095	105047780	in_vitro	in_vivo	14	14	1.98285714	2.59714286	-0.6142857	c(t = -0.5040	0.619	0.91200441	Distal Intergenic	ENSSSCG000	LNPEP	leucyl and cystinyl aminopeptidase [Source:NCBI gene (formerly Entrezgene);Acc:100125826]
chr5	21191062	21192327	1266	*	chr5	21191062	21192327	in_vitro	in_vivo	13	9	1.38076923	1.98222222	-0.601453	c(t = -0.5063	0.619	0.91200441	Promoter (<=1kb)	ENSSSCG000	GDF11	growth differentiation factor 11 [Source:VGNC Symbol;Acc:VGNC:88398]
chr1	145748681	145750021	1341	*	chr1	145748681	145750021	in_vitro	in_vivo	12	9	2.50333333	1.76	0.74333333	c(t = 0.50888	0.619	0.91200441	Distal Intergenic	ENSSSCG000	NFATC1	nuclear factor of activated T cells 1 [Source:NCBI gene (formerly Entrezgene);Acc:397318]
chr4	69615602	69616566	965	*	chr4	69615602	69616566	in_vitro	in_vivo	10	12	2.633	1.70083333	0.93216667	c(t = 0.50804	0.619	0.91200441	Distal Intergenic	ENSSSCG000	ssc-mir-124a	ssc-mir-124a-1 [Source:miRBase;Acc:MI0010680]
chr9	105733077	105733789	713	*	chr9	105733077	105733789	in_vitro	in_vivo	9	12	1.01777778	0.66416667	0.35361111	c(t = 0.50743	0.619	0.91200441	Distal Intergenic	ENSSSCG000	NAMPT	nicotinamide phosphoribosyltransferase [Source:VGNC Symbol;Acc:VGNC:90573]
chr1	1921361	1921991	631	*	chr1	1921361	1921991	in_vitro	in_vivo	9	9	21.0355556	13.6222222	7.41333333	c(t = 0.50505	0.62	0.91244035	Distal Intergenic	ENSSSCG000	ssc-mir-9824	ssc-mir-9824 [Source:miRBase;Acc:MI0031591]
chr7	52985241	52986003	763	*	chr7	52985241	52986003	in_vitro	in_vivo	12	14	4.84666667	3.64428571	1.20238095	c(t = 0.50386	0.62	0.91244035	Distal Intergenic	ENSSSCG000	NMB	neuromedin B [Source:VGNC Symbol;Acc:VGNC:109471]
chr1	175145794	175146674	881	*	chr1	175145794	175146674	in_vitro	in_vivo	9	12	1.85666667	1.32083333	0.53583333	c(t = 0.50525	0.62	0.91244035	Distal Intergenic	ENSSSCG000	FKBP3	FKBP prolyl isomerase 3 [Source:NCBI gene (formerly Entrezgene);Acc:100152728]
chr7	94444735	94446224	1490	*	chr7	94444735	94446224	in_vitro	in_vivo	11	12	1.85272727	1.30166667	0.55106061	c(t = 0.50817	0.62	0.91244035	Distal Intergenic	ENSSSCG000	SYNJ2BP	synaptojanin 2 binding protein [Source:NCBI gene (formerly Entrezgene);Acc:100514718]
chr14	6711388	6712355	968	*	chr14	6711388	6712355	in_vitro	in_vivo	9	15	0.83222222	1.236	-0.4037778	c(t = -0.5035	0.62	0.91244035	Distal Intergenic	ENSSSCG000	CCAR2	cell cycle and apoptosis regulator 2 [Source:NCBI gene (formerly Entrezgene);Acc:100155718]
chr12	1078174	1104596	26423	*	chr12	1078174	1104596	in_vitro	in_vivo	15	16	57.818	53.56375	4.25425	c(t = 0.50009	0.621	0.91244035	Promoter (<=1kb)	ENSSSCG000	SIRT7	sirtuin 7 [Source:VGNC Symbol;Acc:VGNC:92890]
chr2	132535284	132536969	1686	*	chr2	132535284	132536969	in_vitro	in_vivo	14	14	4.37857143	5.54214286	-1.1635714	c(t = -0.5012	0.621	0.91244035	Distal Intergenic	ENSSSCG000	CSF2	colony stimulating factor 2 [Source:NCBI gene (formerly Entrezgene);Acc:397208]
chr2	26579135	26580941	1807	*	chr2	26579135	26580941	in_vitro	in_vivo	13	11	2.81	2.21	0.6	c(t = 0.50182	0.621	0.91244035	Distal Intergenic	ENSSSCG000	CAT	catalase [Source:VGNC Symbol;Acc:VGNC:86208]
chr12	53165575	53166482	908	*	chr12	53165575	53166482	in_vitro	in_vivo	10	11	2.998	2.08636364	0.91163636	c(t = 0.50702	0.621	0.91244035	Distal Intergenic	ENSSSCG000	EFNB3	ephrin B3 [Source:VGNC Symbol;Acc:VGNC:87578]
chr1	219208965	219210478	1514	*	chr1	219208965	219210478	in_vitro	in_vivo	12	11	1.2575	1.99090909	-0.7334091	c(t = -0.5044	0.621	0.91244035	Promoter (<=1kb)	ENSSSCG000	VLDLR	very low density lipoprotein receptor [Source:VGNC Symbol;Acc:VGNC:94832]
chr1	27137470	27138189	720	*	chr1	27137470	27138189	in_vitro	in_vivo	11	12	1.07545455	1.775	-0.6995455	c(t = -0.5030	0.621	0.91244035	Distal Intergenic	ENSSSCG000	IFNGR1	interferon gamma receptor 1 [Source:VGNC Symbol;Acc:VGNC:89041]
chr12	44848301	44848756	456	*	chr12	44848301	44848756	in_vitro	in_vivo	10	10	0.606	0.27	0.336	c(t = 0.50646	0.621	0.91244035	Promoter (<=1kb)	ENSSSCG000	SPAG5	sperm associated antigen 5 [Source:NCBI gene (formerly Entrezgene);Acc:100286810]
chr18	4980080	4981148	1069	*	chr18	4980080	4981148	in_vitro	in_vivo	12	12	22.495	29.9041667	-7.4091667	c(t = -0.4999	0.622	0.91244035	Distal Intergenic	ENSSSCG000	RHEB	Ras homolog, mTORC1 binding [Source:NCBI gene (formerly Entrezgene);Acc:100516050]
chr17	62554486	62556172	1687	*	chr17	62554486	62556172	in_vitro	in_vivo	10	12	16.151	10.3775	5.7735	c(t = 0.50292	0.622	0.91244035	Distal Intergenic	ENSSSCG000	LIME1	Lck interacting transmembrane adaptor 1 [Source:NCBI gene (formerly Entrezgene);Acc:100625216]
chr5	77126152	77127879	1728	*	chr5	77126152	77127879	in_vitro	in_vivo	14	10	5.05214286	3.946	1.10614286	c(t = 0.50040	0.622	0.91244035	Promoter (<=1kb)	ENSSSCG000	SLC38A2	solute carrier family 38 member 2 [Source:VGNC Symbol;Acc:VGNC:93094]
chr14	110539516	110541280	1765	*	chr14	110539516	110541280	in_vitro	in_vivo	14	13	4.02928571	3.35461538	0.67467033	c(t = 0.49943	0.622	0.91244035	Distal Intergenic	ENSSSCG000	GOT1	glutamic-oxaloacetic transaminase 1 [Source:NCBI gene (formerly Entrezgene);Acc:396967]
chrX	3875529	3876417	889	*	chrX	3875529	3876417	in_vitro	in_vivo	9	13	2.71333333	2.11153846	0.60179487	c(t = 0.50415	0.622	0.91244035	Distal Intergenic	ENSSSCG00000038027		patatin like phospholipase domain containing 4 [Source:NCBI gene (formerly Entrezgene);Acc:100294680]
chrX	46002407	46003444	1038	*	chrX	46002407	46003444	in_vitro	in_vivo	13	11	2.85615385	1.88	0.97615385	c(t = 0.50239	0.622	0.91244035	Promoter (<=1kb)	ENSSSCG00000012322		lysine demethylase 5C [Source:NCBI gene (formerly Entrezgene);Acc:100037295]
chr8	18524646	18527052	2407	*	chr8	18524646	18527052	in_vitro	in_vivo	10	16	1.34	1.004375	0.335625	c(t = 0.49916	0.622	0.91244035	Distal Intergenic	ENSSSCG000	DHX15	DEAH-box helicase 15 [Source:NCBI gene (formerly Entrezgene);Acc:100517041]
chr8	6080731	6081588	858	*	chr8	6080731	6081588	in_vitro	in_vivo	14	11	0.53	0.90090909	-0.3709091	c(t = -0.5048	0.622	0.91244035	Distal Intergenic	ENSSSCG000	MSX1	msh homeobox 1 [Source:NCBI gene (formerly Entrezgene);Acc:100302364]
chr11	17947617	17948835	1219	*	chr11	17947617	17948835	in_vitro	in_vivo	11	12	1.18545455	0.88083333	0.30462121	c(t = 0.50029	0.622	0.91244035	Promoter (<=1kb)	ENSSSCG000	KPNA3	karyopherin subunit alpha 3 [Source:VGNC Symbol;Acc:VGNC:89562]
chr13	36221027	36222734	1708	*	chr13	36221027	36222734	in_vitro	in_vivo	11	13	21.88	18.3	3.58	c(t = 0.50093	0.623	0.91244035	Distal Intergenic	ENSSSCG000	SELENOK	selenoprotein K [Source:NCBI gene (formerly Entrezgene);Acc:733604]
chr7	83081756	83086141	4386	*	chr7	83081756	83086141	in_vitro	in_vivo	16	16	6.69625	5.49125	1.205	c(t = 0.49915	0.623	0.91244035	Promoter (<=1kb)	ENSSSCG000	NR2F2	nuclear receptor subfamily 2 group F member 2 [Source:VGNC Symbol;Acc:VGNC:90881]
chr1	169596080	169597223	1144	*	chr1	169596080	169597223	in_vitro	in_vivo	13	12	1.67307692	2.2525	-0.5794231	c(t = -0.4977	0.623	0.91244035	Distal Intergenic	ENSSSCG00000026486		
chr12	46709244	46711071	1828	*	chr12	46709244	46711071	in_vitro	in_vivo	13	11	1.56538462	2.07363636	-0.5082517	c(t = -0.5007	0.623	0.91244035	Distal Intergenic	ENSSSCG000	TRARG1	trafficking regulator of GLUT4 (SLC2A4) 1 [Source:NCBI gene (formerly Entrezgene);Acc:100126201]
chr5	59728149	59729857	1709	*	chr5	59728149	59729857	in_vitro	in_vivo	9	11	2.30777778	1.38272727	0.92505051	c(t = 0.50305	0.623	0.91244035	Distal Intergenic	ENSSSCG000	CDKN1B	cyclin dependent kinase inhibitor 1B [Source:NCBI gene (formerly Entrezgene);Acc:397584]
chr1	100573915	100574796	882	*	chr1	100573915	100574796	in_vitro	in_vivo	13	11	1.09692308	0.82636364	0.27055944	c(t = 0.49831	0.623	0.91244035	Promoter (<=1kb)	ENSSSCG000	SMAD4	SMAD family member 4 [Source:VGNC Symbol;Acc:VGNC:93218]
chr15	137618240	137620045	1806	*	chr15	137618240	137620045	in_vitro	in_vivo	10	10	89.371	86.742	2.629	c(t = 0.50024	0.624	0.91244035	Distal Intergenic	ENSSSCG000	RAMP1	receptor activity modifying protein 1 [Source:NCBI gene (formerly Entrezgene);Acc:397401]
chr3	8836970	8837652	683	*	chr3	8836970	8837652	in_vitro	in_vivo	9	11	15.25	20.7809091	-5.5309091	c(t = -0.4993	0.624	0.91244035	Distal Intergenic	ENSSSCG000	SERPINE1	serpin family E member 1 [Source:NCBI gene (formerly Entrezgene);Acc:396945]
chr3	25476974	25477636	663	*	chr3	25476974	25477636	in_vitro	in_vivo	13	12	11.5653846	17.045	-5.4796154	c(t = -0.4966	0.624	0.91244035	Distal Intergenic	ENSSSCG000	ACSM4	acyl-CoA synthetase medium-chain family member 4 [Source:NCBI gene (formerly Entrezgene);Acc:100512595]
chr11	17681694	17683277	1584	*	chr11	17681694	17683277	in_vitro	in_vivo	15	16	9.178	10.8225	-1.6445	c(t = -0.4954	0.624	0.91244035	Distal Intergenic	NA	NA	NA
chr6	166593803	166595225	1423	*	chr6	166593803	166595225	in_vitro	in_vivo	10	13	9.832	5.69615385	4.13584615	c(t = 0.50427	0.624	0.91244035	Distal Intergenic	ENSSSCG000	DYNLT4	dynein light chain Tctex-type 4 [Source:NCBI gene (formerly Entrezgene);Acc:606747]
chr7	5057871	5060641	2771	*	chr7	5057871	5060641	in_vitro	in_vivo	14	14	2.44	1.95357143	0.48642857	c(t = 0.49577	0.624	0.91244035	Promoter (<=1kb)	ENSSSCG000	BMP6	bone morphogenetic protein 6 [Source:NCBI gene (formerly Entrezgene);Acc:100155536]
chr1	21430049	21430657	609	*	chr1	21430049	21430657	in_vitro	in_vivo	14	10	1.07142857	0.588	0.48342857	c(t = 0.49749	0.624	0.91244035	Distal Intergenic	ENSSSCG000	PLAGL1	PLAG1 like zinc finger 1 [Source:VGNC Symbol;Acc:VGNC:91510]
chr11	1843638	1845055	1418	*	chr11	1843638	1845055	in_vitro	in_vivo	9	11	95.1744444	96.4845455	-1.310101	c(t = -0.5023	0.625	0.91244035	Distal Intergenic	ENSSSCG000	FGF9	fibroblast growth factor 9 [Source:NCBI gene (formerly Entrezgene);Acc:396717]
chr3	1477814	1481295	3482	*	chr3	1477814	1481295	in_vitro	in_vivo	10	12	94.844	95.8858333	-1.0418333	c(t = -0.4969	0.625	0.91244035	Distal Intergenic	ENSSSCG000	MRM2	mitochondrial rRNA methyltransferase 2 [Source:VGNC Symbol;Acc:VGNC:99722]
chr2	151623868	151626792	2925	*	chr2	151623868	151626792	in_vitro	in_vivo	11	10	53.8781818	61.134	-7.2558182	c(t = -0.4982	0.625	0.91244035	Distal Intergenic	ENSSSCG000	MYOZ3	myozenin 3 [Source:VGNC Symbol;Acc:VGNC:90544]
chr1	73124082	73125208	1127	*	chr1	73124082	73125208	in_vitro	in_vivo	10	9	2.149	2.83888889	-0.6898889	c(t = -0.4979	0.625	0.91244035	Distal Intergenic	ENSSSCG000	PDSS2	decaprenyl diphosphate synthase subunit 2 [Source:VGNC Symbol;Acc:VGNC:91291]
chr10	20732446	20733451	1006	*	chr10	20732446	20733451	in_vitro	in_vivo	11	10	3.07	2.423	0.647	c(t = 0.49804	0.625	0.91244035	Distal Intergenic	ENSSSCG000	ssc-mir-181b	ssc-mir-181b-1 [Source:miRBase;Acc:MI0013107]
chr6	3060312	3061127	816	*	chr6	3060312	3061127	in_vitro	in_vivo	12	9	1.11333333	1.65555556	-0.5422222	c(t = -0.5003	0.625	0.91244035	Promoter (<=1kb)	ENSSSCG000	IRF8	interferon regulatory factor 8 [Source:VGNC Symbol;Acc:VGNC:89211]
chr12	53553347	53554403	1057	*	chr12	53553347	53554403	in_vitro	in_vivo	11	10	2.35	1.462	0.888	c(t = 0.50052	0.625	0.91244035	Promoter (<=1kb)	ENSSSCG000	RPL26	ribosomal protein L26 [Source:NCBI gene (formerly Entrezgene);Acc:100627662]
chr4	51511953	51512717	765	*	chr4	51511953	51512717	in_vitro	in_vivo	9	13	1.63777778	1.11384615	0.52393162	c(t = 0.49744	0.625	0.91244035	Distal Intergenic	ENSSSCG000	CA3	carbonic anhydrase 3 [Source:NCBI gene (formerly Entrezgene);Acc:494016]
chr13	189289456	189290299	844	*	chr13	189289456	189290299	in_vitro	in_vivo	9	10	0.26444444	0.588	-0.3235556	c(t = -0.5018	0.625	0.91244035	Distal Intergenic	ENSSSCG000	ssc-mir-155	ssc-mir-155 [Source:miRBase;Acc:MI0015907]
chr10	30975142	30976873	1732	*	chr10	30975142	30976873	in_vitro	in_vivo	11	14	0.37272727	0.55785714	-0.1851299	c(t = -0.4965	0.625	0.91244035	Promoter (<=1kb)	ENSSSCG00000022760		heterous nuclear ribonucleoprotein K [Source:VGNC Symbol;Acc:VGNC:103963]
chr1	256544838	256545500	663	*	chr1	256544838	256545500	in_vitro	in_vivo	10	12	23.215	18.1816667	5.03333333	c(t = 0.49546	0.626	0.91244035	Distal Intergenic	ENSSSCG000	TNC	tenascin C [Source:VGNC Symbol;Acc:VGNC:103192]
chr7	1381041	1381729	689	*	chr7	1381041	1381729	in_vitro	in_vivo	10	10	11.694	17.826	-6.132	c(t = -0.4954	0.626	0.91244035	Distal Intergenic	ENSSSCG000	SERPINB9	serpin peptidase inhibitor, clade B (ovalbumin), member 9 [Source:NCBI gene (formerly Entrezgene);Acc:100126851]
chr18	50698457	50700032	1576	*	chr18	50698457	50700032	in_vitro	in_vivo	12	11	6.46	9.70636364	-3.2463636	c(t = -0.5003	0.626	0.91244035	Distal Intergenic	ENSSSCG000	DDX56	DEAD-box helicase 56 [Source:NCBI gene (formerly Entrezgene);Acc:100513388]
chr3	17351412	17352116	705	*	chr3	17351412	17352116	in_vitro	in_vivo	11	9	5.93181818	8.13111111	-2.1992929	c(t = -0.4986	0.626	0.91244035	Distal Intergenic	ENSSSCG000	KAT8	lysine acetyltransferase 8 [Source:NCBI gene (formerly Entrezgene);Acc:100522367]
chr7	713926	721098	7173	*	chr7	713926	721098	in_vitro	in_vivo	12	15	5.33333333	6.60466667	-1.2713333	c(t = -0.4934	0.626	0.91244035	Distal Intergenic	ENSSSCG000	IRF4	interferon regulatory factor 4 [Source:VGNC Symbol;Acc:VGNC:89207]
chrX	109972753	109973576	824	*	chrX	109972753	109973576	in_vitro	in_vivo	13	9	2.29384615	3.41333333	-1.1194872	c(t = -0.4951	0.626	0.91244035	Distal Intergenic	ENSSSCG000	ssc-mir-363-	ssc-mir-363-1 [Source:miRBase;Acc:MI0014771]
chr2	69339354	69340568	1215	*	chr2	69339354	69340568	in_vitro	in_vivo	9	9	1.31333333	1.97888889	-0.6655556	c(t = -0.4978	0.626	0.91244035	Distal Intergenic	ENSSSCG000	ATG4D	autophagy related 4D cysteine peptidase [Source:VGNC Symbol;Acc:VGNC:85624]
chr1	272379879	272381491	1613	*	chr1	272379879	272381491	in_vitro	in_vivo	9	9	1.03444444	1.71555556	-0.6811111	c(t = -0.4993	0.626	0.91244035	Distal Intergenic	ENSSSCG000	MED27	mediator complex subunit 27 [Source:VGNC Symbol;Acc:VGNC:90117]
chr7	74294797	74295562	766	*	chr7	74294797	74295562	in_vitro	in_vivo	9	13	1.12444444	1.68692308	-0.5624786	c(t = -0.4949	0.626	0.91244035	Distal Intergenic	ENSSSCG000	STXBP6	syntaxin binding protein 6 [Source:NCBI gene (formerly Entrezgene);Acc:100514852]
chr6	168759839	168760605	767	*	chr6	168759839	168760605	in_vitro	in_vivo	9	10	1.01	0.475	0.535	c(t = 0.50406	0.626	0.91244035	Distal Intergenic	ENSSSCG000	CLDN19	claudin 19 [Source:NCBI gene (formerly Entrezgene);Acc:100294682]
chr16	78563230	78565748	2519	*	chr16	78563230	78565748	in_vitro	in_vivo	11	13	10.8063636	15.5161538	-4.7097902	c(t = -0.4945	0.627	0.91244035	Distal Intergenic	ENSSSCG000	TERT	telomerase reverse transcriptase [Source:NCBI gene (formerly Entrezgene);Acc:492280]
chr17	62655314	62668723	13410	*	chr17	62655314	62668723	in_vitro	in_vivo	9	15	18.3477778	14.34	4.00777778	c(t = 0.49323	0.627	0.91244035	Downstream (<=300bp)	ENSSSCG000	LIME1	Lck interacting transmembrane adaptor 1 [Source:NCBI gene (formerly Entrezgene);Acc:100625216]
chr7	21341921	21342922	1002	*	chr7	21341921	21342922	in_vitro	in_vivo	13	12	7.32538462	4.99166667	2.33371795	c(t = 0.49492	0.627	0.91244035	Distal Intergenic	ENSSSCG000	ssc-mir-7857	ssc-mir-7857 [Source:miRBase;Acc:MI0031552]
chr12	16254662	16256264	1603	*	chr12	16254662	16256264	in_vitro	in_vivo	12	11	4.29833333	2.82272727	1.47560606	c(t = 0.49377	0.627	0.91244035	Distal Intergenic	ENSSSCG000	ITGB3	integrin subunit beta 3 [Source:NCBI gene (formerly Entrezgene);Acc:397063]
chr10	9685586	9686078	493	*	chr10	9685586	9686078	in_vitro	in_vivo	9	10	1.17	2.083	-0.913	c(t = -0.4955	0.627	0.91244035	Distal Intergenic	ENSSSCG000	ssc-mir-215	ssc-mir-215 [Source:miRBase;Acc:MI0010687]
chr8	53704624	53704878	255	*	chr8	53704624	53704878	in_vitro	in_vivo	9	9	2.91666667	1.66666667	1.25	c(t = 0.49923	0.627	0.91244035	Distal Intergenic	ENSSSCG000	NPY1R	neuropeptide Y receptor Y1 [Source:VGNC Symbol;Acc:VGNC:90865]
chr1	51784952	51785970	1019	*	chr1	51784952	51785970	in_vitro	in_vivo	13	13	1.99923077	1.43538462	0.56384615	c(t = 0.49326	0.627	0.91244035	Distal Intergenic	ENSSSCG000	ssc-mir-30a	ssc-mir-30a [Source:miRBase;Acc:MI0010688]
chr11	20905352	20906737	1386	*	chr11	20905352	20906737	in_vitro	in_vivo	13	10	1.34153846	0.983	0.35853846	c(t = 0.49352	0.627	0.91244035	Distal Intergenic	ENSSSCG000	ESD	esterase D [Source:VGNC Symbol;Acc:VGNC:87788]
chr8	99782464	99783364	901	*	chr8	99782464	99783364	in_vitro	in_vivo	11	13	1.46090909	0.82692308	0.63398601	c(t = 0.49826	0.627	0.91244035	Distal Intergenic	ENSSSCG000	SPRY1	sprouty RTK signaling antagonist 1 [Source:VGNC Symbol;Acc:VGNC:93424]
chr15	80332101	80333270	1170	*	chr15	80332101	80333270	in_vitro	in_vivo	10	10	1.333	2.026	-0.693	c(t = -0.4930	0.628	0.91244035	Distal Intergenic	ENSSSCG000	CIR1	corepressor interacting with RBPJ, CIR1 [Source:NCBI gene (formerly Entrezgene);Acc:100511415]
chr5	51810809	51811553	745	*	chr5	51810809	51811553	in_vitro	in_vivo	9	15	2.65666667	1.778	0.87866667	c(t = 0.49180	0.628	0.91244035	Promoter (<=1kb)	ENSSSCG000	LDHB	lactate dehydrogenase B [Source:NCBI gene (formerly Entrezgene);Acc:100621540]
chr7	42090252	42091594	1343	*	chr7	42090252	42091594	in_vitro	in_vivo	9	13	1.26111111	1.73076923	-0.4696581	c(t = -0.4927	0.628	0.91244035	Distal Intergenic	ENSSSCG000	CD2AP	CD2 associated protein [Source:VGNC Symbol;Acc:VGNC:86406]
chr4	46340319	46341386	1068	*	chr4	46340319	46341386	in_vitro	in_vivo	11	10	1.12727273	1.536	-0.4087273	c(t = -0.4925	0.628	0.91244035	Distal Intergenic	ENSSSCG000	CALB1	calbindin 1 [Source:NCBI gene (formerly Entrezgene);Acc:100153645]
chr7	31791378	31792157	780	*	chr7	31791378	31792157	in_vitro	in_vivo	11	13	1.05545455	1.43384615	-0.3783916	c(t = -0.4925	0.628	0.91244035	Distal Intergenic	ENSSSCG000	SRPK1	SRSF protein kinase 1 [Source:VGNC Symbol;Acc:VGNC:93462]
chr6	97266959	97267734	776	*	chr6	97266959	97267734	in_vitro	in_vivo	10	11	1.582	1.05909091	0.52290909	c(t = 0.49633	0.628	0.91244035	Promoter (<=1kb)	ENSSSCG000	CIDEA	cell death inducing DFFA like effector a [Source:NCBI gene (formerly Entrezgene);Acc:100127171]
chr18	6081948	6084238	2291	*	chr18	6081948	6084238	in_vitro	in_vivo	10	11	84.622	78.7836364	5.83836364	c(t = 0.49171	0.629	0.91244035	Distal Intergenic	ENSSSCG000	TMUB1	transmembrane and ubiquitin like domain containing 1 [Source:NCBI gene (formerly Entrezgene);Acc:100625342]
chr5	2652774	2655499	2726	*	chr5	2652774	2655499	in_vitro	in_vivo	10	14	36.248	43.5428571	-7.2948571	c(t = -0.4909	0.629	0.91244035	Distal Intergenic	ENSSSCG000	PPARA	peroxisome proliferator activated receptor alpha [Source:NCBI gene (formerly Entrezgene);Acc:397239]
chr7	1052670	1053662	993	*	chr7	1052670	1053662	in_vitro	in_vivo	9	11	21.5866667	28.8472727	-7.2606061	c(t = -0.4917	0.629	0.91244035	Distal Intergenic	ENSSSCG000	SERPINB9	serpin peptidase inhibitor, clade B (ovalbumin), member 9 [Source:NCBI gene (formerly Entrezgene);Acc:100126851]
chr11	811525	812873	1349	*	chr11	811525	812873	in_vitro	in_vivo	14	10	32.4085714	25.169	7.23957143	c(t = 0.49018	0.629	0.91244035	Distal Intergenic	ENSSSCG000	CRYL1	crystallin lambda 1 [Source:VGNC Symbol;Acc:VGNC:87023]
chr13	46203990	46205799	1810	*	chr13	46203990	46205799	in_vitro	in_vivo	13	12	5.60615385	9.11	-3.5038462	c(t = -0.4955	0.629	0.91244035	Distal Intergenic	ENSSSCG000	EOGT	EGF domain specific O-linked N-acetylglucosamine transferase [Source:VGNC Symbol;Acc:VGNC:87718]
chr1	253867748	253868466	719	*	chr1	253867748	253868466	in_vitro	in_vivo	9	9	1.74777778	2.48111111	-0.7333333	c(t = -0.4941	0.629	0.91244035	Promoter (<=1kb)	ENSSSCG000	SLC31A1	solute carrier family 31 member 1 [Source:NCBI gene (formerly Entrezgene);Acc:397186]
chr9	21761132	21761748	617	*	chr9	21761132	21761748	in_vitro	in_vivo	14	9	3.38714286	2.42	0.96714286	c(t = 0.48983	0.629	0.91244035	Distal Intergenic	ENSSSCG000	TYR	tyrosinase [Source:NCBI gene (formerly Entrezgene);Acc:407745]
chr6	89118595	89119726	1132	*	chr6	89118595	89119726	in_vitro	in_vivo	11	10	1.53090909	2.084	-0.5530909	c(t = -0.4912	0.629	0.91244035	Distal Intergenic	ENSSSCG000	HPCA	hippocalcin [Source:VGNC Symbol;Acc:VGNC:88957]
chr7	52829204	52829770	567	*	chr7	52829204	52829770	in_vitro	in_vivo	9	12	3.05555556	1.89583333	1.15972222	c(t = 0.49528	0.629	0.91244035	Distal Intergenic	ENSSSCG000	NMB	neuromedin B [Source:VGNC Symbol;Acc:VGNC:109471]
chr3	7571754	7572478	725	*	chr3	7571754	7572478	in_vitro	in_vivo	10	9	2.682	1.50555556	1.17644444	c(t = 0.49381	0.629	0.91244035	Distal Intergenic	ENSSSCG000	NTAN1	N-terminal asparagine amidase [Source:NCBI gene (formerly Entrezgene);Acc:397107]
chr2	72949592	72950268	677	*	chr2	72949592	72950268	in_vitro	in_vivo	11	10	1.46363636	0.955	0.50863636	c(t = 0.49319	0.629	0.91244035	Distal Intergenic	ENSSSCG000	CAPS	calcyphosine [Source:VGNC Symbol;Acc:VGNC:86180]
chr9	19946277	19947661	1385	*	chr9	19946277	19947661	in_vitro	in_vivo	13	13	0.84461538	0.55	0.29461538	c(t = 0.48949	0.629	0.91244035	Promoter (<=1kb)	ENSSSCG000	PICALM	phosphatidylinositol binding clathrin assembly protein [Source:VGNC Symbol;Acc:VGNC:91415]
chr13	207768768	207771474	2707	*	chr13	207768768	207771474	in_vitro	in_vivo	9	12	92.1966667	94.02	-1.8233333	c(t = -0.4900	0.63	0.91244035	Distal Intergenic	ENSSSCG000	ITGB2	integrin subunit beta 2 [Source:NCBI gene (formerly Entrezgene);Acc:396943]
chr14	140351149	140355996	4848	*	chr14	140351149	140355996	in_vitro	in_vivo	11	13	92.7636364	91.6438462	1.11979021	c(t = 0.48931	0.63	0.91244035	Distal Intergenic	ENSSSCG000	ssc-mir-202	ssc-mir-202 [Source:miRBase;Acc:MI0013157]
chr14	51196713	51197234	522	*	chr14	51196713	51197234	in_vitro	in_vivo	10	10	25.865	18.144	7.721	c(t = 0.49140	0.63	0.91244035	Distal Intergenic	ENSSSCG000	CLDN5	claudin 5 [Source:NCBI gene (formerly Entrezgene);Acc:396997]
chr7	110267352	110268635	1284	*	chr7	110267352	110268635	in_vitro	in_vivo	11	11	1.84636364	2.51545455	-0.6690909	c(t = -0.4911	0.63	0.91244035	Distal Intergenic	ENSSSCG000	SPATA7	spermatosis associated 7 [Source:VGNC Symbol;Acc:VGNC:93389]
chr11	1028823	1030970	2148	*	chr11	1028823	1030970	in_vitro	in_vivo	15	13	2.56866667	2.04923077	0.5194359	c(t = 0.48722	0.63	0.91244035	Distal Intergenic	ENSSSCG000	CRYL1	crystallin lambda 1 [Source:VGNC Symbol;Acc:VGNC:87023]
chr1	13446082	13446677	596	*	chr1	13446082	13446677	in_vitro	in_vivo	11	10	0.99090909	1.484	-0.4930909	c(t = -0.4914	0.63	0.91244035	Promoter (<=1kb)	ENSSSCG000	MTRF1L	mitochondrial translation release factor 1 like [Source:VGNC Symbol;Acc:VGNC:90472]
chr1	26489325	26489968	644	*	chr1	26489325	26489968	in_vitro	in_vivo	9	12	0.97222222	1.46416667	-0.4919444	c(t = -0.4918	0.63	0.91244035	Distal Intergenic	ENSSSCG000	TNFAIP3	TNF alpha induced protein 3 [Source:VGNC Symbol;Acc:VGNC:94252]
chr18	43070753	43071604	852	*	chr18	43070753	43071604	in_vitro	in_vivo	13	11	0.95153846	1.31545455	-0.3639161	c(t = -0.4931	0.63	0.91244035	Distal Intergenic	ENSSSCG000	FKBP14	FKBP prolyl isomerase 14 [Source:NCBI gene (formerly Entrezgene);Acc:100514551]
chr15	85468028	85468933	906	*	chr15	85468028	85468933	in_vitro	in_vivo	9	13	1.35111111	0.94	0.41111111	c(t = 0.49075	0.63	0.91244035	Distal Intergenic	ENSSSCG000	PLEKHA3	pleckstrin homology domain containing A3 [Source:NCBI gene (formerly Entrezgene);Acc:100154010]
chr3	424195	439680	15486	*	chr3	424195	439680	in_vitro	in_vivo	14	14	64.53	69.1471429	-4.6171429	c(t = -0.4865	0.631	0.91244035	Distal Intergenic	ENSSSCG000	ADAP1	ArfGAP with dual PH domains 1 [Source:NCBI gene (formerly Entrezgene);Acc:397453]
chr7	61033806	61034516	711	*	chr7	61033806	61034516	in_vitro	in_vivo	14	11	17.2271429	22.7790909	-5.5519481	c(t = -0.4883	0.631	0.91244035	Distal Intergenic	ENSSSCG000	HEXA	hexosaminidase subunit alpha [Source:VGNC Symbol;Acc:VGNC:111459]
chr11	65229604	65230677	1074	*	chr11	65229604	65230677	in_vitro	in_vivo	13	12	4.67076923	3.68666667	0.98410256	c(t = 0.48617	0.631	0.91244035	Promoter (<=1kb)	ENSSSCG000	DNAJC3	DnaJ heat shock protein family (Hsp40) member C3 [Source:NCBI gene (formerly Entrezgene);Acc:100154166]
chr7	60930586	60931449	864	*	chr7	60930586	60931449	in_vitro	in_vivo	13	11	4.03769231	3.19363636	0.84405594	c(t = 0.48740	0.631	0.91244035	Distal Intergenic	ENSSSCG000	HEXA	hexosaminidase subunit alpha [Source:VGNC Symbol;Acc:VGNC:111459]
chr1	126333999	126335307	1309	*	chr1	126333999	126335307	in_vitro	in_vivo	11	12	1.85272727	1.25	0.60272727	c(t = 0.49104	0.631	0.91244035	Promoter (<=1kb)	ENSSSCG000	AFG2B	AFG2 AAA ATPase homolog B [Source:NCBI gene (formerly Entrezgene);Acc:100156592]
chr6	89256296	89256738	443	*	chr6	89256296	89256738	in_vitro	in_vivo	10	9	2.043	1.04222222	1.00077778	c(t = 0.49010	0.631	0.91244035	Distal Intergenic	ENSSSCG000	HPCA	hippocalcin [Source:VGNC Symbol;Acc:VGNC:88957]
chr2	58517220	58517872	653	*	chr2	58517220	58517872	in_vitro	in_vivo	10	12	0.536	0.82	-0.284	c(t = -0.4884	0.631	0.91244035	Distal Intergenic	ENSSSCG000	TSSK6	testis specific serine kinase 6 [Source:NCBI gene (formerly Entrezgene);Acc:100518240]
chr12	11745469	11745882	414	*	chr12	11745469	11745882	in_vitro	in_vivo	9	9	13.8911111	20.2166667	-6.3255556	c(t = -0.4877	0.632	0.91244035	Exon (NM_001190294/100462758, exon 9 of 13)	ENSSSCG000	WIPI1	WD repeat domain, phosphoinositide interacting 1 [Source:NCBI gene (formerly Entrezgene);Acc:100462758]
chr2	72624706	72626125	1420	*	chr2	72624706	72626125	in_vitro	in_vivo	10	11	10.651	6.61181818	4.03918182	c(t = 0.49324	0.632	0.91244035	Promoter (<=1kb)	ENSSSCG000	SLC25A23	solute carrier family 25 member 23 [Source:VGNC Symbol;Acc:VGNC:93000]
chr18	2547518	2555515	7998	*	chr18	2547518	2555515	in_vitro	in_vivo	14	13	2.77357143	3.37076923	-0.5971978	c(t = -0.4845	0.632	0.91244035	Promoter (1-2kb)	ENSSSCG000	SHH	sonic hedgehog signaling molecule [Source:NCBI gene (formerly Entrezgene);Acc:100512749]
chr5	26964295	26965416	1122	*	chr5	26964295	26965416	in_vitro	in_vivo	12	12	2.68333333	1.71916667	0.96416667	c(t = 0.48706	0.632	0.91244035	Distal Intergenic	NA	NA	NA
chr10	46647290	46647991	702	*	chr10	46647290	46647991	in_vitro	in_vivo	12	10	1.015	1.492	-0.477	c(t = -0.4892	0.632	0.91244035	Distal Intergenic	ENSSSCG000	RPP38	ribonuclease P/MRP subunit p38 [Source:VGNC Symbol;Acc:VGNC:96240]
chr18	17669707	17670410	704	*	chr18	17669707	17670410	in_vitro	in_vivo	13	12	2.24384615	1.29416667	0.94967949	c(t = 0.48588	0.632	0.91244035	Distal Intergenic	ENSSSCG000	ssc-mir-29b-	ssc-mir-29b-1 [Source:miRBase;Acc:MI0002431]
chr9	92270443	92271593	1151	*	chr9	92270443	92271593	in_vitro	in_vivo	13	11	0.95307692	1.27	-0.3169231	c(t = -0.4887	0.632	0.91244035	Distal Intergenic	ENSSSCG000	TRA2A	transformer 2 alpha homolog [Source:VGNC Symbol;Acc:VGNC:94353]
chr4	72881495	72882564	1070	*	chr4	72881495	72882564	in_vitro	in_vivo	13	13	1.51461538	1.11230769	0.40230769	c(t = 0.48645	0.632	0.91244035	Distal Intergenic	ENSSSCG000	SDCBP	syndecan binding protein [Source:VGNC Symbol;Acc:VGNC:92657]
chr9	56798415	56799628	1214	*	chr9	56798415	56799628	in_vitro	in_vivo	12	14	0.6025	0.42642857	0.17607143	c(t = 0.48659	0.632	0.91244035	Distal Intergenic	ENSSSCG000	KCNJ5	potassium inwardly rectifying channel subfamily J member 5 [Source:NCBI gene (formerly Entrezgene);Acc:397448]
chr6	70271506	70272091	586	*	chr6	70271506	70272091	in_vitro	in_vivo	10	12	0.667	0.32083333	0.34616667	c(t = 0.49336	0.632	0.91244035	Distal Intergenic	ENSSSCG000	RBP7	retinol binding protein 7 [Source:NCBI gene (formerly Entrezgene);Acc:100270723]
chr6	165408624	165411589	2966	*	chr6	165408624	165411589	in_vitro	in_vivo	9	10	93.3	91.539	1.761	c(t = 0.48756	0.633	0.91302646	Distal Intergenic	ENSSSCG000	TSPAN1	tetraspanin 1 [Source:VGNC Symbol;Acc:VGNC:94498]
chr6	31747113	31748284	1172	*	chr6	31747113	31748284	in_vitro	in_vivo	12	9	58.2125	48.6288889	9.58361111	c(t = 0.48682	0.633	0.91302646	Promoter (<=1kb)	ENSSSCG000	AKTIP	AKT interacting protein [Source:VGNC Symbol;Acc:VGNC:85233]
chr7	68470444	68471694	1251	*	chr7	68470444	68471694	in_vitro	in_vivo	16	13	2.12125	2.86	-0.73875	c(t = -0.4847	0.633	0.91302646	Distal Intergenic	ENSSSCG000	AP4S1	adaptor related protein complex 4 subunit sigma 1 [Source:VGNC Symbol;Acc:VGNC:85394]
chr15	101210296	101211507	1212	*	chr15	101210296	101211507	in_vitro	in_vivo	10	12	3.658	2.61833333	1.03966667	c(t = 0.48590	0.633	0.91302646	Distal Intergenic	ENSSSCG000	HSPD1	heat shock protein family D (Hsp60) member 1 [Source:NCBI gene (formerly Entrezgene);Acc:492279]
chr3	9994922	9995537	616	*	chr3	9994922	9995537	in_vitro	in_vivo	9	9	1.90555556	1.18444444	0.72111111	c(t = 0.48712	0.633	0.91302646	Distal Intergenic	ENSSSCG000	HSPB1	heat shock protein family B (small) member 1 [Source:NCBI gene (formerly Entrezgene);Acc:493184]
chr4	126020607	126024437	3831	*	chr4	126020607	126024437	in_vitro	in_vivo	12	13	5.195	4.18615385	1.00884615	c(t = 0.48203	0.634	0.91378282	Distal Intergenic	ENSSSCG000	TGFBR3	transforming growth factor beta receptor 3 [Source:VGNC Symbol;Acc:VGNC:93932]
chr14	50020243	50020763	521	*	chr14	50020243	50020763	in_vitro	in_vivo	10	9	2.597	1.58777778	1.00922222	c(t = 0.48483	0.634	0.91378282	Distal Intergenic	ENSSSCG000	VPREB1	V-set pre-B cell surrogate light chain 1 [Source:NCBI gene (formerly Entrezgene);Acc:767627]
chr14	57480634	57482542	1909	*	chr14	57480634	57482542	in_vitro	in_vivo	14	16	0.91428571	1.224375	-0.3100893	c(t = -0.4826	0.634	0.91378282	Distal Intergenic	ENSSSCG000	TSNAX	translin associated factor X [Source:NCBI gene (formerly Entrezgene);Acc:100156036]
chr11	68920709	68921322	614	*	chr11	68920709	68921322	in_vitro	in_vivo	12	12	1.49166667	0.98916667	0.5025	c(t = 0.48321	0.634	0.91378282	Distal Intergenic	ENSSSCG000	TMTC4	transmembrane O-mannosyltransferase targeting cadherins 4 [Source:VGNC Symbol;Acc:VGNC:94244]
chr6	161670164	161671058	895	*	chr6	161670164	161671058	in_vitro	in_vivo	9	9	74.1177778	64.9977778	9.12	c(t = 0.48378	0.635	0.91419539	Distal Intergenic	ENSSSCG000	SPATA6	spermatosis associated 6 [Source:VGNC Symbol;Acc:VGNC:93387]
chr11	2495368	2496221	854	*	chr11	2495368	2496221	in_vitro	in_vivo	9	9	35.1322222	28.25	6.88222222	c(t = 0.48366	0.635	0.91419539	Distal Intergenic	ENSSSCG000	ssc-mir-9814	ssc-mir-9814 [Source:miRBase;Acc:MI0031581]
chr7	84038737	84039072	336	*	chr7	84038737	84039072	in_vitro	in_vivo	12	9	3.735	2.52444444	1.21055556	c(t = 0.48262	0.635	0.91419539	Distal Intergenic	ENSSSCG000	NR2F2	nuclear receptor subfamily 2 group F member 2 [Source:VGNC Symbol;Acc:VGNC:90881]
chr2	28978780	28979391	612	*	chr2	28978780	28979391	in_vitro	in_vivo	11	10	3.25090909	2.212	1.03890909	c(t = 0.48364	0.635	0.91419539	Promoter (<=1kb)	ENSSSCG000	PAX6	paired box 6 [Source:VGNC Symbol;Acc:VGNC:91195]
chr1	138340690	138342678	1989	*	chr1	138340690	138342678	in_vitro	in_vivo	17	10	1.72	1.347	0.373	c(t = 0.48262	0.635	0.91419539	Distal Intergenic	ENSSSCG000	MEF2A	myocyte enhancer factor 2A [Source:NCBI gene (formerly Entrezgene);Acc:100037273]
chr5	48508278	48509352	1075	*	chr5	48508278	48509352	in_vitro	in_vivo	9	10	0.71111111	0.973	-0.2618889	c(t = -0.4837	0.635	0.91419539	Distal Intergenic	ENSSSCG000	ssc-mir-9827	ssc-mir-9827 [Source:miRBase;Acc:MI0031594]
chr3	39087734	39088688	955	*	chr3	39087734	39088688	in_vitro	in_vivo	13	10	2.41846154	1.875	0.54346154	c(t = 0.48044	0.636	0.91450495	Distal Intergenic	ENSSSCG000	ZNF205	zinc finger protein 205 [Source:VGNC Symbol;Acc:VGNC:95204]
chr1	222657832	222659032	1201	*	chr1	222657832	222659032	in_vitro	in_vivo	11	11	2.05545455	1.56363636	0.49181818	c(t = 0.48134	0.636	0.91450495	Distal Intergenic	ENSSSCG000	TJP2	tight junction protein 2 [Source:VGNC Symbol;Acc:VGNC:103188]
chr4	38656142	38656587	446	*	chr4	38656142	38656587	in_vitro	in_vivo	9	9	2.07222222	1.42111111	0.65111111	c(t = 0.48249	0.636	0.91450495	Distal Intergenic	ENSSSCG000	RPL30	ribosomal protein L30 [Source:VGNC Symbol;Acc:VGNC:98849]
chr4	84961771	84962182	412	*	chr4	84961771	84962182	in_vitro	in_vivo	9	10	2.03111111	1.25	0.78111111	c(t = 0.48263	0.636	0.91450495	Promoter (<=1kb)	ENSSSCG000	TMCO1	transmembrane and coiled-coil domains 1 [Source:NCBI gene (formerly Entrezgene);Acc:100151809]
chr9	20123865	20124447	583	*	chr9	20123865	20124447	in_vitro	in_vivo	10	11	0.572	0.95181818	-0.3798182	c(t = -0.4826	0.636	0.91450495	Distal Intergenic	ENSSSCG000	PICALM	phosphatidylinositol binding clathrin assembly protein [Source:VGNC Symbol;Acc:VGNC:91415]
chr6	1044639	1046701	2063	*	chr6	1044639	1046701	in_vitro	in_vivo	10	9	97.533	98.2988889	-0.7658889	c(t = -0.4808	0.637	0.91450495	Distal Intergenic	ENSSSCG000	CYBA	cytochrome b-245 alpha chain [Source:VGNC Symbol;Acc:VGNC:87124]
chr5	1557090	1559138	2049	*	chr5	1557090	1559138	in_vitro	in_vivo	9	13	39.4011111	32.4246154	6.97649573	c(t = 0.48001	0.637	0.91450495	Distal Intergenic	ENSSSCG000	SELENOO	selenoprotein O [Source:NCBI gene (formerly Entrezgene);Acc:100518971]
chr15	137650618	137651875	1258	*	chr15	137650618	137651875	in_vitro	in_vivo	13	10	11.3484615	16.246	-4.8975385	c(t = -0.4792	0.637	0.91450495	Distal Intergenic	ENSSSCG000	RAMP1	receptor activity modifying protein 1 [Source:NCBI gene (formerly Entrezgene);Acc:397401]
chr12	26640492	26641411	920	*	chr12	26640492	26641411	in_vitro	in_vivo	11	12	2.87	3.93583333	-1.0658333	c(t = -0.4796	0.637	0.91450495	Distal Intergenic	ENSSSCG000	LRRC59	leucine rich repeat containing 59 [Source:VGNC Symbol;Acc:VGNC:98082]
chr12	24863771	24864474	704	*	chr12	24863771	24864474	in_vitro	in_vivo	11	9	2.18727273	3.08666667	-0.8993939	c(t = -0.4793	0.637	0.91450495	Distal Intergenic	NA	NA	NA
chr14	51252616	51253259	644	*	chr14	51252616	51253259	in_vitro	in_vivo	9	9	2.05	2.65111111	-0.6011111	c(t = -0.4810	0.637	0.91450495	Distal Intergenic	ENSSSCG000	GP1BB	glycoprotein Ib platelet subunit beta [Source:NCBI gene (formerly Entrezgene);Acc:100155101]
chr5	77645122	77646241	1120	*	chr5	77645122	77646241	in_vitro	in_vivo	13	10	1.29153846	1.88	-0.5884615	c(t = -0.4829	0.637	0.91450495	Promoter (<=1kb)	NA	NA	NA
chr1	221581373	221582232	860	*	chr1	221581373	221582232	in_vitro	in_vivo	10	13	2.474	1.82153846	0.65246154	c(t = 0.48003	0.637	0.91450495	Distal Intergenic	ENSSSCG000	ssc-mir-9823	ssc-mir-9823 [Source:miRBase;Acc:MI0031590]
chr2	135103817	135105118	1302	*	chr2	135103817	135105118	in_vitro	in_vivo	11	13	1.90363636	1.41153846	0.4920979	c(t = 0.48100	0.637	0.91450495	Distal Intergenic	ENSSSCG000	GDF9	growth differentiation factor 9 [Source:NCBI gene (formerly Entrezgene);Acc:414285]
chr1	22164862	22165386	525	*	chr1	22164862	22165386	in_vitro	in_vivo	11	10	0.91363636	0.574	0.33963636	c(t = 0.48033	0.637	0.91450495	Distal Intergenic	ENSSSCG000	PEX3	peroxisomal biosis factor 3 [Source:VGNC Symbol;Acc:VGNC:91327]
chr2	3031238	3043167	11930	*	chr2	3031238	3043167	in_vitro	in_vivo	14	13	93.4764286	95.1346154	-1.6581868	c(t = -0.4796	0.638	0.91457377	Distal Intergenic	ENSSSCG000	FADD	Fas associated via death domain [Source:NCBI gene (formerly Entrezgene);Acc:595129]
chr13	207699324	207703263	3940	*	chr13	207699324	207703263	in_vitro	in_vivo	13	10	83.1953846	76.631	6.56438462	c(t = 0.47999	0.638	0.91457377	Distal Intergenic	ENSSSCG000	ITGB2	integrin subunit beta 2 [Source:NCBI gene (formerly Entrezgene);Acc:396943]
chr2	143004701	143005341	641	*	chr2	143004701	143005341	in_vitro	in_vivo	10	9	12.495	16.58	-4.085	c(t = -0.4791	0.638	0.91457377	Distal Intergenic	ENSSSCG000	DIAPH1	diaphanous related formin 1 [Source:VGNC Symbol;Acc:VGNC:99632]
chr14	104558525	104559078	554	*	chr14	104558525	104559078	in_vitro	in_vivo	9	11	6.35111111	4.95	1.40111111	c(t = 0.47798	0.638	0.91457377	Distal Intergenic	ENSSSCG000	CYP26A1	cytochrome P450 family 26 subfamily A member 1 [Source:VGNC Symbol;Acc:VGNC:103370]
chr12	53779715	53781813	2099	*	chr12	53779715	53781813	in_vitro	in_vivo	12	14	1.64833333	2.19785714	-0.5495238	c(t = -0.4761	0.638	0.91457377	Distal Intergenic	ENSSSCG000	CCDC42	coiled-coil domain containing 42 [Source:NCBI gene (formerly Entrezgene);Acc:100514007]
chr1	8891151	8892204	1054	*	chr1	8891151	8892204	in_vitro	in_vivo	9	9	2.39666667	1.97222222	0.42444444	c(t = 0.48236	0.638	0.91457377	Distal Intergenic	ENSSSCG000	DYNLT1	dynein light chain Tctex-type 1 [Source:NCBI gene (formerly Entrezgene);Acc:100158053]
chr2	8215529	8216413	885	*	chr2	8215529	8216413	in_vitro	in_vivo	12	11	2.38416667	1.78545455	0.59871212	c(t = 0.47804	0.638	0.91457377	Distal Intergenic	ENSSSCG000	ssc-mir-7142	ssc-mir-7142 [Source:miRBase;Acc:MI0023575]
chr1	161983826	161984845	1020	*	chr1	161983826	161984845	in_vitro	in_vivo	11	13	2.16181818	1.58153846	0.58027972	c(t = 0.47757	0.638	0.91457377	Distal Intergenic	ENSSSCG000	ssc-mir-122	ssc-mir-122 [Source:miRBase;Acc:MI0002413]
chr5	22315784	22316589	806	*	chr5	22315784	22316589	in_vitro	in_vivo	11	10	1.71909091	2.384	-0.6649091	c(t = -0.4765	0.639	0.91549497	Distal Intergenic	ENSSSCG000	TAC3	tachykinin 3 [Source:NCBI gene (formerly Entrezgene);Acc:492314]
chr6	45374358	45376015	1658	*	chr6	45374358	45376015	in_vitro	in_vivo	11	10	1.47	2.038	-0.568	c(t = -0.4779	0.639	0.91549497	Distal Intergenic	ENSSSCG000	WDR62	WD repeat domain 62 [Source:VGNC Symbol;Acc:VGNC:94933]
chr13	100807317	100807976	660	*	chr13	100807317	100807976	in_vitro	in_vivo	13	11	1.18846154	0.79090909	0.39755245	c(t = 0.47503	0.639	0.91549497	Distal Intergenic	ENSSSCG000	B3GALNT1	beta-1,3-N-acetylgalactosaminyltransferase 1 [Source:VGNC Symbol;Acc:VGNC:108632]
chr7	52001094	52001984	891	*	chr7	52001094	52001984	in_vitro	in_vivo	14	14	1.59142857	2.21071429	-0.6192857	c(t = -0.4751	0.64	0.91641513	Distal Intergenic	ENSSSCG000	ssc-mir-1839	ssc-mir-1839 [Source:miRBase;Acc:MI0013162]
chr5	82361707	82362452	746	*	chr5	82361707	82362452	in_vitro	in_vivo	11	11	1.87	1.22727273	0.64272727	c(t = 0.47697	0.64	0.91641513	Distal Intergenic	ENSSSCG000	DRAM1	DNA damage regulated autophagy modulator 1 [Source:NCBI gene (formerly Entrezgene);Acc:100462750]
chr13	39789571	39790879	1309	*	chr13	39789571	39790879	in_vitro	in_vivo	13	9	1.31923077	0.85444444	0.46478632	c(t = 0.47975	0.64	0.91641513	Distal Intergenic	ENSSSCG000	ARF4	ADP ribosylation factor 4 [Source:VGNC Symbol;Acc:VGNC:85446]

chr6	53846233	53848232	2000	*	chr6	53846233	53848232	in_vitro	in_vivo	13	12	7.18153846	10.3833333	-3.2017949	c(t = -0.4754	0.641	0.91671683	Distal Intergenic	ENSSSCG000	CYTH2	cytohesin 2 [Source:VGNC Symbol;Acc:VGNC:96962]
chr2	77110936	77111696	761	*	chr2	77110936	77111696	in_vitro	in_vivo	10	9	3.082	2.32111111	0.76088889	c(t = 0.47687	0.641	0.91671683	Distal Intergenic	ENSSSCG000	DAZAP1	DAZ associated protein 1 [Source:NCBI gene (formerly Entrezgene);Acc:100625005]
chr6	128606021	128606633	613	*	chr6	128606021	128606633	in_vitro	in_vivo	12	9	2.92416667	2.11444444	0.80972222	c(t = 0.47464	0.641	0.91671683	Distal Intergenic	ENSSSCG00000003753		PDZK1 interacting protein 1 [Source:NCBI gene (formerly Entrezgene);Acc:414756]
chr16	39770176	39770954	779	*	chr16	39770176	39770954	in_vitro	in_vivo	12	11	0.76333333	1.25909091	-0.4957576	c(t = -0.4750	0.641	0.91671683	Distal Intergenic	ENSSSCG000	ssc-mir-9800	ssc-mir-9800 [Source:miRBase;Acc:MI0031564]
chr4	55738489	55740003	1515	*	chr4	55738489	55740003	in_vitro	in_vivo	12	14	0.81083333	1.13714286	-0.3263095	c(t = -0.4730	0.641	0.91671683	Distal Intergenic	ENSSSCG000	FABP5	fatty acid binding protein 5 [Source:NCBI gene (formerly Entrezgene);Acc:574074]
chr12	47112006	47114417	2412	*	chr12	47112006	47114417	in_vitro	in_vivo	11	12	93.1072727	94.1608333	-1.0535606	c(t = -0.4712	0.642	0.91671683	Distal Intergenic	ENSSSCG000	ABR	ABR activator of RhoGEF and GTPase [Source:VGNC Symbol;Acc:VGNC:84989]
chr5	287741	291306	3566	*	chr5	287741	291306	in_vitro	in_vivo	11	10	92.9290909	90.181	2.74809091	c(t = 0.47275	0.642	0.91671683	Exon (NM_001206372/100517282, exon 12 of 23)	ENSSSCG000	SBF1	SET binding factor 1 [Source:VGNC Symbol;Acc:VGNC:92593]
chr5	126531	127671	1141	*	chr5	126531	127671	in_vitro	in_vivo	10	10	32.726	25.383	7.343	c(t = 0.47412	0.642	0.91671683	Distal Intergenic	ENSSSCG000	ARSA	arylsulfatase A [Source:NCBI gene (formerly Entrezgene);Acc:396973]
chr9	123507802	123508804	1003	*	chr9	123507802	123508804	in_vitro	in_vivo	11	10	17.4927273	21.8	-4.3072727	c(t = -0.4744	0.642	0.91671683	Distal Intergenic	ENSSSCG00000015545		glutamate-ammonia ligase [Source:VGNC Symbol;Acc:VGNC:108587]
chr3	614407	616129	1723	*	chr3	614407	616129	in_vitro	in_vivo	10	13	6.21	7.47	-1.26	c(t = -0.4733	0.642	0.91671683	Promoter (<=1kb)	ENSSSCG000	ADAP1	ArfGAP with dual PH domains 1 [Source:NCBI gene (formerly Entrezgene);Acc:397453]
chr2	5833127	5834113	987	*	chr2	5833127	5834113	in_vitro	in_vivo	10	12	9.111	7.22333333	1.88766667	c(t = 0.47240	0.642	0.91671683	Promoter (<=1kb)	ENSSSCG000	CCS	copper chaperone for superoxide dismutase [Source:NCBI gene (formerly Entrezgene);Acc:414913]
chr18	40531571	40532486	916	*	chr18	40531571	40532486	in_vitro	in_vivo	12	9	3.675	2.70333333	0.97166667	c(t = 0.47273	0.642	0.91671683	Promoter (<=1kb)	ENSSSCG000	FKBP9	FKBP prolyl isomerase 9 [Source:NCBI gene (formerly Entrezgene);Acc:100623924]
chr2	62601688	62602301	614	*	chr2	62601688	62602301	in_vitro	in_vivo	10	11	1.864	1.09636364	0.76763636	c(t = 0.47627	0.642	0.91671683	Distal Intergenic	ENSSSCG000	BRD4	bromodomain containing 4 [Source:VGNC Symbol;Acc:VGNC:85873]
chr12	23434697	23436264	1568	*	chr12	23434697	23436264	in_vitro	in_vivo	11	14	1.28181818	0.96071429	0.3211039	c(t = 0.47213	0.642	0.91671683	Distal Intergenic	ENSSSCG000	PSMB3	proteasome 20S subunit beta 3 [Source:NCBI gene (formerly Entrezgene);Acc:497064]
chr2	76902752	76904006	1255	*	chr2	76902752	76904006	in_vitro	in_vivo	11	9	1.33909091	0.85333333	0.48575758	c(t = 0.47498	0.642	0.91671683	Distal Intergenic	ENSSSCG000	APC2	APC regulator of WNT signaling pathway 2 [Source:NCBI gene (formerly Entrezgene);Acc:100579172]
chr4	902515	905262	2748	*	chr4	902515	905262	in_vitro	in_vivo	9	12	37.8755556	31.6816667	6.19388889	c(t = 0.47354	0.643	0.91695212	Distal Intergenic	ENSSSCG000	GPIHBP1	glycosylphosphatidylinositol anchored high density lipoprotein binding protein 1 [Source:VGNC Symbol;Acc:VGNC:88588]
chr14	137792032	137792771	740	*	chr14	137792032	137792771	in_vitro	in_vivo	9	10	23.7455556	17.761	5.98455556	c(t = 0.47379	0.643	0.91695212	Distal Intergenic	ENSSSCG000	GLRX3	glutaredoxin 3 [Source:VGNC Symbol;Acc:VGNC:88495]
chr13	100252211	100253664	1454	*	chr13	100252211	100253664	in_vitro	in_vivo	13	13	1.58307692	1.97615385	-0.3930769	c(t = -0.4706	0.643	0.91695212	Distal Intergenic	ENSSSCG000	ssc-mir-16-1	ssc-mir-16-1 [Source:miRBase;Acc:MI0008213]
chr1	139494065	139495049	985	*	chr1	139494065	139495049	in_vitro	in_vivo	11	10	1.04272727	1.762	-0.7192727	c(t = -0.4765	0.643	0.91695212	Distal Intergenic	ENSSSCG000	CHSY1	chondroitin sulfate synthase 1 [Source:NCBI gene (formerly Entrezgene);Acc:100520288]
chr4	130297760	130298589	830	*	chr4	130297760	130298589	in_vitro	in_vivo	11	9	0.90454545	1.37888889	-0.4743434	c(t = -0.4738	0.643	0.91695212	Promoter (<=1kb)	ENSSSCG000	ZNHIT6	zinc finger HIT-type containing 6 [Source:VGNC Symbol;Acc:VGNC:98909]
chr6	165874190	165874906	717	*	chr6	165874190	165874906	in_vitro	in_vivo	11	9	0.93636364	1.32888889	-0.3925253	c(t = -0.4732	0.643	0.91695212	Distal Intergenic	ENSSSCG000	MMACHC	metabolism of cobalamin associated C [Source:NCBI gene (formerly Entrezgene);Acc:100513159]
chr3	126905315	126906399	1085	*	chr3	126905315	126906399	in_vitro	in_vivo	9	9	1.84555556	1.28444444	0.56111111	c(t = 0.47580	0.643	0.91695212	Distal Intergenic	ENSSSCG000	ADAM17	ADAM metallopeptidase domain 17 [Source:VGNC Symbol;Acc:VGNC:85065]
chr6	419801	426159	6359	*	chr6	419801	426159	in_vitro	in_vivo	11	13	95.8136364	96.6830769	-0.8694406	c(t = -0.4689	0.644	0.91718681	Distal Intergenic	ENSSSCG00000024974		ribosomal protein L13 [Source:NCBI gene (formerly Entrezgene);Acc:100628232]
chrX	89837168	89838293	1126	*	chrX	89837168	89838293	in_vitro	in_vivo	9	11	8.15555556	11.65	-3.4944444	c(t = -0.4716	0.644	0.91718681	Promoter (<=1kb)	ENSSSCG000	ACSL4	acyl-CoA synthetase long chain family member 4 [Source:VGNC Symbol;Acc:VGNC:85033]
chr1	28566064	28566718	655	*	chr1	28566064	28566718	in_vitro	in_vivo	12	9	1.36	1.86666667	-0.5066667	c(t = -0.4725	0.644	0.91718681	Distal Intergenic	ENSSSCG000	MTFR2	mitochondrial fission regulator 2 [Source:VGNC Symbol;Acc:VGNC:90449]
chr17	28952948	28955916	2969	*	chr17	28952948	28955916	in_vitro	in_vivo	11	14	0.93272727	1.285	-0.3522727	c(t = -0.4684	0.644	0.91718681	Distal Intergenic	ENSSSCG000	THBD	thrombomodulin [Source:NCBI gene (formerly Entrezgene);Acc:100157642]
chr1	269453623	269455957	2335	*	chr1	269453623	269455957	in_vitro	in_vivo	11	14	0.91090909	1.22785714	-0.3169481	c(t = -0.4709	0.644	0.91718681	Distal Intergenic	ENSSSCG000	CRAT	carnitine O-acetyltransferase [Source:VGNC Symbol;Acc:VGNC:86976]
chr2	66710963	66711370	408	*	chr2	66710963	66711370	in_vitro	in_vivo	9	9	1.61666667	1.10111111	0.51555556	c(t = 0.47144	0.644	0.91718681	Distal Intergenic	NA	NA	NA
chr1	243414150	243415727	1578	*	chr1	243414150	243415727	in_vitro	in_vivo	14	14	0.72142857	1.01857143	-0.2971429	c(t = -0.4673	0.644	0.91718681	Distal Intergenic	ENSSSCG000	ERP44	endoplasmic reticulum protein 44 [Source:NCBI gene (formerly Entrezgene);Acc:100192437]
chr1	6725427	6726136	710	*	chr1	6725427	6726136	in_vitro	in_vivo	9	9	20.0244444	11.9655556	8.05888889	c(t = 0.46945	0.645	0.9172739	Intron (NM_001044603/733673, intron 10 of 10)	ENSSSCG000	AGPAT4	1-acylglycerol-3-phosphate O-acyltransferase 4 [Source:VGNC Symbol;Acc:VGNC:85186]
chr2	66095753	66096605	853	*	chr2	66095753	66096605	in_vitro	in_vivo	9	11	2.76888889	1.99818182	0.77070707	c(t = 0.46919	0.645	0.9172739	Distal Intergenic	ENSSSCG000	CALR	calreticulin [Source:NCBI gene (formerly Entrezgene);Acc:100381266]
chr12	47644599	47645452	854	*	chr12	47644599	47645452	in_vitro	in_vivo	10	12	1.991	1.4825	0.5085	c(t = 0.46765	0.645	0.9172739	Distal Intergenic	ENSSSCG000	CRK	CRK proto-onco, adaptor protein [Source:NCBI gene (formerly Entrezgene);Acc:100192444]
chr3	80911600	80912537	938	*	chr3	80911600	80912537	in_vitro	in_vivo	11	10	1.94727273	1.32	0.62727273	c(t = 0.47026	0.645	0.9172739	Distal Intergenic	ENSSSCG000	BCL11A	BCL11 transcription factor A [Source:VGNC Symbol;Acc:VGNC:96562]
chr1	168452080	168453121	1042	*	chr1	168452080	168453121	in_vitro	in_vivo	11	13	0.39	0.64461538	-0.2546154	c(t = -0.4678	0.645	0.9172739	Distal Intergenic	ENSSSCG000	RPLP1	ribosomal protein lateral stalk subunit P1 [Source:NCBI gene (formerly Entrezgene);Acc:100170119]
chr10	53065708	53067189	1482	*	chr10	53065708	53067189	in_vitro	in_vivo	11	9	65.3881818	56.4066667	8.98151515	c(t = 0.46814	0.646	0.9172739	Distal Intergenic	ENSSSCG000	BMI1	BMI1 proto-onco, polycomb ring finger [Source:VGNC Symbol;Acc:VGNC:108265]
chr14	24759860	24760351	492	*	chr14	24759860	24760351	in_vitro	in_vivo	10	11	18.859	27.1372727	-8.2782727	c(t = -0.4663	0.646	0.9172739	Distal Intergenic	ENSSSCG000	PIWIL1	piwi like RNA-mediated silencing 1 [Source:VGNC Symbol;Acc:VGNC:98197]
chr9	32437726	32439106	1381	*	chr9	32437726	32439106	in_vitro	in_vivo	14	15	7.49428571	6.372	1.12228571	c(t = 0.46461	0.646	0.9172739	Distal Intergenic	ENSSSCG000	ssc-mir-9833	ssc-mir-9833 [Source:miRBase;Acc:MI0031602]
chr13	27647771	27648483	713	*	chr13	27647771	27648483	in_vitro	in_vivo	9	11	4.24555556	2.41272727	1.83282828	c(t = 0.47239	0.646	0.9172739	Distal Intergenic	ENSSSCG000	ssc-mir-138	ssc-mir-138 [Source:miRBase;Acc:MI0022131]
chr5	18446496	18448704	2209	*	chr5	18446496	18448704	in_vitro	in_vivo	10	9	1.617	2.37222222	-0.7552222	c(t = -0.4706	0.646	0.9172739	Distal Intergenic	ENSSSCG000	IGFBP6	insulin like growth factor binding protein 6 [Source:VGNC Symbol;Acc:VGNC:89059]
chr15	88376070	88376408	339	*	chr15	88376070	88376408	in_vitro	in_vivo	10	10	3.76	2.345	1.415	c(t = 0.46664	0.646	0.9172739	Promoter (<=1kb)	ENSSSCG000	FRZB	frizzled related protein [Source:VGNC Symbol;Acc:VGNC:96308]
chr4	99379917	99381357	1441	*	chr4	99379917	99381357	in_vitro	in_vivo	11	11	1.49454545	2.10272727	-0.6081818	c(t = -0.4669	0.646	0.9172739	Distal Intergenic	ENSSSCG000	TXNIP	thioredoxin interacting protein [Source:NCBI gene (formerly Entrezgene);Acc:733688]
chr8	43695152	43695855	704	*	chr8	43695152	43695855	in_vitro	in_vivo	11	13	1.77909091	1.22692308	0.55216783	c(t = 0.46947	0.646	0.9172739	Promoter (<=1kb)	ENSSSCG000	CPE	carboxypeptidase E [Source:NCBI gene (formerly Entrezgene);Acc:100037304]
chr14	40601743	40602463	721	*	chr14	40601743	40602463	in_vitro	in_vivo	10	9	1.167	0.79333333	0.37366667	c(t = 0.46858	0.646	0.9172739	Distal Intergenic	ENSSSCG000	ACADS	acyl-CoA dehydrogenase short chain [Source:VGNC Symbol;Acc:VGNC:97862]
chr1	1890448	1891097	650	*	chr1	1890448	1891097	in_vitro	in_vivo	10	9	34.189	42.89	-8.701	c(t = -0.4669	0.647	0.9172739	Distal Intergenic	ENSSSCG000	ssc-mir-9824	ssc-mir-9824 [Source:miRBase;Acc:MI0031591]
chr3	112803312	112804084	773	*	chr3	112803312	112804084	in_vitro	in_vivo	10	12	22.648	17.2508333	5.39716667	c(t = 0.46628	0.647	0.9172739	Distal Intergenic	ENSSSCG000	HADHA	hydroxyacyl-CoA dehydrogenase trifunctional multienzyme complex subunit alpha [Source:NCBI gene (formerly Entrezgene);Acc:397012]
chr17	32825668	32826805	1138	*	chr17	32825668	32826805	in_vitro	in_vivo	9	13	9.85222222	13.7630769	-3.9108547	c(t = -0.4653	0.647	0.9172739	Promoter (<=1kb)	ENSSSCG000	PCED1A	PC-esterase domain containing 1A [Source:VGNC Symbol;Acc:VGNC:95786]
chrX	115206353	115207004	652	*	chrX	115206353	115207004	in_vitro	in_vivo	11	11	8.41727273	11.0127273	-2.5954545	c(t = -0.4662	0.647	0.9172739	Distal Intergenic	ENSSSCG000	ssc-mir-505	ssc-mir-505 [Source:miRBase;Acc:MI0013171]
chr8	75334844	75338199	3356	*	chr8	75334844	75338199	in_vitro	in_vivo	12	14	4.99833333	3.63142857	1.36690476	c(t = 0.46758	0.647	0.9172739	Promoter (<=1kb)	ENSSSCG000	SFRP2	secreted frizzled related protein 2 [Source:NCBI gene (formerly Entrezgene);Acc:100516027]
chrX	98405063	98406116	1054	*	chrX	98405063	98406116	in_vitro	in_vivo	10	11	4.854	2.88181818	1.97218182	c(t = 0.46862	0.647	0.9172739	Distal Intergenic	ENSSSCG000	ATP1B4	ATPase Na+/K+ transporting family member beta 4 [Source:VGNC Symbol;Acc:VGNC:85644]
chr4	88471440	88472205	766	*	chr4	88471440	88472205	in_vitro	in_vivo	10	13	1.599	2.23076923	-0.6317692	c(t = -0.4673	0.647	0.9172739	Distal Intergenic	ENSSSCG00000006350		Fc fragment of IgG, low affinity IIb, receptor (CD32) [Source:NCBI gene (formerly Entrezgene);Acc:613131]
chr2	81613009	81613543	535	*	chr2	81613009	81613543	in_vitro	in_vivo	11	11	2.18181818	1.51545455	0.66636364	c(t = 0.46428	0.647	0.9172739	Distal Intergenic	ENSSSCG000	KIAA1191	KIAA1191 [Source:VGNC Symbol;Acc:VGNC:99783]
chr3	3091195	3092532	1338	*	chr3	3091195	3092532	in_vitro	in_vivo	9	9	26.1633333	32.5544444	-6.3911111	c(t = -0.4660	0.648	0.9172739	Distal Intergenic	ENSSSCG000	AP5Z1	adaptor related protein complex 5 subunit zeta 1 [Source:VGNC Symbol;Acc:VGNC:85397]
chr3	800940	813976	13037	*	chr3	800940	813976	in_vitro	in_vivo	13	13	11.4492308	7.84923077	3.6	c(t = 0.46621	0.648	0.9172739	Distal Intergenic	ENSSSCG000	ssc-mir-339-1	ssc-mir-339-1 [Source:miRBase;Acc:MI0013149]
chr15	45189464	45191360	1897	*	chr15	45189464	45191360	in_vitro	in_vivo	11	16	1.78909091	2.58875	-0.7996591	c(t = -0.4628	0.648	0.9172739	Distal Intergenic	ENSSSCG000	CLDN22	claudin 22 [Source:NCBI gene (formerly Entrezgene);Acc:100294683]
chr7	23064292	23064878	587	*	chr7	23064292	23064878	in_vitro	in_vivo	10	11	3.35	2.24909091	1.10090909	c(t = 0.46399	0.648	0.9172739	Promoter (<=1kb)	ENSSSCG000	RPP21	ribonuclease P/MRP subunit p21 [Source:VGNC Symbol;Acc:VGNC:92431]
chr12	45126833	45127522	690	*	chr12	45126833	45127522	in_vitro	in_vivo	9	12	2.70555556	2.10916667	0.59638889	c(t = 0.46463	0.648	0.9172739	Distal Intergenic	ENSSSCG000	ssc-mir-144	ssc-mir-144 [Source:miRBase;Acc:MI0022132]
chr11	51494201	51494648	448	*	chr11	51494201	51494648	in_vitro	in_vivo	10	12	2.767	2.01416667	0.75283333	c(t = 0.46375	0.648	0.9172739	Distal Intergenic	ENSSSCG000	EDNRB	endothelin receptor type B [Source:VGNC Symbol;Acc:VGNC:87550]
chr13	200695116	200696489	1374	*	chr13	200695116	200696489	in_vitro	in_vivo	12	12	1.21083333	1.62583333	-0.415	c(t = -0.4632	0.648	0.9172739	Promoter (<=1kb)	ENSSSCG000	PIGP	phosphatidylinositol glycan anchor biosynthesis class P [Source:VGNC Symbol;Acc:VGNC:91427]
chr5	18673763	18674702	940	*	chr5	18673763	18674702	in_vitro	in_vivo	10	10	0.947	1.433	-0.486	c(t = -0.4648	0.648	0.9172739	Promoter (<=1kb)	ENSSSCG000	PCBP2	poly(rC) binding protein 2 [Source:VGNC Symbol;Acc:VGNC:103309]
chr7	51877241	51878202	962	*	chr7	51877241	51878202	in_vitro	in_vivo	14	14	1.45142857	1.10785714	0.34357143	c(t = 0.46234	0.648	0.9172739	Distal Intergenic	ENSSSCG000	ssc-mir-1839	ssc-mir-1839 [Source:miRBase;Acc:MI0013162]
chrX	752877	755659	2783	*	chrX	752877	755659	in_vitro	in_vivo	9	10	0.68888889	0.959	-0.2701111	c(t = -0.4643	0.648	0.9172739	Distal Intergenic	ENSSSCG000	ARSL	arylsulfatase E (chondrodysplasia punctata 1) [Source:NCBI gene (formerly Entrezgene);Acc:100627778]
chr13	32029108	32030018	911	*	chr13	32029108	32030018	in_vitro	in_vivo	11	11	0.56454545	0.74181818	-0.1772727	c(t = -0.4631	0.648	0.9172739	Distal Intergenic	ENSSSCG000	AMT	aminomethyltransferase [Source:NCBI gene (formerly Entrezgene);Acc:396679]
chr6	961805	971117	9313	*	chr6	961805	971117	in_vitro	in_vivo	11	10	95.6972727	94.71	0.98727273	c(t = 0.46320	0.649	0.91767525	Distal Intergenic	ENSSSCG000	CYBA	cytochrome b-245 alpha chain [Source:VGNC Symbol;Acc:VGNC:87124]
chr11	4510235	4511193	959	*	chr11	4510235	4511193	in_vitro	in_vivo	12	9	29.7925	36.2688889	-6.4763889	c(t = -0.4629	0.649	0.91767525	Distal Intergenic	ENSSSCG000	GPR12	G protein-coupled receptor 12 [Source:NCBI gene (formerly Entrezgene);Acc:100736641]
chr2	64799891	64800467	577	*	chr2	64799891	64800467	in_vitro	in_vivo	13	14	14.6384615	18.1028571	-3.4643956	c(t = -0.4615	0.649	0.91767525	Distal Intergenic	ENSSSCG000	DNAJB1	DnaJ heat shock protein family (Hsp40) member B1 [Source:NCBI gene (formerly Entrezgene);Acc:396741]
chr8	1258353	1259243	891	*	chr8	1258353	1259243	in_vitro	in_vivo	9	9	1.85888889	1.25666667	0.60222222	c(t = 0.46424	0.649	0.91767525	Distal Intergenic	ENSSSCG000	RNF4	ring finger protein 4 [Source:VGNC Symbol;Acc:VGNC:92392]
chr14	26122267	26123103	837	*	chr14	26122267	26123103	in_vitro	in_vivo	11	14	1.70636364	1.14142857	0.56493506	c(t = 0.46678	0.649	0.91767525	Promoter (<=1kb)	ENSSSCG000	SLC15A4	solute carrier family 15 member 4 [Source:VGNC Symbol;Acc:VGNC:92937]
chr8	103189360	103190375	1016	*	chr8	103189360	103190375	in_vitro	in_vivo	12	15	0.965	0.65933333	0.30566667	c(t = 0.46067	0.649	0.91767525	Distal Intergenic	ENSSSCG000	SMIM43	small integral membrane protein 43 [Source:NCBI gene (formerly Entrezgene);Acc:110262163]
chr12	6337182	6338139	958	*	chr12	6337182	6338139	in_vitro	in_vivo	10	12	30.434	23.3141667	7.11983333	c(t = 0.46100	0.65	0.91780132	Distal Intergenic	ENSSSCG000	FADS6	fatty acid desaturase 6 [Source:VGNC Symbol;Acc:VGNC:87878]
chr6	64186657	64195491	8835	*	chr6	64186657	64195491	in_vitro	in_vivo	12	14	18.7175	23.2378571	-4.5203571	c(t = -0.4603	0.65	0.91780132	Distal Intergenic	ENSSSCG000	PRKCZ	protein kinase C zeta [Source:NCBI gene (formerly Entrezgene);Acc:100533200]
chr1	131032942	131035252	2311	*	chr1	131032942	131035252	in_vitro	in_vivo	13	11	5.60076923	7.13545455	-1.5346853	c(t = -0.4608	0.65	0.91780132	Distal Intergenic	ENSSSCG000	IVD	isovaleryl-CoA dehydrogenase [Source:NCBI gene (formerly Entrezgene);Acc:100156047]
chr17	47625900	47626668	769	*	chr17	47625900	47626668	in_vitro	in_vivo	10	13	2.07	2.70769231	-0.6376923	c(t = -0.4601	0.65	0.91780132	Distal Intergenic	ENSSSCG000	SLPI	secretory leukocyte peptidase inhibitor [Source:NCBI gene (formerly Entrezgene);Acc:396886]
chr14	13944445	13945914	1470	*	chr14	13944445	13945914	in_vitro	in_vivo	14	12	3.265	2.43083333	0.83416667	c(t = 0.46011	0.65	0.91780132	Distal Intergenic	ENSSSCG00000021767		L-threonine dehydrogenase [Source:VGNC Symbol;Acc:VGNC:109162]
chr7	9403574	9404531	958	*	chr7	9403574	9404531	in_vitro	in_vivo	11	12	1.03	0.7625	0.2675	c(t = 0.46095	0.65	0.91780132	Distal Intergenic	ENSSSCG000	SIRT5	sirtuin 5 [Source:NCBI gene (formerly Entrezgene);Acc:100125966]
chr2	7422334	7424934	2601	*	chr2	7422334	7424934	in_vitro	in_vivo	9	11	1.12	0.73272727	0.38727273	c(t = 0.46137	0.65	0.91780132	Promoter (<=1kb)	ENSSSCG000	RASGRP2	RAS guanyl releasing protein 2 [Source:NCBI gene (formerly Entrezgene);Acc:100626093]
chr8	2053912	2069673	15762	*	chr8	2053912	2069673	in_vitro	in_vivo	14	16	56.7385714	62.293125	-5.5545536	c(t = -0.4575	0.651	0.91780132	Distal Intergenic	ENSSSCG000	LRPAP1	LDL receptor related protein associated protein 1 [Source:NCBI gene (formerly Entrezgene);Acc:399654]
chr11	25706077	25707272	1196	*	chr11	25706077	25707272	in_vitro	in_vivo	12	9	48.3408333	55.9044444	-7.5636111	c(t = -0.4596	0.651	0.91780132	Distal Intergenic	ENSSSCG000	MTRF1	mitochondrial translation release factor 1 [Source:VGNC Symbol;Acc:VGNC:90471]
chr7	74979458	74980468	1011	*	chr7	74979458	74980468	in_vitro	in_vivo	13	12	12.3307692	14.5716667	-2.2408974	c(t = -0.4580	0.651	0.91780132	Distal Intergenic	ENSSSCG000	CIDEB	cell death inducing DFFA like effector b [Source:VGNC Symbol;Acc:VGNC:86699]
chr2	58913880	58914904	1025	*	chr2	58913880	58914904	in_vitro	in_vivo	9	10	17.7411111	11.963	5.77811111	c(t = 0.46353	0.651	0.91780132	Distal Intergenic	ENSSSCG000	ARMC6	armadillo repeat containing 6 [Source:VGNC Symbol;Acc:VGNC:108748]
chr2	139781467	139783603	2137	*	chr2	139781467	139783603	in_vitro	in_vivo	10	14	4.194	3.40571429	0.78828571	c(t = 0.46131	0.651	0.91780132	Distal Intergenic	ENSSSCG000	MYOT	myotilin [Source:NCBI gene (formerly Entrezgene);Acc:100101550]
chr6	37668953	37669635	683	*	chr6	37668953	37669635	in_vitro	in_vivo	11	11	4.20272727	3.10363636	1.09909091	c(t = 0.45980	0.651	0.91780132	Distal Intergenic	ENSSSCG000	CBLN1	cerebellin 1 precursor [Source:NCBI gene (formerly Entrezgene);Acc:100127148]
chr10	24109931	24110841	911	*	chr10	24109931	24110841	in_vitro	in_vivo	11	10	2.22272727	2.983	-0.7602727	c(t = -0.4603	0.651	0.91780132	Distal Intergenic	ENSSSCG000	TNNI1	troponin I1, slow skeletal type [Source:VGNC Symbol;Acc:VGNC:95548]
chr7	62442767	62443558	792	*	chr7	62442767	62443558	in_vitro	in_vivo	10	11	1.784	2.52454545	-0.7405455	c(t = -0.4598	0.651	0.91780132	Distal Intergenic	ENSSSCG000	SSTR1	somatostatin receptor 1 [Source:VGNC Symbol;Acc:VGNC:93496]
chr3	23012716	23013586	871	*	chr3	23012716	23013586	in_vitro	in_vivo	10	11	2.89	2.12545455	0.76454545	c(t = 0.46051	0.651	0.91780132	Distal Intergenic	ENSSSCG000	NDUFAB1	NADH:ubiquinone oxidoreductase subunit AB1 [Source:VGNC Symbol;Acc:VGNC:104004]
chrX	41983871	41984692	822	*	chrX	41983871	41984692	in_vitro	in_vivo	11	9	1.19272727	1.79	-0.5972727	c(t = -0.4614	0.652	0.91843064	Distal Intergenic	ENSSSCG000	ARAF	A-Raf proto-onco, serine/threonine kinase [Source:VGNC Symbol;Acc:VGNC:85438]
chrX	13413108	13414053	946	*	chrX	13413108	13414053	in_vitro	in_vivo	11	13	1.95090909	1.43307692	0.51783217	c(t = 0.45801	0.652	0.91843064	Distal Intergenic	ENSSSCG000	SYAP1	synapse associated protein 1 [Source:VGNC Symbol;Acc:VGNC:93648]
chr7	98203443	98204334	892	*	chr7	98203443	98204334	in_vitro	in_vivo	9	10	1.37777778	0.883	0.49477778	c(t = 0.46192	0.652	0.91843064	Distal Intergenic	ENSSSCG000	MLH3	mutL homolog 3 [Source:HGNC Symbol;Acc:HGNC:7128]
chr3	38298201	38299688	1488	*	chr3	38298201	38299688	in_vitro	in_vivo	9	12	94.9044444	96.1208333	-1.2163889	c(t = -0.4593	0.653	0.91843064	Distal Intergenic	ENSSSCG000	TRAP1	TNF receptor associated protein 1 [Source:NCBI gene (formerly Entrezgene);Acc:100515536]
chr2	84133462	84134803	1342	*	chr2	84133462	84134803	in_vitro	in_vivo	11	10	41.1563636	33.005	8.15136364	c(t = 0.45719	0.653	0.91843064	Distal Intergenic	ENSSSCG000	HMGCR	3-hydroxy-3-methylglutaryl-CoA reductase [Source:NCBI gene (formerly Entrezgene);Acc:100144446]
chr12	61506404	61508163	1760	*	chr12	61506404	61508163	in_vitro	in_vivo	10	11	15.872	22.4	-6.528	c(t = -0.4574	0.653	0.91843064	Distal Intergenic	ENSSSCG000	COPS3	COP9 signalosome subunit 3 [Source:VGNC Symbol;Acc:VGNC:86900]
chr2	135151576	135152553	978	*	chr2	135151576	135152553	in_vitro	in_vivo	11	14	21.2463636	15.8678571	5.37850649	c(t = 0.45698	0.653	0.91843064	Distal Intergenic	ENSSSCG000	GDF9	growth differentiation factor 9 [Source:NCBI gene (formerly Entrezgene);Acc:414285]
chr2	75107312	75107943	632	*	chr2	75107312	75107943	in_vitro	in_vivo	9	9	3.46555556	2.25111111	1.21444444	c(t = 0.46216	0.653	0.91843064	Distal Intergenic	ENSSSCG000	FZR1	fizzy and cell division cycle 20 related 1 [Source:VGNC Symbol;Acc:VGNC:88285]
chr6	129635331	129636312	982	*	chr6	129635331	129636312	in_vitro	in_vivo	13	14	2.98	2.03214286	0.94785714	c(t = 0.45583	0.653	0.91843064	Distal Intergenic	ENSSSCG000	UOX	urate oxidase [Source:NCBI gene (formerly Entrezgene);Acc:397510]
chr13	86512636	86513191	556	*	chr13	86512636	86513191	in_vitro	in_vivo	13	11	2.60076923	1.80636364	0.79440559	c(t = 0.45750	0.653	0.91843064	Distal Intergenic	ENSSSCG000	PLSCR1	phospholipid scramblase 1 [Source:NCBI gene (formerly Entrezgene);Acc:100620055]
chr9	70677714	70679035	1322	*	chr9	70677714	70679035	in_vitro	in_vivo	14	12	1.80857143	1.33333333	0.4752381	c(t = 0.45596	0.653	0.91843064	Distal Intergenic	NA	NA	NA
chr8	71181643	71182364	722	*	chr8	71181643	71182364	in_vitro	in_vivo	11	10	1.19727273	0.777	0.42027273	c(t = 0.45644	0.653	0.91843064	Promoter (<=1kb)	ENSSSCG000	RCHY1	ring finger and CHY zinc finger domain containing 1 [Source:VGNC Symbol;Acc:VGNC:92179]
chr15	87158776	87159644	869	*	chr15	87158776	87159644	in_vitro	in_vivo	10	10	0.497	0.294	0.203	c(t = 0.45708	0.653	0.91843064	Distal Intergenic	ENSSSCG000	FRZB	frizzled related protein [Source:VGNC Symbol;Acc:VGNC:96308]
chr3	89803652	89804570	919	*	chr3	89803652	89804570	in_vitro	in_vivo	14	13	15.7107143	21.6623077	-5.9515934	c(t = -0.4544	0.654	0.91866021	Distal Intergenic	ENSSSCG000	FSHR	follicle stimulating hormone receptor [Source:VGNC Symbol;Acc:VGNC:102568]
chr5	15741698	15742570	873	*	chr5	15741698	15742570	in_vitro	in_vivo	9	10	13.2511111	9.396	3.85511111	c(t = 0.45696	0.654	0.91866021	Distal Intergenic	ENSSSCG000	TMBIM6	transmembrane BAX inhibitor motif containing 6 [Source:VGNC Symbol;Acc:VGNC:98374]
chr6	91764062	91765289	1228	*	chr6	91764062	91765289	in_vitro	in_vivo	13	12	4.07538462	4.82416667	-0.7487821	c(t = -0.4536	0.654	0.91866021	Distal Intergenic	NA	NA	NA
chr17	41868902	41869717	816	*	chr17	41868902	41869717	in_vitro	in_vivo	11	13	3.10090909	4.36307692	-1.2621678	c(t = -0.4579	0.654	0.91866021	Distal Intergenic	ENSSSCG000	ADIG	adipogenin [Source:VGNC Symbol;Acc:VGNC:96182]
chr7	36969269	36970476	1208	*	chr7	36969269	36970476	in_vitro	in_vivo	12	10	2.37083333	1.689	0.68183333	c(t = 0.45568	0.654	0.91866021	Distal Intergenic	ENSSSCG000	MED20	mediator complex subunit 20 [Source:NCBI gene (formerly Entrezgene);Acc:100157502]
chr11	4427814	4429094	1281	*	chr11	4427814	4429094	in_vitro	in_vivo	10	12	2.639	1.55333333	1.08566667	c(t = 0.46216	0.654	0.91866021	Promoter (<=1kb)	ENSSSCG000	GPR12	G protein-coupled receptor 12 [Source:NCBI gene (formerly Entrezgene);Acc:100736641]
chr6	82932944	82934589	1646	*	chr6	82932944	82934589	in_vitro	in_vivo	11	13	1.69	1.34384615	0.34615385	c(t = 0.45414	0.654	0.91866021	Promoter (<=1kb)	ENSSSCG000	MACO1	macoilin 1 [Source:NCBI gene (formerly Entrezgene);Acc:654414]
chr6	23277902	23279709	1808	*	chr6	23277902	23279709	in_vitro	in_vivo	12	11	54.545	46.6	7.945	c(t = 0.45397	0.655	0.91911759	Distal Intergenic	ENSSSCG000	CDH11	cadherin 11 [Source:VGNC Symbol;Acc:VGNC:86475]
chr6	20266457	20267415	959	*	chr6	20266457	20267415	in_vitro	in_vivo	9	11	3.01111111	3.84272727	-0.8316162	c(t = -0.4539	0.655	0.91911759	Distal Intergenic	ENSSSCG000	GINS3	GINS complex subunit 3 [Source:VGNC Symbol;Acc:VGNC:88452]
chr1	74626095	74630659	4565	*	chr1	74626095	74630659	in_vitro	in_vivo	16	13	2.2825	1.90769231	0.37480769	c(t = 0.45171	0.655	0.91911759	Promoter (<=1kb)	ENSSSCG000	FOXO3	forkhead box O3 [Source:VGNC Symbol;Acc:VGNC:99715]
chr10	52601008	52601783	776	*	chr10	52601008	52601783	in_vitro	in_vivo	10	9	0.632	1.23444444	-0.6024444	c(t = -0.4604	0.655	0.91911759	Distal Intergenic	ENSSSCG000	BMI1	BMI1 proto-onco, polycomb ring finger [Source:VGNC Symbol;Acc:VGNC:108265]
chr11	12864358	12865079	722	*	chr11	12864358	12865079	in_vitro	in_vivo	12	13	1.25083333	0.90384615	0.34698718	c(t = 0.45268	0.655	0.91911759	Distal Intergenic	ENSSSCG000	ALG5	ALG5 dolichyl-phosphate beta-glucosyltransferase [Source:NCBI gene (formerly Entrezgene);Acc:100154411]
chrX	96950169	96951180	1012	*	chrX	96950169	96951180	in_vitro	in_vivo	10	10	6.06	9.473	-3.413	c(t = -0.4524	0.656	0.91911759	Distal Intergenic	NA	NA	NA
chr7	52793316	52794445	1130	*	chr7	52793316	52794445	in_vitro	in_vivo	13	12	2.06692308	3.04583333	-0.9789103	c(t = -0.4524	0.656	0.91911759	Distal Intergenic	ENSSSCG000	NMB	neuromedin B [Source:VGNC Symbol;Acc:VGNC:109471]
chr4	99306508	99307681	1174	*	chr4	99306508	99307681	in_vitro	in_vivo	9	9	1.88888889	2.66111111	-0.7722222	c(t = -0.4543	0.656	0.91911759	Distal Intergenic	ENSSSCG000	TXNIP	thioredoxin interacting protein [Source:NCBI gene (formerly Entrezgene);Acc:733688]
chr6	67085295	67087615	2321	*	chr6	67085295	67087615	in_vitro	in_vivo	9	10	1.60888889	2.149	-0.5401111	c(t = -0.4536	0.656	0.91911759	Distal Intergenic	ENSSSCG000	RPL22	ribosomal protein L22 [Source:NCBI gene (formerly Entrezgene);Acc:397047]
chr16	74729319	74731345	2027	*	chr16	74729319	74731345	in_vitro	in_vivo	13	11	2.76307692	2.08363636	0.67944056	c(t = 0.45192	0.656	0.91911759	Distal Intergenic	ENSSSCG000	CCT5	chaperonin containing TCP1 subunit 5 [Source:VGNC Symbol;Acc:VGNC:86383]
chr8	5867796	5868250	455	*	chr8	5867796	5868250	in_vitro	in_vivo	11	10	2.79818182	2.037	0.76118182	c(t = 0.45367	0.656	0.91911759	Distal Intergenic	ENSSSCG000	MSX1	msh homeobox 1 [Source:NCBI gene (formerly Entrezgene);Acc:100302364]
chr6	43371051	43372524	1474	*	chr6	43371051	43372524	in_vitro	in_vivo	16	13	1.779375	1.38538462	0.39399038	c(t = 0.45080	0.656	0.91911759	Distal Intergenic	ENSSSCG000	LSM14A	LSM14A mRNA processing body assembly factor [Source:NCBI gene (formerly Entrezgene);Acc:100511455]
chr18	24372544	24372946	403	*	chr18	24372544	24372946	in_vitro	in_vivo	14	11	1.75714286	1.31636364	0.44077922	c(t = 0.45191	0.656	0.91911759	Distal Intergenic	ENSSSCG000	AASS	aminoadipate-semialdehyde synthase [Source:VGNC Symbol;Acc:VGNC:84944]
chr13	22040647	22041017	371	*	chr13	22040647	22041017	in_vitro	in_vivo	9	9	0.50222222	0.75444444	-0.2522222	c(t = -0.4546	0.656	0.91911759	Distal Intergenic	ENSSSCG000	MLH1	mutL homolog 1 [Source:VGNC Symbol;Acc:VGNC:90252]
chr7	1471162	1473096	1935	*	chr7	1471162	1473096	in_vitro	in_vivo	12	12	21.6391667	28.2708333	-6.6316667	c(t = -0.4511	0.657	0.91951284	Distal Intergenic	ENSSSCG000	SERPINB9	serpin peptidase inhibitor, clade B (ovalbumin), member 9 [Source:NCBI gene (formerly Entrezgene);Acc:100126851]
chrX	124427335	124429654	2320	*	chrX	124427335	124429654	in_vitro	in_vivo	10	10	14.747	20.495	-5.748	c(t = -0.4517	0.657	0.91951284	Promoter (<=1kb)	ENSSSCG000	PNCK	pregnancy up-regulated nonubiquitous CaM kinase [Source:NCBI gene (formerly Entrezgene);Acc:100337678]
chr13	207331381	207334112	2732	*	chr13	207331381	207334112	in_vitro	in_vivo	11	12	14.6481818	20.0483333	-5.4001515	c(t = -0.4498	0.657	0.91951284	Distal Intergenic	ENSSSCG000	ITGB2	integrin subunit beta 2 [Source:NCBI gene (formerly Entrezgene);Acc:396943]
chr6	168994003	168995215	1213	*	chr6	168994003	168995215	in_vitro	in_vivo	13	12	2.10153846	1.62333333	0.47820513	c(t = 0.45074	0.657	0.91951284	Distal Intergenic	ENSSSCG000	GUCA2A	guanylate cyclase activator 2A [Source:NCBI gene (formerly Entrezgene);Acc:100301560]
chr7	55219629	55220262	634	*	chr7	55219629	55220262	in_vitro	in_vivo	10	10	2.149	1.315	0.834	c(t = 0.45178	0.657	0.91951284	Distal Intergenic	ENSSSCG000	PLIN1	perilipin 1 [Source:NCBI gene (formerly Entrezgene);Acc:654411]
chr12	25699106	25702044	2939	*	chr12	25699106	25702044	in_vitro	in_vivo	9	14	0.88555556	1.21857143	-0.3330159	c(t = -0.4505	0.657	0.91951284	Distal Intergenic	ENSSSCG000	KAT7	lysine acetyltransferase 7 [Source:VGNC Symbol;Acc:VGNC:89307]
chr8	102147854	102148469	616	*	chr8	102147854	102148469	in_vitro	in_vivo	11	12	12.0981818	7.37916667	4.71901515	c(t = 0.44966	0.658	0.91990722	Promoter (<=1kb)	ENSSSCG000	TRPC3	transient receptor potential cation channel subfamily C member 3 [Source:VGNC Symbol;Acc:VGNC:94461]
chr12	13740935	13742927	1993	*	chr12	13740935	13742927	in_vitro	in_vivo	10	13	3.545	2.90076923	0.64423077	c(t = 0.44927	0.658	0.91990722	Promoter (<=1kb)	ENSSSCG000	PITPNC1	phosphatidylinositol transfer protein cytoplasmic 1 [Source:NCBI gene (formerly Entrezgene);Acc:100233199]
chr8	647613	649721	2109	*	chr8	647613	649721	in_vitro	in_vivo	10	10	3.066	2.341	0.725	c(t = 0.45022	0.658	0.91990722	Distal Intergenic	ENSSSCG000	SLBP	stem-loop binding protein [Source:NCBI gene (formerly Entrezgene);Acc:100513605]
chr9	86511358	86512217	860	*	chr9	86511358	86512217	in_vitro	in_vivo	12	10	1.55416667	1.202	0.35216667	c(t = 0.44922	0.658	0.91990722	Promoter (<=1kb)	ENSSSCG000	AHR	aryl hydrocarbon receptor [Source:NCBI gene (formerly Entrezgene);Acc:396654]
chr5	33525551	33526557	1007	*	chr5	33525551	33526557	in_vitro	in_vivo	11	14	1.05090909	0.79285714	0.25805195	c(t = 0.44933	0.658	0.91990722	Promoter (<=1kb)	ENSSSCG000	CPSF6	cleavage and polyadenylation specific factor 6 [Source:VGNC Symbol;Acc:VGNC:86962]
chr12	27525268	27527091	1824	*	chr12	27525268	27527091	in_vitro	in_vivo	13	13	1.05230769	0.78692308	0.26538462	c(t = 0.44871	0.658	0.91990722	Distal Intergenic	ENSSSCG000	NME2	NME/NM23 nucleoside diphosphate kinase 2 [Source:NCBI gene (formerly Entrezgene);Acc:733683]
chr4	62880758	62881314	557	*	chr4	62880758	62881314	in_vitro	in_vivo	10	11	29.424	37.6309091	-8.2069091	c(t = -0.4486	0.659	0.92041765	Distal Intergenic	ENSSSCG000	RPL7	ribosomal protein L7 [Source:NCBI gene (formerly Entrezgene);Acc:414418]
chr16	36023907	36025757	1851	*	chr16	36023907	36025757	in_vitro	in_vivo	15	14	8.57466667	6.14428571	2.43038095	c(t = 0.44698	0.659	0.92041765	Distal Intergenic	ENSSSCG000	ssc-mir-9799	ssc-mir-9799 [Source:miRBase;Acc:MI0031563]
chr1	205284304	205285295	992	*	chr1	205284304	205285295	in_vitro	in_vivo	12	14	2.155	1.72428571	0.43071429	c(t = 0.44769	0.659	0.92041765	Distal Intergenic	ENSSSCG000	PLIN2	perilipin 2 [Source:VGNC Symbol;Acc:VGNC:91558]
chr15	64528842	64529516	675	*	chr15	64528842	64529516	in_vitro	in_vivo	10	10	2.188	1.32	0.868	c(t = 0.45323	0.659	0.92041765	Distal Intergenic	ENSSSCG000	NR4A2	nuclear receptor subfamily 4 group A member 2 [Source:VGNC Symbol;Acc:VGNC:96451]
chr10	39875244	39875701	458	*	chr10	39875244	39875701	in_vitro	in_vivo	15	15	15.416	20.952	-5.536	c(t = -0.4447	0.66	0.92041765	Distal Intergenic	ENSSSCG000	BAMBI	BMP and activin membrane bound inhibitor [Source:NCBI gene (formerly Entrezgene);Acc:449001]
chr14	77785299	77786954	1656	*	chr14	77785299	77786954	in_vitro	in_vivo	12	13	3.99583333	4.76461538	-0.7687821	c(t = -0.4456	0.66	0.92041765	Distal Intergenic	ENSSSCG000	VDAC2	voltage dependent anion channel 2 [Source:VGNC Symbol;Acc:VGNC:111154]
chr3	38872970	38873894	925	*	chr3	38872970	38873894	in_vitro	in_vivo	12	13	4.3175	3.27615385	1.04134615	c(t = 0.44751	0.66	0.92041765	Distal Intergenic	ENSSSCG000	CLUAP1	clusterin associated protein 1 [Source:VGNC Symbol;Acc:VGNC:86791]
chr4	180269	181031	763	*	chr4	180269	181031	in_vitro	in_vivo	10	12	5.098	3.01166667	2.08633333	c(t = 0.45134	0.66	0.92041765	Distal Intergenic	ENSSSCG000	SLC52A1	solute carrier family 52 member 2 [Source:NCBI gene (formerly Entrezgene);Acc:445519]
chr7	7206130	7207825	1696	*	chr7	7206130	7207825	in_vitro	in_vivo	11	10	1.77545455	2.49	-0.7145455	c(t = -0.4466	0.66	0.92041765	Distal Intergenic	ENSSSCG000	C6orf52	chromosome 7 C6orf52 homolog [Source:VGNC Symbol;Acc:VGNC:86073]
chr6	84293484	84294577	1094	*	chr6	84293484	84294577	in_vitro	in_vivo	12	14	2.4925	1.96071429	0.53178571	c(t = 0.44736	0.66	0.92041765	Promoter (<=1kb)	ENSSSCG000	TRNP1	TMF1 regulated nuclear protein 1 [Source:VGNC Symbol;Acc:VGNC:94456]
chr11	34389652	34390184	533	*	chr11	34389652	34390184	in_vitro	in_vivo	9	11	1.23444444	1.94727273	-0.7128283	c(t = -0.4477	0.66	0.92041765	Distal Intergenic	ENSSSCG000	MTRF1	mitochondrial translation release factor 1 [Source:VGNC Symbol;Acc:VGNC:90471]
chr8	5630881	5636235	5355	*	chr8	5630881	5636235	in_vitro	in_vivo	12	14	2.07333333	1.67714286	0.39619048	c(t = 0.44737	0.66	0.92041765	Promoter (<=1kb)	ENSSSCG000	MSX1	msh homeobox 1 [Source:NCBI gene (formerly Entrezgene);Acc:100302364]
chr9	26166815	26167401	587	*	chr9	26166815	26167401	in_vitro	in_vivo	12	10	1.4725	0.964	0.5085	c(t = 0.44802	0.66	0.92041765	Promoter (<=1kb)	ENSSSCG000	C11orf54	chromosome 9 C11orf54 homolog [Source:VGNC Symbol;Acc:VGNC:86087]
chr6	144576299	144578127	1829	*	chr6	144576299	144578127	in_vitro	in_vivo	9	11	90.0177778	85.4318182	4.5859596	c(t = 0.44695	0.661	0.92041765	Promoter (<=1kb)	ENSSSCG000	DIRAS3	DIRAS family GTPase 3 [Source:NCBI gene (formerly Entrezgene);Acc:733668]
chr16	77844078	77844954	877	*	chr16	77844078	77844954	in_vitro	in_vivo	11	11	13.7172727	19.3127273	-5.5954545	c(t = -0.4456	0.661	0.92041765	Distal Intergenic	ENSSSCG000	TERT	telomerase reverse transcriptase [Source:NCBI gene (formerly Entrezgene);Acc:492280]
chr2	75261397	75263621	2225	*	chr2	75261397	75263621	in_vitro	in_vivo	15	10	8.36066667	12.054	-3.6933333	c(t = -0.4479	0.661	0.92041765	Distal Intergenic	ENSSSCG000	FZR1	fizzy and cell division cycle 20 related 1 [Source:VGNC Symbol;Acc:VGNC:88285]
chr1	211832082	211832578	497	*	chr1	211832082	211832578	in_vitro	in_vivo	10	11	2.021	2.91090909	-0.8899091	c(t = -0.4465	0.661	0.92041765	Distal Intergenic	ENSSSCG000	TYRP1	tyrosinase related protein 1 [Source:VGNC Symbol;Acc:VGNC:94620]
chr11	9737631	9738333	703	*	chr11	9737631	9738333	in_vitro	in_vivo	10	13	3.609	2.65692308	0.95207692	c(t = 0.44587	0.661	0.92041765	Distal Intergenic	ENSSSCG000	RFC3	replication factor C subunit 3 [Source:VGNC Symbol;Acc:VGNC:92233]
chr5	94383682	94384543	862	*	chr5	94383682	94384543	in_vitro	in_vivo	13	12	1.98384615	1.34666667	0.63717949	c(t = 0.44494	0.661	0.92041765	Distal Intergenic	ENSSSCG000	RLIG1	RNA 5'-phosphate and 3'-OH ligase 1 [Source:VGNC Symbol;Acc:VGNC:86040]
chr14	51342553	51343640	1088	*	chr14	51342553	51343640	in_vitro	in_vivo	9	9	1.70222222	1.27444444	0.42777778	c(t = 0.44726	0.661	0.92041765	Promoter (<=1kb)	ENSSSCG000	GNB1L	G protein subunit beta 1 like [Source:VGNC Symbol;Acc:VGNC:88530]
chr13	196802008	196802980	973	*	chr13	196802008	196802980	in_vitro	in_vivo	9	11	1.65	1.19727273	0.45272727	c(t = 0.44639	0.661	0.92041765	Intron (NM_001204775/100533555, intron 1 of 8)	ENSSSCG000	IFNAR2	interferon alpha and beta receptor subunit 2 [Source:NCBI gene (formerly Entrezgene);Acc:100533555]
chr12	14261173	14262086	914	*	chr12	14261173	14262086	in_vitro	in_vivo	11	9	1.55818182	1.12222222	0.4359596	c(t = 0.44760	0.661	0.92041765	Distal Intergenic	ENSSSCG000	KPNA2	karyopherin subunit alpha 2 [Source:VGNC Symbol;Acc:VGNC:89561]
chrX	66764933	66765566	634	*	chrX	66764933	66765566	in_vitro	in_vivo	12	12	7.7125	4.89416667	2.81833333	c(t = 0.44646	0.662	0.92064242	Distal Intergenic	ENSSSCG000	ssc-mir-361	ssc-mir-361 [Source:miRBase;Acc:MI0013145]
chr2	69110774	69114815	4042	*	chr2	69110774	69114815	in_vitro	in_vivo	11	14	3.80727273	2.99214286	0.81512987	c(t = 0.44796	0.662	0.92064242	Distal Intergenic	ENSSSCG000	ICAM1	intercellular adhesion molecule 1 [Source:NCBI gene (formerly Entrezgene);Acc:396750]
chr12	36833800	36835128	1329	*	chr12	36833800	36835128	in_vitro	in_vivo	9	13	2.25222222	2.69769231	-0.4454701	c(t = -0.4451	0.662	0.92064242	Distal Intergenic	ENSSSCG000	TBX4	T-box transcription factor 4 [Source:NCBI gene (formerly Entrezgene);Acc:100620126]
chr5	13754028	13755676	1649	*	chr5	13754028	13755676	in_vitro	in_vivo	13	14	3.12384615	2.41928571	0.70456044	c(t = 0.44320	0.662	0.92064242	Distal Intergenic	ENSSSCG000	CKAP4	cytoskeleton associated protein 4 [Source:NCBI gene (formerly Entrezgene);Acc:100523493]
chr18	48825442	48826295	854	*	chr18	48825442	48826295	in_vitro	in_vivo	10	9	1.467	1.07555556	0.39144444	c(t = 0.44551	0.662	0.92064242	Distal Intergenic	ENSSSCG000	PGAM2	phosphoglycerate mutase 2 [Source:VGNC Symbol;Acc:VGNC:91341]
chr9	124141782	124142490	709	*	chr9	124141782	124142490	in_vitro	in_vivo	9	11	1.61444444	0.90909091	0.70535354	c(t = 0.45054	0.662	0.92064242	Distal Intergenic	ENSSSCG000	NPL	N-acetylneuraminate pyruvate lyase [Source:VGNC Symbol;Acc:VGNC:90851]
chr1	16330729	16331309	581	*	chr1	16330729	16331309	in_vitro	in_vivo	12	12	0.8025	0.59	0.2125	c(t = 0.44257	0.662	0.92064242	Distal Intergenic	ENSSSCG000	PCMT1	protein-L-isoaspartate (D-aspartate) O-methyltransferase [Source:VGNC Symbol;Acc:VGNC:103146]
chr10	57992951	57993564	614	*	chr10	57992951	57993564	in_vitro	in_vivo	9	9	87.8133333	90.5111111	-2.6977778	c(t = -0.4475	0.663	0.92103308	Distal Intergenic	ENSSSCG000	CCNY	cyclin Y [Source:VGNC Symbol;Acc:VGNC:95998]
chr1	1583895	1589690	5796	*	chr1	1583895	1589690	in_vitro	in_vivo	12	11	90.1566667	85.8254545	4.33121212	c(t = 0.44548	0.663	0.92103308	Distal Intergenic	ENSSSCG000	ssc-mir-9824	ssc-mir-9824 [Source:miRBase;Acc:MI0031591]
chrX	124402000	124407184	5185	*	chrX	124402000	124407184	in_vitro	in_vivo	9	13	10.1822222	13.1084615	-2.9262393	c(t = -0.4428	0.663	0.92103308	Distal Intergenic	ENSSSCG000	PNCK	pregnancy up-regulated nonubiquitous CaM kinase [Source:NCBI gene (formerly Entrezgene);Acc:100337678]
chr6	148760692	148761845	1154	*	chr6	148760692	148761845	in_vitro	in_vivo	11	11	3.98	2.67272727	1.30727273	c(t = 0.44448	0.663	0.92103308	Distal Intergenic	ENSSSCG000	EFCAB7	phosphoglucomutase 1 [Source:VGNC Symbol;Acc:VGNC:91355]
chr9	74276618	74277656	1039	*	chr9	74276618	74277656	in_vitro	in_vivo	10	15	3.119	2.59133333	0.52766667	c(t = 0.44097	0.663	0.92103308	Distal Intergenic	ENSSSCG000	COL1A2	collagen type I alpha 2 chain [Source:NCBI gene (formerly Entrezgene);Acc:100626716]
chr5	50970544	50970792	249	*	chr5	50970544	50970792	in_vitro	in_vivo	9	9	1.23444444	0.61777778	0.61666667	c(t = 0.44673	0.663	0.92103308	Distal Intergenic	ENSSSCG000	LDHB	lactate dehydrogenase B [Source:NCBI gene (formerly Entrezgene);Acc:100621540]
chr17	62091279	62095323	4045	*	chr17	62091279	62095323	in_vitro	in_vivo	9	10	94.8966667	93.613	1.28366667	c(t = 0.44355	0.664	0.92158931	Distal Intergenic	ENSSSCG000	ssc-mir-133a	ssc-mir-133a-1 [Source:miRBase;Acc:MI0010681]
chr10	65160121	65161320	1200	*	chr10	65160121	65161320	in_vitro	in_vivo	10	11	2.68	1.94363636	0.73636364	c(t = 0.44413	0.664	0.92158931	Promoter (<=1kb)	ENSSSCG000	GDI2	GDP dissociation inhibitor 2 [Source:NCBI gene (formerly Entrezgene);Acc:414427]
chr3	53080659	53081546	888	*	chr3	53080659	53081546	in_vitro	in_vivo	9	12	0.75666667	1.02583333	-0.2691667	c(t = -0.4408	0.664	0.92158931	Distal Intergenic	ENSSSCG000	IL1R2	interleukin 1 receptor type 2 [Source:VGNC Symbol;Acc:VGNC:89093]
chr6	85411909	85412573	665	*	chr6	85411909	85412573	in_vitro	in_vivo	9	11	0.24666667	0.41090909	-0.1642424	c(t = -0.4413	0.664	0.92158931	Distal Intergenic	ENSSSCG000	ATP5IF1	ATP synthase inhibitory factor subunit 1 [Source:NCBI gene (formerly Entrezgene);Acc:100038002]
chr14	66590192	66590638	447	*	chr14	66590192	66590638	in_vitro	in_vivo	9	11	0.46333333	0.23272727	0.23060606	c(t = 0.44475	0.664	0.92158931	Distal Intergenic	ENSSSCG000	ssc-mir-1296	ssc-mir-1296 [Source:miRBase;Acc:MI0015931]
chrX	117154318	117155760	1443	*	chrX	117154318	117155760	in_vitro	in_vivo	12	15	18.3508333	15.7513333	2.5995	c(t = 0.43973	0.665	0.92264398	Distal Intergenic	ENSSSCG000	ssc-mir-505	ssc-mir-505 [Source:miRBase;Acc:MI0013171]
chr14	107959978	107961420	1443	*	chr14	107959978	107961420	in_vitro	in_vivo	16	13	3.14375	2.07384615	1.06990385	c(t = 0.43956	0.665	0.92264398	Distal Intergenic	ENSSSCG000	OPALIN	oligodendrocytic myelin paranodal and inner loop protein [Source:VGNC Symbol;Acc:VGNC:91046]
chr9	25446895	25447358	464	*	chr9	25446895	25447358	in_vitro	in_vivo	9	9	18.2833333	25.1077778	-6.8244444	c(t = -0.4400	0.666	0.92286513	Distal Intergenic	ENSSSCG000	C11orf54	chromosome 9 C11orf54 homolog [Source:VGNC Symbol;Acc:VGNC:86087]
chr15	33494826	33497218	2393	*	chr15	33494826	33497218	in_vitro	in_vivo	13	15	16.0184615	13.818	2.20046154	c(t = 0.43681	0.666	0.92286513	Distal Intergenic	ENSSSCG000	CLN8	CLN8 transmembrane ER and ERGIC protein [Source:VGNC Symbol;Acc:VGNC:103067]
chr10	23369201	23370528	1328	*	chr10	23369201	23370528	in_vitro	in_vivo	12	9	3.19916667	3.75888889	-0.5597222	c(t = -0.4397	0.666	0.92286513	Distal Intergenic	ENSSSCG000	TNNT2	troponin T2, cardiac type [Source:NCBI gene (formerly Entrezgene);Acc:100622450]
chr7	121564995	121566489	1495	*	chr7	121564995	121566489	in_vitro	in_vivo	9	10	2.79222222	2.111	0.68122222	c(t = 0.43991	0.666	0.92286513	Promoter (<=1kb)	ENSSSCG000	DLK1	delta like non-canonical Notch ligand 1 [Source:VGNC Symbol;Acc:VGNC:87333]
chr11	15876356	15877555	1200	*	chr11	15876356	15877555	in_vitro	in_vivo	12	14	2.61416667	1.92142857	0.6927381	c(t = 0.43840	0.666	0.92286513	Distal Intergenic	ENSSSCG000	SLC25A15	solute carrier family 25 member 15 [Source:VGNC Symbol;Acc:VGNC:92994]
chr6	45418572	45419672	1101	*	chr6	45418572	45419672	in_vitro	in_vivo	10	9	1.787	1.28555556	0.50144444	c(t = 0.44065	0.666	0.92286513	Promoter (<=1kb)	ENSSSCG000	WDR62	WD repeat domain 62 [Source:VGNC Symbol;Acc:VGNC:94933]
chr4	122343235	122343783	549	*	chr4	122343235	122343783	in_vitro	in_vivo	10	10	0.526	0.312	0.214	c(t = 0.43910	0.666	0.92286513	Distal Intergenic	ENSSSCG000	F3	coagulation factor III, tissue factor [Source:VGNC Symbol;Acc:VGNC:87864]
chrX	114954334	114955436	1103	*	chrX	114954334	114955436	in_vitro	in_vivo	9	10	4.53555556	6.805	-2.2694444	c(t = -0.4411	0.667	0.92347185	Distal Intergenic	ENSSSCG000	ssc-mir-505	ssc-mir-505 [Source:miRBase;Acc:MI0013171]
chr14	49870726	49871892	1167	*	chr14	49870726	49871892	in_vitro	in_vivo	10	10	8.013	6.549	1.464	c(t = 0.43792	0.667	0.92347185	Distal Intergenic	ENSSSCG000	MIF	macrophage migration inhibitory factor [Source:NCBI gene (formerly Entrezgene);Acc:397412]
chr12	23915330	23916876	1547	*	chr12	23915330	23916876	in_vitro	in_vivo	10	11	2.008	1.21545455	0.79254545	c(t = 0.44124	0.667	0.92347185	Distal Intergenic	ENSSSCG000	TBX21	T-box transcription factor 21 [Source:NCBI gene (formerly Entrezgene);Acc:100518804]
chr11	2670548	2671138	591	*	chr11	2670548	2671138	in_vitro	in_vivo	12	9	93.5441667	91.9533333	1.59083333	c(t = 0.43841	0.668	0.92347185	Distal Intergenic	ENSSSCG000	ssc-mir-9814	ssc-mir-9814 [Source:miRBase;Acc:MI0031581]
chr16	79159780	79167092	7313	*	chr16	79159780	79167092	in_vitro	in_vivo	10	12	36.872	32.275	4.597	c(t = 0.43633	0.668	0.92347185	Distal Intergenic	ENSSSCG000	TERT	telomerase reverse transcriptase [Source:NCBI gene (formerly Entrezgene);Acc:492280]
chr4	110583453	110584103	651	*	chr4	110583453	110584103	in_vitro	in_vivo	13	12	16.3738462	20.575	-4.2011538	c(t = -0.4341	0.668	0.92347185	Distal Intergenic	ENSSSCG000	GNAI3	G protein subunit alpha i3 [Source:VGNC Symbol;Acc:VGNC:88523]
chr12	45931392	45932234	843	*	chr12	45931392	45932234	in_vitro	in_vivo	13	10	6.09461538	8.654	-2.5593846	c(t = -0.4369	0.668	0.92347185	Distal Intergenic	ENSSSCG000	ssc-mir-423	ssc-mir-423 [Source:miRBase;Acc:MI0013099]
chrX	49980449	49981180	732	*	chrX	49980449	49981180	in_vitro	in_vivo	9	11	3.14555556	5.03272727	-1.8871717	c(t = -0.4366	0.668	0.92347185	Distal Intergenic	ENSSSCG000	ssc-mir-1468	ssc-mir-1468 [Source:miRBase;Acc:MI0022160]
chr2	51127867	51128780	914	*	chr2	51127867	51128780	in_vitro	in_vivo	10	9	2.269	2.85555556	-0.5865556	c(t = -0.4372	0.668	0.92347185	Distal Intergenic	ENSSSCG000	ARF1	ADP ribosylation factor 1 [Source:VGNC Symbol;Acc:VGNC:96793]
chr5	17357887	17359201	1315	*	chr5	17357887	17359201	in_vitro	in_vivo	10	10	3.869	2.85	1.019	c(t = 0.43627	0.668	0.92347185	Distal Intergenic	ENSSSCG000	ACVR1B	activin A receptor type 1B [Source:VGNC Symbol;Acc:VGNC:85056]
chr5	1568113	1570505	2393	*	chr5	1568113	1570505	in_vitro	in_vivo	11	13	2.26272727	1.8	0.46272727	c(t = 0.43509	0.668	0.92347185	Distal Intergenic	ENSSSCG000	SELENOO	selenoprotein O [Source:NCBI gene (formerly Entrezgene);Acc:100518971]
chr1	55859573	55860370	798	*	chr1	55859573	55860370	in_vitro	in_vivo	11	14	1.96363636	1.48285714	0.48077922	c(t = 0.43746	0.668	0.92347185	Distal Intergenic	ENSSSCG000	SMIM8	small integral membrane protein 8 [Source:VGNC Symbol;Acc:VGNC:93256]
chr18	19199394	19199934	541	*	chr18	19199394	19199934	in_vitro	in_vivo	10	11	1.306	0.92818182	0.37781818	c(t = 0.43684	0.668	0.92347185	Distal Intergenic	ENSSSCG000	ssc-mir-182	ssc-mir-182 [Source:miRBase;Acc:MI0022135]
chr6	27922895	27923674	780	*	chr6	27922895	27923674	in_vitro	in_vivo	11	9	9.08909091	6.70555556	2.38353535	c(t = 0.43524	0.669	0.92402318	Distal Intergenic	ENSSSCG000	ssc-mir-328	ssc-mir-328 [Source:miRBase;Acc:MI0013158]
chr4	40375405	40376380	976	*	chr4	40375405	40376380	in_vitro	in_vivo	10	11	1.878	2.51454545	-0.6365455	c(t = -0.4349	0.669	0.92402318	Distal Intergenic	ENSSSCG000	UQCRB	ubiquinol-cytochrome c reductase binding protein [Source:NCBI gene (formerly Entrezgene);Acc:100157621]
chr3	78268111	78269167	1057	*	chr3	78268111	78269167	in_vitro	in_vivo	10	12	0.759	1.12666667	-0.3676667	c(t = -0.4344	0.669	0.92402318	Distal Intergenic	ENSSSCG000	MDH1	malate dehydrogenase 1 [Source:NCBI gene (formerly Entrezgene);Acc:396894]
chr13	31655827	31657327	1501	*	chr13	31655827	31657327	in_vitro	in_vivo	13	14	1.40230769	1.08214286	0.32016484	c(t = 0.43396	0.669	0.92402318	Promoter (<=1kb)	ENSSSCG000	NDUFAF3	NADH:ubiquinone oxidoreductase complex assembly factor 3 [Source:NCBI gene (formerly Entrezgene);Acc:100156018]
chr13	3135054	3135882	829	*	chr13	3135054	3135882	in_vitro	in_vivo	9	12	1.55666667	1.03333333	0.52333333	c(t = 0.43595	0.669	0.92402318	Distal Intergenic	ENSSSCG000	ANKRD28	ankyrin repeat domain 28 [Source:VGNC Symbol;Acc:VGNC:85331]
chr6	1441424	1447378	5955	*	chr6	1441424	1447378	in_vitro	in_vivo	13	11	96.2707692	95.5536364	0.71713287	c(t = 0.43287	0.67	0.92407574	Exon (NM_001244650/100622381, exon 7 of 14)	ENSSSCG000	BANP	BTG3 associated nuclear protein [Source:NCBI gene (formerly Entrezgene);Acc:100622381]
chr1	271528760	271531443	2684	*	chr1	271528760	271531443	in_vitro	in_vivo	9	10	96.0744444	94.839	1.23544444	c(t = 0.43405	0.67	0.92407574	Distal Intergenic	ENSSSCG000	MED27	mediator complex subunit 27 [Source:VGNC Symbol;Acc:VGNC:90117]
chr12	60480743	60495622	14880	*	chr12	60480743	60495622	in_vitro	in_vivo	11	16	70.4209091	75.603125	-5.1822159	c(t = -0.4359	0.67	0.92407574	Distal Intergenic	ENSSSCG000	SLC5A10	solute carrier family 5 member 10 [Source:NCBI gene (formerly Entrezgene);Acc:497235]
chr3	90489274	90489975	702	*	chr3	90489274	90489975	in_vitro	in_vivo	9	9	75.5188889	67.0455556	8.47333333	c(t = 0.43474	0.67	0.92407574	Distal Intergenic	ENSSSCG000	FSHR	follicle stimulating hormone receptor [Source:VGNC Symbol;Acc:VGNC:102568]
chrX	56135109	56135712	604	*	chrX	56135109	56135712	in_vitro	in_vivo	11	9	14.1536364	20.4	-6.2463636	c(t = -0.4359	0.67	0.92407574	Distal Intergenic	ENSSSCG000	ssc-mir-676-1	ssc-mir-676-1 [Source:miRBase;Acc:MI0013152]
chr4	74236715	74237179	465	*	chr4	74236715	74237179	in_vitro	in_vivo	11	11	13.89	11.4118182	2.47818182	c(t = 0.43362	0.67	0.92407574	Distal Intergenic	ENSSSCG000	SDCBP	syndecan binding protein [Source:VGNC Symbol;Acc:VGNC:92657]
chr13	32444696	32445643	948	*	chr13	32444696	32445643	in_vitro	in_vivo	11	10	1.84909091	1.233	0.61609091	c(t = 0.43561	0.67	0.92407574	Distal Intergenic	ENSSSCG000	MON1A	MON1 homolog A, secretory trafficking associated [Source:NCBI gene (formerly Entrezgene);Acc:100626383]
chr10	20039030	20039942	913	*	chr10	20039030	20039942	in_vitro	in_vivo	10	10	1.979	1.153	0.826	c(t = 0.43551	0.67	0.92407574	Distal Intergenic	ENSSSCG000	CAPN2	calpain 2 [Source:NCBI gene (formerly Entrezgene);Acc:397393]
chr14	114522984	114524826	1843	*	chr14	114522984	114524826	in_vitro	in_vivo	9	11	92.2777778	93.9209091	-1.6431313	c(t = -0.4324	0.671	0.92462525	Distal Intergenic	ENSSSCG000	ssc-mir-1307	ssc-mir-1307 [Source:miRBase;Acc:MI0013147]
chr5	43042764	43043651	888	*	chr5	43042764	43043651	in_vitro	in_vivo	12	12	2.7	2.12916667	0.57083333	c(t = 0.43131	0.671	0.92462525	Distal Intergenic	ENSSSCG000	RESF1	retroelement silencing factor 1 [Source:VGNC Symbol;Acc:VGNC:92219]
chr1	228854463	228855381	919	*	chr1	228854463	228855381	in_vitro	in_vivo	13	10	1.66230769	1.388	0.27430769	c(t = 0.43154	0.671	0.92462525	Distal Intergenic	ENSSSCG000	OSTF1	osteoclast stimulating factor 1 [Source:NCBI gene (formerly Entrezgene);Acc:397066]
chr8	78854136	78855695	1560	*	chr8	78854136	78855695	in_vitro	in_vivo	10	15	1.321	0.98133333	0.33966667	c(t = 0.43231	0.671	0.92462525	Distal Intergenic	ENSSSCG000	RPS3A	ribosomal protein S3A [Source:NCBI gene (formerly Entrezgene);Acc:414390]
chr17	4854420	4856027	1608	*	chr17	4854420	4856027	in_vitro	in_vivo	13	13	1.25769231	0.93461538	0.32307692	c(t = 0.42983	0.671	0.92462525	Distal Intergenic	ENSSSCG000	FGF20	fibroblast growth factor 20 [Source:NCBI gene (formerly Entrezgene);Acc:100516301]
chr1	26010763	26011412	650	*	chr1	26010763	26011412	in_vitro	in_vivo	10	10	2.908	2.129	0.779	c(t = 0.43095	0.672	0.92500806	Distal Intergenic	ENSSSCG000	PERP	p53 apoptosis effector related to PMP22 [Source:NCBI gene (formerly Entrezgene);Acc:100513507]
chr8	76148174	76150196	2023	*	chr8	76148174	76150196	in_vitro	in_vivo	14	12	1.225	1.67	-0.445	c(t = -0.4320	0.672	0.92500806	Distal Intergenic	ENSSSCG000	ARFIP1	ADP ribosylation factor interacting protein 1 [Source:VGNC Symbol;Acc:VGNC:85453]
chr2	82660114	82660860	747	*	chr2	82660114	82660860	in_vitro	in_vivo	9	10	0.96888889	1.346	-0.3771111	c(t = -0.4312	0.672	0.92500806	Promoter (<=1kb)	ENSSSCG000	BTF3	basic transcription factor 3 [Source:VGNC Symbol;Acc:VGNC:99597]
chr1	81746066	81746402	337	*	chr1	81746066	81746402	in_vitro	in_vivo	11	11	1.34818182	0.94818182	0.4	c(t = 0.43030	0.672	0.92500806	Distal Intergenic	ENSSSCG000	COL10A1	collagen type X alpha 1 chain [Source:NCBI gene (formerly Entrezgene);Acc:448809]
chr2	82714806	82715584	779	*	chr2	82714806	82715584	in_vitro	in_vivo	11	11	0.47818182	0.79818182	-0.32	c(t = -0.4292	0.672	0.92500806	Promoter (<=1kb)	ENSSSCG000	UTP15	UTP15 small subunit processome component [Source:NCBI gene (formerly Entrezgene);Acc:100515474]
chr6	82400701	82401732	1032	*	chr6	82400701	82401732	in_vitro	in_vivo	9	10	1.25888889	0.75	0.50888889	c(t = 0.43574	0.672	0.92500806	Distal Intergenic	ENSSSCG000	RSRP1	arginine and serine rich protein 1 [Source:VGNC Symbol;Acc:VGNC:92489]
chr13	81545264	81545826	563	*	chr13	81545264	81545826	in_vitro	in_vivo	10	9	28.304	35.7644444	-7.4604444	c(t = -0.4299	0.673	0.92539005	Distal Intergenic	ENSSSCG000	SLC25A36	solute carrier family 25 member 36 [Source:VGNC Symbol;Acc:VGNC:93012]
chr14	46559640	46560857	1218	*	chr14	46559640	46560857	in_vitro	in_vivo	9	12	3.23777778	3.96583333	-0.7280556	c(t = -0.4293	0.673	0.92539005	Distal Intergenic	ENSSSCG000	GAS2L1	growth arrest specific 2 like 1 [Source:NCBI gene (formerly Entrezgene);Acc:100513569]
chr6	109218036	109218513	478	*	chr6	109218036	109218513	in_vitro	in_vivo	14	12	2.23	1.45416667	0.77583333	c(t = 0.42991	0.673	0.92539005	Distal Intergenic	ENSSSCG000	HRH4	histamine receptor H4 [Source:NCBI gene (formerly Entrezgene);Acc:397224]
chr3	8688988	8690214	1227	*	chr3	8688988	8690214	in_vitro	in_vivo	10	13	2	1.10076923	0.89923077	c(t = 0.43437	0.673	0.92539005	Distal Intergenic	ENSSSCG000	ZAN	zonadhesin [Source:NCBI gene (formerly Entrezgene);Acc:397676]
chr13	118614291	118614876	586	*	chr13	118614291	118614876	in_vitro	in_vivo	10	11	0.611	0.93272727	-0.3217273	c(t = -0.4301	0.673	0.92539005	Distal Intergenic	ENSSSCG000	DNAJC19	DnaJ heat shock protein family (Hsp40) member C19 [Source:NCBI gene (formerly Entrezgene);Acc:100154656]
chr1	94294738	94296245	1508	*	chr1	94294738	94296245	in_vitro	in_vivo	12	11	0.67166667	0.89909091	-0.2274242	c(t = -0.4296	0.673	0.92539005	Distal Intergenic	ENSSSCG000	ATP5F1A	ATP synthase F1 subunit alpha [Source:NCBI gene (formerly Entrezgene);Acc:100157880]
chr2	149707567	149711247	3681	*	chr2	149707567	149711247	in_vitro	in_vivo	11	12	97.3854545	96.7025	0.68295455	c(t = 0.42666	0.674	0.92593672	Distal Intergenic	ENSSSCG00000029257		
chr1	271515346	271517258	1913	*	chr1	271515346	271517258	in_vitro	in_vivo	10	10	95.996	94.963	1.033	c(t = 0.42902	0.674	0.92593672	Distal Intergenic	ENSSSCG000	MED27	mediator complex subunit 27 [Source:VGNC Symbol;Acc:VGNC:90117]
chr6	63042934	63043798	865	*	chr6	63042934	63043798	in_vitro	in_vivo	12	9	1.3925	1.88777778	-0.4952778	c(t = -0.4298	0.674	0.92593672	Distal Intergenic	ENSSSCG000	ISG15	ISG15 ubiquitin like modifier [Source:NCBI gene (formerly Entrezgene);Acc:100145895]
chr6	149153739	149156202	2464	*	chr6	149153739	149156202	in_vitro	in_vivo	15	14	2.176	1.87071429	0.30528571	c(t = 0.42645	0.674	0.92593672	Distal Intergenic	ENSSSCG000	EFCAB7	phosphoglucomutase 1 [Source:VGNC Symbol;Acc:VGNC:91355]
chr1	16248942	16249897	956	*	chr1	16248942	16249897	in_vitro	in_vivo	13	13	1.61615385	1.24307692	0.37307692	c(t = 0.42566	0.674	0.92593672	Promoter (<=1kb)	ENSSSCG000	PCMT1	protein-L-isoaspartate (D-aspartate) O-methyltransferase [Source:VGNC Symbol;Acc:VGNC:103146]
chr11	78035940	78036989	1050	*	chr11	78035940	78036989	in_vitro	in_vivo	11	9	20.8827273	27.9088889	-7.0261616	c(t = -0.4275	0.675	0.92631696	Distal Intergenic	ENSSSCG000	F7	coagulation factor VII [Source:NCBI gene (formerly Entrezgene);Acc:733661]
chr16	79550266	79551345	1080	*	chr16	79550266	79551345	in_vitro	in_vivo	9	11	22.0311111	17.0545455	4.97656566	c(t = 0.43101	0.675	0.92631696	Intron (NM_001123206/100144410, intron 2 of 3)	ENSSSCG000	TPPP	tubulin polymerization promoting protein [Source:VGNC Symbol;Acc:VGNC:94344]
chr5	2576934	2577753	820	*	chr5	2576934	2577753	in_vitro	in_vivo	10	9	20.094	16.2044444	3.88955556	c(t = 0.42731	0.675	0.92631696	Distal Intergenic	ENSSSCG000	PPARA	peroxisome proliferator activated receptor alpha [Source:NCBI gene (formerly Entrezgene);Acc:397239]
chr1	123101271	123102994	1724	*	chr1	123101271	123102994	in_vitro	in_vivo	11	9	2.67636364	3.57555556	-0.8991919	c(t = -0.4263	0.675	0.92631696	Promoter (<=1kb)	ENSSSCG000	FBN1	fibrillin 1 [Source:NCBI gene (formerly Entrezgene);Acc:414836]
chr3	110842690	110844376	1687	*	chr3	110842690	110844376	in_vitro	in_vivo	10	11	2.283	1.62727273	0.65572727	c(t = 0.42733	0.675	0.92631696	Promoter (<=1kb)	ENSSSCG000	FOSL2	FOS like 2, AP-1 transcription factor subunit [Source:VGNC Symbol;Acc:VGNC:88192]
chr18	19141825	19142924	1100	*	chr18	19141825	19142924	in_vitro	in_vivo	10	11	1.074	1.39272727	-0.3187273	c(t = -0.4272	0.675	0.92631696	Distal Intergenic	ENSSSCG000	ssc-mir-182	ssc-mir-182 [Source:miRBase;Acc:MI0022135]
chr10	69075270	69078615	3346	*	chr10	69075270	69078615	in_vitro	in_vivo	11	11	96.6818182	95.4890909	1.19272727	c(t = 0.42517	0.676	0.92653112	Distal Intergenic	ENSSSCG000	WDR37	WD repeat domain 37 [Source:VGNC Symbol;Acc:VGNC:96258]
chr12	3857655	3859206	1552	*	chr12	3857655	3859206	in_vitro	in_vivo	9	10	15.9322222	11.112	4.82022222	c(t = 0.42724	0.676	0.92653112	Distal Intergenic	ENSSSCG000	BIRC5	baculoviral IAP repeat containing 5 [Source:VGNC Symbol;Acc:VGNC:85827]
chr8	28801308	28803280	1973	*	chr8	28801308	28803280	in_vitro	in_vivo	14	16	3.17214286	3.7075	-0.5353571	c(t = -0.4216	0.676	0.92653112	Distal Intergenic	ENSSSCG000	ssc-mir-9840	ssc-mir-9840 [Source:miRBase;Acc:MI0031609]
chr3	107840100	107840766	667	*	chr3	107840100	107840766	in_vitro	in_vivo	11	10	1.45727273	1.89	-0.4327273	c(t = -0.4256	0.676	0.92653112	Promoter (<=1kb)	ENSSSCG000	SRD5A2	steroid 5 alpha-reductase 2 [Source:VGNC Symbol;Acc:VGNC:93449]
chr6	37784355	37785375	1021	*	chr6	37784355	37785375	in_vitro	in_vivo	12	12	2.11916667	1.54833333	0.57083333	c(t = 0.42439	0.676	0.92653112	Distal Intergenic	ENSSSCG000	CBLN1	cerebellin 1 precursor [Source:NCBI gene (formerly Entrezgene);Acc:100127148]
chr10	44420973	44422475	1503	*	chr10	44420973	44422475	in_vitro	in_vivo	9	11	1.18444444	0.73545455	0.4489899	c(t = 0.42880	0.676	0.92653112	Distal Intergenic	ENSSSCG000	MRC1	mannose receptor C-type 1 [Source:NCBI gene (formerly Entrezgene);Acc:445003]
chr3	100669138	100670027	890	*	chr3	100669138	100670027	in_vitro	in_vivo	12	10	0.85916667	0.602	0.25716667	c(t = 0.42482	0.676	0.92653112	Distal Intergenic	ENSSSCG000	GALM	galactose mutarotase [Source:NCBI gene (formerly Entrezgene);Acc:399536]
chr4	93690582	93691181	600	*	chr4	93690582	93691181	in_vitro	in_vivo	9	11	8.23111111	9.77909091	-1.5479798	c(t = -0.4249	0.677	0.92690985	Distal Intergenic	ENSSSCG000	RHBG	Rh family B glycoprotein [Source:VGNC Symbol;Acc:VGNC:98845]
chr8	74382126	74383098	973	*	chr8	74382126	74383098	in_vitro	in_vivo	13	15	2.76230769	3.88066667	-1.118359	c(t = -0.4233	0.677	0.92690985	Distal Intergenic	ENSSSCG000	LRAT	lecithin retinol acyltransferase [Source:VGNC Symbol;Acc:VGNC:89798]
chr15	81907694	81910129	2436	*	chr15	81907694	81910129	in_vitro	in_vivo	10	11	2.373	1.88818182	0.48481818	c(t = 0.42375	0.677	0.92690985	Distal Intergenic	NA	NA	NA
chr15	31002575	31005580	3006	*	chr15	31002575	31005580	in_vitro	in_vivo	10	12	0.797	1.015	-0.218	c(t = -0.4228	0.677	0.92690985	Promoter (<=1kb)	ENSSSCG000	INHBB	inhibin subunit beta B [Source:NCBI gene (formerly Entrezgene);Acc:397490]

chr13	19128146	19128617	472	*	chr13	19128146	19128617	in_vitro	in_vivo	9	10	1.07888889	0.772	0.30688889	c(t = 0.42346	0.677	0.92690985	Distal Intergenic	ENSSSCG000	CMTM7	CKLF like MARVEL transmembrane domain containing 7 [Source:VGNC Symbol;Acc:VGNC:103928]
chr7	47788373	47789048	676	*	chr7	47788373	47789048	in_vitro	in_vivo	10	12	1.267	0.76416667	0.50283333	c(t = 0.42478	0.677	0.92690985	Distal Intergenic	ENSSSCG000	DNAJA4	DnaJ heat shock protein family (Hsp40) member A4 [Source:NCBI gene (formerly Entrezgene);Acc:397613]
chr7	13377944	13379039	1096	*	chr7	13377944	13379039	in_vitro	in_vivo	10	13	46.93	39.8461538	7.08384615	c(t = 0.42218	0.678	0.92695784	Distal Intergenic	ENSSSCG000	NHLRC1	NHL repeat containing E3 ubiquitin protein ligase 1 [Source:NCBI gene (formerly Entrezgene);Acc:100152396]
chr5	8780627	8781989	1363	*	chr5	8780627	8781989	in_vitro	in_vivo	11	10	19.1190909	24.968	-5.8489091	c(t = -0.4231	0.678	0.92695784	Distal Intergenic	ENSSSCG000	ATF4	activating transcription factor 4 [Source:NCBI gene (formerly Entrezgene);Acc:100144302]
chr7	61892264	61895402	3139	*	chr7	61892264	61895402	in_vitro	in_vivo	13	15	11.5830769	9.88866667	1.69441026	c(t = 0.42155	0.678	0.92695784	Promoter (<=1kb)	ENSSSCG000	SSTR1	somatostatin receptor 1 [Source:VGNC Symbol;Acc:VGNC:93496]
chr6	63764227	63766004	1778	*	chr6	63764227	63766004	in_vitro	in_vivo	11	10	2.69363636	3.647	-0.9533636	c(t = -0.4220	0.678	0.92695784	Distal Intergenic	ENSSSCG000	TAS1R3	taste 1 receptor member 3 [Source:NCBI gene (formerly Entrezgene);Acc:100127440]
chr6	109235345	109235997	653	*	chr6	109235345	109235997	in_vitro	in_vivo	11	10	1.52909091	1.908	-0.3789091	c(t = -0.4222	0.678	0.92695784	Distal Intergenic	ENSSSCG000	HRH4	histamine receptor H4 [Source:NCBI gene (formerly Entrezgene);Acc:397224]
chr5	5014339	5015649	1311	*	chr5	5014339	5015649	in_vitro	in_vivo	12	10	2.005	1.564	0.441	c(t = 0.42178	0.678	0.92695784	Distal Intergenic	ENSSSCG000	PNPLA3	patatin like phospholipase domain containing 3 [Source:VGNC Symbol;Acc:VGNC:103312]
chr10	42012218	42014866	2649	*	chr10	42012218	42014866	in_vitro	in_vivo	13	14	1.53153846	1.28	0.25153846	c(t = 0.42054	0.678	0.92695784	Distal Intergenic	ENSSSCG000	MTPAP	mitochondrial poly(A) polymerase [Source:VGNC Symbol;Acc:VGNC:96676]
chr13	39523645	39524226	582	*	chr13	39523645	39524226	in_vitro	in_vivo	9	11	0.37	0.60090909	-0.2309091	c(t = -0.4220	0.678	0.92695784	Distal Intergenic	ENSSSCG000	ARF4	ADP ribosylation factor 4 [Source:VGNC Symbol;Acc:VGNC:85446]
chr2	75396091	75397399	1309	*	chr2	75396091	75397399	in_vitro	in_vivo	12	13	24.5108333	30.7038462	-6.1930128	c(t = -0.4199	0.679	0.9275	Distal Intergenic	ENSSSCG000	GNA11	G protein subunit alpha 11 [Source:NCBI gene (formerly Entrezgene);Acc:733588]
chrX	125250525	125251995	1471	*	chrX	125250525	125251995	in_vitro	in_vivo	12	10	8.35833333	12.17	-3.8116667	c(t = -0.4194	0.679	0.9275	Distal Intergenic	ENSSSCG000	DKC1	dyskerin pseudouridine synthase 1 [Source:NCBI gene (formerly Entrezgene);Acc:100216430]
chr7	55575340	55576442	1103	*	chr7	55575340	55576442	in_vitro	in_vivo	10	13	4.078	3.19615385	0.88184615	c(t = 0.42063	0.679	0.9275	Distal Intergenic	ENSSSCG000	ARPIN	actin related protein 2/3 complex inhibitor [Source:VGNC Symbol;Acc:VGNC:111466]
chr13	201008728	201010948	2221	*	chr13	201008728	201010948	in_vitro	in_vivo	10	15	2.327	1.86266667	0.46433333	c(t = 0.42332	0.679	0.9275	Distal Intergenic	ENSSSCG000	PIGP	phosphatidylinositol glycan anchor biosynthesis class P [Source:VGNC Symbol;Acc:VGNC:91427]
chr14	102548166	102549142	977	*	chr14	102548166	102549142	in_vitro	in_vivo	13	15	1.11153846	1.42	-0.3084615	c(t = -0.4180	0.679	0.9275	Promoter (<=1kb)	ENSSSCG000	HTR7	5-hydroxytryptamine receptor 7 [Source:VGNC Symbol;Acc:VGNC:89003]
chr1	119597054	119597567	514	*	chr1	119597054	119597567	in_vitro	in_vivo	9	11	5.39	7.38909091	-1.9990909	c(t = -0.4198	0.68	0.9277117	Distal Intergenic	ENSSSCG000	TMOD3	tropomodulin 3 [Source:NCBI gene (formerly Entrezgene);Acc:397556]
chr2	16657423	16658649	1227	*	chr2	16657423	16658649	in_vitro	in_vivo	10	9	6.619	4.96222222	1.65677778	c(t = 0.42020	0.68	0.9277117	Promoter (<=1kb)	ENSSSCG000	SLC35C1	solute carrier family 35 member C1 [Source:VGNC Symbol;Acc:VGNC:93075]
chr12	59199777	59200868	1092	*	chr12	59199777	59200868	in_vitro	in_vivo	13	16	1.86692308	2.474375	-0.6074519	c(t = -0.4169	0.68	0.9277117	Distal Intergenic	ENSSSCG000	UBB	ubiquitin B [Source:VGNC Symbol;Acc:VGNC:98389]
chr18	10213625	10214692	1068	*	chr18	10213625	10214692	in_vitro	in_vivo	13	14	2.74	2.20071429	0.53928571	c(t = 0.41787	0.68	0.9277117	Distal Intergenic	ENSSSCG000	ZC3HAV1	zinc finger CCCH-type containing, antiviral 1 [Source:VGNC Symbol;Acc:VGNC:95102]
chr12	56384478	56385265	788	*	chr12	56384478	56385265	in_vitro	in_vivo	10	13	0.992	1.27846154	-0.2864615	c(t = -0.4193	0.68	0.9277117	Distal Intergenic	ENSSSCG000	ssc-mir-744	ssc-mir-744 [Source:miRBase;Acc:MI0014769]
chr3	128903431	128904335	905	*	chr3	128903431	128904335	in_vitro	in_vivo	11	14	0.89909091	0.67785714	0.22123377	c(t = 0.41864	0.68	0.9277117	Distal Intergenic	ENSSSCG000	RSAD2	radical S-adenosyl methionine domain containing 2 [Source:NCBI gene (formerly Entrezgene);Acc:396752]
chr1	266345848	266346467	620	*	chr1	266345848	266346467	in_vitro	in_vivo	10	9	0.943	0.60111111	0.34188889	c(t = 0.42006	0.68	0.9277117	Distal Intergenic	ENSSSCG000	RABEPK	Rab9 effector protein with kelch motifs [Source:VGNC Symbol;Acc:VGNC:92045]
chr6	1030633	1038162	7530	*	chr6	1030633	1038162	in_vitro	in_vivo	12	11	87.1008333	90.8754545	-3.7746212	c(t = -0.4193	0.681	0.92796919	Distal Intergenic	ENSSSCG000	CYBA	cytochrome b-245 alpha chain [Source:VGNC Symbol;Acc:VGNC:87124]
chr8	82781892	82782422	531	*	chr8	82781892	82782422	in_vitro	in_vivo	9	10	24.8966667	32.197	-7.3003333	c(t = -0.4184	0.681	0.92796919	Distal Intergenic	ENSSSCG000	SMAD1	SMAD family member 1 [Source:VGNC Symbol;Acc:VGNC:93216]
chr9	103262276	103262984	709	*	chr9	103262276	103262984	in_vitro	in_vivo	12	11	9.27	11.1072727	-1.8372727	c(t = -0.4167	0.681	0.92796919	Distal Intergenic	ENSSSCG000	ARMC10	armadillo repeat containing 10 [Source:NCBI gene (formerly Entrezgene);Acc:100513998]
chr16	961613	962892	1280	*	chr16	961613	962892	in_vitro	in_vivo	13	16	7.33923077	5.3725	1.96673077	c(t = 0.41773	0.681	0.92796919	Distal Intergenic	ENSSSCG000	C1QTNF3	C1q and TNF related 3 [Source:NCBI gene (formerly Entrezgene);Acc:100270814]
chr18	10450424	10451845	1422	*	chr18	10450424	10451845	in_vitro	in_vivo	12	14	1.98	1.59642857	0.38357143	c(t = 0.41636	0.681	0.92796919	Distal Intergenic	ENSSSCG000	ZC3HAV1	zinc finger CCCH-type containing, antiviral 1 [Source:VGNC Symbol;Acc:VGNC:95102]
chr9	139351098	139352554	1457	*	chr9	139351098	139352554	in_vitro	in_vivo	12	9	27.44	20.9944444	6.44555556	c(t = 0.41626	0.682	0.92796919	Intron (NM_214007/397070, intron 1 of 27)	ENSSSCG000	EGFR	epidermal growth factor receptor [Source:NCBI gene (formerly Entrezgene);Acc:397070]
chr18	45327532	45328586	1055	*	chr18	45327532	45328586	in_vitro	in_vivo	9	11	4.32333333	5.42	-1.0966667	c(t = -0.4170	0.682	0.92796919	Distal Intergenic	ENSSSCG000	HOXA13	homeobox A13 [Source:NCBI gene (formerly Entrezgene);Acc:100359352]
chr14	112808281	112808663	383	*	chr14	112808281	112808663	in_vitro	in_vivo	9	9	6.47444444	5.30222222	1.17222222	c(t = 0.41878	0.682	0.92796919	Distal Intergenic	ENSSSCG000	HPS6	HPS6 biogenesis of lysosomal organelles complex 2 subunit 3 [Source:NCBI gene (formerly Entrezgene);Acc:100049675]
chr3	68404159	68404653	495	*	chr3	68404159	68404653	in_vitro	in_vivo	10	9	2.021	3.07666667	-1.0556667	c(t = -0.4200	0.682	0.92796919	Distal Intergenic	ENSSSCG000	DOK1	docking protein 1 [Source:NCBI gene (formerly Entrezgene);Acc:100233179]
chr9	46309009	46310412	1404	*	chr9	46309009	46310412	in_vitro	in_vivo	11	15	2.45909091	2.99733333	-0.5382424	c(t = -0.4142	0.682	0.92796919	Distal Intergenic	ENSSSCG000	HMBS	hydroxymethylbilane synthase [Source:VGNC Symbol;Acc:VGNC:88899]
chr14	108032768	108033881	1114	*	chr14	108032768	108033881	in_vitro	in_vivo	13	14	2.48538462	1.89857143	0.58681319	c(t = 0.41472	0.682	0.92796919	Distal Intergenic	ENSSSCG000	PIK3AP1	phosphoinositide-3-kinase adaptor protein 1 [Source:VGNC Symbol;Acc:VGNC:91435]
chr8	70179688	70180111	424	*	chr8	70179688	70180111	in_vitro	in_vivo	9	13	2.12888889	1.53461538	0.5942735	c(t = 0.41582	0.682	0.92796919	Distal Intergenic	ENSSSCG000	CXCL2	chemokine (C-X-C motif) ligand 2 [Source:NCBI gene (formerly Entrezgene);Acc:414904]
chr6	148912466	148913300	835	*	chr6	148912466	148913300	in_vitro	in_vivo	13	9	0.91153846	1.32555556	-0.4140171	c(t = -0.4201	0.682	0.92796919	Promoter (<=1kb)	ENSSSCG000	EFCAB7	phosphoglucomutase 1 [Source:VGNC Symbol;Acc:VGNC:91355]
chr11	1265112	1266447	1336	*	chr11	1265112	1266447	in_vitro	in_vivo	11	12	1.52363636	1.26916667	0.2544697	c(t = 0.41589	0.682	0.92796919	Distal Intergenic	ENSSSCG000	LATS2	large tumor suppressor kinase 2 [Source:VGNC Symbol;Acc:VGNC:89649]
chr13	203044431	203045313	883	*	chr13	203044431	203045313	in_vitro	in_vivo	10	14	0.682	0.91285714	-0.2308571	c(t = -0.4149	0.682	0.92796919	Promoter (1-2kb)	ENSSSCG000	SH3BGR	SH3 domain binding glutamate rich protein [Source:NCBI gene (formerly Entrezgene);Acc:100626262]
chr18	50852404	50853029	626	*	chr18	50852404	50853029	in_vitro	in_vivo	10	9	6.636	9.17111111	-2.5351111	c(t = -0.4185	0.683	0.92883649	Distal Intergenic	ENSSSCG000	YKT6	YKT6 v-SNARE homolog [Source:NCBI gene (formerly Entrezgene);Acc:100513964]
chr3	16858263	16859102	840	*	chr3	16858263	16859102	in_vitro	in_vivo	9	10	1.80888889	1.344	0.46488889	c(t = 0.41848	0.683	0.92883649	Distal Intergenic	ENSSSCG000	PSPH	phosphoserine phosphatase [Source:VGNC Symbol;Acc:VGNC:91931]
chr15	80645629	80646644	1016	*	chr15	80645629	80646644	in_vitro	in_vivo	11	11	0.41363636	0.64090909	-0.2272727	c(t = -0.4151	0.683	0.92883649	Distal Intergenic	ENSSSCG000	CIR1	corepressor interacting with RBPJ, CIR1 [Source:NCBI gene (formerly Entrezgene);Acc:100511415]
chr14	42995173	42996768	1596	*	chr14	42995173	42996768	in_vitro	in_vivo	12	9	12.0666667	8.84333333	3.22333333	c(t = 0.41676	0.684	0.92920983	Distal Intergenic	ENSSSCG000	CMKLR1	chemerin chemokine-like receptor 1 [Source:VGNC Symbol;Acc:VGNC:86800]
chr15	2499015	2499758	744	*	chr15	2499015	2499758	in_vitro	in_vivo	10	13	4.286	3.11923077	1.16676923	c(t = 0.41315	0.684	0.92920983	Distal Intergenic	ENSSSCG000	RND3	Rho family GTPase 3 [Source:NCBI gene (formerly Entrezgene);Acc:397559]
chr13	196643750	196646226	2477	*	chr13	196643750	196646226	in_vitro	in_vivo	13	14	2.08384615	1.74714286	0.3367033	c(t = 0.41369	0.684	0.92920983	Distal Intergenic	ENSSSCG000	IFNAR2	interferon alpha and beta receptor subunit 2 [Source:NCBI gene (formerly Entrezgene);Acc:100533555]
chr14	50935032	50935881	850	*	chr14	50935032	50935881	in_vitro	in_vivo	9	9	2.11444444	1.63888889	0.47555556	c(t = 0.41506	0.684	0.92920983	Distal Intergenic	ENSSSCG000	TSSK1B	testis specific serine kinase 1B [Source:NCBI gene (formerly Entrezgene);Acc:102159240]
chr1	271336415	271337612	1198	*	chr1	271336415	271337612	in_vitro	in_vivo	9	10	1.06666667	1.505	-0.4383333	c(t = -0.4144	0.684	0.92920983	Distal Intergenic	ENSSSCG000	MED27	mediator complex subunit 27 [Source:VGNC Symbol;Acc:VGNC:90117]
chr7	37555381	37556757	1377	*	chr7	37555381	37556757	in_vitro	in_vivo	10	11	1.065	0.73363636	0.33136364	c(t = 0.41351	0.684	0.92920983	Distal Intergenic	ENSSSCG000	TBCC	tubulin folding cofactor C [Source:NCBI gene (formerly Entrezgene);Acc:100157124]
chr5	9899893	9900516	624	*	chr5	9899893	9900516	in_vitro	in_vivo	10	9	19.173	26.0522222	-6.8792222	c(t = -0.4127	0.685	0.92926729	Exon (NM_001099933/414903, exon 4 of 4)	ENSSSCG000	SOX10	SRY-box transcription factor 10 [Source:VGNC Symbol;Acc:VGNC:93350]
chr12	6773720	6774921	1202	*	chr12	6773720	6774921	in_vitro	in_vivo	9	12	19.3733333	25.535	-6.1616667	c(t = -0.4120	0.685	0.92926729	Distal Intergenic	ENSSSCG000	CD300C	CD300c molecule [Source:NCBI gene (formerly Entrezgene);Acc:100514248]
chr12	44655318	44656366	1049	*	chr12	44655318	44656366	in_vitro	in_vivo	10	15	3.157	2.494	0.663	c(t = 0.41180	0.685	0.92926729	Promoter (1-2kb)	ENSSSCG000	VTN	vitronectin [Source:NCBI gene (formerly Entrezgene);Acc:397192]
chr15	17261964	17262883	920	*	chr15	17261964	17262883	in_vitro	in_vivo	12	15	0.89916667	1.11266667	-0.2135	c(t = -0.4108	0.685	0.92926729	Distal Intergenic	NA	NA	NA
chr13	45400673	45402076	1404	*	chr13	45400673	45402076	in_vitro	in_vivo	12	13	0.74916667	1.08846154	-0.3392949	c(t = -0.4124	0.685	0.92926729	Distal Intergenic	ENSSSCG000	EOGT	EGF domain specific O-linked N-acetylglucosamine transferase [Source:VGNC Symbol;Acc:VGNC:87718]
chr2	87777546	87778470	925	*	chr2	87777546	87778470	in_vitro	in_vivo	12	12	0.68583333	0.86083333	-0.175	c(t = -0.4107	0.685	0.92926729	Distal Intergenic	ENSSSCG000	BHMT2	betaine--homocysteine S-methyltransferase 2 [Source:NCBI gene (formerly Entrezgene);Acc:100523776]
chr2	143132379	143133744	1366	*	chr2	143132379	143133744	in_vitro	in_vivo	10	12	92.889	94.7591667	-1.8701667	c(t = -0.4148	0.686	0.92926729	Distal Intergenic	ENSSSCG000	DIAPH1	diaphanous related formin 1 [Source:VGNC Symbol;Acc:VGNC:99632]
chr2	150535415	150539023	3609	*	chr2	150535415	150539023	in_vitro	in_vivo	9	13	57.6277778	51.3838462	6.24393162	c(t = 0.41125	0.686	0.92926729	Distal Intergenic	ENSSSCG00000032383		
chr2	74018864	74020003	1140	*	chr2	74018864	74020003	in_vitro	in_vivo	11	9	24.9254545	20.3377778	4.58767677	c(t = 0.41176	0.686	0.92926729	Exon (NM_001315738/100623776, exon 2 of 2)	ENSSSCG000	TICAM1	TIR domain containing adaptor molecule 1 [Source:VGNC Symbol;Acc:VGNC:93976]
chr12	57790461	57791645	1185	*	chr12	57790461	57791645	in_vitro	in_vivo	9	10	14.3533333	19.89	-5.5366667	c(t = -0.4112	0.686	0.92926729	Distal Intergenic	ENSSSCG000	COX10	cytochrome c oxidase assembly factor heme A:farnesyltransferase COX10 [Source:NCBI gene (formerly Entrezgene);Acc:100521863]
chr6	56466613	56467377	765	*	chr6	56466613	56467377	in_vitro	in_vivo	12	12	13.2025	17.3541667	-4.1516667	c(t = -0.4104	0.686	0.92926729	Distal Intergenic	ENSSSCG000	ssc-mir-371	ssc-mir-371 [Source:miRBase;Acc:MI0031640]
chr2	141914270	141915266	997	*	chr2	141914270	141915266	in_vitro	in_vivo	10	13	1.977	2.51307692	-0.5360769	c(t = -0.4103	0.686	0.92926729	Distal Intergenic	ENSSSCG000	HBEGF	heparin binding EGF like growth factor [Source:VGNC Symbol;Acc:VGNC:88792]
chr6	138099053	138101520	2468	*	chr6	138099053	138101520	in_vitro	in_vivo	10	13	0.971	1.31769231	-0.3466923	c(t = -0.4108	0.686	0.92926729	Promoter (<=1kb)	ENSSSCG000	LHX8	LIM homeobox 8 [Source:VGNC Symbol;Acc:VGNC:89717]
chr13	202942500	202944237	1738	*	chr13	202942500	202944237	in_vitro	in_vivo	10	12	0.723	1.0075	-0.2845	c(t = -0.4114	0.686	0.92926729	Distal Intergenic	ENSSSCG000	GET1	guided entry of tail-anchored proteins factor 1 [Source:VGNC Symbol;Acc:VGNC:103949]
chr6	63799352	63810586	11235	*	chr6	63799352	63810586	in_vitro	in_vivo	13	14	39.37	43.0978571	-3.7278571	c(t = -0.4072	0.687	0.92926729	Distal Intergenic	ENSSSCG000	TAS1R3	taste 1 receptor member 3 [Source:NCBI gene (formerly Entrezgene);Acc:100127440]
chr2	10894251	10895544	1294	*	chr2	10894251	10895544	in_vitro	in_vivo	10	14	16.721	21.665	-4.944	c(t = -0.4077	0.687	0.92926729	Distal Intergenic	ENSSSCG000	PRPF19	pre-mRNA processing factor 19 [Source:NCBI gene (formerly Entrezgene);Acc:100144769]
chr6	59771690	59774989	3300	*	chr6	59771690	59774989	in_vitro	in_vivo	12	10	11.4	13.585	-2.185	c(t = -0.4086	0.687	0.92926729	Promoter (<=1kb)	ENSSSCG000	U2AF2	U2 small nuclear RNA auxiliary factor 2 [Source:VGNC Symbol;Acc:VGNC:94623]
chr16	63550234	63551139	906	*	chr16	63550234	63551139	in_vitro	in_vivo	10	10	3.05	2.564	0.486	c(t = 0.40992	0.687	0.92926729	Distal Intergenic	ENSSSCG000	FABP6	fatty acid binding protein 6 [Source:NCBI gene (formerly Entrezgene);Acc:397423]
chr1	186035658	186037405	1748	*	chr1	186035658	186037405	in_vitro	in_vivo	13	11	1.42769231	2.01181818	-0.5841259	c(t = -0.4088	0.687	0.92926729	Distal Intergenic	ENSSSCG000	KTN1	kinectin 1 [Source:VGNC Symbol;Acc:VGNC:89608]
chr17	32284301	32285070	770	*	chr17	32284301	32285070	in_vitro	in_vivo	11	11	2.27818182	1.70636364	0.57181818	c(t = 0.41029	0.687	0.92926729	Distal Intergenic	ENSSSCG000	DDRGK1	DDRGK domain containing 1 [Source:NCBI gene (formerly Entrezgene);Acc:100155894]
chr10	9827508	9828386	879	*	chr10	9827508	9828386	in_vitro	in_vivo	10	13	1.576	1.16769231	0.40830769	c(t = 0.41041	0.687	0.92926729	Distal Intergenic	ENSSSCG000	ssc-mir-664	ssc-mir-664 [Source:miRBase;Acc:MI0013123]
chr8	112866640	112868061	1422	*	chr8	112866640	112868061	in_vitro	in_vivo	9	10	1.19444444	0.911	0.28344444	c(t = 0.40977	0.687	0.92926729	Distal Intergenic	ENSSSCG000	EGF	epidermal growth factor [Source:VGNC Symbol;Acc:VGNC:98921]
chr15	47601158	47602201	1044	*	chr15	47601158	47602201	in_vitro	in_vivo	11	13	0.65545455	0.90230769	-0.2468531	c(t = -0.4084	0.687	0.92926729	Promoter (<=1kb)	ENSSSCG000	TM2D2	TM2 domain containing 2 [Source:NCBI gene (formerly Entrezgene);Acc:100154457]
chr1	128489404	128490072	669	*	chr1	128489404	128490072	in_vitro	in_vivo	10	13	0.426	0.23769231	0.18830769	c(t = 0.41255	0.687	0.92926729	Distal Intergenic	ENSSSCG000	ssc-mir-9838	ssc-mir-9838 [Source:miRBase;Acc:MI0031607]
chr6	4242138	4243643	1506	*	chr6	4242138	4243643	in_vitro	in_vivo	9	10	87.8222222	89.13	-1.3077778	c(t = -0.4093	0.688	0.92926729	Distal Intergenic	ENSSSCG000	WFDC1	WAP four-disulfide core domain 1 [Source:VGNC Symbol;Acc:VGNC:94953]
chr2	76396460	76398204	1745	*	chr2	76396460	76398204	in_vitro	in_vivo	9	10	51.8722222	46.199	5.67322222	c(t = 0.40867	0.688	0.92926729	Distal Intergenic	ENSSSCG000	AMH	anti-Mullerian hormone [Source:VGNC Symbol;Acc:VGNC:110572]
chr3	95511776	95512982	1207	*	chr3	95511776	95512982	in_vitro	in_vivo	9	11	10.8722222	14.6081818	-3.7359596	c(t = -0.4107	0.688	0.92926729	Distal Intergenic	ENSSSCG000	SLC3A1	solute carrier family 3 member 1 [Source:NCBI gene (formerly Entrezgene);Acc:100144588]
chr1	126184560	126184926	367	*	chr1	126184560	126184926	in_vitro	in_vivo	10	10	2.174	3.081	-0.907	c(t = -0.4089	0.688	0.92926729	Promoter (<=1kb)	ENSSSCG000	BLOC1S6	biogenesis of lysosomal organelles complex 1 subunit 6 [Source:NCBI gene (formerly Entrezgene);Acc:100049678]
chr2	47582463	47583055	593	*	chr2	47582463	47583055	in_vitro	in_vivo	10	10	1.973	2.908	-0.935	c(t = -0.4077	0.688	0.92926729	Distal Intergenic	ENSSSCG000	DKK3	dickkopf WNT signaling pathway inhibitor 3 [Source:VGNC Symbol;Acc:VGNC:87323]
chr14	129577544	129578376	833	*	chr14	129577544	129578376	in_vitro	in_vivo	11	14	1.30636364	1.685	-0.3786364	c(t = -0.4065	0.688	0.92926729	Distal Intergenic	ENSSSCG000	MCMBP	minichromosome maintenance complex binding protein [Source:VGNC Symbol;Acc:VGNC:90077]
chr8	114145195	114145875	681	*	chr8	114145195	114145875	in_vitro	in_vivo	10	13	1.261	0.98538462	0.27561538	c(t = 0.40725	0.688	0.92926729	Distal Intergenic	ENSSSCG000	ssc-mir-9843	ssc-mir-9843 [Source:miRBase;Acc:MI0031612]
chr12	52418904	52419527	624	*	chr12	52418904	52419527	in_vitro	in_vivo	9	10	0.69111111	0.486	0.20511111	c(t = 0.40851	0.688	0.92926729	Distal Intergenic	ENSSSCG000	ssc-mir-195	ssc-mir-195 [Source:miRBase;Acc:MI0013141]
chrX	124981733	124997206	15474	*	chrX	124981733	124997206	in_vitro	in_vivo	14	13	73.1635714	76.4330769	-3.2695055	c(t = -0.4060	0.689	0.92926729	Distal Intergenic	ENSSSCG000	DNASE1L1	deoxyribonuclease 1 like 1 [Source:NCBI gene (formerly Entrezgene);Acc:654408]
chr2	151451389	151452894	1506	*	chr2	151451389	151452894	in_vitro	in_vivo	9	10	24.9355556	27.932	-2.9964444	c(t = -0.4067	0.689	0.92926729	Distal Intergenic	ENSSSCG000	CD74	CD74 molecule [Source:VGNC Symbol;Acc:VGNC:86428]
chr1	121578365	121579325	961	*	chr1	121578365	121579325	in_vitro	in_vivo	14	12	3.84571429	4.5425	-0.6967857	c(t = -0.4051	0.689	0.92926729	Promoter (<=1kb)	ENSSSCG000	SLC27A2	solute carrier family 27 member 2 [Source:NCBI gene (formerly Entrezgene);Acc:494011]
chr12	18224965	18225842	878	*	chr12	18224965	18225842	in_vitro	in_vivo	9	11	2.25666667	3.03454545	-0.7778788	c(t = -0.4072	0.689	0.92926729	Distal Intergenic	ENSSSCG000	C1QL1	complement C1q like 1 [Source:NCBI gene (formerly Entrezgene);Acc:780424]
chr2	39032310	39033285	976	*	chr2	39032310	39033285	in_vitro	in_vivo	13	13	1.92923077	2.77538462	-0.8461538	c(t = -0.4075	0.689	0.92926729	Distal Intergenic	ENSSSCG000	CSRP3	cysteine and glycine rich protein 3 [Source:NCBI gene (formerly Entrezgene);Acc:100337687]
chr6	100332510	100333670	1161	*	chr6	100332510	100333670	in_vitro	in_vivo	9	13	1.25555556	1.76153846	-0.5059829	c(t = -0.4062	0.689	0.92926729	Distal Intergenic	ENSSSCG000	NDUFV2	NADH:ubiquinone oxidoreductase core subunit V2 [Source:NCBI gene (formerly Entrezgene);Acc:100037991]
chr7	121130954	121133810	2857	*	chr7	121130954	121133810	in_vitro	in_vivo	14	13	1.28642857	1.04923077	0.2371978	c(t = 0.40598	0.689	0.92926729	Distal Intergenic	ENSSSCG000	ssc-mir-345-	ssc-mir-345-1 [Source:miRBase;Acc:MI0013117]
chr18	42651011	42652869	1859	*	chr18	42651011	42652869	in_vitro	in_vivo	15	15	1.25266667	1.04133333	0.21133333	c(t = 0.40478	0.689	0.92926729	Distal Intergenic	ENSSSCG000	MTURN	maturin, neural progenitor differentiation regulator homolog [Source:VGNC Symbol;Acc:VGNC:90476]
chr15	41817389	41818328	940	*	chr15	41817389	41818328	in_vitro	in_vivo	9	16	1.17555556	0.898125	0.27743056	c(t = 0.40578	0.689	0.92926729	Distal Intergenic	ENSSSCG000	CLDN22	claudin 22 [Source:NCBI gene (formerly Entrezgene);Acc:100294683]
chr16	79642034	79643507	1474	*	chr16	79642034	79643507	in_vitro	in_vivo	9	11	15.8877778	11.8836364	4.00414141	c(t = 0.40926	0.69	0.92947415	Distal Intergenic	ENSSSCG000	TPPP	tubulin polymerization promoting protein [Source:VGNC Symbol;Acc:VGNC:94344]
chr9	109556570	109558449	1880	*	chr9	109556570	109558449	in_vitro	in_vivo	13	11	7.05692308	8.71727273	-1.6603497	c(t = -0.4043	0.69	0.92947415	Distal Intergenic	ENSSSCG000	EZH2	enhancer of zeste 2 polycomb repressive complex 2 subunit [Source:VGNC Symbol;Acc:VGNC:87855]
chrX	122394756	122395632	877	*	chrX	122394756	122395632	in_vitro	in_vivo	9	10	6.62333333	8.505	-1.8816667	c(t = -0.4053	0.69	0.92947415	Distal Intergenic	ENSSSCG000	FATE1	fetal and adult testis expressed 1 [Source:NCBI gene (formerly Entrezgene);Acc:396705]
chr1	96728975	96729642	668	*	chr1	96728975	96729642	in_vitro	in_vivo	10	10	1.393	1.919	-0.526	c(t = -0.4058	0.69	0.92947415	Distal Intergenic	ENSSSCG000	SMAD2	SMAD family member 2 [Source:VGNC Symbol;Acc:VGNC:98329]
chr12	42808481	42808974	494	*	chr12	42808481	42808974	in_vitro	in_vivo	9	10	2.50777778	1.546	0.96177778	c(t = 0.40995	0.69	0.92947415	Distal Intergenic	ENSSSCG000	ADAP2	ArfGAP with dual PH domains 2 [Source:VGNC Symbol;Acc:VGNC:85096]
chr5	52737217	52738757	1541	*	chr5	52737217	52738757	in_vitro	in_vivo	13	16	1.10230769	1.354375	-0.2520673	c(t = -0.4035	0.69	0.92947415	Promoter (<=1kb)	ENSSSCG000	PDE3A	phosphodiesterase 3A [Source:NCBI gene (formerly Entrezgene);Acc:396555]
chr11	20995835	20997222	1388	*	chr11	20995835	20997222	in_vitro	in_vivo	10	11	1.43	1.03363636	0.39636364	c(t = 0.40509	0.69	0.92947415	Distal Intergenic	ENSSSCG000	ESD	esterase D [Source:VGNC Symbol;Acc:VGNC:87788]
chr11	77488873	77493328	4456	*	chr11	77488873	77493328	in_vitro	in_vivo	11	12	95.5790909	96.5508333	-0.9717424	c(t = -0.4046	0.691	0.93016903	Distal Intergenic	ENSSSCG000	ANKRD10	ankyrin repeat domain 10 [Source:VGNC Symbol;Acc:VGNC:85322]
chr6	49346321	49349354	3034	*	chr6	49346321	49349354	in_vitro	in_vivo	11	11	6.79818182	5.58454545	1.21363636	c(t = 0.40342	0.691	0.93016903	Promoter (<=1kb)	ENSSSCG000	TGFB1	transforming growth factor beta 1 [Source:VGNC Symbol;Acc:VGNC:98656]
chr9	40988815	40989306	492	*	chr9	40988815	40989306	in_vitro	in_vivo	9	10	6.13333333	4.456	1.67733333	c(t = 0.40541	0.691	0.93016903	Distal Intergenic	ENSSSCG000	DRD2	dopamine receptor D2 [Source:VGNC Symbol;Acc:VGNC:87444]
chr15	121086748	121090759	4012	*	chr15	121086748	121090759	in_vitro	in_vivo	13	15	2.32692308	2.72866667	-0.4017436	c(t = -0.4021	0.691	0.93016903	Promoter (<=1kb)	ENSSSCG000	IHH	Indian hedgehog signaling molecule [Source:VGNC Symbol;Acc:VGNC:96370]
chr10	63751642	63752203	562	*	chr10	63751642	63752203	in_vitro	in_vivo	9	12	23.6911111	17.4158333	6.27527778	c(t = 0.40269	0.692	0.93037439	Distal Intergenic	ENSSSCG000	ITIH2	inter-alpha-trypsin inhibitor heavy chain 2 [Source:NCBI gene (formerly Entrezgene);Acc:396937]
chr9	36375952	36376602	651	*	chr9	36375952	36376602	in_vitro	in_vivo	9	11	2.72777778	3.80727273	-1.0794949	c(t = -0.4028	0.692	0.93037439	Distal Intergenic	ENSSSCG000	SLN	sarcolipin [Source:NCBI gene (formerly Entrezgene);Acc:733627]
chr6	93126629	93128810	2182	*	chr6	93126629	93128810	in_vitro	in_vivo	14	10	1.875	2.778	-0.903	c(t = -0.4081	0.692	0.93037439	Distal Intergenic	ENSSSCG000	ssc-mir-9842	ssc-mir-9842 [Source:miRBase;Acc:MI0031611]
chr2	3607028	3608271	1244	*	chr2	3607028	3608271	in_vitro	in_vivo	11	12	1.96272727	2.62333333	-0.6606061	c(t = -0.4018	0.692	0.93037439	Distal Intergenic	ENSSSCG000	FADD	Fas associated via death domain [Source:NCBI gene (formerly Entrezgene);Acc:595129]
chr9	104274951	104276430	1480	*	chr9	104274951	104276430	in_vitro	in_vivo	13	15	2.69769231	2.242	0.45569231	c(t = 0.40102	0.692	0.93037439	Distal Intergenic	ENSSSCG000	SLC26A5	solute carrier family 26 member 5 [Source:NCBI gene (formerly Entrezgene);Acc:100190993]
chr18	720726	722000	1275	*	chr18	720726	722000	in_vitro	in_vivo	9	10	2.13222222	1.739	0.39322222	c(t = 0.40290	0.692	0.93037439	Distal Intergenic	ENSSSCG000	VIPR2	vasoactive intestinal peptide receptor 2 [Source:NCBI gene (formerly Entrezgene);Acc:100499553]
chr13	28405061	28406674	1614	*	chr13	28405061	28406674	in_vitro	in_vivo	13	13	1.00769231	1.20153846	-0.1938462	c(t = -0.4006	0.692	0.93037439	Distal Intergenic	ENSSSCG000	EXOSC7	exosome component 7 [Source:NCBI gene (formerly Entrezgene);Acc:100511347]
chr10	68157426	68158941	1516	*	chr10	68157426	68158941	in_vitro	in_vivo	10	12	94.138	92.6641667	1.47383333	c(t = 0.40076	0.693	0.93123011	Distal Intergenic	ENSSSCG000	WDR37	WD repeat domain 37 [Source:VGNC Symbol;Acc:VGNC:96258]
chr5	89673144	89674315	1172	*	chr5	89673144	89674315	in_vitro	in_vivo	10	11	4.037	4.92545455	-0.8884545	c(t = -0.4009	0.693	0.93123011	Distal Intergenic	ENSSSCG000	SOCS2	suppressor of cytokine signaling 2 [Source:NCBI gene (formerly Entrezgene);Acc:100037966]
chr11	65203486	65204422	937	*	chr11	65203486	65204422	in_vitro	in_vivo	13	13	2.08769231	1.47923077	0.60846154	c(t = 0.40106	0.693	0.93123011	Distal Intergenic	ENSSSCG000	DNAJC3	DnaJ heat shock protein family (Hsp40) member C3 [Source:NCBI gene (formerly Entrezgene);Acc:100154166]
chr16	79689928	79693238	3311	*	chr16	79689928	79693238	in_vitro	in_vivo	9	12	15.7933333	11.4841667	4.30916667	c(t = 0.40112	0.694	0.93131212	Distal Intergenic	ENSSSCG000	TPPP	tubulin polymerization promoting protein [Source:VGNC Symbol;Acc:VGNC:94344]
chr3	117588293	117594061	5769	*	chr3	117588293	117594061	in_vitro	in_vivo	13	12	2.57692308	2.27916667	0.29775641	c(t = 0.39845	0.694	0.93131212	Distal Intergenic	ENSSSCG000	RHOB	ras homolog family member B [Source:VGNC Symbol;Acc:VGNC:110709]
chr6	94025737	94029112	3376	*	chr6	94025737	94029112	in_vitro	in_vivo	17	14	2.53294118	2.21071429	0.32222689	c(t = 0.39720	0.694	0.93131212	Distal Intergenic	ENSSSCG000	UTP11	UTP11 small subunit processome component [Source:NCBI gene (formerly Entrezgene);Acc:100513019]
chr6	157219736	157220976	1241	*	chr6	157219736	157220976	in_vitro	in_vivo	11	11	2.78363636	2.19181818	0.59181818	c(t = 0.39874	0.694	0.93131212	Distal Intergenic	ENSSSCG000	TMEM61	transmembrane protein 61 [Source:NCBI gene (formerly Entrezgene);Acc:100628096]
chr3	470105	470700	596	*	chr3	470105	470700	in_vitro	in_vivo	10	12	2.324	1.70333333	0.62066667	c(t = 0.39940	0.694	0.93131212	Distal Intergenic	ENSSSCG000	ADAP1	ArfGAP with dual PH domains 1 [Source:NCBI gene (formerly Entrezgene);Acc:397453]
chr4	106758746	106759655	910	*	chr4	106758746	106759655	in_vitro	in_vivo	13	11	1.61461538	1.06454545	0.54916084	c(t = 0.39932	0.694	0.93131212	Distal Intergenic	ENSSSCG000	BCAS2	BCAS2 pre-mRNA processing factor [Source:NCBI gene (formerly Entrezgene);Acc:100154006]
chr8	2131757	2136572	4816	*	chr8	2131757	2136572	in_vitro	in_vivo	10	11	91.957	94.4127273	-2.4557273	c(t = -0.4031	0.695	0.93131212	Exon (NM_001113436/399654, exon 5 of 8)	ENSSSCG000	LRPAP1	LDL receptor related protein associated protein 1 [Source:NCBI gene (formerly Entrezgene);Acc:399654]
chr16	79472981	79484370	11390	*	chr16	79472981	79484370	in_vitro	in_vivo	15	17	48.482	52.5464706	-4.0644706	c(t = -0.3964	0.695	0.93131212	Promoter (<=1kb)	ENSSSCG000	TRIP13	thyroid hormone receptor interactor 13 [Source:NCBI gene (formerly Entrezgene);Acc:100337674]
chr5	22895808	22897067	1260	*	chr5	22895808	22897067	in_vitro	in_vivo	11	11	7.67909091	9.09454545	-1.4154545	c(t = -0.3982	0.695	0.93131212	Distal Intergenic	ENSSSCG000	DDIT3	DNA damage inducible transcript 3 [Source:NCBI gene (formerly Entrezgene);Acc:100240743]
chr7	40459190	40459662	473	*	chr7	40459190	40459662	in_vitro	in_vivo	9	10	4.39666667	3.225	1.17166667	c(t = 0.40209	0.695	0.93131212	Distal Intergenic	ENSSSCG000	CLIC5	chloride intracellular channel 5 [Source:NCBI gene (formerly Entrezgene);Acc:100154589]
chr2	117235160	117236801	1642	*	chr2	117235160	117236801	in_vitro	in_vivo	16	13	3.511875	2.69384615	0.81802885	c(t = 0.39613	0.695	0.93131212	Distal Intergenic	ENSSSCG000	APC	APC regulator of WNT signaling pathway [Source:NCBI gene (formerly Entrezgene);Acc:100517932]
chr3	10132373	10132908	536	*	chr3	10132373	10132908	in_vitro	in_vivo	10	13	2.18	1.65615385	0.52384615	c(t = 0.39751	0.695	0.93131212	Promoter (<=1kb)	ENSSSCG000	MDH2	malate dehydrogenase 2 [Source:NCBI gene (formerly Entrezgene);Acc:397039]
chr4	98561866	98562914	1049	*	chr4	98561866	98562914	in_vitro	in_vivo	10	11	1.856	1.42090909	0.43509091	c(t = 0.39838	0.695	0.93131212	Promoter (<=1kb)	ENSSSCG000	MCL1	MCL1 apoptosis regulator, BCL2 family member [Source:NCBI gene (formerly Entrezgene);Acc:397648]
chr13	26106325	26107113	789	*	chr13	26106325	26107113	in_vitro	in_vivo	11	11	1.22909091	0.82454545	0.40454545	c(t = 0.40053	0.695	0.93131212	Promoter (<=1kb)	ENSSSCG000	SEC22C	SEC22 homolog C, vesicle trafficking protein [Source:VGNC Symbol;Acc:VGNC:92674]
chr6	4503008	4503985	978	*	chr6	4503008	4503985	in_vitro	in_vivo	13	10	1.09	0.81	0.28	c(t = 0.39909	0.695	0.93131212	Distal Intergenic	ENSSSCG000	WFDC1	WAP four-disulfide core domain 1 [Source:VGNC Symbol;Acc:VGNC:94953]
chr2	134533740	134534675	936	*	chr2	134533740	134534675	in_vitro	in_vivo	10	12	1.008	0.75416667	0.25383333	c(t = 0.39849	0.695	0.93131212	Distal Intergenic	ENSSSCG000	PDLIM4	PDZ and LIM domain 4 [Source:VGNC Symbol;Acc:VGNC:91283]
chr12	684037	686086	2050	*	chr12	684037	686086	in_vitro	in_vivo	9	9	68.86	62.0433333	6.81666667	c(t = 0.39716	0.696	0.93183972	Distal Intergenic	ENSSSCG000	WDR45B	WD repeat domain 45B [Source:NCBI gene (formerly Entrezgene);Acc:100462760]
chr3	8012548	8014147	1600	*	chr3	8012548	8014147	in_vitro	in_vivo	12	15	3.535	4.87066667	-1.3356667	c(t = -0.3954	0.696	0.93183972	Distal Intergenic	ENSSSCG000	COPS6	COP9 signalosome subunit 6 [Source:NCBI gene (formerly Entrezgene);Acc:100125954]
chr1	228044376	228045374	999	*	chr1	228044376	228045374	in_vitro	in_vivo	14	13	2.04285714	1.57153846	0.47131868	c(t = 0.39591	0.696	0.93183972	Distal Intergenic	ENSSSCG000	OSTF1	osteoclast stimulating factor 1 [Source:NCBI gene (formerly Entrezgene);Acc:397066]
chr10	10115940	10116655	716	*	chr10	10115940	10116655	in_vitro	in_vivo	9	9	1.00333333	1.41888889	-0.4155556	c(t = -0.3984	0.696	0.93183972	Distal Intergenic	ENSSSCG000	ssc-mir-664	ssc-mir-664 [Source:miRBase;Acc:MI0013123]
chr7	98090793	98091580	788	*	chr7	98090793	98091580	in_vitro	in_vivo	9	10	1.72222222	1.283	0.43922222	c(t = 0.39732	0.696	0.93183972	Promoter (<=1kb)	ENSSSCG000	DLST	dihydrolipoamide S-succinyltransferase [Source:NCBI gene (formerly Entrezgene);Acc:397690]
chr3	127508030	127509045	1016	*	chr3	127508030	127509045	in_vitro	in_vivo	9	12	16.1255556	12.8033333	3.32222222	c(t = 0.39906	0.697	0.93220414	Distal Intergenic	ENSSSCG000	ID2	inhibitor of DNA binding 2 [Source:NCBI gene (formerly Entrezgene);Acc:654298]
chr7	91084423	91085802	1380	*	chr7	91084423	91085802	in_vitro	in_vivo	12	12	1.7875	2.27333333	-0.4858333	c(t = -0.3947	0.697	0.93220414	Distal Intergenic	ENSSSCG000	VTI1B	vesicle transport through interaction with t-SNAREs 1B [Source:NCBI gene (formerly Entrezgene);Acc:404692]
chr17	48053946	48054332	387	*	chr17	48053946	48054332	in_vitro	in_vivo	9	9	0.85444444	1.41111111	-0.5566667	c(t = -0.3964	0.697	0.93220414	Distal Intergenic	ENSSSCG000	TNNC2	troponin C2, fast skeletal type [Source:NCBI gene (formerly Entrezgene);Acc:414905]
chr6	96362346	96363492	1147	*	chr6	96362346	96363492	in_vitro	in_vivo	13	11	0.82692308	1.22272727	-0.3958042	c(t = -0.3970	0.697	0.93220414	Distal Intergenic	ENSSSCG000	HPCAL4	hippocalcin like 4 [Source:VGNC Symbol;Acc:VGNC:88959]
chr4	51210805	51211815	1011	*	chr4	51210805	51211815	in_vitro	in_vivo	9	12	1.57555556	1.19083333	0.38472222	c(t = 0.39581	0.697	0.93220414	Promoter (<=1kb)	ENSSSCG000	ssc-mir-9858	ssc-mir-9858 [Source:miRBase;Acc:MI0031636]
chr3	94862873	94865218	2346	*	chr3	94862873	94865218	in_vitro	in_vivo	13	12	1.34230769	1.0525	0.28980769	c(t = 0.39435	0.697	0.93220414	Distal Intergenic	ENSSSCG000	EPAS1	endothelial PAS domain protein 1 [Source:NCBI gene (formerly Entrezgene);Acc:100037272]
chr4	1173647	1177902	4256	*	chr4	1173647	1177902	in_vitro	in_vivo	14	11	94.2578571	93.2163636	1.04149351	c(t = 0.39255	0.698	0.93260677	Distal Intergenic	ENSSSCG000	GPIHBP1	glycosylphosphatidylinositol anchored high density lipoprotein binding protein 1 [Source:VGNC Symbol;Acc:VGNC:88588]
chr14	30697949	30699753	1805	*	chr14	30697949	30699753	in_vitro	in_vivo	13	12	1.31615385	1.67916667	-0.3630128	c(t = -0.3930	0.698	0.93260677	Distal Intergenic	ENSSSCG000	HPD	4-hydroxyphenylpyruvate dioxygenase [Source:VGNC Symbol;Acc:VGNC:88960]
chr7	28631781	28632706	926	*	chr7	28631781	28632706	in_vitro	in_vivo	12	13	1.93416667	1.49846154	0.43570513	c(t = 0.39243	0.698	0.93260677	Distal Intergenic	ENSSSCG000	PRIM2	DNA primase subunit 2 [Source:VGNC Symbol;Acc:VGNC:91796]
chr6	70494763	70496137	1375	*	chr6	70494763	70496137	in_vitro	in_vivo	12	13	1.2525	0.97692308	0.27557692	c(t = 0.39408	0.698	0.93260677	Distal Intergenic	ENSSSCG000	RBP7	retinol binding protein 7 [Source:NCBI gene (formerly Entrezgene);Acc:100270723]
chr13	14969365	14970736	1372	*	chr13	14969365	14970736	in_vitro	in_vivo	10	13	1.033	0.71769231	0.31530769	c(t = 0.39772	0.698	0.93260677	Distal Intergenic	ENSSSCG000	TOP2B	DNA topoisomerase II beta [Source:NCBI gene (formerly Entrezgene);Acc:100156319]
chr13	207014963	207022884	7922	*	chr13	207014963	207022884	in_vitro	in_vivo	14	14	65.1507143	69.4442857	-4.2935714	c(t = -0.3915	0.699	0.93260677	Distal Intergenic	ENSSSCG000	AGPAT3	1-acylglycerol-3-phosphate O-acyltransferase 3 [Source:VGNC Symbol;Acc:VGNC:85185]
chr7	36993509	36994681	1173	*	chr7	36993509	36994681	in_vitro	in_vivo	12	14	18.655	16.0235714	2.63142857	c(t = 0.39205	0.699	0.93260677	Distal Intergenic	ENSSSCG000	MED20	mediator complex subunit 20 [Source:NCBI gene (formerly Entrezgene);Acc:100157502]
chr12	2169000	2169895	896	*	chr12	2169000	2169895	in_vitro	in_vivo	9	9	10.2911111	7.96444444	2.32666667	c(t = 0.39458	0.699	0.93260677	Distal Intergenic	ENSSSCG000	SLC26A11	solute carrier family 26 member 11 [Source:NCBI gene (formerly Entrezgene);Acc:100191010]
chr5	6540259	6541390	1132	*	chr5	6540259	6541390	in_vitro	in_vivo	12	10	9.60833333	7.339	2.26933333	c(t = 0.39343	0.699	0.93260677	Promoter (1-2kb)	ENSSSCG000	CYP2D6	cytochrome P450 family 2 subfamily D member 6 [Source:NCBI gene (formerly Entrezgene);Acc:397687]
chr2	80636648	80637889	1242	*	chr2	80636648	80637889	in_vitro	in_vivo	9	9	9.44555556	6.61444444	2.83111111	c(t = 0.39535	0.699	0.93260677	Promoter (<=1kb)	ENSSSCG000	MXD3	MAX dimerization protein 3 [Source:VGNC Symbol;Acc:VGNC:100175]
chrX	41579422	41581075	1654	*	chrX	41579422	41581075	in_vitro	in_vivo	16	12	5.34125	3.8725	1.46875	c(t = 0.39176	0.699	0.93260677	Distal Intergenic	ENSSSCG000	RGN	regucalcin [Source:VGNC Symbol;Acc:VGNC:92256]
chr18	43725045	43728293	3249	*	chr18	43725045	43728293	in_vitro	in_vivo	14	16	1.89071429	2.183125	-0.2924107	c(t = -0.3909	0.699	0.93260677	Distal Intergenic	ENSSSCG000	JAZF1	JAZF zinc finger 1 [Source:VGNC Symbol;Acc:VGNC:89280]
chr14	11051298	11052014	717	*	chr14	11051298	11052014	in_vitro	in_vivo	10	15	1.342	1.81466667	-0.4726667	c(t = -0.3922	0.699	0.93260677	Distal Intergenic	ENSSSCG000	EPHX2	epoxide hydrolase 2 [Source:NCBI gene (formerly Entrezgene);Acc:414425]
chr11	77354566	77356438	1873	*	chr11	77354566	77356438	in_vitro	in_vivo	11	13	1.61	1.19307692	0.41692308	c(t = 0.39254	0.699	0.93260677	Promoter (<=1kb)	ENSSSCG000	ANKRD10	ankyrin repeat domain 10 [Source:VGNC Symbol;Acc:VGNC:85322]
chr8	135843582	135845875	2294	*	chr8	135843582	135845875	in_vitro	in_vivo	12	14	2.35083333	2.91857143	-0.5677381	c(t = -0.3898	0.7	0.93280735	Distal Intergenic	ENSSSCG000	ENOPH1	enolase-phosphatase 1 [Source:VGNC Symbol;Acc:VGNC:98922]
chr1	239841469	239844092	2624	*	chr1	239841469	239844092	in_vitro	in_vivo	12	14	2.705	2.19857143	0.50642857	c(t = 0.39062	0.7	0.93280735	Distal Intergenic	ENSSSCG000	NANS	N-acetylneuraminate synthase [Source:NCBI gene (formerly Entrezgene);Acc:100155883]
chr4	16100711	16101728	1018	*	chr4	16100711	16101728	in_vitro	in_vivo	11	14	2.43454545	1.84642857	0.58811688	c(t = 0.39015	0.7	0.93280735	Distal Intergenic	ENSSSCG000	FBXO32	F-box protein 32 [Source:NCBI gene (formerly Entrezgene);Acc:733657]
chr1	165762593	165764190	1598	*	chr1	165762593	165764190	in_vitro	in_vivo	12	9	1.2475	1.68	-0.4325	c(t = -0.3909	0.7	0.93280735	Distal Intergenic	ENSSSCG000	CALML4	calmodulin like 4 [Source:NCBI gene (formerly Entrezgene);Acc:100519528]
chr10	46899323	46900133	811	*	chr10	46899323	46900133	in_vitro	in_vivo	11	13	1.18	0.89153846	0.28846154	c(t = 0.39159	0.7	0.93280735	Promoter (<=1kb)	ENSSSCG000	SUV39H2	SUV39H2 histone lysine methyltransferase [Source:VGNC Symbol;Acc:VGNC:95841]
chr3	59202158	59202510	353	*	chr3	59202158	59202510	in_vitro	in_vivo	10	11	0.455	0.74272727	-0.2877273	c(t = -0.3913	0.7	0.93280735	Distal Intergenic	ENSSSCG000	MAT2A	methionine adenosyltransferase 2A [Source:NCBI gene (formerly Entrezgene);Acc:100312974]
chr3	9971552	9972238	687	*	chr3	9971552	9972238	in_vitro	in_vivo	10	12	0.766	0.55	0.216	c(t = 0.39258	0.7	0.93280735	Distal Intergenic	ENSSSCG000	ZP3	zona pellucida glycoprotein 3 [Source:NCBI gene (formerly Entrezgene);Acc:396927]
chr6	1466746	1468614	1869	*	chr6	1466746	1468614	in_vitro	in_vivo	9	9	93.5	91.8777778	1.62222222	c(t = 0.39205	0.701	0.93333073	Intron (NM_001244650/100622381, intron 3 of 12)	ENSSSCG000	BANP	BTG3 associated nuclear protein [Source:NCBI gene (formerly Entrezgene);Acc:100622381]
chr9	11640897	11641384	488	*	chr9	11640897	11641384	in_vitro	in_vivo	11	10	2.31	3.306	-0.996	c(t = -0.3922	0.701	0.93333073	Distal Intergenic	ENSSSCG000	MYO7A	myosin VIIA [Source:NCBI gene (formerly Entrezgene);Acc:397373]
chr1	228141619	228142555	937	*	chr1	228141619	228142555	in_vitro	in_vivo	11	9	2.87181818	2.15222222	0.71959596	c(t = 0.39193	0.701	0.93333073	Promoter (<=1kb)	ENSSSCG000	OSTF1	osteoclast stimulating factor 1 [Source:NCBI gene (formerly Entrezgene);Acc:397066]
chr3	125233179	125234859	1681	*	chr3	125233179	125234859	in_vitro	in_vivo	10	13	1.833	1.51615385	0.31684615	c(t = 0.38979	0.701	0.93333073	Distal Intergenic	ENSSSCG000	LPIN1	lipin 1 [Source:VGNC Symbol;Acc:VGNC:89793]
chr8	31810090	31810756	667	*	chr8	31810090	31810756	in_vitro	in_vivo	11	13	0.96909091	1.50846154	-0.5393706	c(t = -0.3904	0.701	0.93333073	Distal Intergenic	ENSSSCG000	CHRNA9	cholinergic receptor nicotinic alpha 9 subunit [Source:VGNC Symbol;Acc:VGNC:86671]
chr10	11389173	11391250	2078	*	chr10	11389173	11391250	in_vitro	in_vivo	11	13	17.28	21.4769231	-4.1969231	c(t = -0.3876	0.702	0.93348122	Distal Intergenic	ENSSSCG000	FH	fumarate hydratase [Source:VGNC Symbol;Acc:VGNC:96300]
chr6	67253146	67254500	1355	*	chr6	67253146	67254500	in_vitro	in_vivo	11	10	3.13	4.359	-1.229	c(t = -0.3913	0.702	0.93348122	Distal Intergenic	ENSSSCG000	ICMT	isoprenylcysteine carboxyl methyltransferase [Source:NCBI gene (formerly Entrezgene);Acc:100514410]
chr7	27206490	27207015	526	*	chr7	27206490	27207015	in_vitro	in_vivo	12	10	2.37416667	1.623	0.75116667	c(t = 0.38825	0.702	0.93348122	Distal Intergenic	ENSSSCG000	HCRTR2	hypocretin receptor 2 [Source:NCBI gene (formerly Entrezgene);Acc:397428]
chr16	48403253	48405229	1977	*	chr16	48403253	48405229	in_vitro	in_vivo	13	13	2.17923077	1.45230769	0.72692308	c(t = 0.38972	0.702	0.93348122	Distal Intergenic	ENSSSCG000	CARTPT	CART prepropeptide [Source:NCBI gene (formerly Entrezgene);Acc:397252]
chr16	22500357	22501324	968	*	chr16	22500357	22501324	in_vitro	in_vivo	9	11	1.35888889	0.96272727	0.39616162	c(t = 0.38899	0.702	0.93348122	Distal Intergenic	ENSSSCG000	SLC1A3	solute carrier family 1 member 3 [Source:VGNC Symbol;Acc:VGNC:92965]
chr3	22586632	22587064	433	*	chr3	22586632	22587064	in_vitro	in_vivo	9	10	0.19111111	0.345	-0.1538889	c(t = -0.3901	0.702	0.93348122	Distal Intergenic	ENSSSCG000	NDUFAB1	NADH:ubiquinone oxidoreductase subunit AB1 [Source:VGNC Symbol;Acc:VGNC:104004]
chr6	682046	685083	3038	*	chr6	682046	685083	in_vitro	in_vivo	10	10	90.917	88.988	1.929	c(t = 0.38968	0.703	0.93348122	Distal Intergenic	ENSSSCG000	GALNS	galactosamine (N-acetyl)-6-sulfatase [Source:VGNC Symbol;Acc:VGNC:97061]
chr9	3113686	3115223	1538	*	chr9	3113686	3115223	in_vitro	in_vivo	11	11	19.9663636	15.7763636	4.19	c(t = 0.38715	0.703	0.93348122	Distal Intergenic	ENSSSCG000	MRPL17	mitochondrial ribosomal protein L17 [Source:NCBI gene (formerly Entrezgene);Acc:100512482]
chr5	96784699	96787194	2496	*	chr5	96784699	96787194	in_vitro	in_vivo	12	14	2.2075	2.84928571	-0.6417857	c(t = -0.3861	0.703	0.93348122	Distal Intergenic	ENSSSCG000	MGAT4C	MGAT4 family member C [Source:NCBI gene (formerly Entrezgene);Acc:414851]
chr14	80105372	80107116	1745	*	chr14	80105372	80107116	in_vitro	in_vivo	12	13	1.355	1.72153846	-0.3665385	c(t = -0.3865	0.703	0.93348122	Promoter (<=1kb)	ENSSSCG000	KCNMA1	potassium calcium-activated channel subfamily M alpha 1 [Source:VGNC Symbol;Acc:VGNC:107403]
chr9	107273419	107273912	494	*	chr9	107273419	107273912	in_vitro	in_vivo	9	11	1.24888889	0.88181818	0.36707071	c(t = 0.38783	0.703	0.93348122	Distal Intergenic	ENSSSCG000	SLC26A3	solute carrier family 26 member 3 [Source:VGNC Symbol;Acc:VGNC:93028]
chr14	77925135	77926926	1792	*	chr14	77925135	77926926	in_vitro	in_vivo	13	12	3.53615385	2.84083333	0.69532051	c(t = 0.38665	0.704	0.93348122	Distal Intergenic	ENSSSCG000	VDAC2	voltage dependent anion channel 2 [Source:VGNC Symbol;Acc:VGNC:111154]
chr8	82044704	82047409	2706	*	chr8	82044704	82047409	in_vitro	in_vivo	13	13	3.04307692	2.40923077	0.63384615	c(t = 0.38508	0.704	0.93348122	Distal Intergenic	ENSSSCG000	SLC10A7	solute carrier family 10 member 7 [Source:VGNC Symbol;Acc:VGNC:92919]
chr7	54655696	54656083	388	*	chr7	54655696	54656083	in_vitro	in_vivo	10	10	1.46	2.044	-0.584	c(t = -0.3878	0.704	0.93348122	Distal Intergenic	ENSSSCG000	MFGE8	milk fat globule EGF and factor V/VIII domain containing [Source:VGNC Symbol;Acc:VGNC:109463]
chr17	62173544	62175164	1621	*	chr17	62173544	62175164	in_vitro	in_vivo	10	9	2.459	1.94111111	0.51788889	c(t = 0.38697	0.704	0.93348122	Distal Intergenic	ENSSSCG000	ssc-mir-133a	ssc-mir-133a-1 [Source:miRBase;Acc:MI0010681]
chr3	16983564	16983970	407	*	chr3	16983564	16983970	in_vitro	in_vivo	9	9	1.23444444	1.85666667	-0.6222222	c(t = -0.3876	0.704	0.93348122	Distal Intergenic	ENSSSCG000	PSPH	phosphoserine phosphatase [Source:VGNC Symbol;Acc:VGNC:91931]
chr8	80303281	80306754	3474	*	chr8	80303281	80306754	in_vitro	in_vivo	13	14	1.33461538	1.06214286	0.27247253	c(t = 0.38487	0.704	0.93348122	Distal Intergenic	ENSSSCG000	EDNRA	endothelin receptor type A [Source:VGNC Symbol;Acc:VGNC:87549]
chr12	429815	433187	3373	*	chr12	429815	433187	in_vitro	in_vivo	9	9	92.8155556	94.0288889	-1.2133333	c(t = -0.3856	0.705	0.93348122	Intron (NM_001244105/100512127, intron 7 of 38)	ENSSSCG000	TBCD	tubulin folding cofactor D [Source:NCBI gene (formerly Entrezgene);Acc:100512127]
chr12	50679635	50680728	1094	*	chr12	50679635	50680728	in_vitro	in_vivo	9	12	11.3922222	14.0608333	-2.6686111	c(t = -0.3857	0.705	0.93348122	Distal Intergenic	ENSSSCG000	ASPA	aspartoacylase [Source:NCBI gene (formerly Entrezgene);Acc:100142661]
chrX	125464598	125465313	716	*	chrX	125464598	125465313	in_vitro	in_vivo	11	10	4.10090909	5.362	-1.2610909	c(t = -0.3883	0.705	0.93348122	Distal Intergenic	ENSSSCG000	VBP1	VHL binding protein 1 [Source:VGNC Symbol;Acc:VGNC:104090]
chr7	49622181	49624296	2116	*	chr7	49622181	49624296	in_vitro	in_vivo	13	10	2.87846154	3.736	-0.8575385	c(t = -0.3864	0.705	0.93348122	Distal Intergenic	ENSSSCG000	TLNRD1	talin rod domain containing 1 [Source:NCBI gene (formerly Entrezgene);Acc:100152002]
chr3	39212725	39215863	3139	*	chr3	39212725	39215863	in_vitro	in_vivo	9	10	4.58666667	3.489	1.09766667	c(t = 0.38690	0.705	0.93348122	Distal Intergenic	ENSSSCG000	PKMYT1	protein kinase, membrane associated tyrosine/threonine 1 [Source:VGNC Symbol;Acc:VGNC:91480]
chr2	59883195	59885347	2153	*	chr2	59883195	59885347	in_vitro	in_vivo	11	14	2.26090909	2.89071429	-0.6298052	c(t = -0.3840	0.705	0.93348122	Promoter (<=1kb)	ENSSSCG000	SLC5A5	solute carrier family 5 member 5 [Source:NCBI gene (formerly Entrezgene);Acc:399542]
chr15	121579998	121581177	1180	*	chr15	121579998	121581177	in_vitro	in_vivo	10	15	2.434	1.73466667	0.69933333	c(t = 0.38555	0.705	0.93348122	Promoter (<=1kb)	ENSSSCG000	INHA	inhibin subunit alpha [Source:VGNC Symbol;Acc:VGNC:96372]
chr3	55833708	55834719	1012	*	chr3	55833708	55834719	in_vitro	in_vivo	9	12	1.34888889	1.70583333	-0.3569444	c(t = -0.3841	0.705	0.93348122	Distal Intergenic	ENSSSCG000	INPP4A	inositol polyphosphate-4-phosphatase type I A [Source:VGNC Symbol;Acc:VGNC:89140]
chr15	73772164	73774142	1979	*	chr15	73772164	73774142	in_vitro	in_vivo	15	13	1.90133333	1.51153846	0.38979487	c(t = 0.38300	0.705	0.93348122	Distal Intergenic	ENSSSCG000	PHOSPHO2	phosphatase, orphan 2 [Source:VGNC Symbol;Acc:VGNC:96172]
chr6	94813769	94814765	997	*	chr6	94813769	94814765	in_vitro	in_vivo	11	10	2.02	1.374	0.646	c(t = 0.38612	0.705	0.93348122	Promoter (<=1kb)	ENSSSCG000	MYCBP	MYC binding protein [Source:VGNC Symbol;Acc:VGNC:110209]
chr11	14576315	14576754	440	*	chr11	14576315	14576754	in_vitro	in_vivo	10	13	0.77	1.16230769	-0.3923077	c(t = -0.3838	0.705	0.93348122	Distal Intergenic	ENSSSCG000	FOXO1	forkhead box O1 [Source:NCBI gene (formerly Entrezgene);Acc:397077]
chr1	75457154	75458615	1462	*	chr1	75457154	75458615	in_vitro	in_vivo	10	11	1.204	0.92272727	0.28127273	c(t = 0.38525	0.705	0.93348122	Distal Intergenic	ENSSSCG000	PPIL6	peptidylprolyl isomerase like 6 [Source:VGNC Symbol;Acc:VGNC:98216]
chr3	6296096	6296671	576	*	chr3	6296096	6296671	in_vitro	in_vivo	13	10	1.10692308	0.876	0.23092308	c(t = 0.38345	0.705	0.93348122	Distal Intergenic	ENSSSCG000	ARPC1A	actin related protein 2/3 complex subunit 1A [Source:VGNC Symbol;Acc:VGNC:106444]
chr3	17635541	17636522	982	*	chr3	17635541	17636522	in_vitro	in_vivo	10	10	1.27	0.755	0.515	c(t = 0.38488	0.705	0.93348122	Distal Intergenic	ENSSSCG000	PHKG2	phosphorylase kinase catalytic subunit gamma 2 [Source:VGNC Symbol;Acc:VGNC:91395]
chr9	32810811	32812363	1553	*	chr9	32810811	32812363	in_vitro	in_vivo	10	15	0.915	0.64066667	0.27433333	c(t = 0.38477	0.705	0.93348122	Distal Intergenic	ENSSSCG000	ssc-mir-9833	ssc-mir-9833 [Source:miRBase;Acc:MI0031602]
chr2	150847445	150848911	1467	*	chr2	150847445	150848911	in_vitro	in_vivo	9	12	59.22	53.2733333	5.94666667	c(t = 0.38303	0.706	0.93367923	Distal Intergenic	ENSSSCG000	ssc-mir-378-	ssc-mir-378-1 [Source:miRBase;Acc:MI0013088]
chr6	1017855	1020221	2367	*	chr6	1017855	1020221	in_vitro	in_vivo	9	9	16.9266667	22.1166667	-5.19	c(t = -0.3840	0.706	0.93367923	Exon (NM_214267/397507, exon 2 of 9)	ENSSSCG000	CYBA	cytochrome b-245 alpha chain [Source:VGNC Symbol;Acc:VGNC:87124]
chr2	69221282	69222047	766	*	chr2	69221282	69222047	in_vitro	in_vivo	10	11	4.838	5.93	-1.092	c(t = -0.3837	0.706	0.93367923	Distal Intergenic	ENSSSCG000	CDC37	cell division cycle 37, HSP90 cochaperone [Source:VGNC Symbol;Acc:VGNC:86450]
chr1	106751265	106753045	1781	*	chr1	106751265	106753045	in_vitro	in_vivo	17	13	2.70176471	2.12769231	0.5740724	c(t = 0.38193	0.706	0.93367923	Distal Intergenic	ENSSSCG000	FECH	ferrochelatase [Source:VGNC Symbol;Acc:VGNC:88081]
chr1	67293185	67295006	1822	*	chr1	67293185	67295006	in_vitro	in_vivo	12	13	1.64666667	2.10846154	-0.4617949	c(t = -0.3819	0.706	0.93367923	Promoter (<=1kb)	ENSSSCG000	SIM1	SIM bHLH transcription factor 1 [Source:VGNC Symbol;Acc:VGNC:92876]
chr16	32805229	32806373	1145	*	chr16	32805229	32806373	in_vitro	in_vivo	9	11	1.69	1.24909091	0.44090909	c(t = 0.38738	0.706	0.93367923	Promoter (<=1kb)	ENSSSCG000	FST	follistatin [Source:NCBI gene (formerly Entrezgene);Acc:445002]
chr7	44777346	44777707	362	*	chr7	44777346	44777707	in_vitro	in_vivo	9	9	0.88555556	1.22888889	-0.3433333	c(t = -0.3859	0.706	0.93367923	Distal Intergenic	ENSSSCG000	TFAP2B	transcription factor AP-2 beta [Source:VGNC Symbol;Acc:VGNC:93909]
chr8	334587	339736	5150	*	chr8	334587	339736	in_vitro	in_vivo	10	13	73.837	68.8615385	4.97546154	c(t = 0.38177	0.707	0.93375322	Distal Intergenic	ENSSSCG000	ATP5ME	ATP synthase membrane subunit e [Source:NCBI gene (formerly Entrezgene);Acc:396950]
chr5	7151324	7152905	1582	*	chr5	7151324	7152905	in_vitro	in_vivo	9	11	29.4544444	23.9890909	5.46535354	c(t = 0.38308	0.707	0.93375322	Distal Intergenic	ENSSSCG000	ACO2	aconitase 2 [Source:NCBI gene (formerly Entrezgene);Acc:396999]
chr6	27806858	27809982	3125	*	chr6	27806858	27809982	in_vitro	in_vivo	15	12	4.664	6.0225	-1.3585	c(t = -0.3807	0.707	0.93375322	Distal Intergenic	ENSSSCG000	ssc-mir-328	ssc-mir-328 [Source:miRBase;Acc:MI0013158]
chr17	53459034	53460098	1065	*	chr17	53459034	53460098	in_vitro	in_vivo	11	14	1.27090909	0.99714286	0.27376623	c(t = 0.38068	0.707	0.93375322	Promoter (<=1kb)	ENSSSCG00000007481	ZFP64	zinc finger protein [Source:VGNC Symbol;Acc:VGNC:95720]
chr13	2451960	2452616	657	*	chr13	2451960	2452616	in_vitro	in_vivo	13	13	1.04692308	0.80846154	0.23846154	c(t = 0.38088	0.707	0.93375322	Promoter (<=1kb)	ENSSSCG000	CAPN7	calpain 7 [Source:NCBI gene (formerly Entrezgene);Acc:100037936]
chr18	25569224	25570091	868	*	chr18	25569224	25570091	in_vitro	in_vivo	9	14	1.08111111	0.69714286	0.38396825	c(t = 0.38191	0.707	0.93375322	Distal Intergenic	ENSSSCG000	ING3	inhibitor of growth family member 3 [Source:VGNC Symbol;Acc:VGNC:89132]
chr12	6272395	6273172	778	*	chr12	6272395	6273172	in_vitro	in_vivo	12	12	21.6208333	27.2075	-5.5866667	c(t = -0.3800	0.708	0.93375322	Distal Intergenic	ENSSSCG000	MRPL58	mitochondrial ribosomal protein L58 [Source:NCBI gene (formerly Entrezgene);Acc:100512432]
chr12	61057557	61060930	3374	*	chr12	61057557	61060930	in_vitro	in_vivo	11	11	16.3763636	20.2609091	-3.8845455	c(t = -0.3798	0.708	0.93375322	Distal Intergenic	ENSSSCG000	COPS3	COP9 signalosome subunit 3 [Source:VGNC Symbol;Acc:VGNC:86900]
chr17	46637264	46638235	972	*	chr17	46637264	46638235	in_vitro	in_vivo	12	13	5.2675	6.28384615	-1.0163462	c(t = -0.3796	0.708	0.93375322	Distal Intergenic	ENSSSCG000	FITM2	fat storage inducing transmembrane protein 2 [Source:NCBI gene (formerly Entrezgene);Acc:100158013]
chr6	47273410	47274483	1074	*	chr6	47273410	47274483	in_vitro	in_vivo	11	12	2.70181818	3.365	-0.6631818	c(t = -0.3799	0.708	0.93375322	Distal Intergenic	ENSSSCG000	YIF1B	Yip1 interacting factor homolog B, membrane trafficking protein [Source:VGNC Symbol;Acc:VGNC:95026]
chr6	52097966	52102292	4327	*	chr6	52097966	52102292	in_vitro	in_vivo	14	14	3.12428571	2.56571429	0.55857143	c(t = 0.37863	0.708	0.93375322	Distal Intergenic	ENSSSCG000	QPCTL	glutaminyl-peptide cyclotransferase like [Source:VGNC Symbol;Acc:VGNC:92027]
chr1	43213639	43215201	1563	*	chr1	43213639	43215201	in_vitro	in_vivo	11	12	3.00727273	2.4475	0.55977273	c(t = 0.38101	0.708	0.93375322	Distal Intergenic	ENSSSCG000	PLN	phospholamban [Source:NCBI gene (formerly Entrezgene);Acc:397421]
chr13	81849655	81850739	1085	*	chr13	81849655	81850739	in_vitro	in_vivo	10	9	3.145	2.28555556	0.85944444	c(t = 0.38154	0.708	0.93375322	Distal Intergenic	ENSSSCG000	SLC25A36	solute carrier family 25 member 36 [Source:VGNC Symbol;Acc:VGNC:93012]
chr8	130590015	130591273	1259	*	chr8	130590015	130591273	in_vitro	in_vivo	13	9	1.55538462	1.27888889	0.27649573	c(t = 0.37979	0.708	0.93375322	Distal Intergenic	NA	NA	NA
chr3	38646395	38647349	955	*	chr3	38646395	38647349	in_vitro	in_vivo	9	11	0.72555556	0.97909091	-0.2535354	c(t = -0.3816	0.708	0.93375322	Distal Intergenic	ENSSSCG000	DNASE1	deoxyribonuclease 1 [Source:VGNC Symbol;Acc:VGNC:102564]
chr4	39112474	39113715	1242	*	chr4	39112474	39113715	in_vitro	in_vivo	14	12	0.775	0.56083333	0.21416667	c(t = 0.37872	0.708	0.93375322	Promoter (<=1kb)	ENSSSCG000	MTDH	metadherin [Source:VGNC Symbol;Acc:VGNC:90439]
chrX	43327581	43329137	1557	*	chrX	43327581	43329137	in_vitro	in_vivo	15	12	21.7746667	24.7383333	-2.9636667	c(t = -0.3774	0.709	0.93411023	Distal Intergenic	ENSSSCG000	FOXP3	forkhead box P3 [Source:VGNC Symbol;Acc:VGNC:88223]
chr1	1062562	1063584	1023	*	chr1	1062562	1063584	in_vitro	in_vivo	9	12	18.3444444	23.8491667	-5.5047222	c(t = -0.3781	0.709	0.93411023	Distal Intergenic	ENSSSCG000	ssc-mir-9815	ssc-mir-9815 [Source:miRBase;Acc:MI0031582]
chr3	95330662	95332058	1397	*	chr3	95330662	95332058	in_vitro	in_vivo	11	14	12.4136364	8.53142857	3.88220779	c(t = 0.37972	0.709	0.93411023	Distal Intergenic	ENSSSCG000	SLC3A1	solute carrier family 3 member 1 [Source:NCBI gene (formerly Entrezgene);Acc:100144588]
chr11	5303589	5305575	1987	*	chr11	5303589	5305575	in_vitro	in_vivo	11	16	6.15272727	7.00625	-0.8535227	c(t = -0.3781	0.709	0.93411023	Promoter (<=1kb)	ENSSSCG000	PDX1	pancreatic and duodenal homeobox 1 [Source:NCBI gene (formerly Entrezgene);Acc:397288]
chr1	120026205	120027143	939	*	chr1	120026205	120027143	in_vitro	in_vivo	14	11	1.50642857	1.85454545	-0.3481169	c(t = -0.3774	0.709	0.93411023	Distal Intergenic	ENSSSCG000	TMOD3	tropomodulin 3 [Source:NCBI gene (formerly Entrezgene);Acc:397556]
chr4	79652011	79653257	1247	*	chr4	79652011	79653257	in_vitro	in_vivo	12	11	1.48333333	1.15363636	0.32969697	c(t = 0.37846	0.709	0.93411023	Promoter (<=1kb)	ENSSSCG000	UBE2V2	ubiquitin conjugating enzyme E2 V2 [Source:NCBI gene (formerly Entrezgene);Acc:100627960]
chrX	16422566	16423662	1097	*	chrX	16422566	16423662	in_vitro	in_vivo	13	15	10.5430769	8.41333333	2.12974359	c(t = 0.37593	0.71	0.93494688	Distal Intergenic	ENSSSCG000	EIF1AX	eukaryotic translation initiation factor 1A X-linked [Source:NCBI gene (formerly Entrezgene);Acc:100522912]
chr3	5462768	5464355	1588	*	chr3	5462768	5464355	in_vitro	in_vivo	10	12	2.353	1.69833333	0.65466667	c(t = 0.37738	0.71	0.93494688	Distal Intergenic	ENSSSCG000	AIMP2	aminoacyl tRNA synthetase complex interacting multifunctional protein 2 [Source:NCBI gene (formerly Entrezgene);Acc:100514707]
chr9	103179345	103179944	600	*	chr9	103179345	103179944	in_vitro	in_vivo	11	14	1.26909091	0.84928571	0.41980519	c(t = 0.37717	0.71	0.93494688	Promoter (<=1kb)	ENSSSCG000	ARMC10	armadillo repeat containing 10 [Source:NCBI gene (formerly Entrezgene);Acc:100513998]
chr4	594210	601345	7136	*	chr4	594210	601345	in_vitro	in_vivo	11	16	33.4354545	36.986875	-3.5514205	c(t = -0.3760	0.711	0.93517689	Distal Intergenic	ENSSSCG000	HSF1	heat shock transcription factor 1 [Source:VGNC Symbol;Acc:VGNC:88993]
chr6	51568190	51570131	1942	*	chr6	51568190	51570131	in_vitro	in_vivo	11	11	14.8227273	18.86	-4.0372727	c(t = -0.3777	0.711	0.93517689	Distal Intergenic	ENSSSCG000	BLOC1S3	biogenesis of lysosomal organelles complex 1 subunit 3 [Source:NCBI gene (formerly Entrezgene);Acc:100049671]
chr7	30318381	30321801	3421	*	chr7	30318381	30321801	in_vitro	in_vivo	11	12	6.93727273	5.365	1.57227273	c(t = 0.37803	0.711	0.93517689	Promoter (<=1kb)	ENSSSCG000	HMGA1	high mobility group AT-hook 1 [Source:VGNC Symbol;Acc:VGNC:103070]
chr3	18141143	18141740	598	*	chr3	18141143	18141740	in_vitro	in_vivo	9	13	1.83444444	2.38076923	-0.5463248	c(t = -0.3755	0.711	0.93517689	Distal Intergenic	ENSSSCG000	CDIPT	CDP-diacylglycerol--inositol 3-phosphatidyltransferase [Source:VGNC Symbol;Acc:VGNC:86494]
chr13	80788702	80790363	1662	*	chr13	80788702	80790363	in_vitro	in_vivo	13	10	2.29769231	1.893	0.40469231	c(t = 0.37571	0.711	0.93517689	Distal Intergenic	ENSSSCG000	RBP1	retinol binding protein 1 [Source:VGNC Symbol;Acc:VGNC:107167]
chr3	77478427	77479506	1080	*	chr3	77478427	77479506	in_vitro	in_vivo	11	12	1.85363636	1.53083333	0.32280303	c(t = 0.37517	0.711	0.93517689	Distal Intergenic	ENSSSCG000	UGP2	UDP-glucose pyrophosphorylase 2 [Source:VGNC Symbol;Acc:VGNC:98400]
chr17	59031030	59035381	4352	*	chr17	59031030	59035381	in_vitro	in_vivo	12	16	16.5291667	18.47125	-1.9420833	c(t = -0.3728	0.712	0.93517689	Promoter (<=1kb)	ENSSSCG00000007520	GNAS	complex locus [Source:VGNC Symbol;Acc:VGNC:110580]
chr18	43183443	43184771	1329	*	chr18	43183443	43184771	in_vitro	in_vivo	10	10	16.445	13.601	2.844	c(t = 0.37470	0.712	0.93517689	Distal Intergenic	ENSSSCG000	FKBP14	FKBP prolyl isomerase 14 [Source:NCBI gene (formerly Entrezgene);Acc:100514551]
chr2	8854552	8857177	2626	*	chr2	8854552	8857177	in_vitro	in_vivo	10	12	3.501	4.19416667	-0.6931667	c(t = -0.3761	0.712	0.93517689	Promoter (1-2kb)	NA	NA	NA
chr14	31311952	31313443	1492	*	chr14	31311952	31313443	in_vitro	in_vivo	11	12	3.20545455	2.7025	0.50295455	c(t = 0.37465	0.712	0.93517689	Distal Intergenic	ENSSSCG000	ORAI1	ORAI calcium release-activated calcium modulator 1 [Source:NCBI gene (formerly Entrezgene);Acc:100154923]
chr13	87731852	87733591	1740	*	chr13	87731852	87733591	in_vitro	in_vivo	12	11	1.26166667	1.70818182	-0.4465152	c(t = -0.3763	0.712	0.93517689	Distal Intergenic	ENSSSCG000	PLSCR1	phospholipid scramblase 1 [Source:NCBI gene (formerly Entrezgene);Acc:100620055]
chr17	52376723	52378327	1605	*	chr17	52376723	52378327	in_vitro	in_vivo	12	15	1.225	1.50733333	-0.2823333	c(t = -0.3730	0.712	0.93517689	Promoter (<=1kb)	ENSSSCG000	DPM1	dolichyl-phosphate mannosyltransferase subunit 1, catalytic [Source:VGNC Symbol;Acc:VGNC:96235]
chr6	77455286	77456997	1712	*	chr6	77455286	77456997	in_vitro	in_vivo	10	12	1.585	1.31	0.275	c(t = 0.37474	0.712	0.93517689	Distal Intergenic	ENSSSCG000	ALDH4A1	aldehyde dehydrogenase 4 family member A1 [Source:NCBI gene (formerly Entrezgene);Acc:100820830]
chr17	58837881	58840028	2148	*	chr17	58837881	58840028	in_vitro	in_vivo	12	13	1.44583333	1.11538462	0.33044872	c(t = 0.37469	0.712	0.93517689	Promoter (<=1kb)	ENSSSCG000	STX16	syntaxin 16 [Source:VGNC Symbol;Acc:VGNC:110223]
chr3	69939122	69939719	598	*	chr3	69939122	69939719	in_vitro	in_vivo	9	9	1.46444444	1.10888889	0.35555556	c(t = 0.37672	0.712	0.93517689	Distal Intergenic	ENSSSCG000	CCT7	chaperonin containing TCP1 subunit 7 [Source:NCBI gene (formerly Entrezgene);Acc:100322871]
chr6	163626520	163627050	531	*	chr6	163626520	163627050	in_vitro	in_vivo	9	9	15.1833333	21.7133333	-6.53	c(t = -0.3751	0.713	0.93601042	Distal Intergenic	ENSSSCG000	SPATA6	spermatosis associated 6 [Source:VGNC Symbol;Acc:VGNC:93387]
chr4	100426056	100426416	361	*	chr4	100426056	100426416	in_vitro	in_vivo	10	12	3.356	2.635	0.721	c(t = 0.37322	0.713	0.93601042	Distal Intergenic	ENSSSCG000	PRKAB2	protein kinase AMP-activated non-catalytic subunit beta 2 [Source:VGNC Symbol;Acc:VGNC:91799]
chr2	49804088	49805061	974	*	chr2	49804088	49805061	in_vitro	in_vivo	11	13	2.02727273	1.50692308	0.52034965	c(t = 0.37356	0.713	0.93601042	Distal Intergenic	ENSSSCG000	ADM	adrenomedullin [Source:NCBI gene (formerly Entrezgene);Acc:397195]
chr1	253927877	253928249	373	*	chr1	253927877	253928249	in_vitro	in_vivo	10	10	48.02	41.896	6.124	c(t = 0.37185	0.714	0.93655616	Distal Intergenic	ENSSSCG000	SLC31A1	solute carrier family 31 member 1 [Source:NCBI gene (formerly Entrezgene);Acc:397186]

chr18	55644071	55646271	2201	*	chr18	55644071	55646271	in_vitro	in_vivo	13	14	3.59230769	3.00428571	0.58802198	c(t = 0.37320	0.714	0.93655616	Distal Intergenic	ENSSSCG000	RARRES2	retinoic acid receptor responder 2 [Source:VGNC Symbol;Acc:VGNC:92098]
chr1	27379801	27381599	1799	*	chr1	27379801	27381599	in_vitro	in_vivo	13	11	2.88692308	2.23090909	0.65601399	c(t = 0.37094	0.714	0.93655616	Distal Intergenic	ENSSSCG000	IFNGR1	interferon gamma receptor 1 [Source:VGNC Symbol;Acc:VGNC:89041]
chr1	109921476	109922526	1051	*	chr1	109921476	109922526	in_vitro	in_vivo	14	14	6.65071429	8.04142857	-1.3907143	c(t = -0.3688	0.715	0.93655616	Distal Intergenic	ENSSSCG000	ssc-mir-190a	ssc-mir-190a [Source:miRBase;Acc:MI0022136]
chr6	166956387	166957587	1201	*	chr6	166956387	166957587	in_vitro	in_vivo	11	13	5.93545455	7.74615385	-1.8106993	c(t = -0.3699	0.715	0.93655616	Distal Intergenic	ENSSSCG000	DMAP1	DNA methyltransferase 1 associated protein 1 [Source:VGNC Symbol;Acc:VGNC:87344]
chr7	62473501	62474872	1372	*	chr7	62473501	62474872	in_vitro	in_vivo	13	15	5.06769231	4.13133333	0.93635897	c(t = 0.36979	0.715	0.93655616	Distal Intergenic	ENSSSCG000	SSTR1	somatostatin receptor 1 [Source:VGNC Symbol;Acc:VGNC:93496]
chr3	21467228	21468151	924	*	chr3	21467228	21468151	in_vitro	in_vivo	9	12	1.70666667	2.44333333	-0.7366667	c(t = -0.3704	0.715	0.93655616	Distal Intergenic	ENSSSCG000	AQP8	aquaporin 8 [Source:NCBI gene (formerly Entrezgene);Acc:100127152]
chr3	41478996	41480288	1293	*	chr3	41478996	41480288	in_vitro	in_vivo	10	10	2.585	2.191	0.394	c(t = 0.37072	0.715	0.93655616	Distal Intergenic	ENSSSCG000	NME4	NME/NM23 nucleoside diphosphate kinase 4 [Source:VGNC Symbol;Acc:VGNC:90792]
chr2	65869519	65870444	926	*	chr2	65869519	65870444	in_vitro	in_vivo	11	12	2.73818182	2.14583333	0.59234848	c(t = 0.37148	0.715	0.93655616	Distal Intergenic	ENSSSCG000	TRMT1	tRNA methyltransferase 1 [Source:VGNC Symbol;Acc:VGNC:94441]
chr6	34758408	34759244	837	*	chr6	34758408	34759244	in_vitro	in_vivo	14	11	1.18357143	1.54454545	-0.360974	c(t = -0.3721	0.715	0.93655616	Distal Intergenic	ENSSSCG000	NOD2	nucleotide binding oligomerization domain containing 2 [Source:NCBI gene (formerly Entrezgene);Acc:100125838]
chr7	117942825	117943793	969	*	chr7	117942825	117943793	in_vitro	in_vivo	12	11	1.105	1.43363636	-0.3286364	c(t = -0.3726	0.715	0.93655616	Promoter (<=1kb)	ENSSSCG000	VRK1	VRK serine/threonine kinase 1 [Source:VGNC Symbol;Acc:VGNC:98408]
chr4	124992359	124993150	792	*	chr4	124992359	124993150	in_vitro	in_vivo	11	13	1.91909091	1.42538462	0.49370629	c(t = 0.37183	0.715	0.93655616	Distal Intergenic	ENSSSCG000	TGFBR3	transforming growth factor beta receptor 3 [Source:VGNC Symbol;Acc:VGNC:93932]
chr17	29993251	29995607	2357	*	chr17	29993251	29995607	in_vitro	in_vivo	9	11	1.74555556	1.36272727	0.38282828	c(t = 0.37116	0.715	0.93655616	Distal Intergenic	ENSSSCG000	THBD	thrombomodulin [Source:NCBI gene (formerly Entrezgene);Acc:100157642]
chr5	77075136	77077707	2572	*	chr5	77075136	77077707	in_vitro	in_vivo	13	13	5.10769231	4.06461538	1.04307692	c(t = 0.36824	0.716	0.93738671	Distal Intergenic	ENSSSCG000	SLC38A2	solute carrier family 38 member 2 [Source:VGNC Symbol;Acc:VGNC:93094]
chr6	39669756	39674751	4996	*	chr6	39669756	39674751	in_vitro	in_vivo	13	14	3.31615385	3.74642857	-0.4302747	c(t = -0.3684	0.716	0.93738671	Distal Intergenic	ENSSSCG000	SLC7A9	solute carrier family 7 member 9 [Source:VGNC Symbol;Acc:VGNC:93177]
chr7	39262796	39263469	674	*	chr7	39262796	39263469	in_vitro	in_vivo	9	12	0.74222222	1.02416667	-0.2819444	c(t = -0.3687	0.716	0.93738671	Distal Intergenic	ENSSSCG000	HSP90AB1	heat shock protein 90 alpha family class B member 1 [Source:NCBI gene (formerly Entrezgene);Acc:396742]
chr5	2663156	2664645	1490	*	chr5	2663156	2664645	in_vitro	in_vivo	10	11	66.399	70.9427273	-4.5437273	c(t = -0.3675	0.717	0.93773741	Distal Intergenic	ENSSSCG000	PPARA	peroxisome proliferator activated receptor alpha [Source:NCBI gene (formerly Entrezgene);Acc:397239]
chr12	57794926	57795618	693	*	chr12	57794926	57795618	in_vitro	in_vivo	9	10	22.2622222	16.5	5.76222222	c(t = 0.36797	0.717	0.93773741	Distal Intergenic	ENSSSCG000	COX10	cytochrome c oxidase assembly factor heme A:farnesyltransferase COX10 [Source:NCBI gene (formerly Entrezgene);Acc:100521863]
chrX	106546949	106552118	5170	*	chrX	106546949	106552118	in_vitro	in_vivo	10	12	7.376	9.24166667	-1.8656667	c(t = -0.3683	0.717	0.93773741	Distal Intergenic	ENSSSCG000	AIFM1	apoptosis inducing factor mitochondria associated 1 [Source:VGNC Symbol;Acc:VGNC:85198]
chr7	55574166	55574503	338	*	chr7	55574166	55574503	in_vitro	in_vivo	9	9	5.34555556	6.52333333	-1.1777778	c(t = -0.3699	0.717	0.93773741	Distal Intergenic	ENSSSCG000	ARPIN	actin related protein 2/3 complex inhibitor [Source:VGNC Symbol;Acc:VGNC:111466]
chr6	141958895	141960439	1545	*	chr6	141958895	141960439	in_vitro	in_vivo	15	13	1.542	1.79846154	-0.2564615	c(t = -0.3671	0.717	0.93773741	Promoter (<=1kb)	ENSSSCG000	PTGER3	prostaglandin E receptor 3 [Source:VGNC Symbol;Acc:VGNC:98225]
chr11	45017941	45019189	1249	*	chr11	45017941	45019189	in_vitro	in_vivo	10	11	1.701	1.38272727	0.31827273	c(t = 0.36820	0.717	0.93773741	Distal Intergenic	ENSSSCG000	KLF5	KLF transcription factor 5 [Source:NCBI gene (formerly Entrezgene);Acc:100038005]
chr12	59224094	59226275	2182	*	chr12	59224094	59226275	in_vitro	in_vivo	10	12	93.565	92.0008333	1.56416667	c(t = 0.36685	0.718	0.938118	Distal Intergenic	ENSSSCG000	UBB	ubiquitin B [Source:VGNC Symbol;Acc:VGNC:98389]
chrX	7912158	7913021	864	*	chrX	7912158	7913021	in_vitro	in_vivo	11	10	31.8318182	25.417	6.41481818	c(t = 0.36720	0.718	0.938118	Distal Intergenic	ENSSSCG000	AMELY	amelogenin X-linked [Source:NCBI gene (formerly Entrezgene);Acc:396940]
chr11	24963221	24964857	1637	*	chr11	24963221	24964857	in_vitro	in_vivo	11	14	3.71090909	3.155	0.55590909	c(t = 0.36684	0.718	0.938118	Distal Intergenic	ENSSSCG000	RGCC	regulator of cell cycle [Source:VGNC Symbol;Acc:VGNC:92251]
chr10	51840434	51842125	1692	*	chr10	51840434	51842125	in_vitro	in_vivo	9	12	1.46555556	1.9025	-0.4369444	c(t = -0.3664	0.718	0.938118	Distal Intergenic	ENSSSCG000	PIP4K2A	phosphatidylinositol-5-phosphate 4-kinase type 2 alpha [Source:VGNC Symbol;Acc:VGNC:96485]
chr5	82543639	82544743	1105	*	chr5	82543639	82544743	in_vitro	in_vivo	10	9	0.841	1.10333333	-0.2623333	c(t = -0.3684	0.718	0.938118	Distal Intergenic	ENSSSCG000	DRAM1	DNA damage regulated autophagy modulator 1 [Source:NCBI gene (formerly Entrezgene);Acc:100462750]
chr16	79236312	79273117	36806	*	chr16	79236312	79273117	in_vitro	in_vivo	14	15	46.4214286	43.16	3.26142857	c(t = 0.36376	0.719	0.938118	Promoter (<=1kb)	ENSSSCG000	TERT	telomerase reverse transcriptase [Source:NCBI gene (formerly Entrezgene);Acc:492280]
chr12	40492457	40493372	916	*	chr12	40492457	40493372	in_vitro	in_vivo	9	10	21.67	27.472	-5.802	c(t = -0.3656	0.719	0.938118	Distal Intergenic	ENSSSCG000	CCL1	C-C motif chemokine ligand 1 [Source:NCBI gene (formerly Entrezgene);Acc:100310829]
chr15	137060331	137060856	526	*	chr15	137060331	137060856	in_vitro	in_vivo	11	9	12.9718182	16.81	-3.8381818	c(t = -0.3695	0.719	0.938118	Distal Intergenic	ENSSSCG000	ssc-mir-7141	ssc-mir-7141 [Source:miRBase;Acc:MI0023574]
chr7	38266320	38266789	470	*	chr7	38266320	38266789	in_vitro	in_vivo	10	12	4.113	3.395	0.718	c(t = 0.36681	0.719	0.938118	Distal Intergenic	ENSSSCG000	SLC22A7	solute carrier family 22 member 7 [Source:VGNC Symbol;Acc:VGNC:92983]
chr12	32889701	32892082	2382	*	chr12	32889701	32892082	in_vitro	in_vivo	15	15	1.54266667	1.82666667	-0.284	c(t = -0.3636	0.719	0.938118	Promoter (<=1kb)	ENSSSCG000	NOG	noggin [Source:NCBI gene (formerly Entrezgene);Acc:574073]
chr8	10960676	10962003	1328	*	chr8	10960676	10962003	in_vitro	in_vivo	9	14	2.1	1.49285714	0.60714286	c(t = 0.36696	0.719	0.938118	Distal Intergenic	ENSSSCG000	CD38	CD38 molecule [Source:NCBI gene (formerly Entrezgene);Acc:100511702]
chr5	68975619	68976141	523	*	chr5	68975619	68976141	in_vitro	in_vivo	11	10	1.10363636	1.422	-0.3183636	c(t = -0.3657	0.719	0.938118	Distal Intergenic	ENSSSCG000	ADIPOR2	adiponectin receptor 2 [Source:VGNC Symbol;Acc:VGNC:85141]
chr1	7679156	7680433	1278	*	chr1	7679156	7680433	in_vitro	in_vivo	12	12	1.09083333	0.82	0.27083333	c(t = 0.36433	0.719	0.938118	Promoter (<=1kb)	ENSSSCG000	SOD2	superoxide dismutase 2 [Source:NCBI gene (formerly Entrezgene);Acc:100154319]
chr6	34477929	34478833	905	*	chr6	34477929	34478833	in_vitro	in_vivo	11	10	0.69	0.509	0.181	c(t = 0.36501	0.719	0.938118	Distal Intergenic	ENSSSCG000	NOD2	nucleotide binding oligomerization domain containing 2 [Source:NCBI gene (formerly Entrezgene);Acc:100125838]
chr2	302040	304603	2564	*	chr2	302040	304603	in_vitro	in_vivo	10	13	10.743	8.58153846	2.16146154	c(t = 0.36351	0.72	0.93862595	Distal Intergenic	NA	NA	NA
chr4	41747497	41748473	977	*	chr4	41747497	41748473	in_vitro	in_vivo	9	12	3.08333333	3.73083333	-0.6475	c(t = -0.3641	0.72	0.93862595	Distal Intergenic	ENSSSCG000	CCNE2	cyclin E2 [Source:VGNC Symbol;Acc:VGNC:86355]
chr12	13390546	13392376	1831	*	chr12	13390546	13392376	in_vitro	in_vivo	10	15	1.845	2.634	-0.789	c(t = -0.3633	0.72	0.93862595	Distal Intergenic	ENSSSCG000	CACNG1	calcium voltage-gated channel auxiliary subunit gamma 1 [Source:VGNC Symbol;Acc:VGNC:86124]
chr5	15347034	15347926	893	*	chr5	15347034	15347926	in_vitro	in_vivo	11	13	2.23545455	1.78307692	0.45237762	c(t = 0.36371	0.72	0.93862595	Distal Intergenic	ENSSSCG000	TUBA1A	tubulin alpha 1a [Source:NCBI gene (formerly Entrezgene);Acc:100135051]
chr10	26635265	26638370	3106	*	chr10	26635265	26638370	in_vitro	in_vivo	13	13	1.06769231	0.84	0.22769231	c(t = 0.36211	0.72	0.93862595	Distal Intergenic	ENSSSCG000	ssc-mir-24-2	ssc-mir-24-2 [Source:miRBase;Acc:MI0013111]
chr6	82533691	82534381	691	*	chr6	82533691	82534381	in_vitro	in_vivo	11	9	37.9045455	43.9588889	-6.0543434	c(t = -0.3622	0.721	0.93913305	Distal Intergenic	ENSSSCG000	RSRP1	arginine and serine rich protein 1 [Source:VGNC Symbol;Acc:VGNC:92489]
chr12	38455064	38456542	1479	*	chr12	38455064	38456542	in_vitro	in_vivo	10	13	3.453	2.67153846	0.78146154	c(t = 0.36462	0.721	0.93913305	Distal Intergenic	ENSSSCG000	MRM1	mitochondrial rRNA methyltransferase 1 [Source:NCBI gene (formerly Entrezgene);Acc:100513493]
chr6	164112343	164115439	3097	*	chr6	164112343	164115439	in_vitro	in_vivo	12	15	1.82833333	1.52933333	0.299	c(t = 0.36293	0.721	0.93913305	Distal Intergenic	ENSSSCG000	CYP4A24	cytochrome P450 family 4 subfamily A member 24 [Source:NCBI gene (formerly Entrezgene);Acc:403326]
chr8	84390065	84392097	2033	*	chr8	84390065	84392097	in_vitro	in_vivo	12	14	1.2975	1.06214286	0.23535714	c(t = 0.36173	0.721	0.93913305	Distal Intergenic	ENSSSCG000	ANAPC10	anaphase promoting complex subunit 10 [Source:VGNC Symbol;Acc:VGNC:98912]
chr1	221217783	221219169	1387	*	chr1	221217783	221219169	in_vitro	in_vivo	12	12	0.80166667	1.04666667	-0.245	c(t = -0.3623	0.721	0.93913305	Distal Intergenic	ENSSSCG000	ssc-mir-9823	ssc-mir-9823 [Source:miRBase;Acc:MI0031590]
chr7	1182087	1183688	1602	*	chr7	1182087	1183688	in_vitro	in_vivo	10	13	60.852	66.25	-5.398	c(t = -0.3602	0.722	0.93963929	Distal Intergenic	ENSSSCG000	SERPINB9	serpin peptidase inhibitor, clade B (ovalbumin), member 9 [Source:NCBI gene (formerly Entrezgene);Acc:100126851]
chr16	30169994	30170501	508	*	chr16	30169994	30170501	in_vitro	in_vivo	9	12	12.7466667	10.5775	2.16916667	c(t = 0.36428	0.722	0.93963929	Distal Intergenic	ENSSSCG000	CCL28	C-C motif chemokine ligand 28 [Source:NCBI gene (formerly Entrezgene);Acc:554303]
chrX	9504396	9505628	1233	*	chrX	9504396	9505628	in_vitro	in_vivo	13	10	5.71615385	4.904	0.81215385	c(t = 0.36297	0.722	0.93963929	Promoter (<=1kb)	ENSSSCG000	PRPS2	phosphoribosyl pyrophosphate synthetase 2 [Source:NCBI gene (formerly Entrezgene);Acc:100510993]
chr16	34477954	34478908	955	*	chr16	34477954	34478908	in_vitro	in_vivo	9	9	0.64111111	1.05777778	-0.4166667	c(t = -0.3648	0.722	0.93963929	Distal Intergenic	ENSSSCG000	GZMA	granzyme A (granzyme 1, cytotoxic T-lymphocyte-associated serine esterase 3) [Source:NCBI gene (formerly Entrezgene);Acc:100526762]
chr13	79139249	79140095	847	*	chr13	79139249	79140095	in_vitro	in_vivo	10	10	0.556	0.765	-0.209	c(t = -0.3615	0.722	0.93963929	Distal Intergenic	ENSSSCG000	FAIM	Fas apoptotic inhibitory molecule [Source:VGNC Symbol;Acc:VGNC:87882]
chr1	72699835	72701157	1323	*	chr1	72699835	72701157	in_vitro	in_vivo	13	12	17.3284615	15.2133333	2.11512821	c(t = 0.35849	0.723	0.94014467	Distal Intergenic	ENSSSCG000	ATG5	autophagy related 5 [Source:VGNC Symbol;Acc:VGNC:103029]
chr2	134783026	134783799	774	*	chr2	134783026	134783799	in_vitro	in_vivo	9	12	4.27666667	5.74166667	-1.465	c(t = -0.3601	0.723	0.94014467	Promoter (<=1kb)	ENSSSCG000	IRF1	interferon regulatory factor 1 [Source:VGNC Symbol;Acc:VGNC:89203]
chr10	65704464	65705713	1250	*	chr10	65704464	65705713	in_vitro	in_vivo	12	10	1.32833333	1.75	-0.4216667	c(t = -0.3590	0.723	0.94014467	Distal Intergenic	ENSSSCG000	AKR1E2	aldo-keto reductase family 1 member E2 [Source:VGNC Symbol;Acc:VGNC:96003]
chr2	61138907	61139628	722	*	chr2	61138907	61139628	in_vitro	in_vivo	9	11	1.11	1.61272727	-0.5027273	c(t = -0.3631	0.723	0.94014467	Distal Intergenic	ENSSSCG000	KLF2	KLF transcription factor 2 [Source:NCBI gene (formerly Entrezgene);Acc:100174959]
chr1	265705595	265706138	544	*	chr1	265705595	265706138	in_vitro	in_vivo	11	11	0.39909091	0.26	0.13909091	c(t = 0.35989	0.723	0.94014467	Distal Intergenic	ENSSSCG000	ARPC5L	actin related protein 2/3 complex subunit 5 like [Source:NCBI gene (formerly Entrezgene);Acc:100154462]
chr13	206625803	206629648	3846	*	chr13	206625803	206629648	in_vitro	in_vivo	14	13	20.8007143	24.4830769	-3.6823626	c(t = -0.3567	0.724	0.9404902	Distal Intergenic	ENSSSCG000	PDXK	pyridoxal kinase [Source:NCBI gene (formerly Entrezgene);Acc:396983]
chr7	75734903	75735631	729	*	chr7	75734903	75735631	in_vitro	in_vivo	10	13	6.471	9.27	-2.799	c(t = -0.3584	0.724	0.9404902	Distal Intergenic	ENSSSCG000	PABPN1	poly(A) binding protein nuclear 1 [Source:NCBI gene (formerly Entrezgene);Acc:100155260]
chr15	44398214	44398954	741	*	chr15	44398214	44398954	in_vitro	in_vivo	9	13	12.7311111	8.05384615	4.67726496	c(t = 0.36052	0.724	0.9404902	Distal Intergenic	ENSSSCG000	CLDN22	claudin 22 [Source:NCBI gene (formerly Entrezgene);Acc:100294683]
chr4	640101	642338	2238	*	chr4	640101	642338	in_vitro	in_vivo	9	15	6.76555556	5.50933333	1.25622222	c(t = 0.35778	0.724	0.9404902	Distal Intergenic	ENSSSCG000	HSF1	heat shock transcription factor 1 [Source:VGNC Symbol;Acc:VGNC:88993]
chr1	8105882	8107639	1758	*	chr1	8105882	8107639	in_vitro	in_vivo	9	13	4.28	3.33923077	0.94076923	c(t = 0.36252	0.724	0.9404902	Distal Intergenic	ENSSSCG000	TAGAP	T cell activation RhoGTPase activating protein [Source:NCBI gene (formerly Entrezgene);Acc:100155114]
chr4	77399198	77400174	977	*	chr4	77399198	77400174	in_vitro	in_vivo	11	13	2.48818182	3.21615385	-0.727972	c(t = -0.3591	0.724	0.9404902	Distal Intergenic	ENSSSCG000	ATP6V1H	ATPase H+ transporting V1 subunit H [Source:VGNC Symbol;Acc:VGNC:85677]
chr7	25046231	25046545	315	*	chr7	25046231	25046545	in_vitro	in_vivo	9	10	3.38555556	4.859	-1.4734444	c(t = -0.3587	0.725	0.9405445	Exon (NM_001206441/733650, exon 11 of 12)	ENSSSCG000	HLA-DOB	MHC class II, DO beta [Source:NCBI gene (formerly Entrezgene);Acc:100135049]
chr2	49893147	49895032	1886	*	chr2	49893147	49895032	in_vitro	in_vivo	13	12	2.34076923	2.81083333	-0.4700641	c(t = -0.3590	0.725	0.9405445	Distal Intergenic	ENSSSCG000	ADM	adrenomedullin [Source:NCBI gene (formerly Entrezgene);Acc:397195]
chr1	18512149	18513201	1053	*	chr1	18512149	18513201	in_vitro	in_vivo	11	12	2.08090909	1.60666667	0.47424242	c(t = 0.35775	0.725	0.9405445	Distal Intergenic	ENSSSCG000	RAB32	RAB32, member RAS oncogene family [Source:NCBI gene (formerly Entrezgene);Acc:100144496]
chr8	41855519	41856728	1210	*	chr8	41855519	41856728	in_vitro	in_vivo	11	12	1.02818182	1.32083333	-0.2926515	c(t = -0.3565	0.725	0.9405445	Distal Intergenic	ENSSSCG000	KIT	KIT proto-oncogene, receptor tyrosine kinase [Source:NCBI gene (formerly Entrezgene);Acc:396810]
chr4	76846693	76847844	1152	*	chr4	76846693	76847844	in_vitro	in_vivo	9	11	1.29333333	0.96727273	0.32606061	c(t = 0.35768	0.725	0.9405445	Distal Intergenic	ENSSSCG000	MRPL15	mitochondrial ribosomal protein L15 [Source:VGNC Symbol;Acc:VGNC:98820]
chr12	26154626	26156039	1414	*	chr12	26154626	26156039	in_vitro	in_vivo	9	11	1.04222222	0.69272727	0.34949495	c(t = 0.35980	0.725	0.9405445	Distal Intergenic	ENSSSCG000	TAC4	tachykinin 4 [Source:VGNC Symbol;Acc:VGNC:96765]
chr3	10227582	10228278	697	*	chr3	10227582	10228278	in_vitro	in_vivo	9	10	95.8877778	96.798	-0.9102222	c(t = -0.3577	0.726	0.9405445	Intron (NM_001129959/100170114, intron 2 of 15)	ENSSSCG000	POR	cytochrome p450 oxidoreductase [Source:VGNC Symbol;Acc:VGNC:109477]
chr17	53426806	53427748	943	*	chr17	53426806	53427748	in_vitro	in_vivo	11	12	65.4809091	71.58	-6.0990909	c(t = -0.3558	0.726	0.9405445	Exon (NM_001113449/100155190, exon 6 of 6)	ENSSSCG00000007481		ZFP64 zinc finger protein [Source:VGNC Symbol;Acc:VGNC:95720]
chrX	122663206	122663526	321	*	chrX	122663206	122663526	in_vitro	in_vivo	9	9	14.5222222	18.5811111	-4.0588889	c(t = -0.3581	0.726	0.9405445	Distal Intergenic	ENSSSCG000	FATE1	fetal and adult testis expressed 1 [Source:NCBI gene (formerly Entrezgene);Acc:396705]
chr6	27793774	27795459	1686	*	chr6	27793774	27795459	in_vitro	in_vivo	11	11	8.11545455	6.37909091	1.73636364	c(t = 0.35766	0.726	0.9405445	Distal Intergenic	ENSSSCG000	ssc-mir-328	ssc-mir-328 [Source:miRBase;Acc:MI0013158]
chr6	83806362	83806932	571	*	chr6	83806362	83806932	in_vitro	in_vivo	11	9	3.69454545	4.75222222	-1.0576768	c(t = -0.3567	0.726	0.9405445	Promoter (<=1kb)	ENSSSCG000	LIN28A	lin-28 homolog A [Source:NCBI gene (formerly Entrezgene);Acc:100142662]
chr2	69224020	69225918	1899	*	chr2	69224020	69225918	in_vitro	in_vivo	11	14	3.48636364	4.06928571	-0.5829221	c(t = -0.3576	0.726	0.9405445	Distal Intergenic	ENSSSCG000	CDC37	cell division cycle 37, HSP90 cochaperone [Source:VGNC Symbol;Acc:VGNC:86450]
chr8	2439347	2445488	6142	*	chr8	2439347	2445488	in_vitro	in_vivo	10	12	2.203	2.62083333	-0.4178333	c(t = -0.3554	0.726	0.9405445	Distal Intergenic	ENSSSCG000	LRPAP1	LDL receptor related protein associated protein 1 [Source:NCBI gene (formerly Entrezgene);Acc:399654]
chr6	12056546	12057839	1294	*	chr6	12056546	12057839	in_vitro	in_vivo	13	13	1.43692308	1.79538462	-0.3584615	c(t = -0.3555	0.726	0.9405445	Distal Intergenic	ENSSSCG000	COG4	component of oligomeric golgi complex 4 [Source:NCBI gene (formerly Entrezgene);Acc:100625346]
chr9	135078513	135079304	792	*	chr9	135078513	135079304	in_vitro	in_vivo	10	11	0.5	0.76727273	-0.2672727	c(t = -0.3555	0.726	0.9405445	Promoter (<=1kb)	ENSSSCG00000038506		CD46 molecule, complement regulatory protein [Source:NCBI gene (formerly Entrezgene);Acc:396922]
chr6	46303777	46304288	512	*	chr6	46303777	46304288	in_vitro	in_vivo	11	12	1.08636364	0.7375	0.34886364	c(t = 0.35610	0.726	0.9405445	Distal Intergenic	ENSSSCG000	COX7A1	cytochrome c oxidase subunit 7A1 [Source:NCBI gene (formerly Entrezgene);Acc:399685]
chrX	98104023	98106270	2248	*	chrX	98104023	98106270	in_vitro	in_vivo	11	14	10.1172727	8.35857143	1.7587013	c(t = 0.35391	0.727	0.94072992	Distal Intergenic	ENSSSCG000	NKAP	NFKB activating protein [Source:VGNC Symbol;Acc:VGNC:90756]
chr7	69527301	69528450	1150	*	chr7	69527301	69528450	in_vitro	in_vivo	11	11	4.24	3.10272727	1.13727273	c(t = 0.35428	0.727	0.94072992	Distal Intergenic	ENSSSCG000	AP4S1	adaptor related protein complex 4 subunit sigma 1 [Source:VGNC Symbol;Acc:VGNC:85394]
chr5	9526388	9527488	1101	*	chr5	9526388	9527488	in_vitro	in_vivo	9	11	2.35333333	1.89	0.46333333	c(t = 0.35558	0.727	0.94072992	Distal Intergenic	ENSSSCG000	KDELR3	KDEL endoplasmic reticulum protein retention receptor 3 [Source:NCBI gene (formerly Entrezgene);Acc:100514523]
chr14	47799140	47800198	1059	*	chr14	47799140	47800198	in_vitro	in_vivo	9	9	0.86333333	1.20222222	-0.3388889	c(t = -0.3557	0.727	0.94072992	Distal Intergenic	ENSSSCG000	SMTN	smoothelin [Source:NCBI gene (formerly Entrezgene);Acc:414369]
chr4	40403207	40403765	559	*	chr4	40403207	40403765	in_vitro	in_vivo	9	9	0.82222222	1.19111111	-0.3688889	c(t = -0.3560	0.727	0.94072992	Promoter (<=1kb)	ENSSSCG000	UQCRB	ubiquinol-cytochrome c reductase binding protein [Source:NCBI gene (formerly Entrezgene);Acc:100157621]
chr17	53071517	53072567	1051	*	chr17	53071517	53072567	in_vitro	in_vivo	12	13	0.7875	1.06615385	-0.2786538	c(t = -0.3541	0.727	0.94072992	Promoter (<=1kb)	ENSSSCG000	ATP9A	ATPase phospholipid transporting 9A (putative) [Source:NCBI gene (formerly Entrezgene);Acc:100136901]
chr1	98568735	98569606	872	*	chr1	98568735	98569606	in_vitro	in_vivo	9	11	1.11	0.80272727	0.30727273	c(t = 0.35616	0.727	0.94072992	Distal Intergenic	ENSSSCG000	SMAD7	SMAD family member 7 [Source:VGNC Symbol;Acc:VGNC:93221]
chr14	26477645	26478128	484	*	chr14	26477645	26478128	in_vitro	in_vivo	9	9	10.1911111	13.5388889	-3.3477778	c(t = -0.3543	0.728	0.9409149	Distal Intergenic	ENSSSCG000	SLC15A4	solute carrier family 15 member 4 [Source:VGNC Symbol;Acc:VGNC:92937]
chr10	52445273	52447376	2104	*	chr10	52445273	52447376	in_vitro	in_vivo	11	12	6.79272727	9.6075	-2.8147727	c(t = -0.3531	0.728	0.9409149	Distal Intergenic	ENSSSCG000	SPAG6	sperm associated antigen 6 [Source:NCBI gene (formerly Entrezgene);Acc:100286811]
chr6	53520750	53521796	1047	*	chr6	53520750	53521796	in_vitro	in_vivo	10	10	4.906	6.309	-1.403	c(t = -0.3543	0.728	0.9409149	Distal Intergenic	ENSSSCG000	SULT2A1	sulfotransferase family 2A member 1 [Source:VGNC Symbol;Acc:VGNC:93613]
chr16	51085996	51086754	759	*	chr16	51085996	51086754	in_vitro	in_vivo	9	10	1.80777778	2.38	-0.5722222	c(t = -0.3546	0.728	0.9409149	Distal Intergenic	ENSSSCG000	STC2	stanniocalcin 2 [Source:NCBI gene (formerly Entrezgene);Acc:100126236]
chr4	93412610	93413575	966	*	chr4	93412610	93413575	in_vitro	in_vivo	12	10	2.59833333	1.962	0.63633333	c(t = 0.35226	0.728	0.9409149	Distal Intergenic	ENSSSCG000	CRABP2	cellular retinoic acid binding protein 2 [Source:NCBI gene (formerly Entrezgene);Acc:100155151]
chr15	71246985	71247783	799	*	chr15	71246985	71247783	in_vitro	in_vivo	9	12	1.49888889	1.90666667	-0.4077778	c(t = -0.3524	0.728	0.9409149	Distal Intergenic	ENSSSCG000	IFIH1	interferon induced with helicase C domain 1 [Source:VGNC Symbol;Acc:VGNC:98042]
chr1	197308857	197309902	1046	*	chr1	197308857	197309902	in_vitro	in_vivo	11	15	2.14454545	1.67733333	0.46721212	c(t = 0.35349	0.728	0.9409149	Distal Intergenic	ENSSSCG000	ssc-mir-9832	ssc-mir-9832 [Source:miRBase;Acc:MI0031601]
chr13	28318321	28319419	1099	*	chr13	28318321	28319419	in_vitro	in_vivo	13	9	5.11230769	4.38777778	0.72452991	c(t = 0.35146	0.729	0.94154398	Distal Intergenic	ENSSSCG000	EXOSC7	exosome component 7 [Source:NCBI gene (formerly Entrezgene);Acc:100511347]
chr7	67593481	67594700	1220	*	chr7	67593481	67594700	in_vitro	in_vivo	10	13	2.179	2.62	-0.441	c(t = -0.3519	0.729	0.94154398	Distal Intergenic	ENSSSCG000	GPR33	G protein-coupled receptor 33 [Source:NCBI gene (formerly Entrezgene);Acc:100157823]
chr9	50334890	50335881	992	*	chr9	50334890	50335881	in_vitro	in_vivo	10	10	1.539	1.16	0.379	c(t = 0.35248	0.729	0.94154398	Distal Intergenic	ENSSSCG000	HSPA8	heat shock protein family A (Hsp70) member 8 [Source:NCBI gene (formerly Entrezgene);Acc:100511890]
chr15	139019330	139028805	9476	*	chr15	139019330	139028805	in_vitro	in_vivo	12	13	92.435	91.6584615	0.77653846	c(t = 0.34904	0.73	0.94154398	Distal Intergenic	ENSSSCG000	ssc-mir-149	ssc-mir-149 [Source:miRBase;Acc:MI0016620]
chr2	76416948	76418289	1342	*	chr2	76416948	76418289	in_vitro	in_vivo	11	12	34.6290909	30.7291667	3.89992424	c(t = 0.34969	0.73	0.94154398	Promoter (1-2kb)	ENSSSCG000	AMH	anti-Mullerian hormone [Source:VGNC Symbol;Acc:VGNC:110572]
chr5	987893	989083	1191	*	chr5	987893	989083	in_vitro	in_vivo	9	11	14	18.3436364	-4.3436364	c(t = -0.3505	0.73	0.94154398	Distal Intergenic	ENSSSCG000	SELENOO	selenoprotein O [Source:NCBI gene (formerly Entrezgene);Acc:100518971]
chr2	81464735	81465377	643	*	chr2	81464735	81465377	in_vitro	in_vivo	10	11	5.154	6.55181818	-1.3978182	c(t = -0.3499	0.73	0.94154398	Distal Intergenic	ENSSSCG000	HIGD2A	HIG1 hypoxia inducible domain family member 2A [Source:NCBI gene (formerly Entrezgene);Acc:100037984]
chr12	1443238	1448907	5670	*	chr12	1443238	1448907	in_vitro	in_vivo	10	14	2.68	3.18428571	-0.5042857	c(t = -0.3491	0.73	0.94154398	Distal Intergenic	ENSSSCG000	TEPSIN	TEPSIN adaptor related protein complex 4 accessory protein [Source:NCBI gene (formerly Entrezgene);Acc:100626379]
chr7	121416404	121418153	1750	*	chr7	121416404	121418153	in_vitro	in_vivo	11	15	2.14363636	2.61	-0.4663636	c(t = -0.3501	0.73	0.94154398	Distal Intergenic	ENSSSCG000	ssc-mir-9830	ssc-mir-9830 [Source:miRBase;Acc:MI0031599]
chr3	95524407	95525169	763	*	chr3	95524407	95525169	in_vitro	in_vivo	10	10	1.971	2.484	-0.513	c(t = -0.3512	0.73	0.94154398	Distal Intergenic	ENSSSCG000	SLC3A1	solute carrier family 3 member 1 [Source:NCBI gene (formerly Entrezgene);Acc:100144588]
chr3	55171007	55171496	490	*	chr3	55171007	55171496	in_vitro	in_vivo	9	10	3.14888889	2.412	0.73688889	c(t = 0.35394	0.73	0.94154398	Distal Intergenic	ENSSSCG000	MGAT4A	alpha-1,3-mannosyl-glycoprotein 4-beta-N-acetylglucosaminyltransferase A [Source:VGNC Symbol;Acc:VGNC:108158]
chr15	80253320	80254158	839	*	chr15	80253320	80254158	in_vitro	in_vivo	11	13	1.57818182	2.08692308	-0.5087413	c(t = -0.3494	0.731	0.94154398	Distal Intergenic	ENSSSCG000	CIR1	corepressor interacting with RBPJ, CIR1 [Source:NCBI gene (formerly Entrezgene);Acc:100511415]
chr8	26943355	26943601	247	*	chr8	26943355	26943601	in_vitro	in_vivo	10	12	1.556	2.0775	-0.5215	c(t = -0.3482	0.731	0.94154398	Distal Intergenic	ENSSSCG000	ssc-mir-9840	ssc-mir-9840 [Source:miRBase;Acc:MI0031609]
chr6	18334808	18335569	762	*	chr6	18334808	18335569	in_vitro	in_vivo	11	10	1.64181818	1.29	0.35181818	c(t = 0.34835	0.731	0.94154398	Distal Intergenic	ENSSSCG000	CDH1	cadherin 1 [Source:NCBI gene (formerly Entrezgene);Acc:100048953]
chr9	125612334	125613955	1622	*	chr9	125612334	125613955	in_vitro	in_vivo	11	11	1.35636364	1.07090909	0.28545455	c(t = 0.34868	0.731	0.94154398	Distal Intergenic	ENSSSCG000	NIBAN1	niban apoptosis regulator 1 [Source:NCBI gene (formerly Entrezgene);Acc:100516994]
chr7	23199567	23200503	937	*	chr7	23199567	23200503	in_vitro	in_vivo	10	11	96.817	96.1436364	0.67336364	c(t = 0.34819	0.732	0.94154398	Exon (NM_001123106/100144456, exon 5 of 20)	ENSSSCG000	DHX16	DEAH-box helicase 16 [Source:VGNC Symbol;Acc:VGNC:87287]
chr13	208309823	208313242	3420	*	chr13	208309823	208313242	in_vitro	in_vivo	9	13	27.9388889	32.3515385	-4.4126496	c(t = -0.3485	0.732	0.94154398	Distal Intergenic	ENSSSCG000	ITGB2	integrin subunit beta 2 [Source:NCBI gene (formerly Entrezgene);Acc:396943]
chr3	115175707	115178114	2408	*	chr3	115175707	115178114	in_vitro	in_vivo	14	14	6.35357143	5.45071429	0.90285714	c(t = 0.34692	0.732	0.94154398	Distal Intergenic	ENSSSCG000	NCOA1	nuclear receptor coactivator 1 [Source:VGNC Symbol;Acc:VGNC:90615]
chrX	14885213	14886585	1373	*	chrX	14885213	14886585	in_vitro	in_vivo	12	11	2.77083333	3.60454545	-0.8337121	c(t = -0.3480	0.732	0.94154398	Distal Intergenic	ENSSSCG000	EIF1AX	eukaryotic translation initiation factor 1A X-linked [Source:NCBI gene (formerly Entrezgene);Acc:100522912]
chr9	20488379	20489238	860	*	chr9	20488379	20489238	in_vitro	in_vivo	9	11	1.94888889	2.46545455	-0.5165657	c(t = -0.3479	0.732	0.94154398	Promoter (<=1kb)	ENSSSCG000	ME3	malic enzyme 3 [Source:VGNC Symbol;Acc:VGNC:90097]
chr16	77705122	77706352	1231	*	chr16	77705122	77706352	in_vitro	in_vivo	9	11	3.21666667	2.38818182	0.82848485	c(t = 0.34847	0.732	0.94154398	Distal Intergenic	ENSSSCG000	TERT	telomerase reverse transcriptase [Source:NCBI gene (formerly Entrezgene);Acc:492280]
chr7	62470714	62471948	1235	*	chr7	62470714	62471948	in_vitro	in_vivo	11	14	2.77818182	2.14571429	0.63246753	c(t = 0.34682	0.732	0.94154398	Distal Intergenic	ENSSSCG000	SSTR1	somatostatin receptor 1 [Source:VGNC Symbol;Acc:VGNC:93496]
chr14	77766940	77767823	884	*	chr14	77766940	77767823	in_vitro	in_vivo	10	12	2.887	2.14416667	0.74283333	c(t = 0.34667	0.732	0.94154398	Promoter (<=1kb)	ENSSSCG000	VDAC2	voltage dependent anion channel 2 [Source:VGNC Symbol;Acc:VGNC:111154]
chr2	16254127	16254874	748	*	chr2	16254127	16254874	in_vitro	in_vivo	9	11	2.28111111	1.79	0.49111111	c(t = 0.34821	0.732	0.94154398	Distal Intergenic	ENSSSCG000	MDK	midkine [Source:VGNC Symbol;Acc:VGNC:100167]
chr6	63868765	63871291	2527	*	chr6	63868765	63871291	in_vitro	in_vivo	11	10	90.5754545	92.298	-1.7225455	c(t = -0.3463	0.733	0.94154398	Distal Intergenic	ENSSSCG000	PRKCZ	protein kinase C zeta [Source:NCBI gene (formerly Entrezgene);Acc:100533200]
chr13	207803099	207816077	12979	*	chr13	207803099	207816077	in_vitro	in_vivo	9	16	74.5788889	76.8725	-2.2936111	c(t = -0.3459	0.733	0.94154398	Distal Intergenic	ENSSSCG000	ITGB2	integrin subunit beta 2 [Source:NCBI gene (formerly Entrezgene);Acc:396943]
chr8	6127401	6128503	1103	*	chr8	6127401	6128503	in_vitro	in_vivo	10	11	12.071	10.39	1.681	c(t = 0.34605	0.733	0.94154398	Distal Intergenic	ENSSSCG000	MSX1	msh homeobox 1 [Source:NCBI gene (formerly Entrezgene);Acc:100302364]
chr13	70416666	70418059	1394	*	chr13	70416666	70418059	in_vitro	in_vivo	14	12	2.60571429	2.16416667	0.44154762	c(t = 0.34472	0.733	0.94154398	Promoter (<=1kb)	ENSSSCG000	CHCHD4	coiled-coil-helix-coiled-coil-helix domain containing 4 [Source:NCBI gene (formerly Entrezgene);Acc:100154663]
chr6	118085515	118087978	2464	*	chr6	118085515	118087978	in_vitro	in_vivo	12	11	1.37833333	1.68272727	-0.3043939	c(t = -0.3456	0.733	0.94154398	Distal Intergenic	ENSSSCG000	ZNF24	zinc finger protein 24 [Source:VGNC Symbol;Acc:VGNC:98422]
chr17	14475784	14476416	633	*	chr17	14475784	14476416	in_vitro	in_vivo	13	14	1.50692308	1.20714286	0.29978022	c(t = 0.34465	0.733	0.94154398	Distal Intergenic	ENSSSCG000	CHGB	chromogranin B [Source:NCBI gene (formerly Entrezgene);Acc:397154]
chr4	130644796	130645857	1062	*	chr4	130644796	130645857	in_vitro	in_vivo	11	10	1.26272727	0.971	0.29172727	c(t = 0.34640	0.733	0.94154398	Promoter (<=1kb)	ENSSSCG000	BCL10	BCL10 immune signaling adaptor [Source:NCBI gene (formerly Entrezgene);Acc:100125346]
chr1	71104373	71105157	785	*	chr1	71104373	71105157	in_vitro	in_vivo	11	12	1.22363636	0.95833333	0.26530303	c(t = 0.34621	0.733	0.94154398	Promoter (<=1kb)	ENSSSCG000	HACE1	HECT domain and ankyrin repeat containing E3 ubiquitin protein ligase 1 [Source:VGNC Symbol;Acc:VGNC:88769]
chr1	168751572	168752101	530	*	chr1	168751572	168752101	in_vitro	in_vivo	11	11	1.11454545	0.84545455	0.26909091	c(t = 0.34657	0.733	0.94154398	Promoter (<=1kb)	ENSSSCG00000026486		
chr6	164281905	164283637	1733	*	chr6	164281905	164283637	in_vitro	in_vivo	9	9	0.58333333	0.82	-0.2366667	c(t = -0.3480	0.733	0.94154398	Distal Intergenic	ENSSSCG000	CYP4A24	cytochrome P450 family 4 subfamily A member 24 [Source:NCBI gene (formerly Entrezgene);Acc:403326]
chr9	77851588	77852297	710	*	chr9	77851588	77852297	in_vitro	in_vivo	9	10	0.71888889	0.538	0.18088889	c(t = 0.34679	0.733	0.94154398	Promoter (<=1kb)	ENSSSCG000	MIOS	meiosis regulator for oocyte development [Source:VGNC Symbol;Acc:VGNC:90234]
chr8	466162	467527	1366	*	chr8	466162	467527	in_vitro	in_vivo	9	11	13.3833333	16.2981818	-2.9148485	c(t = -0.3456	0.734	0.94154398	Distal Intergenic	ENSSSCG000	SLBP	stem-loop binding protein [Source:NCBI gene (formerly Entrezgene);Acc:100513605]
chr2	88679798	88680393	596	*	chr2	88679798	88680393	in_vitro	in_vivo	10	11	10.846	8.51363636	2.33236364	c(t = 0.34727	0.734	0.94154398	Distal Intergenic	ENSSSCG000	CMYA5	cardiomyopathy associated 5 [Source:VGNC Symbol;Acc:VGNC:86809]
chrX	102640285	102641229	945	*	chrX	102640285	102641229	in_vitro	in_vivo	12	12	6.6425	8.50666667	-1.8641667	c(t = -0.3454	0.734	0.94154398	Distal Intergenic	ENSSSCG000	ssc-mir-2483	ssc-mir-2483 [Source:miRBase;Acc:MI0022167]
chr2	137623221	137624119	899	*	chr2	137623221	137624119	in_vitro	in_vivo	9	12	3.00222222	2.48416667	0.51805556	c(t = 0.34531	0.734	0.94154398	Promoter (<=1kb)	ENSSSCG000	MACROH2A1	macroH2A.1 histone [Source:VGNC Symbol;Acc:VGNC:109808]
chr12	60162022	60163293	1272	*	chr12	60162022	60163293	in_vitro	in_vivo	10	9	1.941	2.43222222	-0.4912222	c(t = -0.3461	0.734	0.94154398	Distal Intergenic	ENSSSCG000	SLC5A10	solute carrier family 5 member 10 [Source:NCBI gene (formerly Entrezgene);Acc:497235]
chr7	5606148	5606871	724	*	chr7	5606148	5606871	in_vitro	in_vivo	12	13	1.6225	2.15307692	-0.5305769	c(t = -0.3454	0.734	0.94154398	Distal Intergenic	ENSSSCG000	EEF1E1	eukaryotic translation elongation factor 1 epsilon 1 [Source:VGNC Symbol;Acc:VGNC:109188]
chr6	161156918	161157899	982	*	chr6	161156918	161157899	in_vitro	in_vivo	9	11	1.11333333	1.59272727	-0.4793939	c(t = -0.3446	0.734	0.94154398	Distal Intergenic	ENSSSCG000	COA7	cytochrome c oxidase assembly factor 7 [Source:NCBI gene (formerly Entrezgene);Acc:100627075]
chr8	53210340	53211231	892	*	chr8	53210340	53211231	in_vitro	in_vivo	10	13	1.347	1.13615385	0.21084615	c(t = 0.34488	0.734	0.94154398	Distal Intergenic	ENSSSCG000	NPY1R	neuropeptide Y receptor Y1 [Source:VGNC Symbol;Acc:VGNC:90865]
chr17	55155671	55156354	684	*	chr17	55155671	55156354	in_vitro	in_vivo	9	10	1.53444444	1.075	0.45944444	c(t = 0.34961	0.734	0.94154398	Promoter (<=1kb)	ENSSSCG000	PFDN4	prefoldin subunit 4 [Source:NCBI gene (formerly Entrezgene);Acc:100144532]
chr6	149160941	149161931	991	*	chr6	149160941	149161931	in_vitro	in_vivo	12	13	1.25	0.88846154	0.36153846	c(t = 0.34491	0.734	0.94154398	Distal Intergenic	ENSSSCG000	EFCAB7	phosphoglucomutase 1 [Source:VGNC Symbol;Acc:VGNC:91355]
chr16	75228389	75232390	4002	*	chr16	75228389	75232390	in_vitro	in_vivo	10	11	92.693	91.1063636	1.58663636	c(t = 0.34395	0.735	0.94188344	Distal Intergenic	ENSSSCG000	CCT5	chaperonin containing TCP1 subunit 5 [Source:VGNC Symbol;Acc:VGNC:86383]
chr13	31990619	31991906	1288	*	chr13	31990619	31991906	in_vitro	in_vivo	9	13	4.33555556	5.18538462	-0.8498291	c(t = -0.3427	0.735	0.94188344	Promoter (<=1kb)	ENSSSCG000	RHOA	ras homolog family member A [Source:VGNC Symbol;Acc:VGNC:92284]
chr13	75605313	75606138	826	*	chr13	75605313	75606138	in_vitro	in_vivo	11	9	1.95090909	2.60777778	-0.6568687	c(t = -0.3447	0.735	0.94188344	Promoter (<=1kb)	ENSSSCG000	ANAPC13	anaphase promoting complex subunit 13 [Source:VGNC Symbol;Acc:VGNC:85294]
chr2	15244782	15245341	560	*	chr2	15244782	15245341	in_vitro	in_vivo	11	9	2.42454545	1.67333333	0.75121212	c(t = 0.34436	0.735	0.94188344	3' UTR	ENSSSCG000	SPI1	Spi-1 proto-onco [Source:VGNC Symbol;Acc:VGNC:98335]
chr17	57160293	57167005	6713	*	chr17	57160293	57167005	in_vitro	in_vivo	13	16	1.78538462	1.565625	0.21975962	c(t = 0.34259	0.735	0.94188344	Promoter (<=1kb)	ENSSSCG000	TFAP2C	transcription factor AP-2 gamma [Source:NCBI gene (formerly Entrezgene);Acc:100144525]
chr14	36063132	36064853	1722	*	chr14	36063132	36064853	in_vitro	in_vivo	12	12	1.62583333	1.3275	0.29833333	c(t = 0.34228	0.735	0.94188344	Distal Intergenic	ENSSSCG000	FBXO21	F-box protein 21 [Source:VGNC Symbol;Acc:VGNC:88035]
chr15	138401203	138413278	12076	*	chr15	138401203	138413278	in_vitro	in_vivo	9	11	33.2811111	39.38	-6.0988889	c(t = -0.3433	0.736	0.94206529	Distal Intergenic	ENSSSCG000	RAMP1	receptor activity modifying protein 1 [Source:NCBI gene (formerly Entrezgene);Acc:397401]
chr7	60956661	60957210	550	*	chr7	60956661	60957210	in_vitro	in_vivo	9	10	16.3066667	20.735	-4.4283333	c(t = -0.3432	0.736	0.94206529	Distal Intergenic	ENSSSCG000	HEXA	hexosaminidase subunit alpha [Source:VGNC Symbol;Acc:VGNC:111459]
chr4	126922280	126923086	807	*	chr4	126922280	126923086	in_vitro	in_vivo	9	9	11.9722222	8.47	3.50222222	c(t = 0.34612	0.736	0.94206529	Distal Intergenic	ENSSSCG000	GBP2	guanylate binding protein 2, interferon-inducible [Source:NCBI gene (formerly Entrezgene);Acc:100153137]
chr12	15224576	15225924	1349	*	chr12	15224576	15225924	in_vitro	in_vivo	14	14	3.185	2.78285714	0.40214286	c(t = 0.34024	0.736	0.94206529	Promoter (<=1kb)	ENSSSCG000	LIMD2	LIM domain containing 2 [Source:VGNC Symbol;Acc:VGNC:89726]
chr7	70660561	70661776	1216	*	chr7	70660561	70661776	in_vitro	in_vivo	9	14	1.74333333	1.29571429	0.44761905	c(t = 0.34429	0.736	0.94206529	Distal Intergenic	ENSSSCG000	AP4S1	adaptor related protein complex 4 subunit sigma 1 [Source:VGNC Symbol;Acc:VGNC:85394]
chr8	32969750	32971245	1496	*	chr8	32969750	32971245	in_vitro	in_vivo	14	11	1.51357143	1.13818182	0.37538961	c(t = 0.34081	0.736	0.94206529	Distal Intergenic	ENSSSCG000	TMEM33	transmembrane protein 33 [Source:VGNC Symbol;Acc:VGNC:94171]
chr13	40167266	40167698	433	*	chr13	40167266	40167698	in_vitro	in_vivo	10	10	1.576	1.034	0.542	c(t = 0.34227	0.736	0.94206529	Distal Intergenic	ENSSSCG000	PDHB	pyruvate dehydrogenase E1 subunit beta [Source:NCBI gene (formerly Entrezgene);Acc:100516042]
chr7	63488461	63490788	2328	*	chr7	63488461	63490788	in_vitro	in_vivo	12	12	12.7008333	10.65	2.05083333	c(t = 0.34118	0.737	0.94271721	Promoter (<=1kb)	ENSSSCG000	NKX2-1	NK2 homeobox 1 [Source:VGNC Symbol;Acc:VGNC:90767]
chr6	49063425	49063723	299	*	chr6	49063425	49063723	in_vitro	in_vivo	9	10	3.34333333	4.149	-0.8056667	c(t = -0.3424	0.737	0.94271721	Promoter (<=1kb)	ENSSSCG000	CYP2A19	cytochrome P450 family 2 subfamily A member 19 [Source:NCBI gene (formerly Entrezgene);Acc:403149]
chr2	135068794	135069486	693	*	chr2	135068794	135069486	in_vitro	in_vivo	10	11	0.238	0.39545455	-0.1574545	c(t = -0.3411	0.737	0.94271721	Distal Intergenic	ENSSSCG000	IL4	interleukin 4 [Source:NCBI gene (formerly Entrezgene);Acc:397225]
chr6	28428091	28428458	368	*	chr6	28428091	28428458	in_vitro	in_vivo	9	10	0.55555556	0.333	0.22255556	c(t = 0.34360	0.737	0.94271721	Distal Intergenic	ENSSSCG000	THAP11	THAP domain containing 11 [Source:NCBI gene (formerly Entrezgene);Acc:100520959]
chr17	58875136	58877690	2555	*	chr17	58875136	58877690	in_vitro	in_vivo	12	12	27.7508333	31.6883333	-3.9375	c(t = -0.3384	0.738	0.94305454	Promoter (<=1kb)	ENSSSCG000	NPEPL1	aminopeptidase like 1 [Source:VGNC Symbol;Acc:VGNC:95798]
chr5	9318455	9319208	754	*	chr5	9318455	9319208	in_vitro	in_vivo	10	12	15.072	12.7541667	2.31783333	c(t = 0.33922	0.738	0.94305454	Distal Intergenic	ENSSSCG00000043051		
chr15	53686823	53688571	1749	*	chr15	53686823	53688571	in_vitro	in_vivo	11	15	1.76545455	2.106	-0.3405455	c(t = -0.3389	0.738	0.94305454	Distal Intergenic	ENSSSCG000	UBXN8	UBX domain protein 8 [Source:VGNC Symbol;Acc:VGNC:96239]
chr10	52580626	52583166	2541	*	chr10	52580626	52583166	in_vitro	in_vivo	14	13	1.69214286	2.06153846	-0.3693956	c(t = -0.3396	0.738	0.94305454	Distal Intergenic	ENSSSCG000	SPAG6	sperm associated antigen 6 [Source:NCBI gene (formerly Entrezgene);Acc:100286811]
chr1	53875374	53877030	1657	*	chr1	53875374	53877030	in_vitro	in_vivo	11	13	2.07545455	1.69307692	0.38237762	c(t = 0.33967	0.738	0.94305454	Distal Intergenic	ENSSSCG000	OOEP	oocyte expressed protein [Source:NCBI gene (formerly Entrezgene);Acc:100152355]
chr14	20420267	20421437	1171	*	chr14	20420267	20421437	in_vitro	in_vivo	10	13	1.445	1.18692308	0.25807692	c(t = 0.33951	0.738	0.94305454	Distal Intergenic	ENSSSCG000	HMGB2	high mobility group box 2 [Source:VGNC Symbol;Acc:VGNC:88902]
chr17	44012717	44013722	1006	*	chr17	44012717	44013722	in_vitro	in_vivo	9	13	7.49777778	5.92692308	1.5708547	c(t = 0.34115	0.739	0.94401845	Distal Intergenic	ENSSSCG000	LPIN3	lipin 3 [Source:NCBI gene (formerly Entrezgene);Acc:100170771]
chr3	119987541	119989431	1891	*	chr3	119987541	119989431	in_vitro	in_vivo	12	13	0.87166667	1.09461538	-0.2229487	c(t = -0.3378	0.739	0.94401845	Promoter (<=1kb)	ENSSSCG000	KCNS3	potassium voltage-gated channel modifier subfamily S member 3 [Source:VGNC Symbol;Acc:VGNC:89387]
chr4	387482	396374	8893	*	chr4	387482	396374	in_vitro	in_vivo	10	14	95.016	94.4635714	0.55242857	c(t = 0.33595	0.74	0.94451088	Distal Intergenic	ENSSSCG000	SLC52A1	solute carrier family 52 member 2 [Source:NCBI gene (formerly Entrezgene);Acc:445519]
chr6	127890204	127890909	706	*	chr6	127890204	127890909	in_vitro	in_vivo	12	12	2.4625	3.05833333	-0.5958333	c(t = -0.3374	0.74	0.94451088	Distal Intergenic	ENSSSCG000	HSBP1L1	heat shock factor binding protein 1 like 1 [Source:NCBI gene (formerly Entrezgene);Acc:100192206]
chr7	70641892	70642304	413	*	chr7	70641892	70642304	in_vitro	in_vivo	10	9	2.315	1.84111111	0.47388889	c(t = 0.33669	0.74	0.94451088	Distal Intergenic	ENSSSCG000	AP4S1	adaptor related protein complex 4 subunit sigma 1 [Source:VGNC Symbol;Acc:VGNC:85394]
chr13	134352914	134353564	651	*	chr13	134352914	134353564	in_vitro	in_vivo	9	10	1.96555556	1.4	0.56555556	c(t = 0.33808	0.74	0.94451088	Promoter (<=1kb)	ENSSSCG000	RUBCN	rubicon autophagy regulator [Source:VGNC Symbol;Acc:VGNC:92509]
chr1	128305408	128305965	558	*	chr1	128305408	128305965	in_vitro	in_vivo	12	11	0.9875	0.78545455	0.20204545	c(t = 0.33599	0.74	0.94451088	Promoter (<=1kb)	ENSSSCG000	ssc-mir-9838	ssc-mir-9838 [Source:miRBase;Acc:MI0031607]
chr3	85649742	85650030	289	*	chr3	85649742	85650030	in_vitro	in_vivo	9	10	0.60555556	1	-0.3944444	c(t = -0.3374	0.741	0.94547319	Promoter (<=1kb)	ENSSSCG000	PPP4R3B	protein phosphatase 4 regulatory subunit 3B [Source:NCBI gene (formerly Entrezgene);Acc:100512224]
chr6	105659896	105660477	582	*	chr6	105659896	105660477	in_vitro	in_vivo	9	10	0.64444444	0.843	-0.1985556	c(t = -0.3362	0.741	0.94547319	Promoter (<=1kb)	ENSSSCG000	TYMS	thymidylate synthetase [Source:NCBI gene (formerly Entrezgene);Acc:100627209]
chr18	16514597	16516188	1592	*	chr18	16514597	16516188	in_vitro	in_vivo	13	10	3.36538462	2.918	0.44738462	c(t = 0.33365	0.742	0.94596384	Distal Intergenic	ENSSSCG000	CHCHD3	coiled-coil-helix-coiled-coil-helix domain containing 3 [Source:VGNC Symbol;Acc:VGNC:110298]
chr1	217441152	217441831	680	*	chr1	217441152	217441831	in_vitro	in_vivo	11	10	2.25090909	2.88	-0.6290909	c(t = -0.3349	0.742	0.94596384	Promoter (<=1kb)	ENSSSCG000	SLC1A1	solute carrier family 1 member 1 [Source:NCBI gene (formerly Entrezgene);Acc:397003]
chr2	135283198	135284237	1040	*	chr2	135283198	135284237	in_vitro	in_vivo	13	10	1.75384615	2.463	-0.7091538	c(t = -0.3371	0.742	0.94596384	Distal Intergenic	ENSSSCG000	LEAP2	liver enriched antimicrobial peptide 2 [Source:NCBI gene (formerly Entrezgene);Acc:396687]
chr13	96495881	96497553	1673	*	chr13	96495881	96497553	in_vitro	in_vivo	14	14	1.12214286	1.37857143	-0.2564286	c(t = -0.3326	0.742	0.94596384	Distal Intergenic	ENSSSCG000	SSR3	signal sequence receptor subunit 3 [Source:VGNC Symbol;Acc:VGNC:93492]
chr2	71546573	71547633	1061	*	chr2	71546573	71547633	in_vitro	in_vivo	10	13	0.92	0.73076923	0.18923077	c(t = 0.33370	0.742	0.94596384	Distal Intergenic	ENSSSCG000	PET100	PET100 cytochrome c oxidase chaperone [Source:NCBI gene (formerly Entrezgene);Acc:100037981]
chr10	24931375	24931837	463	*	chr10	24931375	24931837	in_vitro	in_vivo	10	12	3.458	4.39916667	-0.9411667	c(t = -0.3322	0.743	0.94676753	Promoter (<=1kb)	ENSSSCG000	CYB5R1	cytochrome b5 reductase 1 [Source:VGNC Symbol;Acc:VGNC:95584]
chr5	22020741	22021950	1210	*	chr5	22020741	22021950	in_vitro	in_vivo	12	10	2.27083333	2.745	-0.4741667	c(t = -0.3342	0.743	0.94676753	Distal Intergenic	ENSSSCG000	PRIM1	DNA primase subunit 1 [Source:VGNC Symbol;Acc:VGNC:103313]
chr3	93853422	93854576	1155	*	chr3	93853422	93854576	in_vitro	in_vivo	11	12	0.91818182	0.69416667	0.22401515	c(t = 0.33446	0.743	0.94676753	Promoter (<=1kb)	ENSSSCG000	SOCS5	suppressor of cytokine signaling 5 [Source:VGNC Symbol;Acc:VGNC:93332]
chr18	807891	810241	2351	*	chr18	807891	810241	in_vitro	in_vivo	11	12	20.9118182	25.1583333	-4.2465152	c(t = -0.3304	0.744	0.94709955	Distal Intergenic	ENSSSCG000	VIPR2	vasoactive intestinal peptide receptor 2 [Source:NCBI gene (formerly Entrezgene);Acc:100499553]
chr7	57972943	57974944	2002	*	chr7	57972943	57974944	in_vitro	in_vivo	13	11	26.5607692	22.37	4.19076923	c(t = 0.33095	0.744	0.94709955	Distal Intergenic	ENSSSCG000	IMP3	IMP U3 small nucleolar ribonucleoprotein 3 [Source:NCBI gene (formerly Entrezgene);Acc:100151831]
chr6	52544610	52545973	1364	*	chr6	52544610	52545973	in_vitro	in_vivo	12	11	4.92333333	4.34636364	0.5769697	c(t = 0.33033	0.744	0.94709955	Distal Intergenic	ENSSSCG000	CALM3	calmodulin 3 [Source:NCBI gene (formerly Entrezgene);Acc:396838]
chr15	32202023	32202821	799	*	chr15	32202023	32202821	in_vitro	in_vivo	12	11	1.28666667	0.94727273	0.33939394	c(t = 0.33058	0.744	0.94709955	Distal Intergenic	NA	NA	NA
chr17	5596713	5598257	1545	*	chr17	5596713	5598257	in_vitro	in_vivo	10	15	0.625	0.91666667	-0.2916667	c(t = -0.3311	0.744	0.94709955	Distal Intergenic	ENSSSCG000	SLC7A2	solute carrier family 7 member 2 [Source:VGNC Symbol;Acc:VGNC:95506]
chr1	141886964	141888154	1191	*	chr1	141886964	141888154	in_vitro	in_vivo	14	13	0.6	0.76461538	-0.1646154	c(t = -0.3305	0.744	0.94709955	Promoter (<=1kb)	ENSSSCG000	UBE3A	ubiquitin protein ligase E3A [Source:VGNC Symbol;Acc:VGNC:94652]
chr12	61073558	61100387	26830	*	chr12	61073558	61100387	in_vitro	in_vivo	14	14	95.1	94.4535714	0.64642857	c(t = 0.32969	0.745	0.94774458	Distal Intergenic	ENSSSCG000	COPS3	COP9 signalosome subunit 3 [Source:VGNC Symbol;Acc:VGNC:86900]
chr2	141282120	141285396	3277	*	chr2	141282120	141285396	in_vitro	in_vivo	13	14	3.40692308	3.77357143	-0.3666484	c(t = -0.3303	0.745	0.94774458	Distal Intergenic	ENSSSCG000	SPATA24	spermatosis associated 24 [Source:VGNC Symbol;Acc:VGNC:93381]
chr7	707240	710819	3580	*	chr7	707240	710819	in_vitro	in_vivo	12	13	3.83166667	3.08384615	0.74782051	c(t = 0.32928	0.745	0.94774458	Distal Intergenic	ENSSSCG000	IRF4	interferon regulatory factor 4 [Source:VGNC Symbol;Acc:VGNC:89207]
chr13	193294008	193294677	670	*	chr13	193294008	193294677	in_vitro	in_vivo	10	13	0.991	0.73923077	0.25176923	c(t = 0.32949	0.745	0.94774458	Distal Intergenic	ENSSSCG000	CLDN17	claudin 17 [Source:NCBI gene (formerly Entrezgene);Acc:100294681]
chr12	60575854	60581450	5597	*	chr12	60575854	60581450	in_vitro	in_vivo	12	10	22.3125	25.874	-3.5615	c(t = -0.3278	0.746	0.94807508	Distal Intergenic	ENSSSCG000	SREBF1	sterol regulatory element binding transcription factor 1 [Source:VGNC Symbol;Acc:VGNC:99083]
chr13	206229591	206232109	2519	*	chr13	206229591	206232109	in_vitro	in_vivo	11	12	4.75909091	5.6375	-0.8784091	c(t = -0.3278	0.746	0.94807508	Distal Intergenic	ENSSSCG000	PDXK	pyridoxal kinase [Source:NCBI gene (formerly Entrezgene);Acc:396983]
chr6	100853824	100854720	897	*	chr6	100853824	100854720	in_vitro	in_vivo	12	11	1.51666667	1.75	-0.2333333	c(t = -0.3277	0.746	0.94807508	Distal Intergenic	ENSSSCG000	NDUFV2	NADH:ubiquinone oxidoreductase core subunit V2 [Source:NCBI gene (formerly Entrezgene);Acc:100037991]
chr14	139953892	139955866	1975	*	chr14	139953892	139955866	in_vitro	in_vivo	16	15	1.91875	1.634	0.28475	c(t = 0.32732	0.746	0.94807508	Distal Intergenic	ENSSSCG000	GLRX3	glutaredoxin 3 [Source:VGNC Symbol;Acc:VGNC:88495]
chr1	8243705	8244469	765	*	chr1	8243705	8244469	in_vitro	in_vivo	11	10	1.40363636	1.07	0.33363636	c(t = 0.32930	0.746	0.94807508	Distal Intergenic	ENSSSCG000	TAGAP	T cell activation RhoGTPase activating protein [Source:NCBI gene (formerly Entrezgene);Acc:100155114]
chr8	55700106	55701441	1336	*	chr8	55700106	55701441	in_vitro	in_vivo	11	14	1.04727273	0.815	0.23227273	c(t = 0.32860	0.746	0.94807508	Distal Intergenic	ENSSSCG00000008898		HOP homeobox [Source:VGNC Symbol;Acc:VGNC:88932]
chrX	98324759	98325389	631	*	chrX	98324759	98325389	in_vitro	in_vivo	10	13	3.658	4.86923077	-1.2112308	c(t = -0.3286	0.747	0.94824827	Distal Intergenic	ENSSSCG000	NKAP	NFKB activating protein [Source:VGNC Symbol;Acc:VGNC:90756]
chr2	67913082	67913453	372	*	chr2	67913082	67913453	in_vitro	in_vivo	9	9	2.76	3.63555556	-0.8755556	c(t = -0.3282	0.747	0.94824827	Distal Intergenic	ENSSSCG00000023331		ubiquitin like 5 [Source:VGNC Symbol;Acc:VGNC:111109]
chr2	2116223	2118192	1970	*	chr2	2116223	2118192	in_vitro	in_vivo	13	13	2.06769231	2.51153846	-0.4438462	c(t = -0.3263	0.747	0.94824827	Distal Intergenic	ENSSSCG000	NAP1L4	nucleosome assembly protein 1 like 4 [Source:VGNC Symbol;Acc:VGNC:90577]
chr8	71940207	71940775	569	*	chr8	71940207	71940775	in_vitro	in_vivo	10	12	2.417	1.84166667	0.57533333	c(t = 0.32760	0.747	0.94824827	Distal Intergenic	ENSSSCG000	SCARB2	scavenger receptor class B member 2 [Source:VGNC Symbol;Acc:VGNC:92614]
chr1	56491665	56493354	1690	*	chr1	56491665	56493354	in_vitro	in_vivo	14	12	1.25928571	1.47833333	-0.2190476	c(t = -0.3273	0.747	0.94824827	Distal Intergenic	ENSSSCG000	SPACA1	sperm acrosome associated 1 [Source:NCBI gene (formerly Entrezgene);Acc:100156797]
chr8	116031498	116032106	609	*	chr8	116031498	116032106	in_vitro	in_vivo	9	13	0.74111111	1.06230769	-0.3211966	c(t = -0.3274	0.747	0.94824827	Distal Intergenic	ENSSSCG000	AIMP1	aminoacyl tRNA synthetase complex interacting multifunctional protein 1 [Source:VGNC Symbol;Acc:VGNC:85202]
chr18	55877654	55878015	362	*	chr18	55877654	55878015	in_vitro	in_vivo	11	12	0.65	0.4675	0.1825	c(t = 0.32704	0.747	0.94824827	Distal Intergenic	ENSSSCG000	RARRES2	retinoic acid receptor responder 2 [Source:VGNC Symbol;Acc:VGNC:92098]
chr13	18760773	18763325	2553	*	chr13	18760773	18763325	in_vitro	in_vivo	14	15	1.51642857	1.74666667	-0.2302381	c(t = -0.3250	0.748	0.94904738	Distal Intergenic	ENSSSCG000	CMTM7	CKLF like MARVEL transmembrane domain containing 7 [Source:VGNC Symbol;Acc:VGNC:103928]
chr18	19441272	19442220	949	*	chr18	19441272	19442220	in_vitro	in_vivo	9	14	1.29555556	1.69357143	-0.3980159	c(t = -0.3260	0.748	0.94904738	Distal Intergenic	ENSSSCG000	OPN1SW	opsin 1, short wave sensitive [Source:NCBI gene (formerly Entrezgene);Acc:397164]
chr1	19806493	19807441	949	*	chr1	19806493	19807441	in_vitro	in_vivo	12	12	1.14666667	1.39083333	-0.2441667	c(t = -0.3253	0.748	0.94904738	Distal Intergenic	ENSSSCG000	RAB32	RAB32, member RAS oncogene family [Source:NCBI gene (formerly Entrezgene);Acc:100144496]
chr14	139003294	139003884	591	*	chr14	139003294	139003884	in_vitro	in_vivo	9	9	26.6522222	32.0166667	-5.3644444	c(t = -0.3259	0.749	0.94985992	Distal Intergenic	ENSSSCG000	GLRX3	glutaredoxin 3 [Source:VGNC Symbol;Acc:VGNC:88495]
chr12	3393064	3394744	1681	*	chr12	3393064	3394744	in_vitro	in_vivo	9	10	91.6666667	90.238	1.42866667	c(t = 0.32348	0.75	0.94985992	Distal Intergenic	ENSSSCG00000017164		TIMP metallopeptidase inhibitor 2 [Source:VGNC Symbol;Acc:VGNC:93995]
chr13	94417378	94418098	721	*	chr13	94417378	94418098	in_vitro	in_vivo	10	11	3.519	2.83909091	0.67990909	c(t = 0.32353	0.75	0.94985992	Promoter (<=1kb)	ENSSSCG000	GPR149	G protein-coupled receptor 149 [Source:VGNC Symbol;Acc:VGNC:88605]
chr17	10984234	10986168	1935	*	chr17	10984234	10986168	in_vitro	in_vivo	13	15	2.37	2.80066667	-0.4306667	c(t = -0.3223	0.75	0.94985992	Distal Intergenic	NA	NA	NA
chr5	30110688	30111601	914	*	chr5	30110688	30111601	in_vitro	in_vivo	9	10	2.72	1.911	0.809	c(t = 0.32555	0.75	0.94985992	Distal Intergenic	ENSSSCG000	LEMD3	LEM domain containing 3 [Source:NCBI gene (formerly Entrezgene);Acc:100511508]
chr3	9316574	9318990	2417	*	chr3	9316574	9318990	in_vitro	in_vivo	11	14	1.69727273	1.49857143	0.1987013	c(t = 0.32312	0.75	0.94985992	Distal Intergenic	ENSSSCG000	CLDN15	claudin 15 [Source:VGNC Symbol;Acc:VGNC:86732]
chr14	80376402	80377726	1325	*	chr14	80376402	80377726	in_vitro	in_vivo	14	14	1.57714286	1.27285714	0.30428571	c(t = 0.32191	0.75	0.94985992	Distal Intergenic	ENSSSCG000	KCNMA1	potassium calcium-activated channel subfamily M alpha 1 [Source:VGNC Symbol;Acc:VGNC:107403]
chr12	43336650	43338239	1590	*	chr12	43336650	43338239	in_vitro	in_vivo	11	10	1.47181818	1.2	0.27181818	c(t = 0.32399	0.75	0.94985992	Promoter (<=1kb)	ENSSSCG000	ssc-mir-193a	ssc-mir-193a [Source:miRBase;Acc:MI0013113]
chr12	44603763	44604519	757	*	chr12	44603763	44604519	in_vitro	in_vivo	10	11	0.769	1.17818182	-0.4091818	c(t = -0.3229	0.75	0.94985992	Distal Intergenic	ENSSSCG000	VTN	vitronectin [Source:NCBI gene (formerly Entrezgene);Acc:397192]
chr14	22689194	22689930	737	*	chr14	22689194	22689930	in_vitro	in_vivo	9	11	1.41111111	1.05454545	0.35656566	c(t = 0.32497	0.75	0.94985992	Distal Intergenic	ENSSSCG000	PIWIL1	piwi like RNA-mediated silencing 1 [Source:VGNC Symbol;Acc:VGNC:98197]
chr8	63602562	63605057	2496	*	chr8	63602562	63605057	in_vitro	in_vivo	11	14	0.68454545	0.82285714	-0.1383117	c(t = -0.3234	0.75	0.94985992	Distal Intergenic	NA	NA	NA
chr11	2683894	2685672	1779	*	chr11	2683894	2685672	in_vitro	in_vivo	9	10	91.3266667	93.454	-2.1273333	c(t = -0.3256	0.751	0.95018686	Distal Intergenic	ENSSSCG000	ssc-mir-9814	ssc-mir-9814 [Source:miRBase;Acc:MI0031581]
chr1	7446389	7448785	2397	*	chr1	7446389	7448785	in_vitro	in_vivo	9	10	76.7433333	70.691	6.05233333	c(t = 0.32251	0.751	0.95018686	Intron (NM_001244473/397214, intron 2 of 47)	ENSSSCG000	IGF2R	insulin like growth factor 2 receptor [Source:NCBI gene (formerly Entrezgene);Acc:397214]
chr12	25441758	25443317	1560	*	chr12	25441758	25443317	in_vitro	in_vivo	11	10	2.28545455	3.076	-0.7905455	c(t = -0.3223	0.751	0.95018686	Distal Intergenic	ENSSSCG00000048918		beta-1,4-N-acetyl-galactosaminyltransferase 2 [Source:NCBI gene (formerly Entrezgene);Acc:100621328]
chr2	70136909	70137561	653	*	chr2	70136909	70137561	in_vitro	in_vivo	12	11	2.4	3.02272727	-0.6227273	c(t = -0.3219	0.751	0.95018686	Distal Intergenic	ENSSSCG000	PRKCSH	protein kinase C substrate 80K-H [Source:VGNC Symbol;Acc:VGNC:91810]
chr7	22824216	22825620	1405	*	chr7	22824216	22825620	in_vitro	in_vivo	14	14	2.75642857	2.26357143	0.49285714	c(t = 0.32059	0.751	0.95018686	Promoter (<=1kb)	NA	NA	NA
chr3	37927810	37928769	960	*	chr3	37927810	37928769	in_vitro	in_vivo	9	9	0.71	1.03333333	-0.3233333	c(t = -0.3252	0.751	0.95018686	Distal Intergenic	ENSSSCG000	HMOX2	heme oxygenase 2 [Source:VGNC Symbol;Acc:VGNC:88912]
chr6	17817894	17818303	410	*	chr6	17817894	17818303	in_vitro	in_vivo	11	10	2.35363636	1.877	0.47663636	c(t = 0.32102	0.752	0.95082593	Promoter (<=1kb)	ENSSSCG000	DERPC	DERPC proline and glycine rich nuclear protein [Source:NCBI gene (formerly Entrezgene);Acc:116621588]
chr1	189497552	189498578	1027	*	chr1	189497552	189498578	in_vitro	in_vivo	14	12	1.28285714	1.52166667	-0.2388095	c(t = -0.3194	0.752	0.95082593	Distal Intergenic	ENSSSCG000	SIX1	SIX homeobox 1 [Source:NCBI gene (formerly Entrezgene);Acc:100156847]
chr9	24772322	24775029	2708	*	chr9	24772322	24775029	in_vitro	in_vivo	13	11	1.58307692	1.32454545	0.25853147	c(t = 0.32129	0.752	0.95082593	Distal Intergenic	ENSSSCG000	C11orf54	chromosome 9 C11orf54 homolog [Source:VGNC Symbol;Acc:VGNC:86087]
chr4	42777304	42778381	1078	*	chr4	42777304	42778381	in_vitro	in_vivo	12	14	1.34416667	0.975	0.36916667	c(t = 0.32212	0.752	0.95082593	Distal Intergenic	ENSSSCG000	CCNE2	cyclin E2 [Source:VGNC Symbol;Acc:VGNC:86355]
chr10	52224014	52225324	1311	*	chr10	52224014	52225324	in_vitro	in_vivo	9	13	2.36777778	1.63461538	0.73316239	c(t = 0.32213	0.753	0.95130774	Promoter (<=1kb)	ENSSSCG000	PIP4K2A	phosphatidylinositol-5-phosphate 4-kinase type 2 alpha [Source:VGNC Symbol;Acc:VGNC:96485]
chr3	68540267	68541093	827	*	chr3	68540267	68541093	in_vitro	in_vivo	10	9	2.308	1.52555556	0.78244444	c(t = 0.32339	0.753	0.95130774	Distal Intergenic	ENSSSCG000	DOK1	docking protein 1 [Source:NCBI gene (formerly Entrezgene);Acc:100233179]
chr1	37911315	37912718	1404	*	chr1	37911315	37912718	in_vitro	in_vivo	14	12	1.24714286	1.50083333	-0.2536905	c(t = -0.3193	0.753	0.95130774	Distal Intergenic	ENSSSCG000	HEY2	hes related family bHLH transcription factor with YRPW motif 2 [Source:VGNC Symbol;Acc:VGNC:88864]
chr5	93300974	93304140	3167	*	chr5	93300974	93304140	in_vitro	in_vivo	14	13	0.75714286	0.91076923	-0.1536264	c(t = -0.3179	0.753	0.95130774	Promoter (<=1kb)	ENSSSCG000	DUSP6	dual specificity phosphatase 6 [Source:NCBI gene (formerly Entrezgene);Acc:100622968]
chr6	111235080	111236870	1791	*	chr6	111235080	111236870	in_vitro	in_vivo	14	12	0.55142857	0.41083333	0.14059524	c(t = 0.31908	0.753	0.95130774	Distal Intergenic	ENSSSCG000	KCTD1	potassium channel tetramerization domain containing 1 [Source:VGNC Symbol;Acc:VGNC:98482]
chrX	497500	502064	4565	*	chrX	497500	502064	in_vitro	in_vivo	11	13	32.3281818	28.8053846	3.5227972	c(t = 0.31756	0.754	0.95178876	Distal Intergenic	ENSSSCG000	ARSL	arylsulfatase E (chondrodysplasia punctata 1) [Source:NCBI gene (formerly Entrezgene);Acc:100627778]
chr8	135901729	135902564	836	*	chr8	135901729	135902564	in_vitro	in_vivo	9	9	12.8022222	17.2688889	-4.4666667	c(t = -0.3189	0.754	0.95178876	Distal Intergenic	ENSSSCG000	ENOPH1	enolase-phosphatase 1 [Source:VGNC Symbol;Acc:VGNC:98922]
chr6	52634601	52635934	1334	*	chr6	52634601	52635934	in_vitro	in_vivo	12	11	10.5283333	14.1654545	-3.6371212	c(t = -0.3179	0.754	0.95178876	Distal Intergenic	ENSSSCG000	AP2S1	adaptor related protein complex 2 subunit sigma 1 [Source:VGNC Symbol;Acc:VGNC:96909]
chr13	9213718	9214343	626	*	chr13	9213718	9214343	in_vitro	in_vivo	10	13	2.704	2.25153846	0.45246154	c(t = 0.31804	0.754	0.95178876	Distal Intergenic	ENSSSCG000	UBE2E2	ubiquitin conjugating enzyme E2 E2 [Source:VGNC Symbol;Acc:VGNC:111205]
chr1	130201315	130202136	822	*	chr1	130201315	130202136	in_vitro	in_vivo	10	11	1.032	1.28636364	-0.2543636	c(t = -0.3190	0.754	0.95178876	Distal Intergenic	ENSSSCG000	DLL4	delta like canonical Notch ligand 4 [Source:NCBI gene (formerly Entrezgene);Acc:100152163]
chr3	8170598	8171414	817	*	chr3	8170598	8171414	in_vitro	in_vivo	10	9	2.157	2.64222222	-0.4852222	c(t = -0.3178	0.755	0.95211273	Distal Intergenic	ENSSSCG000	PVRIG	PVR related immunoglobulin domain containing [Source:VGNC Symbol;Acc:VGNC:92008]
chr1	2643078	2644080	1003	*	chr1	2643078	2644080	in_vitro	in_vivo	9	10	1.88333333	1.479	0.40433333	c(t = 0.31811	0.755	0.95211273	Distal Intergenic	ENSSSCG000	SFT2D1	SFT2 domain containing 1 [Source:NCBI gene (formerly Entrezgene);Acc:100513321]
chr8	104560041	104560778	738	*	chr8	104560041	104560778	in_vitro	in_vivo	9	16	1.81888889	1.25	0.56888889	c(t = 0.31568	0.755	0.95211273	Distal Intergenic	ENSSSCG000	MYOZ2	myozenin 2 [Source:NCBI gene (formerly Entrezgene);Acc:733663]
chr13	72195523	72196384	862	*	chr13	72195523	72196384	in_vitro	in_vivo	10	13	1.436	1.18307692	0.25292308	c(t = 0.31690	0.755	0.95211273	Distal Intergenic	ENSSSCG000	SEC61A1	SEC61 translocon subunit alpha 1 [Source:VGNC Symbol;Acc:VGNC:92682]
chr9	129054886	129056417	1532	*	chr9	129054886	129056417	in_vitro	in_vivo	10	9	1.085	0.89444444	0.19055556	c(t = 0.31695	0.755	0.95211273	Distal Intergenic	ENSSSCG000	SMYD2	SET and MYND domain containing 2 [Source:VGNC Symbol;Acc:VGNC:93273]
chr15	101510441	101511469	1029	*	chr15	101510441	101511469	in_vitro	in_vivo	10	14	0.86	0.66071429	0.19928571	c(t = 0.31841	0.755	0.95211273	Distal Intergenic	ENSSSCG000	MOBKL3	MOB family member 4, phocein [Source:NCBI gene (formerly Entrezgene);Acc:613127]
chr8	264947	268067	3121	*	chr8	264947	268067	in_vitro	in_vivo	9	14	93.1855556	92.1142857	1.07126984	c(t = 0.31448	0.756	0.95274844	Distal Intergenic	ENSSSCG000	ATP5ME	ATP synthase membrane subunit e [Source:NCBI gene (formerly Entrezgene);Acc:396950]
chr2	150854603	150860625	6023	*	chr2	150854603	150860625	in_vitro	in_vivo	13	11	85.2223077	87.5409091	-2.3186014	c(t = -0.3144	0.756	0.95274844	Distal Intergenic	ENSSSCG000	ssc-mir-378-	ssc-mir-378-1 [Source:miRBase;Acc:MI0013088]
chr2	49082751	49083432	682	*	chr2	49082751	49083432	in_vitro	in_vivo	12	10	23.2216667	25.426	-2.2043333	c(t = -0.3164	0.756	0.95274844	Distal Intergenic	ENSSSCG000	RNF141	ring finger protein 141 [Source:VGNC Symbol;Acc:VGNC:98292]
chr3	40092032	40093430	1399	*	chr3	40092032	40093430	in_vitro	in_vivo	11	14	1.31545455	1.015	0.30045455	c(t = 0.31637	0.756	0.95274844	Distal Intergenic	ENSSSCG000	MSRB1	methionine sulfoxide reductase B1 [Source:NCBI gene (formerly Entrezgene);Acc:100037965]
chr4	2548351	2552917	4567	*	chr4	2548351	2552917	in_vitro	in_vivo	11	11	88.5818182	86.4172727	2.16454545	c(t = 0.31363	0.757	0.9532271	Distal Intergenic	NA	NA	NA

chr14	140823626	140827068	3443	*	chr14	140823626	140827068	in_vitro	in_vivo	11	12	26.3572727	30.8683333	-4.5110606	c(t = -0.3140	0.757	0.9532271	Distal Intergenic	ENSSSCG000	ssc-mir-202	ssc-mir-202 [Source:miRBase;Acc:MI0013157]
chr6	54078981	54081394	2414	*	chr6	54078981	54081394	in_vitro	in_vivo	11	11	7.16818182	8.74272727	-1.5745455	c(t = -0.3142	0.757	0.9532271	Promoter (<=1kb)	ENSSSCG000	FUT1	fucosyltransferase 1 (H blood group) [Source:NCBI gene (formerly Entrezgene);Acc:397138]
chr1	39752288	39752975	688	*	chr1	39752288	39752975	in_vitro	in_vivo	10	13	2.818	2.24	0.578	c(t = 0.31293	0.757	0.9532271	Distal Intergenic	ENSSSCG000	FABP7	fatty acid binding protein 7 [Source:NCBI gene (formerly Entrezgene);Acc:574075]
chr6	708446	709366	921	*	chr6	708446	709366	in_vitro	in_vivo	9	9	2.17666667	1.75555556	0.42111111	c(t = 0.31551	0.757	0.9532271	Distal Intergenic	ENSSSCG000	GALNS	galactosamine (N-acetyl)-6-sulfatase [Source:VGNC Symbol;Acc:VGNC:97061]
chr2	151500357	151504158	3802	*	chr2	151500357	151504158	in_vitro	in_vivo	14	12	97.1435714	96.5716667	0.57190476	c(t = 0.31226	0.758	0.95354886	Distal Intergenic	ENSSSCG000	CD74	CD74 molecule [Source:VGNC Symbol;Acc:VGNC:86428]
chr16	78596030	78597580	1551	*	chr16	78596030	78597580	in_vitro	in_vivo	11	14	20.0827273	23.75	-3.6672727	c(t = -0.3122	0.758	0.95354886	Distal Intergenic	ENSSSCG000	TERT	telomerase reverse transcriptase [Source:NCBI gene (formerly Entrezgene);Acc:492280]
chr15	78201394	78204425	3032	*	chr15	78201394	78204425	in_vitro	in_vivo	9	14	2.20111111	2.84285714	-0.641746	c(t = -0.3120	0.758	0.95354886	Distal Intergenic	ENSSSCG000	DYNC1I2	dynein cytoplasmic 1 intermediate chain 2 [Source:NCBI gene (formerly Entrezgene);Acc:100154566]
chr13	124699810	124700734	925	*	chr13	124699810	124700734	in_vitro	in_vivo	10	13	2.08	1.68076923	0.39923077	c(t = 0.31212	0.758	0.95354886	Distal Intergenic	ENSSSCG000	ADIPOQ	adiponectin, C1Q and collagen domain containing [Source:NCBI gene (formerly Entrezgene);Acc:397660]
chr14	40003385	40004961	1577	*	chr14	40003385	40004961	in_vitro	in_vivo	15	15	1.09933333	0.90933333	0.19	c(t = 0.31099	0.758	0.95354886	Distal Intergenic	ENSSSCG000	ALDH2	aldehyde dehydrogenase 2 family member [Source:NCBI gene (formerly Entrezgene);Acc:733685]
chr18	42265975	42266967	993	*	chr18	42265975	42266967	in_vitro	in_vivo	9	9	0.85666667	0.69	0.16666667	c(t = 0.31406	0.758	0.95354886	Promoter (<=1kb)	ENSSSCG000	CRHR2	corticotropin releasing hormone receptor 2 [Source:VGNC Symbol;Acc:VGNC:86994]
chr12	3642794	3645076	2283	*	chr12	3642794	3645076	in_vitro	in_vivo	12	12	3.85083333	3.33083333	0.52	c(t = 0.31062	0.759	0.95418207	Promoter (<=1kb)	ENSSSCG000	SOCS3	suppressor of cytokine signaling 3 [Source:VGNC Symbol;Acc:VGNC:99052]
chr3	10270499	10271163	665	*	chr3	10270499	10271163	in_vitro	in_vivo	9	11	1.85222222	2.33	-0.4777778	c(t = -0.3121	0.759	0.95418207	Promoter (<=1kb)	ENSSSCG000	POR	cytochrome p450 oxidoreductase [Source:VGNC Symbol;Acc:VGNC:109477]
chr18	37948106	37949106	1001	*	chr18	37948106	37949106	in_vitro	in_vivo	10	11	2.71	2.13727273	0.57272727	c(t = 0.31176	0.759	0.95418207	Distal Intergenic	ENSSSCG000	FKBP9	FKBP prolyl isomerase 9 [Source:NCBI gene (formerly Entrezgene);Acc:100623924]
chr1	194504337	194505535	1199	*	chr1	194504337	194505535	in_vitro	in_vivo	12	13	1.49166667	1.21230769	0.27935897	c(t = 0.31124	0.759	0.95418207	Promoter (<=1kb)	ENSSSCG000	PPP2R5E	protein phosphatase 2 regulatory subunit B'epsilon [Source:NCBI gene (formerly Entrezgene);Acc:100525377]
chr1	148033510	148035128	1619	*	chr1	148033510	148035128	in_vitro	in_vivo	11	13	15.75	19.4107692	-3.6607692	c(t = -0.3099	0.76	0.95419049	Distal Intergenic	ENSSSCG000	MBP	myelin basic protein [Source:NCBI gene (formerly Entrezgene);Acc:414286]
chr11	50738346	50738794	449	*	chr11	50738346	50738794	in_vitro	in_vivo	9	10	12.16	10.101	2.059	c(t = 0.30999	0.76	0.95419049	Distal Intergenic	ENSSSCG000	EDNRB	endothelin receptor type B [Source:VGNC Symbol;Acc:VGNC:87550]
chr3	125664936	125666798	1863	*	chr3	125664936	125666798	in_vitro	in_vivo	14	11	9.63642857	7.04181818	2.59461039	c(t = 0.30942	0.76	0.95419049	Distal Intergenic	ENSSSCG000	PDIA6	protein disulfide isomerase family A member 6 [Source:NCBI gene (formerly Entrezgene);Acc:100499564]
chr2	59061265	59065222	3958	*	chr2	59061265	59065222	in_vitro	in_vivo	15	15	2.47933333	2.74866667	-0.2693333	c(t = -0.3079	0.76	0.95419049	Distal Intergenic	ENSSSCG000	TMEM59L	transmembrane protein 59 like [Source:VGNC Symbol;Acc:VGNC:94193]
chr13	98592071	98592720	650	*	chr13	98592071	98592720	in_vitro	in_vivo	9	9	1.44777778	1.84333333	-0.3955556	c(t = -0.3111	0.76	0.95419049	Distal Intergenic	ENSSSCG000	SHOX2	short stature homeobox 2 [Source:VGNC Symbol;Acc:VGNC:107170]
chr8	38181419	38182302	884	*	chr8	38181419	38182302	in_vitro	in_vivo	9	10	2.42777778	1.707	0.72077778	c(t = 0.31112	0.76	0.95419049	Distal Intergenic	ENSSSCG000	ZAR1	zygote arrest 1 [Source:VGNC Symbol;Acc:VGNC:95051]
chr2	140345948	140346757	810	*	chr2	140345948	140346757	in_vitro	in_vivo	9	12	0.92888889	1.27833333	-0.3494444	c(t = -0.3101	0.76	0.95419049	Distal Intergenic	ENSSSCG000	CDC25C	cell division cycle 25C [Source:NCBI gene (formerly Entrezgene);Acc:100144474]
chr2	79549530	79550197	668	*	chr2	79549530	79550197	in_vitro	in_vivo	11	12	0.93545455	1.15833333	-0.2228788	c(t = -0.3095	0.76	0.95419049	Distal Intergenic	ENSSSCG000	PROP1	PROP paired-like homeobox 1 [Source:VGNC Symbol;Acc:VGNC:91834]
chr15	138606808	138608430	1623	*	chr15	138606808	138608430	in_vitro	in_vivo	9	10	9.25888889	12.028	-2.7691111	c(t = -0.3094	0.761	0.95421562	Distal Intergenic	ENSSSCG000	ssc-mir-149	ssc-mir-149 [Source:miRBase;Acc:MI0016620]
chr1	246277536	246278736	1201	*	chr1	246277536	246278736	in_vitro	in_vivo	11	14	9.89454545	8.04428571	1.85025974	c(t = 0.30850	0.761	0.95421562	Promoter (<=1kb)	ENSSSCG000	ABCA1	ATP binding cassette subfamily A member 1 [Source:NCBI gene (formerly Entrezgene);Acc:100152112]
chr7	53145843	53147229	1387	*	chr7	53145843	53147229	in_vitro	in_vivo	11	15	2.64454545	3.34666667	-0.7021212	c(t = -0.3084	0.761	0.95421562	Distal Intergenic	ENSSSCG000	BLM	BLM RecQ like helicase [Source:VGNC Symbol;Acc:VGNC:96569]
chr6	70240879	70242137	1259	*	chr6	70240879	70242137	in_vitro	in_vivo	9	10	2.32666667	2.873	-0.5463333	c(t = -0.3102	0.761	0.95421562	Distal Intergenic	ENSSSCG000	RBP7	retinol binding protein 7 [Source:NCBI gene (formerly Entrezgene);Acc:100270723]
chr17	57674353	57676560	2208	*	chr17	57674353	57676560	in_vitro	in_vivo	10	15	1.54	1.29466667	0.24533333	c(t = 0.30926	0.761	0.95421562	Promoter (<=1kb)	ENSSSCG000	BMP7	bone morphotic protein 7 [Source:VGNC Symbol;Acc:VGNC:95491]
chr2	39704712	39706115	1404	*	chr2	39704712	39706115	in_vitro	in_vivo	13	12	1.31615385	1.08666667	0.22948718	c(t = 0.30793	0.761	0.95421562	Distal Intergenic	ENSSSCG000	CSRP3	cysteine and glycine rich protein 3 [Source:NCBI gene (formerly Entrezgene);Acc:100337687]
chr1	270363751	270364715	965	*	chr1	270363751	270364715	in_vitro	in_vivo	13	9	11.9176923	10.5366667	1.38102564	c(t = 0.31005	0.762	0.95421562	Distal Intergenic	ENSSSCG000	FUBP3	far upstream element binding protein 3 [Source:VGNC Symbol;Acc:VGNC:103093]
chr8	1311415	1313369	1955	*	chr8	1311415	1313369	in_vitro	in_vivo	10	13	9.184	7.18384615	2.00015385	c(t = 0.30969	0.762	0.95421562	Distal Intergenic	ENSSSCG000	RNF4	ring finger protein 4 [Source:VGNC Symbol;Acc:VGNC:92392]
chr7	23264897	23266935	2039	*	chr7	23264897	23266935	in_vitro	in_vivo	10	12	4.619	5.40333333	-0.7843333	c(t = -0.3075	0.762	0.95421562	Promoter (<=1kb)	ENSSSCG000	FLOT1	flotillin 1 [Source:VGNC Symbol;Acc:VGNC:88158]
chr13	53184365	53185078	714	*	chr13	53184365	53185078	in_vitro	in_vivo	11	11	4.28545455	3.53909091	0.74636364	c(t = 0.30762	0.762	0.95421562	Distal Intergenic	ENSSSCG000	MITF	melanocyte inducing transcription factor [Source:VGNC Symbol;Acc:VGNC:90243]
chr5	13757297	13757858	562	*	chr5	13757297	13757858	in_vitro	in_vivo	9	12	3.09555556	2.58083333	0.51472222	c(t = 0.30793	0.762	0.95421562	Distal Intergenic	ENSSSCG000	CKAP4	cytoskeleton associated protein 4 [Source:NCBI gene (formerly Entrezgene);Acc:100523493]
chr10	60240679	60241734	1056	*	chr10	60240679	60241734	in_vitro	in_vivo	10	14	0.908	1.13714286	-0.2291429	c(t = -0.3069	0.762	0.95421562	Distal Intergenic	ENSSSCG000	CDC123	cell division cycle 123 [Source:VGNC Symbol;Acc:VGNC:95819]
chr12	11882643	11883052	410	*	chr12	11882643	11883052	in_vitro	in_vivo	11	11	1.25	0.97	0.28	c(t = 0.30683	0.762	0.95421562	Distal Intergenic	ENSSSCG000	AMZ2	archaelysin family metallopeptidase 2 [Source:VGNC Symbol;Acc:VGNC:85292]
chr13	134363488	134364475	988	*	chr13	134363488	134364475	in_vitro	in_vivo	13	12	0.60076923	0.8075	-0.2067308	c(t = -0.3070	0.762	0.95421562	Distal Intergenic	ENSSSCG000	RUBCN	rubicon autophagy regulator [Source:VGNC Symbol;Acc:VGNC:92509]
chr2	41758052	41759160	1109	*	chr2	41758052	41759160	in_vitro	in_vivo	11	11	92.3854545	93.5209091	-1.1354545	c(t = -0.3059	0.763	0.95421562	Distal Intergenic	ENSSSCG000	RPS13	ribosomal protein S13 [Source:NCBI gene (formerly Entrezgene);Acc:100513520]
chrX	121557758	121558787	1030	*	chrX	121557758	121558787	in_vitro	in_vivo	12	9	13.22	16.4822222	-3.2622222	c(t = -0.3059	0.763	0.95421562	Distal Intergenic	ENSSSCG000	FATE1	fetal and adult testis expressed 1 [Source:NCBI gene (formerly Entrezgene);Acc:396705]
chr11	78517538	78519023	1486	*	chr11	78517538	78519023	in_vitro	in_vivo	11	9	20.6127273	16.4111111	4.20161616	c(t = 0.30574	0.763	0.95421562	3' UTR	ENSSSCG000	F10	coagulation factor X [Source:NCBI gene (formerly Entrezgene);Acc:733662]
chr9	89758729	89759434	706	*	chr9	89758729	89759434	in_vitro	in_vivo	11	10	4.78636364	5.697	-0.9106364	c(t = -0.3063	0.763	0.95421562	Distal Intergenic	ENSSSCG000	ITGB8	integrin subunit beta 8 [Source:VGNC Symbol;Acc:VGNC:89246]
chr6	44597007	44598242	1236	*	chr6	44597007	44598242	in_vitro	in_vivo	10	10	2.614	3.086	-0.472	c(t = -0.3066	0.763	0.95421562	Distal Intergenic	ENSSSCG000	FXYD3	FXYD domain containing ion transport regulator 3 [Source:VGNC Symbol;Acc:VGNC:97060]
chr14	76304615	76305376	762	*	chr14	76304615	76305376	in_vitro	in_vivo	11	9	3.21090909	2.44222222	0.76868687	c(t = 0.30647	0.763	0.95421562	Distal Intergenic	ENSSSCG000	MYOZ1	myozenin 1 [Source:VGNC Symbol;Acc:VGNC:90542]
chr1	191211383	191211920	538	*	chr1	191211383	191211920	in_vitro	in_vivo	11	9	2.60636364	2.01555556	0.59080808	c(t = 0.30680	0.763	0.95421562	Distal Intergenic	ENSSSCG000	ssc-mir-7-1	ssc-mir-7-1 [Source:miRBase;Acc:MI0022122]
chr7	65302608	65303417	810	*	chr7	65302608	65303417	in_vitro	in_vivo	13	13	1.73692308	1.37384615	0.36307692	c(t = 0.30495	0.763	0.95421562	Distal Intergenic	ENSSSCG000	SNX6	sorting nexin 6 [Source:NCBI gene (formerly Entrezgene);Acc:100154775]
chr3	47600680	47601550	871	*	chr3	47600680	47601550	in_vitro	in_vivo	12	14	0.92083333	1.15	-0.2291667	c(t = -0.3044	0.763	0.95421562	Distal Intergenic	ENSSSCG00000008137		LIM zinc finger domain containing 1 [Source:VGNC Symbol;Acc:VGNC:110285]
chr2	97703420	97704033	614	*	chr2	97703420	97704033	in_vitro	in_vivo	9	9	0.32666667	0.47666667	-0.15	c(t = -0.3069	0.763	0.95421562	Distal Intergenic	ENSSSCG000	MEF2C	myocyte enhancer factor 2C [Source:VGNC Symbol;Acc:VGNC:90127]
chr5	540054	547557	7504	*	chr5	540054	547557	in_vitro	in_vivo	13	16	91.2146154	90.278125	0.93649038	c(t = 0.30373	0.764	0.95484469	Distal Intergenic	ENSSSCG000	SELENOO	selenoprotein O [Source:NCBI gene (formerly Entrezgene);Acc:100518971]
chr14	57971856	57972318	463	*	chr14	57971856	57972318	in_vitro	in_vivo	11	10	5.28090909	4.424	0.85690909	c(t = 0.30502	0.764	0.95484469	Distal Intergenic	ENSSSCG000	TSNAX	translin associated factor X [Source:NCBI gene (formerly Entrezgene);Acc:100156036]
chr12	57049778	57050930	1153	*	chr12	57049778	57050930	in_vitro	in_vivo	12	12	3.3375	2.8175	0.52	c(t = 0.30413	0.764	0.95484469	Distal Intergenic	ENSSSCG000	MYOCD	myocardin [Source:NCBI gene (formerly Entrezgene);Acc:396571]
chr18	29284596	29285571	976	*	chr18	29284596	29285571	in_vitro	in_vivo	12	10	0.98333333	0.812	0.17133333	c(t = 0.30372	0.764	0.95484469	Promoter (<=1kb)	ENSSSCG000	ST7	suppression of tumorigenicity 7 [Source:NCBI gene (formerly Entrezgene);Acc:100125380]
chr2	118728925	118731416	2492	*	chr2	118728925	118731416	in_vitro	in_vivo	14	16	4.90714286	4.25125	0.65589286	c(t = 0.30206	0.765	0.95485969	Distal Intergenic	ENSSSCG00000038141		toll like receptor adaptor molecule 2 [Source:NCBI gene (formerly Entrezgene);Acc:100520421]
chr1	12121457	12122489	1033	*	chr1	12121457	12122489	in_vitro	in_vivo	14	12	1.87357143	1.47416667	0.39940476	c(t = 0.30295	0.765	0.95485969	Distal Intergenic	ENSSSCG000	CLDN20	claudin 20 [Source:NCBI gene (formerly Entrezgene);Acc:100153817]
chr13	22298564	22299416	853	*	chr13	22298564	22299416	in_vitro	in_vivo	13	10	1.31307692	1.098	0.21507692	c(t = 0.30235	0.765	0.95485969	Distal Intergenic	ENSSSCG000	MLH1	mutL homolog 1 [Source:VGNC Symbol;Acc:VGNC:90252]
chr3	113228885	113229770	886	*	chr3	113228885	113229770	in_vitro	in_vivo	10	11	0.899	1.09272727	-0.1937273	c(t = -0.3032	0.765	0.95485969	Distal Intergenic	ENSSSCG000	RAB10	RAB10, member RAS oncogene family [Source:NCBI gene (formerly Entrezgene);Acc:100526104]
chr6	165670840	165671536	697	*	chr6	165670840	165671536	in_vitro	in_vivo	10	9	1.3	1.05666667	0.24333333	c(t = 0.30423	0.765	0.95485969	Distal Intergenic	ENSSSCG000	AKR1A1	aldo-keto reductase family 1 member A1 [Source:VGNC Symbol;Acc:VGNC:85229]
chr3	69656940	69658505	1566	*	chr3	69656940	69658505	in_vitro	in_vivo	16	13	1.123125	0.99923077	0.12389423	c(t = 0.30244	0.765	0.95485969	Distal Intergenic	ENSSSCG000	CCT7	chaperonin containing TCP1 subunit 7 [Source:NCBI gene (formerly Entrezgene);Acc:100322871]
chr13	27006107	27006931	825	*	chr13	27006107	27006931	in_vitro	in_vivo	9	14	0.55222222	0.68142857	-0.1292063	c(t = -0.3031	0.765	0.95485969	Promoter (<=1kb)	ENSSSCG000	ABHD5	abhydrolase domain containing 5, lysophosphatidic acid acyltransferase [Source:NCBI gene (formerly Entrezgene);Acc:497624]
chr7	4911994	4914594	2601	*	chr7	4911994	4914594	in_vitro	in_vivo	12	11	87.8158333	90.4572727	-2.6414394	c(t = -0.3026	0.766	0.95485969	Distal Intergenic	ENSSSCG000	BMP6	bone morphogenetic protein 6 [Source:NCBI gene (formerly Entrezgene);Acc:100155536]
chr3	520101	549800	29700	*	chr3	520101	549800	in_vitro	in_vivo	15	16	60.93	62.965625	-2.035625	c(t = -0.3008	0.766	0.95485969	3' UTR	ENSSSCG000	ADAP1	ArfGAP with dual PH domains 1 [Source:NCBI gene (formerly Entrezgene);Acc:397453]
chr18	50728428	50729797	1370	*	chr18	50728428	50729797	in_vitro	in_vivo	11	9	16.59	13.4733333	3.11666667	c(t = 0.30269	0.766	0.95485969	Distal Intergenic	ENSSSCG000	DDX56	DEAD-box helicase 56 [Source:NCBI gene (formerly Entrezgene);Acc:100513388]
chr6	42987793	42989153	1361	*	chr6	42987793	42989153	in_vitro	in_vivo	11	10	3.73727273	3.292	0.44527273	c(t = 0.30232	0.766	0.95485969	Distal Intergenic	ENSSSCG000	SLC7A9	solute carrier family 7 member 9 [Source:VGNC Symbol;Acc:VGNC:93177]
chr15	46266605	46267846	1242	*	chr15	46266605	46267846	in_vitro	in_vivo	12	11	2.65333333	2.24454545	0.40878788	c(t = 0.30207	0.766	0.95485969	Distal Intergenic	ENSSSCG000	LRP2BP	LRP2 binding protein [Source:VGNC Symbol;Acc:VGNC:96080]
chr4	89343225	89343934	710	*	chr4	89343225	89343934	in_vitro	in_vivo	9	9	1.47333333	2.22222222	-0.7488889	c(t = -0.3043	0.766	0.95485969	Distal Intergenic	ENSSSCG000	USF1	upstream transcription factor 1 [Source:VGNC Symbol;Acc:VGNC:98893]
chr8	137610714	137612493	1780	*	chr8	137610714	137612493	in_vitro	in_vivo	12	12	1.34333333	1.75833333	-0.415	c(t = -0.3027	0.766	0.95485969	Distal Intergenic	ENSSSCG000	BMP3	bone morphogenetic protein 3 [Source:NCBI gene (formerly Entrezgene);Acc:100512974]
chr3	113044609	113045780	1172	*	chr3	113044609	113045780	in_vitro	in_vivo	12	11	0.98666667	1.27090909	-0.2842424	c(t = -0.3020	0.766	0.95485969	Distal Intergenic	ENSSSCG000	RAB10	RAB10, member RAS oncogene family [Source:NCBI gene (formerly Entrezgene);Acc:100526104]
chr9	53008584	53009504	921	*	chr9	53008584	53009504	in_vitro	in_vivo	9	10	1.68111111	1.222	0.45911111	c(t = 0.30481	0.766	0.95485969	Distal Intergenic	ENSSSCG000	PUS3	pseudouridine synthase 3 [Source:VGNC Symbol;Acc:VGNC:98228]
chr6	64664948	64665830	883	*	chr6	64664948	64665830	in_vitro	in_vivo	10	11	22.298	27.2181818	-4.9201818	c(t = -0.3004	0.767	0.95508648	Distal Intergenic	ENSSSCG000	PRXL2B	peroxiredoxin like 2B [Source:VGNC Symbol;Acc:VGNC:98543]
chr3	42599421	42600106	686	*	chr3	42599421	42600106	in_vitro	in_vivo	12	12	13.7233333	11.445	2.27833333	c(t = 0.30215	0.767	0.95508648	Distal Intergenic	ENSSSCG000	CARD19	caspase recruitment domain family member 19 [Source:VGNC Symbol;Acc:VGNC:86188]
chrX	44179770	44180381	612	*	chrX	44179770	44180381	in_vitro	in_vivo	9	9	8.50888889	6.56888889	1.94	c(t = 0.30188	0.767	0.95508648	Distal Intergenic	ENSSSCG000	CLCN5	chloride voltage-gated channel 5 [Source:NCBI gene (formerly Entrezgene);Acc:397263]
chr2	69320157	69321007	851	*	chr2	69320157	69321007	in_vitro	in_vivo	9	10	4.93888889	6.017	-1.0781111	c(t = -0.3031	0.767	0.95508648	Promoter (<=1kb)	ENSSSCG000	ATG4D	autophagy related 4D cysteine peptidase [Source:VGNC Symbol;Acc:VGNC:85624]
chr14	43304842	43305734	893	*	chr14	43304842	43305734	in_vitro	in_vivo	12	13	1.59666667	1.87307692	-0.2764103	c(t = -0.3003	0.767	0.95508648	Distal Intergenic	ENSSSCG000	HPS4	HPS4 biosis of lysosomal organelles complex 3 subunit 2 [Source:VGNC Symbol;Acc:VGNC:88966]
chr6	68964084	68965011	928	*	chr6	68964084	68965011	in_vitro	in_vivo	10	9	94.474	95.6833333	-1.2093333	c(t = -0.3003	0.768	0.95508648	Distal Intergenic	ENSSSCG000	PARK7	Parkinsonism associated deglycase [Source:NCBI gene (formerly Entrezgene);Acc:780404]
chrX	44542462	44543414	953	*	chrX	44542462	44543414	in_vitro	in_vivo	12	13	6.09416667	7.73769231	-1.6435256	c(t = -0.2981	0.768	0.95508648	Distal Intergenic	ENSSSCG000	BMP15	bone morphogenetic protein 15 [Source:NCBI gene (formerly Entrezgene);Acc:448811]
chr4	32701018	32702438	1421	*	chr4	32701018	32702438	in_vitro	in_vivo	12	14	2.5525	2.17642857	0.37607143	c(t = 0.29813	0.768	0.95508648	Promoter (<=1kb)	ENSSSCG000	LRP12	LDL receptor related protein 12 [Source:VGNC Symbol;Acc:VGNC:89818]
chr2	4223585	4224737	1153	*	chr2	4223585	4224737	in_vitro	in_vivo	10	12	2.104	1.725	0.379	c(t = 0.29873	0.768	0.95508648	Distal Intergenic	ENSSSCG000	CPT1A	carnitine palmitoyltransferase 1A [Source:NCBI gene (formerly Entrezgene);Acc:399527]
chr7	29835431	29836888	1458	*	chr7	29835431	29836888	in_vitro	in_vivo	10	9	1.226	1.49555556	-0.2695556	c(t = -0.2992	0.768	0.95508648	Distal Intergenic	ENSSSCG000	UQCC2	ubiquinol-cytochrome c reductase complex assembly factor 2 [Source:NCBI gene (formerly Entrezgene);Acc:100153818]
chr1	184881571	184882509	939	*	chr1	184881571	184882509	in_vitro	in_vivo	13	16	1.77461538	1.47625	0.29836538	c(t = 0.29817	0.768	0.95508648	Promoter (<=1kb)	ENSSSCG000	KTN1	kinectin 1 [Source:VGNC Symbol;Acc:VGNC:89608]
chr13	48486781	48487497	717	*	chr13	48486781	48487497	in_vitro	in_vivo	10	12	1.757	1.43666667	0.32033333	c(t = 0.29952	0.768	0.95508648	Distal Intergenic	ENSSSCG000	EOGT	EGF domain specific O-linked N-acetylglucosamine transferase [Source:VGNC Symbol;Acc:VGNC:87718]
chr15	82426263	82427281	1019	*	chr15	82426263	82427281	in_vitro	in_vivo	9	11	1.20222222	0.94818182	0.2540404	c(t = 0.29889	0.768	0.95508648	Distal Intergenic	ENSSSCG000	MTX2	metaxin 2 [Source:VGNC Symbol;Acc:VGNC:96427]
chr1	225040937	225041906	970	*	chr1	225040937	225041906	in_vitro	in_vivo	9	11	1.03333333	0.8	0.23333333	c(t = 0.30008	0.768	0.95508648	Distal Intergenic	ENSSSCG000	ABHD17B	abhydrolase domain containing 17B, depalmitoylase [Source:VGNC Symbol;Acc:VGNC:84977]
chr4	71264	72500	1237	*	chr4	71264	72500	in_vitro	in_vivo	11	9	95.6045455	94.5488889	1.05565657	c(t = 0.29918	0.769	0.95508648	Distal Intergenic	ENSSSCG000	SLC52A1	solute carrier family 52 member 2 [Source:NCBI gene (formerly Entrezgene);Acc:445519]
chr5	77793767	77794493	727	*	chr5	77793767	77794493	in_vitro	in_vivo	9	9	23.6522222	18.47	5.18222222	c(t = 0.29965	0.769	0.95508648	Distal Intergenic	NA	NA	NA
chr7	25153492	25155223	1732	*	chr7	25153492	25155223	in_vitro	in_vivo	9	14	3.59777778	4.28071429	-0.6829365	c(t = -0.2977	0.769	0.95508648	5' UTR	ENSSSCG000	BRD2	bromodomain containing 2 [Source:VGNC Symbol;Acc:VGNC:85872]
chr4	100729960	100730834	875	*	chr4	100729960	100730834	in_vitro	in_vivo	10	12	2.582	1.88833333	0.69366667	c(t = 0.29924	0.769	0.95508648	Distal Intergenic	ENSSSCG000	PRKAB2	protein kinase AMP-activated non-catalytic subunit beta 2 [Source:VGNC Symbol;Acc:VGNC:91799]
chr7	41530278	41531194	917	*	chr7	41530278	41531194	in_vitro	in_vivo	10	12	1.937	1.51166667	0.42533333	c(t = 0.29842	0.769	0.95508648	Promoter (<=1kb)	ENSSSCG000	PLA2G7	phospholipase A2 group VII [Source:VGNC Symbol;Acc:VGNC:91501]
chrX	106659892	106661951	2060	*	chrX	106659892	106661951	in_vitro	in_vivo	13	14	12.8046154	10.2607143	2.5439011	c(t = 0.29547	0.77	0.95508648	Distal Intergenic	ENSSSCG000	AIFM1	apoptosis inducing factor mitochondria associated 1 [Source:VGNC Symbol;Acc:VGNC:85198]
chr18	47992514	47994405	1892	*	chr18	47992514	47994405	in_vitro	in_vivo	12	13	2.725	2.38076923	0.34423077	c(t = 0.29616	0.77	0.95508648	Promoter (<=1kb)	ENSSSCG000	NPY	neuropeptide Y [Source:NCBI gene (formerly Entrezgene);Acc:397304]
chr6	80903219	80903918	700	*	chr6	80903219	80903918	in_vitro	in_vivo	13	11	1.56769231	1.88181818	-0.3141259	c(t = -0.2967	0.77	0.95508648	Promoter (<=1kb)	ENSSSCG000	KDM1A	lysine demethylase 1A [Source:VGNC Symbol;Acc:VGNC:89408]
chr13	73831627	73832446	820	*	chr13	73831627	73832446	in_vitro	in_vivo	10	11	1.184	1.54454545	-0.3605455	c(t = -0.2982	0.77	0.95508648	Distal Intergenic	ENSSSCG000	ACKR4	atypical chemokine receptor 4 [Source:NCBI gene (formerly Entrezgene);Acc:100037292]
chr13	79245677	79246486	810	*	chr13	79245677	79246486	in_vitro	in_vivo	10	12	1.427	1.19416667	0.23283333	c(t = 0.29666	0.77	0.95508648	Distal Intergenic	ENSSSCG000	FAIM	Fas apoptotic inhibitory molecule [Source:VGNC Symbol;Acc:VGNC:87882]
chr1	54596435	54598726	2292	*	chr1	54596435	54598726	in_vitro	in_vivo	14	14	1.31214286	1.115	0.19714286	c(t = 0.29528	0.77	0.95508648	Distal Intergenic	ENSSSCG000	CGA	glycoprotein hormones, alpha polypeptide [Source:VGNC Symbol;Acc:VGNC:86613]
chrX	110115644	110117059	1416	*	chrX	110115644	110117059	in_vitro	in_vivo	9	11	5.94666667	7.92	-1.9733333	c(t = -0.2974	0.771	0.95508648	Promoter (1-2kb)	ENSSSCG000	ssc-mir-106a	ssc-mir-106a [Source:miRBase;Acc:MI0002412]
chr1	31674342	31675578	1237	*	chr1	31674342	31675578	in_vitro	in_vivo	13	14	3.29692308	3.82214286	-0.5252198	c(t = -0.2945	0.771	0.95508648	Promoter (<=1kb)	ENSSSCG000	CCN2	cellular communication network factor 2 [Source:NCBI gene (formerly Entrezgene);Acc:100152141]
chr7	25242220	25243129	910	*	chr7	25242220	25243129	in_vitro	in_vivo	11	10	4.26090909	3.125	1.13590909	c(t = 0.29564	0.771	0.95508648	Promoter (<=1kb)	ENSSSCG000	RXRB	retinoid X receptor beta [Source:VGNC Symbol;Acc:VGNC:92529]
chr7	4511482	4514211	2730	*	chr7	4511482	4514211	in_vitro	in_vivo	13	15	2.35153846	1.95	0.40153846	c(t = 0.29463	0.771	0.95508648	Distal Intergenic	ENSSSCG000	SSR1	signal sequence receptor subunit 1 [Source:NCBI gene (formerly Entrezgene);Acc:100152689]
chr15	76088477	76089243	767	*	chr15	76088477	76089243	in_vitro	in_vivo	10	10	2.242	1.833	0.409	c(t = 0.29706	0.771	0.95508648	Distal Intergenic	ENSSSCG000	PHOSPHO2	phosphatase, orphan 2 [Source:VGNC Symbol;Acc:VGNC:96172]
chr9	88289290	88290780	1491	*	chr9	88289290	88290780	in_vitro	in_vivo	16	11	1.0775	1.32363636	-0.2461364	c(t = -0.2958	0.771	0.95508648	Distal Intergenic	ENSSSCG000	POLR1F	RNA polymerase I subunit F [Source:NCBI gene (formerly Entrezgene);Acc:100517907]
chr6	168049461	168050267	807	*	chr6	168049461	168050267	in_vitro	in_vivo	10	10	1.378	1.052	0.326	c(t = 0.29572	0.771	0.95508648	Distal Intergenic	ENSSSCG000	CDC20	cell division cycle 20 [Source:VGNC Symbol;Acc:VGNC:86444]
chr5	73407874	73408592	719	*	chr5	73407874	73408592	in_vitro	in_vivo	11	11	0.23	0.30909091	-0.0790909	c(t = -0.2953	0.771	0.95508648	Distal Intergenic	ENSSSCG000	ADAMTS20	ADAM metallopeptidase with thrombospondin type 1 motif 20 [Source:VGNC Symbol;Acc:VGNC:85082]
chr7	55213715	55214827	1113	*	chr7	55213715	55214827	in_vitro	in_vivo	9	10	96.36	95.528	0.832	c(t = 0.29404	0.772	0.95508648	Distal Intergenic	ENSSSCG000	PLIN1	perilipin 1 [Source:NCBI gene (formerly Entrezgene);Acc:654411]
chr6	61324232	61325231	1000	*	chr6	61324232	61325231	in_vitro	in_vivo	9	9	12.3144444	16.0366667	-3.7222222	c(t = -0.2945	0.772	0.95508648	Distal Intergenic	ENSSSCG000	GALP	galanin like peptide [Source:NCBI gene (formerly Entrezgene);Acc:396772]
chr6	53675504	53676422	919	*	chr6	53675504	53676422	in_vitro	in_vivo	13	9	5.19538462	4.42222222	0.77316239	c(t = 0.29448	0.772	0.95508648	Distal Intergenic	ENSSSCG000	EMP3	epithelial membrane protein 3 [Source:VGNC Symbol;Acc:VGNC:87690]
chr1	119407272	119408048	777	*	chr1	119407272	119408048	in_vitro	in_vivo	10	10	2.941	2.353	0.588	c(t = 0.29434	0.772	0.95508648	Distal Intergenic	ENSSSCG000	ARPP19	cAMP regulated phosphoprotein 19 [Source:NCBI gene (formerly Entrezgene);Acc:397362]
chr14	88534131	88535045	915	*	chr14	88534131	88535045	in_vitro	in_vivo	13	9	2.74076923	2.28222222	0.45854701	c(t = 0.29365	0.772	0.95508648	Distal Intergenic	ENSSSCG00000010370		annexin A8 [Source:NCBI gene (formerly Entrezgene);Acc:100155930]
chr4	109785411	109787362	1952	*	chr4	109785411	109787362	in_vitro	in_vivo	15	15	1.89733333	2.234	-0.3366667	c(t = -0.2929	0.772	0.95508648	Distal Intergenic	ENSSSCG000	PROK1	prokineticin 1 [Source:NCBI gene (formerly Entrezgene);Acc:100359362]
chr13	132430708	132431716	1009	*	chr13	132430708	132431716	in_vitro	in_vivo	10	10	2.244	1.886	0.358	c(t = 0.29418	0.772	0.95508648	Distal Intergenic	ENSSSCG000	HES1	hes family bHLH transcription factor 1 [Source:NCBI gene (formerly Entrezgene);Acc:100499567]
chr6	80034909	80035919	1011	*	chr6	80034909	80035919	in_vitro	in_vivo	11	10	1.23272727	1.499	-0.2662727	c(t = -0.2946	0.772	0.95508648	Distal Intergenic	ENSSSCG00000030015		
chr10	26632850	26633333	484	*	chr10	26632850	26633333	in_vitro	in_vivo	9	10	0.58222222	0.428	0.15422222	c(t = 0.29439	0.772	0.95508648	Distal Intergenic	ENSSSCG000	ssc-mir-24-2	ssc-mir-24-2 [Source:miRBase;Acc:MI0013111]
chr14	138781217	138783237	2021	*	chr14	138781217	138783237	in_vitro	in_vivo	12	12	96.1758333	96.6491667	-0.4733333	c(t = -0.2922	0.773	0.95508648	Distal Intergenic	ENSSSCG000	GLRX3	glutaredoxin 3 [Source:VGNC Symbol;Acc:VGNC:88495]
chr4	124958464	124959182	719	*	chr4	124958464	124959182	in_vitro	in_vivo	12	11	8.06833333	10.4309091	-2.3625758	c(t = -0.2916	0.773	0.95508648	Distal Intergenic	ENSSSCG000	TGFBR3	transforming growth factor beta receptor 3 [Source:VGNC Symbol;Acc:VGNC:93932]
chr6	170232291	170233522	1232	*	chr6	170232291	170233522	in_vitro	in_vivo	9	10	3.45888889	4.18	-0.7211111	c(t = -0.2936	0.773	0.95508648	Distal Intergenic	ENSSSCG000	ssc-mir-9829	ssc-mir-9829 [Source:miRBase;Acc:MI0031598]
chr7	63487212	63487951	740	*	chr7	63487212	63487951	in_vitro	in_vivo	9	11	4.70666667	3.91636364	0.79030303	c(t = 0.29546	0.773	0.95508648	Promoter (<=1kb)	ENSSSCG000	NKX2-1	NK2 homeobox 1 [Source:VGNC Symbol;Acc:VGNC:90767]
chr13	26035796	26037110	1315	*	chr13	26035796	26037110	in_vitro	in_vivo	10	12	4.872	3.81083333	1.06116667	c(t = 0.29385	0.773	0.95508648	Promoter (<=1kb)	ENSSSCG000	VIPR1	vasoactive intestinal peptide receptor 1 [Source:VGNC Symbol;Acc:VGNC:94827]
chr14	112129173	112130185	1013	*	chr14	112129173	112130185	in_vitro	in_vivo	10	13	2.025	2.41615385	-0.3911538	c(t = -0.2930	0.773	0.95508648	Distal Intergenic	ENSSSCG000	KAZALD1	Kazal type serine peptidase inhibitor domain 1 [Source:NCBI gene (formerly Entrezgene);Acc:100624902]
chr3	39274545	39275811	1267	*	chr3	39274545	39275811	in_vitro	in_vivo	10	11	2.74	2.27272727	0.46727273	c(t = 0.29235	0.773	0.95508648	Promoter (<=1kb)	ENSSSCG000	SRRM2	serine/arginine repetitive matrix 2 [Source:VGNC Symbol;Acc:VGNC:93468]
chr11	5486502	5487984	1483	*	chr11	5486502	5487984	in_vitro	in_vivo	10	15	1.214	1.44533333	-0.2313333	c(t = -0.2918	0.773	0.95508648	Distal Intergenic	ENSSSCG000	CDX2	caudal type homeobox 2 [Source:NCBI gene (formerly Entrezgene);Acc:100127132]
chr2	12530913	12532327	1415	*	chr2	12530913	12532327	in_vitro	in_vivo	9	12	1.00666667	0.80416667	0.2025	c(t = 0.29284	0.773	0.95508648	Distal Intergenic	ENSSSCG000	MPEG1	macrophage expressed 1 [Source:NCBI gene (formerly Entrezgene);Acc:100516953]
chr6	57964410	57965514	1105	*	chr6	57964410	57965514	in_vitro	in_vivo	9	12	0.51888889	0.70916667	-0.1902778	c(t = -0.2932	0.773	0.95508648	Promoter (<=1kb)	ENSSSCG000	PPP2R1A	protein phosphatase 2 scaffold subunit Aalpha [Source:NCBI gene (formerly Entrezgene);Acc:397088]
chr12	60891851	60894496	2646	*	chr12	60891851	60894496	in_vitro	in_vivo	10	11	91.091	89.9309091	1.16009091	c(t = 0.29103	0.774	0.95508648	Promoter (<=1kb)	ENSSSCG00000018055		phosphatidylethanolamine N-methyltransferase [Source:VGNC Symbol;Acc:VGNC:91311]
chr6	50437768	50439086	1319	*	chr6	50437768	50439086	in_vitro	in_vivo	11	9	81.1390909	78.7277778	2.41131313	c(t = 0.29198	0.774	0.95508648	Distal Intergenic	ENSSSCG000	IRGC	immunity related GTPase cinema [Source:NCBI gene (formerly Entrezgene);Acc:100462753]
chr13	189199673	189200588	916	*	chr13	189199673	189200588	in_vitro	in_vivo	10	12	3.21	2.54416667	0.66583333	c(t = 0.29086	0.774	0.95508648	Distal Intergenic	ENSSSCG000	ssc-mir-155	ssc-mir-155 [Source:miRBase;Acc:MI0015907]
chr1	269995271	269995975	705	*	chr1	269995271	269995975	in_vitro	in_vivo	9	10	2.63888889	2	0.63888889	c(t = 0.29315	0.774	0.95508648	Distal Intergenic	ENSSSCG000	TOR1A	torsin family 1 member A [Source:NCBI gene (formerly Entrezgene);Acc:100125953]
chr6	60830789	60831791	1003	*	chr6	60830789	60831791	in_vitro	in_vivo	11	11	1.55818182	1.94636364	-0.3881818	c(t = -0.2922	0.774	0.95508648	Distal Intergenic	ENSSSCG000	GALP	galanin like peptide [Source:NCBI gene (formerly Entrezgene);Acc:396772]
chr12	10362205	10363245	1041	*	chr12	10362205	10363245	in_vitro	in_vivo	11	12	2.43090909	1.92583333	0.50507576	c(t = 0.29251	0.774	0.95508648	Distal Intergenic	ENSSSCG000	KCNJ2	potassium inwardly rectifying channel subfamily J member 2 [Source:VGNC Symbol;Acc:VGNC:89357]
chr13	11358483	11360380	1898	*	chr13	11358483	11360380	in_vitro	in_vivo	11	12	1.51181818	1.84333333	-0.3315152	c(t = -0.2903	0.774	0.95508648	Distal Intergenic	ENSSSCG00000037030		ribosomal protein L15 [Source:NCBI gene (formerly Entrezgene);Acc:100620094]
chr4	42985012	42985771	760	*	chr4	42985012	42985771	in_vitro	in_vivo	11	13	0.88	1.08538462	-0.2053846	c(t = -0.2902	0.774	0.95508648	Distal Intergenic	ENSSSCG000	CCNE2	cyclin E2 [Source:VGNC Symbol;Acc:VGNC:86355]
chr9	97114341	97115086	746	*	chr9	97114341	97115086	in_vitro	in_vivo	9	13	1.34666667	1.02923077	0.3174359	c(t = 0.29144	0.774	0.95508648	Distal Intergenic	ENSSSCG000	CACNA2D1	calcium voltage-gated channel auxiliary subunit alpha2delta 1 [Source:VGNC Symbol;Acc:VGNC:86120]
chr1	14910298	14912886	2589	*	chr1	14910298	14912886	in_vitro	in_vivo	9	9	35.1222222	30.2977778	4.82444444	c(t = 0.29054	0.775	0.95508648	Distal Intergenic	ENSSSCG000	ARMT1	acidic residue methyltransferase 1 [Source:NCBI gene (formerly Entrezgene);Acc:100157036]
chr14	74885628	74886832	1205	*	chr14	74885628	74886832	in_vitro	in_vivo	11	12	22.3736364	26.8558333	-4.482197	c(t = -0.2898	0.775	0.95508648	Distal Intergenic	ENSSSCG000	PSAP	prosaposin [Source:VGNC Symbol;Acc:VGNC:109480]
chrX	125188874	125190678	1805	*	chrX	125188874	125190678	in_vitro	in_vivo	12	13	22.17	18.8253846	3.34461538	c(t = 0.28880	0.775	0.95508648	Distal Intergenic	ENSSSCG000	DKC1	dyskerin pseudouridine synthase 1 [Source:NCBI gene (formerly Entrezgene);Acc:100216430]
chr12	57613004	57613634	631	*	chr12	57613004	57613634	in_vitro	in_vivo	10	10	19.521	15.734	3.787	c(t = 0.29161	0.775	0.95508648	Distal Intergenic	ENSSSCG000	ELAC2	elaC ribonuclease Z 2 [Source:NCBI gene (formerly Entrezgene);Acc:100520804]
chr2	138352144	138353776	1633	*	chr2	138352144	138353776	in_vitro	in_vivo	10	15	7.551	6.50866667	1.04233333	c(t = 0.29103	0.775	0.95508648	Distal Intergenic	ENSSSCG000	TRPC7	transient receptor potential cation channel subfamily C member 7 [Source:NCBI gene (formerly Entrezgene);Acc:100271727]
chr1	169801455	169802431	977	*	chr1	169801455	169802431	in_vitro	in_vivo	11	11	3.39181818	4.03454545	-0.6427273	c(t = -0.2914	0.775	0.95508648	Distal Intergenic	ENSSSCG000	PNN	pinin, desmosome associated protein [Source:NCBI gene (formerly Entrezgene);Acc:100526149]
chr14	135196530	135197449	920	*	chr14	135196530	135197449	in_vitro	in_vivo	10	11	1.92	1.54545455	0.37454545	c(t = 0.29143	0.775	0.95508648	Distal Intergenic	ENSSSCG000	UROS	uroporphyrinogen III synthase [Source:VGNC Symbol;Acc:VGNC:94735]
chr2	88022630	88024393	1764	*	chr2	88022630	88024393	in_vitro	in_vivo	12	15	1.36333333	1.15933333	0.204	c(t = 0.28892	0.775	0.95508648	Distal Intergenic	ENSSSCG000	BHMT	betaine--homocysteine S-methyltransferase [Source:NCBI gene (formerly Entrezgene);Acc:397371]
chr18	33422978	33424202	1225	*	chr18	33422978	33424202	in_vitro	in_vivo	10	13	0.909	1.10307692	-0.1940769	c(t = -0.2901	0.775	0.95508648	Distal Intergenic	ENSSSCG000	IFRD1	interferon related developmental regulator 1 [Source:NCBI gene (formerly Entrezgene);Acc:493185]
chr9	139277140	139277482	343	*	chr9	139277140	139277482	in_vitro	in_vivo	10	9	19.16	23.2988889	-4.1388889	c(t = -0.2890	0.776	0.95508648	Distal Intergenic	ENSSSCG000	EGFR	epidermal growth factor receptor [Source:NCBI gene (formerly Entrezgene);Acc:397070]
chr5	87864128	87865086	959	*	chr5	87864128	87865086	in_vitro	in_vivo	12	12	14.6416667	16.4625	-1.8208333	c(t = -0.2884	0.776	0.95508648	Distal Intergenic	ENSSSCG000	ssc-mir-331	ssc-mir-331 [Source:miRBase;Acc:MI0013142]
chr3	37752846	37754881	2036	*	chr3	37752846	37754881	in_vitro	in_vivo	9	12	4.44	6.055	-1.615	c(t = -0.2885	0.776	0.95508648	Distal Intergenic	ENSSSCG000	CDIP1	cell death inducing p53 target 1 [Source:VGNC Symbol;Acc:VGNC:86493]
chr5	757124	758679	1556	*	chr5	757124	758679	in_vitro	in_vivo	10	12	2.051	2.40333333	-0.3523333	c(t = -0.2887	0.776	0.95508648	Distal Intergenic	ENSSSCG000	SELENOO	selenoprotein O [Source:NCBI gene (formerly Entrezgene);Acc:100518971]
chr17	49527991	49528589	599	*	chr17	49527991	49528589	in_vitro	in_vivo	9	11	1.61666667	1.96545455	-0.3487879	c(t = -0.2889	0.776	0.95508648	Promoter (<=1kb)	ENSSSCG000	NCOA3	nuclear receptor coactivator 3 [Source:NCBI gene (formerly Entrezgene);Acc:100135659]
chr1	39868299	39868836	538	*	chr1	39868299	39868836	in_vitro	in_vivo	11	12	2.09363636	1.665	0.42863636	c(t = 0.28818	0.776	0.95508648	Distal Intergenic	ENSSSCG000	FABP7	fatty acid binding protein 7 [Source:NCBI gene (formerly Entrezgene);Acc:574075]
chr13	96651679	96652236	558	*	chr13	96651679	96652236	in_vitro	in_vivo	9	12	1.26222222	1.65416667	-0.3919444	c(t = -0.2901	0.776	0.95508648	Distal Intergenic	ENSSSCG000	SSR3	signal sequence receptor subunit 3 [Source:VGNC Symbol;Acc:VGNC:93492]
chr2	136777835	136781105	3271	*	chr2	136777835	136781105	in_vitro	in_vivo	11	10	1.36363636	1.116	0.24763636	c(t = 0.28902	0.776	0.95508648	Distal Intergenic	ENSSSCG000	CDKN2AIPNL	CDKN2A interacting protein N-terminal like [Source:NCBI gene (formerly Entrezgene);Acc:100513343]
chr2	27558961	27560250	1290	*	chr2	27558961	27560250	in_vitro	in_vivo	13	16	1.13307692	0.939375	0.19370192	c(t = 0.28692	0.776	0.95508648	Distal Intergenic	ENSSSCG000	CSTF3	cleavage stimulation factor subunit 3 [Source:NCBI gene (formerly Entrezgene);Acc:100622040]
chr8	9162201	9162625	425	*	chr8	9162201	9162625	in_vitro	in_vivo	9	11	1.24333333	1.61	-0.3666667	c(t = -0.2880	0.777	0.95587019	Distal Intergenic	ENSSSCG000	RAB28	RAB28, member RAS onco family [Source:VGNC Symbol;Acc:VGNC:98955]
chr8	40157178	40157974	797	*	chr8	40157178	40157974	in_vitro	in_vivo	11	11	0.59454545	0.78545455	-0.1909091	c(t = -0.2869	0.777	0.95587019	Distal Intergenic	ENSSSCG000	PDGFRA	platelet derived growth factor receptor alpha [Source:NCBI gene (formerly Entrezgene);Acc:100627123]
chr2	150414375	150415847	1473	*	chr2	150414375	150415847	in_vitro	in_vivo	11	12	94.2918182	93.4791667	0.81265152	c(t = 0.28522	0.778	0.95587019	Distal Intergenic	ENSSSCG000	AFAP1L1	actin filament associated protein 1 like 1 [Source:NCBI gene (formerly Entrezgene);Acc:100513463]
chr3	111834636	111836825	2190	*	chr3	111834636	111836825	in_vitro	in_vivo	12	11	5.765	6.68454545	-0.9195455	c(t = -0.2856	0.778	0.95587019	3' UTR	ENSSSCG000	TRIM54	tripartite motif containing 54 [Source:NCBI gene (formerly Entrezgene);Acc:100689265]
chr13	69723218	69724571	1354	*	chr13	69723218	69724571	in_vitro	in_vivo	9	12	4.57555556	3.7975	0.77805556	c(t = 0.28734	0.778	0.95587019	Distal Intergenic	ENSSSCG000	CCDC174	coiled-coil domain containing 174 [Source:NCBI gene (formerly Entrezgene);Acc:100689257]
chr12	45504906	45505816	911	*	chr12	45504906	45505816	in_vitro	in_vivo	12	9	2.54833333	2.07777778	0.47055556	c(t = 0.28632	0.778	0.95587019	Distal Intergenic	NA	NA	NA
chr8	73303508	73305680	2173	*	chr8	73303508	73305680	in_vitro	in_vivo	13	15	1.67307692	1.42133333	0.25174359	c(t = 0.28495	0.778	0.95587019	Distal Intergenic	ENSSSCG000	NPY2R	neuropeptide Y receptor Y2 [Source:VGNC Symbol;Acc:VGNC:109473]
chr7	34183141	34183666	526	*	chr7	34183141	34183666	in_vitro	in_vivo	10	12	1.097	0.79833333	0.29866667	c(t = 0.28652	0.778	0.95587019	Distal Intergenic	ENSSSCG000	BTBD9	BTB domain containing 9 [Source:VGNC Symbol;Acc:VGNC:85909]
chr6	18155551	18156168	618	*	chr6	18155551	18156168	in_vitro	in_vivo	9	9	1.03666667	0.74111111	0.29555556	c(t = 0.28701	0.778	0.95587019	Promoter (<=1kb)	ENSSSCG000	CDH1	cadherin 1 [Source:NCBI gene (formerly Entrezgene);Acc:100048953]
chr12	33396669	33397852	1184	*	chr12	33396669	33397852	in_vitro	in_vivo	9	13	79.1911111	82.9146154	-3.7235043	c(t = -0.2849	0.779	0.95587019	Promoter (<=1kb)	ENSSSCG000	AKAP1	A-kinase anchoring protein 1 [Source:NCBI gene (formerly Entrezgene);Acc:100533549]
chr17	62634898	62643370	8473	*	chr17	62634898	62643370	in_vitro	in_vivo	11	9	28.2772727	26.4466667	1.83060606	c(t = 0.28479	0.779	0.95587019	Distal Intergenic	ENSSSCG000	LIME1	Lck interacting transmembrane adaptor 1 [Source:NCBI gene (formerly Entrezgene);Acc:100625216]
chr8	111720688	111722637	1950	*	chr8	111720688	111722637	in_vitro	in_vivo	9	12	26.5433333	23.3508333	3.1925	c(t = 0.28520	0.779	0.95587019	3' UTR	ENSSSCG000	PITX2	paired like homeodomain 2 [Source:VGNC Symbol;Acc:VGNC:91467]
chr6	59372963	59374160	1198	*	chr6	59372963	59374160	in_vitro	in_vivo	12	10	6.8775	8.685	-1.8075	c(t = -0.2844	0.779	0.95587019	Distal Intergenic	ENSSSCG000	TNNI3	troponin I3, cardiac type [Source:NCBI gene (formerly Entrezgene);Acc:100049696]
chrX	84732512	84733090	579	*	chrX	84732512	84733090	in_vitro	in_vivo	10	10	9.131	6.573	2.558	c(t = 0.28467	0.779	0.95587019	Distal Intergenic	ENSSSCG000	PLP1	proteolipid protein 1 [Source:NCBI gene (formerly Entrezgene);Acc:397029]
chr13	5396386	5398195	1810	*	chr13	5396386	5398195	in_vitro	in_vivo	10	10	4.645	5.211	-0.566	c(t = -0.2851	0.779	0.95587019	Distal Intergenic	ENSSSCG000	RAB5A	RAB5A, member RAS onco family [Source:VGNC Symbol;Acc:VGNC:108687]
chr15	81093552	81094380	829	*	chr15	81093552	81094380	in_vitro	in_vivo	9	10	3.25222222	2.645	0.60722222	c(t = 0.28536	0.779	0.95587019	Promoter (<=1kb)	ENSSSCG000	ATP5MC3	ATP synthase membrane subunit c locus 3 [Source:NCBI gene (formerly Entrezgene);Acc:100513629]
chr7	55766067	55767221	1155	*	chr7	55766067	55767221	in_vitro	in_vivo	11	10	1.65181818	1.367	0.28481818	c(t = 0.28530	0.779	0.95587019	Distal Intergenic	ENSSSCG000	IDH2	isocitrate dehydrogenase (NADP(+)) 2 [Source:NCBI gene (formerly Entrezgene);Acc:397603]
chr6	63667510	63668757	1248	*	chr6	63667510	63668757	in_vitro	in_vivo	10	10	1.509	1.306	0.203	c(t = 0.28569	0.779	0.95587019	Distal Intergenic	ENSSSCG000	TAS1R3	taste 1 receptor member 3 [Source:NCBI gene (formerly Entrezgene);Acc:100127440]
chr15	132426010	132426786	777	*	chr15	132426010	132426786	in_vitro	in_vivo	11	12	0.78181818	1.04583333	-0.2640152	c(t = -0.2849	0.779	0.95587019	Distal Intergenic	ENSSSCG000	PTMA	prothymosin alpha [Source:VGNC Symbol;Acc:VGNC:106455]
chr5	81366579	81367544	966	*	chr5	81366579	81367544	in_vitro	in_vivo	11	9	2.14090909	2.65444444	-0.5135354	c(t = -0.2854	0.78	0.95633376	Distal Intergenic	ENSSSCG000	IGF1	insulin like growth factor 1 [Source:VGNC Symbol;Acc:VGNC:98044]
chr6	96576483	96578016	1534	*	chr6	96576483	96578016	in_vitro	in_vivo	14	12	2.11928571	2.64916667	-0.529881	c(t = -0.2830	0.78	0.95633376	Distal Intergenic	ENSSSCG000	CIDEA	cell death inducing DFFA like effector a [Source:NCBI gene (formerly Entrezgene);Acc:100127171]
chr11	78708263	78709912	1650	*	chr11	78708263	78709912	in_vitro	in_vivo	9	13	2.54555556	2.20384615	0.3417094	c(t = 0.28465	0.78	0.95633376	Promoter (<=1kb)	ENSSSCG000	TMCO3	transmembrane and coiled-coil domains 3 [Source:VGNC Symbol;Acc:VGNC:94050]
chr13	124623866	124624403	538	*	chr13	124623866	124624403	in_vitro	in_vivo	11	11	2.25909091	1.78363636	0.47545455	c(t = 0.28342	0.78	0.95633376	Distal Intergenic	ENSSSCG000	ADIPOQ	adiponectin, C1Q and collagen domain containing [Source:NCBI gene (formerly Entrezgene);Acc:397660]
chr1	207347157	207348202	1046	*	chr1	207347157	207348202	in_vitro	in_vivo	11	9	1.42818182	1.09666667	0.33151515	c(t = 0.28326	0.78	0.95633376	Distal Intergenic	ENSSSCG000	PSIP1	PC4 and SFRS1 interacting protein 1 [Source:VGNC Symbol;Acc:VGNC:91898]
chr1	150975185	150976009	825	*	chr1	150975185	150976009	in_vitro	in_vivo	10	10	1.955	2.6	-0.645	c(t = -0.2819	0.781	0.95649163	Promoter (1-2kb)	ENSSSCG000	CBLN2	cerebellin 2 [Source:VGNC Symbol;Acc:VGNC:86226]
chr3	78897752	78898613	862	*	chr3	78897752	78898613	in_vitro	in_vivo	12	14	2.4475	2.09428571	0.35321429	c(t = 0.28213	0.781	0.95649163	Distal Intergenic	ENSSSCG000	TMEM17	transmembrane protein 17 [Source:NCBI gene (formerly Entrezgene);Acc:100627459]
chr13	148578091	148579394	1304	*	chr13	148578091	148579394	in_vitro	in_vivo	12	13	2.10333333	1.79846154	0.30487179	c(t = 0.28209	0.781	0.95649163	Distal Intergenic	ENSSSCG000	CD96	CD96 molecule [Source:VGNC Symbol;Acc:VGNC:86437]
chr3	83337181	83337746	566	*	chr3	83337181	83337746	in_vitro	in_vivo	10	13	2.006	1.64153846	0.36446154	c(t = 0.28312	0.781	0.95649163	Distal Intergenic	ENSSSCG000	ssc-mir-9789	ssc-mir-9789 [Source:miRBase;Acc:MI0031551]
chr13	73900426	73901282	857	*	chr13	73900426	73901282	in_vitro	in_vivo	11	13	1.30090909	1.58615385	-0.2852448	c(t = -0.2818	0.781	0.95649163	Distal Intergenic	ENSSSCG000	ACKR4	atypical chemokine receptor 4 [Source:NCBI gene (formerly Entrezgene);Acc:100037292]
chr7	97197718	97198438	721	*	chr7	97197718	97198438	in_vitro	in_vivo	10	10	1.487	1.204	0.283	c(t = 0.28200	0.781	0.95649163	Distal Intergenic	ENSSSCG000	LIN52	lin-52 DREAM MuvB core complex component [Source:NCBI gene (formerly Entrezgene);Acc:100157325]
chr14	40624197	40624645	449	*	chr14	40624197	40624645	in_vitro	in_vivo	11	9	0.69909091	0.91666667	-0.2175758	c(t = -0.2826	0.781	0.95649163	Distal Intergenic	ENSSSCG000	ACADS	acyl-CoA dehydrogenase short chain [Source:VGNC Symbol;Acc:VGNC:97862]
chr6	52526446	52527330	885	*	chr6	52526446	52527330	in_vitro	in_vivo	9	12	10.8166667	13.0066667	-2.19	c(t = -0.2808	0.782	0.95650738	Distal Intergenic	ENSSSCG000	CALM3	calmodulin 3 [Source:NCBI gene (formerly Entrezgene);Acc:396838]
chr4	127900648	127901696	1049	*	chr4	127900648	127901696	in_vitro	in_vivo	12	14	13.6258333	10.5907143	3.03511905	c(t = 0.28040	0.782	0.95650738	Distal Intergenic	ENSSSCG000	GBP1	guanylate binding protein 1, interferon-inducible [Source:NCBI gene (formerly Entrezgene);Acc:100151938]
chr5	67412103	67412765	663	*	chr5	67412103	67412765	in_vitro	in_vivo	10	10	1.379	1.74	-0.361	c(t = -0.2810	0.782	0.95650738	Promoter (<=1kb)	ENSSSCG000	WASHC1	WASH complex subunit 1 [Source:NCBI gene (formerly Entrezgene);Acc:100512536]
chr5	73496341	73497649	1309	*	chr5	73496341	73497649	in_vitro	in_vivo	14	11	1.24857143	1.43363636	-0.1850649	c(t = -0.2794	0.782	0.95650738	Distal Intergenic	ENSSSCG000	ADAMTS20	ADAM metallopeptidase with thrombospondin type 1 motif 20 [Source:VGNC Symbol;Acc:VGNC:85082]
chr7	53639893	53641617	1725	*	chr7	53639893	53641617	in_vitro	in_vivo	15	16	1.64133333	1.4325	0.20883333	c(t = 0.27966	0.782	0.95650738	Promoter (<=1kb)	ENSSSCG000	ZXDC	ZXD family zinc finger C [Source:VGNC Symbol;Acc:VGNC:95343]
chr9	109260511	109261426	916	*	chr9	109260511	109261426	in_vitro	in_vivo	9	9	1.53444444	1.14777778	0.38666667	c(t = 0.28289	0.782	0.95650738	Promoter (<=1kb)	ENSSSCG000	PDIA4	protein disulfide isomerase family A member 4 [Source:NCBI gene (formerly Entrezgene);Acc:100522203]
chr1	261407092	261407943	852	*	chr1	261407092	261407943	in_vitro	in_vivo	10	10	0.684	1	-0.316	c(t = -0.2820	0.782	0.95650738	Distal Intergenic	ENSSSCG000	RAB14	RAB14, member RAS onco family [Source:VGNC Symbol;Acc:VGNC:98238]
chr5	1230211	1231838	1628	*	chr5	1230211	1231838	in_vitro	in_vivo	10	11	26.363	22.48	3.883	c(t = 0.27943	0.783	0.95650738	Distal Intergenic	ENSSSCG000	SELENOO	selenoprotein O [Source:NCBI gene (formerly Entrezgene);Acc:100518971]
chr10	17437860	17438677	818	*	chr10	17437860	17438677	in_vitro	in_vivo	10	12	7.783	9.37583333	-1.5928333	c(t = -0.2805	0.783	0.95650738	Distal Intergenic	ENSSSCG000	DESI2	desumoylating isopeptidase 2 [Source:VGNC Symbol;Acc:VGNC:96037]
chr2	117837619	117838340	722	*	chr2	117837619	117838340	in_vitro	in_vivo	12	13	2.55083333	3.08769231	-0.536859	c(t = -0.2792	0.783	0.95650738	Distal Intergenic	ENSSSCG000	APC	APC regulator of WNT signaling pathway [Source:NCBI gene (formerly Entrezgene);Acc:100517932]
chr9	389501	390604	1104	*	chr9	389501	390604	in_vitro	in_vivo	10	12	3.267	2.7925	0.4745	c(t = 0.28055	0.783	0.95650738	Distal Intergenic	NA	NA	NA
chr1	87116492	87118485	1994	*	chr1	87116492	87118485	in_vitro	in_vivo	10	12	1.412	1.65166667	-0.2396667	c(t = -0.2787	0.783	0.95650738	Distal Intergenic	ENSSSCG000	HMGN3	high mobility group nucleosomal binding domain 3 [Source:VGNC Symbol;Acc:VGNC:88908]
chr8	32966867	32967385	519	*	chr8	32966867	32967385	in_vitro	in_vivo	10	9	1.158	1.47777778	-0.3197778	c(t = -0.2804	0.783	0.95650738	Distal Intergenic	ENSSSCG000	TMEM33	transmembrane protein 33 [Source:VGNC Symbol;Acc:VGNC:94171]
chr7	19585798	19586629	832	*	chr7	19585798	19586629	in_vitro	in_vivo	9	12	0.66111111	0.86916667	-0.2080556	c(t = -0.2797	0.783	0.95650738	Distal Intergenic	ENSSSCG000	ACOT13	acyl-CoA thioesterase 13 [Source:VGNC Symbol;Acc:VGNC:85018]
chr15	140267909	140269704	1796	*	chr15	140267909	140269704	in_vitro	in_vivo	9	10	70.0822222	66.818	3.26422222	c(t = 0.27896	0.784	0.95650738	Distal Intergenic	ENSSSCG000	GAL3ST2	galactose-3-O-sulfotransferase 2 [Source:NCBI gene (formerly Entrezgene);Acc:396666]
chr17	58658835	58660418	1584	*	chr17	58658835	58660418	in_vitro	in_vivo	15	13	26.6806667	30.7523077	-4.071641	c(t = -0.2772	0.784	0.95650738	Distal Intergenic	ENSSSCG000	VAPB	VAMP associated protein B and C [Source:VGNC Symbol;Acc:VGNC:108743]
chr15	139493758	139495902	2145	*	chr15	139493758	139495902	in_vitro	in_vivo	9	10	11.7688889	14.286	-2.5171111	c(t = -0.2781	0.784	0.95650738	Distal Intergenic	ENSSSCG000	ssc-mir-149	ssc-mir-149 [Source:miRBase;Acc:MI0016620]
chrX	30614273	30615320	1048	*	chrX	30614273	30615320	in_vitro	in_vivo	13	13	5.40538462	6.59692308	-1.1915385	c(t = -0.2773	0.784	0.95650738	Distal Intergenic	ENSSSCG00000028148		dystrophin [Source:NCBI gene (formerly Entrezgene);Acc:497636]
chr3	78361282	78362614	1333	*	chr3	78361282	78362614	in_vitro	in_vivo	10	13	2.05	2.37769231	-0.3276923	c(t = -0.2773	0.784	0.95650738	Distal Intergenic	ENSSSCG000	MDH1	malate dehydrogenase 1 [Source:NCBI gene (formerly Entrezgene);Acc:396894]
chr5	83935730	83936450	721	*	chr5	83935730	83936450	in_vitro	in_vivo	10	11	2.403	2.06090909	0.34209091	c(t = 0.27869	0.784	0.95650738	Distal Intergenic	ENSSSCG000	NR1H4	nuclear receptor subfamily 1 group H member 4 [Source:VGNC Symbol;Acc:VGNC:90872]
chr15	25606000	25606675	676	*	chr15	25606000	25606675	in_vitro	in_vivo	10	9	2.096	1.62	0.476	c(t = 0.27928	0.784	0.95650738	Distal Intergenic	ENSSSCG000	BIN1	bridging integrator 1 [Source:VGNC Symbol;Acc:VGNC:95475]
chr8	4122231	4123579	1349	*	chr8	4122231	4123579	in_vitro	in_vivo	10	10	1.005	1.231	-0.226	c(t = -0.2790	0.784	0.95650738	Distal Intergenic	ENSSSCG000	MAN2B2	mannosidase alpha class 2B member 2 [Source:VGNC Symbol;Acc:VGNC:98100]
chr5	11365174	11366712	1539	*	chr5	11365174	11366712	in_vitro	in_vivo	10	15	0.935	0.734	0.201	c(t = 0.27854	0.784	0.95650738	Distal Intergenic	ENSSSCG000	TXN2	thioredoxin 2 [Source:VGNC Symbol;Acc:VGNC:94602]
chr14	132482479	132483634	1156	*	chr14	132482479	132483634	in_vitro	in_vivo	13	11	0.44846154	0.64909091	-0.2006294	c(t = -0.2793	0.784	0.95650738	Distal Intergenic	ENSSSCG000	ssc-mir-4331	ssc-mir-4331-2 [Source:miRBase;Acc:MI0033415]
chr1	271209245	271210659	1415	*	chr1	271209245	271210659	in_vitro	in_vivo	11	9	92.1936364	93.4088889	-1.2152525	c(t = -0.2771	0.785	0.95652301	Distal Intergenic	ENSSSCG000	FUBP3	far upstream element binding protein 3 [Source:VGNC Symbol;Acc:VGNC:103093]
chr1	146002484	146004019	1536	*	chr1	146002484	146004019	in_vitro	in_vivo	10	10	3.418	4.265	-0.847	c(t = -0.2773	0.785	0.95652301	Promoter (<=1kb)	ENSSSCG000	NFATC1	nuclear factor of activated T cells 1 [Source:NCBI gene (formerly Entrezgene);Acc:397318]
chr12	36874832	36878466	3635	*	chr12	36874832	36878466	in_vitro	in_vivo	14	15	4.69071429	4.03666667	0.65404762	c(t = 0.27588	0.785	0.95652301	Distal Intergenic	ENSSSCG000	TBX2	T-box transcription factor 2 [Source:NCBI gene (formerly Entrezgene);Acc:100622658]
chr1	260756116	260757365	1250	*	chr1	260756116	260757365	in_vitro	in_vivo	13	14	1.55769231	1.73857143	-0.1808791	c(t = -0.2760	0.785	0.95652301	Distal Intergenic	ENSSSCG000	C5	complement C5 [Source:VGNC Symbol;Acc:VGNC:103041]
chr8	7422932	7423939	1008	*	chr8	7422932	7423939	in_vitro	in_vivo	11	14	1.64272727	1.39571429	0.24701299	c(t = 0.27671	0.785	0.95652301	Distal Intergenic	ENSSSCG000	RAB28	RAB28, member RAS onco family [Source:VGNC Symbol;Acc:VGNC:98955]
chr2	32645107	32646001	895	*	chr2	32645107	32646001	in_vitro	in_vivo	12	12	1.08083333	0.87583333	0.205	c(t = 0.27646	0.785	0.95652301	Promoter (<=1kb)	ENSSSCG000	BDNF	brain derived neurotrophic factor [Source:VGNC Symbol;Acc:VGNC:85795]
chr4	481394	486945	5552	*	chr4	481394	486945	in_vitro	in_vivo	14	14	25.8192857	24.1257143	1.69357143	c(t = 0.27463	0.786	0.95652301	Promoter (<=1kb)	ENSSSCG000	HSF1	heat shock transcription factor 1 [Source:VGNC Symbol;Acc:VGNC:88993]
chr13	145630044	145631177	1134	*	chr13	145630044	145631177	in_vitro	in_vivo	11	13	18.4427273	22.1846154	-3.7418881	c(t = -0.2746	0.786	0.95652301	Distal Intergenic	ENSSSCG000	ATP6V1A	ATPase H+ transporting V1 subunit A [Source:NCBI gene (formerly Entrezgene);Acc:445531]
chrX	122707368	122709996	2629	*	chrX	122707368	122709996	in_vitro	in_vivo	15	16	6.836	8.373125	-1.537125	c(t = -0.2744	0.786	0.95652301	Distal Intergenic	ENSSSCG000	FATE1	fetal and adult testis expressed 1 [Source:NCBI gene (formerly Entrezgene);Acc:396705]
chr12	52613339	52615262	1924	*	chr12	52613339	52615262	in_vitro	in_vivo	11	14	6.35	7.03857143	-0.6885714	c(t = -0.2745	0.786	0.95652301	Promoter (<=1kb)	ENSSSCG000	CLDN7	claudin 7 [Source:VGNC Symbol;Acc:VGNC:97930]
chr8	135895289	135896616	1328	*	chr8	135895289	135896616	in_vitro	in_vivo	11	12	4.36818182	3.53166667	0.83651515	c(t = 0.27579	0.786	0.95652301	Distal Intergenic	ENSSSCG000	ENOPH1	enolase-phosphatase 1 [Source:VGNC Symbol;Acc:VGNC:98922]
chr7	662737	665750	3014	*	chr7	662737	665750	in_vitro	in_vivo	13	14	2.99	2.60214286	0.38785714	c(t = 0.27527	0.786	0.95652301	Distal Intergenic	ENSSSCG000	IRF4	interferon regulatory factor 4 [Source:VGNC Symbol;Acc:VGNC:89207]
chr6	63050636	63051918	1283	*	chr6	63050636	63051918	in_vitro	in_vivo	9	11	1.29222222	1.69545455	-0.4032323	c(t = -0.2765	0.786	0.95652301	Distal Intergenic	ENSSSCG000	ISG15	ISG15 ubiquitin like modifier [Source:NCBI gene (formerly Entrezgene);Acc:100145895]
chr1	235592390	235593062	673	*	chr1	235592390	235593062	in_vitro	in_vivo	14	12	0.58428571	0.72666667	-0.142381	c(t = -0.2752	0.786	0.95652301	Distal Intergenic	ENSSSCG000	VCP	valosin containing protein [Source:VGNC Symbol;Acc:VGNC:109420]
chr1	73188281	73190609	2329	*	chr1	73188281	73190609	in_vitro	in_vivo	13	9	42.4715385	46.1155556	-3.6440171	c(t = -0.2738	0.787	0.95652301	Distal Intergenic	ENSSSCG000	PDSS2	decaprenyl diphosphate synthase subunit 2 [Source:VGNC Symbol;Acc:VGNC:91291]
chrX	43236707	43237168	462	*	chrX	43236707	43237168	in_vitro	in_vivo	11	10	8.34	10.736	-2.396	c(t = -0.2744	0.787	0.95652301	Distal Intergenic	ENSSSCG000	PLP2	proteolipid protein 2 [Source:NCBI gene (formerly Entrezgene);Acc:100512511]
chr13	117404593	117405057	465	*	chr13	117404593	117405057	in_vitro	in_vivo	13	11	4.56923077	3.54181818	1.02741259	c(t = 0.27358	0.787	0.95652301	Promoter (<=1kb)	ENSSSCG000	MFN1	mitofusin 1 [Source:VGNC Symbol;Acc:VGNC:90178]
chr18	17834573	17835185	613	*	chr18	17834573	17835185	in_vitro	in_vivo	9	9	3.35	2.71444444	0.63555556	c(t = 0.27539	0.787	0.95652301	Distal Intergenic	ENSSSCG000	ssc-mir-29b-	ssc-mir-29b-1 [Source:miRBase;Acc:MI0002431]
chr11	25064551	25065102	552	*	chr11	25064551	25065102	in_vitro	in_vivo	11	12	1.71272727	2.17416667	-0.4614394	c(t = -0.2754	0.787	0.95652301	Distal Intergenic	ENSSSCG000	RGCC	regulator of cell cycle [Source:VGNC Symbol;Acc:VGNC:92251]
chr10	52571382	52572306	925	*	chr10	52571382	52572306	in_vitro	in_vivo	11	12	1.39181818	1.57916667	-0.1873485	c(t = -0.2742	0.787	0.95652301	Promoter (<=1kb)	ENSSSCG000	SPAG6	sperm associated antigen 6 [Source:NCBI gene (formerly Entrezgene);Acc:100286811]
chr3	51511195	51511646	452	*	chr3	51511195	51511646	in_vitro	in_vivo	10	14	1.985	1.49	0.495	c(t = 0.27479	0.787	0.95652301	Distal Intergenic	ENSSSCG000	SLC9A2	solute carrier family 9 member A2 [Source:NCBI gene (formerly Entrezgene);Acc:100101921]
chr14	125108130	125108920	791	*	chr14	125108130	125108920	in_vitro	in_vivo	10	9	1.908	1.44888889	0.45911111	c(t = 0.27530	0.787	0.95652301	Distal Intergenic	ENSSSCG000	ADRB1	adrenoceptor beta 1 [Source:NCBI gene (formerly Entrezgene);Acc:397355]
chr1	66515495	66516311	817	*	chr1	66515495	66516311	in_vitro	in_vivo	12	9	1.06416667	1.39888889	-0.3347222	c(t = -0.2753	0.787	0.95652301	Distal Intergenic	ENSSSCG000	CCNC	cyclin C [Source:VGNC Symbol;Acc:VGNC:103046]
chr10	15722311	15724133	1823	*	chr10	15722311	15724133	in_vitro	in_vivo	12	11	0.9625	0.73818182	0.22431818	c(t = 0.27317	0.787	0.95652301	Distal Intergenic	ENSSSCG000	SCCPDH	saccharopine dehydrogenase (putative) [Source:NCBI gene (formerly Entrezgene);Acc:100512485]
chr11	4552039	4555416	3378	*	chr11	4552039	4555416	in_vitro	in_vivo	12	11	59.1025	62.7445455	-3.6420455	c(t = -0.2723	0.788	0.95668925	Distal Intergenic	ENSSSCG000	GPR12	G protein-coupled receptor 12 [Source:NCBI gene (formerly Entrezgene);Acc:100736641]
chr6	77356960	77357551	592	*	chr6	77356960	77357551	in_vitro	in_vivo	9	10	22.6244444	18.007	4.61744444	c(t = 0.27389	0.788	0.95668925	Exon (NM_001267894/100913086, exon 3 of 6)	ENSSSCG000	TAS1R2	taste 1 receptor member 2 [Source:VGNC Symbol;Acc:VGNC:93741]
chr17	47387366	47387911	546	*	chr17	47387366	47387911	in_vitro	in_vivo	9	10	2.78222222	3.788	-1.0057778	c(t = -0.2743	0.788	0.95668925	Distal Intergenic	ENSSSCG000	TOMM34	translocase of outer mitochondrial membrane 34 [Source:VGNC Symbol;Acc:VGNC:95647]
chr4	75645455	75646908	1454	*	chr4	75645455	75646908	in_vitro	in_vivo	12	10	2.255	1.841	0.414	c(t = 0.27314	0.788	0.95668925	Distal Intergenic	ENSSSCG000	SDR16C5	short chain dehydrogenase/reductase family 16C member 5 [Source:VGNC Symbol;Acc:VGNC:98853]
chr5	34002835	34004074	1240	*	chr5	34002835	34004074	in_vitro	in_vivo	9	13	1.25888889	1.56846154	-0.3095726	c(t = -0.2719	0.788	0.95668925	Distal Intergenic	ENSSSCG000	CCT2	chaperonin containing TCP1 subunit 2 [Source:VGNC Symbol;Acc:VGNC:86380]
chr9	25688585	25689000	416	*	chr9	25688585	25689000	in_vitro	in_vivo	10	11	0.623	0.45454545	0.16845455	c(t = 0.27269	0.788	0.95668925	Distal Intergenic	ENSSSCG000	C11orf54	chromosome 9 C11orf54 homolog [Source:VGNC Symbol;Acc:VGNC:86087]
chr11	77750875	77756110	5236	*	chr11	77750875	77756110	in_vitro	in_vivo	12	14	24.1458333	21.0885714	3.0572619	c(t = 0.27139	0.789	0.95668925	Distal Intergenic	ENSSSCG000	ANKRD10	ankyrin repeat domain 10 [Source:VGNC Symbol;Acc:VGNC:85322]
chr14	140762892	140767627	4736	*	chr14	140762892	140767627	in_vitro	in_vivo	11	11	17.7818182	21.0418182	-3.26	c(t = -0.2718	0.789	0.95668925	Distal Intergenic	ENSSSCG000	ssc-mir-202	ssc-mir-202 [Source:miRBase;Acc:MI0013157]
chr7	60009073	60011310	2238	*	chr7	60009073	60011310	in_vitro	in_vivo	11	15	5.18909091	5.724	-0.5349091	c(t = -0.2714	0.789	0.95668925	Distal Intergenic	ENSSSCG000	BBS4	Bardet-Biedl syndrome 4 [Source:NCBI gene (formerly Entrezgene);Acc:100513212]
chr4	109494617	109496524	1908	*	chr4	109494617	109496524	in_vitro	in_vivo	13	12	3.90846154	4.70333333	-0.7948718	c(t = -0.2722	0.789	0.95668925	Distal Intergenic	ENSSSCG000	KCNA2	potassium voltage-gated channel subfamily A member 2 [Source:VGNC Symbol;Acc:VGNC:89325]
chr6	169598340	169599978	1639	*	chr6	169598340	169599978	in_vitro	in_vivo	14	12	3.02142857	3.47083333	-0.4494048	c(t = -0.2711	0.789	0.95668925	Distal Intergenic	ENSSSCG000	ssc-mir-4338	ssc-mir-4338 [Source:miRBase;Acc:MI0015933]
chr1	100107346	100109196	1851	*	chr1	100107346	100109196	in_vitro	in_vivo	12	12	2.63	3.03416667	-0.4041667	c(t = -0.2708	0.789	0.95668925	Distal Intergenic	ENSSSCG000	MRO	maestro [Source:VGNC Symbol;Acc:VGNC:103127]
chr2	142165206	142167017	1812	*	chr2	142165206	142167017	in_vitro	in_vivo	13	15	1.29538462	1.672	-0.3766154	c(t = -0.2710	0.789	0.95668925	Promoter (<=1kb)	ENSSSCG000	ANKHD1	ankyrin repeat and KH domain containing 1 [Source:NCBI gene (formerly Entrezgene);Acc:100512270]
chr9	109245078	109246212	1135	*	chr9	109245078	109246212	in_vitro	in_vivo	10	11	23.292	19.4972727	3.79472727	c(t = 0.26968	0.79	0.95668925	Distal Intergenic	ENSSSCG000	PDIA4	protein disulfide isomerase family A member 4 [Source:NCBI gene (formerly Entrezgene);Acc:100522203]
chr7	121389442	121391655	2214	*	chr7	121389442	121391655	in_vitro	in_vivo	11	11	19.3618182	16.4945455	2.86727273	c(t = 0.26990	0.79	0.95668925	Distal Intergenic	ENSSSCG000	ssc-mir-9830	ssc-mir-9830 [Source:miRBase;Acc:MI0031599]
chr4	111293476	111294186	711	*	chr4	111293476	111294186	in_vitro	in_vivo	11	10	4.94272727	5.965	-1.0222727	c(t = -0.2699	0.79	0.95668925	Distal Intergenic	ENSSSCG000	SLC25A24	solute carrier family 25 member 24 [Source:NCBI gene (formerly Entrezgene);Acc:100518780]
chr7	76062697	76065080	2384	*	chr7	76062697	76065080	in_vitro	in_vivo	13	13	3.35846154	2.95923077	0.39923077	c(t = 0.26943	0.79	0.95668925	Distal Intergenic	ENSSSCG000	HAUS4	HAUS augmin like complex subunit 4 [Source:VGNC Symbol;Acc:VGNC:111456]
chr2	4234633	4237293	2661	*	chr2	4234633	4237293	in_vitro	in_vivo	12	13	2.11333333	2.4	-0.2866667	c(t = -0.2699	0.79	0.95668925	Distal Intergenic	ENSSSCG000	CPT1A	carnitine palmitoyltransferase 1A [Source:NCBI gene (formerly Entrezgene);Acc:399527]
chr1	203059343	203060423	1081	*	chr1	203059343	203060423	in_vitro	in_vivo	13	9	1.33076923	1.62444444	-0.2936752	c(t = -0.2706	0.79	0.95668925	Promoter (1-2kb)	ENSSSCG000	SLC24A2	solute carrier family 24 member 2 [Source:VGNC Symbol;Acc:VGNC:92987]
chr7	107815704	107816752	1049	*	chr7	107815704	107816752	in_vitro	in_vivo	10	13	1.508	1.17230769	0.33569231	c(t = 0.27083	0.79	0.95668925	Distal Intergenic	ENSSSCG000	GALC	galactosylceramidase [Source:VGNC Symbol;Acc:VGNC:88324]

chr6	44150156	44151845	1690	*	chr6	44150156	44151845	in_vitro	in_vivo	16	13	0.98625	1.10461538	-0.1183654	c(t = -0.2694	0.79	0.95668925	Distal Intergenic	ENSSSCG000	GPI	glucose-6-phosphate isomerase [Source:NCBI gene (formerly Entrezgene);Acc:397602]
chr7	68592709	68593944	1236	*	chr7	68592709	68593944	in_vitro	in_vivo	14	15	0.97857143	0.79933333	0.1792381	c(t = 0.26990	0.79	0.95668925	Distal Intergenic	ENSSSCG000	AP4S1	adaptor related protein complex 4 subunit sigma 1 [Source:VGNC Symbol;Acc:VGNC:85394]
chr7	59452068	59452518	451	*	chr7	59452068	59452518	in_vitro	in_vivo	9	11	1.01	0.70454545	0.30545455	c(t = 0.27236	0.79	0.95668925	Distal Intergenic	ENSSSCG000	CYP11A1	cytochrome P450 family 11 subfamily A member 1 [Source:VGNC Symbol;Acc:VGNC:103363]
chr12	3516970	3520191	3222	*	chr12	3516970	3520191	in_vitro	in_vivo	9	10	91.47	90.031	1.439	c(t = 0.27074	0.791	0.95699543	Distal Intergenic	ENSSSCG000	SOCS3	suppressor of cytokine signaling 3 [Source:VGNC Symbol;Acc:VGNC:99052]
chrX	41835426	41836890	1465	*	chrX	41835426	41836890	in_vitro	in_vivo	15	9	6.368	7.92777778	-1.5597778	c(t = -0.2699	0.791	0.95699543	Distal Intergenic	ENSSSCG000	RGN	regucalcin [Source:VGNC Symbol;Acc:VGNC:92256]
chr14	132696466	132698886	2421	*	chr14	132696466	132698886	in_vitro	in_vivo	9	13	1.61666667	1.87846154	-0.2617949	c(t = -0.2686	0.791	0.95699543	Distal Intergenic	ENSSSCG000	ssc-mir-4331	ssc-mir-4331-2 [Source:miRBase;Acc:MI0033415]
chr16	49188755	49189973	1219	*	chr16	49188755	49189973	in_vitro	in_vivo	13	11	1.94230769	1.62181818	0.32048951	c(t = 0.26954	0.791	0.95699543	Promoter (<=1kb)	ENSSSCG000	DRD1	dopamine receptor D1 [Source:VGNC Symbol;Acc:VGNC:87443]
chr2	147569161	147570944	1784	*	chr2	147569161	147570944	in_vitro	in_vivo	9	11	1.98444444	1.54272727	0.44171717	c(t = 0.26993	0.791	0.95699543	Distal Intergenic	ENSSSCG000	PPP2R2B	protein phosphatase 2 regulatory subunit Bbeta [Source:VGNC Symbol;Acc:VGNC:100336]
chr1	66538862	66540450	1589	*	chr1	66538862	66540450	in_vitro	in_vivo	11	10	1.53454545	1.288	0.24654545	c(t = 0.26914	0.791	0.95699543	Distal Intergenic	ENSSSCG000	CCNC	cyclin C [Source:VGNC Symbol;Acc:VGNC:103046]
chr17	56868608	56869358	751	*	chr17	56868608	56869358	in_vitro	in_vivo	10	11	12.59	15.7490909	-3.1590909	c(t = -0.2679	0.792	0.95730104	Promoter (<=1kb)	ENSSSCG000	MC3R	melanocortin 3 receptor [Source:NCBI gene (formerly Entrezgene);Acc:100141404]
chrX	125148477	125150848	2372	*	chrX	125148477	125150848	in_vitro	in_vivo	12	9	17.0091667	13.8277778	3.18138889	c(t = 0.26761	0.792	0.95730104	Distal Intergenic	ENSSSCG000	DKC1	dyskerin pseudouridine synthase 1 [Source:NCBI gene (formerly Entrezgene);Acc:100216430]
chr4	109121693	109122815	1123	*	chr4	109121693	109122815	in_vitro	in_vivo	9	12	2.88111111	2.27416667	0.60694444	c(t = 0.26813	0.792	0.95730104	Distal Intergenic	ENSSSCG000	DRAM2	DNA damage regulated autophagy modulator 2 [Source:VGNC Symbol;Acc:VGNC:87437]
chr8	11083949	11084606	658	*	chr8	11083949	11084606	in_vitro	in_vivo	10	13	2.233	1.95	0.283	c(t = 0.26680	0.792	0.95730104	Promoter (<=1kb)	ENSSSCG000	CD38	CD38 molecule [Source:NCBI gene (formerly Entrezgene);Acc:100511702]
chr12	37474881	37475664	784	*	chr12	37474881	37475664	in_vitro	in_vivo	9	9	1.70888889	1.19111111	0.51777778	c(t = 0.26947	0.792	0.95730104	Distal Intergenic	ENSSSCG000	CA4	carbonic anhydrase 4 [Source:NCBI gene (formerly Entrezgene);Acc:100511956]
chr3	100886889	100887924	1036	*	chr3	100886889	100887924	in_vitro	in_vivo	14	14	1.15428571	0.95785714	0.19642857	c(t = 0.26646	0.792	0.95730104	Distal Intergenic	ENSSSCG000	GALM	galactose mutarotase [Source:NCBI gene (formerly Entrezgene);Acc:399536]
chr16	79579040	79580558	1519	*	chr16	79579040	79580558	in_vitro	in_vivo	10	13	95.578	94.6861538	0.89184615	c(t = 0.26607	0.793	0.95775656	Distal Intergenic	ENSSSCG000	TPPP	tubulin polymerization promoting protein [Source:VGNC Symbol;Acc:VGNC:94344]
chr17	39840431	39842044	1614	*	chr17	39840431	39842044	in_vitro	in_vivo	10	10	9.342	10.972	-1.63	c(t = -0.2669	0.793	0.95775656	Distal Intergenic	ENSSSCG000	MYL9	myosin light chain 9 [Source:NCBI gene (formerly Entrezgene);Acc:100157760]
chr6	30204223	30205976	1754	*	chr6	30204223	30205976	in_vitro	in_vivo	10	11	2.151	2.50545455	-0.3544545	c(t = -0.2661	0.793	0.95775656	Distal Intergenic	ENSSSCG000	MMP2	matrix metallopeptidase 2 [Source:NCBI gene (formerly Entrezgene);Acc:397391]
chr1	158288183	158289173	991	*	chr1	158288183	158289173	in_vitro	in_vivo	11	12	2.36363636	1.98666667	0.3769697	c(t = 0.26617	0.793	0.95775656	Distal Intergenic	ENSSSCG000	SERPINB10	serpin family B member 10 [Source:NCBI gene (formerly Entrezgene);Acc:100154994]
chr12	19871452	19872100	649	*	chr12	19871452	19872100	in_vitro	in_vivo	9	9	0.63888889	0.48777778	0.15111111	c(t = 0.26707	0.793	0.95775656	Distal Intergenic	ENSSSCG000	RPL27	ribosomal protein L27 [Source:NCBI gene (formerly Entrezgene);Acc:100037995]
chr16	19949972	19952082	2111	*	chr16	19949972	19952082	in_vitro	in_vivo	13	14	12.3461538	11.2464286	1.09972527	c(t = 0.26380	0.794	0.95791052	Distal Intergenic	ENSSSCG000	C1QTNF3	C1q and TNF related 3 [Source:NCBI gene (formerly Entrezgene);Acc:100270814]
chrX	125484930	125485786	857	*	chrX	125484930	125485786	in_vitro	in_vivo	14	10	4.60928571	3.594	1.01528571	c(t = 0.26503	0.794	0.95791052	Promoter (<=1kb)	ENSSSCG000	VBP1	VHL binding protein 1 [Source:VGNC Symbol;Acc:VGNC:104090]
chr18	48692960	48694220	1261	*	chr18	48692960	48694220	in_vitro	in_vivo	11	13	1.96272727	2.45153846	-0.4888112	c(t = -0.2653	0.794	0.95791052	Distal Intergenic	ENSSSCG000	PGAM2	phosphoglycerate mutase 2 [Source:VGNC Symbol;Acc:VGNC:91341]
chr7	93795720	93797379	1660	*	chr7	93795720	93797379	in_vitro	in_vivo	13	12	1.96307692	2.43666667	-0.4735897	c(t = -0.2658	0.794	0.95791052	Distal Intergenic	ENSSSCG000	SYNJ2BP	synaptojanin 2 binding protein [Source:NCBI gene (formerly Entrezgene);Acc:100514718]
chr4	19617029	19617846	818	*	chr4	19617029	19617846	in_vitro	in_vivo	11	10	1.43909091	1.78	-0.3409091	c(t = -0.2655	0.794	0.95791052	Promoter (<=1kb)	ENSSSCG000	MAL2	mal, T cell differentiation protein 2 [Source:VGNC Symbol;Acc:VGNC:98818]
chr2	126349628	126350465	838	*	chr2	126349628	126350465	in_vitro	in_vivo	9	12	1.28222222	0.985	0.29722222	c(t = 0.26524	0.794	0.95791052	Distal Intergenic	NA	NA	NA
chr6	70701354	70702243	890	*	chr6	70701354	70702243	in_vitro	in_vivo	10	11	0.963	0.73090909	0.23209091	c(t = 0.26565	0.794	0.95791052	Promoter (<=1kb)	ENSSSCG000	CENPS	apoptosis-inducing, TAF9-like domain 1 [Source:NCBI gene (formerly Entrezgene);Acc:100522376]
chr12	59386778	59389477	2700	*	chr12	59386778	59389477	in_vitro	in_vivo	10	12	96.005	95.4925	0.5125	c(t = 0.26296	0.795	0.95791392	Distal Intergenic	ENSSSCG000	UBB	ubiquitin B [Source:VGNC Symbol;Acc:VGNC:98389]
chr3	45759806	45761447	1642	*	chr3	45759806	45761447	in_vitro	in_vivo	11	15	10.4563636	11.7353333	-1.2789697	c(t = -0.2636	0.795	0.95791392	Promoter (<=1kb)	ENSSSCG000	ADRA2B	adrenoceptor alpha 2B [Source:NCBI gene (formerly Entrezgene);Acc:641353]
chr2	16163546	16164358	813	*	chr2	16163546	16164358	in_vitro	in_vivo	9	10	3.44222222	4.534	-1.0917778	c(t = -0.2660	0.795	0.95791392	Distal Intergenic	ENSSSCG000	MDK	midkine [Source:VGNC Symbol;Acc:VGNC:100167]
chr3	4591909	4593049	1141	*	chr3	4591909	4593049	in_vitro	in_vivo	11	12	3.83363636	3.35083333	0.48280303	c(t = 0.26325	0.795	0.95791392	Distal Intergenic	ENSSSCG000	RAC1	Rac family small GTPase 1 [Source:VGNC Symbol;Acc:VGNC:92047]
chr1	187474846	187475463	618	*	chr1	187474846	187475463	in_vitro	in_vivo	9	11	1.70222222	2.10454545	-0.4023232	c(t = -0.2640	0.795	0.95791392	Distal Intergenic	ENSSSCG000	ARID4A	AT-rich interaction domain 4A [Source:VGNC Symbol;Acc:VGNC:85509]
chr6	8468271	8469918	1648	*	chr6	8468271	8469918	in_vitro	in_vivo	13	9	1.83538462	1.51777778	0.31760684	c(t = 0.26628	0.795	0.95791392	Distal Intergenic	ENSSSCG000	BCO1	beta-carotene oxygenase 1 [Source:VGNC Symbol;Acc:VGNC:85789]
chr2	84536783	84538393	1611	*	chr2	84536783	84538393	in_vitro	in_vivo	10	14	1.333	1.11928571	0.21371429	c(t = 0.26424	0.795	0.95791392	Distal Intergenic	ENSSSCG000	HMGCR	3-hydroxy-3-methylglutaryl-CoA reductase [Source:NCBI gene (formerly Entrezgene);Acc:100144446]
chrX	6295308	6296818	1511	*	chrX	6295308	6296818	in_vitro	in_vivo	9	11	1.08666667	0.84818182	0.23848485	c(t = 0.26497	0.795	0.95791392	Distal Intergenic	ENSSSCG00000038027		patatin like phospholipase domain containing 4 [Source:NCBI gene (formerly Entrezgene);Acc:100294680]
chr3	40334399	40336468	2070	*	chr3	40334399	40336468	in_vitro	in_vivo	9	9	85.0355556	88.2355556	-3.2	c(t = -0.2643	0.796	0.95806735	Distal Intergenic	ENSSSCG000	JPT2	Jupiter microtubule associated homolog 2 [Source:NCBI gene (formerly Entrezgene);Acc:474165]
chr12	54639953	54641102	1150	*	chr12	54639953	54641102	in_vitro	in_vivo	9	11	84.6188889	86.6754545	-2.0565657	c(t = -0.2631	0.796	0.95806735	Distal Intergenic	ENSSSCG000	GLP2R	glucagon like peptide 2 receptor [Source:VGNC Symbol;Acc:VGNC:88490]
chr4	1031192	1038901	7710	*	chr4	1031192	1038901	in_vitro	in_vivo	9	13	85.8666667	84.2	1.66666667	c(t = 0.26312	0.796	0.95806735	Distal Intergenic	ENSSSCG000	GPIHBP1	glycosylphosphatidylinositol anchored high density lipoprotein binding protein 1 [Source:VGNC Symbol;Acc:VGNC:88588]
chr11	74187491	74188582	1092	*	chr11	74187491	74188582	in_vitro	in_vivo	11	14	9.91818182	12.6278571	-2.7096753	c(t = -0.2622	0.796	0.95806735	Exon (NM_001114286/100135673, exon 5 of 5)	ENSSSCG000	EFNB2	ephrin B2 [Source:NCBI gene (formerly Entrezgene);Acc:100135673]
chrX	110385773	110387768	1996	*	chrX	110385773	110387768	in_vitro	in_vivo	13	9	9.79923077	11.4322222	-1.6329915	c(t = -0.2620	0.796	0.95806735	Promoter (<=1kb)	ENSSSCG000	ssc-mir-503	ssc-mir-503 [Source:miRBase;Acc:MI0010684]
chr2	28977009	28978277	1269	*	chr2	28977009	28978277	in_vitro	in_vivo	10	13	1.799	1.38461538	0.41438462	c(t = 0.26481	0.796	0.95806735	Promoter (<=1kb)	ENSSSCG000	PAX6	paired box 6 [Source:VGNC Symbol;Acc:VGNC:91195]
chr8	128952248	128953634	1387	*	chr8	128952248	128953634	in_vitro	in_vivo	11	12	1.41909091	1.11333333	0.30575758	c(t = 0.26193	0.796	0.95806735	Distal Intergenic	NA	NA	NA
chr11	77525697	77535180	9484	*	chr11	77525697	77535180	in_vitro	in_vivo	13	15	92.8815385	93.6246667	-0.7431282	c(t = -0.2616	0.797	0.95823241	Distal Intergenic	ENSSSCG000	ANKRD10	ankyrin repeat domain 10 [Source:VGNC Symbol;Acc:VGNC:85322]
chr1	273515258	273520515	5258	*	chr1	273515258	273520515	in_vitro	in_vivo	15	13	94.6133333	93.6130769	1.00025641	c(t = 0.26010	0.797	0.95823241	Distal Intergenic	ENSSSCG000	ssc-mir-9812	ssc-mir-9812 [Source:miRBase;Acc:MI0031579]
chr12	52722065	52723814	1750	*	chr12	52722065	52723814	in_vitro	in_vivo	9	11	6.59555556	7.98818182	-1.3926263	c(t = -0.2609	0.797	0.95823241	Distal Intergenic	ENSSSCG000	SLC2A4	solute carrier family 2 member 4 [Source:NCBI gene (formerly Entrezgene);Acc:396754]
chr3	16393458	16394286	829	*	chr3	16393458	16394286	in_vitro	in_vivo	11	11	2.38181818	2.00272727	0.37909091	c(t = 0.26109	0.797	0.95823241	Distal Intergenic	ENSSSCG000	TPST1	tyrosylprotein sulfotransferase 1 [Source:VGNC Symbol;Acc:VGNC:94352]
chr13	133420065	133420509	445	*	chr13	133420065	133420509	in_vitro	in_vivo	10	11	0.606	0.78636364	-0.1803636	c(t = -0.2612	0.797	0.95823241	Distal Intergenic	ENSSSCG000	SLC51A	solute carrier family 51 subunit alpha [Source:VGNC Symbol;Acc:VGNC:93138]
chr2	12473076	12474333	1258	*	chr2	12473076	12474333	in_vitro	in_vivo	10	11	20.466	23.6627273	-3.1967273	c(t = -0.2602	0.798	0.95823241	Distal Intergenic	ENSSSCG000	MPEG1	macrophage expressed 1 [Source:NCBI gene (formerly Entrezgene);Acc:100516953]
chr5	28817384	28817959	576	*	chr5	28817384	28817959	in_vitro	in_vivo	12	12	2.04	1.81333333	0.22666667	c(t = 0.25917	0.798	0.95823241	Distal Intergenic	ENSSSCG000	TBK1	TANK binding kinase 1 [Source:VGNC Symbol;Acc:VGNC:93787]
chr1	186044353	186045145	793	*	chr1	186044353	186045145	in_vitro	in_vivo	11	11	1.45545455	1.79636364	-0.3409091	c(t = -0.2599	0.798	0.95823241	Distal Intergenic	ENSSSCG000	KTN1	kinectin 1 [Source:VGNC Symbol;Acc:VGNC:89608]
chr9	36970006	36970546	541	*	chr9	36970006	36970546	in_vitro	in_vivo	10	10	2.365	1.758	0.607	c(t = 0.26200	0.798	0.95823241	Distal Intergenic	ENSSSCG000	ATM	ATM serine/threonine kinase [Source:VGNC Symbol;Acc:VGNC:108572]
chr8	54909123	54910495	1373	*	chr8	54909123	54910495	in_vitro	in_vivo	11	14	1.06363636	1.21642857	-0.1527922	c(t = -0.2591	0.798	0.95823241	Distal Intergenic	ENSSSCG00000008898		HOP homeobox [Source:VGNC Symbol;Acc:VGNC:88932]
chr3	1577859	1582589	4731	*	chr3	1577859	1582589	in_vitro	in_vivo	11	13	96.2281818	95.7530769	0.4751049	c(t = 0.25753	0.799	0.95823241	Distal Intergenic	ENSSSCG000	NUDT1	nudix hydrolase 1 [Source:NCBI gene (formerly Entrezgene);Acc:100624718]
chr18	50825079	50826159	1081	*	chr18	50825079	50826159	in_vitro	in_vivo	9	14	95.98	95.3214286	0.65857143	c(t = 0.25791	0.799	0.95823241	Distal Intergenic	ENSSSCG000	DDX56	DEAD-box helicase 56 [Source:NCBI gene (formerly Entrezgene);Acc:100513388]
chr11	78147966	78150551	2586	*	chr11	78147966	78150551	in_vitro	in_vivo	9	12	23.4133333	21.1858333	2.2275	c(t = 0.25903	0.799	0.95823241	Distal Intergenic	ENSSSCG000	F7	coagulation factor VII [Source:NCBI gene (formerly Entrezgene);Acc:733661]
chr3	131194022	131194733	712	*	chr3	131194022	131194733	in_vitro	in_vivo	10	10	10.687	8.308	2.379	c(t = 0.25880	0.799	0.95823241	Distal Intergenic	ENSSSCG000	RNASEH1	ribonuclease H1 [Source:NCBI gene (formerly Entrezgene);Acc:100626539]
chr6	30511879	30517265	5387	*	chr6	30511879	30517265	in_vitro	in_vivo	14	14	2.38785714	2.69285714	-0.305	c(t = -0.2575	0.799	0.95823241	Distal Intergenic	ENSSSCG000	MMP2	matrix metallopeptidase 2 [Source:NCBI gene (formerly Entrezgene);Acc:397391]
chr15	105970099	105971793	1695	*	chr15	105970099	105971793	in_vitro	in_vivo	9	13	2.93888889	2.41923077	0.51965812	c(t = 0.26044	0.799	0.95823241	Promoter (<=1kb)	ENSSSCG000	BMPR2	bone morphogenetic protein receptor type 2 [Source:NCBI gene (formerly Entrezgene);Acc:100127483]
chr11	18543025	18544465	1441	*	chr11	18543025	18544465	in_vitro	in_vivo	10	17	2.063	2.35058824	-0.2875882	c(t = -0.2580	0.799	0.95823241	Distal Intergenic	ENSSSCG000	PHF11	PHD finger protein 11 [Source:VGNC Symbol;Acc:VGNC:91377]
chr7	63356804	63357669	866	*	chr7	63356804	63357669	in_vitro	in_vivo	10	14	1.906	1.57785714	0.32814286	c(t = 0.25891	0.799	0.95823241	Distal Intergenic	ENSSSCG000	NKX2-1	NK2 homeobox 1 [Source:VGNC Symbol;Acc:VGNC:90767]
chr1	95808512	95810086	1575	*	chr1	95808512	95810086	in_vitro	in_vivo	13	15	1.13538462	1.40333333	-0.2679487	c(t = -0.2582	0.799	0.95823241	Distal Intergenic	ENSSSCG000	HAUS1	HAUS augmin like complex subunit 1 [Source:NCBI gene (formerly Entrezgene);Acc:100152640]
chr2	25421461	25423558	2098	*	chr2	25421461	25423558	in_vitro	in_vivo	14	14	1.415	1.25857143	0.15642857	c(t = 0.25711	0.799	0.95823241	Distal Intergenic	ENSSSCG000	PAMR1	peptidase domain containing associated with muscle reration 1 [Source:VGNC Symbol;Acc:VGNC:91164]
chr6	9870450	9871124	675	*	chr6	9870450	9871124	in_vitro	in_vivo	9	10	0.69555556	0.839	-0.1434444	c(t = -0.2591	0.799	0.95823241	Distal Intergenic	ENSSSCG000	BCO1	beta-carotene oxygenase 1 [Source:VGNC Symbol;Acc:VGNC:85789]
chr2	69056036	69057505	1470	*	chr2	69056036	69057505	in_vitro	in_vivo	9	13	4.22666667	4.94538462	-0.7187179	c(t = -0.2570	0.8	0.95823241	Promoter (<=1kb)	ENSSSCG000	S1PR2	sphingosine-1-phosphate receptor 2 [Source:NCBI gene (formerly Entrezgene);Acc:100511551]
chr9	18495719	18496007	289	*	chr9	18495719	18496007	in_vitro	in_vivo	9	9	3.73111111	3.19222222	0.53888889	c(t = 0.25724	0.8	0.95823241	Distal Intergenic	ENSSSCG000	TMEM126B	transmembrane protein 126B [Source:NCBI gene (formerly Entrezgene);Acc:100626990]
chr8	39162846	39163641	796	*	chr8	39162846	39163641	in_vitro	in_vivo	10	12	2.279	2.69916667	-0.4201667	c(t = -0.2565	0.8	0.95823241	Promoter (<=1kb)	ENSSSCG000	SPATA18	spermatosis associated 18 [Source:VGNC Symbol;Acc:VGNC:98966]
chr17	51500232	51500962	731	*	chr17	51500232	51500962	in_vitro	in_vivo	9	10	2.4	2.014	0.386	c(t = 0.25720	0.8	0.95823241	Distal Intergenic	ENSSSCG000	RNF114	ring finger protein 114 [Source:NCBI gene (formerly Entrezgene);Acc:414917]
chr16	30305486	30307132	1647	*	chr16	30305486	30307132	in_vitro	in_vivo	13	15	1.16384615	1.32533333	-0.1614872	c(t = -0.2561	0.8	0.95823241	Distal Intergenic	ENSSSCG000	ITGA2	integrin subunit alpha 2 [Source:VGNC Symbol;Acc:VGNC:89234]
chr1	166162430	166163527	1098	*	chr1	166162430	166163527	in_vitro	in_vivo	15	11	1.29866667	1.07090909	0.22775758	c(t = 0.25656	0.8	0.95823241	Distal Intergenic	ENSSSCG000	CLN6	CLN6 transmembrane ER protein [Source:VGNC Symbol;Acc:VGNC:86771]
chr9	107456054	107457038	985	*	chr9	107456054	107457038	in_vitro	in_vivo	11	12	0.75181818	1.04166667	-0.2898485	c(t = -0.2586	0.8	0.95823241	Distal Intergenic	ENSSSCG000	SLC26A3	solute carrier family 26 member 3 [Source:VGNC Symbol;Acc:VGNC:93028]
chr2	6334922	6335847	926	*	chr2	6334922	6335847	in_vitro	in_vivo	9	10	1.11111111	0.789	0.32211111	c(t = 0.25875	0.8	0.95823241	Distal Intergenic	ENSSSCG000	CATSPER1	cation channel sperm associated 1 [Source:NCBI gene (formerly Entrezgene);Acc:100689223]
chr3	7527612	7529392	1781	*	chr3	7527612	7529392	in_vitro	in_vivo	11	11	96.0709091	95.6090909	0.46181818	c(t = 0.25539	0.801	0.95823241	Exon (NM_214042/397107, exon 4 of 11)	ENSSSCG000	NTAN1	N-terminal asparagine amidase [Source:NCBI gene (formerly Entrezgene);Acc:397107]
chr16	76214405	76216904	2500	*	chr16	76214405	76216904	in_vitro	in_vivo	12	13	92.5858333	93.4230769	-0.8372436	c(t = -0.2559	0.801	0.95823241	Distal Intergenic	ENSSSCG000	TERT	telomerase reverse transcriptase [Source:NCBI gene (formerly Entrezgene);Acc:492280]
chr5	64116264	64117021	758	*	chr5	64116264	64117021	in_vitro	in_vivo	10	15	10.427	9.07466667	1.35233333	c(t = 0.25486	0.801	0.95823241	Distal Intergenic	ENSSSCG000	NOP2	NOP2 nucleolar protein [Source:VGNC Symbol;Acc:VGNC:103306]
chr4	103152941	103154322	1382	*	chr4	103152941	103154322	in_vitro	in_vivo	11	13	2.32818182	2.01769231	0.31048951	c(t = 0.25558	0.801	0.95823241	Promoter (<=1kb)	ENSSSCG000	TENT5C	terminal nucleotidyltransferase 5C [Source:VGNC Symbol;Acc:VGNC:93875]
chr10	51453624	51455597	1974	*	chr10	51453624	51455597	in_vitro	in_vivo	13	13	1.41384615	1.56923077	-0.1553846	c(t = -0.2552	0.801	0.95823241	Distal Intergenic	ENSSSCG000	PIP4K2A	phosphatidylinositol-5-phosphate 4-kinase type 2 alpha [Source:VGNC Symbol;Acc:VGNC:96485]
chr1	118898169	118900672	2504	*	chr1	118898169	118900672	in_vitro	in_vivo	13	12	1.16461538	1.37416667	-0.2095513	c(t = -0.2552	0.801	0.95823241	Distal Intergenic	ENSSSCG000	ARPP19	cAMP regulated phosphoprotein 19 [Source:NCBI gene (formerly Entrezgene);Acc:397362]
chr7	36742369	36743989	1621	*	chr7	36742369	36743989	in_vitro	in_vivo	9	10	1.45888889	1.243	0.21588889	c(t = 0.25666	0.801	0.95823241	Distal Intergenic	ENSSSCG000	TREM1	triggering receptor expressed on myeloid cells 1 [Source:NCBI gene (formerly Entrezgene);Acc:396601]
chr3	114363502	114364476	975	*	chr3	114363502	114364476	in_vitro	in_vivo	9	10	1.01222222	1.205	-0.1927778	c(t = -0.2569	0.801	0.95823241	Distal Intergenic	ENSSSCG000	NCOA1	nuclear receptor coactivator 1 [Source:VGNC Symbol;Acc:VGNC:90615]
chr5	13067201	13069039	1839	*	chr5	13067201	13069039	in_vitro	in_vivo	12	13	0.94916667	0.82615385	0.12301282	c(t = 0.25470	0.801	0.95823241	Distal Intergenic	ENSSSCG000	RTCB	RNA 2',3'-cyclic phosphate and 5'-OH ligase [Source:NCBI gene (formerly Entrezgene);Acc:733658]
chr6	160155919	160156669	751	*	chr6	160155919	160156669	in_vitro	in_vivo	9	12	0.84777778	0.66	0.18777778	c(t = 0.25583	0.801	0.95823241	Distal Intergenic	ENSSSCG000	COA7	cytochrome c oxidase assembly factor 7 [Source:NCBI gene (formerly Entrezgene);Acc:100627075]
chr14	137135502	137136488	987	*	chr14	137135502	137136488	in_vitro	in_vivo	11	9	22.6690909	26.6244444	-3.9553535	c(t = -0.2550	0.802	0.95853344	Distal Intergenic	ENSSSCG000	GLRX3	glutaredoxin 3 [Source:VGNC Symbol;Acc:VGNC:88495]
chr2	77407809	77408858	1050	*	chr2	77407809	77408858	in_vitro	in_vivo	9	13	24.5444444	21.2215385	3.32290598	c(t = 0.25421	0.802	0.95853344	Distal Intergenic	ENSSSCG000	KISS1R	KISS1 receptor [Source:NCBI gene (formerly Entrezgene);Acc:733704]
chr7	672609	674520	1912	*	chr7	672609	674520	in_vitro	in_vivo	10	13	22.435	19.33	3.105	c(t = 0.25400	0.802	0.95853344	Distal Intergenic	ENSSSCG000	IRF4	interferon regulatory factor 4 [Source:VGNC Symbol;Acc:VGNC:89207]
chr12	57238024	57238731	708	*	chr12	57238024	57238731	in_vitro	in_vivo	11	11	1.78636364	1.47909091	0.30727273	c(t = 0.25468	0.802	0.95853344	Promoter (<=1kb)	ENSSSCG000	ELAC2	elaC ribonuclease Z 2 [Source:NCBI gene (formerly Entrezgene);Acc:100520804]
chr7	51650573	51652422	1850	*	chr7	51650573	51652422	in_vitro	in_vivo	14	16	0.91	1.036875	-0.126875	c(t = -0.2533	0.802	0.95853344	Distal Intergenic	ENSSSCG000	ssc-mir-1839	ssc-mir-1839 [Source:miRBase;Acc:MI0013162]
chr9	116149009	116149919	911	*	chr9	116149009	116149919	in_vitro	in_vivo	10	9	1.089	0.79333333	0.29566667	c(t = 0.25528	0.802	0.95853344	Distal Intergenic	ENSSSCG000	SERPINC1	serpin family C member 1 [Source:NCBI gene (formerly Entrezgene);Acc:100125972]
chr11	9542941	9544354	1414	*	chr11	9542941	9544354	in_vitro	in_vivo	13	11	95.3938462	95.8363636	-0.4425175	c(t = -0.2524	0.803	0.9588339	Distal Intergenic	ENSSSCG000	RFC3	replication factor C subunit 3 [Source:VGNC Symbol;Acc:VGNC:92233]
chr14	58962893	58963740	848	*	chr14	58962893	58963740	in_vitro	in_vivo	9	9	2.24	2.66888889	-0.4288889	c(t = -0.2538	0.803	0.9588339	Distal Intergenic	ENSSSCG000	TSNAX	translin associated factor X [Source:NCBI gene (formerly Entrezgene);Acc:100156036]
chr4	78980064	78982453	2390	*	chr4	78980064	78982453	in_vitro	in_vivo	11	14	1.79181818	2.14714286	-0.3553247	c(t = -0.2525	0.803	0.9588339	Distal Intergenic	ENSSSCG000	ssc-mir-9839	ssc-mir-9839 [Source:miRBase;Acc:MI0031608]
chr1	232355066	232357619	2554	*	chr1	232355066	232357619	in_vitro	in_vivo	14	12	1.68785714	1.97583333	-0.2879762	c(t = -0.2530	0.803	0.9588339	Distal Intergenic	ENSSSCG000	GNAQ	G protein subunit alpha q [Source:NCBI gene (formerly Entrezgene);Acc:654405]
chr2	6436925	6438224	1300	*	chr2	6436925	6438224	in_vitro	in_vivo	12	12	0.88727273	1.08083333	-0.1935606	c(t = -0.2536	0.803	0.9588339	Distal Intergenic	ENSSSCG000	FIBP	FGF1 intracellular binding protein [Source:NCBI gene (formerly Entrezgene);Acc:100525557]
chr18	26046766	26047902	1137	*	chr18	26046766	26047902	in_vitro	in_vivo	11	13	1.02181818	0.83076923	0.19104895	c(t = 0.25219	0.803	0.9588339	Promoter (<=1kb)	ENSSSCG000	TSPAN12	tetraspanin 12 [Source:VGNC Symbol;Acc:VGNC:94501]
chr1	11443546	11443993	448	*	chr1	11443546	11443993	in_vitro	in_vivo	9	9	13.5811111	9.87666667	3.70444444	c(t = 0.25191	0.804	0.9591375	Distal Intergenic	ENSSSCG000	TFB1M	transcription factor B1, mitochondrial [Source:VGNC Symbol;Acc:VGNC:93913]
chr11	68765374	68766032	659	*	chr11	68765374	68766032	in_vitro	in_vivo	11	14	4.98272727	5.64285714	-0.6601299	c(t = -0.2511	0.804	0.9591375	Distal Intergenic	ENSSSCG000	TMTC4	transmembrane O-mannosyltransferase targeting cadherins 4 [Source:VGNC Symbol;Acc:VGNC:94244]
chr9	57016991	57018863	1873	*	chr9	57016991	57018863	in_vitro	in_vivo	12	12	3.86333333	3.285	0.57833333	c(t = 0.25159	0.804	0.9591375	Distal Intergenic	ENSSSCG000	KCNJ5	potassium inwardly rectifying channel subfamily J member 5 [Source:NCBI gene (formerly Entrezgene);Acc:397448]
chr15	131079829	131080884	1056	*	chr15	131079829	131080884	in_vitro	in_vivo	10	10	1.382	1.637	-0.255	c(t = -0.2524	0.804	0.9591375	Distal Intergenic	ENSSSCG000	ITM2C	integral membrane protein 2C [Source:NCBI gene (formerly Entrezgene);Acc:100144481]
chr1	235757794	235758441	648	*	chr1	235757794	235758441	in_vitro	in_vivo	12	11	1.26833333	1.46545455	-0.1971212	c(t = -0.2509	0.804	0.9591375	Distal Intergenic	ENSSSCG000	VCP	valosin containing protein [Source:VGNC Symbol;Acc:VGNC:109420]
chr9	55856551	55857308	758	*	chr9	55856551	55857308	in_vitro	in_vivo	11	11	28.9118182	25.3536364	3.55818182	c(t = 0.24964	0.805	0.9591375	Exon (NM_214225/397448, exon 2 of 3)	ENSSSCG000	KCNJ5	potassium inwardly rectifying channel subfamily J member 5 [Source:NCBI gene (formerly Entrezgene);Acc:397448]
chr13	79934599	79935266	668	*	chr13	79934599	79935266	in_vitro	in_vivo	11	9	22.0372727	18.3622222	3.67505051	c(t = 0.25146	0.805	0.9591375	Distal Intergenic	ENSSSCG000	FOXL2	forkhead box L2 [Source:NCBI gene (formerly Entrezgene);Acc:100622956]
chr3	39519762	39520483	722	*	chr3	39519762	39520483	in_vitro	in_vivo	9	10	1.68888889	2.206	-0.5171111	c(t = -0.2503	0.805	0.9591375	Distal Intergenic	ENSSSCG000	ECI1	enoyl-CoA delta isomerase 1 [Source:NCBI gene (formerly Entrezgene);Acc:100738177]
chr11	70942945	70943786	842	*	chr11	70942945	70943786	in_vitro	in_vivo	10	14	1.93	1.45142857	0.47857143	c(t = 0.25100	0.805	0.9591375	Distal Intergenic	ENSSSCG000	BIVM	basic, immunoglobulin-like variable motif containing [Source:VGNC Symbol;Acc:VGNC:109180]
chr1	83140628	83140982	355	*	chr1	83140628	83140982	in_vitro	in_vivo	11	13	0.83909091	1.04230769	-0.2032168	c(t = -0.2504	0.805	0.9591375	Distal Intergenic	ENSSSCG000	CALHM6	calcium homeostasis modulator family member 6 [Source:VGNC Symbol;Acc:VGNC:86147]
chr6	98813877	98815468	1592	*	chr6	98813877	98815468	in_vitro	in_vivo	12	13	1.1075	0.95923077	0.14826923	c(t = 0.25040	0.805	0.9591375	Distal Intergenic	ENSSSCG000	NDUFV2	NADH:ubiquinone oxidoreductase core subunit V2 [Source:NCBI gene (formerly Entrezgene);Acc:100037991]
chr9	7765835	7770277	4443	*	chr9	7765835	7770277	in_vitro	in_vivo	10	14	16.841	18.7357143	-1.8947143	c(t = -0.2491	0.806	0.9591375	Distal Intergenic	ENSSSCG000	P2RY6	pyrimidinergic receptor P2Y6 [Source:VGNC Symbol;Acc:VGNC:91128]
chr12	18863365	18864843	1479	*	chr12	18863365	18864843	in_vitro	in_vivo	13	12	12.1076923	10.1925	1.91519231	c(t = 0.24936	0.806	0.9591375	Exon (NM_213998/397059, exon 2 of 30)	ENSSSCG000	ITGA2B	integrin subunit alpha 2b [Source:NCBI gene (formerly Entrezgene);Acc:397059]
chrX	97735028	97736268	1241	*	chrX	97735028	97736268	in_vitro	in_vivo	9	10	6.16555556	4.962	1.20355556	c(t = 0.24957	0.806	0.9591375	Promoter (<=1kb)	ENSSSCG000	PGRMC1	progesterone receptor membrane component 1 [Source:NCBI gene (formerly Entrezgene);Acc:396946]
chr2	7059135	7060015	881	*	chr2	7059135	7060015	in_vitro	in_vivo	9	11	4.17444444	3.53727273	0.63717172	c(t = 0.25039	0.806	0.9591375	Distal Intergenic	ENSSSCG000	FAU	FAU ubiquitin like and ribosomal protein S30 fusion [Source:NCBI gene (formerly Entrezgene);Acc:396977]
chr5	438724	440351	1628	*	chr5	438724	440351	in_vitro	in_vivo	10	11	2.988	2.53909091	0.44890909	c(t = 0.25021	0.806	0.9591375	Distal Intergenic	ENSSSCG000	MAPK12	mitogen-activated protein kinase 12 [Source:VGNC Symbol;Acc:VGNC:89998]
chr4	92964552	92965390	839	*	chr4	92964552	92965390	in_vitro	in_vivo	10	10	2.571	2.101	0.47	c(t = 0.24982	0.806	0.9591375	Distal Intergenic	ENSSSCG000	MRPL24	mitochondrial ribosomal protein L24 [Source:VGNC Symbol;Acc:VGNC:90357]
chr6	75015290	75016443	1154	*	chr6	75015290	75016443	in_vitro	in_vivo	13	12	1.24384615	1.46	-0.2161538	c(t = -0.2487	0.806	0.9591375	Distal Intergenic	ENSSSCG000	HSPB7	heat shock protein family B (small) member 7 [Source:NCBI gene (formerly Entrezgene);Acc:100627988]
chr6	160910980	160911803	824	*	chr6	160910980	160911803	in_vitro	in_vivo	10	9	1.748	1.42555556	0.32244444	c(t = 0.24933	0.806	0.9591375	Distal Intergenic	ENSSSCG000	COA7	cytochrome c oxidase assembly factor 7 [Source:NCBI gene (formerly Entrezgene);Acc:100627075]
chr14	37739214	37741050	1837	*	chr14	37739214	37741050	in_vitro	in_vivo	11	10	1.46	1.256	0.204	c(t = 0.24972	0.806	0.9591375	Distal Intergenic	ENSSSCG000	OAS2	2'-5'-oligoadenylate synthetase 2 [Source:VGNC Symbol;Acc:VGNC:91007]
chr6	96733074	96734372	1299	*	chr6	96733074	96734372	in_vitro	in_vivo	13	12	0.74923077	0.92416667	-0.1749359	c(t = -0.2500	0.806	0.9591375	Distal Intergenic	ENSSSCG000	CIDEA	cell death inducing DFFA like effector a [Source:NCBI gene (formerly Entrezgene);Acc:100127171]
chr12	50347175	50348936	1762	*	chr12	50347175	50348936	in_vitro	in_vivo	12	13	0.935	0.81538462	0.11961538	c(t = 0.24835	0.806	0.9591375	Distal Intergenic	ENSSSCG000	ASPA	aspartoacylase [Source:NCBI gene (formerly Entrezgene);Acc:100142661]
chr9	1190082	1191393	1312	*	chr9	1190082	1191393	in_vitro	in_vivo	11	9	97.3872727	97.8211111	-0.4338384	c(t = -0.2478	0.807	0.95943611	Distal Intergenic	NA	NA	NA
chr5	64037168	64037879	712	*	chr5	64037168	64037879	in_vitro	in_vivo	10	10	4.174	4.871	-0.697	c(t = -0.2486	0.807	0.95943611	Distal Intergenic	ENSSSCG000	NOP2	NOP2 nucleolar protein [Source:VGNC Symbol;Acc:VGNC:103306]
chr9	102347985	102349338	1354	*	chr9	102347985	102349338	in_vitro	in_vivo	13	15	3.65076923	4.366	-0.7152308	c(t = -0.2473	0.807	0.95943611	Distal Intergenic	ENSSSCG000	FGL2	fibrinogen like 2 [Source:NCBI gene (formerly Entrezgene);Acc:448808]
chr17	34268317	34268910	594	*	chr17	34268317	34268910	in_vitro	in_vivo	9	12	3.12444444	2.62083333	0.50361111	c(t = 0.24750	0.807	0.95943611	Distal Intergenic	ENSSSCG000	ssc-mir-9798	ssc-mir-9798 [Source:miRBase;Acc:MI0031562]
chr1	16139115	16140322	1208	*	chr1	16139115	16140322	in_vitro	in_vivo	13	13	2.00615385	2.34384615	-0.3376923	c(t = -0.2472	0.807	0.95943611	Distal Intergenic	ENSSSCG000	ULBP1	UL16 binding protein 1 [Source:NCBI gene (formerly Entrezgene);Acc:445523]
chr14	109210483	109211206	724	*	chr14	109210483	109211206	in_vitro	in_vivo	10	9	0.623	0.82777778	-0.2047778	c(t = -0.2494	0.807	0.95943611	Distal Intergenic	ENSSSCG000	SFRP5	secreted frizzled related protein 5 [Source:NCBI gene (formerly Entrezgene);Acc:100153176]
chr2	57906563	57907267	705	*	chr2	57906563	57907267	in_vitro	in_vivo	10	13	26.321	29.5284615	-3.2074615	c(t = -0.2465	0.808	0.95958584	Distal Intergenic	ENSSSCG000	MGAT1	alpha-1,3-mannosyl-glycoprotein 2-beta-N-acetylglucosaminyltransferase [Source:VGNC Symbol;Acc:VGNC:96738]
chr7	21606199	21607174	976	*	chr7	21606199	21607174	in_vitro	in_vivo	10	10	2.576	3.227	-0.651	c(t = -0.2464	0.808	0.95958584	Distal Intergenic	ENSSSCG000	ssc-mir-7857	ssc-mir-7857 [Source:miRBase;Acc:MI0031552]
chr3	11705727	11707084	1358	*	chr3	11705727	11707084	in_vitro	in_vivo	11	14	2.24545455	1.92785714	0.3175974	c(t = 0.24614	0.808	0.95958584	Distal Intergenic	ENSSSCG000	NCF1	neutrophil cytosolic factor 1 [Source:NCBI gene (formerly Entrezgene);Acc:100134857]
chr17	33869383	33870103	721	*	chr17	33869383	33870103	in_vitro	in_vivo	9	10	1.95666667	1.652	0.30466667	c(t = 0.24883	0.808	0.95958584	Promoter (<=1kb)	ENSSSCG000	NSFL1C	NSFL1 cofactor [Source:VGNC Symbol;Acc:VGNC:95704]
chr14	60309351	60310615	1265	*	chr14	60309351	60310615	in_vitro	in_vivo	14	14	1.40142857	1.56	-0.1585714	c(t = -0.2450	0.808	0.95958584	Distal Intergenic	ENSSSCG000	ACTA1	actin alpha 1, skeletal muscle [Source:NCBI gene (formerly Entrezgene);Acc:100154254]
chr8	70417749	70418154	406	*	chr8	70417749	70418154	in_vitro	in_vivo	9	10	1.19888889	1.017	0.18188889	c(t = 0.24872	0.808	0.95958584	Promoter (<=1kb)	ENSSSCG000	AREG	amphiregulin [Source:NCBI gene (formerly Entrezgene);Acc:397668]
chr14	59239880	59240743	864	*	chr14	59239880	59240743	in_vitro	in_vivo	11	10	0.51727273	0.41	0.10727273	c(t = 0.24711	0.808	0.95958584	Promoter (<=1kb)	ENSSSCG000	GNPAT	glyceronephosphate O-acyltransferase [Source:NCBI gene (formerly Entrezgene);Acc:100156253]
chr14	141383149	141384393	1245	*	chr14	141383149	141384393	in_vitro	in_vivo	9	12	93.5277778	92.2491667	1.27861111	c(t = 0.24623	0.809	0.96003166	Distal Intergenic	ENSSSCG000	ECHS1	enoyl-CoA hydratase, short chain 1 [Source:NCBI gene (formerly Entrezgene);Acc:100156927]
chr10	66762223	66767794	5572	*	chr10	66762223	66767794	in_vitro	in_vivo	14	10	22.4014286	19.219	3.18242857	c(t = 0.24552	0.809	0.96003166	Distal Intergenic	ENSSSCG000	KLF6	KLF transcription factor 6 [Source:NCBI gene (formerly Entrezgene);Acc:100174961]
chr9	52065293	52066017	725	*	chr9	52065293	52066017	in_vitro	in_vivo	11	12	4.8	4.21	0.59	c(t = 0.24455	0.809	0.96003166	Distal Intergenic	ENSSSCG000	STT3A	STT3 oligosaccharyltransferase complex catalytic subunit A [Source:VGNC Symbol;Acc:VGNC:93581]
chr13	68995202	68997022	1821	*	chr13	68995202	68997022	in_vitro	in_vivo	9	13	2.49888889	2.20384615	0.29504274	c(t = 0.24525	0.809	0.96003166	Distal Intergenic	ENSSSCG000	H1-8	H1.8 linker histone [Source:VGNC Symbol;Acc:VGNC:109773]
chr2	14896103	14896842	740	*	chr2	14896103	14896842	in_vitro	in_vivo	12	11	0.6575	0.78636364	-0.1288636	c(t = -0.2447	0.809	0.96003166	Distal Intergenic	ENSSSCG000	NDUFS3	NADH:ubiquinone oxidoreductase core subunit S3 [Source:NCBI gene (formerly Entrezgene);Acc:100510948]
chr3	30271289	30274164	2876	*	chr3	30271289	30274164	in_vitro	in_vivo	9	13	60.8755556	56.1923077	4.68324786	c(t = 0.24458	0.81	0.9603286	Distal Intergenic	ENSSSCG000	CPPED1	calcineurin like phosphoesterase domain containing 1 [Source:NCBI gene (formerly Entrezgene);Acc:100627178]
chr13	71278113	71278928	816	*	chr13	71278113	71278928	in_vitro	in_vivo	10	10	1.691	2.148	-0.457	c(t = -0.2448	0.81	0.9603286	Distal Intergenic	ENSSSCG000	GP9	glycoprotein IX platelet [Source:VGNC Symbol;Acc:VGNC:88567]
chr9	56259186	56262318	3133	*	chr9	56259186	56262318	in_vitro	in_vivo	15	13	1.53466667	1.71769231	-0.1830256	c(t = -0.2426	0.81	0.9603286	Distal Intergenic	ENSSSCG000	KCNJ5	potassium inwardly rectifying channel subfamily J member 5 [Source:NCBI gene (formerly Entrezgene);Acc:397448]
chr14	1164890	1167342	2453	*	chr14	1164890	1167342	in_vitro	in_vivo	11	14	1.84	1.60142857	0.23857143	c(t = 0.24386	0.81	0.9603286	Promoter (<=1kb)	ENSSSCG000	GADD45G	growth arrest and DNA damage inducible gamma [Source:NCBI gene (formerly Entrezgene);Acc:100152997]
chr3	127243271	127244510	1240	*	chr3	127243271	127244510	in_vitro	in_vivo	11	14	0.94	1.14857143	-0.2085714	c(t = -0.2431	0.81	0.9603286	Distal Intergenic	ENSSSCG000	ID2	inhibitor of DNA binding 2 [Source:NCBI gene (formerly Entrezgene);Acc:654298]
chr2	119939806	119940499	694	*	chr2	119939806	119940499	in_vitro	in_vivo	11	11	0.83545455	0.99181818	-0.1563636	c(t = -0.2433	0.81	0.9603286	Promoter (<=1kb)	ENSSSCG000	CDO1	cysteine dioxygenase type 1 [Source:NCBI gene (formerly Entrezgene);Acc:100312964]
chr12	5143700	5144765	1066	*	chr12	5143700	5144765	in_vitro	in_vivo	9	11	2.98222222	2.51363636	0.46858586	c(t = 0.24433	0.811	0.96032933	Distal Intergenic	ENSSSCG000	ACOX1	acyl-CoA oxidase 1 [Source:VGNC Symbol;Acc:VGNC:85020]
chr15	45763125	45764483	1359	*	chr15	45763125	45764483	in_vitro	in_vivo	13	13	1.80846154	2.13923077	-0.3307692	c(t = -0.2418	0.811	0.96032933	Distal Intergenic	ENSSSCG000	CASP3	caspase 3 [Source:NCBI gene (formerly Entrezgene);Acc:397244]
chr4	121040994	121042213	1220	*	chr4	121040994	121042213	in_vitro	in_vivo	12	11	1.74	2.08454545	-0.3445455	c(t = -0.2428	0.811	0.96032933	Distal Intergenic	ENSSSCG000	DPYD	dihydropyrimidine dehydrogenase [Source:VGNC Symbol;Acc:VGNC:98787]
chr2	81505759	81506436	678	*	chr2	81505759	81506436	in_vitro	in_vivo	9	11	1.30222222	1.56	-0.2577778	c(t = -0.2431	0.811	0.96032933	Distal Intergenic	ENSSSCG000	ssc-mir-1271	ssc-mir-1271 [Source:miRBase;Acc:MI0015922]
chrX	13229442	13230607	1166	*	chrX	13229442	13230607	in_vitro	in_vivo	11	10	1.22090909	1.427	-0.2060909	c(t = -0.2430	0.811	0.96032933	Distal Intergenic	ENSSSCG000	SYAP1	synapse associated protein 1 [Source:VGNC Symbol;Acc:VGNC:93648]
chr18	23721766	23722891	1126	*	chr18	23721766	23722891	in_vitro	in_vivo	14	15	1.00714286	1.202	-0.1948571	c(t = -0.2413	0.811	0.96032933	Distal Intergenic	ENSSSCG000	SPAM1	sperm adhesion molecule 1 [Source:NCBI gene (formerly Entrezgene);Acc:397074]
chr3	40875414	40878691	3278	*	chr3	40875414	40878691	in_vitro	in_vivo	11	12	95.1245455	96.04	-0.9154545	c(t = -0.2408	0.812	0.96032933	Distal Intergenic	NA	NA	NA
chr9	37613688	37615256	1569	*	chr9	37613688	37615256	in_vitro	in_vivo	11	14	63.5818182	59.2414286	4.34038961	c(t = 0.24080	0.812	0.96032933	Distal Intergenic	ENSSSCG000	RDX	radixin [Source:NCBI gene (formerly Entrezgene);Acc:494457]
chr11	25643532	25644840	1309	*	chr11	25643532	25644840	in_vitro	in_vivo	13	17	9.37846154	11.2441176	-1.8656561	c(t = -0.2397	0.812	0.96032933	Distal Intergenic	ENSSSCG000	MTRF1	mitochondrial translation release factor 1 [Source:VGNC Symbol;Acc:VGNC:90471]
chr17	61624006	61628500	4495	*	chr17	61624006	61628500	in_vitro	in_vivo	12	13	10.7225	9.06846154	1.65403846	c(t = 0.24068	0.812	0.96032933	Distal Intergenic	ENSSSCG000	PSMA7	proteasome 20S subunit alpha 7 [Source:NCBI gene (formerly Entrezgene);Acc:100626738]
chr6	18234872	18236102	1231	*	chr6	18234872	18236102	in_vitro	in_vivo	10	12	2.893	2.44333333	0.44966667	c(t = 0.24164	0.812	0.96032933	Distal Intergenic	ENSSSCG000	CDH1	cadherin 1 [Source:NCBI gene (formerly Entrezgene);Acc:100048953]
chr16	37951390	37952216	827	*	chr16	37951390	37952216	in_vitro	in_vivo	11	12	1.46181818	1.7725	-0.3106818	c(t = -0.2415	0.812	0.96032933	Distal Intergenic	ENSSSCG000	ssc-mir-582	ssc-mir-582 [Source:miRBase;Acc:MI0022154]
chr2	3488327	3490870	2544	*	chr2	3488327	3490870	in_vitro	in_vivo	11	12	1.90181818	1.6075	0.29431818	c(t = 0.24091	0.812	0.96032933	Distal Intergenic	ENSSSCG000	FADD	Fas associated via death domain [Source:NCBI gene (formerly Entrezgene);Acc:595129]
chr5	53690269	53691802	1534	*	chr5	53690269	53691802	in_vitro	in_vivo	10	10	1.847	1.508	0.339	c(t = 0.24097	0.812	0.96032933	Distal Intergenic	ENSSSCG000	PLCZ1	phospholipase C zeta 1 [Source:VGNC Symbol;Acc:VGNC:103310]
chr1	268590476	268592165	1690	*	chr1	268590476	268592165	in_vitro	in_vivo	11	10	1.19363636	1.381	-0.1873636	c(t = -0.2419	0.812	0.96032933	Distal Intergenic	ENSSSCG000	LCN2	lipocalin 2 [Source:VGNC Symbol;Acc:VGNC:89662]
chr4	111579846	111580520	675	*	chr4	111579846	111580520	in_vitro	in_vivo	10	10	1.168	0.995	0.173	c(t = 0.24203	0.812	0.96032933	Promoter (<=1kb)	ENSSSCG000	SLC25A24	solute carrier family 25 member 24 [Source:NCBI gene (formerly Entrezgene);Acc:100518780]
chr10	24092819	24093710	892	*	chr10	24092819	24093710	in_vitro	in_vivo	10	12	15.399	18.2625	-2.8635	c(t = -0.2403	0.813	0.960625	Distal Intergenic	ENSSSCG000	TNNI1	troponin I1, slow skeletal type [Source:VGNC Symbol;Acc:VGNC:95548]
chr16	50399523	50400218	696	*	chr16	50399523	50400218	in_vitro	in_vivo	11	11	3.62272727	3.22	0.40272727	c(t = 0.23989	0.813	0.960625	Distal Intergenic	ENSSSCG000	STC2	stanniocalcin 2 [Source:NCBI gene (formerly Entrezgene);Acc:100126236]
chr14	40822078	40823079	1002	*	chr14	40822078	40823079	in_vitro	in_vivo	9	13	1.73666667	2.03846154	-0.3017949	c(t = -0.2414	0.813	0.960625	Distal Intergenic	ENSSSCG000	HNF1A	HNF1 homeobox A [Source:NCBI gene (formerly Entrezgene);Acc:574067]
chr3	39602775	39603672	898	*	chr3	39602775	39603672	in_vitro	in_vivo	10	11	1.347	1.05909091	0.28790909	c(t = 0.24168	0.813	0.960625	Distal Intergenic	ENSSSCG000	ECI1	enoyl-CoA delta isomerase 1 [Source:NCBI gene (formerly Entrezgene);Acc:100738177]
chr10	20089390	20090329	940	*	chr10	20089390	20090329	in_vitro	in_vivo	10	13	1.218	1.04692308	0.17107692	c(t = 0.24000	0.813	0.960625	Distal Intergenic	ENSSSCG000	CAPN2	calpain 2 [Source:NCBI gene (formerly Entrezgene);Acc:397393]
chr13	124387499	124387918	420	*	chr13	124387499	124387918	in_vitro	in_vivo	13	9	0.50153846	0.68	-0.1784615	c(t = -0.2427	0.813	0.960625	Promoter (<=1kb)	ENSSSCG000	TBCCD1	TBCC domain containing 1 [Source:VGNC Symbol;Acc:VGNC:93784]
chr4	47074381	47074947	567	*	chr4	47074381	47074947	in_vitro	in_vivo	9	10	1.24333333	1.479	-0.2356667	c(t = -0.2388	0.814	0.96150982	Distal Intergenic	ENSSSCG000	DECR1	2,4-dienoyl-CoA reductase 1 [Source:NCBI gene (formerly Entrezgene);Acc:503544]
chr14	39932587	39933254	668	*	chr14	39932587	39933254	in_vitro	in_vivo	10	9	1.23	0.94666667	0.28333333	c(t = 0.23996	0.814	0.96150982	Promoter (<=1kb)	ENSSSCG000	ALDH2	aldehyde dehydrogenase 2 family member [Source:NCBI gene (formerly Entrezgene);Acc:733685]
chr7	24147765	24149419	1655	*	chr7	24147765	24149419	in_vitro	in_vivo	10	12	29.03	25.4716667	3.55833333	c(t = 0.23679	0.815	0.96150982	Exon (NM_001123204/445520, exon 3 of 50)	ENSSSCG000	TNXB	tenascin XB [Source:NCBI gene (formerly Entrezgene);Acc:445520]
chrX	37103339	37104671	1333	*	chrX	37103339	37104671	in_vitro	in_vivo	11	13	14.42	17.0384615	-2.6184615	c(t = -0.2366	0.815	0.96150982	Distal Intergenic	ENSSSCG000	DDX3X	DEAD-box helicase 3 X-linked [Source:NCBI gene (formerly Entrezgene);Acc:100515940]
chrX	54823111	54823947	837	*	chrX	54823111	54823947	in_vitro	in_vivo	11	10	8.26363636	9.673	-1.4093636	c(t = -0.2369	0.815	0.96150982	Distal Intergenic	ENSSSCG000	AR	androgen receptor [Source:VGNC Symbol;Acc:VGNC:103903]
chr7	97531982	97534247	2266	*	chr7	97531982	97534247	in_vitro	in_vivo	13	13	2.25615385	1.96615385	0.29	c(t = 0.23709	0.815	0.96150982	Distal Intergenic	ENSSSCG000	VRTN	vertebrae development associated [Source:NCBI gene (formerly Entrezgene);Acc:100157734]
chr2	3629827	3637855	8029	*	chr2	3629827	3637855	in_vitro	in_vivo	14	13	1.50214286	1.68307692	-0.1809341	c(t = -0.2361	0.815	0.96150982	Distal Intergenic	ENSSSCG000	FADD	Fas associated via death domain [Source:NCBI gene (formerly Entrezgene);Acc:595129]
chr7	92643585	92645583	1999	*	chr7	92643585	92645583	in_vitro	in_vivo	9	13	1.75888889	1.47538462	0.28350427	c(t = 0.23757	0.815	0.96150982	Promoter (<=1kb)	ENSSSCG000	ACTN1	actinin alpha 1 [Source:NCBI gene (formerly Entrezgene);Acc:100513412]
chr1	191223712	191224535	824	*	chr1	191223712	191224535	in_vitro	in_vivo	9	9	1.17333333	1.46222222	-0.2888889	c(t = -0.2379	0.815	0.96150982	Distal Intergenic	ENSSSCG000	ssc-mir-7-1	ssc-mir-7-1 [Source:miRBase;Acc:MI0022122]
chr1	43003337	43004496	1160	*	chr1	43003337	43004496	in_vitro	in_vivo	13	15	1.08846154	0.92733333	0.16112821	c(t = 0.23764	0.815	0.96150982	Distal Intergenic	ENSSSCG000	MAN1A1	mannosidase alpha class 1A member 1 [Source:VGNC Symbol;Acc:VGNC:89966]
chr11	78764547	78774083	9537	*	chr11	78764547	78774083	in_vitro	in_vivo	11	15	96.9436364	96.7046667	0.2389697	c(t = 0.23565	0.816	0.96209519	Distal Intergenic	ENSSSCG000	ATP4B	ATPase H+/K+ transporting subunit beta [Source:VGNC Symbol;Acc:VGNC:85655]
chr12	12815190	12816109	920	*	chr12	12815190	12816109	in_vitro	in_vivo	9	10	1.61777778	1.351	0.26677778	c(t = 0.23603	0.816	0.96209519	Distal Intergenic	ENSSSCG000	CACNG1	calcium voltage-gated channel auxiliary subunit gamma 1 [Source:VGNC Symbol;Acc:VGNC:86124]
chr8	71982974	71984242	1269	*	chr8	71982974	71984242	in_vitro	in_vivo	10	12	6.758	7.93583333	-1.1778333	c(t = -0.2356	0.817	0.96209519	Distal Intergenic	ENSSSCG000	SCARB2	scavenger receptor class B member 2 [Source:VGNC Symbol;Acc:VGNC:92614]
chr12	26335198	26337241	2044	*	chr12	26335198	26337241	in_vitro	in_vivo	11	12	5.38363636	4.635	0.74863636	c(t = 0.23461	0.817	0.96209519	Distal Intergenic	ENSSSCG000	SGCA	sarcoglycan alpha [Source:VGNC Symbol;Acc:VGNC:92788]
chr2	102589884	102591675	1792	*	chr2	102589884	102591675	in_vitro	in_vivo	14	10	2.25	2.623	-0.373	c(t = -0.2346	0.817	0.96209519	Distal Intergenic	ENSSSCG00000039731		glutaredoxin [Source:NCBI gene (formerly Entrezgene);Acc:397463]
chr3	80745668	80746507	840	*	chr3	80745668	80746507	in_vitro	in_vivo	9	9	2.68666667	2.23111111	0.45555556	c(t = 0.23658	0.817	0.96209519	Distal Intergenic	ENSSSCG000	ssc-mir-9811	ssc-mir-9811 [Source:miRBase;Acc:MI0031578]
chrX	35887979	35891043	3065	*	chrX	35887979	35891043	in_vitro	in_vivo	15	14	2.46266667	2.19357143	0.26909524	c(t = 0.23442	0.817	0.96209519	Distal Intergenic	ENSSSCG000	DDX3X	DEAD-box helicase 3 X-linked [Source:NCBI gene (formerly Entrezgene);Acc:100515940]
chr7	30421467	30422569	1103	*	chr7	30421467	30422569	in_vitro	in_vivo	9	10	1.52666667	1.87333333	-0.3466667	c(t = -0.2359	0.817	0.96209519	Promoter (<=1kb)	ENSSSCG000	NUDT3	nudix hydrolase 3 [Source:NCBI gene (formerly Entrezgene);Acc:100737442]
chr10	60854359	60855387	1029	*	chr10	60854359	60855387	in_vitro	in_vivo	12	11	2.08583333	1.80545455	0.28037879	c(t = 0.23462	0.817	0.96209519	Distal Intergenic	ENSSSCG000	CDC123	cell division cycle 123 [Source:VGNC Symbol;Acc:VGNC:95819]
chr1	108514583	108515849	1267	*	chr1	108514583	108515849	in_vitro	in_vivo	13	9	1.96461538	1.70222222	0.26239316	c(t = 0.23409	0.817	0.96209519	Distal Intergenic	ENSSSCG000	LACTB	lactamase beta [Source:VGNC Symbol;Acc:VGNC:89614]
chr7	86123771	86124734	964	*	chr7	86123771	86124734	in_vitro	in_vivo	9	11	1.82	1.51545455	0.30454545	c(t = 0.23618	0.817	0.96209519	Promoter (<=1kb)	NA	NA	NA
chr8	104886198	104886520	323	*	chr8	104886198	104886520	in_vitro	in_vivo	9	10	0.88666667	1.131	-0.2443333	c(t = -0.2350	0.817	0.96209519	Distal Intergenic	ENSSSCG000	MYOZ2	myozenin 2 [Source:NCBI gene (formerly Entrezgene);Acc:733663]
chr9	136487690	136488647	958	*	chr9	136487690	136488647	in_vitro	in_vivo	9	11	5.62888889	5.03181818	0.59707071	c(t = 0.23423	0.818	0.96209519	Distal Intergenic	ENSSSCG000	DDC	dopa decarboxylase [Source:VGNC Symbol;Acc:VGNC:87204]
chr16	18840853	18843384	2532	*	chr16	18840853	18843384	in_vitro	in_vivo	10	13	3.047	3.49153846	-0.4445385	c(t = -0.2325	0.818	0.96209519	Distal Intergenic	ENSSSCG000	C1QTNF3	C1q and TNF related 3 [Source:NCBI gene (formerly Entrezgene);Acc:100270814]
chr18	47297949	47299127	1179	*	chr18	47297949	47299127	in_vitro	in_vivo	12	12	3.1575	2.79666667	0.36083333	c(t = 0.23256	0.818	0.96209519	Distal Intergenic	ENSSSCG000	CYCS	cytochrome c, somatic [Source:NCBI gene (formerly Entrezgene);Acc:100170131]
chr4	76855136	76857370	2235	*	chr4	76855136	76857370	in_vitro	in_vivo	13	12	1.99384615	1.80416667	0.18967949	c(t = 0.23215	0.818	0.96209519	Distal Intergenic	ENSSSCG000	MRPL15	mitochondrial ribosomal protein L15 [Source:VGNC Symbol;Acc:VGNC:98820]
chr9	109384316	109385974	1659	*	chr9	109384316	109385974	in_vitro	in_vivo	13	13	1.59538462	1.34769231	0.24769231	c(t = 0.23359	0.818	0.96209519	Promoter (<=1kb)	ENSSSCG000	EZH2	enhancer of zeste 2 polycomb repressive complex 2 subunit [Source:VGNC Symbol;Acc:VGNC:87855]
chr15	47625194	47625749	556	*	chr15	47625194	47625749	in_vitro	in_vivo	13	10	1.31307692	1.106	0.20707692	c(t = 0.23371	0.818	0.96209519	Distal Intergenic	ENSSSCG000	TM2D2	TM2 domain containing 2 [Source:NCBI gene (formerly Entrezgene);Acc:100154457]
chr14	76499184	76500131	948	*	chr14	76499184	76500131	in_vitro	in_vivo	13	11	0.91461538	1.06363636	-0.149021	c(t = -0.2332	0.818	0.96209519	Distal Intergenic	ENSSSCG000	CHCHD1	coiled-coil-helix-coiled-coil-helix domain containing 1 [Source:VGNC Symbol;Acc:VGNC:86625]
chr5	39362890	39363761	872	*	chr5	39362890	39363761	in_vitro	in_vivo	10	12	0.534	0.67666667	-0.1426667	c(t = -0.2334	0.818	0.96209519	Promoter (<=1kb)	ENSSSCG000	NAP1L1	nucleosome assembly protein 1 like 1 [Source:VGNC Symbol;Acc:VGNC:90576]
chr6	135524289	135525145	857	*	chr6	135524289	135525145	in_vitro	in_vivo	9	10	1.58777778	1.99	-0.4022222	c(t = -0.2335	0.819	0.96282928	Distal Intergenic	ENSSSCG000	PIGK	phosphatidylinositol glycan anchor biosynthesis class K [Source:NCBI gene (formerly Entrezgene);Acc:606750]
chr5	21656444	21657276	833	*	chr5	21656444	21657276	in_vitro	in_vivo	10	9	1.016	0.82888889	0.18711111	c(t = 0.23273	0.819	0.96282928	Distal Intergenic	ENSSSCG000	CS	citrate synthase [Source:VGNC Symbol;Acc:VGNC:103225]
chr15	54094484	54094974	491	*	chr15	54094484	54094974	in_vitro	in_vivo	9	11	0.30888889	0.43272727	-0.1238384	c(t = -0.2329	0.819	0.96282928	Distal Intergenic	ENSSSCG000	UBXN8	UBX domain protein 8 [Source:VGNC Symbol;Acc:VGNC:96239]
chrX	41863912	41864470	559	*	chrX	41863912	41864470	in_vitro	in_vivo	11	10	94.4763636	95.185	-0.7086364	c(t = -0.2315	0.82	0.96297085	Distal Intergenic	ENSSSCG000	RGN	regucalcin [Source:VGNC Symbol;Acc:VGNC:92256]
chr15	139512761	139514938	2178	*	chr15	139512761	139514938	in_vitro	in_vivo	9	14	13.6222222	16.18	-2.5577778	c(t = -0.2309	0.82	0.96297085	Distal Intergenic	ENSSSCG000	ssc-mir-149	ssc-mir-149 [Source:miRBase;Acc:MI0016620]
chr4	62610903	62611910	1008	*	chr4	62610903	62611910	in_vitro	in_vivo	9	13	7.29666667	6.21076923	1.08589744	c(t = 0.23054	0.82	0.96297085	Distal Intergenic	ENSSSCG000	RPL7	ribosomal protein L7 [Source:NCBI gene (formerly Entrezgene);Acc:414418]
chr13	32106678	32107554	877	*	chr13	32106678	32107554	in_vitro	in_vivo	9	13	6.36888889	5.81076923	0.55811966	c(t = 0.23005	0.82	0.96297085	Distal Intergenic	ENSSSCG000	APEH	acylaminoacyl-peptide hydrolase [Source:VGNC Symbol;Acc:VGNC:85408]
chr13	204914103	204915079	977	*	chr13	204914103	204915079	in_vitro	in_vivo	10	10	1.836	2.044	-0.208	c(t = -0.2312	0.82	0.96297085	Distal Intergenic	ENSSSCG000	MX1	MX dynamin like GTPase 1 [Source:NCBI gene (formerly Entrezgene);Acc:397128]
chr17	11817384	11818697	1314	*	chr17	11817384	11818697	in_vitro	in_vivo	11	12	1.02727273	1.1775	-0.1502273	c(t = -0.2302	0.82	0.96297085	Distal Intergenic	ENSSSCG000	SMIM19	small integral membrane protein 19 [Source:NCBI gene (formerly Entrezgene);Acc:100152445]
chr11	1864369	1864735	367	*	chr11	1864369	1864735	in_vitro	in_vivo	9	10	97.9577778	97.424	0.53377778	c(t = 0.22975	0.821	0.96297085	Distal Intergenic	ENSSSCG000	FGF9	fibroblast growth factor 9 [Source:NCBI gene (formerly Entrezgene);Acc:396717]
chr6	75110090	75112120	2031	*	chr6	75110090	75112120	in_vitro	in_vivo	13	12	86.14	87.7808333	-1.6408333	c(t = -0.2291	0.821	0.96297085	Distal Intergenic	ENSSSCG000	HSPB7	heat shock protein family B (small) member 7 [Source:NCBI gene (formerly Entrezgene);Acc:100627988]
chr11	793094	794813	1720	*	chr11	793094	794813	in_vitro	in_vivo	12	14	12.6566667	14.0207143	-1.3640476	c(t = -0.2285	0.821	0.96297085	Distal Intergenic	ENSSSCG000	CRYL1	crystallin lambda 1 [Source:VGNC Symbol;Acc:VGNC:87023]
chr14	658699	659709	1011	*	chr14	658699	659709	in_vitro	in_vivo	10	10	5.36666667	4.63	0.73666667	c(t = 0.22940	0.821	0.96297085	Distal Intergenic	ENSSSCG000	SPIN1	spindlin 1 [Source:NCBI gene (formerly Entrezgene);Acc:100337684]
chr10	48178041	48179143	1103	*	chr10	48178041	48179143	in_vitro	in_vivo	9	9	2.04333333	2.35333333	-0.31	c(t = -0.2305	0.821	0.96297085	Distal Intergenic	ENSSSCG000	PRPF18	pre-mRNA processing factor 18 [Source:VGNC Symbol;Acc:VGNC:108278]
chr1	75522280	75523303	1024	*	chr1	75522280	75523303	in_vitro	in_vivo	12	10	1.7425	2.078	-0.3355	c(t = -0.2302	0.821	0.96297085	Distal Intergenic	ENSSSCG000	PPIL6	peptidylprolyl isomerase like 6 [Source:VGNC Symbol;Acc:VGNC:98216]
chr5	45745265	45745867	603	*	chr5	45745265	45745867	in_vitro	in_vivo	15	9	2.296	1.92111111	0.37488889	c(t = 0.22934	0.821	0.96297085	Distal Intergenic	ENSSSCG000	PTHLH	parathyroid hormone like hormone [Source:VGNC Symbol;Acc:VGNC:91963]
chr7	75788706	75789477	772	*	chr7	75788706	75789477	in_vitro	in_vivo	10	9	1.19	1.45222222	-0.2622222	c(t = -0.2294	0.821	0.96297085	Distal Intergenic	ENSSSCG000	PABPN1	poly(A) binding protein nuclear 1 [Source:NCBI gene (formerly Entrezgene);Acc:100155260]
chr1	150709729	150712378	2650	*	chr1	150709729	150712378	in_vitro	in_vivo	14	16	1.355	1.199375	0.155625	c(t = 0.22879	0.821	0.96297085	Distal Intergenic	ENSSSCG000	CBLN2	cerebellin 2 [Source:VGNC Symbol;Acc:VGNC:86226]
chrX	41192997	41194732	1736	*	chrX	41192997	41194732	in_vitro	in_vivo	13	13	8.35846154	9.46384615	-1.1053846	c(t = -0.2273	0.822	0.96355552	Distal Intergenic	ENSSSCG000	RGN	regucalcin [Source:VGNC Symbol;Acc:VGNC:92256]
chr14	7277105	7278156	1052	*	chr14	7277105	7278156	in_vitro	in_vivo	12	10	7.4825	9.395	-1.9125	c(t = -0.2297	0.822	0.96355552	Distal Intergenic	ENSSSCG000	PEBP4	phosphatidylethanolamine binding protein 4 [Source:VGNC Symbol;Acc:VGNC:91303]
chr6	145533230	145534315	1086	*	chr6	145533230	145534315	in_vitro	in_vivo	13	11	1.78461538	1.51181818	0.2727972	c(t = 0.22815	0.822	0.96355552	Distal Intergenic	ENSSSCG000	IL23R	interleukin 23 receptor [Source:VGNC Symbol;Acc:VGNC:89100]
chr11	71141729	71142494	766	*	chr11	71141729	71142494	in_vitro	in_vivo	12	10	1.21166667	1.511	-0.2993333	c(t = -0.2297	0.822	0.96355552	Distal Intergenic	ENSSSCG000	BIVM	basic, immunoglobulin-like variable motif containing [Source:VGNC Symbol;Acc:VGNC:109180]
chr18	24885648	24886171	524	*	chr18	24885648	24886171	in_vitro	in_vivo	10	9	4.885	5.61444444	-0.7294444	c(t = -0.2274	0.823	0.96413948	Distal Intergenic	ENSSSCG000	AASS	aminoadipate-semialdehyde synthase [Source:VGNC Symbol;Acc:VGNC:84944]
chr12	17305010	17306888	1879	*	chr12	17305010	17306888	in_vitro	in_vivo	13	11	3.71153846	4.20727273	-0.4957343	c(t = -0.2263	0.823	0.96413948	Promoter (<=1kb)	ENSSSCG000	CRHR1	corticotropin releasing hormone receptor 1 [Source:VGNC Symbol;Acc:VGNC:98983]
chr3	1115368	1121883	6516	*	chr3	1115368	1121883	in_vitro	in_vivo	12	15	2.75583333	2.52933333	0.2265	c(t = 0.22695	0.823	0.96413948	Distal Intergenic	ENSSSCG000	ssc-mir-9817	ssc-mir-9817 [Source:miRBase;Acc:MI0031584]
chr1	112620426	112620975	550	*	chr1	112620426	112620975	in_vitro	in_vivo	11	12	1.02181818	1.1875	-0.1656818	c(t = -0.2270	0.823	0.96413948	Distal Intergenic	ENSSSCG000	CCNB2	cyclin B2 [Source:VGNC Symbol;Acc:VGNC:86350]
chr5	15504467	15505487	1021	*	chr5	15504467	15505487	in_vitro	in_vivo	10	11	32.013	28.5645455	3.44845455	c(t = 0.22571	0.824	0.96428202	Distal Intergenic	ENSSSCG000	TMBIM6	transmembrane BAX inhibitor motif containing 6 [Source:VGNC Symbol;Acc:VGNC:98374]
chr13	73351510	73352464	955	*	chr13	73351510	73352464	in_vitro	in_vivo	9	13	21.7277778	18.83	2.89777778	c(t = 0.22623	0.824	0.96428202	Distal Intergenic	ENSSSCG000	ACKR4	atypical chemokine receptor 4 [Source:NCBI gene (formerly Entrezgene);Acc:100037292]
chr9	106707480	106708021	542	*	chr9	106707480	106708021	in_vitro	in_vivo	11	9	9.04727273	7.46333333	1.58393939	c(t = 0.22612	0.824	0.96428202	Exon (NM_213939/396979, exon 2 of 11)	ENSSSCG000	PIK3CG	phosphatidylinositol-4,5-bisphosphate 3-kinase catalytic subunit gamma [Source:VGNC Symbol;Acc:VGNC:91443]
chr16	79831730	79832805	1076	*	chr16	79831730	79832805	in_vitro	in_vivo	9	12	4.86666667	4.16416667	0.7025	c(t = 0.22546	0.824	0.96428202	Distal Intergenic	ENSSSCG000	TPPP	tubulin polymerization promoting protein [Source:VGNC Symbol;Acc:VGNC:94344]
chr5	85257946	85259133	1188	*	chr5	85257946	85259133	in_vitro	in_vivo	9	10	1.84111111	2.148	-0.3068889	c(t = -0.2262	0.824	0.96428202	Promoter (<=1kb)	ENSSSCG000	IKBIP	IKBKB interacting protein [Source:VGNC Symbol;Acc:VGNC:103291]
chr14	71928137	71928911	775	*	chr14	71928137	71928911	in_vitro	in_vivo	10	11	1.673	1.45090909	0.22209091	c(t = 0.22580	0.824	0.96428202	Distal Intergenic	ENSSSCG000	TET1	tet methylcytosine dioxygenase 1 [Source:NCBI gene (formerly Entrezgene);Acc:100625859]
chr18	5347507	5348008	502	*	chr18	5347507	5348008	in_vitro	in_vivo	12	9	1.2275	1.02	0.2075	c(t = 0.22507	0.824	0.96428202	Distal Intergenic	ENSSSCG000	RHEB	Ras homolog, mTORC1 binding [Source:NCBI gene (formerly Entrezgene);Acc:100516050]
chr11	3895603	3896365	763	*	chr11	3895603	3896365	in_vitro	in_vivo	10	11	51.95	47.6945455	4.25545455	c(t = 0.22384	0.825	0.96471774	Distal Intergenic	ENSSSCG000	GPR12	G protein-coupled receptor 12 [Source:NCBI gene (formerly Entrezgene);Acc:100736641]
chr1	100698084	100699377	1294	*	chr1	100698084	100699377	in_vitro	in_vivo	15	11	1.72933333	2.03545455	-0.3061212	c(t = -0.2252	0.825	0.96471774	Distal Intergenic	ENSSSCG000	SMAD4	SMAD family member 4 [Source:VGNC Symbol;Acc:VGNC:93218]
chr6	143273292	143274888	1597	*	chr6	143273292	143274888	in_vitro	in_vivo	9	12	1.37083333	1.55333333	-0.1825	c(t = -0.2239	0.825	0.96471774	Distal Intergenic	NA	NA	NA
chr1	149583701	149584576	876	*	chr1	149583701	149584576	in_vitro	in_vivo	10	10	1.29	1.041	0.249	c(t = 0.22399	0.825	0.96471774	Promoter (<=1kb)	ENSSSCG000	CNDP2	carnosine dipeptidase 2 [Source:VGNC Symbol;Acc:VGNC:86812]
chr15	95015169	95016061	893	*	chr15	95015169	95016061	in_vitro	in_vivo	12	13	1.1	0.98153846	0.11846154	c(t = 0.22390	0.825	0.96471774	Distal Intergenic	ENSSSCG000	MFSD6	major facilitator superfamily domain containing 6 [Source:VGNC Symbol;Acc:VGNC:96052]
chr6	53224809	53225569	761	*	chr6	53224809	53225569	in_vitro	in_vivo	11	10	14.5890909	16.817	-2.2279091	c(t = -0.2232	0.826	0.96515281	Distal Intergenic	ENSSSCG000	NAPA	NSF attachment protein alpha [Source:VGNC Symbol;Acc:VGNC:90578]
chr17	27269522	27270941	1420	*	chr17	27269522	27270941	in_vitro	in_vivo	11	14	3.09909091	2.76714286	0.33194805	c(t = 0.22189	0.826	0.96515281	Distal Intergenic	ENSSSCG000	SEC23B	SEC23 homolog B, COPII coat complex component [Source:VGNC Symbol;Acc:VGNC:95490]
chr1	149856300	149857052	753	*	chr1	149856300	149857052	in_vitro	in_vivo	9	9	1.69666667	2.14888889	-0.4522222	c(t = -0.2254	0.826	0.96515281	Distal Intergenic	ENSSSCG000	CYB5A	cytochrome b5 type A [Source:VGNC Symbol;Acc:VGNC:96727]
chr14	347531	349457	1927	*	chr14	347531	349457	in_vitro	in_vivo	11	11	2.44363636	2.14181818	0.30181818	c(t = 0.22379	0.826	0.96515281	Distal Intergenic	ENSSSCG000	SPIN1	spindlin 1 [Source:NCBI gene (formerly Entrezgene);Acc:100337684]
chr7	100777121	100777675	555	*	chr7	100777121	100777675	in_vitro	in_vivo	12	10	1.7	1.975	-0.275	c(t = -0.2225	0.826	0.96515281	Distal Intergenic	ENSSSCG000	SLIRP	SRA stem-loop interacting RNA binding protein [Source:NCBI gene (formerly Entrezgene);Acc:100037987]
chr2	137211423	137212953	1531	*	chr2	137211423	137212953	in_vitro	in_vivo	9	14	2.64555556	2.34357143	0.30198413	c(t = 0.22178	0.827	0.96540567	Distal Intergenic	ENSSSCG000	PITX1	paired like homeodomain 1 [Source:NCBI gene (formerly Entrezgene);Acc:100689266]
chr7	91413305	91414020	716	*	chr7	91413305	91414020	in_vitro	in_vivo	14	13	1.47714286	1.31230769	0.16483516	c(t = 0.22136	0.827	0.96540567	Promoter (<=1kb)	ENSSSCG000	VTI1B	vesicle transport through interaction with t-SNAREs 1B [Source:NCBI gene (formerly Entrezgene);Acc:404692]
chr9	60807057	60807845	789	*	chr9	60807057	60807845	in_vitro	in_vivo	10	11	0.852	1.02454545	-0.1725455	c(t = -0.2221	0.827	0.96540567	Promoter (<=1kb)	ENSSSCG000	ACAD8	acyl-CoA dehydrogenase family member 8 [Source:NCBI gene (formerly Entrezgene);Acc:100627341]
chr7	110704695	110705805	1111	*	chr7	110704695	110705805	in_vitro	in_vivo	10	10	1.132	1.02	0.112	c(t = 0.22119	0.827	0.96540567	Distal Intergenic	ENSSSCG000	SPATA7	spermatosis associated 7 [Source:VGNC Symbol;Acc:VGNC:93389]
chr15	95512881	95514473	1593	*	chr15	95512881	95514473	in_vitro	in_vivo	13	12	0.58	0.7	-0.12	c(t = -0.2212	0.827	0.96540567	Distal Intergenic	ENSSSCG000	STAT1	signal transducer and activator of transcription 1 [Source:VGNC Symbol;Acc:VGNC:95523]
chr6	71980744	71981720	977	*	chr6	71980744	71981720	in_vitro	in_vivo	[illegible]	[illegible]	2.81153846	3.41	-0.5984615	[illegible]	0.828	0.96540567	Distal Intergenic	ENSSSCG000	NPPB	natriuretic peptide B [Source:NCBI gene (formerly Entrezgene);Acc:396844]
chrX	114573064	114575504	2442	*	chrX	114573064	114575504	in_vitro	in_vivo	[illegible]	[illegible]	1.78888889	1.31076923	0.47811966	[illegible]	0.828	0.96540567	Promoter (<=1kb)	ENSSSCG000	ssc-mir-505	ssc-mir-505 [Source:miRBase;Acc:MI0003171]
chr7	2247574	2249740	2167	*	chr7	2247574	2249740	in_vitro	in_vivo	[illegible]	[illegible]	1.17909091	1.53866667	-0.3595758	[illegible]	0.828	0.96540567	Promoter (<=1kb)	ENSSSCG000	ECI2	enoyl-CoA delta isomerase 2 [Source:VGNC Symbol;Acc:VGNC:87532]
chr13	87756673	87757239	567	*	chr13	87756673	87757239	in_vitro	in_vivo	[illegible]	[illegible]	1.68888889	1.99272727	-0.3038384	[illegible]	0.828	0.96540567	Distal Intergenic	ENSSSCG000	PLSCR1	phospholipid scramblase 1 [Source:NCBI gene (formerly Entrezgene);Acc:100620055]
chr18	9172277	9173200	924	*	chr18	9172277	9173200	in_vitro	in_vivo	[illegible]	[illegible]	1.30909091	1.656	-0.3469091	[illegible]	0.828	0.96540567	Distal Intergenic	ENSSSCG000	NDUFB2	NADH:ubiquinone oxidoreductase subunit B2 [Source:NCBI gene (formerly Entrezgene);Acc:100153895]

chr9	48095052	48095440	389	*	chr9	48095052	48095440	in_vitro	in_vivo	12	14	1.77833333	1.555	0.22333333	c(t = 0.21905	0.828	0.96540567	Distal Intergenic	ENSSSCG000	ssc-mir-9818	ssc-mir-9818 [Source:miRBase;Acc:MI0031585]
chr3	12039478	12040198	721	*	chr3	12039478	12040198	in_vitro	in_vivo	9	12	0.92555556	1.17166667	-0.2461111	c(t = -0.2213	0.828	0.96540567	Distal Intergenic	ENSSSCG000	NCF1	neutrophil cytosolic factor 1 [Source:NCBI gene (formerly Entrezgene);Acc:100134857]
chr7	37823256	37825208	1953	*	chr7	37823256	37825208	in_vitro	in_vivo	10	11	1.27	1.08636364	0.18363636	c(t = 0.22048	0.828	0.96540567	Promoter (<=1kb)	ENSSSCG000	TBCC	tubulin folding cofactor C [Source:NCBI gene (formerly Entrezgene);Acc:100157124]
chr18	3674927	3675280	354	*	chr18	3674927	3675280	in_vitro	in_vivo	10	9	26.875	23.2444444	3.63055556	c(t = 0.21947	0.829	0.96540567	Distal Intergenic	ENSSSCG000	INSIG1	insulin induced gene 1 [Source:NCBI gene (formerly Entrezgene);Acc:403162]
chr6	77025319	77026163	845	*	chr6	77025319	77026163	in_vitro	in_vivo	10	10	25.93	22.66	3.27	c(t = 0.21927	0.829	0.96540567	Distal Intergenic	ENSSSCG000	TAS1R2	taste 1 receptor member 2 [Source:VGNC Symbol;Acc:VGNC:93741]
chr14	133375700	133376748	1049	*	chr14	133375700	133376748	in_vitro	in_vivo	10	12	3.568	4.23916667	-0.6711667	c(t = -0.2188	0.829	0.96540567	Distal Intergenic	ENSSSCG00000010734		ornithine aminotransferase [Source:NCBI gene (formerly Entrezgene);Acc:100156865]
chr1	113932640	113934074	1435	*	chr1	113932640	113934074	in_vitro	in_vivo	12	15	2.25666667	1.96733333	0.28933333	c(t = 0.21960	0.829	0.96540567	Distal Intergenic	ENSSSCG000	AQP9	aquaporin 9 [Source:NCBI gene (formerly Entrezgene);Acc:100127153]
chr10	41773304	41774241	938	*	chr10	41773304	41774241	in_vitro	in_vivo	10	10	1.736	1.432	0.304	c(t = 0.21996	0.829	0.96540567	Distal Intergenic	ENSSSCG000	MTPAP	mitochondrial poly(A) polymerase [Source:VGNC Symbol;Acc:VGNC:96676]
chr7	59733358	59733873	516	*	chr7	59733358	59733873	in_vitro	in_vivo	12	9	1.24083333	1.04777778	0.19305556	c(t = 0.21915	0.829	0.96540567	Distal Intergenic	ENSSSCG000	CYP11A1	cytochrome P450 family 11 subfamily A member 1 [Source:VGNC Symbol;Acc:VGNC:103363]
chrX	108178642	108179934	1293	*	chrX	108178642	108179934	in_vitro	in_vivo	12	16	6.39	5.64125	0.74875	c(t = 0.21636	0.83	0.96540567	Promoter (<=1kb)	ENSSSCG000	STK26	serine/threonine kinase 26 [Source:NCBI gene (formerly Entrezgene);Acc:100233205]
chr5	13274974	13276303	1330	*	chr5	13274974	13276303	in_vitro	in_vivo	13	14	1.48230769	1.24642857	0.23587912	c(t = 0.21808	0.83	0.96540567	Distal Intergenic	ENSSSCG000	RTCB	RNA 2',3'-cyclic phosphate and 5'-OH ligase [Source:NCBI gene (formerly Entrezgene);Acc:733658]
chr1	234368306	234371169	2864	*	chr1	234368306	234371169	in_vitro	in_vivo	14	13	0.98714286	1.19	-0.2028571	c(t = -0.2180	0.83	0.96540567	Distal Intergenic	ENSSSCG000	VCP	valosin containing protein [Source:VGNC Symbol;Acc:VGNC:109420]
chr1	21265506	21266441	936	*	chr1	21265506	21266441	in_vitro	in_vivo	12	12	0.62916667	0.52583333	0.10333333	c(t = 0.21756	0.83	0.96540567	Distal Intergenic	ENSSSCG000	PLAGL1	PLAG1 like zinc finger 1 [Source:VGNC Symbol;Acc:VGNC:91510]
chr12	60206934	60212780	5847	*	chr12	60206934	60212780	in_vitro	in_vivo	9	11	89.4622222	87.7263636	1.73585859	c(t = 0.21658	0.831	0.96540567	Distal Intergenic	ENSSSCG000	SLC5A10	solute carrier family 5 member 10 [Source:NCBI gene (formerly Entrezgene);Acc:497235]
chr6	100290707	100291580	874	*	chr6	100290707	100291580	in_vitro	in_vivo	9	12	27.0588889	30.995	-3.9361111	c(t = -0.2164	0.831	0.96540567	Distal Intergenic	ENSSSCG000	NDUFV2	NADH:ubiquinone oxidoreductase core subunit V2 [Source:NCBI gene (formerly Entrezgene);Acc:100037991]
chr17	19627230	19630558	3329	*	chr17	19627230	19630558	in_vitro	in_vivo	13	14	3.47384615	3.09642857	0.37741758	c(t = 0.21580	0.831	0.96540567	Distal Intergenic	ENSSSCG000	TMX4	thioredoxin related transmembrane protein 4 [Source:NCBI gene (formerly Entrezgene);Acc:100627887]
chr10	39932209	39933996	1788	*	chr10	39932209	39933996	in_vitro	in_vivo	9	13	1.73888889	2.03769231	-0.2988034	c(t = -0.2185	0.831	0.96540567	Distal Intergenic	ENSSSCG000	BAMBI	BMP and activin membrane bound inhibitor [Source:NCBI gene (formerly Entrezgene);Acc:449001]
chr6	84426731	84427126	396	*	chr6	84426731	84427126	in_vitro	in_vivo	11	10	2.11454545	1.856	0.25854545	c(t = 0.21643	0.831	0.96540567	Promoter (<=1kb)	ENSSSCG000	SLC9A1	solute carrier family 9 member A1 [Source:NCBI gene (formerly Entrezgene);Acc:397458]
chr9	77509110	77510115	1006	*	chr9	77509110	77510115	in_vitro	in_vivo	15	12	1.34133333	1.56333333	-0.222	c(t = -0.2160	0.831	0.96540567	Distal Intergenic	ENSSSCG000	ASNS	asparagine synthetase (glutamine-hydrolyzing) [Source:NCBI gene (formerly Entrezgene);Acc:100312961]
chr15	80927063	80927872	810	*	chr15	80927063	80927872	in_vitro	in_vivo	11	12	1.49454545	1.2525	0.24204545	c(t = 0.21680	0.831	0.96540567	Distal Intergenic	ENSSSCG000	ATP5MC3	ATP synthase membrane subunit c locus 3 [Source:NCBI gene (formerly Entrezgene);Acc:100513629]
chr4	16137117	16137426	310	*	chr4	16137117	16137426	in_vitro	in_vivo	9	11	1.19	0.89818182	0.29181818	c(t = 0.21779	0.831	0.96540567	Distal Intergenic	ENSSSCG000	DERL1	derlin 1 [Source:NCBI gene (formerly Entrezgene);Acc:100626802]
chr7	682322	683388	1067	*	chr7	682322	683388	in_vitro	in_vivo	9	14	20.0255556	23.3471429	-3.3215873	c(t = -0.2148	0.832	0.96540567	Distal Intergenic	ENSSSCG000	IRF4	interferon regulatory factor 4 [Source:VGNC Symbol;Acc:VGNC:89207]
chr14	7347995	7348317	323	*	chr14	7347995	7348317	in_vitro	in_vivo	9	9	13.9744444	17.1688889	-3.1944444	c(t = -0.2150	0.832	0.96540567	Distal Intergenic	ENSSSCG000	PEBP4	phosphatidylethanolamine binding protein 4 [Source:VGNC Symbol;Acc:VGNC:91303]
chr16	79683353	79686819	3467	*	chr16	79683353	79686819	in_vitro	in_vivo	10	10	15.859	13.523	2.336	c(t = 0.21578	0.832	0.96540567	Distal Intergenic	ENSSSCG000	TPPP	tubulin polymerization promoting protein [Source:VGNC Symbol;Acc:VGNC:94344]
chr7	75209848	75210581	734	*	chr7	75209848	75210581	in_vitro	in_vivo	9	10	2.47555556	2.976	-0.5004444	c(t = -0.2171	0.832	0.96540567	Distal Intergenic	ENSSSCG000	PCK2	phosphoenolpyruvate carboxykinase 2, mitochondrial [Source:VGNC Symbol;Acc:VGNC:91223]
chr14	19824182	19825871	1690	*	chr14	19824182	19825871	in_vitro	in_vivo	13	15	1.80384615	2.078	-0.2741538	c(t = -0.2138	0.832	0.96540567	Distal Intergenic	ENSSSCG000	HMGB2	high mobility group box 2 [Source:VGNC Symbol;Acc:VGNC:88902]
chr13	123057942	123059185	1244	*	chr13	123057942	123059185	in_vitro	in_vivo	13	15	1.50307692	1.69533333	-0.1922564	c(t = -0.2147	0.832	0.96540567	Distal Intergenic	ENSSSCG000	TRA2B	transformer 2 beta homolog [Source:VGNC Symbol;Acc:VGNC:94354]
chr4	55450791	55451521	731	*	chr4	55450791	55451521	in_vitro	in_vivo	11	12	1.25818182	1.56583333	-0.3076515	c(t = -0.2146	0.832	0.96540567	Distal Intergenic	ENSSSCG000	FABP5	fatty acid binding protein 5 [Source:NCBI gene (formerly Entrezgene);Acc:574074]
chr4	60636986	60637817	832	*	chr4	60636986	60637817	in_vitro	in_vivo	10	9	1.779	1.50555556	0.27344444	c(t = 0.21573	0.832	0.96540567	Distal Intergenic	ENSSSCG000	LY96	lymphocyte antigen 96 [Source:VGNC Symbol;Acc:VGNC:89907]
chr3	26760321	26761137	817	*	chr3	26760321	26761137	in_vitro	in_vivo	10	13	1.691	1.4	0.291	c(t = 0.21664	0.832	0.96540567	Promoter (<=1kb)	ENSSSCG000	SMG1	SMG1 nonsense mediated mRNA decay associated PI3K related kinase [Source:VGNC Symbol;Acc:VGNC:93248]
chr13	21670215	21671034	820	*	chr13	21670215	21671034	in_vitro	in_vivo	11	12	0.97181818	1.11083333	-0.1390152	c(t = -0.2149	0.832	0.96540567	Distal Intergenic	ENSSSCG000	MLH1	mutL homolog 1 [Source:VGNC Symbol;Acc:VGNC:90252]
chr1	127237848	127238624	777	*	chr1	127237848	127238624	in_vitro	in_vivo	12	12	1.315	1.04166667	0.27333333	c(t = 0.21560	0.832	0.96540567	Distal Intergenic	ENSSSCG000	B2M	beta-2-microglobulin [Source:NCBI gene (formerly Entrezgene);Acc:397033]
chr9	52840952	52842827	1876	*	chr9	52840952	52842827	in_vitro	in_vivo	13	12	95.4615385	95.9116667	-0.4501282	c(t = -0.2131	0.833	0.96540567	Distal Intergenic	ENSSSCG000	ACRV1	acrosomal vesicle protein 1 [Source:VGNC Symbol;Acc:VGNC:108568]
chr14	140363981	140372357	8377	*	chr14	140363981	140372357	in_vitro	in_vivo	12	14	58.545	56.1471429	2.39785714	c(t = 0.21364	0.833	0.96540567	Distal Intergenic	ENSSSCG000	ssc-mir-202	ssc-mir-202 [Source:miRBase;Acc:MI0013157]
chr18	29706626	29707082	457	*	chr18	29706626	29707082	in_vitro	in_vivo	12	9	23.6183333	27.0277778	-3.4094444	c(t = -0.2147	0.833	0.96540567	Promoter (<=1kb)	ENSSSCG000	CAV2	caveolin 2 [Source:NCBI gene (formerly Entrezgene);Acc:100125375]
chr7	1376213	1377255	1043	*	chr7	1376213	1377255	in_vitro	in_vivo	10	10	13.482	11.039	2.443	c(t = 0.21367	0.833	0.96540567	Distal Intergenic	ENSSSCG000	SERPINB9	serpin peptidase inhibitor, clade B (ovalbumin), member 9 [Source:NCBI gene (formerly Entrezgene);Acc:100126851]
chr16	22960646	22966384	5739	*	chr16	22960646	22966384	in_vitro	in_vivo	14	16	6.35285714	7.050625	-0.6977679	c(t = -0.2140	0.833	0.96540567	Distal Intergenic	ENSSSCG000	SLC1A3	solute carrier family 1 member 3 [Source:VGNC Symbol;Acc:VGNC:92965]
chr18	55683884	55685170	1287	*	chr18	55683884	55685170	in_vitro	in_vivo	10	15	3.628	3.32666667	0.30133333	c(t = 0.21283	0.833	0.96540567	Distal Intergenic	ENSSSCG000	RARRES2	retinoic acid receptor responder 2 [Source:VGNC Symbol;Acc:VGNC:92098]
chr10	53332155	53332915	761	*	chr10	53332155	53332915	in_vitro	in_vivo	10	10	2.746	3.225	-0.479	c(t = -0.2144	0.833	0.96540567	Distal Intergenic	ENSSSCG000	BMI1	BMI1 proto-onco, polycomb ring finger [Source:VGNC Symbol;Acc:VGNC:108265]
chrX	39952952	39954983	2032	*	chrX	39952952	39954983	in_vitro	in_vivo	14	15	2.54571429	2.81133333	-0.265619	c(t = -0.2126	0.833	0.96540567	Distal Intergenic	ENSSSCG000	ssc-mir-221	ssc-mir-221 [Source:miRBase;Acc:MI0008221]
chr13	44480610	44482171	1562	*	chr13	44480610	44482171	in_vitro	in_vivo	11	14	1.79727273	2.12214286	-0.3248701	c(t = -0.2134	0.833	0.96540567	Distal Intergenic	ENSSSCG00000033189		family with sequence similarity 107 member A [Source:NCBI gene (formerly Entrezgene);Acc:100516512]
chr9	124847356	124848681	1326	*	chr9	124847356	124848681	in_vitro	in_vivo	10	10	1.367	1.599	-0.232	c(t = -0.2143	0.833	0.96540567	Promoter (<=1kb)	ENSSSCG000	ARPC5	actin related protein 2/3 complex subunit 5 [Source:NCBI gene (formerly Entrezgene);Acc:100174965]
chr6	32493517	32495415	1899	*	chr6	32493517	32495415	in_vitro	in_vivo	12	10	1.83333333	1.482	0.35133333	c(t = 0.21392	0.833	0.96540567	Distal Intergenic	ENSSSCG000	AKTIP	AKT interacting protein [Source:VGNC Symbol;Acc:VGNC:85233]
chr12	24895897	24896422	526	*	chr12	24895897	24896422	in_vitro	in_vivo	9	10	1.66666667	1.284	0.38266667	c(t = 0.21632	0.833	0.96540567	Distal Intergenic	NA	NA	NA
chr11	5623444	5624326	883	*	chr11	5623444	5624326	in_vitro	in_vivo	10	12	18.037	21.0925	-3.0555	c(t = -0.2124	0.834	0.96554534	Distal Intergenic	ENSSSCG000	CDX2	caudal type homeobox 2 [Source:NCBI gene (formerly Entrezgene);Acc:100127132]
chr14	139218810	139219652	843	*	chr14	139218810	139219652	in_vitro	in_vivo	9	9	17.4944444	20.3044444	-2.81	c(t = -0.2130	0.834	0.96554534	Distal Intergenic	ENSSSCG000	GLRX3	glutaredoxin 3 [Source:VGNC Symbol;Acc:VGNC:88495]
chr2	137200153	137201951	1799	*	chr2	137200153	137201951	in_vitro	in_vivo	10	12	4.577	4.22	0.357	c(t = 0.21352	0.834	0.96554534	Exon (NM_001244370/100689266, exon 3 of 3)	ENSSSCG000	PITX1	paired like homeodomain 1 [Source:NCBI gene (formerly Entrezgene);Acc:100689266]
chr13	128619162	128619908	747	*	chr13	128619162	128619908	in_vitro	in_vivo	9	12	2.52444444	2.0725	0.45194444	c(t = 0.21261	0.834	0.96554534	Distal Intergenic	ENSSSCG000	OSTN	osteocrin [Source:NCBI gene (formerly Entrezgene);Acc:100049691]
chr7	4862363	4864192	1830	*	chr7	4862363	4864192	in_vitro	in_vivo	12	15	1.69416667	1.504	0.19016667	c(t = 0.21195	0.834	0.96554534	Distal Intergenic	ENSSSCG000	RIOK1	RIO kinase 1 [Source:VGNC Symbol;Acc:VGNC:92315]
chr2	84996007	84998365	2359	*	chr2	84996007	84998365	in_vitro	in_vivo	10	15	1.209	1.43533333	-0.2263333	c(t = -0.2129	0.834	0.96554534	Distal Intergenic	ENSSSCG000	HMGCR	3-hydroxy-3-methylglutaryl-CoA reductase [Source:NCBI gene (formerly Entrezgene);Acc:100144446]
chr13	175349565	175349971	407	*	chr13	175349565	175349971	in_vitro	in_vivo	9	10	0.65111111	0.857	-0.2058889	c(t = -0.2143	0.834	0.96554534	Distal Intergenic	NA	NA	NA
chr6	45360401	45361493	1093	*	chr6	45360401	45361493	in_vitro	in_vivo	13	12	6.55923077	5.68416667	0.8750641	c(t = 0.21219	0.835	0.96626638	Distal Intergenic	ENSSSCG000	TYROBP	transmembrane immune signaling adaptor TYROBP [Source:VGNC Symbol;Acc:VGNC:94619]
chr2	69716999	69717938	940	*	chr2	69716999	69717938	in_vitro	in_vivo	10	10	0.947	1.25	-0.303	c(t = -0.2122	0.835	0.96626638	Distal Intergenic	ENSSSCG000	LDLR	low density lipoprotein receptor [Source:VGNC Symbol;Acc:VGNC:89671]
chr2	48666252	48667499	1248	*	chr2	48666252	48667499	in_vitro	in_vivo	11	13	0.85272727	0.98384615	-0.1311189	c(t = -0.2112	0.835	0.96626638	Distal Intergenic	ENSSSCG000	RNF141	ring finger protein 141 [Source:VGNC Symbol;Acc:VGNC:98292]
chr12	539658	549252	9595	*	chr12	539658	549252	in_vitro	in_vivo	12	13	80.72	78.5823077	2.13769231	c(t = 0.20994	0.836	0.96669576	3' UTR	ENSSSCG000	WDR45B	WD repeat domain 45B [Source:NCBI gene (formerly Entrezgene);Acc:100462760]
chr10	64801362	64803940	2579	*	chr10	64801362	64803940	in_vitro	in_vivo	9	11	2.53666667	2.20454545	0.33212121	c(t = 0.21088	0.836	0.96669576	Distal Intergenic	ENSSSCG000	IL2RA	interleukin 2 receptor subunit alpha [Source:VGNC Symbol;Acc:VGNC:108275]
chr14	105829724	105830991	1268	*	chr14	105829724	105830991	in_vitro	in_vivo	12	13	2.39166667	2.18307692	0.20858974	c(t = 0.20955	0.836	0.96669576	Distal Intergenic	ENSSSCG000	NOC3L	NOC3 like DNA replication regulator [Source:VGNC Symbol;Acc:VGNC:90808]
chr8	12967909	12969766	1858	*	chr8	12967909	12969766	in_vitro	in_vivo	14	14	1.27357143	1.43785714	-0.1642857	c(t = -0.2092	0.836	0.96669576	Promoter (<=1kb)	ENSSSCG000	LCORL	ligand dependent nuclear receptor corepressor like [Source:VGNC Symbol;Acc:VGNC:98937]
chr7	89395609	89397302	1694	*	chr7	89395609	89397302	in_vitro	in_vivo	12	13	1.23666667	1.08923077	0.1474359	c(t = 0.20926	0.836	0.96669576	Distal Intergenic	ENSSSCG000	FUT8	fucosyltransferase 8 [Source:VGNC Symbol;Acc:VGNC:109455]
chr10	68547517	68548822	1306	*	chr10	68547517	68548822	in_vitro	in_vivo	9	9	96.4411111	96.9666667	-0.5255556	c(t = -0.2097	0.837	0.96697147	Distal Intergenic	ENSSSCG000	WDR37	WD repeat domain 37 [Source:VGNC Symbol;Acc:VGNC:96258]
chr3	132417965	132420446	2482	*	chr3	132417965	132420446	in_vitro	in_vivo	10	13	20.504	23.7976923	-3.2936923	c(t = -0.2088	0.837	0.96697147	Distal Intergenic	ENSSSCG000	TPO	thyroid peroxidase [Source:VGNC Symbol;Acc:VGNC:94341]
chr14	37597685	37600021	2337	*	chr14	37597685	37600021	in_vitro	in_vivo	11	12	4.12545455	3.55083333	0.57462121	c(t = 0.20825	0.837	0.96697147	Distal Intergenic	ENSSSCG000	OAS2	2'-5'-oligoadenylate synthetase 2 [Source:VGNC Symbol;Acc:VGNC:91007]
chr1	106743459	106745118	1660	*	chr1	106743459	106745118	in_vitro	in_vivo	14	14	1.665	1.49642857	0.16857143	c(t = 0.20860	0.837	0.96697147	Distal Intergenic	ENSSSCG000	FECH	ferrochelatase [Source:VGNC Symbol;Acc:VGNC:88081]
chr7	98575075	98576108	1034	*	chr7	98575075	98576108	in_vitro	in_vivo	10	10	1.036	1.178	-0.142	c(t = -0.2091	0.837	0.96697147	Distal Intergenic	ENSSSCG000	BATF	basic leucine zipper ATF-like transcription factor [Source:NCBI gene (formerly Entrezgene);Acc:100624723]
chr7	9742228	9743367	1140	*	chr7	9742228	9743367	in_vitro	in_vivo	10	13	87.611	88.6438462	-1.0328462	c(t = -0.2072	0.838	0.96697147	Distal Intergenic	ENSSSCG000	SIRT5	sirtuin 5 [Source:NCBI gene (formerly Entrezgene);Acc:100125966]
chr15	76900712	76904429	3718	*	chr15	76900712	76904429	in_vitro	in_vivo	11	14	5.53454545	6.26714286	-0.7325974	c(t = -0.2080	0.838	0.96697147	Distal Intergenic	ENSSSCG000	GAD1	glutamate decarboxylase 1 [Source:VGNC Symbol;Acc:VGNC:96312]
chr4	94673131	94675547	2417	*	chr4	94673131	94675547	in_vitro	in_vivo	10	12	6.089	5.43166667	0.65733333	c(t = 0.20776	0.838	0.96697147	Promoter (<=1kb)	ENSSSCG000	EFNA1	ephrin A1 [Source:VGNC Symbol;Acc:VGNC:98789]
chrX	35865025	35872125	7101	*	chrX	35865025	35872125	in_vitro	in_vivo	13	13	2.64692308	2.89076923	-0.2438462	c(t = -0.2062	0.838	0.96697147	Distal Intergenic	ENSSSCG000	MID1IP1	MID1 interacting protein 1 [Source:VGNC Symbol;Acc:VGNC:90214]
chr13	205151961	205153154	1194	*	chr13	205151961	205153154	in_vitro	in_vivo	9	12	3.14222222	2.71916667	0.42305556	c(t = 0.20781	0.838	0.96697147	Distal Intergenic	ENSSSCG000	MX1	MX dynamin like GTPase 1 [Source:NCBI gene (formerly Entrezgene);Acc:397128]
chr7	78029035	78029667	633	*	chr7	78029035	78029667	in_vitro	in_vivo	11	9	1.91909091	2.29555556	-0.3764646	c(t = -0.2076	0.838	0.96697147	Distal Intergenic	ENSSSCG000	NDRG2	NDRG family member 2 [Source:VGNC Symbol;Acc:VGNC:90632]
chr9	131767722	131768304	583	*	chr9	131767722	131768304	in_vitro	in_vivo	11	10	1.79454545	2.238	-0.4434545	c(t = -0.2074	0.838	0.96697147	Distal Intergenic	ENSSSCG000	SLC30A1	solute carrier family 30 member 1 [Source:NCBI gene (formerly Entrezgene);Acc:503660]
chr6	18901027	18901862	836	*	chr6	18901027	18901862	in_vitro	in_vivo	9	10	1.27222222	1.071	0.20122222	c(t = 0.20756	0.838	0.96697147	Distal Intergenic	ENSSSCG000	NLRC5	NLR family CARD domain containing 5 [Source:VGNC Symbol;Acc:VGNC:90782]
chr15	32064289	32065560	1272	*	chr15	32064289	32065560	in_vitro	in_vivo	12	13	1.03833333	0.87230769	0.16602564	c(t = 0.20664	0.838	0.96697147	Distal Intergenic	ENSSSCG000	SCTR	secretin receptor [Source:VGNC Symbol;Acc:VGNC:95482]
chrX	124674202	124694808	20607	*	chrX	124674202	124694808	in_vitro	in_vivo	13	16	70	68.059375	1.940625	c(t = 0.20581	0.839	0.9673991	Distal Intergenic	ENSSSCG000	RENBP	renin binding protein [Source:NCBI gene (formerly Entrezgene);Acc:396934]
chrX	111903498	111904810	1313	*	chrX	111903498	111904810	in_vitro	in_vivo	10	13	9.78	8.17307692	1.60692308	c(t = 0.20634	0.839	0.9673991	Distal Intergenic	ENSSSCG00000012703		RNA binding motif protein X-linked [Source:NCBI gene (formerly Entrezgene);Acc:100158042]
chr2	47385475	47386601	1127	*	chr2	47385475	47386601	in_vitro	in_vivo	13	12	2.50461538	2.16583333	0.33878205	c(t = 0.20506	0.839	0.9673991	Promoter (<=1kb)	ENSSSCG000	DKK3	dickkopf WNT signaling pathway inhibitor 3 [Source:VGNC Symbol;Acc:VGNC:87323]
chr8	56230501	56233159	2659	*	chr8	56230501	56233159	in_vitro	in_vivo	13	14	1.46	1.67428571	-0.2142857	c(t = -0.2054	0.839	0.9673991	Distal Intergenic	ENSSSCG000	IGFBP7	insulin like growth factor binding protein 7 [Source:NCBI gene (formerly Entrezgene);Acc:100302573]
chr10	8168922	8170178	1257	*	chr10	8168922	8170178	in_vitro	in_vivo	11	10	1.29272727	1.119	0.17372727	c(t = 0.20668	0.839	0.9673991	Distal Intergenic	ENSSSCG000	SPATA17	spermatosis associated 17 [Source:VGNC Symbol;Acc:VGNC:96065]
chr9	950448	951554	1107	*	chr9	950448	951554	in_vitro	in_vivo	9	10	57.7988889	55.079	2.71988889	c(t = 0.20481	0.84	0.96764732	Distal Intergenic	NA	NA	NA
chr9	25462529	25463237	709	*	chr9	25462529	25463237	in_vitro	in_vivo	10	11	26.14	22.9381818	3.20181818	c(t = 0.20508	0.84	0.96764732	Distal Intergenic	ENSSSCG000	C11orf54	chromosome 9 C11orf54 homolog [Source:VGNC Symbol;Acc:VGNC:86087]
chr17	61205975	61207695	1721	*	chr17	61205975	61207695	in_vitro	in_vivo	9	9	12.8388889	15.5933333	-2.7544444	c(t = -0.2051	0.84	0.96764732	Distal Intergenic	ENSSSCG000	PSMA7	proteasome 20S subunit alpha 7 [Source:NCBI gene (formerly Entrezgene);Acc:100626738]
chr15	24675994	24677600	1607	*	chr15	24675994	24677600	in_vitro	in_vivo	15	16	2.556	2.315625	0.240375	c(t = 0.20391	0.84	0.96764732	Distal Intergenic	ENSSSCG000	MARCO	macrophage receptor with collagenous structure [Source:NCBI gene (formerly Entrezgene);Acc:100516298]
chr4	110047090	110049989	2900	*	chr4	110047090	110049989	in_vitro	in_vivo	12	14	1.4625	1.29714286	0.16535714	c(t = 0.20548	0.84	0.96764732	Distal Intergenic	ENSSSCG000	AHCYL1	adenosylhomocysteinase like 1 [Source:VGNC Symbol;Acc:VGNC:97873]
chr8	1166394	1166779	386	*	chr8	1166394	1166779	in_vitro	in_vivo	9	10	35.5555556	32.627	2.92855556	c(t = 0.20389	0.841	0.96764732	Distal Intergenic	ENSSSCG000	RNF4	ring finger protein 4 [Source:VGNC Symbol;Acc:VGNC:92392]
chrX	124832646	124833424	779	*	chrX	124832646	124833424	in_vitro	in_vivo	9	11	19.37	16.4827273	2.88727273	c(t = 0.20434	0.841	0.96764732	Distal Intergenic	ENSSSCG00000029523		
chr6	51507631	51508619	989	*	chr6	51507631	51508619	in_vitro	in_vivo	10	9	9.635	7.69111111	1.94388889	c(t = 0.20353	0.841	0.96764732	Distal Intergenic	ENSSSCG000	BLOC1S3	biogenesis of lysosomal organelles complex 1 subunit 3 [Source:NCBI gene (formerly Entrezgene);Acc:100049671]
chr9	132747107	132748683	1577	*	chr9	132747107	132748683	in_vitro	in_vivo	11	12	2.22545455	2.47083333	-0.2453788	c(t = -0.2030	0.841	0.96764732	Distal Intergenic	ENSSSCG000	IRF6	interferon regulatory factor 6 [Source:VGNC Symbol;Acc:VGNC:89209]
chr14	14380495	14381038	544	*	chr14	14380495	14381038	in_vitro	in_vivo	10	11	1.765	1.5	0.265	c(t = 0.20460	0.841	0.96764732	Distal Intergenic	ENSSSCG00000021767		L-threonine dehydrogenase [Source:VGNC Symbol;Acc:VGNC:109162]
chr13	89221068	89221965	898	*	chr13	89221068	89221965	in_vitro	in_vivo	11	12	1.77636364	1.44416667	0.33219697	c(t = 0.20415	0.841	0.96764732	Distal Intergenic	ENSSSCG000	HPS3	HPS3 biosis of lysosomal organelles complex 2 subunit 1 [Source:VGNC Symbol;Acc:VGNC:88965]
chr6	35515022	35516574	1553	*	chr6	35515022	35516574	in_vitro	in_vivo	10	13	1.001	1.17384615	-0.1728462	c(t = -0.2040	0.841	0.96764732	Promoter (<=1kb)	ENSSSCG000	CBLN1	cerebellin 1 precursor [Source:NCBI gene (formerly Entrezgene);Acc:100127148]
chr4	115311178	115311798	621	*	chr4	115311178	115311798	in_vitro	in_vivo	9	9	3.92	4.55777778	-0.6377778	c(t = -0.2027	0.842	0.96764732	Distal Intergenic	ENSSSCG00000036331		amylase, alpha 2B (pancreatic) [Source:NCBI gene (formerly Entrezgene);Acc:397397]
chr6	51798432	51799074	643	*	chr6	51798432	51799074	in_vitro	in_vivo	9	9	3.83888889	3.29222222	0.54666667	c(t = 0.20288	0.842	0.96764732	Promoter (<=1kb)	ENSSSCG000	ERCC1	ERCC excision repair 1, endonuclease non-catalytic subunit [Source:NCBI gene (formerly Entrezgene);Acc:100286808]
chr5	8985749	8988292	2544	*	chr5	8985749	8988292	in_vitro	in_vivo	9	11	2.04444444	2.39636364	-0.3519192	c(t = -0.2039	0.842	0.96764732	Distal Intergenic	ENSSSCG000	RPL3	ribosomal protein L3 [Source:NCBI gene (formerly Entrezgene);Acc:396768]
chr1	220833585	220834718	1134	*	chr1	220833585	220834718	in_vitro	in_vivo	13	13	2.14846154	1.88615385	0.26230769	c(t = 0.20204	0.842	0.96764732	Distal Intergenic	ENSSSCG000	DMRT1	doublesex and mab-3 related transcription factor 1 [Source:NCBI gene (formerly Entrezgene);Acc:397199]
chr13	45355619	45356865	1247	*	chr13	45355619	45356865	in_vitro	in_vivo	11	13	1.68545455	1.48461538	0.20083916	c(t = 0.20232	0.842	0.96764732	Distal Intergenic	ENSSSCG00000033189		family with sequence similarity 107 member A [Source:NCBI gene (formerly Entrezgene);Acc:100516512]
chr3	38753131	38753975	845	*	chr3	38753131	38753975	in_vitro	in_vivo	11	11	0.61454545	0.48545455	0.12909091	c(t = 0.20187	0.842	0.96764732	Promoter (<=1kb)	ENSSSCG000	CLUAP1	clusterin associated protein 1 [Source:VGNC Symbol;Acc:VGNC:86791]
chr17	8448663	8449435	773	*	chr17	8448663	8449435	in_vitro	in_vivo	11	11	0.37909091	0.28363636	0.09545455	c(t = 0.20161	0.842	0.96764732	Distal Intergenic	ENSSSCG000	FAT1	FAT atypical cadherin 1 [Source:NCBI gene (formerly Entrezgene);Acc:100154043]
chr13	206732916	206736160	3245	*	chr13	206732916	206736160	in_vitro	in_vivo	9	13	96.2444444	95.6807692	0.56367521	c(t = 0.20197	0.843	0.96764732	Distal Intergenic	ENSSSCG000	CSTB	cystatin B [Source:NCBI gene (formerly Entrezgene);Acc:100038020]
chr12	5348536	5349556	1021	*	chr12	5348536	5349556	in_vitro	in_vivo	10	10	73.445	76.111	-2.666	c(t = -0.2007	0.843	0.96764732	Distal Intergenic	ENSSSCG000	ACOX1	acyl-CoA oxidase 1 [Source:VGNC Symbol;Acc:VGNC:85020]
chr3	126364771	126365657	887	*	chr3	126364771	126365657	in_vitro	in_vivo	10	11	2.844	2.47363636	0.37036364	c(t = 0.20074	0.843	0.96764732	Distal Intergenic	ENSSSCG000	KLF11	KLF transcription factor 11 [Source:NCBI gene (formerly Entrezgene);Acc:100174954]
chr18	51801914	51802967	1054	*	chr18	51801914	51802967	in_vitro	in_vivo	10	12	2.191	1.9175	0.2735	c(t = 0.20128	0.843	0.96764732	Distal Intergenic	ENSSSCG000	POLD2	DNA polymerase delta 2, accessory subunit [Source:VGNC Symbol;Acc:VGNC:91628]
chr3	1938771	1939733	963	*	chr3	1938771	1939733	in_vitro	in_vivo	9	11	2.06666667	1.77909091	0.28757576	c(t = 0.20077	0.843	0.96764732	Distal Intergenic	ENSSSCG000	AMZ1	archaelysin family metallopeptidase 1 [Source:NCBI gene (formerly Entrezgene);Acc:100337670]
chr1	29936361	29936806	446	*	chr1	29936361	29936806	in_vitro	in_vivo	10	9	0.213	0.28444444	-0.0714444	c(t = -0.2010	0.843	0.96764732	Distal Intergenic	ENSSSCG000	SGK1	serum/glucocorticoid regulated kinase 1 [Source:VGNC Symbol;Acc:VGNC:92793]
chr5	7122012	7123142	1131	*	chr5	7122012	7123142	in_vitro	in_vivo	9	9	92.1311111	92.77	-0.6388889	c(t = -0.1997	0.844	0.96764732	Distal Intergenic	ENSSSCG000	ACO2	aconitase 2 [Source:NCBI gene (formerly Entrezgene);Acc:396999]
chr15	121253797	121254795	999	*	chr15	121253797	121254795	in_vitro	in_vivo	9	12	17.18	15.3483333	1.83166667	c(t = 0.19940	0.844	0.96764732	Exon (NM_001190275/100155182, exon 14 of 14)	ENSSSCG000	ATG9A	autophagy related 9A [Source:VGNC Symbol;Acc:VGNC:99703]
chr8	72586768	72589318	2551	*	chr8	72586768	72589318	in_vitro	in_vivo	12	14	3.81416667	4.22642857	-0.4122619	c(t = -0.2001	0.844	0.96764732	Distal Intergenic	ENSSSCG000	SCARB2	scavenger receptor class B member 2 [Source:VGNC Symbol;Acc:VGNC:92614]
chr1	220755398	220756149	752	*	chr1	220755398	220756149	in_vitro	in_vivo	9	13	3.39777778	3.91153846	-0.5137607	c(t = -0.1997	0.844	0.96764732	Distal Intergenic	ENSSSCG000	DMRT1	doublesex and mab-3 related transcription factor 1 [Source:NCBI gene (formerly Entrezgene);Acc:397199]
chr14	14888158	14889983	1826	*	chr14	14888158	14889983	in_vitro	in_vivo	12	12	4.0775	3.6475	0.43	c(t = 0.19998	0.844	0.96764732	Promoter (<=1kb)	ENSSSCG000	GATA4	GATA binding protein 4 [Source:VGNC Symbol;Acc:VGNC:88365]
chr4	88972183	88973295	1113	*	chr4	88972183	88973295	in_vitro	in_vivo	9	12	2.62666667	3.11083333	-0.4841667	c(t = -0.2004	0.844	0.96764732	Promoter (<=1kb)	ENSSSCG000	HSPA6	heat shock protein family A (Hsp70) member 6 [Source:NCBI gene (formerly Entrezgene);Acc:396906]
chr8	72790378	72791560	1183	*	chr8	72790378	72791560	in_vitro	in_vivo	10	11	2.497	2.93272727	-0.4357273	c(t = -0.2001	0.844	0.96764732	Distal Intergenic	ENSSSCG000	SCARB2	scavenger receptor class B member 2 [Source:VGNC Symbol;Acc:VGNC:92614]
chr8	111707791	111708951	1161	*	chr8	111707791	111708951	in_vitro	in_vivo	10	13	2.593	2.32153846	0.27146154	c(t = 0.20062	0.844	0.96764732	Distal Intergenic	ENSSSCG000	PITX2	paired like homeodomain 2 [Source:VGNC Symbol;Acc:VGNC:91467]
chr14	115586951	115588442	1492	*	chr14	115586951	115588442	in_vitro	in_vivo	12	12	1.91416667	2.19333333	-0.2791667	c(t = -0.2001	0.844	0.96764732	Distal Intergenic	ENSSSCG000	GSTO1	glutathione S-transferase omega 1 [Source:NCBI gene (formerly Entrezgene);Acc:397117]
chr13	33893073	33893917	845	*	chr13	33893073	33893917	in_vitro	in_vivo	12	9	1.38916667	1.85222222	-0.4630556	c(t = -0.2)	0.844	0.96764732	Distal Intergenic	ENSSSCG000	ABHD14A	abhydrolase domain containing 14A [Source:VGNC Symbol;Acc:VGNC:84973]
chr2	119340294	119342176	1883	*	chr2	119340294	119342176	in_vitro	in_vivo	13	14	1.04692308	0.92357143	0.12335165	c(t = 0.19855	0.844	0.96764732	Distal Intergenic	ENSSSCG00000038141		toll like receptor adaptor molecule 2 [Source:NCBI gene (formerly Entrezgene);Acc:100520421]
chr9	72503079	72503885	807	*	chr9	72503079	72503885	in_vitro	in_vivo	9	13	0.75777778	0.89384615	-0.1360684	c(t = -0.2007	0.844	0.96764732	Distal Intergenic	ENSSSCG000	CYP51A1	cytochrome P450 family 51 subfamily A member 1 [Source:VGNC Symbol;Acc:VGNC:103377]
chr2	64718792	64719237	446	*	chr2	64718792	64719237	in_vitro	in_vivo	12	9	0.64083333	0.85444444	-0.2136111	c(t = -0.2)	0.844	0.96764732	Distal Intergenic	ENSSSCG000	DNAJB1	DnaJ heat shock protein family (Hsp40) member B1 [Source:NCBI gene (formerly Entrezgene);Acc:396741]
chr14	140474613	140476912	2300	*	chr14	140474613	140476912	in_vitro	in_vivo	9	13	49.2811111	46.6776923	2.6034188	c(t = 0.19762	0.845	0.96792741	Distal Intergenic	ENSSSCG000	ssc-mir-202	ssc-mir-202 [Source:miRBase;Acc:MI0013157]
chr7	1442670	1444133	1464	*	chr7	1442670	1444133	in_vitro	in_vivo	9	14	15.1988889	18.0242857	-2.8253968	c(t = -0.1975	0.845	0.96792741	Distal Intergenic	ENSSSCG000	SERPINB9	serpin peptidase inhibitor, clade B (ovalbumin), member 9 [Source:NCBI gene (formerly Entrezgene);Acc:100126851]
chr14	59515615	59517402	1788	*	chr14	59515615	59517402	in_vitro	in_vivo	12	13	2.6925	3.14	-0.4475	c(t = -0.1983	0.845	0.96792741	Distal Intergenic	ENSSSCG000	ARV1	ARV1 homolog, fatty acid homeostasis modulator [Source:VGNC Symbol;Acc:VGNC:85554]
chr14	98105400	98106098	699	*	chr14	98105400	98106098	in_vitro	in_vivo	9	11	1.82777778	2.15363636	-0.3258586	c(t = -0.1987	0.845	0.96792741	Intron (NM_001044574/733640, intron 3 of 17)	ENSSSCG000	DKK1	dickkopf WNT signaling pathway inhibitor 1 [Source:NCBI gene (formerly Entrezgene);Acc:100157640]
chr18	23019343	23021410	2068	*	chr18	23019343	23021410	in_vitro	in_vivo	15	14	1.9	1.75214286	0.14785714	c(t = 0.19758	0.845	0.96792741	Distal Intergenic	ENSSSCG000	POT1	protection of telomeres 1 [Source:VGNC Symbol;Acc:VGNC:104032]
chr2	70895286	70896292	1007	*	chr2	70895286	70896292	in_vitro	in_vivo	9	11	1.11111111	1.38	-0.2688889	c(t = -0.1985	0.845	0.96792741	Promoter (<=1kb)	ENSSSCG000	RAB11B	RAB11B, member RAS oncogene family [Source:NCBI gene (formerly Entrezgene);Acc:100513782]
chr4	93644735	93645818	1084	*	chr4	93644735	93645818	in_vitro	in_vivo	9	12	5.59555556	4.93416667	0.66138889	c(t = 0.19710	0.846	0.968207	Distal Intergenic	NA	NA	NA
chr9	39071991	39072897	907	*	chr9	39071991	39072897	in_vitro	in_vivo	11	15	4.92818182	4.434	0.49418182	c(t = 0.19667	0.846	0.968207	Distal Intergenic	ENSSSCG000	POU2AF1	POU class 2 homeobox associating factor 1 [Source:NCBI gene (formerly Entrezgene);Acc:100512063]
chr7	52957673	52958196	524	*	chr7	52957673	52958196	in_vitro	in_vivo	11	11	1.19909091	1.51545455	-0.3163636	c(t = -0.1977	0.846	0.968207	Distal Intergenic	ENSSSCG000	NMB	neuromedin B [Source:VGNC Symbol;Acc:VGNC:109471]
chr7	7250484	7251598	1115	*	chr7	7250484	7251598	in_vitro	in_vivo	10	11	1.523	1.33636364	0.18663636	c(t = 0.19653	0.846	0.968207	Distal Intergenic	ENSSSCG000	C6orf52	chromosome 7 C6orf52 homolog [Source:VGNC Symbol;Acc:VGNC:86073]
chr6	92081172	92082180	1009	*	chr6	92081172	92082180	in_vitro	in_vivo	10	11	0.841	0.98454545	-0.1435455	c(t = -0.1980	0.846	0.968207	Promoter (<=1kb)	NA	NA	NA
chr2	144301001	144302311	1311	*	chr2	144301001	144302311	in_vitro	in_vivo	9	9	0.40777778	0.33222222	0.07555556	c(t = 0.19770	0.846	0.968207	Distal Intergenic	ENSSSCG000	NDFIP1	Nedd4 family interacting protein 1 [Source:NCBI gene (formerly Entrezgene);Acc:100628119]
chrX	8873567	8874982	1416	*	chrX	8873567	8874982	in_vitro	in_vivo	11	12	8.11363636	9.27583333	-1.162197	c(t = -0.1958	0.847	0.96890871	Distal Intergenic	ENSSSCG000	MSL3	MSL complex subunit 3 [Source:NCBI gene (formerly Entrezgene);Acc:100526025]
chr1	159020692	159023249	2558	*	chr1	159020692	159023249	in_vitro	in_vivo	13	14	1.94	1.69357143	0.24642857	c(t = 0.19560	0.847	0.96890871	Distal Intergenic	ENSSSCG000	RNF152	ring finger protein 152 [Source:VGNC Symbol;Acc:VGNC:92364]
chr6	101210679	101212112	1434	*	chr6	101210679	101212112	in_vitro	in_vivo	9	13	1.13777778	1.00307692	0.13470085	c(t = 0.19492	0.847	0.96890871	Distal Intergenic	ENSSSCG000	TGIF1	TGFB induced factor homeobox 1 [Source:VGNC Symbol;Acc:VGNC:93935]
chrX	124565641	124568413	2773	*	chrX	124565641	124568413	in_vitro	in_vivo	12	13	8.25166667	9.21923077	-0.9675641	c(t = -0.1937	0.848	0.96890871	Distal Intergenic	ENSSSCG00000024676		SRSF protein kinase 3 [Source:VGNC Symbol;Acc:VGNC:93464]
chr6	67433294	67435163	1870	*	chr6	67433294	67435163	in_vitro	in_vivo	13	12	2.25615385	2.47583333	-0.2196795	c(t = -0.1946	0.848	0.96890871	Distal Intergenic	ENSSSCG000	ICMT	isoprenylcysteine carboxyl methyltransferase [Source:NCBI gene (formerly Entrezgene);Acc:100514410]
chr15	103157324	103160319	2996	*	chr15	103157324	103160319	in_vitro	in_vivo	13	12	2.51307692	2.2775	0.23557692	c(t = 0.19337	0.848	0.96890871	Distal Intergenic	ENSSSCG000	SPATS2L	spermatosis associated serine rich 2 like [Source:VGNC Symbol;Acc:VGNC:96047]
chr3	19368161	19368900	740	*	chr3	19368161	19368900	in_vitro	in_vivo	10	10	2	1.556	0.444	c(t = 0.19497	0.848	0.96890871	Distal Intergenic	ENSSSCG000	IL4R	interleukin 4 receptor [Source:VGNC Symbol;Acc:VGNC:89110]
chr1	145058991	145060013	1023	*	chr1	145058991	145060013	in_vitro	in_vivo	13	10	1.17384615	1.311	-0.1371538	c(t = -0.1942	0.848	0.96890871	Distal Intergenic	ENSSSCG000	NFATC1	nuclear factor of activated T cells 1 [Source:NCBI gene (formerly Entrezgene);Acc:397318]
chr13	129626233	129626907	675	*	chr13	129626233	129626907	in_vitro	in_vivo	11	14	1.01181818	0.88	0.13181818	c(t = 0.19367	0.848	0.96890871	Distal Intergenic	ENSSSCG000	OSTN	osteocrin [Source:NCBI gene (formerly Entrezgene);Acc:100049691]
chr3	7590897	7591405	509	*	chr3	7590897	7591405	in_vitro	in_vivo	10	9	0.323	0.42777778	-0.1047778	c(t = -0.1954	0.848	0.96890871	Distal Intergenic	ENSSSCG000	NTAN1	N-terminal asparagine amidase [Source:NCBI gene (formerly Entrezgene);Acc:397107]
chr11	18461026	18461665	640	*	chr11	18461026	18461665	in_vitro	in_vivo	9	9	0.35888889	0.27111111	0.08777778	c(t = 0.19515	0.848	0.96890871	Distal Intergenic	ENSSSCG000	PHF11	PHD finger protein 11 [Source:VGNC Symbol;Acc:VGNC:91377]
chrX	90419291	90420441	1151	*	chrX	90419291	90420441	in_vitro	in_vivo	11	12	11.5763636	13.06	-1.4836364	c(t = -0.1936	0.849	0.96918672	Distal Intergenic	NA	NA	NA
chrX	21068360	21069742	1383	*	chrX	21068360	21069742	in_vitro	in_vivo	15	10	9.67	8.628	1.042	c(t = 0.19310	0.849	0.96918672	Distal Intergenic	ENSSSCG000	SAT1	spermidine/spermine N1-acetyltransferase 1 [Source:NCBI gene (formerly Entrezgene);Acc:397645]
chr7	54906320	54906934	615	*	chr7	54906320	54906934	in_vitro	in_vivo	10	9	2.356	1.97555556	0.38044444	c(t = 0.19378	0.849	0.96918672	Distal Intergenic	ENSSSCG000	ssc-mir-9-3	ssc-mir-9-3 [Source:miRBase;Acc:MI0022123]
chr1	71157820	71159121	1302	*	chr1	71157820	71159121	in_vitro	in_vivo	12	9	1.81583333	1.54666667	0.26916667	c(t = 0.19267	0.849	0.96918672	Distal Intergenic	ENSSSCG000	LIN28B	lin-28 homolog B [Source:VGNC Symbol;Acc:VGNC:89729]
chr2	62184508	62185060	553	*	chr2	62184508	62185060	in_vitro	in_vivo	9	11	0.79333333	1.00181818	-0.2084848	c(t = -0.1929	0.849	0.96918672	Distal Intergenic	ENSSSCG000	PGLYRP2	peptidoglycan recognition protein 2 [Source:NCBI gene (formerly Entrezgene);Acc:396557]
chr1	74180994	74181488	495	*	chr1	74180994	74181488	in_vitro	in_vivo	10	11	0.676	0.58363636	0.09236364	c(t = 0.19259	0.849	0.96918672	Distal Intergenic	ENSSSCG000	FOXO3	forkhead box O3 [Source:VGNC Symbol;Acc:VGNC:99715]
chr4	130701345	130703206	1862	*	chr4	130701345	130703206	in_vitro	in_vivo	12	13	2.28166667	2.55230769	-0.270641	c(t = -0.1914	0.85	0.96959748	Distal Intergenic	ENSSSCG000	BCL10	BCL10 immune signaling adaptor [Source:NCBI gene (formerly Entrezgene);Acc:100125346]
chr13	61438682	61439620	939	*	chr13	61438682	61439620	in_vitro	in_vivo	11	11	1.62909091	1.87909091	-0.25	c(t = -0.1916	0.85	0.96959748	Distal Intergenic	ENSSSCG000	BHLHE40	basic helix-loop-helix family member e40 [Source:VGNC Symbol;Acc:VGNC:85814]
chr7	56083681	56084941	1261	*	chr7	56083681	56084941	in_vitro	in_vivo	17	12	0.99058824	1.135	-0.1444118	c(t = -0.1921	0.85	0.96959748	Distal Intergenic	ENSSSCG000	IDH2	isocitrate dehydrogenase (NADP(+)) 2 [Source:NCBI gene (formerly Entrezgene);Acc:397603]
chr9	46335813	46336618	806	*	chr9	46335813	46336618	in_vitro	in_vivo	11	12	0.89909091	0.74666667	0.15242424	c(t = 0.19195	0.85	0.96959748	Distal Intergenic	ENSSSCG000	HMBS	hydroxymethylbilane synthase [Source:VGNC Symbol;Acc:VGNC:88899]
chr1	98133052	98135881	2830	*	chr1	98133052	98135881	in_vitro	in_vivo	10	12	6.583	7.33	-0.747	c(t = -0.1901	0.851	0.96959748	Distal Intergenic	ENSSSCG000	SMAD7	SMAD family member 7 [Source:VGNC Symbol;Acc:VGNC:93221]
chr9	114262673	114263476	804	*	chr9	114262673	114263476	in_vitro	in_vivo	10	14	4.198	3.67928571	0.51871429	c(t = 0.19066	0.851	0.96959748	Distal Intergenic	ENSSSCG000	METTL13	methyltransferase 13, eEF1A lysine and N-terminal methyltransferase [Source:NCBI gene (formerly Entrezgene);Acc:100512915]
chr5	63992430	63993354	925	*	chr5	63992430	63993354	in_vitro	in_vivo	9	9	1.50666667	1.82888889	-0.3222222	c(t = -0.1908	0.851	0.96959748	Distal Intergenic	ENSSSCG000	LAG3	lymphocyte activating 3 [Source:NCBI gene (formerly Entrezgene);Acc:100125962]
chr4	6920350	6921454	1105	*	chr4	6920350	6921454	in_vitro	in_vivo	11	11	1.93909091	1.70181818	0.23727273	c(t = 0.19015	0.851	0.96959748	Distal Intergenic	ENSSSCG000	ssc-mir-30d	ssc-mir-30d [Source:miRBase;Acc:MI0013091]
chr15	106776444	106777193	750	*	chr15	106776444	106777193	in_vitro	in_vivo	13	9	1.44153846	1.66111111	-0.2195726	c(t = -0.1899	0.851	0.96959748	Distal Intergenic	ENSSSCG000	CYP20A1	cytochrome P450 family 20 subfamily A member 1 [Source:NCBI gene (formerly Entrezgene);Acc:100124408]
chr7	32544002	32544521	520	*	chr7	32544002	32544521	in_vitro	in_vivo	9	9	1.37777778	1.64	-0.2622222	c(t = -0.1920	0.851	0.96959748	Distal Intergenic	ENSSSCG000	PI16	peptidase inhibitor 16 [Source:NCBI gene (formerly Entrezgene);Acc:100155010]
chr18	42924955	42926405	1451	*	chr18	42924955	42926405	in_vitro	in_vivo	10	14	1.979	1.625	0.354	c(t = 0.19149	0.851	0.96959748	Distal Intergenic	ENSSSCG000	FKBP14	FKBP prolyl isomerase 14 [Source:NCBI gene (formerly Entrezgene);Acc:100514551]
chr14	54891100	54892064	965	*	chr14	54891100	54892064	in_vitro	in_vivo	12	14	1.67833333	1.41785714	0.26047619	c(t = 0.18940	0.851	0.96959748	Distal Intergenic	ENSSSCG000	LGALS8	galectin 8 [Source:VGNC Symbol;Acc:VGNC:89697]
chr12	7759931	7760892	962	*	chr12	7759931	7760892	in_vitro	in_vivo	13	13	1.54076923	1.32846154	0.21230769	c(t = 0.19040	0.851	0.96959748	Distal Intergenic	ENSSSCG000	SSTR2	somatostatin receptor 2 [Source:VGNC Symbol;Acc:VGNC:99086]
chrX	36270874	36271717	844	*	chrX	36270874	36271717	in_vitro	in_vivo	11	13	10.9390909	9.47769231	1.4613986	c(t = 0.18922	0.852	0.96973045	Distal Intergenic	ENSSSCG000	DDX3X	DEAD-box helicase 3 X-linked [Source:NCBI gene (formerly Entrezgene);Acc:100515940]
chr12	48141180	48143222	2043	*	chr12	48141180	48143222	in_vitro	in_vivo	13	14	5.92230769	6.62785714	-0.7055495	c(t = -0.1879	0.852	0.96973045	Distal Intergenic	NA	NA	NA
chr8	112520547	112521147	601	*	chr8	112520547	112521147	in_vitro	in_vivo	9	10	2.97555556	3.412	-0.4364444	c(t = -0.1899	0.852	0.96973045	Distal Intergenic	ENSSSCG000	EGF	epidermal growth factor [Source:VGNC Symbol;Acc:VGNC:98921]
chr17	31216827	31217846	1020	*	chr17	31216827	31217846	in_vitro	in_vivo	10	12	1.555	1.385	0.17	c(t = 0.18964	0.852	0.96973045	Distal Intergenic	ENSSSCG000	ADRA1D	adrenoceptor alpha 1D [Source:NCBI gene (formerly Entrezgene);Acc:552898]
chr6	28195064	28196719	1656	*	chr6	28195064	28196719	in_vitro	in_vivo	13	12	0.98846154	1.08833333	-0.0998718	c(t = -0.1887	0.852	0.96973045	Distal Intergenic	ENSSSCG000	CTCF	CCCTC-binding factor [Source:VGNC Symbol;Acc:VGNC:87057]
chr1	131364891	131366213	1323	*	chr1	131364891	131366213	in_vitro	in_vivo	14	13	0.82428571	0.97384615	-0.1495604	c(t = -0.1893	0.852	0.96973045	Distal Intergenic	ENSSSCG000	THBS1	thrombospondin 1 [Source:NCBI gene (formerly Entrezgene);Acc:492313]
chrX	515628	517578	1951	*	chrX	515628	517578	in_vitro	in_vivo	12	13	0.9475	0.83153846	0.11596154	c(t = 0.18903	0.852	0.96973045	Distal Intergenic	ENSSSCG000	ARSL	arylsulfatase E (chondrodysplasia punctata 1) [Source:NCBI gene (formerly Entrezgene);Acc:100627778]
chr2	809081	865008	55928	*	chr2	809081	865008	in_vitro	in_vivo	16	15	38.705	40.1033333	-1.3983333	c(t = -0.1871	0.853	0.97000666	Promoter (<=1kb)	ENSSSCG000	TOLLIP	toll interacting protein [Source:NCBI gene (formerly Entrezgene);Acc:100625123]
chr3	132811513	132812686	1174	*	chr3	132811513	132812686	in_vitro	in_vivo	9	12	9.00777778	8.0775	0.93027778	c(t = 0.18824	0.853	0.97000666	Distal Intergenic	ENSSSCG000	SH3YL1	SH3 and SYLF domain containing 1 [Source:VGNC Symbol;Acc:VGNC:92834]
chr18	18975057	18978573	3517	*	chr18	18975057	18978573	in_vitro	in_vivo	12	16	4.26833333	3.895	0.37333333	c(t = 0.18742	0.853	0.97000666	Distal Intergenic	ENSSSCG000	ssc-mir-183	ssc-mir-183 [Source:miRBase;Acc:MI0002439]
chr13	72655608	72656519	912	*	chr13	72655608	72656519	in_vitro	in_vivo	11	9	2.08454545	1.83111111	0.25343434	c(t = 0.18737	0.853	0.97000666	Distal Intergenic	ENSSSCG000	MGLL	monoglyceride lipase [Source:VGNC Symbol;Acc:VGNC:90199]
chr4	81936650	81937943	1294	*	chr4	81936650	81937943	in_vitro	in_vivo	10	13	1.92	1.56153846	0.35846154	c(t = 0.18830	0.853	0.97000666	Promoter (<=1kb)	ENSSSCG000	ATP1B1	ATPase Na+/K+ transporting subunit beta 1 [Source:NCBI gene (formerly Entrezgene);Acc:396898]
chr8	130527432	130528038	607	*	chr8	130527432	130528038	in_vitro	in_vivo	10	10	1.011	0.878	0.133	c(t = 0.18920	0.853	0.97000666	Promoter (<=1kb)	NA	NA	NA
chr3	17400132	17401529	1398	*	chr3	17400132	17401529	in_vitro	in_vivo	9	11	72.9944444	70.02	2.97444444	c(t = 0.18639	0.854	0.97028238	Distal Intergenic	ENSSSCG000	KAT8	lysine acetyltransferase 8 [Source:NCBI gene (formerly Entrezgene);Acc:100522367]
chr15	138612709	138614596	1888	*	chr15	138612709	138614596	in_vitro	in_vivo	11	11	30.1690909	33.0518182	-2.8827273	c(t = -0.1857	0.854	0.97028238	Distal Intergenic	ENSSSCG000	ssc-mir-149	ssc-mir-149 [Source:miRBase;Acc:MI0016620]
chr8	1244066	1245211	1146	*	chr8	1244066	1245211	in_vitro	in_vivo	9	9	14.2166667	16.2933333	-2.0766667	c(t = -0.1878	0.854	0.97028238	Distal Intergenic	ENSSSCG000	RNF4	ring finger protein 4 [Source:VGNC Symbol;Acc:VGNC:92392]
chr13	200641940	200643216	1277	*	chr13	200641940	200643216	in_vitro	in_vivo	11	12	2.90090909	3.16666667	-0.2657576	c(t = -0.1861	0.854	0.97028238	Distal Intergenic	ENSSSCG000	PIGP	phosphatidylinositol glycan anchor biosynthesis class P [Source:VGNC Symbol;Acc:VGNC:91427]
chr9	9069664	9070551	888	*	chr9	9069664	9070551	in_vitro	in_vivo	12	13	2.66583333	2.34769231	0.31814103	c(t = 0.18643	0.854	0.97028238	Distal Intergenic	ENSSSCG000	POLD3	DNA polymerase delta 3, accessory subunit [Source:NCBI gene (formerly Entrezgene);Acc:100626052]
chr1	1697971	1699606	1636	*	chr1	1697971	1699606	in_vitro	in_vivo	12	13	2.4925	2.26923077	0.22326923	c(t = 0.18606	0.854	0.97028238	Distal Intergenic	ENSSSCG000	ssc-mir-9824	ssc-mir-9824 [Source:miRBase;Acc:MI0031591]
chr2	76108786	76111628	2843	*	chr2	76108786	76111628	in_vitro	in_vivo	13	14	1.18384615	1.35214286	-0.1682967	c(t = -0.1846	0.855	0.97127494	Distal Intergenic	ENSSSCG000	OAZ1	ornithine decarboxylase antizyme 1 [Source:NCBI gene (formerly Entrezgene);Acc:397034]
chr6	95811302	95812427	1126	*	chr6	95811302	95812427	in_vitro	in_vivo	9	13	2.10555556	1.88846154	0.21709402	c(t = 0.18364	0.856	0.97197991	Distal Intergenic	ENSSSCG000	HPCAL4	hippocalcin like 4 [Source:VGNC Symbol;Acc:VGNC:88959]
chr1	160024176	160025282	1107	*	chr1	160024176	160025282	in_vitro	in_vivo	12	13	0.9125	1.04307692	-0.1305769	c(t = -0.1840	0.856	0.97197991	Distal Intergenic	ENSSSCG000	RNF152	ring finger protein 152 [Source:VGNC Symbol;Acc:VGNC:92364]
chrX	12396938	12398110	1173	*	chrX	12396938	12398110	in_vitro	in_vivo	12	12	1.06333333	0.95666667	0.10666667	c(t = 0.18384	0.856	0.97197991	Distal Intergenic	ENSSSCG000	ACE2	angiotensin converting enzyme 2 [Source:VGNC Symbol;Acc:VGNC:85008]
chr1	68060778	68061348	571	*	chr1	68060778	68061348	in_vitro	in_vivo	10	10	2.483	2.878	-0.395	c(t = -0.1830	0.857	0.97210996	Distal Intergenic	ENSSSCG000	SIM1	SIM bHLH transcription factor 1 [Source:VGNC Symbol;Acc:VGNC:92876]
chr3	5719818	5721843	2026	*	chr3	5719818	5721843	in_vitro	in_vivo	10	16	2.178	2.4075	-0.2295	c(t = -0.1824	0.857	0.97210996	Distal Intergenic	ENSSSCG000	KPNA7	karyopherin subunit alpha 7 [Source:VGNC Symbol;Acc:VGNC:89566]
chr7	88410551	88411228	678	*	chr7	88410551	88411228	in_vitro	in_vivo	9	13	2.15666667	1.89230769	0.26435897	c(t = 0.18366	0.857	0.97210996	Distal Intergenic	ENSSSCG000	GPX2	glutathione peroxidase 2 [Source:NCBI gene (formerly Entrezgene);Acc:767625]
chr1	266074692	266075521	830	*	chr1	266074692	266075521	in_vitro	in_vivo	9	10	1.39444444	1.56	-0.1655556	c(t = -0.1836	0.857	0.97210996	Distal Intergenic	ENSSSCG000	RABEPK	Rab9 effector protein with kelch motifs [Source:VGNC Symbol;Acc:VGNC:92045]
chr7	90346310	90348377	2068	*	chr7	90346310	90348377	in_vitro	in_vivo	14	14	1.21428571	1.355	-0.1407143	c(t = -0.1814	0.857	0.97210996	Promoter (<=1kb)	ENSSSCG000	GPHN	gephyrin [Source:VGNC Symbol;Acc:VGNC:88587]
chr5	21372860	21373883	1024	*	chr5	21372860	21373883	in_vitro	in_vivo	12	9	0.79833333	0.95444444	-0.1561111	c(t = -0.1824	0.857	0.97210996	Distal Intergenic	ENSSSCG000	CDK2	cyclin dependent kinase 2 [Source:NCBI gene (formerly Entrezgene);Acc:100154715]
chr15	101337752	101338679	928	*	chr15	101337752	101338679	in_vitro	in_vivo	10	10	0.65	0.759	-0.109	c(t = -0.1828	0.857	0.97210996	Distal Intergenic	ENSSSCG000	HSPD1	heat shock protein family D (Hsp60) member 1 [Source:NCBI gene (formerly Entrezgene);Acc:492279]
chrX	41773146	41774594	1449	*	chrX	41773146	41774594	in_vitro	in_vivo	11	11	5.46636364	4.77181818	0.69454545	c(t = 0.18100	0.858	0.97252655	Distal Intergenic	ENSSSCG000	RGN	regucalcin [Source:VGNC Symbol;Acc:VGNC:92256]
chr14	23818	24236	419	*	chr14	23818	24236	in_vitro	in_vivo	13	12	3.60615385	4.305	-0.6988462	c(t = -0.1811	0.858	0.97252655	Promoter (<=1kb)	ENSSSCG00000009578		cyclin dependent kinase 20 [Source:NCBI gene (formerly Entrezgene);Acc:100157041]
chr14	114304516	114305162	647	*	chr14	114304516	114305162	in_vitro	in_vivo	13	11	1.82769231	1.57363636	0.25405594	c(t = 0.18047	0.858	0.97252655	Distal Intergenic	ENSSSCG000	ssc-mir-1307	ssc-mir-1307 [Source:miRBase;Acc:MI0013147]
chr15	67320099	67321158	1060	*	chr15	67320099	67321158	in_vitro	in_vivo	13	13	1.25230769	1.41384615	-0.1615385	c(t = -0.1813	0.858	0.97252655	Distal Intergenic	ENSSSCG000	ITGB6	integrin subunit beta 6 [Source:VGNC Symbol;Acc:VGNC:98054]
chr7	104188994	104189982	989	*	chr7	104188994	104189982	in_vitro	in_vivo	11	13	1.14181818	1.30846154	-0.1666434	c(t = -0.1810	0.858	0.97252655	Distal Intergenic	ENSSSCG000	TSHR	thyroid stimulating hormone receptor [Source:VGNC Symbol;Acc:VGNC:94491]
chr7	78040293	78042912	2620	*	chr7	78040293	78042912	in_vitro	in_vivo	13	12	17.4738462	18.855	-1.3811538	c(t = -0.1790	0.859	0.97307126	Distal Intergenic	ENSSSCG000	NDRG2	NDRG family member 2 [Source:VGNC Symbol;Acc:VGNC:90632]
chr2	134595028	134596049	1022	*	chr2	134595028	134596049	in_vitro	in_vivo	11	12	3.13	3.51083333	-0.3808333	c(t = -0.1799	0.859	0.97307126	Promoter (<=1kb)	ENSSSCG000	SLC22A4	solute carrier family 22 member 4 [Source:VGNC Symbol;Acc:VGNC:92981]
chr14	20144643	20145219	577	*	chr14	20144643	20145219	in_vitro	in_vivo	10	9	0.857	1.00444444	-0.1474444	c(t = -0.1798	0.859	0.97307126	Distal Intergenic	ENSSSCG000	HMGB2	high mobility group box 2 [Source:VGNC Symbol;Acc:VGNC:88902]
chr1	55614659	55615222	564	*	chr1	55614659	55615222	in_vitro	in_vivo	10	13	0.959	0.83538462	0.12361538	c(t = 0.17931	0.859	0.97307126	Distal Intergenic	ENSSSCG000	CGA	glycoprotein hormones, alpha polypeptide [Source:VGNC Symbol;Acc:VGNC:86613]
chrX	6447379	6448031	653	*	chrX	6447379	6448031	in_vitro	in_vivo	11	11	95.1536364	94.6054545	0.54818182	c(t = 0.17935	0.86	0.97307126	Distal Intergenic	ENSSSCG000	AMELY	amelogenin X-linked [Source:NCBI gene (formerly Entrezgene);Acc:396940]
chr4	130835185	130836399	1215	*	chr4	130835185	130836399	in_vitro	in_vivo	9	11	2.29333333	2.02272727	0.27060606	c(t = 0.18064	0.86	0.97307126	Distal Intergenic	ENSSSCG000	BCL10	BCL10 immune signaling adaptor [Source:NCBI gene (formerly Entrezgene);Acc:100125346]
chr17	41688742	41693852	5111	*	chr17	41688742	41693852	in_vitro	in_vivo	14	14	1.83357143	1.97714286	-0.1435714	c(t = -0.1778	0.86	0.97307126	Distal Intergenic	ENSSSCG000	ADIG	adipogenin [Source:VGNC Symbol;Acc:VGNC:96182]
chr17	43410581	43413952	3372	*	chr17	43410581	43413952	in_vitro	in_vivo	12	14	1.89166667	1.78714286	0.10452381	c(t = 0.17820	0.86	0.97307126	Distal Intergenic	ENSSSCG000	ZHX3	zinc fingers and homeoboxes 3 [Source:VGNC Symbol;Acc:VGNC:95717]
chr1	204941763	204942837	1075	*	chr1	204941763	204942837	in_vitro	in_vivo	11	11	1.93909091	1.69181818	0.24727273	c(t = 0.17857	0.86	0.97307126	Distal Intergenic	ENSSSCG000	PLIN2	perilipin 2 [Source:VGNC Symbol;Acc:VGNC:91558]
chr11	44186113	44186923	811	*	chr11	44186113	44186923	in_vitro	in_vivo	9	10	1.70333333	1.478	0.22533333	c(t = 0.17953	0.86	0.97307126	Distal Intergenic	ENSSSCG000	KLF5	KLF transcription factor 5 [Source:NCBI gene (formerly Entrezgene);Acc:100038005]
chr1	109825419	109827118	1700	*	chr1	109825419	109827118	in_vitro	in_vivo	13	14	1.40615385	1.25928571	0.14686813	c(t = 0.17897	0.86	0.97307126	Distal Intergenic	ENSSSCG000	ssc-mir-190a	ssc-mir-190a [Source:miRBase;Acc:MI0022136]
chr10	46449825	46450578	754	*	chr10	46449825	46450578	in_vitro	in_vivo	9	9	1.14777778	0.96333333	0.18444444	c(t = 0.17910	0.86	0.97307126	Distal Intergenic	ENSSSCG000	RPP38	ribonuclease P/MRP subunit p38 [Source:VGNC Symbol;Acc:VGNC:96240]
chr13	201935711	201936265	555	*	chr13	201935711	201936265	in_vitro	in_vivo	9	10	29.4577778	32.502	-3.0442222	c(t = -0.1782	0.861	0.97318496	Distal Intergenic	ENSSSCG000	ERG	ETS transcription factor ERG [Source:VGNC Symbol;Acc:VGNC:87768]
chr8	138324432	138325266	835	*	chr8	138324432	138325266	in_vitro	in_vivo	9	10	25.6566667	22.446	3.21066667	c(t = 0.17822	0.861	0.97318496	Distal Intergenic	ENSSSCG000	BMP3	bone morphogenetic protein 3 [Source:NCBI gene (formerly Entrezgene);Acc:100512974]
chr1	236340984	236341976	993	*	chr1	236340984	236341976	in_vitro	in_vivo	10	10	11.879	13.338	-1.459	c(t = -0.1775	0.861	0.97318496	Distal Intergenic	ENSSSCG00000005311		CD72 molecule [Source:VGNC Symbol;Acc:VGNC:86427]
chr11	15493998	15495028	1031	*	chr11	15493998	15495028	in_vitro	in_vivo	15	11	1.874	2.20181818	-0.3278182	c(t = -0.1777	0.861	0.97318496	Promoter (<=1kb)	ENSSSCG000	SLC25A15	solute carrier family 25 member 15 [Source:VGNC Symbol;Acc:VGNC:92994]
chr17	1061056	1061989	934	*	chr17	1061056	1061989	in_vitro	in_vivo	10	11	1.527	1.70818182	-0.1811818	c(t = -0.1772	0.861	0.97318496	Distal Intergenic	ENSSSCG000	CLDN23	claudin 23 [Source:NCBI gene (formerly Entrezgene);Acc:100156235]
chr7	5362742	5363527	786	*	chr7	5362742	5363527	in_vitro	in_vivo	9	13	1.70666667	1.43307692	0.27358974	c(t = 0.17844	0.861	0.97318496	Promoter (<=1kb)	ENSSSCG000	BLOC1S5	biogenesis of lysosomal organelles complex 1 subunit 5 [Source:NCBI gene (formerly Entrezgene);Acc:100049677]
chr7	42769310	42770064	755	*	chr7	42769310	42770064	in_vitro	in_vivo	10	9	1.245	1.01	0.235	c(t = 0.17730	0.861	0.97318496	Distal Intergenic	ENSSSCG000	CD2AP	CD2 associated protein [Source:VGNC Symbol;Acc:VGNC:86406]
chr5	69106945	69107740	796	*	chr5	69106945	69107740	in_vitro	in_vivo	10	9	25.263	27.71	-2.447	c(t = -0.1769	0.862	0.97318496	Distal Intergenic	ENSSSCG000	ADIPOR2	adiponectin receptor 2 [Source:VGNC Symbol;Acc:VGNC:85141]
chr3	32160124	32161103	980	*	chr3	32160124	32161103	in_vitro	in_vivo	9	9	6.64	7.55333333	-0.9133333	c(t = -0.1764	0.862	0.97318496	Distal Intergenic	ENSSSCG000	NUBP1	NUBP iron-sulfur cluster assembly factor 1, cytosolic [Source:VGNC Symbol;Acc:VGNC:90943]
chr4	94728919	94730101	1183	*	chr4	94728919	94730101	in_vitro	in_vivo	10	9	5.166	4.55666667	0.60933333	c(t = 0.17739	0.862	0.97318496	Distal Intergenic	ENSSSCG000	EFNA1	ephrin A1 [Source:VGNC Symbol;Acc:VGNC:98789]
chr18	50142809	50144592	1784	*	chr18	50142809	50144592	in_vitro	in_vivo	11	10	2.36545455	2.193	0.17245455	c(t = 0.17710	0.862	0.97318496	Distal Intergenic	ENSSSCG000	IGFBP1	insulin like growth factor binding protein 1 [Source:NCBI gene (formerly Entrezgene);Acc:397270]
chr9	2561612	2562448	837	*	chr9	2561612	2562448	in_vitro	in_vivo	9	9	2.57555556	2.12333333	0.45222222	c(t = 0.17674	0.862	0.97318496	Distal Intergenic	ENSSSCG000	MRPL17	mitochondrial ribosomal protein L17 [Source:NCBI gene (formerly Entrezgene);Acc:100512482]
chr14	90725361	90725983	623	*	chr14	90725361	90725983	in_vitro	in_vivo	9	10	1.74	1.545	0.195	c(t = 0.17690	0.862	0.97318496	Distal Intergenic	ENSSSCG000	ZFAND4	zinc finger AN1-type containing 4 [Source:VGNC Symbol;Acc:VGNC:95136]
chr6	45576768	45577500	733	*	chr6	45576768	45577500	in_vitro	in_vivo	10	9	1.127	0.94	0.187	c(t = 0.17667	0.862	0.97318496	Distal Intergenic	ENSSSCG000	COX7A1	cytochrome c oxidase subunit 7A1 [Source:NCBI gene (formerly Entrezgene);Acc:399685]
chr1	190205909	190207045	1137	*	chr1	190205909	190207045	in_vitro	in_vivo	13	12	1.03153846	0.91583333	0.11570513	c(t = 0.17591	0.862	0.97318496	Distal Intergenic	ENSSSCG000	SLC38A6	solute carrier family 38 member 6 [Source:VGNC Symbol;Acc:VGNC:93098]
chr4	4801093	4801680	588	*	chr4	4801093	4801680	in_vitro	in_vivo	9	11	31.8233333	28.7872727	3.03606061	c(t = 0.17538	0.863	0.97372635	Distal Intergenic	ENSSSCG000	ago-02	argonaute RISC catalytic component 2 [Source:NCBI gene (formerly Entrezgene);Acc:100155245]
chr5	10252259	10253385	1127	*	chr5	10252259	10253385	in_vitro	in_vivo	9	10	2.91666667	2.556	0.36066667	c(t = 0.17607	0.863	0.97372635	Distal Intergenic	ENSSSCG000	LGALS2	galectin 2 [Source:NCBI gene (formerly Entrezgene);Acc:100217393]
chr7	113045174	113046323	1150	*	chr7	113045174	113046323	in_vitro	in_vivo	13	13	1.22076923	1.03153846	0.18923077	c(t = 0.17521	0.863	0.97372635	Distal Intergenic	ENSSSCG00000002446		ataxin 3 [Source:NCBI gene (formerly Entrezgene);Acc:100144422]
chr18	40538769	40539574	806	*	chr18	40538769	40539574	in_vitro	in_vivo	11	15	0.80727273	0.89466667	-0.0873939	c(t = -0.1748	0.863	0.97372635	Distal Intergenic	ENSSSCG000	FKBP9	FKBP prolyl isomerase 9 [Source:NCBI gene (formerly Entrezgene);Acc:100623924]
chr13	205349595	205353178	3584	*	chr13	205349595	205353178	in_vitro	in_vivo	12	11	90.4708333	88.3490909	2.12174242	c(t = 0.17405	0.864	0.97372635	Distal Intergenic	ENSSSCG000	TFF3	trefoil factor 3 [Source:NCBI gene (formerly Entrezgene);Acc:100627480]
chr8	1989078	1992742	3665	*	chr8	1989078	1992742	in_vitro	in_vivo	11	13	74.6890909	72.4023077	2.28678322	c(t = 0.17291	0.864	0.97372635	Distal Intergenic	ENSSSCG000	LRPAP1	LDL receptor related protein associated protein 1 [Source:NCBI gene (formerly Entrezgene);Acc:399654]
chr3	101208841	101209896	1056	*	chr3	101208841	101209896	in_vitro	in_vivo	11	14	2.32727273	2.11357143	0.2137013	c(t = 0.17322	0.864	0.97372635	Distal Intergenic	ENSSSCG000	GALM	galactose mutarotase [Source:NCBI gene (formerly Entrezgene);Acc:399536]
chr1	139014427	139015538	1112	*	chr1	139014427	139015538	in_vitro	in_vivo	11	13	2.03272727	1.84923077	0.1834965	c(t = 0.17348	0.864	0.97372635	Distal Intergenic	ENSSSCG000	MEF2A	myocyte enhancer factor 2A [Source:NCBI gene (formerly Entrezgene);Acc:100037273]
chr3	101382691	101383327	637	*	chr3	101382691	101383327	in_vitro	in_vivo	12	12	1.95083333	1.74333333	0.2075	c(t = 0.17345	0.864	0.97372635	Distal Intergenic	ENSSSCG000	GALM	galactose mutarotase [Source:NCBI gene (formerly Entrezgene);Acc:399536]
chr15	126710939	126711986	1048	*	chr15	126710939	126711986	in_vitro	in_vivo	11	9	1.87272727	1.57777778	0.29494949	c(t = 0.17431	0.864	0.97372635	Distal Intergenic	ENSSSCG000	SERPINE2	serpin family E member 2 [Source:NCBI gene (formerly Entrezgene);Acc:397542]
chr14	50868898	50869592	695	*	chr14	50868898	50869592	in_vitro	in_vivo	10	12	1.032	1.15833333	-0.1263333	c(t = -0.1736	0.864	0.97372635	Distal Intergenic	ENSSSCG000	TSSK1B	testis specific serine kinase 1B [Source:NCBI gene (formerly Entrezgene);Acc:102159240]
chr2	18954339	18954870	532	*	chr2	18954339	18954870	in_vitro	in_vivo	10	10	0.798	0.672	0.126	c(t = 0.17391	0.864	0.97372635	Distal Intergenic	ENSSSCG000	ssc-mir-670	ssc-mir-670 [Source:miRBase;Acc:MI0033398]
chr2	137766010	137767326	1317	*	chr2	137766010	137767326	in_vitro	in_vivo	9	11	61.5677778	57.9481818	3.61959596	c(t = 0.17310	0.865	0.97377658	Distal Intergenic	ENSSSCG000	CXCL14	C-X-C motif chemokine ligand 14 [Source:NCBI gene (formerly Entrezgene);Acc:494467]
chr2	142641101	142642772	1672	*	chr2	142641101	142642772	in_vitro	in_vivo	11	13	54.4581818	51.5538462	2.90433566	c(t = 0.17204	0.865	0.97377658	Distal Intergenic	ENSSSCG000	CD14	CD14 molecule [Source:NCBI gene (formerly Entrezgene);Acc:100037938]
chr4	93462039	93464265	2227	*	chr4	93462039	93464265	in_vitro	in_vivo	14	11	5.71785714	6.38090909	-0.6630519	c(t = -0.1715	0.865	0.97377658	Distal Intergenic	ENSSSCG000	GPATCH4	G-patch domain containing 4 [Source:NCBI gene (formerly Entrezgene);Acc:100152732]
chr1	66544736	66546420	1685	*	chr1	66544736	66546420	in_vitro	in_vivo	13	12	1.15076923	1.25166667	-0.1008974	c(t = -0.1721	0.865	0.97377658	Distal Intergenic	ENSSSCG000	CCNC	cyclin C [Source:VGNC Symbol;Acc:VGNC:103046]
chr15	29713751	29714200	450	*	chr15	29713751	29714200	in_vitro	in_vivo	9	10	1.37	1.178	0.192	c(t = 0.17323	0.865	0.97377658	Distal Intergenic	ENSSSCG000	NIFK	nucleolar protein interacting with the FHA domain of MKI67 [Source:VGNC Symbol;Acc:VGNC:99725]
chr6	46436091	46436833	743	*	chr6	46436091	46436833	in_vitro	in_vivo	14	12	0.93142857	1.05333333	-0.1219048	c(t = -0.1718	0.865	0.97377658	Distal Intergenic	ENSSSCG000	PPP1R14A	protein phosphatase 1 regulatory inhibitor subunit 14A [Source:VGNC Symbol;Acc:VGNC:98539]
chr3	1397064	1398811	1748	*	chr3	1397064	1398811	in_vitro	in_vivo	12	11	95.8208333	95.4363636	0.3844697	c(t = 0.17094	0.866	0.97377658	Distal Intergenic	ENSSSCG000	MRM2	mitochondrial rRNA methyltransferase 2 [Source:VGNC Symbol;Acc:VGNC:99722]
chr4	429062	430184	1123	*	chr4	429062	430184	in_vitro	in_vivo	10	10	15.424	13.885	1.539	c(t = 0.17160	0.866	0.97377658	Distal Intergenic	ENSSSCG000	SLC52A1	solute carrier family 52 member 2 [Source:NCBI gene (formerly Entrezgene);Acc:445519]
chr5	1499294	1505647	6354	*	chr5	1499294	1505647	in_vitro	in_vivo	12	15	14.1125	12.756	1.3565	c(t = 0.17018	0.866	0.97377658	Distal Intergenic	ENSSSCG000	SELENOO	selenoprotein O [Source:NCBI gene (formerly Entrezgene);Acc:100518971]
chr6	54601535	54602403	869	*	chr6	54601535	54602403	in_vitro	in_vivo	10	9	3.137	3.42	-0.283	c(t = -0.1716	0.866	0.97377658	Distal Intergenic	ENSSSCG000	FCGRT	Fc gamma receptor and transporter [Source:VGNC Symbol;Acc:VGNC:88064]
chr3	114577465	114578101	637	*	chr3	114577465	114578101	in_vitro	in_vivo	9	12	1.96222222	2.255	-0.2927778	c(t = -0.1728	0.866	0.97377658	Distal Intergenic	ENSSSCG000	NCOA1	nuclear receptor coactivator 1 [Source:VGNC Symbol;Acc:VGNC:90615]
chr9	13010266	13011011	746	*	chr9	13010266	13011011	in_vitro	in_vivo	11	10	1.62090909	2	-0.3790909	c(t = -0.1720	0.866	0.97377658	Distal Intergenic	ENSSSCG000	NDUFC2	NADH:ubiquinone oxidoreductase subunit C2 [Source:NCBI gene (formerly Entrezgene);Acc:100513030]

chr3	49680923	49681740	818	*	chr3	49680923	49681740	in_vitro	in_vivo	12	9	0.90583333	1.06444444	-0.1586111	c(t = -0.1711	0.866	0.97377658	Promoter (<=1kb)	ENSSSCG000	MRPS9	mitochondrial ribosomal protein S9 [Source:VGNC Symbol;Acc:VGNC:90401]
chr12	4822075	4822841	767	*	chr12	4822075	4822841	in_vitro	in_vivo	9	13	0.68666667	0.80769231	-0.1210256	c(t = -0.1707	0.866	0.97377658	Distal Intergenic	ENSSSCG00000036592		serine and arginine rich splicing factor 2 [Source:VGNC Symbol;Acc:VGNC:99085]
chr3	22382499	22383619	1121	*	chr3	22382499	22383619	in_vitro	in_vivo	11	14	0.47818182	0.57214286	-0.093961	c(t = -0.1709	0.866	0.97377658	Distal Intergenic	ENSSSCG000	NDUFAB1	NADH:ubiquinone oxidoreductase subunit AB1 [Source:VGNC Symbol;Acc:VGNC:104004]
chr8	108022127	108022763	637	*	chr8	108022127	108022763	in_vitro	in_vivo	11	11	11.4463636	13.2018182	-1.7554545	c(t = -0.1705	0.867	0.97377658	Distal Intergenic	ENSSSCG000	UGT8	UDP glycosyltransferase 8 [Source:VGNC Symbol;Acc:VGNC:98401]
chr1	250539837	250540682	846	*	chr1	250539837	250540682	in_vitro	in_vivo	13	10	1.17307692	1.065	0.10807692	c(t = 0.16923	0.867	0.97377658	Distal Intergenic	ENSSSCG000	PALM2AKAP	PALM2-AKAP2 fusion [Source:NCBI gene (formerly Entrezgene);Acc:100152369]
chr4	77673204	77674074	871	*	chr4	77673204	77674074	in_vitro	in_vivo	9	10	1.12777778	0.954	0.17377778	c(t = 0.17155	0.867	0.97377658	Distal Intergenic	ENSSSCG000	ATP6V1H	ATPase H+ transporting V1 subunit H [Source:VGNC Symbol;Acc:VGNC:85677]
chr7	100525837	100526208	372	*	chr7	100525837	100526208	in_vitro	in_vivo	10	12	32.957	30.5383333	2.41866667	c(t = 0.16845	0.868	0.97377658	Distal Intergenic	ENSSSCG000	GSTZ1	glutathione S-transferase zeta 1 [Source:VGNC Symbol;Acc:VGNC:88727]
chr14	141386757	141388633	1877	*	chr14	141386757	141388633	in_vitro	in_vivo	10	12	3.321	3.64583333	-0.3248333	c(t = -0.1681	0.868	0.97377658	Distal Intergenic	ENSSSCG000	ECHS1	enoyl-CoA hydratase, short chain 1 [Source:NCBI gene (formerly Entrezgene);Acc:100156927]
chr8	137552359	137555287	2929	*	chr8	137552359	137555287	in_vitro	in_vivo	12	11	3.0475	2.78909091	0.25840909	c(t = 0.16846	0.868	0.97377658	Distal Intergenic	ENSSSCG000	BMP3	bone morphogenetic protein 3 [Source:NCBI gene (formerly Entrezgene);Acc:100512974]
chr3	68883410	68884014	605	*	chr3	68883410	68884014	in_vitro	in_vivo	10	10	2.566	2.243	0.323	c(t = 0.16952	0.868	0.97377658	Distal Intergenic	ENSSSCG000	SLC4A5	solute carrier family 4 member 5 [Source:NCBI gene (formerly Entrezgene);Acc:100620628]
chr17	58594620	58595577	958	*	chr17	58594620	58595577	in_vitro	in_vivo	12	10	1.9825	2.219	-0.2365	c(t = -0.1689	0.868	0.97377658	Promoter (<=1kb)	ENSSSCG000	VAPB	VAMP associated protein B and C [Source:VGNC Symbol;Acc:VGNC:108743]
chr5	7454228	7455433	1206	*	chr5	7454228	7455433	in_vitro	in_vivo	11	14	2.16363636	1.98714286	0.17649351	c(t = 0.16816	0.868	0.97377658	Distal Intergenic	ENSSSCG000	MCHR1	melanin concentrating hormone receptor 1 [Source:VGNC Symbol;Acc:VGNC:103300]
chr7	51844505	51845368	864	*	chr7	51844505	51845368	in_vitro	in_vivo	13	14	1.28692308	1.44428571	-0.1573626	c(t = -0.1679	0.868	0.97377658	Distal Intergenic	ENSSSCG000	ssc-mir-1839	ssc-mir-1839 [Source:miRBase;Acc:MI0013162]
chr16	4270937	4271831	895	*	chr16	4270937	4271831	in_vitro	in_vivo	11	10	1.36727273	1.185	0.18227273	c(t = 0.16863	0.868	0.97377658	Distal Intergenic	ENSSSCG000	C1QTNF3	C1q and TNF related 3 [Source:NCBI gene (formerly Entrezgene);Acc:100270814]
chr1	121145799	121146765	967	*	chr1	121145799	121146765	in_vitro	in_vivo	9	10	0.41111111	0.503	-0.0918889	c(t = -0.1681	0.868	0.97377658	Distal Intergenic	ENSSSCG000	SLC27A2	solute carrier family 27 member 2 [Source:NCBI gene (formerly Entrezgene);Acc:494011]
chr12	1273158	1284128	10971	*	chr12	1273158	1284128	in_vitro	in_vivo	10	13	94.479	94.8261538	-0.3471538	c(t = -0.1673	0.869	0.97377658	Distal Intergenic	ENSSSCG000	ARHGDIA	Rho GDP dissociation inhibitor alpha [Source:VGNC Symbol;Acc:VGNC:85483]
chr11	2695360	2697261	1902	*	chr11	2695360	2697261	in_vitro	in_vivo	9	9	89.0511111	88.3555556	0.69555556	c(t = 0.16816	0.869	0.97377658	Distal Intergenic	ENSSSCG000	ssc-mir-9814	ssc-mir-9814 [Source:miRBase;Acc:MI0031581]
chr9	402033	403734	1702	*	chr9	402033	403734	in_vitro	in_vivo	9	11	36.4722222	33.6063636	2.86585859	c(t = 0.16732	0.869	0.97377658	Distal Intergenic	NA	NA	NA
chr8	1938721	1939456	736	*	chr8	1938721	1939456	in_vitro	in_vivo	9	9	11.8122222	13.7555556	-1.9433333	c(t = -0.1673	0.869	0.97377658	Distal Intergenic	ENSSSCG000	HTT	huntingtin [Source:VGNC Symbol;Acc:VGNC:89006]
chr4	79685812	79687585	1774	*	chr4	79685812	79687585	in_vitro	in_vivo	11	14	7.45363636	6.98714286	0.46649351	c(t = 0.16666	0.869	0.97377658	Distal Intergenic	ENSSSCG000	UBE2V2	ubiquitin conjugating enzyme E2 V2 [Source:NCBI gene (formerly Entrezgene);Acc:100627960]
chr5	6677594	6678627	1034	*	chr5	6677594	6678627	in_vitro	in_vivo	9	11	4.98888889	5.61363636	-0.6247475	c(t = -0.1683	0.869	0.97377658	Promoter (<=1kb)	ENSSSCG000	CENPM	centromere protein M [Source:VGNC Symbol;Acc:VGNC:86553]
chr13	120727493	120728296	804	*	chr13	120727493	120728296	in_vitro	in_vivo	10	12	1.872	1.71333333	0.15866667	c(t = 0.16758	0.869	0.97377658	Distal Intergenic	ENSSSCG000	B3GNT5	UDP-GlcNAc:betaGal beta-1,3-N-acetylglucosaminyltransferase 5 [Source:VGNC Symbol;Acc:VGNC:85726]
chr13	207042304	207051651	9348	*	chr13	207042304	207051651	in_vitro	in_vivo	12	14	70.4408333	68.4407143	2.00011905	c(t = 0.16605	0.87	0.97377658	Distal Intergenic	ENSSSCG000	AGPAT3	1-acylglycerol-3-phosphate O-acyltransferase 3 [Source:VGNC Symbol;Acc:VGNC:85185]
chr9	1899886	1900506	621	*	chr9	1899886	1900506	in_vitro	in_vivo	9	10	34.5955556	31.908	2.68755556	c(t = 0.16622	0.87	0.97377658	Distal Intergenic	NA	NA	NA
chr18	16583902	16584958	1057	*	chr18	16583902	16584958	in_vitro	in_vivo	11	10	27.8654545	25.27	2.59545455	c(t = 0.16617	0.87	0.97377658	Distal Intergenic	ENSSSCG000	CHCHD3	coiled-coil-helix-coiled-coil-helix domain containing 3 [Source:VGNC Symbol;Acc:VGNC:110298]
chrX	124788042	124789546	1505	*	chrX	124788042	124789546	in_vitro	in_vivo	9	15	9.14777778	8.394	0.75377778	c(t = 0.16618	0.87	0.97377658	Distal Intergenic	ENSSSCG00000029523		
chr2	47027443	47028141	699	*	chr2	47027443	47028141	in_vitro	in_vivo	11	12	8.25454545	7.71	0.54454545	c(t = 0.16593	0.87	0.97377658	Distal Intergenic	ENSSSCG000	TEAD1	TEA domain transcription factor 1 [Source:VGNC Symbol;Acc:VGNC:93853]
chr11	616168	618166	1999	*	chr11	616168	618166	in_vitro	in_vivo	12	16	2.30416667	2.55875	-0.2545833	c(t = -0.1650	0.87	0.97377658	Distal Intergenic	ENSSSCG000	CRYL1	crystallin lambda 1 [Source:VGNC Symbol;Acc:VGNC:87023]
chr6	128249721	128251207	1487	*	chr6	128249721	128251207	in_vitro	in_vivo	11	14	2.18909091	1.95214286	0.23694805	c(t = 0.16574	0.87	0.97377658	Distal Intergenic	ENSSSCG000	HSBP1L1	heat shock factor binding protein 1 like 1 [Source:NCBI gene (formerly Entrezgene);Acc:100192206]
chr2	26826645	26827220	576	*	chr2	26826645	26827220	in_vitro	in_vivo	11	9	1.59454545	1.82444444	-0.229899	c(t = -0.1659	0.87	0.97377658	Distal Intergenic	ENSSSCG000	CAT	catalase [Source:VGNC Symbol;Acc:VGNC:86208]
chr6	62894335	62895489	1155	*	chr6	62894335	62895489	in_vitro	in_vivo	10	12	1.743	1.5825	0.1605	c(t = 0.16599	0.87	0.97377658	Distal Intergenic	ENSSSCG000	ISG15	ISG15 ubiquitin like modifier [Source:NCBI gene (formerly Entrezgene);Acc:100145895]
chr10	12528260	12529239	980	*	chr10	12528260	12529239	in_vitro	in_vivo	13	12	1.24153846	1.38166667	-0.1401282	c(t = -0.1659	0.87	0.97377658	Distal Intergenic	ENSSSCG000	DEGS1	delta 4-desaturase, sphingolipid 1 [Source:VGNC Symbol;Acc:VGNC:95590]
chr6	84310267	84310895	629	*	chr6	84310267	84310895	in_vitro	in_vivo	10	14	1.112	1.31285714	-0.2008571	c(t = -0.1659	0.87	0.97377658	Distal Intergenic	ENSSSCG000	TRNP1	TMF1 regulated nuclear protein 1 [Source:VGNC Symbol;Acc:VGNC:94456]
chr13	124582406	124583887	1482	*	chr13	124582406	124583887	in_vitro	in_vivo	9	10	1.04555556	0.936	0.10955556	c(t = 0.16630	0.87	0.97377658	Promoter (<=1kb)	ENSSSCG000	EIF4A2	eukaryotic translation initiation factor 4A2 [Source:VGNC Symbol;Acc:VGNC:87624]
chr9	91199311	91199771	461	*	chr9	91199311	91199771	in_vitro	in_vivo	12	12	0.65083333	0.55416667	0.09666667	c(t = 0.16540	0.87	0.97377658	Distal Intergenic	ENSSSCG000	IL6	interleukin 6 [Source:NCBI gene (formerly Entrezgene);Acc:399500]
chr7	96900640	96901311	672	*	chr7	96900640	96901311	in_vitro	in_vivo	11	12	1.23	1.38416667	-0.1541667	c(t = -0.1653	0.871	0.97445398	Distal Intergenic	ENSSSCG000	PSEN1	presenilin 1 [Source:VGNC Symbol;Acc:VGNC:91897]
chr9	116402109	116403094	986	*	chr9	116402109	116403094	in_vitro	in_vivo	10	12	0.87	0.72583333	0.14416667	c(t = 0.16523	0.871	0.97445398	Distal Intergenic	ENSSSCG000	SERPINC1	serpin family C member 1 [Source:NCBI gene (formerly Entrezgene);Acc:100125972]
chr3	4900213	4901272	1060	*	chr3	4900213	4901272	in_vitro	in_vivo	10	13	0.501	0.42	0.081	c(t = 0.16477	0.871	0.97445398	Distal Intergenic	ENSSSCG000	RAC1	Rac family small GTPase 1 [Source:VGNC Symbol;Acc:VGNC:92047]
chr2	8286187	8288147	1961	*	chr2	8286187	8288147	in_vitro	in_vivo	12	15	10.2666667	9.36933333	0.89733333	c(t = 0.16219	0.872	0.97445398	Distal Intergenic	ENSSSCG000	ssc-mir-7142	ssc-mir-7142 [Source:miRBase;Acc:MI0023575]
chr4	1687114	1688811	1698	*	chr4	1687114	1688811	in_vitro	in_vivo	12	11	3.74166667	4.11090909	-0.3692424	c(t = -0.1633	0.872	0.97445398	Distal Intergenic	ENSSSCG000	GPIHBP1	glycosylphosphatidylinositol anchored high density lipoprotein binding protein 1 [Source:VGNC Symbol;Acc:VGNC:88588]
chr13	206710182	206711099	918	*	chr13	206710182	206711099	in_vitro	in_vivo	9	11	2.00888889	1.75454545	0.25434343	c(t = 0.16472	0.872	0.97445398	Promoter (<=1kb)	ENSSSCG000	CSTB	cystatin B [Source:NCBI gene (formerly Entrezgene);Acc:100038020]
chr13	14214709	14215973	1265	*	chr13	14214709	14215973	in_vitro	in_vivo	11	9	1.20363636	1.34	-0.1363636	c(t = -0.1634	0.872	0.97445398	Distal Intergenic	ENSSSCG000	TOP2B	DNA topoisomerase II beta [Source:NCBI gene (formerly Entrezgene);Acc:100156319]
chr3	126086488	126087785	1298	*	chr3	126086488	126087785	in_vitro	in_vivo	9	13	1.29888889	1.13692308	0.16196581	c(t = 0.16357	0.872	0.97445398	Promoter (1-2kb)	ENSSSCG000	ODC1	ornithine decarboxylase 1 [Source:VGNC Symbol;Acc:VGNC:104014]
chr3	83536426	83537170	745	*	chr3	83536426	83537170	in_vitro	in_vivo	9	10	1.20222222	1.072	0.13022222	c(t = 0.16421	0.872	0.97445398	Distal Intergenic	ENSSSCG000	ssc-mir-9789	ssc-mir-9789 [Source:miRBase;Acc:MI0031551]
chr18	33220109	33221410	1302	*	chr18	33220109	33221410	in_vitro	in_vivo	11	12	1.20818182	1.06416667	0.14401515	c(t = 0.16380	0.872	0.97445398	Promoter (<=1kb)	ENSSSCG000	IFRD1	interferon related developmental regulator 1 [Source:NCBI gene (formerly Entrezgene);Acc:493185]
chr14	99928232	99930152	1921	*	chr14	99928232	99930152	in_vitro	in_vivo	13	14	1.20384615	1.02071429	0.18313187	c(t = 0.16349	0.872	0.97445398	Promoter (<=1kb)	ENSSSCG00000010440		phosphatase and tensin homolog [Source:VGNC Symbol;Acc:VGNC:111132]
chrX	43028110	43029717	1608	*	chrX	43028110	43029717	in_vitro	in_vivo	12	12	5.74666667	6.53666667	-0.79	c(t = -0.1616	0.873	0.97500436	Distal Intergenic	ENSSSCG000	PQBP1	polyglutamine binding protein 1 [Source:VGNC Symbol;Acc:VGNC:91767]
chr6	103196682	103197290	609	*	chr6	103196682	103197290	in_vitro	in_vivo	9	10	6.09333333	5.327	0.76633333	c(t = 0.16308	0.873	0.97500436	Distal Intergenic	ENSSSCG000	TGIF1	TGFB induced factor homeobox 1 [Source:VGNC Symbol;Acc:VGNC:93935]
chr13	73276181	73276931	751	*	chr13	73276181	73276931	in_vitro	in_vivo	9	10	1.30111111	1.168	0.13311111	c(t = 0.16275	0.873	0.97500436	Distal Intergenic	ENSSSCG000	ACKR4	atypical chemokine receptor 4 [Source:NCBI gene (formerly Entrezgene);Acc:100037292]
chr4	68968953	68969807	855	*	chr4	68968953	68969807	in_vitro	in_vivo	13	9	0.92538462	1.08333333	-0.1579487	c(t = -0.1623	0.873	0.97500436	Distal Intergenic	ENSSSCG000	ARMC1	armadillo repeat containing 1 [Source:NCBI gene (formerly Entrezgene);Acc:100156048]
chr5	553453	556123	2671	*	chr5	553453	556123	in_vitro	in_vivo	11	11	93.8581818	93.1472727	0.71090909	c(t = 0.16119	0.874	0.9752708	Distal Intergenic	ENSSSCG000	SELENOO	selenoprotein O [Source:NCBI gene (formerly Entrezgene);Acc:100518971]
chr16	78904131	78907213	3083	*	chr16	78904131	78907213	in_vitro	in_vivo	12	13	25.8683333	23.8676923	2.00064103	c(t = 0.16020	0.874	0.9752708	Distal Intergenic	ENSSSCG000	TERT	telomerase reverse transcriptase [Source:NCBI gene (formerly Entrezgene);Acc:492280]
chr14	58237469	58238478	1010	*	chr14	58237469	58238478	in_vitro	in_vivo	11	10	19.0809091	16.86	2.22090909	c(t = 0.16121	0.874	0.9752708	Distal Intergenic	ENSSSCG000	TSNAX	translin associated factor X [Source:NCBI gene (formerly Entrezgene);Acc:100156036]
chr9	52350003	52352512	2510	*	chr9	52350003	52352512	in_vitro	in_vivo	12	12	2.695	2.43583333	0.25916667	c(t = 0.16082	0.874	0.9752708	Distal Intergenic	ENSSSCG000	STT3A	STT3 oligosaccharyltransferase complex catalytic subunit A [Source:VGNC Symbol;Acc:VGNC:93581]
chr7	75776644	75778034	1391	*	chr7	75776644	75778034	in_vitro	in_vivo	12	14	1.68833333	1.84571429	-0.157381	c(t = -0.1598	0.874	0.9752708	Distal Intergenic	ENSSSCG000	PABPN1	poly(A) binding protein nuclear 1 [Source:NCBI gene (formerly Entrezgene);Acc:100155260]
chr12	38874853	38875589	737	*	chr12	38874853	38875589	in_vitro	in_vivo	9	10	1.24222222	1.427	-0.1847778	c(t = -0.1605	0.874	0.9752708	Distal Intergenic	ENSSSCG000	ACACA	acetyl-CoA carboxylase alpha [Source:VGNC Symbol;Acc:VGNC:84997]
chr6	167081150	167085120	3971	*	chr6	167081150	167085120	in_vitro	in_vivo	12	15	70.435	72.0906667	-1.6556667	c(t = -0.1584	0.875	0.97567832	3' UTR	ENSSSCG000	DMAP1	DNA methyltransferase 1 associated protein 1 [Source:VGNC Symbol;Acc:VGNC:87344]
chr18	48780040	48781132	1093	*	chr18	48780040	48781132	in_vitro	in_vivo	9	10	8.03444444	8.948	-0.9135556	c(t = -0.1603	0.875	0.97567832	Distal Intergenic	ENSSSCG000	PGAM2	phosphoglycerate mutase 2 [Source:VGNC Symbol;Acc:VGNC:91341]
chrX	43613429	43614121	693	*	chrX	43613429	43614121	in_vitro	in_vivo	13	9	7.63923077	8.75555556	-1.1163248	c(t = -0.1592	0.875	0.97567832	Distal Intergenic	ENSSSCG000	ssc-mir-532	ssc-mir-532 [Source:miRBase;Acc:MI0013150]
chr13	54180458	54180813	356	*	chr13	54180458	54180813	in_vitro	in_vivo	9	10	0.92777778	1.08	-0.1522222	c(t = -0.1594	0.875	0.97567832	Distal Intergenic	ENSSSCG000	MITF	melanocyte inducing transcription factor [Source:VGNC Symbol;Acc:VGNC:90243]
chr16	74270342	74271308	967	*	chr16	74270342	74271308	in_vitro	in_vivo	14	11	0.46857143	0.53090909	-0.0623377	c(t = -0.1594	0.875	0.97567832	Distal Intergenic	ENSSSCG000	CCT5	chaperonin containing TCP1 subunit 5 [Source:VGNC Symbol;Acc:VGNC:86383]
chr3	10669539	10670313	775	*	chr3	10669539	10670313	in_vitro	in_vivo	9	10	18.3122222	20.723	-2.4107778	c(t = -0.1585	0.876	0.97617269	Distal Intergenic	ENSSSCG000	TRIM50	tripartite motif containing 50 [Source:NCBI gene (formerly Entrezgene);Acc:397383]
chr1	119020758	119021964	1207	*	chr1	119020758	119021964	in_vitro	in_vivo	10	12	2.067	1.91083333	0.15616667	c(t = 0.15857	0.876	0.97617269	Distal Intergenic	ENSSSCG000	ARPP19	cAMP regulated phosphoprotein 19 [Source:NCBI gene (formerly Entrezgene);Acc:397362]
chr10	12431302	12432071	770	*	chr10	12431302	12432071	in_vitro	in_vivo	13	15	1.14846154	1.02866667	0.11979487	c(t = 0.15719	0.876	0.97617269	Promoter (<=1kb)	ENSSSCG000	FH	fumarate hydratase [Source:VGNC Symbol;Acc:VGNC:96300]
chr6	185797	194125	8329	*	chr6	185797	194125	in_vitro	in_vivo	11	14	95.1890909	95.62	-0.4309091	c(t = -0.1582	0.877	0.97617269	Distal Intergenic	ENSSSCG000	MC1R	melanocortin 1 receptor [Source:NCBI gene (formerly Entrezgene);Acc:494018]
chr4	2656158	2657565	1408	*	chr4	2656158	2657565	in_vitro	in_vivo	11	11	2.17363636	2.41545455	-0.2418182	c(t = -0.1568	0.877	0.97617269	Distal Intergenic	NA	NA	NA
chr10	29322146	29322939	794	*	chr10	29322146	29322939	in_vitro	in_vivo	11	11	2.42090909	2.13363636	0.28727273	c(t = 0.15671	0.877	0.97617269	Distal Intergenic	ENSSSCG000	SLC28A3	solute carrier family 28 member 3 [Source:VGNC Symbol;Acc:VGNC:95810]
chr1	86163524	86164535	1012	*	chr1	86163524	86164535	in_vitro	in_vivo	10	9	1.492	1.66111111	-0.1691111	c(t = -0.1581	0.877	0.97617269	Distal Intergenic	ENSSSCG000	BCKDHB	branched chain keto acid dehydrogenase E1 subunit beta [Source:VGNC Symbol;Acc:VGNC:85777]
chr18	11704611	11705655	1045	*	chr18	11704611	11705655	in_vitro	in_vivo	12	13	1.34666667	1.22538462	0.12128205	c(t = 0.15591	0.877	0.97617269	Distal Intergenic	ENSSSCG000	PTN	pleiotrophin [Source:VGNC Symbol;Acc:VGNC:91965]
chr5	56817276	56818084	809	*	chr5	56817276	56818084	in_vitro	in_vivo	11	10	0.78	0.68	0.1	c(t = 0.15770	0.877	0.97617269	Distal Intergenic	ENSSSCG000	MGST1	microsomal glutathione S-transferase 1 [Source:VGNC Symbol;Acc:VGNC:103301]
chr7	60047988	60049537	1550	*	chr7	60047988	60049537	in_vitro	in_vivo	12	12	82.08	84.0416667	-1.9616667	c(t = -0.1551	0.878	0.97617269	Distal Intergenic	ENSSSCG000	BBS4	Bardet-Biedl syndrome 4 [Source:NCBI gene (formerly Entrezgene);Acc:100513212]
chr13	3781968	3783126	1159	*	chr13	3781968	3783126	in_vitro	in_vivo	9	14	24.8188889	27.3264286	-2.5075397	c(t = -0.1553	0.878	0.97617269	Distal Intergenic	ENSSSCG000	DPH3	diphthamide biosynthesis 3 [Source:NCBI gene (formerly Entrezgene);Acc:100156362]
chr10	14467113	14469509	2397	*	chr10	14467113	14469509	in_vitro	in_vivo	12	12	2.68583333	2.50916667	0.17666667	c(t = 0.15496	0.878	0.97617269	Distal Intergenic	ENSSSCG000	PSEN2	presenilin 2 [Source:VGNC Symbol;Acc:VGNC:96508]
chr6	56068107	56068836	730	*	chr6	56068107	56068836	in_vitro	in_vivo	9	10	2.48777778	2.233	0.25477778	c(t = 0.15640	0.878	0.97617269	Distal Intergenic	ENSSSCG000	ssc-mir-935	ssc-mir-935 [Source:miRBase;Acc:MI0013156]
chr12	18526214	18527346	1133	*	chr12	18526214	18527346	in_vitro	in_vivo	12	11	1.90916667	2.21363636	-0.3044697	c(t = -0.1563	0.878	0.97617269	Distal Intergenic	ENSSSCG000	GJC1	gap junction protein gamma 1 [Source:VGNC Symbol;Acc:VGNC:88468]
chr5	69460564	69462204	1641	*	chr5	69460564	69462204	in_vitro	in_vivo	10	13	1.928	1.70230769	0.22569231	c(t = 0.15668	0.878	0.97617269	Distal Intergenic	ENSSSCG000	ADA2	adenosine deaminase 2 [Source:NCBI gene (formerly Entrezgene);Acc:397079]
chr4	68287351	68288355	1005	*	chr4	68287351	68288355	in_vitro	in_vivo	13	11	1.38615385	1.26909091	0.11706294	c(t = 0.15590	0.878	0.97617269	Distal Intergenic	ENSSSCG000	CRH	corticotropin releasing hormone [Source:VGNC Symbol;Acc:VGNC:86992]
chr5	6733234	6733918	685	*	chr5	6733234	6733918	in_vitro	in_vivo	9	9	89.0822222	89.7555556	-0.6733333	c(t = -0.1551	0.879	0.97617269	Distal Intergenic	ENSSSCG000	CENPM	centromere protein M [Source:VGNC Symbol;Acc:VGNC:86553]
chr10	68012843	68014067	1225	*	chr10	68012843	68014067	in_vitro	in_vivo	10	12	23.97	26.2616667	-2.2916667	c(t = -0.1536	0.879	0.97617269	Distal Intergenic	ENSSSCG000	WDR37	WD repeat domain 37 [Source:VGNC Symbol;Acc:VGNC:96258]
chr17	62687177	62689176	2000	*	chr17	62687177	62689176	in_vitro	in_vivo	9	10	16.8577778	18.842	-1.9842222	c(t = -0.1552	0.879	0.97617269	Distal Intergenic	ENSSSCG000	LIME1	Lck interacting transmembrane adaptor 1 [Source:NCBI gene (formerly Entrezgene);Acc:100625216]
chrX	109419911	109422213	2303	*	chrX	109419911	109422213	in_vitro	in_vivo	12	12	9.27916667	8.015	1.26416667	c(t = 0.15424	0.879	0.97617269	Distal Intergenic	ENSSSCG000	ssc-mir-363-	ssc-mir-363-1 [Source:miRBase;Acc:MI0014771]
chrX	34763275	34765592	2318	*	chrX	34763275	34765592	in_vitro	in_vivo	15	14	7.16333333	6.41285714	0.75047619	c(t = 0.15328	0.879	0.97617269	Promoter (<=1kb)	ENSSSCG000	MID1IP1	MID1 interacting protein 1 [Source:VGNC Symbol;Acc:VGNC:90214]
chr10	58075410	58076488	1079	*	chr10	58075410	58076488	in_vitro	in_vivo	9	10	4.50111111	3.851	0.65011111	c(t = 0.15649	0.879	0.97617269	Distal Intergenic	ENSSSCG000	CCNY	cyclin Y [Source:VGNC Symbol;Acc:VGNC:95998]
chr7	26141234	26142263	1030	*	chr7	26141234	26142263	in_vitro	in_vivo	12	11	2.66833333	2.44727273	0.22106061	c(t = 0.15428	0.879	0.97617269	Distal Intergenic	ENSSSCG000	HCRTR2	hypocretin receptor 2 [Source:NCBI gene (formerly Entrezgene);Acc:397428]
chr17	48966571	48967457	887	*	chr17	48966571	48967457	in_vitro	in_vivo	10	10	1.67	1.467	0.203	c(t = 0.15591	0.879	0.97617269	Distal Intergenic	ENSSSCG000	SLC35C2	solute carrier family 35 member C2 [Source:VGNC Symbol;Acc:VGNC:95831]
chr13	50979300	50980272	973	*	chr13	50979300	50980272	in_vitro	in_vivo	12	12	1.62083333	1.375	0.24583333	c(t = 0.15416	0.879	0.97617269	Distal Intergenic	ENSSSCG000	MITF	melanocyte inducing transcription factor [Source:VGNC Symbol;Acc:VGNC:90243]
chr8	103927226	103927903	678	*	chr8	103927226	103927903	in_vitro	in_vivo	12	14	1.52666667	1.35428571	0.17238095	c(t = 0.15451	0.879	0.97617269	Distal Intergenic	ENSSSCG000	FABP2	fatty acid binding protein 2 [Source:NCBI gene (formerly Entrezgene);Acc:595106]
chr6	87825080	87825809	730	*	chr6	87825080	87825809	in_vitro	in_vivo	9	12	1.20333333	1.10916667	0.09416667	c(t = 0.15482	0.879	0.97617269	Distal Intergenic	ENSSSCG000	FABP3	fatty acid binding protein 3 [Source:NCBI gene (formerly Entrezgene);Acc:399532]
chr17	38255633	38256157	525	*	chr17	38255633	38256157	in_vitro	in_vivo	9	9	0.95888889	0.79333333	0.16555556	c(t = 0.15535	0.879	0.97617269	Distal Intergenic	ENSSSCG000	ACSS2	acyl-CoA synthetase short chain family member 2 [Source:VGNC Symbol;Acc:VGNC:95665]
chr12	36803215	36803917	703	*	chr12	36803215	36803917	in_vitro	in_vivo	9	11	23.8111111	21.5927273	2.21838384	c(t = 0.15339	0.88	0.97629565	Exon (NM_001246249/100620126, exon 10 of 10)	ENSSSCG000	TBX4	T-box transcription factor 4 [Source:NCBI gene (formerly Entrezgene);Acc:100620126]
chrX	124483800	124487109	3310	*	chrX	124483800	124487109	in_vitro	in_vivo	11	14	20.3436364	18.755	1.58863636	c(t = 0.15241	0.88	0.97629565	Distal Intergenic	ENSSSCG00000024676		SRSF protein kinase 3 [Source:VGNC Symbol;Acc:VGNC:93464]
chr7	23995901	23997382	1482	*	chr7	23995901	23997382	in_vitro	in_vivo	9	11	9.69777778	10.2618182	-0.5640404	c(t = -0.1525	0.88	0.97629565	Promoter (<=1kb)	ENSSSCG000	ZBTB12	zinc finger and BTB domain containing 12 [Source:VGNC Symbol;Acc:VGNC:95060]
chr17	47488984	47490140	1157	*	chr17	47488984	47490140	in_vitro	in_vivo	15	14	6.65466667	6.23285714	0.42180952	c(t = 0.15240	0.88	0.97629565	Distal Intergenic	ENSSSCG00000035053		peptidase inhibitor 3 [Source:NCBI gene (formerly Entrezgene);Acc:396757]
chr6	167357347	167358754	1408	*	chr6	167357347	167358754	in_vitro	in_vivo	9	9	3.47	3.82	-0.35	c(t = -0.1529	0.88	0.97629565	Distal Intergenic	ENSSSCG000	KLF17	KLF transcription factor 17 [Source:NCBI gene (formerly Entrezgene);Acc:100302639]
chr4	34221012	34222788	1777	*	chr4	34221012	34222788	in_vitro	in_vivo	12	14	2.36833333	2.51428571	-0.1459524	c(t = -0.1528	0.88	0.97629565	Distal Intergenic	ENSSSCG000	AZIN1	antizyme inhibitor 1 [Source:VGNC Symbol;Acc:VGNC:85712]
chr1	111374964	111376856	1893	*	chr1	111374964	111376856	in_vitro	in_vivo	13	11	1.83846154	1.70636364	0.1320979	c(t = 0.15314	0.88	0.97629565	Distal Intergenic	ENSSSCG000	ANXA2	annexin A2 [Source:VGNC Symbol;Acc:VGNC:103027]
chr14	140962216	140967477	5262	*	chr14	140962216	140967477	in_vitro	in_vivo	9	11	26.1833333	28.6072727	-2.4239394	c(t = -0.1522	0.881	0.976841	Distal Intergenic	ENSSSCG000	ssc-mir-202	ssc-mir-202 [Source:miRBase;Acc:MI0013157]
[truncated: 219,528 more chars]
